# Atom-level machine learning of protein-glycan interactions and cross-chiral recognition in glycobiology

**DOI:** 10.1126/sciadv.adx6373

**Published:** 2025-12-05

**Authors:** Eric J. Carpenter, Chuanhao Peng, Simatsidk Haregu, Nicholas Twells, Logan Woudstra, Amika Sood, Jonathan Cartmell, Robert J. Woods, Lara K. Mahal, Sheng-Kai Wang, Russell Greiner, Ratmir Derda

**Affiliations:** ^1^ Department of Chemistry, University of Alberta, Edmonton, Alberta T6G 2G2, Canada.; ^2^ Department of Computing Science, University of Alberta, Edmonton, Alberta T6G 2E8, Canada.; ^3^ Complex Carbohydrate Research Center, University of Georgia, Athens, GA 30602, USA.; ^4^ Department of Chemistry, National Tsing Hua University, Hsinchu 30013, Taiwan.; ^5^ Frontier Research Center on Fundamental and Applied Sciences of Matters, National Tsing Hua University, Hsinchu 30013, Taiwan.; ^6^ Alberta Machine Intelligence Institute (AMII), 1101-10065 Jasper Avenue, Edmonton, Alberta T5J 3B1, Canada.

## Abstract

We describe a machine-learned (ML) model, MCNet, which predicts interactions between proteins and glycans. MCNet predicted quantitative interactions between glycan-binding proteins (GBPs) and enantiomers of common glycans, which were not part of the original training datasets. l-glycans are rare in nature but are important in consideration of safety of putative mirror-image life-forms. Current ML models that predict properties of glycans from their monosaccharide composition cannot extrapolate properties of mirror glycans. Instead, MCNet uses an atom-level description of the glycan to output an estimate of binding to GBPs. MCNet is trained using data from glycan microarrays and affinity measurements unified using a “fraction bound” parameter. Trained MCNet predicted unexpected binding of l-glucose to some fucose-binding GBPs. Both glycan and lectin arrays conformed these predictions. ML models akin to MCNet reach beyond traditional glycobiology and make it possible to anticipate interaction between biomolecules in mirror-life forms and present-day life-forms.

## Teaser

Machine learning algorithms, validated by experiment, can effectively investigate the safety of glycans from putative mirror life.

## INTRODUCTION

Glycans are major building blocks of life and major constituents of the glycocalyx that coats cells in all kingdoms of life (*1*). Recognition of glycans by glycan-binding proteins (GBPs or lectins) is critical for cell-to-cell communication, immune responses, self versus nonself recognition (*2*), pathological responses like tumorigenesis (*2*), and normal physiological processes like fertilization (*3*, *4*). Unlike DNA, RNA, or proteins, glycans form linear and branched oligomers with diverse stereochemistry of the monomers and linkages, giving rise to a vast “feasible complexity” of glycans (*5*); that is, the number of feasible glycan structures is orders of magnitude higher than for similarly sized nucleotides or proteins, even for small structures (*6*, *7*). However, estimates of “biologically relevant complexity”—the number of distinct glycan structures synthesized by a given organism—are smaller by many orders of magnitude. For example, the human glycome is estimated to be limited to ~7000 structures created by known glycosynthetic enzymes (*8*). Reinforcing this dichotomy in the numbers of possible glycans, inquiries in glycobiology are skewed to glycans of biologically relevant complexity. Such glycans dominate glycan microarrays, like those produced by the Consortium for Functional Glycomics (CFG) (*9*). Data from CFG and related glycan arrays are used for training of machine learning (ML) models that can then predict protein-glycan interaction (*10*). The resulting ML models, again, are focused on predicting interactions between the biologically relevant glycan motifs and well-understood lectins in their training data. Some researchers may question the need to study “biologically irrelevant” protein-glycan recognition, while others (*11*) teach the need to break away from dogmatic paradigms. For example, the elegant report of Canner and Gray used ML models to ask the fundamental question, what is a glycan binding protein (*12*–*14*), and uncovered the intriguing possibility that many organisms contain GBPs that have not yet been acknowledged as such. An interesting example are C-type lectins; it is not known how many of the C-type lectins actually bind to either traditional or nontraditional glycans (*15*, *16*). Here, we train ML models that predict quantitative glycan-GBP interactions; however, our primary goal is to break away from the limitations of working complexity and instead examine binding in (i) the feasible complexity of glycans and (ii) glycomimetic (GM) structures.

Emerging open-ended questions from the feasible complexity pertain to enantiomers of known glycans. Such glycans would be produced by mirror-image life, for which the plausibility of laboratory construction has been discussed for the past few decades (*17*). The feasibility of creating mirror life was boosted by the recent synthesis of critical d-enzymes such as d-DNA-ligase, d-RNA polymerase, and enantiomeric ribozymes (*18*–*20*), as well as enzymes for ligation of d-proteins (*21*). The pioneering work of Kent and co-workers experimentally proved that enzymes made of d-amino acids do not recognize peptide and peptidomimetic substrates of the natural enzyme, but they effectively process the enantiomers of natural substrates (*22*). Mirror-image phage display (*23*) showed that l-peptides evolved to recognize mirror-image d-proteins do not bind to l-proteins; however, the mirror d-peptides do bind to l-proteins. The resistance of mirror-image proteins (*24*) and enantiomeric aptamers (*18*–*20*) to degradation by natural peptidases and nucleases in vivo is well established. These combined observations lead to a general notion of an absence of cross-chiral recognition between peptides, nucleotides, and metabolites. This limited cross-chiral recognition is an important consideration raised in a 2024 report in *Science*, cautioning that if mirror-life forms were constructed, they may evade many aspects of immune recognition, response, and ecological control that are present in conventional-chiral organisms (*25*). A follow-up commentary (*26*) discussed the overwhelming abundance of glycans, the critical role of glycan-GBP interactions in self versus nonself determination, and prevalence of mirror-image forms (enantiomers) of monosaccharide building blocks in nature. We refined this argument by analyzing 19,266 glycans across all kingdoms of life compiled by Bojar *et al.* (*27*) used to predict the taxonomic origin of glycans (*27*–*29*). From 947 glycan monomers in this set, only 85 + 85 can be matched as bona fide enantiomeric pairs (fig. S1). d-hexoses—mannose (Man), glucose (Glc), galactose (Gal), and their *N*-acetylated derivatives (e.g., GlcNAc) are abundant in the glycocalyx of most organisms. l-hexose counterparts are rare in nature, while the NAc-derivatives have never been detected (fig. S1). In contrast, both l and d forms are naturally abundant for rhamnose (Rha), xylose (Xyl), fucose (Fuc), and its *N*-acetyl derivative (FucNAc). It is possible that l/d imbalance in such datasets stems from the observation bias. Nature—containing many more prokaryotes than eukaryotes—may be much richer in l-glycans than purported by current knowledge sets of glycans, the latter being eukaryotic centric and even human centric.

**Fig. 1.**
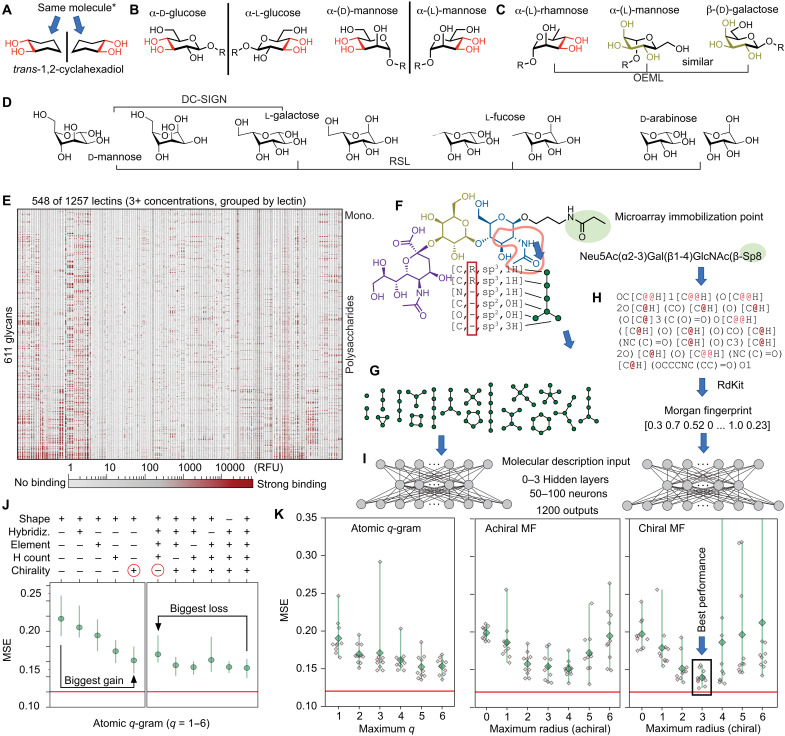
ML of glycan-GBP interactions from all-atomic description. (**A** to **D**) Examples of preservation of stereochemical motifs between enantiomers, rare l-glycans, and naturally abundant d-glycans. (**E**) Summary of the glycan:GBP binding data from CFG used for ML. (**F** to **H**) Each glycan is converted to counts of (G) AQG or (H) MF. (F) Features of nodes in AQG include element, chiral state, hybridization, attached hydrogen count, and overall shape. (**I**) Either MQG or MF counts are used as input into a fully connected neural network that is trained to produce binding strength estimates at its outputs. (**J**) MSE is used to measure the performance of models using different features in the AQG nodes. Models trained using AQG with only a minimal set of features rank importance of chirality > H-count > element > hybridization. Similarly, systematic removal of features from AQG show that removal of chirality diminishes performance, whereas removal of hybridization, element identity, or H-count is inconsequential. (**K**) Comparison of AQG of size 1 to 6 and MF or *r* = 0 to 6 show that AQG with six atoms achieve best performance in AQG, and chiral MF3 as best across all representations. There is a marked improvement in models trained using chiral versus achiral MF3. Each point is an MSE measured in a separate training fold; the vertical green line shows mean and range of MSE; the red line denotes the performance of GlyNet with monosaccharide embedding.

Mirror-image glycans would be one of the first entities encountered by our immune systems in interactions with putative mirror-image life-forms (*26*) or present-day life-forms that use such mirror-image building blocks; however, our understanding of cross-chiral recognition between proteins and glycans is very limited. For example, the interaction of 1,2-bis-equatorial diol of d-mannose with Ca^2+^ in C-type lectins is at the heart of the immune response elicited by these lectins (*15*, *16*). Recognition of biologically rare l-mannose (*30*) is rarely studied (*31*), but the core recognition motif of d- and l-mannose is mirror symmetric, 1,2-bis-equatorial diol. Reflection of this diol produces the same 1,2-bis-equatorial diol (Fig. 1, A and B). Antibodies to l-rhamnose (6-deoxy-l-mannose) are abundant in human serum (*25*), but recognition of l-mannose by l-rhamnose–binding proteins is poorly understood. The spatial arrangement of three adjacent hydroxyls in l-mannose mimics one in d-galactose (Fig. 1C). There are scarce reports of l-mannose serving as a ligand for galactose-binding proteins (*31*), and complementary reports that l-galactose is recognized by the d-mannose–binding immune lectin Dendritic Cell-Specific Intercellular adhesion molecule-3-Grabbing Non-integrin (DC-SIGN) (*26*). *Osmerus eperlanus mordax* [*sic*] lectins recognize the monosaccharides l-Rha, l-Man, and d-Gal (*30*), while *Ralstonia solanacearum* lectin (RSL) recognizes another set, l-Fuc, l-Gal, d-Ara, and d-Man (Fig. 1D) (*32*). In turn, l-galactose, the enantiomer of common galactose, and a close analog of natural l-fucose was reported as a viable substrate of α1,2-l-fucosyltransferase (*33*). Further, the discovery of l-glucosidases points to possible use of l-glucose in glycoconjugates and the existence of lectins for their recognition (*30*, *34*). A parsimonious interpretation of this binding behavior is that the structural similarities between monosaccharides and the co-occurrence of these building blocks in nature incentivizes lectin evolution. This critical difference in symmetry inversion between glycans and other biological oligomers highlights that cross-chiral glycan-GBP interaction is more plastic than cross-chiral recognition between polypeptides, oligonucleotides, and metabolites (*25*). We propose that ML models that effectively embed atomic composition and chirality of atoms can improve evaluation of cross-chiral recognition between glycans and GBPs and unify our understanding of traditional glycans and the “rare glycans” observed in the microbial world.

Outside of the traditional domain of mammalian N- and O-linked glycans, data for glycan-GBP recognition are not systematic. Even in a human glycome, recognition of heparin sulfate proteoglycans and mucins is only starting to emerge (*35*, *36*). Rules for recognition by GBPs for plant glycans (*37*) or eubacterial glycans are built progressively using complex organic synthesis of glycan arrays (*38*–*41*) and specialized lectin arrays (*42*, *43*). *N*-glycans in Archaea contain “exotic” structures, i.e., those that have different architectures and subunits when compared to commonly studied mammalian or plant *N*-glycans (*44*). Intriguingly, many orthologous functions, including speciation in sexually reproducing Archaea have been proposed to be driven by interactions of these exotic glycans with as yet uncharacterized GBPs (*44*). A barrier toward integrating this information is the ever-increasing, but still incomplete, list of all monosaccharide building blocks across all kingdoms of life. Mirror inversion exacerbates the problem by effectively doubling the complexity. ML models that represent glycans strictly as collections of carbohydrate monomers (*27*, *29*, *45*) cannot extrapolate properties of any glycans that contain monosaccharides that are not included in the model’s encoding and training. GLAMOUR (*28*), GNNGLY (*46*), and GIFFLAR (*47*) models are based on atom-level descriptions and hybrid monomer-atom level descriptions of glycans. Such models can extrapolate properties of uncommon building blocks, but an imbalance between “common” and “rare” building blocks in training datasets (fig. S1) may or may not make it difficult for these models to predict of binding properties of molecules containing the rare monosaccharides. Monomer level descriptions are also not trivial to apply to arbitrary chemical structures in datasets like BindingDB (*48*) or ChEMBL (*49*). Here, we examined the possibility of completely foregoing monosaccharide-level descriptions and predicting GBP-binding properties of glycans from only their atom-level structures.

Atom-level structural insights such as crystallography and molecular dynamics are workhorses in understanding glycan-protein interactions (*50*). These investigations show that the specific conformations of the glycan and of the protein determine the glycan-GBP recognition (*51*). This realization led to development of methods that estimate binding properties by computing conformations of proteins and glycans to find optimal matches (e.g., by docking) (*50*, *52*). Such calculations approximate thermodynamic values by intensive computations of the ensemble of all conformers and are contrasted by Anfinsen’s thermodynamic hypothesis that “the folded structure is determined [solely] by the amino acid sequence” (*53*). This hypothesis is reinforced by the 2024 Nobel Prize for ML models that predict folded structures from the primary sequence (*54*). Subsequent predictions of structure of protein-ligand complexes (*55*) suggest that ML models can predict strength of interactions using only the internal atomic connectivity of the two partners. Here, we examine the plausibility of lightweight ML models that predict binding of protein to glycan from the mere atomic connectivity of glycan. A caveat in the Anfinsen hypothesis is the role of the environment; Anfinsen’s ribonuclease (RNAse) adopted two distinct conformations in folding and denaturing buffers (*53*). Similarly, all molecular interactions depend on the environment, but successful ML models for protein-glycan interactions have been trained (*27*, *56*–*58*) by focusing on data from nearly uniform binding environments (similar temperature, buffer, and pH). Concentration of binding partners is an important consideration because the binding tends to zero as the concentration tends to zero. Most ML models predict binding of glycans to proteins at fixed concentrations (*27*, *45*, *47*, *59*, *60*). We incorporate the concentration into the training explicitly to allow merger or data from different sources and to predict fraction bound (*f*) at a given concentration. We paraphrase the dream of “I want to know if this oligosaccharide motif binds to this GBP,” to the more finely detailed request, “Given a GBP and a glycan of a definitive atomic composition, I want to know the fraction of glycan, *f*, bound to this GBP.” Our manuscript shows the plausibility of training a model that takes a glycan, GBP, and the concentration, as input and returns the associated fraction bound. Such model predicted hitherto unknown interactions of rare enantiomers of glycans with common GBPs, and we validated these predictions experimentally. In a head-to-head comparison with state-of-the art models, we observe that some of these interactions were not anticipated by models that predict interactions of proteins with glycans (*27*, *47*) and models that predict interactions of protein with generic small molecules (*61*, *62*). A model that forgoes monosaccharide composition can readily incorporate properties of glycans across all kingdoms of life as well as documented effects of spacers, linkers, and scaffolds on which glycans are displayed (figs. S2 and S3). It also provides the first important glimpses into cross-chiral recognition between glycans and GBPs.

## RESULTS

### Embedding of atomic representations of glycans

As in previous reports (*9*, *27*, *45*, *59*), the core dataset for the training of ML models was binding of GBP to glycans on microarrays produced by the CFG. We specifically used data from Mammalian Printed Array version 5. In short, these data describe performance of 611 glyco-conjugates printed in discrete spots on the glass plate exposed to 1257 fluorescently labeled GBP compositions and imaged by fluorescent scanner. The amount of protein bound to each glycan is reported in relative fluorescent units (RFUs, Fig. 1E). These data have been used to train classifiers to predict binder versus nonbinder (*58*, *63*, *64*) or similar binary decisions (e.g., has motif/does not have motif) (*57*, *65*) and regression-type models with continuous valued (numeric) outputs (*27*, *45*, *47*, *59*, *60*). We previously trained a neural network, GlyNet, to predict 1257 continuous RFU values when given a glycan structure represented by decomposing the structures to constituent polysaccharide *q*-grams (MQG) (*59*). To test whether atom-level representations of glycans can predict qualitative interactions with GBP, we converted the 611 glycoconjugates to two types of representations of molecular structures, Morgan fingerprints (MF) and atom *q*-grams (AQG, Fig. 1, F to H). AQGs, inspired by genomic *k*-mer statistical methods (*66*, *67*) allowed us to draw an explainable parallel between monosaccharide-tree embedding (*59*) and the atom-level embedding. Replacing the MQG inputs of the GlyNet architecture with MF or AQG inputs gave rise to several neural network architectures with two to three hidden layers and 50 to 100 neurons per layer (Fig. 1I); each architecture was optimized using Adam (*68*) and tested in 10-fold cross-validation (with respect to glycans) for their ability to predict the RFU values for lectins given an MF or AQG representation of CFG glycans.

Figure 1 (J and K) presents the performance for each neural network model after selecting an optimal number of hidden layers, nodes in hidden layers, and weight decay parameters. The distribution of glycans to the 10 cross-validation folds was identical for all models and identical to that of the previously published GlyNet (*59*); hence, the distributions of mean squared errors (MSEs) across the folds is comparable between all models with the MSE of the GlyNet model (red line in Fig. 1K) and by proxy to many other state-of-the art models (*27*, *56*–*58*). A simple, explainable representation of glycans using AQG of size 6 yielded MSE = 0.15, which was only modestly worse than GlyNet with its monomer-level embedding (MSE = 0.12). Systematic subtraction or addition of atom feature labels such as graph topology, element, hybridization, chirality, number of attached hydrogens (H-count), and chirality of the node (R, S, or none) rank chirality as the most important feature and H-count as the least important atom features (Fig. 1J). Deletion of atom identities or their hybridization (proxy for bond orders) was inconsequential to predictions. Describing glycans by featureless graphs with six nodes yielded MSE = 0.22, but adding only chirality information yielded MSE = 0.16 (Fig. 1J). Both observations show an expected critical role of chirality in predicting binding of glycans to lectins. Intriguingly, we observed that we could reasonably predict glycan-protein interactions by representing glycans as graphs only labeled with the chirality of each node. This observation shows how glycan-GBP recognition differs from the interactions of proteins with classical small molecules that contain few to no chiral centers.

MFs of radius ≤3 and including chirality (MF3_c_) yielded a performance comparable to that of GlyNet (Fig. 1K). Elimination of chirality in the MFs led to a notable decrease in performance (Fig. 1K). Decreasing the maximum radius below 3 was clearly detrimental, whereas increasing it above 3 (from 1385-dimensional to 3142-dimensional vectors at *r* ≤ 4) also decreased median performance and creates increased fluctuations between the folds. Both observations may be due to the small size of the dataset. The ML models that use MF as inputs and predict performance over 1257 lectins can be tuned to have similar performance as existing, published models trained in this dataset (fig. S4). This observation clearly demonstrated that it is possible to predict glycan-binding performance by replacing a domain-specific monomer composition with universal atom-level representation. A simple learning architecture like fully connected neural networks can be trained even over a relatively small dataset of 600 glycans despite the relatively large number of atoms in some glycans (500 nonhydrogen atoms).

### Expansion of training beyond microarray datasets

**Fig. 2.**
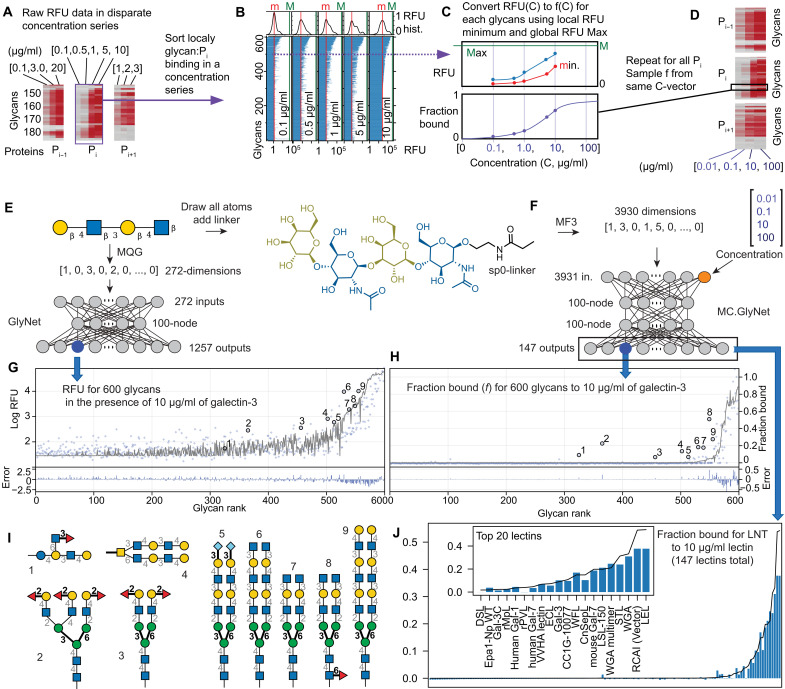
Workflow of data in training of MCNet. (**A**) A total of 147 lectins tested at multiple concentrations in the CFG v5 data were identified. (**B**) Microarray RFU distributions were processed to extract a background and maximum that were then (**C**) used to rescale into a fraction bound and (**D**) resampled at multiple concentrations. We compared (**E**) our prior monosaccharide tree–based RFU model to (**F**) an atom-graph (includes spacer structures) model using MF and protein concentrations. The results (**G** and **H**, respectively) show similar trends. In particular, (**I**) a group of glycans predicted by the MF Net model to bind more strongly to Gal-3 than the CFG data, overlaps significantly (6 of 10) with over-predictions in the prior GlyNet model, and are of structures reported (*76*, *77*) to have binding. (**J**) Model predictions for binding of LNT to the 147 lectins.

Atom-based representation makes it possible to combine learning for natural glycans and “unnatural” GM compounds, which have structures that are not composed of monosaccharides. To test this hypothesis, we selected 274 GM structures with reported binding strength (association constants, *K*_a_) to galectins-1, -3, and -7 manually curated from 42 publications (see tables S1, S2, and S5). We embedded the GM structures using MF3_c_ and attempted to train a multioutput neural network that predicted RFU values in some outputs and *K*_a_ values for other outputs. The training failed, likely because of the disparate structure of the datasets; RFU values were available for CFG glycans, but not GMs, while *K*a data were available only for the GMs but not the CFG glycans (see Supplementary Discussion and figs. S5 to S7). Arthur and coworkers previously extrapolated dissociation constant (*K*_d_) surrogates from the RFU values, but this demonstration was limited to one class of arrays and its ability to yield ML-compatible merger of disparate data types is unclear (*69*). In contrast, Haab and coworkers elegantly merged binding data from disparate experimental sources in the CarboGrove (*56*) dataset and accompanying ML model, by focusing on specific motifs in dose response curves (*65*, *70*). Inspired by Haab’s success with dose-response curves, we elected to convert both RFU and K_a_ to a series of values describing the fraction, *f*, of the glycans bound to the GBP at a concentrations, *C* (Fig. 2, A to D, and figs. S6 to S8). *K*_a_ values are trivially converted to *f*(*C*) via the Henderson-Hasselbalch equation (fig. S7). For the CFG glycans, *f* was interpolated between the available concentrations using an array-specific minimum and a global maximum RFU (Fig. 2, A to D, detailed in fig. S6 and data S2). The *f* value is suited to learning because it is always in the (0, 1) region. It allows the qualitative dichotomy where *f* ≈ 0 and *f* ≈ 1 are equivalent to “no binding” and “yes binding”; it also brings an important reminder that such assessment can be made only when concentration is specified. Last, *f* has an intuitive interpretation [e.g., *f* = 0.5 at a concentration nearing *K*_a_, median inhibitory concentration (IC_50_), or median effective concentration (EC_50_)], although such generalization needs to be done with caution as highlighted in numerous publications (*71*–*75*).

To test *f* as a predictive output, we trained a fully connected neural network, *Net*, where ***f*** = *Net*(**M**, *C*) takes inputs **M** and *C*, the MF3 description vector and the concentration, respectively, and returns the resultant fractions bound, ***f***. This architecture, referred to as MCNet, has 3931 inputs, 1 for protein concentration (*C*) and the 3930-dimensional **M**-vector MF3_c_, and the 147-dimensional output ***f*** vector has one value for each of the 147 lectins from the CFG dataset (Fig. 2F). Models were trained on CFG glycans using the same 10-fold cross-validation structure as above with one addition; all concentrations were in the same hold-out fold. MSEs from MCNet cannot be compared directly to the MSE of models trained on RFU (fig. S4) or *z* scores (*27*, *47*), but assessment can be made on individual predictions of glycan-binding profiles for galectins in Fig. 2 (G versus H) and all other lectins juxtaposed with predictions made by a monosaccharide-based model GlyNet (or LectinOracle; see data S9). In addition to comparison of predictions made for microarray data in held-out datasets, we compare the ability of MCNet, and LectinOracle and other models to extrapolate beyond microarray data to enantiomeric glycans (see “Mirror image glycan extrapolation” section).

Of 599 glycans, MCNet predicted ~10 with modest upward deviation from the predicted trend (i.e., “false-positive binders”; Fig. 2H). Examining individual structures, we noted that 7 of the 10 glycans contain definitive galectin-binding motifs (LacNAc repeats; Fig. 2I), and 6 of the 10 of these had the same over-predicted property in the prior GlyNet model, many more than random chance would predict. Atom-level model MCNet and GlyNet are apparently learning galectin-binding features from binding of other glycans (Fig. 2, E to H), although MCNet is looking at smaller groups of atoms and GlyNet at the groups of monosaccharides. Glycans 2 and 3 contained terminal LacNAc with a α-2 fucosylation on the galactose (group H; Fig. 2I); however α–2 fucosylated lactose has been reported to bind more strongly than lactose by Klassen and co-workers (*76*), and the α–2 fucosylated LacNAc was confirmed as ligand for galectin-1 by Surolia and co-workers (table S1) (*77*). A 2022 run of galectin-3 binding to CFG arrays by Arthur, Stowel, and co-workers (data not used in MCNet training) confirmed that group-H glycans on CFG arrays are modest, but detectable, binders to galectin-3 (*69*). The same glycans (2 and 3) were predicted to have an order of magnitude higher RFU than the “ground truth” by the GlyNet model (Fig. 2G). We suspect that these glycans are experimental false negatives of the glycan microarray measurements rather than false positives of the ML model. Thus, the atom-level MCNet not only achieved the desired performance but also highlighted experimental false-negative observations in the CFG dataset. This is not to criticize the CFG dataset but rather is a reminder of the well-established notion that the experimental uncertainty defines a natural upper limit to the predictive performance of ML models (*75*).

**Fig. 3.**
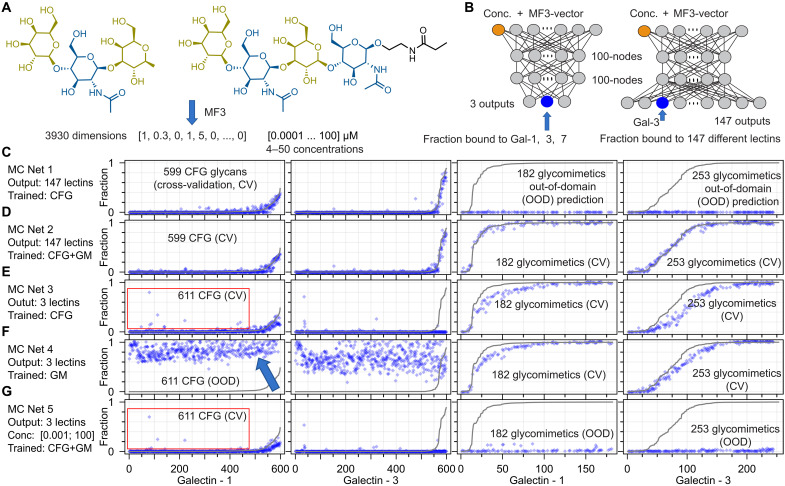
Predicted binding for the CFG glycans and the GMs across different model classes. All plots are at ~10 μg/ml. (**A**) Both glycans and GM compounds can be embedded as MF3 vector. (**B**) MCNet architectures with either 3 or 147 outputs were used in these tests. (**C**) MCNet1 trained using CFG glycan data (analogous to Fig. 2, F and H) predict binding of CFG glycans to galectins in held-out datasets but failed to predicts binding of GMs to galectins. (**D**) MCNet2 trained on GM data exhibits reasonable prediction of GM-galectin binding but fails in predicting binding properties of the CFG glycans. (**E**) MCNet3 trained using both datasets exhibits the desired predictions and exhibits improvement in predictions of GM:galectin interactions when compared to MCNet2, trained only on GM data, or (**F**) to MCNet4, trained only on galectin-binding data of GM and CFG compounds. (**G**) MCNet5 trained only on galectin:CFG data makes inferior predictions for binding of CFG glycans to galectin-3 and it only learns some trends in galectin-1:CFG dataset.

We tested the ability of the MCNet architecture to make out-of-domain predictions. MCNet1 was trained only on CFG data and was unable to predict any binding for GM molecules (Fig. 3C). Training model MCNet2 only on the GM dataset made it possible to predict the binding of GM compounds in held-out folds, but MCNet2 was completely wrong in predicting the binding of CFG glycans to galectins (Fig. 3D). In contrast, the MCNet3 model (trained on both GM and CFG data) effectively predicted GM binding to Gal-1, -3, and -7 for held out GM folds and to all 147-lectins for held-out CFG folds (Fig. 3E and data S10). Glycan and GM recognition are nonoverlapping, and knowledge of one does not provide knowledge suitable for the other. Conversely, blending of datasets is positively synergistic; accuracy of the binding prediction of GMs to galectins and even for CFG glycans to the 144 other lectins increased for the MCNet3 model, trained using both CFG and GM molecules. We note that the number of compounds increased from 611 in CFG to 885 in the combined CFG and GM dataset. The increase in accuracy is more than would be naïvely expected from a mere 45% increase in the data size.

As a control, model MCNet4, trained on all GM, but only on the subset of CFG data describing binding to galectins-1, -3, and -7, was also able to predict binding for held out GM and CFG datasets, albeit with worse accuracy than MCNet3 (Fig. 3G). This observation illustrates how knowledge from seemingly unrelated glycan-GBP interactions, used in training of MCNet3, enhanced the accuracy of prediction of glycan-galectin interactions. Another control MCNet5 trained only on a subset of CFG data describing binding to galectins-1, -3, and -7 to CFG glycans, failed to predict GM binding (Fig. 3G). We noticed that prediction of galectin-3 binding deteriorated in MCNet4 and 5, but galectin-1 predictions remained similar to MCNet1 and 3 (Fig. 3, F and G). It is tempting to blame the ML architecture for these failures, but the reasons are likely more fundamental. MCNet1 exhibits lower MSE than MCNet3 (fig. S11), and yet it fails to extrapolate from CFG to GM datasets (Fig. 3C), indicating that the rules of protein recognition by chiral hydrophilic sp^3^-centers in CFG dataset and achiral hydrophobic sp^2^-centers in GM dataset cannot be extrapolated from one another. This observation is a reinforcement of well-known notion that value of trained ML models should be assessed not only on MSEs but also on performance in outside-of-domain datasets.

We compared the performance of MCNet architectures to the performance of models that use different learning architectures or different datasets. In the same dataset of glycans (G) and using the same 10-fold partitioning, a Random Forest (RF) architecture yielded similar MSEs, while eXtreme Gradient Boosting (XGBoost) (*78*) produced improved MSEs on the MCNet1 task (fig. S11). This comparison is not trivial to extend to G + GM datasets (i.e., MCNet3) because missing data points in the G + GM dataset necessitate substantial imputation of filler values. We demonstrated expansion to a larger number of lectins as well as to microarray data measured only on one concentration. In training on an expanded dataset that included lectins measured only in one concentration, the change MSE was only minor (fig. S11). Inspired by LectinOracle (*27*, *47*), we converted the MCNet multioutput architecture to a model that takes a protein representation (Evolutionary Scale Modeling), glycan representation (MF3_c_), and concentration as inputs to calculate the fraction bound. A preliminary exploration of architectures (fully connected neural networks, RF, and XGBoost) using the same loss function as MCNet (MSE calculated in the same glycan-based partitioning as MCNet) produced an MSE comparable to MCNet’s (fig. S11). We note that a true power of protein-aware models—extrapolation to proteins beyond proteins included in the training dataset—are beyond the scope and experimental data collected here and will be evaluated elsewhere.

### Out-of-domain extrapolation of binding properties to other datasets

In principle, MCNet architectures are capable of extrapolating lectin binding not only to glycan structures but also to any molecule (e.g., molecules from BindingDB; see fig. S12). High-level observations mirror the observations already made in Fig. 3. Specifically, MCNet1, trained only on CFG glycans, predicts zero binding performance for all molecules that are not bona fide glycans. Despite these deficiencies, MCNet1 effectively identifies glycans that are reasonable predictions as ligands for specific lectins. As there are currently no systematic ground truths for these predictions, only some of these predictions can be evaluated. An interesting case is the bacterial glycans, which are not part of the training set. MCNet predicts that galectins-1 and -3 interact with Gal-GalNAc oligomers, structures reminiscent of the *Escherichia coli* O127 outer polysaccharide and the *Klebsiella pneumoniae* O-antigens and other bacterial polysaccharides reported by Drickamer and co-workers (*42*), Kiessling, Imperiali and co-workers (*79*), and Cummings, Arthur, Stowell and co-workers (*80*, *81*). Such data provide a baseline benchmark for all future predictions or experimental validations (see data S1).

### Mirror image glycan extrapolation

**Fig. 4.**
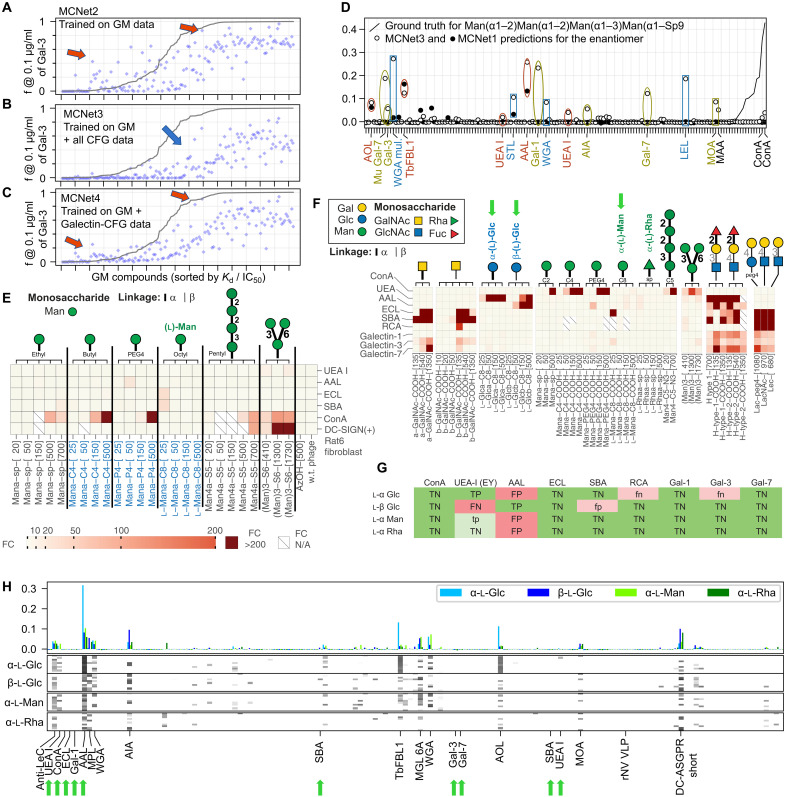
Prediction of cross-chiral recognition by MCNet. (**A** to **C**) Solid line describes the ground truth, the binding of GM compounds to galectin-3. Blue dots are the predicted binding strengths of the GM-enantiomers. Expert knowledge of GM–galectin-3 interactions dictates that all predictions should be below the solid line (i.e., the enantiomer’s activity is lower than the activity of the parental compound). Only MCNet3 (B) produced answers aligned with this “expert knowledge” of cross-chiral recognition. Galectins-1 and -7 exhibit similar trends (fig. S13). (**D**) MCNet3 predictions of binding of 147 lectins to α-l-Glc, β-l-Glc, α-l-Man, and α-l-Rha monomers displayed on a glycan array by a two-carbon linker (a.k.a., “Sp0” in CFG notation). Predictions for lectins denoted with green arrows [ConA, UEA-I (EY Labs), AAL, ECL, SBA, galectins -1, -3, and -7] have been validated using monomers displayed with liquid glycan array (**E** and **F**). These confirmed the binding of l-Glc to AAL and to UEA-I and a lack of binding of all other lectins. (E) Experimental confirmation of the cross-chiral recognition. l-mannose, displayed at 25 to 500 copies on M13 virions fails to bind to ConA; however, glycans containing d-mannose exhibit expected binding to the same liquid array. (F) Among nine lectins, the two canonical fucose-binders bind to l-Glc. The rightmost seven columns are d-sugars as positive controls. More details are shown in fig. S15. (**G**) Numeric values (fraction bound-ML model predictions) and fold change (LiGA) were thresholded to classify each as binding or nonbinding for comparison. Mean predictions were taken over by the models from the cross-validation hold-out fold. Notably, 28 of the 36 predictions agree between the two. (**H**) Details of fraction bound predictions for four l-sugars. Mean values are shown in colored bars (top) and values for individual cross-validation folds in heatmaps (bottom).

Inversion of all stereocenters in every molecule yields enantiomers of the glycans and GM compounds. We tested the ability of MCNet to predict recognition between lectins and the enantiomers of the molecules from the CFG and GM datasets. Such observations have no ground truth, only an “expert opinion.” For example, with 100% certainty, we can assert that every GM compound should bind to galectins-1, -3, and -7 much better than the enantiomer of the same compound. The absence of cross-chiral recognition arises from the well-understood structural role of the chiral centers around the galactose ring. Not all MCNet models predicted a drop in cross-chiral recognition. MCNet2 trained only on GM compounds placed the activity of enantiomers at random, either above or below the performance of the parental compounds (Fig. 4A). This observation is recurrent in the ML literature, and other reports have observed that chirality is one of the most difficult concepts for ML models to understand (*82*). In contrast, an MCNet3 model trained on GM and CFG data—the latter contains numerous examples of epimers and diastereomers—was able to predict that nearly all enantiomers of GM compounds are less active than parental compounds (Fig. 4B). It was important to include data for binding to all 147 lectins; MCNet4 trained using data for 300 GM and 600 CFG glycans binding only to galectins-1, -3, and -7 has diminished understanding of cross-chiral recognition (Fig. 4C) when compared to MCNet3 (Fig. 4B and fig. S13). MCNet appears to agglomerate the knowledge from various proteins effectively. Indirect knowledge of how epimerization of individual atoms influences glycan-protein recognition helps MCNet3 to produce the right answers for out-of-domain cross-chiral recognition.

Epimerization may yield another common glycan (e.g., changing a glucose into mannose), but it may also give rise to one typically considered unusual or rare (e.g., β-Glc to α-l-Ido). We examined the ability of MCNet to predict binding strength for epimers of existing glycans (i.e., molecules that differ from the original by the inversion of one chiral center; see data S11, pages 983 to 1594). MCNet 1 and 3 generally agree on their predictions with patterns of strongly bound lectins common to both models, but some predictions diverge where binding of the parent glycan is weak. As expected, for many glycans, both MCNet models showed epimerizations in small glycans that destroy recognition by lectins of the original glycan while often having no effect in larger glycans; see data S11. Further summary of observations is available in the Supplementary Materials.

We then tested the ability of MCNet to predict properties of enantiomers of the CFG glycans. A ground truth for these does not exist because it is unreasonable to propose manufacturing the CFG arrays with 600 enantiomers of existing glycans. However, P. Kim’s mirror-image paradigm (*83*) postulates that the binding of l-lectins to the enantiomers of CFG glycans can be measured by probing d-lectins against the existing CFG array. Unfortunately, synthesizing C-type lectins (DC-SIGN, DC-SIGNR, and Langerin) from all d-amino acids is not trivial, and our attempts are still ongoing. Nevertheless, we established the experimental ground truth for these observations first by measuring binding of several lectins to various valences of l-mannose and d-mannose in the same liquid glycan array (*84*). Unlike d-mannose, l-mannose was completely inactive in binding to every calcium-dependent lectin tested [concanavalin A (ConA) and DC-SIGN on the surface of live cells]. Both MCNet1 and MCNet3 were able to corroborate these observations. For Man(α1-6)Man(β-Sp10 and Man(α1-2)Man(α1-2)Man(α1-3)Man(α-Sp9), both MCNet1 and MCNet3 predict that the mirror forms of the glycans have a large decrease in binding to ConA (Fig. 4D). MCNet predicted that a tetra-mannose enantiomer is recognized by lectins that traditionally bind galactose (galectin, MOA, and AIA); fucose (AOL, TbFBL-1, and UEA-I); and GlcNAc, GlcNAc oligomers, and LacDiNAc structures (Stl, WGA, and LEL). Liquid Glycan Array (LiGA) experiments observed only a weak binding between l-Man and prototypical d-Gal–binding lectins (Fig. 4, E and F). We could not validate whether tetra-l-mannose have binding properties not exhibited by l-Man because such validation requires complex synthesis of oligo-l-mannose. Nevertheless, the initial observations with l-monomers prompted us to expand liquid glycan array tests to l-monomers that can be accessed synthetically. Testing the binding of with α- and β-l-Glc, α-l-Man, and α-l-Rha to eight lectins—ConA, UEA-I, AAL, ECL, SBA, galectins-1, -3, and -7—unveiled unexpected binding of l-Glc to the classical fucose binding lectins UEA and AAL (Fig. 4F and figs. S13 and S15), and this binding was anticipated by MCNet3 (Fig. 4, G and H). We also observed no binding of ConA, ECL, SBA, galectins-1, -3, and -7 to any of the four l-monomers, the lack of such binding as was also anticipated by MCNet3 (Fig. 4, G and H). To further explore interactions between l-Glc and fucose binding lectins, we confirmed binding to UEA and AAL in clonal binding assays (fig. S14). We then constructed a third version of LiGA that contained α- and β-l-glucose, mannose and control β- d-glucose displayed at several densities (fig. S16). This experiment reconfirmed prior observations that l-glucose–conjugated phage binds to UEA-I, AAL, and yet another fucose-binding lectin RSL in the presence of many other fucose-modified glycoconjugates admixed in the same liquid array (fig. S16). In the same array, neither the phage modified by enantiomer d-glucose nor the diastereomer d-galactose bind to UEA-I, AAL, and RSL even when the density of their display was increased to four to five times that of the displayed density of l-glucose. All the LiGA data we report here (see figs. S16 and S17) measure l-Glc binding in the presence of multiple, and more numerous, fucose containing structures. The observed binding occurs despite competition with the canonically preferred fucose epitopes (fig. S16).

To test whether binding of l-Glc to fucose-binding lectins is generalizable, we used a previously reported array of 103 lectins (*85*). This array contains 11 lectins with the ability to recognize fucose: SNA-II, PhoSL, PSA, AAL, AOL, PA-IIL, LcH (2), AAL (2), LTL (2), TJA-II (2), and UEA-I (3), where numbers in brackets indicates the number of vendors for each lectin. Figure S17 describes synthesis of fluorescently labeled glycoconjugates used on lectin arrays. Figures S18 to S20 outline quality control (QC) of this lectin microarray with fluorescently labeled M13 bacteriophage modified by Man3 trisaccharide (figs. S18 and S20) or fucose-containing trisaccharide (H-type 1) (fig. S19). QC with H-type 1 glycoconjugates confirmed functional performance of AOL, TJA-II (Aniara and Medicago), AAL (Vector and Medicago), UEA-I (GeneTex), and UEA-I (Vector), but we observed that UEA-I (BioWorld), LcH, PSA, LTL, SNA-II, and PhoSL did not responded to H-type 1 (fig. S19). This observation was not unexpected as LcH, PSA, and PhoSL bind core fucose and LTL binds Lewis X. These calibrated lectin microarray experiments confirmed binding of β-l-glucose to AOL, AAL for both suppliers (Vector and Medicago), and weak binding to UEA-I from GeneTex but not the UEA-I from Vector or TJA-II, which bound to H-1 glycan. In the same setting, α-l-glucose only bound to UEA-I (GeneTex) but not AAL, AOL, TJA-II, or UEA-I from Vector (fig. S19). The binding profile of β- l-glucose was distinct from that of β-d-glucose conjugates, further confirming the role of chirality (fig. S19). Concerns about batch variability of UEA-I in the lectin array can be mitigated because LiGA (figs. S15 and S16) and clonal assays (fig. S14) confirmed that α-l-Glc and β-l-Glc bind to UEA-I from a fourth supplier (Sigma-Aldrich). LiGA also confirmed that β-l-glucose (but not α-l-glucose) binds to AAL (figs. S25 and S16). Both LiGA and lectin array confirmed lack of interaction between β-d-glucose and UEA, or AAL (fig. S16). The same calibrated lectin array highlighted distinct interactions of α-l-Man and α-d-Man with 103 lectins. QC with Man-3 conjugates confirmed functionality of printed ConA, GNA, PSA, LcH, NPL, and other Man-binding lectins (figs. S20 and S21). Figure S25 provides a summary of binding results anticipated by MCNet juxtaposed to measurements on 34 lectins on the lectin array. Only 3 of 34 predictions were incorrect for both α- and β-l-Glc, which compares well with the two mismatches between the Man-3 glycan array data and lectin array data. In addition to compelling data by LiGA and lectin arrays, structural superpositioning visualizes how fucose fits into the binding site of the known structures of UEA-I (fig. S22) and AAL (fig. S23), and how these lectins can accommodate l-glucose in the same binding site. Inhibition of l-Glc binding to UEA-I by soluble fucose further confirms that l-Glc and l-Fuc occupy the same binding site, in UEA-I (fig. S24).

Encouraged by agreement between out-of-domain extrapolations made by MCNet and experimental confirmations in two formats, liquid glycan array (Fig. 4, E to G, and figs. S15 and S16) and lectin array (figs. S18 to S21 and S25), we tested whether published ML architectures could produce the same predictions. We applied this comparison to models that can represent the rare l-glycans. ML models that represent glycans as collections of atoms, in theory, could be trained to predict binding for these rare glycans. As detailed in Supplementary Discussion, we deprioritized GNNGLY (*46*), GLAMOUR (*28*) models, and models from the GlycanML benchmark paper (*29*) and focused on LectinOracle-SweetNet (*27*) and LectinOracle-GIFFLAR (*47*). We evaluated two additional models reported recently to yield best-in-class performance on BindingDB datasets. Specifically, AI Bind (*61*) outperformed other models in predicting probabilities of protein-ligand interactions, whereas the CNN model from the PEER benchmark (*62*) delivered the best estimates of *K*_d_ from BindingDB. Continuous outputs from five models—fraction bound, *z* scores, binding probabilities, and log(*K*_d_)—do not match the data types of LiGA nor that of the lectin arrays (fig. S26, detailed data in “Comparison to other Models” folder in Supplementary Methods). However, applying a uniform threshold to 66 experimental observations made by both techniques across 11 lectins and 6 glycans in LiGA and lectin array identified 9 of 66 “binders” and 58 of 66 “nonbinders.” To convert continuous values to classification, we applied logical thresholds to predictions from MCNet (*f* > 0), LectinOracle, and GIFFLAR (*z* > 0) and adjusted the threshold to PEER-CNN and AI Bind to yield similar ratio between binders and nonbinders (fig. S26). Application of 11 independent evaluations (fig. S26) demonstrated that MCNet3 is leading in 5 evaluations (Jaccard Index, F1 score, NPV, Balanced accuracy, and Informedness) followed by LectinOracle_SweetNet (*4*) and LectinOracle_GIFFLAR. We attribute the relatively poor performance of AI Bind (*82*) to chirality issues because MolFormer-XL, used as a molecular encoder, in AI Bind is not sensitive to stereochemical information, which makes the published, trained version of AI Bind problematic for this task. The relative insensitivity of the PEER-CNN model to changes in stereochemistry of ligand (fig. S27) might originate from the low contribution of bona fide enantiomers and diastereomers in the BindDB training set. The distinct behaviors of stereoisomers predicted by MCNet and a close performance by LectinOracle models highlight the power of combined datasets, such as the CFG array dataset and the galectin-binding GM for training of ML models that are capable of out-of-domain prediction for cross-chiral recognition. The sheer number of chiral centers, diastereomers, and near-stereoisomers provides rich training ground for training models focused on predictions of the effect of chirality rather than the effect of addition and substation of atoms and functional groups.

## DISCUSSION

Here, we put contemporary understanding of protein-glycan interactions to a test by aiming to solve biologically irrelevant questions. We investigate whether it is possible to predict the binding ability of nonnatural and rare glycans, such as the enantiomers of natural carbohydrates. Such extrapolation is not without its benefits. Glycans across all kingdoms of life appear to be tightly linked to speciation. Species recognize each other and make decision such as “friend” and “foe” by integrating data from glycan-protein interactions; glycan-based pathogen-associated molecular patterns and damage-associated molecular patterns are central to our immunity. The prediction of glycan-GBP interaction strengths offers a universally comparable property and give rises to taxonomy and speciation orthogonal to the traditional 16*S*/18*S* RNA and DNA taxonomy (*86*). Synthesis of mirror-image life-forms is not the question of “if” but “when” (*17*, *87*). These new species will not use entirely unknown glycans but rather the well-defined enantiomers of known glycans. It is important to understand how this new class of species coated with mirror glycans will be interpreted using canonical protein receptors. Will they be adequately recognized by self/nonself recognition receptors? Our manuscript does not resolve this question but builds the ML framework for addressing such question with a higher degree of confidence.

The atoms and their connections within a glycan appears to be necessary and sufficient for lightweight ML models to predict the recognition of that glycan by protein receptors. Identities and connectivities of atoms relate to the Kolmogorov complexity of a molecule; it defines the molecule and is sufficient to compute its properties. For example, quantum mechanics–based techniques can estimate electronic structure and a Boltzmann ensemble of conformations from mere atomic connectivity. ML models can be trained using atomic coordinates; but such models produce properties of specific conformers. All the conformations need to be computed, and then integration over the Boltzmann-weighted ensemble yields a property of the molecule. We believe that thermodynamic scalars such as binding strengths between glycans and proteins at a given concentration can be predicted without explicit knowledge of (all) conformations using only atom connectivity. This observation generalizes the well-accepted Anfinsen’s hypothesis across the entire domain of molecular interactions. It can be phrased simply as “atomic connectivity of the interacting partners are necessary and sufficient to determine the strength of the binding between these partners*.*”

We find it productive to reexamine what are perhaps the most bare-bone and interpretable representations of molecules, the AQG counts. Predictions of glycan properties from molecules represented with *q*-grams or MF of one atom are, in retrospect, expected because the 611 CFG glycans are rather diverse. There are only a few isomeric glycans and no enantiomeric glycans in the CFG collection. The binding of large glycans can be correlated with the “expanded elemental composition” of such glycans without any consideration for topology. Mere memorization of composition is a risk in learning from datasets that contain molecules with diverse sets of atoms. The true challenge for molecular interaction is binding prediction for bona fide isomers and stereoisomers. MCNet3 can predict binding to a diverse set of diastereomeric glycans, enabling a first exploration of mirror-recognition glycobiology. We noticed, however that MCNet struggles to predict obvious expert knowledge such as “binding of galectin-3 to LacNAc must be ablated when carbons 2, 3, 4, or 5 are epimerized in either ring,” (see pages 50 and 51 in data S11). This is “obvious knowledge” for a glycobiologist, yet it is not clear how to integrate such obvious expert knowledge into the ML training without exhaustive synthesis and measurement of all epimers of LacNAc.

The inability of l-mannose to bind to lectins that bind d-mannose (Fig. 4) was anticipated by Goldstein *et al.* in 1965 (*88*) The dichotomy between l- and d-mannose may be rationalized on the basis of the relation of l-mannose and l-rhamnose. l-rhamnose–binding lectins do not recognize d-mannose (*89*–*91*) and d-mannose–binding proteins rarely bind to l-rhamnose. However, counterexamples are known; some lectins bind mannose and rhamnose equally (*92*). A report of Tatibouët and co-workers measured the binding of a multivalent bovine serum albumin (BSA)– l-Rha_17_ conjugate to ConA and DC-SIGN with IC_50_ of 3 and 0.6 μM, while BSA- d-Man_16_ achieved similar activity (IC_50_ of 0.2 and 0.6 μM). Hence, even extrapolating between l-mannose, l-rhamnose, and d-mannose may not be obvious to human experts and needs to be supported by experimental validation. The binding of l-glucose to some lectins that canonically recognize fucose is another experimental observation that was anticipated by MCNet. A minor discrepancy in alpha versus beta anomers of l-glucose (Fig. 4H versus fig. S14) may be a result of a mismatch in predictions (extrapolated from glass-array data) and testing (made in liquid array). Part of the problem may be inability of glass-based CFG array to detect binding of glycans to monosaccharides (Fig. 1E). It is possible that signals from monosaccharides are overshadowed by stronger binding to a larger oligosaccharide on the same array. Suboptimal presentation of glycans on the glass surface has been shown to be improved in LiGA, where density of the glycan can be varied systematically (*84*). Integration of LiGA data and CFG data via a universal “fraction bound” will reintroduce critical information about glycan-lectin interactions. An agglomeration of such measurements would form an effective ML training dataset for learning about the role of stereochemistry in glycan recognition and in overall molecular recognition. Embedding an atom-level description of glycans not only helps with data integration but also gives the first glimpse into the unseen mirror-universe of glycans; such bizarre mirror-image monomers have never been considered by any ML model due to lack of any training data. These observations will help to improve understanding of the risks associated with the synthesis of living mirror-image cells (*25*).

### Steps that need to be taken for the findings to be applied

We proposed that total chemical synthesis of a lectin made of d-amino acids and testing of such a lectin on a CFG microarray could test many predictions made here. Testing of the extended array of l-glycans (monomers and dimers) on a lectin array can further validate the observations and expand the set beyond l-glucose and l-mannose. Computational docking (*93*–*98*) and superimposing structures (figs. S22 and S23) is another fast way to test the predicted interactions between enantiomeric glycans and common lectins. Such techniques could rapidly advance exploration of cross-chiral recognition in glycobiology.

### Limitations

Learning from the glass-based glycan array and testing on the bacteriophage-based LiGA or glass-based lectin array is not ideal. Correlations between CFG glycan array and LiGA have been reported (*84*); similarly, correlation of lectin behaviors on glass-based glycan and lectin arrays is known. MCNet3 is trained on glass-surface display of glycans which is largely silent for all tested monosaccharides (Fig. 1E). In contrast, the bacteriophage display of glycans can detect monosaccharide binding to lectins in LiGA and in lectin glycan arrays after appropriate tuning of surface density. Incorporation of both LiGA data and lectin array data into the future training of ML models is a critical next step.

We anticipated that learning the dose-response should not be different from learning parameters of the curves that describes them. The models described here did not always learn the monotonously increasing sigmoidal nature of the binding curves that might be “obvious to the human domain expert” (fig. S28). Models were trained using *f* values in concentrations between 10^−5^ and 100 μM for each molecule (fig. S28). Still, for many GM compounds, models failed to predict an obvious boundary condition: *f* must become zero at infinitely low concentrations. Instead, for many GM compounds, *f* plateaued at arbitrary values. Predictions for some molecules were monotonous, whereas for others, we observed unexpected bumps and dips reminiscent of overfitting the monotonous ground truth with a complex-shaped high-degree polynomial. We anticipate that these problems will be resolved in future algorithms that learn *f* from discrete binding values.

The ability to represent a structure does not imply the ability to predict its binding accurately. The AQG approach has an obvious out-of-domain deficiency; if the CFG and GM datasets do not contain an element (e.g., Br), how can a model even know how to interpret bromine atoms in the 38,956 bioactive molecules that contain bromine (fig. S29)? Any molecule with a bromine atom is a cold-start problem. An analogous rationale can be made for groups of atoms (functional groups) that are absent from the training dataset. For example, the databases GlyTouCan and BindingDB contain molecules with cyclopropane rings and other functionalities (fig. S30) that are completely absent from CFG and the GM training datasets (table S2). The chirality of individual atoms presents another “cold start problem,” and in the absence of any examples of chiral differences, the ML model (e.g., MCNet2) cannot make any meaningful predictions for inversion of stereochemistry. Chemical intuition can explain the properties of bromine from trends in periodic table (e.g., H, F, Cl, Br, I, etc.) from simple features like count of electrons and trends in the periodic table. Chemical intuition suggests that ML models could take into the account electronic structure of atoms and functional groups (orbital structure, polarizability, and Milliken charge) instead of their linguistic description (bromine, chlorine, cyclopropane, etc.). Such models might be able to overcome some cold start problems. Kelly and co-workers profiled 357 quantum mechanical descriptors to predict those that contribute the most to protein-glycan interactions (*99*), and bioinformatic analysis by Kiessling and Woolfson identified electronic properties in both the glycan and the protein side chains that these interactions (*100*). However, simple electronic structure theory cannot explain long-range effects caused by chirality. The effect of chirality on molecular recognition is easier to teach by example. This observation highlights critical importance of datasets emanating from glycans, glycobiology, and protein-glycan interactions for global efforts in design of chirality aware ML models of molecular recognition. Integration of datasets that describe protein-glycan interactions into any ML models introduces the most complete description of the effect of local changes in chirality on the overall molecular recognition; no other fields offer such exhaustive coverage of the properties of diastereomers. Our work helps such integration by proposing strategies for integration of disparate quantitative data via universal fraction bound and encouraging all-atomic molecular representation in the field forged on a monomer-centric alphabet.

## MATERIALS AND METHODS

### Comparison of MF and AQG encodings using previously published RFU data

Two types of embedding—AQGs and MFs—were used to train models that take these atom-level molecular representations of glycans as inputs and predict RFU values for 1257 lectin compositions as output (Fig. 1I). AQG models did not use “bond order” directly; in its place, we used the hybridization of atoms (nodes), e.g., sp., sp^2^, and sp^3^. To differentiate between *q*-grams with and without edges to other neighboring atoms, we included a count of attached hydrogen atoms. Both AQGs and MFs were generated using RDKit from SMILES string descriptions of glycans, which included the anomeric configuration and the linker structure, as defined in CFG datasets (Fig. 1F). To represent the structure of glycans immobilized on glass, we included the carboxylic acid portion from the microarray immobilization point (Fig. 1F). SMILES structures, in turn, were generated using custom python code. Both the code and one-to-one correspondence of SMILES and condensed International Union of Pure Applied Chemistry names are available on GitHub.com in derdalab/MCNet/SMILES-CFG611+GM.txt.

Training was performed using 10-fold cross-validation, and hold-out sets were selected as in the previous GlyNet paper (*59*). In our prior work, we assigned molecules with the same glycan structures to the same train/test groups. We continue to do this although the current models are now sensitive to the differences in the linker/spacer structures attached to them. We compared MSE (loss) of models with different levels of details in the atoms/nodes, different maximum subgraph sizes *q* ≤ 1 to 6, numbers of hidden layers, nodes in hidden layers, and choices of the weight decay parameter, and compared atom-level structure results to our prior monosaccharide-level work, GlyNet (*59*).

#### Training and evaluation of the models

We explored variations in the maximum sizes of the graphlets and in the per atom features. Adding more features and larger graphlets gave better results (Fig. 1J), except for the largest radii (4 and greater) of MFs. The atom-level models are comparable to the monosaccharide ones on the same CFG dataset in our prior work. Especially for the MF-based ones, MSE performance is comparable. Not all the hyperparameters are equally good, but we can choose the acceptable ones. In particular, the models are sensitive to the number of hidden layers, the size of the hidden layers, choice of atom labels, and the types of graphlets used. We further trained 20 + 18 models (20: Fig. 1K; 18: fig. S4) that used MFs as input and aimed to predict RFU values for 1257 lectin compositions as output. Training was performed using Adam (*68*) with its default settings (PyTorch implementation). In addition to fully connected neural networks, we tested RF and XGBoost, and the results are described in fig. S4. The MSE values of atom-level models are comparable to previously published GlyNet (*59*) and SweetNet (*45*) models that learn protein binding from monosaccharide-level embedding in the same CFG dataset. For the MF-based models, MSE performance is comparable and in case of XGBoost even exceeds the performance of previously published models (fig. S4).

#### Preprocessing of glycan microarray data to convert to fraction bound

Glycan microarray data and associated metadata were downloaded from the CFG website (https://functionalglycomics.org). From tables containing spot intensities, we took the difference between the spot foreground and background values before taking means over the six replica spots to get an RFU for each glycoconjugate. For each microarray, we examined the distribution of the RFU values using Kernel Density Estimation (KDE). We did this with Gaussian kernels and a bandwidth of 0.2 in scikit-learn (*101*). We then identified the peak formed by non-/weak-binding glycans. This peak is the maximum of the KDE curve below 4000 RFU.

We then converted the RFU values to fractions bound by linearly transforming them so that this peak was at zero and the maximum across all arrays (65,536) was at unity. Values below the peak would rescale to negative numbers and were changed to zero. The microarray metadata were then examined, and those arrays with the same cbpID, sample description, and investigator-1 fields were grouped together. We then restricted ourselves to groups with at least three different concentrations.

#### Glycomimetic data

For the GM molecules, data were extracted from published literature. The specific publication used to collect these molecules are listed in table S5, and low-resolution structures of these GM molecules are displayed in table S2.

#### Preprocessing and merger of disparate datasets via fraction bound

We identified 147 cases where the multiple CFG arrays were run on the same protein but with at least three different input concentrations. We did not use the clamping of the bottom third to zero as in our prior report. Instead, we identified an average minimum RFU signal (see fig. S8) from abundance peaks that we take to correspond to the nonbinding condition. We then estimate the fraction bound of the glycan by rescaling each glycoconjugate’s mean RFU (across flowcell replicates). The per experiment RFU minimum becomes 0, and the maximum RFU experimentally observed across all data becomes 1. Values above and below this range were clamped to 1 and 0, respectively. For these 147 cases, we calculated fractions bound from CFG array results.

For each glycoconjugate, we took the fractions bound at different concentrations and extrapolated these to a set of concentrations. In most cases, this was 0.1, 1.0, 10.0, and 100 μg/ml. However, we also explored a denser set with 50 concentrations between 0.01 and 100 μM. Concentrations are from the set {1, 1.5, 2, 3, 4, 5, 7} × 10^{−5, −4, −3, −2, −1, 0, 1}^ and 100 μM. Note that each molecule was provided as input multiple times, once for each concentration.

In cases where we had a published *K*_a_ or *K*_d_ value, such as the GM compounds, fractions bound were calculated from the published *K*_a_/*K*_d_ values at the same set of concentrations using the Henderson-Hasselbalch equation (fig. S9). We then trained models with these concentration-molecule inputs with the combined fractions bound (fig. S10) as the desired outputs.

#### Training of the MCNet models

We paired MF encoding (chiral, maximum radius 3) and concentration as input to train three types of models: (i) fully connected neural network; (ii) XGBoost; and (iii) RF. Training was performed using Adam (*68*) with 0.0001 weight decay and 0.001 learning rate. MSE results from the training are described in fig. S11. Trained models are available on GitHub in the MCNet/Models directory.

#### Training of protein-aware models

At the request of the reviewer, we demonstrated that the MCNet architecture can be converted to a protein-aware model with no loss in performance. To this end, we paired MF encoding (chiral, radius 3) with ESMc2 encoding of protein and concentration as input to train three types of models: (i) fully connected neural network; (ii) XGBoost (*78*); and (iii) RF (*102*). These models were collectively referred to as PMCf models. Training was performed using Adam (*68*) with 0.0001 weight decay and 0.001 learning rate. MSE results from the training are described in fig. S10C alongside the MSE values of the MCNet models trained on similar data. Trained models are available on GitHub in the models directory.

#### Using published models for comparison

Comparison to two types of published models was used in this publication.

1. **ProteinGlycan ML:** We examined all models disclosed in peer-review literature or preprints that have been trained for specific task of predictions quantitative interaction between glycans and proteins and selected LectinOracle and GIFFLAR for comparison based on criteria detailed in Supplementary text. Reported/predicted values are typically Z′ transformed values from glycan microarrays. Values greater than 0 are interpreted as binding, whereas Z′ < 0 denotes lack of binding. An exact comparison to fraction bound is not trivial but feasible; see discussion in Supplementary text.

2. **BindingDB ML:** We examined a small subset of models disclosed either in in peer-review literature or preprints that have been trained for specific task of predictions of quantitative of interactions between small molecules and proteins reported in BindingDB. We elected to use models from the PEER benchmark and AI-Bind as detailed in Supplementary text. Reported/predicted values are typically as –log(*K*_d_) or normalized binding probability. In many cases, these values formally are identical to concentration at which fraction bound of the protein is *f* = 0.5. The binding probabilities and log(*K*_d_) cannot be compared exactly but they have the same tendency: Higher number denotes stronger binding. They can be used for internal comparison, e.g., a model predicts that protein P binds stronger to glycan A than to glycan B. For details see Supplementary text.

#### Comparison task

It is not possible to compare the literature-reported accuracies directly because the type of measurements, types of data, folds, and assessment of training varies greatly across different publications. Instead, we elected a uniform task in which each model was tasked to predict interactions between a series of glycans (provided as SMILES strings) and proteins (provided as amino acid strings). Specifics are provided in the Supplementary Materials in 267 Lectins x 76 glycans.xlsx within the data S1. From these predictions, we focused our analysis on 6 definitive glycans and 11 definitive GBPs for which we acquired exhaustive experimental measurements using liquid glycan arrays and lectin arrays. See “MCNet vs 4 other models.xlsx” in the same location.

As some models predict thermodynamic values (*K*_d_), others predict transformed assay outputs (Z′), and others predict binding probabilities, the exact comparison to the ground truth in the task is also not possible. Applying a uniform threshold to measurements from liquid glycan array (FC > 1.5) and glycan array (fluorescent intensity >1000) and using OR function to combine the data further, we converted 6 × 11 = 66 measurements to 9 positive (P) and 57 negative (N) binding values. We further applied thresholds to the continuous predictions made by SweetNet_LectinOracle, GIFFLAR_LectinOracle, CNN_PEER_Benchmark, and AI-Bind, as described in the individual sections (see Supplementary text), to convert these predictions to P and N classifications as well. Converting both experimental data and predictions to classifications made it possible to count true positive (TP), false positive (FP), false negative (FN), and true negative (TN) values. We then used these to compute a set of 11 distinct quality metrics:

1. Jaccard Index = TP / (TP + FN + FP)

2. Accuracy = (TP + TN) / (P + N)

3. F1 score = 2 × TP / (2 × TP + FP + FN)

4. True-positive rate (TRP) = TP / (TP + FN)

5. True-negative rate (TNP), recall or sensitivity = TN / (FP + TN)

6. Positive predictive value (PPV) = TP / (TP + FP)

7. Negative predictive value (NPV) = TN / (TN + FN)

8. Balanced accuracy = (TPR + TNR) / 2

9. Informedness = TPR + TNR − 1

10. Matthews correlation coefficient (MCC) = SQRT(TPR × TNR × PPV × NPV) – SQRT[(1 – TPR) × (1 – TNR) × (1 – NPV) × (1 – PPV)]

### Biochemical methods

#### Materials and general laboratory information

Tetramethylrhodamine *N*-hydroxysuccinimide (TAMRA-NHS) ester (5-isomer, #27120) was obtained from Lumiprobe. ConA (#C2010), *Erythrina cristagalli* lectin (ECL, #L5390), NHS (#130672), and *N*-(3-dimethylaminopropyl)-*N*′-ethylcarbodiimide hydrochloride (EDC, #E7750) were purchased from Sigma-Aldrich. DC-SIGN(+), langerin(+), and parental Raji cells, along with purified DC-SIGN ECDs, were provided by C. Rademacher (University of Vienna). DNA primers were ordered from Integrated DNA Technologies. Zeba spin desalting columns (7 kDa MWCO, 0.5 ml, #89883) and additional biochemical reagents were from Thermo Fisher Scientific. HBS buffer [20 mM Hepes, 150 mM NaCl, and 2 mM CaCl_2_ (pH 7.4)] and phosphate-buffered saline (PBS) [137 mM NaCl, 10 mM Na_2_HPO_4_, and 2.7 mM KCl (pH 7.4)] were prepared and sterilized by 0.22-μm filtration.

#### LiGA experiments

LiGA experiments were performed as described in prior reports (*84*, *103*, *104*). The most substantial changes were the addition of phages decorated by l-glycans to the array mixture. The datasets used and brief summaries of them are listed in table S4. Experimental details for production of such l-glycans, modification of phage, and LiGA assays are provided below.

#### Chemical modification of phage clones with glycans to build LiGA components

SDB phages [10^12^ to 10^13^ plaque-forming unit (PFU)/ml in 1:1 PBS:glycerol] were mixed with dibenzocyclooctyne (DBCO) NHS ester (50 mM in *N*,*N*′-dimethylformamide) to a final concentration of 0.2 to 2.0 mM, typically achieving 5 to 50% pVIII modification after 45 min. Conjugated clones were purified with Zeba spin desalting columns (7-kDa MWCO) per the manufacturer’s instructions. Azido-glycans (10 mM in nuclease-free water) were then added to a final concentration of 2 mM and incubated overnight at 4°C. Reactions were monitored by matrix-assisted laser desorption/ionization–time-of-flight mass spectrometry (MALDI-TOF MS), with additional azido-glycan and extended incubation if residual pVIII-DBCO was detected. Final conjugates were purified with Zeba columns and then stored at 4°C or as 50% glycerol stocks at −20°C.

Alternatively, carboxylate glycans were conjugated via EDC/NHS coupling following a protocol adapted from the Gildersleeve group (*105*). Carboxylate glycans (15 mM in water) were activated by incubation with equimolar EDC hydrochloride (60 mM, freshly prepared) and NHS (60 mM, freshly prepared) at room temperature for 1 hour. The resulting glycan-NHS esters were added to SDB phages in borate buffer (pH 8.0) and incubated for 45 min. at room temperature. Glycophages were purified with Zeba spin columns and stored at 4°C or as 50% glycerol stocks at −20°C.

#### Analysis of glycosylation of phage samples by MALDI-TOF MS

MALDI-TOF MS spectra were acquired on an AB Sciex Voyager Elite (v5.0) with a 337-nm pulsed nitrogen laser in positive ion mode. A two-layer sinapinic acid matrix was used: Layer 1 (10 mg/ml in acetone-methanol, 4:1) and layer 2 [10 mg/ml in acetonitrile-water, 1:1, with 0.1% trifluoroacetic acid (TFA)]. Samples were prepared by depositing 0.7 μl of layer 1 on MALDI target plate, drying, and then adding 1.5 μl of the mixture of phages in PBS and layer 2 (1:2, v/v). Spots were washed with 0.1% TFA. The modified-to-unmodified pVIII ratio was quantified using the plotONEmaldi.m MATLAB script adopted from the previous publications and available in data S5. An example of analysis is presented in fig. S14. Raw data files for MALDI spectra are also available in the same archive. Summaries of analyzed MALDI spectra are included in the Supplementary Materials.

#### Binding of the LiGA to protein-coated plates

LiGA was prepared by combining equal volumes of the desired monoclonal glycophages in a single tube. The mixture was characterized by titering and deep sequencing before use in binding experiments. To prepare the assay plate, 50 μl of protein solution (50 μg/ml in PBS) was added to each well of a 96-well plate (Corning, #CLS3369), sealed with tape (Thermo Fisher Scientific, #15036), and incubated overnight at 4°C. The next day, the wells were washed three times with washing buffer (HBS supplemented with 0.1% Tween-20), blocked with 50 μl of blocking solution (HBS with 1% BSA) for 1 hour at room temperature, and washed again three times with washing buffer. LiGA solution (50 μl, 10^9^ PFU/ml in HBS) was then added to each well and incubated for 1 hour at room temperature. The wells were subsequently washed three times with washing buffer to remove unbound phage. For elution, 50 μl of HCl (pH 2.0) was added to each well and incubated for 9 min at room temperature. The eluates were transferred into 1.5-ml microcentrifuge tubes containing 25 μl of 5× Phusion HF buffer (NEB, #M0530S) to neutralize the acid. The neutralized samples were then used for titer determination and as templates for subsequent PCR analysis.

#### Binding of the LiGA to lectins on the cell surface

This protocol follows a published protocol (*84*). Briefly, lectin-positive and negative mammalian cells at confluence were detached from culture flasks, pelleted at 300*g* for 5 min., and resuspended in wash buffer (HBS with 0.1% BSA) at a density of 2 × 10^6^ cells/ml. Aliquots of 500 μl were transferred into fluorescence-activated cell sorting tubes (Corning, #352054), centrifuged at 300*g* for 5 min. at 4°C, and the cell pellets were resuspended in 100 μl of LiGA solution (10^9^ PFU/ml in HBS with 2% BSA). The samples were incubated on ice for 1 hour to allow binding.

Following incubation, cells were washed three times with cold (4°C) wash buffer and once with cold HBS, centrifuging at 300*g* for 5 min at 4°C between each wash. The final pellets were resuspended in lysis buffer (0.1 mg/ml RNase A in 1× HF buffer) and lysed by vigorous vortexing. Lysates were then centrifuged at 2000*g* for 3 min, and the supernatants were transferred to 1.5-ml microcentrifuge tubes containing 1 μl of proteinase K (10 mg/ml). The mixture was incubated at 55°C for 10 min and subsequently heat-denatured at 95°C for another 10 min.

After a final centrifugation at 21,100*g* for 3 min, the supernatants were collected into fresh 1.5-ml microcentrifuge tubes. These recovered phage solutions were used for quantitative polymerase chain reaction (qPCR) and served as templates for subsequent PCR analysis.

#### Preparation of TAMRA-labeled glycophages for lectin array experiments

Glycans were conjugated to M13 phages by following a protocol adapted from the Gildersleeve group. Each monoclonal glycophage was subsequently labeled with 0.1 mM TAMRA NHS ester to enable fluorescent detection in lectin microarrays. Briefly, carboxylate glycans (15 mM stock in water) were mixed with an equimolar amount of EDC hydrochloride (60 mM stock in water; freshly prepared) and NHS (60 mM stock in water; freshly prepared) and incubated at room temperature for 1 hour to generate glycan-NHS esters. The activated glycans were then added to the phage solution (1× borate buffer, pH 8.0) and incubated at room temperature for 45 min. Glycophages were purified using Zeba spin columns (7 K MWCO) and the glycosylation of the phage pVIII capsid protein was verified by MALDI-TOF MS. Purified glycophages (in 1× borate buffer, pH 8.0) were then incubated with 0.1 mM TAMRA-NHS ester at room temperature for 1 hour, followed by purification with Zeba spin columns. The extent of fluorescent labeling was assessed by MALDI-TOF MS. Last, the labeled phages were mixed with an equal volume of glycerol and stored at −20°C until use in lectin microarray experiments. Analysis of the conjugates by MALDI is described in fig. S17 and in “One-Page-One-MALDI_CP_SH.docx” within the data S5. The raw data files for MALDI spectra are in the same file.

#### Binding of TAMRA-labeled glycophages to lectin microarray

TAMRA-labeled glycophages were hybridized to the lectin microarray (fig. S18A) manufactured in Mahal laboratory, and all the details can be found in the MIRAGE tables (tables S7 and S8). Briefly, vacuum-sealed lectin microarray slides stored at −20°C were first placed at room temperature until thawed and then opened and submerged for 1 hour in a 50 mM ethanolamine, 100 mM boric acid buffer at room temperature under gentle agitation. Slides were washed for 5 min in 0.01% PBST at room temperature under gentle agitation and then washed for 5 min in 1 × PBS at room temperature under gentle agitation before being dried in a slide spinner. Fifty microliters of each TAMRA-labeled glycophage sample was added to a lectin microarray with 50 μl 0.01% PBST (with 0.1 mM Ca^2+^) and hybridized for 1 hour at 30°C under agitation. Hybridization solution was then removed, and arrays were washed with 150 μl of 0.01% PBST twice—once for 1 min at room temperature under agitation and then for 5 min at room temperature under agitation. Arrays were then washed with 150 μl of 1 × PBS twice—once for 1 min at room temperature under agitation and then for 10 min at room temperature under agitation. Last, the slides were briefly dipped in deionized water and dried in a slide spinner. Slides were scanned in a Genepix 4400A Microarray Scanner at 532 nm and fluorescence values extracted using Genepix Pro 7 software. For each sample run on the lectin microarrays, active binding was assessed by filtering out background-subtracted fluorescence values lower than our 1000 AU cutoff. Results from all experiment are summarized in figs. S18 to S22.

### Synthetic methods

#### General methods

Methanol (MeOH), hexanes, toluene, acetic acid (HOAc), acetonitrile, and ethyl acetate (EtOAc) were supplied by Thermo Fisher Scientific and were of ACS grade or high-performance liquid chromatography (HPLC) grade. Argon was of 4.9 grade (Linde). Second Generation Grubbs Catalyst (500 mg; Umicore, M204) was purchased from Sigma-Aldrich (St. Louis, MO), and handled under air but stored dry, or solvated, only under oxygen-free argon. 7-octenoic acid was supplied by Aaron Chemicals LLC (San Diego, CA). Ten % Pd/C was from Strem Metals (Newburyport, MA). Allyl glycoside precursors were provided by GlycoNet (Canadian Glycomics Network, Edmonton, Alberta; https://canadianglycomics.ca/ services/request-services-plus/) and showed ^1^H spectra consistent with literature. Olefin cross metathesis reactions were conducted in threaded vials or tubes sealed with fluoropolymer-lined septa. Relevant solvents were dried in bulk (static incubation with argon-filled molecular sieves), and then portions were sparged of residual oxygen with argon at time of use. Ten % Pd/C was removed from reactions by filtration or centrifugation, and the initial filtrates or supernatants were augmented with a rinse of the same solvent to extract residual product from catalyst and vessels. HPLC C18 separations were carried out on a 10 mm–by–250 mm XBridge column packed with 10 μm/100 Å C18 (Waters, Bedford MA) at a flow rate of 10 ml/min with constant 0.1% HOAc as mobile phase modifier and a linear gradient from 5 to 35% acetonitrile in for water over 30 min. Under these conditions, 8-carboxyoctyl tetra-ols and tri-ols eluted after around 17 to 18 and 19 to 20 min, respectively. The ^1^H nuclear magnetic resonance spectra of all final products are listed on figs. S31 to S33, and structures of intermediates are shown in fig. S34.

#### *Synthesis of 8-(methoxycarbonyl)octyl 2,3,4,6-tetra-O-acetyl* α*-l-glucopyranoside (K) and 8-(methoxycarbonyl)octyl 2,3,4,6-tetra-O-acetyl* β*-l-glucopyranoside (L)*

Under argon, a solution of 420 mg of methyl 7-octenoate (2.7 mmol, 4.9 eq) and 20 mg of M204 (24 μmol and 0.04 eq) in 5 ml of 1:1 acetic acid:toluene was magnetically stirred under argon for one hour to preform **P** (dimethyl tetradec-7-ene-1,14-dioate) in situ. Then, a fresh solution of 10 mg of M204 (12 μmol and 0.02 eq) and a mixture of both allyl 2,3,4,6-tetra-*O*-acetyl-l-glucopyranoside anomers (7:3 α:β anomers, 214 mg; 386 μmol α-anomer, 165 μmol β-anomer) in 5 ml of toluene was added at once. The solution was stirred at room temperature and concentrated by evacuating the headspace through a restrictive orifice. After 1 hour, the volume had diminished to 1 ml, and 40 mg of M204 in 1 ml of toluene was added while continuing to concentrate. After a further 95 min, silica gel separation (25 to 40% EtOAc in hexanes) recovered some of the residual **P** (190 mg 1.2 mmol, and 2.2 eq) and separated as a mixture 185 mg of unsaturated glycosides. One hundred and sixty-two milligrams of this, proportional to 338-μmol α- and 145-μmol β-allyl glycoside starting materials, was dissolved in 7 ml of MeOH, and 50 mg 10% Pd/C (46 μmol and 0.15 eq) was added. After bubbling the suspension with hydrogen for 2 hours, the catalyst was removed. High-performance normal phase separation on a 7.8 mm–by–300 mm Waters μPorasil HPLC column eluted at 10 ml/min with a gradient of diethyl ether in hexanes (linear from 10 to 60% over 50 min) first gave 67 mg of pure methoxycarbonyloctyl α-l-glucoside peracetate **K** (129 μmol, 38% over two steps), and further elution afforded 34 mg of pure methoxycarbonyloctyl β-l-glucoside peracetate **L** (65 μmol, 45% over two steps).

#### *Synthesis of 9-(*α*-l-glucopyranosyloxy) nonanoic acid (M)*

**K** (67 mg and 129 μmol) was deprotected by treatment with NaOMe (200 μl 1 M, 200 μmol, and 1.5 eq) in excess MeOH overnight, followed by concentration to dryness and redissolution in 3 ml of 9:1 MeOH:water. After hydrolysis of the methyl ester, the solution was treated with H^+^ resin, filtered and concentrated. HPLC purification gave 35 mg of **M** (104 μmol, 81%).

#### *Synthesis of 9-(*β*-l-glucopyranosyloxy) nonanoic acid (N)*

**L** (34 mg, 66 μmol) was deprotected by treatment with NaOMe (100 μl 1 M, 100 μmol, and 1.5 eq) in excess MeOH overnight, followed by concentration to dryness and redissolution in 3 ml of 9:1 MeOH:water. After hydrolysis of the methyl ester, the solution was treated with H^+^ resin, filtered, and concentrated. HPLC purification gave 10 mg **N** (30 μmol, 45%).

#### *Synthesis of 9-(*α*-l-mannopyranosyloxy) nonanoic acid (O)*

8-methoxycarbonyloctyl α-l-mannopyranoside was synthesized by the method known for its D enantiomer (*106*), only starting from l-mannose.

## Supplementary Materials

### The PDF file includes:

Supplementary Text Figs. S1 to S34 Tables S1 to S10 Legends for data S1 to S5 Data S6 to S11 References
