# Supplementary material for: Atom-level machine learning of protein-glycan interactions and cross-chiral recognition in glycobiology
Source: Sci Adv. 2025 Dec 5;11(49):eadx6373. doi: 10.1126/sciadv.adx6373 (PMC12680057; doi:10.1126/sciadv.adx6373)
Supplement: Supplementary file 1 — Supplementary Text Figs. S1 to S34 Tables S1 to S10 Legends for data S1 to S5 Data S6 to S11 References [file sciadv.adx6373_sm.pdf]

Science Advances
AAAS

Supplementary Materials for

Atom-level machine learning of protein-glycan interactions and cross-chiral recognition in glycobiology

Eric J. Carpenter *et al.*

Corresponding author: Ratmir Derda, ratmir@ualberta.ca

Sci. Adv. **11**, eadx6373 (2025)
DOI: 10.1126/sciadv.adx6373

The PDF file includes:

Supplementary Text
Figs. S1 to S34
Tables S1 to S10
Legends for data S1 to S5
Data S6 to S11
References

Other Supplementary Material for this manuscript includes the following:

Data S1 to S5

Supplementary Text

Discussion of benefits of concentration series vs. traditional binding outcomes estimated by single value.

MCNet employs concentration, *C*, as an input variable. The use of *C* as an input causes the merger of multiple copies of a sample at various concentrations into a concentration series or a "dose response". Although extrapolation of $f(C)$ from K_d values is trivial (**Figure S9**) the reverse is not true: not every dose-response observation can be reliable converted to K_d. Additional insights can be drawn concentration series that cannot be drawn from a single parameter (e.g., K_d). For example, it is not trivial to extrapolate to *bona fide* K_d from many assays which report incomplete titrations. In such cases, the values are reported as inequality (e.g., K_d > minimum value), but explicit use of the fraction bound can dissect partial formation of complexes from no observable complex formation.
A second benefit of f is an explicit boundary condition, $f = 0$ when $C = 0$ which provides additional datapoints for the ML training. We observe that without this boundary condition, ML makes $f > 0$ predictions at $C = 0$ (**Figure S28**). Other benefits come from with non-linear dose-responses:
i) The implicit incorporation of cooperativity (steeper dose-response curve), and
ii) The possibility of incorporating non-linear effect responses such as steric occlusion or the Hook effect, both describing apparent decrease in binding with an increase in concentration.

Three dimensional analysis of L-Glucose/Fucose binding to UEA-I and AAL.

The predictions made by the MCNet3 model about the binding of L-Glucose to fucose-binding lectins UEA-I and AAL are structurally supported by superimposition analyses of L-Glucose to the L-Fucose within their crystal structures 1JXN and 1IUC, respectively. The superposition was performed ensuring that the L-glucose anomeric carbon (C1) could extend into a polysaccharide chain without steric hindrance from the protein.

UEA-I: The crystal structure of UEA-I (1JXN) bound to its native ligand is shown in panel A of **Figure S27**. Superimposing α-L-Glucose (Panel B) and β-L-Glucose (Panel C) demonstrates their potential to mimic the native fucose's binding pose.

As shown in Table S8, native Fucose in UEA-I forms H-bonds with GLU 44 (OE2) and O2/O3, GLY 105 (N) and O3, ASP 87 (OD2) and O4, and ASN 134 (ND2) and O4. Superimposed α-L-Glucose retains similar interactions with GLU 44 (OE2) at O2/O3, GLY 105 (N) at O3, and ASP 87 (OD1, OD2) at O4. β-L-Glucose (Panel C) forms the same H-bonds with the protein as α-L-Glucose.

AAL: The crystal structure of AAL (1IUC) bound to its native ligand is shown in panel A of **Figure S28**. Superimposing of α-L-Glucose and β-L-Glucose onto α-L-Fucose was performed is shown in panel B and C respectively.

H-bond analysis of AAL (Table S9) shows native Fucose interacts with GLU 36 (OE1, OE2), TRP 97 (thNE1), and ARG 24 (NE, NH2) at O3, O4, and O5. Both superimposed α-L-Glucose

and β-L-Glucose maintain similar H-bond patterns as GLU 36 (OE1, OE2) and TRP 97 (NE1) interact with O3, and ARG 24 (NE, NH2) interacts with O5. This consistency further supports L-glucose's engagement with fucose-binding proteins.

The superimposition of α-L-Glucose into the AAL binding site indicates possible binding with favorable hydrogen bonding, which contrasts with the experimental results from the Liquid Glycan Array (LiGA) (Figure 4F). This difference can occur because poses obtained through superimposition based on structural similarity provide only a snapshot of potential binding. They do not consider the dynamic nature of protein-ligand interactions in the solution. Thus, the most energetically favorable binding pose might differ from the geometrically superimposed one. Additionally, experimental conditions in LiGA may vary from those in crystallography. Minor variations in buffer composition, pH, or temperature during the determination of crystal structure could also affect binding.

Details of third-party models used for comparison

SweetNet_LectinOracle and GIFFLAR_LectinOracle. We reached out to Daniel Bojar and Roman Joeres to coordinate calculations of binding predictions for selected glycans and glycan-binding proteins using two models:

(1) The original published version of LectinOracle (*27*) comprised of SweetNet paired with ESM1b to predict glycan-protein interaction (denoted here as SweetNet_LectinOracle). In short, SweetNet is a graph convolutional neural network that uses graph representation learning to compute computer-interpretable embeddings for glycans.

(2) GIFFLAR (*47*) is an upgraded encoder for glycans that combines monomer and atomic representation of glycans. It takes IUPAC-condensed notations and computes computer-interpretable embeddings. GIFFLAR paired with ESM2_t33 to encode lectins yields an updated version of LectinOracle (denoted here as GIFFLAR_LectinOracle).

Trained versions of SweetNet_LectinOracle and GIFFLAR_ LectinOracle were used to predict the binding of glycan monomers (IUPAC or SMILES respectively) against the glycan-binding proteins which were used to train the LectinOracle model. See https://github.com/BojarLab/glycowork/blob/master/glycowork/glycan_data The glycan-binding proteins included into the evaluation of "Mirror image glycan extrapolation" task, are a subset of this list.

Predictions from both models are in *z*-scores, higher number represents stronger binding. Positive *z*-scores are considered binders. We note that neither SweetNet (*27*) nor GIFFLAR (*47*) encode the structure of the linker that connects glycan to the surface of the array, nor the stereochemistry of the reducing end. Predictions, thus, do not differentiate between the alpha and beta anomers of glucose (or any other monomers). Nevertheless, both models output predictions for the L and D versions of glycan monomers and the results are shown in **Figure S26** and "**GIFFLAR Predictions.xlsx**", and "**MCNet vs 4 other models.xlsx**"

We thank both Daniel Bojar and Roman Joeres for extensive communication and help with making these predictions.

GLAMOUR The GLAMOUR model published by Rafael Gomez-Bombarelli lab (*28*) was successfully used for several classification tasks, but it was not employed for prediction of protein-glycan interactions. We reached out to the authors of the GLAMOUR publications to train such a model from scratch and make prediction of binding of glycans and glycan-binding proteins. Due to time constrains, the results from GLAMOUR are not included in this publication and will be presented in the future report. Nevertheless, we thank Tatem Rios, Soojung Yang, Katharina Jager in the Rafael Gomez-Bombarelli lab at MIT for their valuable feedback.

GNNGLY The published model was used for several classification tasks, but it was not employed to predict protein-glycan interactions (*46*). We reached out to the authors of GLYGNN publications for consultation but received no reply. Hence, we decided that GLYGNN would not be part of the current comparison.

GlycanML benchmark models The publication (*29*) describes a series of tasks related to properties of glycans, datasets for such tasks and models trained on these datasets. We examined the report and noted that glycans used in the protein-glycan binding tasks were (a) tokenized sequences derived from the IUPAC-condensed sequence, or (b) graphs at the monosaccharide level of detail. However, neither (a) nor (b) included stereochemical designations (L- or D-prefixes). As a result, it is unclear whether models trained using GlycanML protein-glycan binding benchmark data interpret L-glycans appropriately. We reached out to the authors of GlycanML publications for consultation but received no reply. As a result, none of the models reported in the GlycanML benchmark publication were part of the comparison.

PEER benchmark At the time of the resubmission of this article to peer review (August 19, 2025) the GitHub repository associated with the PEER benchmark publication (*62*) contained code sufficient for training of the models but it did not offer trained models. We tried to train all models available for protein-ligand interaction on an Nvidia 2070 with 8 GB of memory. CNN was the only architecture we could train. LSTM, Transformer, ResNet, ProtBERT (unfixed weights), and ESM-1b (fixed and unfixed weights) all produced out of memory errors. It may be feasible to train ProtBERT with fixed weights, but it would take significant computational time, and it would be unlikely to yield the best performance, as CNN is reported as having the best performance for the BindingDB task in the PEER benchmark. CNN, thus, is a good representative model. We believe that the CNN is trained correctly as we observed a similar RMSE (1.480) to the one reported in the publication (1.497). The trained model was used to extrapolate the binding for glycans (from SMILES strings) and glycan-binding proteins from amino acid sequences. Predictions from the CNN model in the PEER benchmark are reported as negative logarithm of Kd (pKd), a higher number is stronger binding. The results from prediction for glycan and lectins are displayed in **Figures S26, S27**, and "PEER Predictions.xlsx", and "MCNet vs 4 other models.xlsx" within **Supplementary Data S1**.

AI-Bind Predictions by the trained version of AI Bind (*61*) were performed by the original authors of the model (Hao Xu and Giulia Menichettiand, Harvard) using a supplied table (see file 267 Lectins x 76 glycans.xlsx) of 76 glycans and 267 glycan-binding proteins. The model trained by the authors uses ProtT5 to encode proteins and MoLFormer-XL to encode molecules, both kept fixed (frozen). This setup—particularly MoLFormer-XL—is not sensitive to stereochemical information, which may make it less than ideal for prediction the evaluation of binding properties of stereoisomers and diastereomers of glycans. AIBind is trained as a binary classification model, 1 for binding and 0 for non-binding, but the output is a continuous binding probability. Two binding probabilities (e.g., 0.01 vs. 0.03) for the same protein may in principle

be interpreted as relative ranking indicators. Further details of binding probability are described in the original publication. Binding probabilities computed by AI-Bind for L-glycans and lectins are displayed in **Figure S26**, “AI Bind Predictions.xlsx”, and “MCNet vs 4 other models.xlsx” We thank Hao Xu and Giulia Menichettiand (Harvard) for their help with these predictions.

Figure S1. Enantiomeric pairs in nature (continued on the next page)

A.

Rank	Name	SMILE..count	E-count	Enantiomer	E-Rank	SMILE of enantiomer
4	Gal	OC(O1)[C@H](O)[C@@H](O)[C@@H](O)[C@H]1CO........9761	22	L-Gal	132	OC(O1)[C@@H](O)[C@H](O)[C@H](O)[C@@H]1CO
5	GlcNAc	CC(N[C@@H]1[C@@H](O)[C@H](O)[C@@H](CO)OC1O)=O........8802	0	na	na	na
6	Glc	OC(O1)[C@H](O)[C@@H](O)[C@H](O)[C@H]1CO........7051	3	L-Glc	475	OC(O1)[C@@H](O)[C@H](O)[C@@H](O)[C@@H]1CO
7	Man	OC(O1)[C@@H](O)[C@@H](O)[C@H](O)[C@H]1CO........6452	37	L-Man	104	OC(O1)[C@H](O)[C@H](O)[C@@H](O)[C@@H]1CO
8	Fuc	C[C@H]1[C@@H](O)[C@@H](O)[C@H](O)C(O)O1........4153	420	D-Fuc	20	C[C@@H]1[C@H](O)[C@H](O)[C@@H](O)C(O)O1
9	GalNAc	OC(O1)[C@H](NC(C)=O)[C@@H](O)[C@@H](O)[C@H]1CO........3468	1	L-GalNAc	856	OC(O1)[C@@H](NC(C)=O)[C@H](O)[C@H](O)[C@@H]1CO
10	Rha	C[C@H]1[C@H](O)[C@@H](O)[C@@H](O)C(O)O1........2411	258	D-Rha	29	C[C@@H]1[C@@H](O)[C@H](O)[C@H](O)C(O)O1
11	NeuNAc	O=C(O)C1(O)O[C@@H]([C@H](O)[C@H](O)CO)[C@H](NC(C)=O)[C@@H](O)C1........2249	0	na	na	na
12	Kdo	O=C(O)C1(O)O[C@H]([C@H](O)CO)[C@H](O)[C@H](O)C1........1565	0	na	na	na
13	LDManHep	OC(O1)[C@@H](O)[C@@H](O)[C@H](O)[C@H]1[C@@H](O)CO........1397	1	DLManHep	718	OC(O1)[C@H](O)[C@H](O)[C@@H](O)[C@@H]1[C@H](O)CO
14	GlcA	O[C@H]1C(O)O[C@H](C(O)=O)[C@@H](O)[C@@H]1O........1350	2	L-GlcA	605	O[C@@H]1C(O)O[C@@H](C(O)=O)[C@H](O)[C@H]1O
15	Xyl	OC1[C@H](O)[C@@H](O)[C@H](O)CO1........1274	29	L-Xyl	112	OC1[C@@H](O)[C@H](O)[C@@H](O)CO1
16	GlcN	OC(O1)[C@H](N)[C@@H](O)[C@H](O)[C@H]1CO........867	0	na	na	na
17	GalA	O[C@H]1C(O)O[C@H](C(O)=O)[C@H](O)[C@@H]1O........560	0	na	na	na
18	Galf	OC(O1)[C@H](O)[C@@H](O)[C@@H]1[C@H](O)CO........500	0	na	na	na
19	NeuNGc	O=C(O)C1(O)O[C@@H]([C@H](O)[C@H](O)CO)[C@H](NC(CO)=O)[C@@H](O)C1........421	0	na	na	na
20	D-Fuc	C[C@@H]1[C@H](O)[C@H](O)[C@@H](O)C(O)O1........420	4153	Fuc	8	C[C@H]1[C@@H](O)[C@@H](O)[C@H](O)C(O)O1
21	GlcOPN	OC(O1)[C@H](N)[C@@H](O)[C@H](OP(O)(O)=O)[C@H]1CO........400	0	na	na	na
22	QuiNAc	C[C@@H]1[C@@H](O)[C@H](O)[C@@H](NC(C)=O)C(O)O1........345	45	L-QuiNAc	90	C[C@H]1[C@H](O)[C@@H](O)[C@H](NC(C)=O)C(O)O1
23	LDManHepOPEtn	OC1O[C@@H]([C@H]([C@@H]([C@@H]1O)O)O)[C@@H](OP(OCCN)(O)=O)CO........337	0	na	na	na
24	DDManHep	OC(O1)[C@@H](O)[C@@H](O)[C@H](O)[C@H]1[C@H](O)CO........331	0	na	na	na
25	LDManHepOP	OC1O[C@H]([C@H](CO)O)[C@@H](OP(O)(O)=O)[C@H](O)[C@@H]1O........311	0	na	na	na
26	Araf	OC(O1)[C@H](O)[C@@H](O)[C@@H]1CO........308	80	D-Araf	69	OC(O1)[C@@H](O)[C@H](O)[C@H]1CO
27	GlcNAcOS	OC(O1)[C@H](NC(C)=O)[C@@H](O)[C@H](O)[C@H]1COS(=O)(O)=O........303	0	na	na	na
28	GalOS	OC(O1)[C@H](O)[C@@H](O)[C@@H](O)[C@H]1COS(=O)(O)=O........264	8	L-GalOS	258	OC(O1)[C@@H](O)[C@H](O)[C@H](O)[C@@H]1COS(=O)(O)=
29	D-Rha	C[C@@H]1[C@@H](O)[C@H](O)[C@H](O)C(O)O1........258	2411	Rha	10	C[C@H]1[C@H](O)[C@@H](O)[C@@H](O)C(O)O1
30	Gro-ol	OCC(O)CO........251	251	Gro-ol	30	OCC(O)CO
31	Ara	OC1[C@H](O)[C@@H](O)[C@@H](O)CO1........247	56	D-Ara	81	OC1[C@@H](O)[C@H](O)[C@H](O)CO1
32	Kdn	O=C(O)C1(O)O[C@@H]([C@H](O)[C@H](O)CO)[C@H](O)[C@@H](O)C1........242	0	na	na	na
33	Fruf	OCC(O1)(O)[C@@H](O)[C@H](O)[C@H]1CO........235	2	L-Fruf	604	OCC(O1)(O)[C@H](O)[C@@H](O)[C@@H]1CO
34	D-FucNAc	C[C@@H]1[C@H](O)[C@H](O)[C@@H](NC(C)=O)C(O)O1........198	148	FucNAc	42	C[C@H]1[C@@H](O)[C@@H](O)[C@H](NC(C)=O)C(O)O1
36	GalN	OC(O1)[C@H](N)[C@@H](O)[C@@H](O)[C@H]1CO........190	0	na	na	na
37	KdoOP	O=C(O)C1(O)O[C@H]([C@H](O)CO)[C@H](O)[C@H](OP(O)(O)=O)C1........182	0	na	na	na
38	GalOMe	OC(O1)[C@H](O)[C@@H](OC)[C@@H](O)[C@H]1CO........161	0	na	na	na
39	GlcOAc	OC(O1)[C@H](O)[C@@H](O)[C@H](O)[C@H]1COC(C)=O........153	0	na	na	na
40	ManNAc	CC(N[C@H]1[C@@H](O)[C@H](O)[C@@H](CO)OC1O)=O........152	0	na	na	na
41	AraN	OC1[C@H](O)[C@@H](O)[C@@H](N)CO1........148	4	D-AraN	377	OC1[C@@H](O)[C@H](O)[C@H](N)CO1
42	FucNAc	C[C@H]1[C@@H](O)[C@@H](O)[C@H](NC(C)=O)C(O)O1........148	198	D-FucNAc	34	C[C@@H]1[C@H](O)[C@H](O)[C@@H](NC(C)=O)C(O)O1
43	RhaOMe	C[C@H]1[C@H](O)[C@@H](O)[C@@H](OC)C(O)O1........139	38	D-RhaOMe	101	C[C@@H]1[C@@H](O)[C@H](O)[C@H](OC)C(O)O1
44	ManOP	OC(O1)[C@@H](O)[C@@H](O)[C@H](O)[C@H]1COP(O)(O)=O........133	0	na	na	na
45	GlcOMeA	O[C@H]1C(O)O[C@H](C(OC)=O)[C@@H](O)[C@@H]1O........128	0	na	na	na
46	KdoOPPEtn	O=C(O)C1(O)O[C@H]([C@H](O)CO)[C@H](O)[C@H](OP(OP(OCCN)(O)=O)(O)=O)C1........127	0	na	na	na
47	RhaOAc	C[C@H]1[C@H](O)[C@@H](O)[C@@H](OC(C)=O)C(O)O1........126	0	na	na	na

B

Rank	Name	SMILE	Count	eCount	eSMILE	eName	eRank
4	Gal	OC(O1)[C@H](O)[C@@H](O)[C@@H](O)[C@H]1CO	9761	22	OC(O1)[C@@H](O)[C@H](O)[C@H](O)[C@@H]1CO	L-Gal	132
6	Glc	OC(O1)[C@H](O)[C@@H](O)[C@H](O)[C@H]1CO	7051	3	OC(O1)[C@@H](O)[C@H](O)[C@@H](O)[C@@H]1CO	L-Glc	475
7	Man	OC(O1)[C@@H](O)[C@@H](O)[C@H](O)[C@H]1CO	6452	37	OC(O1)[C@H](O)[C@H](O)[C@@H](O)[C@@H]1CO	L-Man	104
8	Fuc	C[C@H]1[C@@H](O)[C@@H](O)[C@H](O)C(O)O1	4153	420	C[C@@H]1[C@H](O)[C@H](O)[C@@H](O)C(O)O1	D-Fuc	20
9	GalNAc	OC(O1)[C@H](NC(C)=O)[C@@H](O)[C@@H](O)[C@H]1CO	3468	1	OC(O1)[C@@H](NC(C)=O)[C@H](O)[C@H](O)[C@@H]1CO	L-GalNAc	856
10	Rha	C[C@H]1[C@H](O)[C@@H](O)[C@@H](O)C(O)O1	2411	258	C[C@@H]1[C@@H](O)[C@H](O)[C@H](O)C(O)O1	D-Rha	29
13	LDManHep	OC(O1)[C@@H](O)[C@@H](O)[C@H](O)[C@H]1[C@@H](O)CO	1397	1	OC(O1)[C@H](O)[C@H](O)[C@@H](O)[C@@H]1[C@H](O)CO	DLManHep	718
14	GlcA	O[C@H]1C(O)O[C@H](C(O)=O)[C@@H](O)[C@@H]1O	1350	2	O[C@@H]1C(O)O[C@@H](C(O)=O)[C@H](O)[C@H]1O	L-GlcA	605
15	Xyl	OC1[C@H](O)[C@@H](O)[C@H](O)CO1	1274	29	OC1[C@@H](O)[C@H](O)[C@@H](O)CO1	L-Xyl	112
22	QuiNAc	C[C@@H]1[C@@H](O)[C@H](O)[C@@H](NC(C)=O)C(O)O1	345	45	C[C@H]1[C@H](O)[C@@H](O)[C@H](NC(C)=O)C(O)O1	L-QuiNAc	90
26	Araf	OC(O1)[C@H](O)[C@@H](O)[C@@H]1CO	308	80	OC(O1)[C@@H](O)[C@H](O)[C@H]1CO	D-Araf	69
28	GalOS	OC(O1)[C@H](O)[C@@H](O)[C@@H](O)[C@H]1COS(=O)(O)=O	264	8	OC(O1)[C@@H](O)[C@H](O)[C@H](O)[C@@H]1COS(=O)(O)=O	L-GalOS	258
31	Ara	OC1[C@H](O)[C@@H](O)[C@@H](O)CO1	247	56	OC1[C@@H](O)[C@H](O)[C@H](O)CO1	D-Ara	81
33	Fruf	OCC(O1)(O)[C@@H](O)[C@H](O)[C@H]1CO	235	2	OCC(O1)(O)[C@H](O)[C@@H](O)[C@@H]1CO	L-Fruf	604
34	D-FucNAc	C[C@@H]1[C@H](O)[C@H](O)[C@@H](NC(C)=O)C(O)O1	198	148	C[C@H]1[C@@H](O)[C@@H](O)[C@H](NC(C)=O)C(O)O1	FucNAc	42
41	AraN	OC1[C@H](O)[C@@H](O)[C@@H](N)CO1	148	4	OC1[C@@H](O)[C@H](O)[C@H](N)CO1	D-AraN	377
43	RhaOMe	C[C@H]1[C@H](O)[C@@H](O)[C@@H](OC)C(O)O1	139	38	C[C@@H]1[C@@H](O)[C@H](O)[C@H](OC)C(O)O1	D-RhaOMe	101
48	Ribf	OC(O1)[C@H](O)[C@H](O)[C@H]1CO	121	1	OC(O1)[C@@H](O)[C@@H](O)[C@@H]1CO	L-Ribf	869
61	ManOMe	OC(O1)[C@@H](O)[C@@H](OC)[C@H](O)[C@H]1CO	85	20	OC(O1)[C@H](O)[C@H](OC)[C@@H](O)[C@@H]1CO	L-ManOMe	145
64	Qui	C[C@@H]1[C@@H](O)[C@H](O)[C@@H](O)C(O)O1	84	2	C[C@H]1[C@H](O)[C@@H](O)[C@H](O)C(O)O1	L-Qui	611
66	DigOMe	C[C@@H]1[C@@H](O)[C@@H](OC)CC(O)O1	83	10	C[C@H]1[C@H](O)[C@H](OC)CC(O)O1	L-DigOMe	221
70	2,5-Anhydro-Man	O=C[C@@H](O1)[C@@H](O)[C@H](O)[C@H]1CO	79	2	O=C[C@H](O1)[C@H](O)[C@@H](O)[C@@H]1CO	2,5-Anhydro-L-Man	498
72	6dTal	C[C@H]1[C@@H](O)[C@@H](O)[C@@H](O)C(O)O1	70	10	C[C@@H]1[C@H](O)[C@H](O)[C@H](O)C(O)O1	D-6dTal	214
73	IdoA	O[C@H]1C(O)O[C@@H](C(O)=O)[C@@H](O)[C@@H]1O	70	4	O[C@@H]1C(O)O[C@H](C(O)=O)[C@H](O)[C@H]1O	D-IdoA	381
74	Abe	C[C@@H]1[C@H](O)C[C@@H](O)C(O)O1	68	41	C[C@H]1[C@@H](O)C[C@H](O)C(O)O1	Col	97
78	GalNAcA	OC1O[C@H](C(O)=O)[C@H](O)[C@H](O)[C@H]1NC(C)=O	62	23	OC1O[C@@H](C(O)=O)[C@@H](O)[C@@H](O)[C@@H]1NC(C)=O	L-GalNAcA	128
80	GalAN	N[C@H]1C(O)O[C@H](C(O)=O)[C@H](O)[C@@H]1O	57	1	N[C@@H]1C(O)O[C@@H](C(O)=O)[C@@H](O)[C@H]1O	L-GalAN	855
84	L-ThrHexA4en	OC1[C@H](O)[C@@H](O)C(O)=C(C(O)=O)O1	53	3	OC1[C@@H](O)[C@H](O)C(O)=C(C(O)=O)O1	D-ThrHexA4en	444
89	D-Apif	OC1[C@@H](O)[C@@](CO)(O)CO1	45	3	OC1[C@H](O)[C@](CO)(O)CO1	Apif	431
91	Ole	C[C@@H]1[C@@H](O)[C@H](OC)CC(O)O1	45	5	C[C@H]1[C@H](O)[C@@H](OC)CC(O)O1	L-Ole	358
100	6dTalOAc	C[C@H]1[C@@H](O)[C@@H](O)[C@@H](OC(C)=O)C(O)O1	38	8	C[C@@H]1[C@H](O)[C@H](O)[C@H](OC(C)=O)C(O)O1	D-6dTalOAc	248
106	D-6dAraHexOMe	CC1[C@@H](O)[C@@H](O)[C@H](OC)C(O)O1	32	5	CC1[C@H](O)[C@H](O)[C@@H](OC)C(O)O1	L-6dAraHexOMe	356
113	3,6-Anhydro-L-Gal	O=C[C@@H](O)[C@H]1[C@H](O)[C@@H](O)CO1	28	8	O=C[C@H](O)[C@@H]1[C@@H](O)[C@H](O)CO1	3,6-Anhydro-Gal	245
123	XylHex	OC1OC(CO)[C@@H](O)[C@H](O)[C@H]1O	25	7	OC1OC(CO)[C@H](O)[C@@H](O)[C@@H]1O	L-XylHex	282
129	Ery	OC1[C@H](O)[C@H](O)CO1	22	1	OC1[C@@H](O)[C@@H](O)CO1	L-Ery	853
137	Rha-ol	C[C@H](O)[C@H](O)[C@@H](O)[C@@H](O)CO	22	2	C[C@@H](O)[C@@H](O)[C@H](O)[C@H](O)CO	D-Rha-ol	530
144	L-Aco	C[C@H]1[C@H](O)[C@@H](OC)[C@@H](O)C(O)O1	20	6	C[C@@H]1[C@@H](O)[C@H](OC)[C@H](O)C(O)O1	Aco	294
146	6dTalOMe	C[C@H]1[C@@H](O)[C@@H](OC)[C@@H](O)C(O)O1	19	10	C[C@@H]1[C@H](O)[C@H](OC)[C@H](O)C(O)O1	D-6dTalOMe	215
151	LyxHex	OC(O1)[C@@H](O)[C@@H](O)[C@H](O)C1CO	19	9	OC(O1)[C@H](O)[C@H](O)[C@@H](O)C1CO	L-LyxHex	240
158	L-GulNAcA	OC1O[C@@H](C(O)=O)[C@@H](O)[C@H](O)[C@@H]1NC(C)=O	18	5	OC1O[C@H](C(O)=O)[C@H](O)[C@@H](O)[C@H]1NC(C)=O	GulNAcA	352
163	D-FucNBut	C[C@@H]1[C@H](O)[C@H](O)[C@@H](NC(CCC)=O)C(O)O1	17	5	C[C@H]1[C@@H](O)[C@@H](O)[C@H](NC(CCC)=O)C(O)O1	FucNBut	335
164	FucN	C[C@H]1[C@@H](O)[C@@H](O)[C@H](N)C(O)O1	17	13	C[C@@H]1[C@H](O)[C@H](O)[C@@H](N)C(O)O1	D-FucN	187

C.

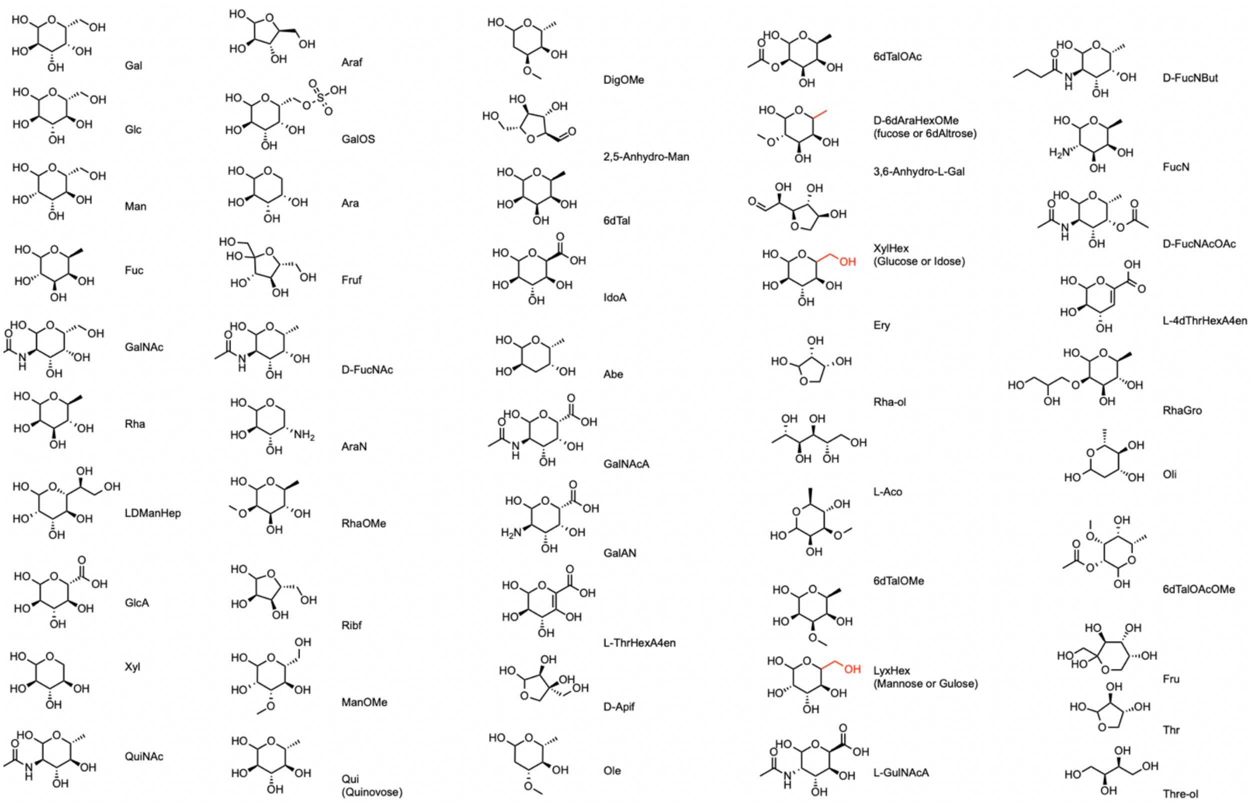

Fig. S1: The relative abundance of L- vs. D-enantiomers in nature.

(A) All Monomers and some enantiomers_BOTH.xlsx,
(B) uniqueSMILEs_count_enantio_onlyONE.xlsx and
(C) all monomers that have enantiomers 1-50.cdxml
All three files are shared in the Supplementary data "/Glycan Enantiomer Analysis" folder

The tables in (A-B) originate from data for 19,299 glycans reported by Bojar, Collins et al. (*60*) and subsequently used to train LectinOracle models (*27*), GIFFLAR model (*47*), GNNGLY model(*46*) and models described in GlycanML benchmark (*29*). In a subsequent publication, Rafael Gomez-Bombarelli and co-workers parsed the 19,299 glycans dataset into a re-curated list of 19,147 glycans represented as uniform "SMILES-MONOMERS-BONDS text files" and employed these files to train GLAMOUR models (*28*). The latter .txt files were downloaded from:
https://github.com/learningmatter-mit/GLAMOUR/tree/main/dataset
To produce the master table in (A) the downloaded data was analyzed using MatLab script that counted the occurrences and occurrence-based ranking of all reported monomers in all 19,147 txt files. The same script extracted the corresponding SMILE strings of each monomer, produced SMILE strings of enantiomers of said monomer and attempted to match the enantiomer to all SMILES in the same dataset. Due to symmetry of enantiomers, the matching happened twice for L and D glycans: Examples of such symmetric match can be seen in for Fucose (rank 8) and D-fucose (Rank 20). Some monomers in the dataset contained no stereocenters and these

asymmetric monomers were flagged and excluded from further analysis (e.g., see Gro-ol monomer, Rank 30).
From a total of 950 unique monomers, and excluding achiral monomers, both enantiomer were found a subset of 85+85 monomers. For these monomers, a sub-table with 85 entries was produced (B). This table describes the rank of the more abundant glycan (Rank), the rank of its enantiomer (eRank), the counts for both monomer and its enantiomer in the original dataset (Count and eCount), and the SMILE strings of both monomer and the enantiomer. The SMILE strings of the top 50 matched entries were checked manually. At least one entry in this table was confirmed as erroneous entry: A singular L-GalNAc entry was mapped to entry in GlyCosmos, https://glytoucan.org/Structures/Glycans/G71619RX which in turn can be mapped to CarbBank https://www.genome.jp/dbget-bin/www_bget?carbbank+18502 pointing to the original publication (*107*). The comparison and analysis of the publication (*107*) confirms that L-GlcNAc is an erroneous entry:

CarbBank and Glycosmos entry: Entry in the original publication:

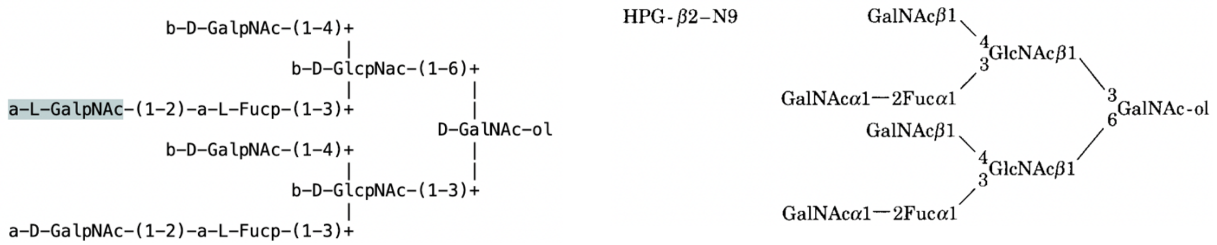

Further analysis of entries titled D-6dAraHexOMe (rank 106), XylHex (rank 123) and LyxHex (rank 151) in ChemDraw rendering of SMILE strings (C) highlighted a missing stereochemical information for c6 carbon and as such they cannot be assigned to a specific natural monomer with certainty. These entries are highlighted red in panel C. Beyond several hand-curated entries, many entries have not been carefully analyzed and it is possible that typos exist in other entries. However, this preliminary analysis indicates that not all glycan building blocks have corresponding enantiomers in nature. Enantiomers have been described for <20% of all natural monomers and >50% of most abundant monomers have no reported enantiomers. In many cases, the enantiomer of abundant glycan can be classified as "rare" due to low frequency of reports of such monomer in the literature.

All data and scripts employed to produce this analysis are available in Supplementary data "/Glycan Enantiomer Analysis" folder

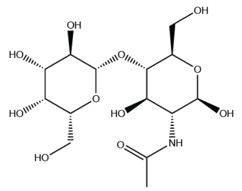

unmodified glycan (LAcNAc)
Used for ITC, ESI-MS

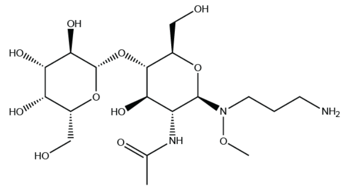

N-alkyl-methoxyamine bi-functional linkers
For microarrays
dot:10.1007/s10719-017-9785-4 M.S.M. Timmer and coworkers

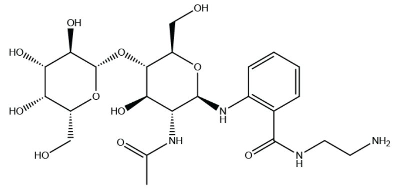

2-amino-N-(2-amino-ethyl)-benzamide (AEAB)
For microarrays
doi: 10.1016/j.chembiol.2008.11.004 R. Cummings and coworkers

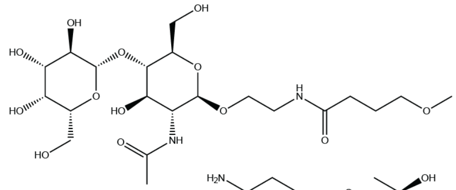

CFG Glass Array Sp0

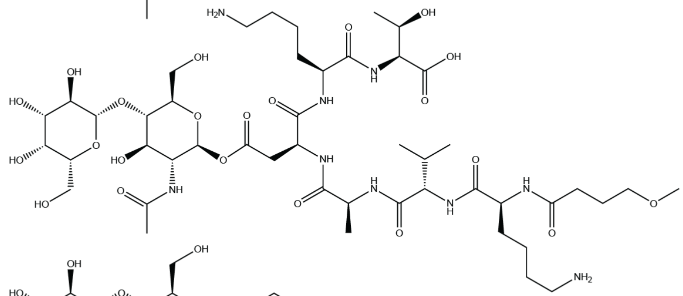

CFG Glass Array Sp24

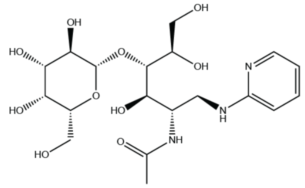

frontal affinity chromatography with
fluorescence detection (FAC-FD) pyridylaminated (PA)

Fig. S2. A diversity of chemical structures are used to represent the same glycan.

Here the same glycan (LacNAc) is shown with different linkage structures employed in various measurement assays. Notice that the linker may be compatible in size to (or even larger than) the disaccharide.

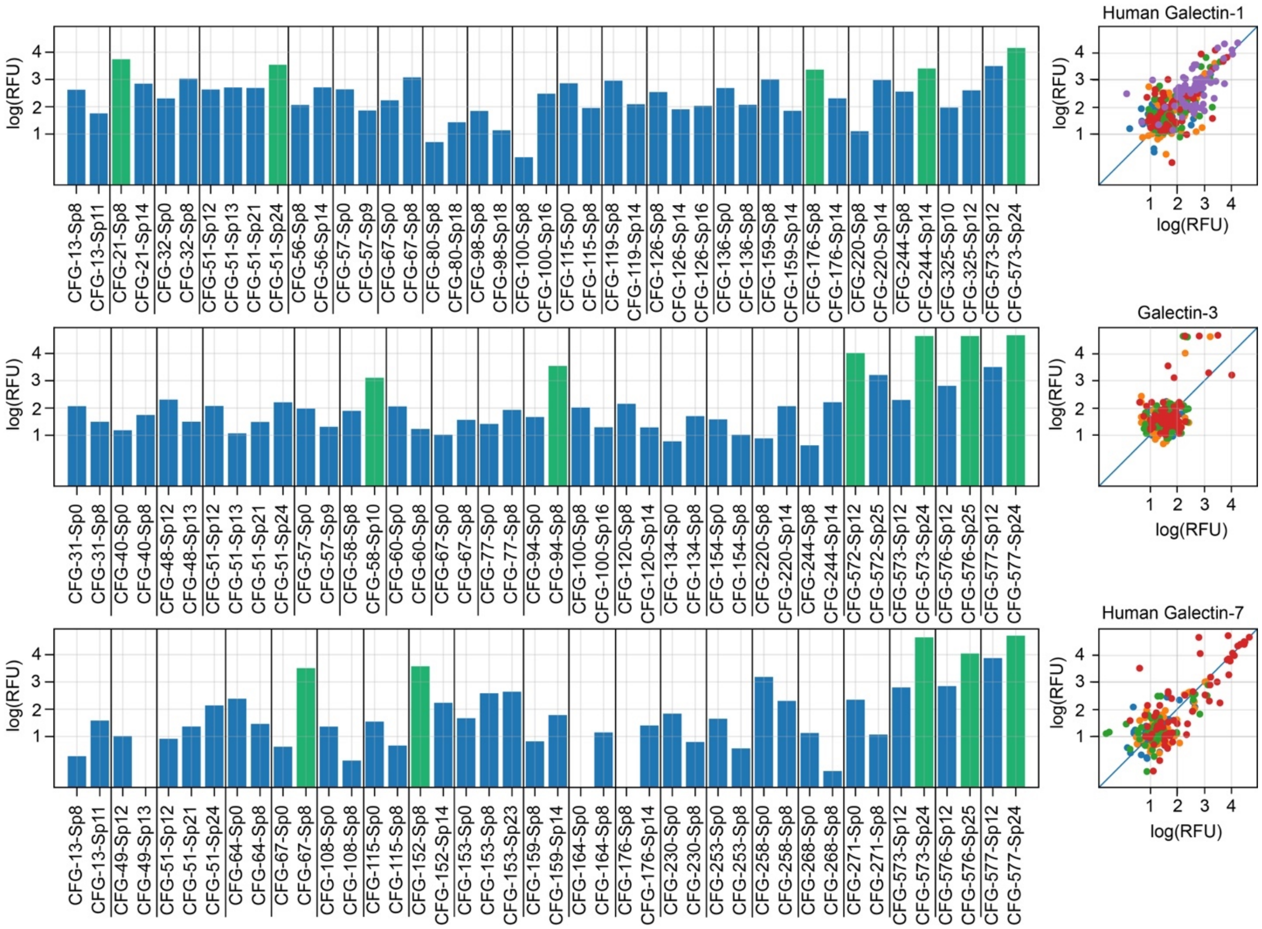

Fig. S3. Effect of different linkages on the binding among glycans in the CFG data.

At left blue bars show the reported binding (in RFU) for twenty glycans with multiple spacers. At right the scatterplots plot the RFUs of pairs with identical glycans but differing spacers. Different colours represent different concentrations of the galectins.

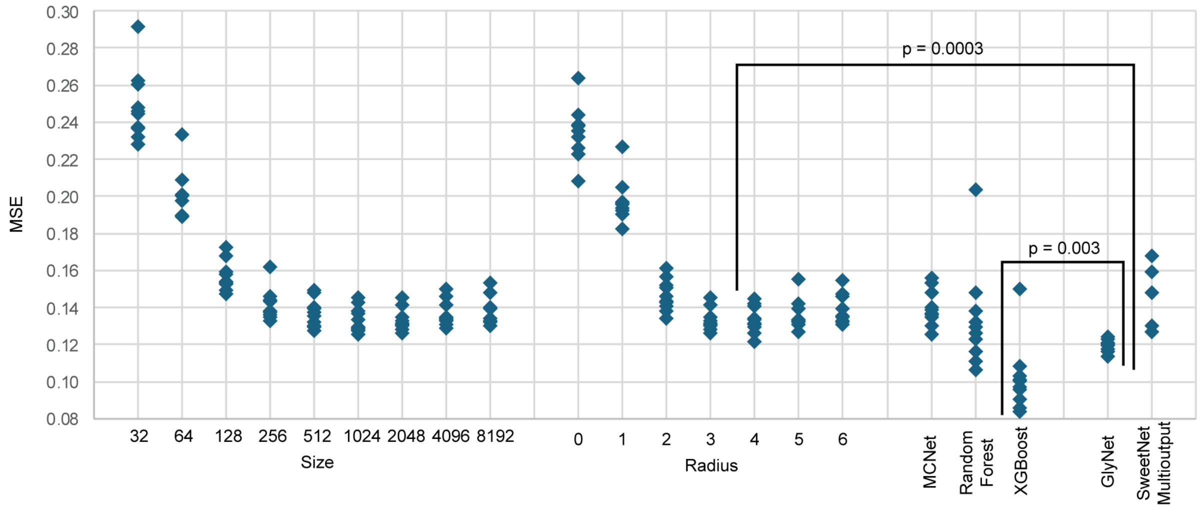

Fig. S4. Fine tuning of Morgan fingerprint (MF) models.

The leftmost 9 entries show performance, of atom-level alternative models wrapping the features into vectors modulo various sizes are tested (at optimal radius 3). Performance is reported as MSE calculated using the same folds as models in Figure 1. The fine-tuning points to 2048 as the most optimal size. Using this size, modification of radius yields MSE that approaches the MSE of GlyNet and is indistinguishable from MSE of SweetNet in the same task. Replacing the fully connected neural network architecture with Random Forest yields an MSE indistinguishable from that of GlyNet. MSE of a model that uses XGBoost significantly (p = 0.003, Mann-Whitney U-Test) exceeds the performance of GlyNet on the same cross-validation dataset. These results show that appropriately tuned Morgan fingerprint-based models can exceed the performance of monomer-based models on the same task (prediction of RFU values for protein-glycan interactions).

A

	No Confident Zeros		Assymetric Pseudo-zeros		Symmetric Pseudo-zeros	
alpha-Gal - Sp8	n/a	1.4	**3.0**	1.4	3.0	1.4
beta-Glc - Sp8	n/a	1.3	**3.0**	1.3	3.0	1.3
beta-Man - Sp8	n/a	2.2	n/a	2.2	n/a	2.2
13-saccharide	n/a	4.0	n/a	4.0	n/a	4.0
Neu5Gc + above	n/a	1.5	**3.0**	1.5	3.0	1.5
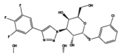	7.3	n/a	7.3	n/a	7.3	n/a
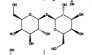	4.3	n/a	4.3	n/a	4.3	n/a
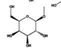	2.4	n/a	2.4	n/a	2.4	**2.0**

B

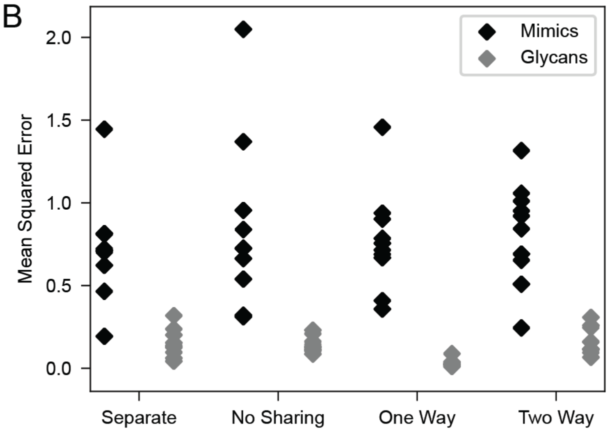

Fig. S5. Pseudo-labeling RFU and Kd datasets with "confident zeros".

An initial attempt to merge RFU and Kd values in one model, output two numbers, one for each type. The training loss could only be calculated and backpropagated from the one output for which we had a ground truth value. We expected that an internal result would be translated into the two output formats. Since the two measures of binding describe the same physical systems, we reasoned that if a known RFU was low (non-binding) we could confidently know that the Kd was near zero (also non-binding) and vice versa. We used such pseudo-zeros to enlarge the set of ground truth values. (A) Example of data that combines RFU values for CFG glycans (small value = no binding) and log(Ka) values for glycomimetic (GM) compounds (small log(Ka) = strong binding). In asymmetric (one-way) pseudo-labeling, any compound that has low log(RFU) value was also assigned a low log(Ka) value (i.e., compounds that show no binding in CFG microarrays are very likely to possess a very low Ka). In symmetric (two-way) pseudo-labeling, any compound that has low log(Ka) value was also assigned a low log(RFU) value. (B) Distributions of losses (MSE) for models trained on the two datasets, i) results from two separate models, ii) one model with both datasets, iii) one model with both datasets and asymmetric pseudo-labelling, and iv) one model with both datasets and symmetric pseudo-labelling.

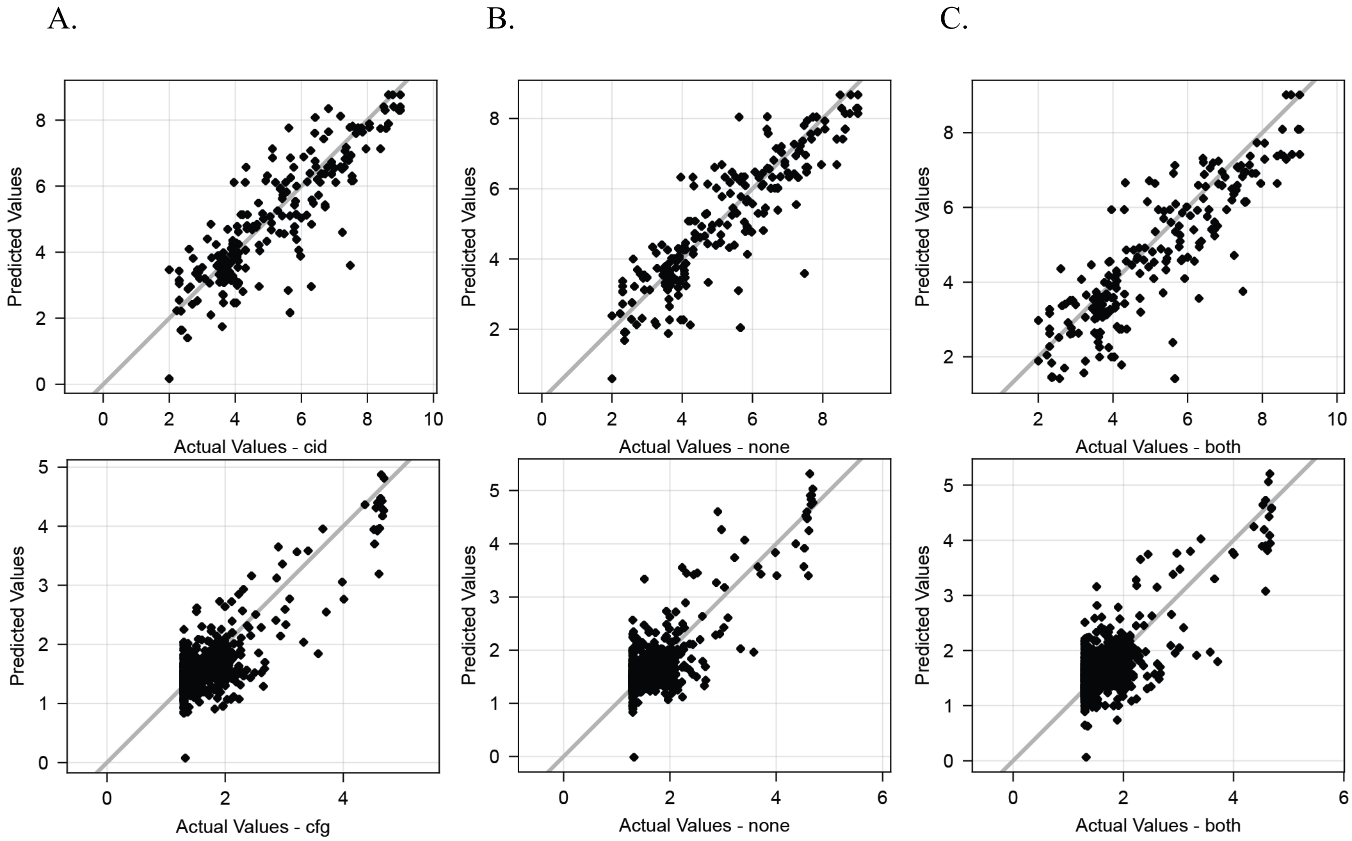

Fig. S6. Plots of predicted vs. actual values (on hold-out sets) of CFG glycans and glycomimetics.

Binding to Galectin-3 sample (upper panels) and the GM compounds binding to Galectin-3 (lower panels). (A) Model1 trained on original RFU-Kd data; (B) Model2 trained on data with asymmetric pseudo-labels; (C) Model3 trained on data with symmetric pseudo-labels. All three models are reproducing the data but show no sensitivity to any additional pseudo-labels.

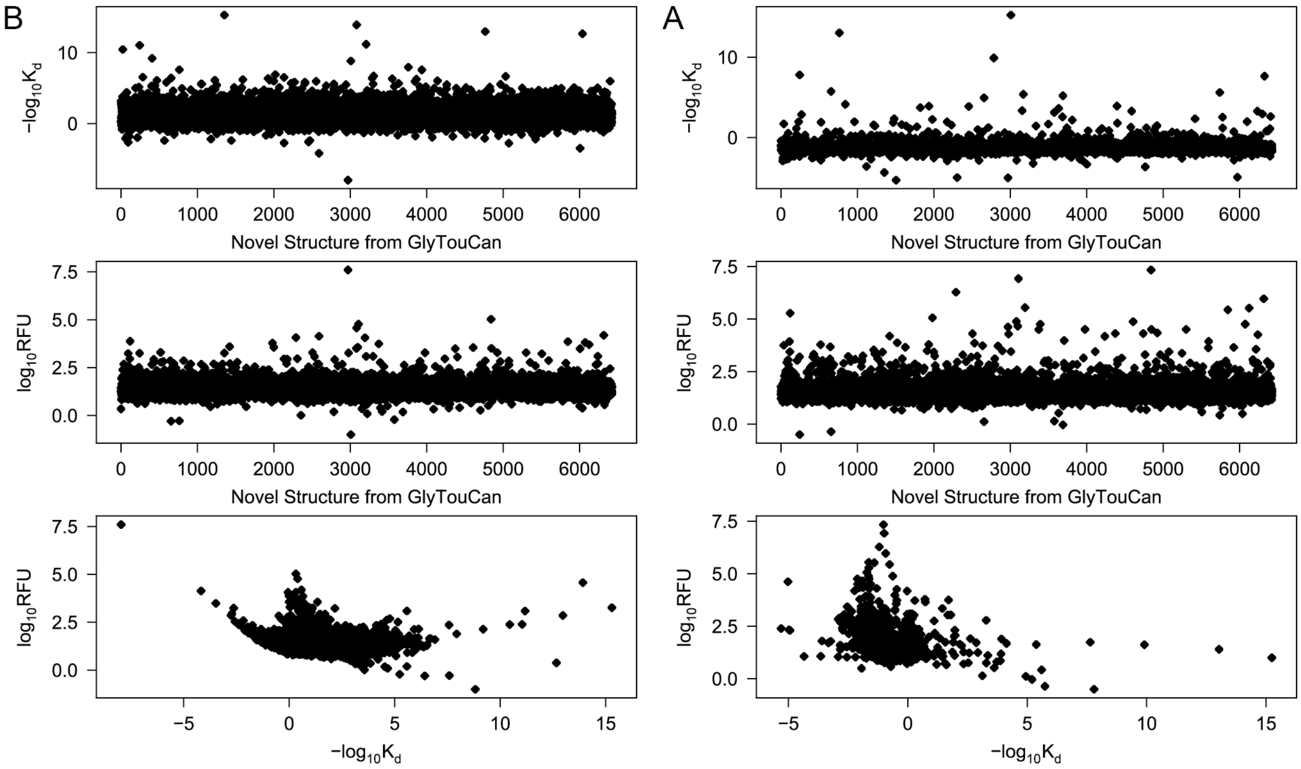

Fig. S7. Two model trained on combined RFU+Kd data.

(A) with pseudolabels (symmetric) and (B) without pseudolabels. Models were tested for their ability to predict binding of glycans from GlyTouCan to Galectin-3. Although models can make predictions, there exists no anticipated direct correlation between predicted log(RFU) and predicted log(Ka). The trend is exactly the opposite: compounds with high log(RFU) (i.e., strong binders in microarray) are predicted to have low Ka (not a binders) and compounds with high Ka (strong binders) are predicted to have no detectable RFU signal on microarray.

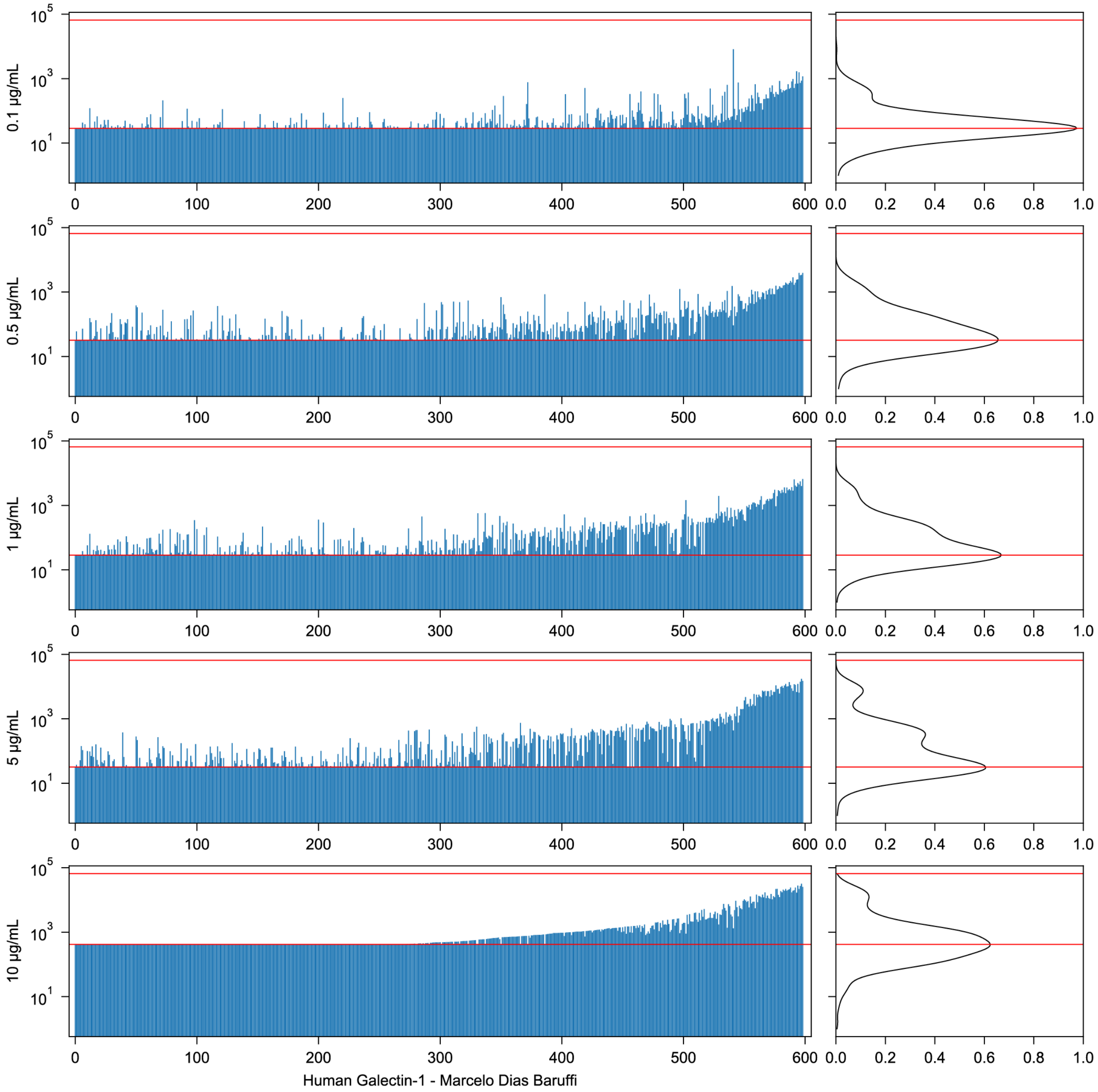

Fig. S8. Conversion of glycan microarray RFU values to fractions bound.

Shown are RFU data from five arrays with different concentrations of Galectin-1. In each of the five, many of the RFUs are clustered about a low value. This is shown as peaks in the kernel density estimation plots (right of figure). The levels of these peaks (lower red lines) are taken as background RFUs, and assumed to be non-binding ($f = 0$). The maximum RFU across all samples (upper red lines) is taken as full binding ($f = 1$).

Some Henderson–Hasselbalch Mathematics

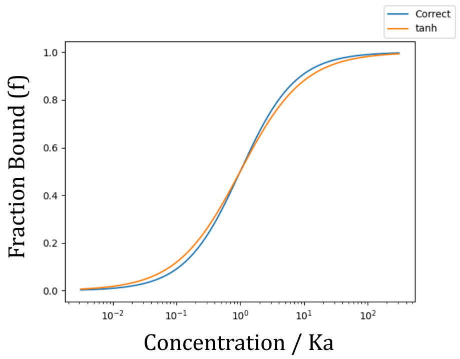

At Concentration = Ka:
f = 1 / (1 + Ka/Ka) = 0.5

At C = 10 Ka: f = 0.909,
tanh sigmoid = 0.881

$X + Y \rightleftharpoons XY$	reaction
$K_a = \frac{1}{K_d} = \frac{[X][Y]}{[XY]}$	kinetic equation
$[Y_0] = [Y] + [XY]$	conservation of mass
$f = \frac{[XY]}{[Y_0]}$	fraction of Y bound
$1 = \frac{[Y]}{[Y_0]} + f$	fraction unbound
$\frac{K_a}{[X]} = \frac{[Y][Y_0]}{[XY][Y_0]} = \frac{1-f}{f}$	rearranged with substitutions
$f = \frac{1}{1 + K_a/[X]} = \left(1 + 10^{-\log K_d}/[X]\right)^{-1}$	solved for f using Ka/Kd and [X]

Fig. S9. Mathematics of the Henderson-Hasselbalch equation.

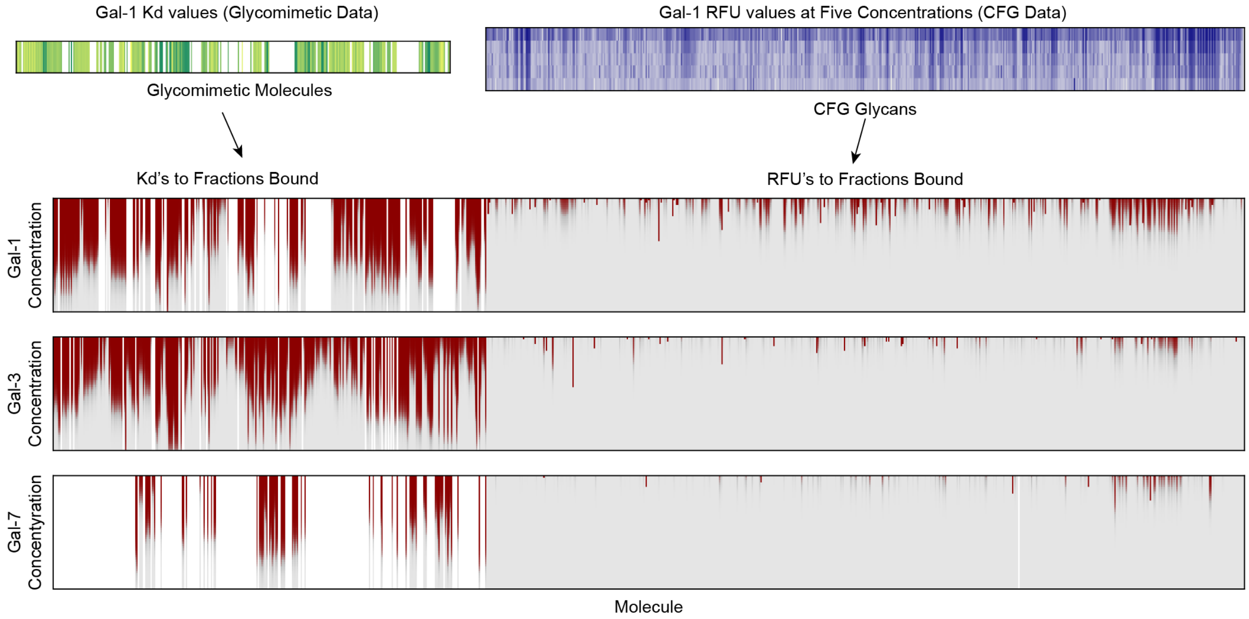

Fig. S10. Overview of the merger of data from multiple sources to unified fractions bound.

Here Kd values for Galectin-1 binding to the glycomimetic molecules and RFU values for five Galectin-1 CFG arrays are converted into set of fractions bound, *f*s, across the 50 concentrations (vertical axes). These are then combined into a single set across all the molecules. Unknown (missing) values propagate through the processing and produce holes in the results (white columns). Also shown are the results for Galectins-3 and 7.

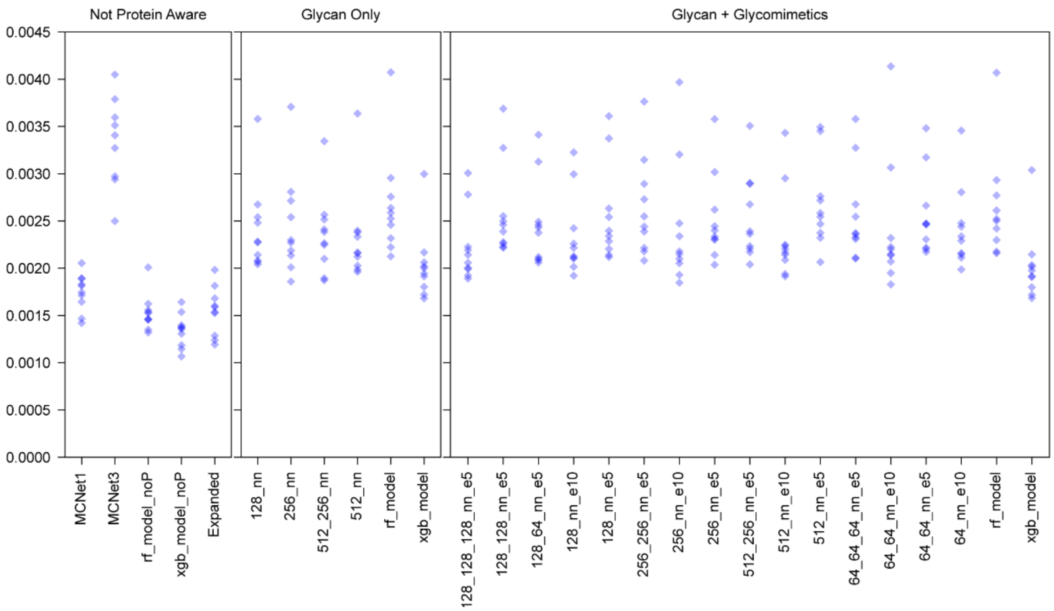

Fig. S11. Neural network implementation against other simple machine learning techniques.

All values are MSEs from different cross-validation hold-out folds and these folds are identical to those used in Figure 3.

Values in "**Not protein aware**" box have MCNet1 and MCNet3 performance from Figure 3 as benchmark. We find that supplying the MCNet1-data to a RandomForest algorithm (scikit-learn, "rf_model_noP") produces about the same performance as measured by MSE. Supplying the MCNet1-data to XGBoost ("xgb_model_noP") produces a model with a lower MSE ($p = 0.0001$ by Mann-Whitney U-test). Despite optimal performance of XGBoost, we continue to use neural networks because training on MCNet3 data for multiple lectins in parallel is incompatible with XGBoost libraries which do not tolerate gaps in the output data for training. Data labeled "Expanded" describes MSE performance of MCNet1-like model trained on expanded dataset of lectins that included some values measured only at one concentration.

Values in "**Glycan only**" box present MSEs from neural networks, RF and XGboost models that employ proteins representation (ESMc2), glycan representation ($MF3_c$) and concentration (C) to calculate the fraction bound for the same glycans used in training of MCNet1; training set included 267 lectins (147 lectins from MCNet1, excluding 20 lectins for which protein sequences are not know + 150 additional lectins).

Values in "**Glycan+Glycomimetics**" describe MSE for neural networks, RF and XGboost models that employ proteins representation (ESMc2), glycan representation ($MF3_c$) and concentration (C) to calculate the fraction bound for a collection of glycans and glycomimetic compounds used in training of MCNet3; training set included 267 lectins (147 lectins from MCNet1, excluding 20 lectins for which protein sequences are not know + 150 additional lectins). Labels with numbers in the names are models that use neural networks with specific number of neurons in different layers (64_64_64 is three layers, 64 neurons each); "rf_model" and "xgf_model" are RF and XGBoost. Expansion from 147 lectins to yield MSEs still comparable to MSE of MCNet Although the MSE values are comparable to

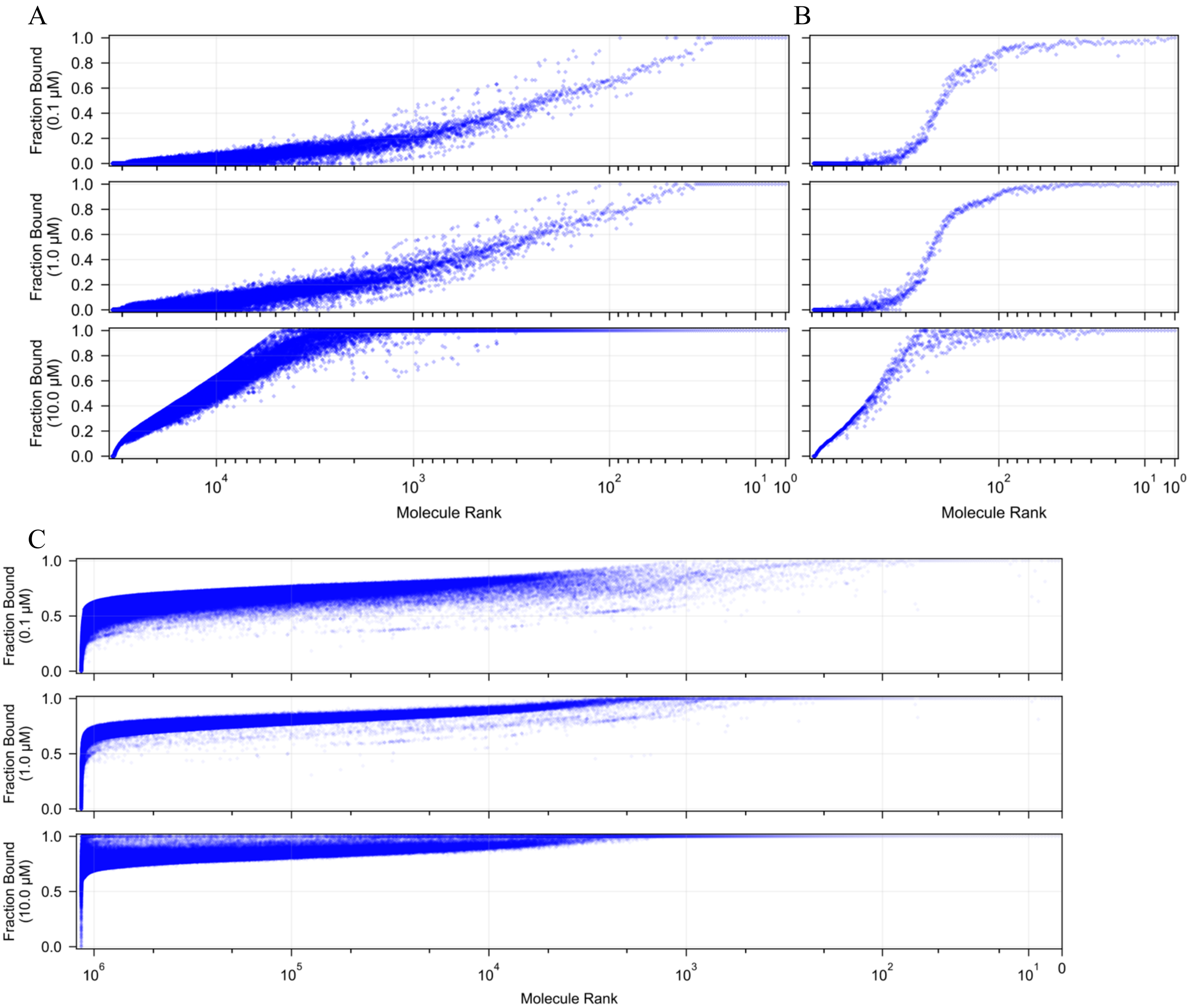

Fig. S12. Predictions of binding of GlyTouCan and CFG glycans to Galectin-1.

A) GlyToucan and B) CFG. The molecules have been ranked by the sums of the fractions bound across the three concentrations (0.1 µM, 1.0 µM, and 10 µM). A non-linear axis is used to emphasize the strongest binders. C). Predicted binding over the BindingDB set, by Model MCNet at 0.1 µM concentration. See SMILES-GlyTouCan.txt for corresponding molecule identifiers. In contrast to panels A and B, nearly all one million small molecules are predicted to have $f > 0.5$ at 0.1 µM concentration.

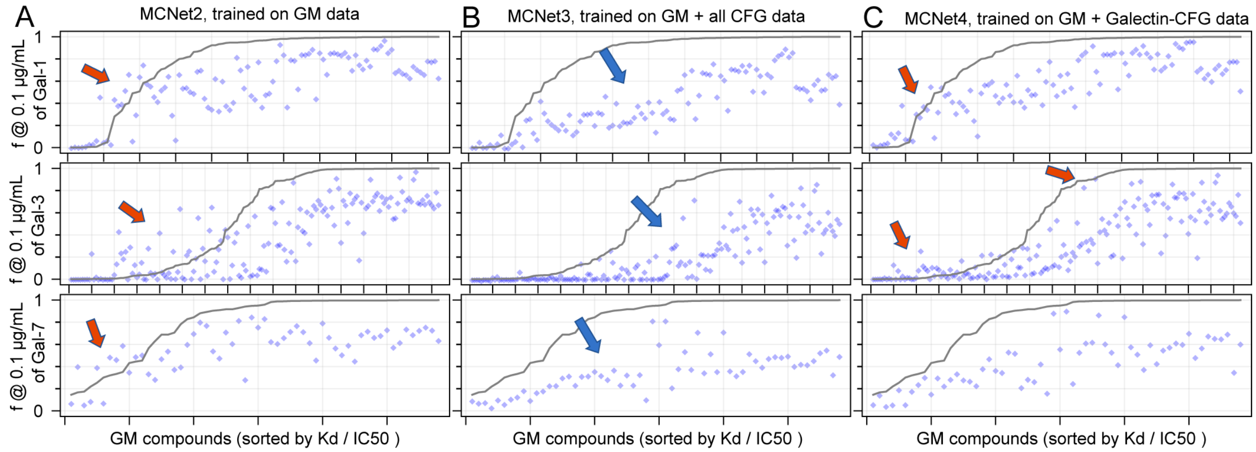

Fig. S13. Prediction of cross-chiral recognition by MCNet.

Solid lines describe the ground truth: binding of glycomimetic compounds to Galectins-1, 3 and 7. Blue dots are predicted binding strength of the GM-enantiomer. Expert knowledge of GM-galectin interactions dictates that all predictions should be below the solid line (i.e., activity of enantiomer must be lower than the activity of the parental compound). Only MCNet3 (**B**) produced answers aligned with the "expert knowledge" of cross-chiral recognition.

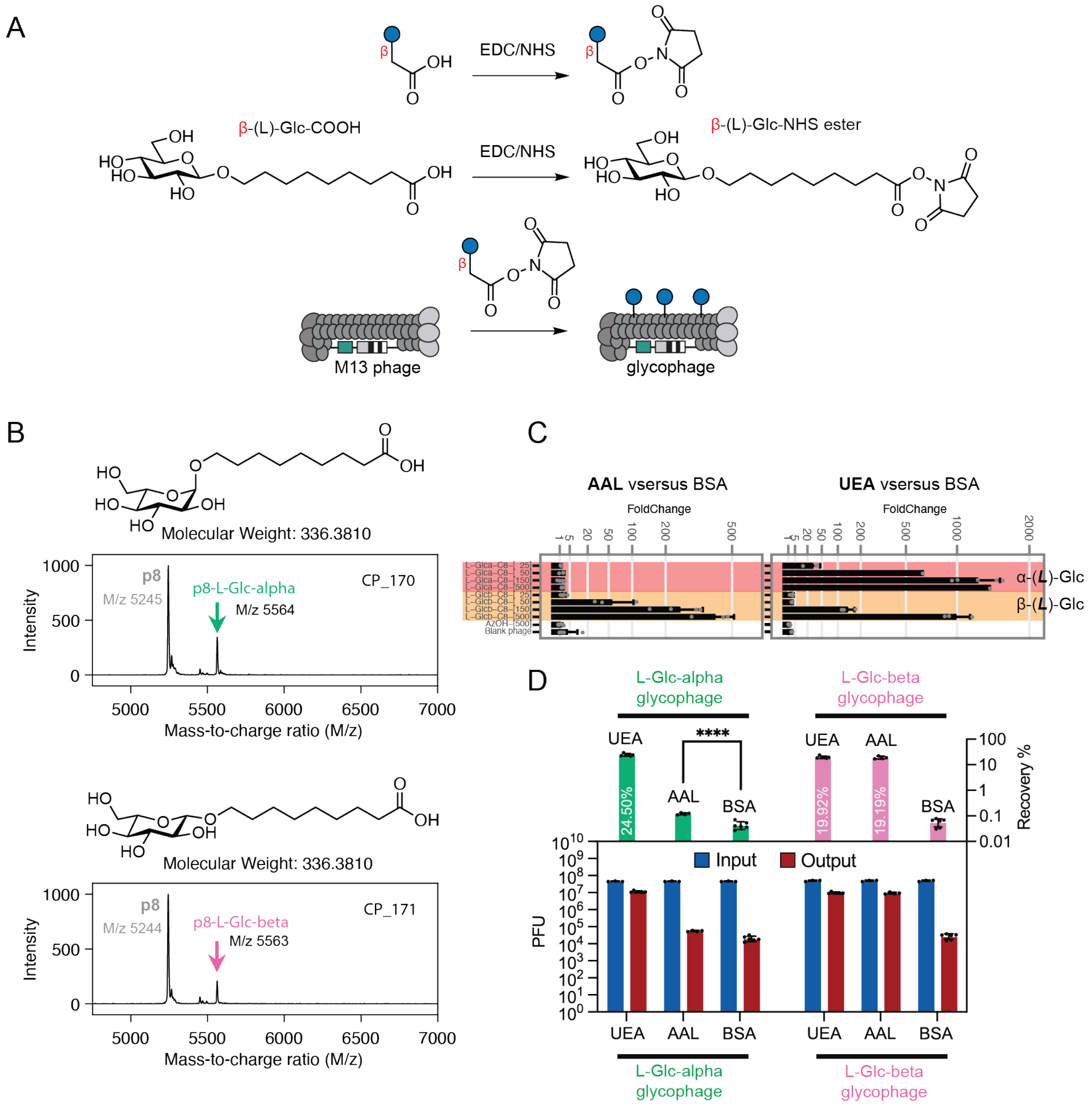

Fig. S14. Experimental data showing binding of monoclonal α-L-Glc and β-L-Glc conjugated glycophages to UEA and AAL.

(A) Reaction scheme of conjugating L-Glc to phages; (B) MALDI-TOF MS spectra of α-L-Glc and β-L-Glc conjugated glycophages; (C) LiGA binding profile shows α-L-Glc and β-L-Glc conjugated glycophages density-dependently interact with UEA-I and AAL; (D) Recovery of monoclonal α-L-Glc and β-L-Glc conjugated glycophages after binding to UEA-I and AAL-coated plates. n = 3 input, n = 4 output (AAL), n = 6 output (UEA and BSA).

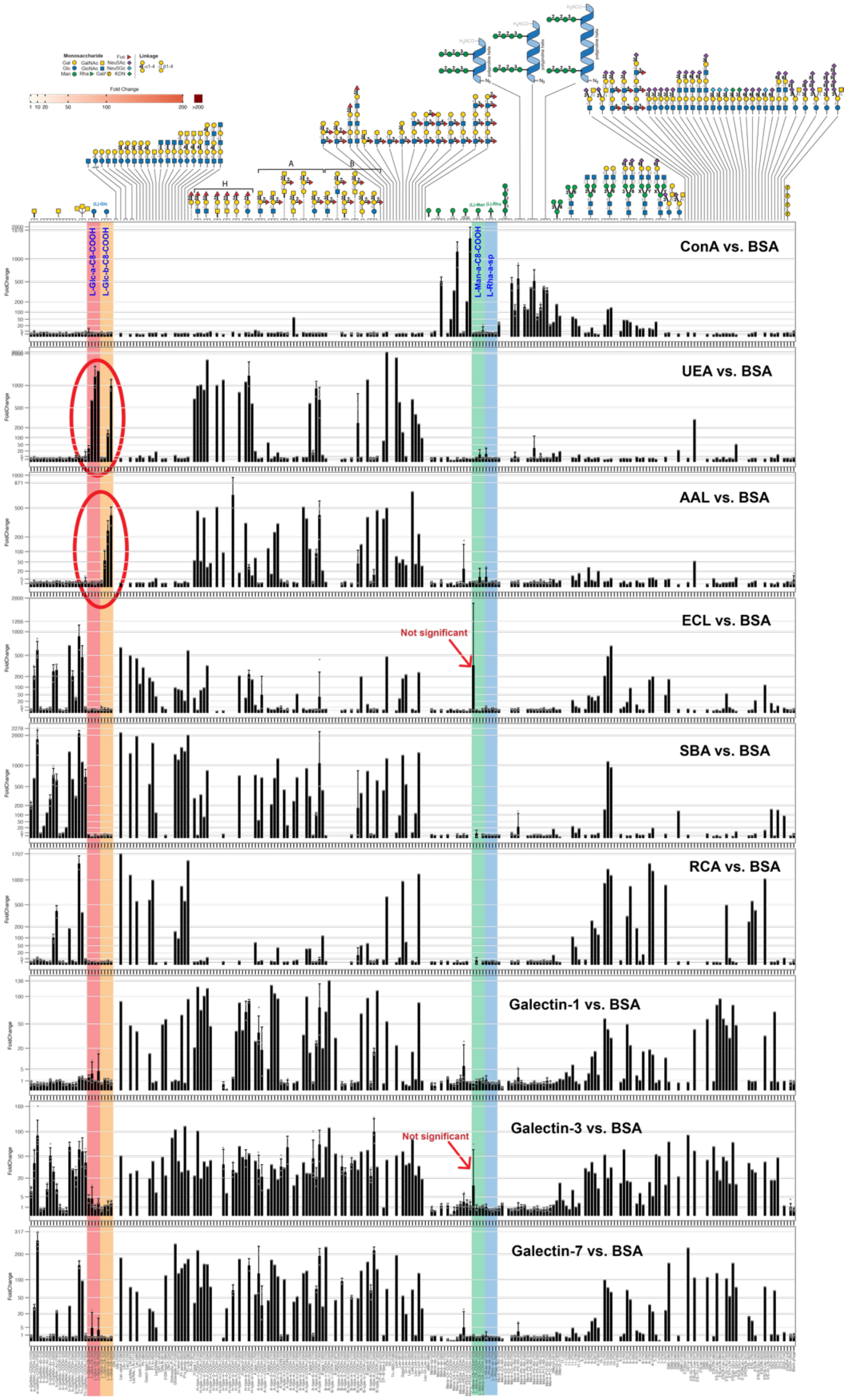

Fig. S15. Experimental data showing binding of a Liquid Glycan Array to eight lectins.

Displays of four L-sugars (α-L-Glc, β-L-Glc, α-L-Man, α-L-Rha) in the coloured columns show substantial binding (large fold-changes) for the L-glucoses when presented to the lectins UEA-I and AAL (red circles) but not in the other cases. For details of experiment see **Figure S18**.

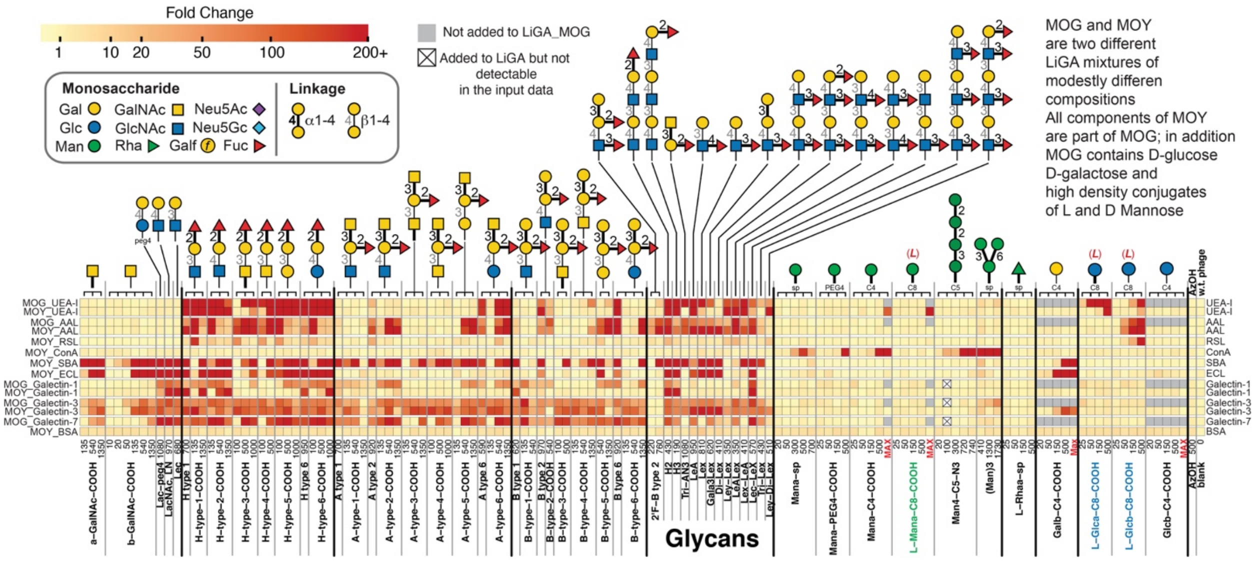

Fig. S16. Heatmap of glycan-binding profiles of lectins to LiGA that contains diverse L and D monomers.

Colors in the heatmap represent fold change (FC) ratio of normalized Output and Input reads of glycan-associated DNA barcode, as determined by Next Generation Sequencing and processed by EdgeR Differential Enrichment analysis repurposed for phage-displayed libraries (*108*) and described in published LiGA protocol (*103*). **Input** is produced by PCR amplification and NGS analysi of LiGA mixture prior to selection. **Output** is produced by incubating the LiGA mixture in the 4-5 wells coated by the said lectin, followed by wash of unbound glycophage, elution and PCR and NGS. Higher FC represents stronger binding. A Control protein, BSA, exhibits fold change in low single digit range for all glycans. Data is acquired using two independent LiGA mixtures of nested composition: LiGA_MOG and LiGA_MOY: see note in the upper right corner All three Fuc-binding lectins we tested (UEA-I, AAL, and RSL) showed varying degrees of interaction with blood group glycans (HBGs) and Lewis-type glycans, consistent with their known specificities. Some HBG-conjugated glycophages, specifically HBG type 3 and HBG type 4 did not interact with UEA-I, AAL, nor RSL. However, as we used a combination of lectins to benchmark the binding, we noted that HGB-3 and HGB-4 bind to Galactose-binding lectin SBA; the same SBA lectin recognizes Gal and GalNAc but not Man and Glc monomers as expected from its glycan-binding profile. Lack of binding of fucose-binding lectins to HGB-3 and HGB-4, therefore, represents a valid preference of these lectins for HBG architecture and it is not due to inappropriate display of glycans or lectins in this assay. Once the quality control of all glycans is confirmed, we noted that all three fucose-binding lectins (UEA-I, AAL, and RSL) displayed strong binding to L-glucose-conjugated phages. Binding to phages modified by enantiomer D-glucose or diastereomer D-galactose to the same UEA-I, AAL, and RSL lectins was not observed even when density of both glycan was ramped up to modify all 2700 coat proteins (denoted here as "Max" density). Ramping up the density of L-alpha-mannose and D-alpha-mannose to the same "Max"-density unveiled additional observation: L-alpha-mannose interacts with UEA-I but not

AAL, and RSL and not classical Mannose-binding protein ConA. In contrast, D-alpha-mannose interacts with ConA at a range of densities and interacts weakly UEA-I at a “Max” density.

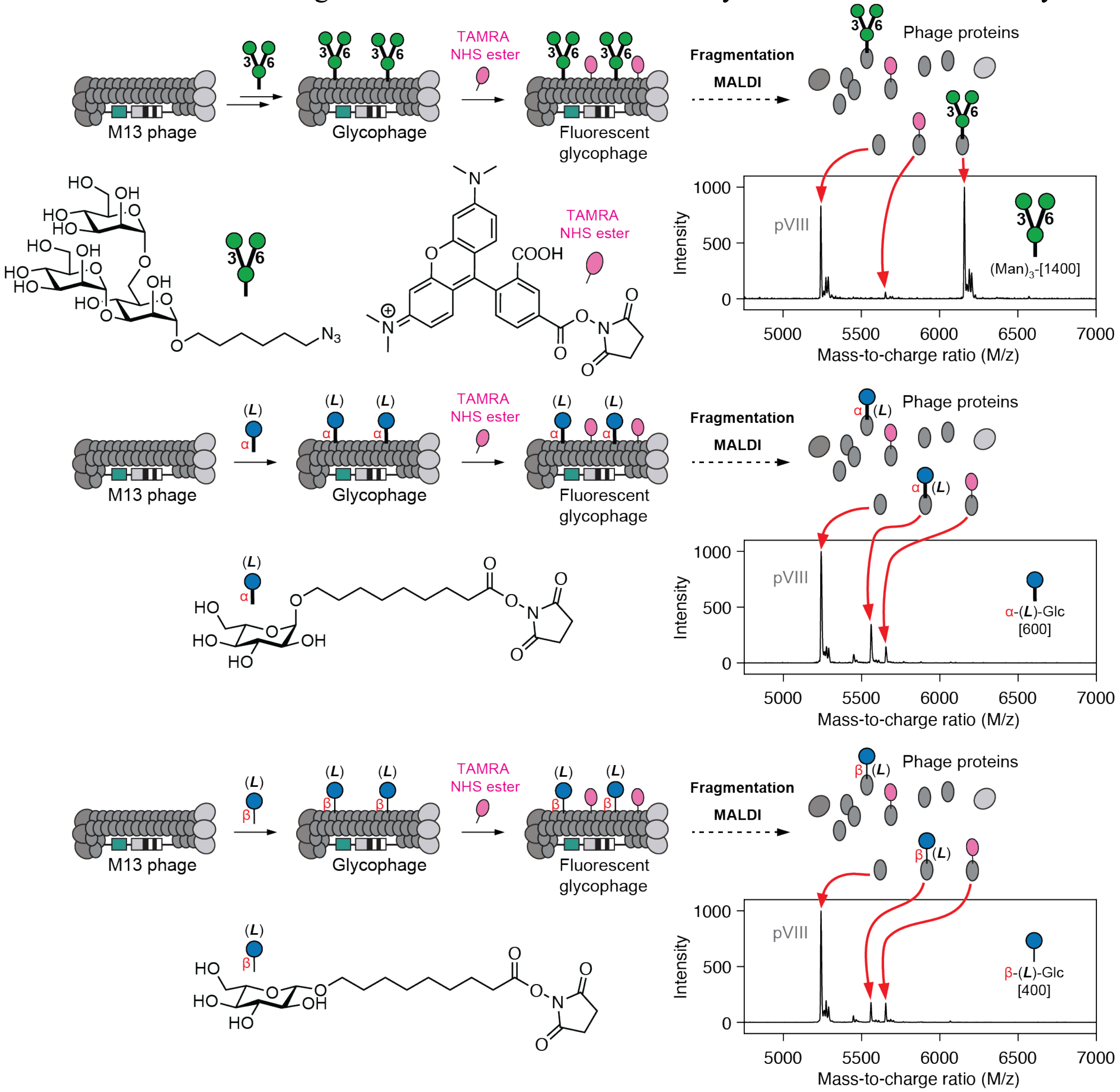

Fig. S17. Generation of fluorescent glycophages for lectin array binding experiments.

Reaction scheme for conjugating glycans and TAMRA to phages to produce TAMRA-labeled glycophages, and MALDI-TOF MS spectra of TAMRA-labeled (Man)3-, α-L-Glc-, and β-L-Glc-conjugated glycophages.

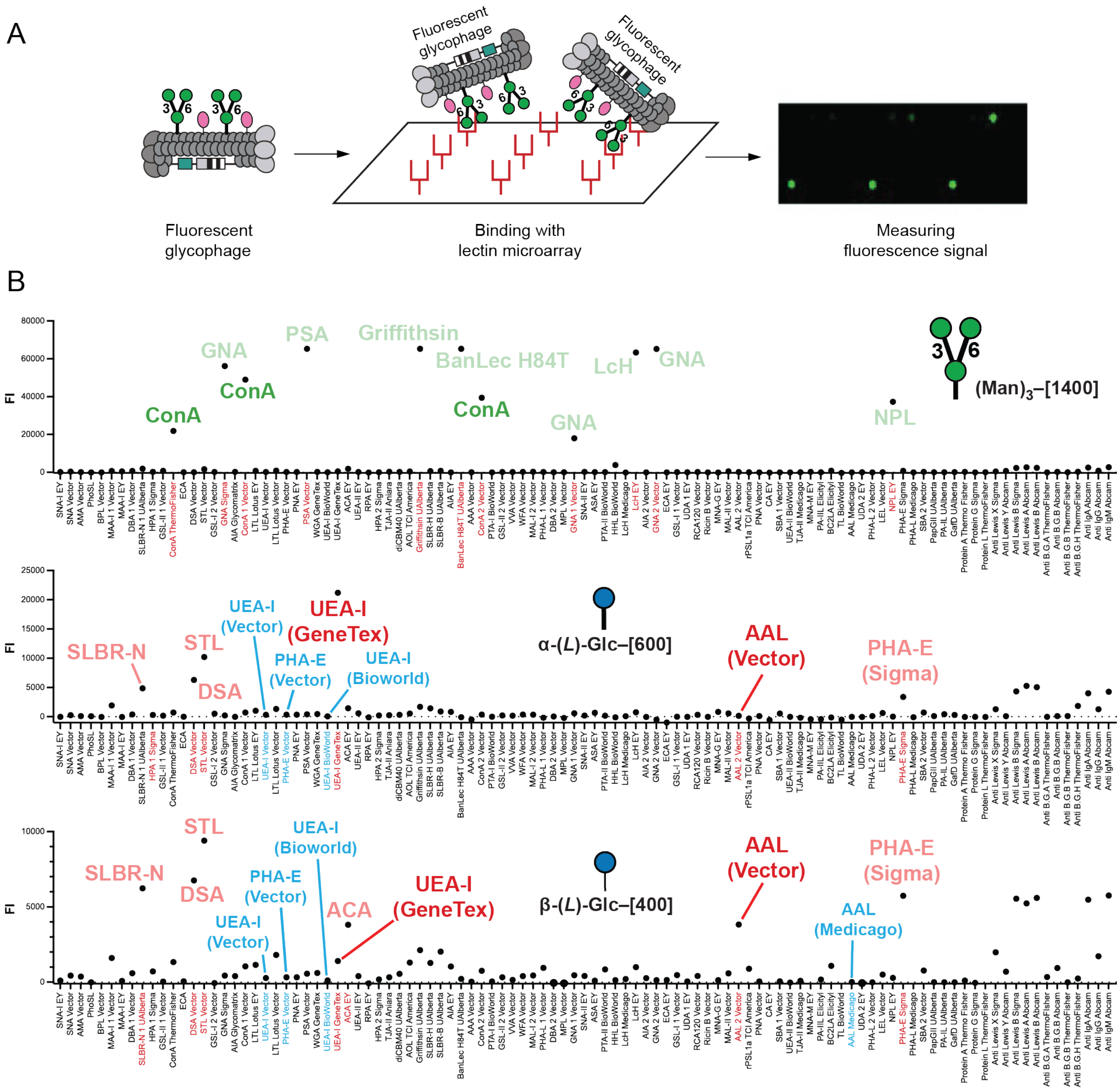

Fig. S18. Experimental data showing binding of fluorescent glycophages to lectin microarray.

(A) Scheme of binding of fluorescent glycophages to lectin microarray; (B) Fluorescence signal of TAMRA-labeled (Man)3-, α-L-Glc-, and β-L-Glc-conjugated glycophages to lectins. TAMRA-labeled (Man)3-conjugated glycophage only shows binding with Mannose-binding lectins. TAMRA-labeled α-L-Glc-conjugated glycophage shows interaction with UEA-I from GeneTex (red) but not AAL which correlates with the LiGA binding profile. TAMRA-labeled β-L-Glc-conjugated glycophage shows interaction with both UEA-I (GeneTex) and AAL (Vector), but not from other suppliers (blue labels).

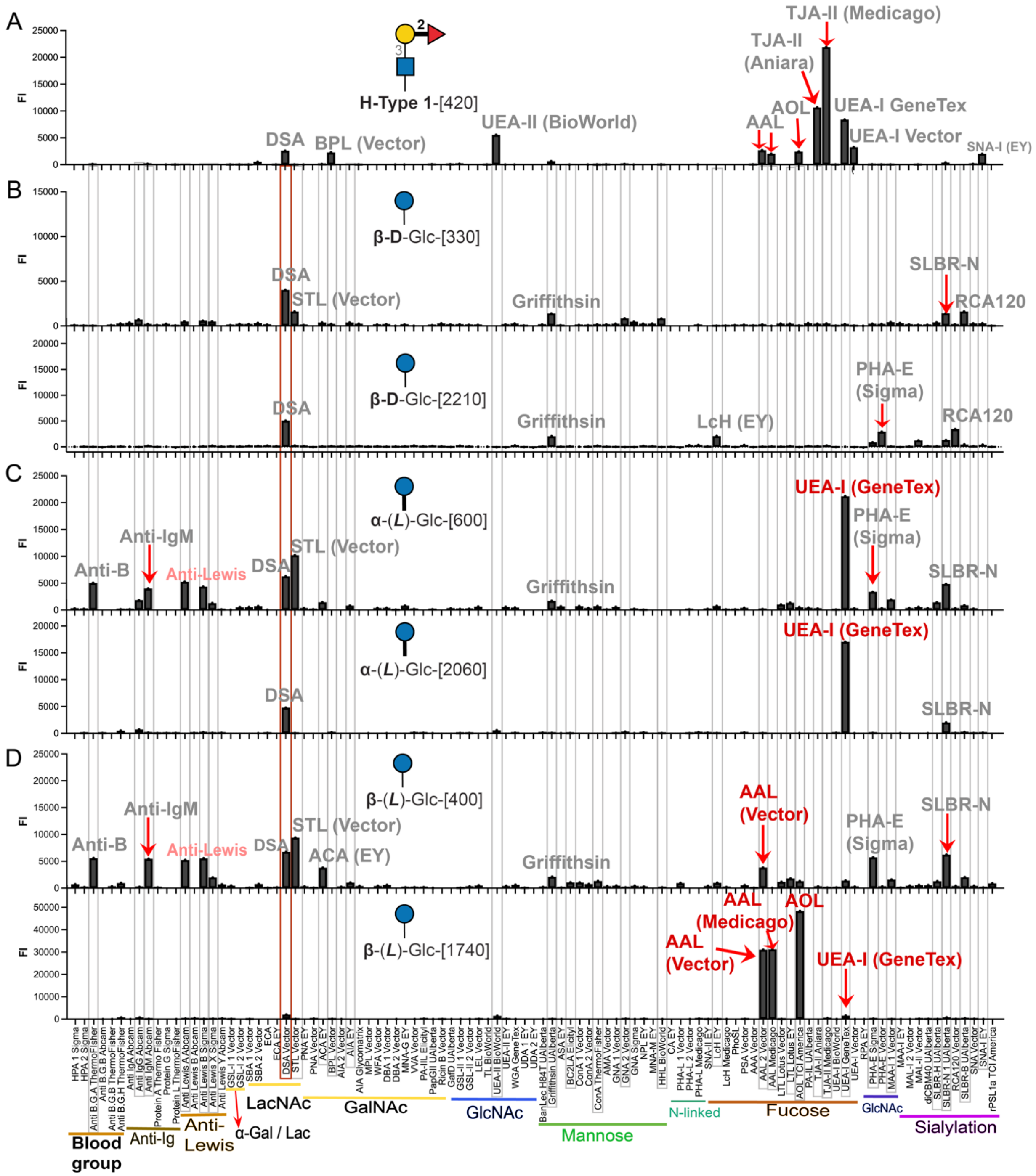

Fig. S19. Lectin microarray experimental data showing binding of fluorescent glycophages to lectins.

Fluorescence signals of TAMRA-labeled glycophages are shown for (A) H-type 1 (control for fucose-binding lectins), and low and high displayed densities of β-D-Glc (B), α-L-Glc (C), and β-L-Glc (D). The TAMRA-labeled α-L-Glc -conjugated glycophages showed strong binding to UEA-I (GeneTex) and β-L-Glc to AAL, AOL and weak binding to UEA-I (GeneTex). The H-type I control demonstrates functional binding to multiple fucose-binding lectins.

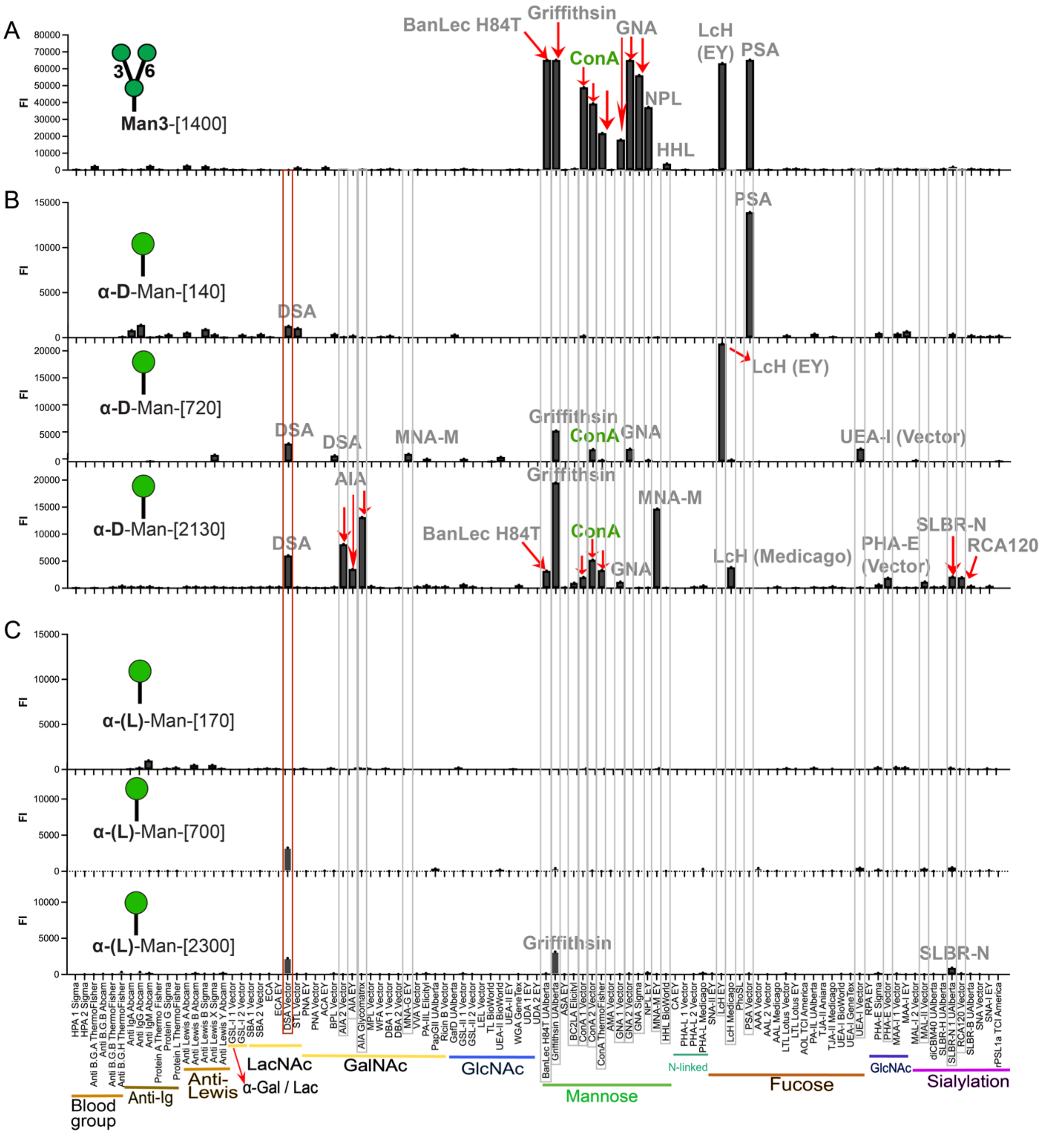

Fig. S20. Lectin microarray experimental data showing binding of fluorescent glycophages to lectins.

Fluorescence signals of TAMRA-labeled glycophages are shown for (A) the control Man-3-conjugated glycophage, (B) various displayed densities of D-Mannose and (C) L-Man-conjugated glycophages. The TAMRA-labeled Man-3 control glycophage exhibits binding for mannose-binding lectins including LcH and PSA.

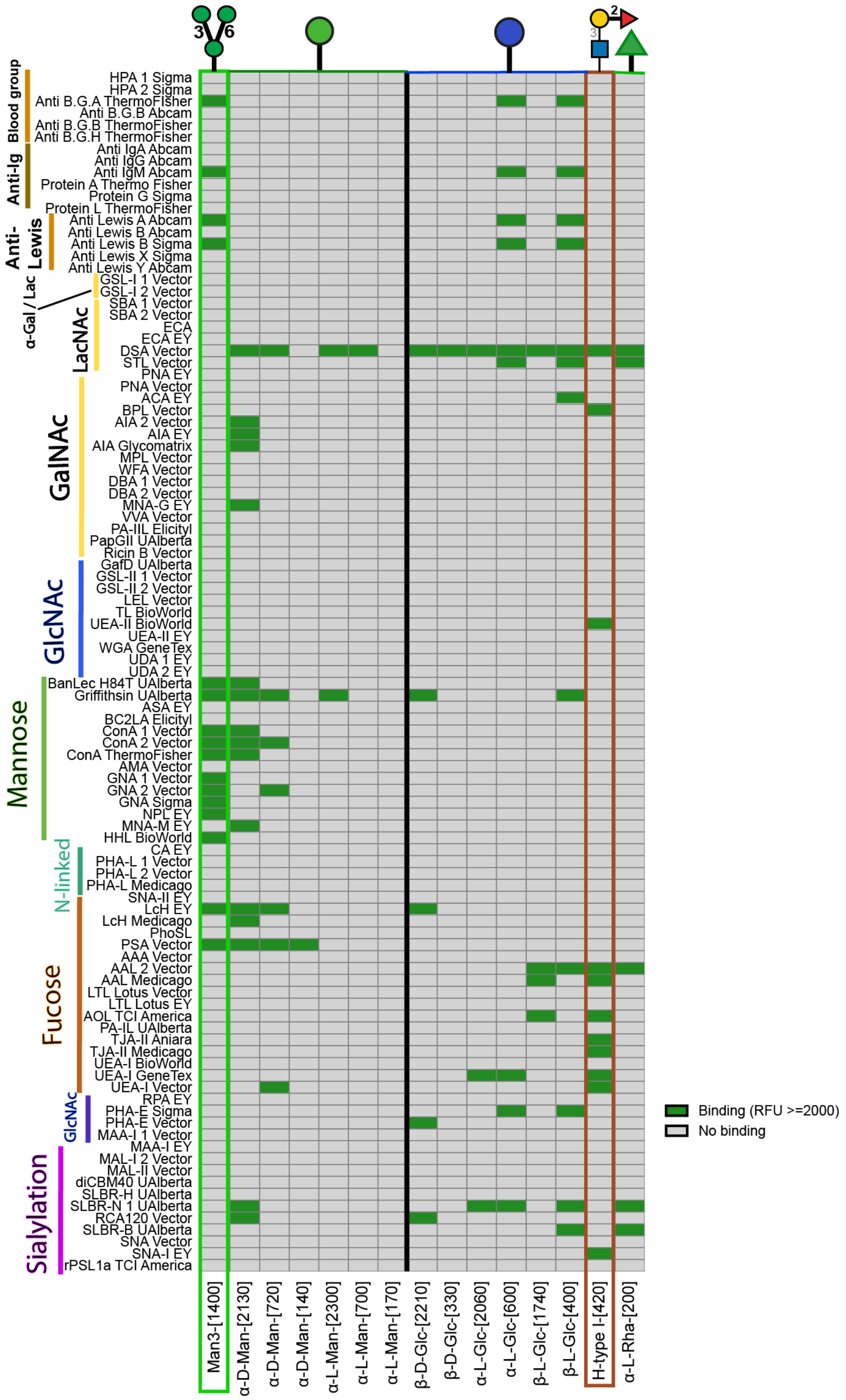

Fig. S21. Lectin microarray data summary showing binding profiles of fluorescently labeled glyco-phages to various lectins.

TAMRA-labeled glycophages were tested against the lectin array to assess binding specificity. These glycophages displayed either low (140-720 copies per phage) or high (1400-2300 copies per phage) densities of glycans (both D and L α-Man, α-L-Glc, β-L-Glc, β-D-Glc, α-L-Rha with (Man)3 and H-type 1 as controls).

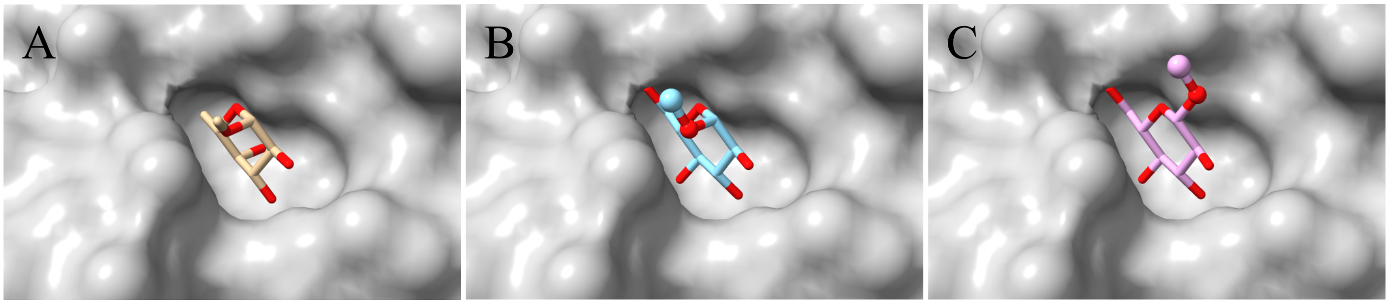

Fig. S22. Structural Analysis of L-Glucose Superimposition in Crystal Structure of UEA-I.

A. Crystal structure of UEA-I (PDB ID: 1JXN) showing the protein as surface (grey) and fucose ligand as sticks (brown sticks). B. Alignment of α-L-Glucose (blue sticks) into the UEA-1 binding site from superimposition of the α-L-Glucose onto the α-L-Fucose ligand. C. Alignment of α-L-Glucose (pink sticks) into the UEA-1 binding site from superimposition of the β-L-Glucose onto the α-L-Fucose ligand. The oxygen atoms are in red and the O-methyl group at the anomeric position is shown as balls.

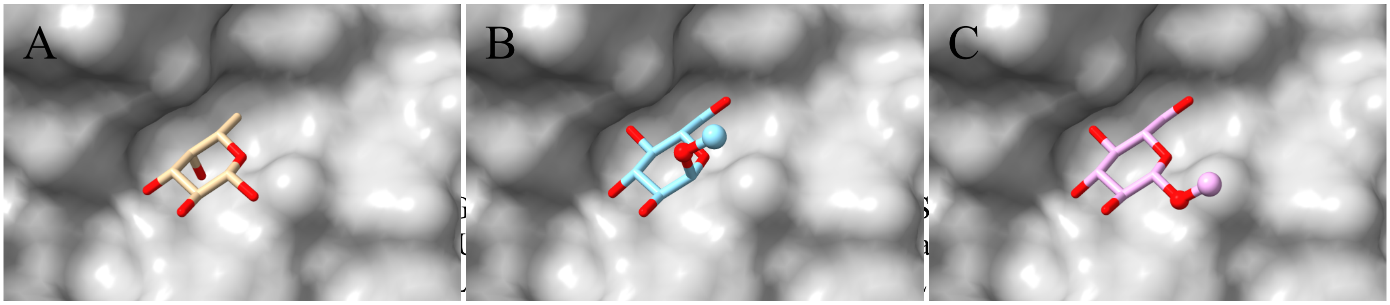

Fig. S23. Structural Analysis of L-Glucose Superimposition in Crystal Structure of AAL.

A. Crystal structure AAL (PDB ID: 1IUC) showing the protein as surface and fucose as sticks (brown sticks). B. Alignment of α-L-Glucose (blue sticks) into the AAL binding site from superimposition of the α-L-Glucose onto the α-L-Fucose ligand. C. Alignment of α-L-Glucose (pink sticks) into the AAL binding site from superimposition of the β-L-Glucose onto the α-L-Fucose ligand. The oxygen atoms are in red and the O-methyl group at the anomeric position is shown as balls.

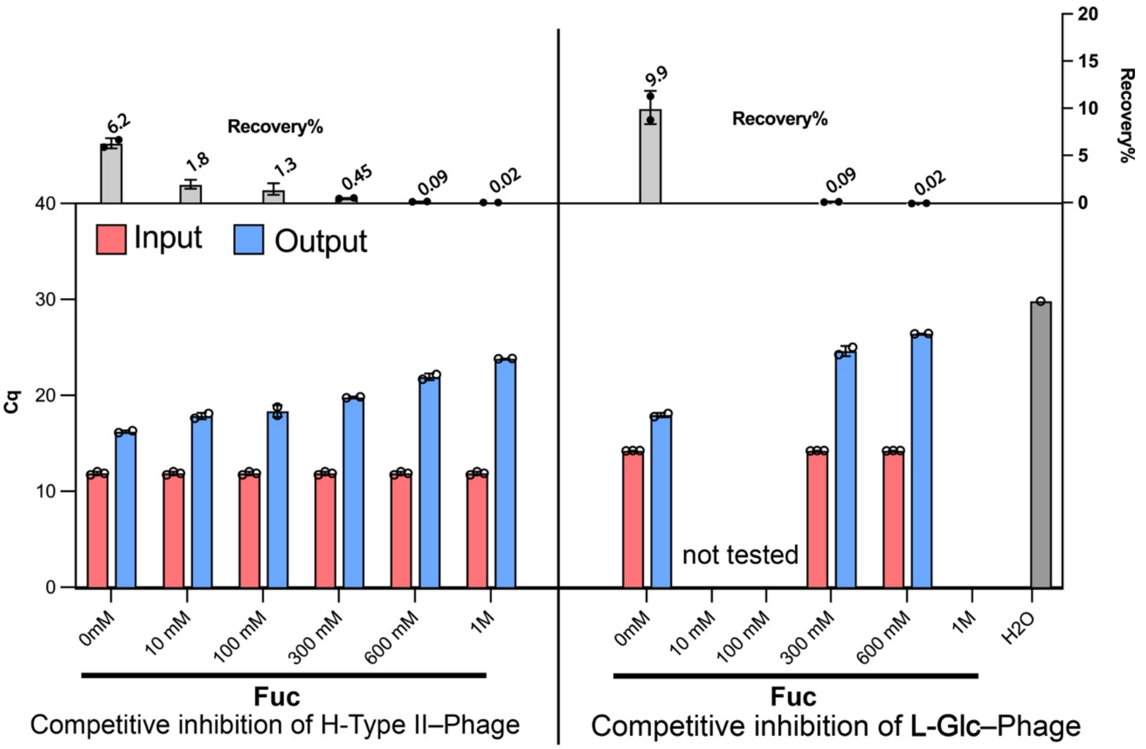

Fig. S24. Soluble fucose inhibits interactions between UEA-I and phages that display H2 glycan or L-Glucose.

UEA-I was coated on the surface and incubated with clonal phages that display H2 glycan or L-Glucose. Phage was then eluted using acidic buffer, and the number of eluted phage particles was measured by qPCR. In the absence of inhibitor, 5-6% of both phage was retained on the surface. When incubation was done in the presence of soluble fucose, the retention of phage dropped progressively and in a dose-dependent fashion. The assays demonstrate that the same amount of Fucose (e.g., 300 mM) inhibits L-Glc (9.9% → 0.09% i.e. 110x inhibition) more readily than H2 glycan (6.2% → 0.45%, 14x inhibition). Such observation is expected because interaction of Fucose-containing trisaccharide with UEA-I is likely to be stronger than interaction of UEA-I with L-Glc

Lectin	α-L-Glc		β-L-Glc		Man-3	
	Pred.	Exp.	Pred.	Exp.	Pred.	Exp.
SNA	–	–	–	–	–	–
UEA-I	–	–	–	–	–	–
ConA	–	–	–	–	+	+
AAL	+	–	+	+	–	–
MPL	–	–	+	–	–	–
WGA	–	–	–	–	–	–
AIA	+	–	+	–	–	–
CA	–	–	–	–	–	–
GSL-II	–	–	–	–	–	–
GSL-I	–	–	–	–	–	–
DBA	–	–	–	–	–	–
HHL	–	–	–	–	+	+
HPA	–	–	–	–	–	–
LcH	–	–	–	–	–	+
LEL	–	–	–	–	–	–
MAL-I	–	–	–	–	–	–
MAA-I	–	–	–	–	–	–

Lectin	α-L-Glc		β-L-Glc		Man-3	
	Pred.	Exp.	Pred.	Exp.	Pred.	Exp.
PHA-E	–	–	–	–	–	–
MLA-II	–	–	–	–	–	–
PHA-L	–	–	–	–	–	–
RCA-I	–	–	–	–	–	–
PNA	–	–	–	–	–	–
PSA	–	–	–	–	–	+
SBA	–	–	–	–	–	–
STL	+	+	–	+	–	–
TJA-II	–	–	–	–	–	–
TL	–	–	–	–	–	–
UDA	–	–	–	–	–	–
UEA-II	–	–	–	–	–	–
PA-IL	–	–	–	–	–	–
AOL	+	–	–	–	–	–
BPL	–	–	–	–	–	–
GNA	–	–	–	–	+	+
MNA-M	–	–	–	–	–	–

Fig. S25. Comparison of experimental lectin microarray data with model predictions made by MCNet3.

Numeric values are thresholded to classify each lectin as binding (+) or not binding (–). Besides the model predictions for alpha- and beta-L-glucose, the ground-truth values for Man-3 are shown to compare the consistency of the CFG glycan array and lectin-array results. For some lectins, such as UEA-I and PHA-E lectin-array lots from different suppliers were discordant. In these cases data shown is for lots that match the supplier used to train the MCNet.

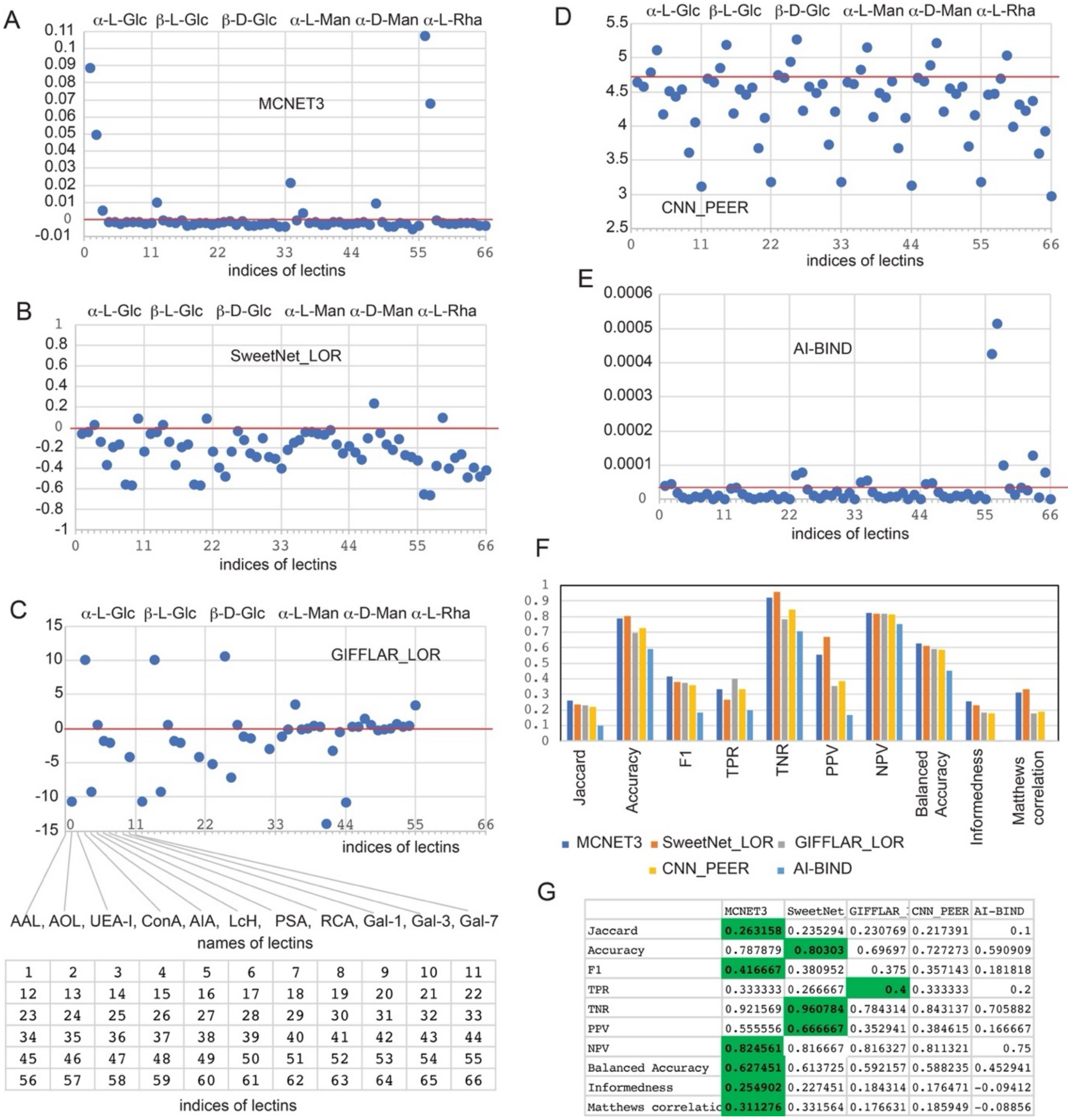

1	2	3	4	5	6	7	8	9	10	11
12	13	14	15	16	17	18	19	20	21	22
23	24	25	26	27	28	29	30	31	32	33
34	35	36	37	38	39	40	41	42	43	44
45	46	47	48	49	50	51	52	53	54	55
56	57	58	59	60	61	62	63	64	65	66

	MCNET3	SweetNet_	GIFFLAR_	CNN_PEER	AI-BIND
Jaccard	0.263158	0.235294	0.230769	0.217391	0.1
Accuracy	0.787879	0.80303	0.69697	0.727273	0.590909
F1	0.416667	0.380952	0.375	0.357143	0.181818
TPR	0.333333	0.266667	0.4	0.333333	0.2
TNR	0.921569	0.960784	0.784314	0.843137	0.705882
PPV	0.555556	0.666667	0.352941	0.384615	0.166667
NPV	0.824561	0.816667	0.816327	0.811321	0.75
Balanced Accuracy	0.627451	0.613725	0.592157	0.588235	0.452941
Informedness	0.254902	0.227451	0.184314	0.176471	-0.09412
Matthews correlatio	0.311276	0.331564	0.176631	0.185949	-0.08856

Fig. S26. Comparison of the predictions of binding for 6 glycans and 11 lectins.

Predictions made by (a) MCNet to predictions made by (B) LectinOracle_SweetNet (*27*); (C) LectinOracle_GIFFLAR (*47*); (D) CNN model from PEER Benchmark (*62*); (E) AI-BIND model (*61*). Names of lectins and their distribution to 66 indices is described in (C). Each panel contains a threshold (red line) that separates Positive from Negative values (binned predictions that represent “binding” vs. “non binding”). (F) Calculation of 10 metric values that compare classification of ground truth, based on Liquid Glycan Array and Lectin Array and classification of binned predictions. (G) Table summarizing the computed values. Model ranked #1 in each metrics is highlighted green. MCNet3 is leading in 5 categories (Jaccard Index, F1 score, NPV, Balanced accuracy, and Informedness) followed by LectinOracle_SweetNet (*27*) and LectinOracle_GIFFLAR . Details of calculations are available in “Comparison to other Models/MCNet vs 4 other models.xlsx”

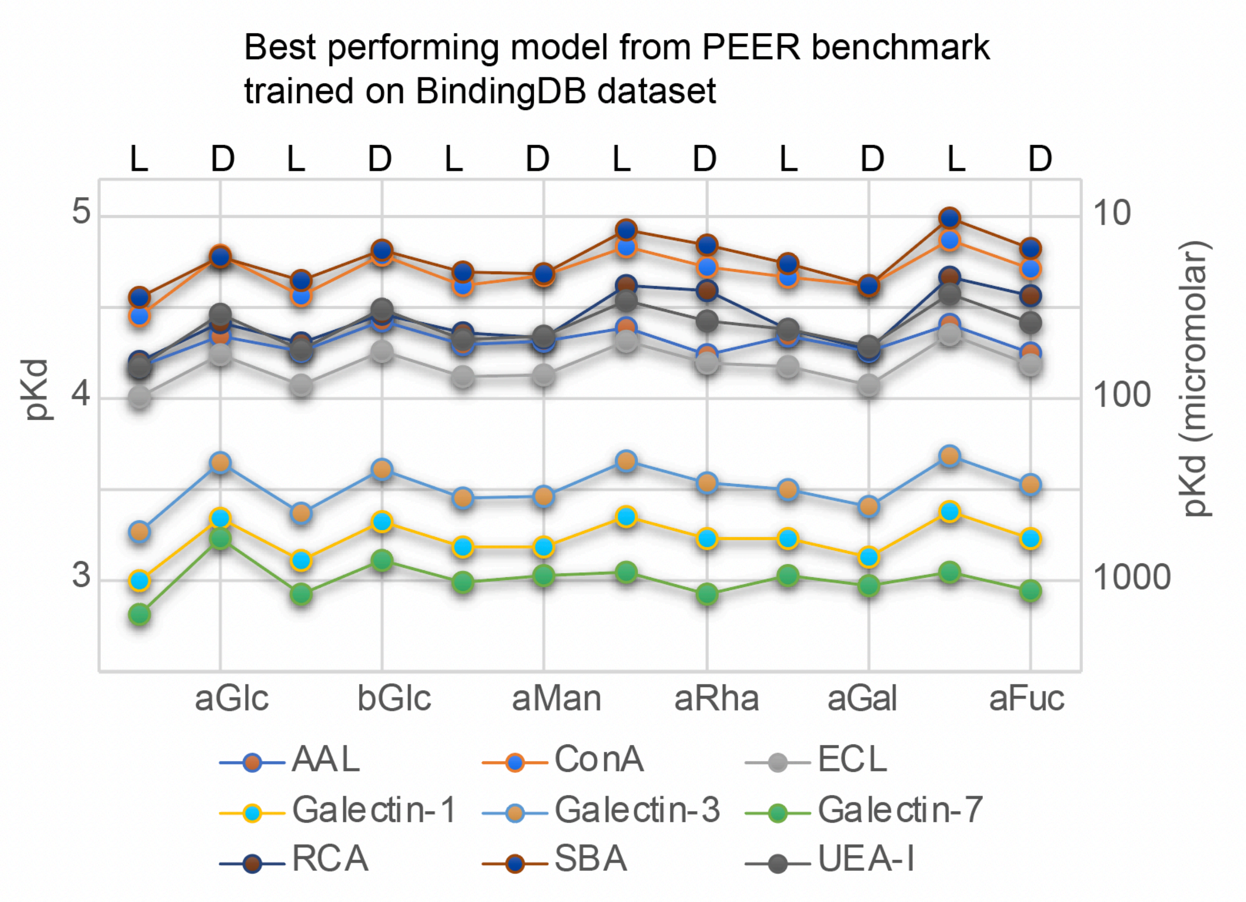

Fig. S27. Predictions made by CNN model trained on data from PEER benchmark originating from BindingDB dataset exhibit low sensitivity to changes in stereochemistry of ligand.

Although the predicted responses for L and D enantiomers of glycans are not the same, the trends in responses appear to be relatively insensitive to the identity of 12 tested stereoisomers or near-isomers (Rha and Fuc are 6-deoxy variants of hexoses) when examined across 9 different lectins.

The differences in predictions between L and D isomers do not translate to ground truth: Many of these interactions (ConA:D-Man, SBA:D-Gal) are known binders that we have confirmed to be very sensitive to the stereochemistry of hexose (e.g., L-Man does not bind to ConA). The model fails to capture these differences nor do they predict the newly discovered interactions of L-Glc but not D-Glc with AAL and UEA.

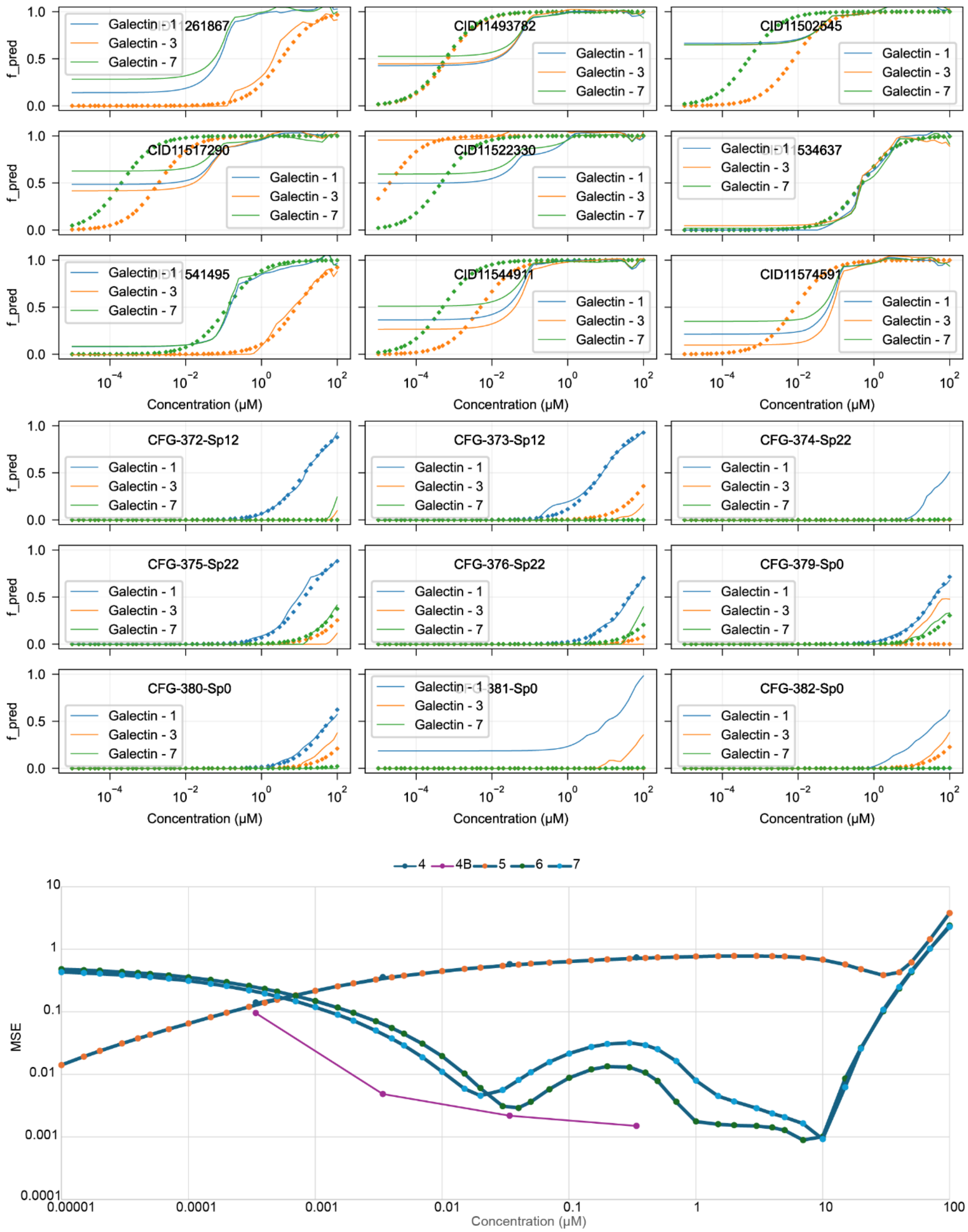

Fig. S28. The prediction performance of the MCNet3 model as a function of concentration.

Shown at top are predicted fractions bound to Galectin-1, -3, and -7 (solid curves) against the data points used to train the model (diamonds). While the CFG glycans (top 3 rows) in general reproduce the curves, the glycomimetics (middle 3 rows) have significant mismatches, although there are some exceptions. The MSE loss over the hold-out molecules varies with the concentration.

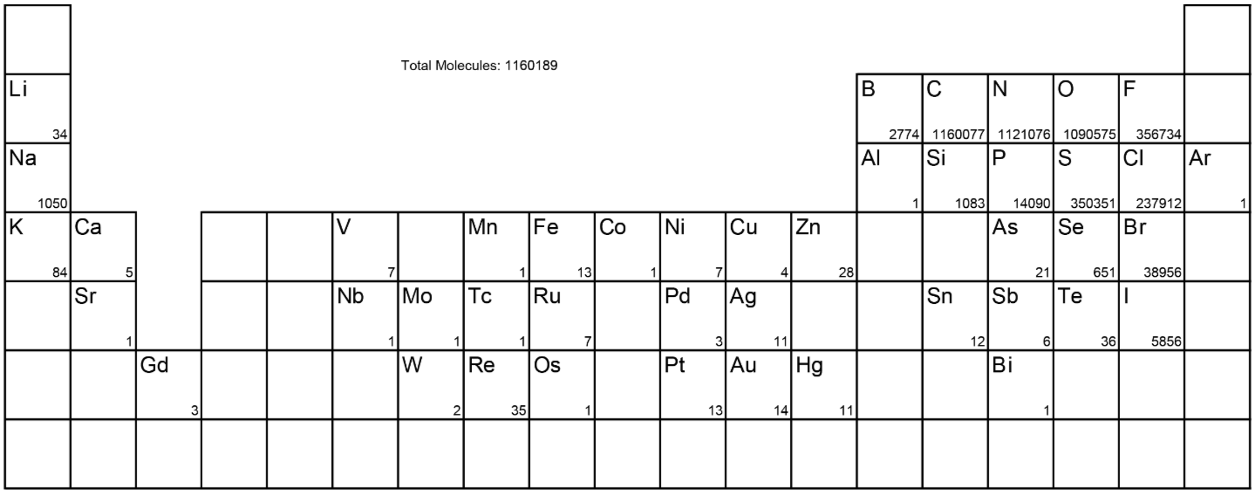

Fig. S29. The elements in the BindingDB molecules.

Numbers in the boxes are the count of molecules containing at least one atom of that element.

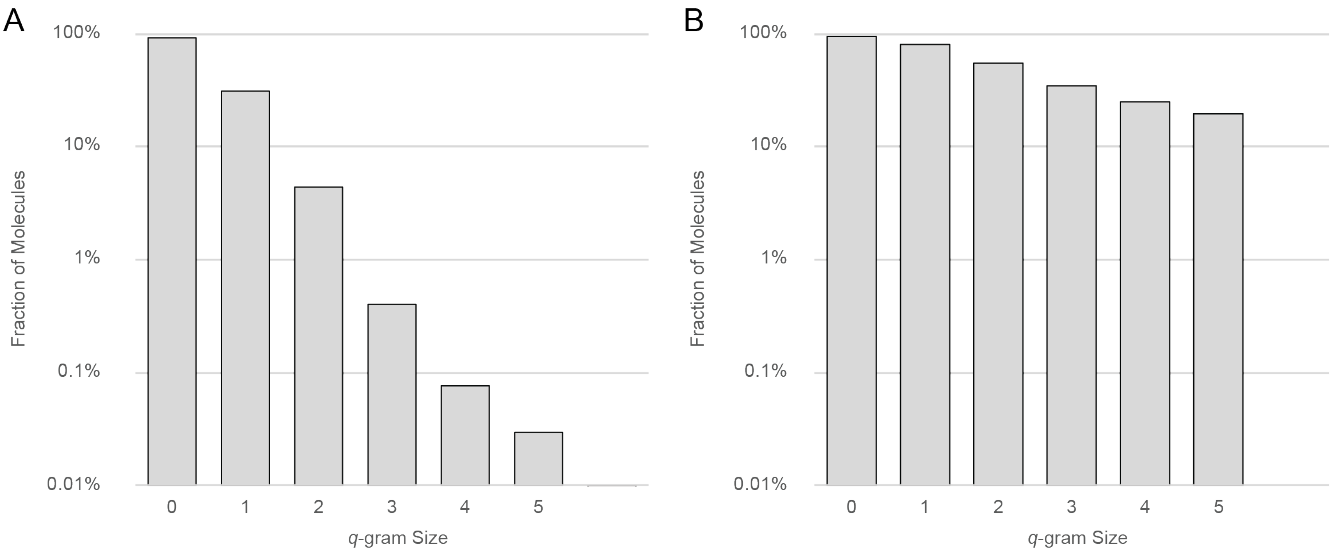

Fig. S30. Effect of *q*-gram size on the representability of molecules.

The fraction of molecules for A) the BindingDB dataset and B) the GlyTouCan dataset that can be represented using only q-grams present in a training set comprised of the combined CFG glycan and glycomimetic molecule sets. Notice that the glycans in GlyTouCan have greater similarity to the training set than the assorted small molecules of BindDB as evidenced by slower drop in the fraction of representable molecules as the maximal q-gram size increases.

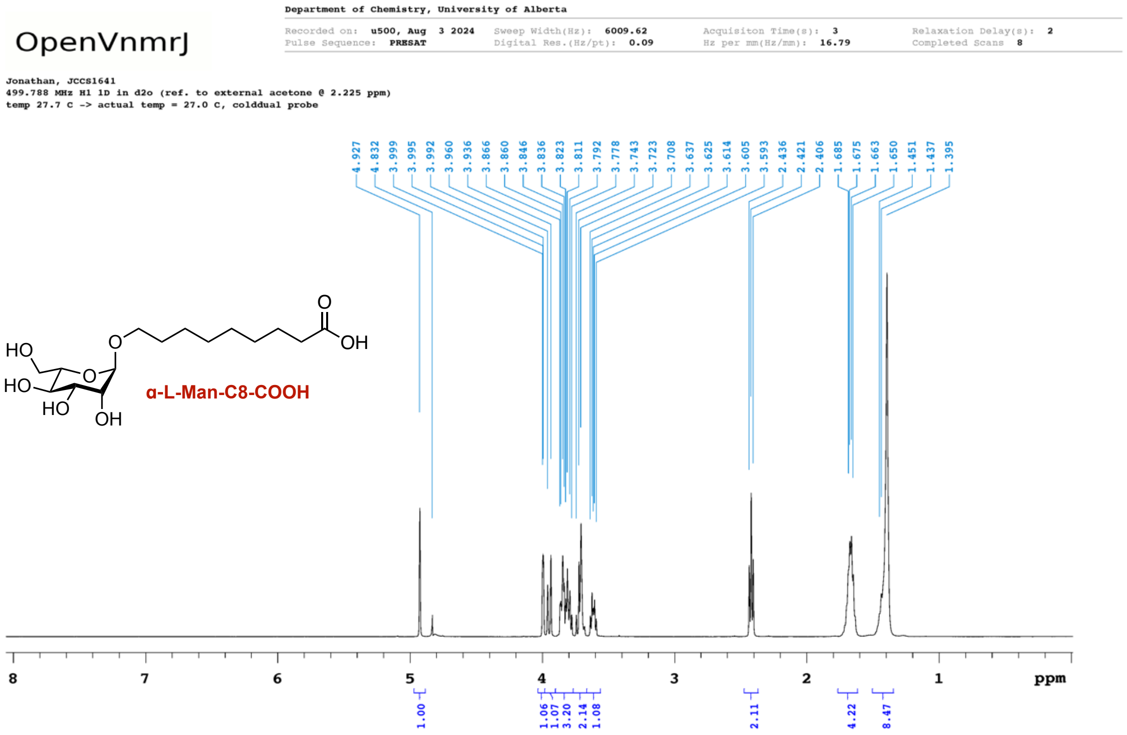

Fig S31. NMR characterization for a-L-Man-C8.

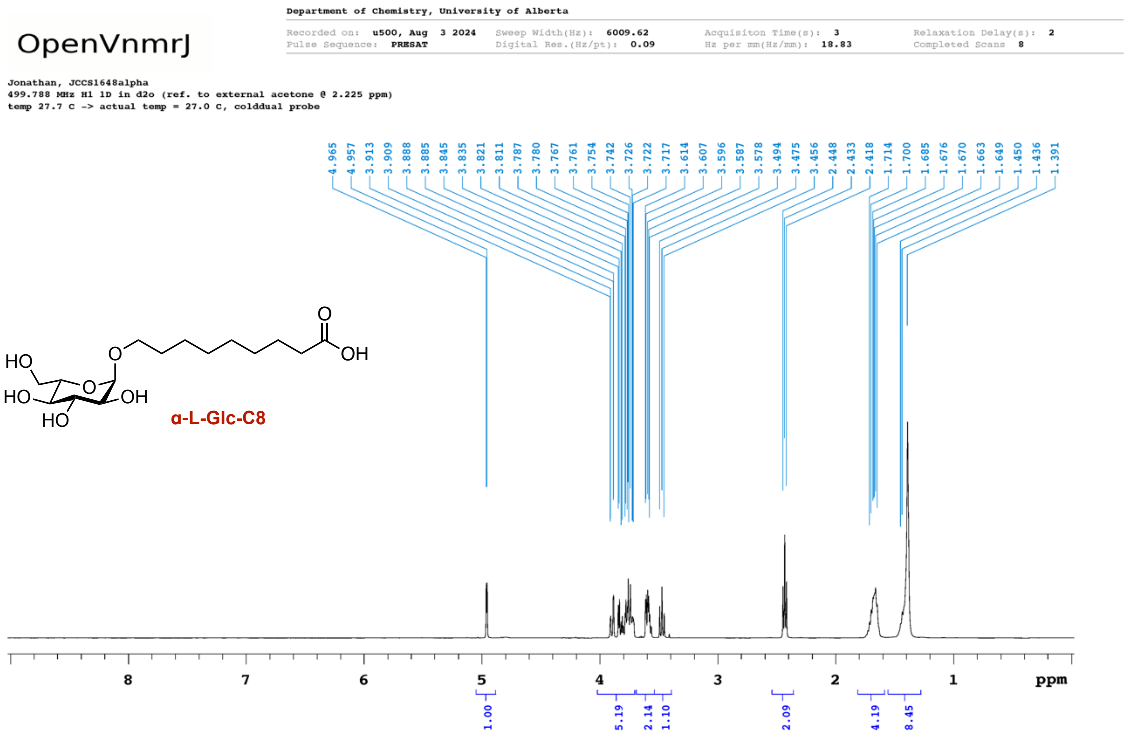

Fig S32. NMR characterization for a-L-Glc-C8.

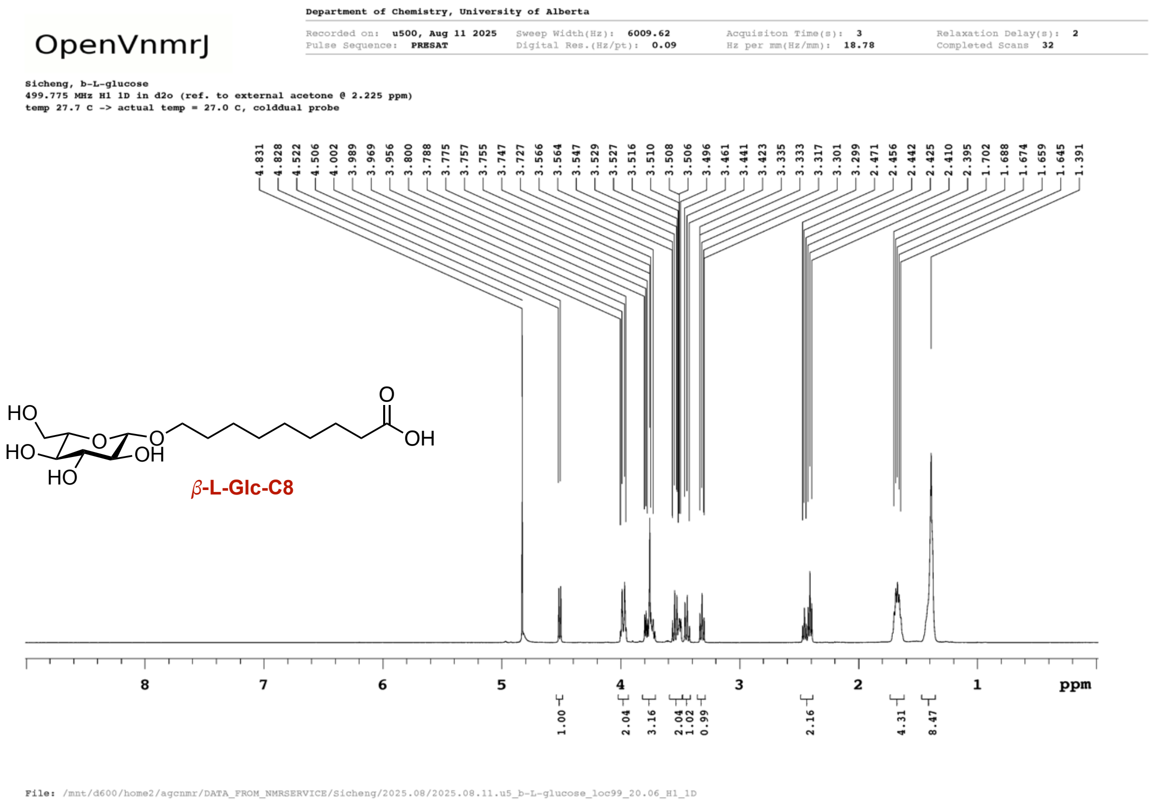

Fig S33. NMR characterization for β-L-Glc-C8.

Target	Glycan	R	R’	R”
**M**	α-L-Glc*P*	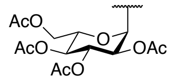	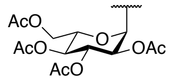	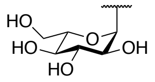
**N**	β-L-Glc*P*	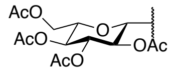	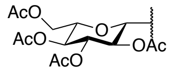	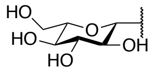
**O**	α-L-Man*P*	N/A	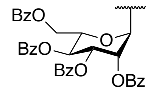	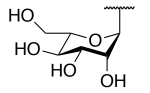

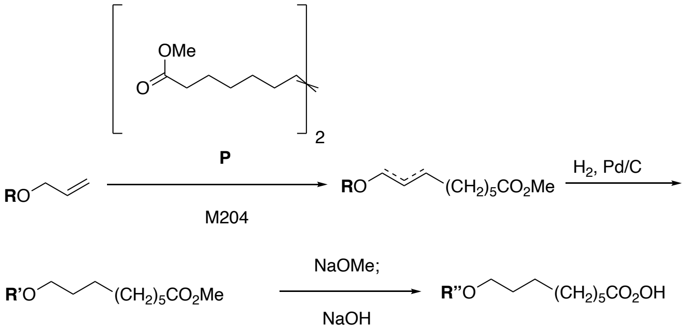

Figure S34: Synthetic intermediates and targets.

CFG code	1	3	7	Name	Gal-1<br>300	 287	Gal-3<br>300	 287	Gal-7<br>300	 287	IUPAC
154-Sp0	51	28	4	Lactose		6100	802	1160	2200	4900	Gal(β1-4)Glc(β
154-Sp8	48	35	16								
134-Sp0	246	16	8	LacNAc-I<br>LNB	9000		8200<br>1500	2880			Gal(β1-3)GlcNAc(β
134-Sp8	397	38	12								
153-Sp0	200	42	4	LacNAc-II<br>LacNAc	11000	24000	19000<br>5238	37000<br>8645	1700	6300	Gal(β1-4)GlcNAc(β
153-Sp8	281	49	8								
153-Sp23	276	8	0								
				ThiodiGal<br>TDG	18000		9800<br>1780	1930		4000	Gal(β1-1β)Gal
147-Sp0	823	43	10	DiLacNAc	7400		35000			7400	Gal(β1-4)GlcNAc(β1-3)Gal(β1-4)GlcNAc(β
146-Sp0	1002	8999	18	TriLacNAc	22000		63000			6500	Gal(β1-4)GlcNAc(β1-3)Gal(β1-4)GlcNAc(β1-3)Gal(β1-4)GlcNAc(β
133-Sp10	305	31	48	Lacto-N-tetraose<br>LNTet	10000		26000<br>8040	18200			Gal(β1-3)GlcNAc(β1-3)Gal(β1-4)Glc(β
148-Sp0	838	61	23	Lacto-N-neo-tetraose							Gal(β1-4)GlcNAc(β1-3)Gal(β1-4)Glc(β
148-Sp8	960	25	49								
258-Sp0	177	44	7	2,3-Sialyl LacNAc		23000		33000			Neu5Ac(α2-3)Gal(β1-4)GlcNAc(β
258-Sp8	328	35	5								
268-Sp0	025	26	13	2,6-Sialyl LacNAc							Neu5Ac(α2-6)Gal(β1-4)GlcNAc(β
268-Sp8	13	18	-5								
270-Sp0	55	28	31	2,6-Sialyl DiLacNAc			32000	48000			Neu5Ac(α2-6)Gal(β1-4)GlcNAc(β1-3)Gal(β1-4)GlcNAc(β
				2,6-Sialyl Lacto-N-neo-tetraose				21000			Neu5Ac(α2-6)Gal(β1-4)GlcNAc(β1-3)Gal(β1-4)Glc(β
166-Sp0	37	390	-5	Trisaccharide				8800			GlcNAc(β1-3)Gal(β1-4)Glc(β
				C2-DiLacNAc	7400		21000				Gal(β1-4)GlcNAc(β1-)[Gal(β1-4)GlcNAc(β1-)](CH2)2
				C3-DiLacNAc	15000		23000				Gal(β1-4)GlcNAc(β1-)[Gal(β1-4)GlcNAc(β1-)](CH2)3
				C4-DiLacNAc	12000		22000				Gal(β1-4)GlcNAc(β1-)[Gal(β1-4)GlcNAc(β1-)](CH2)4
				Lacto-N-hexaose<br>LNhex		18000	65000<br>23277	147000<br>34309			Gal(β1-4)GlcNAc(β1-6)[Gal(β1-4)GlcNAc(β1-3)]Gal(β1-4)Glc(β
				2,4-Pentasaccharide		31000	26000	61000			Gal(β1-4)GlcNAc(β1-4)[Gal(β1-4)GlcNAc(β1-2)]Gal(β
				2,6-Pentasaccharide		12000	22000	61000			Gal(β1-4)GlcNAc(β1-6)[Gal(β1-4)GlcNAc(β1-2)]Gal(β
				3,6-Pentasaccharide		25000	27000	53000			Gal(β1-4)GlcNAc(β1-6)[Gal(β1-4)GlcNAc(β1-3)]Gal(β
				4,6-Pentasaccharide		51000	25000	59000			Gal(β1-4)GlcNAc(β1-6)[Gal(β1-4)GlcNAc(β1-4)]Gal(β
				2'FL			2420	2310			Fuc(a1-4)Gal(β1-4)Glc(β
				A-Tri			18672	22928			Gal(a1-4)Gal(β1-4)GlcNAc(β
				B-tri			13391	28200			GalNAc(a1-3)[Fuca1-2)]Gal(β
				A-tetra			13830	43100			GalNAc(a1-3)[Fuca1-2)]Gal(β1-4)Glc(β

Table S1. Thermodynamic galectin binding data from Brewer's and Surolia's groups. Column #2 lists RFU values of the glycans in CFG array binding at 5.0 µg/mL of Galectins 1, 3, and 7. Columns 4-5-6 list Ka values (M^{-1}) measured by ITC. Yellow highlights values are from Surolia and co-workers.

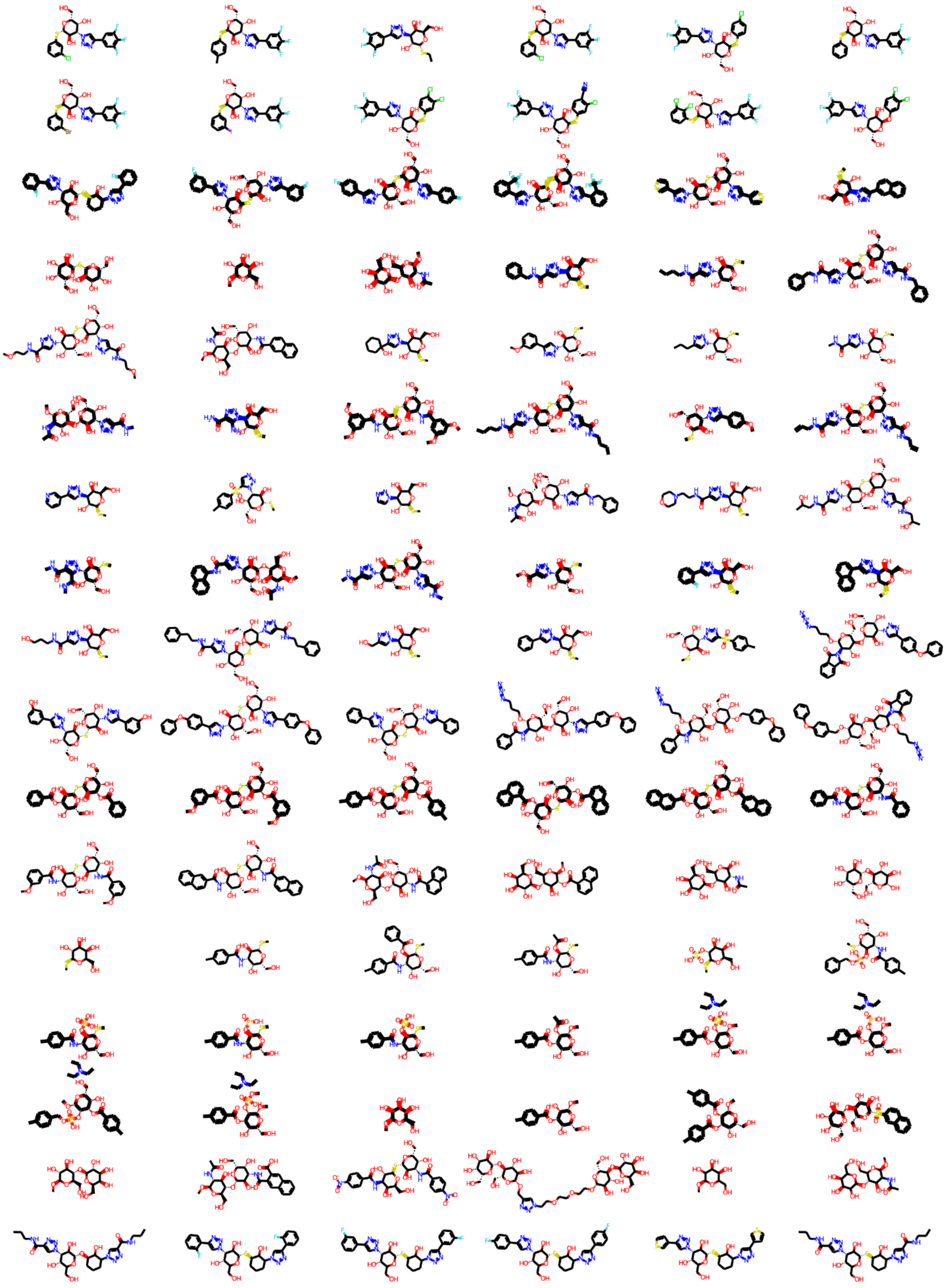

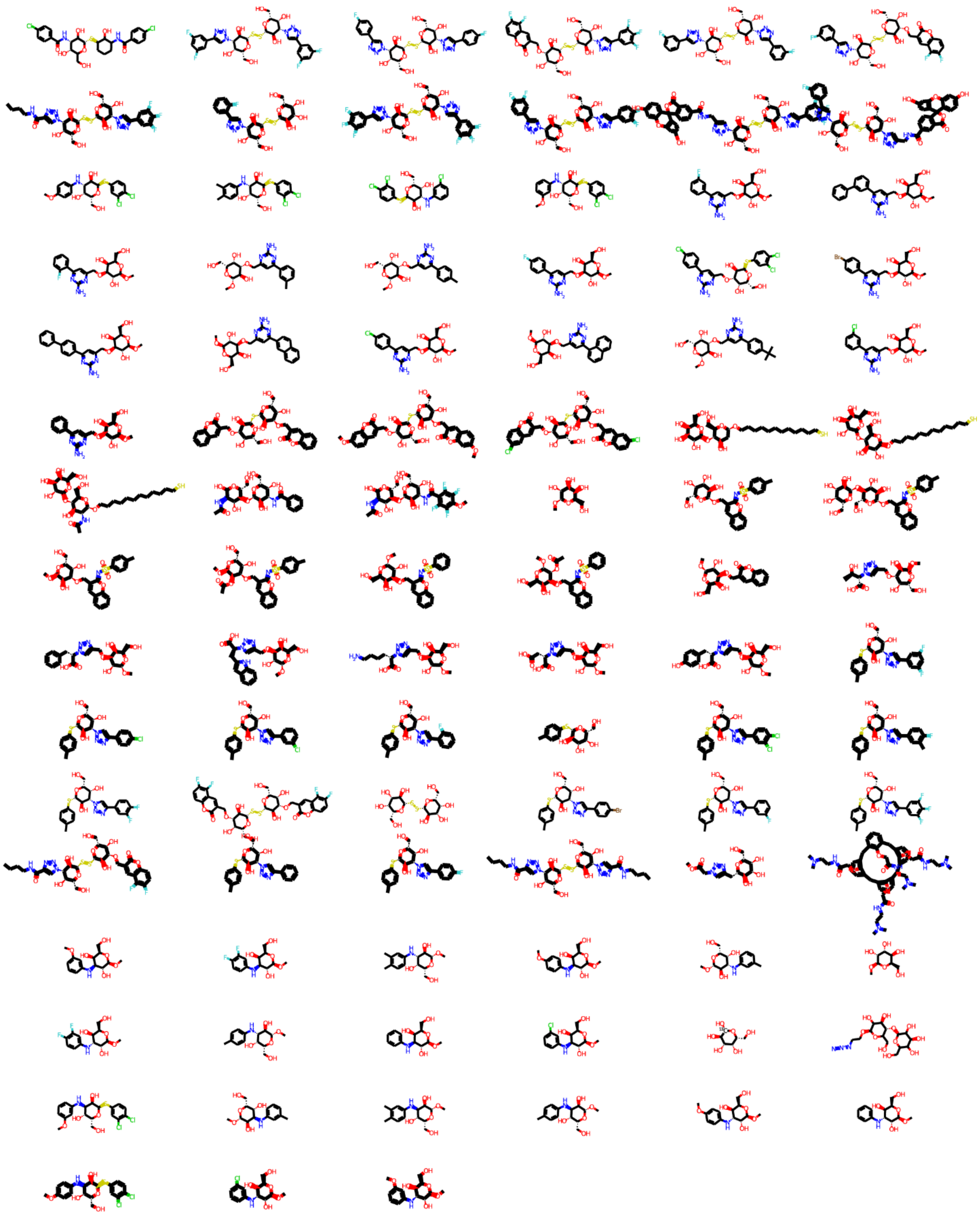

Table S2. Structures of glycomimetic molecules.

#	N	cbpId	Investigator	Protein Description	Vendor	Lot
1	9	cbp_2281	Lara K. Mahal	Sambucus Nigra Agglutinin	Vector Labs	W 0425
2	7		Nicolai Bovin	Anti-H(O) lectin 1		
3	7	cbp_2787	Lara K. Mahal	Ulex Europaeus Agglutinin I	EY Labs	290224-1
4	6	cbp_2554	Lara K. Mahal	Concanavalin A	EY Labs	290210-5
5	6	cbp_2456	Lara K. Mahal	Erythrina cristagalli lectin	Vector Labs	X 0219
6	5	cbp_hum_Stlect_00116	Marcelo Dias Baruffi	Human Galectin1		
7	5	cbp_1304	Marcelo Dias Baruffi	Mouse Galectin1		
8	5	cbp_2450	Lara K. Mahal	Aleuria Aurantia Lectin	Vector	W0119
9	5	cbp_2469	Lara K. Mahal	Maclura Pomifera Lectin	Vector Labs	W 0201
10	5	cbp_2390	Lara K. Mahal	Wheat Germ Agglutinin	Vector Labs	X 0103
11	5	cbp_2553	Lara K. Mahal	Wheat Germ Agglutinin	EY labs	290227-5
12	5	cbp_2685	Lara K. Mahal	Cholera Toxin B	Sigma	lOOM4099V
13	5	cbp_hum_Stlect_00118	Richard D. Cummings	Galectin-3		
14	4	cbp_1608	Elizabeth M. Meiering	ThreeFoil		
15	4	cbp_2440	Karel Bezouska	Natural killer cell receptor – NKR-P1C		
16	4	cbp_2452	Lara K. Mahal	Amaranthus caudatus Lectin	Vector	W1106
17	4	cbp_2561	Lara K. Mahal	Jacalin lectin (Artocarpus integrifolia)	EY Labs	281129-1
18	4	cbp_1478	Janko Kos	Mono1-CNL Clitocybe nebularis Ricin B-like Lectin		
19	4	cbp_1478	Janko Kos	Clitocybe nebularis Ricin B-like lectin		
20	4	cbp_1622	Bidadi Venkataram Prasad	P11-VP8*		
21	4	cbp_2689	Kinjiro Morimoto	Pseudomonas fluorescens agglutinin		
22	4	cbp_2493	Lara K. Mahal	Colchicum autumnale lectin EY_290516-1	EY Labs	290516-1
23	4	cbp_2541	Lara K. Mahal	Caragana arborescens Agglutinin	EY Labs	290521-1
24	4	cbp_2453	Lara K. Mahal	Concanavalin A	Vector Labs	W 0828
25	4	cbp_2460	Lara K. Mahal	Griffonia simplicifolia Lectin II	Vector Labs	V 0715
26	4	cbp_2459	Lara K. Mahal	Griffonia Simplicifolia Lectin I	Vector Labs	V 1208
27	4	cbp_2458	Lara K. Mahal	Galanthus Nivalis Lectin Vector_W0719	Vector Labs	W 0719
28	4	cbp_2457	Lara K. Mahal	Euonymus Europaeus Lectin Vector_W1010	Vector Labs	W 1010
29	4	cbp_2455	Lara K. Mahal	Datura stramonium lectin	Vector Labs	W 1027
30	4	cbp_2454	Lara K. Mahal	Dolichos Biflorus Agglutinin	Vector Labs	W 0725
31	4	cbp_2462	Lara K. Mahal	Hippeastrum rutilum lectin	Vector Labs	U 0513
32	4	cbp_2544	Lara K. Mahal	Hippeastrum rutilum lectin	EY Labs	290601-1
33	4	cbp_2770	Lara K. Mahal	Helix pomatia agglutinin	Sigma	028K3810V
34	4	cbp_2542	Lara K. Mahal	Cytisus scoparius Lectin	EY Labs	290607-1
35	4	cbp_2461	Lara K. Mahal	Griffonia Simplicifolia Lectin I Isolectin B4	Vector Labs	W 1028
36	4	cbp_2464	Lara K. Mahal	Lens Culinaris Agglutinin	Vector Labs	W 1218

37	4	cbp_2511	Lara K. Mahal	Lens culinaris Agglutinin EY_292014-1	EY Labs	291204-1
38	4	cbp_2465	Lara K. Mahal	Lycopersicon Esculentum Lectin	Vector Labs	W 0424
39	4	cbp_2470	Lara K. Mahal	Narcissus Pseudonarcissus Lectin	Vector Labs	W 1120
40	4	cbp_2466	Lara K. Mahal	Lotus Tetragonolobus Lectin Vector_X0412	Vector Labs	X 0412
41	4	cbp_2467	Lara K. Mahal	Maackia Amurensis Lectin I Vector_W0224	Vector Labs	W 0224
42	4	cbp_2555	Lara K. Mahal	Maackia amurensis Lectin	EY Labs	290224-5
43	4	cbp_2471	Lara K. Mahal	Phaseolus vulgaris lectin E	Vector Labs	W 0514
44	4	cbp_2774	Lara K. Mahal	Maackia amurensis agglutinin (MAA)	Seikagaku Bio	22101C
45	4	cbp_2468	Lara K. Mahal	Maackia Amurensis Lectin II	Vector Labs	W 1014
46	4	cbp_2558	Lara K. Mahal	Phaseolus vulgaris lectin E	EY Labs	281005-2
47	4	cbp_2522	Lara K. Mahal	Pisum sativum Lectin	EY Labs	280S0S-1
48	4	cbp_2472	Lara K. Mahal	Phytohemagglutinin-L	Vector Labs	W 1031
49	4	cbp_2547	Lara K. Mahal	Psophocarpus tetragonolobus lectin	EY Labs	290606-1
50	4	cbp_2475	Lara K. Mahal	Psophocarpus Tetragonolobus Lectin I	Vector Labs	W 1011
51	4	cbp_2476	Lara K. Mahal	Psophocarpus tetragonolobus (Winged bean) Lectin II	Vector Labs	B 0906
52	4	cbp_2276	Lara K. Mahal	Ricinus Communis Agglutinin I	Vector Labs	W 0423
53	4	cbp_2473	Lara K. Mahal	Peanut Agglutinin	Vector Labs	W 0530
54	4	cbp_2474	Lara K. Mahal	Pisum Sativum Agglutinin	Vector Labs	V 1101
55	4	cbp_2478	Lara K. Mahal	Soybean Agglutinin	Vector Labs	V 1024
56	4	cbp_2479	Lara K. Mahal	Solanum tuberosum lectin	Vector Labd	V 0808
57	4	cbp_2550	Lara K. Mahal	Trichosanthes Japonica Agglutinin I	Seikagaku Bio.	P04401
58	4	cbp_2551	Lara K. Mahal	Trichosanthes Japonica Agglutinin II	Seikagaku Bio.	P04Y01
59	4	cbp_2531	Lara K. Mahal	Tulip lectin	EY Labs	290521-1
60	4	cbp_2548	Lara K. Mahal	Urtica dioica Lectin	EY Labs	29021-1
61	4	cbp_2533	Lara K. Mahal	Ulex europaeus agglutinin-II	EY Labs	290521-2
62	4	cbp_2482	Lara K. Mahal	Vicia Villosa Lectin	Vector Labs	X 0130
63	4	cbp_2483	Lara K. Mahal	Wisteria Floribunda Lectin	Vector Labs	W 0703
64	4	cbp_2784	Lara K. Mahal	Pseudomonas aeruginosa-I galactophilic lectin	Sigma	049k4052
65	4	cbp_2788	Lara K. Mahal	Wheat Germ Agglutinin	Seikagaku Bio.	82306
66	4	cbp_2690	Miriam Dwek	Native Helix pomatia agglutinin		
67	4	cbp_2703	Jatinder Singh	Sauromatum guttatum (venosum) lectin		
68	4	cbp_2602	Jatinder Singh	Artocarpus hypargyreus lectin		
69	4	cbp_2751	Shashikala Inamdar	Sclerotium Rolfsii lectin variant 1		
70	4	cbp_1606	Janko Kos	Macrolepiota procera lectin		

71	4	cbp_2905	Anne Imberty	Terfezia boudieri fucose binding lectin 1		
72	4	cbp_2937	Markus Kuenzler	Coprinopsis cinerea Ricin B lectin (ORF: CC1G-10077)		
73	4	cbp_2916	Yvette Van Kooyk	Macrophage Galactose-Type C-Type Lectin Family 6 Member B		
74	4	cbp_2917	Yvette Van Kooyk	Macrophage Galactose-Type C-Type Lectin 6A, Histidine 262 Threonine Variant		
75	4	cbp_hum_Ctlect_292	Yvette Van Kooyk	Macrophage Galactose-Type C-Type Lectin 6A		
76	4	cbp_2951	Rongsheng Jin	HA70 complex-Alexa		
77	4	cbp_2390	Brian Haab	Wheat Germ Agglutinin (WGA)	Vector Labs	X 0103
78	4	cbp_3020	Anne Imberty	Psathyrella velutina Lectin		
79	4	cbp_3061	Rich Olson	rPVL		
80	4	cbp_pla_other_334	Carole A. Bewley	Banana lectin		
81	4	cbp_3129	Pavel Lukyanov	Crenomytilus grayanus lectin		
82	4	cbp_hum_Stlect_00118	Richard D. Cummings	Galactin-3		
83	4	cbp_3484	Richard D. Cummings	Human Galectin-7		
84	4	cbp_3486	Richard D. Cummings	Mouse Galactin-7		
85	3		Brian Haab	Carbohydrate antigen 19-9 CA19.9-1B844		
86	3		Brian Haab	CA19.9-9L426		
87	3		Brian Haab	CA19.9-121SLE		
88	3		Hans-Jörg Bühring	IPS-K-4A2B8		
89	3	cbp_1672	Marie Hanigan	Microvirin		
90	3		Hiroto Kawashima	ab5		
91	3	cbp_2552	Lara K. Mahal	Aspergillus oryzae lectin	TCI America	HB3QM
92	3	cbp_2487	Lara K. Mahal	Agaricus bisporus agglutinin	EY Labs	290511-1
93	3	cbp_1478	Janko Kos	Mono2W-CNL		
94	3	cbp_2451	Lara K. Mahal	Bauhinia Purpurea Lectin	Vector Labs	V 0519
95	3	cbp_2506	Lara K. Mahal	Griffonia simplicifolia Lectin II	EY Labs	290516-2
96	3	cbp_2505	Lara K. Mahal	Euonymus europaeus lectin	EY Labs	280420-2
97	3	cbp_2507	Lara K. Mahal	Galanthus Nivalis Lectin	EY Labs	280407-2
98	3	cbp_2509	Lara K. Mahal	Hippeastrum hybrid agglutinin	EY Labs	280707-1
99	3	cbp_2560	Lara K. Mahal	Phytohemagglutinin-L	EY Labs	241205-2
100	3	cbp_2563	Lara K. Mahal	Peanut Agglutinin	EY Labs	290224-2
101	3	cbp_2562	Lara K. Mahal	Soybean Agglutinin	EY Labs	280828-2
102	3	cbp_2480	Lara K. Mahal	Styphnolobium japonicum Lectin	Vector Labs	W 0612
103	3	cbp_2481	Lara K. Mahal	Ulex Europaeus Agglutinin I	Vector Labs	V 1207
104	3	cbp_2549	Lara K. Mahal	Vicia graminea lectin	EY Labs	290601-2
105	3	cbp_2687	Lara K. Mahal	Wheat Germ Agglutinin	Sigma	110M4002V
106	3	cbp_2789	Lara K. Mahal	Morus nigra Agglutinin	EY Labs	

107	3	cbp_2790	Lara K. Mahal	Marasmius oreades Agglutinin	EY Labs	290802-1
108	3	cbp_1716	Rich Olson	WT-VCC		
109	3	cbp_hum_Ctlect_228	Irma Van Die	ME5(detected with a-HUD)		
110	3	cbp_1477	Hui Sun	Aleuria Aurantia Lectin		
111	3	cbp_2445	José M. Mancheño	Laetiporus sulphureus lectin		
112	3	cbp_2446	José M. Mancheño	laminin-binding lectin		
113	3	cbp_hum_Ctlect_228	Irma Van Die	ME7(detected with a-HUD)		
114	3	cbp_2733	John Fraser	SSL0-488		
115	3	cbp_2744	Zeev Pancer	VLRB.aGPA.23		
116	3	cbp_1714	Robert L. Atmar	rNV VLP-Cy3		
117	3	cbp_2904	Anne Imberty	AFCNVH		
118	3	cbp_2903	Anne Imberty	Hypnea musciformis lectin		
119	3		Lara K. Mahal	#8 Lewis B (T218) (Abcam)		
120	3		Lara K. Mahal	#15 Sialyl Lewis X (BD)		
121	3		Lara K. Mahal	#14 Sialyl Lewis X (Calbio)		
122	3	cbp_hum_Stlect_00116	Francoise Guerlesquin	hGal-1-488+BCR		
123	3	cbp_2938	Anne Imberty	Terfezia boudieri fucose binding lectin 2		
124	3	cbp_hum_Ctlect_217	Kurt Drickamer	MGL 416		
125	3	cbp_2948	Anne Imberty	Bryothamnion triquetrum lectin		
126	3		John Kearney	GAC78		
127	3	cbp_2984	Gerard Zurawski	DC-ASGPR short		
128	3		Luc Teyton	Sp Serotype 14.2		
129	3		Luc Teyton	Sp Serotype 14.15		
130	3	cbp_2750	Shashikala Inamdar	Recombinant Sclerotium rolfsii lectin		
131	3	cbp_oth_Other_933	Shashikala Inamdar	Sarcalumenin lectin		
132	3	cbp_hum_Stlect_00116	Linda Baum	Galectin-1		
133	3	cbp_3021	Horacio Heras	PsSC		
134	3	cbp_3074	Nobuyuki Matoba	Avaren-Fc		
135	3	cbp_3134	Janko Kos	CnSepL		
136	3		Brian Haab	CA19-9 Ab (DRG)		
137	3	cbp_mou_Stlect_203	Oscar Campetella	Wild Type Galectin-8		
138	3	cbp_3151	Markus Kuenzler	Coprinopsis cinerea lectin 1		
139	3	cbp_3239	Almudena Fernández Briera	PhoSPL001		
140	3	cbp_oth_other_339	Ronnie Willaert	Epithelial adhesin 1 Epa1-Np mutant Y228W		
141	3	cbp_oth_other_339	Ronnie Willaert	Epithelial adhesin 1 Epa1-Np mutant Y228A		
142	3	cbp_oth_other_339	Ronnie Willaert	Epithelial adhesin 1 Epa1-Np mutant E227G		
143	3	cbp_oth_other_339	Ronnie Willaert	Epithelial adhesin 1 Epa1-Np mutant E227A		

144	3	cbp_oth_other_339	Ronnie Willaert	Epithelial adhesin 1 Epa1-Np WT		
145	3	cbp_3345	Janko Kos	Clytia nigra Mannose-Binding Lectin		
146	3	cbp_3245	Shashikala Inamdar	Dioclea bicolor Lectin		
147	3		Kate Rittenhouse-Olson	Antibody		

Table S3. List of lectin multi-concentration groups (N = number of concentrations).

Dataset	Target	Immobilized on (Catalog No.)
20250131-87MOGaalPAN-IF	AAL (Vector)	Corning® High Bind Microplate (Millipore Sigma #CLS3369)
20250131-87MOGueaPAN-IF	UEA-I (Sigma)	
20250131-87MOGcaPAN-IF	ConA (Sigma)	
20250131-87MOGeaaPAN-IF	ECL (Sigma)	
20250131-87MOGzfaPAN-IF	SBA (Sigma)	
20250131-87MOGbsPAN-IF	BSA (reference; Sigma)	
20250131-87MOGgoPANN-IF	Galectin-1 (GlycoGenetics)	Pierce™ Nickel Coated Plates (ThermoFisher #15342)
20250718-87MOYgoPANN-IF		
20250131-87MOGgaPANN-IF	Galectin-3 (GlycoGenetics)	
20250718-87MOYgaPANN-IF		
20250131-87MOGgsPANN-IF	Galectin-7 (GlycoGenetics)	
20250131-87MOGbwPANN-IF	Untreated well (reference)	
20241030-87MEGaalPAN-IF	AAL (Vector)	Corning® High Bind Microplate (Millipore Sigma #CLS3369)
20241030-87MEGueaPAN-IF	UEA-I (Sigma)	
20241030-87MEGcaPAN-IF	ConA (Sigma)	
20241030-87MEGeaaPAN-IF	ECL (Sigma)	
20241030-87MEGzfaPAN-IF	SBA (Sigma)	
20241030-87MEGbsPAN-IF	BSA (reference; Sigma)	
20250716-87MOYaalPAN-IF	AAL (Vector)	Corning® High Bind Microplate (Millipore Sigma #CLS3369)
20250801-87MOYueaPAN-GL	UEA-I (Sigma)	
20250718-87MOYrslPAN-IF	RSL (Elicityl)	
20250716-87MOYcaPAN-IF	ConA (Sigma)	
20250716-87MOYeaaPAN-IF	ECL (Sigma)	
20250718-87MOYzfaPAN-IF	SBA (Sigma)	
20250716-87MOYbsPAN-IF	BSA (reference; Sigma)	
20241030-87MEGooNA-IF	No target	Naïve LiGA MEG
20250131-87MOGooNA-IF	No target	Naïve LiGA MOG
20250716-87MOYooNA-EG	No target	Naïve LiGA MOY

Table S4. LiGA data used in this work.

The listed datasets are publicly available at https://48hd.cloud. The original source/vendor of each lectin has been indicated inside parentheses.

109	134	https://doi.org/10.1016/j.bmc.2010.05.040
110	87	https://doi.org/10.1021/acsmedchemlett.9b00396
111	72	https://doi.org/10.1021/acs.jmedchem.7b01626
112	62	https://doi.org/10.1021/jm801077j
113	57	https://doi.org/10.1016/j.bmcl.2014.05.063
114	39	https://doi.org/10.1039/C9MD00183B
115	36	https://doi.org/10.1021/acs.jmedchem.6b00957
116	28	https://doi.org/10.1016/j.bmcl.2010.12.049
117	25	https://doi.org/10.1016/j.bmcl.2008.05.066
118	17	https://doi.org/10.1021/jm301677r
119	17	https://pubmed.ncbi.nlm.nih.gov/11921396/ (doi not found)
120	12	https://doi.org/10.1002/cmdc.201700744
121	10	https://doi.org/10.1016/j.bmc.2008.06.044
122	9	https://doi.org/10.1016/j.bmc.2015.04.044
123	9	https://doi.org/10.1021/jm701266y
124	5	https://doi.org/10.1002/cbic.201600285
125	3	https://pubs.acs.org/doi/10.1021/jm500325k
126	2	https://doi.org/10.1093/glycob/cwm026
127	1	https://doi.org/10.1021/jm300014q

Table S5. Galectin inhibitor data from literature.

	**Description***
**1. Sample: Glycan-containing sample (e.g. glycan, glycoprotein, cell lysate, cell, glycopeptide, etc.)**	
Description of Sample	Glycophage constructs
Sample preparation protocol	Glycophage construct samples were labeled with TAMRA prior to analysis on the lectin microarrays, and thus required no sample preparation.
Labeling protocol for sample detection	Samples are labelled with TAMRA.
Two-color reference (if used)	N/A
Assay protocol	Lectin microarrays are blocked with blocking buffer (50 mM ethanolamine and 100 mM boric acid) for one hour at room temperature. Slides are rinsed once with PBST (0.01%) and once with 1X PBS for 5 minutes each, then dried using a slide spinner. Each slide is mounted on a 24-well format hybridization cassette (Arrayit), in which each well contains a subarray. To each well, 50 uL of sample was added, then diluted with PBST to reach the final volume (100uL) and concentration (0.005%). Slides are incubated in a hybridization oven on a rocker tray for one hour at 30°C in the dark. After hybridization, arrays are washed with PBST (0.01%) once for one minute, then once for five minutes. Arrays are lastly washed once with PBS for 1 minute, then once for ten minutes. Once finished, slides are removed from the cassette and briefly immersed in ultrapure water, then dried using a slide spinner.
**2. Lectin Library**	
General description of the lectin library used in the array	Lectin microarrays are generated in house.
List of lectins and/or glycan-binding proteins, their source, concentration, and buffer	Please see **Supplemental Table S6**.
Modification of lectins	N/A

3. Immobilization Surface; e.g., Microarray Slide	
Immobilization surface	Nexterion Slide H Barcoded 3D Hydrogel Coated.
Manufacturer	Schott North America.
Custom preparation of the surface	N/A
**4. Array Production**	
Description of Arrayer	Nano-Plotter 2.1 piezoelectric printer (GeSim, Germany) with cooled microwell plate holder and cooled printing deck.
Lectin deposition	Triplicates of each lectin are printed onto each subarray.
Printing conditions	Dilute lectins to the pre-determined concentrations in the print buffer (final concentration of print buffer: 1 mM monosaccharide in PBS, 5 ng/mL Atto 532; Please see Supplemental Table 1 for the concentrations of lectins). Load the mixed solution to the microplate. Before printing, check the humidity of the print chamber. The humidity should be kept around 50% throughout the entire print. Ensure both microwell plate holder and printing deck are cooled. Adjust the cooling temperature based on ambient temperature and the temperature of the cooled slide deck surface, preventing moisture building up inside the print chamber. Once printing is complete, allow the slides to dry for at least one hour before vacuum sealing and storage at -20°C.
Array layout	Each microarray contains 21 subarrays (3 columns and 7 rows). In each subarray, triplicates of a lectin are printed, with six lectins in each row. The number of columns is 18, and the row number depends on how many lectin probes are printed on the arrays (i.e. 132 lectins require 22 rows).
Quality control	The well-characterized glycoproteins glycophorin, ovalbumin, asialofetuin, 1:1 A549:HEK293T cell lysate, human serum, fetuin, and RNase B are used for quality assurance of the printed microarrays.
**5. Detector and Data Processing**	
Instrument (scanner, flow cytometer)	Fluorescent Slide Scanner GenePix 4400A (Molecular Devices).

Instrument settings	A low resolution preview scan of the slide is performed to adjust photomultiplier tube (PMT) gain for the channel used (TAMRA: 532nm) so that the signals are not saturated and within the linear detection range. After adjusting PMT gain, slides are scanned in the given channel at 5 µm resolution.
Image analysis software	GenePix Pro 7 (Molecular Devices).
Data processing and statistical analysis	Extracted data is processed for quality checks using Grubbs' outlier test with $\alpha = 0.05$. Log2 values of the average signals are median-normalized over the individual subarray in each channel.
**6. Lectin Microarray Data Presentation**	
Data presentation and interpretation	Raw values output by Genepix Pro 7 software were thresholded by a cut-off of >2000 AU.
**7. Data Location**	
Data Location	https://doi.org/10.7303/syn65918063

Table S6. Lectins used in microarrays.

Lectin	Species/Origin	Print Conc. (µg/mL)	Rough Specificity /Inhibitory monosaccharide	Vendor/Source
AAA	*Anguilla anguilla agglutinin*	2000	Fucose	Vector
AAL	*Aleuria aurantia*	2000	Fucose	Medicago/Vector
ACA	*Amaranthus Caudatus*	2000	Gal-β1,3-GalNAc	EY
AIA	*Artocarpus integrifolia*	2000	β1,3-GalNAc	EY/Glycomatrix/Vector
AMA	*Allium moly*	2000	Oligo mannose	Vector
Anti-B.G. Lewis A	MAb mouse IgG1 [7LE]	undiluted	Lewis A	Abcam
Anti-B.G. Lewis B	MAb mouse IgM [2-25LE]	undiluted	Lewis B	Abcam/Sigma
Anti-B.G. Lewis X	MAb mouse IgM [P12]	undiluted	Lewis X	Sigma
Anti-B.G. Lewis Y	MAb mouse IgM [F3]	undiluted	Lewis Y	Abcam
Anti-Blood Group A	MAb mouse IgM [HE-193]	undiluted	Blood Group A	Thermo Fisher
Anti-Blood Group B	MAb mouse IgM [HEB-29]	undiluted	Blood Group B	Abcam/ Thermo Fisher
Anti-Blood Group H	MAb mouse IgG3 [17-206]	undiluted	Blood Group H	Thermo Fisher
Anti-B.G. Sialyl Lewis A	MAb mouse IgG1 [GT933]	undiluted	Sialyl Lewis A	GeneTex
Anti-IgA	MAb IgG1 [KT41]	undiluted	IgA	Abcam
Anti-IgG	MAb IgG2a [KT131]	undiluted	IgG	Abcam
Anti-IgM	MAb IgG1 [KT16]	undiluted	IgM	Abcam
AOL	*Aspergillus oryzae*	2000	Fucose	TCI America
ASA	*Allium sativum*	2000	Mannose	EY
BanLec H84T	*Musa acuminate*	2000	High Mannose	Generated in house
BC2L-A	*Burkholderia cenocepacia*	2000	Mannose	Elicityl
BPA/BPL	*Bauhinia purpurea*	2000	β-Gal / β-GalNAc	Vector

CA	*Colchicum autumnale*	2000	Bi-antennary N-linked glycans	EY
ConA	*Canavalia ensiformis*	2000	Tri-mannose core	Thermo Fisher/Vector
DBA	*Dolichos biflorus*	2000	GalNAc	Vector
diCBM40	engineered NanI from *Clostridium perfrin gens*	1500	α Sialylation	Generated in house
DSA	*Datura stramonium*	2000	LacNAc	Vector
ECA	*Erythrina cristagalli*	2000	LacNAc	EY/Vector
GafD	*Escherichia coli*	2000	GlcNAc	Generated in house
GNA/GNL	*Galanthus nivalis*	2000	Oligo mannose	Sigma/Vector
GS/GSL-I	*Griffonia simplicifoia-I*	2000	α-Gal / Lac	Vector
GS/GSL-II	*Griffonia simplicifoia-II*	2000	GlcNAc	Vector
HHL	*Hippeastrum hybrid*	2000	Oligo/High mannose	BioWorld
HPA	*Helix pomatia*	2000	Blood Group A	Sigma
LcH	*Lens culinaris*	2000	Core Fucose	EY/Medicago
LEA/LEL	*Lycopersicon esculentum*	2000	GlcNAc	Vector
LTL Lotus	*Lotus tetragonolobus*	2000	Fucose	EY/Vector
MAA/MAL-I	*Maackia amurensis-I*	2000	Sialylation/Sulfation	EY/Vector
MAA/MAL-II	*Maackia amurensis-II*	2000	Sialylation/Sulfation	Vector
MNA-G	*Morus nigra Morniga G*	2000	GalNAc	EY
MNA-M	*Morus nigra Morniga M*	2000	Oligo mannose / Gal	EY
MPA/MPL	*Maclura pomifera*	2000	β1,3-GalNAc	Vector
NPA/NPL	*Narcissus pseudonarcissus*	2000	Oligo mannose	EY

PA-IL	*Pseudomonas aeruginosa*	2000	Gal	Generated in house
PA-IIL	*Pseudomonas aeruginosa*	2000	Fucose	Elicityl
PapGII	*Escherichia coli*	2000	Gal	Generated in house
PHA-E	*Phaseolus vulgaris Erythroagglutinin*	2000	Bisecting GlcNAc	Sigma/Vector
PHA-L	*Phaseolus vulgaris Leukoagglutinin*	2000	β1,6 Branching N-Link glycans	Medicago/Vector
PhoSL	*Pholiota squarrosa*	2000	Core Fucose	Bio Basic
PNA	*Arachis hyogaea*	2000	Gal-β1,3-GalNAc	EY/Vector
Recombinant Protein A	*Staphylococcus aureus*	2000	Immunoglobulins	Thermo Fisher
Recombinant Protein G	*Streptococcus* Group G	2000	Immunoglobulins	Sigma
Recombinant Protein L	*Peptostreptococcus magnus*	2000	Immunoglobulins	Thermo Fisher
PSA	*Pisum sativum*	2000	Core Fucose	Vector
PSL	*Polyporus squamosus*	2000	α2,6 sialylation	TCI America
PTA/PTL-I	*Psophocarpus tetragonolobus-I*	2000	α2 Fucose	BioWorld
PTA/PTL-II	*Psophocarpus tetragonolobus-II*	2000	α2 Fucose	BioWorld
RCA120	*Ricinus Communis Agglutinin I*	2000	Gal / Lac	Vector
rGRFT	*Griffithsia*	1700	High mannose	Generated in house
Ricin B Chain	*Ricinus communis*	2000	Gal	Vector
RCA120	*Ricinus communis*	2000	Gal	Vector
RPA	*Robinia pseudoacacia*	2000	Multiantennary N-glycans with bisecting GlcNAc	EY
SBA	*Glycine max*	2000	LacdiNAc	Vector
SLBR-B	*Streptococcus gordonii M99*	2300	α2,6 sialylation	Generated in house
SLBR-H	*Streptococcus gordonii DL1*	2000	α2,3 sialylation	Generated in house
SLBR-N	*Streptococcus gordonii UB10712*	2000	α2,3 sialylation	Generated in house

SNA	*Sambucus nigra*	2000	α2,6 sialylation	EY/Vector
SNA-II	*Sambucus nigra-II*	2000	α2 Fucose /oligo mannose	EY
STL	*Solanum tuberosum Agglutinin*	2000	LacNAc	Vector
TJA-II	*Trichosanthes japonica-II*	2000	α2 Fucose	Aniara Diagnostica/Medicago
TL	*Tulipa sp.*	2000	GlcNAc	BioWorld
UDA	*Urtica dioica*	2000	GlcNAc / Oligo mannose	EY
UEA-I	*Ulex europaaeus-I*	2000	α2 Fucose	BioWorld/GeneTex/Vector
UEA-II	*Ulex europaaeus-II*	2000	GlcNAc	EY/BioWorld
VVA	*Vicia villosa*	2000	Terminal GalNAc	Vector
WFA	*Wisteria floribunda*	2000	GalNAc-β1,4	Vector
WGA	*Triticum vulgare*	2000	GlcNAc	GeneTex

Table S7. Lectin microarray information.

	Protein			
	Residue Number	Residue Name	Atom Name	Ligand
α-L-Fuc	44	GLU	Oε2	O2
	44	GLU	Oε2	O3
	105	GLY	N	O3
	87	ASP	Oδ2	O4
	134	ASN	Nδ2	O4
α-L-Glc	44	GLU	Oε2	O2
	44	GLU	Oε2	O3
	105	GLY	N	O3
	87	ASP	Oδ1	O4
	87	ASP	Oδ2	O4
β-L-Glc	44	GLU	Oε2	O2
	44	GLU	Oε2	O3
	105	GLY	N	O3
	87	ASP	Oδ1	O4
	87	ASP	Oδ2	O4

Table S8. Hydrogen bond interactions between protein and ligands in UEA-I (PDB ID: 1JX).

	Protein			
	Residue Number	Residue Name	Atom Name	Ligand
α-L-Fuc	36	GLU	Oε1	O3
	36	GLU	Oε2	O3
	97	TRP	Nε1	O3
	**24**	**ARG**	**Nε**	**O4**
	**36**	**GLU**	**Oε1**	**O4**
	**36**	**GLU**	**Oε2**	**O4**
	24	ARG	Nε	O5
	24	ARG	NH2	O5
α-L-Glc	36	GLU	Oε1	O3
	36	GLU	Oε2	O3
	97	TRP	Nε1	O3
	24	ARG	Nε	O5
	24	ARG	NH2	O5
β-L-Glc	36	GLU	Oε1	O3
	36	GLU	Oε2	O3
	97	TRP	Nε1	O3
	24	ARG	Nε	O5
	24	ARG	NH2	O5

Table S9. Hydrogen bond interactions between protein and ligands in AAL (PDB ID: 1IUC).

	Train Set	Type	protein aware	Description
MCNet1	599 glycan	Neural Net	No	
MCNet3	611 Glycan + GM	Neural Net	No	
rf_model_noP	599 glycan	Random Forest	No	
GlyNET_MF3	611 glycan	Neural Net	No	RFU – Radius 3
xgb_model_noP	599 glycan	XG Boost	No	
128_nn	Glycan	Neural Net	Yes	1L 128
256_nn	Glycan	Neural Net	Yes	1L 256
512_256_nn	Glycan	Neural Net	Yes	2L 512, 256
512_nn	Glycan	Neural Net	Yes	1L 512
rf_model	Glycan	Random Forest	Yes	RF
xgb_model	Glycan	XG Boost	Yes	XGB
128_128_128_nn_e5	Glycan + GM	Neural Net	Yes	3L: 128, 128, 128
128_128_nn_e5	Glycan + GM	Neural Net	Yes	2L: 128, 128
128_64_nn_e5	Glycan + GM	Neural Net	Yes	2L: 128, 64
128_nn_e10	Glycan + GM	Neural Net	Yes	1L: 128 E10
128_nn_e5	Glycan + GM	Neural Net	Yes	1L: 128
256_256_nn_e5	Glycan + GM	Neural Net	Yes	2L: 256, 256
256_nn_e10	Glycan + GM	Neural Net	Yes	1L: 256 E10
256_nn_e5	Glycan + GM	Neural Net	Yes	1L: 256
512_256_nn_e5	Glycan + GM	Neural Net	Yes	2L 512, 256
512_nn_e10	Glycan + GM	Neural Net	Yes	1L 512, E10
512_nn_e5	Glycan + GM	Neural Net	Yes	1L: 512
64_64_64_nn_e5	Glycan + GM	Neural Net	Yes	3L: 64, 64, 64
64_64_nn_e10	Glycan + GM	Neural Net	Yes	2L: 64, 64 E10
64_64_nn_e5	Glycan + GM	Neural Net	Yes	2L: 64, 64
64_nn_e10	Glycan + GM	Neural Net	Yes	1L 64 E10
rf_model	Glycan + GM	Random Forest	Yes	RF
xgb_model	Glycan + GM	XG Boost	Yes	XGB
LectinOracle	Bojar's set		Yes	z-Scores
GIFFLAR	Bojar's set		Yes	z-Scores
AI Bind	BindingDB		Yes	
PEER CNN	BindingDB		Yes	

Table S10. Models used in the present work.

Data S1.
ZIP archive containing tables of predictions from various models.

Data S2.
ZIP archive containing various data files and MatLab scripts for the enantiomer statistics.

Data S3.
Table of fluorescence data from lectin microarrays–1st set.

Data S4.
Table of fluorescence data from lectin microarrays–2nd set.

Data S5.
ZIP archive containing MALDI MS spectra data.

Data S6. (pages 61–83)
Figures presenting binding predictions for the enantiomers of mannose-rich lectins.

Data S7. (pages 84–131)
Plots of the MALDI MS spectra and associated metadata.

Data S8. (pages 132–237)
Plots of model predictions as the concentration varies.

Data S9. (pages 238–384)
Plots of predictions from the MCNet1, MCNet3, and LectinOracle models for the various lectins.

Data S10. (pages 385–983)
Plots of predictions from the MCNet models for the CFG glycans.

Data S11. (pages 983–1594)
Heatmaps showing predicted binding from MCNet1 (top) and MCNet3 (bottom) for the epimers of the CFG glycans.

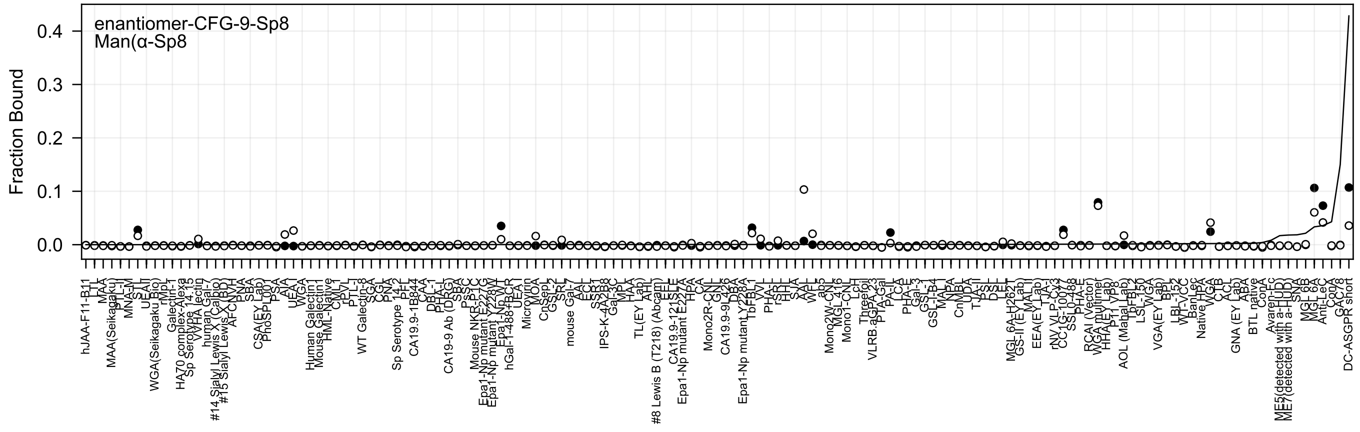
enantiomer-CFG-9-Sp8
Man(α-Sp8
Fraction Bound
0.4
0.3
0.2
0.1
0.0

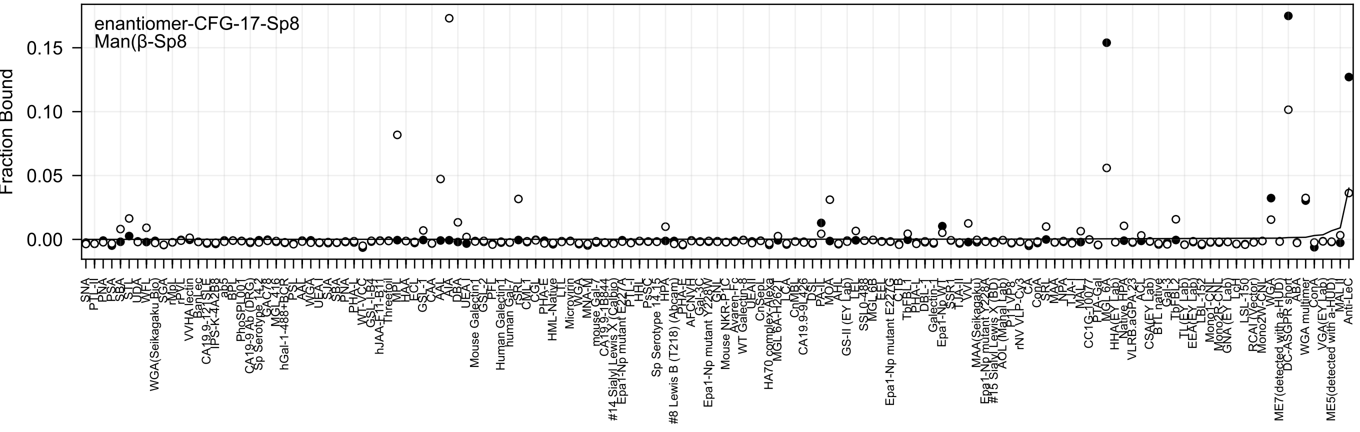

enantiomer-CFG-17-Sp8
Man(β-Sp8
Fraction Bound
0.15
0.10
0.05
0.00

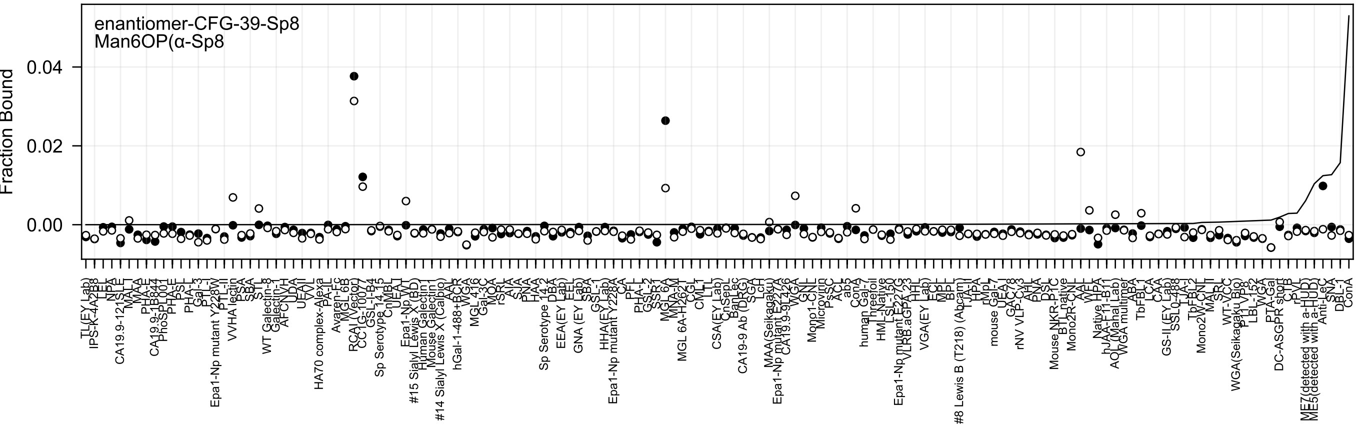
enantiomer-CFG-39-Sp8
Man6OP(α-Sp8
Fraction Bound
0.04
0.02
0.00

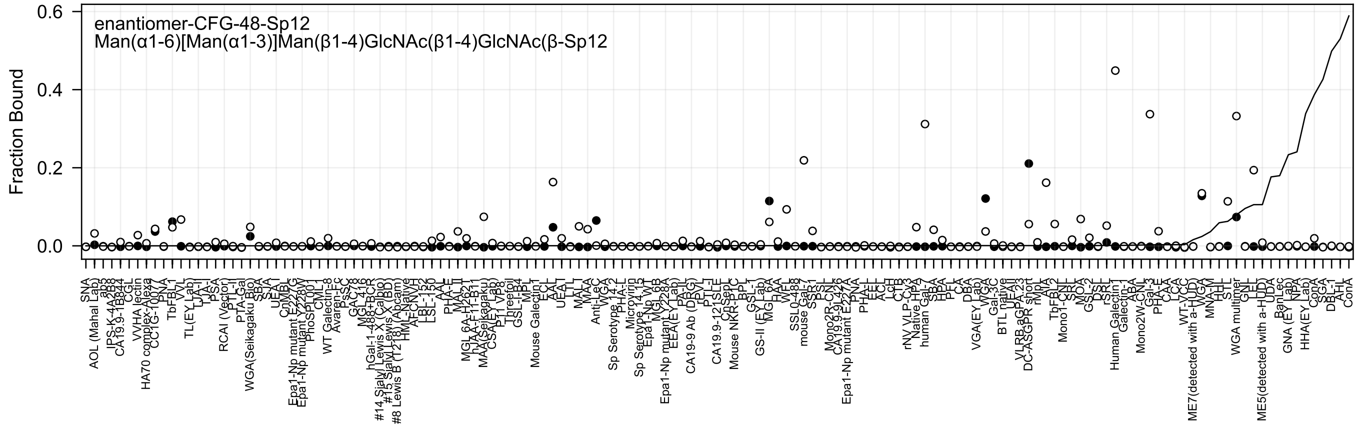

enantiomer-CFG-48-Sp12
Man(α1-6)[Man(α1-3)]Man(β1-4)GlcNAc(β1-4)GlcNAc(β-Sp12
Fraction Bound
0.6
0.4
0.2
0.0
SNA
AOL (Mahal Lab)
ab2
IPS-K-4A2B8
CA19.9-1B844
CGL
VVHA lectin
HA70 complex-Alexa
CC1G-10077
PNA
TbFBL1
VVL
TL(EY Lab)
LJA-I
TJA-I
PSA
RCAI (Vector)
PTL-II
PTA-Gal
WGA(Seikagaku Bio)
SBA
SJA
UEA-I
CnMBL
Epa1-Np mutant E227G
Epa1-Np mutant Y228W
PhoSL001
CML
WT Galectin-8
Avaren-Fc
PsSC
GAC78
MGL-416
hGal-1-488+BCR
#14 Sialyl Lewis X (Calbio)
#15 Sialyl Lewis X (BD)
#8 Lewis B (T218) (Abcam)
HML-Native
AFCNVH
LBL-152
LSL-150
AAL
PHA-E
MAL-II
MGL 6A-H2621
hJAA-F11-B11
MAA(Seikagaku)
CSA(EY Lab)
P11 VP8
Threefoil
GSL-I-B4
MPL
Mouse Galectin1
ECL
AAT
UEA-I
LTL
MAL-I
MAA
Anti-LeC
WGA
Sp Serotype 14.2
PHA-L
Microvirin
Sp Serotype 14.15
Epa1-Np WT
MGL 6B
Epa1-Np mutant Y228A
EEA(EY Lab)
PA-IL
CA19-9 Ab (DRG)
rPVL
PTL-I
CA19.9-121SLE
CnSep
Mouse NKR-P1C
BPL
GSL-1
GS-II (EY Lab)
MGL 6A
HAA
WFL
SSL0-488
mouse Gal-7
SSR1
PSL
Mono2R-CN
CA19.9-9L426
Epa1-Np mutant E227A
PNA
PHA-L
EEL
ACL
LcH
CTB
rNV VLP-Cy3
Native Hpa
human Gal-7
SBA
HPA
PFL
CA
DBA
VGA(EY Lab)
WGA
Gal-3C
BTL native
UEA-II
VLRB aGPA.23
DC-ASGPR short
rMpL
AIA
TbFBL2
Mono1-CN
SRL
MOA
GSL-2
DSL
rSRL
Human Galectin1
Galectin-1
ABA
Mono2W-CN
Gal-3
PHA-E
CAA
LCA
WT-VCC
ME7(detected with a-HUD)
WGA
MNA-M
HHL
STL
WGA multimer
GNL
LEL
ME5(detected with a-HUD)
UDA
BanLec
GNA (EY Lab)
NPA
HHA(EY Lab)
ConA
SSGA
DBL-1
AHL
ConA

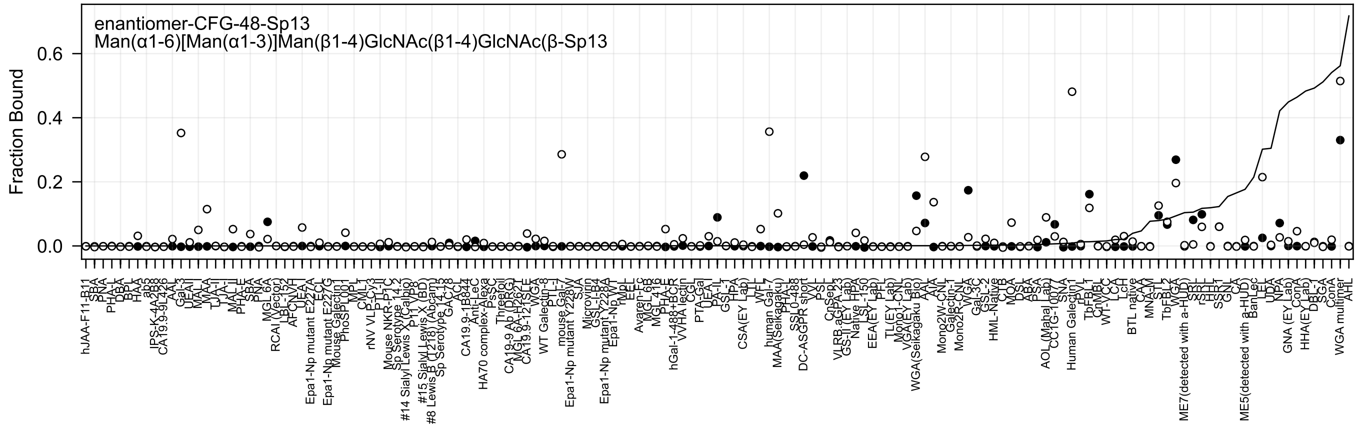

enantiomer-CFG-48-Sp13
Man(α1-6)[Man(α1-3)]Man(β1-4)GlcNAc(β1-4)GlcNAc(β-Sp13
Fraction Bound
0.0
0.2
0.4
0.6
hJAA-F11-B11
SBA
PNA
PHA-L
DBA
BPL
HAA
ab5
IPS-K-4A2B8
CA19.9-9L426
AAL
Gal-3
UEAI
MAL-I
MAA
LTA-II
TJA-I
MAL-II
PHA-E
SBA
PNA
MGL 6A
RCAI (Vector)
LBL-152
AFCNVH
UEA-I
Epa1-Np mutant E227A
ECL
Epa1-Np mutant E227G
Mouse Galectin1
PhoSL001
MPL
CML1
rNV VLP-Cy3
PTL-II
Mouse NKR-P1C
Sp Serotype 14.2
#14 Sialyl Lewis X (Calbio)
P11 YP8
#15 Sialyl Lewis X (BD)
#8 Lewis B (T218) (Abcam)
Sp Serotype 14.15
GAC78
ACL
CA19.9-1B844
Anti-LeC
HA70 complex-Alexa
PsSC
Threefoil
CA19-9 Ab (DRG)
MGL 6A-H267T
CA19.9-121SLE
WGA
WT Galectin-8
PTL-I
mouse Gal-7
Epa1-Np mutant Y228W
SJA
Microvirin
GSL-I-B4
Epa1-Np mutant Y228A
Epa1-Np WT
rMPL
EEL
Avaren-Fc
MGL 6B
MGL 416
PHA-E
hGal-1-488+BCR
VWHA lectin
CGL
PTA-Gal
UEA-I
PA-IL
GS-I
HPA
CSA(EY Lab)
LTL
WFL
human Gal-7
MAA(Seikagaku)
PHA-L
SSL0-488
DC-ASGPR short
VVL
PSL
CnSep
VLRB.aGPA.23
GS-II (EY Lab)
Native HPA
LSL-150
EEA(EY Lab)
PFL
TL(EY Lab)
Mono1-CNL
VGA(EY Lab)
WGA(Seikagaku Bio)
AAL
AIA
Mono2W-CNL
Galectin-1
Mono2R-CNL
WGA
Gal-3C
GSL-2
HML-Native
CTB
MOA
DSL
ABA
PSA
AOL (Mahal Lab)
CC1G-10077
SNA
Human Galectin1
PVL
TbF-BL1
CnMBL
WT-VCC
LCA
LcH
BTL native
CAA
MNA-M
STL
TbF-BL2
WGA
ME7(detected with a-HUD)
SRL
rSRL
HHL
SSR1
GNL
CA
ME5(detected with a-HUD)
BanLec
LEL
UDA
NPA
GNA (EY Lab)
ConA
HHA(EY Lab)
DBL-1
SGA
ConA
WGA multimer
AHL

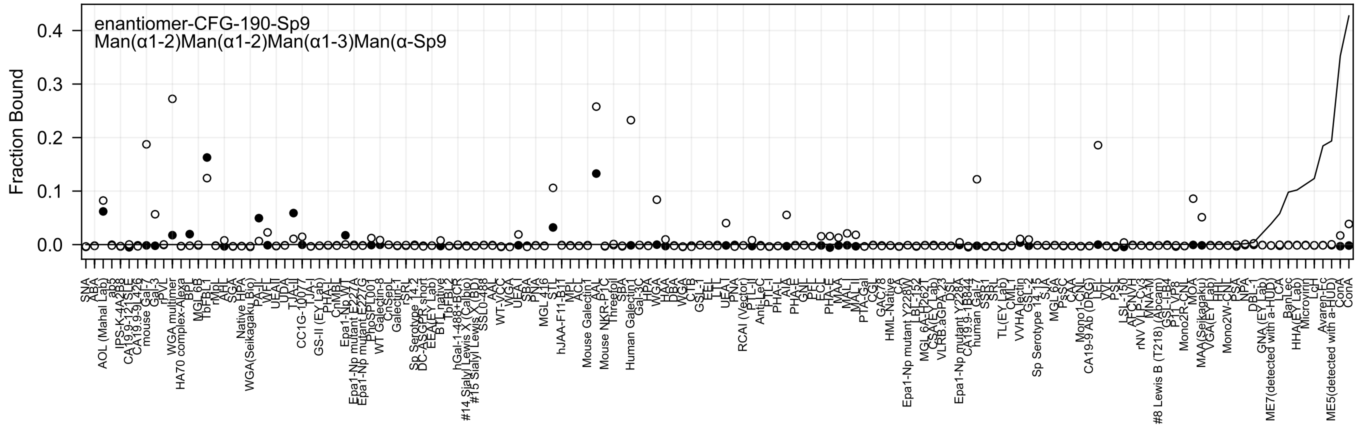
enantiomer-CFG-190-Sp9
Man(α1-2)Man(α1-2)Man(α1-3)Man(α-Sp9
Fraction Bound
0.0
0.1
0.2
0.3
0.4
SNA
ABA
AOL (Mahal Lab)
ab3
IPS-K-4A2B8
CA19.9-121SLE
CA19.9-9L426
mouse Gal-7
Gal-3
rPVL
WGA multimer
HA70 complex-Alexa
BPL
MGL 6B
TbFBL1
rMpL
AHL
SGA
Native HPA
WGA(Seikagaku Bio)
PA-IL
WFL
UEA-II
UDA
TJA-II
CC1G-1007
TJA-I
GS-II (EY Lab)
PHA-L
CnMBL
Epa1-Np WT
Epa1-Np mutant E227A
Epa1-Np mutant E227G
PhoSL001
WT Galectin-8
CnSepL
Galectin-1
rSRL
Sp Serotype 14.2
DC-ASGPR short
EEA(EY Lab)
BTL native
TbFBL2
hGal-1-488+BCR
#14 Sialyl Lewis X (Calbio)
#15 Sialyl Lewis X (BD)
SSL0-488
AAL
WT-VCC
WT-WGA
UEA-I
SBA
PNA
MGL 416
STL
hJAA-F11-B11
MPL
ACL
Mouse Galectin1
AAL
Mouse NKR-P1C
Threefoil
SBA
Human Galectin1
Gal-3C
HPA
WGA
HAA
DBA
WGA
CTB
GSL-1
EEL
LEL
UEA-I
PNA
RCAI (Vector)
PTL-II
Anti-LeC
PTL-I
PHA-L
AIA
PHA-E
GNL
PFL
ECL
PHA-E
MAA
MAL-I
MAL-II
PTA-Gal
CGL
GAC78
HML-Native
CA
Epa1-Np mutant Y228W
LBL-152
MGL 6A-H262T
CSA(EY Lab)
VLRB.aGPA.23
DSL
Epa1-Np mutant Y228A
CA19.9-1B844
human Gal-7
SSR-1
SRL
TL(EY Lab)
CML
VVHA lectin
GSL-2
Sp Serotype 14.15
SJA
MGL 6A
PsSC
CAA
Mono1-CNT
CA19-9 Ab (DRG)
LEL
VVL
PSL
LSL-150
AFCNVH
rNV VLP-CV3
MNA-M
#8 Lewis B (T218) (Abcam)
GSL-IB4
P1-VP8
Mono2R-CNT
MOA
MAA(Seikagaku)
VGA(EY Lab)
HHL
Mono2W-CNT
PSA
NPA
DBL-1
GNA (EY Lab)
ME7(detected with a-HUD)
LCA
BanLec
HHA(EY Lab)
Microvirin
LcH
Avaren-Fc
ME5(detected with a-HUD)
ConA
ConA

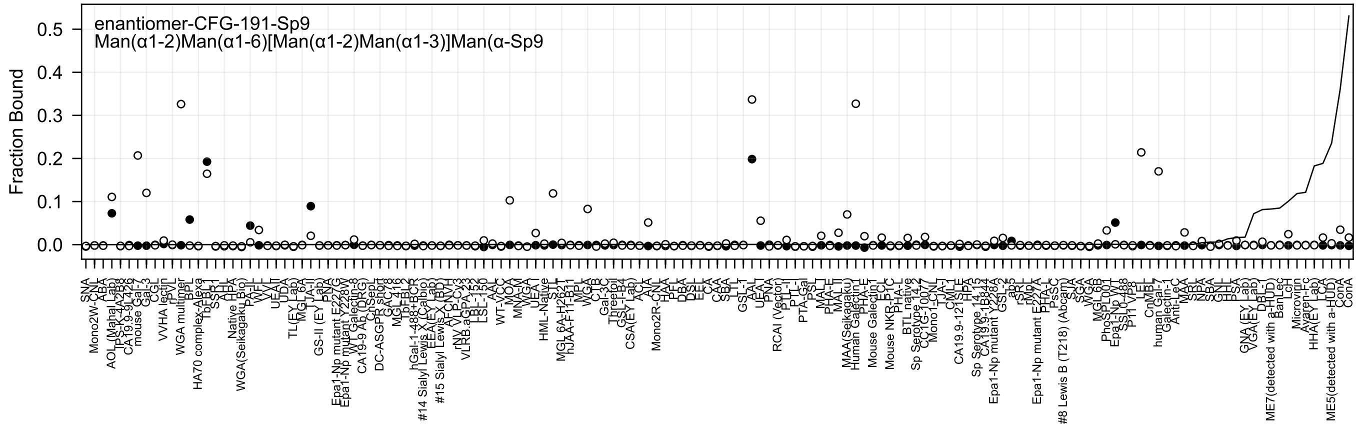

enantiomer-CFG-191-Sp9
Man(α1-2)Man(α1-6)[Man(α1-2)Man(α1-3)]Man(α-Sp9
Fraction Bound
0.0
0.1
0.2
0.3
0.4
0.5

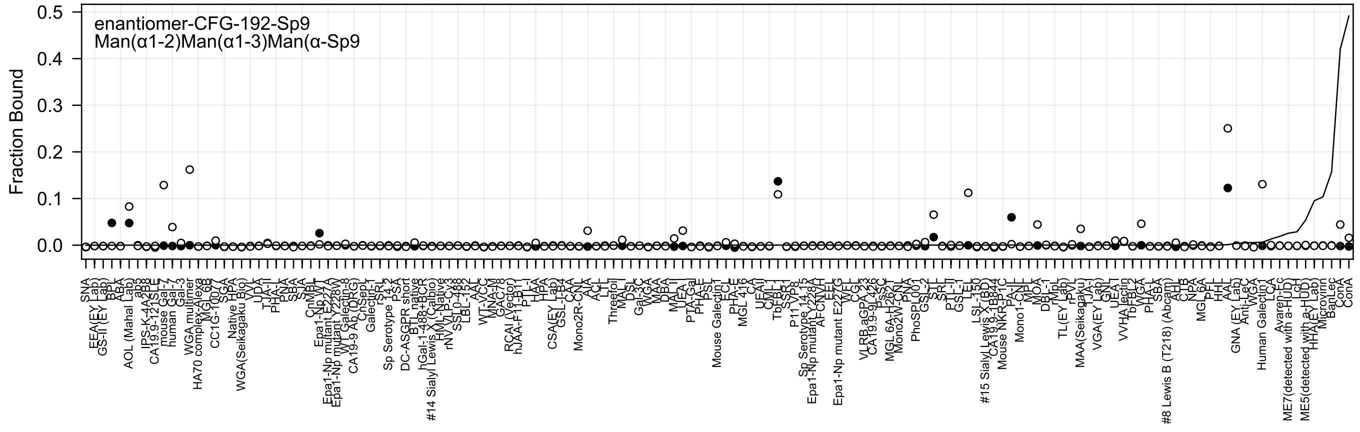
enantiomer-CFG-192-Sp9
Man(α1-2)Man(α1-3)Man(α-Sp9
Fraction Bound
0.5
0.4
0.3
0.2
0.1
0.0

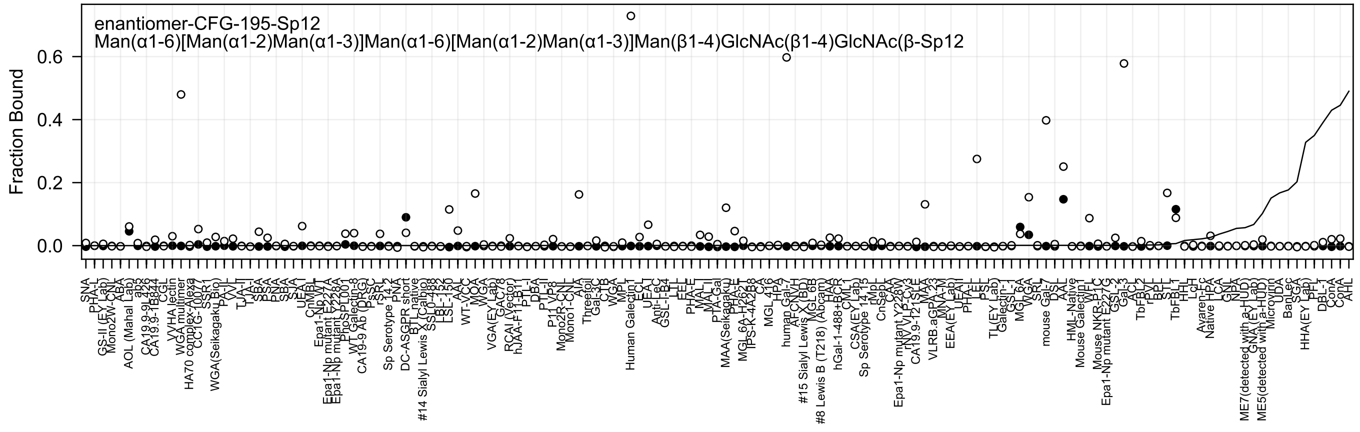
enantiomer-CFG-195-Sp12
Man(α1-6)[Man(α1-2)Man(α1-3)]Man(α1-6)[Man(α1-2)Man(α1-3)]Man(β1-4)GlcNAc(β1-4)GlcNAc(β-Sp12
Fraction Bound
0.0
0.2
0.4
0.6

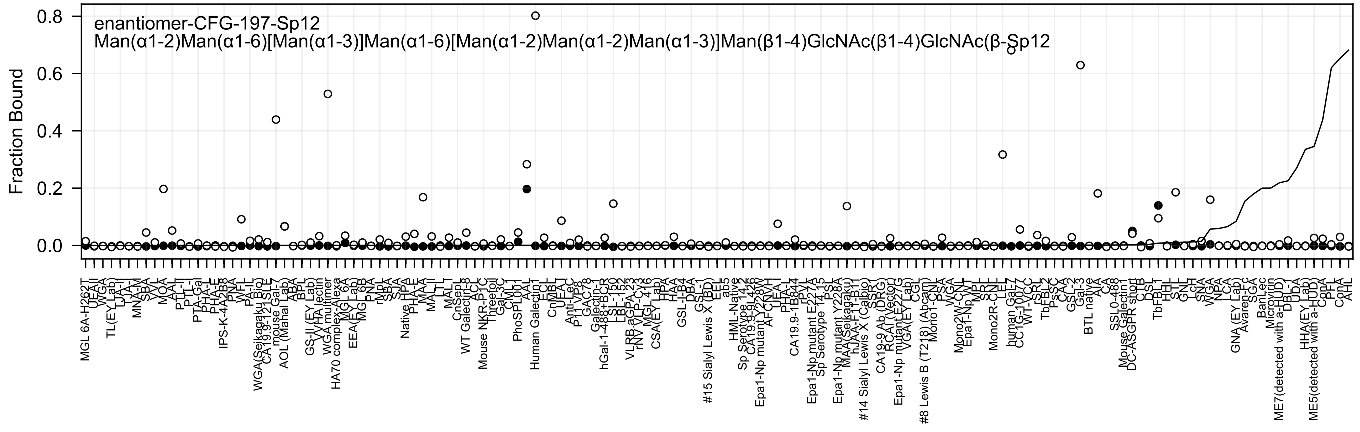

enantiomer-CFG-197-Sp12
Man(α1-2)Man(α1-6)[Man(α1-3)]Man(α1-6)[Man(α1-2)Man(α1-2)Man(α1-3)]Man(β1-4)GlcNAc(β1-4)GlcNAc(β-Sp12
Fraction Bound
0.0
0.2
0.4
0.6
0.8

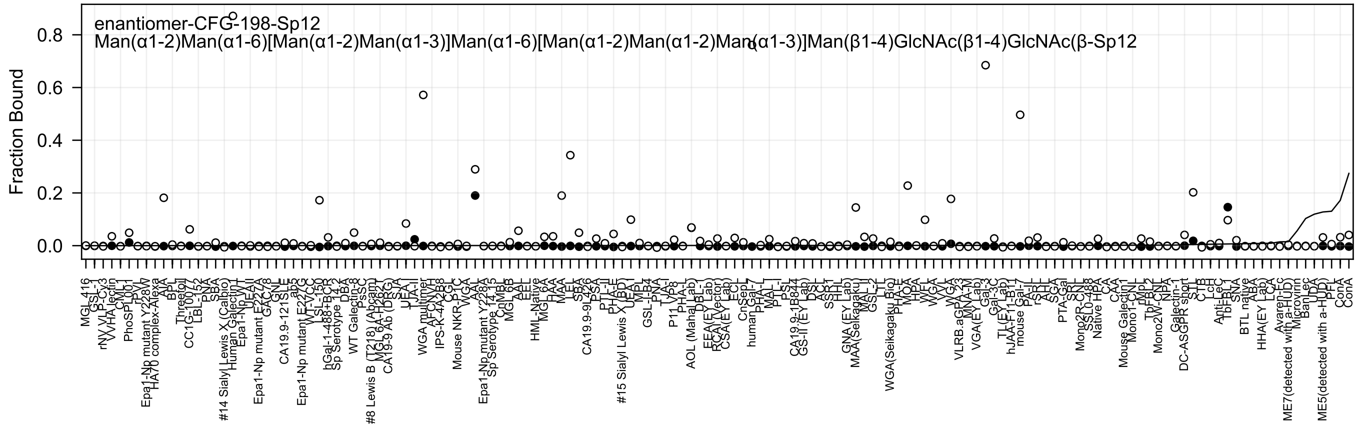
enantiomer-CFG-198-Sp12
Man(α1-2)Man(α1-6)[Man(α1-2)Man(α1-3)]Man(α1-6)[Man(α1-2)Man(α1-2)Man(α1-3)]Man(β1-4)GlcNAc(β1-4)GlcNAc(β-Sp12
Fraction Bound
0.0
0.2
0.4
0.6
0.8

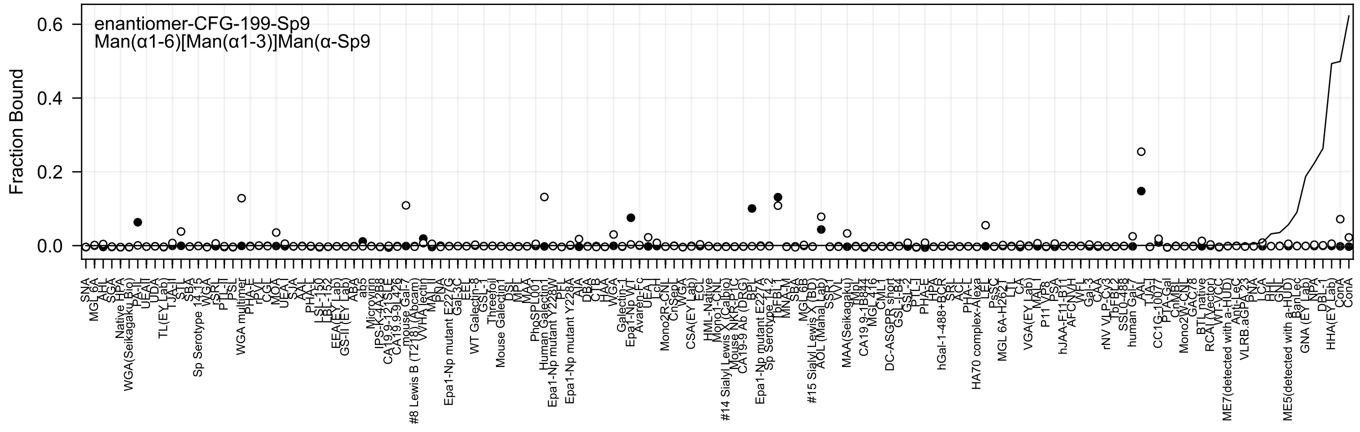

enantiomer-CFG-199-Sp9
Man(α1-6)[Man(α1-3)]Man(α-Sp9
Fraction Bound
0.6
0.4
0.2
0.0

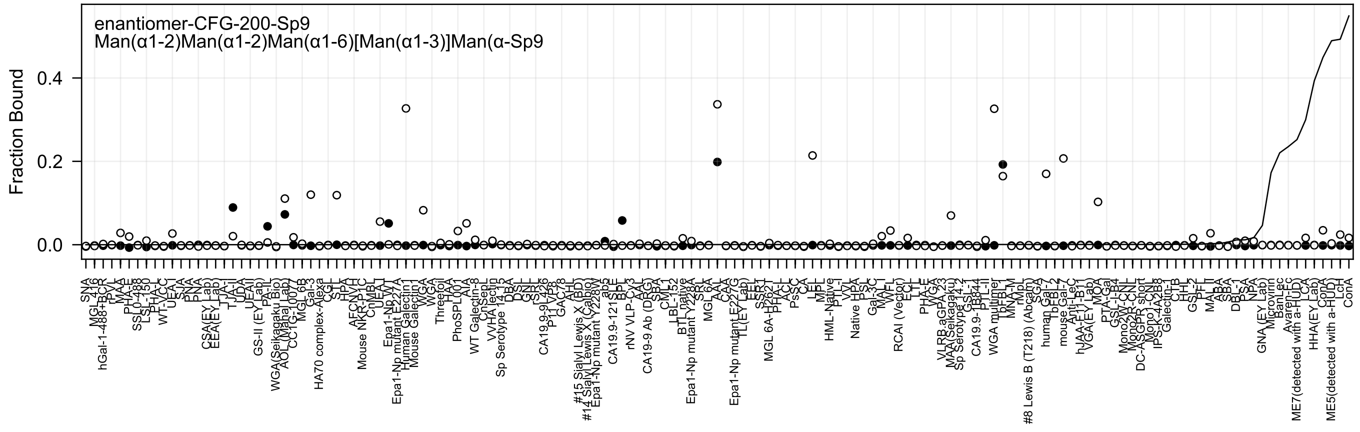
enantiomer-CFG-200-Sp9
Man(α1-2)Man(α1-2)Man(α1-6)[Man(α1-3)]Man(α-Sp9
Fraction Bound
0.4
0.2
0.0

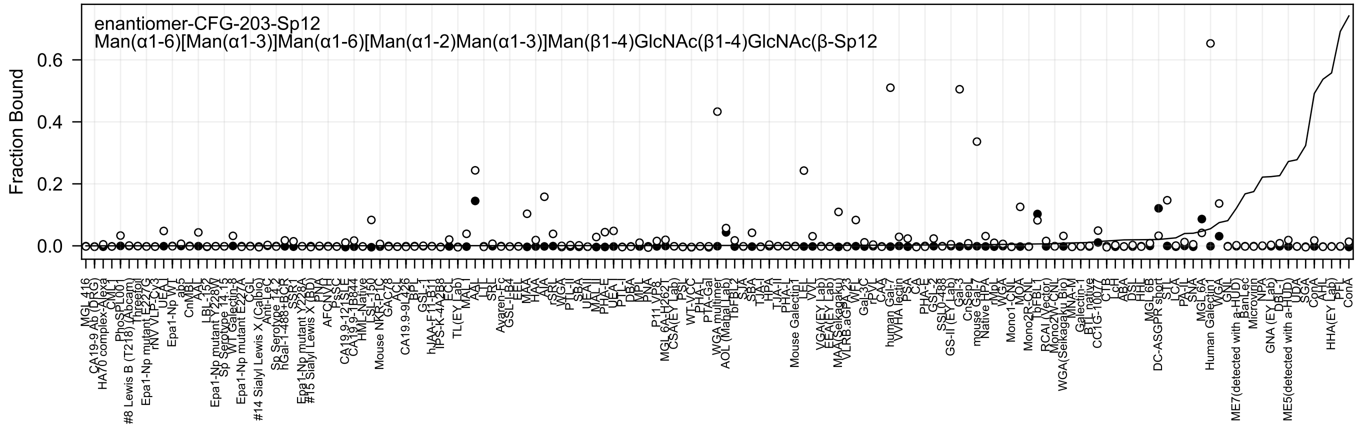
enantiomer-CFG-203-Sp12
Man(α1-6)[Man(α1-3)]Man(α1-6)[Man(α1-2)Man(α1-3)]Man(β1-4)GlcNAc(β1-4)GlcNAc(β-Sp12
Fraction Bound
0.6
0.4
0.2
0.0

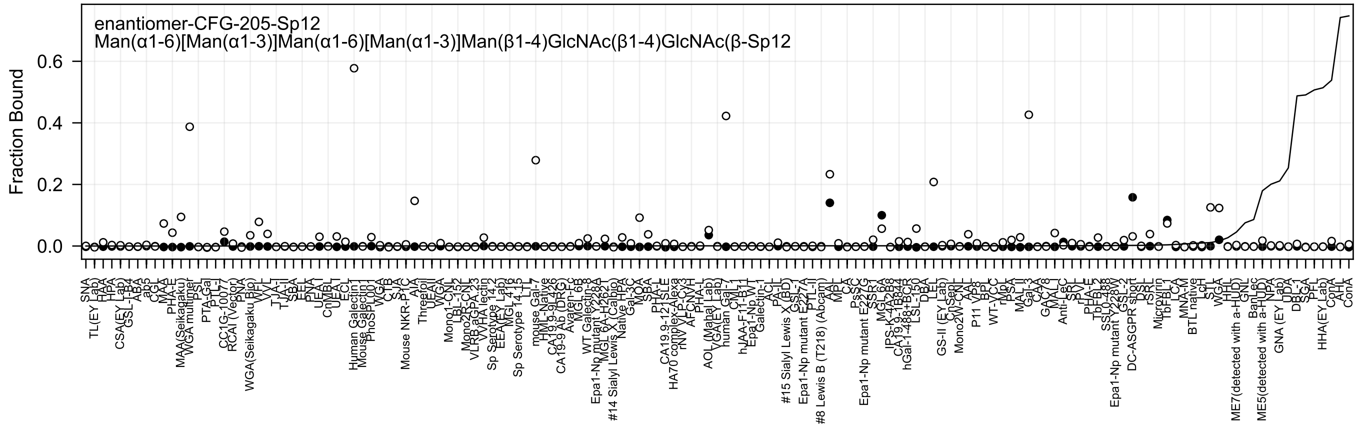

enantiomer-CFG-205-Sp12
Man(α1-6)[Man(α1-3)]Man(α1-6)[Man(α1-3)]Man(β1-4)GlcNAc(β1-4)GlcNAc(β-Sp12
Fraction Bound
0.0
0.2
0.4
0.6

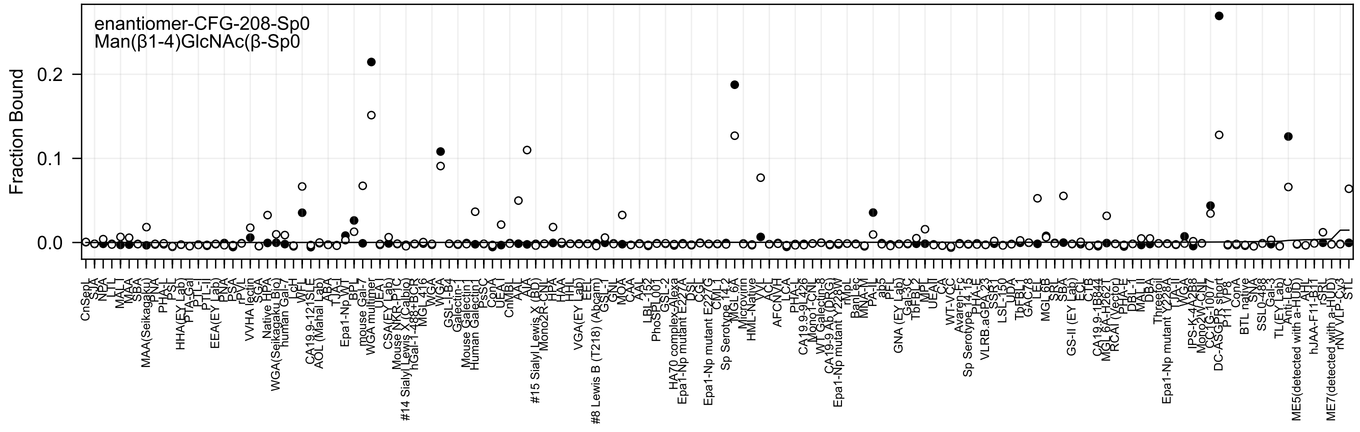

enantiomer-CFG-208-Sp0
Man(β1-4)GlcNAc(β-Sp0
Fraction Bound
0.2
0.1
0.0

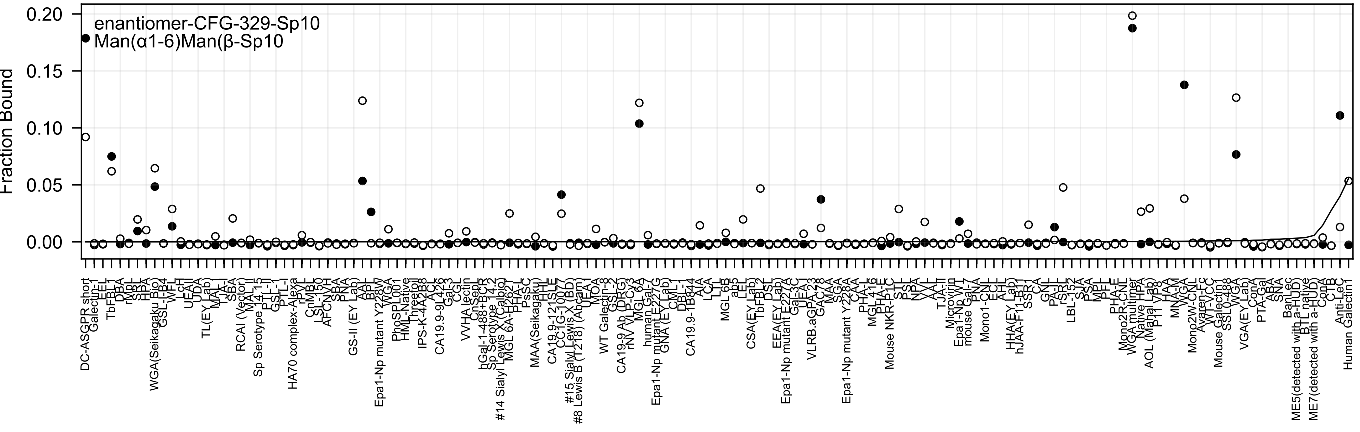
enantiomer-CFG-329-Sp10
Man(α1-6)Man(β-Sp10
Fraction Bound
0.20
0.15
0.10
0.05
0.00

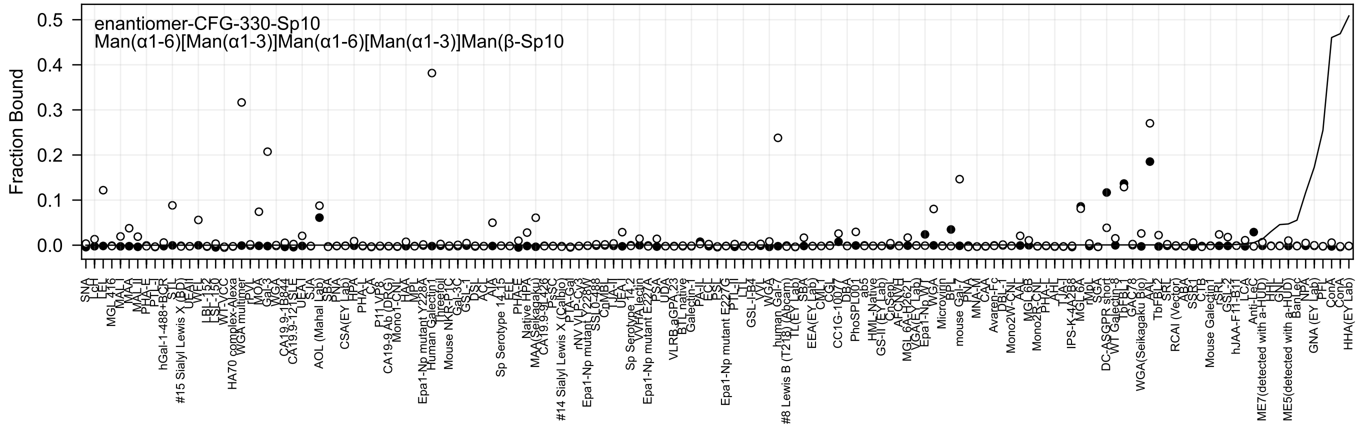

enantiomer-CFG-330-Sp10
Man(α1-6)[Man(α1-3)]Man(α1-6)[Man(α1-3)]Man(β-Sp10
Fraction Bound

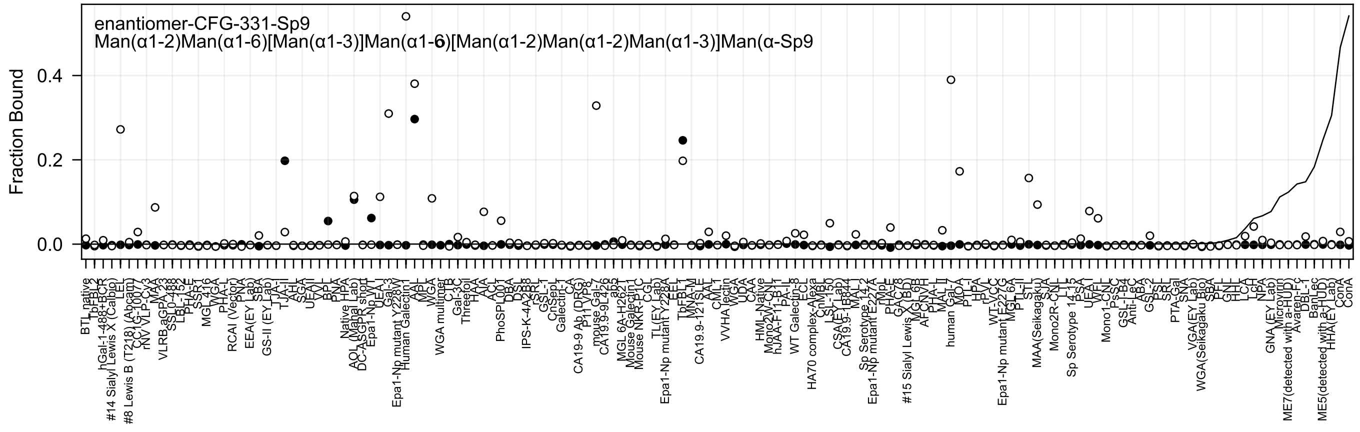

enantiomer-CFG-331-Sp9
Man(α1-2)Man(α1-6)[Man(α1-3)]Man(α1-6)[Man(α1-2)Man(α1-2)Man(α1-3)]Man(α-Sp9
Fraction Bound

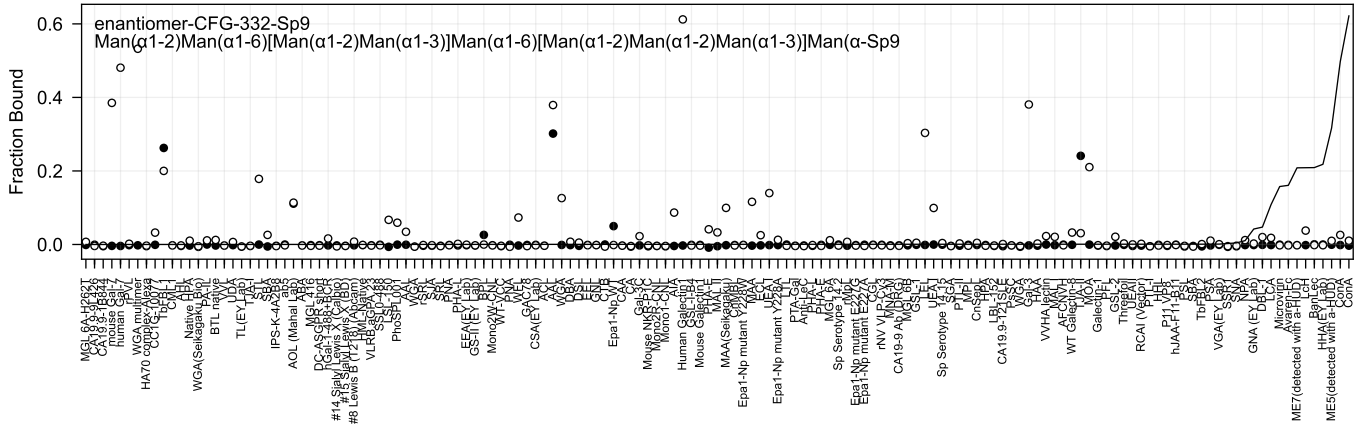

enantiomer-CFG-332-Sp9
Man(α1-2)Man(α1-6)[Man(α1-2)Man(α1-3)]Man(α1-6)[Man(α1-2)Man(α1-2)Man(α1-3)]Man(α-Sp9
Fraction Bound
0.6
0.4
0.2
0.0

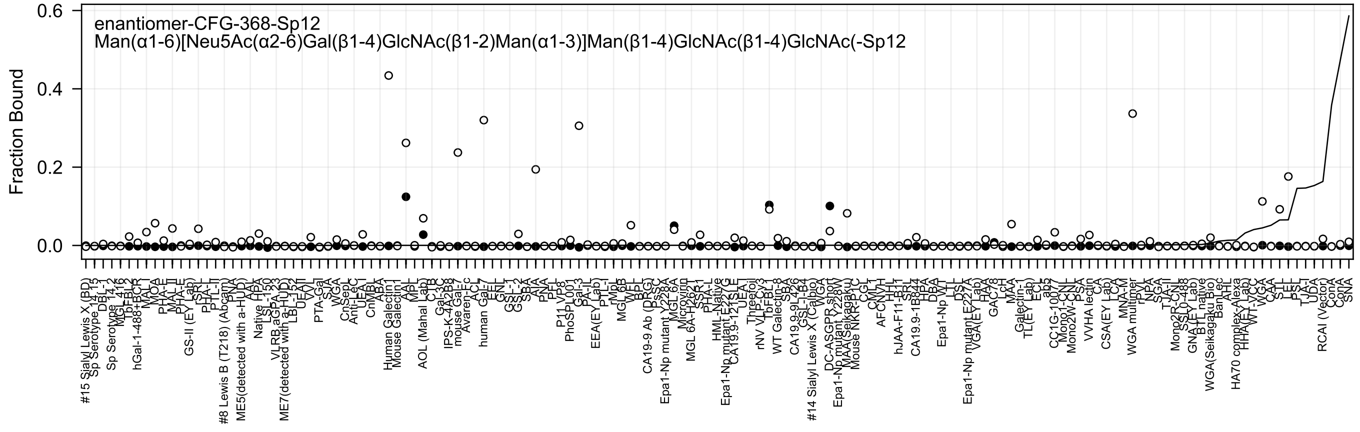

enantiomer-CFG-368-Sp12
Man(α1-6)[Neu5Ac(α2-6)Gal(β1-4)GlcNAc(β1-2)Man(α1-3)]Man(β1-4)GlcNAc(β1-4)GlcNAc(-Sp12
Fraction Bound
0.0
0.2
0.4
0.6

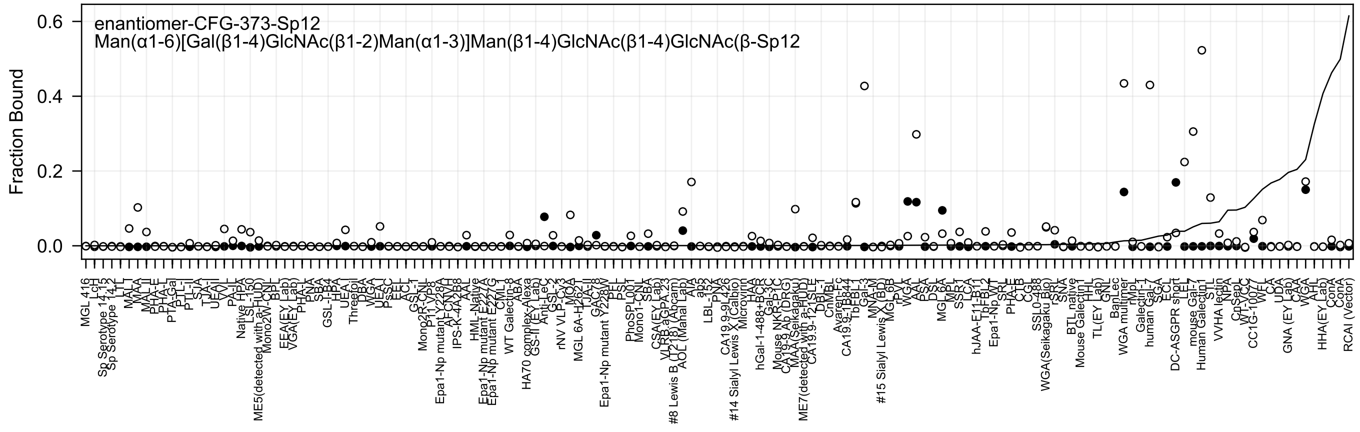
enantiomer-CFG-373-Sp12
Man(α1-6)[Gal(β1-4)GlcNAc(β1-2)Man(α1-3)]Man(β1-4)GlcNAc(β1-4)GlcNAc(β-Sp12
Fraction Bound
0.0
0.2
0.4
0.6

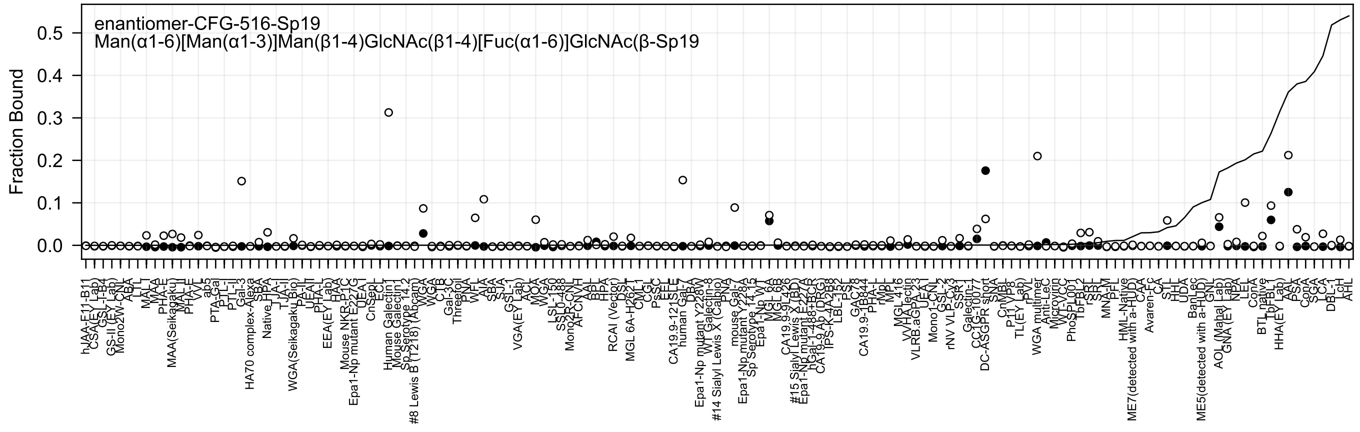
enantiomer-CFG-516-Sp19
Man(α1-6)[Man(α1-3)]Man(β1-4)GlcNAc(β1-4)[Fuc(α1-6)]GlcNAc(β-Sp19
Fraction Bound
0.0
0.1
0.2
0.3
0.4
0.5

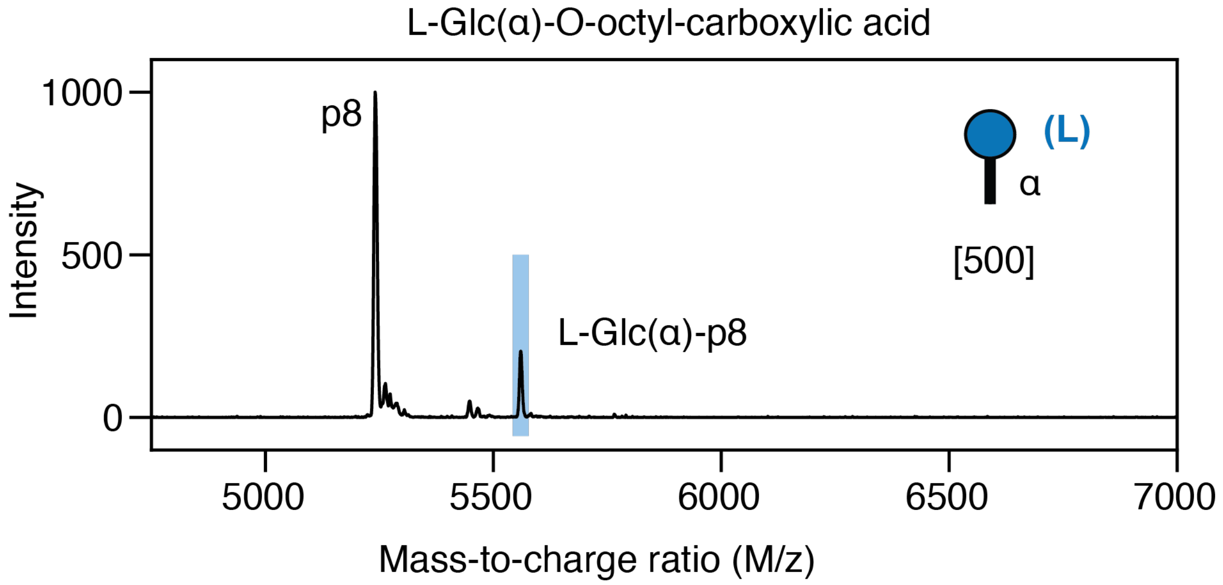

MALDI-TOF spectra of glycophage: L-Glca-C8-COOH - [500]
MSDB number: CP_MSDB_25
Sequencing File: 20250716-87MOYooNA-EG
Barcode:
AAAAAACTACTCTTTGCTATTCCACTGGTGGTACCTTTCTATTCTCACTCGAGTGTTGA
GAAGAATGATCAAAAAACATATCATGCGGGAGGGGGT
Axis Name: L-Glca-C8 - [500]
IUPAC: L-Glc(α)-O-octyl-carboxylic acid
Common Name: L-Glca-C8
Glytoucan ID: NA
Compound Number: L-Glca
MALDI File: CP_MSDB_25_L-Glca-C8-COOH.txt

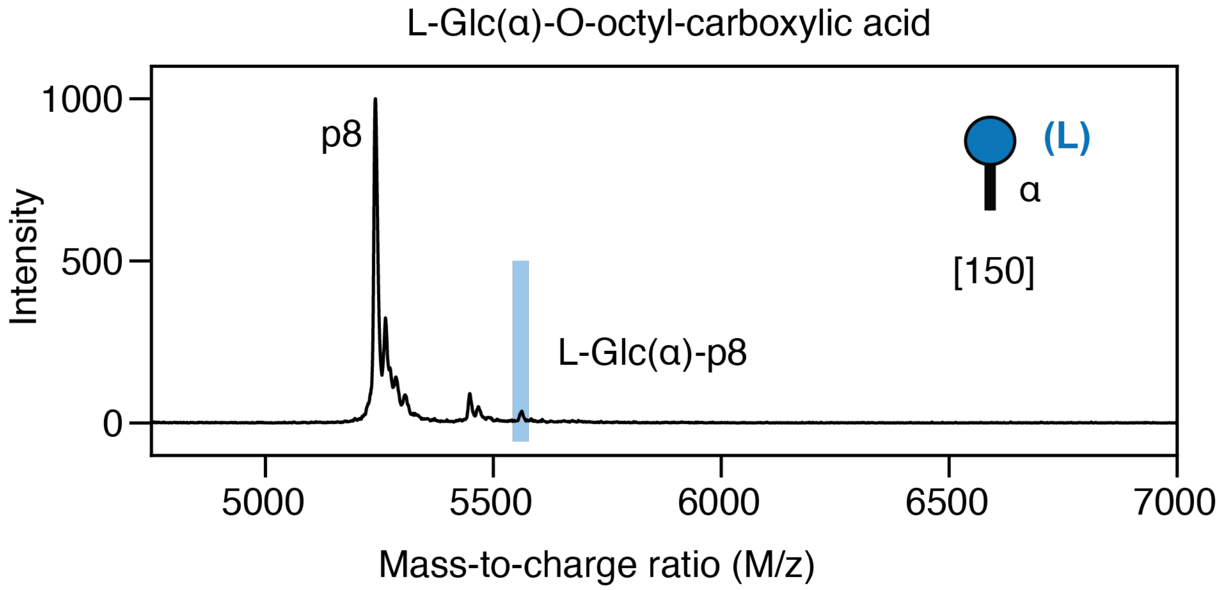

MALDI-TOF spectra of glycophage: L-Glca-C8-COOH - [150]
MSDB number: CP_MSDB_26
Sequencing File: 20250716-87MOYooNA-EG
Barcode:
AAAAAACTACTCTTCGCGATCCCACTGGTGGTACCTTTCTATTCTCACTCGAGCGTCG
AAAAGAACGATCAAAAAACTTATCACGCAGGGGGCGGA
Axis Name: L-Glca-C8 - [150]
IUPAC: L-Glc(α)-O-octyl-carboxylic acid
Common Name: L-Glca-C8
Glytoucan ID: NA
Compound Number: L-Glca
MALDI File: CP_MSDB_26_L-Glca-C8-COOH.txt

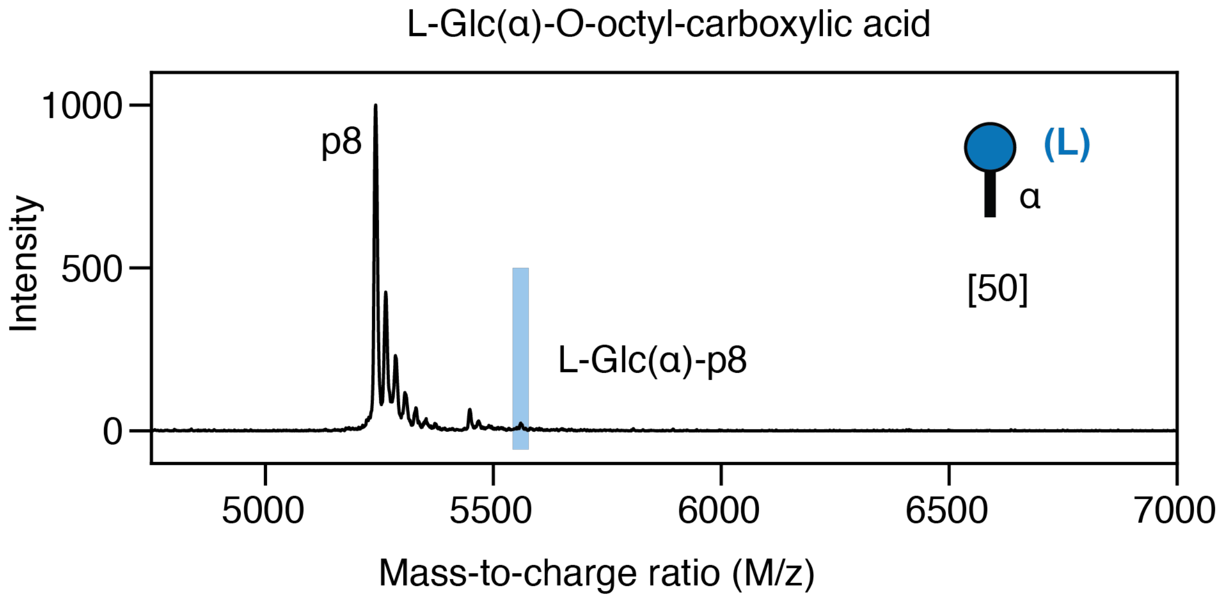

MALDI-TOF spectra of glycophage: L-Glca-C8-COOH - [50]
MSDB number: CP_MSDB_27
Sequencing File: 20250716-87MOYooNA-EG
Barcode:
AAAAAACTTCTGTTTGCCATTCCTCTGGTGGTACCTTTCTATTCTCACTCGAGTGTCGA
AAAAAATGACCAGAAGACTTACCACGCTGGGGGGGT
Axis Name: L-Glca-C8 - [50]
IUPAC: L-Glc(α)-O-octyl-carboxylic acid
Common Name: L-Glca-C8
Glytoucan ID: NA
Compound Number: L-Glca
MALDI File: CP_MSDB_27_L-Glca-C8-COOH.txt

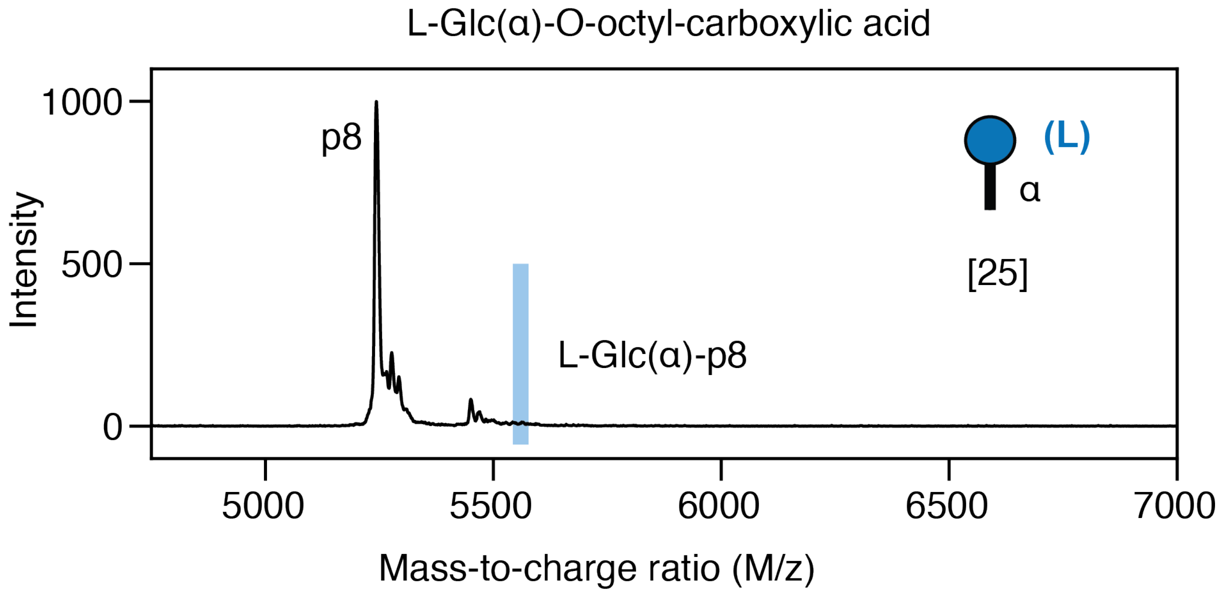

MALDI-TOF spectra of glycophage: L-Glca-C8-COOH - [25]
MSDB number: CP_MSDB_28
Sequencing File: 20250716-87MOYooNA-EG
Barcode:
AAAAAACTGCTCTTTGCGATACCACTGGTGGTACCTTTCTATTCTCACTCGAGCGTCG
AAAAAAATGATCAAAAGACATACCACGCGGGAGGTGGT
Axis Name: L-Glca-C8 - [25]
IUPAC: L-Glc(α)-O-octyl-carboxylic acid
Common Name: L-Glca-C8
Glytoucan ID: NA
Compound Number: L-Glca
MALDI File: CP_MSDB_28_L-Glca-C8-COOH.txt

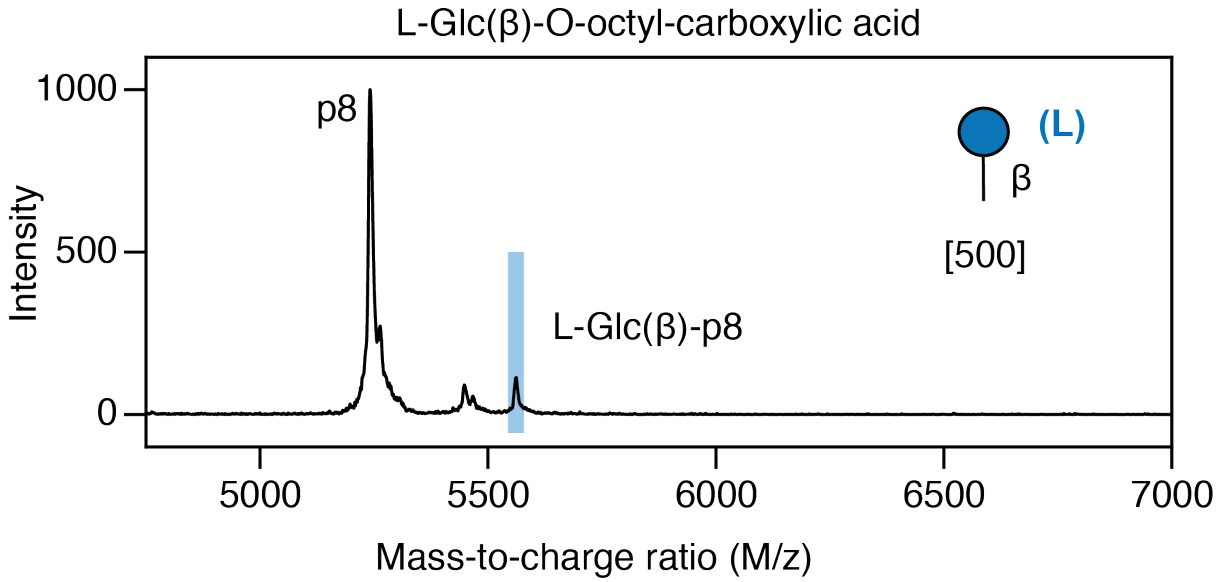

MALDI-TOF spectra of glycophage: L-Glcb-C8-COOH - [500]
MSDB number: CP_MSDB_29
Sequencing File: 20250716-87MOYooNA-EG
Barcode:
AAAAAACTACTGTTCGCAATCCCGCTAGTGGTACCTTTCTATTCTCACTCGAGTGTAG
AGAAGAATGATCAAAAGACGTATCATGCGGGGGGGGGA
Axis Name: L-Glcb-C8 - [500]
IUPAC: L-Glc(β)-O-octyl-carboxylic acid
Common Name: L-Glcb-C8
Glytoucan ID: NA
Compound Number: L-Glcb
MALDI File: CP_MSDB_29_L-GIcb-C8-COOH.txt

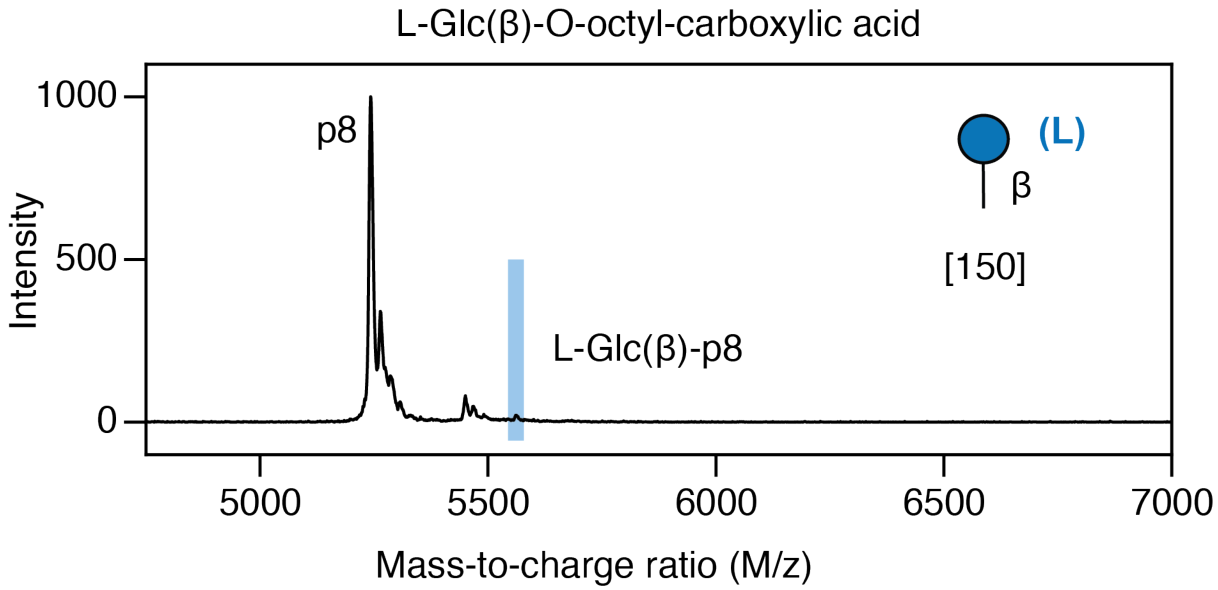

MALDI-TOF spectra of glycophage: L-Glcb-C8-COOH - [150]
MSDB number: CP_MSDB_30
Sequencing File: 20250716-87MOYooNA-EG
Barcode:
AAAAAACTGCTTTTTGCCATACCGCTTGTGGTACCTTTCTATTCTCACTCGAGTGTAGA
GAAAAACGATCAGAAAACGTATCATGCAGGGGGAGGG
Axis Name: L-Glcb-C8 - [150]
IUPAC: L-Glc(β)-O-octyl-carboxylic acid
Common Name: L-Glcb-C8
Glytoucan ID: NA
Compound Number: L-Glcb
MALDI File: CP_MSDB_30_L-GIcb-C8-COOH.txt

L-Glc(β)-O-octyl-carboxylic acid

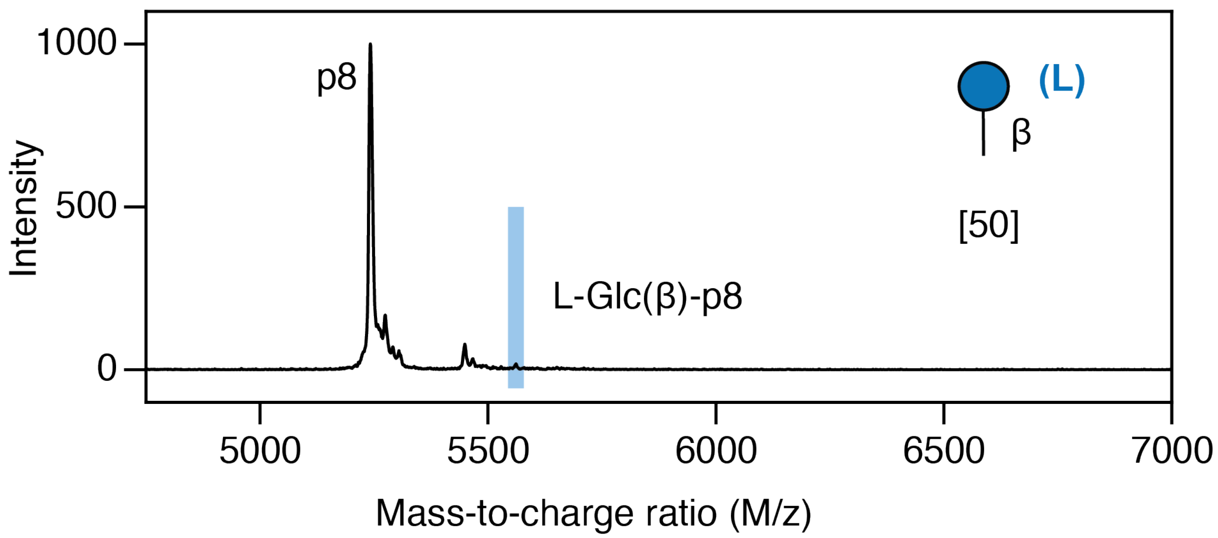

MALDI-TOF spectra of glycophage: L-Glcb-C8-COOH - [50]
MSDB number: CP_MSDB_31
Sequencing File: 20250716-87MOYooNA-EG
Barcode:
AAAAAACTACTGTTTGCGATCCCTCTGGTGGTACCTTTCTATTCTCACTCGAGTGTTG
AGAAAAATGATCAGAAGACTTACCACGCGGGGGGAGGA
Axis Name: L-Glcb-C8 - [50]
IUPAC: L-Glc(β)-O-octyl-carboxylic acid
Common Name: L-Glcb-C8
Glytoucan ID: NA
Compound Number: L-Glcb
MALDI File: CP_MSDB_31_L-GIcb-C8-COOH.txt

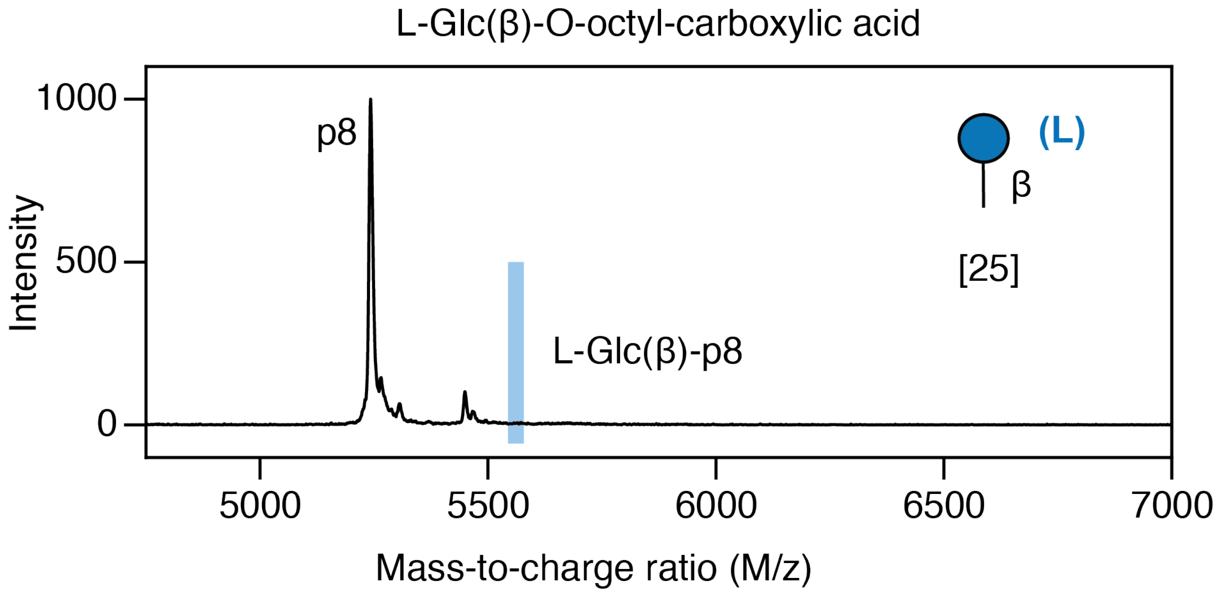

MALDI-TOF spectra of glycophage: L-Glcb-C8-COOH - [25]
MSDB number: CP_MSDB_32
Sequencing File: 20250716-87MOYooNA-EG
Barcode:
AAAAAACTTCTCTTCGCAATCCCGCTTGTGGTACCTTTCTATTCTCACTCGAGTGTTG
AAAAAAATGATCAAAAAACATATCATGCTGGGGGTGGT
Axis Name: L-Glcb-C8 - [25]
IUPAC: L-Glc(β)-O-octyl-carboxylic acid
Common Name: L-Glcb-C8
Glytoucan ID: NA
Compound Number: L-Glcb
MALDI File: CP_MSDB_32_L-GIcb-C8-COOH.txt

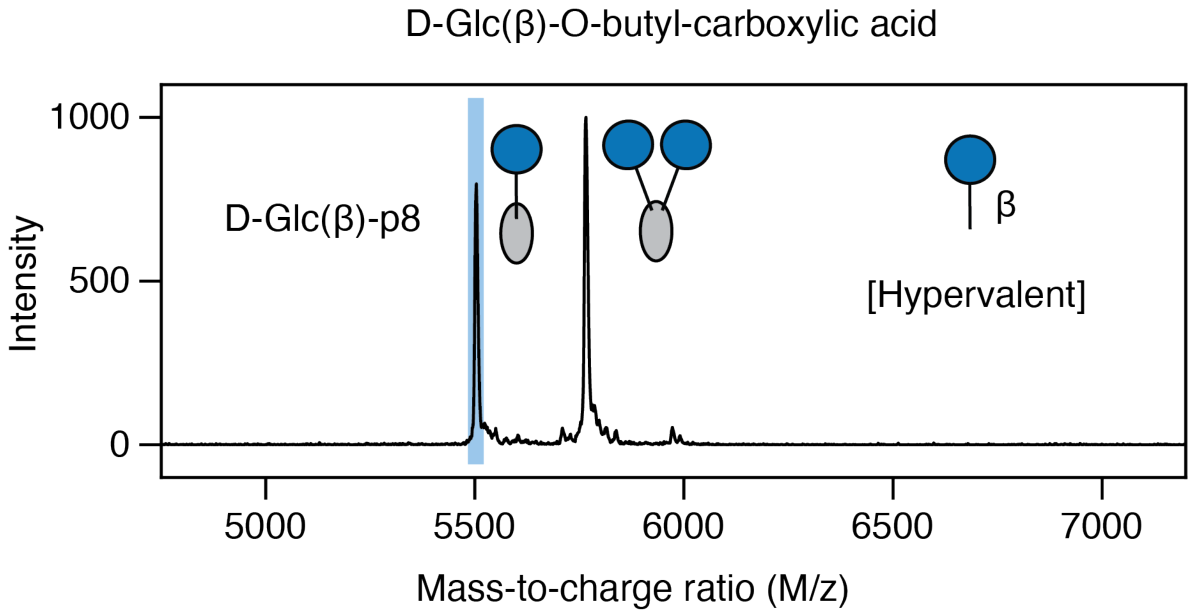

MALDI-TOF spectra of glycophage: D-Glcb-C4-COOH - [MAX]
MSDB number: CP_MSDB_75
Sequencing File: 20250716-87MOYooNA-EG
Barcode:
AAAAAACTGCTGTTTGCTATACCCCTGGTGGTACCTTTCTATTCTCACTCGAGCGTCG
AAAAGAACGACCAAAAAACATATCACGCAGGGGGCGGA
Axis Name: D-Glcb-C4 - [MAX]
IUPAC: D-Glc(β)-O-butyl-carboxylic acid
Common Name: D-Glcb-C4
Glytoucan ID: NA
Compound Number: D-Glcb
MALDI File: CP_MSDB_75--beta-D-Glc-C4-COOH_Hypervalent.txt

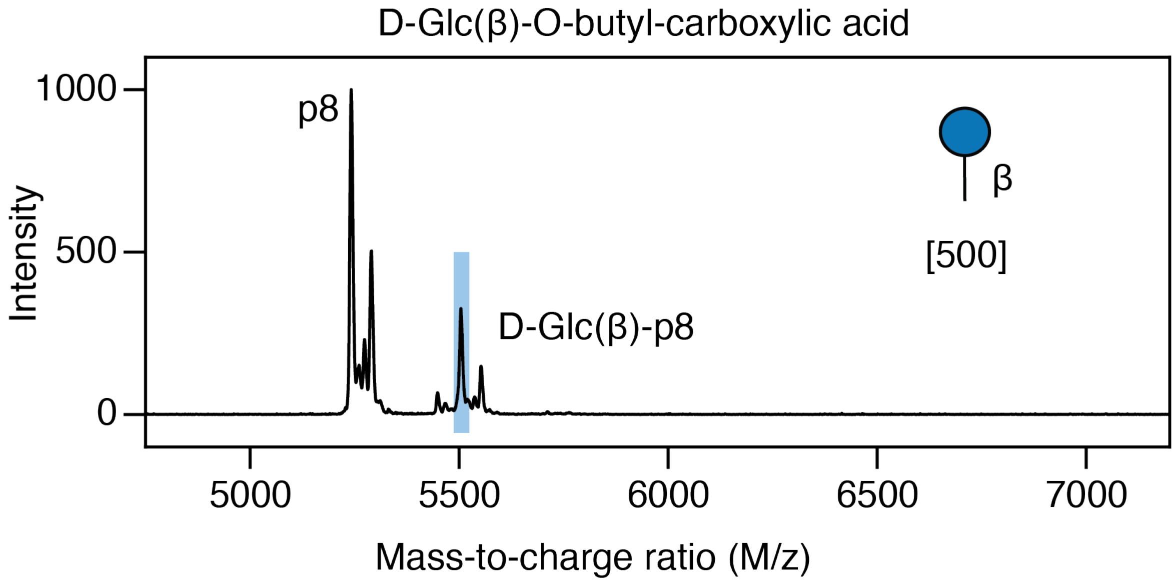

MALDI-TOF spectra of glycophage: D-Glcb-C4-COOH - [500]
MSDB number: CP_MSDB_67
Sequencing File: 20250716-87MOYooNA-EG
Barcode:
AAAAAACTACTGTTTGCGATCCCACTCGTGGTACCTTTCTATTCTCACTCGAGTGTTG
AGAAGAATGATCAGAAGACTTATCATGCTGGCGGCGGT
Axis Name: D-Glcb-C4 - [500]
IUPAC: D-Glc(β)-O-butyl-carboxylic acid
Common Name: D-Glcb-C4
Glytoucan ID: NA
Compound Number: D-Glcb
MALDI File: CP_MSDB_67_beta-D-GIc-C4-COOH_0.4mM.txt

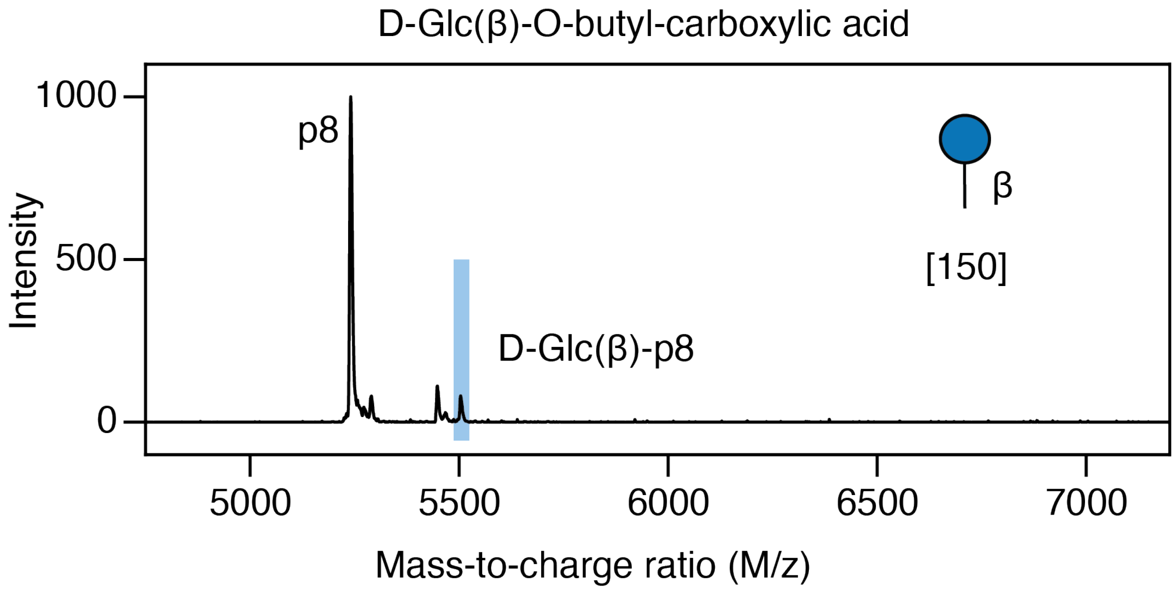

MALDI-TOF spectra of glycophage: D-Glcb-C4-COOH - [150]
MSDB number: CP_MSDB_66
Sequencing File: 20250716-87MOYooNA-EG
Barcode:
AAAAAACTGCTGTTTGCGATCCCGCTGGTGGTACCTTTCTATTCTCACTCGAGTGTAG
AGAAAAACGACCAAAAGACGTATCACGCAGGTGGGGGG
Axis Name: D-Glcb-C4 - [150]
IUPAC: D-Glc(β)-O-butyl-carboxylic acid
Common Name: D-Glcb-C4
Glytoucan ID: NA
Compound Number: D-Glcb
MALDI File: CP_MSDB_66_beta-D-GIc-C4-COOH_0.12mM.txt

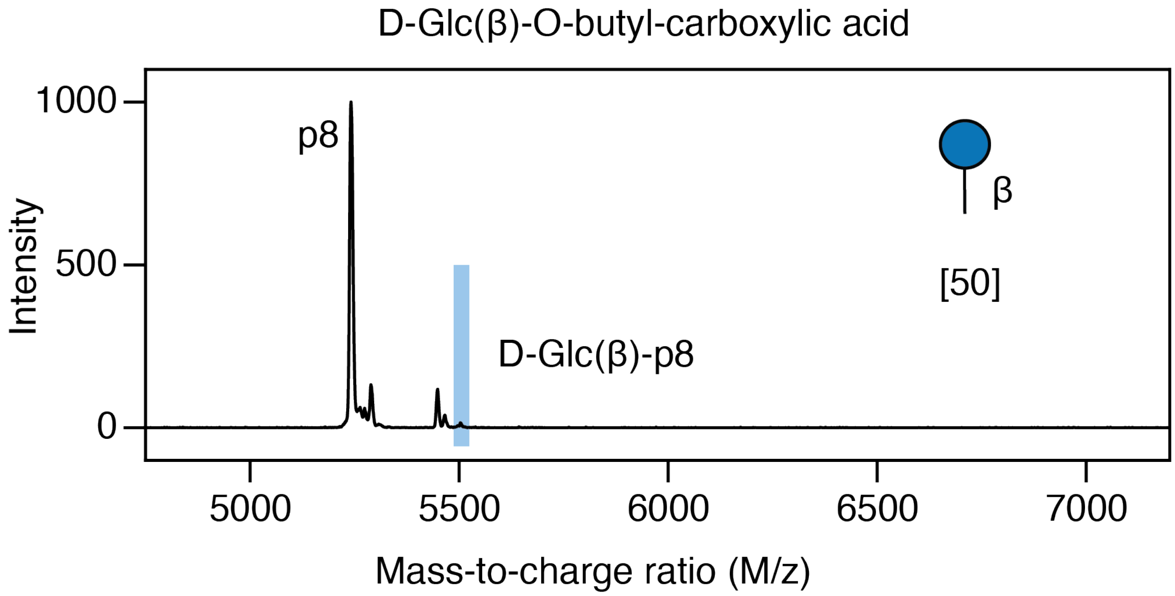

MALDI-TOF spectra of glycophage: D-Glcb-C4-COOH - [50]
MSDB number: CP_MSDB_65
Sequencing File: 20250716-87MOYooNA-EG
Barcode:
AAAAAACTGCTGTTCGCGATACCCCTGGTGGTACCTTTCTATTCTCACTCGAGTGTTG
AGAAAAATGACCAGAAAACCTATCATGCAGGCGGGGGG
Axis Name: D-Glcb-C4 - [50]
IUPAC: D-Glc(β)-O-butyl-carboxylic acid
Common Name: D-Glcb-C4
Glytoucan ID: NA
Compound Number: D-Glcb
MALDI File: CP_MSDB_65_beta-D-Glc-C4-COOH_0.04mM.txt

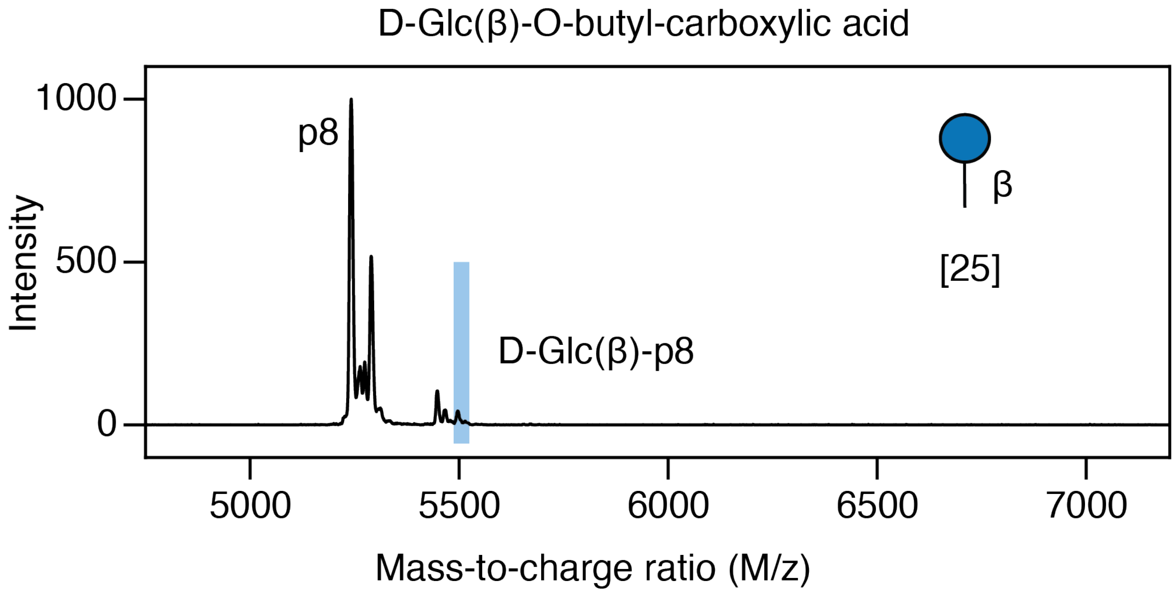

MALDI-TOF spectra of glycophage: D-Glcb-C4-COOH - [25]
MSDB number: CP_MSDB_63
Sequencing File: 20250716-87MOYooNA-EG
Barcode:
AAAAAACTACTGTTCGCGATCCCTCTCGTGGTACCTTTCTATTCTCACTCGAGTGTAG
AAAAAAACGATCAGAAGACTTATCATGCTGGCGGAGGC
Axis Name: D-Glcb-C4 - [25]
IUPAC: D-Glc(β)-O-butyl-carboxylic acid
Common Name: D-Glcb-C4
Glytoucan ID: NA
Compound Number: D-Glcb
MALDI File: CP_MSDB_63_beta-D-GIc-C4-COOH_0.016mM.txt

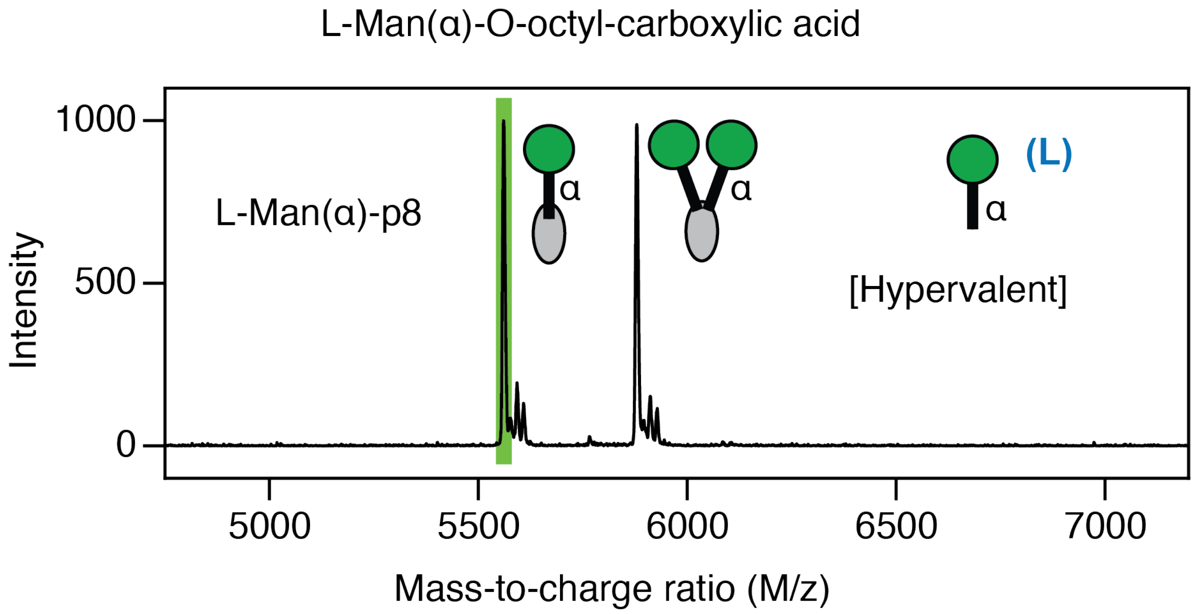

MALDI-TOF spectra of glycophage: L-Mana-C8-COOH - [MAX]
MSDB number: CP_MSDB_68
Sequencing File: 20250716-87MOYooNA-EG
Barcode:
AAAAAACTTCTCTTTGCGATTCCGCTGGTGGTACCTTTCTATTCTCACTCGAGCGTGG
AAAAGAACGATCAAAAGACGTACCATGCAGGGGGAGGG
Axis Name: L-Mana-C8-COOH - [MAX]
IUPAC: L-Man(α)-O-octyl-carboxylic acid
Common Name: L-Mana-C8
Glytoucan ID: NA
Compound Number: L-Mana
MALDI File: CP_MSDB_68_alpha-L-Man-C8-COOH_Max.txt

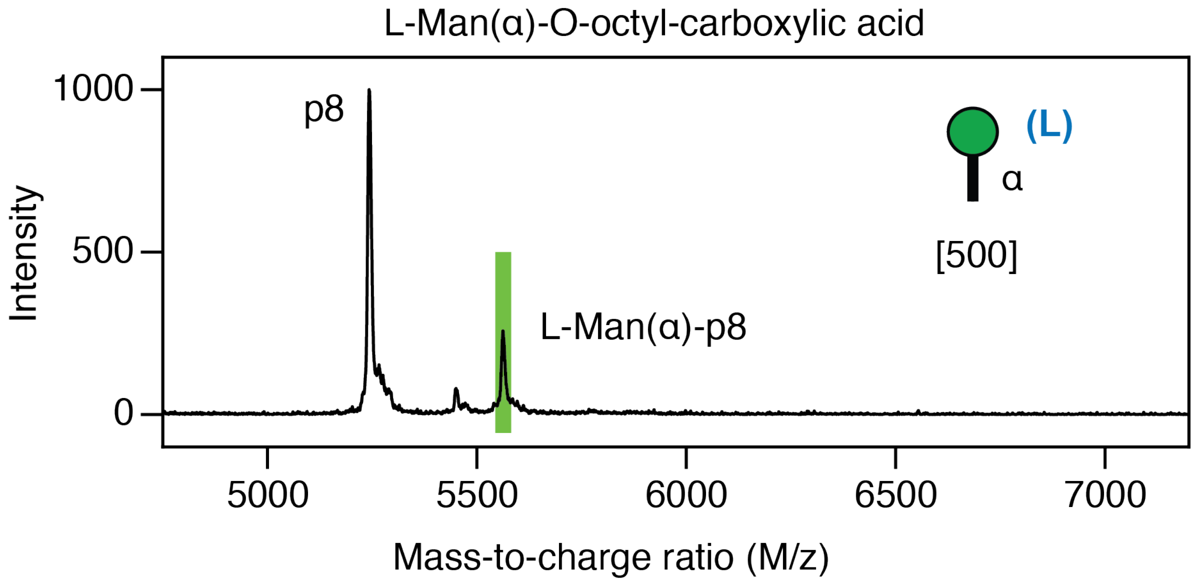

MALDI-TOF spectra of glycophage: L-Mana-C8-COOH - [500]
MSDB number: CP_MSDB_12
Sequencing File: 20250716-87MOYooNA-EG
Barcode:
AAAAAACTACTGTTCGCAATACCTCTCGTGGTACCTTTCTATTCTCACTCGAGCGTAG
AAAAAAATGACCAAAAGACGTATCATGCTGGTGGCGGA
Axis Name: L-Mana-C8 - [500]
IUPAC: L-Man(α)-O-octyl-carboxylic acid
Common Name: L-Mana-C8
Glytoucan ID: NA
Compound Number: L-Mana
MALDI File: CP_MSDB_12_L-Man-C8-COOH_500.txt

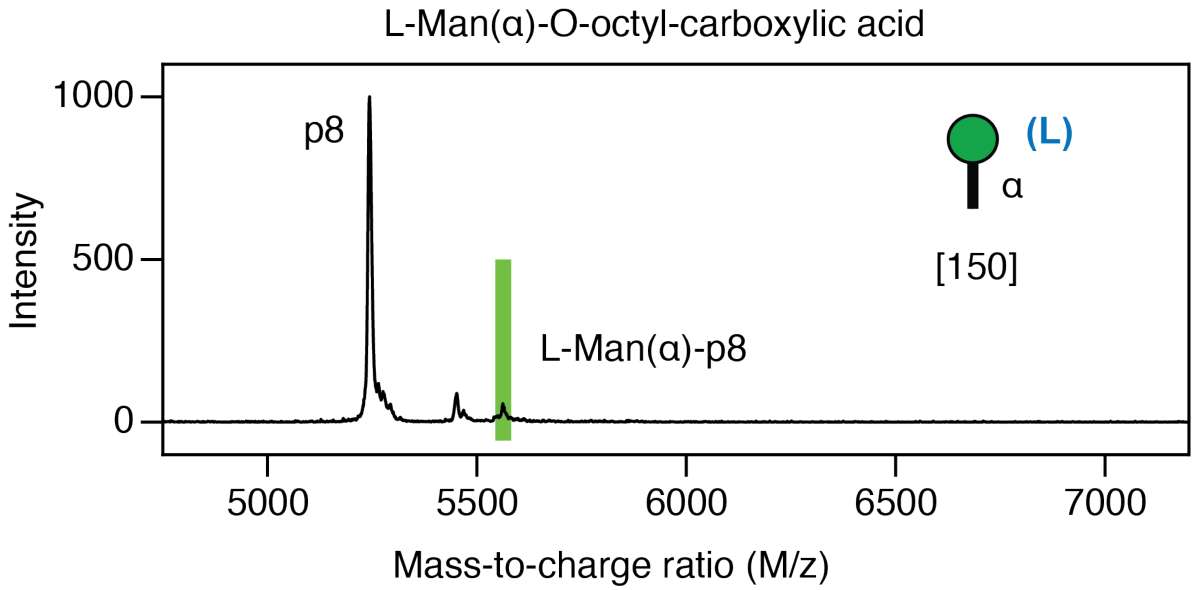

MALDI-TOF spectra of glycophage: L-Mana-C8-COOH - [150]
MSDB number: CP_MSDB_11
Sequencing File: 20250716-87MOYooNA-EG
Barcode:
AAAAAACTGCTGTTTGCGATCCCGCTAGTGGTACCTTTCTATTCTCACTCGAGCGTGG
AGAAGAATGATCAAAAAACGTATCACGCCGGCGGTGGG
Axis Name: L-Mana-C8 - [150]
IUPAC: L-Man(α)-O-octyl-carboxylic acid
Common Name: L-Mana-C8
Glytoucan ID: NA
Compound Number: L-Mana
MALDI File: CP_MSDB_11_L-Man-C8-COOH_150.txt

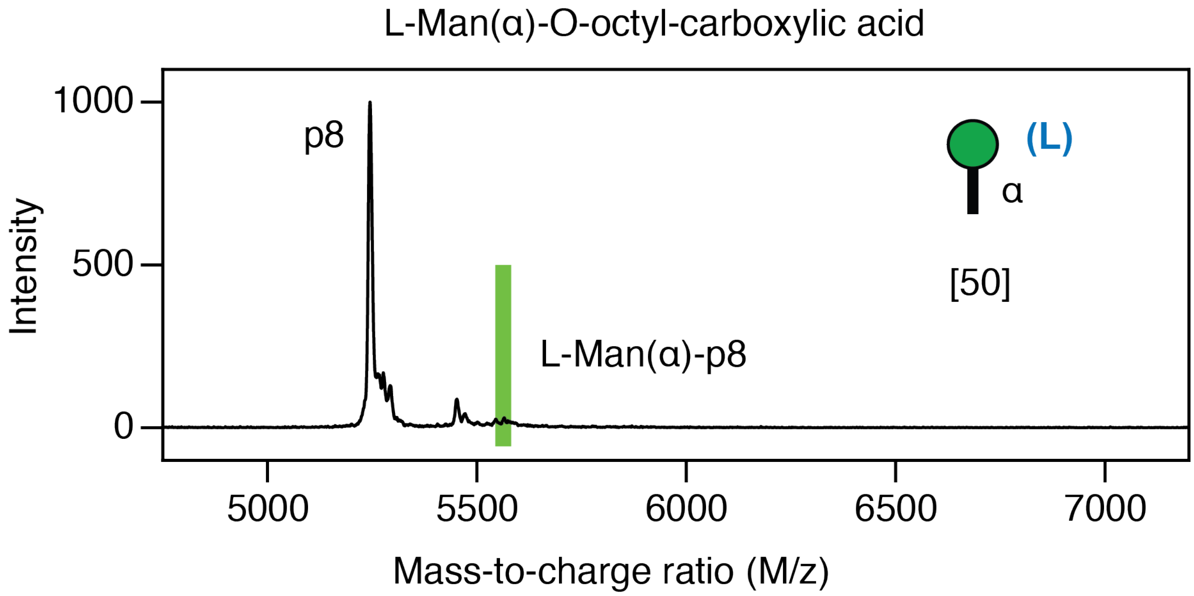

MALDI-TOF spectra of glycophage: L-Mana-C8-COOH - [50]
MSDB number: CP_MSDB_10
Sequencing File: 20250716-87MOYooNA-EG
Barcode:
AAAAAACTGCTGTTTGCGATCCCGCTTGTGGTACCTTTCTATTCTCACTCGAGTGTGG
AGAAGAACGATCAGAAGACCTATCATGCCGGAGGAGGT
Axis Name: L-Mana-C8 - [50]
IUPAC: L-Man(α)-O-octyl-carboxylic acid
Common Name: L-Mana-C8
Glytoucan ID: NA
Compound Number: L-Mana
MALDI File: CP_MSDB_10_L-Man-C8-COOH_50.txt

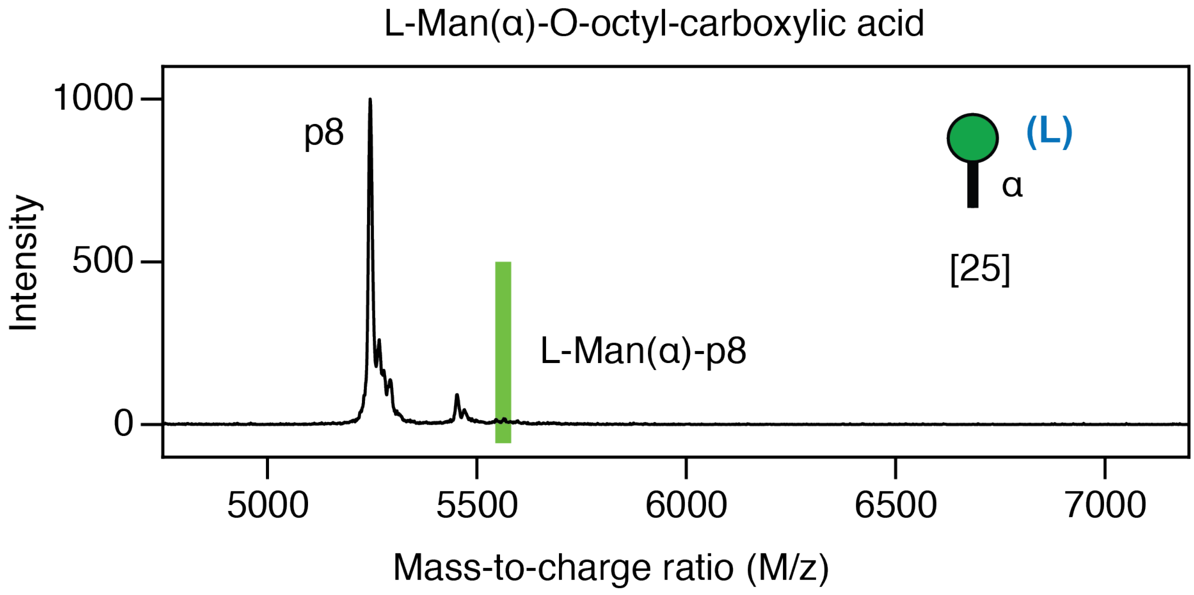

MALDI-TOF spectra of glycophage: L-Mana-C8-COOH - [25]
MSDB number: CP_MSDB_9
Sequencing File: 20250716-87MOYooNA-EG
Barcode:
AAAAAACTACTCTTTGCGATACCACTTGTGGTACCTTTCTATTCTCACTCGAGTGTCG
AAAAAAATGACCAAAAGACCTACCATGCCGGGGGTGGT
Axis Name: L-Mana-C8 - [25]
IUPAC: L-Man(α)-O-octyl-carboxylic acid
Common Name: L-Mana-C8
Glytoucan ID: NA
Compound Number: L-Mana
MALDI File: CP_MSDB_9_L-Man-C8-COOH_25.txt

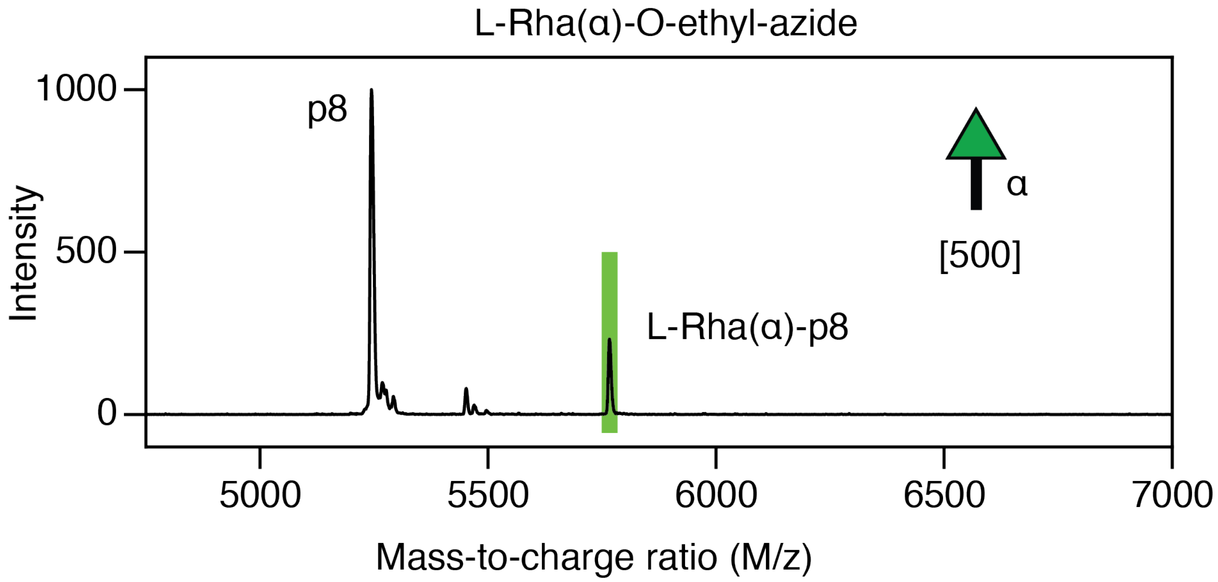

MALDI-TOF spectra of glycophage: L-Rhaa-sp - [500]
MSDB number: CP_MSDB_33
Sequencing File: 20250716-87MOYooNA-EG
Barcode:
AAAAAACTTCTTTTCGCCATACCGCTAGTGGTACCTTTCTATTCTCACTCGAGCGTGG
AGAAGAATGATCAAAAAACTTATCACGCAGGTGGAGGA
Axis Name: L-Rhaa-sp - [500]
IUPAC: L-Rha(α)-O-ethyl-azide
Common Name: L-Rhaa-sp
Glytoucan ID: NA
Compound Number: L-Rha
MALDI File: CP_MSDB_33_DBCO_L-Rha-Sp.txt

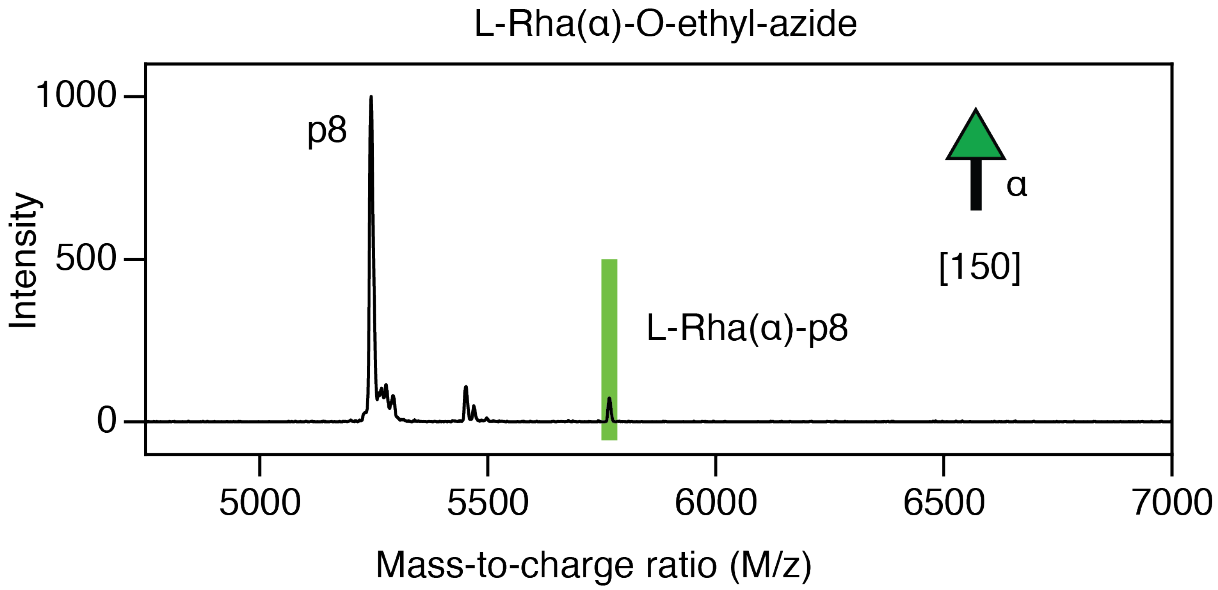

MALDI-TOF spectra of glycophage: L-Rhaa-sp - [150]
MSDB number: CP_MSDB_36
Sequencing File: 20250716-87MOYooNA-EG
Barcode:
AAAAAACTTCTCTTTGCGATACCCCTTGTGGTACCTTTCTATTCTCACTCGAGTGTCGA
GAAAAATGACCAGAAGACGTACCACGCGGGGGGCGGT
Axis Name: L-Rhaa-sp - [150]
IUPAC: L-Rha(α)-O-ethyl-azide
Common Name: L-Rhaa-sp
Glytoucan ID: NA
Compound Number: L-Rha
MALDI File: CP_MSDB_36_DBCO_L-Rha-Sp.txt

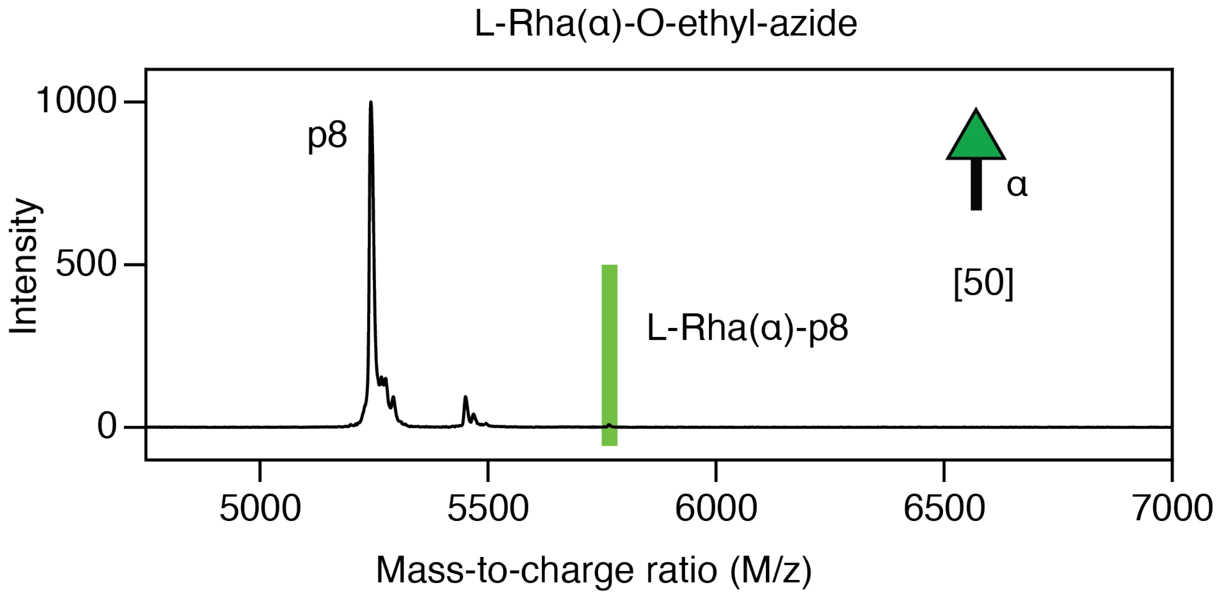

MALDI-TOF spectra of glycophage: L-Rhaa-sp - [50]
MSDB number: CP_MSDB_35
Sequencing File: 20250716-87MOYooNA-EG
Barcode:
AAAAAATTATTATTCGCAATTCCTTTAGTGGTACCTTTCTATTCTCACTCGAGCGTTGA
AAAAAACGACCAGAAAACCTATCATGCAGGTGGGGG
Axis Name: L-Rhaa-sp - [50]
IUPAC: L-Rha(α)-O-ethyl-azide
Common Name: L-Rhaa-sp
Glytoucan ID: NA
Compound Number: L-Rha
MALDI File: CP_MSDB_35_DBCO_L-Rha-Sp.txt

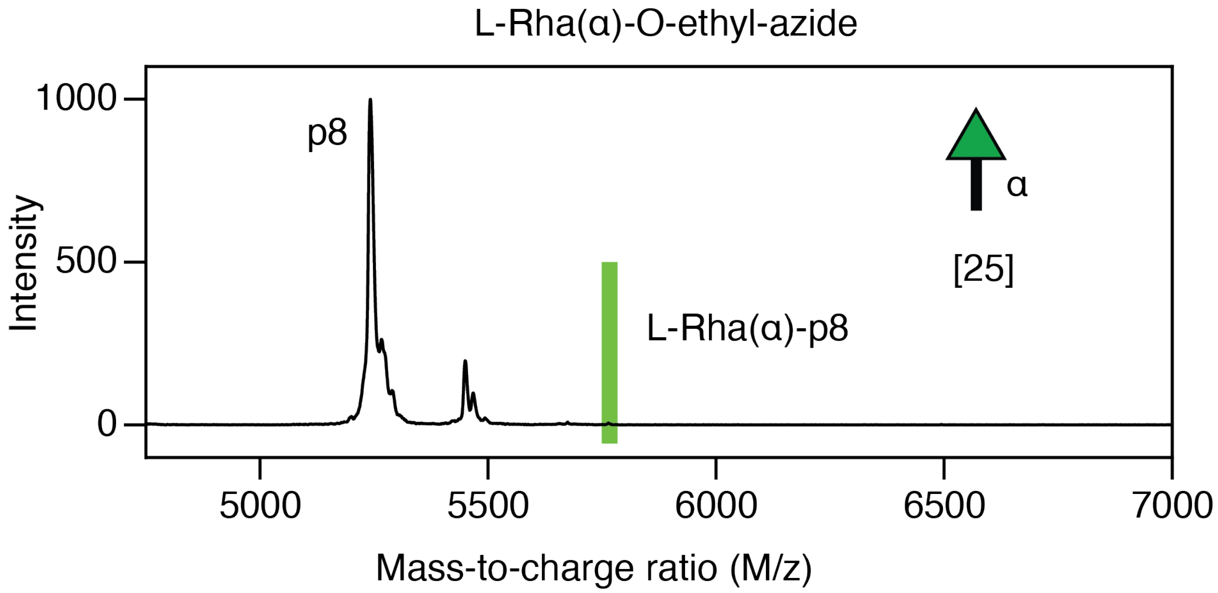

MALDI-TOF spectra of glycophage: L-Rhaa-sp - [25]
MSDB number: CP_MSDB_34
Sequencing File: 20250716-87MOYooNA-EG
Barcode:
AAAAAACTTCTATTCGCAATCCCTCTGGTGGTACCTTTCTATTCTCACTCGAGCGTTGA
GAAGAACGATCAAAAGACGTATCACGCAGGGGGGGGG
Axis Name: L-Rhaa-sp - [25]
IUPAC: L-Rha(α)-O-ethyl-azide
Common Name: L-Rhaa-sp
Glytoucan ID: NA
Compound Number: L-Rha
MALDI File: CP_MSDB_34_DBCO_L-Rha-Sp.txt

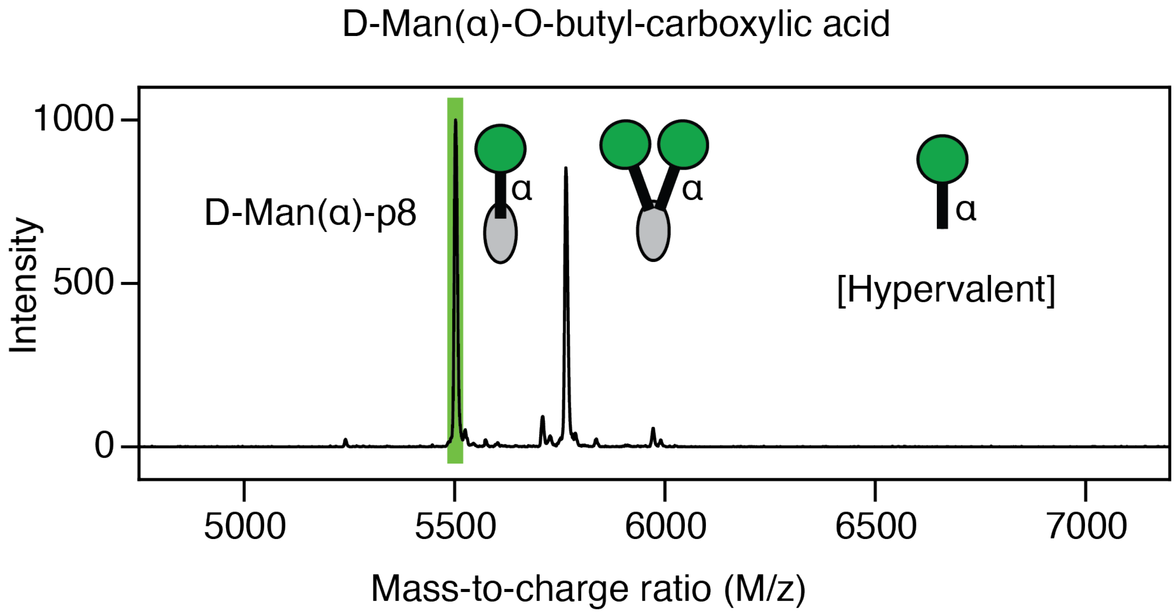

MALDI-TOF spectra of glycophage: D-Mana-C4-COOH - [MAX]
MSDB number: CP_MSDB_69
Sequencing File: 20250716-87MOYooNA-EG
Barcode:
AAAAAACTGCTATTCGCTATCCCGCTAGTGGTACCTTTCTATTCTCACTCGAGTGTTGA GAAGAACGACCAAAAGACATATCATGCGGGAGGTGGC
Axis Name: D-Mana-C4-COOH - [MAX]
IUPAC: D-Man(α)-O-butyl-carboxylic acid
Common Name: D-Mana-C4
Glytoucan ID: NA
Compound Number: D-Mana
MALDI File: CP_MSDB_69_alpha-D-Man-C4-COOH_Max.txt

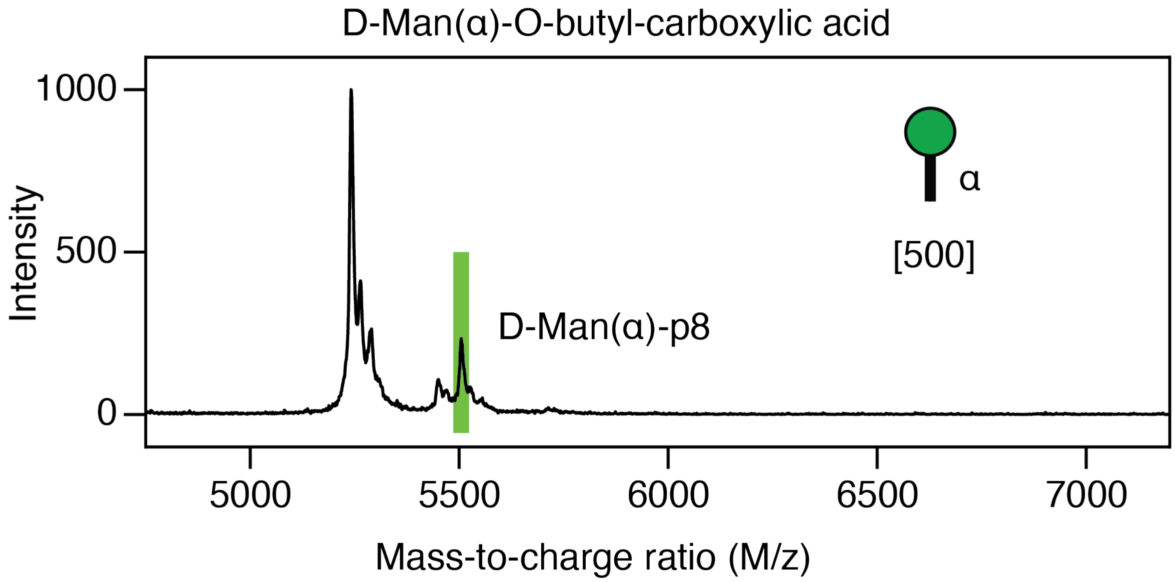

MALDI-TOF spectra of glycophage: D-Mana-C4 - [500]
MSDB number: CP_MSDB_4
Sequencing File: 20250716-87MOYooNA-EG
Barcode:
AAAAAACTACTGTTCGCTATCCCGCTGGTGGTACCTTTCTATTCTCACTCGAGTGTGG
AAAAGAATGATCAGAAAACTTACCATGCCGGTGGCGGG
Axis Name: D-Mana-C4 - [500]
IUPAC: D-Man(α)-O-butyl-carboxylic acid
Common Name: D-Mana-C4
Glytoucan ID: NA
Compound Number: D-Mana
MALDI File: CP_MSDB_4_Mana-C4-COOH_500.txt

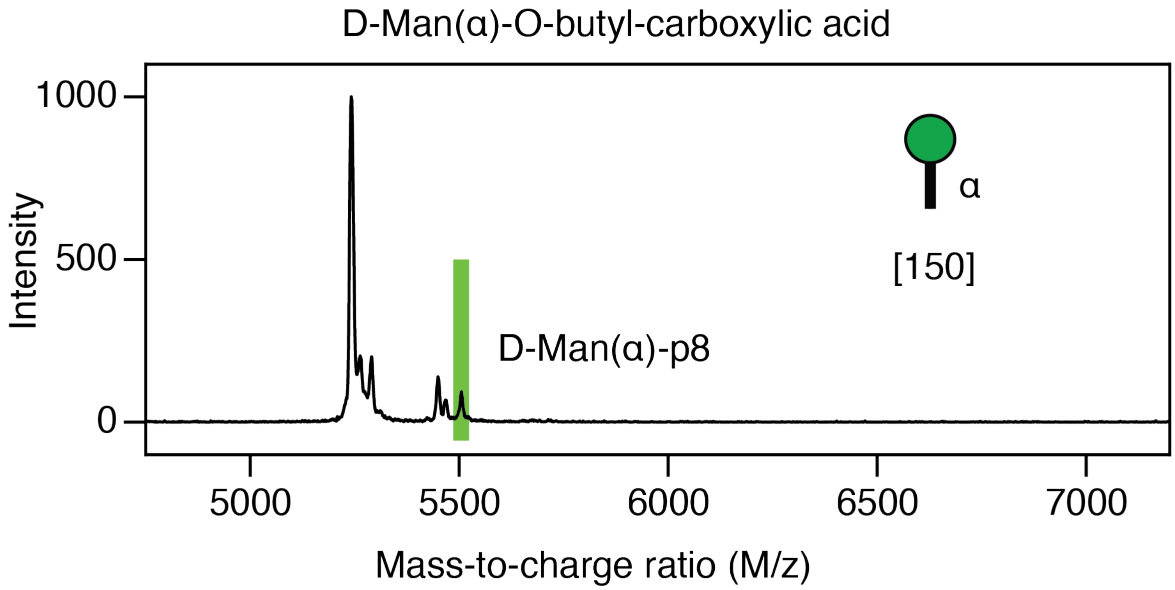

MALDI-TOF spectra of glycophage: D-Mana-C4 - [150]
MSDB number: CP_MSDB_3
Sequencing File: 20250716-87MOYooNA-EG
Barcode:
AAAAAACTGCTCTTTGCTATTCCCCTGGTGGTACCTTTCTATTCTCACTCGAGCGTGG
AAAAAAACGATCAGAAAACATACCATGCTGGCGGAGGA
Axis Name: D-Mana-C4 - [150]
IUPAC: D-Man(α)-O-butyl-carboxylic acid
Common Name: D-Mana-C4
Glytoucan ID: NA
Compound Number: D-Mana
MALDI File: CP_MSDB_3_Mana-C4-COOH_150.txt

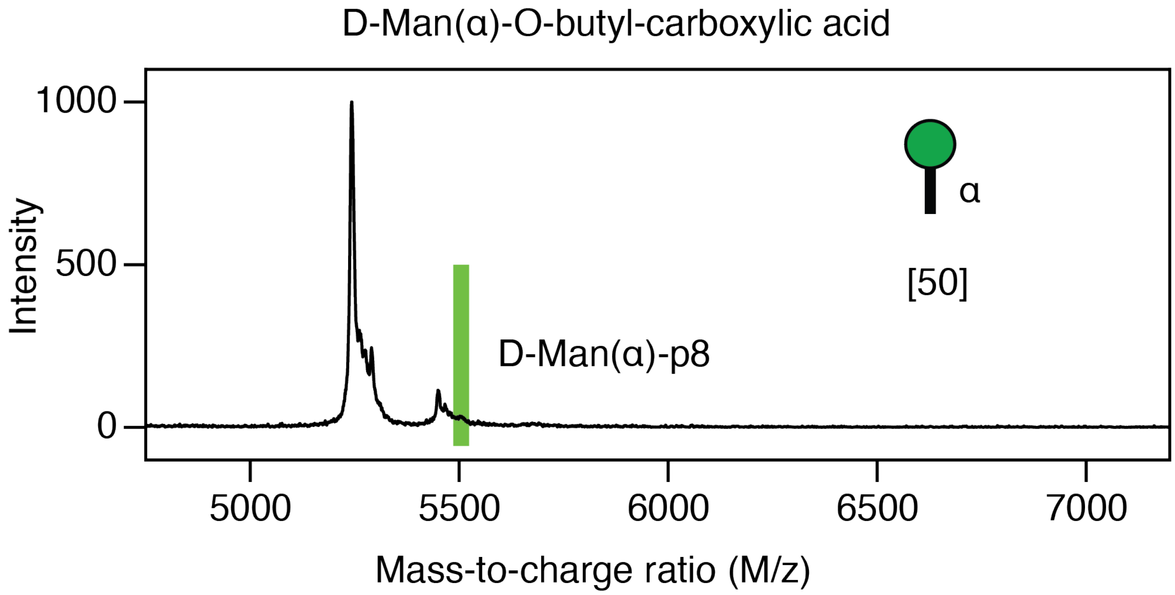

MALDI-TOF spectra of glycophage: D-Mana-C4 - [50]
MSDB number: CP_MSDB_2
Sequencing File: 20250716-87MOYooNA-EG
Barcode:
AAAAAACTACTTTTCGCCATTCCGCTAGTGGTACCTTTCTATTCTCACTCGAGTGTTGA
GAAAAATGATCAAAAGACTTATCACGCGGGCGGCGGA
Axis Name: D-Mana-C4 - [50]
IUPAC: D-Man(α)-O-butyl-carboxylic acid
Common Name: D-Mana-C4
Glytoucan ID: NA
Compound Number: D-Mana
MALDI File: CP_MSDB_2_Mana-C4-COOH_50.txt

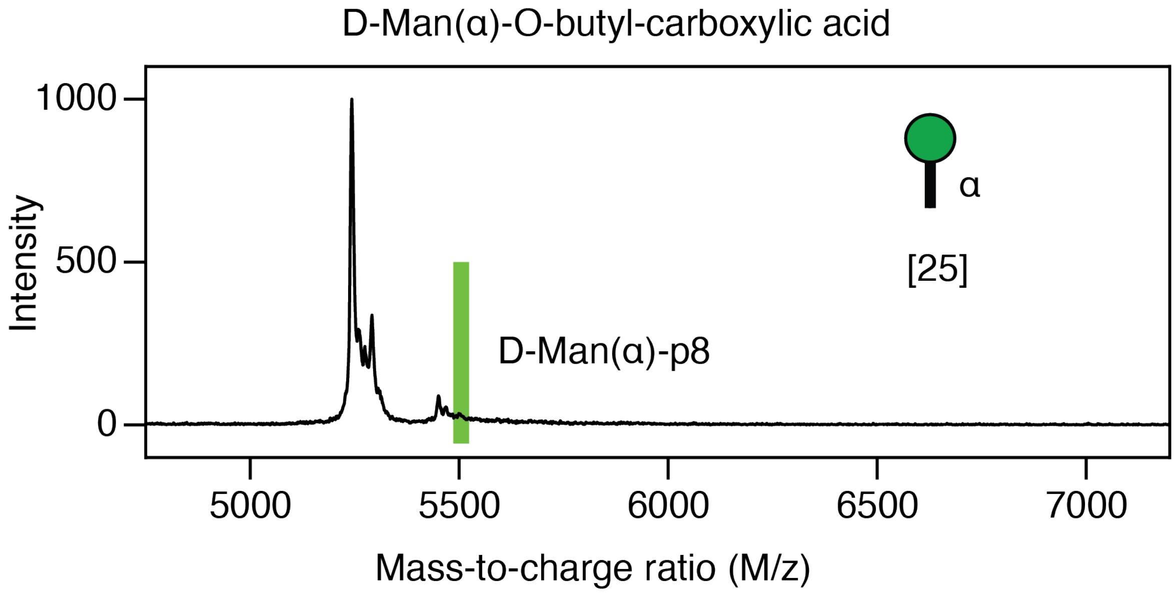

MALDI-TOF spectra of glycophage: D-Mana-C4 - [25]
MSDB number: CP_MSDB_1
Sequencing File: 20250716-87MOYooNA-EG
Barcode:
AAAAAACTTCTGTTCGCGATACCGCTGGTGGTACCTTTCTATTCTCACTCGAGCGTCG
AAAAAAATGACCAAAAGACTTATCATGCAGGGGGCGGA
Axis Name: D-Mana-C4 - [25]
IUPAC: D-Man(α)-O-butyl-carboxylic acid
Common Name: D-Mana-C4
Glytoucan ID: NA
Compound Number: D-Mana
MALDI File: CP_MSDB_1_Mana-C4-COOH_25.txt

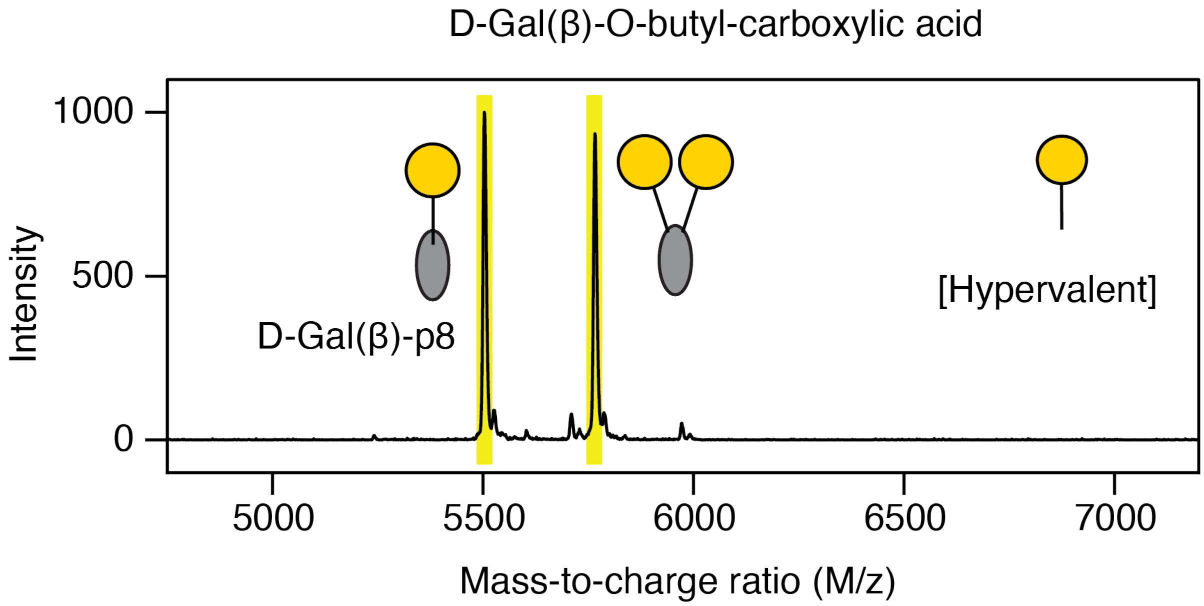

MALDI-TOF spectra of glycophage: D-Galb-C4-COOH - [MAX]
MSDB number: CP_MSDB_62
Sequencing File: 20250716-87MOYooNA-EG
Barcode:
AAAAAACTACTGTTTGCTATTCCTCTGGTGGTACCTTTCTATTCTCACTCGAGCGTAGA
GAAGAATGATCAAAAAACCTATCATGCAGGCGGCGGA
Axis Name: D-Galb-C4-COOH - [MAX]
IUPAC: D-Gal(β)-O-butyl-carboxylic acid
Common Name: D-Galb-C4
Glytoucan ID: NA
Compound Number: D-Gal-b
MALDI File: CP_MSDB_62_beta-D-Gal-C4-COOH_Max.txt

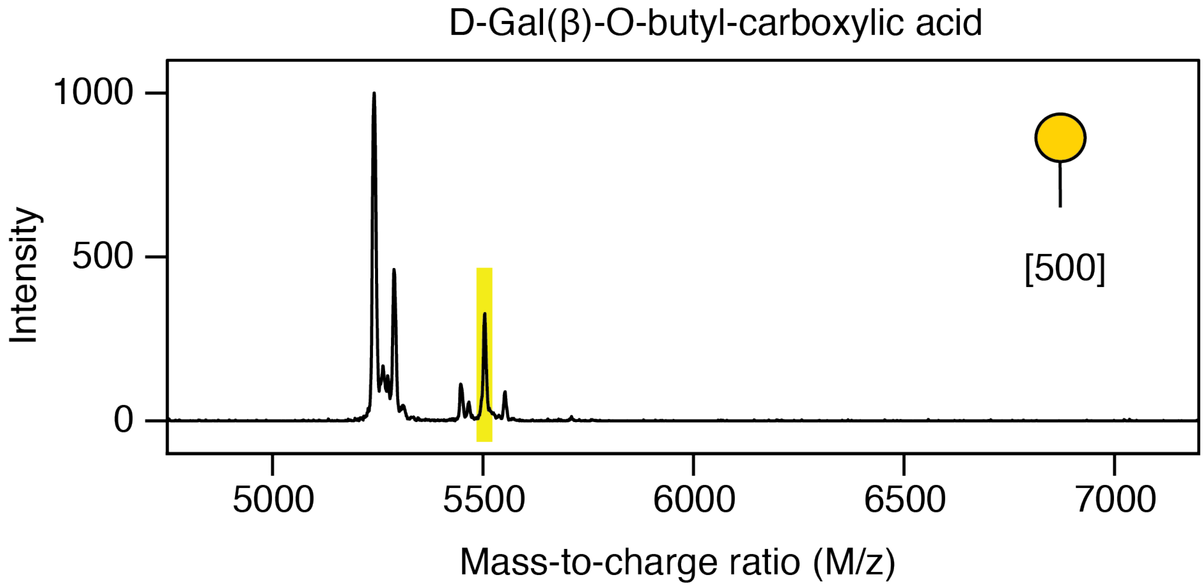

MALDI-TOF spectra of glycophage: D-Galb-C4-COOH - [500]
MSDB number: CP_MSDB_61
Sequencing File: 20250716-87MOYooNA-EG
Barcode:
AAAAAACTACTGTTCGCAATCCCGCTAGTGGTACCTTTCTATTCTCACTCGAGTGTCG
AAAAGAACGACCAAAAGACATATCATGCAGGCGGAGGT
Axis Name: D-Galb-C4-COOH - [500]
IUPAC: D-Gal(β)-O-butyl-carboxylic acid
Common Name: D-Galb-C4
Glytoucan ID: NA
Compound Number: D-Gal-b
MALDI File: CP_MSDB_61_beta-D-Gal-C4-COOH_0.4mM.txt

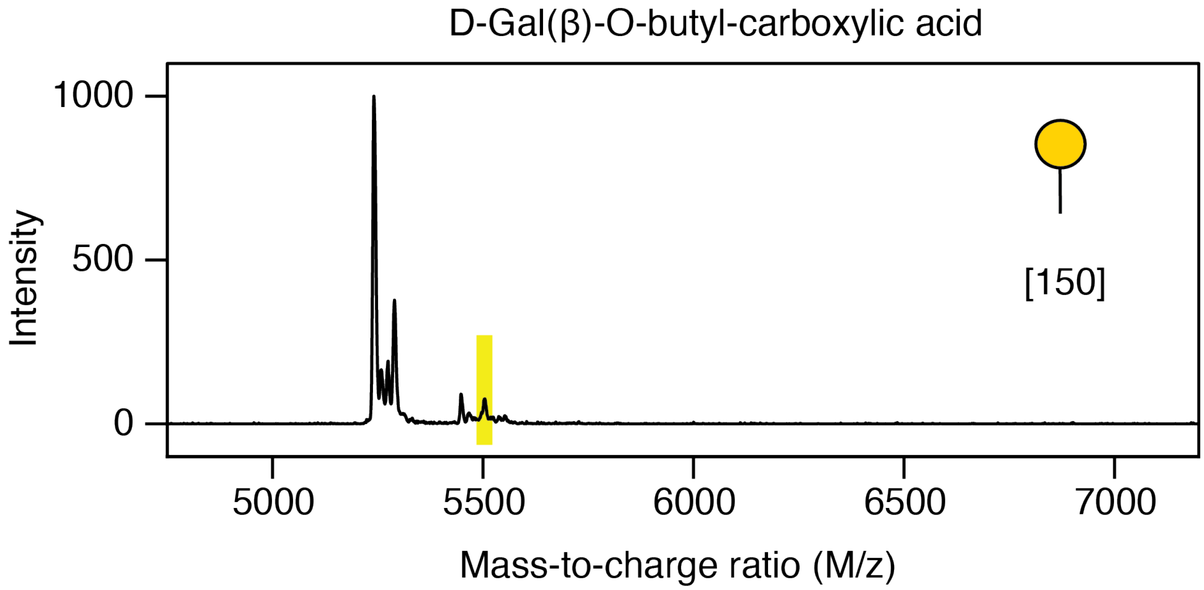

MALDI-TOF spectra of glycophage: D-Galb-C4-COOH - [150]
MSDB number: CP_MSDB_60
Sequencing File: 20250716-87MOYooNA-EG
Barcode:
AAAAAACTACTATTTGCGATCCCCCTAGTGGTACCTTTCTATTCTCACTCGAGCGTGGA
AAAAAACGATCAAAAGACGTACCATGCAGGAGGAGGC
Axis Name: D-Galb-C4-COOH - [150]
IUPAC: D-Gal(β)-O-butyl-carboxylic acid
Common Name: D-Galb-C4
Glytoucan ID: NA
Compound Number: D-Gal-b
MALDI File: CP_MSDB_60_beta-D-Gal-C4-COOH_0.12mM.txt

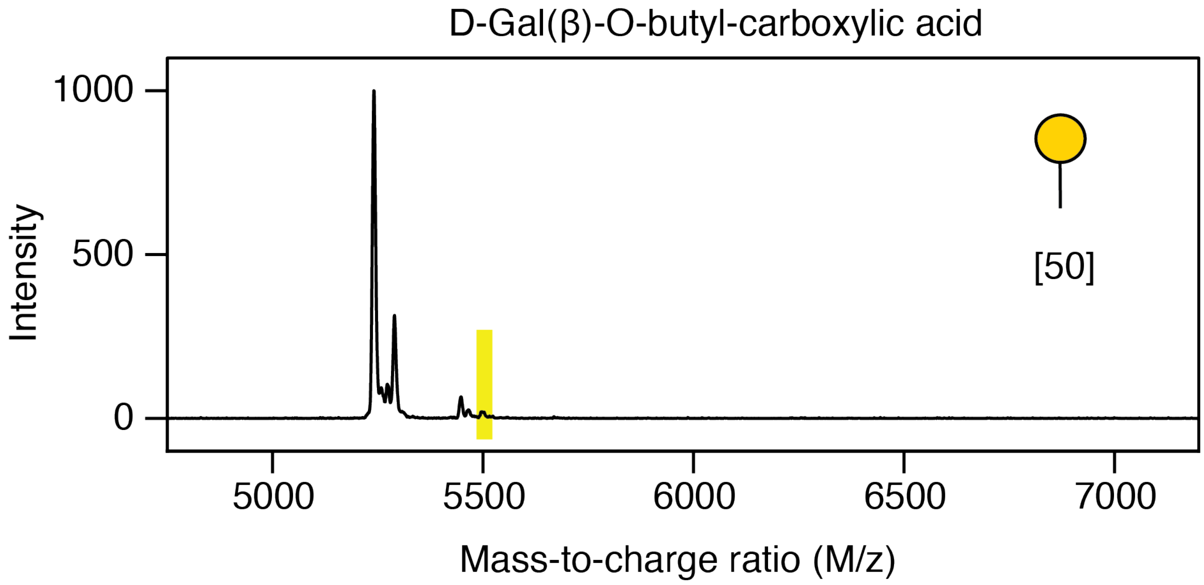

MALDI-TOF spectra of glycophage: D-Galb-C4-COOH - [50]
MSDB number: CP_MSDB_59
Sequencing File: 20250716-87MOYooNA-EG
Barcode:
AAAAAACTACTGTTTGCCATTCCCCTGGTGGTACCTTTCTATTCTCACTCGAGTGTTGA
GAAGAACGATCAGAAGACCTATCACGCAGGGGGCGGG
Axis Name: D-Galb-C4-COOH - [50]
IUPAC: D-Gal(β)-O-butyl-carboxylic acid
Common Name: D-Galb-C4
Glytoucan ID: NA
Compound Number: D-Gal-b
MALDI File: CP_MSDB_59_beta-D-Gal-C4-COOH_0.04mM.txt

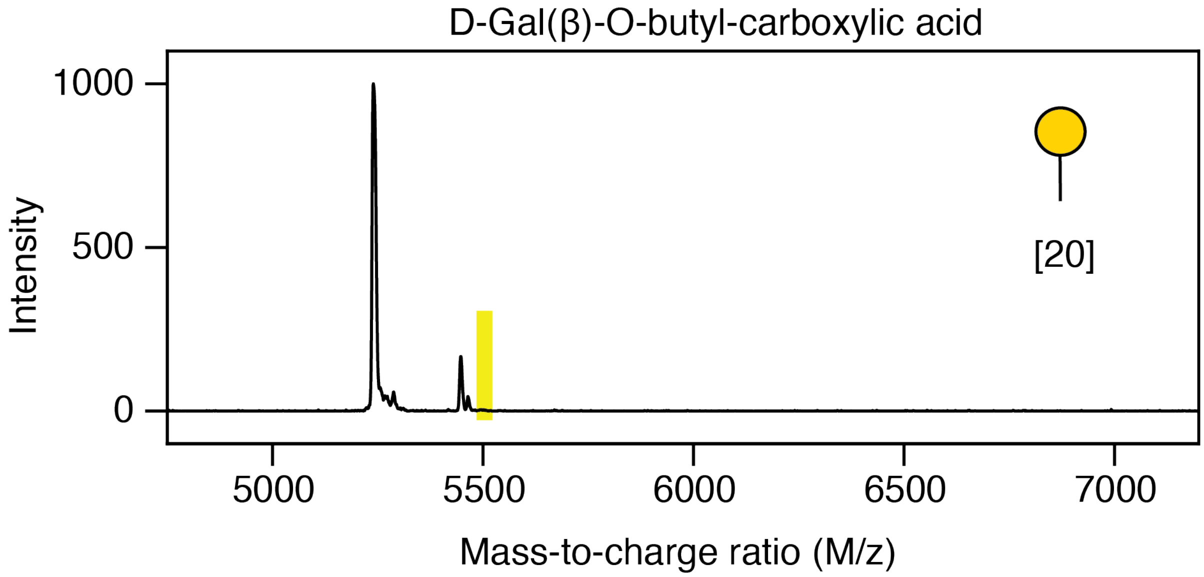

MALDI-TOF spectra of glycophage: D-Galb-C4-COOH - [20]
MSDB number: CP_MSDB_58
Sequencing File: 20250716-87MOYooNA-EG
Barcode:
AAAAAACTACTGTTCGCGATCCCACTGGTGGTACCTTTCTATTCTCACTCGAGTGTGG
AAAAAAACGATCAGAAAACGTACCATGCTGGTGGCGGG
Axis Name: D-Galb-C4-COOH - [20]
IUPAC: D-Gal(β)-O-butyl-carboxylic acid
Common Name: D-Galb-C4
Glytoucan ID: NA
Compound Number: D-Gal-b
MALDI File: CP_MSDB_58_beta-D-Gal-C4-COOH_0.016mM.txt

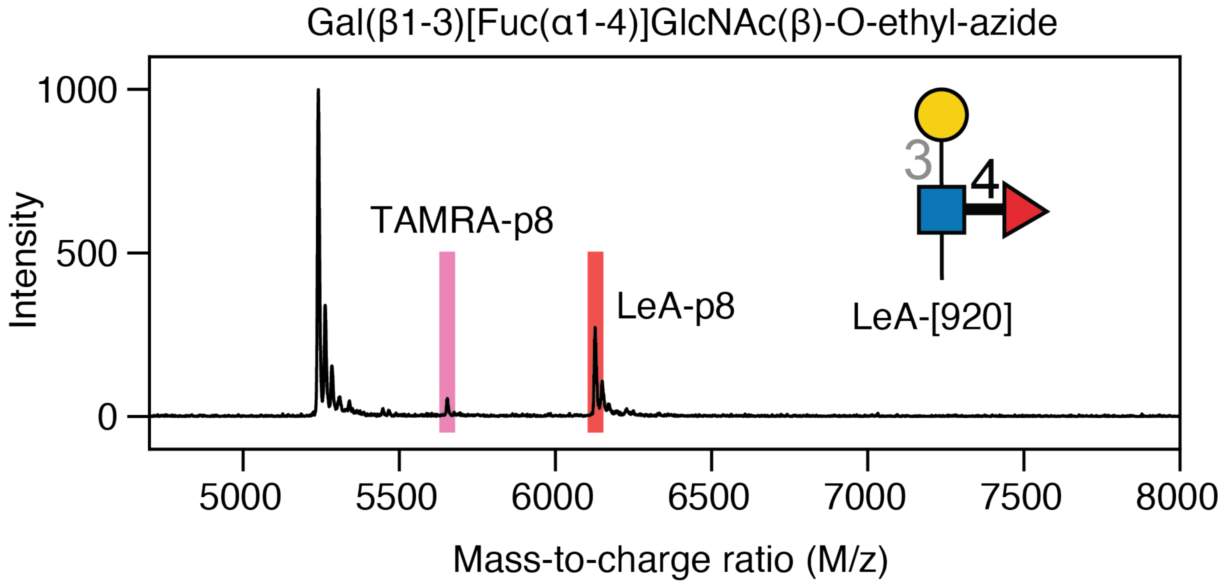

MALDI-TOF spectra of glycophage: LeA-Sp - [920]
SDB number: SDB114
Fluorescent Labeling condition: Labelled in 1X Borate buffer (pH 8.0) with 0.1 mM TAMRA-NHS ester at room temperature for 3 hours.
IUPAC: Gal(β1-3)[Fuc(α1-4)]GlcNAc(β)-O-ethyl-azide
MALDI File: LeA-920_0.1 mM TAMRA-NHS_3 h.txt
Used in: Lectin Microarray Hybridization

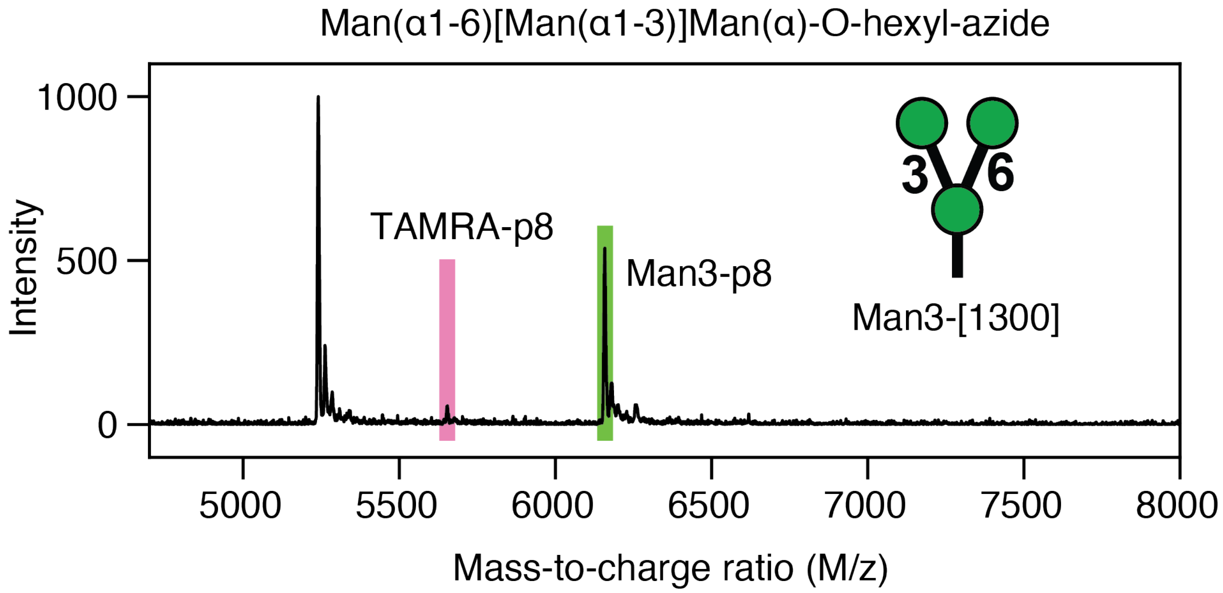

MALDI-TOF spectra of glycophage: (Man)3-S6 - [1300]
SDB number: SDB121
Fluorescent Labeling condition: Labelled in 1X Borate buffer (pH 8.0) with 0.1 mM TAMRA-NHS ester at room temperature for 3 hours.
IUPAC: Man(α1-6)[Man(α1-3)]Man(α)-O-hexyl-azide
MALDI File: Man3-1300_0.1 mM TAMRA-NHS_3 h.txt
Used in: Lectin Microarray Hybridization

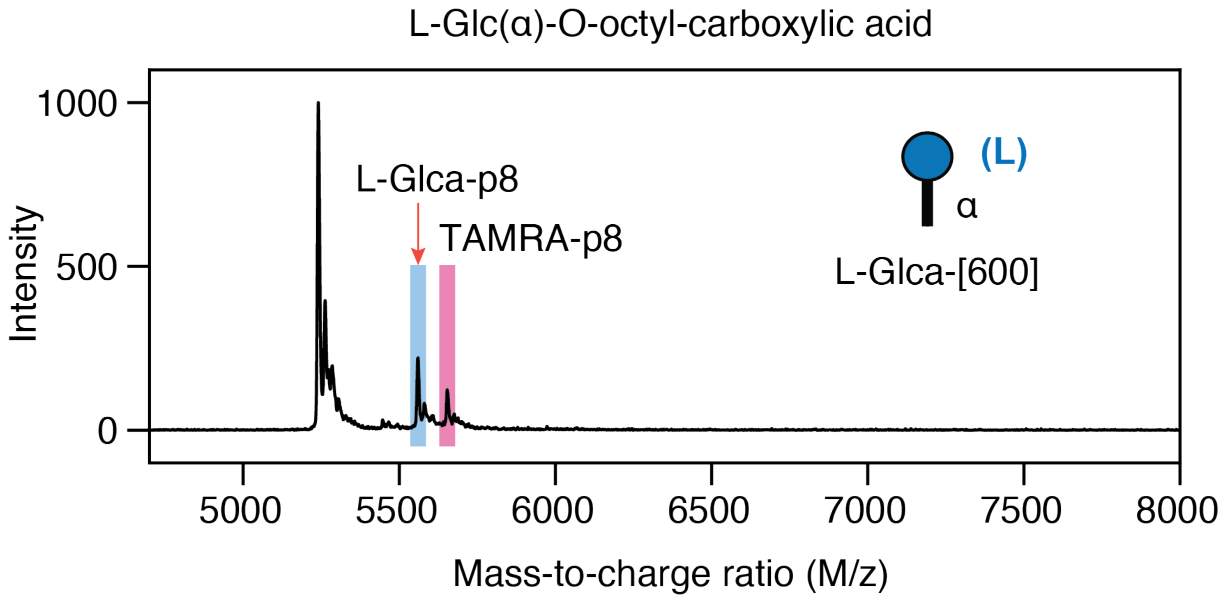

MALDI-TOF spectra of glycophage: L-Glca-C8-COOH - [600]
SDB number: CP_170
Fluorescent Labeling condition: Labelled in 1X Borate buffer (pH 8.0) with 0.1 mM TAMRA-NHS ester at room temperature for 3 hours.
IUPAC: L-Glc(α)-O-octyl-carboxylic acid
MALDI File: CP_170_L-Glc-alpha-600_0.1 mM TAMRA-NHS_3 h.txt
Used in: Lectin Microarray Hybridization

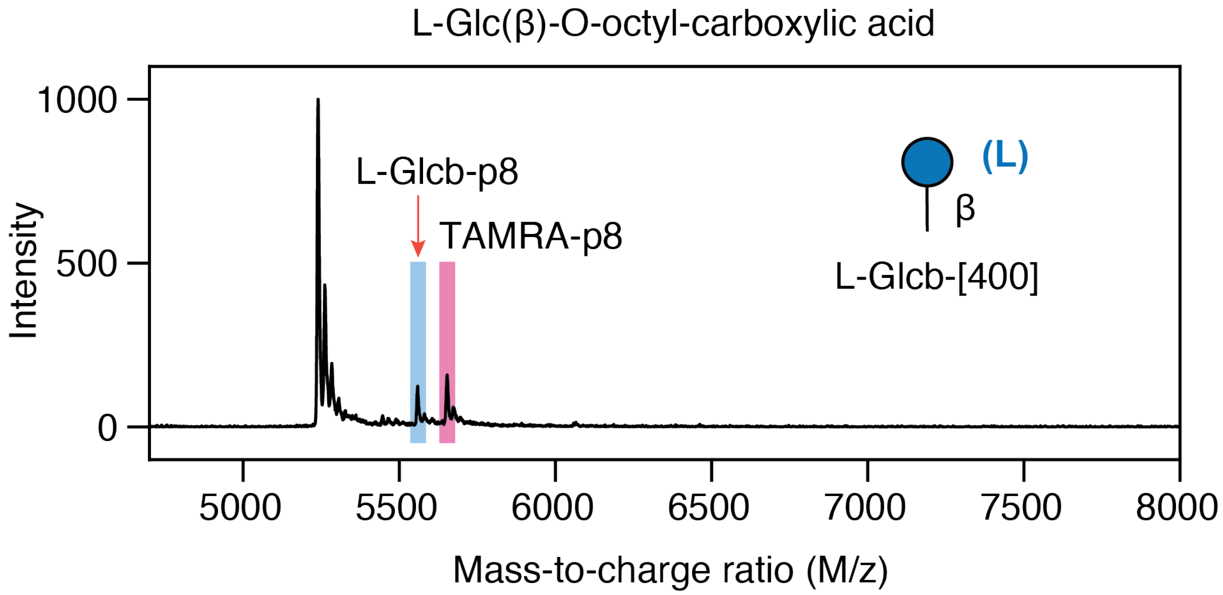

MALDI-TOF spectra of glycophage: L-Glcb-C8-COOH - [400]
SDB number: CP_171
Fluorescent Labeling condition: Labelled in 1X Borate buffer (pH 8.0) with 0.1 mM TAMRA-NHS ester at room temperature for 3 hours.
IUPAC: L-Glc(β)-O-octyl-carboxylic acid
MALDI File: CP_171_L-Glc-beta-400_0.1 mM TAMRA-NHS_3 h.txt
Used in: Lectin Microarray Hybridization

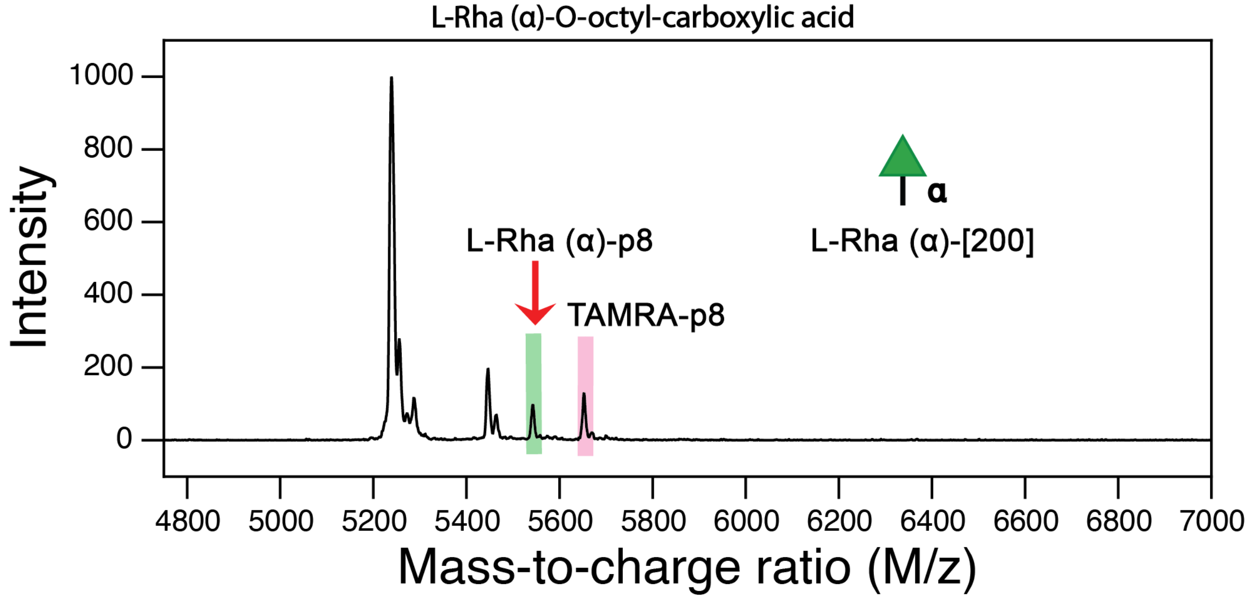

MALDI-TOF spectra of glycophage: L-Rha(a)-C8-COOH - [200]
SDB number: TA_132
Fluorescent Labeling condition: Labelled in 1X Borate buffer (pH 8.0) with 0.1 mM TAMRA-NHS ester at room temperature for 3 hours.
IUPAC: L-Rha (α)-O-octyl-carboxylic acid
MALDI File: TA_132_L-Rha-alpha-200_0.1 mM TAMRA-NHS_3 h.txt
Used in: Lectin Microarray Hybridization

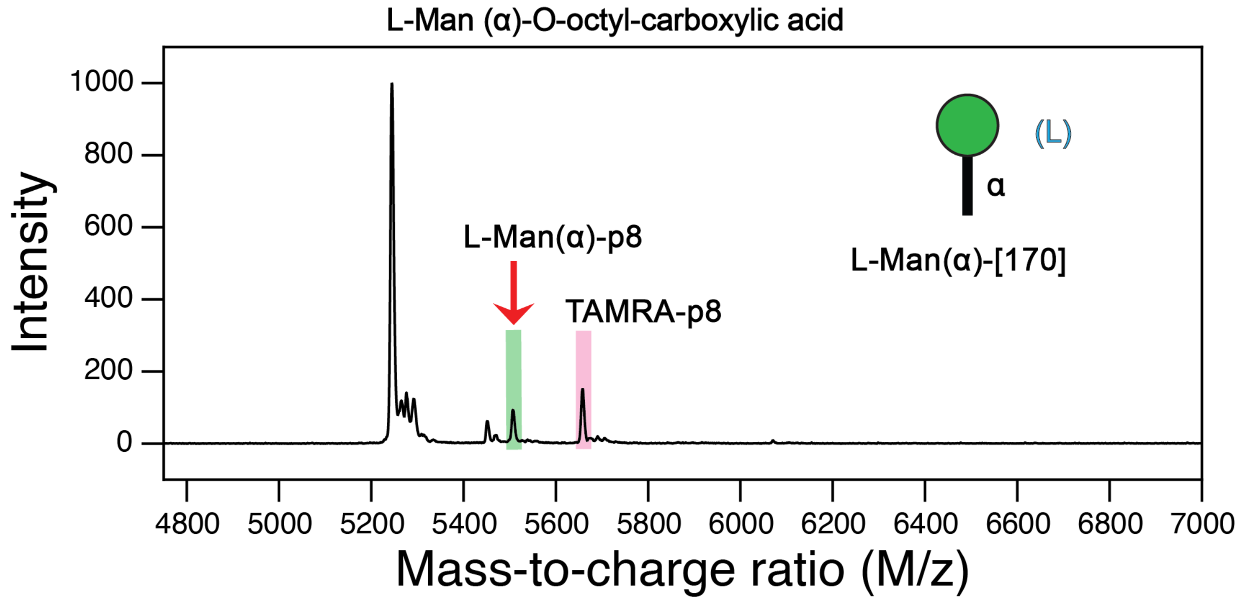

MALDI-TOF spectra of glycophage: L-Man(a)-C8-COOH - [170]
SDB number: TA_133
Fluorescent Labeling condition: Labelled in 1X Borate buffer (pH 8.0) with 0.1 mM TAMRA-NHS ester at room temperature for 3 hours.
IUPAC: L-Man (α)-O-octyl-carboxylic acid
MALDI File: TA_133_L-Man-alpha-170_0.1 mM TAMRA-NHS_3 h.txt
Used in: Lectin Microarray Hybridization

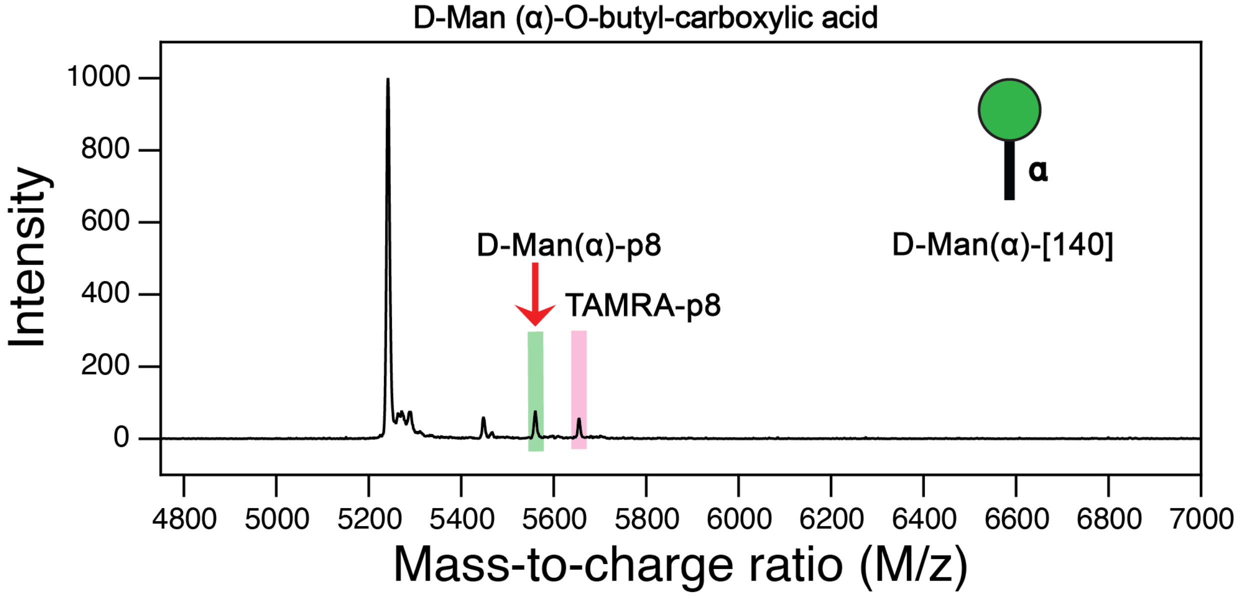

MALDI-TOF spectra of glycophage: D-Man(a)-C4-COOH - [140]
SDB number: TA_134
Fluorescent Labeling condition: Labelled in 1X Borate buffer (pH 8.0) with 0.1 mM TAMRA-NHS ester at room temperature for 3 hours.
IUPAC: D-Man (α)-O-butyl-carboxylic acid
MALDI File: TA_134_D-Man-alpha-140_0.1 mM TAMRA-NHS_3 h.txt
Used in: Lectin Microarray Hybridization

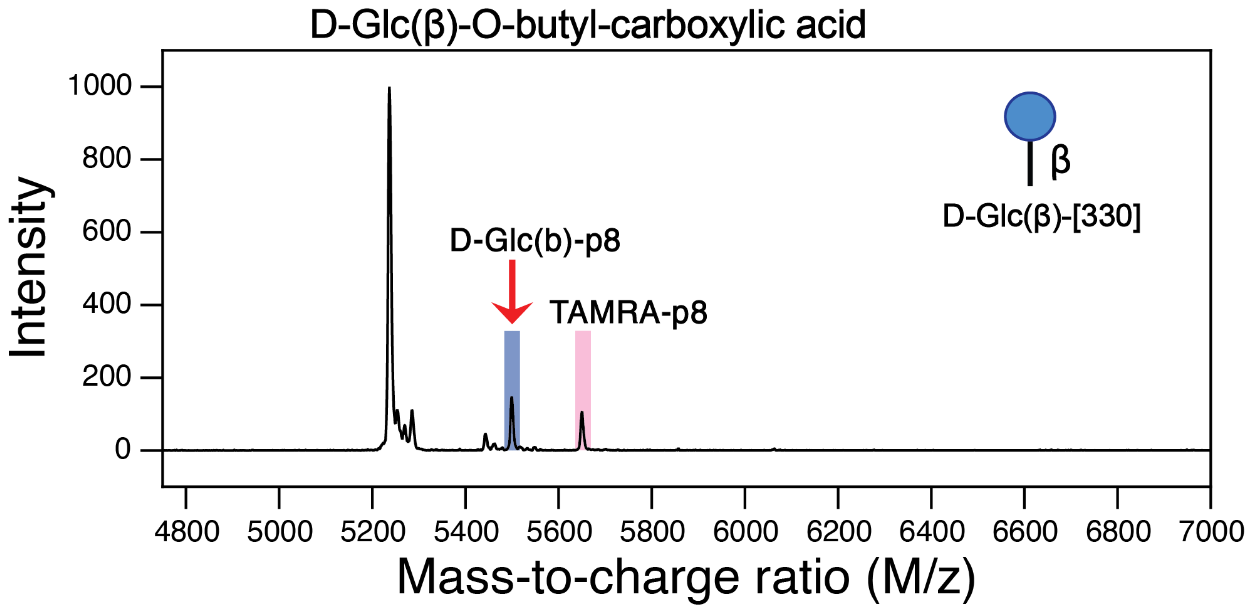

MALDI-TOF spectra of glycophage: D-Glcb-C4-COOH - [300]
SDB number: TA_135
Fluorescent Labeling condition: Labelled in 1X Borate buffer (pH 8.0) with 0.1 mM TAMRA-NHS ester at room temperature for 3 hours.
IUPAC: D-Glc(β)-O-butyl-carboxylic acid
MALDI File: TA_135_D-Glc-beta-330_0.1 mM TAMRA-NHS_3 h.txt
Used in: Lectin Microarray Hybridization

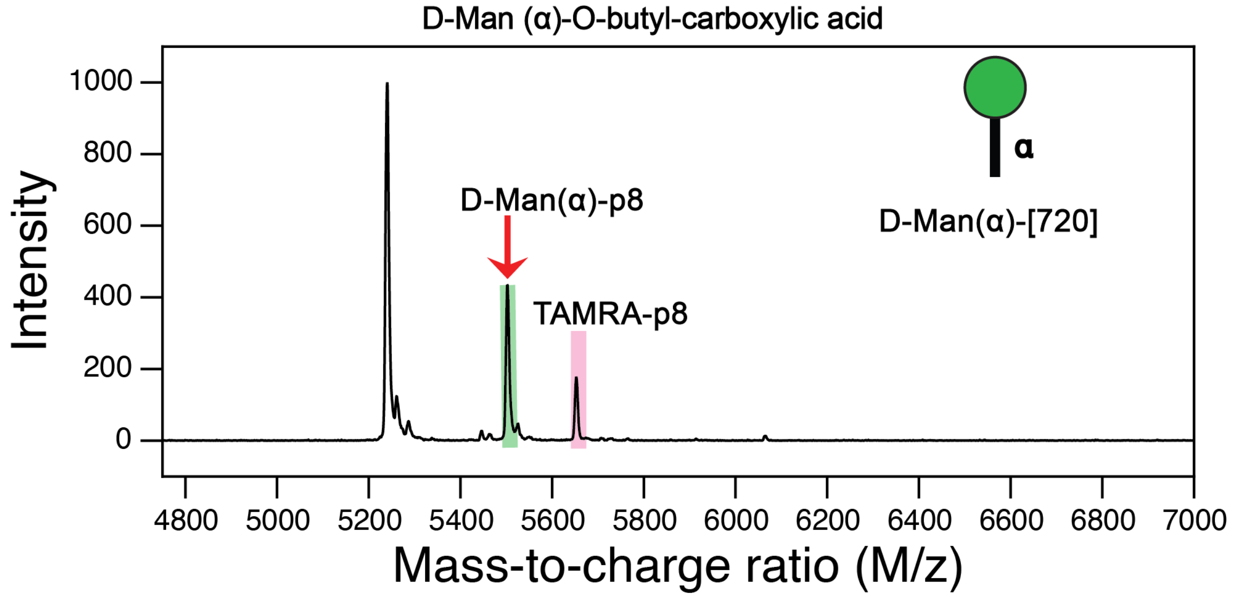

MALDI-TOF spectra of glycophage: D-Man(a)-C4-COOH - [720]
SDB number: SDB1
Fluorescent Labeling condition: Labelled in 1X Borate buffer (pH 8.0) with 0.1 mM TAMRA-NHS ester at room temperature for 3 hours.
IUPAC: D-Man (α)-O-butyl-carboxylic acid
MALDI File: SDB1_D-Man-alpha-720_0.1 mM TAMRA-NHS_3 h.txt
Used in: Lectin Microarray Hybridization

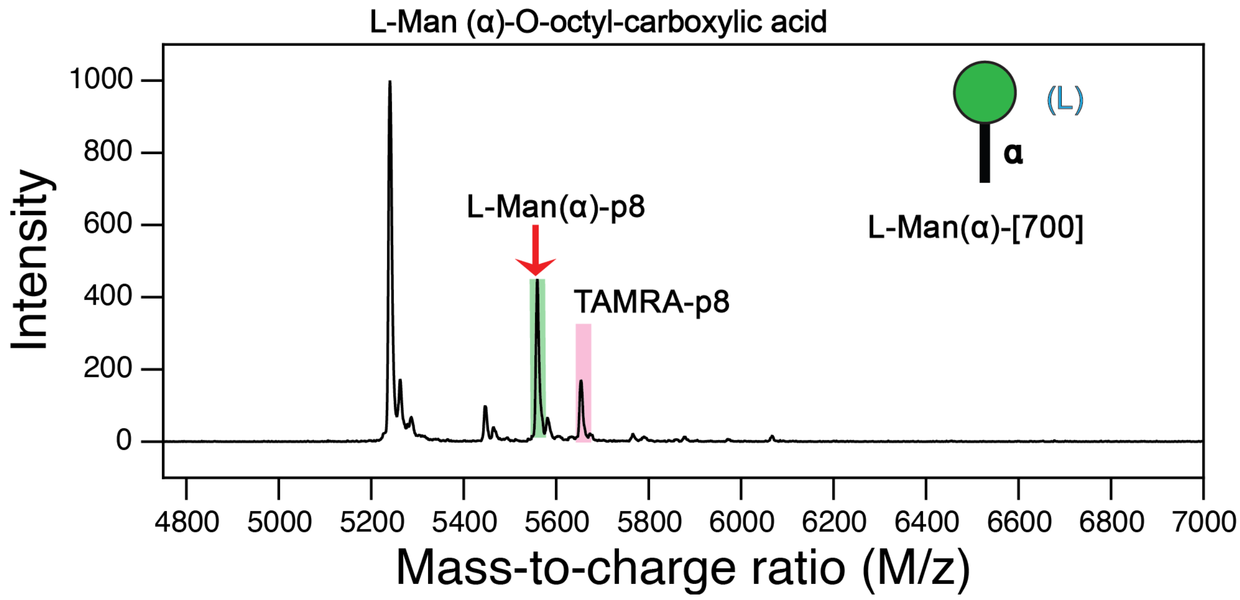

MALDI-TOF spectra of glycophage: L-Man(a)-C8-COOH - [700]
SDB number: SDB1
Fluorescent Labeling condition: Labelled in 1X Borate buffer (pH 8.0) with 0.1 mM TAMRA-NHS ester at room temperature for 3 hours.
IUPAC: L-Man (α)-O-octyl-carboxylic acid
MALDI File: SDB1_L-Man-alpha-700_0.1 mM TAMRA-NHS_3 h.txt
Used in: Lectin Microarray Hybridization

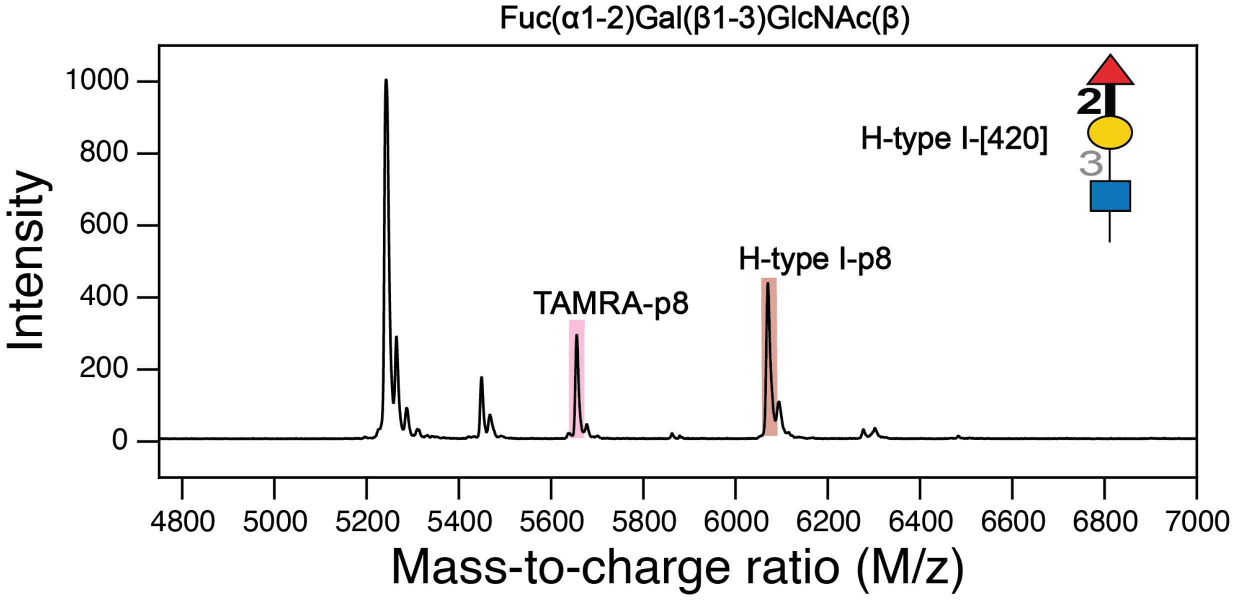

MALDI-TOF spectra of glycophage: H-type I-C8-COOH - [420]
SDB number: SDB1
Fluorescent Labeling condition: Labelled in 1X Borate buffer (pH 8.0) with 0.1 mM TAMRA-NHS ester at room temperature for 3 hours.
IUPAC: Fuc(α1-2)Gal(β1-3)GlcNAc(β)-O-octyl-carboxylic acid
MALDI File: SDB1_H-type-I-420_0.1 mM TAMRA-NHS_3 h.txt
Used in: Lectin Microarray Hybridization

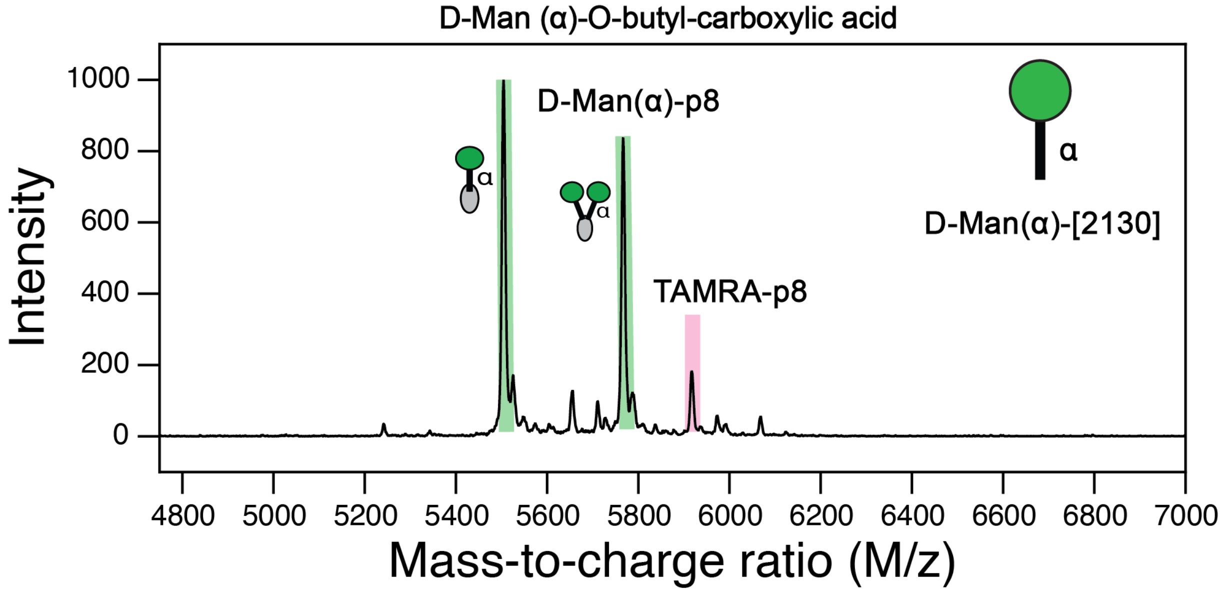

MALDI-TOF spectra of glycophage: D-Man(a)-C4-COOH - [2130]
SDB number: SDB1
Fluorescent Labeling condition: Labelled in 1X Borate buffer (pH 8.0) with 0.1 mM TAMRA-NHS ester at room temperature for 3 hours.
IUPAC: D-Man (α)-O-butyl-carboxylic acid
MALDI File: SBD1_D-Man-alpha-2130_0.1 mM TAMRA-NHS_3 h.txt
Used in: Lectin Microarray Hybridization

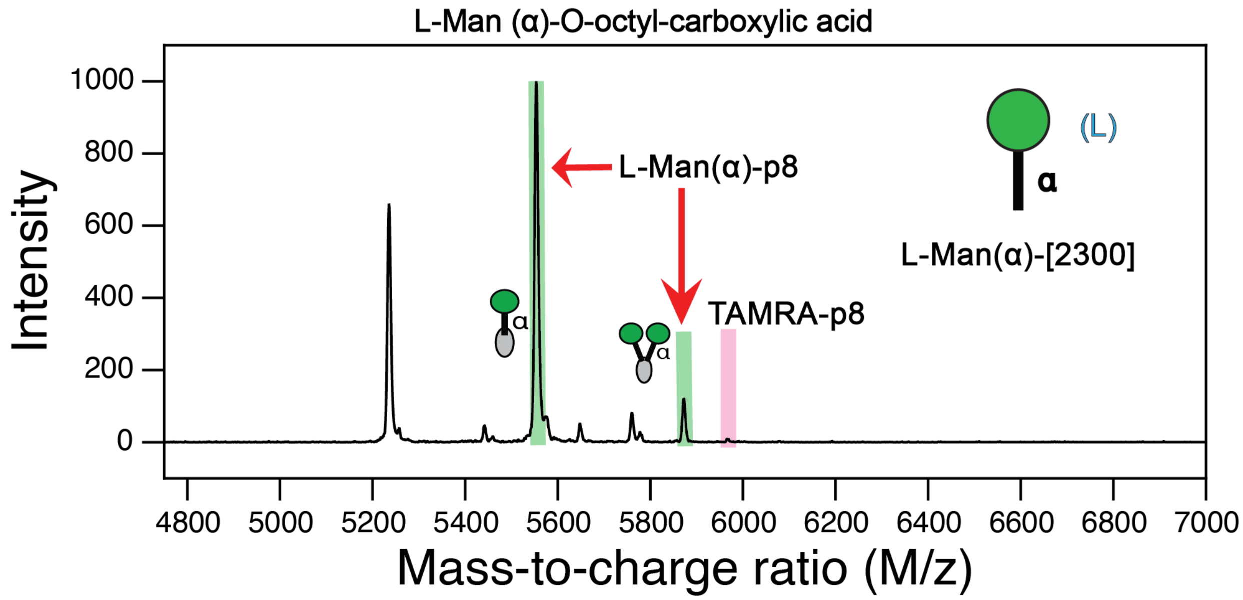

MALDI-TOF spectra of glycophage: L-Man(a)-C8-COOH - [2300]
SDB number: SDB1
Fluorescent Labeling condition: Labelled in 1X Borate buffer (pH 8.0) with 0.1 mM TAMRA-NHS ester at room temperature for 3 hours.
IUPAC: L-Man (α)-O-octyl-carboxylic acid
MALDI File: SDB1_L-Man-alpha-2300_0.1 mM TAMRA-NHS_3 h.txt
Used in: Lectin Microarray Hybridization

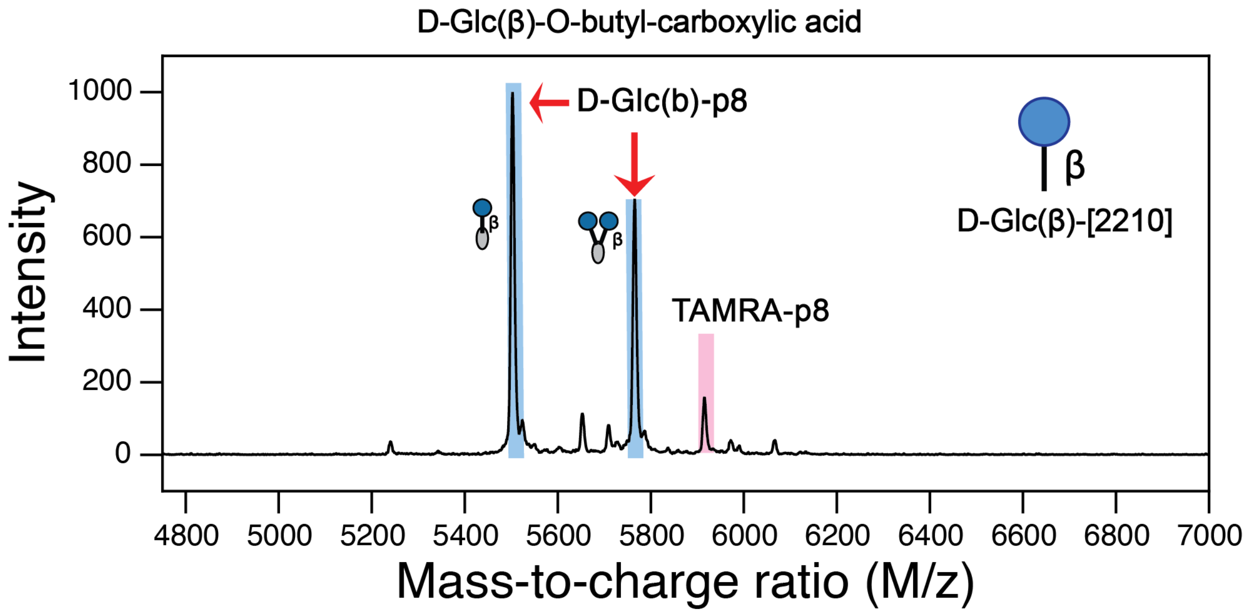

MALDI-TOF spectra of glycophage: D-Glcb-C4-COOH - [2210]
SDB number: SDB1
Fluorescent Labeling condition: Labelled in 1X Borate buffer (pH 8.0) with 0.1 mM TAMRA-NHS ester at room temperature for 3 hours.
IUPAC: D-Glc(β)-O-butyl-carboxylic acid
MALDI File: SDB1_D-Glc-beta-2210_0.1 mM TAMRA-NHS_3 h.txt
Used in: Lectin Microarray Hybridization

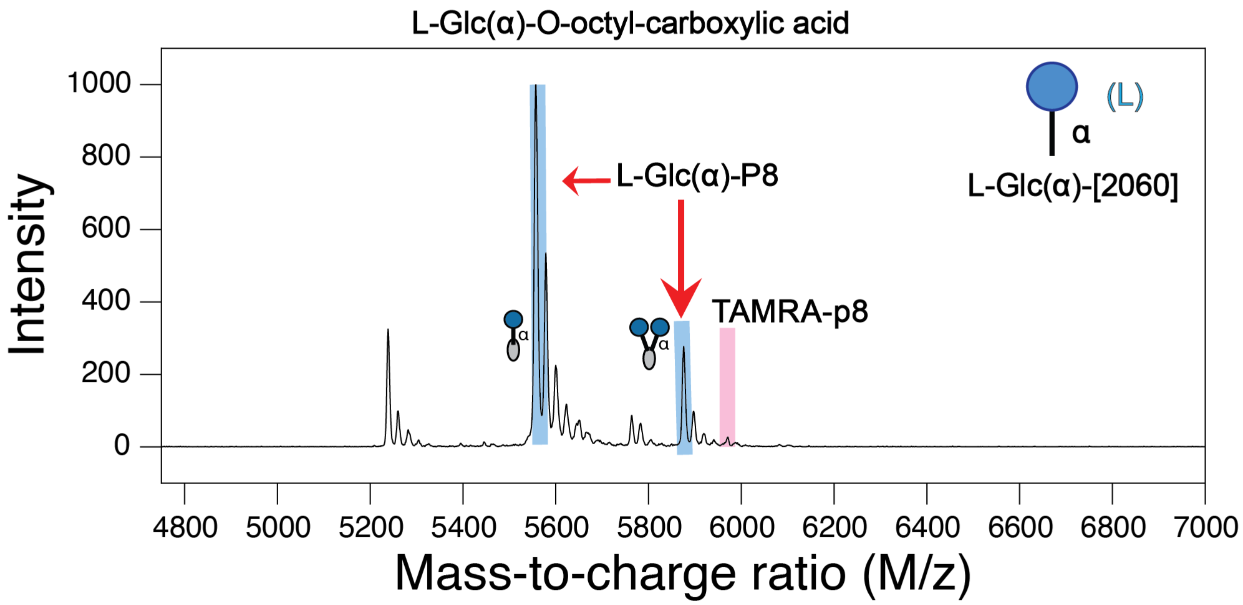

MALDI-TOF spectra of glycophage: L-Glca-C8-COOH - [2060]
SDB number: SDB1
Fluorescent Labeling condition: Labelled in 1X Borate buffer (pH 8.0) with 0.1 mM TAMRA-NHS ester at room temperature for 3 hours.
IUPAC: L-Glc(α)-O-octyl-carboxylic acid
MALDI File: SDB1_L-Glc-beta-2060_0.1 mM TAMRA-NHS_3 h.txt
Used in: Lectin Microarray Hybridization

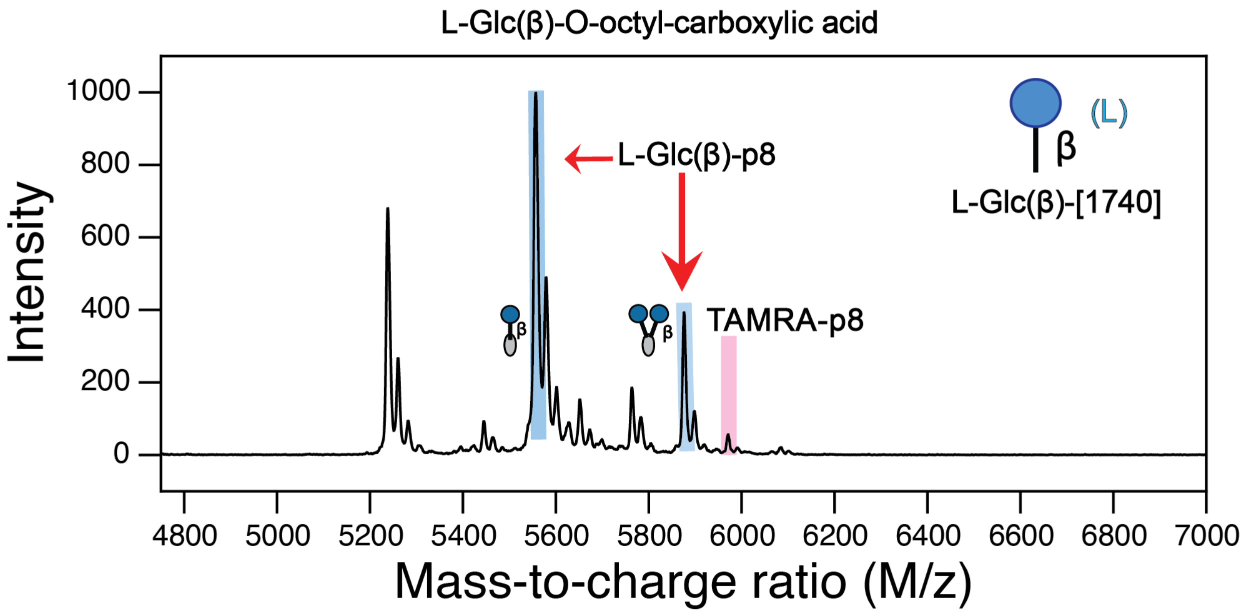

MALDI-TOF spectra of glycophage: L-GlcB-C8-COOH - [1740]
SDB number: SDB1
Fluorescent Labeling condition: Labelled in 1X Borate buffer (pH 8.0) with 0.1 mM TAMRA-NHS ester at room temperature for 3 hours.
IUPAC: L-Glc(β)-O-octyl-carboxylic acid
MALDI File: SDB1_L-Glc-beta-1740_0.1 mM TAMRA-NHS_3 h.txt
Used in: Lectin Microarray Hybridization

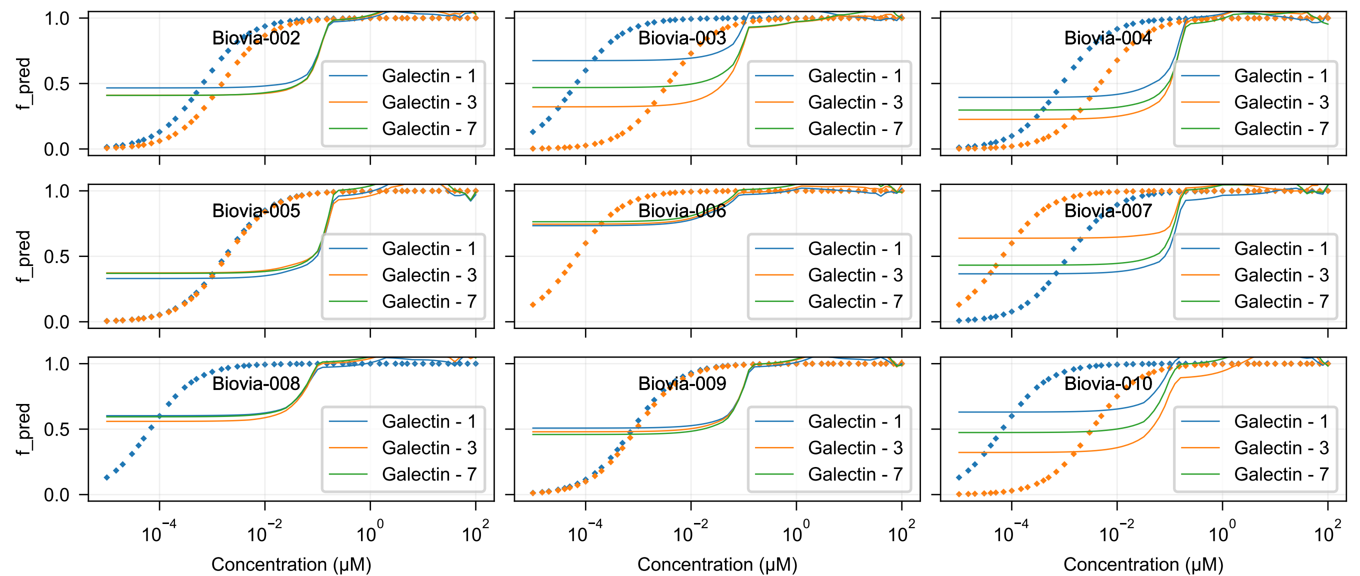

f_pred
1.0
0.5
0.0
Biovia-002
Biovia-003
Biovia-004
Biovia-005
Biovia-006
Biovia-007
Biovia-008
Biovia-009
Biovia-010
Galectin - 1
Galectin - 3
Galectin - 7
10^{-4}
10^{-2}
10^{0}
10^{2}
Concentration (μM)

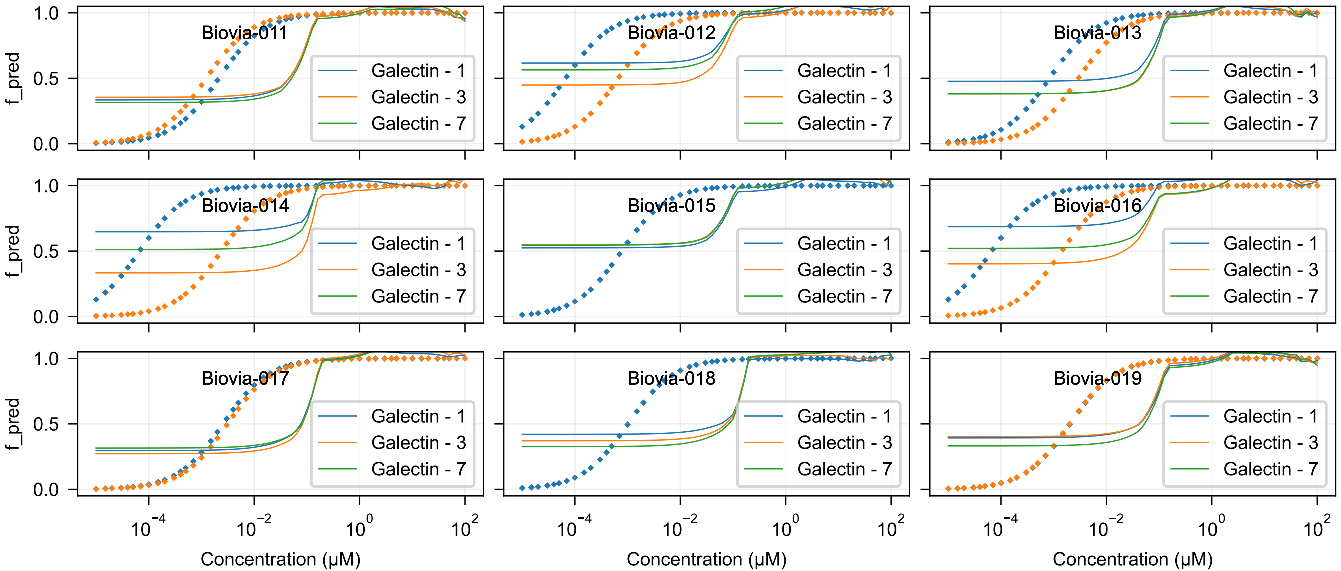

Biovia-011
Biovia-012
Biovia-013
Biovia-014
Biovia-015
Biovia-016
Biovia-017
Biovia-018
Biovia-019
Galectin - 1
Galectin - 3
Galectin - 7
f_pred
0.0
0.5
1.0
10^{-4}
10^{-2}
10^{0}
10^{2}
Concentration (μM)

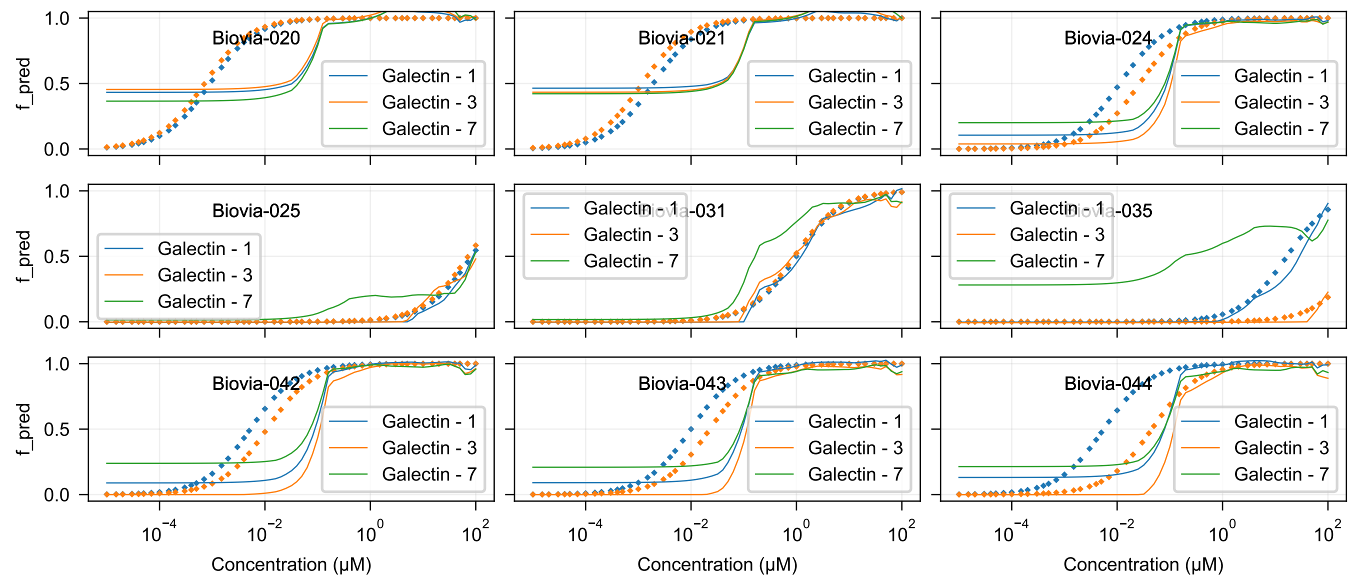
Biovia-020
Biovia-021
Biovia-024
Biovia-025
Biovia-031
Biovia-035
Biovia-042
Biovia-043
Biovia-044
Galectin - 1
Galectin - 3
Galectin - 7
f_pred
Concentration (μM)
0.0
0.5
1.0
10^{-4}
10^{-2}
10^{0}
10^{2}

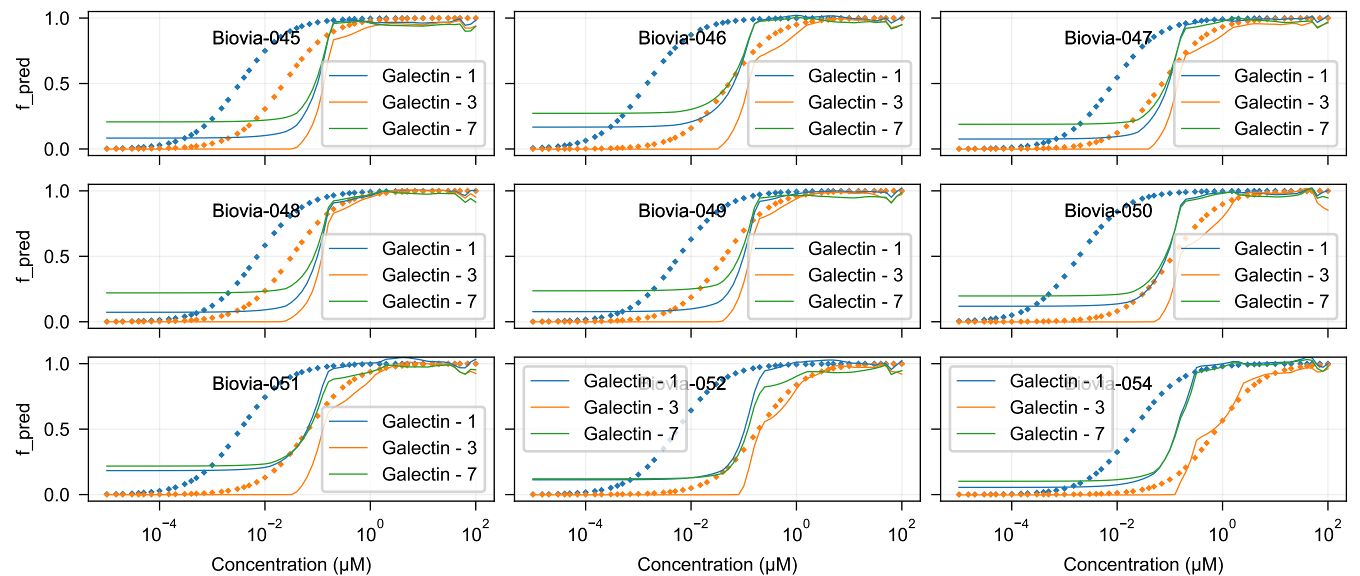
Biovia-045
Biovia-046
Biovia-047
Biovia-048
Biovia-049
Biovia-050
Biovia-051
Biovia-052
Biovia-054
Galectin - 1
Galectin - 3
Galectin - 7
f_pred
Concentration (μM)

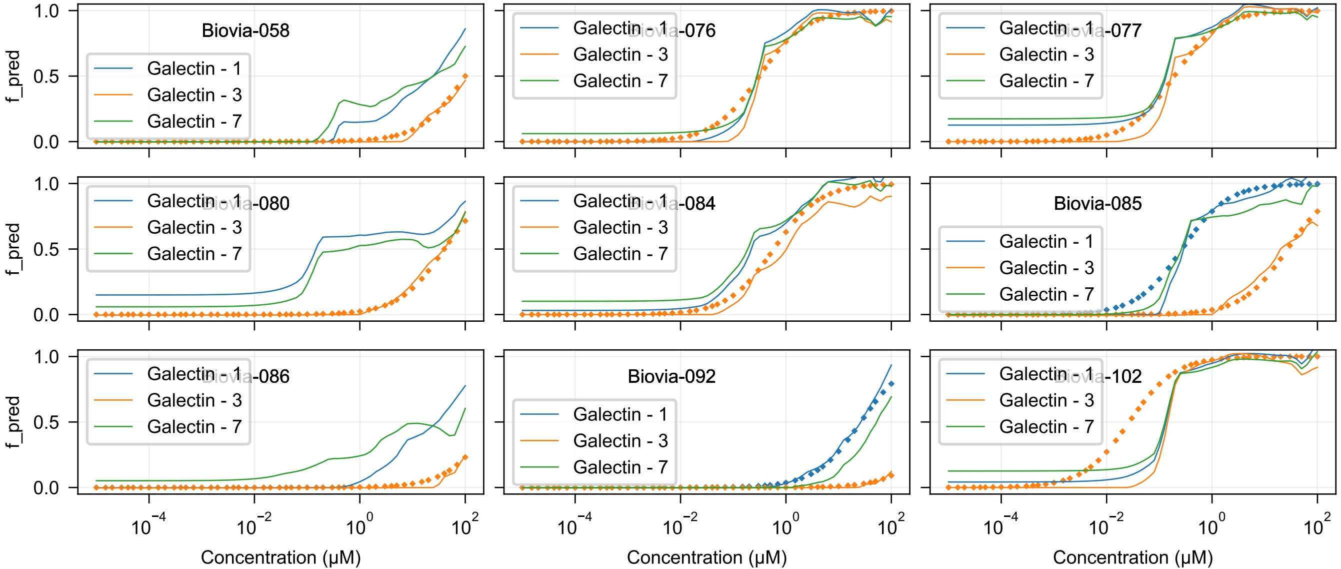
Biovia-058
Biovia-076
Biovia-077
Biovia-080
Biovia-084
Biovia-085
Biovia-086
Biovia-092
Biovia-102
Galectin - 1
Galectin - 3
Galectin - 7
f_pred
Concentration (μM)

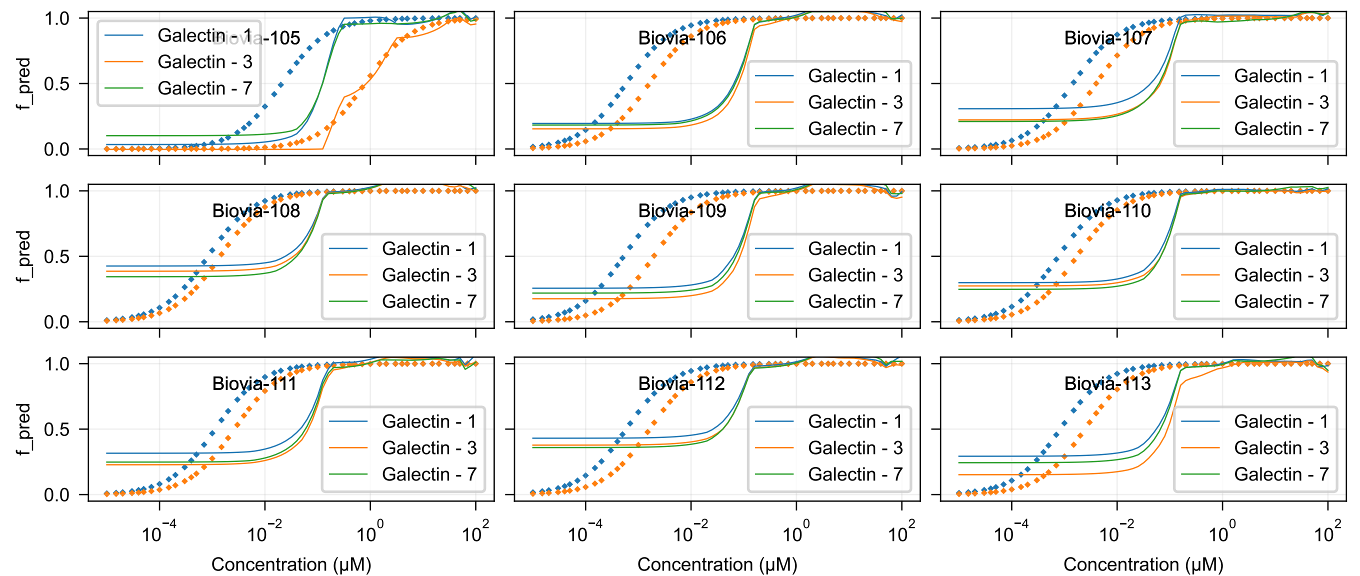

Biovia-105
Biovia-106
Biovia-107
Biovia-108
Biovia-109
Biovia-110
Biovia-111
Biovia-112
Biovia-113
Galectin - 1
Galectin - 3
Galectin - 7
f_pred
Concentration (μM)

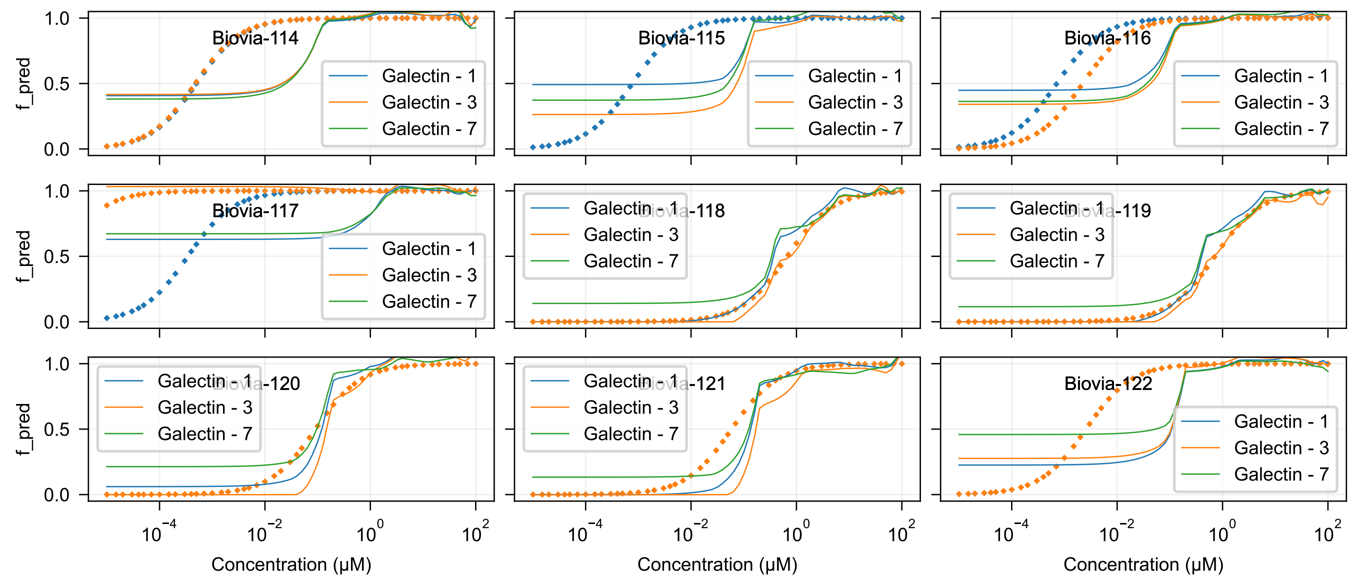
Biovia-114
Biovia-115
Biovia-116
Biovia-117
Biovia-118
Biovia-119
Biovia-120
Biovia-121
Biovia-122
Galectin - 1
Galectin - 3
Galectin - 7
f_pred
1.0
0.5
0.0
10^{-4}
10^{-2}
10^{0}
10^{2}
Concentration (μM)

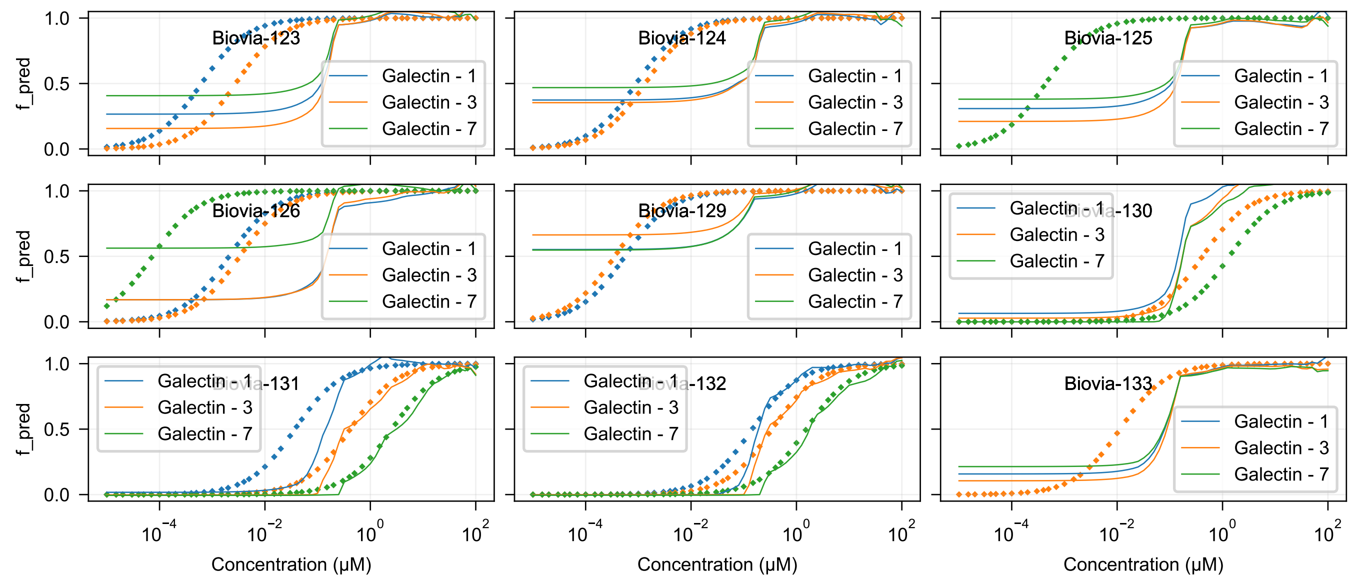

Biovia-123
Biovia-124
Biovia-125
Biovia-126
Biovia-129
Biovia-130
Biovia-131
Biovia-132
Biovia-133
Galectin - 1
Galectin - 3
Galectin - 7
f_pred
Concentration (μM)

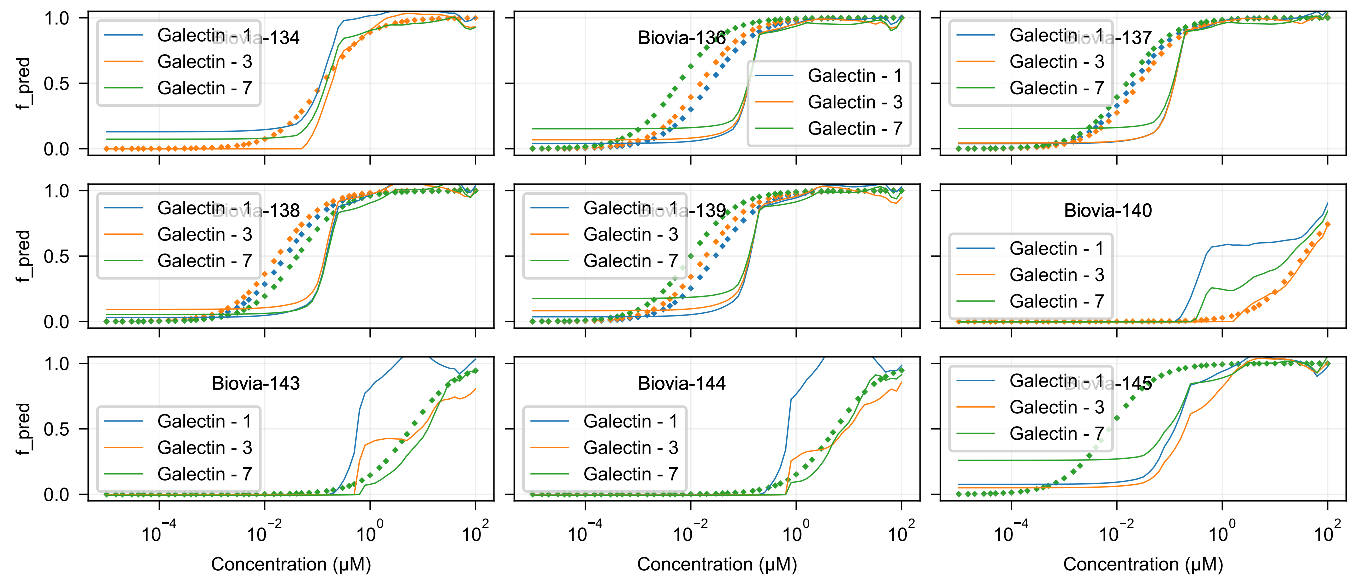

Biovia-134
Biovia-136
Biovia-137
Biovia-138
Biovia-139
Biovia-140
Biovia-143
Biovia-144
Biovia-145
Galectin - 1
Galectin - 3
Galectin - 7
f_pred
Concentration (μM)

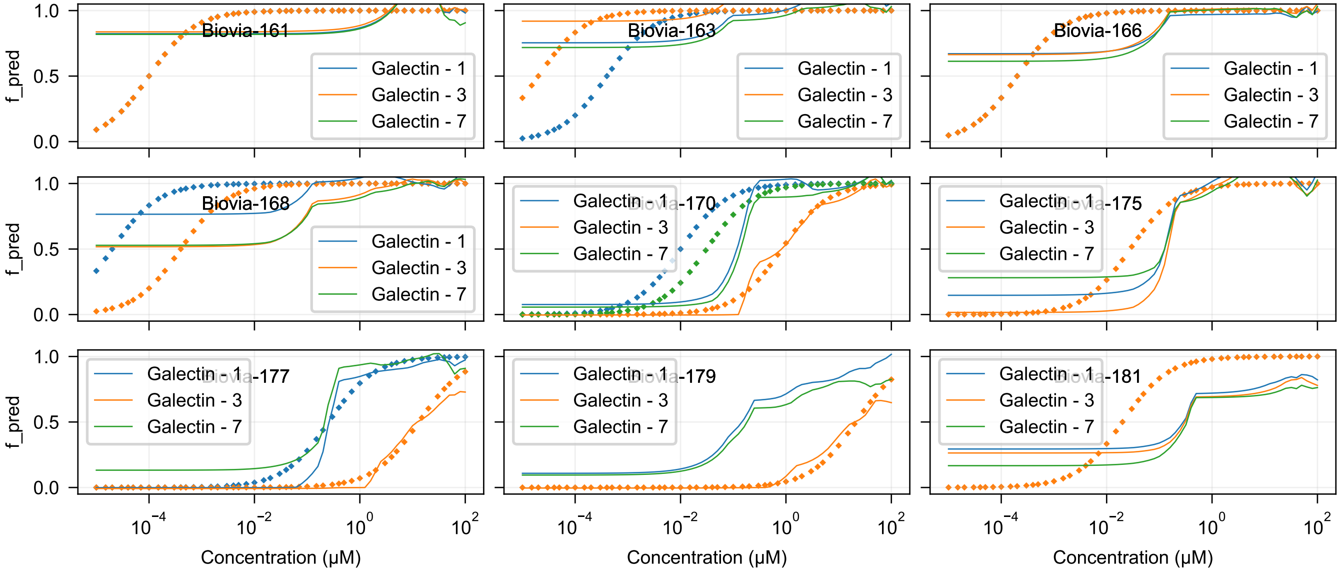
Biovia-161
Biovia-163
Biovia-166
Biovia-168
Biovia-170
Biovia-175
Biovia-177
Biovia-179
Biovia-181
Galectin - 1
Galectin - 3
Galectin - 7
f_pred
1.0
0.5
0.0
10^{-4}
10^{-2}
10^{0}
10^{2}
Concentration (μM)

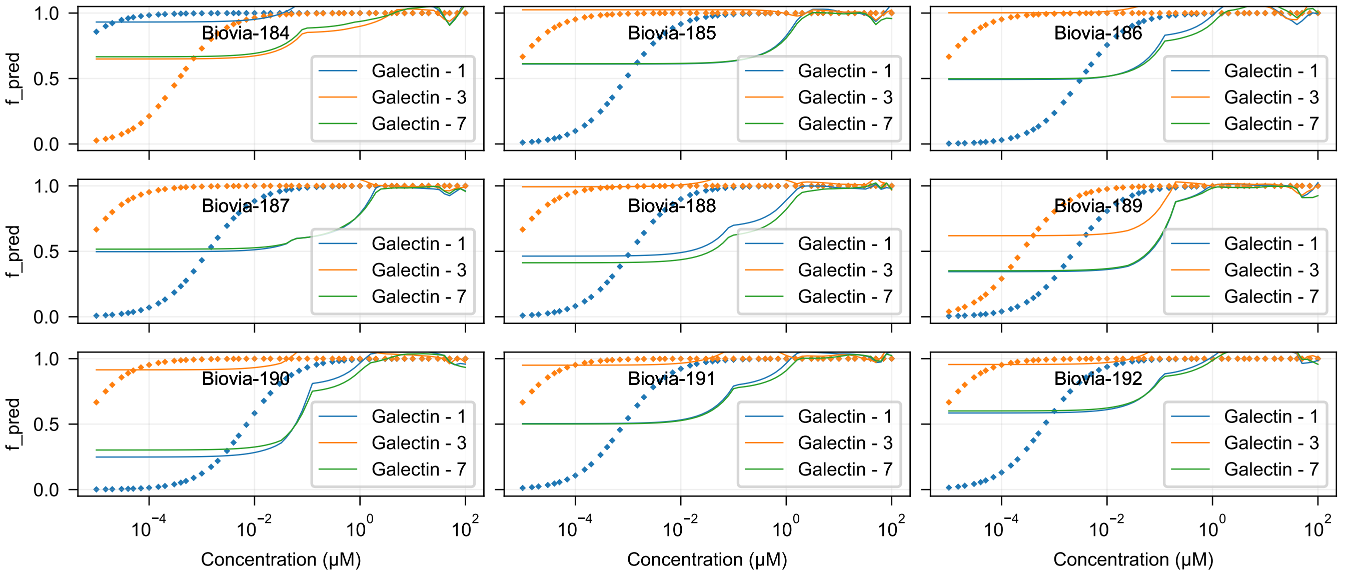

Biovia-184
Biovia-185
Biovia-186
Biovia-187
Biovia-188
Biovia-189
Biovia-190
Biovia-191
Biovia-192
Galectin - 1
Galectin - 3
Galectin - 7
f_pred
Concentration (μM)

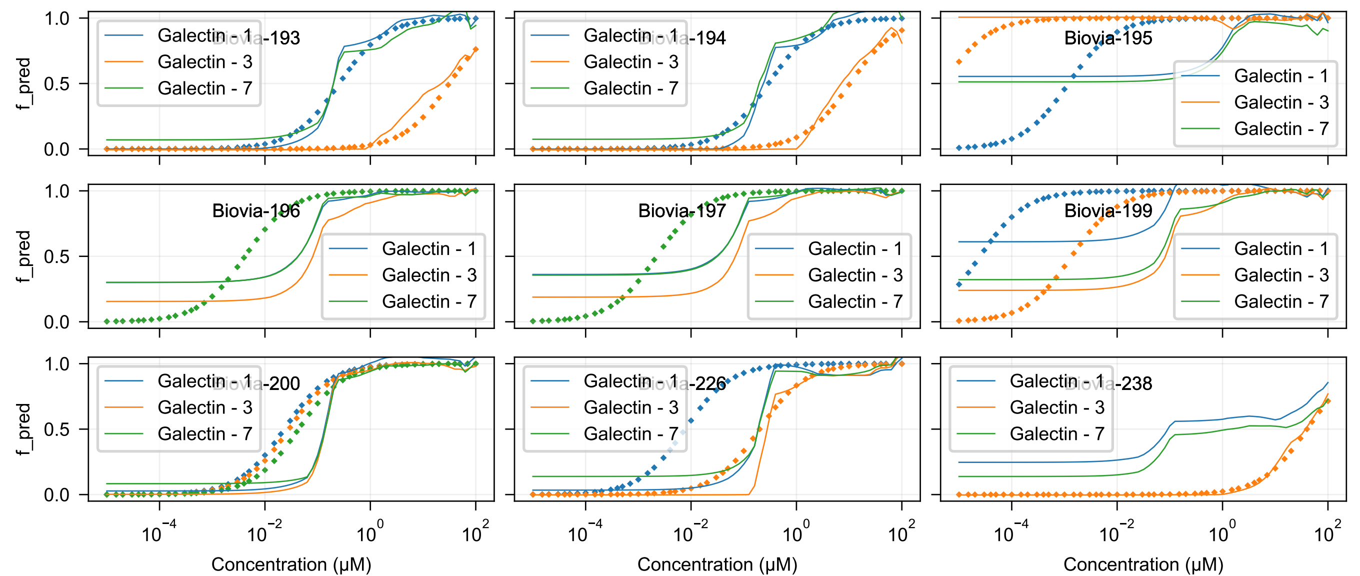

Biovia-193
Biovia-194
Biovia-195
Biovia-196
Biovia-197
Biovia-199
Biovia-200
Biovia-226
Biovia-238
Galectin - 1
Galectin - 3
Galectin - 7
f_pred
Concentration (μM)

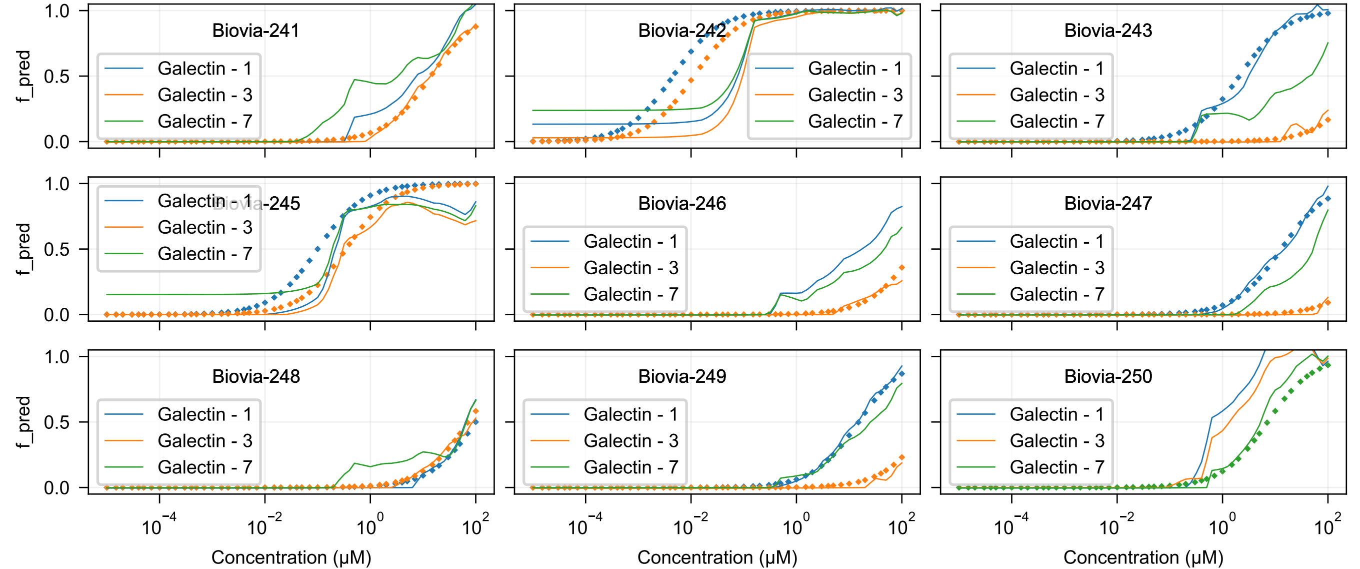
Biovia-241
Biovia-242
Biovia-243
Biovia-245
Biovia-246
Biovia-247
Biovia-248
Biovia-249
Biovia-250
Galectin - 1
Galectin - 3
Galectin - 7
f_pred
1.0
0.5
0.0
10^{-4}
10^{-2}
10^{0}
10^{2}
Concentration (μM)

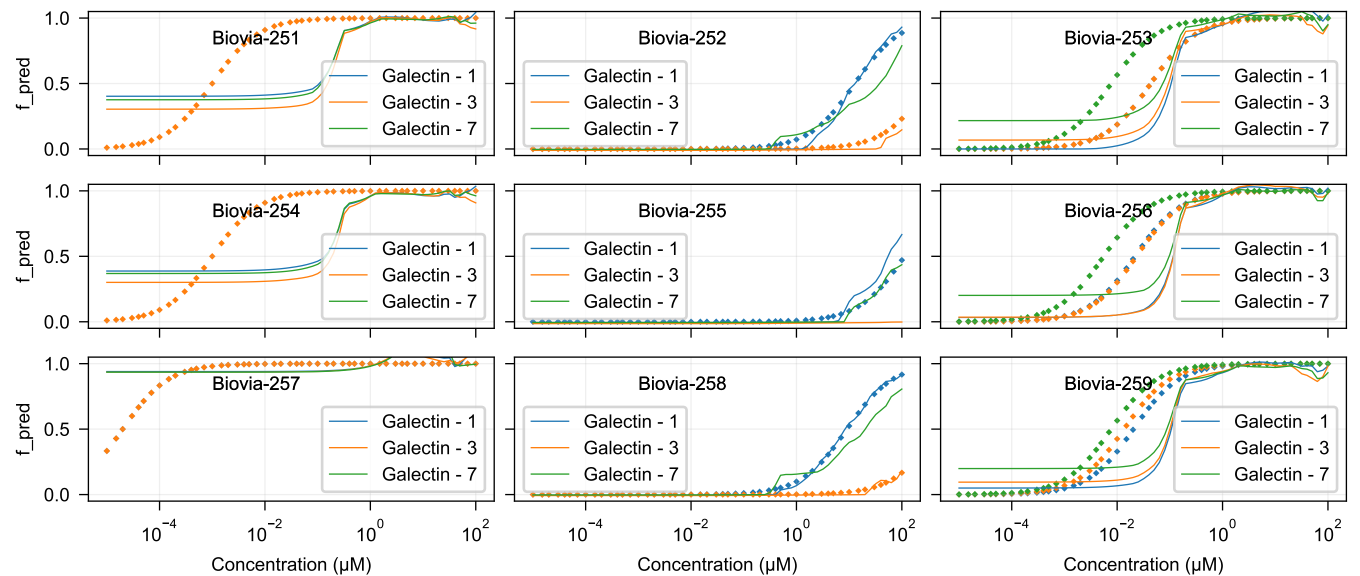
Biovia-251
Biovia-252
Biovia-253
Biovia-254
Biovia-255
Biovia-256
Biovia-257
Biovia-258
Biovia-259
Galectin - 1
Galectin - 3
Galectin - 7
f_pred
Concentration (μM)
1.0
0.5
0.0
10^{-4}
10^{-2}
10^{0}
10^{2}

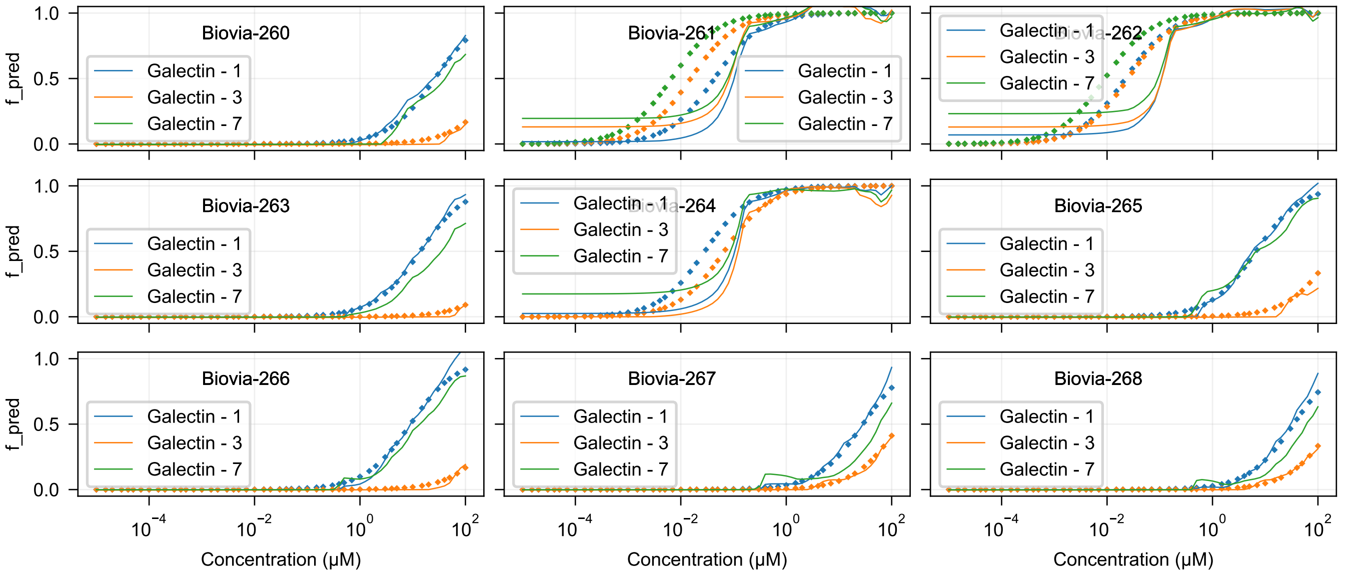
Biovia-260
Biovia-261
Biovia-262
Biovia-263
Biovia-264
Biovia-265
Biovia-266
Biovia-267
Biovia-268
Galectin - 1
Galectin - 3
Galectin - 7
f_pred
Concentration (μM)

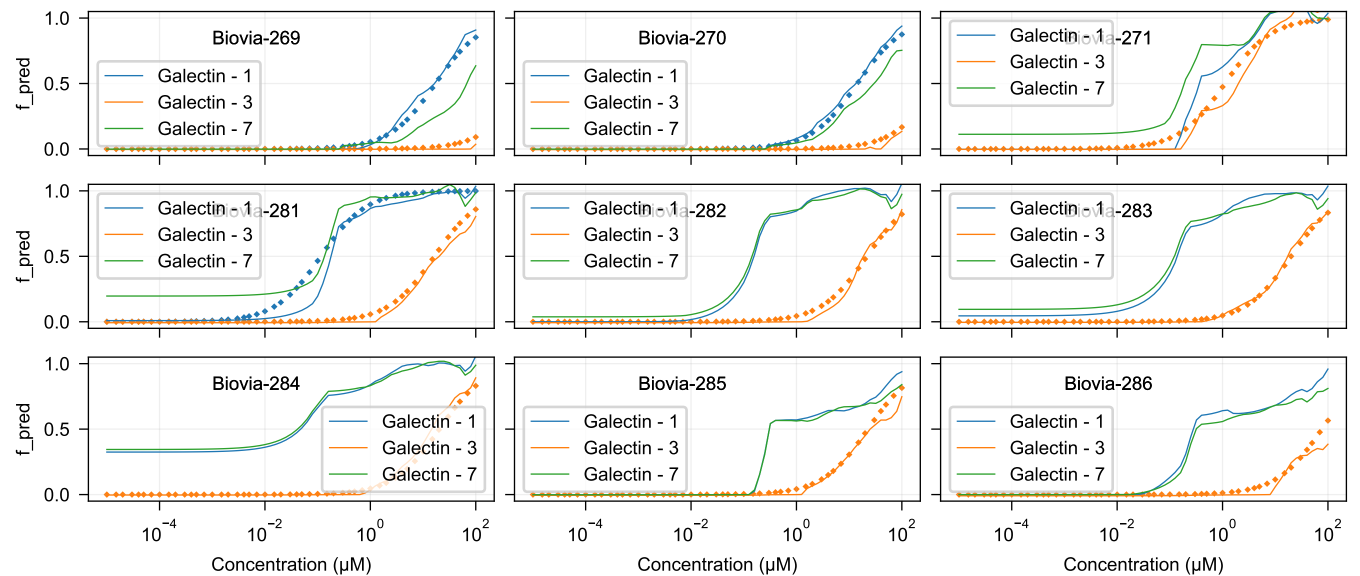

Biovia-269
Biovia-270
Biovia-271
Biovia-281
Biovia-282
Biovia-283
Biovia-284
Biovia-285
Biovia-286
Galectin - 1
Galectin - 3
Galectin - 7
f_pred
Concentration (μM)

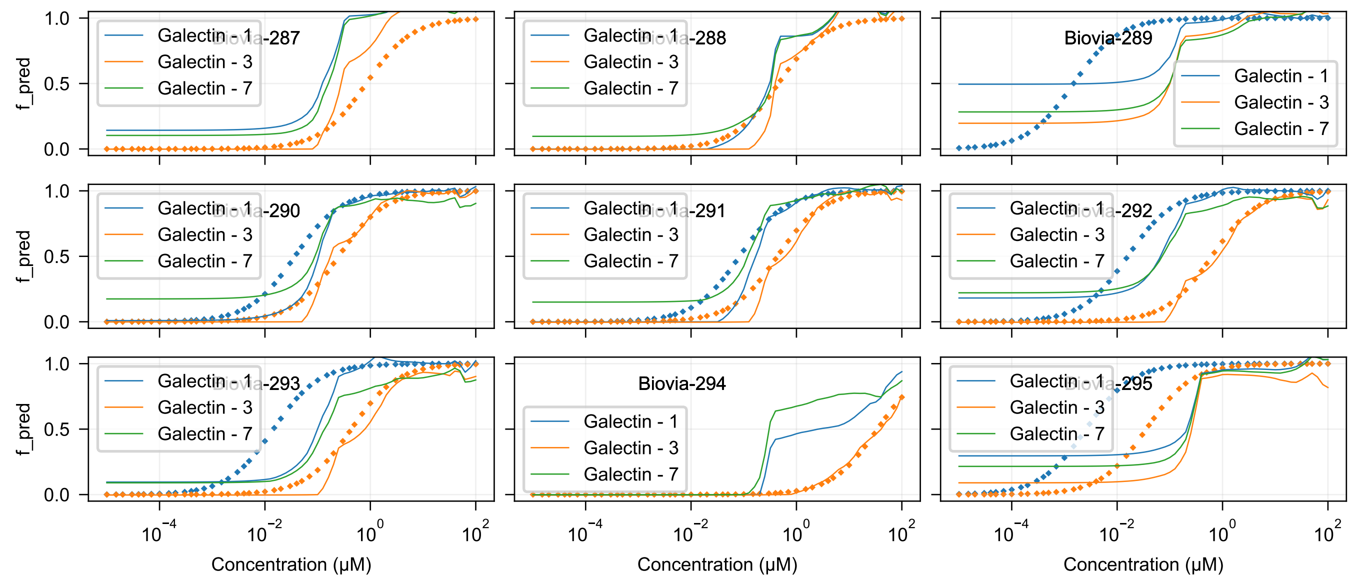

Biovia-287
Biovia-288
Biovia-289
Biovia-290
Biovia-291
Biovia-292
Biovia-293
Biovia-294
Biovia-295
Galectin - 1
Galectin - 3
Galectin - 7
f_pred
1.0
0.5
0.0
10^{-4}
10^{-2}
10^{0}
10^{2}
Concentration (μM)

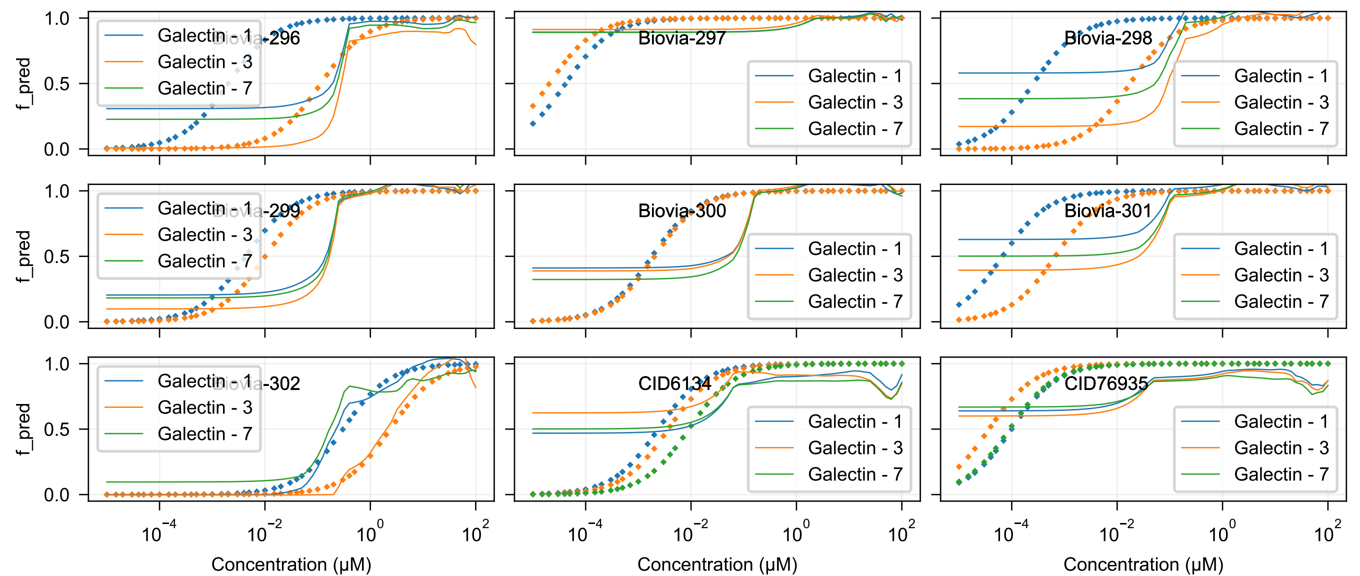

Biovia-296
Biovia-297
Biovia-298
Biovia-299
Biovia-300
Biovia-301
Biovia-302
CID6134
CID76935
Galectin - 1
Galectin - 3
Galectin - 7
f_pred
Concentration (μM)

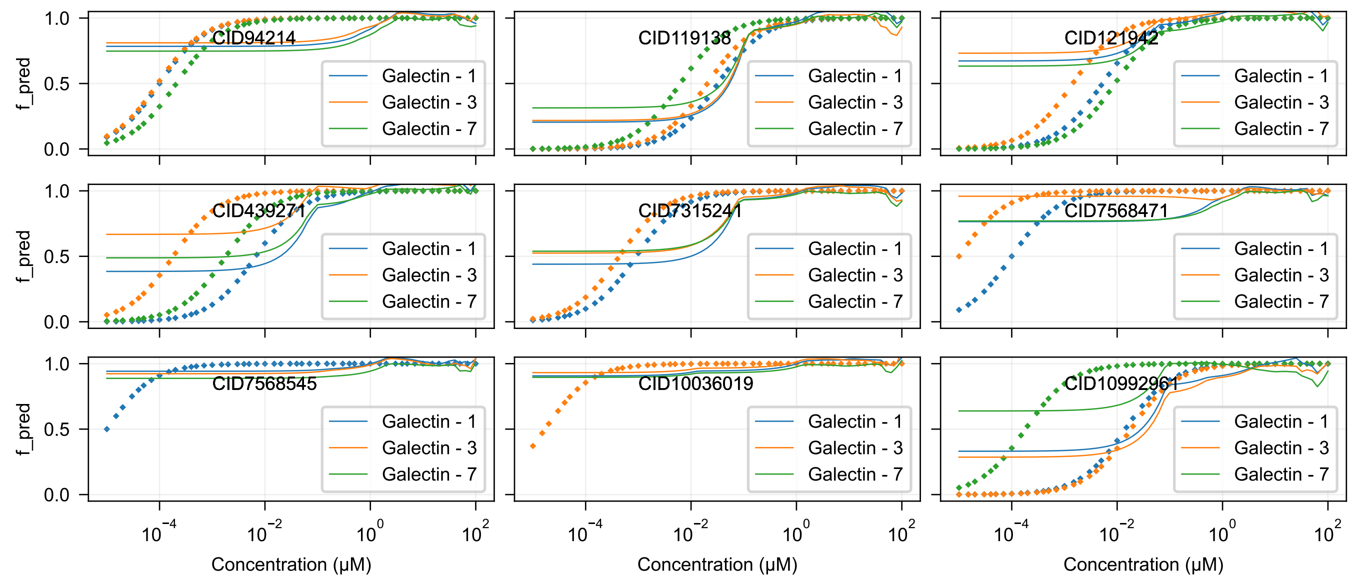

CID94214
CID119138
CID121942
CID439271
CID7315241
CID7568471
CID7568545
CID10036019
CID10992961
Galectin - 1
Galectin - 3
Galectin - 7
f_pred
Concentration (μM)

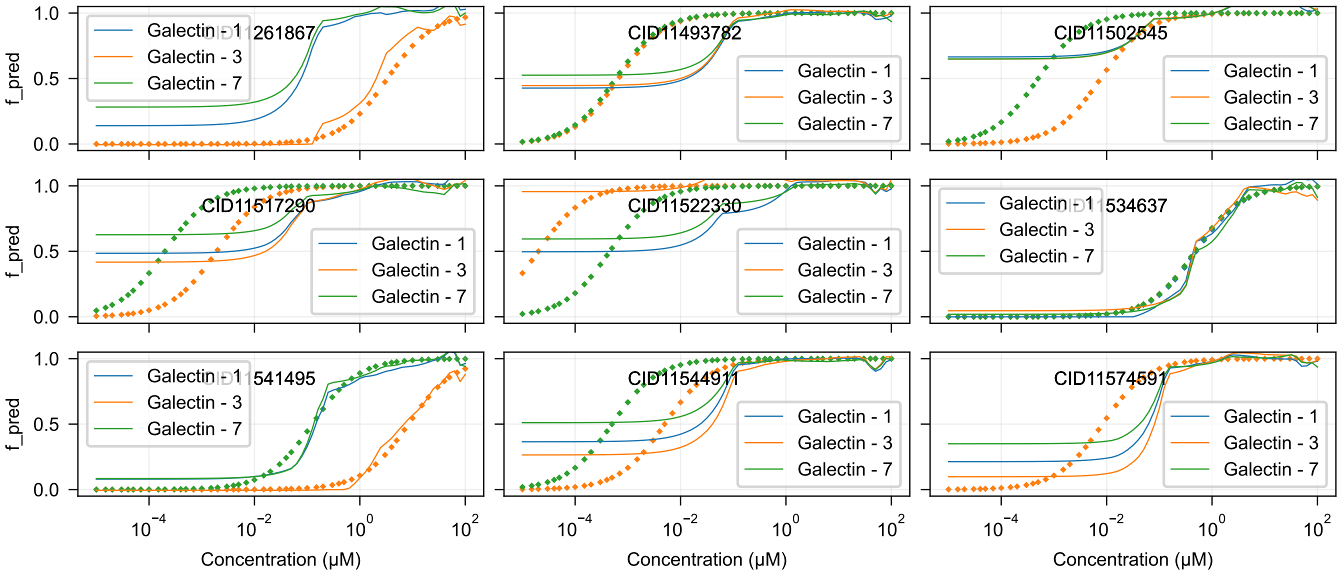

CID11261867
CID11493782
CID11502545
CID11517290
CID11522330
CID11534637
CID11541495
CID11544911
CID11574591
Galectin - 1
Galectin - 3
Galectin - 7
f_pred
Concentration (μM)

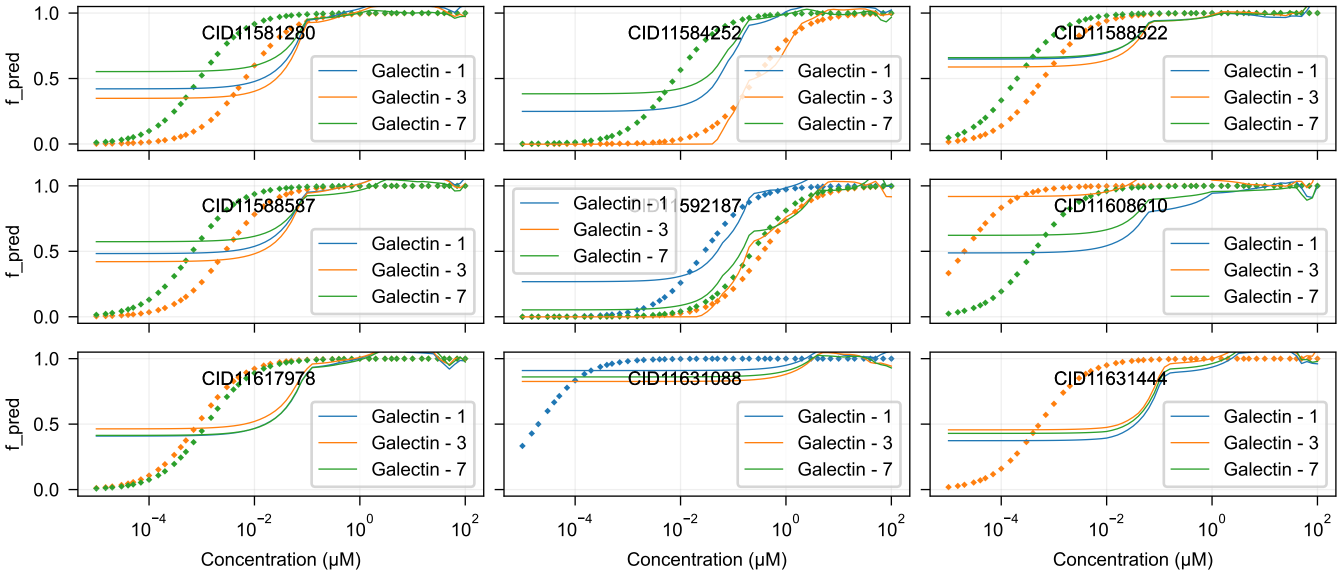
CID11581280
CID11584252
CID11588522
CID11588587
CID11592187
CID11608610
CID11617978
CID11631088
CID11631444
Galectin - 1
Galectin - 3
Galectin - 7
f_pred
Concentration (μM)

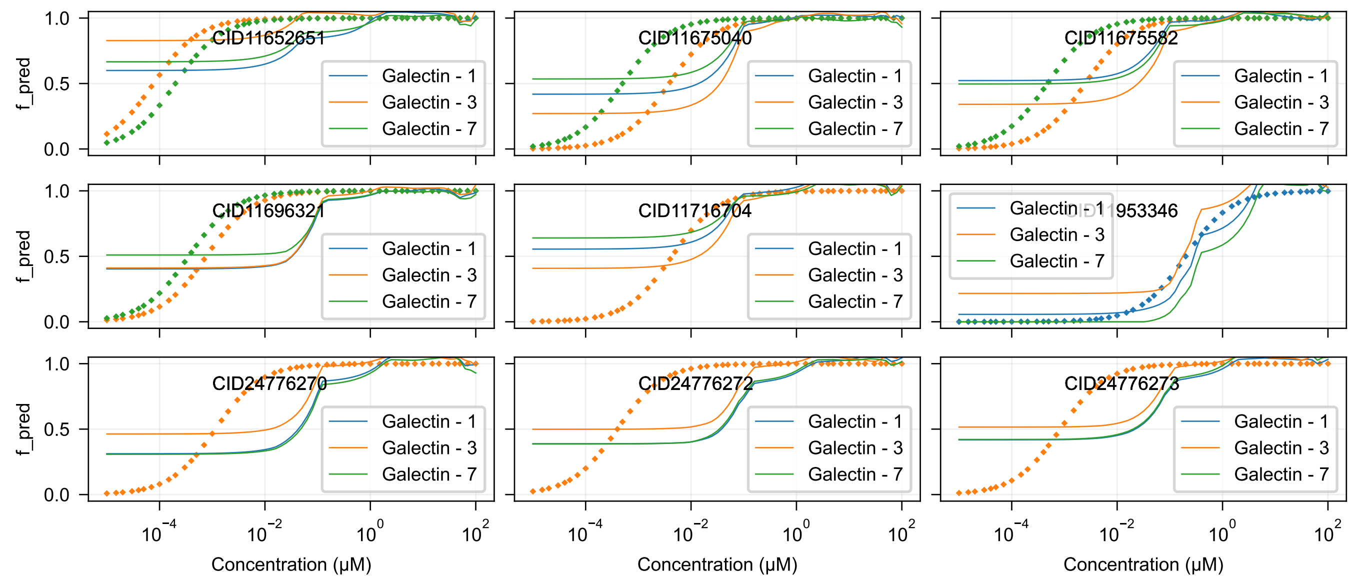

CID11652651
CID11675040
CID11675582
CID11696321
CID11716704
CID11953346
CID24776270
CID24776272
CID24776273
Galectin - 1
Galectin - 3
Galectin - 7
f_pred
Concentration (μM)

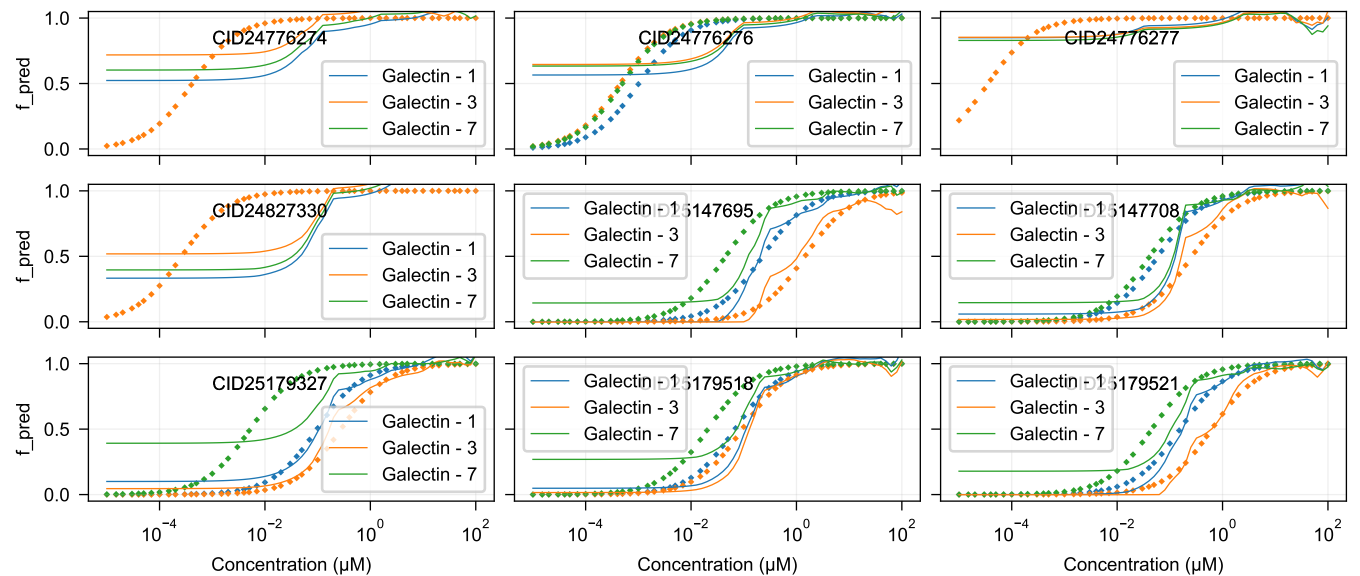

CID24776274
CID24776276
CID24776277
CID24827330
CID25147695
CID25147708
CID25179327
CID25179518
CID25179521
Galectin - 1
Galectin - 3
Galectin - 7
f_pred
1.0
0.5
0.0
10^{-4}
10^{-2}
10^{0}
10^{2}
Concentration (μM)

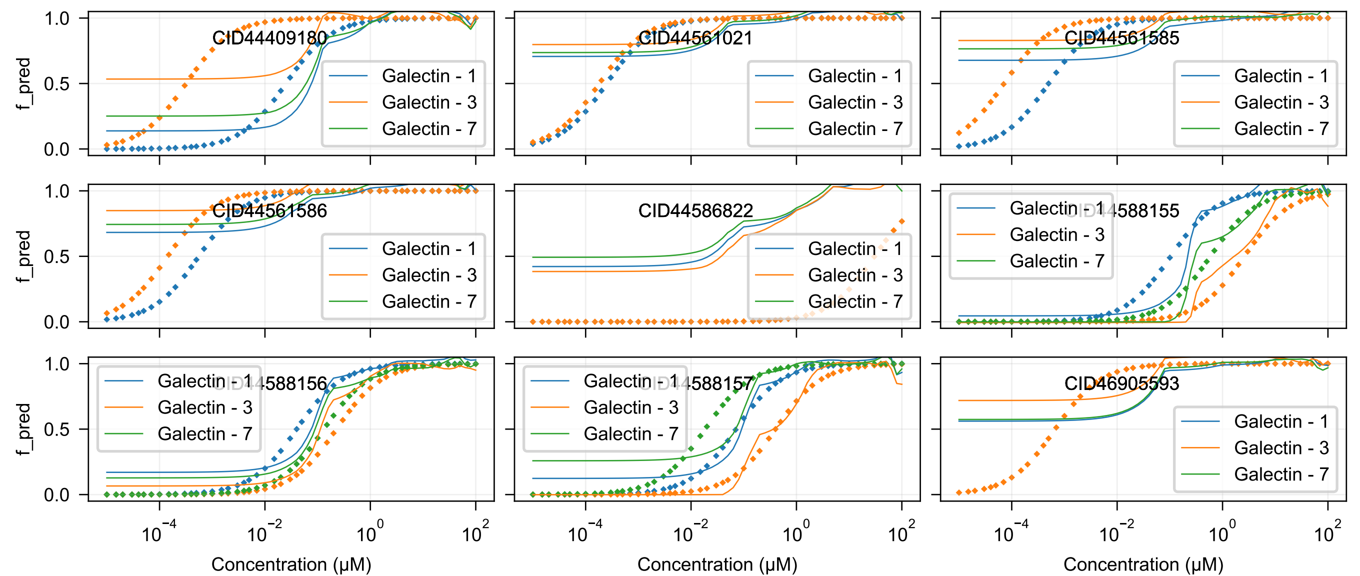

CID44409180
CID44561021
CID44561585
CID44561586
CID44586822
CID44588155
CID44588156
CID44588157
CID46905593
Galectin - 1
Galectin - 3
Galectin - 7
f_pred
Concentration (μM)

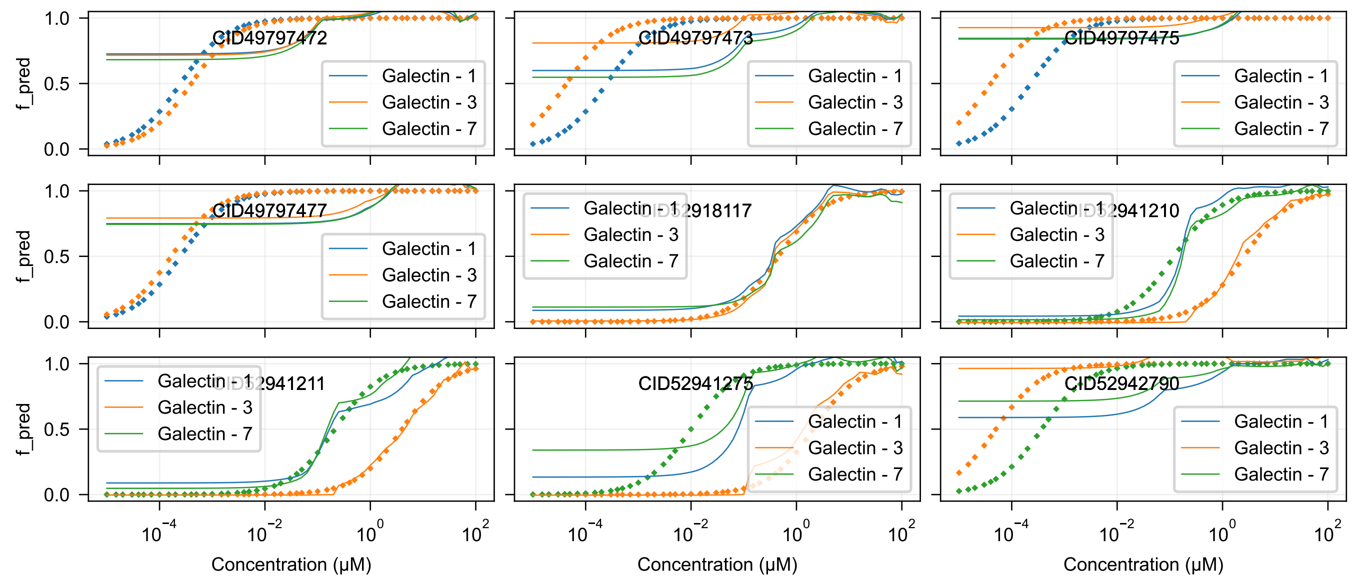
CID49797472
CID49797473
CID49797475
CID49797477
CID52918117
CID52941210
CID52941211
CID52941275
CID52942790
Galectin - 1
Galectin - 3
Galectin - 7
f_pred
Concentration (μM)

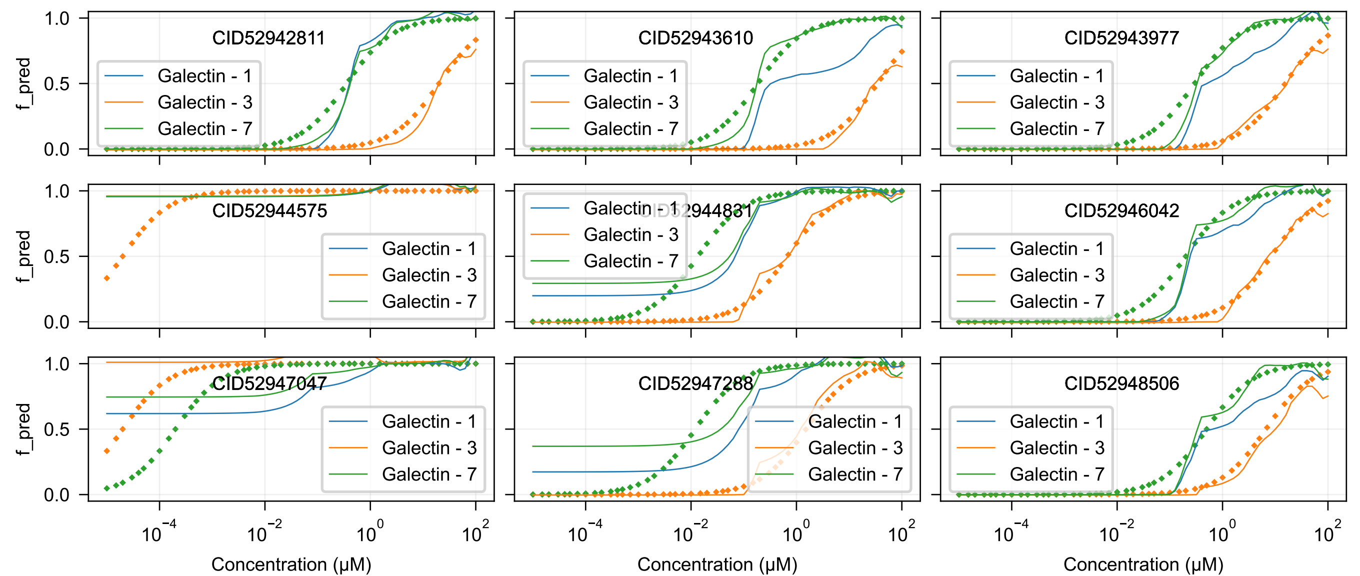
CID52942811
CID52943610
CID52943977
CID52944575
CID52944831
CID52946042
CID52947047
CID52947288
CID52948506
Galectin - 1
Galectin - 3
Galectin - 7
f_pred
Concentration (μM)

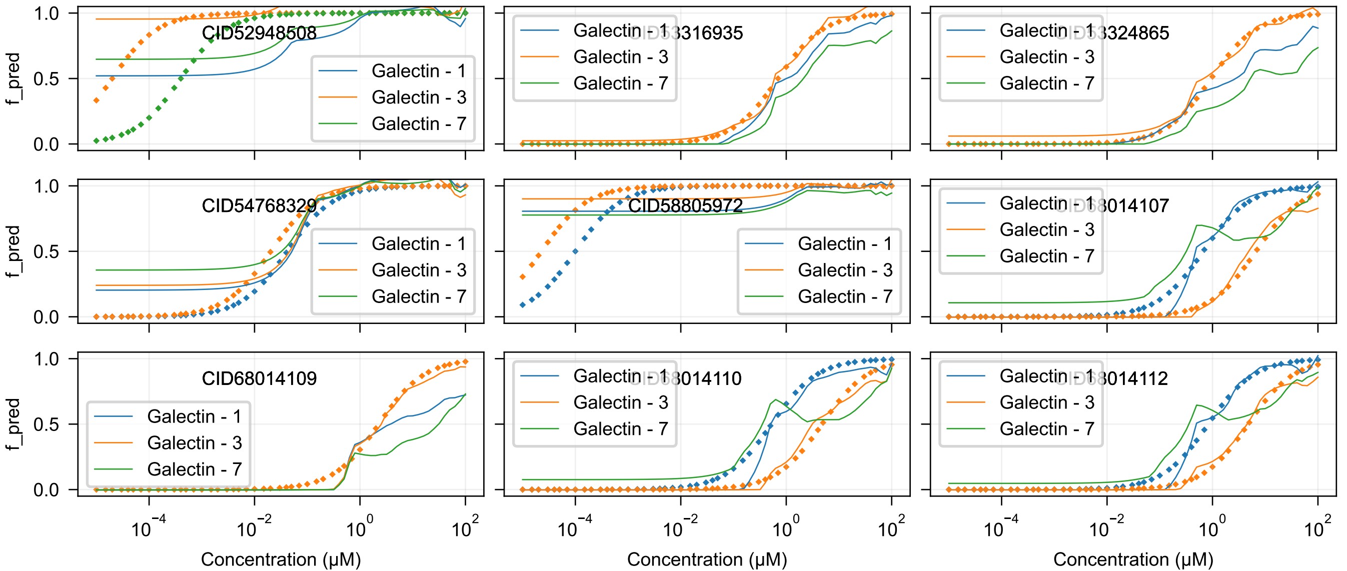

CID52948508
CID54768329
CID58805972
CID68014109
Galectin - 1
Galectin - 3
Galectin - 7
f_pred
Concentration (μM)

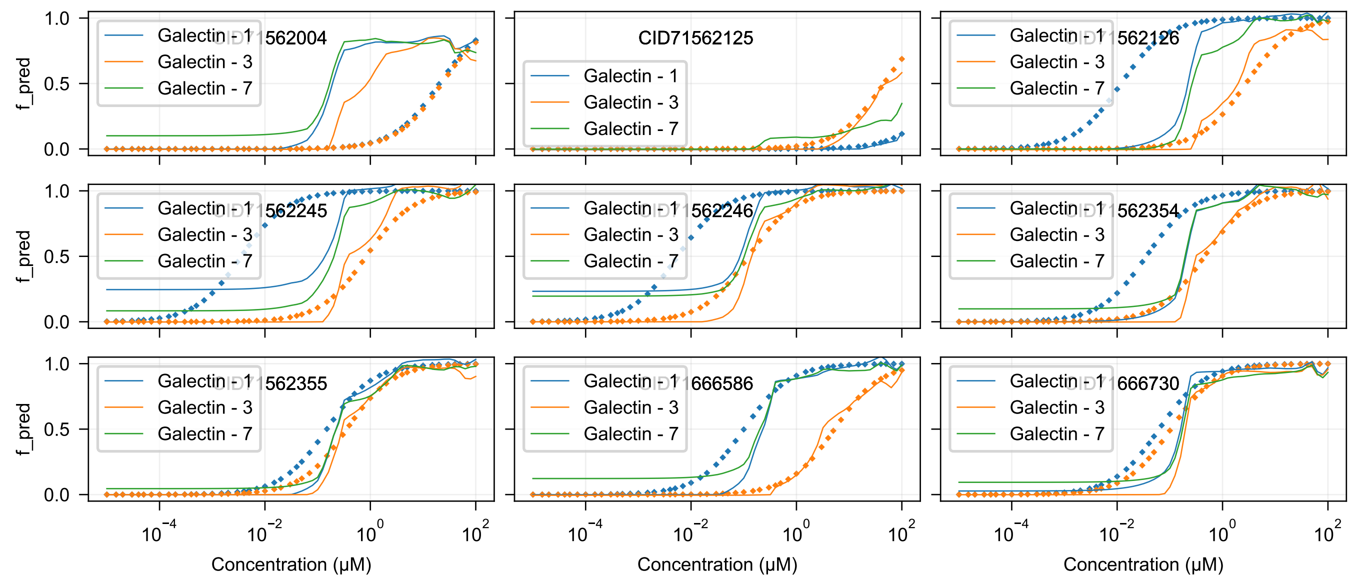

CID71562004
CID71562125
CID71562126
CID71562245
CID71562246
CID71562354
CID71562355
CID71666586
CID71666730
Galectin - 1
Galectin - 3
Galectin - 7
f_pred
Concentration (μM)

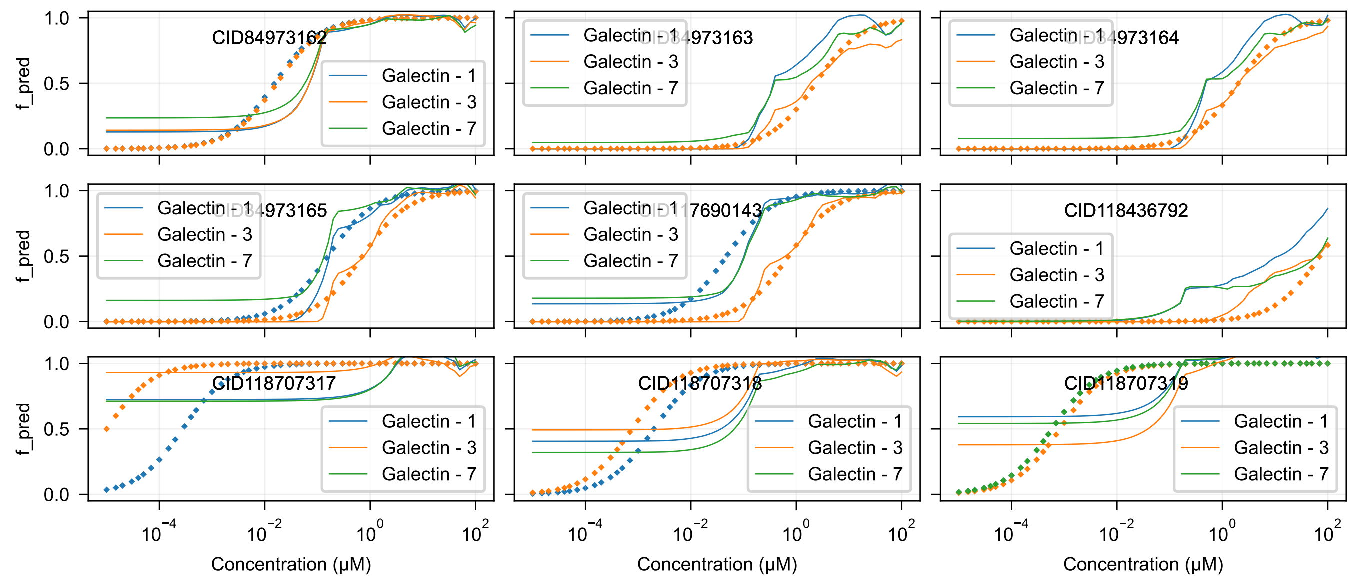
CID84973162
CID84973163
CID84973164
CID84973165
CID117690143
CID118436792
CID118707317
CID118707318
CID118707319
Galectin - 1
Galectin - 3
Galectin - 7
f_pred
Concentration (μM)

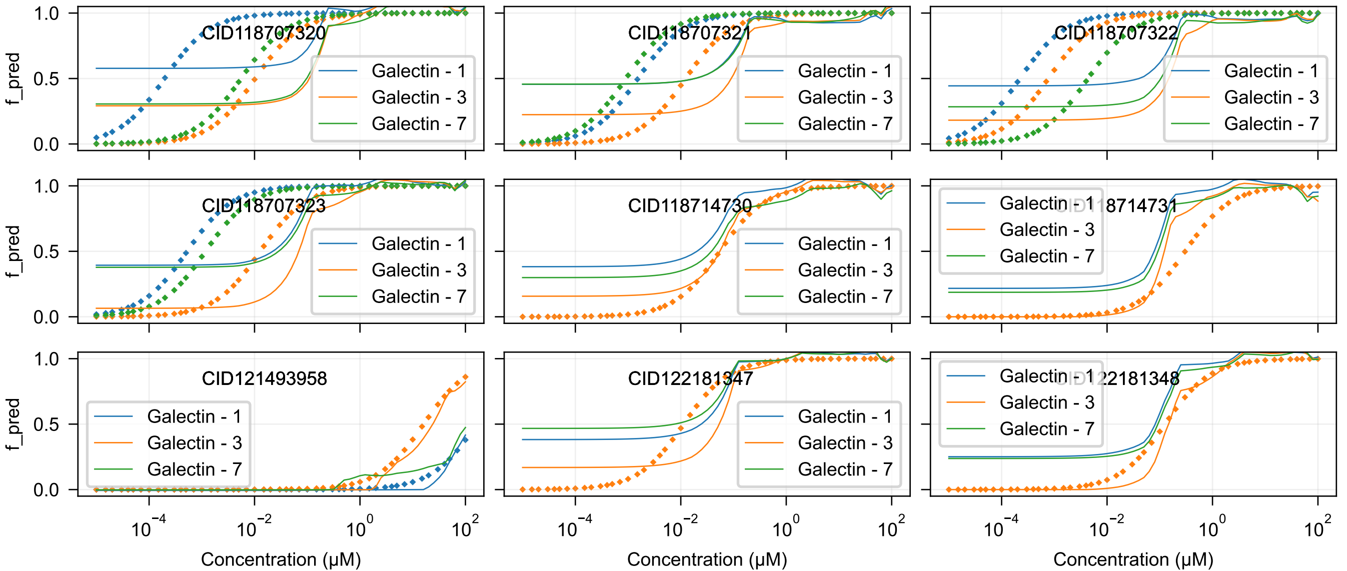

CID118707320
CID118707321
CID118707322
CID118707323
CID118714730
CID118714731
CID121493958
CID122181347
CID122181348
Galectin - 1
Galectin - 3
Galectin - 7
f_pred
Concentration (μM)

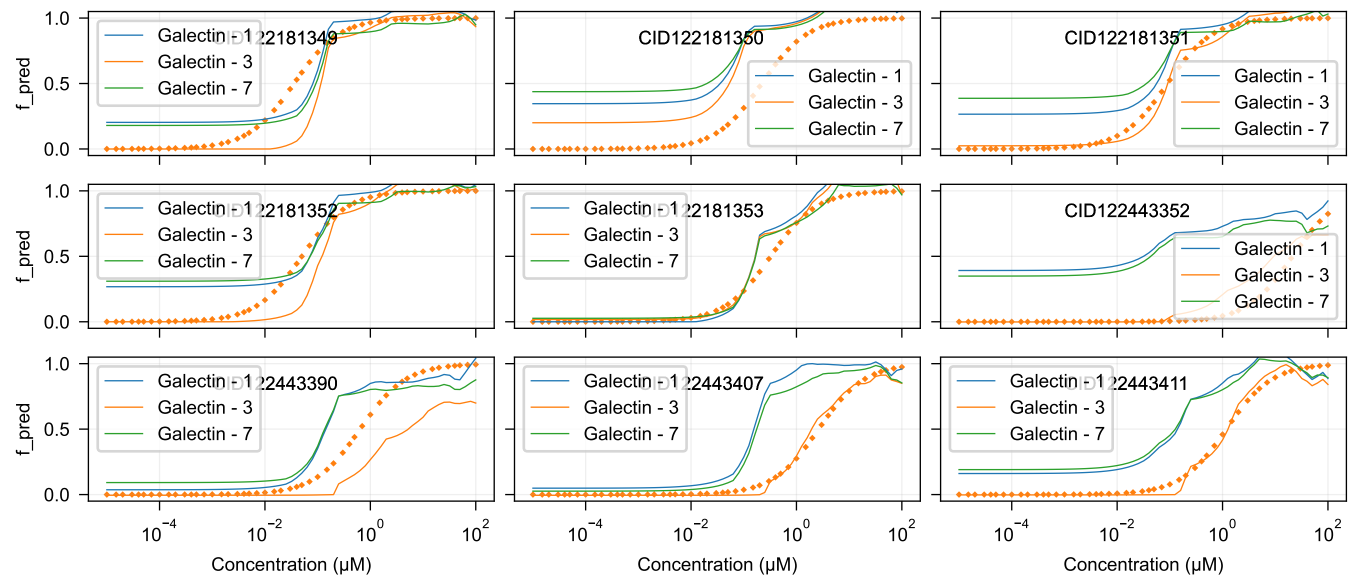
CID122181349
CID122181350
CID122181351
CID122181352
CID122181353
CID122443352
CID122443390
CID122443407
CID122443411
Galectin - 1
Galectin - 3
Galectin - 7
f_pred
0.0
0.5
1.0
10^{-4}
10^{-2}
10^{0}
10^{2}
Concentration (μM)

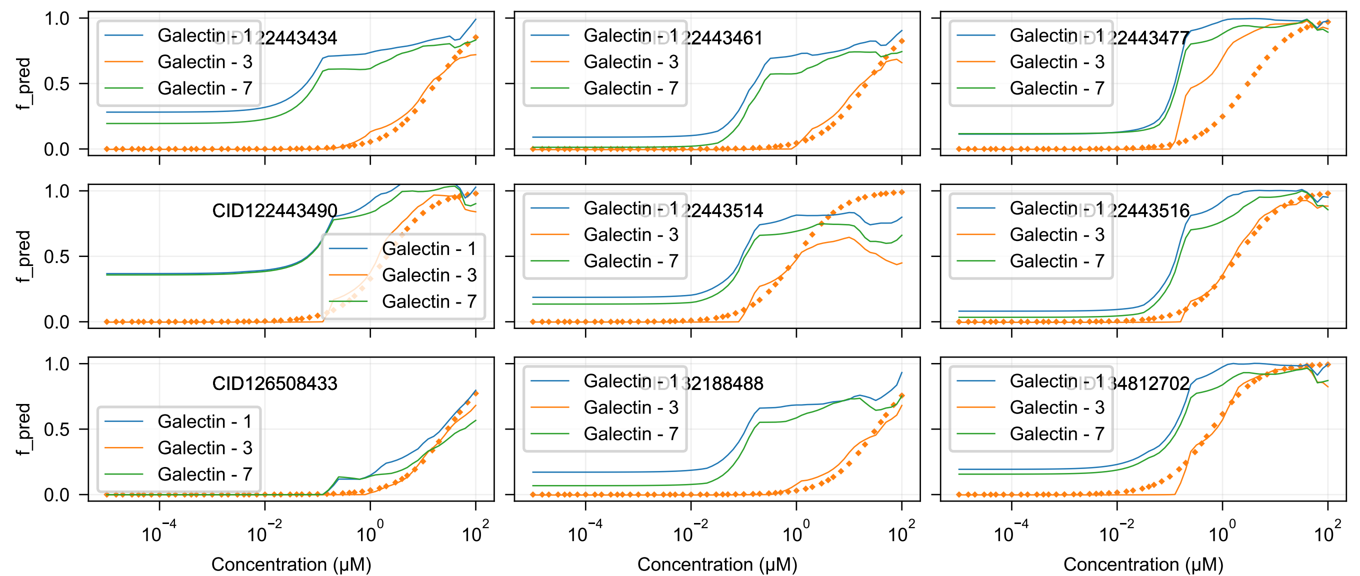

CID122443434
CID122443461
CID122443477
CID122443490
CID122443514
CID122443516
CID126508433
CID132188488
CID134812702
Galectin - 1
Galectin - 3
Galectin - 7
f_pred
1.0
0.5
0.0
10^{-4}
10^{-2}
10^{0}
10^{2}
Concentration (μM)

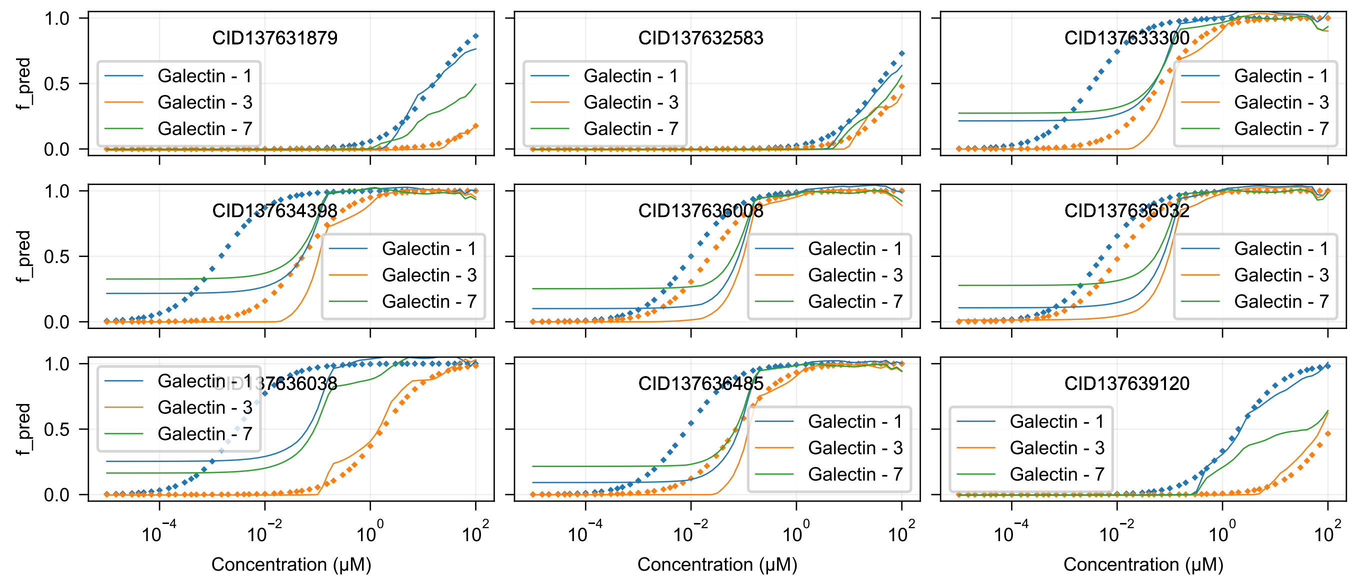

CID137631879
CID137632583
CID137633300
CID137634398
CID137636008
CID137636032
CID137636038
CID137636485
CID137639120
Galectin - 1
Galectin - 3
Galectin - 7
f_pred
1.0
0.5
0.0
10^{-4}
10^{-2}
10^{0}
10^{2}
Concentration (μM)

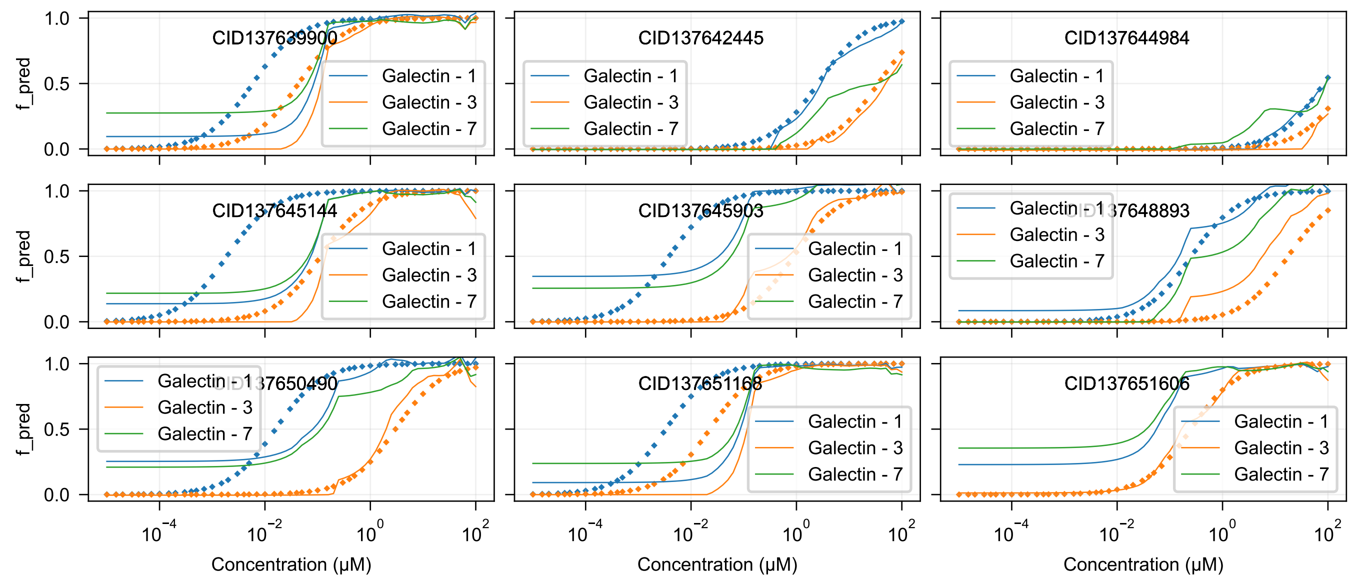

CID137639900
CID137642445
CID137644984
CID137645144
CID137645903
CID137648893
CID137650490
CID137651168
CID137651606
Galectin - 1
Galectin - 3
Galectin - 7
f_pred
1.0
0.5
0.0
10^{-4}
10^{-2}
10^{0}
10^{2}
Concentration (μM)

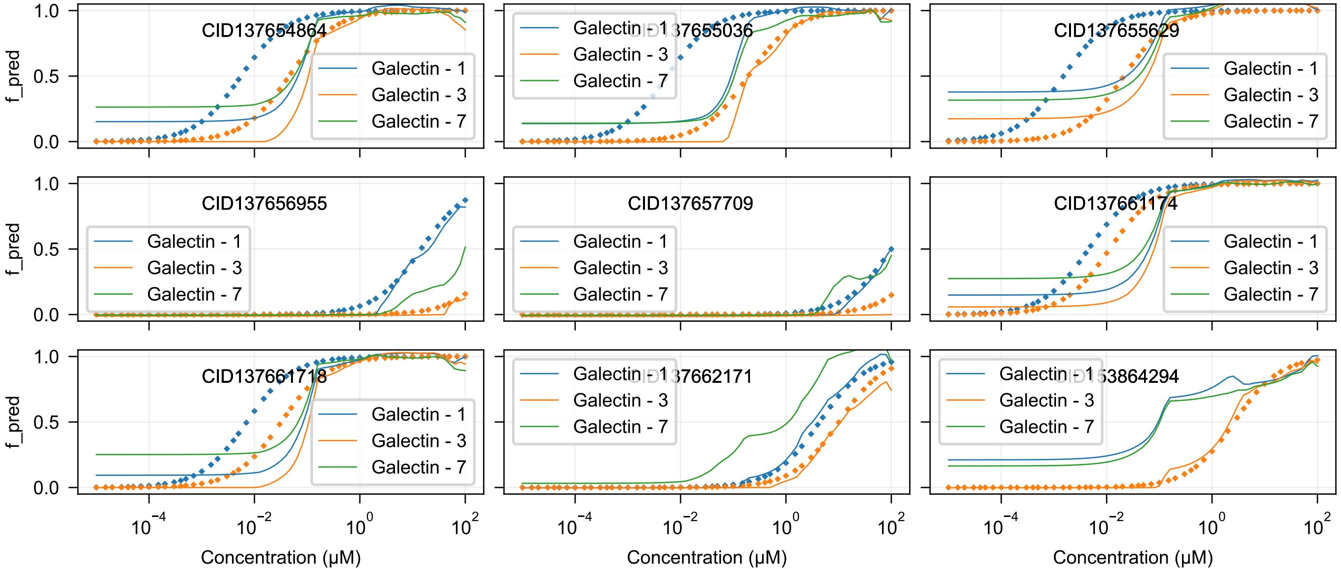

f_pred
1.0
0.5
0.0
CID137654864
CID137655036
CID137655629
CID137656955
CID137657709
CID137661174
CID137661718
CID137662171
CID153864294
Galectin - 1
Galectin - 3
Galectin - 7
10^{-4}
10^{-2}
10^{0}
10^{2}
Concentration (μM)

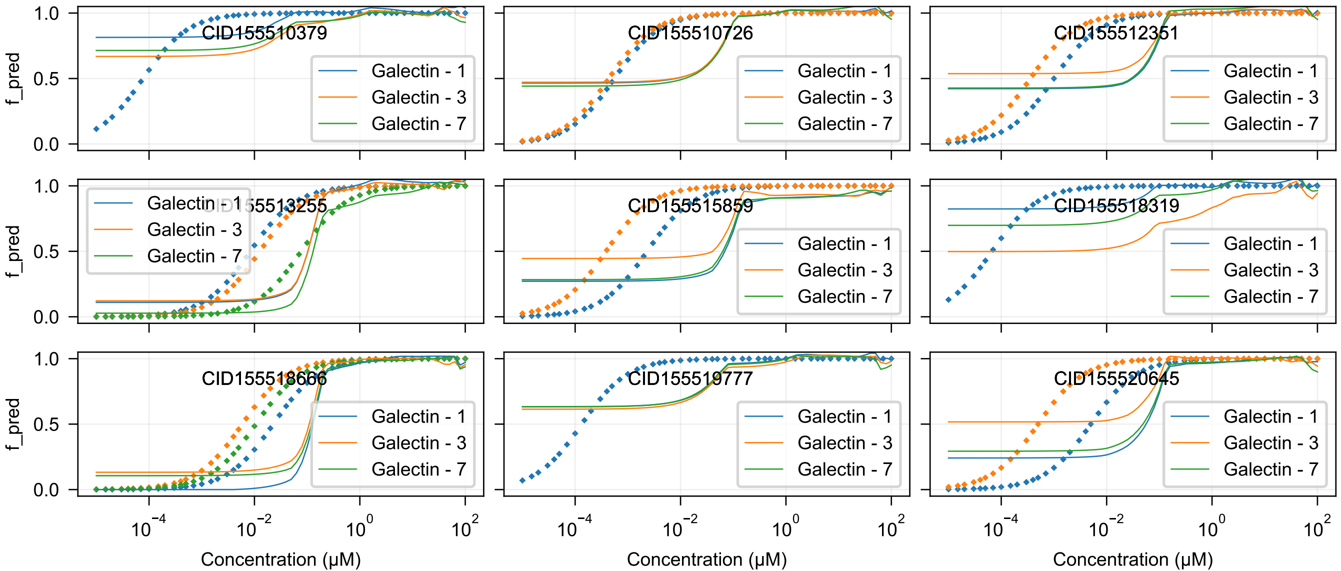

CID155510379
CID155510726
CID155512351
CID155513255
CID155515859
CID155518319
CID155518666
CID155519777
CID155520645
Galectin - 1
Galectin - 3
Galectin - 7
f_pred
Concentration (μM)

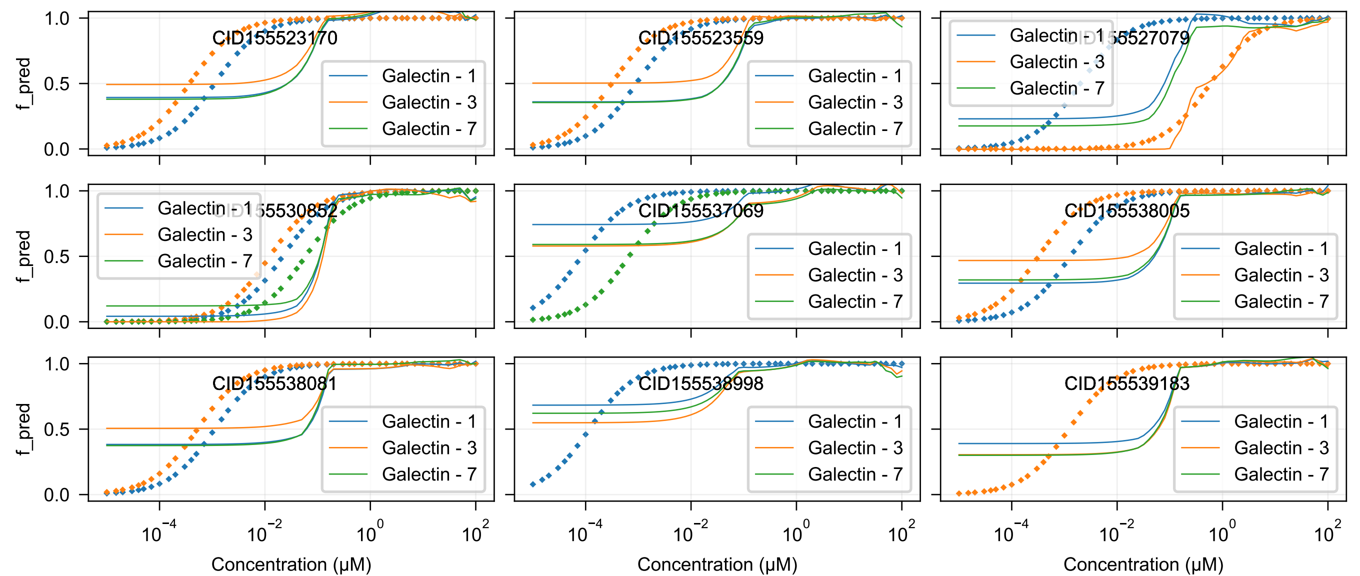

CID155523170
CID155523559
CID155527079
CID155530852
CID155537069
CID155538005
CID155538081
CID155538998
CID155539183
Galectin - 1
Galectin - 3
Galectin - 7
f_pred
Concentration (μM)

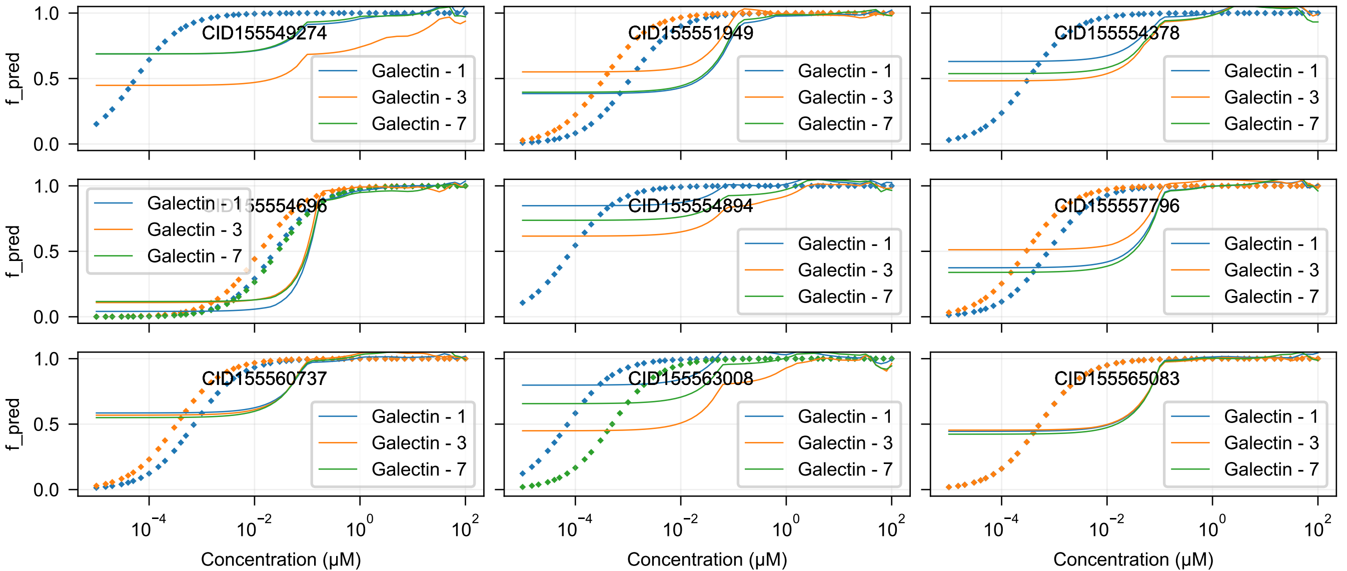
CID155549274
CID155551949
CID155554378
CID155554696
CID155554894
CID155557796
CID155560737
CID155563008
CID155565083
Galectin - 1
Galectin - 3
Galectin - 7
f_pred
Concentration (μM)

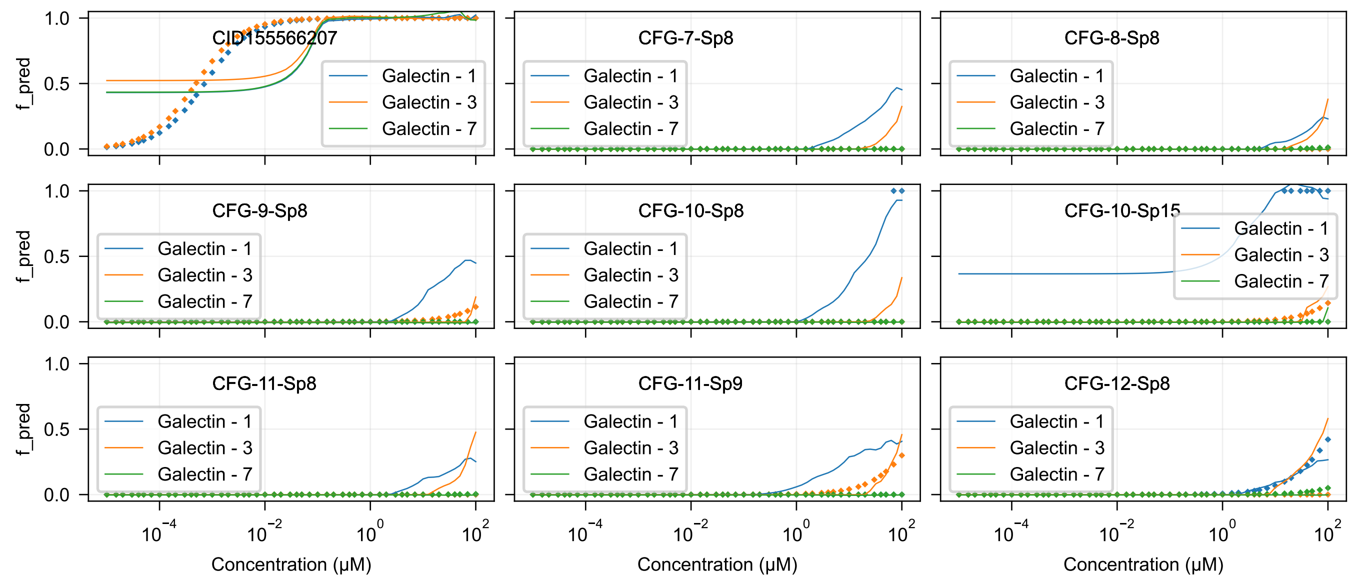
CID155566207
CFG-7-Sp8
CFG-8-Sp8
CFG-9-Sp8
CFG-10-Sp8
CFG-10-Sp15
CFG-11-Sp8
CFG-11-Sp9
CFG-12-Sp8
Galectin - 1
Galectin - 3
Galectin - 7
f_pred
0.0
0.5
1.0
10^{-4}
10^{-2}
10^{0}
10^{2}
Concentration (μM)

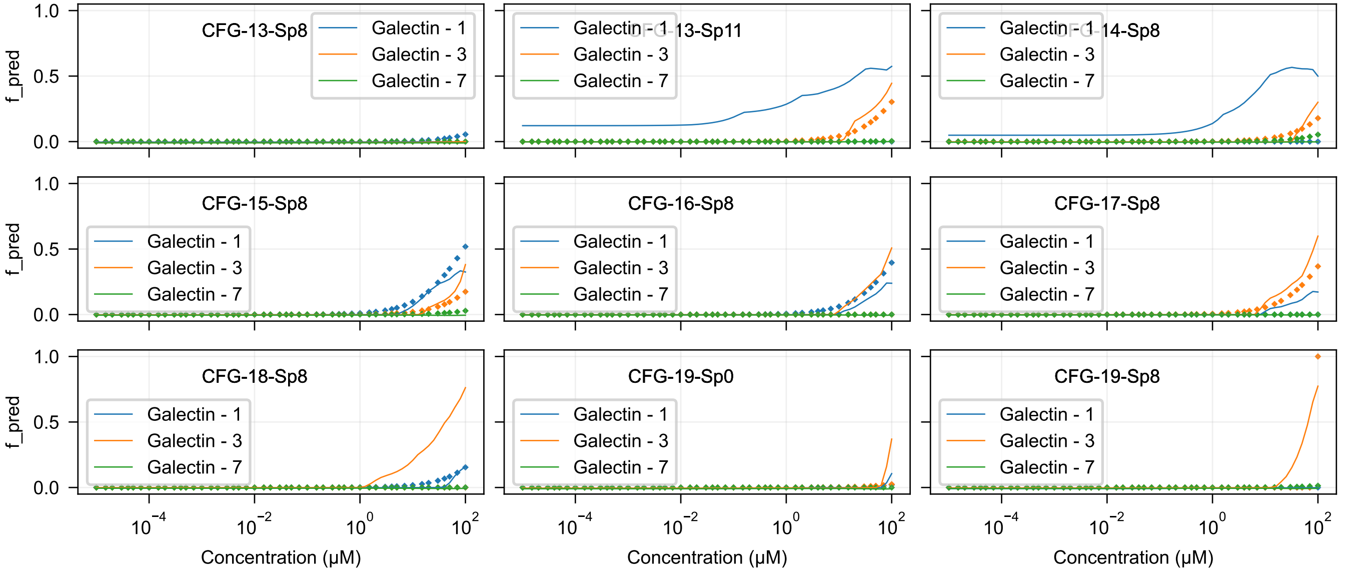

CFG-13-Sp8
CFG-13-Sp11
CFG-14-Sp8
CFG-15-Sp8
CFG-16-Sp8
CFG-17-Sp8
CFG-18-Sp8
CFG-19-Sp0
CFG-19-Sp8
Galectin - 1
Galectin - 3
Galectin - 7
f_pred
0.0
0.5
1.0
10^{-4}
10^{-2}
10^{0}
10^{2}
Concentration (μM)

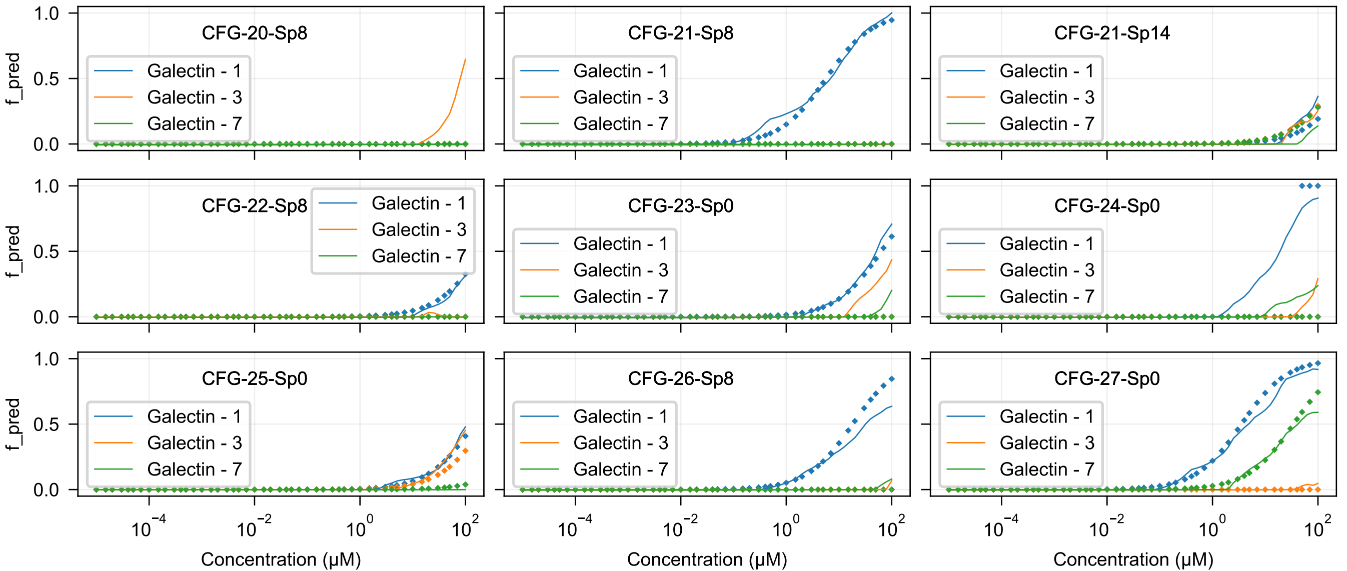

CFG-20-Sp8
CFG-21-Sp8
CFG-21-Sp14
CFG-22-Sp8
CFG-23-Sp0
CFG-24-Sp0
CFG-25-Sp0
CFG-26-Sp8
CFG-27-Sp0
Galectin - 1
Galectin - 3
Galectin - 7
f_pred
1.0
0.5
0.0
10^{-4}
10^{-2}
10^{0}
10^{2}
Concentration (μM)

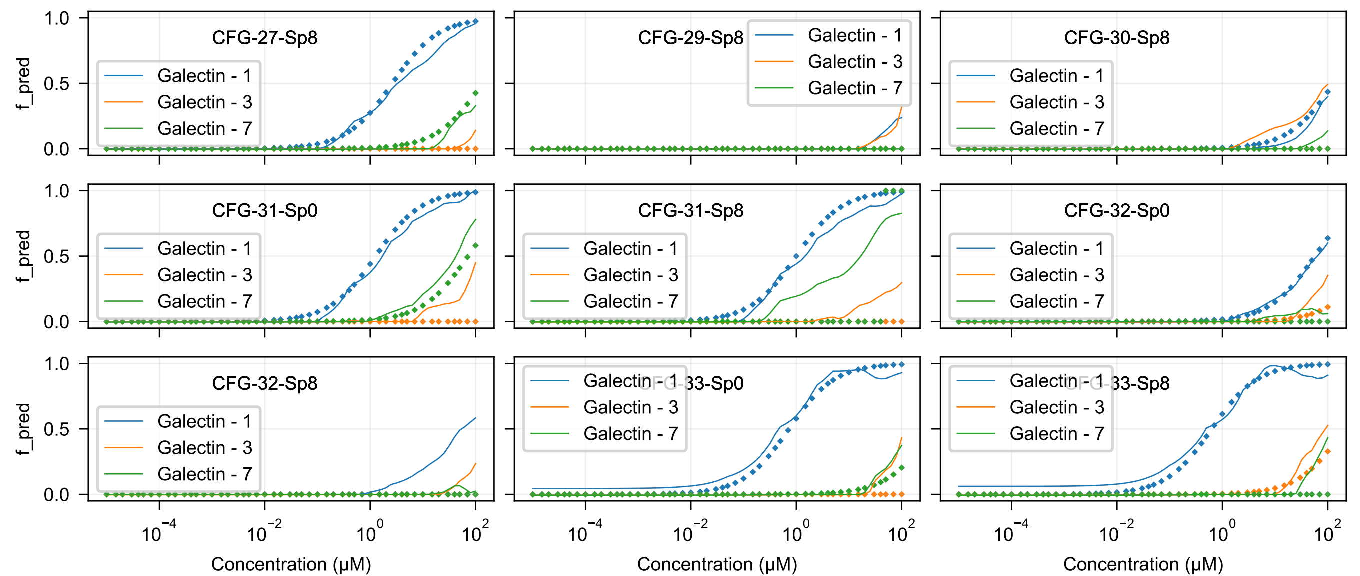
CFG-27-Sp8
CFG-29-Sp8
CFG-30-Sp8
CFG-31-Sp0
CFG-31-Sp8
CFG-32-Sp0
CFG-32-Sp8
CFG-33-Sp0
CFG-33-Sp8
Galectin - 1
Galectin - 3
Galectin - 7
f_pred
1.0
0.5
0.0
10^{-4}
10^{-2}
10^{0}
10^{2}
Concentration (μM)

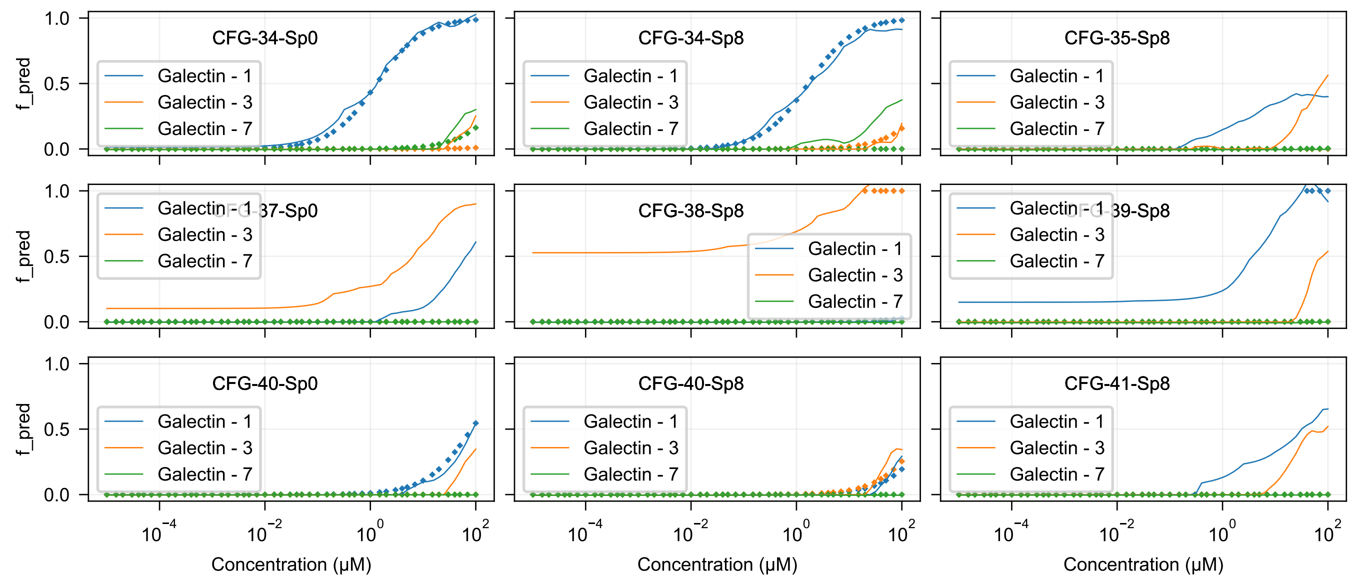

CFG-34-Sp0
CFG-34-Sp8
CFG-35-Sp8
CFG-37-Sp0
CFG-38-Sp8
CFG-39-Sp8
CFG-40-Sp0
CFG-40-Sp8
CFG-41-Sp8
Galectin - 1
Galectin - 3
Galectin - 7
f_pred
0.0
0.5
1.0
10^{-4}
10^{-2}
10^{0}
10^{2}
Concentration (μM)

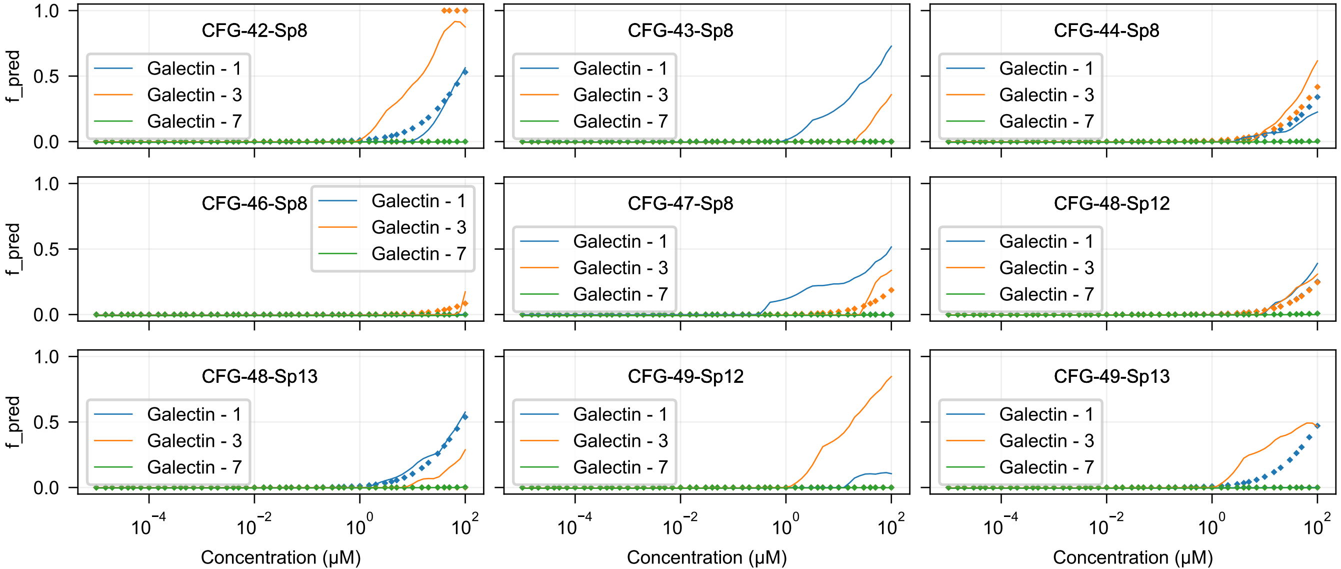

CFG-42-Sp8
CFG-43-Sp8
CFG-44-Sp8
CFG-46-Sp8
CFG-47-Sp8
CFG-48-Sp12
CFG-48-Sp13
CFG-49-Sp12
CFG-49-Sp13
Galectin - 1
Galectin - 3
Galectin - 7
f_pred
0.0
0.5
1.0
10^{-4}
10^{-2}
10^{0}
10^{2}
Concentration (μM)

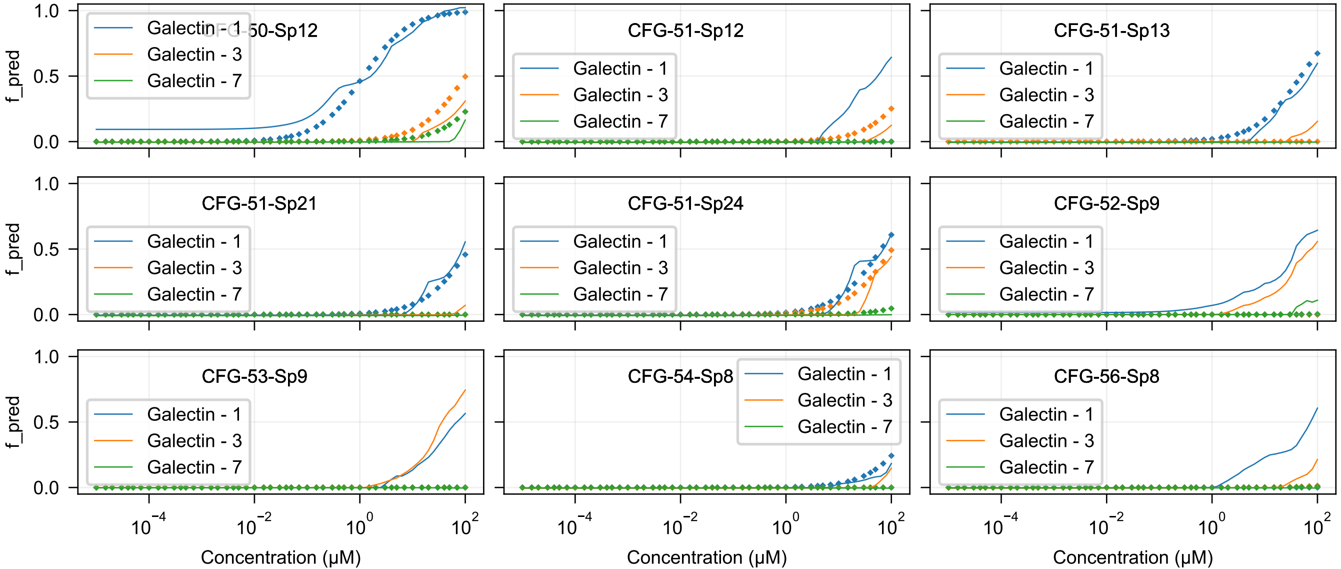

CFG-50-Sp12
CFG-51-Sp12
CFG-51-Sp13
CFG-51-Sp21
CFG-51-Sp24
CFG-52-Sp9
CFG-53-Sp9
CFG-54-Sp8
CFG-56-Sp8
Galectin - 1
Galectin - 3
Galectin - 7
f_pred
Concentration (μM)

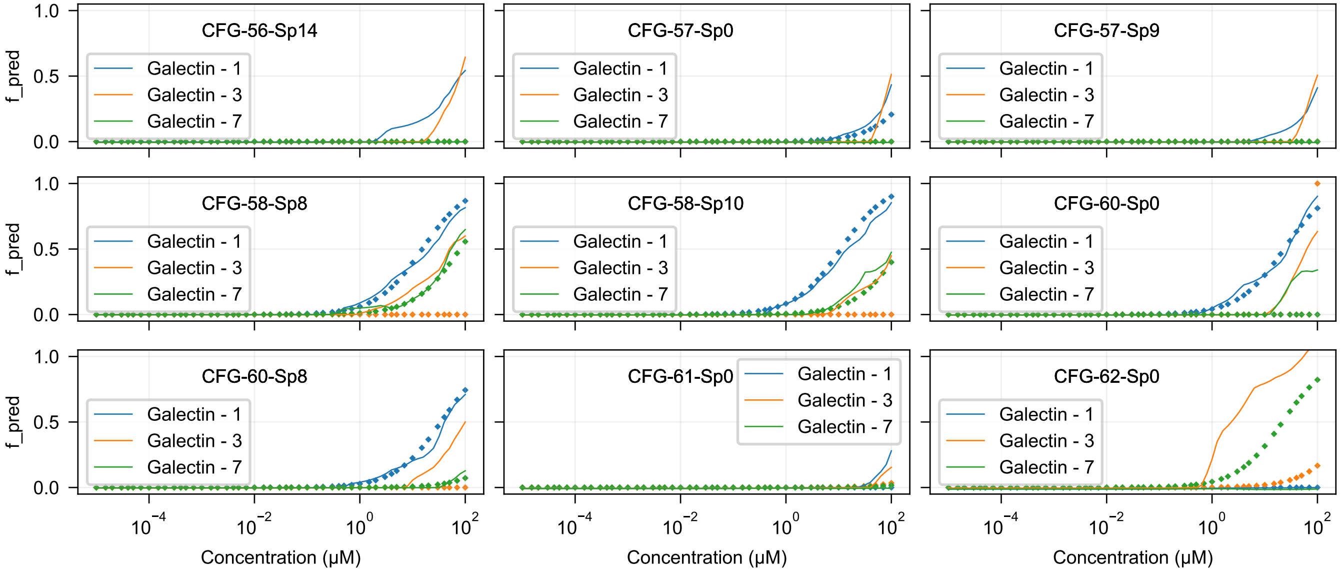
CFG-56-Sp14
CFG-57-Sp0
CFG-57-Sp9
CFG-58-Sp8
CFG-58-Sp10
CFG-60-Sp0
CFG-60-Sp8
CFG-61-Sp0
CFG-62-Sp0
Galectin - 1
Galectin - 3
Galectin - 7
f_pred
1.0
0.5
0.0
10^{-4}
10^{-2}
10^{0}
10^{2}
Concentration (μM)

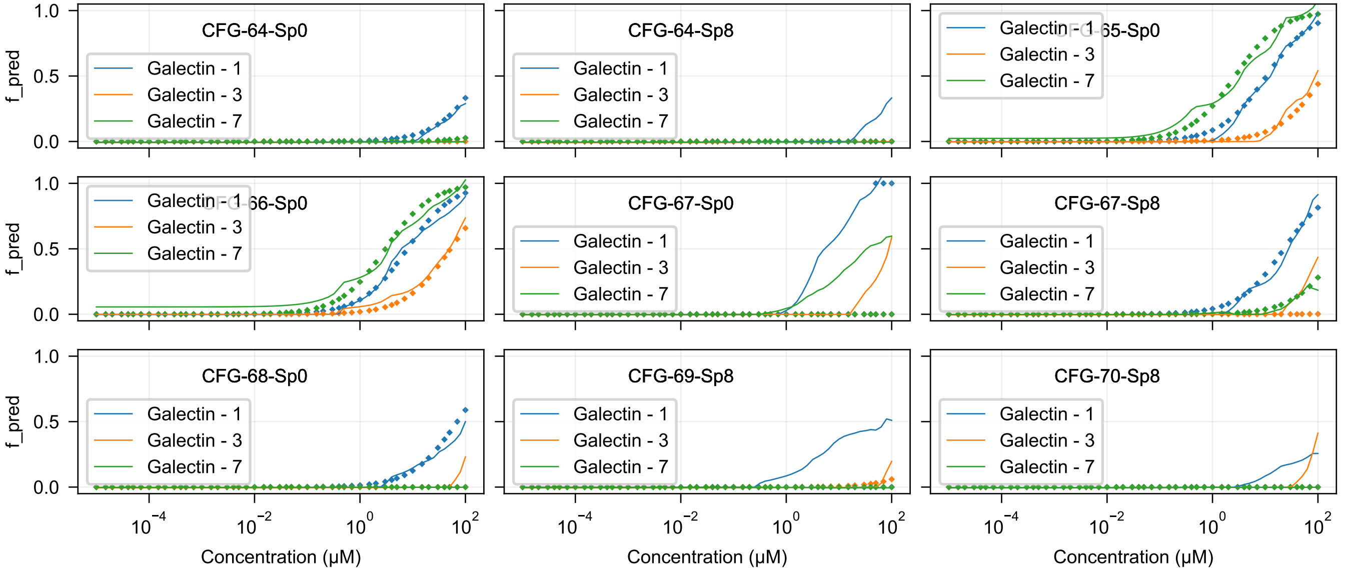
CFG-64-Sp0
CFG-64-Sp8
CFG-65-Sp0
CFG-66-Sp0
CFG-67-Sp0
CFG-67-Sp8
CFG-68-Sp0
CFG-69-Sp8
CFG-70-Sp8
Galectin - 1
Galectin - 3
Galectin - 7
f_pred
1.0
0.5
0.0
10^{-4}
10^{-2}
10^{0}
10^{2}
Concentration (μM)

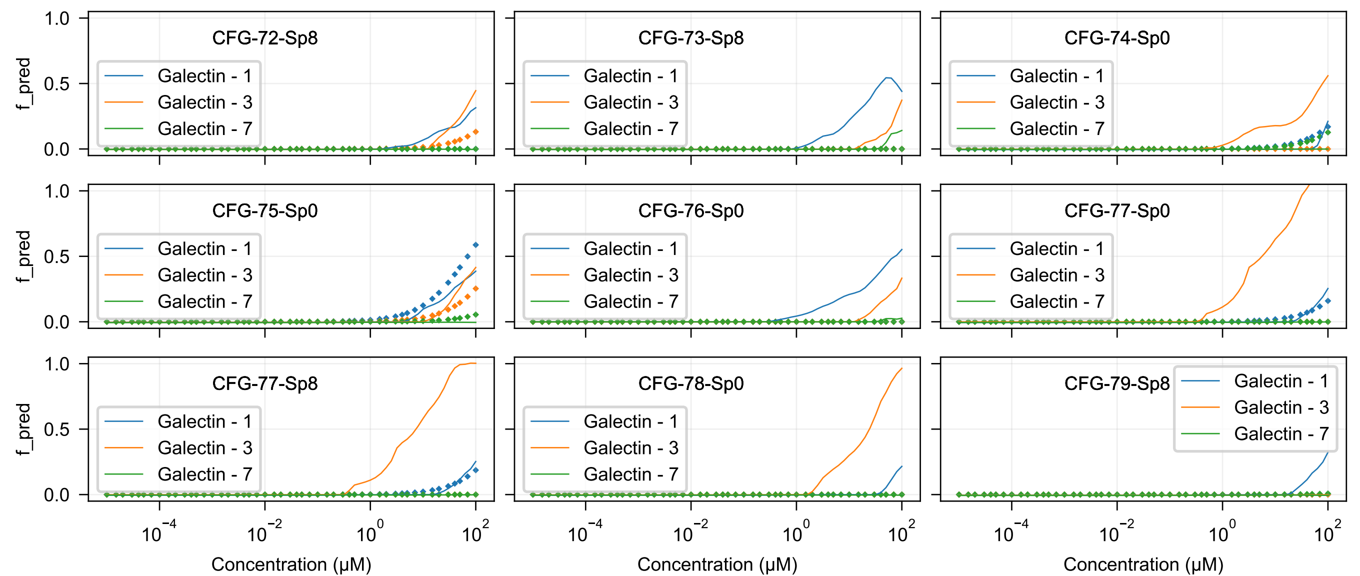

CFG-72-Sp8
CFG-73-Sp8
CFG-74-Sp0
CFG-75-Sp0
CFG-76-Sp0
CFG-77-Sp0
CFG-77-Sp8
CFG-78-Sp0
CFG-79-Sp8
Galectin - 1
Galectin - 3
Galectin - 7
f_pred
0.0
0.5
1.0
10^{-4}
10^{-2}
10^{0}
10^{2}
Concentration (μM)

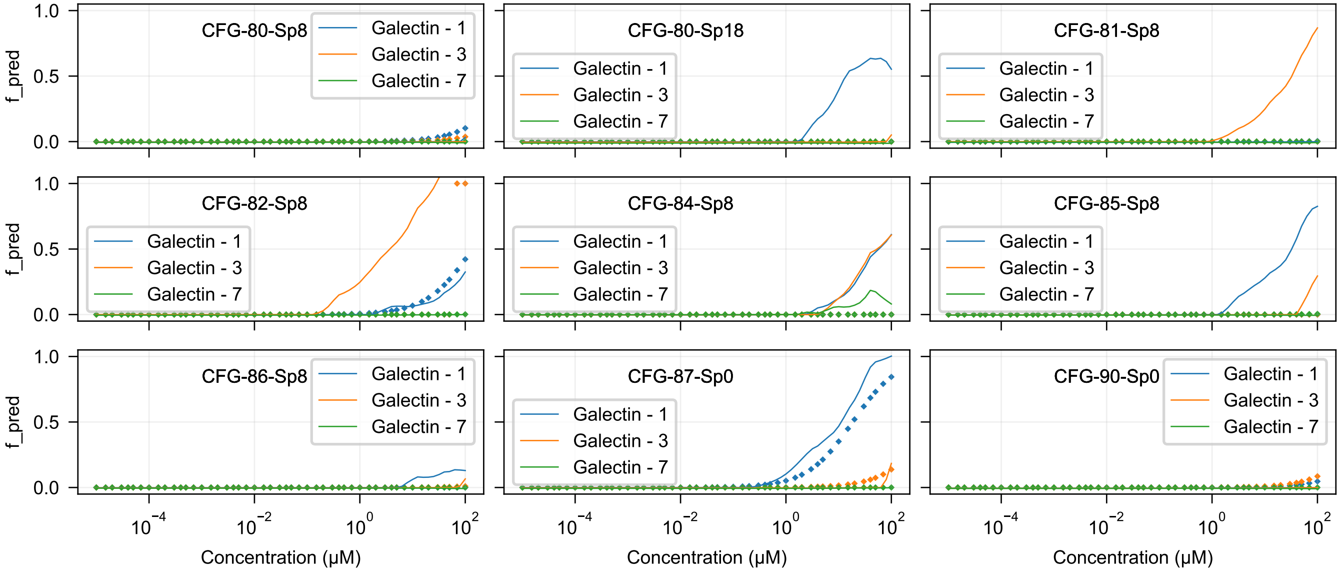

CFG-80-Sp8
CFG-80-Sp18
CFG-81-Sp8
CFG-82-Sp8
CFG-84-Sp8
CFG-85-Sp8
CFG-86-Sp8
CFG-87-Sp0
CFG-90-Sp0
Galectin - 1
Galectin - 3
Galectin - 7
f_pred
Concentration (μM)

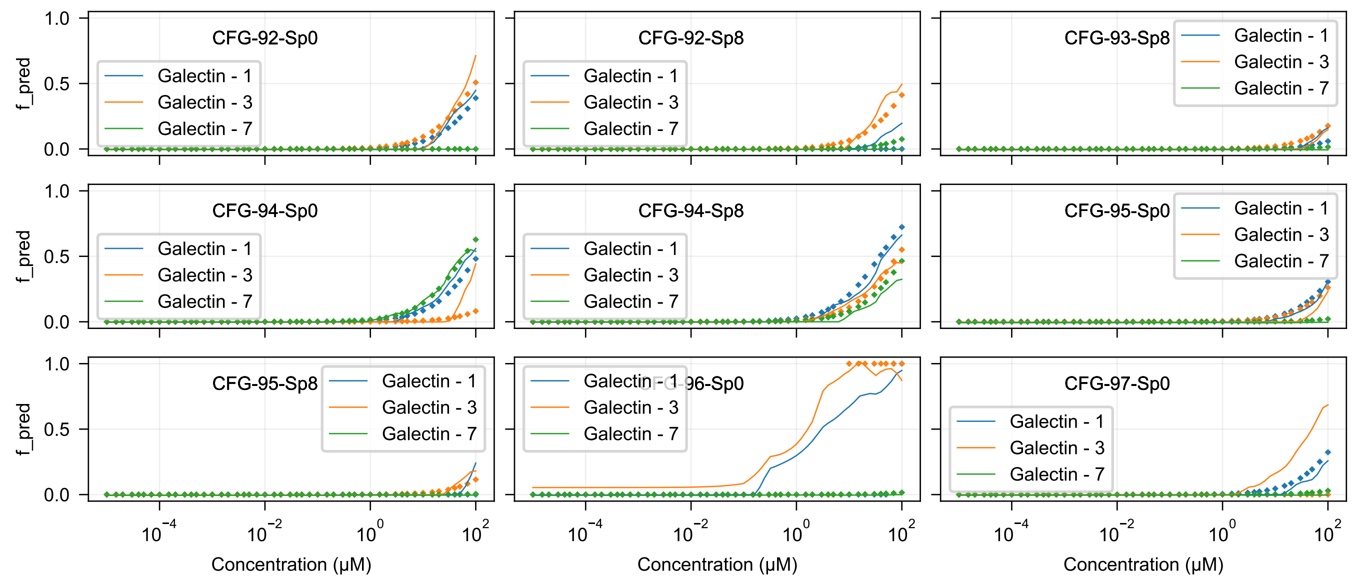

CFG-92-Sp0
CFG-92-Sp8
CFG-93-Sp8
CFG-94-Sp0
CFG-94-Sp8
CFG-95-Sp0
CFG-95-Sp8
CFG-96-Sp0
CFG-97-Sp0
Galectin - 1
Galectin - 3
Galectin - 7
f_pred
Concentration (μM)

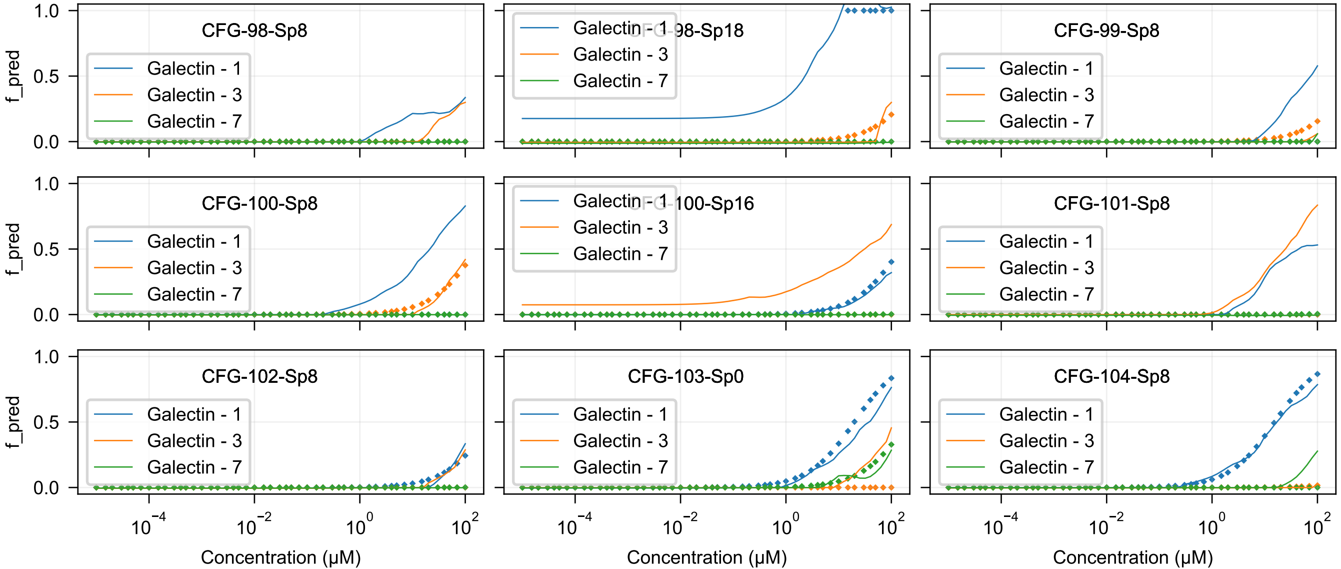

CFG-98-Sp8
CFG-98-Sp18
CFG-99-Sp8
CFG-100-Sp8
CFG-100-Sp16
CFG-101-Sp8
CFG-102-Sp8
CFG-103-Sp0
CFG-104-Sp8
Galectin - 1
Galectin - 3
Galectin - 7
f_pred
Concentration (μM)

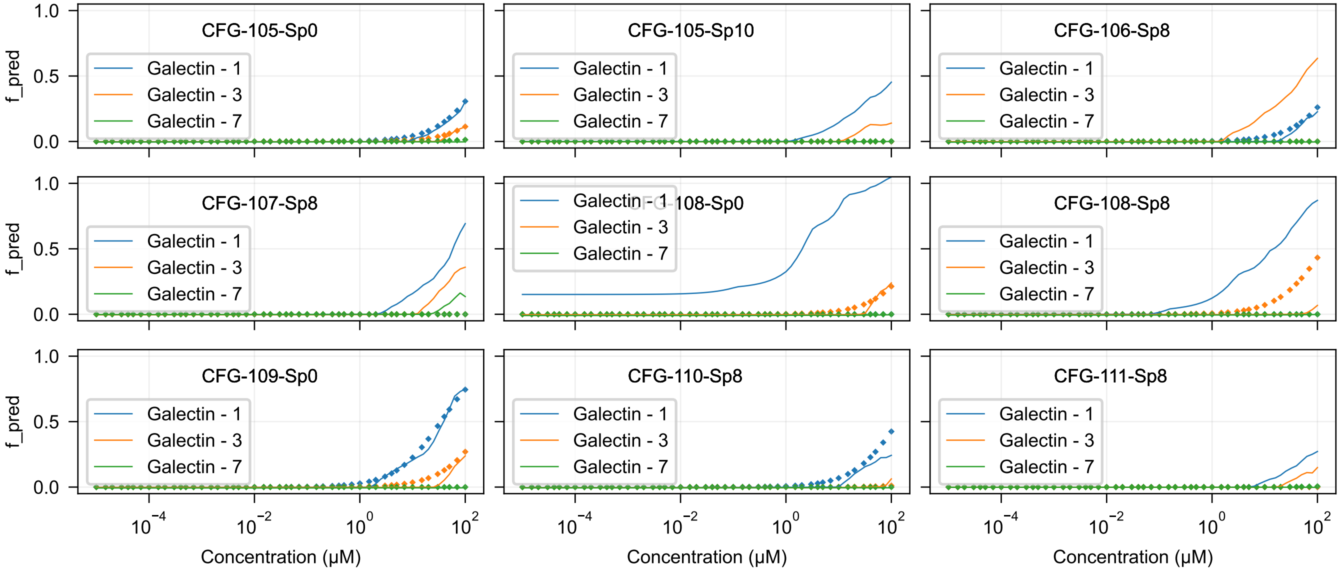
CFG-105-Sp0
CFG-105-Sp10
CFG-106-Sp8
CFG-107-Sp8
CFG-108-Sp0
CFG-108-Sp8
CFG-109-Sp0
CFG-110-Sp8
CFG-111-Sp8
Galectin - 1
Galectin - 3
Galectin - 7
f_pred
Concentration (μM)

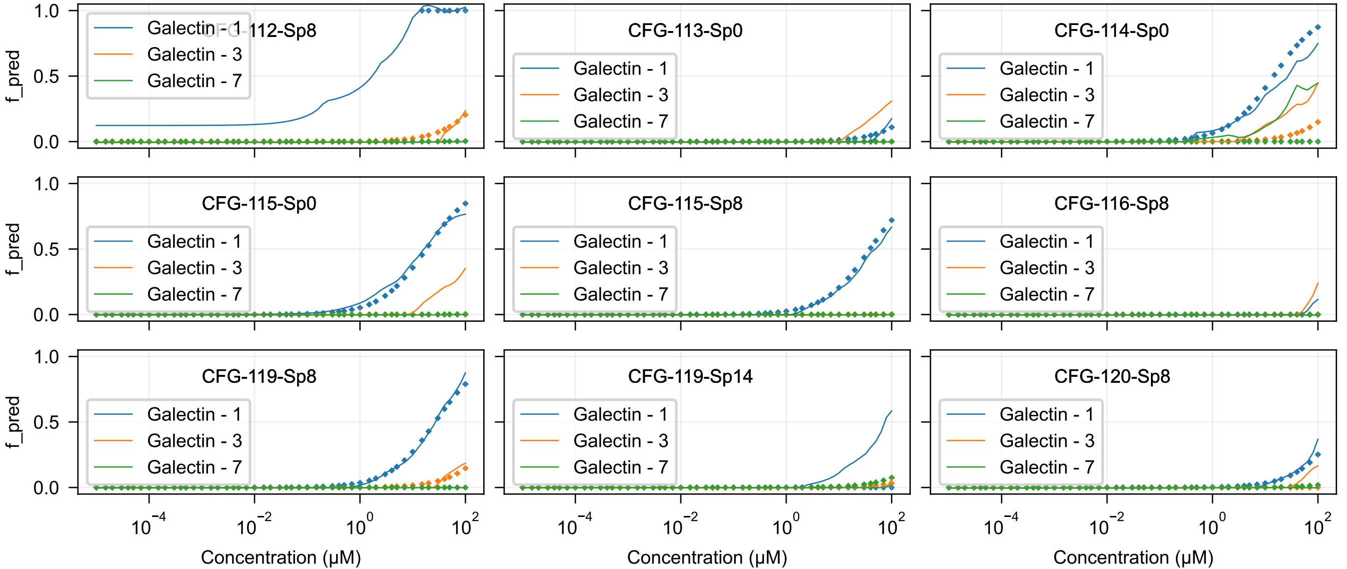
CFG-112-Sp8
CFG-113-Sp0
CFG-114-Sp0
CFG-115-Sp0
CFG-115-Sp8
CFG-116-Sp8
CFG-119-Sp8
CFG-119-Sp14
CFG-120-Sp8
Galectin - 1
Galectin - 3
Galectin - 7
f_pred
Concentration (μM)

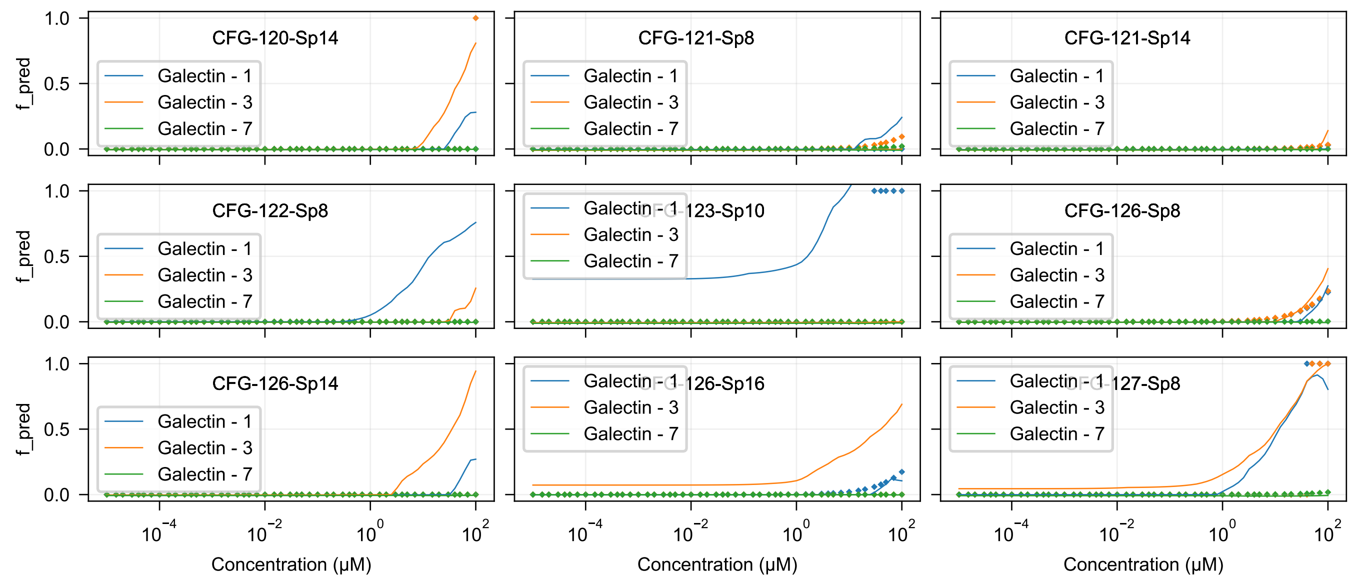

CFG-120-Sp14
CFG-121-Sp8
CFG-121-Sp14
CFG-122-Sp8
CFG-123-Sp10
CFG-126-Sp8
CFG-126-Sp14
CFG-126-Sp16
CFG-127-Sp8
Galectin - 1
Galectin - 3
Galectin - 7
f_pred
Concentration (μM)

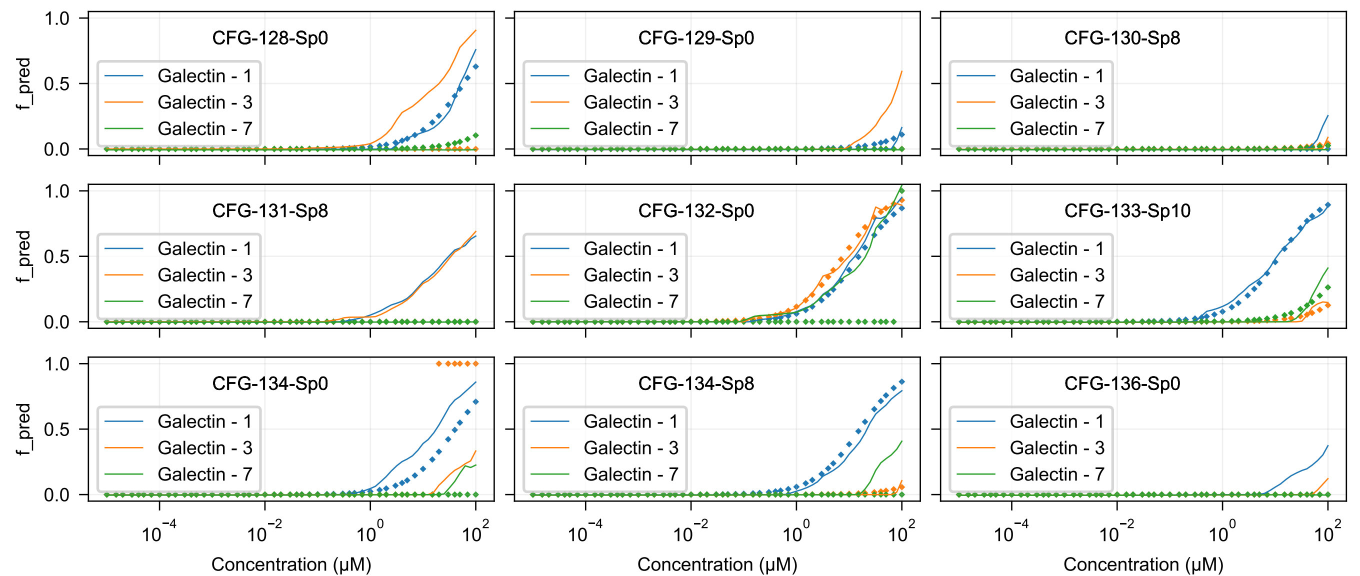

CFG-128-Sp0
CFG-129-Sp0
CFG-130-Sp8
CFG-131-Sp8
CFG-132-Sp0
CFG-133-Sp10
CFG-134-Sp0
CFG-134-Sp8
CFG-136-Sp0
Galectin - 1
Galectin - 3
Galectin - 7
f_pred
Concentration (μM)

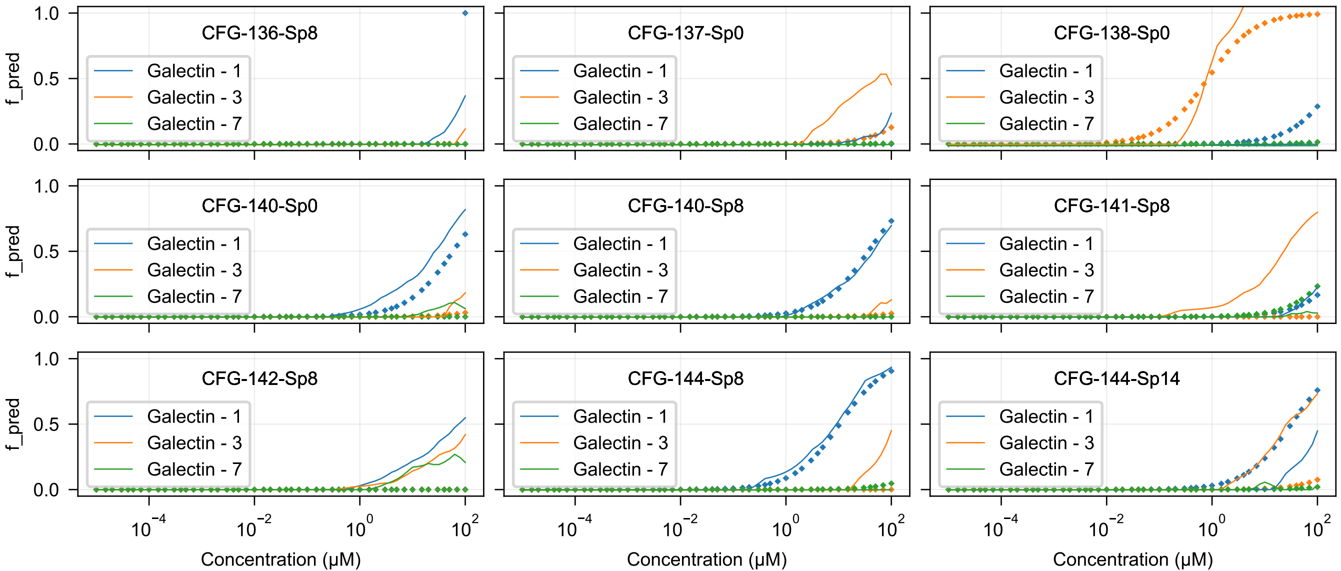

CFG-136-Sp8
CFG-137-Sp0
CFG-138-Sp0
CFG-140-Sp0
CFG-140-Sp8
CFG-141-Sp8
CFG-142-Sp8
CFG-144-Sp8
CFG-144-Sp14
Galectin - 1
Galectin - 3
Galectin - 7
f_pred
Concentration (μM)

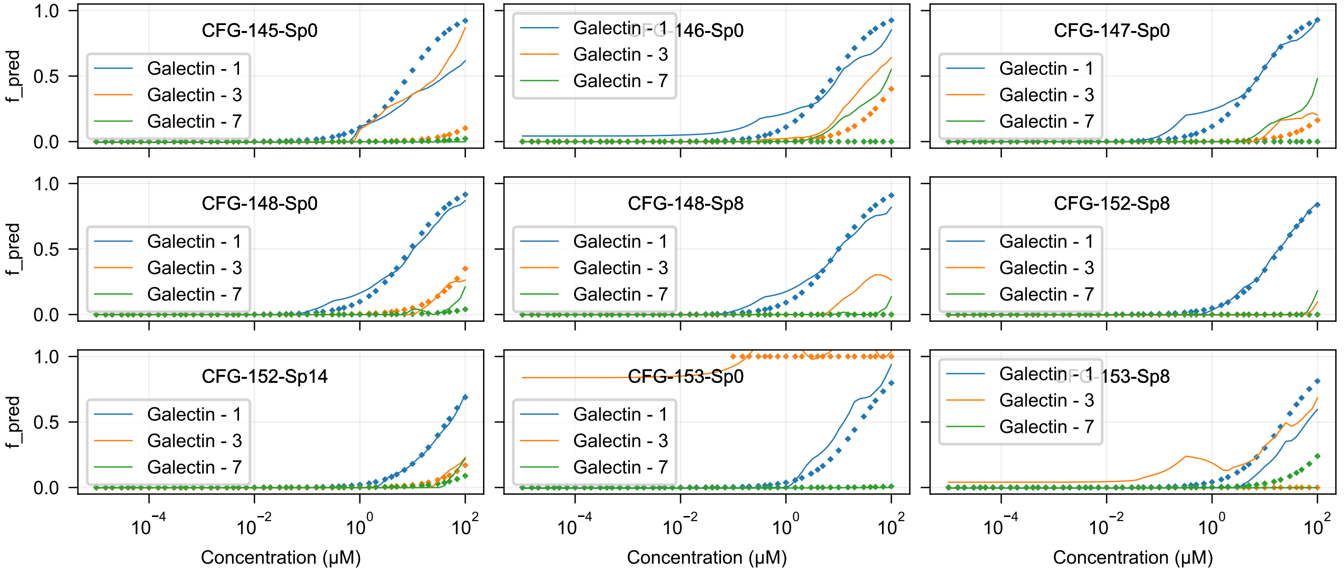
CFG-145-Sp0
CFG-146-Sp0
CFG-147-Sp0
CFG-148-Sp0
CFG-148-Sp8
CFG-152-Sp8
CFG-152-Sp14
CFG-153-Sp0
CFG-153-Sp8
Galectin - 1
Galectin - 3
Galectin - 7
f_pred
Concentration (μM)

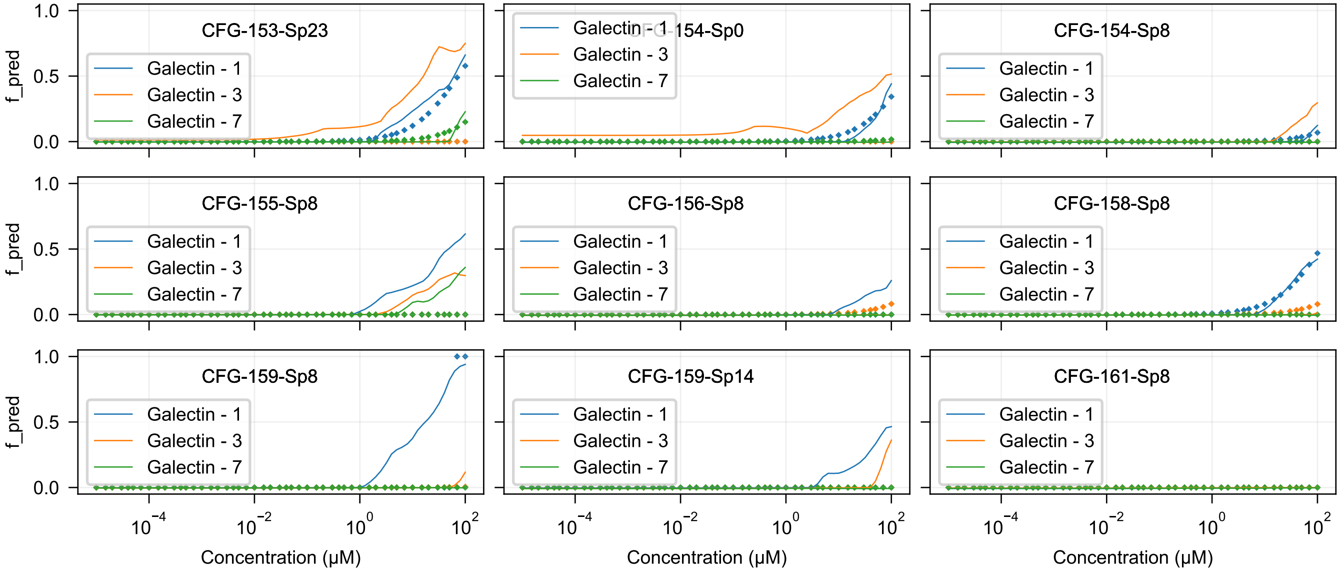
CFG-153-Sp23
CFG-154-Sp0
CFG-154-Sp8
CFG-155-Sp8
CFG-156-Sp8
CFG-158-Sp8
CFG-159-Sp8
CFG-159-Sp14
CFG-161-Sp8
Galectin - 1
Galectin - 3
Galectin - 7
f_pred
1.0
0.5
0.0
10^{-4}
10^{-2}
10^{0}
10^{2}
Concentration (μM)

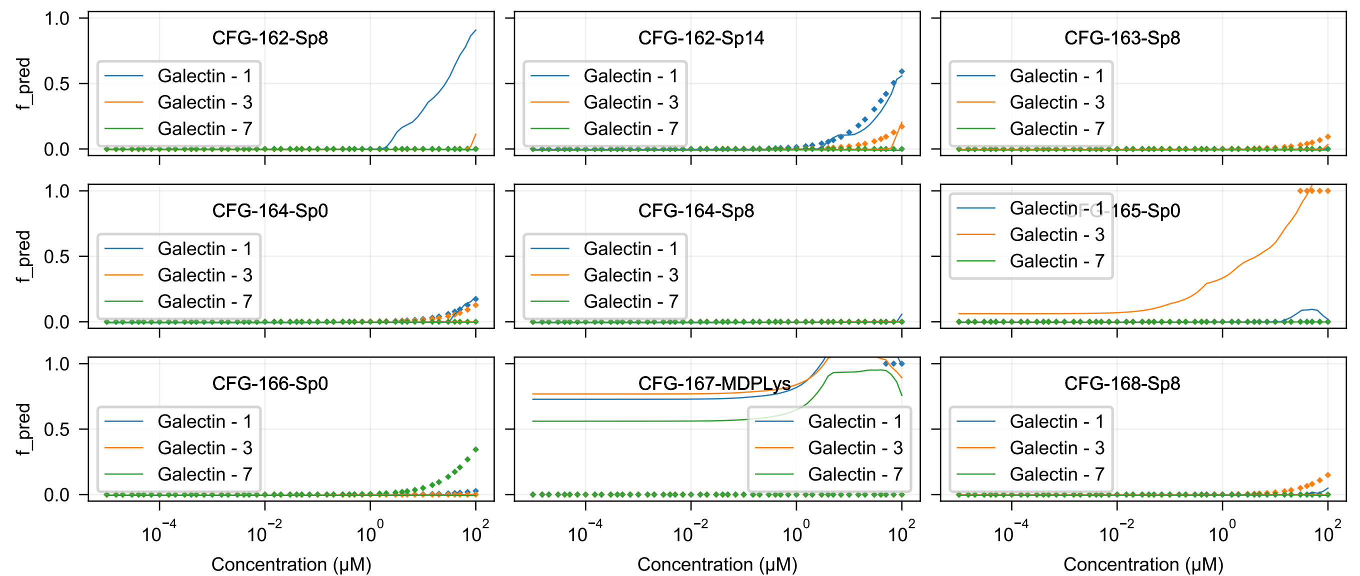

CFG-162-Sp8
CFG-162-Sp14
CFG-163-Sp8
CFG-164-Sp0
CFG-164-Sp8
CFG-165-Sp0
CFG-166-Sp0
CFG-167-MDPLys
CFG-168-Sp8
Galectin - 1
Galectin - 3
Galectin - 7
f_pred
Concentration (μM)

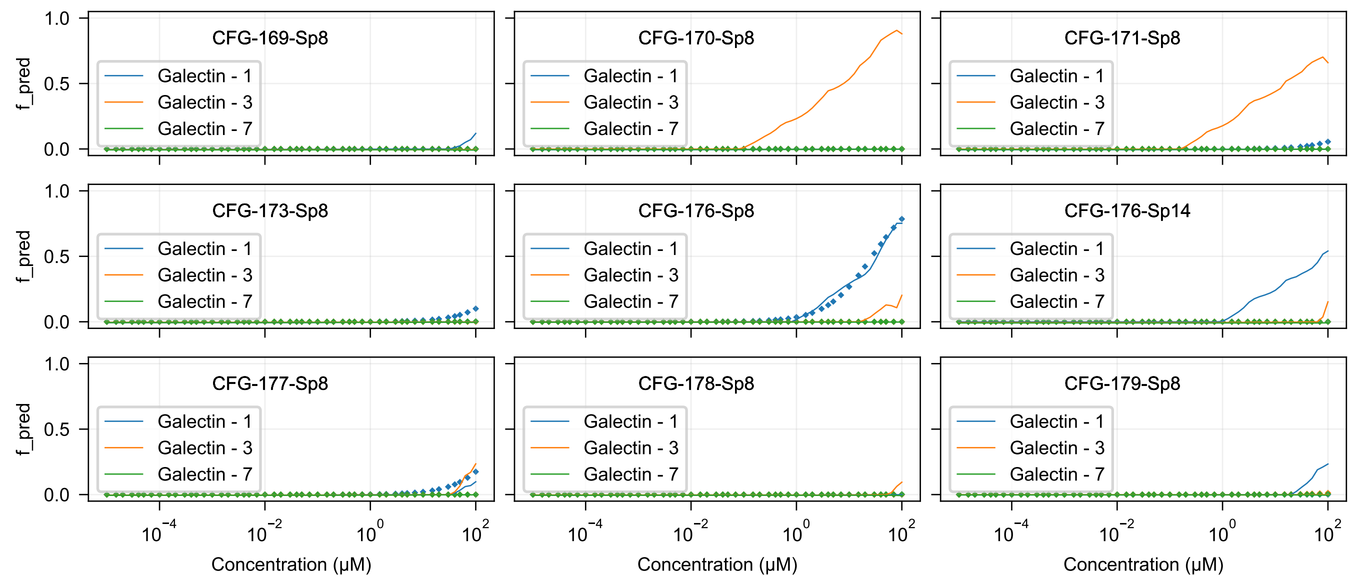
CFG-169-Sp8
CFG-170-Sp8
CFG-171-Sp8
CFG-173-Sp8
CFG-176-Sp8
CFG-176-Sp14
CFG-177-Sp8
CFG-178-Sp8
CFG-179-Sp8
Galectin - 1
Galectin - 3
Galectin - 7
f_pred
Concentration (μM)

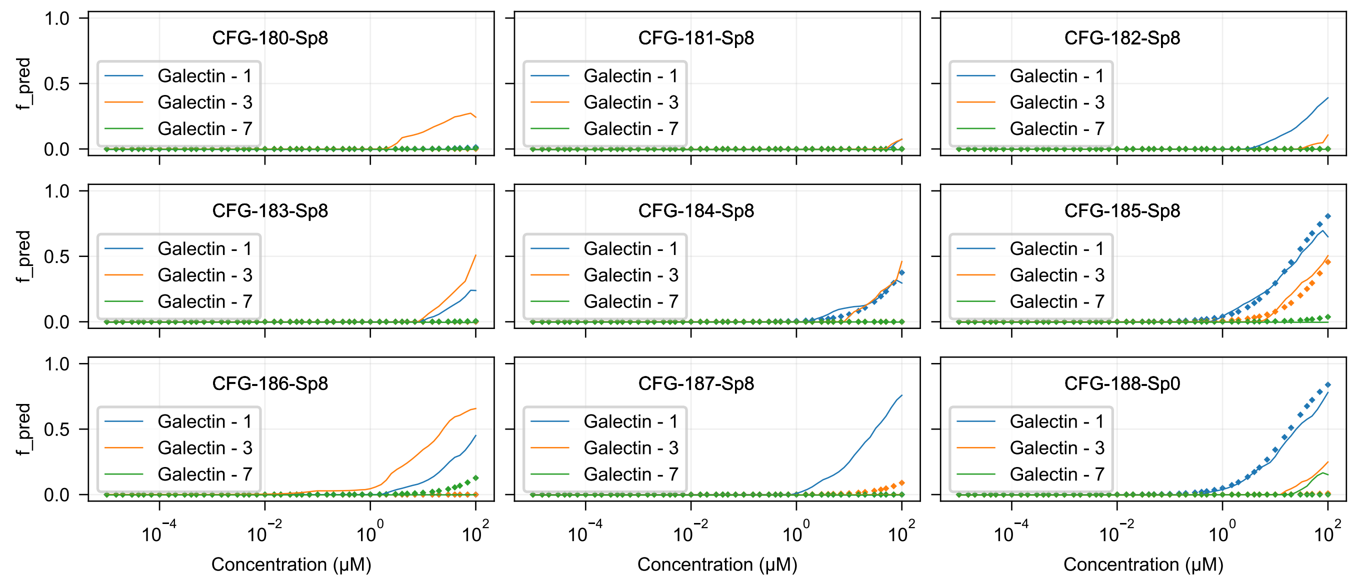

CFG-180-Sp8
CFG-181-Sp8
CFG-182-Sp8
CFG-183-Sp8
CFG-184-Sp8
CFG-185-Sp8
CFG-186-Sp8
CFG-187-Sp8
CFG-188-Sp0
Galectin - 1
Galectin - 3
Galectin - 7
f_pred
0.0
0.5
1.0
10^{-4}
10^{-2}
10^{0}
10^{2}
Concentration (μM)

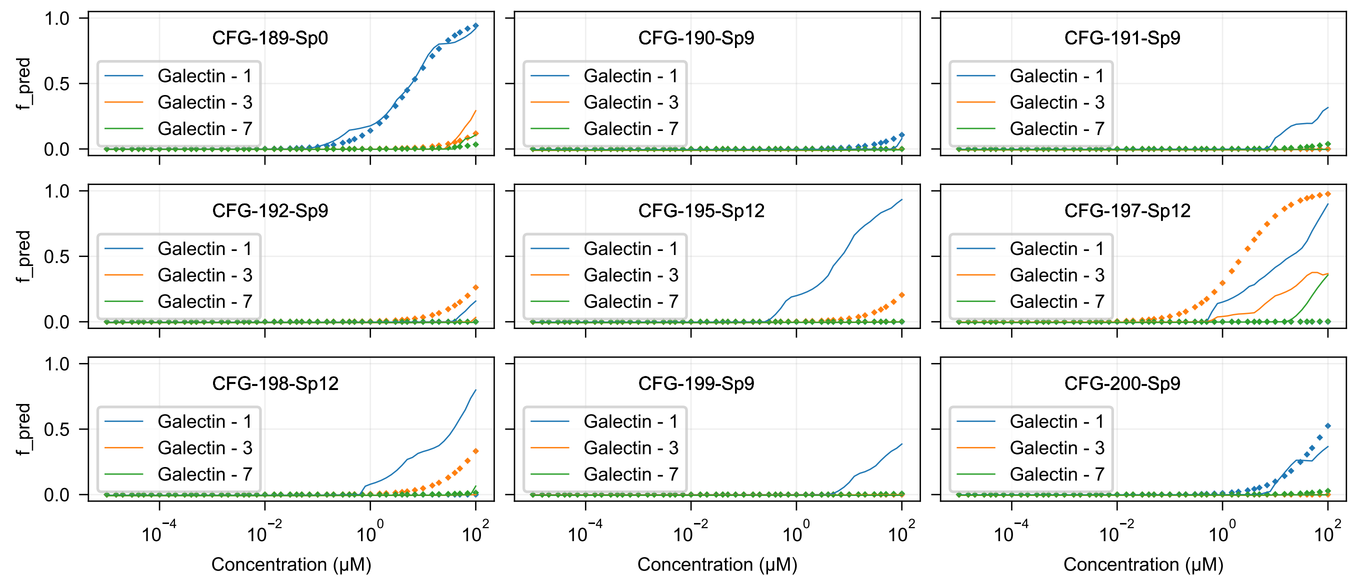
CFG-189-Sp0
CFG-190-Sp9
CFG-191-Sp9
CFG-192-Sp9
CFG-195-Sp12
CFG-197-Sp12
CFG-198-Sp12
CFG-199-Sp9
CFG-200-Sp9
Galectin - 1
Galectin - 3
Galectin - 7
f_pred
1.0
0.5
0.0
10^{-4}
10^{-2}
10^{0}
10^{2}
Concentration (μM)

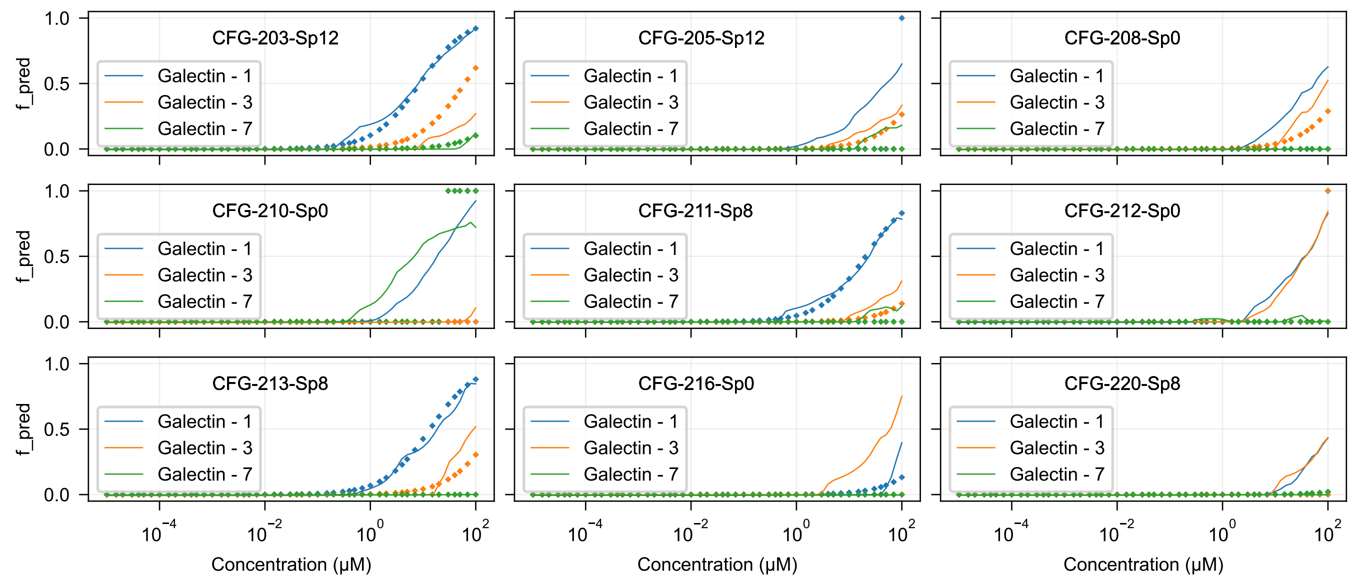
CFG-203-Sp12
CFG-205-Sp12
CFG-208-Sp0
CFG-210-Sp0
CFG-211-Sp8
CFG-212-Sp0
CFG-213-Sp8
CFG-216-Sp0
CFG-220-Sp8
Galectin - 1
Galectin - 3
Galectin - 7
f_pred
Concentration (μM)

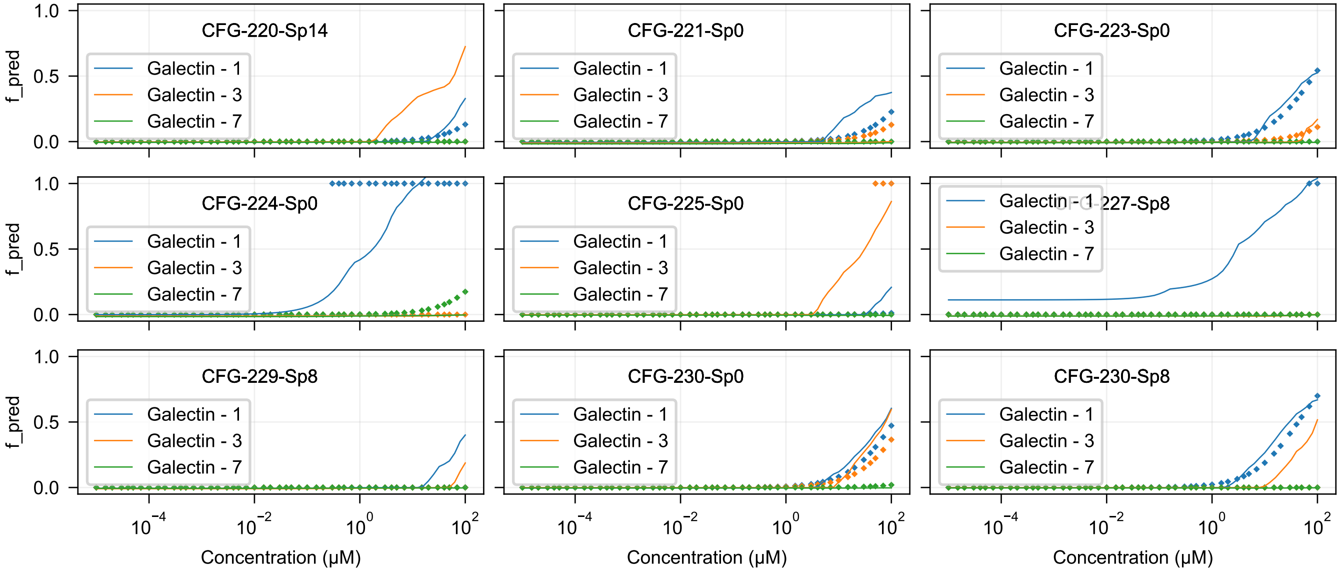

CFG-220-Sp14
CFG-221-Sp0
CFG-223-Sp0
CFG-224-Sp0
CFG-225-Sp0
CFG-227-Sp8
CFG-229-Sp8
CFG-230-Sp0
CFG-230-Sp8
Galectin - 1
Galectin - 3
Galectin - 7
f_pred
1.0
0.5
0.0
10^{-4}
10^{-2}
10^{0}
10^{2}
Concentration (μM)

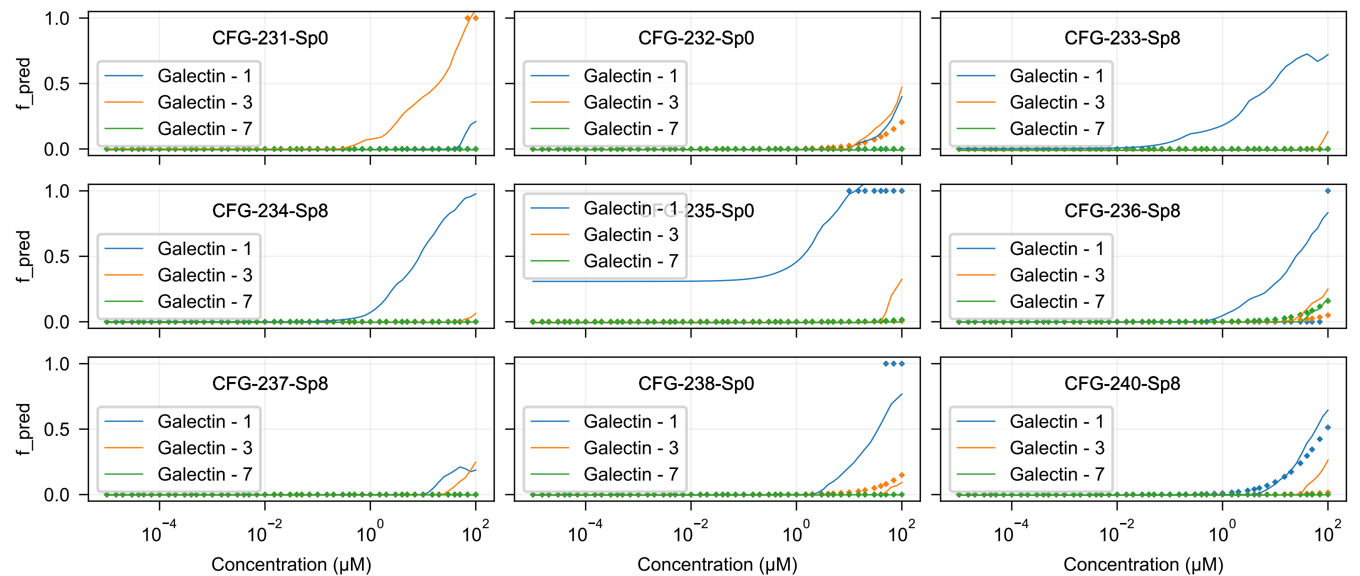

CFG-231-Sp0
CFG-232-Sp0
CFG-233-Sp8
CFG-234-Sp8
CFG-235-Sp0
CFG-236-Sp8
CFG-237-Sp8
CFG-238-Sp0
CFG-240-Sp8
Galectin - 1
Galectin - 3
Galectin - 7
f_pred
1.0
0.5
0.0
10^{-4}
10^{-2}
10^{0}
10^{2}
Concentration (μM)

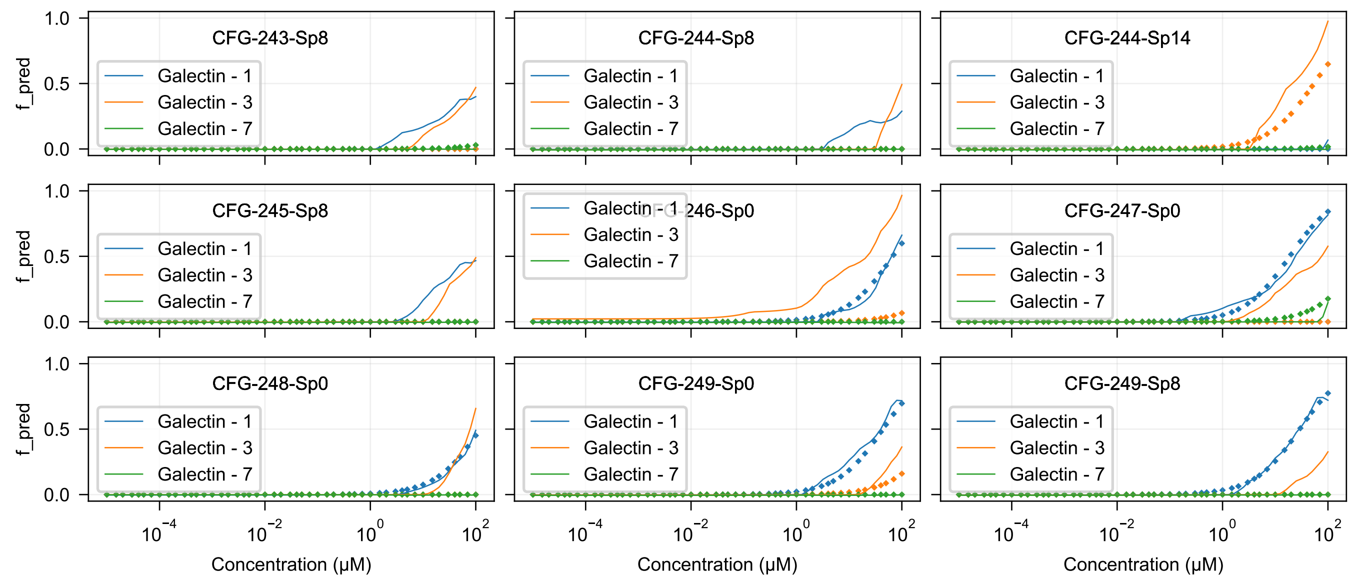

CFG-243-Sp8
CFG-244-Sp8
CFG-244-Sp14
CFG-245-Sp8
CFG-246-Sp0
CFG-247-Sp0
CFG-248-Sp0
CFG-249-Sp0
CFG-249-Sp8
Galectin - 1
Galectin - 3
Galectin - 7
f_pred
Concentration (μM)

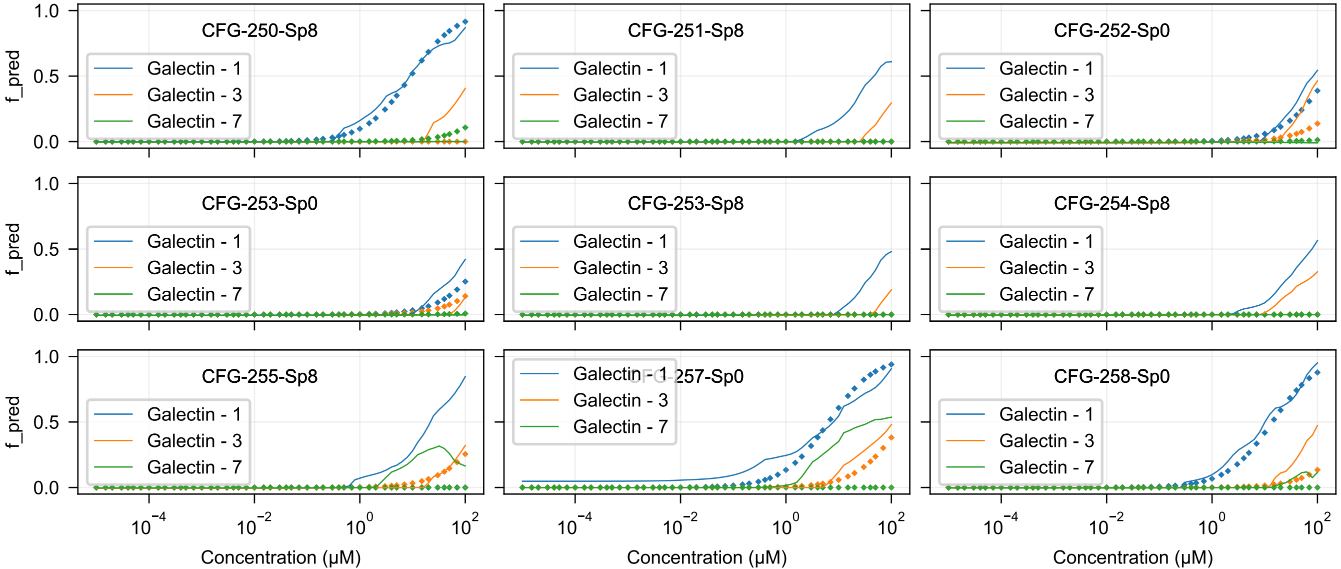

CFG-250-Sp8
CFG-251-Sp8
CFG-252-Sp0
CFG-253-Sp0
CFG-253-Sp8
CFG-254-Sp8
CFG-255-Sp8
CFG-257-Sp0
CFG-258-Sp0
Galectin - 1
Galectin - 3
Galectin - 7
f_pred
1.0
0.5
0.0
10^{-4}
10^{-2}
10^{0}
10^{2}
Concentration (μM)

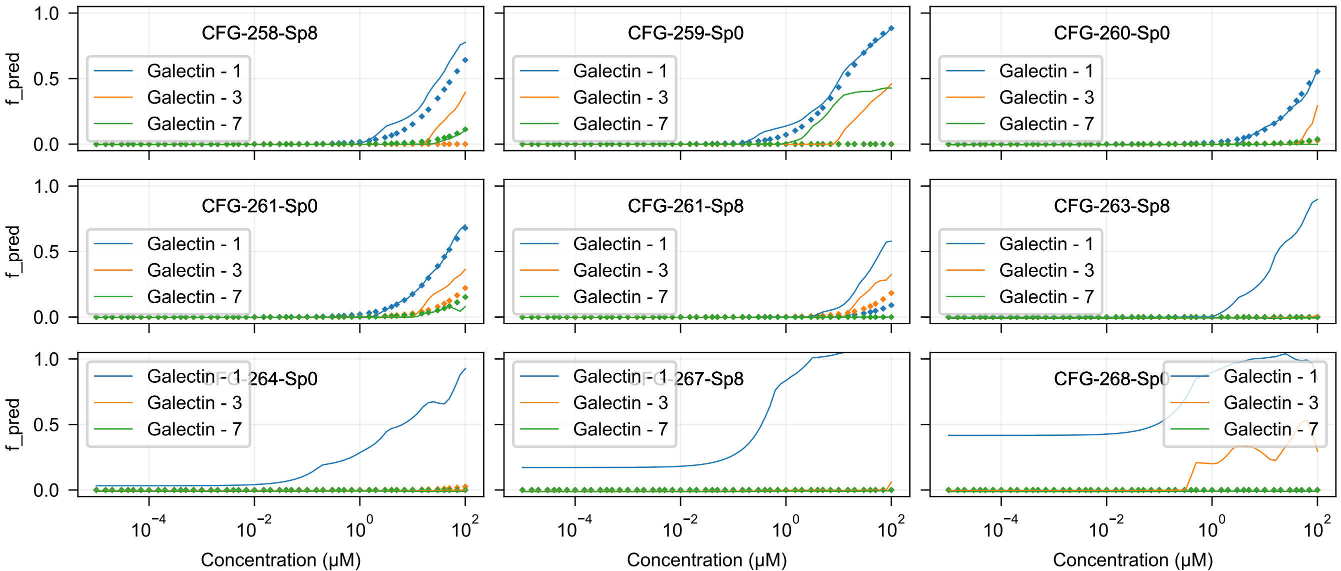
CFG-258-Sp8
CFG-259-Sp0
CFG-260-Sp0
CFG-261-Sp0
CFG-261-Sp8
CFG-263-Sp8
CFG-264-Sp0
CFG-267-Sp8
CFG-268-Sp0
Galectin - 1
Galectin - 3
Galectin - 7
f_pred
Concentration (μM)

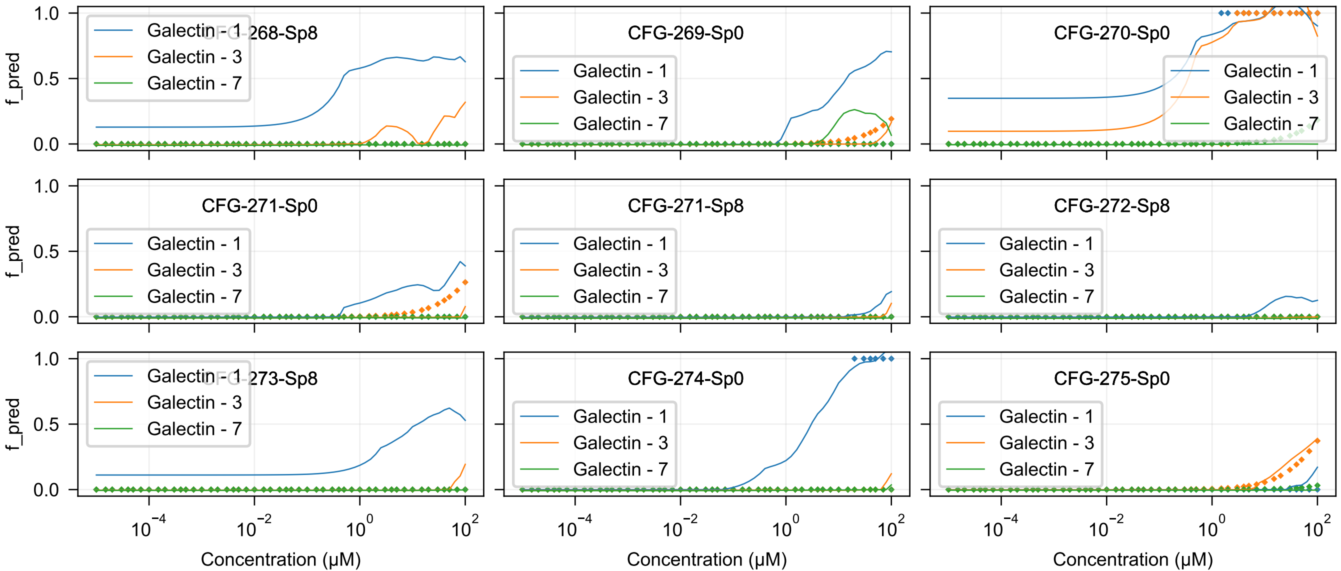

CFG-268-Sp8
CFG-269-Sp0
CFG-270-Sp0
CFG-271-Sp0
CFG-271-Sp8
CFG-272-Sp8
CFG-273-Sp8
CFG-274-Sp0
CFG-275-Sp0
Galectin - 1
Galectin - 3
Galectin - 7
f_pred
1.0
0.5
0.0
10^{-4}
10^{-2}
10^{0}
10^{2}
Concentration (μM)

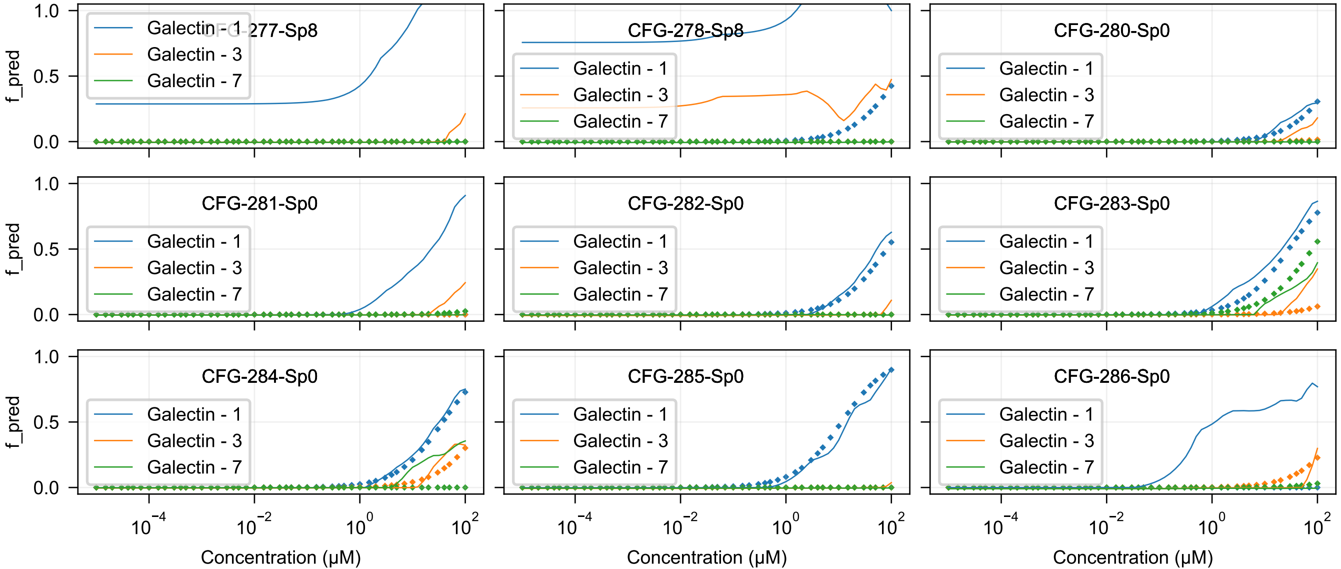

CFG-277-Sp8
CFG-278-Sp8
CFG-280-Sp0
CFG-281-Sp0
CFG-282-Sp0
CFG-283-Sp0
CFG-284-Sp0
CFG-285-Sp0
CFG-286-Sp0
Galectin - 1
Galectin - 3
Galectin - 7
f_pred
1.0
0.5
0.0
10^{-4}
10^{-2}
10^{0}
10^{2}
Concentration (μM)

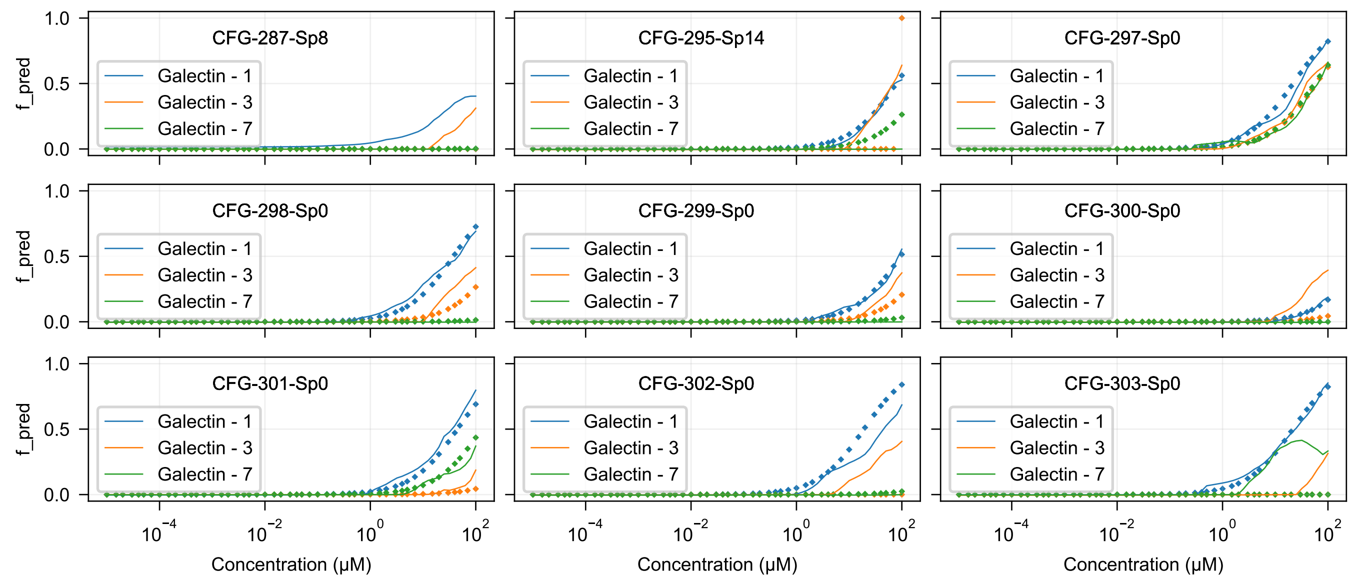

CFG-287-Sp8
CFG-295-Sp14
CFG-297-Sp0
CFG-298-Sp0
CFG-299-Sp0
CFG-300-Sp0
CFG-301-Sp0
CFG-302-Sp0
CFG-303-Sp0
Galectin - 1
Galectin - 3
Galectin - 7
f_pred
1.0
0.5
0.0
10^-4
10^-2
10^0
10^2
Concentration (μM)

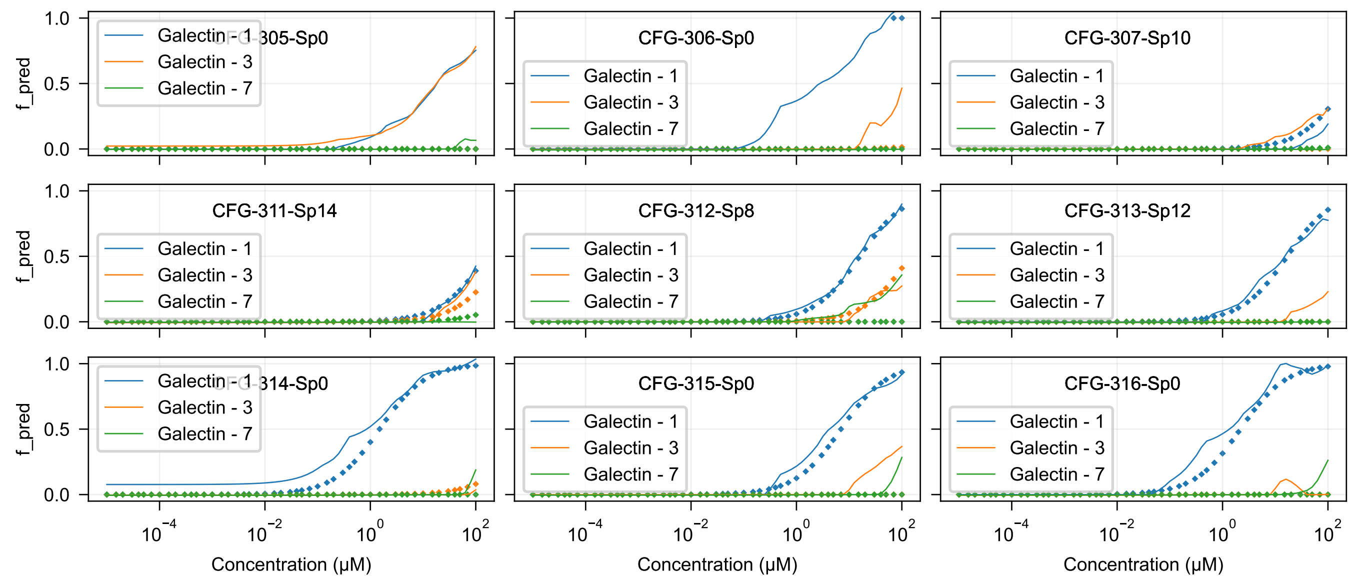
CFG-305-Sp0
CFG-306-Sp0
CFG-307-Sp10
CFG-311-Sp14
CFG-312-Sp8
CFG-313-Sp12
CFG-314-Sp0
CFG-315-Sp0
CFG-316-Sp0
Galectin - 1
Galectin - 3
Galectin - 7
f_pred
Concentration (μM)

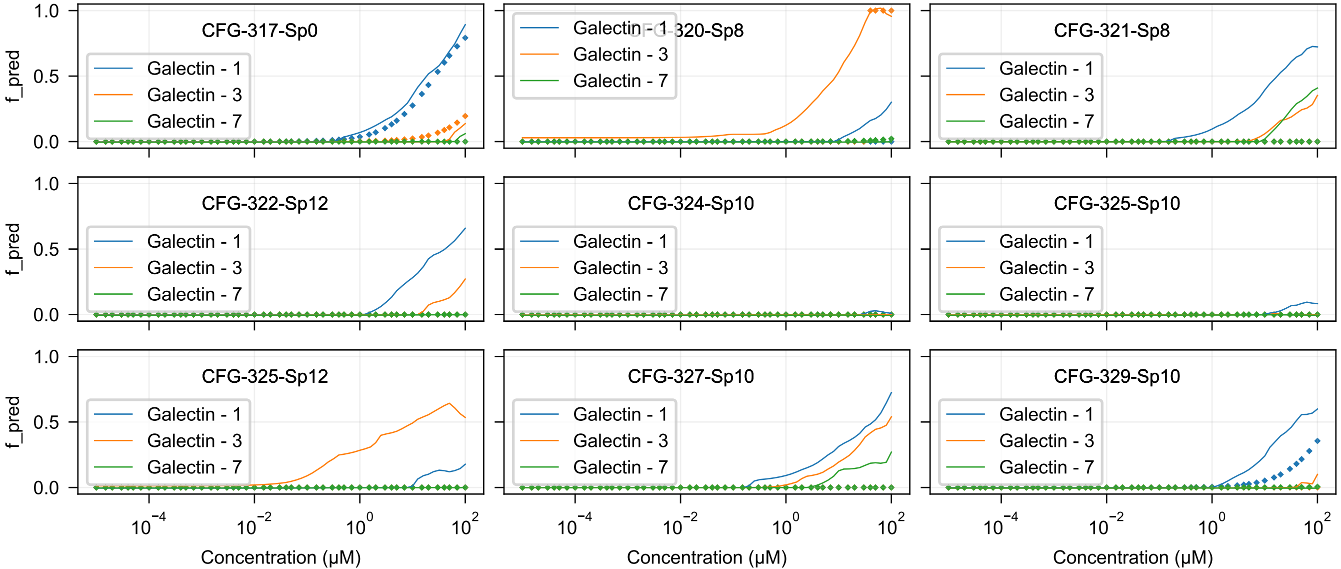

CFG-317-Sp0
CFG-320-Sp8
CFG-321-Sp8
CFG-322-Sp12
CFG-324-Sp10
CFG-325-Sp10
CFG-325-Sp12
CFG-327-Sp10
CFG-329-Sp10
Galectin - 1
Galectin - 3
Galectin - 7
f_pred
Concentration (μM)

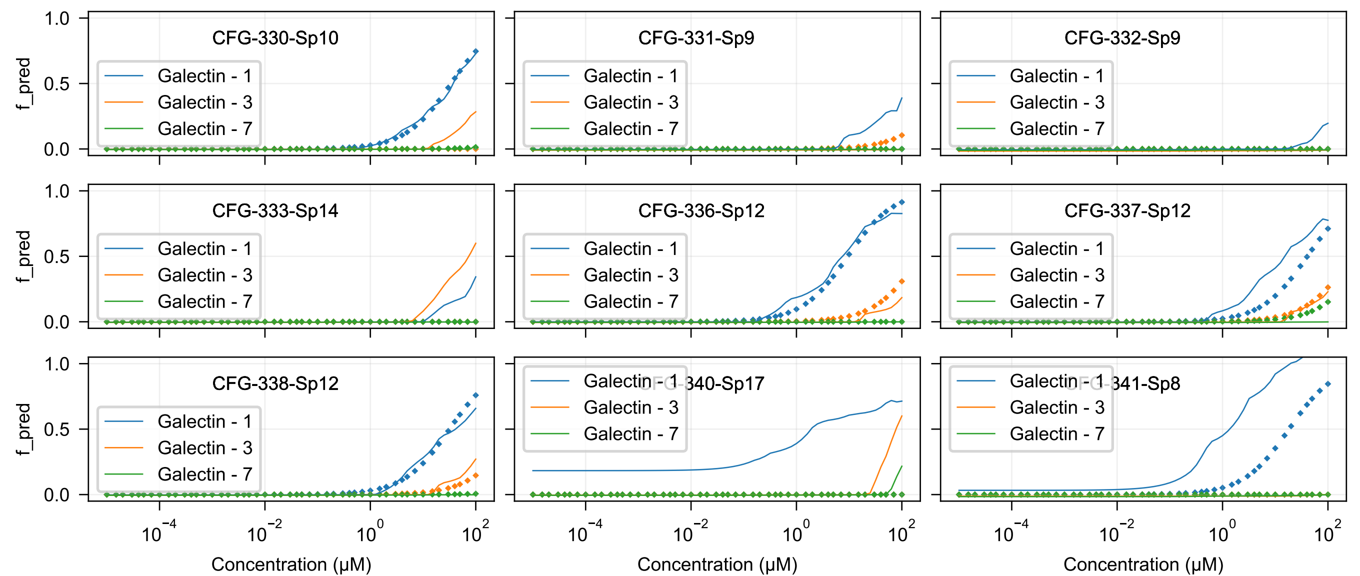
CFG-330-Sp10
CFG-331-Sp9
CFG-332-Sp9
CFG-333-Sp14
CFG-336-Sp12
CFG-337-Sp12
CFG-338-Sp12
CFG-340-Sp17
CFG-341-Sp8
Galectin - 1
Galectin - 3
Galectin - 7
f_pred
Concentration (μM)
0.0
0.5
1.0
10^{-4}
10^{-2}
10^{0}
10^{2}

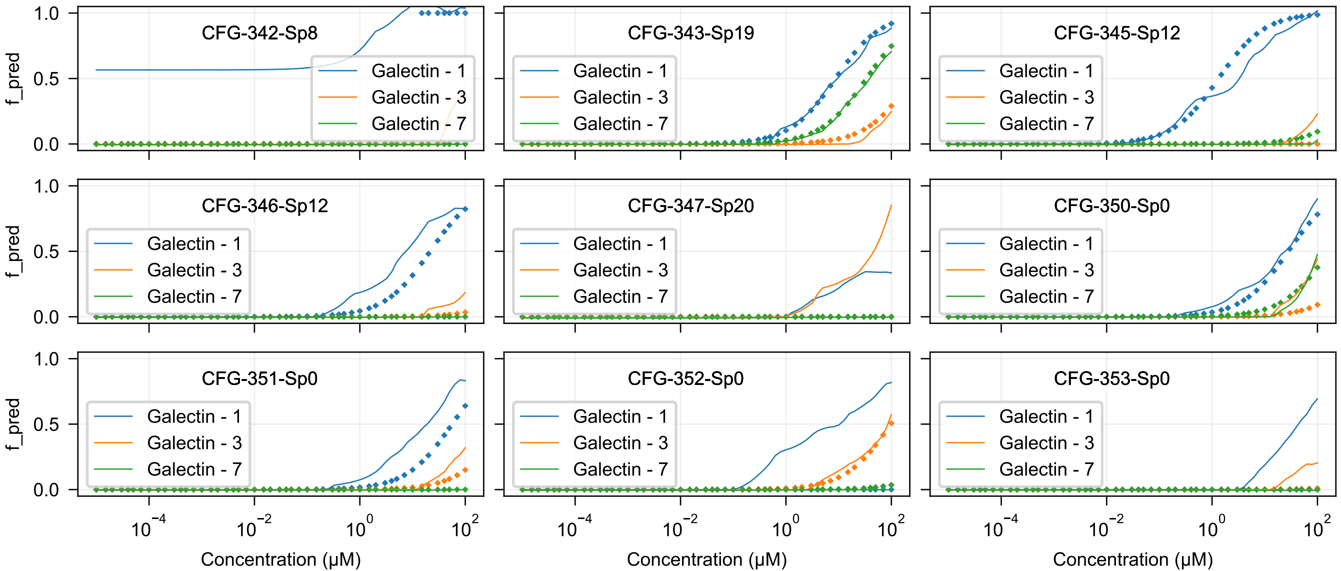

CFG-342-Sp8
CFG-343-Sp19
CFG-345-Sp12
CFG-346-Sp12
CFG-347-Sp20
CFG-350-Sp0
CFG-351-Sp0
CFG-352-Sp0
CFG-353-Sp0
Galectin - 1
Galectin - 3
Galectin - 7
f_pred
1.0
0.5
0.0
10^{-4}
10^{-2}
10^{0}
10^{2}
Concentration (μM)

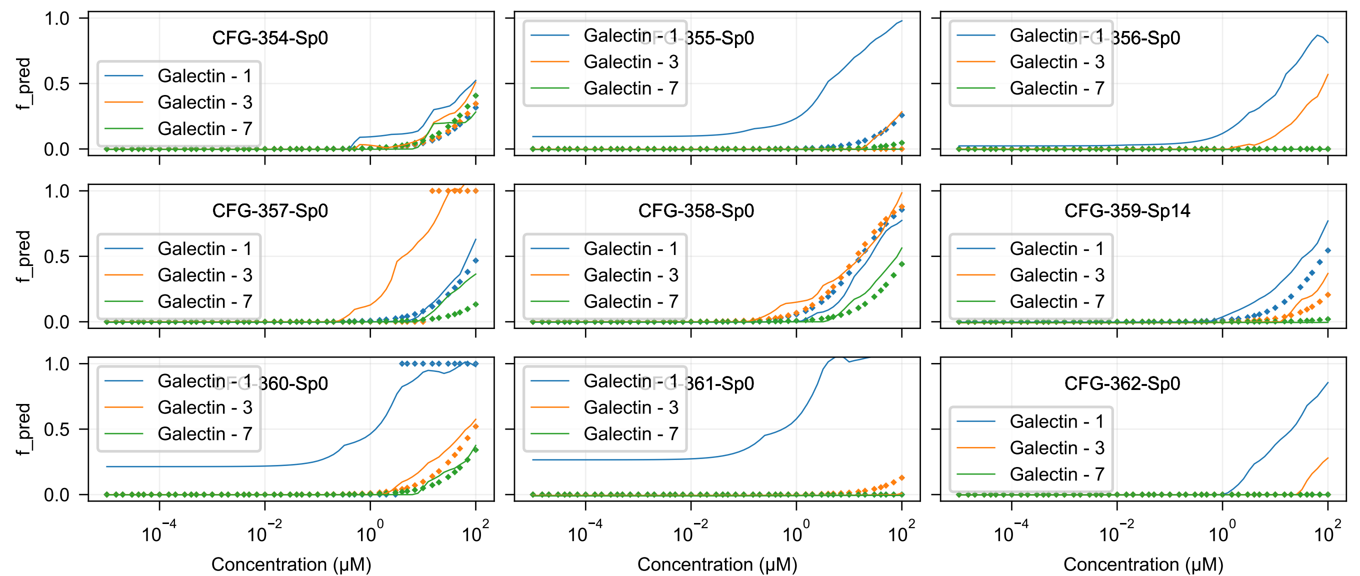
CFG-354-Sp0
CFG-355-Sp0
CFG-356-Sp0
CFG-357-Sp0
CFG-358-Sp0
CFG-359-Sp14
CFG-360-Sp0
CFG-361-Sp0
CFG-362-Sp0
Galectin - 1
Galectin - 3
Galectin - 7
f_pred
1.0
0.5
0.0
10^{-4}
10^{-2}
10^{0}
10^{2}
Concentration (μM)

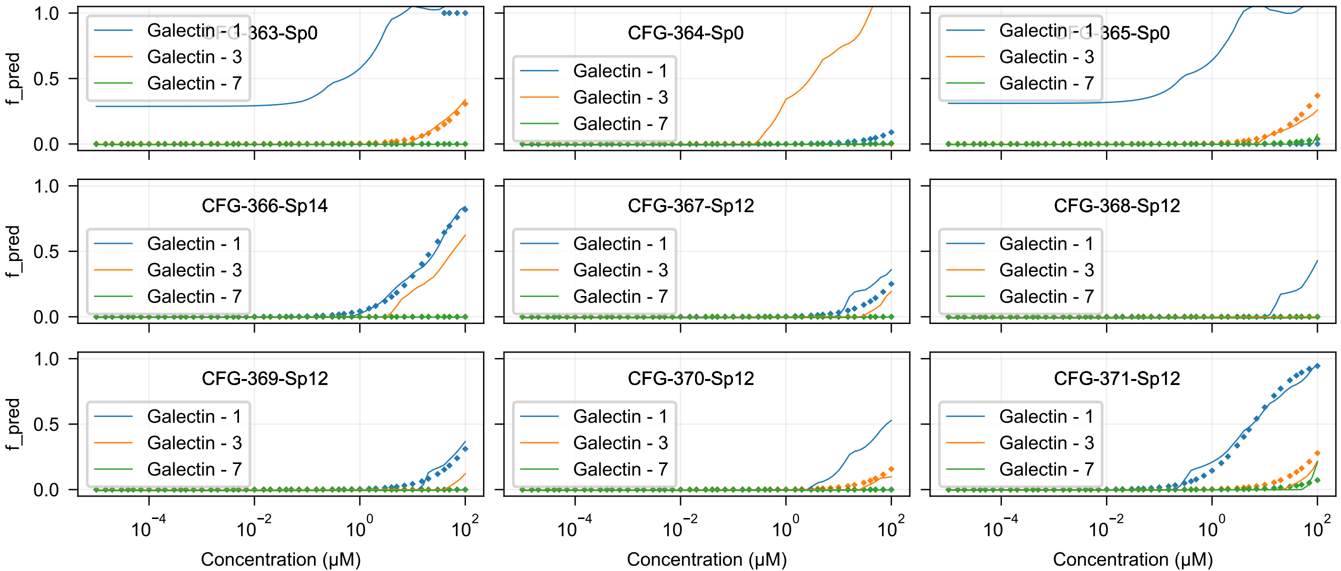

CFG-363-Sp0
CFG-364-Sp0
CFG-365-Sp0
CFG-366-Sp14
CFG-367-Sp12
CFG-368-Sp12
CFG-369-Sp12
CFG-370-Sp12
CFG-371-Sp12
Galectin - 1
Galectin - 3
Galectin - 7
f_pred
Concentration (μM)

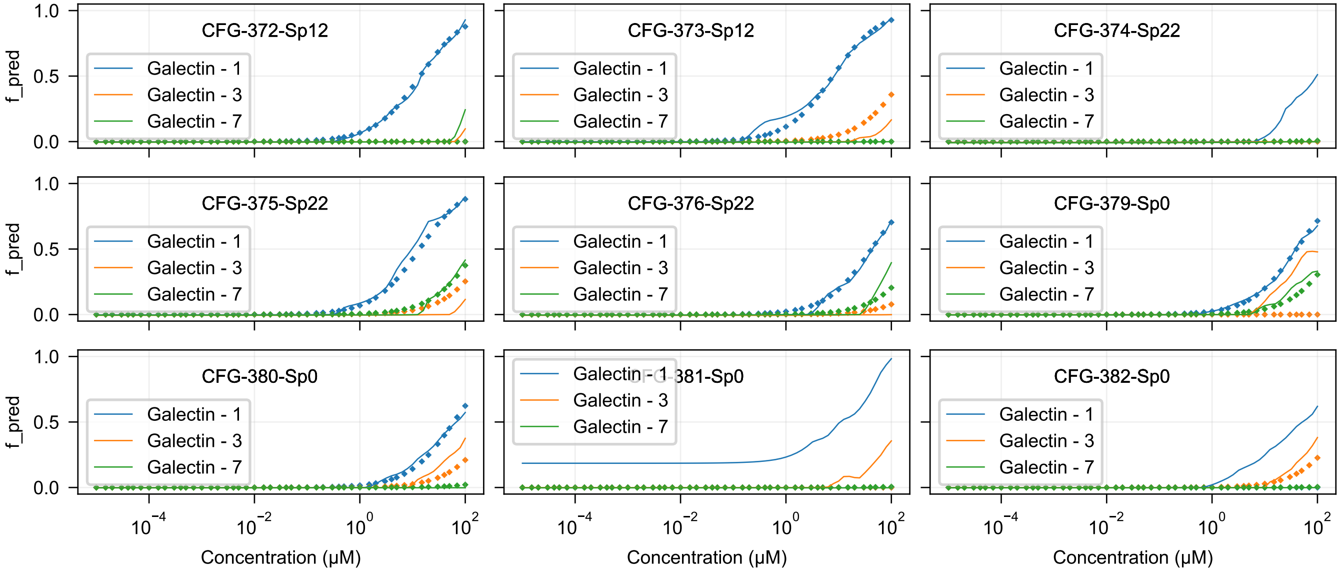
CFG-372-Sp12
CFG-373-Sp12
CFG-374-Sp22
CFG-375-Sp22
CFG-376-Sp22
CFG-379-Sp0
CFG-380-Sp0
CFG-381-Sp0
CFG-382-Sp0
Galectin - 1
Galectin - 3
Galectin - 7
f_pred
1.0
0.5
0.0
10^{-4}
10^{-2}
10^{0}
10^{2}
Concentration (μM)

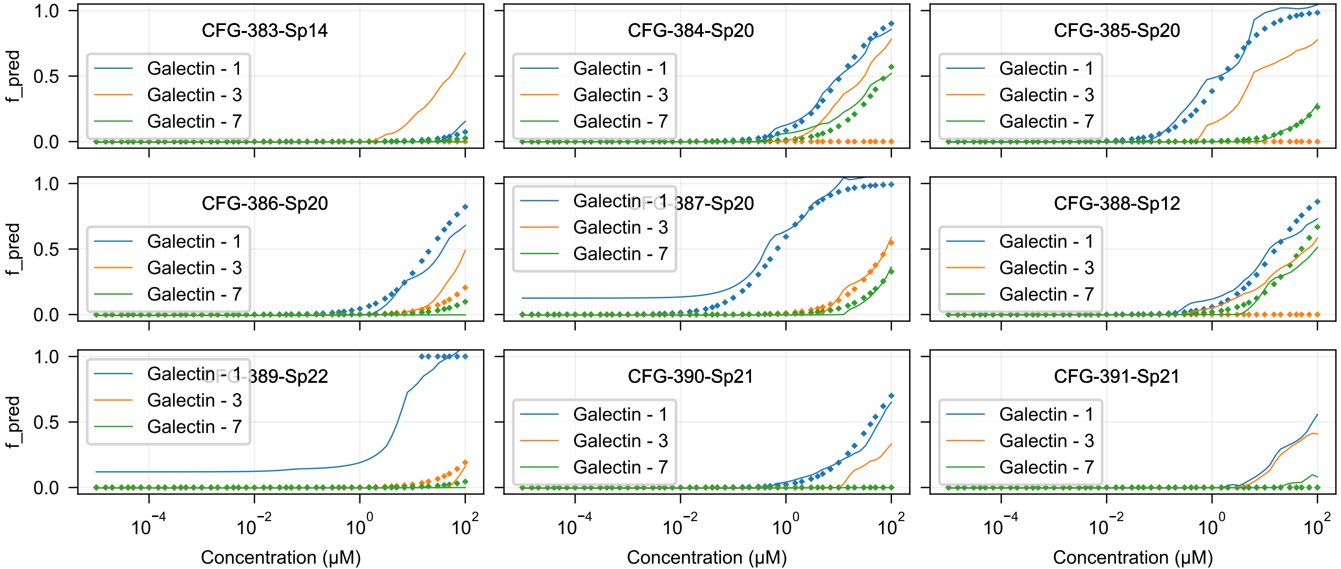

CFG-383-Sp14
CFG-384-Sp20
CFG-385-Sp20
CFG-386-Sp20
CFG-387-Sp20
CFG-388-Sp12
CFG-389-Sp22
CFG-390-Sp21
CFG-391-Sp21
Galectin - 1
Galectin - 3
Galectin - 7
f_pred
1.0
0.5
0.0
10^{-4}
10^{-2}
10^{0}
10^{2}
Concentration (μM)

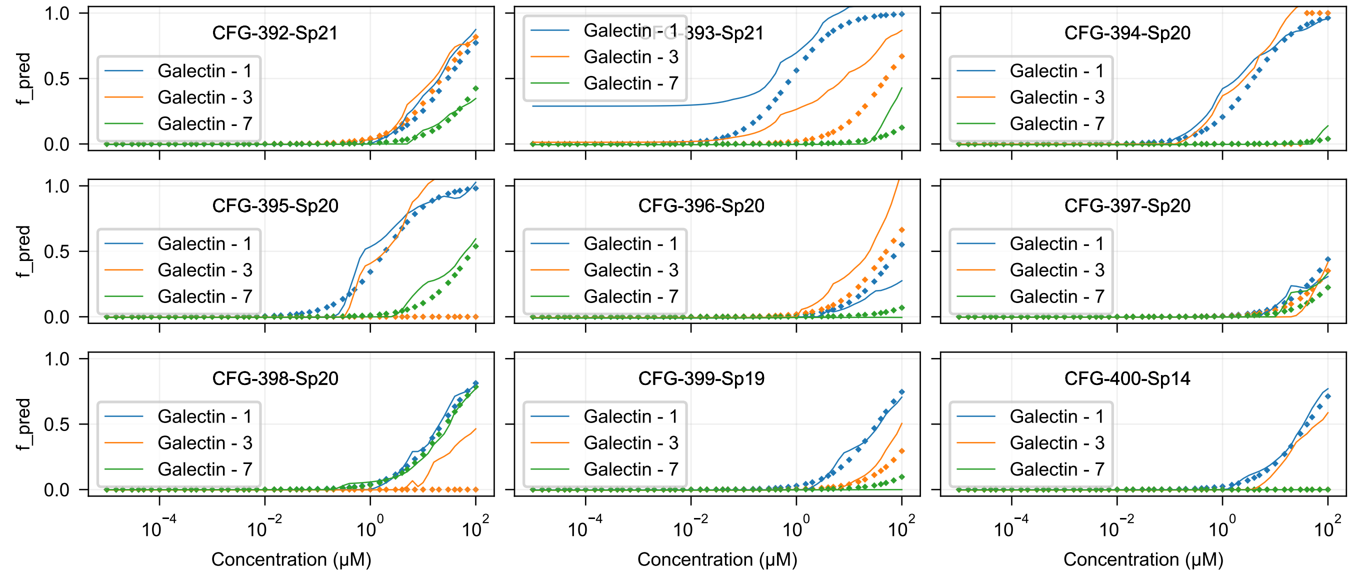
CFG-392-Sp21
CFG-393-Sp21
CFG-394-Sp20
CFG-395-Sp20
CFG-396-Sp20
CFG-397-Sp20
CFG-398-Sp20
CFG-399-Sp19
CFG-400-Sp14
Galectin - 1
Galectin - 3
Galectin - 7
f_pred
1.0
0.5
0.0
10^{-4}
10^{-2}
10^{0}
10^{2}
Concentration (μM)

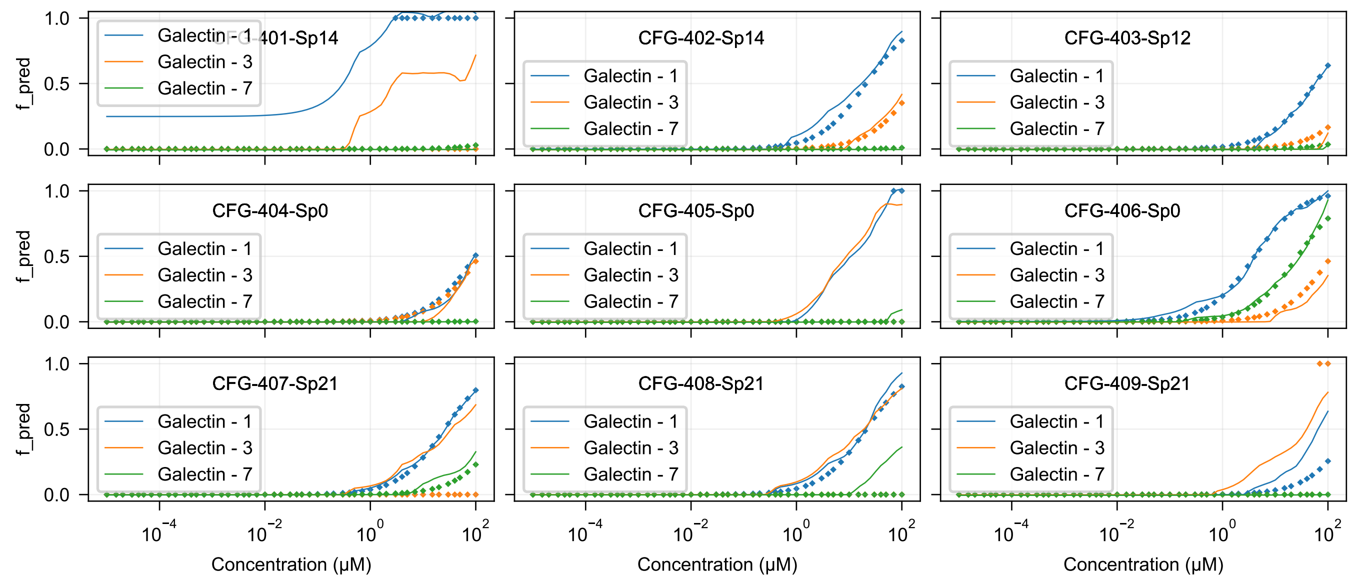

CFG-401-Sp14
CFG-402-Sp14
CFG-403-Sp12
CFG-404-Sp0
CFG-405-Sp0
CFG-406-Sp0
CFG-407-Sp21
CFG-408-Sp21
CFG-409-Sp21
Galectin - 1
Galectin - 3
Galectin - 7
f_pred
Concentration (μM)

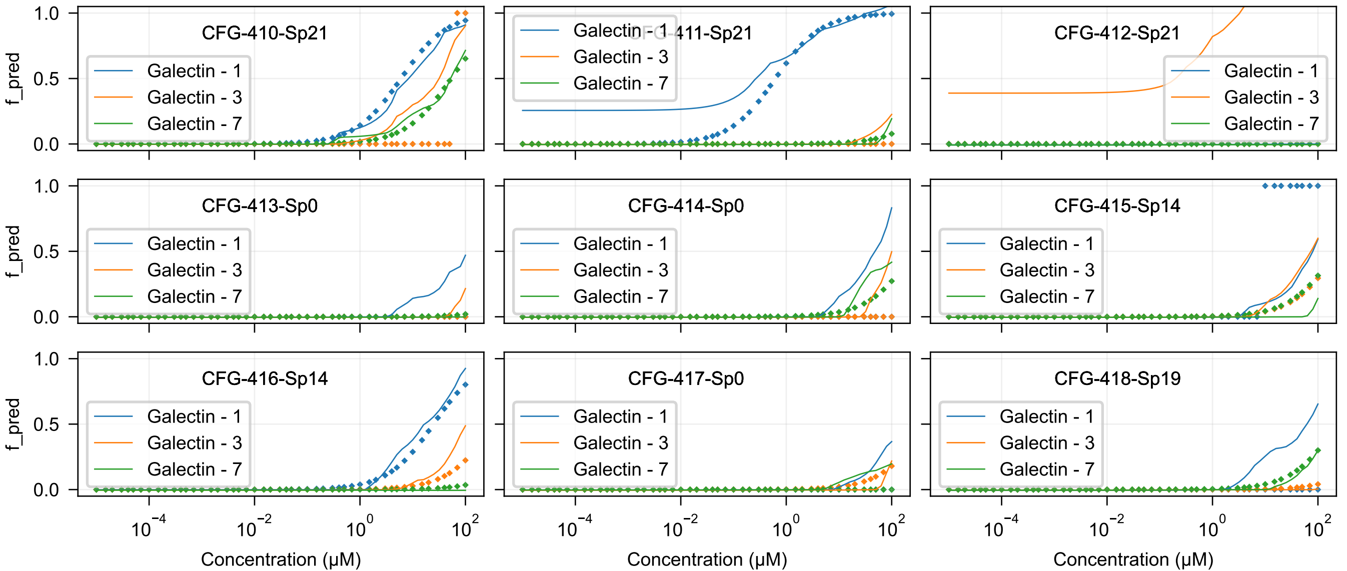

CFG-410-Sp21
CFG-411-Sp21
CFG-412-Sp21
CFG-413-Sp0
CFG-414-Sp0
CFG-415-Sp14
CFG-416-Sp14
CFG-417-Sp0
CFG-418-Sp19
Galectin - 1
Galectin - 3
Galectin - 7
f_pred
1.0
0.5
0.0
10^{-4}
10^{-2}
10^{0}
10^{2}
Concentration (μM)

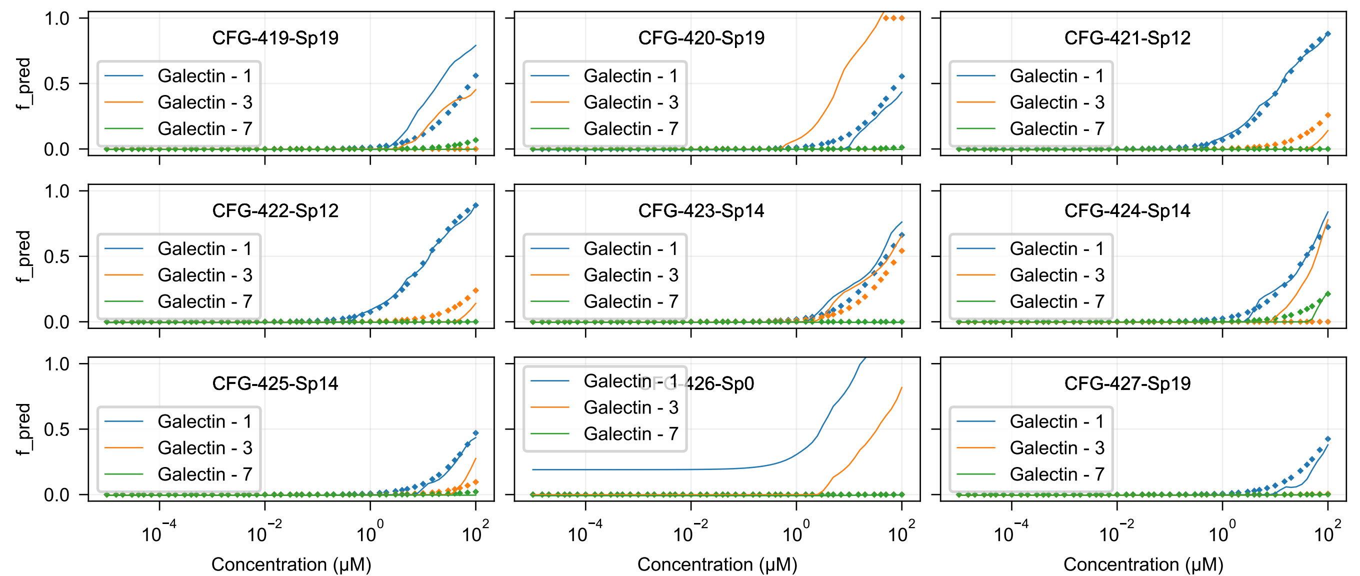

CFG-419-Sp19
CFG-420-Sp19
CFG-421-Sp12
CFG-422-Sp12
CFG-423-Sp14
CFG-424-Sp14
CFG-425-Sp14
CFG-426-Sp0
CFG-427-Sp19
Galectin - 1
Galectin - 3
Galectin - 7
f_pred
1.0
0.5
0.0
10^{-4}
10^{-2}
10^{0}
10^{2}
Concentration (μM)

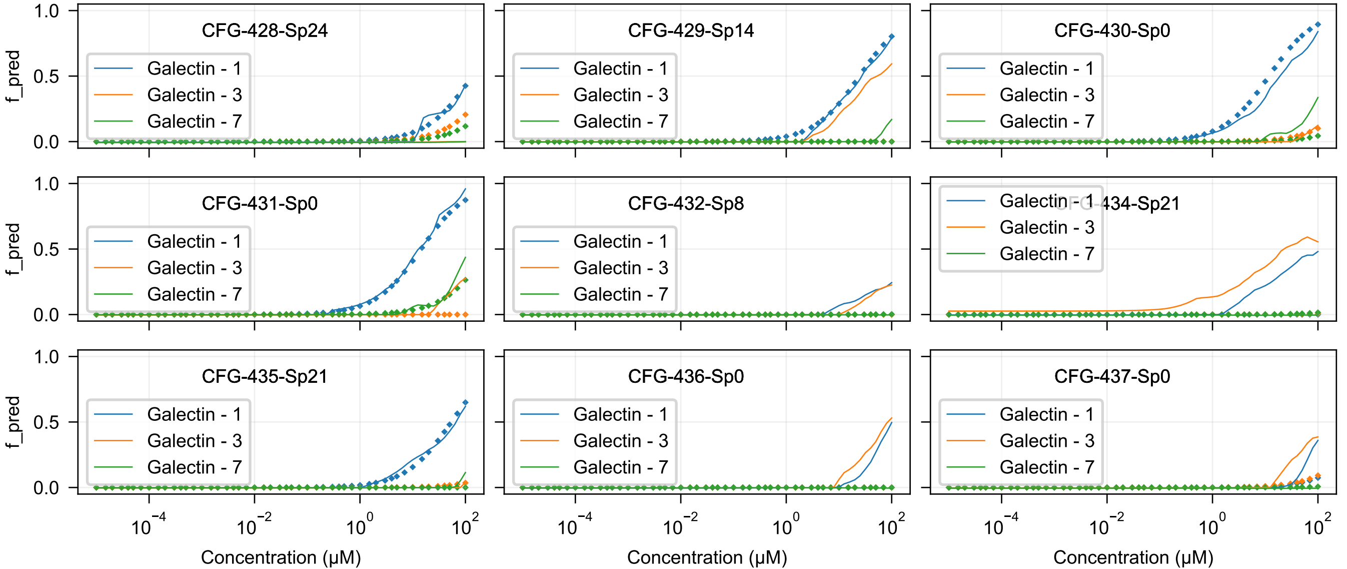

CFG-428-Sp24
CFG-429-Sp14
CFG-430-Sp0
CFG-431-Sp0
CFG-432-Sp8
CFG-434-Sp21
CFG-435-Sp21
CFG-436-Sp0
CFG-437-Sp0
Galectin - 1
Galectin - 3
Galectin - 7
f_pred
1.0
0.5
0.0
10^{-4}
10^{-2}
10^{0}
10^{2}
Concentration (μM)

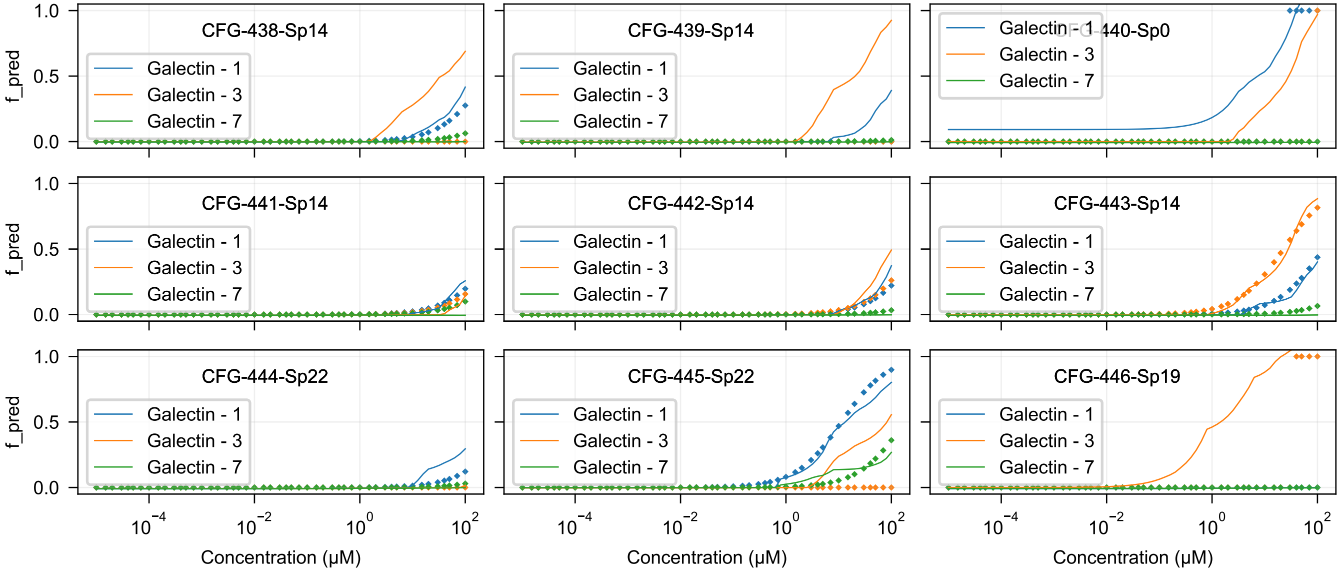

CFG-438-Sp14
CFG-439-Sp14
CFG-440-Sp0
CFG-441-Sp14
CFG-442-Sp14
CFG-443-Sp14
CFG-444-Sp22
CFG-445-Sp22
CFG-446-Sp19
Galectin - 1
Galectin - 3
Galectin - 7
f_pred
1.0
0.5
0.0
10^{-4}
10^{-2}
10^{0}
10^{2}
Concentration (μM)

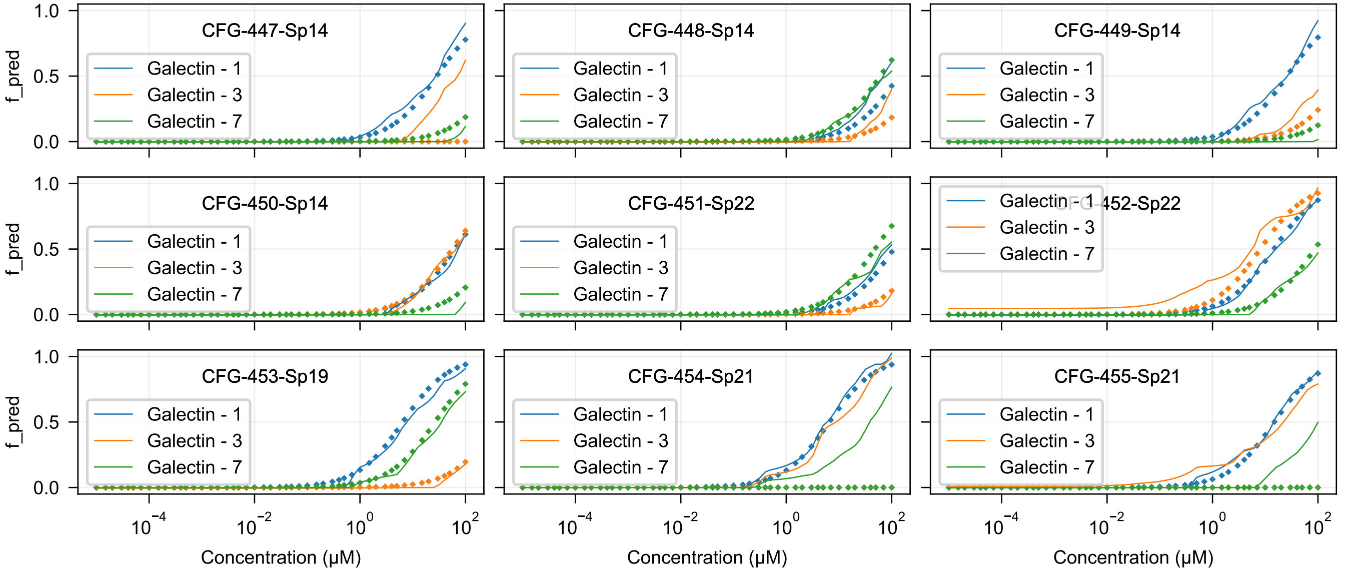

CFG-447-Sp14
CFG-448-Sp14
CFG-449-Sp14
CFG-450-Sp14
CFG-451-Sp22
CFG-452-Sp22
CFG-453-Sp19
CFG-454-Sp21
CFG-455-Sp21
Galectin - 1
Galectin - 3
Galectin - 7
f_pred
1.0
0.5
0.0
10^{-4}
10^{-2}
10^{0}
10^{2}
Concentration (μM)

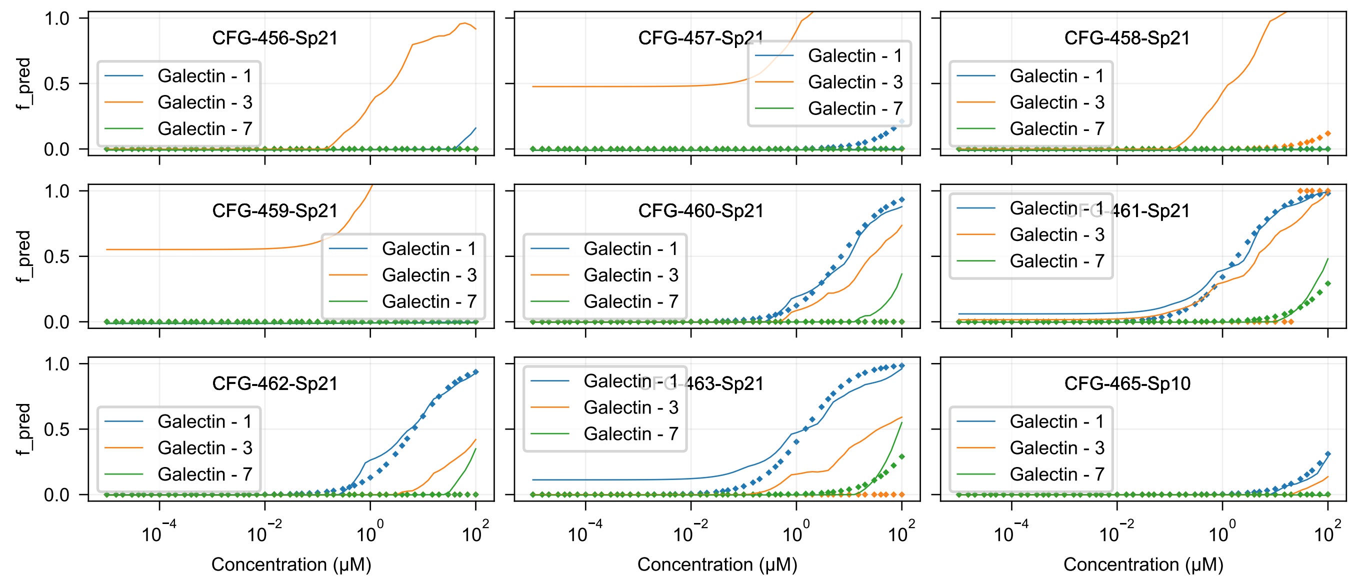

CFG-456-Sp21
CFG-457-Sp21
CFG-458-Sp21
CFG-459-Sp21
CFG-460-Sp21
CFG-461-Sp21
CFG-462-Sp21
CFG-463-Sp21
CFG-465-Sp10
Galectin - 1
Galectin - 3
Galectin - 7
f_pred
Concentration (μM)

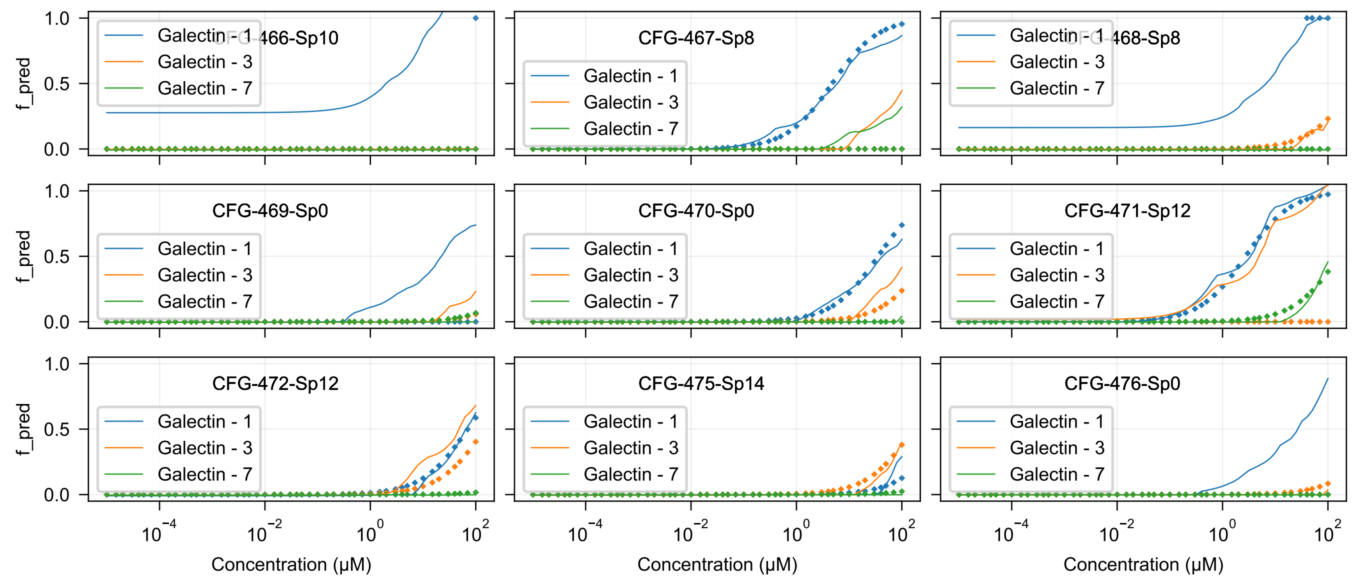

CFG-466-Sp10
CFG-467-Sp8
CFG-468-Sp8
CFG-469-Sp0
CFG-470-Sp0
CFG-471-Sp12
CFG-472-Sp12
CFG-475-Sp14
CFG-476-Sp0
Galectin - 1
Galectin - 3
Galectin - 7
f_pred
Concentration (μM)

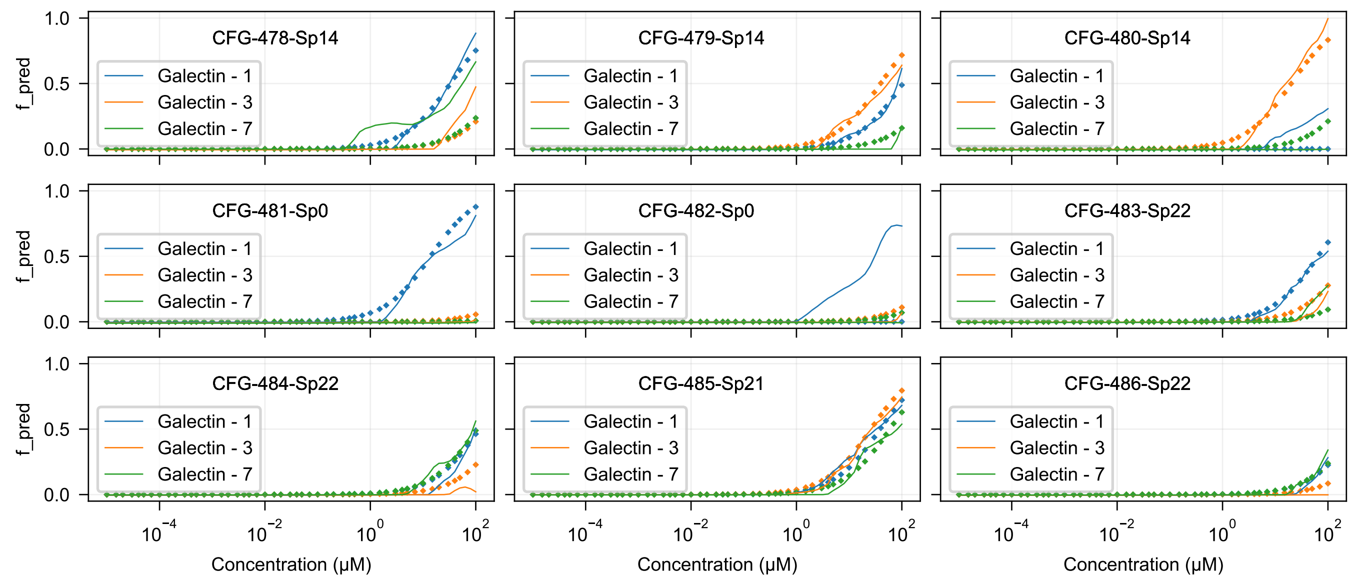
CFG-478-Sp14
CFG-479-Sp14
CFG-480-Sp14
CFG-481-Sp0
CFG-482-Sp0
CFG-483-Sp22
CFG-484-Sp22
CFG-485-Sp21
CFG-486-Sp22
Galectin - 1
Galectin - 3
Galectin - 7
f_pred
1.0
0.5
0.0
10^{-4}
10^{-2}
10^{0}
10^{2}
Concentration (μM)

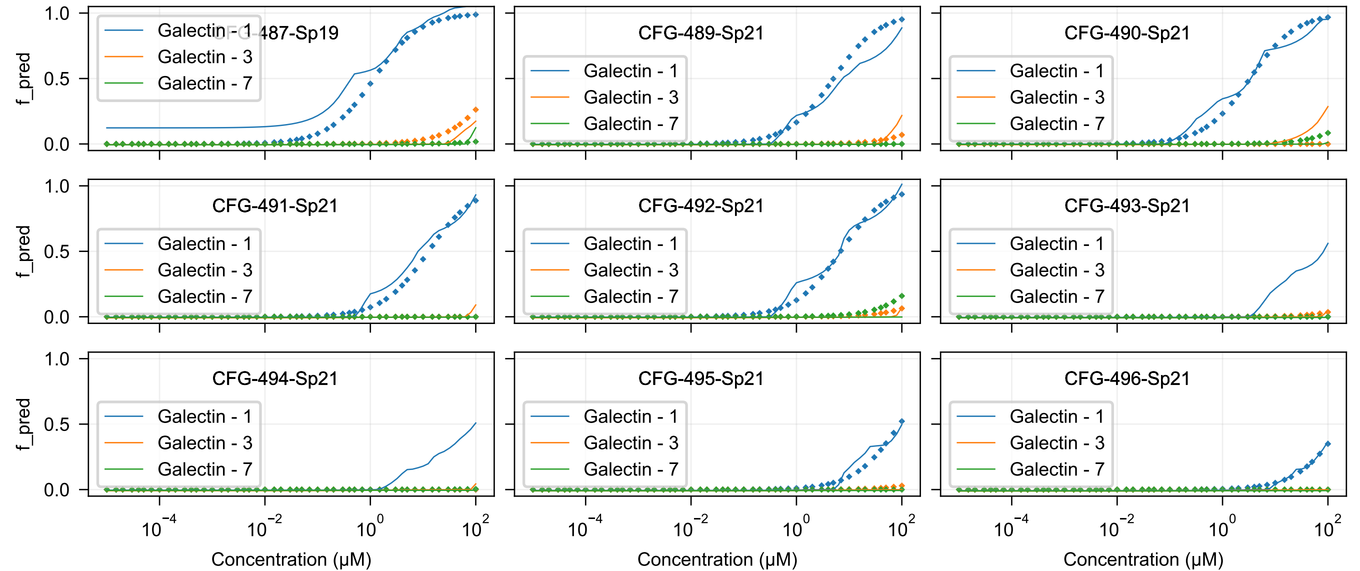
CFG-487-Sp19
CFG-489-Sp21
CFG-490-Sp21
CFG-491-Sp21
CFG-492-Sp21
CFG-493-Sp21
CFG-494-Sp21
CFG-495-Sp21
CFG-496-Sp21
Galectin - 1
Galectin - 3
Galectin - 7
f_pred
0.0
0.5
1.0
10^{-4}
10^{-2}
10^{0}
10^{2}
Concentration (μM)

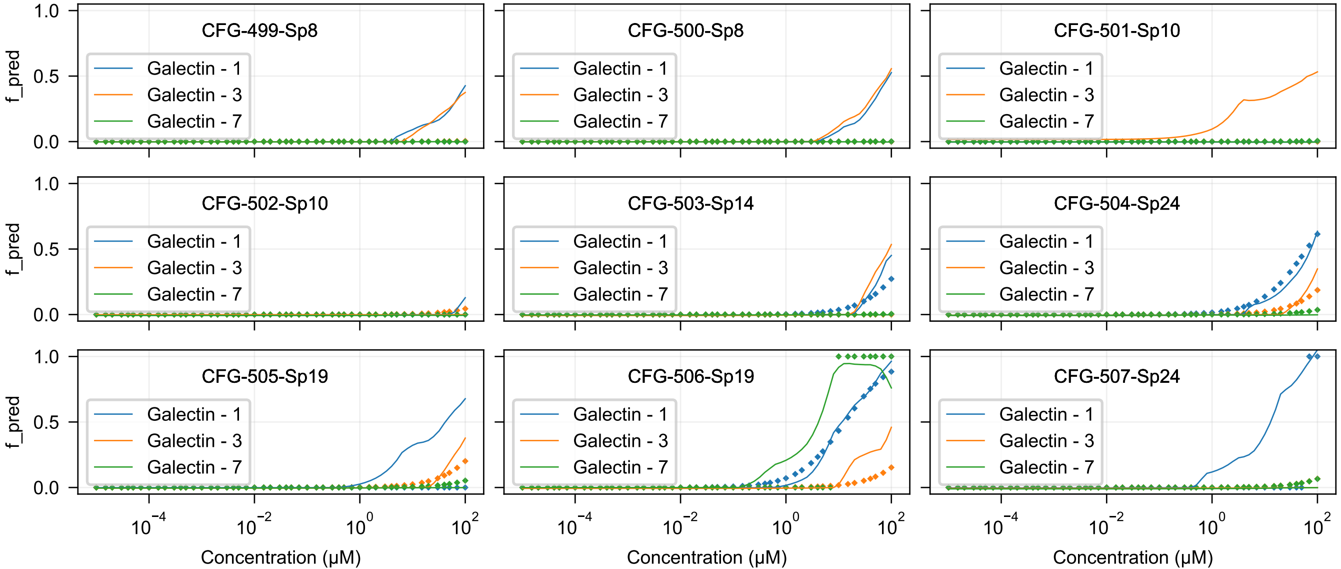

CFG-499-Sp8
CFG-500-Sp8
CFG-501-Sp10
CFG-502-Sp10
CFG-503-Sp14
CFG-504-Sp24
CFG-505-Sp19
CFG-506-Sp19
CFG-507-Sp24
Galectin - 1
Galectin - 3
Galectin - 7
f_pred
1.0
0.5
0.0
10^{-4}
10^{-2}
10^{0}
10^{2}
Concentration (μM)

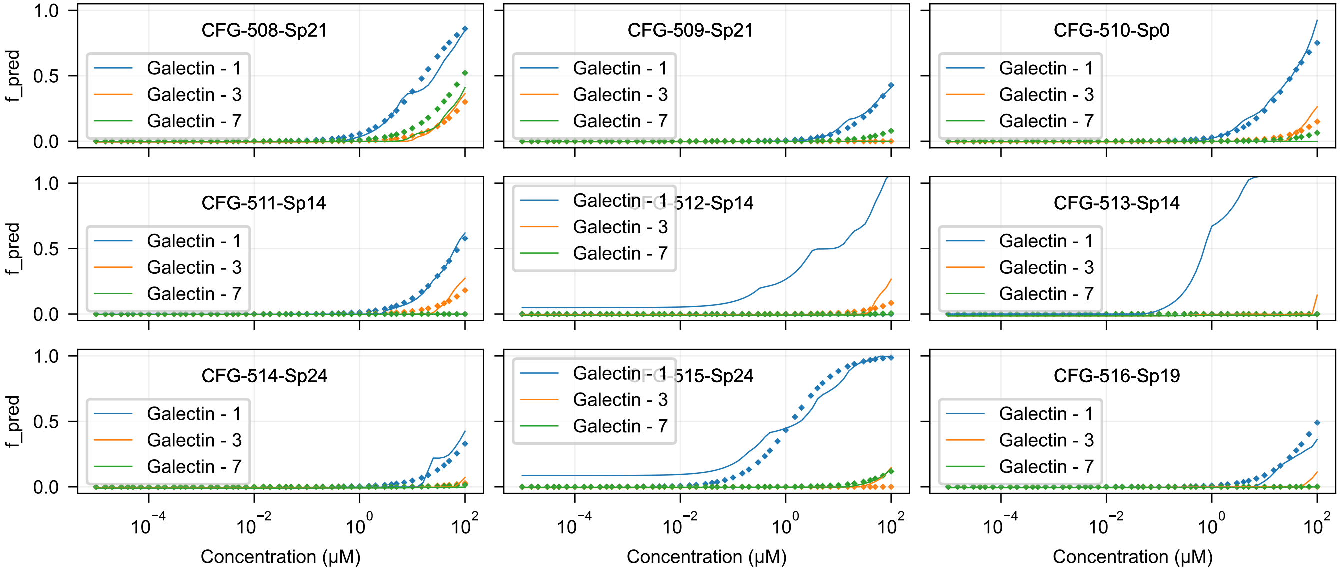

CFG-508-Sp21
CFG-509-Sp21
CFG-510-Sp0
CFG-511-Sp14
CFG-512-Sp14
CFG-513-Sp14
CFG-514-Sp24
CFG-515-Sp24
CFG-516-Sp19
Galectin - 1
Galectin - 3
Galectin - 7
f_pred
Concentration (μM)
0.0
0.5
1.0
10^{-4}
10^{-2}
10^{0}
10^{2}

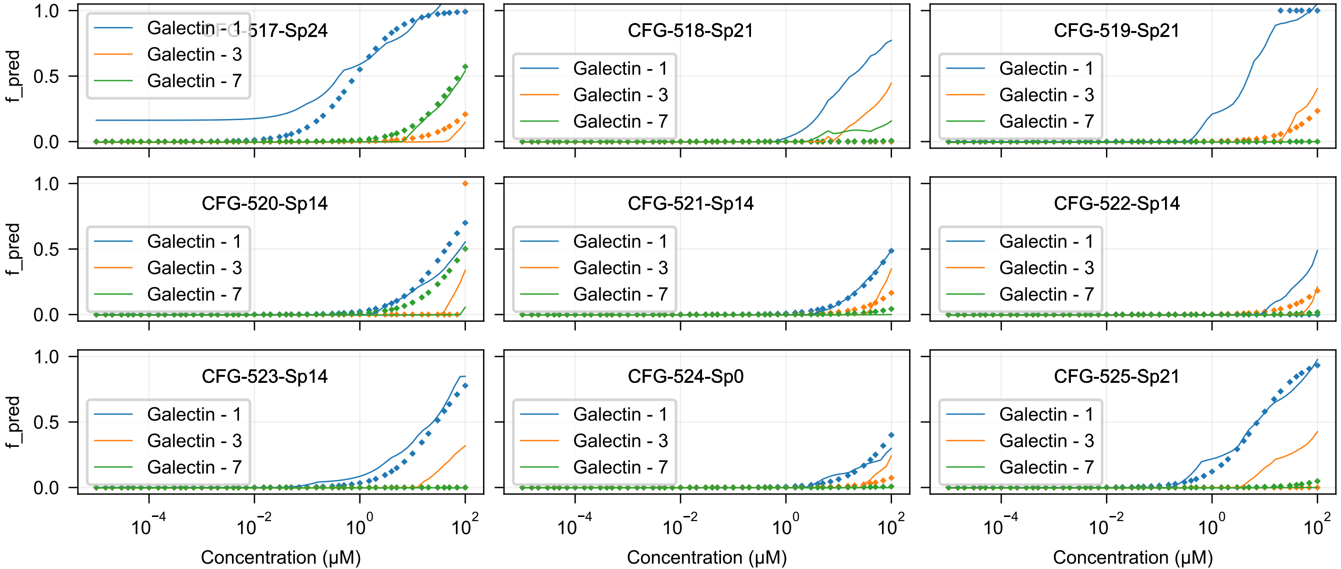
CFG-517-Sp24
CFG-518-Sp21
CFG-519-Sp21
CFG-520-Sp14
CFG-521-Sp14
CFG-522-Sp14
CFG-523-Sp14
CFG-524-Sp0
CFG-525-Sp21
Galectin - 1
Galectin - 3
Galectin - 7
f_pred
Concentration (μM)
1.0
0.5
0.0
10^{-4}
10^{-2}
10^{0}
10^{2}

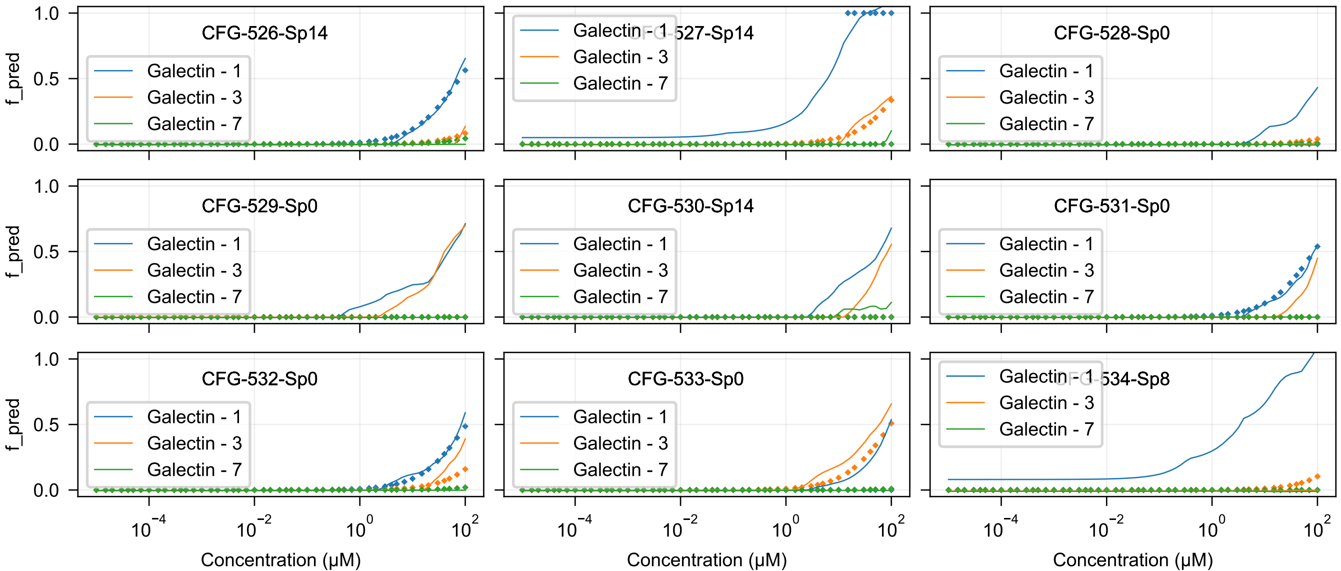

CFG-526-Sp14
CFG-527-Sp14
CFG-528-Sp0
CFG-529-Sp0
CFG-530-Sp14
CFG-531-Sp0
CFG-532-Sp0
CFG-533-Sp0
CFG-534-Sp8
Galectin - 1
Galectin - 3
Galectin - 7
f_pred
1.0
0.5
0.0
10^{-4}
10^{-2}
10^{0}
10^{2}
Concentration (μM)

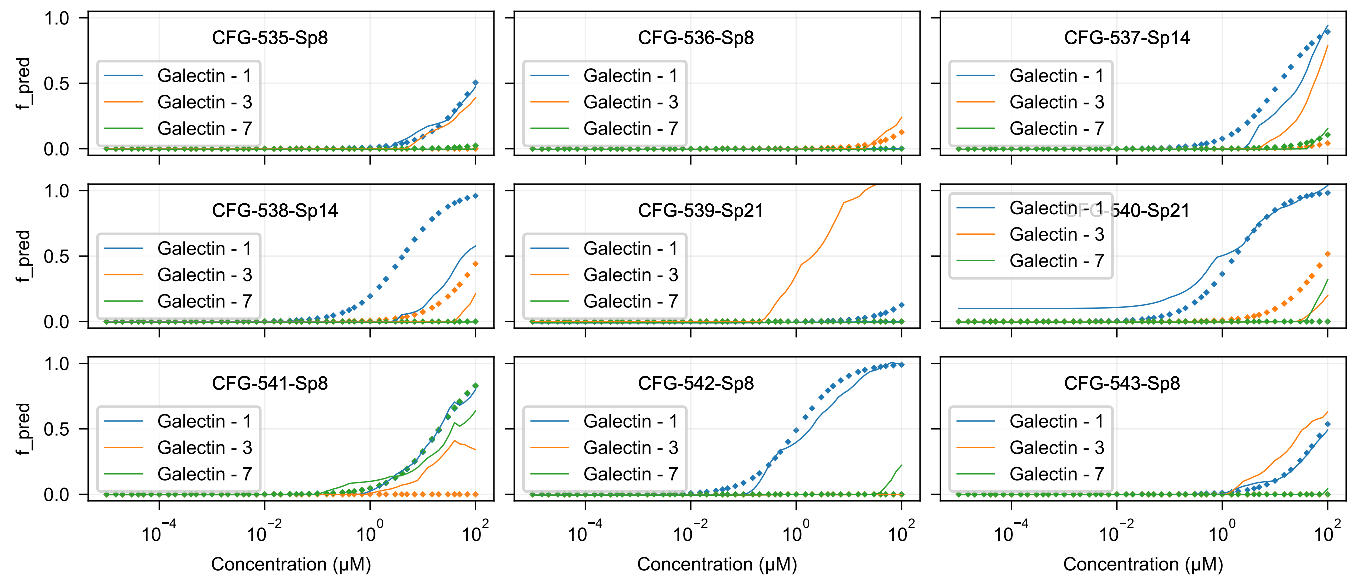
CFG-535-Sp8
CFG-536-Sp8
CFG-537-Sp14
CFG-538-Sp14
CFG-539-Sp21
CFG-540-Sp21
CFG-541-Sp8
CFG-542-Sp8
CFG-543-Sp8
Galectin - 1
Galectin - 3
Galectin - 7
f_pred
Concentration (μM)
1.0
0.5
0.0
10^{-4}
10^{-2}
10^{0}
10^{2}

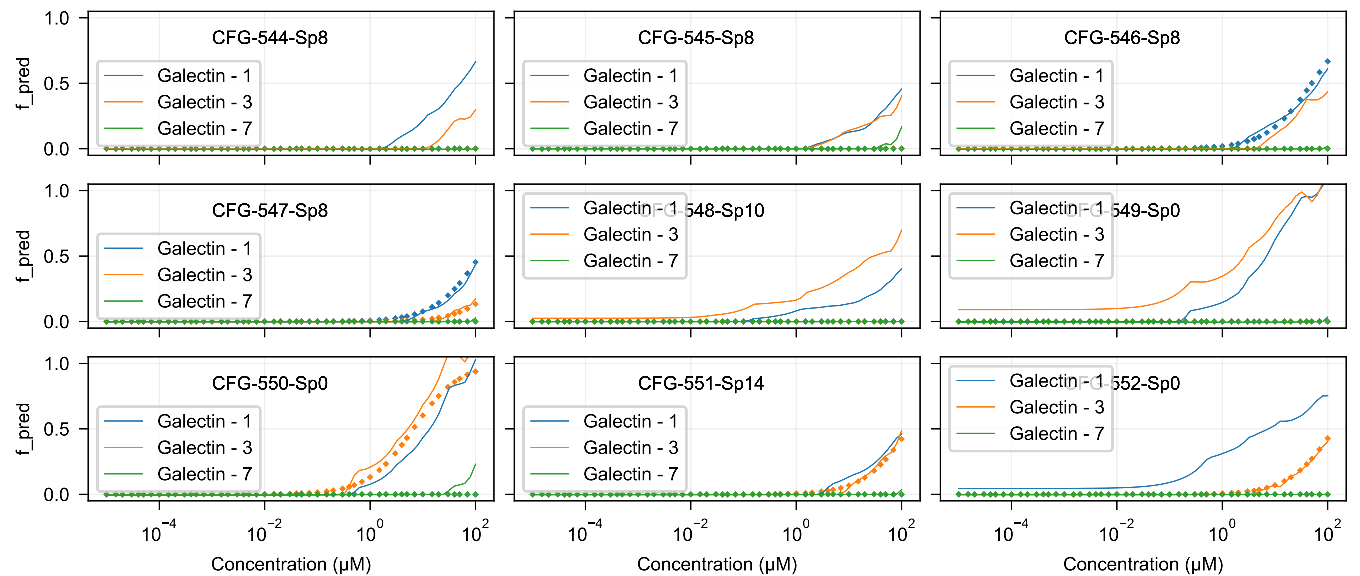
CFG-544-Sp8
CFG-545-Sp8
CFG-546-Sp8
CFG-547-Sp8
CFG-548-Sp10
CFG-549-Sp0
CFG-550-Sp0
CFG-551-Sp14
CFG-552-Sp0
Galectin - 1
Galectin - 3
Galectin - 7
f_pred
1.0
0.5
0.0
10^{-4}
10^{-2}
10^{0}
10^{2}
Concentration (μM)

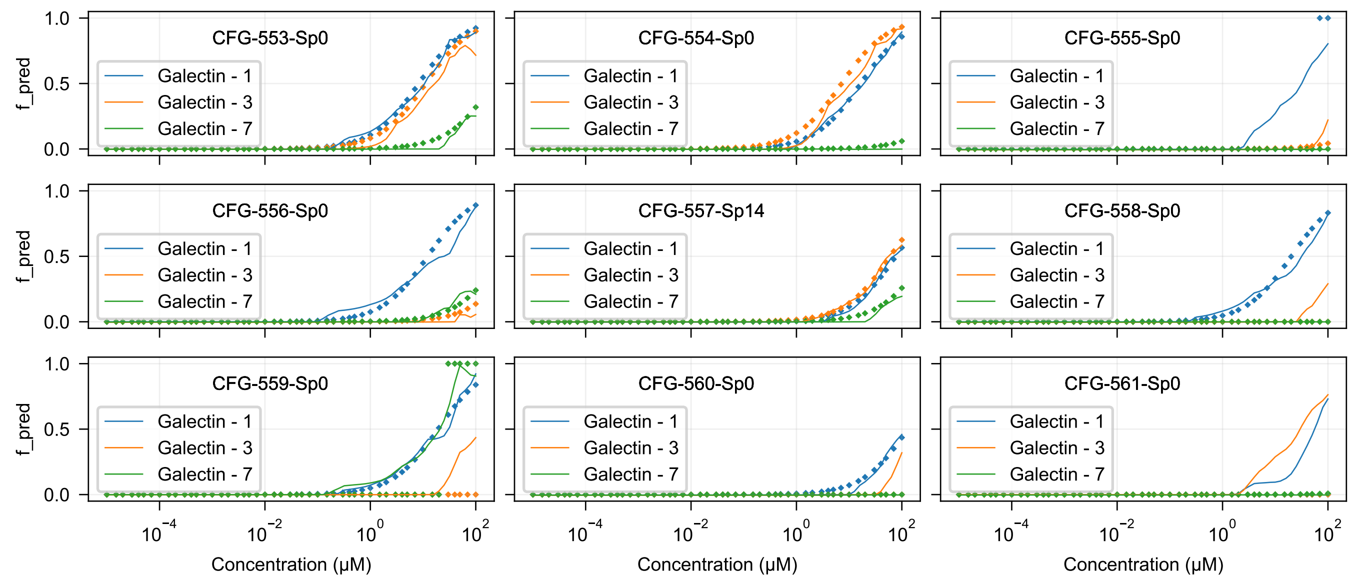

CFG-553-Sp0
CFG-554-Sp0
CFG-555-Sp0
CFG-556-Sp0
CFG-557-Sp14
CFG-558-Sp0
CFG-559-Sp0
CFG-560-Sp0
CFG-561-Sp0
Galectin - 1
Galectin - 3
Galectin - 7
f_pred
1.0
0.5
0.0
10^{-4}
10^{-2}
10^{0}
10^{2}
Concentration (μM)

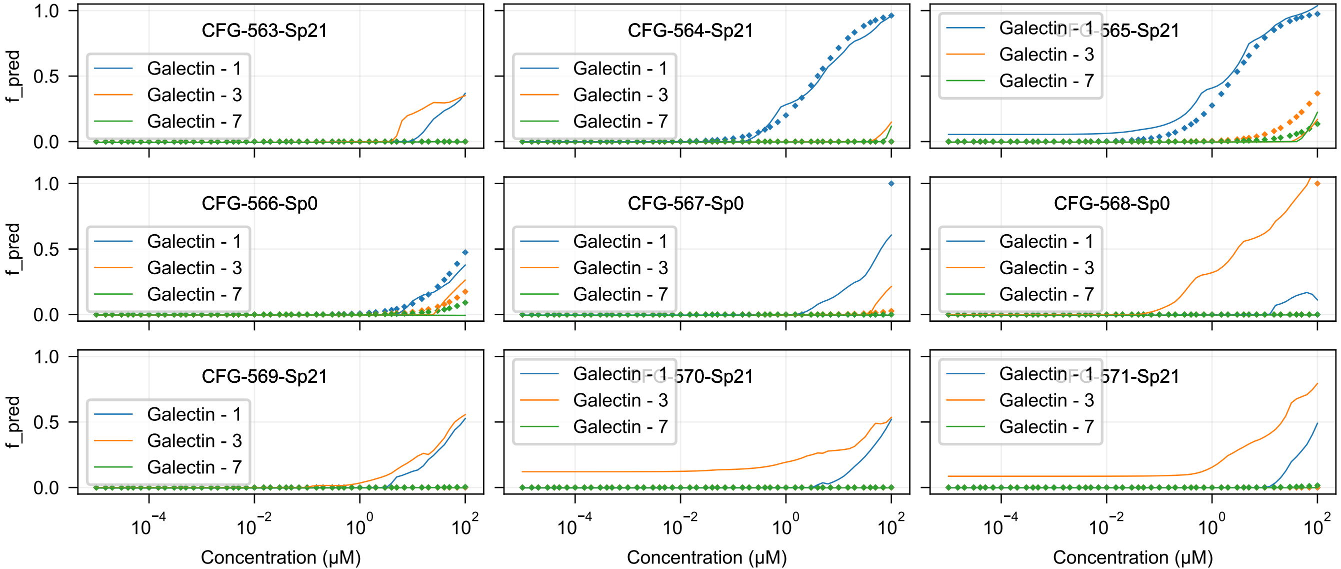
CFG-563-Sp21
CFG-564-Sp21
CFG-565-Sp21
CFG-566-Sp0
CFG-567-Sp0
CFG-568-Sp0
CFG-569-Sp21
CFG-570-Sp21
CFG-571-Sp21
Galectin - 1
Galectin - 3
Galectin - 7
f_pred
1.0
0.5
0.0
10^{-4}
10^{-2}
10^{0}
10^{2}
Concentration (μM)

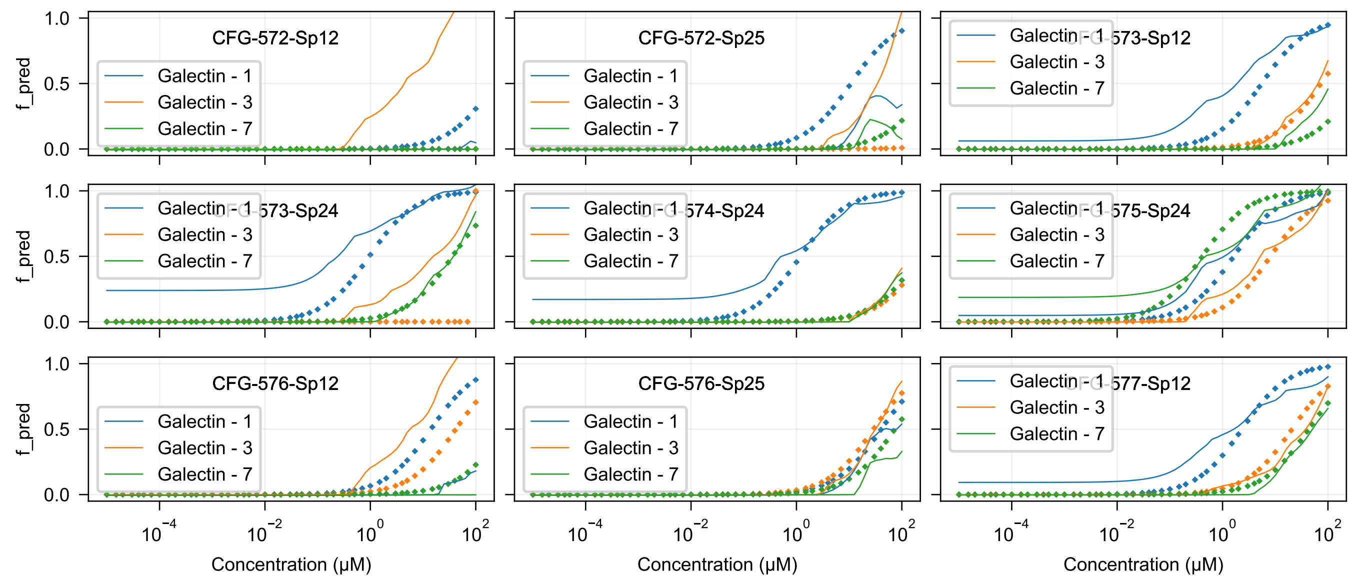
CFG-572-Sp12
CFG-572-Sp25
CFG-573-Sp12
CFG-573-Sp24
CFG-574-Sp24
CFG-575-Sp24
CFG-576-Sp12
CFG-576-Sp25
CFG-577-Sp12
Galectin - 1
Galectin - 3
Galectin - 7
f_pred
Concentration (μM)
0.0
0.5
1.0
10^{-4}
10^{-2}
10^{0}
10^{2}

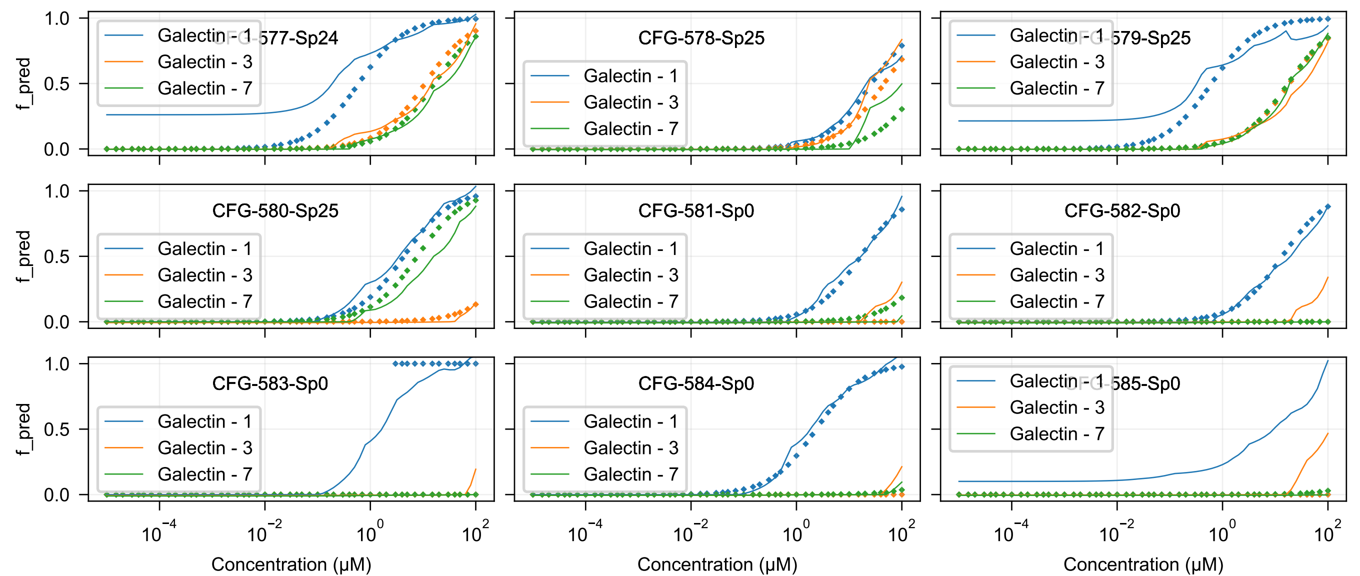

CFG-577-Sp24
CFG-578-Sp25
CFG-579-Sp25
CFG-580-Sp25
CFG-581-Sp0
CFG-582-Sp0
CFG-583-Sp0
CFG-584-Sp0
CFG-585-Sp0
Galectin - 1
Galectin - 3
Galectin - 7
f_pred
Concentration (μM)

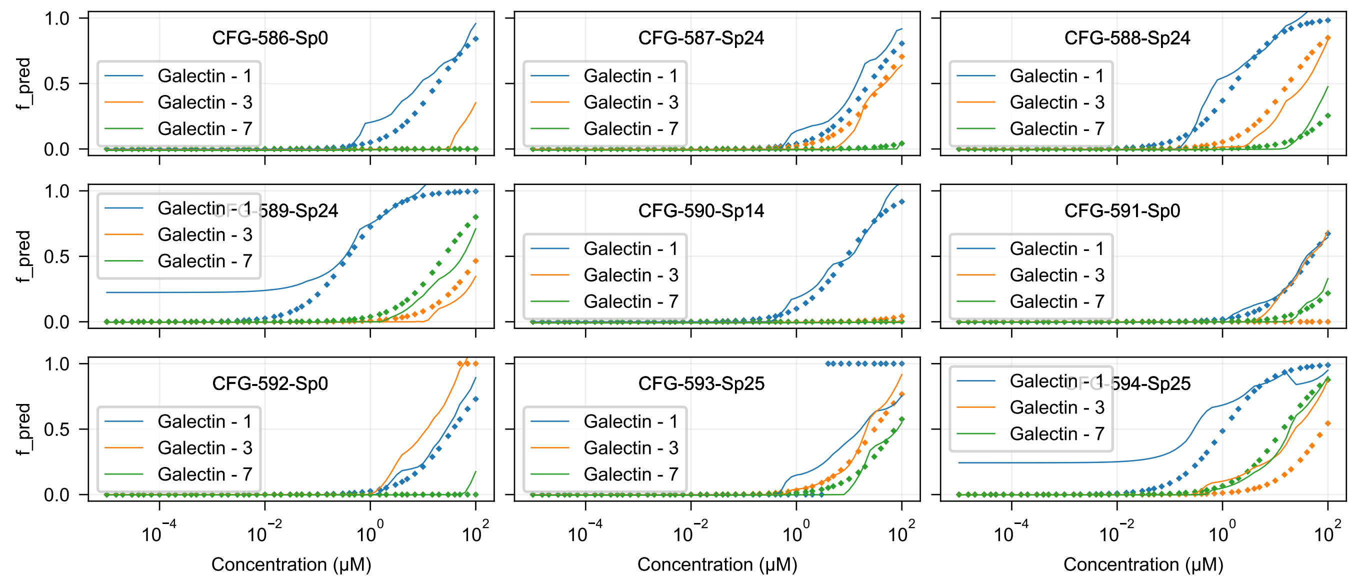
CFG-586-Sp0
CFG-587-Sp24
CFG-588-Sp24
CFG-589-Sp24
CFG-590-Sp14
CFG-591-Sp0
CFG-592-Sp0
CFG-593-Sp25
CFG-594-Sp25
Galectin - 1
Galectin - 3
Galectin - 7
f_pred
1.0
0.5
0.0
10^{-4}
10^{-2}
10^{0}
10^{2}
Concentration (μM)

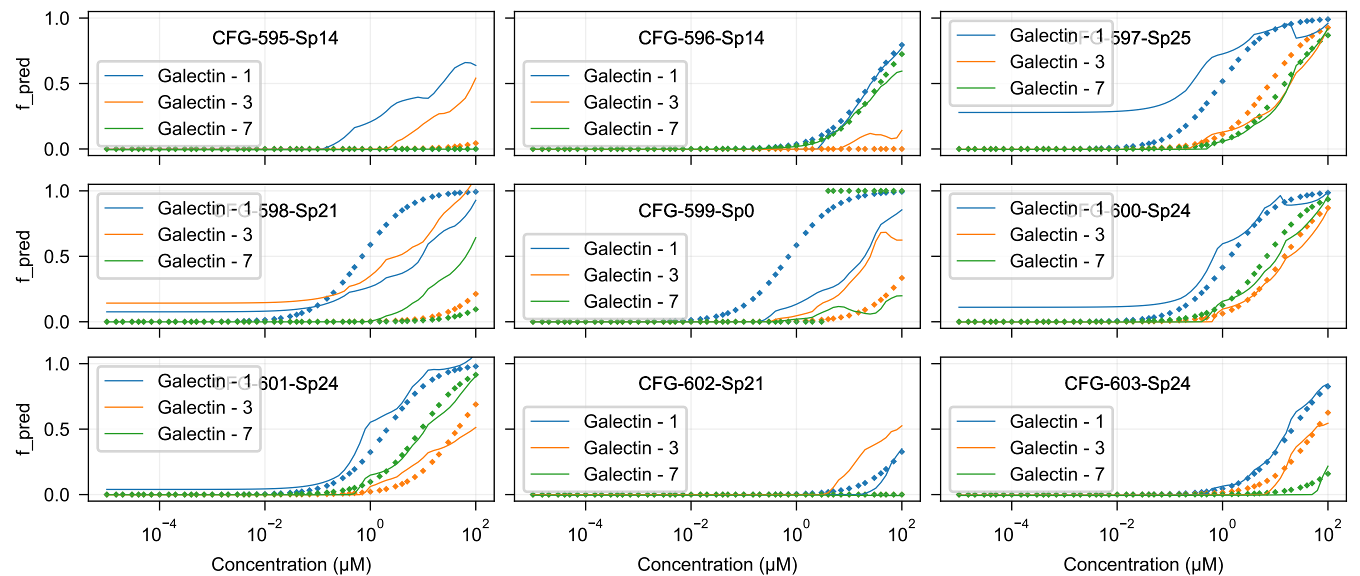

CFG-595-Sp14
CFG-596-Sp14
CFG-597-Sp25
CFG-598-Sp21
CFG-599-Sp0
CFG-600-Sp24
CFG-601-Sp24
CFG-602-Sp21
CFG-603-Sp24
Galectin - 1
Galectin - 3
Galectin - 7
f_pred
0.0
0.5
1.0
10^{-4}
10^{-2}
10^{0}
10^{2}
Concentration (μM)

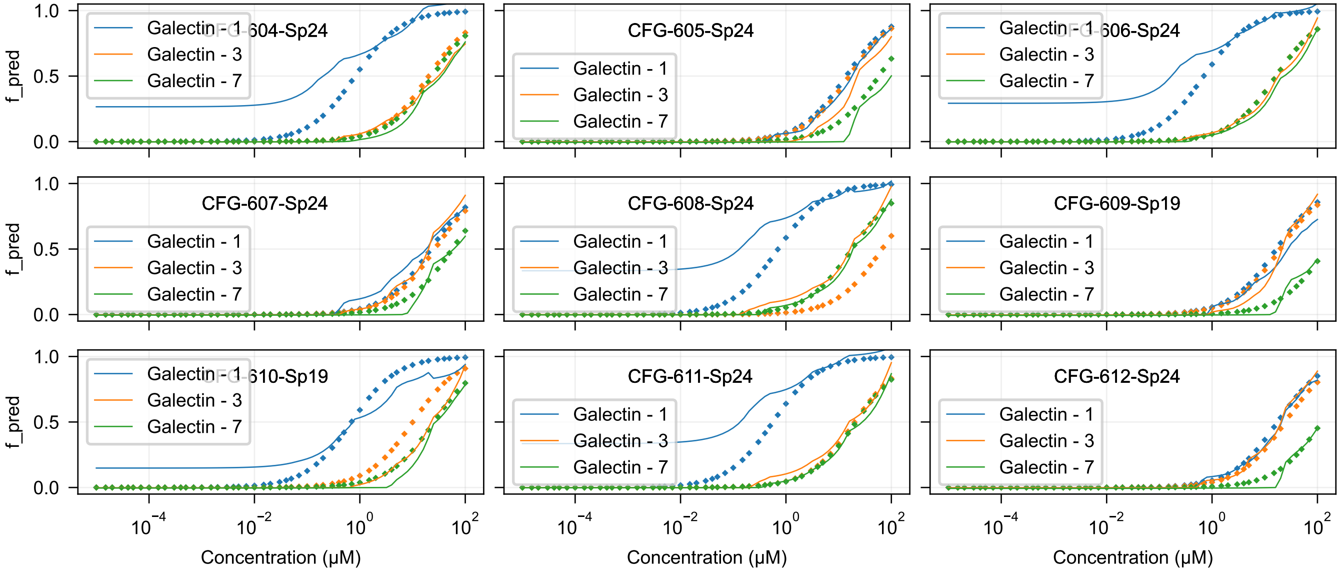
CFG-604-Sp24
CFG-605-Sp24
CFG-606-Sp24
CFG-607-Sp24
CFG-608-Sp24
CFG-609-Sp19
CFG-610-Sp19
CFG-611-Sp24
CFG-612-Sp24
Galectin - 1
Galectin - 3
Galectin - 7
f_pred
Concentration (μM)

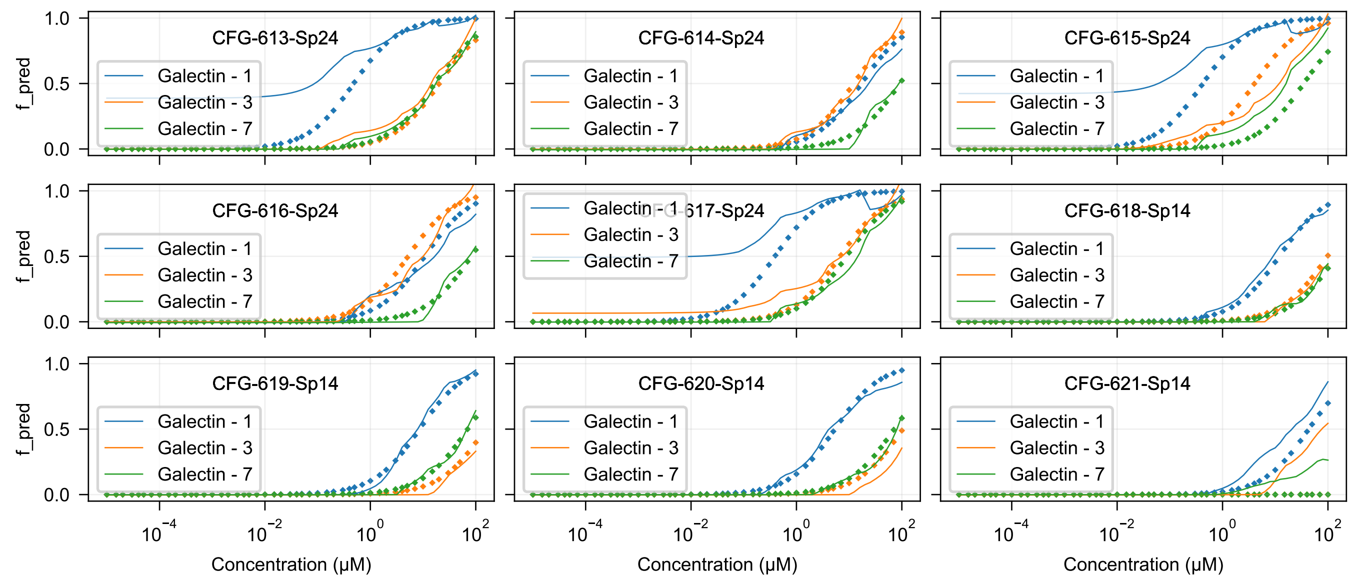

CFG-613-Sp24
CFG-614-Sp24
CFG-615-Sp24
CFG-616-Sp24
CFG-617-Sp24
CFG-618-Sp14
CFG-619-Sp14
CFG-620-Sp14
CFG-621-Sp14
Galectin - 1
Galectin - 3
Galectin - 7
f_pred
1.0
0.5
0.0
10^{-4}
10^{-2}
10^{0}
10^{2}
Concentration (μM)

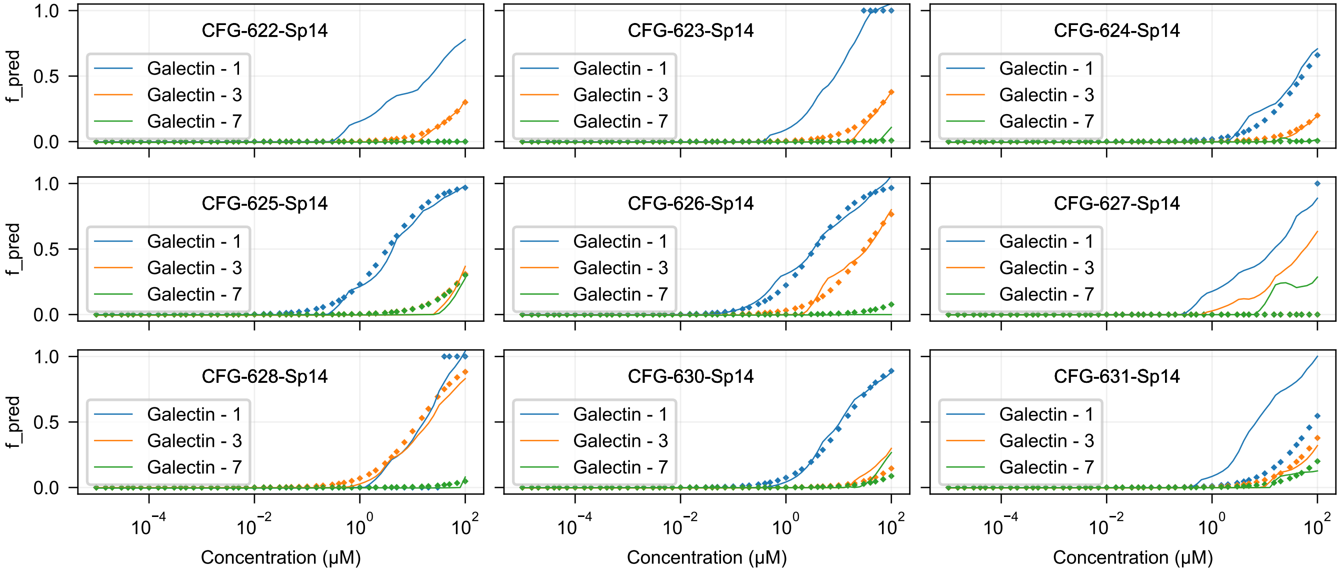

CFG-622-Sp14
CFG-623-Sp14
CFG-624-Sp14
CFG-625-Sp14
CFG-626-Sp14
CFG-627-Sp14
CFG-628-Sp14
CFG-630-Sp14
CFG-631-Sp14
Galectin - 1
Galectin - 3
Galectin - 7
f_pred
1.0
0.5
0.0
10^{-4}
10^{-2}
10^{0}
10^{2}
Concentration (μM)

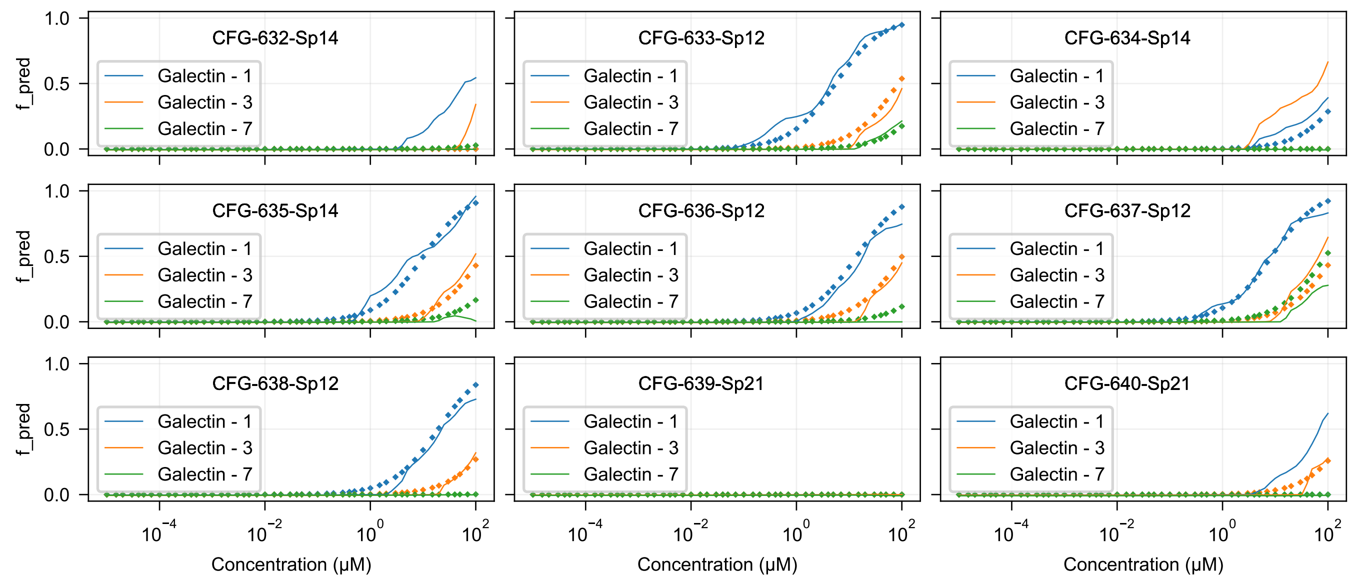

CFG-632-Sp14
CFG-633-Sp12
CFG-634-Sp14
CFG-635-Sp14
CFG-636-Sp12
CFG-637-Sp12
CFG-638-Sp12
CFG-639-Sp21
CFG-640-Sp21
Galectin - 1
Galectin - 3
Galectin - 7
f_pred
Concentration (μM)

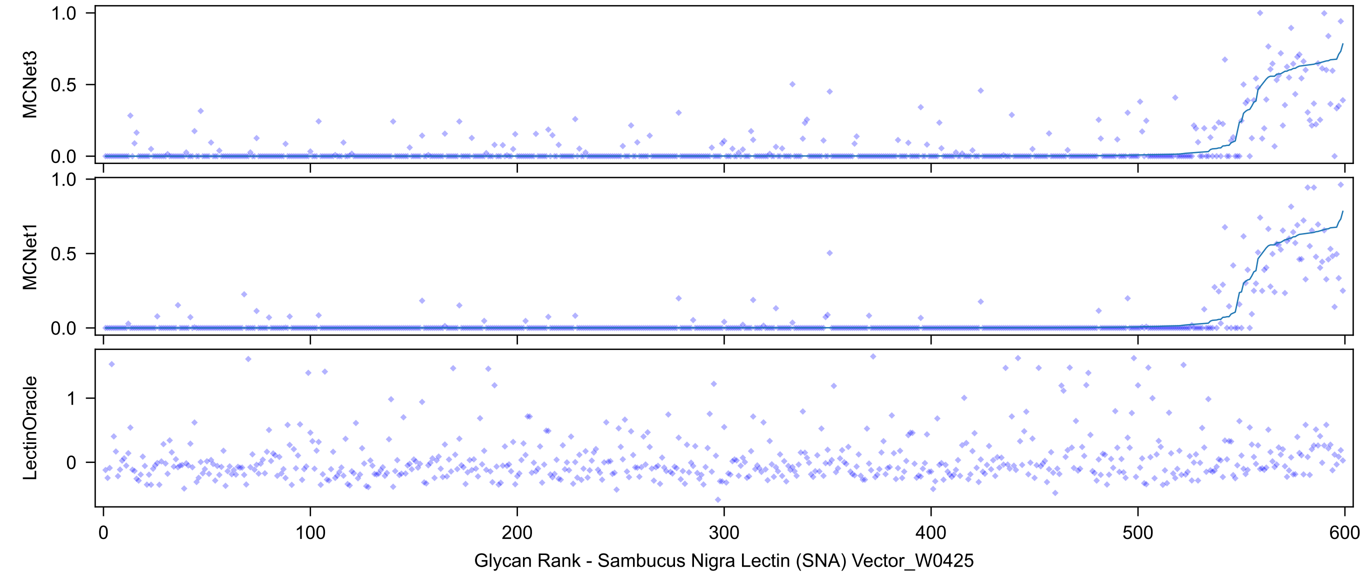
MCNet3
MCNet1
LectinOracle
1.0
0.5
0.0
1
0
0
100
200
300
400
500
600
Glycan Rank - Sambucus Nigra Lectin (SNA) Vector_W0425

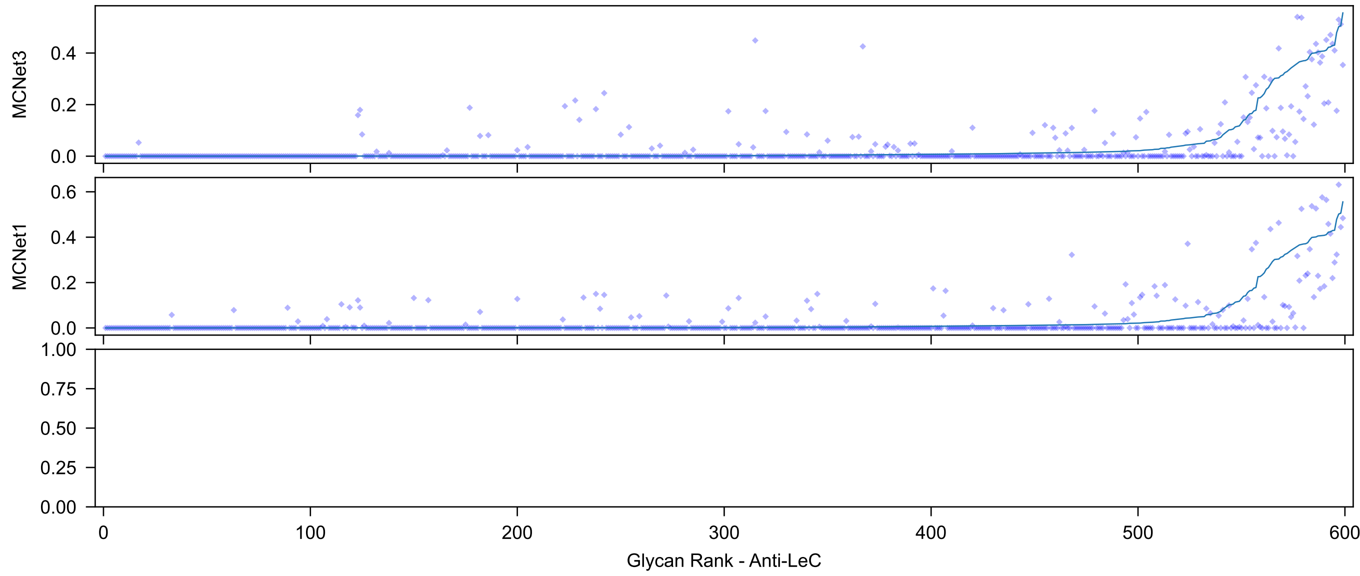

MCNet3
0.4
0.2
0.0
MCNet1
0.6
0.4
0.2
0.0
1.00
0.75
0.50
0.25
0.00
0
100
200
300
400
500
600
Glycan Rank - Anti-LeC

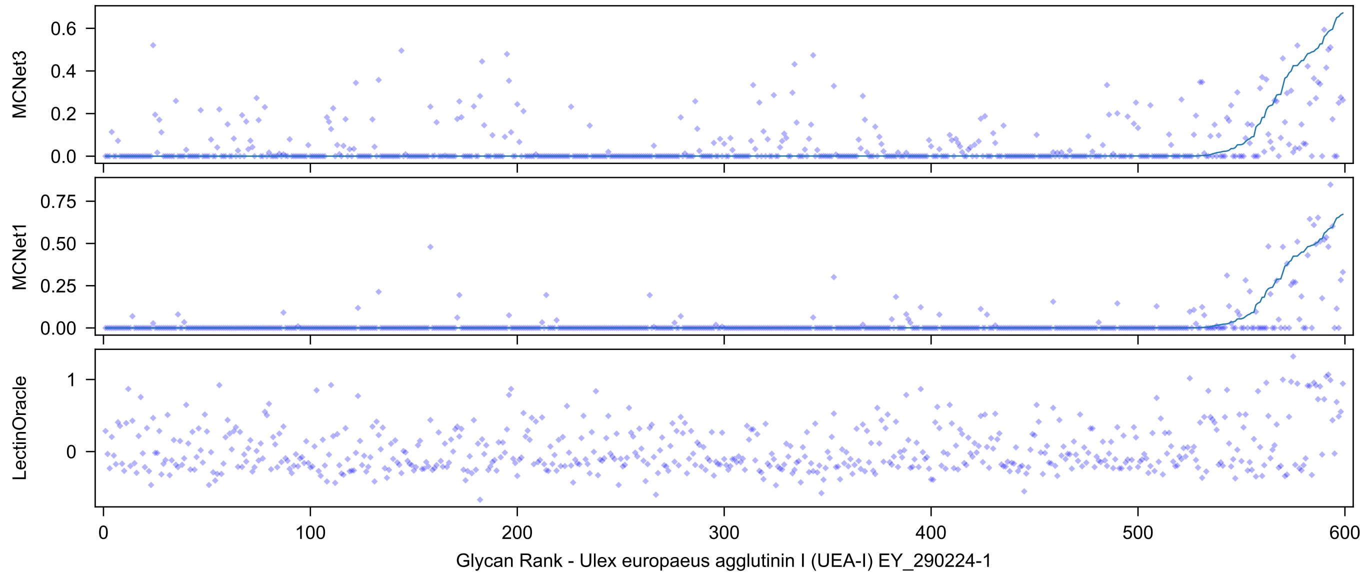
MCNet3
MCNet1
LectinOracle
0.6
0.4
0.2
0.0
0.75
0.50
0.25
0.00
1
0
0
100
200
300
400
500
600
Glycan Rank - Ulex europaeus agglutinin I (UEA-I) EY_290224-1

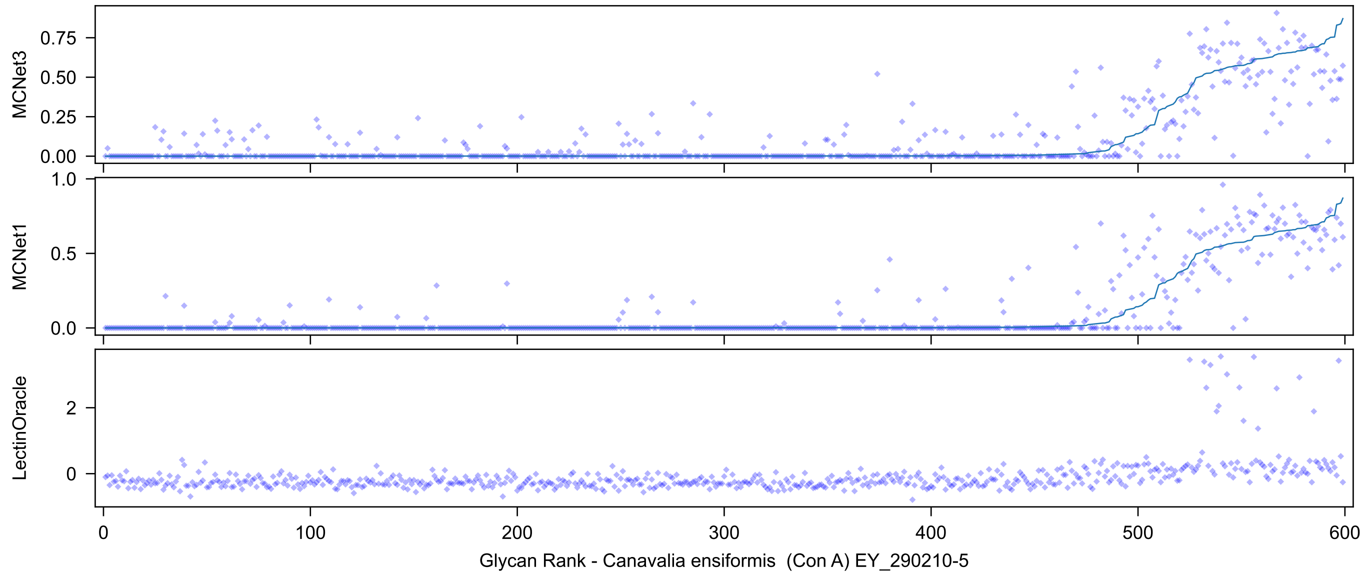

MCNet3
0.00
0.25
0.50
0.75
MCNet1
0.0
0.5
1.0
LectinOracle
0
2
0
100
200
300
400
500
600
Glycan Rank - Canavalia ensiformis (Con A) EY_290210-5

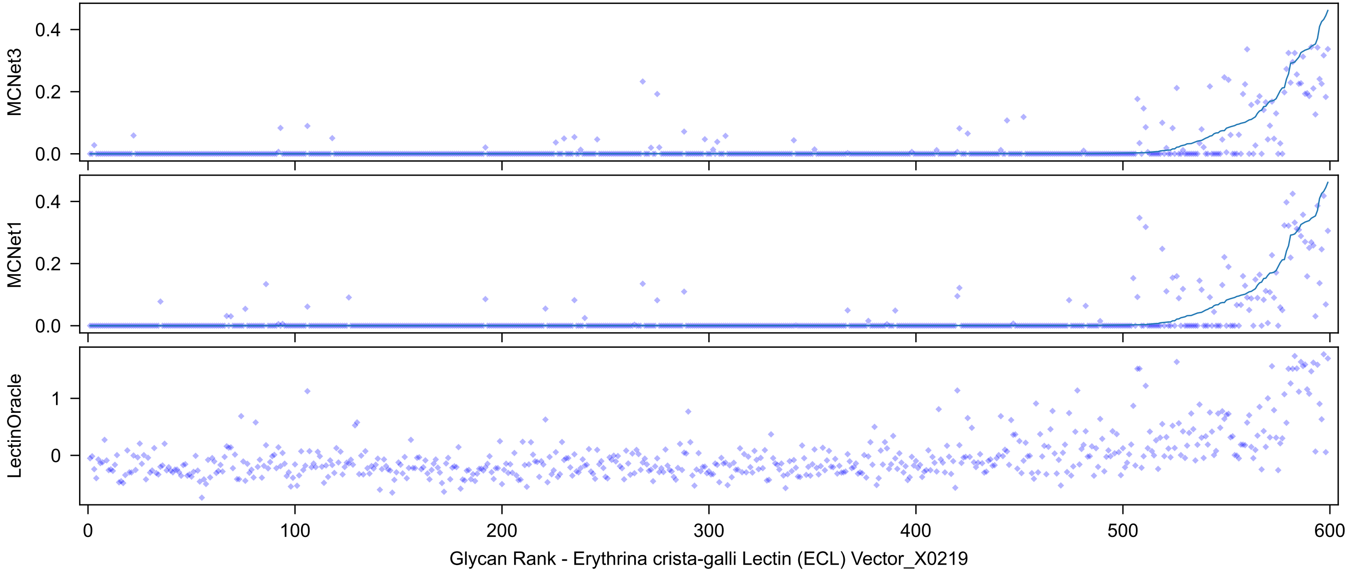
MCNet3
0.4
0.2
0.0
MCNet1
0.4
0.2
0.0
LectinOracle
1
0
0
100
200
300
400
500
600
Glycan Rank - Erythrina crista-galli Lectin (ECL) Vector_X0219

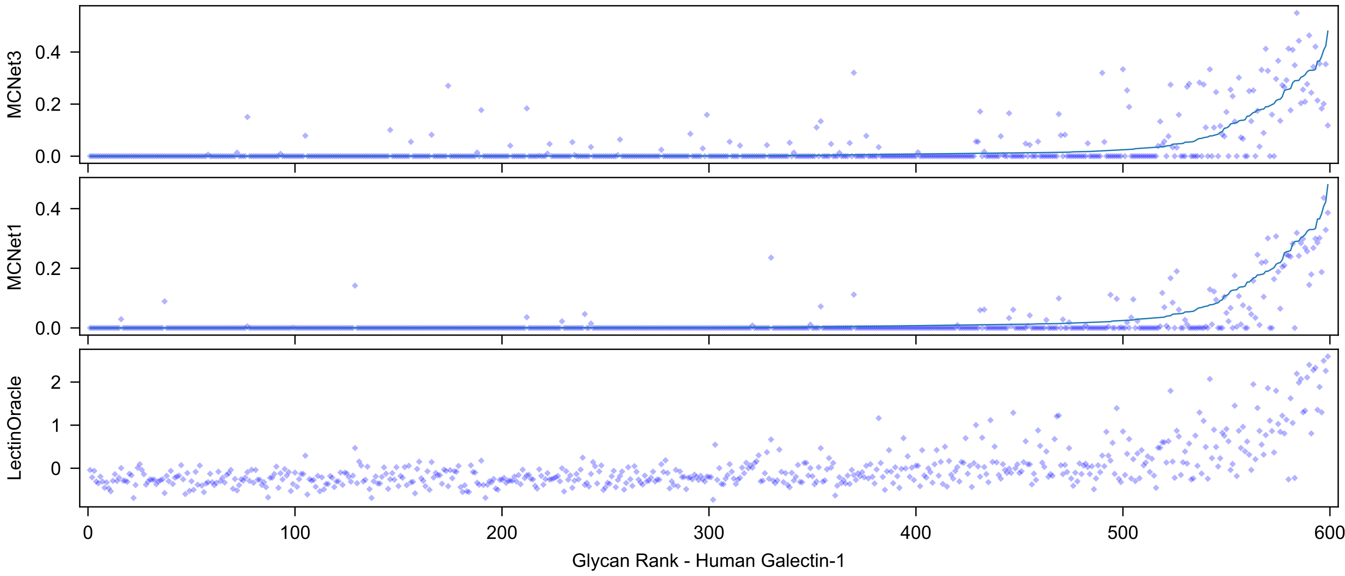
MCNet3
0.4
0.2
0.0
MCNet1
0.4
0.2
0.0
LectinOracle
2
1
0
0
100
200
300
400
500
600
Glycan Rank - Human Galectin-1

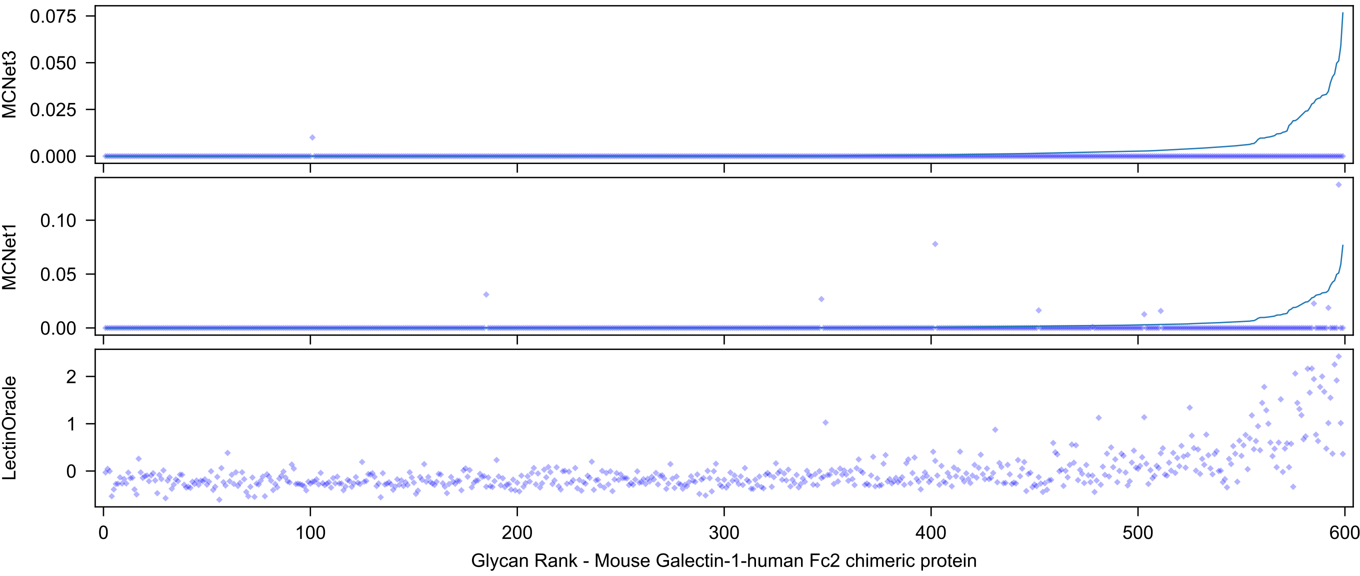

MCNet3
0.075
0.050
0.025
0.000
MCNet1
0.10
0.05
0.00
LectinOracle
2
1
0
0
100
200
300
400
500
600
Glycan Rank - Mouse Galectin-1-human Fc2 chimeric protein

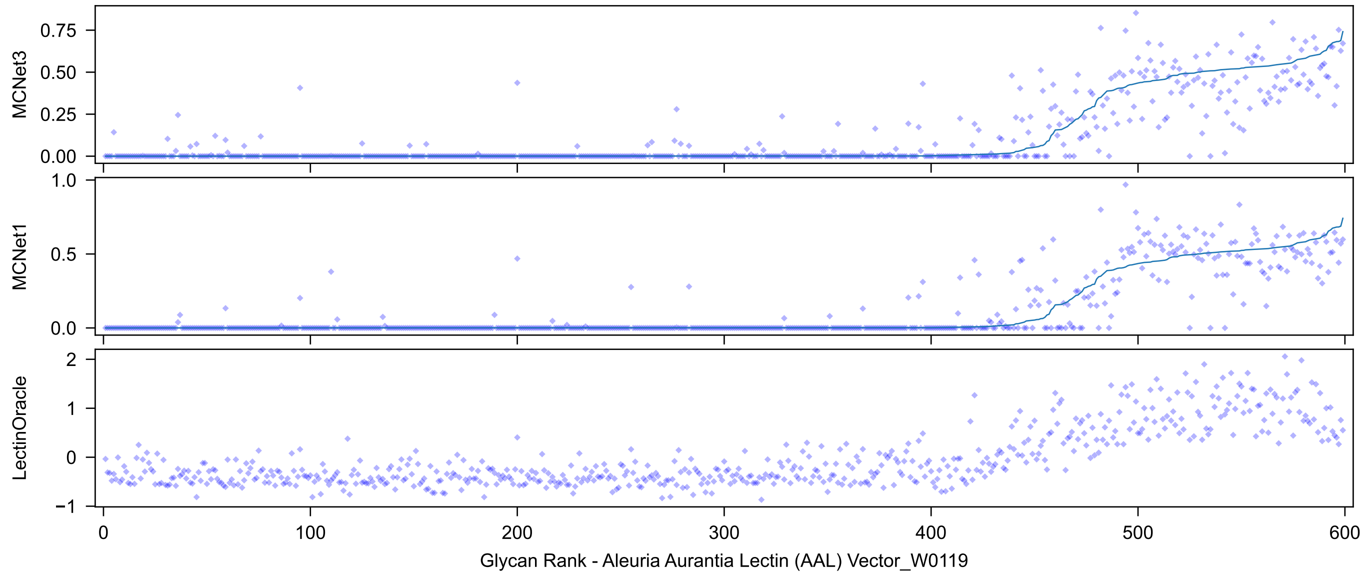
MCNet3
0.00
0.25
0.50
0.75
MCNet1
0.0
0.5
1.0
LectinOracle
−1
0
1
2
0
100
200
300
400
500
600
Glycan Rank - Aleuria Aurantia Lectin (AAL) Vector_W0119

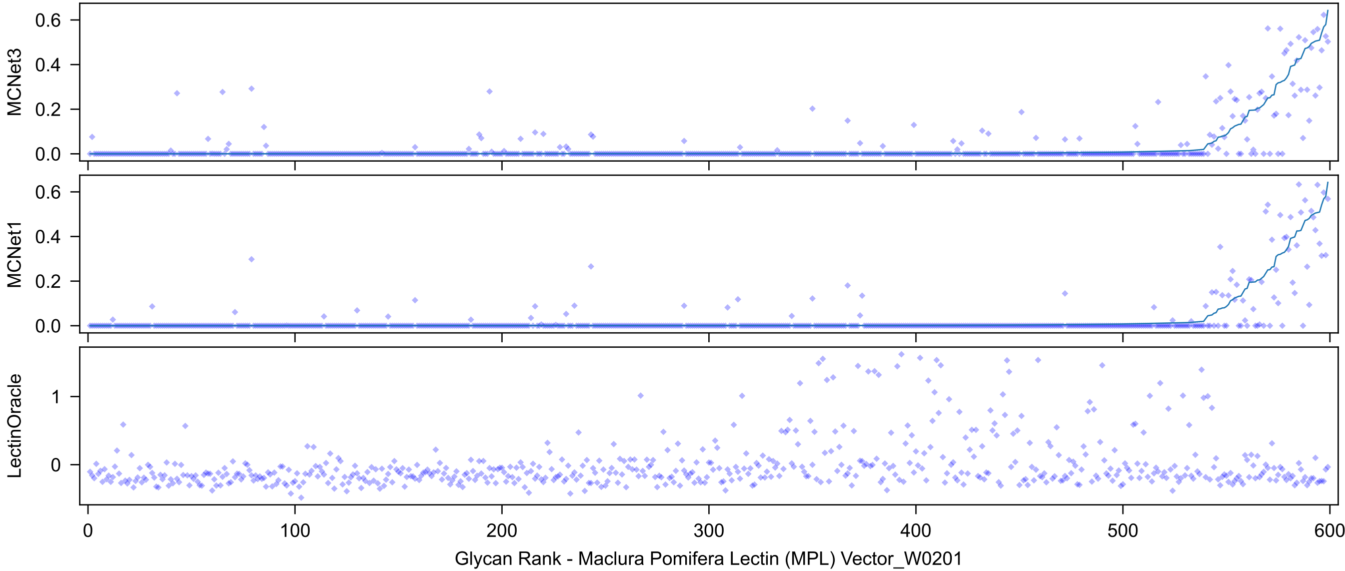

MCNet3
MCNet1
LectinOracle
Glycan Rank - Maclura Pomifera Lectin (MPL) Vector_W0201

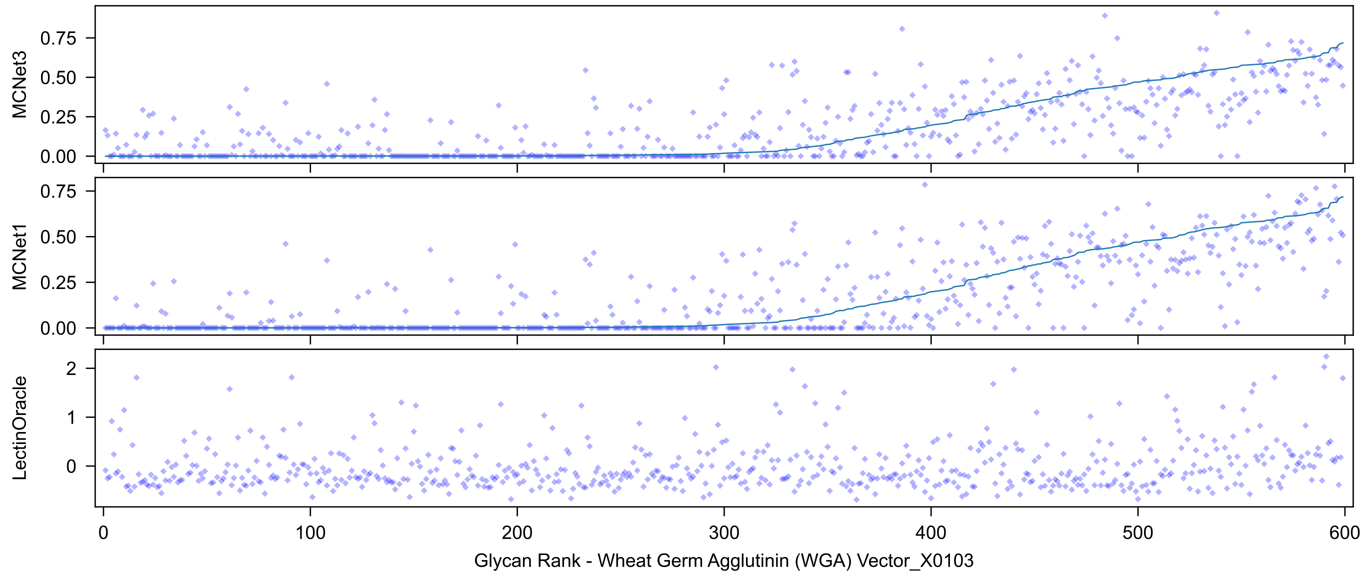

MCNet3
0.00
0.25
0.50
0.75
MCNet1
0.00
0.25
0.50
0.75
LectinOracle
0
1
2
0
100
200
300
400
500
600
Glycan Rank - Wheat Germ Agglutinin (WGA) Vector_X0103

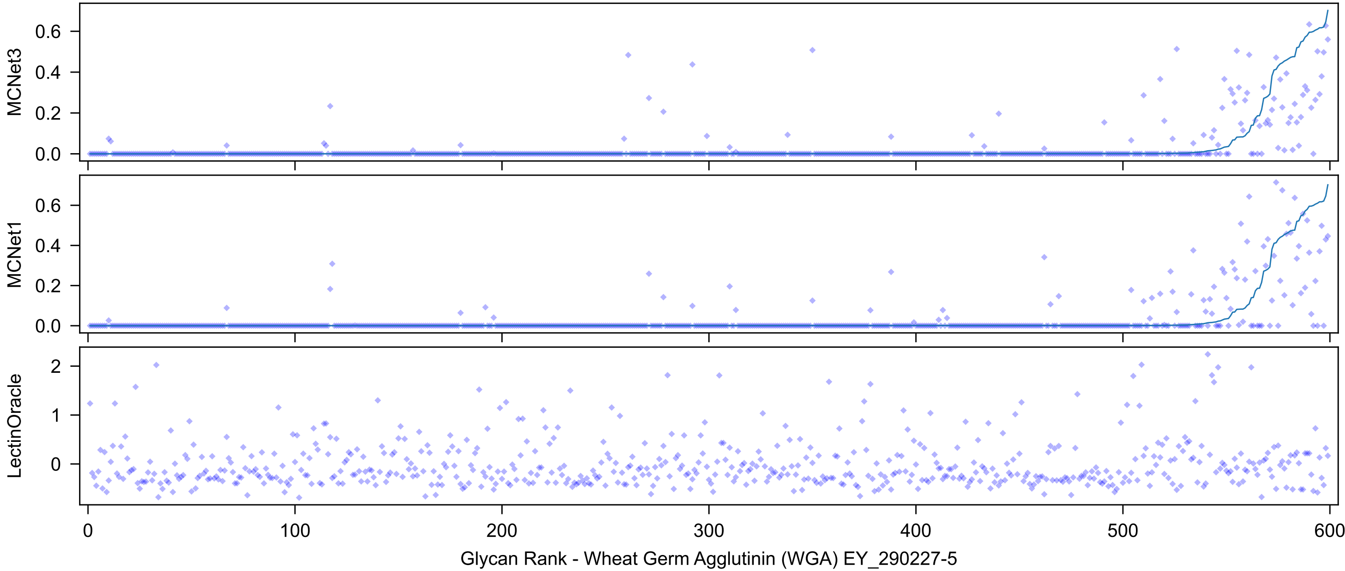

MCNet3
MCNet1
LectinOracle
0.0
0.2
0.4
0.6
0
1
2
0
100
200
300
400
500
600
Glycan Rank - Wheat Germ Agglutinin (WGA) EY_290227-5

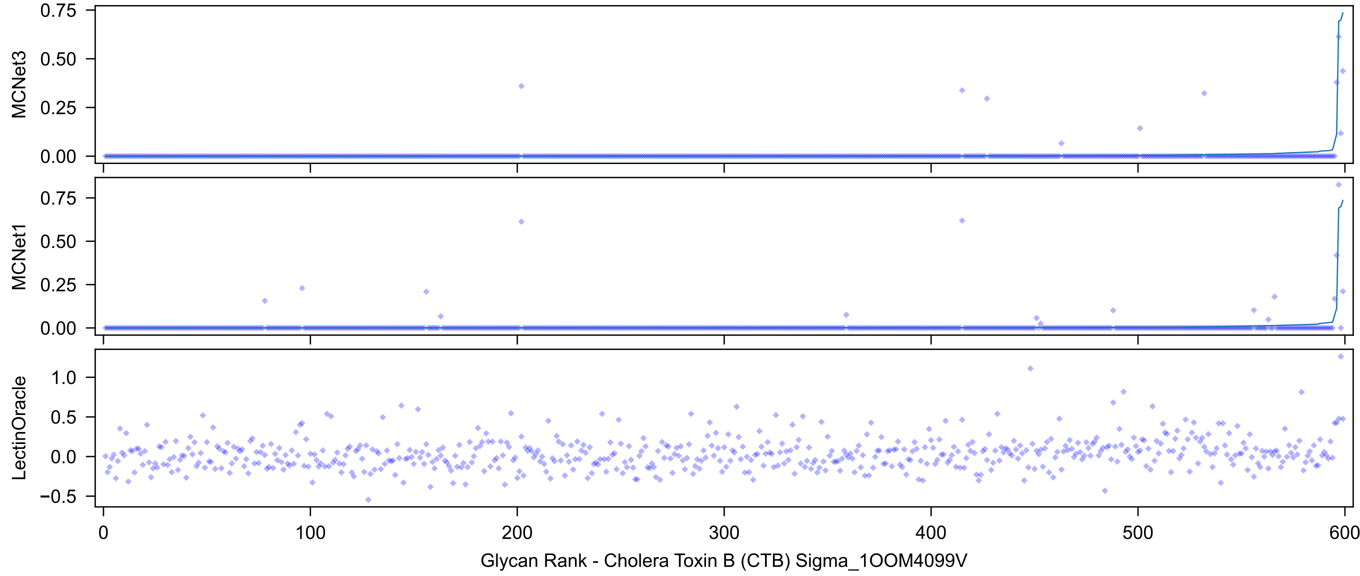
MCNet3
MCNet1
LectinOracle
0.75
0.50
0.25
0.00
1.0
0.5
0.0
−0.5
0
100
200
300
400
500
600
Glycan Rank - Cholera Toxin B (CTB) Sigma_1OOM4099V

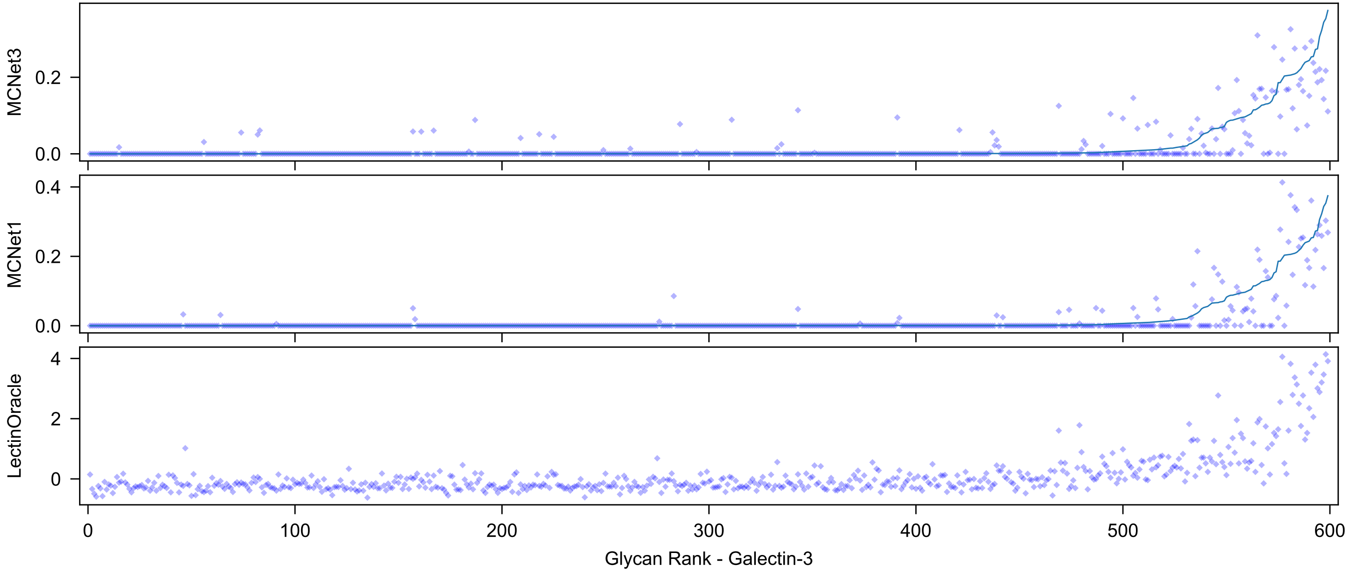

MCNet3
0.2
0.0
MCNet1
0.4
0.2
0.0
LectinOracle
4
2
0
0
100
200
300
400
500
600
Glycan Rank - Galectin-3

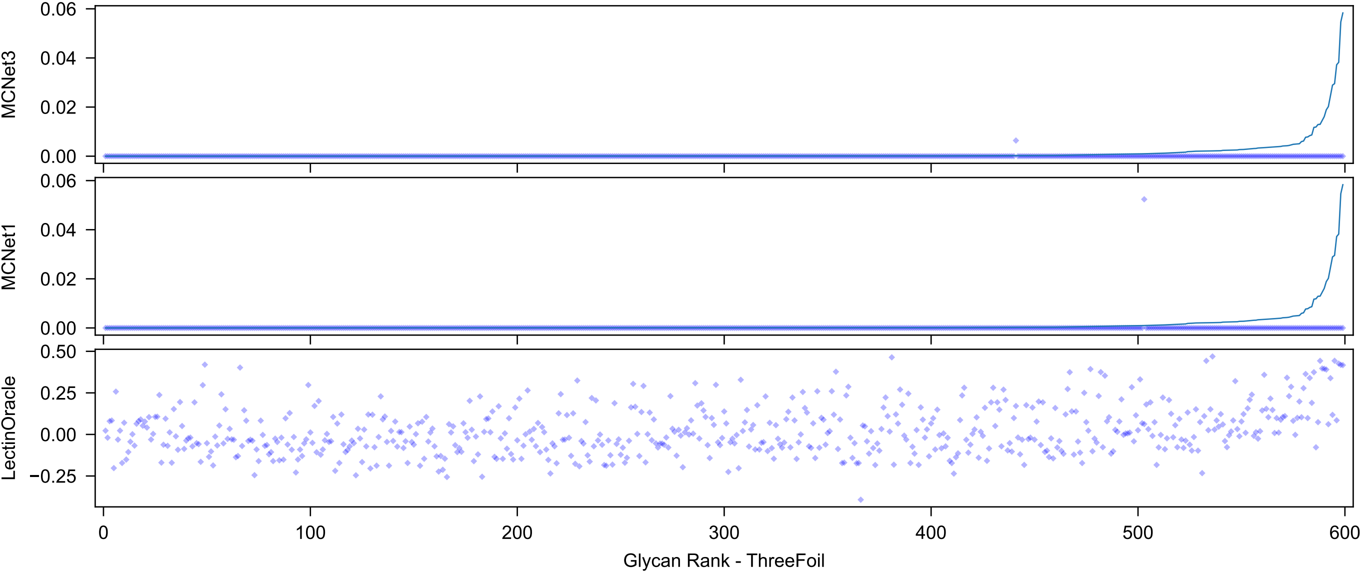

MCNet3
MCNet1
LectinOracle
0.06
0.04
0.02
0.00
0.50
0.25
−0.25
0
100
200
300
400
500
600
Glycan Rank - ThreeFoil

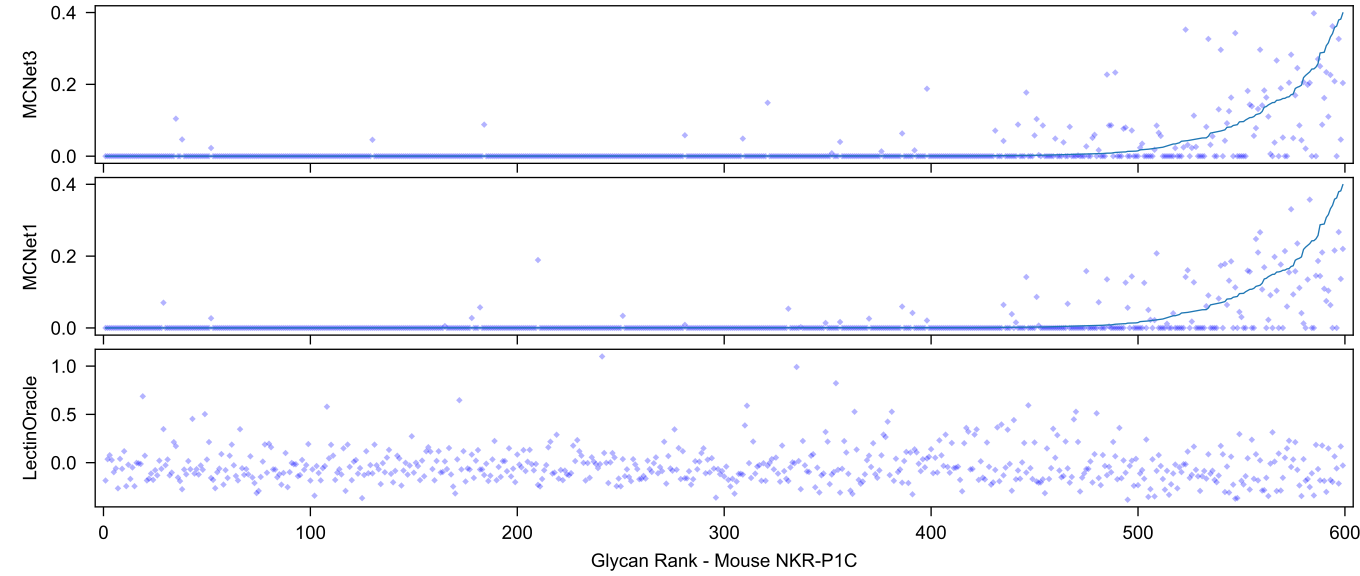

MCNet3
MCNet1
LectinOracle
0.0
0.2
0.4
0.5
1.0
0
100
200
300
400
500
600
Glycan Rank - Mouse NKR-P1C

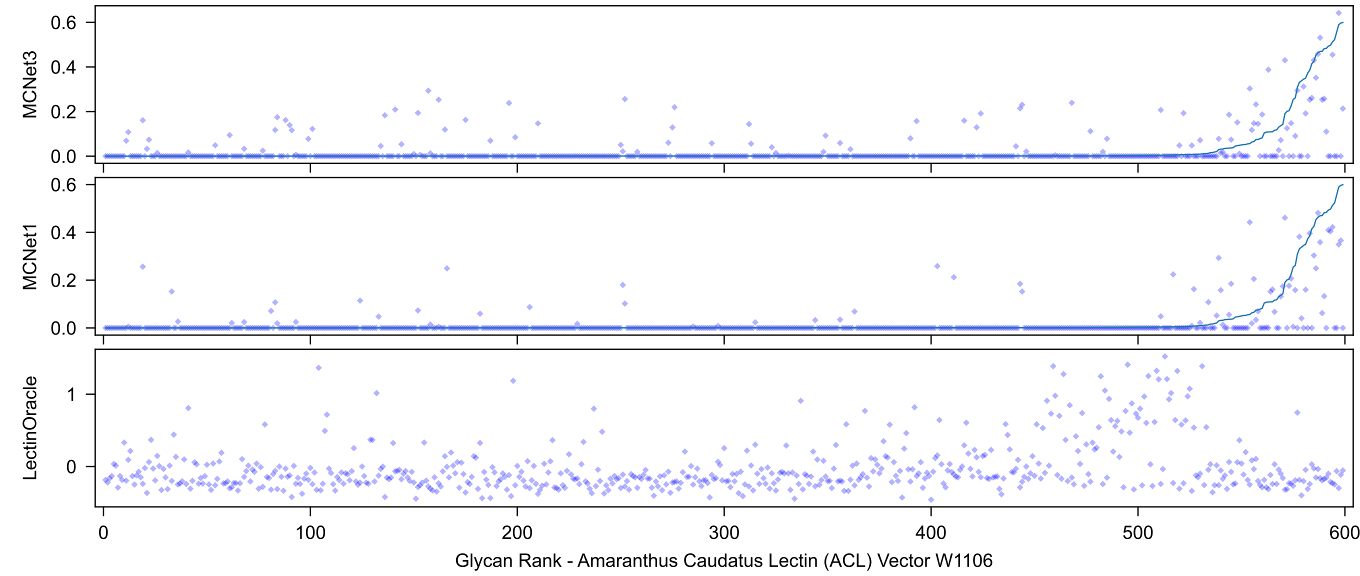
MCNet3
MCNet1
LectinOracle
0.6
0.4
0.2
0.0
1
0
0
100
200
300
400
500
600
Glycan Rank - Amaranthus Caudatus Lectin (ACL) Vector W1106

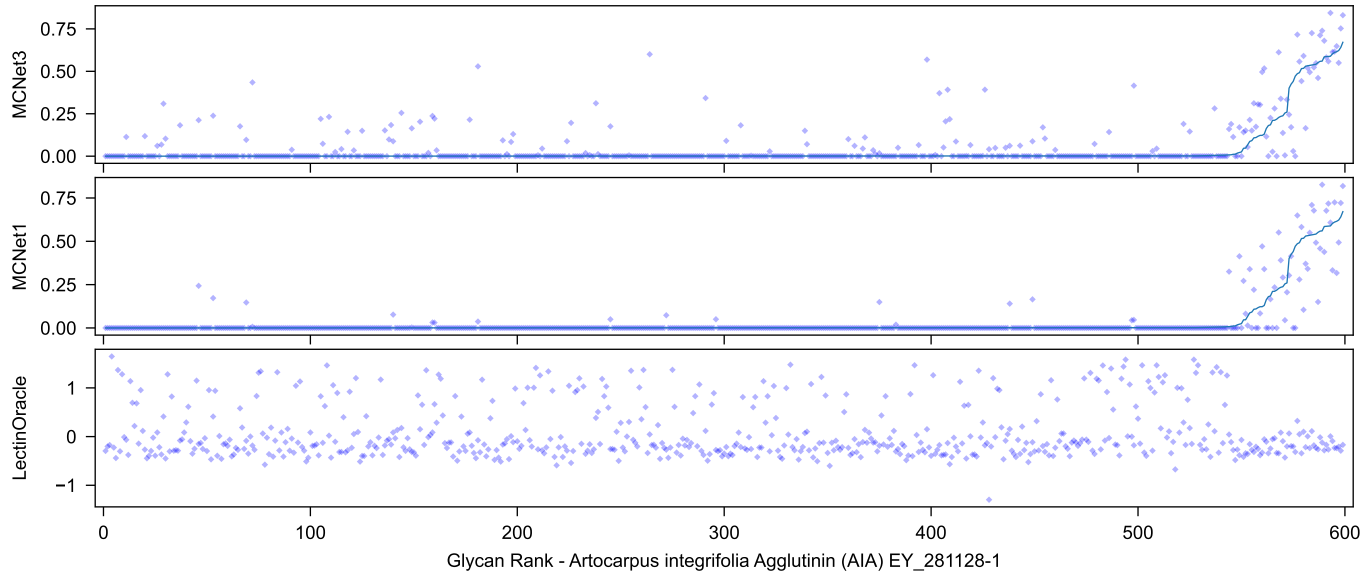

MCNet3
MCNet1
LectinOracle
0.00
0.25
0.50
0.75
−1
0
1
0
100
200
300
400
500
600
Glycan Rank - Artocarpus integrifolia Agglutinin (AIA) EY_281128-1

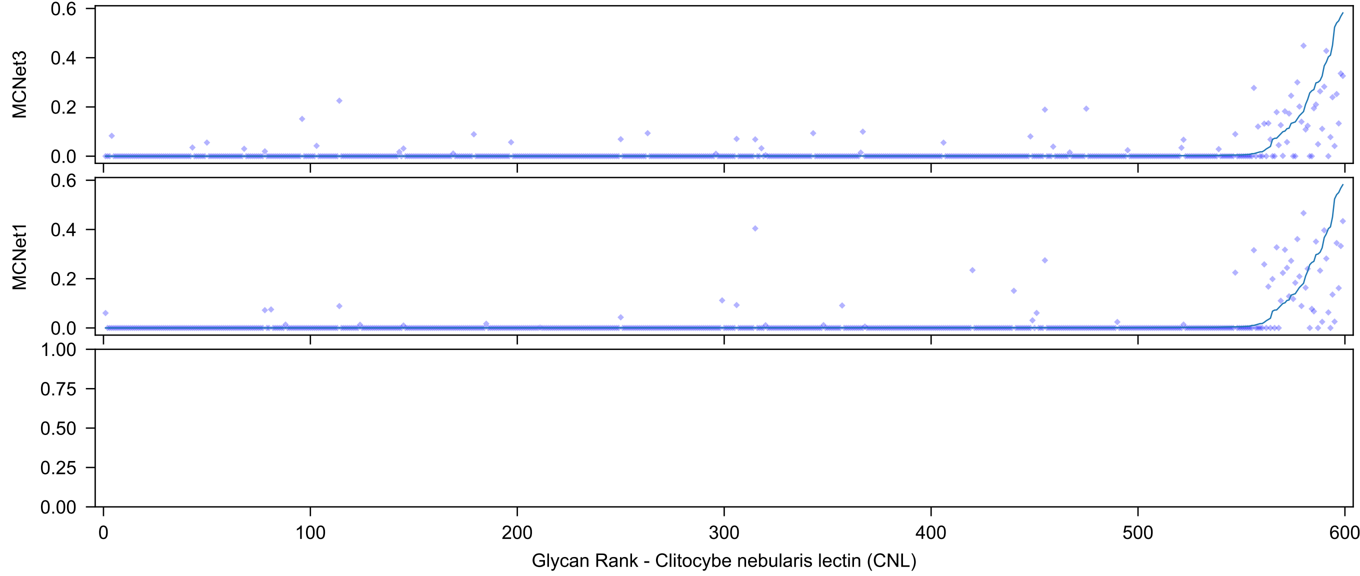
MCNet3
MCNet1
0.6
0.4
0.2
0.0
1.00
0.75
0.50
0.25
0.00
0
100
200
300
400
500
600
Glycan Rank - Clitocybe nebularis lectin (CNL)

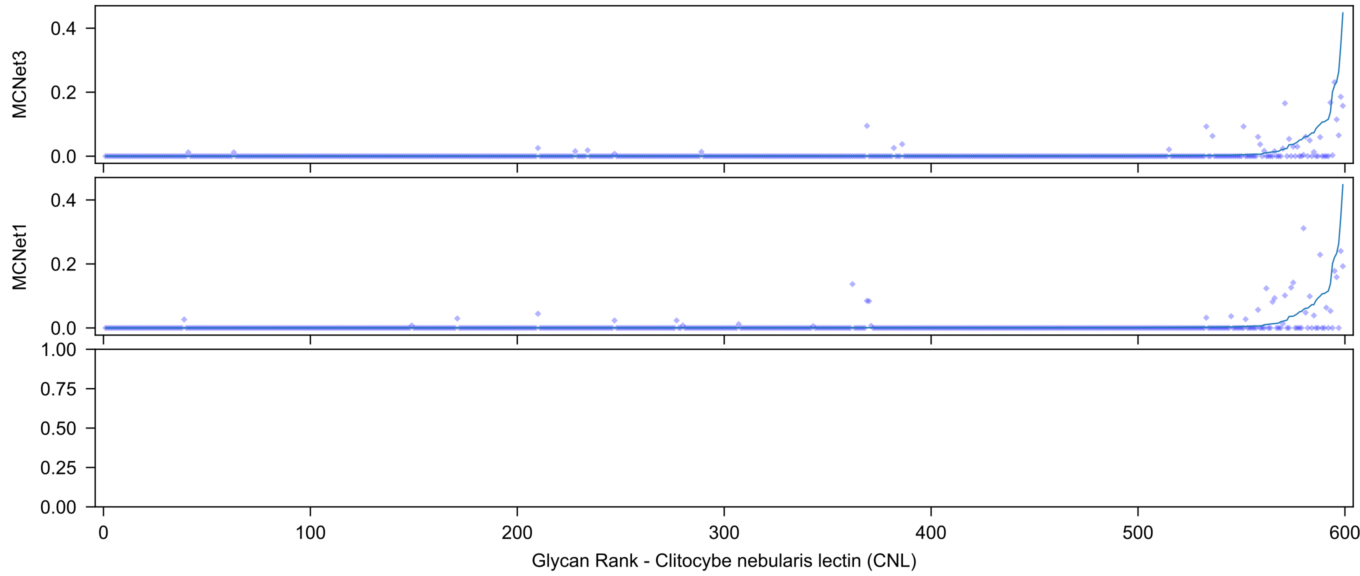

MCNet3
MCNet1
0.4
0.2
0.0
1.00
0.75
0.50
0.25
0.00
0
100
200
300
400
500
600
Glycan Rank - Clitocybe nebularis lectin (CNL)

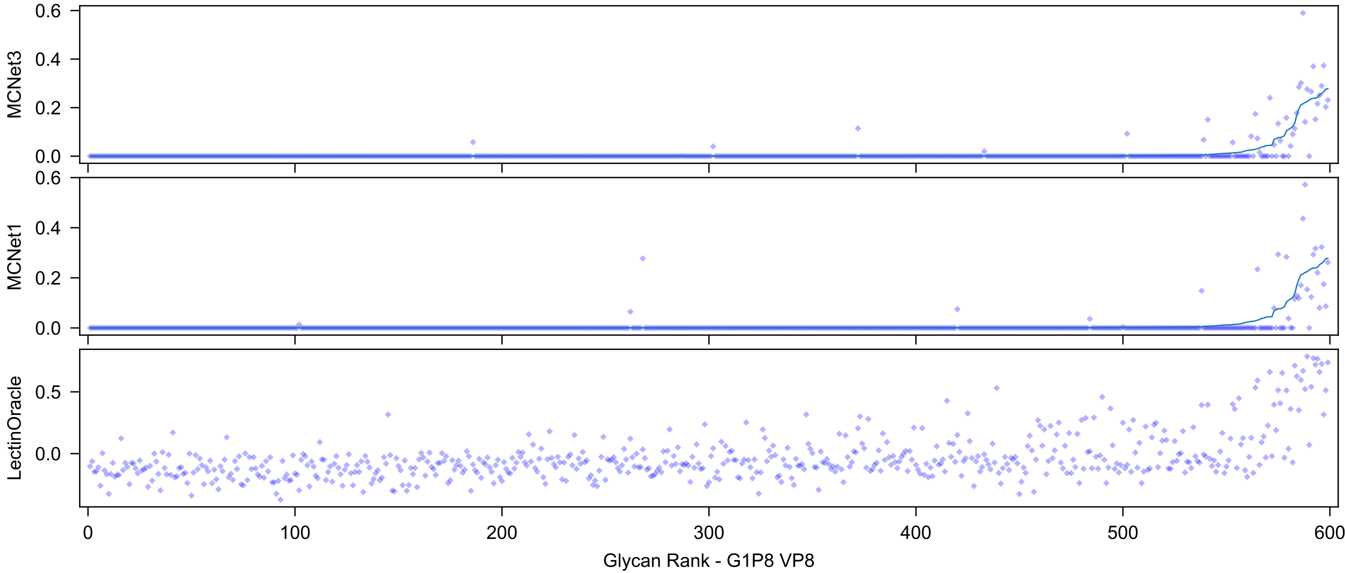

MCNet3
MCNet1
LectinOracle
0.6
0.4
0.2
0.0
0.5
0
100
200
300
400
500
600
Glycan Rank - G1P8 VP8

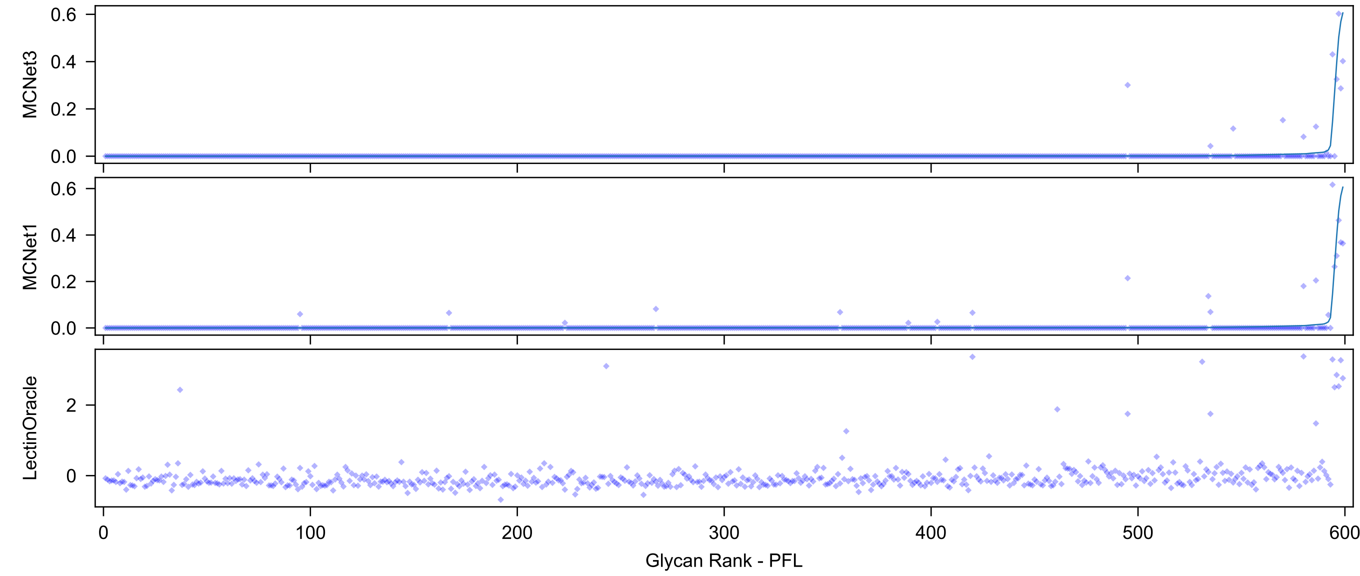

MCNet3
MCNet1
LectinOracle
0.6
0.4
0.2
0.0
2
0
0
100
200
300
400
500
600
Glycan Rank - PFL

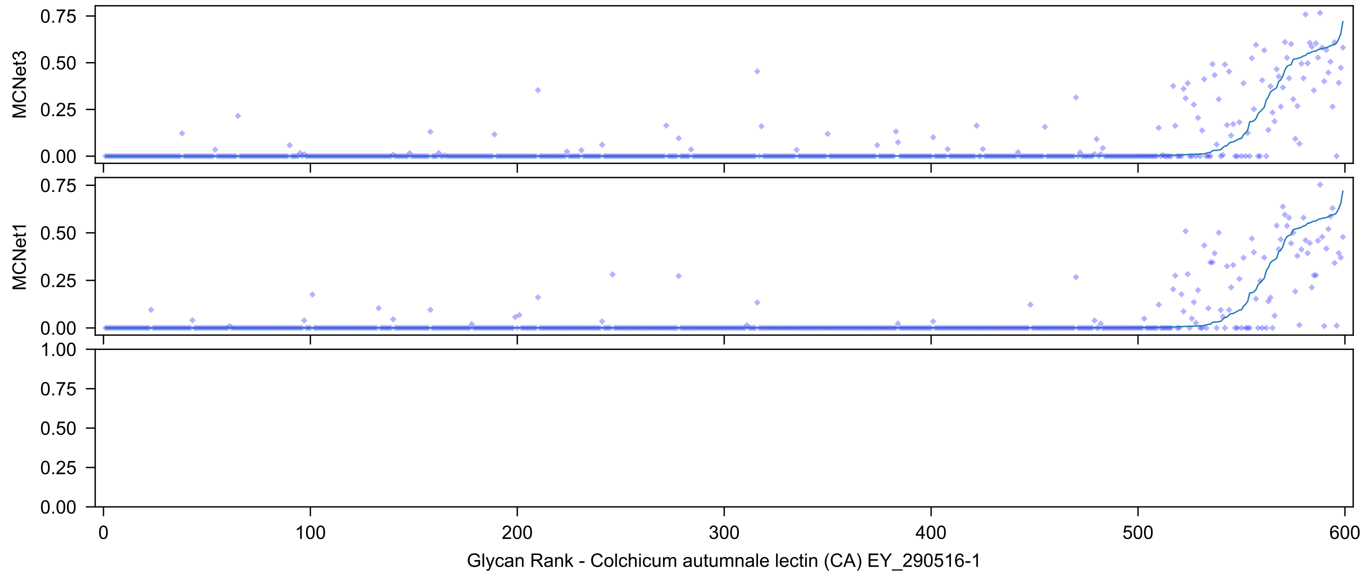

MCNet3
MCNet1
0.00
0.25
0.50
0.75
1.00
0
100
200
300
400
500
600
Glycan Rank - Colchicum autumnale lectin (CA) EY_290516-1

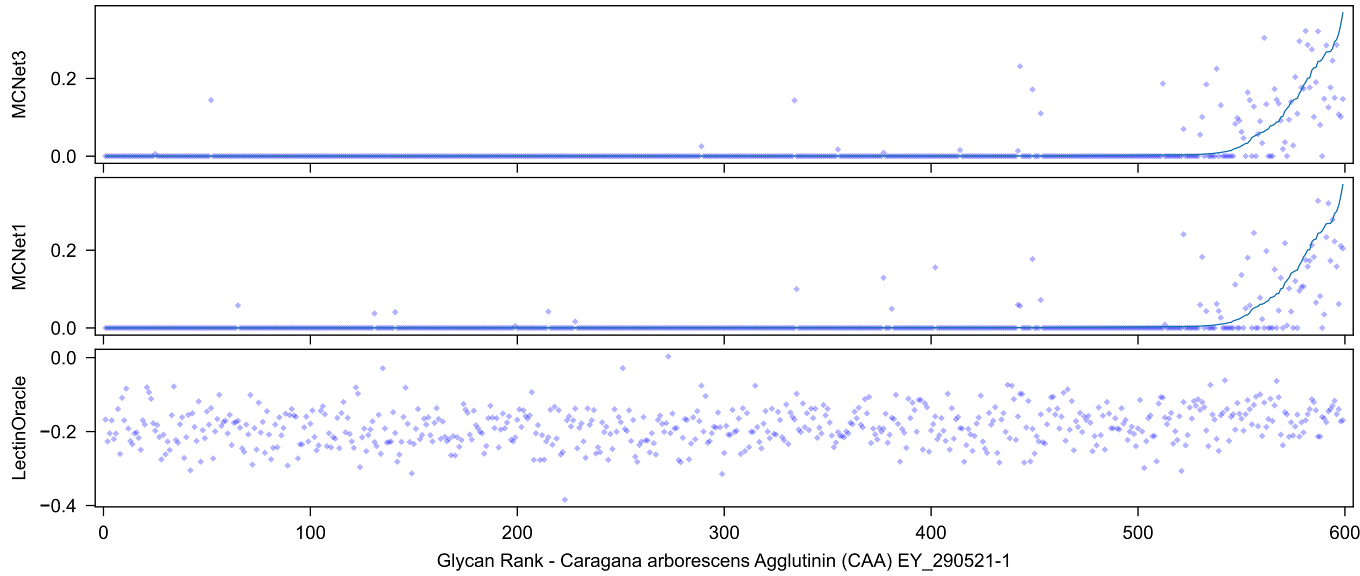

MCNet3
0.2
0.0
MCNet1
0.2
0.0
LectinOracle
0.0
−0.2
−0.4
0
100
200
300
400
500
600
Glycan Rank - Caragana arborescens Agglutinin (CAA) EY_290521-1

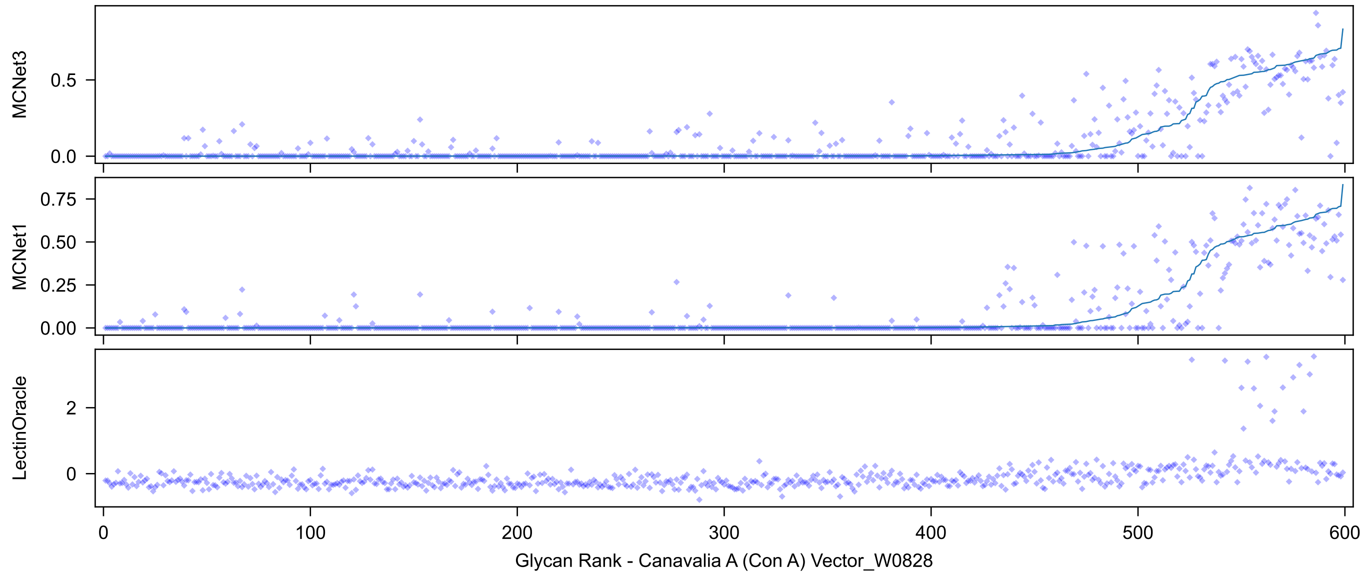
MCNet3
0.5
0.0
MCNet1
0.75
0.50
0.25
0.00
LectinOracle
2
0
0
100
200
300
400
500
600
Glycan Rank - Canavalia A (Con A) Vector_W0828

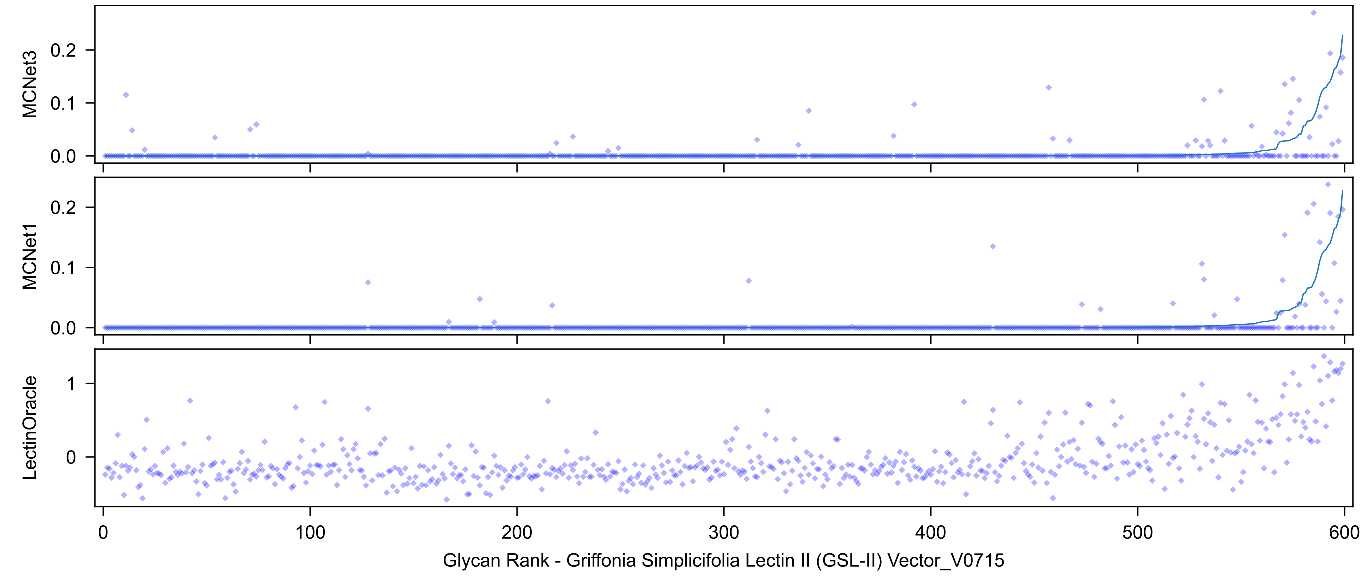

MCNet3
0.2
0.1
0.0
MCNet1
0.2
0.1
0.0
LectinOracle
1
0
0
100
200
300
400
500
600
Glycan Rank - Griffonia Simplicifolia Lectin II (GSL-II) Vector_V0715

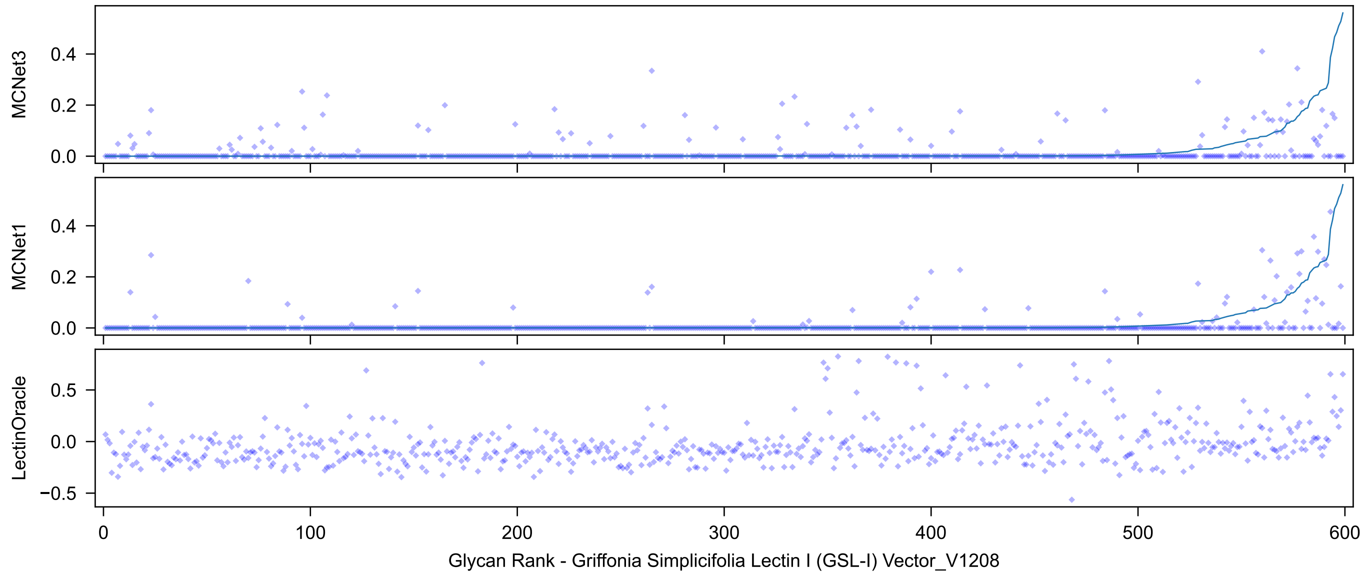
MCNet3
MCNet1
LectinOracle
0.4
0.2
0.0
0.5
0.0
−0.5
0
100
200
300
400
500
600
Glycan Rank - Griffonia Simplicifolia Lectin I (GSL-I) Vector_V1208

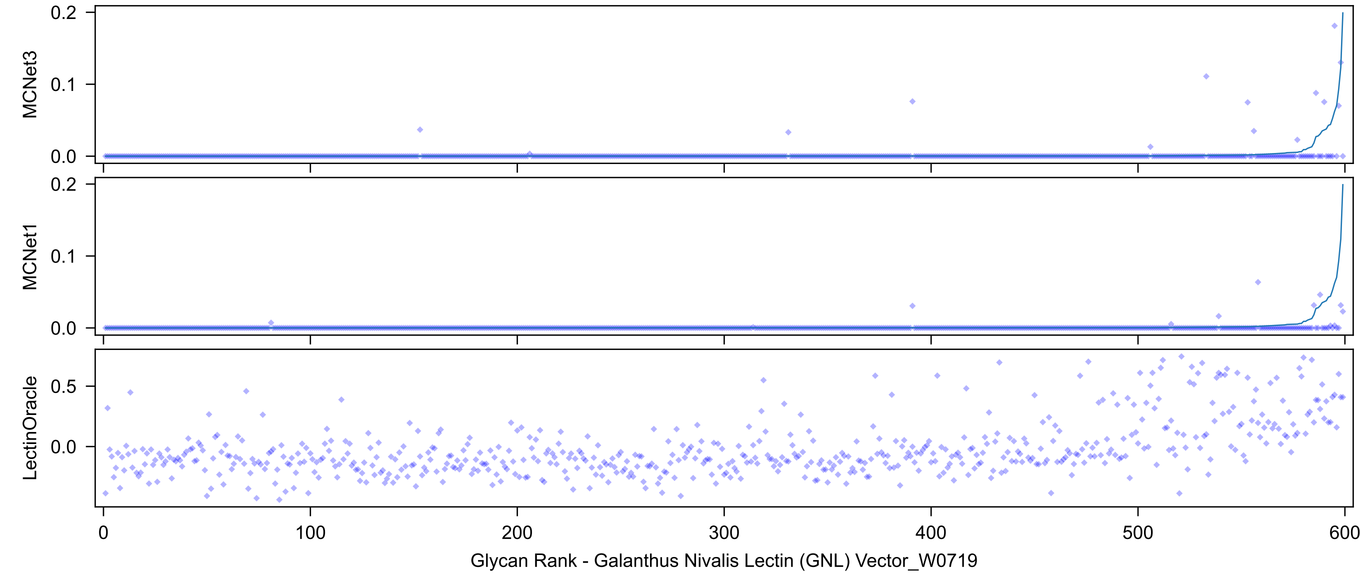

MCNet3
MCNet1
LectinOracle
0.2
0.1
0.0
0.5
0
100
200
300
400
500
600
Glycan Rank - Galanthus Nivalis Lectin (GNL) Vector_W0719

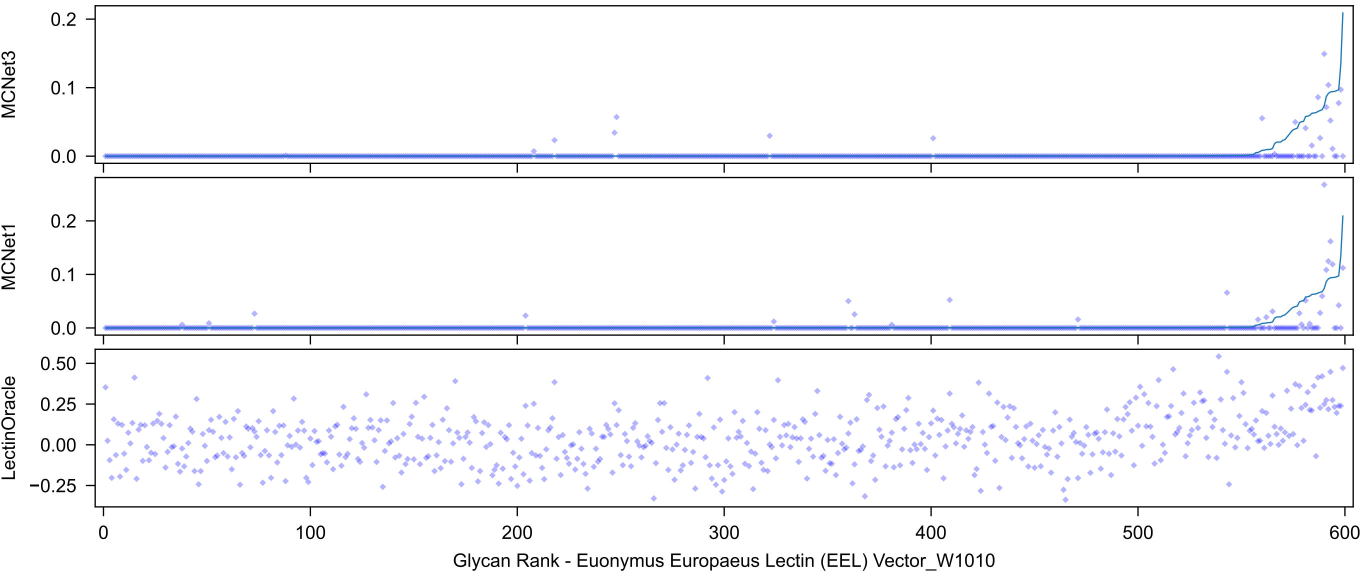

MCNet3
MCNet1
LectinOracle
0.2
0.1
0.0
0.50
0.25
0.00
−0.25
0
100
200
300
400
500
600
Glycan Rank - Euonymus Europaeus Lectin (EEL) Vector_W1010

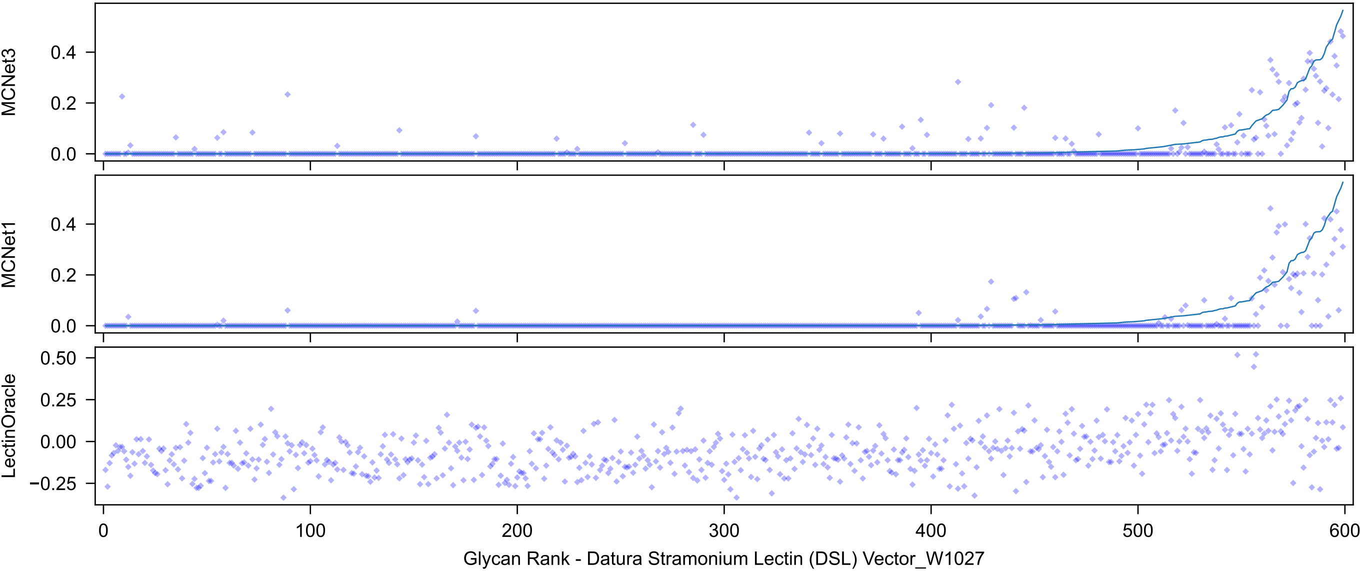

MCNet3
0.4
0.2
0.0
MCNet1
0.4
0.2
0.0
LectinOracle
0.50
0.25
0.00
−0.25
0
100
200
300
400
500
600
Glycan Rank - Datura Stramonium Lectin (DSL) Vector_W1027

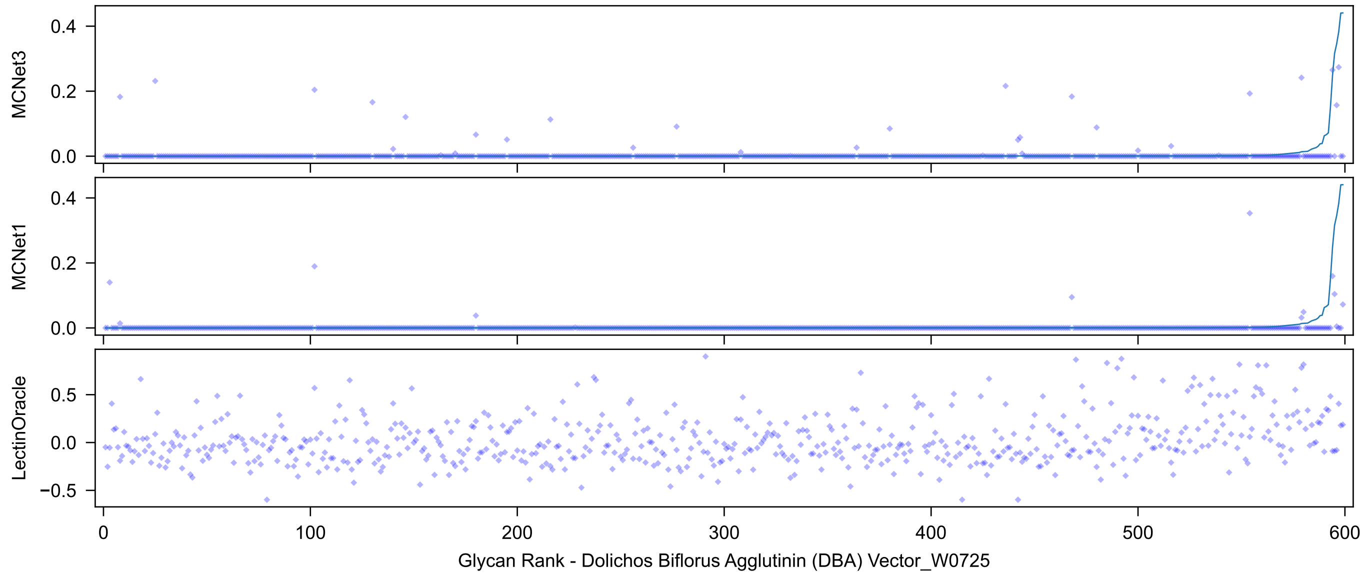

MCNet3
0.4
0.2
0.0
MCNet1
0.4
0.2
0.0
LectinOracle
0.5
0.0
−0.5
0
100
200
300
400
500
600
Glycan Rank - Dolichos Biflorus Agglutinin (DBA) Vector_W0725

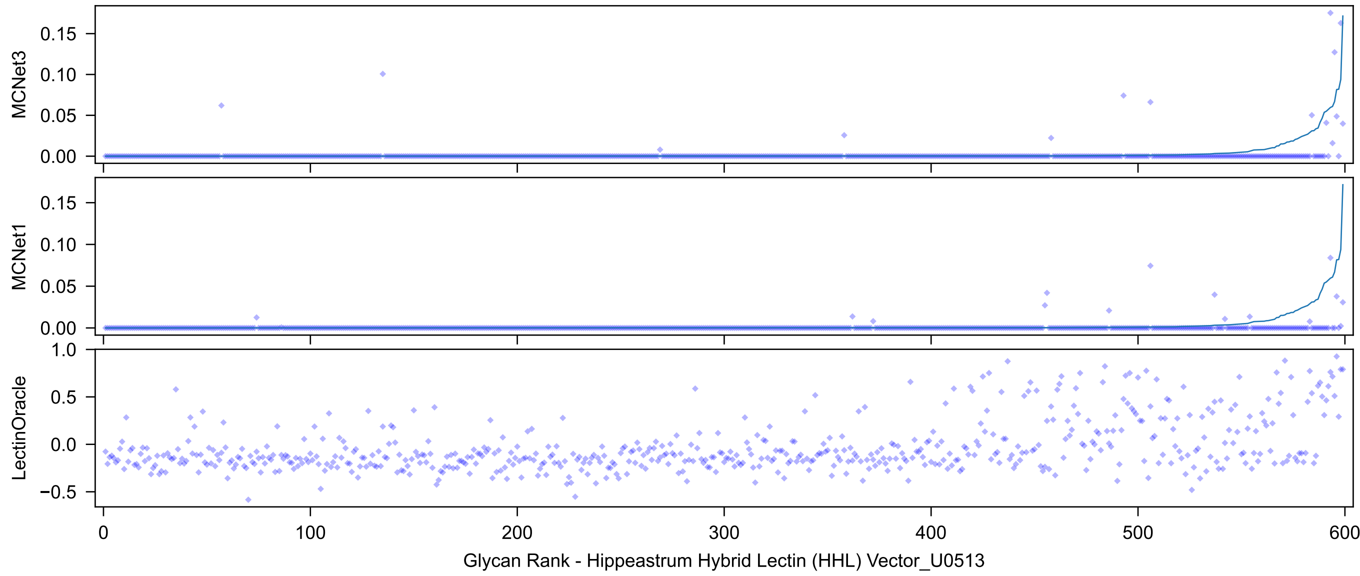

MCNet3
MCNet1
LectinOracle
0.15
0.10
0.05
0.00
1.0
0.5
0.0
−0.5
0
100
200
300
400
500
600
Glycan Rank - Hippeastrum Hybrid Lectin (HHL) Vector_U0513

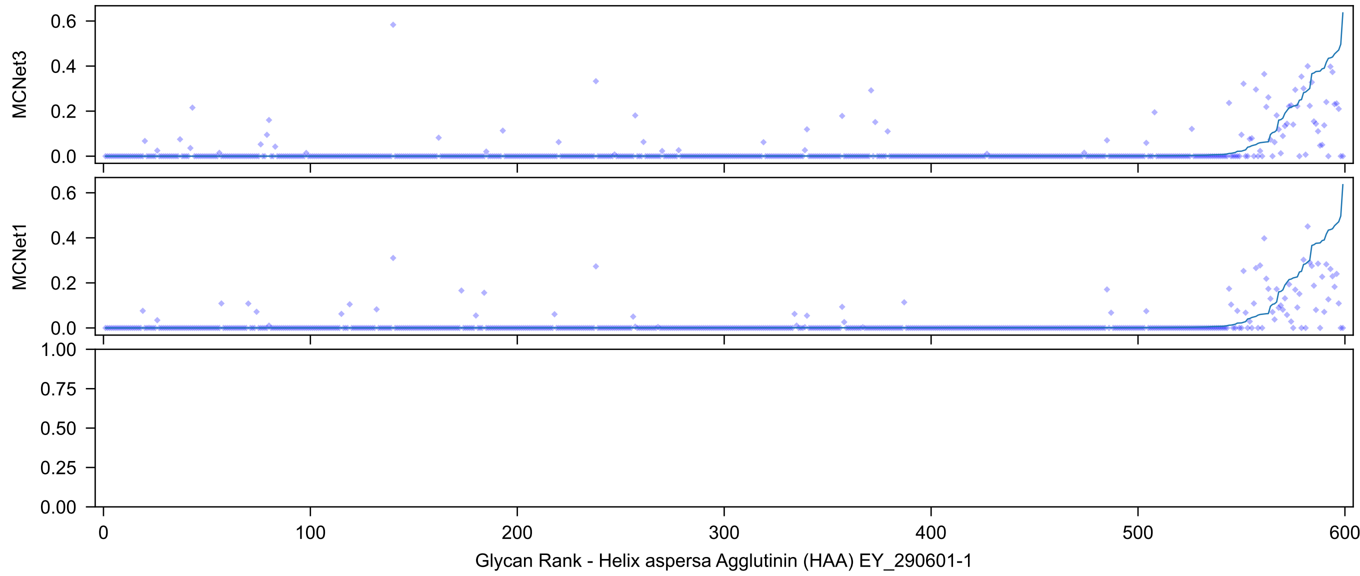

MCNet3
MCNet1
0.6
0.4
0.2
0.0
1.00
0.75
0.50
0.25
0.00
0
100
200
300
400
500
600
Glycan Rank - Helix aspersa Agglutinin (HAA) EY_290601-1

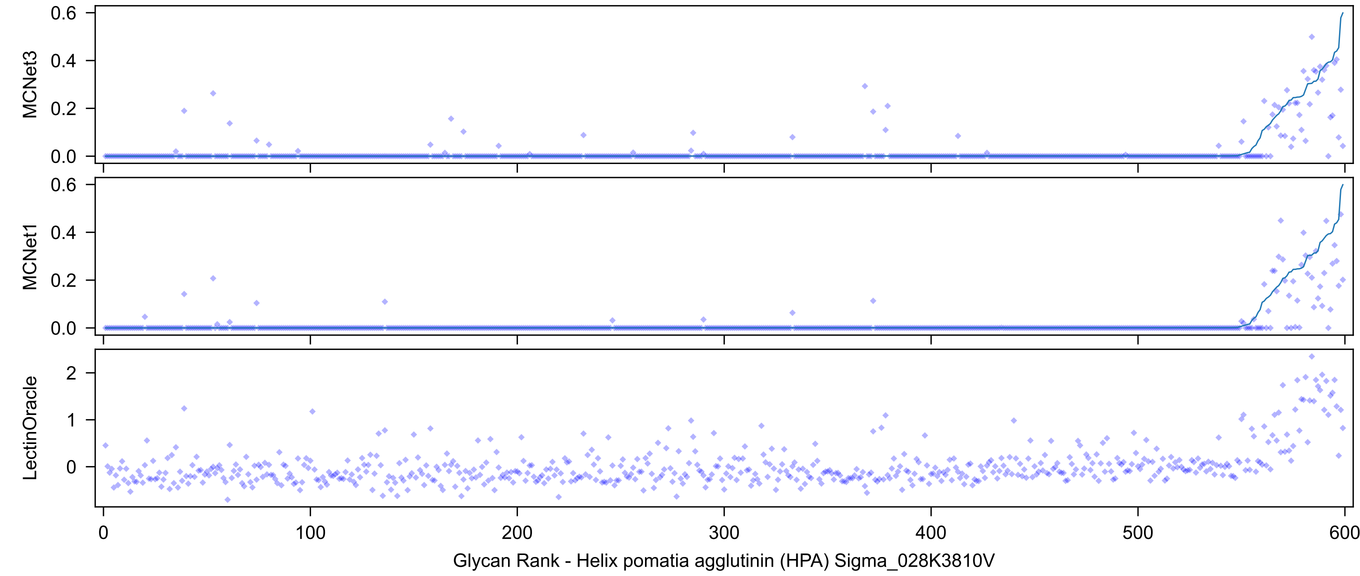
MCNet3
MCNet1
LectinOracle
0.0
0.2
0.4
0.6
0
1
2
0
100
200
300
400
500
600
Glycan Rank - Helix pomatia agglutinin (HPA) Sigma_028K3810V

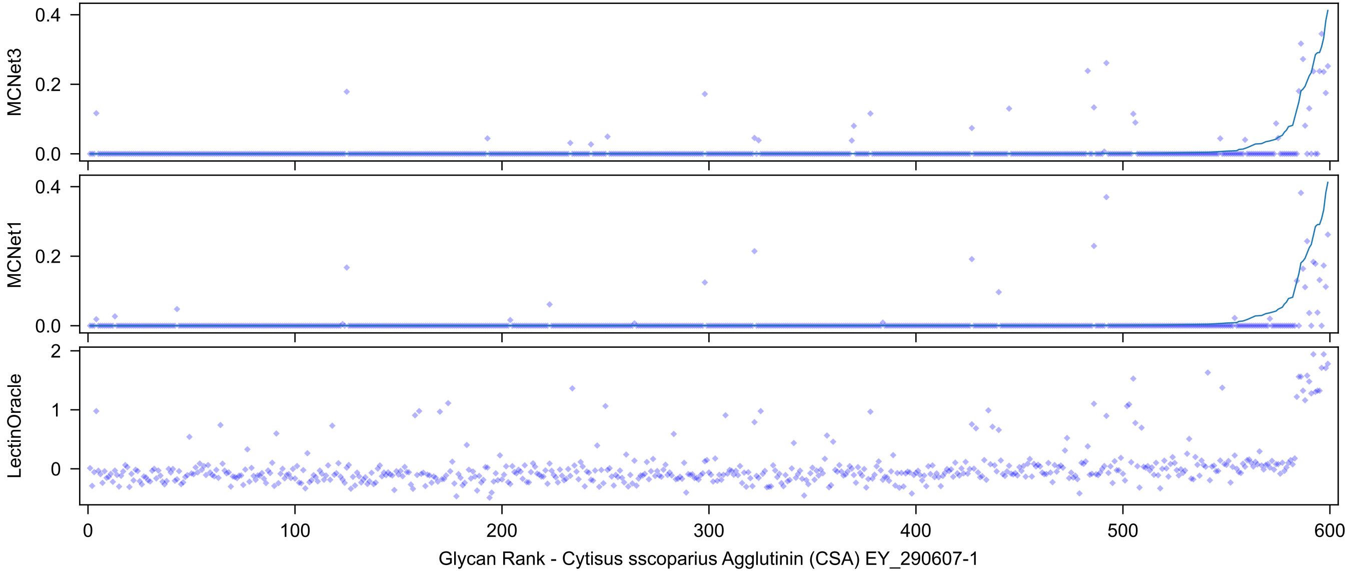

MCNet3
MCNet1
LectinOracle
0.4
0.2
0.0
2
1
0
0
100
200
300
400
500
600
Glycan Rank - Cytisus sscoparius Agglutinin (CSA) EY_290607-1

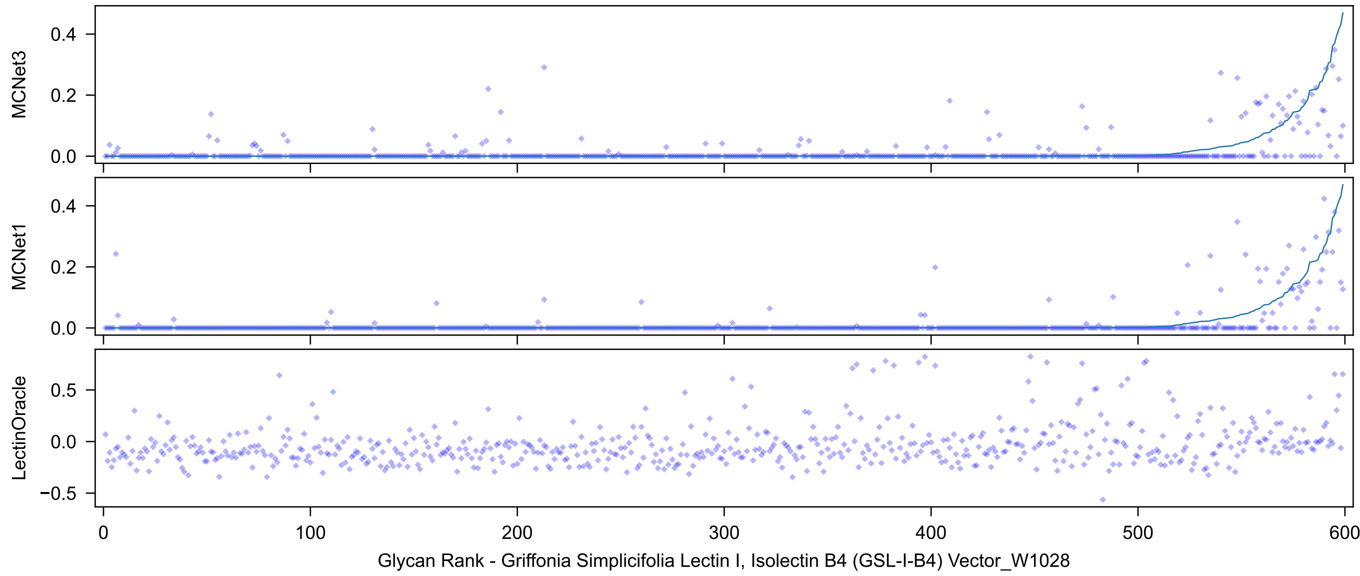
MCNet3
MCNet1
LectinOracle
0.4
0.2
0.0
0.5
−0.5
0
100
200
300
400
500
600
Glycan Rank - Griffonia Simplicifolia Lectin I, Isolectin B4 (GSL-I-B4) Vector_W1028

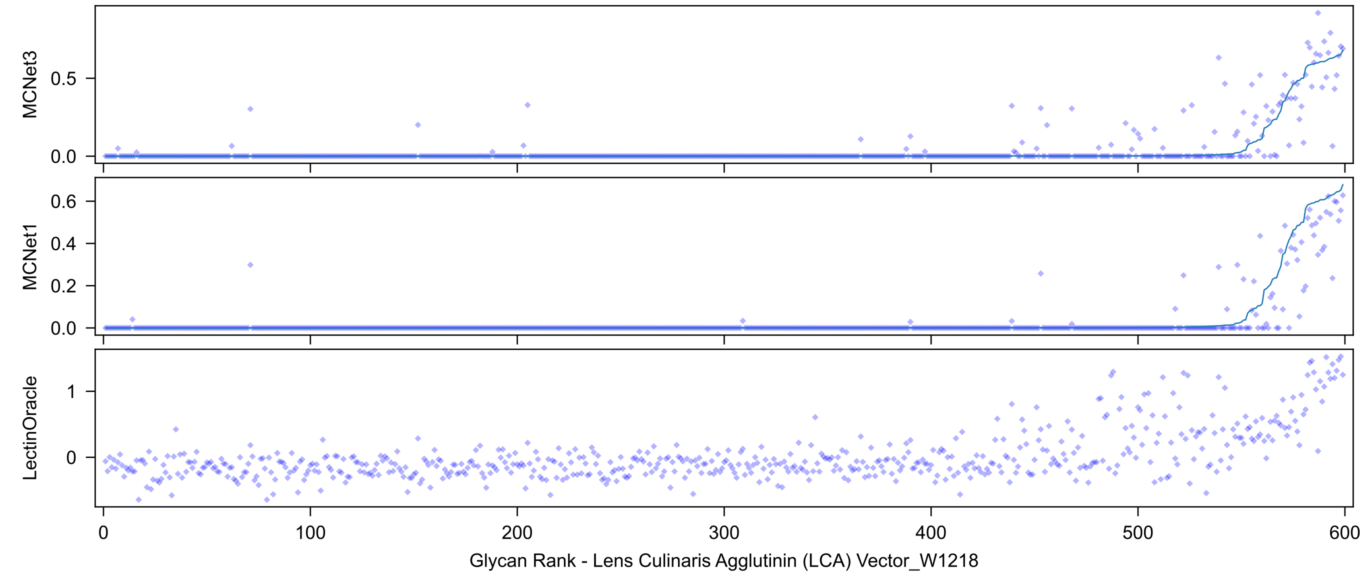

MCNet3
0.5
0.0
MCNet1
0.6
0.4
0.2
0.0
LectinOracle
1
0
0
100
200
300
400
500
600
Glycan Rank - Lens Culinaris Agglutinin (LCA) Vector_W1218

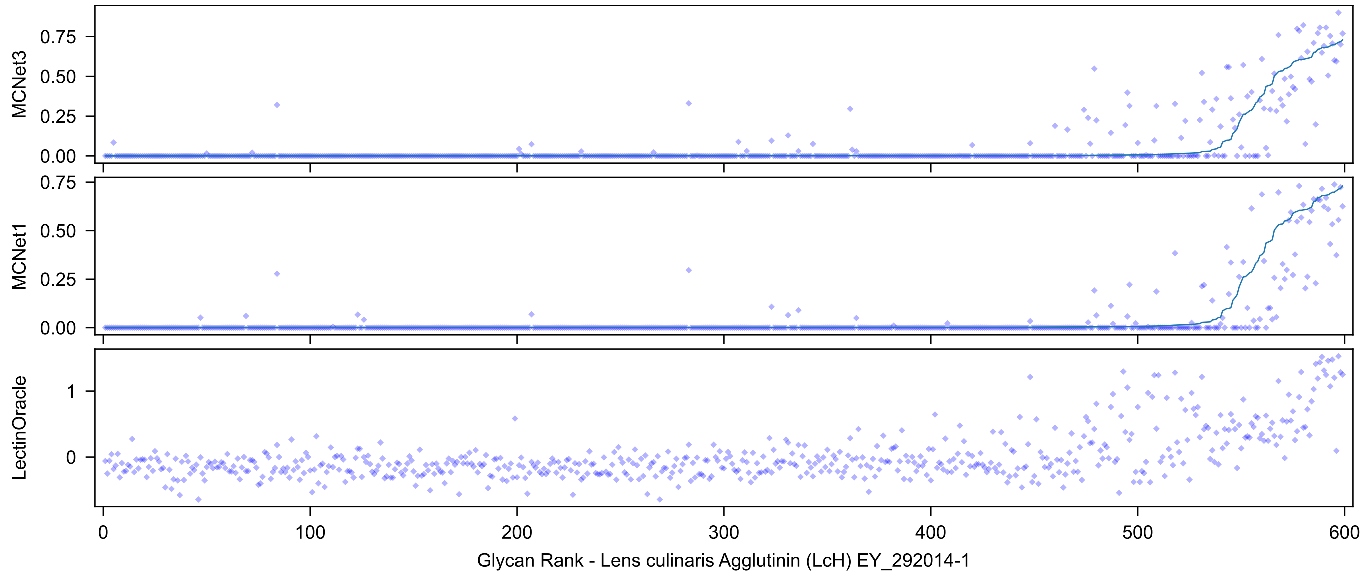

MCNet3
0.75
0.50
0.25
0.00
MCNet1
0.75
0.50
0.25
0.00
LectinOracle
1
0
0
100
200
300
400
500
600
Glycan Rank - Lens culinaris Agglutinin (LcH) EY_292014-1

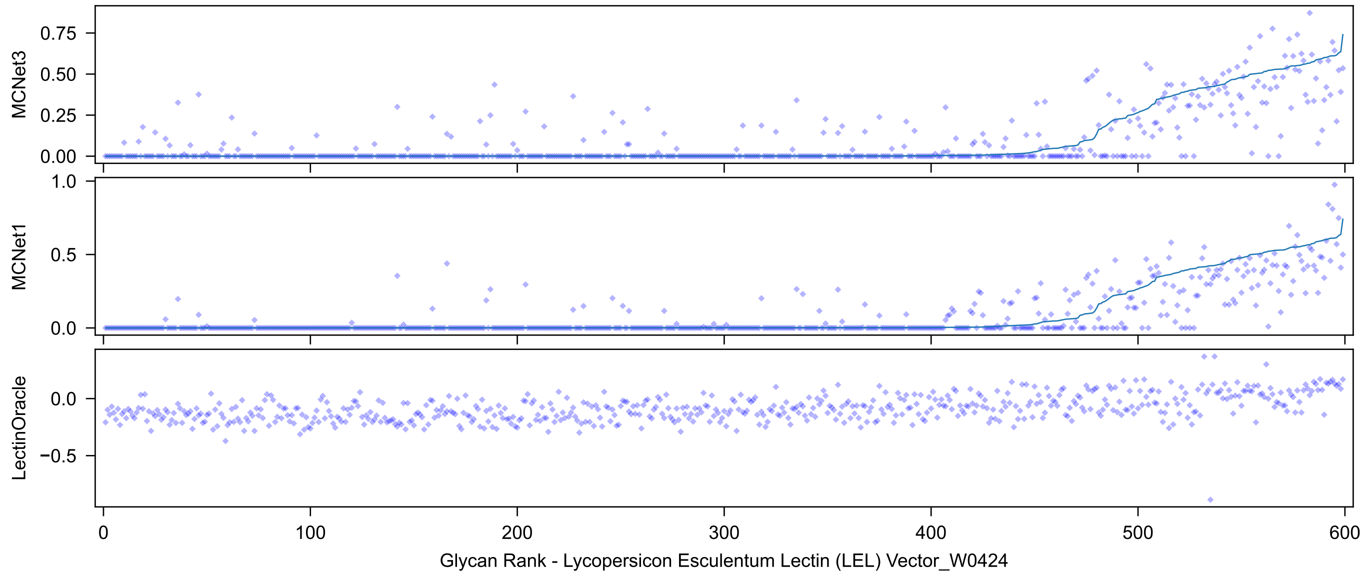

MCNet3
0.00
0.25
0.50
0.75
MCNet1
0.0
0.5
1.0
LectinOracle
−0.5
0.0
0
100
200
300
400
500
600
Glycan Rank - Lycopersicon Esculentum Lectin (LEL) Vector_W0424

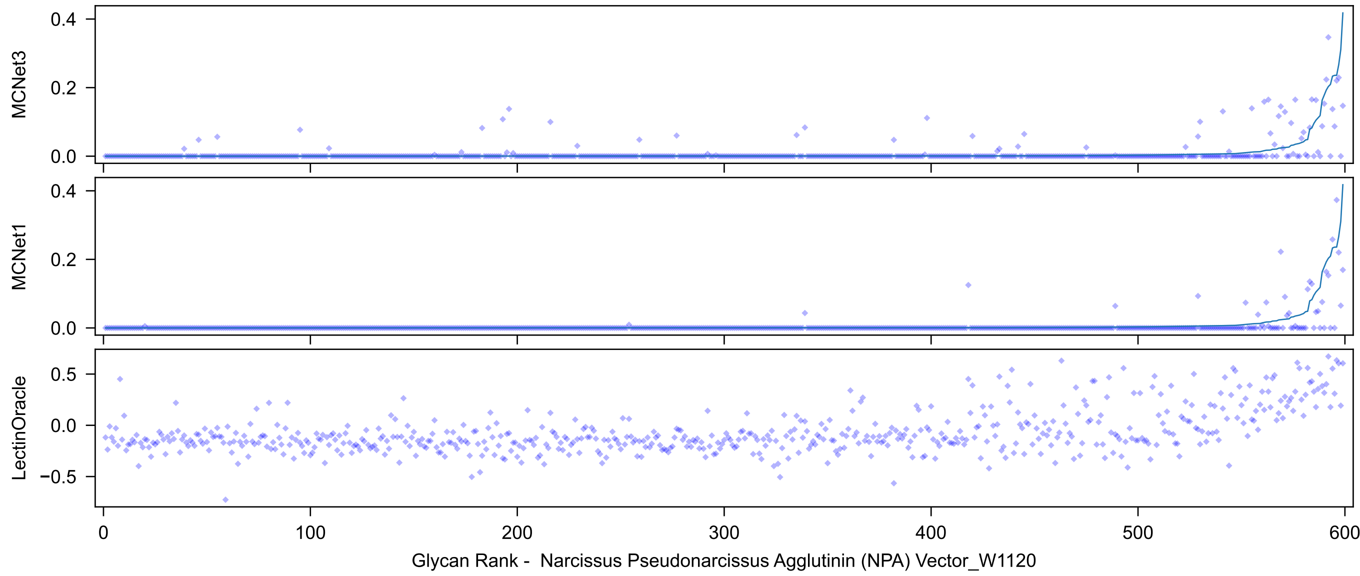

MCNet3
MCNet1
LectinOracle
0.4
0.2
0.0
0.5
−0.5
0
100
200
300
400
500
600
Glycan Rank - Narcissus Pseudonarcissus Agglutinin (NPA) Vector_W1120

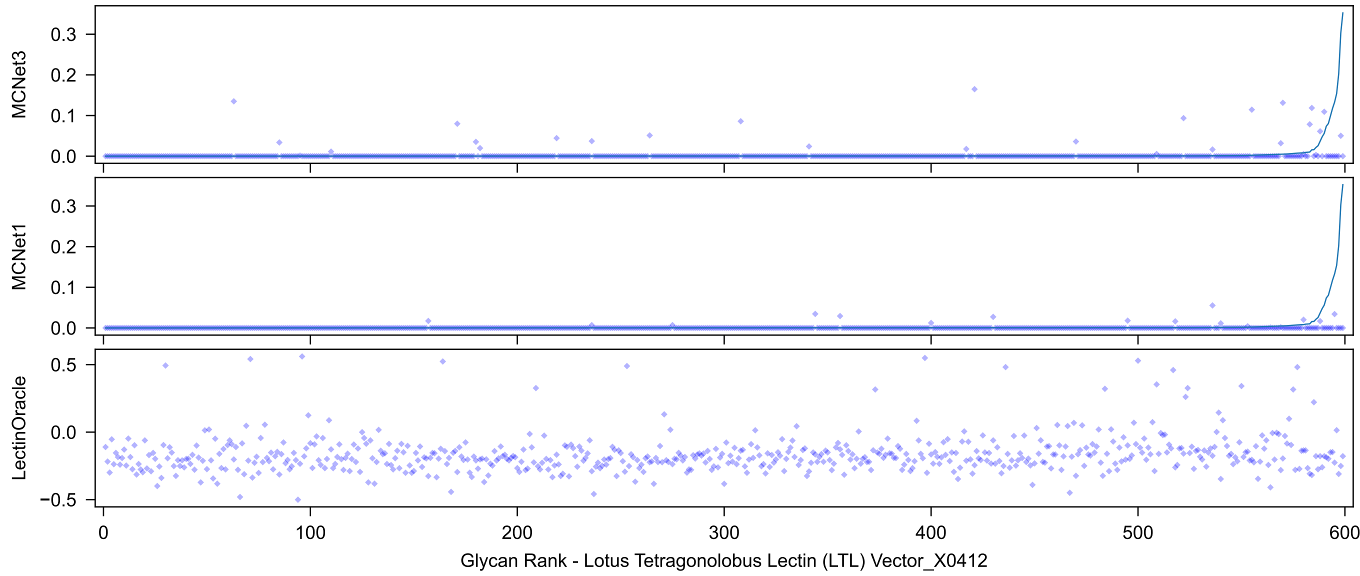

MCNet3
MCNet1
LectinOracle
0.3
0.2
0.1
0.0
0.5
0.0
−0.5
0
100
200
300
400
500
600
Glycan Rank - Lotus Tetragonolobus Lectin (LTL) Vector_X0412

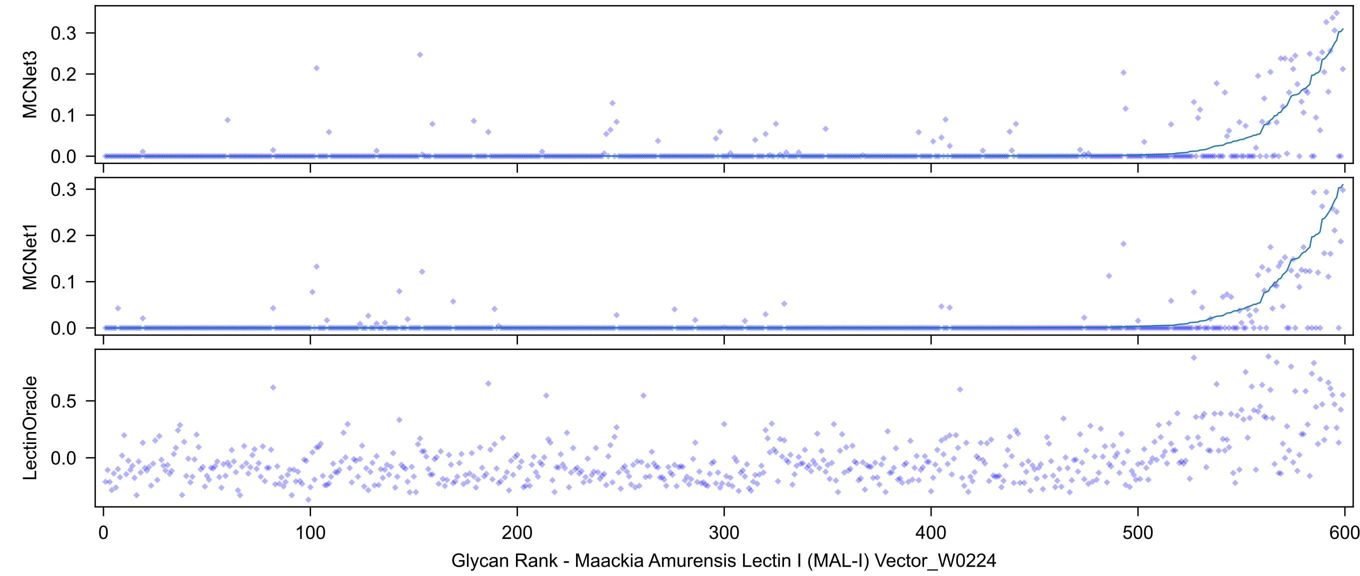

MCNet3
MCNet1
LectinOracle
0.0
0.1
0.2
0.3
0.5
0
100
200
300
400
500
600
Glycan Rank - Maackia Amurensis Lectin I (MAL-I) Vector_W0224

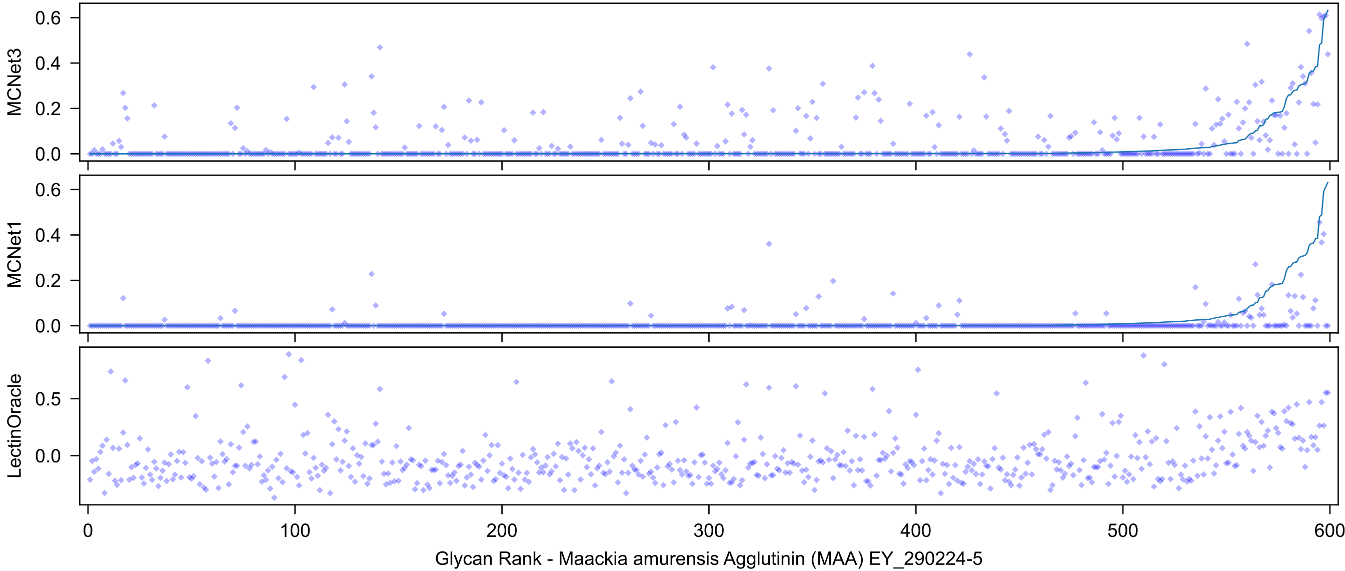

MCNet3
MCNet1
LectinOracle
0.0
0.2
0.4
0.6
0.5
0
100
200
300
400
500
600
Glycan Rank - Maackia amurensis Agglutinin (MAA) EY_290224-5

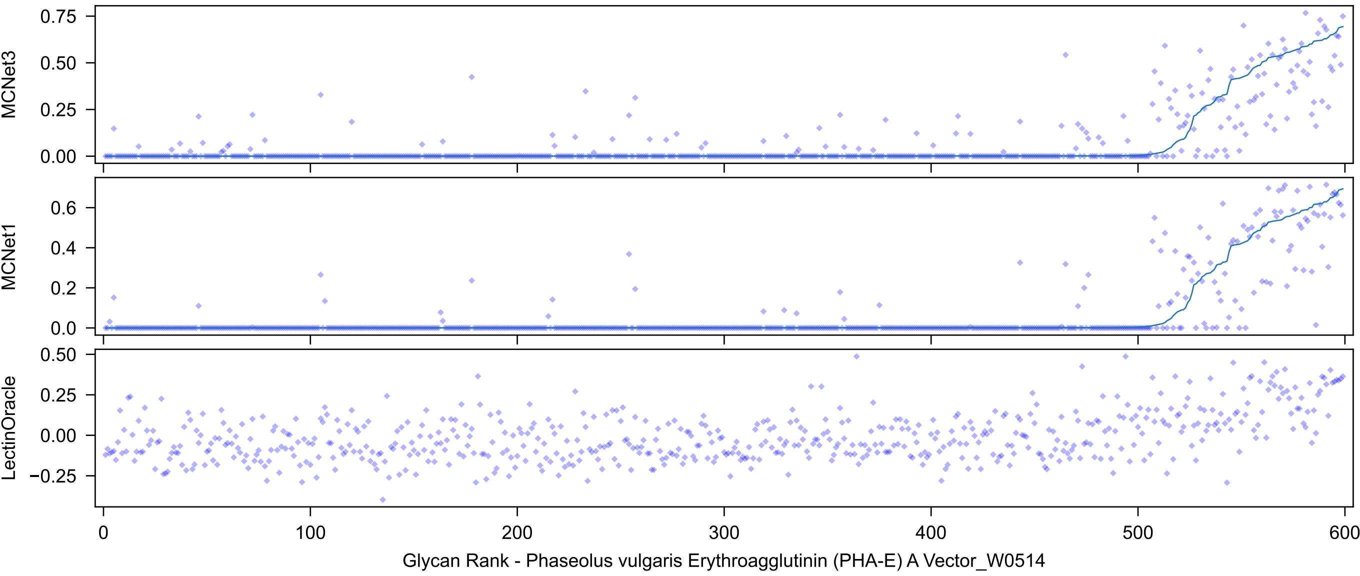

MCNet3
MCNet1
LectinOracle
0.75
0.50
0.25
0.00
0.6
0.4
0.2
0.0
0.50
0.25
0.00
−0.25
0
100
200
300
400
500
600
Glycan Rank - Phaseolus vulgaris Erythroagglutinin (PHA-E) A Vector_W0514

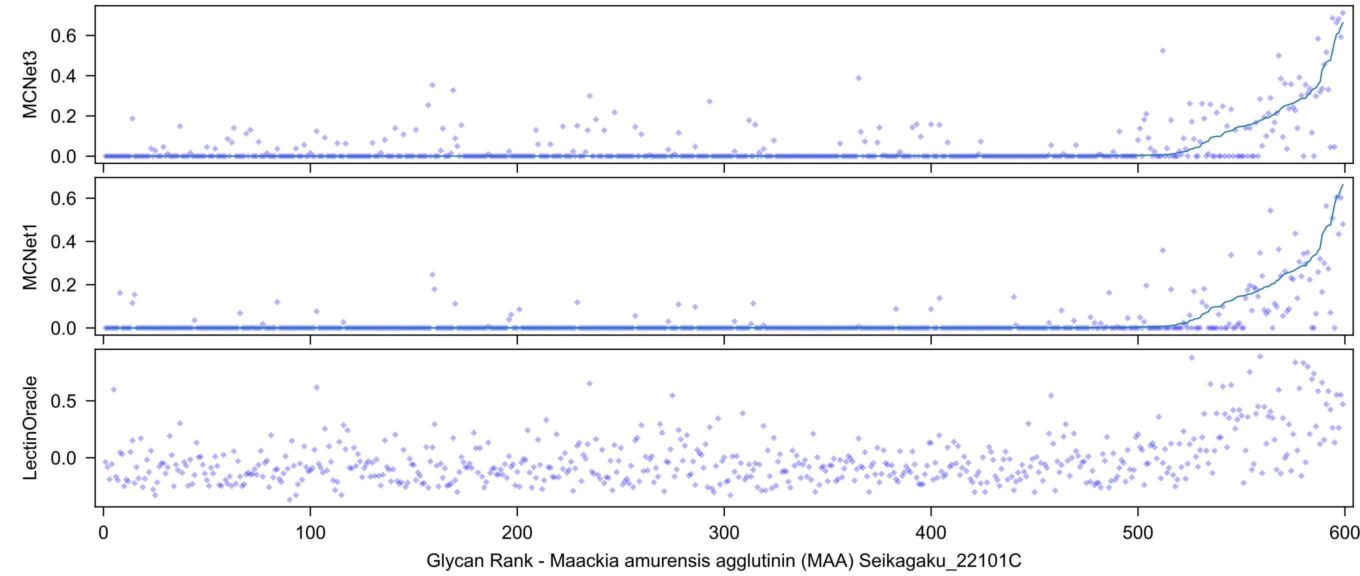
MCNet3
MCNet1
LectinOracle
0.0
0.2
0.4
0.6
0.5
0
100
200
300
400
500
600
Glycan Rank - Maackia amurensis agglutinin (MAA) Seikagaku_22101C

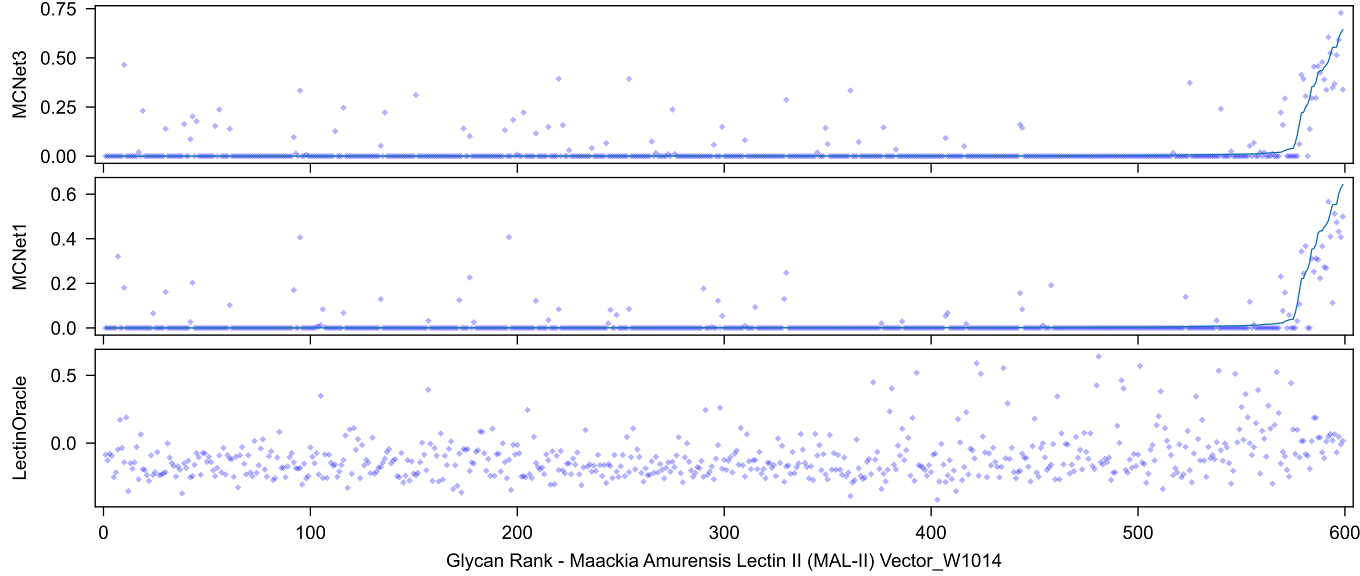
MCNet3
0.75
0.50
0.25
0.00
MCNet1
0.6
0.4
0.2
0.0
LectinOracle
0.5
0.0
0
100
200
300
400
500
600
Glycan Rank - Maackia Amurensis Lectin II (MAL-II) Vector_W1014

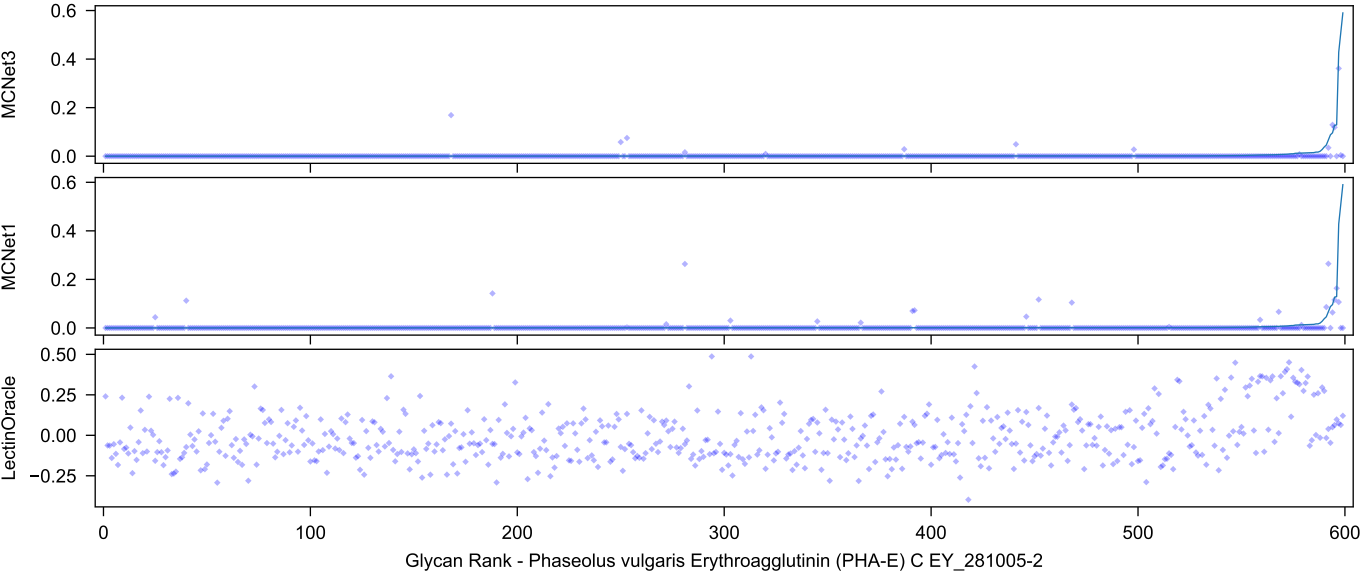

MCNet3
MCNet1
LectinOracle
0.6
0.4
0.2
0.0
0.50
0.25
0.00
−0.25
0
100
200
300
400
500
600
Glycan Rank - Phaseolus vulgaris Erythroagglutinin (PHA-E) C EY_281005-2

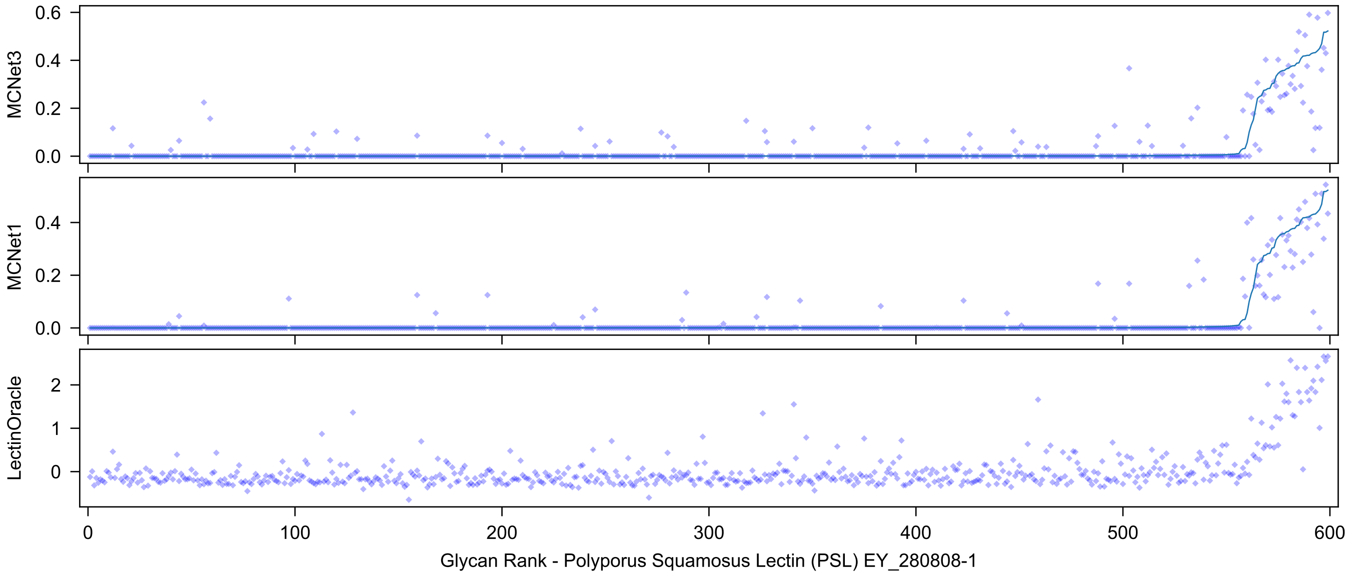
MCNet3
MCNet1
LectinOracle
0.0
0.2
0.4
0.6
0
1
2
100
200
300
400
500
600
Glycan Rank - Polyporus Squamosus Lectin (PSL) EY_280808-1

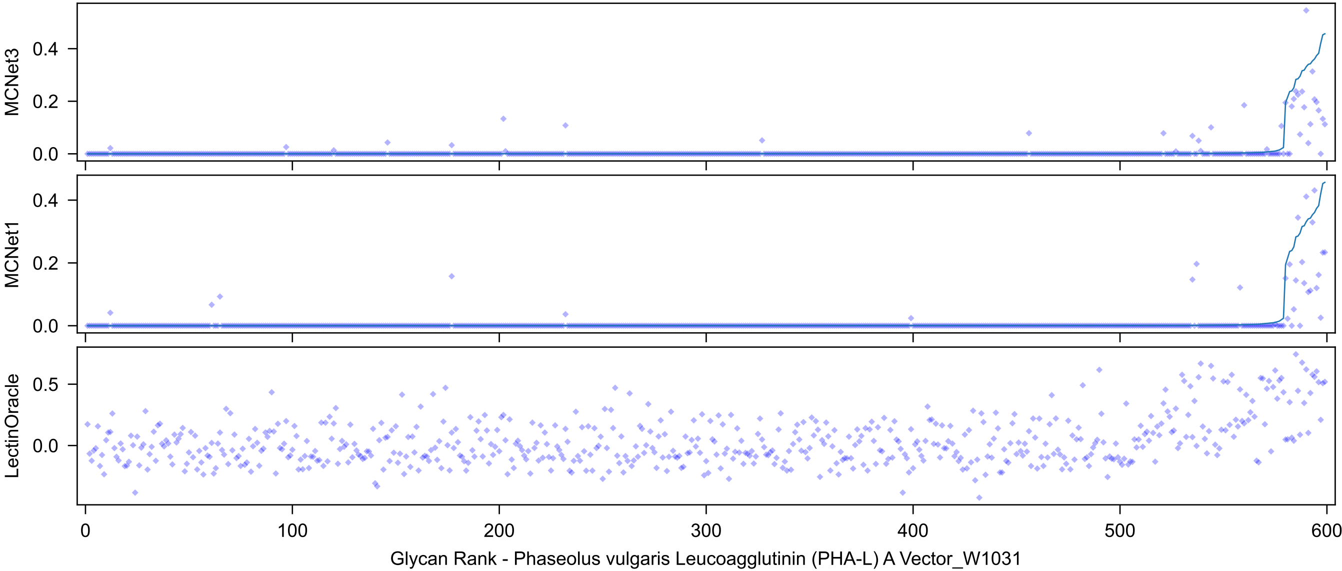

MCNet3
0.4
0.2
0.0
MCNet1
0.4
0.2
0.0
LectinOracle
0.5
0.0
0
100
200
300
400
500
600
Glycan Rank - Phaseolus vulgaris Leucoagglutinin (PHA-L) A Vector_W1031

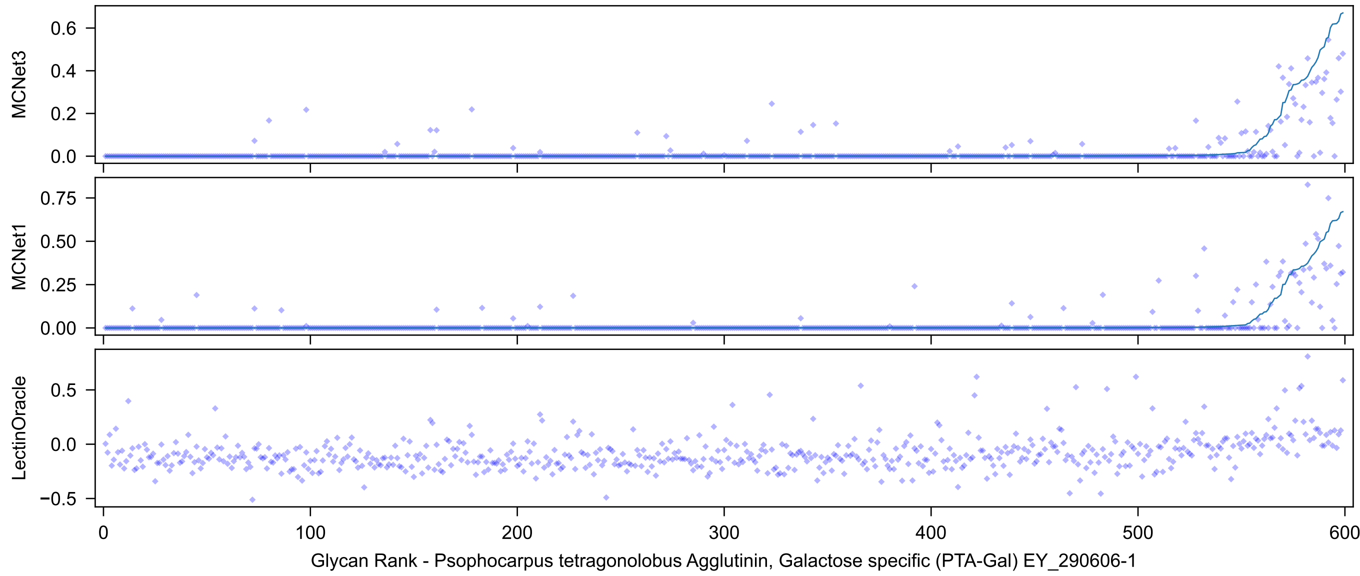

MCNet3
MCNet1
LectinOracle
0.6
0.4
0.2
0.0
0.75
0.50
0.25
0.00
0.5
0.0
−0.5
0
100
200
300
400
500
600
Glycan Rank - Psophocarpus tetragonolobus Agglutinin, Galactose specific (PTA-Gal) EY_290606-1

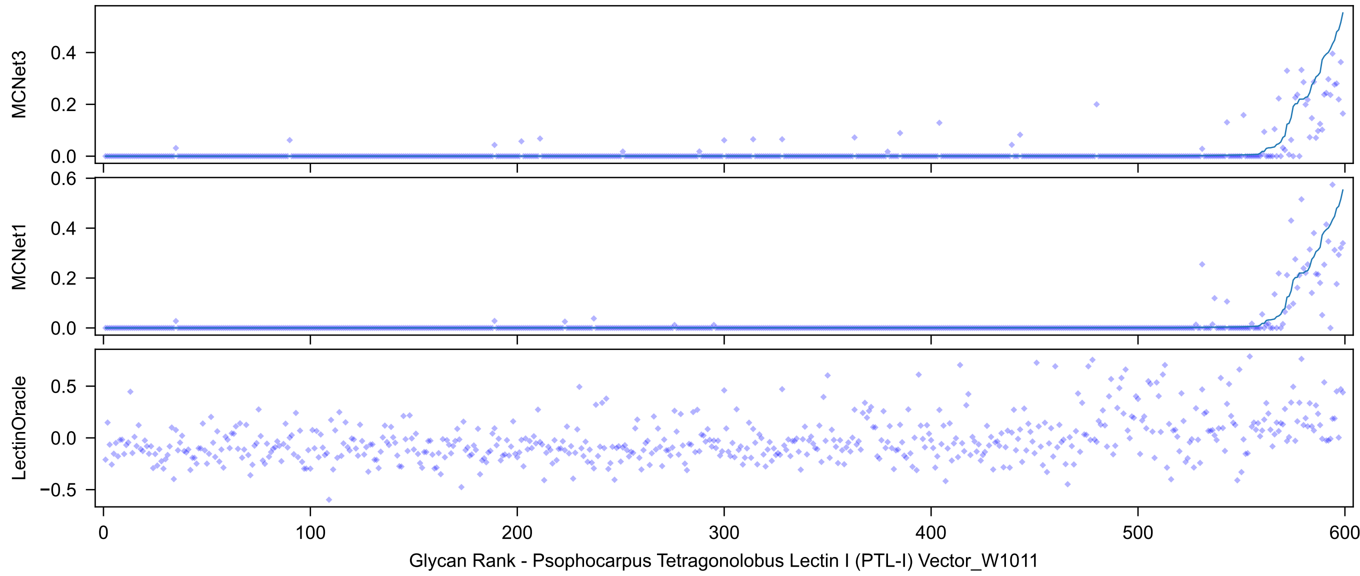

MCNet3
0.4
0.2
0.0
0.6
MCNet1
0.4
0.2
0.0
LectinOracle
0.5
0.0
−0.5
0
100
200
300
400
500
600
Glycan Rank - Psophocarpus Tetragonolobus Lectin I (PTL-I) Vector_W1011

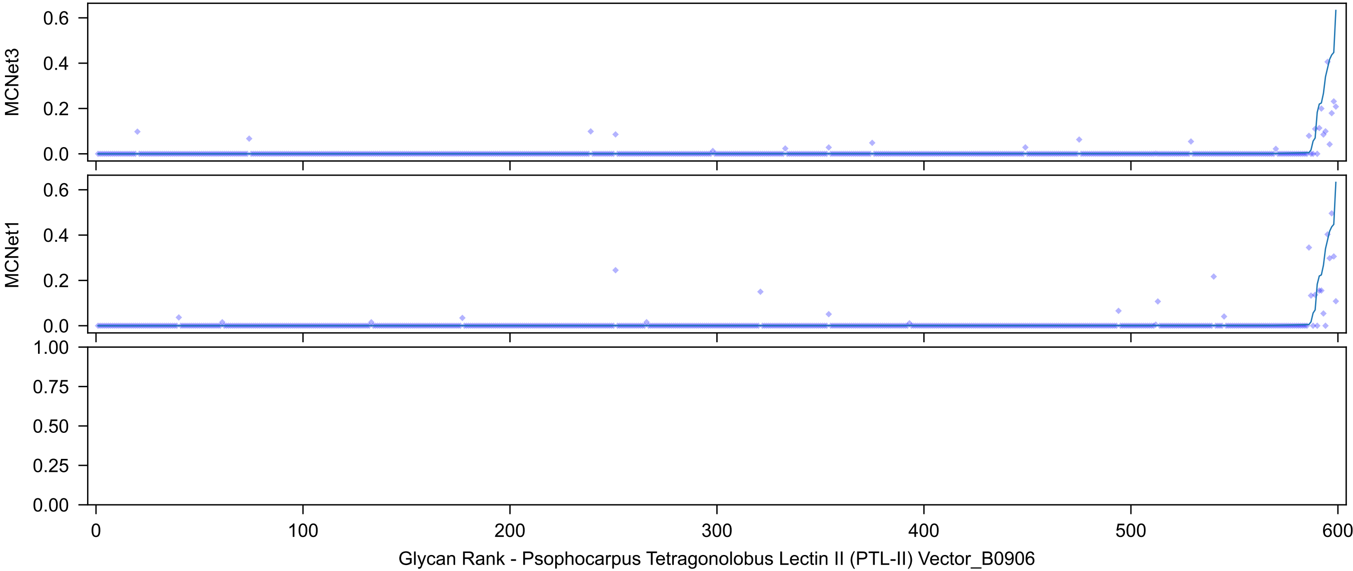
MCNet3
MCNet1
0.6
0.4
0.2
0.0
1.00
0.75
0.50
0.25
0.00
0
100
200
300
400
500
600
Glycan Rank - Psophocarpus Tetragonolobus Lectin II (PTL-II) Vector_B0906

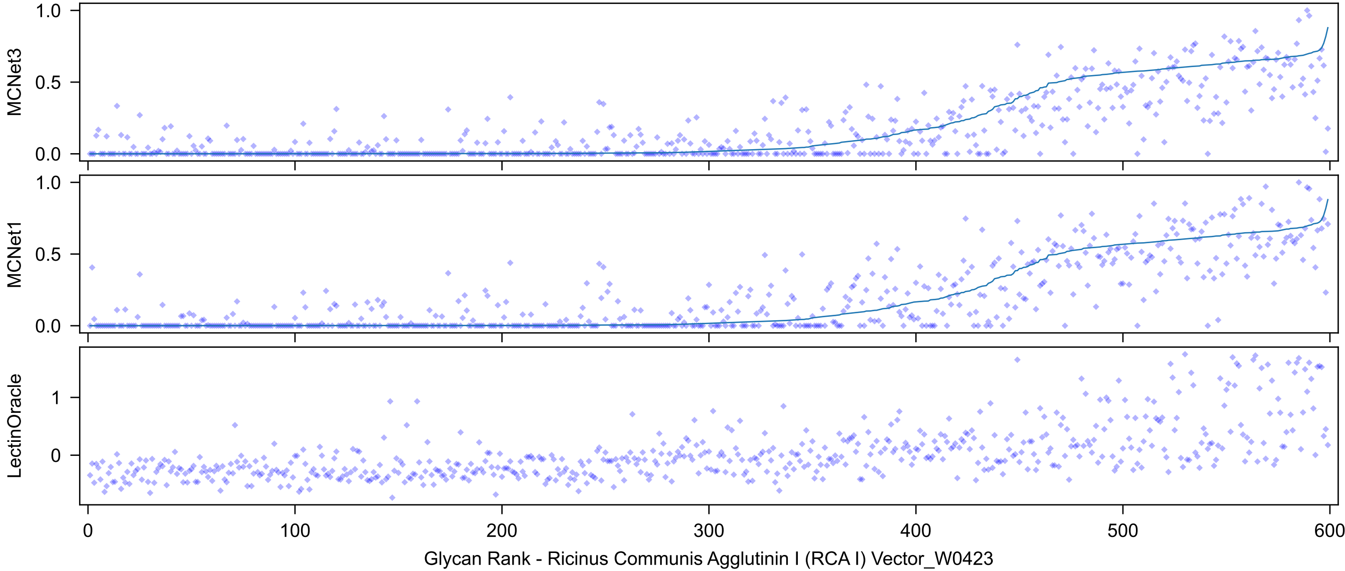
MCNet3
MCNet1
LectinOracle
1.0
0.5
0.0
1
0
0
100
200
300
400
500
600
Glycan Rank - Ricinus Communis Agglutinin I (RCA I) Vector_W0423

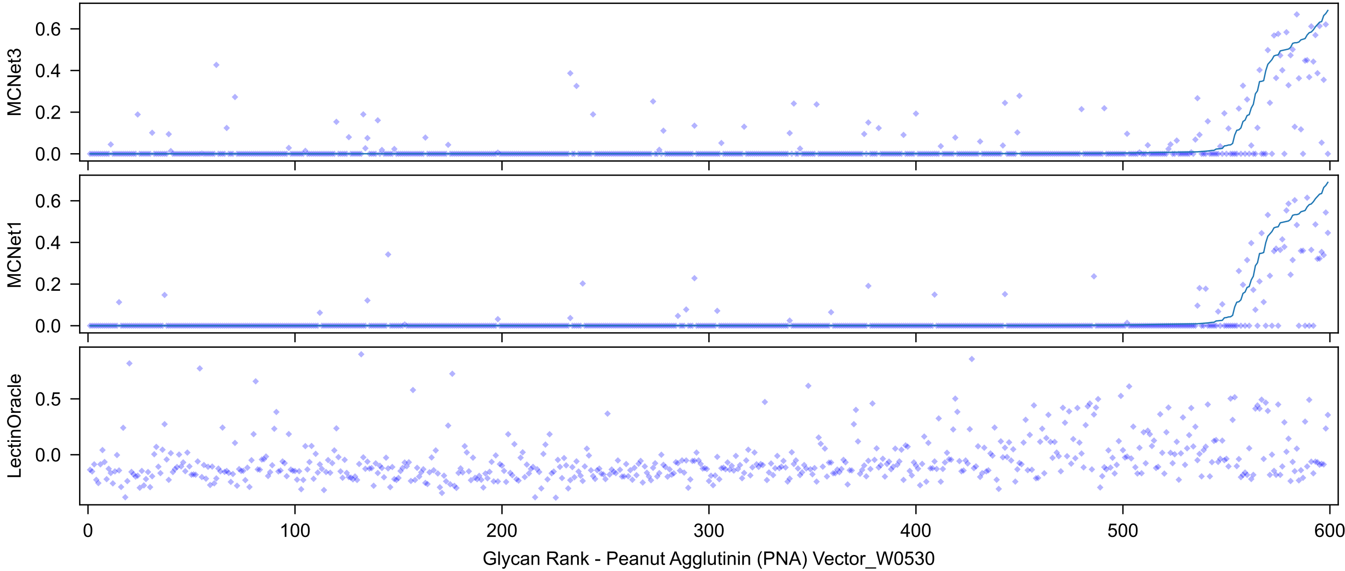

MCNet3
MCNet1
LectinOracle
0.0
0.2
0.4
0.6
0.5
0
100
200
300
400
500
600
Glycan Rank - Peanut Agglutinin (PNA) Vector_W0530

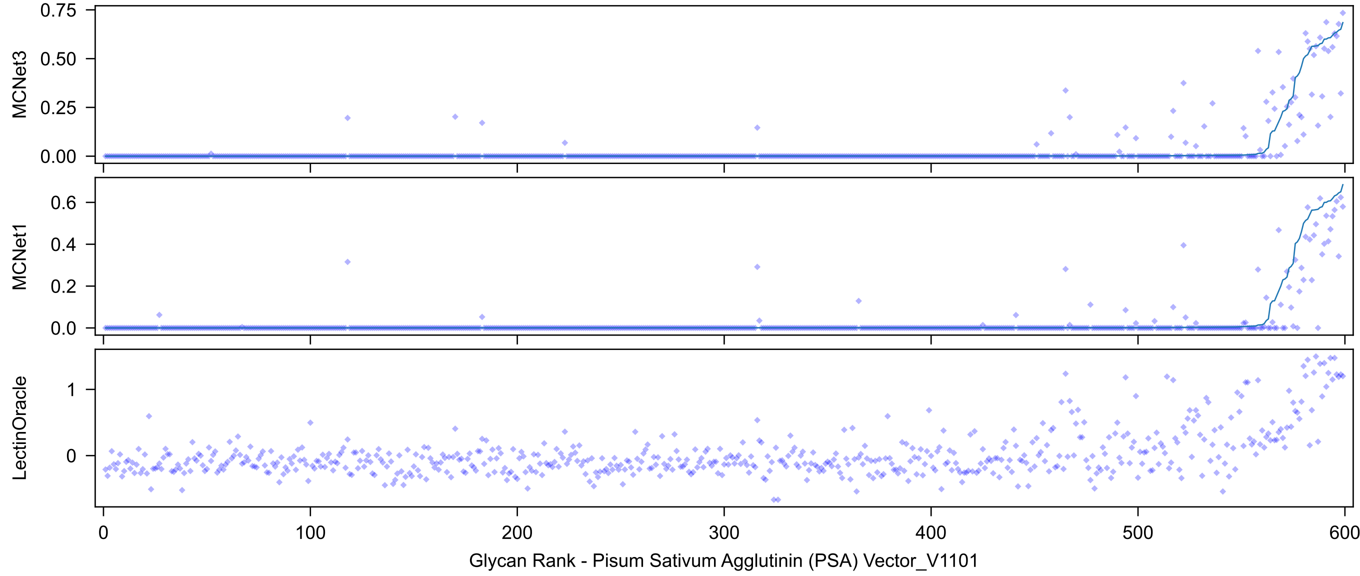

0.75
0.50
0.25
0.00
MCNet3
0.6
0.4
0.2
0.0
MCNet1
1
0
LectinOracle
0
100
200
300
400
500
600
Glycan Rank - Pisum Sativum Agglutinin (PSA) Vector_V1101

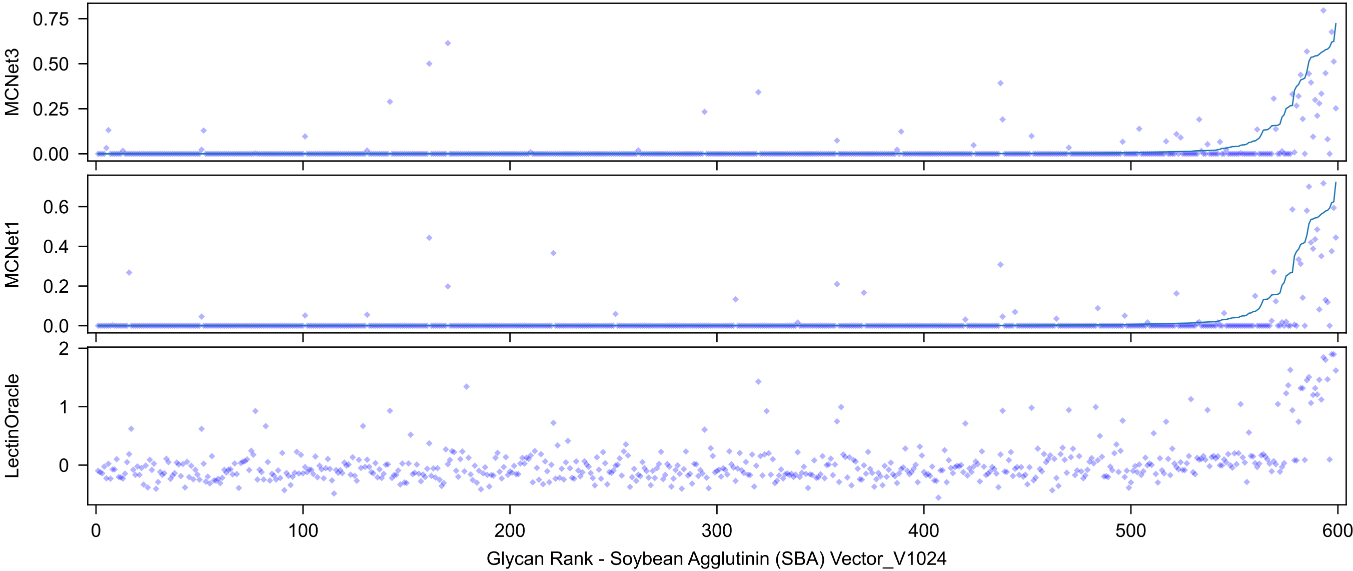

MCNet3
0.75
0.50
0.25
0.00
MCNet1
0.6
0.4
0.2
0.0
LectinOracle
2
1
0
0
100
200
300
400
500
600
Glycan Rank - Soybean Agglutinin (SBA) Vector_V1024

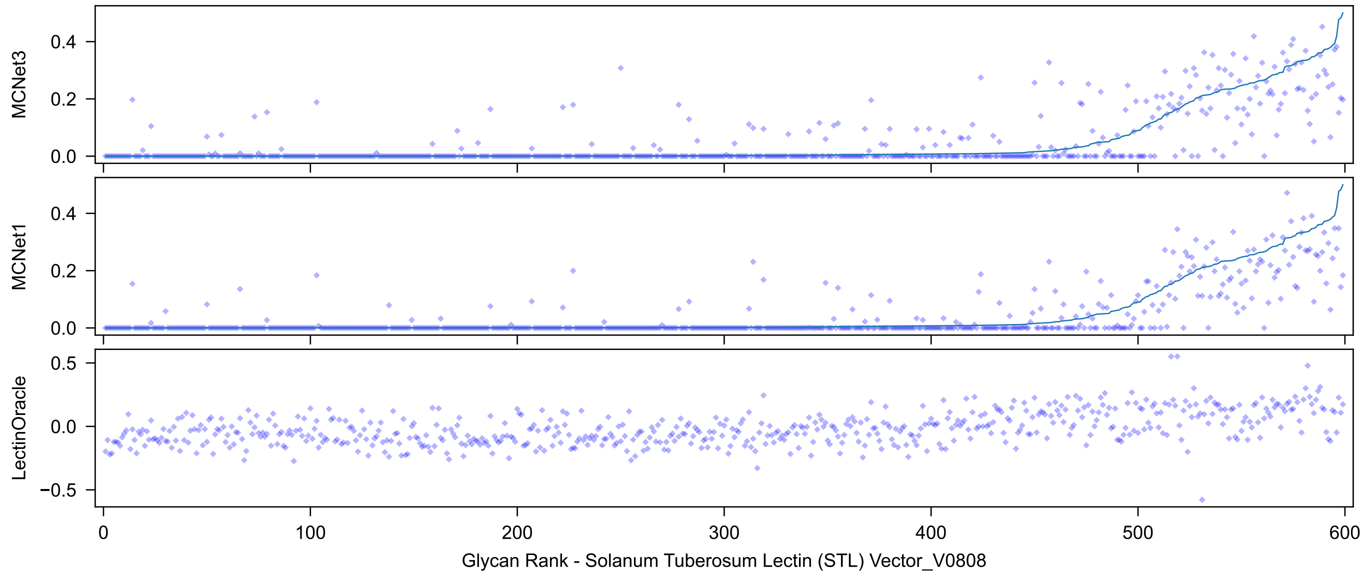
MCNet3
MCNet1
LectinOracle
0.0
0.2
0.4
0.5
−0.5
0
100
200
300
400
500
600
Glycan Rank - Solanum Tuberosum Lectin (STL) Vector_V0808

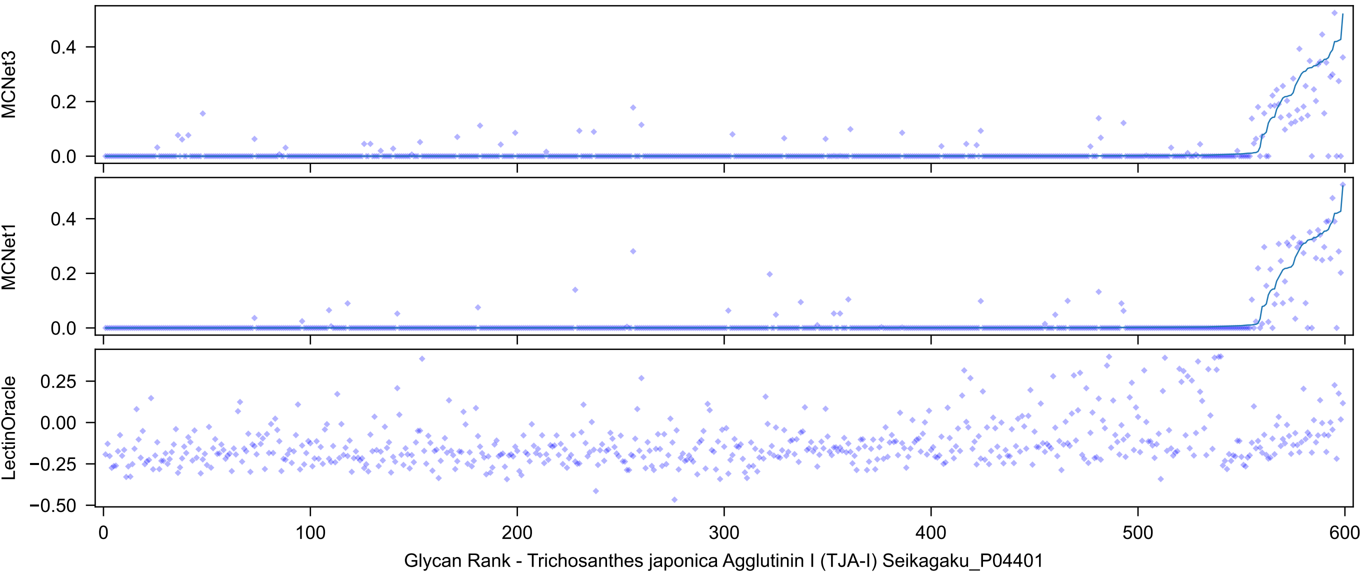
MCNet3
0.4
0.2
0.0
MCNet1
0.4
0.2
0.0
LectinOracle
0.25
0.00
−0.25
−0.50
0
100
200
300
400
500
600
Glycan Rank - Trichosanthes japonica Agglutinin I (TJA-I) Seikagaku_P04401

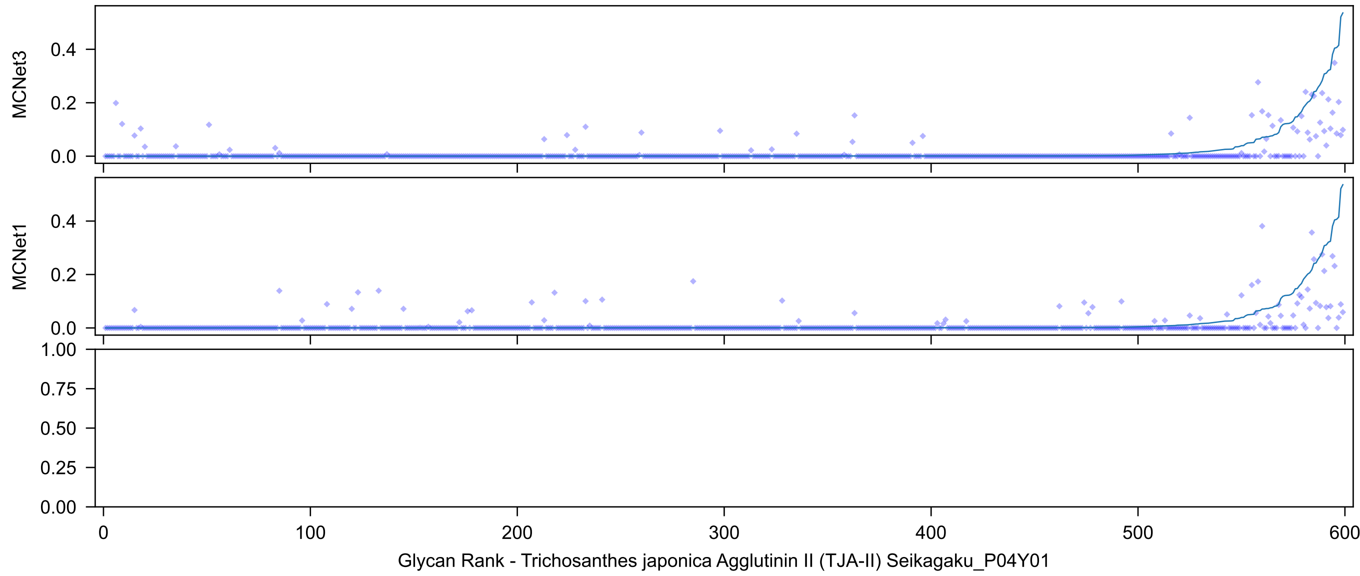

MCNet3
MCNet1
0.0
0.2
0.4
0.00
0.25
0.50
0.75
1.00
0
100
200
300
400
500
600
Glycan Rank - Trichosanthes japonica Agglutinin II (TJA-II) Seikagaku_P04Y01

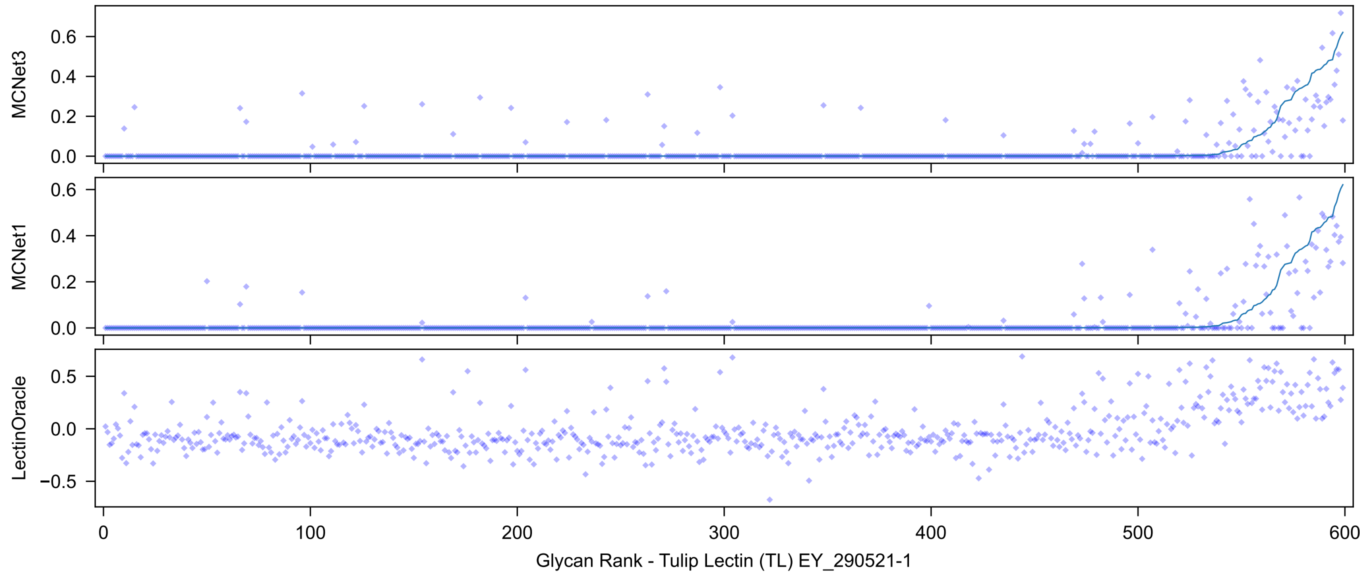

MCNet3
MCNet1
LectinOracle
0.6
0.4
0.2
0.0
0.5
0.0
−0.5
0
100
200
300
400
500
600
Glycan Rank - Tulip Lectin (TL) EY_290521-1

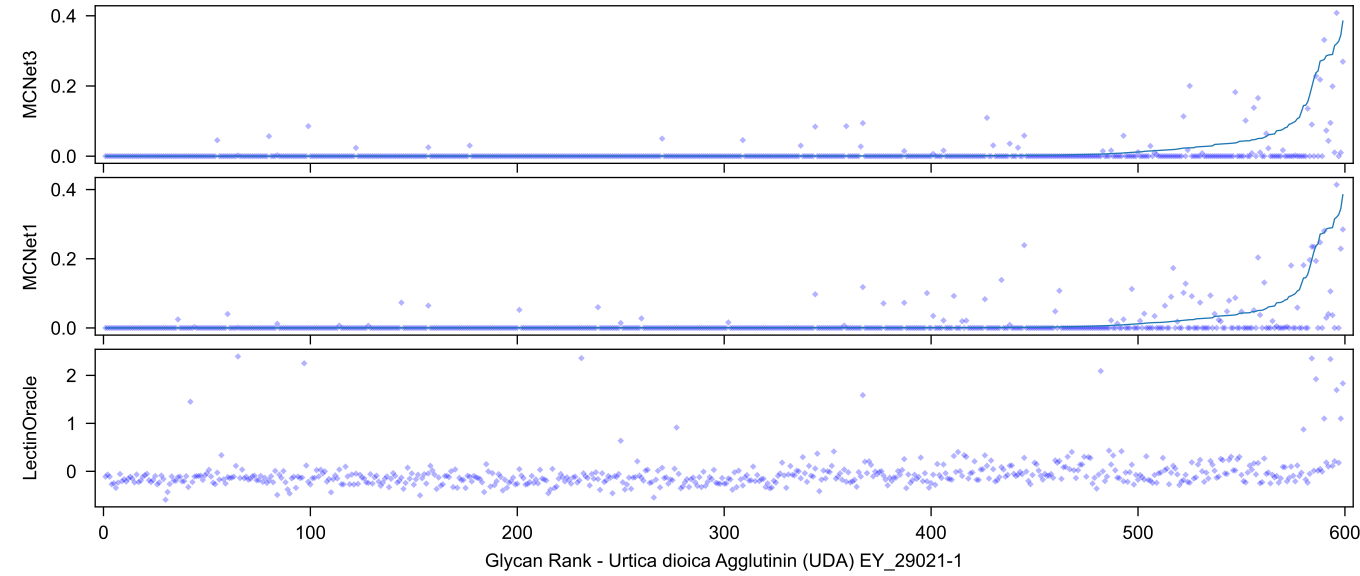

MCNet3
MCNet1
LectinOracle
0.4
0.2
0.0
2
1
0
100
200
300
400
500
600
Glycan Rank - Urtica dioica Agglutinin (UDA) EY_29021-1

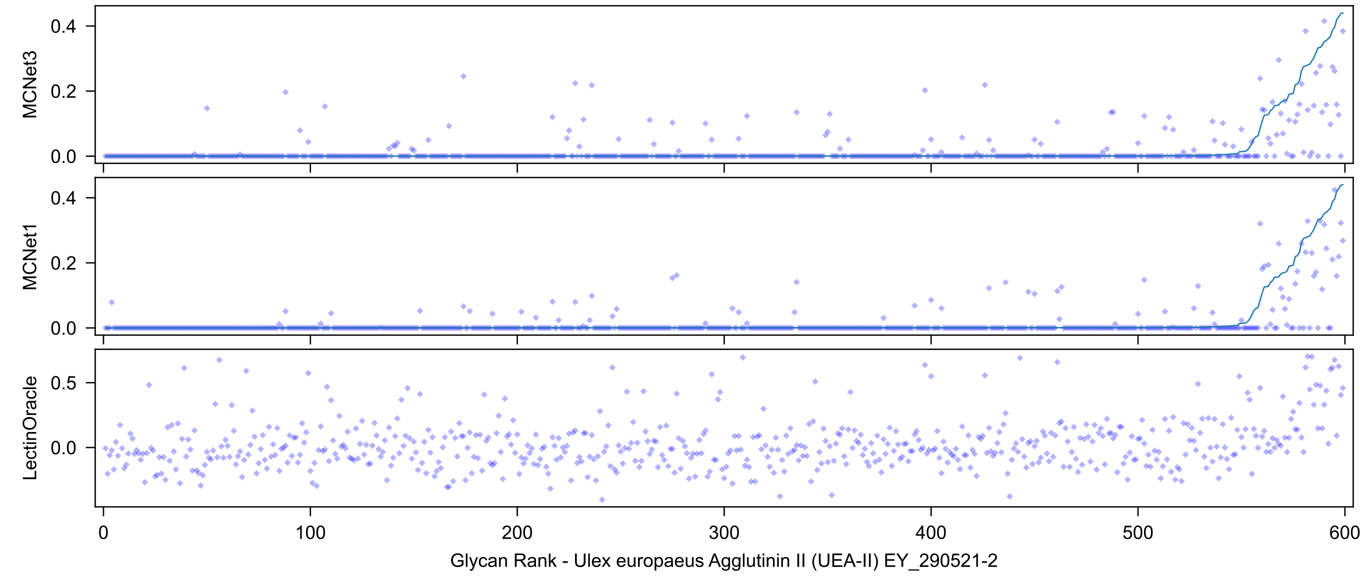

MCNet3
MCNet1
LectinOracle
0.4
0.2
0.0
0.5
0
100
200
300
400
500
600
Glycan Rank - Ulex europaeus Agglutinin II (UEA-II) EY_290521-2

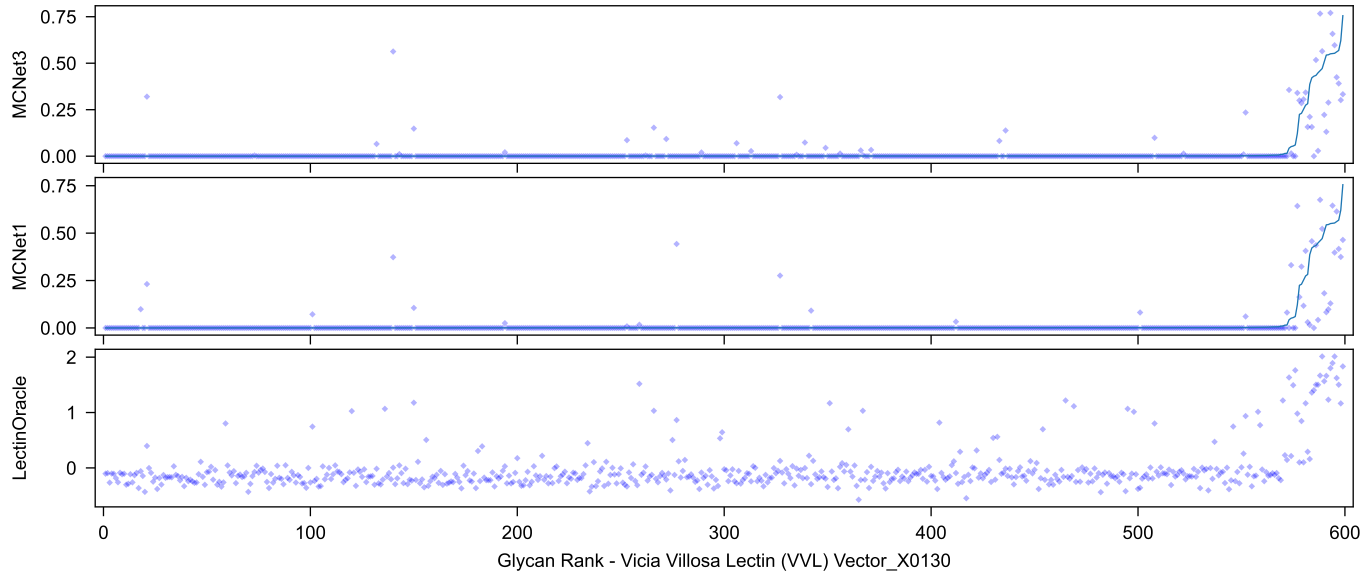

MCNet3
MCNet1
LectinOracle
0.00
0.25
0.50
0.75
0
1
2
0
100
200
300
400
500
600
Glycan Rank - Vicia Villosa Lectin (VVL) Vector_X0130

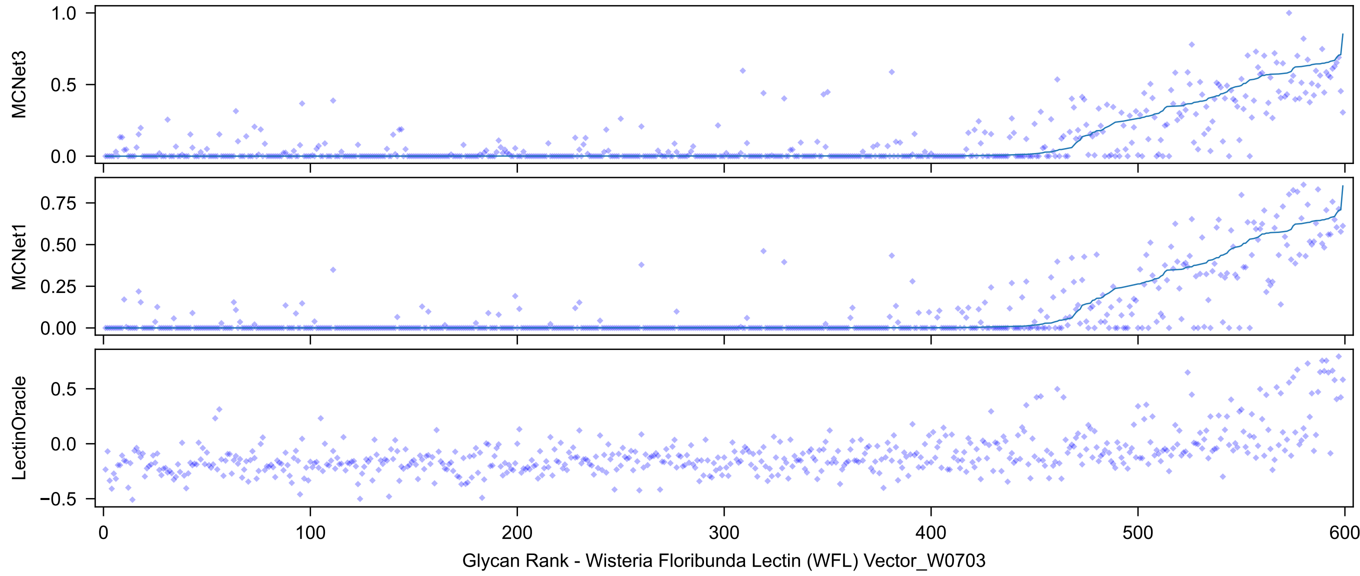

MCNet3
MCNet1
LectinOracle
1.0
0.5
0.0
0.75
0.50
0.25
0.00
−0.5
0
100
200
300
400
500
600
Glycan Rank - Wisteria Floribunda Lectin (WFL) Vector_W0703

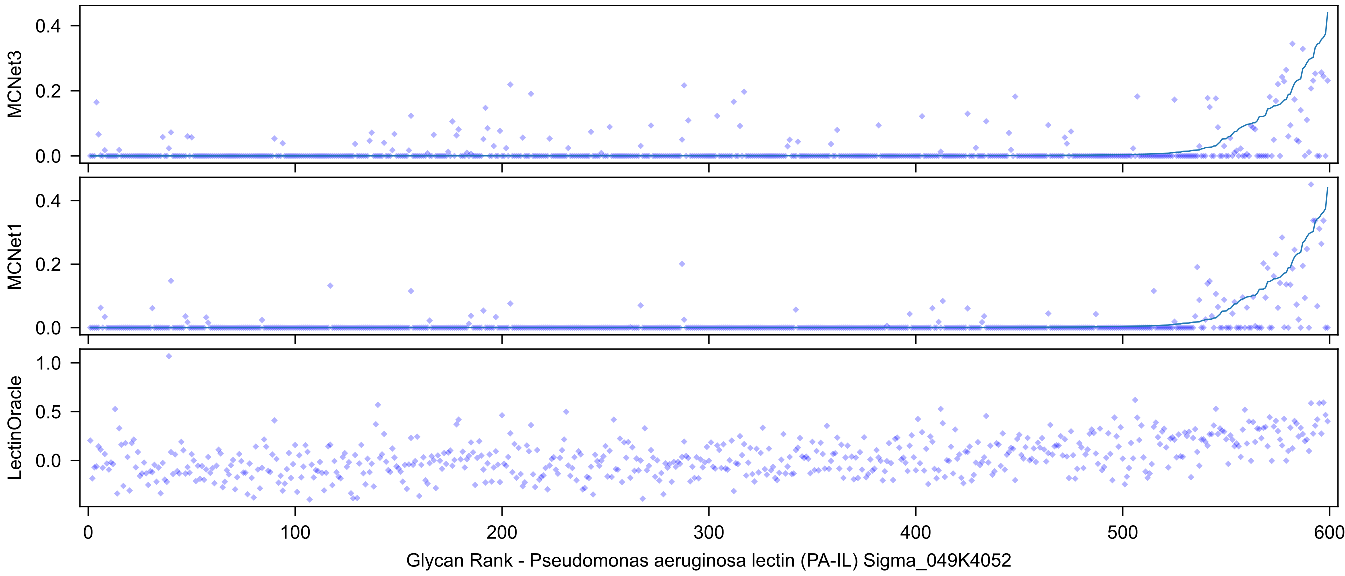
MCNet3
0.4
0.2
0.0
MCNet1
0.4
0.2
0.0
LectinOracle
1.0
0.5
0.0
0
100
200
300
400
500
600
Glycan Rank - Pseudomonas aeruginosa lectin (PA-IL) Sigma_049K4052

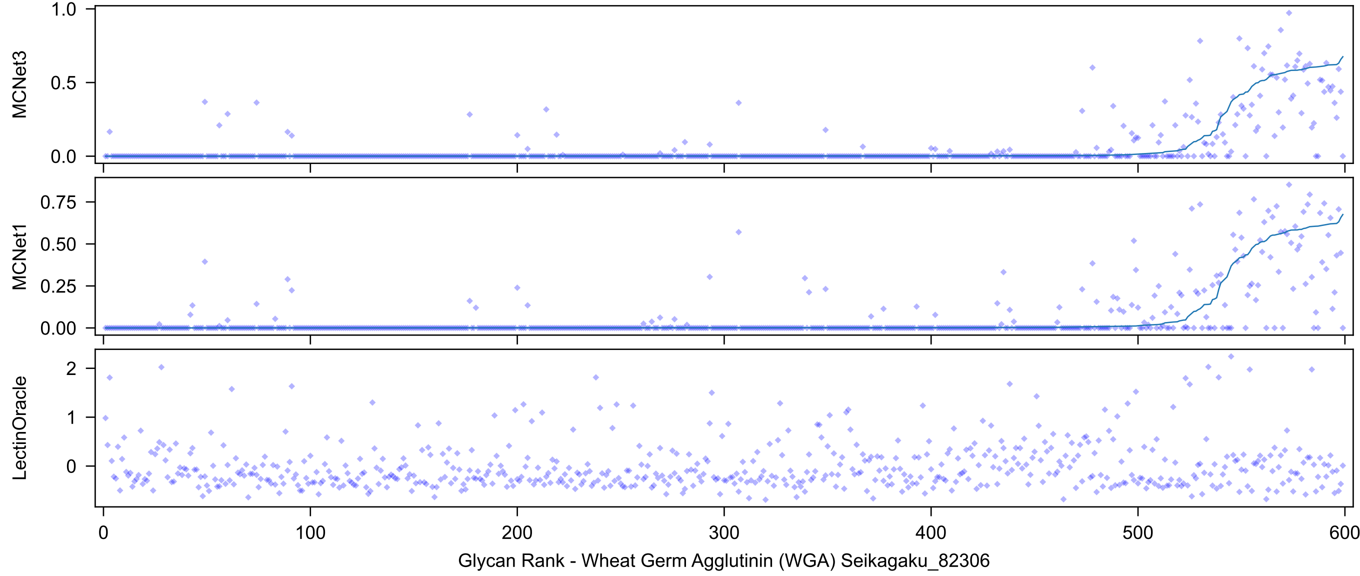

MCNet3
MCNet1
LectinOracle
1.0
0.5
0.0
0.75
0.50
0.25
0.00
2
1
0
0
100
200
300
400
500
600
Glycan Rank - Wheat Germ Agglutinin (WGA) Seikagaku_82306

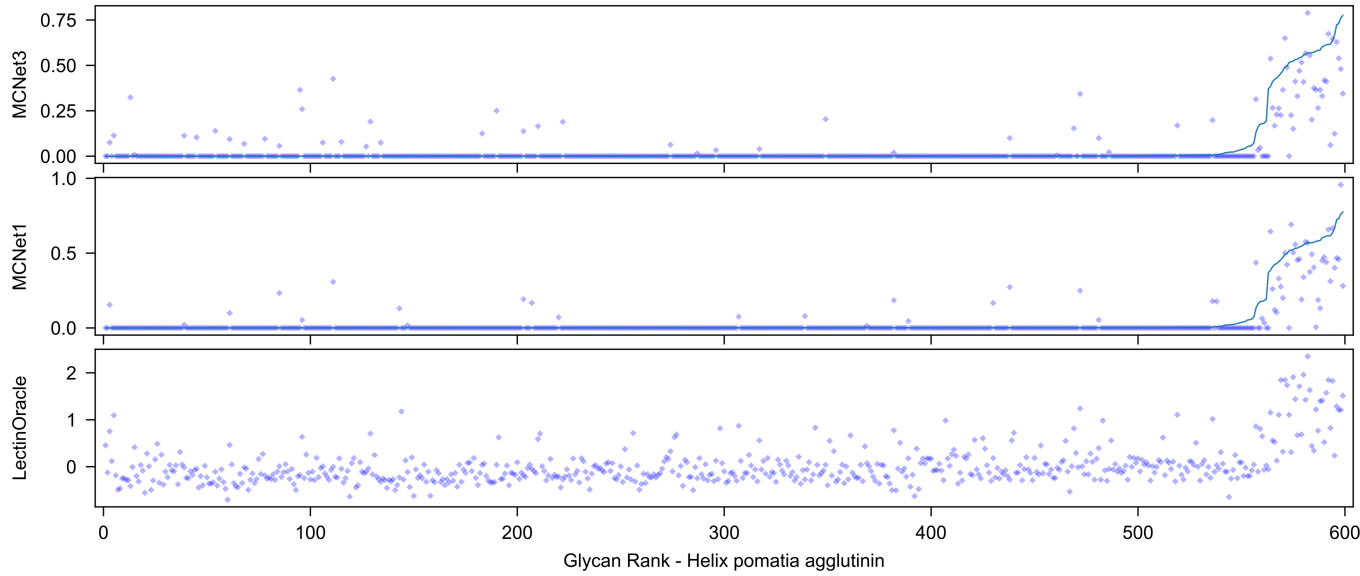

MCNet3
0.75
0.50
0.25
0.00
MCNet1
1.0
0.5
0.0
LectinOracle
2
1
0
0
100
200
300
400
500
600
Glycan Rank - Helix pomatia agglutinin

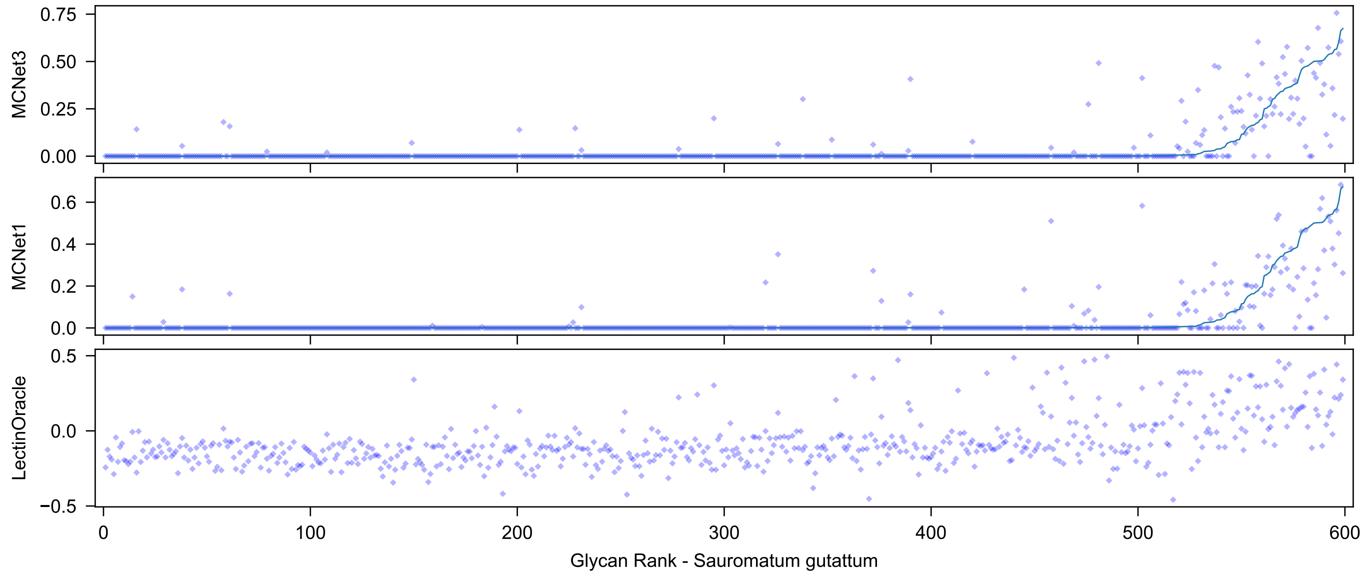

MCNet3
MCNet1
LectinOracle
Glycan Rank - Sauromatum gutattum
0.75
0.50
0.25
0.00
0.6
0.4
0.2
0.0
0.5
0.0
−0.5
0
100
200
300
400
500
600

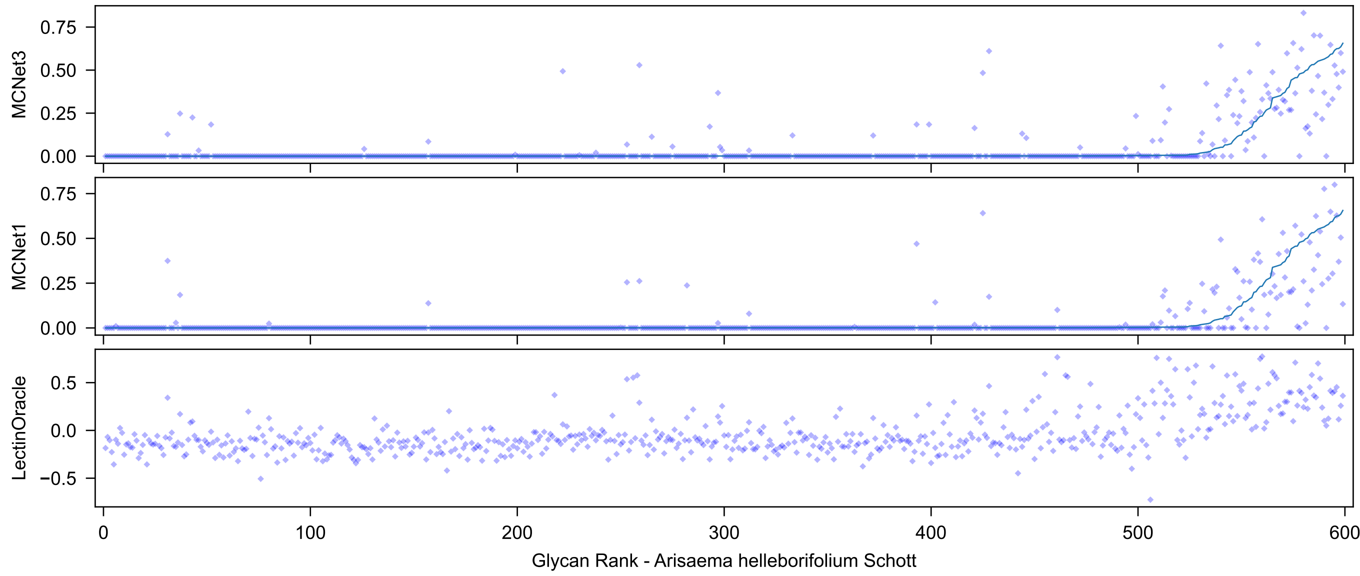

MCNet3
MCNet1
LectinOracle
0.00
0.25
0.50
0.75
−0.5
0.0
0.5
0
100
200
300
400
500
600
Glycan Rank - Arisaema helleborifolium Schott

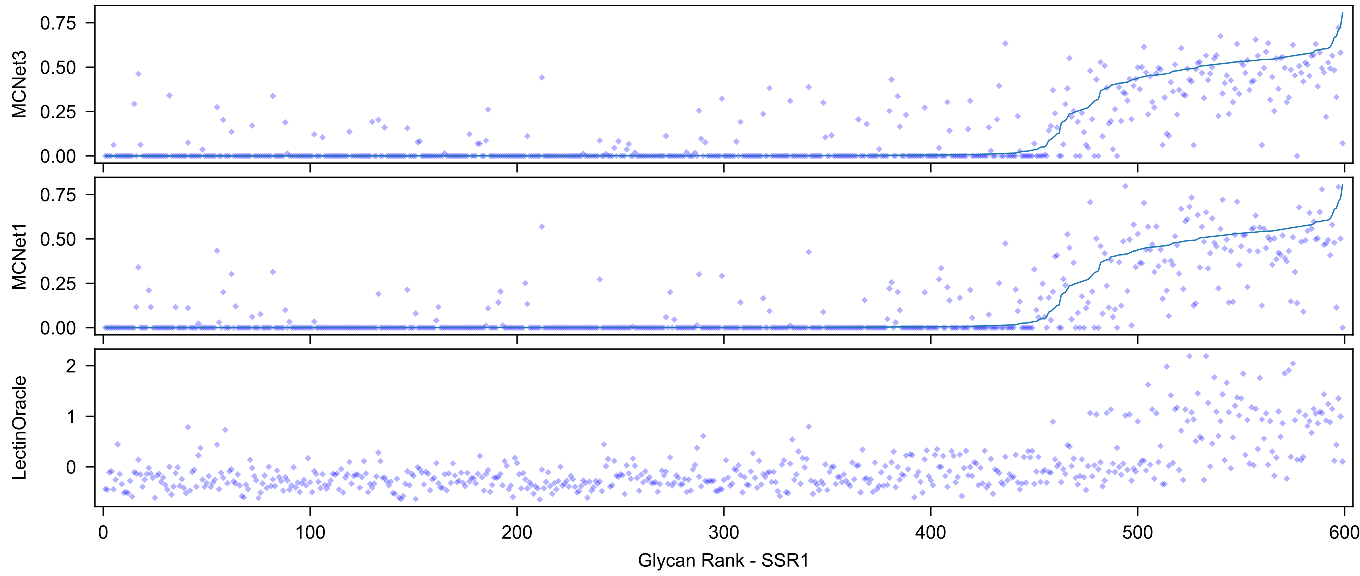

MCNet3
0.75
0.50
0.25
0.00
MCNet1
0.75
0.50
0.25
0.00
LectinOracle
2
1
0
0
100
200
300
400
500
600
Glycan Rank - SSR1

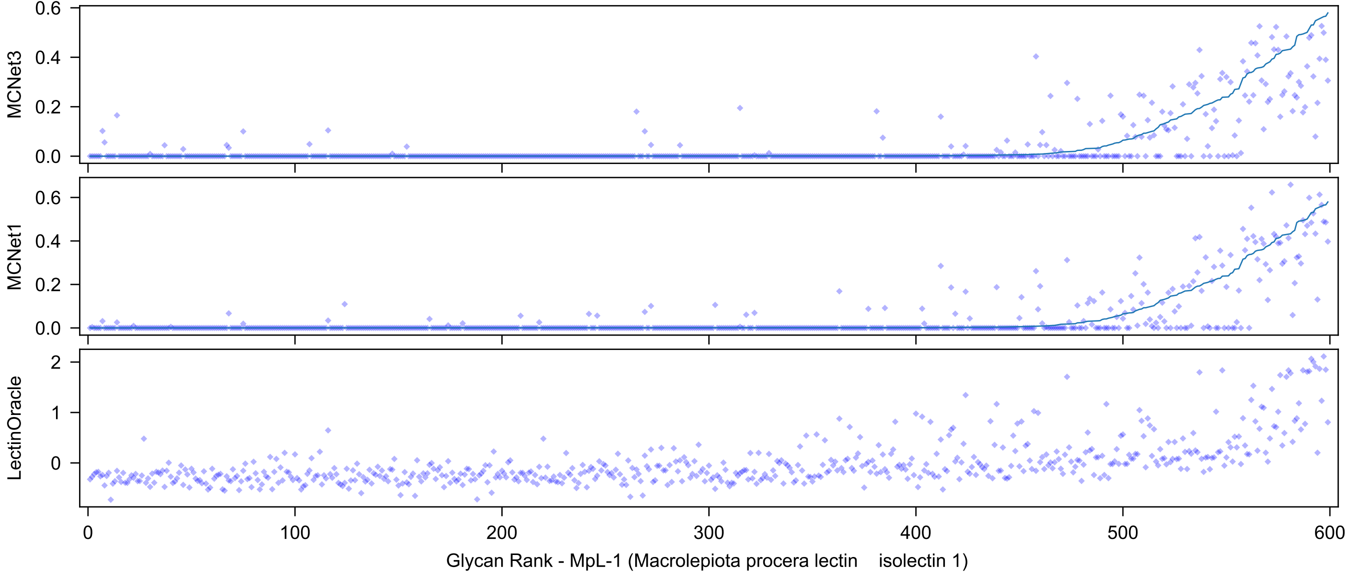

MCNet3
MCNet1
LectinOracle
0.0
0.2
0.4
0.6
0
1
2
0
100
200
300
400
500
600
Glycan Rank - MpL-1 (Macrolepiota procera lectin isolectin 1)

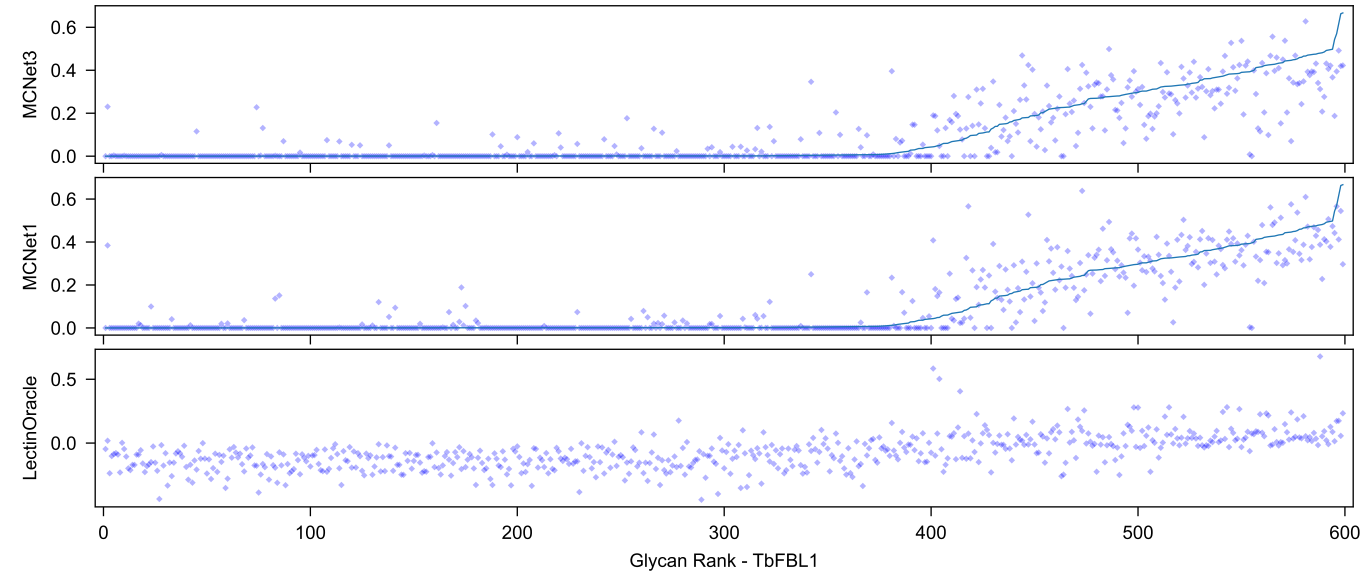
MCNet3
MCNet1
LectinOracle
0.6
0.4
0.2
0.0
0.5
0.0
0
100
200
300
400
500
600
Glycan Rank - TbFBL1

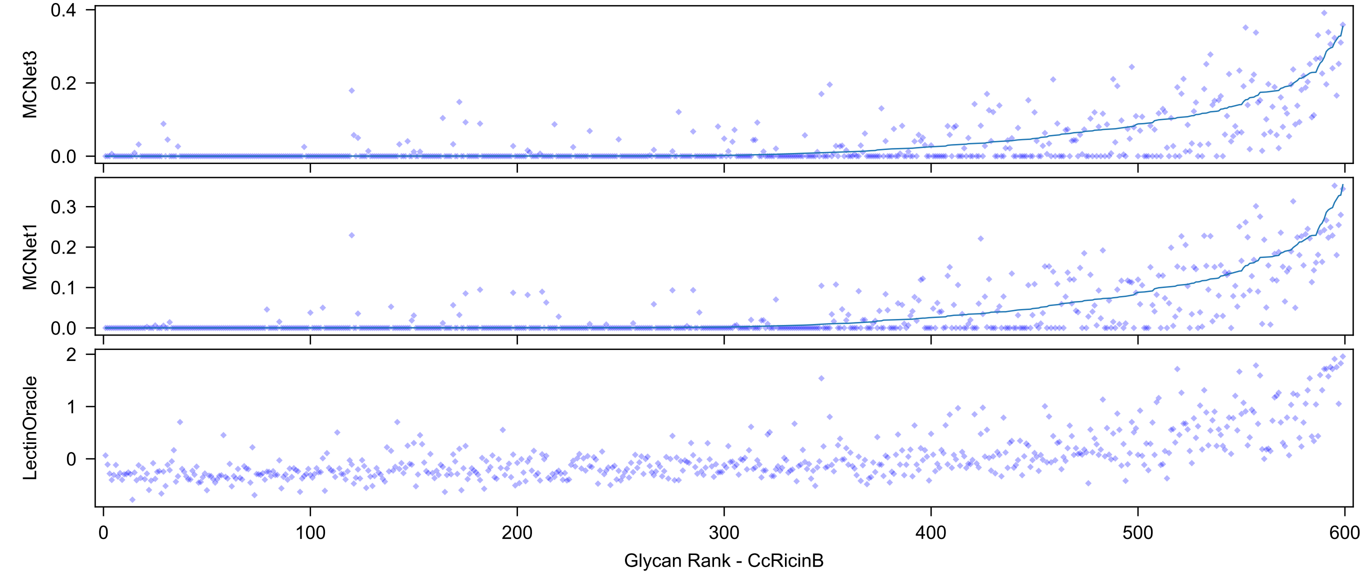

MCNet3
0.4
0.2
0.0
MCNet1
0.3
0.2
0.1
0.0
LectinOracle
2
1
0
0
100
200
300
400
500
600
Glycan Rank - CcRicinB

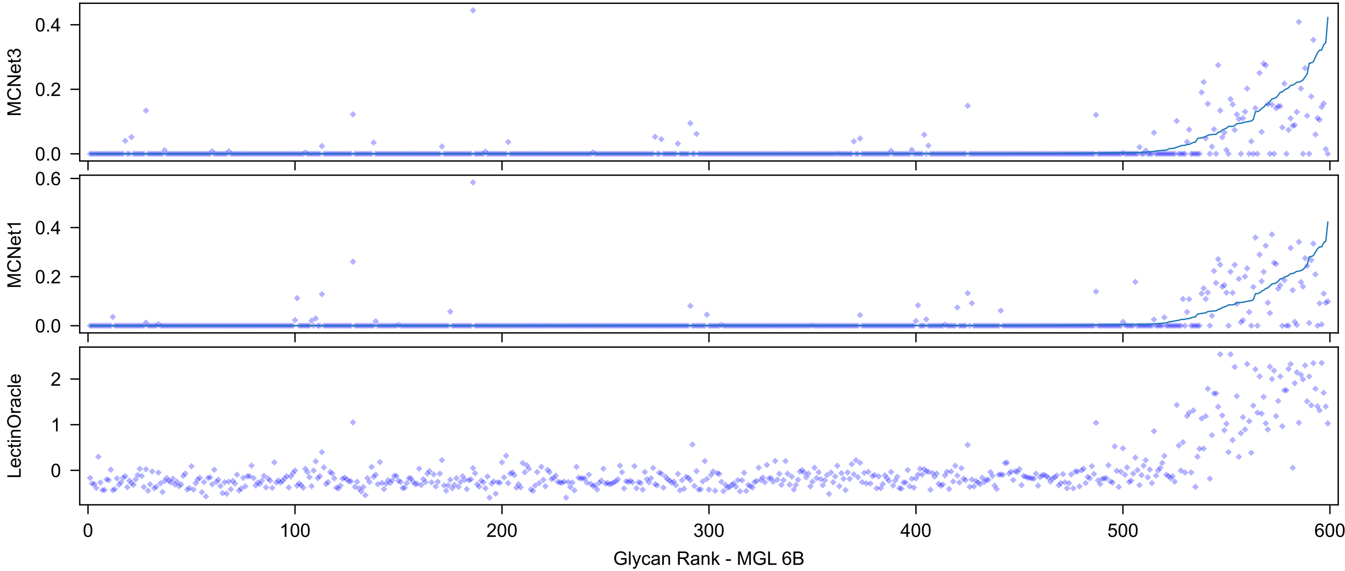

MCNet3
MCNet1
LectinOracle
0.0
0.2
0.4
0.6
0
1
2
0
100
200
300
400
500
600
Glycan Rank - MGL 6B

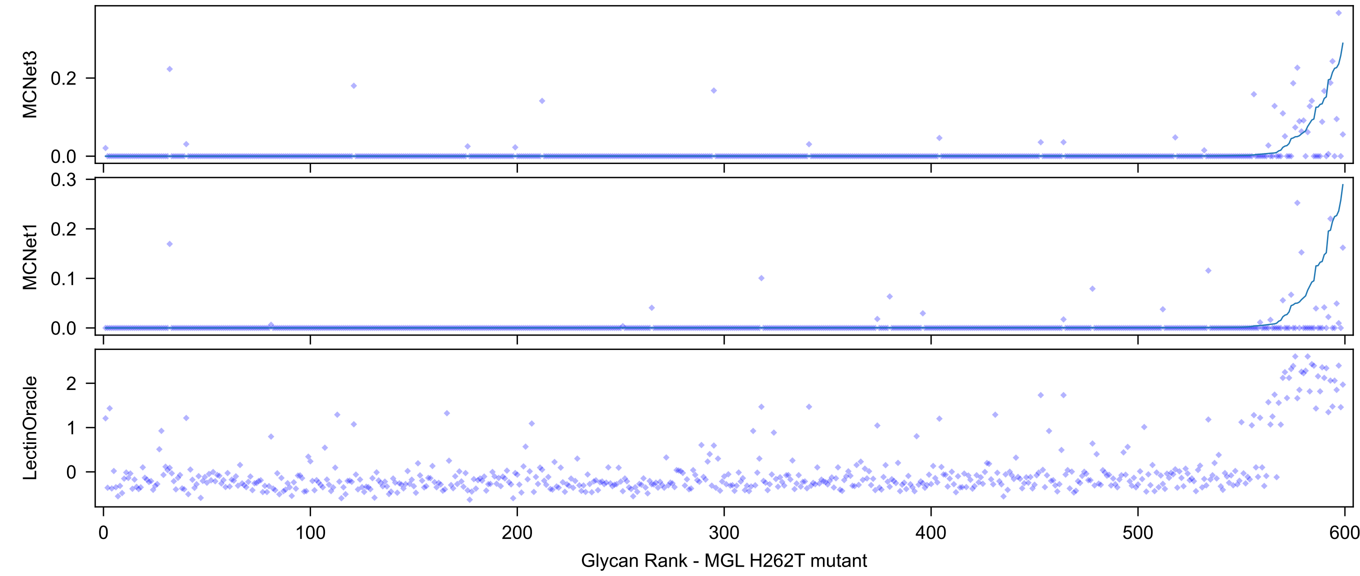

MCNet3
MCNet1
LectinOracle
0.0
0.2
0.1
0.3
0
1
2
100
200
300
400
500
600
Glycan Rank - MGL H262T mutant

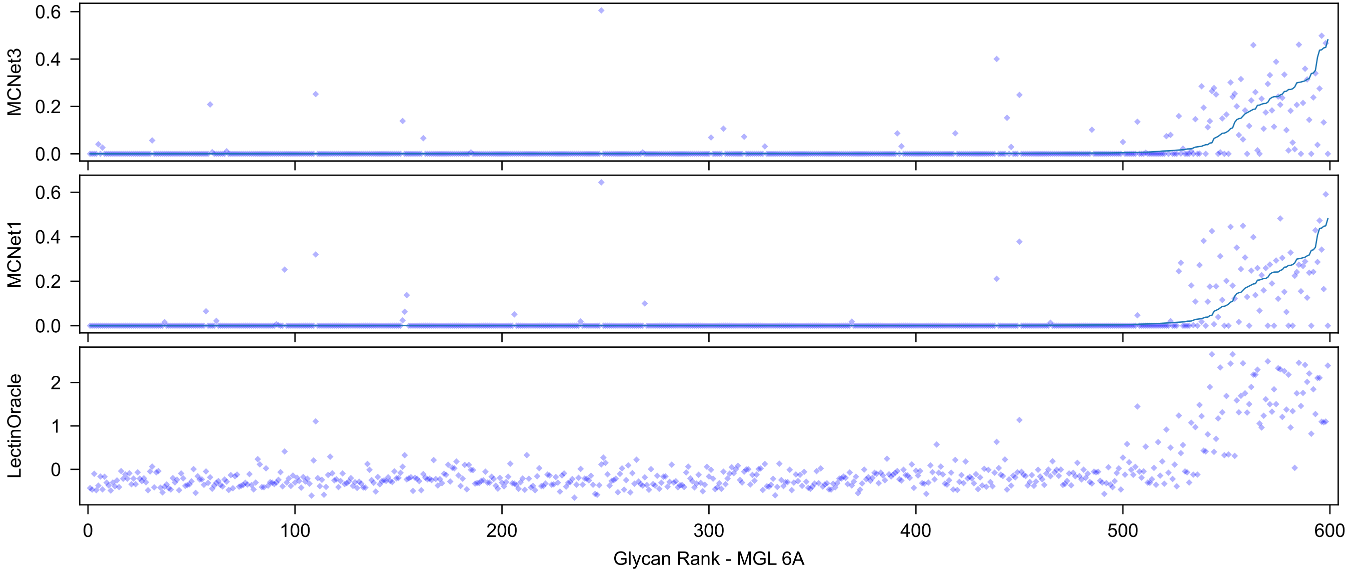

MCNet3
MCNet1
LectinOracle
0.6
0.4
0.2
0.0
2
1
0
0
100
200
300
400
500
600
Glycan Rank - MGL 6A

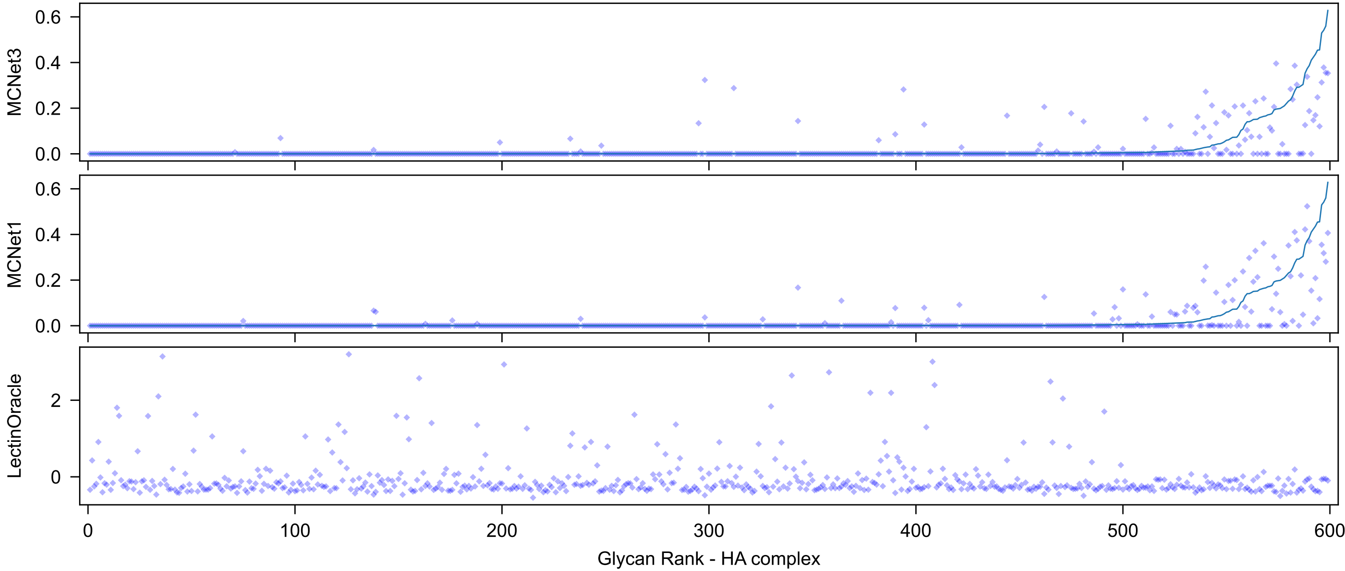

MCNet3
MCNet1
LectinOracle
Glycan Rank - HA complex
0.6
0.4
0.2
0.0
2
0
0
100
200
300
400
500
600

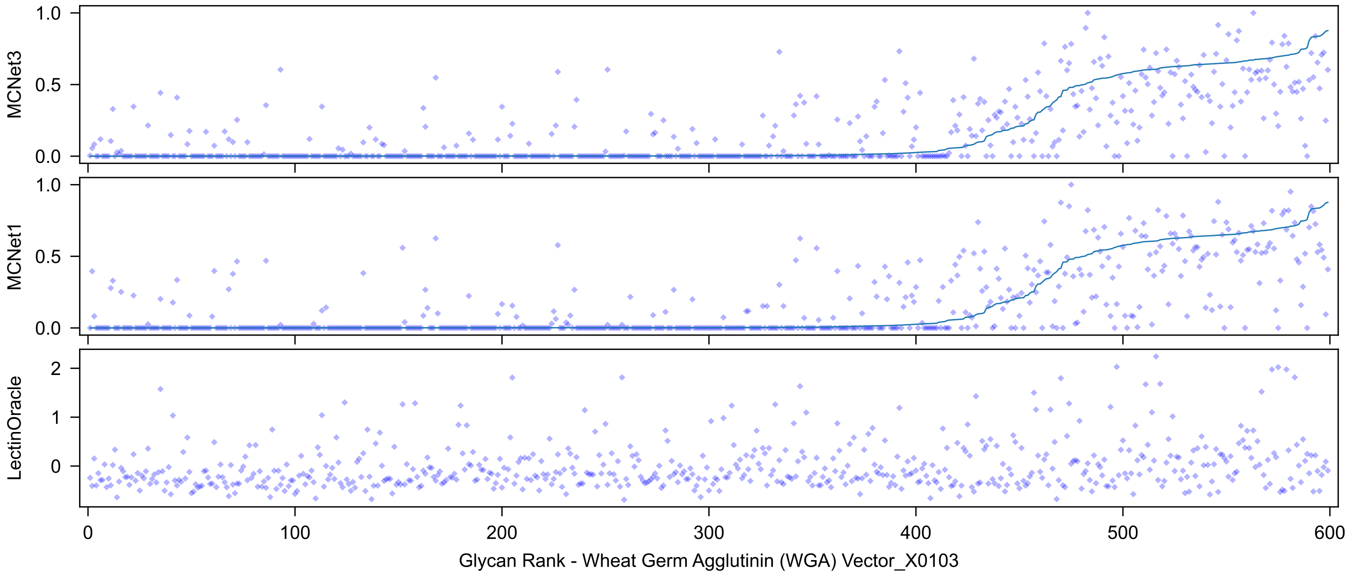

MCNet3
MCNet1
LectinOracle
1.0
0.5
0.0
2
1
0
0
100
200
300
400
500
600
Glycan Rank - Wheat Germ Agglutinin (WGA) Vector_X0103

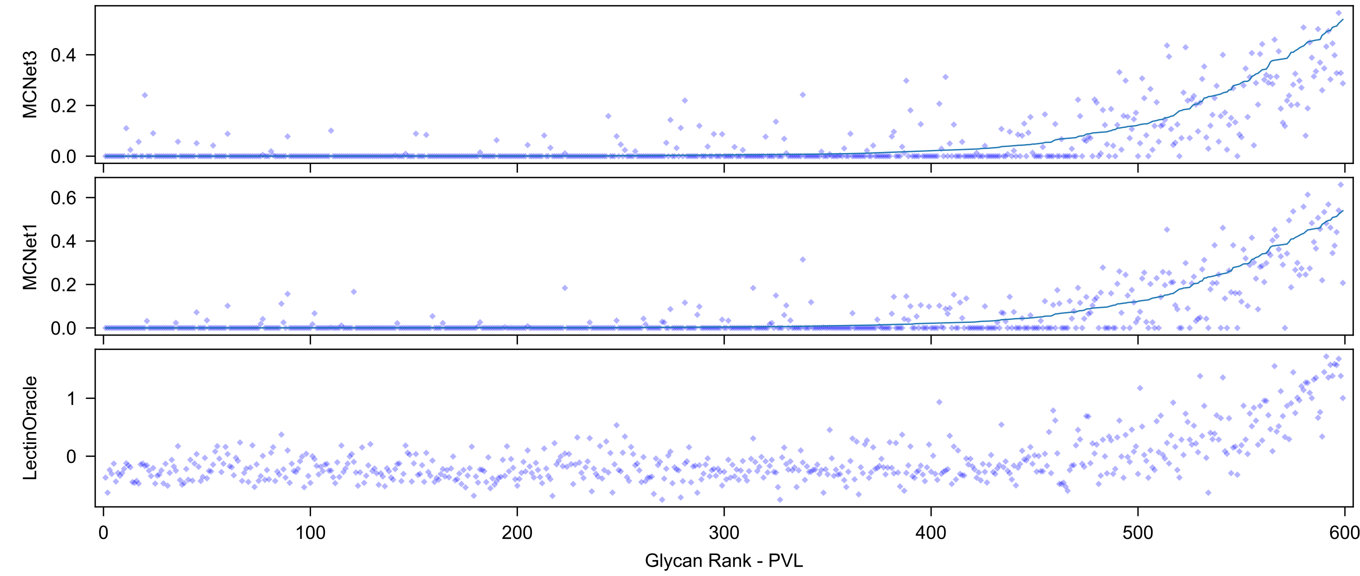

MCNet3
0.4
0.2
0.0
MCNet1
0.6
0.4
0.2
0.0
LectinOracle
1
0
0
100
200
300
400
500
600
Glycan Rank - PVL

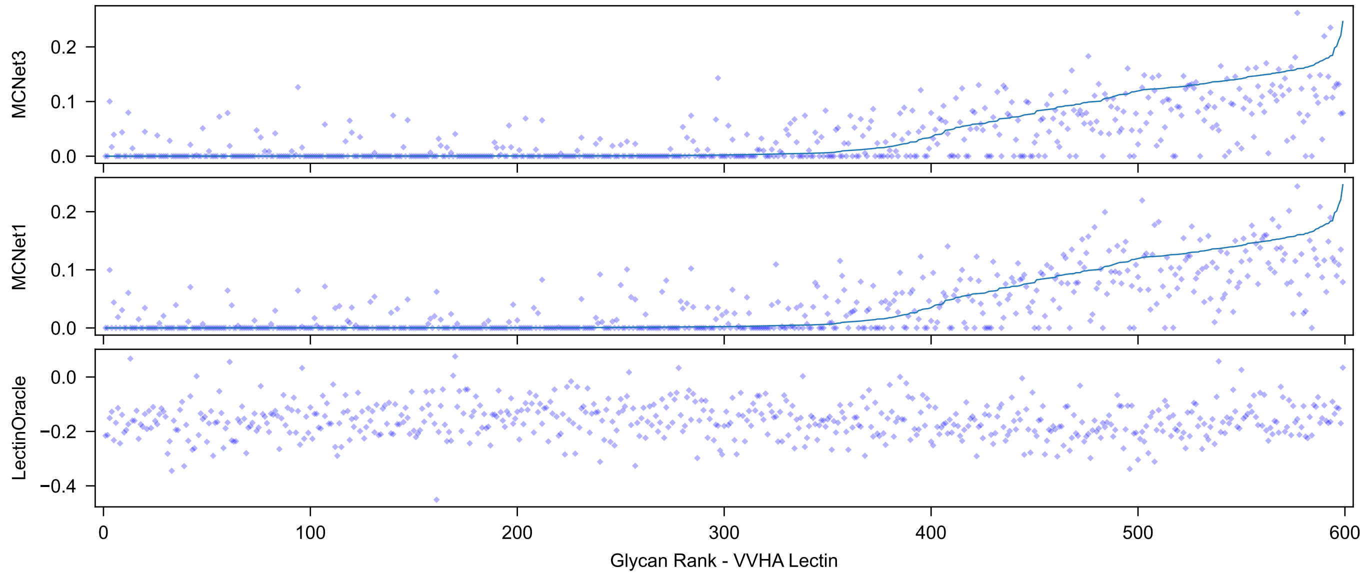

MCNet3
MCNet1
LectinOracle
0.0
0.1
0.2
−0.4
−0.2
0
100
200
300
400
500
600
Glycan Rank - VVHA Lectin

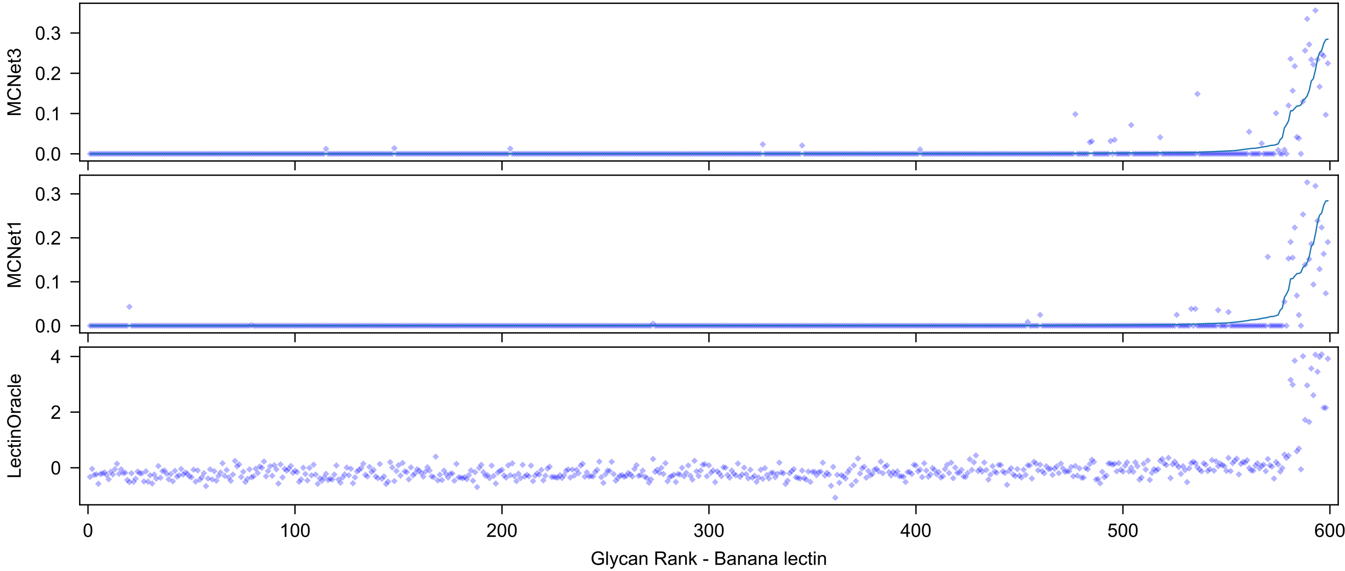

MCNet3
0.0
0.1
0.2
0.3
MCNet1
0.0
0.1
0.2
0.3
LectinOracle
0
2
4
0
100
200
300
400
500
600
Glycan Rank - Banana lectin

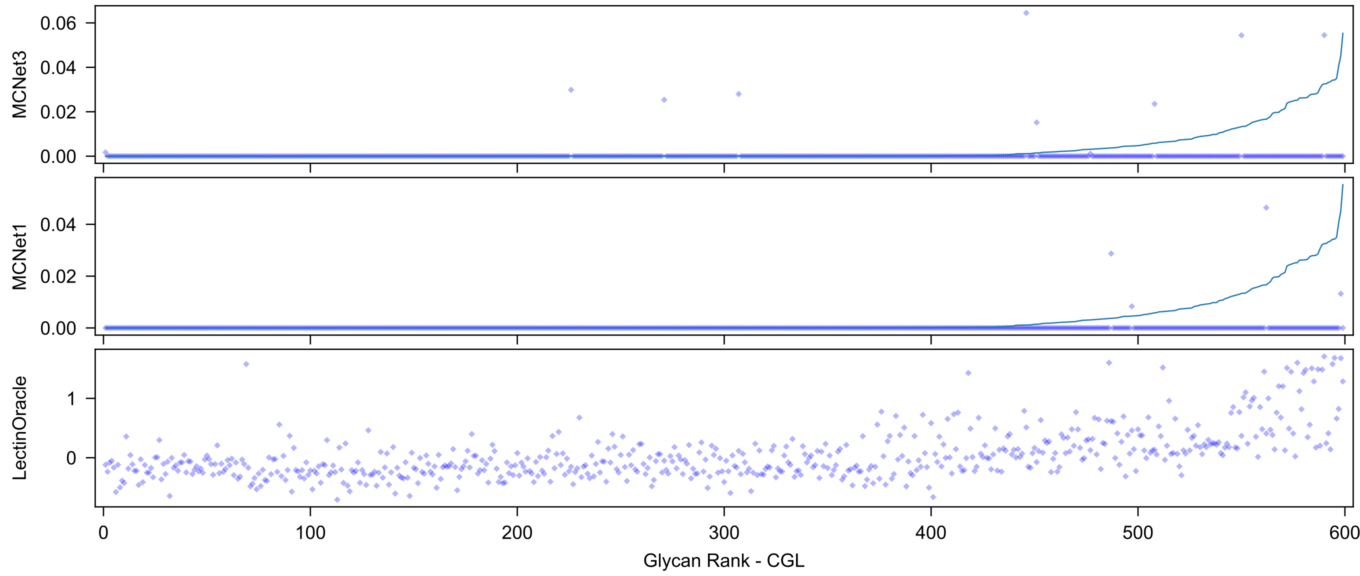
MCNet3
0.06
0.04
0.02
0.00
MCNet1
0.04
0.02
0.00
LectinOracle
1
0
0
100
200
300
400
500
600
Glycan Rank - CGL

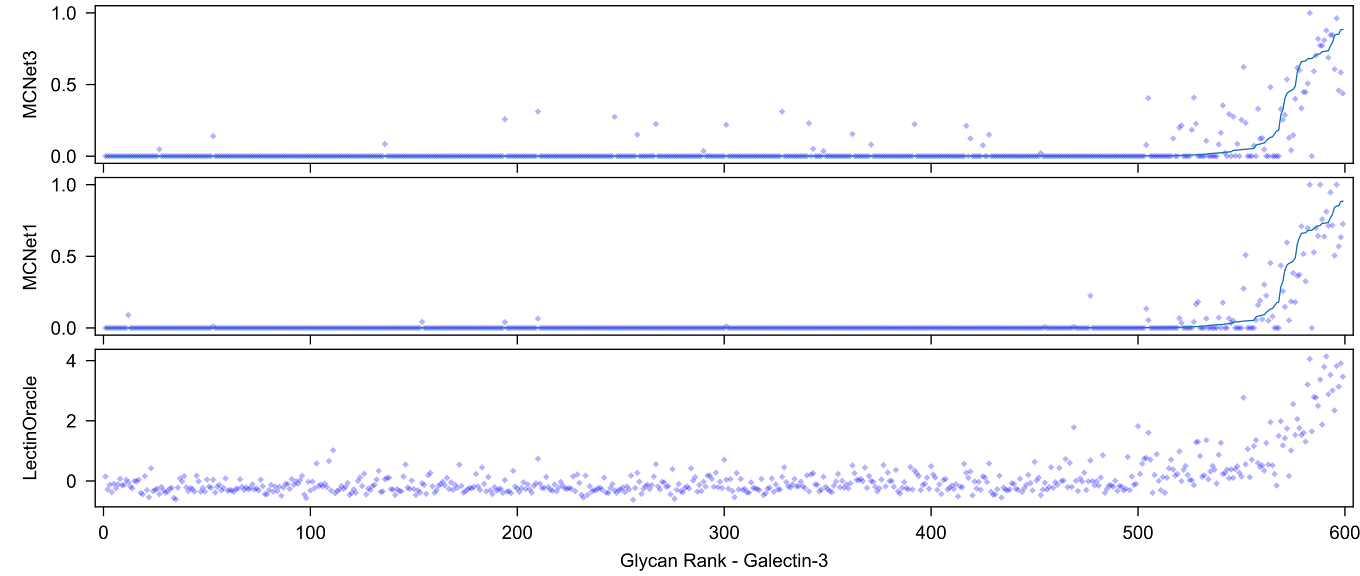
MCNet3
MCNet1
LectinOracle
Glycan Rank - Galectin-3
1.0
0.5
0.0
4
2
0
100
200
300
400
500
600

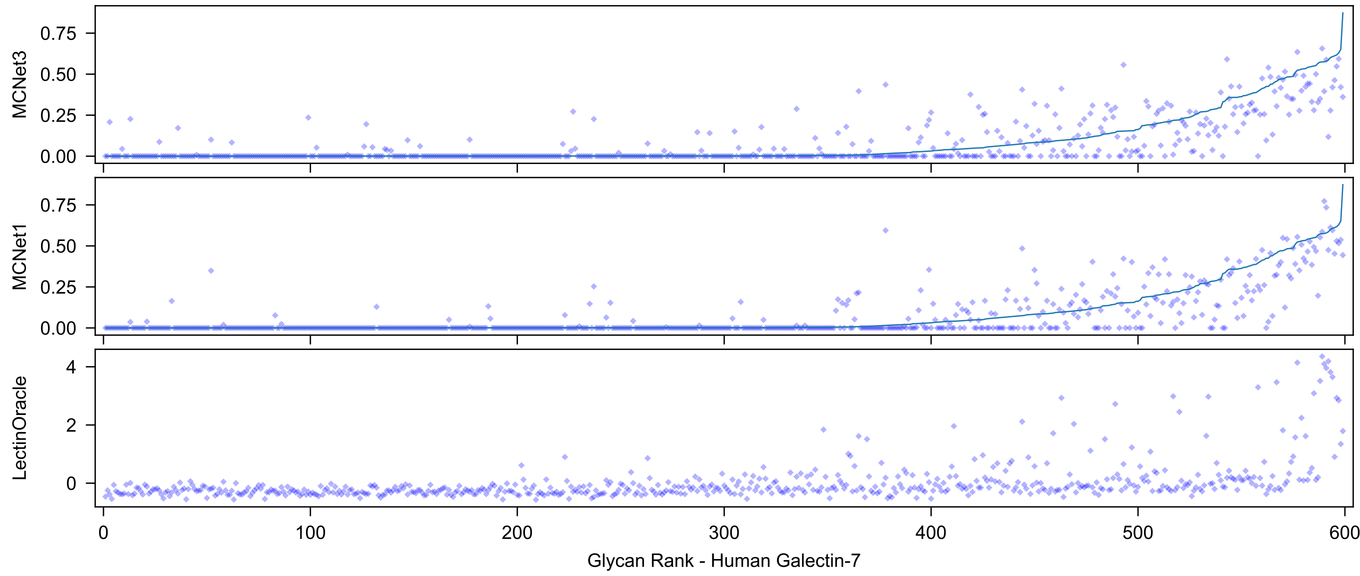
MCNet3
MCNet1
LectinOracle
0.00
0.25
0.50
0.75
0
2
4
0
100
200
300
400
500
600
Glycan Rank - Human Galectin-7

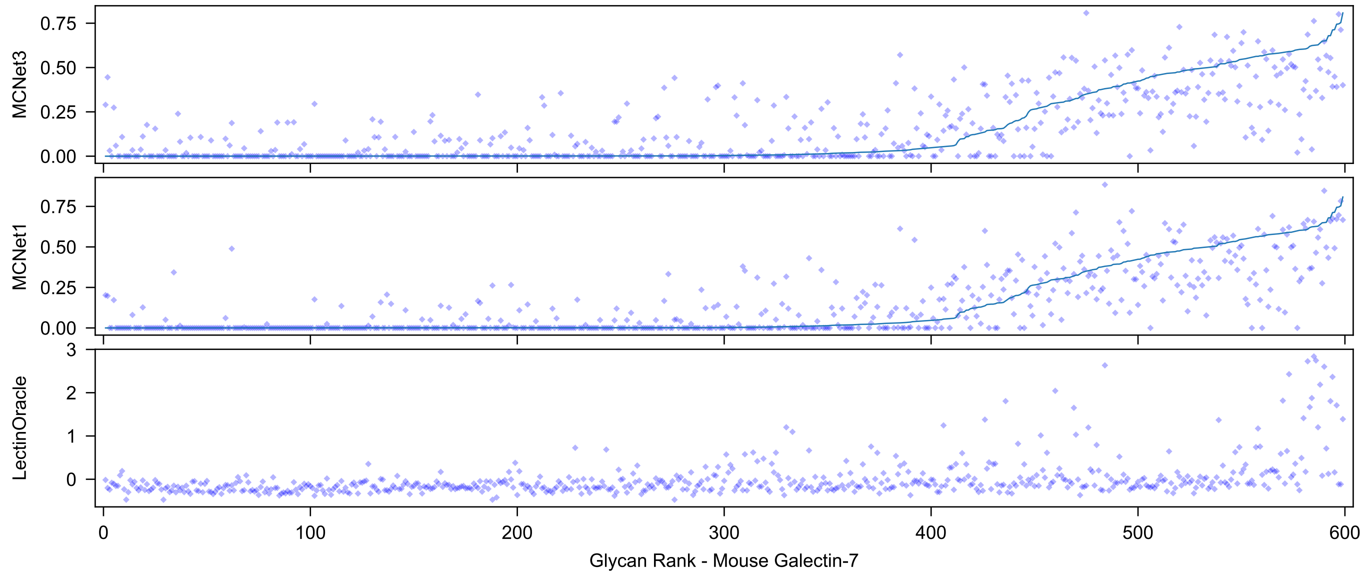

MCNet3
0.75
0.50
0.25
0.00
MCNet1
0.75
0.50
0.25
0.00
LectinOracle
3
2
1
0
0
100
200
300
400
500
600
Glycan Rank - Mouse Galectin-7

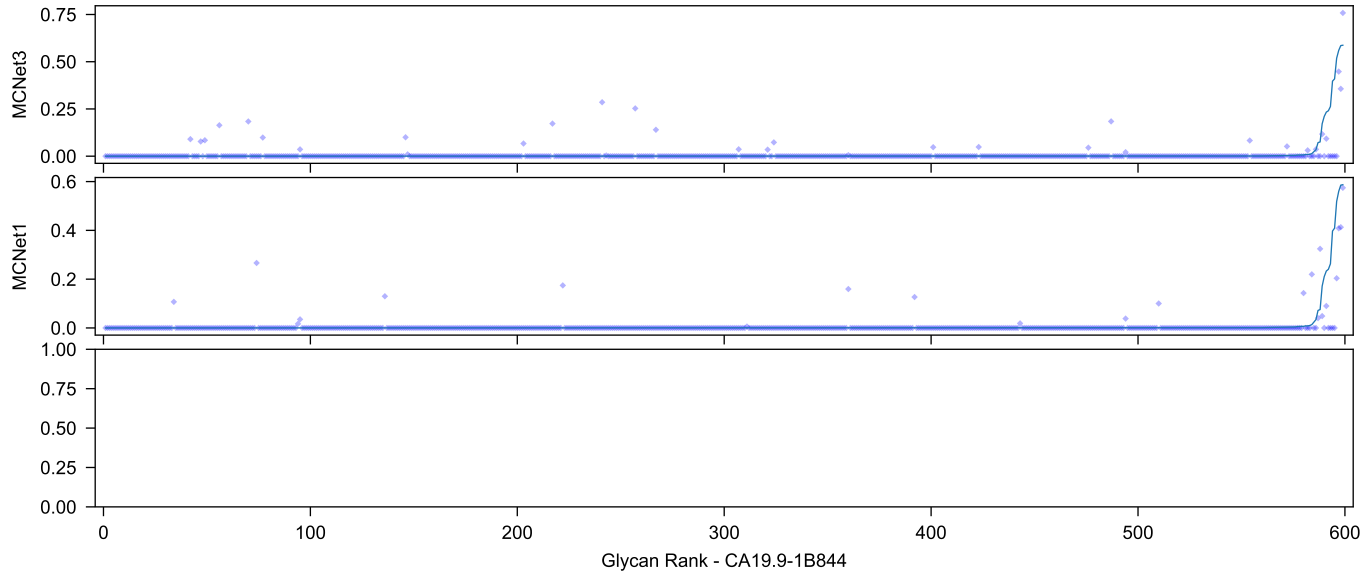
MCNet3
0.75
0.50
0.25
0.00
MCNet1
0.6
0.4
0.2
0.0
1.00
0.75
0.50
0.25
0.00
0
100
200
300
400
500
600
Glycan Rank - CA19.9-1B844

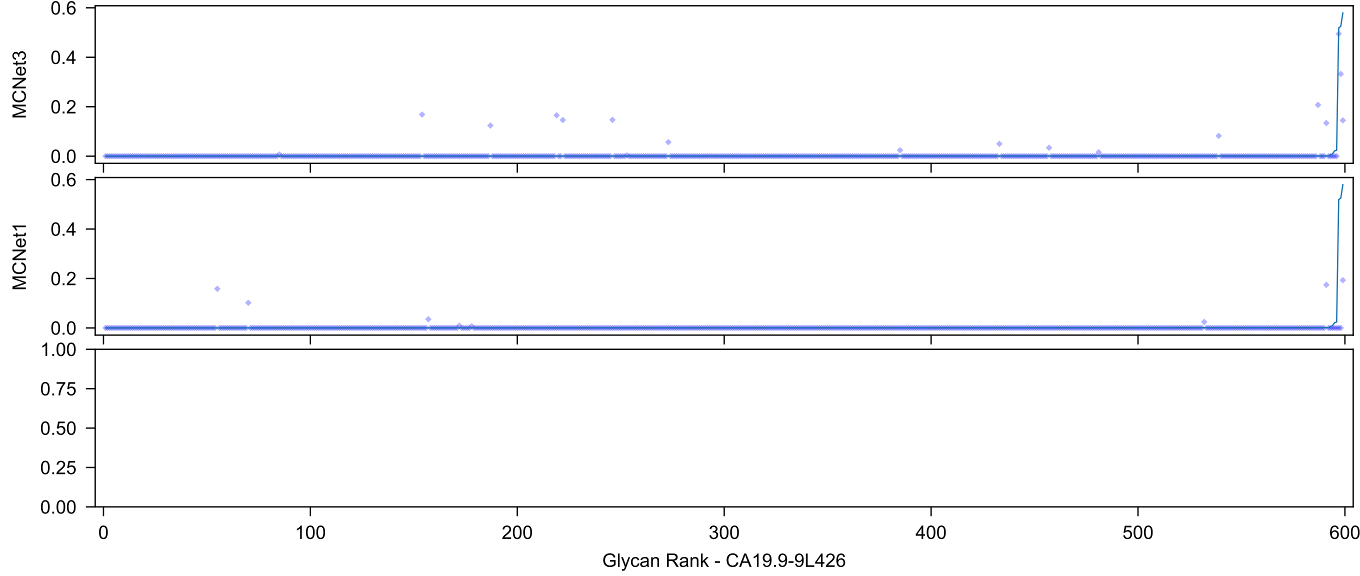

MCNet3
MCNet1
0.6
0.4
0.2
0.0
1.00
0.75
0.50
0.25
0.00
0
100
200
300
400
500
600
Glycan Rank - CA19.9-9L426

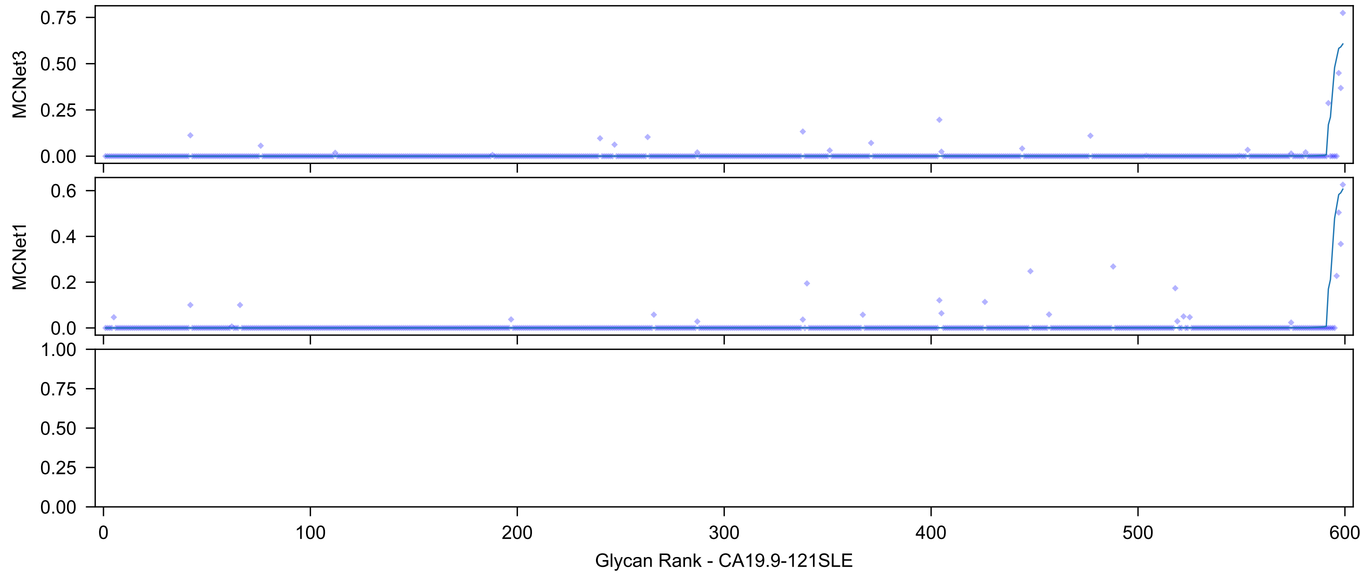
MCNet3
0.75
0.50
0.25
0.00
MCNet1
0.6
0.4
0.2
0.0
1.00
0.75
0.50
0.25
0.00
0
100
200
300
400
500
600
Glycan Rank - CA19.9-121SLE

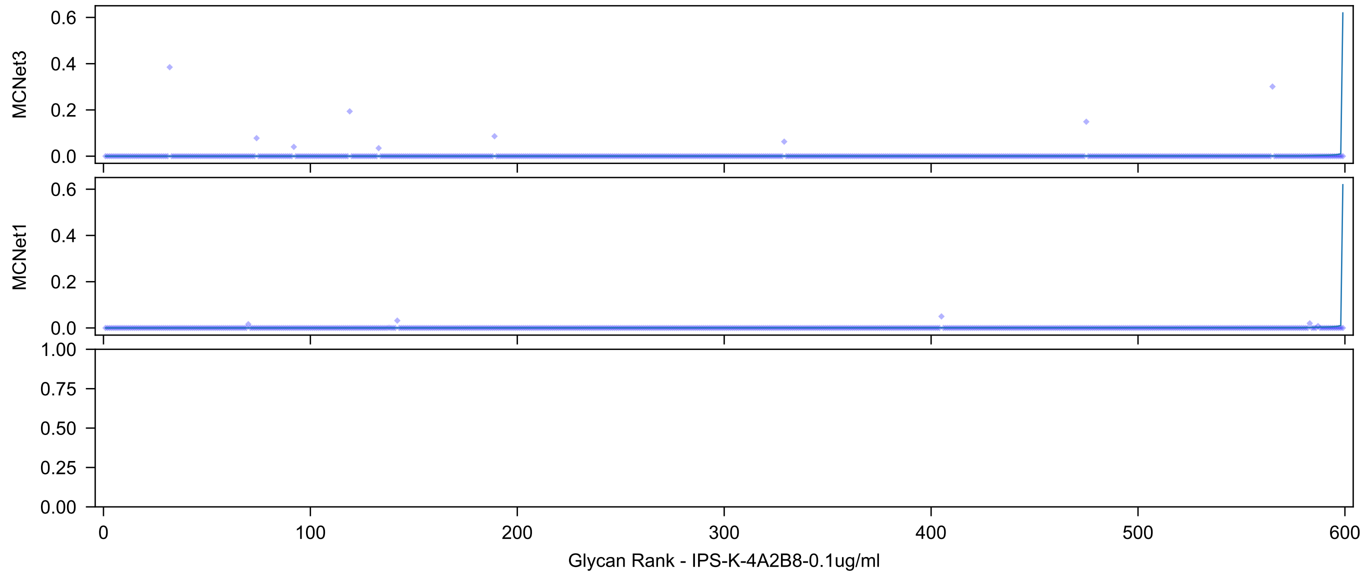

MCNet3
MCNet1
0.0
0.2
0.4
0.6
0.00
0.25
0.50
0.75
1.00
0
100
200
300
400
500
600
Glycan Rank - IPS-K-4A2B8-0.1ug/ml

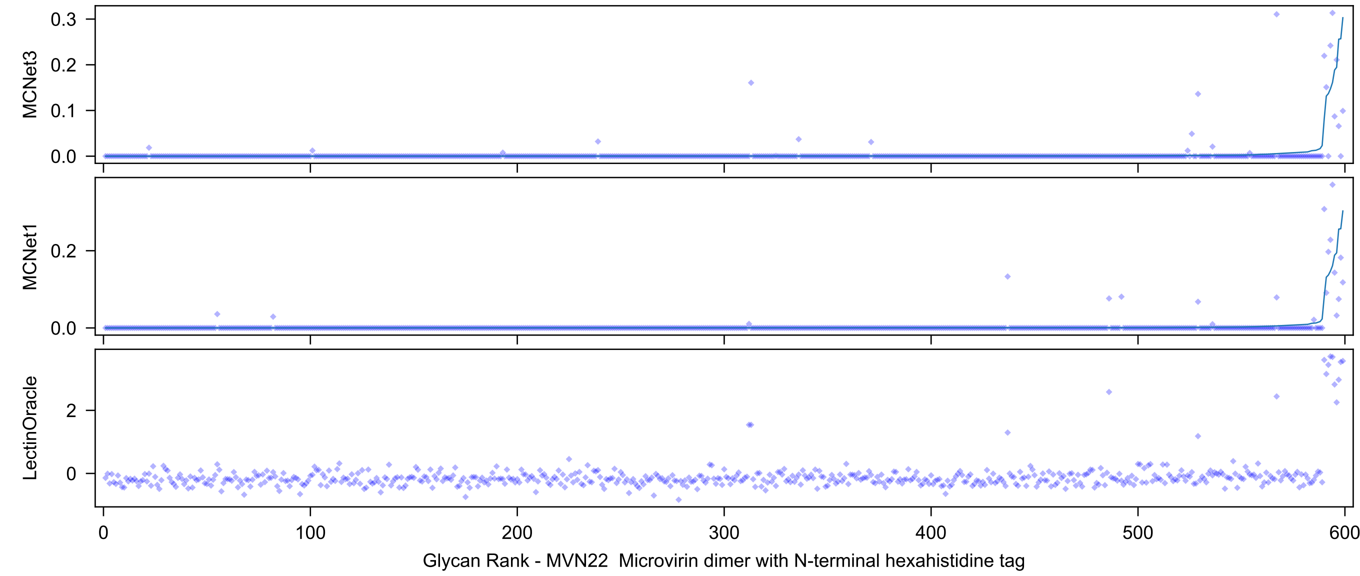
MCNet3
MCNet1
LectinOracle
0.3
0.2
0.1
0.0
0.2
0.0
2
0
0
100
200
300
400
500
600
Glycan Rank - MVN22 Microvirin dimer with N-terminal hexahistidine tag

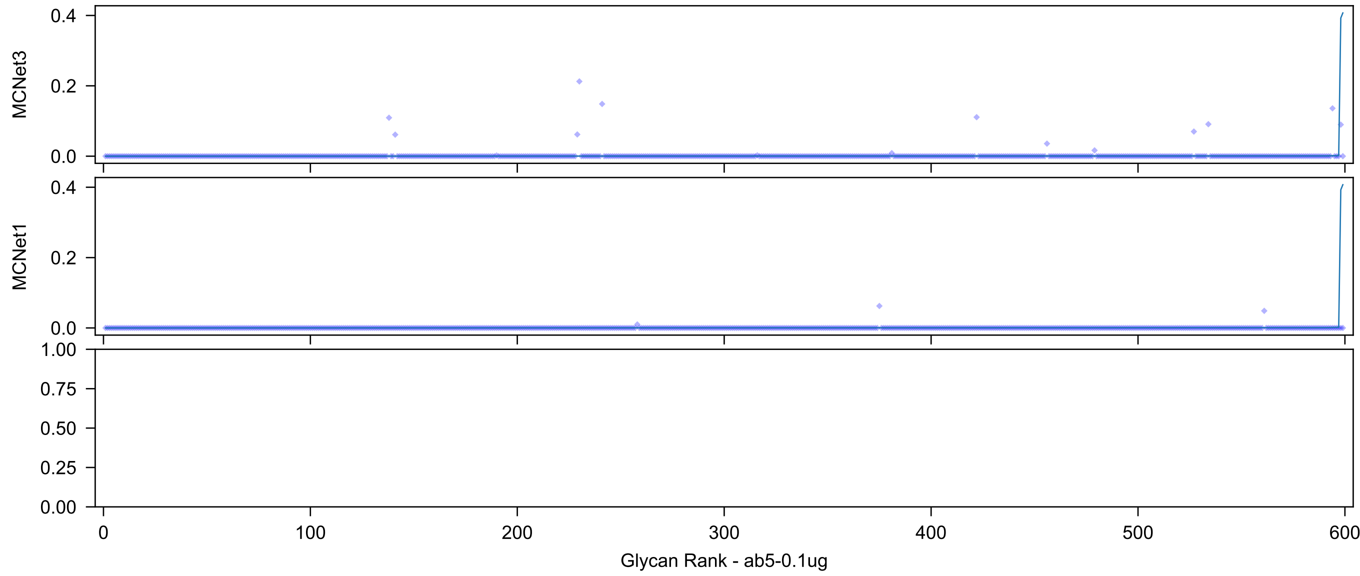

MCNet3
MCNet1
0.4
0.2
0.0
1.00
0.75
0.50
0.25
0.00
0
100
200
300
400
500
600
Glycan Rank - ab5-0.1ug

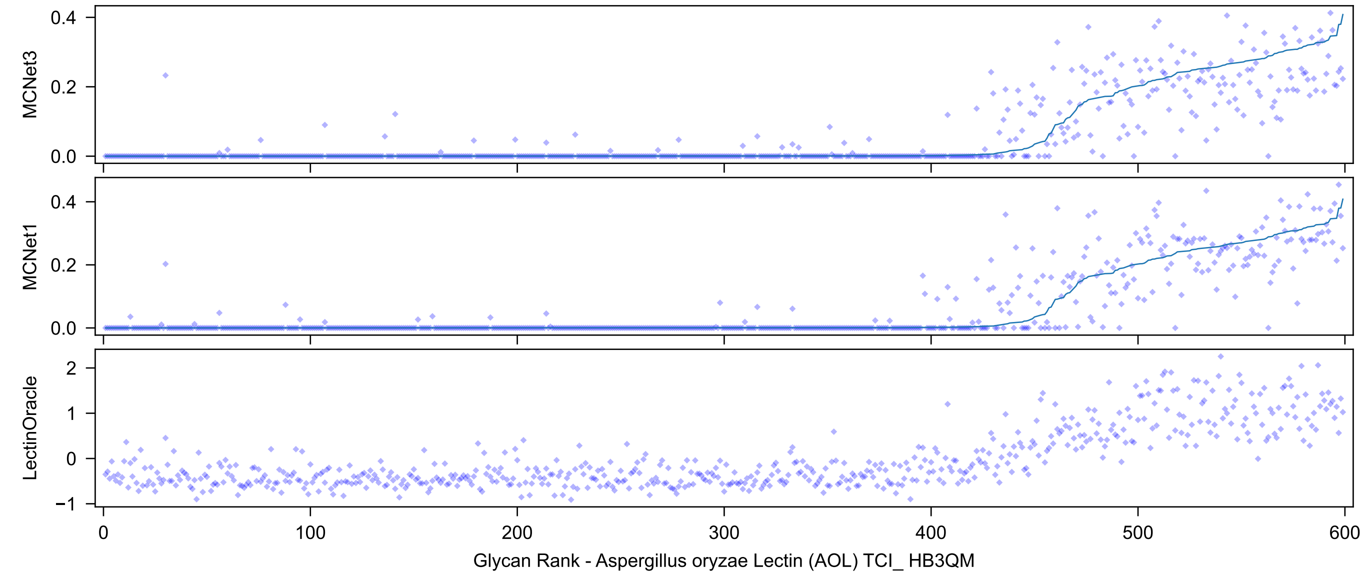

MCNet3
MCNet1
LectinOracle
0.4
0.2
0.0
2
1
0
−1
0
100
200
300
400
500
600
Glycan Rank - Aspergillus oryzae Lectin (AOL) TCI_ HB3QM

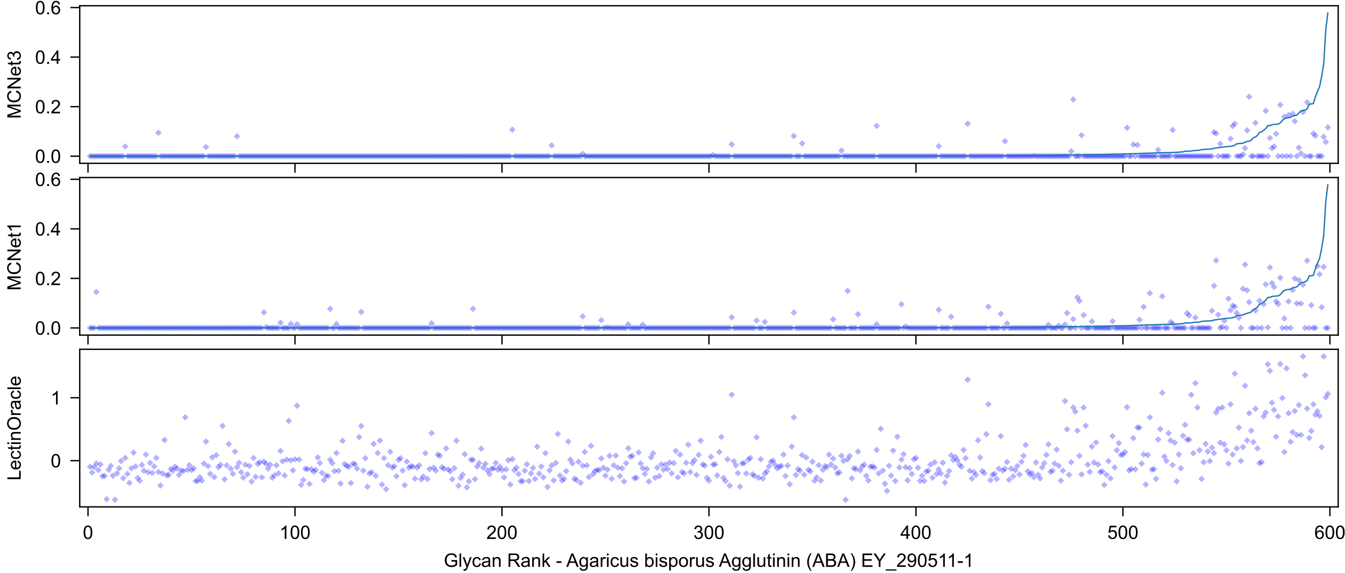

MCNet3
MCNet1
LectinOracle
0.0
0.2
0.4
0.6
0
1
0
100
200
300
400
500
600
Glycan Rank - Agaricus bisporus Agglutinin (ABA) EY_290511-1

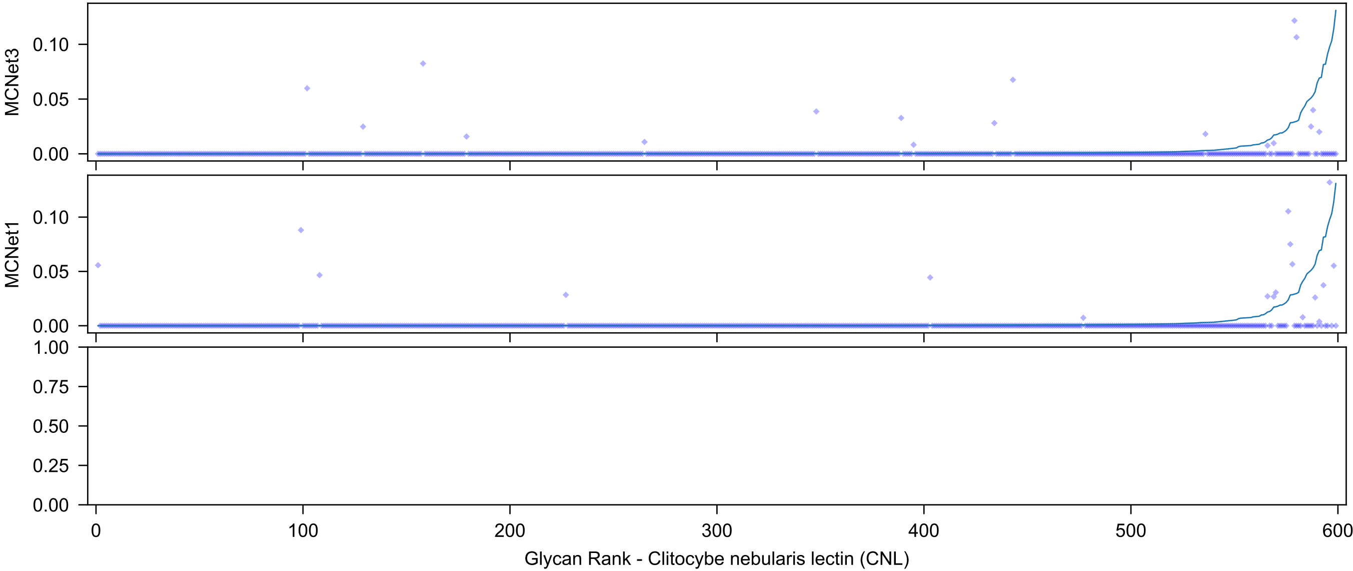

MCNet3
0.10
0.05
0.00
MCNet1
0.10
0.05
0.00
1.00
0.75
0.50
0.25
0.00
0
100
200
300
400
500
600
Glycan Rank - Clitocybe nebularis lectin (CNL)

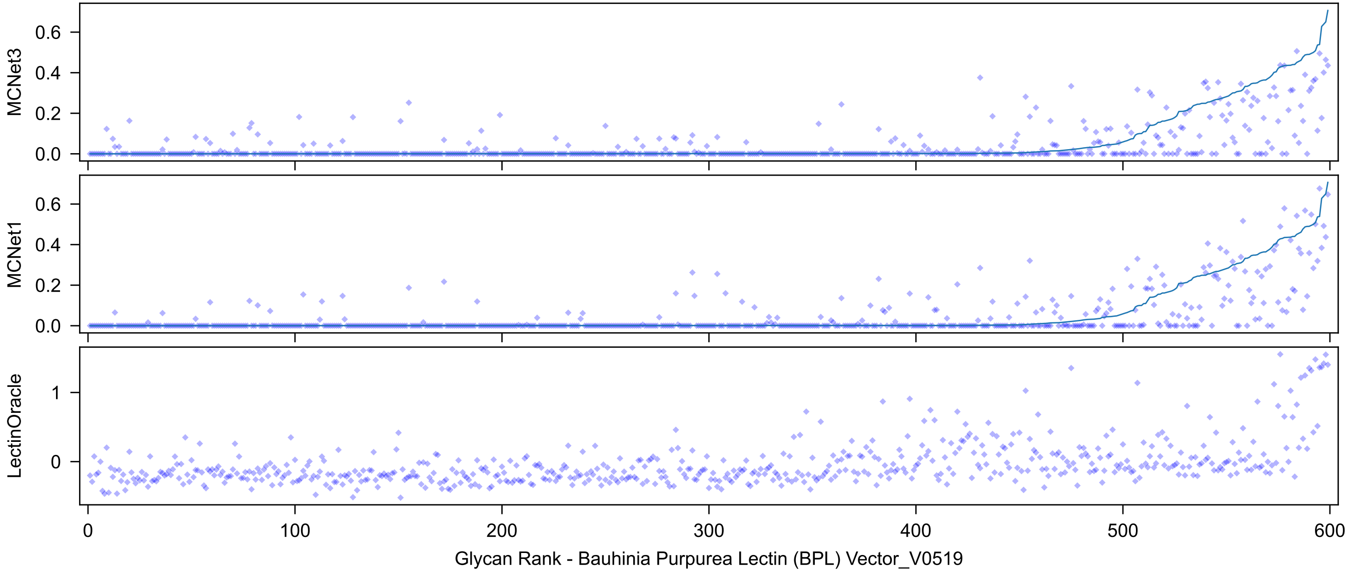
MCNet3
MCNet1
LectinOracle
0.0
0.2
0.4
0.6
0
1
0
100
200
300
400
500
600
Glycan Rank - Bauhinia Purpurea Lectin (BPL) Vector_V0519

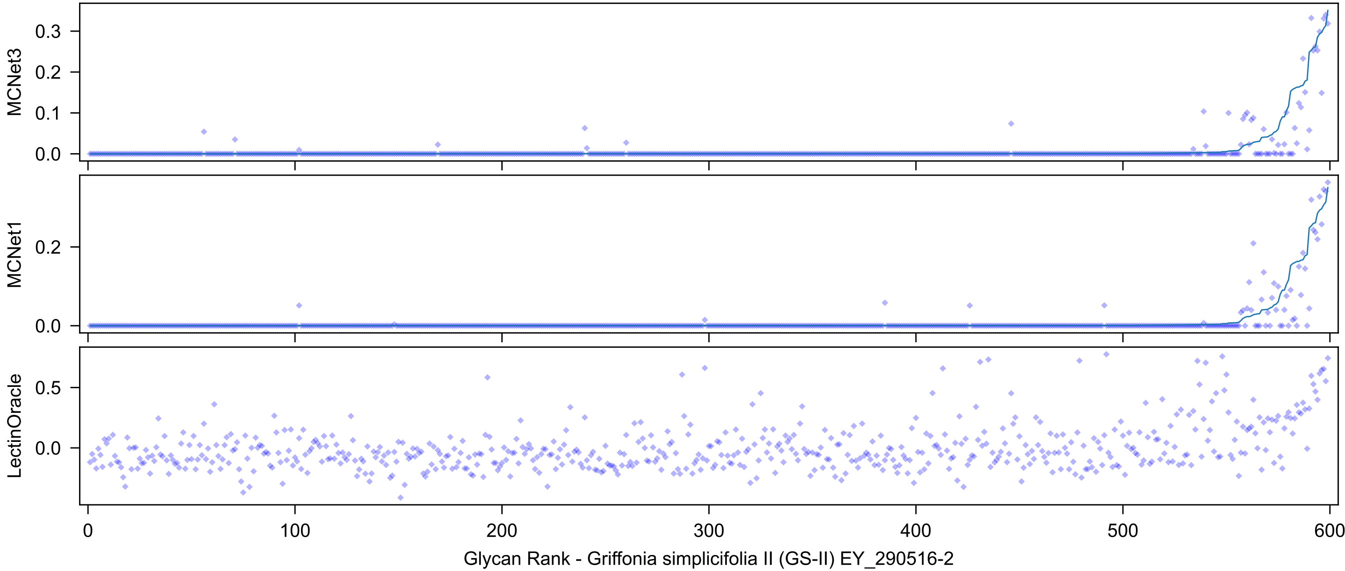

MCNet3
0.3
0.2
0.1
0.0
MCNet1
0.2
0.0
LectinOracle
0.5
0.0
0
100
200
300
400
500
600
Glycan Rank - Griffonia simplicifolia II (GS-II) EY_290516-2

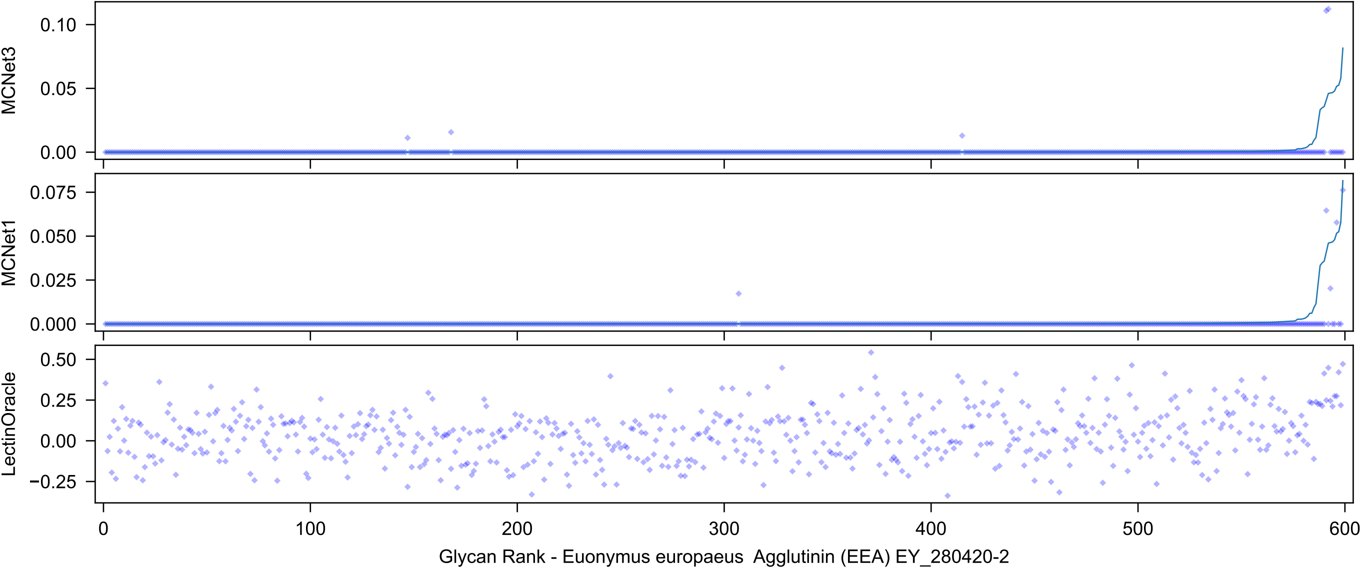

MCNet3
0.10
0.05
0.00
MCNet1
0.075
0.050
0.025
0.000
LectinOracle
0.50
0.25
0.00
−0.25
0
100
200
300
400
500
600
Glycan Rank - Euonymus europaeus Agglutinin (EEA) EY_280420-2

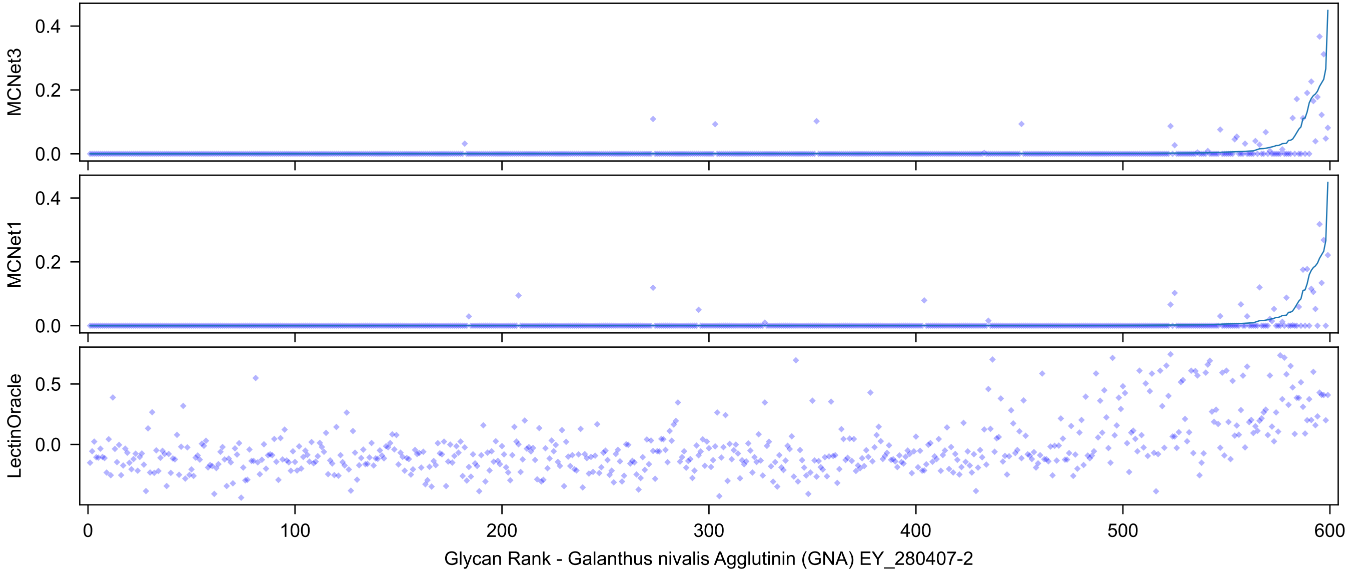

MCNet3
MCNet1
LectinOracle
0.4
0.2
0.0
0.5
0
100
200
300
400
500
600
Glycan Rank - Galanthus nivalis Agglutinin (GNA) EY_280407-2

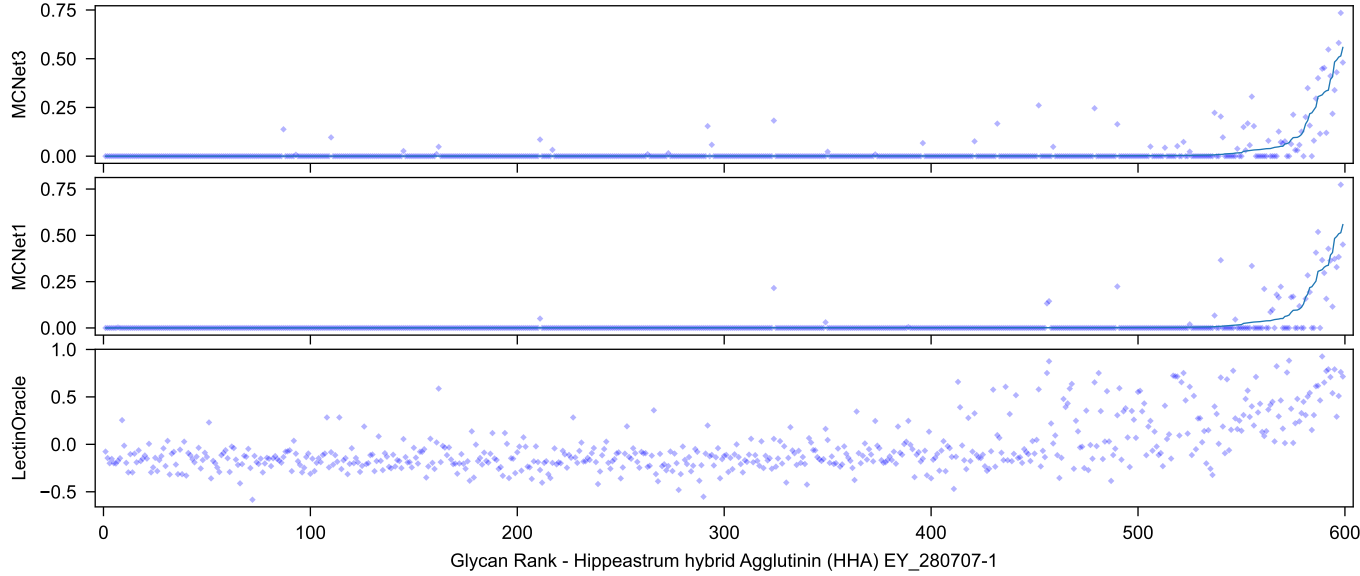

MCNet3
MCNet1
LectinOracle
0.75
0.50
0.25
0.00
1.0
0.5
0.0
−0.5
0
100
200
300
400
500
600
Glycan Rank - Hippeastrum hybrid Agglutinin (HHA) EY_280707-1

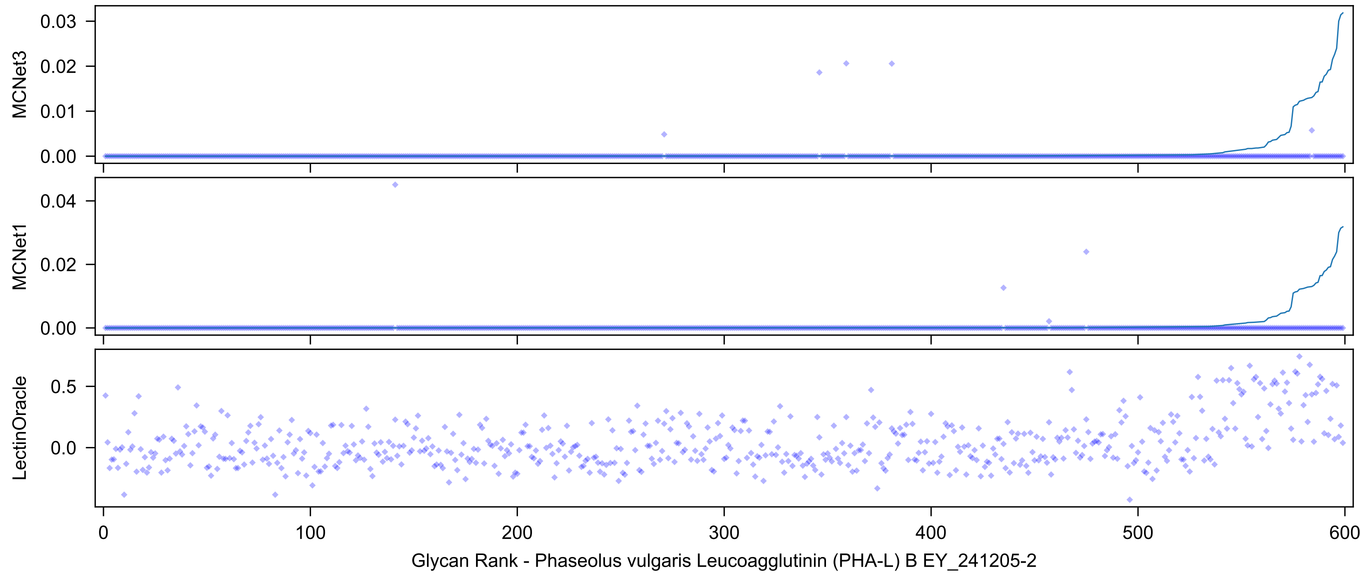

MCNet3
0.03
0.02
0.01
0.00
MCNet1
0.04
0.02
0.00
LectinOracle
0.5
0.0
0
100
200
300
400
500
600
Glycan Rank - Phaseolus vulgaris Leucoagglutinin (PHA-L) B EY_241205-2

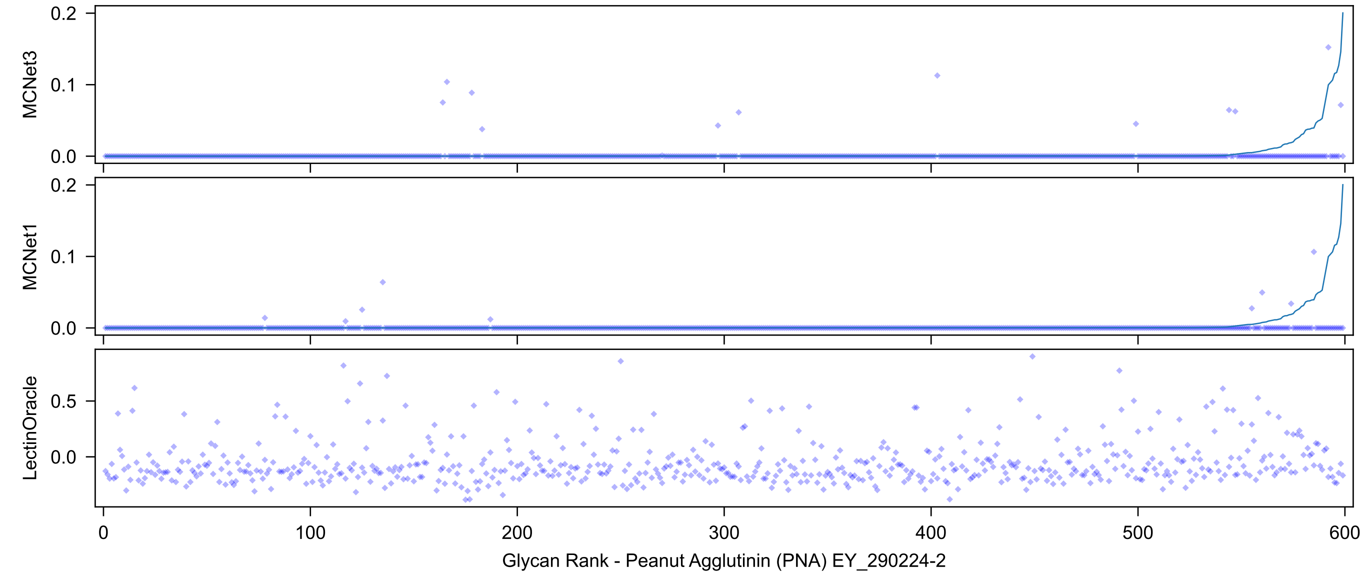

MCNet3
MCNet1
LectinOracle
0.2
0.1
0.0
0.5
0
100
200
300
400
500
600
Glycan Rank - Peanut Agglutinin (PNA) EY_290224-2

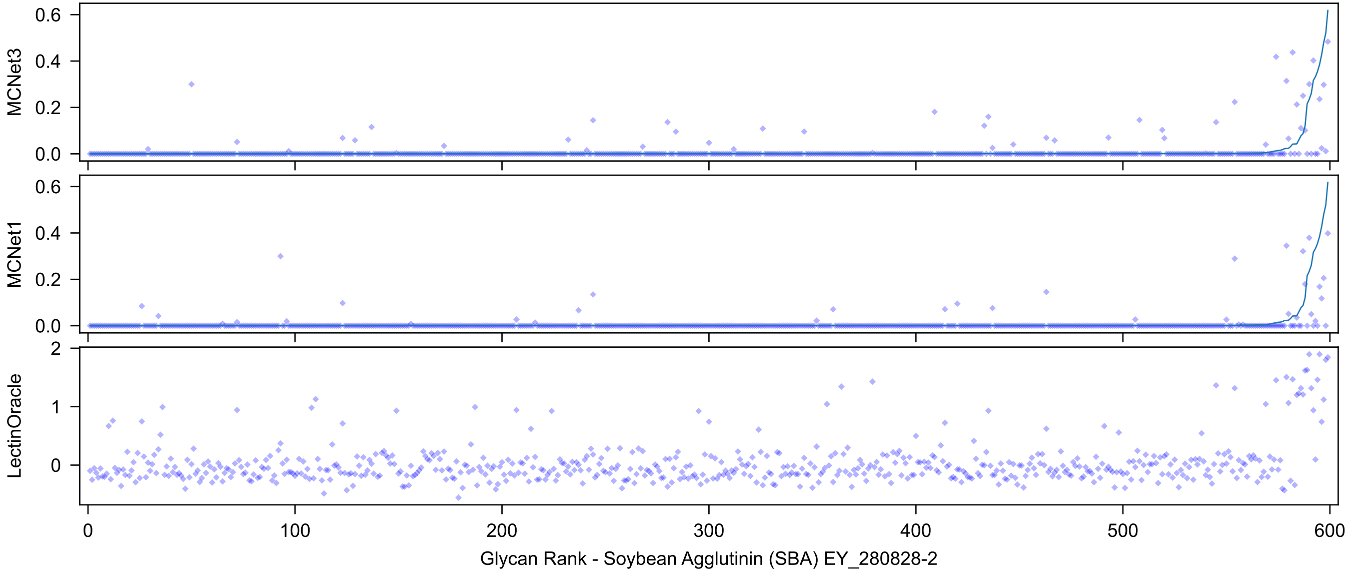

MCNet3
MCNet1
LectinOracle
0.6
0.4
0.2
0.0
2
1
0
0
100
200
300
400
500
600
Glycan Rank - Soybean Agglutinin (SBA) EY_280828-2

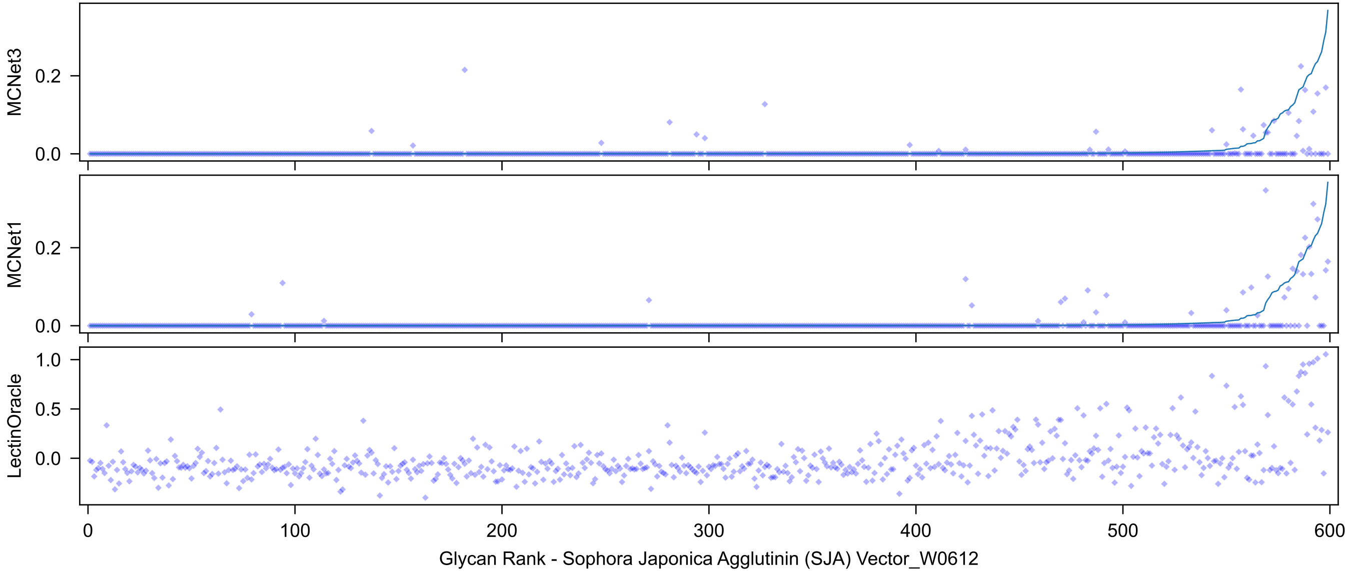
MCNet3
MCNet1
LectinOracle
0.0
0.2
0.0
0.2
0.0
0.5
1.0
0
100
200
300
400
500
600
Glycan Rank - Sophora Japonica Agglutinin (SJA) Vector_W0612

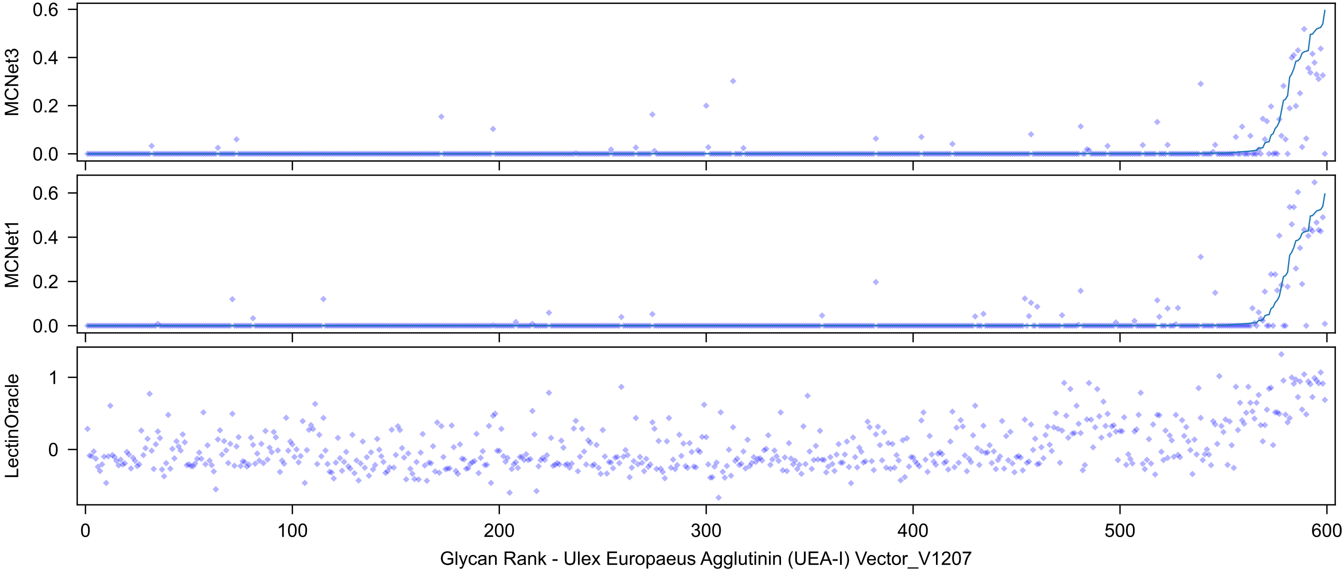

MCNet3
MCNet1
LectinOracle
0.0
0.2
0.4
0.6
0
1
0
100
200
300
400
500
600
Glycan Rank - Ulex Europaeus Agglutinin (UEA-I) Vector_V1207

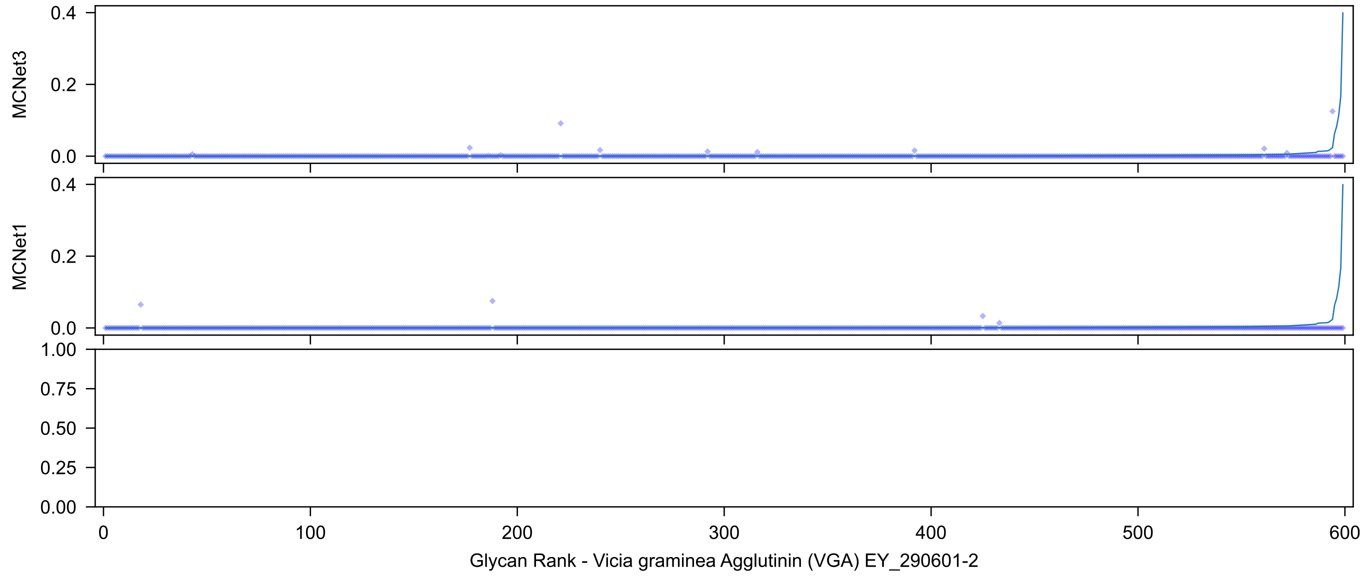
MCNet3
0.4
0.2
0.0
MCNet1
0.4
0.2
0.0
1.00
0.75
0.50
0.25
0.00
0
100
200
300
400
500
600
Glycan Rank - Vicia graminea Agglutinin (VGA) EY_290601-2

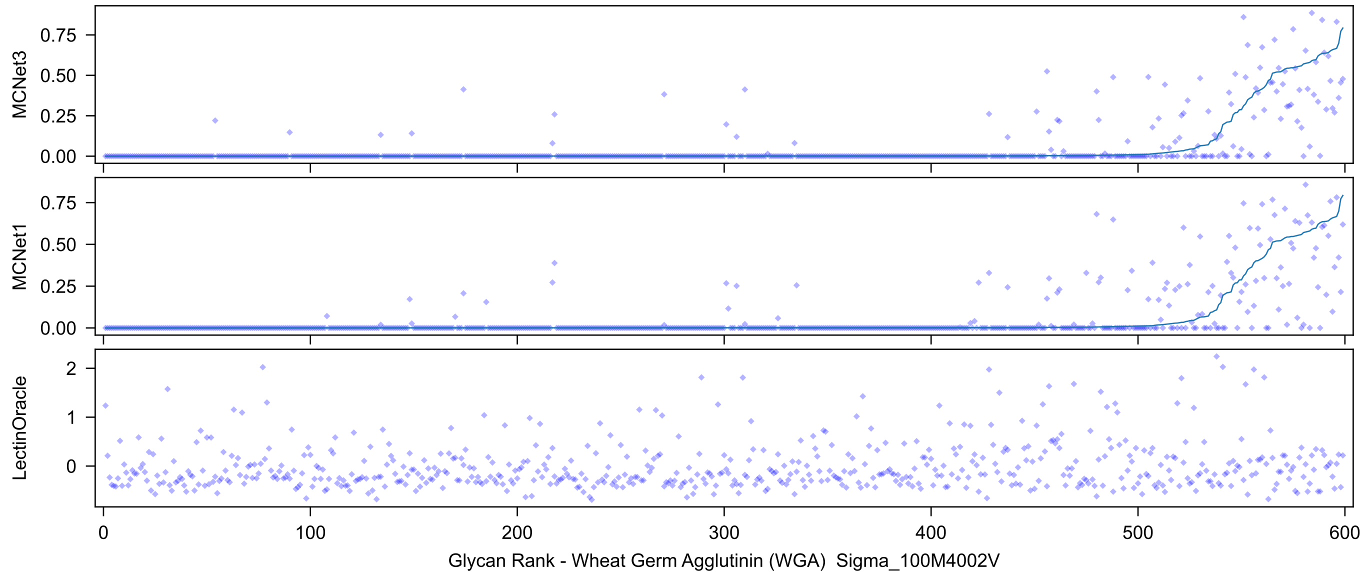

MCNet3
0.00
0.25
0.50
0.75
MCNet1
0.00
0.25
0.50
0.75
LectinOracle
0
1
2
0
100
200
300
400
500
600
Glycan Rank - Wheat Germ Agglutinin (WGA) Sigma_100M4002V

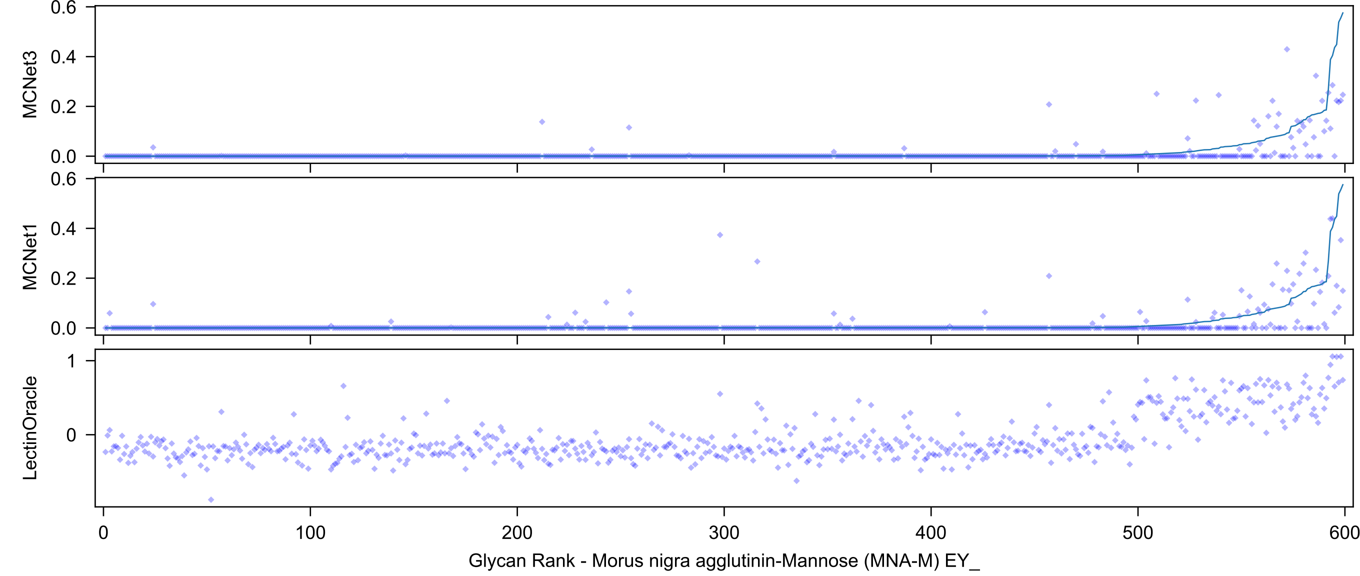
MCNet3
MCNet1
LectinOracle
0.6
0.4
0.2
0.0
1
0
0
100
200
300
400
500
600
Glycan Rank - Morus nigra agglutinin-Mannose (MNA-M) EY_

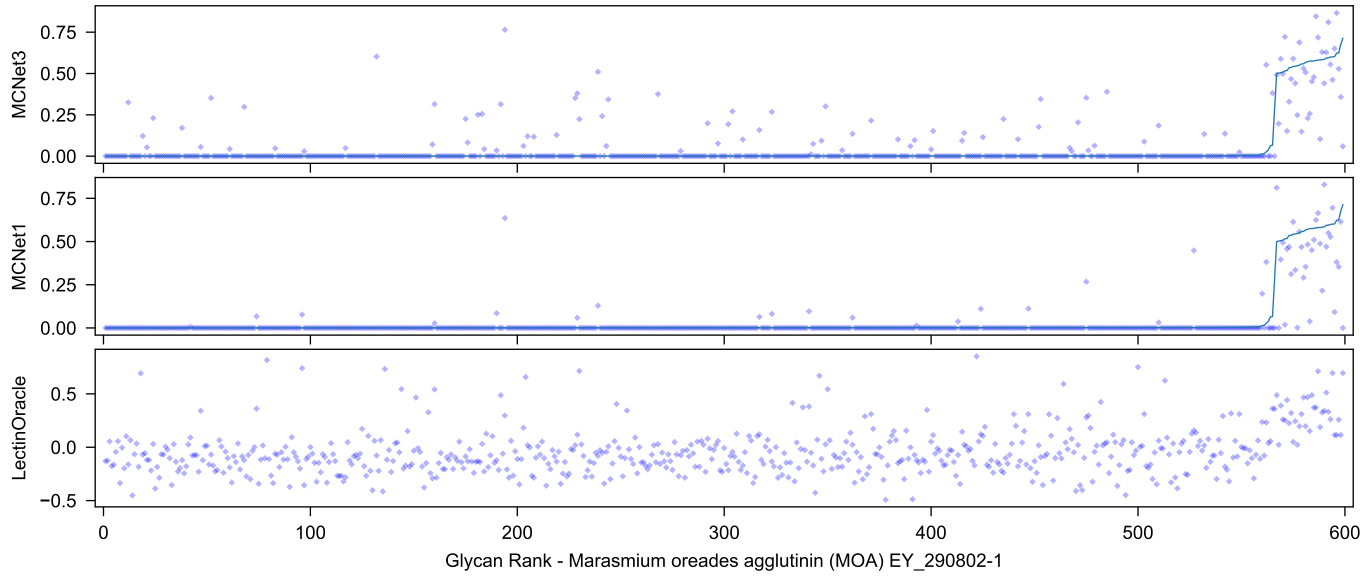
MCNet3
0.00
0.25
0.50
0.75
MCNet1
0.00
0.25
0.50
0.75
LectinOracle
−0.5
0.0
0.5
0
100
200
300
400
500
600
Glycan Rank - Marasmium oreades agglutinin (MOA) EY_290802-1

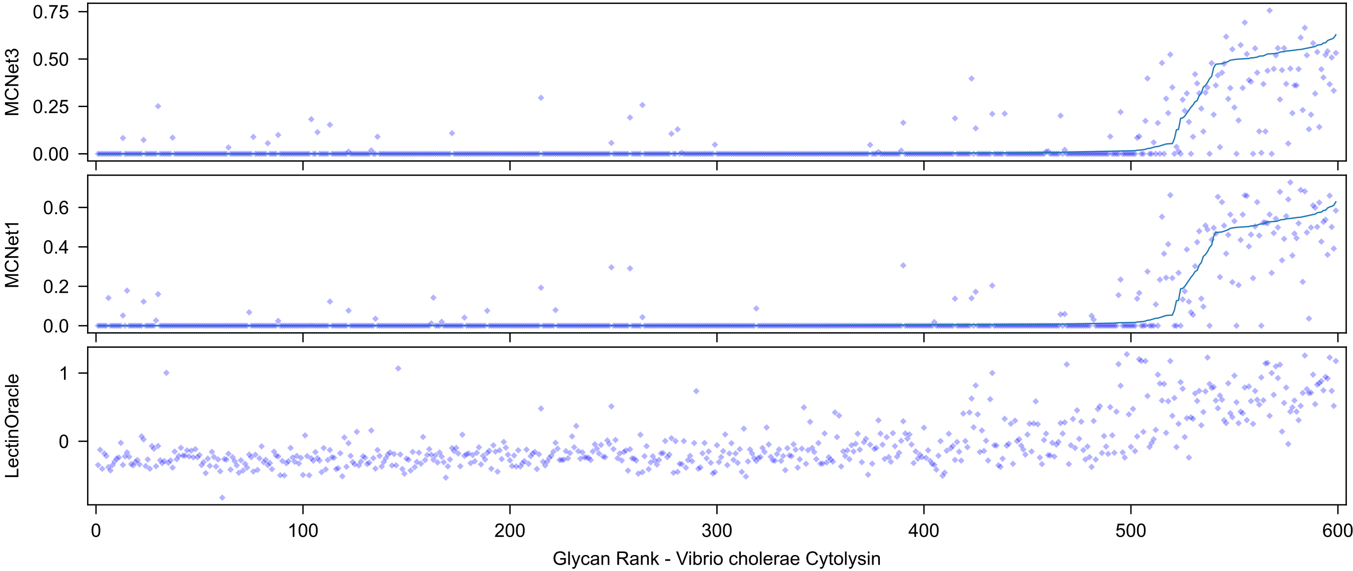

MCNet3
0.75
0.50
0.25
0.00
MCNet1
0.6
0.4
0.2
0.0
LectinOracle
1
0
0
100
200
300
400
500
600
Glycan Rank - Vibrio cholerae Cytolysin

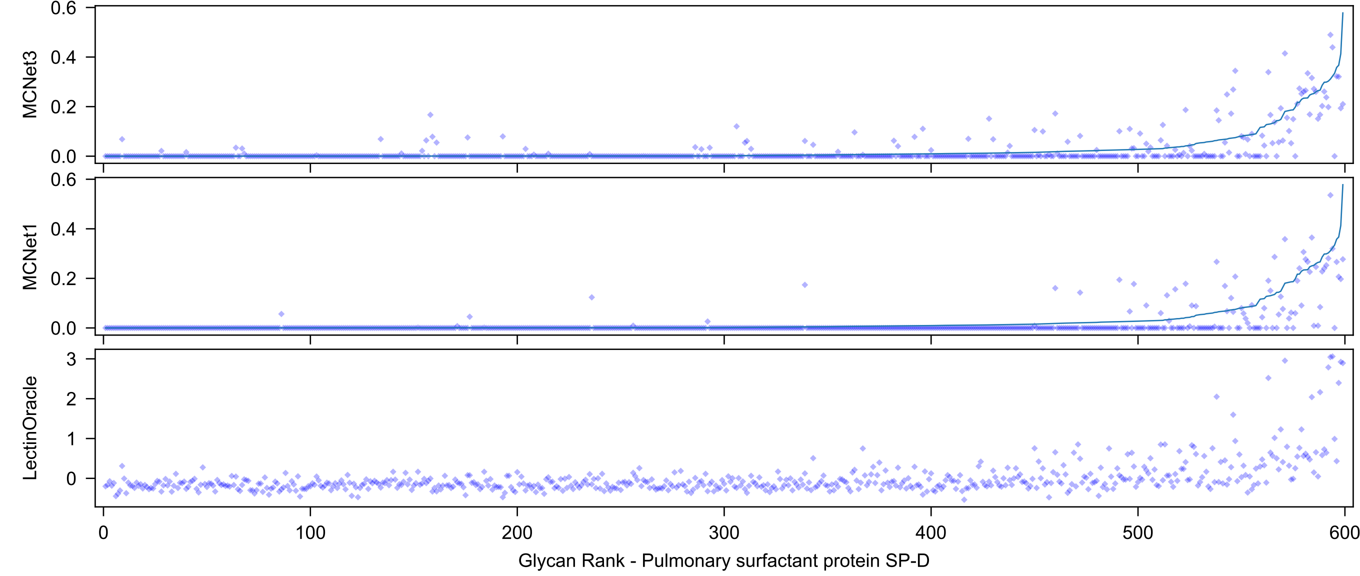

MCNet3
MCNet1
LectinOracle
0.0
0.2
0.4
0.6
0
1
2
3
0
100
200
300
400
500
600
Glycan Rank - Pulmonary surfactant protein SP-D

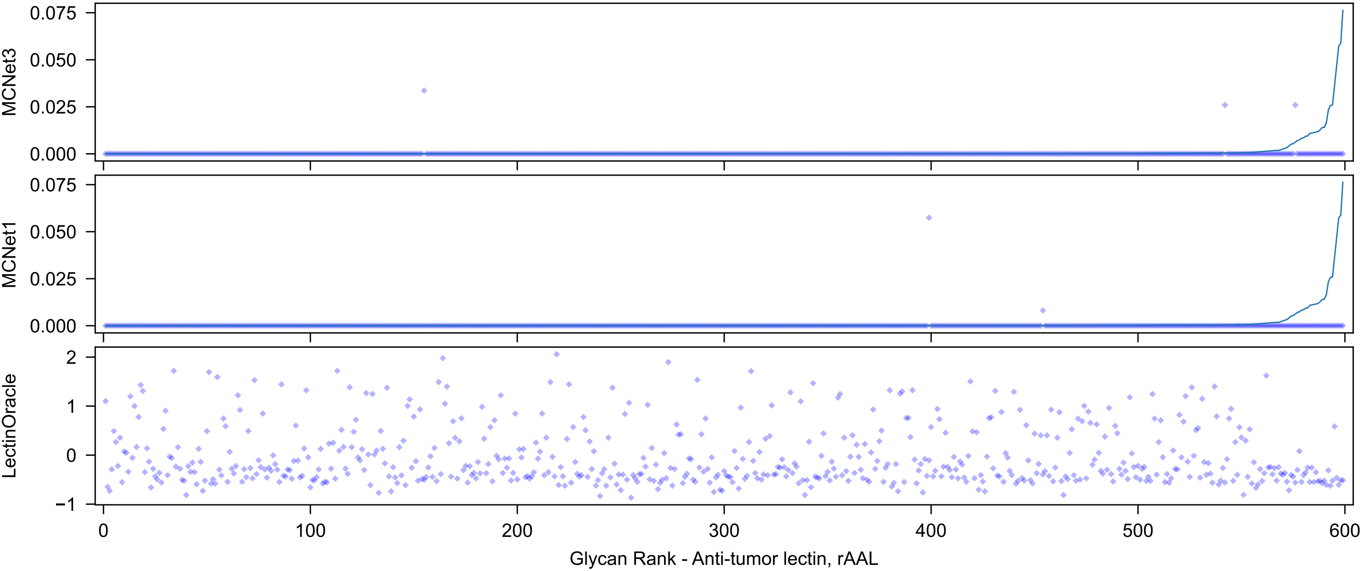

MCNet3
0.075
0.050
0.025
0.000
MCNet1
0.075
0.050
0.025
0.000
LectinOracle
2
1
0
−1
0
100
200
300
400
500
600
Glycan Rank - Anti-tumor lectin, rAAL

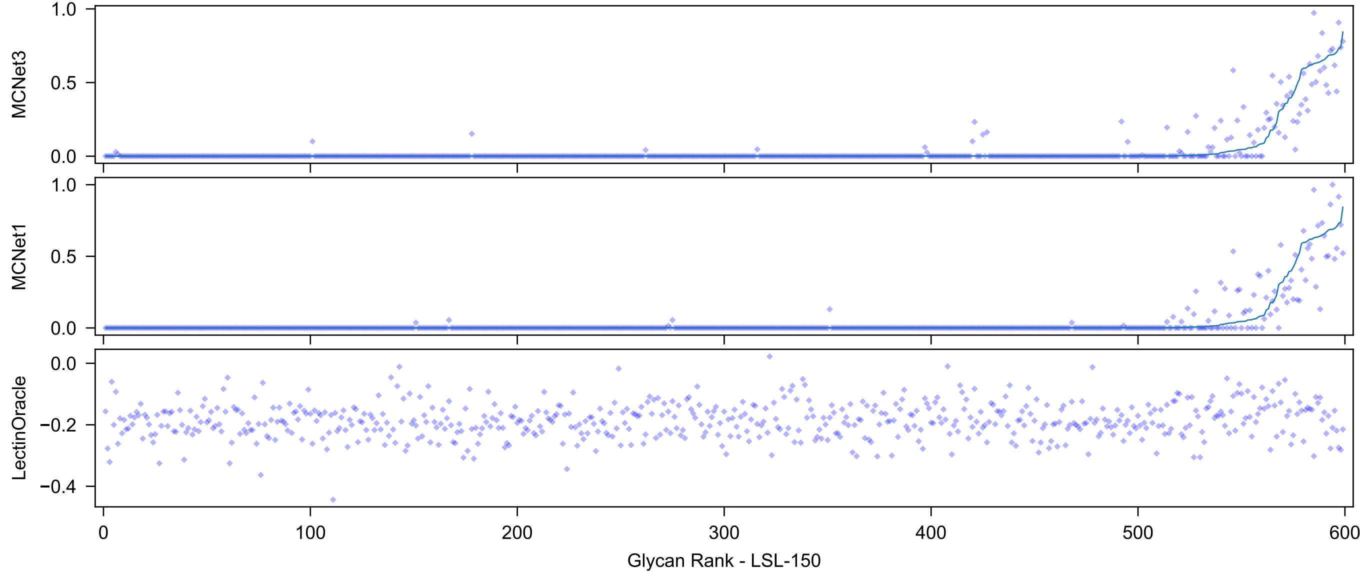
MCNet3
MCNet1
LectinOracle
1.0
0.5
0.0
−0.2
−0.4
0
100
200
300
400
500
600
Glycan Rank - LSL-150

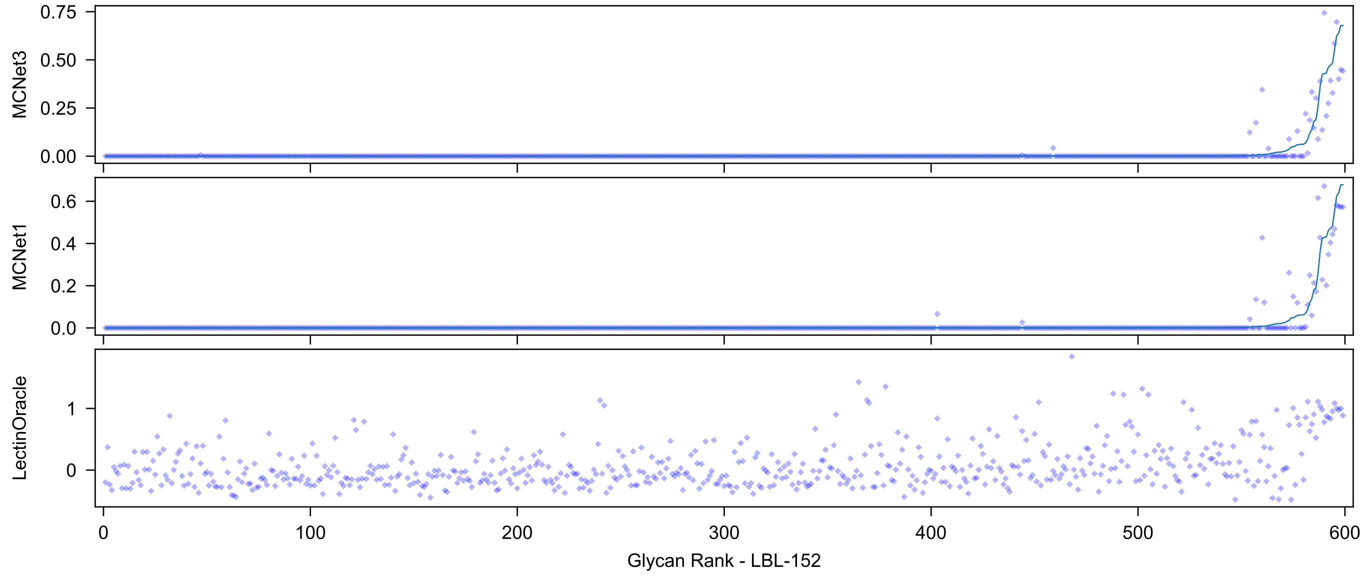

MCNet3
0.75
0.50
0.25
0.00
MCNet1
0.6
0.4
0.2
0.0
LectinOracle
1
0
0
100
200
300
400
500
600
Glycan Rank - LBL-152

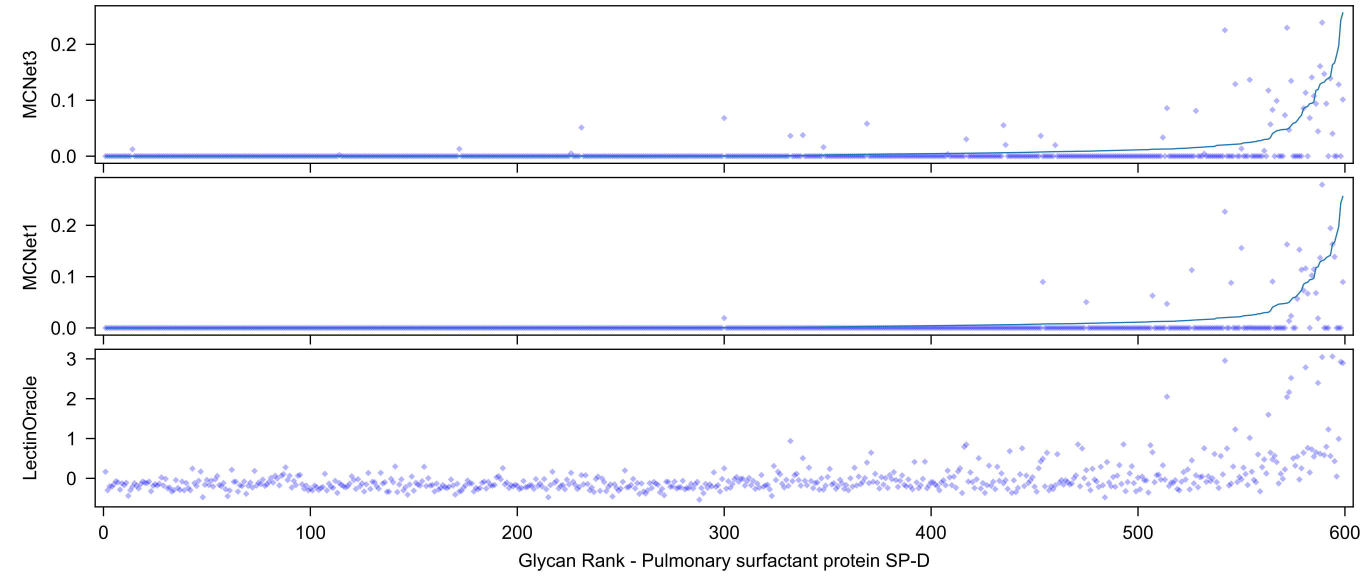
MCNet3
0.0
0.1
0.2
MCNet1
0.0
0.1
0.2
LectinOracle
0
1
2
3
0
100
200
300
400
500
600
Glycan Rank - Pulmonary surfactant protein SP-D

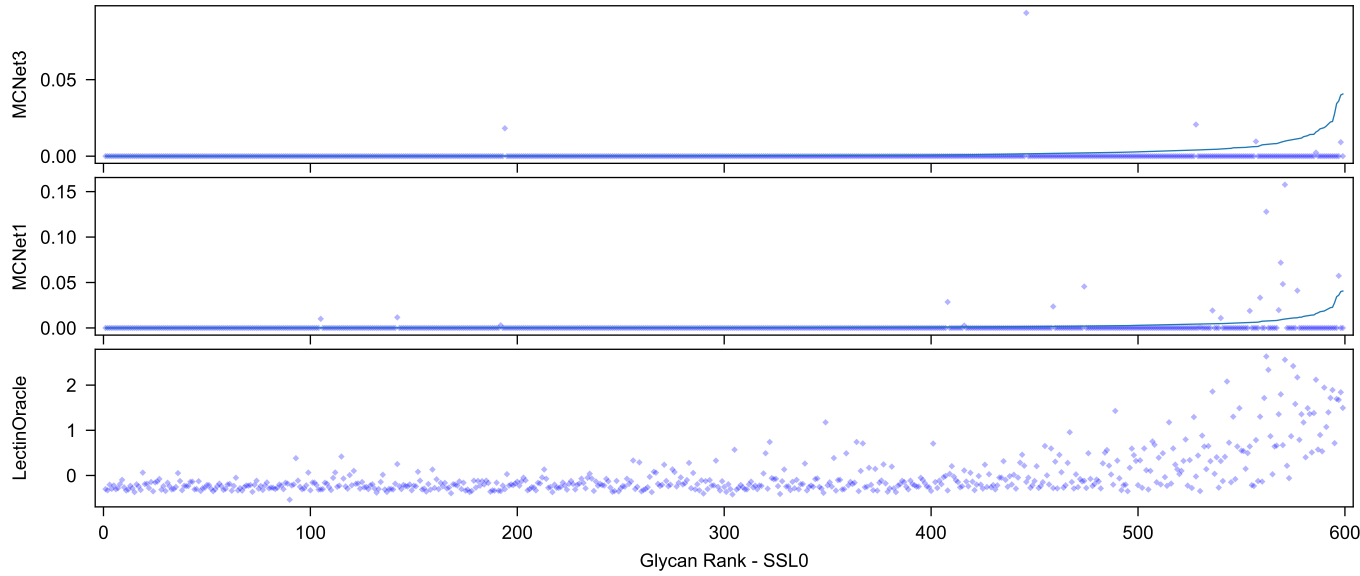

MCNet3
0.05
0.00
MCNet1
0.15
0.10
0.05
0.00
LectinOracle
2
1
0
0
100
200
300
400
500
600
Glycan Rank - SSL0

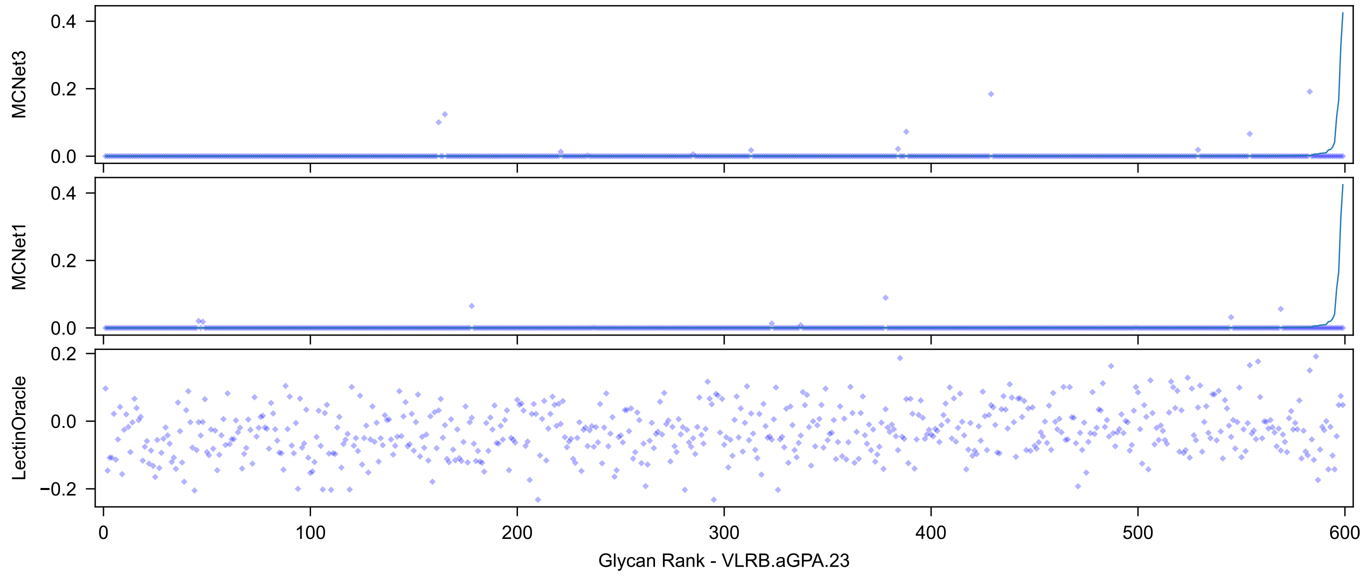
MCNet3
0.4
0.2
0.0
MCNet1
0.4
0.2
0.0
LectinOracle
0.2
0.0
−0.2
0
100
200
300
400
500
600
Glycan Rank - VLRB.aGPA.23

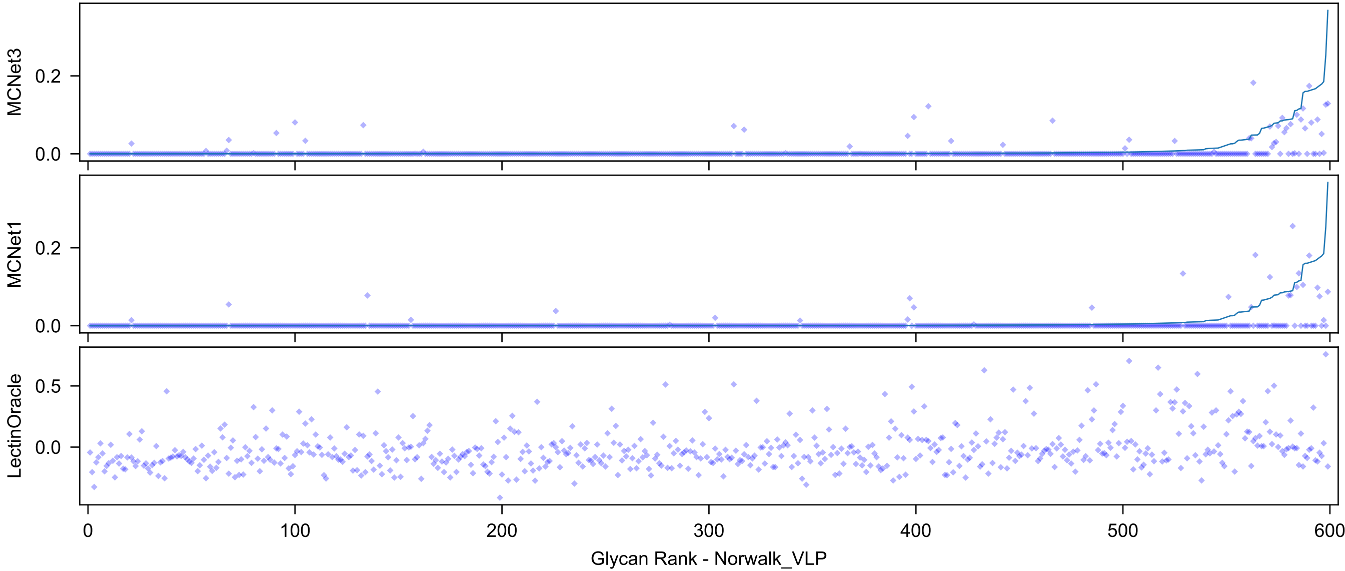

MCNet3
MCNet1
LectinOracle
0.0
0.2
0.0
0.5
0
100
200
300
400
500
600
Glycan Rank - Norwalk_VLP

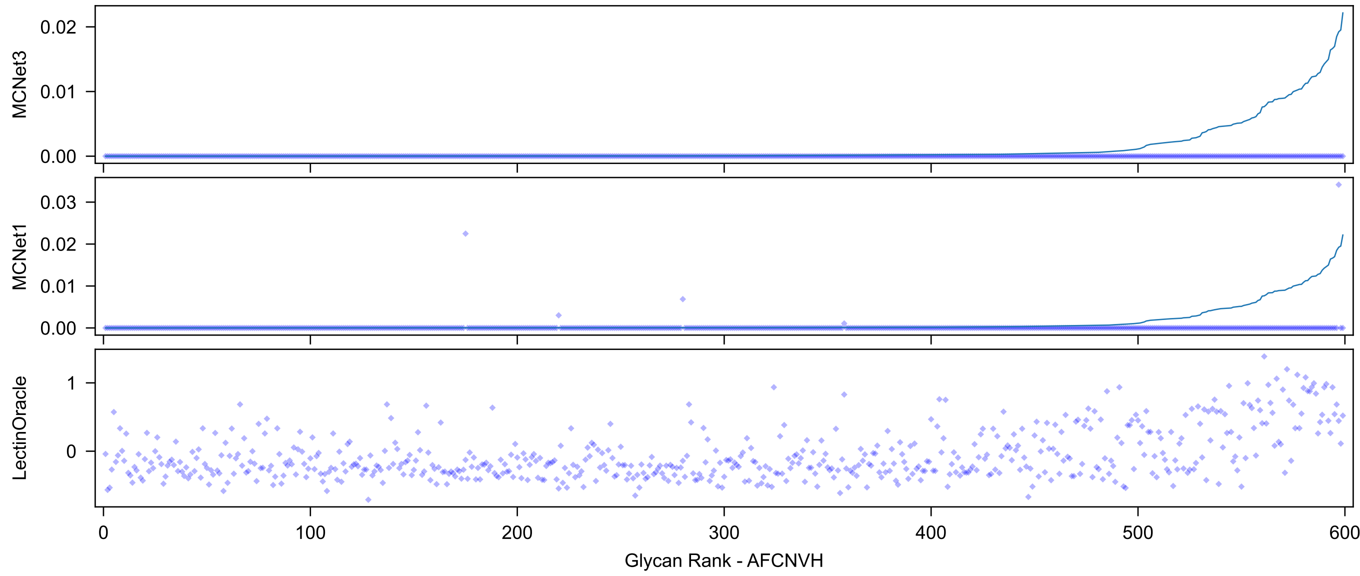

MCNet3
0.02
0.01
0.00
MCNet1
0.03
0.02
0.01
0.00
LectinOracle
1
0
0
100
200
300
400
500
600
Glycan Rank - AFCNVH

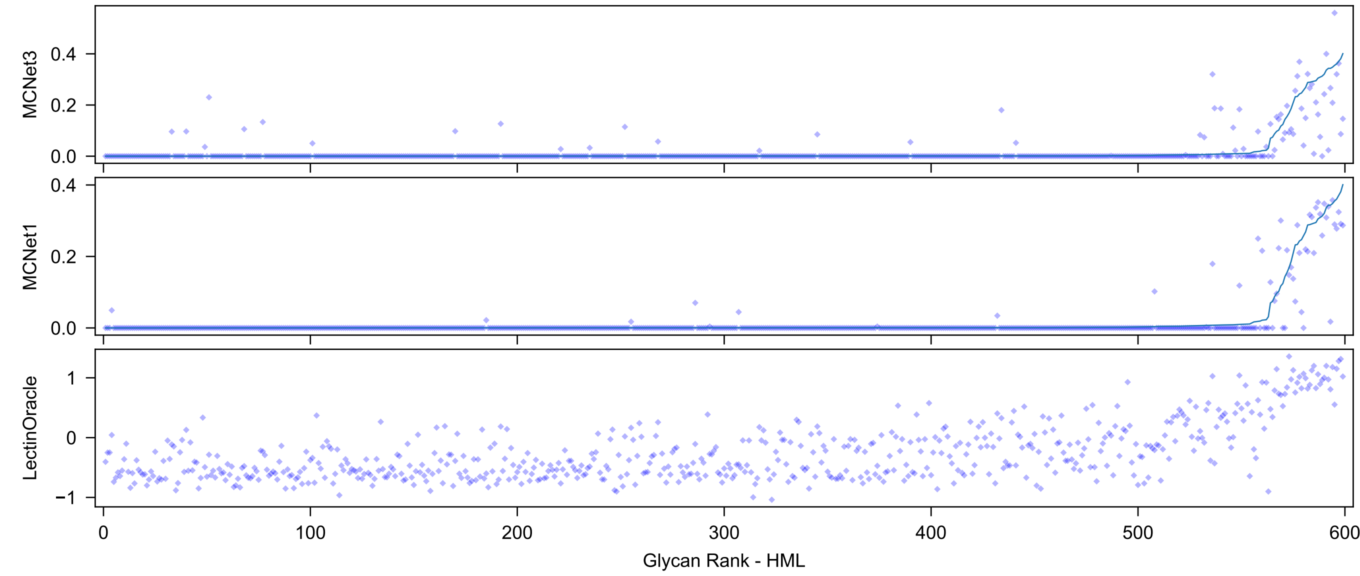

MCNet3
MCNet1
LectinOracle
0.4
0.2
0.0
1
0
−1
0
100
200
300
400
500
600
Glycan Rank - HML

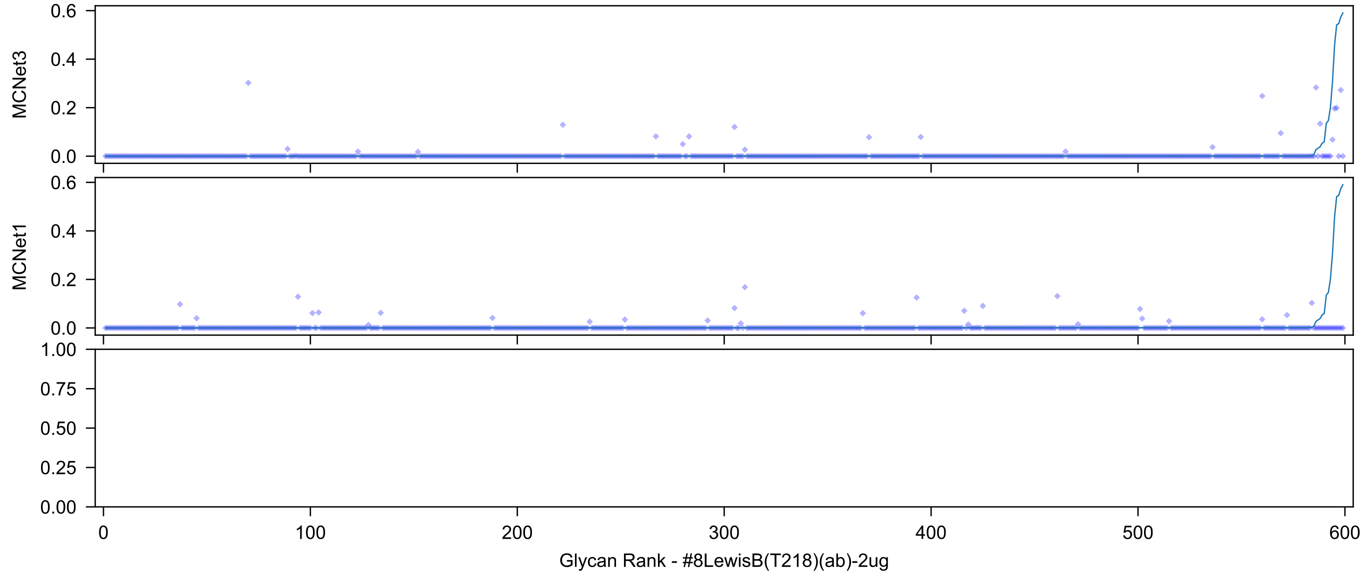

MCNet3
MCNet1
0.0
0.2
0.4
0.6
0.00
0.25
0.50
0.75
1.00
0
100
200
300
400
500
600
Glycan Rank - #8LewisB(T218)(ab)-2ug

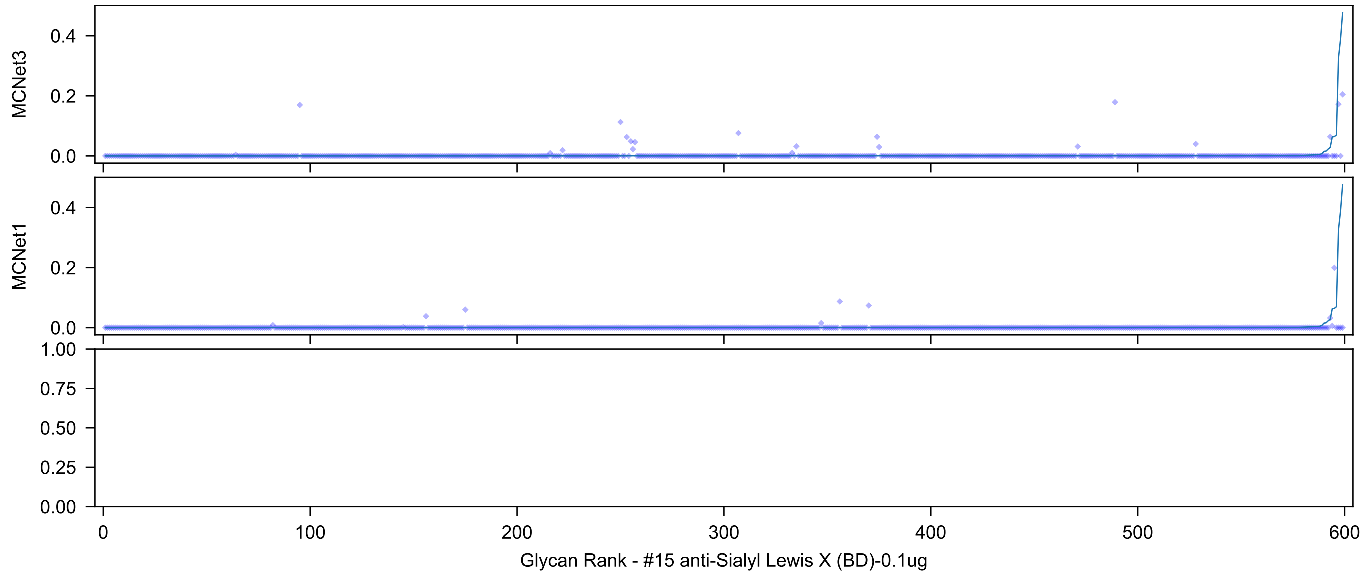
MCNet3
MCNet1
0.4
0.2
0.0
1.00
0.75
0.50
0.25
0.00
0
100
200
300
400
500
600
Glycan Rank - #15 anti-Sialyl Lewis X (BD)-0.1ug

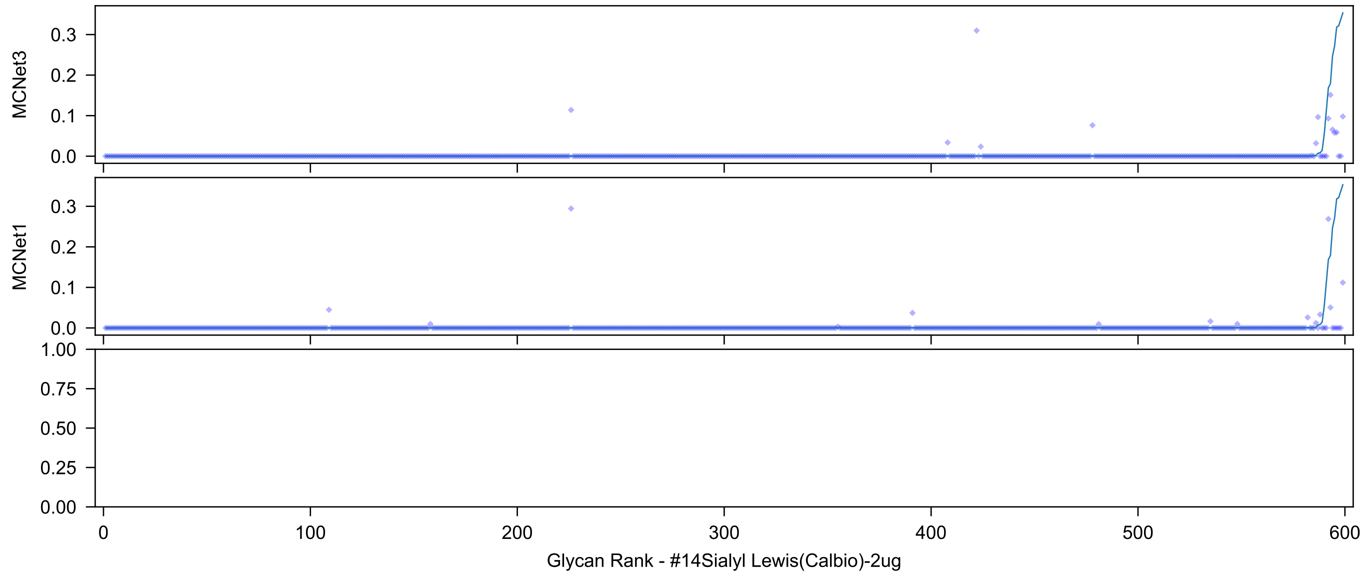

MCNet3
0.3
0.2
0.1
0.0
MCNet1
0.3
0.2
0.1
0.0
1.00
0.75
0.50
0.25
0.00
0
100
200
300
400
500
600
Glycan Rank - #14Sialyl Lewis(Calbio)-2ug

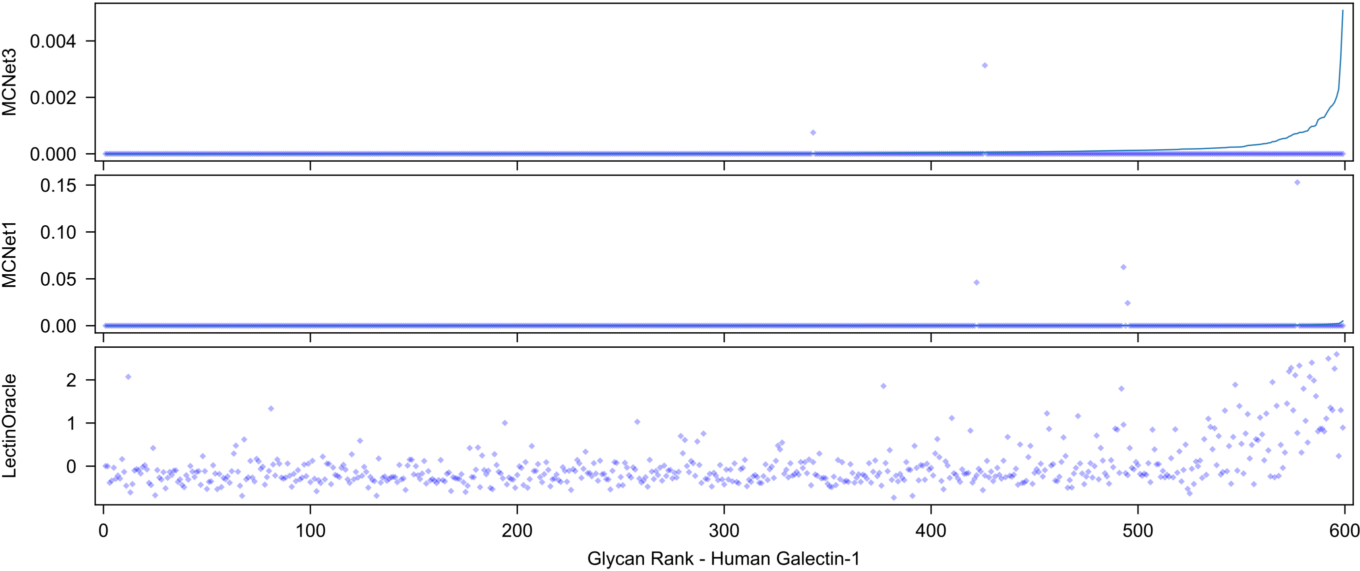

MCNet3
0.004
0.002
0.000
MCNet1
0.15
0.10
0.05
0.00
LectinOracle
2
1
0
0
100
200
300
400
500
600
Glycan Rank - Human Galectin-1

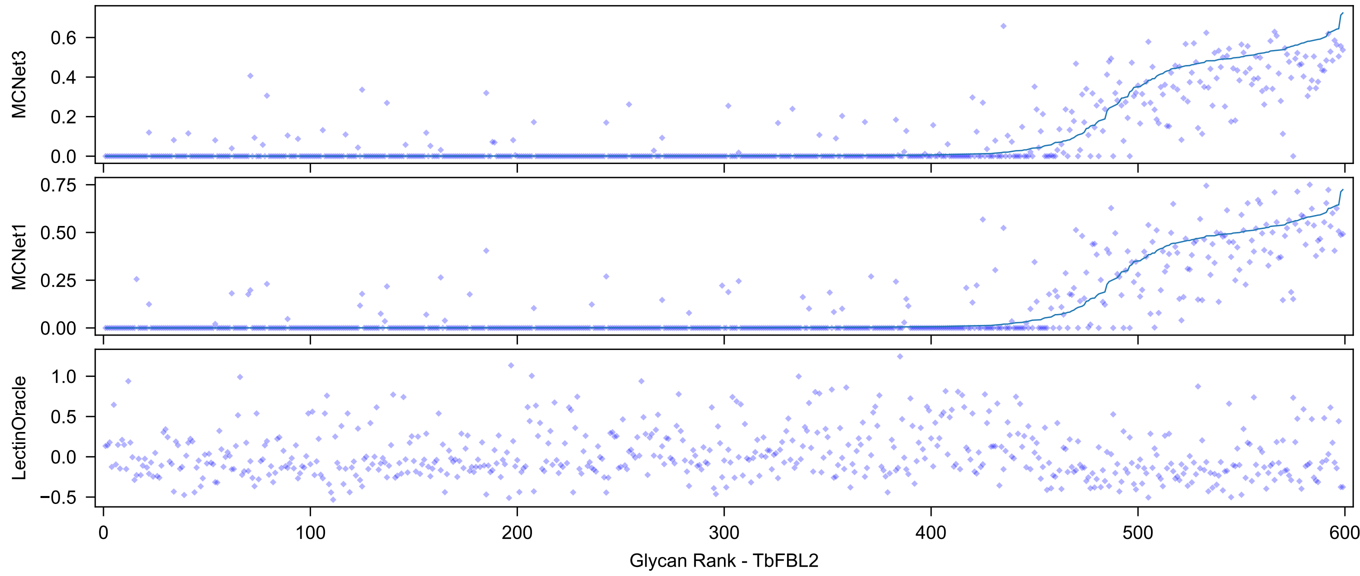
MCNet3
MCNet1
LectinOracle
Glycan Rank - TbFBL2
0.0
0.2
0.4
0.6
0.00
0.25
0.50
0.75
−0.5
0.0
0.5
1.0
0
100
200
300
400
500
600

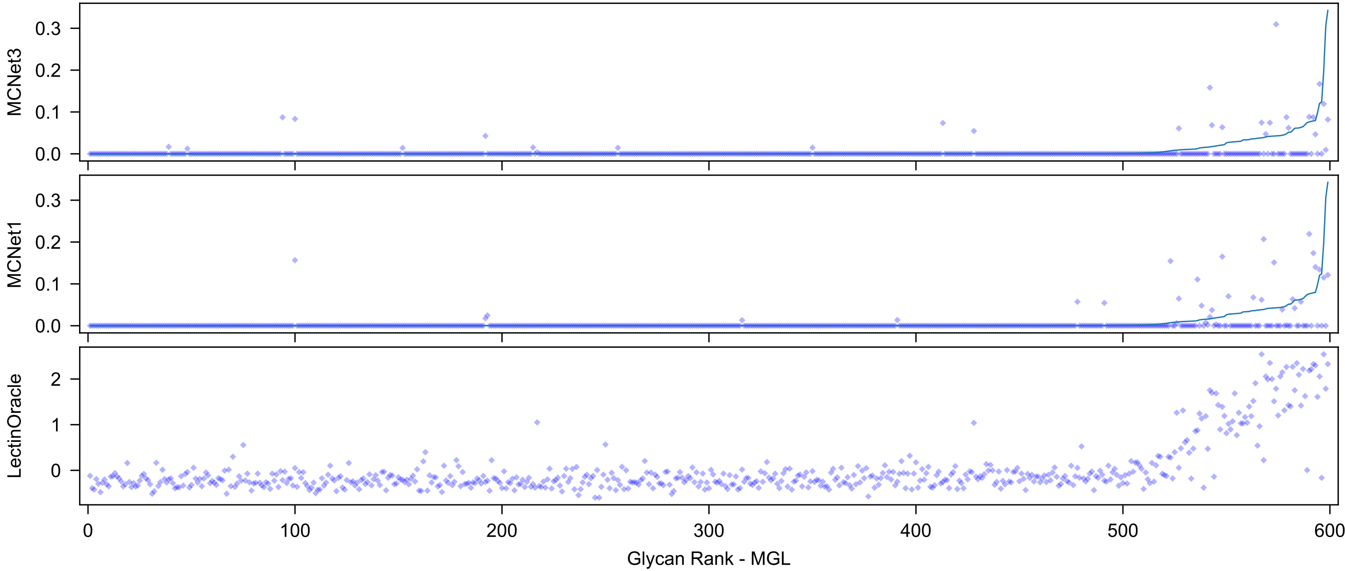

MCNet3
MCNet1
LectinOracle
0.0
0.1
0.2
0.3
0
1
2
0
100
200
300
400
500
600
Glycan Rank - MGL

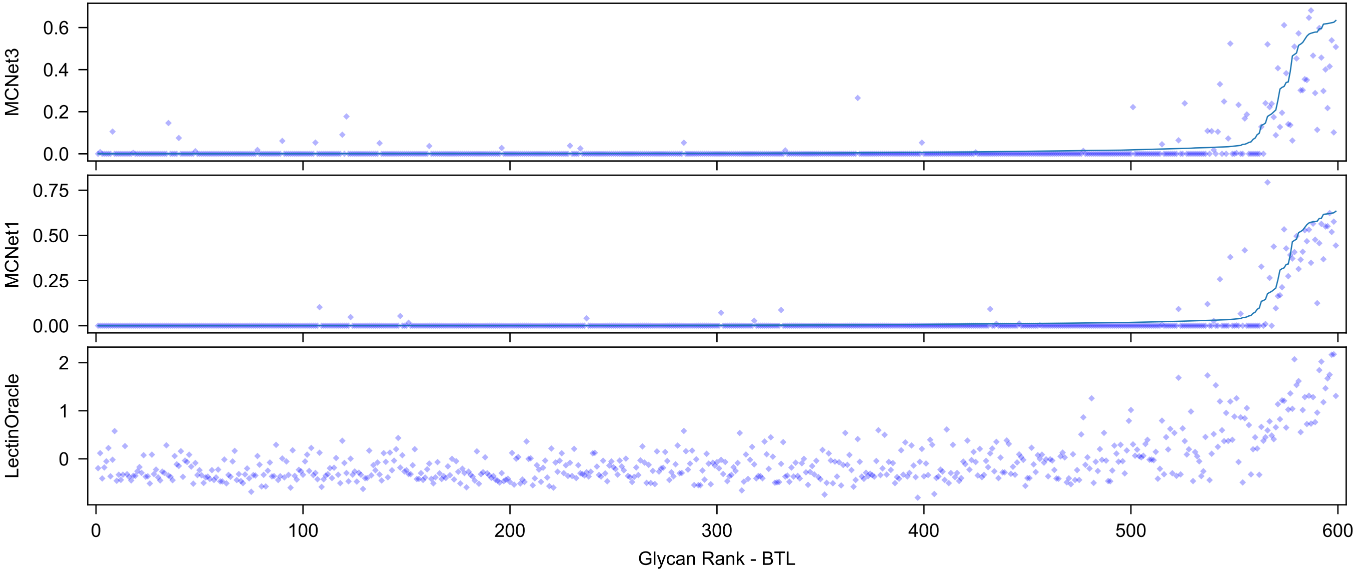
MCNet3
0.0
0.2
0.4
0.6
MCNet1
0.00
0.25
0.50
0.75
LectinOracle
0
1
2
0
100
200
300
400
500
600
Glycan Rank - BTL

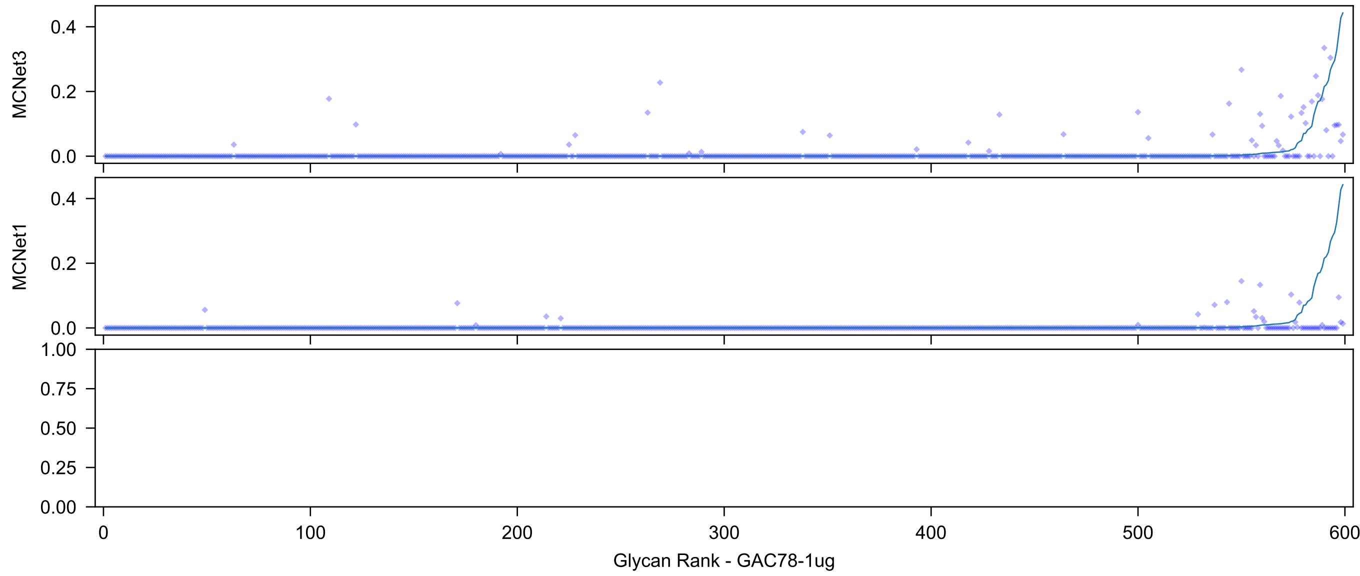
MCNet3
MCNet1
0.0
0.2
0.4
0.00
0.25
0.50
0.75
1.00
0
100
200
300
400
500
600
Glycan Rank - GAC78-1ug

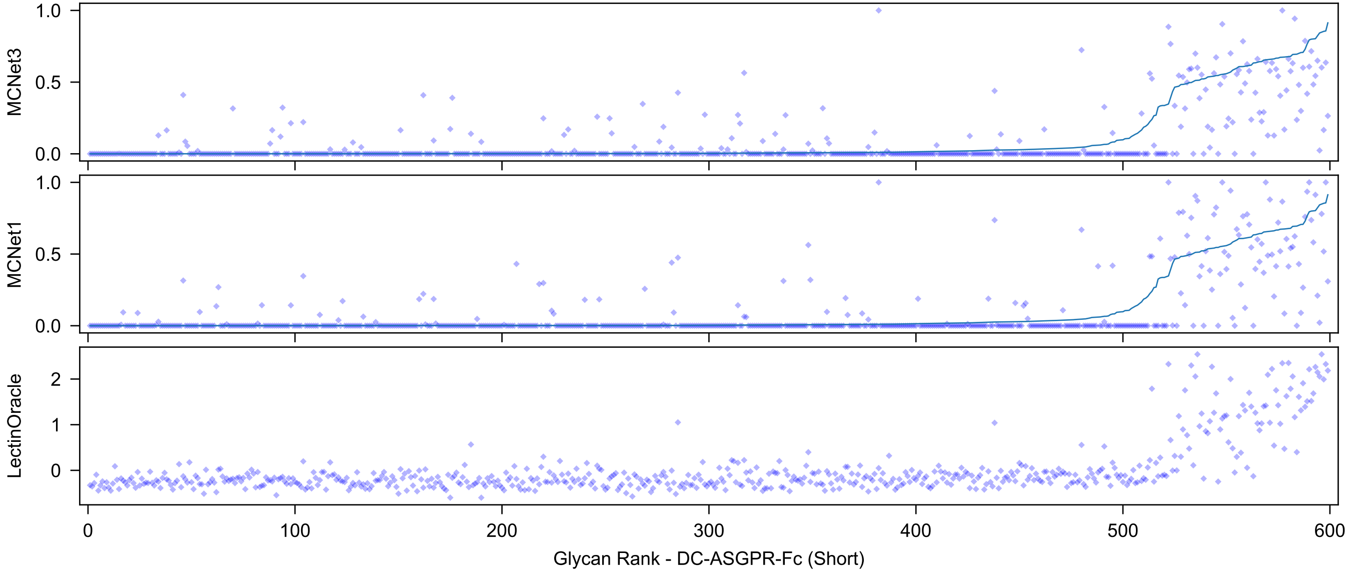
MCNet3
1.0
0.5
0.0
MCNet1
1.0
0.5
0.0
LectinOracle
2
1
0
0
100
200
300
400
500
600
Glycan Rank - DC-ASGPR-Fc (Short)

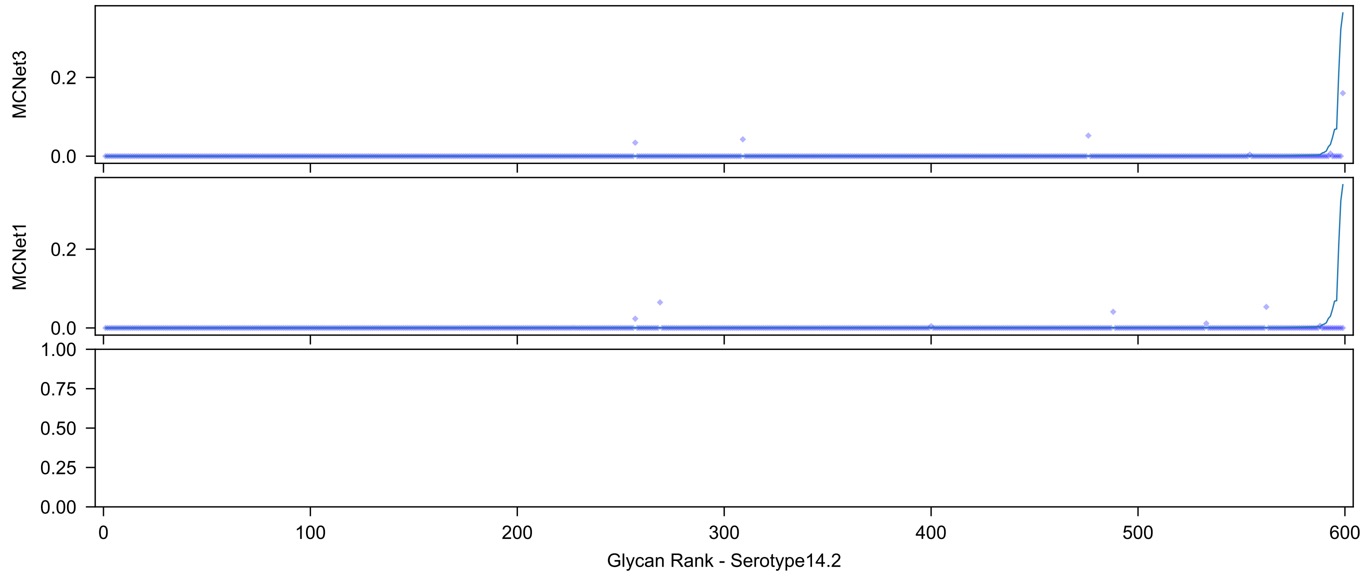
MCNet3
0.2
0.0
MCNet1
0.2
0.0
1.00
0.75
0.50
0.25
0.00
0
100
200
300
400
500
600
Glycan Rank - Serotype14.2

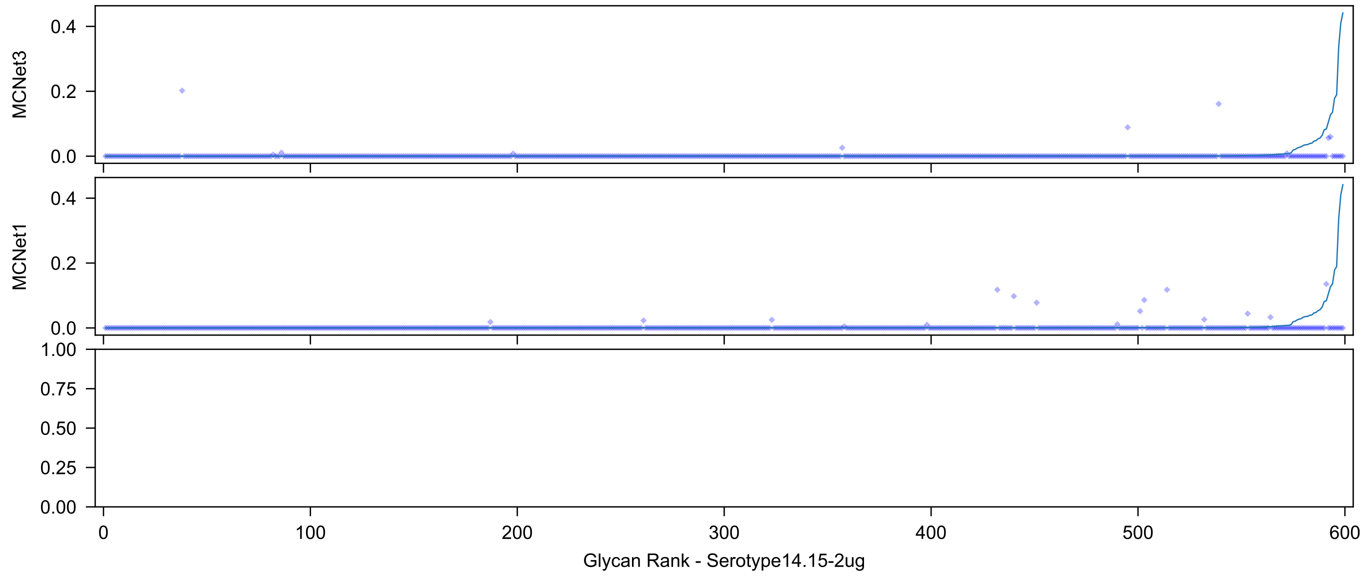

MCNet3
MCNet1
0.4
0.2
0.0
1.00
0.75
0.50
0.25
0.00
0
100
200
300
400
500
600
Glycan Rank - Serotype14.15-2ug

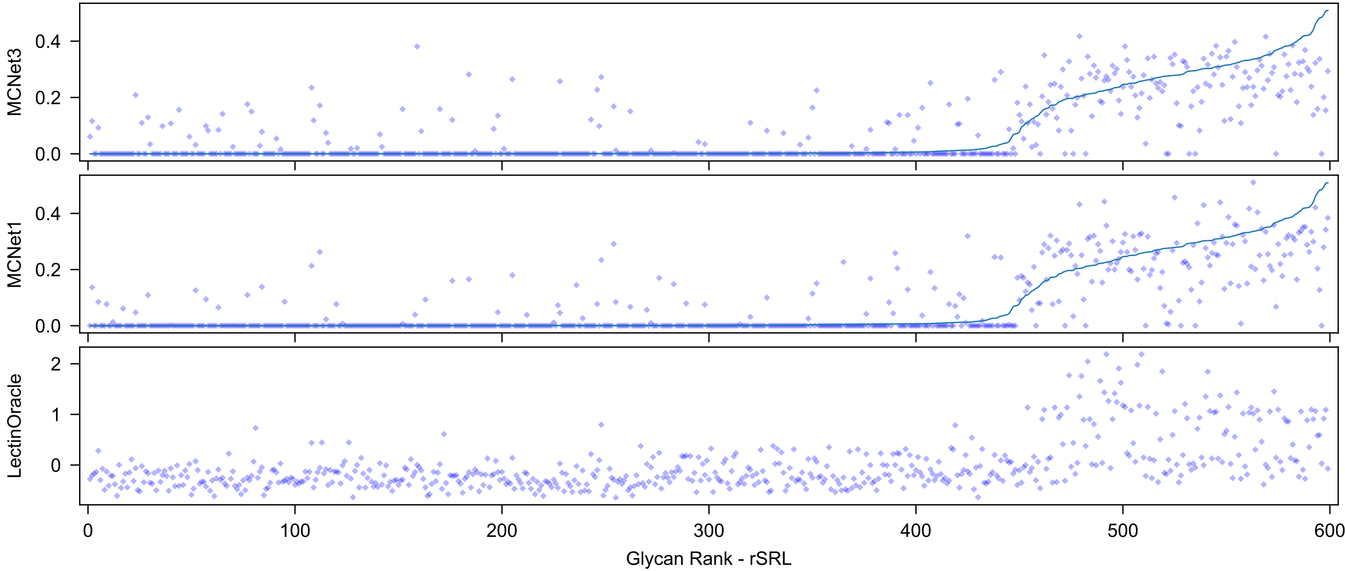

MCNet3
MCNet1
LectinOracle
0.0
0.2
0.4
0
1
2
0
100
200
300
400
500
600
Glycan Rank - rSRL

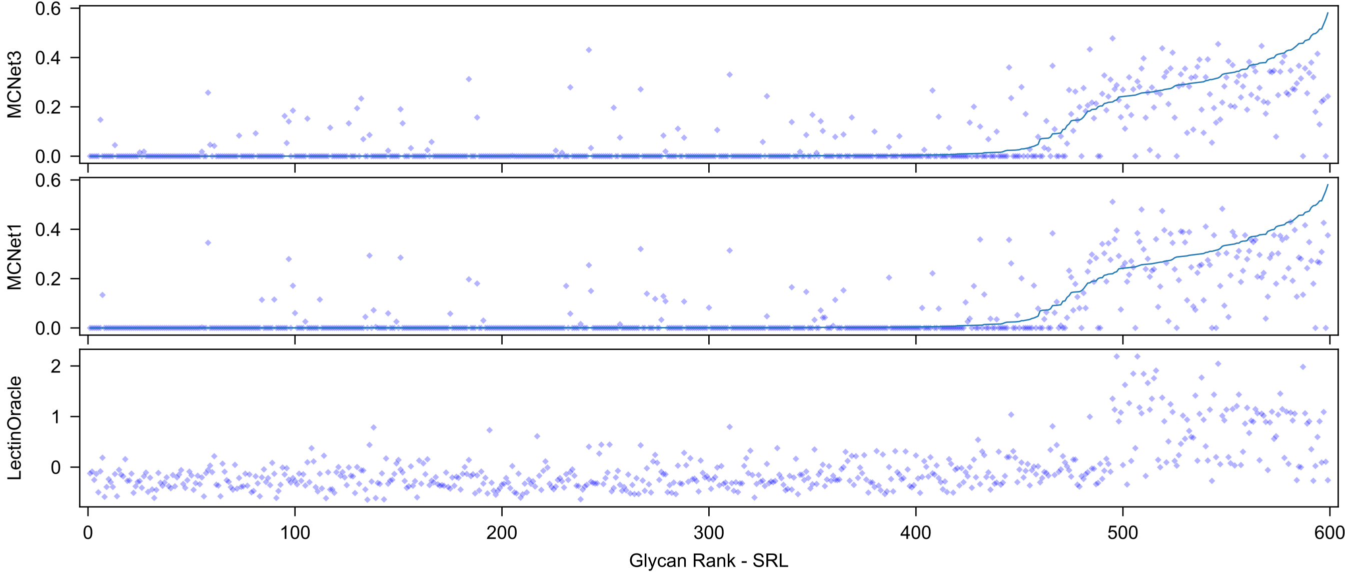

0.6
0.4
0.2
0.0
MCNet3
0.6
0.4
0.2
0.0
MCNet1
2
1
0
LectinOracle
0
100
200
300
400
500
600
Glycan Rank - SRL

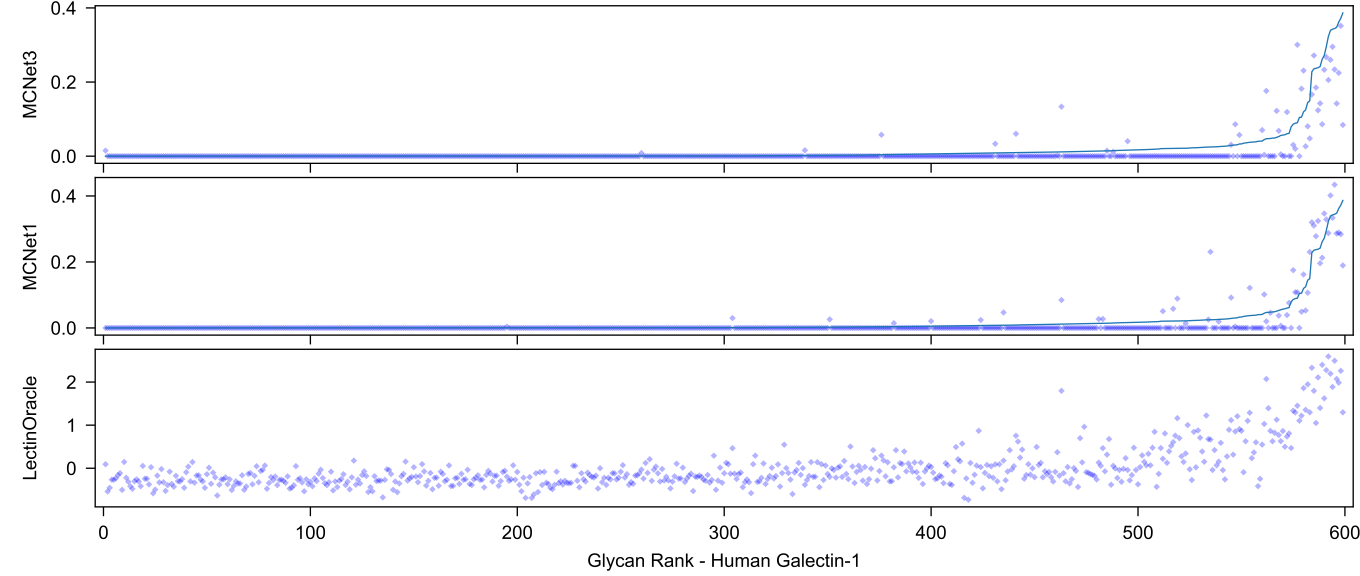

MCNet3
0.4
0.2
0.0
MCNet1
0.4
0.2
0.0
LectinOracle
2
1
0
0
100
200
300
400
500
600
Glycan Rank - Human Galectin-1

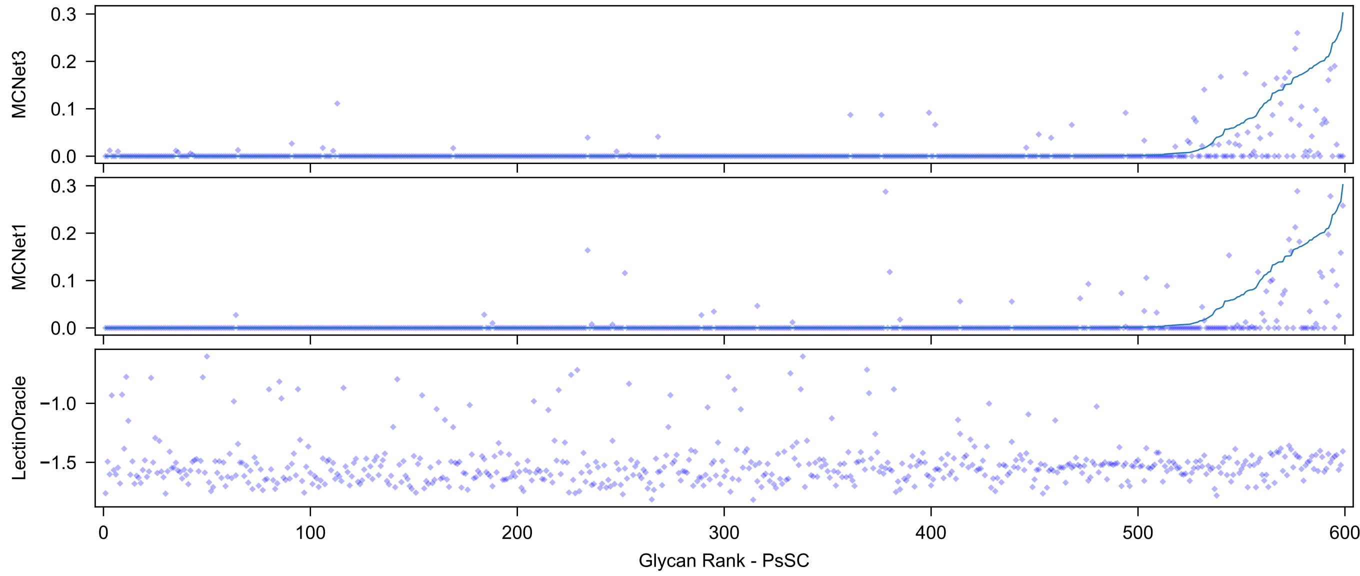

MCNet3
MCNet1
LectinOracle
0.3
0.2
0.1
0.0
−1.0
−1.5
0
100
200
300
400
500
600
Glycan Rank - PsSC

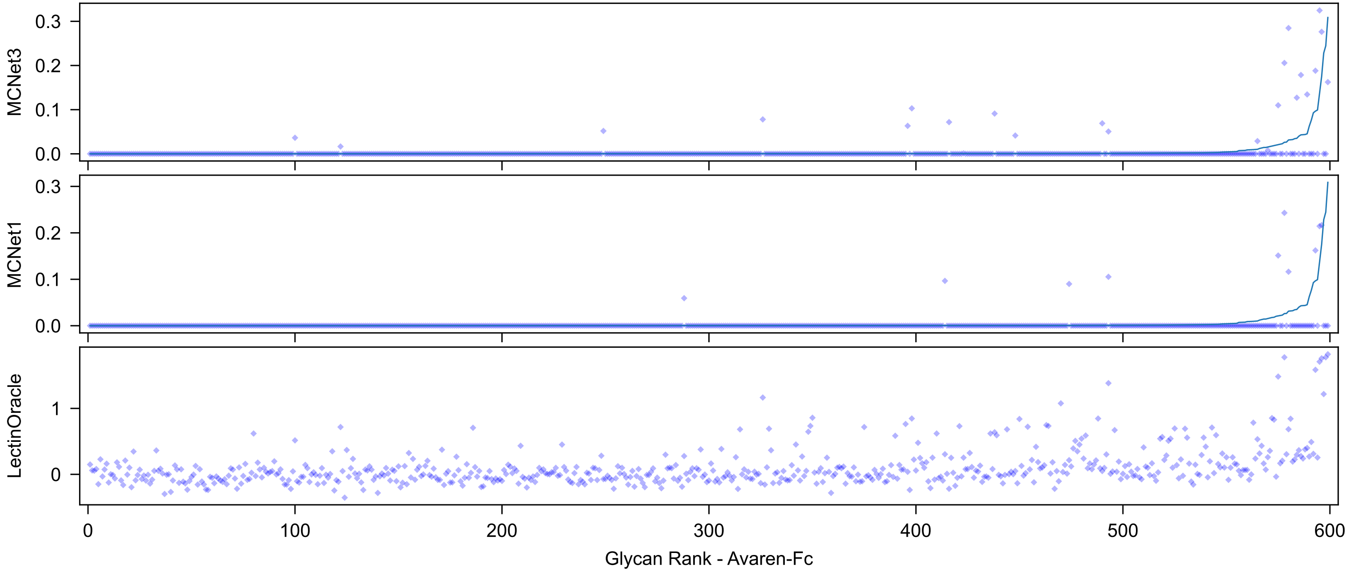

MCNet3
MCNet1
LectinOracle
0.0
0.1
0.2
0.3
0
1
0
100
200
300
400
500
600
Glycan Rank - Avaren-Fc

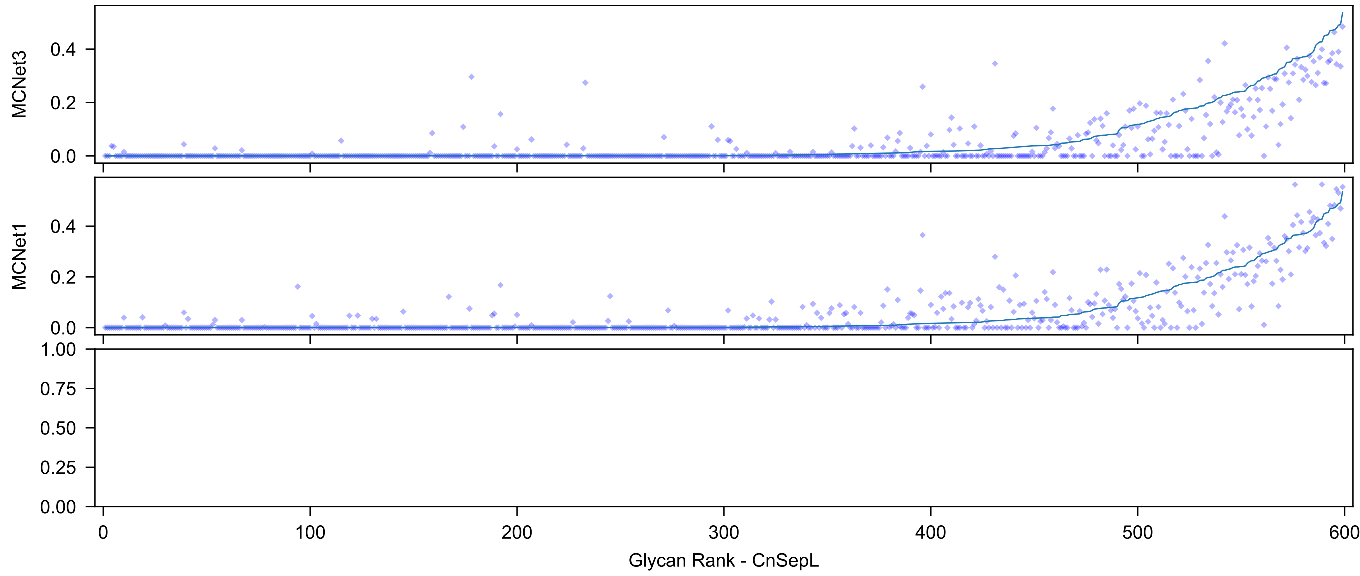

MCNet3
MCNet1
0.0
0.2
0.4
0.00
0.25
0.50
0.75
1.00
0
100
200
300
400
500
600
Glycan Rank - CnSepL

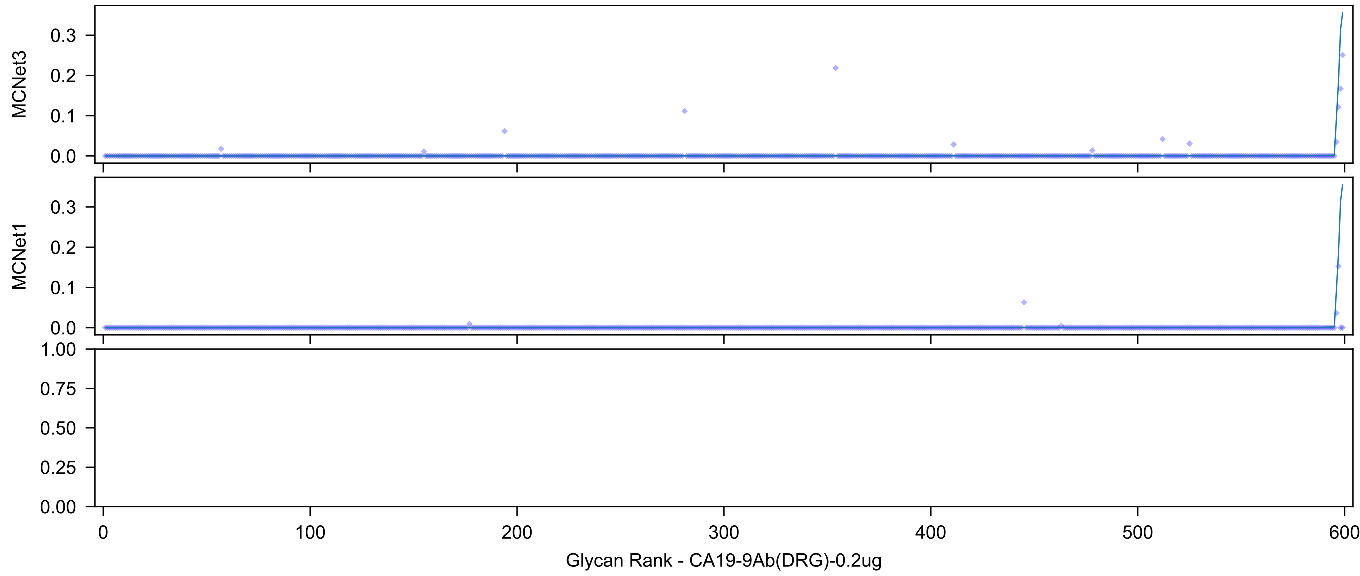

MCNet3
MCNet1
0.0
0.1
0.2
0.3
0.00
0.25
0.50
0.75
1.00
0
100
200
300
400
500
600
Glycan Rank - CA19-9Ab(DRG)-0.2ug

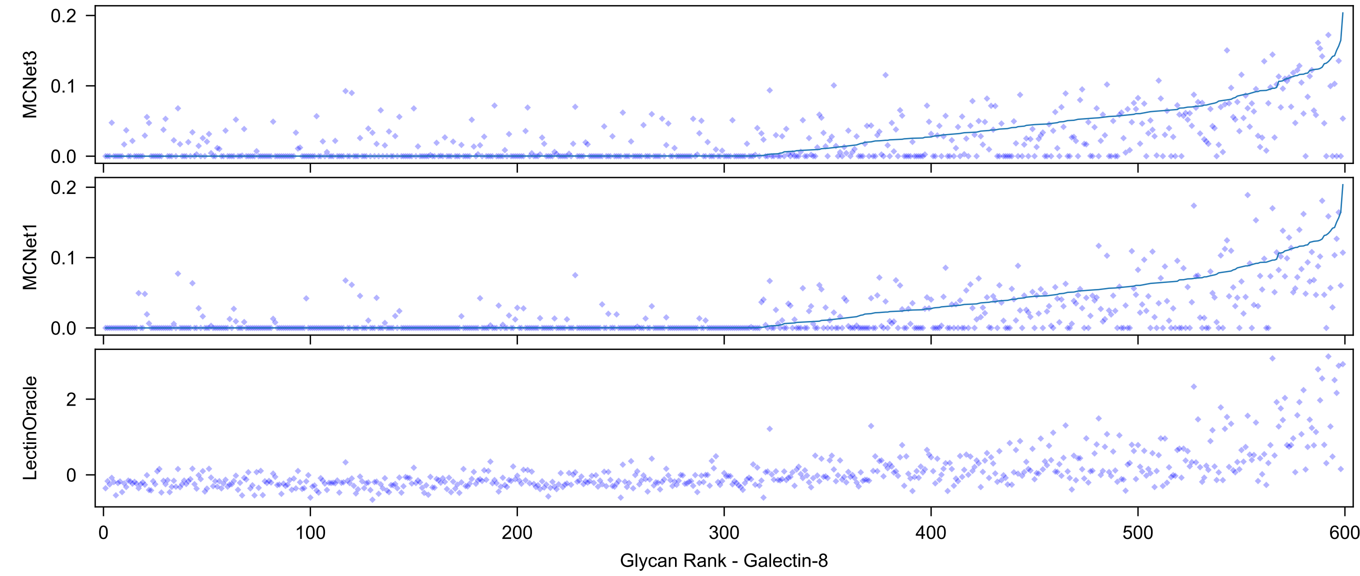

MCNet3
MCNet1
LectinOracle
0.2
0.1
0.0
2
0
0
100
200
300
400
500
600
Glycan Rank - Galectin-8

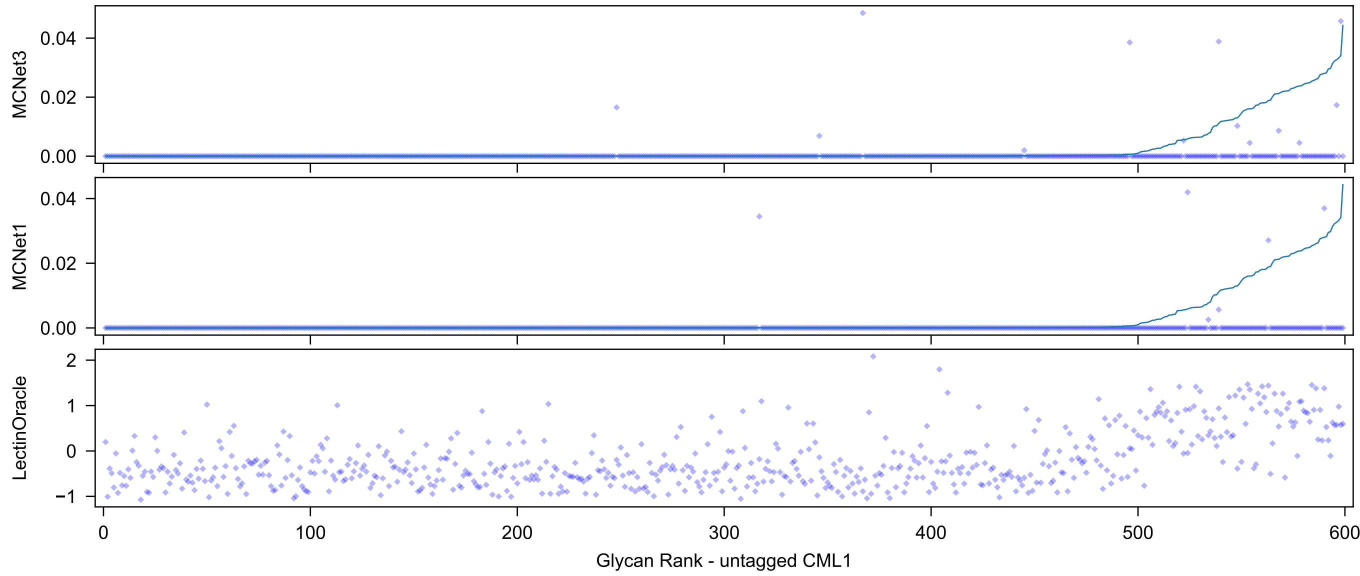

MCNet3
0.04
0.02
0.00
MCNet1
0.04
0.02
0.00
LectinOracle
2
1
0
−1
0
100
200
300
400
500
600
Glycan Rank - untagged CML1

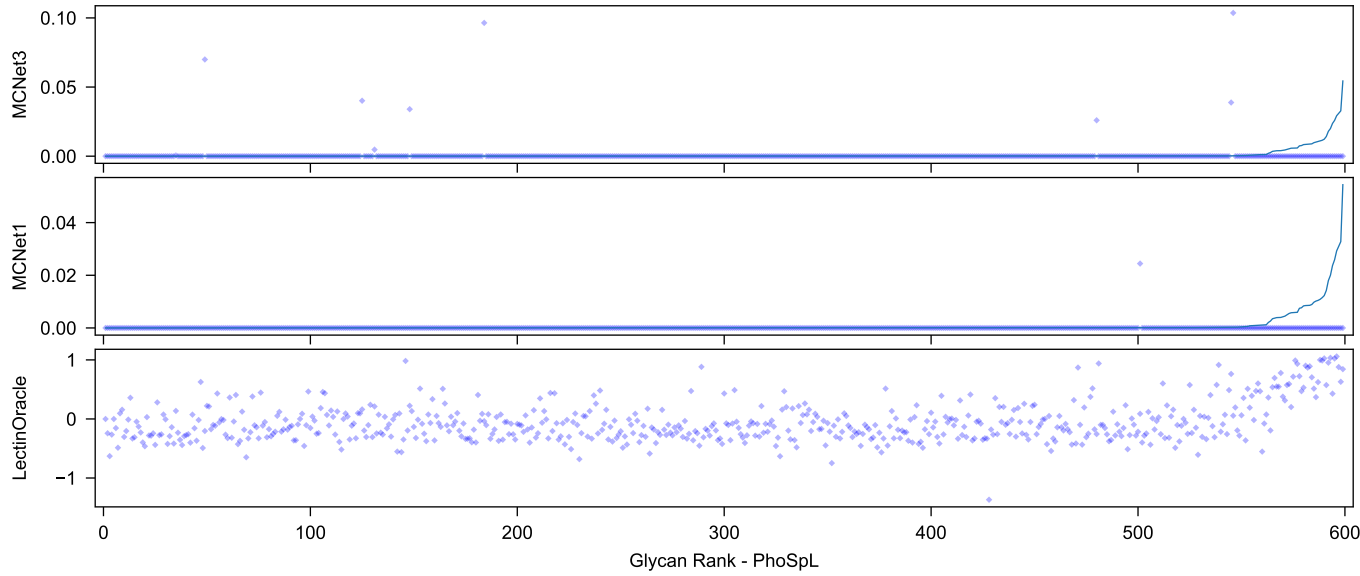
MCNet3
0.10
0.05
0.00
MCNet1
0.04
0.02
0.00
LectinOracle
1
0
−1
0
100
200
300
400
500
600
Glycan Rank - PhoSpL

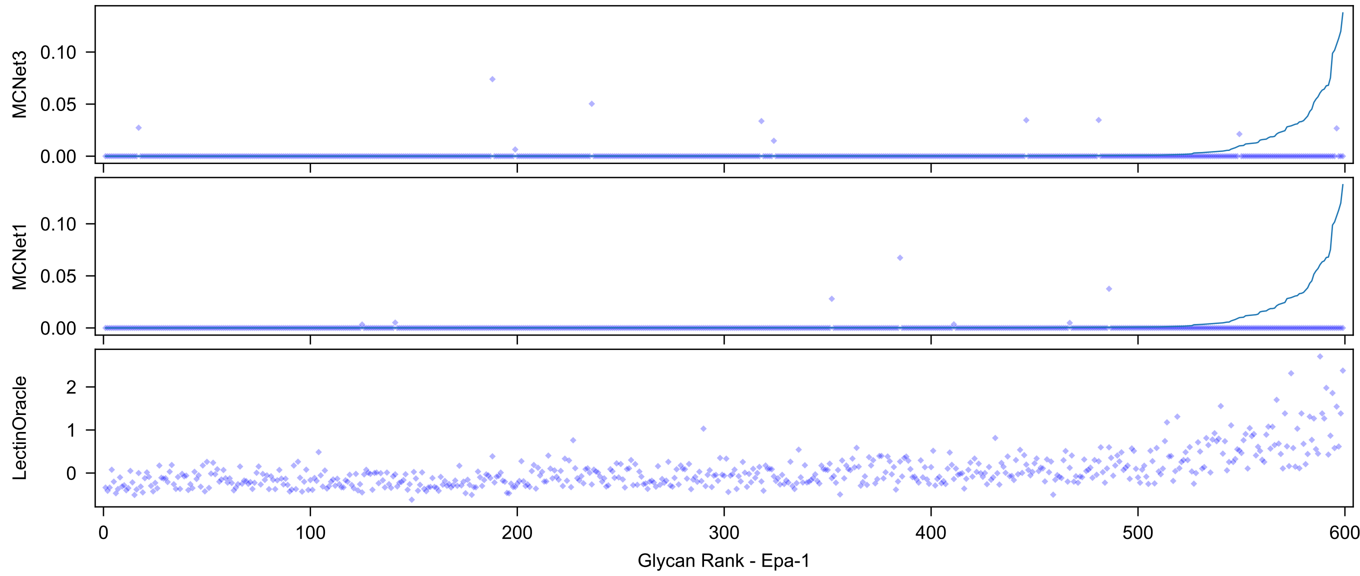

MCNet3
0.10
0.05
0.00
MCNet1
0.10
0.05
0.00
LectinOracle
2
1
0
0
100
200
300
400
500
600
Glycan Rank - Epa-1

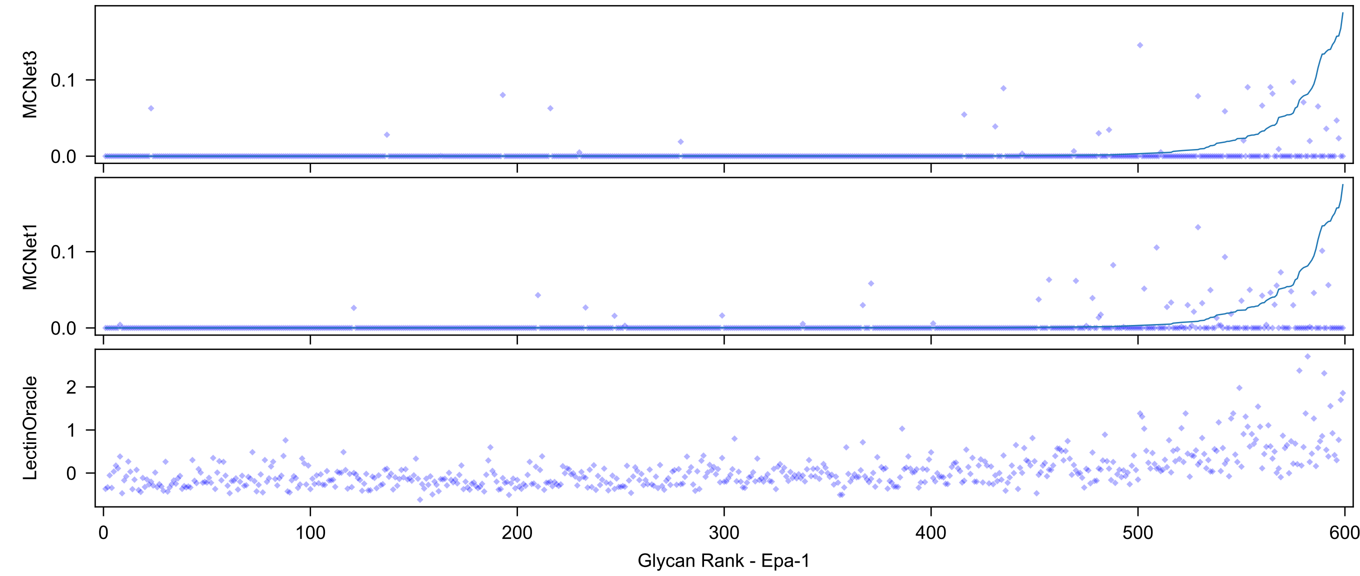
MCNet3
0.1
0.0
MCNet1
0.1
0.0
LectinOracle
2
1
0
0
100
200
300
400
500
600
Glycan Rank - Epa-1

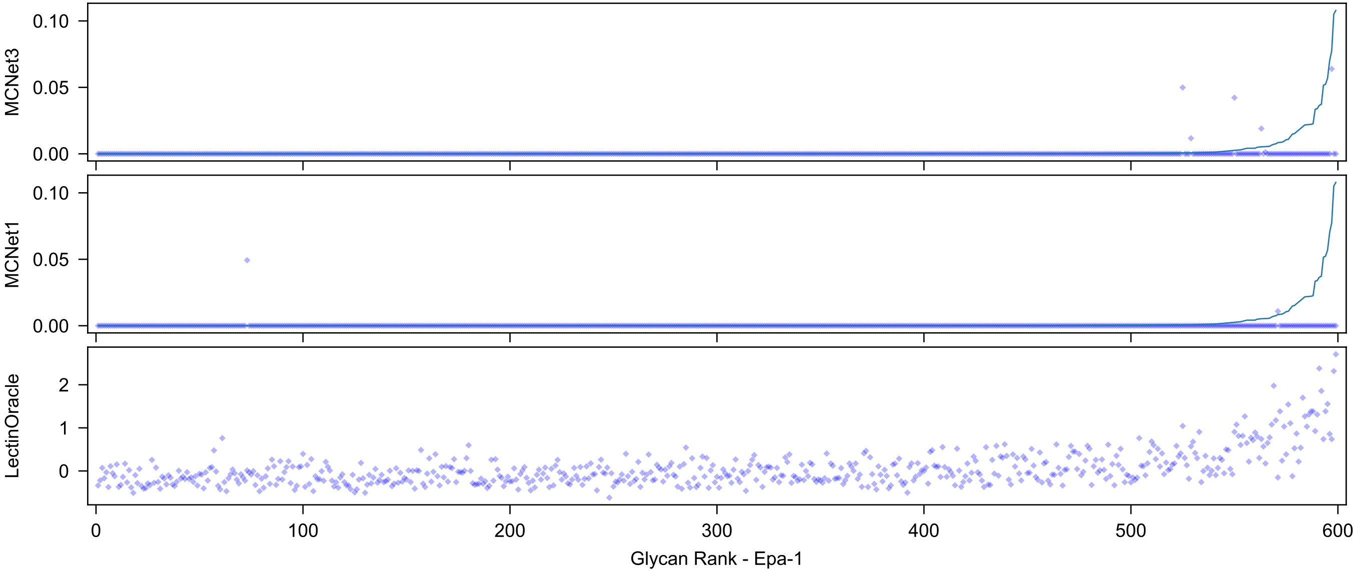

MCNet3
MCNet1
LectinOracle
0.10
0.05
0.00
2
1
0
0
100
200
300
400
500
600
Glycan Rank - Epa-1

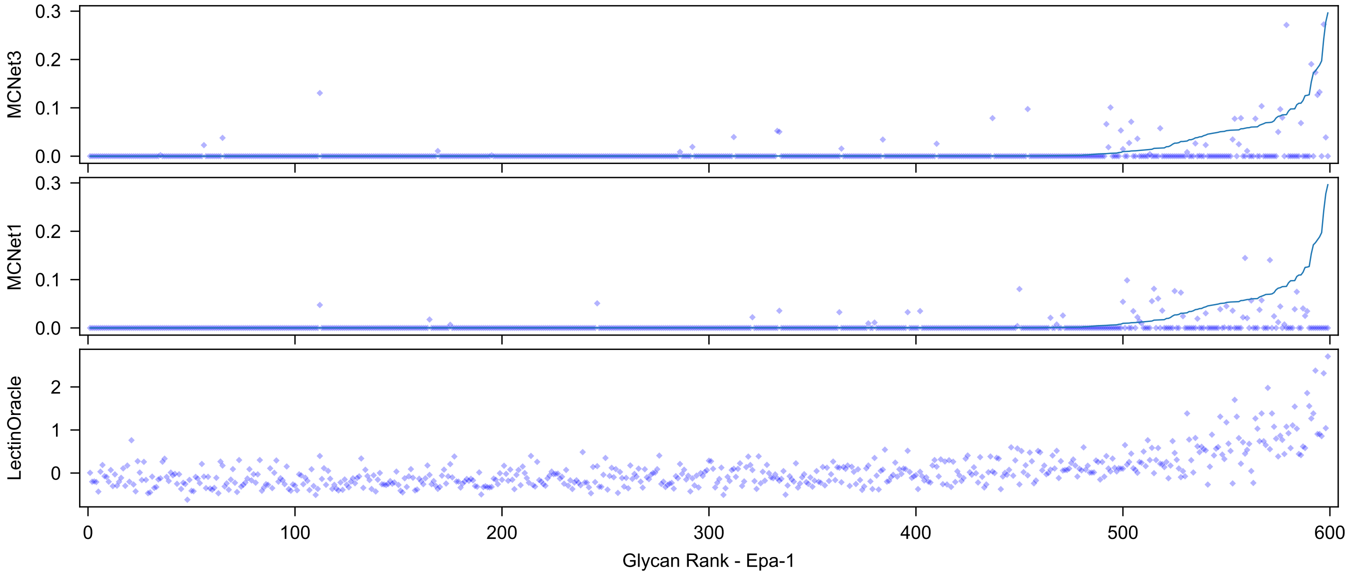
MCNet3
MCNet1
LectinOracle
0.0
0.1
0.2
0.3
0
1
2
0
100
200
300
400
500
600
Glycan Rank - Epa-1

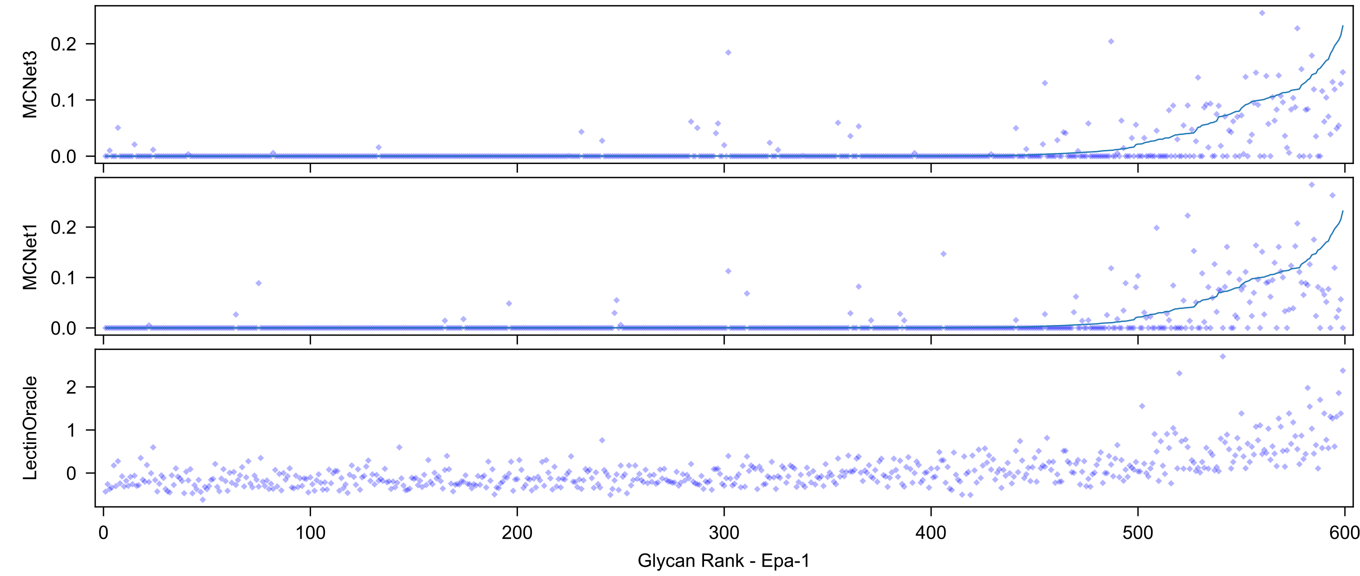

MCNet3
MCNet1
LectinOracle
0.0
0.1
0.2
0
1
2
0
100
200
300
400
500
600
Glycan Rank - Epa-1

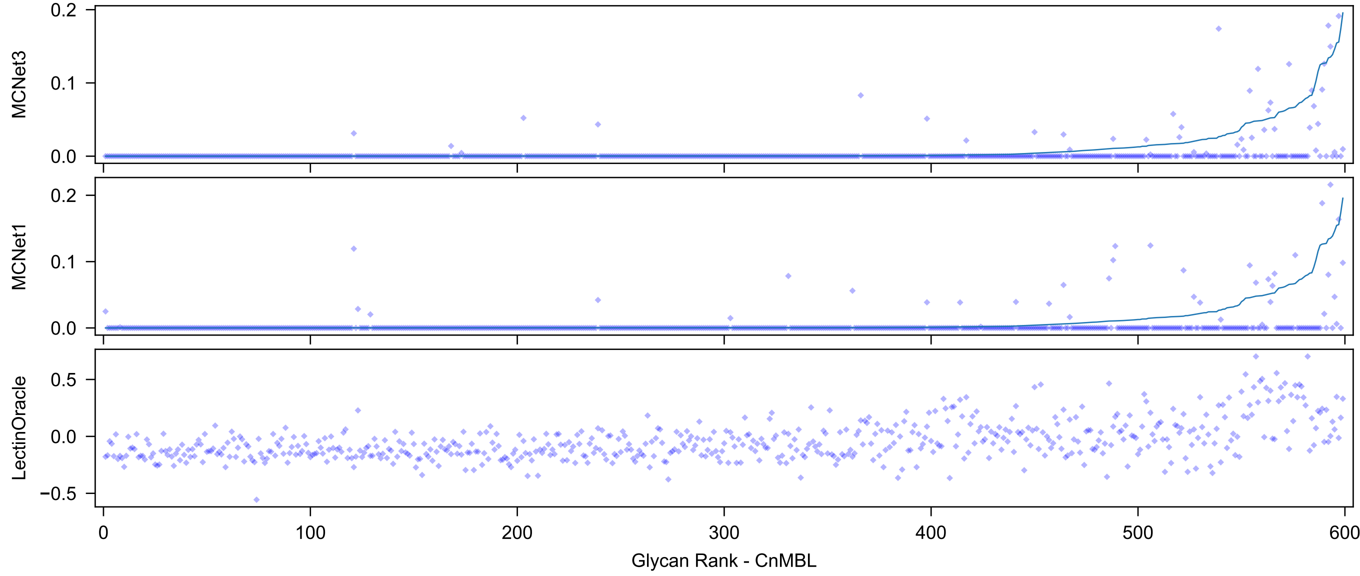

MCNet3
0.2
0.1
0.0
MCNet1
0.2
0.1
0.0
LectinOracle
0.5
0.0
−0.5
0
100
200
300
400
500
600
Glycan Rank - CnMBL

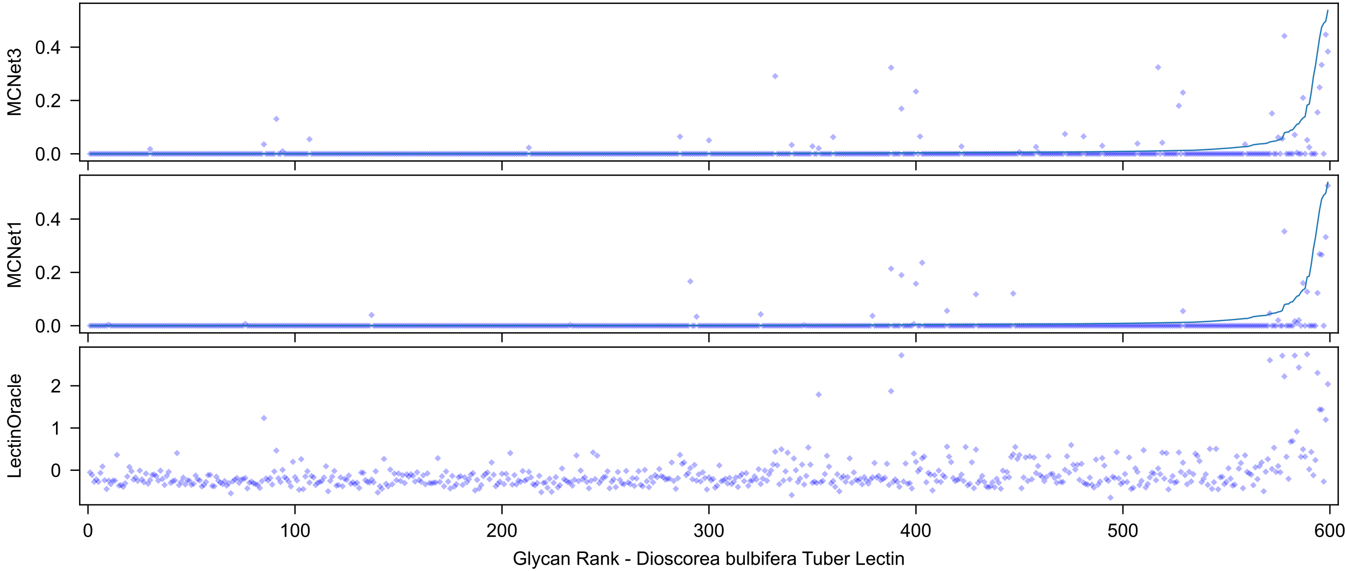

MCNet3
MCNet1
LectinOracle
0.0
0.2
0.4
0.0
0.2
0.4
0
1
2
0
100
200
300
400
500
600
Glycan Rank - Dioscorea bulbifera Tuber Lectin

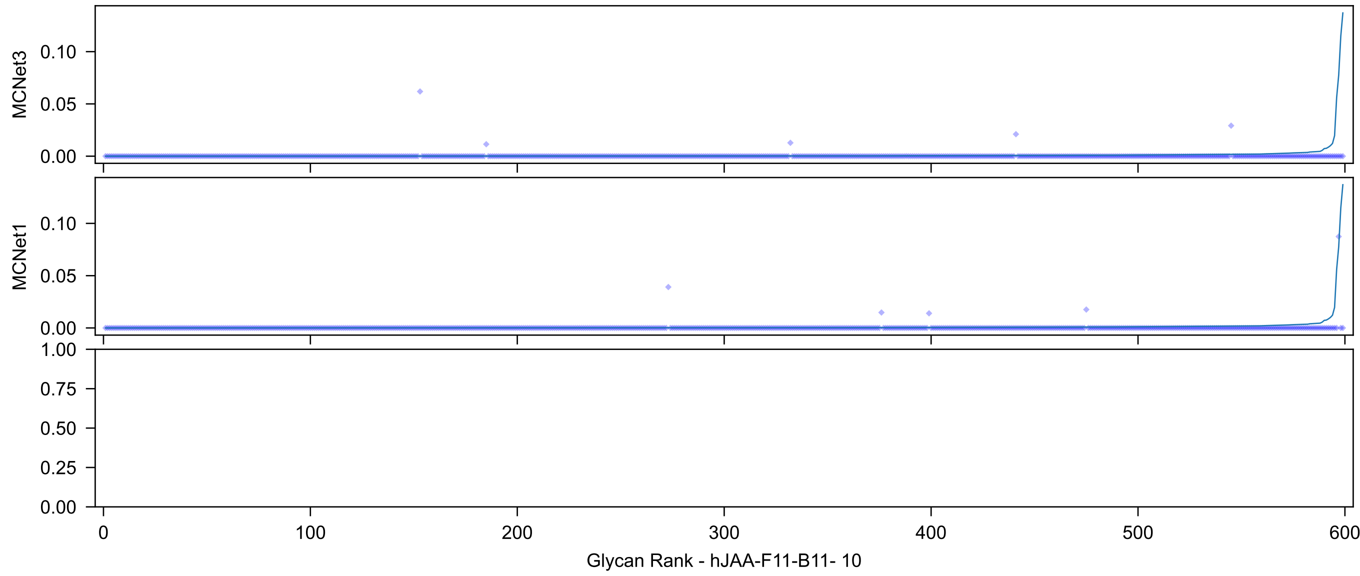

MCNet3
MCNet1
0.10
0.05
0.00
1.00
0.75
0.50
0.25
0
100
200
300
400
500
600
Glycan Rank - hJAA-F11-B11- 10

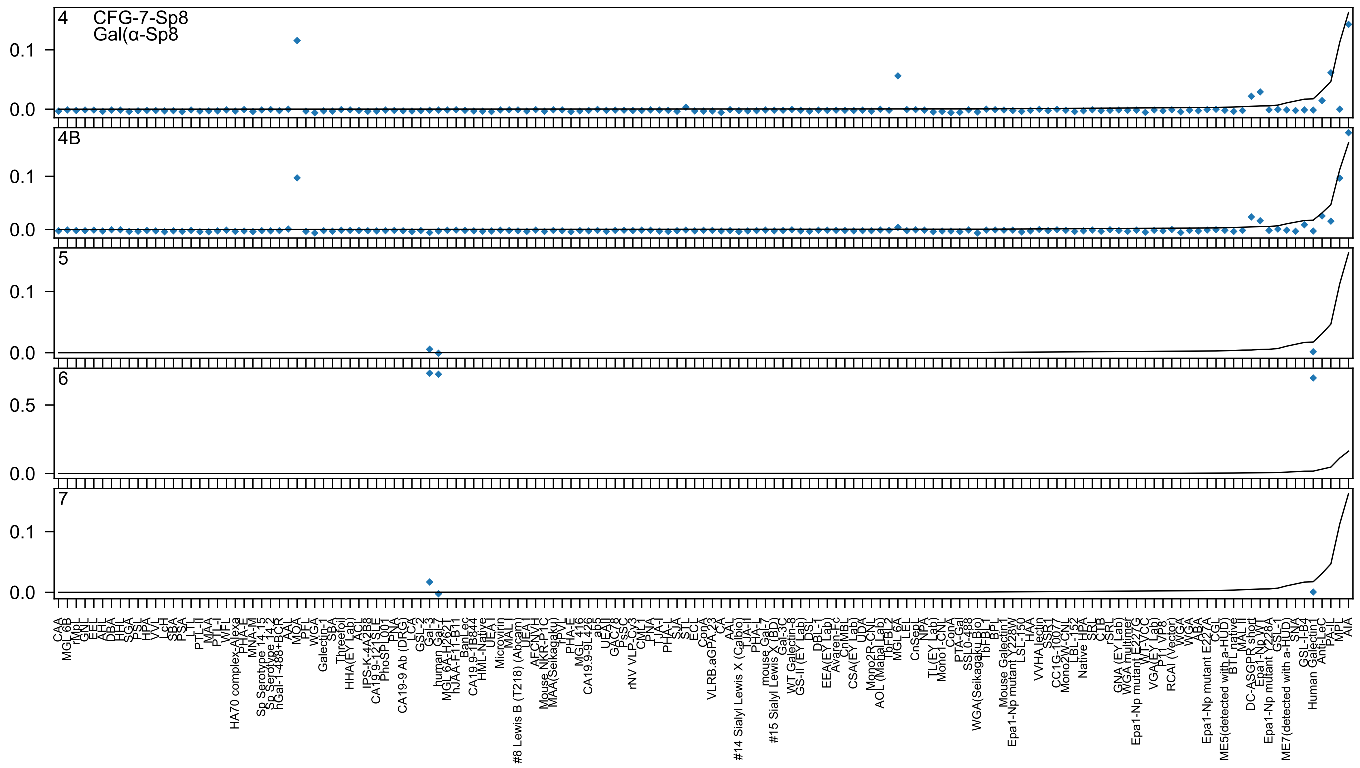
4
CFG-7-Sp8
Gal(α-Sp8
4B
5
6
7

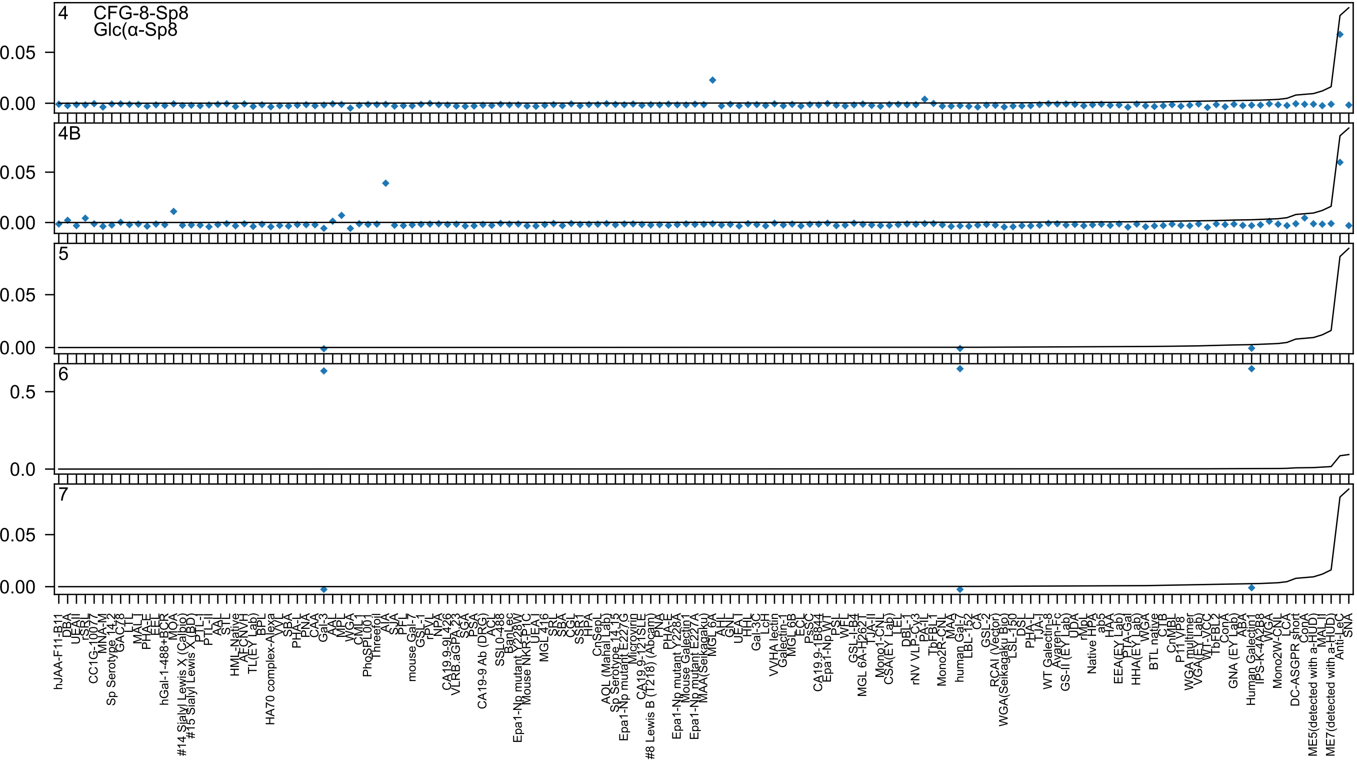

4
CFG-8-Sp8
Glc(α-Sp8
4B
5
6
7

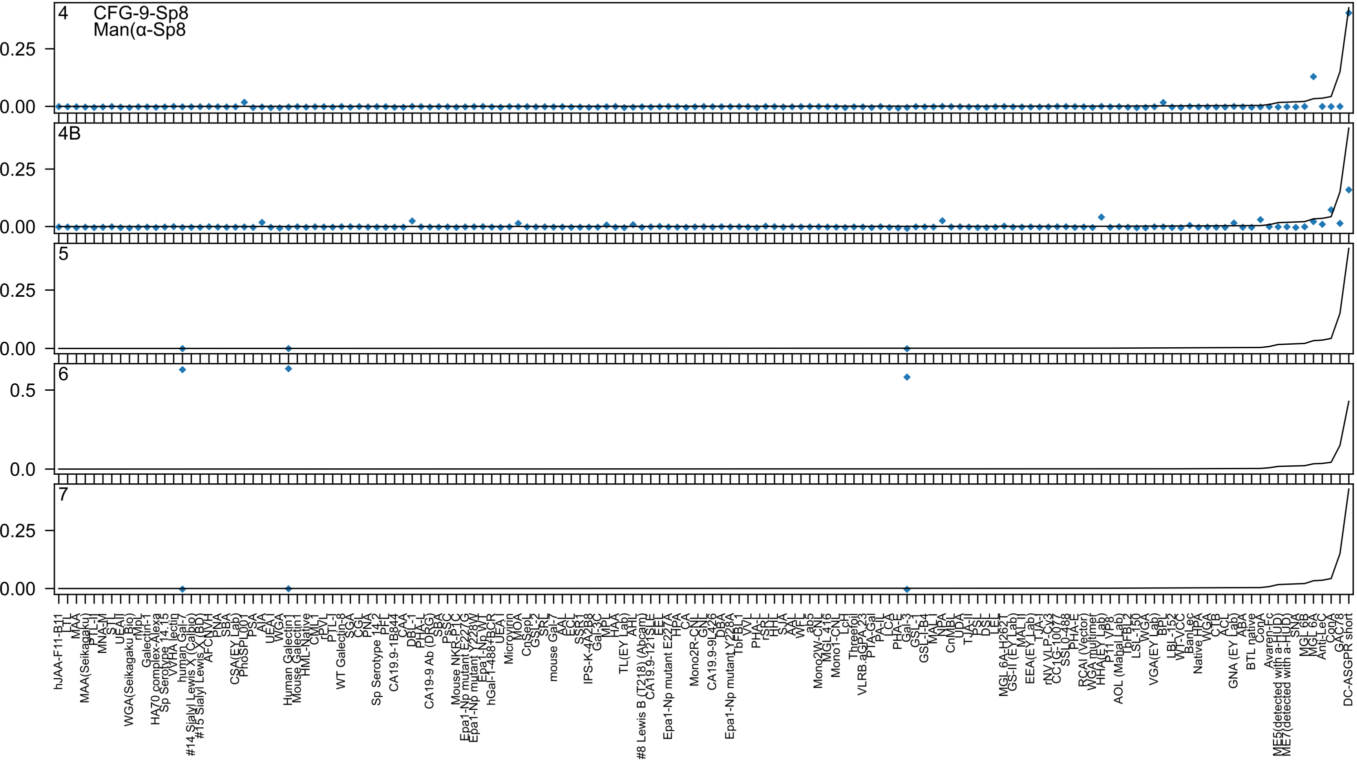

4 CFG-9-Sp8
Man(α-Sp8
4B
5
6
7
0.25
0.00
0.5
0.0

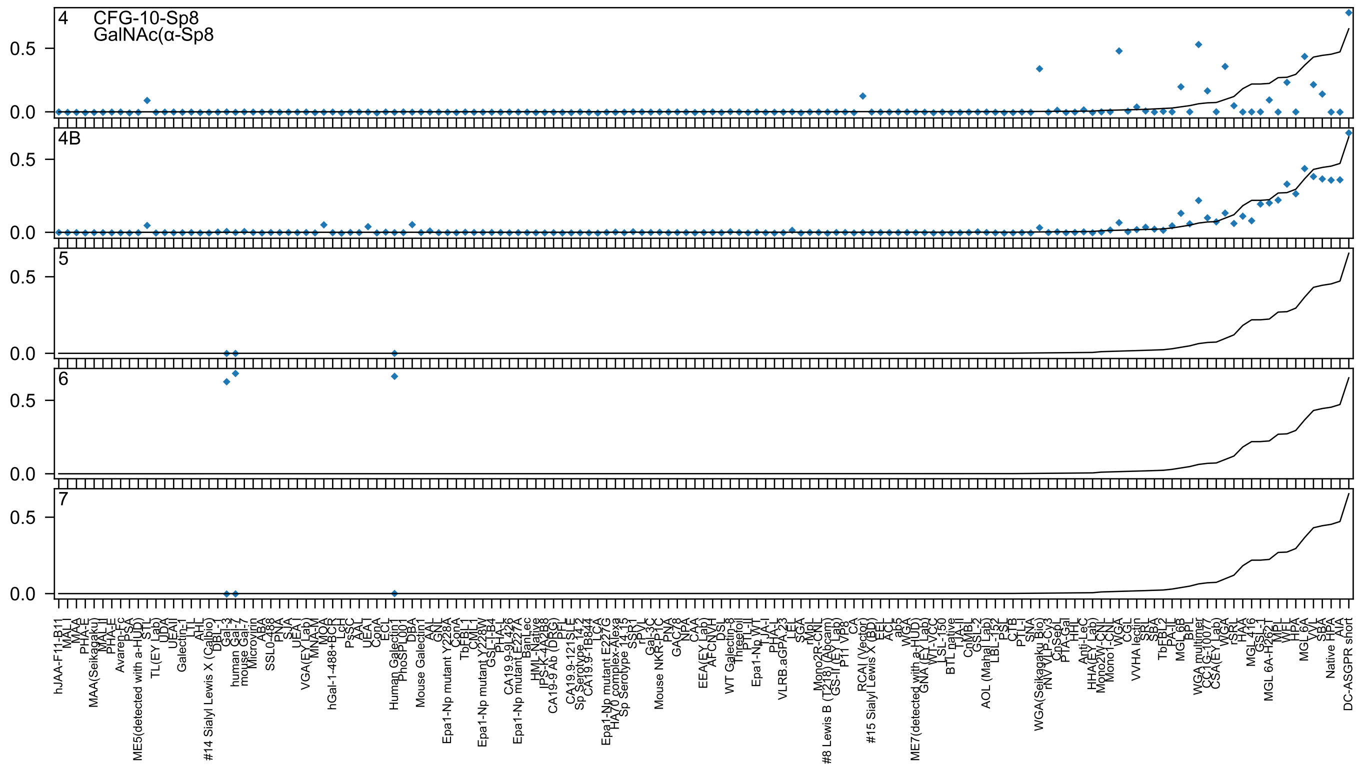

4
CFG-10-Sp8
GalNAc(α-Sp8
4B
5
6
7
0.5
0.0

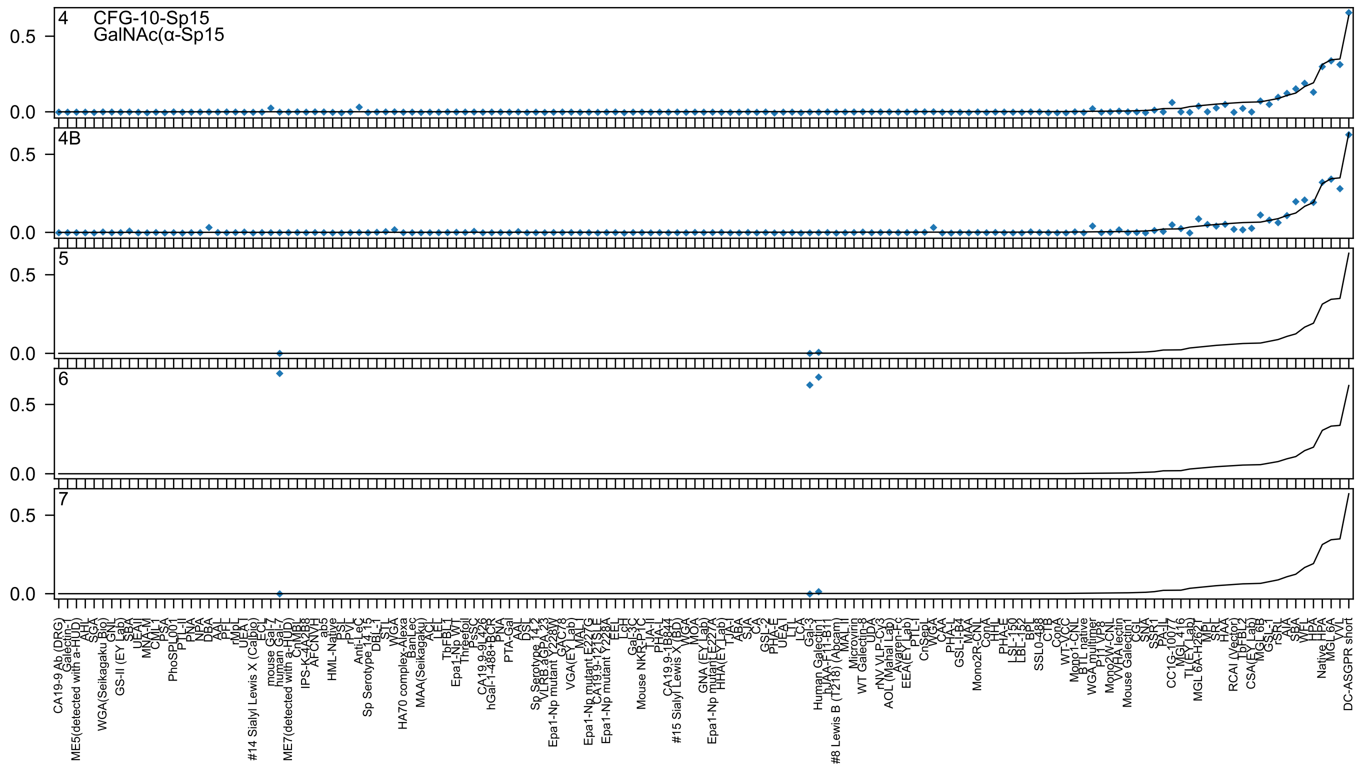
4 CFG-10-Sp15
GalNAc(α-Sp15
4B
5
6
7

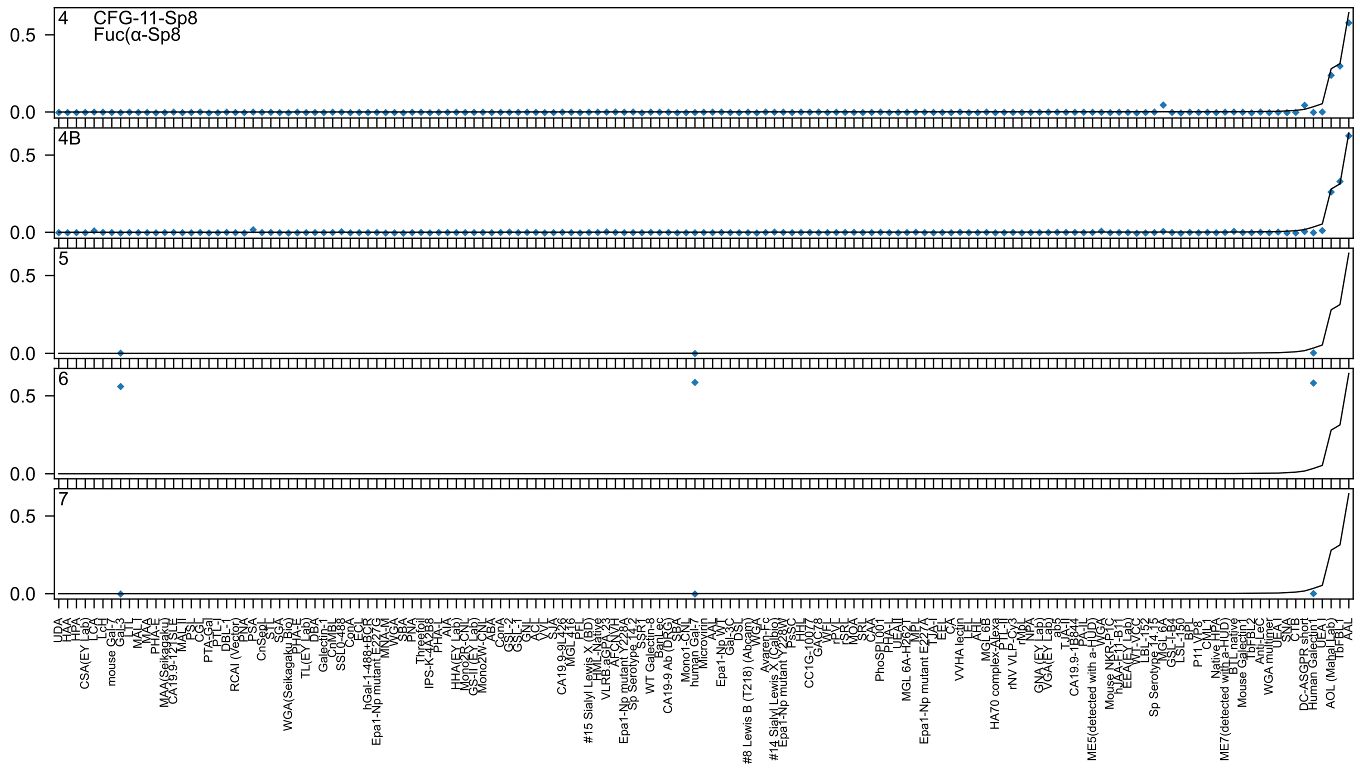
4
CFG-11-Sp8
Fuc(α-Sp8
4B
5
6
7

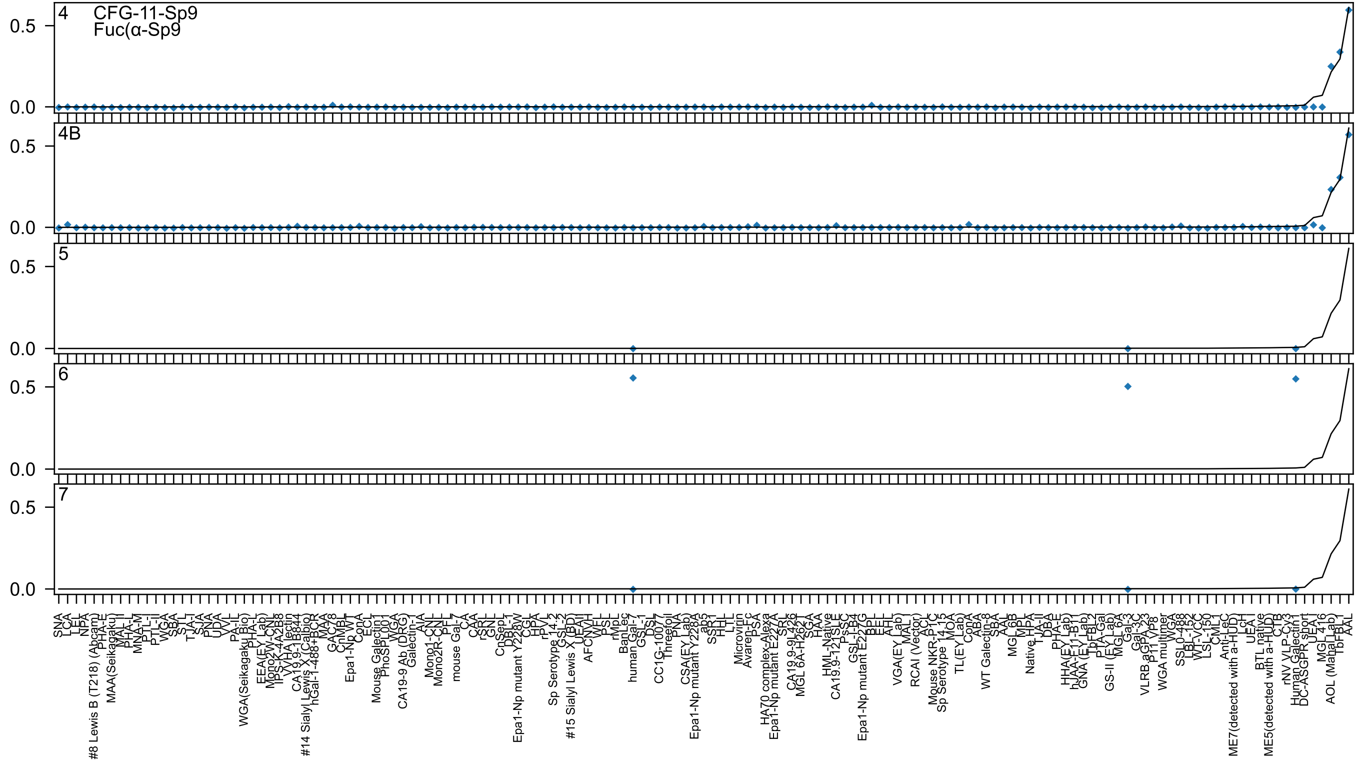
4
CFG-11-Sp9
Fuc(α-Sp9
4B
5
6
7
0.5
0.0

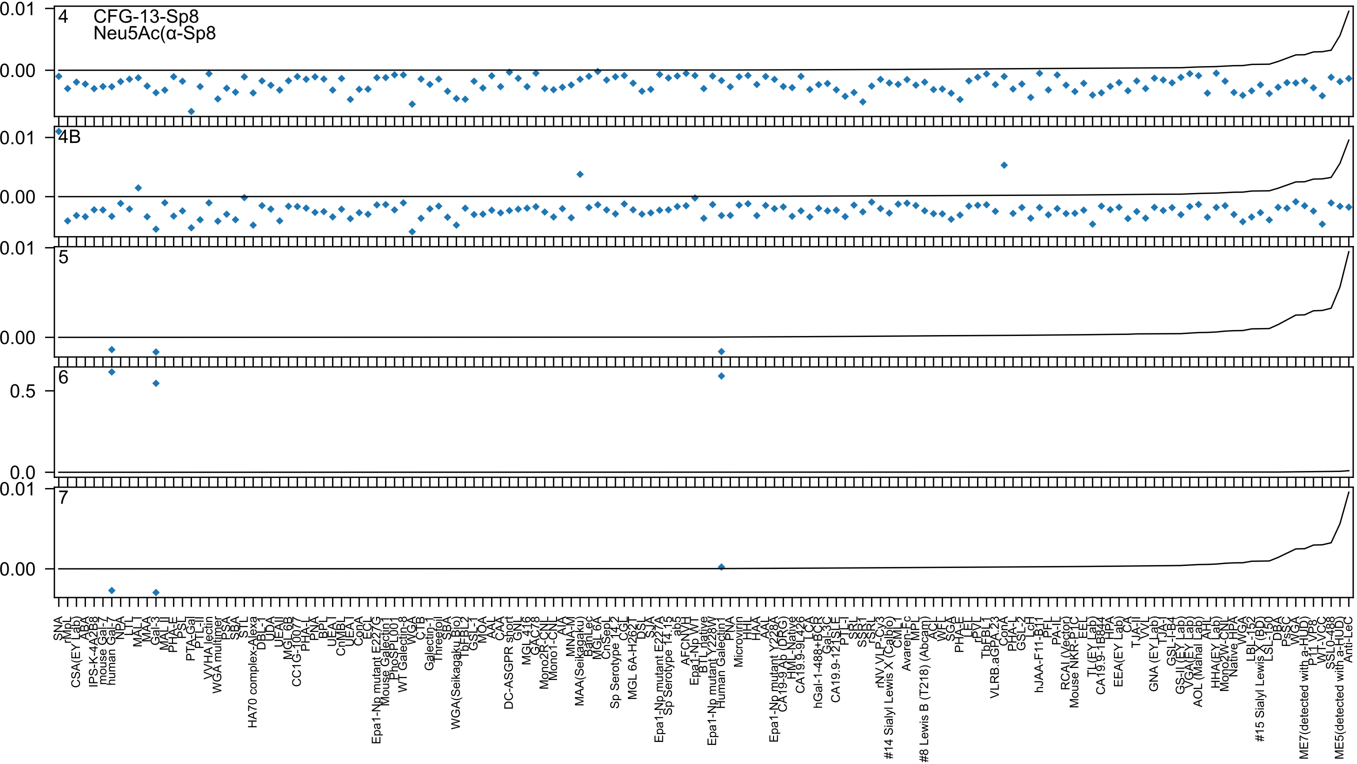
4 CFG-13-Sp8
Neu5Ac(α-Sp8
0.01
0.00
4B
0.01
0.00
5
0.01
0.00
6
0.5
0.0
7
0.01
0.00
SNA
rMpL
CSA(EY Lab)
ABA
IPS-K-4A2B8
mouse Gal-7
human Gal-7
NPA
LTL
MAL-I
MAA
Gal-3
MAL_II
PHA-E
PSL
PTA-Gal
PTL-II
VVHA lectin
WGA multimer
PSA
SBA
STL
HA70 complex-Alexa
DBL-1
UDA
UEA-II
MGL 6B
CC1G-10077
PHA-L
PNA
BPL
UEA-I
CnMBL
UEA-I
ConA
ECL
Epa1-Np mutant E227G
Mouse Galectin1
PhoSL001
WT Galectin-8
WGA
CTB
Galectin-1
Threefoil
SBA
WGA(Seikagaku Bio)
TbFBL2
GSL-I
MOA
AAL
CAA
DC-ASGPR short
GNL
MGL 416
GAC78
Mono2R-CNL
Mono1-CNL
AIA
MNA-M
MAA(Seikagaku)
BanLec
MGL 6A
CnSep1
Sp Serotype 14.2
CGL
MGL 6A-H262T
DSL
SJA
Epa1-Np mutant E227A
Sp Serotype 14.15
ab5
AFCNVH
Epa1-Np WT
BTL native
Epa1-Np mutant Y228W
Human Galectin1
PNA
Microvirin
HHL
HAA
AAL
Epa1-Np mutant Y228A
CA19-9Ab (DRG)
HML-Native
CA19.9-9L426
LCA
hGal-1-488+BCR
Gal-3C
CA19.9-121SLE
PTL-I
SRL
SSR1
rSRL
rNV VLP-Cy3
#14 Sialyl Lewis X (Calbio)
CML
Avaren-Fc
MPL
#8 Lewis B (T218) (Abcam)
ACL
WFL
SGA
PHA-E
LEL
rPVL
TbFBL1
VLRB.aGPA.23
ConA
PHA-L
GSL-2
LcH
hJAA-F11-B11
PFL
PA-IL
RCA I (Vector)
Mouse NKR-P1C
EEL
TL(EY Lab)
CA19.9-1B8A4
HPA
EEA(EY Lab)
CA
TJA-II
VVL
GNA (EY Lab)
TJA-I
GSL-I-B4
GS-II (EY Lab)
VGA(EY Lab)
AOL (Mahal Lab)
AHL
HHA(EY Lab)
Mono2W-CNL
Native HPA
WGA
LBL-152
#15 Sialyl Lewis X (BD)
LSL-150
DBA
PSSC
WGA
ME7(detected with a-HUD)
P11 VP8
WT-VCC
SSL0-488
ME5(detected with a-HUD)
Anti-LeC

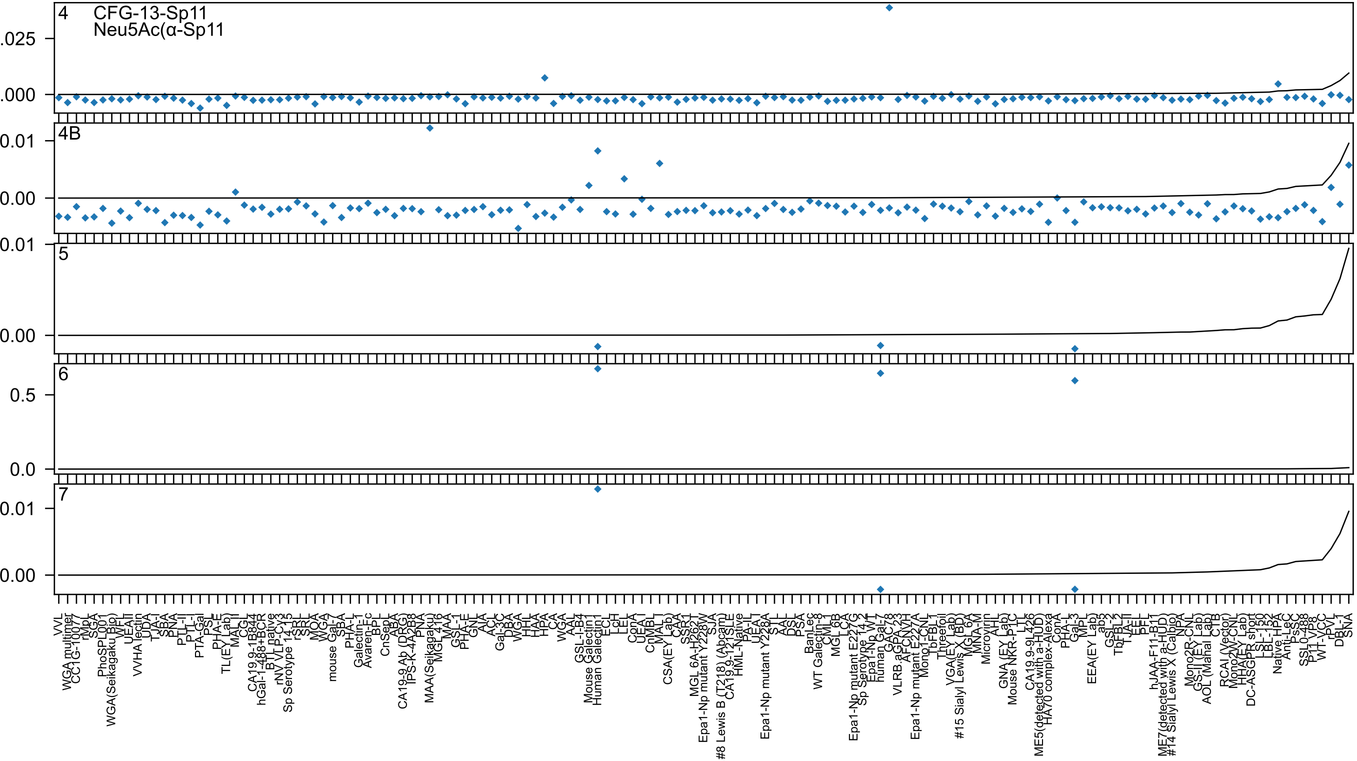

4
CFG-13-Sp11
Neu5Ac(α-Sp11
.025
.000
4B
0.01
0.00
5
0.01
0.00
6
0.5
0.0
7
0.01
0.00

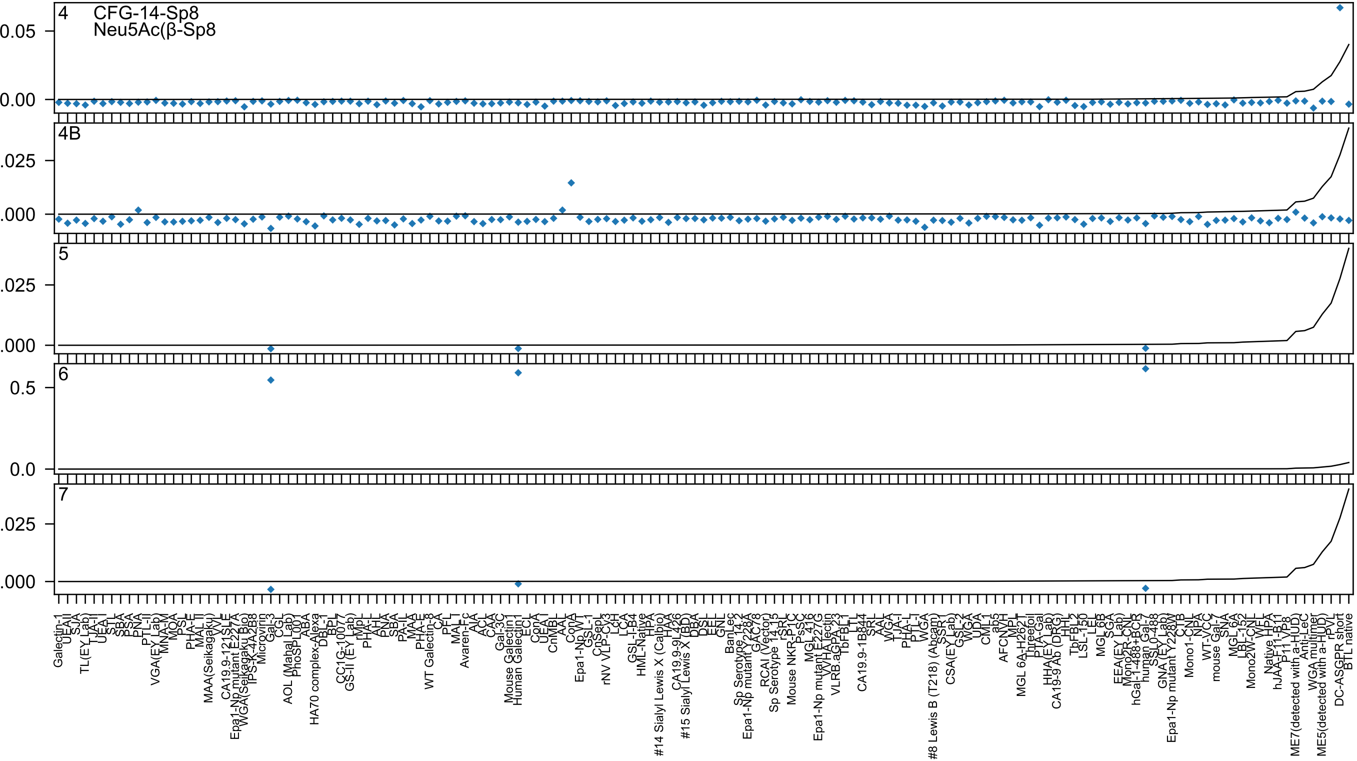

4
CFG-14-Sp8
Neu5Ac(β-Sp8
4B
5
6
7
0.05
0.00
.025
.000
0.5
0.0

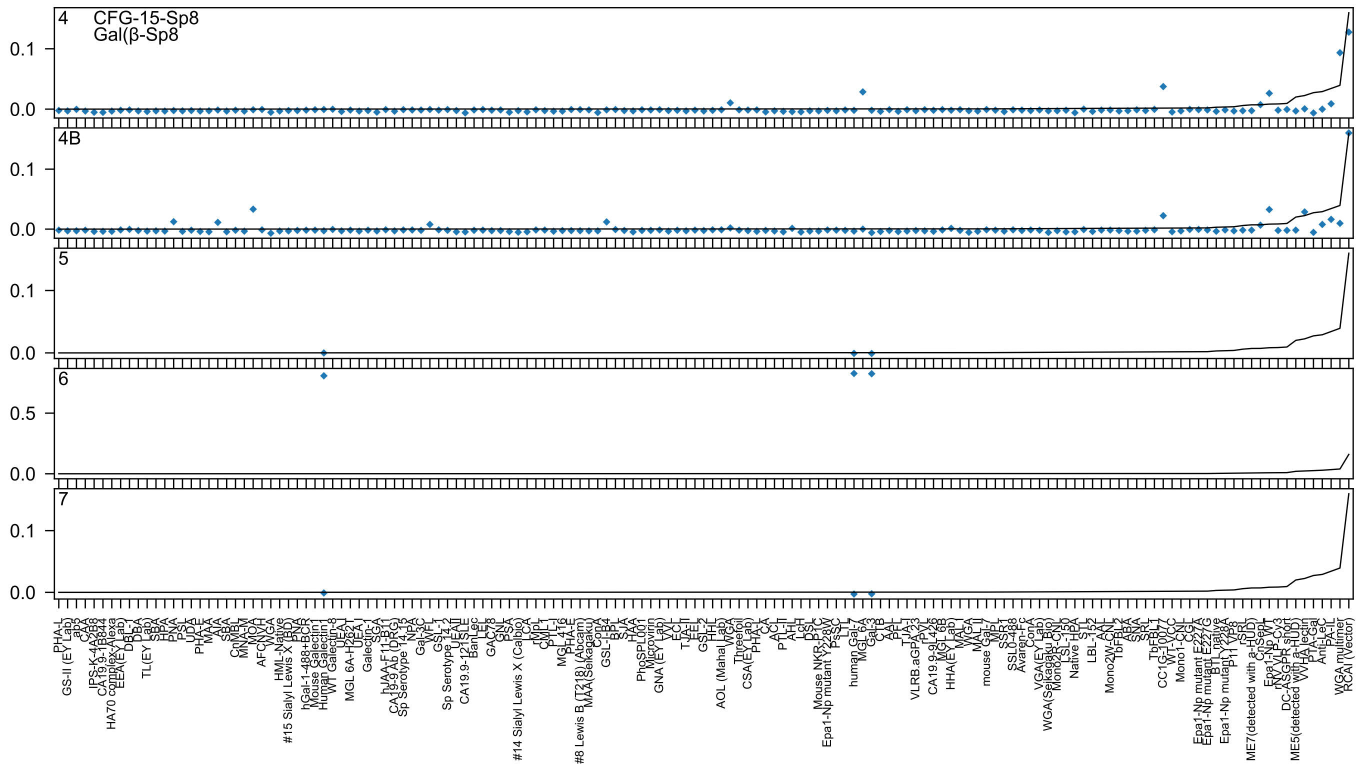
4
CFG-15-Sp8
Gal(β-Sp8
4B
5
6
7

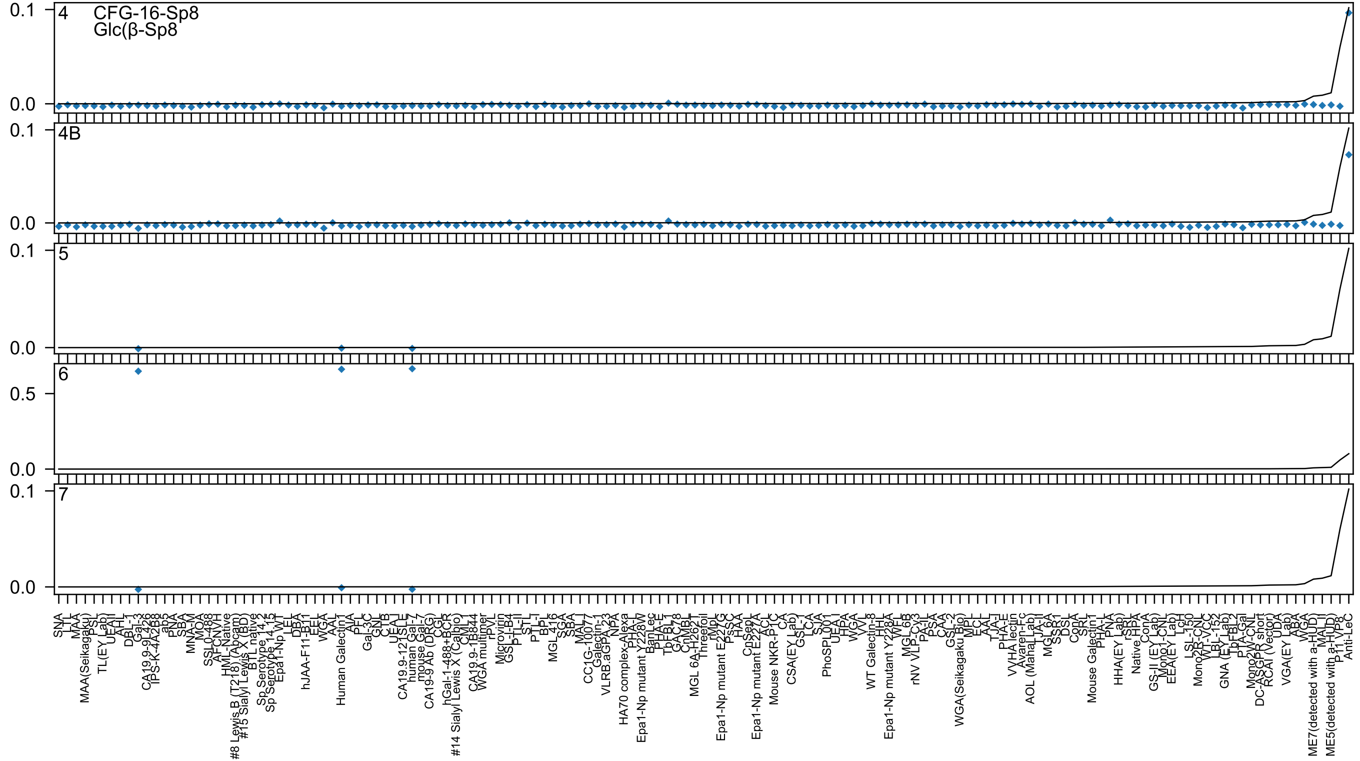
4
CFG-16-Sp8
Glc(β-Sp8
4B
5
6
7

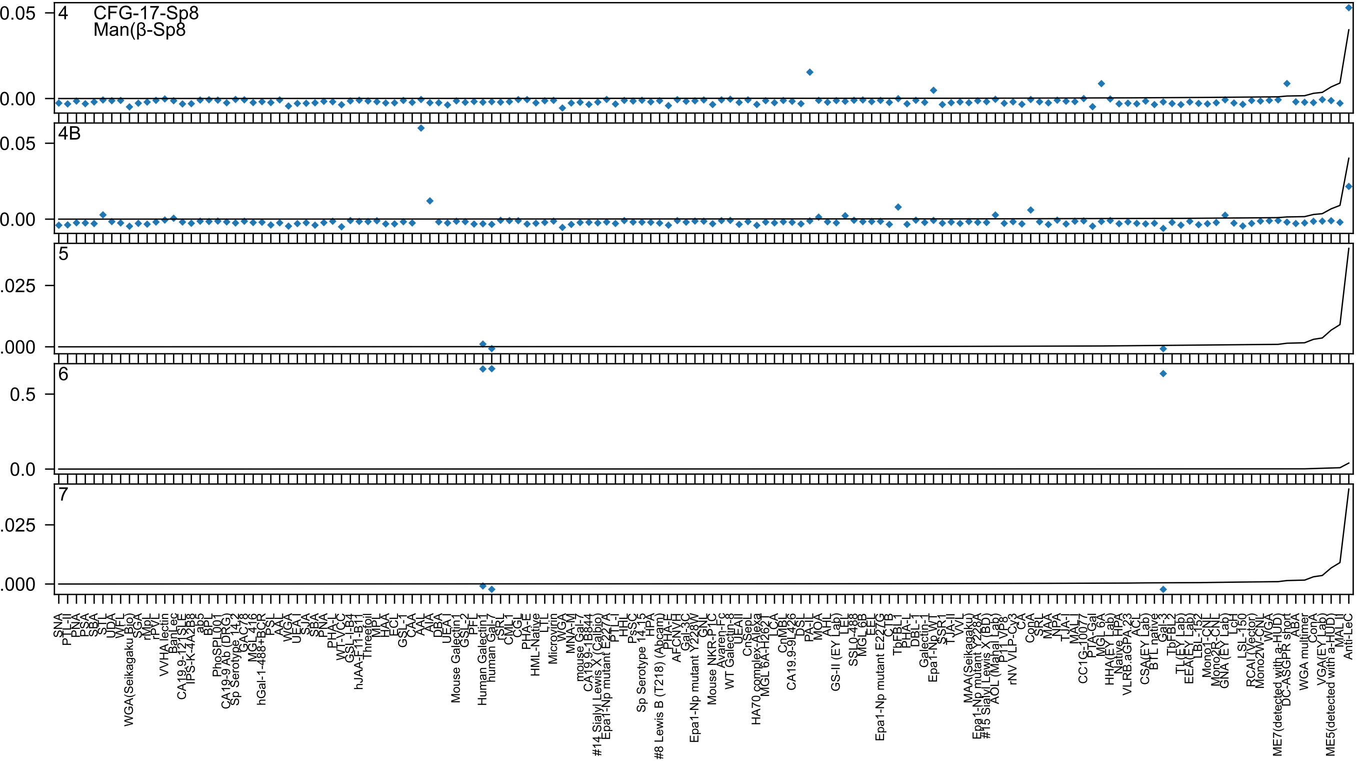

4
CFG-17-Sp8
Man(β-Sp8
4B
5
6
7

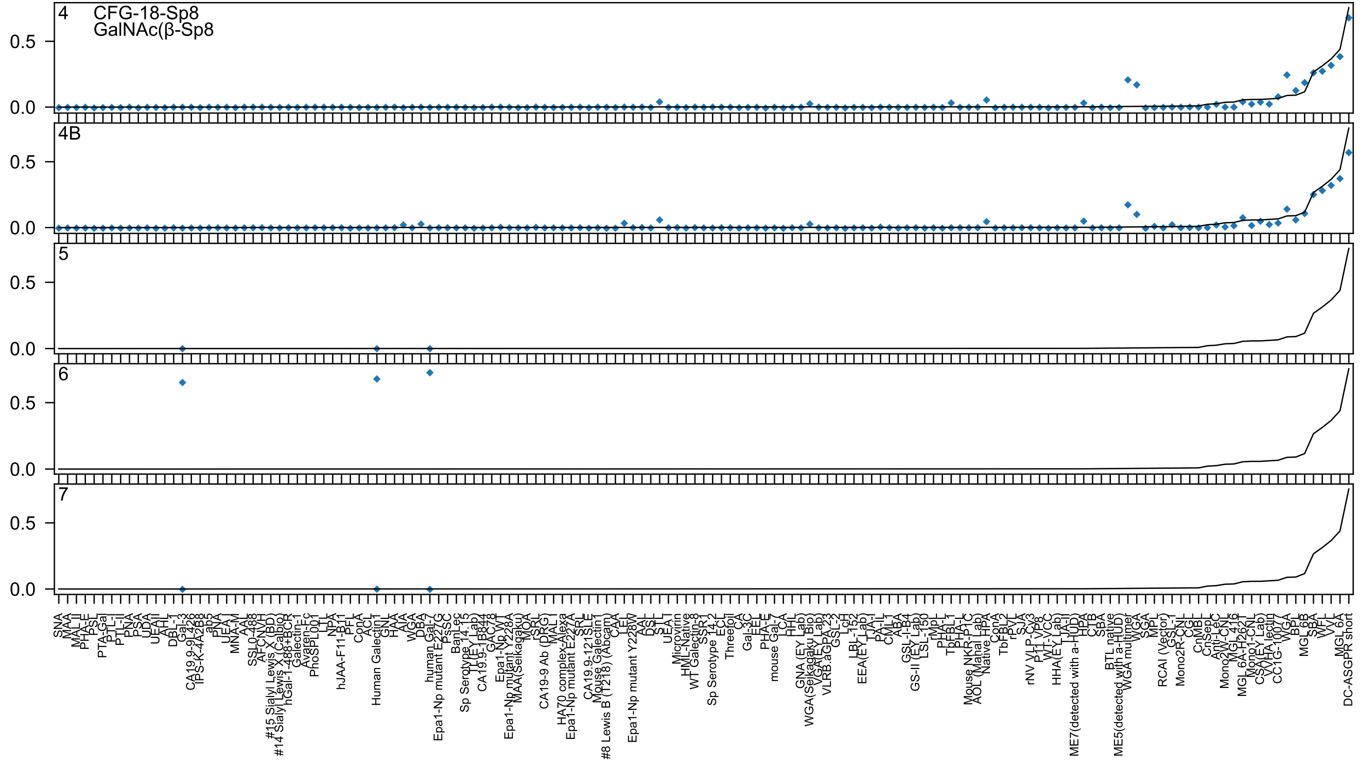
4
CFG-18-Sp8
GalNAc(β-Sp8
4B
5
6
7

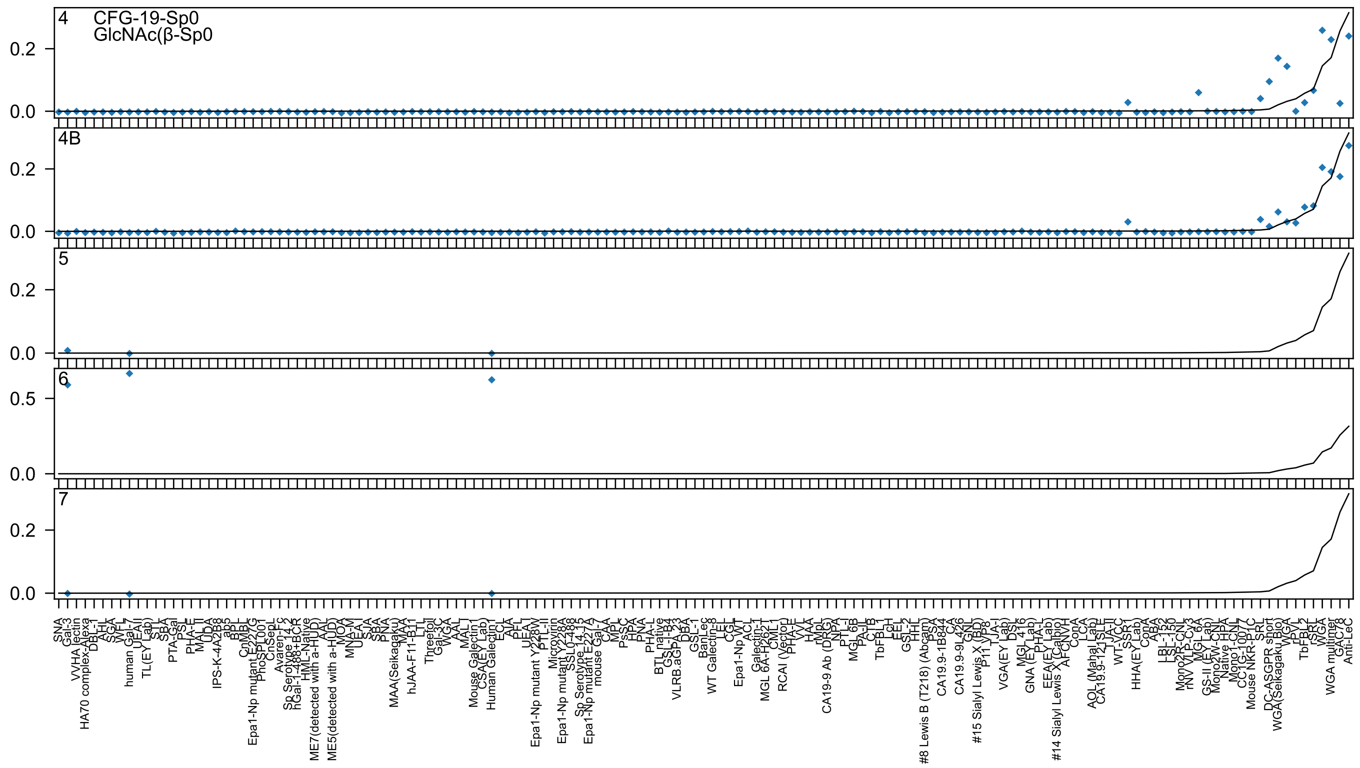

4
CFG-19-Sp0
GlcNAc(β-Sp0
4B
5
6
7
0.0
0.2
0.5

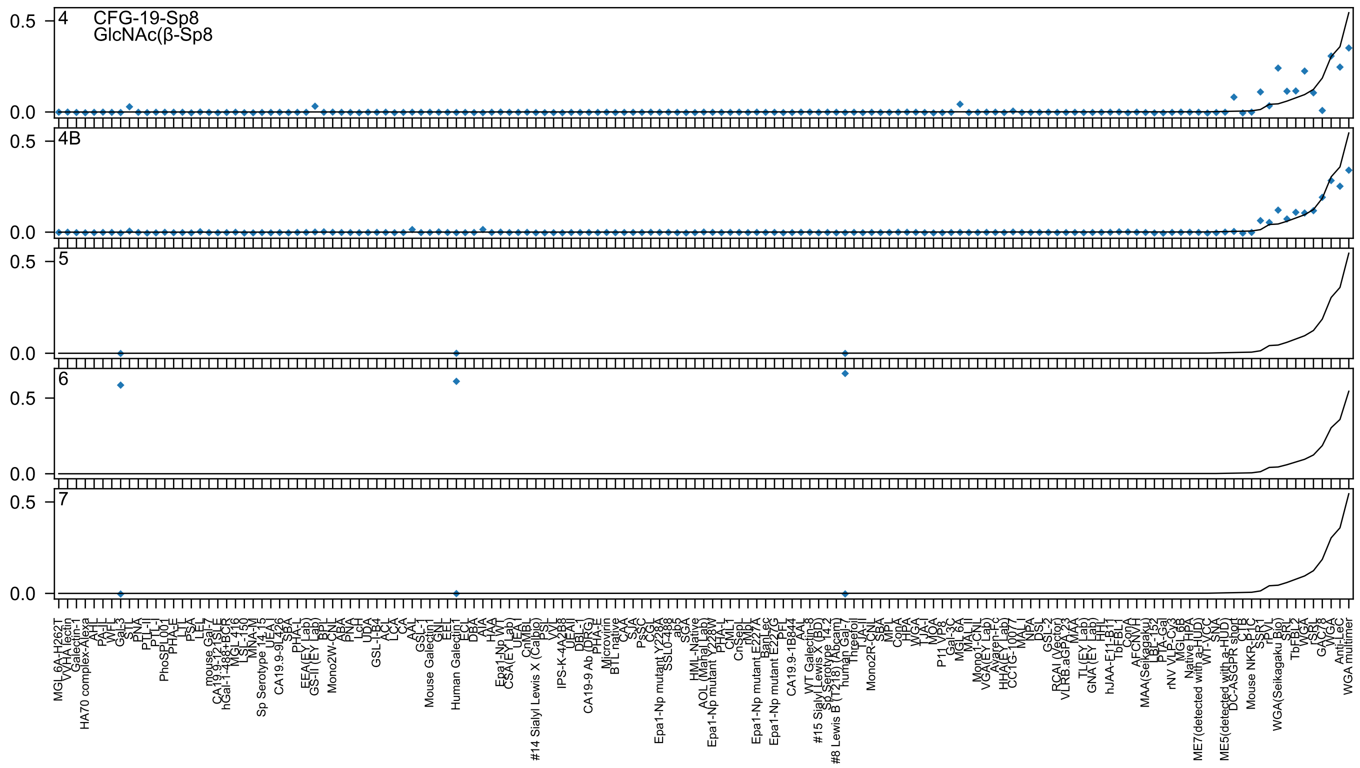
4 CFG-19-Sp8
GlcNAc(β-Sp8
4B
5
6
7
0.5
0.0
MGL 6A-H262T
Galectin-1
HA70 complex-Alexa
Gal-3
PhoSL001
mouse Gal-7
Sp Serotype 14.15
Mono2W-CNL
Mouse Galectin1
Human Galectin1
#14 Sialyl Lewis X (Calbio)
CA19-9 Ab (DRG)
Epa1-Np mutant Y228A
Epa1-Np mutant E227A
Epa1-Np mutant E227G
#8 Lewis B (T218) (Abcam)
human Gal-7
Mono2R-CNL
MAA(Seikagaku)
ME7(detected with a-HUD)
ME5(detected with a-HUD)
DC-ASGPR short
Mouse NKR-P1C
WGA(Seikagaku Bio)
WGA multimer

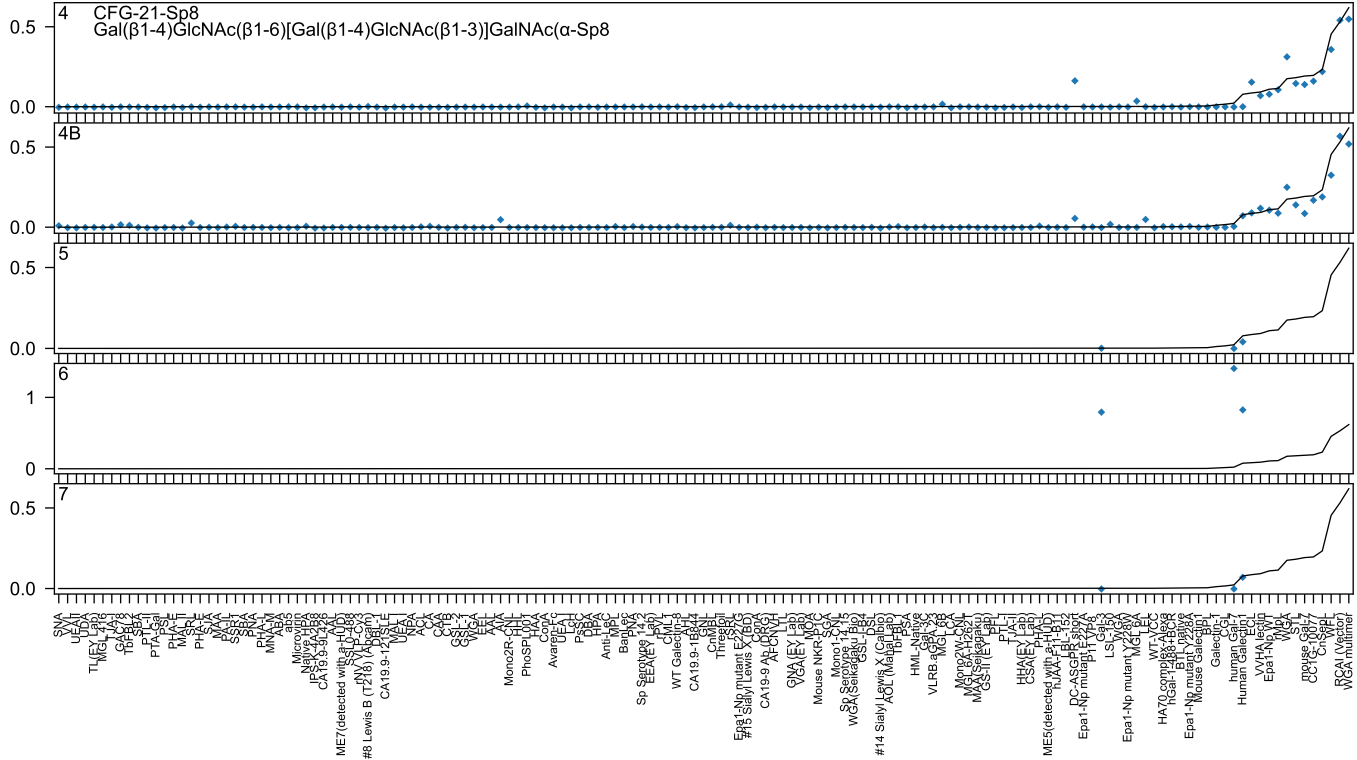
4
CFG-21-Sp8
Gal(β1-4)GlcNAc(β1-6)[Gal(β1-4)GlcNAc(β1-3)]GalNAc(α-Sp8
4B
5
6
7
0.5
0.0
1
0

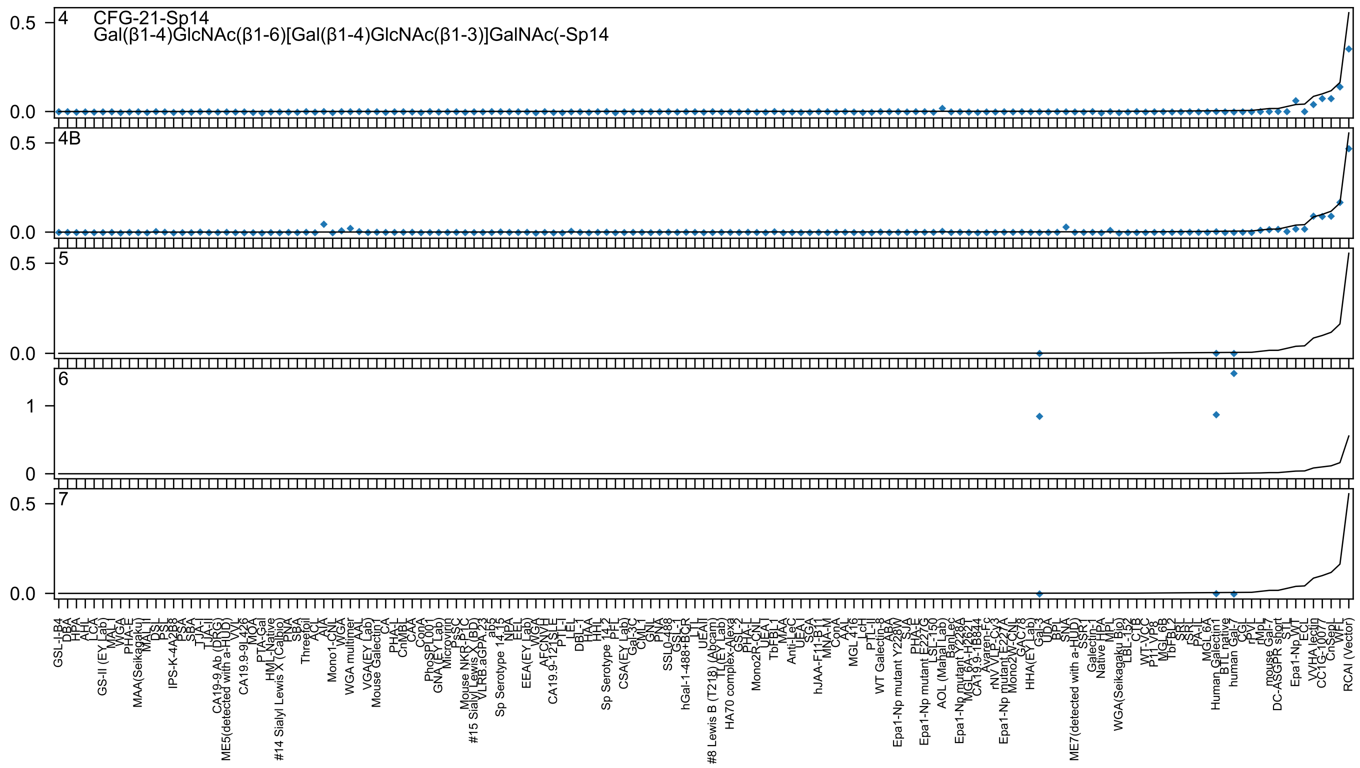

4
CFG-21-Sp14
Gal(β1-4)GlcNAc(β1-6)[Gal(β1-4)GlcNAc(β1-3)]GalNAc(-Sp14
4B
5
6
7
GSL-I-B4
RCAI (Vector)

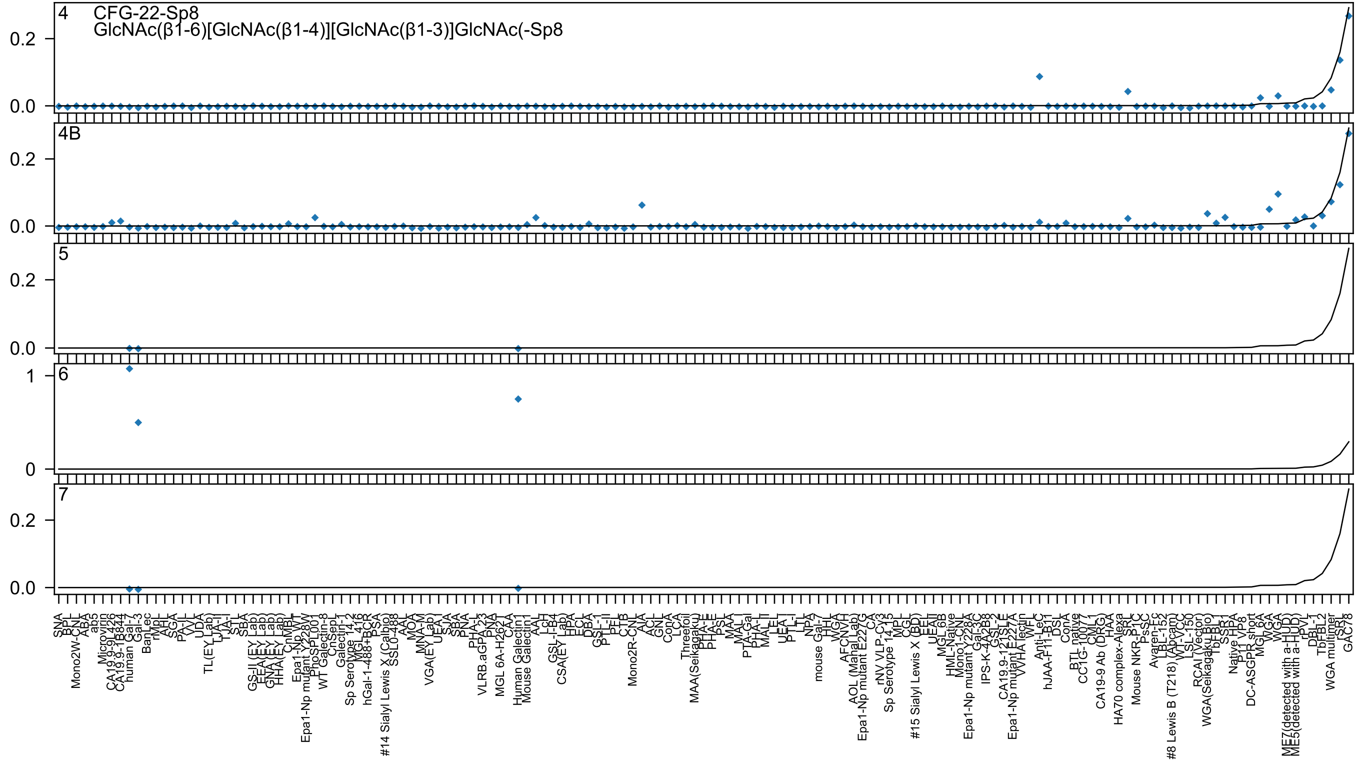

4 CFG-22-Sp8
GlcNAc(β1-6)[GlcNAc(β1-4)][GlcNAc(β1-3)]GlcNAc(-Sp8
4B
5
6
7

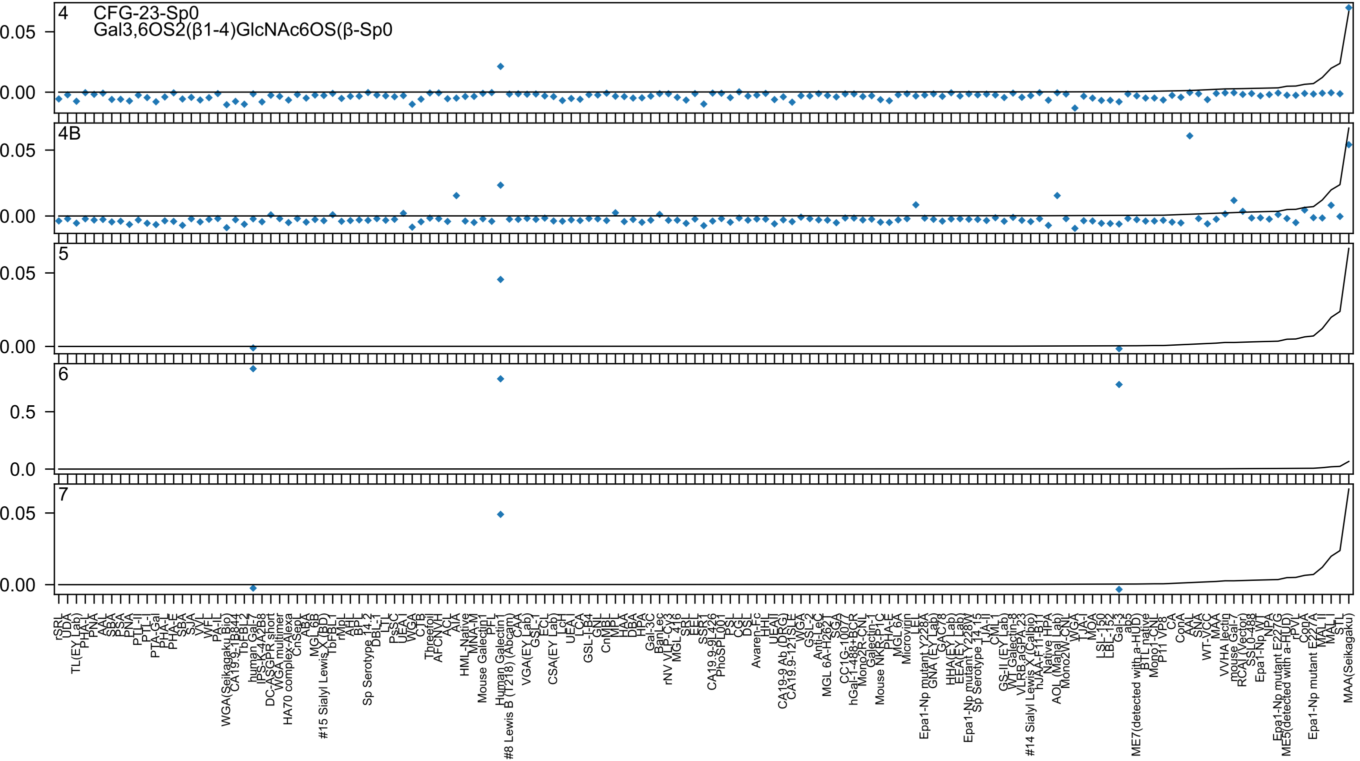

4
CFG-23-Sp0
Gal3,6OS2(β1-4)GlcNAc6OS(β-Sp0
4B
5
6
7
0.05
0.00
0.5
0.0

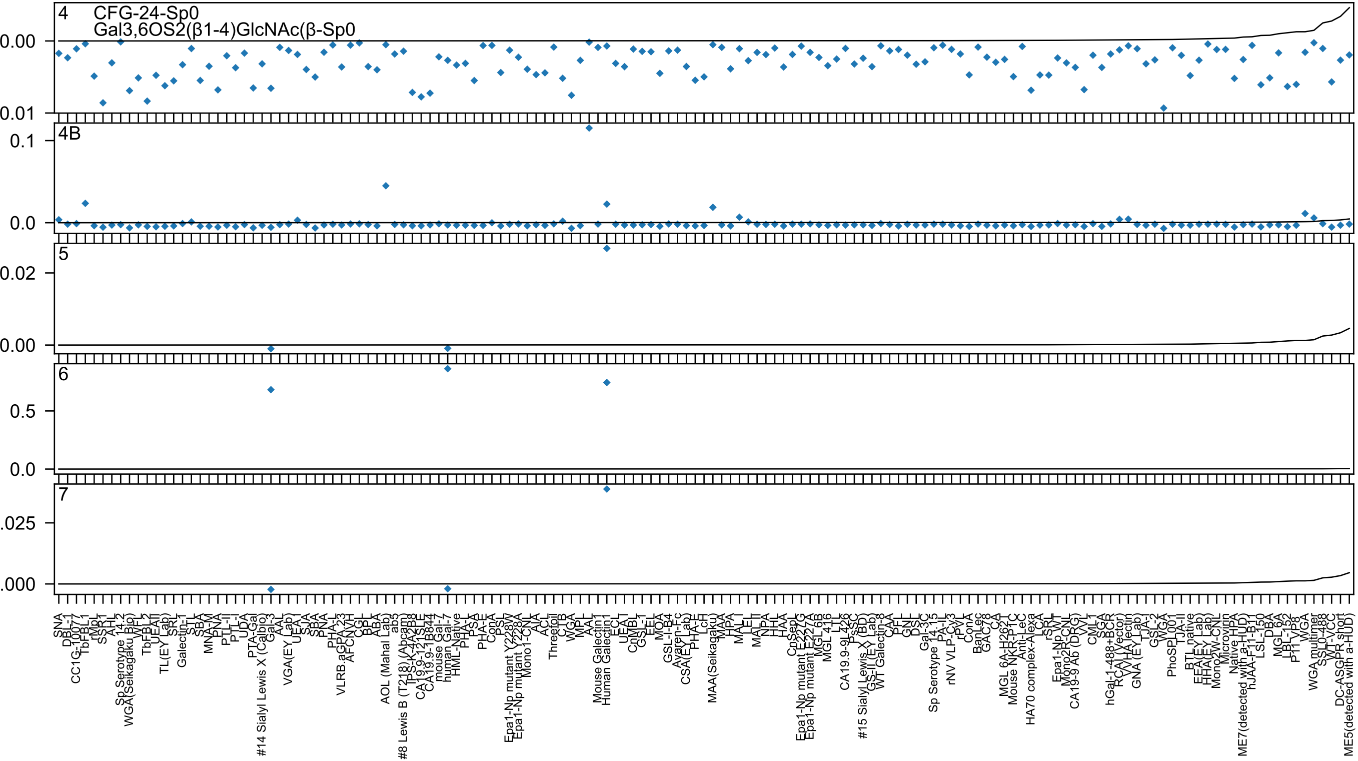
4 CFG-24-Sp0
Gal3,6OS2(β1-4)GlcNAc(β-Sp0
4B
5
6
7

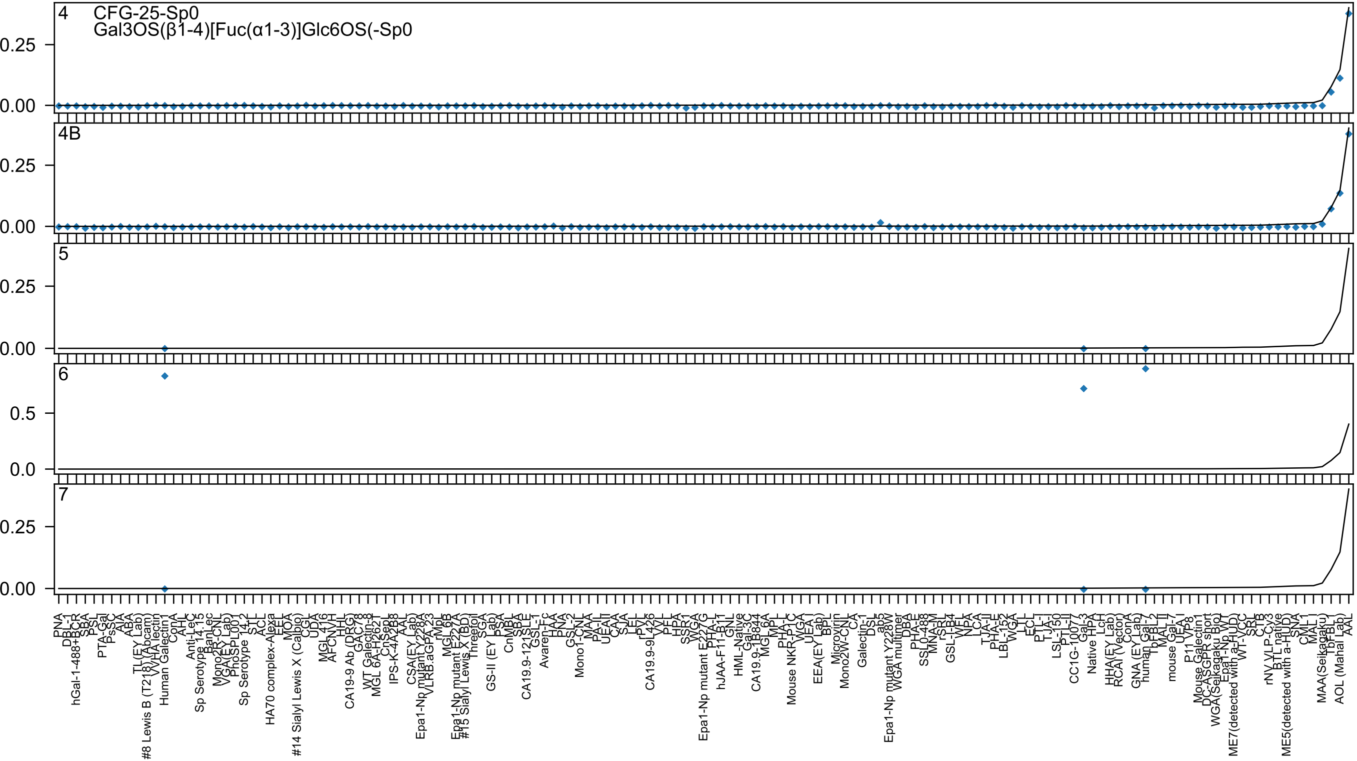
4
CFG-25-Sp0
Gal3OS(β1-4)[Fuc(α1-3)]Glc6OS(-Sp0
0.25
0.00
4B
0.25
0.00
5
0.25
0.00
6
0.5
0.0
7
0.25
0.00

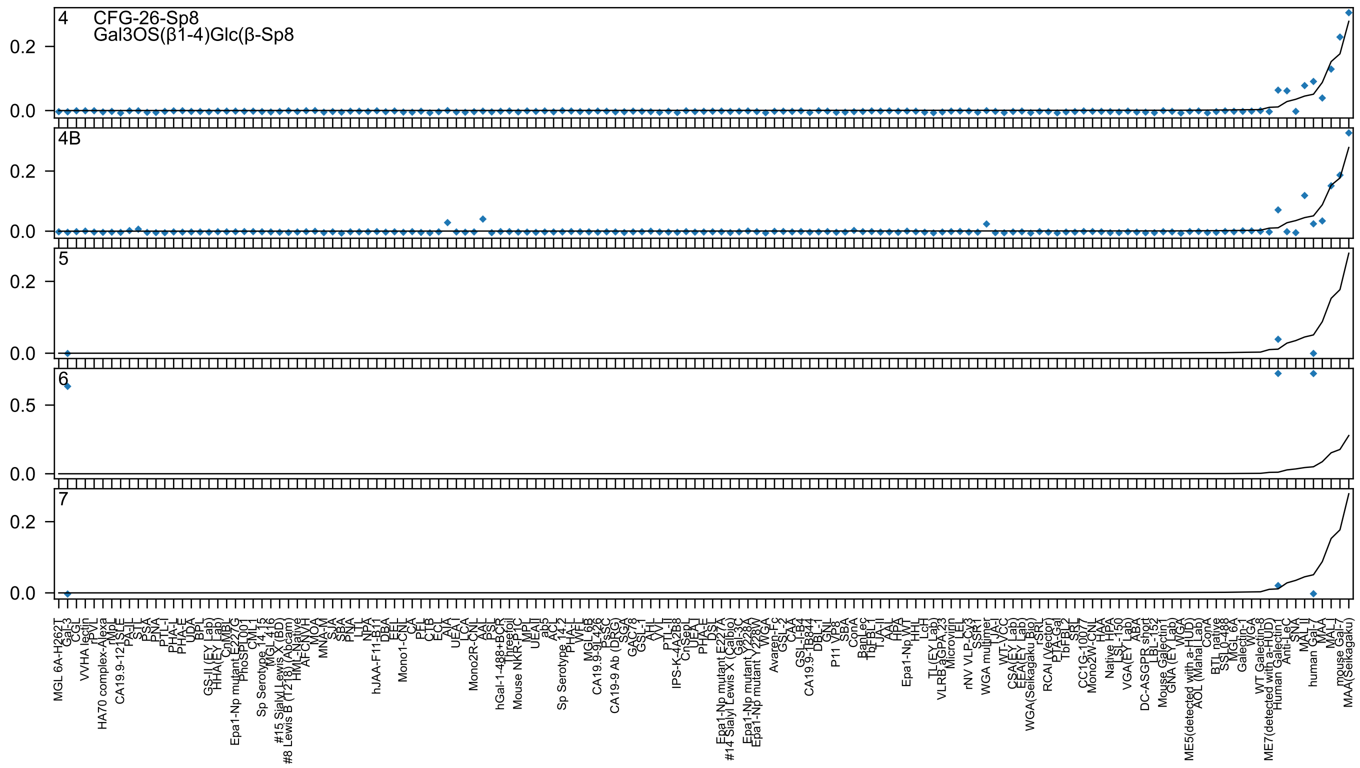
4
CFG-26-Sp8
Gal3OS(β1-4)Glc(β-Sp8
4B
5
6
7
0.2
0.0
0.5

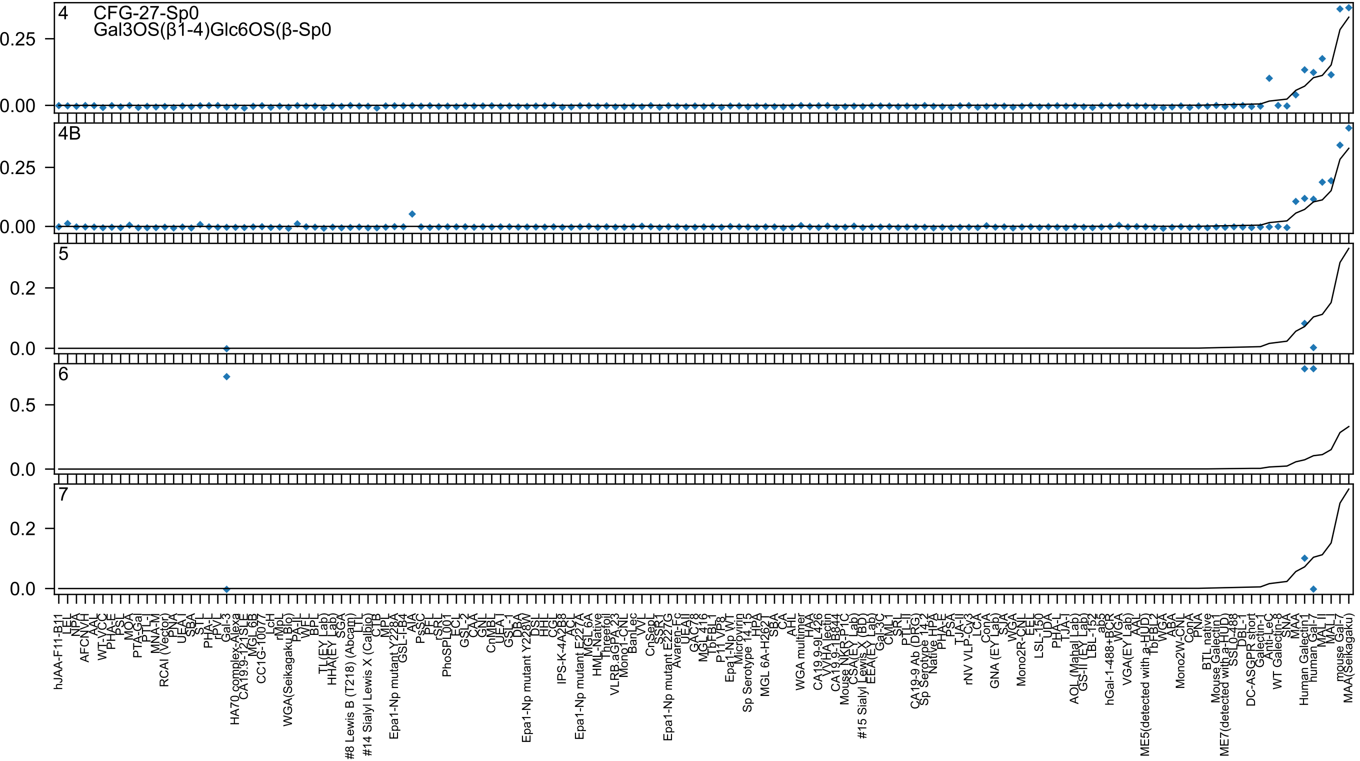

CFG-27-Sp0
Gal3OS(β1-4)Glc6OS(β-Sp0
4
4B
5
6
7

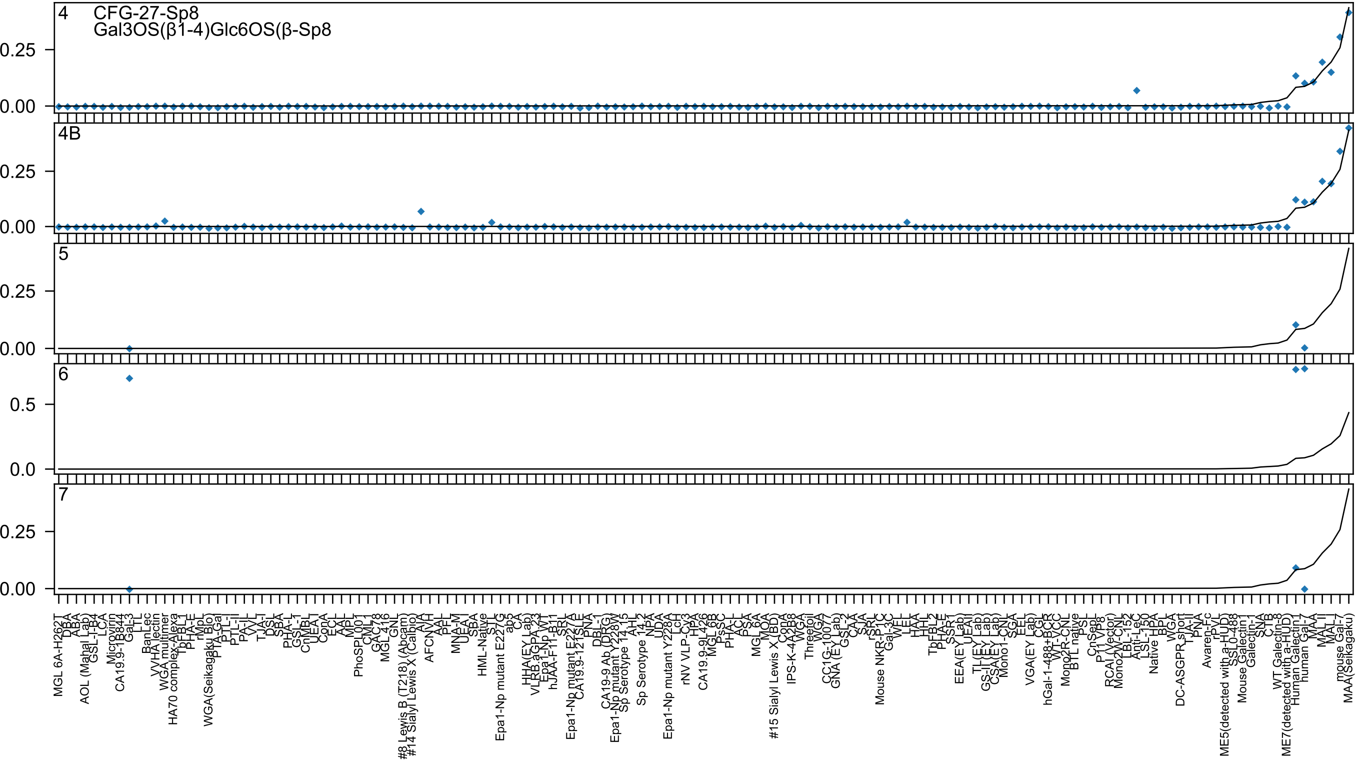

4
CFG-27-Sp8
Gal3OS(β1-4)Glc6OS(β-Sp8
4B
5
6
7
0.25
0.00
0.5
0.0

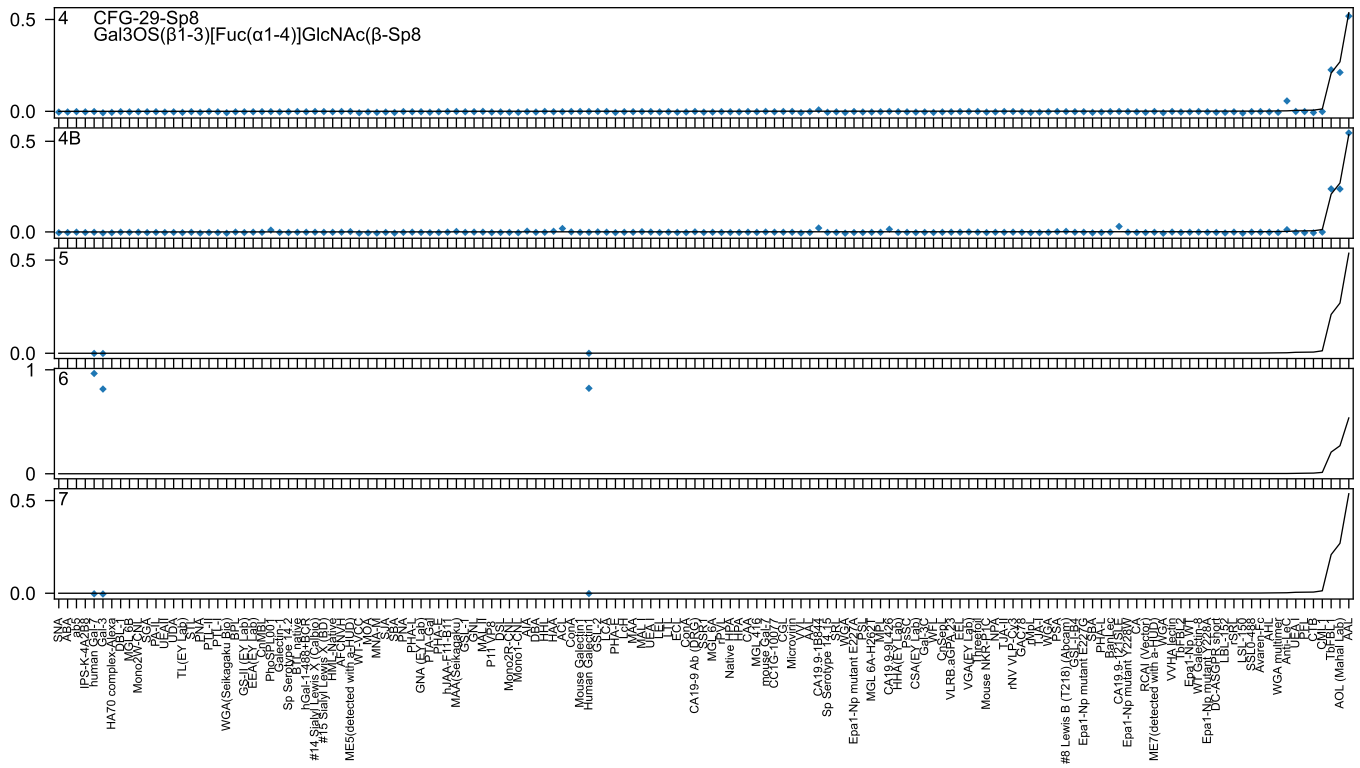
4 CFG-29-Sp8
Gal3OS(β1-3)[Fuc(α1-4)]GlcNAc(β-Sp8
4B
5
6
7

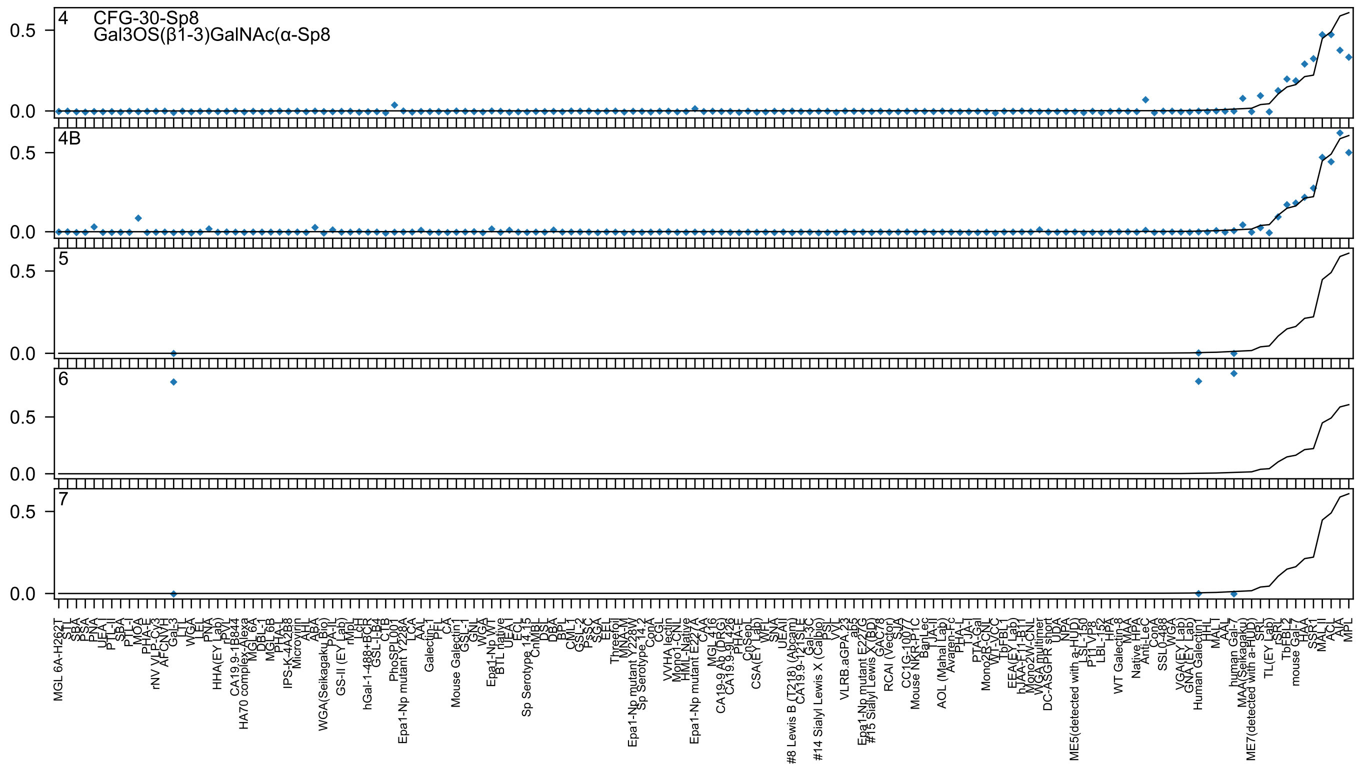
4
CFG-30-Sp8
Gal3OS(β1-3)GalNAc(α-Sp8
4B
5
6
7
0.5
0.0

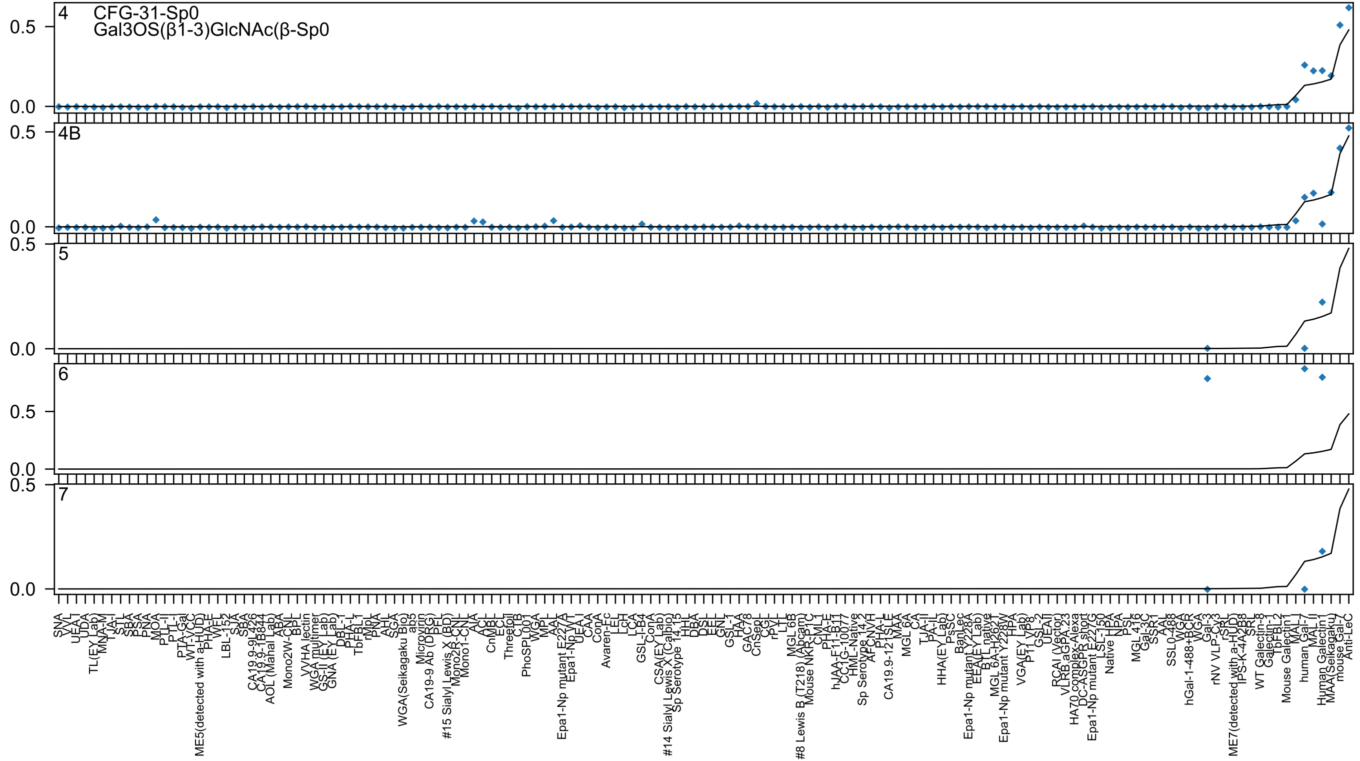
4
CFG-31-Sp0
Gal3OS(β1-3)GlcNAc(β-Sp0
4B
5
6
7

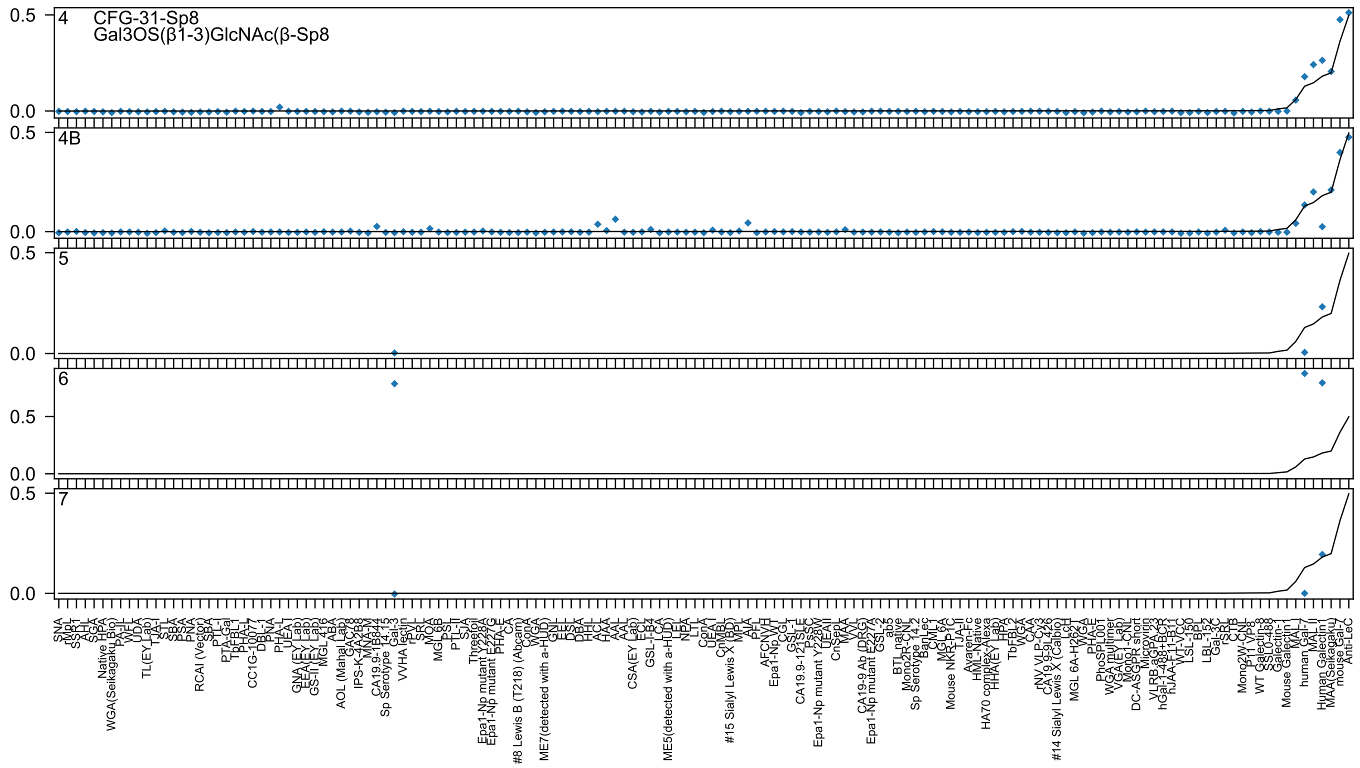

CFG-31-Sp8
Gal3OS(β1-3)GlcNAc(β-Sp8
4
4B
5
6
7
0.5
0.0

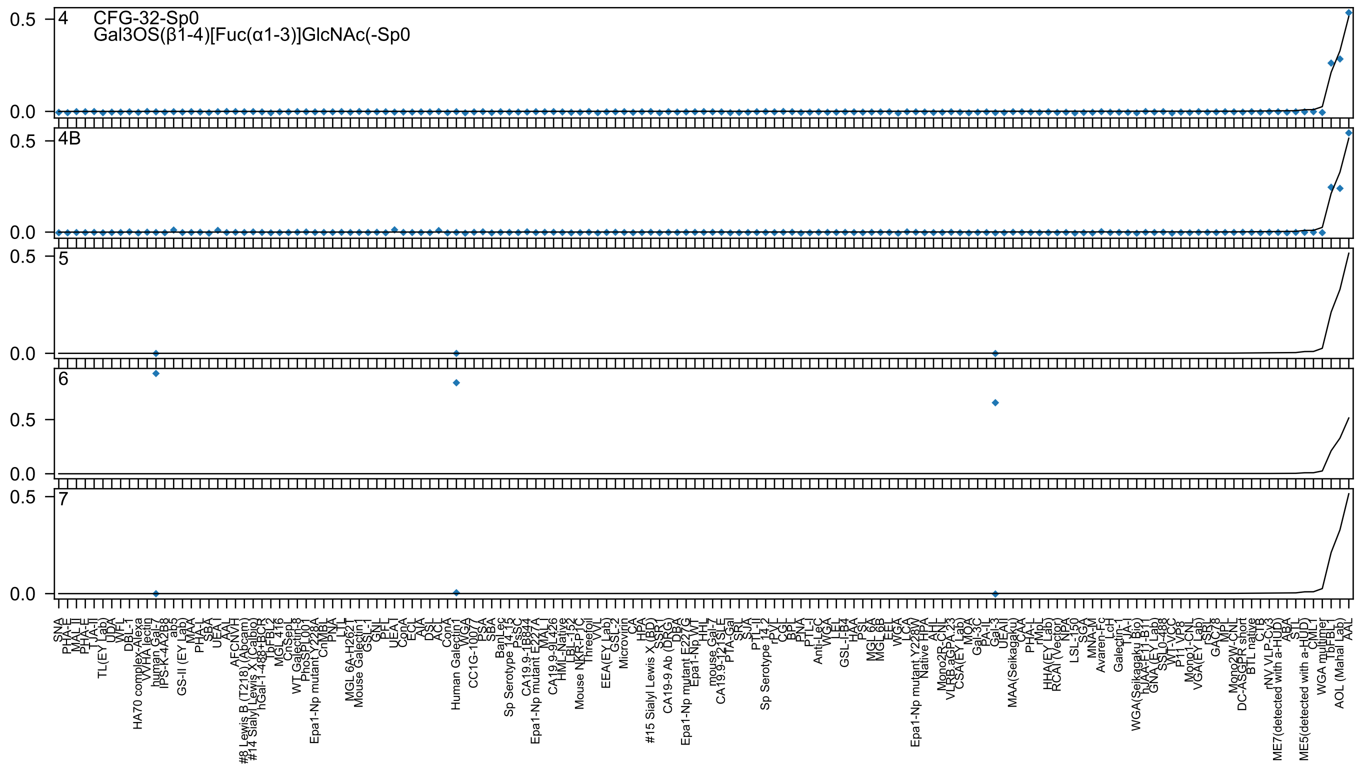
4 CFG-32-Sp0
Gal3OS(β1-4)[Fuc(α1-3)]GlcNAc(-Sp0
4B
5
6
7
0.5
0.0

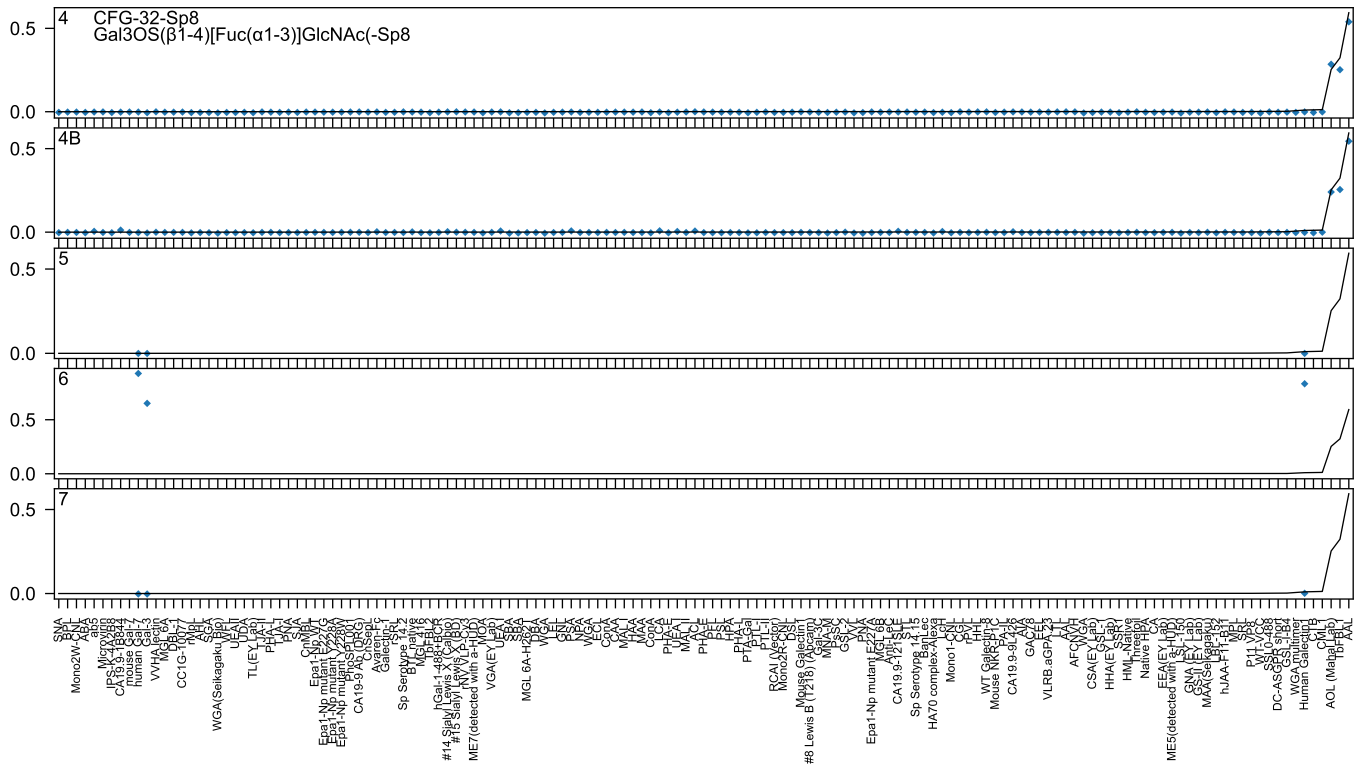

4
CFG-32-Sp8
Gal3OS(β1-4)[Fuc(α1-3)]GlcNAc(-Sp8
4B
5
6
7
0.5
0.0

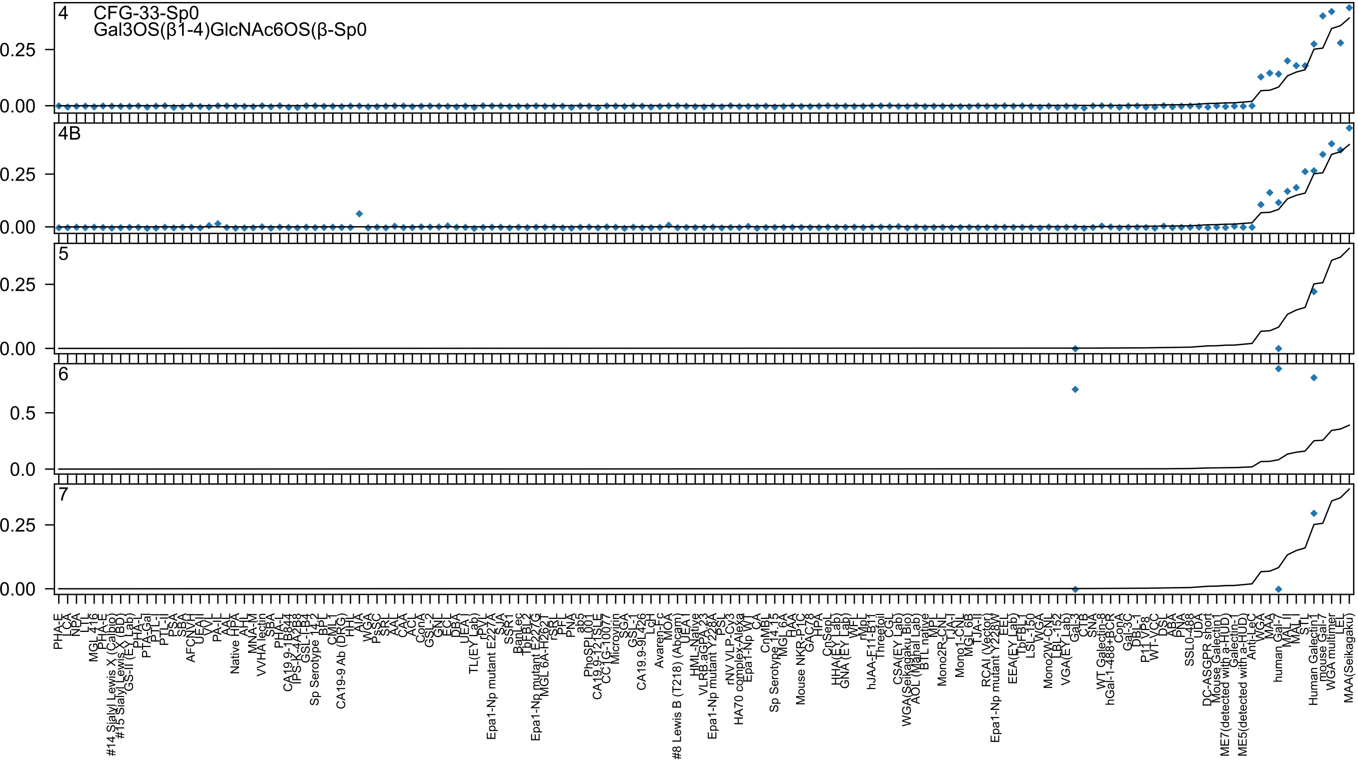

CFG-33-Sp0
Gal3OS(β1-4)GlcNAc6OS(β-Sp0
4
4B
5
6
7

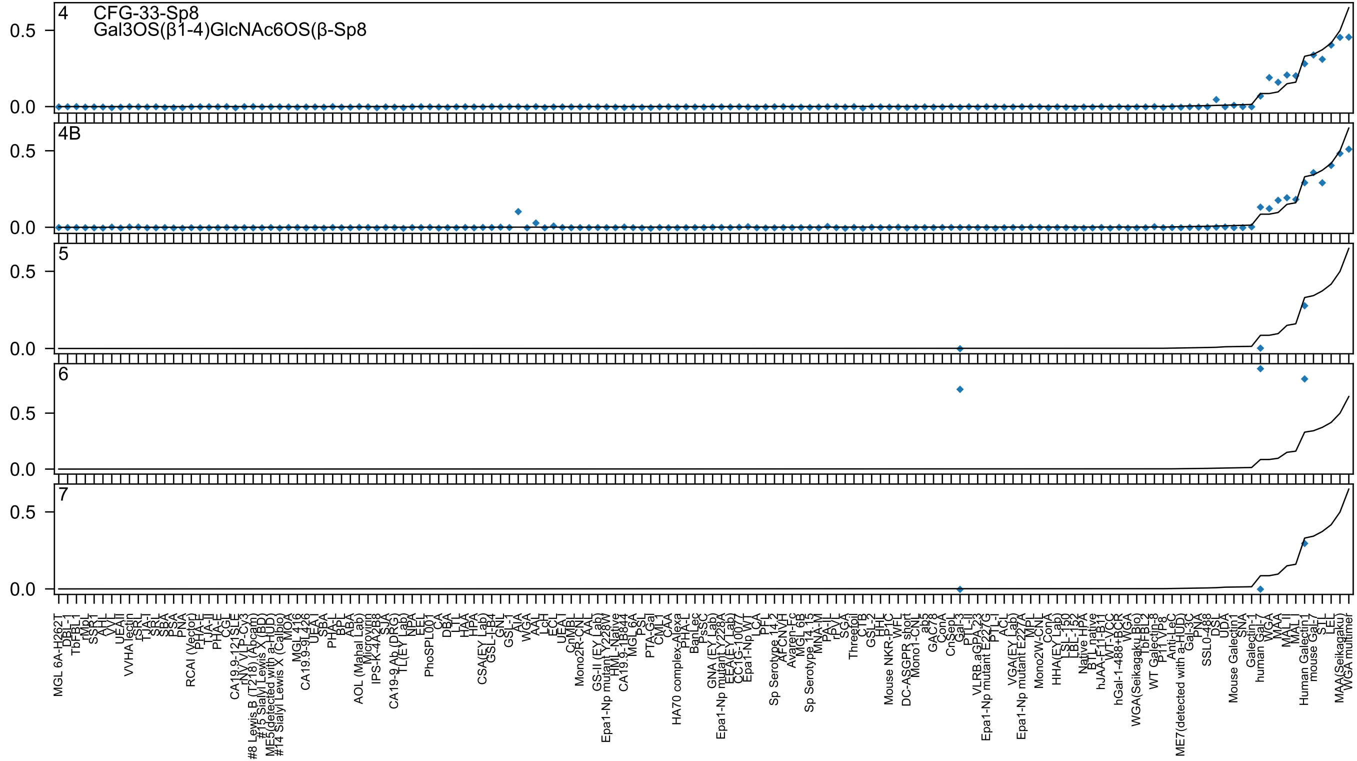

4 CFG-33-Sp8
Gal3OS(β1-4)GlcNAc6OS(β-Sp8
4B
5
6
7

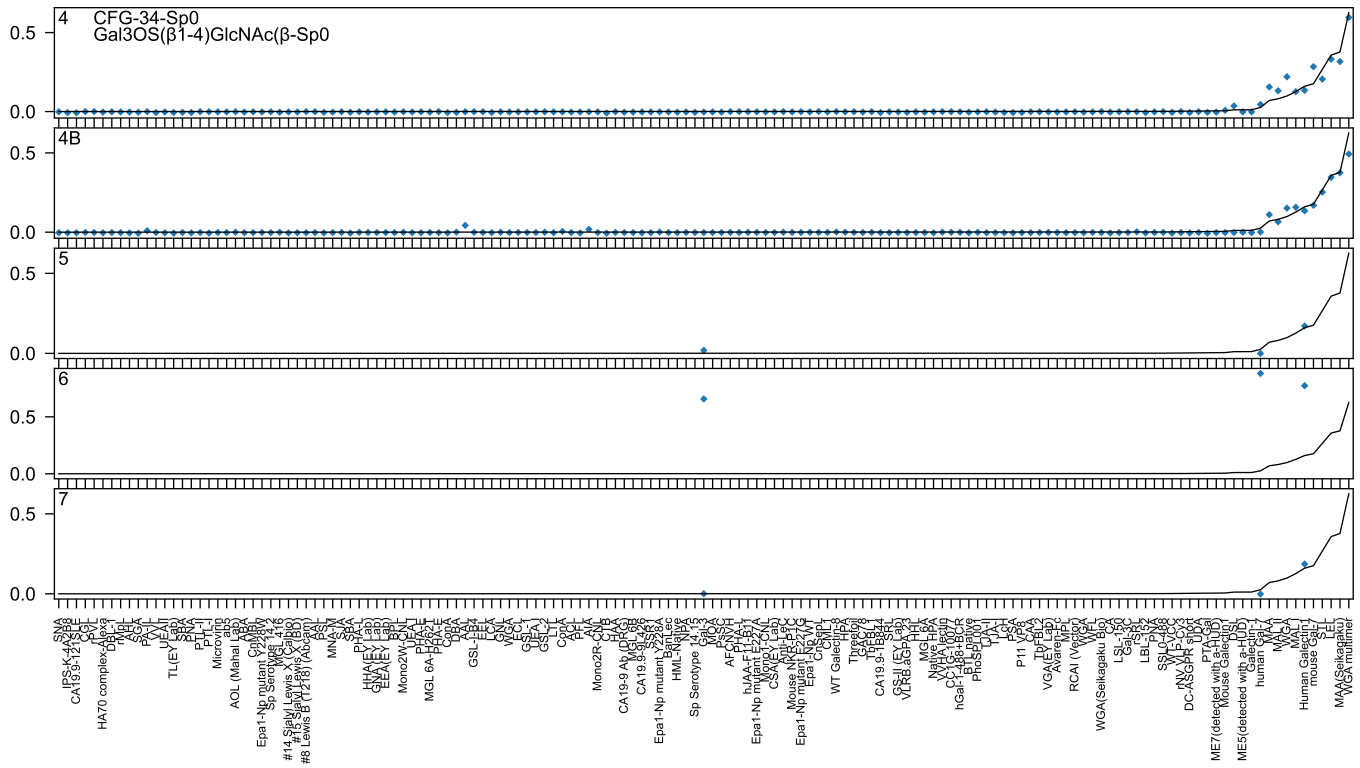

4 CFG-34-Sp0
Gal3OS(β1-4)GlcNAc(β-Sp0
4B
5
6
7
0.5
0.0

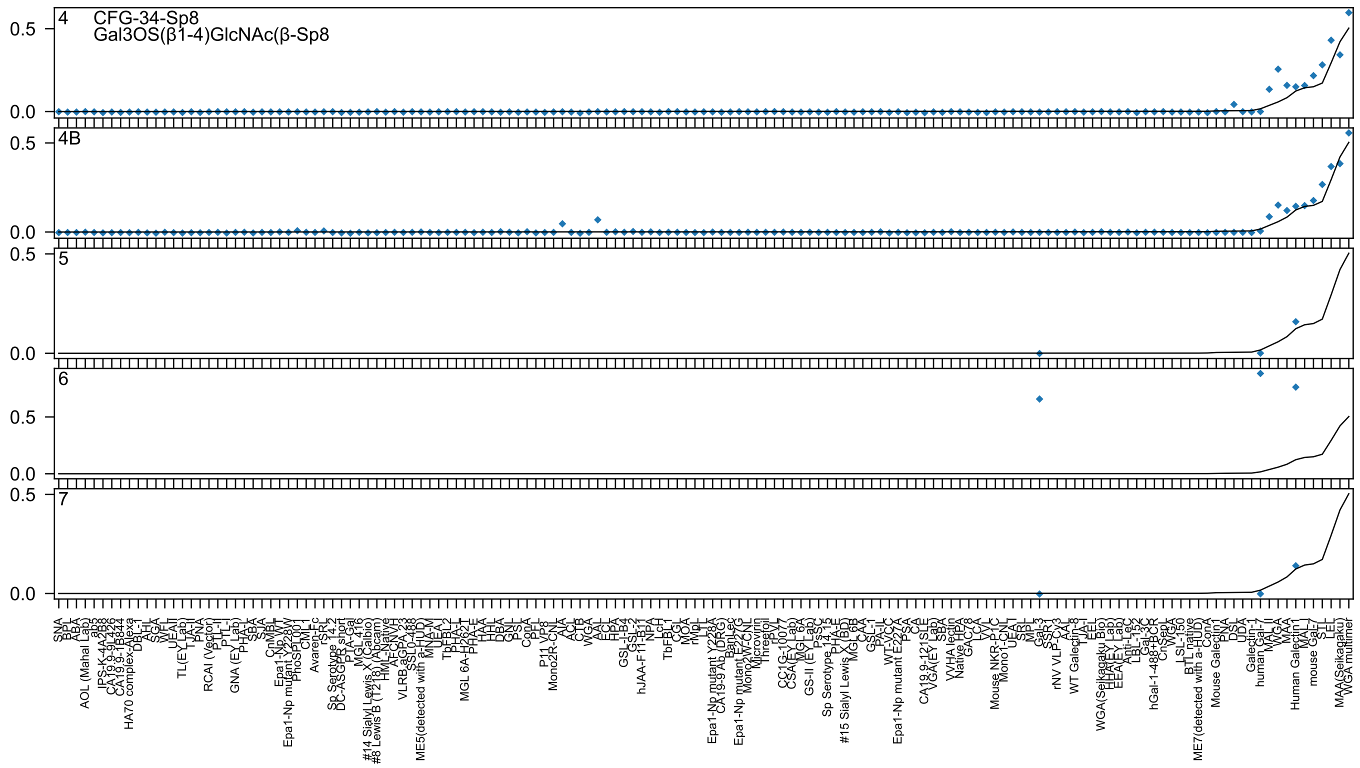
4 CFG-34-Sp8
Gal3OS(β1-4)GlcNAc(β-Sp8
4B
5
6
7

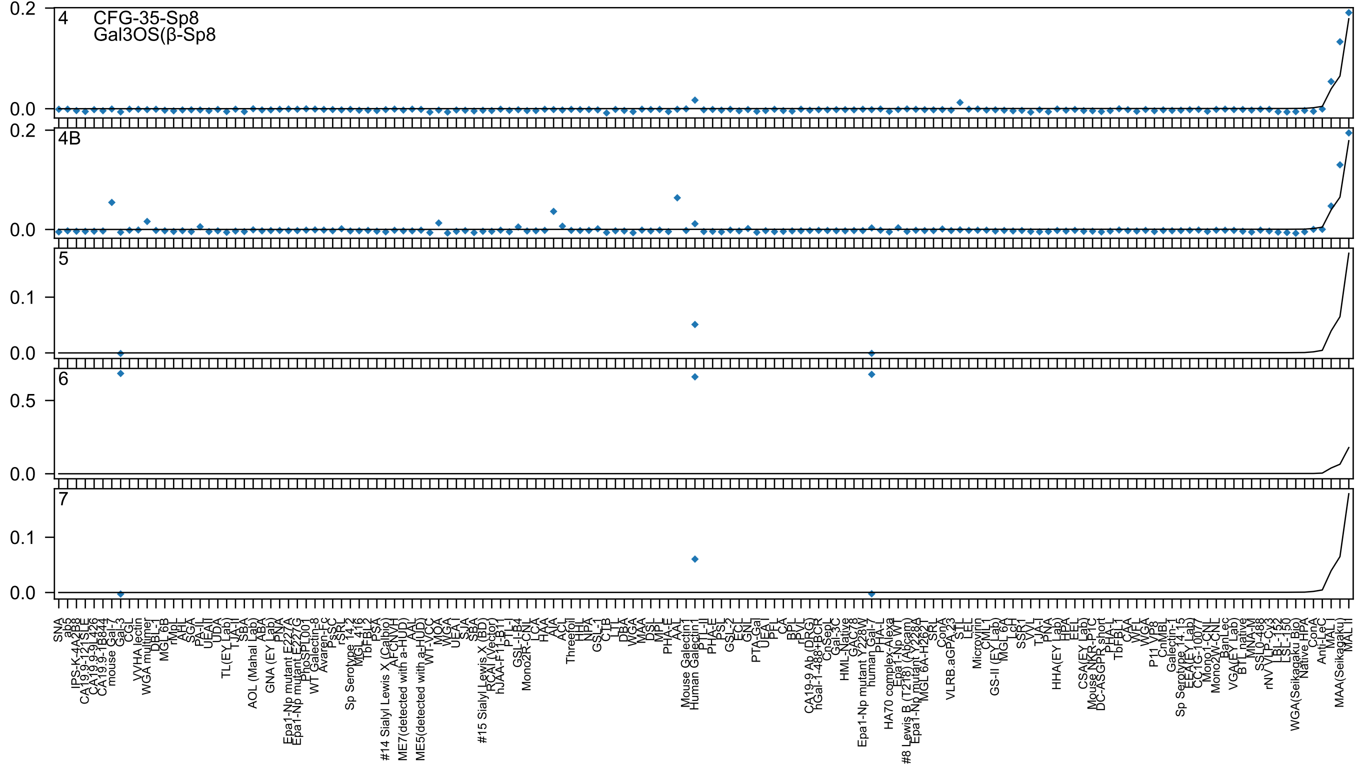
4 CFG-35-Sp8
Gal3OS(β-Sp8
4B
5
6
7
0.0
0.1
0.2
0.5
SNA
ab5
IPS-K-4A2B8
CA19.9-121SLE
CA19.9-9L426
CA19.9-1B844
mouse Gal-7
Gal-3
CGL
VVHA lectin
WGA multimer
DBL-1
MGL 6B
rMpL
AHL
SGA
PA-IL
UEA-II
UDA
TL(EY Lab)
TJA-II
SBA
AOL (Mahal Lab)
ABA
GNA (EY Lab)
PNA
Epa1-Np mutant E227A
Epa1-Np mutant E227G
PhoSL001
WT Galectin-8
Avaren-Fc
PsSC
rSRL
Sp Serotype 14.2
MGL 416
TbFBL2
PSA
#14 Sialyl Lewis X (Calbio)
AFCNVH
ME7(detected with a-HUD)
AAL
ME5(detected with a-HUD)
WT-VCC
MOA
WGA
UEA-I
SJA
SBA
#15 Sialyl Lewis X (BD)
RCAI (Vector)
hJAA-F11-B11
PTL-I
GSL-I-B4
Mono2R-CNL
LCA
HAA
AIA
ACL
Threefoil
HHL
NPA
GSL-1
CTB
LTL
DBA
WGA
MAA
DSL
MPL
PHA-E
AAL
Mouse Galectin1
Human Galectin1
PTL-II
PHA-E
PSL
GSL-2
ECL
GNL
PTA-Gal
UEA-I
PFL
CA
BPL
rPVL
CA19-9 Ab (DRG)
hGal-1-488+BCR
CnSepL
Gal-3C
HML-Native
GAC78
Epa1-Np mutant Y228W
human Gal-7
PHA-L
HA70 complex-Alexa
Epa1-Np WT
#8 Lewis B (T218) (Abcam)
Epa1-Np mutant Y228A
MGL 6A-H262T
SRL
ConA
VLRB.aGPA.23
STL
LEL
Microvirin
CML1
GS-II (EY Lab)
MGL 6A
LcH
SSA1
VVL
TJA-I
PNA
HHA(EY Lab)
HPA
EEL
CSA(EY Lab)
Mouse NKR-P1C
DC-ASGPR short
PHA-L
TbFBL1
CAA
WFL
WGA
P11 VP8
CnMBL
Galectin-1
Sp Serotype 14.15
EEA(EY Lab)
CC1G-1007
Mono1-CNL
Mono2W-CNL
BanLec
VGA(EY Lab)
BTL native
MNA-M
SSL0-488
rNV VLP-Cy3
LBL-152
LSL-150
WGA(Seikagaku Bio)
Native HPA
ConA
Anti-LeC
MAL I
MAA(Seikagaku)
MAL II

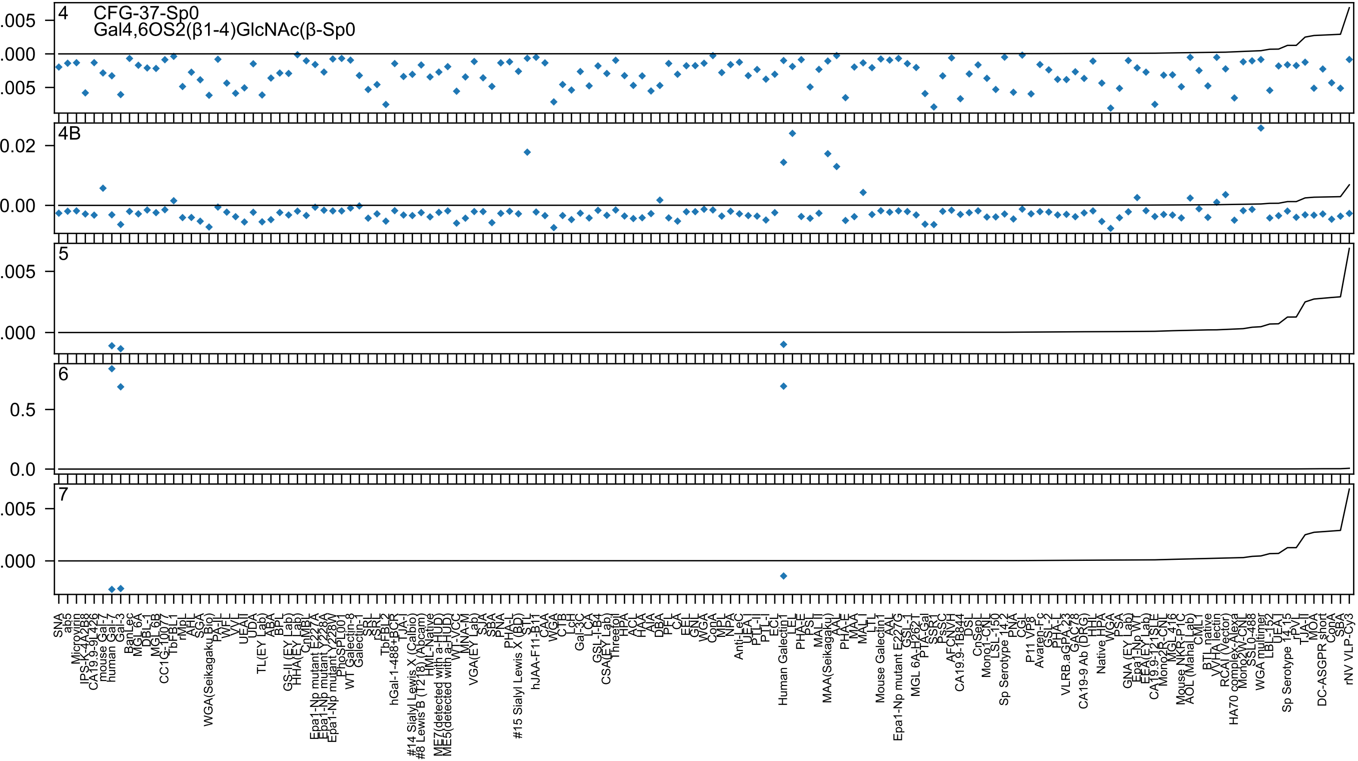
4
CFG-37-Sp0
Gal4,6OS2(β1-4)GlcNAc(β-Sp0
4B
5
6
7

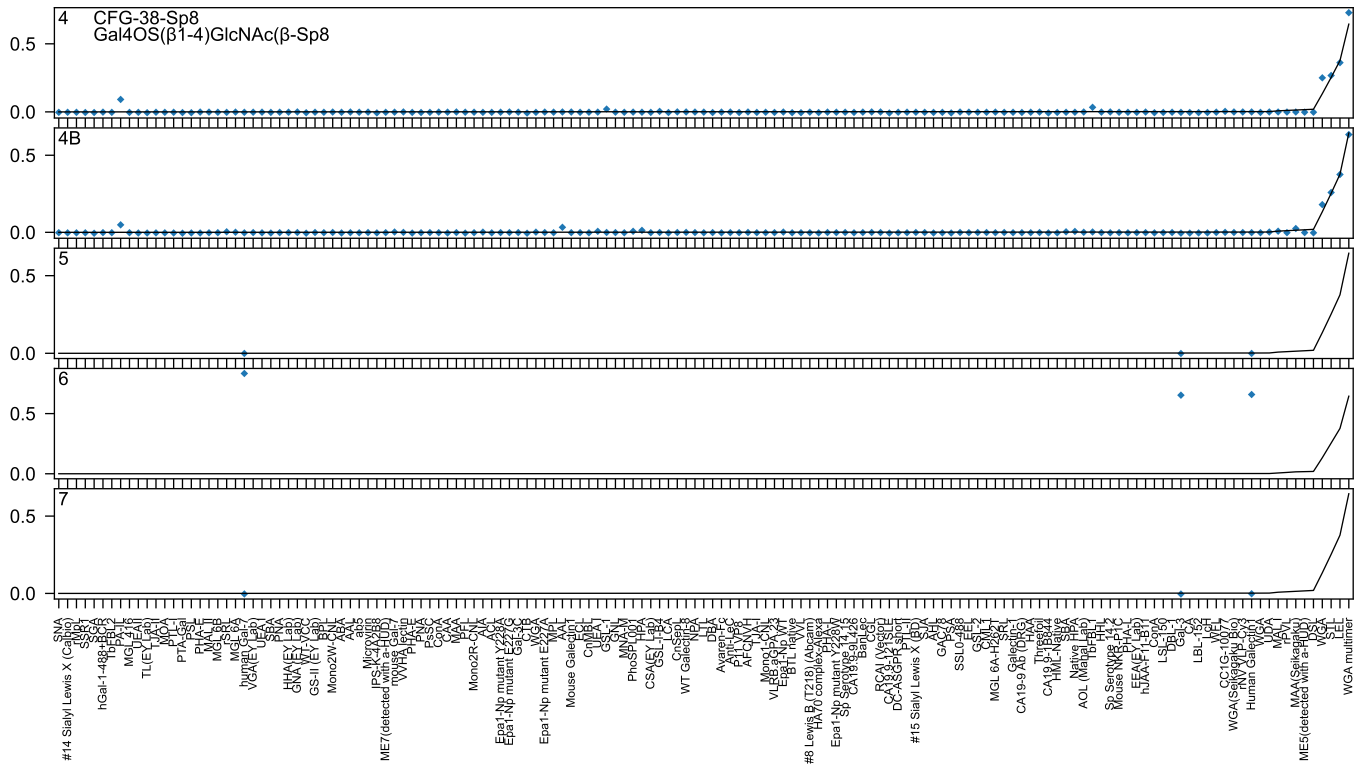

4
CFG-38-Sp8
Gal4OS(β1-4)GlcNAc(β-Sp8
4B
5
6
7
0.0
0.5
SNA
#14 Sialyl Lewis X (Calbio)
rMPL
SSR1
SGA
hGal-1-488+BCR
TbFBL2
PA-IL
MGL 416
UEAII
TL(EY Lab)
TJA-II
MOA
PTL-I
PTA-Gal
PSL
PHA-E
MAL II
MGL 6B
rSRL
MGL 6A
human Gal-7
VGA(EY Lab)
UEA I
SBA
PNA
HHA(EY Lab)
GNA (EY Lab)
WT-VCC
GS-II (EY Lab)
BPL
Mono2W-CNL
ABA
AAL
ab5
Microvirin
IPS-K-4A2B8
ME7(detected with a-HUD)
mouse Gal-7
WHA lectin
PHA-E
PNA
PsSC
ConA
CAA
MAA
PFL
Mono2R-CNL
AIA
ACL
Epa1-Np mutant Y228A
Epa1-Np mutant E227G
Gal-3C
CTB
WGA
Epa1-Np mutant E227A
MPL
AAT
Mouse Galectin1
ECL
CnMBL
UEA I
GSL-I
GNL
MNA-M
PhoSL001
HPA
CSA(EY Lab)
GSL-I-B4
LCA
CnSep
WT Galectin-8
NPA
LTL
DBA
Avaren-Fc
Anti-LeC
P1IVP8
AFC
NVH
TJA-I
Mono1-CNL
VLRB.aGPA.23
Epa1-Np WT
BTL native
VVL
#8 Lewis B (T218) (Abcam)
HA70 complex-Alexa
PHA-L
Epa1-Np mutant Y228W
Sp Serotype 14.15
CA19.9.9L426
BanLec
CGL
RCAI (Vector)
CA19.9-121SLE
DC-ASGPR short
PTL-II
#15 Sialyl Lewis X (BD)
SJA
AH
GAC78
PSA
SSL0-488
EEL
GSL-2
CML-1
MGL 6A-H262T
SRL
Galectin-1
CA19-9 Ab (DRG)
HAA
Threefoil
CA19.9-1B844
HML-Native
SBA
Native HPA
AOL (Manal Lab)
TbFBL1
HHL
Sp Serotype 14.2
Mouse NKR-P1C
PHA-L
EEA(EY Lab)
hJAA-F11-B1
ConA
LSL-150
DBL-1
Gal-3
CA
LBL-152
LcH
WFL
CC1G-10077
WGA(Seikagaku Bio)
rNV VLP-Cy3
Human Galectin1
WGA
UDA
MAL I
rPVL
MAA(Seikagaku)
ME5(detected with a-HUD)
DSL
WGA
STL
LEL
WGA multimer

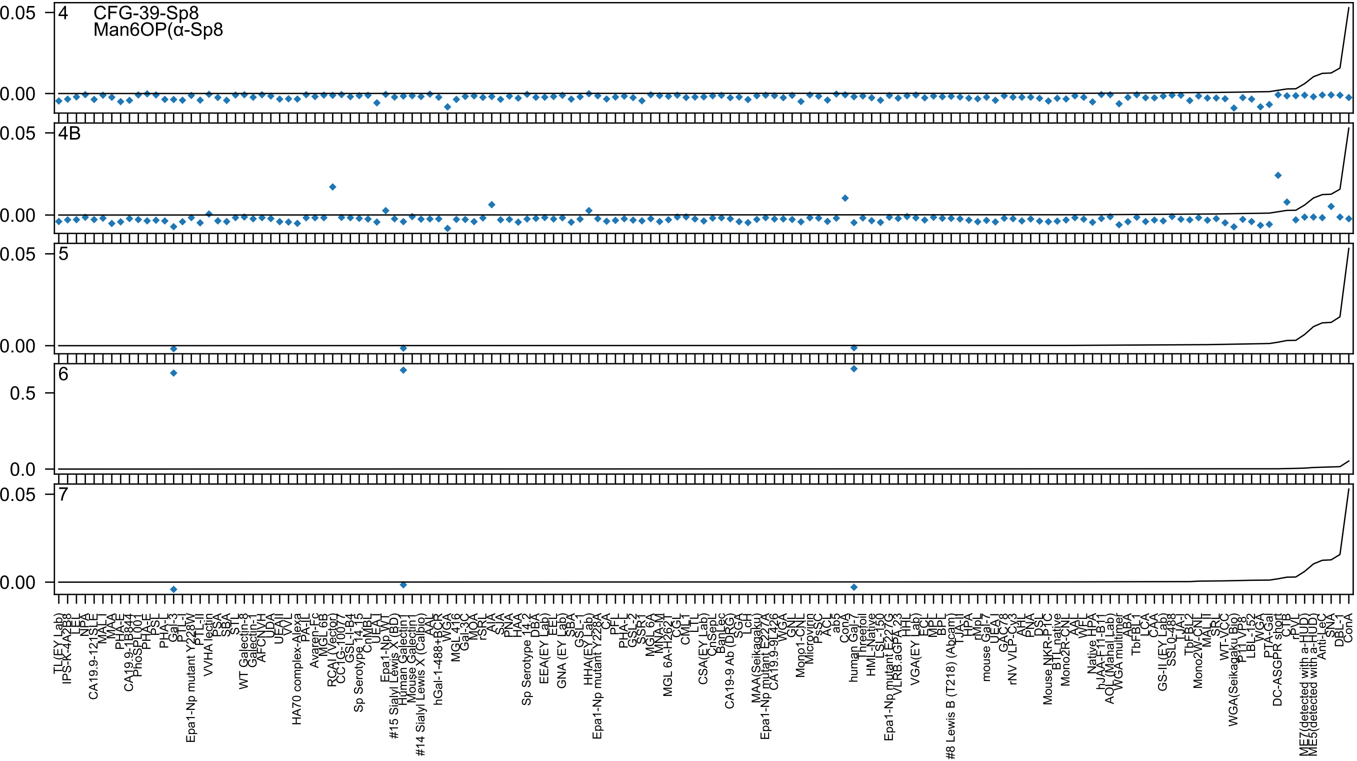

4
CFG-39-Sp8
Man6OP(α-Sp8
4B
5
6
7
0.05
0.00
0.5
0.0

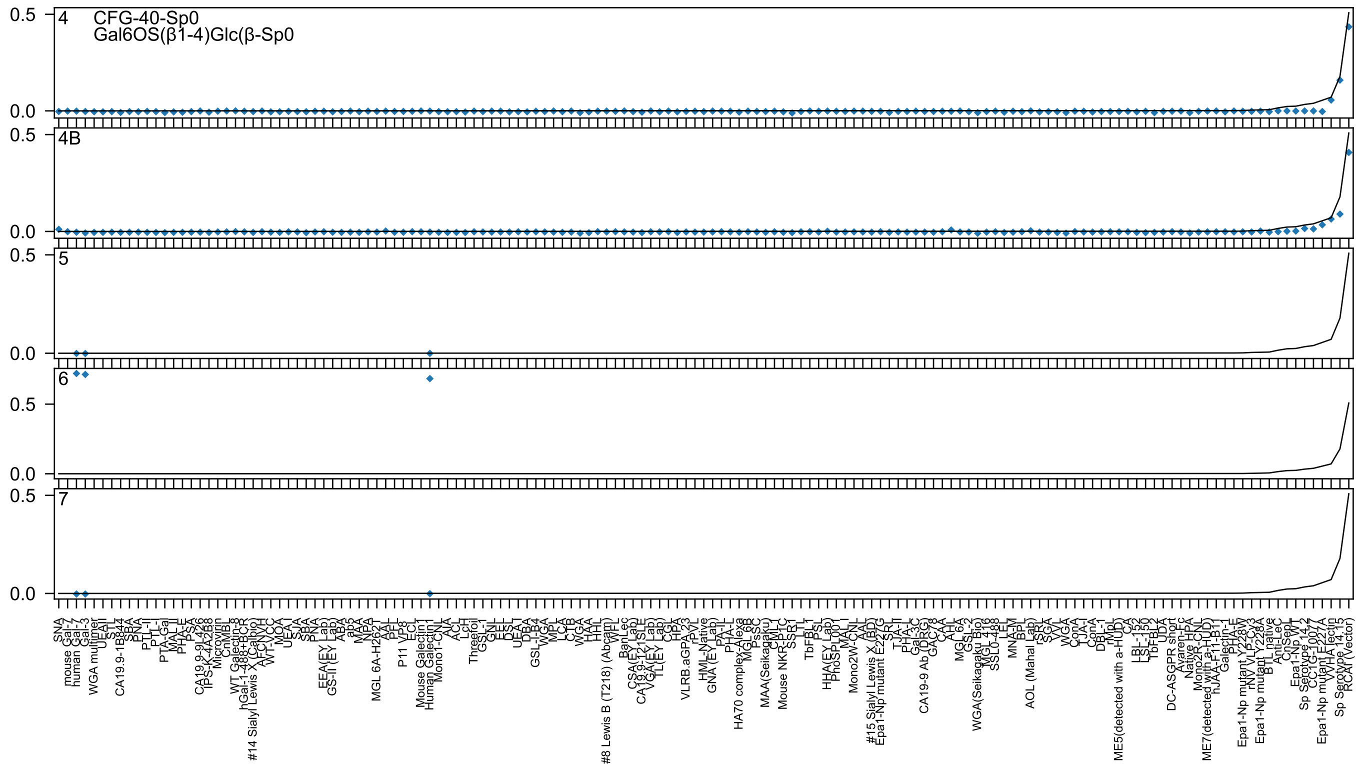

CFG-40-Sp0
Gal6OS(β1-4)Glc(β-Sp0
4
4B
5
6
7
0.5
0.0

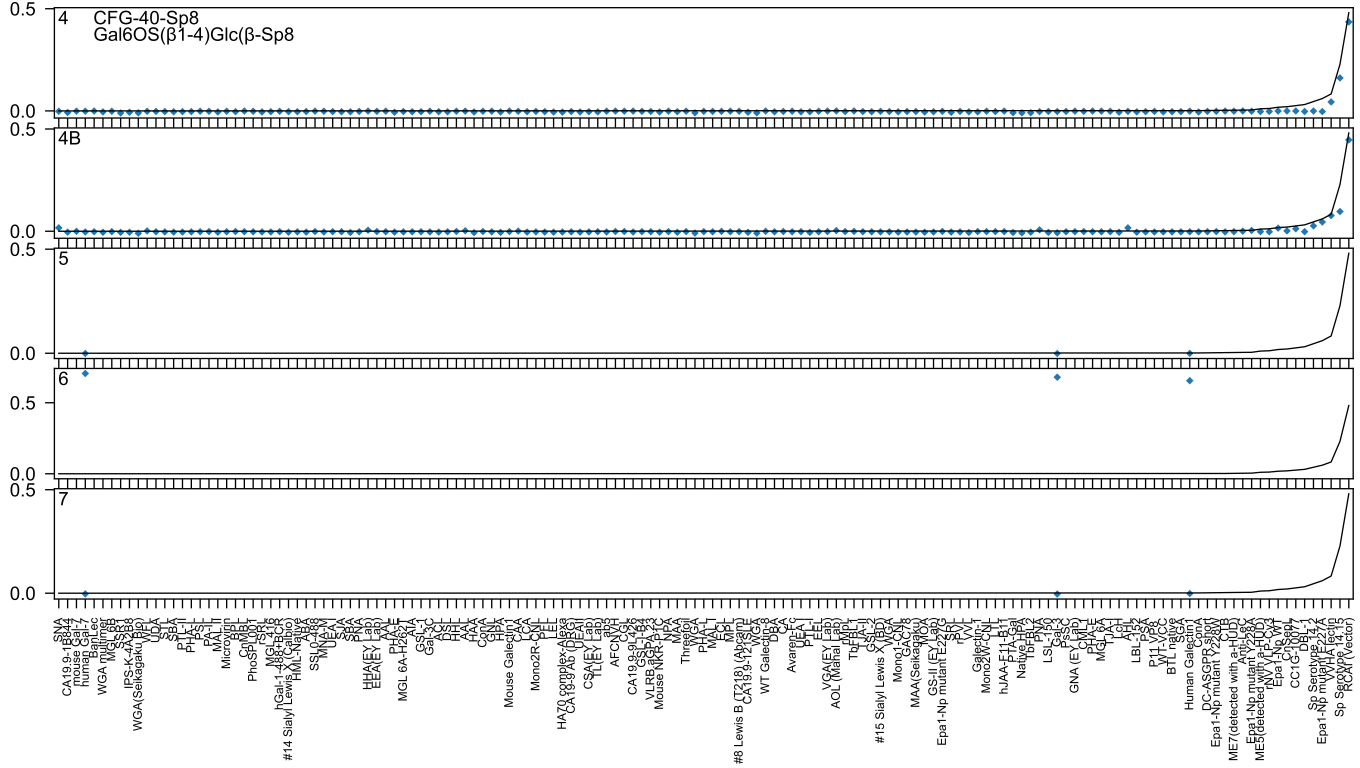
4 CFG-40-Sp8
Gal6OS(β1-4)Glc(β-Sp8
4B
5
6
7

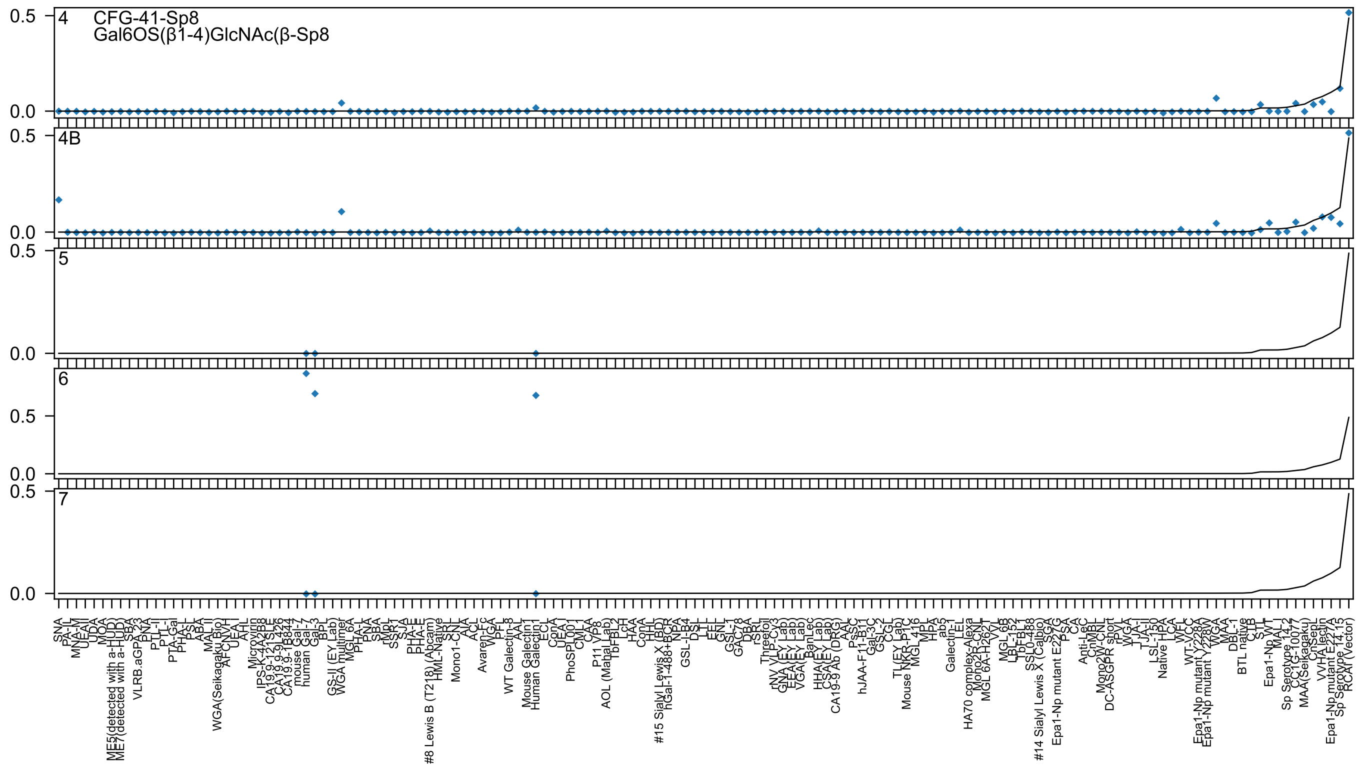

4
CFG-41-Sp8
Gal6OS(β1-4)GlcNAc(β-Sp8
4B
5
6
7

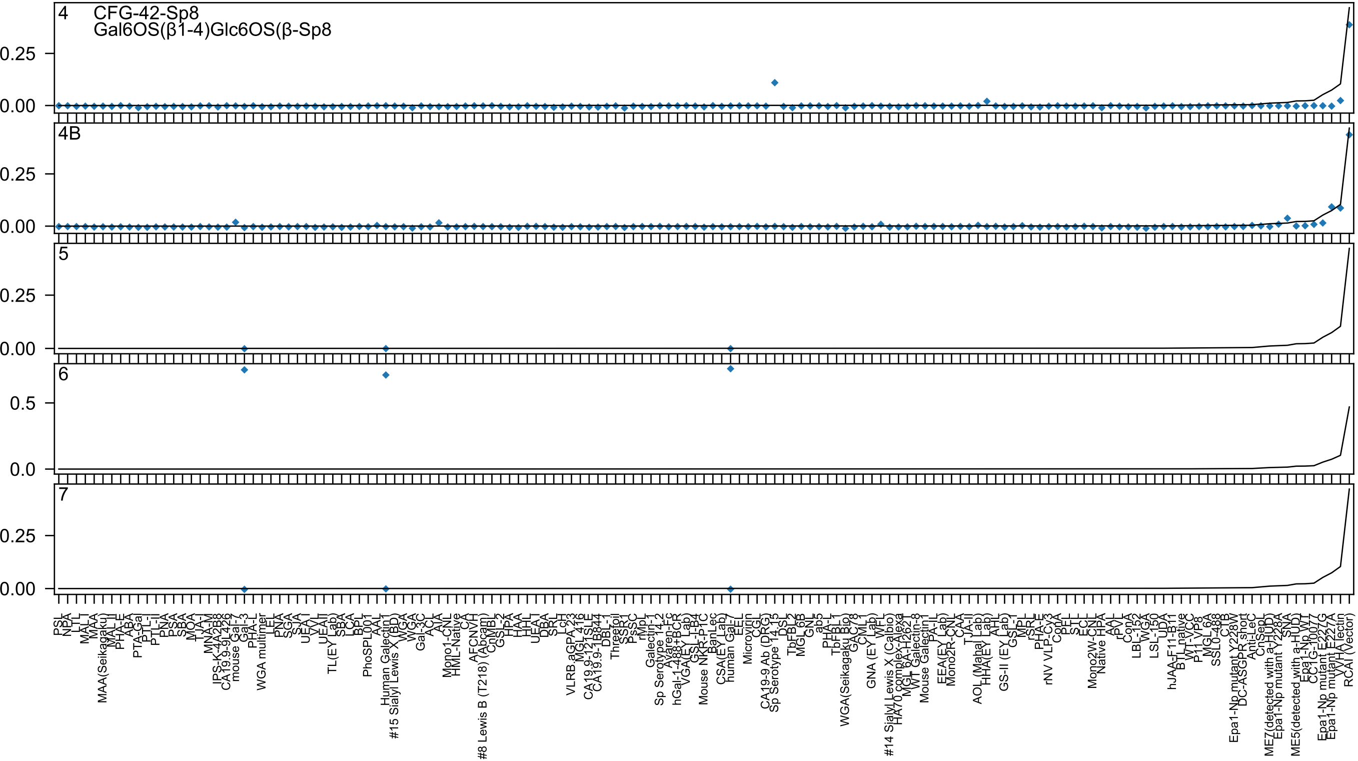
4
CFG-42-Sp8
Gal6OS(β1-4)Glc6OS(β-Sp8
4B
5
6
7

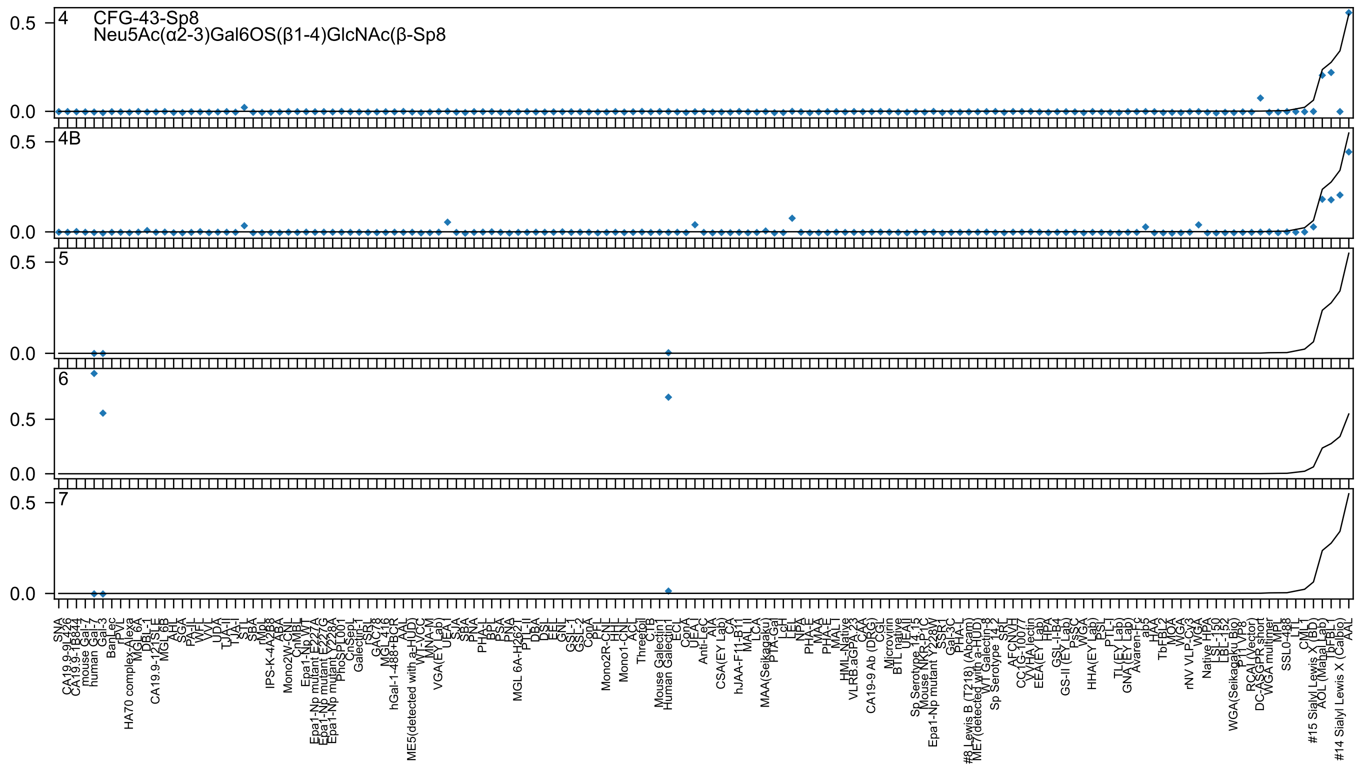
CFG-43-Sp8
Neu5Ac(α2-3)Gal6OS(β1-4)GlcNAc(β-Sp8
4
4B
5
6
7
0.5
0.0

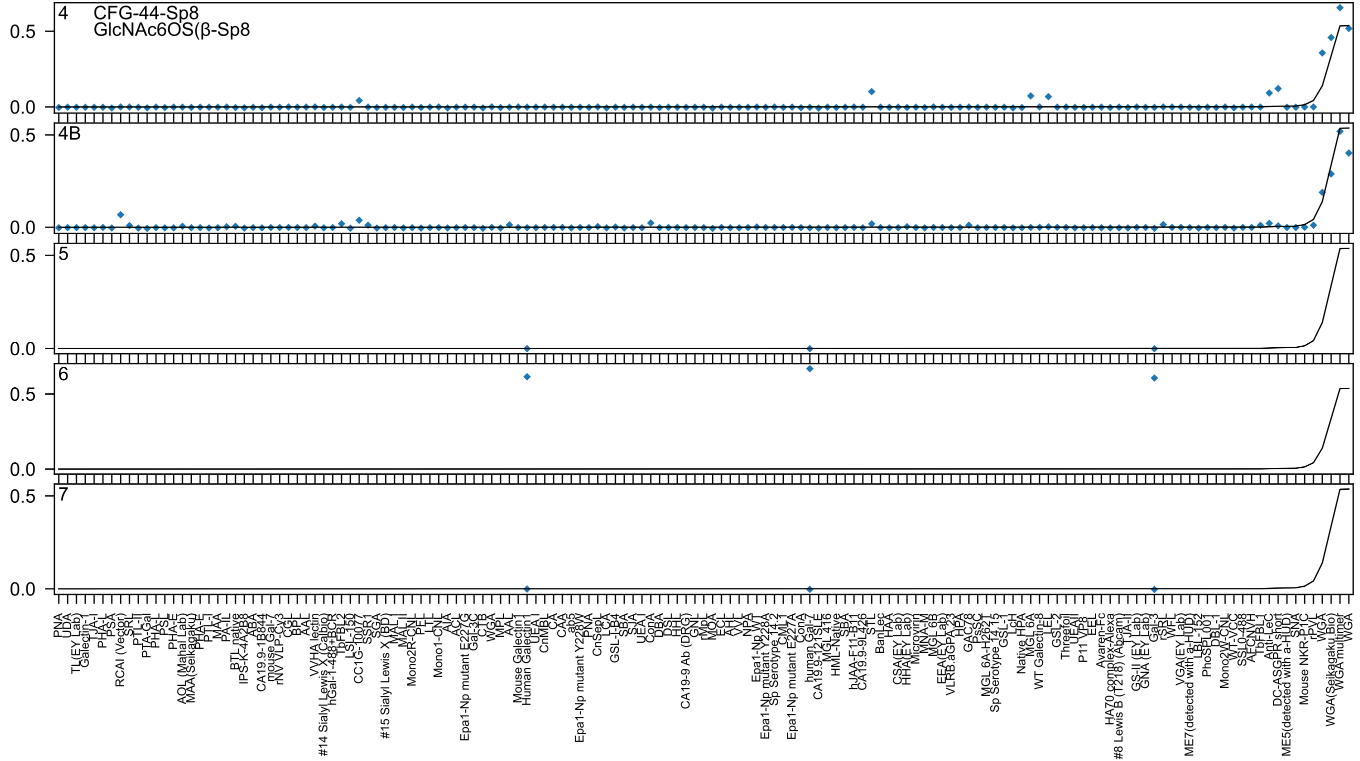
4 CFG-44-Sp8
GlcNAc6OS(β-Sp8
4B
5
6
7
0.5
0.0

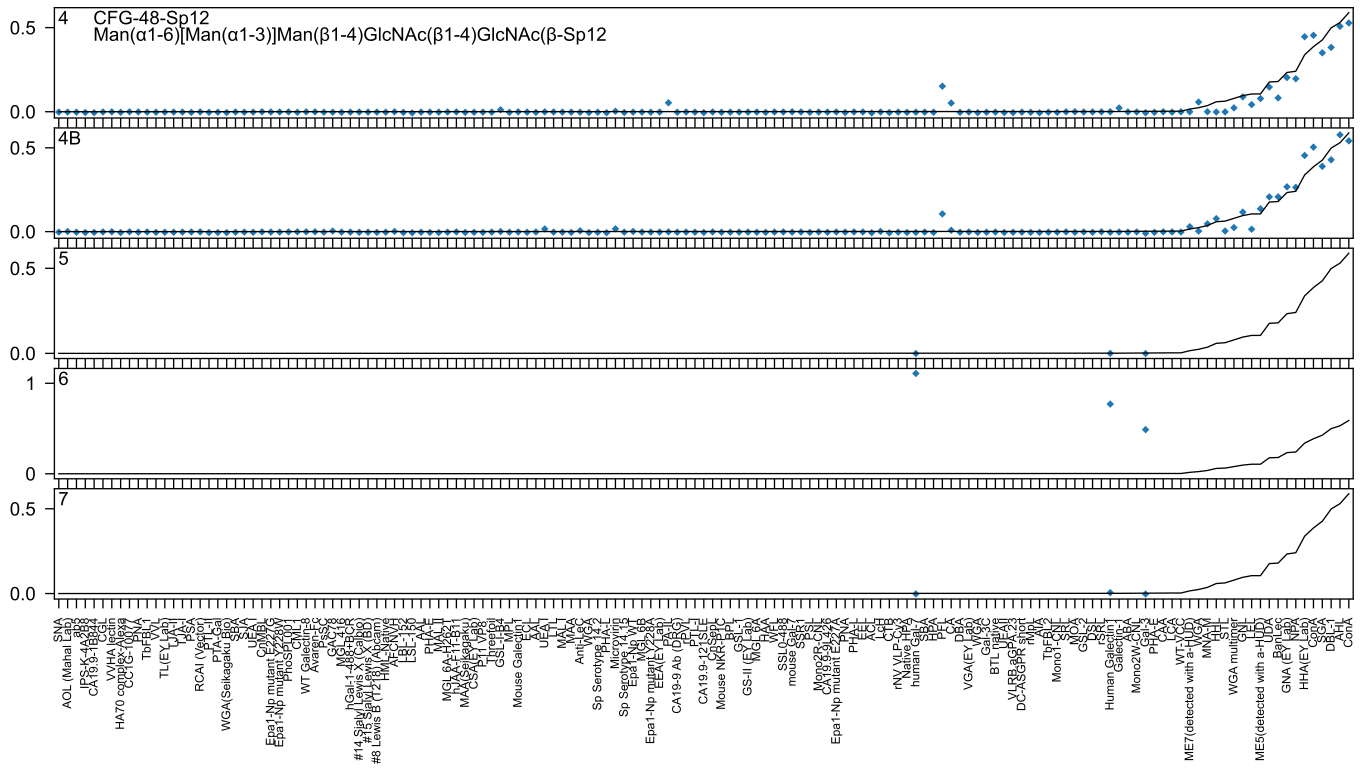

4 CFG-48-Sp12
Man(α1-6)[Man(α1-3)]Man(β1-4)GlcNAc(β1-4)GlcNAc(β-Sp12
4B
5
6
7

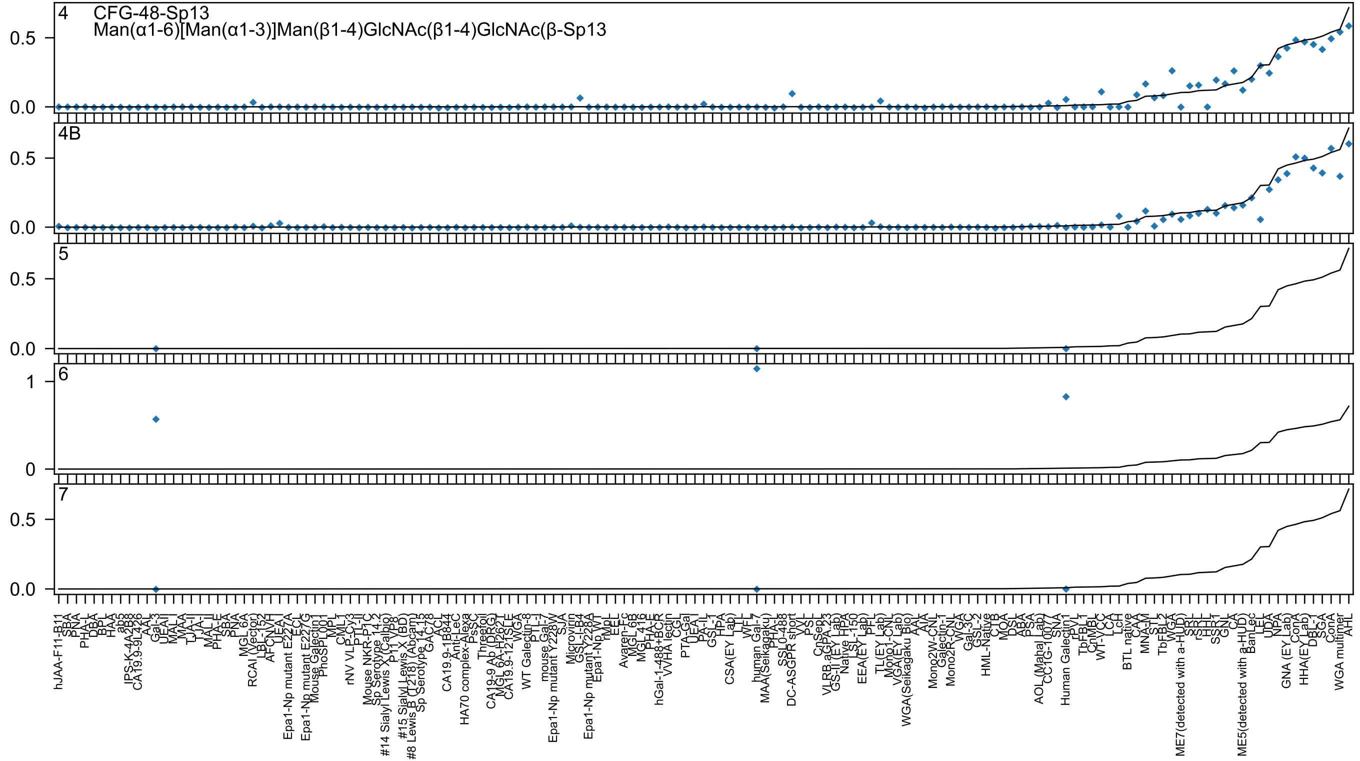

4 CFG-48-Sp13
Man(α1-6)[Man(α1-3)]Man(β1-4)GlcNAc(β1-4)GlcNAc(β-Sp13
4B
5
6
7

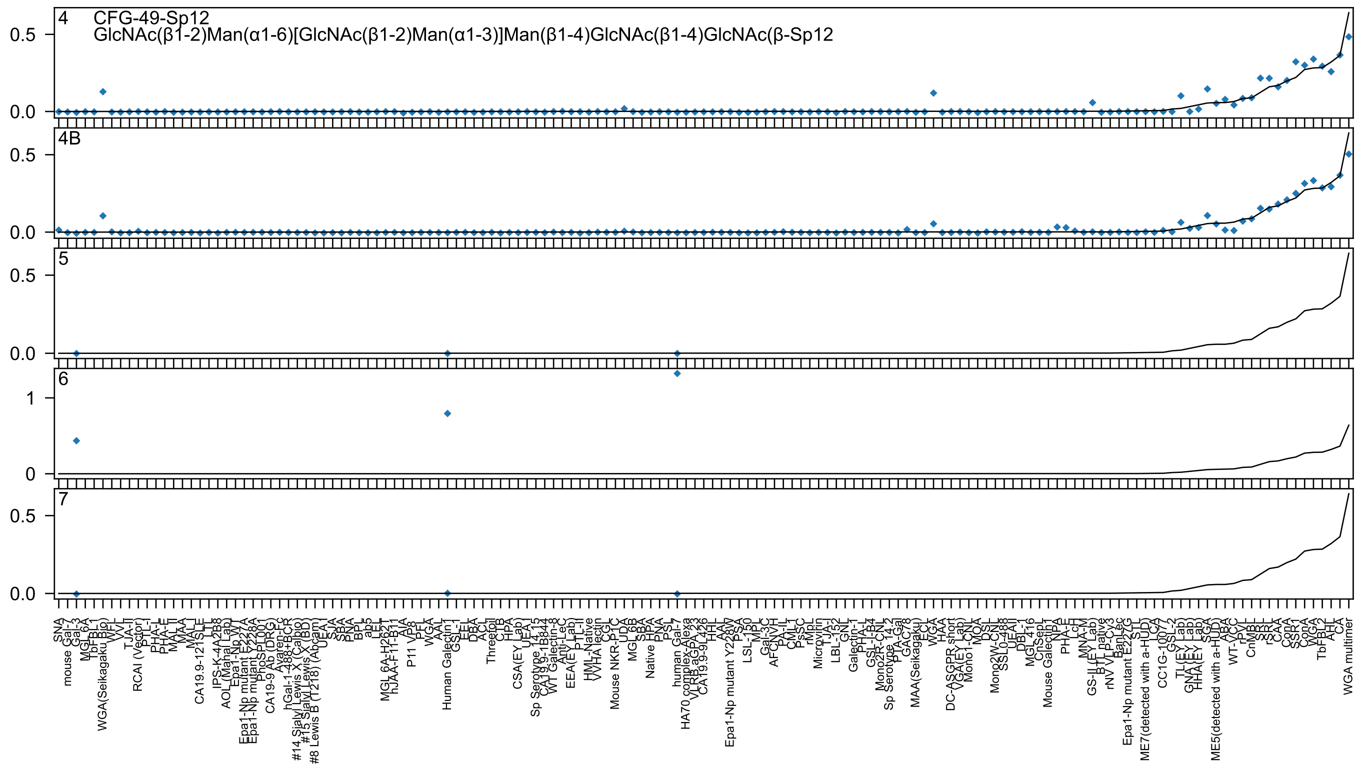
4 CFG-49-Sp12
GlcNAc(β1-2)Man(α1-6)[GlcNAc(β1-2)Man(α1-3)]Man(β1-4)GlcNAc(β1-4)GlcNAc(β-Sp12
4B
5
6
7
0.0
0.5
0
1
SNA
mouse Gal-7
Gal-3
MGL 6A
TbFBL1
WGA(Seikagaku Bio)
WFL
VVL
TJA-II
RCAI (Vector)
PTL-I
PHA-L
PHA-E
MAL II
MAA
MAL I
CA19.9-121SLE
LTL
IPS-K-4A2B8
AOL (Mahal Lab)
Epa1-Np WT
Epa1-Np mutant E227A
Epa1-Np mutant Y228A
PhoSL001
CA19-9 Ab (DRG)
Avaren-Fc
hGal-1-488+BCR
#14 Sialyl Lewis X (Calbio)
#15 Sialyl Lewis X (BD)
#8 Lewis B (T218) (Abcam)
UEA-I
SJA
SBA
PNA
BPL
ab5
LEL
MGL 6A-H262T
hJAA-F11-B11
AIA
P11 VP8
PFL
WGA
AAL
Human Galectin1
GSL-I
EEL
DBA
ACL
Threefoil
CTB
HPA
CSA(EY Lab)
UEA-I
Sp Serotype 14.15
CA19.9-1B844
WT Galectin-8
Anti-LeC
EEA(EY Lab)
PTL-II
HML-Native
VVHA lectin
CGL
Mouse NKR-P1C
UDA
MGL 6B
SBA
Native HPA
PNA
PSL
human Gal-7
HA70 complex-Alexa
VLRB.aGPA.23
CA19.9-9L426
HHL
AAL
Epa1-Np mutant Y228W
PSA
LSL-150
MPL
Gal-3C
AFCNVH
PA-IL
CML-I
PsSC
rMpL
Microvirin
TJA-I
LBL-152
GNL
Galectin-1
PHA-L
GSL-I-B4
Mono2R-CNL
Sp Serotype 14.2
PTA-Gal
GAC78
MAA(Seikagaku)
ECL
WGA
HAA
DC-ASGPR short
VGA(EY Lab)
Mono1-CNL
MOA
DSL
Mono2W-CNL
SSL0-488
UEAII
DBL-1
MGL 416
CnSep
Mouse Galectin1
NPA
PHA-E
LcH
MNA-M
GS-II (EY Lab)
BTL native
rNV VLP-Cy3
BanLec
Epa1-Np mutant E227G
STL
ME7(detected with a-HUD)
LCA
CC1G-10077
GSL-2
TL(EY Lab)
GNA(EY Lab)
HHA(EY Lab)
SGA
ME5(detected with a-HUD)
ABA
WT-VCC
rPVL
CnMBL
SRL
rSRL
CAA
ConA
SSR1
ConA
WGA
TbFBL2
AHL
CA
WGA multimer

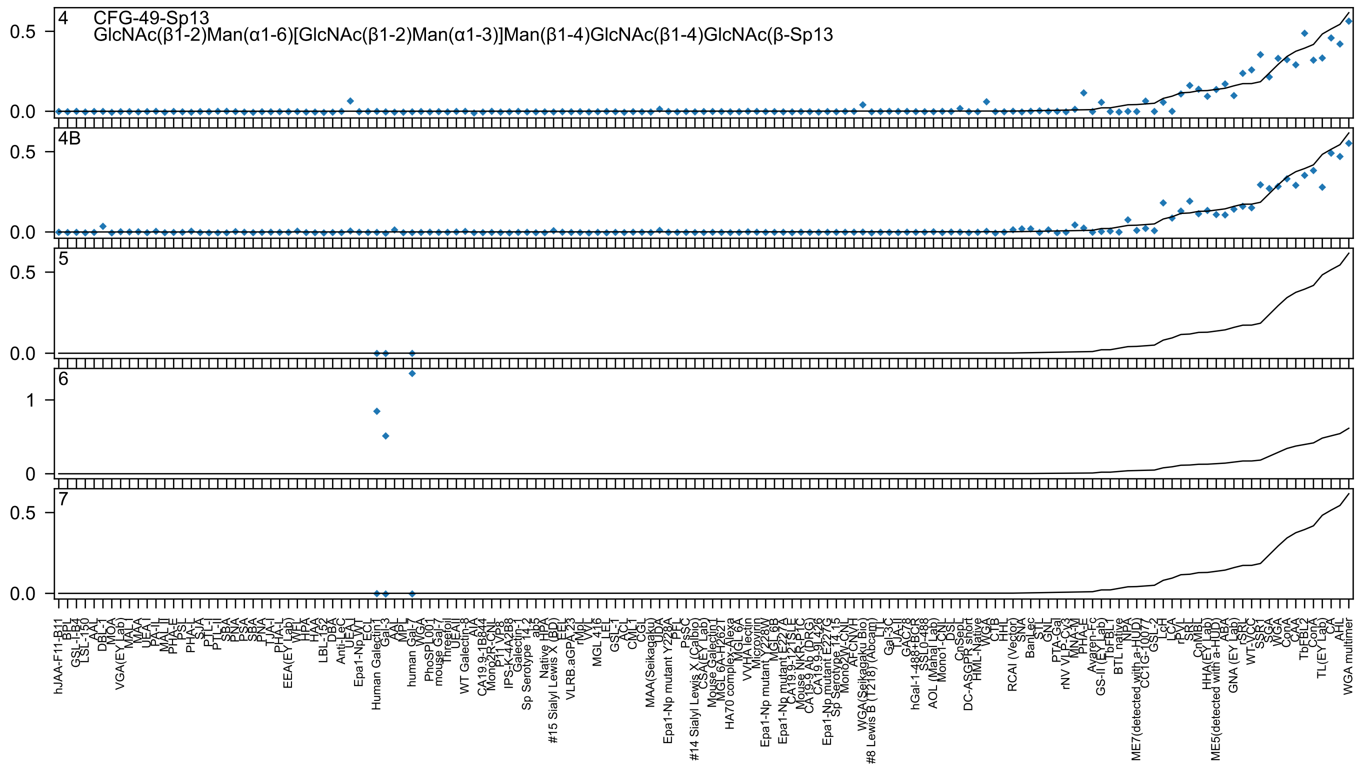

CFG-49-Sp13
GlcNAc(β1-2)Man(α1-6)[GlcNAc(β1-2)Man(α1-3)]Man(β1-4)GlcNAc(β1-4)GlcNAc(β-Sp13
4
4B
5
6
7

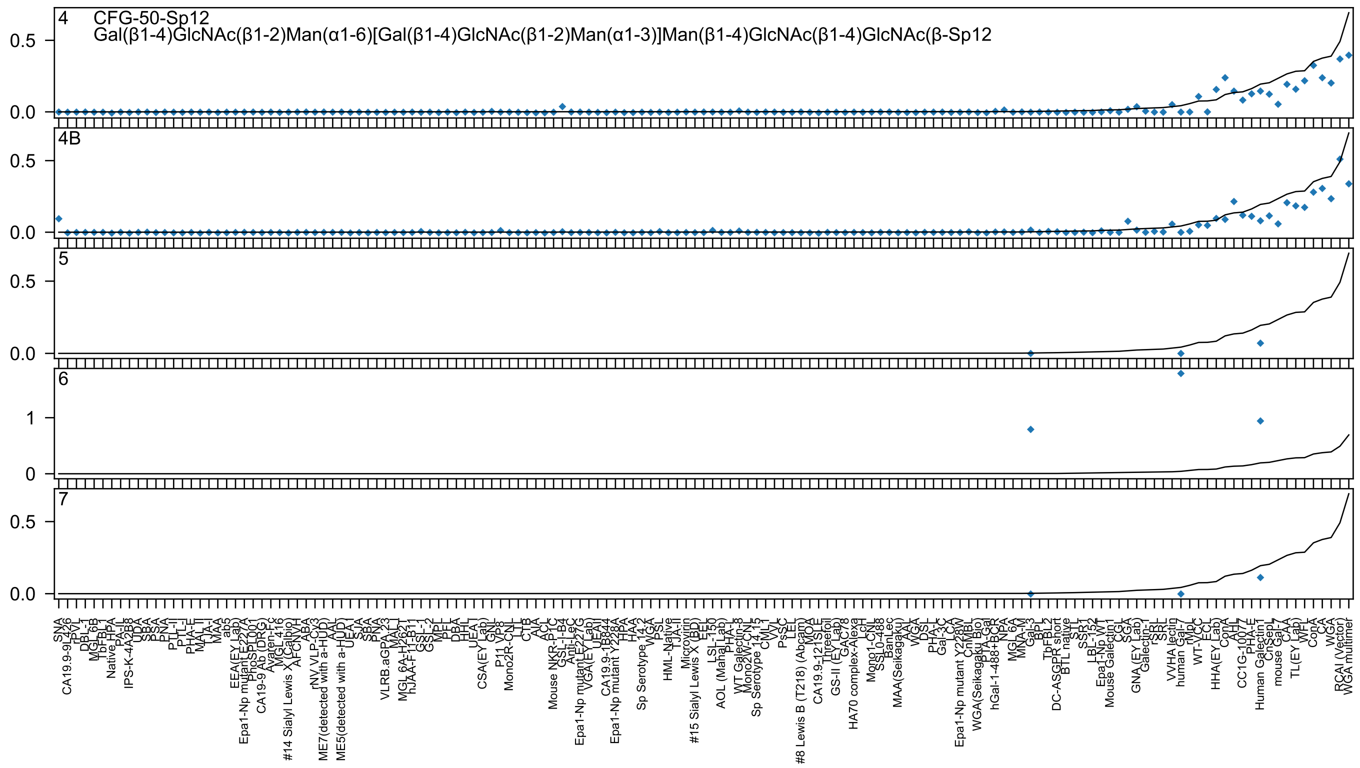
4
CFG-50-Sp12
Gal(β1-4)GlcNAc(β1-2)Man(α1-6)[Gal(β1-4)GlcNAc(β1-2)Man(α1-3)]Man(β1-4)GlcNAc(β1-4)GlcNAc(β-Sp12
0.5
0.0
4B
0.5
0.0
5
0.5
0.0
6
1
0
7
0.5
0.0
SNA
CA19.9-9L426
rPVL
DBL-1
MGL 6B
TbFBL1
Native HPA
PA-IL
IPS-K-4A2B8
UDA
SBA
PSA
PNA
PTL-II
PTL-I
PHA-E
MAL-II
TJA-I
MAA
ab5
EEA(EY Lab)
Epa1-Np mutant E227A
PhoSL001
CA19-9 Ab (DRG)
Avaren-Fc
MGL 416
#14 Sialyl Lewis X (Calbio)
AFCNVH
ABA
rNV VLP-Cy3
ME7(detected with a-HUD)
AAL
ME5(detected with a-HUD)
UEA-I
SJA
SBA
PNA
VLRB.aGPA.23
MAL-I
MGL 6A-H262T
hJAA-F11-B11
GSL-I
GSL-2
MPL
PFL
DBA
HHL
UEA-I
CSA(EY Lab)
GNL
P11 VP8
Mono2R-CNL
LTL
CTB
AIA
ACL
Mouse NKR-P1C
GSL-I-B4
Anti-LeC
Epa1-Np mutant E227G
VGA(EY Lab)
UEAII
CA19.9-1B844
Epa1-Np mutant Y228A
HPA
HAA
Sp Serotype 14.2
WGA
PSL
HML-Native
TJA-II
Microvirin
#15 Sialyl Lewis X (BD)
EEL
LSL-150
AOL (Mahal Lab)
PHA-L
WT Galectin-8
Mono2W-CNL
Sp Serotype 14.15
CML-I
VVL
PsSC
LEL
#8 Lewis B (T218) (Abcam)
MOA
CA19.9-121SLE
Threefoil
GS-II (EY Lab)
GAC78
HA70 complex-Alexa
LcH
Mono1-CNL
SSL0-488
BanLec
MAA(Seikagaku)
AAA
WGA
DSL
PHA-L
Gal-3C
LCA
Epa1-Np mutant Y228W
CnMBL
WGA(Seikagaku Bio)
PTA-Gal
hGal-1-488+BCR
NPA
MGL 6A
MNA-M
Gal-3
BPL
TbFBL2
DC-ASGPR short
BTL native
STL
SSR1
LBL-152
Epa1-Np WT
Mouse Galectin1
CGL
SGA
GNA (EY Lab)
Galectin-1
rSRL
SRL
VVHA lectin
human Gal-7
rMpL
WT-VCC
ECL
HHA(EY Lab)
ConA
AHL
CC1G-10077
PHA-E
Human Galectin1
CnSep1
mouse Gal-7
CAA
TL(EY Lab)
WFL
ConA
CA
WGA
RCAI (Vector)
WGA multimer

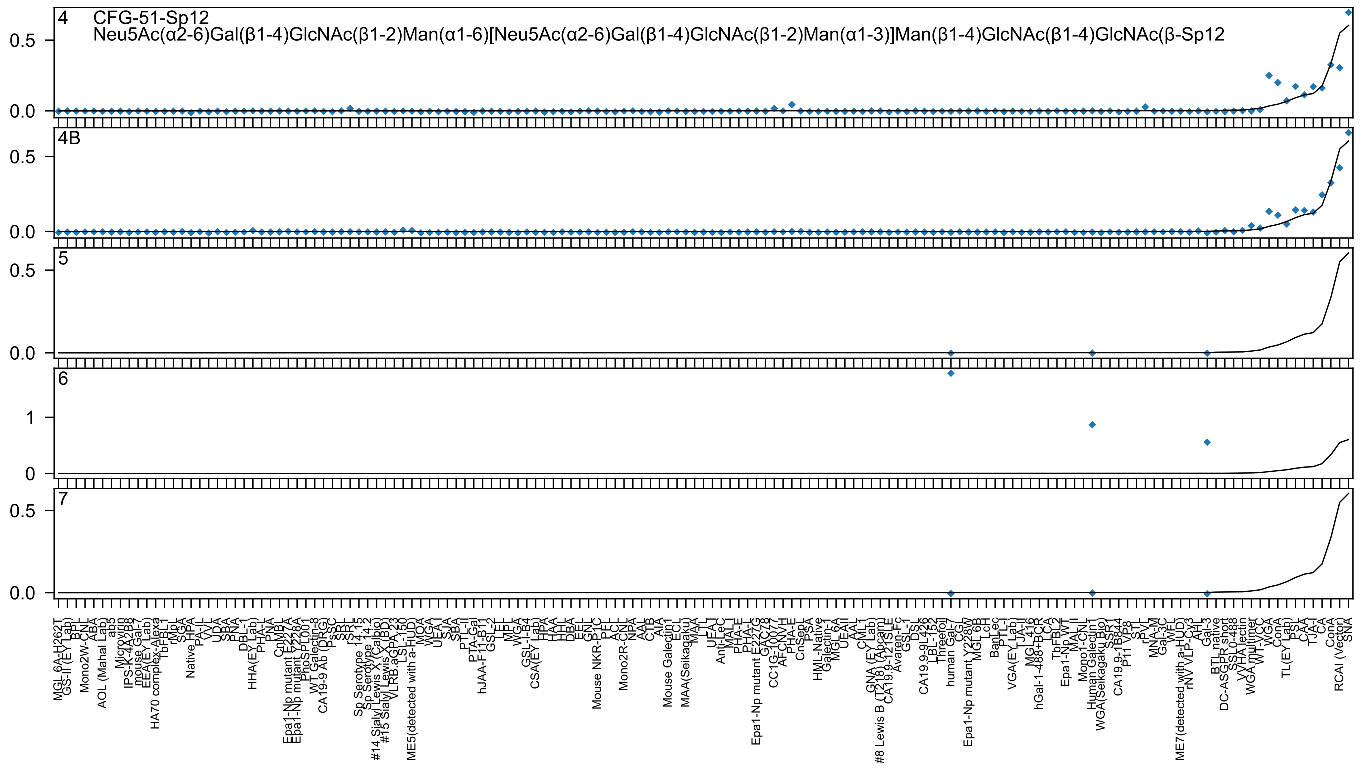
4 CFG-51-Sp12
Neu5Ac(α2-6)Gal(β1-4)GlcNAc(β1-2)Man(α1-6)[Neu5Ac(α2-6)Gal(β1-4)GlcNAc(β1-2)Man(α1-3)]Man(β1-4)GlcNAc(β1-4)GlcNAc(β-Sp12
4B
5
6
7

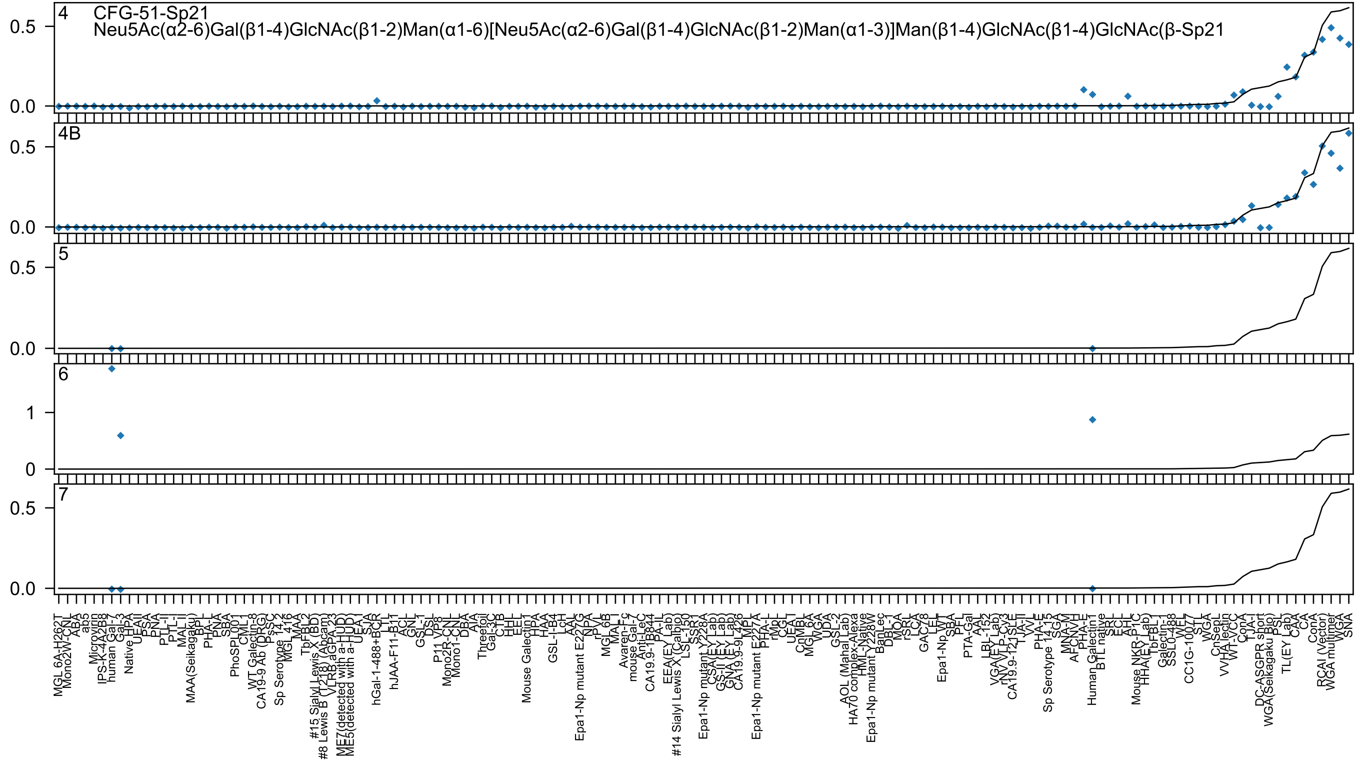

4 CFG-51-Sp21
Neu5Ac(α2-6)Gal(β1-4)GlcNAc(β1-2)Man(α1-6)[Neu5Ac(α2-6)Gal(β1-4)GlcNAc(β1-2)Man(α1-3)]Man(β1-4)GlcNAc(β1-4)GlcNAc(β-Sp21
4B
5
6
7

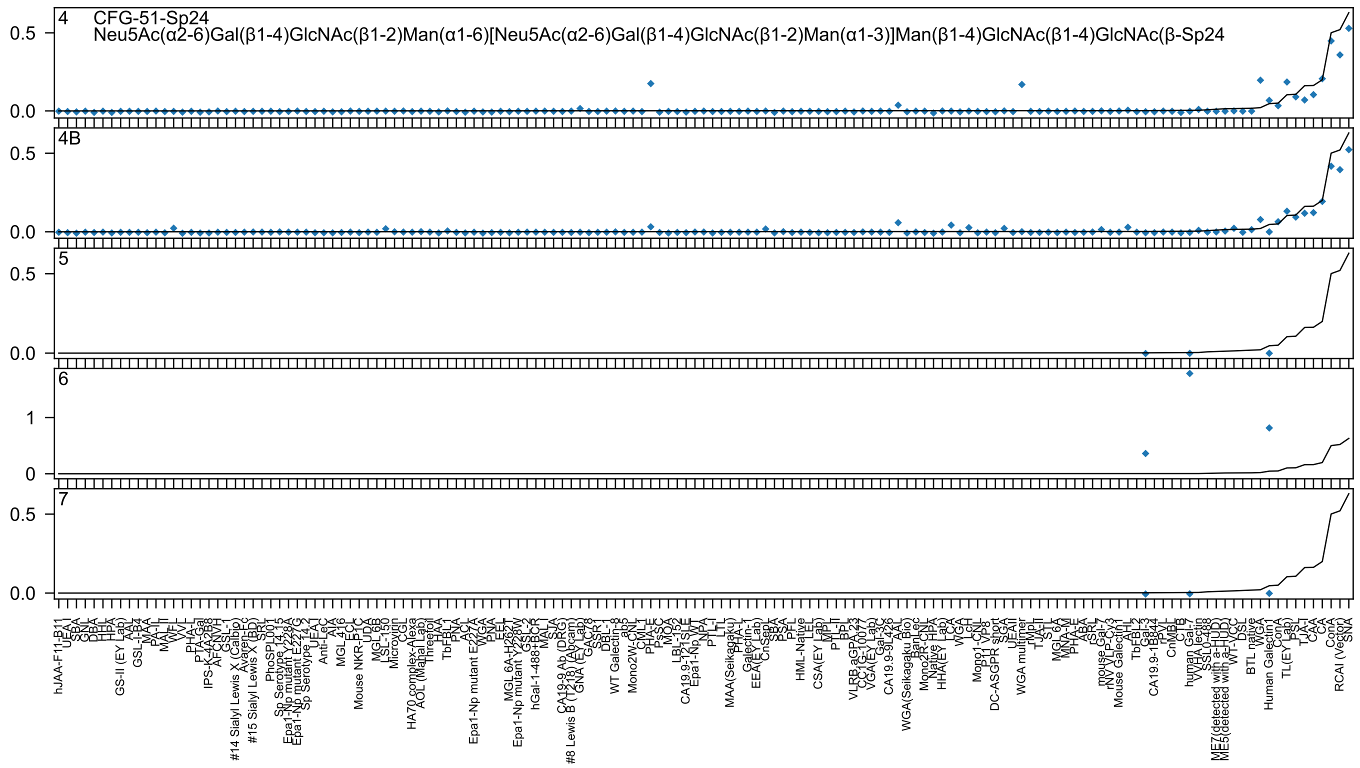

4 CFG-51-Sp24
Neu5Ac(α2-6)Gal(β1-4)GlcNAc(β1-2)Man(α1-6)[Neu5Ac(α2-6)Gal(β1-4)GlcNAc(β1-2)Man(α1-3)]Man(β1-4)GlcNAc(β1-4)GlcNAc(β-Sp24
4B
5
6
7
0.5
0.0
1
0

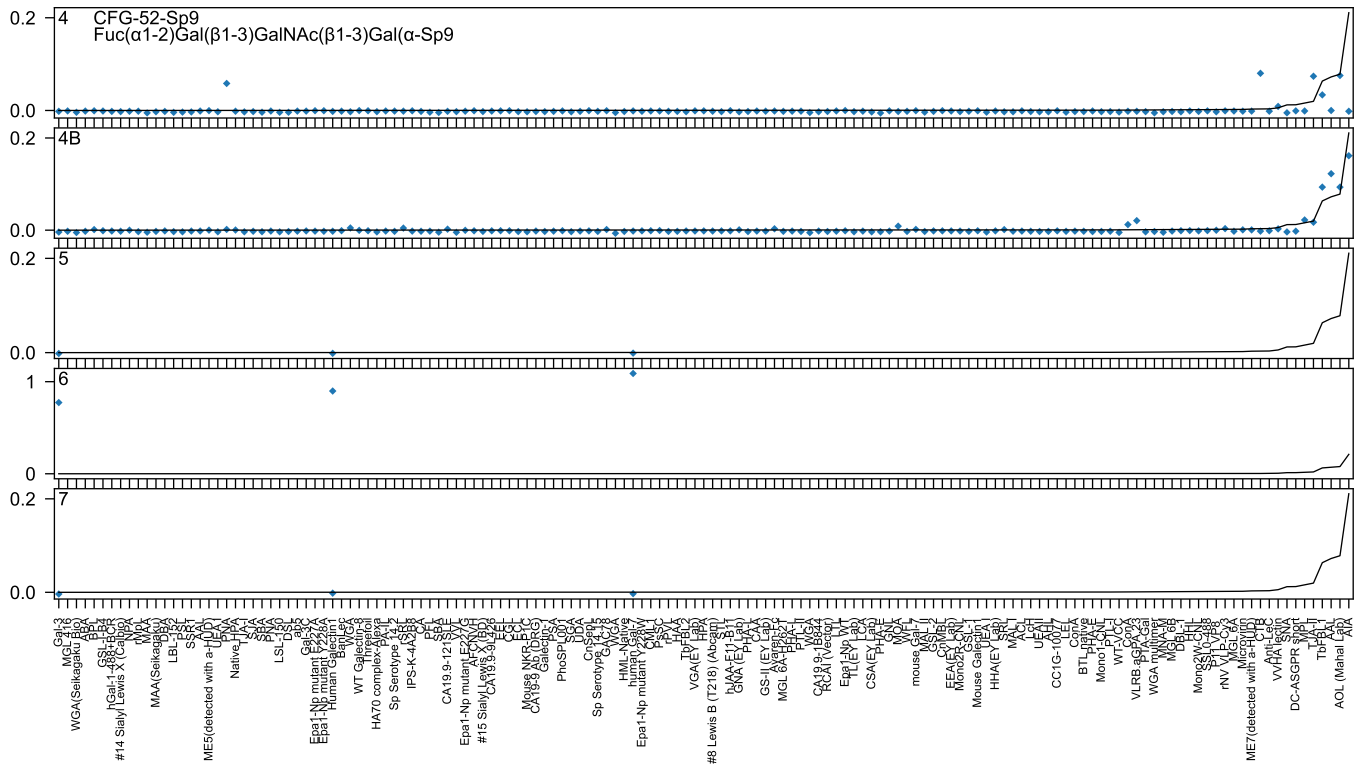

4 CFG-52-Sp9
Fuc(α1-2)Gal(β1-3)GalNAc(β1-3)Gal(α-Sp9
4B
5
6
7

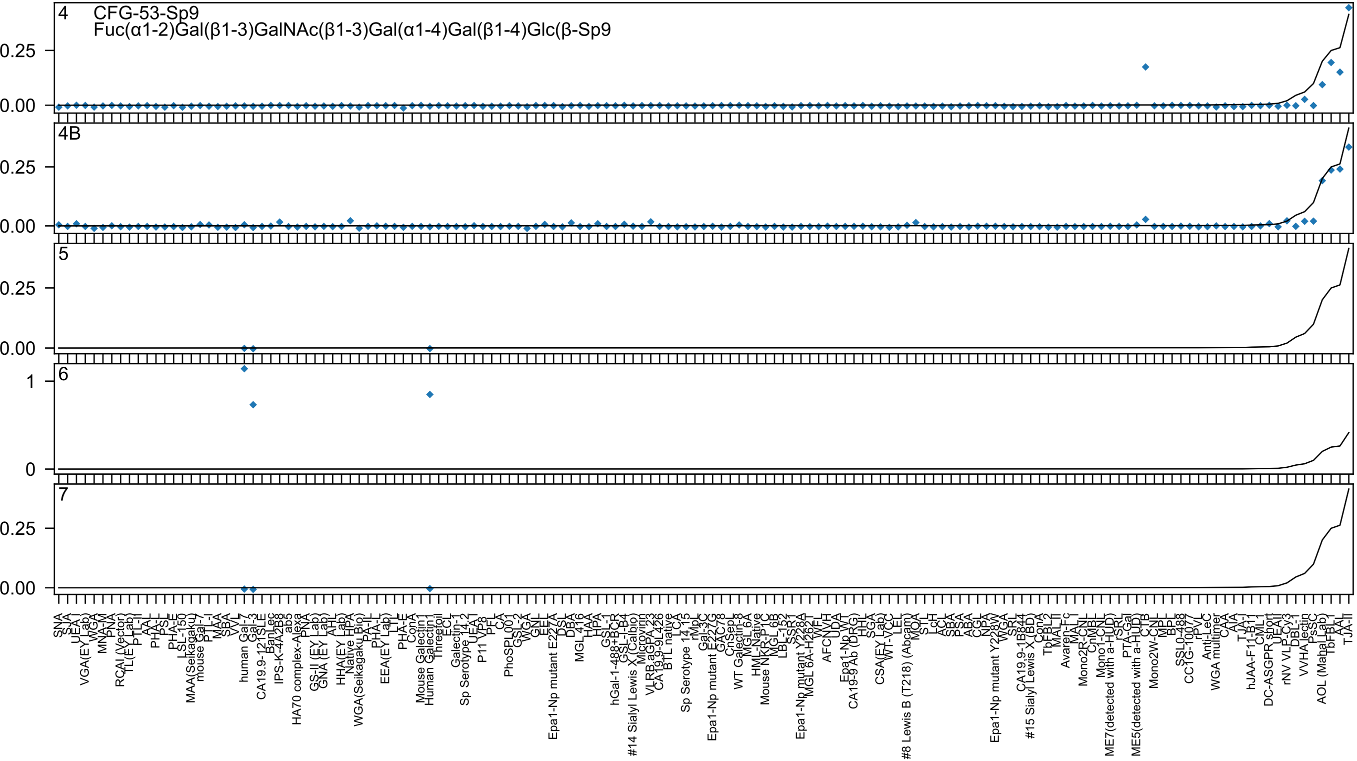

4
CFG-53-Sp9
Fuc(α1-2)Gal(β1-3)GalNAc(β1-3)Gal(α1-4)Gal(β1-4)Glc(β-Sp9
0.25
0.00
4B
0.25
0.00
5
0.25
0.00
6
1
0
7
0.25
0.00
SNA
SJA
UEA-I
VGA(EY Lab)
WGA
MNA-M
PNA
RCAI (Vector)
TL(EY Lab)
PTL-II
AAL
PHA-L
PSL
PHA-E
LSL-150
MAA(Seikagaku)
mouse Gal-7
PTL-I
MAA
SBA
VVL
human Gal-7
Gal-3
CA19.9-121SLE
BanLec
IPS-K-4A2B8
ab5
HA70 complex-Alexa
PNA
GS-II (EY Lab)
GNA (EY Lab)
AHL
HHA(EY Lab)
Native HPA
WGA(Seikagaku Bio)
PA-II
PHA-L
EEA(EY Lab)
LTL
PHA-E
ConA
Mouse Galectin1
Human Galectin1
Threefoil
ECL
Galectin-1
Sp Serotype 14.2
UEA-I
P11 VP8
PFL
CA
PhoSPL001
GSL-2
WGA
GNL
EEL
Epa1-Np mutant E227A
DSL
DBA
MGL 416
HAA
HPA
GSL-1
hGal-1-488+BCR
GSL-I-B4
#14 Sialyl Lewis X (Calbio)
Microvirin
VLRB aGPA.23
CA19.9-9L426
BTL native
LCA
Sp Serotype 14.15
rMpL
Gal-3C
Epa1-Np mutant E227G
GAC78
CnSep
WT Galectin-8
MGL 6A
HML-Native
Mouse NKR-P1C
MGL 6B
LBL-152
SSR1
Epa1-Np mutant Y228A
MGL 6A-H262T
WFL
AFCNVH
UDA
Epa1-Np WT
CA19-9 Ab (DRG)
HHL
SGA
CSA(EY Lab)
WT-VCC
LEL
#8 Lewis B (T218) (Abcam)
MOA
STL
LcH
ACL
SBA
PSA
ABA
CGL
NPA
Epa1-Np mutant Y228W
WGA
SRL
CA19.9-1B844
#15 Sialyl Lewis X (BD)
ConA
TbFBL2
MAL II
MAL I
Avaren-Fc
Mono2R-CNL
CnMBL
Mono1-CNL
ME7(detected with a-HUD)
rSRL
PTA-Gal
ME5(detected with a-HUD)
CTB
Mono2W-CNL
MPL
BPL
SSL0-488
CC1G-1007
rPVL
Anti-LeC
WGA multimer
CAA
AIA
TJA-I
hJAA-F11-B11
CML1
DC-ASGPR short
UEA II
rNV VLP-Cy3
DBL-1
VVHA lectin
PsSC
AOL (Mahal Lab)
TbFBL1
AAL
TJA-II

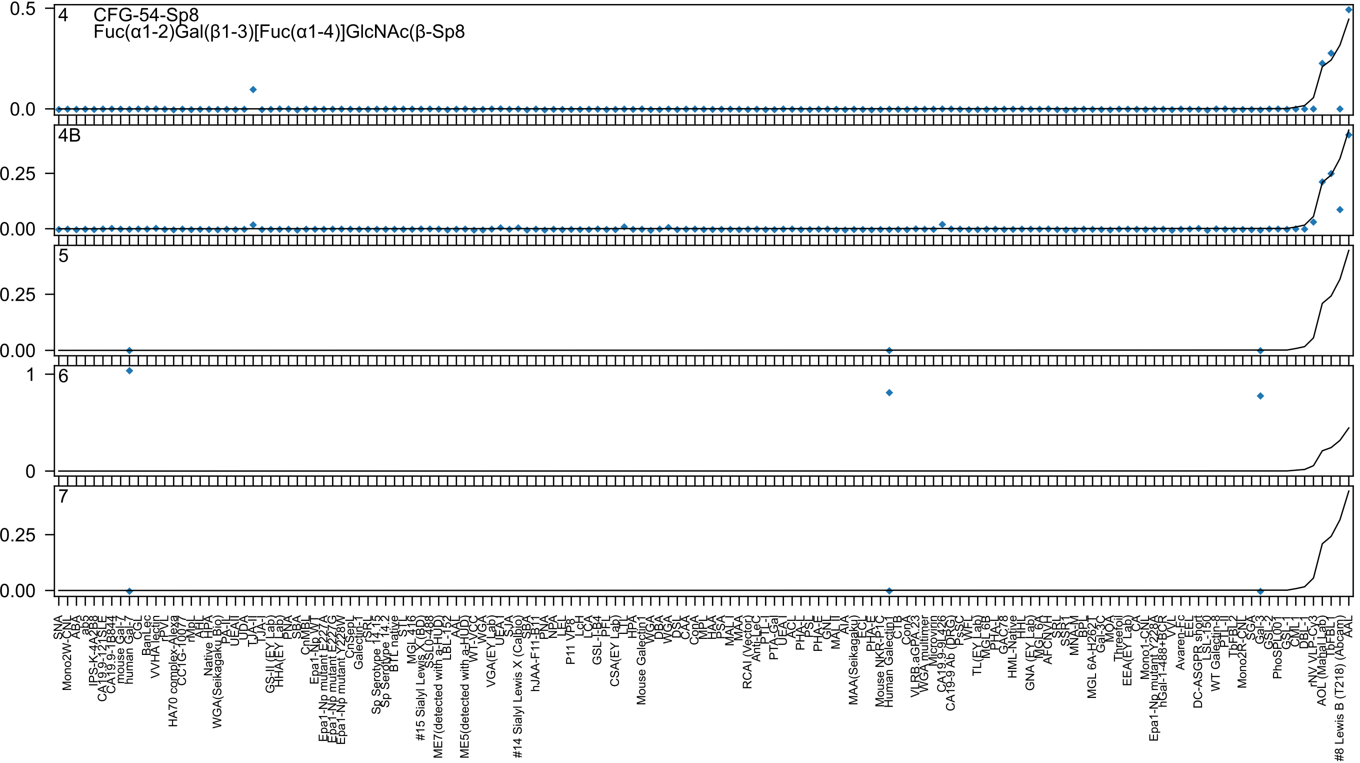
CFG-54-Sp8
Fuc(α1-2)Gal(β1-3)[Fuc(α1-4)]GlcNAc(β-Sp8
4
4B
5
6
7

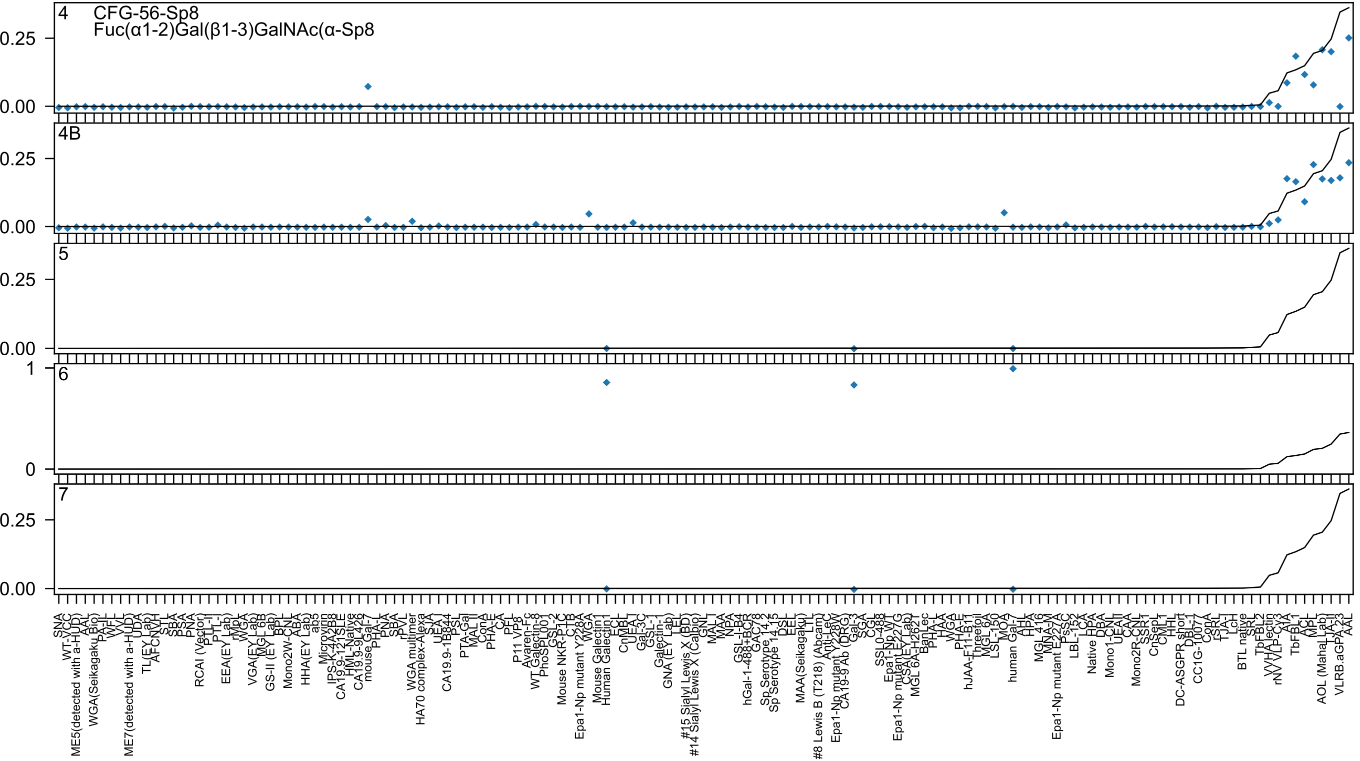

4
CFG-56-Sp8
Fuc(α1-2)Gal(β1-3)GalNAc(α-Sp8
4B
5
6
7
0.25
0.00
1
0

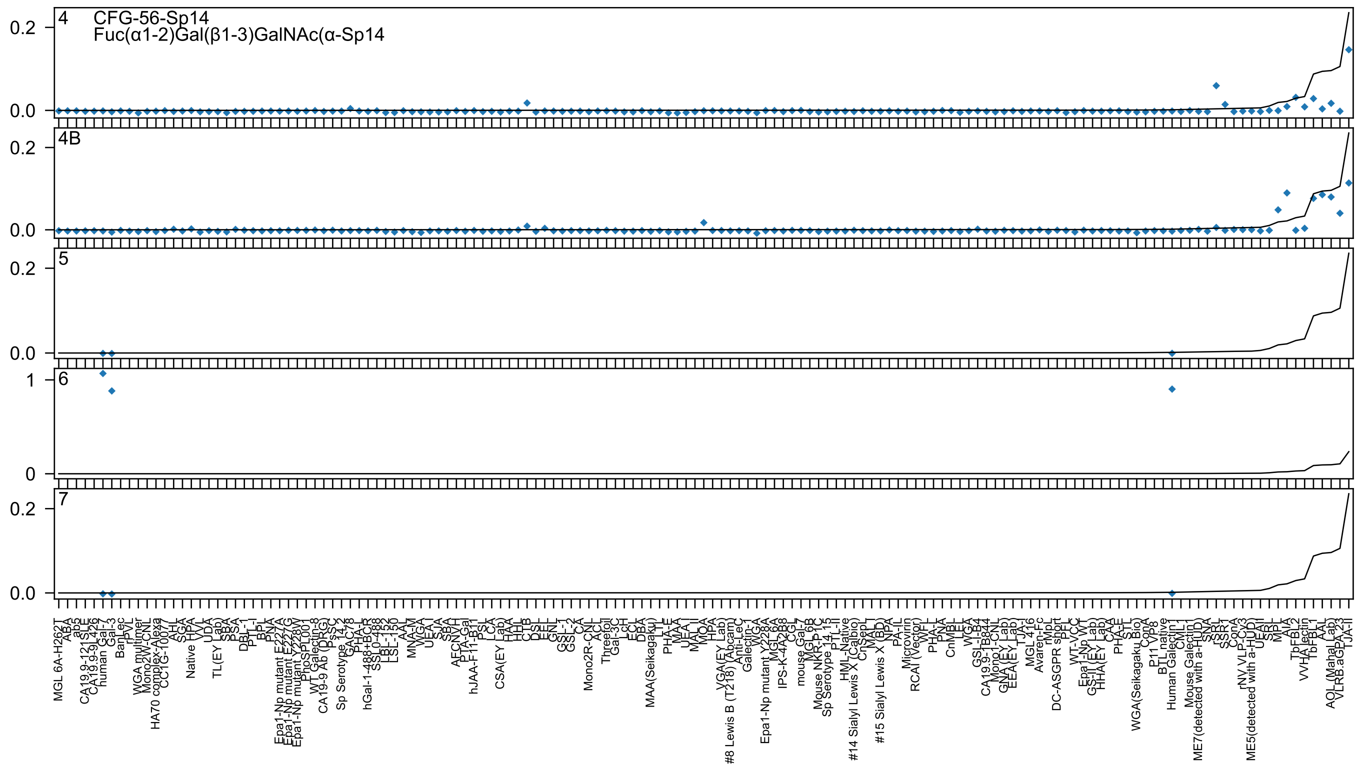
4 CFG-56-Sp14
Fuc(α1-2)Gal(β1-3)GalNAc(α-Sp14
4B
5
6
7

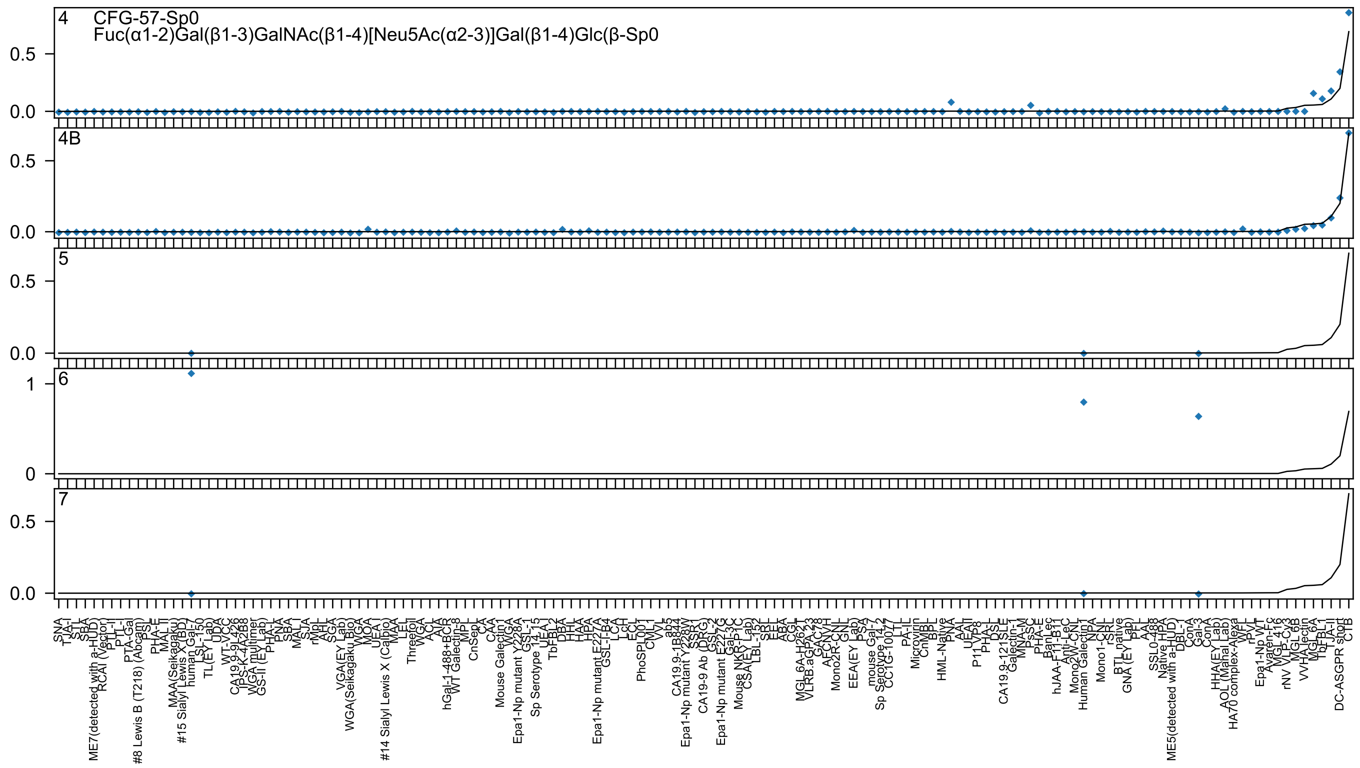

4
CFG-57-Sp0
Fuc(α1-2)Gal(β1-3)GalNAc(β1-4)[Neu5Ac(α2-3)]Gal(β1-4)Glc(β-Sp0
4B
5
6
7

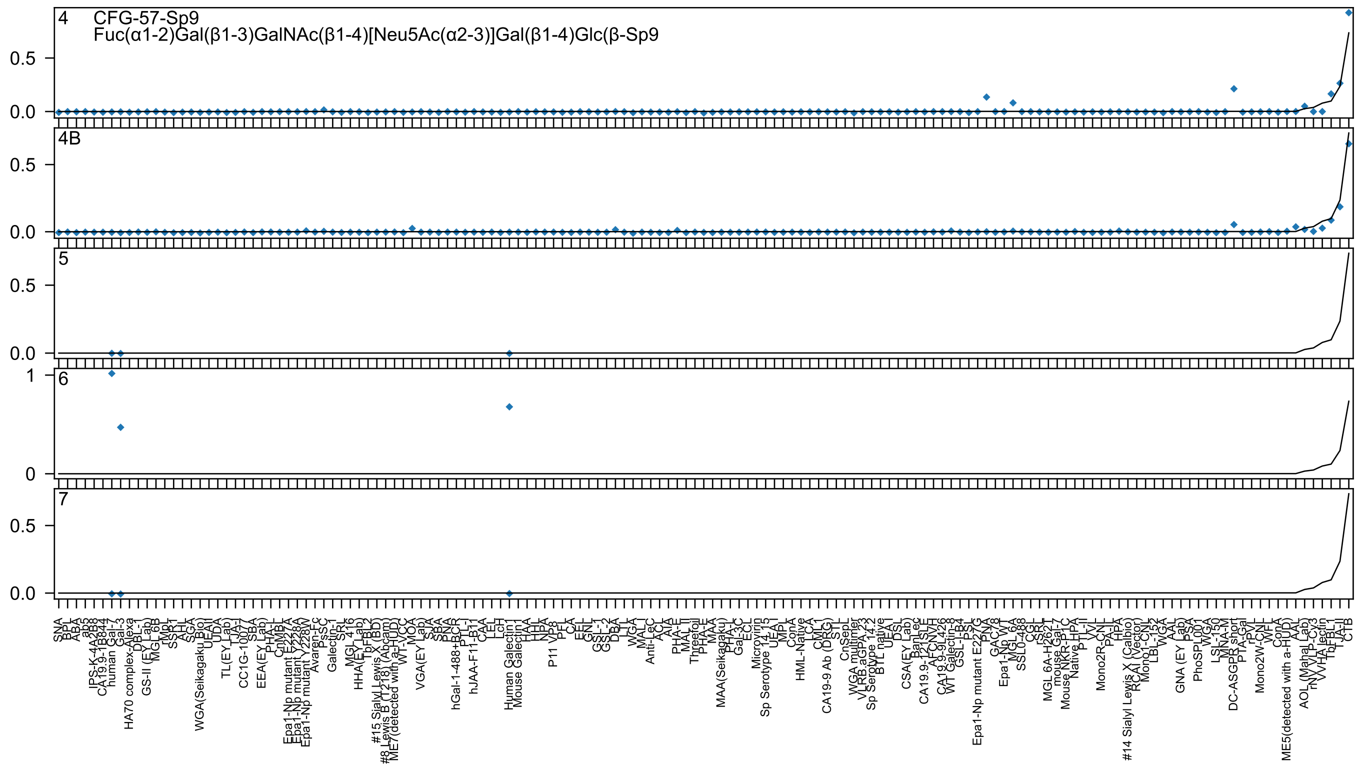
4
CFG-57-Sp9
Fuc(α1-2)Gal(β1-3)GalNAc(β1-4)[Neu5Ac(α2-3)]Gal(β1-4)Glc(β-Sp9
4B
5
6
7
0.0
0.5
0
1

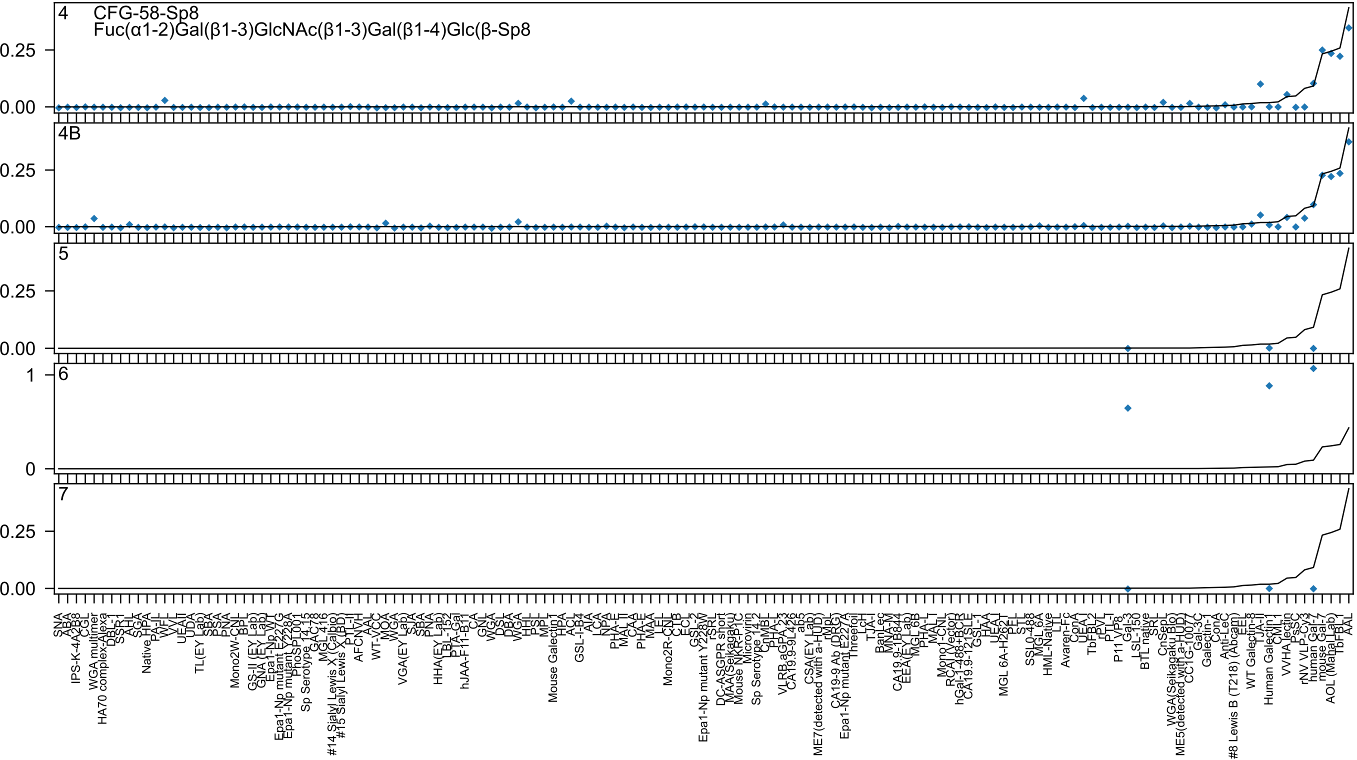

4
CFG-58-Sp8
Fuc(α1-2)Gal(β1-3)GlcNAc(β1-3)Gal(β1-4)Glc(β-Sp8
0.25
0.00
4B
0.25
0.00
5
0.25
0.00
6
1
0
7
0.25
0.00
SNA
ABA
IPS-K-4A2B8
CGL
WGA multimer
HA70 complex-Alexa
DBL-1
SSR-1
AHL
SGA
Native HPA
PA-IL
WFL
VVL
UEAII
UDA
TL(EY Lab)
SBA
PSA
PNA
Mono2W-CNL
BPL
GS-II (EY Lab)
GNA (EY Lab)
Epa1-Np WT
Epa1-Np mutant E227G
Epa1-Np mutant Y228A
PhoSL001
Sp Serotype 14.15
GAC78
MGL 416
#14 Sialyl Lewis X (Calbio)
#15 Sialyl Lewis X (BD)
PTL-II
AFCNVH
AAL
WT-VCC
MOA
WGA
VGA(EY Lab)
SJA
SBA
PNA
HHA(EY Lab)
LBL-152
PTA-Gal
hJAA-F11-B11
CA
GNL
WGA
DSL
DBA
WGA
HHL
PSL
MPL
Mouse Galectin1
HPA
ACL
GSL-I-B4
AIA
LCA
NPA
PHA-E
MAL II
CAA
PHA-E
MAA
LEL
Mono2R-CNL
CTB
ECL
GSL-2
Epa1-Np mutant Y228W
rSRL
DC-ASGPR short
MAA(Seikagaku)
Mouse NKR-P1C
Microvirin
Sp Serotype 14.2
CnMBL
PHA-L
VLRB aGPA.23
CA19.9-9L426
ab5
CSA(EY Lab)
ME7(detected with a-HUD)
rMpL
CA19-9 Ab (DRG)
Epa1-Np mutant E227A
Threefoil
LcH
TJA-I
BanLec
MNA-M
CA19.9-1B844
EEA(EY Lab)
MGL 6B
PHA-L
MAL I
Mono1-CNL
RCAI (Vector)
hGal-1-488+BCR
CA19.9-121SLE
GSL-1
HAA
UEA I
MGL 6A-H262T
PFL
STL
SSL0-488
MGL 6A
HML-Native
LTL
Avaren-Fc
ConA
UEA I
TbFBL2
rPVL
PTL-I
P11 VP8
Gal-3
LSL-150
BTL native
SRL
CnSepL
WGA(Seikagaku Bio)
ME5(detected with a-HUD)
CC1G-10077
Gal-3C
Galectin-1
ConA
Anti-LeC
#8 Lewis B (T218) (Abcam)
EEL
WT Galectin-8
TJA-II
Human Galectin1
CML-1
VVHA lectin
PsSC
rNV VLP-Cy3
human Gal-7
mouse Gal-7
AOL (Magal Lab)
TbFBL1
AAL

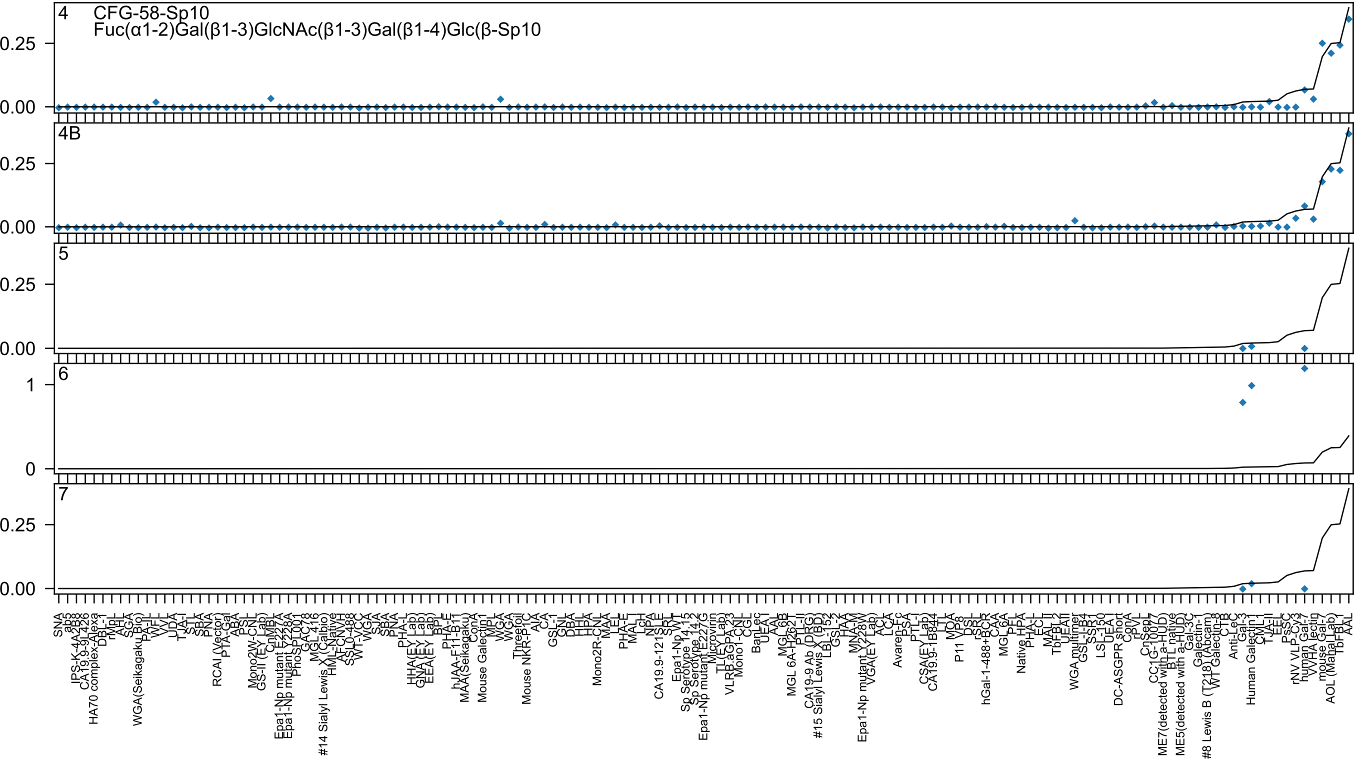
4 CFG-58-Sp10
Fuc(α1-2)Gal(β1-3)GlcNAc(β1-3)Gal(β1-4)Glc(β-Sp10
4B
5
6
7
0.25
0.00
1
0

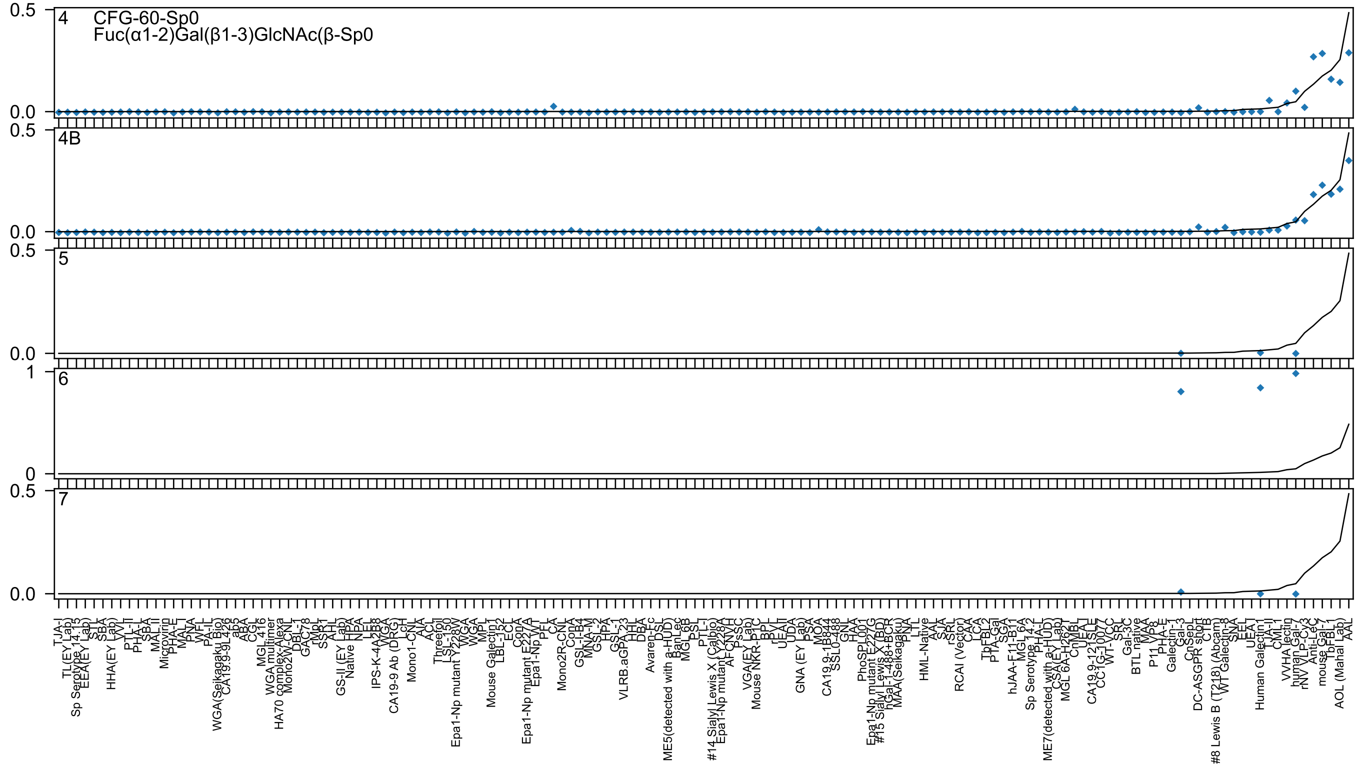

4
CFG-60-Sp0
Fuc(α1-2)Gal(β1-3)GlcNAc(β-Sp0
4B
5
6
7
0.5
0.0
1
0

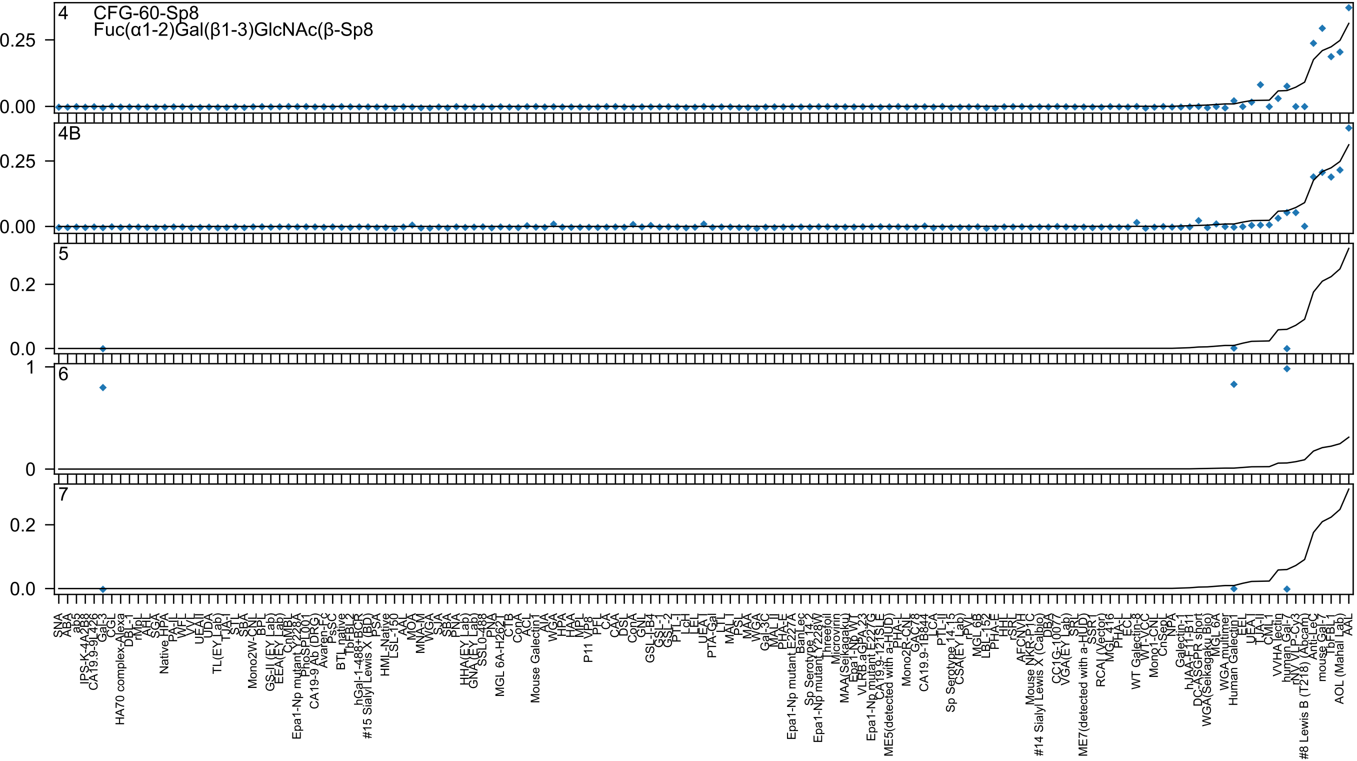

4 CFG-60-Sp8
Fuc(α1-2)Gal(β1-3)GlcNAc(β-Sp8
4B
5
6
7

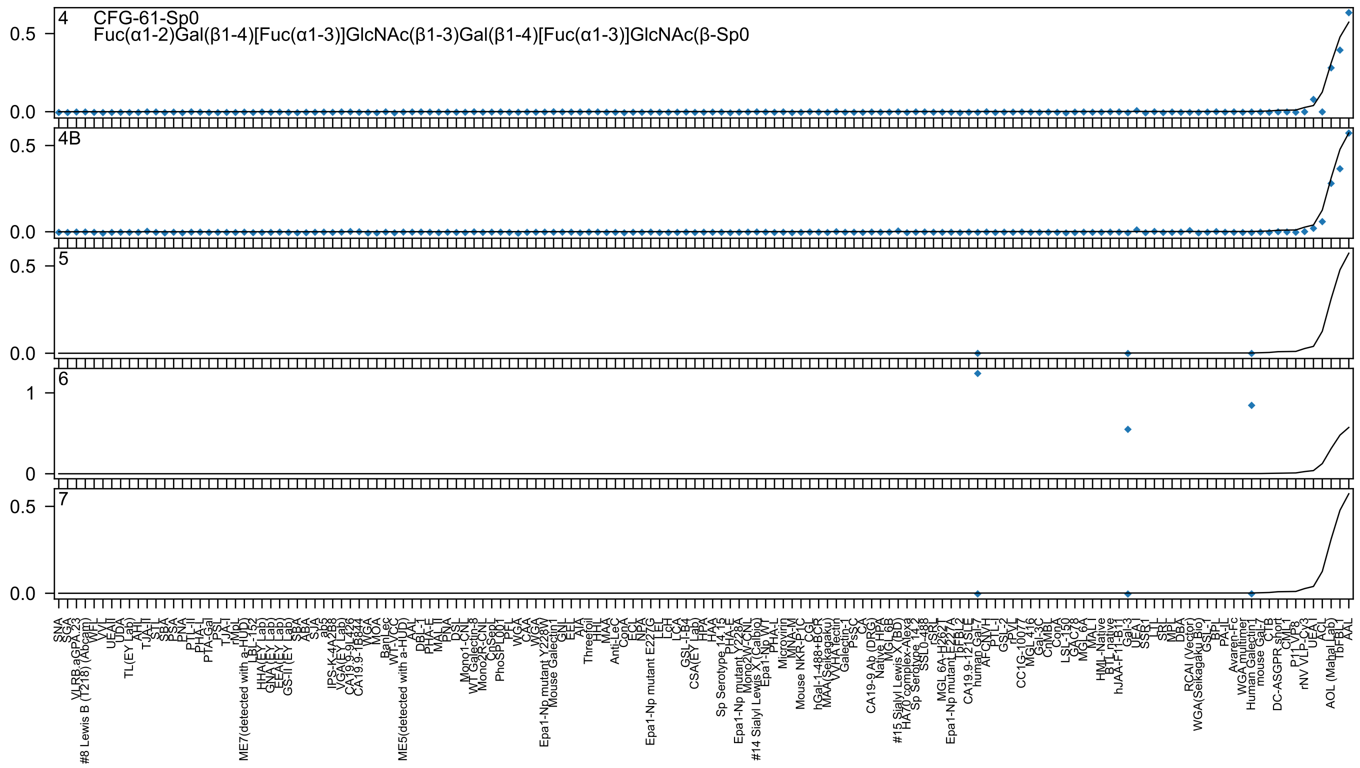
4 CFG-61-Sp0
Fuc(α1-2)Gal(β1-4)[Fuc(α1-3)]GlcNAc(β1-3)Gal(β1-4)[Fuc(α1-3)]GlcNAc(β-Sp0
4B
5
6
7
0.5
0.0
1
0
SNA
SGA
VLRB.aGPA.23
#8 Lewis B (T218) (Abcam)
WFL
VVL
UEAII
UDA
TL(EY Lab)
AHL
TJA-II
STL
SBA
PSA
PNA
PTL-II
PHA-L
PTA-Gal
PSL
TJA-I
rMpL
ME7(detected with a-HUD)
LBL-152
HHA(EY Lab)
GNA(EY Lab)
EEA(EY Lab)
GS-II(EY Lab)
SBA
ABA
SJA
ab5
IPS-K-4A2B8
VGA(EY Lab)
CA19.9-9L426
CA19.9-1B844
WGA
MOA
BanLec
WT-VCC
ME5(detected with a-HUD)
AAL
DBL-I
PHA-E
MAL II
PNA
DSL
Mono1-CNL
WT Galectin-8
Mono2R-CNL
CnSepL
PhoSL001
PFL
WGA
CAA
WGA
Epa1-Np mutant Y228W
Mouse Galectin1
GNL
EEL
AIA
Threefoil
HHL
MAA
Anti-LeC
ConA
ECL
NPA
Epa1-Np mutant E227G
LEL
LcH
LCA
GSL-I-B4
CSA(EY Lab)
HPA
HAA
Sp Serotype 14.15
PHA-E
Epa1-Np mutant Y228A
Mono2W-CNL
#14 Sialyl Lewis X (Calbio)
Epa1-Np WT
PHA-L
Microvirin
MNA-M
Mouse NKR-P1C
CGL
hGal-1-488+BCR
MAA(Seikagaku)
VVHA lectin
Galectin-1
PsSC
CA
CA19-9 Ab (DRG)
Native HPA
MGL 6B
#15 Sialyl Lewis X (BD)
HA70 complex-Alexa
Sp Serotype 14.2
SSL0-488
rSRL
MGL 6A-H262T
Epa1-Np mutant E227A
TbFBL2
CA19.9-121SLE
human Gal-7
AFCNVH
PTL-I
GSL-2
rPVL
CC1G-10077
MGL 416
Gal-3C
CnMBL
ConA
LSL-150
GAC78
MGL 6A
MAL I
HML-Native
BTL native
hJAA-F11-B11
Gal-3
UEA
SSR1
LTL
SRL
MPL
DBA
RCAI (Vector)
WGA(Seikagaku Bio)
GSL-I
BPL
PA-IL
Avaren-Fc
WGA multimer
Human Galectin1
mouse Gal-7
CTB
DC-ASGPR short
CML1
P11 VP8
rNV VLP-Cy3
UEA-I
ACL
AOL (Mahal Lab)
TbFBL1
AAL

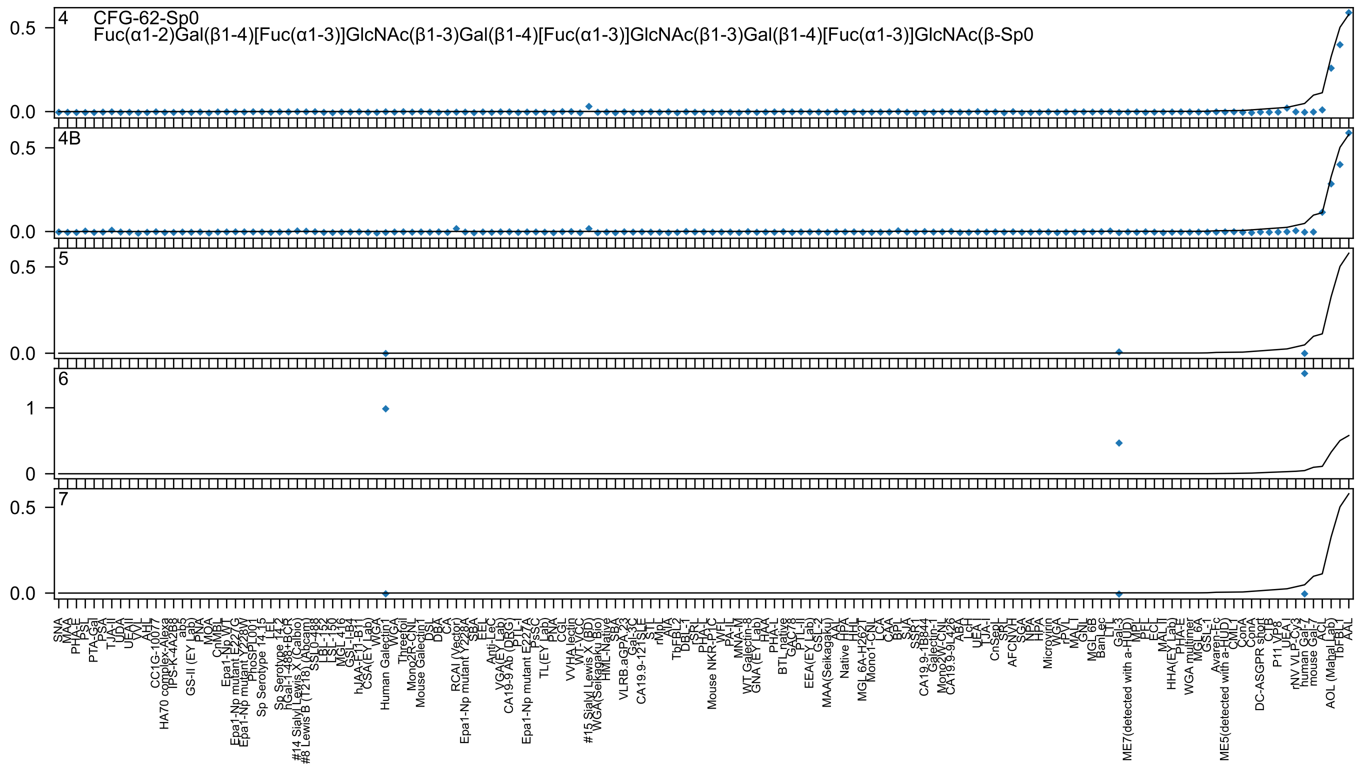
4
CFG-62-Sp0
Fuc(α1-2)Gal(β1-4)[Fuc(α1-3)]GlcNAc(β1-3)Gal(β1-4)[Fuc(α1-3)]GlcNAc(β1-3)Gal(β1-4)[Fuc(α1-3)]GlcNAc(β-Sp0
4B
5
6
7

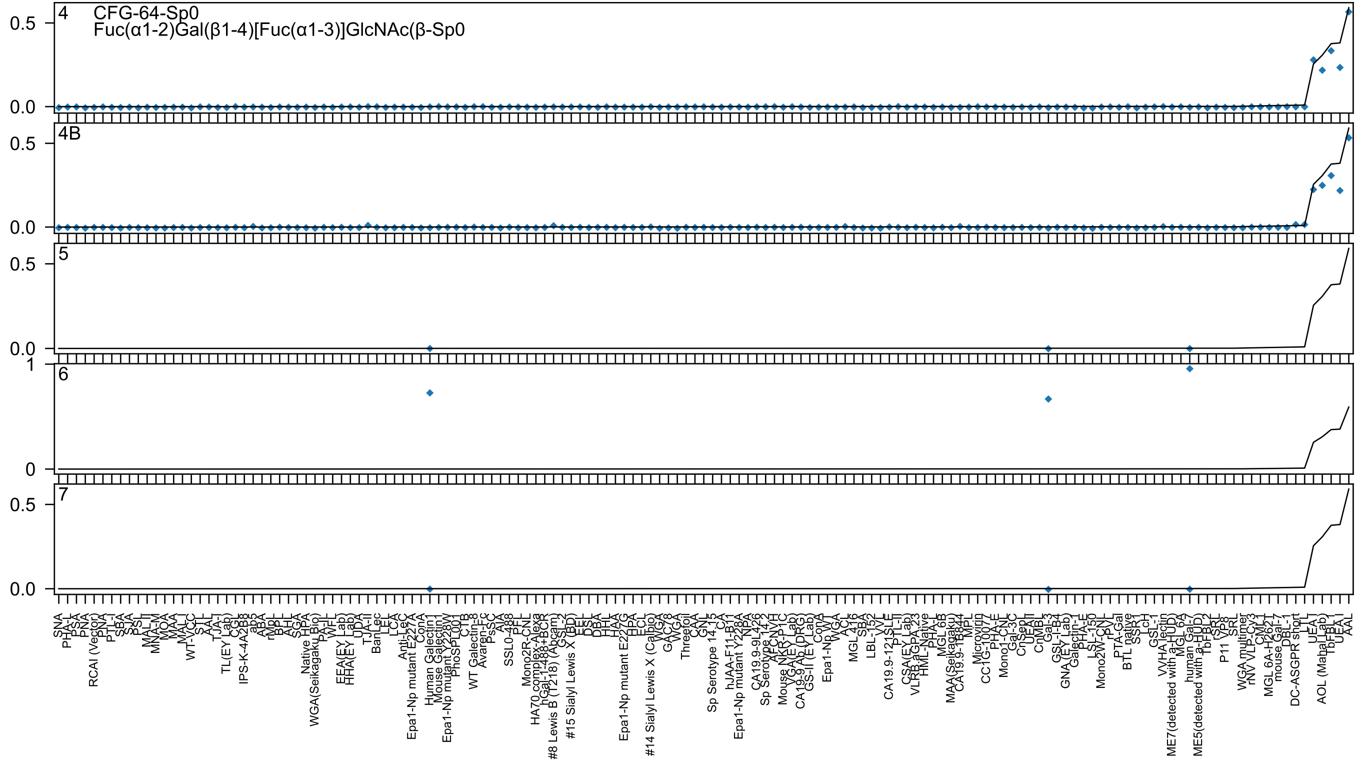
4
CFG-64-Sp0
Fuc(α1-2)Gal(β1-4)[Fuc(α1-3)]GlcNAc(β-Sp0
4B
5
6
7
0.5
0.0
1
0
SNA
PHA-L
PSA
PNA
RCAI (Vector)
PNA
PTL-I
SBA
SJA
PSL
MAL-II
MNA-M
MOA
MAA
MAL-I
WT-VCC
STL
AAL
TJA-I
TL(EY Lab)
CGL
IPS-K-4A2B8
ab5
ABA
rMpL
BPL
AHL
SGA
Native HPA
WGA(Seikagaku Bio)
PA-IL
WFL
EEA(EY Lab)
HHA(EY Lab)
UDA
TJA-II
BanLec
LEL
LCA
Anti-LeC
Epa1-Np mutant E227A
ConA
Human Galectin1
Mouse Galectin1
Epa1-Np mutant Y228W
PhoSL001
CTB
WT Galectin-8
Avaren-Fc
PsSC
AIA
SSL0-488
PFL
Mono2R-CNL
HA70 complex-Alexa
hGal-1-488+BCR
#8 Lewis B (T218) (Abcam)
GSL-2
#15 Sialyl Lewis X (BD)
EEL
DSL
DBA
HHL
HAA
Epa1-Np mutant E227G
HPA
ECL
#14 Sialyl Lewis X (Calbio)
WGA
GAC78
WGA
Threefoil
CAA
GNL
Sp Serotype 14.15
CA
hJAA-F11-B11
Epa1-Np mutant Y228A
NPA
CA19.9-9L426
Sp Serotype 14.2
AFCNVH
Mouse NKR-P1C
VGA(EY Lab)
CA19-9 Ab (DRG)
GS-II (EY Lab)
ConA
Epa1-Np WT
WGA
ACL
MGL 416
SBA
LBL-152
VVL
CA19.9-121SLE
PTL-II
CSA(EY Lab)
VLRB.aGPA.23
HML-Native
PHA-L
MGL 6B
MAA(Seikagaku)
CA19.9-1B844
MPL
Microvirin
CC1G-10077
PHA-E
Mono1-CNL
Gal-3C
CnSep
UEAII
CnMBL
Gal-3
GSL-I-B4
GNA (EY Lab)
Galectin-1
PHA-E
LSL-150
Mono2W-CNL
rPVL
PTA-Gal
BTL native
SSR1
LcH
GSL-1
VVHA lectin
ME7(detected with a-HUD)
MGL 6A
human Gal-7
ME5(detected with a-HUD)
TbFBL2
rSRL
P11 VP8
SRL
WGA multimer
rNV VLP-Cy3
CML1
MGL 6A-H262T
mouse Gal-7
DBL-1
DC-ASGPR short
LTL
UEA1
AOL (Mahal Lab)
TbFBL1
UEA I
AAL

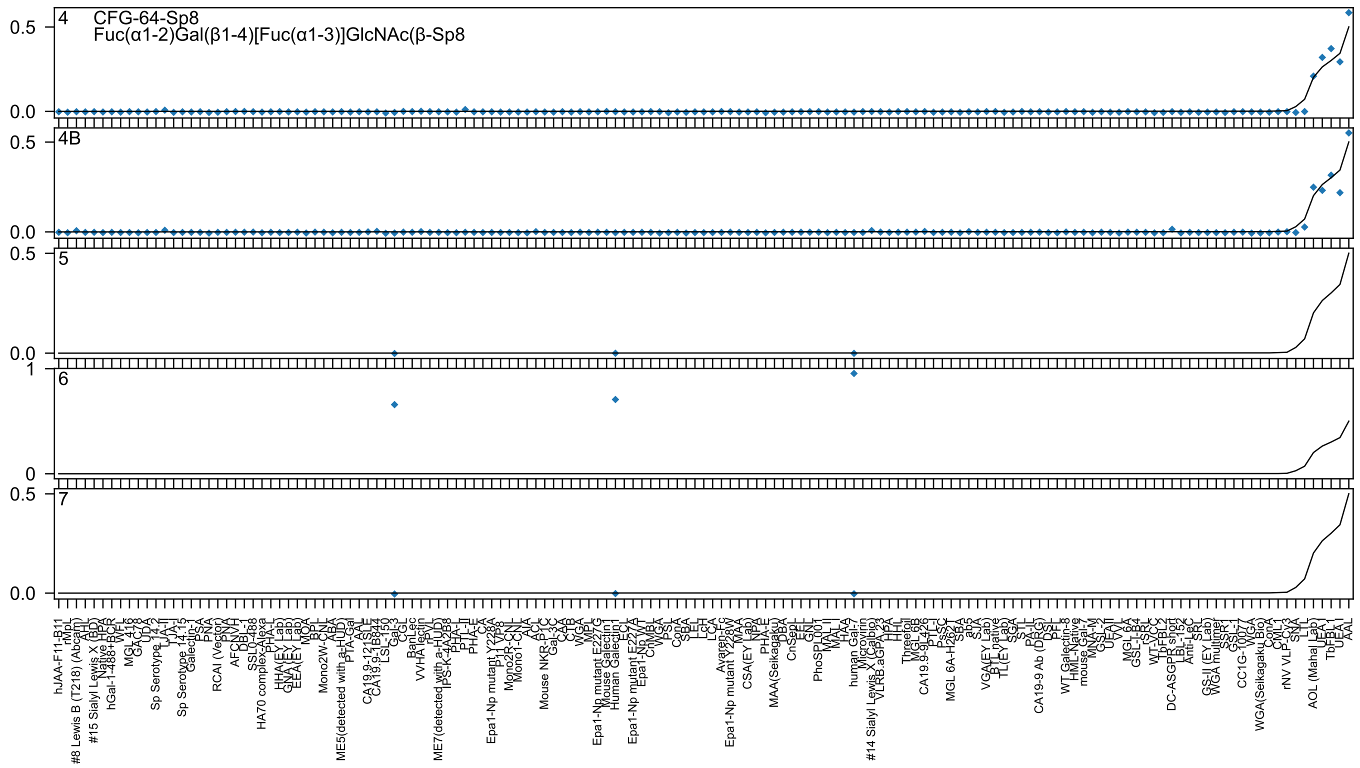

4 CFG-64-Sp8
Fuc(α1-2)Gal(β1-4)[Fuc(α1-3)]GlcNAc(β-Sp8
4B
5
6
7

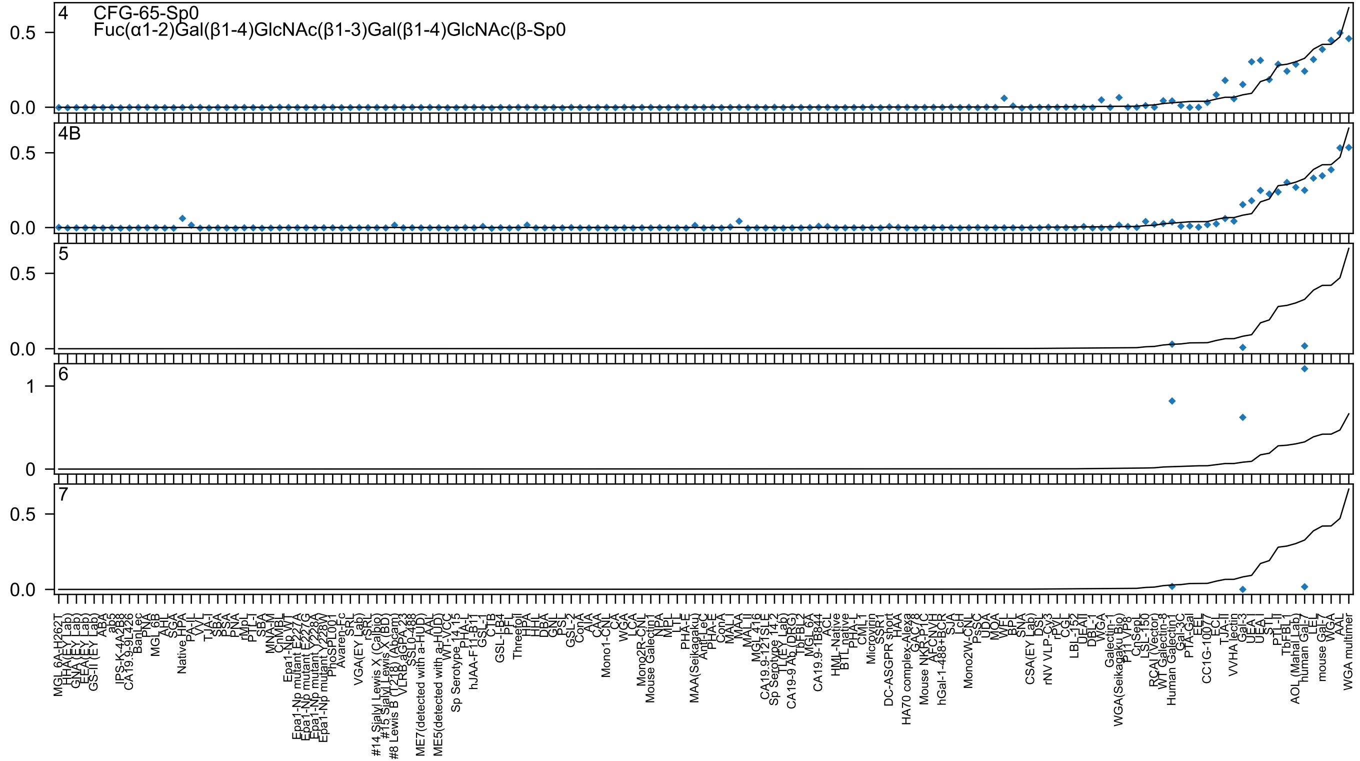

4 CFG-65-Sp0
Fuc(α1-2)Gal(β1-4)GlcNAc(β1-3)Gal(β1-4)GlcNAc(β-Sp0
4B
5
6
7

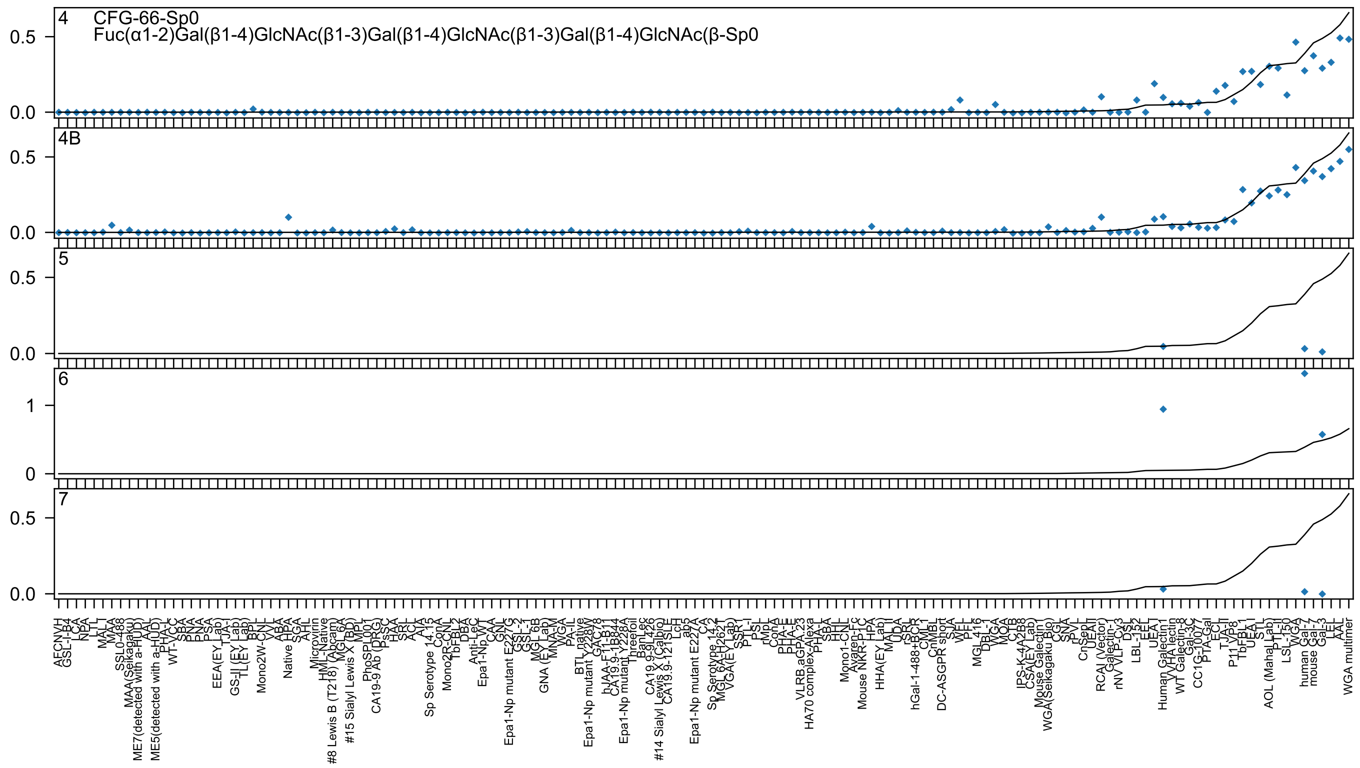
4 CFG-66-Sp0
Fuc(α1-2)Gal(β1-4)GlcNAc(β1-3)Gal(β1-4)GlcNAc(β1-3)Gal(β1-4)GlcNAc(β-Sp0
4B
5
6
7
0.0
0.5
0
1
AFCNVH
GSL-I-B4
LCA
NPA
LTL
MAL
MAA
SSL0-488
MAA(Seikagaku)
ME7(detected with a-HUD)
AAL
ME5(detected with a-HUD)
PHA-L
WT-VCC
SBA
PNA
PNA
PSA
EEA(EY_Lab)
TJA-I
GS-II (EY_Lab)
TL(EY_Lab)
BPL
Mono2W-CNL
VVL
ABA
Native HPA
SGA
AHL
Microvirin
HML-Native
#8 Lewis B (T218) (Abcam)
MGL 6A
#15 Sialyl Lewis X (BD)
MPL
PhoSPL001
CA19-9 Ab (DRG)
PsSC
HAA
SRL
ACL
AIA
Sp Serotype 14.15
ConA
Mono2R-CNL
TbFBL2
DBA
Anti-LeC
Epa1-Np WT
CAA
GNL
Epa1-Np mutant E227G
GSL-2
GSL-1
MGL 6B
GNA (EY_Lab)
MNA-M
WGA
PA-IL
BTL native
Epa1-Np mutant Y228W
GAC78
hJAA-F11-B11
CA19.9-1B844
Epa1-Np mutant Y228A
Threefoil
BanLec
CA19.9-9L426
#14 Sialyl Lewis X (Calbio)
CA19.9-121SLE
LcH
ab5
Epa1-Np mutant E227A
CA
Sp Serotype 14.2
MGL 6A-H262T
VGA(EY_Lab)
SSR1
PTL-I
PSL
rMpL
ConA
PHA-E
PHA-E
VLRB.aGPA.23
HA70 complex-Alexa
PHA-L
SBA
HHL
Mono1-CNL
Avaren-Fc
Mouse NKR-P1C
HPA
HHA(EY_Lab)
MAL II
UDA
rSRL
hGal-1-488+BCR
CML1
CnMBL
DC-ASGPR short
SJA
WFL
PFL
MGL 416
DBL-1
WGA
MOA
CTB
IPS-K-4A2B8
CSA(EY_Lab)
Mouse Galectin1
WGA(Seikagaku Bio)
CGL
SNA
rPVL
CnSep
UEAII
RCAI (Vector)
Galectin-1
rNV VLP-Cy3
DSL
LBL-152
EEL
UEA I
Human Galectin1
VVHA lectin
WT Galectin-8
Gal-3C
CC1G-10077
PTA-Gal
ECL
TJA-II
P11 VP8
TbFBL1
UEA I
STL
AOL (Mahal Lab)
PTL-II
LSL-150
WGA
human Gal-7
mouse Gal-3
LEL
AAL
WGA multimer

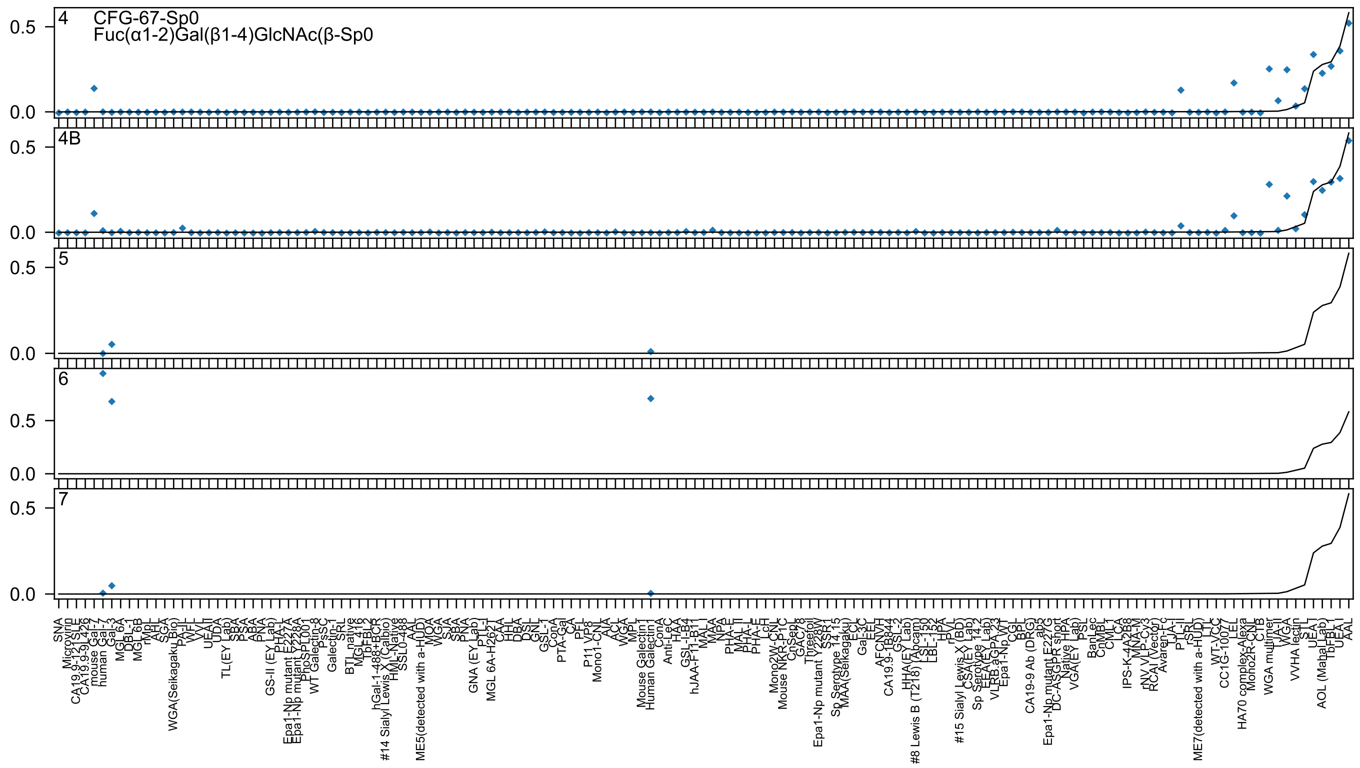
4
CFG-67-Sp0
Fuc(α1-2)Gal(β1-4)GlcNAc(β-Sp0
4B
5
6
7
0.5
0.0

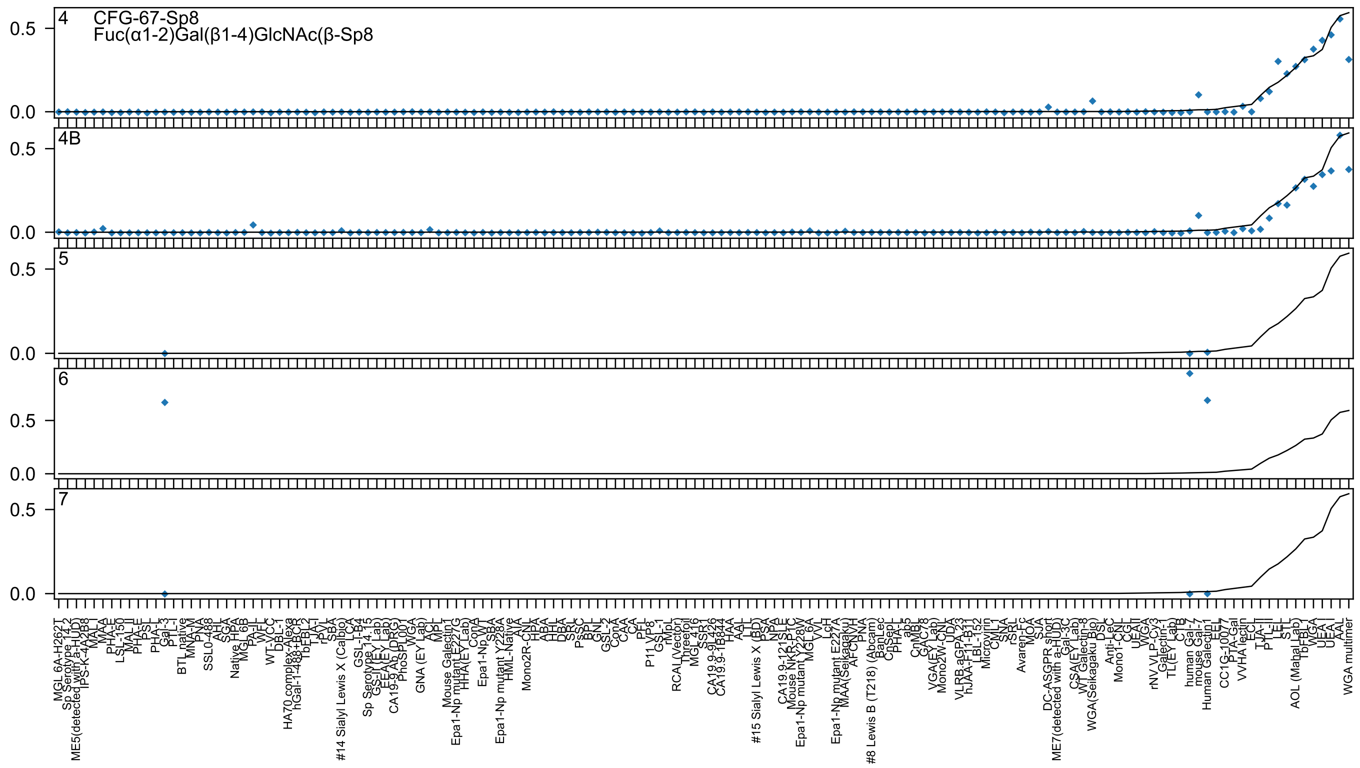

4
CFG-67-Sp8
Fuc(α1-2)Gal(β1-4)GlcNAc(β-Sp8
4B
5
6
7
0.5
0.0

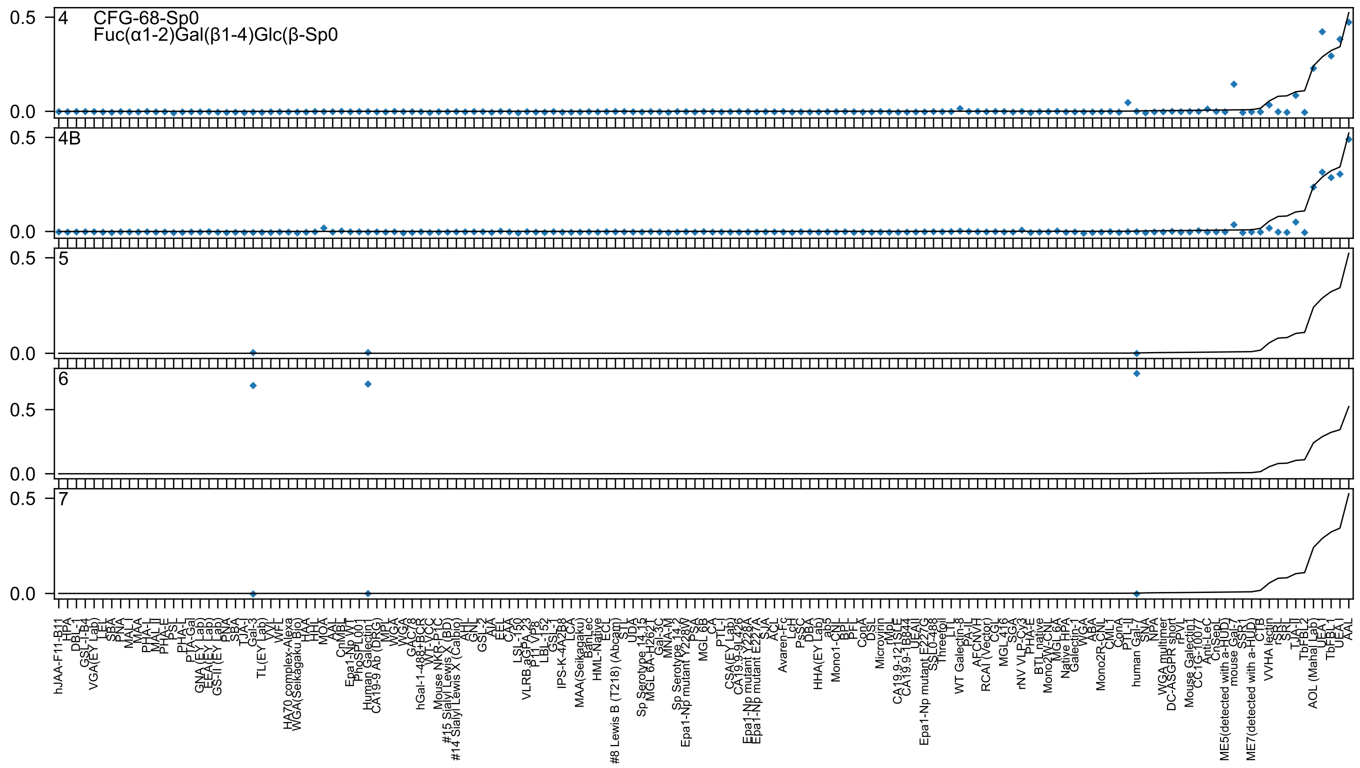
4
CFG-68-Sp0
Fuc(α1-2)Gal(β1-4)Glc(β-Sp0
4B
5
6
7
0.5
0.0
hJAA-F11-B11
HPA
DBL-1
GSL-I-B4
VGA(EY Lab)
LEA
SBA
PNA
MAL-I
MAA
PHA-L
MAL-II
PHA-E
PSL
PHA-L
PTA-Gal
GNA (EY Lab)
EEA(EY Lab)
GS-II (EY Lab)
PNA
SBA
TJA-I
Gal-3
TL(EY Lab)
VVL
WFL
HA70 complex-Alexa
WGA(Seikagaku Bio)
HAA
HHL
MOA
AAL
CnMBL
Epa1-Np WT
PhoSL001
Human Galectin1
CA19-9 Ab (DRG)
MPL
WGA
WGA
GAC78
hGal-1-488+BCR
WT-YCC
Mouse NKR-P1C
#15 Sialyl Lewis X (BD)
#14 Sialyl Lewis X (Calbio)
AHL
GNL
GSL-2
AIA
EEL
CAA
LSL-150
VLRB.aGPA.23
P11 VP8
LBL-152
GSL-1
IPS-K-4A2B8
LCA
MAA(Seikagaku)
BanLec
HML-Native
ECL
#8 Lewis B (T218) (Abcam)
STL
UDA
Sp Serotype 14.15
MGL 6A-H267T
Gal-3C
MNA-M
Sp Serotype 14.2
Epa1-Np mutant Y228W
PSA
MGL 6B
CA
PTL-I
CSA(EY Lab)
CA19.9-9L426
Epa1-Np mutant Y228A
Epa1-Np mutant E227A
SJA
ACL
Avaren-Fc
LcH
PsSC
DBA
HHA(EY Lab)
ab5
Mono1-CNL
BPL
PFL
ConA
DSL
Microvirin
rMpL
CA19.9-121SLE
CA19.9-1B844
UEAII
Epa1-Np mutant E227G
SSL0-488
Threefoil
LTL
WT Galectin-8
PA-IL
AFCNVH
RCAI (Vector)
CGL
MGL 416
SGA
rNV VLP-Cy3
PHA-E
BTL native
Mono2W-CNL
MGL 6A
Native HPA
Galectin-1
WGA
ABA
Mono2R-CNL
CML1
ConA
PTL-II
human Gal-7
SNA
NPA
WGA multimer
DC-ASGPR short
rPVL
Mouse Galectin1
CC1G-10077
Anti-LeC
CnSep1
ME5(detected with a-HUD)
mouse Gal-7
SSR1
ME7(detected with a-HUD)
CTB
VVHA lectin
rSRL
SRL
TJA-II
TbFBL2
AOL (Mahal Lab)
UEA-I
TbFBL1
UEA-I
AAL

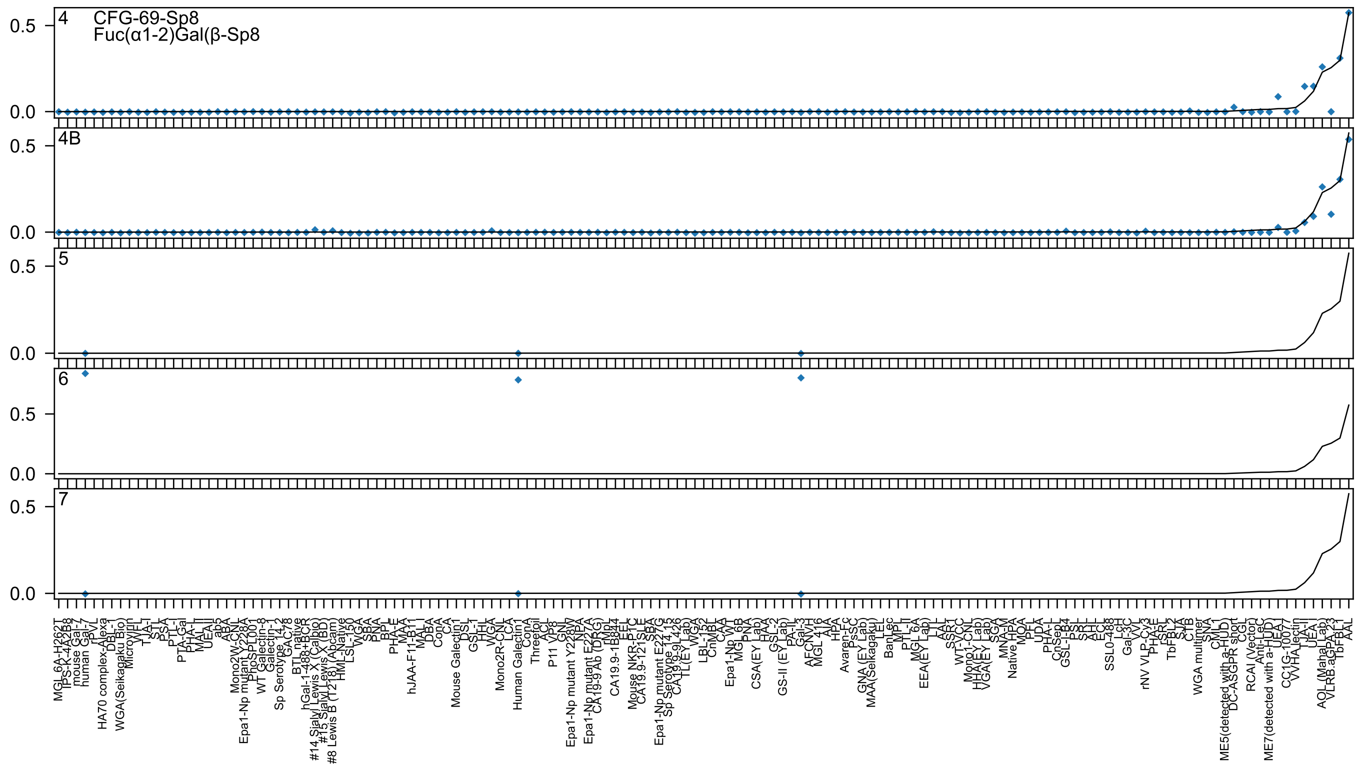
4
CFG-69-Sp8
Fuc(α1-2)Gal(β-Sp8
4B
5
6
7
0.5
0.0

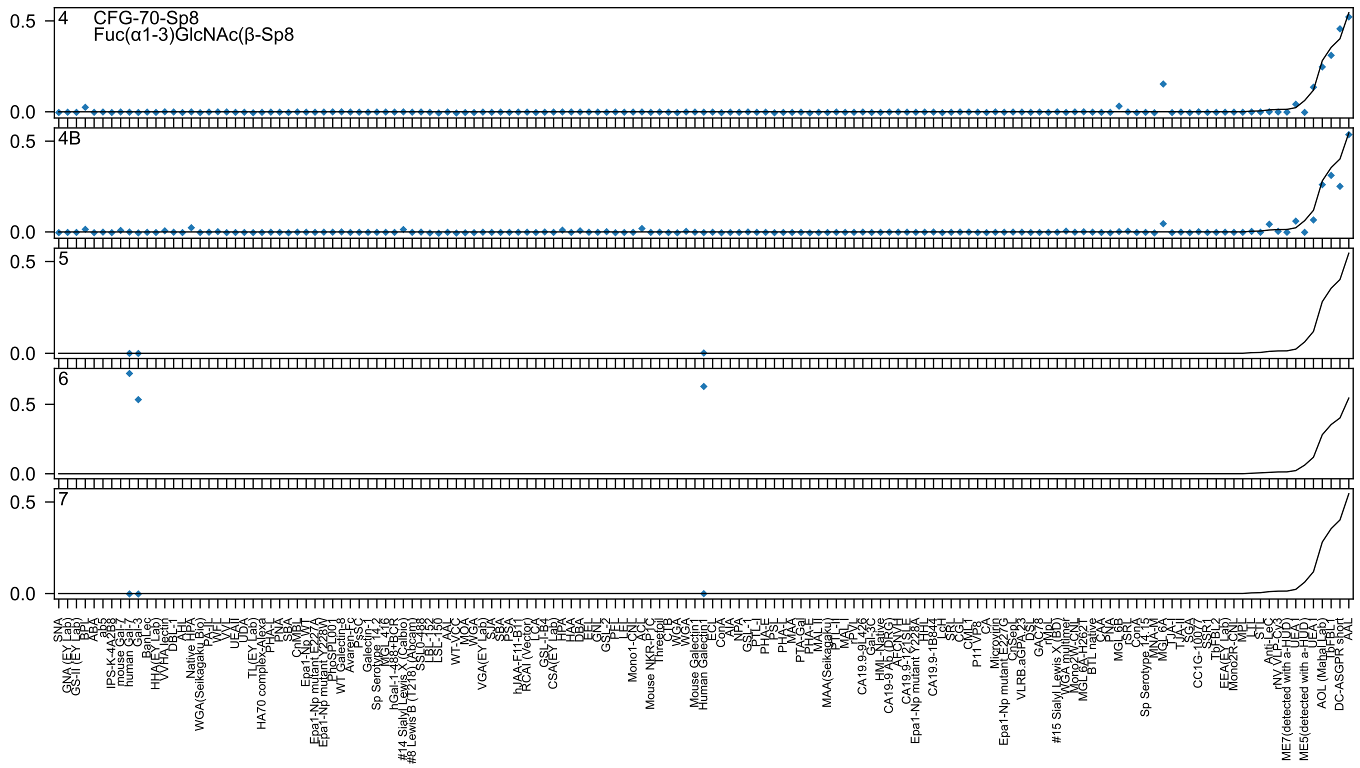
4
CFG-70-Sp8
Fuc(α1-3)GlcNAc(β-Sp8
4B
5
6
7

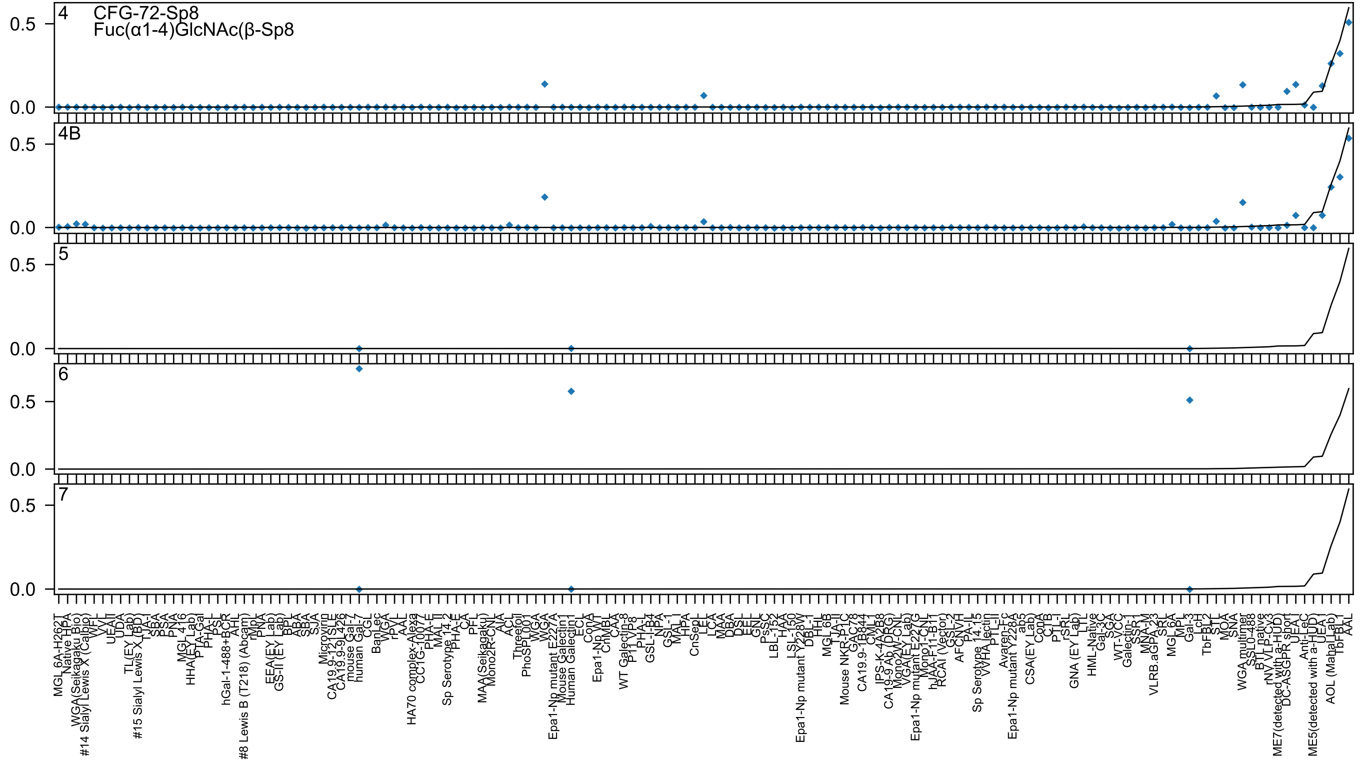

4 CFG-72-Sp8
Fuc(α1-4)GlcNAc(β-Sp8
4B
5
6
7
0.5
0.0

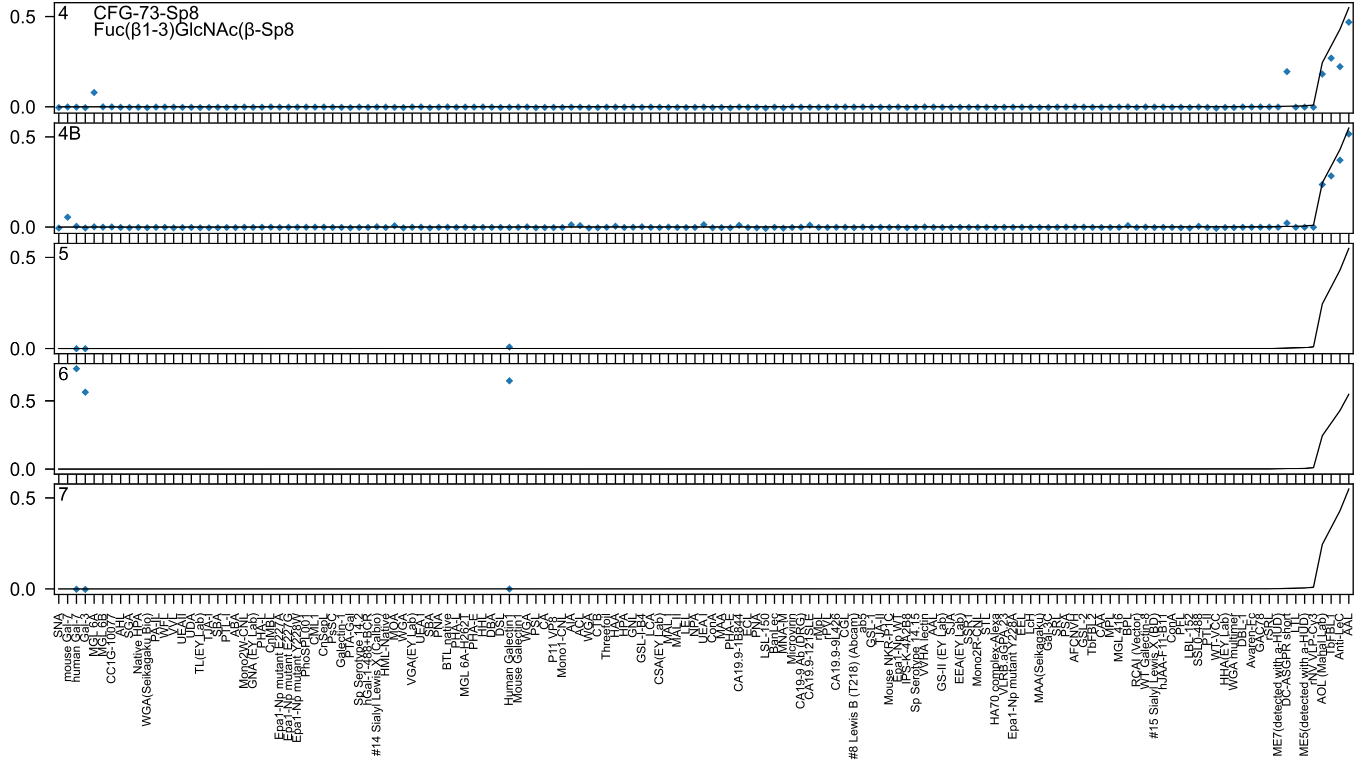

4
CFG-73-Sp8
Fuc(β1-3)GlcNAc(β-Sp8
4B
5
6
7
0.5
0.0

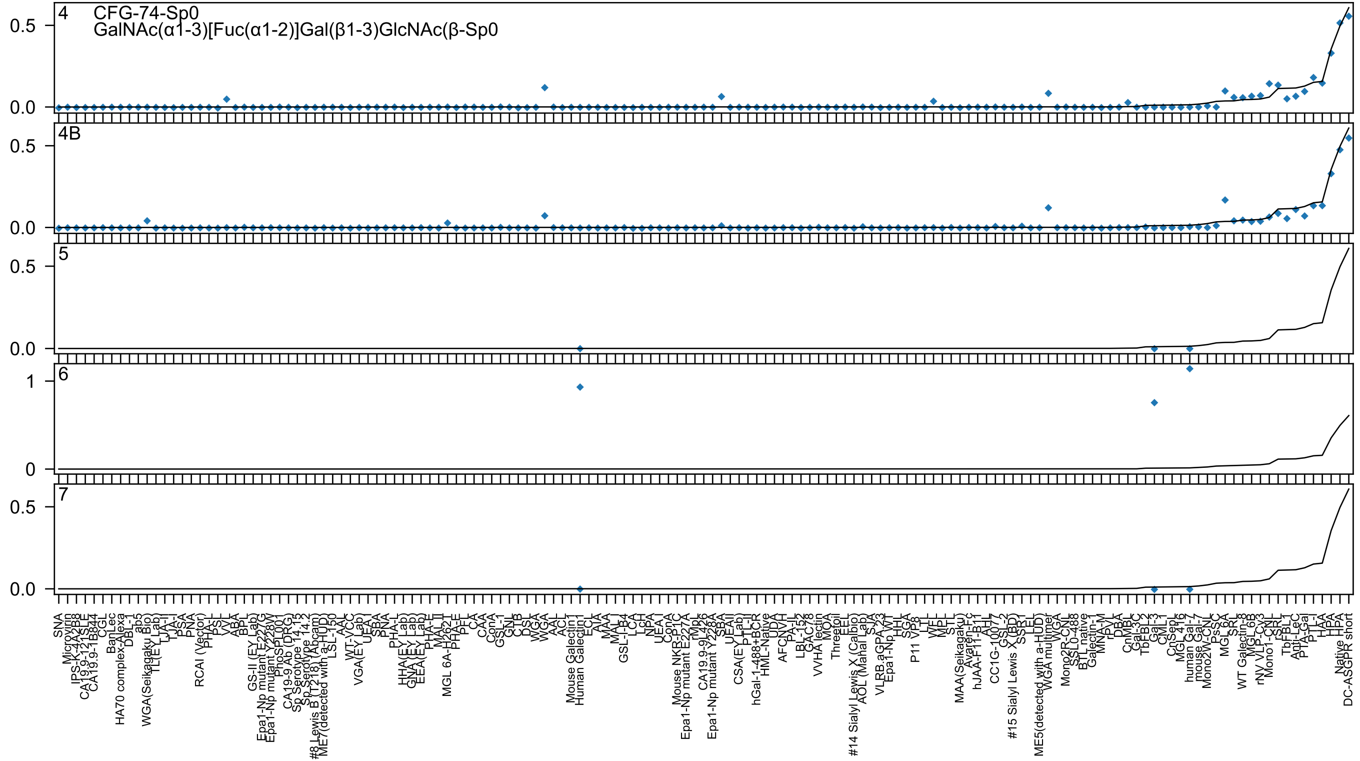

4
CFG-74-Sp0
GalNAc(α1-3)[Fuc(α1-2)]Gal(β1-3)GlcNAc(β-Sp0
4B
5
6
7

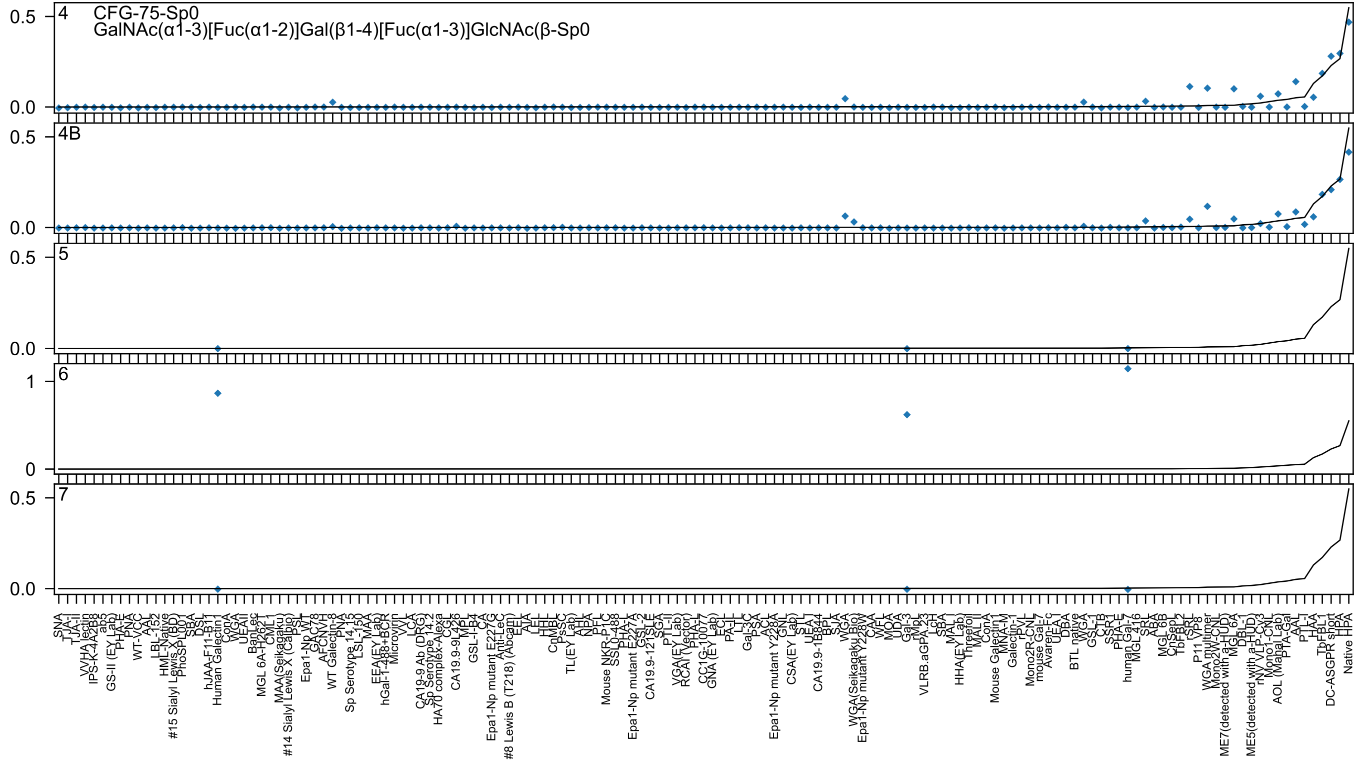

4 CFG-75-Sp0
GalNAc(α1-3)[Fuc(α1-2)]Gal(β1-4)[Fuc(α1-3)]GlcNAc(β-Sp0
4B
5
6
7

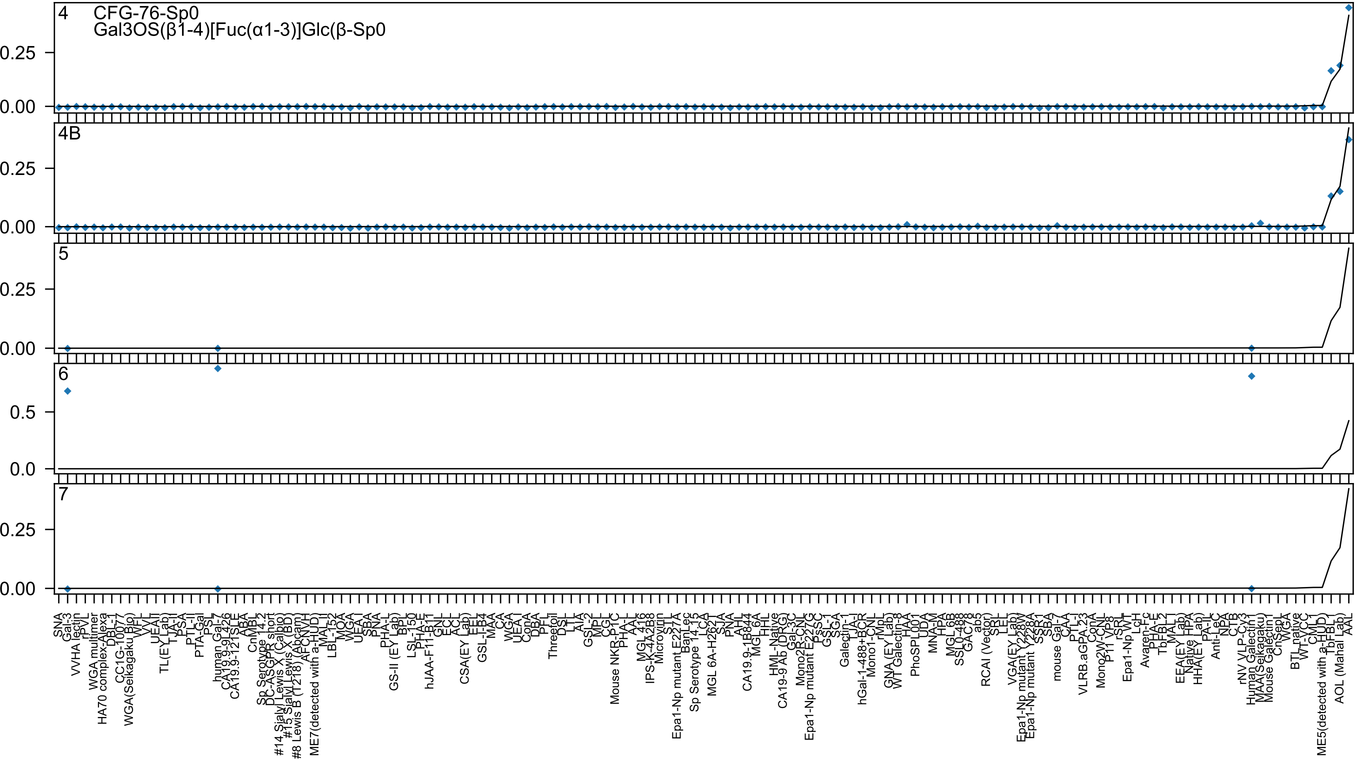

4
CFG-76-Sp0
Gal3OS(β1-4)[Fuc(α1-3)]Glc(β-Sp0
4B
5
6
7

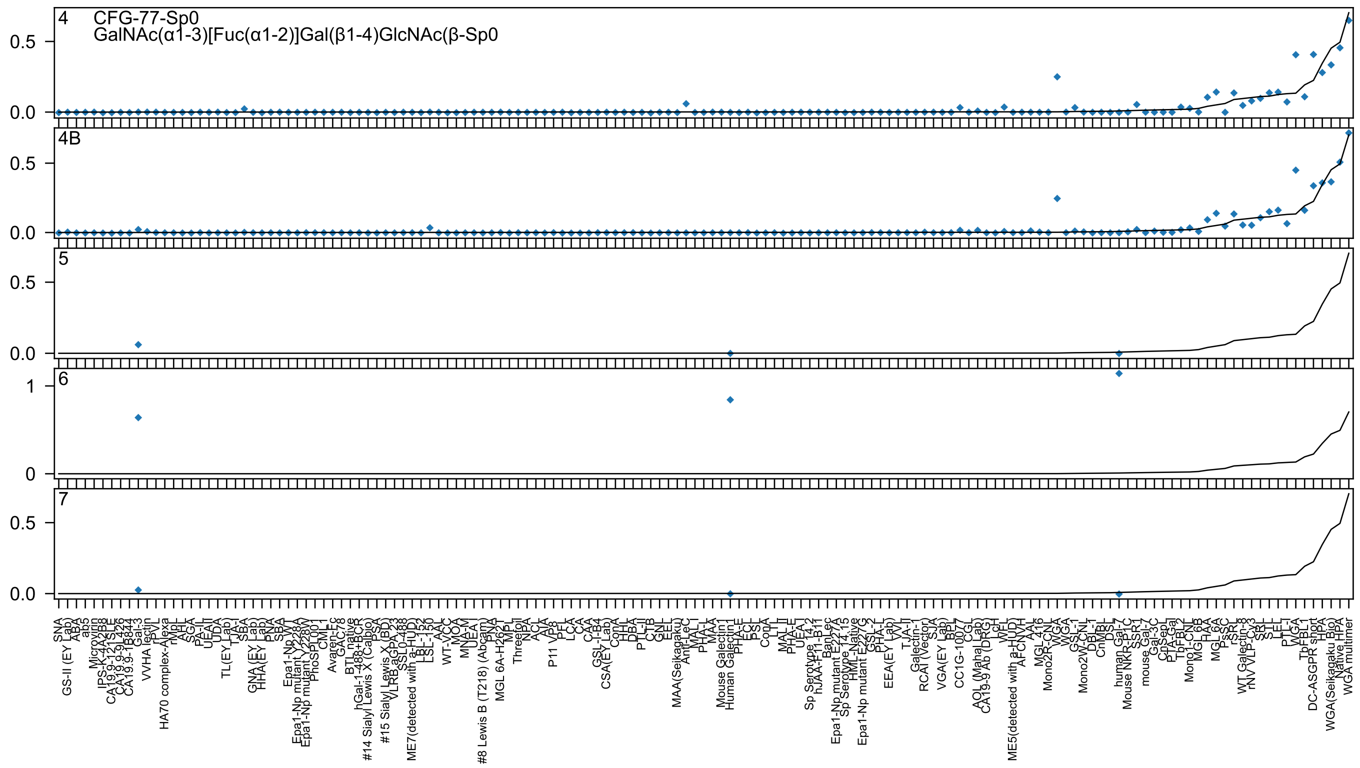

4
CFG-77-Sp0
GalNAc(α1-3)[Fuc(α1-2)]Gal(β1-4)GlcNAc(β-Sp0
4B
5
6
7

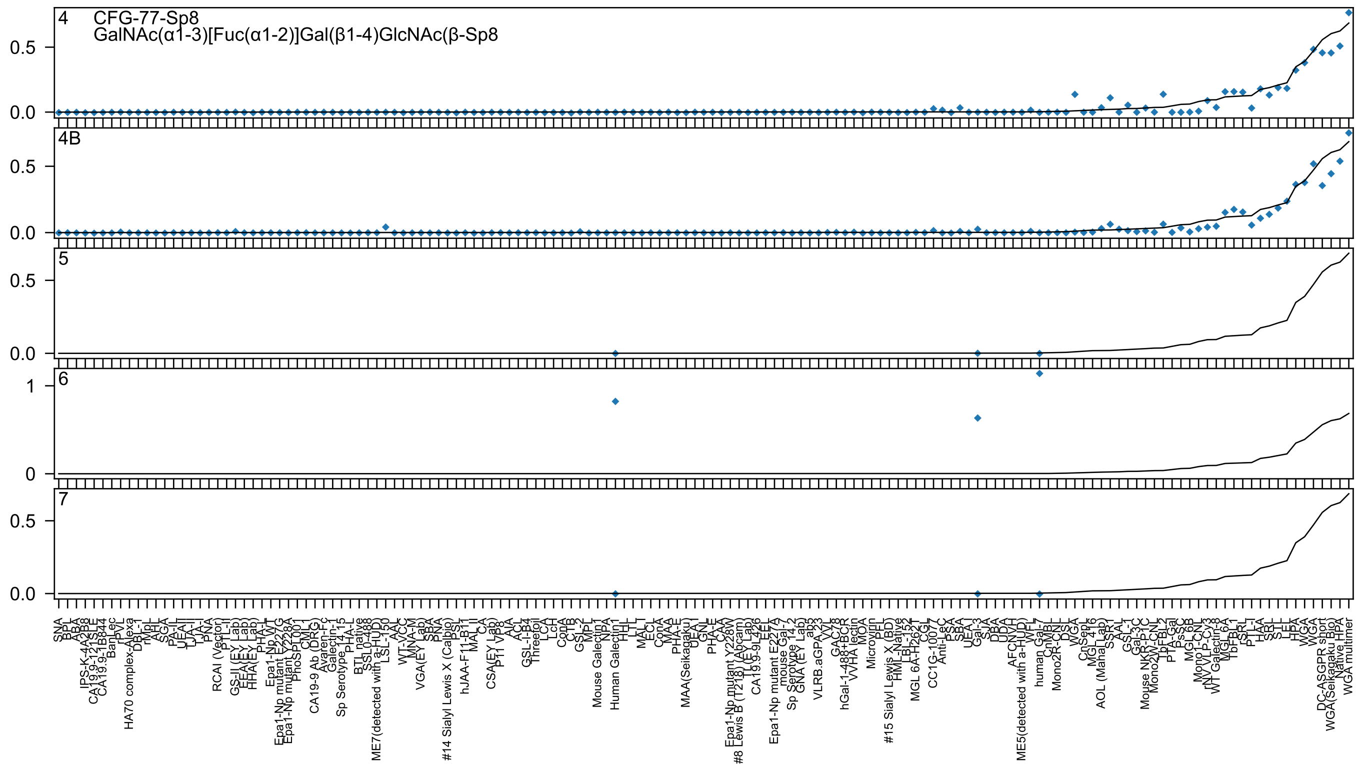
4
CFG-77-Sp8
GalNAc(α1-3)[Fuc(α1-2)]Gal(β1-4)GlcNAc(β-Sp8
4B
5
6
7

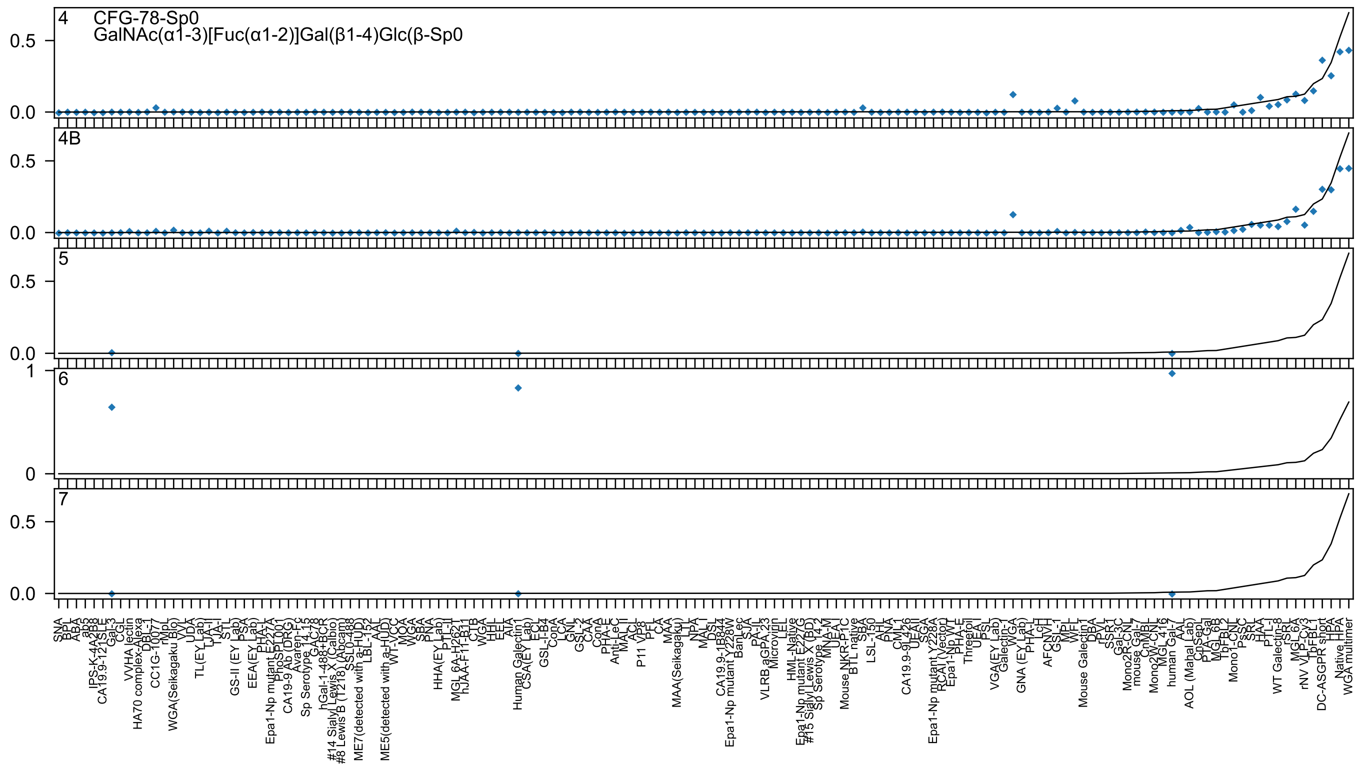

4 CFG-78-Sp0
GalNAc(α1-3)[Fuc(α1-2)]Gal(β1-4)Glc(β-Sp0
4B
5
6
7

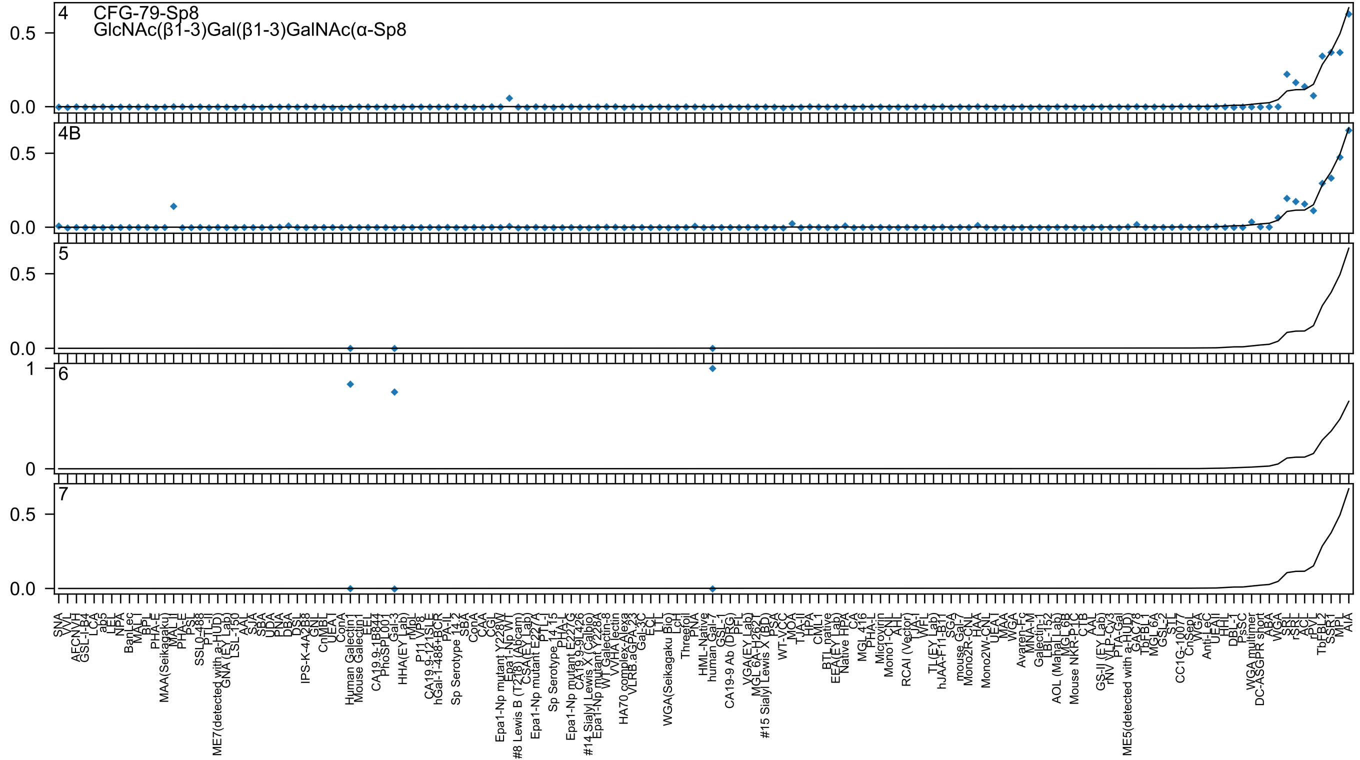

4
CFG-79-Sp8
GlcNAc(β1-3)Gal(β1-3)GalNAc(α-Sp8
4B
5
6
7

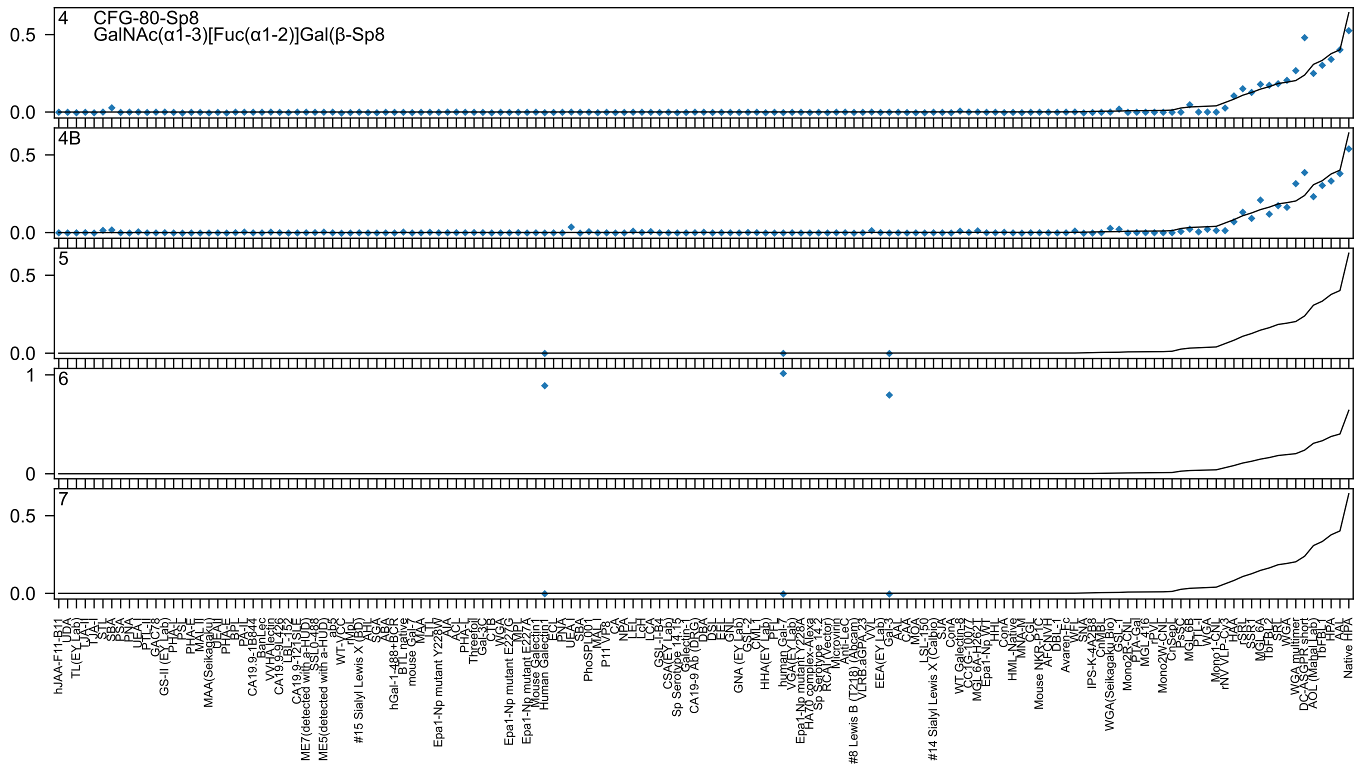
4
CFG-80-Sp8
GalNAc(α1-3)[Fuc(α1-2)]Gal(β-Sp8
0.5
0.0
4B
0.5
0.0
5
0.5
0.0
6
1
0
7
0.5
0.0

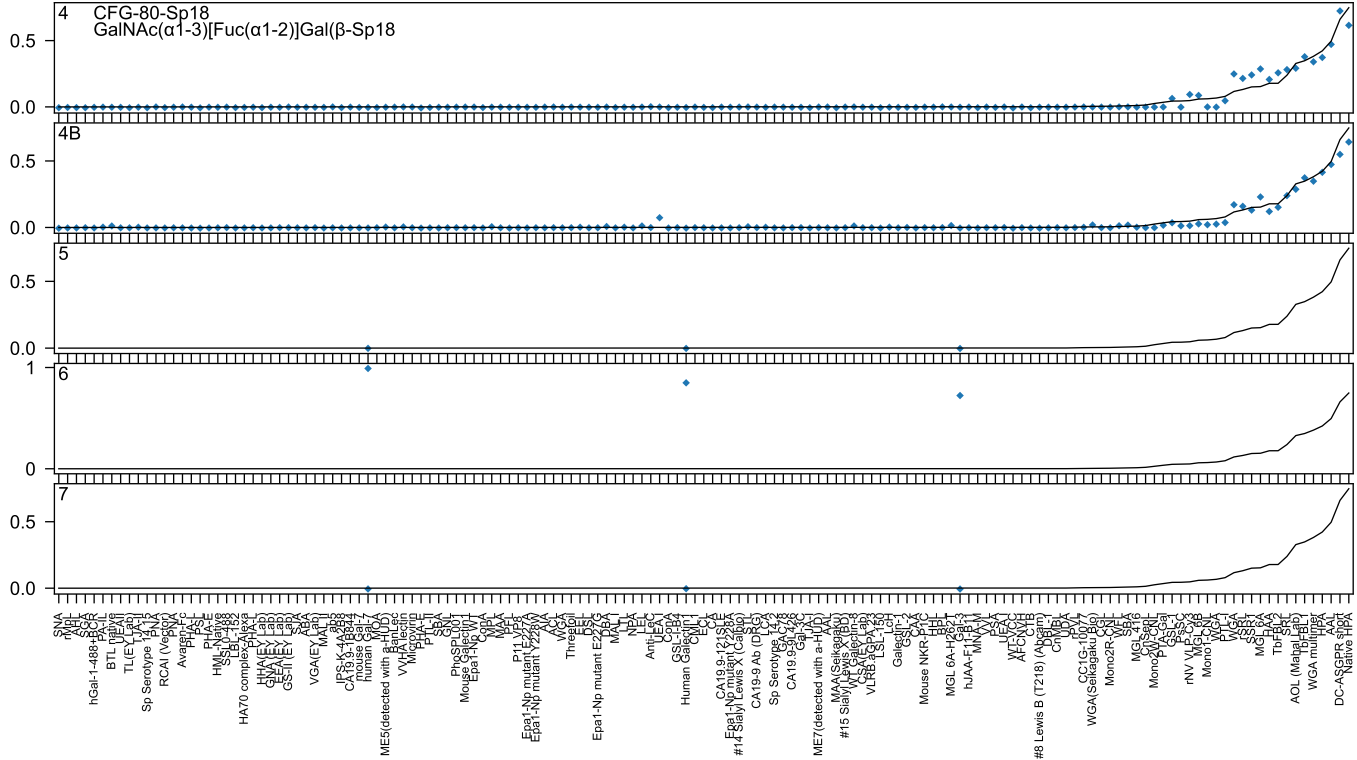
4
CFG-80-Sp18
GalNAc(α1-3)[Fuc(α1-2)]Gal(β-Sp18
4B
5
6
7

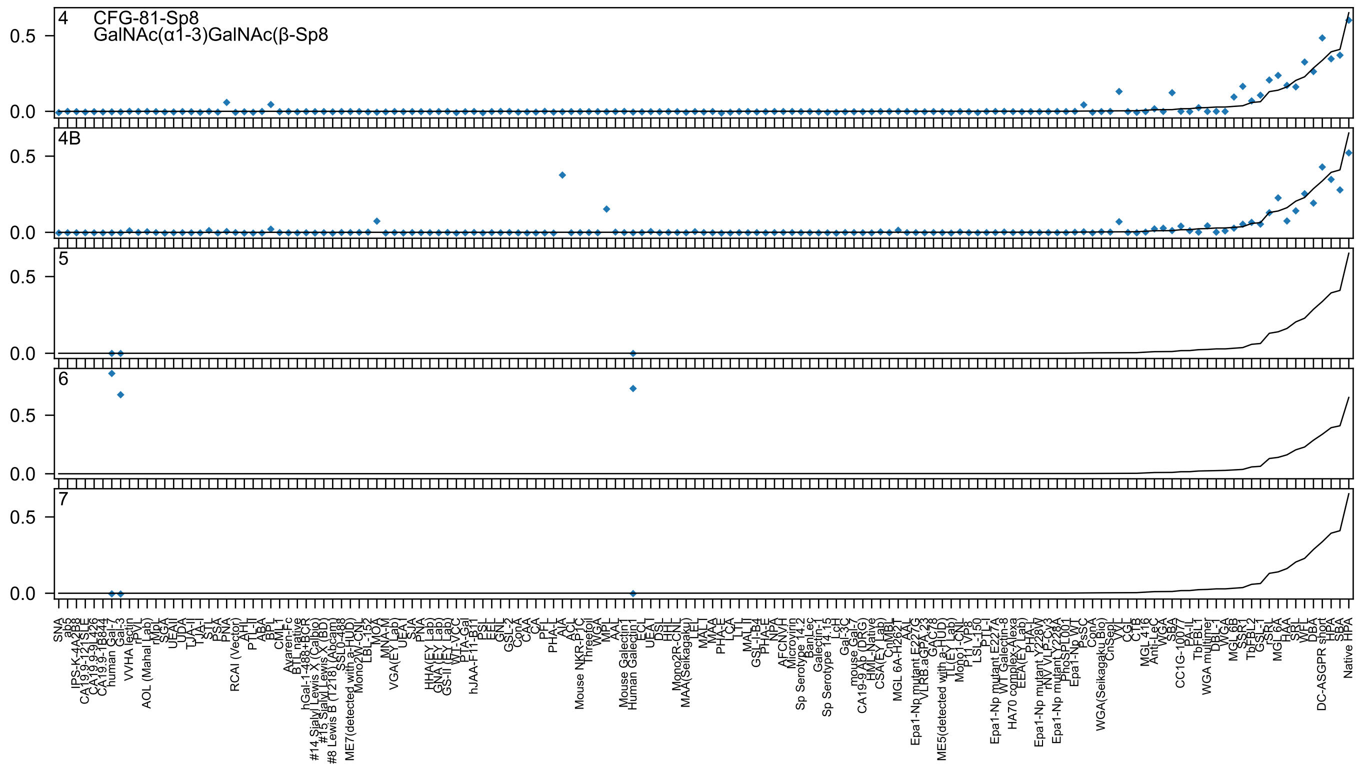
4 CFG-81-Sp8
GalNAc(α1-3)GalNAc(β-Sp8
4B
5
6
7

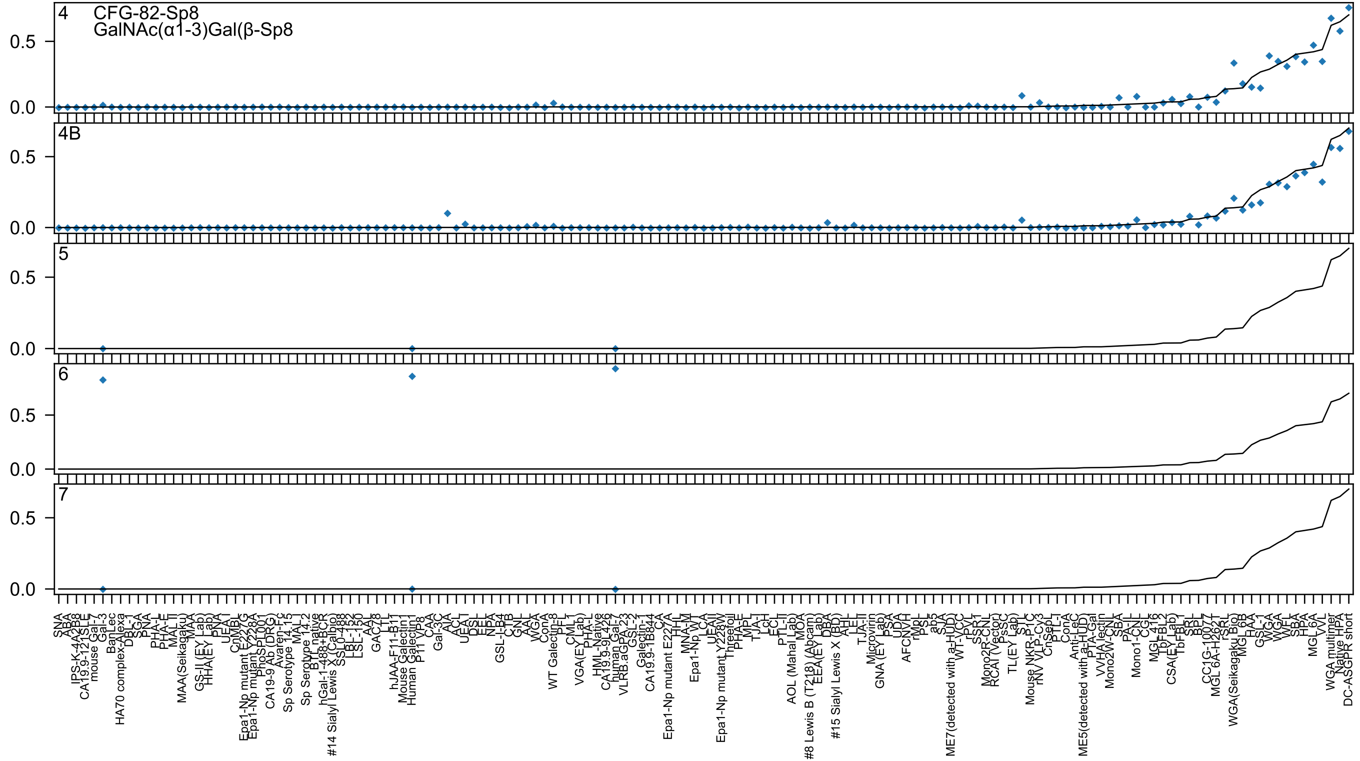
4
CFG-82-Sp8
GalNAc(α1-3)Gal(β-Sp8
4B
5
6
7
0.5
0.0

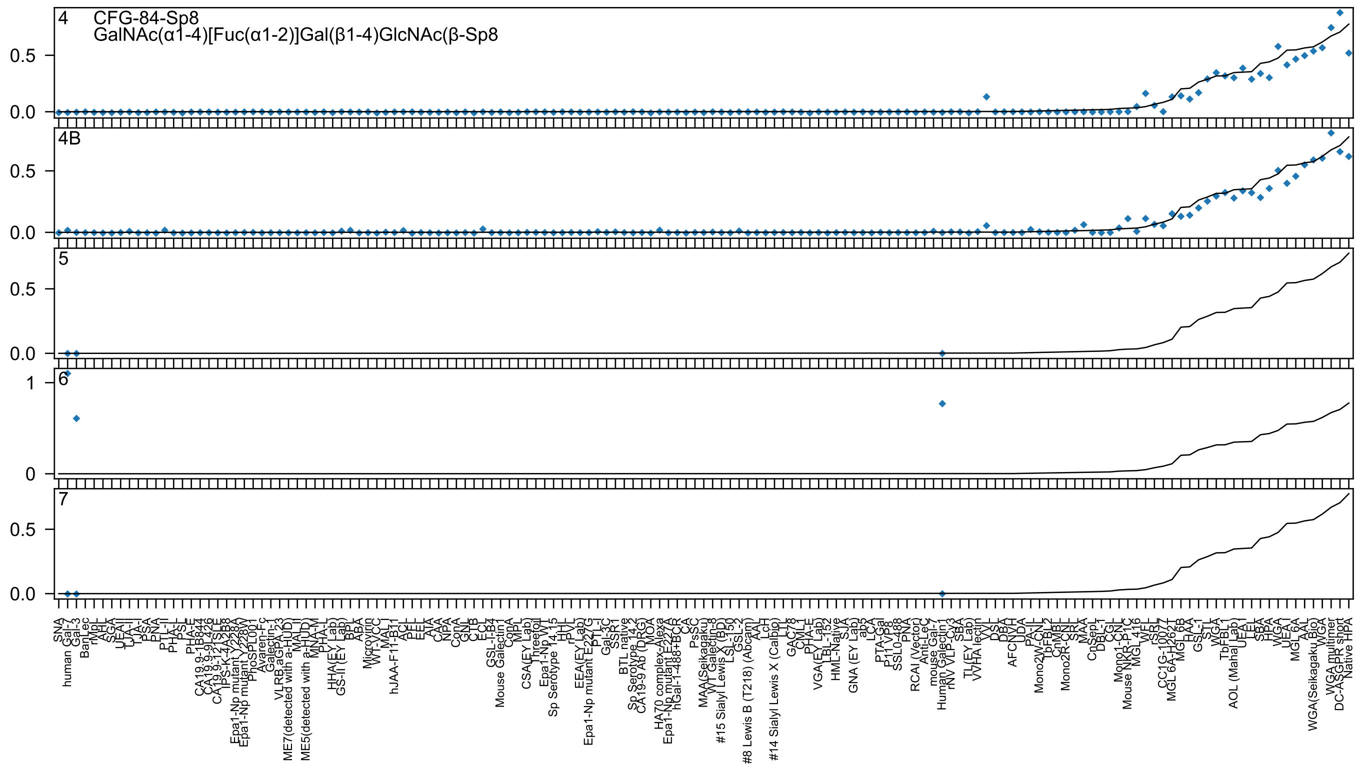
4
CFG-84-Sp8
GalNAc(α1-4)[Fuc(α1-2)]Gal(β1-4)GlcNAc(β-Sp8
4B
5
6
7

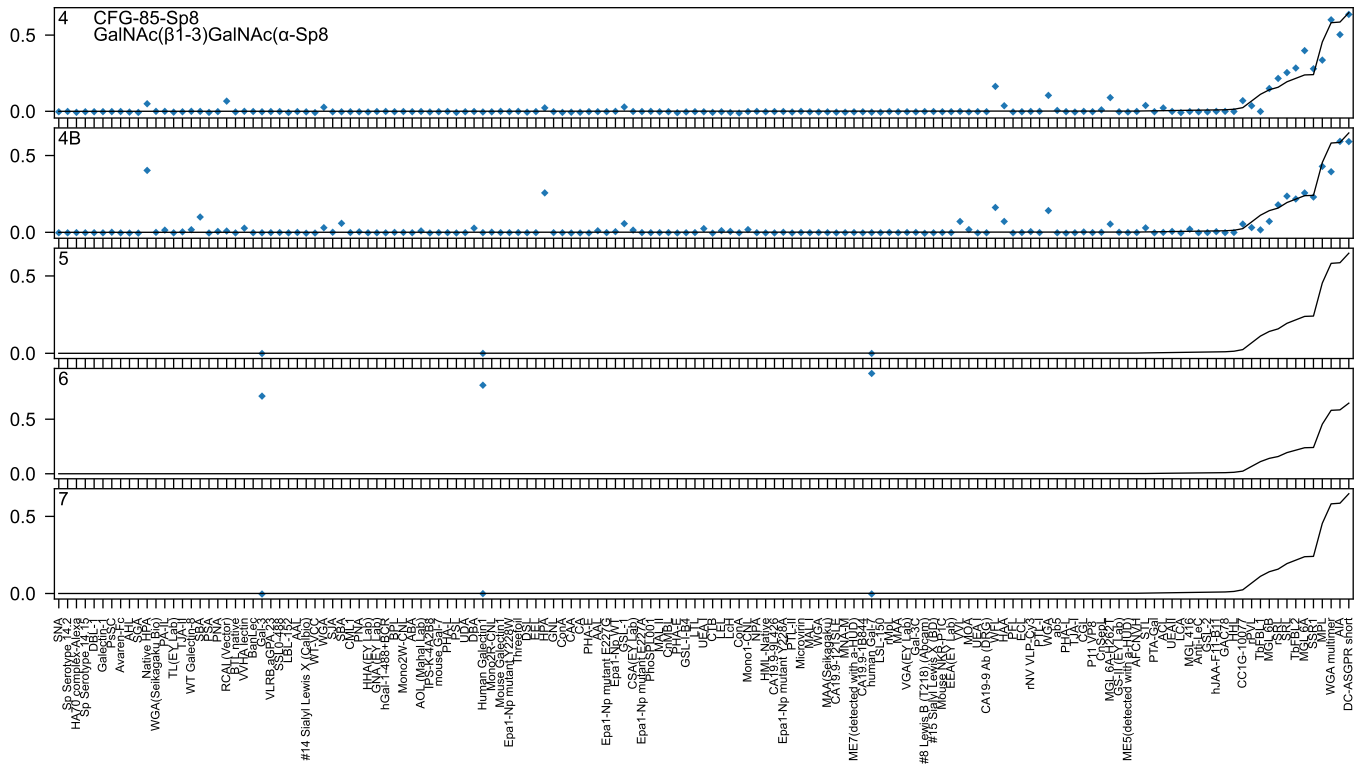
4
CFG-85-Sp8
GalNAc(β1-3)GalNAc(α-Sp8
4B
5
6
7
0.5
0.0
SNA
WGA multimer
AIA
DC-ASGPR short

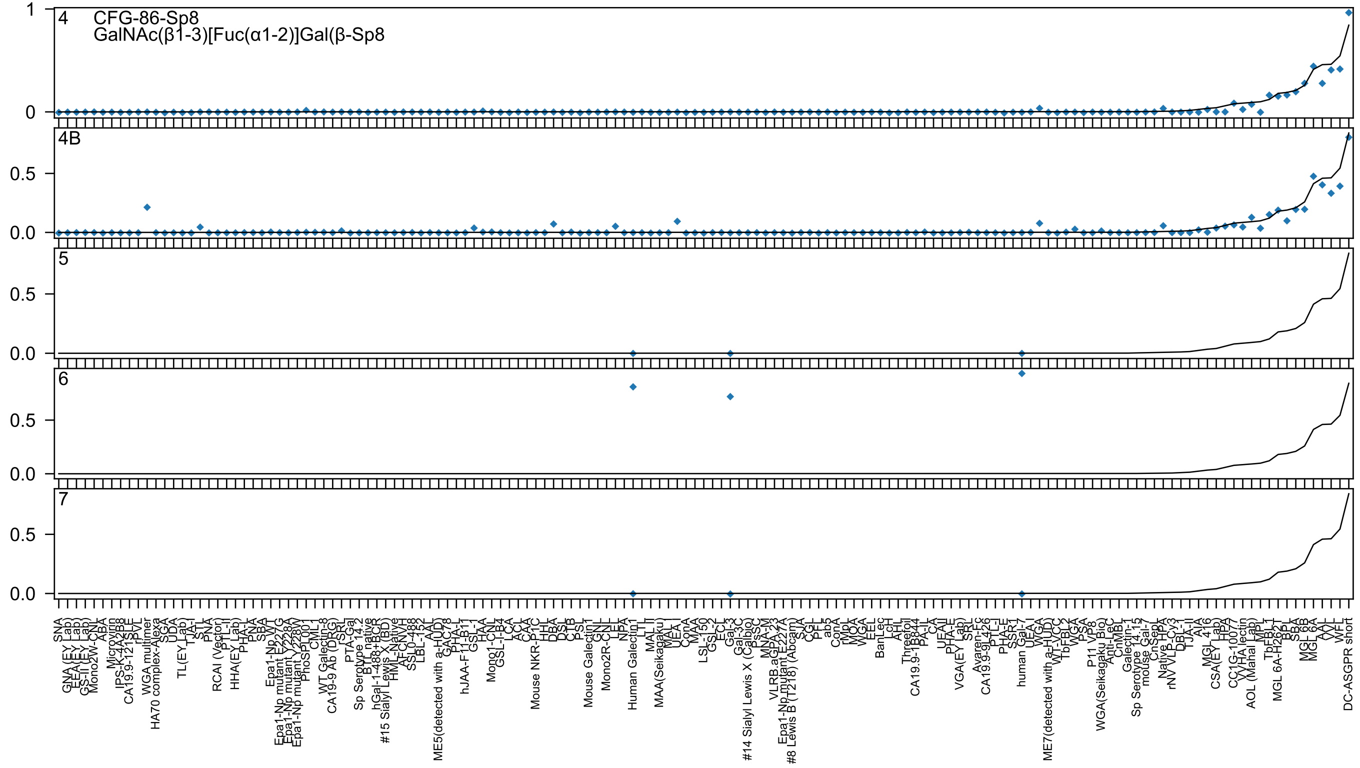
CFG-86-Sp8
GalNAc(β1-3)[Fuc(α1-2)]Gal(β-Sp8
4
4B
5
6
7
SNA
WGA multimer
HA70 complex-Alexa
Human Galectin1
Gal-3
human Gal-7
DC-ASGPR short

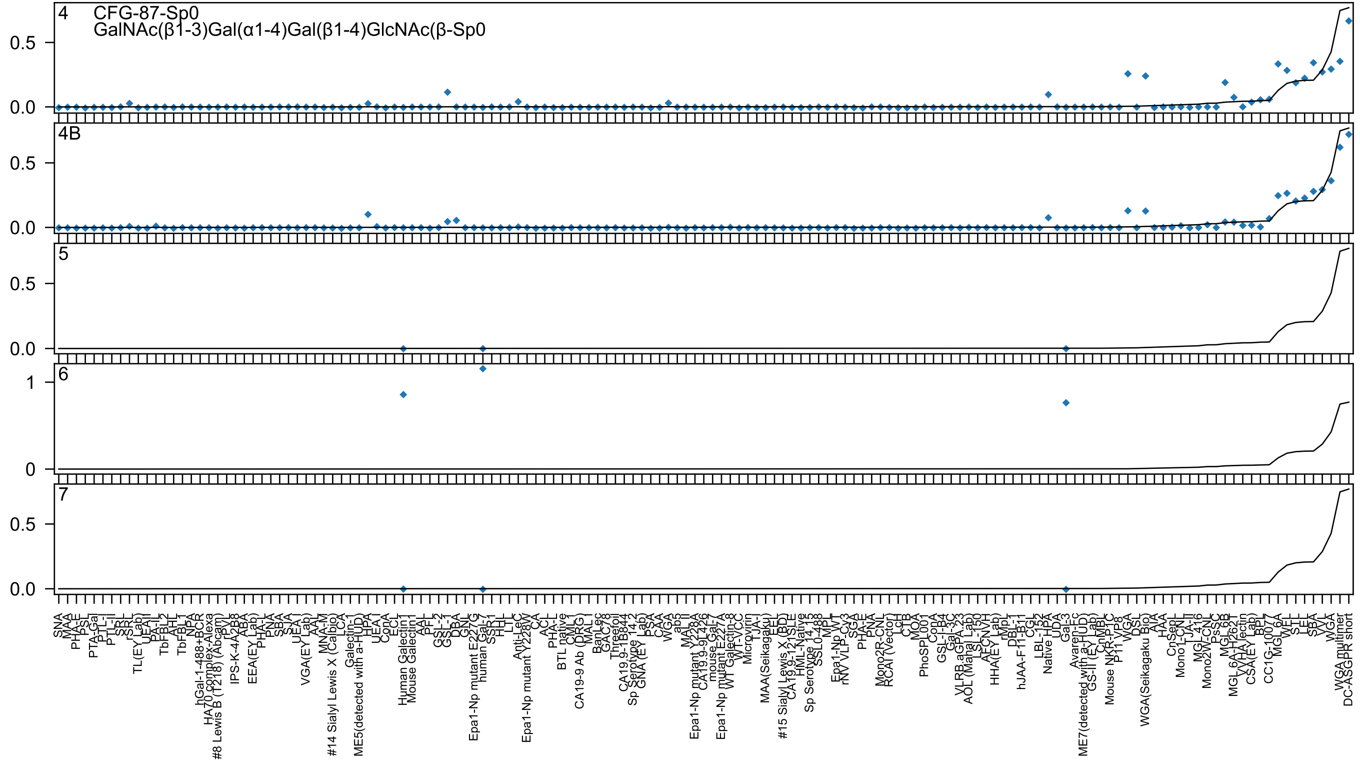

4
CFG-87-Sp0
GalNAc(β1-3)Gal(α1-4)Gal(β1-4)GlcNAc(β-Sp0
4B
5
6
7

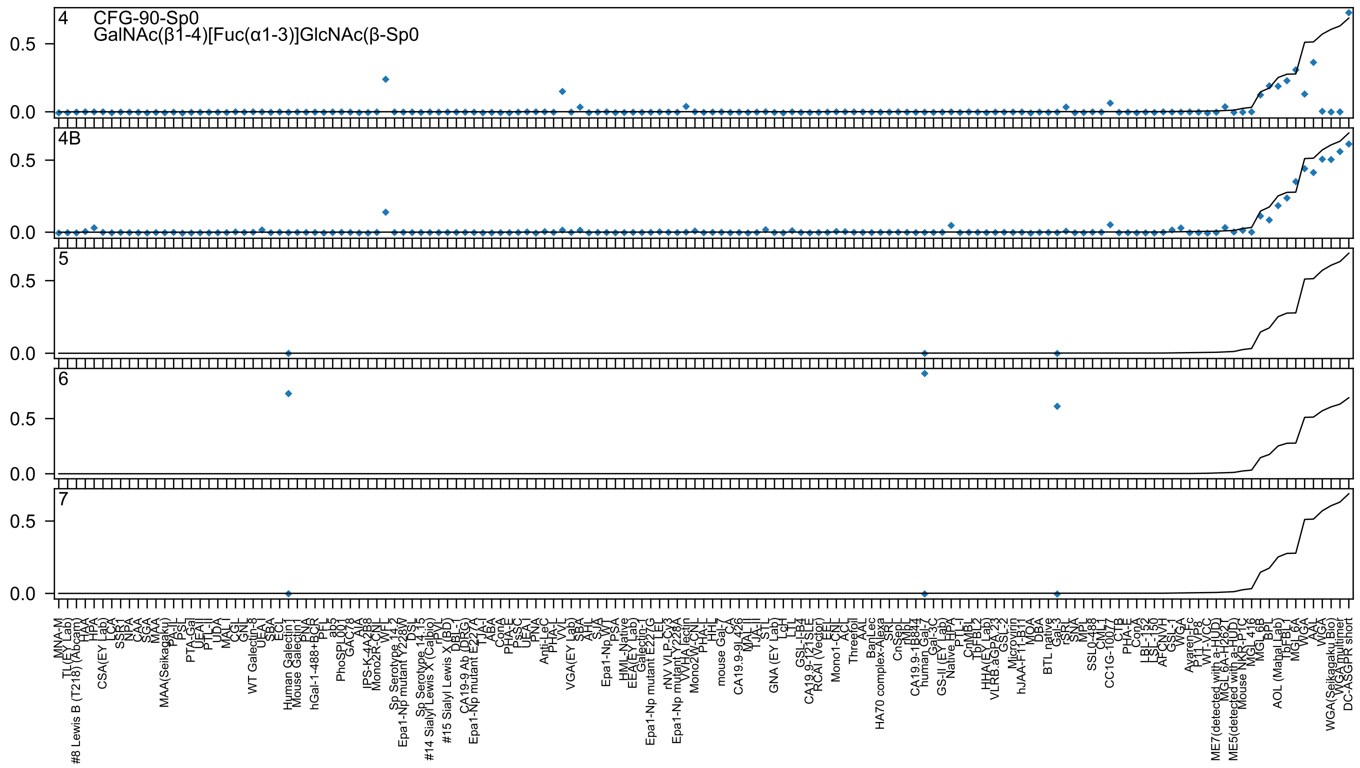

4 CFG-90-Sp0
GalNAc(β1-4)[Fuc(α1-3)]GlcNAc(β-Sp0
4B
5
6
7
0.5
0.0

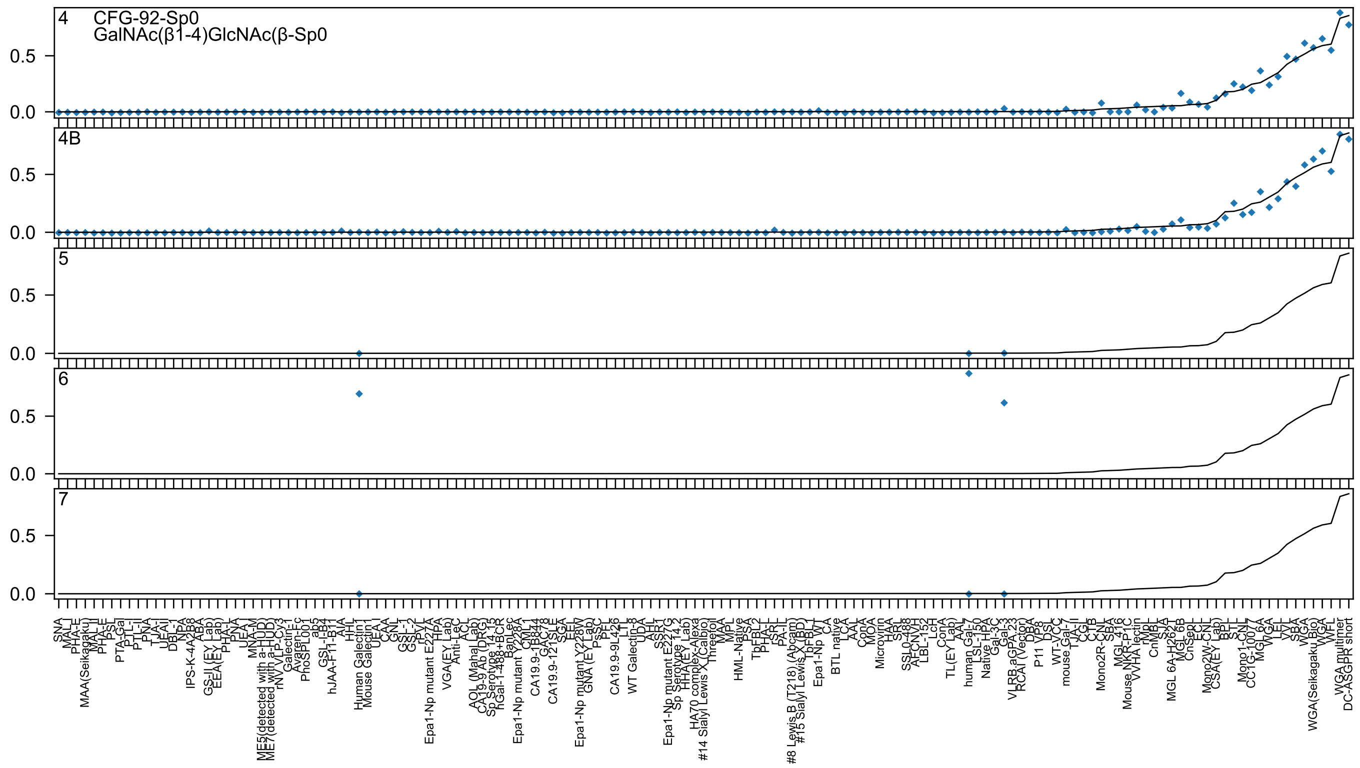
4 CFG-92-Sp0
GalNAc(β1-4)GlcNAc(β-Sp0
4B
5
6
7
0.0
0.5

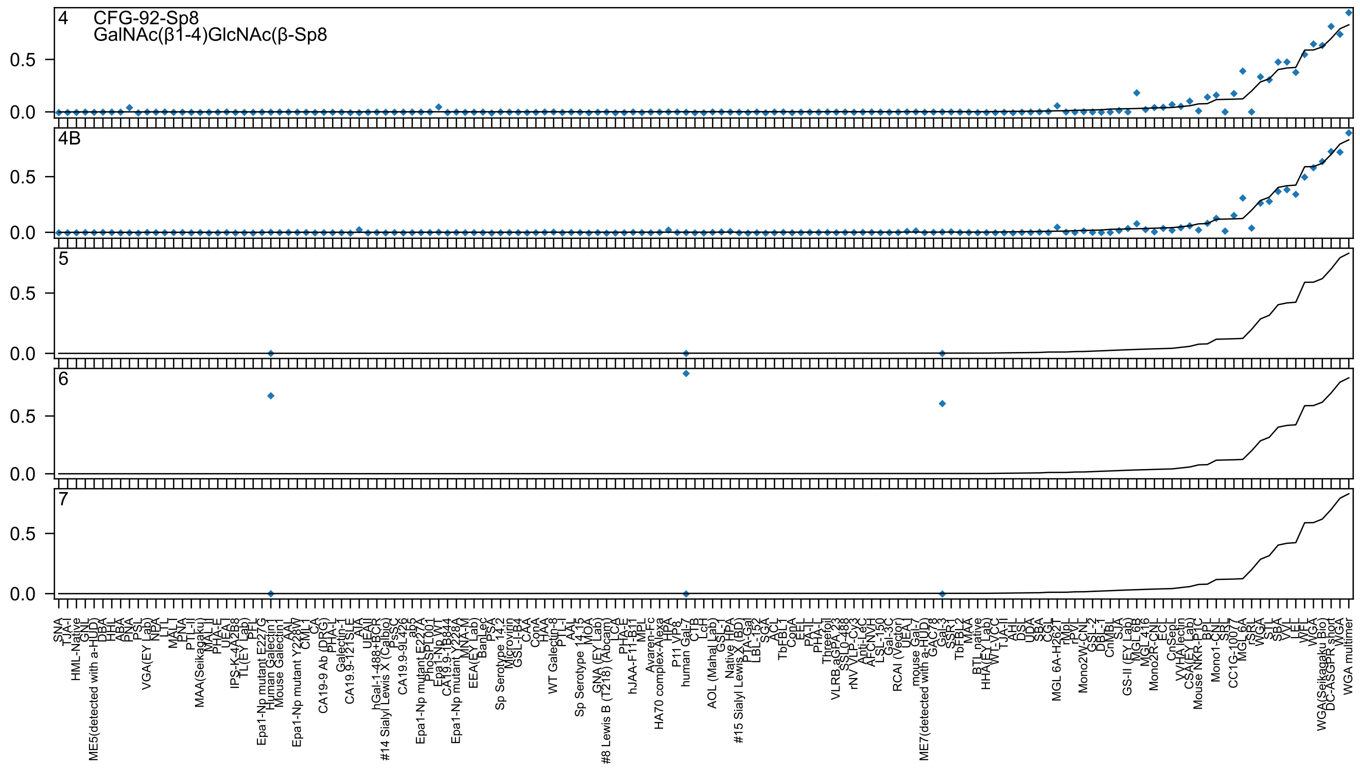
4
CFG-92-Sp8
GalNAc(β1-4)GlcNAc(β-Sp8
4B
5
6
7
0.5
0.0

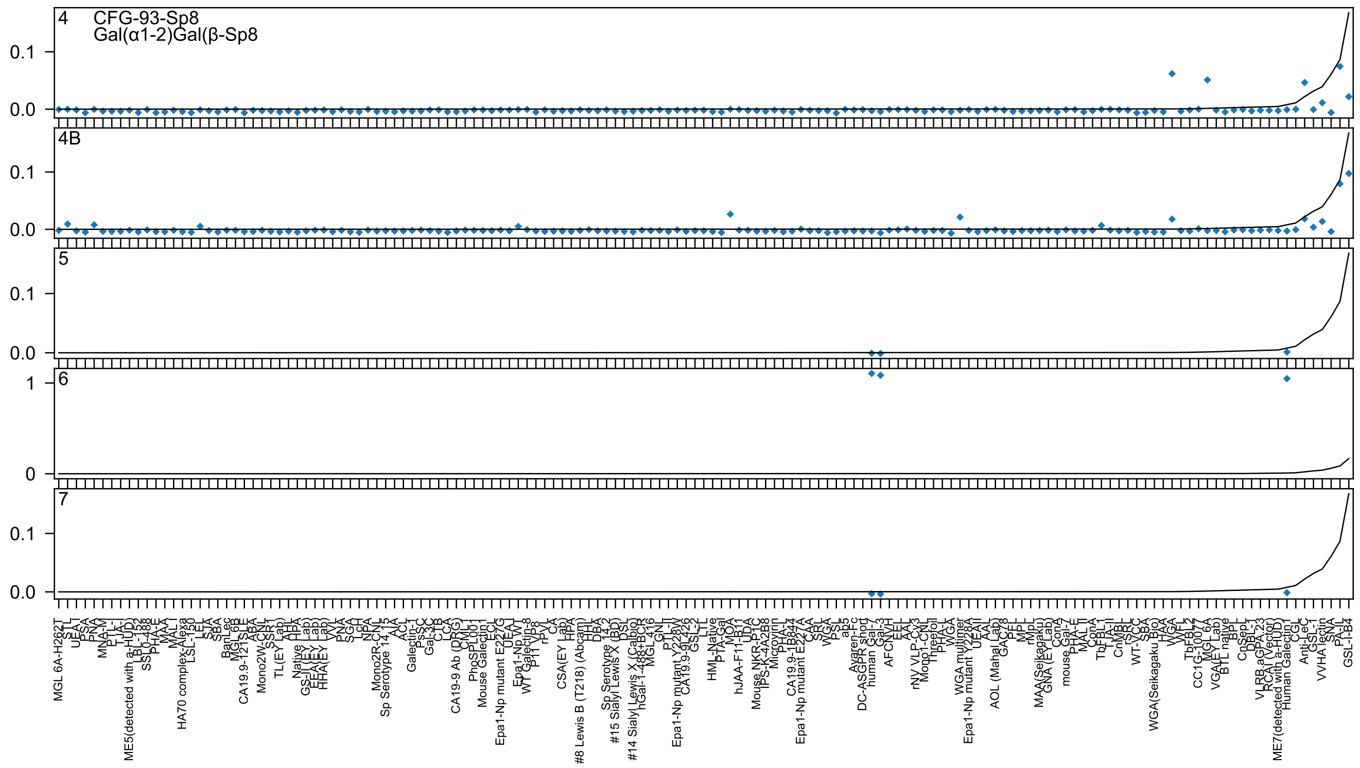
4
CFG-93-Sp8
Gal(α1-2)Gal(β-Sp8
4B
5
6
7
0.1
0.0
1
0
MGL 6A-H262T
STL
UEA-I
PSA
PNA
MNA-M
PTL-I
TJA-I
ME5(detected with a-HUD)
LBL-152
SSL0-488
PHA-E
MAA
MAL I
HA70 complex-Alexa
LSL-150
LEL
SJA
SBA
BanLec
MGL 6B
CA19.9-121SLE
ABA
Mono2W-CNL
SSR1
TL(EY Lab)
AHL
Native HPA
GS-II (EY Lab)
EEA(EY Lab)
HHA(EY Lab)
VVL
PNA
SGA
LcH
NPA
Mono2R-CNL
Sp Serotype 14_15
AIA
ACL
Galectin-1
PsSC
Gal-3C
CTB
LCA
CA19-9 Ab (DRG)
CML
PhoSL001
Mouse Galectin1
ECL
Epa1-Np mutant E227G
UEA-I
Epa1-Np WT
WT Galectin-8
P11 VP8
rPVL
CA
CSA(EY Lab)
HPA
#8 Lewis B (T218) (Abcam)
HHL
DBA
Sp Serotype 14_2
#15 Sialyl Lewis X (BD)
DSL
#14 Sialyl Lewis X (Calbio)
hGal-1-488+BCR
MGL 416
GNL
PTL-II
Epa1-Np mutant Y228W
CA19.9-9L426
GSL-2
LTL
HML-Native
PTA-Gal
MOA
hJAA-F11-B11
UDA
Mouse NKR-P1C
IPS-K-4A2B8
Microvirin
PHA-L
CA19.9-1B844
Epa1-Np mutant E227A
CAA
SRL
WGA
PSL
ab5
Avaren-Fc
DC-ASGPR short
human Gal-7
Gal-3
AFCNVH
EEL
AAL
rNV VLP-Cv3
Mono1-CNL
Threefoil
PHA-L
WGA
WGA multimer
Epa1-Np mutant Y228A
UEAII
AAL
AOL (Mahal Lab)
GAC78
PFL
MPL
rMpL
MAA(Seikagaku)
GNA (EY Lab)
ConA
mouse Gal-7
PHA-E
MAL II
ConA
TbFBL1
TJA-II
CnMBL
rSRL
WT-VCC
SBA
WGA(Seikagaku Bio)
HAA
WGA
WFL
TbFBL2
CC1G-10077
MGL 6A
VGA(EY Lab)
BTL native
BPL
CnSep1
DBL-1
VLRB.aGPA.23
RCA I (Vector)
ME7(detected with a-HUD)
Human Galectin1
CGL
Anti-LeC
GSL-1
VVHA lectin
SNA
PA-IL
GSL-I-B4

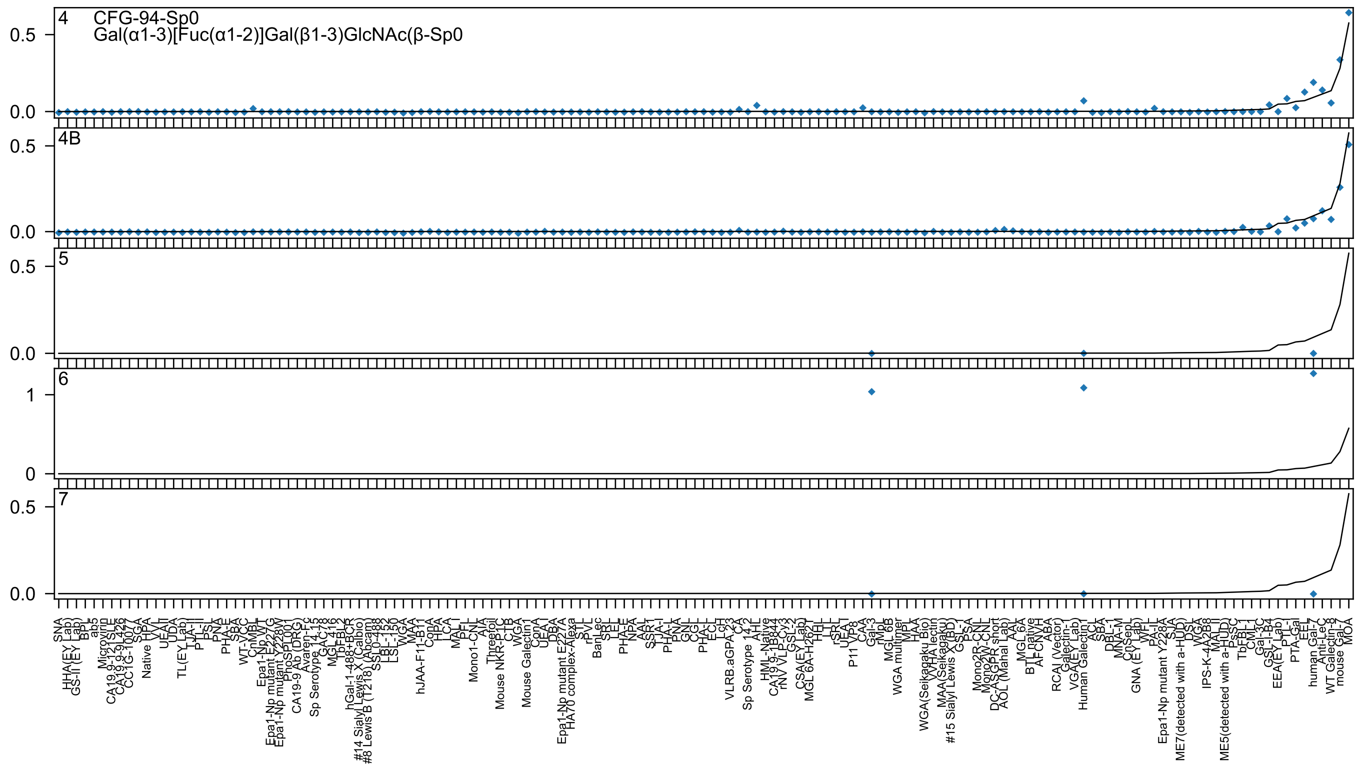

4 CFG-94-Sp0
Gal(α1-3)[Fuc(α1-2)]Gal(β1-3)GlcNAc(β-Sp0
4B
5
6
7

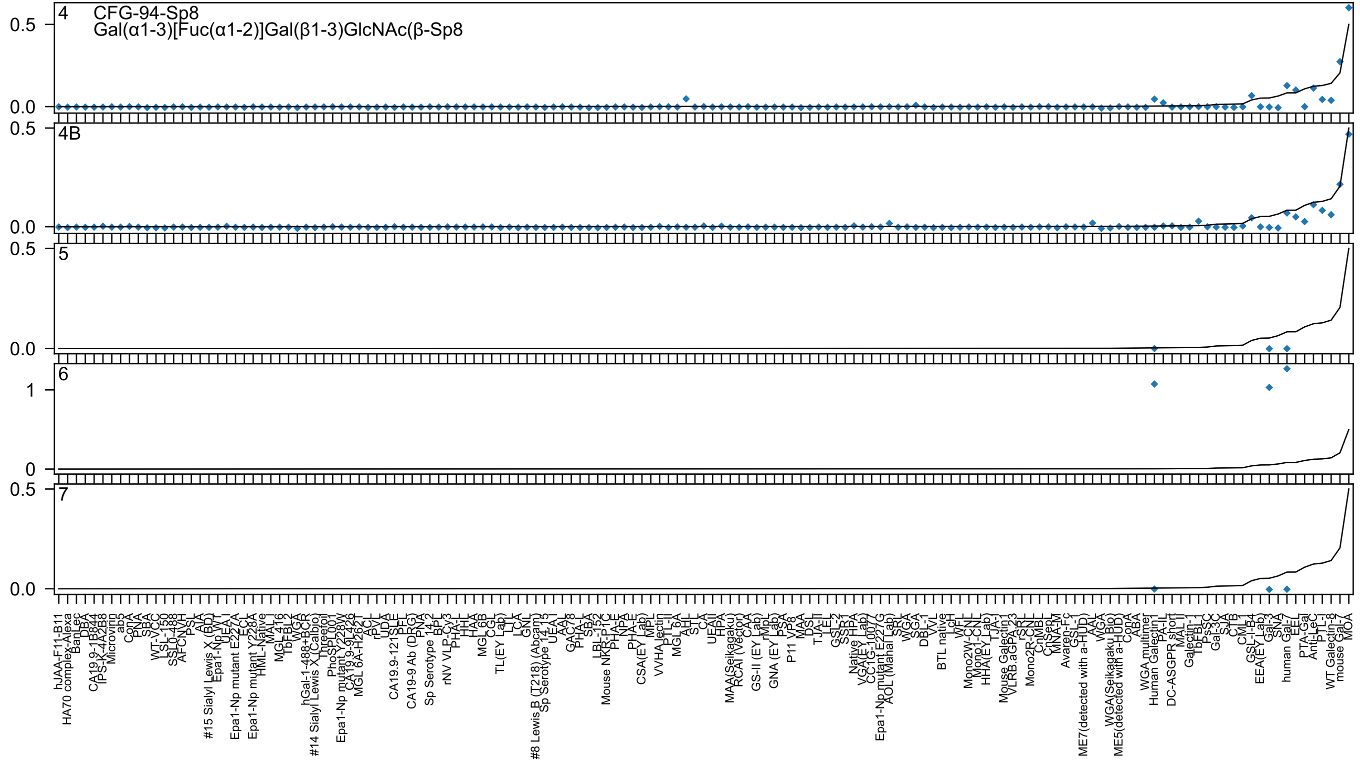

4 CFG-94-Sp8
Gal(α1-3)[Fuc(α1-2)]Gal(β1-3)GlcNAc(β-Sp8
4B
5
6
7

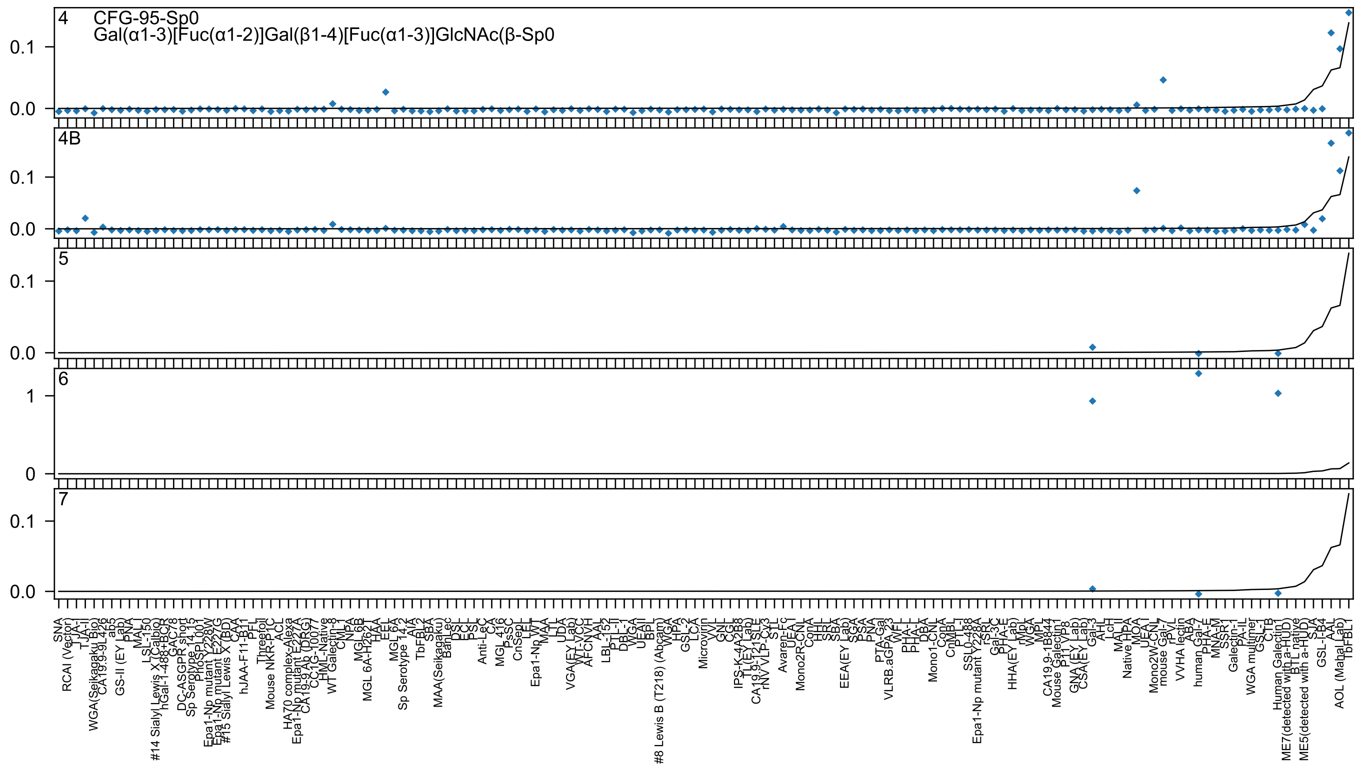

4
CFG-95-Sp0
Gal(α1-3)[Fuc(α1-2)]Gal(β1-4)[Fuc(α1-3)]GlcNAc(β-Sp0
4B
5
6
7

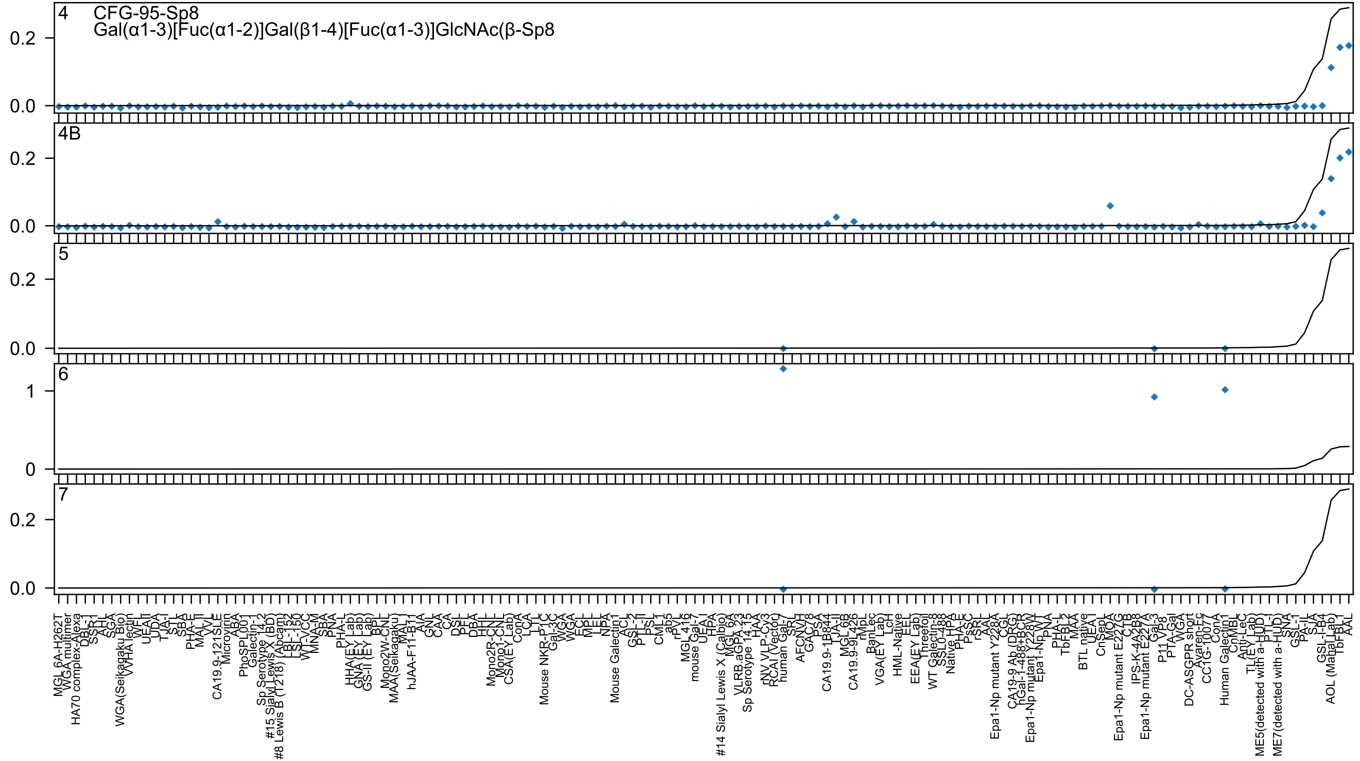
4
CFG-95-Sp8
Gal(α1-3)[Fuc(α1-2)]Gal(β1-4)[Fuc(α1-3)]GlcNAc(β-Sp8
4B
5
6
7
0.0
0.2
0
1

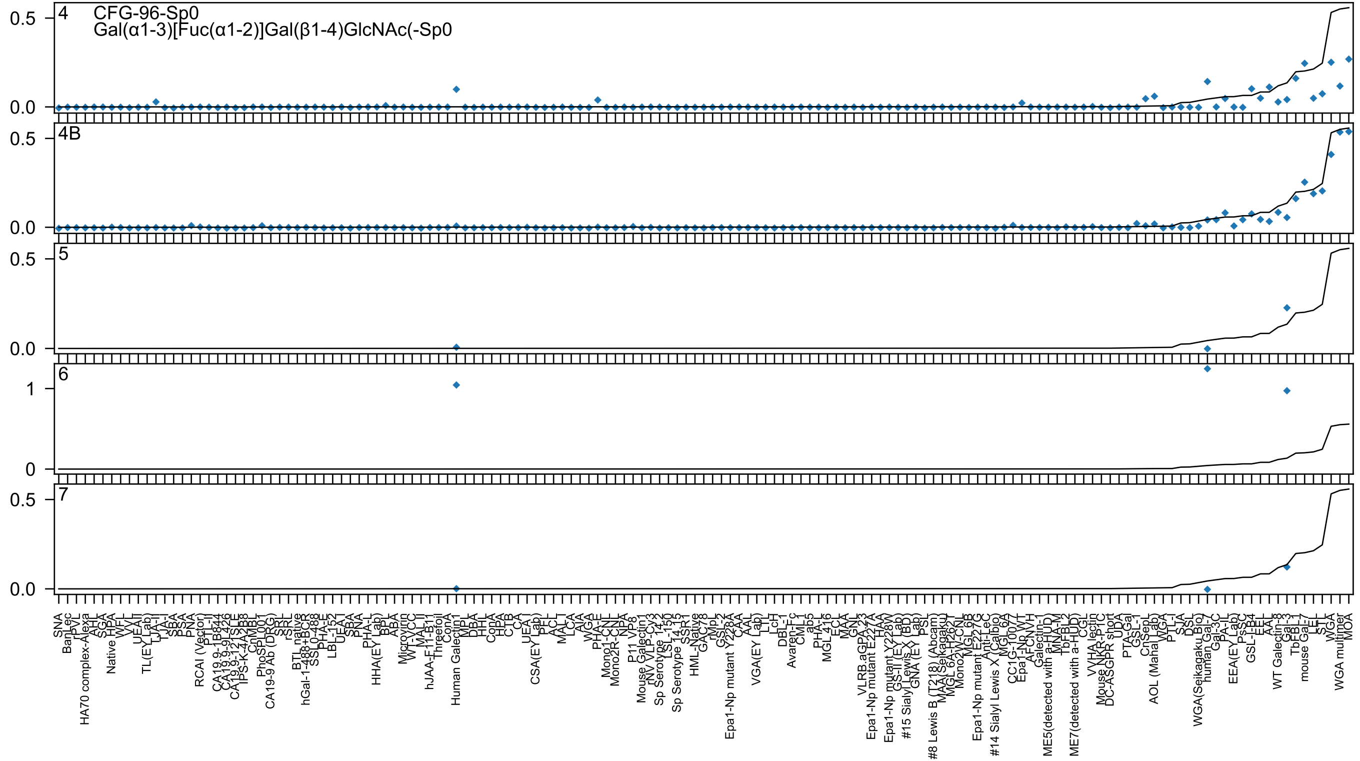
4
CFG-96-Sp0
Gal(α1-3)[Fuc(α1-2)]Gal(β1-4)GlcNAc(-Sp0
4B
5
6
7

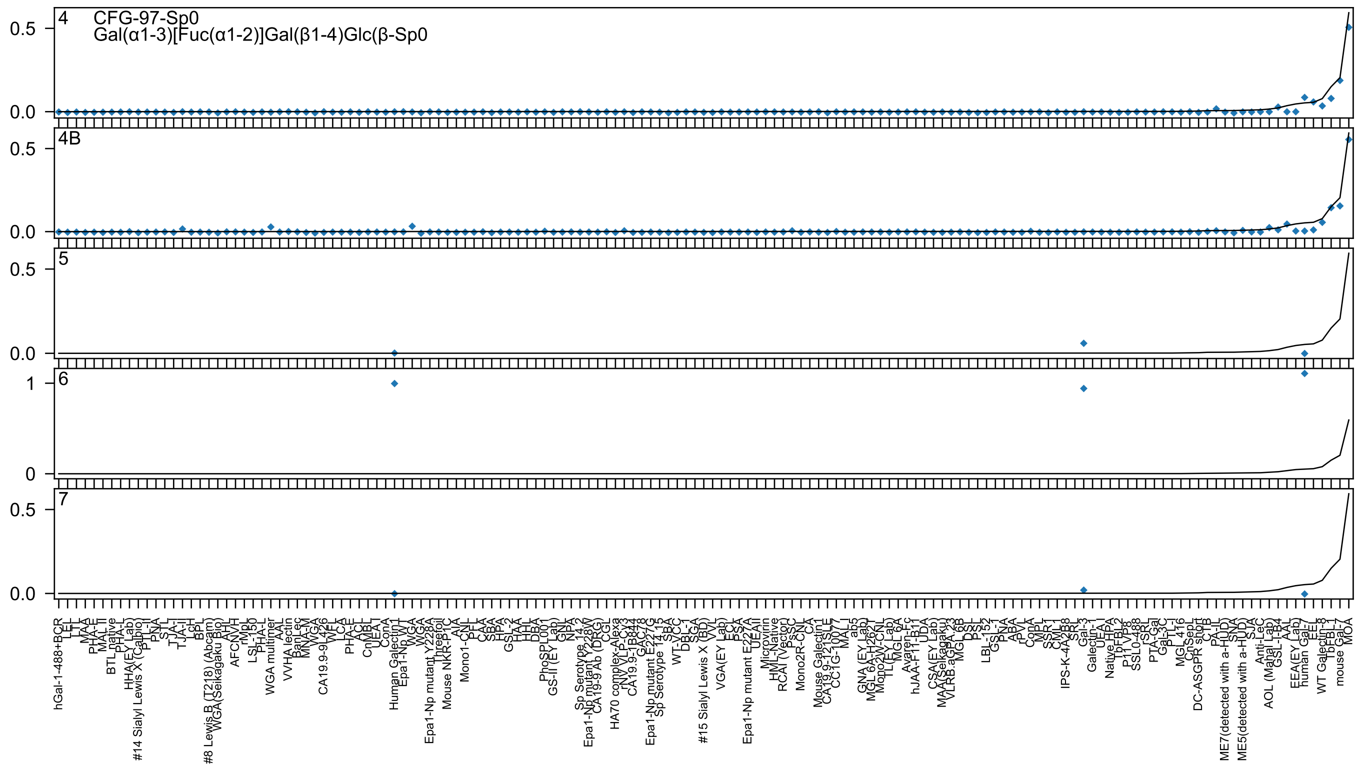
4 CFG-97-Sp0
Gal(α1-3)[Fuc(α1-2)]Gal(β1-4)Glc(β-Sp0
4B
5
6
7

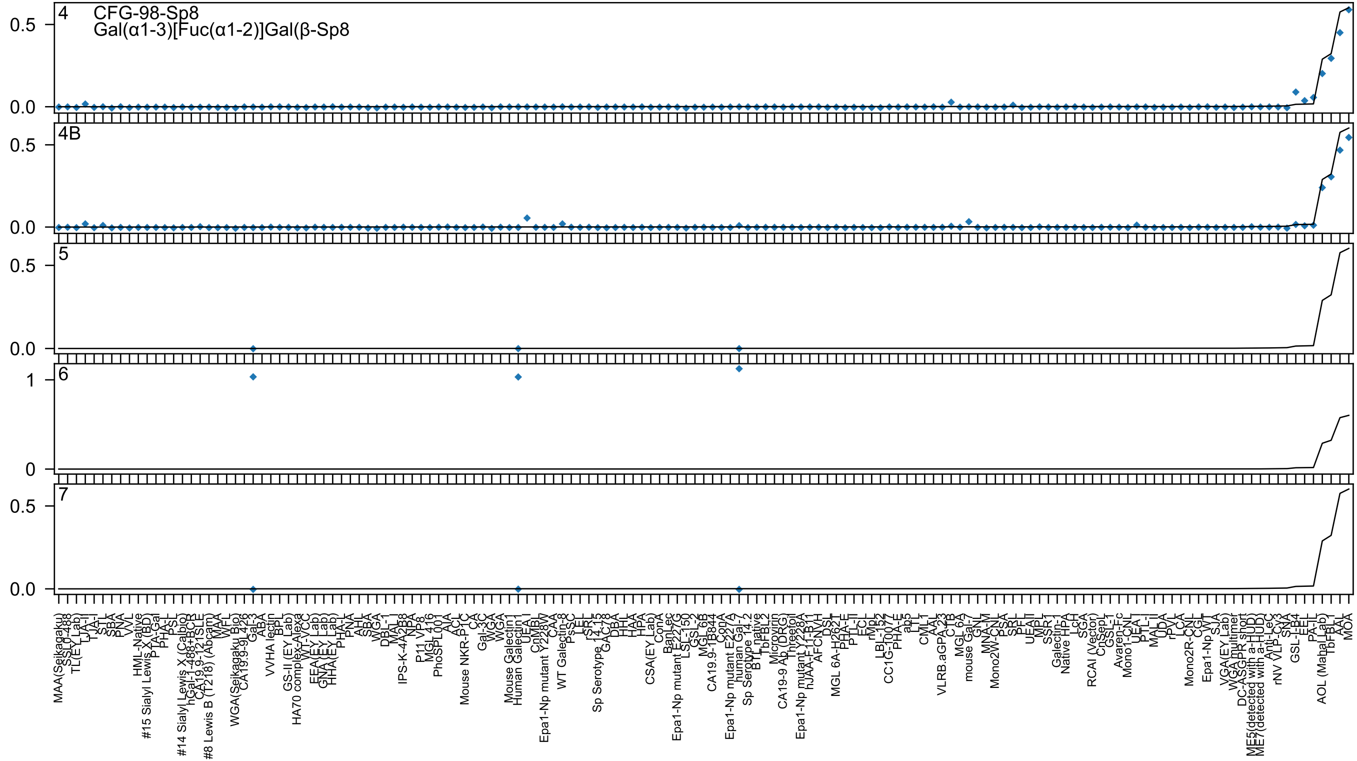

4
CFG-98-Sp8
Gal(α1-3)[Fuc(α1-2)]Gal(β-Sp8
4B
5
6
7

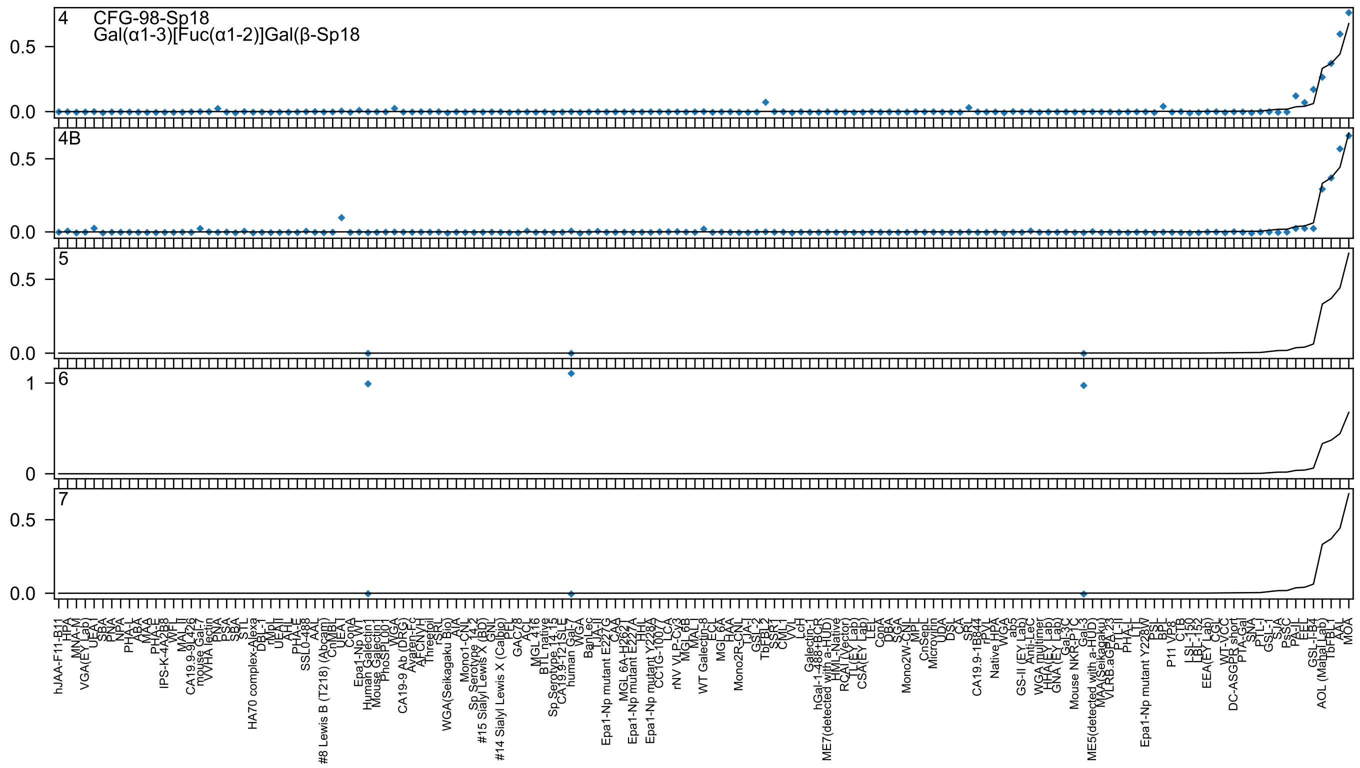
4
CFG-98-Sp18
Gal(α1-3)[Fuc(α1-2)]Gal(β-Sp18
4B
5
6
7

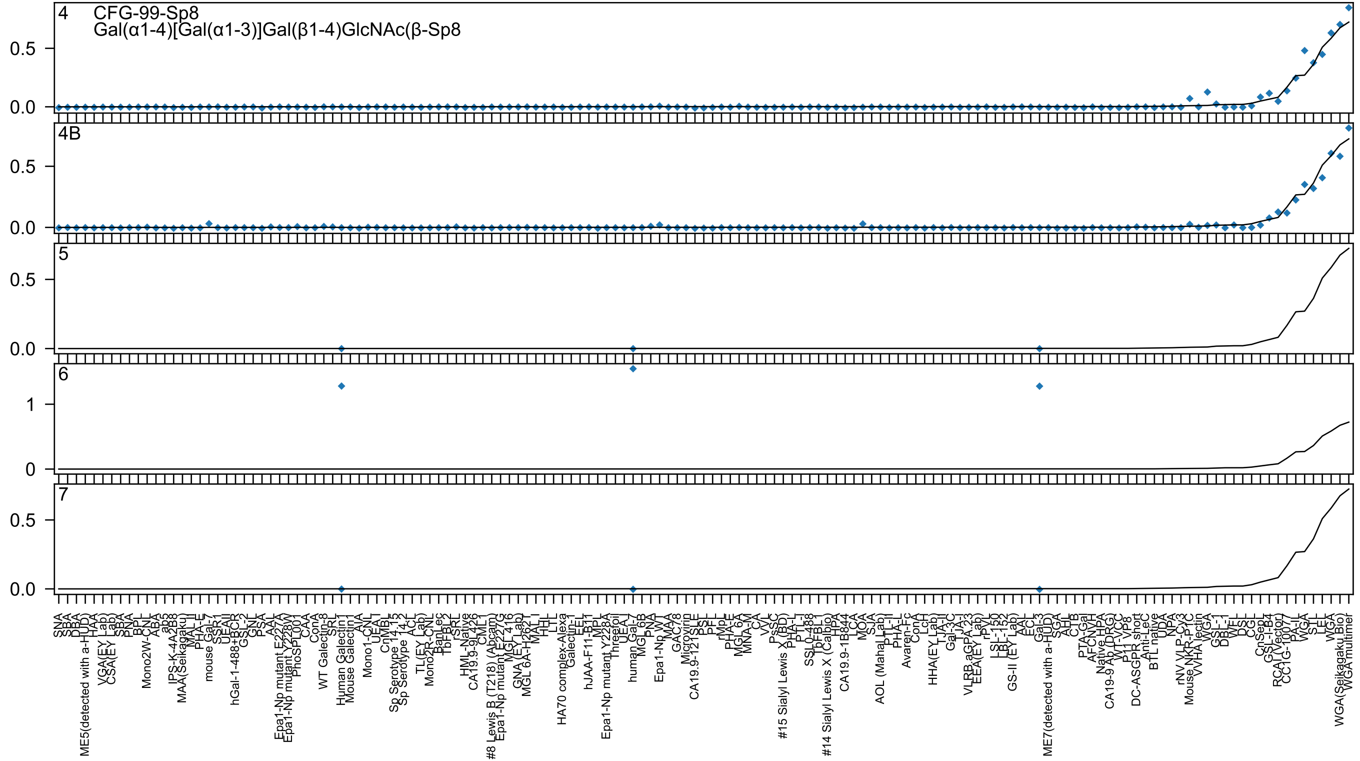
4 CFG-99-Sp8
Gal(α1-4)[Gal(α1-3)]Gal(β1-4)GlcNAc(β-Sp8
4B
5
6
7

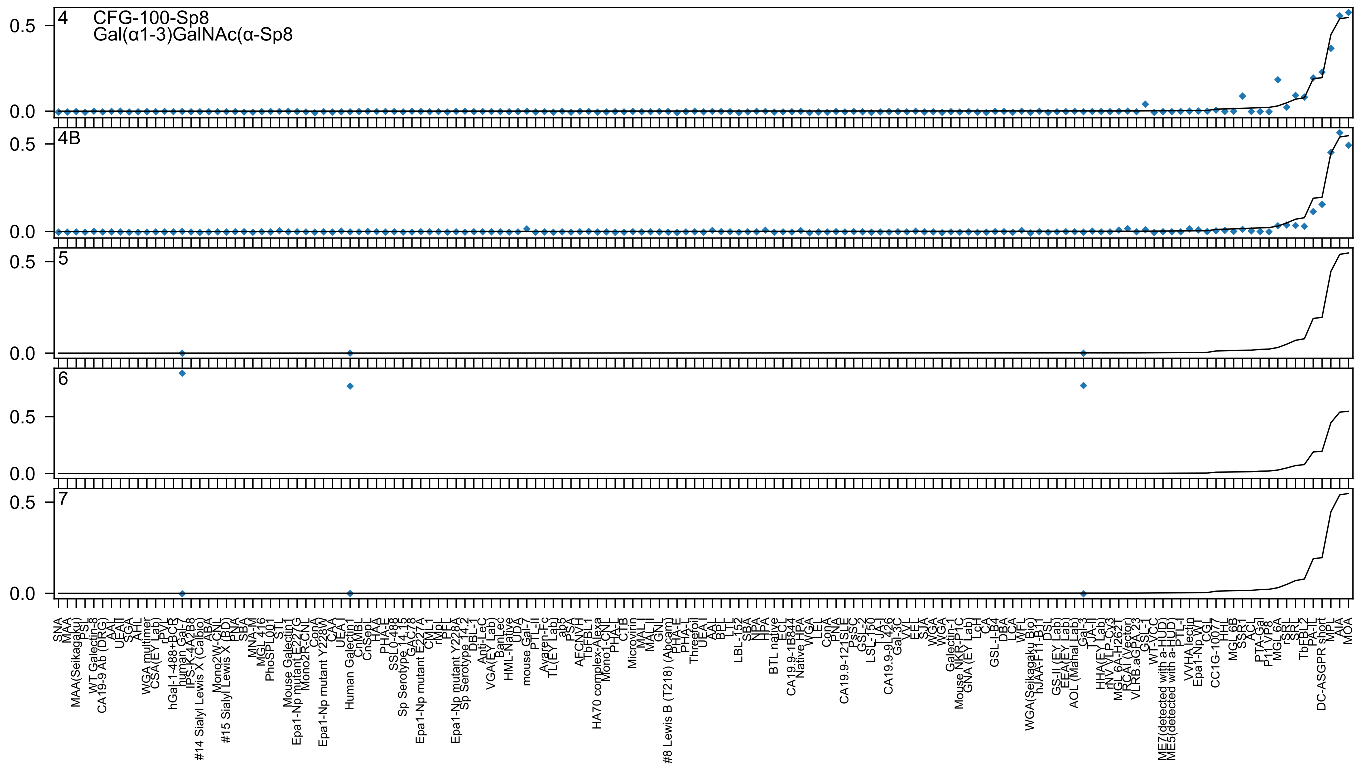
4
CFG-100-Sp8
Gal(α1-3)GalNAc(α-Sp8
4B
5
6
7
0.5
0.0

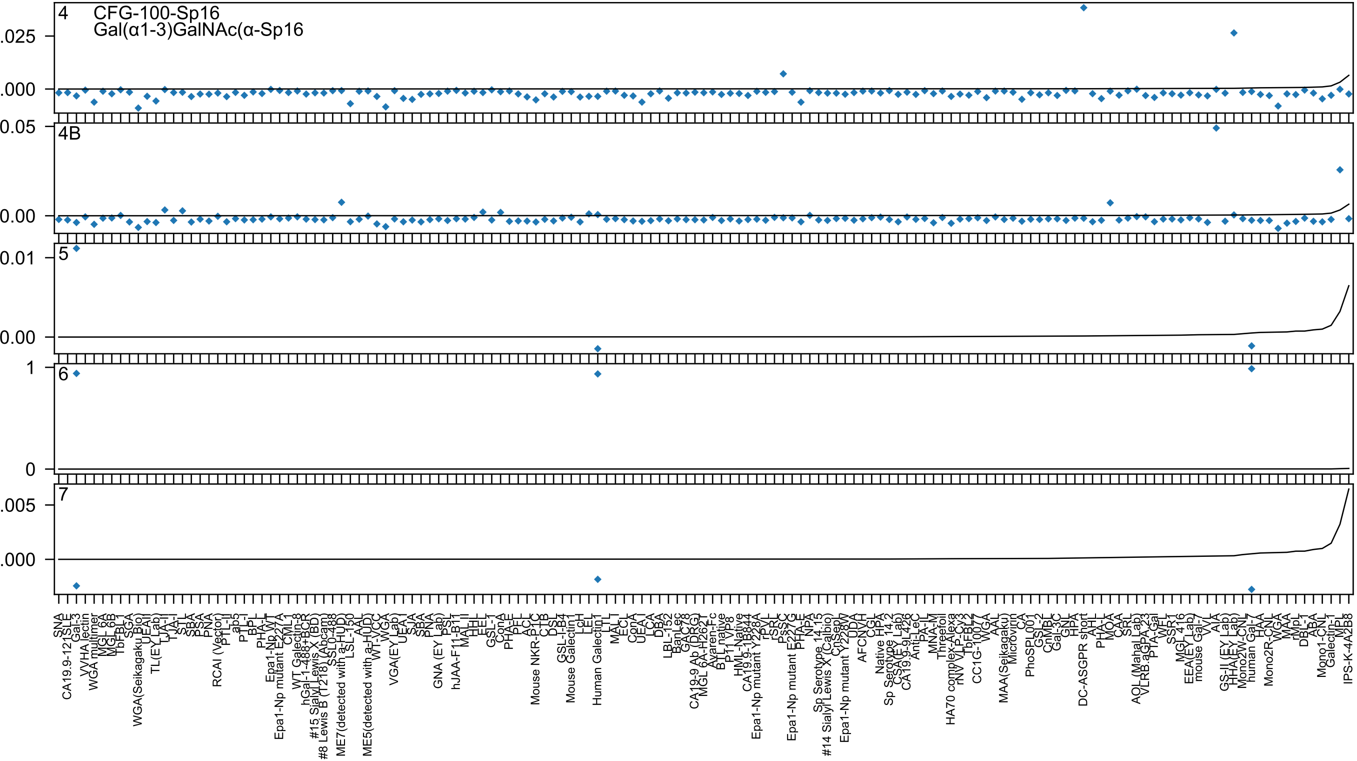

4 CFG-100-Sp16
Gal(α1-3)GalNAc(α-Sp16
4B
5
6
7

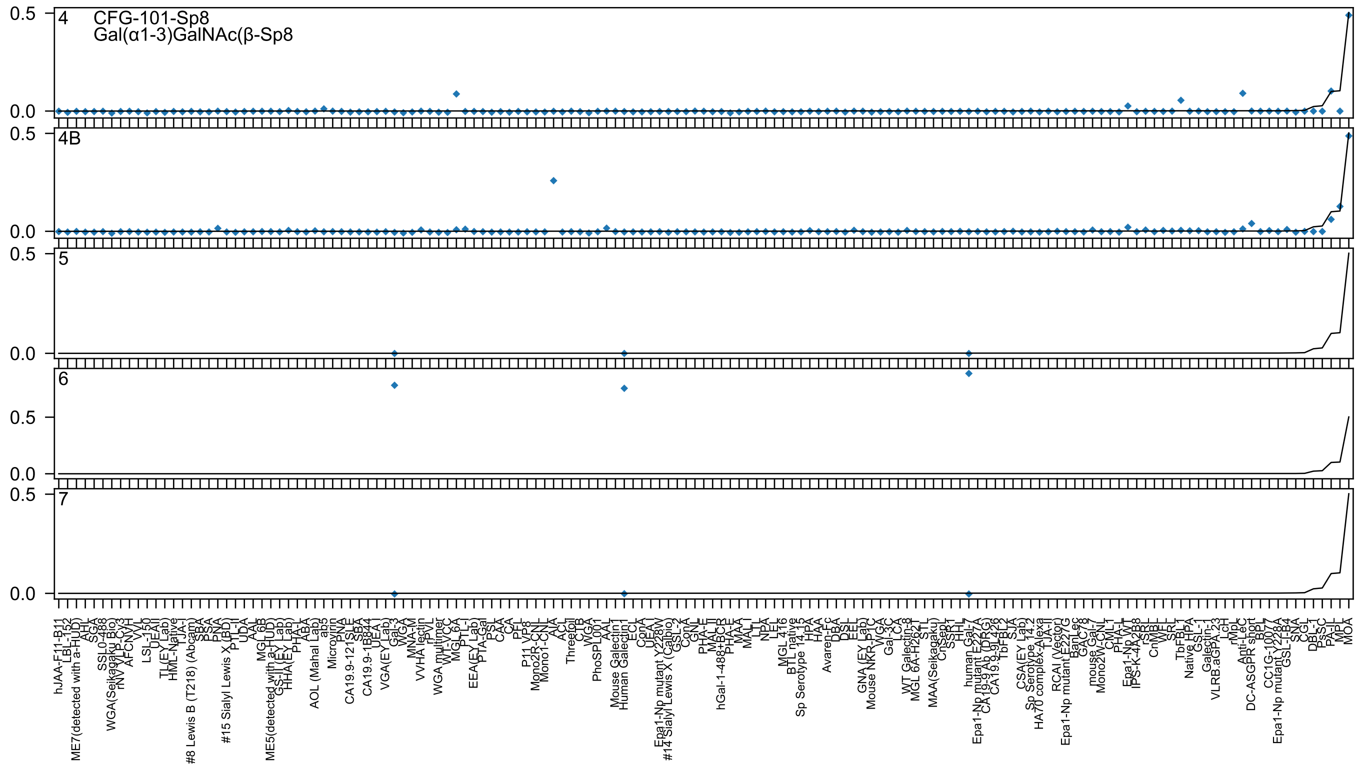
4
CFG-101-Sp8
Gal(α1-3)GalNAc(β-Sp8
4B
5
6
7
0.5
0.0

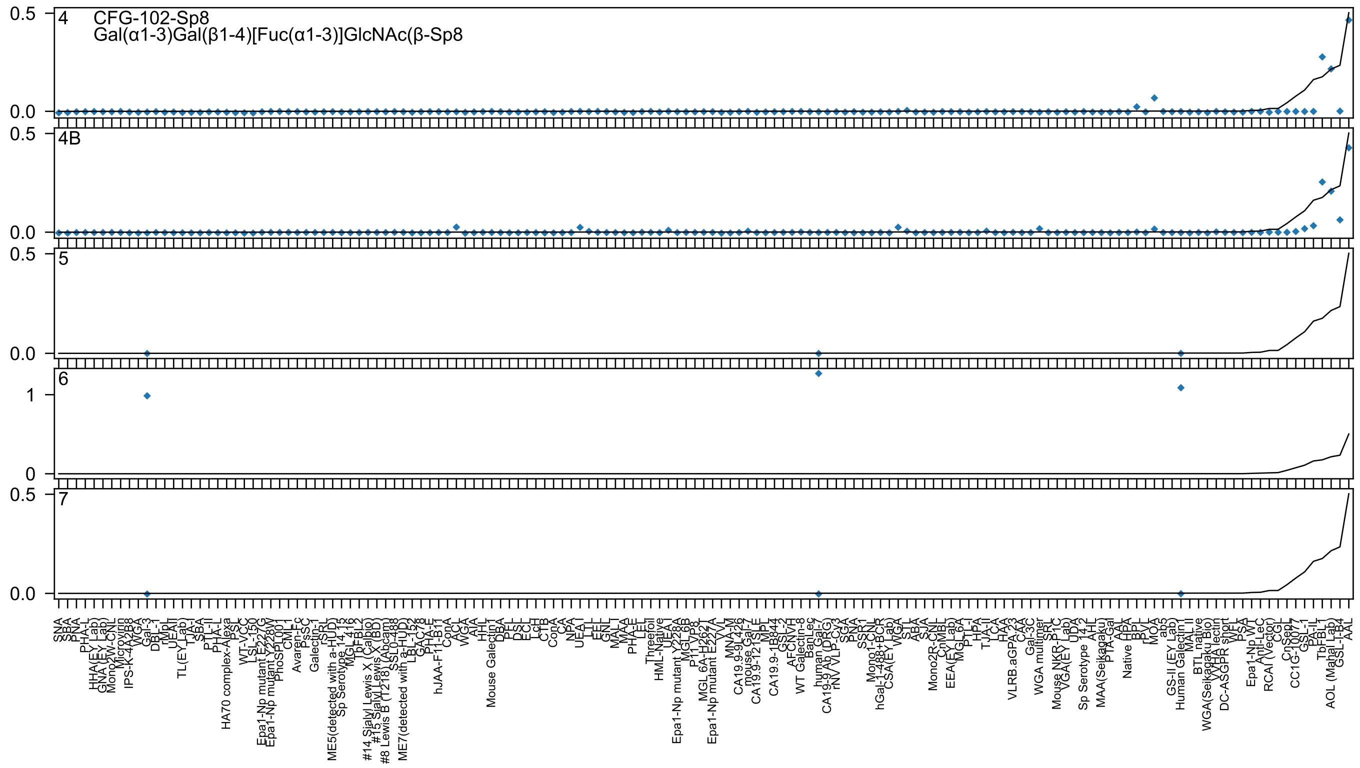

4
CFG-102-Sp8
Gal(α1-3)Gal(β1-4)[Fuc(α1-3)]GlcNAc(β-Sp8
4B
5
6
7

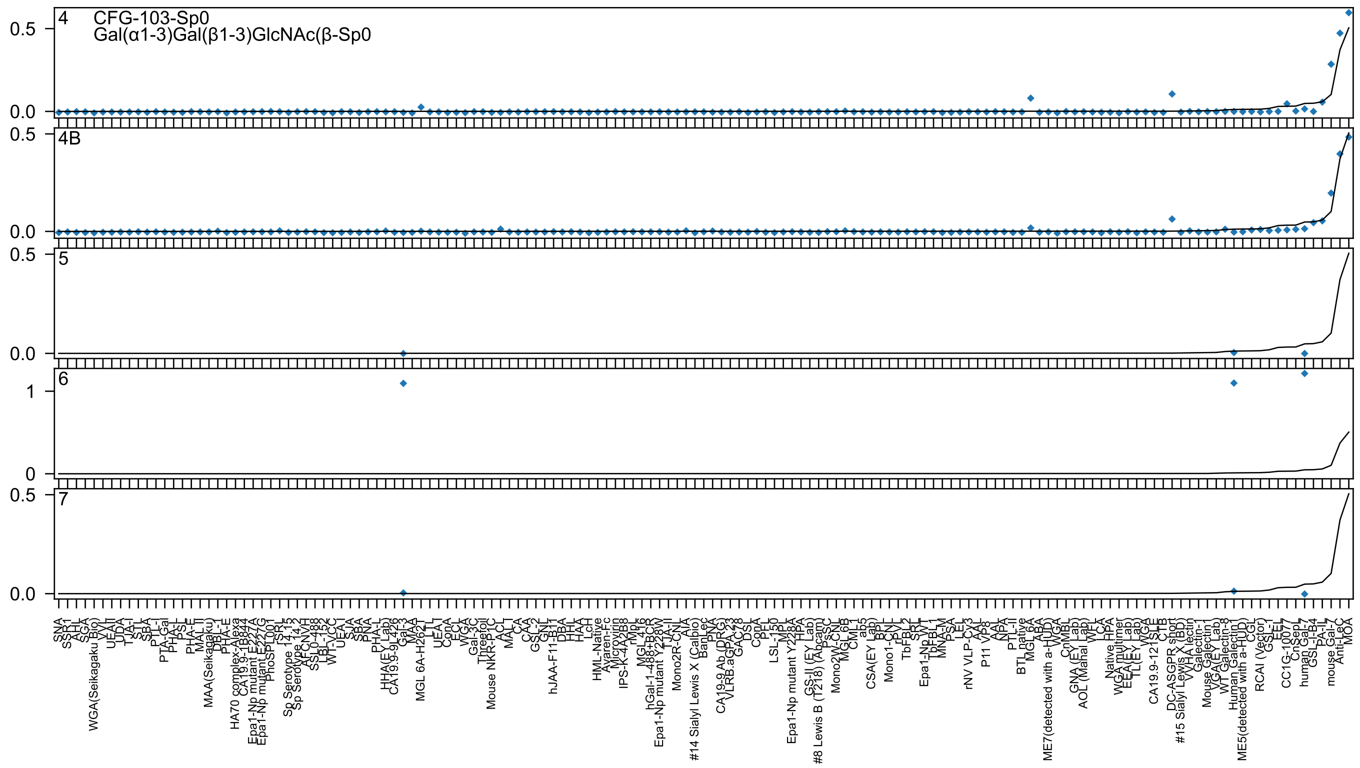
4
CFG-103-Sp0
Gal(α1-3)Gal(β1-3)GlcNAc(β-Sp0
4B
5
6
7

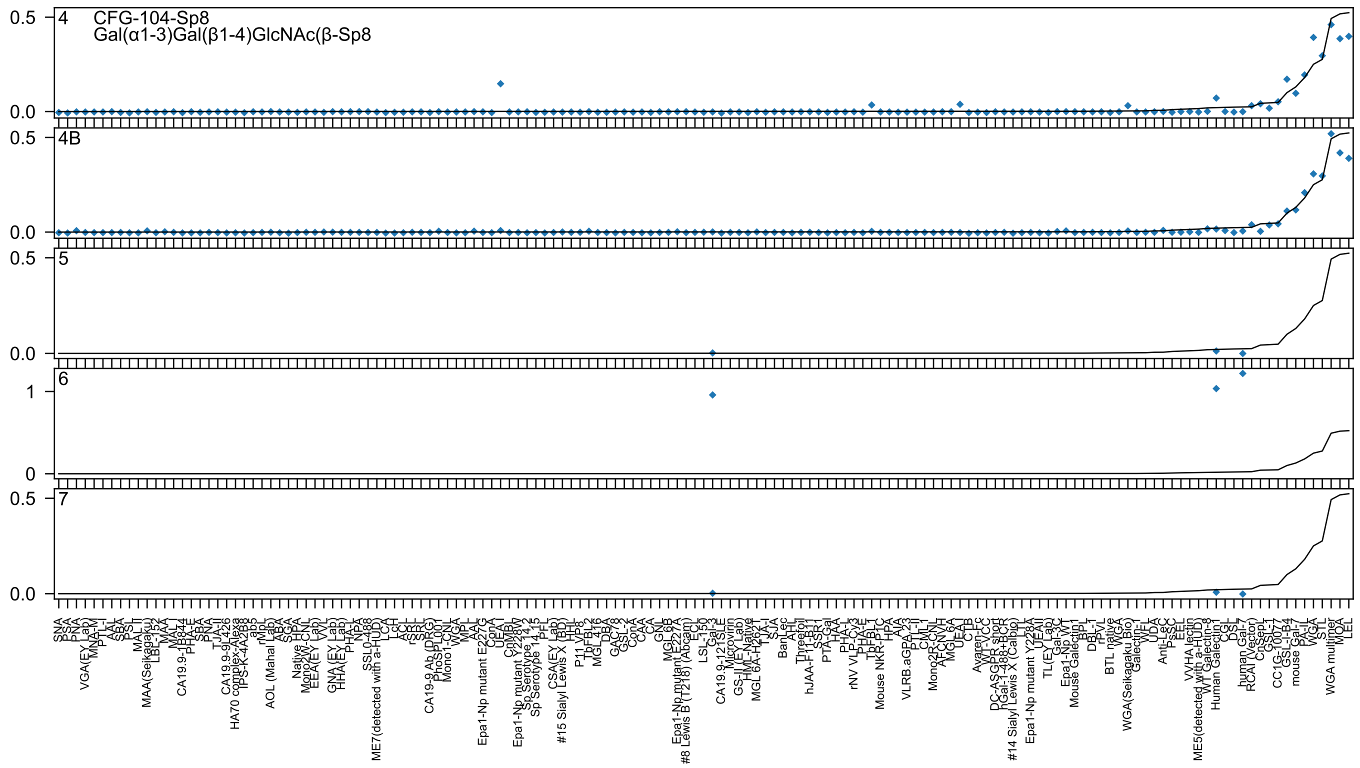

CFG-104-Sp8
Gal(α1-3)Gal(β1-4)GlcNAc(β-Sp8

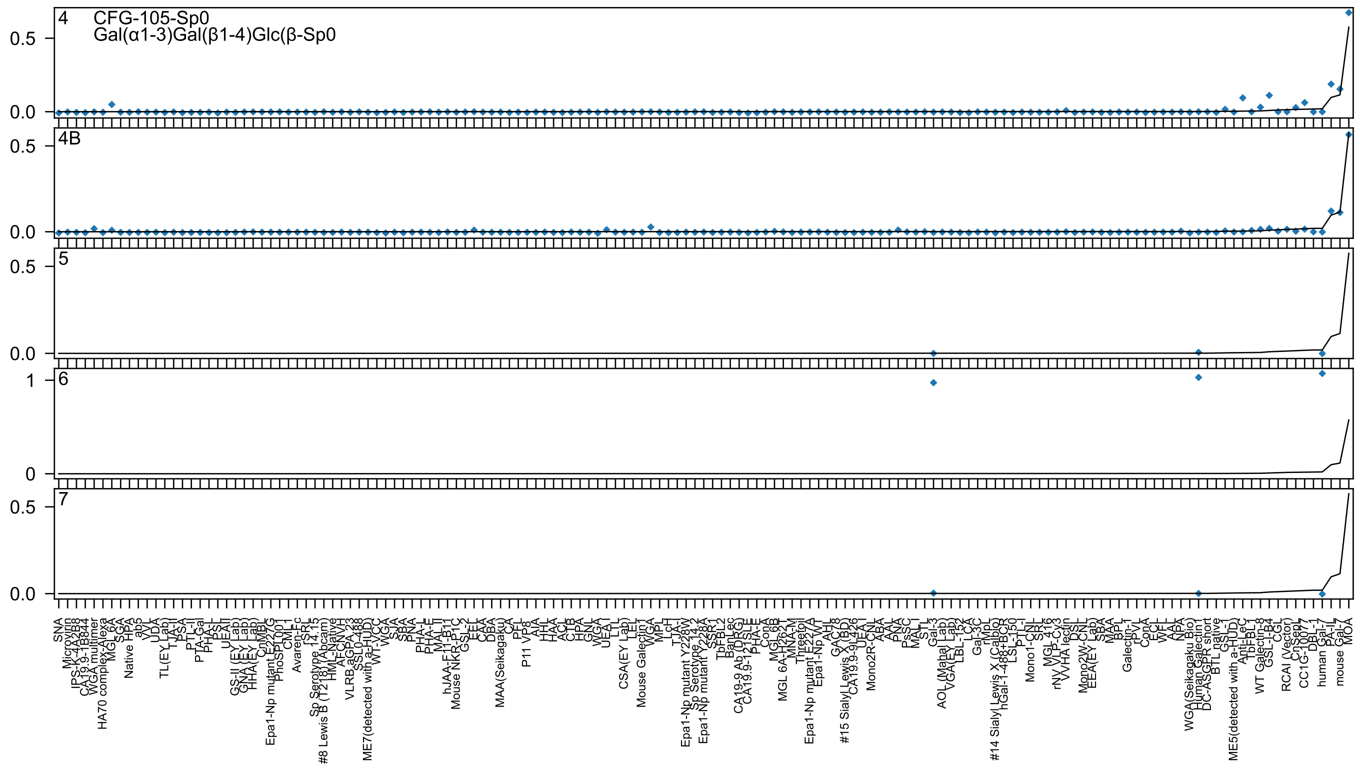

4 CFG-105-Sp0
Gal(α1-3)Gal(β1-4)Glc(β-Sp0
4B
5
6
7

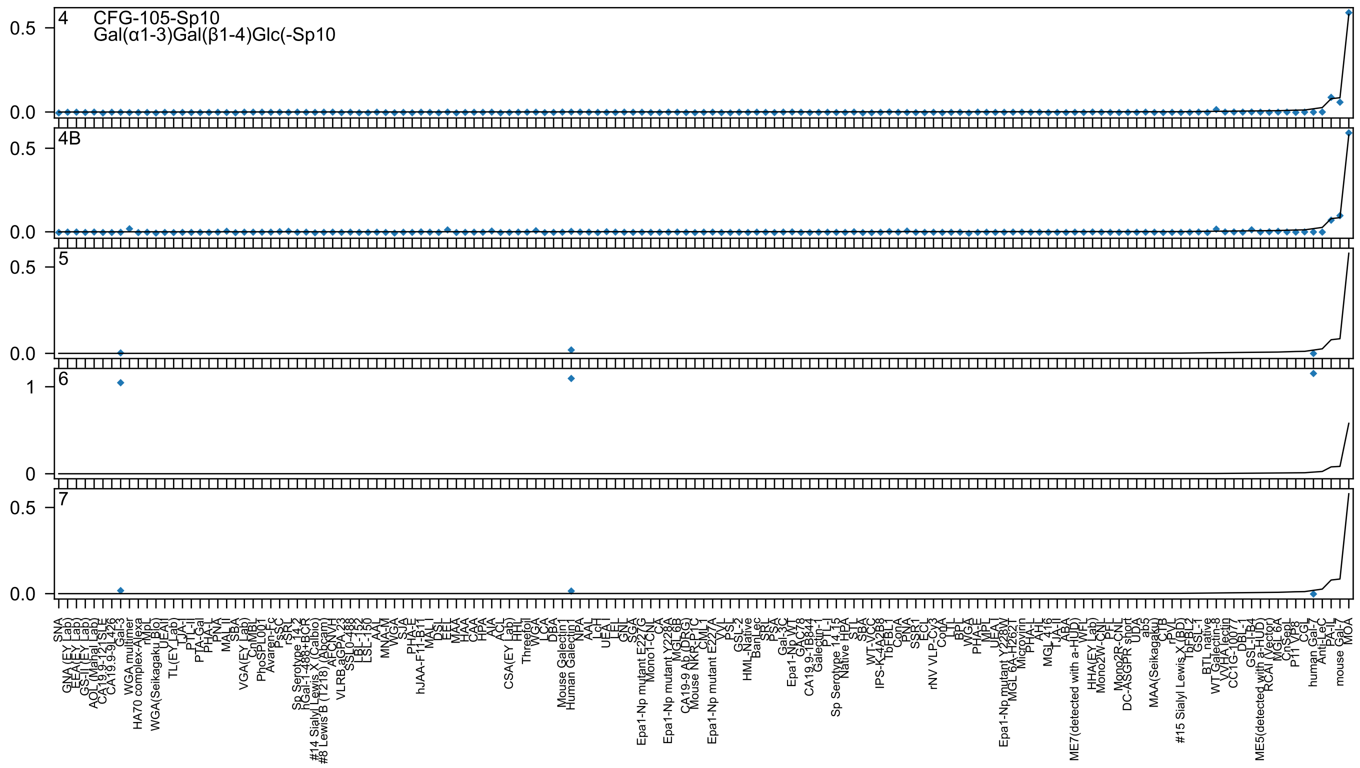

4
CFG-105-Sp10
Gal(α1-3)Gal(β1-4)Glc(-Sp10
4B
5
6
7
0.5
0.0
1
0

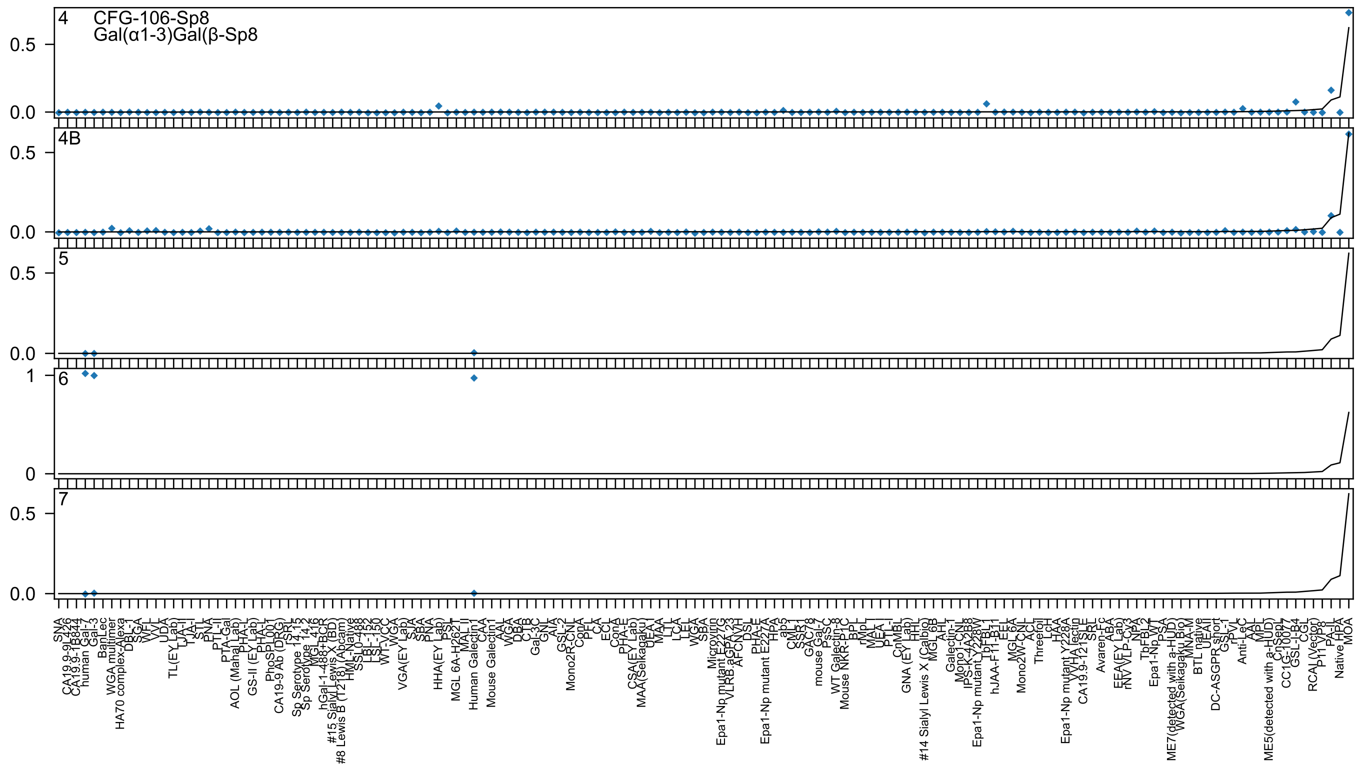
4
CFG-106-Sp8
Gal(α1-3)Gal(β-Sp8
4B
5
6
7
0.5
0.0
1
0
SNA
CA19.9-9L426
CA19.9-1B844
human Gal-7
Gal-3
BanLec
WGA multimer
HA70 complex-Alexa
DBL-1
SGA
WFL
VVL
UDA
TL(EY Lab)
LJA-II
TJA-I
STL
PNA
PTL-II
PTA-Gal
AOL (Mahal Lab)
PHA-L
GS-II (EY Lab)
PHA-L
PhoSL001
CA19-9 Ab (DRG)
TSRL
Sp Serotype 14.15
Sp Serotype 14.2
MGL 416
hGal-1-488+BCR
#15 Sialyl Lewis X (BD)
#8 Lewis B (T218) (Abcam)
HML-Native
SSL0-488
LBL-152
LSL-150
WT-VCC
WGA
VGA(EY Lab)
SJA
SBA
PNA
HHA(EY Lab)
PSL
MGL 6A-H262T
MAL-II
Human Galectin1
CAA
Mouse Galectin1
AAL
WGA
DBA
CTB
Gal-3C
GNL
AIA
GSL-2
Mono2R-CNL
ConA
PFL
CA
ECL
ConA
PHA-E
CSA(EY Lab)
MAA(Selkagaku)
UEA-I
MAA
LTL
LCA
LEL
WGA
SBA
Microvirin
Epa1-Np mutant E227G
VLRB.aGPA.23
AFCNVH
DSL
PHA-E
Epa1-Np mutant E227A
HPA
ab5
CML1
SSR1
GAC78
mouse Gal-7
PsSC
WT Galectin-8
Mouse NKR-P1C
BPL
rMpL
MAL-I
UEA-I
PTL-I
CnMBL
GNA (EY Lab)
HHL
#14 Sialyl Lewis X (Calbio)
MGL 6B
AHL
Galectin-1
Mono1-CNL
IPS-K-4A2B8
Epa1-Np mutant Y228W
TbFBL1
hJAA-F11-B11
EEL
MGL 6A
Mono2W-CNL
ACL
Threefoil
LcH
HAA
Epa1-Np mutant Y228A
VVHA lectin
CA19.9-121SLE
SRL
Avaren-Fc
ABA
EEA(EY Lab)
rNV VLP-Cy3
NPA
TbFBL2
Epa1-Np WT
PSA
ME7(detected with a-HUD)
WGA(Seikagaku Bio)
MNA-M
BTL native
UEAII
DC-ASGPR short
GSL-1
rPVL
Anti-LeC
AAL
MPL
ME5(detected with a-HUD)
CnSepL
CC1G-10077
GSL-I-B4
CGL
RCAI (Vector)
P11 VP8
PA-IL
Native HPA
MOA

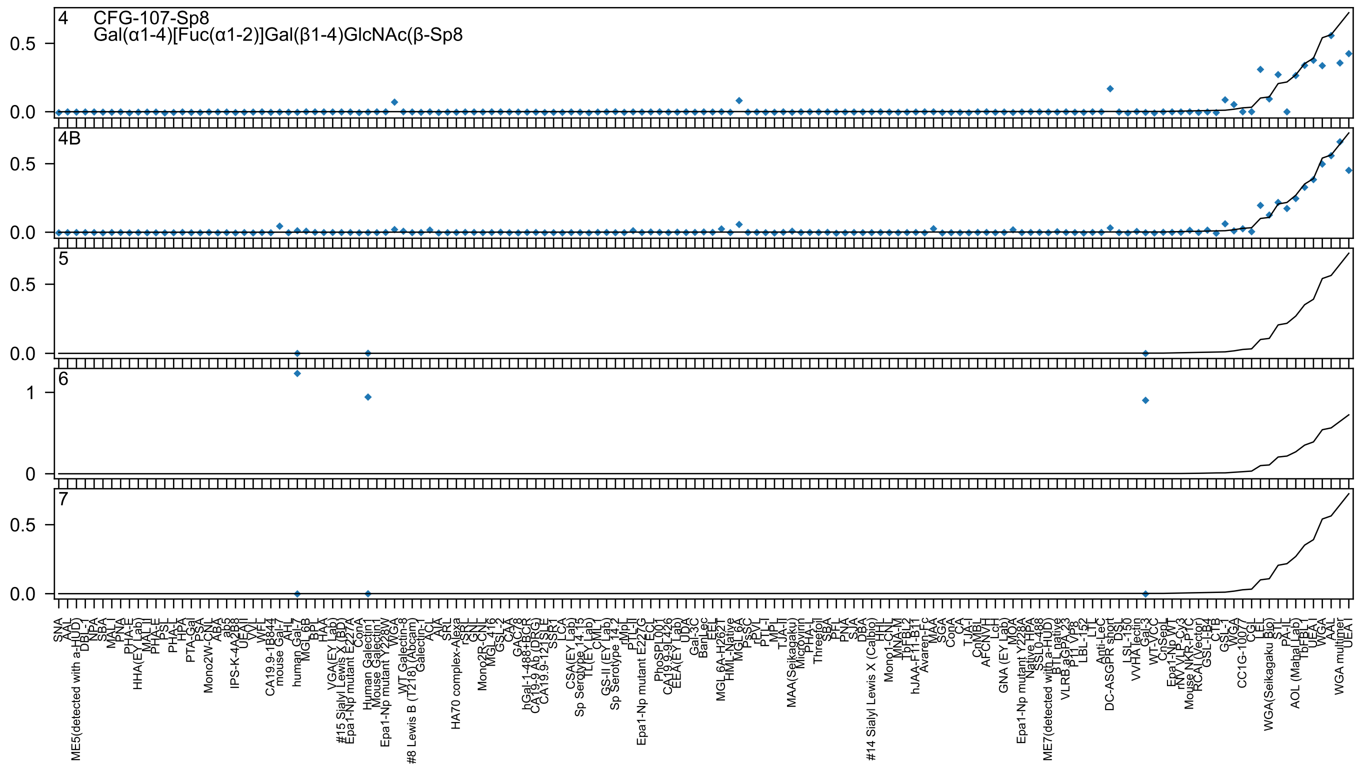
4
CFG-107-Sp8
Gal(α1-4)[Fuc(α1-2)]Gal(β1-4)GlcNAc(β-Sp8
4B
5
6
7

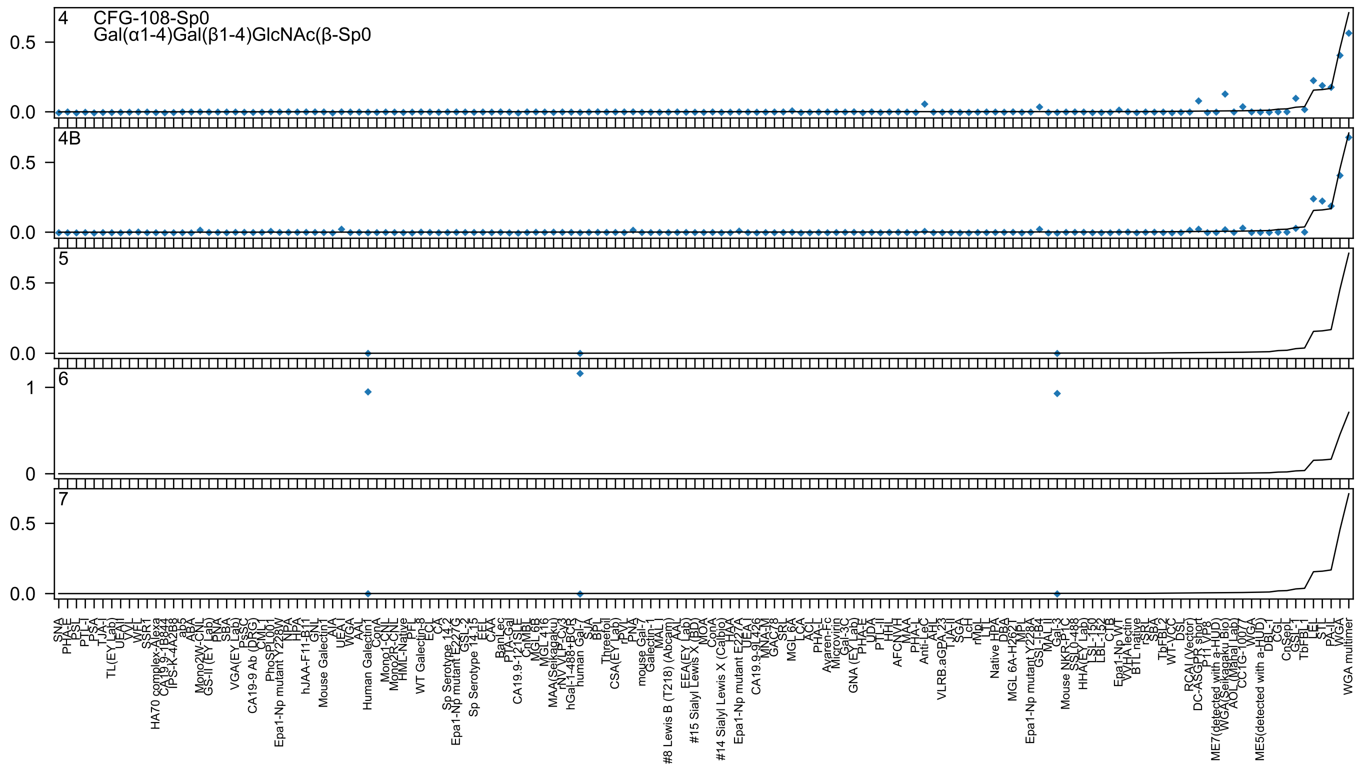
4 CFG-108-Sp0
Gal(α1-4)Gal(β1-4)GlcNAc(β-Sp0
4B
5
6
7

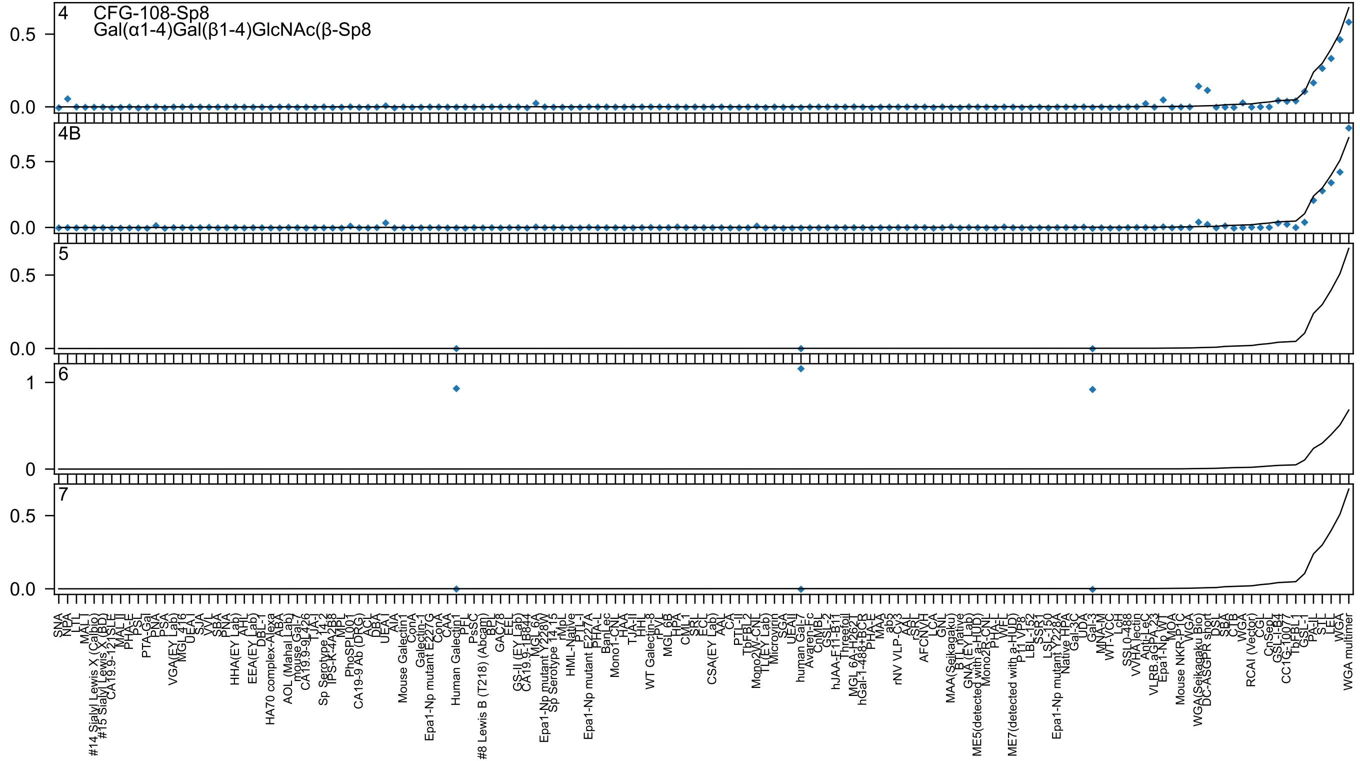

4 CFG-108-Sp8
Gal(α1-4)Gal(β1-4)GlcNAc(β-Sp8
4B
5
6
7

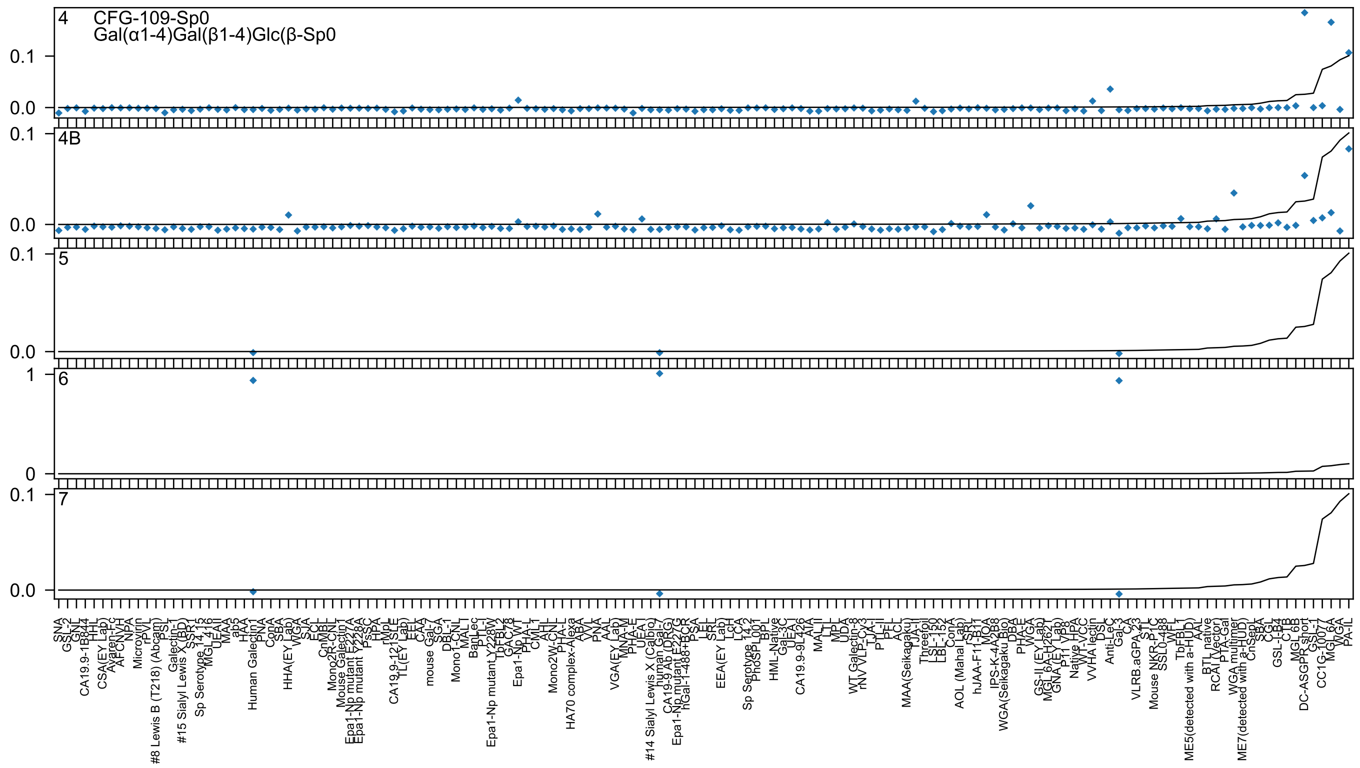
4
CFG-109-Sp0
Gal(α1-4)Gal(β1-4)Glc(β-Sp0
4B
5
6
7
0.1
0.0
1
0
SNA
GSL-2
GNL
CA19.9-1B844
HHL
CSA(EY Lab)
Avaren-Fc
AFCNVH
NPA
Microvirin
rPVL
#8 Lewis B (T218) (Abcam)
PSL
Galectin-1
#15 Sialyl Lewis X (BD)
SSR1
Sp Serotype 14 13
MGL 4 16
UEAII
MAA
ab5
HAA
Human Galectin1
PNA
ConA
SBA
HHA(EY Lab)
WGA
SJA
ECL
CnMBL
Mono2R-CNL
Mouse Galectin1
Epa1-Np mutant E227A
Epa1-Np mutant Y228A
PsSC
HPA
rMpL
CA19.9-121SLE
TL(EY Lab)
EEL
CAA
mouse Gal-7
SGA
DBL-1
Mono1-CNL
MAL
BanLec
PTL-I
Epa1-Np mutant Y228W
TbFBL2
GAC78
Epa1-Np WT
PHA-L
CML1
AHL
Mono2W-CNL
PHA-L
HA70 complex-Alexa
ABA
VVL
PNA
AAL
VGA(EY Lab)
MNA-M
PHA-E
UEA-I
#14 Sialyl Lewis X (Calbio)
human Gal-7
CA19-9 Ab (DRG)
Epa1-Np mutant E227G
hGal-1-488+BCR
PSA
LEL
SRL
EEA(EY Lab)
LcH
LCA
Sp Serotype 14 2
PhoSL001
BPL
HML-Native
Gal-3C
UEA I
CA19.9-9L426
AIA
MAL II
LTL
MPL
UDA
WT Galectin-8
rNV VLP-Cy3
TJA-I
PTL-II
PFL
ACL
MAA(Seikagaku)
TJA-II
Threefoil
LSL-150
LBL-152
ConA
AOL (Mahal Lab)
rSRL
hJAA-F11-B11
MOA
IPS-K-4A2B8
WGA(Seikagaku Bio)
DBA
PHA-E
WGA
GS-II (EY Lab)
MGL 6A-H262T
GNA (EY Lab)
P11 VP8
Native HPA
WT-VCC
VVHA lectin
DSL
Anti-LeC
Gal-3
CA
VLRB.aGPA.23
ST
Mouse NKR-P1C
SSL0-488
WFL
TbFBL1
ME5(detected with a-HUD)
AAL
BTL native
RCAI (Vector)
PTA-Gal
WGA multimer
ME7(detected with a-HUD)
CnSept
SBA
CGL
GSL-I-B4
CTB
MGL 6B
DC-ASGPR short
GSL-1
CC1G-10077
MGL 6A
WGA
PA-IL

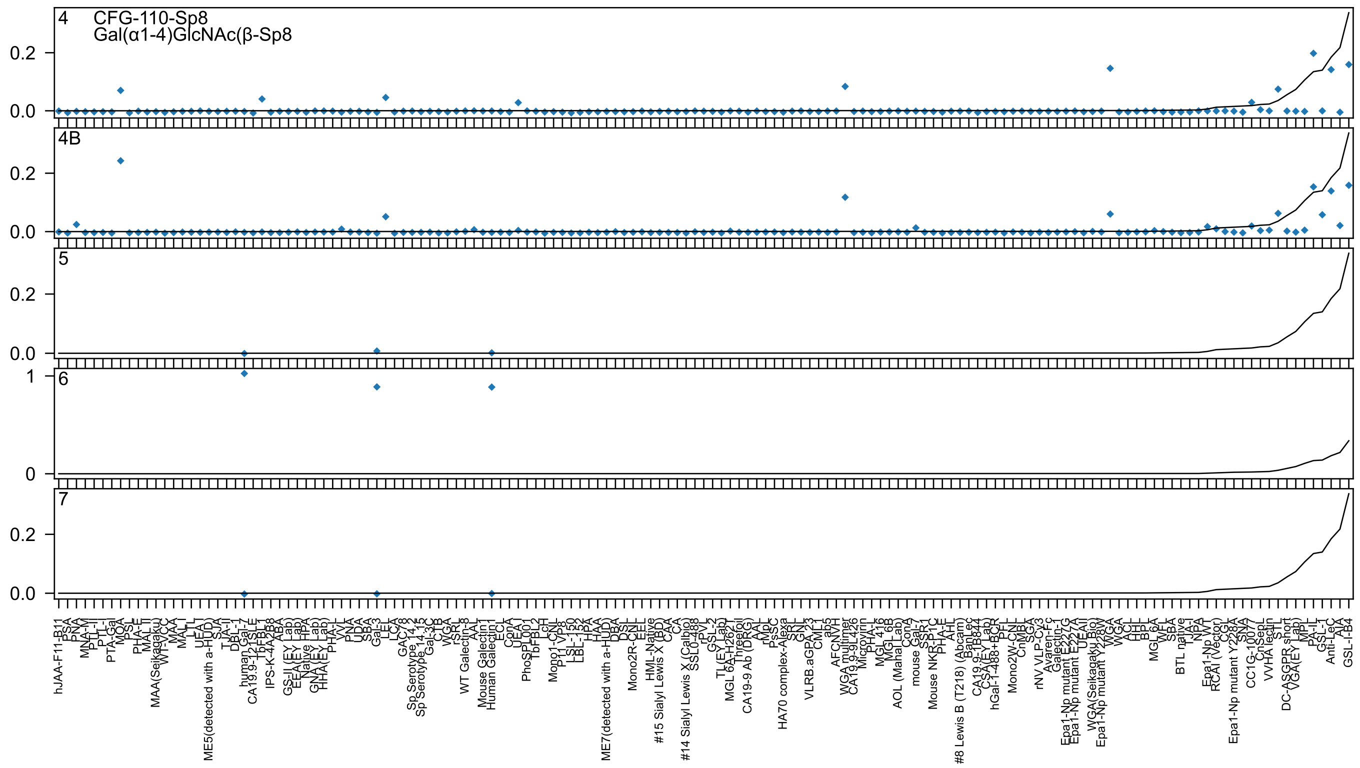

4 CFG-110-Sp8
Gal(α1-4)GlcNAc(β-Sp8
4B
5
6
7

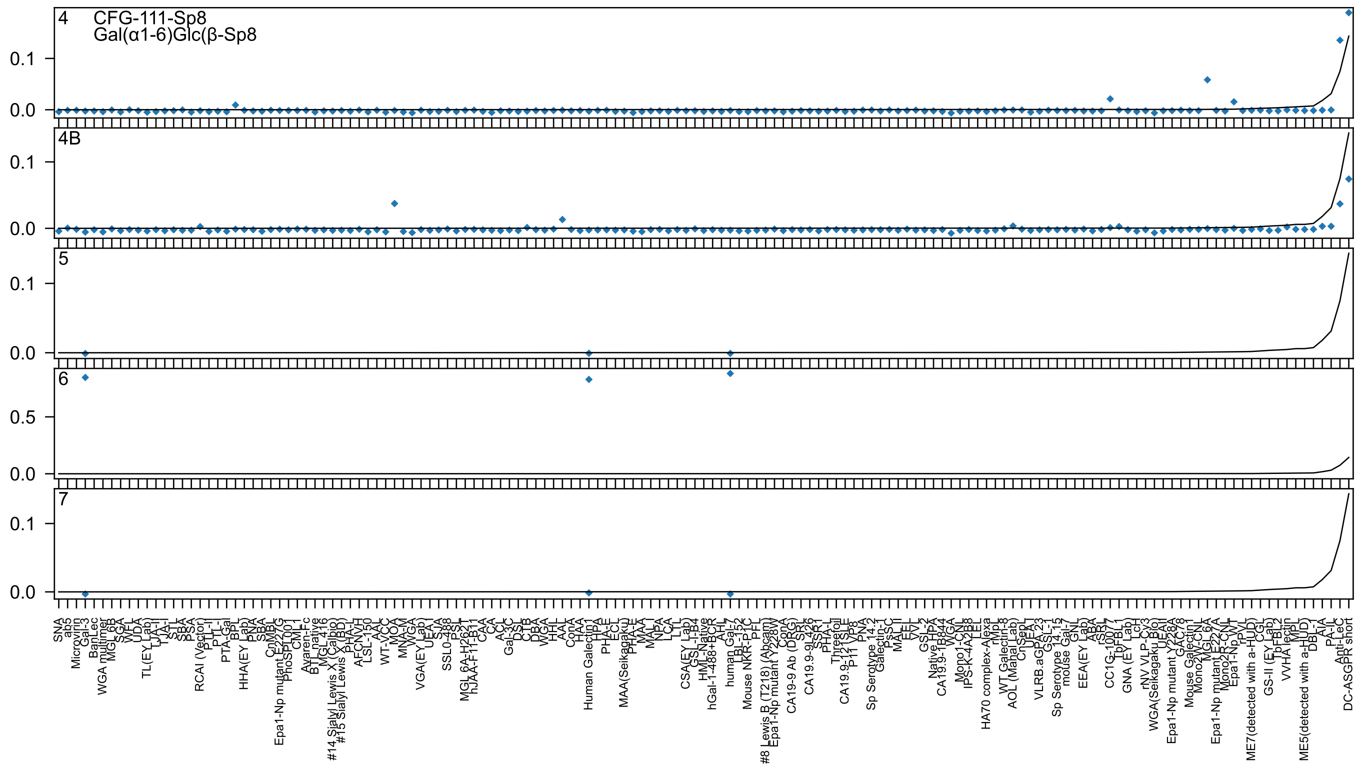

4
CFG-111-Sp8
Gal(α1-6)Glc(β-Sp8
4B
5
6
7
0.1
0.0
0.5

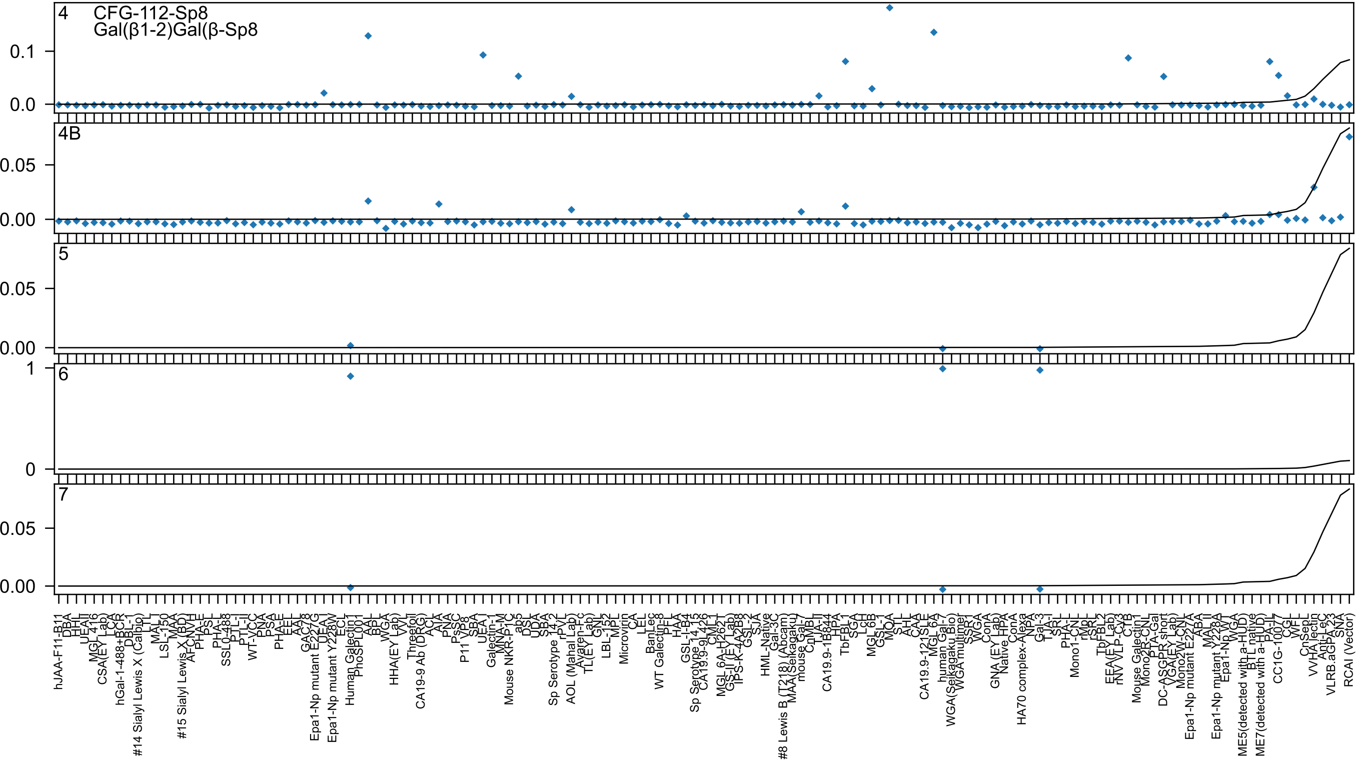
4
CFG-112-Sp8
Gal(β1-2)Gal(β-Sp8
0.1
0.0
4B
0.05
0.00
5
0.05
0.00
6
1
0
7
0.05
0.00

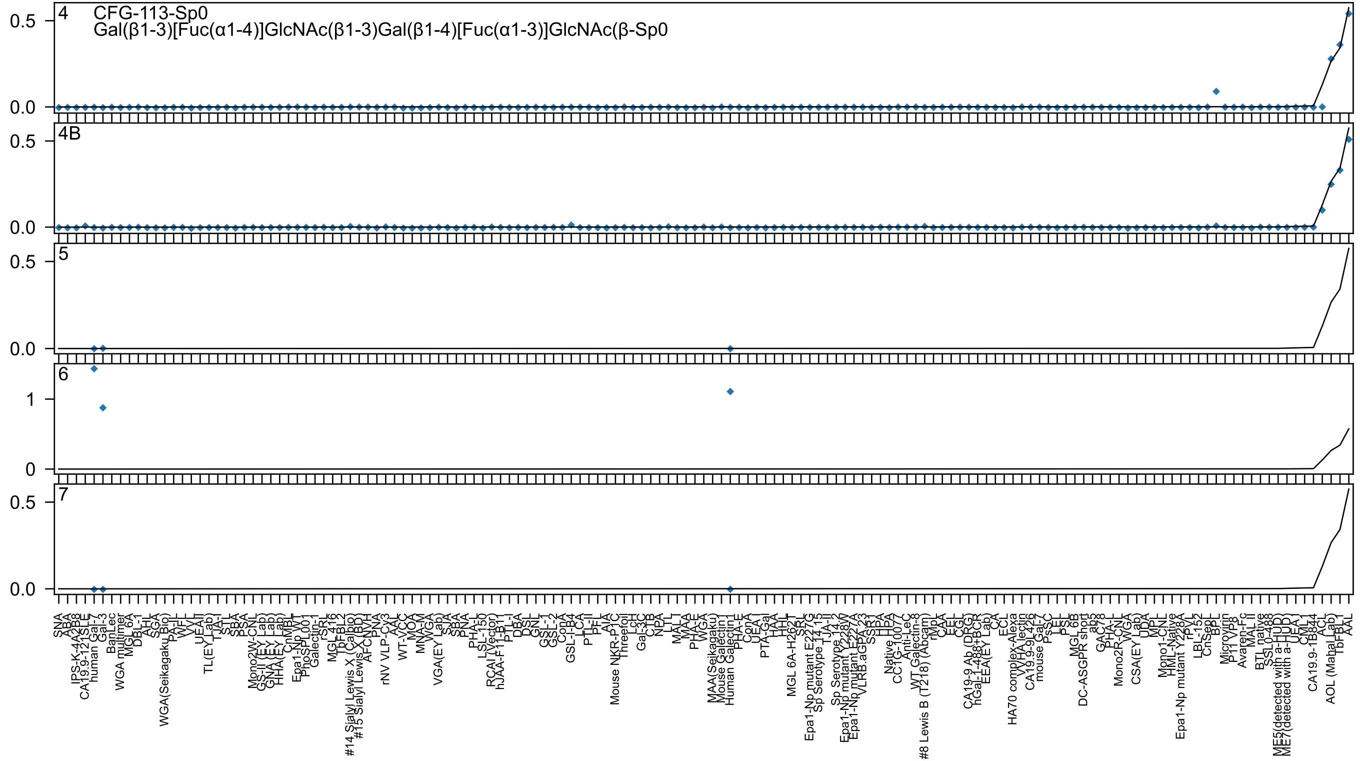

4
CFG-113-Sp0
Gal(β1-3)[Fuc(α1-4)]GlcNAc(β1-3)Gal(β1-4)[Fuc(α1-3)]GlcNAc(β-Sp0
4B
5
6
7

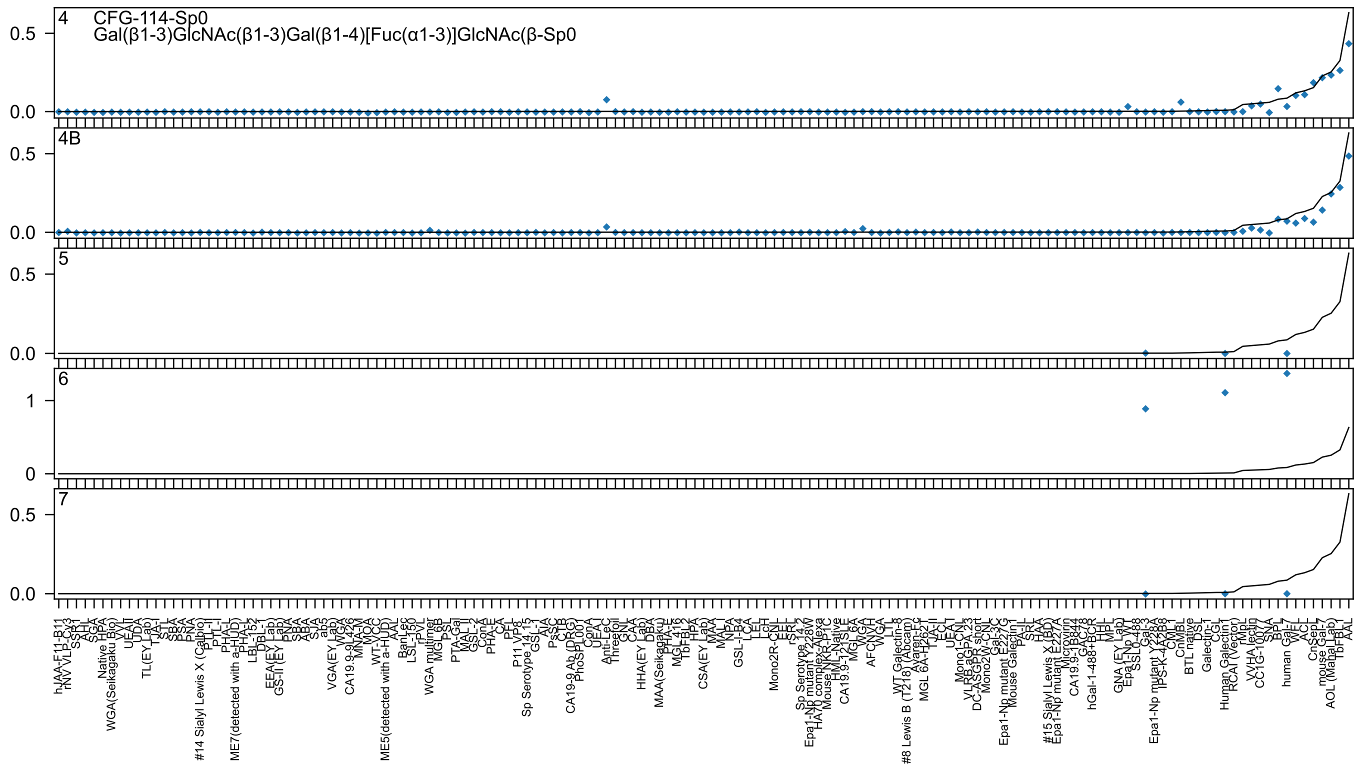
4 CFG-114-Sp0
Gal(β1-3)GlcNAc(β1-3)Gal(β1-4)[Fuc(α1-3)]GlcNAc(β-Sp0
4B
5
6
7

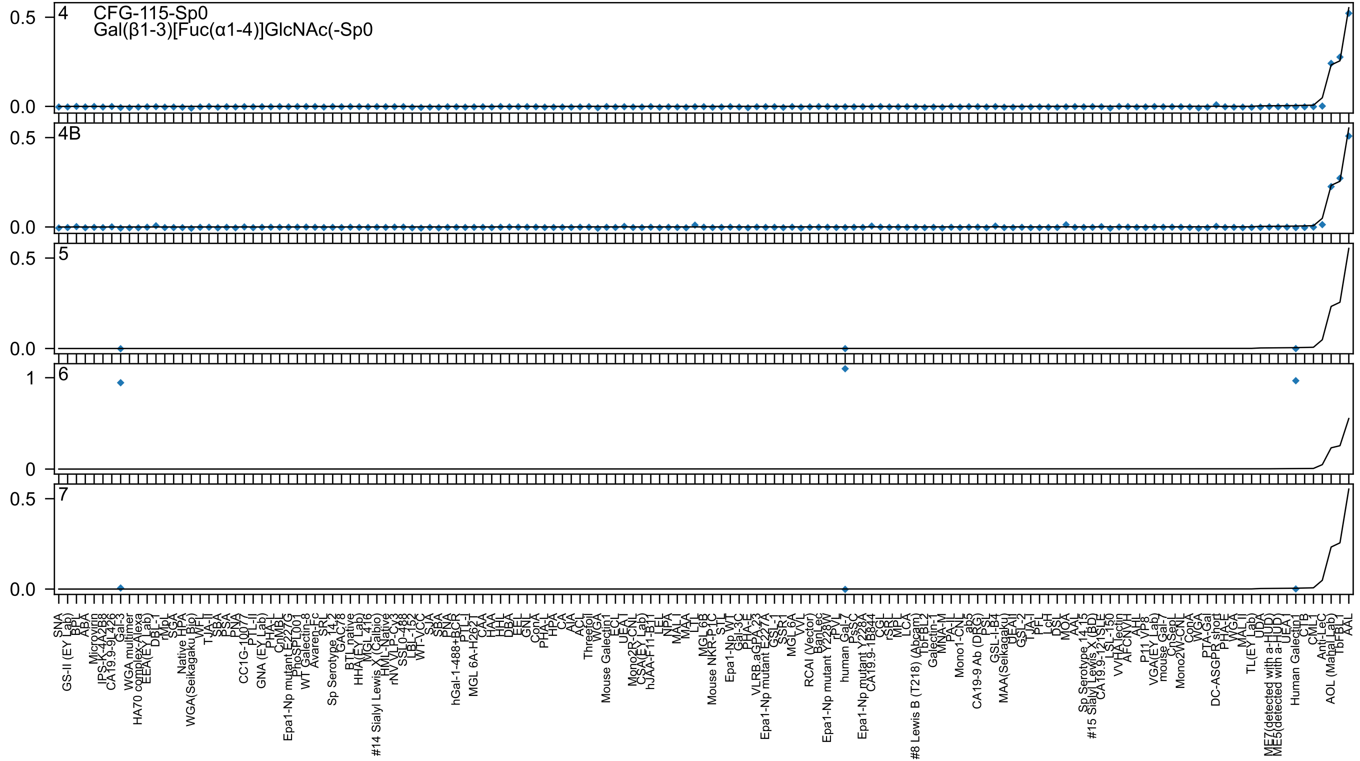

CFG-115-Sp0
Gal(β1-3)[Fuc(α1-4)]GlcNAc(-Sp0
4
4B
5
6
7

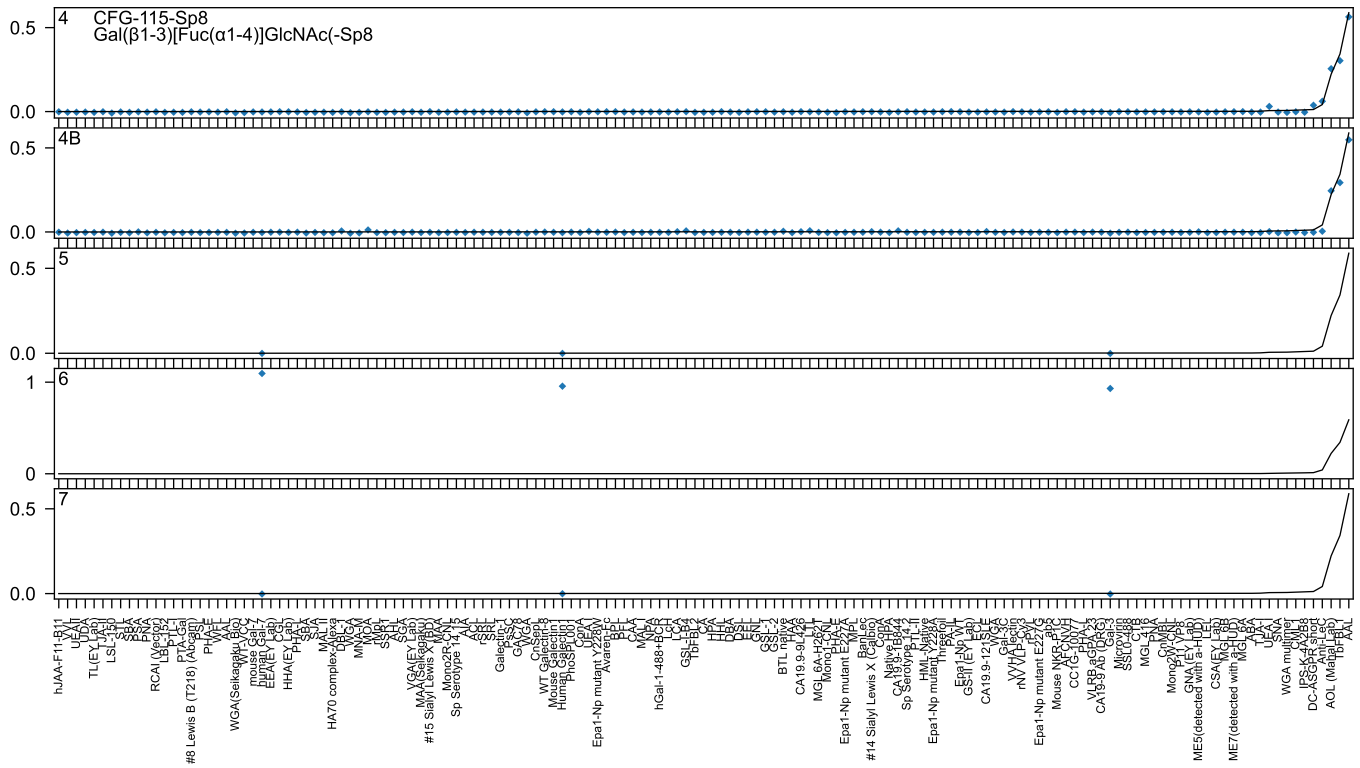
4
CFG-115-Sp8
Gal(β1-3)[Fuc(α1-4)]GlcNAc(-Sp8
4B
5
6
7
0.5
0.0
1
0

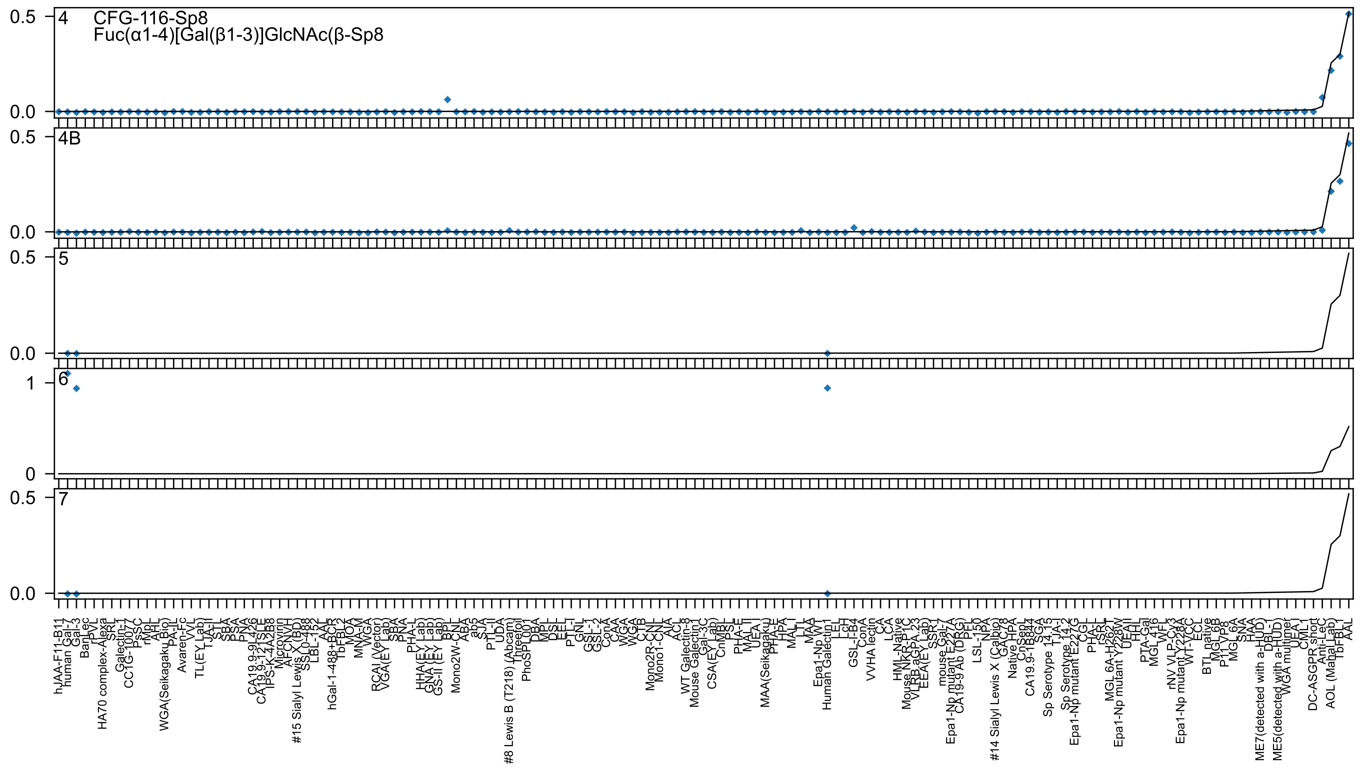

4
CFG-116-Sp8
Fuc(α1-4)[Gal(β1-3)]GlcNAc(β-Sp8
4B
5
6
7

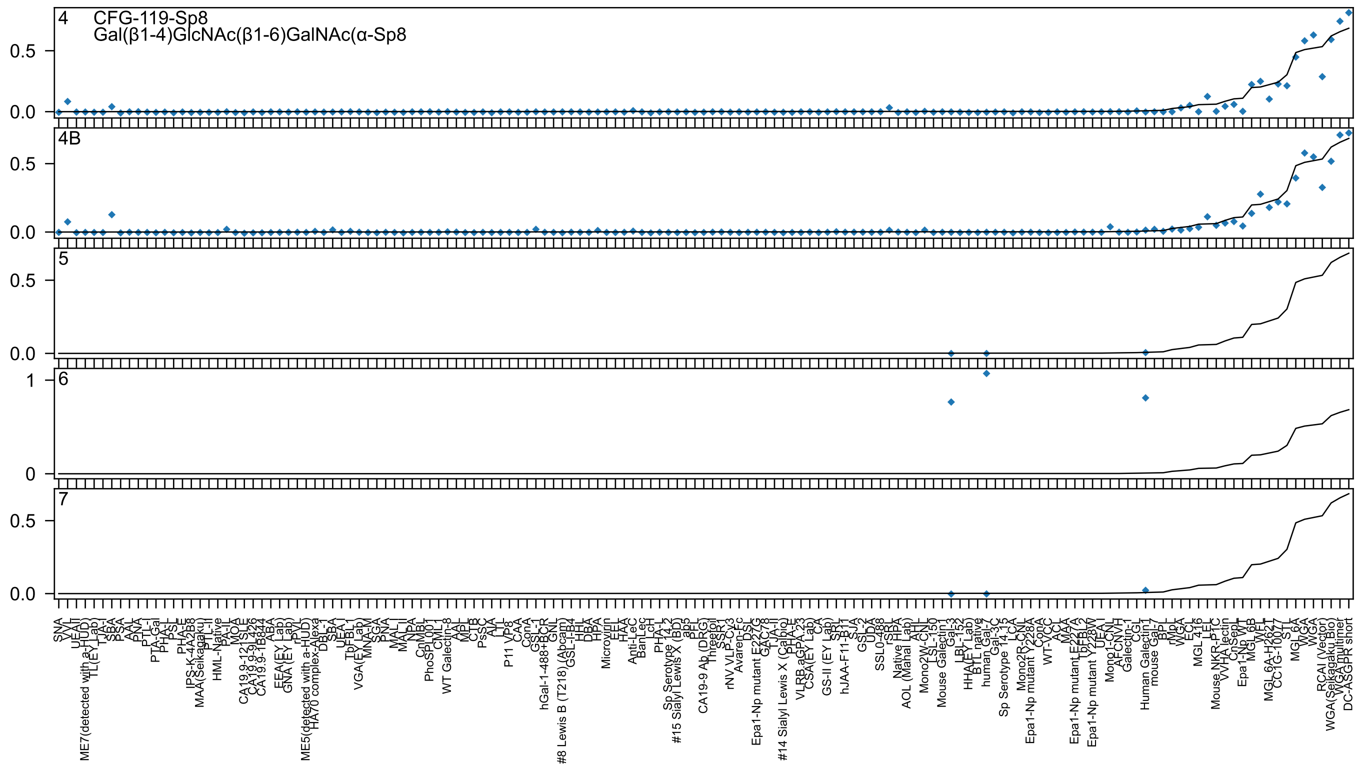
4
CFG-119-Sp8
Gal(β1-4)GlcNAc(β1-6)GalNAc(α-Sp8
4B
5
6
7
0.5
0.0
1
0

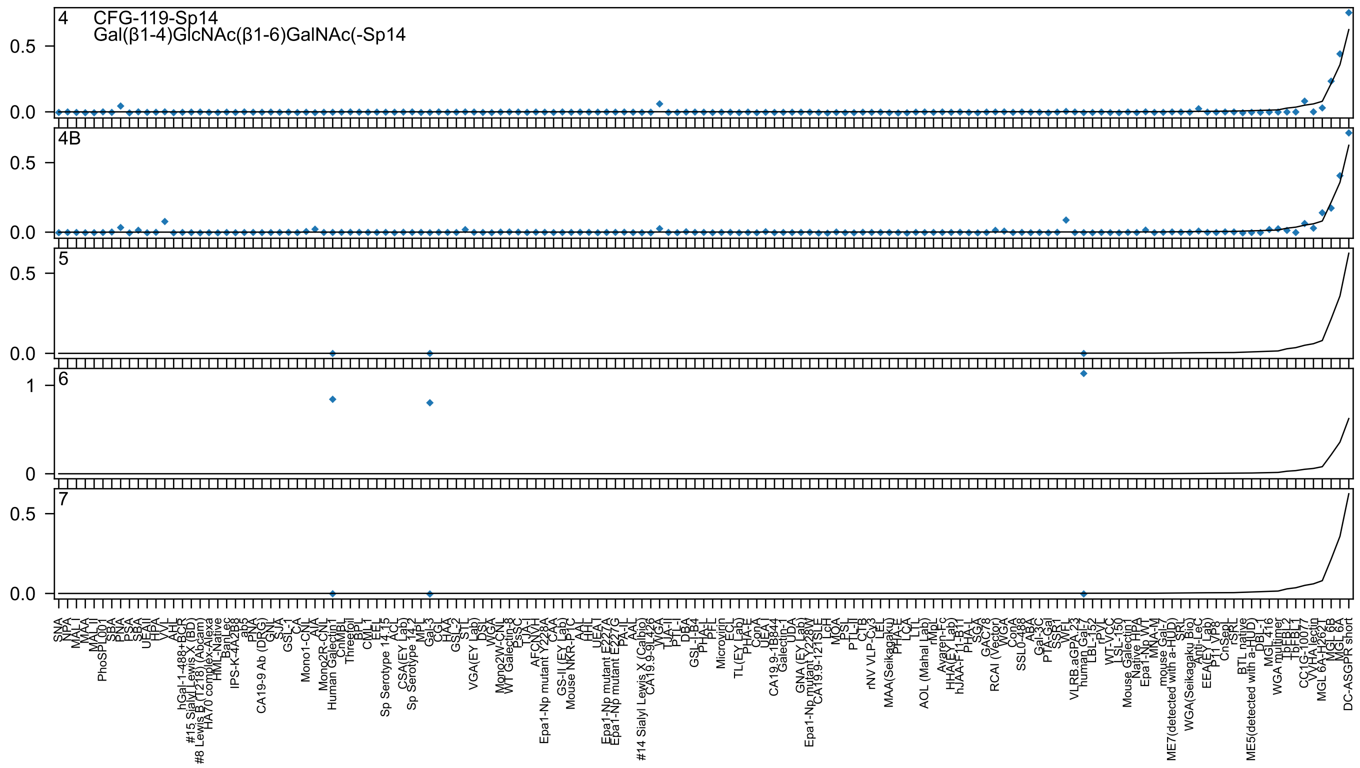

4
CFG-119-Sp14
Gal(β1-4)GlcNAc(β1-6)GalNAc(-Sp14
4B
5
6
7

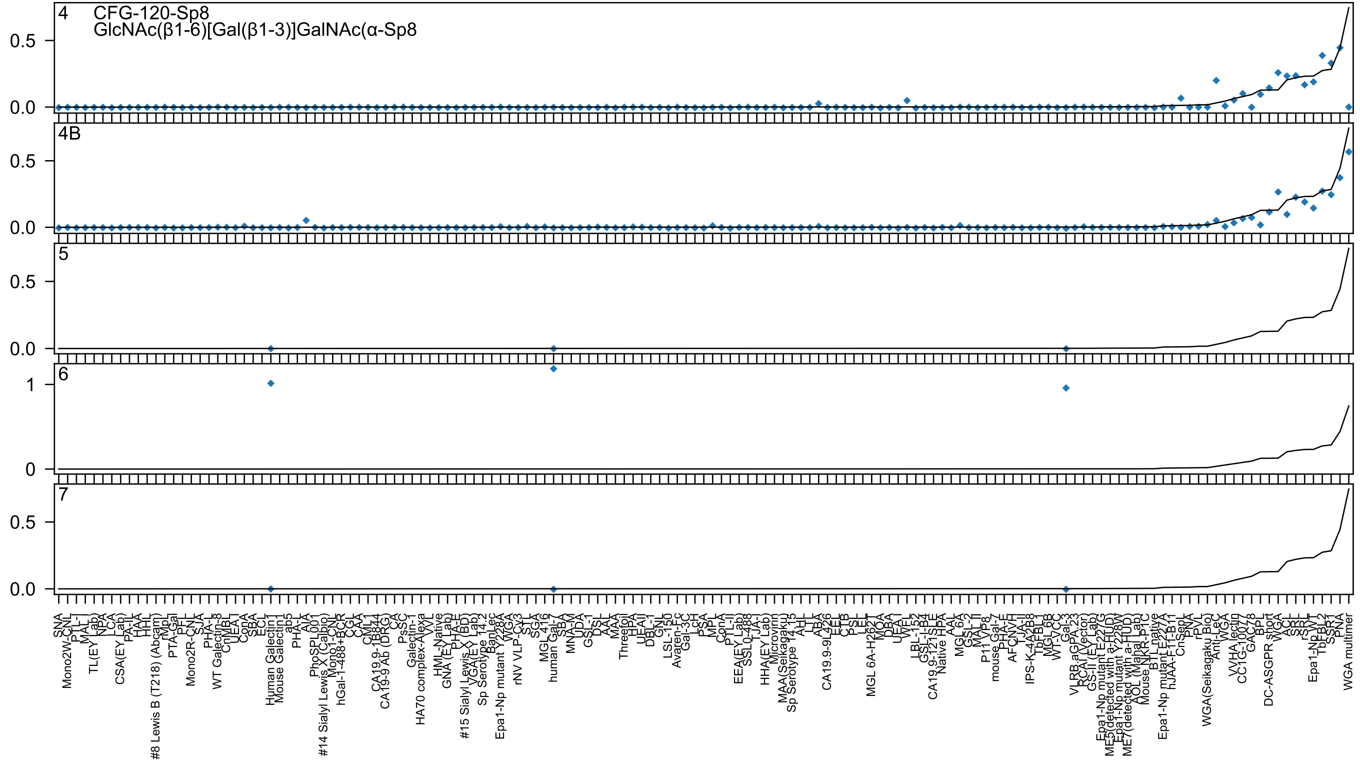

4 CFG-120-Sp8
GlcNAc(β1-6)[Gal(β1-3)]GalNAc(α-Sp8
4B
5
6
7

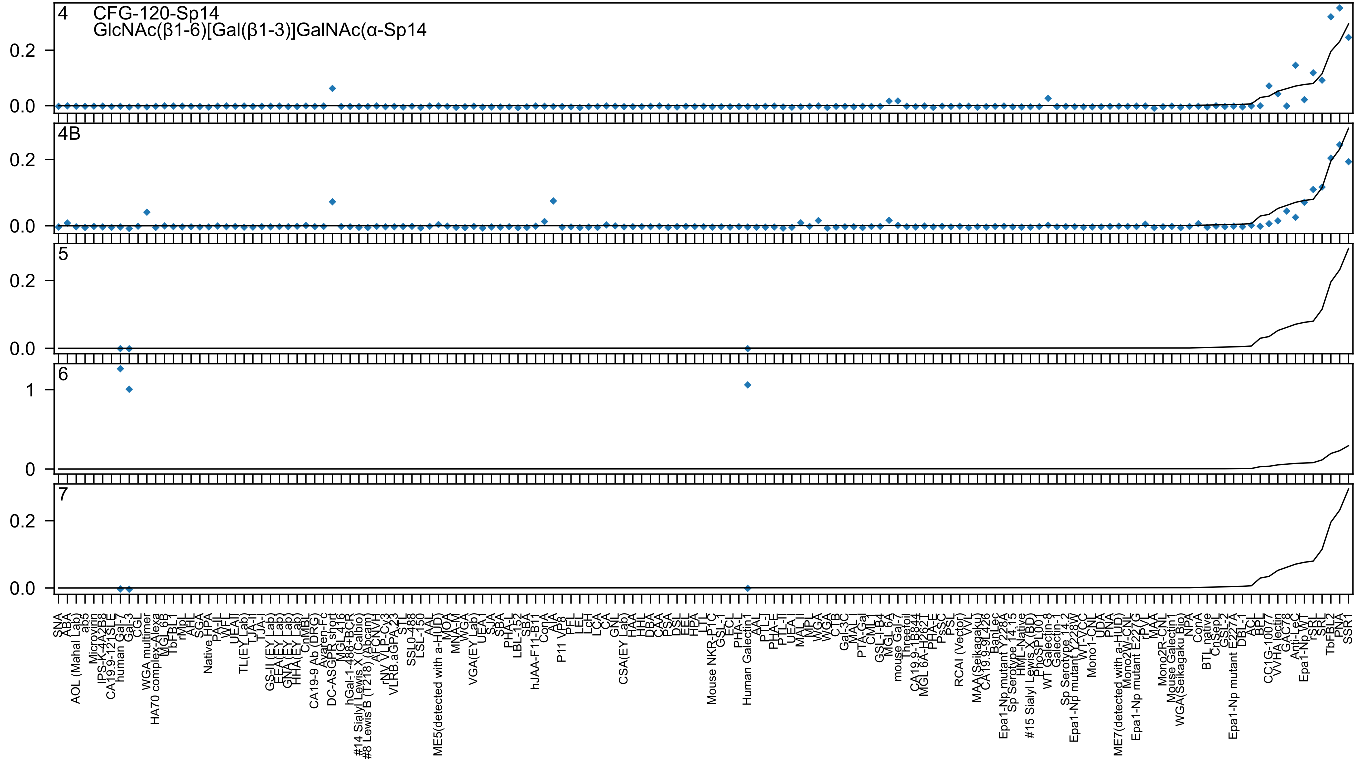
4 CFG-120-Sp14
GlcNAc(β1-6)[Gal(β1-3)]GalNAc(α-Sp14
4B
5
6
7

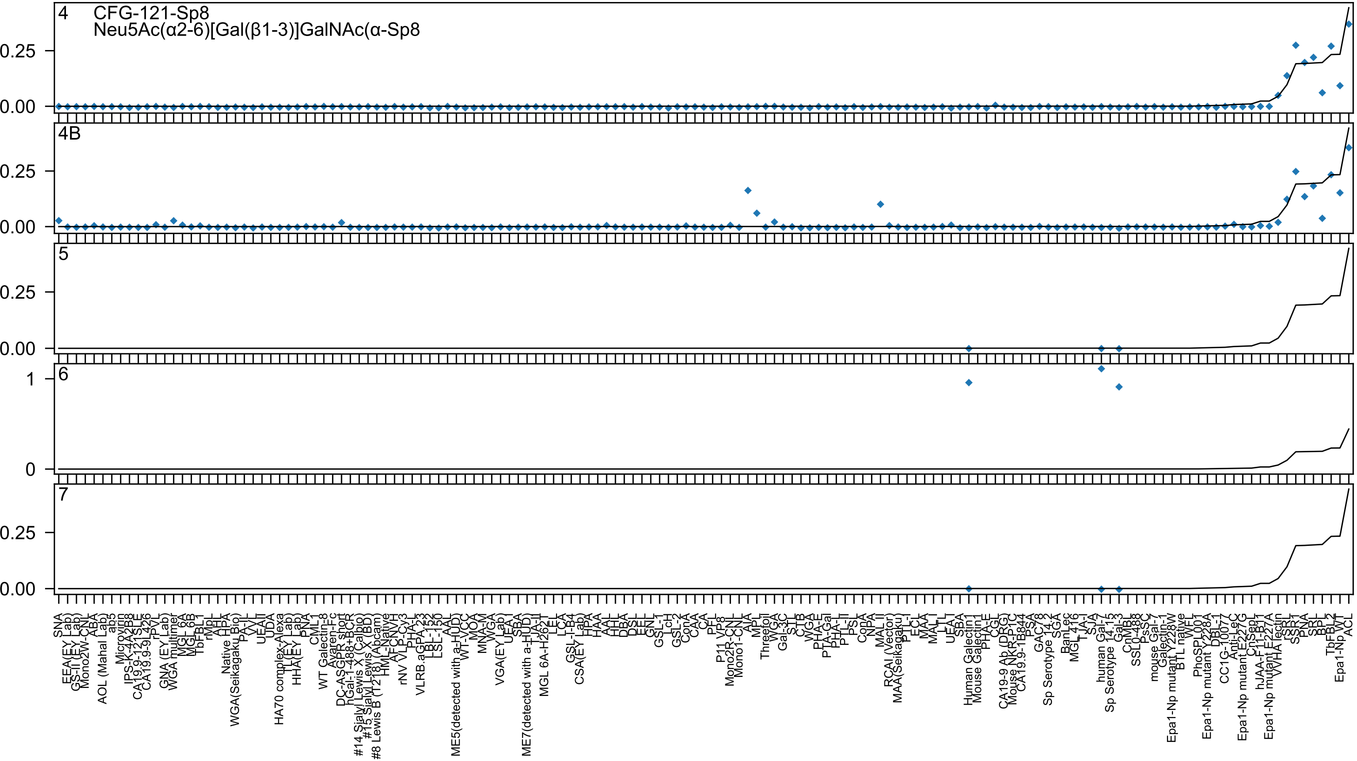

4
CFG-121-Sp8
Neu5Ac(α2-6)[Gal(β1-3)]GalNAc(α-Sp8
4B
5
6
7
0.25
0.00
1
0
SNA
EEA(EY Lab)
GS-II (EY Lab)
Mono2W-CNL
ABA
AOL (Mahal Lab)
ab5
Microvirin
IPS-K-4A2B8
CA19.9-121SLE
CA19.9-9L426
rPVL
GNA (EY Lab)
WGA multimer
MGL 6A
MGL 6B
TbFBL1
rMpL
AAL
Native HPA
WGA(Seikagaku Bio)
PA-IL
VVL
UEA II
UDA
HA70 complex-Alexa
TL(EY Lab)
HHA(EY Lab)
PNA
CML1
WT Galectin-8
Avaren-Fc
DC-ASGPR short
hGal-1-488+BCR
#14 Sialyl Lewis X (Calbio)
#15 Sialyl Lewis X (BD)
#8 Lewis B (T218) (Abcam)
HML-Native
AFCNVH
rNV VLP-Cy3
PHA-L
VLRB.aGPA.23
LBL-152
LSL-150
AAL
ME5(detected with a-HUD)
WT-VCC
MOA
MNA-M
WGA
VGA(EY Lab)
UEA I
SBA
ME7(detected with a-HUD)
TJA-II
MGL 6A-H262L
LEL
LCA
GSL-I-B4
CSA(EY Lab)
HPA
HAA
AAL
HHL
DBA
DSL
EEL
GNL
GSL-I
LcH
GSL-2
ConA
CAA
CA
PFL
P11 VP8
Mono2R-CNL
Mono1-CNL
AIA
MPL
Threefoil
WGA
Gal-3C
STL
CTB
WGA
PHA-E
PTA-Gal
PHA-L
PTL-II
PSL
ConA
NPA
MAL II
RCAI (Vector)
MAA(Seikagaku)
PTL-I
ECL
MAA
MAL I
LTL
UEA I
SBA
Human Galectin1
Mouse Galectin1
PHA-E
CGL
CA19-9 Ab (DRG)
Mouse NKR-P1C
CA19.9-1B844
PSA
GAC78
Sp Serotype 14.2
SGA
BanLec
MGL 416
TJA-I
SJA
human Gal-7
Sp Serotype 14.5
Gal-3
CnMBL
SSL0-488
PsSC
mouse Gal-7
Galectin-1
Epa1-Np mutant Y228W
BTL native
WFL
PhoSL001
Epa1-Np mutant Y228A
DBL-1
CC1G-1007
Anti-LeC
Epa1-Np mutant E227G
CnSept
hJAA-F11-B11
Epa1-Np mutant E227A
VVHA lectin
rSRL
SSR1
PNA
SRL
BPL
TbFBL2
Epa1-Np WT
ACL

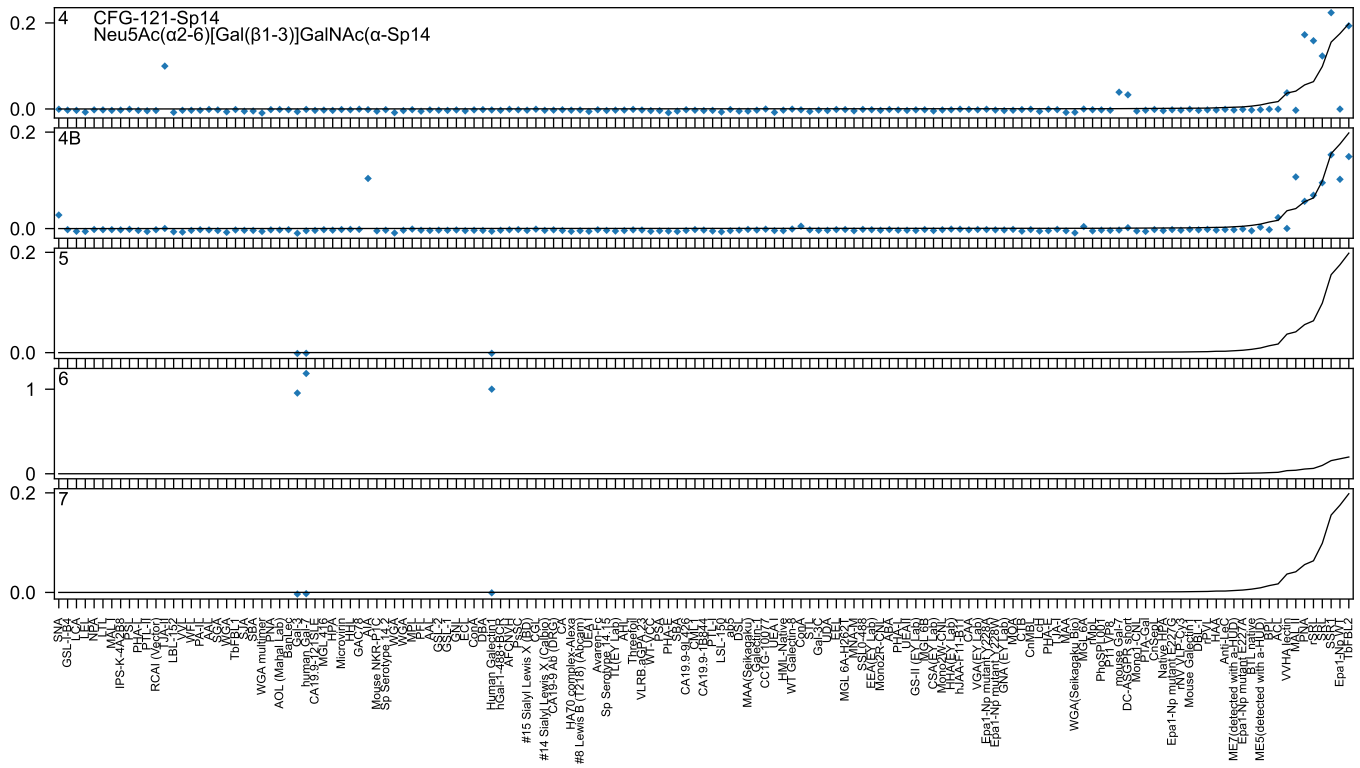
4
CFG-121-Sp14
Neu5Ac(α2-6)[Gal(β1-3)]GalNAc(α-Sp14
4B
5
6
7
0.2
0.0
1
0

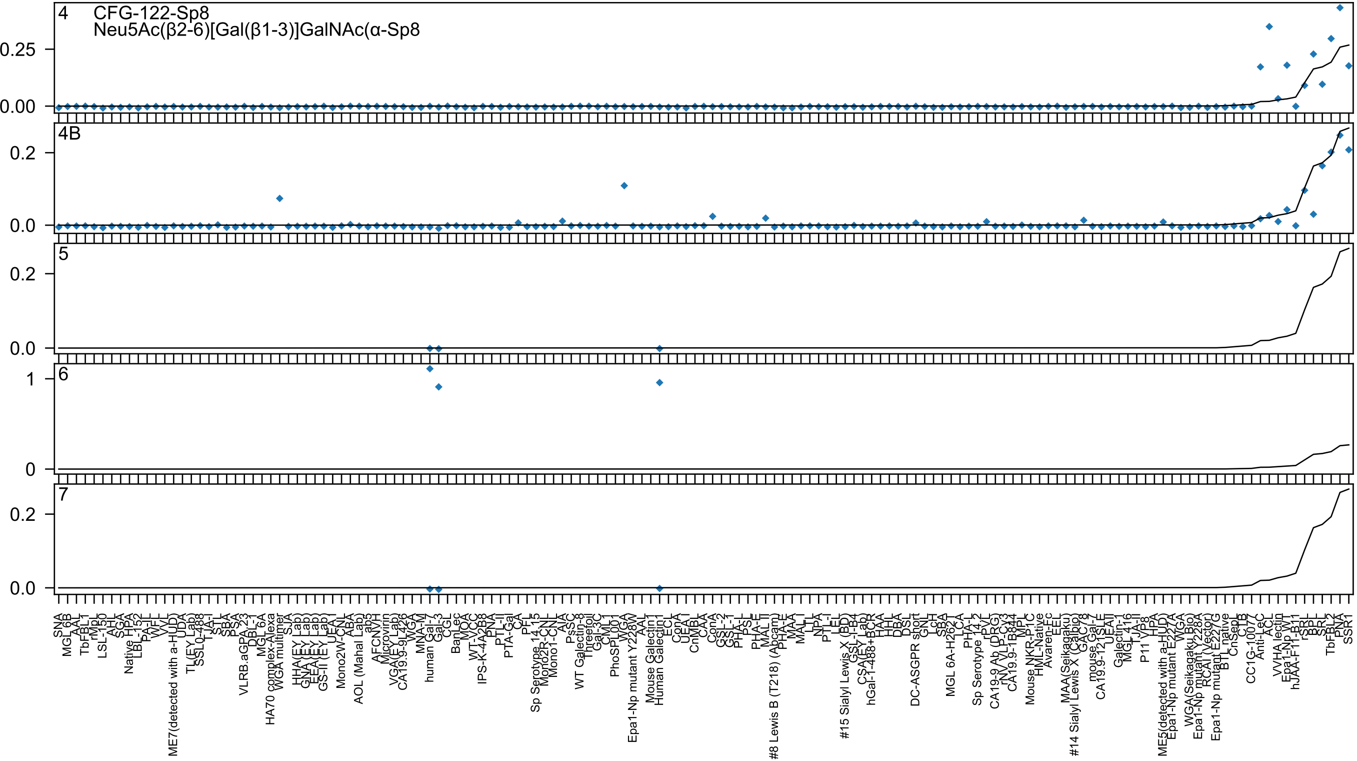

4 CFG-122-Sp8
Neu5Ac(β2-6)[Gal(β1-3)]GalNAc(α-Sp8
4B
5
6
7
0.25
0.00
0.2
0.0
1
0

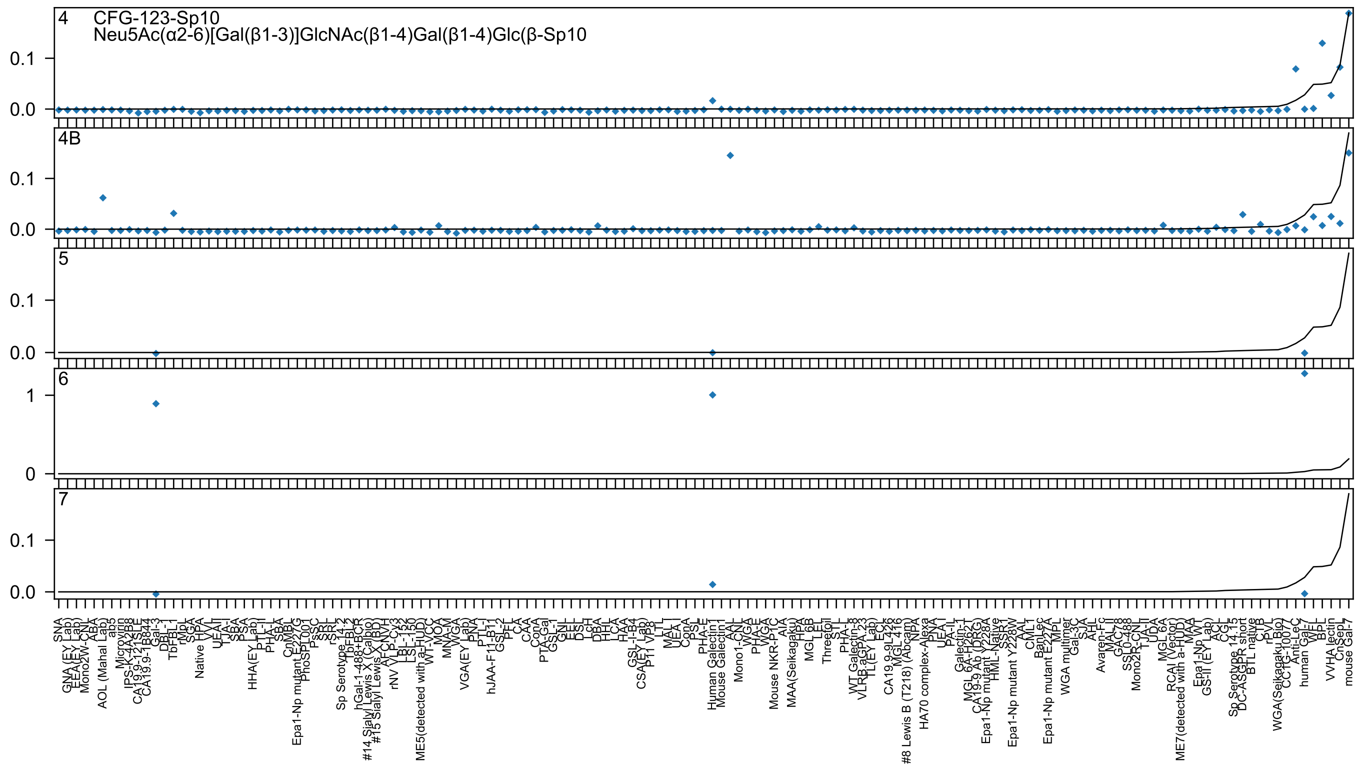

CFG-123-Sp10
Neu5Ac(α2-6)[Gal(β1-3)]GlcNAc(β1-4)Gal(β1-4)Glc(β-Sp10
4
4B
5
6
7

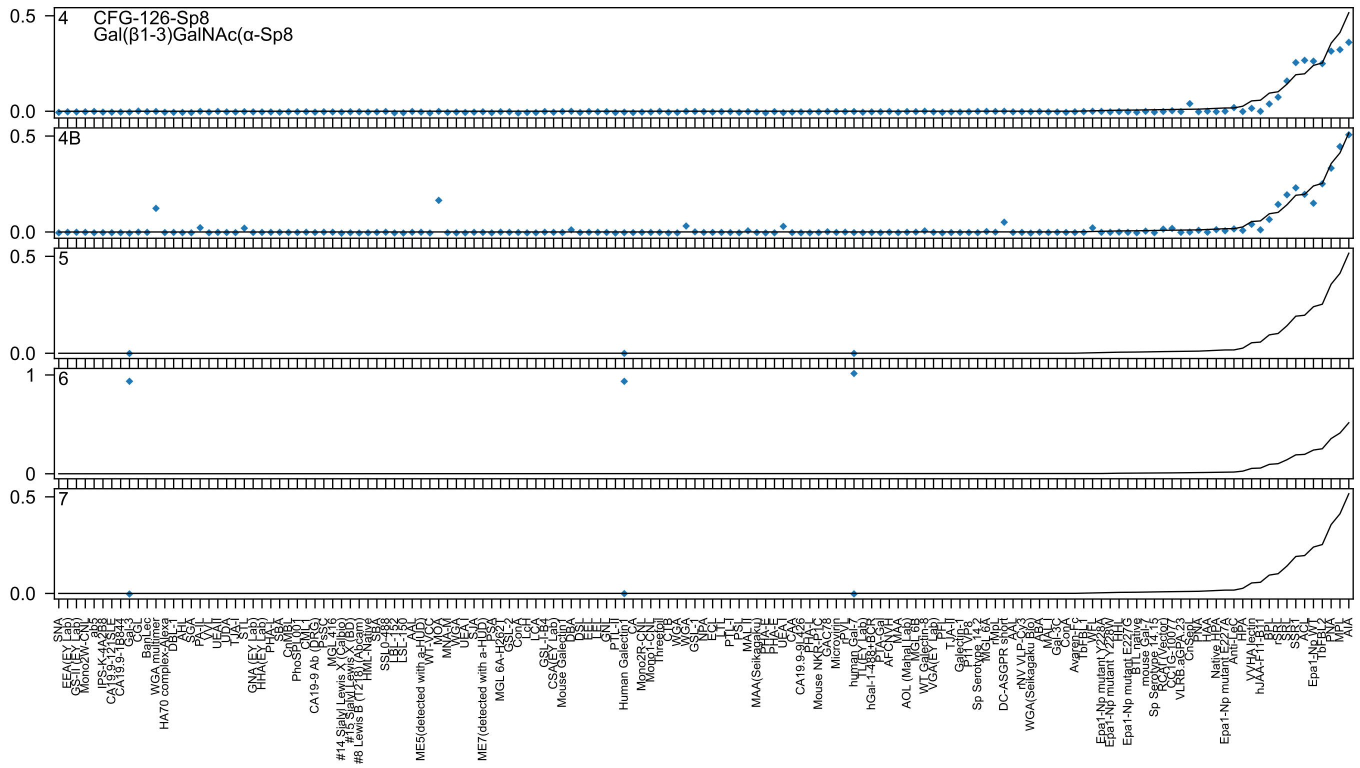
4
CFG-126-Sp8
Gal(β1-3)GalNAc(α-Sp8
4B
5
6
7

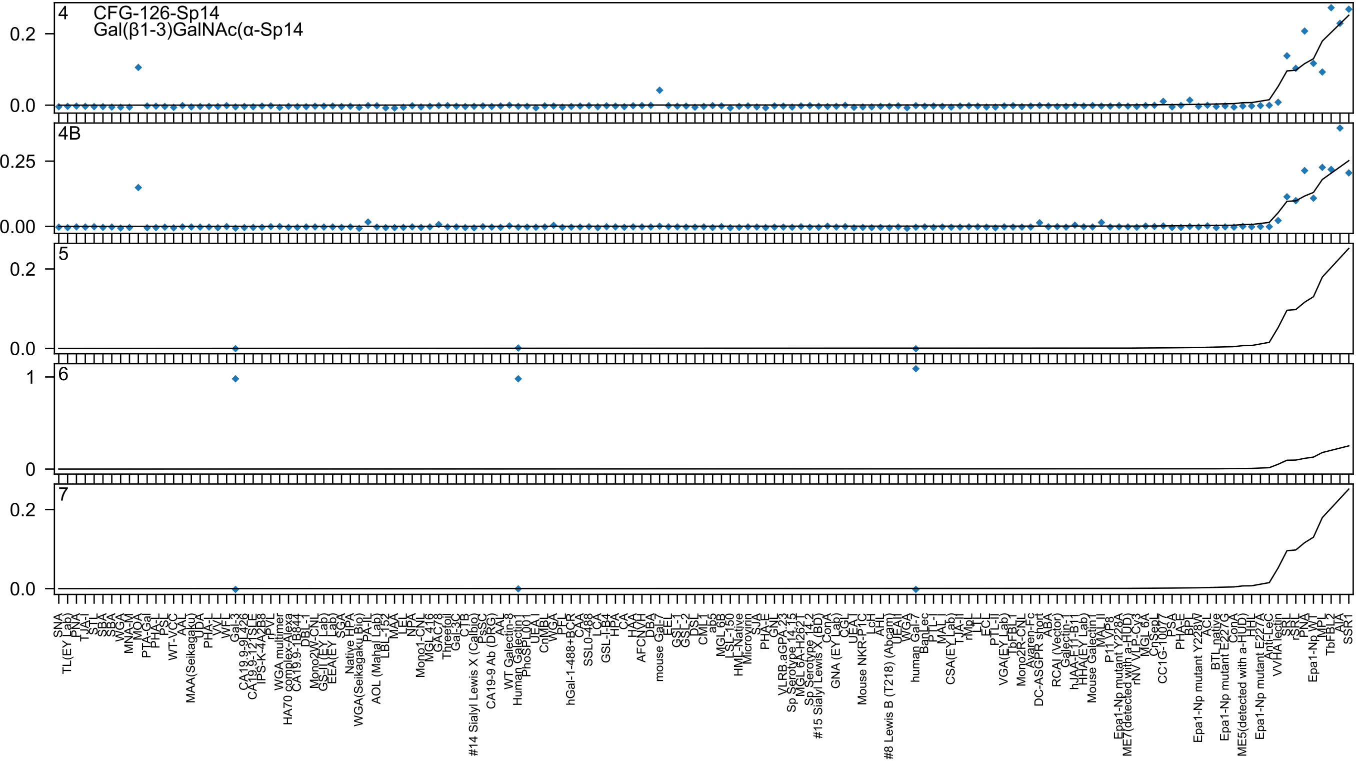

4
CFG-126-Sp14
Gal(β1-3)GalNAc(α-Sp14
4B
5
6
7

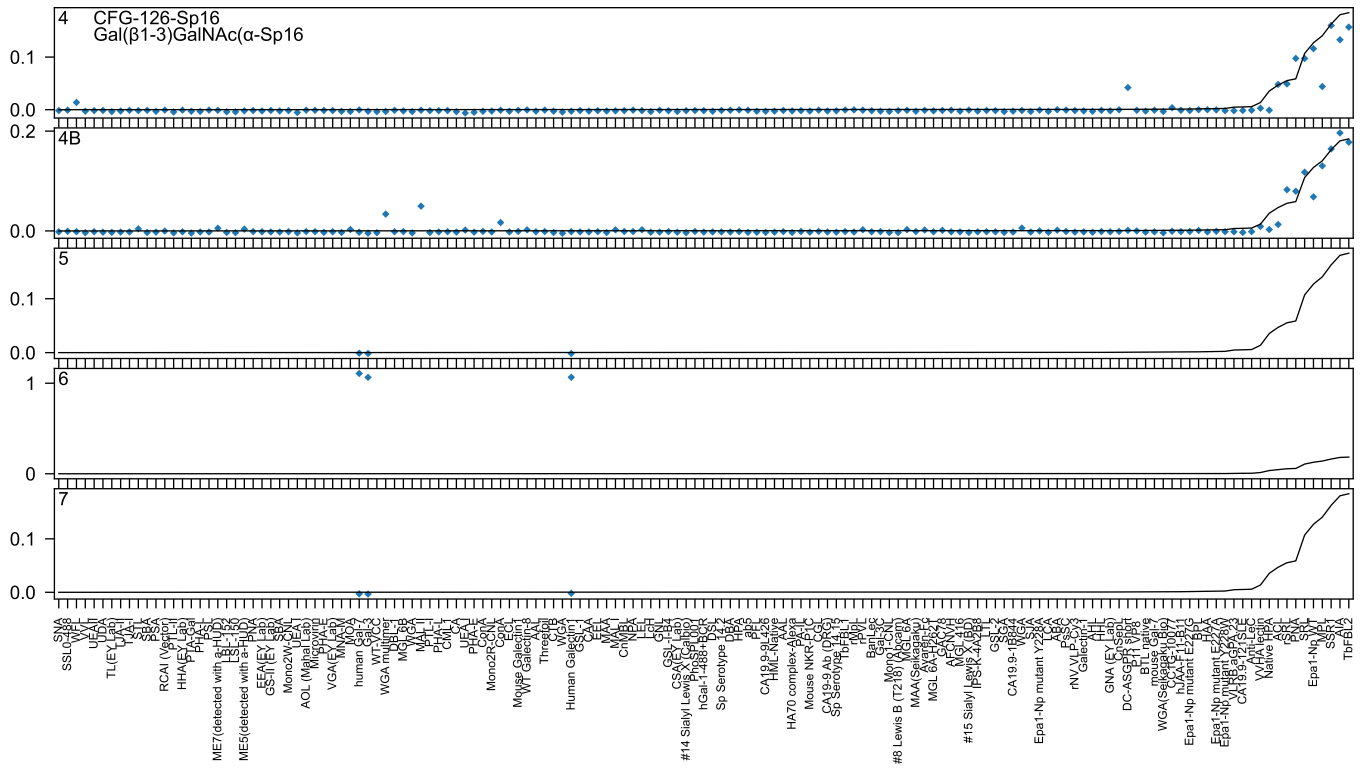

4
CFG-126-Sp16
Gal(β1-3)GalNAc(α-Sp16
4B
5
6
7
0.0
0.1
0.2
0
1
SNA
SSL0-488
human Gal-7
Gal-3
Human Galectin1
TbFBL2

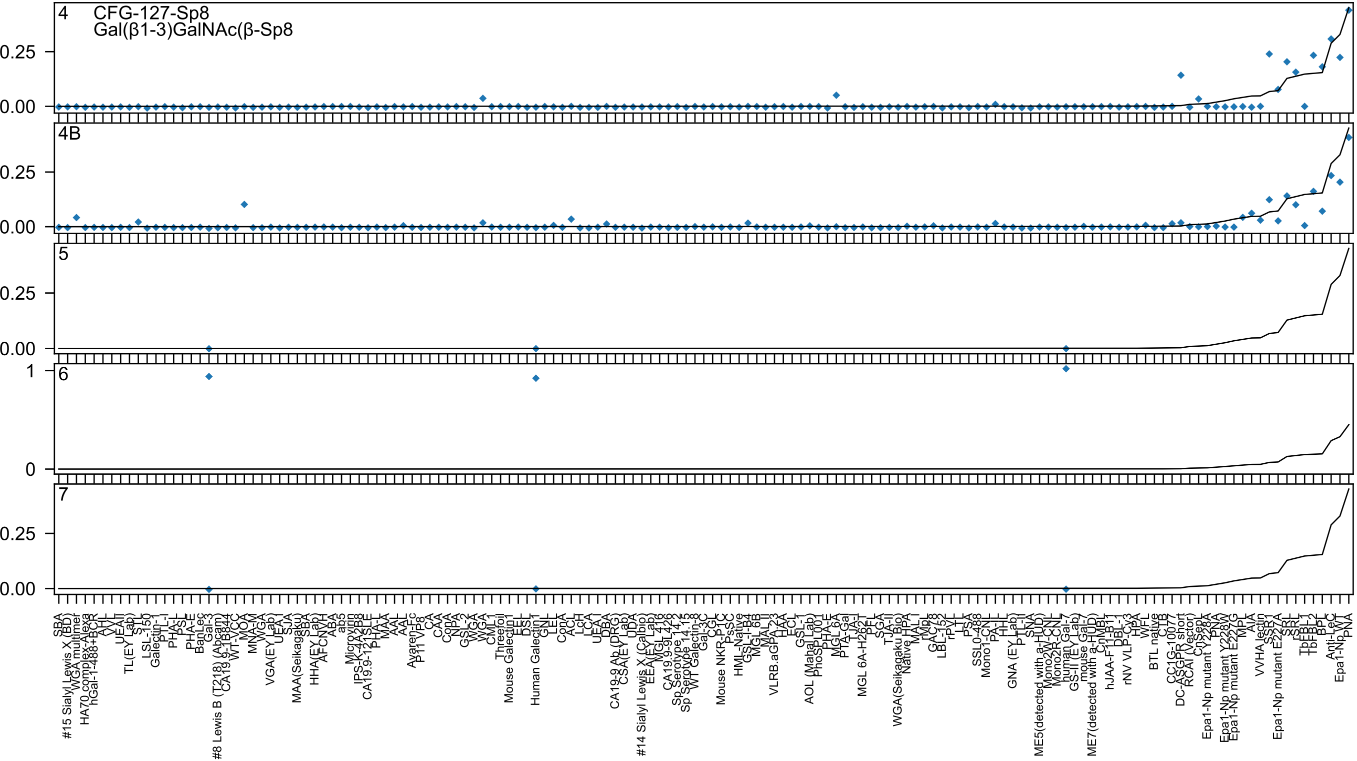

4
CFG-127-Sp8
Gal(β1-3)GalNAc(β-Sp8
4B
5
6
7
0.25
0.00
1
0

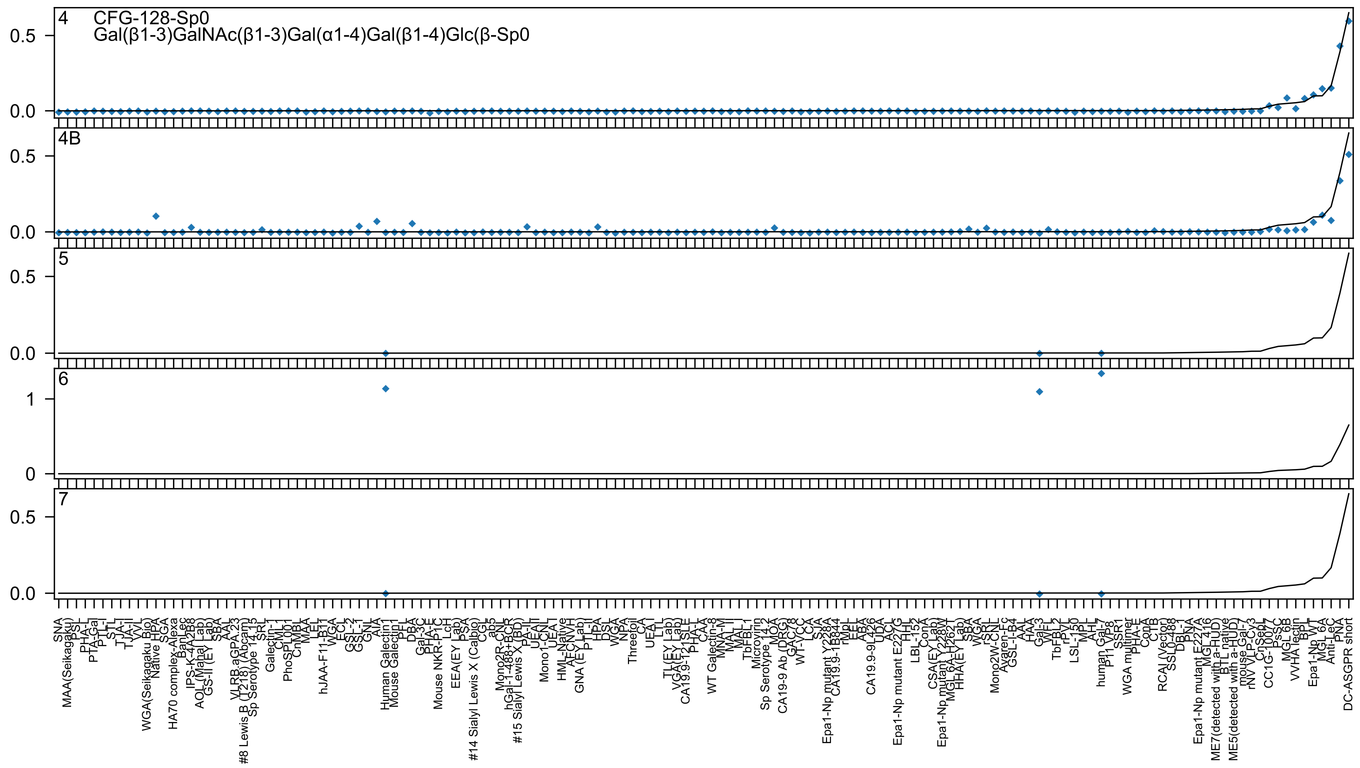

4 CFG-128-Sp0
Gal(β1-3)GalNAc(β1-3)Gal(α1-4)Gal(β1-4)Glc(β-Sp0
4B
5
6
7

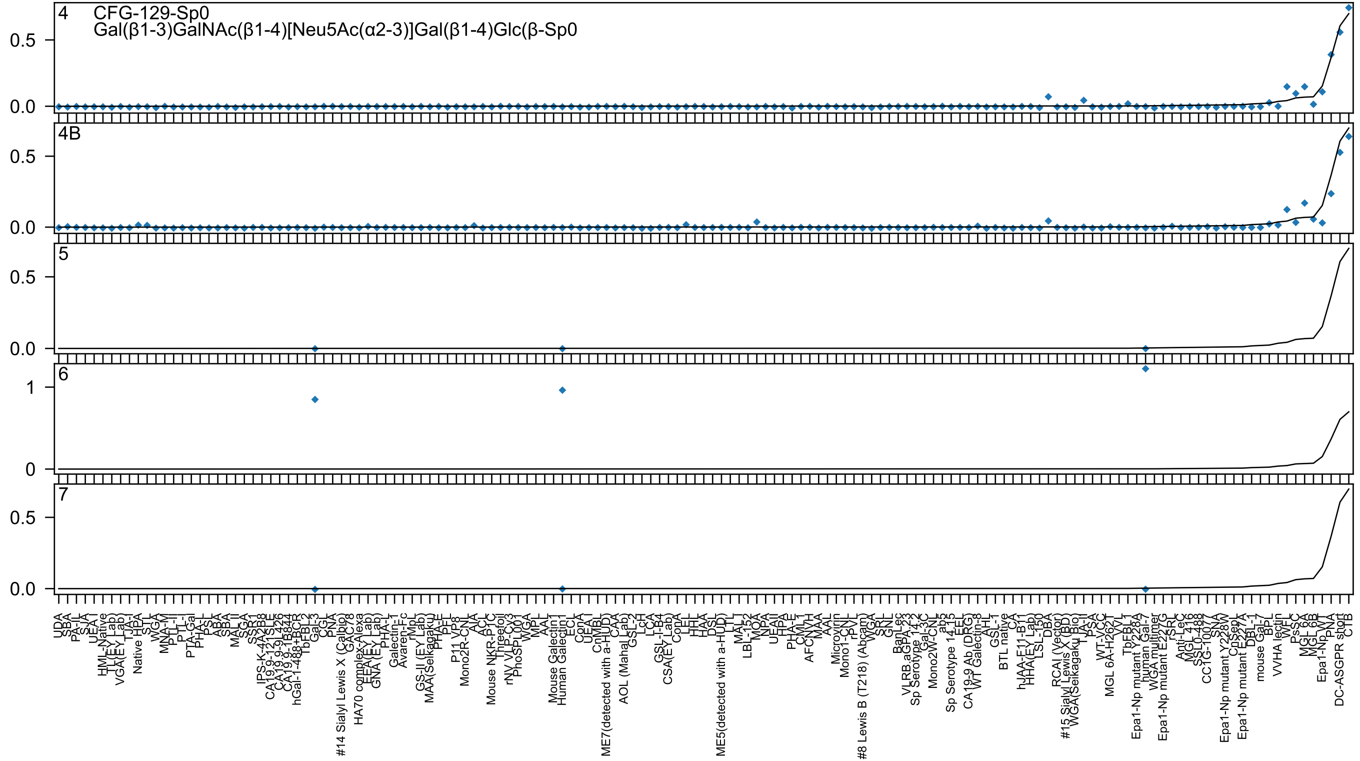

4
CFG-129-Sp0
Gal(β1-3)GalNAc(β1-4)[Neu5Ac(α2-3)]Gal(β1-4)Glc(β-Sp0
4B
5
6
7

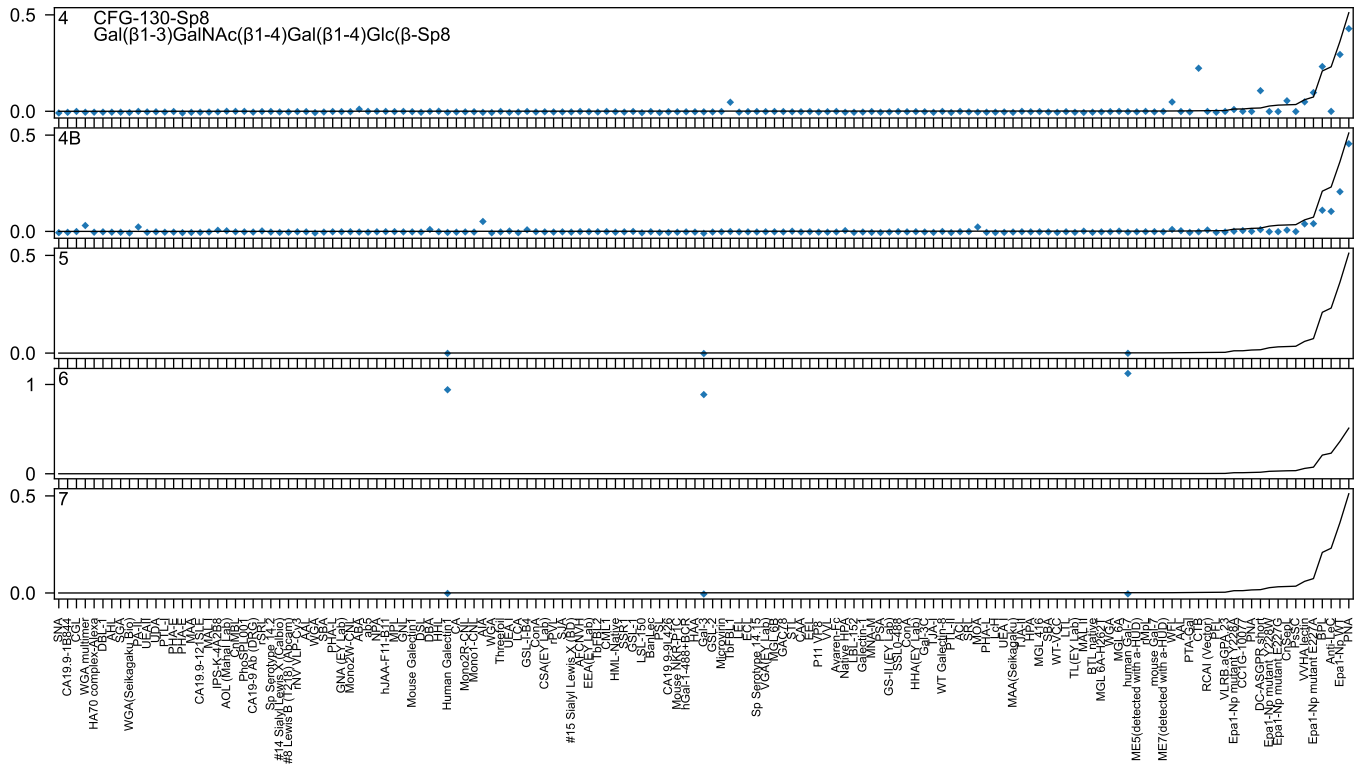

4
CFG-130-Sp8
Gal(β1-3)GalNAc(β1-4)Gal(β1-4)Glc(β-Sp8
4B
5
6
7

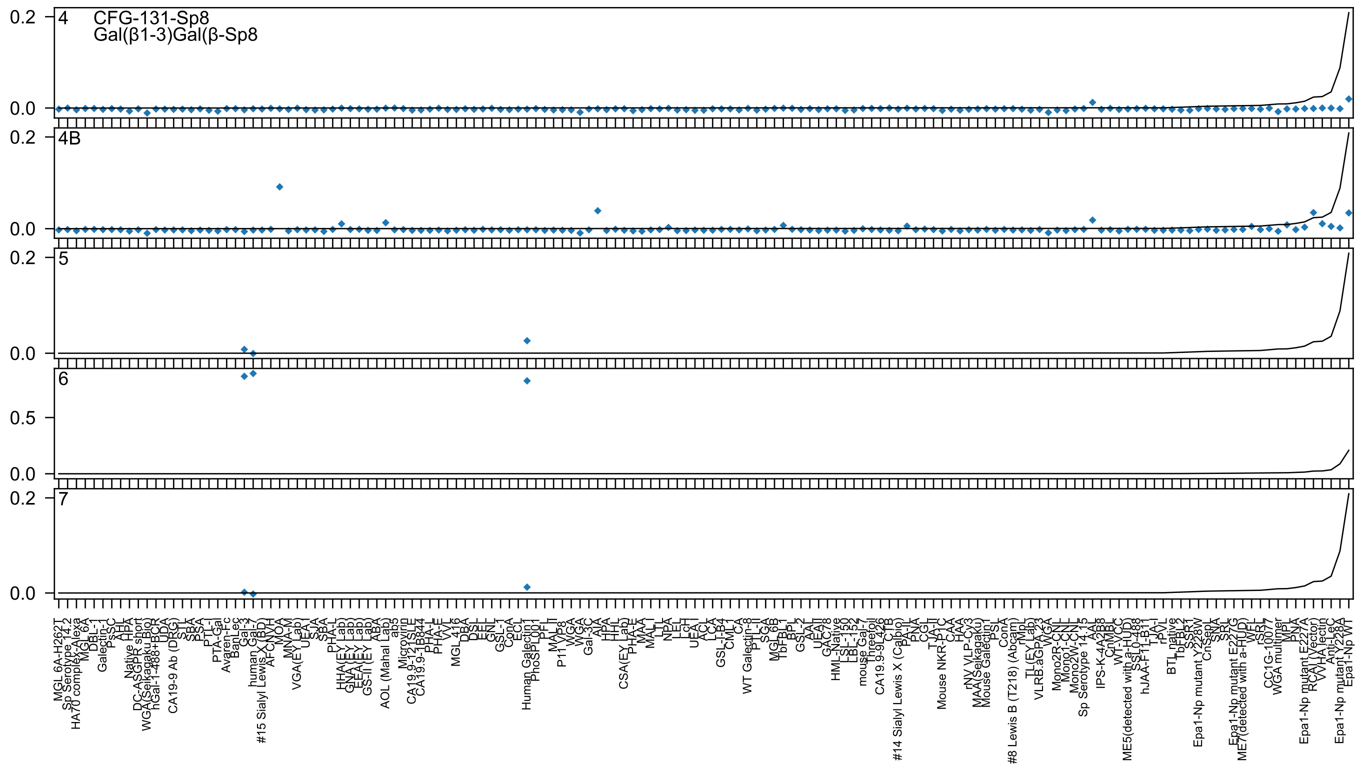
4
CFG-131-Sp8
Gal(β1-3)Gal(β-Sp8
4B
5
6
7

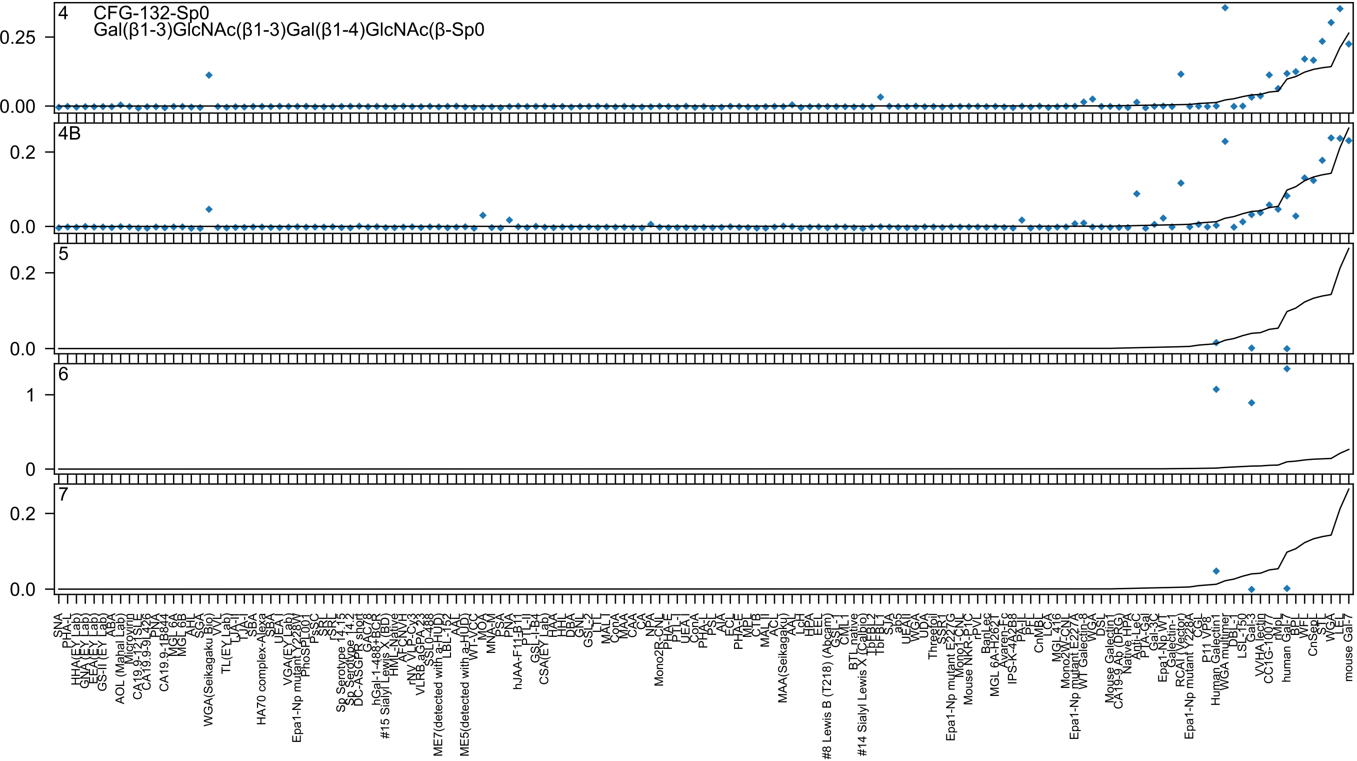
CFG-132-Sp0
Gal(β1-3)GlcNAc(β1-3)Gal(β1-4)GlcNAc(β-Sp0

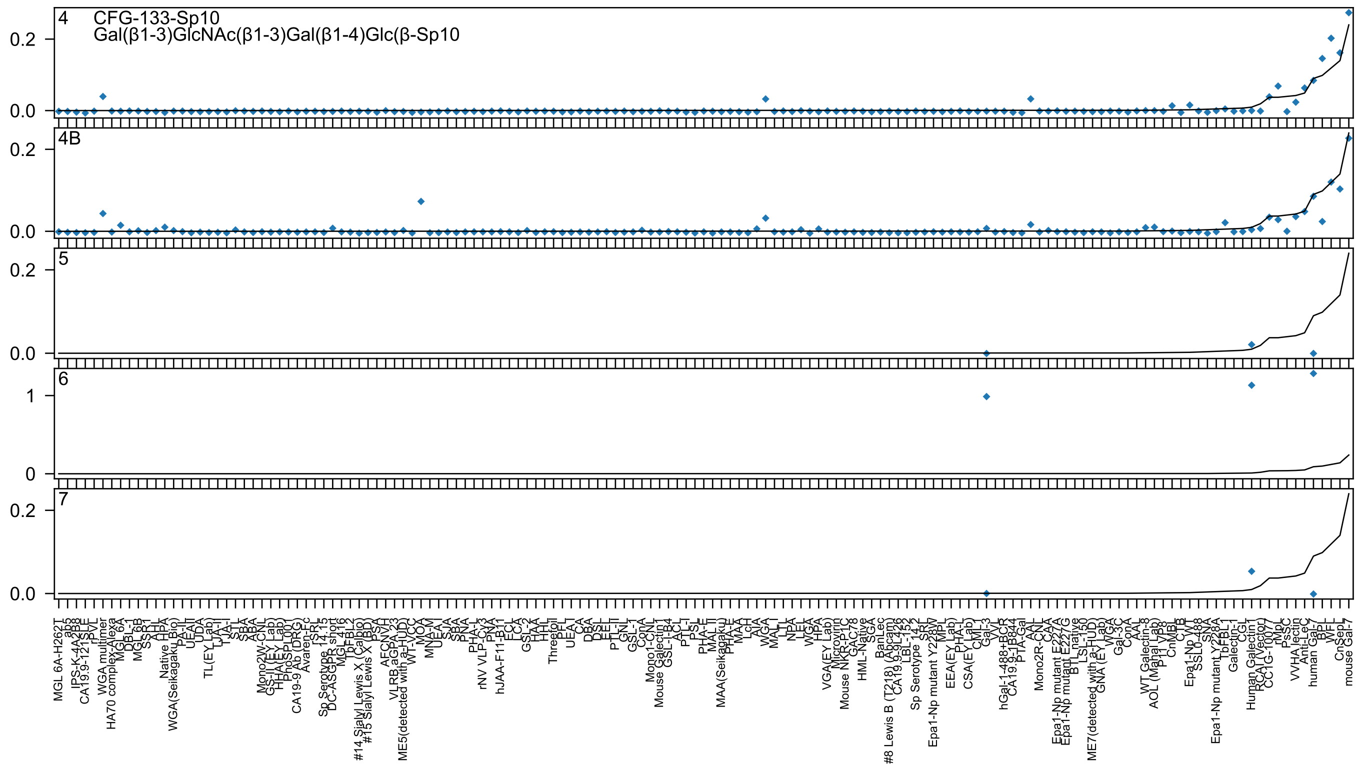
4
CFG-133-Sp10
Gal(β1-3)GlcNAc(β1-3)Gal(β1-4)Glc(β-Sp10
4B
5
6
7

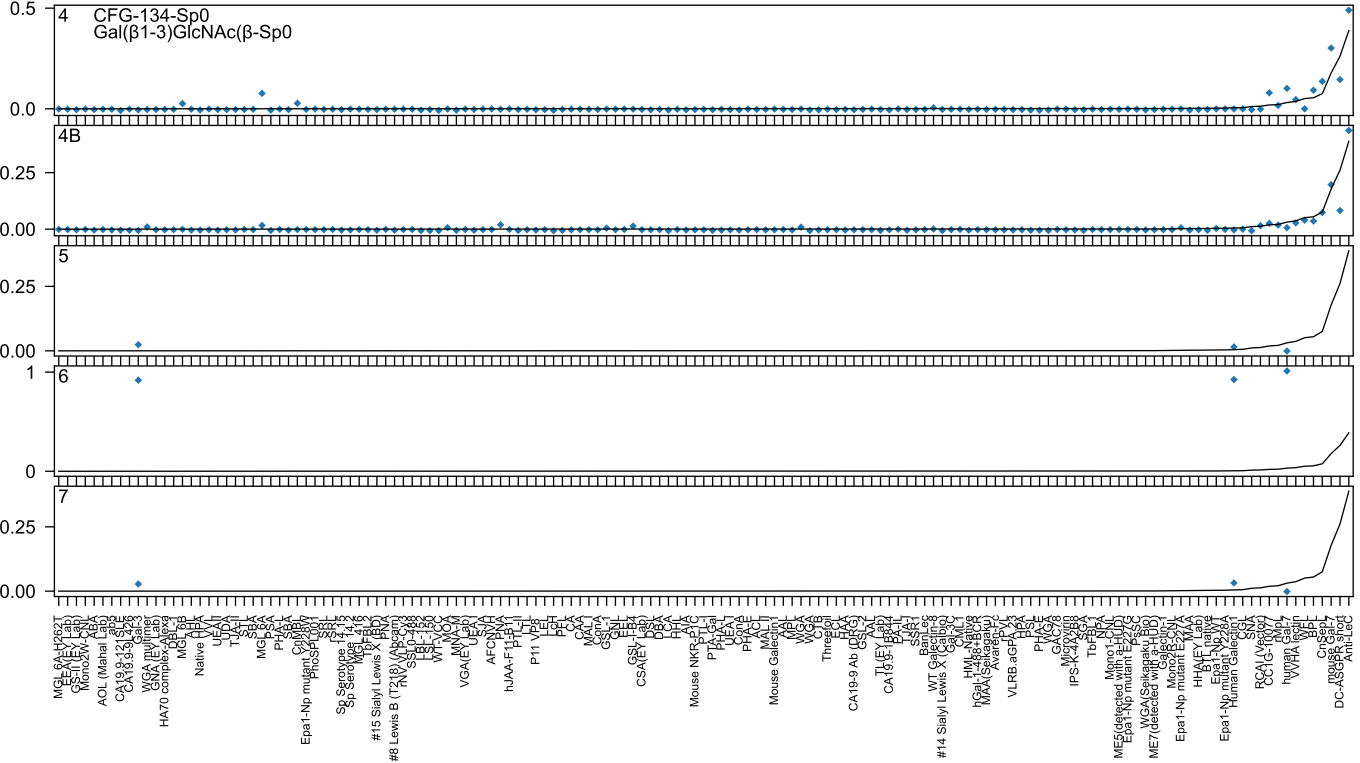

CFG-134-Sp0
Gal(β1-3)GlcNAc(β-Sp0

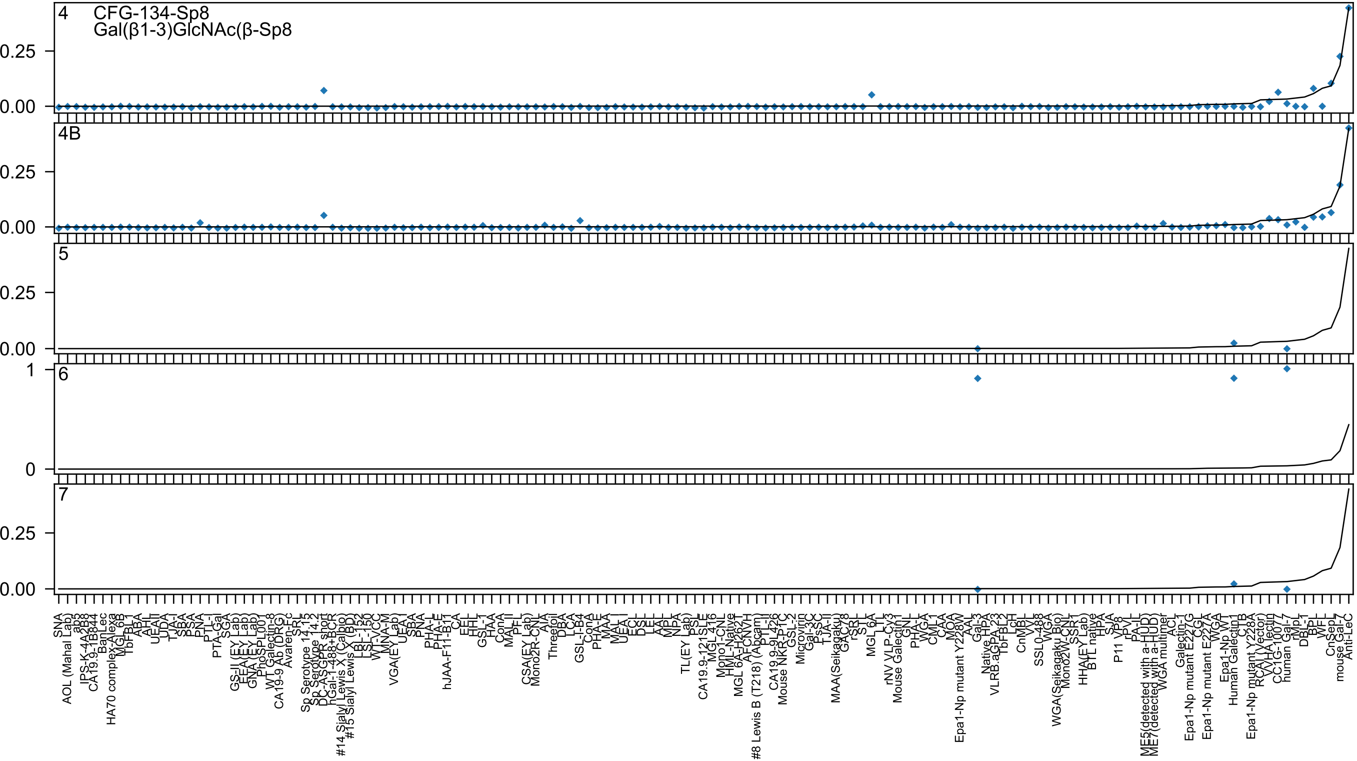
4
CFG-134-Sp8
Gal(β1-3)GlcNAc(β-Sp8
4B
5
6
7
0.25
0.00
1
0

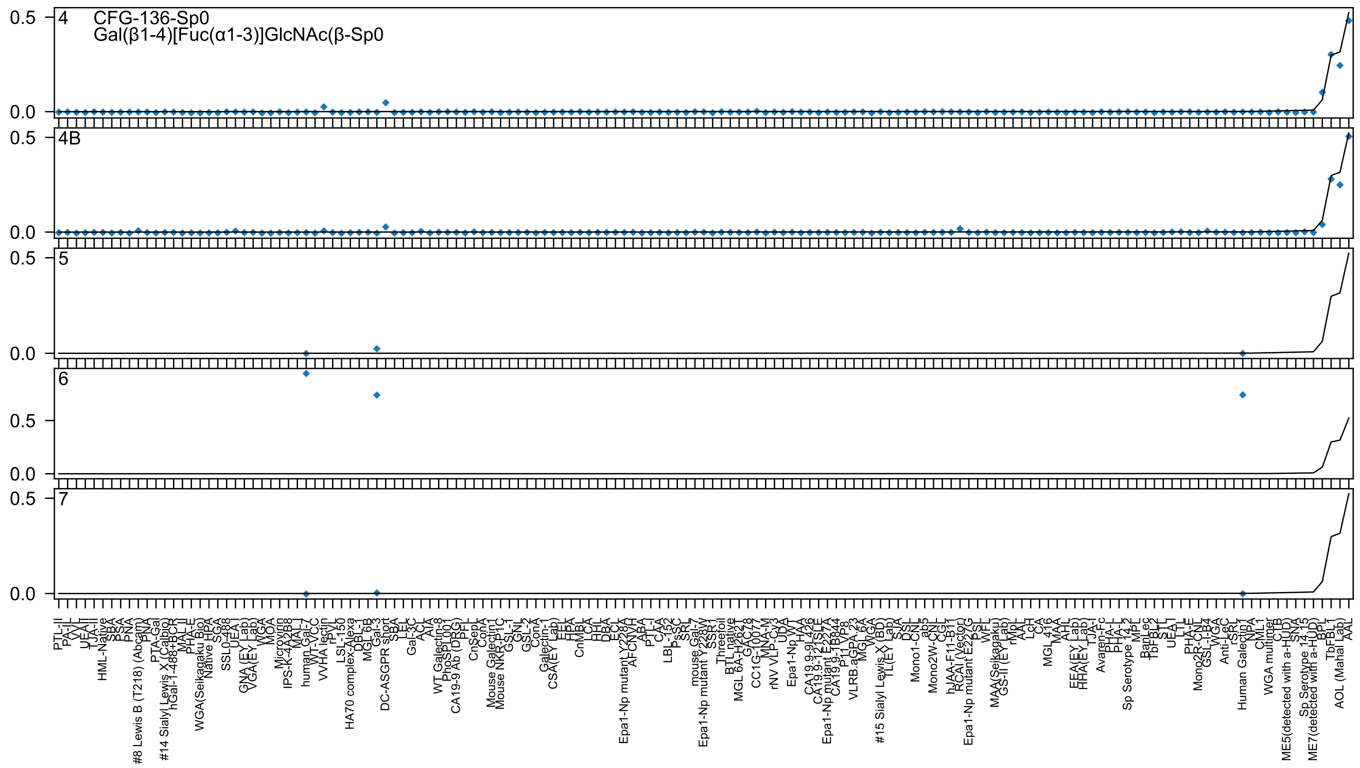
4
CFG-136-Sp0
Gal(β1-4)[Fuc(α1-3)]GlcNAc(β-Sp0
4B
5
6
7
0.5
0.0

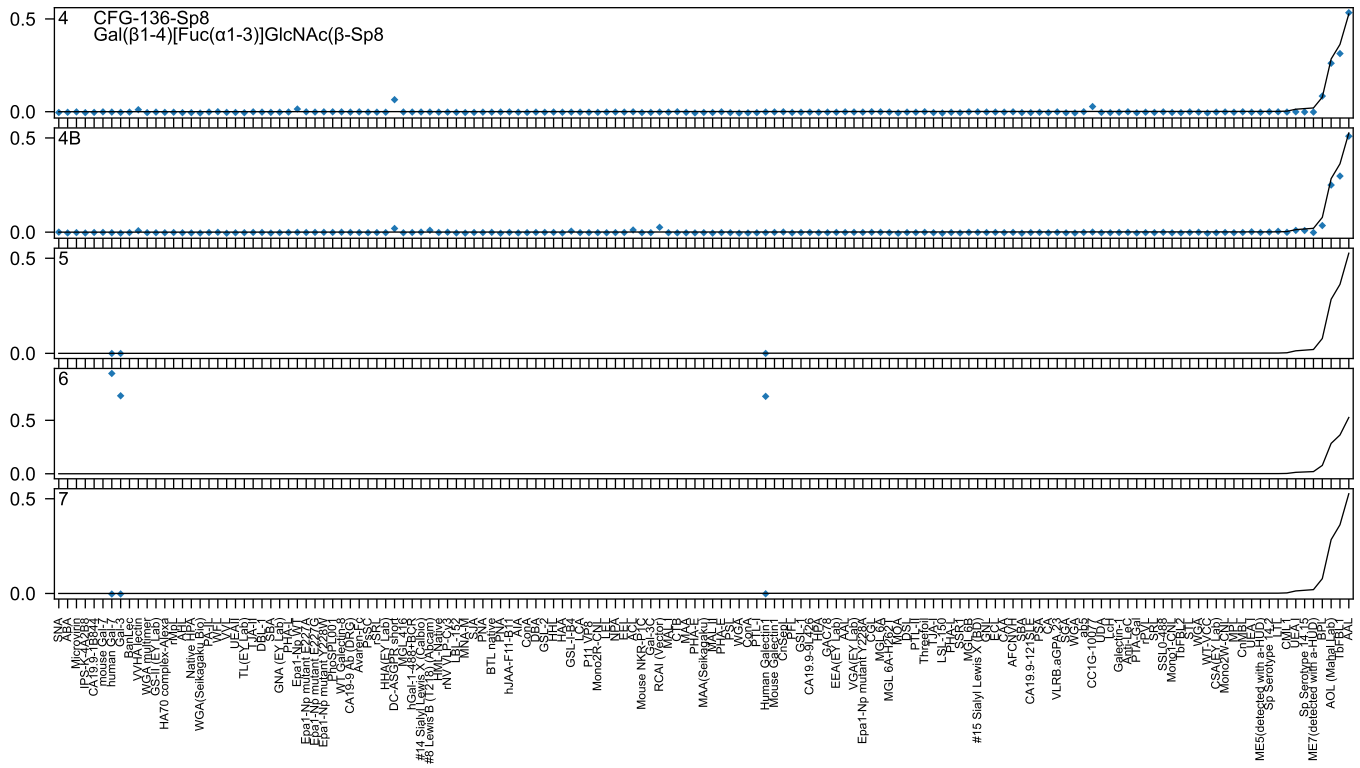
4
CFG-136-Sp8
Gal(β1-4)[Fuc(α1-3)]GlcNAc(β-Sp8
4B
5
6
7
0.5
0.0

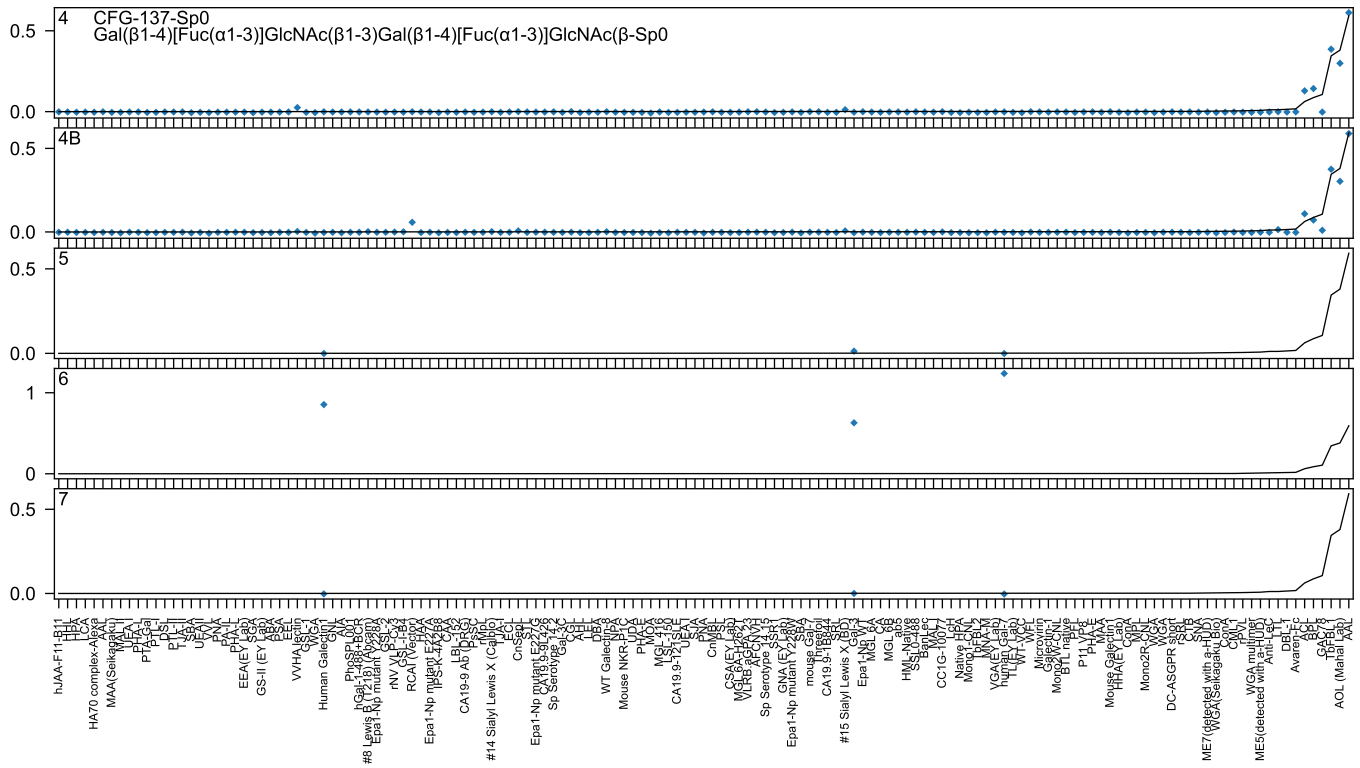

4 CFG-137-Sp0
Gal(β1-4)[Fuc(α1-3)]GlcNAc(β1-3)Gal(β1-4)[Fuc(α1-3)]GlcNAc(β-Sp0
4B
5
6
7

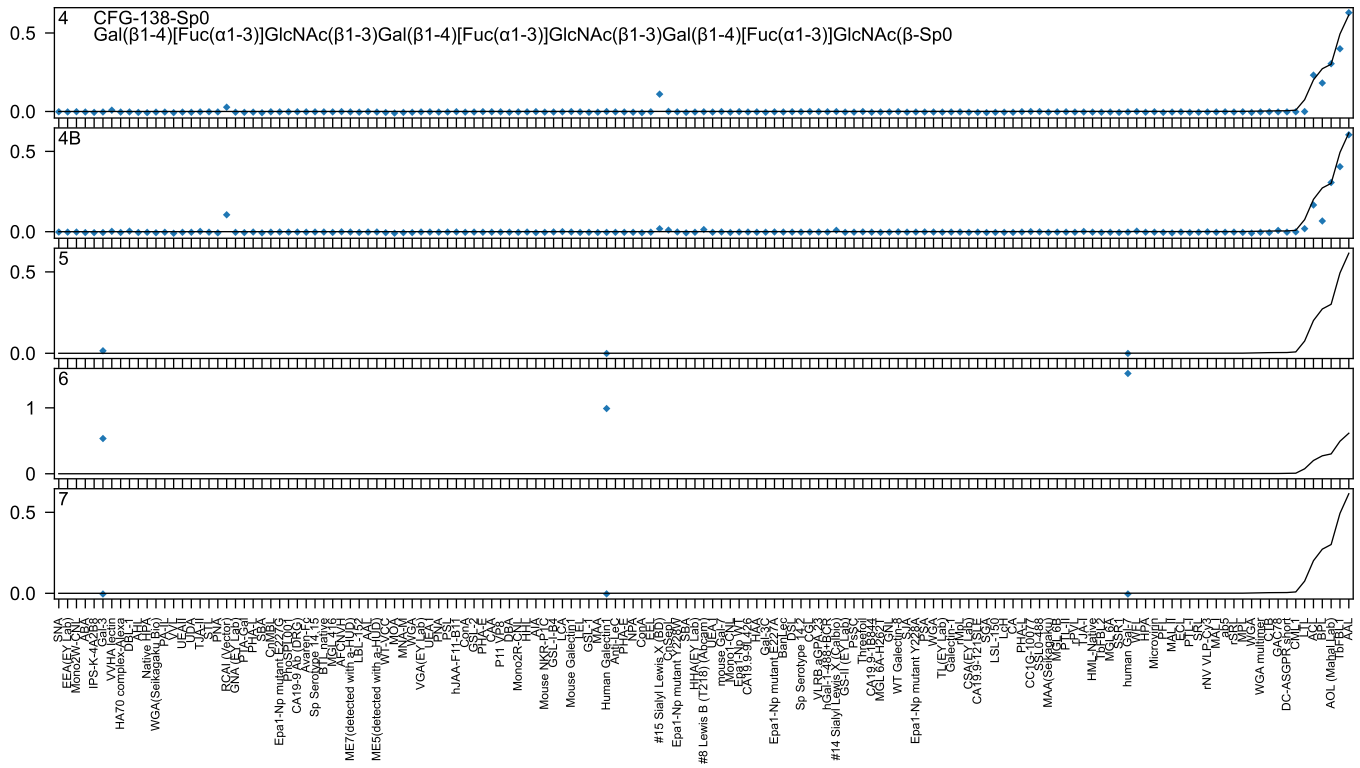
4
CFG-138-Sp0
Gal(β1-4)[Fuc(α1-3)]GlcNAc(β1-3)Gal(β1-4)[Fuc(α1-3)]GlcNAc(β1-3)Gal(β1-4)[Fuc(α1-3)]GlcNAc(β-Sp0
4B
5
6
7

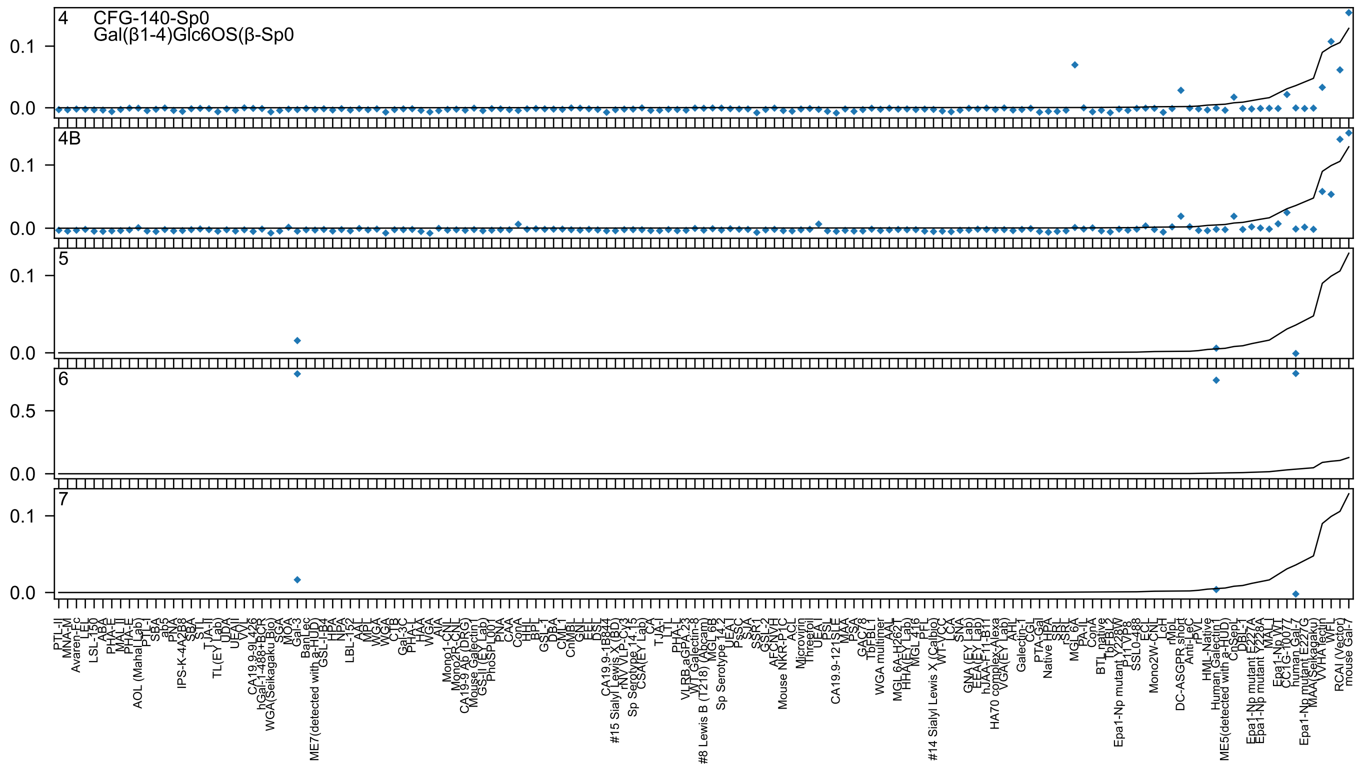

4
CFG-140-Sp0
Gal(β1-4)Glc6OS(β-Sp0
4B
5
6
7

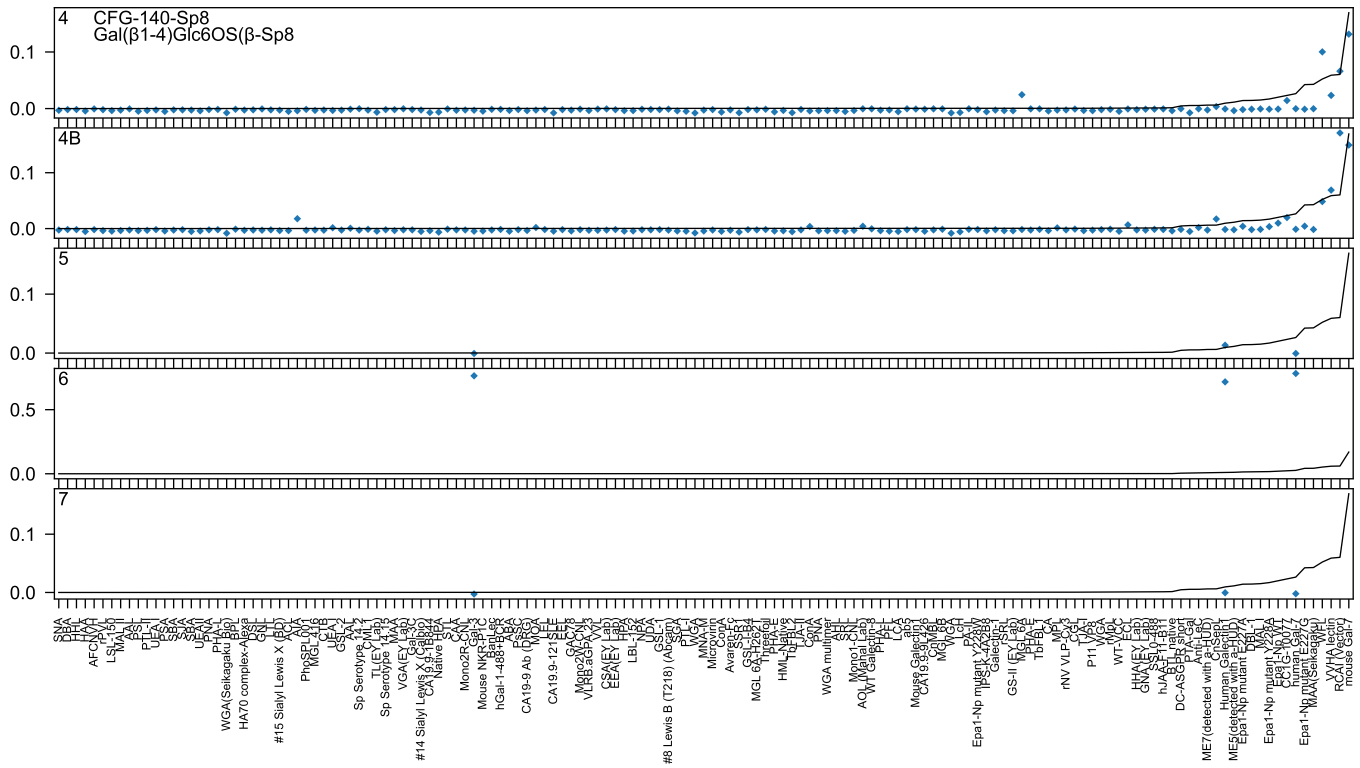

4
CFG-140-Sp8
Gal(β1-4)Glc6OS(β-Sp8
4B
5
6
7

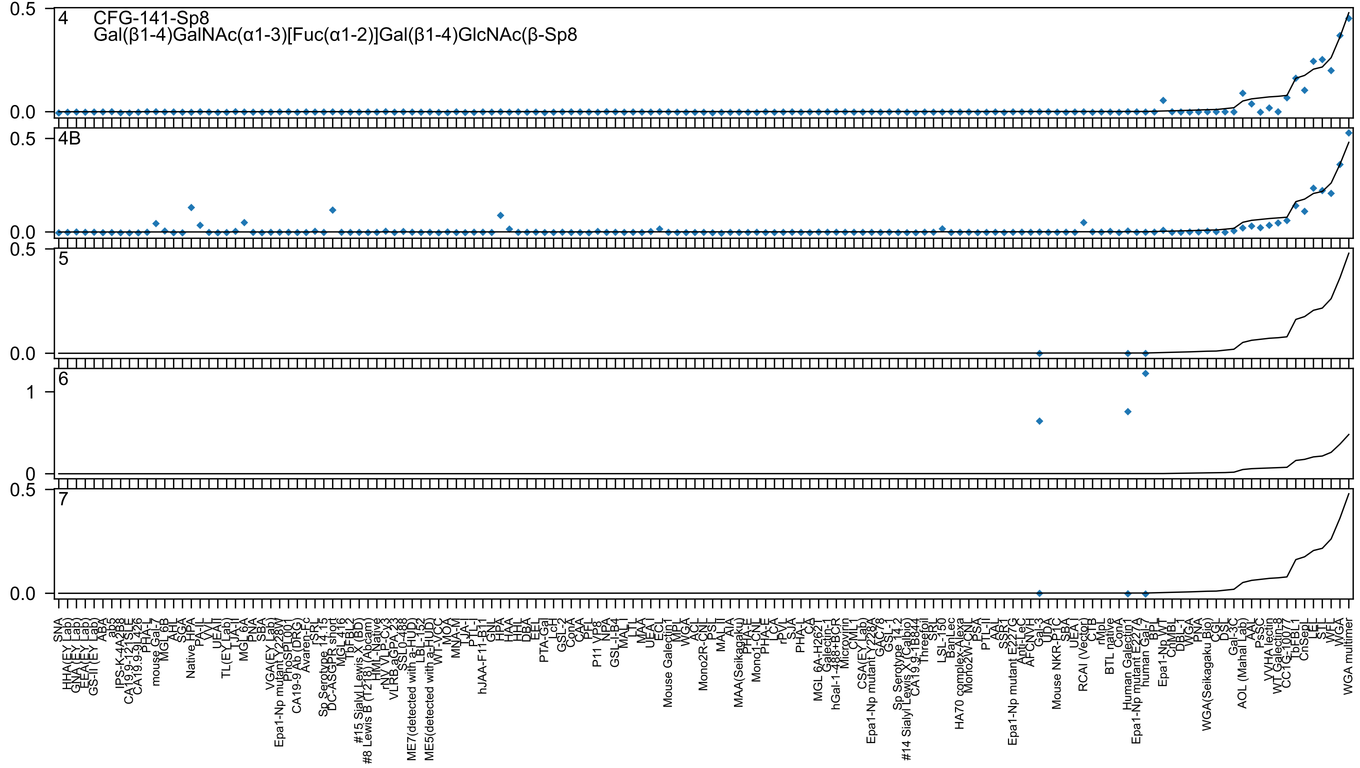
4 CFG-141-Sp8
Gal(β1-4)GalNAc(α1-3)[Fuc(α1-2)]Gal(β1-4)GlcNAc(β-Sp8
4B
5
6
7

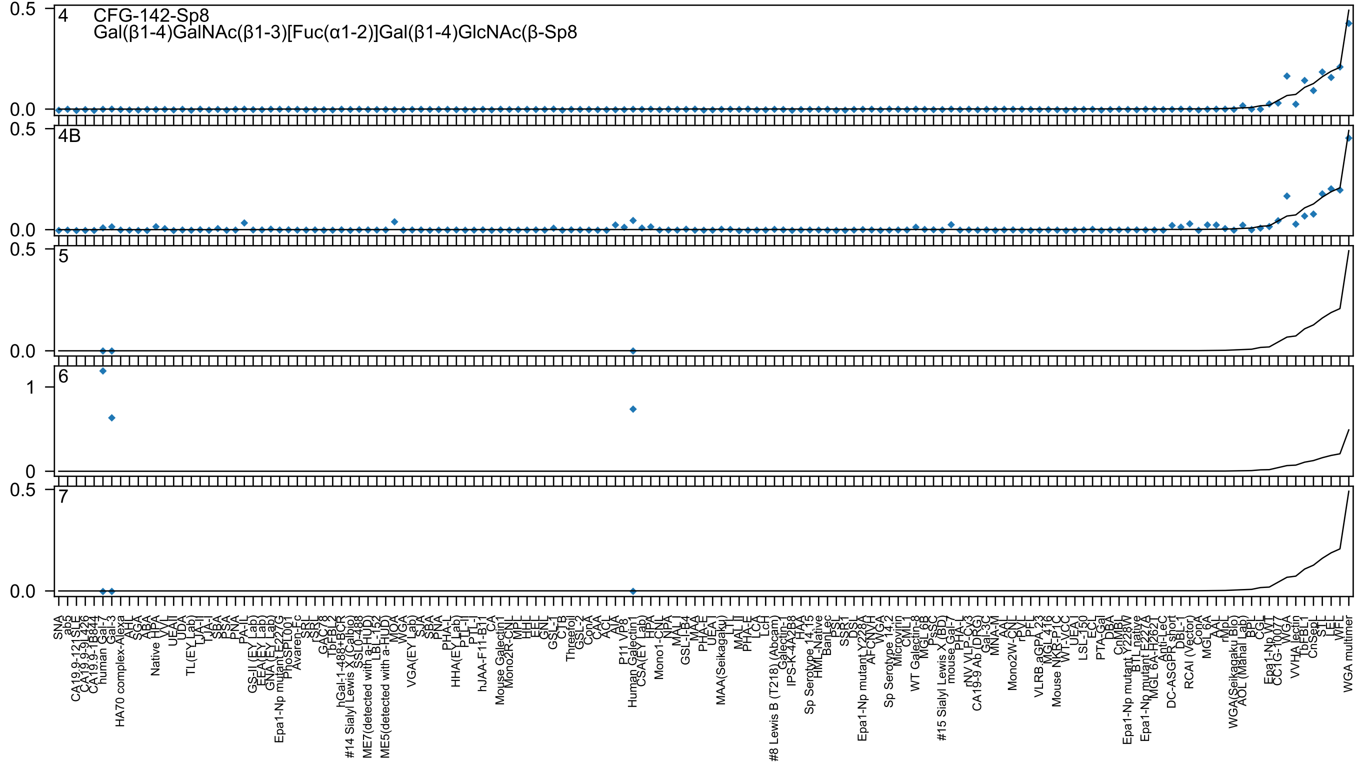

CFG-142-Sp8
Gal(β1-4)GalNAc(β1-3)[Fuc(α1-2)]Gal(β1-4)GlcNAc(β-Sp8
4
4B
5
6
7

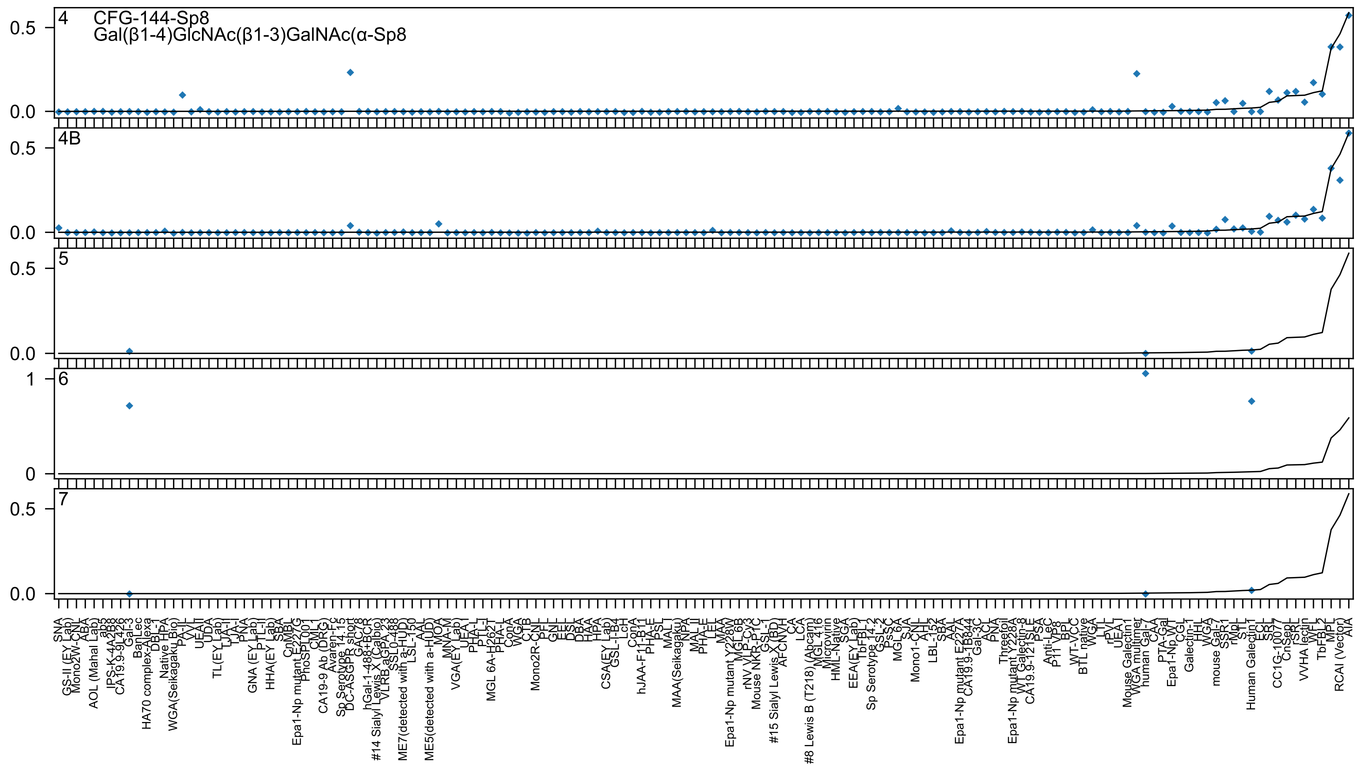
CFG-144-Sp8
Gal(β1-4)GlcNAc(β1-3)GalNAc(α-Sp8

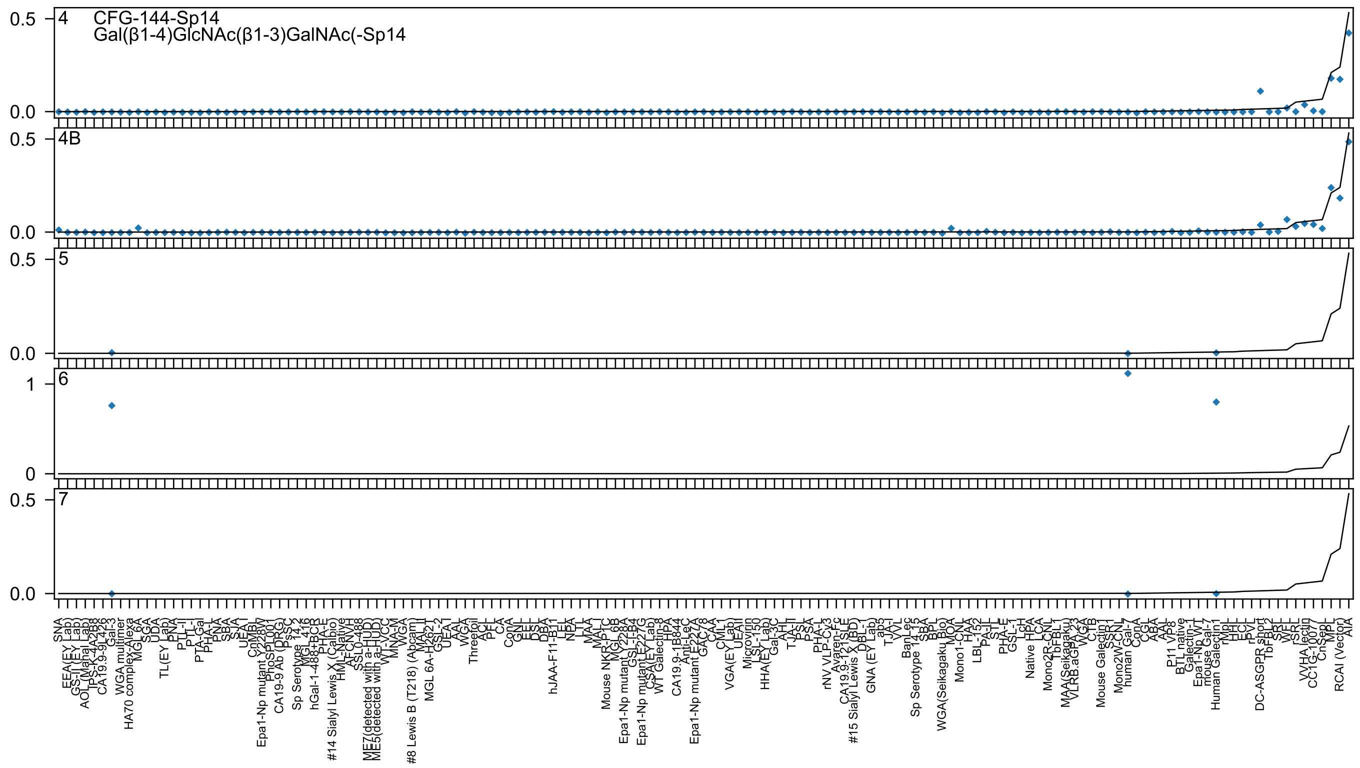
4 CFG-144-Sp14
Gal(β1-4)GlcNAc(β1-3)GalNAc(-Sp14
4B
5
6
7

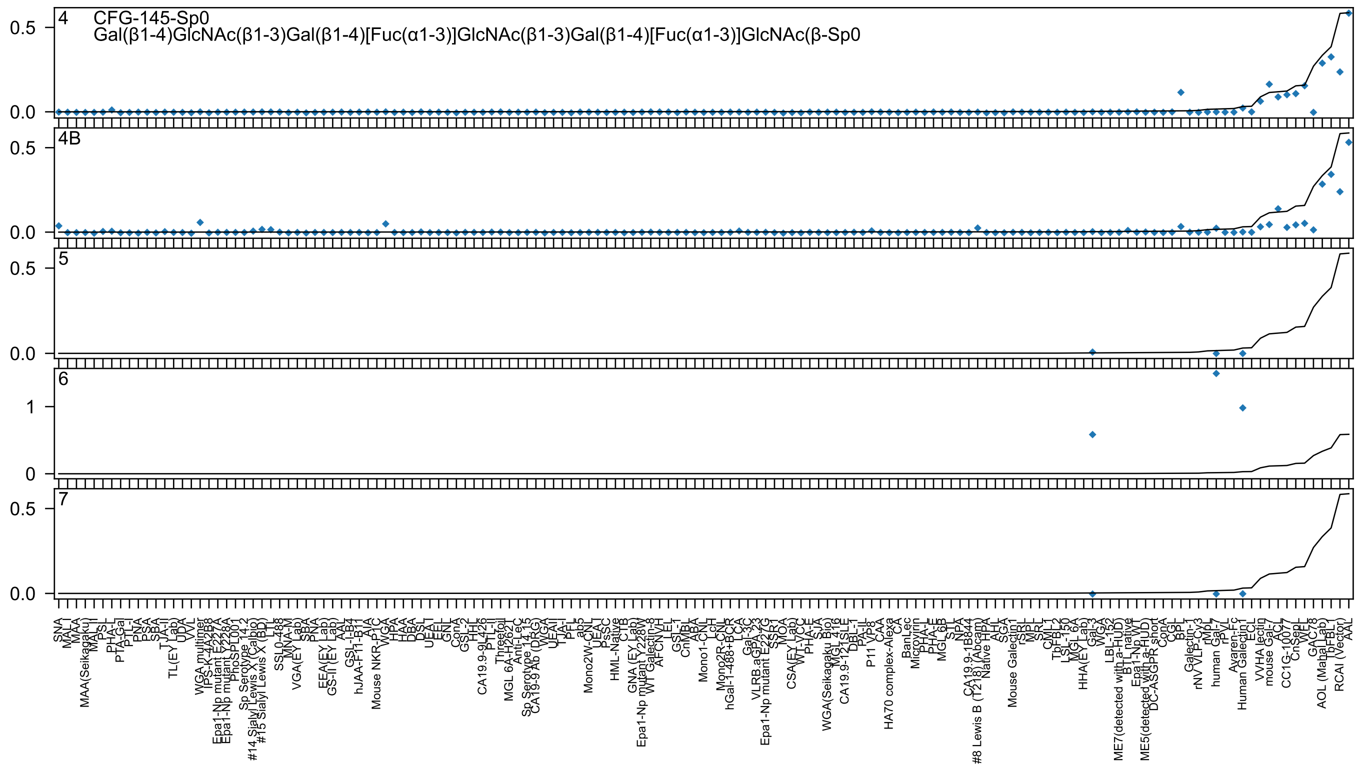

4 CFG-145-Sp0
Gal(β1-4)GlcNAc(β1-3)Gal(β1-4)[Fuc(α1-3)]GlcNAc(β1-3)Gal(β1-4)[Fuc(α1-3)]GlcNAc(β-Sp0
4B
5
6
7

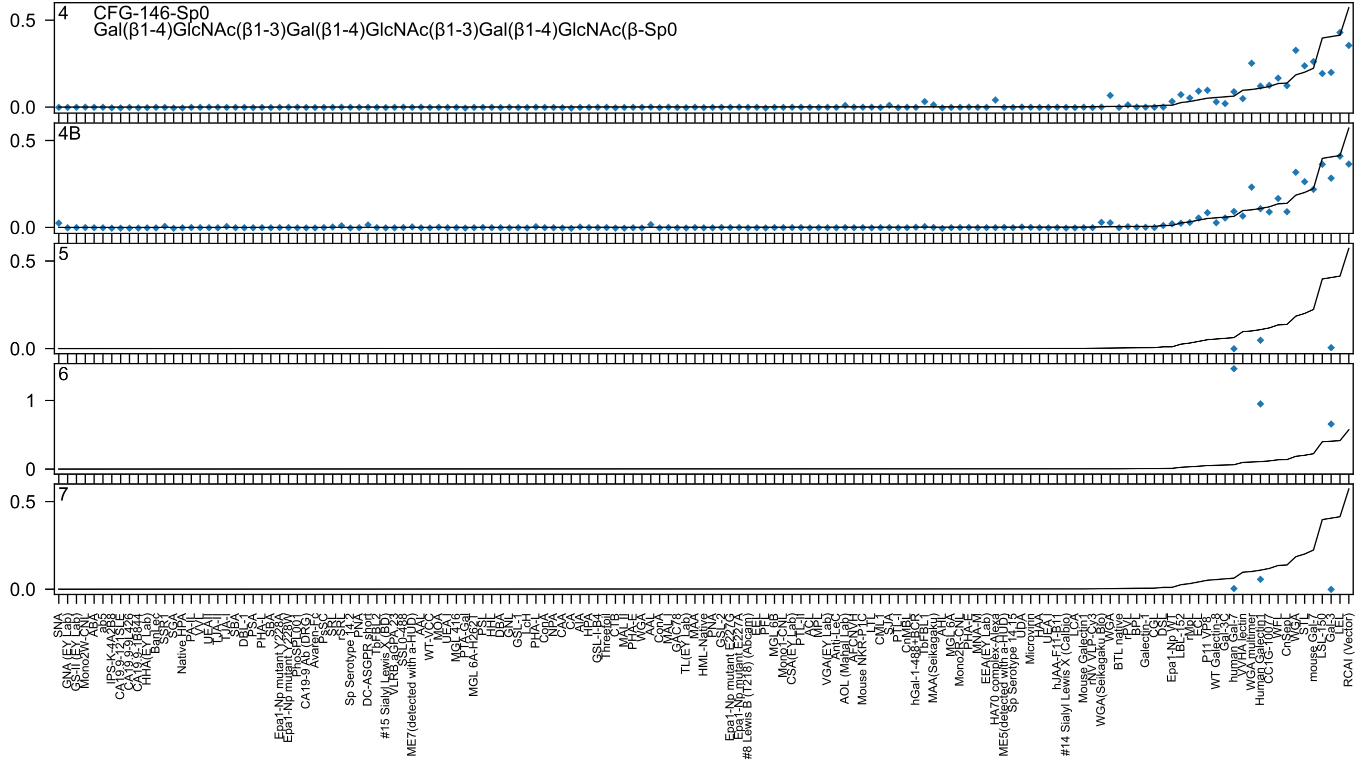

4 CFG-146-Sp0
Gal(β1-4)GlcNAc(β1-3)Gal(β1-4)GlcNAc(β1-3)Gal(β1-4)GlcNAc(β-Sp0
4B
5
6
7

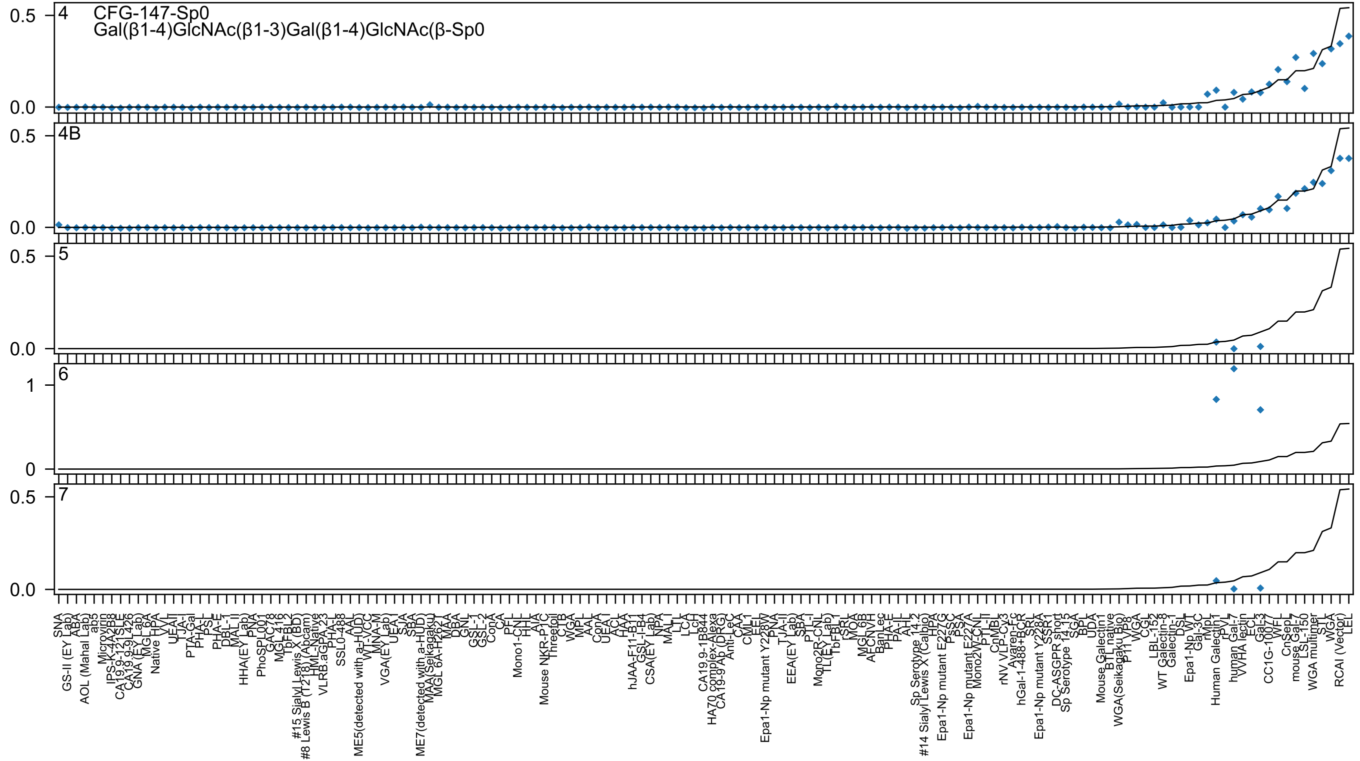

4
CFG-147-Sp0
Gal(β1-4)GlcNAc(β1-3)Gal(β1-4)GlcNAc(β-Sp0
4B
5
6
7
0.0
0.5
0
1
SNA
GS-II (EY Lab)
ABA
AOL (Mahal Lab)
ab5
Microvirin
IPS-K-4A2B8
CA19.9-121SLE
CA19.9-9L426
GNA (EY Lab)
MGL 6A
Native HPA
VVL
UEAII
TJA-I
PTA-Gal
PHA-L
PSL
PHA-E
DBL-1
MAL-II
HHA(EY Lab)
PNA
PhoSL001
GAC78
MGL 416
TbFBL2
#15 Sialyl Lewis X (BD)
#8 Lewis B (T218) (Abcam)
HML-Native
VLRB.aGPA.23
PHA-L
SSL0-488
AAL
ME5(detected with a-HUD)
WT-VCC
MNA-M
VGA(EY Lab)
UEA-I
SJA
SBA
ME7(detected with a-HUD)
MAA(Seikagaku)
MGL 6A-H262T
MAA
DBA
GNL
GSL-1
GSL-2
ConA
CA
PFL
Mono1-CNL
HHL
AIA
Mouse NKR-P1C
Threefoil
CTB
WGA
MPL
AAL
ConA
UEA-I
ACL
HAA
hJAA-F11-B11
GSL-I-B4
CSA(EY Lab)
NPA
MAL-I
LTL
LCA
LcH
CA19.9-1B844
HA70 complex-Alexa
CA19-9 Ab (DRG)
Anti-LeC
CAA
CVL-1
EEL
Epa1-Np mutant Y228W
PNA
TJA-II
EEA(EY Lab)
SBA
PTL-I
Mono2R-CNL
TL(EY Lab)
TbFBL1
rSRL
MOA
MGL 6B
AFCNVH
BanLec
PHA-E
PA-IL
AAH
Sp Serotype 14.2
#14 Sialyl Lewis X (Calbio)
HPA
Epa1-Np mutant E227G
PsSC
PSA
Epa1-Np mutant E227A
Mono2W-CNL
PTL-II
CnMBL
rNV VLP-Cy3
Avaren-Fc
hGal-1-488+BCR
SRL
Epa1-Np mutant Y228A
SSR1
DC-ASGPR short
Sp Serotype 14.15
SGA
BPL
UDA
Mouse Galectin1
BTL native
WGA(Seikagaku Bio)
P11 VP8
WGA
CGL
LBL-152
WT Galectin-8
Galectin-1
DSL
Epa1-Np WT
Gal-3C
rMpL
Human Galectin1
rPVL
human Gal-7
VVHA lectin
ECL
Gal-3
CC1G-1007
WFL
CnSep7
mouse Gal-7
LSL-150
WGA multimer
STL
WGA
RCAI (Vector)
LEL

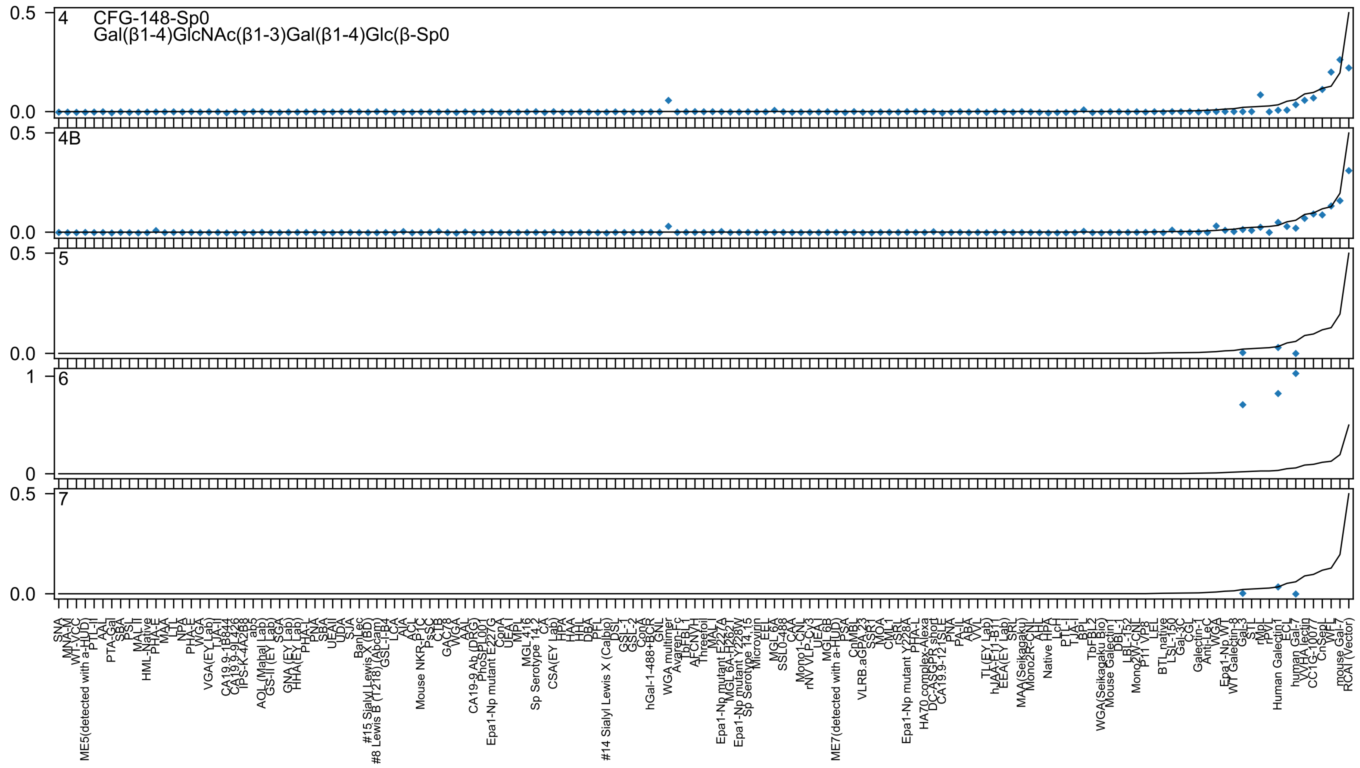

4 CFG-148-Sp0
Gal(β1-4)GlcNAc(β1-3)Gal(β1-4)Glc(β-Sp0
4B
5
6
7
0.5
0.0
1
0

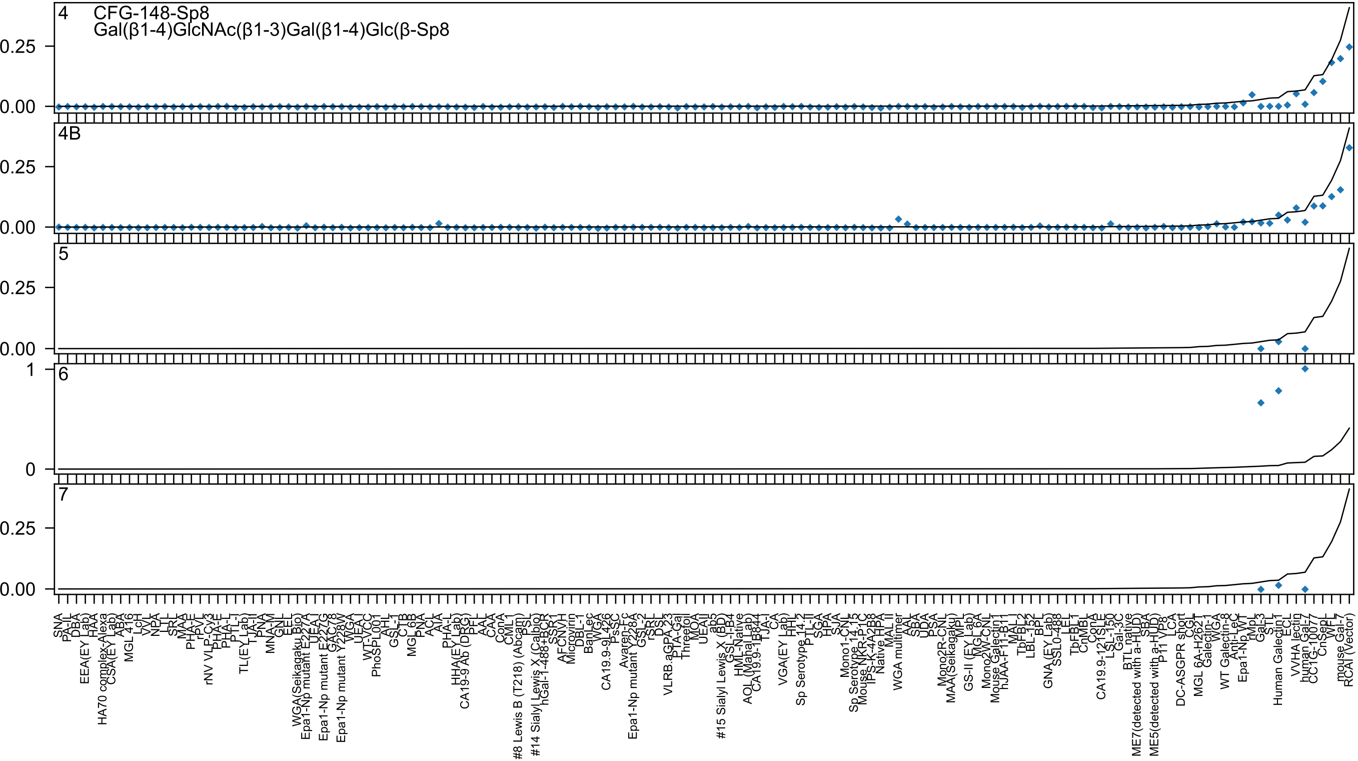
CFG-148-Sp8
Gal(β1-4)GlcNAc(β1-3)Gal(β1-4)Glc(β-Sp8

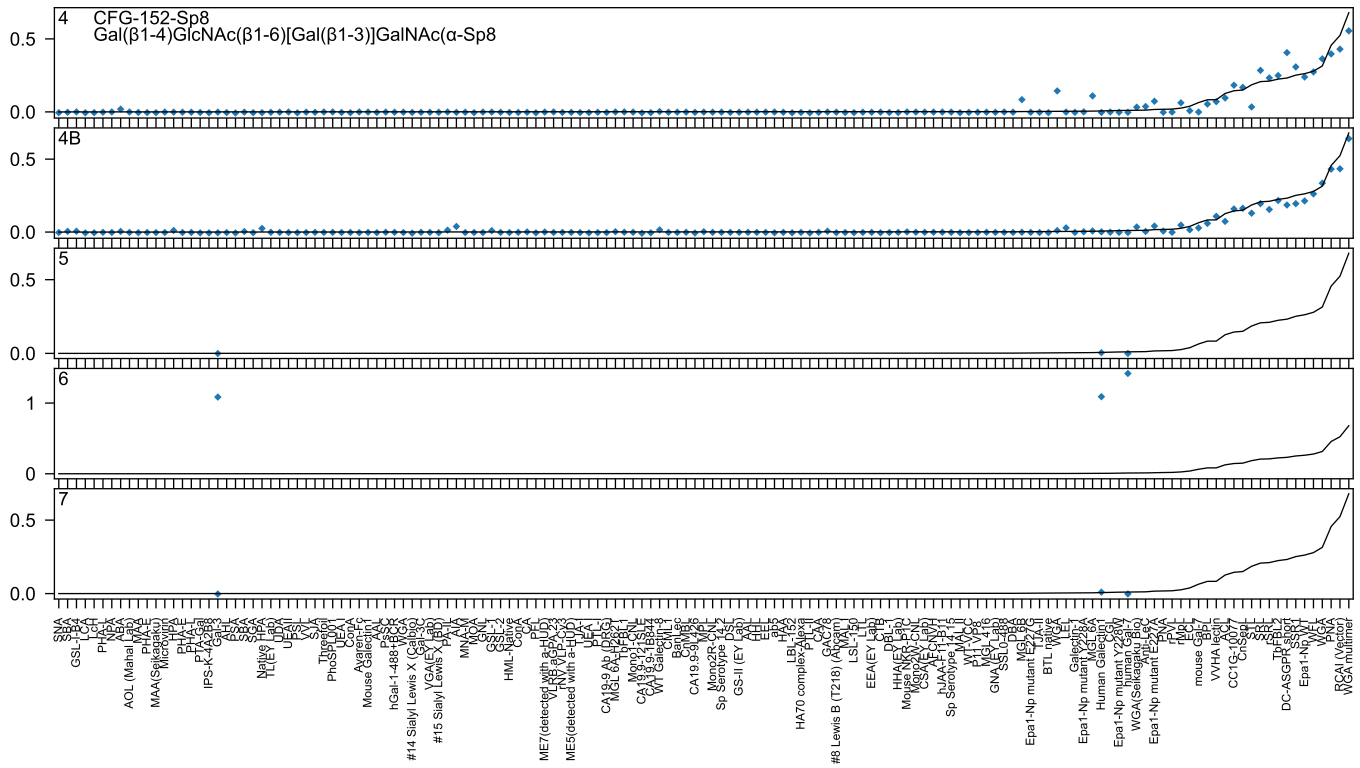

4 CFG-152-Sp8
Gal(β1-4)GlcNAc(β1-6)[Gal(β1-3)]GalNAc(α-Sp8
4B
5
6
7

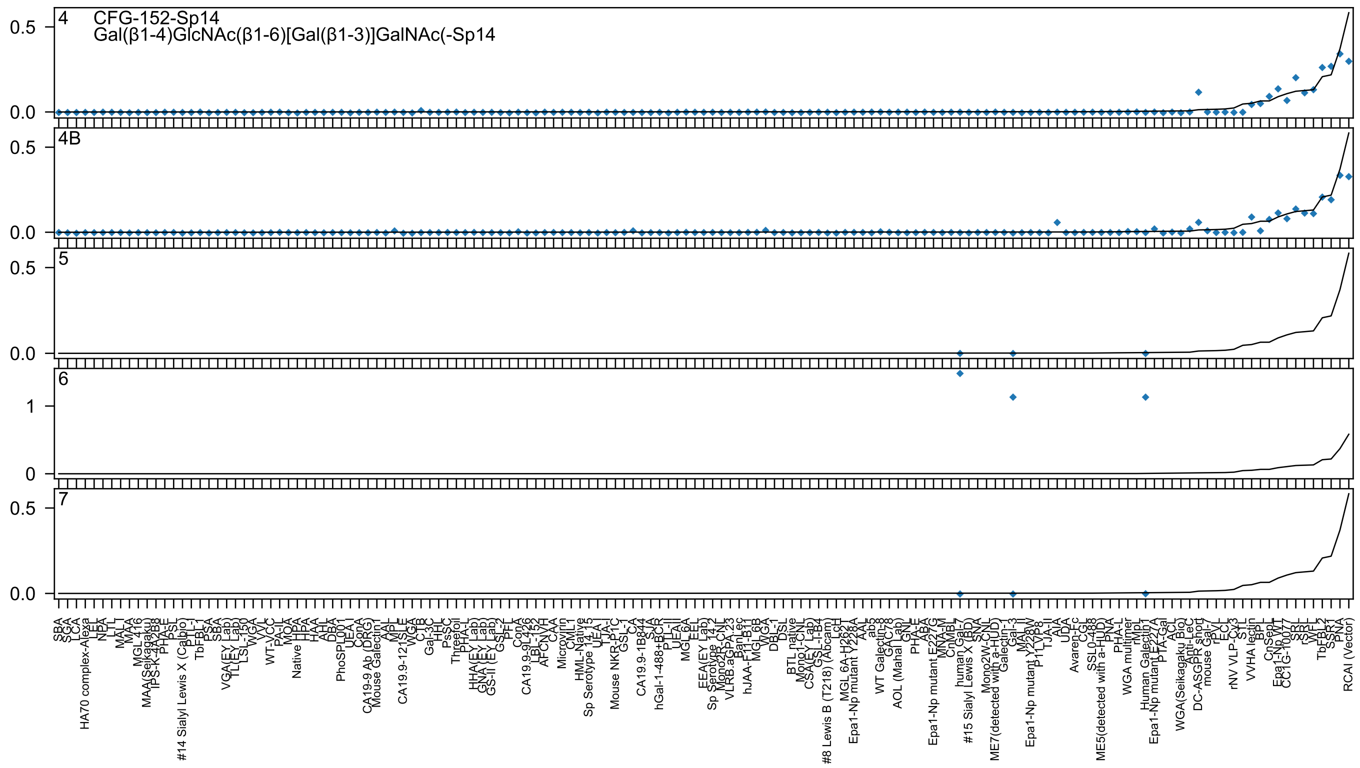
4
CFG-152-Sp14
Gal(β1-4)GlcNAc(β1-6)[Gal(β1-3)]GalNAc(-Sp14
4B
5
6
7
0.5
0.0
1
0
SBA
SGA
LCA
HA70 complex-Alexa
LEL
NPA
LTL
MAL-I
MAA
MGL 416
MAA(Seikagaku)
IPS-K-4A2B8
PHA-E
PSL
#14 Sialyl Lewis X (Calbio)
PTL-I
TbFBL-1
PSA
SBA
VGA(EY Lab)
TL(EY Lab)
LSL-150
WGA
VVL
WT-VCC
PA-IL
MOA
Native HPA
HPA
HAA
AHL
DBA
PhoSL
UEA-I
ConA
CA19-9 Ab (DRG)
Mouse Galectin1
AAL
MPL
CA19.9-121SLE
WGA
CTB
Gal-3C
HHL
PsSC
Threefoil
PHA-L
HHA(EY Lab)
GNA (EY Lab)
GS-II (EY Lab)
GSL-2
PFL
ConA
CA19.9-9L426
LBL-152
AFCNVH
CAA
Microvirin
CML1
HML-Native
Sp Serotype 14.15
UEA-I
TJA-I
Mouse NKR-P1C
GSL-1
CA
CA19.9-1B844
SJA
hGal-1-488+BCR
PTL-II
UEA-II
MGL 6A
EEL
EEA(EY Lab)
Sp Serotype 14.2
Mono2R-CNL
VLRB.aGPA.23
BanLec
hJAA-F11-B1
MGL 6B
WGA
DBL-1
DSL
BTL native
Mono1-CNL
CSA(EY Lab)
GSL-I-B4
#8 Lewis B (T218) (Abcam)
LcH
MGL 6A-H262T
Epa1-Np mutant Y228A
AAL
ab5
WT Galectin-8
GAC78
AOL (Mahal Lab)
GNL
PHA-E
ABA
Epa1-Np mutant E227G
MNA-M
CnMBL
human Gal-7
#15 Sialyl Lewis X (BD)
SNA
Mono2W-CNL
ME7(detected with a-HUD)
Galectin-1
Gal-3
MAL-II
Epa1-Np mutant Y228W
P1LVP8
TJA-II
AIA
UDA
Avaren-Fc
CGL
SSL0-488
ME5(detected with a-HUD)
PNA
PHA-L
WGA multimer
rMpL
Human Galectin1
Epa1-Np mutant E227A
PTA-Gal
ACL
WGA(Seikagaku Bio)
Anti-LeC
DC-ASGPR short
mouse Gal-7
rPVL
ECL
rNV VLP-Cy3
STL
VVHA lectin
BPL
CnSep
Epa1-Np WT
CC1G-10077
SRL
rSRL
WFL
TbFBL2
SSR1
PNA
RCAI (Vector)

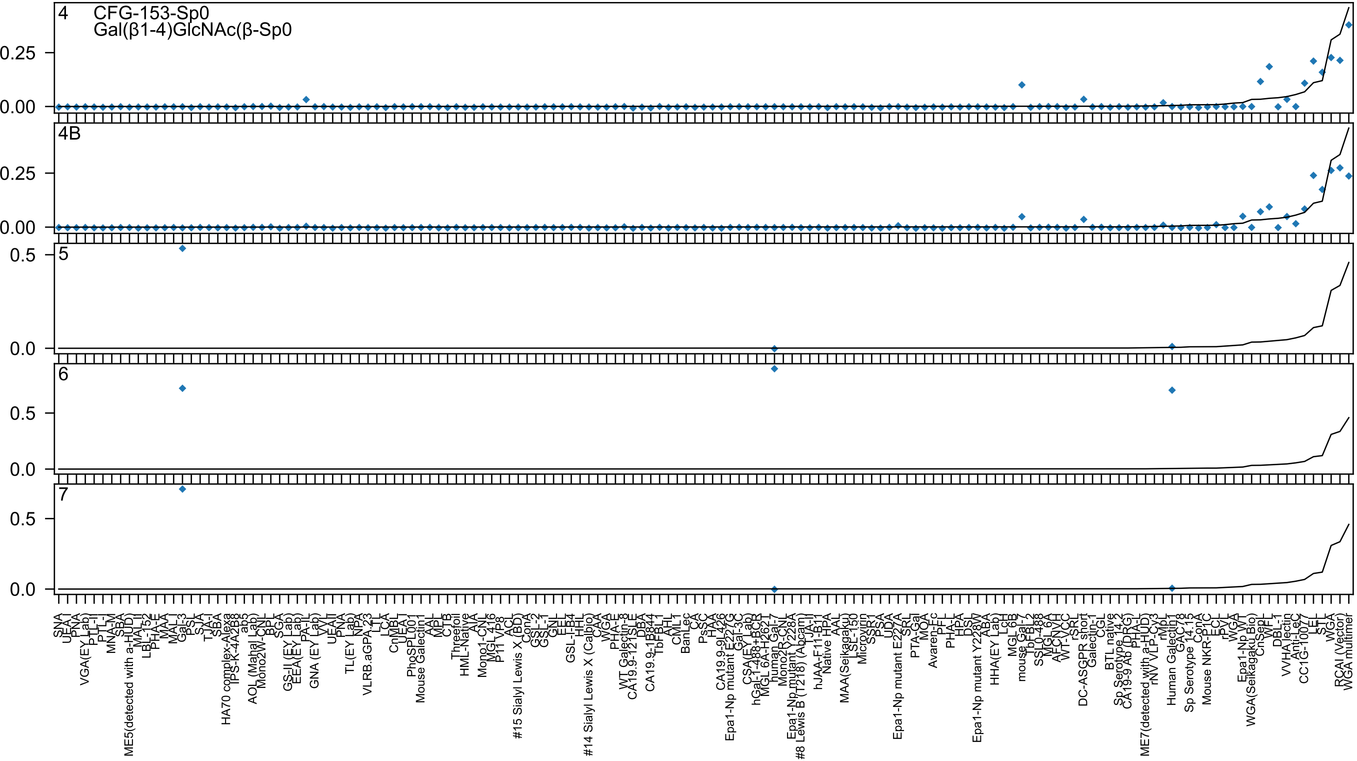
4 CFG-153-Sp0
Gal(β1-4)GlcNAc(β-Sp0
4B
5
6
7

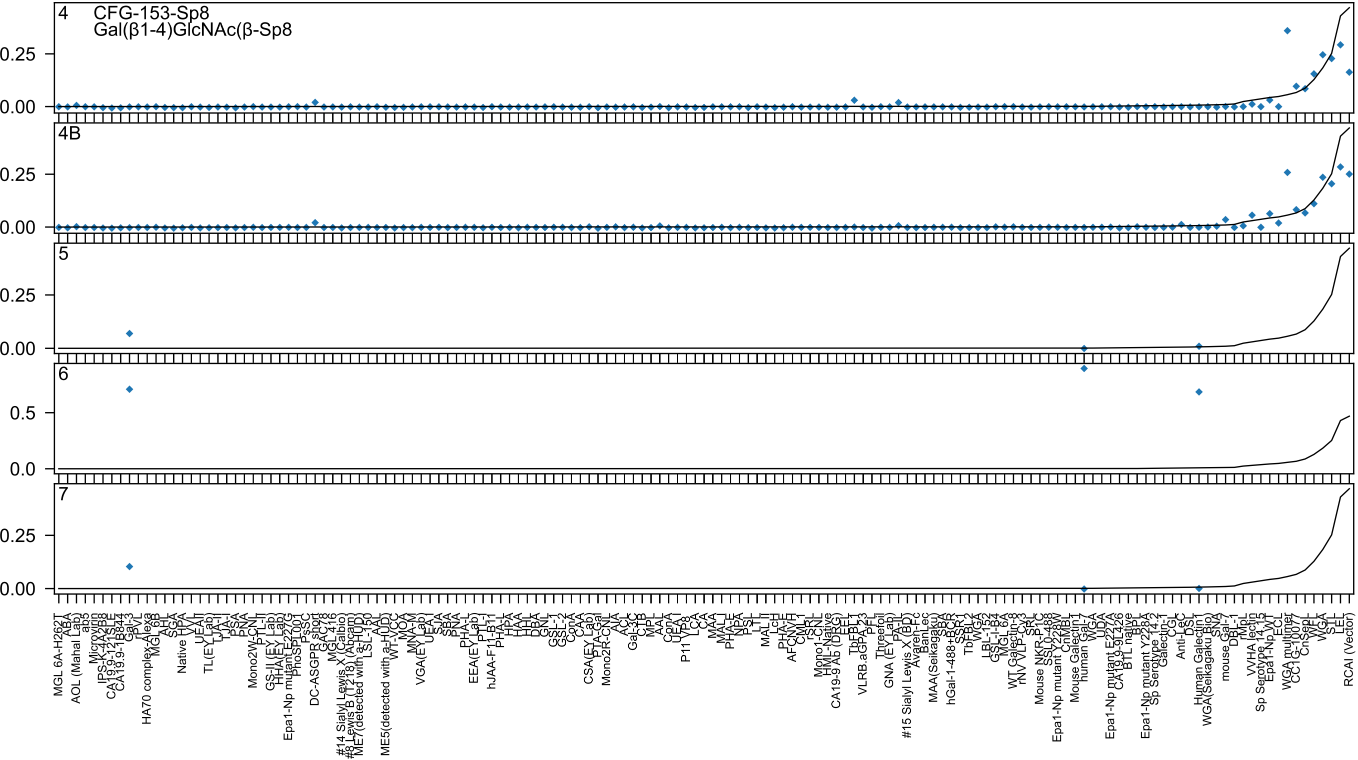
4
CFG-153-Sp8
Gal(β1-4)GlcNAc(β-Sp8
4B
5
6
7

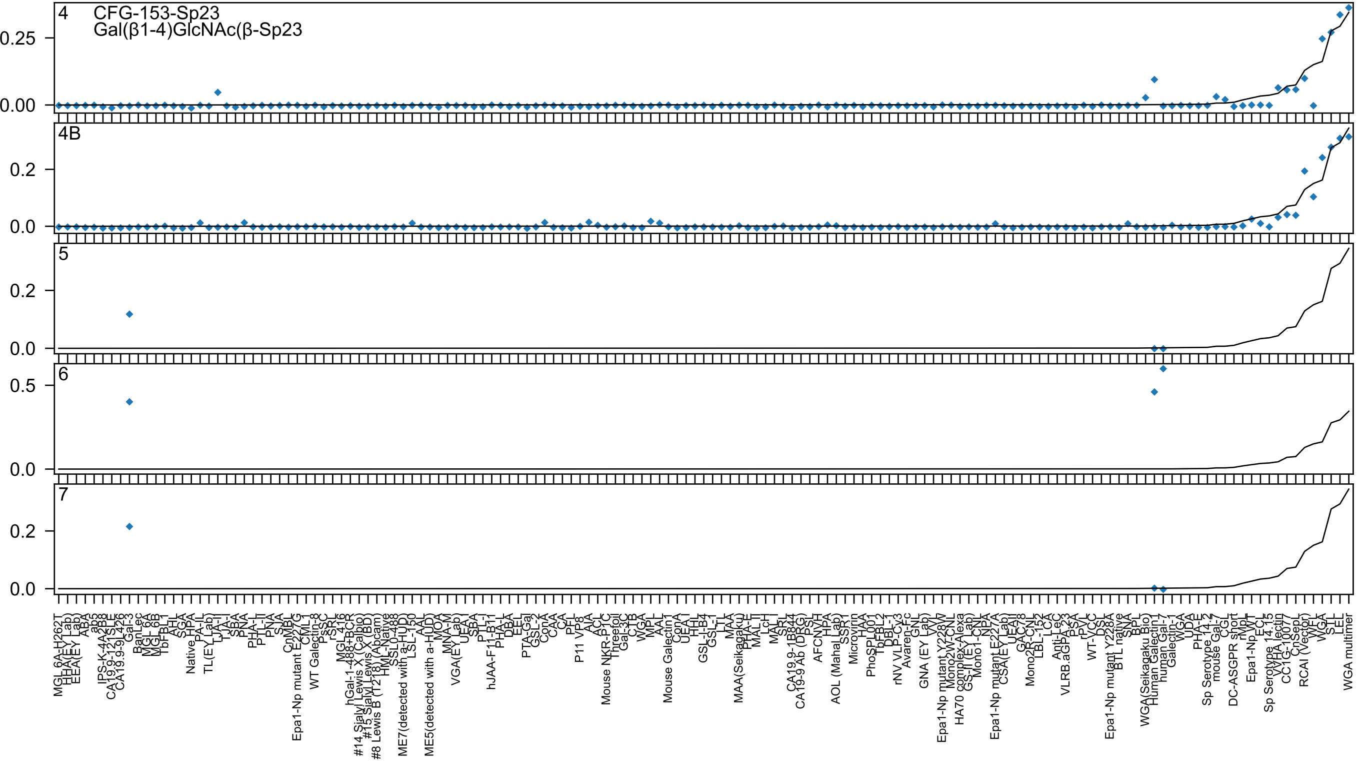

4
CFG-153-Sp23
Gal(β1-4)GlcNAc(β-Sp23
4B
5
6
7

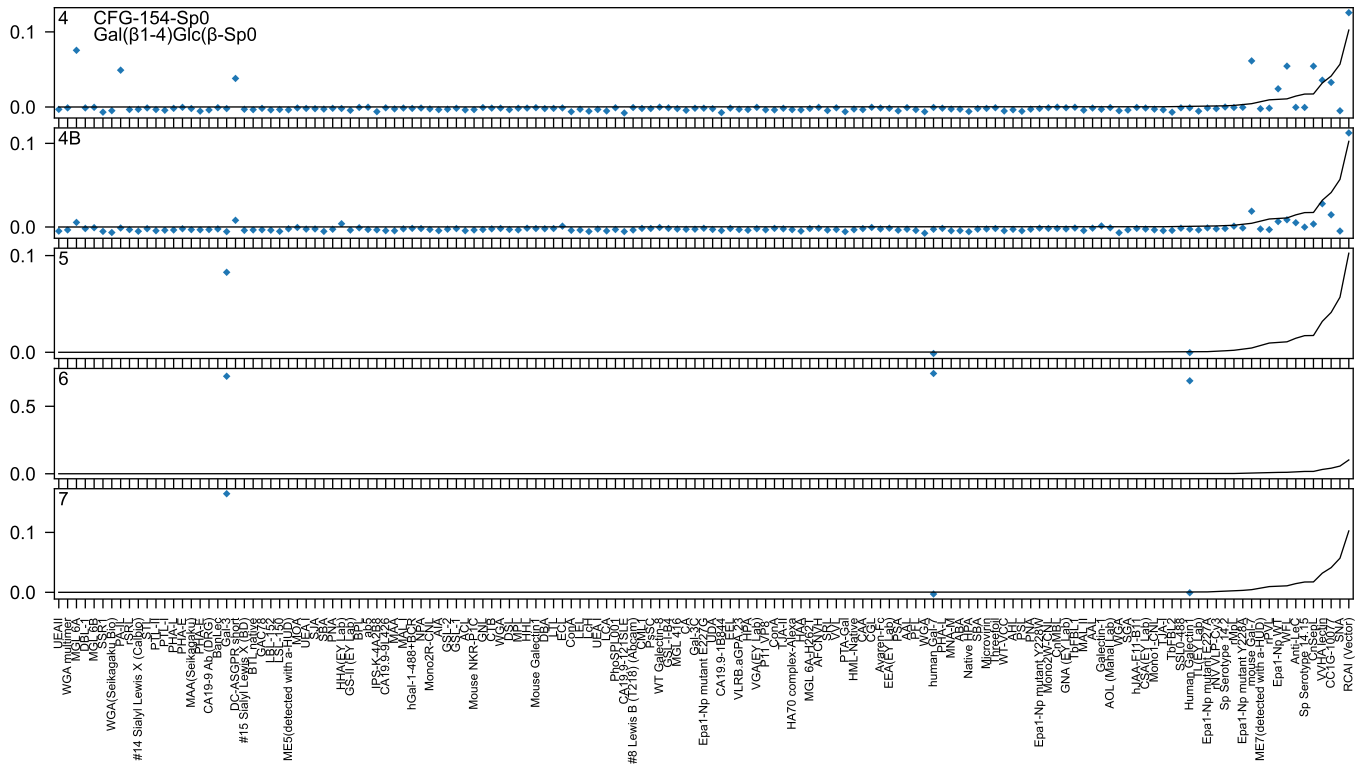
4
CFG-154-Sp0
Gal(β1-4)Glc(β-Sp0
4B
5
6
7
0.1
0.0
0.5

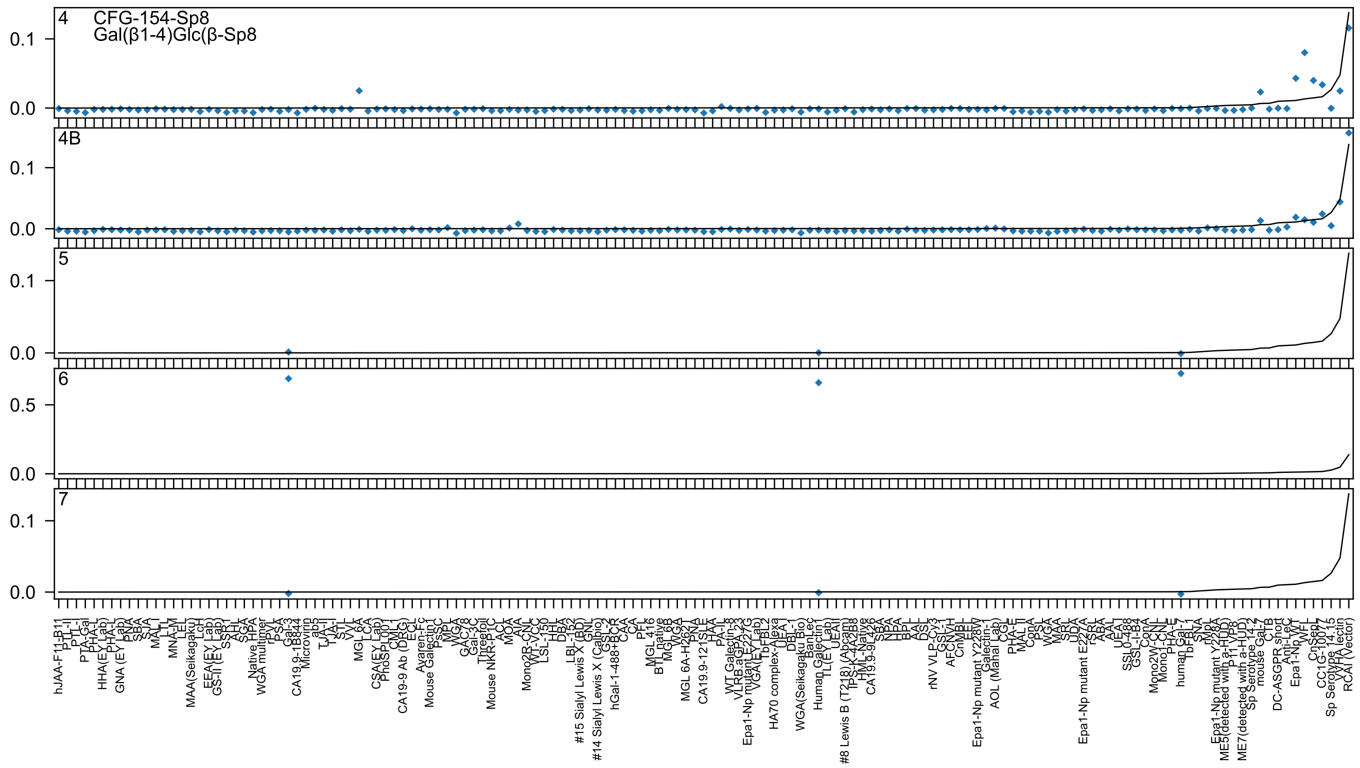

4
CFG-154-Sp8
Gal(β1-4)Glc(β-Sp8
4B
5
6
7
0.1
0.0
0.5

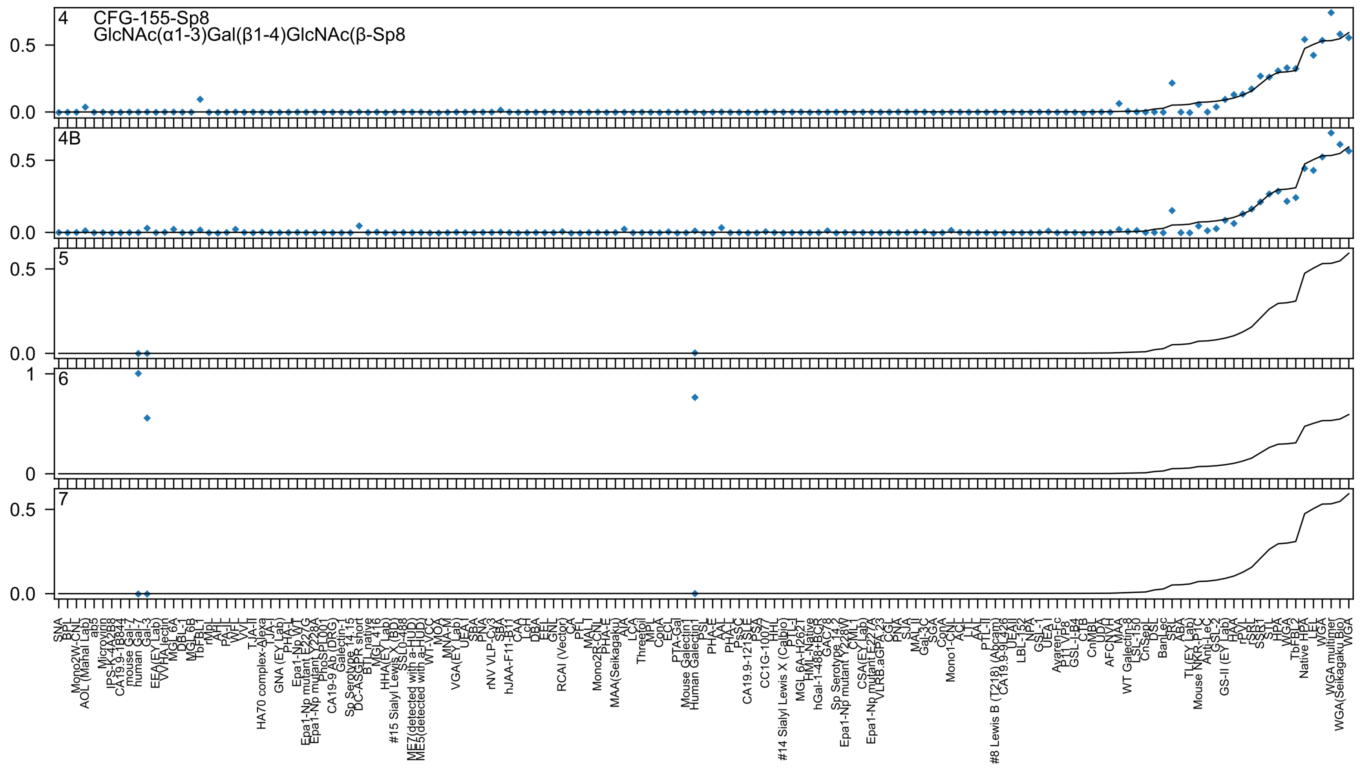

4
CFG-155-Sp8
GlcNAc(α1-3)Gal(β1-4)GlcNAc(β-Sp8
4B
5
6
7

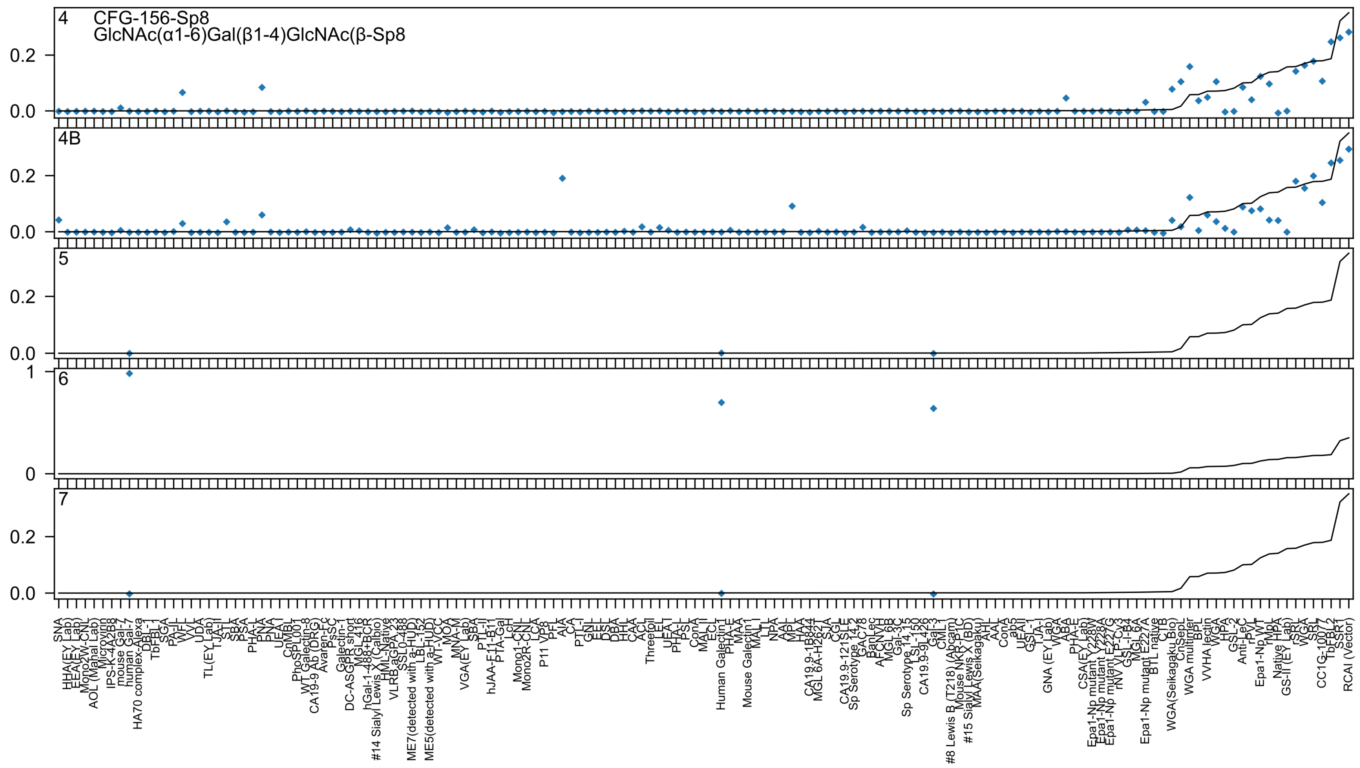

4
CFG-156-Sp8
GlcNAc(α1-6)Gal(β1-4)GlcNAc(β-Sp8
4B
5
6
7
0.0
0.2
0
1

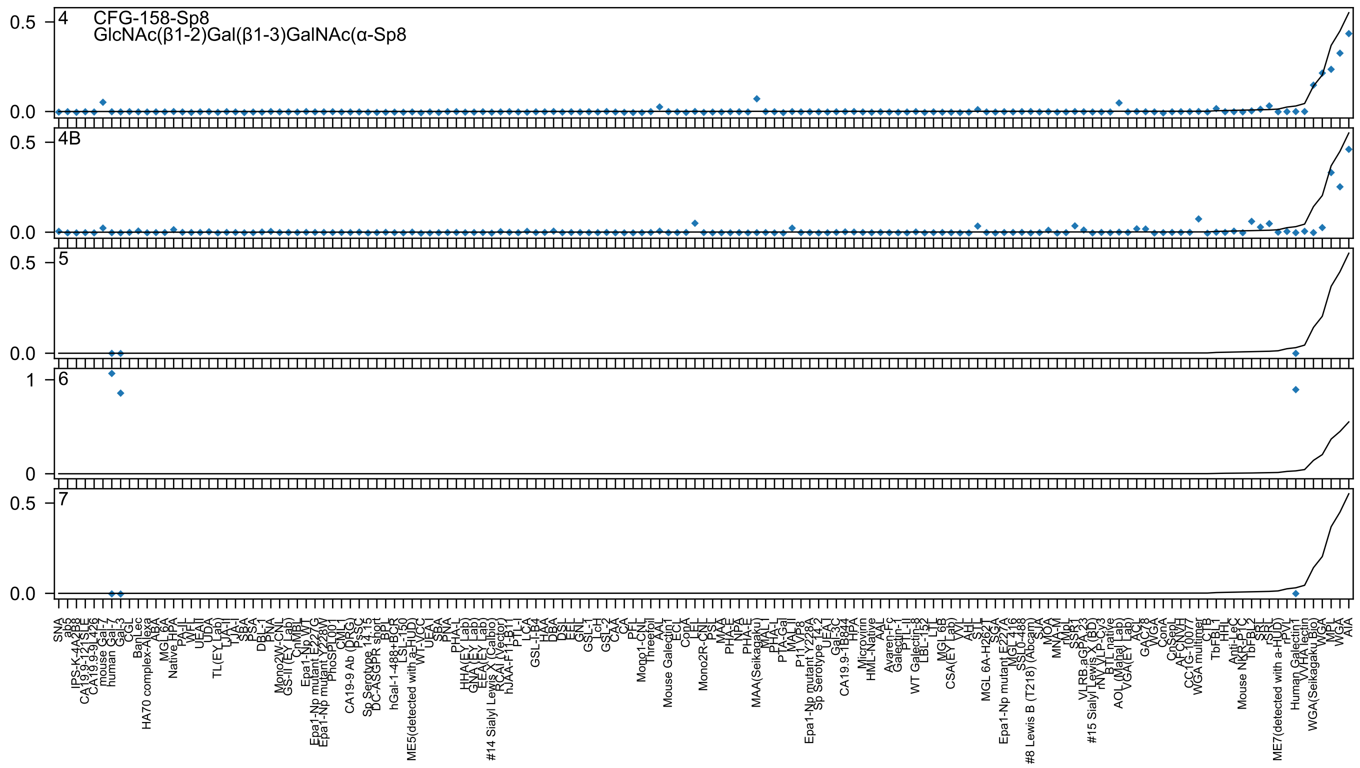
4
CFG-158-Sp8
GlcNAc(β1-2)Gal(β1-3)GalNAc(α-Sp8
4B
5
6
7

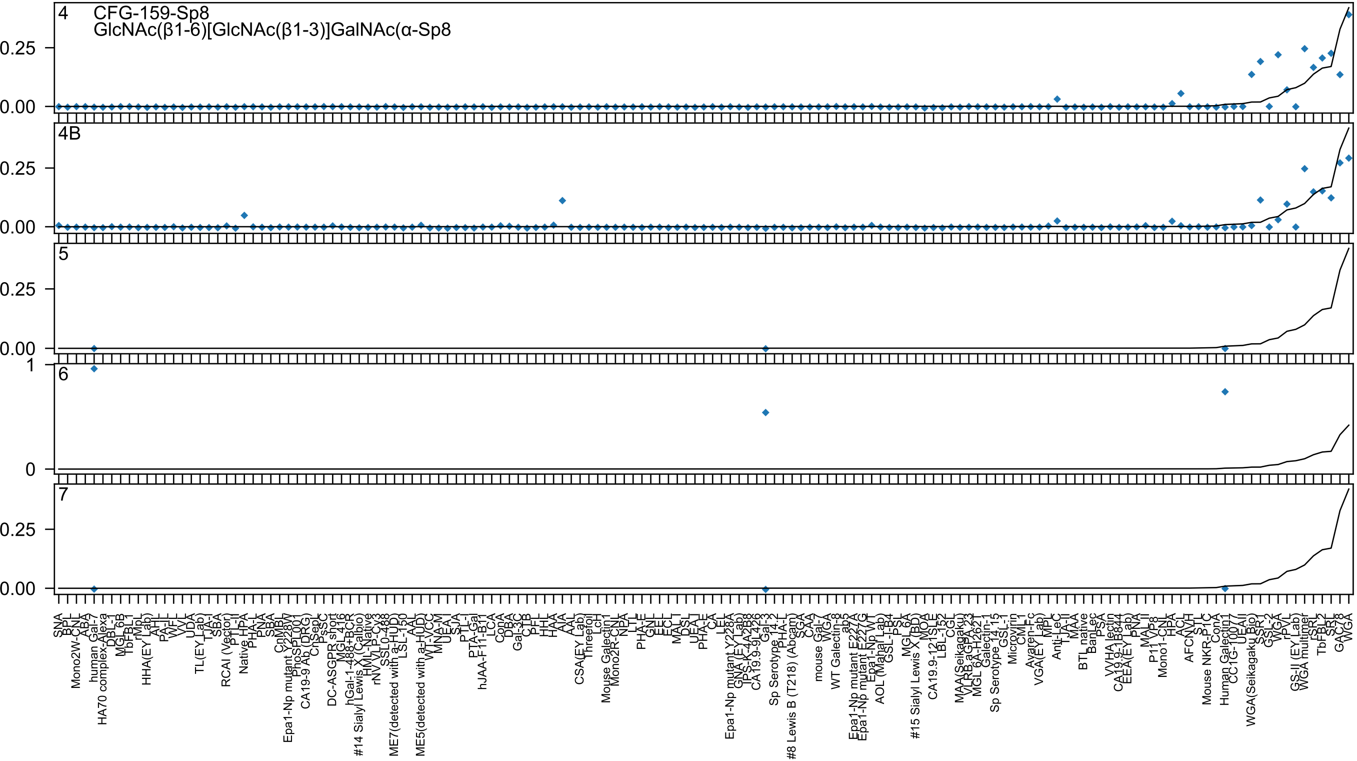
4
CFG-159-Sp8
GlcNAc(β1-6)[GlcNAc(β1-3)]GalNAc(α-Sp8
4B
5
6
7

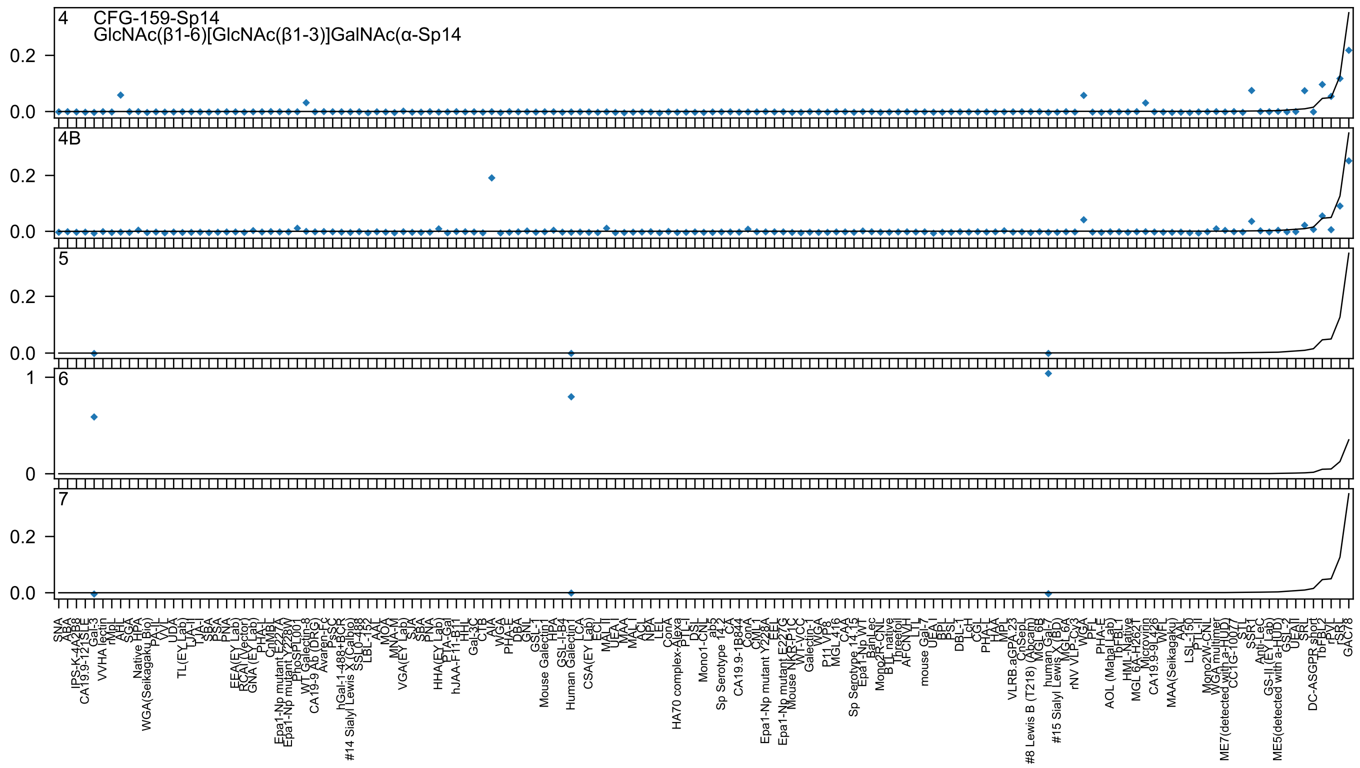

4 CFG-159-Sp14
GlcNAc(β1-6)[GlcNAc(β1-3)]GalNAc(α-Sp14
4B
5
6
7

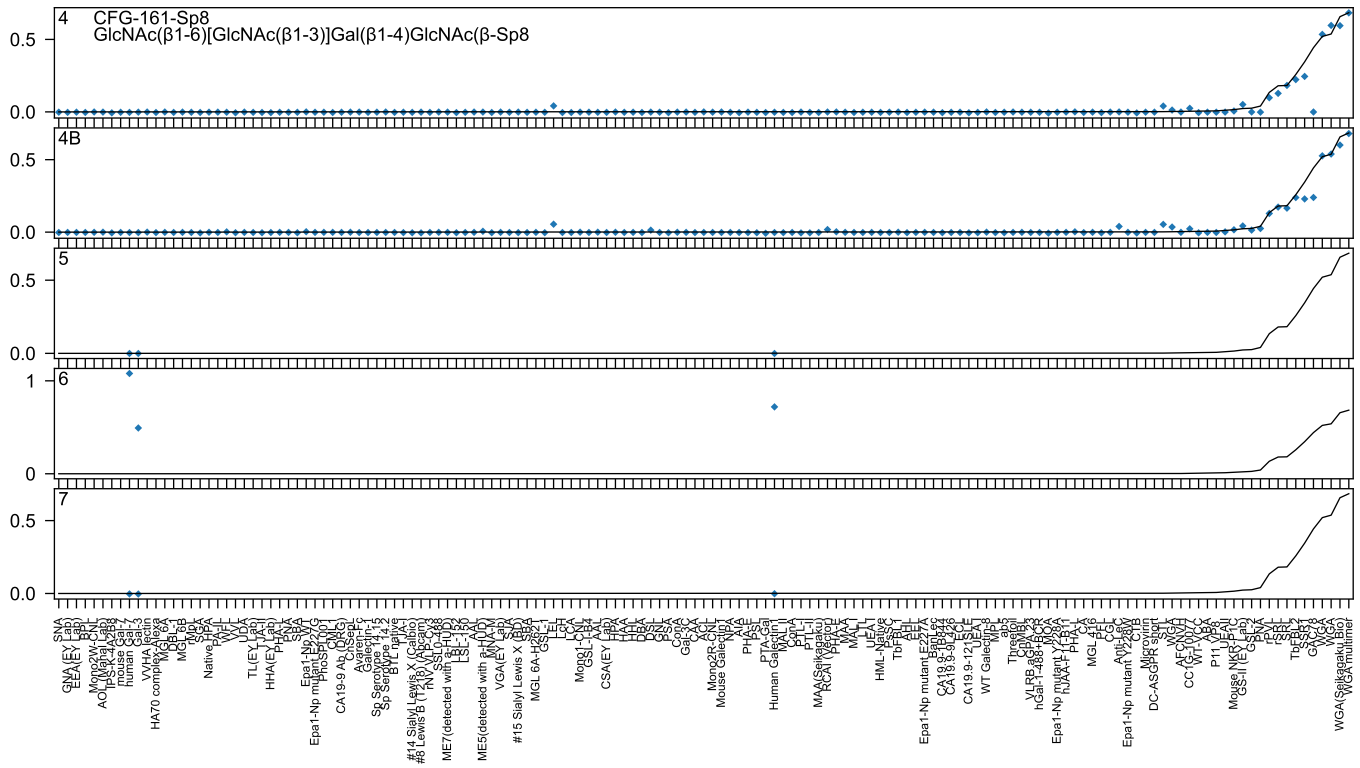

4
CFG-161-Sp8
GlcNAc(β1-6)[GlcNAc(β1-3)]Gal(β1-4)GlcNAc(β-Sp8
4B
5
6
7
0.0
0.5
0
1
SNA
GNA(EY Lab)
EEA(EY Lab)
BPL
Mono2W-CNL
AOL(Mahal Lab)
TPS-K-4A2B8
mouse Gal-7
human Gal-7
Gal-3
VVHA lectin
HA70 complex-Alexa
MGL 6A
DBL-1
MGL 6B
rMpL
SGA
Native HPA
PA-IL
WFL
VVL
UDA
TL(EY Lab)
TJA-II
HHA(EY Lab)
PHA-L
PNA
SBA
Epa1-Np WT
Epa1-Np mutant E227G
PhoSL001
CML1
CA19-9 Ab (DRG)
ChSepL
Avaren-Fc
Galectin-1
Sp Serotype 14.1.5
Sp Serotype 14.4.2
BTL native
TJA-I
#14 Sialyl Lewis X (Calbio)
#8 Lewis B (T218) (Abcam)
rNV
VLP-GX3
SSL0-488
ME7(detected with a-HUD)
LBL-152
LSL-150
AAL
ME5(detected with a-HUD)
MNA-M
VGA(EY Lab)
SJA
#15 Sialyl Lewis X (BD)
SBA
MGL 6A-H262T
GSL-1
LEL
LcH
LCA
Mono1-CNL
GSL-I-B4
AAL
CSA(EY Lab)
HPA
HAA
HHL
DBA
DSL
GNL
PSA
ConA
Gal-3C
CAA
ACL
Mono2R-CNL
Mouse Galectin1
NPA
AIA
PHA-E
PSL
PTA-Gal
Human Galectin1
MAL II
ConA
PTL-I
PTL-II
MAA(Seikagaku)
RCAI (Vector)
PHA-E
MAA
MAL I
LTL
UEA
HML-Native
PsSC
TbFBL1
AHL
EEL
Epa1-Np mutant E227A
BanLec
CA19.9-1B844
CA19.9-9L426
ECL
CA19.9-121SLE
UEA I
WT Galectin-8
MPL
ab5
Threefoil
CnMBL
VLRB.aGPA.23
hGal-1-488+BCR
MOA
Epa1-Np mutant Y228A
hJAA-F11-B11
PHA-L
CA
MGL 416
PFL
CGL
Anti-LeC
Epa1-Np mutant Y228W
CTB
Microvirin
DC-ASGPR short
STL
WGA
AFCNVH
CC1G-10077
WT-VCC
ABA
P11 VP8
UEAII
Mouse NKR-P1C
GS-II (EY Lab)
GSL-2
PNA
rPVL
rSRL
SRL
TbFBL2
SSR1
GAC78
WGA
WGA(Seikagaku Bio)
WGA multimer

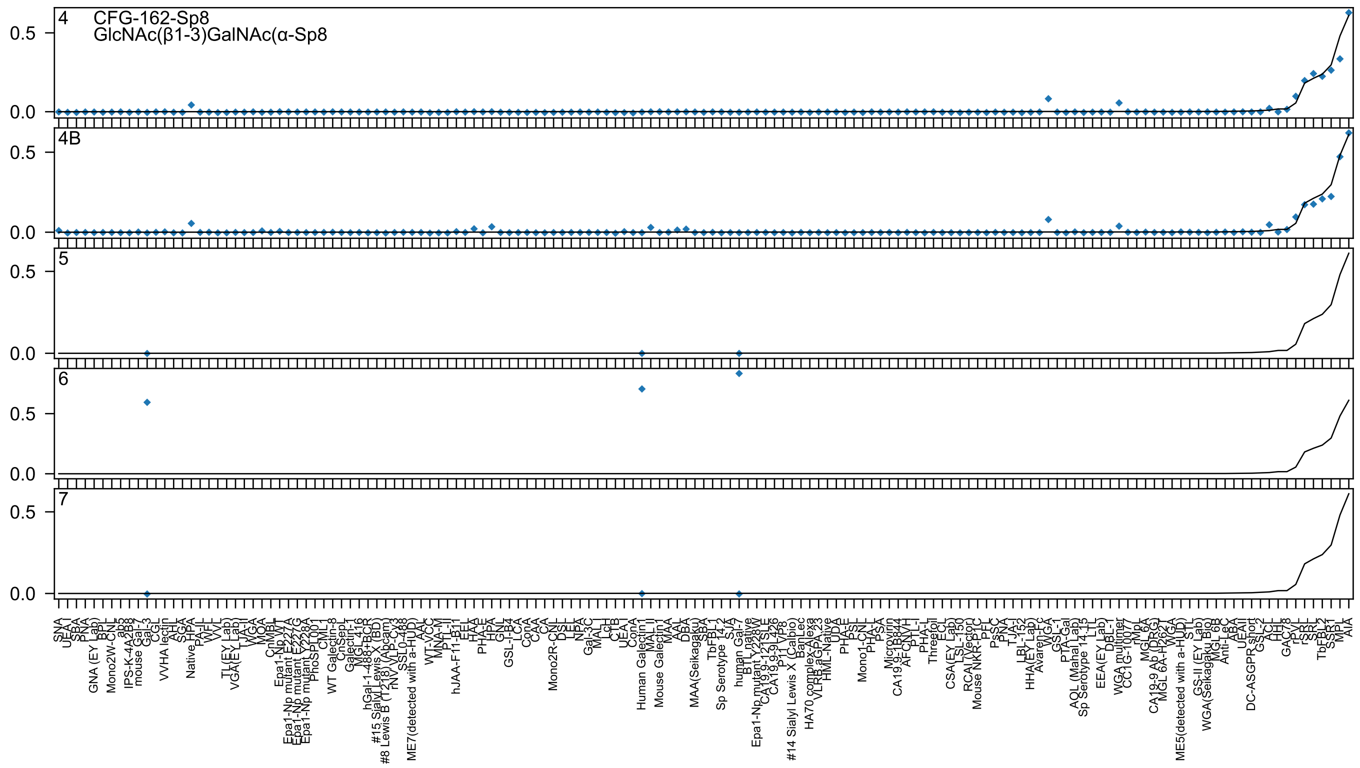

4
CFG-162-Sp8
GlcNAc(β1-3)GalNAc(α-Sp8
4B
5
6
7
0.5
0.0

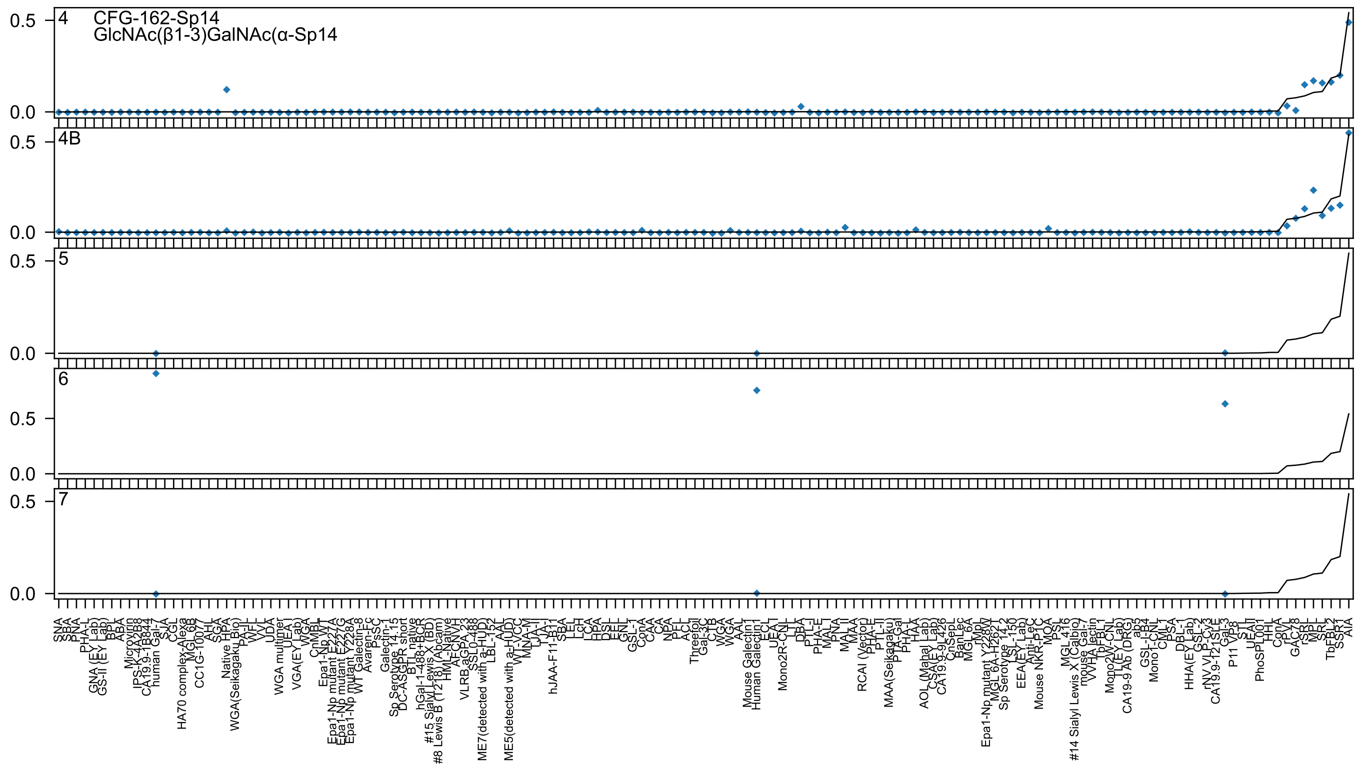

4
CFG-162-Sp14
GlcNAc(β1-3)GalNAc(α-Sp14
4B
5
6
7

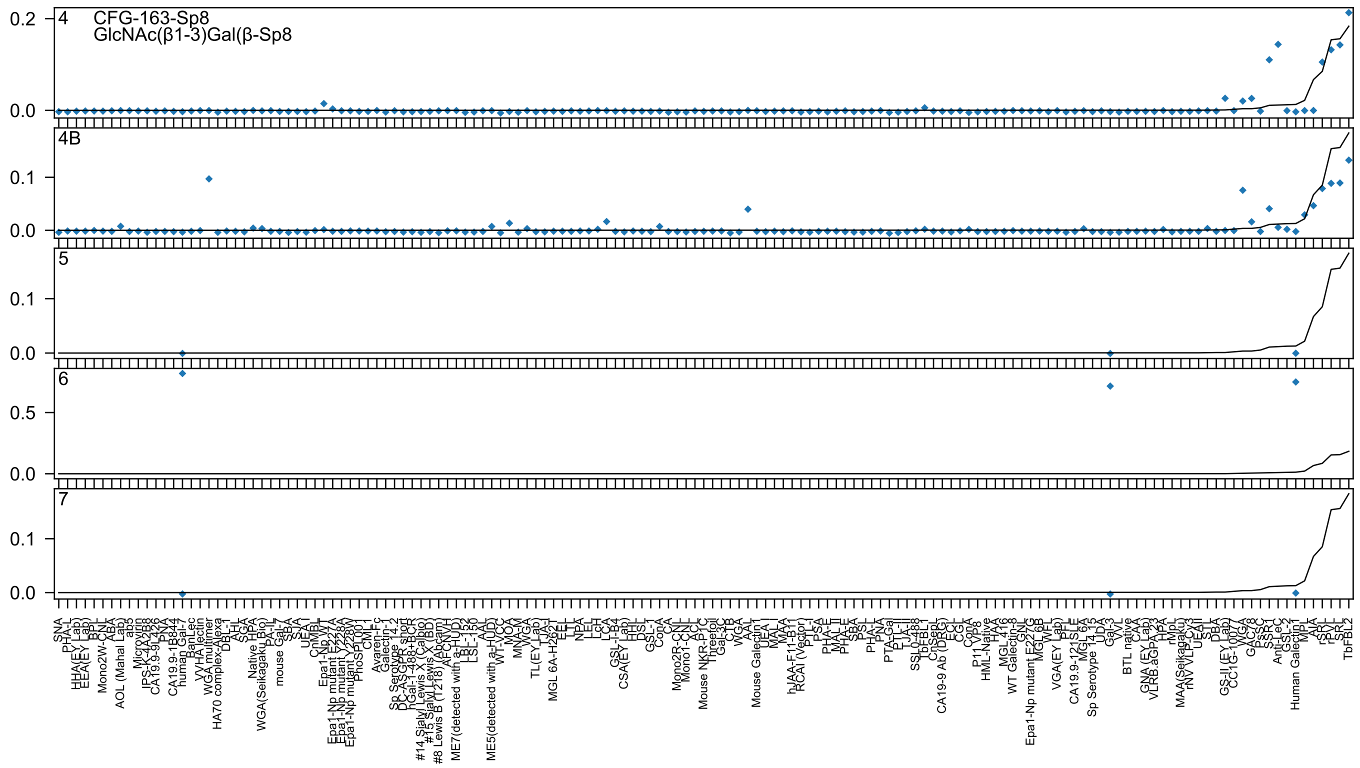

4
CFG-163-Sp8
GlcNAc(β1-3)Gal(β-Sp8
4B
5
6
7

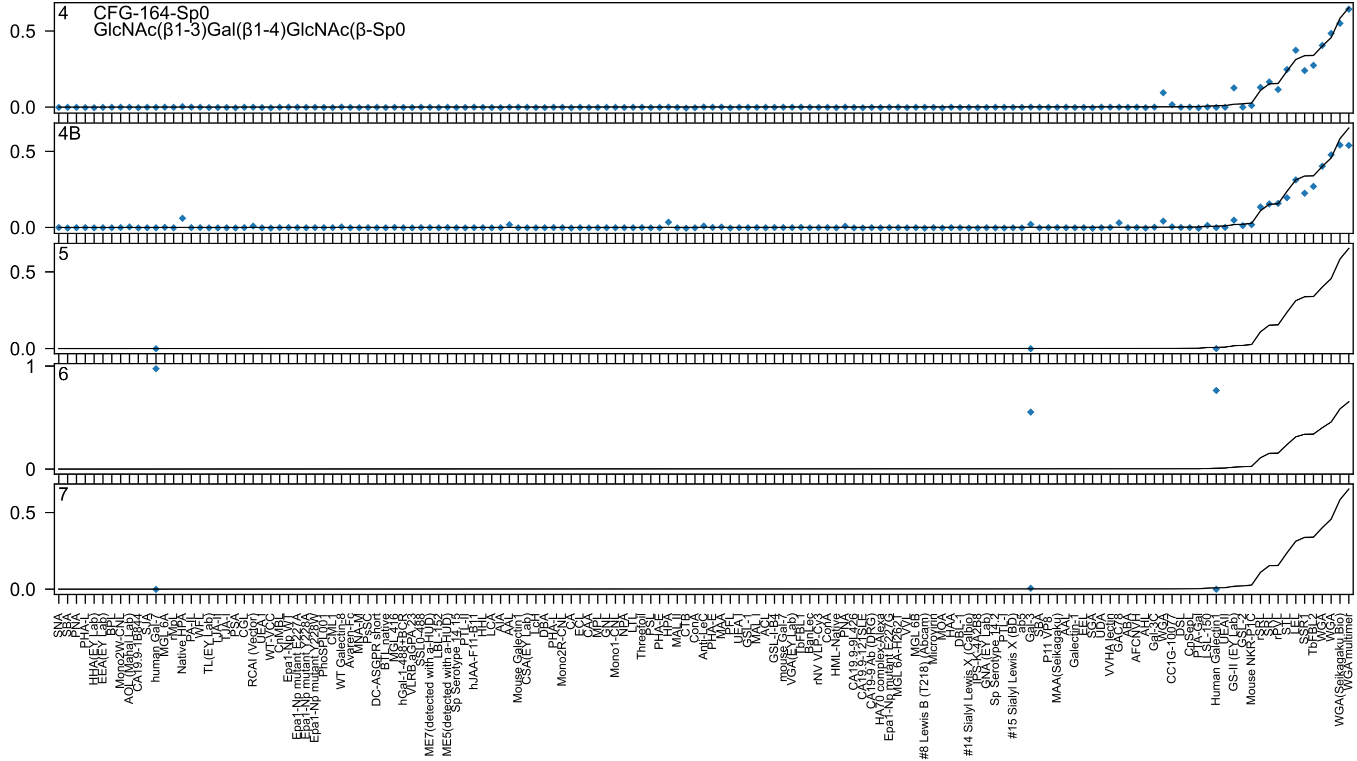

4 CFG-164-Sp0
GlcNAc(β1-3)Gal(β1-4)GlcNAc(β-Sp0
4B
5
6
7
SNA
SBA
PNA
PHA-L
HHA(EY Lab)
EEA(EY Lab)
BPL
Mono2W-CNL
AOL (Mahal Lab)
CA19.9-1B844
SJA
human Gal-7
MGL 6A
rMpL
Native HPA
PA-IL
WFL
TL(EY Lab)
TJA-II
TJA-I
PSA
CGL
RCAI (Vector)
UEA-I
WT-VCC
CnMBL
Epa1-Np WT
Epa1-Np mutant E227A
Epa1-Np mutant Y228A
Epa1-Np mutant Y228W
PhoSL001
CML1
WT Galectin-8
Avaren-Fc
MNA-M
PsSC
DC-ASGPR short
BTL native
MGL 416
hGal-1-488+BCR
VLRB.aGPA.23
SSL0-488
ME7(detected with a-HUD)
LBL-152
ME5(detected with a-HUD)
Sp Serotype 14.15
PTL-II
hJAA-F11-B1
HHL
LCA
AIA
AAL
Mouse Galectin1
CSA(EY Lab)
LcH
DBA
PHA-L
Mono2R-CNL
CA
ECL
CAA
MPL
GNL
Mono1-CNL
NPA
LTL
Threefoil
PSL
PHA-E
HPA
MAL II
CTB
ConA
Anti-LeC
PHA-E
MAA
PFL
UEA-I
GSL-1
MAL-I
ACL
GSL-I-B4
mouse Gal-7
VGA(EY Lab)
TbFBL1
BanLec
rNV VLP-Cy3
ConA
HML-Native
PNA
CA19.9-9L426
CA19.9-121SLE
HA70 complex-Alexa
CA19-9 Ab (DRG)
Epa1-Np mutant E227G
MGL 6A-H262T
VVL
MGL 6B
#8 Lewis B (T218) (Abcam)
Microvirin
MOA
HAA
DBL-1
#14 Sialyl Lewis X (Calbio
IPS-K-4A2B8
GNA (EY Lab)
Sp Serotype 14.2
PTL-I
#15 Sialyl Lewis X (BD)
ab3
Gal-3
SBA
P11 VP8
MAA(Seikagaku)
AAL
Galectin-1
EEL
SGA
UDA
VVHA lectin
GAC78
ABA
AFCNVH
AHL
Gal-3C
WGA
CC1G-10077
DSL
CnSepL
PTA-Gal
LSL-150
Human Galectin1
UEAII
GS-II (EY Lab)
GSL-2
Mouse NKR-P1C
rSRL
SRL
rPVL
STL
LEL
SSR1
TbFBL2
WGA
VGA
WGA(Seikagaku Bio)
WGA multimer

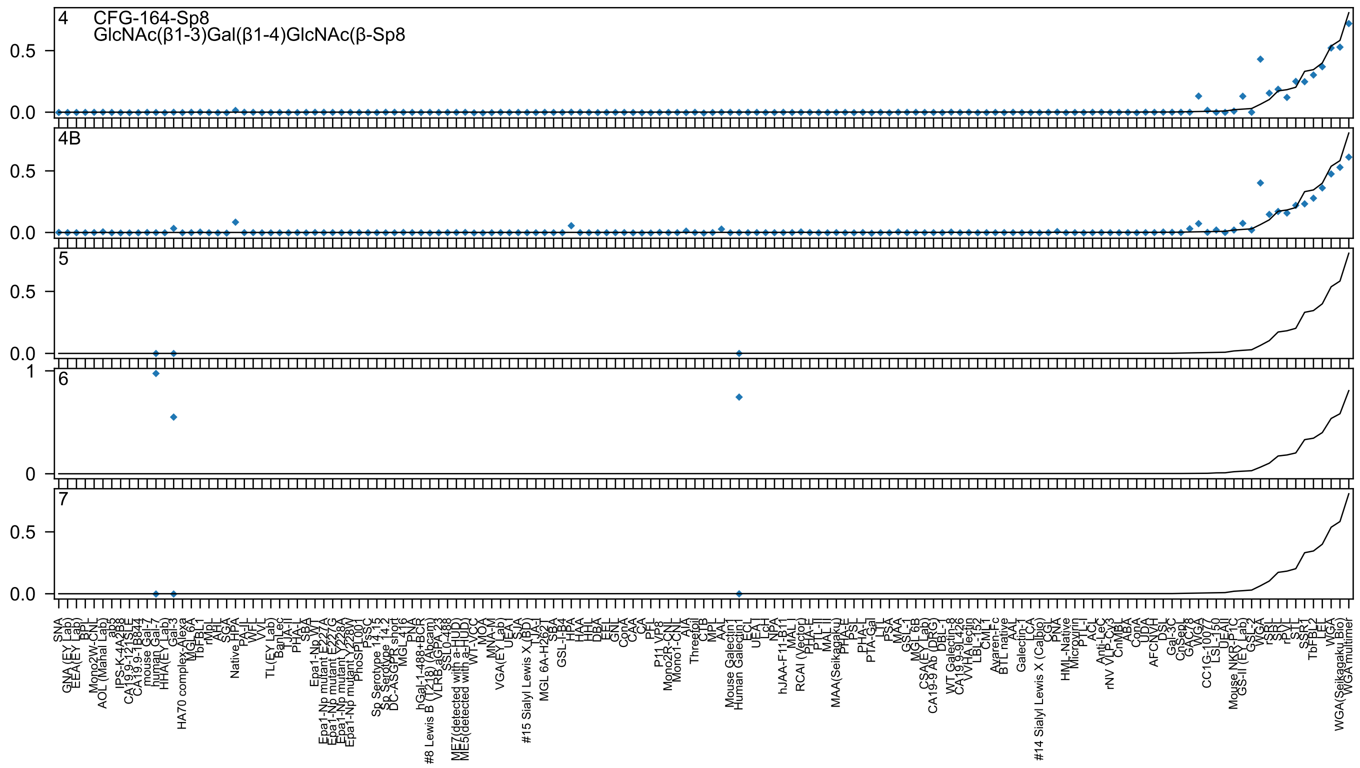

4
CFG-164-Sp8
GlcNAc(β1-3)Gal(β1-4)GlcNAc(β-Sp8
4B
5
6
7
0.0
0.5
0
1

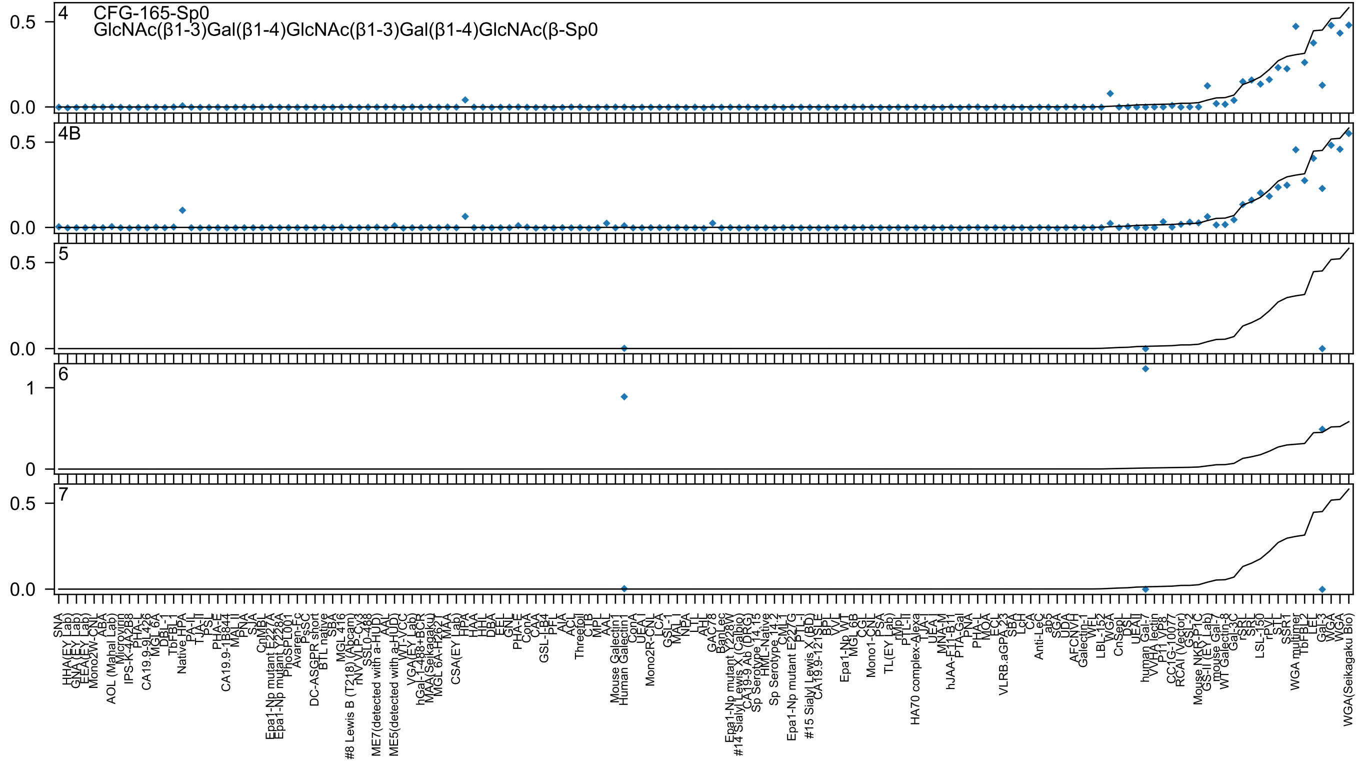

CFG-165-Sp0
GlcNAc(β1-3)Gal(β1-4)GlcNAc(β1-3)Gal(β1-4)GlcNAc(β-Sp0
4
4B
5
6
7

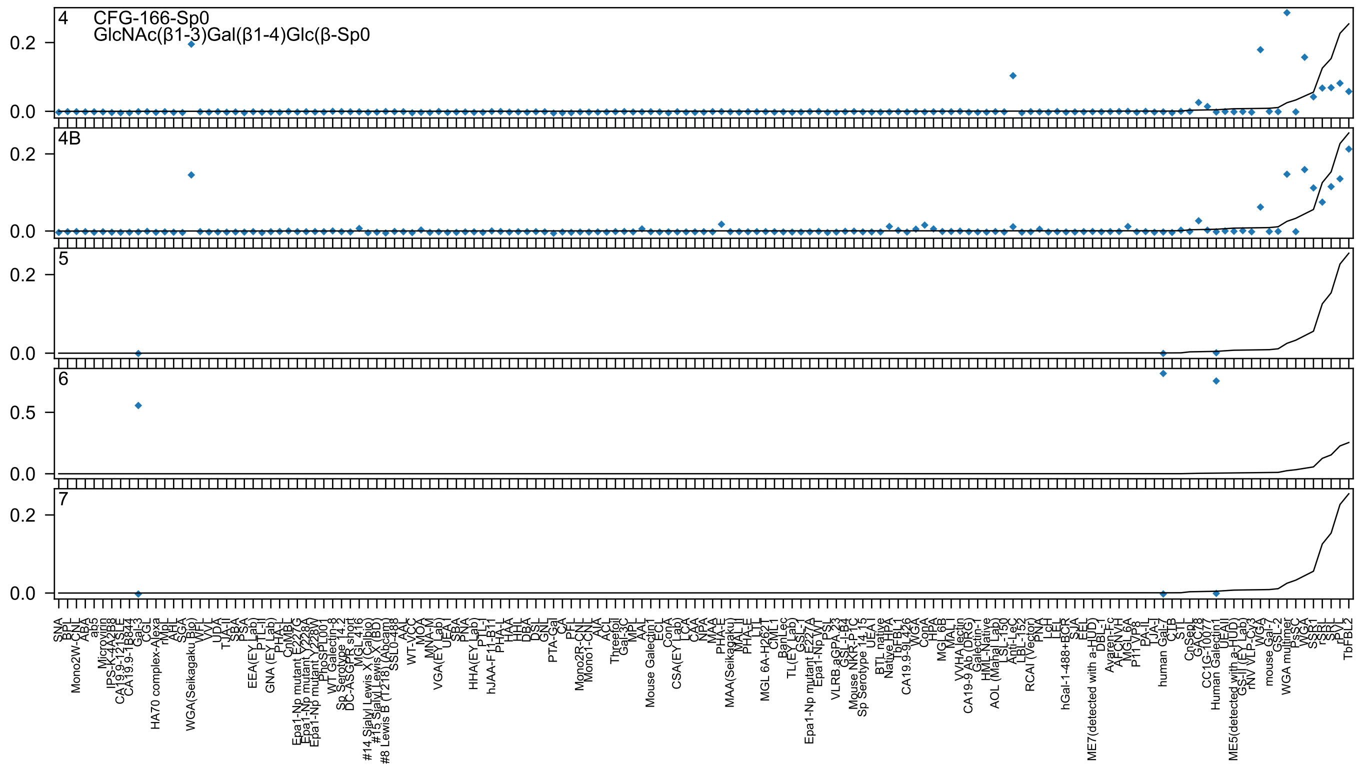
4
CFG-166-Sp0
GlcNAc(β1-3)Gal(β1-4)Glc(β-Sp0
4B
5
6
7
0.0
0.2
0.5

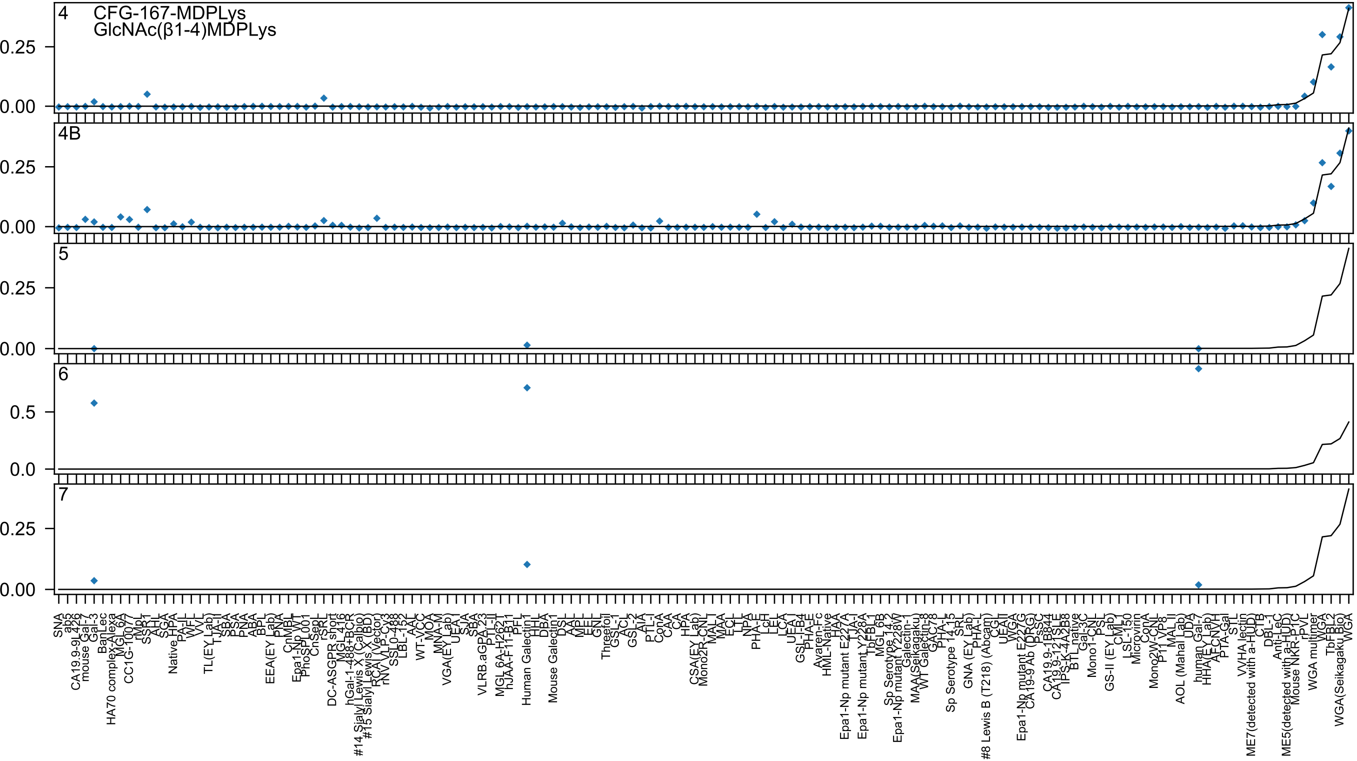

4
CFG-167-MDPLys
GlcNAc(β1-4)MDPLys
4B
5
6
7

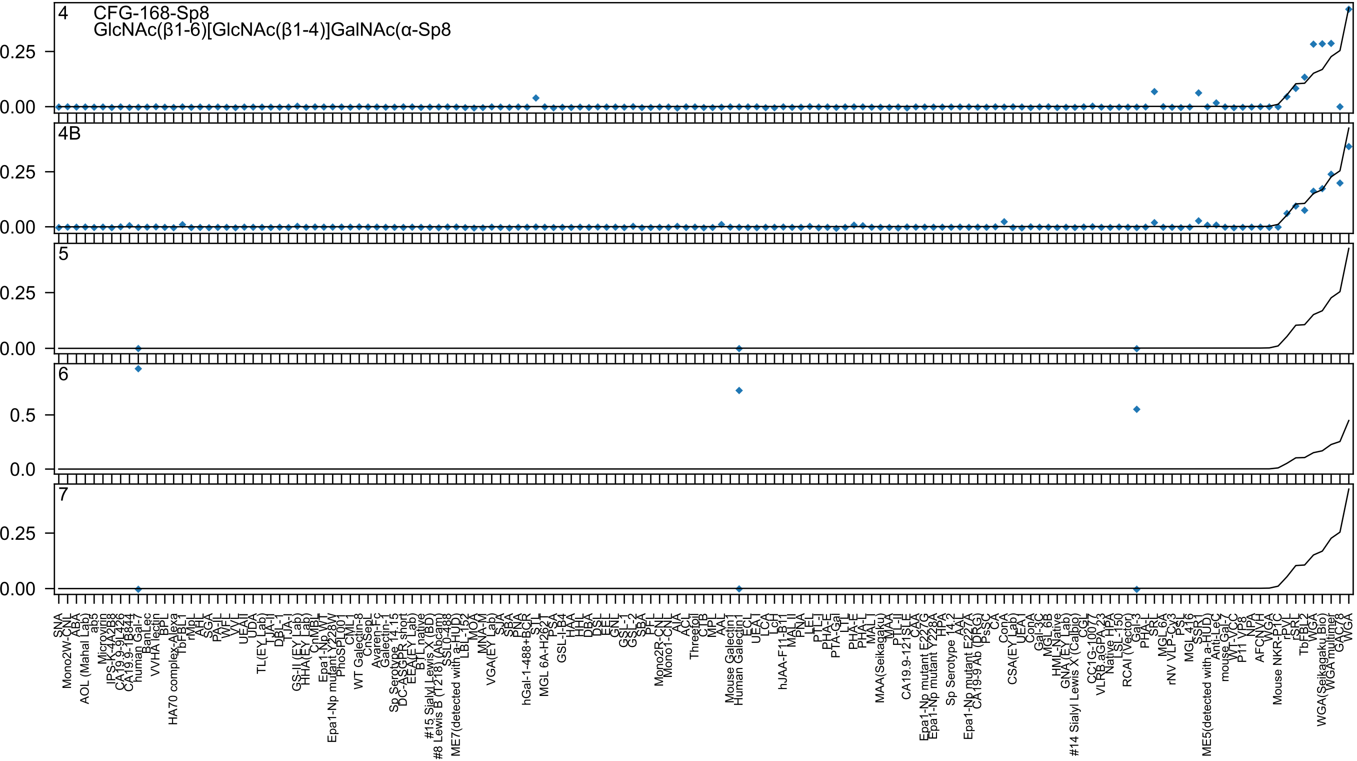
4
CFG-168-Sp8
GlcNAc(β1-6)[GlcNAc(β1-4)]GalNAc(α-Sp8
4B
5
6
7

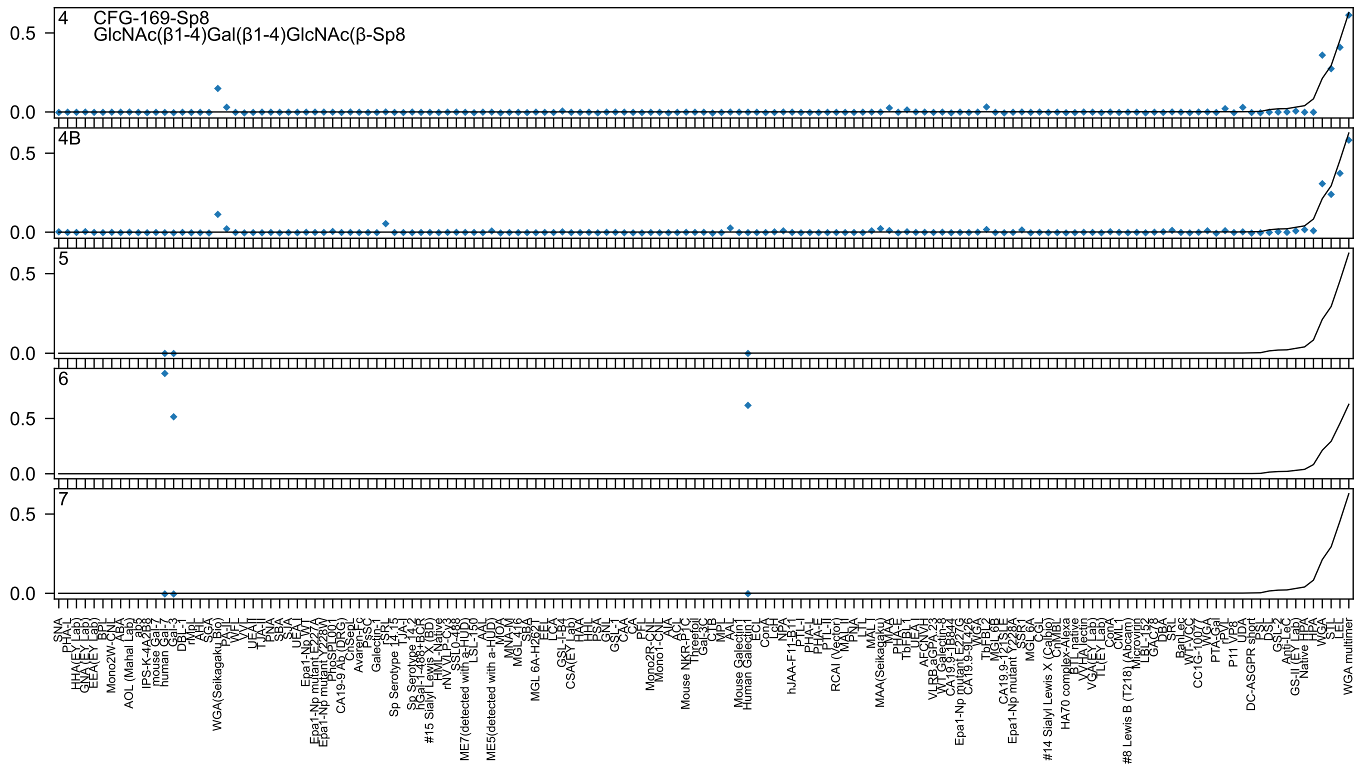
4 CFG-169-Sp8
GlcNAc(β1-4)Gal(β1-4)GlcNAc(β-Sp8
4B
5
6
7

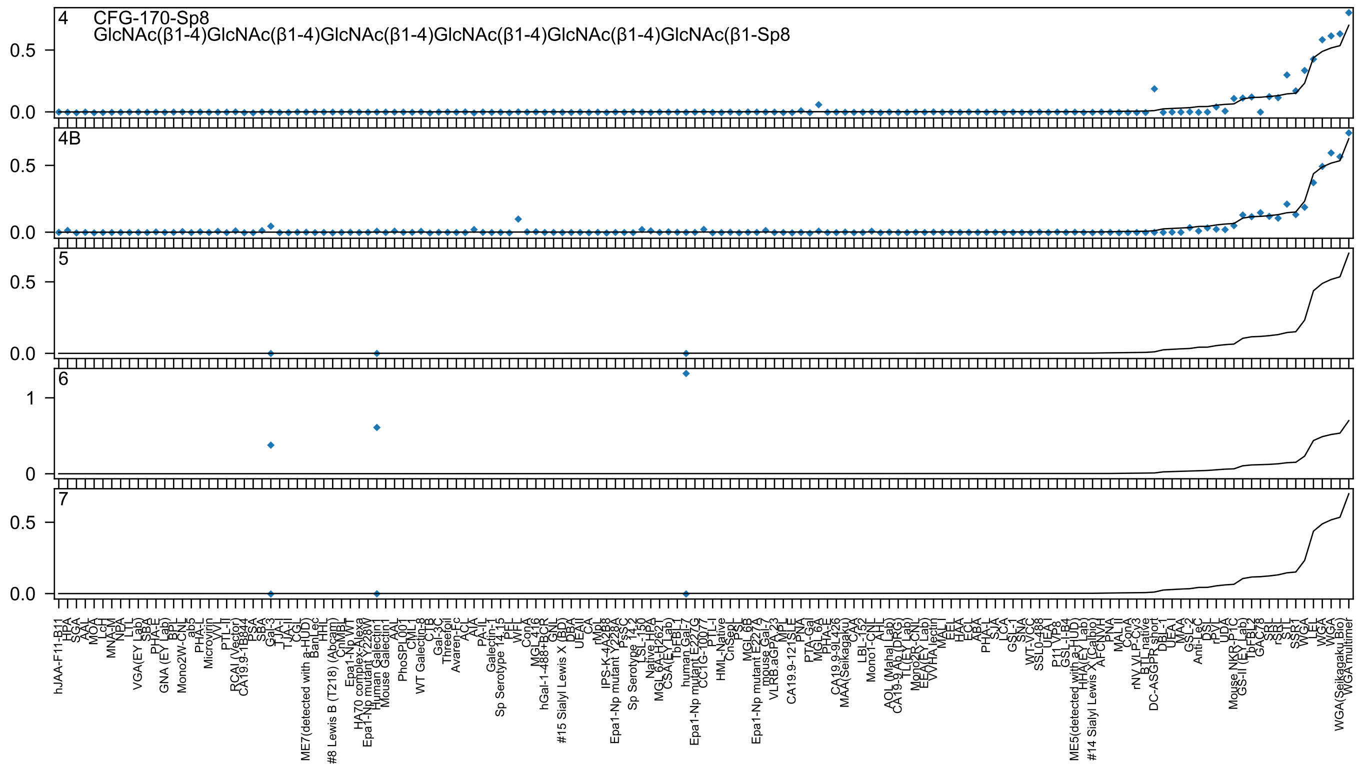

4
CFG-170-Sp8
GlcNAc(β1-4)GlcNAc(β1-4)GlcNAc(β1-4)GlcNAc(β1-4)GlcNAc(β1-4)GlcNAc(β1-Sp8
4B
5
6
7

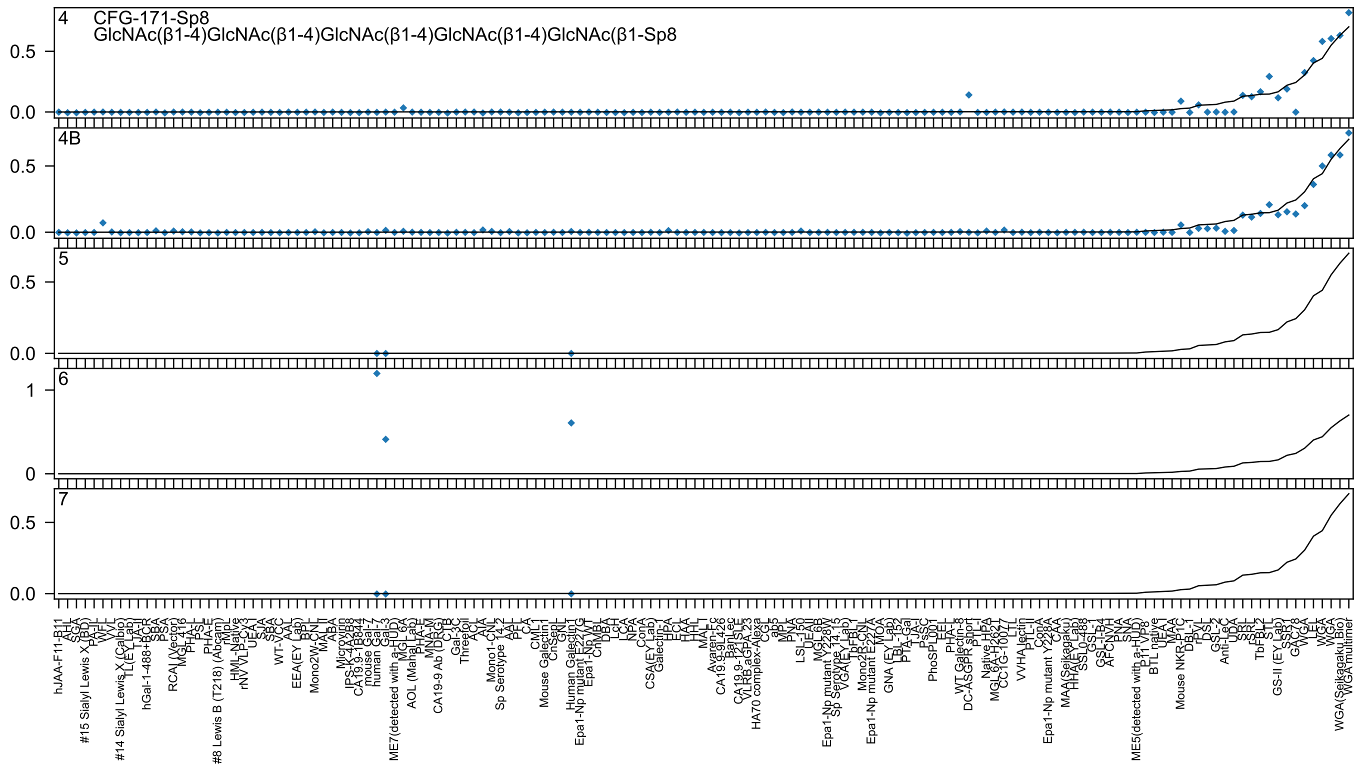
4 CFG-171-Sp8
GlcNAc(β1-4)GlcNAc(β1-4)GlcNAc(β1-4)GlcNAc(β1-4)GlcNAc(β1-Sp8
4B
5
6
7

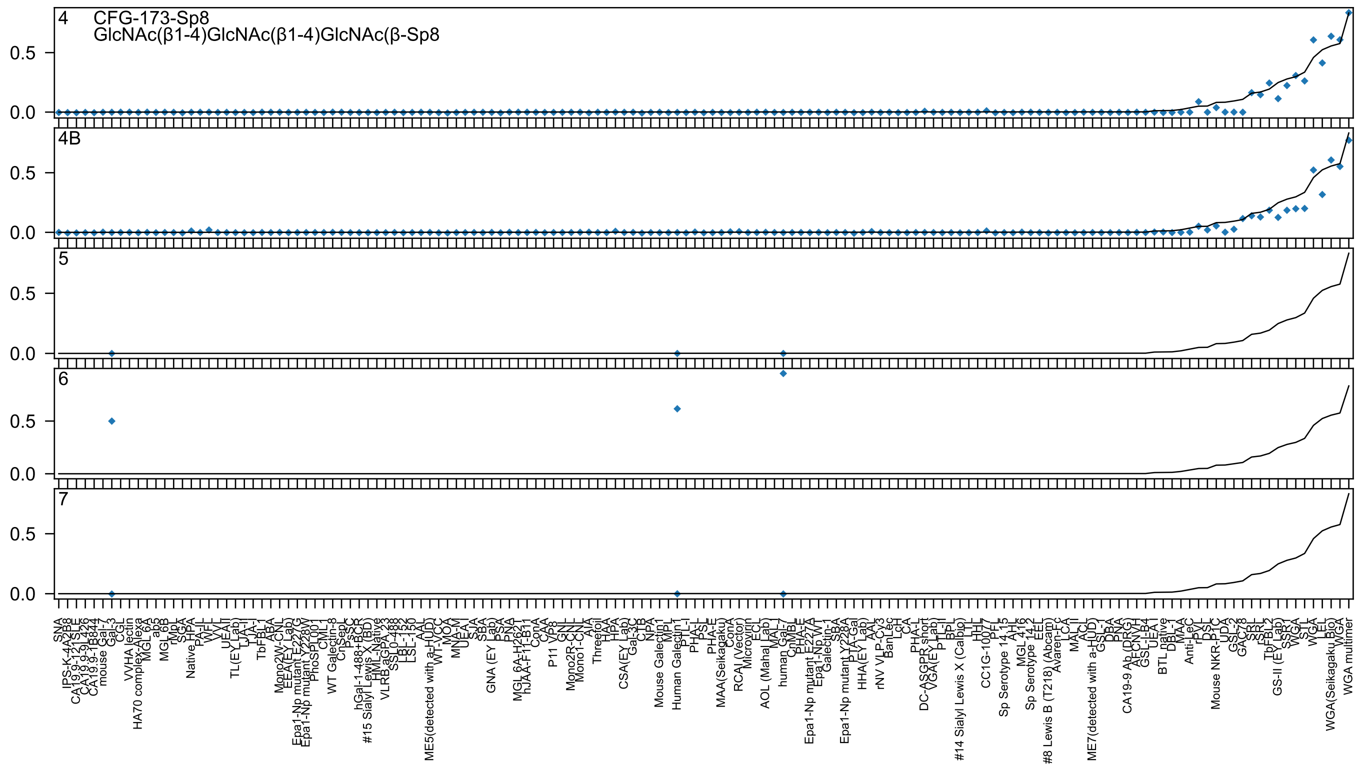

4
CFG-173-Sp8
GlcNAc(β1-4)GlcNAc(β1-4)GlcNAc(β-Sp8
4B
5
6
7
0.5
0.0
SNA
IPS-K-4A2B8
CA19.9-121SLE
CA19.9-9L426
CA19.9-1B844
mouse Gal-7
Gal-3
CGL
VVHA lectin
HA70 complex-Alexa
MGL 6A
ab5
MGL 6B
rMpL
SGA
Native HPA
PA-IL
WFL
VVL
UEAII
TL(EY Lab)
LJA-I
TbFBL1
ABA
Mono2W-CNL
EEA(EY Lab)
Epa1-Np mutant E227G
Epa1-Np mutant Y228W
PhoSL001
CML1
WT Galectin-8
CnSepL
PsSC
hGal-1-488+BCR
#15 Sialyl Lewis X (BD)
HML-Native
VLRB.aGPA.23
SSL0-488
LBL-152
LSL-150
AAL
ME5(detected with a-HUD)
WT-VCC
MOA
MNA-M
UEA-I
SJA
SBA
GNA (EY Lab)
PSA
PNA
MGL 6A-H262T
hJAA-F11-B11
ConA
CAA
P11 VP8
GNL
Mono2R-CNL
Mono1-CNL
AIA
Threefoil
HAA
HPA
CSA(EY Lab)
Gal-3C
CTB
NPA
Mouse Galectin1
MPL
Human Galectin1
PTL-I
PHA-L
PSL
PHA-E
MAA(Seikagaku)
ConA
RCAI (Vector)
Microvirin
ECL
AOL (Mahal Lab)
MAL-I
human Gal-7
CnMBL
PHA-E
Epa1-Np mutant E227A
Epa1-Np WT
Galectin-1
SBA
Epa1-Np mutant Y228A
PTA-Gal
HHA(EY Lab)
AAL
rNV VLP-Cy3
BanLec
LcH
CA
PHA-L
DC-ASGPR short
VGA(EY Lab)
PTL-II
BPL
#14 Sialyl Lewis X (Calbio)
LTL
HHL
CC1G-10077
PFL
Sp Serotype 14.15
AH-L
MGL 416
Sp Serotype 14.2
EEL
#8 Lewis B (T218) (Abcam)
Avaren-Fc
LCA
MAL II
ACL
ME7(detected with a-HUD)
GSL-I
DBA
PNA
CA19-9 Ab (DRG)
AFCNVH
GSL-I-B4
UEA I
BTL native
DBL-1
MAA
Anti-LeC
rPVL
DSL
Mouse NKR-P1C
UDA
GSL-2
GAC78
SRL
rSRL
TbFBL2
GS-II (EY Lab)
SSR-1
WGA
STL
LEL
WGA(Seikagaku Bio)
WGA
WGA multimer

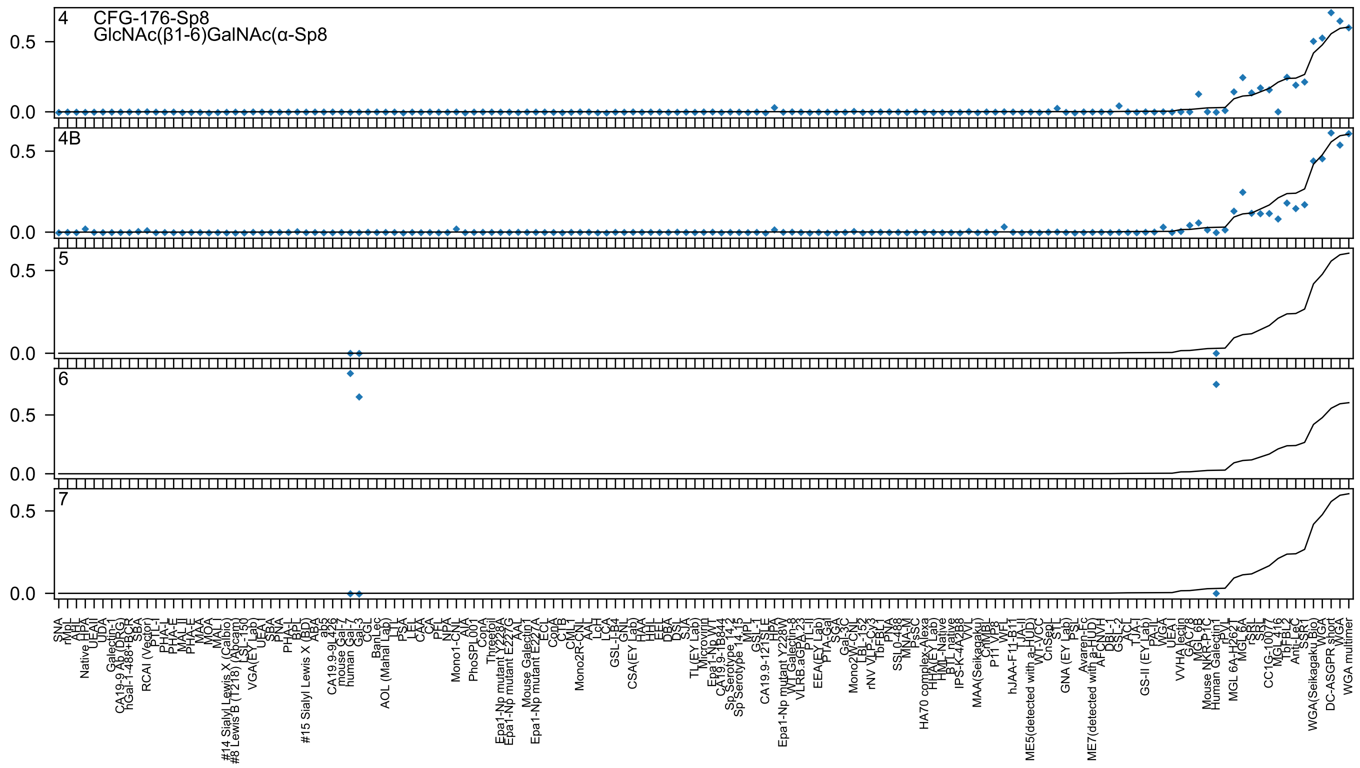
4 CFG-176-Sp8
GlcNAc(β1-6)GalNAc(α-Sp8
4B
5
6
7
0.5
0.0

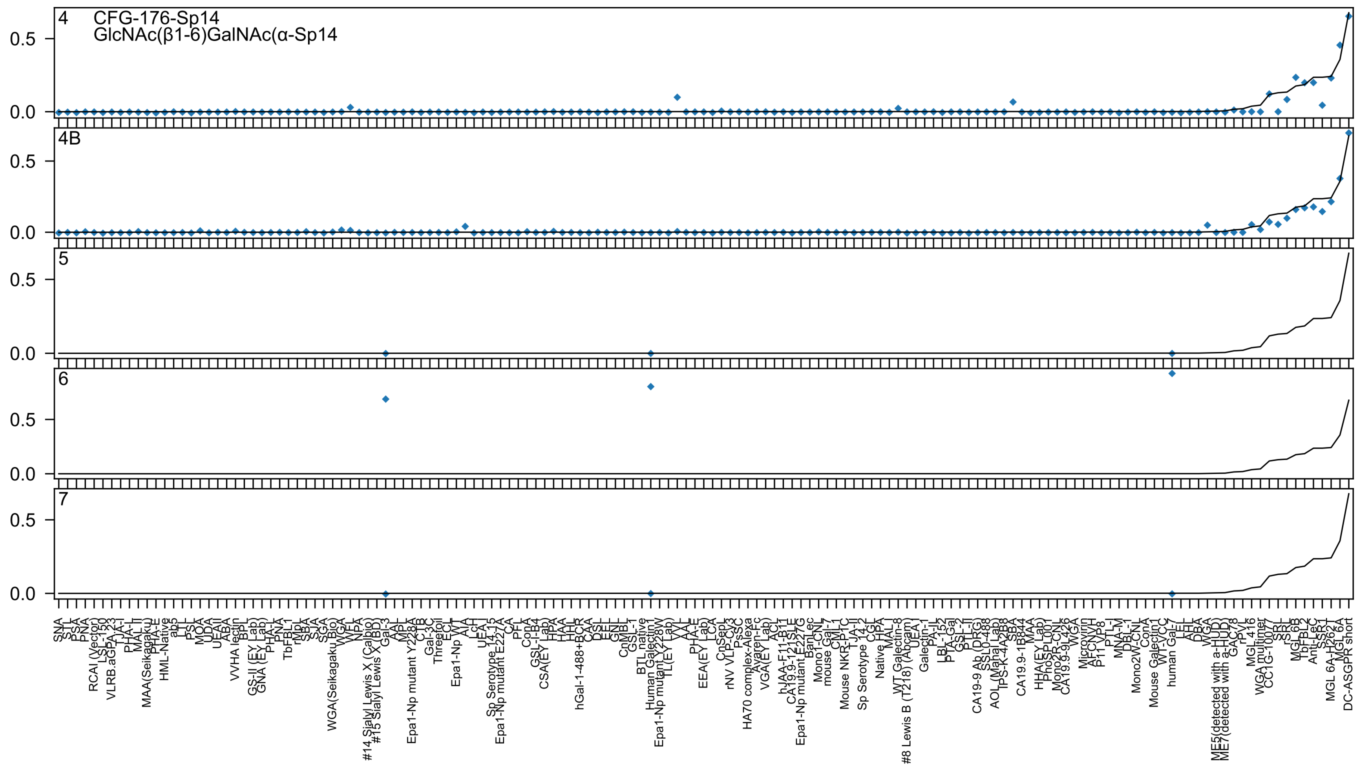

4 CFG-176-Sp14
GlcNAc(β1-6)GalNAc(α-Sp14
4B
5
6
7

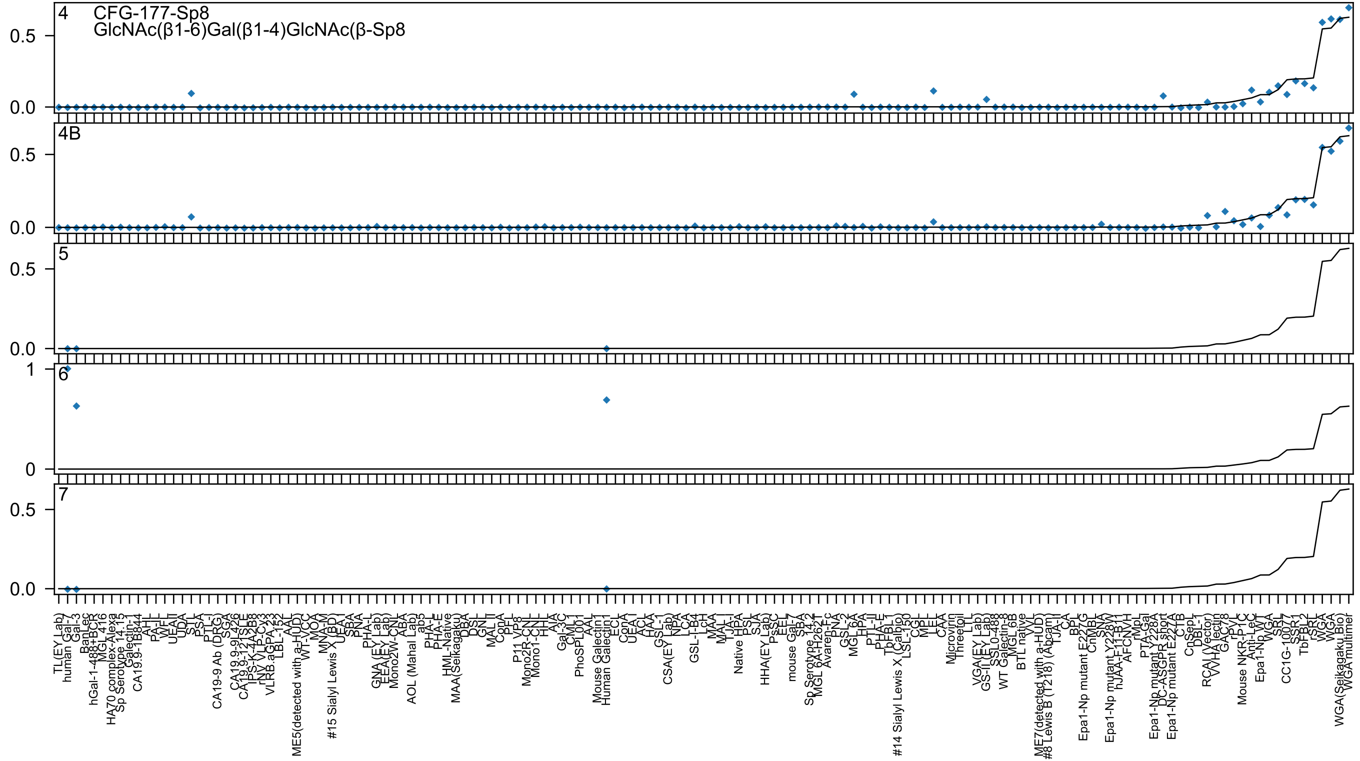
4 CFG-177-Sp8
GlcNAc(β1-6)Gal(β1-4)GlcNAc(β-Sp8
4B
5
6
7
0.5
0.0
1
0

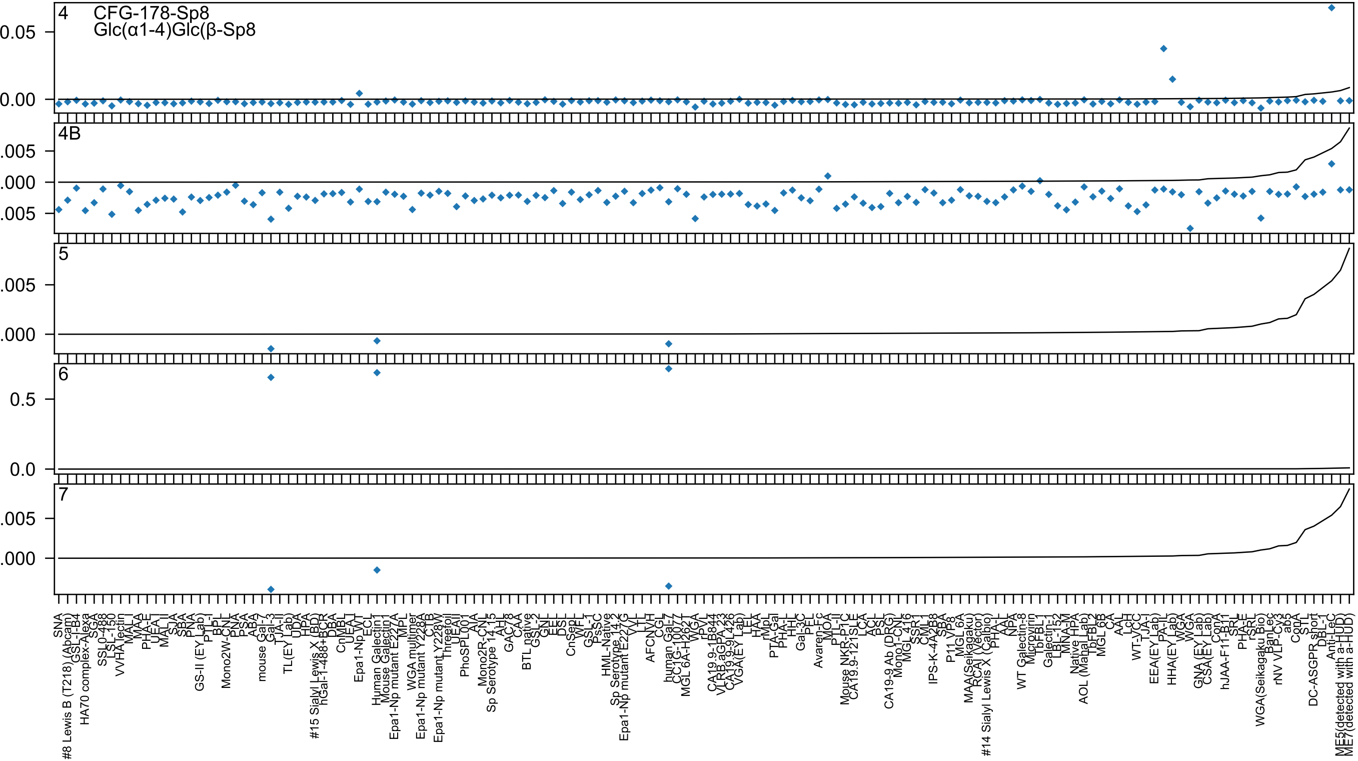

4
CFG-178-Sp8
Glc(α1-4)Glc(β-Sp8
4B
5
6
7

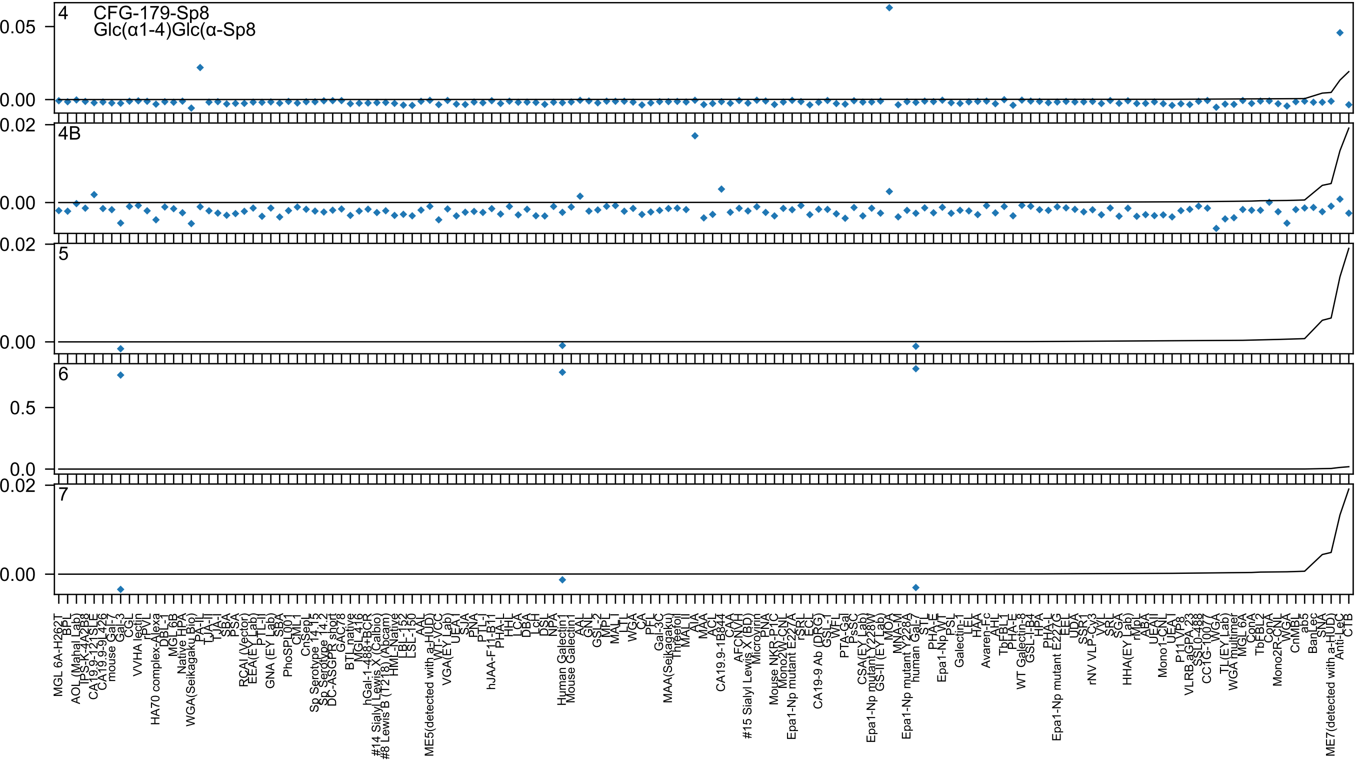
4
CFG-179-Sp8
Glc(α1-4)Glc(α-Sp8
0.05
0.00
4B
0.02
0.00
5
0.02
0.00
6
0.5
0.0
7
0.02
0.00

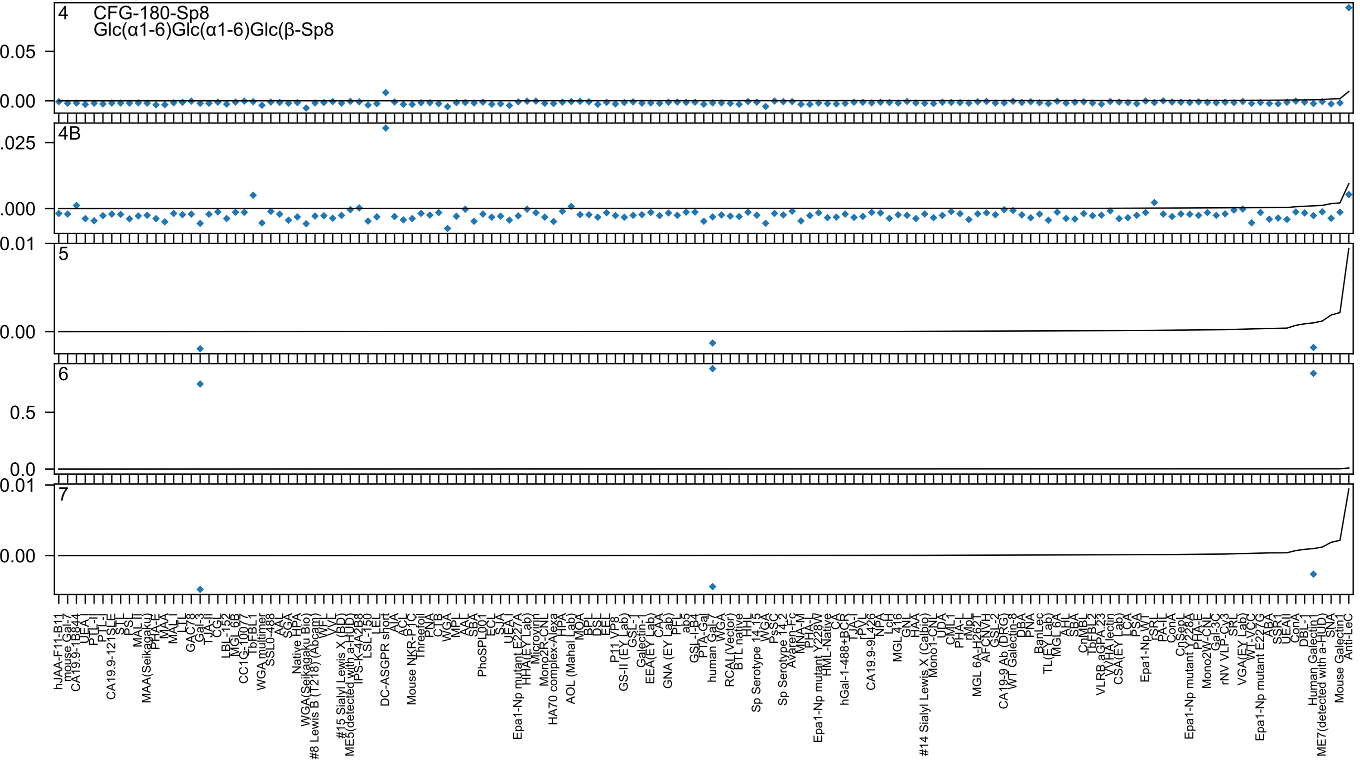

CFG-180-Sp8
Glc(α1-6)Glc(α1-6)Glc(β-Sp8
4
4B
5
6
7

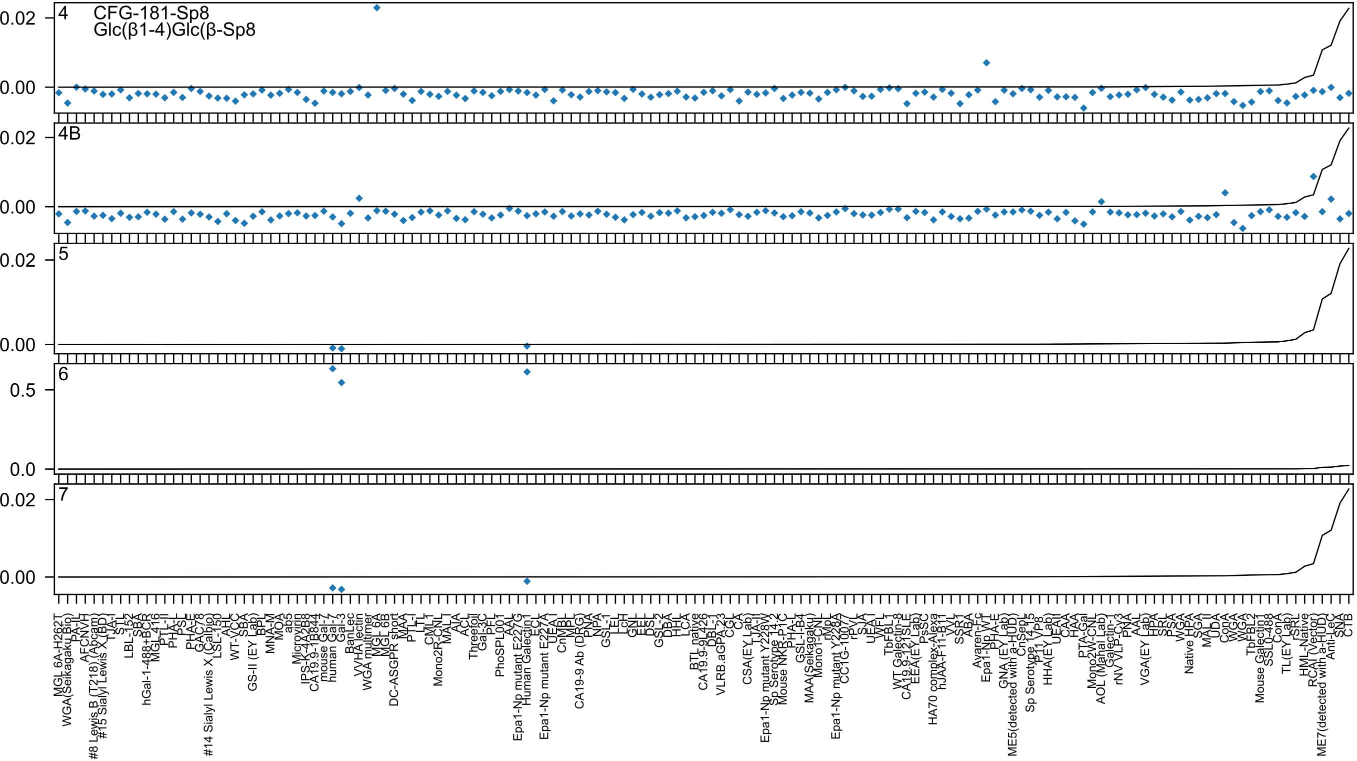
4
CFG-181-Sp8
Glc(β1-4)Glc(β-Sp8
4B
5
6
7
0.02
0.00
0.5
0.0

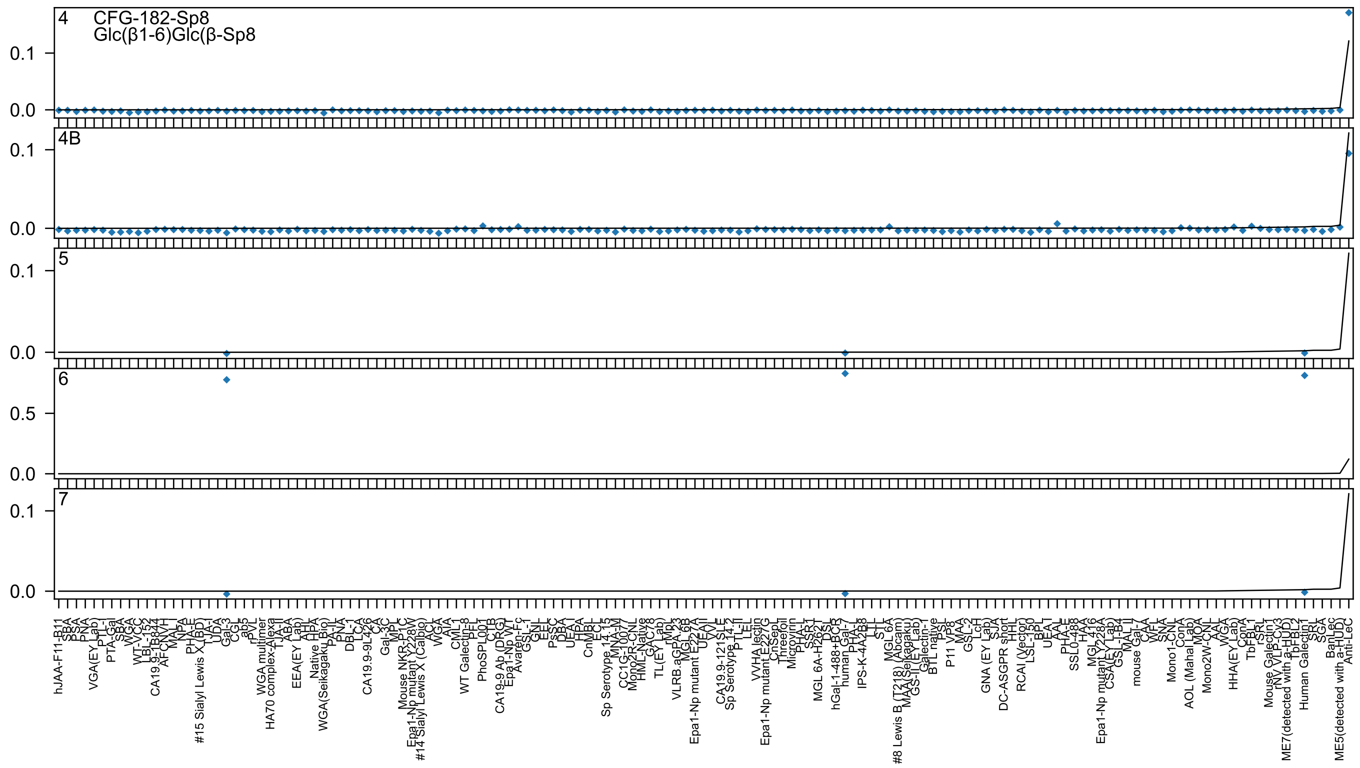
4 CFG-182-Sp8
Glc(β1-6)Glc(β-Sp8
4B
5
6
7

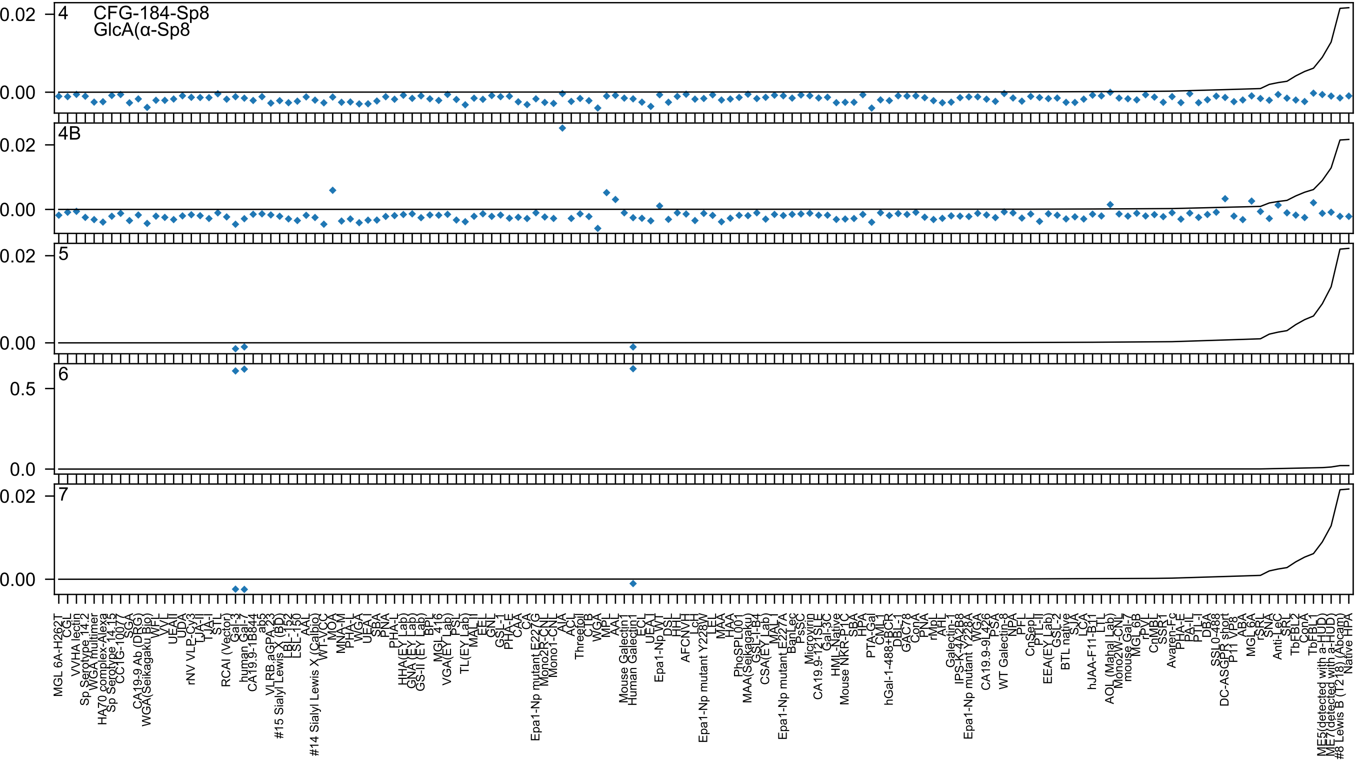

4
CFG-184-Sp8
GlcA(α-Sp8
4B
5
6
7
0.02
0.00
0.5
0.0
MGL 6A-H262T
CGL
VVHA lectin
Sp Serotype 14.2
WGA multimer
HA70 complex-Alexa
Sp Serotype 14.13
CC1G-10077
SGA
CA19-9 Ab (DRG)
WGA(Seikagaku Bio)
WFL
VVL
UEAII
UDA
rNV VLP-Cy3
TJA-II
TJA-I
STL
RCAI (Vector)
Gal-3
human Gal-7
CA19.9-1B844
ab5
VLRB.aGPA.23
#15 Sialyl Lewis X (BD)
LBL-152
LSL-150
AAL
#14 Sialyl Lewis X (Calbio)
WT-VCC
MOA
MNA-M
PHA-L
WGA
UEA-I
SBA
PNA
PHA-L
HHA(EY Lab)
GNA (EY Lab)
GS-II (EY Lab)
BPL
MGL 416
VGA(EY Lab)
PSL
TL(EY Lab)
MAL-II
EEL
GNL
GSL-1
PHA-E
CAA
CA
Epa1-Np mutant E227G
Mono2R-CNL
Mono1-CNL
AIA
ACL
Threefoil
CTB
WGA
MPL
AAL
Mouse Galectin1
Human Galectin1
ECL
UEA-I
Epa1-Np WT
DSL
HHL
AFCNVH
LcH
Epa1-Np mutant Y228W
LEL
MAA
HAA
PhoSL001
MAA(Seikagaku)
GSL-I-B4
CSA(EY Lab)
MAL-I
Epa1-Np mutant E227A
BanLec
PsSC
Microvirin
CA19.9-121SLE
Gal-3C
HML-Native
Mouse NKR-P1C
SBA
HPA
PTA-Gal
CML-I
hGal-1-488+BCR
DBL-1
GAC78
ConA
PNA
rMpL
AHL
Galectin-1
IPS-K-4A2B8
Epa1-Np mutant Y228A
WGA
CA19.9-9L426
PSA
WT Galectin-8
NPA
PFL
CnSepL
PTL-II
EEA(EY Lab)
GSL-2
BTL native
SJA
LCA
hJAA-F11-B11
LTL
AOL (Mahal Lab)
Mono2W-CNL
mouse Gal-7
MGL 6B
rPVL
CnMBL
SSR1
Avaren-Fc
PHA-E
PA-IL
PTL-I
DBA
SSL0-488
DC-ASGPR short
P11 VP8
ABA
MGL 6A
rSRL
SNA
Anti-LeC
SRL
TbFBL2
ConA
TbFBL1
ME5(detected with a-HUD)
ME7(detected with a-HUD)
#8 Lewis B (T218) (Abcam)
Native HPA

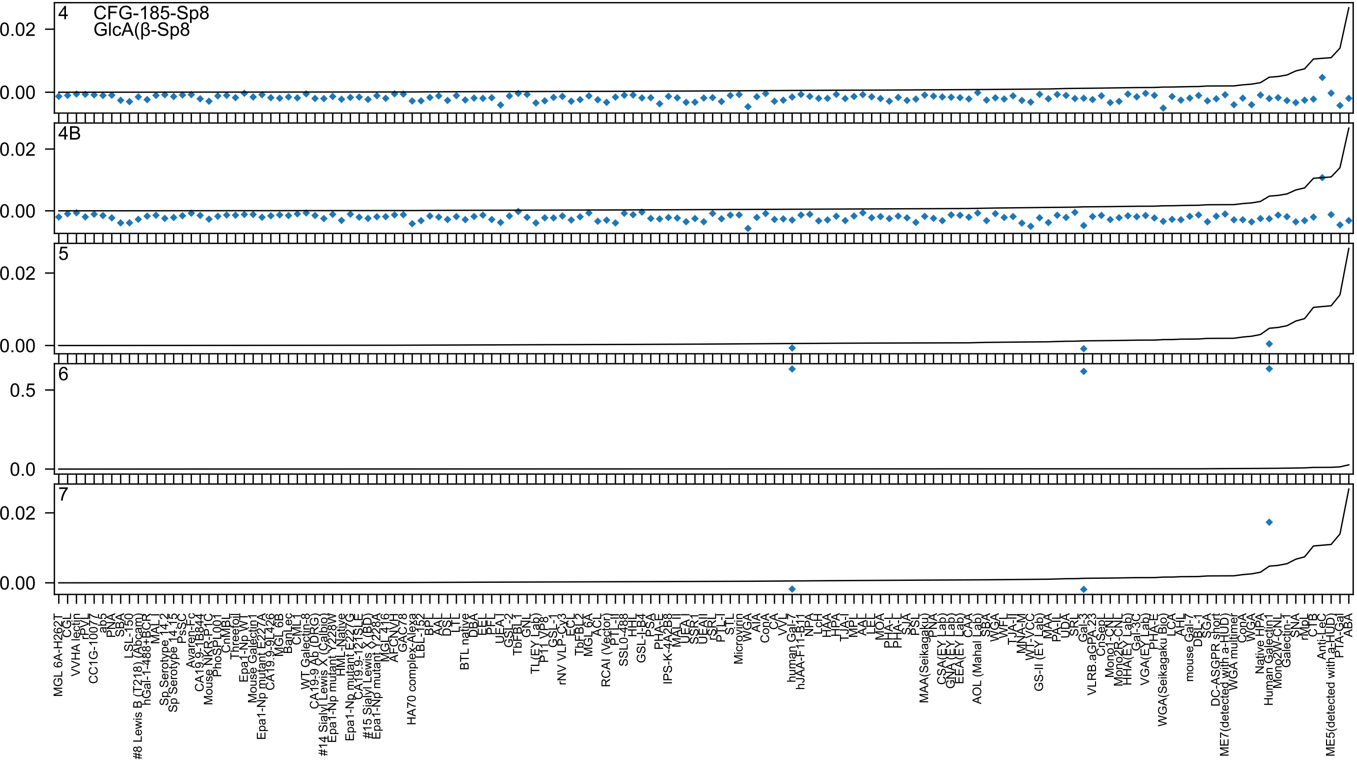

4
CFG-185-Sp8
GlcA(β-Sp8
4B
5
6
7
0.02
0.00
0.5
0.0

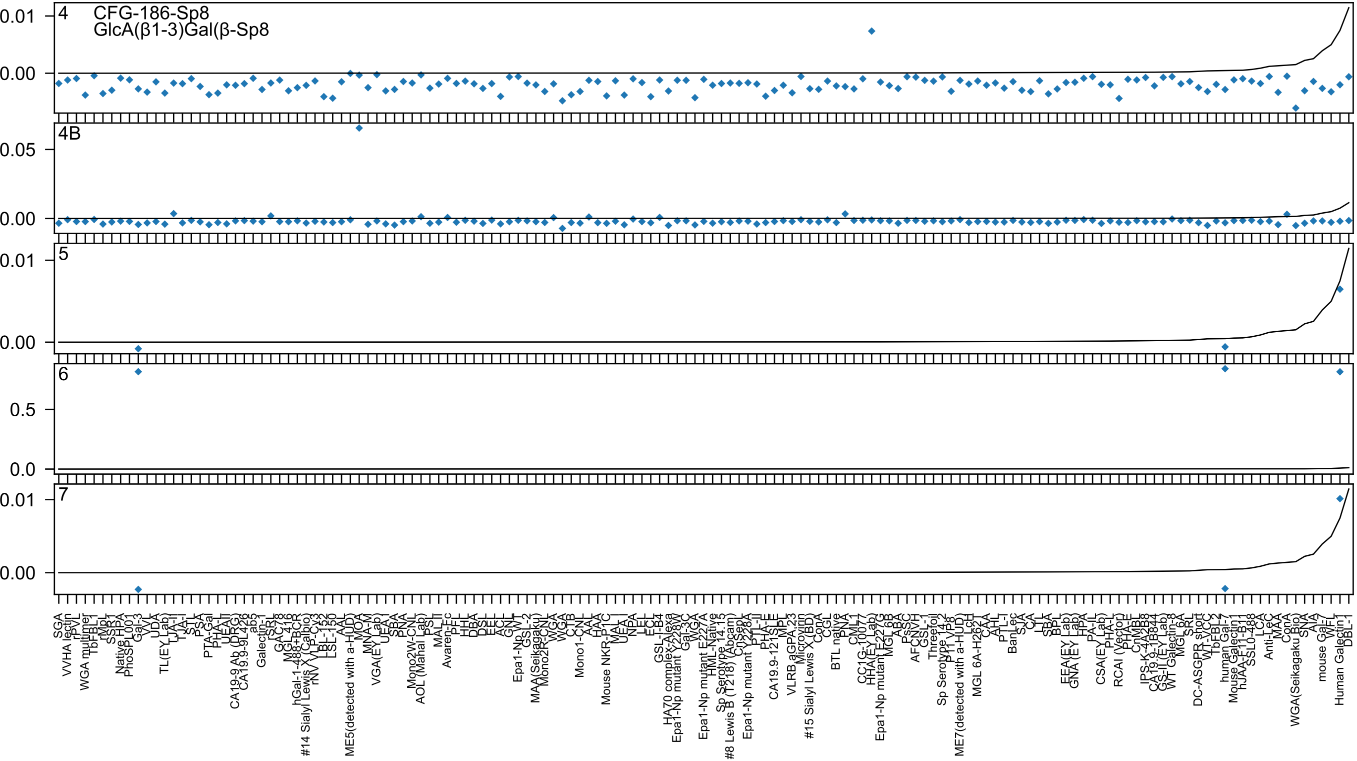
4
CFG-186-Sp8
GlcA(β1-3)Gal(β-Sp8
0.01
0.00
4B
0.05
0.00
5
0.01
0.00
6
0.5
0.0
7
0.01
0.00
SGA
VVHA lectin
rPVL
WGA multimer
TbFBL1
rMpL
Native HPA
PhoSPL001
Gal-3
VVL
UDA
TL(EY Lab)
TJA-II
TJA-I
ST
PSA
PTA-Gal
PHA-L
UEA-II
CA19-9 Ab (DRG)
CA19.9-9L426
ab5
Galectin-1
rSRL
GAC78
MGL416
hGal-1-488+BCR
#14 Sialyl Lewis X (Calbio)
rNV VLP-Cy3
LBL-152
LSL-150
AAL
ME5(detected with a-HUD)
MOA
MNA-M
VGA(EY Lab)
UEA-I
SBA
PNA
Mono2W-CNL
AOL (Mahal Lab)
PSL
MAL II
Avaren-Fc
PFL
HHL
DBA
DSL
EEL
ACL
GNL
Epa1-Np WT
GSL-2
MAA(Seikagaku)
Mono2R-CNL
WGA
WGA
CTB
Mono1-CNL
AAL
HAA
Mouse NKR-P1C
MAL I
UEA I
NPA
LEL
ECL
GSL-I-B4
HA70 complex-Alexa
Epa1-Np mutant Y228W
Gal-3C
WGA
Epa1-Np mutant E227A
HML-Native
Sp Serotype 14.15
#8 Lewis B (T218) (Abcam)
CnSep
Epa1-Np mutant Y228A
PTL-II
PHA-E
CA19.9-121SLE
MPL
VLRB.aGPA.23
Microvirin
#15 Sialyl Lewis X (BD)
ConA
CGL
BTL native
PNA
CML1
CC1G-10077
HHA(EY Lab)
Epa1-Np mutant E227G
MGL 6B
ABA
PsSC
AFCNVH
GSL-1
Threefoil
Sp Serotype 14.2
P11 VP8
ME7(detected with a-HUD)
LcH
MGL 6A-H262T
CAA
AHL
PTL-I
BanLec
SJA
CA
LTL
SBA
BPL
EEA(EY Lab)
GNA (EY Lab)
HPA
PA-IL
CSA(EY Lab)
PHA-L
RCAI (Vector)
PHA-E
CnMBL
IPS-K-4A2B8
CA19.9-1B844
GS-II (EY Lab)
WT Galectin-8
MGL 6A
SRL
DC-ASGPR short
WT-VCC
TbFBL2
human Gal-7
Mouse Galectin1
hJAA-F11-B1
SSL0-488
LCA
Anti-LeC
MAA
ConA
WGA(Seikagaku Bio)
SNA
AIA
mouse Gal-7
WFL
Human Galectin1
DBL-1

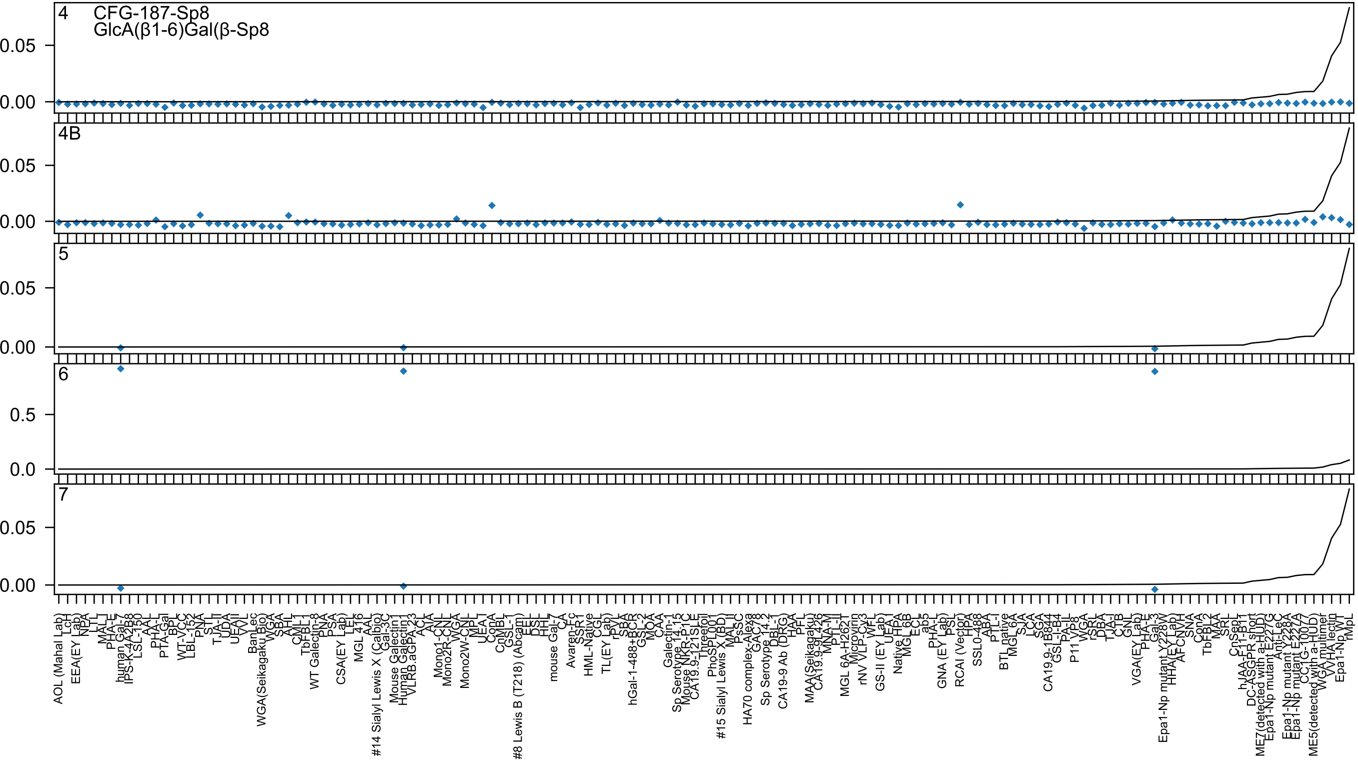
4
CFG-187-Sp8
GlcA(β1-6)Gal(β-Sp8
4B
5
6
7
0.05
0.00
0.5
0.0

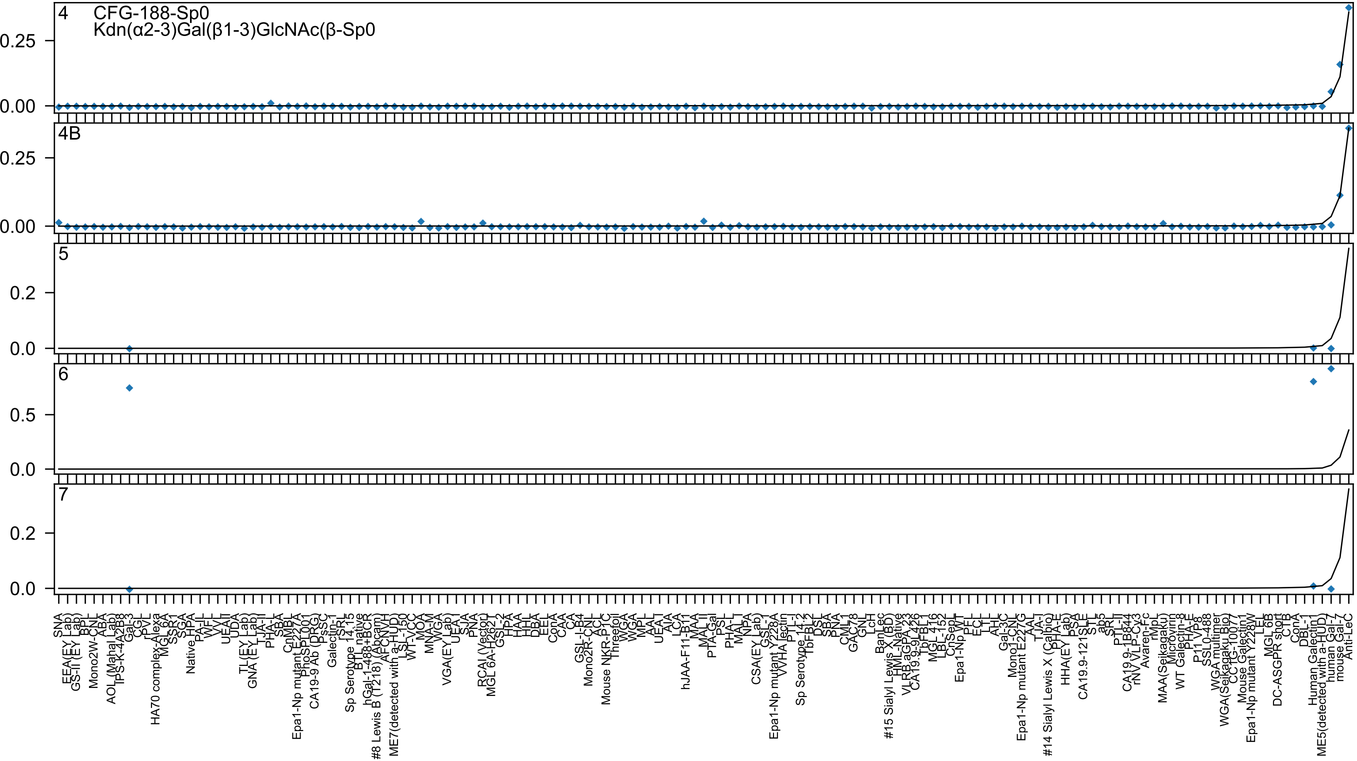

4
CFG-188-Sp0
Kdn(α2-3)Gal(β1-3)GlcNAc(β-Sp0
4B
5
6
7
0.25
0.00
0.2
0.0
0.5

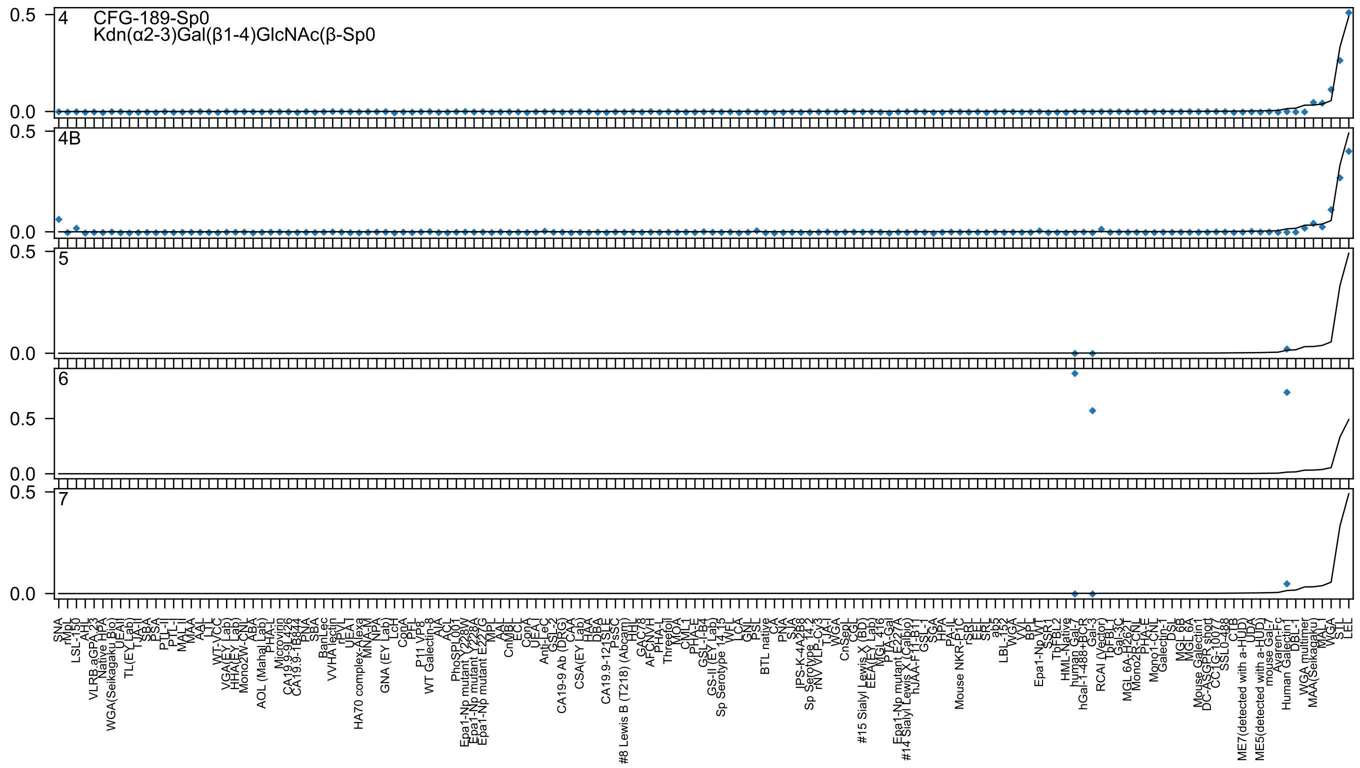

4
CFG-189-Sp0
Kdn(α2-3)Gal(β1-4)GlcNAc(β-Sp0
4B
5
6
7
0.5
0.0

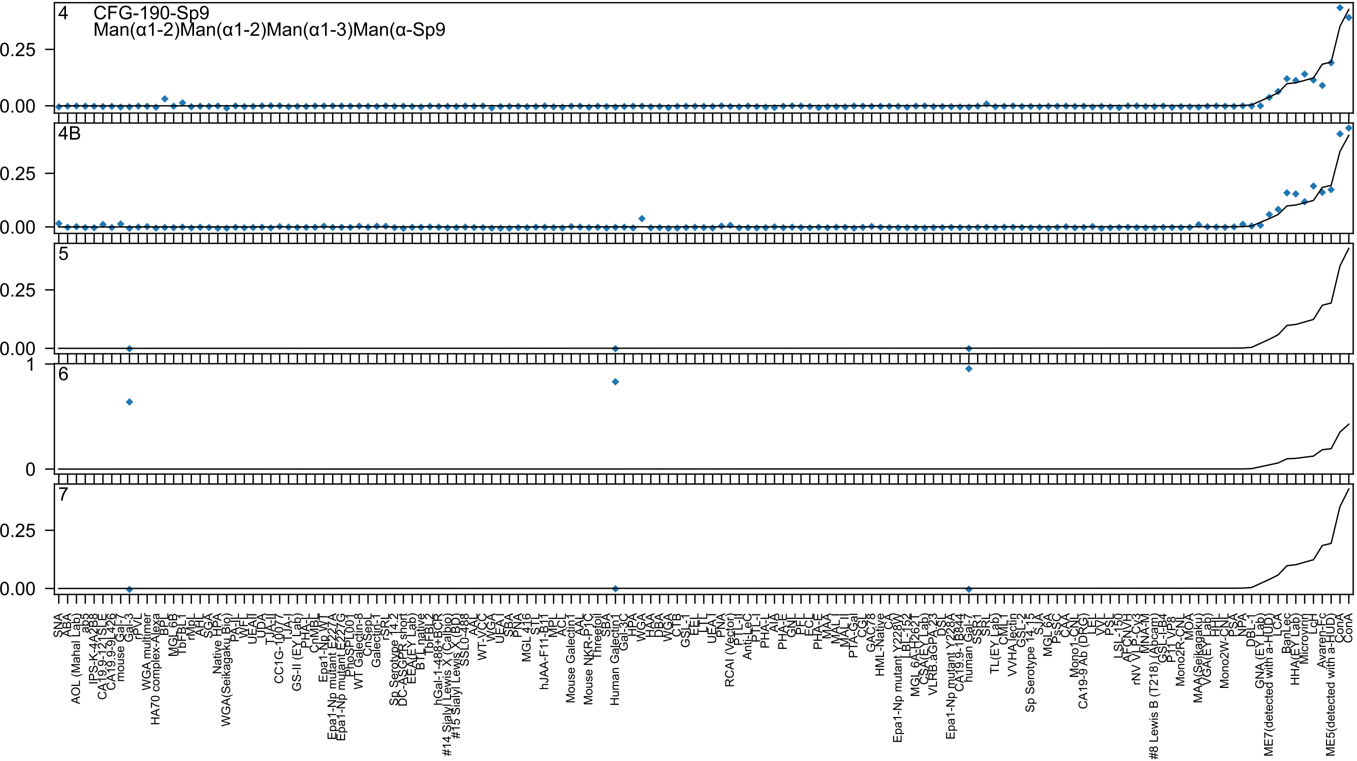

CFG-190-Sp9
Man(α1-2)Man(α1-2)Man(α1-3)Man(α-Sp9

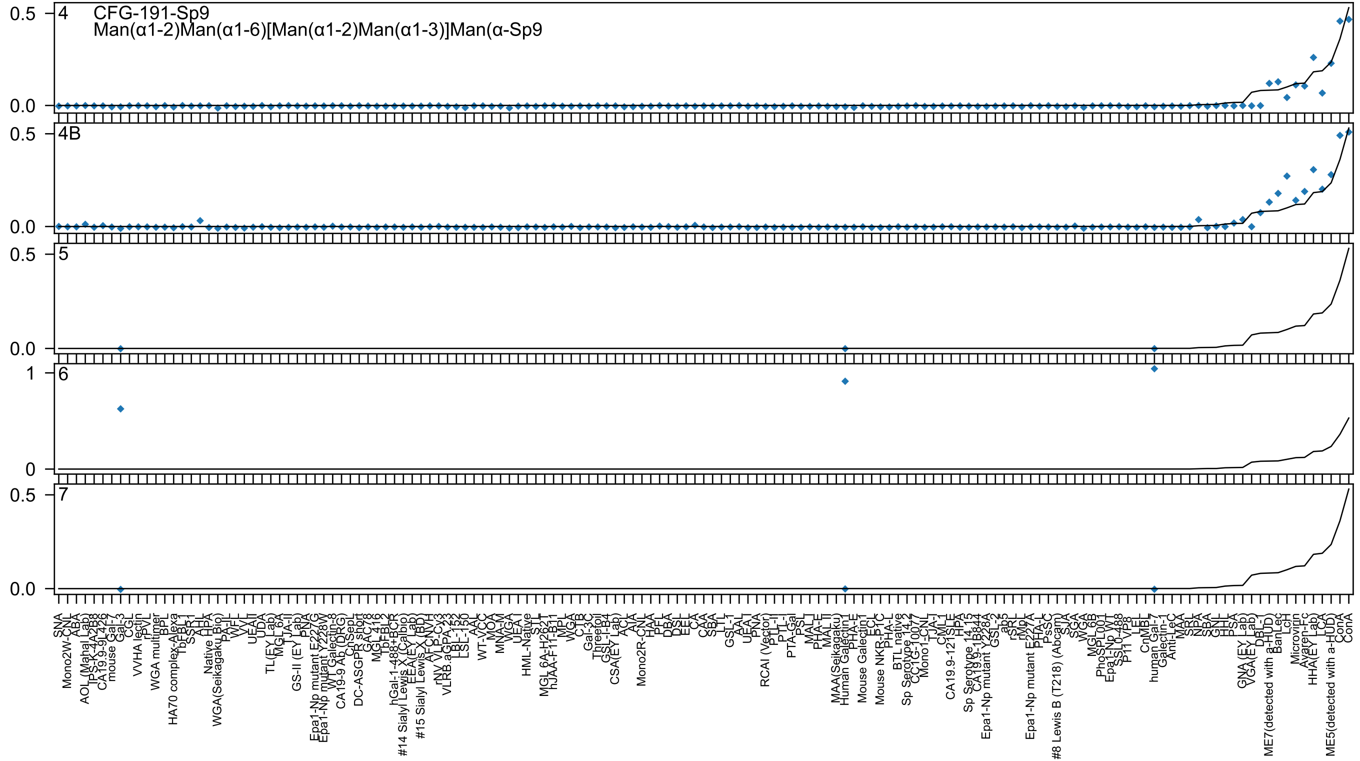
CFG-191-Sp9
Man(α1-2)Man(α1-6)[Man(α1-2)Man(α1-3)]Man(α-Sp9
4
4B
5
6
7

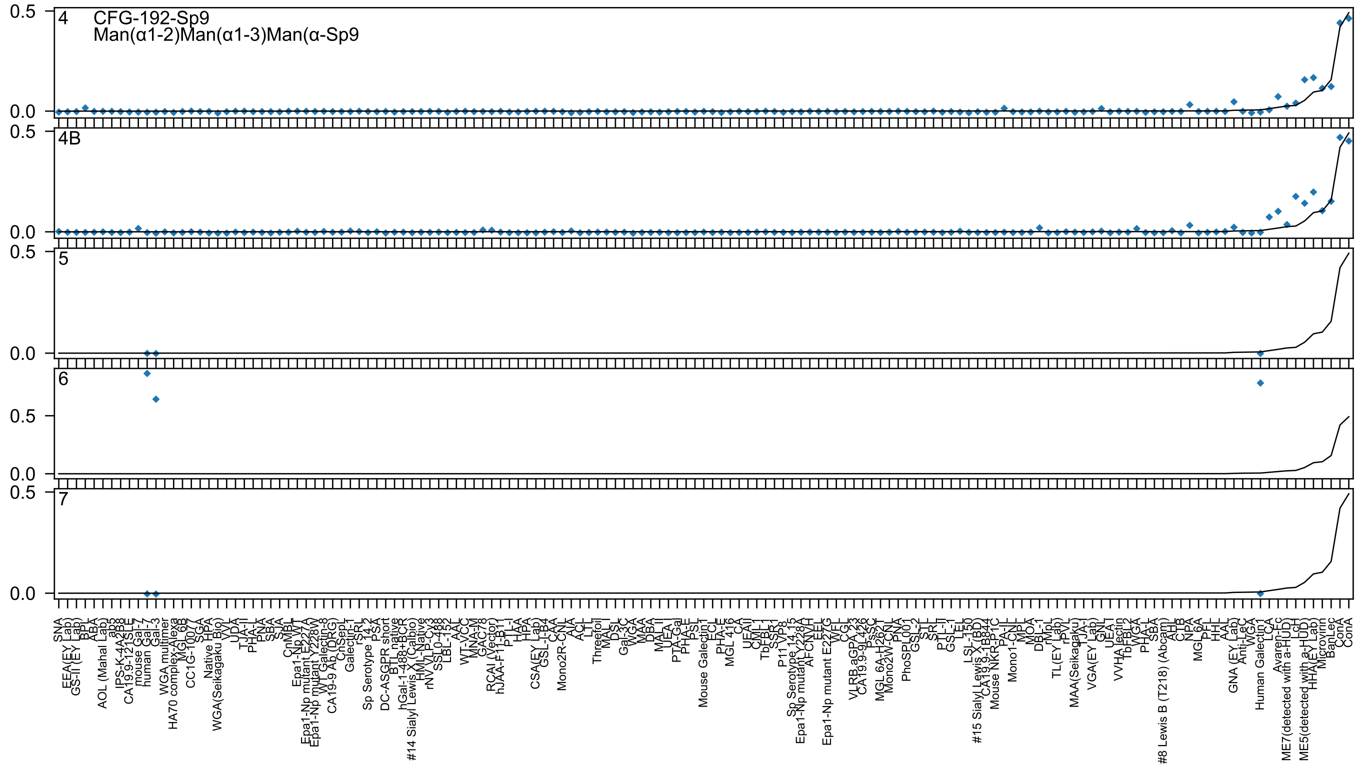

4
CFG-192-Sp9
Man(α1-2)Man(α1-3)Man(α-Sp9
4B
5
6
7
0.5
0.0
SNA
ConA

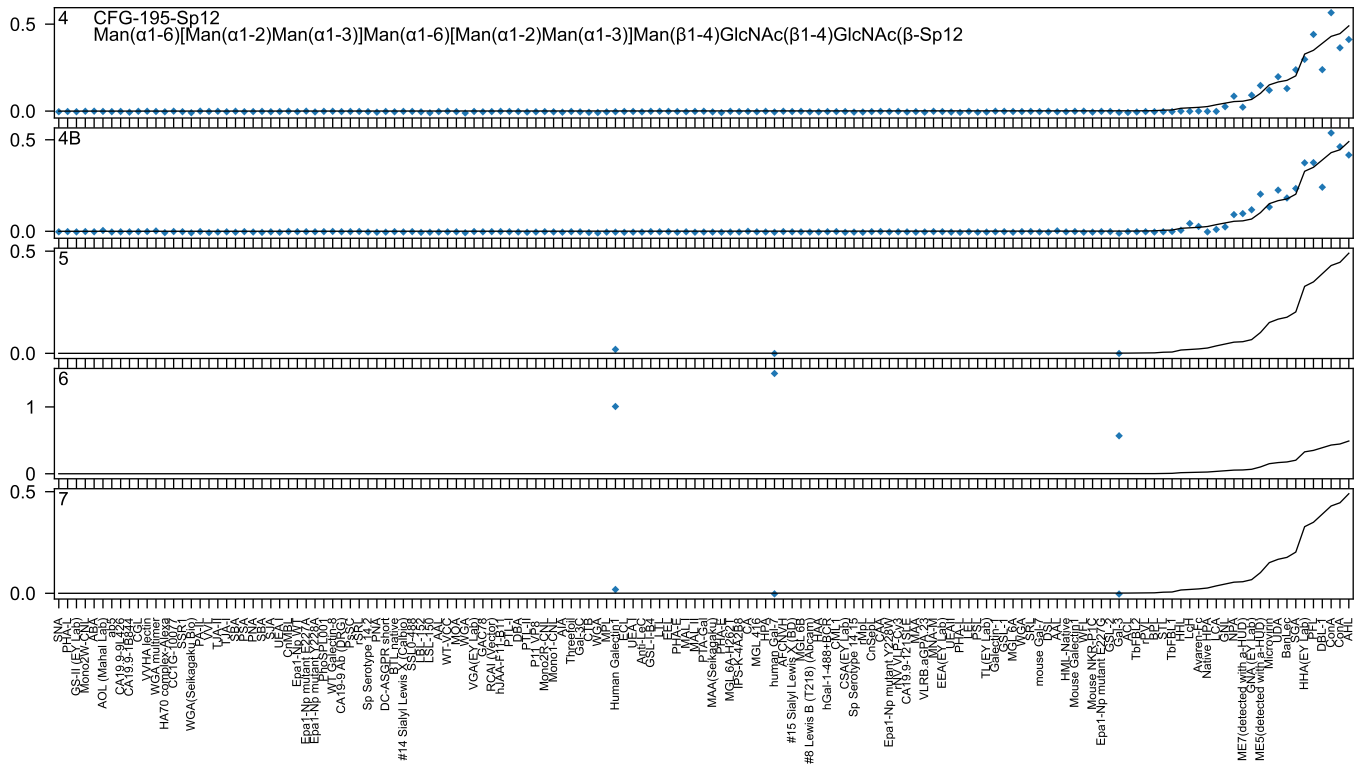
4
CFG-195-Sp12
Man(α1-6)[Man(α1-2)Man(α1-3)]Man(α1-6)[Man(α1-2)Man(α1-3)]Man(β1-4)GlcNAc(β1-4)GlcNAc(β-Sp12
4B
5
6
7
0.5
0.0
1
0
SNA
PHA-L
GS-II (EY Lab)
Mono2W-CNL
ABA
AOL (Mahal Lab)
ab5
CA19.9-9L426
CA19.9-1B844
CGL
VVHA lectin
WGA multimer
HA70 complex-Alexa
CC1G-10077
SSR1
WGA(Seikagaku Bio)
PA-IL
VVL
TJA-II
TJA-I
SBA
PSA
PNA
SBA
SJA
UEA-I
CnMBL
Epa1-Np WT
Epa1-Np mutant E227A
Epa1-Np mutant Y228A
PhoSL001
WT Galectin-8
CA19-9 Ab (DRG)
PsSC
rSRL
Sp Serotype 14.2
PNA
DC-ASGPR short
BTL native
#14 Sialyl Lewis X (Calbio)
SSL0-488
LBL-152
LSL-150
AAL
WT-VCC
MOA
WGA
VGA(EY Lab)
GAC78
RCAI (Vector)
hJAA-F11-B11
PTL-I
DBA
PTL-II
P11 VP8
Mono2R-CNL
Mono1-CNL
AIA
Threefoil
Gal-3C
CTB
WGA
MPL
Human Galectin1
ECL
UEAI
Anti-LeC
GSL-I-B4
LTL
EEL
PHA-E
MAL-I
MAL-II
PTA-Gal
MAA(Seikagaku)
PHA-E
MGL 6A-H262T
IPS-K-4A2B8
CA
MGL 416
HPA
human Gal-7
AFCNVH
#15 Sialyl Lewis X (BD)
MGL 6B
#8 Lewis B (T218) (Abcam)
HAA
hGal-1-488+BCR
CML1
CSA(EY Lab)
Sp Serotype 14.15
rMpL
CnSeb
CAA
Epa1-Np mutant Y228W
rNV VLP-Cy3
CA19.9-121SLE
MAA
VLRB.aGPA.23
MNA-M
EEA(EY Lab)
UEAII
PHA-L
LEL
PSL
TL(EY Lab)
Galectin-1
GSL-I
MGL 6A
WGA
SRL
mouse Gal-7
DSL
AAL
HML-Native
Mouse Galectin1
WFL
Mouse NKR-P1C
Epa1-Np mutant E227G
GSL-2
Gal-3
ACL
TbFBL2
rPVL
BPL
STL
TbFBL1
HHL
LcH
Avaren-Fc
Native HPA
LCA
GNL
NPA
ME7(detected with a-HUD)
GNA (EY Lab)
ME5(detected with a-HUD)
Microvirin
UDA
BanLec
SGA
HHA(EY Lab)
PFL
DBL-1
ConA
ConA
AHL

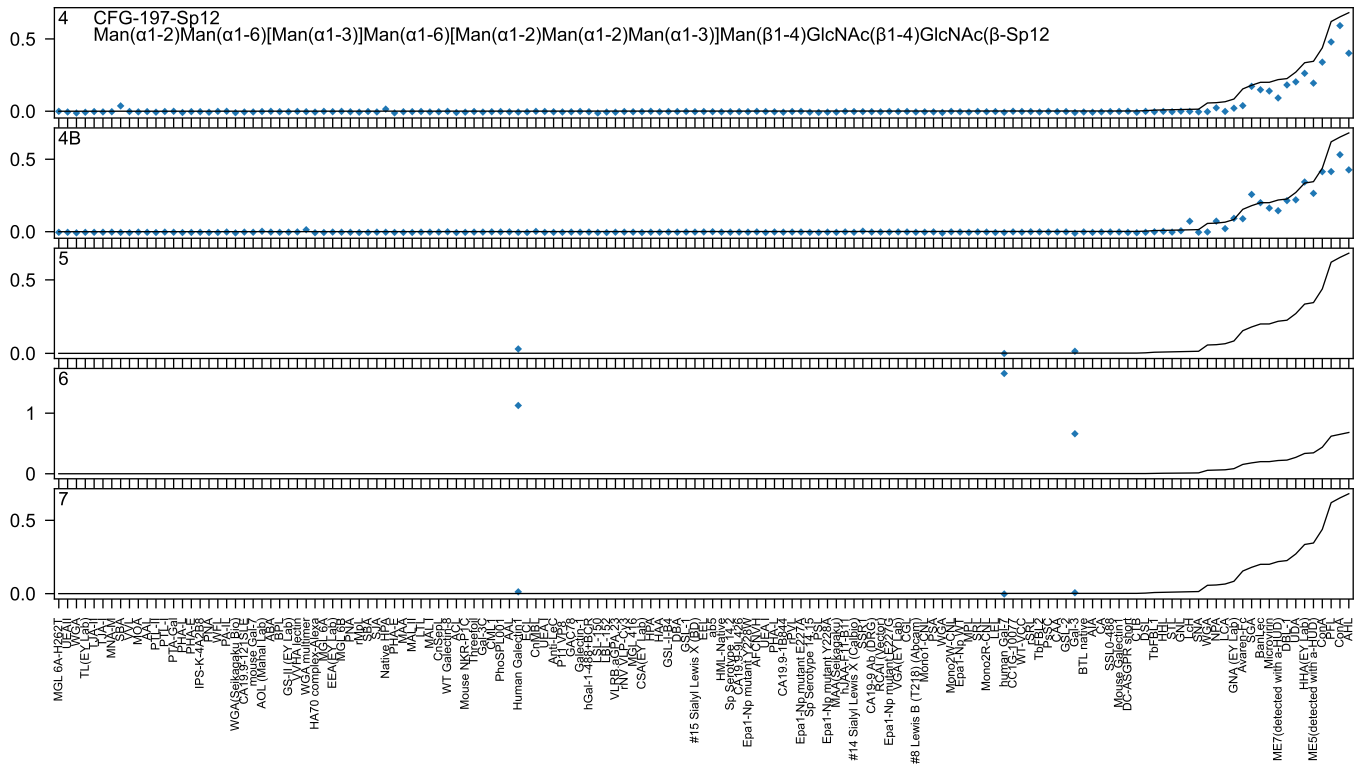
4
CFG-197-Sp12
Man(α1-2)Man(α1-6)[Man(α1-3)]Man(α1-6)[Man(α1-2)Man(α1-2)Man(α1-3)]Man(β1-4)GlcNAc(β1-4)GlcNAc(β-Sp12
4B
5
6
7
0.0
0.5
0
1
MGL 6A-H262T
UEA-II
WGA
TL(EY Lab)
TJA-I
TJA-II
MNA-M
SBA
VVL
MOA
AAL
PTL-II
PTL-I
PTA-Gal
PHA-L
PHA-E
IPS-K-4A2B8
PNA
WFL
PA-IL
WGA(Seikagaku Bio)
CA19.9-121SLE
mouse Gal-7
AOL (Mahal Lab)
ABA
BPL
GS-II (EY Lab)
VVHA lectin
WGA multimer
HA70 complex-Alexa
MGL 6A
EEA(EY Lab)
MGL 6B
PNA
rMpL
SBA
SJA
Native HPA
PHA-E
MAA
MAL II
LTL
MAL I
CnSept
WT Galectin-8
ACL
Mouse NKR-P1C
Threefoil
Gal-3C
CML1
PhoSPL001
AAL
Human Galectin1
ECL
CnMBL
UEA1
Anti-LeC
P11VP8
GAC78
Galectin-1
hGal-1-488+BCR
LSL-150
LBL-152
VLRB.aGPA.23
rNV VLP-Cy3
MGL 416
CSA(EY Lab)
HPA
HAA
GSL-I-B4
DBA
GSL-I
#15 Sialyl Lewis X (BD)
EEL
ab5
HML-Native
Sp Serotype 14.2
CA19.9 9L426
Epa1-Np mutant Y228W
AFCNVH
UEA-I
PHA-L
CA19.9-1B844
PPYL
Epa1-Np mutant E227A
Sp Serotype 14.15
PSL
Epa1-Np mutant Y228A
MAA(Seikagaku)
hJAA-F11-B11
#14 Sialyl Lewis X (Calbio)
SSR1
CA19-9 Ab (DRG)
RCAI (Vector)
Epa1-Np mutant E227G
VGA(EY Lab)
CGL
#8 Lewis B (T218) (Abcam)
Mono1-CNL
PSA
WGA
Mono2W-CNL
Epa1-Np WT
MPL
SRL
Mono2R-CNL
LEL
human Gal-7
CC1G-10077
WT-VCC
rSRL
TbFBL2
PsSC
CAA
GSL-2
Gal-3
BTL native
AIA
CA
SSL0-488
Mouse Galectin1
DC-ASGPR short
CTB
DSL
TbFBL1
HHL
STL
GNL
LcH
SNA
WGA
NPA
LCA
GNA (EY Lab)
Avaren-Fc
SGA
BanLec
Microvirin
ME7(detected with a-HUD)
DBL-1
UDA
HHA(EY Lab)
ME5(detected with a-HUD)
CanA
PFL
ConA
AHL

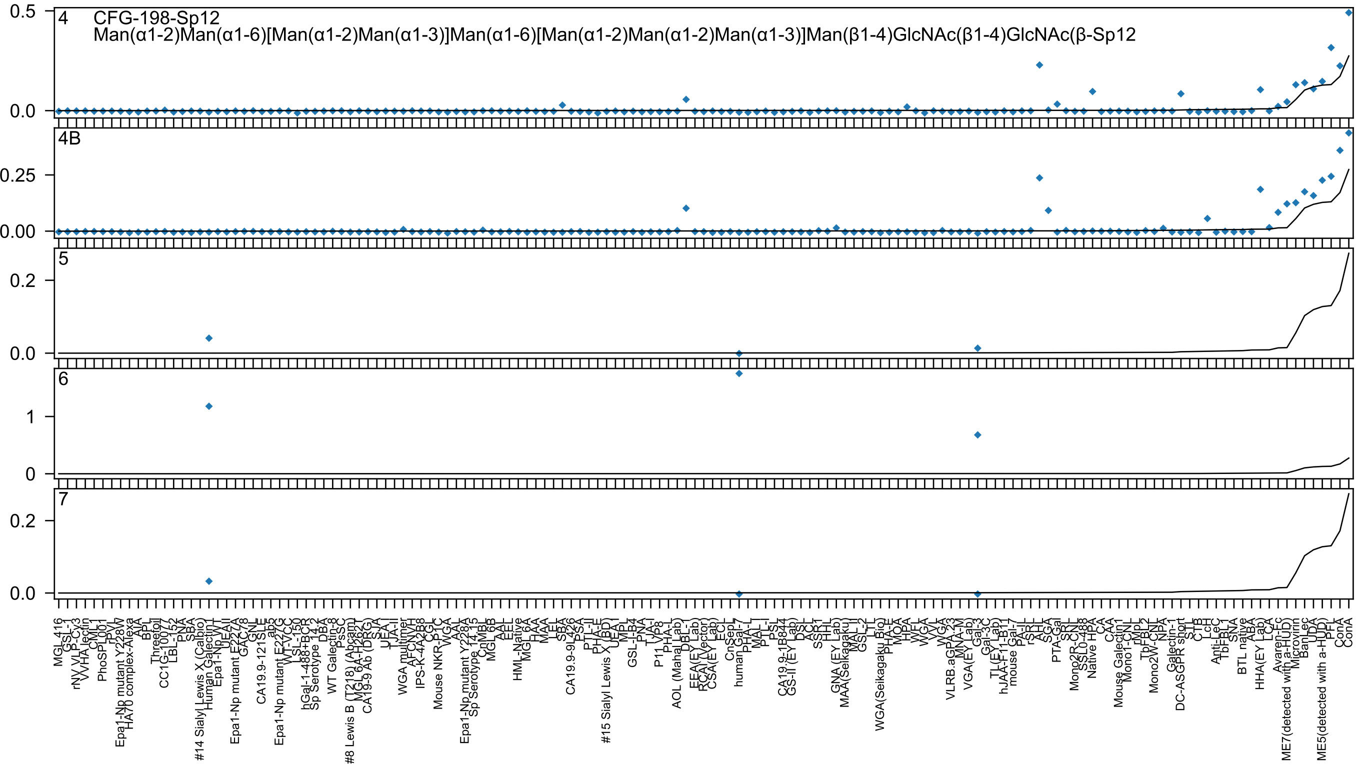
CFG-198-Sp12
Man(α1-2)Man(α1-6)[Man(α1-2)Man(α1-3)]Man(α1-6)[Man(α1-2)Man(α1-2)Man(α1-3)]Man(β1-4)GlcNAc(β1-4)GlcNAc(β-Sp12
4
4B
5
6
7

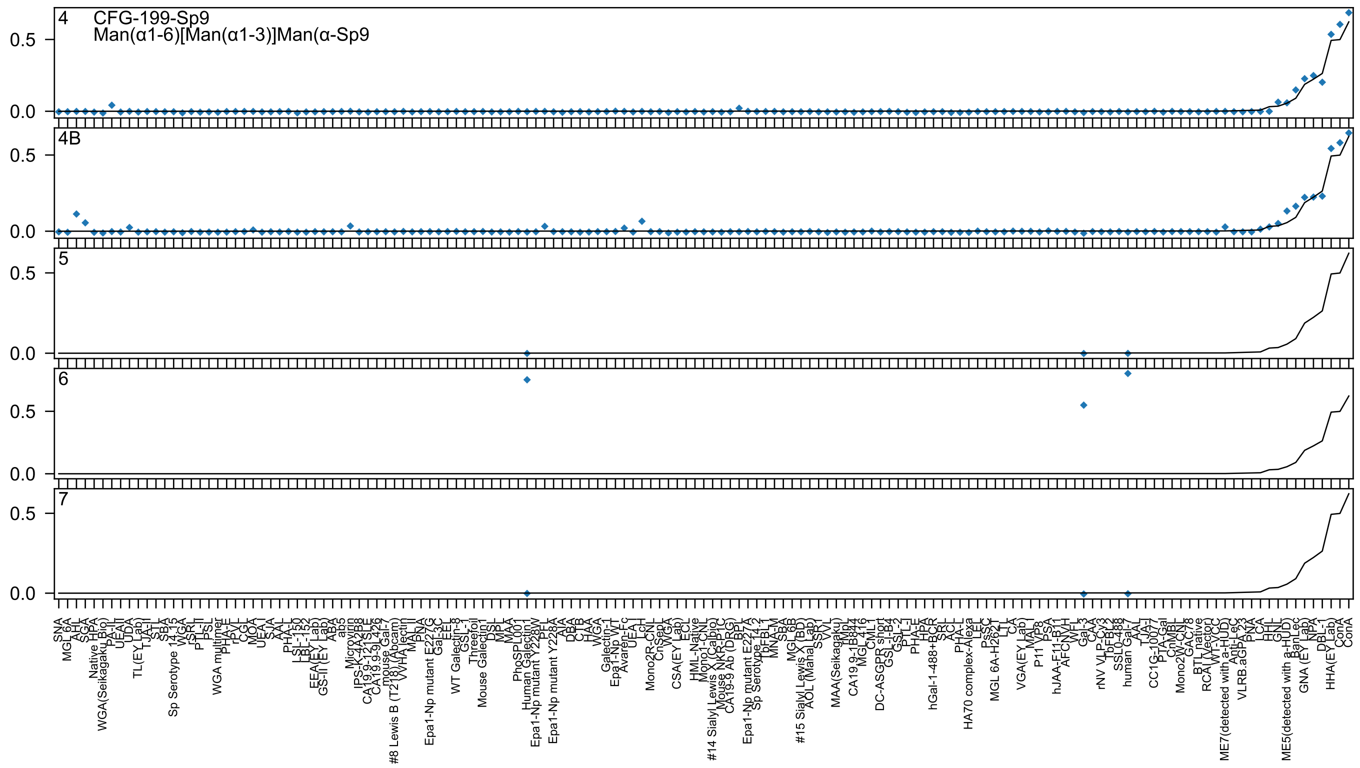
4
CFG-199-Sp9
Man(α1-6)[Man(α1-3)]Man(α-Sp9
4B
5
6
7
0.0
0.5
SNA
MGL 6A
AHL
SGA
Native HPA
WGA(Seikagaku Bio)
PA-II
UEA-II
UDA
TL(EY Lab)
TJA-II
STL
SBA
Sp Serotype 14.15
WGA
rSRL
PTL-II
PSL
WGA multimer
PHA-E
rPVL
CGL
MOA
UEA-I
SJA
AAL
PHA-L
LSL-150
LBL-152
EEA(EY Lab)
GS-II (EY Lab)
ABA
ab5
Microvirin
IPS-K-4A2B8
CA19.9-121SLE
CA19.9-9L426
mouse Gal-7
#8 Lewis B (T218) (Abcam)
WHA lectin
MAL II
PNA
Epa1-Np mutant E227G
Gal-3C
EEL
WT Galectin-8
GSL-I
Threefoil
Mouse Galectin1
DSL
MPL
MAA
PhoSL001
Human Galectin1
Epa1-Np mutant Y228W
PFL
Epa1-Np mutant Y228A
AIA
DBA
CTB
HAA
WGA
Galectin-1
Epa1-Np WT
Avaren-Fc
UEA-I
LcH
Mono2R-CNL
CnSep
WGA
CSA(EY Lab)
ECL
HML-Native
Mono1-CNL
#14 Sialyl Lewis X (Calbio)
Mouse NKR-P1C
CA19-9 Ab (DRG)
BPL
Epa1-Np mutant E227A
Sp Serotype 14.2
TbFBL1
MNA-M
SBA
MGL 6B
#15 Sialyl Lewis X (BD)
AOL (Mahal Lab)
SSR1
VVL
MAA(Seikagaku)
TMp
CA19.9-1B844
MGL 416
CML1
DC-ASGPR short
GSL-I-B4
GSL-2
PTL-I
PHA-E
HPA
hGal-1-488+BCR
SRL
ACL
PHA-L
HA70 complex-Alexa
LEL
PsSC
MGL 6A-H262T
LTL
CA
VGA(EY Lab)
MAL I
P11 VP8
PSA
hJAA-F11-B11
AFCNVH
WFL
Gal-3
CAA
rNV VLP-Cy3
TbFBL2
SSL0-488
human Gal-7
AAL
TJA-I
CC1G-10077
PTA-Gal
CnMBL
Mono2W-CNL
GAC78
BTL native
RCA I (Vector)
WT-VCC
ME7(detected with a-HUD)
Anti-LeC
VLRB.aGPA.23
PNA
LCA
HHL
GNL
ME5(detected with a-HUD)
BanLec
GNA (EY Lab)
NPA
DBL-1
HHA(EY Lab)
ConA
ConA

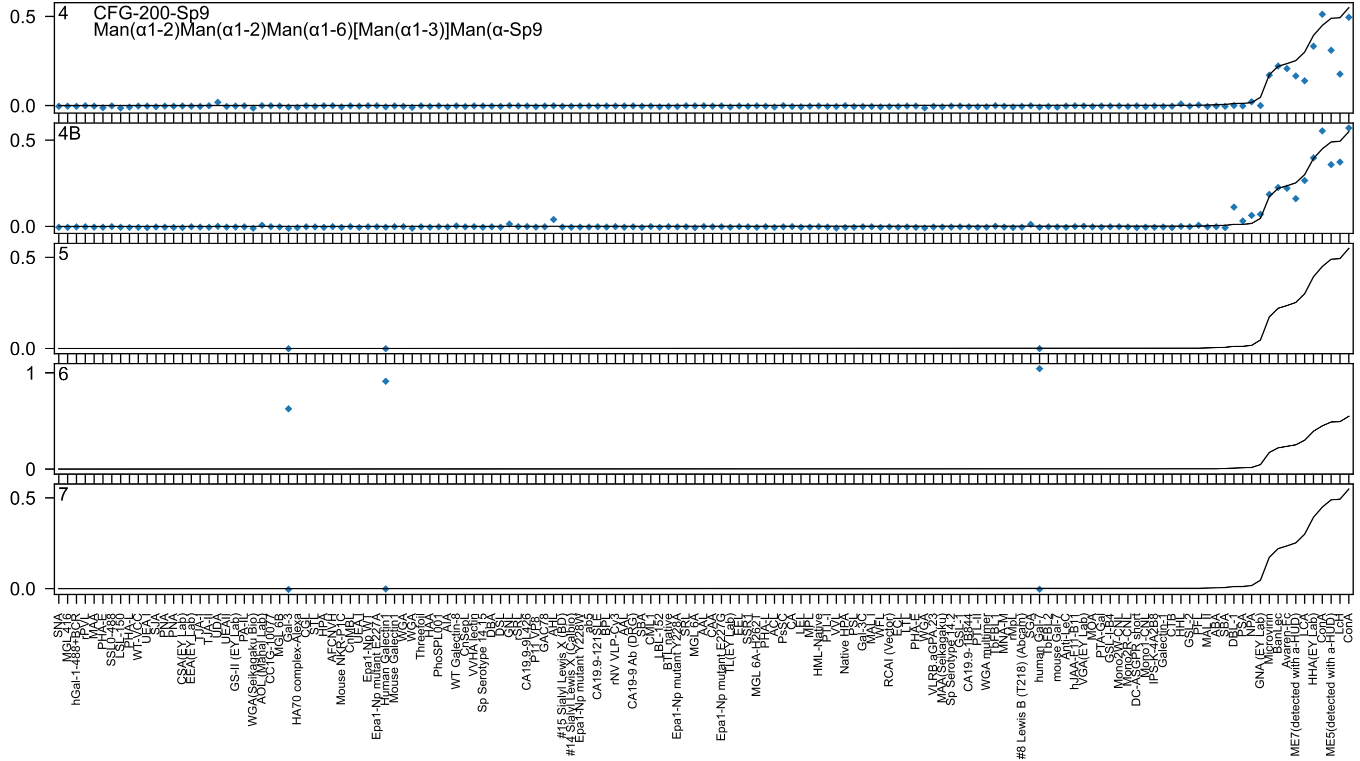

4
CFG-200-Sp9
Man(α1-2)Man(α1-2)Man(α1-6)[Man(α1-3)]Man(α-Sp9
4B
5
6
7
0.5
0.0
1
0

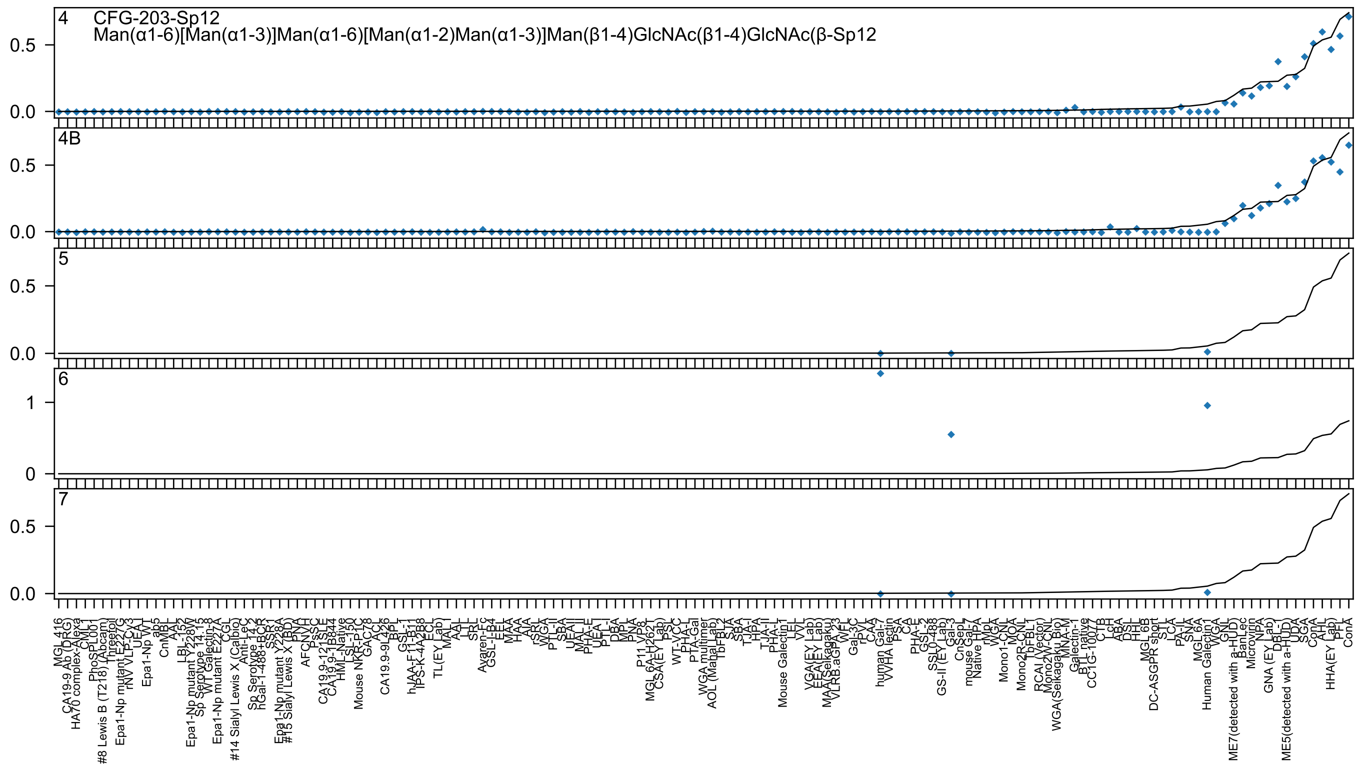

CFG-203-Sp12
Man(α1-6)[Man(α1-3)]Man(α1-6)[Man(α1-2)Man(α1-3)]Man(β1-4)GlcNAc(β1-4)GlcNAc(β-Sp12
4
4B
5
6
7

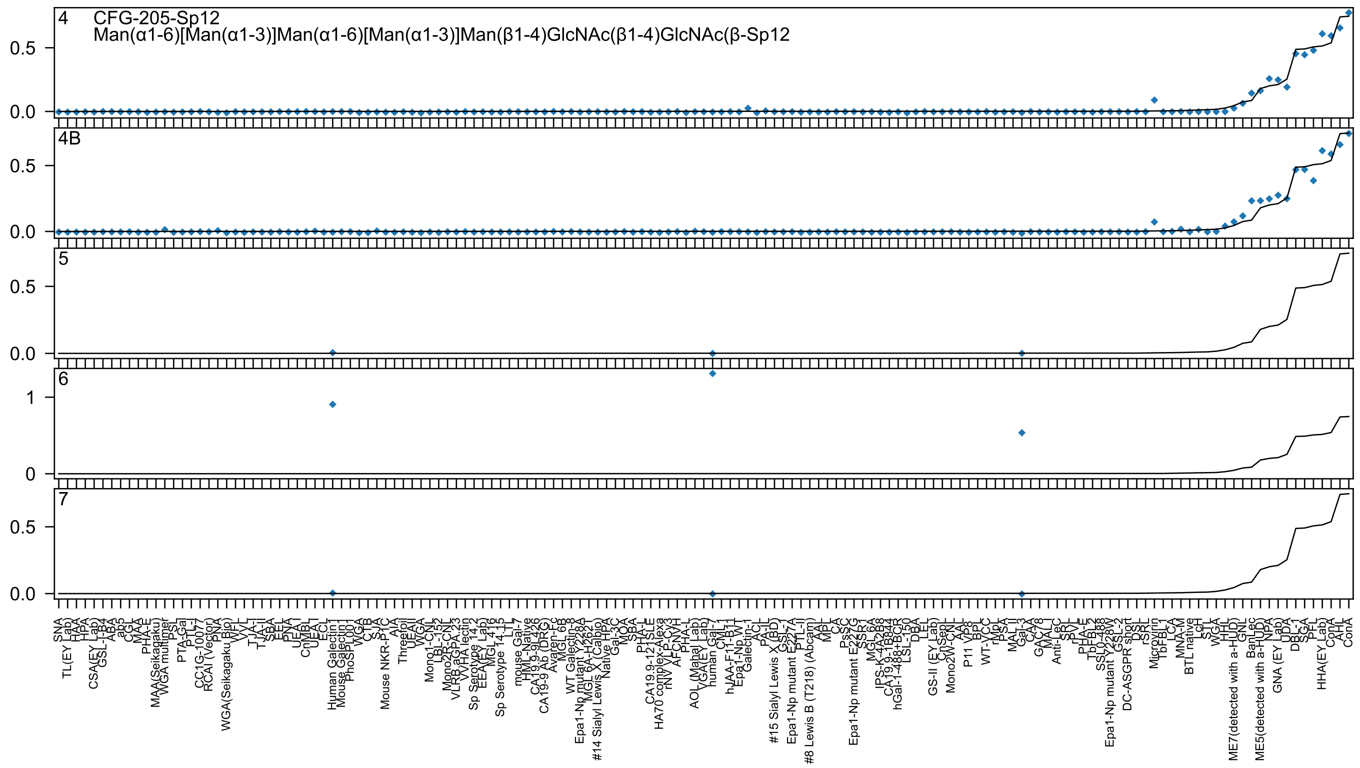

CFG-205-Sp12
Man(α1-6)[Man(α1-3)]Man(α1-6)[Man(α1-3)]Man(β1-4)GlcNAc(β1-4)GlcNAc(β-Sp12
4
4B
5
6
7

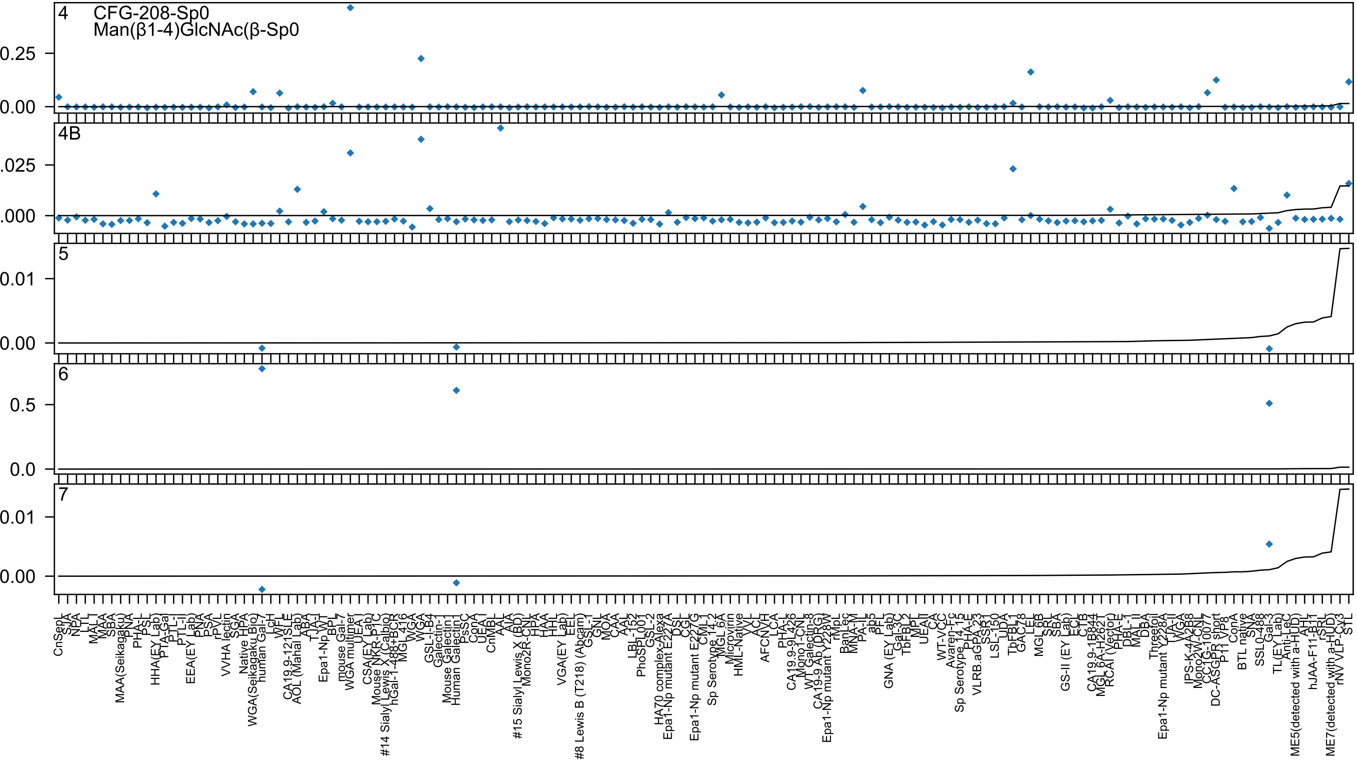

4 CFG-208-Sp0
Man(β1-4)GlcNAc(β-Sp0
4B
5
6
7

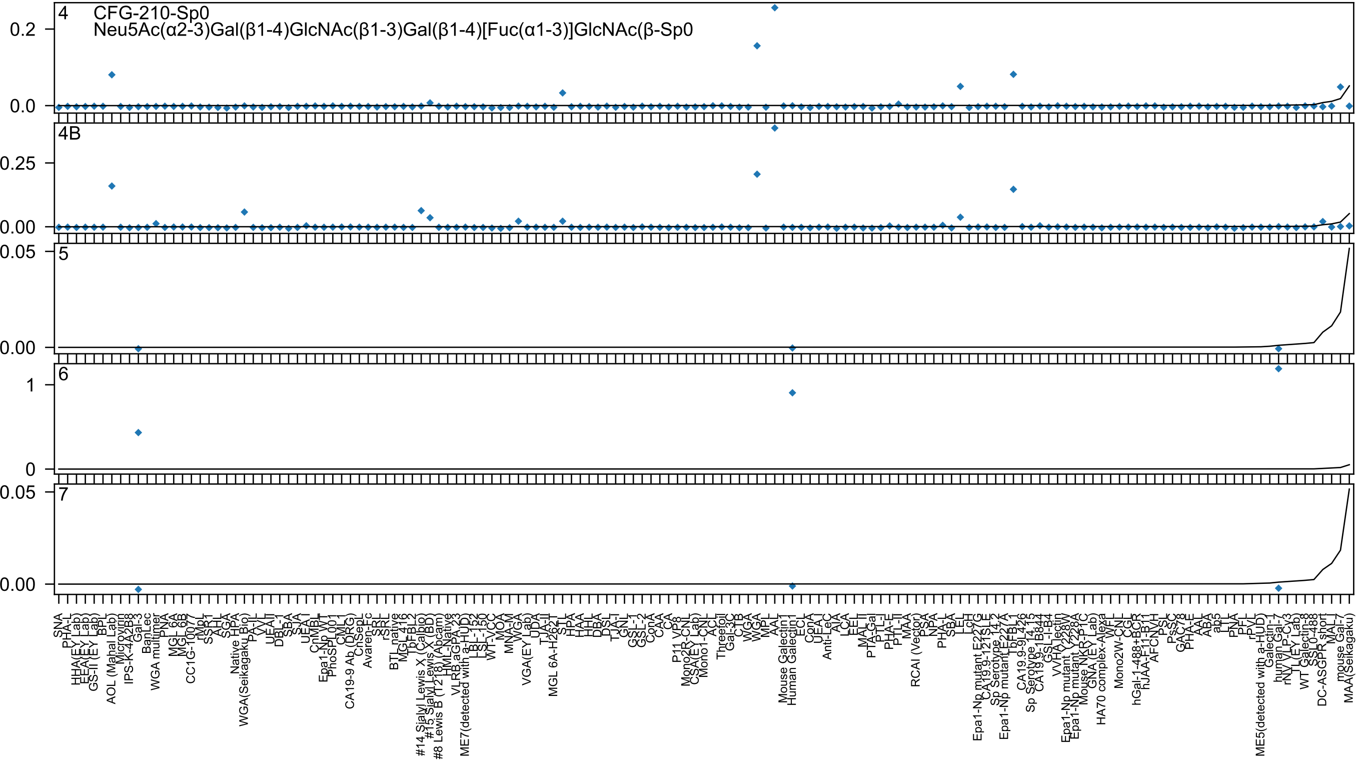

4
CFG-210-Sp0
Neu5Ac(α2-3)Gal(β1-4)GlcNAc(β1-3)Gal(β1-4)[Fuc(α1-3)]GlcNAc(β-Sp0
4B
5
6
7

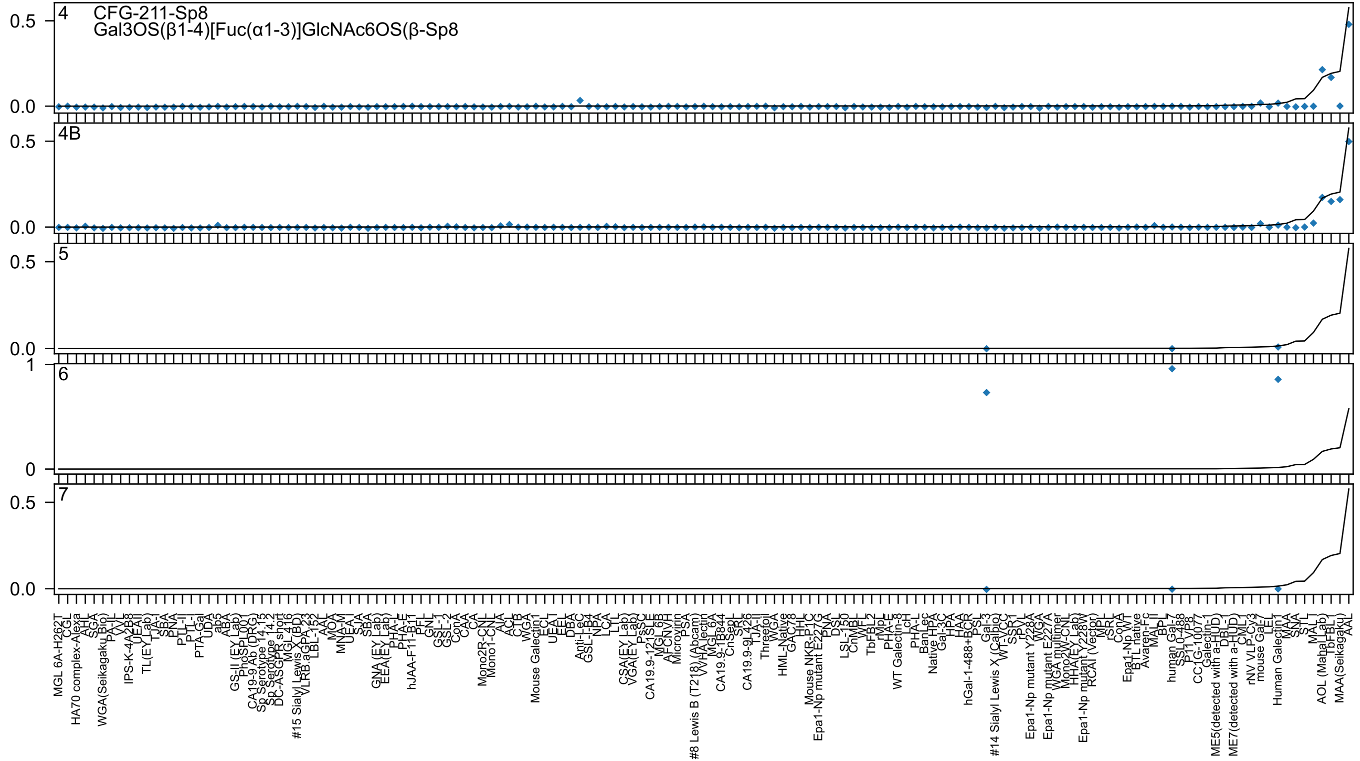
4 CFG-211-Sp8
Gal3OS(β1-4)[Fuc(α1-3)]GlcNAc6OS(β-Sp8
4B
5
6
7
0.5
0.0
1
0
MGL 6A-H262T
CGL
HA70 complex-Alexa
AAL
MAA(Seikagaku)
TbFBL1
AOL (Mahal Lab)
MAL I
STL
SNA
MAA
Human Galectin1
LEL
mouse Gal-7
rNV VLP-Cy3
human Gal-7
#14 Sialyl Lewis X (Calbio)
Gal-3
#15 Sialyl Lewis X (BD)
#8 Lewis B (T218) (Abcam)

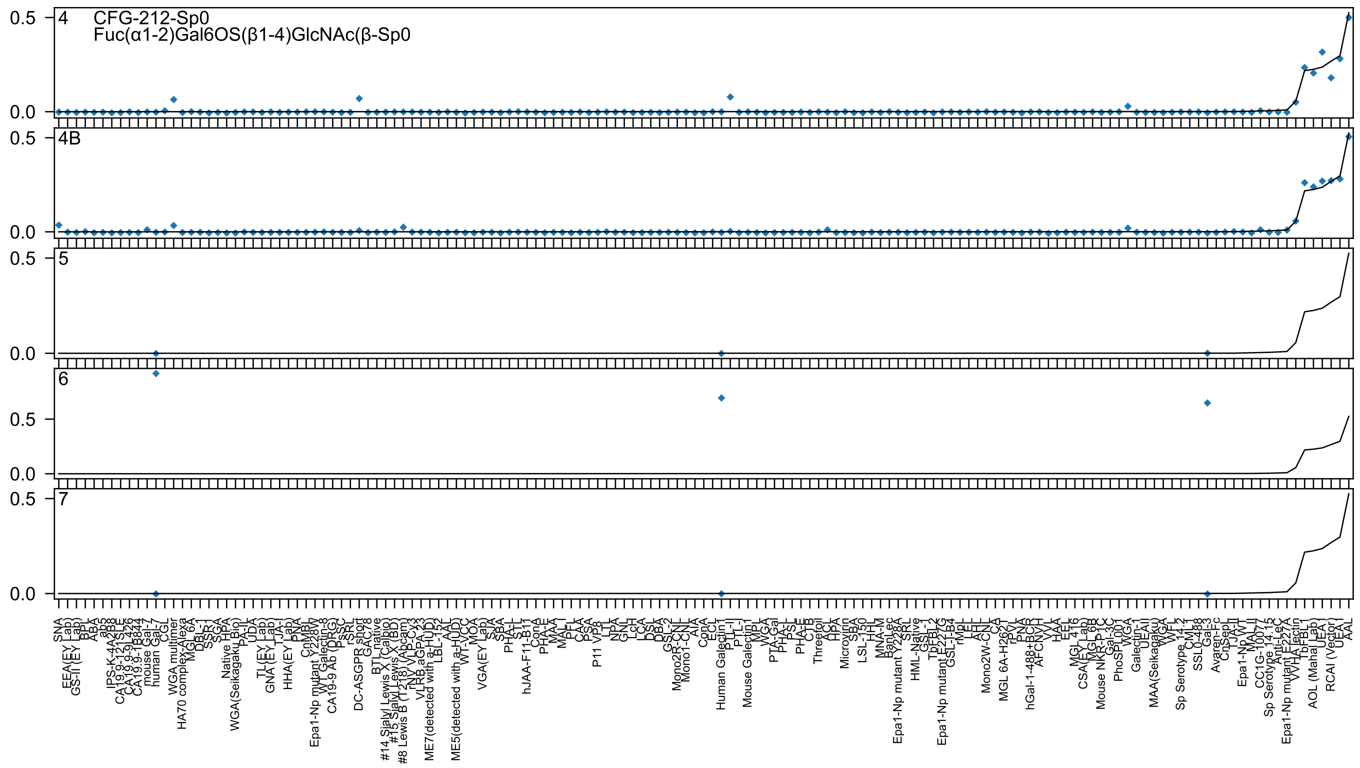

4
CFG-212-Sp0
Fuc(α1-2)Gal6OS(β1-4)GlcNAc(β-Sp0
4B
5
6
7
0.5
0.0

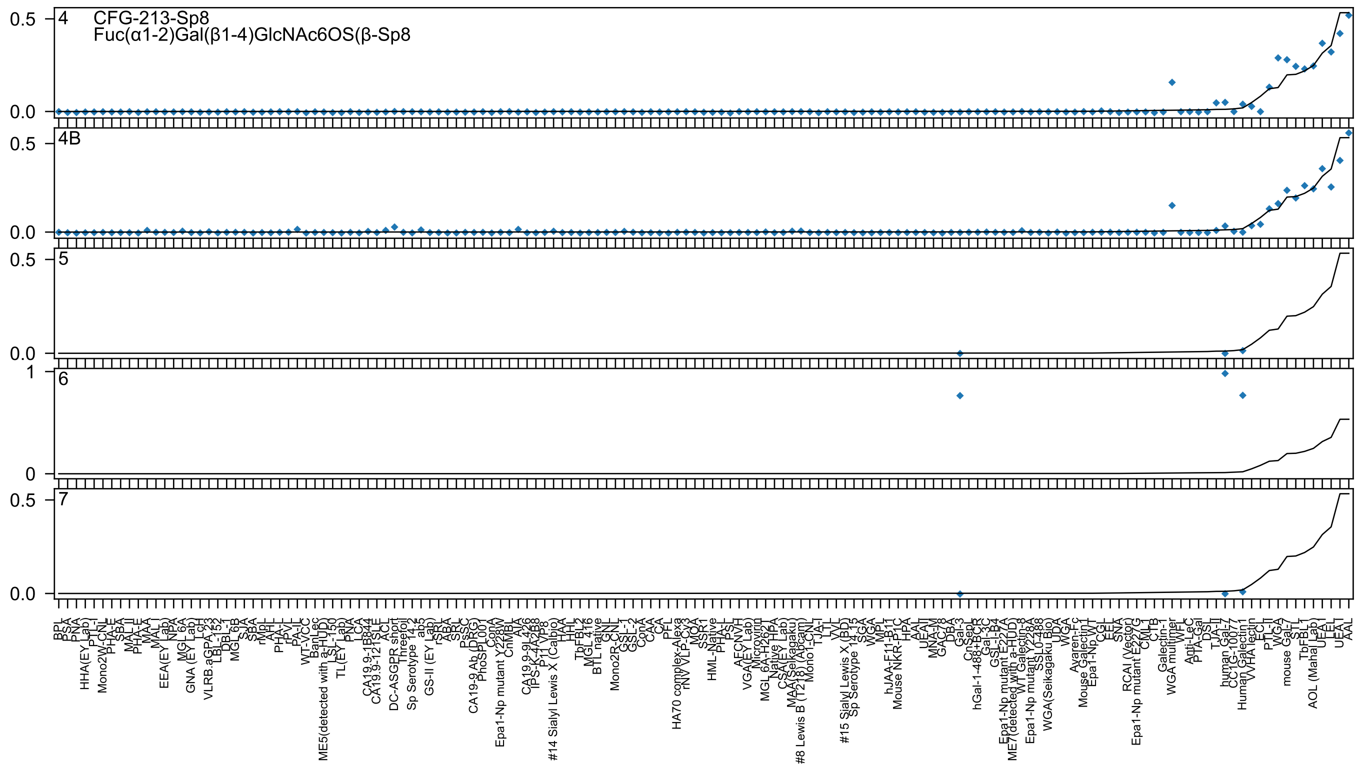

CFG-213-Sp8
Fuc(α1-2)Gal(β1-4)GlcNAc6OS(β-Sp8
4
4B
5
6
7

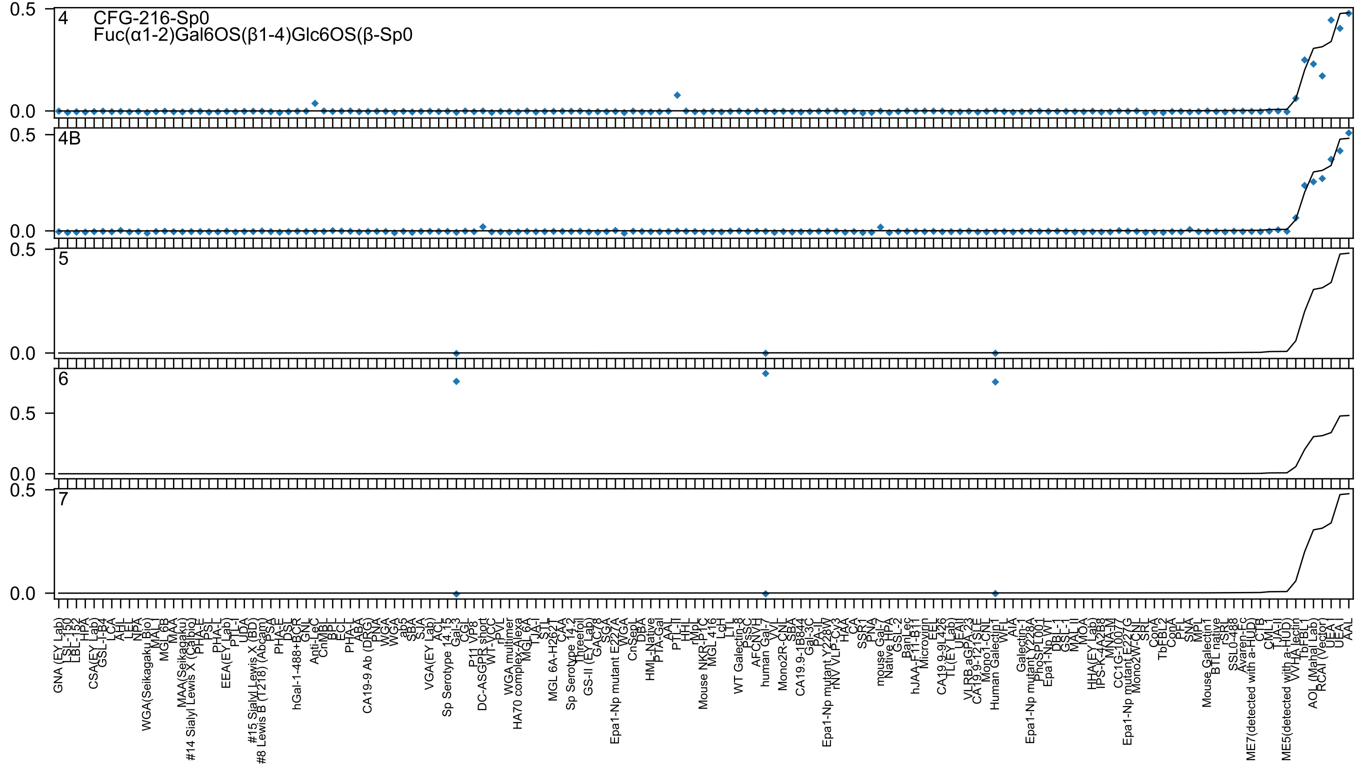

4
CFG-216-Sp0
Fuc(α1-2)Gal6OS(β1-4)Glc6OS(β-Sp0
4B
5
6
7
0.5
0.0
GNA (EY Lab)
LSL-150
LBL-152
HPA
CSA(EY Lab)
GSL-I-B4
LCA
AHL
LEL
NPA
WGA(Seikagaku Bio)
MAL
MGL 6B
MAA
MAA(Seikagaku)
#14 Sialyl Lewis X (Calbio)
PHA-E
PSL
PHA-L
EEA(EY Lab)
PTL-I
UDA
#15 Sialyl Lewis X (BD)
#8 Lewis B (T218) (Abcam)
PSA
PHA-E
DSL
hGal-1-488+BCR
GNL
Anti-LeC
CnMBP
BPL
ECL
PHA-L
ABA
CA19-9 Ab (DRG)
PNA
WGA
WGA
ab5
SBA
SJA
VGA(EY Lab)
ACL
Sp Serotype 14.15
Gal-3
CGL
VP8
P11 VP8
DC-ASGPR short
WT-VCC
rPVL
WGA multimer
HA70 complex-Alexa
MGL 6A
TJA-I
STL
MGL 6A-H262T
CAA
Sp Serotype 14.2
Threefoil
GS-II (EY Lab)
GAC78
SGA
Epa1-Np mutant E227A
WGA
CnSep
DBA
HML-Native
PTA-Gal
AAL
PTL-II
HHL
rMpL
Mouse NKR-P1C
MGL 416
LcH
LTL
WT Galectin-8
PsSC
AFCNVH
human Gal-7
VVL
Mono2R-CNL
SBA
CA19.9-1B844
Gal-3C
PA-IL
Epa1-Np mutant Y228W
rNV VLP-CV3
HAA
CA
SSR1
PNA
mouse Gal-7
Native HPA
GSL-2
BanLec
hJAA-F11-B11
Microvirin
EEL
CA19.9-9L426
TL(EY Lab)
UEAII
VLRB aGPA.23
CA19.9-121SLE
Mono1-CNL
Human Galectin1
WFL
AIA
Galectin-1
Epa1-Np mutant Y228A
PhoSL001
Epa1-Np WT
DBL-1
GSL-1
MAL II
MOA
HHA(EY Lab)
IPS-K-4A2B8
MNA-M
CC1G-10077
Epa1-Np mutant E227G
Mono2W-CNL
SRL
ConA
TbFBL2
ConA
PFL
SNA
MPL
Mouse Galectin1
BTL native
rSRL
SSL0-488
Avaren-Fc
ME7(detected with a-HUD)
CTB
CML-1
TJA-II
ME5(detected with a-HUD)
WHA lectin
TbFBL1
AOL (Mahal Lab)
RCAI (Vector)
UEA I
UEA I
AAL

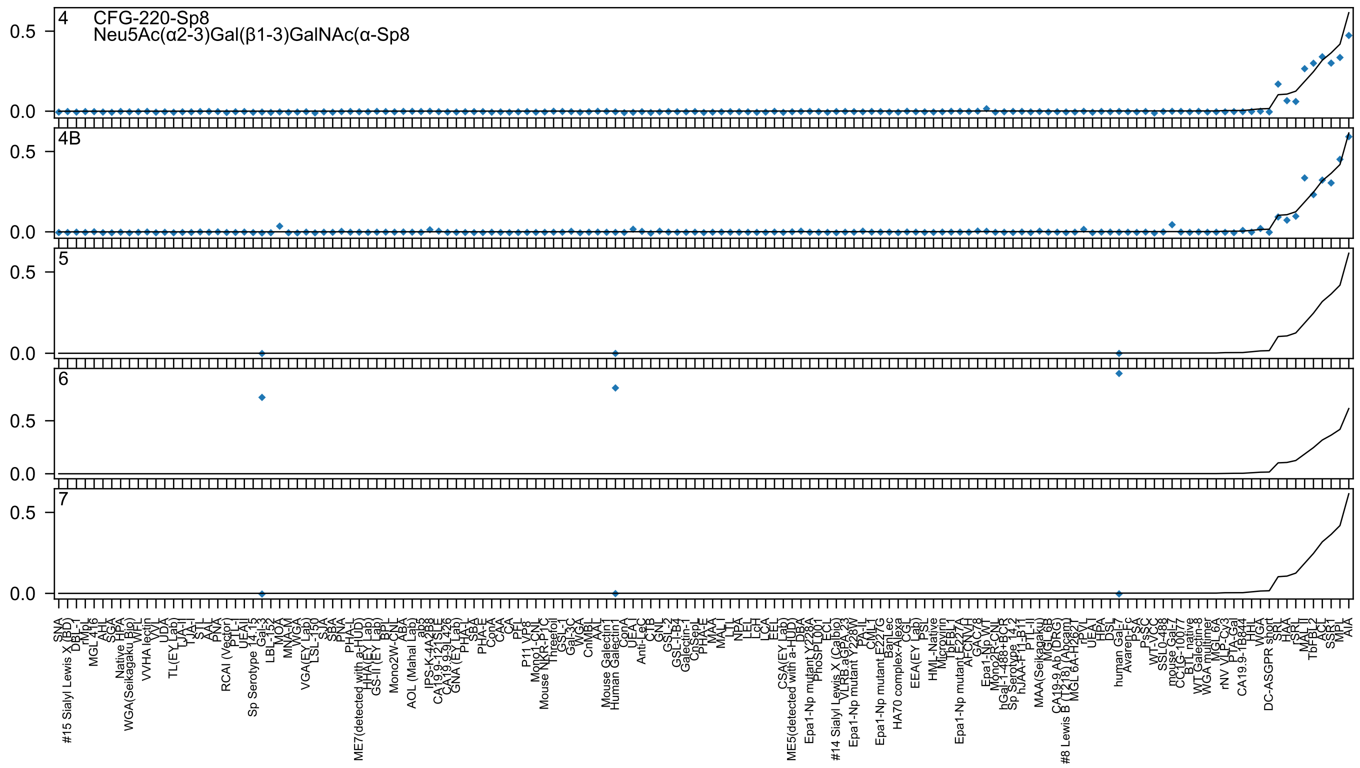

4
CFG-220-Sp8
Neu5Ac(α2-3)Gal(β1-3)GalNAc(α-Sp8
4B
5
6
7
0.5
0.0

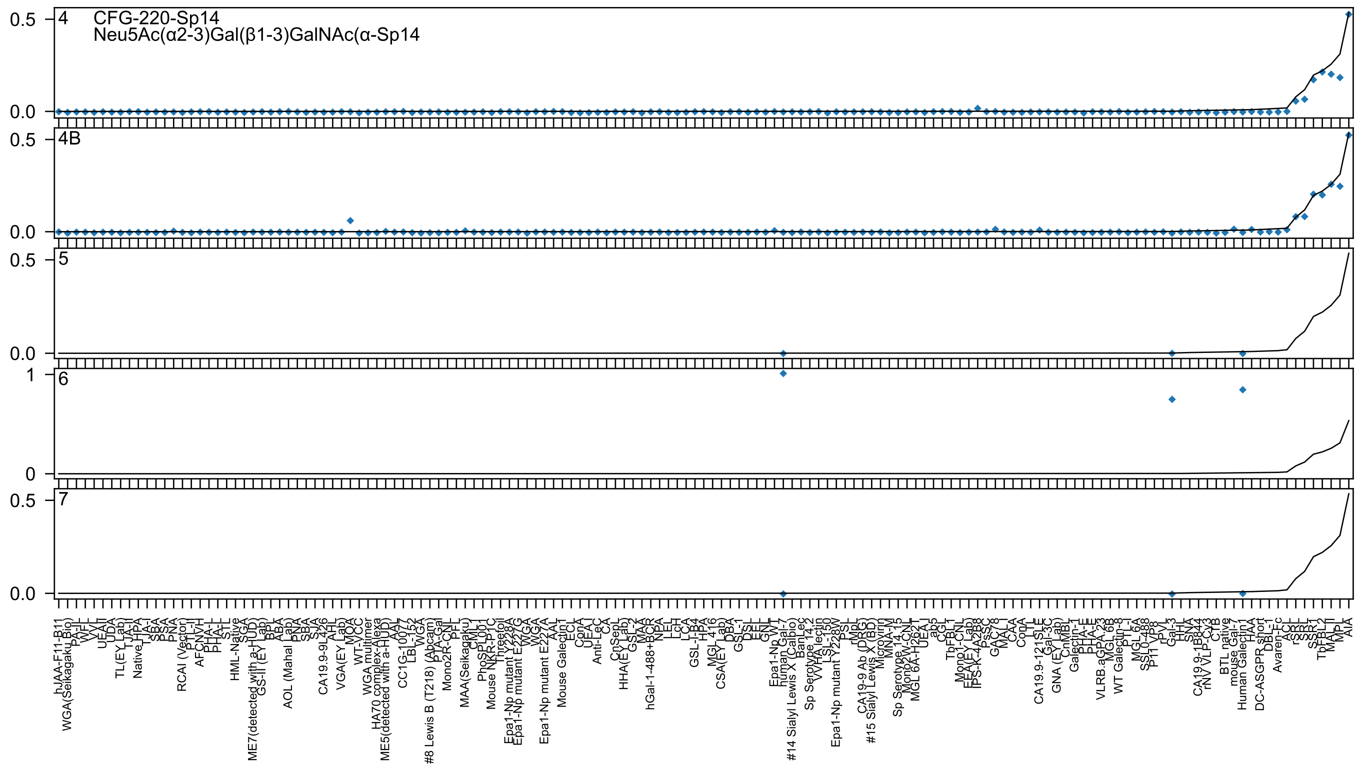

CFG-220-Sp14
Neu5Ac(α2-3)Gal(β1-3)GalNAc(α-Sp14
4
4B
5
6
7
0.5
0.0
1
0

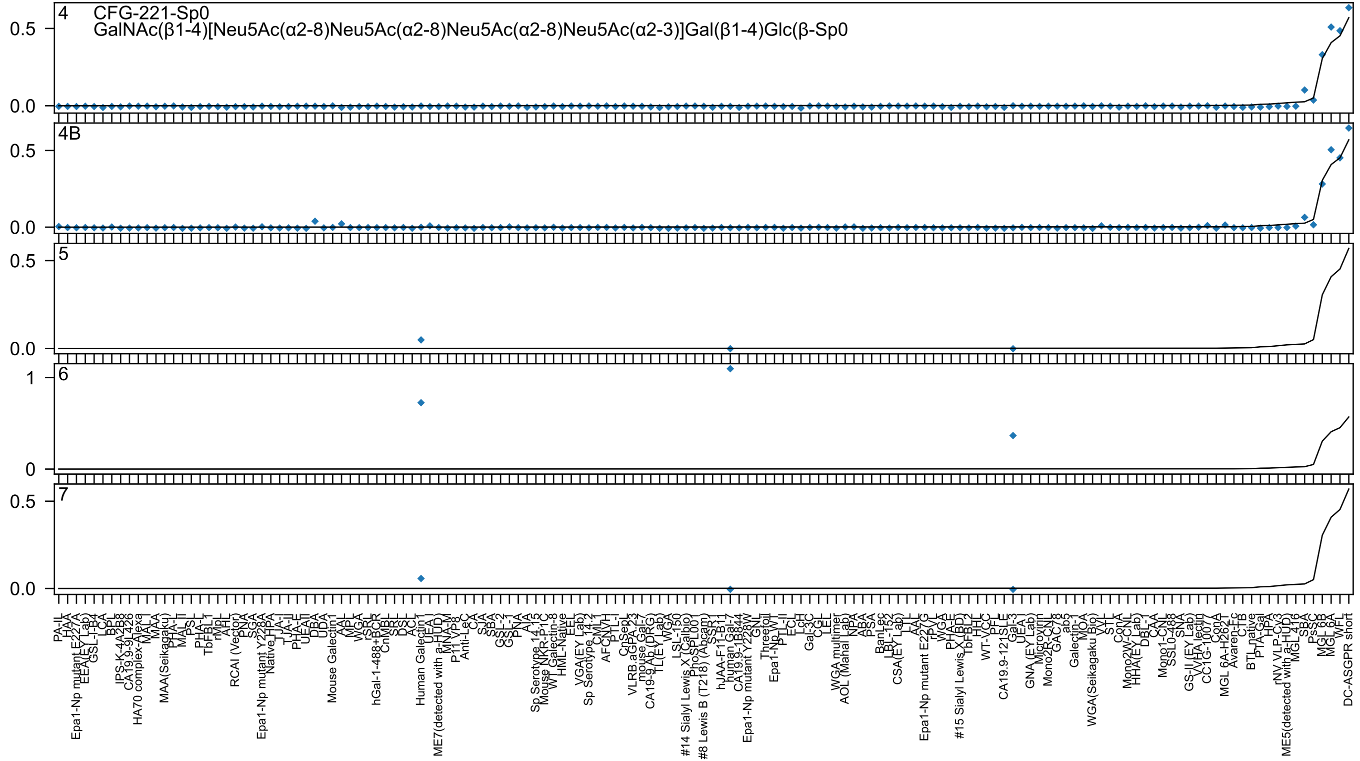
4 CFG-221-Sp0
GalNAc(β1-4)[Neu5Ac(α2-8)Neu5Ac(α2-8)Neu5Ac(α2-8)Neu5Ac(α2-3)]Gal(β1-4)Glc(β-Sp0
4B
5
6
7

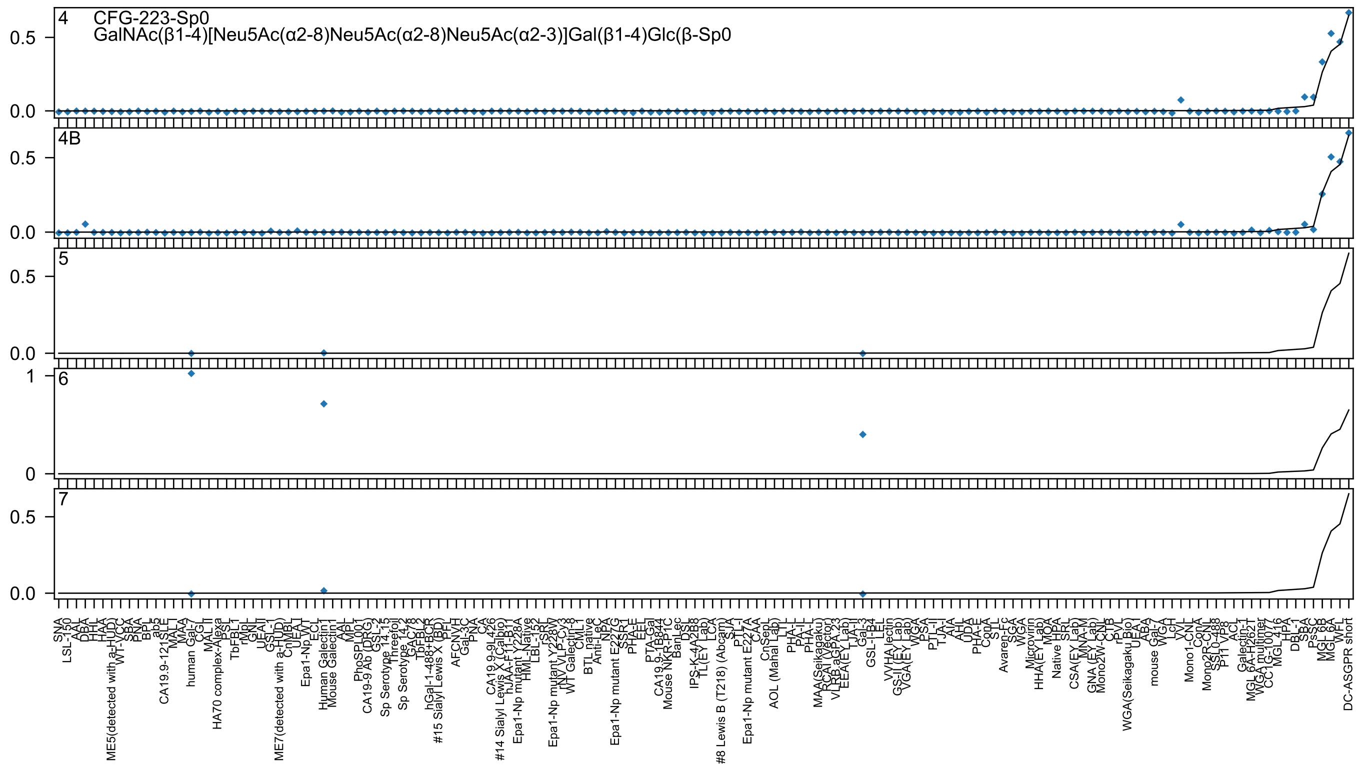
4
CFG-223-Sp0
GalNAc(β1-4)[Neu5Ac(α2-8)Neu5Ac(α2-8)Neu5Ac(α2-3)]Gal(β1-4)Glc(β-Sp0
4B
5
6
7

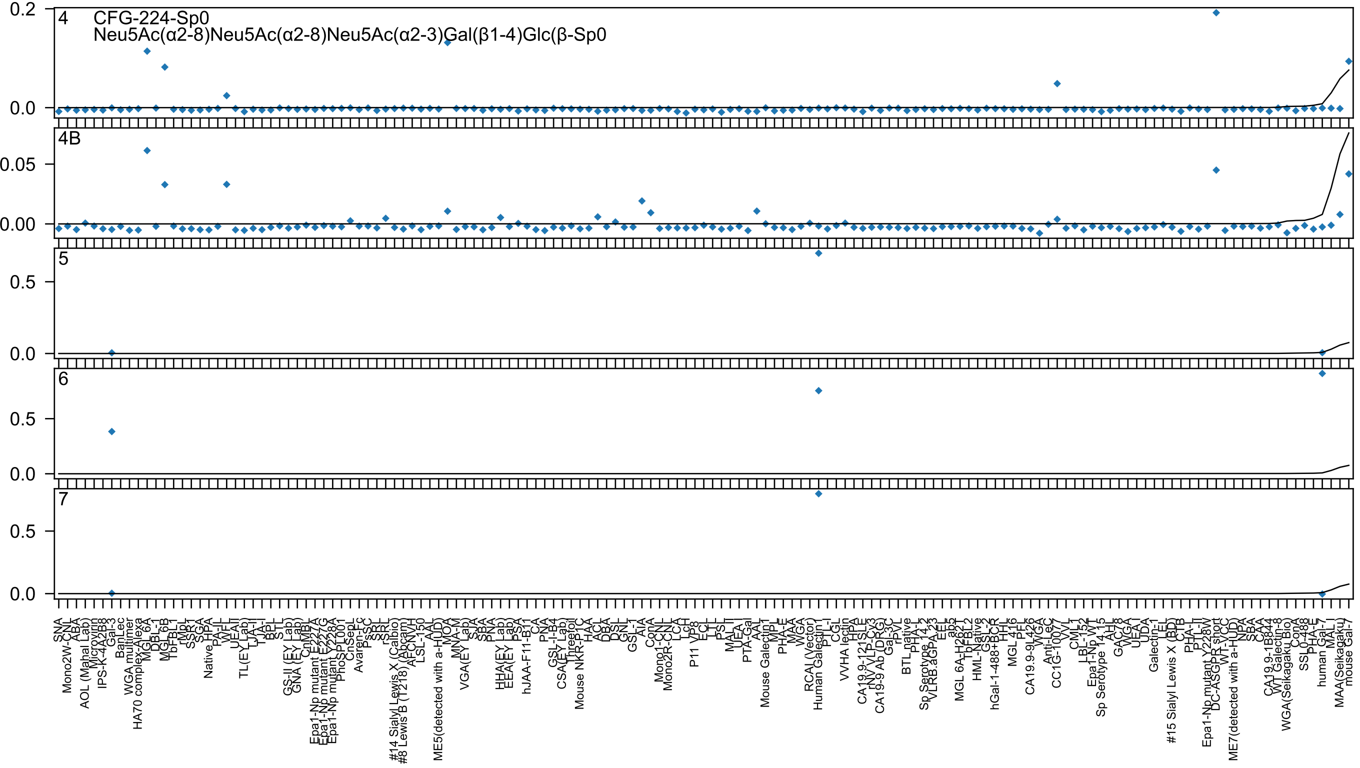
CFG-224-Sp0
Neu5Ac(α2-8)Neu5Ac(α2-8)Neu5Ac(α2-3)Gal(β1-4)Glc(β-Sp0
4
4B
5
6
7

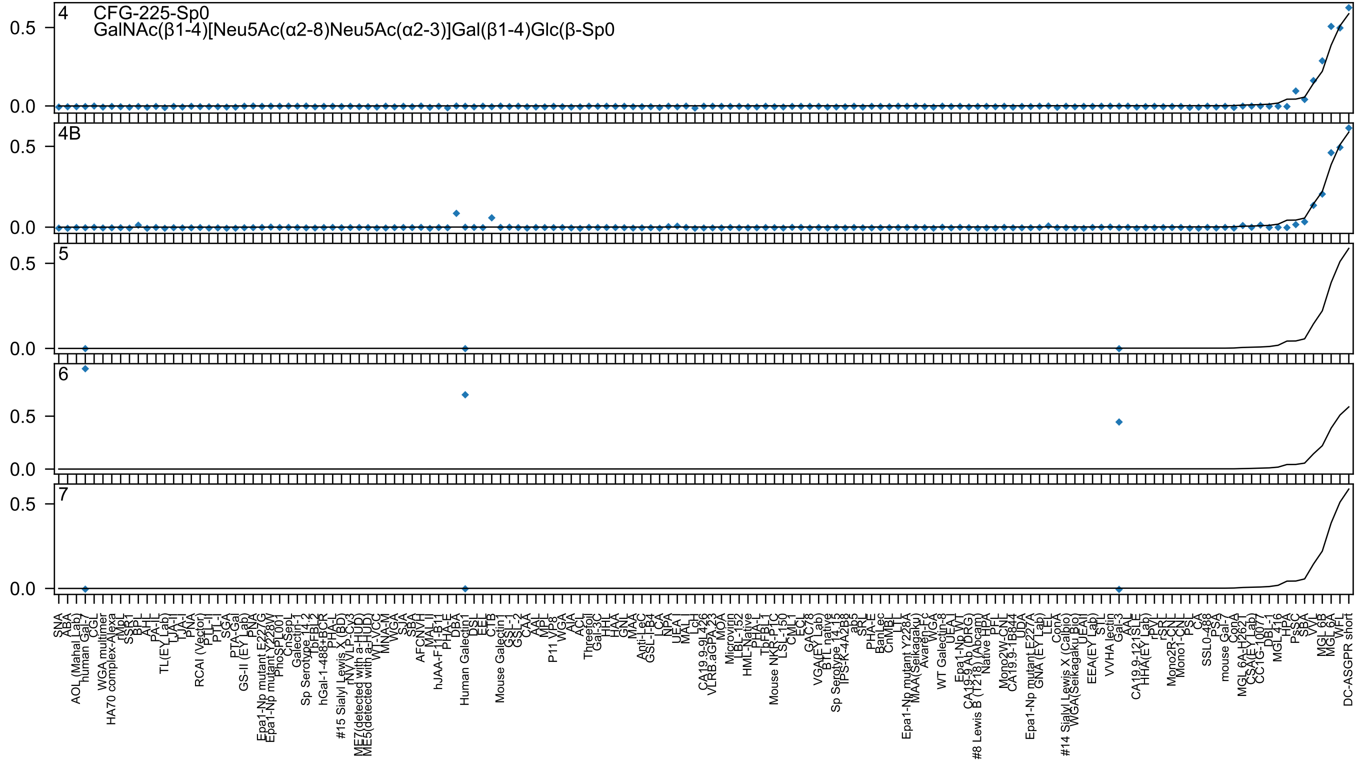

4
CFG-225-Sp0
GalNAc(β1-4)[Neu5Ac(α2-8)Neu5Ac(α2-3)]Gal(β1-4)Glc(β-Sp0
4B
5
6
7
0.5
0.0

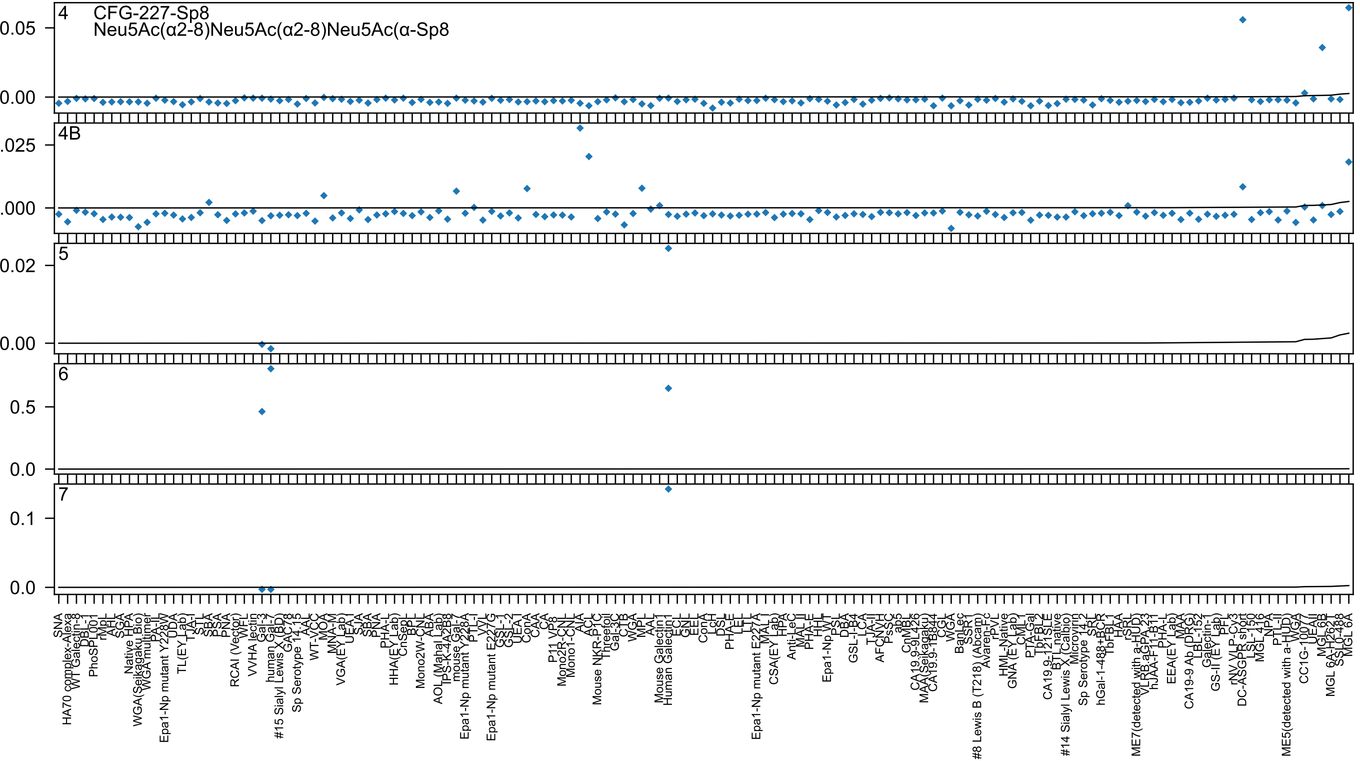
4
CFG-227-Sp8
Neu5Ac(α2-8)Neu5Ac(α2-8)Neu5Ac(α-Sp8
4B
5
6
7

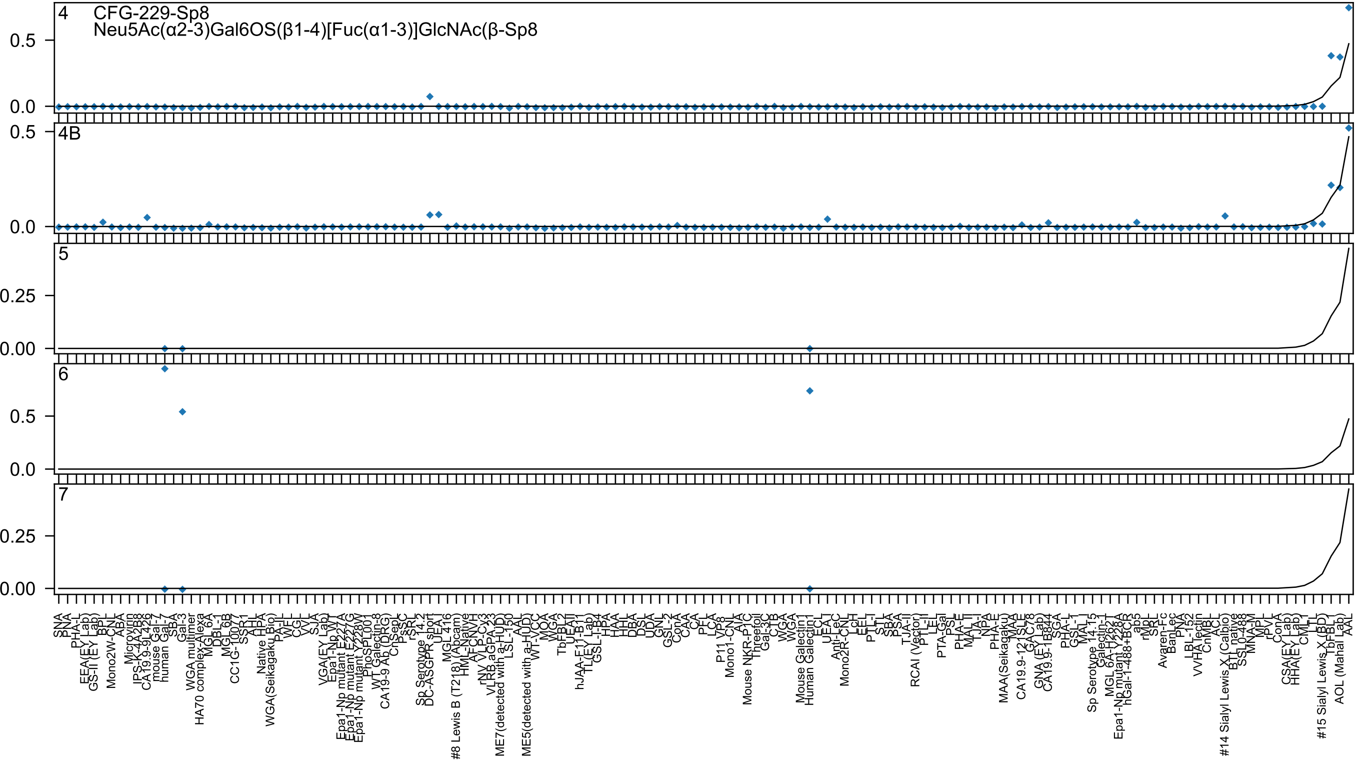

4 CFG-229-Sp8
Neu5Ac(α2-3)Gal6OS(β1-4)[Fuc(α1-3)]GlcNAc(β-Sp8
4B
5
6
7

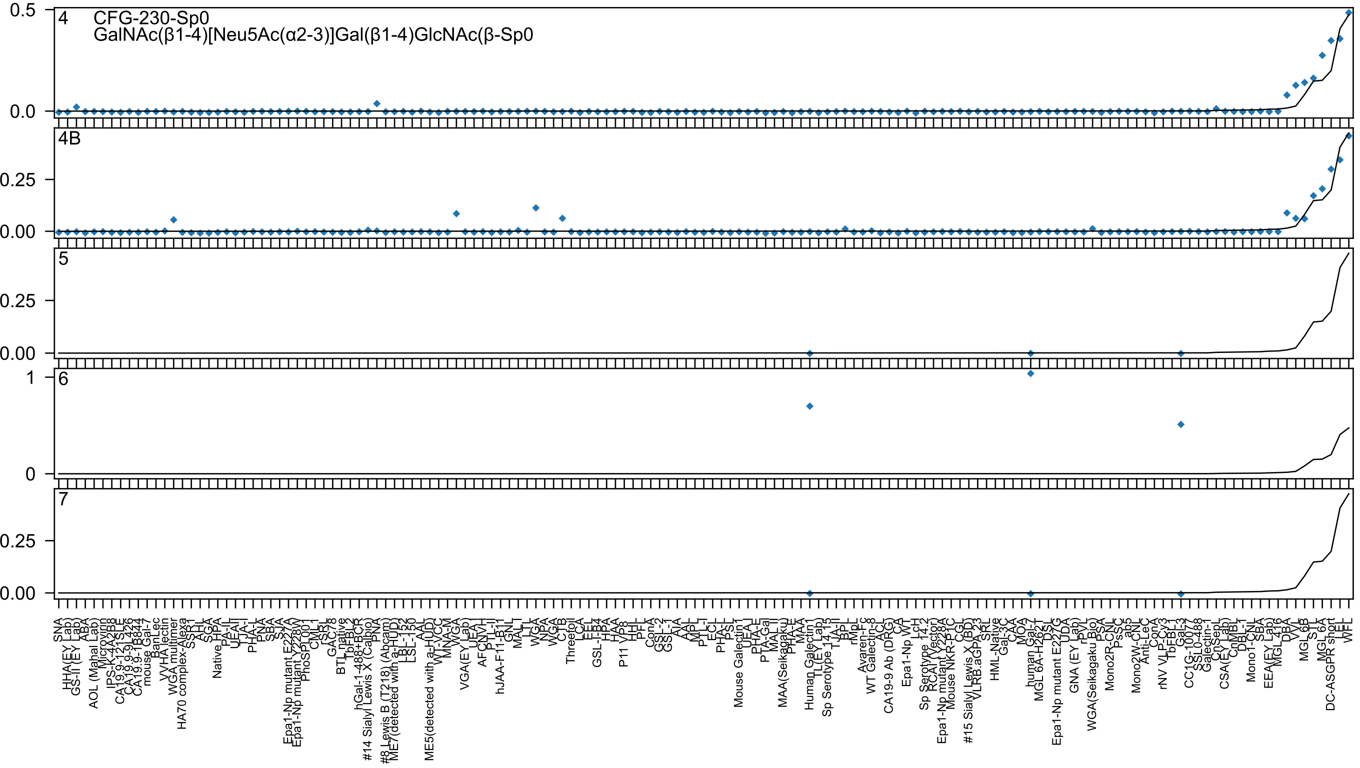
CFG-230-Sp0
GalNAc(β1-4)[Neu5Ac(α2-3)]Gal(β1-4)GlcNAc(β-Sp0
4
4B
5
6
7

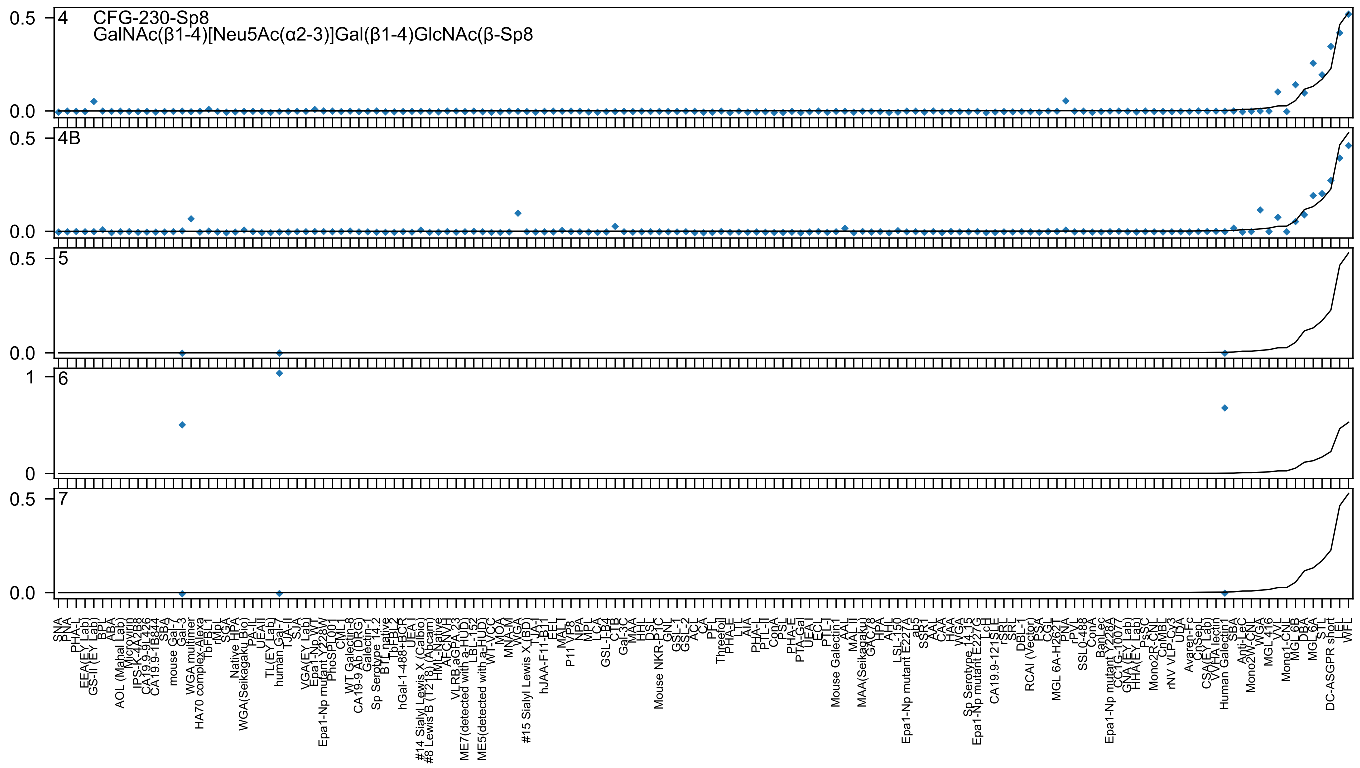
CFG-230-Sp8
GalNAc(β1-4)[Neu5Ac(α2-3)]Gal(β1-4)GlcNAc(β-Sp8
4
4B
5
6
7

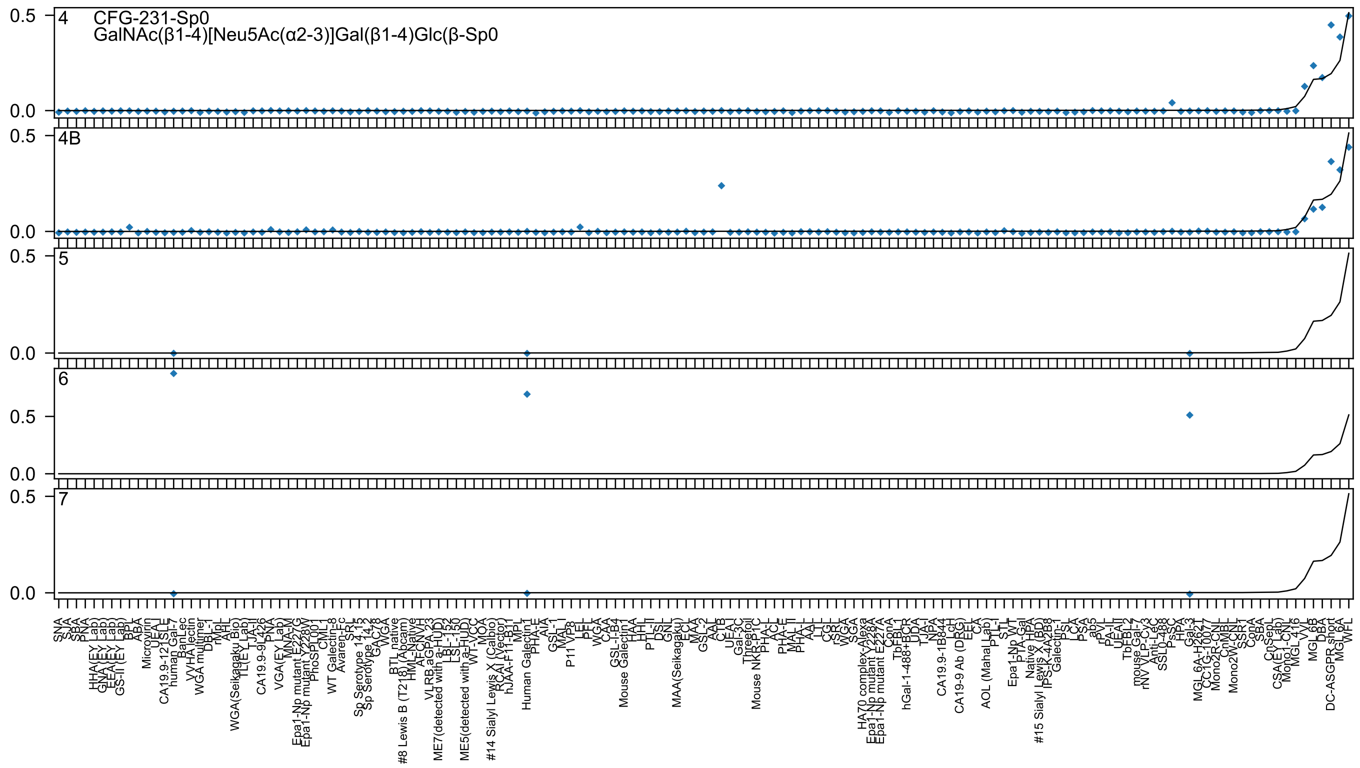

4
CFG-231-Sp0
GalNAc(β1-4)[Neu5Ac(α2-3)]Gal(β1-4)Glc(β-Sp0
4B
5
6
7

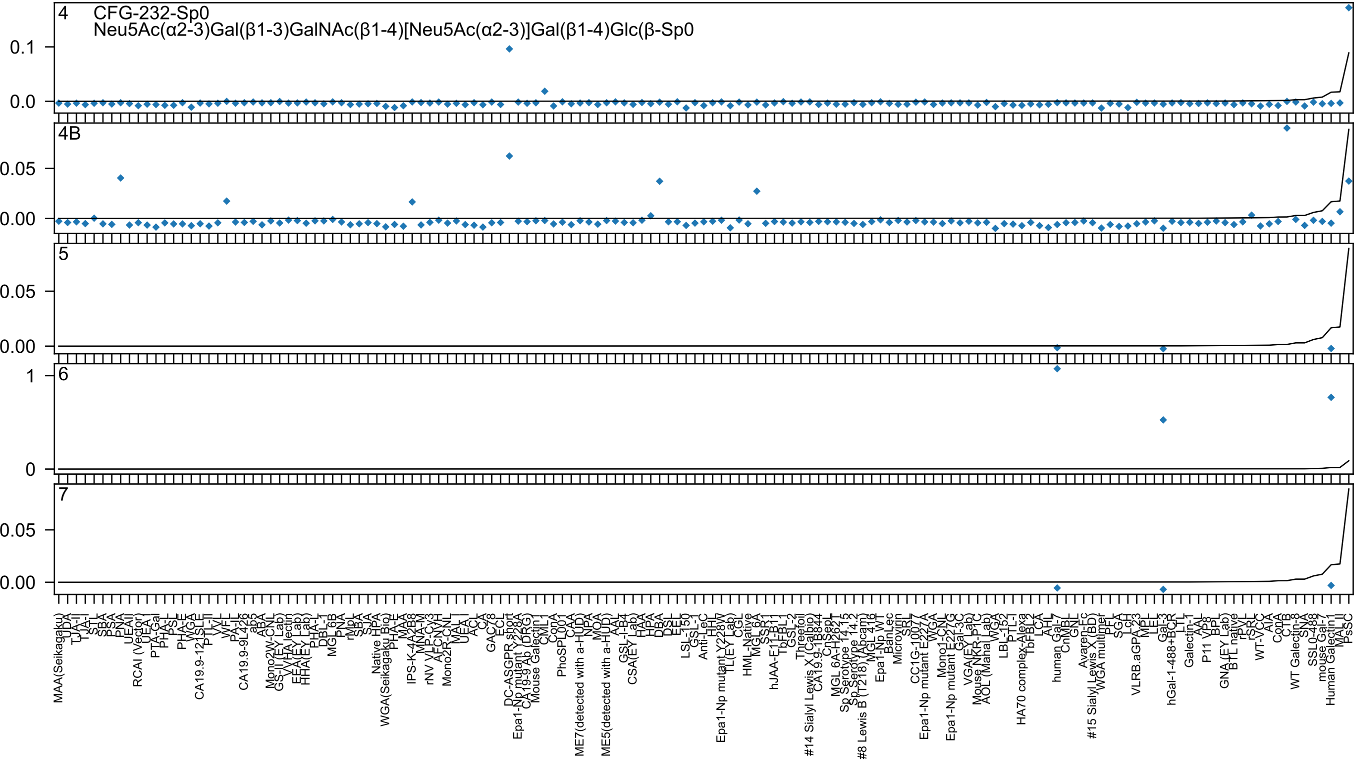

4 CFG-232-Sp0
Neu5Ac(α2-3)Gal(β1-3)GalNAc(β1-4)[Neu5Ac(α2-3)]Gal(β1-4)Glc(β-Sp0
4B
5
6
7

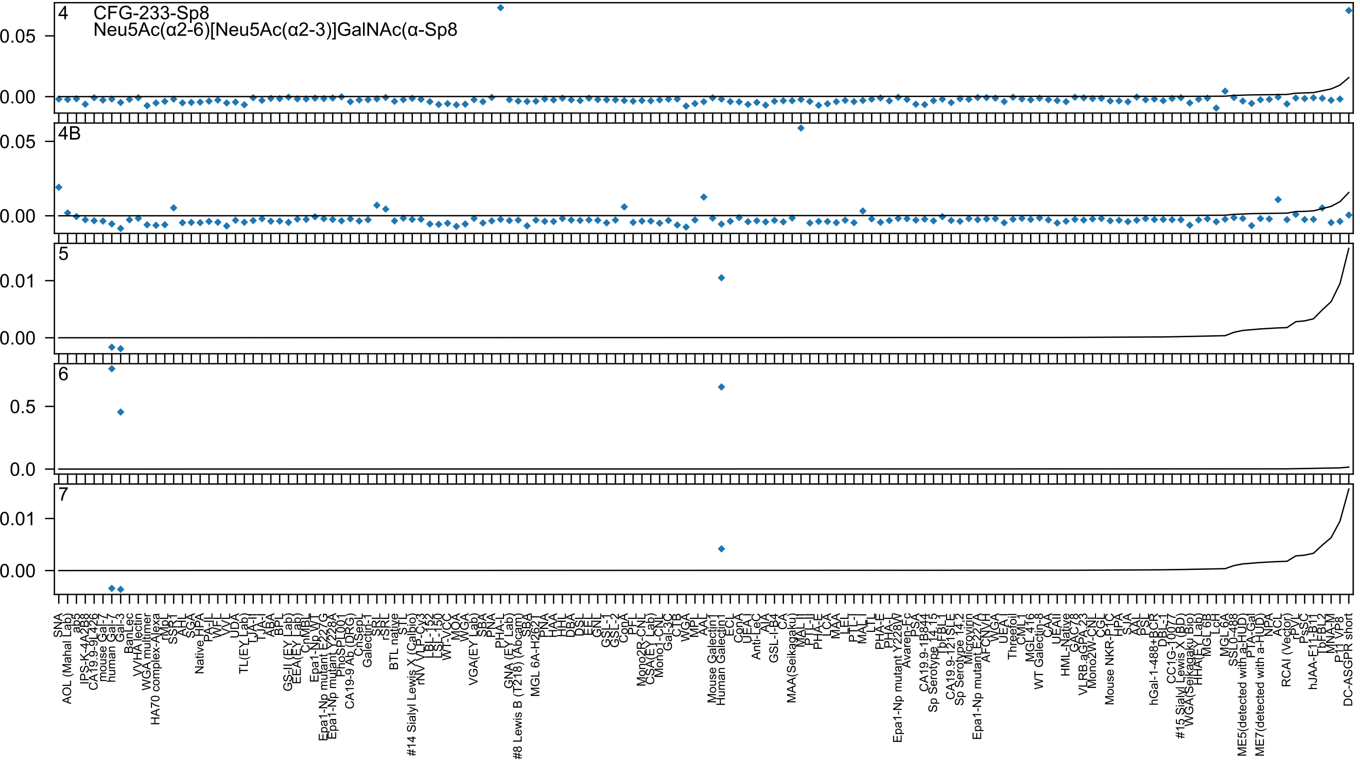
4
CFG-233-Sp8
Neu5Ac(α2-6)[Neu5Ac(α2-3)]GalNAc(α-Sp8
0.05
0.00
4B
5
0.01
6
0.5
0.0
7
SNA
AOL (Mahal Lab)
ab5
IPS-K-4A2B8
CA19.9-9L426
mouse Gal-7
human Gal-7
Gal-3
BanLec
VVHA lectin
WGA multimer
HA70 complex-Alexa
rMpL
SSR1
AHL
SGA
Native HPA
PA-IL
WFL
VVL
UDA
TL(EY Lab)
TJA-II
TJA-I
ABA
BPL
GS-II (EY Lab)
EEA(EY Lab)
CnMBL
Epa1-Np WT
Epa1-Np mutant E227G
Epa1-Np mutant Y228A
PhoSL001
CA19-9 Ab (DRG)
ChSep1
Galectin-1
SRL
rSRL
BTL native
STL
#14 Sialyl Lewis X (Calbio)
rNV VLP-Cy3
LBL-152
LSL-150
WT-VCC
MOA
WGA
VGA(EY Lab)
SBA
PNA
PHA-L
GNA (EY Lab)
#8 Lewis B (T218) (Abcam)
SBA
MGL 6A-H262T
PNA
HAA
HHL
DBA
DSL
EEL
GNL
GSL-1
GSL-2
ConA
PFL
Mono2R-CNL
CSA(EY Lab)
Mono1-CNL
Gal-3C
CTB
WGA
MPL
AAL
Mouse Galectin1
Human Galectin1
ECL
ConA
UEA-I
Anti-LeC
AIA
GSL-I-B4
CA
MAA(Seikagaku)
MAL-II
PTL-II
PHA-E
LCA
MAA
LEL
PTL-I
MAL-I
LTL
PHA-E
PHA-L
Epa1-Np mutant Y228W
Avaren-Fc
PSA
CA19.9-1B844
Sp Serotype 14.15
TbFBL1
CA19.9-121SLE
Sp Serotype 14.2
Microvirin
Epa1-Np mutant E227A
AFCNVH
WGA
UEA
Threefoil
CML1
MGL 416
WT Galectin-8
CAA
UEAII
HML-Native
GAC78
VLRB.aGPA.23
Mono2W-CNL
CGL
Mouse NKR-P1C
HPA
SJA
AAL
PSL
hGal-1-488+BCR
DBL-1
CC1G-10077
#15 Sialyl Lewis X (BD)
WGA(Seikagaku Bio)
HHA(EY Lab)
MGL 6B
LcH
MGL6A
SSL0-488
ME5(detected with a-HUD)
PTA-Gal
ME7(detected with a-HUD)
NPA
ACL
RCAI (Vector)
rPVL
PsSC
hJAA-E11-B11
TbFBL2
MNA-M
P11 VP8
DC-ASGPR short

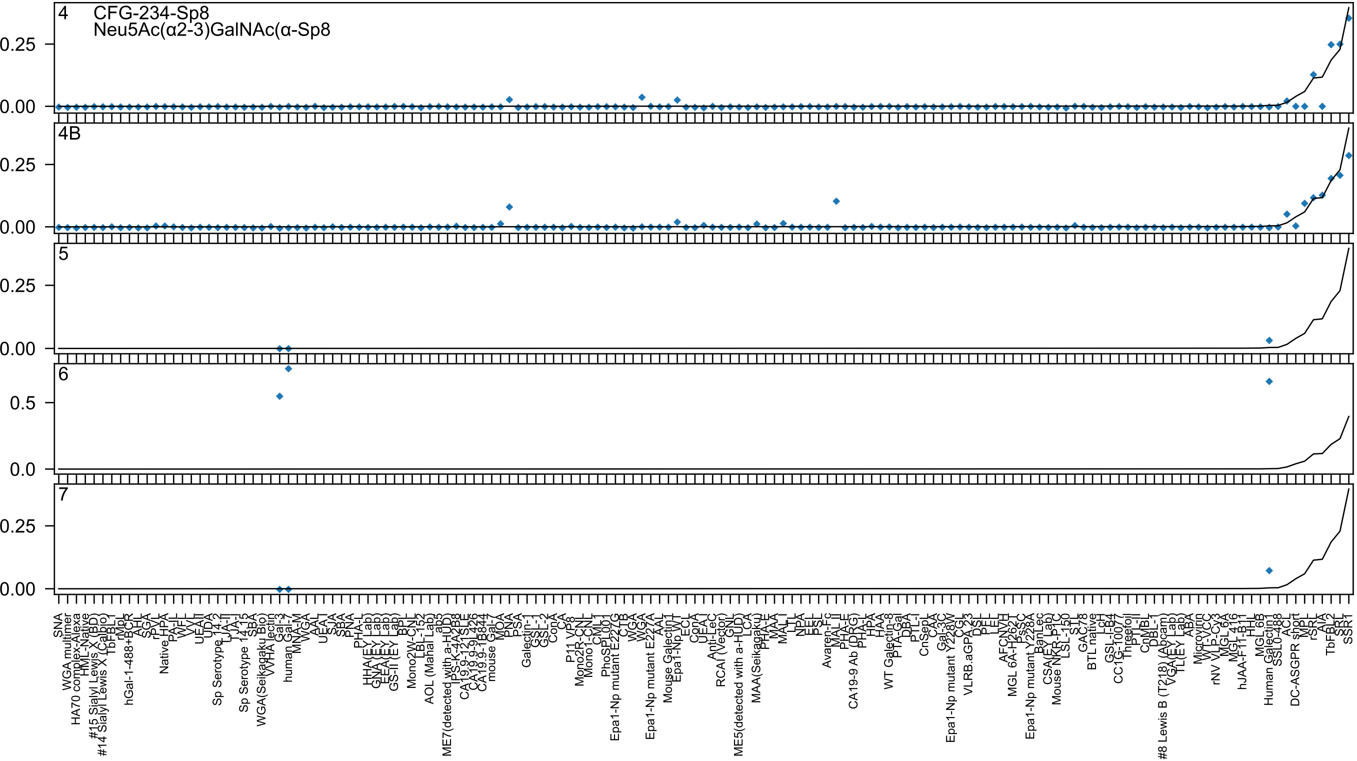

4
CFG-234-Sp8
Neu5Ac(α2-3)GalNAc(α-Sp8
0.25
0.00
4B
0.25
0.00
5
0.25
0.00
6
0.5
0.0
7
0.25
0.00

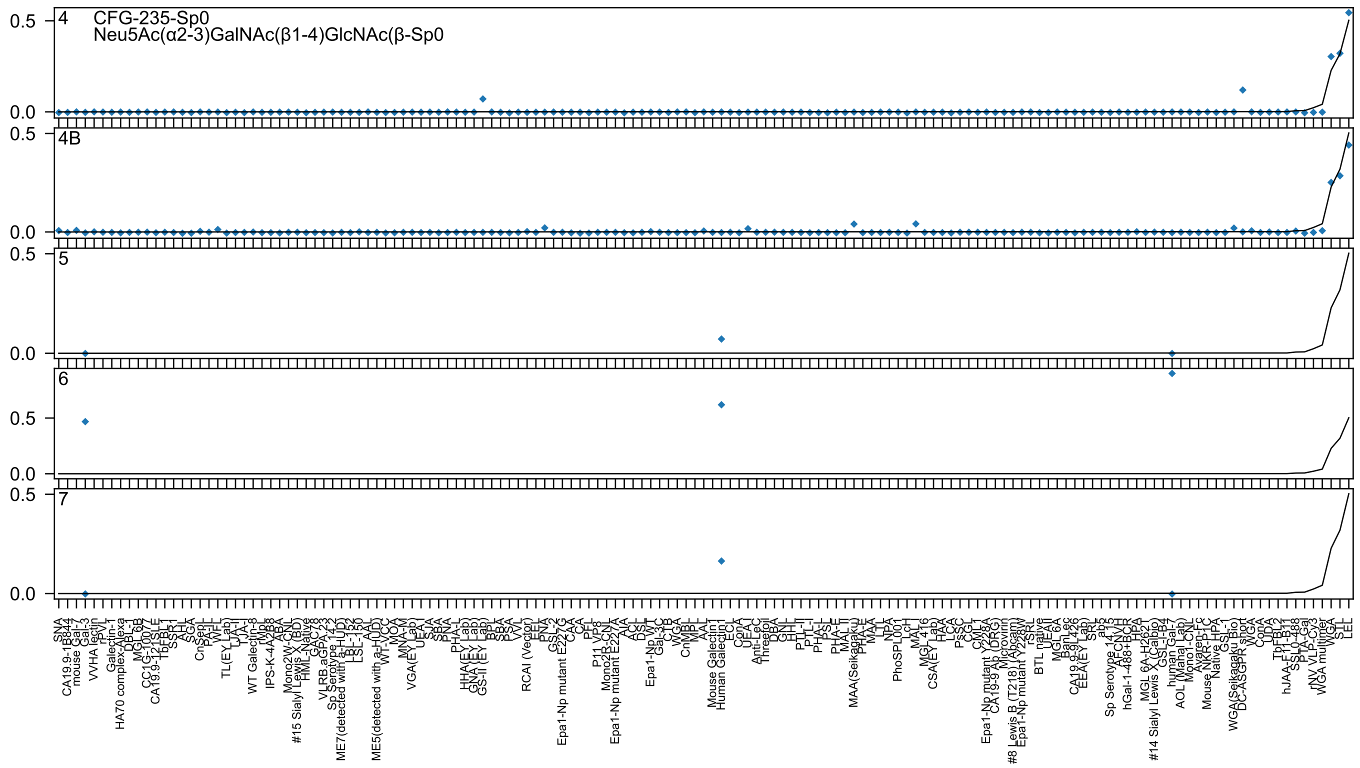
4
CFG-235-Sp0
Neu5Ac(α2-3)GalNAc(β1-4)GlcNAc(β-Sp0
4B
5
6
7
0.5
0.0

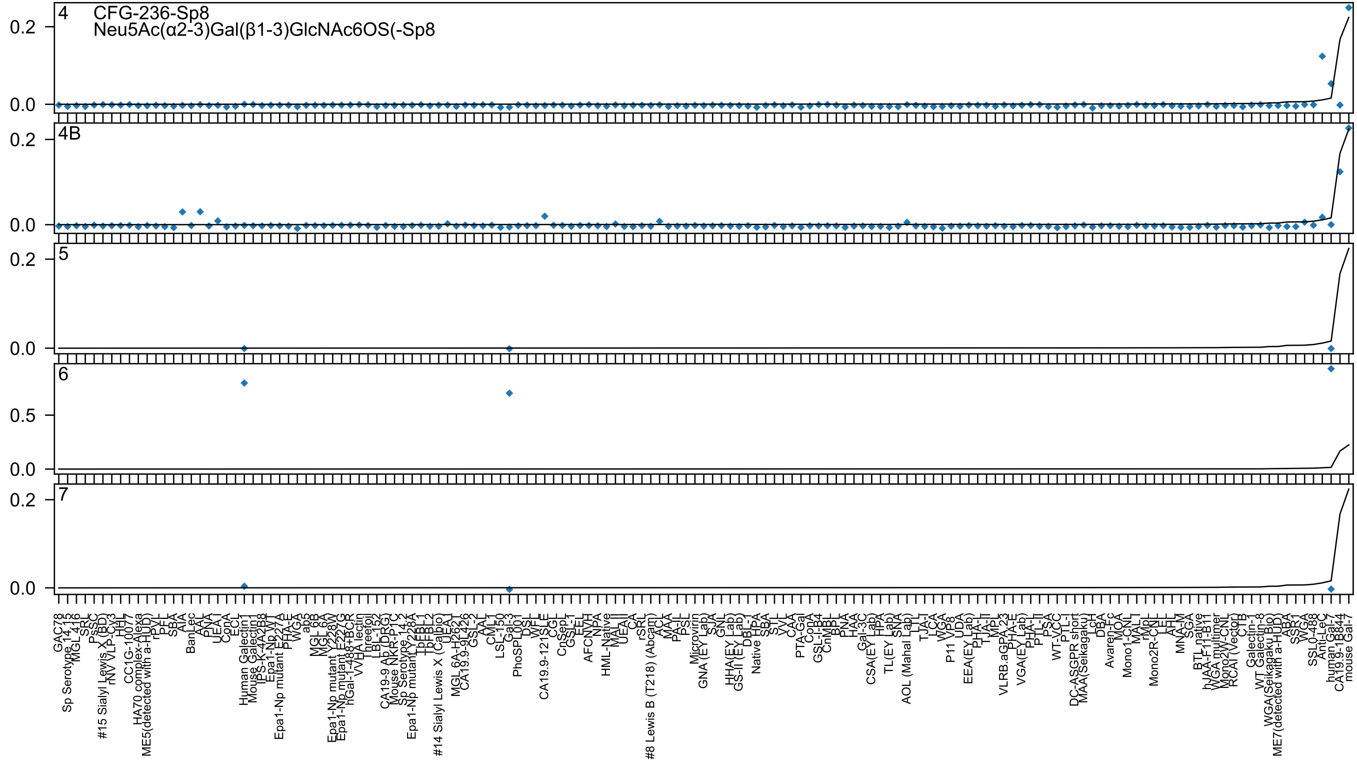
4
CFG-236-Sp8
Neu5Ac(α2-3)Gal(β1-3)GlcNAc6OS(-Sp8
4B
5
6
7

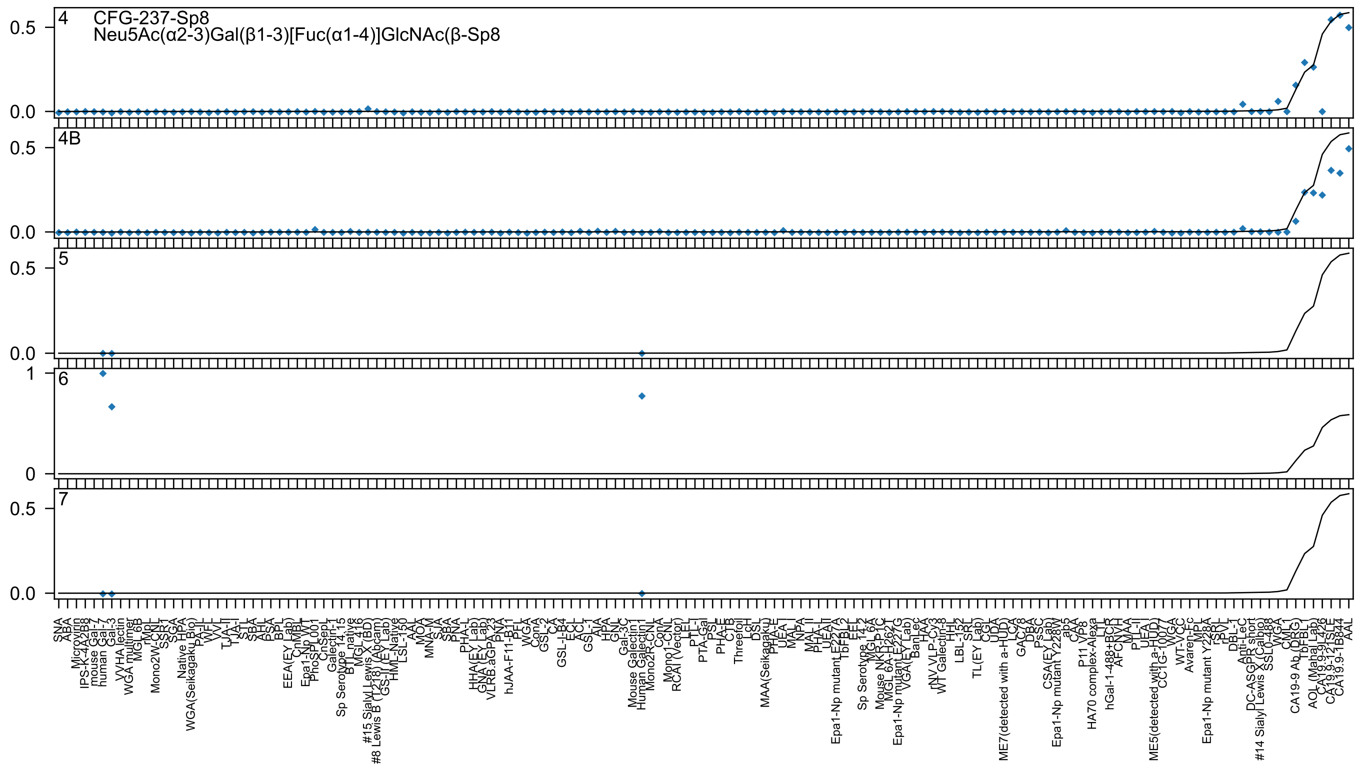

4
CFG-237-Sp8
Neu5Ac(α2-3)Gal(β1-3)[Fuc(α1-4)]GlcNAc(β-Sp8
4B
5
6
7

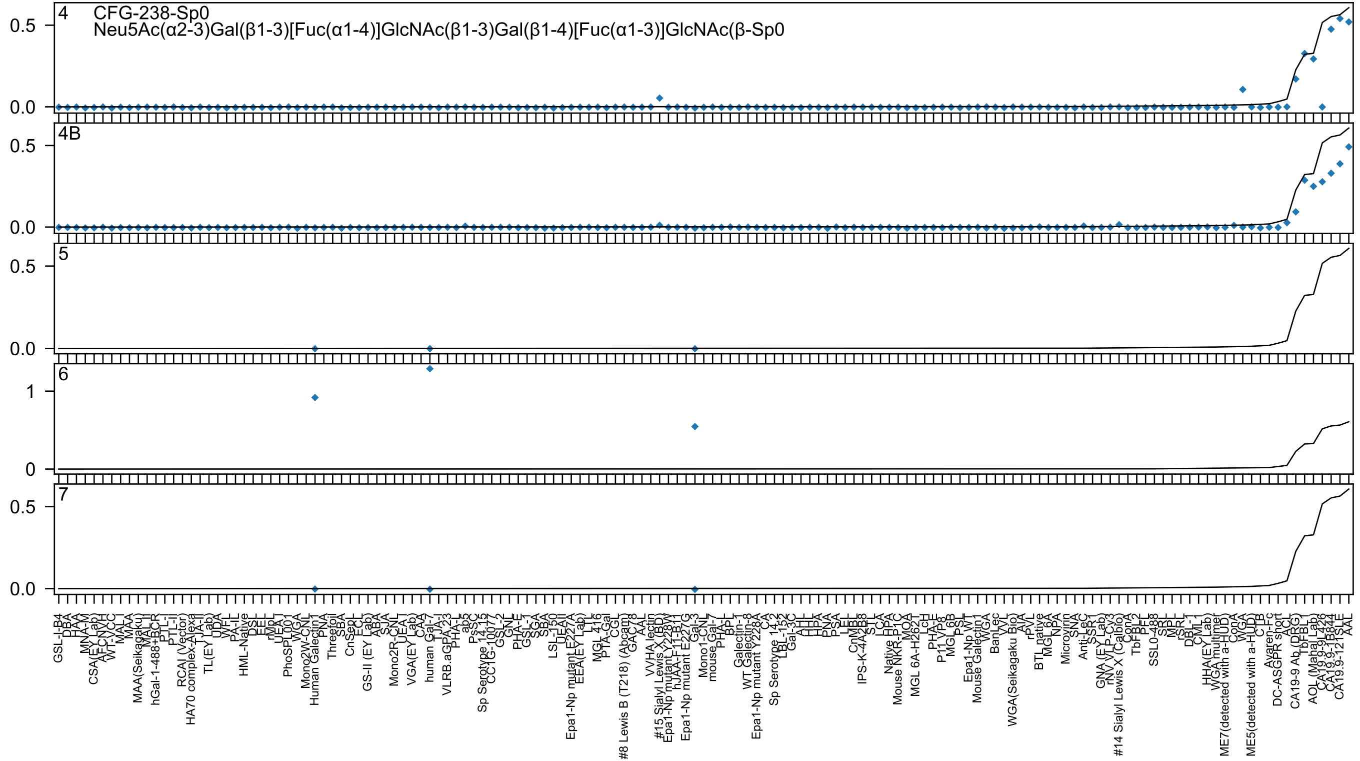

4
CFG-238-Sp0
Neu5Ac(α2-3)Gal(β1-3)[Fuc(α1-4)]GlcNAc(β1-3)Gal(β1-4)[Fuc(α1-3)]GlcNAc(β-Sp0
4B
5
6
7

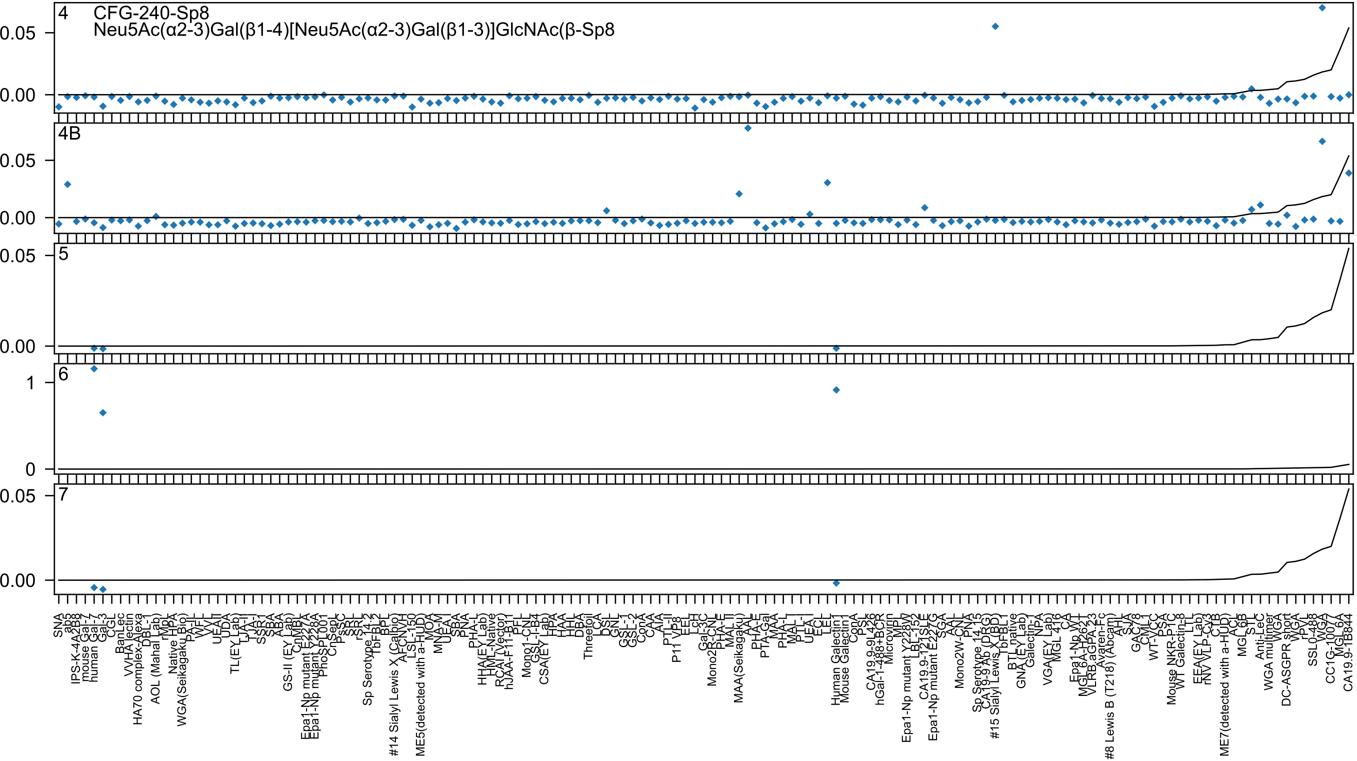

4 CFG-240-Sp8
Neu5Ac(α2-3)Gal(β1-4)[Neu5Ac(α2-3)Gal(β1-3)]GlcNAc(β-Sp8
4B
5
6
7

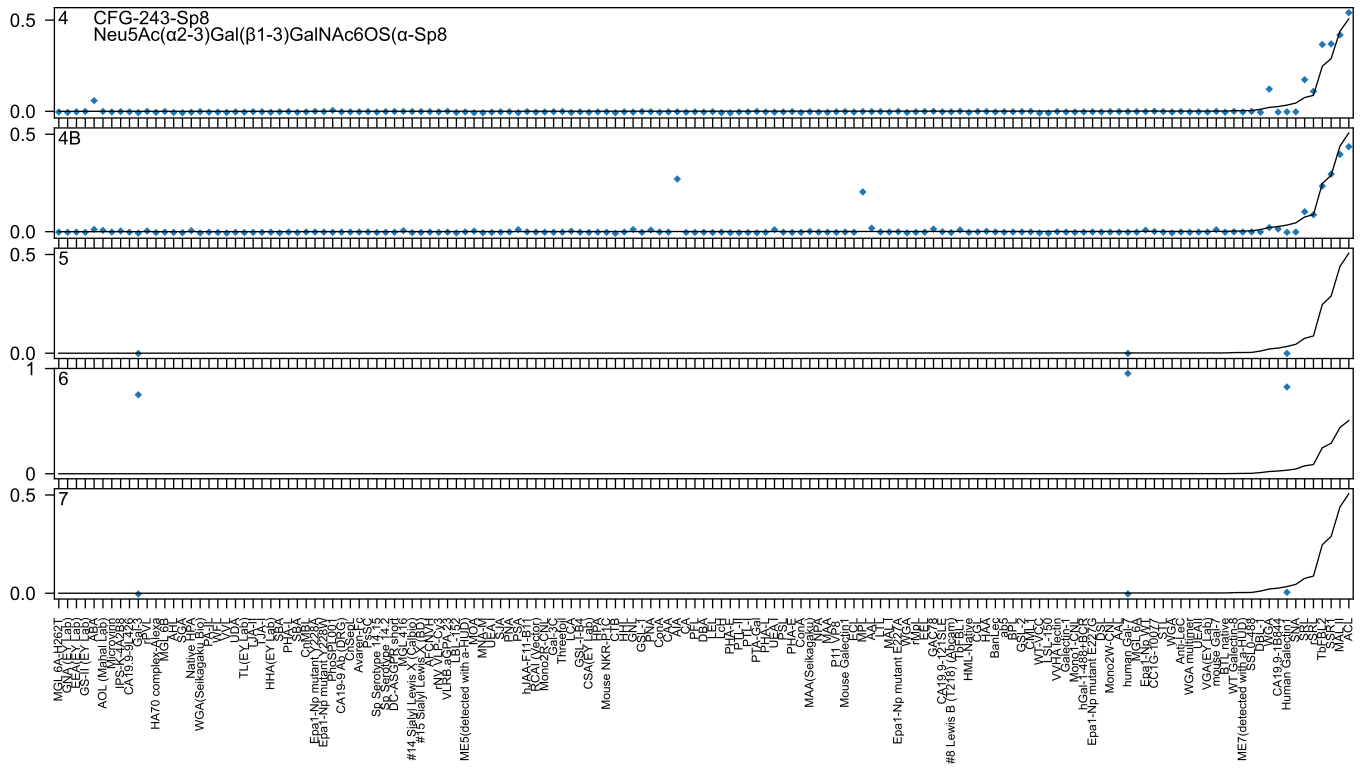

CFG-243-Sp8
Neu5Ac(α2-3)Gal(β1-3)GalNAc6OS(α-Sp8
4
4B
5
6
7

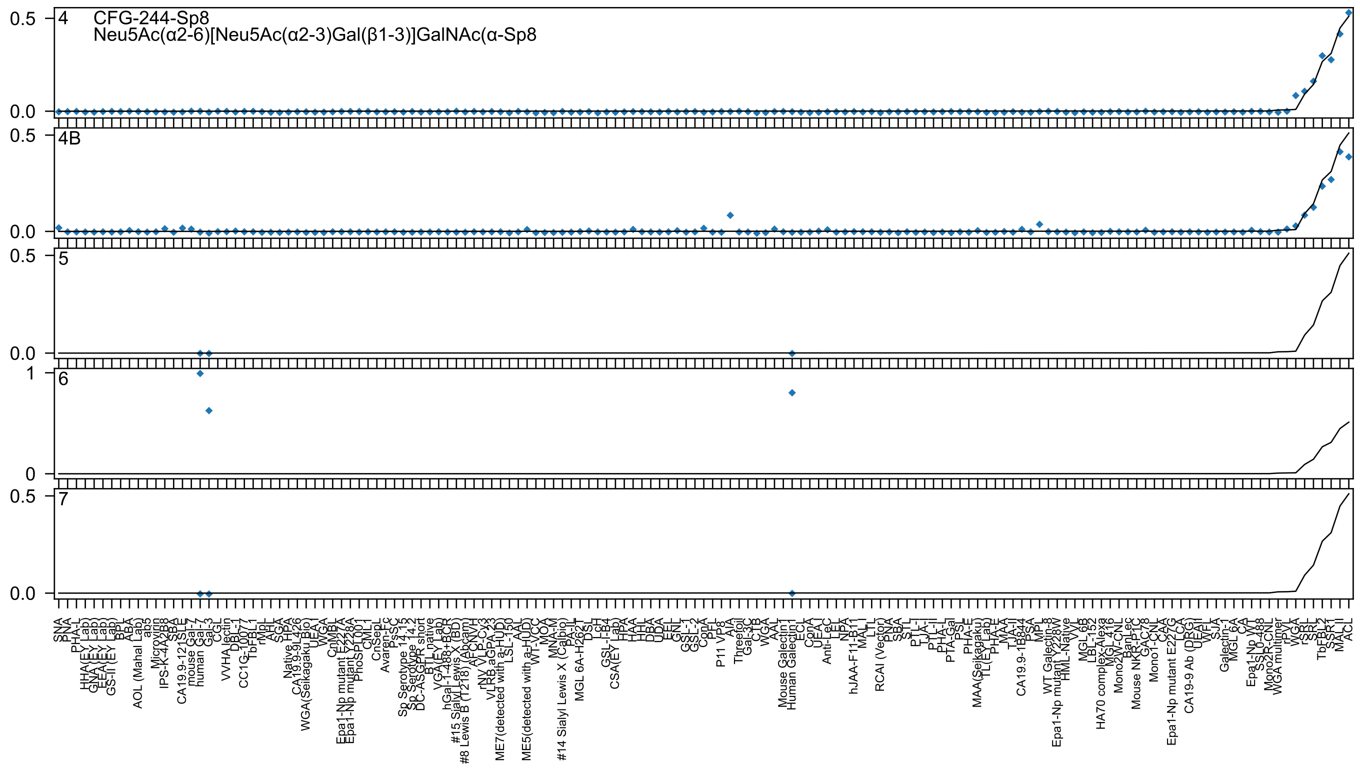

4
CFG-244-Sp8
Neu5Ac(α2-6)[Neu5Ac(α2-3)Gal(β1-3)]GalNAc(α-Sp8
4B
5
6
7

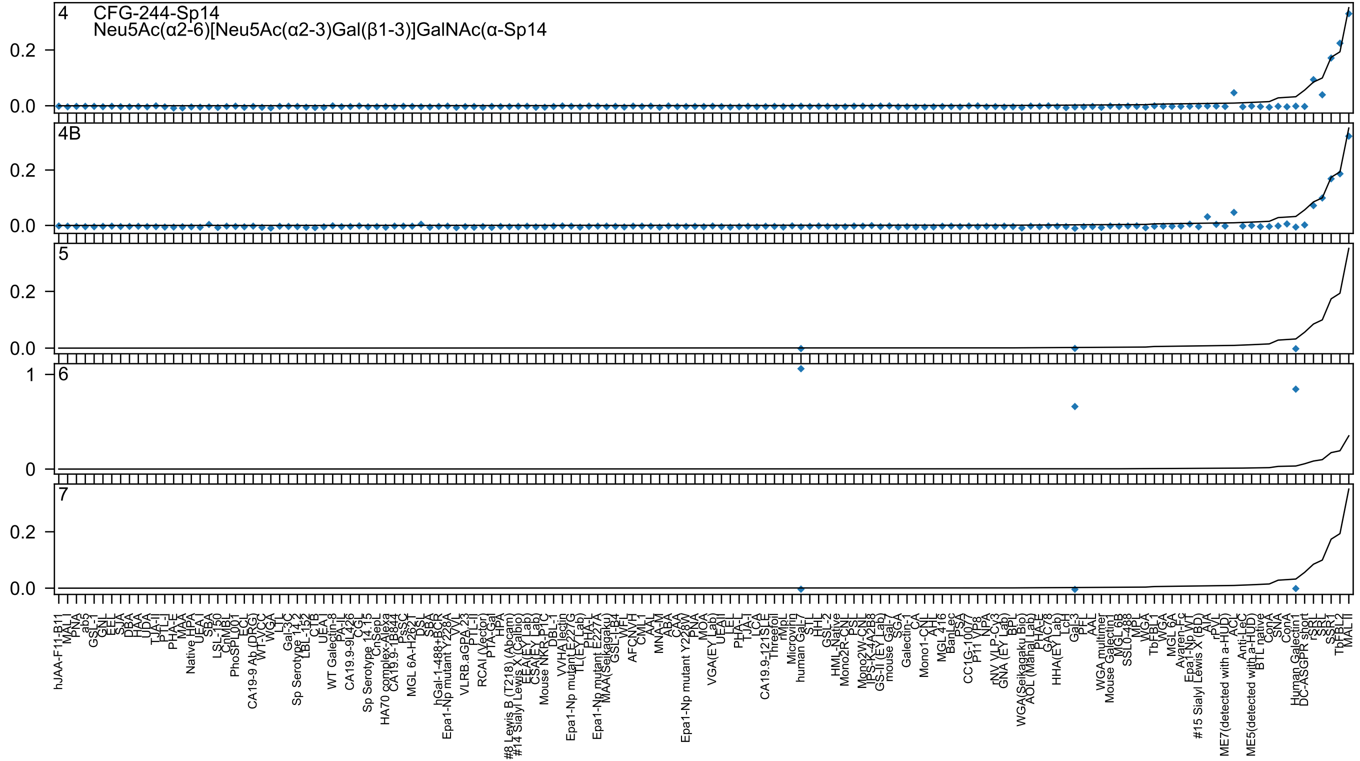
4 CFG-244-Sp14
Neu5Ac(α2-6)[Neu5Ac(α2-3)Gal(β1-3)]GalNAc(α-Sp14
4B
5
6
7
0.0
0.2
0
1
hJAA-F11-B11
MAL I
PNA
ab5
GSL-I
GNL
EEL
SJA
DBA
HAA
UDA
TJA-II
PTL-I
PHA-E
MAA
Native HPA
UEA I
SBA
LSL-150
CnMBL
PhoSL001
ECL
CA19-9 Ab (DRG)
WT-VCC
WGA
LTL
Gal-3C
Sp Serotype 14.2
LBL-152
CTB
UEA I
WT Galectin-8
PA-IL
CA19.9-9L426
CGL
Sp Serotype 14.15
CnSepL
HA70 complex-Alexa
CA19.9-1B844
PsSC
MGL 6A-H262T
DSL
SBA
hGal-1-488+BCR
Epa1-Np mutant Y228A
VVL
VLRB.aGPA.23
PTL-II
RCAI (Vector)
PTA-Gal
HPA
#8 Lewis B (T218) (Abcam)
#14 Sialyl Lewis X (Calbio)
EEA(EY Lab)
CSA(EY Lab)
Mouse NKR-P1C
DBL-1
VVHA lectin
Epa1-Np mutant E227G
TL(EY Lab)
PHA-L
Epa1-Np mutant E227A
MAA(Seikagaku)
GSL-I-B4
WFL
AFCNVH
CML1
AAL
MNA-M
ABA
CAA
Epa1-Np mutant Y228W
PNA
MOA
VGA(EY Lab)
UEAII
LEL
PHA-L
TJA-I
LCA
CA19.9-121SLE
Threefoil
rMpL
Microvirin
human Gal-7
STL
HHL
GSL-2
HML-Native
Mono2R-CNL
PSL
Mono2W-CNL
IPS-K-4A2B8
GS-II (EY Lab)
mouse Gal-7
SGA
Galectin-1
CA
Mono1-CNL
AHL
MGL 416
BanLec
PSA
CC1G-10077
P11 VP8
NPA
rNV VLP-Cy3
GNA (EY Lab)
BPL
WGA(Seikagaku Bio)
AOL (Manai Lab)
PHA-E
GAC78
HHA(EY Lab)
LcH
Gal-3
PFL
AAL
WGA multimer
Mouse Galectin1
MGL 6B
SSL0-488
MPL
WGA
TbFBL1
WGA
MGL 6A
Avaren-Fc
Epa1-Np WT
#15 Sialyl Lewis X (BD)
AIA
rPVL
ME7(detected with a-HUD)
ACL
Anti-LeC
ME5(detected with a-HUD)
BTL native
ConA
SNA
ConA
Human Galectin1
DC-ASGPR short
rSRL
SRL
SSR1
TbFBL2
MAL II

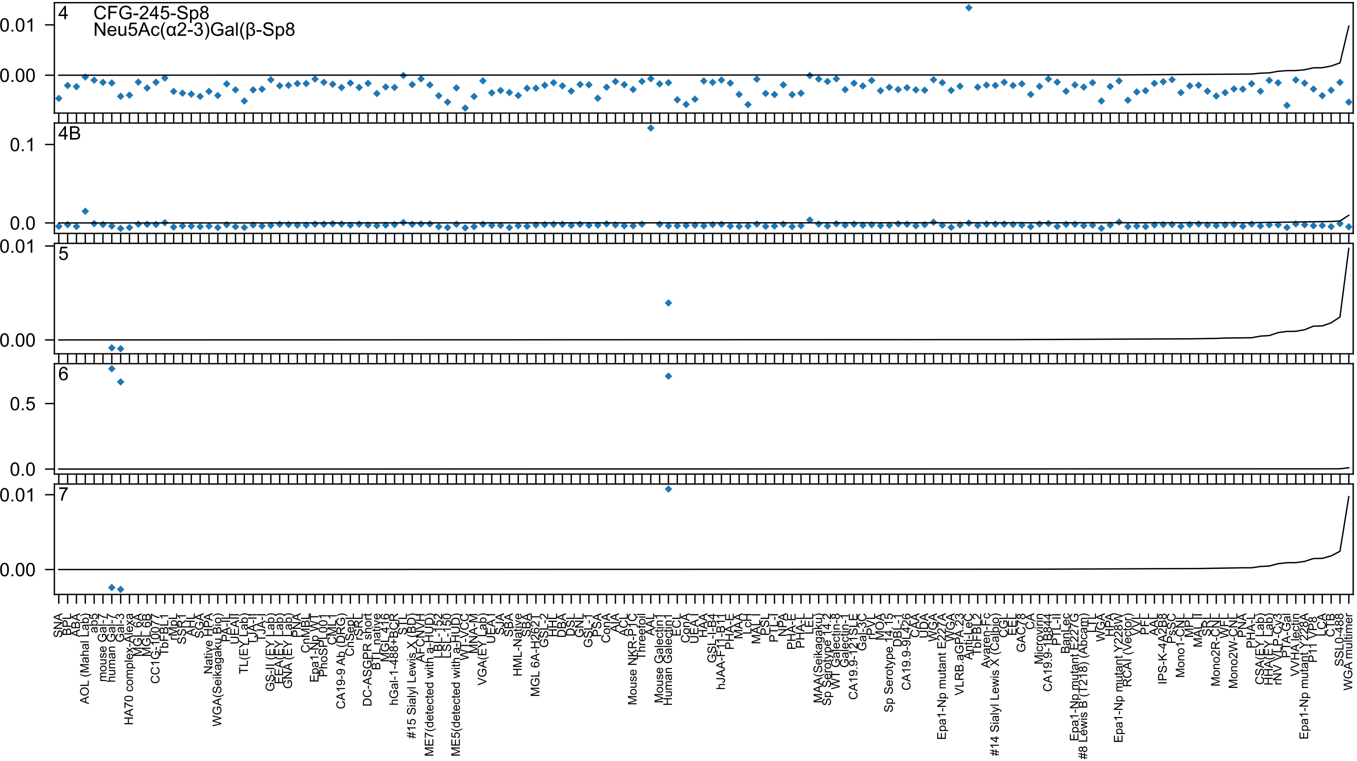

4
CFG-245-Sp8
Neu5Ac(α2-3)Gal(β-Sp8
4B
5
6
7

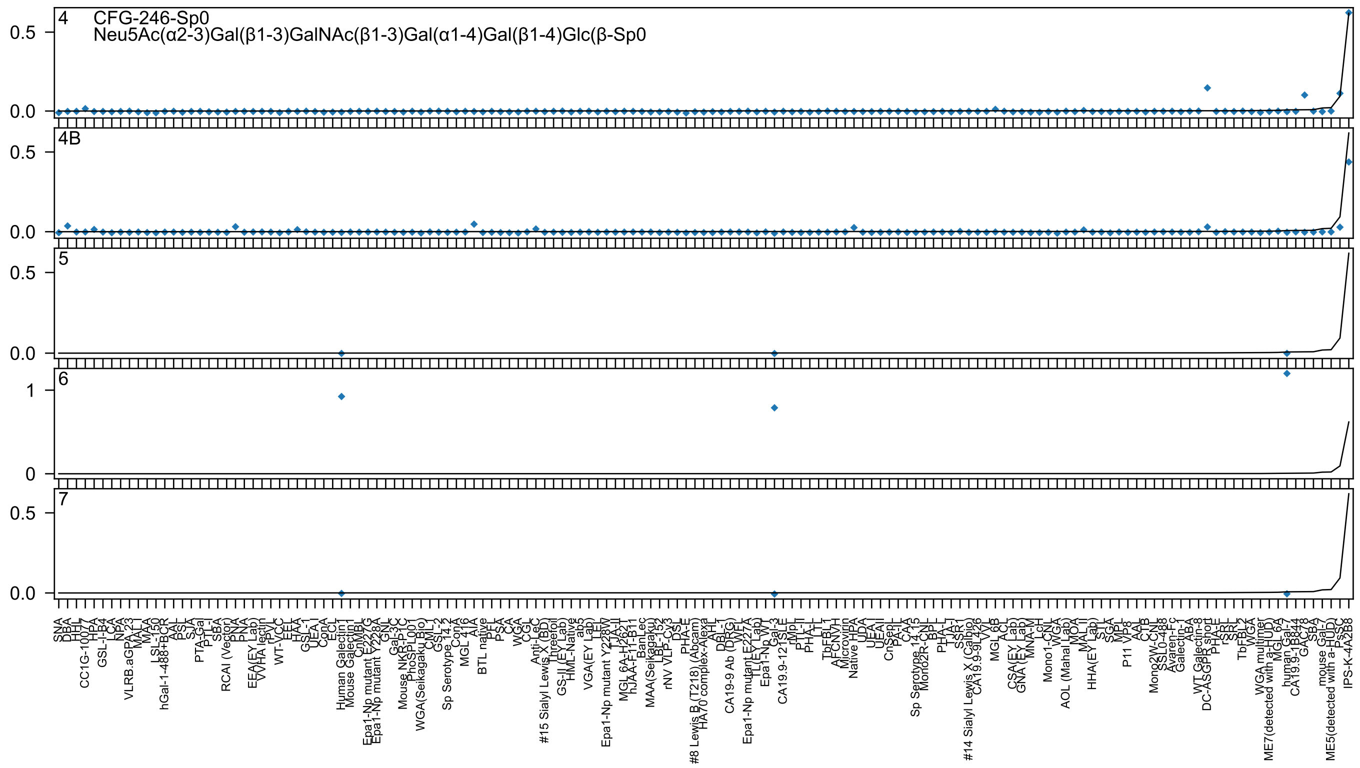
4
CFG-246-Sp0
Neu5Ac(α2-3)Gal(β1-3)GalNAc(β1-3)Gal(α1-4)Gal(β1-4)Glc(β-Sp0
4B
5
6
7

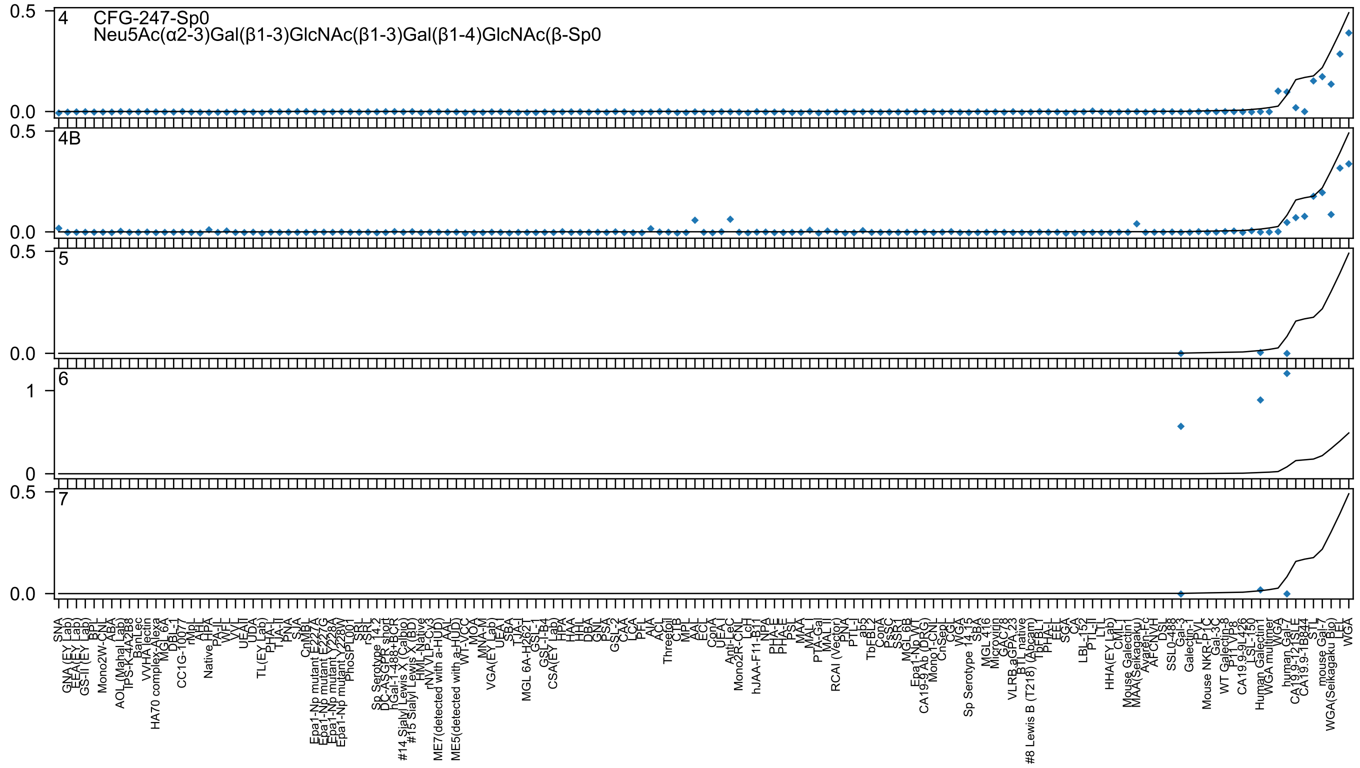

4
CFG-247-Sp0
Neu5Ac(α2-3)Gal(β1-3)GlcNAc(β1-3)Gal(β1-4)GlcNAc(β-Sp0
4B
5
6
7
0.5
0.0
1
0

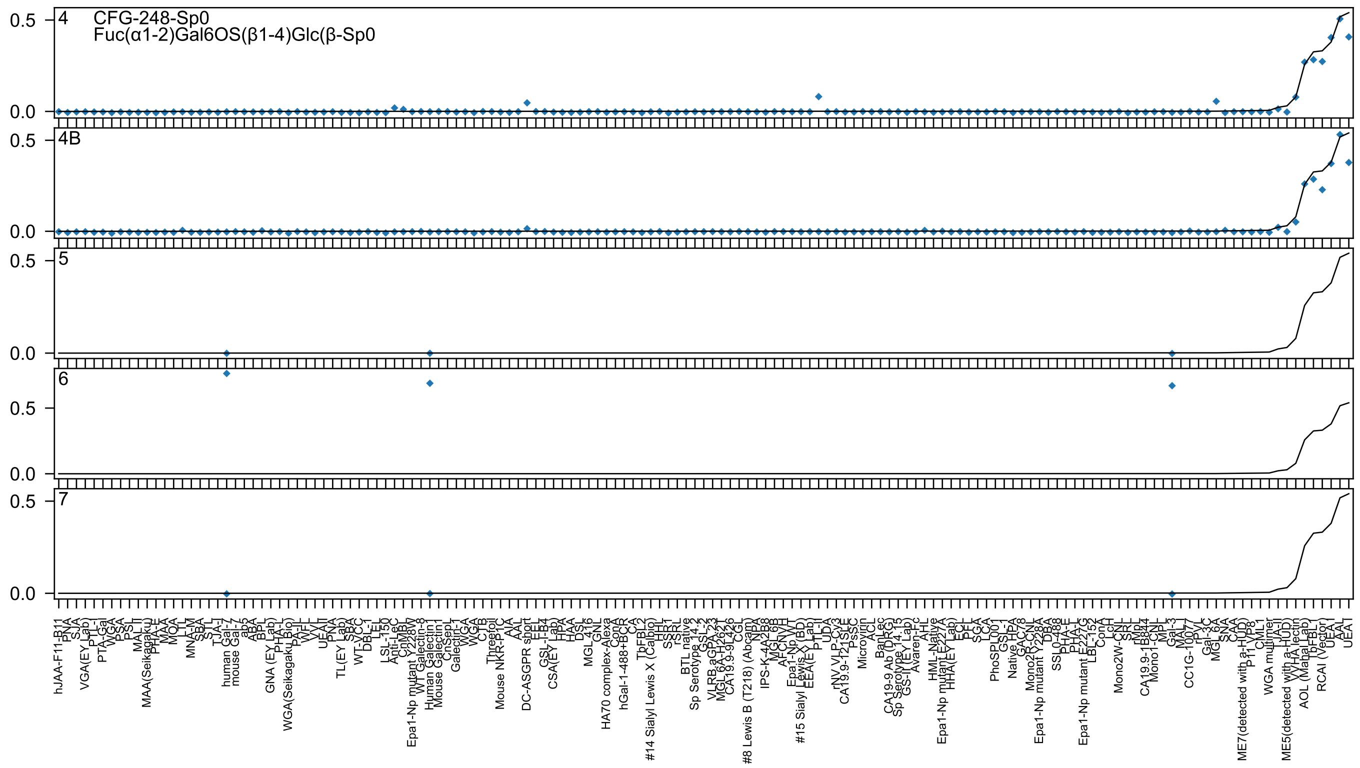
4
CFG-248-Sp0
Fuc(α1-2)Gal6OS(β1-4)Glc(β-Sp0
4B
5
6
7
0.5
0.0

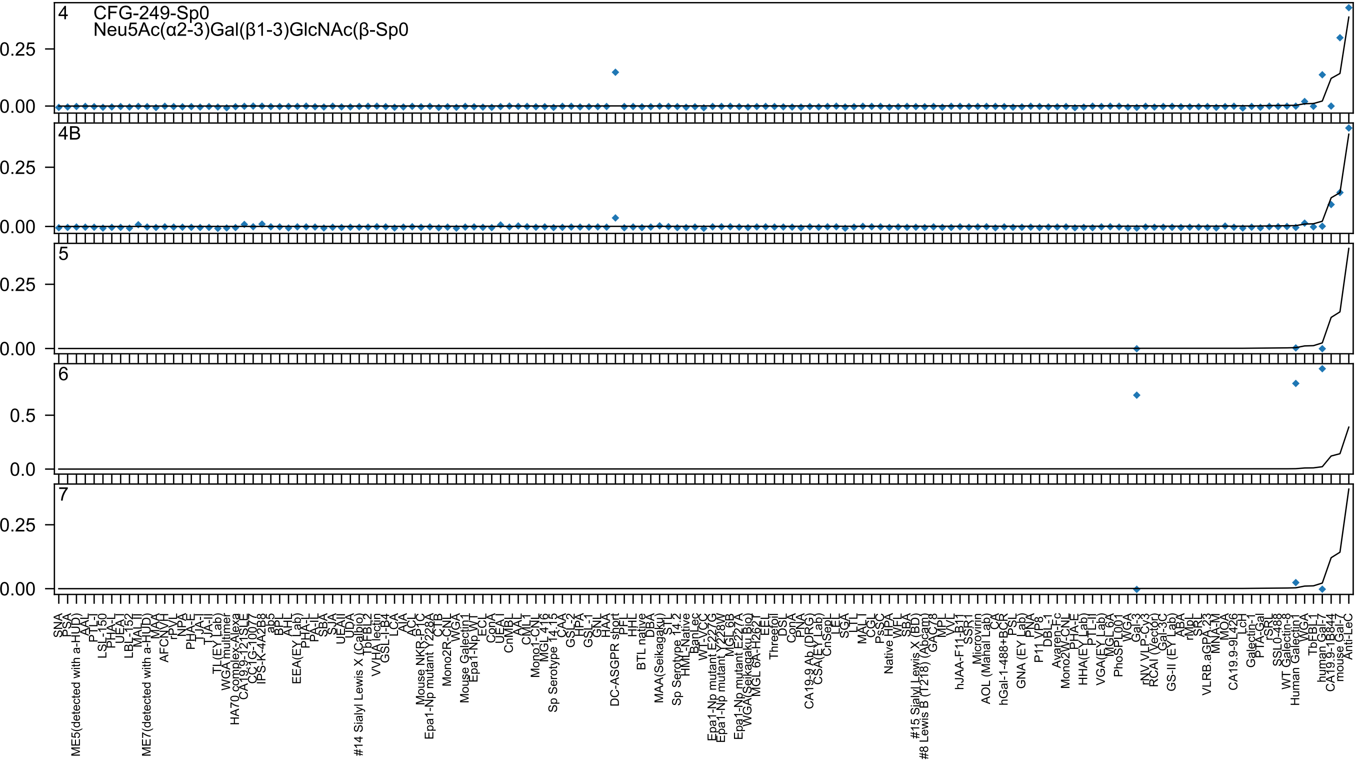

4
CFG-249-Sp0
Neu5Ac(α2-3)Gal(β1-3)GlcNAc(β-Sp0
4B
5
6
7

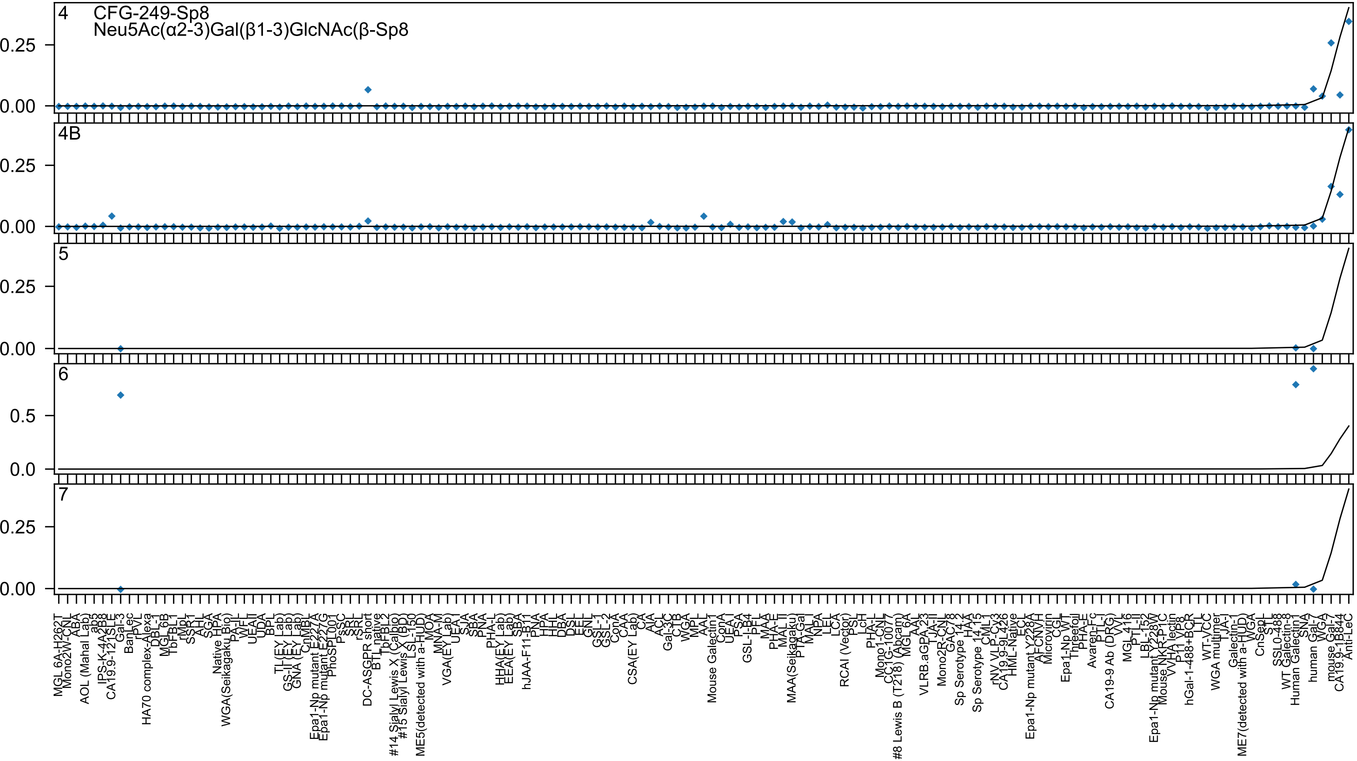

4
CFG-249-Sp8
Neu5Ac(α2-3)Gal(β1-3)GlcNAc(β-Sp8
4B
5
6
7
0.25
0.00
0.5
0.0
MGL 6A-H262T
Mono2W-CNL
ABA
AOL (Mahal Lab)
ab5
IPS-K-4A2B8
CA19.9-121SLE
Gal-3
BanLec
rPVL
HA70 complex-Alexa
DBL-1
MGL 6B
TbFBL1
rMpL
SSBT
SGA
Native HPA
WGA(Seikagaku Bio)
PA-II
WFL
UEA-II
UDA
BPL
TL(EY Lab)
GS-II(EY Lab)
GNA (EY Lab)
CnMBL
Epa1-Np mutant E227A
Epa1-Np mutant E227G
PhoSL001
PsSC
SRL
rSRL
DC-ASGPR short
BTL native
TbFBL2
#14 Sialyl Lewis X (Calbio)
#15 Sialyl Lewis X (BD)
LSL-150
ME5(detected with a-HUD)
MOA
MNA-M
VGA(EY Lab)
UEA-I
STA
SBA
PNA
PHA-L
HHA(EY Lab)
EEA(EY Lab)
SBA
hJAA-F11-B11
PNA
HPA
HHL
DBA
DSL
EEL
GNL
GSL-I
GSL-2
ConA
CAA
CSA(EY Lab)
CA
AIA
ACL
Gal-3C
CTB
WGA
MPL
AAL
Mouse Galectin1
ConA
UEA-I
PSA
GSL-I-B4
PFL
MAA
PHA-E
MAL-II
MAA(Seikagaku)
PTA-Gal
MAL-I
NPA
LEA
LCA
RCAI (Vector)
PSL
LcH
PHA-L
Mono1-CNL
CC1G-10077
#8 Lewis B (T218) (Abcam)
MGL 6A
AAL
VLRB.aGPA.23
TJA-II
Mono2R-CNL
GAC78
Sp Serotype 14.2
HAA
Sp Serotype 14.15
CML1
rNV VLP-Cy3
CA19.9-9L426
HML-Native
ECL
Epa1-Np mutant Y228A
Microvirin
CGL
Epa1-Np WT
Threefoil
PHA-E
Avaren-Fc
PTL-I
CA19-9 Ab (DRG)
VVL
MGL 416
PTL-II
LBL-152
Epa1-Np mutant Y228W
Mouse NKR-P1C
VVHA lectin
P11 VP8
hGal-1-488+BCR
LTL
WT-VCC
WGA multimer
TJA-I
Galectin-1
ME7(detected with a-HUD)
WGA
CnSepL
ST
SSL0-488
WT Galectin-8
Human Galectin1
SNA
human Gal-7
WGA
mouse Gal-7
CA19.9-1B844
Anti-LeC

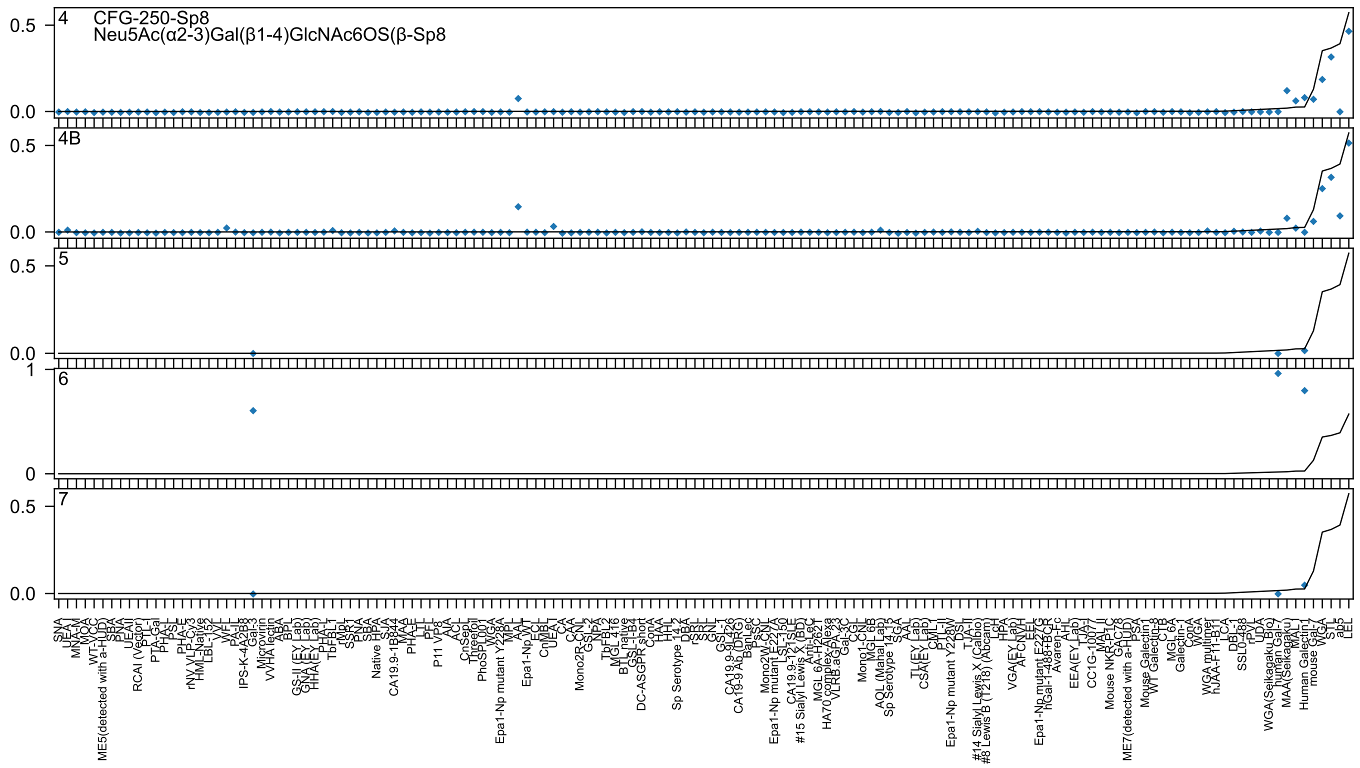

4
CFG-250-Sp8
Neu5Ac(α2-3)Gal(β1-4)GlcNAc6OS(β-Sp8
4B
5
6
7

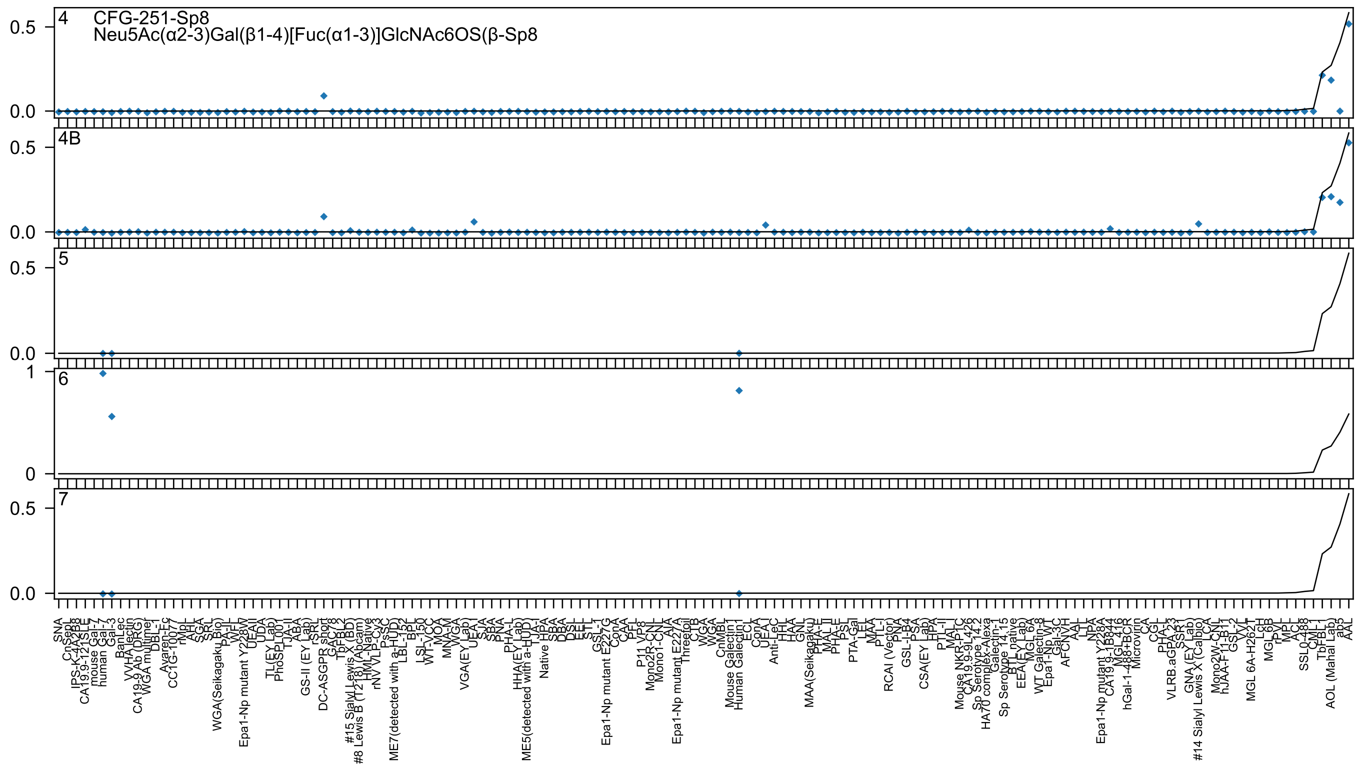
CFG-251-Sp8
Neu5Ac(α2-3)Gal(β1-4)[Fuc(α1-3)]GlcNAc6OS(β-Sp8
4
4B
5
6
7

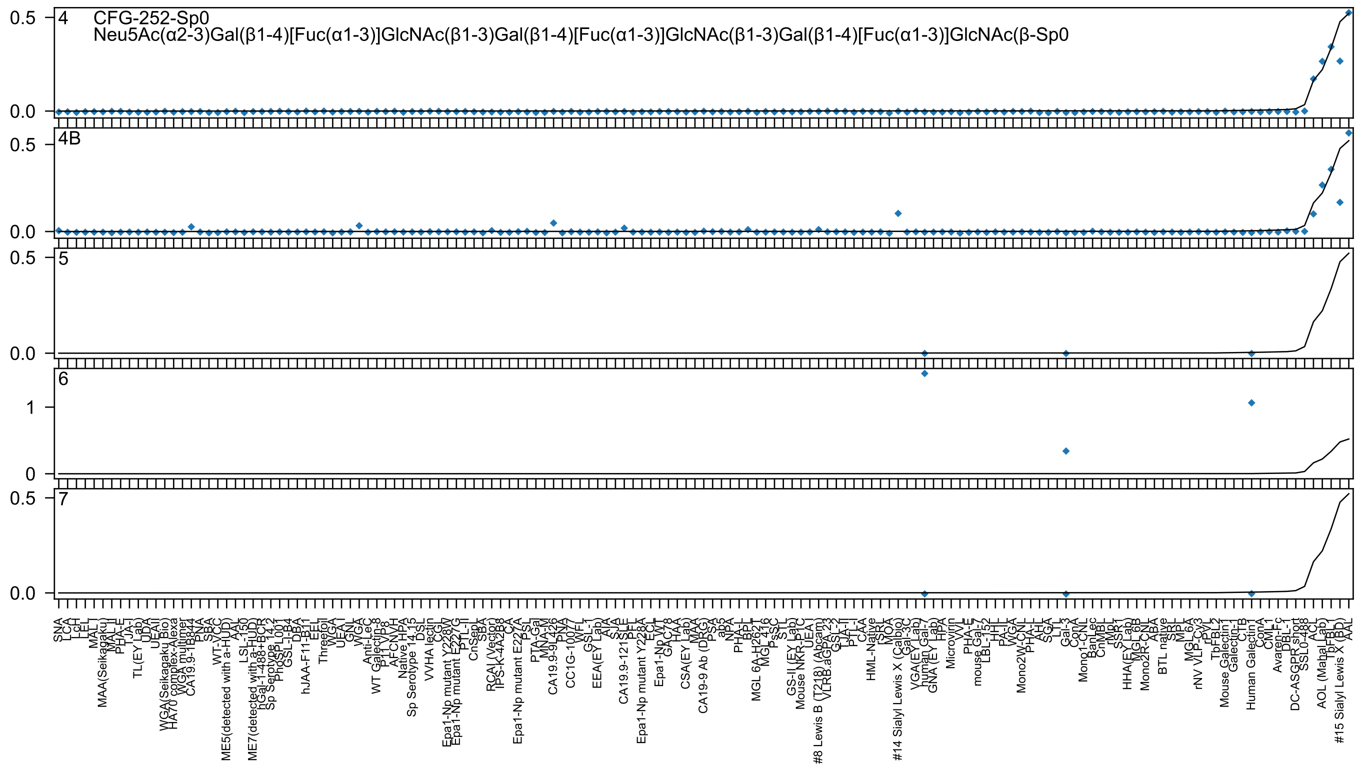

4 CFG-252-Sp0
Neu5Ac(α2-3)Gal(β1-4)[Fuc(α1-3)]GlcNAc(β1-3)Gal(β1-4)[Fuc(α1-3)]GlcNAc(β1-3)Gal(β1-4)[Fuc(α1-3)]GlcNAc(β-Sp0
4B
5
6
7

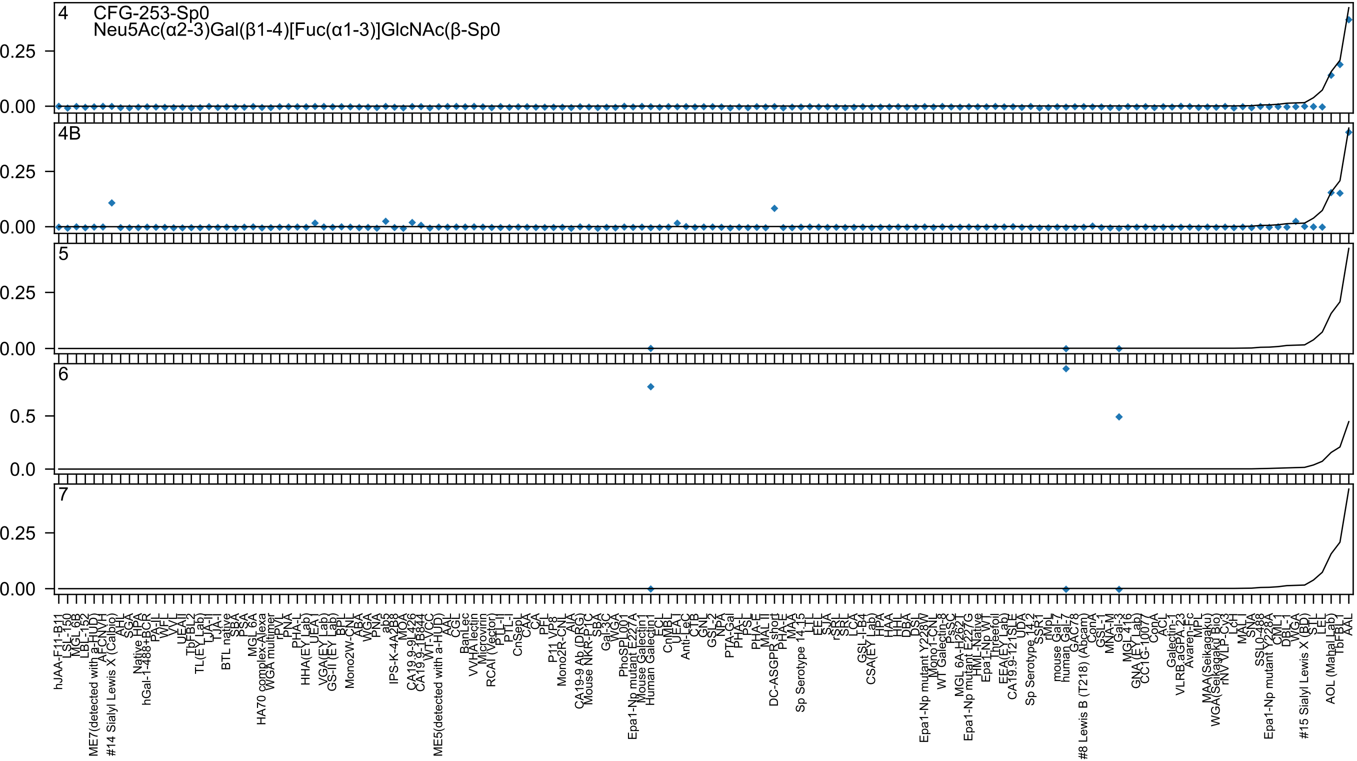
4
CFG-253-Sp0
Neu5Ac(α2-3)Gal(β1-4)[Fuc(α1-3)]GlcNAc(β-Sp0
4B
5
6
7
hJAA-F11-B11
#14 Sialyl Lewis X (Calbio)
Human Galectin1
DC-ASGPR short
human Gal-7
Gal-3
#15 Sialyl Lewis X (BD)
AOL (Mahal Lab)
TbFBL1
AAL

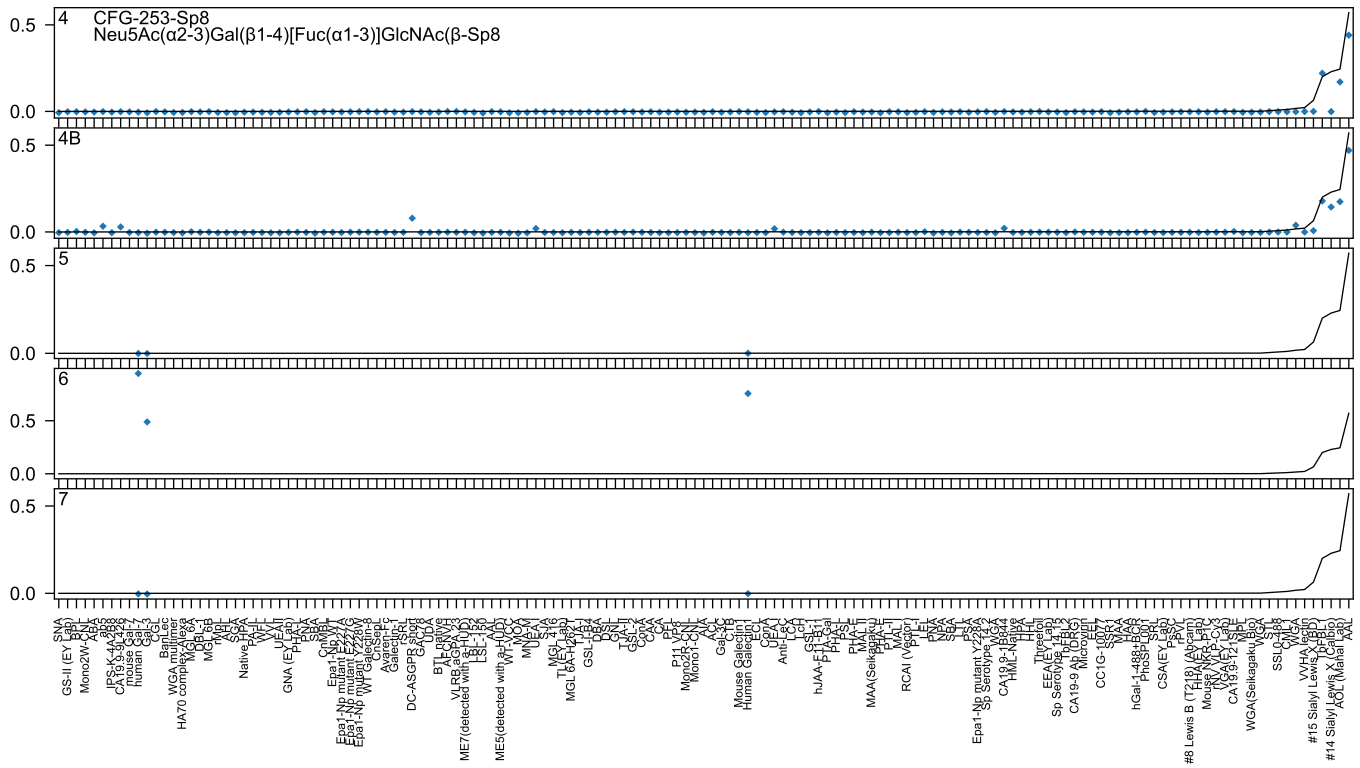
4
CFG-253-Sp8
Neu5Ac(α2-3)Gal(β1-4)[Fuc(α1-3)]GlcNAc(β-Sp8
4B
5
6
7

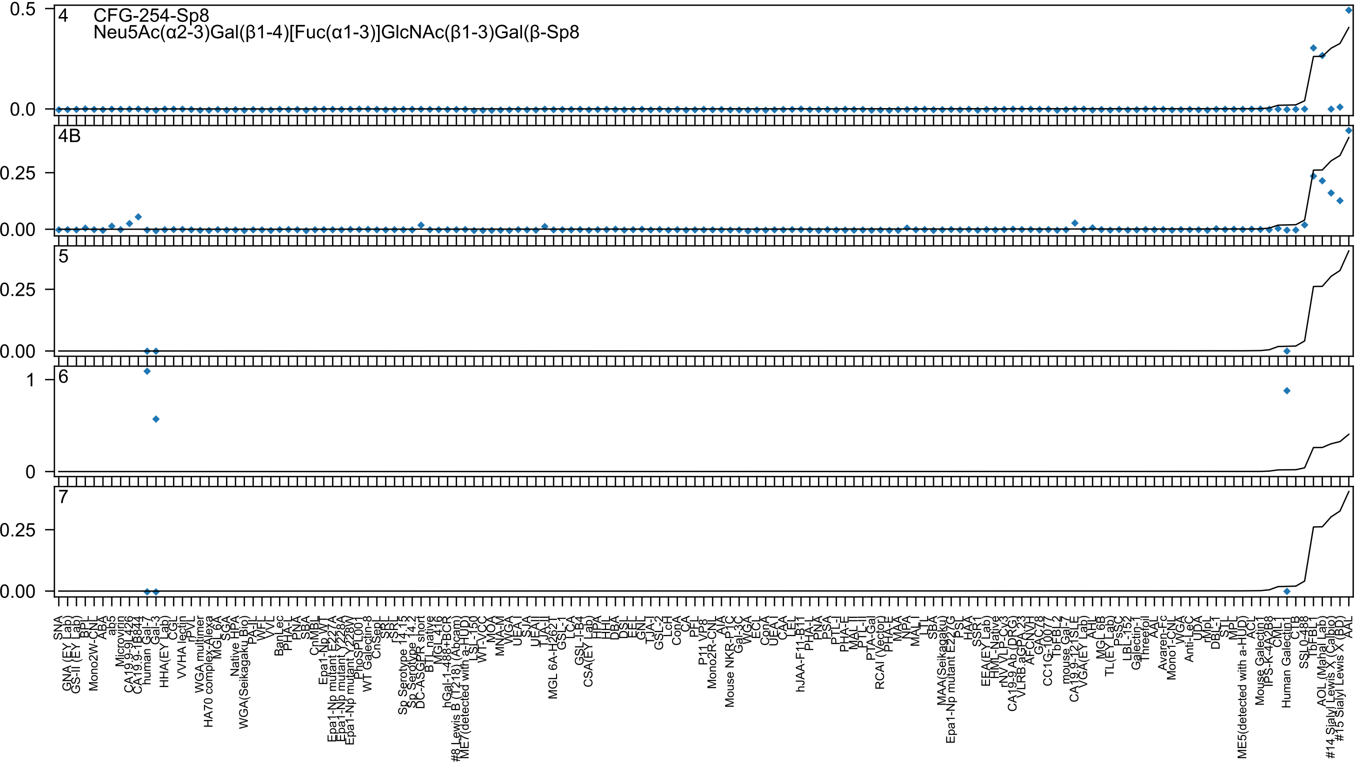

4
CFG-254-Sp8
Neu5Ac(α2-3)Gal(β1-4)[Fuc(α1-3)]GlcNAc(β1-3)Gal(β-Sp8
0.5
0.0
4B
0.25
0.00
5
0.25
0.00
6
1
0
7
0.25
0.00
SNA
GNA (EY Lab)
GS-II (EY Lab)
BPL
Mono2W-CNL
ABA
ab5
Microvirin
CA19.9-9L426
CA19.9-1B844
human Gal-7
Gal-3
HHA(EY Lab)
CGL
VVHA lectin
rPVL
WGA multimer
HA70 complex-Alexa
MGL 6A
SGA
Native HPA
WGA(Seikagaku Bio)
PA-IL
WFL
VVL
BanLec
PHA-L
PNA
SBA
CnMBL
Epa1-Np WT
Epa1-Np mutant E227A
Epa1-Np mutant Y228A
Epa1-Np mutant Y228W
PhoSL001
WT Galectin-8
CnSepL
SRL
rSRL
Sp Serotype 14.15
Sp Serotype 14.2
DC-ASGPR short
BTL native
MGL 416
hGal-1-488+BCR
#8 Lewis B (T218) (Abcam)
ME7(detected with a-HUD)
LSL-150
WT-VCC
MOA
MNA-M
WGA
UEA-I
SJA
UEA-II
TJA-II
MGL 6A-H2621
GSL-I
LCA
GSL-I-B4
CSA(EY Lab)
HPA
HHL
DBA
DSL
EEL
GNL
TJA-I
GSL-2
LcH
ConA
CA
PFL
P11 VP8
Mono2R-CNL
AIA
Mouse NKR-P1C
Gal-3C
WGA
ECL
ConA
UEA-I
CAA
LEL
hJAA-F11-B11
PHA-L
PNA
PSA
PTL-I
PHA-E
MAL-II
PTL-II
PTA-Gal
RCAI (Vector)
PHA-E
MAA
NPA
MAL-I
LTL
SBA
MAA(Seikagaku)
Epa1-Np mutant E227G
PSL
HAA
SSR1
EEA(EY Lab)
HML-Native
rNV VLP-Cy3
CA19-9 Ab (DRG)
VLRB.aGPA.23
AFCNVH
GAC78
CC1G-1007
TbFBL-2
mouse Gal-7
CA19.9-121SLE
VGA(EY Lab)
AHL
MGL 6B
TL(EY Lab)
PsSC
LBL-152
Galectin-1
Threefoil
AAL
Avaren-Fc
Mono1-CNL
WGA
Anti-LeC
UDA
rMpL
DBL-1
STL
MPL
ME5(detected with a-HUD)
ACL
Mouse Galectin1
IPS-K-4A2B8
CML-1
Human Galectin1
CTB
SSL0-488
TbFBL1
AOL (Mahal Lab)
#14 Sialyl Lewis X (Calbio)
#15 Sialyl Lewis X (BD)
AAL

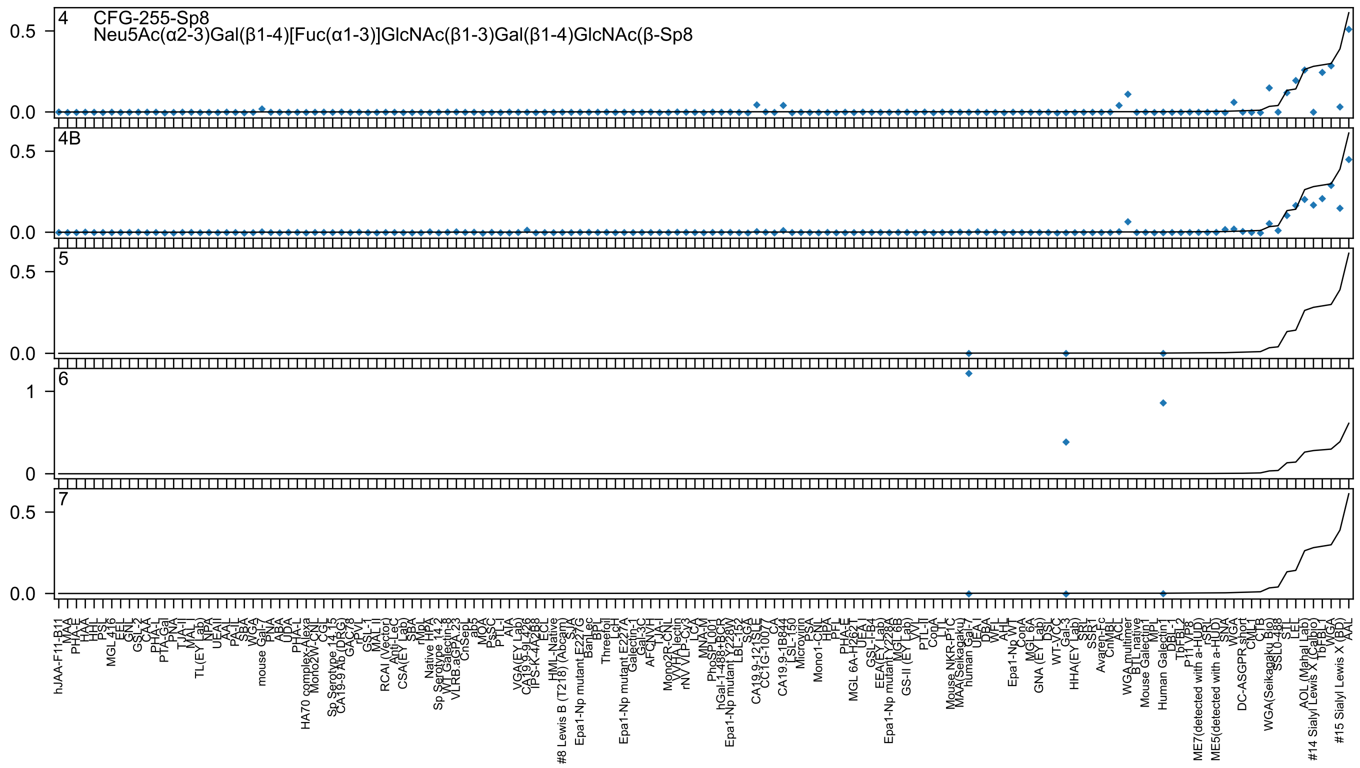

4 CFG-255-Sp8
Neu5Ac(α2-3)Gal(β1-4)[Fuc(α1-3)]GlcNAc(β1-3)Gal(β1-4)GlcNAc(β-Sp8
4B
5
6
7

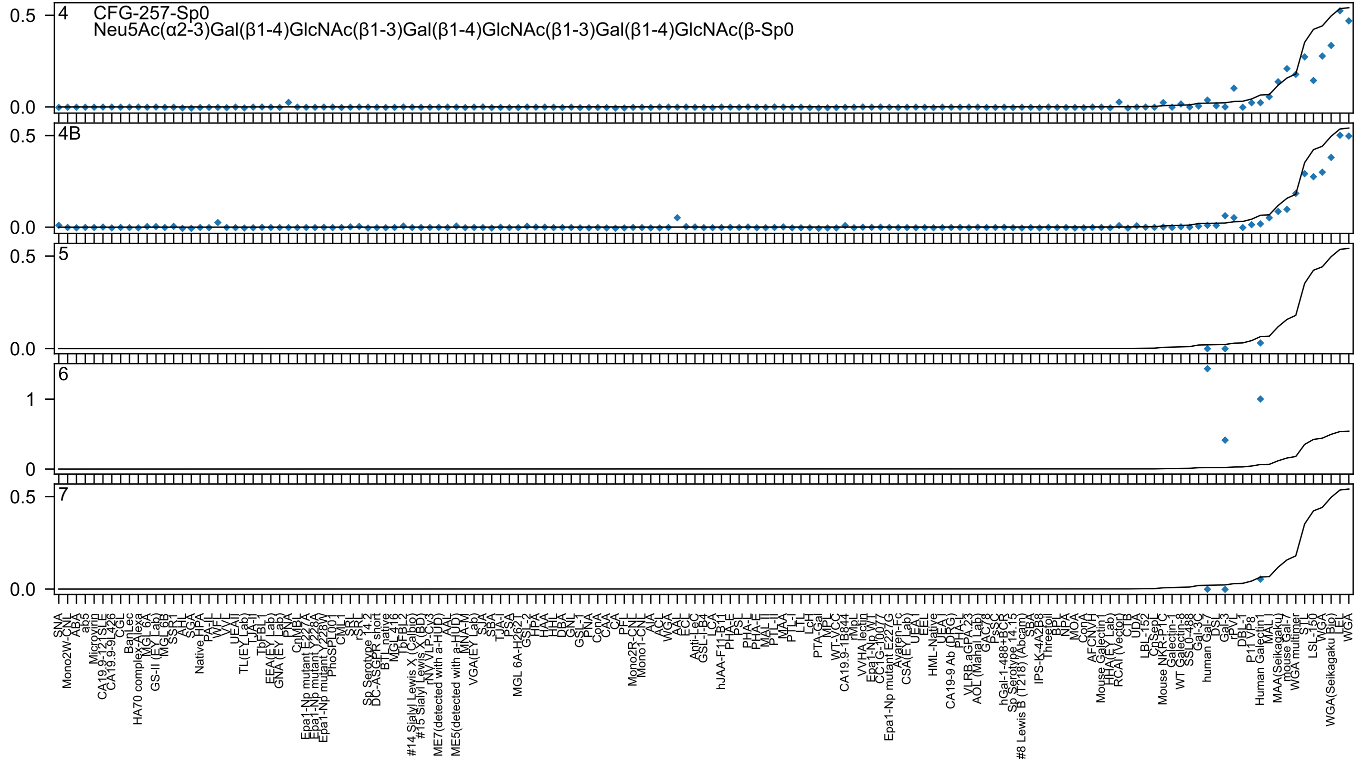

4
CFG-257-Sp0
Neu5Ac(α2-3)Gal(β1-4)GlcNAc(β1-3)Gal(β1-4)GlcNAc(β1-3)Gal(β1-4)GlcNAc(β-Sp0
4B
5
6
7

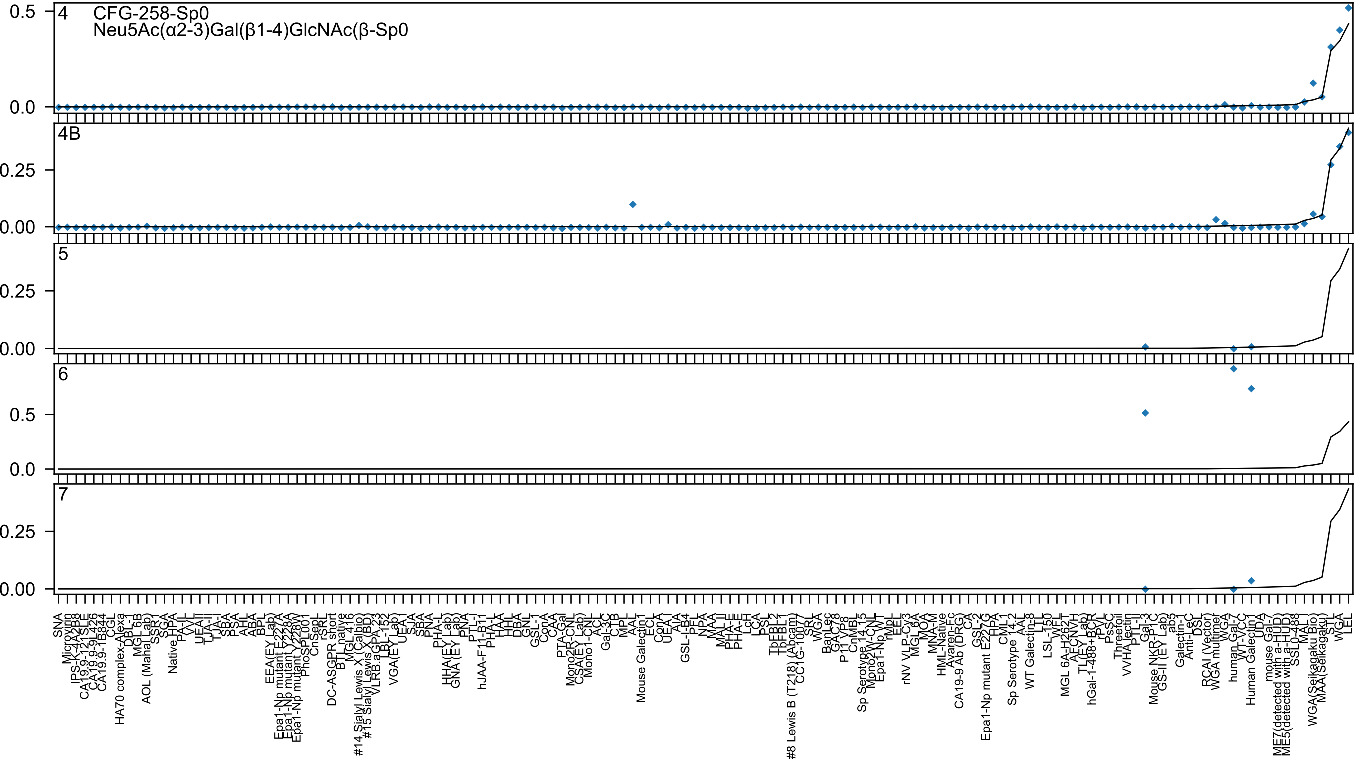
CFG-258-Sp0
Neu5Ac(α2-3)Gal(β1-4)GlcNAc(β-Sp0
4
4B
5
6
7

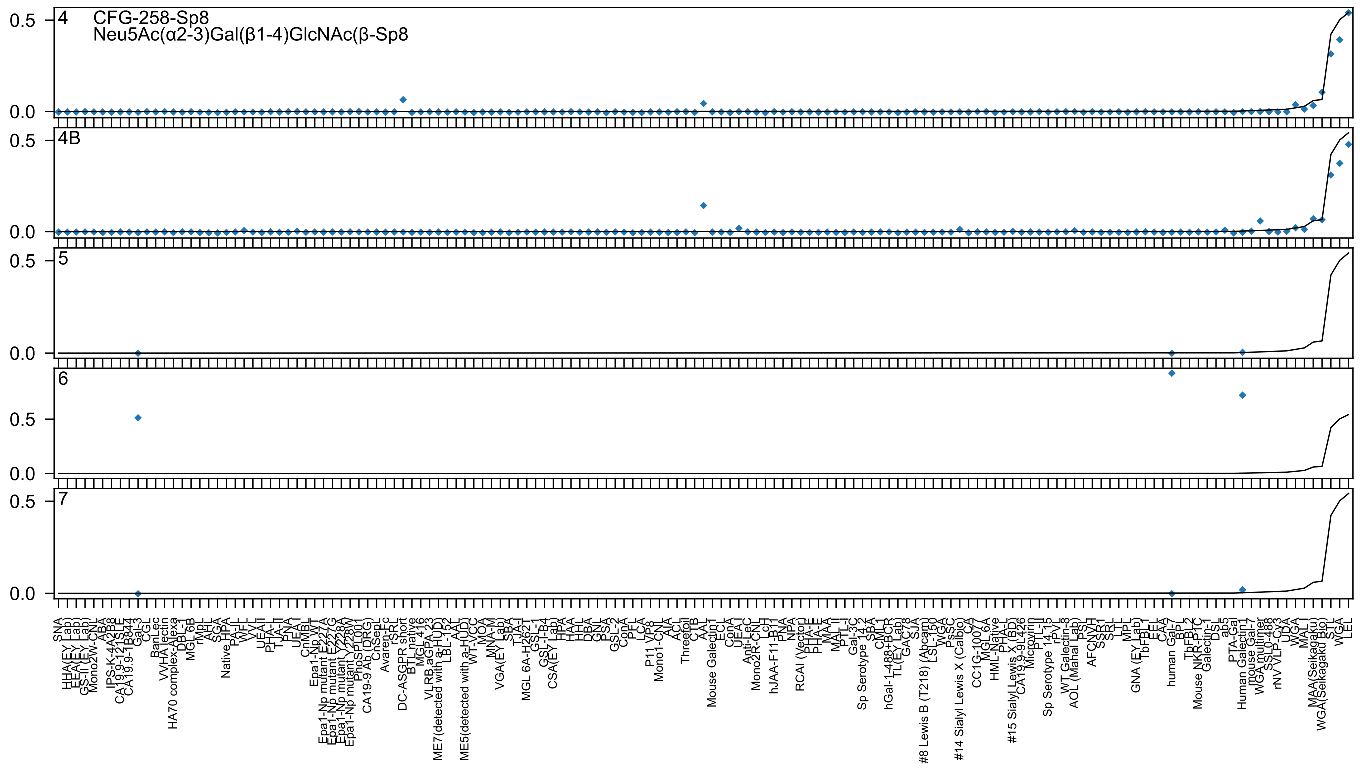

CFG-258-Sp8
Neu5Ac(α2-3)Gal(β1-4)GlcNAc(β-Sp8
4
4B
5
6
7

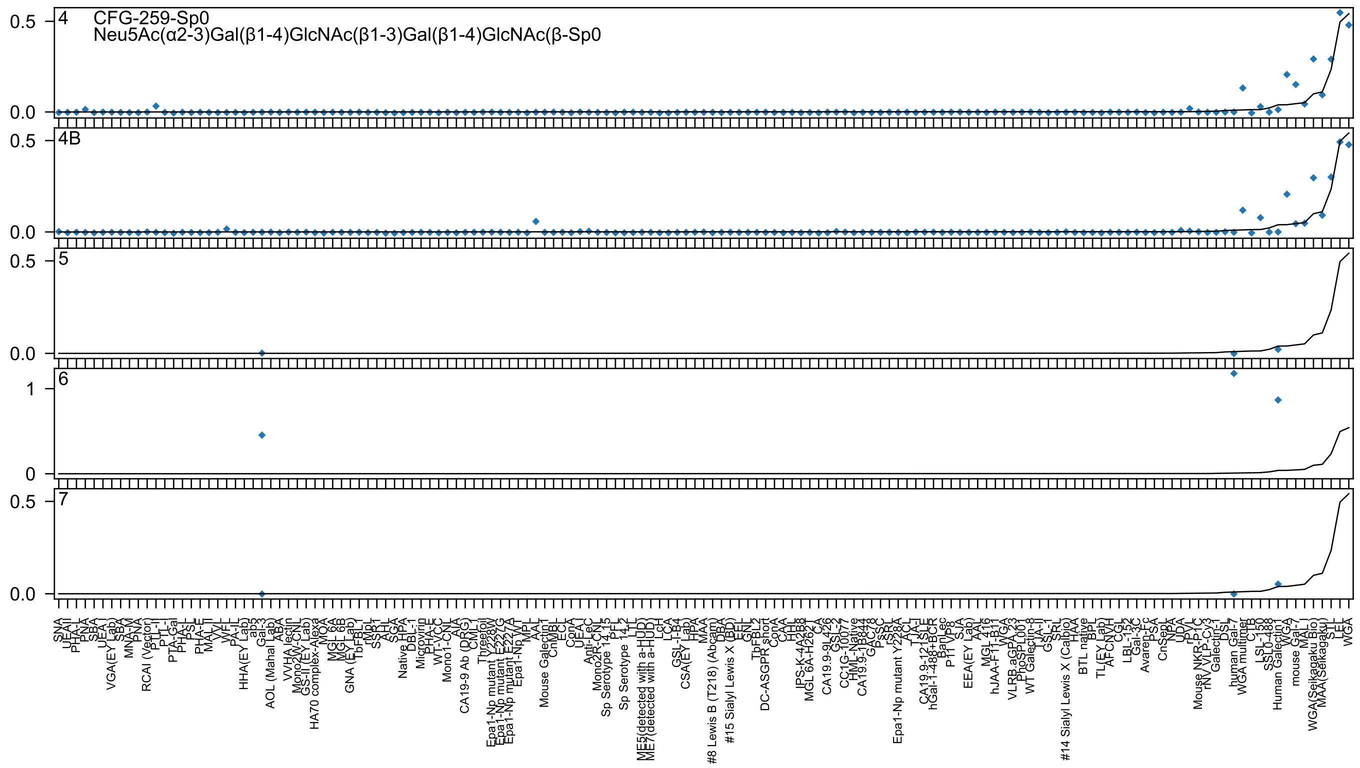
4
CFG-259-Sp0
Neu5Ac(α2-3)Gal(β1-4)GlcNAc(β1-3)Gal(β1-4)GlcNAc(β-Sp0
4B
5
6
7
0.5
0.0
1
0

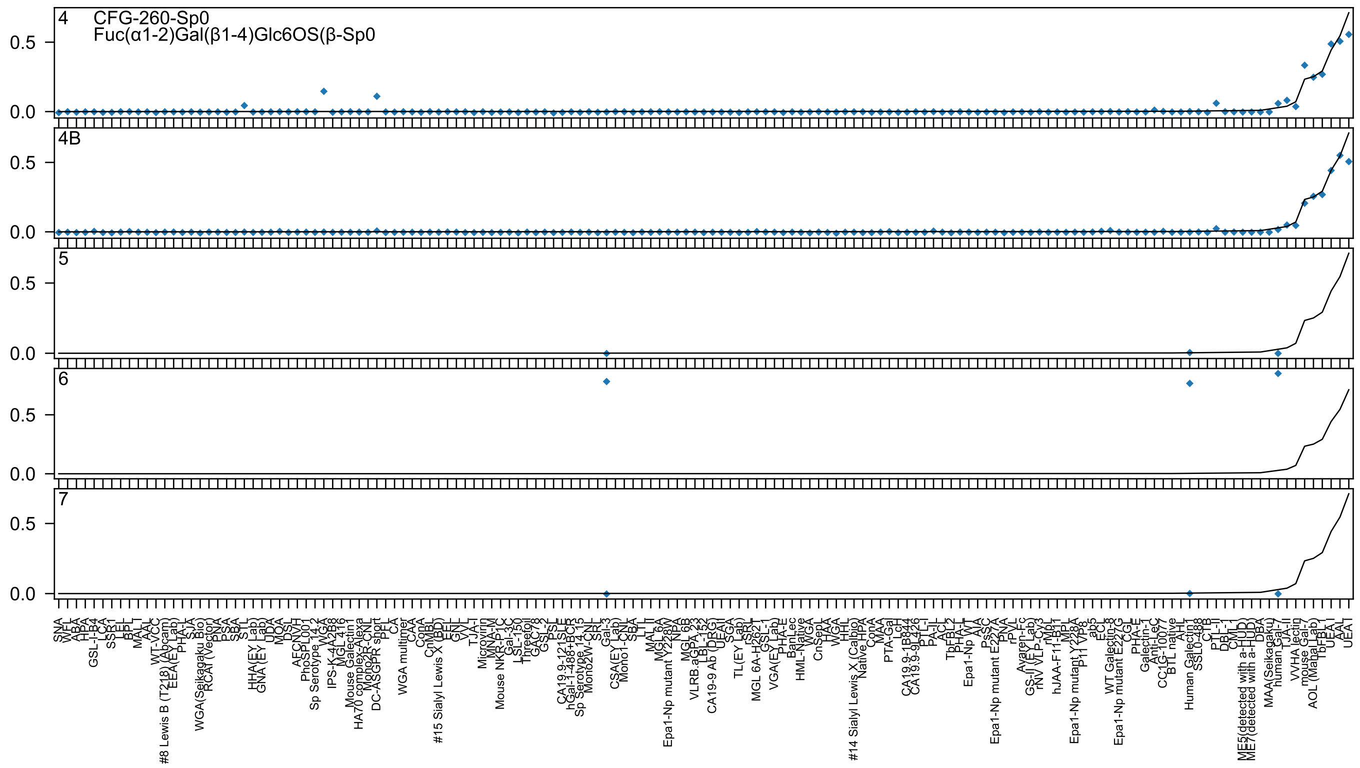
4 CFG-260-Sp0
Fuc(α1-2)Gal(β1-4)Glc6OS(β-Sp0
4B
5
6
7

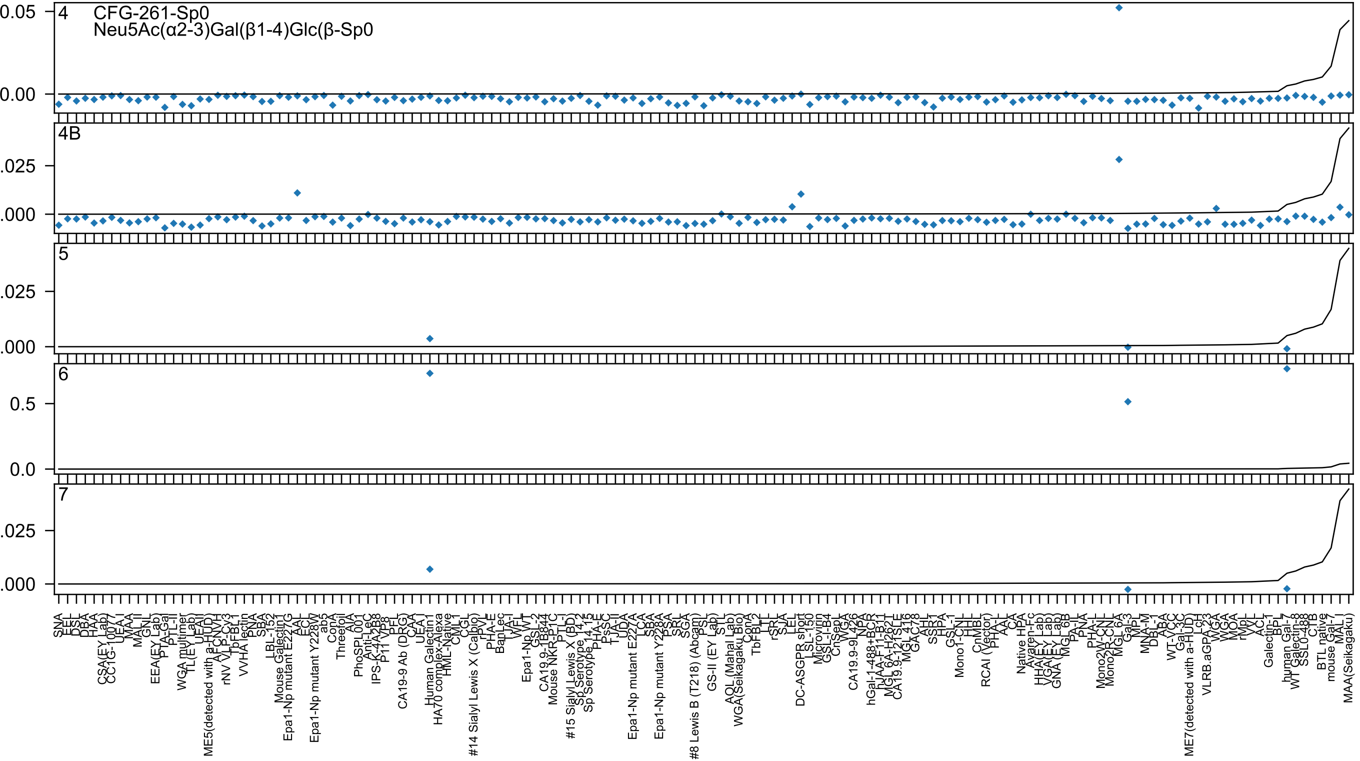
CFG-261-Sp0
Neu5Ac(α2-3)Gal(β1-4)Glc(β-Sp0

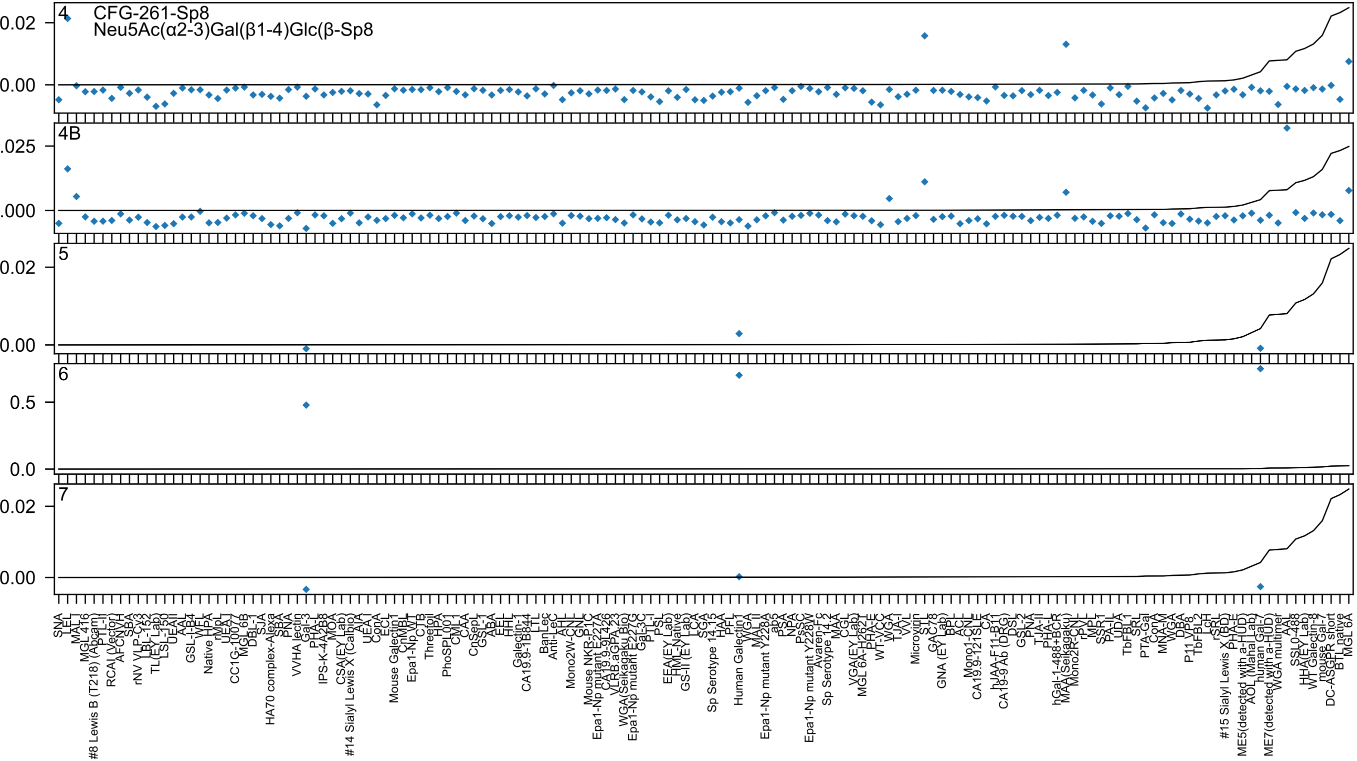
4
CFG-261-Sp8
Neu5Ac(α2-3)Gal(β1-4)Glc(β-Sp8
4B
5
6
7

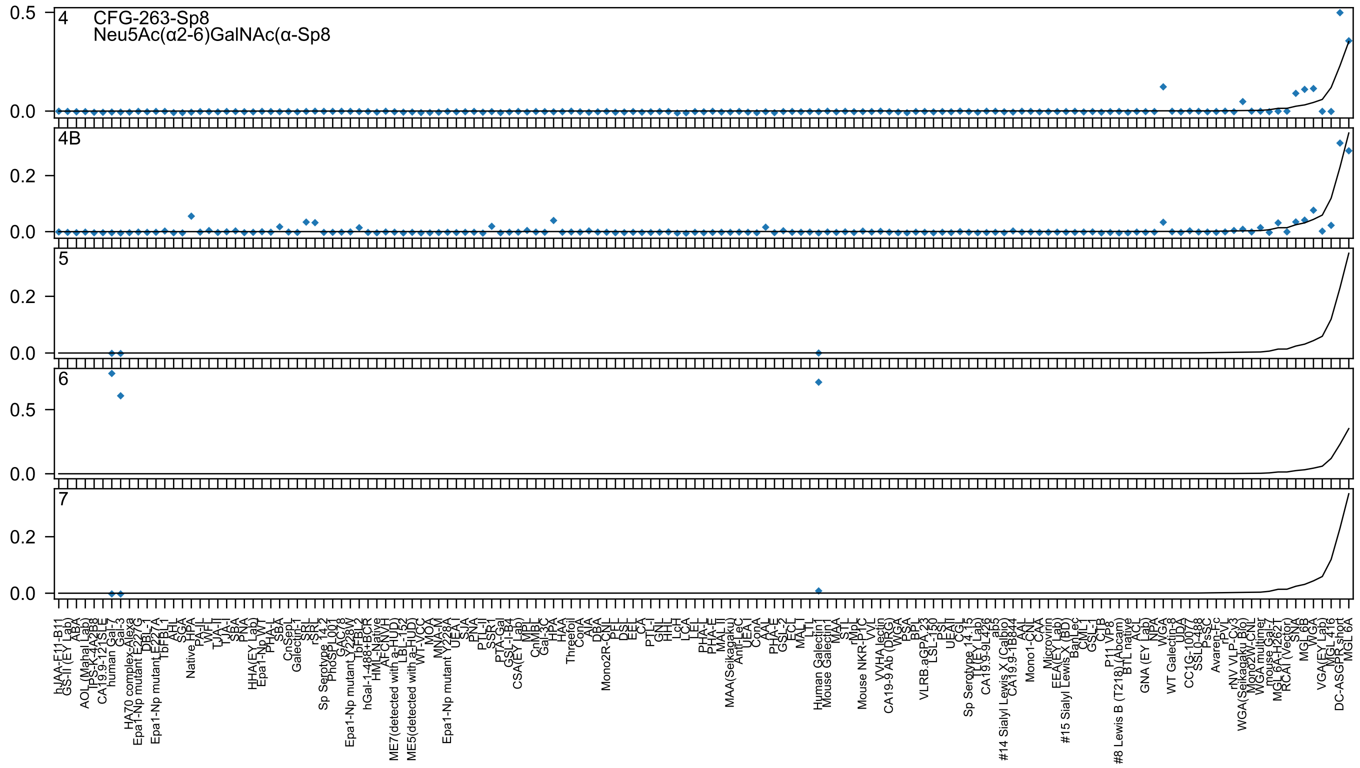
4
CFG-263-Sp8
Neu5Ac(α2-6)GalNAc(α-Sp8
4B
5
6
7

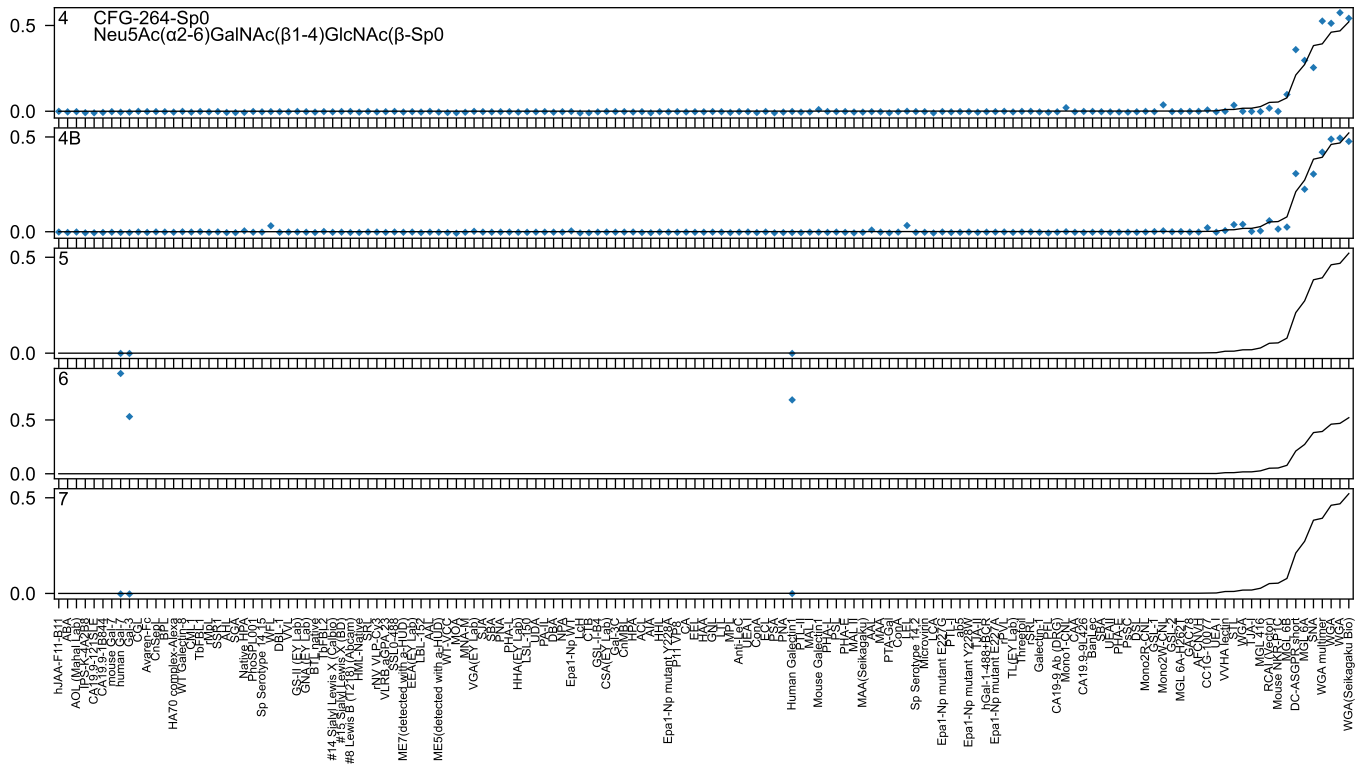

4
CFG-264-Sp0
Neu5Ac(α2-6)GalNAc(β1-4)GlcNAc(β-Sp0
4B
5
6
7

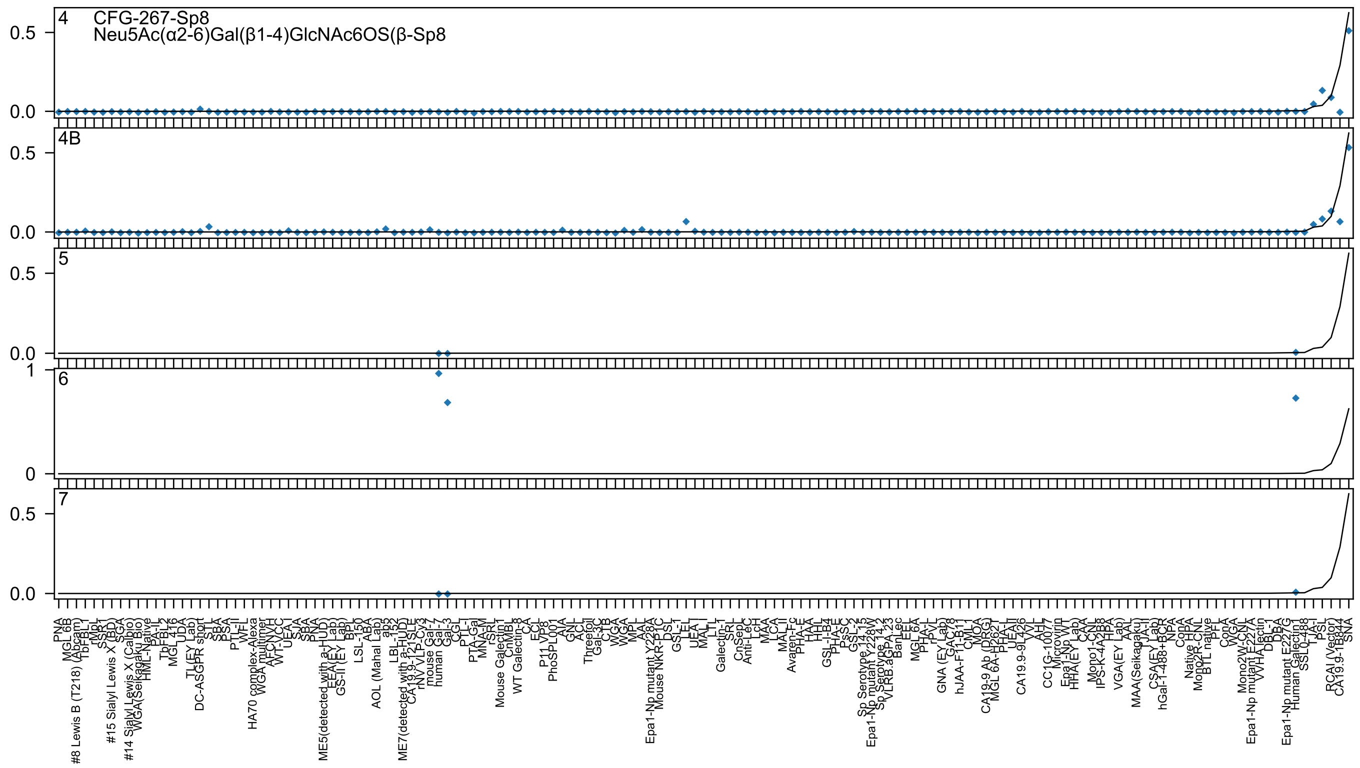

4 CFG-267-Sp8
Neu5Ac(α2-6)Gal(β1-4)GlcNAc6OS(β-Sp8
4B
5
6
7

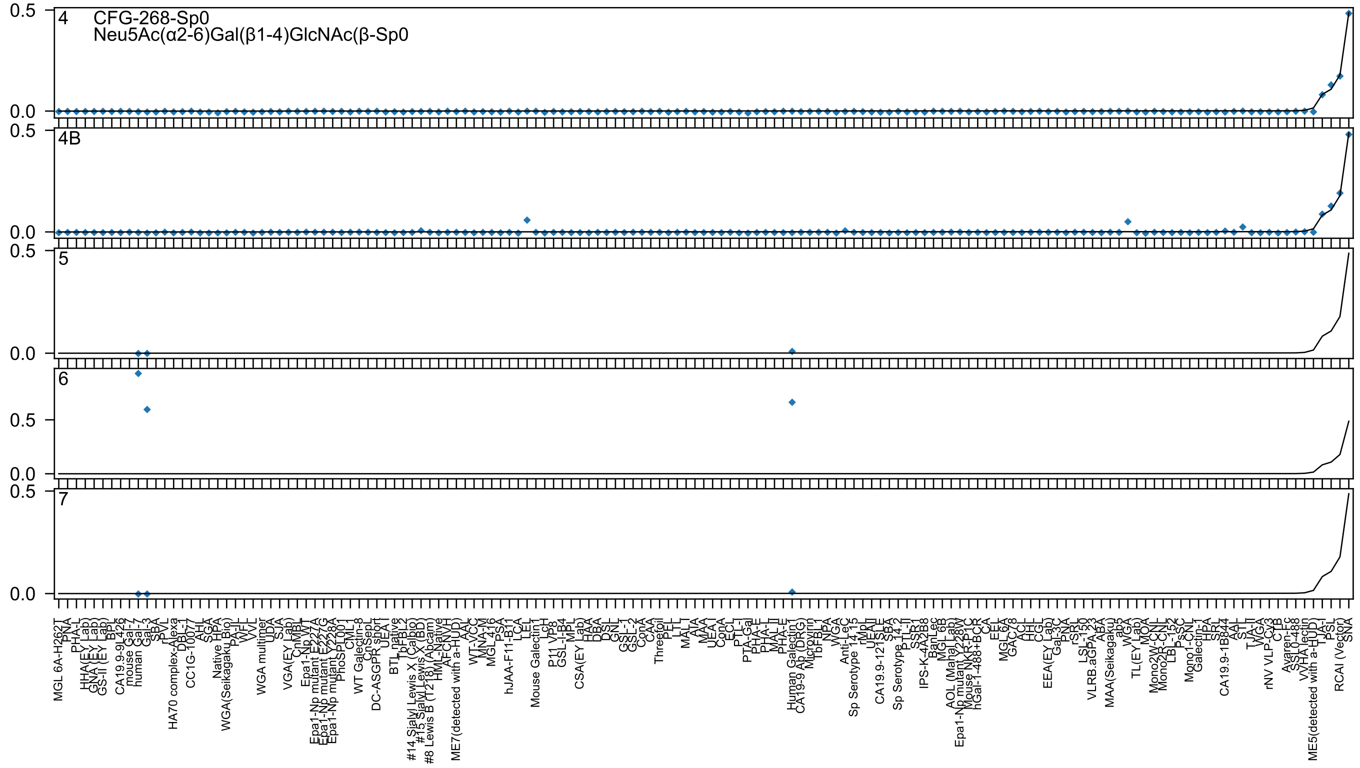

4
CFG-268-Sp0
Neu5Ac(α2-6)Gal(β1-4)GlcNAc(β-Sp0
4B
5
6
7
0.0
0.5

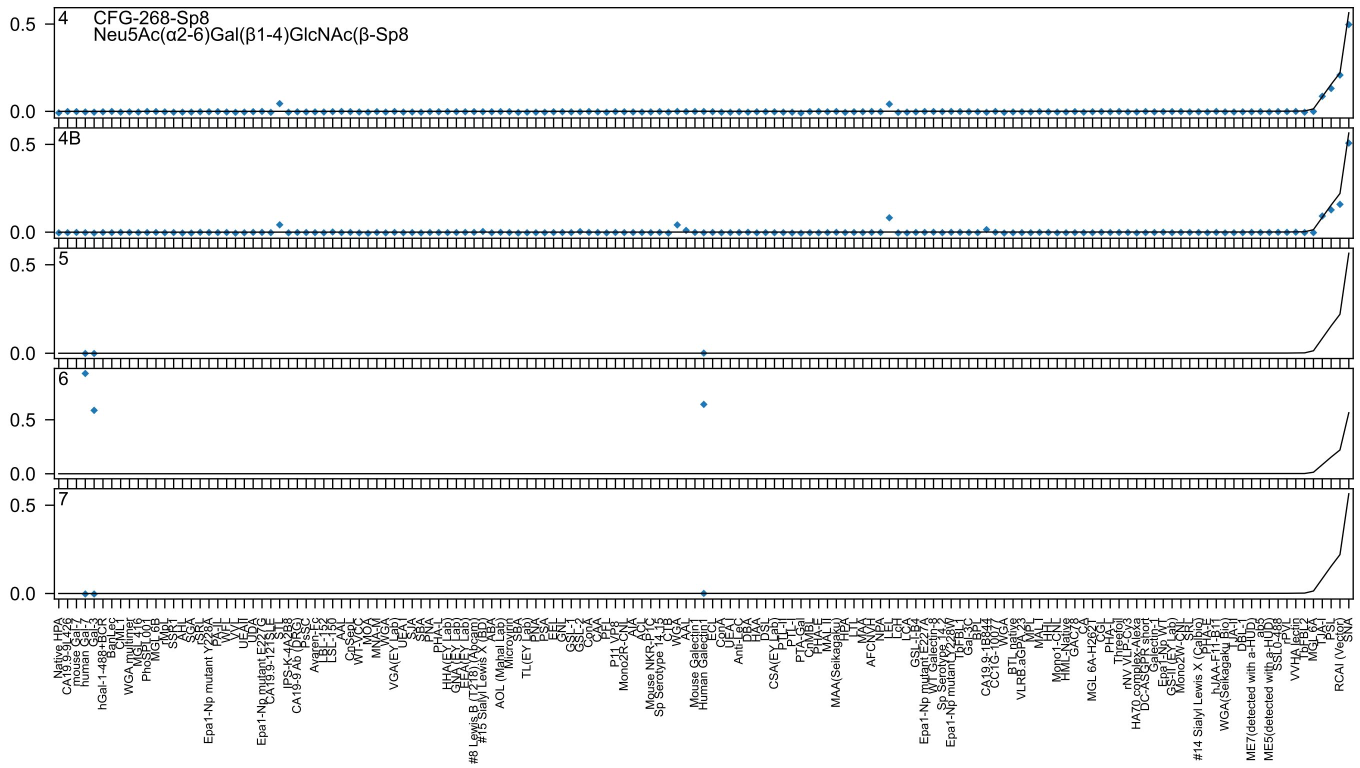
4
CFG-268-Sp8
Neu5Ac(α2-6)Gal(β1-4)GlcNAc(β-Sp8
4B
5
6
7

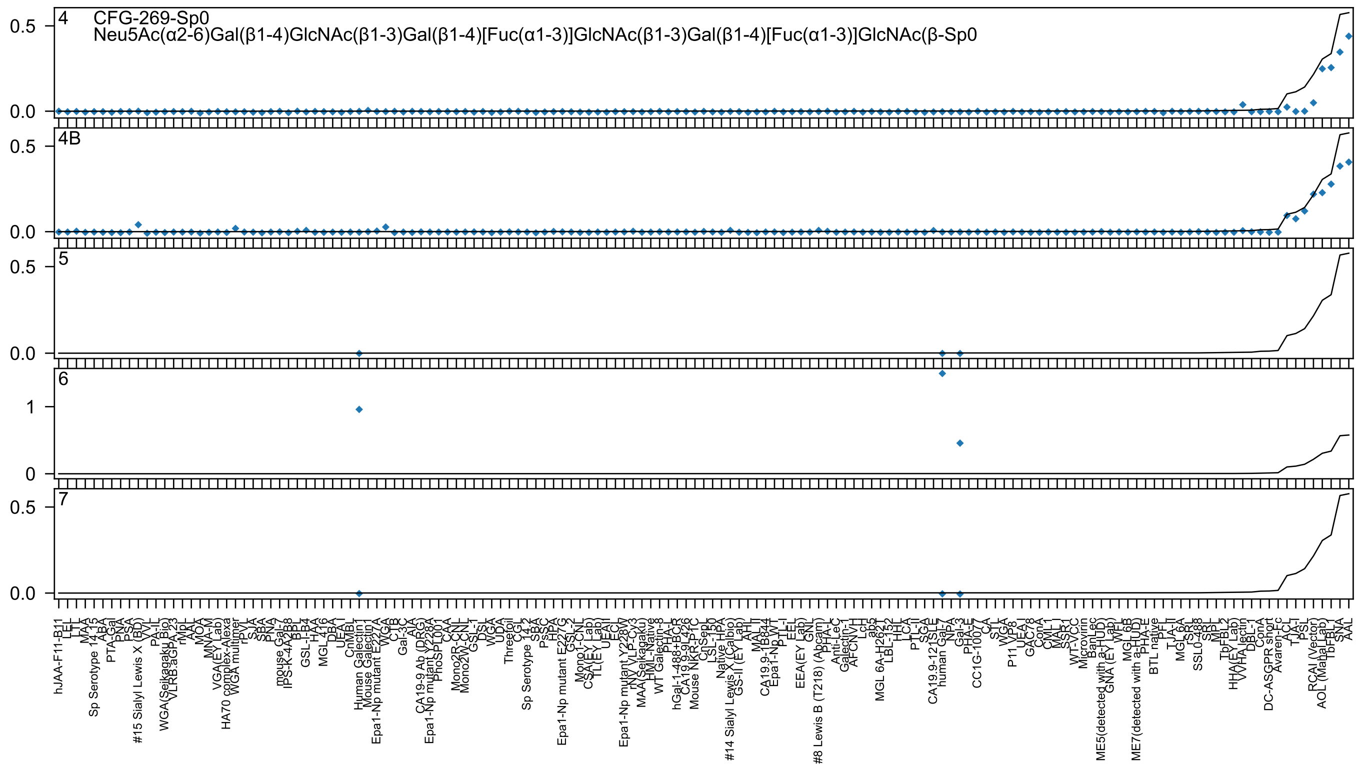

4
CFG-269-Sp0
Neu5Ac(α2-6)Gal(β1-4)GlcNAc(β1-3)Gal(β1-4)[Fuc(α1-3)]GlcNAc(β1-3)Gal(β1-4)[Fuc(α1-3)]GlcNAc(β-Sp0
4B
5
6
7
0.5
0.0
1
0

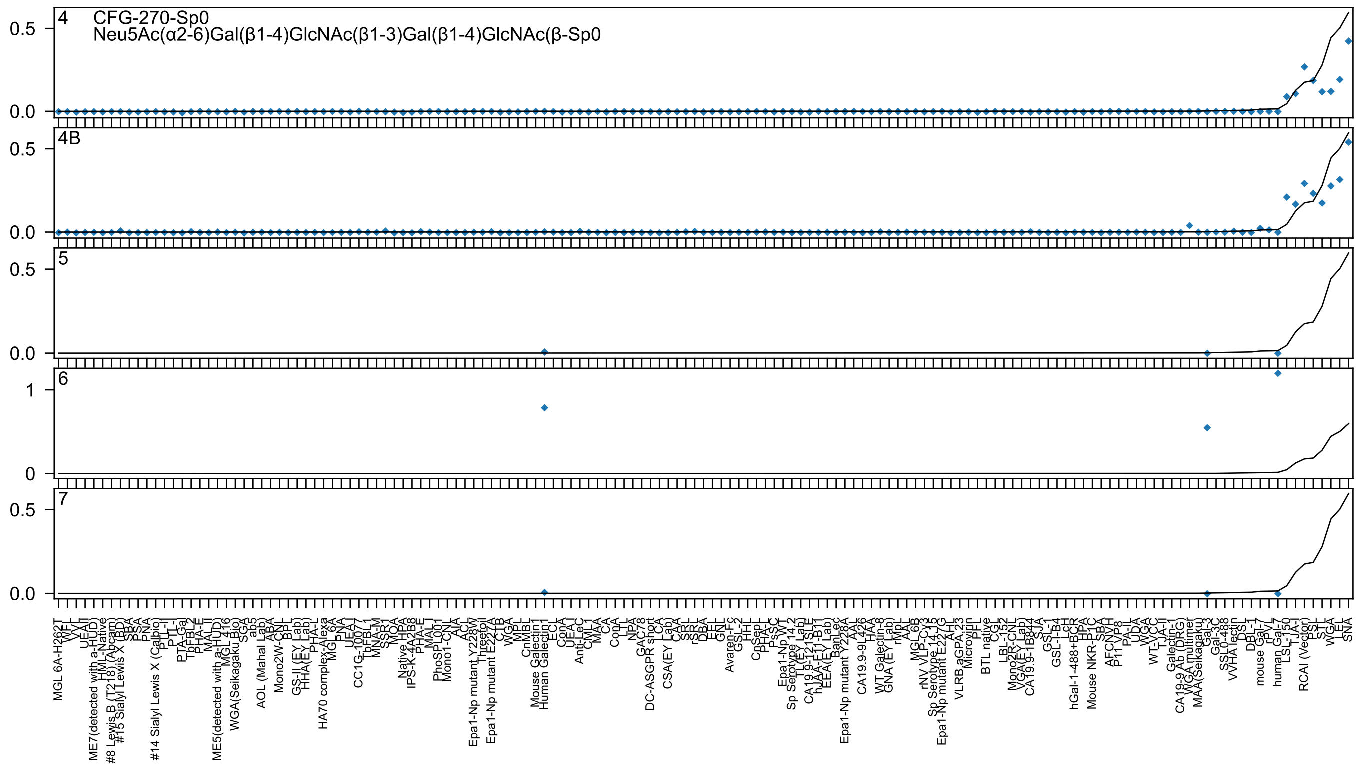

CFG-270-Sp0
Neu5Ac(α2-6)Gal(β1-4)GlcNAc(β1-3)Gal(β1-4)GlcNAc(β-Sp0
4
4B
5
6
7

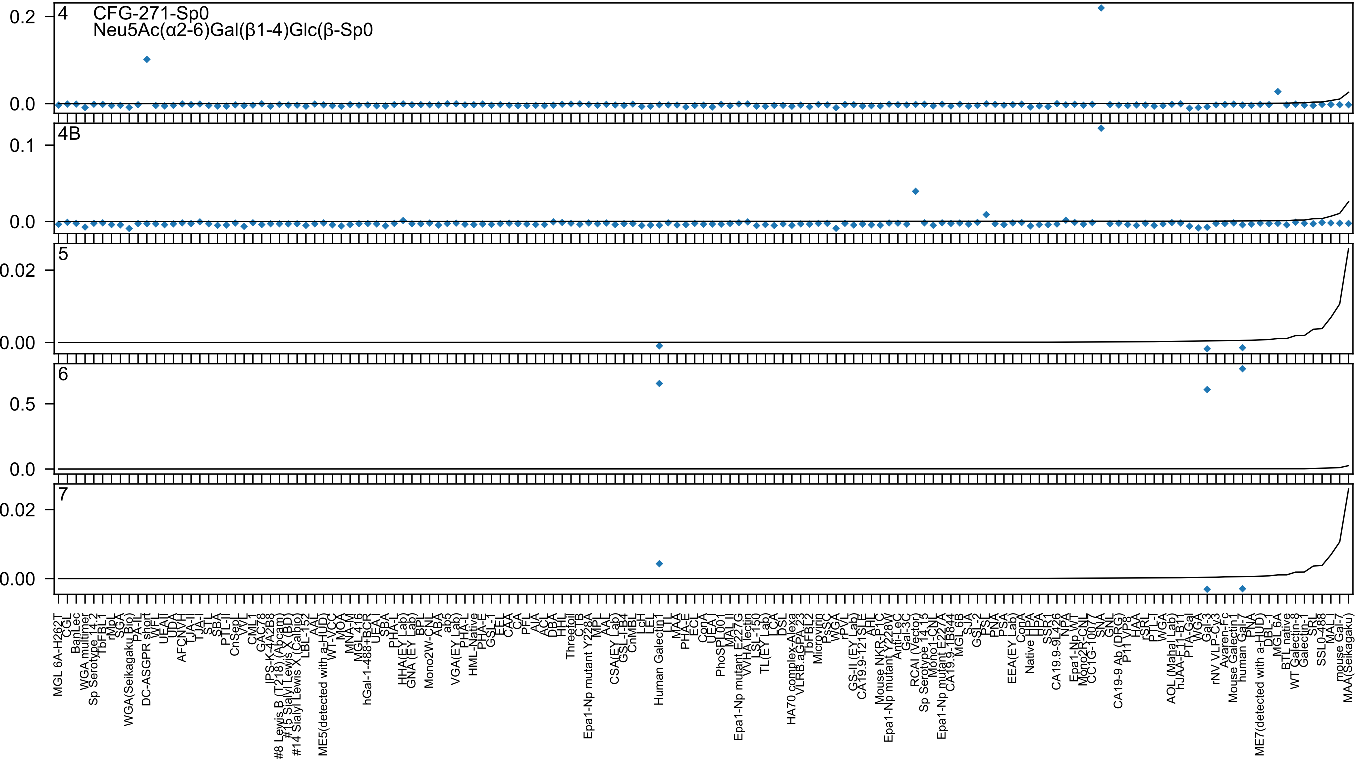

4
CFG-271-Sp0
Neu5Ac(α2-6)Gal(β1-4)Glc(β-Sp0
4B
5
6
7

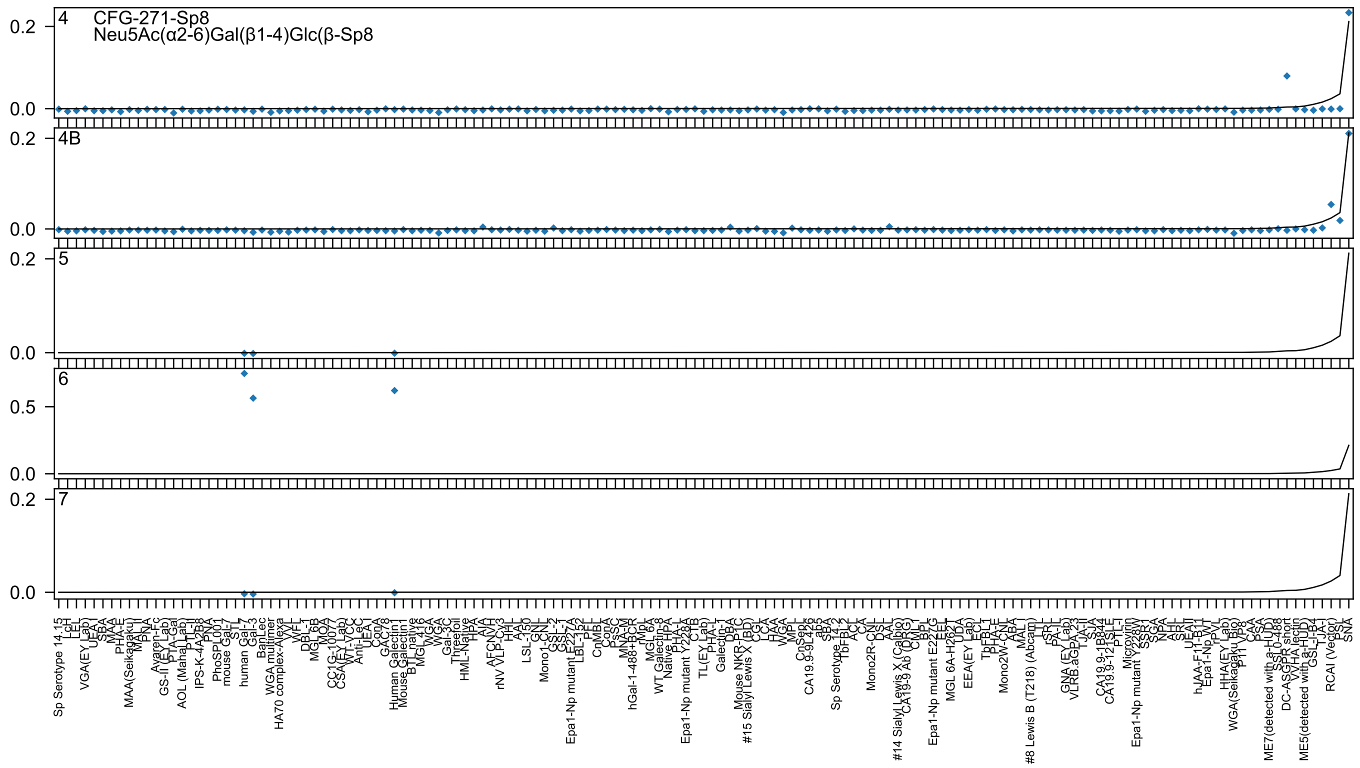
CFG-271-Sp8
Neu5Ac(α2-6)Gal(β1-4)Glc(β-Sp8
4
4B
5
6
7

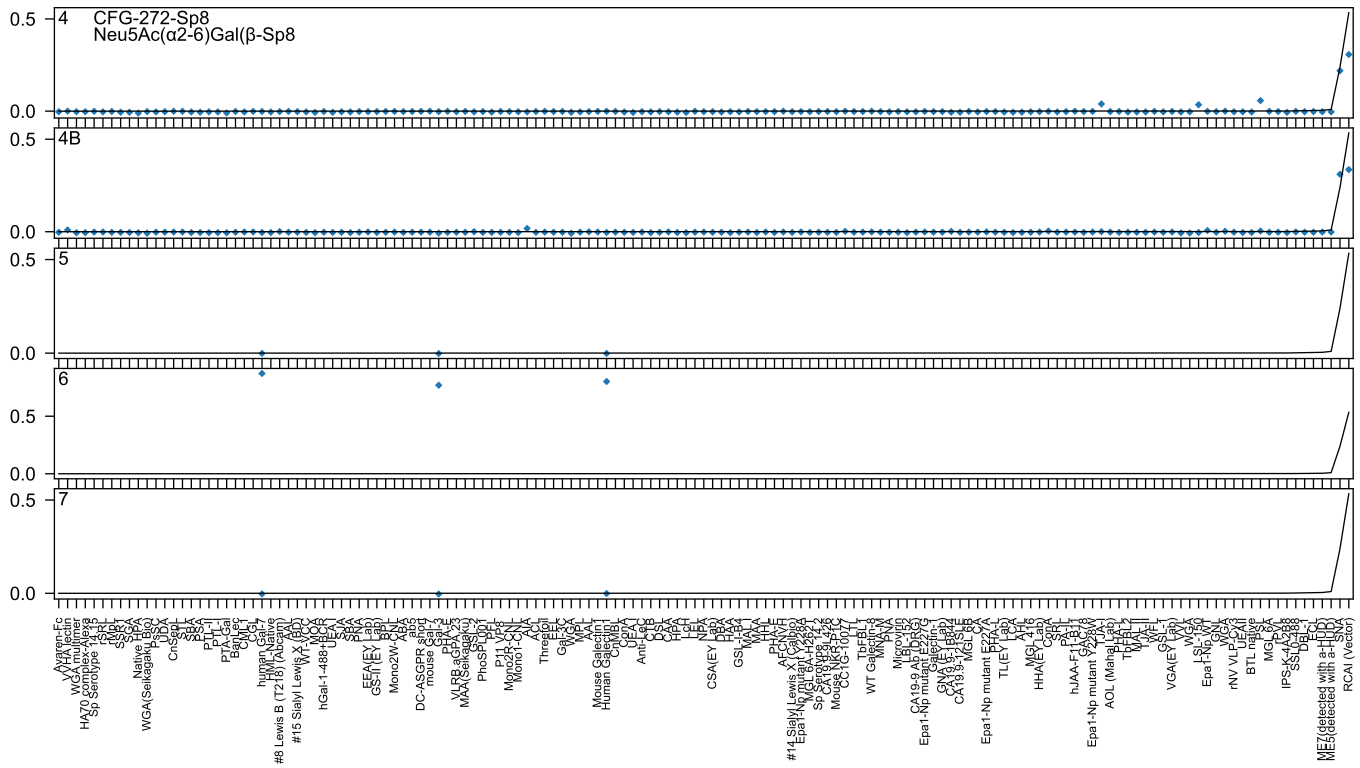

4
CFG-272-Sp8
Neu5Ac(α2-6)Gal(β-Sp8
4B
5
6
7
0.5
0.0

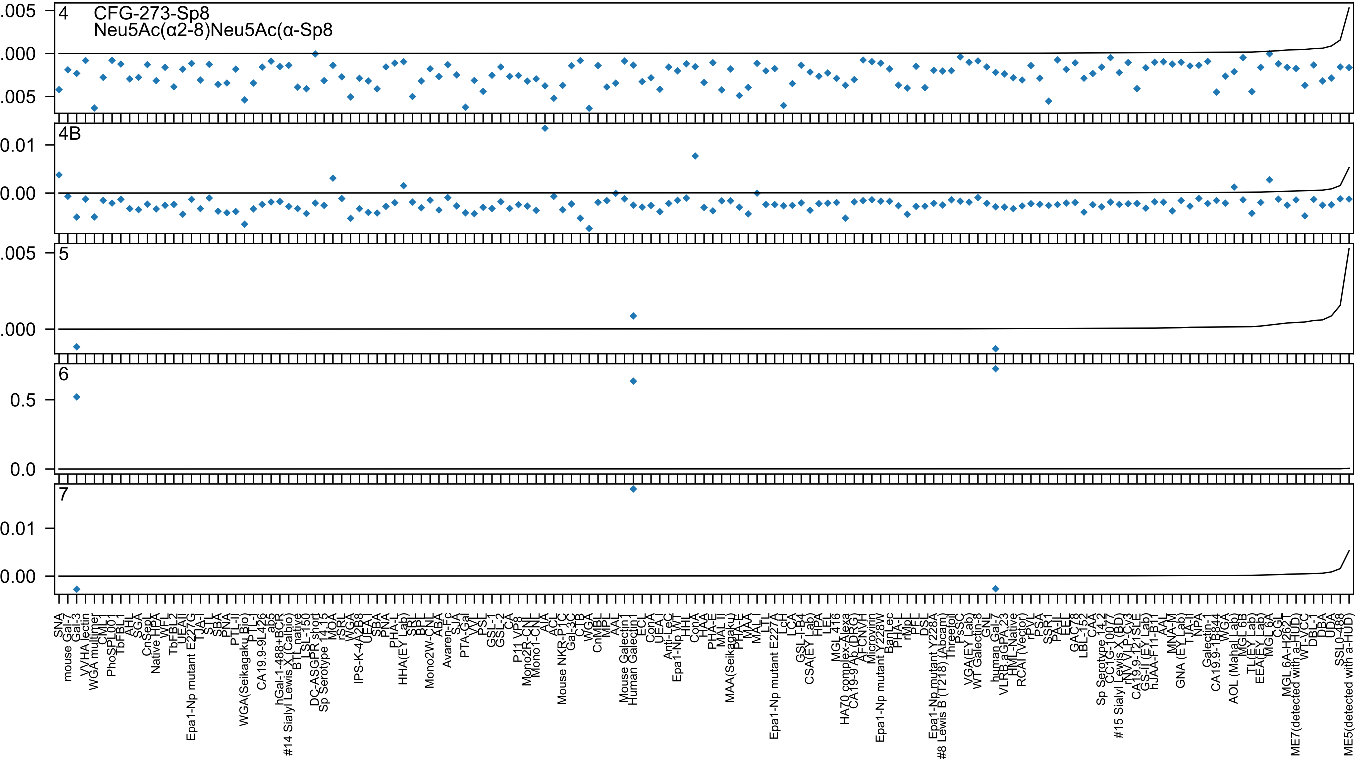

CFG-273-Sp8
Neu5Ac(α2-8)Neu5Ac(α-Sp8
4
4B
5
6
7

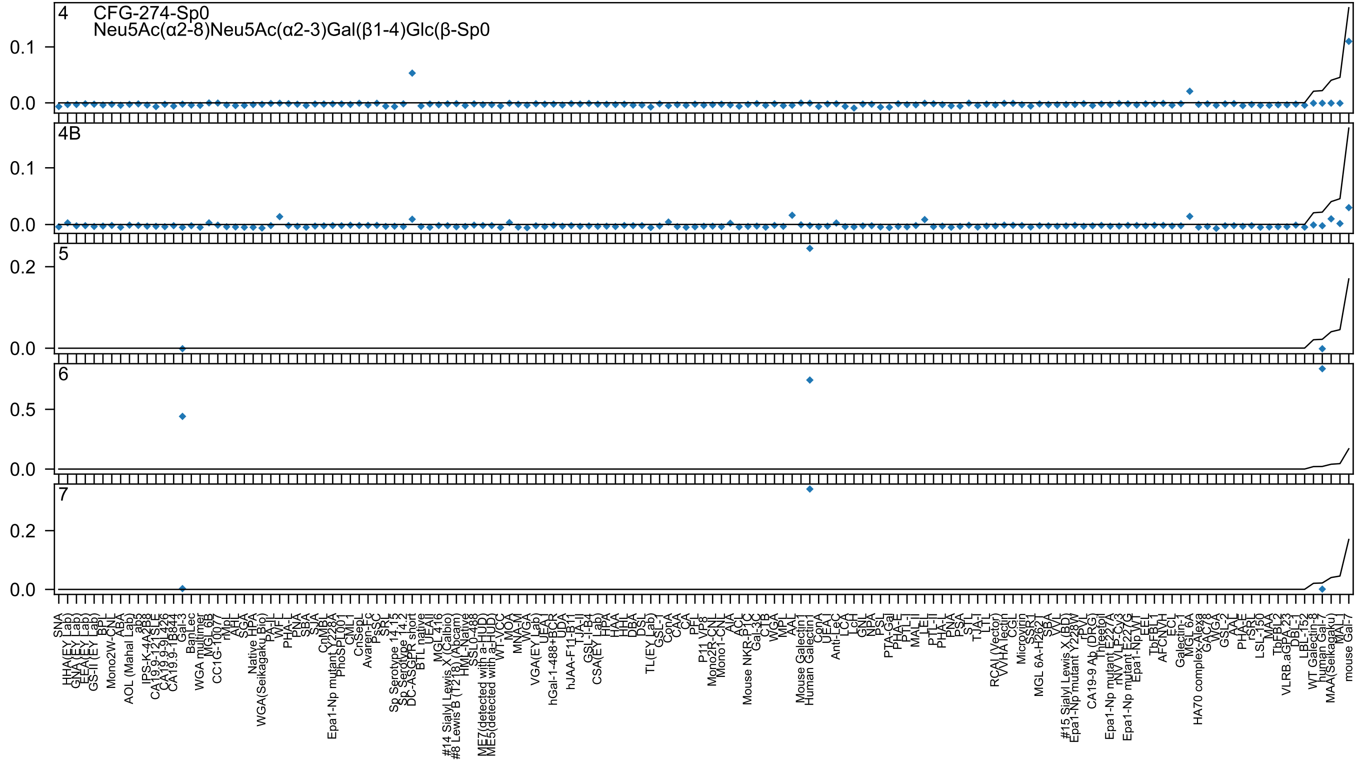
4
CFG-274-Sp0
Neu5Ac(α2-8)Neu5Ac(α2-3)Gal(β1-4)Glc(β-Sp0
4B
5
6
7
0.0
0.1
0.2
0.5
SNA
HHA(EY Lab)
GNA(EY Lab)
EEA(EY Lab)
GS-II (EY Lab)
BPL
Mono2W-CNL
ABA
AOL (Mahal Lab)
ab3
IPS-K-4A2B8
CA19.9-121SLE
CA19.9-9L426
CA19.9-1B844
Gal-3
BanLec
WGA multimer
MGL 6B
CC1G-1007
rMpL
AHL
SGA
Native HPA
WGA(Seikagaku Bio)
PA-IL
WFL
PHA-L
PNA
SBA
SJA
CnMBL
Epa1-Np mutant Y228A
PhoSL001
CML1
CnSepL
Avaren-Fc
PsSC
SRL
Sp Serotype 14.15
Sp Serotype 14.2
DC-ASGPR short
BTL native
UEAII
MGL 416
#14 Sialyl Lewis X (Calbio)
#8 Lewis B (T218) (Abcam)
HML-Native
SSL0-488
ME7(detected with a-HUD)
ME5(detected with a-HUD)
WT-VCC
MOA
MNA-M
WGA
VGA(EY Lab)
UEA-I
hGal-1-488+BCR
UDA
hJAA-F11-B11
TJA-II
GSL-I-B4
CSA(EY Lab)
HPA
HAA
HHL
DBA
DSL
TL(EY Lab)
GSL-I
ConA
CAA
CA
PFL
P11 VP8
Mono2R-CNL
Mono1-CNL
AIA
ACL
Mouse NKR-P1C
Gal-3C
CTB
WGA
MPL
AAL
Mouse Galectin1
Human Galectin1
ConA
UEA-I
Anti-LeC
LCA
LcH
GNL
NPA
PSL
PTA-Gal
PHA-E
PTL-I
MAL-II
LEL
PTL-II
PHA-L
PNA
PSA
STL
TJA-I
LTL
RCAI (Vector)
WHA lectin
CGL
Microvirin
SSR1
MGL 6A-H262
SBA
VVL
#15 Sialyl Lewis X (BD)
Epa1-Np mutant Y228W
rPVL
CA19-9 Ab (DRG)
Threefoil
Epa1-Np mutant E227A
rNV VLP-Cy3
Epa1-Np mutant E227G
Epa1-Np WT
EEL
TbFBL1
AFCNVH
ECL
Galectin-1
MGL 6A
HA70 complex-Alexa
GAC78
WGA
GSL-2
AAL
PHA-E
rSRL
LSL-150
MAA
TbFBL2
VLRB.aGPA.23
DBL-1
LBL-152
WT Galectin-8
human Gal-7
MAA(Seikagaku)
MAL-I
mouse Gal-7

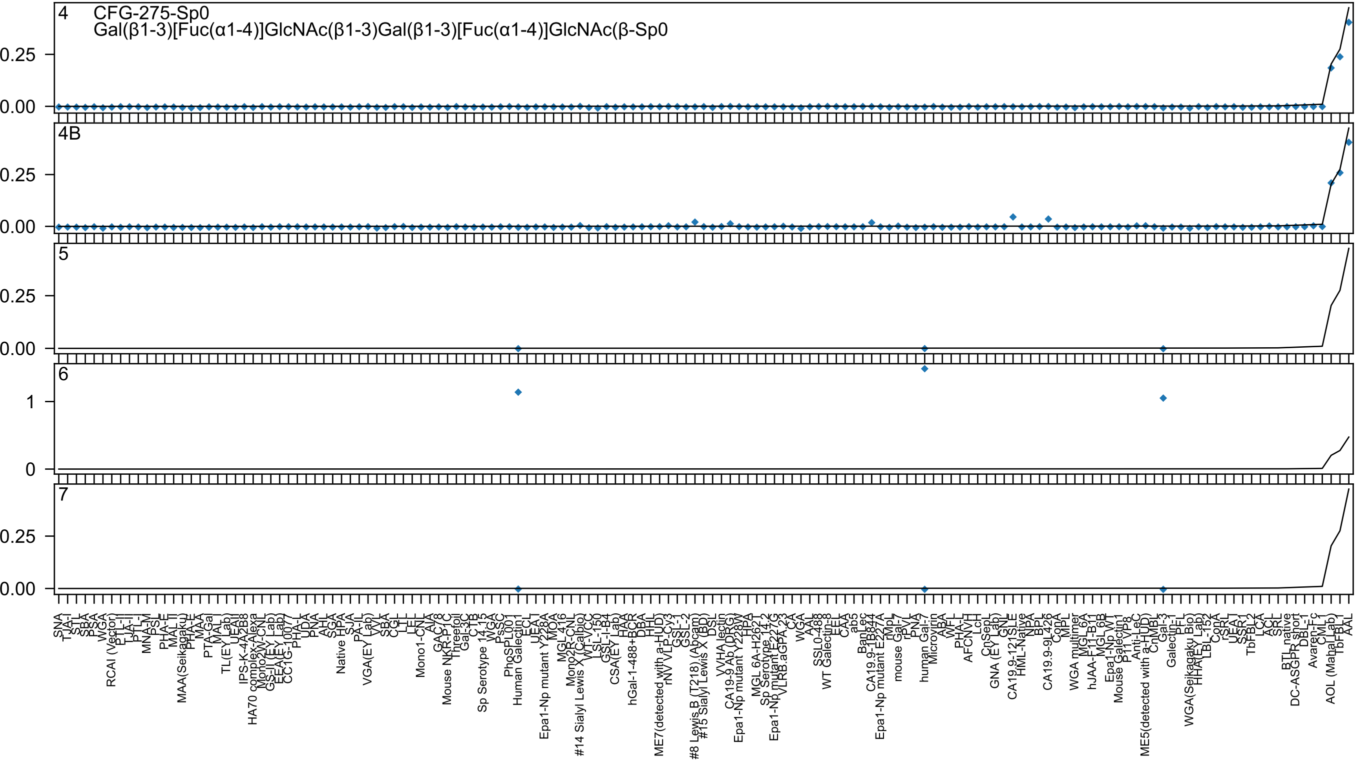
4
CFG-275-Sp0
Gal(β1-3)[Fuc(α1-4)]GlcNAc(β1-3)Gal(β1-3)[Fuc(α1-4)]GlcNAc(β-Sp0
0.25
0.00
4B
5
6
1
0
7

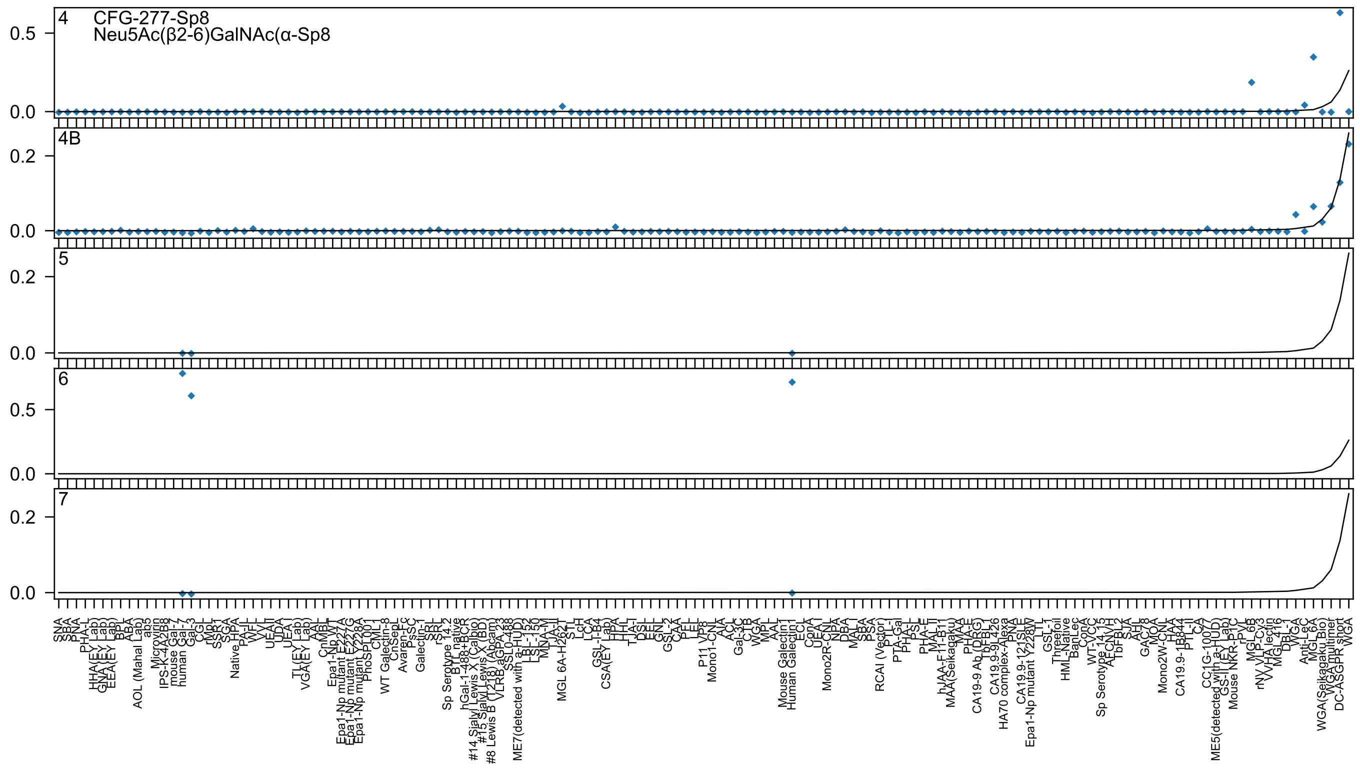

4
CFG-277-Sp8
Neu5Ac(β2-6)GalNAc(α-Sp8
4B
5
6
7
0.5
0.2
0.0

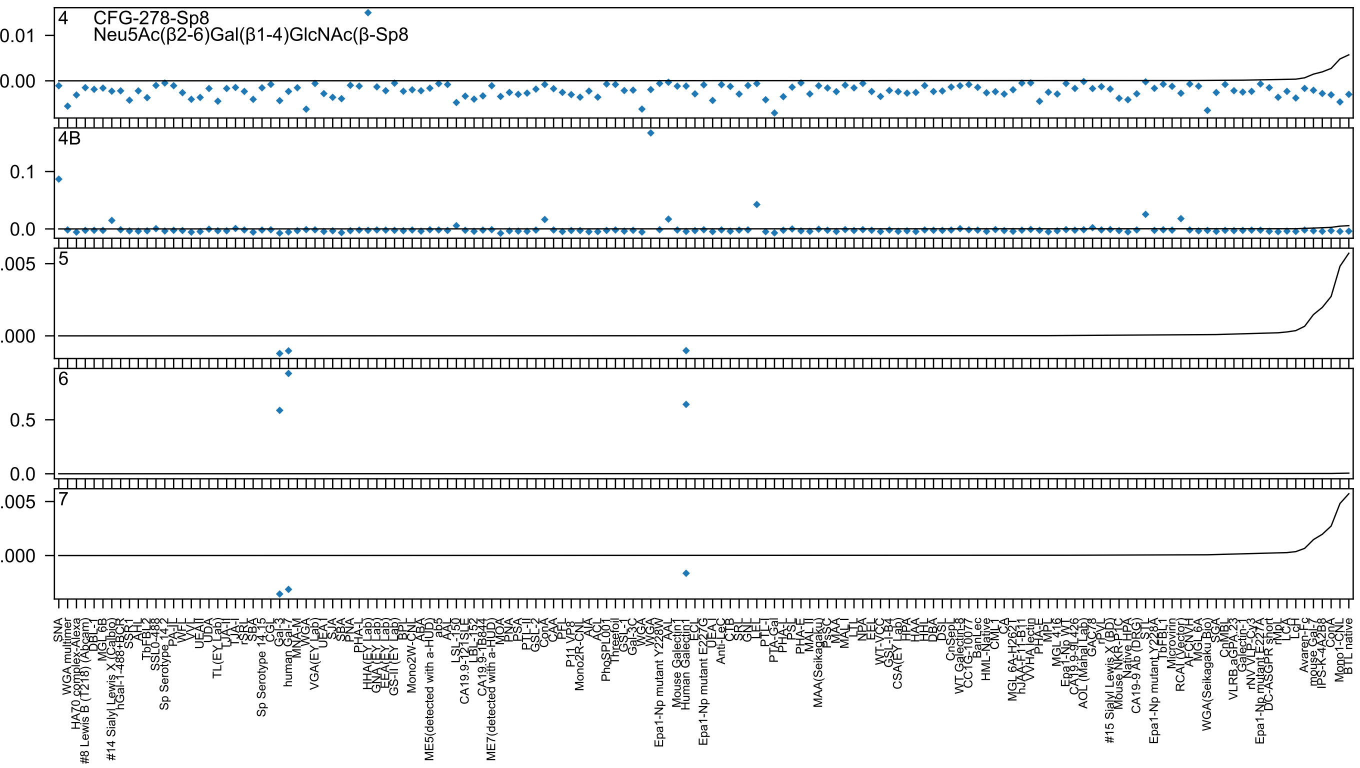

CFG-278-Sp8
Neu5Ac(β2-6)Gal(β1-4)GlcNAc(β-Sp8
4
4B
5
6
7

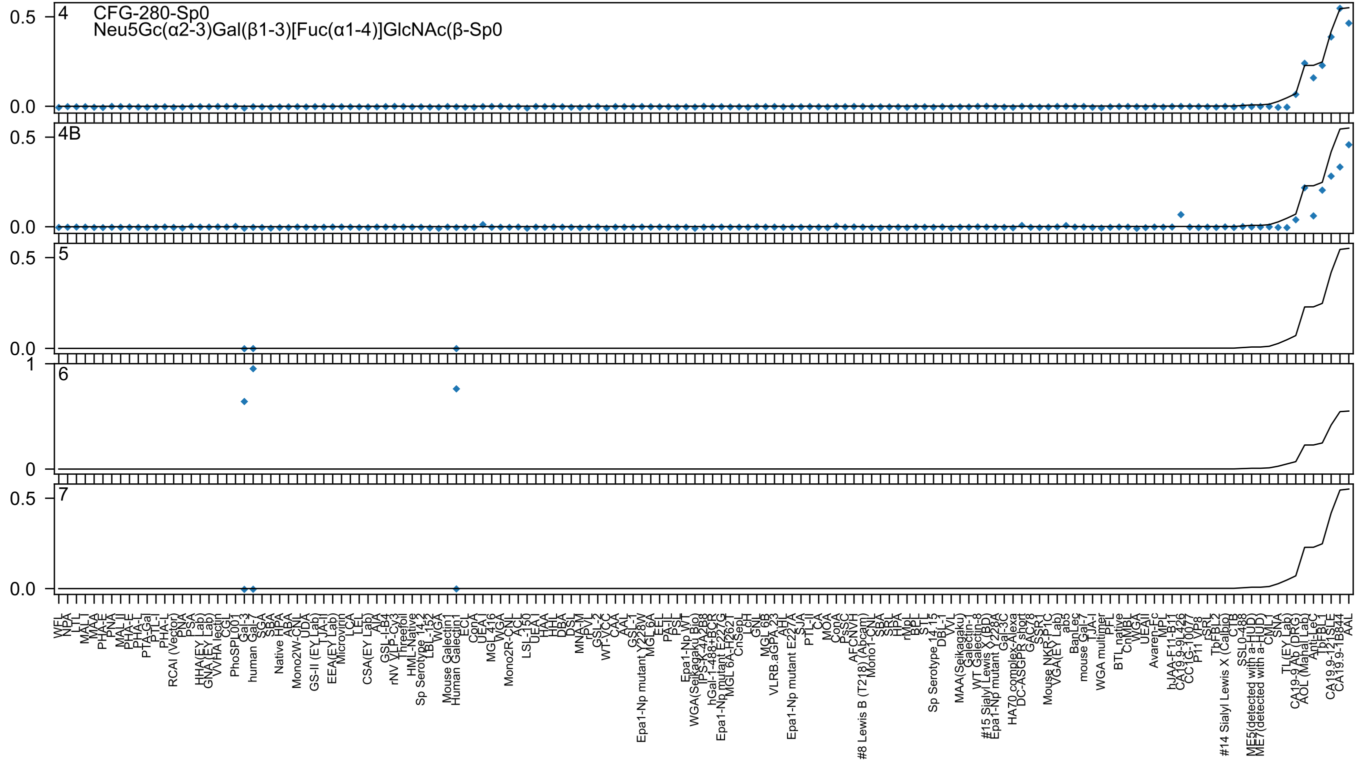
4 CFG-280-Sp0
Neu5Gc(α2-3)Gal(β1-3)[Fuc(α1-4)]GlcNAc(β-Sp0
4B
5
6
7

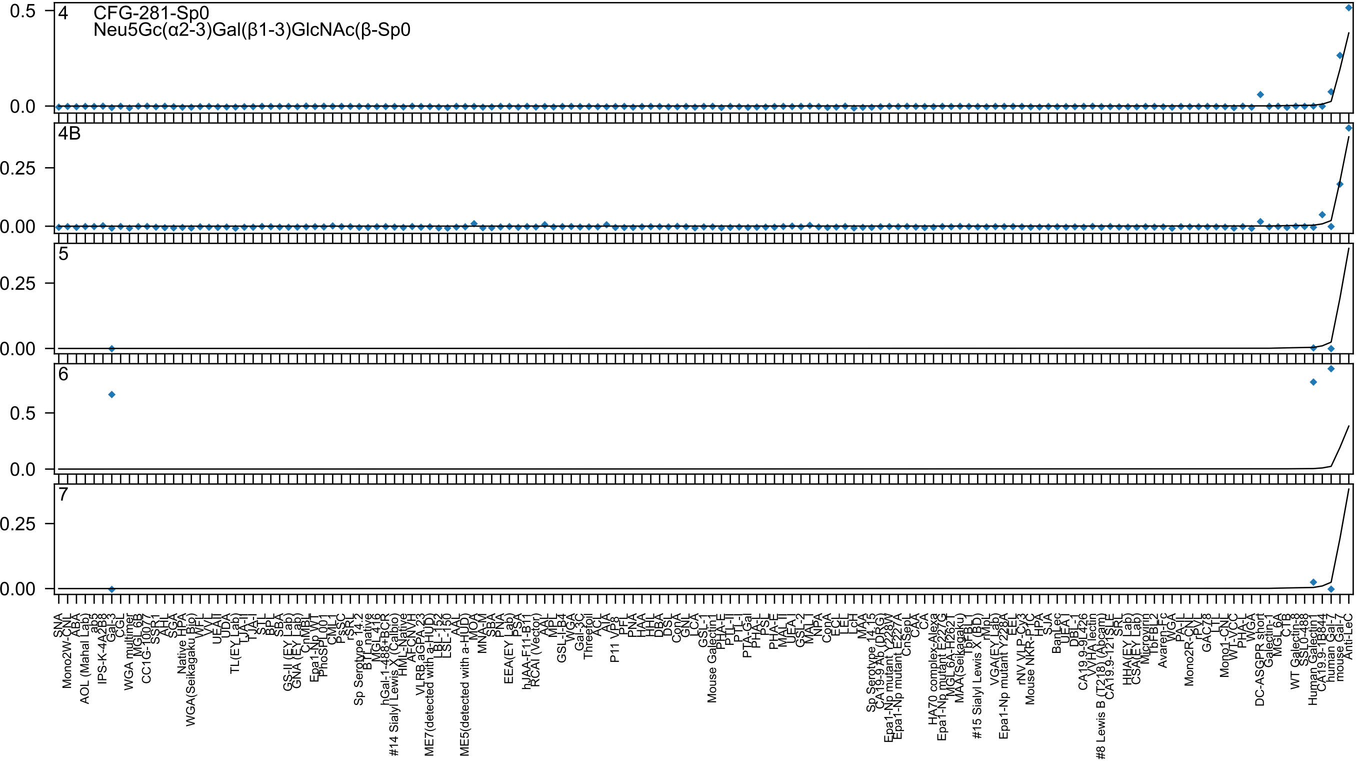

4
CFG-281-Sp0
Neu5Gc(α2-3)Gal(β1-3)GlcNAc(β-Sp0
4B
5
6
7

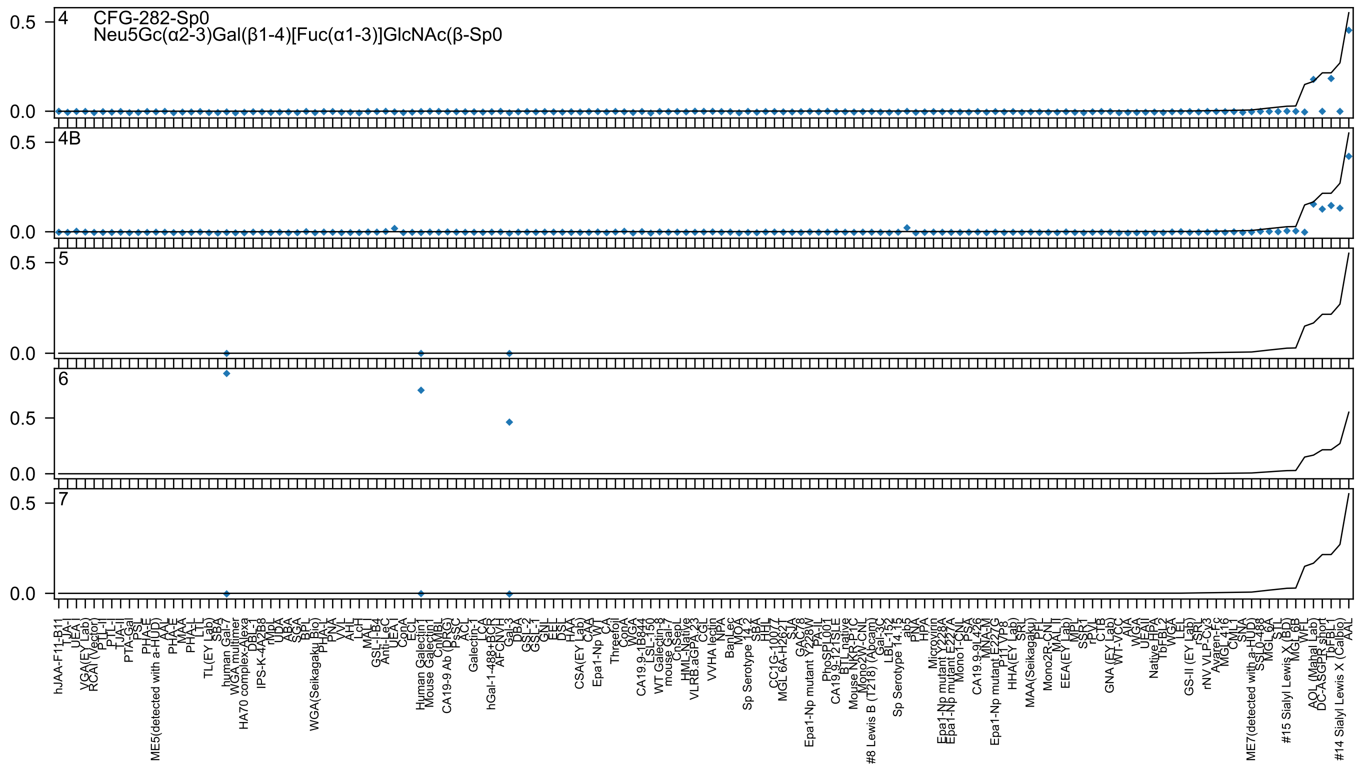

CFG-282-Sp0
Neu5Gc(α2-3)Gal(β1-4)[Fuc(α1-3)]GlcNAc(β-Sp0
4
4B
5
6
7

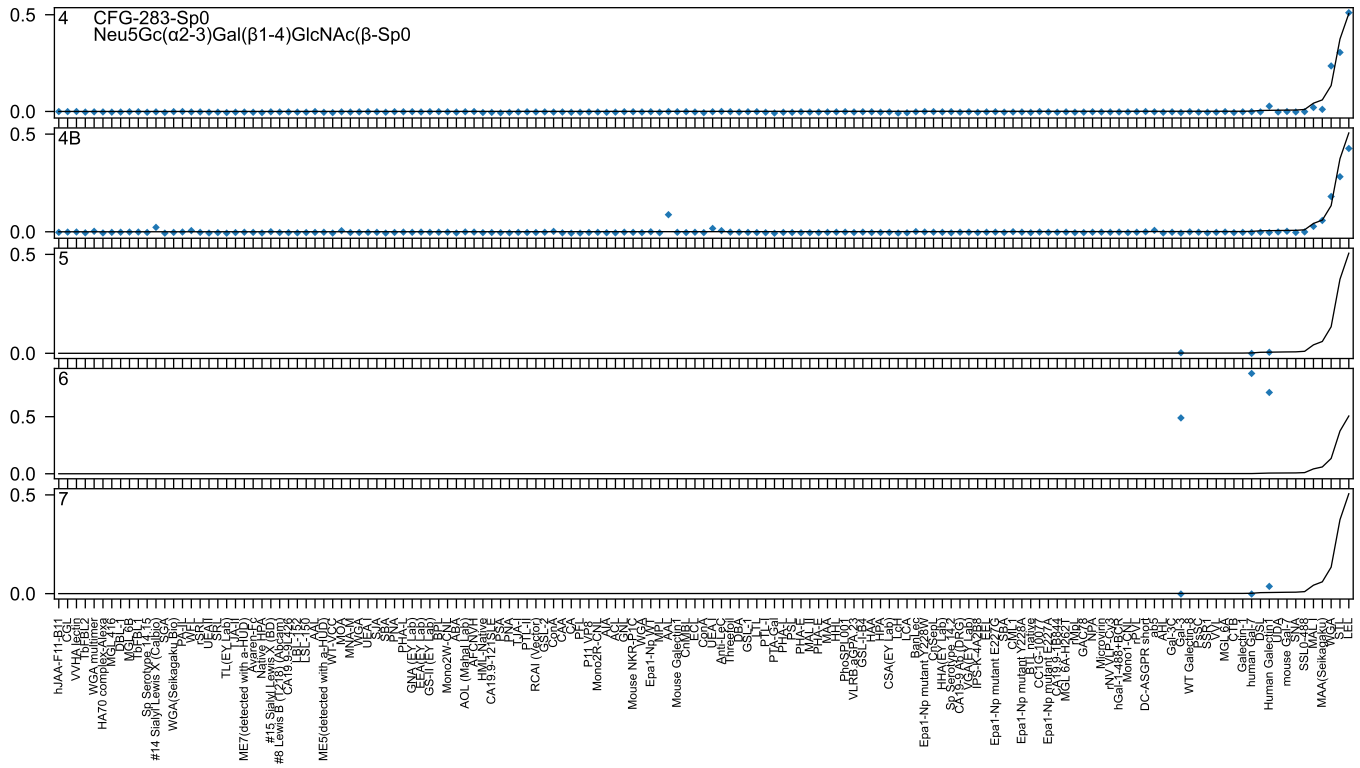
CFG-283-Sp0
Neu5Gc(α2-3)Gal(β1-4)GlcNAc(β-Sp0
4
4B
5
6
7

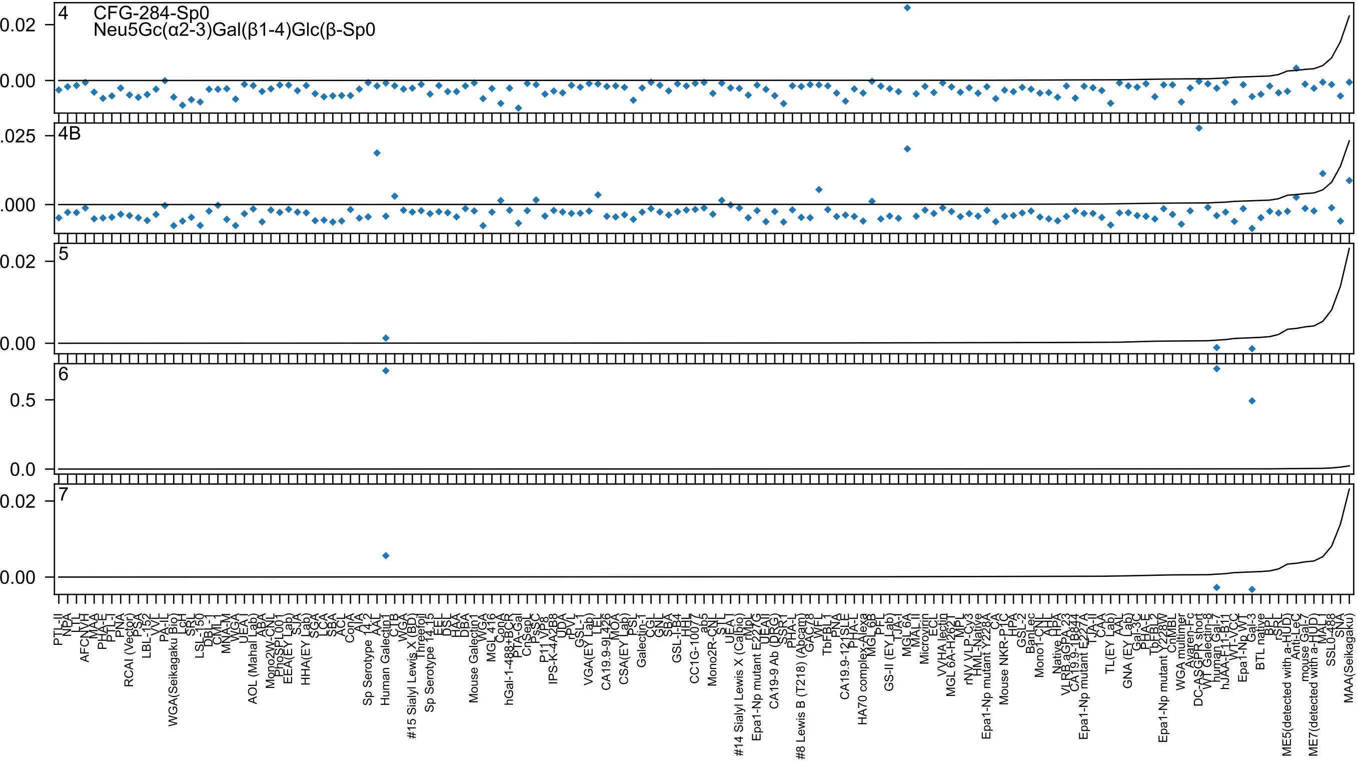

4 CFG-284-Sp0
Neu5Gc(α2-3)Gal(β1-4)Glc(β-Sp0
0.02
0.00
4B
.025
.000
5
0.02
0.00
6
0.5
0.0
7
0.02
0.00
PTL-II
NPA
LTL
AFCNVH
MAA
PHA-E
PTL-I
PNA
RCAI (Vector)
PSA
LBL-152
VVL
PA-IL
WGA(Seikagaku Bio)
LcH
SRL
LSL-150
DBL-1
CML-1
MNA-M
WGA
UEA-I
AOL (Mahal Lab)
ABA
Mono2W-CNL
PhoSL001
EEA(EY Lab)
SJA
HHA(EY Lab)
SGA
LCA
SBA
ACL
ConA
AIA
Sp Serotype 14.2
AAT
Human Galectin1
CTB
WGA
#15 Sialyl Lewis X (BD)
Threefoil
Sp Serotype 14.15
EEL
DSL
HAA
DBA
Mouse Galectin1
WGA
MGL 416
ConA
hGal-1-488+BCR
PTA-Gal
CnSep1
PsSC
P11 VP8
IPS-K-4A2B8
UDA
rPVL
GSL-1
VGA(EY Lab)
LEL
CA19.9-9L426
MOA
CSA(EY Lab)
PSL
Galectin-1
CGL
GNL
SBA
GSL-I-B4
HHL
CC1G-10077
ab5
Mono2R-CNL
STL
UEA-I
#14 Sialyl Lewis X (Calbio)
rMpL
Epa1-Np mutant E227G
UEAII
CA19-9 Ab (DRG)
SSR1
PHA-L
#8 Lewis B (T218) (Abcam)
GAC78
WFL
TbFBL1
PNA
CA19.9-121SLE
PHA-L
HA70 complex-Alexa
MGL 6B
PFL
GS-II (EY Lab)
TJA-I
MGL 6A
MAL II
Microvirin
ECL
VVHA lectin
MGL 6A-H262T
MPL
rNV VLP-Cy3
HML-Native
Epa1-Np mutant Y228A
CA
Mouse NKR-P1C
HPA
GSL-2
BanLec
Mono1-CNL
AAL
Native HPA
VLRB.aGPA.23
CA19.9-1B844
Epa1-Np mutant E227A
TJA-II
CAA
TL(EY Lab)
AAL
GNA (EY Lab)
Gal-3C
PHA-E
TbFBL2
Epa1-Np mutant Y228W
CnMBL
WGA multimer
Avaren-Fc
DC-ASGPR short
WT Galectin-8
human Gal-7
hJAA-F11-B11
WT-VCC
Epa1-Np WT
Gal-3
BTL native
BPL
rSRL
ME5(detected with a-HUD)
Anti-LeC
mouse Gal-7
ME7(detected with a-HUD)
MAL I
SSL0-488
SNA
MAA(Seikagaku)

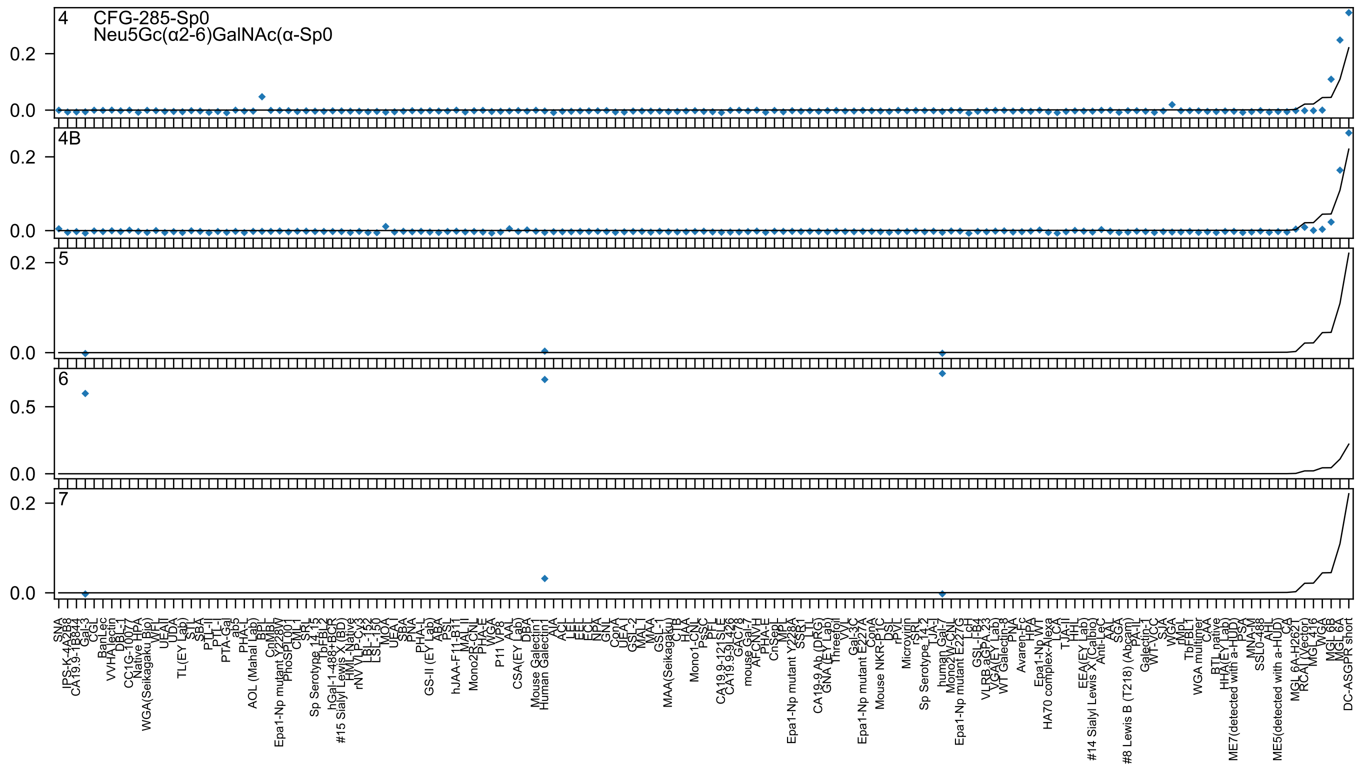
4
CFG-285-Sp0
Neu5Gc(α2-6)GalNAc(α-Sp0
4B
5
6
7

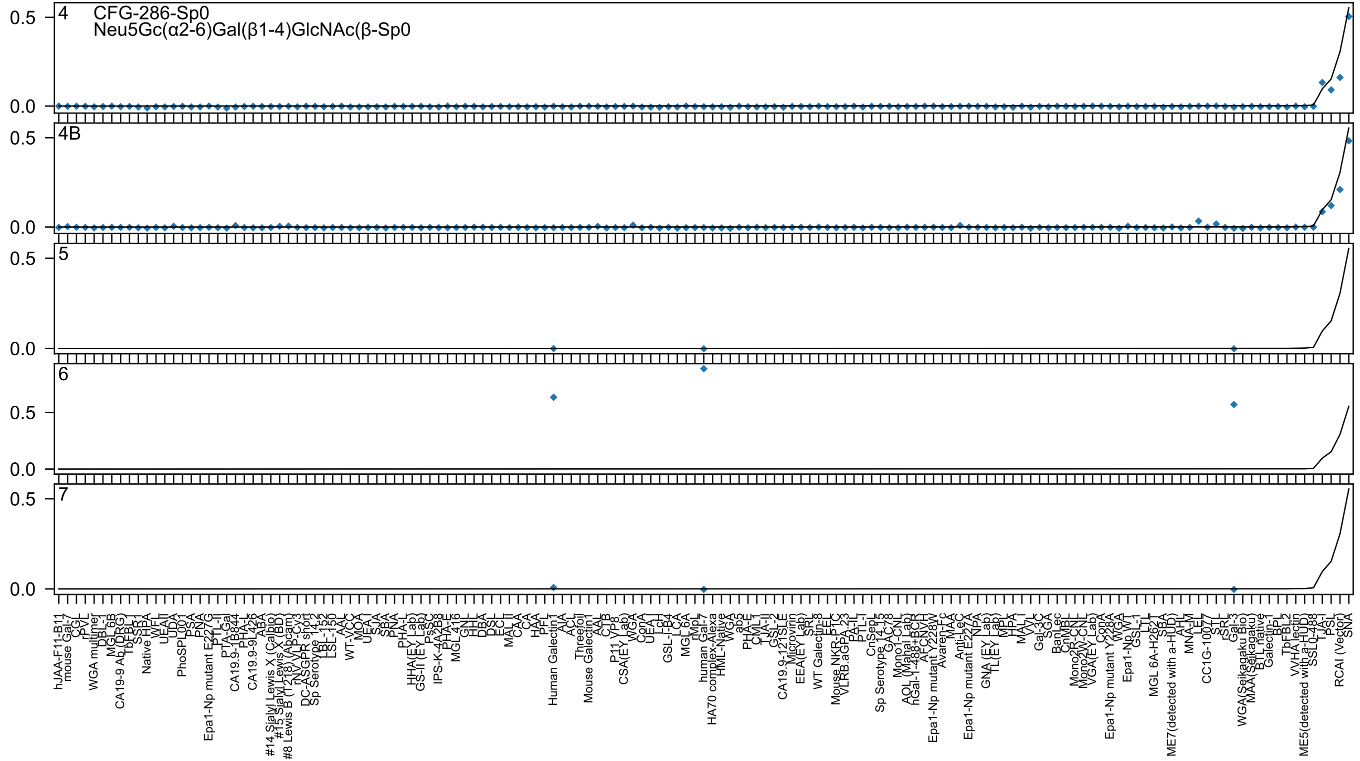
4
CFG-286-Sp0
Neu5Gc(α2-6)Gal(β1-4)GlcNAc(β-Sp0
4B
5
6
7
0.5
0.0
hJAA-F11-B11
mouse Gal-7
CGL
rPVL
WGA multimer
DBL-I
MGL 6B
CA19-9 Ab (DRG)
TbFBL1
SSR1
Native HPA
WFL
UEA-II
UDA
PhoSPL001
PSA
PNA
Epa1-Np mutant E227G
PTL-II
PTA-Gal
CA19.9-1B844
PHA-L
CA19.9-9L426
ABA
#14 Sialyl Lewis X (Calbio)
#15 Sialyl Lewis X (BD)
#8 Lewis B (T218) (Abcam)
rNV-VLP-Cy3
DC-ASGPR short
Sp Serotype 14.2
LBL-152
LSL-150
AAL
WT-VCC
MOA
UEA-I
SJA
SBA
PNA
PHA-L
HHA(EY Lab)
GS-II (EY Lab)
PsSC
IPS-K-4A2B8
PHA-E
MGL 416
GNL
HHL
DBA
DSL
ECL
MAL II
CAA
CA
HAA
PFL
Human Galectin1
AIA
ACL
Threefoil
Mouse Galectin1
AAL
CTB
P11 VP8
CSA(EY Lab)
WGA
ConA
UEA-I
LcH
GSL-I-B4
LCA
MGL 6A
rMpL
human Gal-7
HA70 complex-Alexa
HML-Native
WGA
ab5
PHA-E
CML-I
TJA-II
GSL-2
CA19.9-121SLE
Microvirin
EEA(EY Lab)
SRL
EEL
WT Galectin-8
Mouse NKR-P1C
VLRB.aGPA.23
PA-IL
PTL-I
CnSeph
Sp Serotype 14-78
Mono1-CNL
AOL (Manal Lab)
hGal-1-488+BCR
Epa1-Np mutant Y228W
Avaren-Fc
MAA
Anti-LeC
Epa1-Np mutant E227A
NPA
GNA (EY Lab)
TL(EY Lab)
MPL
HPA
MAL-I
VVL
Gal-3C
SGA
BanLec
CnMBL
Mono2R-CNL
Mono2W-CNL
VGA(EY Lab)
ConA
Epa1-Np mutant Y228A
WGA
Epa1-Np WT
GSL-I
LTL
MGL 6A-H262T
SBA
ME7(detected with a-HUD)
AHL
MNA-M
LEL
CC1G-10077
STL
rSRL
Gal-3
WGA(Seikagaku Bio)
MAA(Seikagaku)
BTL native
Galectin-1
BPL
TbFBL2
VVHA lectin
ME5(detected with a-HUD)
SSL0-488
TJA-I
PSL
RCAI (Vector)
SNA

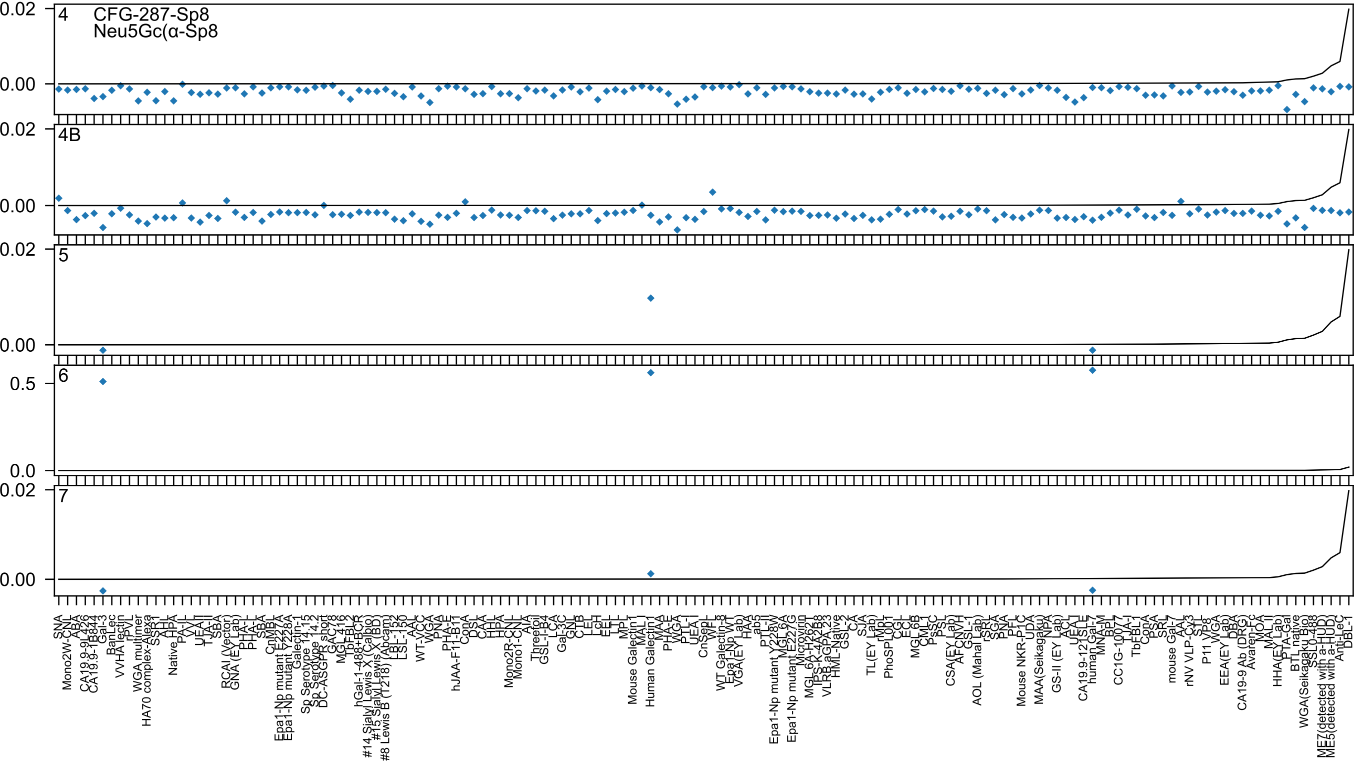

4
CFG-287-Sp8
Neu5Gc(α-Sp8
4B
5
6
7

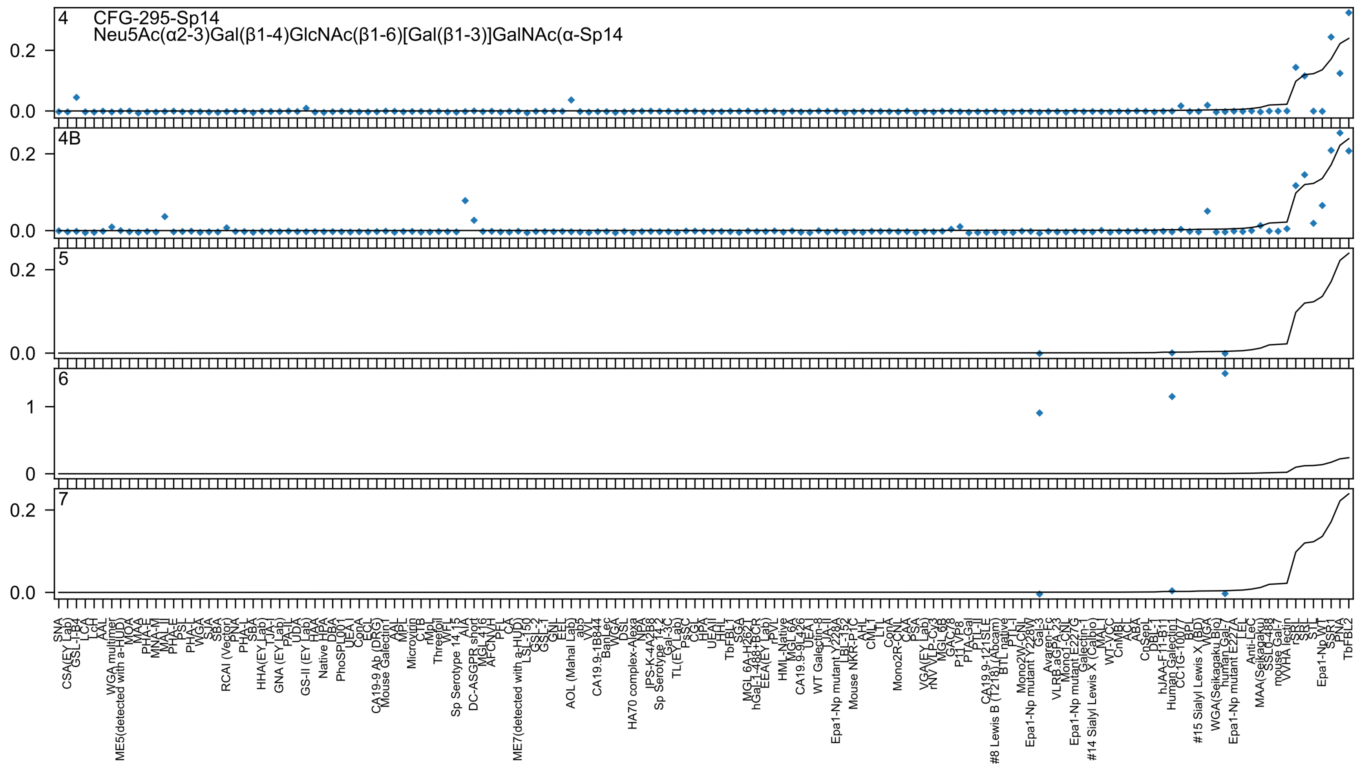
4 CFG-295-Sp14
Neu5Ac(α2-3)Gal(β1-4)GlcNAc(β1-6)[Gal(β1-3)]GalNAc(α-Sp14
4B
5
6
7

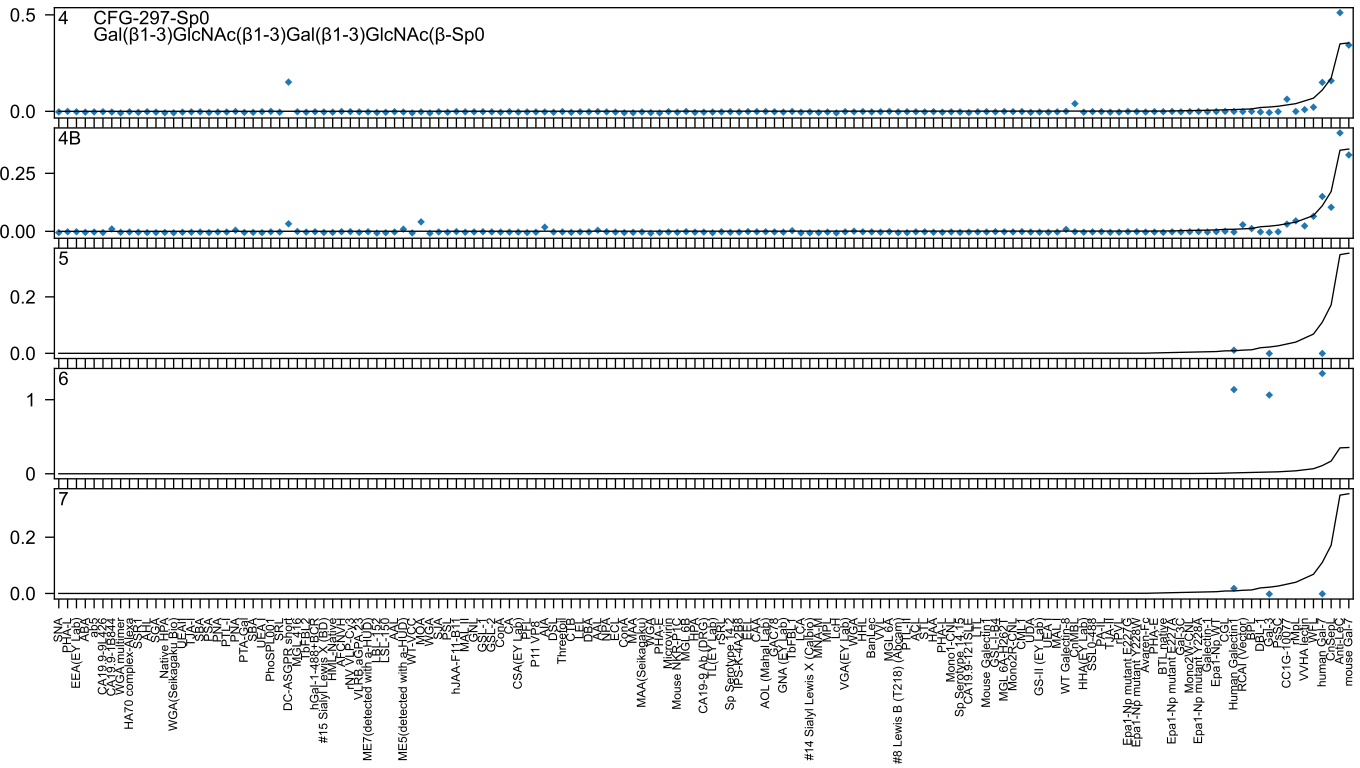
4
CFG-297-Sp0
Gal(β1-3)GlcNAc(β1-3)Gal(β1-3)GlcNAc(β-Sp0
4B
5
6
7

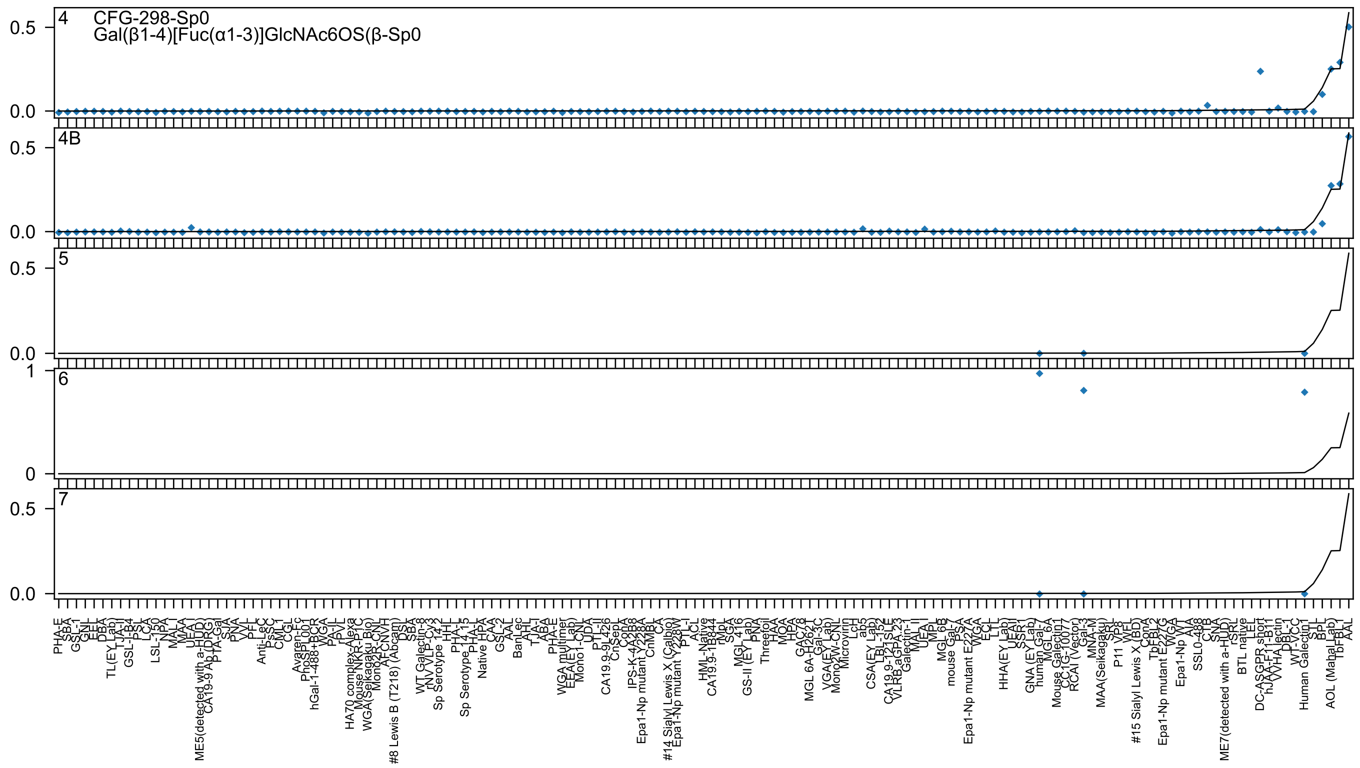

4
CFG-298-Sp0
Gal(β1-4)[Fuc(α1-3)]GlcNAc6OS(β-Sp0
4B
5
6
7
0.5
0.0
1
0

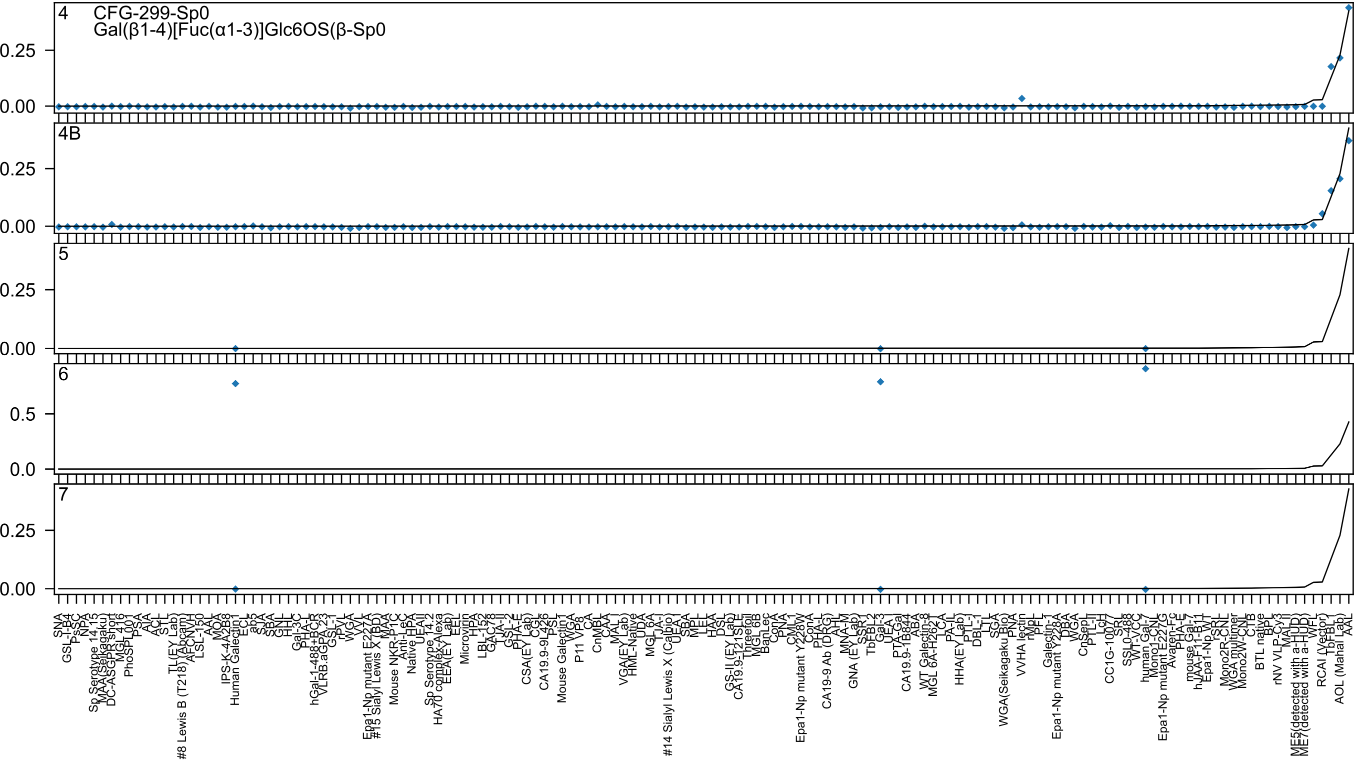
4
CFG-299-Sp0
Gal(β1-4)[Fuc(α1-3)]Glc6OS(β-Sp0
4B
5
6
7
0.25
0.00
0.5
0.0

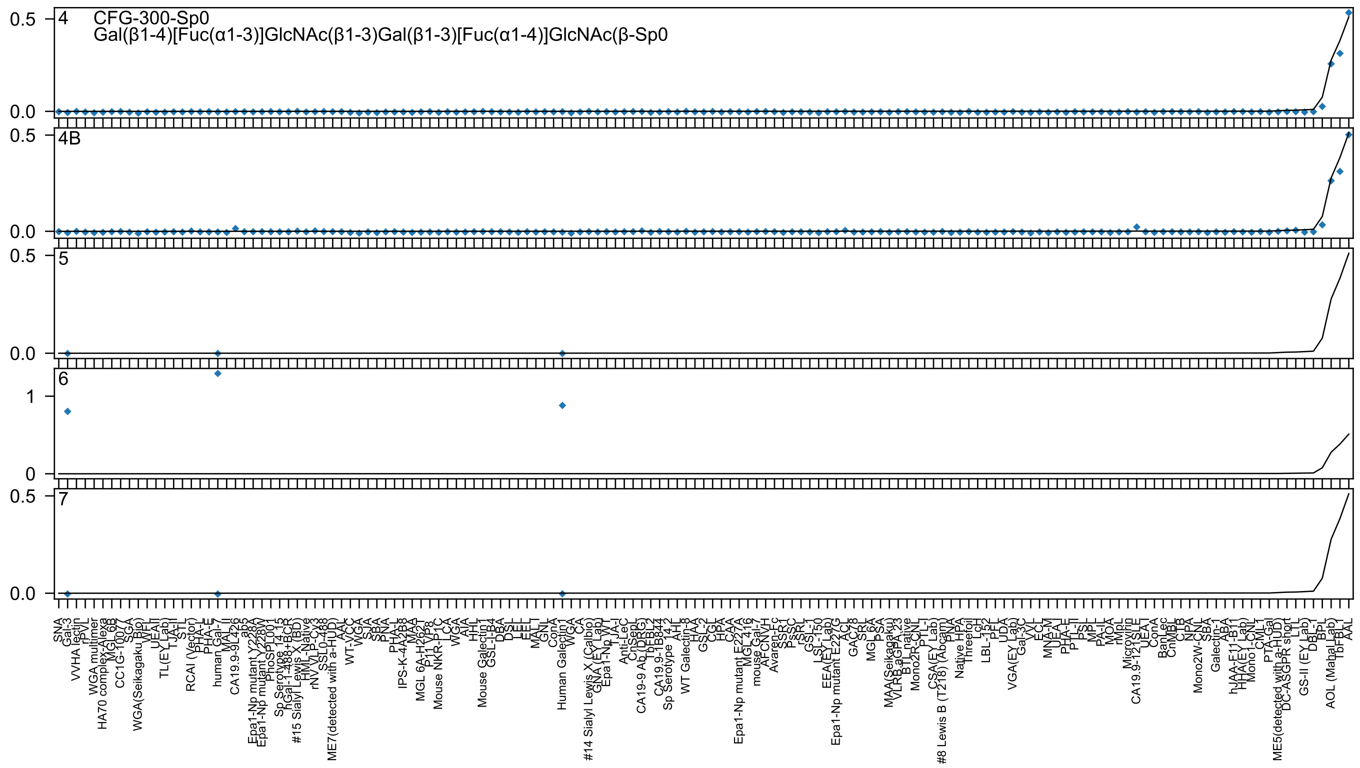
CFG-300-Sp0
Gal(β1-4)[Fuc(α1-3)]GlcNAc(β1-3)Gal(β1-3)[Fuc(α1-4)]GlcNAc(β-Sp0
4
4B
5
6
7

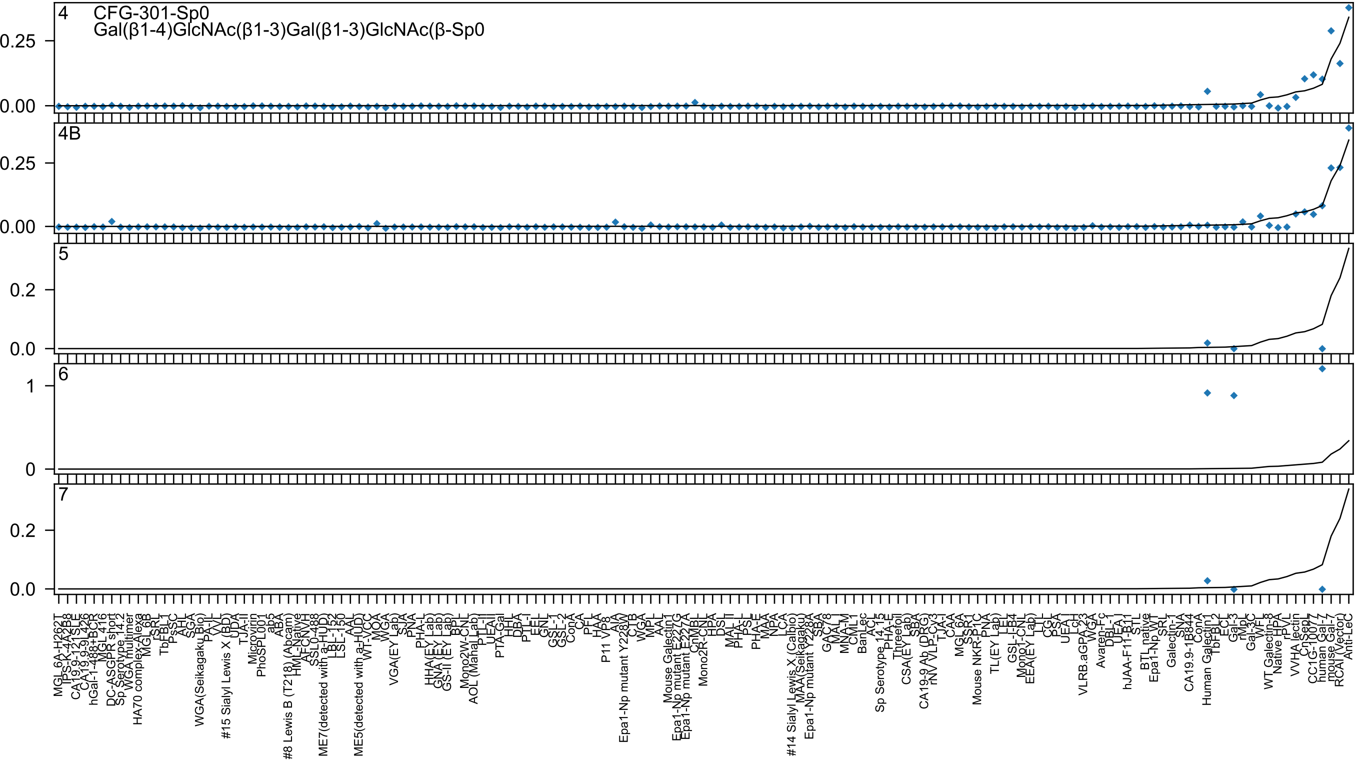
4
CFG-301-Sp0
Gal(β1-4)GlcNAc(β1-3)Gal(β1-3)GlcNAc(β-Sp0
4B
5
6
7
0.25
0.00
0.2
0.0
1
0
MGL_6A-H262T
CA19.9-121SLE
hGal-1-488+BCR
MGL_416
DC-ASGPR short
Sp_Serotype_14.2
WGA multimer
HA70 complex-Alexa
MGL_6B
TbFBL1
PsSC
AHL
SGA
WGA(Seikagaku_Bio)
#15 Sialyl Lewis X (BD)
UDA
TJA-II
Microvirin
PhoSL001
ab5
ABA
#8 Lewis B (T218) (Abcam)
HML-Native
AFCNVH
SSL0-488
ME7(detected with a-HUD)
LSL-150
AAL
ME5(detected with a-HUD)
WT-VCC
MOA
WGA
VGA(EY Lab)
SJA
PNA
PHA-L
HHA(EY Lab)
GNA (EY Lab)
GS-II (EY Lab)
BPL
Mono2W-CNL
AOL (Mana Lab)
PTL-II
UEAII
PTA-Gal
HHL
DBA
PTL-I
EEL
GNL
GSL-I
GSL-2
ConA
CA
PFL
HAA
P11 VP8
AIA
Epa1-Np mutant Y228W
CTB
WGA
MPL
AAL
Mouse Galectin1
Epa1-Np mutant E227G
Epa1-Np mutant E227A
CnMBL
Mono2R-CNL
HPA
DSL
MAL_II
PHA-L
PSL
PHA-E
MAA
NPA
LCA
#14 Sialyl Lewis X (Calbio)
MAA(Seikagaku)
Epa1-Np mutant Y228A
SBA
GAC78
MAL_I
MNA-M
CML1
BanLec
ACL
Sp Serotype_14.15
PHA-E
Threefoil
CSA(EY Lab)
SBA
CA19-9 Ab (DRG)
rNV VLP-CY3
TJA-I
CAA
MGL_6A
SSR1
Mouse NKR-P1C
PNA
TL(EY Lab)
LEL
GSL-I-B4
Mono1-CNL
EEA(EY Lab)
LTL
CGL
PSA
UEA I
LcH
VLRB.aGPA.23
WGA
Avaren-Fc
DBL-1
UEA I
hJAA-F11-B11
STL
BTL native
Epa1-Np_WT
SRL
Galectin-1
SNA
CA19.9-1B844
ConA
Human Galectin1
TbFBL2
ECL
Gal-3
rMpL
Gal-3C
WFL
WT Galectin-8
Native HPA
rPVL
VVHA lectin
CnSep1
CC1G-10077
human Gal-7
mouse Gal-7
RCAI (Vector)
Anti-LeC

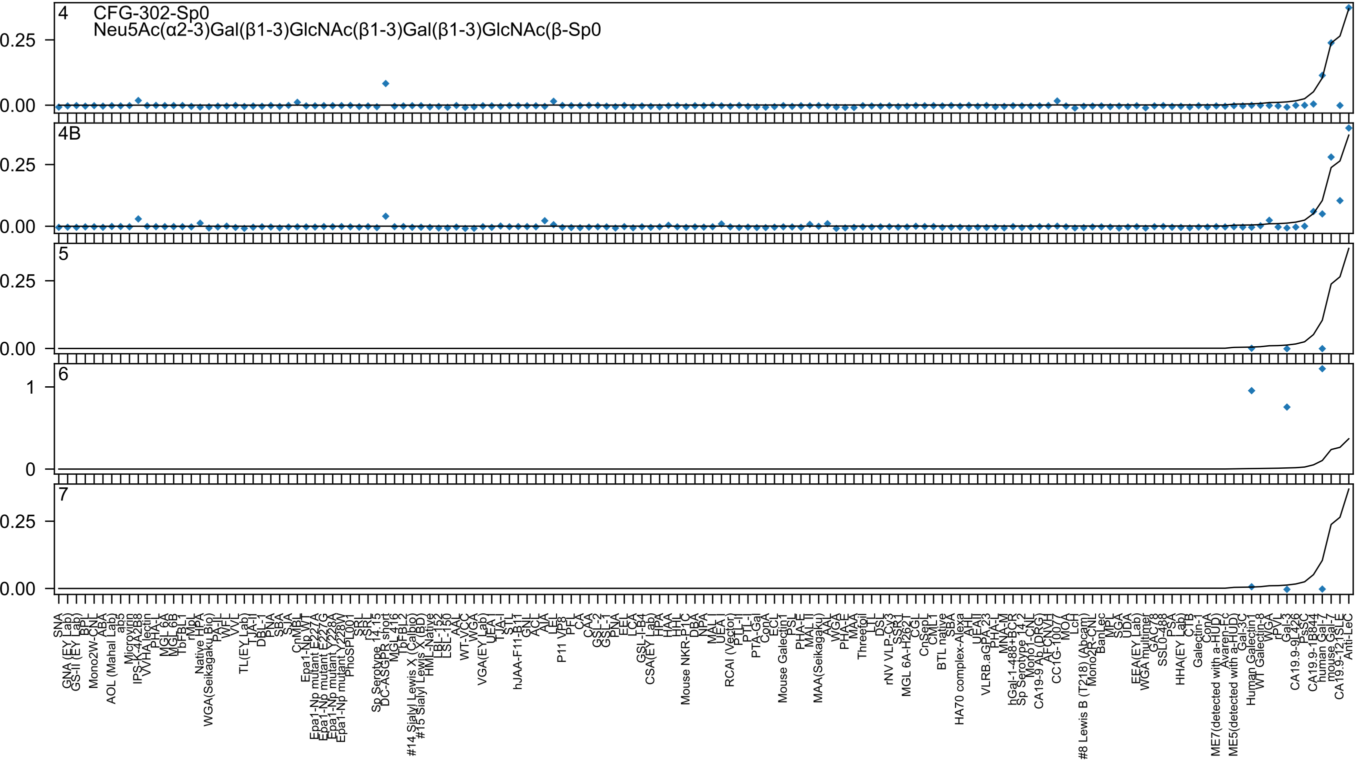
4
CFG-302-Sp0
Neu5Ac(α2-3)Gal(β1-3)GlcNAc(β1-3)Gal(β1-3)GlcNAc(β-Sp0
4B
5
6
7
0.25
0.00
1
0

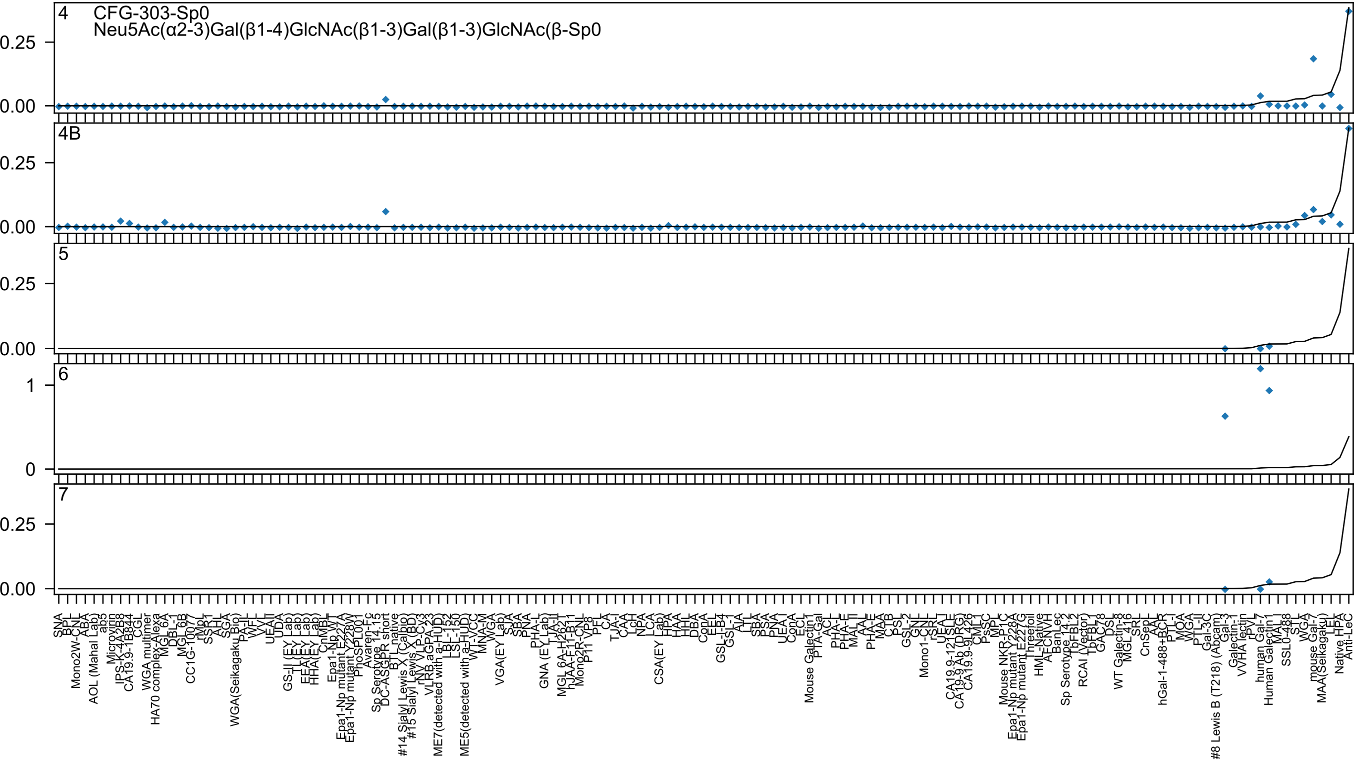
4
CFG-303-Sp0
Neu5Ac(α2-3)Gal(β1-4)GlcNAc(β1-3)Gal(β1-3)GlcNAc(β-Sp0
4B
5
6
7
0.25
0.00
1
0

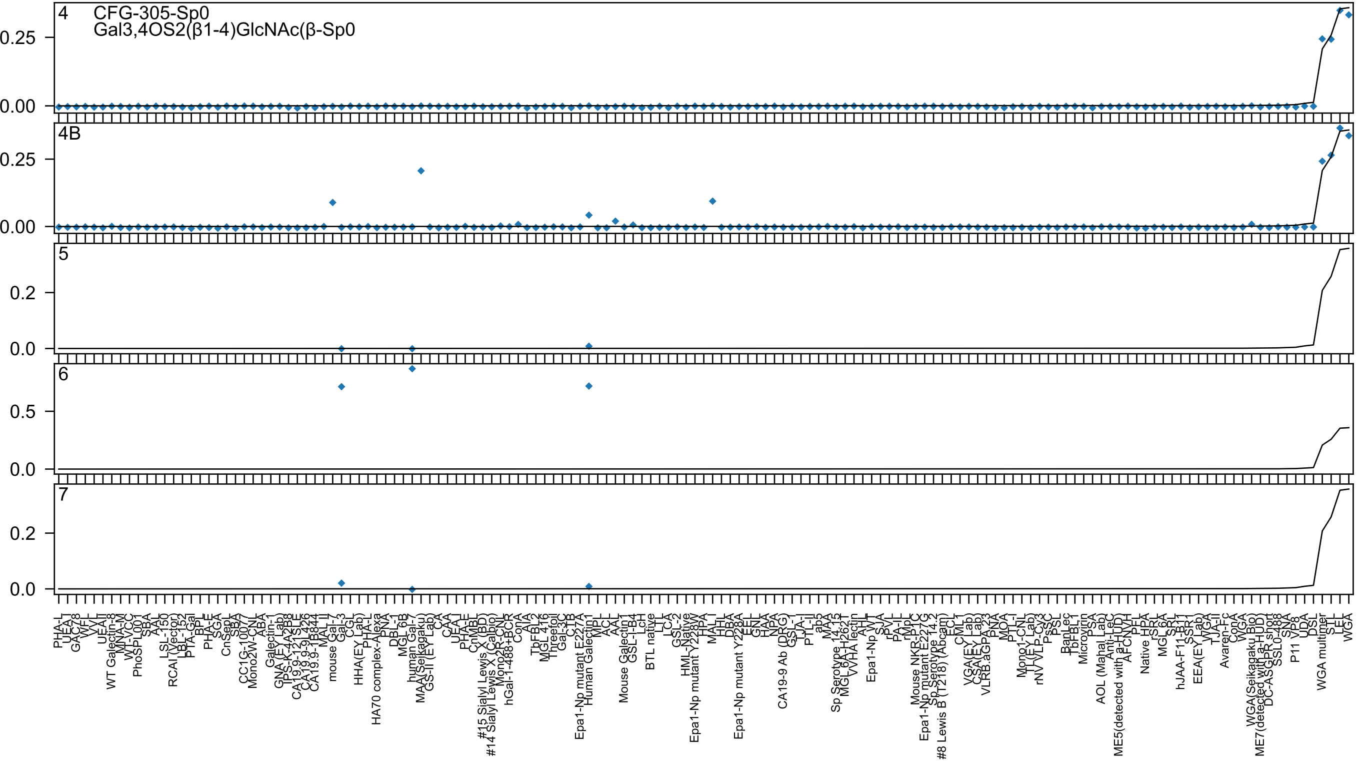
4
CFG-305-Sp0
Gal3,4OS2(β1-4)GlcNAc(β-Sp0
0.25
0.00
4B
0.25
0.00
5
0.2
0.0
6
0.5
0.0
7
0.2
0.0
PHA-L
UEA-I
GAC78
WFL
VVL
UEA-I
WT Galectin-8
MNA-M
WT-VCC
PhoSL001
SBA
AAL
LSL-150
RCAI (Vector)
LBL-152
PTA-Gal
BPL
PHA-E
SGA
CnSepL
SBA
CC1G-10077
Mono2W-CNL
ABA
Galectin-1
GNA (EY Lab)
IPS-K-4A2B8
CA19.9-121SLE
CA19.9-9L426
CA19.9-1B844
MAL II
mouse Gal-7
Gal-3
CGL
HHA(EY Lab)
PHA-L
HA70 complex-Alexa
PNA
DBL-1
MGL 6B
human Gal-7
MAA(Seikagaku)
GS-II (EY Lab)
CA
CAA
UEA-I
PHA-E
CnMBL
#15 Sialyl Lewis X (BD)
#14 Sialyl Lewis X (Calbio)
Mono2R-CNL
hGal-1-488+BCR
ConA
AIA
TbFBL2
MGL 416
Threefoil
Gal-3C
CTB
Epa1-Np mutant E227A
Human Galectin1
MPL
ACL
AAL
Mouse Galectin1
GSL-I-B4
LcH
BTL native
LTL
LCA
GSL-2
HML-Native
Epa1-Np mutant Y228W
HPA
MAL I
HHL
DBA
Epa1-Np mutant Y228A
EEL
GNL
HAA
NPA
CA19-9 Ab (DRG)
GSL-1
TJA-I
PTL-II
ab5
MAA
Sp Serotype 14.15
MGL 6A-H262T
VVHA lectin
AHL
Epa1-Np WT
SJA
rPVL
PA-IL
rMpL
Mouse NKR-P1C
Epa1-Np mutant E227G
Sp Serotype 14.2
#8 Lewis B (T218) (Abcam)
ECL
CML1
VGA(EY Lab)
CSA(EY Lab)
VLRB.aGPA.23
PNA
MOA
PTL-I
Mono1-CNL
TL(EY Lab)
rNV VLP-Cy3
PsSC
PSL
BanLec
TbFBL1
Microvirin
PSA
AOL (Mahal Lab)
Anti-LeC
ME5(detected with a-HUD)
AFCNVH
PFL
Native HPA
rSRL
MGL 6A
SRL
hJAA-F11-B11
SSR1
EEA(EY Lab)
WGA
TJA-II
Avaren-Fc
ConA
WGA
WGA(Seikagaku Bio)
ME7(detected with a-HUD)
DC-ASGPR short
SSL0-488
SNA
P11 VP8
UDA
DSL
WGA multimer
STL
LEL
WGA

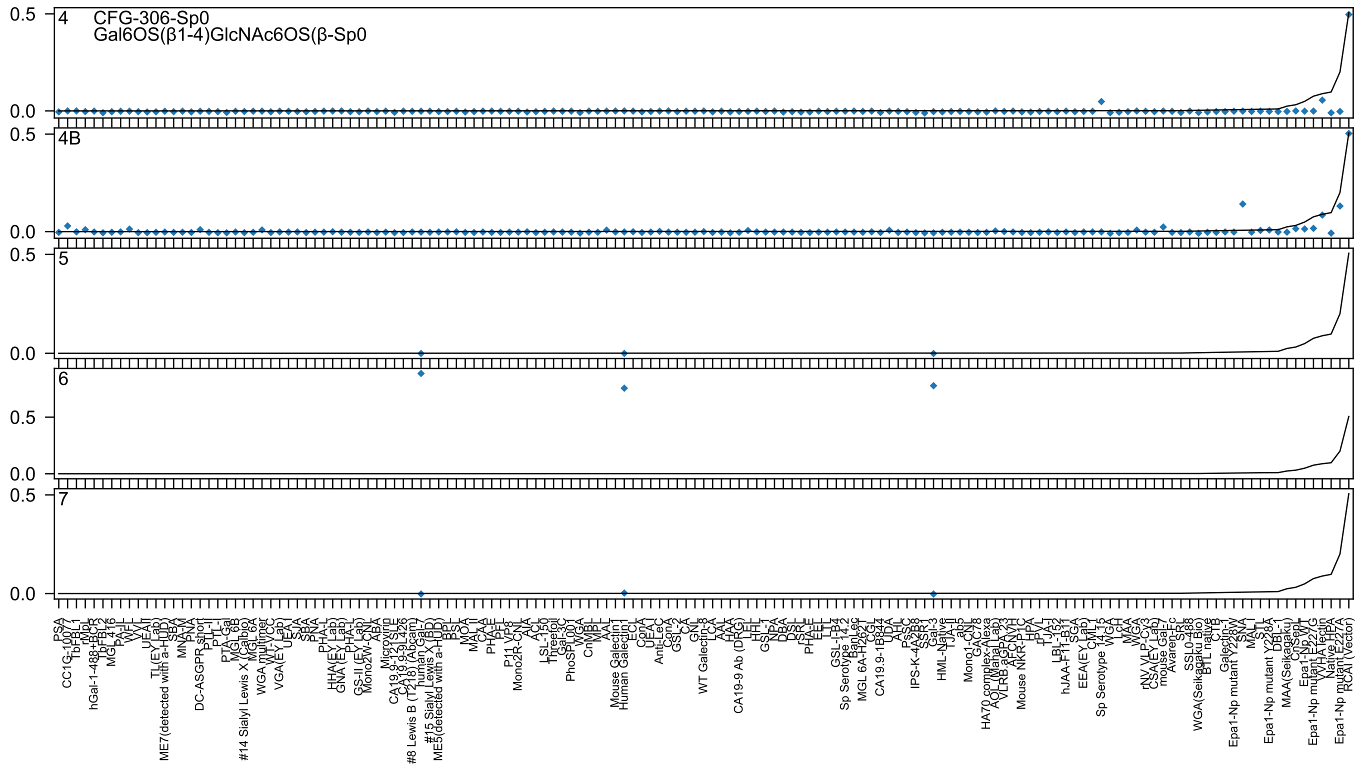
4 CFG-306-Sp0
Gal6OS(β1-4)GlcNAc6OS(β-Sp0
4B
5
6
7

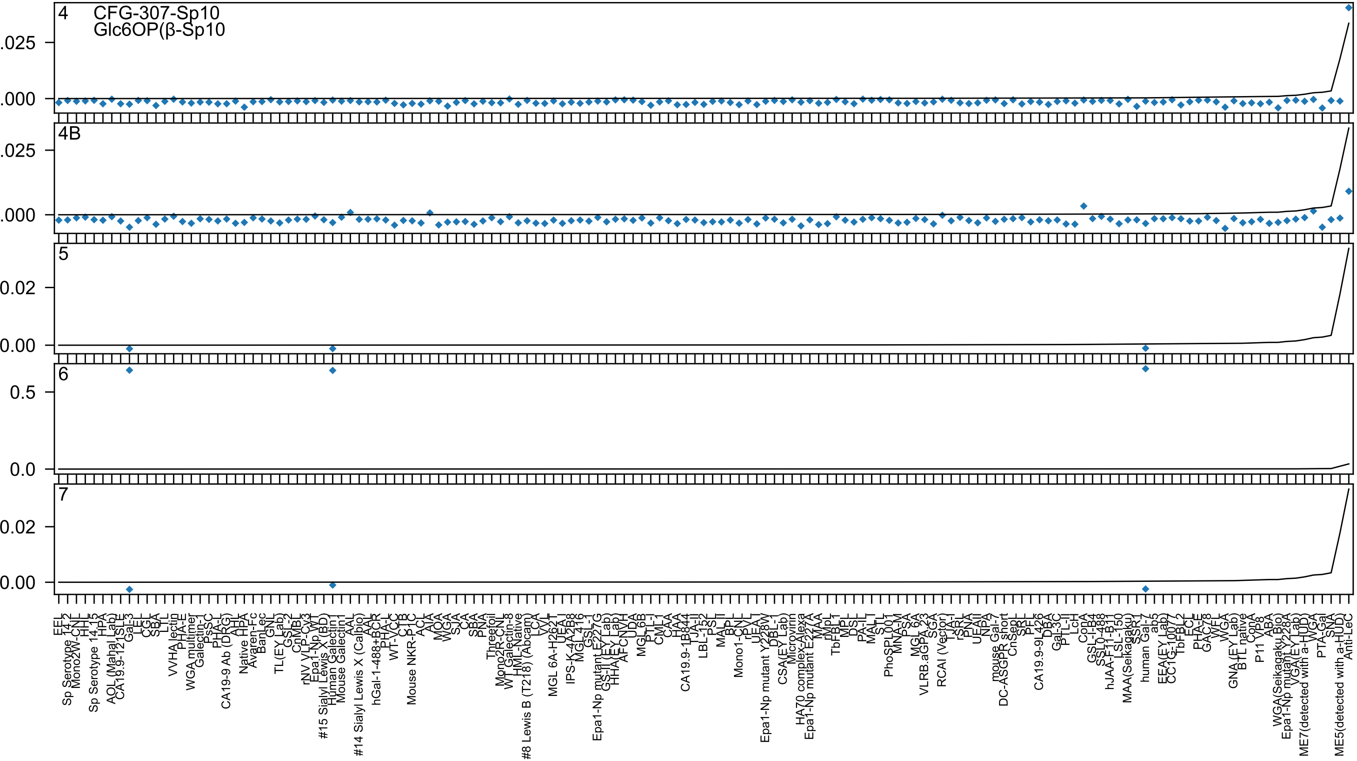

4 CFG-307-Sp10
Glc6OP(β-Sp10
4B
5
6
7

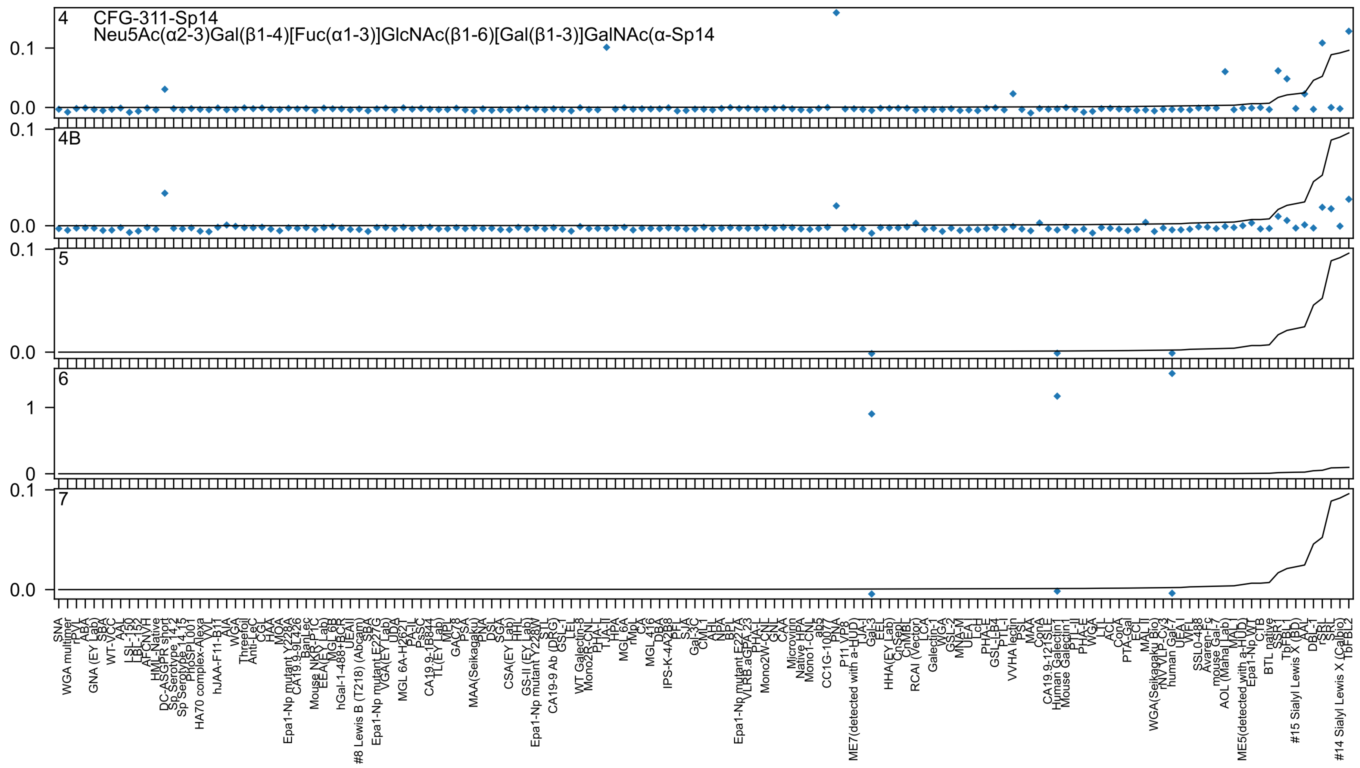

4
CFG-311-Sp14
Neu5Ac(α2-3)Gal(β1-4)[Fuc(α1-3)]GlcNAc(β1-6)[Gal(β1-3)]GalNAc(α-Sp14
4B
5
6
7

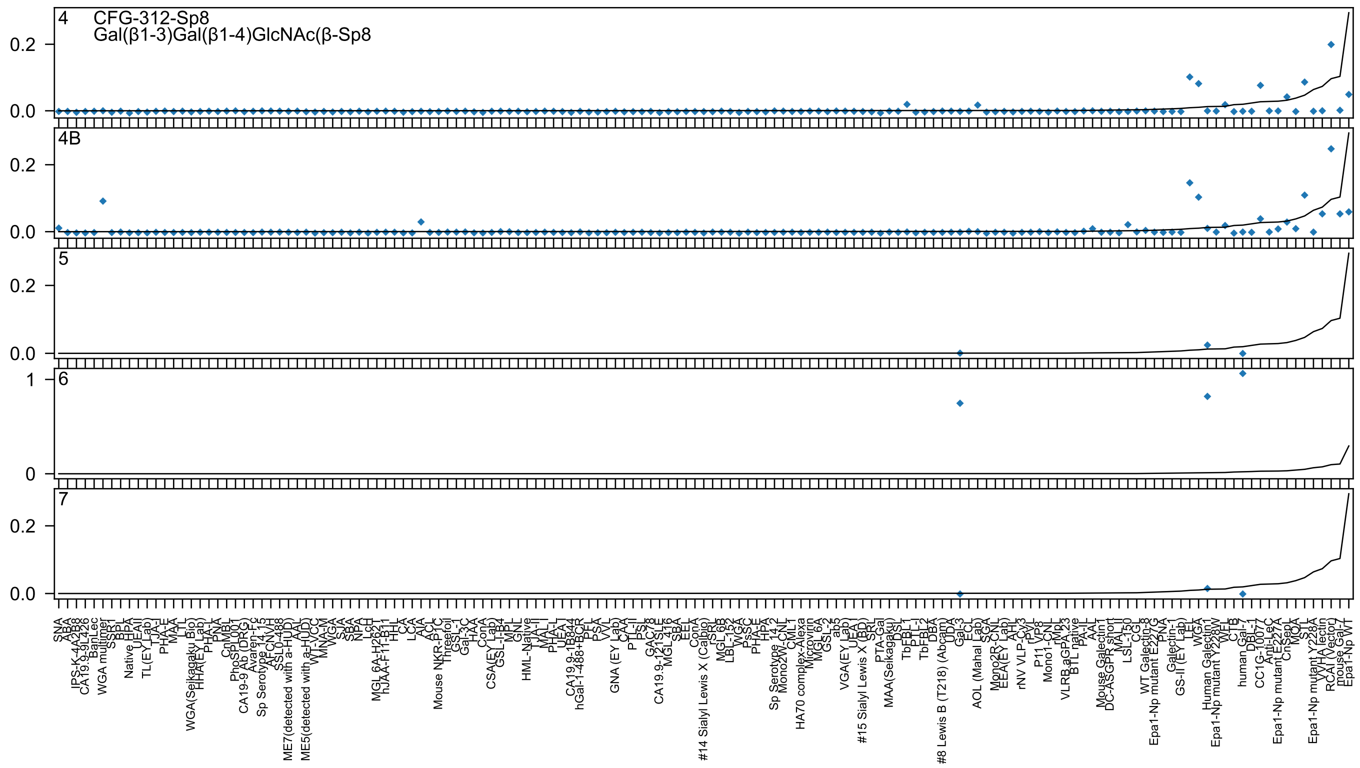
CFG-312-Sp8
Gal(β1-3)Gal(β1-4)GlcNAc(β-Sp8
4
4B
5
6
7

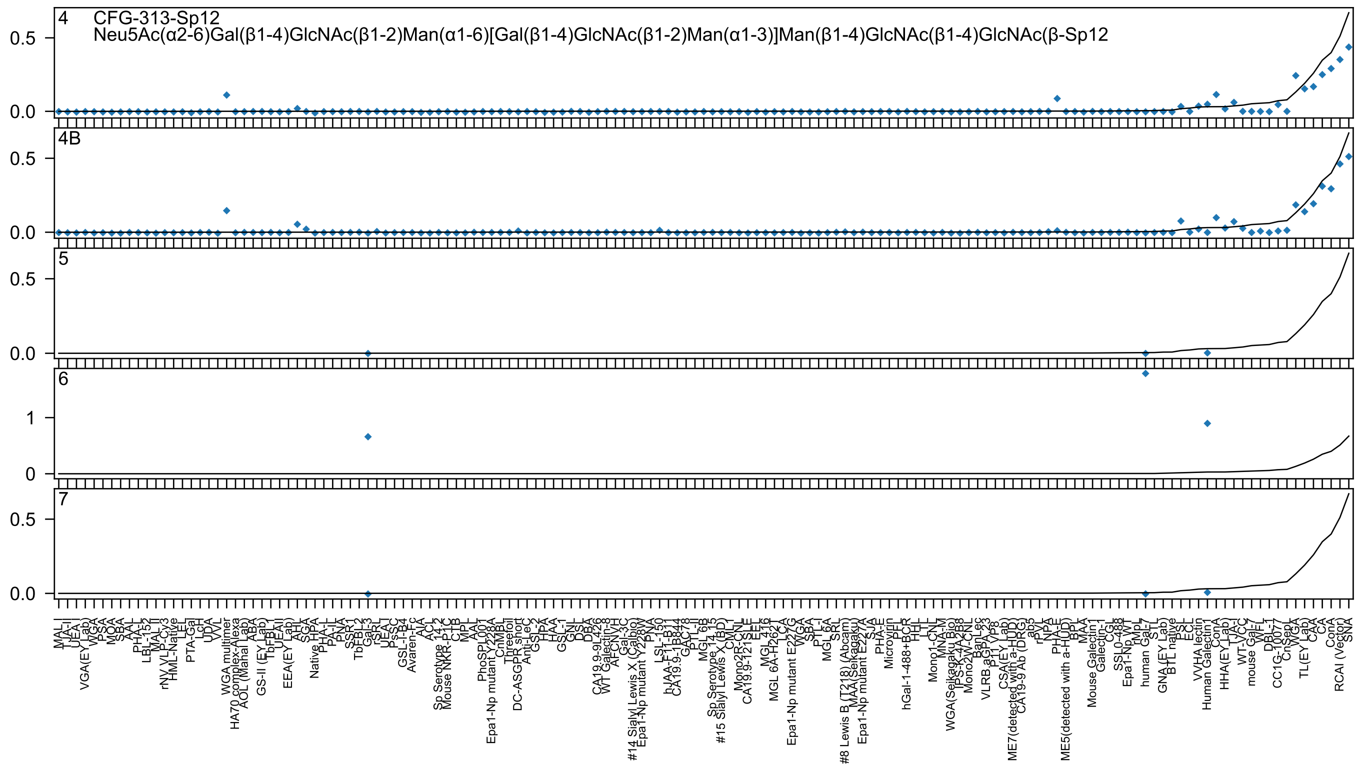

4 CFG-313-Sp12
Neu5Ac(α2-6)Gal(β1-4)GlcNAc(β1-2)Man(α1-6)[Gal(β1-4)GlcNAc(β1-2)Man(α1-3)]Man(β1-4)GlcNAc(β1-4)GlcNAc(β-Sp12
4B
5
6
7

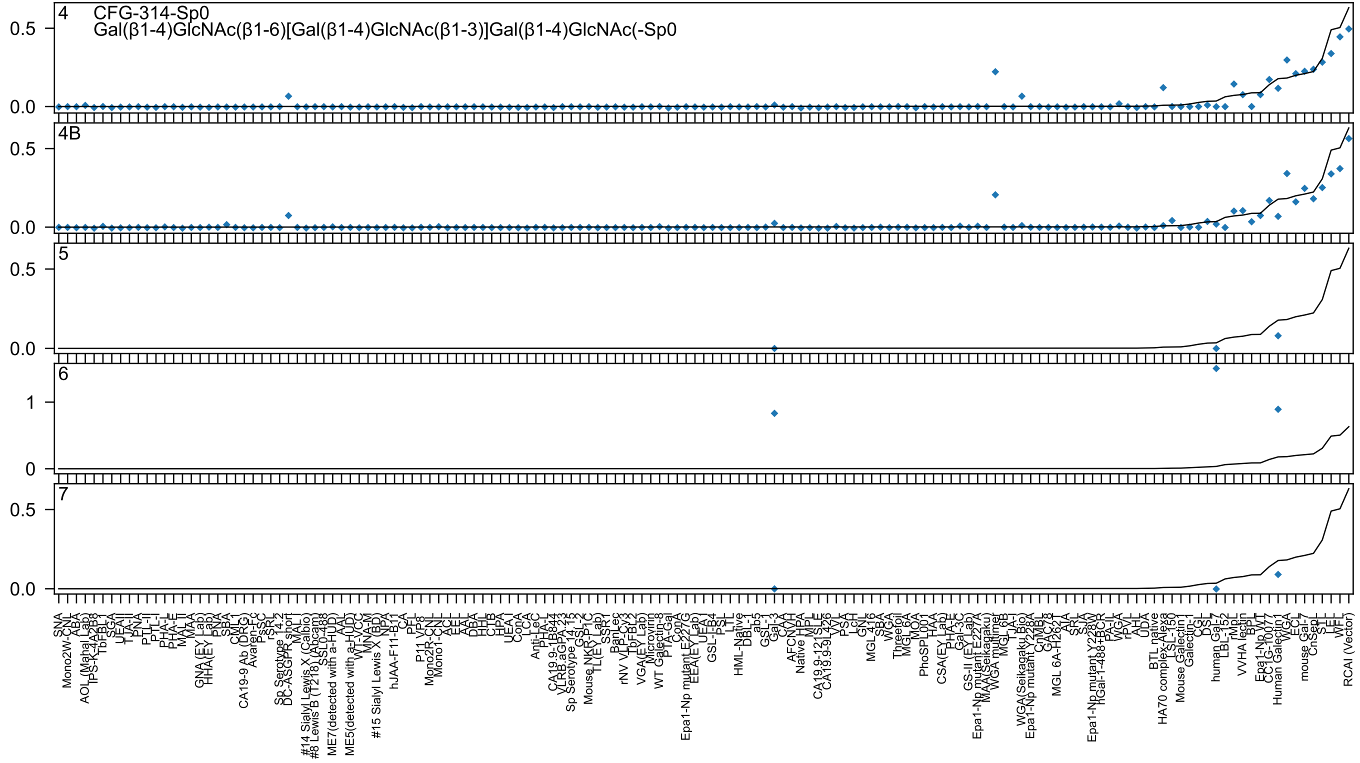
4
CFG-314-Sp0
Gal(β1-4)GlcNAc(β1-6)[Gal(β1-4)GlcNAc(β1-3)]Gal(β1-4)GlcNAc(-Sp0
4B
5
6
7

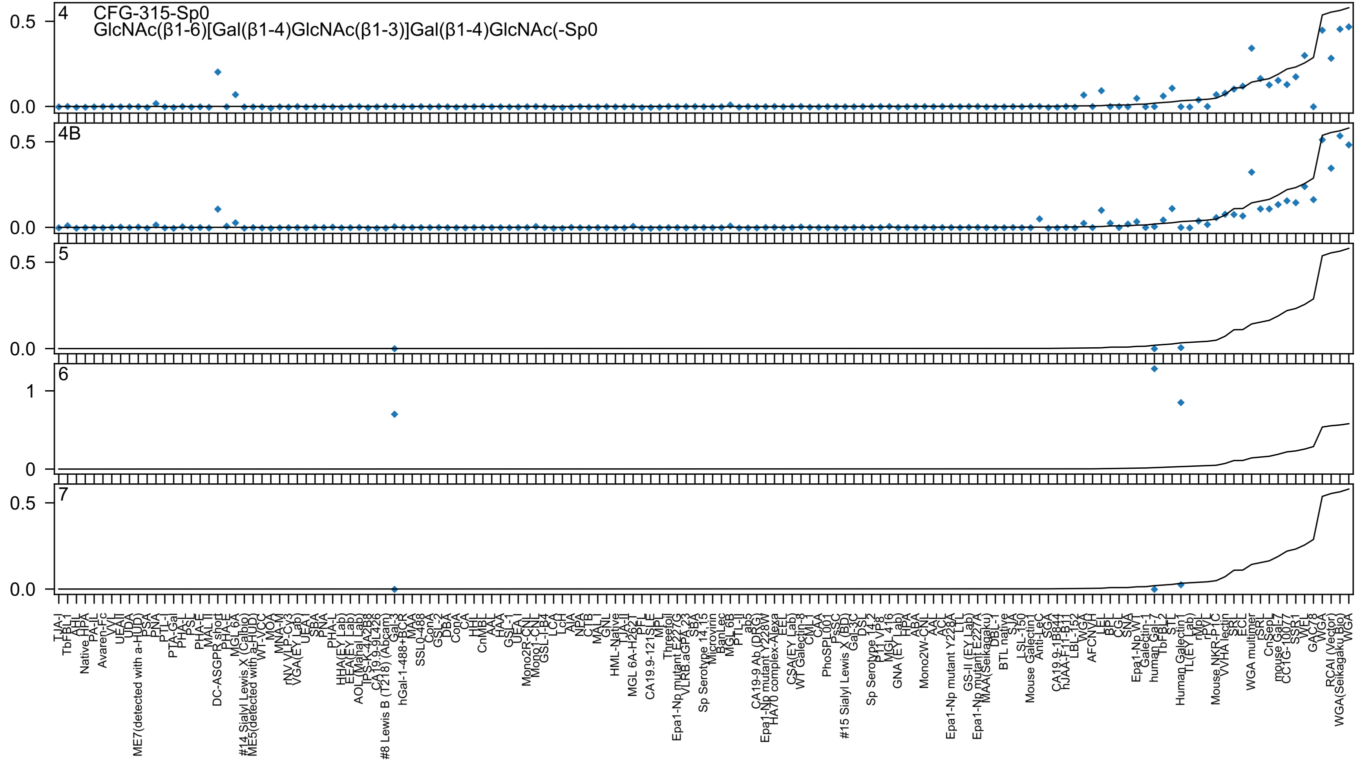

4
CFG-315-Sp0
GlcNAc(β1-6)[Gal(β1-4)GlcNAc(β1-3)]Gal(β1-4)GlcNAc(-Sp0
4B
5
6
7

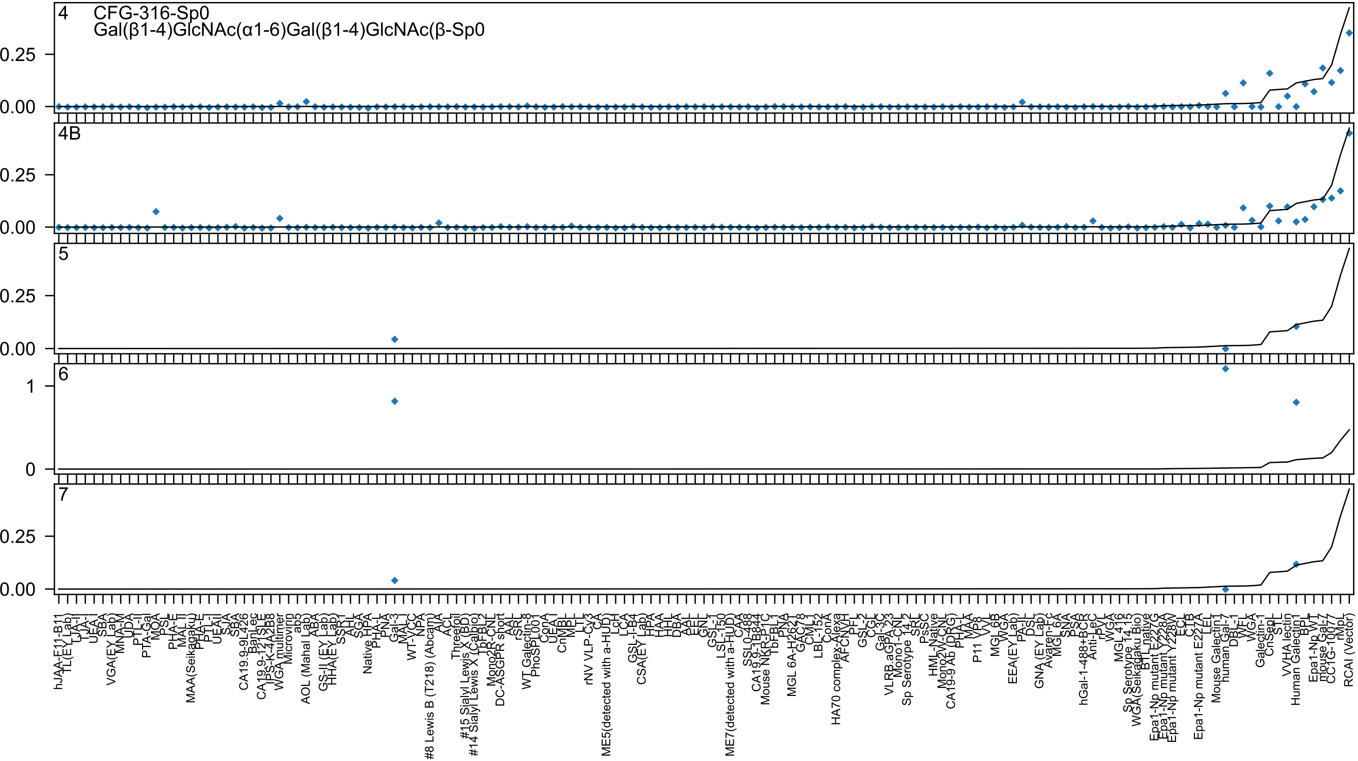

4
CFG-316-Sp0
Gal(β1-4)GlcNAc(α1-6)Gal(β1-4)GlcNAc(β-Sp0
4B
5
6
7

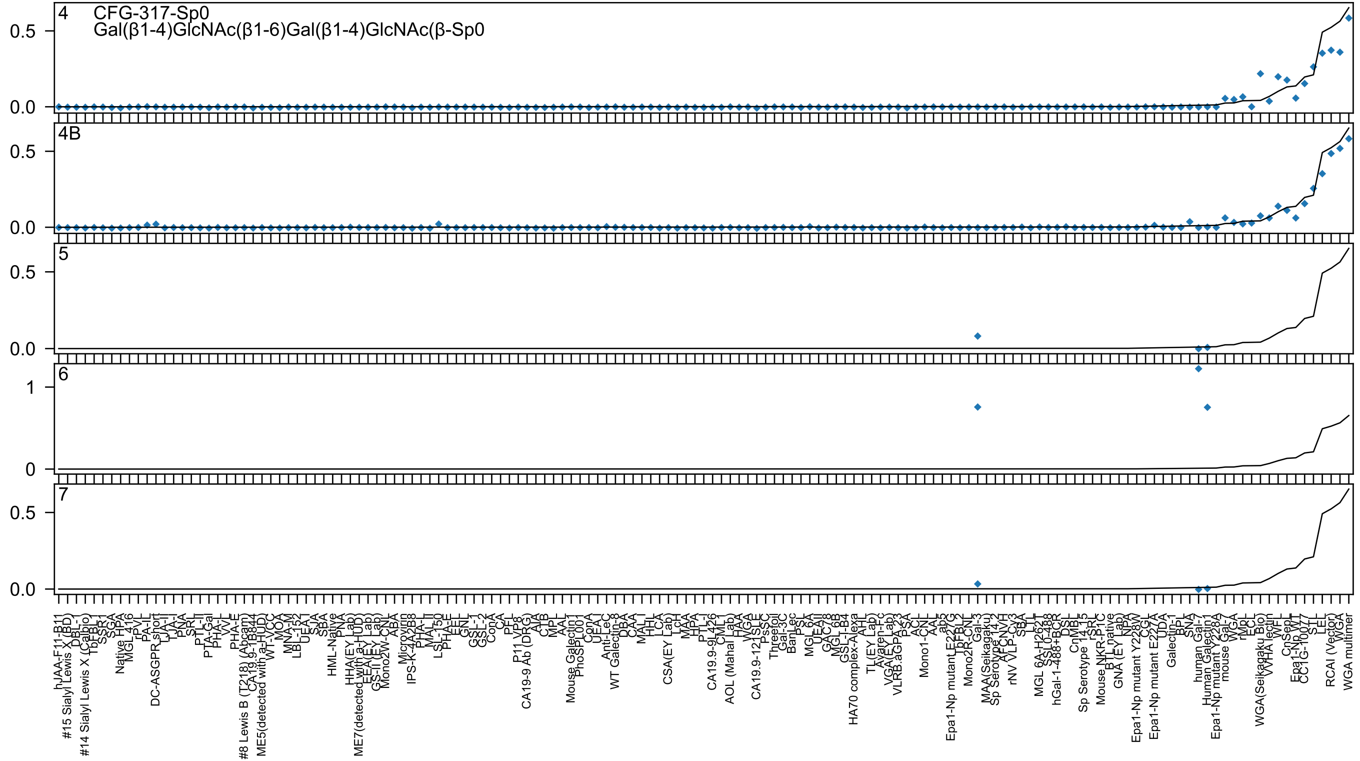

4 CFG-317-Sp0
Gal(β1-4)GlcNAc(β1-6)Gal(β1-4)GlcNAc(β-Sp0
4B
5
6
7

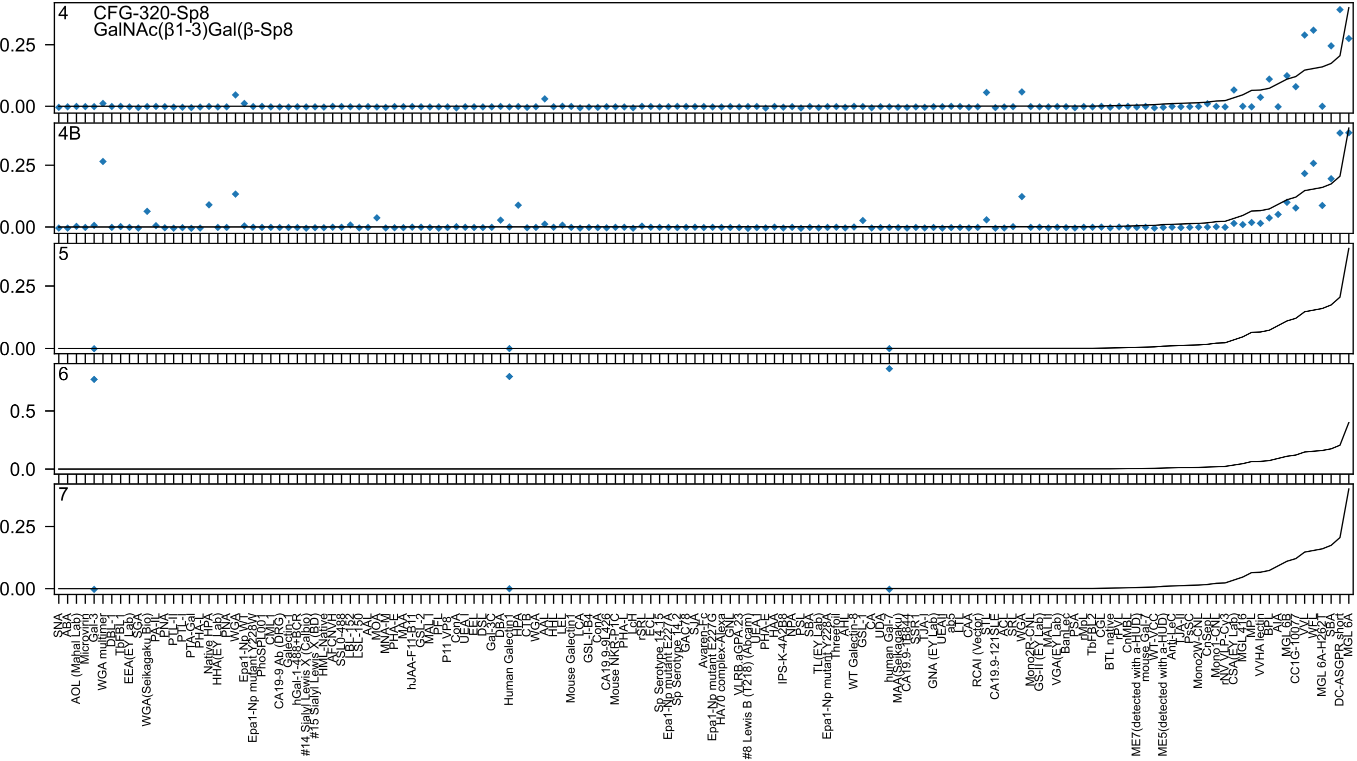
4
CFG-320-Sp8
GalNAc(β1-3)Gal(β-Sp8
4B
5
6
7
SNA
DC-ASGPR_short
MGL 6A

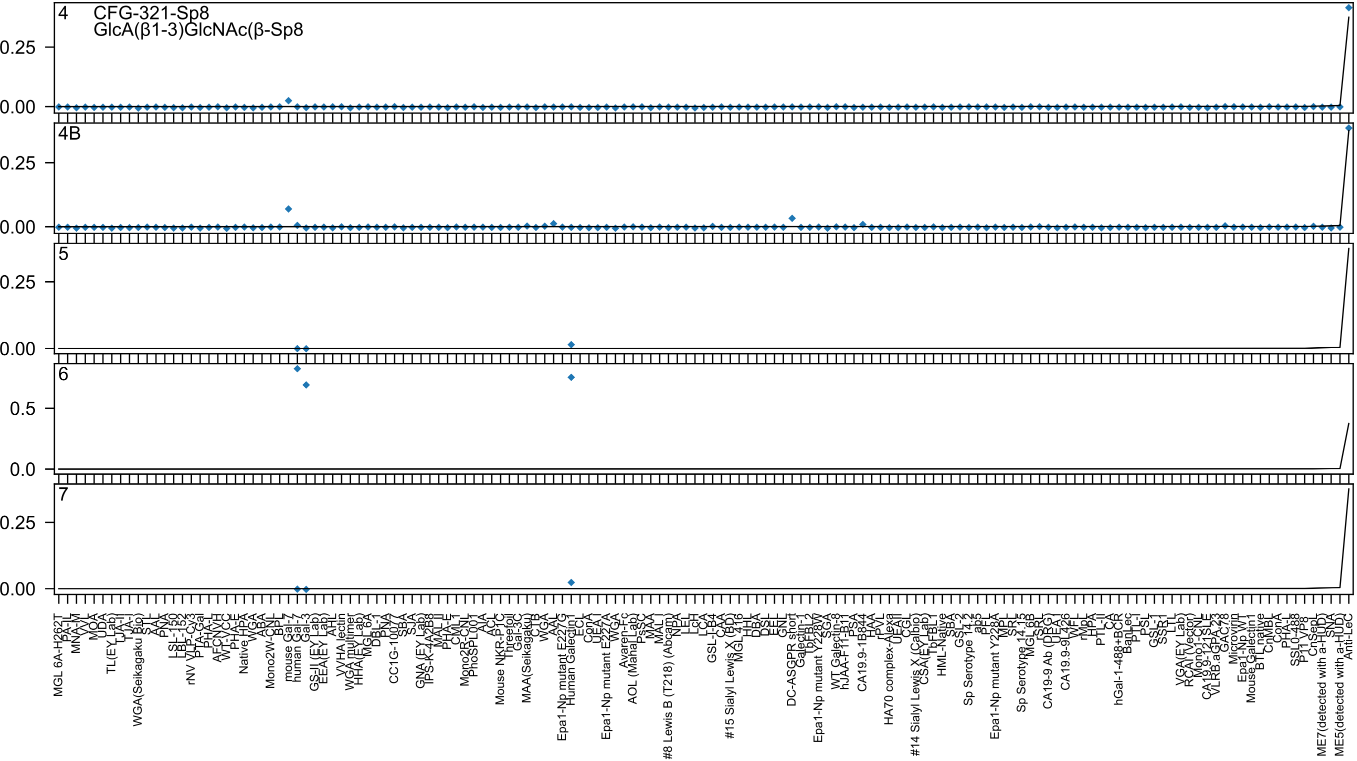

4
CFG-321-Sp8
GlcA(β1-3)GlcNAc(β-Sp8
4B
5
6
7
0.25
0.00
0.5
0.0

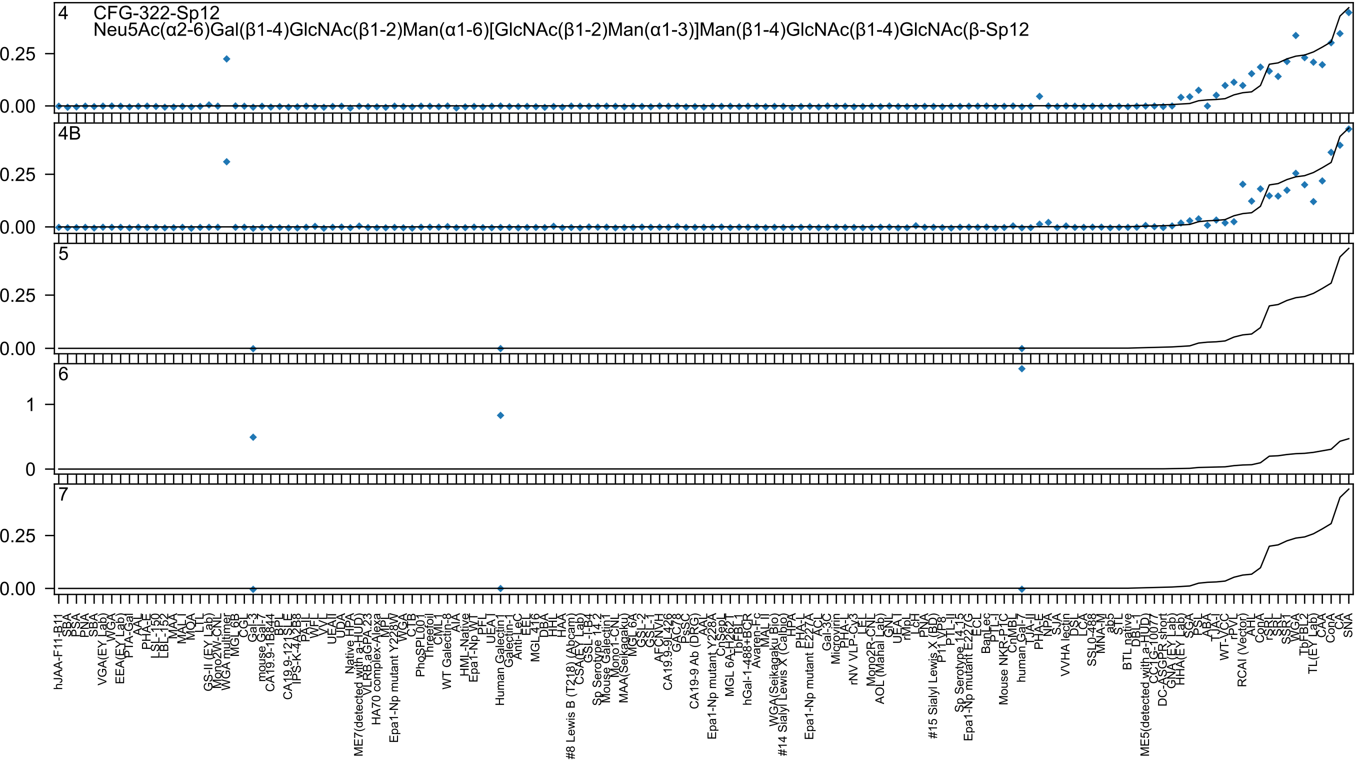
4
CFG-322-Sp12
Neu5Ac(α2-6)Gal(β1-4)GlcNAc(β1-2)Man(α1-6)[GlcNAc(β1-2)Man(α1-3)]Man(β1-4)GlcNAc(β1-4)GlcNAc(β-Sp12
4B
5
6
7

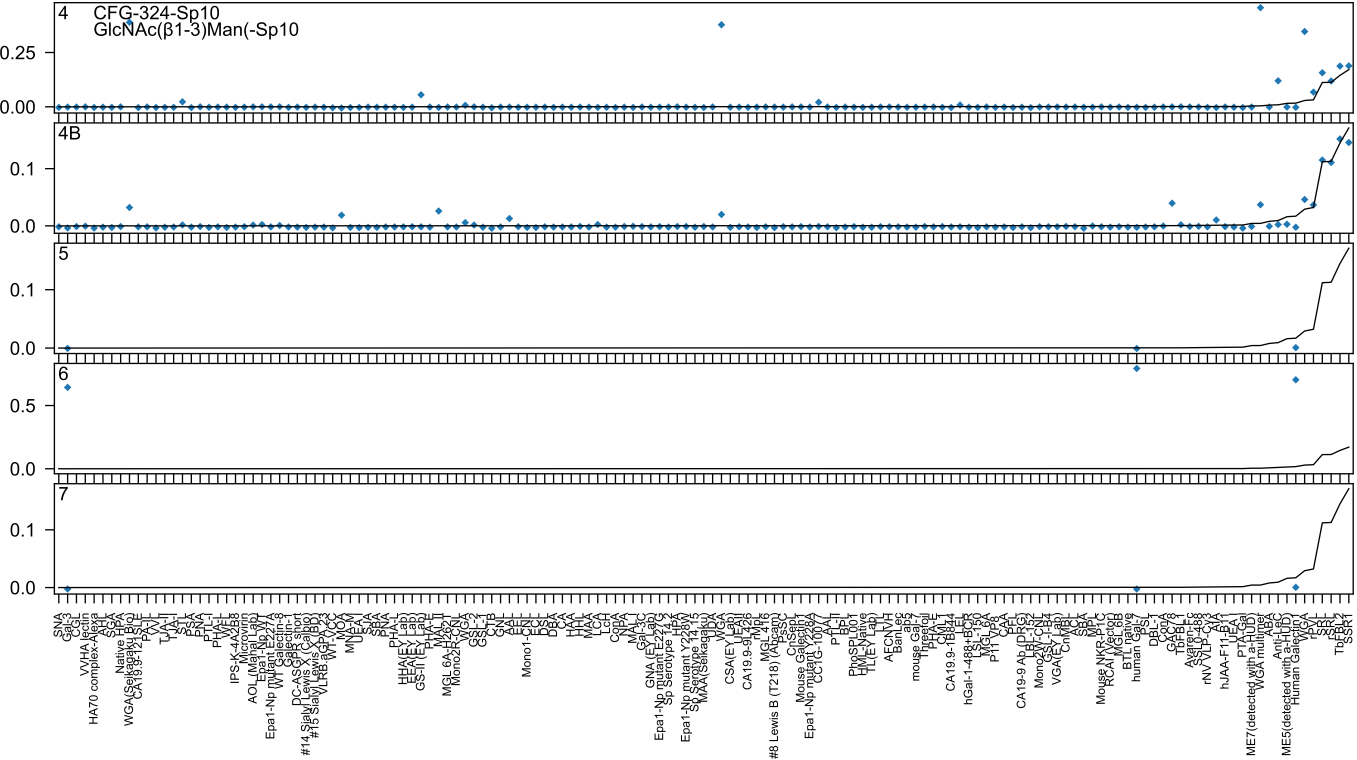

4
CFG-324-Sp10
GlcNAc(β1-3)Man(-Sp10
0.25
0.00
4B
0.1
0.0
5
0.1
0.0
6
0.5
0.0
7
0.1
0.0

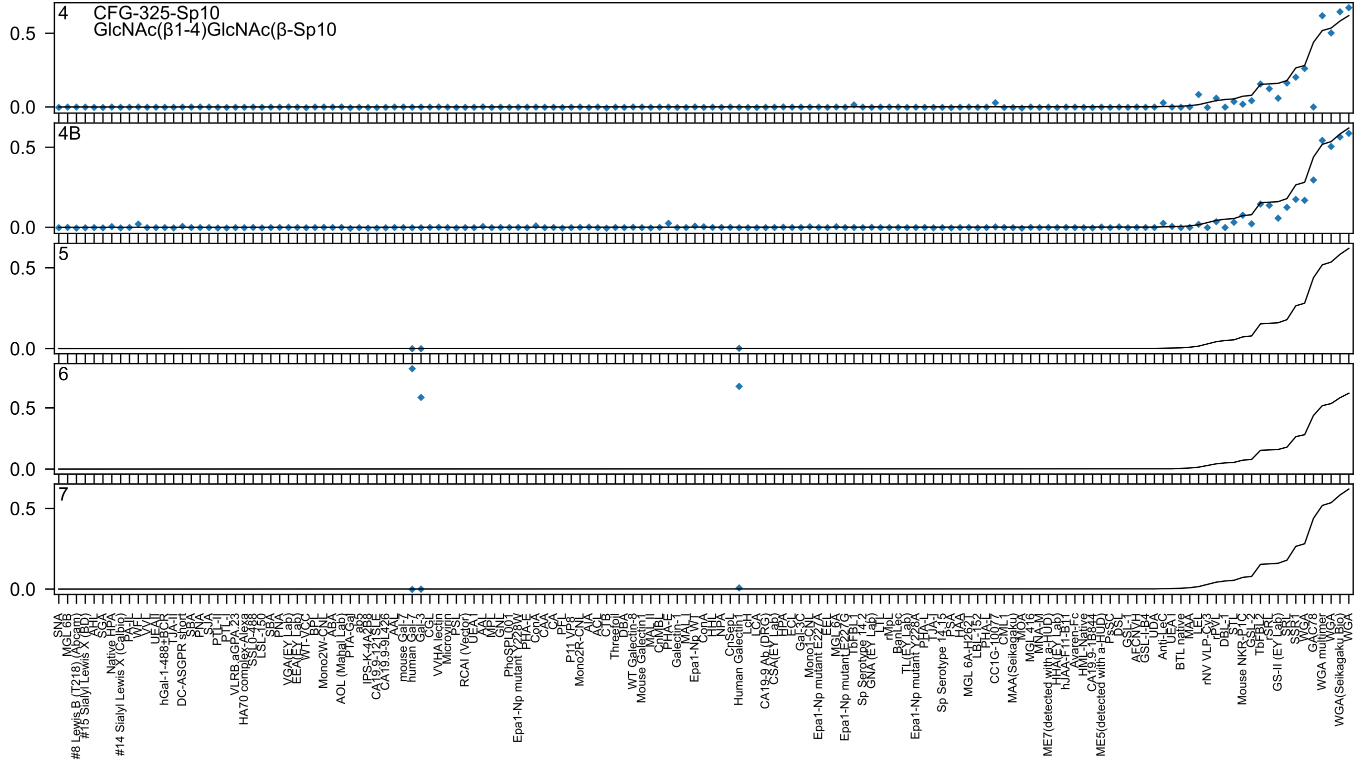
4
CFG-325-Sp10
GlcNAc(β1-4)GlcNAc(β-Sp10
4B
5
6
7
0.5
0.0

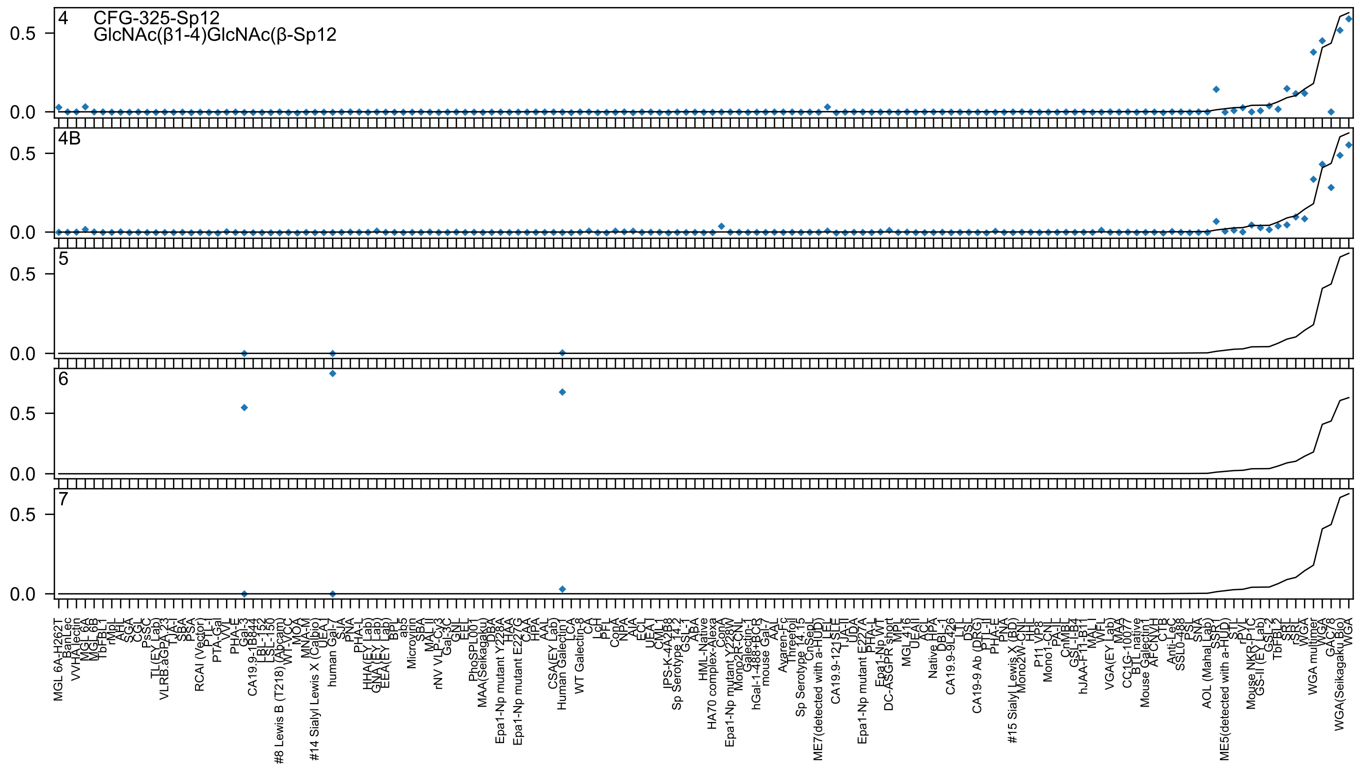
4
CFG-325-Sp12
GlcNAc(β1-4)GlcNAc(β-Sp12
4B
5
6
7
0.5
0.0

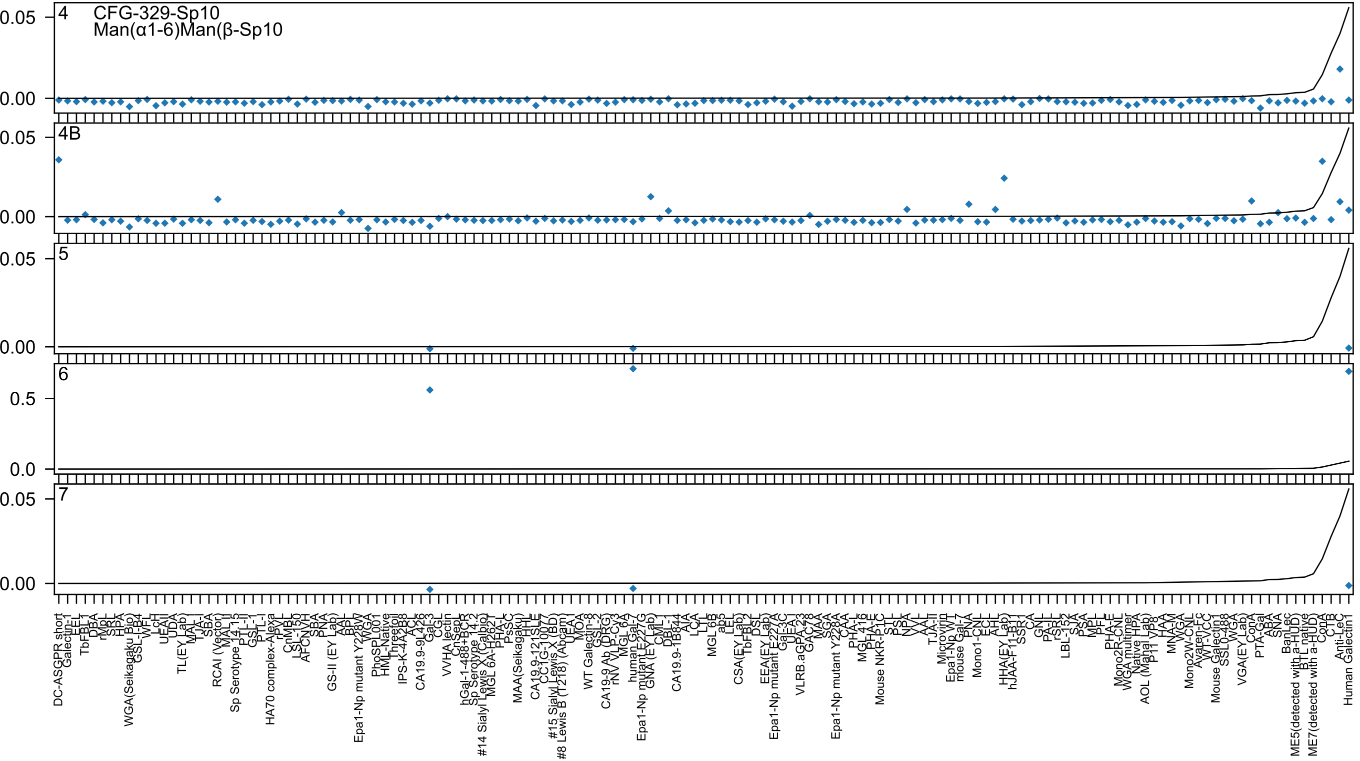

4 CFG-329-Sp10
Man(α1-6)Man(β-Sp10
4B
5
6
7
0.05
0.00
0.5
0.0

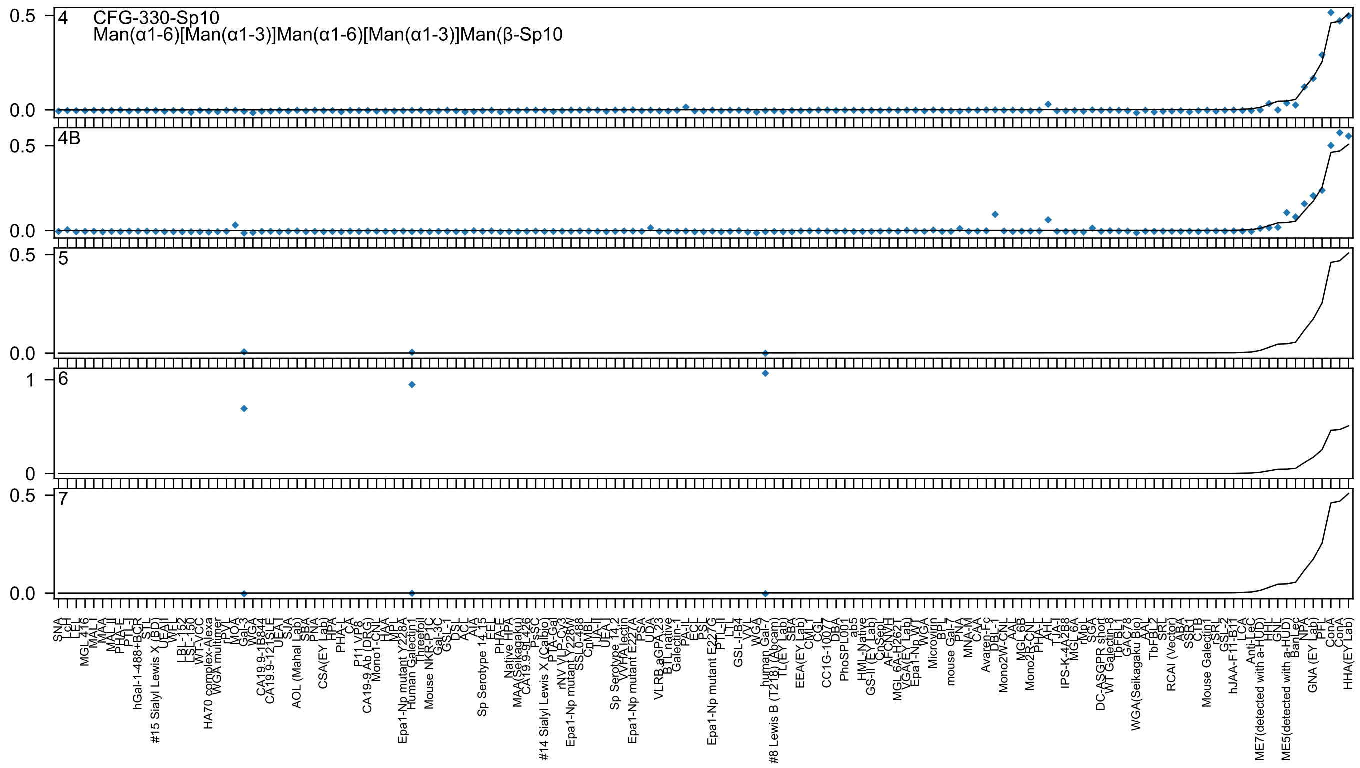
4
CFG-330-Sp10
Man(α1-6)[Man(α1-3)]Man(α1-6)[Man(α1-3)]Man(β-Sp10
4B
5
6
7
0.5
0.0
1
0

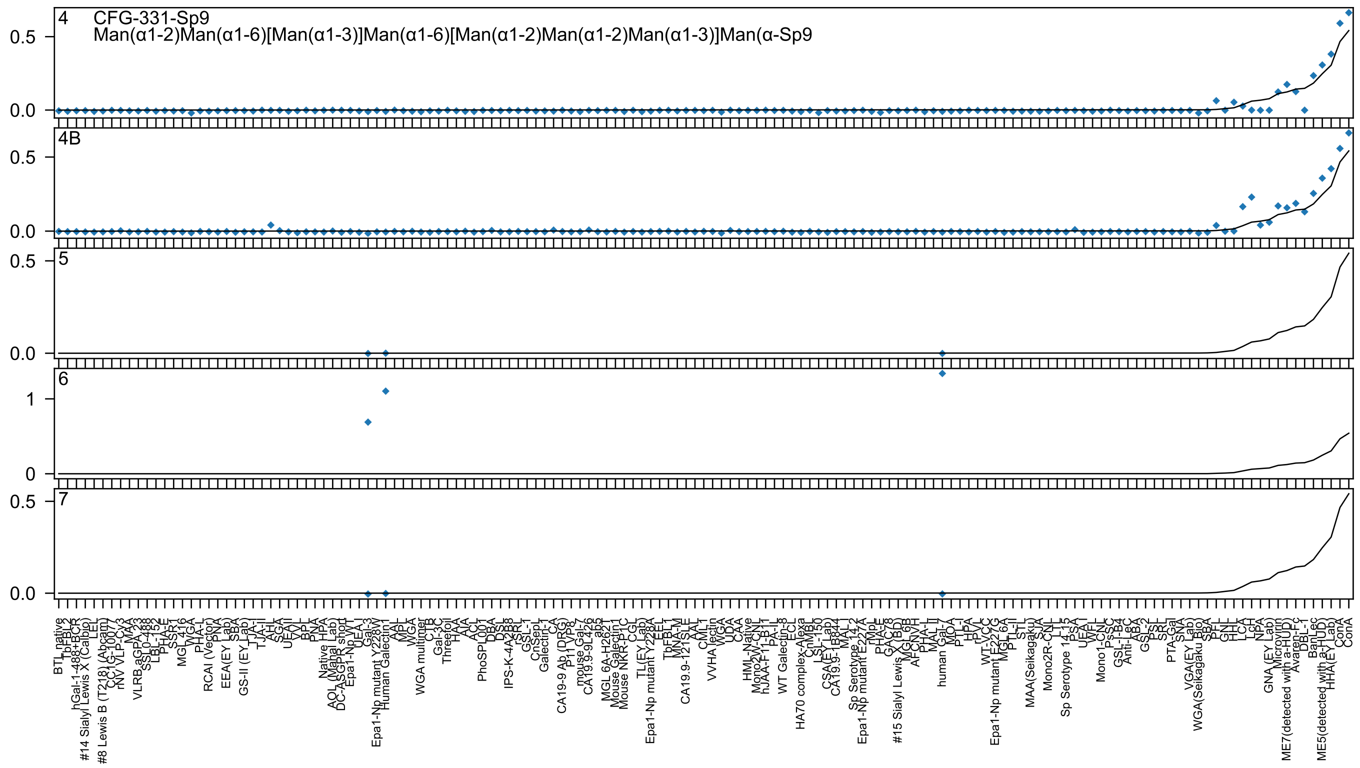
4 CFG-331-Sp9
Man(α1-2)Man(α1-6)[Man(α1-3)]Man(α1-6)[Man(α1-2)Man(α1-2)Man(α1-3)]Man(α-Sp9
4B
5
6
7

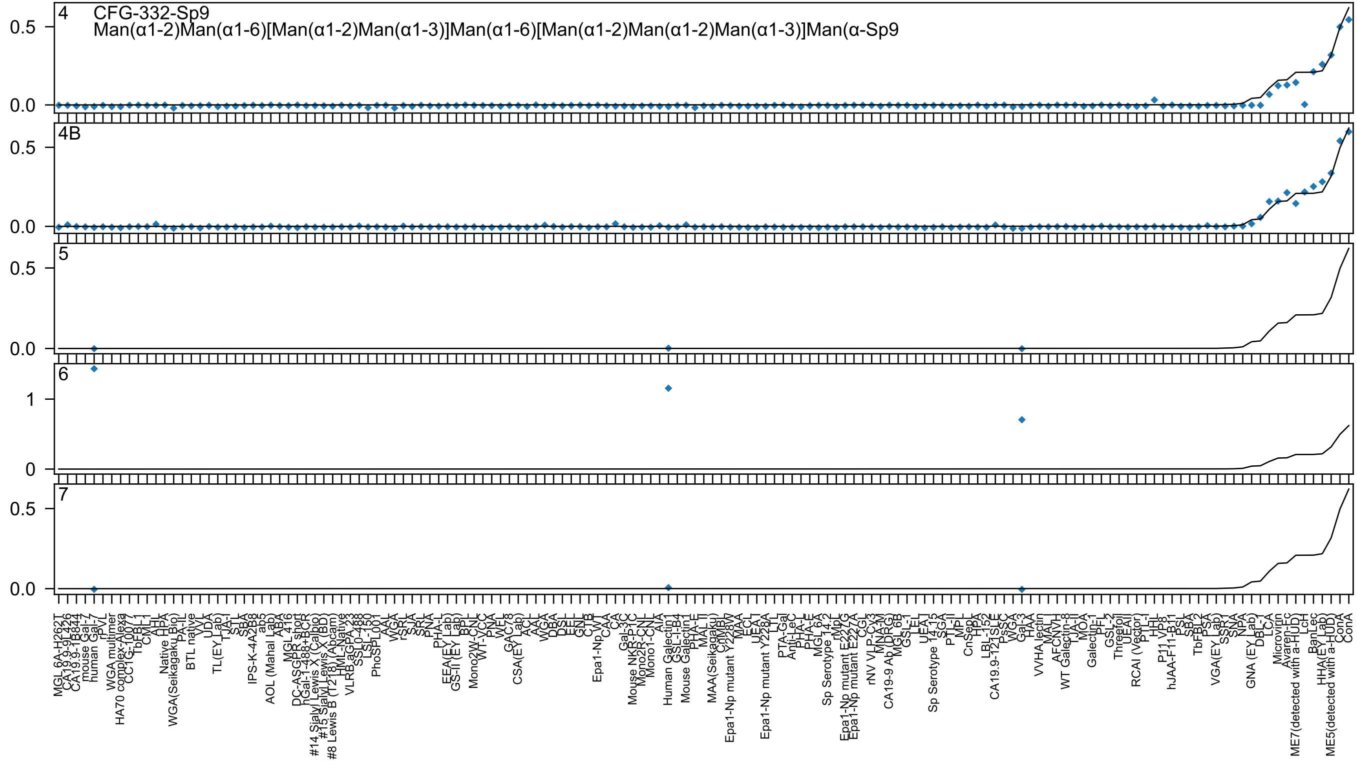

4
CFG-332-Sp9
Man(α1-2)Man(α1-6)[Man(α1-2)Man(α1-3)]Man(α1-6)[Man(α1-2)Man(α1-2)Man(α1-3)]Man(α-Sp9
4B
5
6
7

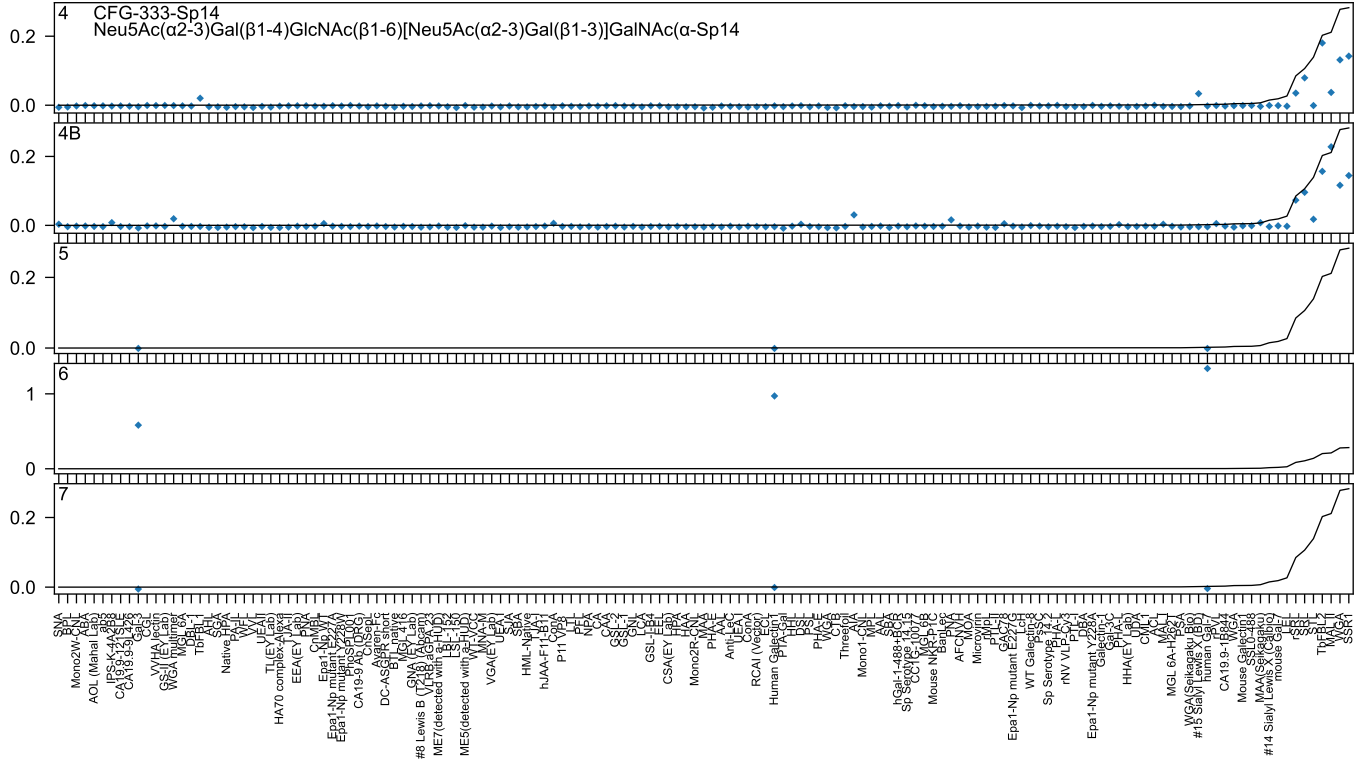

4 CFG-333-Sp14
Neu5Ac(α2-3)Gal(β1-4)GlcNAc(β1-6)[Neu5Ac(α2-3)Gal(β1-3)]GalNAc(α-Sp14
4B
5
6
7

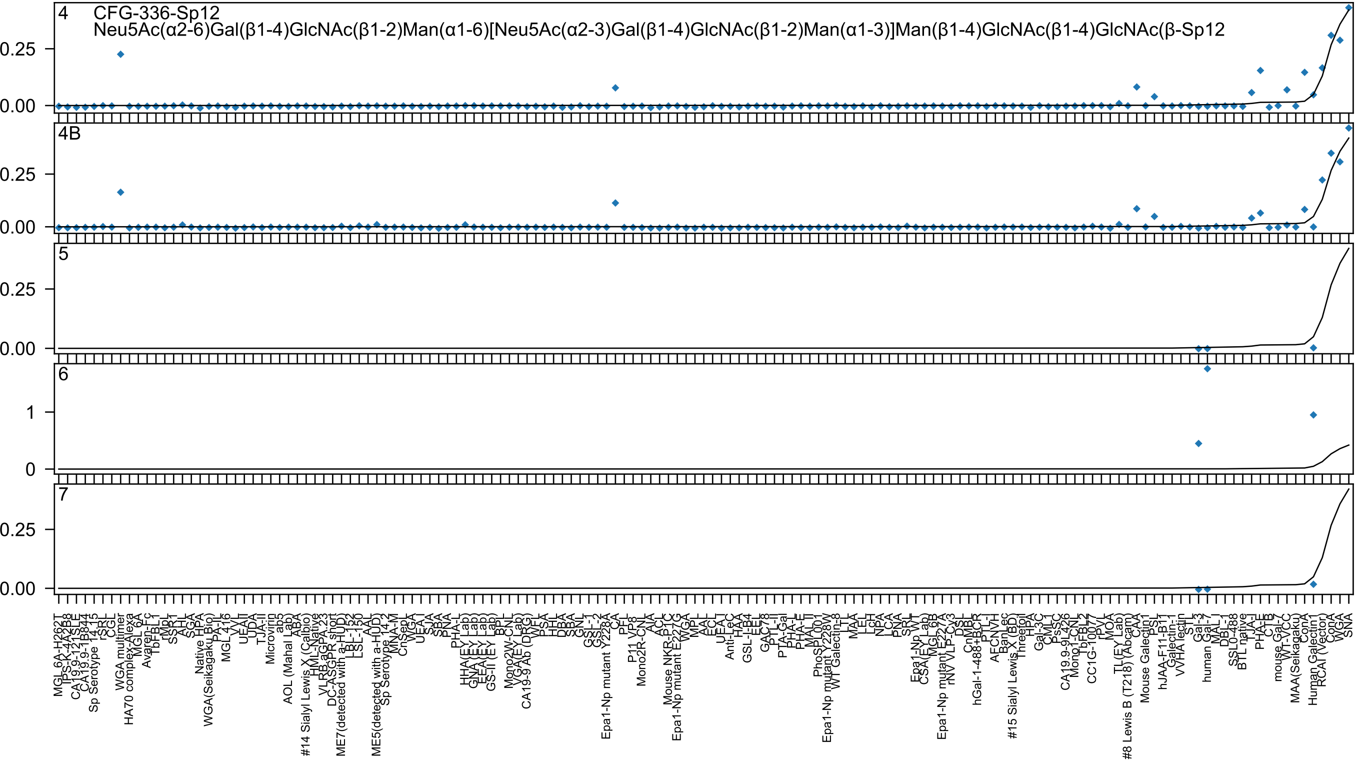

4 CFG-336-Sp12
Neu5Ac(α2-6)Gal(β1-4)GlcNAc(β1-2)Man(α1-6)[Neu5Ac(α2-3)Gal(β1-4)GlcNAc(β1-2)Man(α1-3)]Man(β1-4)GlcNAc(β1-4)GlcNAc(β-Sp12
4B
5
6
7

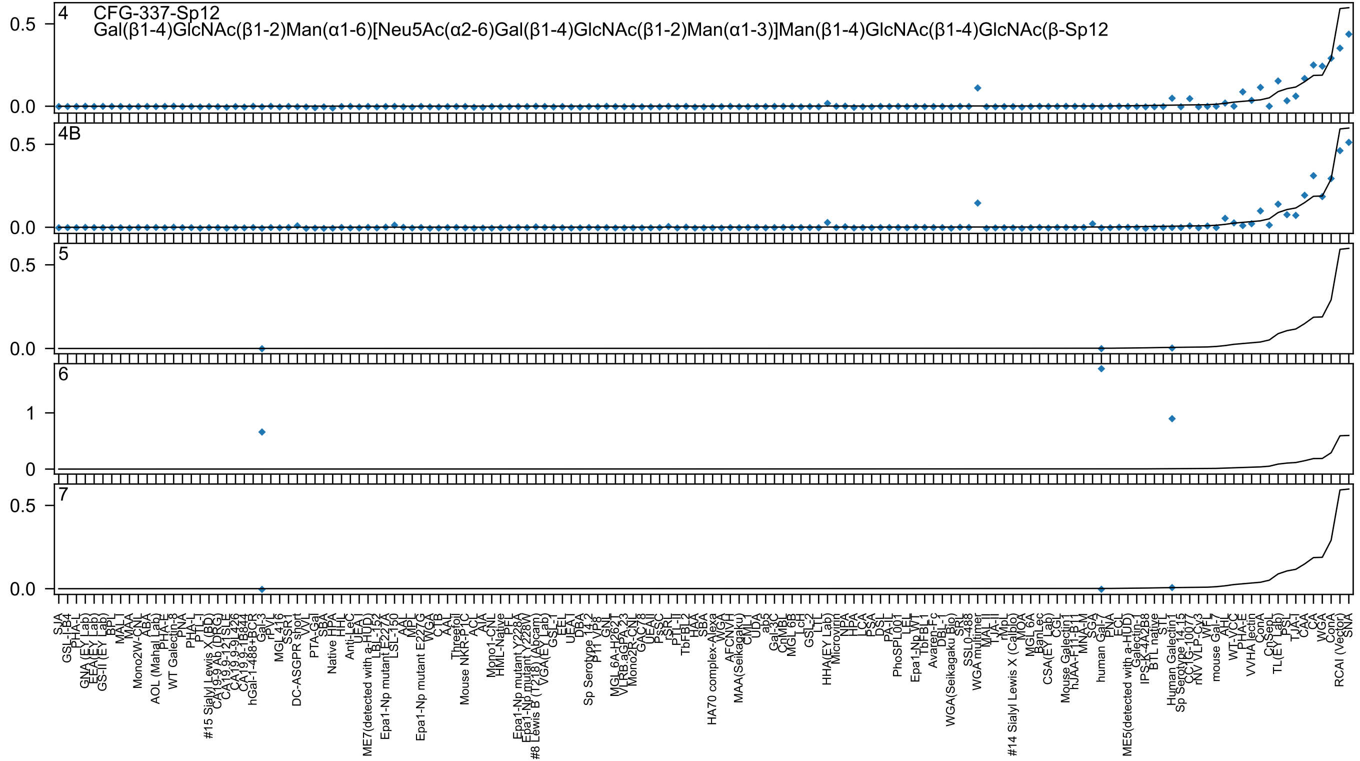

4
CFG-337-Sp12
Gal(β1-4)GlcNAc(β1-2)Man(α1-6)[Neu5Ac(α2-6)Gal(β1-4)GlcNAc(β1-2)Man(α1-3)]Man(β1-4)GlcNAc(β1-4)GlcNAc(β-Sp12
4B
5
6
7
0.5
0.0
1
0
SJA
GSL-I-B4
PHA-L
GNA(EY Lab)
EEA(EY Lab)
GS-II (EY Lab)
BPL
MAL-I
MAA
Mono2W-CNL
ABA
AOL (Mahal Lab)
PHA-E
WT Galectin-8
PNA
PHA-L
PTL-I
#15 Sialyl Lewis X (BD)
CA19-9 Ab (DRG)
CA19.9-121SLE
CA19.9-9L426
CA19.9-1B844
hGal-1-488+BCR
Gal-3
rPVL
MGL 416
SSR1
DC-ASGPR short
VVL
PTA-Gal
SBA
Native HPA
HHL
Anti-LeC
UEA-I
ME7(detected with a-HUD)
LBL-152
Epa1-Np mutant E227A
LSL-150
AAL
MPL
Epa1-Np mutant E227G
WGA
CTB
AAL
Threefoil
Mouse NKR-P1C
ACL
AIA
Mono1-CNL
HML-Native
PFL
Epa1-Np mutant Y228A
Epa1-Np mutant Y228W
#8 Lewis B (T218) (Abcam)
VGA(EY Lab)
GSL-I
EEL
UEA-I
DBA
Sp Serotype 14.2
P1 VP8
GNL
MGL 6A-H262T
VLRB.aGPA.23
Mono2R-CNL
GAC78
UEA-II
PsSC
rSRL
PTL-II
TbFBL2
HAA
SBA
HA70 complex-Alexa
WGA
AFCNVH
MAA(Seikagaku)
CML-I
UDA
ab5
Gal-3C
CnMBL
MGL 6B
LcH
GSL-2
LTL
HHA(EY Lab)
Microvirin
NPA
HPA
LCA
PSA
DSL
PA-IL
PhoSPL001
LEL
Epa1-Np WT
TbFBL1
Avaren-Fc
DBL-1
WGA(Seikagaku Bio)
SRL
SSL0-488
WGA multimer
MAL-II
TJA-II
rMpL
#14 Sialyl Lewis X (Calbio)
MOA
MGL 6A
BanLec
CSA(EY Lab)
CGL
Mouse Galectin1
hJAA-F11-B11
MNA-M
SGA
human Gal-7
PNA
ECL
ME5(detected with a-HUD)
Galectin-1
IPS-K-4A2B8
BTL native
STL
Human Galectin1
Sp Serotype 14.15
CCIG-1007
rNV VLP-Cy3
WFL
mouse Gal-7
AHL
WT-VCC
PHA-E
VVHA lectin
ConA
CnSepL
TL(EY Lab)
PSL
TJA-I
CAA
CA
WGA
ConA
RCAI (Vector)
SNA

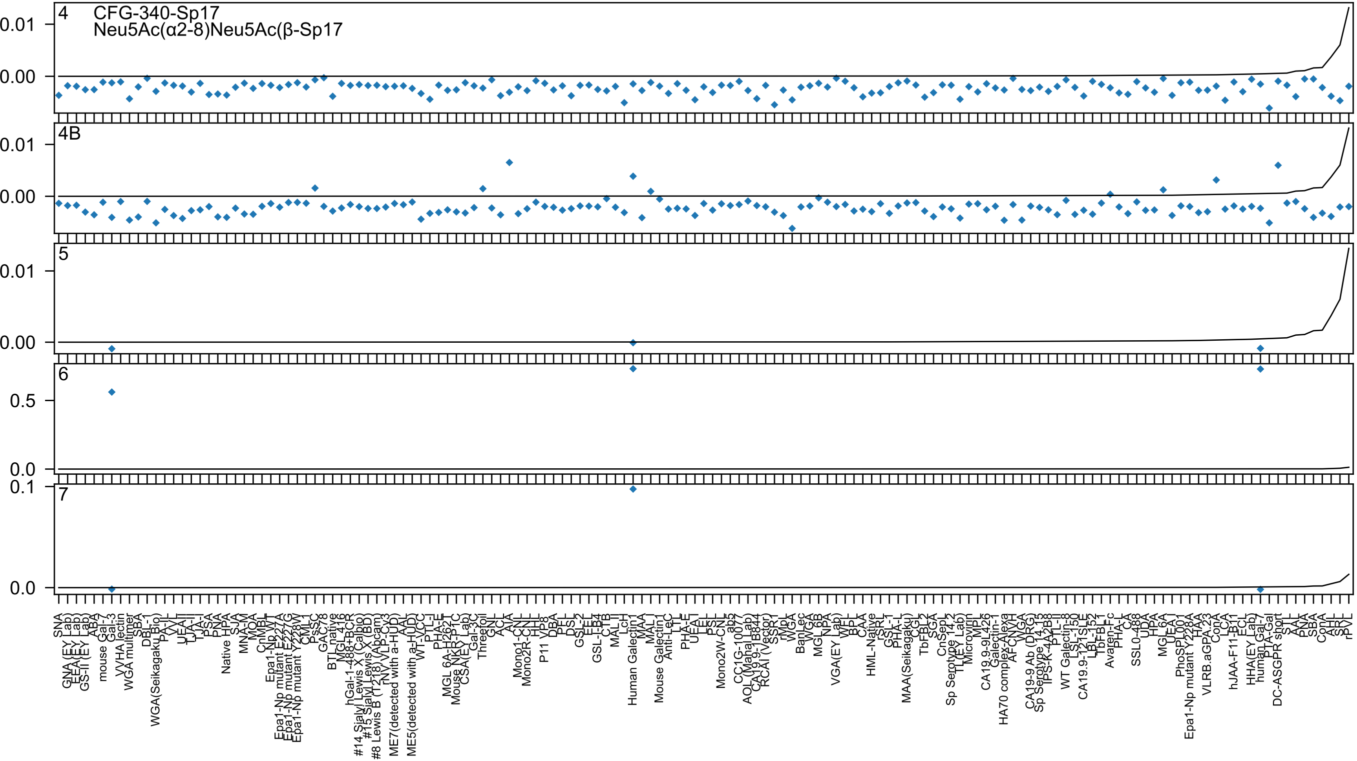

CFG-340-Sp17
Neu5Ac(α2-8)Neu5Ac(β-Sp17
4
4B
5
6
7

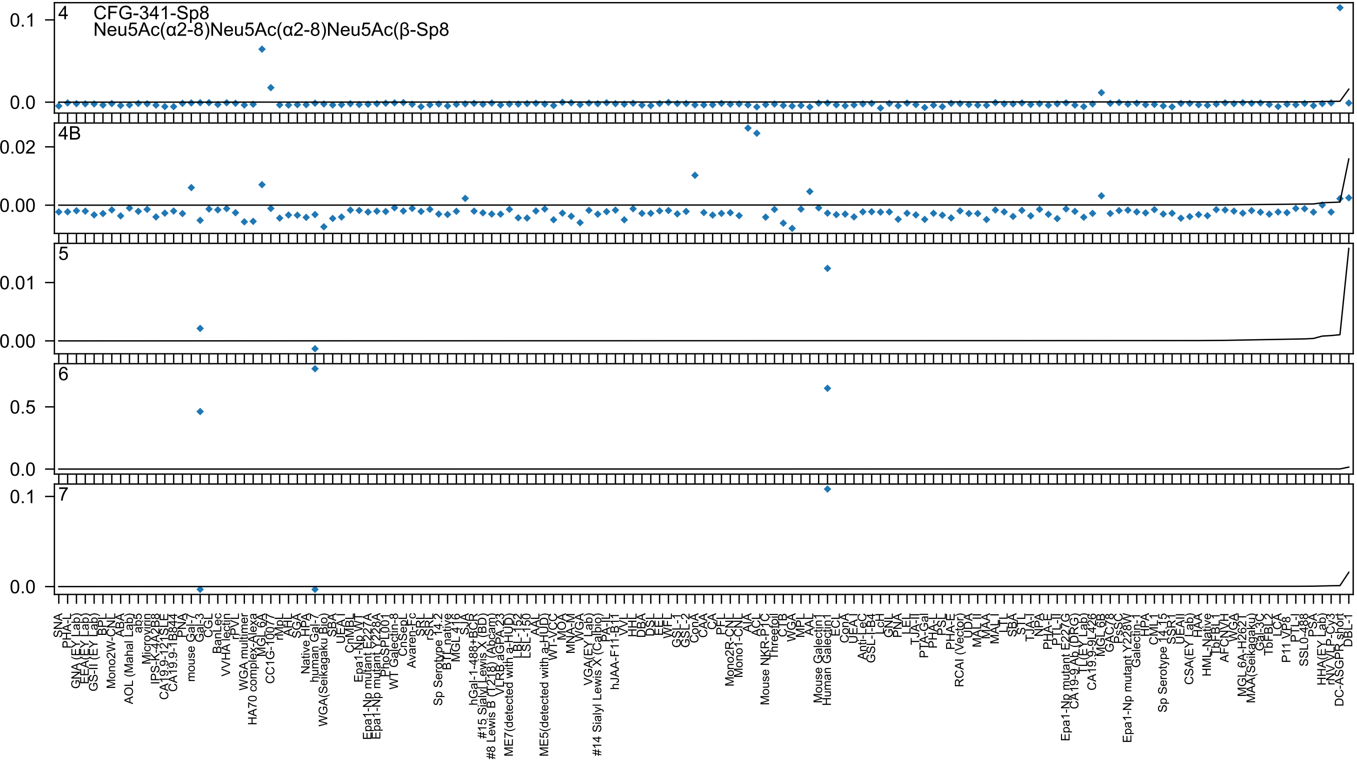

CFG-341-Sp8
Neu5Ac(α2-8)Neu5Ac(α2-8)Neu5Ac(β-Sp8
4
4B
5
6
7

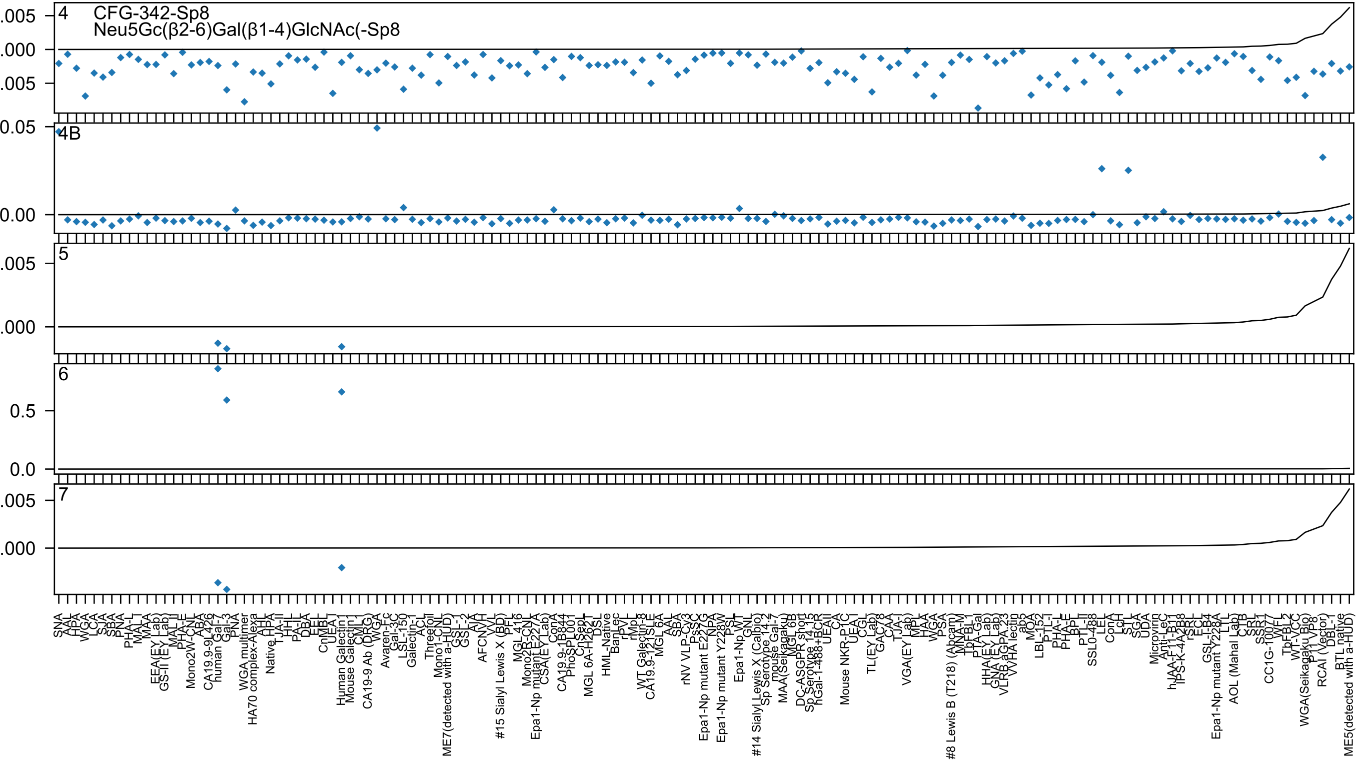
4
CFG-342-Sp8
Neu5Gc(β2-6)Gal(β1-4)GlcNAc(-Sp8
.005
.000
.005
4B
0.05
0.00
5
.005
.000
6
0.5
0.0
7
.005
.000
SNA
AAL
HPA
WGA
LCA
SJA
SBA
PNA
PHA-L
MAL-I
MAA
EEA(EY Lab)
GS-II (EY Lab)
MAL II
PHA-E
Mono2W-CN1
ABA
CA19.9-9L426
human Gal-7
Gal-3
PNA
WGA multimer
HA70 complex-Alexa
AH
Native HPA
TJA-II
HHL
PA-IL
DBA
EEL
CnMBL
UEA I
Human Galectin1
Mouse Galectin1
CML-I
CA19-9 Ab (DRG)
WGA
Avaren-Fc
Gal-3C
LSL-150
Galectin-1
ACL
Threefoil
Mono1-CN1
ME7(detected with a-HUD)
GSL-2
AIA
AFCNVH
VVL
#15 Sialyl Lewis X (BD)
PFL
MGL416
Mono2R-CN1
Epa1-Np mutant E227A
CSA(EY Lab)
ConA
CA19.9-1B844
PhoSL001
CnSepL
MGL 6A-H262T
DSL
HML-Native
BanLec
rPVL
rMpL
WT Galectin-8
CA19.9-121SLE
MGL 6A
AAL
SBA
rNV VLP-Cy3
PsSC
Epa1-Np mutant E227G
NPA
Epa1-Np mutant Y228W
PSL
Epa1-Np WT
GNL
#14 Sialyl Lewis X (Calbio)
Sp Serotype 14.2
mouse Gal-7
MAA(Seikagaku)
MGL 6B
DC-ASGPR short
Sp Serotype 14.15
hGal-1-488+BCR
UEAII
CA
Mouse NKR-P1C
UEA I
CGL
TL(EY Lab)
GAC78
CAA
TJA-I
VGA(EY Lab)
MPL
HAA
WGA
PSA
#8 Lewis B (T218) (Abcam)
MNA-M
TbFBL1
PTA-Gal
HHA(EY Lab)
GNA (EY Lab)
VLRB aGPA.23
VVHA lectin
ab5
MOA
LBL-152
PTL-I
PHA-L
PHA-E
BPL
PTL-II
SSL0-488
LEA
ConA
LcH
STL
SGA
UDA
Microvirin
Anti-LeC
hJAA-F11-B11
IPS-K-4A2B8
rSRL
ECL
GSL-I-B4
Epa1-Np mutant Y228A
LTL
AOL (Mahal Lab)
CTB
SRL
SSR1
CC1G-10077
WFL
TbFBL2
WT-VCC
WGA(Seikagaku Bio)
P11 VP8
RCAI (Vector)
DBL-I
BTL native
ME5(detected with a-HUD)

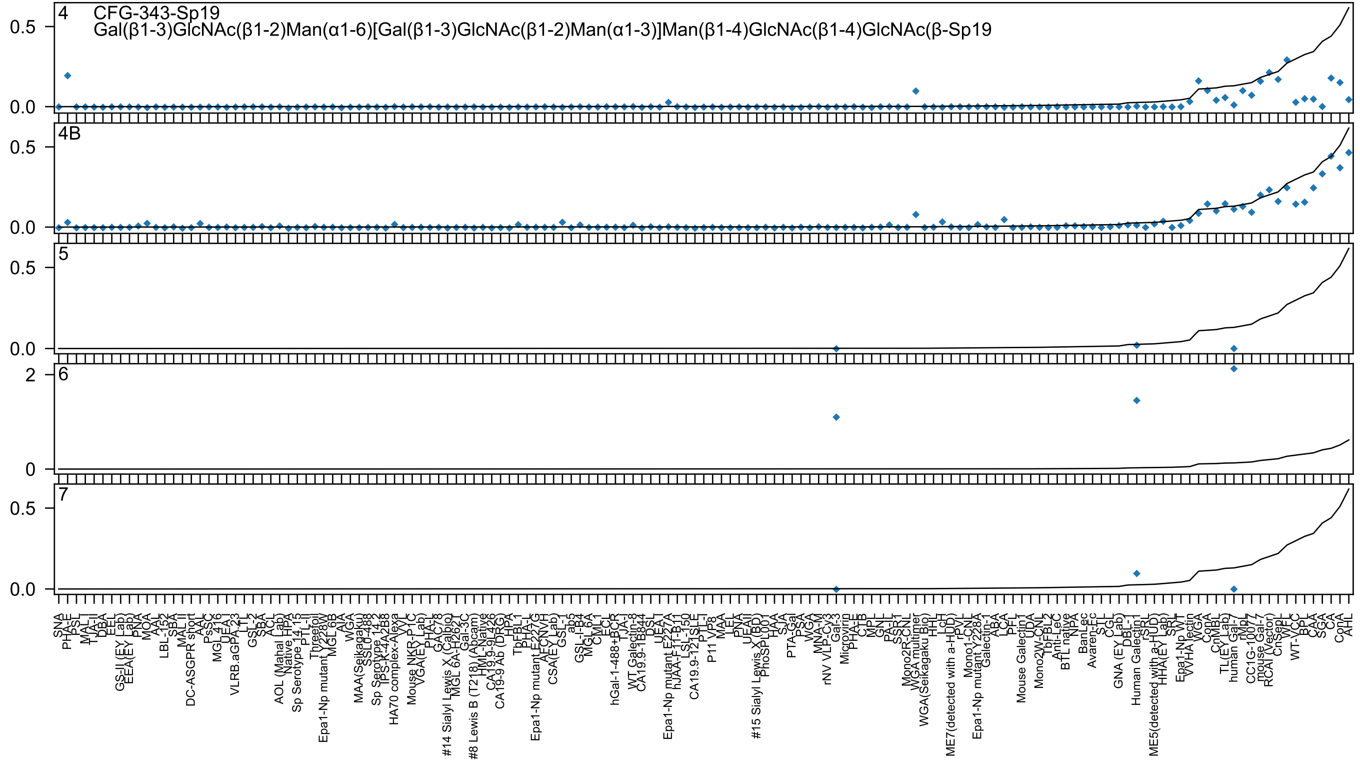
4
CFG-343-Sp19
Gal(β1-3)GlcNAc(β1-2)Man(α1-6)[Gal(β1-3)GlcNAc(β1-2)Man(α1-3)]Man(β1-4)GlcNAc(β1-4)GlcNAc(β-Sp19
4B
5
6
7
0.0
0.5
0
2
SNA
PHA-E
PSL
MAL-I
TJA-II
DBA
EEL
GS-II (EY Lab)
EEA(EY Lab)
PNA
MOA
AAL
LBL-152
SBA
MAL II
DC-ASGPR short
AAL
PsSC
MGL 416
UEA I
VLRB.aGPA.23
LTL
GSL-2
SBA
ACL
AOL (Mahal Lab)
Native HPA
Sp Serotype 14.15
PTL-II
Threefoil
Epa1-Np mutant Y228W
MGL 6B
AIA
WGA
MAA(Seikagaku)
SSL0-488
Sp Serotype 14.2
TPS-K-4A2B8
HA70 complex-Alexa
VVL
Mouse NKR-P1C
VGA(EY Lab)
PHA-L
GAC78
#14 Sialyl Lewis X (Calbio)
MGL 6A-H262
Gal-3C
#8 Lewis B (T218) (Abcam)
HML-Native
CA19.9-9L426
CA19-9 Ab (DRG)
HPA
TbFBL1
PHA-L
Epa1-Np mutant E227G
AFCNVH
CSA(EY Lab)
GSL-I
ab5
GSL-I-B4
MGL 6A
CML1
ECL
hGal-1-488+BCR
TJA-I
WT Galectin-8
CA19.9-1B844
DSL
UEA I
Epa1-Np mutant E227A
hJAA-F11-B11
LSL-150
CA19.9-121SLE
PTL-I
P11 VP8
MAA
LEL
PNA
UEAII
#15 Sialyl Lewis X (BD)
PhoSL001
HAA
SJA
PTA-Gal
PSA
WGA
MNA-M
rNV VLP-Cy3
Gal-3
Microvirin
PHA-E
CTB
MPL
GNL
PA-IL
SSR1
Mono2R-CNL
WGA multimer
WGA(Seikagaku Bio)
HHL
LcH
ME7(detected with a-HUD)
rPVL
Mono1-CNL
Epa1-Np mutant Y228A
Galectin-1
ABA
LCA
PFL
Mouse Galectin1
UDA
Mono2W-CNL
TbFBL2
Anti-LeC
BTL native
NPA
BanLec
Avaren-Fc
STL
CGL
GNA (EY Lab)
DBL-1
Human Galectin1
rSRL
ME5(detected with a-HUD)
HHA(EY Lab)
SRL
Epa1-Np WT
VVHA lectin
WGA
ConA
CnMBL
TL(EY Lab)
human Gal-7
rNpL
CC1G-10077
mouse Gal-7
RCAI (Vector)
CnSepL
WFL
WT-VCC
BPL
CAA
SGA
CA
ConA
AHL

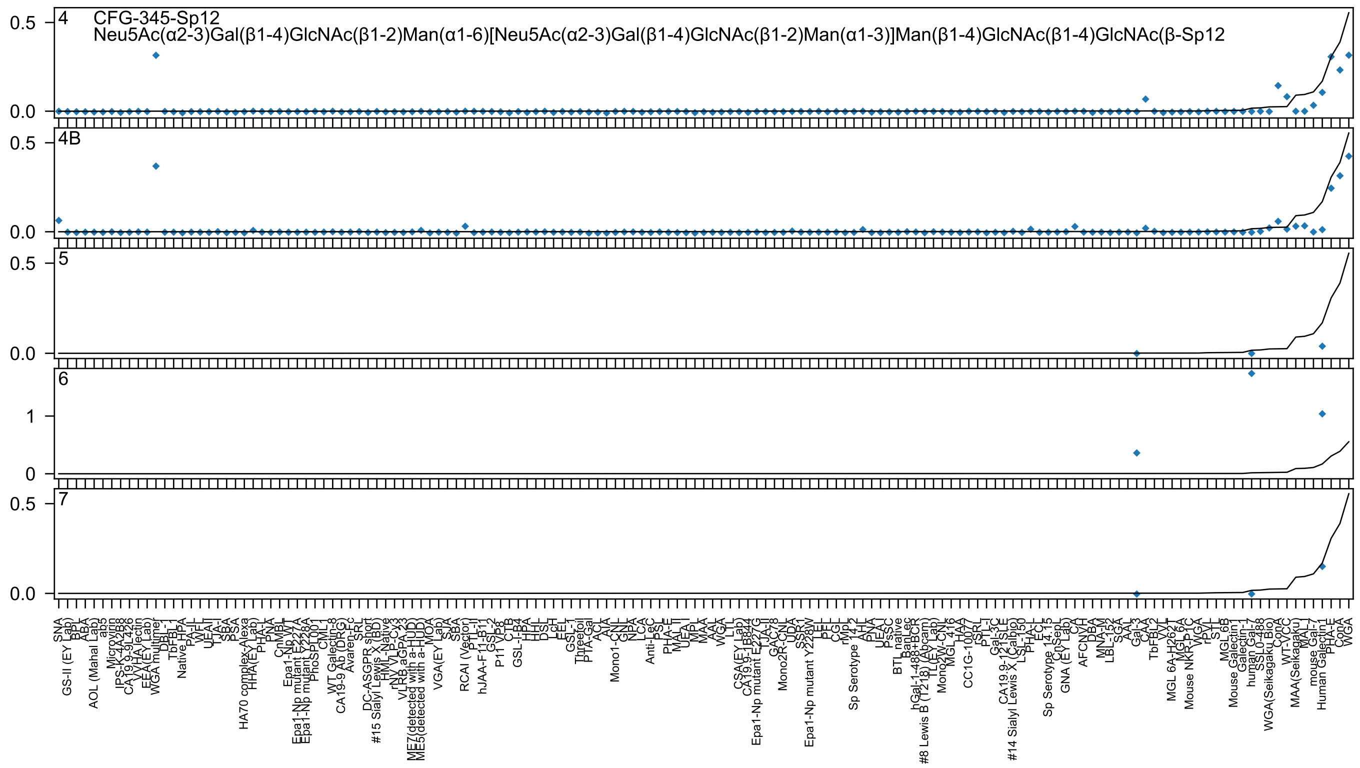

4
CFG-345-Sp12
Neu5Ac(α2-3)Gal(β1-4)GlcNAc(β1-2)Man(α1-6)[Neu5Ac(α2-3)Gal(β1-4)GlcNAc(β1-2)Man(α1-3)]Man(β1-4)GlcNAc(β1-4)GlcNAc(β-Sp12
4B
5
6
7
0.5
0.0
1
0

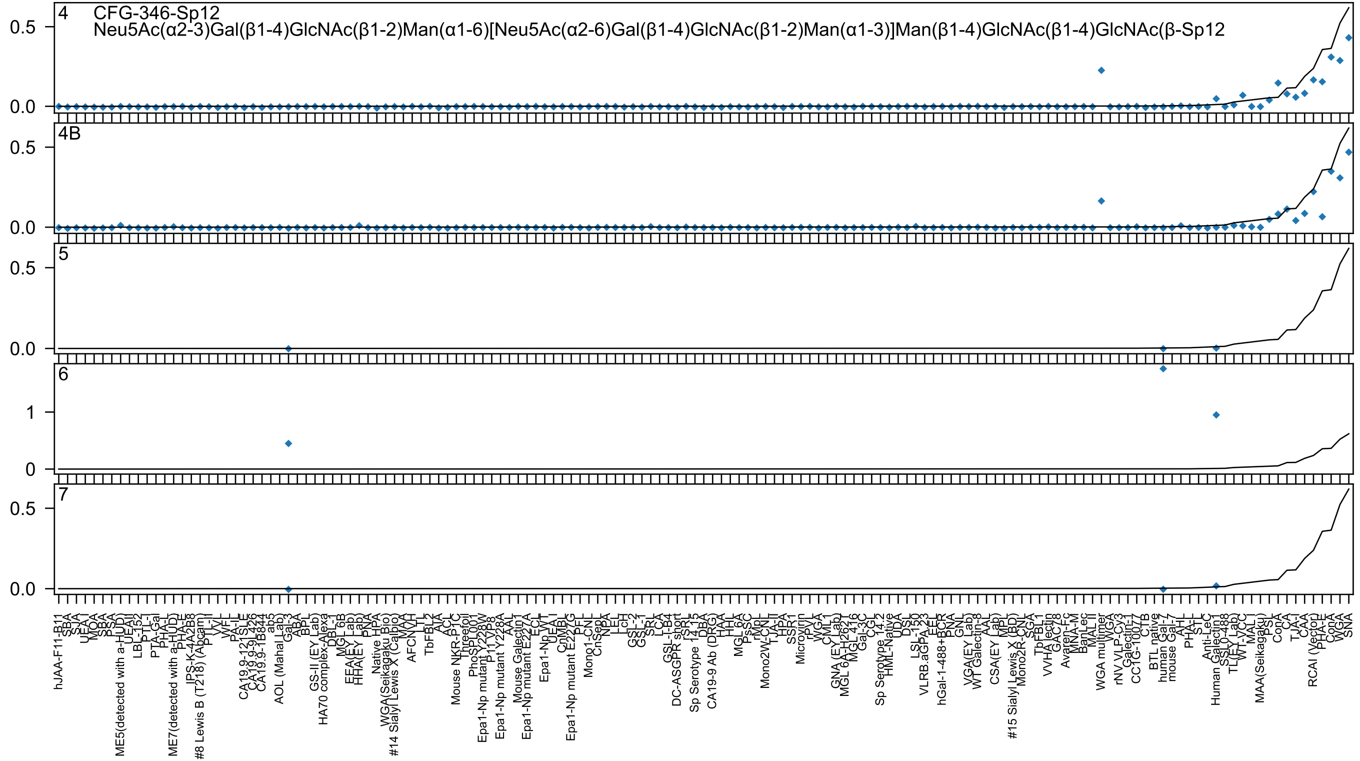
4 CFG-346-Sp12
Neu5Ac(α2-3)Gal(β1-4)GlcNAc(β1-2)Man(α1-6)[Neu5Ac(α2-6)Gal(β1-4)GlcNAc(β1-2)Man(α1-3)]Man(β1-4)GlcNAc(β1-4)GlcNAc(β-Sp12
4B
5
6
7

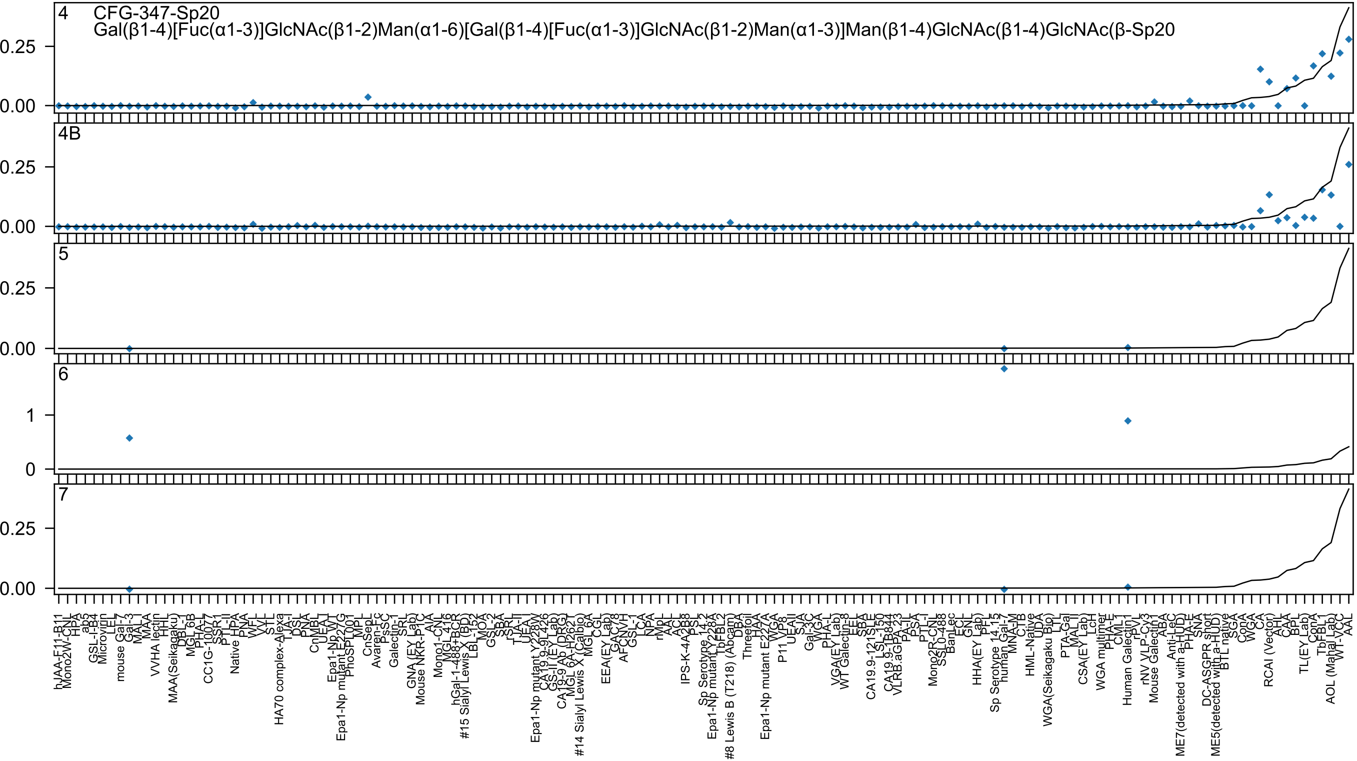
4 CFG-347-Sp20
Gal(β1-4)[Fuc(α1-3)]GlcNAc(β1-2)Man(α1-6)[Gal(β1-4)[Fuc(α1-3)]GlcNAc(β1-2)Man(α1-3)]Man(β1-4)GlcNAc(β1-4)GlcNAc(β-Sp20
4B
5
6
7

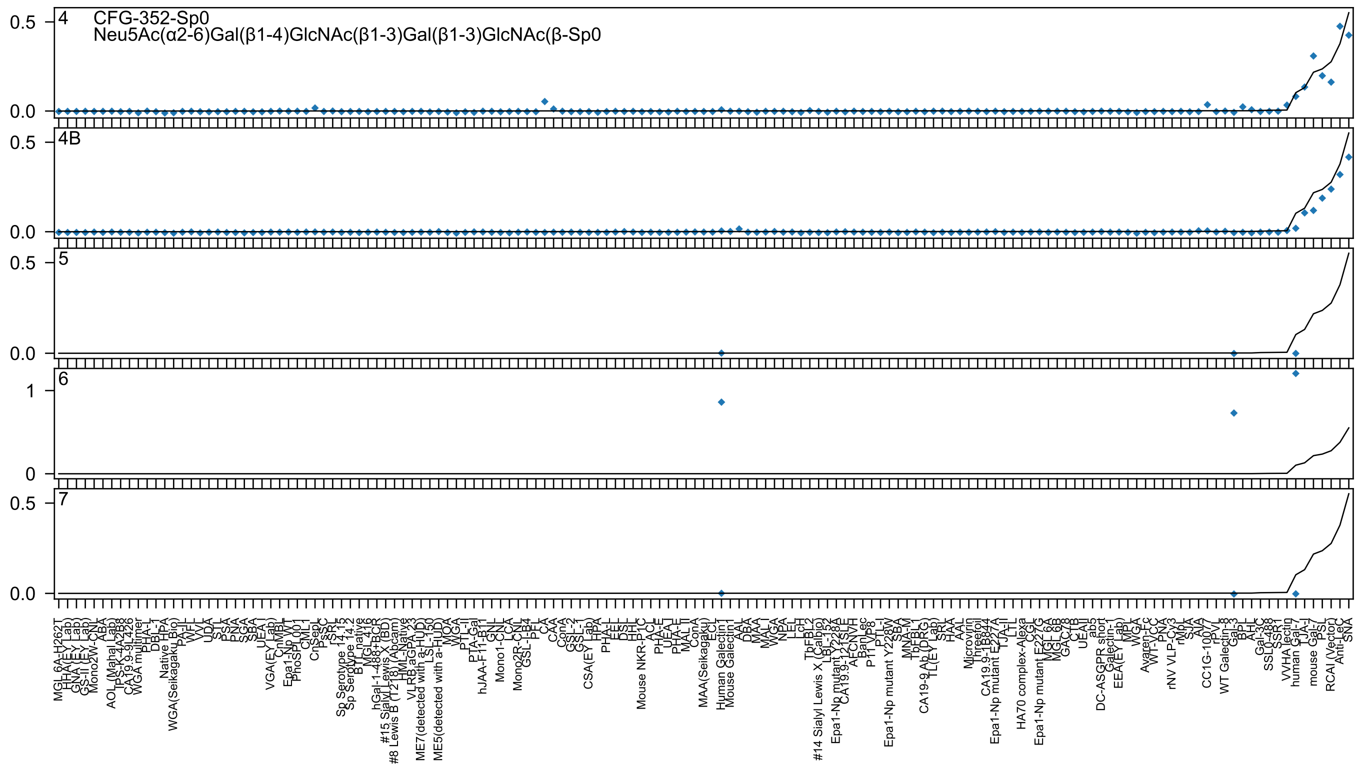
4
CFG-352-Sp0
Neu5Ac(α2-6)Gal(β1-4)GlcNAc(β1-3)Gal(β1-3)GlcNAc(β-Sp0
4B
5
6
7

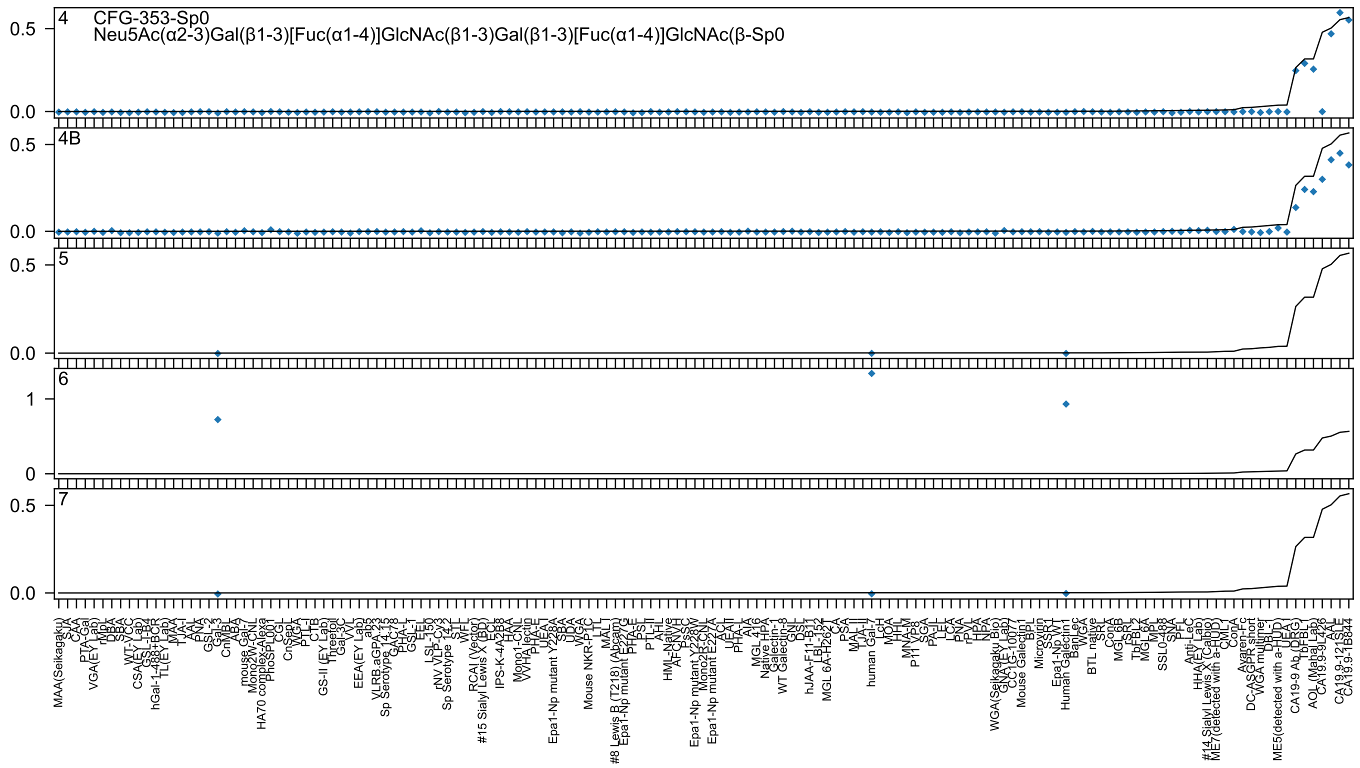

4
CFG-353-Sp0
Neu5Ac(α2-3)Gal(β1-3)[Fuc(α1-4)]GlcNAc(β1-3)Gal(β1-3)[Fuc(α1-4)]GlcNAc(β-Sp0
4B
5
6
7
0.0
0.5
0
1
MAA(Seikagaku)
SJA
CAA
PTA-Gal
VGA(EY Lab)
DBA
SBA
WT-VCC
CSA(EY Lab)
GSL-I-B4
hGal-1-488+BCR
TL(EY Lab)
MAA
TJA-I
AAL
PNA
GSL-2
Gal-3
CnMBL
ABA
mouse Gal-7
Mono2W-CNL
HA70 complex-Alexa
PhoSL001
CGL
CnSept
WGA
PTL-I
CTB
GS-II (EY Lab)
Threefoil
Gal-3C
VVL
EEA(EY Lab)
VLRB.aGPA.23
Sp Serotype 14.15
GAC78
PHA-L
GSL-1
EEL
LSL-150
rNV VLP-Cy3
Sp Serotype 14.2
STL
WFL
RCAI (Vector)
#15 Sialyl Lewis X (BD)
ECL
IPS-K-4A2B8
HAA
Mono1-CNL
WHA lectin
PHA-E
UEA-I
Epa1-Np mutant Y228A
SBA
UDA
WGA
Mouse NKR-P1C
LTL
MAL I
#8 Lewis B (T218) (Abcam)
Epa1-Np mutant E227G
PHA-E
PSL
PTL-II
AHL
HML-Native
AFCNVH
PsSC
Epa1-Np mutant Y228W
Mono2R-CNL
Epa1-Np mutant E227A
ACL
UEAII
PHA-L
AIA
MGL 416
Native HPA
Galectin-1
Galectin-8
WT Galectin-8
GNL
DSL
hJAA-F11-B11
LBL-152
MGL 6A-H262A
CA
PSA
MAL-II
TJA-II
human Gal-7
LcH
MOA
HHL
MNA-M
P11 VP8
SGA
PA-IL
LEL
LCA
PNA
rPVL
HPA
NPA
WGA(Seikagaku Bio)
GNA(EY Lab)
CC1G-10077
Mouse Galectin1
BPL
Microvirin
SSR1
Epa1-Np WT
Human Galectin1
BanLec
WGA
BTL native
SRL
ConA
MGL 6B
rSRL
TbFBL2
MGL 6A
MPL
SSL0-488
SNA
PFL
Anti-LeC
HHA(EY Lab)
#14 Sialyl Lewis X (Calbio)
ME7(detected with a-HUD)
CML
ConA
Avaren-Fc
DC-ASGPR short
WGA multimer
DBL-I
ME5(detected with a-HUD)
UEA-I
CA19-9 Ab (DRG)
TbFBL1
AOL (Mahal Lab)
CA19.9-9L426
AAL
CA19.9-121SLE
CA19.9-1B844

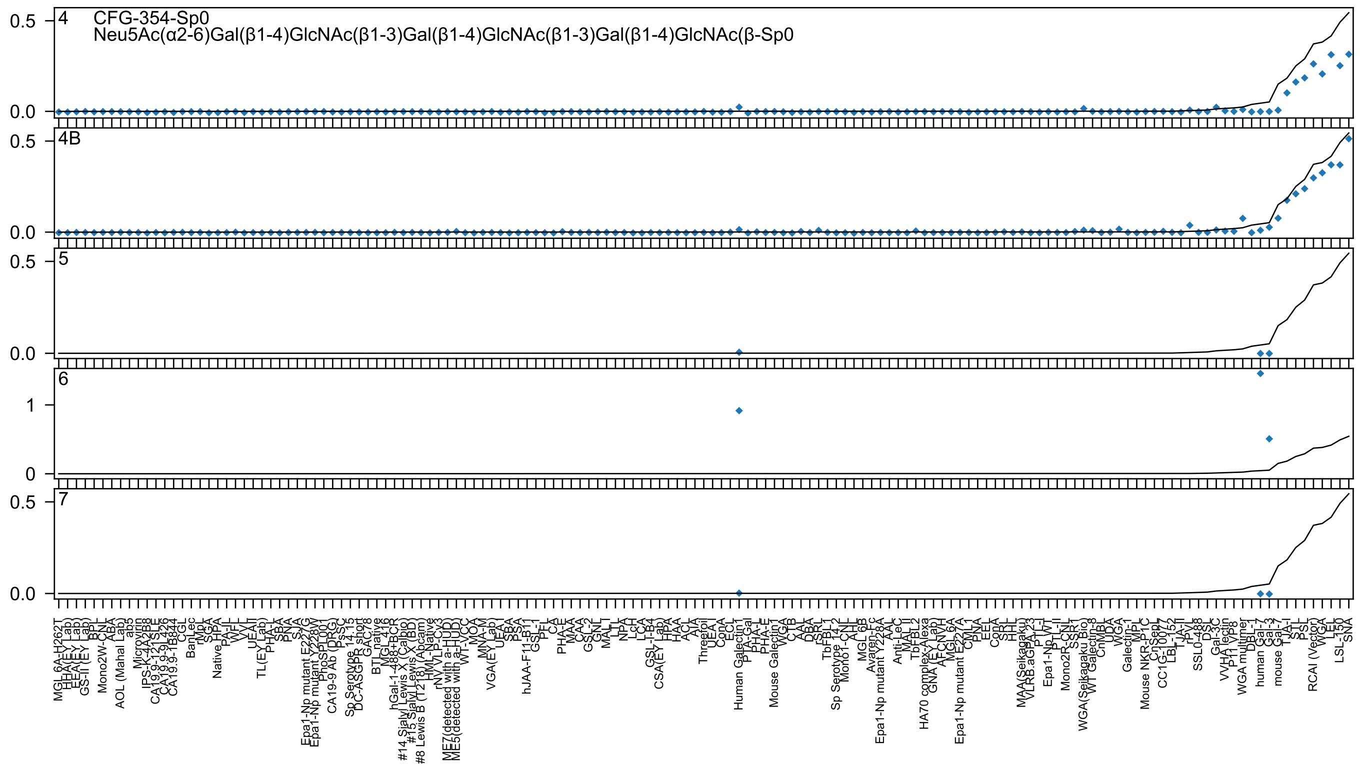

4
CFG-354-Sp0
Neu5Ac(α2-6)Gal(β1-4)GlcNAc(β1-3)Gal(β1-4)GlcNAc(β1-3)Gal(β1-4)GlcNAc(β-Sp0
4B
5
6
7
0.5
0.0
1
0

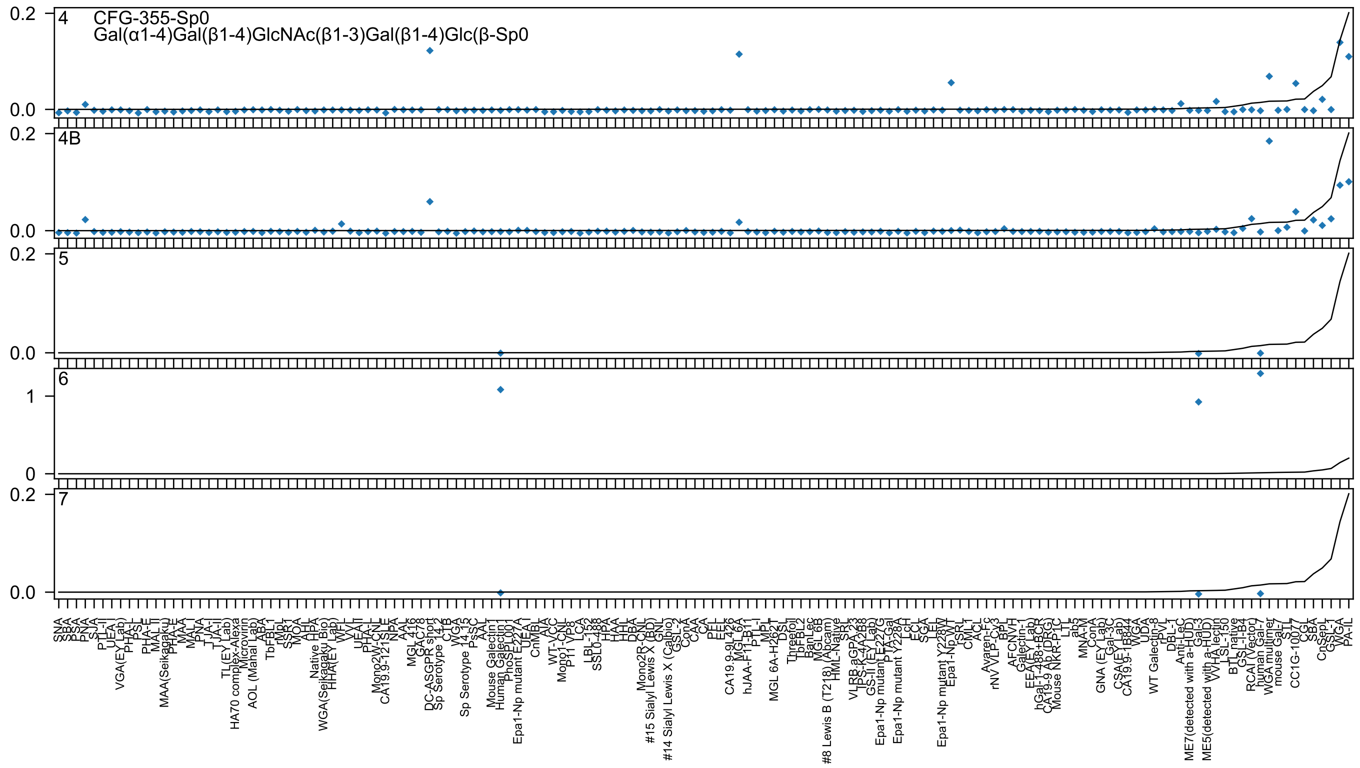

4
CFG-355-Sp0
Gal(α1-4)Gal(β1-4)GlcNAc(β1-3)Gal(β1-4)Glc(β-Sp0
4B
5
6
7

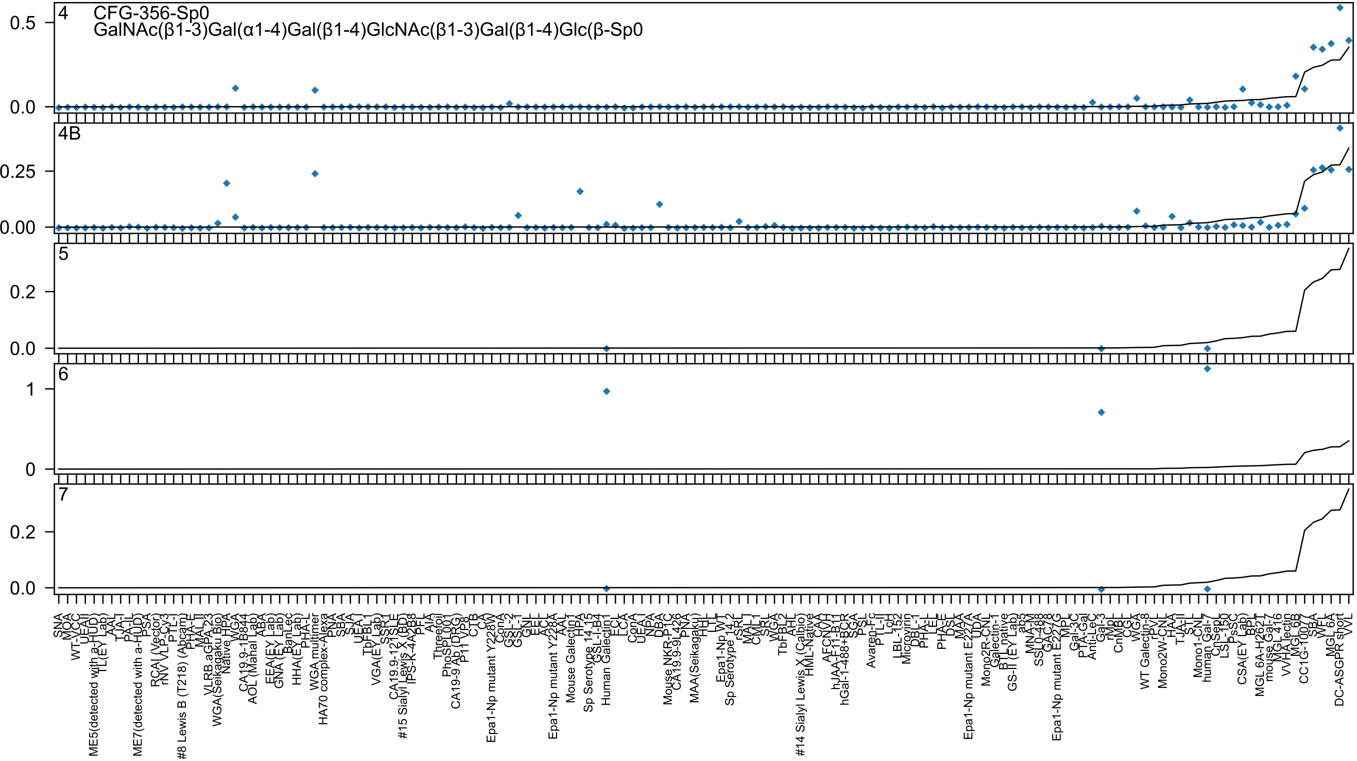

4
CFG-356-Sp0
GalNAc(β1-3)Gal(α1-4)Gal(β1-4)GlcNAc(β1-3)Gal(β1-4)Glc(β-Sp0
4B
5
6
7

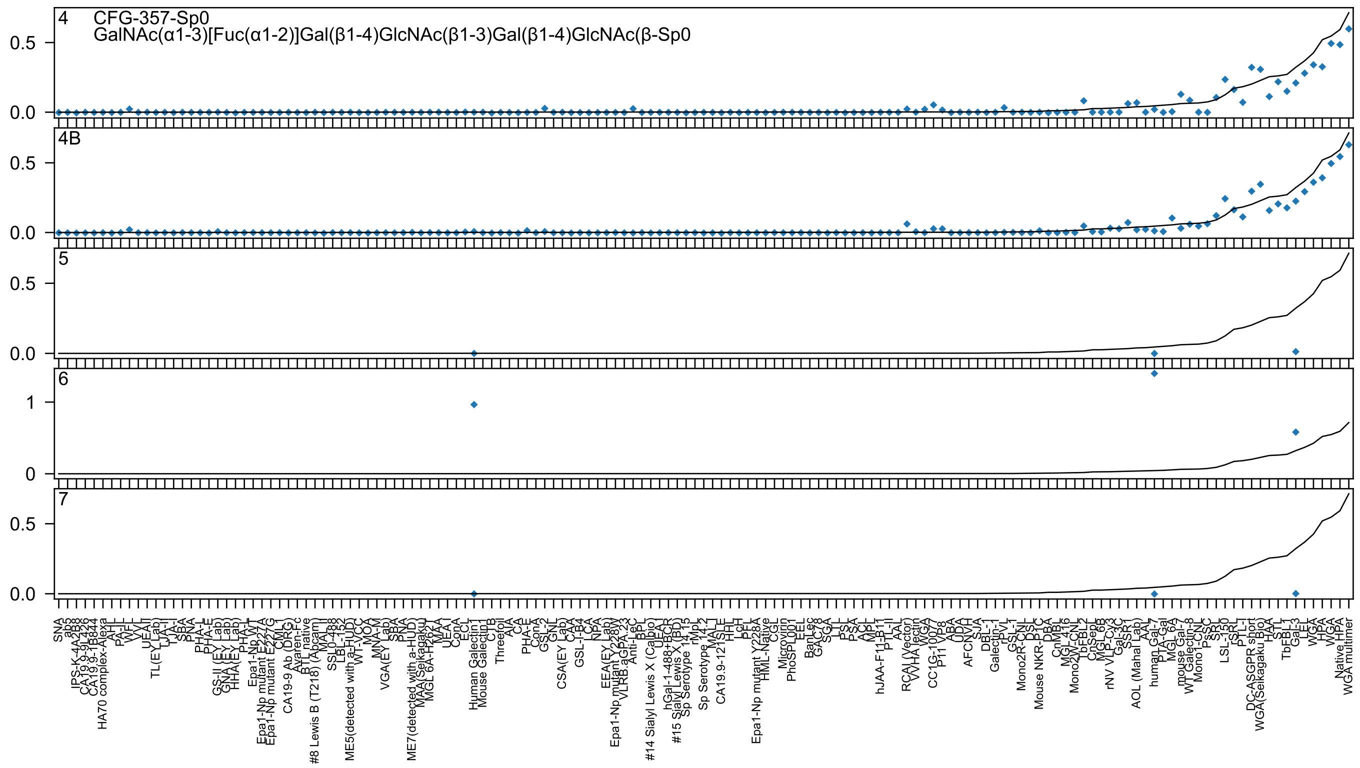

4 CFG-357-Sp0
GalNAc(α1-3)[Fuc(α1-2)]Gal(β1-4)GlcNAc(β1-3)Gal(β1-4)GlcNAc(β-Sp0
4B
5
6
7
0.0
0.5
0
1
SNA
abs
IPS-K-4A2B8
CA19.9-9L426
CA19.9-1B844
HA70 complex-Alexa
AHL
PA-IL
WFL
VVL
UEAII
TL(EY Lab)
TJA-II
TJA-I
SBA
PNA
PHA-L
PHA-E
GS-II (EY Lab)
GNA (EY Lab)
HHA(EY Lab)
PHA-L
Epa1-Np WT
Epa1-Np mutant E227A
Epa1-Np mutant E227G
CML
CA19-9 Ab (DRG)
Avaren-Fc
BTL native
#8 Lewis B (T218) (Abcam)
MAL
SSL0-488
LBL-152
ME5(detected with a-HUD)
WT-VCC
MOA
MNA-M
VGA(EY Lab)
SBA
PNA
ME7(detected with a-HUD)
MAA(Seikagaku)
MGL 6A-H262T
MAA
UEA-I
ConA
ECL
Human Galectin1
Mouse Galectin1
CTB
Threefoil
AIA
CA
PHA-E
ConA
GSL-2
GNL
CSA(EY Lab)
CAA
GSL-I-B4
LCA
NPA
EEA(EY Lab)
Epa1-Np mutant Y228W
VLRB.aGPA.23
Anti-LeC
BPL
UEA-I
#14 Sialyl Lewis X (Calbio)
hGal-1-488+BCR
#15 Sialyl Lewis X (BD)
Sp Serotype 14.15
rMpL
Sp Serotype 14.2
MAL-I
CA19.9-121SLE
HHL
LcH
PFL
Epa1-Np mutant Y228A
HML-Native
CGL
Microvirin
PhoSL001
EEL
BanLec
GAC78
SGA
LTL
PSL
PSA
ACL
MPL
hJAA-F11-B11
PTL-II
AAL
RCAI (Vector)
VVHA lectin
WGA
CC1G-10077
P11 VP8
ABA
UDA
AFCNVH
SJA
DBL-1
Galectin-1
rPVL
GSL-1
Mono2R-CNL
DSL
Mouse NKR-P1C
DBA
CnMBL
MGL-416
Mono2W-CNL
TbFBL2
CnSep1
MGL 6B
rNV VLP-Cy3
Gal-3C
SSR1
AOL (Mahal Lab)
AAL
human Gal-7
PTA-Gal
MGL 6A
mouse Gal-7
WT Galectin-8
Mono1-CNL
PsSC
SRL
LSL-150
rSRL
PTL-I
DC-ASGPR short
WGA(Seikagaku Bio)
HAA
STL
TbFBL1
Gal-3
LEL
WGA
HPA
WGA
Native HPA
WGA multimer

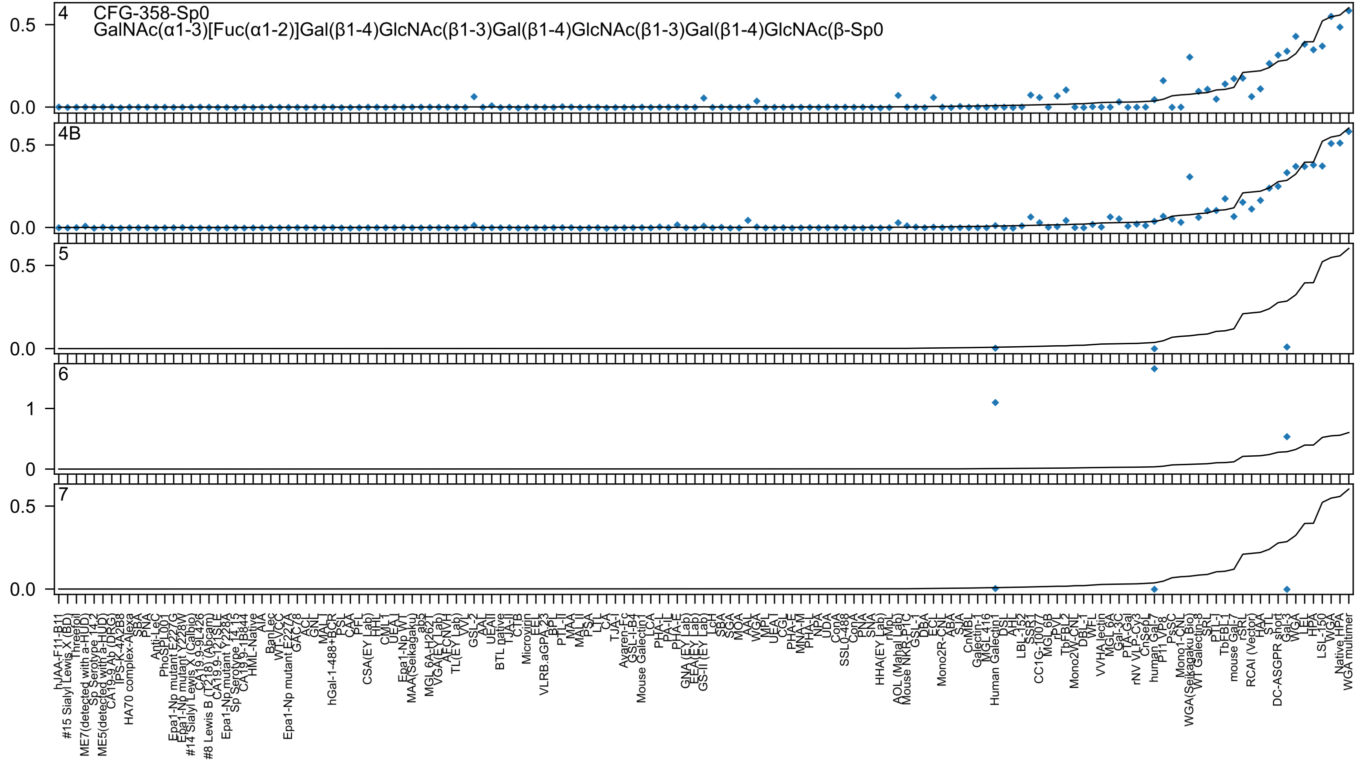

4
CFG-358-Sp0
GalNAc(α1-3)[Fuc(α1-2)]Gal(β1-4)GlcNAc(β1-3)Gal(β1-4)GlcNAc(β1-3)Gal(β1-4)GlcNAc(β-Sp0
4B
5
6
7

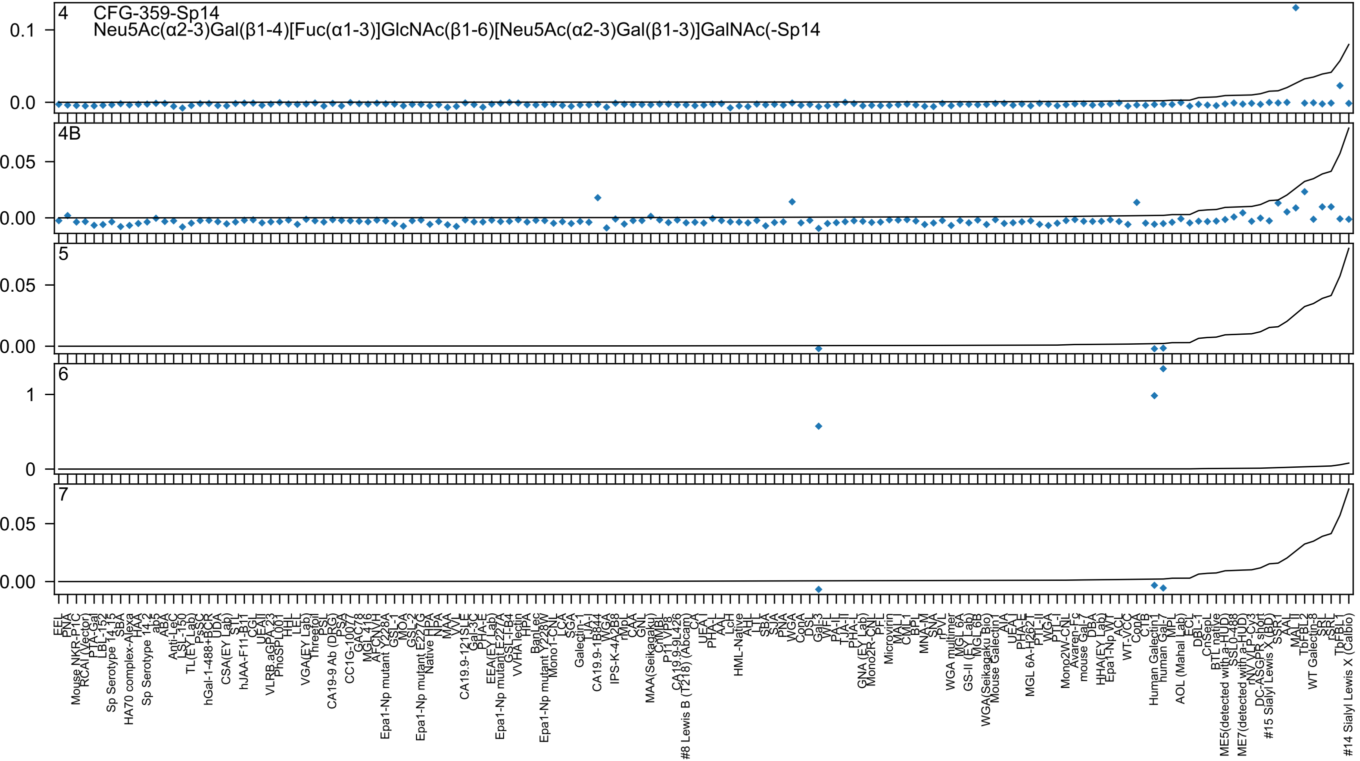
4 CFG-359-Sp14
Neu5Ac(α2-3)Gal(β1-4)[Fuc(α1-3)]GlcNAc(β1-6)[Neu5Ac(α2-3)Gal(β1-3)]GalNAc(-Sp14
4B
5
6
7

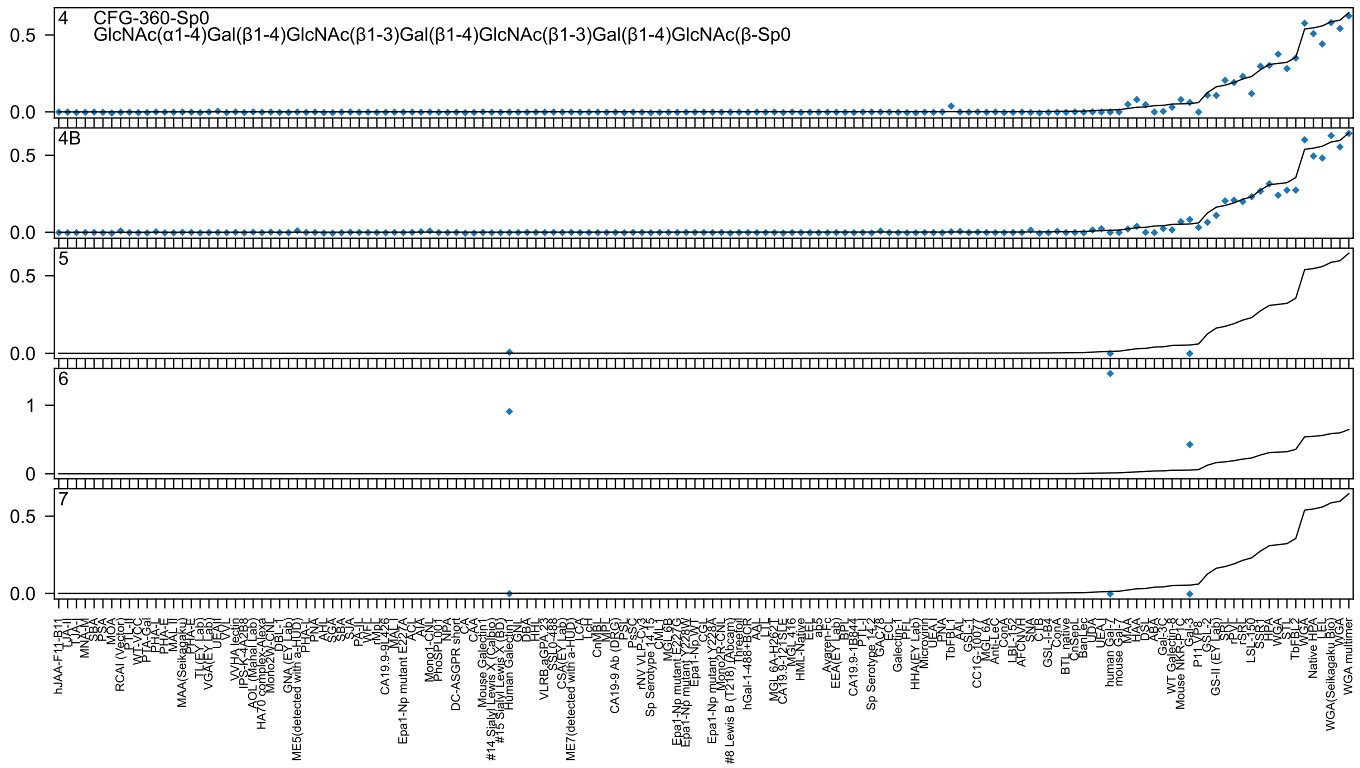

4 CFG-360-Sp0
GlcNAc(α1-4)Gal(β1-4)GlcNAc(β1-3)Gal(β1-4)GlcNAc(β1-3)Gal(β1-4)GlcNAc(β-Sp0
4B
5
6
7

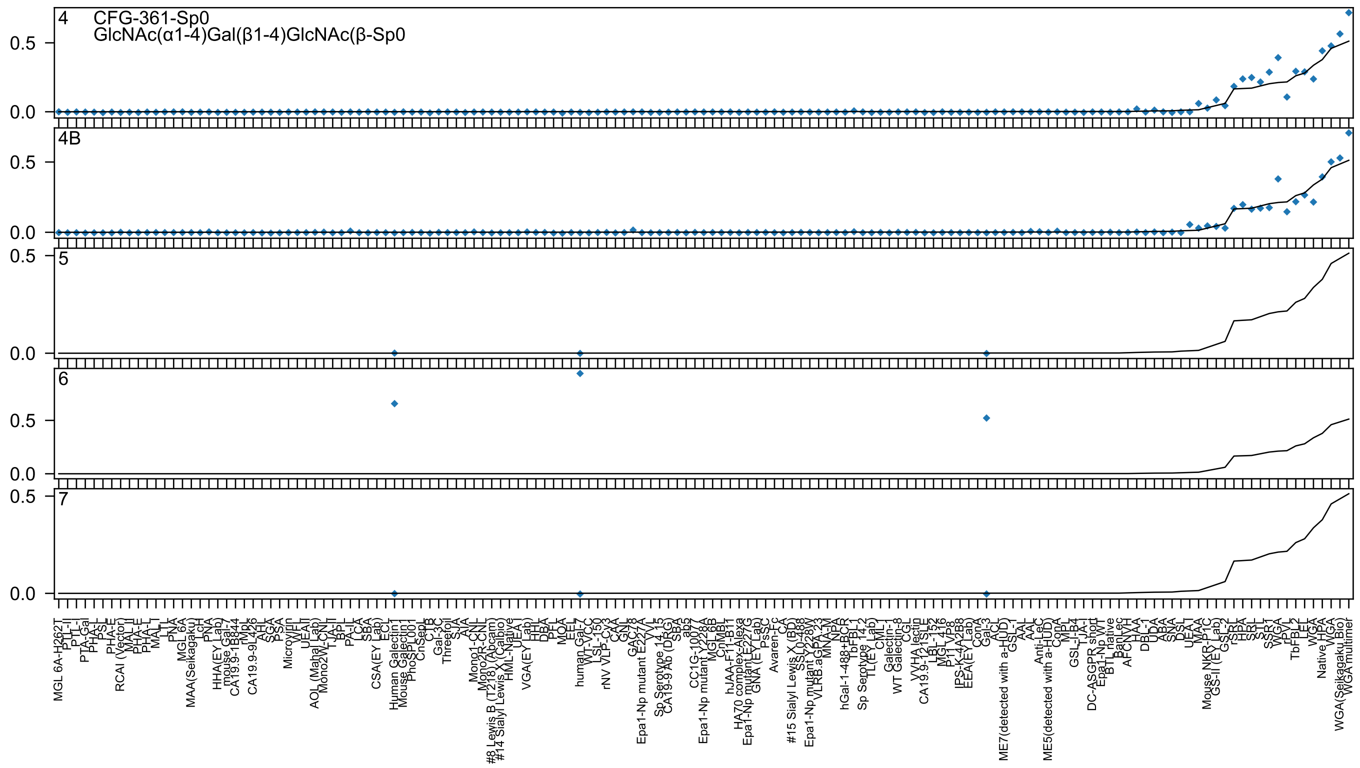
4
CFG-361-Sp0
GlcNAc(α1-4)Gal(β1-4)GlcNAc(β-Sp0
4B
5
6
7
0.5
0.0

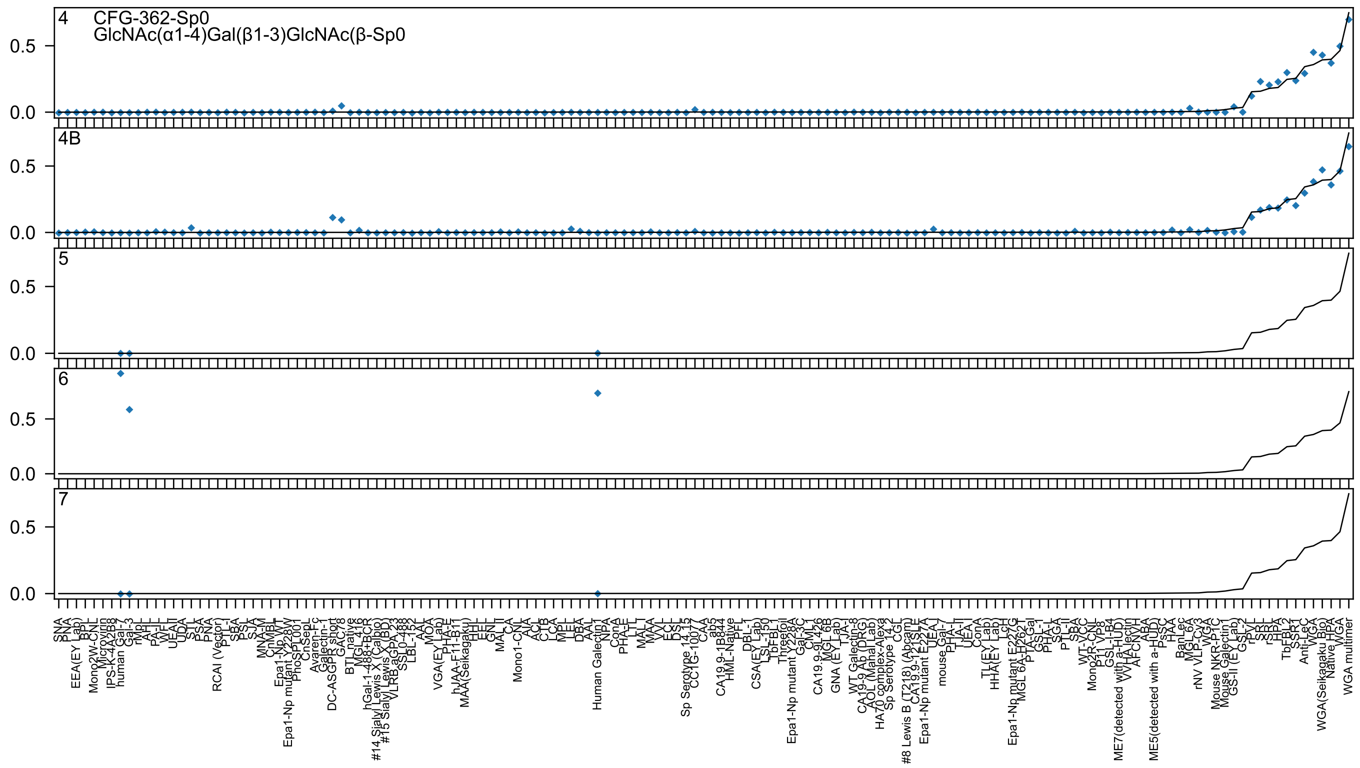
4
CFG-362-Sp0
GlcNAc(α1-4)Gal(β1-3)GlcNAc(β-Sp0
4B
5
6
7
0.5
0.0

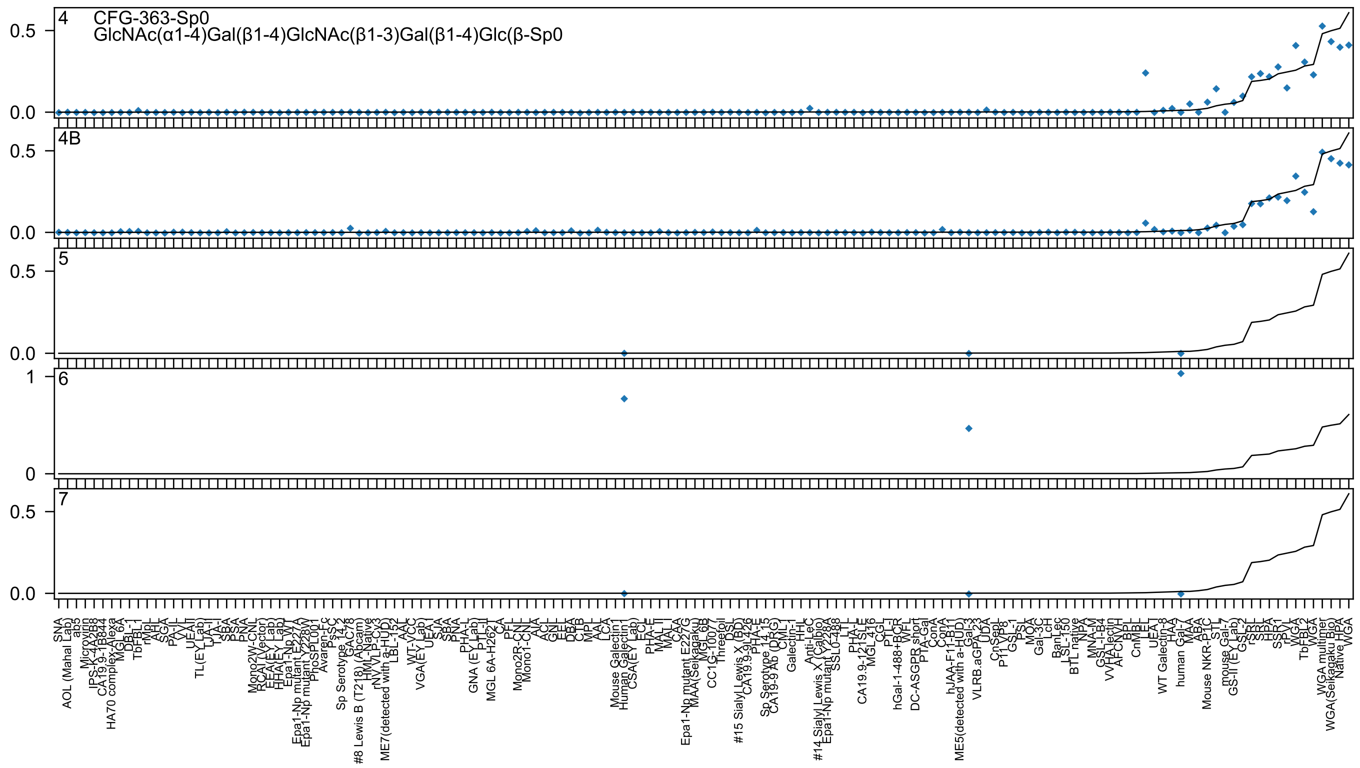

4 CFG-363-Sp0
GlcNAc(α1-4)Gal(β1-4)GlcNAc(β1-3)Gal(β1-4)Glc(β-Sp0
4B
5
6
7

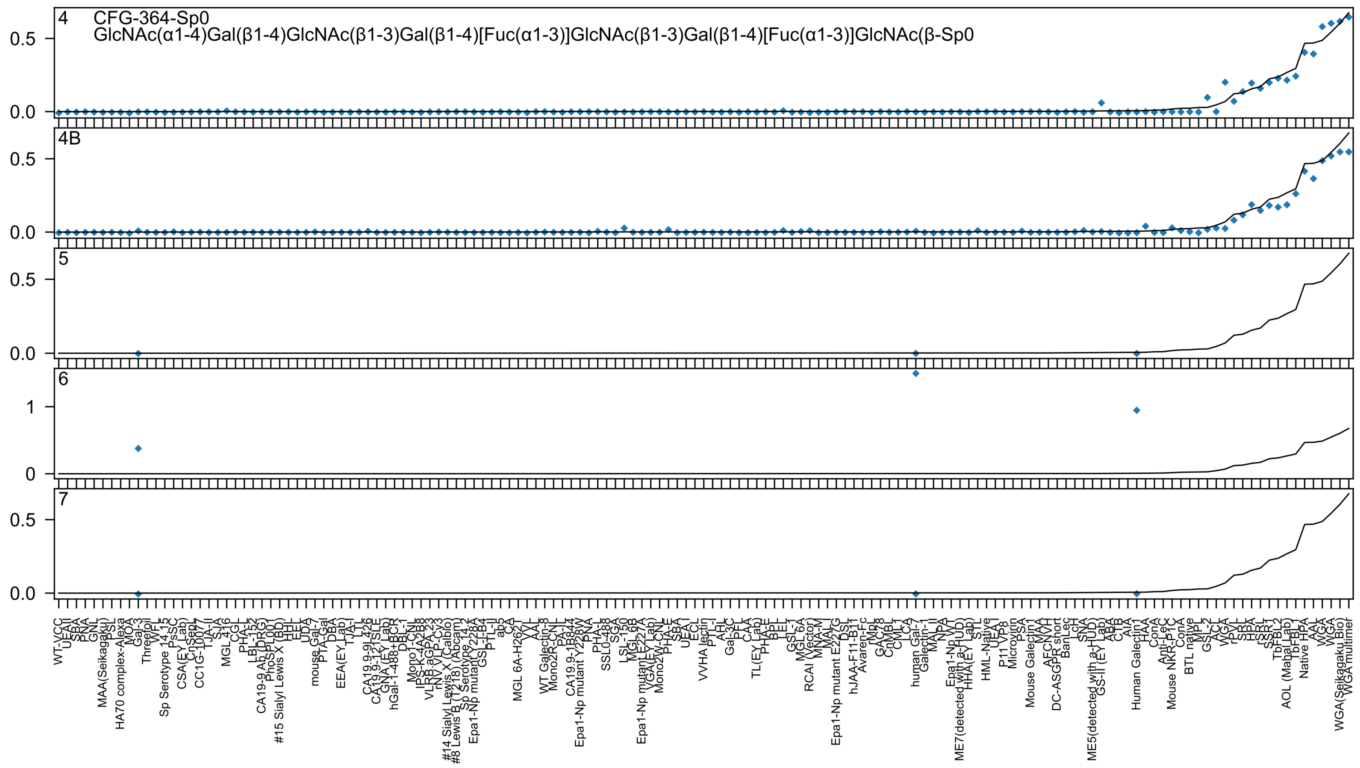

4 CFG-364-Sp0
GlcNAc(α1-4)Gal(β1-4)GlcNAc(β1-3)Gal(β1-4)[Fuc(α1-3)]GlcNAc(β1-3)Gal(β1-4)[Fuc(α1-3)]GlcNAc(β-Sp0
4B
5
6
7

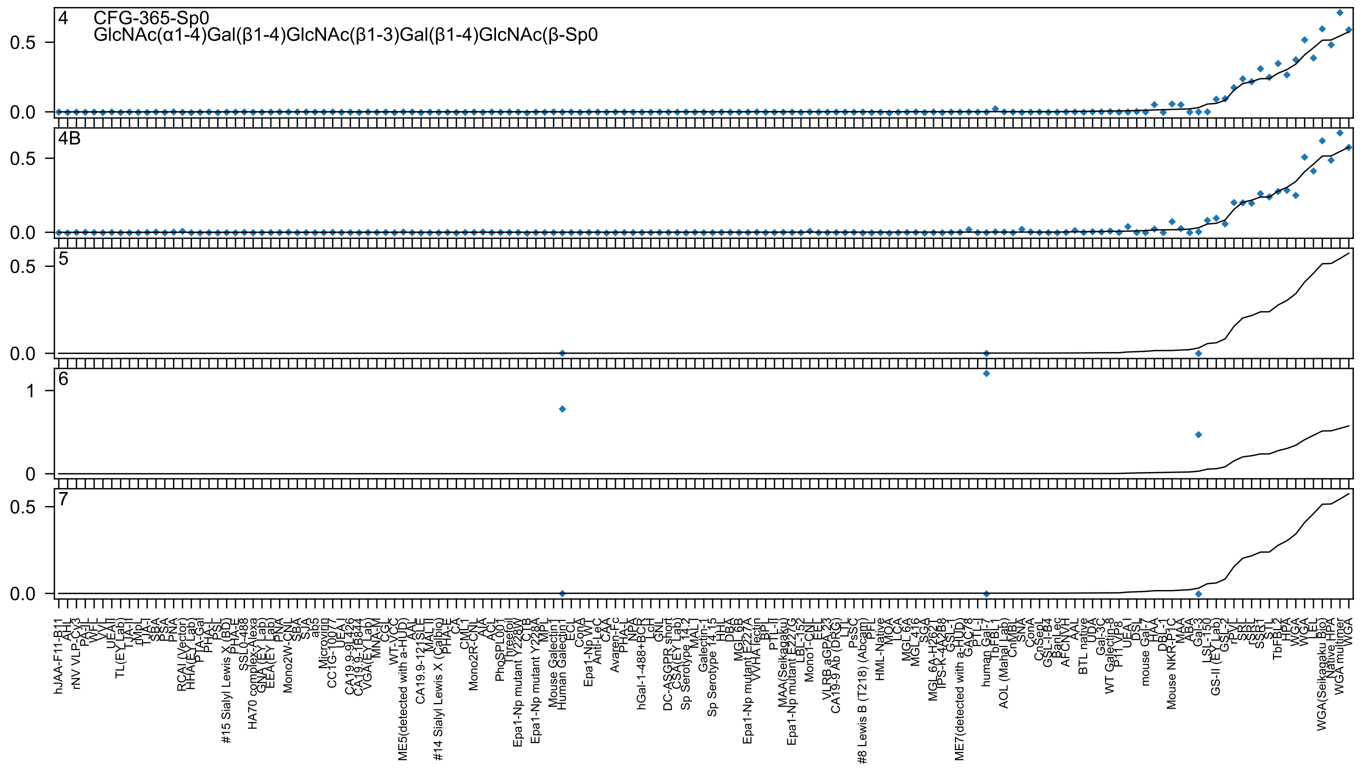

4 CFG-365-Sp0
GlcNAc(α1-4)Gal(β1-4)GlcNAc(β1-3)Gal(β1-4)GlcNAc(β-Sp0
4B
5
6
7

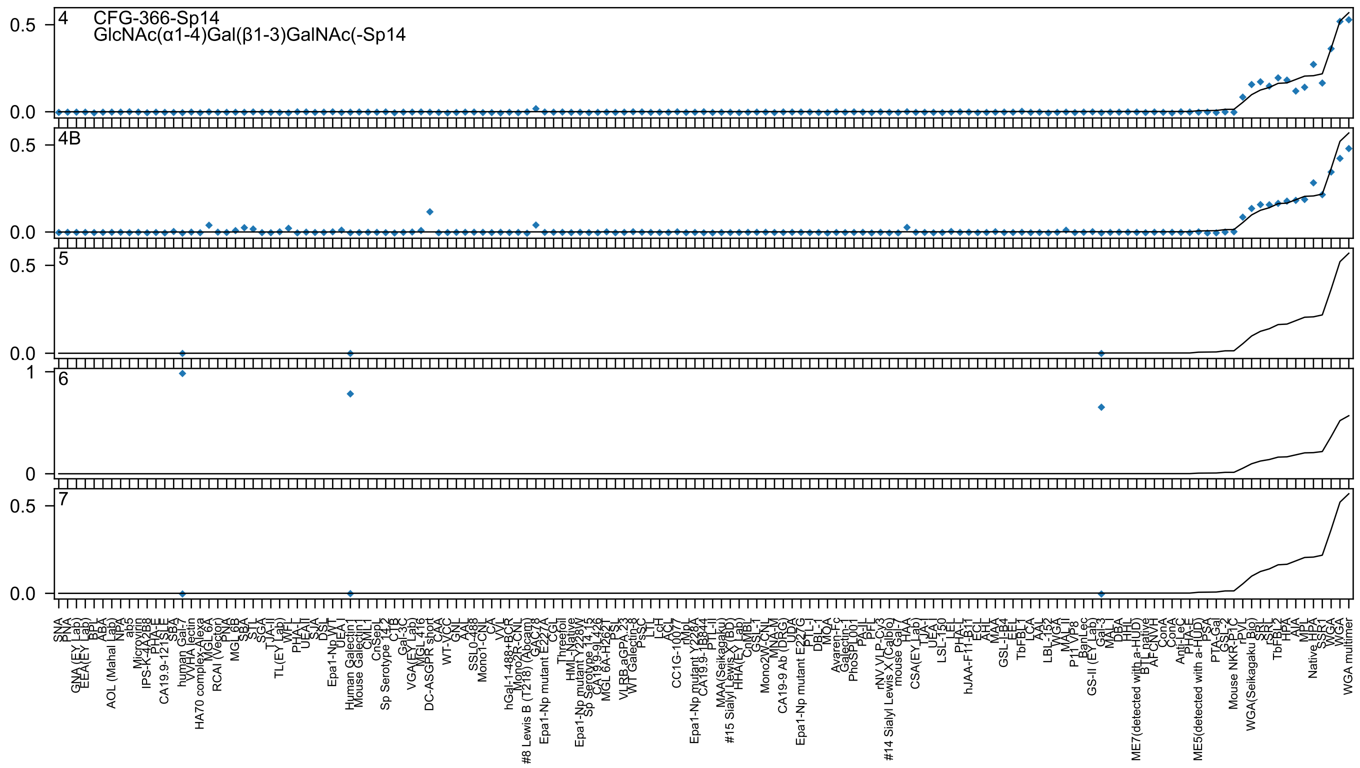
4 CFG-366-Sp14
GlcNAc(α1-4)Gal(β1-3)GalNAc(-Sp14
4B
5
6
7

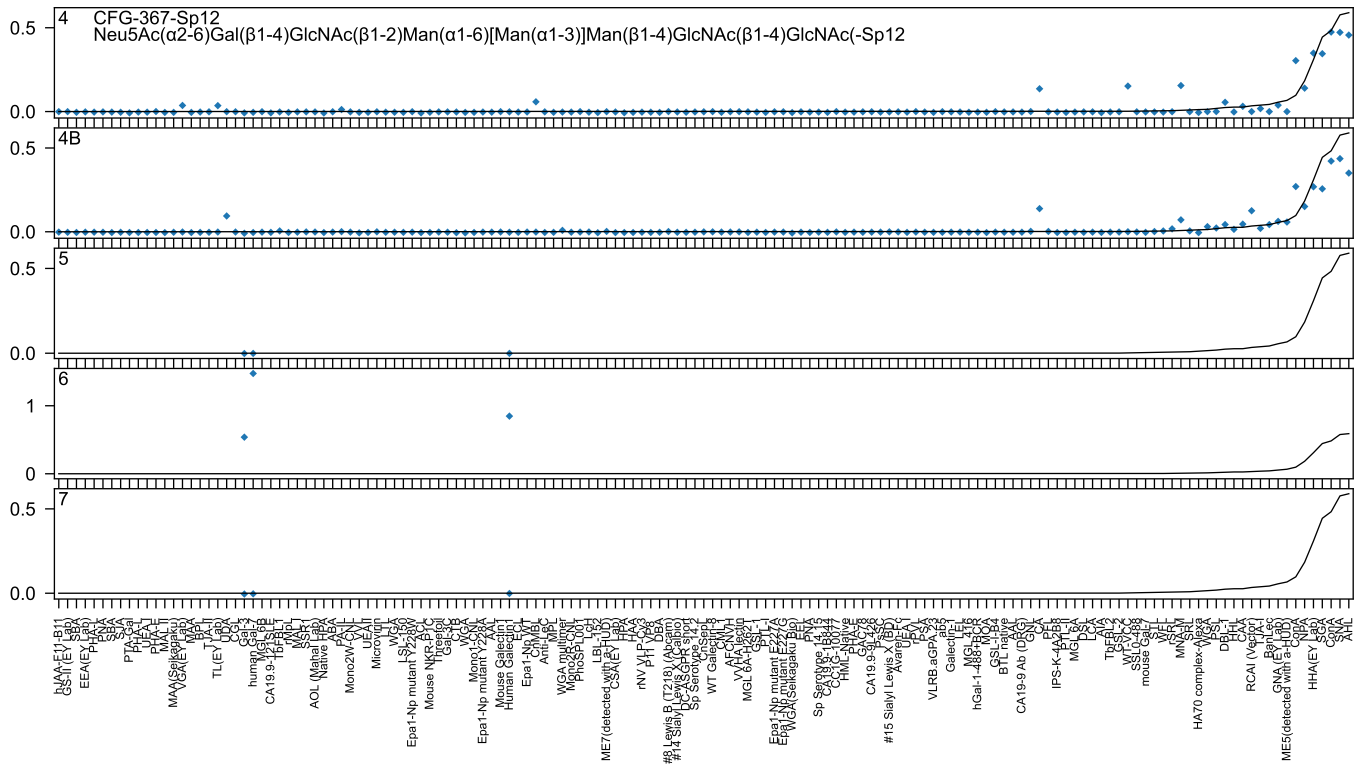

4
CFG-367-Sp12
Neu5Ac(α2-6)Gal(β1-4)GlcNAc(β1-2)Man(α1-6)[Man(α1-3)]Man(β1-4)GlcNAc(β1-4)GlcNAc(-Sp12
4B
5
6
7

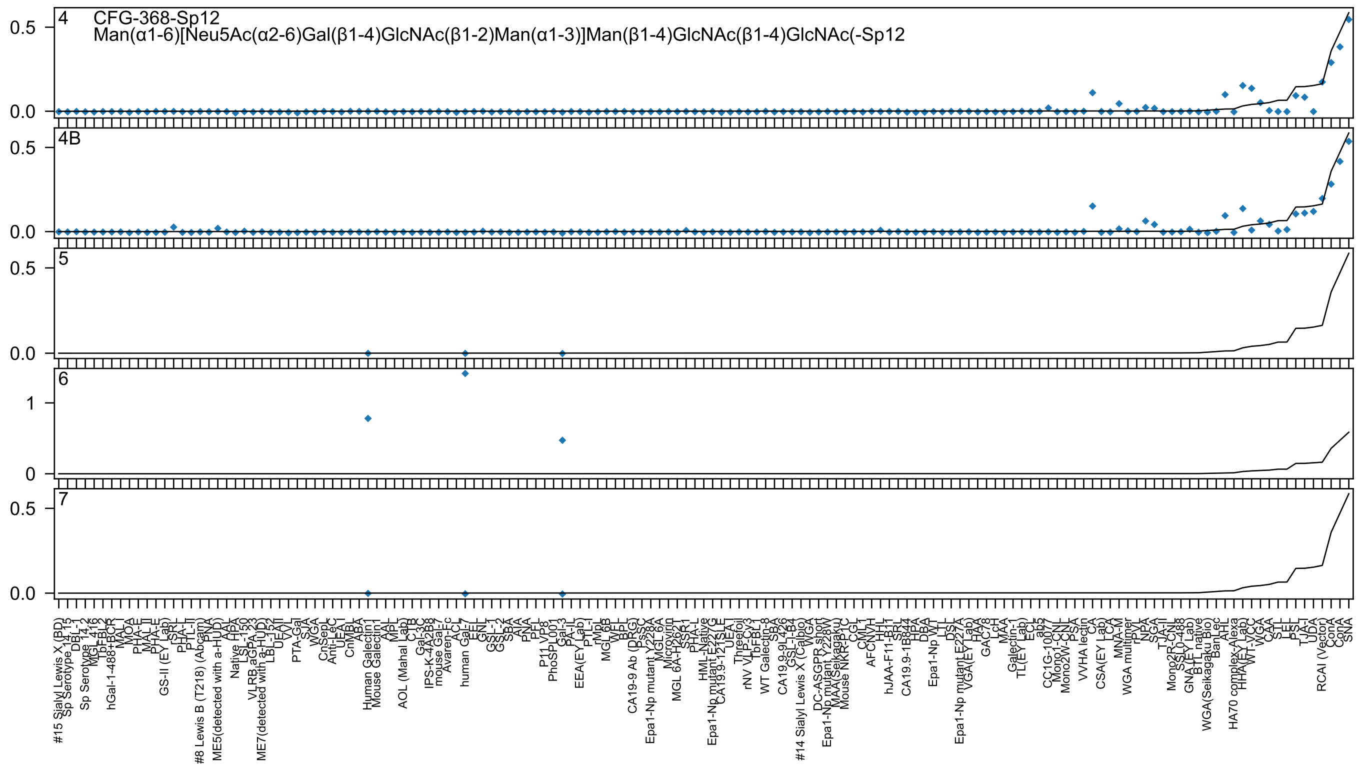

CFG-368-Sp12
Man(α1-6)[Neu5Ac(α2-6)Gal(β1-4)GlcNAc(β1-2)Man(α1-3)]Man(β1-4)GlcNAc(β1-4)GlcNAc(-Sp12
4
4B
5
6
7

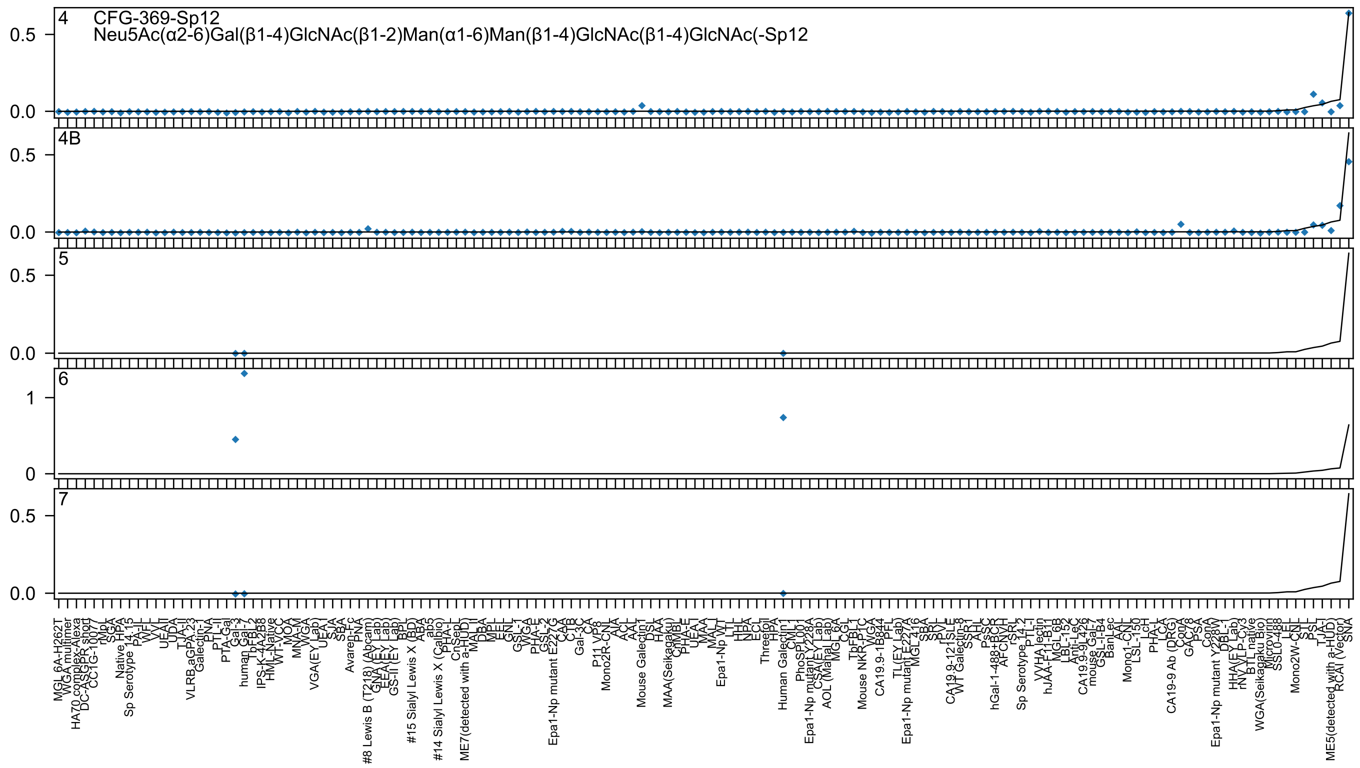
4 CFG-369-Sp12
Neu5Ac(α2-6)Gal(β1-4)GlcNAc(β1-2)Man(α1-6)Man(β1-4)GlcNAc(β1-4)GlcNAc(-Sp12
4B
5
6
7

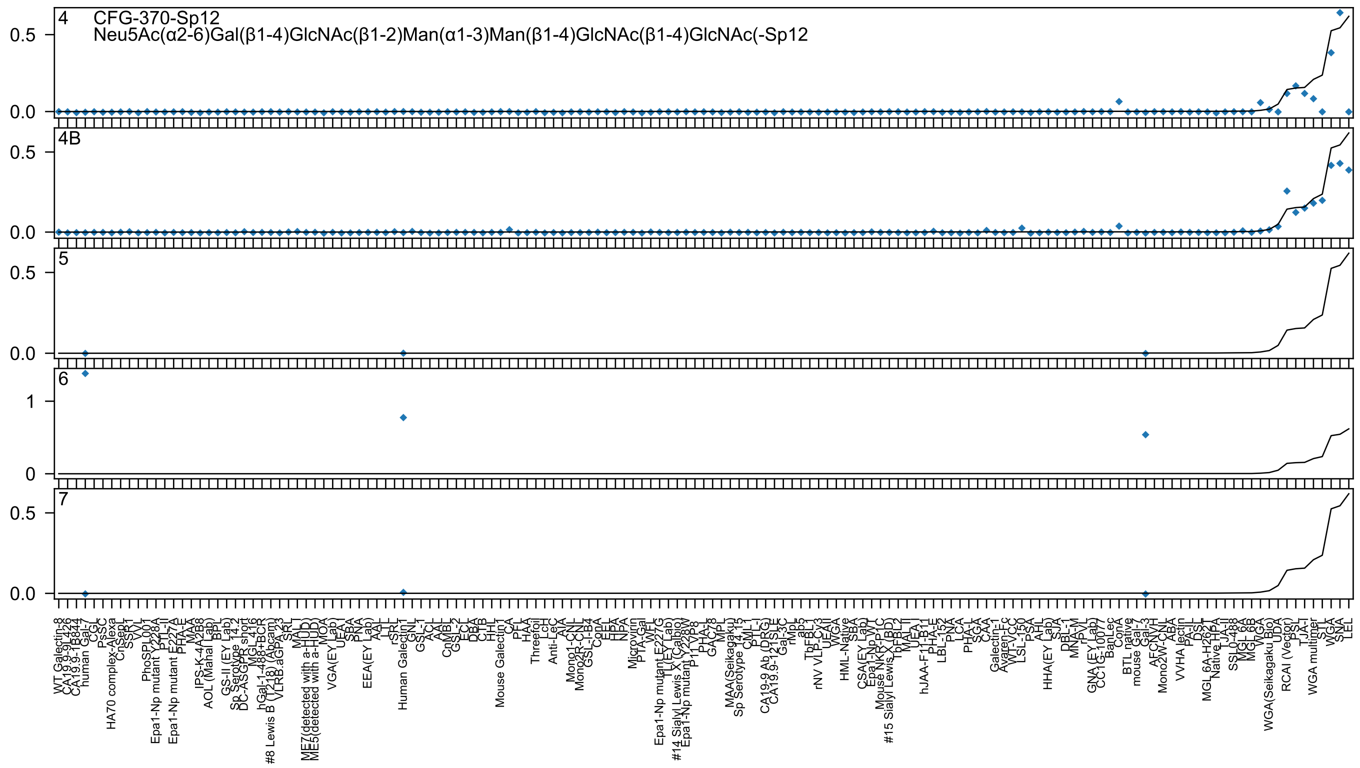
4
CFG-370-Sp12
Neu5Ac(α2-6)Gal(β1-4)GlcNAc(β1-2)Man(α1-3)Man(β1-4)GlcNAc(β1-4)GlcNAc(-Sp12
4B
5
6
7

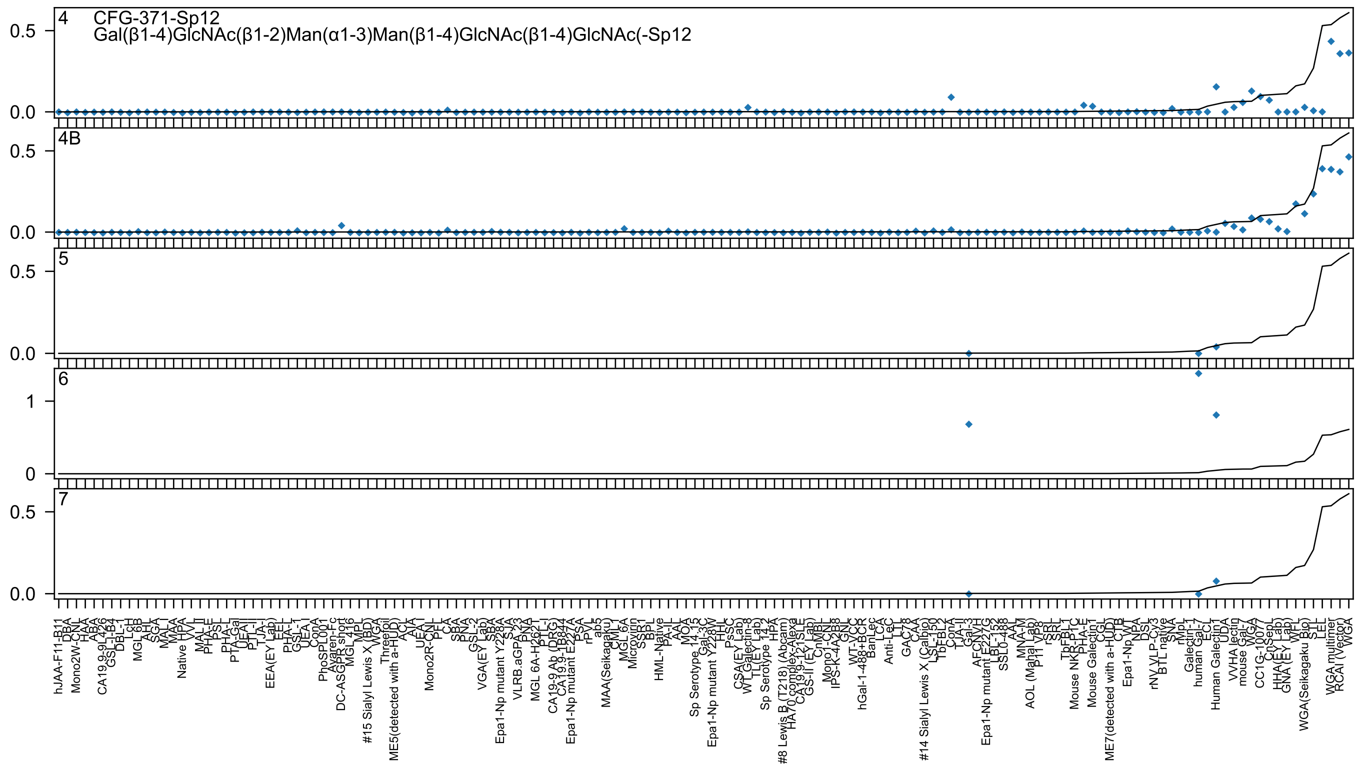

4
CFG-371-Sp12
Gal(β1-4)GlcNAc(β1-2)Man(α1-3)Man(β1-4)GlcNAc(β1-4)GlcNAc(-Sp12
4B
5
6
7
0.0
0.5
0
1
hJAA-F11-B11
DBA
Mono2W-CNL
HAA
ABA
CA19.9-9L426
GSL-I-B4
DBL-1
LcH
MGL 6B
AHL
SGA
MAL I
MAA
Native HPA
VVL
MAL II
PHA-E
PSL
PHA-L
PTA-Gal
UEA II
PTL-II
TJA-I
EEA(EY Lab)
EEL
PHA-L
GSL-1
UEA I
ConA
PhoSPL001
Avaren-Fc
DC-ASGPR short
MGL 416
MPL
#15 Sialyl Lewis X (BD)
WGA
Threefoil
ME5(detected with a-HUD)
ACL
AIA
UEA I
Mono2R-CNL
PFL
CA
SBA
PNA
GSL-2
VGA(EY Lab)
SBA
Epa1-Np mutant Y228A
SJA
VLRB.aGPA.23
PNA
MGL 6A-H262T
PTL-I
CA19-9 Ab (DRG)
CA19.9-1B844
Epa1-Np mutant E227A
PSA
rPVL
ab5
MAA(Seikagaku)
CML1
MGL 6A
Microvirin
SSR1
BPL
HML-Native
PA-IL
AAL
MOA
Sp Serotype 14.15
Gal-3C
Epa1-Np mutant Y228W
HHL
PsSC
CSA(EY Lab)
WT Galectin-8
TL(EY Lab)
Sp Serotype 14.2
HPA
#8 Lewis B (T218) (Abcam)
HA70 complex-Alexa
CA19.9-121SLE
GS-II (EY Lab)
CnMBL
Mono1-CNL
IPS-K-4A2B8
GNL
WT-VCC
hGal-1-488+BCR
BanLec
LCA
Anti-LeC
LTL
GAC78
CAA
#14 Sialyl Lewis X (Calbio)
LSL-150
TbFBL2
ConA
TJA-II
Gal-3
AFCNVH
Epa1-Np mutant E227G
LBL-152
SSL0-488
AAL
MNA-M
AOL (Mahal Lab)
P11 VP8
rSRL
TbFBL1
Mouse NKR-P1C
PHA-E
Mouse Galectin1
CGL
ME7(detected with a-HUD)
CTB
Epa1-Np WT
NPA
DSL
rNV VLP-Cy3
BTL native
SNA
rMpL
Galectin-1
human Gal-7
ECL
Human Galectin1
UDA
VVHA lectin
mouse Gal-7
WGA
CC1G-1007
CnSep
HHA(EY Lab)
GNA (EY Lab)
WFL
WGA(Seikagaku Bio)
STL
LEL
WGA multimer
RCAI (Vector)
WGA

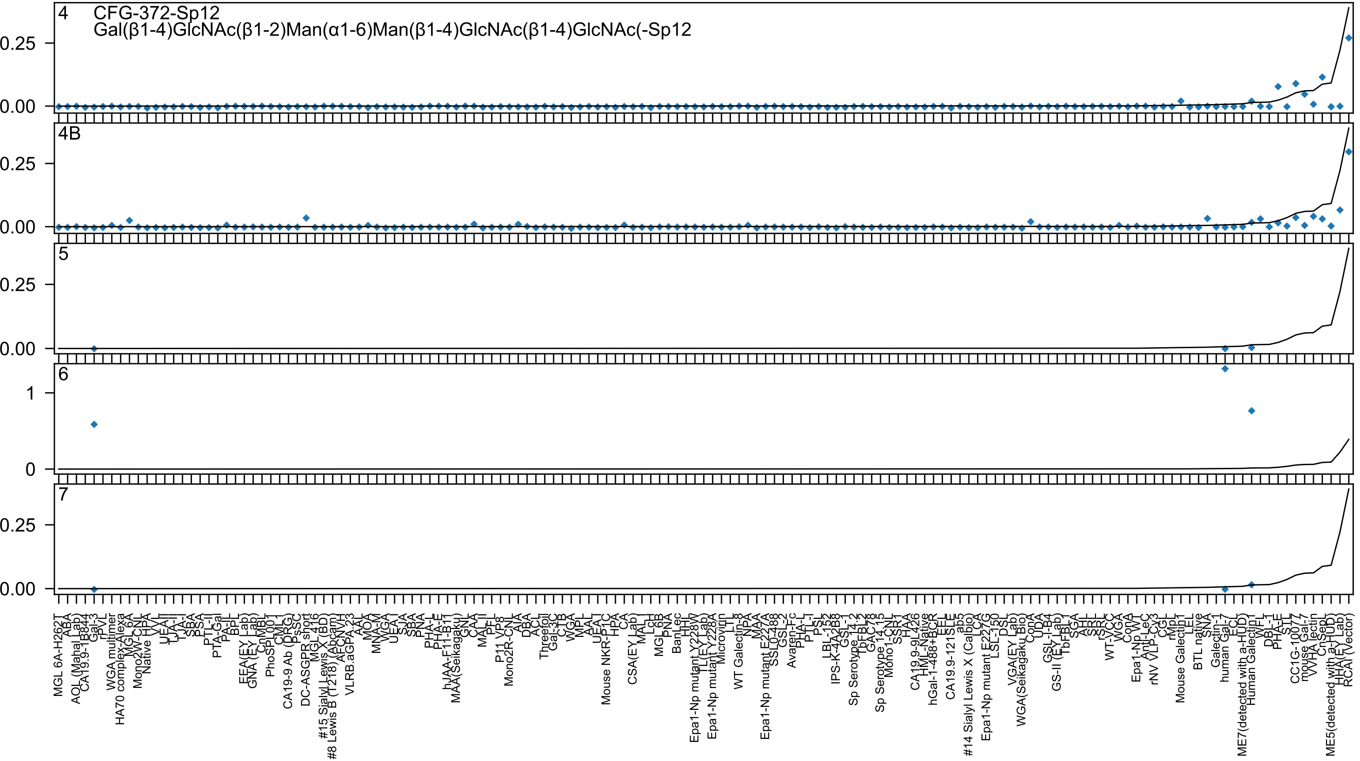

4
CFG-372-Sp12
Gal(β1-4)GlcNAc(β1-2)Man(α1-6)Man(β1-4)GlcNAc(β1-4)GlcNAc(-Sp12
4B
5
6
7
0.25
0.00
1
0

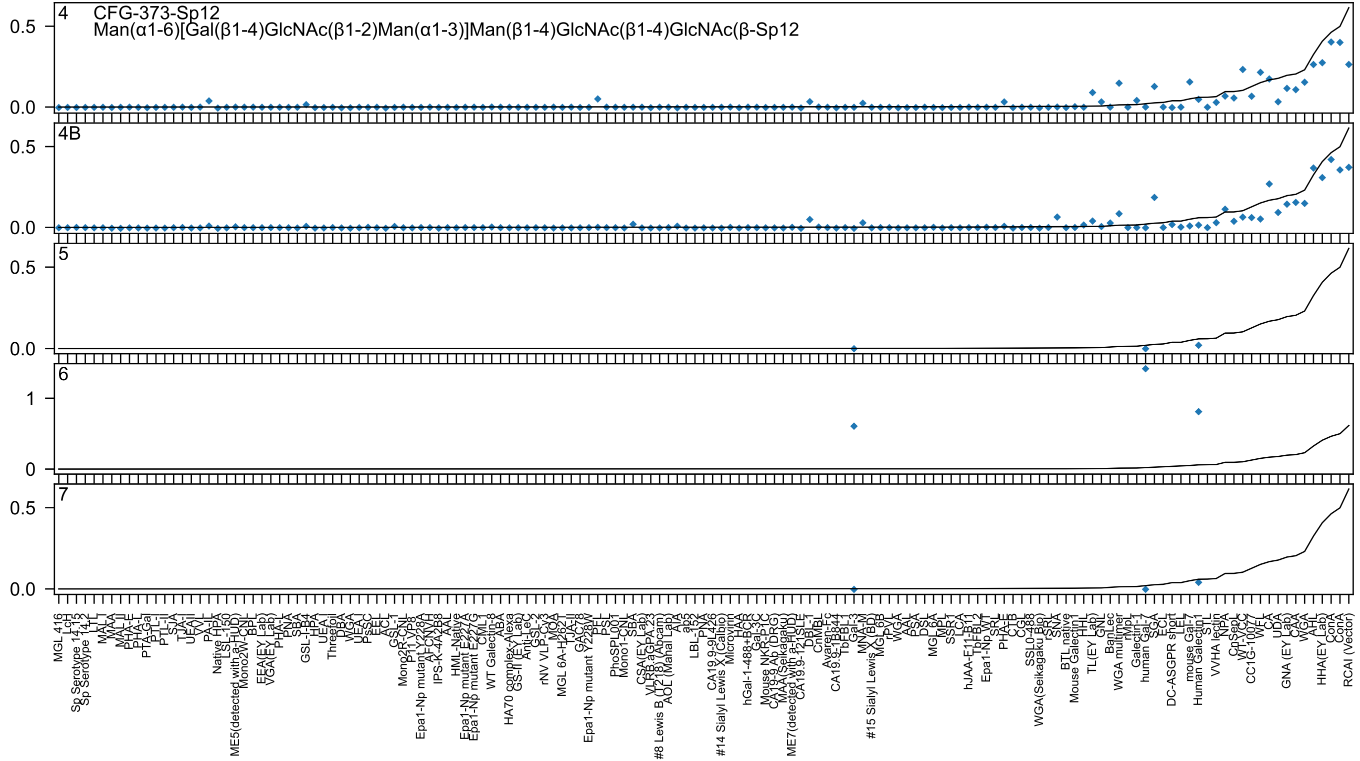
4
CFG-373-Sp12
Man(α1-6)[Gal(β1-4)GlcNAc(β1-2)Man(α1-3)]Man(β1-4)GlcNAc(β1-4)GlcNAc(β-Sp12
4B
5
6
7

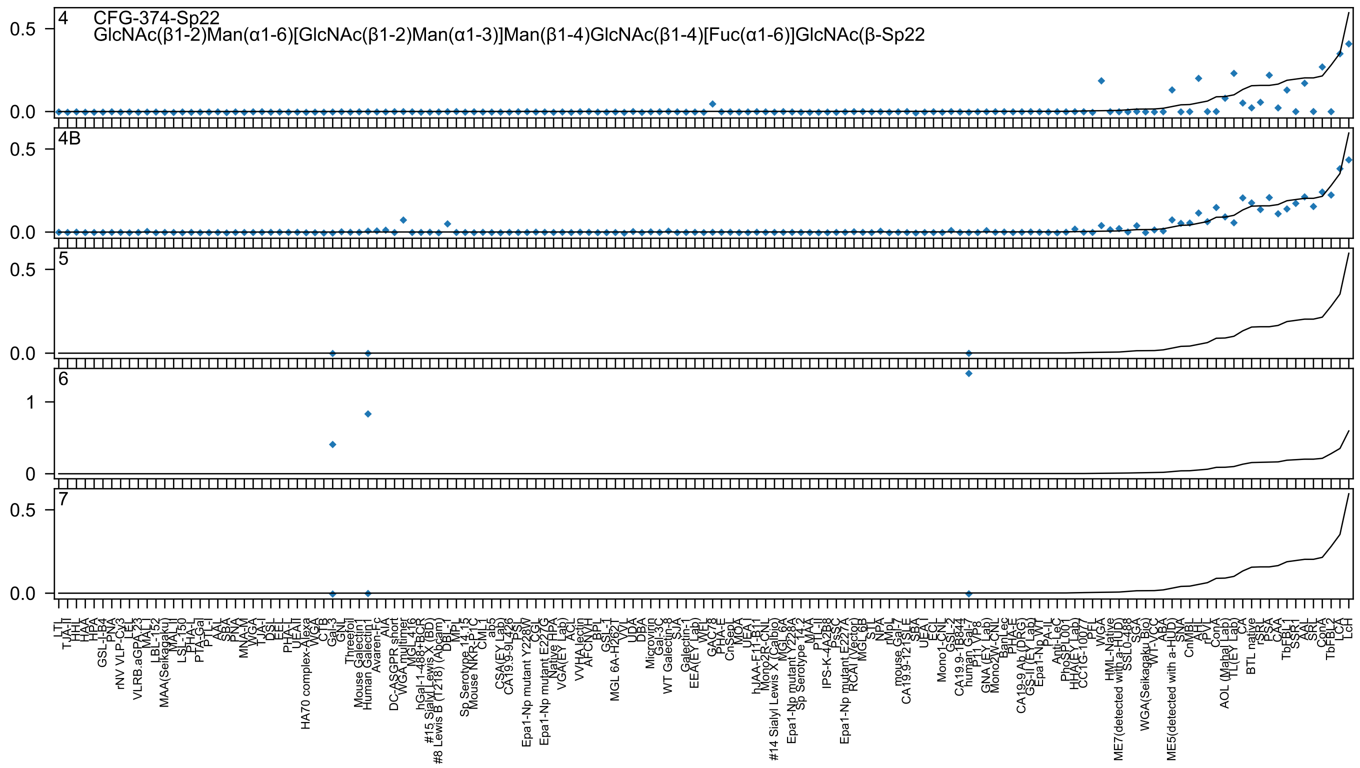
4 CFG-374-Sp22
GlcNAc(β1-2)Man(α1-6)[GlcNAc(β1-2)Man(α1-3)]Man(β1-4)GlcNAc(β1-4)[Fuc(α1-6)]GlcNAc(β-Sp22
4B
5
6
7

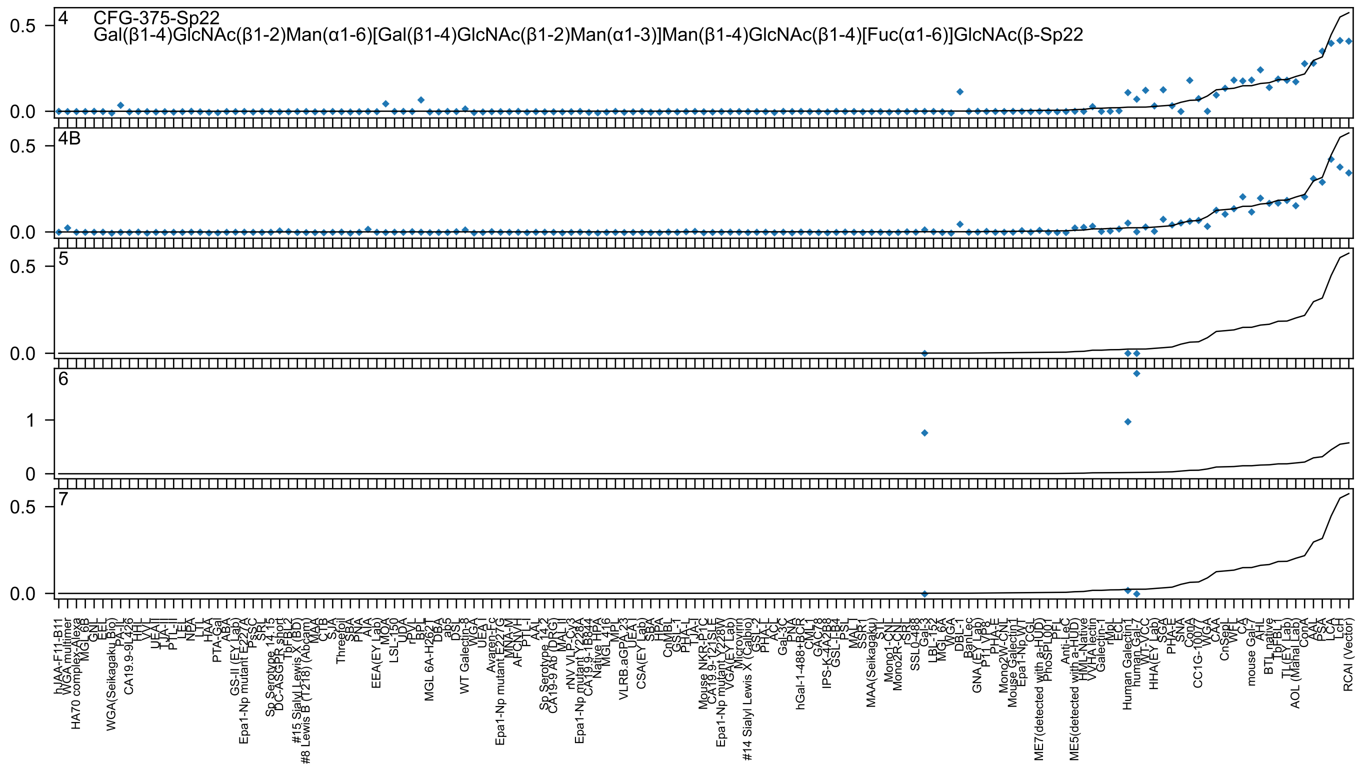

4 CFG-375-Sp22
Gal(β1-4)GlcNAc(β1-2)Man(α1-6)[Gal(β1-4)GlcNAc(β1-2)Man(α1-3)]Man(β1-4)GlcNAc(β1-4)[Fuc(α1-6)]GlcNAc(β-Sp22
4B
5
6
7

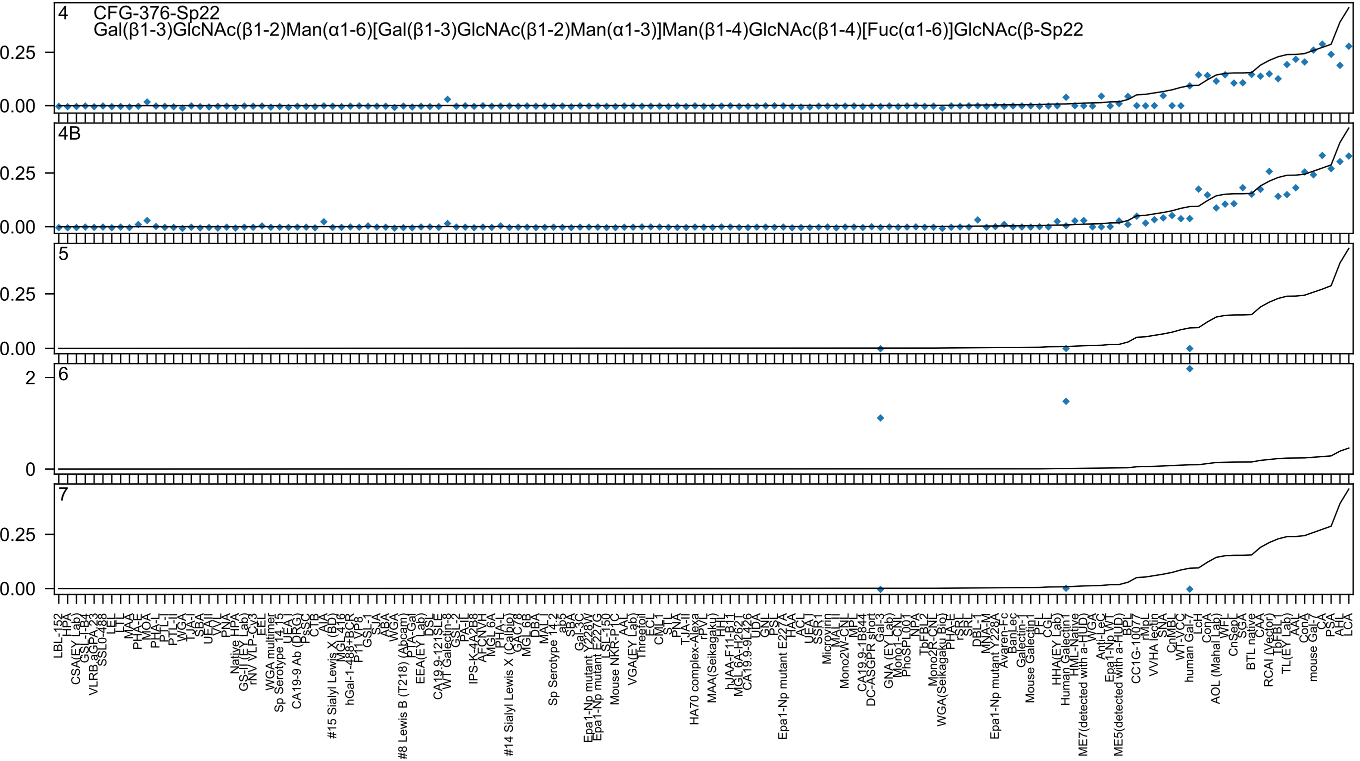

4 CFG-376-Sp22
Gal(β1-3)GlcNAc(β1-2)Man(α1-6)[Gal(β1-3)GlcNAc(β1-2)Man(α1-3)]Man(β1-4)GlcNAc(β1-4)[Fuc(α1-6)]GlcNAc(β-Sp22
4B
5
6
7
0.25
0.00
2
0

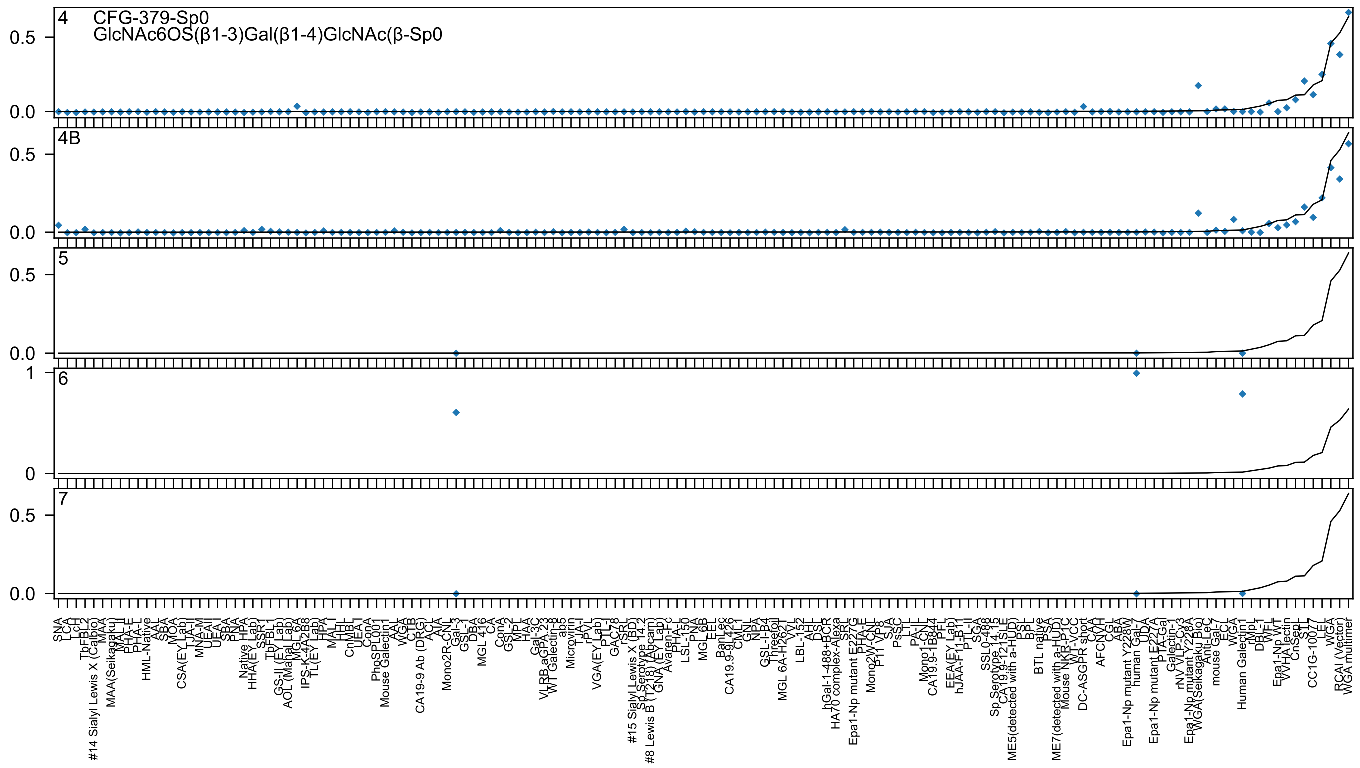

4
CFG-379-Sp0
GlcNAc6OS(β1-3)Gal(β1-4)GlcNAc(β-Sp0
4B
5
6
7
0.0
0.5
0
1
SNA
LCA
LcH
TbFBL2
#14 Sialyl Lewis X (Calbio)
MAA
MAA(Seikagaku)
MAL II
PHA-E
PHA-L
HML-Native
AAL
SBA
MOA
CSA(EY Lab)
TJA-II
MNA-M
UEAII
UEA I
SBA
PNA
Native HPA
HHA(EY Lab)
SSR1
TbFBL1
GS-II (EY Lab)
AOL (Manal Lab)
MGL 6A
IPS-K-4A2B8
TL(EY Lab)
HPA
MAL I
HHL
CnMBL
UEA I
ConA
PhoSL001
Mouse Galectin 1
AAL
WGA
CTB
CA19-9 Ab (DRG)
ACL
AIA
Mono2R-CNL
Gal-3
GSL-I
DBA
MGL 416
CA
ConA
GSL-2
MPL
HAA
Gal-3C
VLRB.aGPA.23
WT Galectin-8
ab5
Microvirin
TJA-I
rPVL
VGA(EY Lab)
PTL-I
GAC78
rSRL
#15 Sialyl Lewis X (BD)
Sp Serotype 14.2
#8 Lewis B (T218) (Abcam)
GNA (EY Lab)
Avaren-Fc
PHA-L
LSL-150
PNA
MGL 6B
EEL
BanLec
CA19.9-9L426
CML
GNL
NPA
GSL-I-B4
Threefoil
MGL 6A-H262T
VVL
LBL-152
AHL
DSL
hGal-1-488+BCR
HA70 complex-Alexa
SRL
Epa1-Np mutant E227G
PHA-E
Mono2W-CNL
P11 VP8
SJA
PsSC
LTL
PA-IL
Mono1-CNL
CA19.9-1B844
PFL
EEA(EY Lab)
hJAA-F11-B11
PTL-II
SGA
SSL0-488
Sp Serotype 14.15
CA19.9-121SLE
ME5(detected with a-HuD)
BSL
BPL
BTL native
PSA
ME7(detected with a-HuD)
Mouse NKR-P1C
WT-VCC
DC-ASGPR short
CAA
AFCNYH
CGL
ABA
Epa1-Np mutant Y228W
human Gal-7
UDA
Epa1-Np mutant E227A
PTA-Gal
Galectin-1
rNV VLP-Cy3
Epa1-Np mutant Y228A
WGA(Seikagaku Bio)
Anti-LeC
mouse Gal-7
ECL
WGA
Human Galectin1
rMpL
DBL-1
WFL
Epa1-Np WT
VVHA lectin
CnSepL
STL
CC1G-10077
LEL
WGA
RCAI (Vector)
WGA multimer

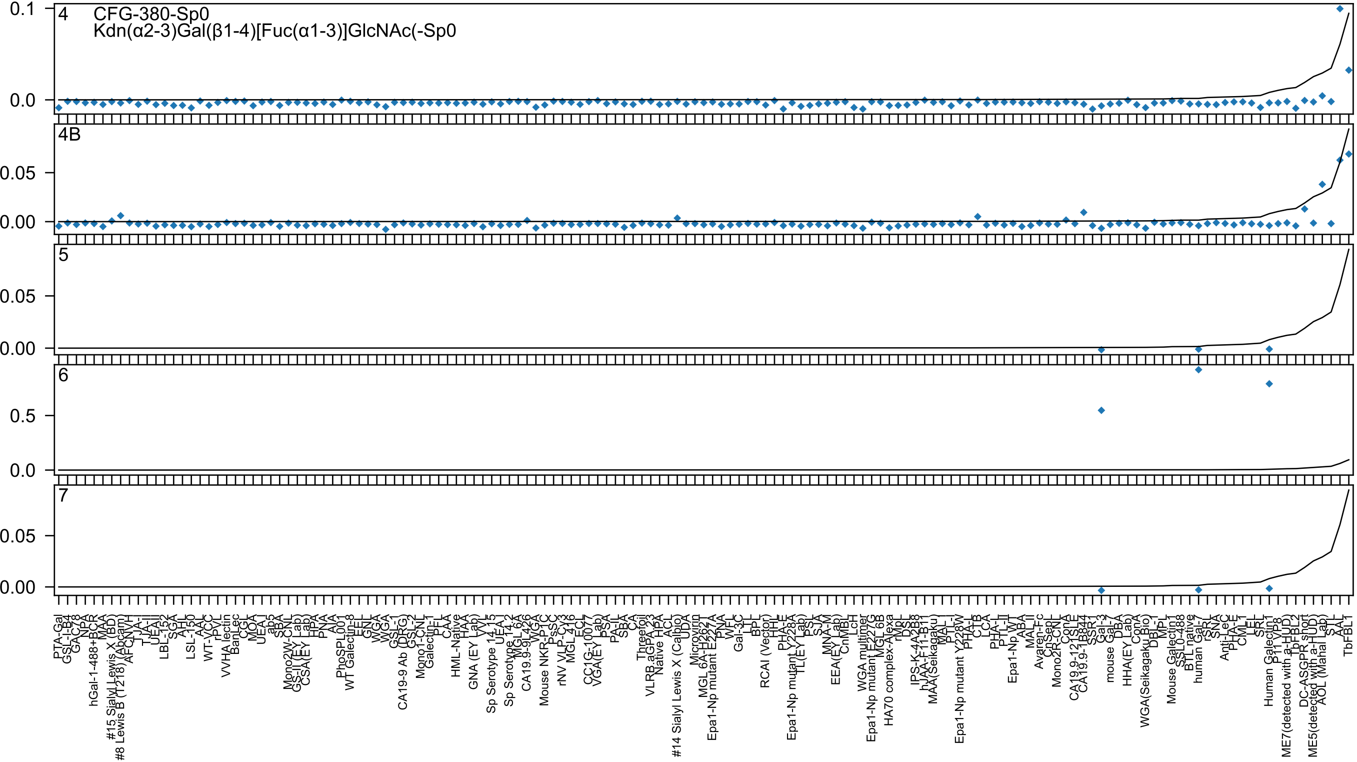

CFG-380-Sp0
Kdn(α2-3)Gal(β1-4)[Fuc(α1-3)]GlcNAc(-Sp0

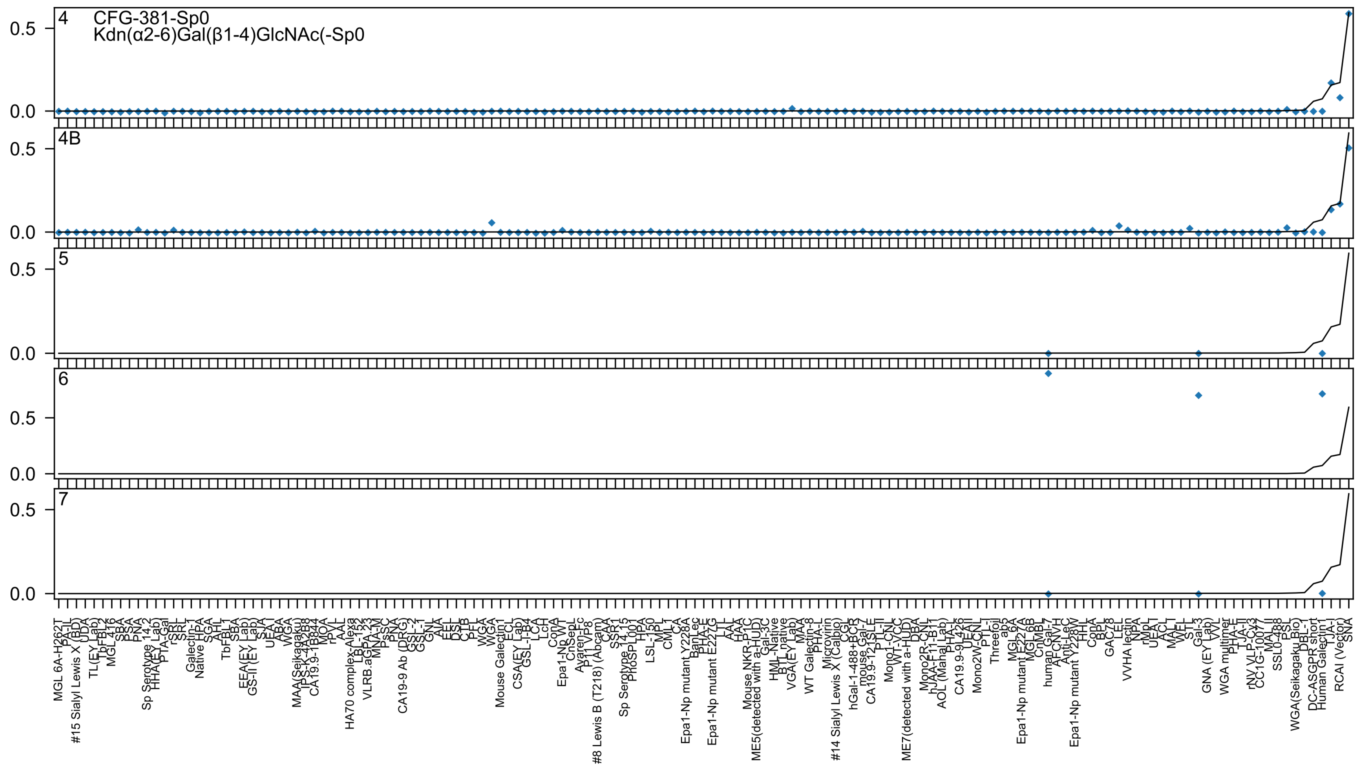

4
CFG-381-Sp0
Kdn(α2-6)Gal(β1-4)GlcNAc(-Sp0
4B
5
6
7

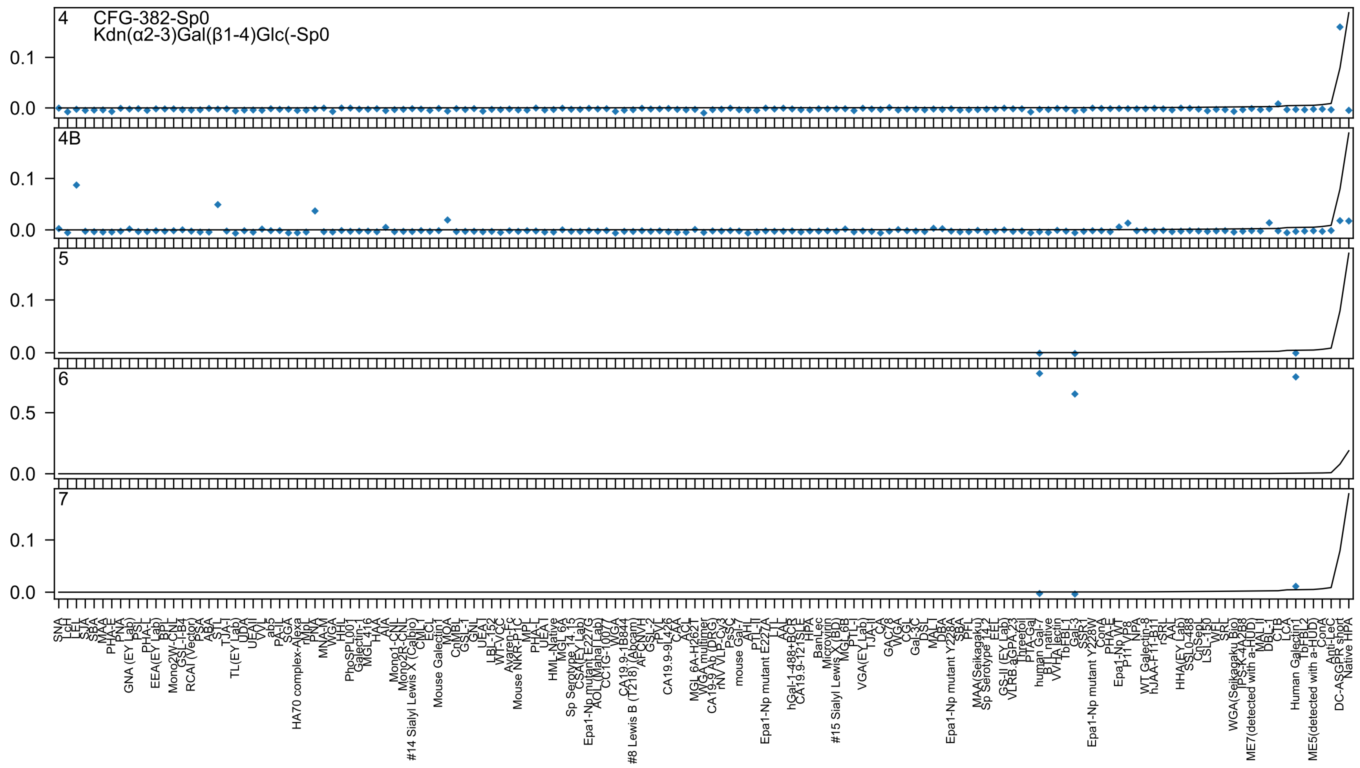

4 CFG-382-Sp0
Kdn(α2-3)Gal(β1-4)Glc(-Sp0
4B
5
6
7

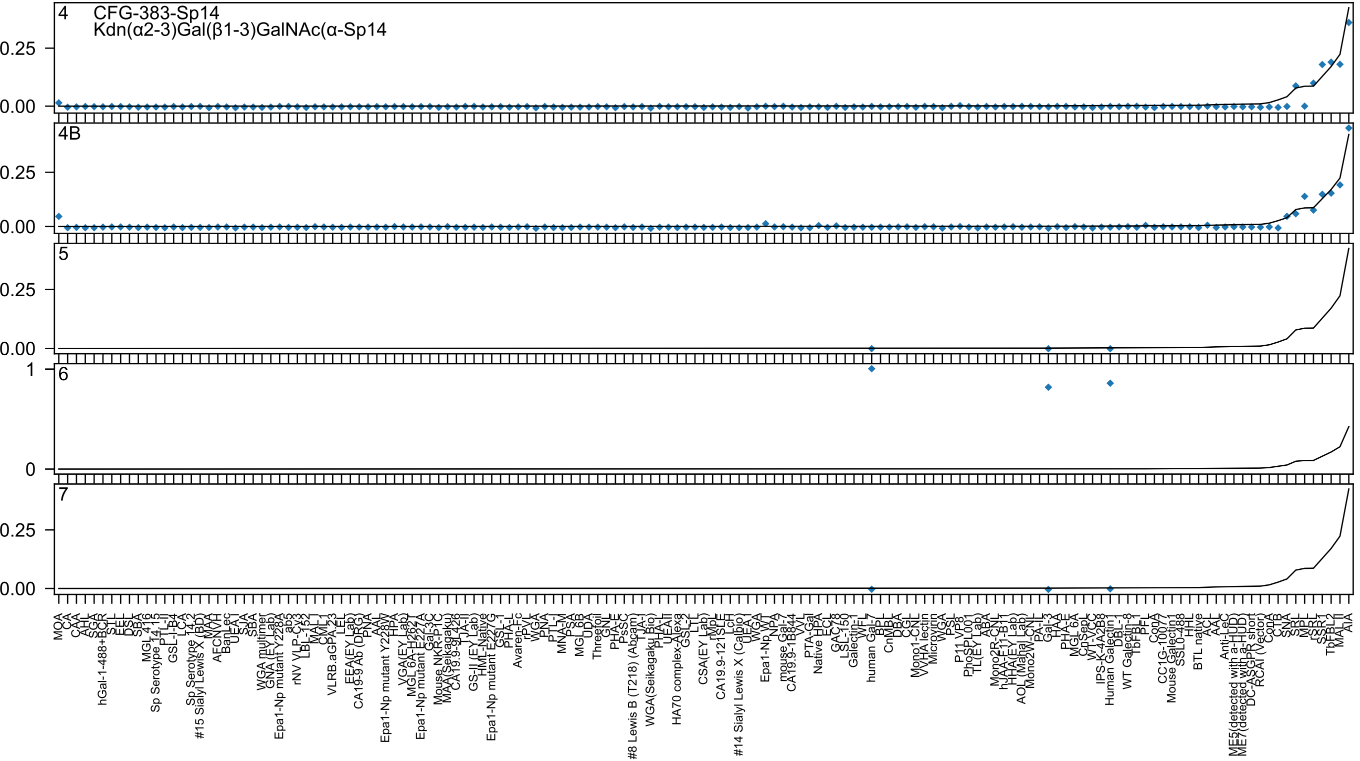

4
CFG-383-Sp14
Kdn(α2-3)Gal(β1-3)GalNAc(α-Sp14
4B
5
6
7
0.25
0.00
1
0

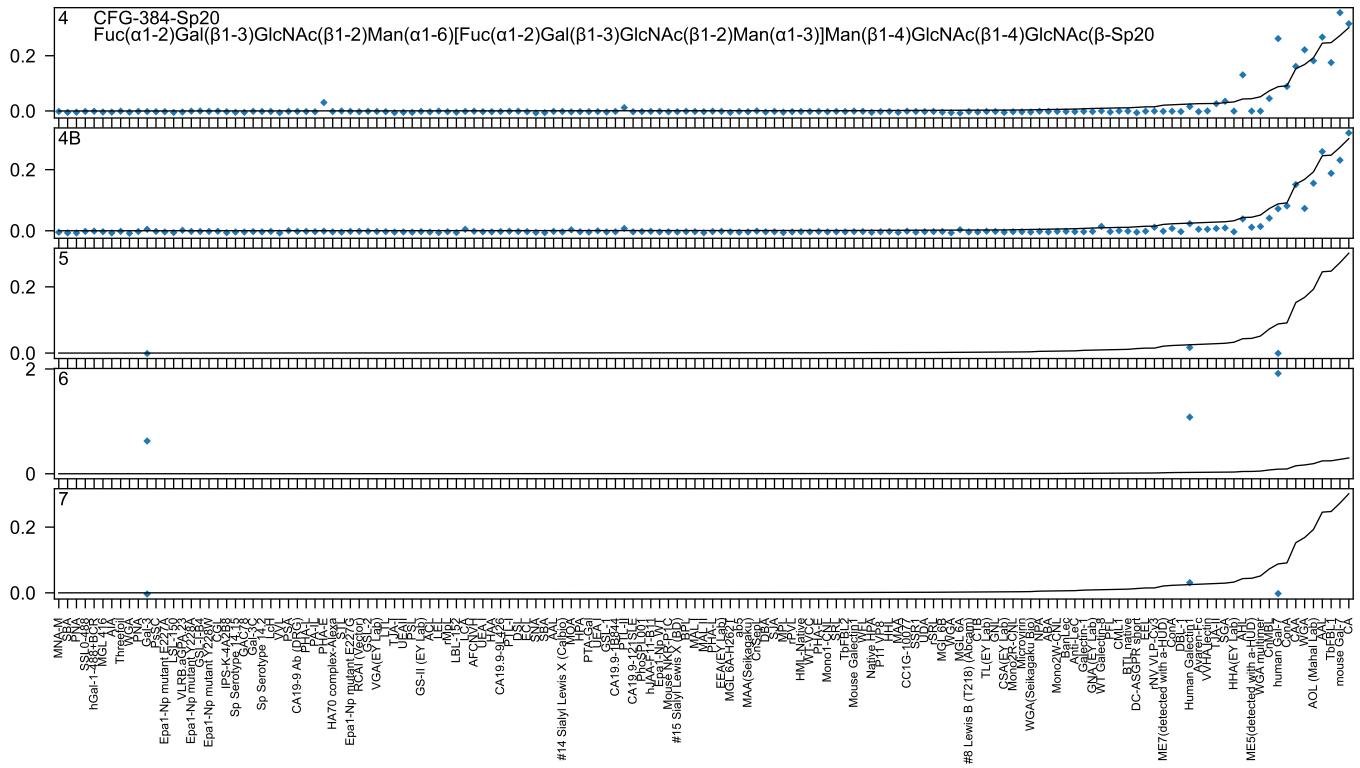

4 CFG-384-Sp20
Fuc(α1-2)Gal(β1-3)GlcNAc(β1-2)Man(α1-6)[Fuc(α1-2)Gal(β1-3)GlcNAc(β1-2)Man(α1-3)]Man(β1-4)GlcNAc(β1-4)GlcNAc(β-Sp20
4B
5
6
7
0.2
0.0
2
0

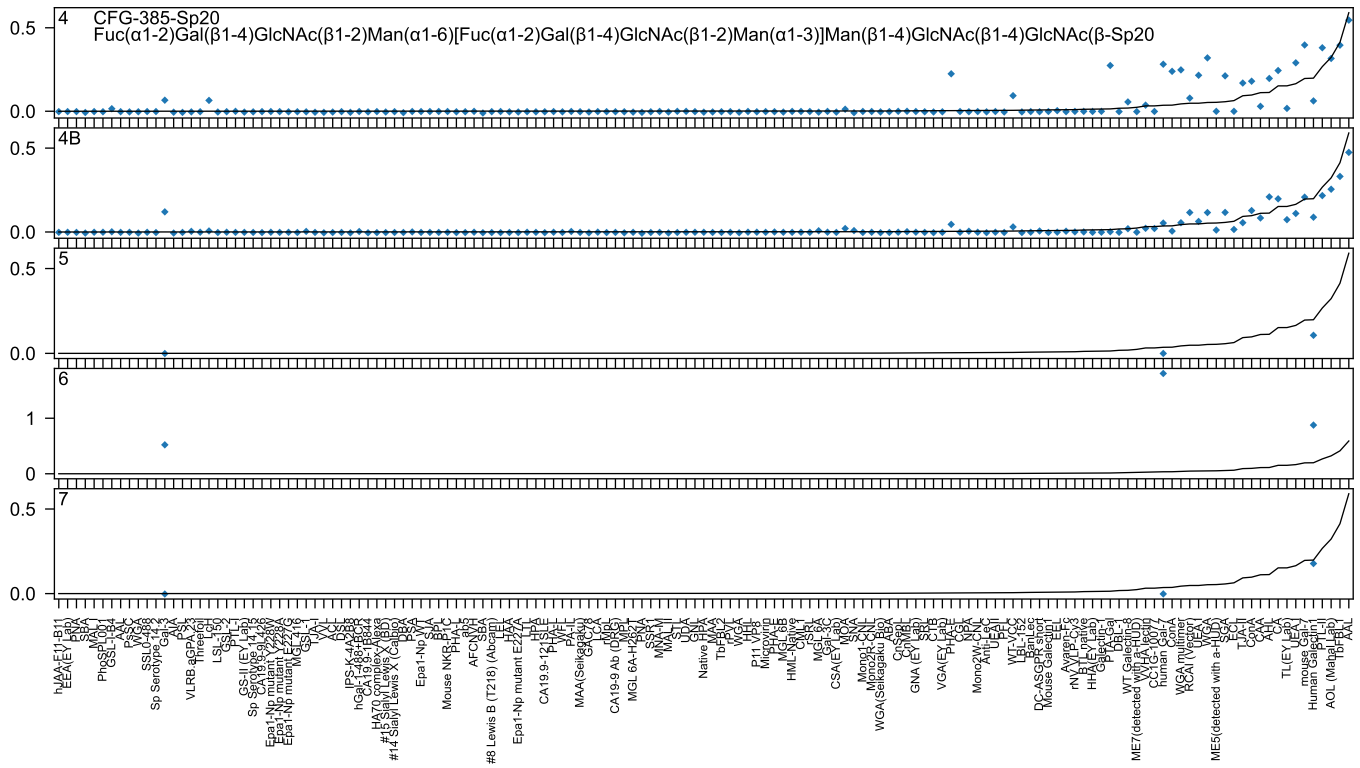

4
CFG-385-Sp20
Fuc(α1-2)Gal(β1-4)GlcNAc(β1-2)Man(α1-6)[Fuc(α1-2)Gal(β1-4)GlcNAc(β1-2)Man(α1-3)]Man(β1-4)GlcNAc(β1-4)GlcNAc(β-Sp20
4B
5
6
7
0.0
0.5
0
1
hJAA-F11-B11
EEA(EY Lab)
PNA
SBA
MAL
PhoSL001
GSL-I-B4
AAL
PsSC
WGA
SSL0-488
Sp Serotype 14-2
Gal-3
AIA
PSL
VLRB.aGPA.23
Threefoil
LcH
LSL-150
GSL-2
PTL-I
GS-II (EY Lab)
Sp Serotype 14-15
CA19.9-9L426
Epa1-Np mutant Y228W
Epa1-Np mutant Y228A
Epa1-Np mutant E227G
MGL416
GSL-1
TJA-I
VVL
ACL
DSL
IPS-K-4A2B8
hGal-1-488+BCR
CA19.9-1B844
HA70 complex-Alexa
#15 Sialyl Lewis X (BD)
#14 Sialyl Lewis X (Calbio)
DBA
PSA
Epa1-Np WT
SJA
BPL
Mouse NKR-P1C
PHA-L
ab5
AFCN-H
SBA
#8 Lewis B (T218) (Abcam)
LEL
HAA
Epa1-Np mutant E227A
LTL
HPA
CA19.9-121SLE
PHA-L
WFL
PA-IL
MAA(Seikagaku)
GAC78
LCA
rMPL
CA19-9 Ab (DRG)
MPL
MGL 6A-H262T
PNA
SSR1
MNA-M
MAL II
STL
UDA
GNL
HPA
Native HPA
MAA
TbFBL2
rPVL
WGA
HHL
P11 VP8
Microvirin
PHA-E
MGL 6B
HML-Native
CVL1
rSRL
MGL 6A
Gal-3C
CSA(EY Lab)
MOA
SNA
Mono1-CNL
Mono2R-CNL
WGA(Seikagaku Bio)
ABA
CnSepL
CnMBL
GNA (EY Lab)
SRL
CTB
VGA(EY Lab)
PHA-E
CGL
NPA
Mono2W-CNL
Anti-LeC
UEAII
PFL
WT-VCC
LBL-152
BanLec
DC-ASGPR short
Mouse Galectin1
EEL
Avaren-Fc
rNV VLP-Cy3
BTL native
HHA(EY Lab)
Galectin-1
PTA-Gal
DBL-1
WT Galectin-8
ME7(detected with a-HUD)
VVHA lectin
CC1G-10077
human Gal-7
ConA
WGA multimer
RCAI (Vector)
UEA I
WGA
ME5(detected with a-HUD)
SGA
ECL
TJA-II
ConA
CAA
AHL
CA
TL(EY Lab)
UEA-I
mouse Gal-7
Human Galectin1
PTL-II
AOL (Mahal Lab)
TbFBL1
AAL

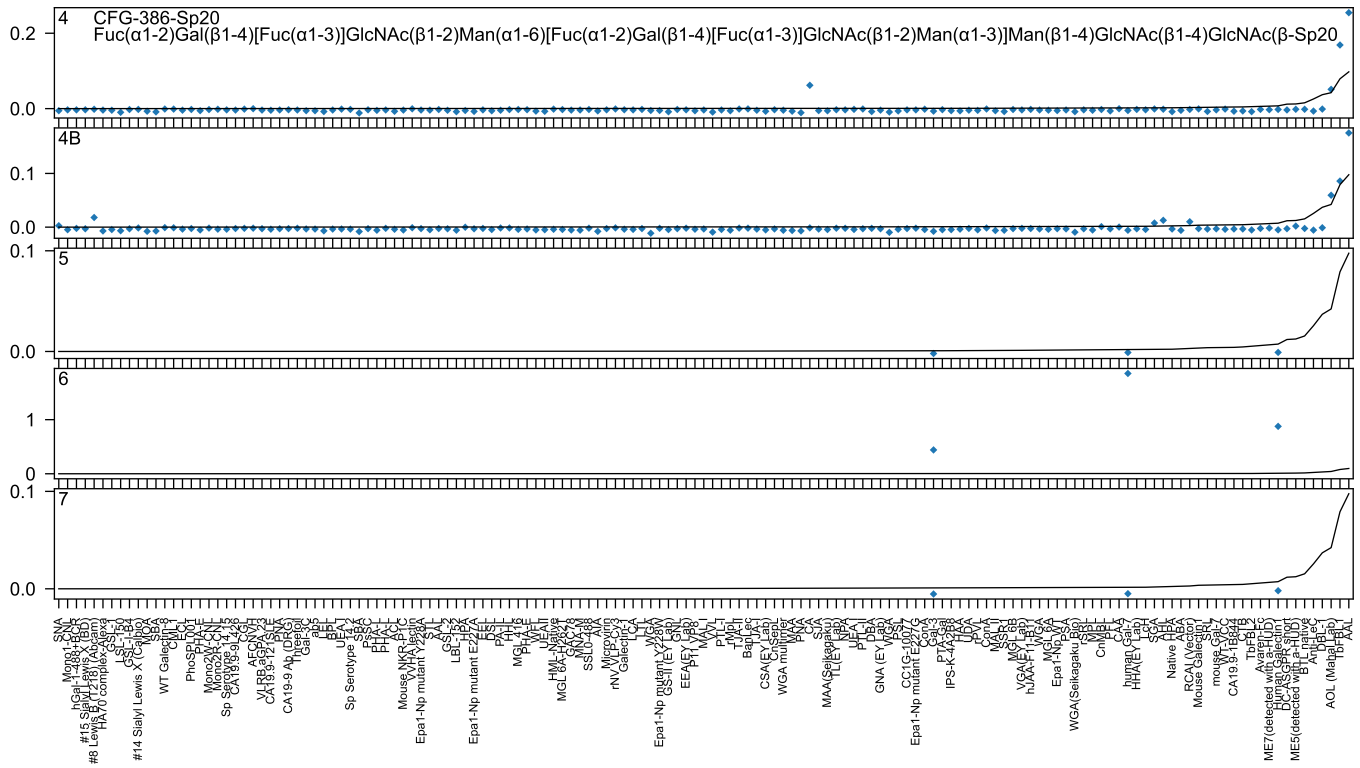

4
CFG-386-Sp20
Fuc(α1-2)Gal(β1-4)[Fuc(α1-3)]GlcNAc(β1-2)Man(α1-6)[Fuc(α1-2)Gal(β1-4)[Fuc(α1-3)]GlcNAc(β1-2)Man(α1-3)]Man(β1-4)GlcNAc(β1-4)GlcNAc(β-Sp20
4B
5
6
7

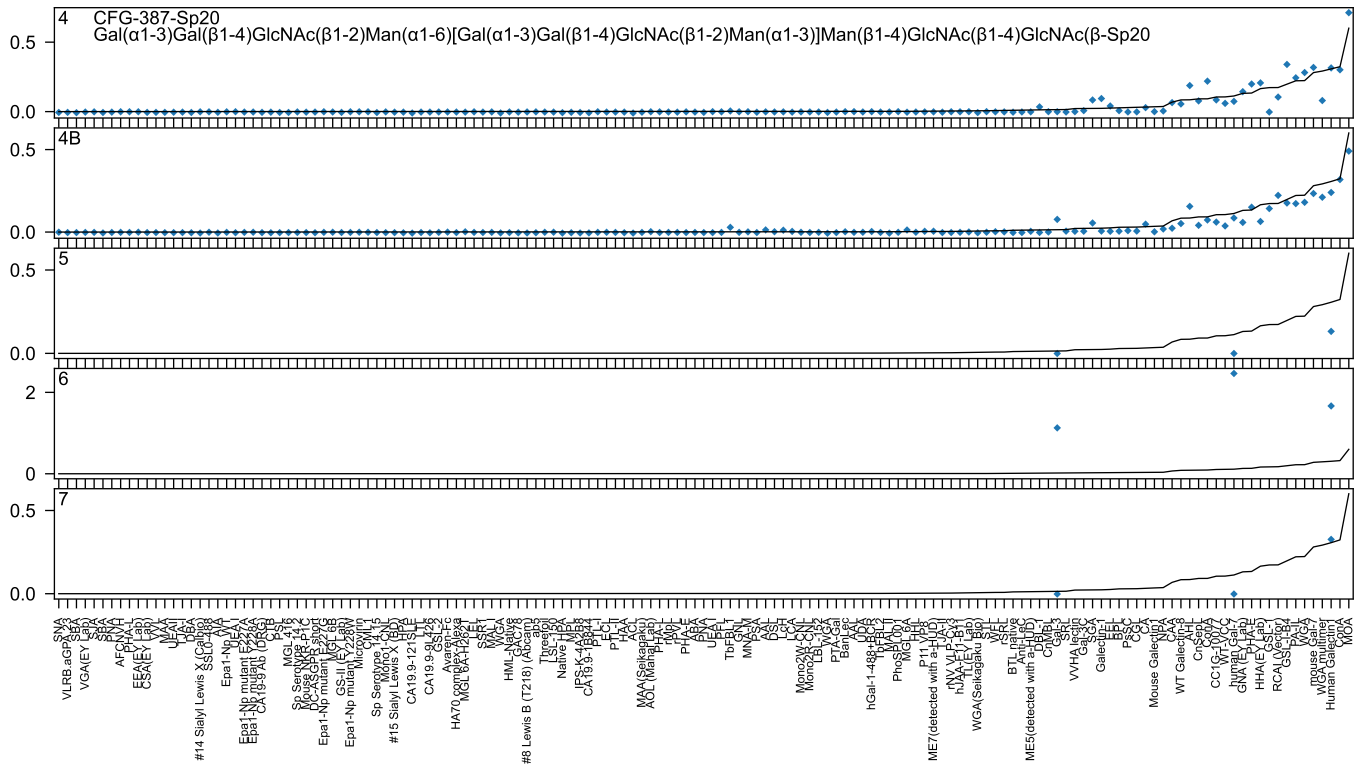
4 CFG-387-Sp20
Gal(α1-3)Gal(β1-4)GlcNAc(β1-2)Man(α1-6)[Gal(α1-3)Gal(β1-4)GlcNAc(β1-2)Man(α1-3)]Man(β1-4)GlcNAc(β1-4)GlcNAc(β-Sp20
4B
5
6
7

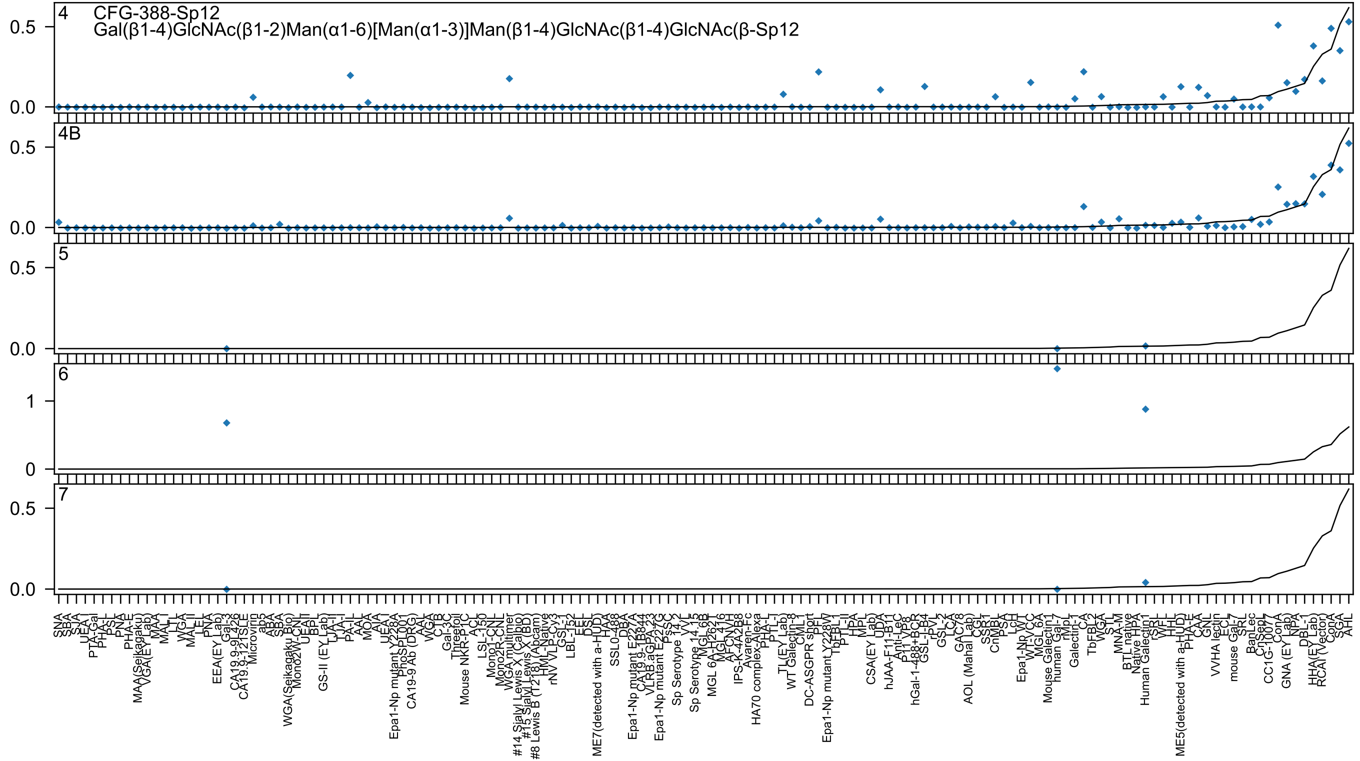

4
CFG-388-Sp12
Gal(β1-4)GlcNAc(β1-2)Man(α1-6)[Man(α1-3)]Man(β1-4)GlcNAc(β1-4)GlcNAc(β-Sp12
4B
5
6
7

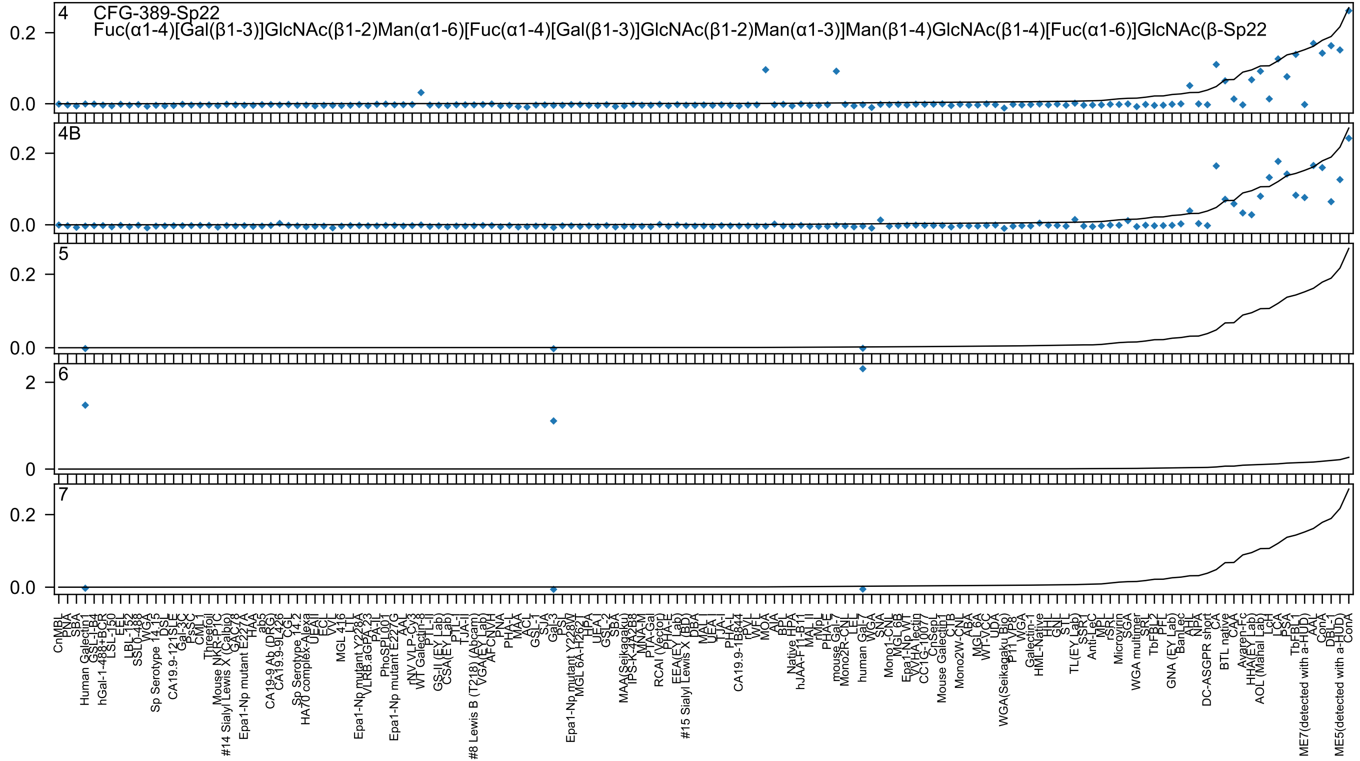
4 CFG-389-Sp22
Fuc(α1-4)[Gal(β1-3)]GlcNAc(β1-2)Man(α1-6)[Fuc(α1-4)[Gal(β1-3)]GlcNAc(β1-2)Man(α1-3)]Man(β1-4)GlcNAc(β1-4)[Fuc(α1-6)]GlcNAc(β-Sp22
4B
5
6
7
0.2
0.0
2
0

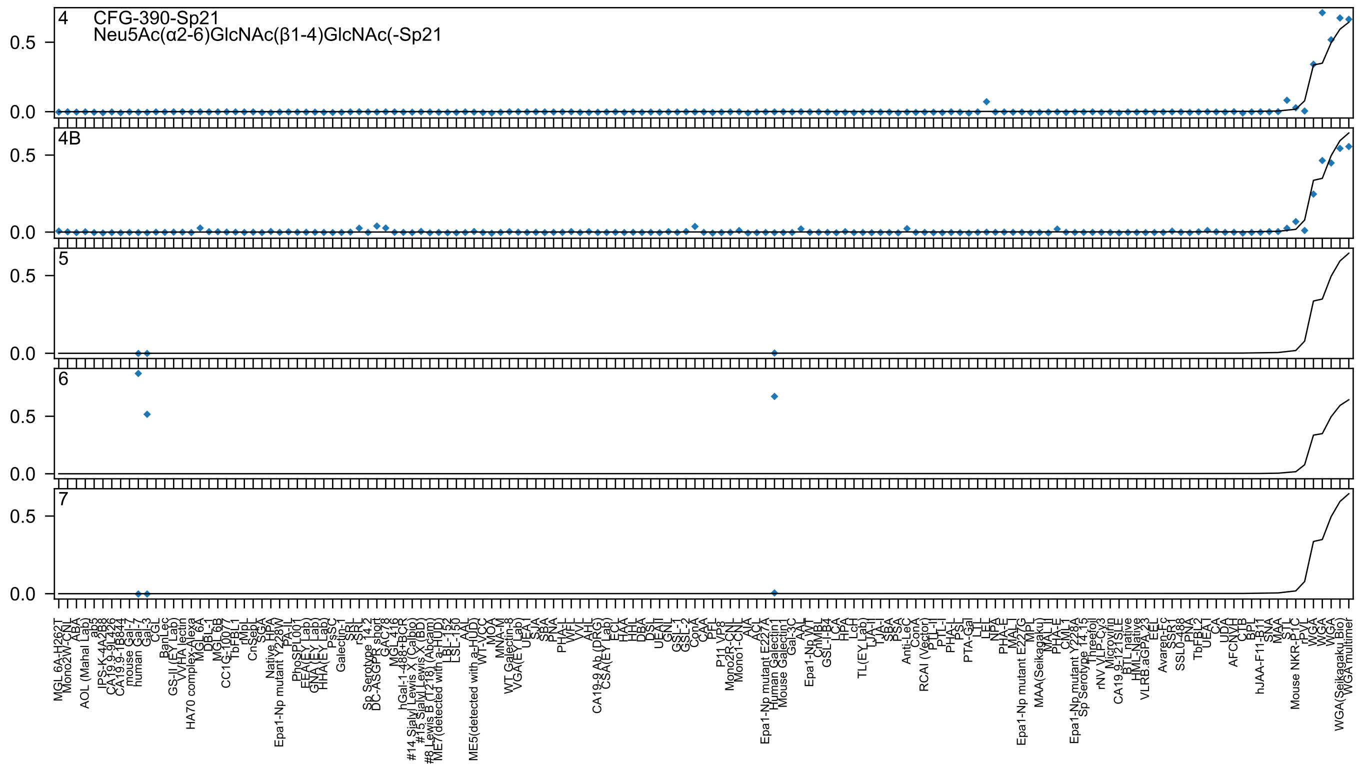

4
CFG-390-Sp21
Neu5Ac(α2-6)GlcNAc(β1-4)GlcNAc(-Sp21
4B
5
6
7
0.5
0.0

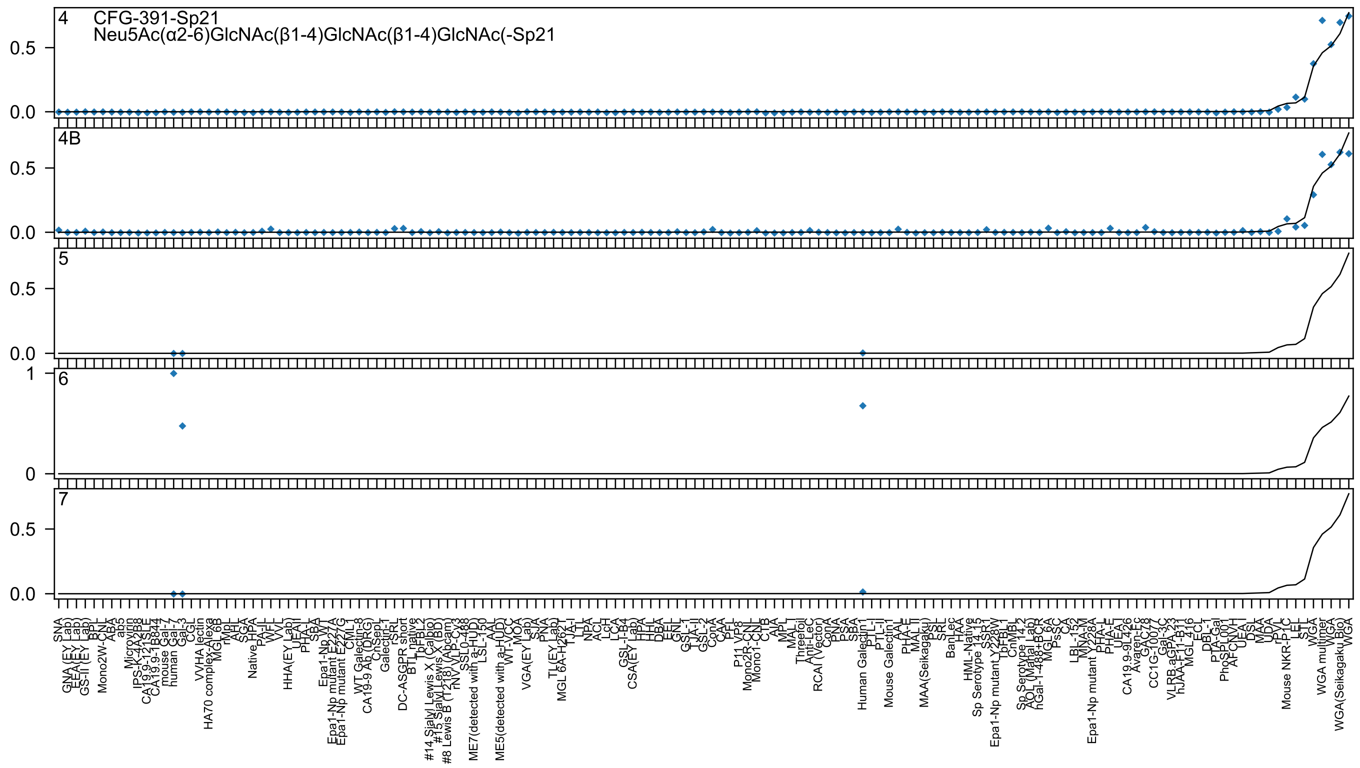

4 CFG-391-Sp21
Neu5Ac(α2-6)GlcNAc(β1-4)GlcNAc(β1-4)GlcNAc(-Sp21
4B
5
6
7

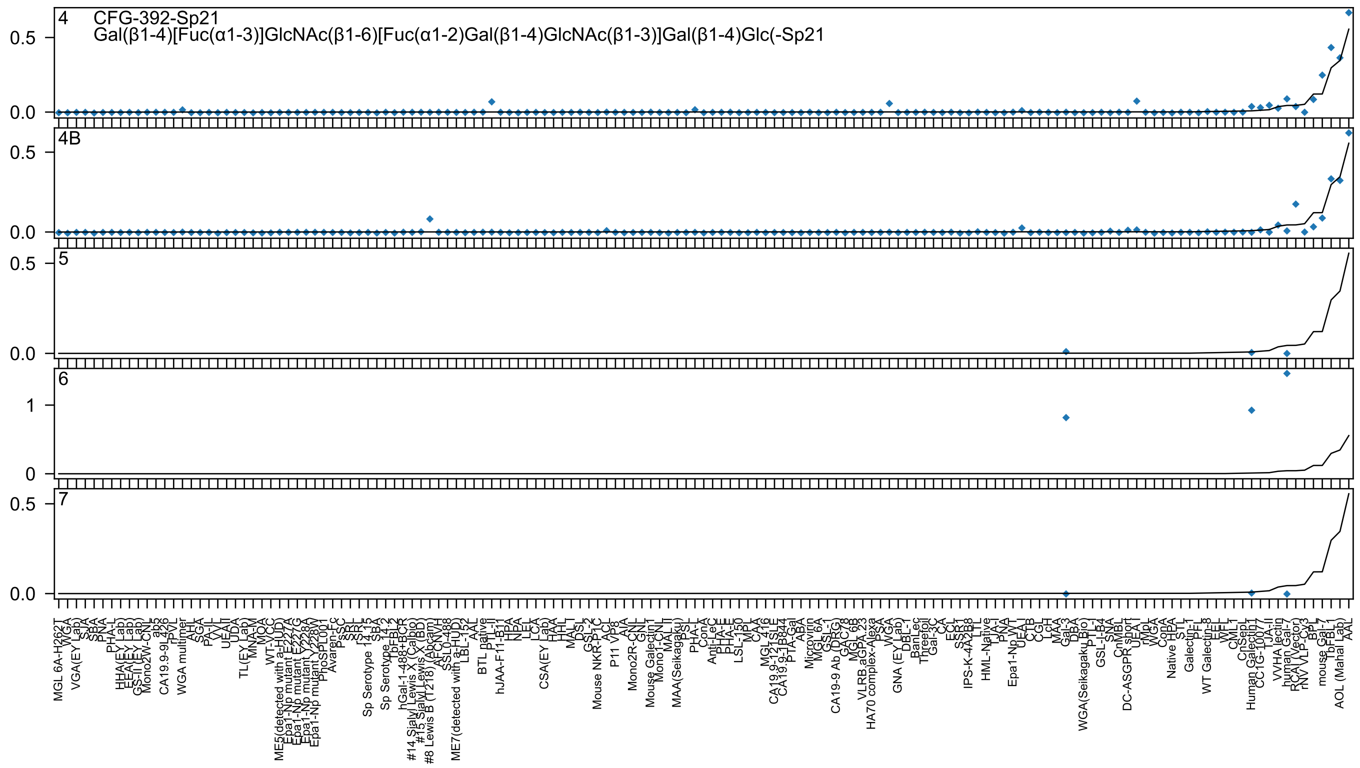

4 CFG-392-Sp21
Gal(β1-4)[Fuc(α1-3)]GlcNAc(β1-6)[Fuc(α1-2)Gal(β1-4)GlcNAc(β1-3)]Gal(β1-4)Glc(-Sp21
4B
5
6
7

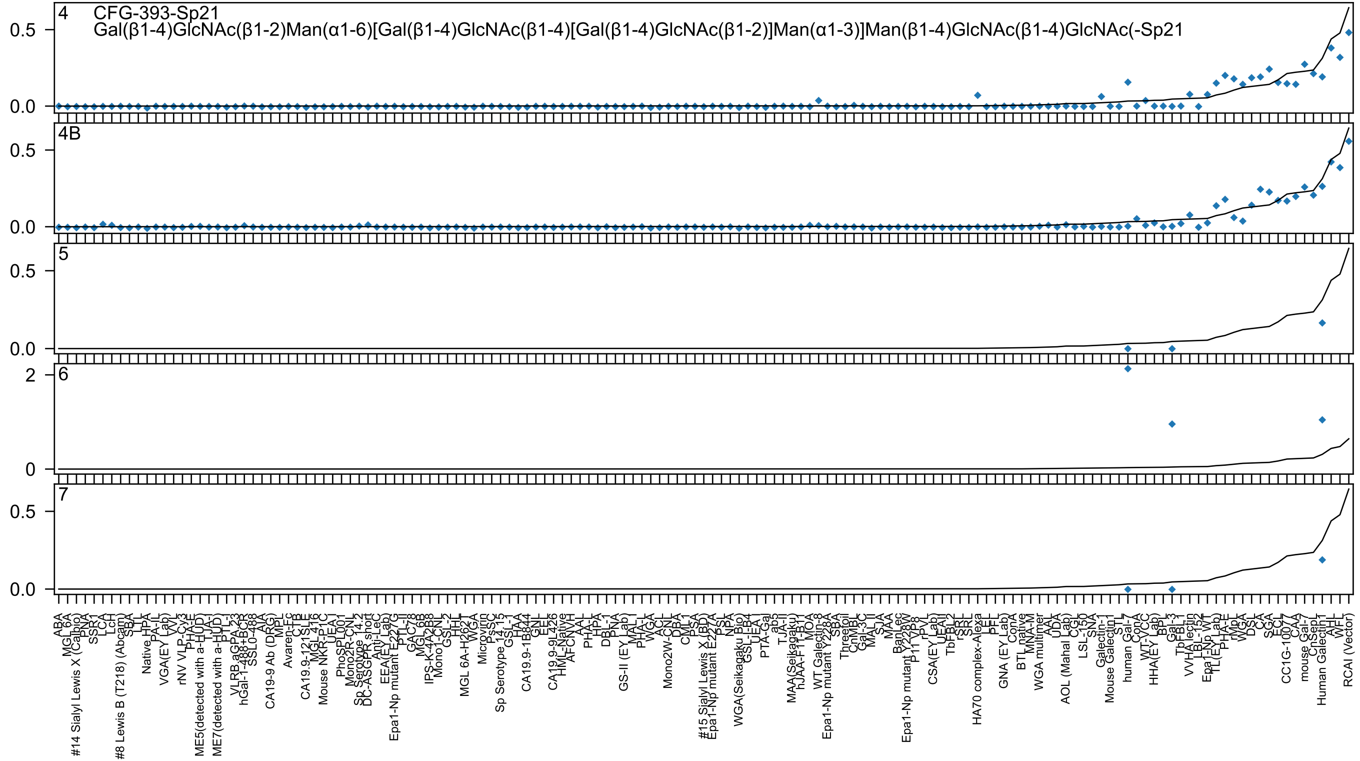
4 CFG-393-Sp21
Gal(β1-4)GlcNAc(β1-2)Man(α1-6)[Gal(β1-4)GlcNAc(β1-4)[Gal(β1-4)GlcNAc(β1-2)]Man(α1-3)]Man(β1-4)GlcNAc(β1-4)GlcNAc(-Sp21
4B
5
6
7
0.0
0.5
0
2
ABA
MGL 6A
#14 Sialyl Lewis X (Calbio)
PNA
SSR1
LCA
LcH
#8 Lewis B (T218) (Abcam)
SBA
LTL
Native HPA
PA-IL
VGA(EY Lab)
WVL
rNV VLP-Cy3
PHA-E
ME5(detected with a-HUD)
TJA-I
ME7(detected with a-HUD)
PTL-I
VLRB aGPA.23
hGal-1-488+BCR
SSL0-488
AIA
CA19-9 Ab (DRG)
MPL
Avaren-Fc
CTB
CA19.9-121SLE
MGL 416
Mouse NKR-P1C
UEA I
PhoSL001
Mono2R-CNL
Sp Serotype 14.2
DC-ASGPR short
Anti-LeC
EEA(EY Lab)
Epa1-Np mutant E227G
PTL-II
GAC78
MGL 6B
IPS-K-4A2B8
Mono1-CNL
GSL-2
HHL
MGL 6A-H262T
WGA
Microvirin
PsSC
Sp Serotype 14.15
GSL-1
HAA
CA19.9-1B844
GNL
EEL
CA19.9-9L426
HML-Native
AFCNVH
AAL
PHA-L
HPA
DBL-1
PNA
GS-II (EY Lab)
MAL I
PHA-L
WGA
ACL
Mono2W-CNL
DBA
CML1
PSA
#15 Sialyl Lewis X (BD)
Epa1-Np mutant E227A
PSL
NPA
WGA(Seikagaku Bio)
GSL-I-B4
UEA I
PTA-Gal
ab5
TJA-II
MAA(Seikagaku)
hJAA-F1T-B11
MOA
WT Galectin-8
Epa1-Np mutant Y228A
SBA
Threetoil
CnMBL
Gal-3C
MAL II
SJA
MAA
BanLec
Epa1-Np mutant Y228W
P11 VP8
rPVL
CSA(EY Lab)
UEAII
TbFBL2
rSRL
SRL
HA70 complex-Alexa
LEL
PFL
GNA (EY Lab)
ConA
BTL native
MNA-M
WGA multimer
AAL
UDA
AOL (Mahal Lab)
CGL
LSL-150
SNA
Galectin-1
Mouse Galectin-1
STL
human Gal-7
ConA
WT-YCC
HHA(EY Lab)
BPL
Gal-3
TbFBL1
VVHA lectin
LBL-152
Epa1-Np WT
TL(EY Lab)
PHA-E
rMpL
WGA
DSL
CA
SGA
ECL
CC1G-10077
CAA
mouse Gal-7
CnSepL
Human Galectin-1
AHL
WFL
RCAI (Vector)

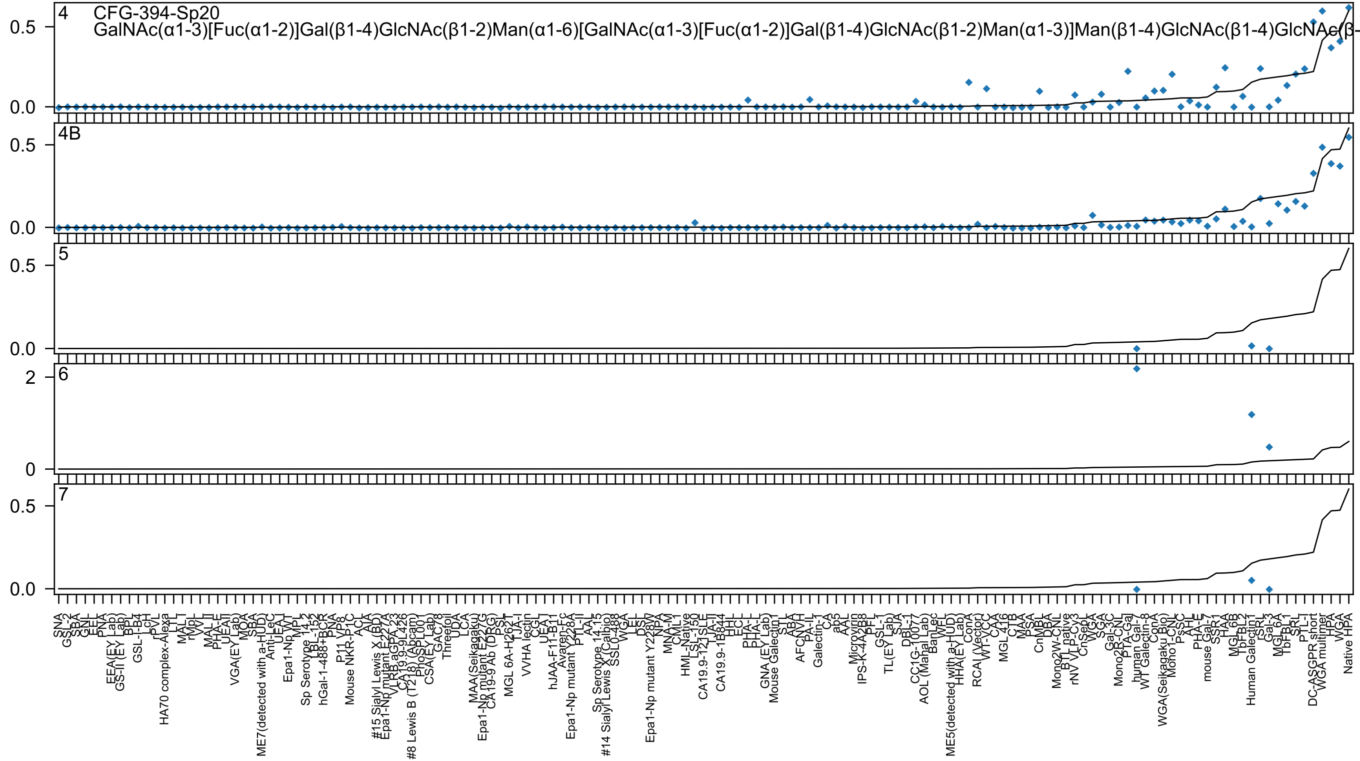

4 CFG-394-Sp20
GalNAc(α1-3)[Fuc(α1-2)]Gal(β1-4)GlcNAc(β1-2)Man(α1-6)[GalNAc(α1-3)[Fuc(α1-2)]Gal(β1-4)GlcNAc(β1-2)Man(α1-3)]Man(β1-4)GlcNAc(β1-4)GlcNAc(β-
4B
5
6
7
0.0
0.5
0
2

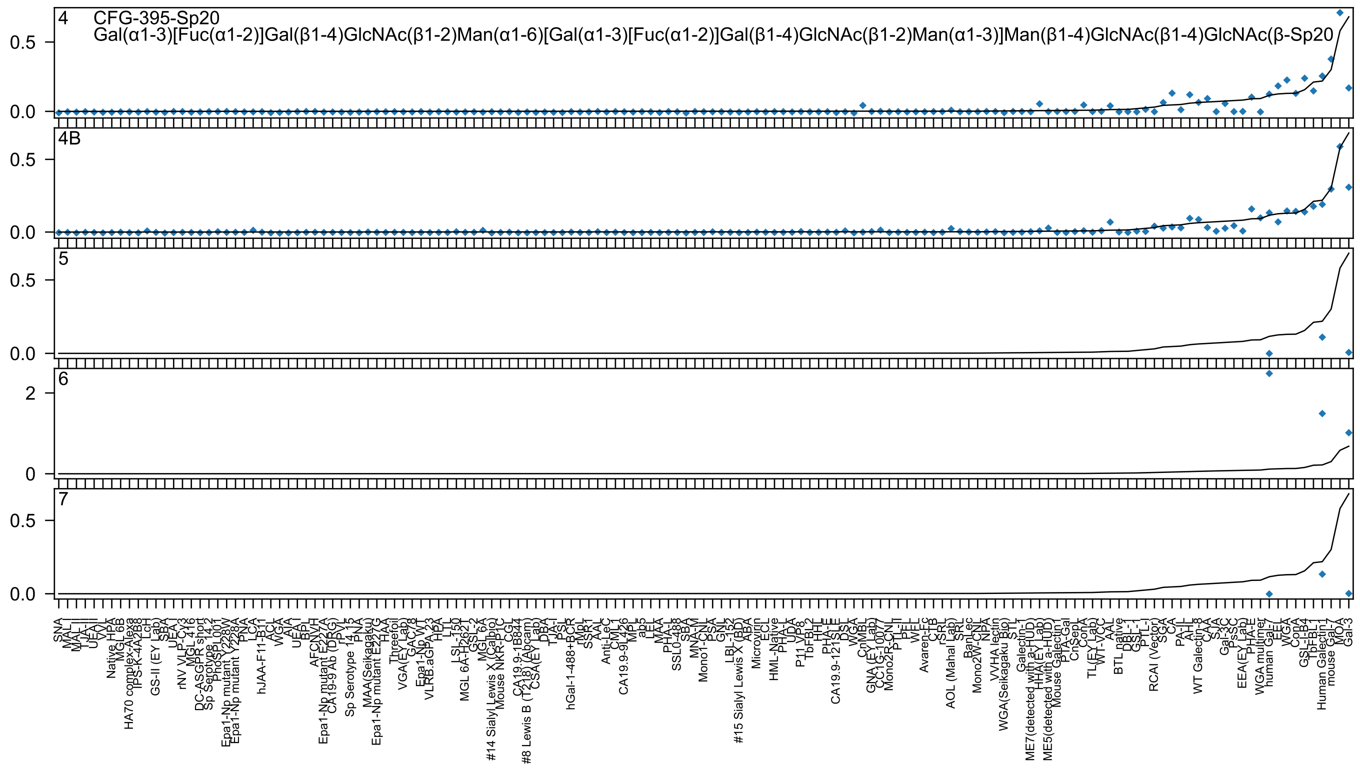

4 CFG-395-Sp20
Gal(α1-3)[Fuc(α1-2)]Gal(β1-4)GlcNAc(β1-2)Man(α1-6)[Gal(α1-3)[Fuc(α1-2)]Gal(β1-4)GlcNAc(β1-2)Man(α1-3)]Man(β1-4)GlcNAc(β1-4)GlcNAc(β-Sp20
4B
5
6
7

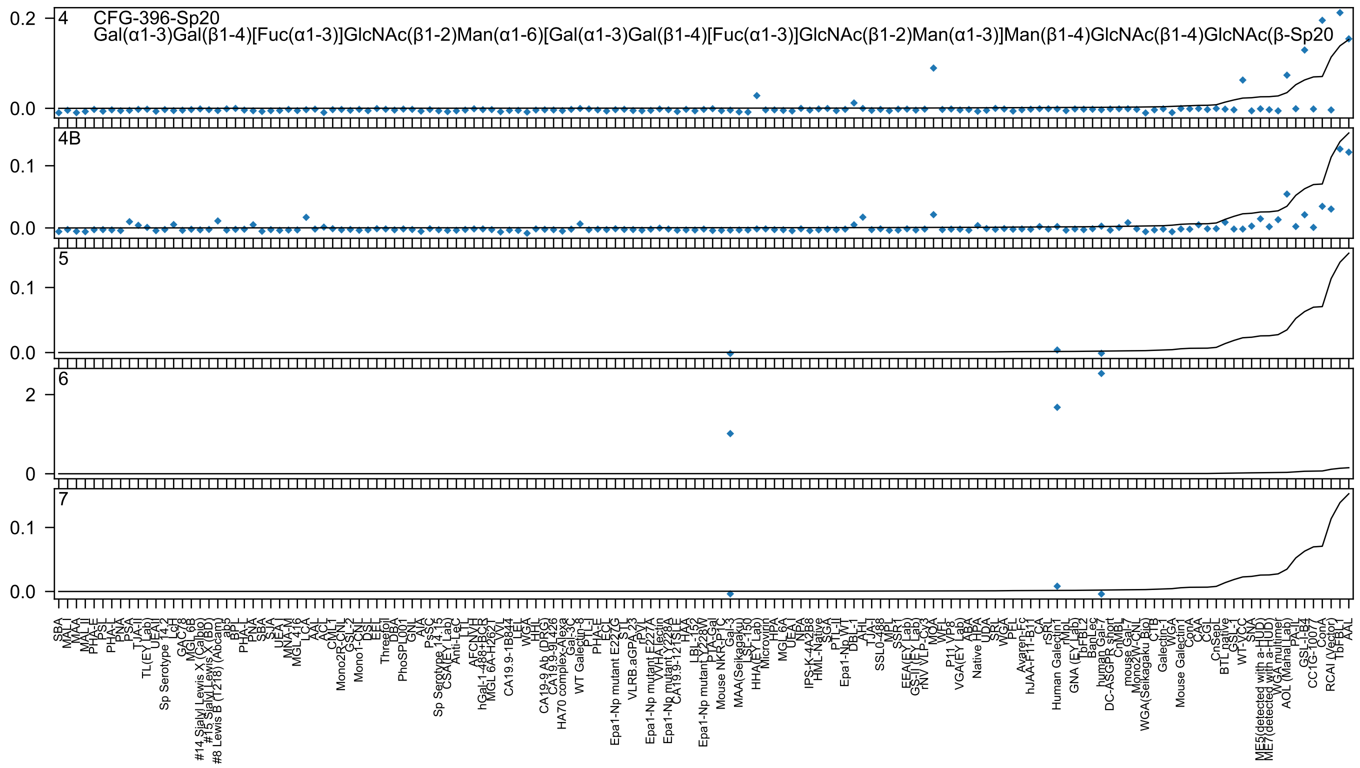
4
CFG-396-Sp20
Gal(α1-3)Gal(β1-4)[Fuc(α1-3)]GlcNAc(β1-2)Man(α1-6)[Gal(α1-3)Gal(β1-4)[Fuc(α1-3)]GlcNAc(β1-2)Man(α1-3)]Man(β1-4)GlcNAc(β1-4)GlcNAc(β-Sp20
4B
5
6
7

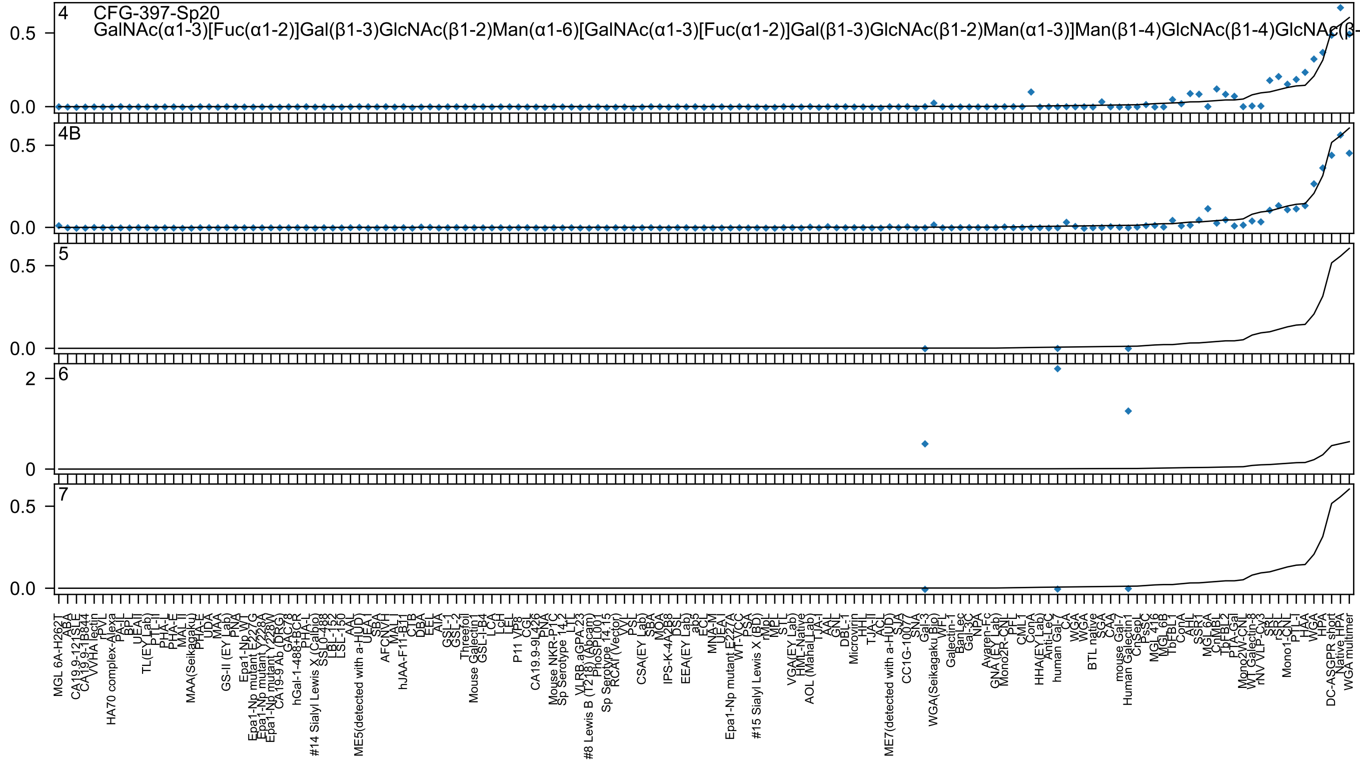
4 CFG-397-Sp20
GalNAc(α1-3)[Fuc(α1-2)]Gal(β1-3)GlcNAc(β1-2)Man(α1-6)[GalNAc(α1-3)[Fuc(α1-2)]Gal(β1-3)GlcNAc(β1-2)Man(α1-3)]Man(β1-4)GlcNAc(β1-4)GlcNAc(β-
4B
5
6
7
0.0
0.5
0
2
MGL 6A-H262T
ABA
CA19.9-121SLE
CA19.9-1B844
WHA lectin
rPVL
HA70 complex-Alexa
PA-IL
BPL
UEAII
TL(EY Lab)
PTL-II
PHA-L
PHA-E
MAL II
MAA(Seikagaku)
PHA-E
UDA
MAA
GS-II (EY Lab)
PNA
Epa1-Np WT
Epa1-Np mutant E227G
Epa1-Np mutant Y228A
Epa1-Np mutant Y228W
CA19-9 Ab (DRG)
GAC78
hGal-1-488+BCR
PHA-L
#14 Sialyl Lewis X (Calbio)
SSL0-488
LBL-152
LSL-150
AAL
ME5(detected with a-HUD)
UEA-I
SBA
AFCNVH
MAL-I
hJAA-F11-B11
CTB
DBA
EEL
AIA
GSL-1
GSL-2
Threefoil
Mouse Galectin1
GSL-I-B4
LCA
LcH
LEL
P11 VP8
CGL
CA19.9-9L426
PNA
Mouse NKR-P1C
Sp Serotype 14.2
LTL
VLRB.aGPA.23
#8 Lewis B (T218) (Abcam)
PhoSL001
Sp Serotype 14.15
RCAI (Vector)
VVL
PSL
CSA(EY Lab)
SBA
MOA
IPS-K-4A2B8
DSL
EEA(EY Lab)
ab5
ECL
MNA-M
UEA-I
Epa1-Np mutant E227A
WT-VYCC
PSA
#15 Sialyl Lewis X (BD)
rMpL
MPL
STL
VGA(EY Lab)
HML-Native
AOL (Mahal Lab)
TJA-I
AAL
GNL
DBL-1
Microvirin
HHL
TJA-II
ACL
ME7(detected with a-HUD)
SJA
CC1G-10077
SNA
Gal-3
WGA(Seikagaku Bio)
WFL
Galectin-1
BanLec
Gal-3C
NPA
Avaren-Fc
GNA (EY Lab)
Mono2R-CNL
PEL
CML1
ConA
HHA(EY Lab)
Anti-LeC
human Gal-7
CA
WGA
WGA
BTL native
SGA
CAA
mouse Gal-7
Human Galectin1
CnSep1
PsSC
MGL 416
MGL 6B
TbFBL1
ConA
AHL
SSR1
MGL 6A
CnMBL
TbFBL2
PA-Gal
Mono2W-CNL
WT Galectin-8
rNV VLP-Cy3
SRL
rSRL
Mono1-CNL
PTL-I
HAA
WGA
HPA
DC-ASGPR short
Native HPA
WGA multimer

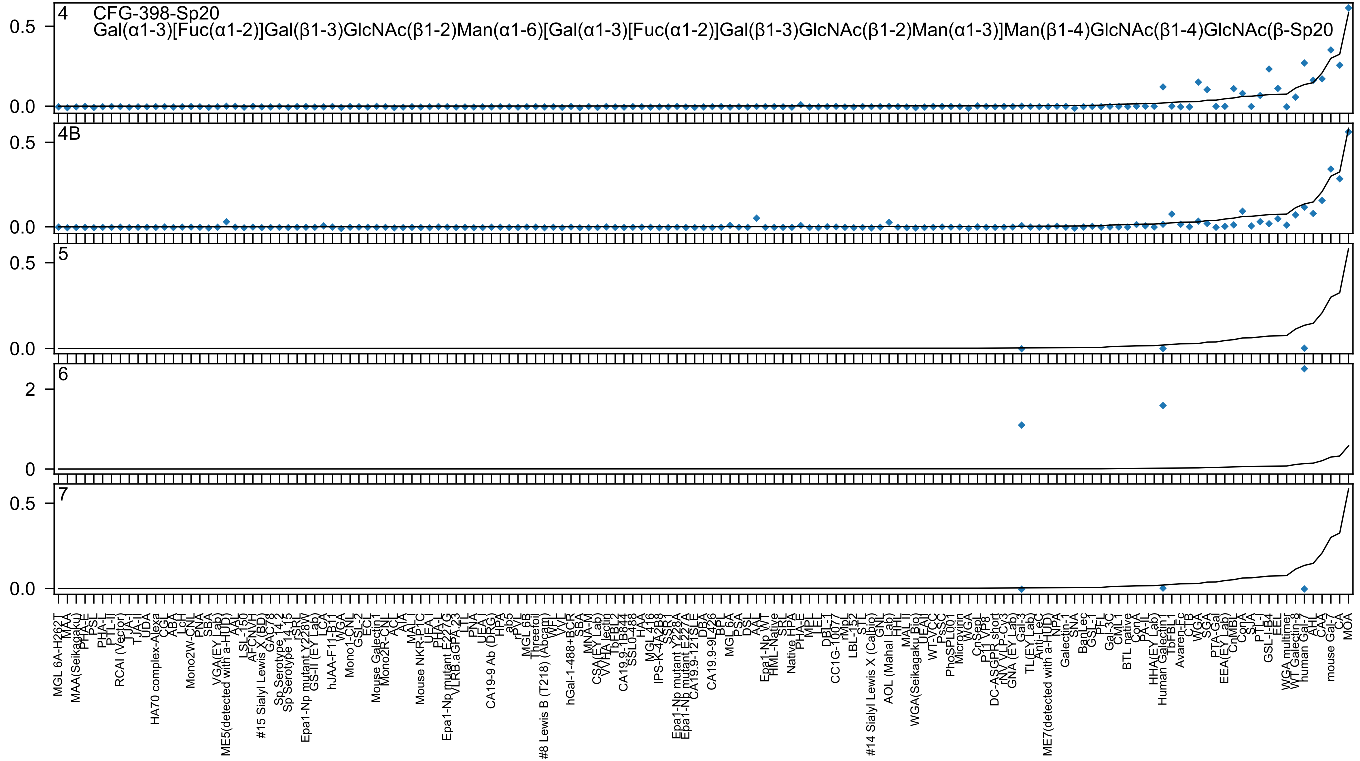

4
CFG-398-Sp20
Gal(α1-3)[Fuc(α1-2)]Gal(β1-3)GlcNAc(β1-2)Man(α1-6)[Gal(α1-3)[Fuc(α1-2)]Gal(β1-3)GlcNAc(β1-2)Man(α1-3)]Man(β1-4)GlcNAc(β1-4)GlcNAc(β-Sp20
4B
5
6
7
MGL 6A-H262T
MAA(Seikagaku)
RCAI (Vector)
HA70 complex-Alexa
Mono2W-CNL
VGA(EY Lab)
ME5(detected with a-HUD)
#15 Sialyl Lewis X (BD)
Sp Serotype 14
Epa1-Np mutant Y228W
GS-II (EY Lab)
hJAA-F11-B11
Mono1-CNL
Mouse Galectin1
Mono2R-CNL
Mouse NKR-P1C
Epa1-Np mutant E227G
VLRB.aGPA.23
CA19-9 Ab (DRG)
#8 Lewis B (T218) (Abcam)
hGal-1-488+BCR
CSA(EY Lab)
CA19.9-1B844
SSL0-488
IPS-K-4A2B8
Epa1-Np mutant Y228A
Epa1-Np mutant E227A
CA19.9-121SLE
CA19.9-9L426
Epa1-Np WT
HML-Native
Native HPA
CC1G-10077
LBL-152
#14 Sialyl Lewis X (Calbio)
AOL (Mahal Lab)
WGA(Seikagaku Bio)
WT-VCC
PhoSPL001
Microvirin
DC-ASGPR short
GNA (EY Lab)
TL(EY Lab)
ME7(detected with a-HUD)
Galectin-1
BanLec
BTL native
HHA(EY Lab)
Human Galectin1
Avaren-Fc
EEA(EY Lab)
WGA multimer
WT Galectin-8
human Gal-7
mouse Gal-7
MOA

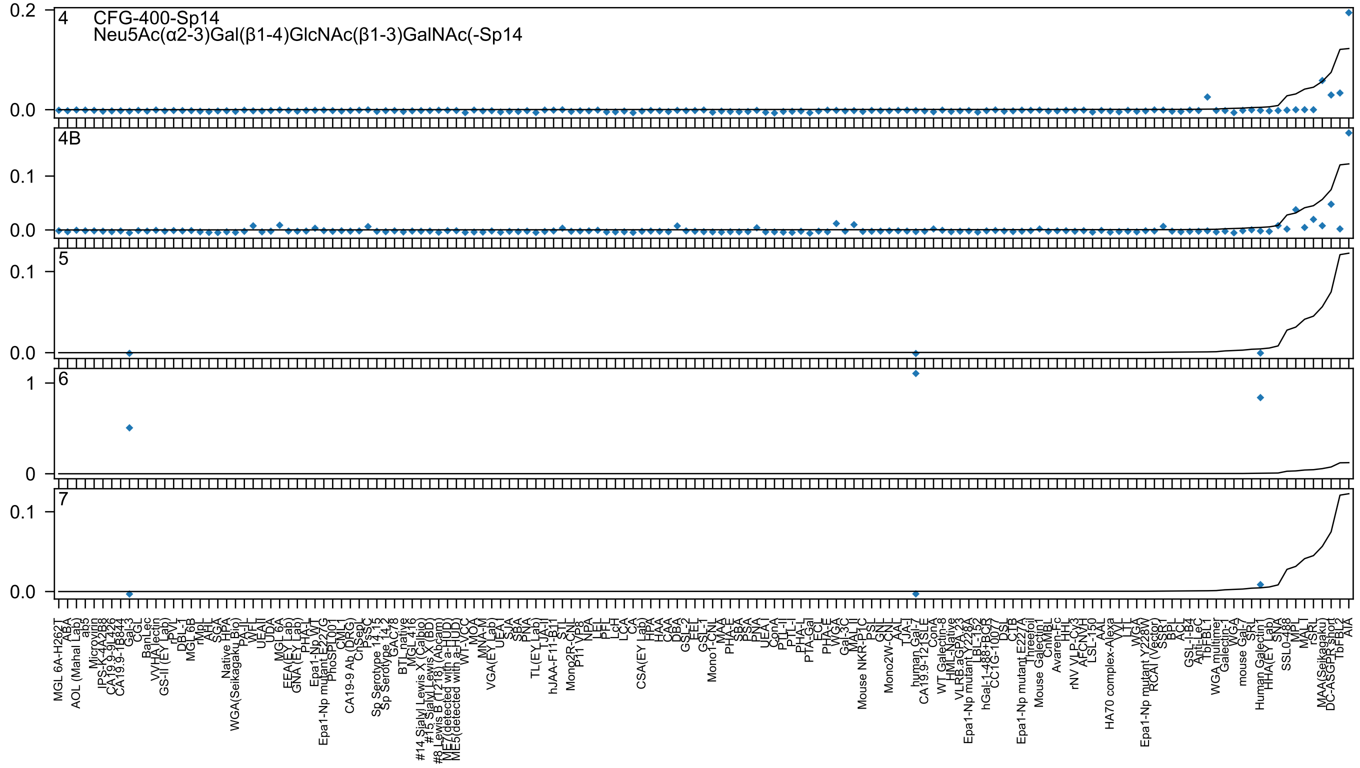

4
CFG-400-Sp14
Neu5Ac(α2-3)Gal(β1-4)GlcNAc(β1-3)GalNAc(-Sp14
4B
5
6
7

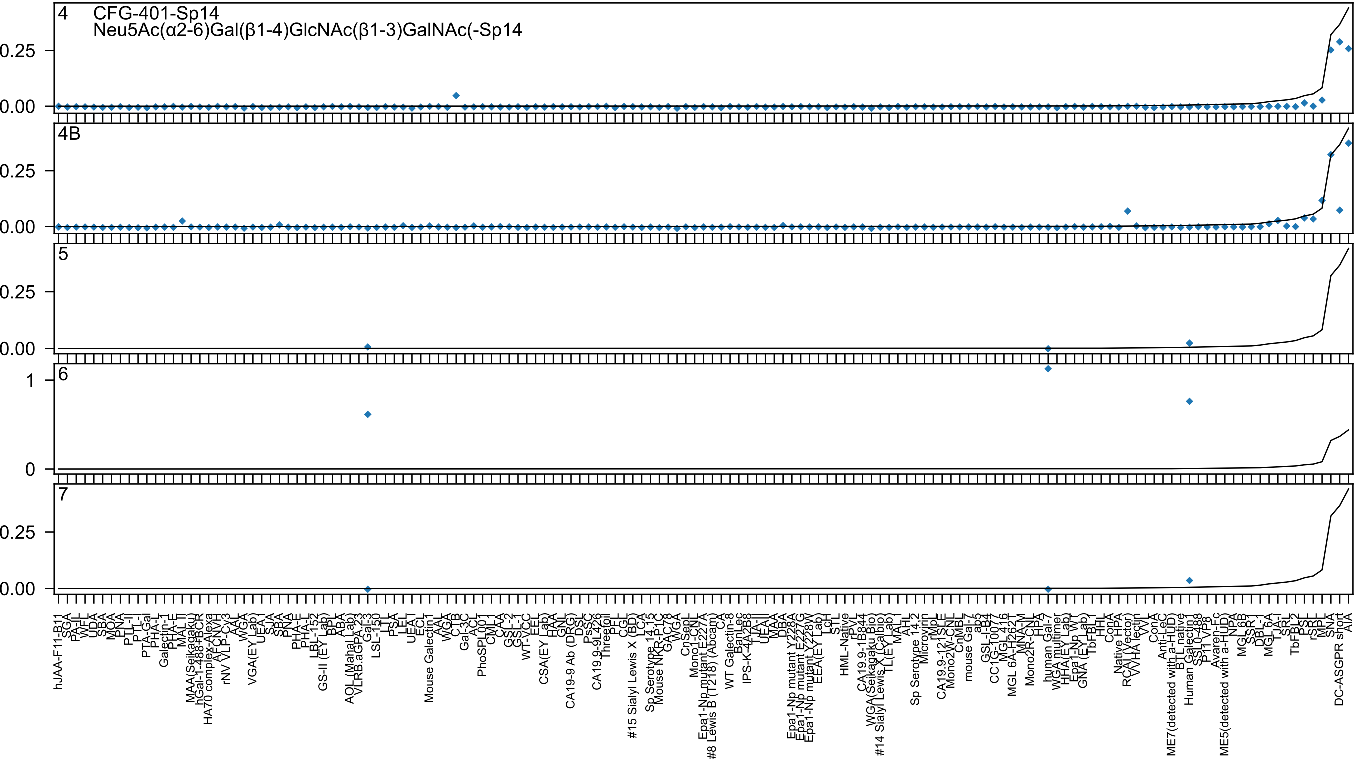
4
CFG-401-Sp14
Neu5Ac(α2-6)Gal(β1-4)GlcNAc(β1-3)GalNAc(-Sp14
4B
5
6
7
0.25
0.00
1
0

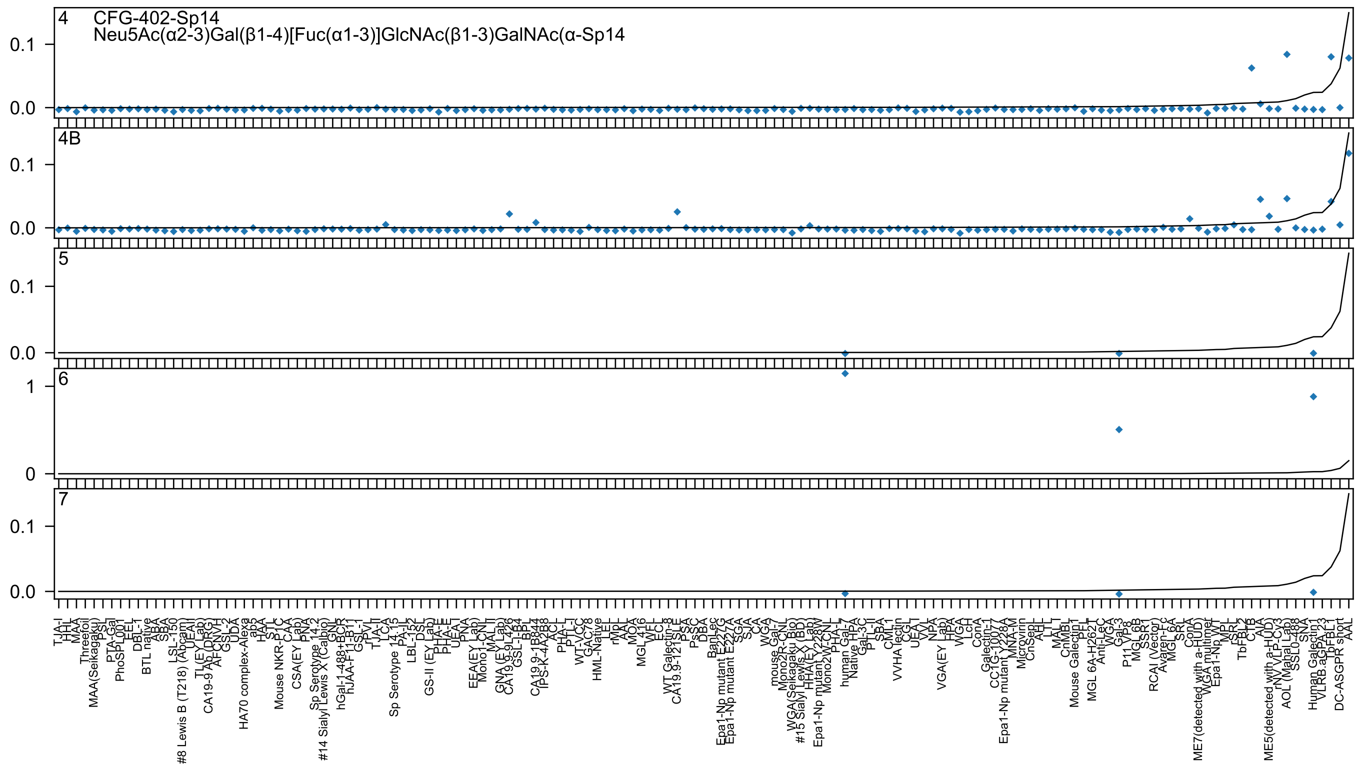

4 CFG-402-Sp14
Neu5Ac(α2-3)Gal(β1-4)[Fuc(α1-3)]GlcNAc(β1-3)GalNAc(α-Sp14
4B
5
6
7

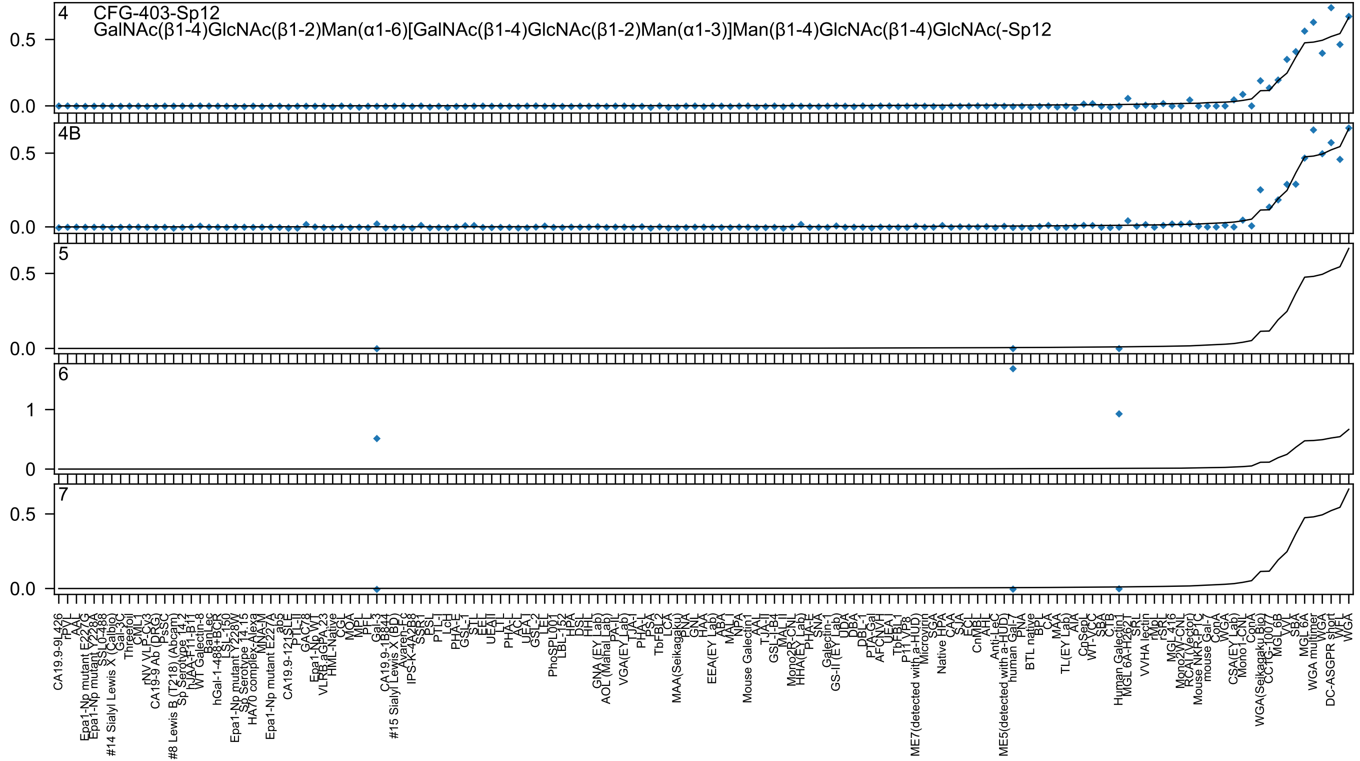

4
CFG-403-Sp12
GalNAc(β1-4)GlcNAc(β1-2)Man(α1-6)[GalNAc(β1-4)GlcNAc(β1-2)Man(α1-3)]Man(β1-4)GlcNAc(β1-4)GlcNAc(-Sp12
0.5
0.0
4B
0.5
0.0
5
0.5
0.0
6
1
0
7
0.5
0.0
CA19.9-9L426
rPVL
AAL
Epa1-Np mutant E227G
Epa1-Np mutant Y228A
SSL0-488
#14 Sialyl Lewis X (Calbio)
Gal-3C
Threefoil
CML1
rNV VLP-Cy3
CA19-9 Ab (DRG)
PsSC
#8 Lewis B (T218) (Abcam)
Sp Serotype 14.2
hJAA-F11-B11
WT Galectin-8
BanLec
hGal-1-488+BCR
LSL-150
Epa1-Np mutant Y228W
Sp Serotype 14.15
HA70 complex-Alexa
MNA-M
Epa1-Np mutant E227A
ab5
CA19.9-121SLE
PTL-II
GAC78
Epa1-Np WT
VLRB aGPA.23
HML-Native
CGL
MOA
MPL
PFL
Gal-3
CA19.9-1B844
#15 Sialyl Lewis X (BD)
Avaren-Fc
IPS-K-4A2B8
SSR1
PSL
PTL-I
LcH
PHA-E
GSL-I
STL
EEL
UEA II
LTL
PHA-L
ACL
UEA-I
GSL-2
LEL
PhoSPL001
LBL-152
HPA
DSL
HHL
GNA (EY Lab)
AOL (Mahal Lab)
PA-IL
VGA(EY Lab)
TJA-I
PHA-L
PSA
TbFBL2
LCA
MAA(Seikagaku)
PNA
GNL
HAA
EEA(EY Lab)
ABA
MAL I
NPA
Mouse Galectin1
AAL
TJA-II
GSL-I-B4
MAL II
Mono2R-CNL
HHA(EY Lab)
PHA-E
SNA
Galectin-1
GS-II (EY Lab)
UDA
DBA
DBL-1
PTA-Gal
AFCNVH
UEA I
TbFBL1
P11 VP8
ME7(detected with a-HUD)
Microvirin
SGA
Native HPA
CAA
SJA
ECL
CnMBL
AHL
Anti-LeC
ME5(detected with a-HUD)
human Gal-7
PNA
BTL native
BPL
CA
MAA
TL(EY Lab)
AIA
CnSep
WT-VCC
SBA
CTB
Human Galectin1
MGL 6A-H262T
SRL
VVHA lectin
rMpL
rSRL
MGL 416
Mono2W-CNL
RCA I(Vector)
Mouse NKR-P1C
mouse Gal-7
ConA
WGA
CSA(EY Lab)
Mono1-CNL
ConA
WGA(Seikagaku Bio)
CCTG-1007
MGL 6B
VVL
SBA
MGL 6A
WGA multimer
WGA
DC-ASGPR short
WFL
WGA

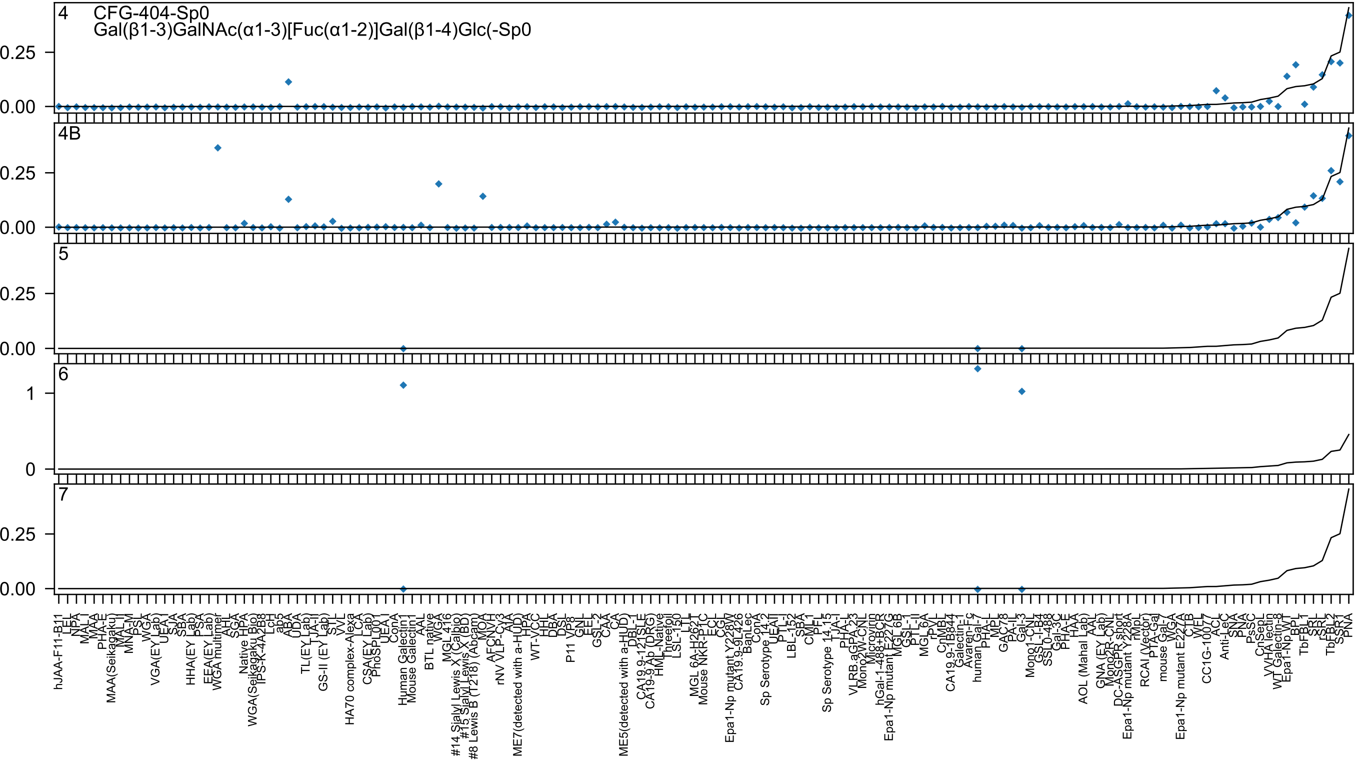
4
CFG-404-Sp0
Gal(β1-3)GalNAc(α1-3)[Fuc(α1-2)]Gal(β1-4)Glc(-Sp0
4B
5
6
7
0.25
0.00
1
0

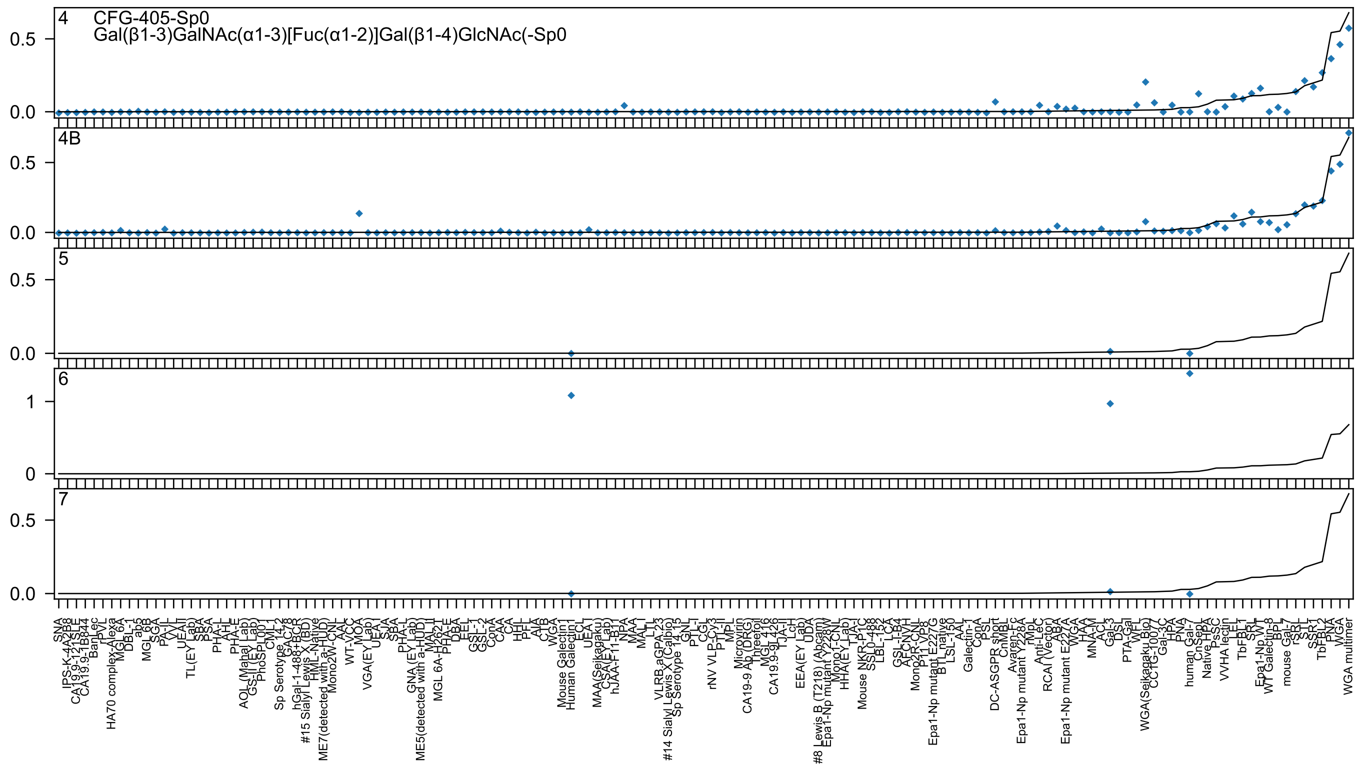

4 CFG-405-Sp0
Gal(β1-3)GalNAc(α1-3)[Fuc(α1-2)]Gal(β1-4)GlcNAc(-Sp0
4B
5
6
7

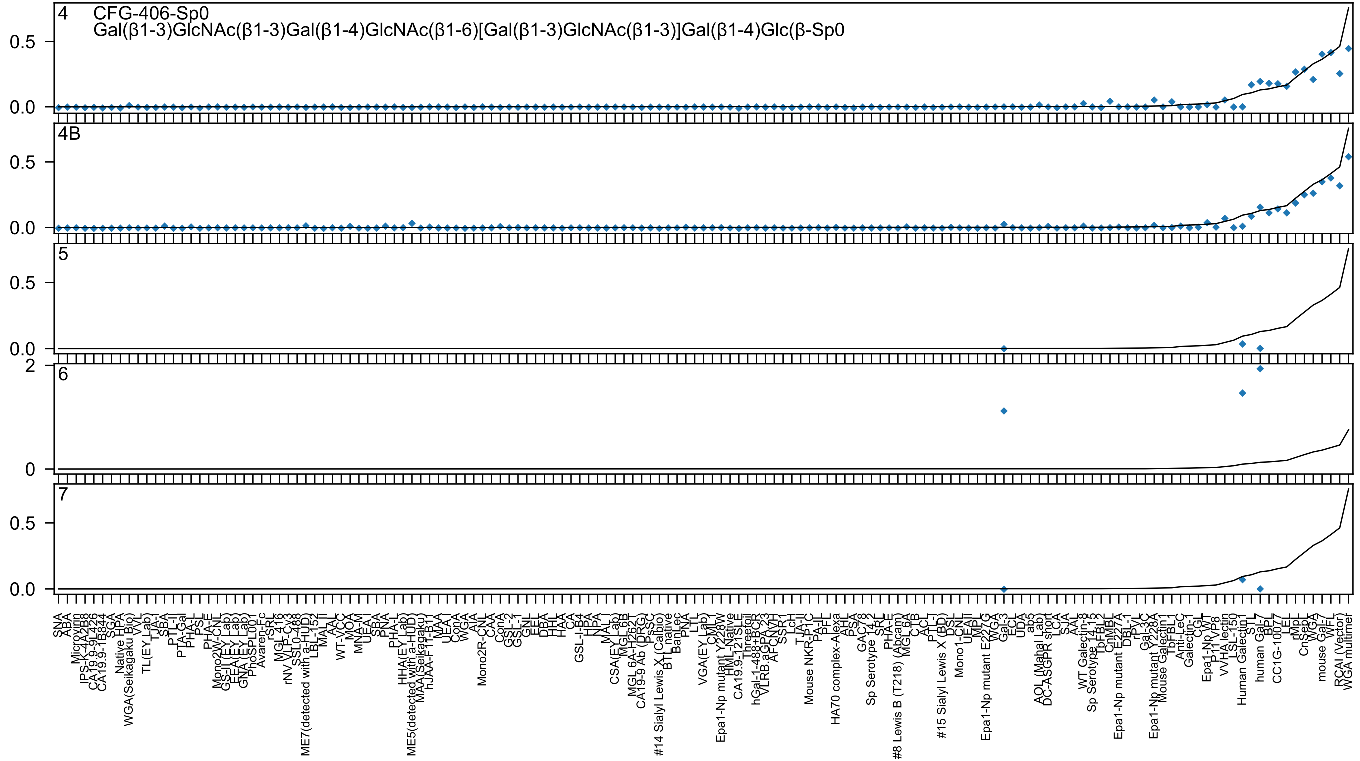

4
CFG-406-Sp0
Gal(β1-3)GlcNAc(β1-3)Gal(β1-4)GlcNAc(β1-6)[Gal(β1-3)GlcNAc(β1-3)]Gal(β1-4)Glc(β-Sp0
4B
5
6
7

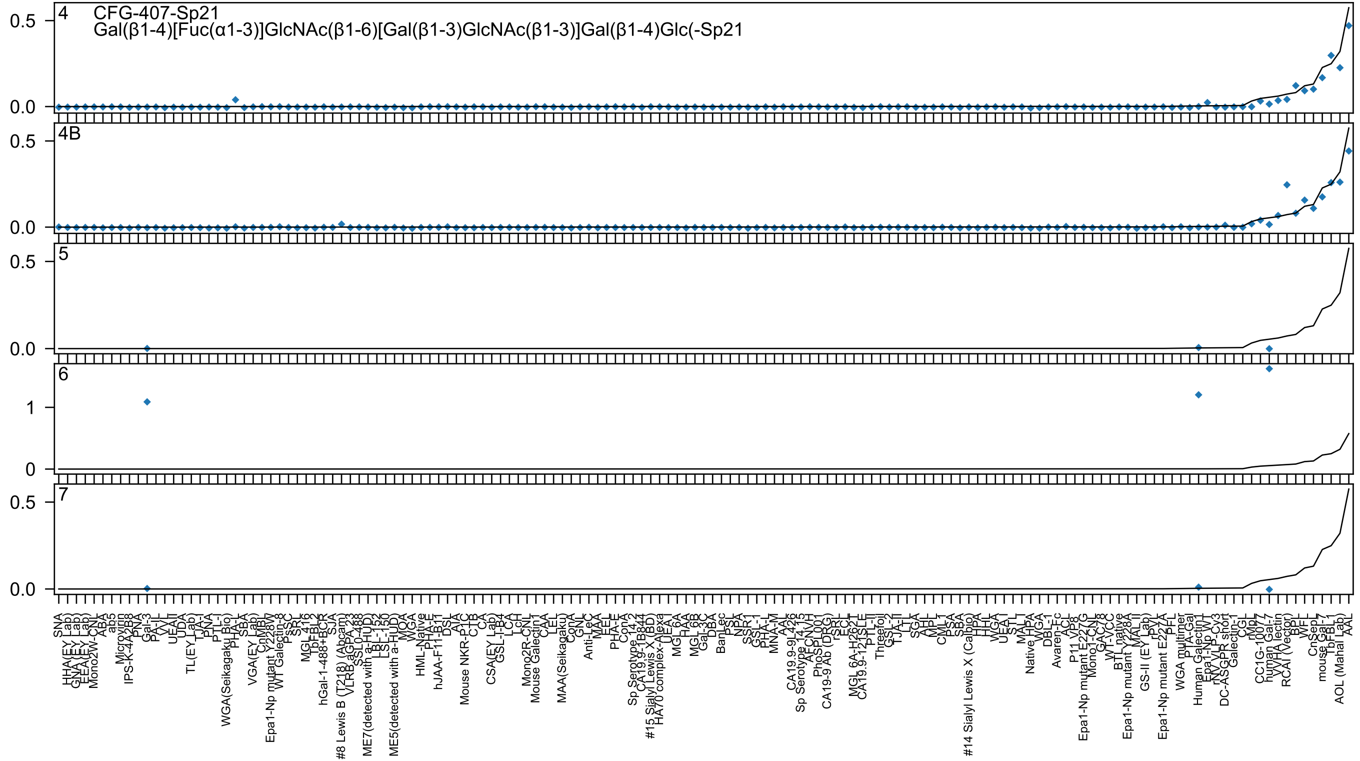

4
CFG-407-Sp21
Gal(β1-4)[Fuc(α1-3)]GlcNAc(β1-6)[Gal(β1-3)GlcNAc(β1-3)]Gal(β1-4)Glc(-Sp21
4B
5
6
7

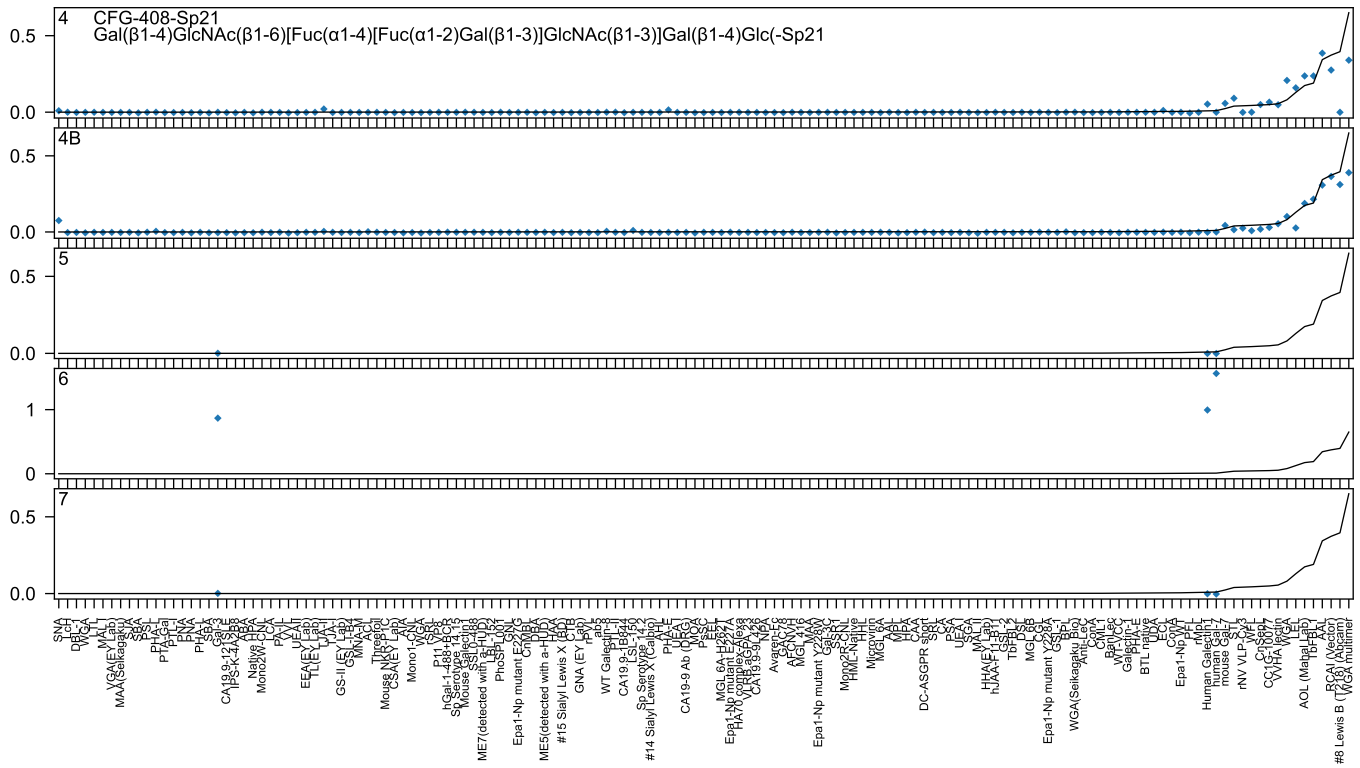
4 CFG-408-Sp21
Gal(β1-4)GlcNAc(β1-6)[Fuc(α1-4)[Fuc(α1-2)Gal(β1-3)]GlcNAc(β1-3)]Gal(β1-4)Glc(-Sp21
4B
5
6
7

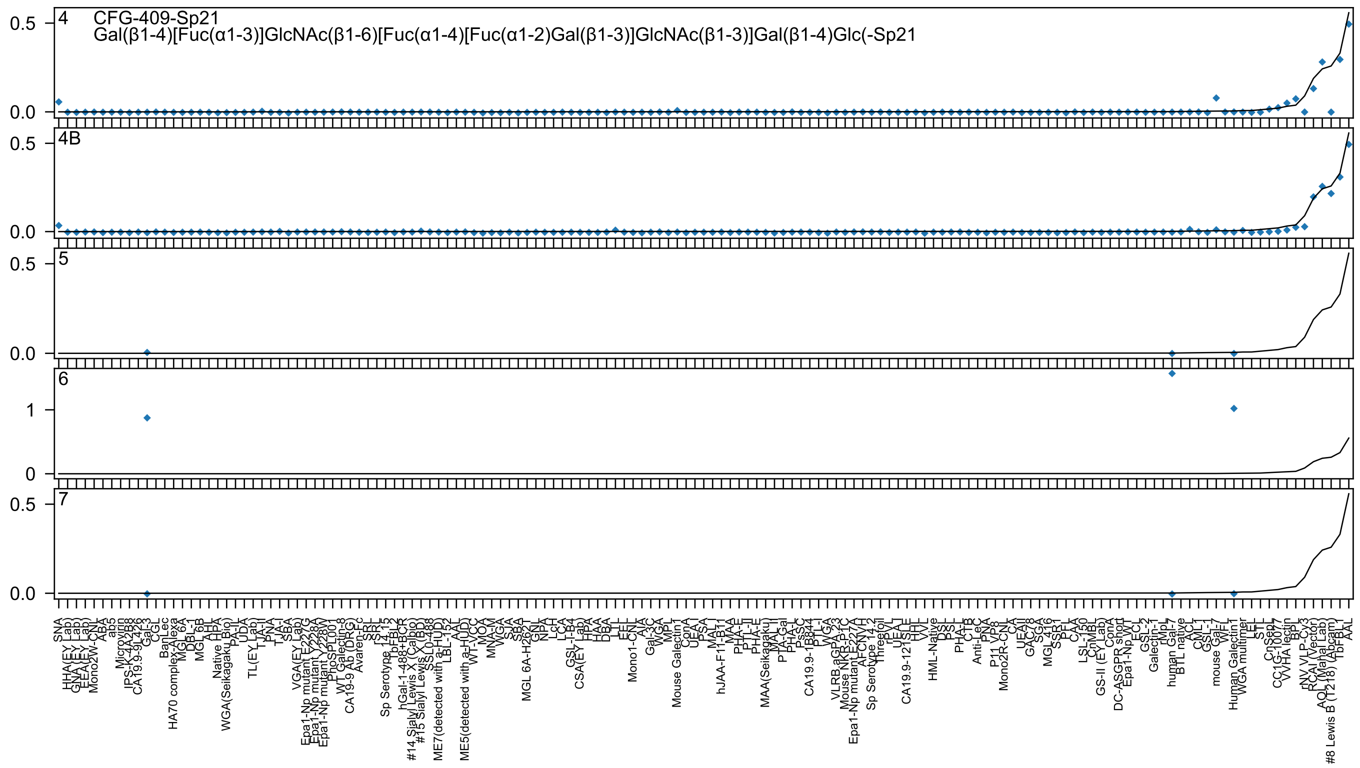

CFG-409-Sp21
Gal(β1-4)[Fuc(α1-3)]GlcNAc(β1-6)[Fuc(α1-4)[Fuc(α1-2)Gal(β1-3)]GlcNAc(β1-3)]Gal(β1-4)Glc(-Sp21
4
4B
5
6
7

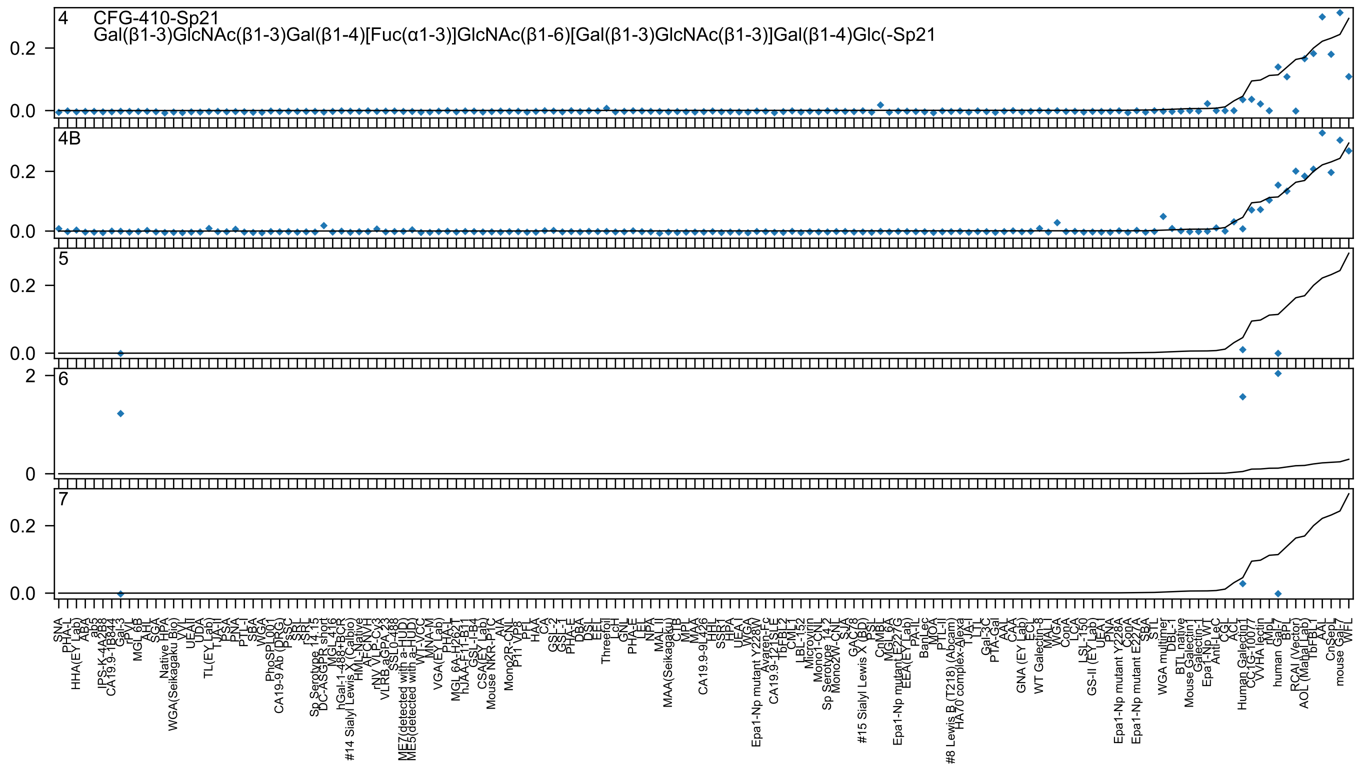

4 CFG-410-Sp21
Gal(β1-3)GlcNAc(β1-3)Gal(β1-4)[Fuc(α1-3)]GlcNAc(β1-6)[Gal(β1-3)GlcNAc(β1-3)]Gal(β1-4)Glc(-Sp21
4B
5
6
7

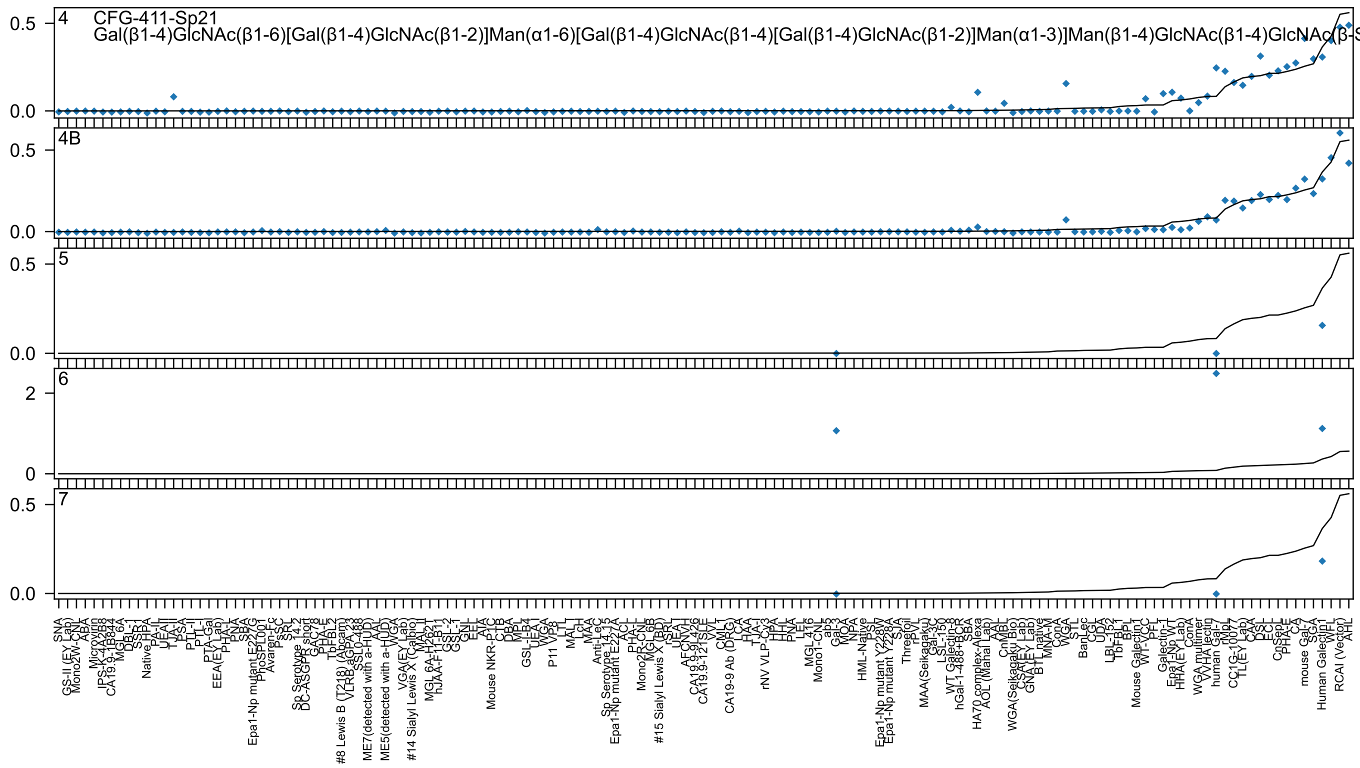
4
CFG-411-Sp21
Gal(β1-4)GlcNAc(β1-6)[Gal(β1-4)GlcNAc(β1-2)]Man(α1-6)[Gal(β1-4)GlcNAc(β1-4)[Gal(β1-4)GlcNAc(β1-2)]Man(α1-3)]Man(β1-4)GlcNAc(β1-4)GlcNAc(β-S
4B
5
6
7

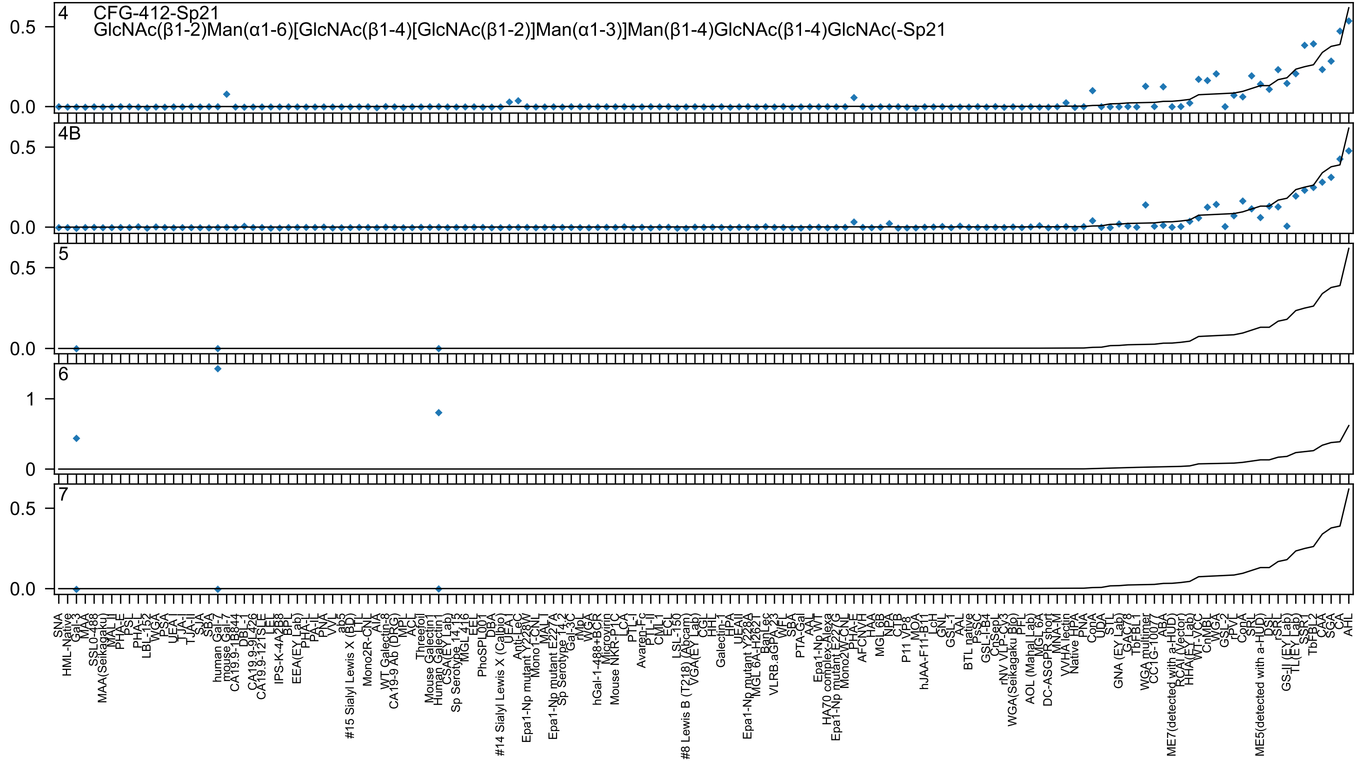

4 CFG-412-Sp21
GlcNAc(β1-2)Man(α1-6)[GlcNAc(β1-4)[GlcNAc(β1-2)]Man(α1-3)]Man(β1-4)GlcNAc(β1-4)GlcNAc(-Sp21
4B
5
6
7

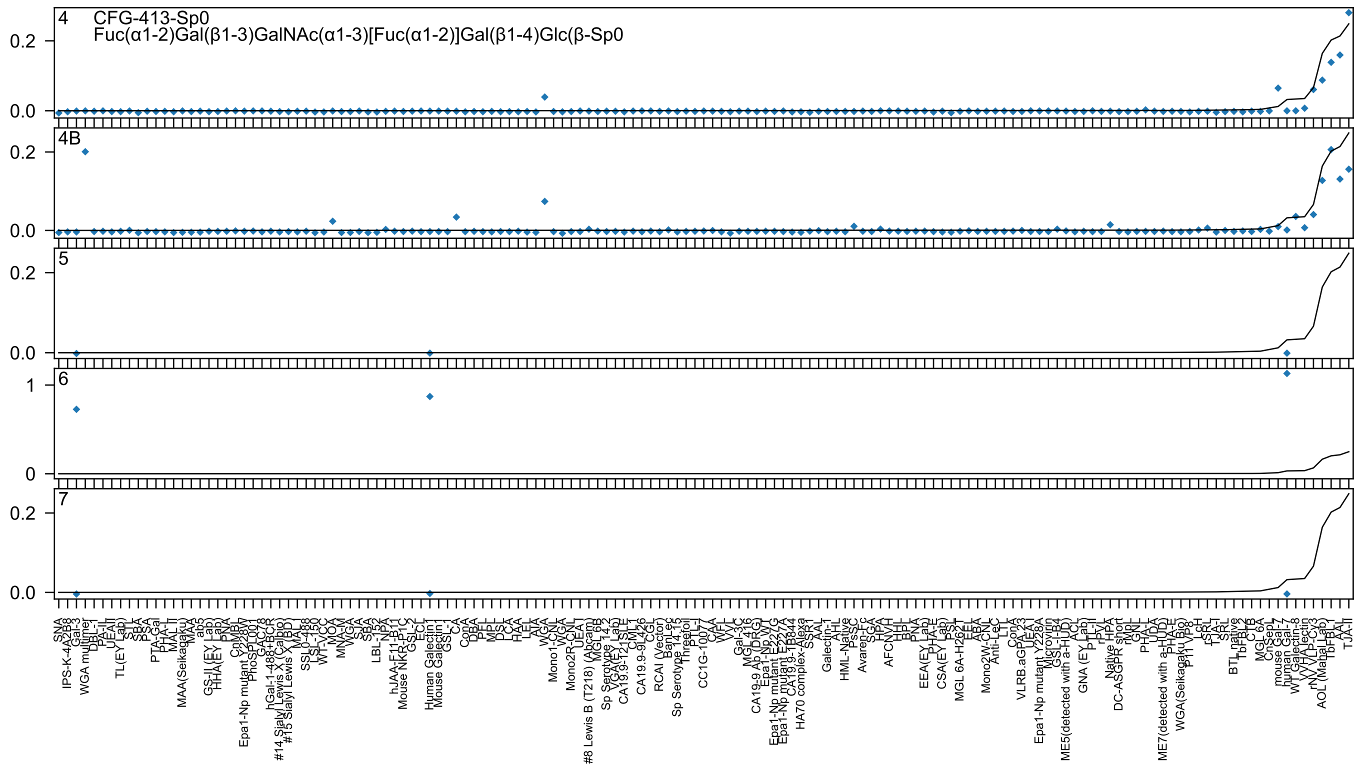

4 CFG-413-Sp0
Fuc(α1-2)Gal(β1-3)GalNAc(α1-3)[Fuc(α1-2)]Gal(β1-4)Glc(β-Sp0
4B
5
6
7

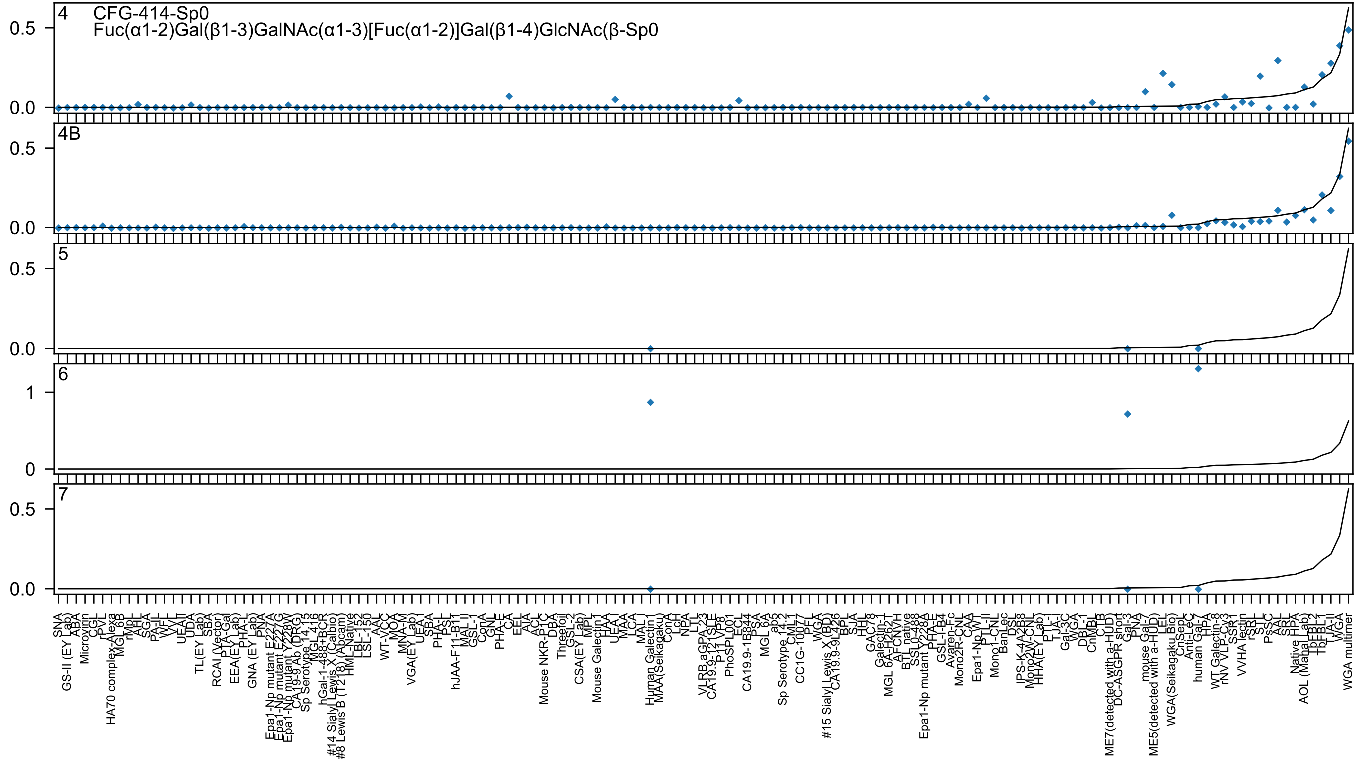

4
CFG-414-Sp0
Fuc(α1-2)Gal(β1-3)GalNAc(α1-3)[Fuc(α1-2)]Gal(β1-4)GlcNAc(β-Sp0
4B
5
6
7

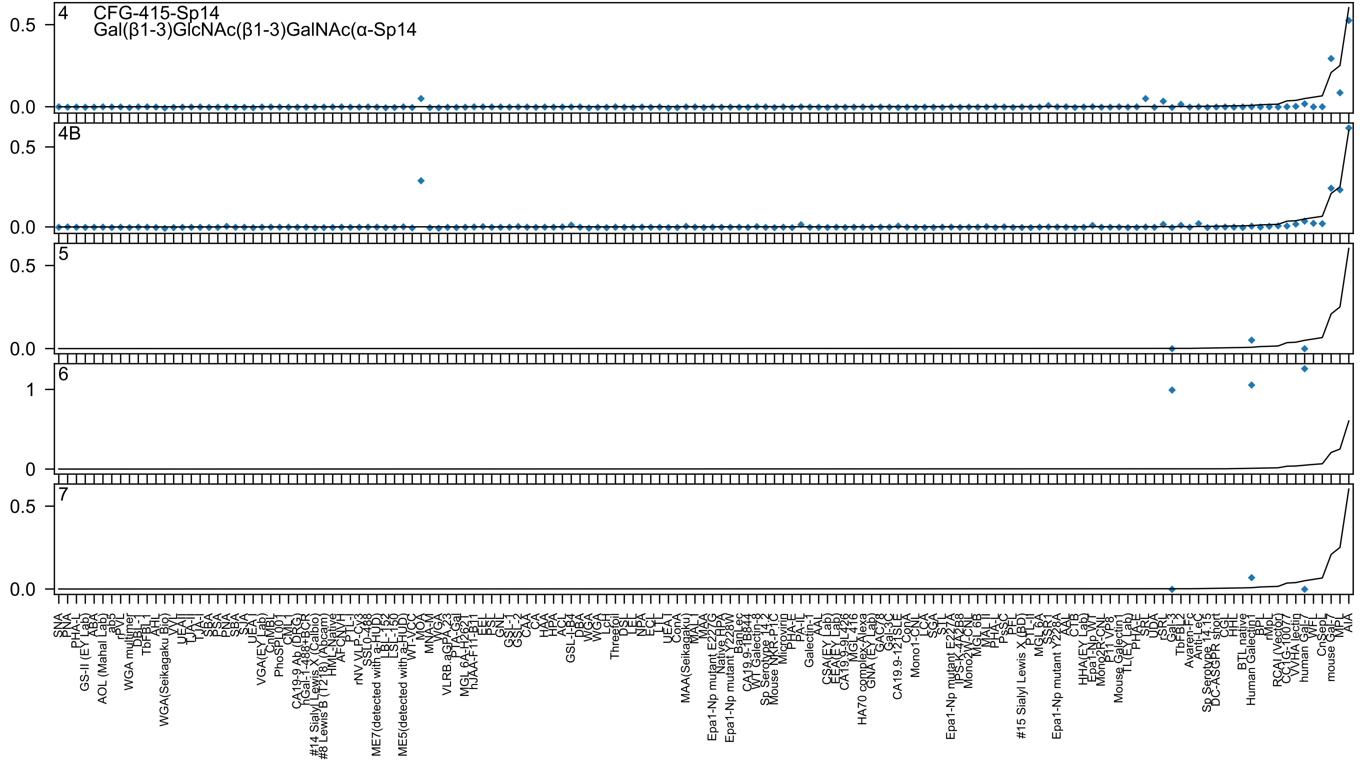
4
CFG-415-Sp14
Gal(β1-3)GlcNAc(β1-3)GalNAc(α-Sp14
4B
5
6
7
0.5
0.0
1
0

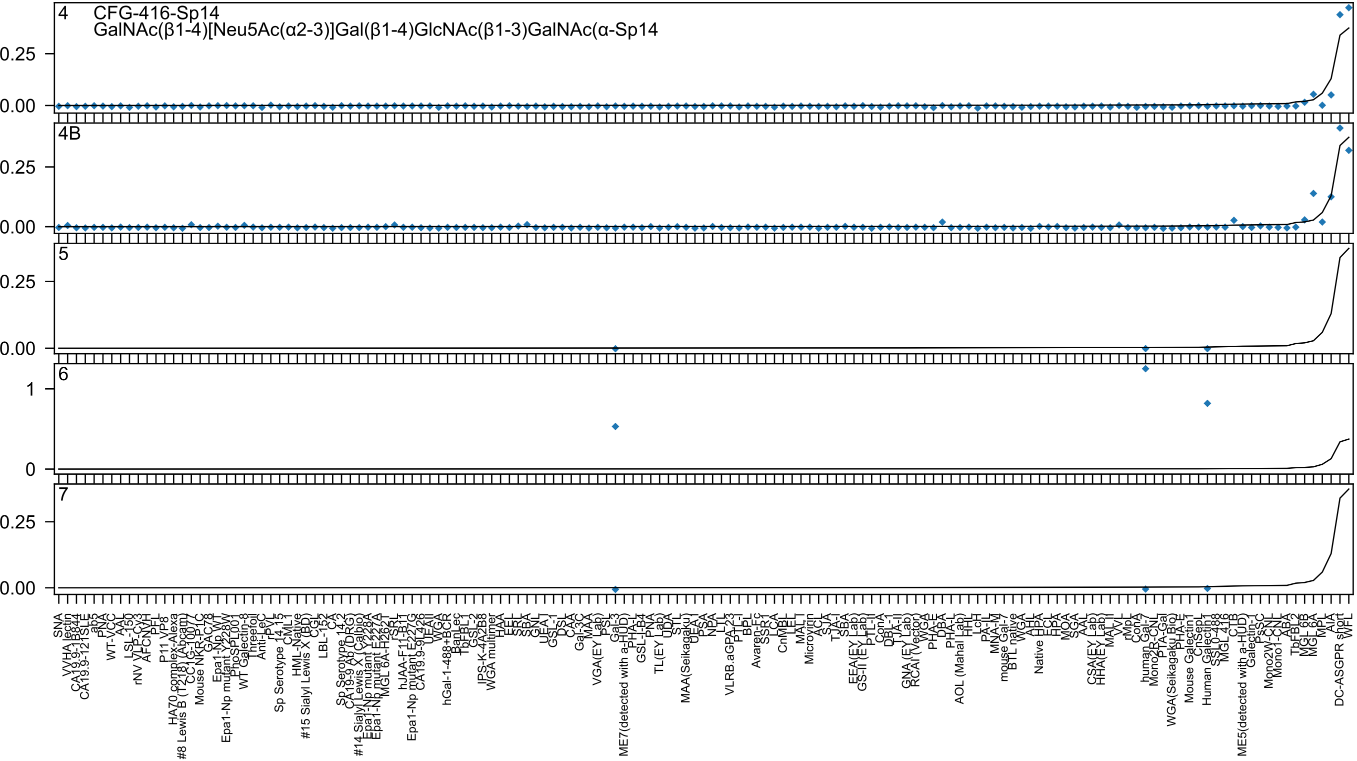
4
CFG-416-Sp14
GalNAc(β1-4)[Neu5Ac(α2-3)]Gal(β1-4)GlcNAc(β1-3)GalNAc(α-Sp14
4B
5
6
7
0.25
0.00
1
0

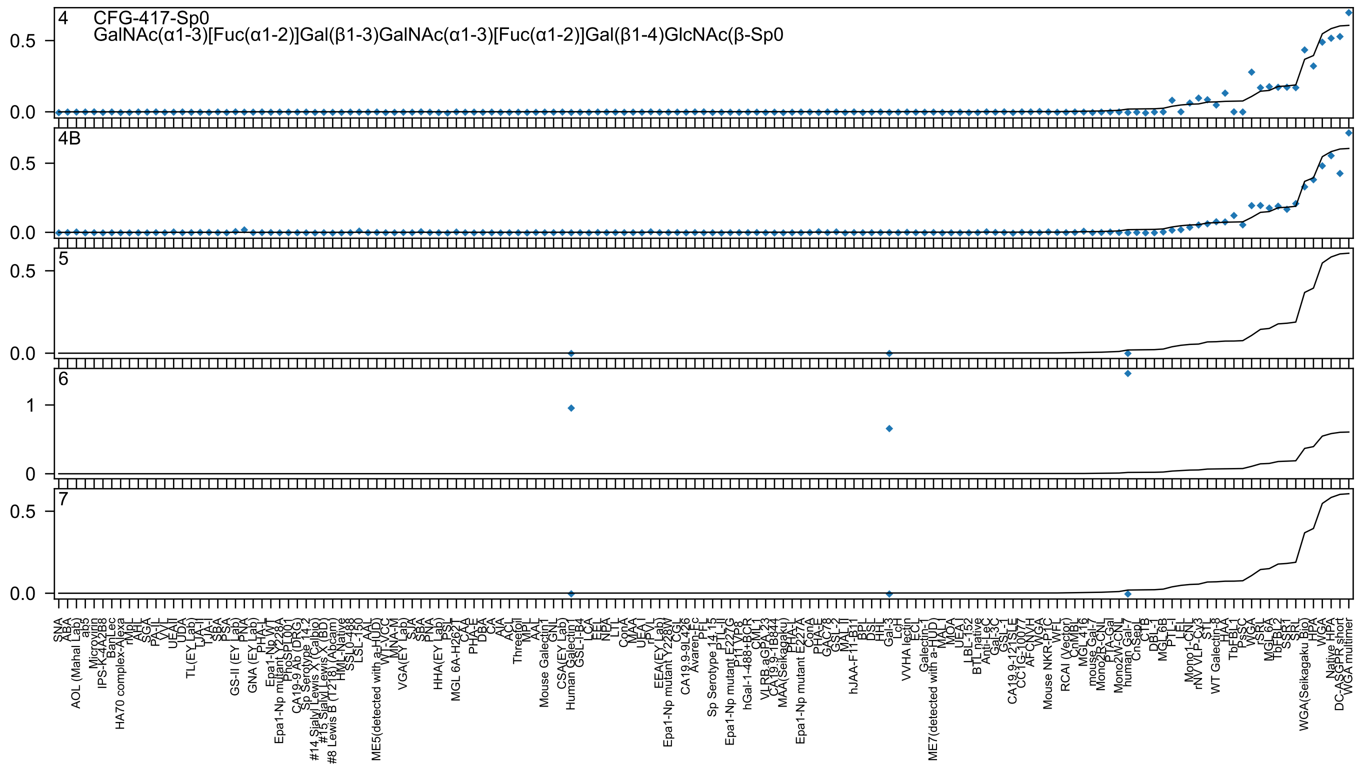

CFG-417-Sp0
GalNAc(α1-3)[Fuc(α1-2)]Gal(β1-3)GalNAc(α1-3)[Fuc(α1-2)]Gal(β1-4)GlcNAc(β-Sp0

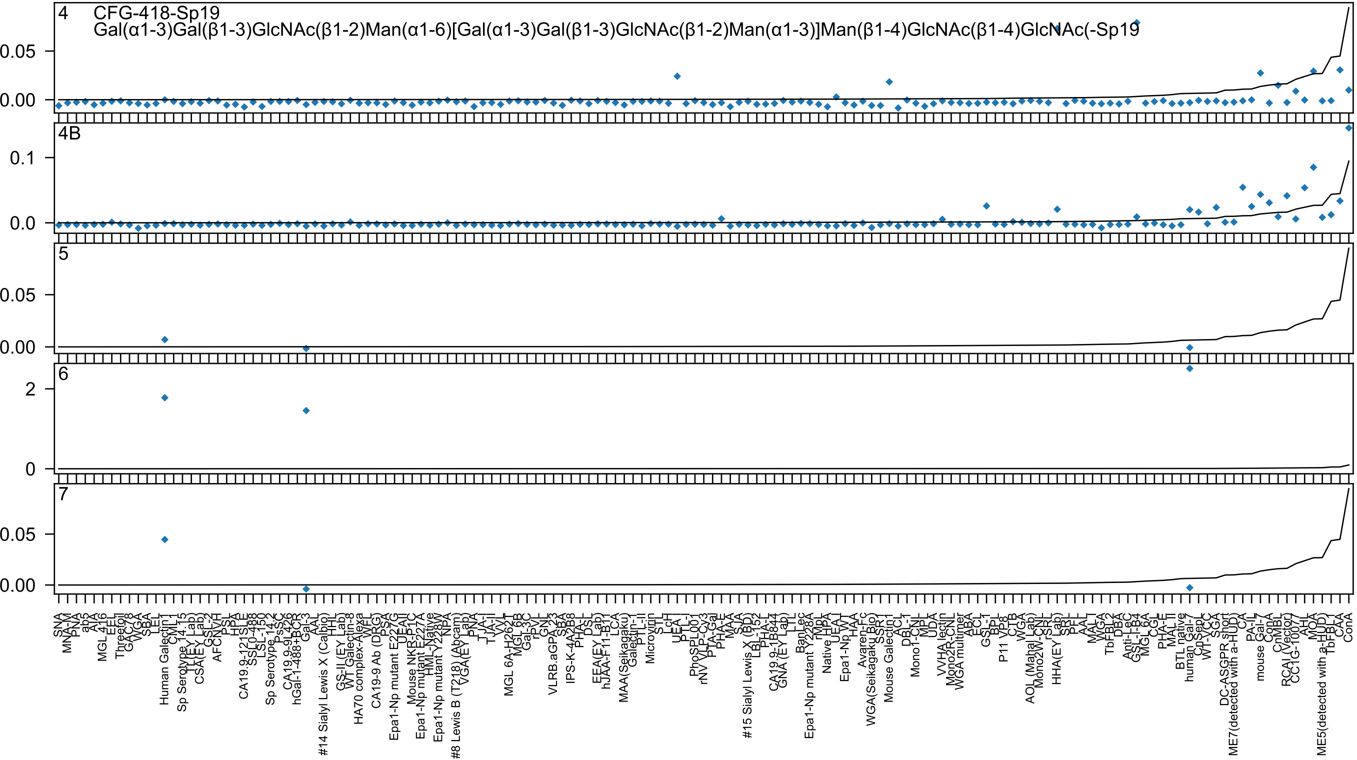
4 CFG-418-Sp19
Gal(α1-3)Gal(β1-3)GlcNAc(β1-2)Man(α1-6)[Gal(α1-3)Gal(β1-3)GlcNAc(β1-2)Man(α1-3)]Man(β1-4)GlcNAc(β1-4)GlcNAc(-Sp19
4B
5
6
7

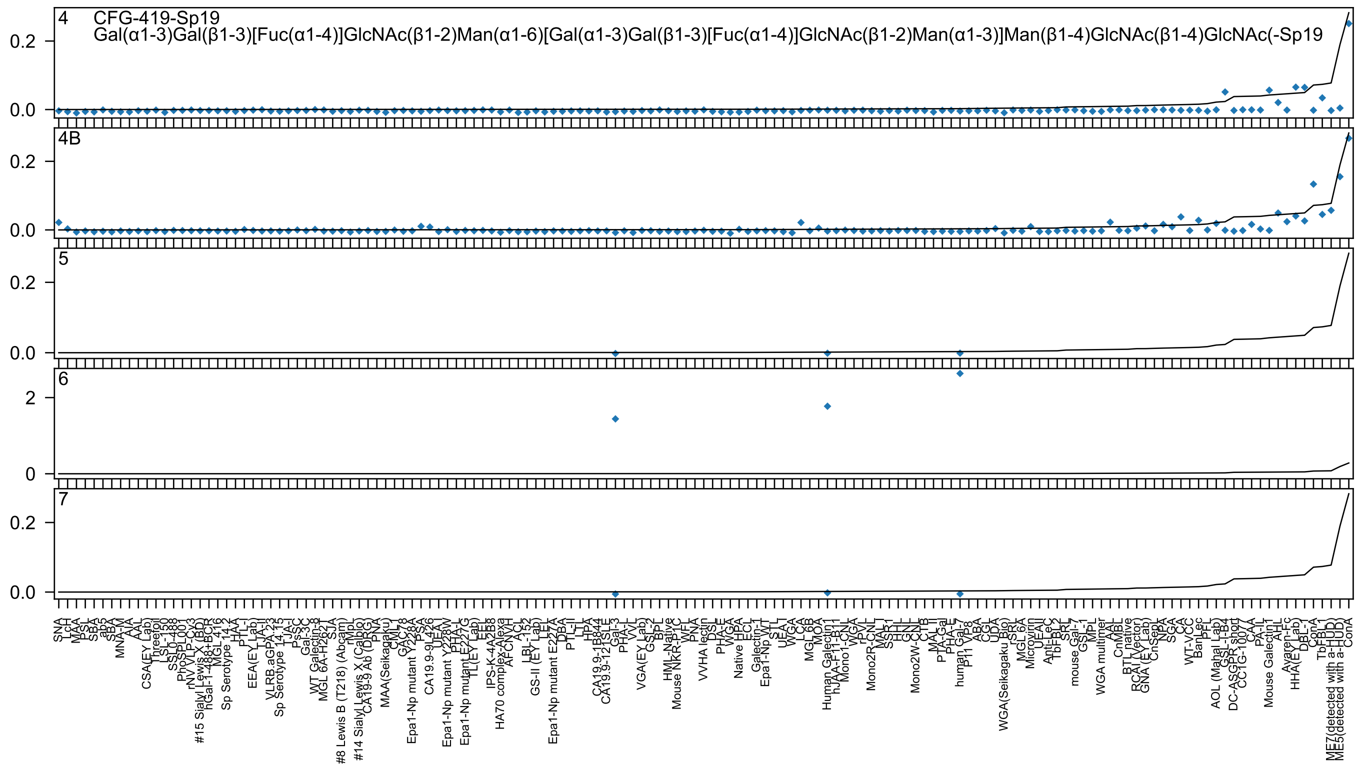

4
CFG-419-Sp19
Gal(α1-3)Gal(β1-3)[Fuc(α1-4)]GlcNAc(β1-2)Man(α1-6)[Gal(α1-3)Gal(β1-3)[Fuc(α1-4)]GlcNAc(β1-2)Man(α1-3)]Man(β1-4)GlcNAc(β1-4)GlcNAc(-Sp19
4B
5
6
7

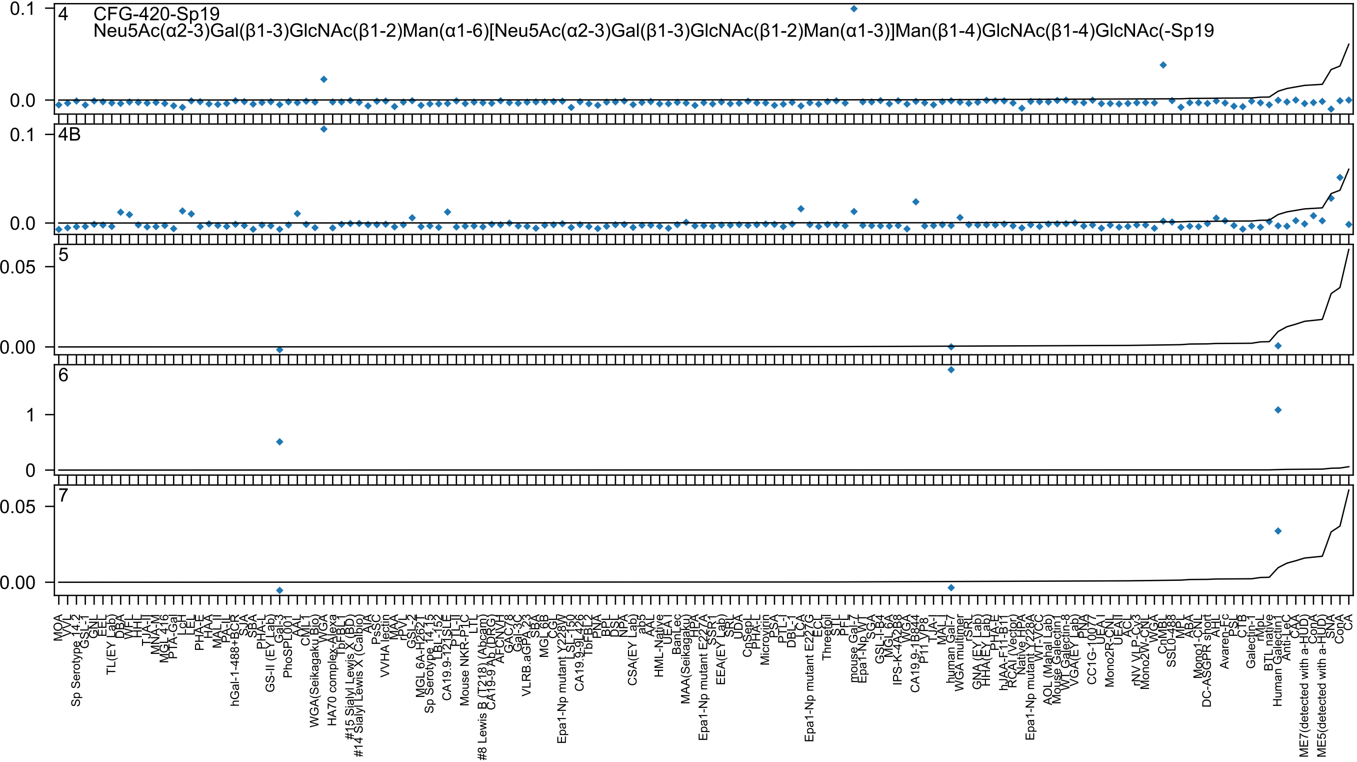

4 CFG-420-Sp19
Neu5Ac(α2-3)Gal(β1-3)GlcNAc(β1-2)Man(α1-6)[Neu5Ac(α2-3)Gal(β1-3)GlcNAc(β1-2)Man(α1-3)]Man(β1-4)GlcNAc(β1-4)GlcNAc(-Sp19
4B
5
6
7

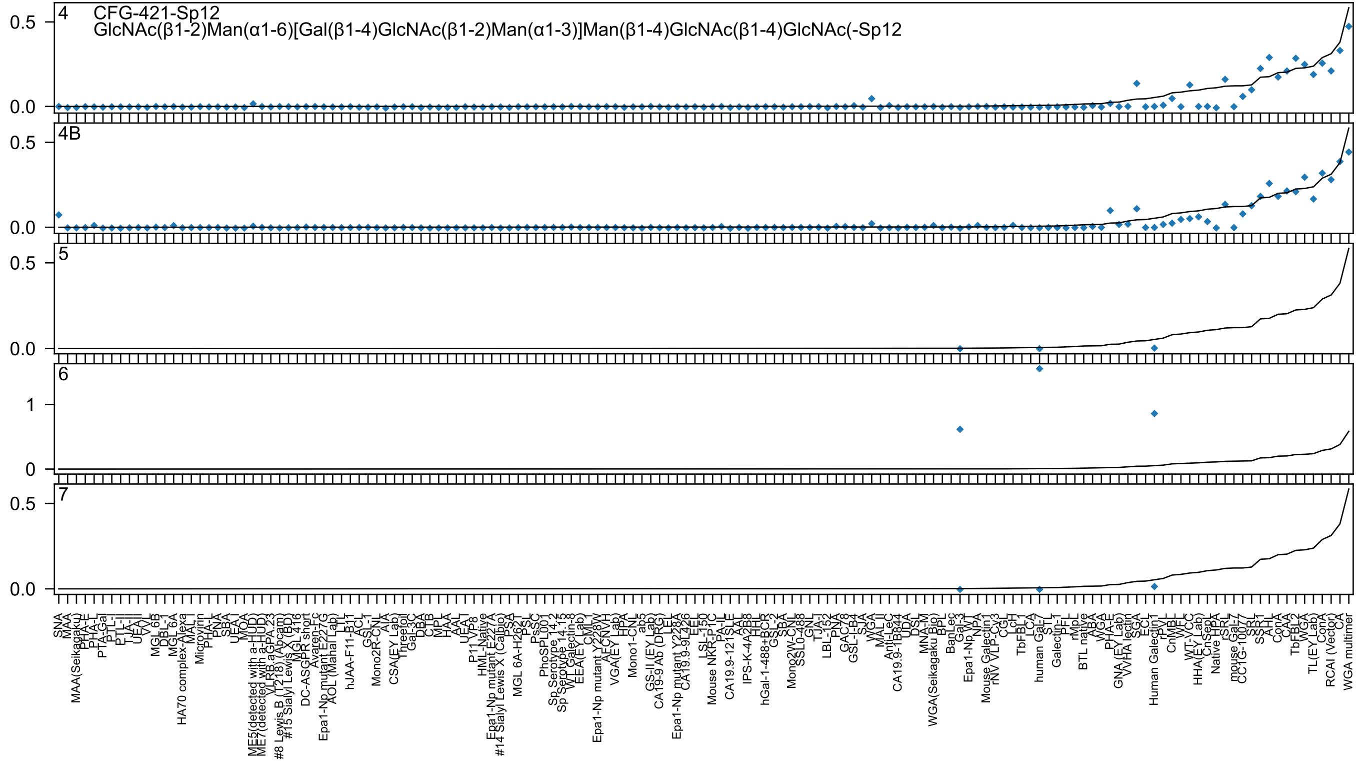

4
CFG-421-Sp12
GlcNAc(β1-2)Man(α1-6)[Gal(β1-4)GlcNAc(β1-2)Man(α1-3)]Man(β1-4)GlcNAc(β1-4)GlcNAc(-Sp12
4B
5
6
7

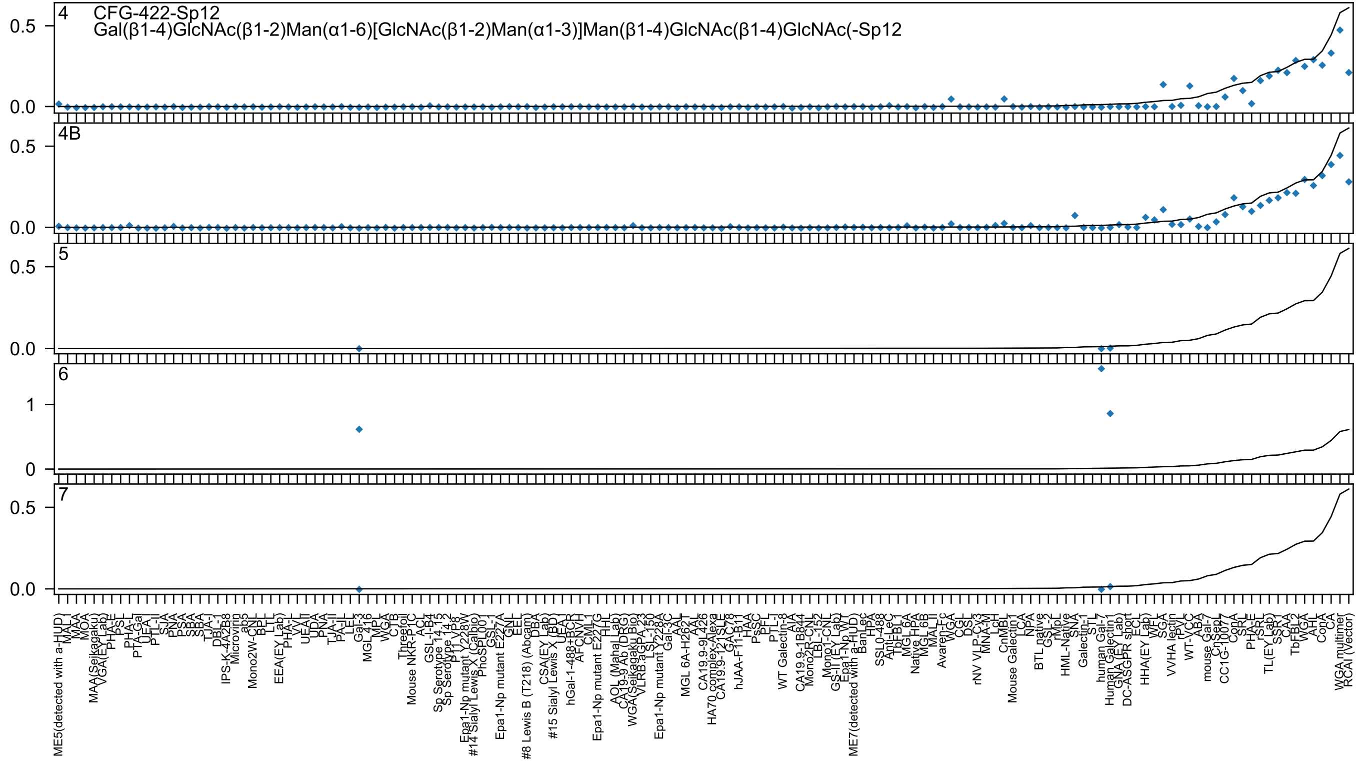
4 CFG-422-Sp12
Gal(β1-4)GlcNAc(β1-2)Man(α1-6)[GlcNAc(β1-2)Man(α1-3)]Man(β1-4)GlcNAc(β1-4)GlcNAc(-Sp12
4B
5
6
7
0.5
0.0
1
0

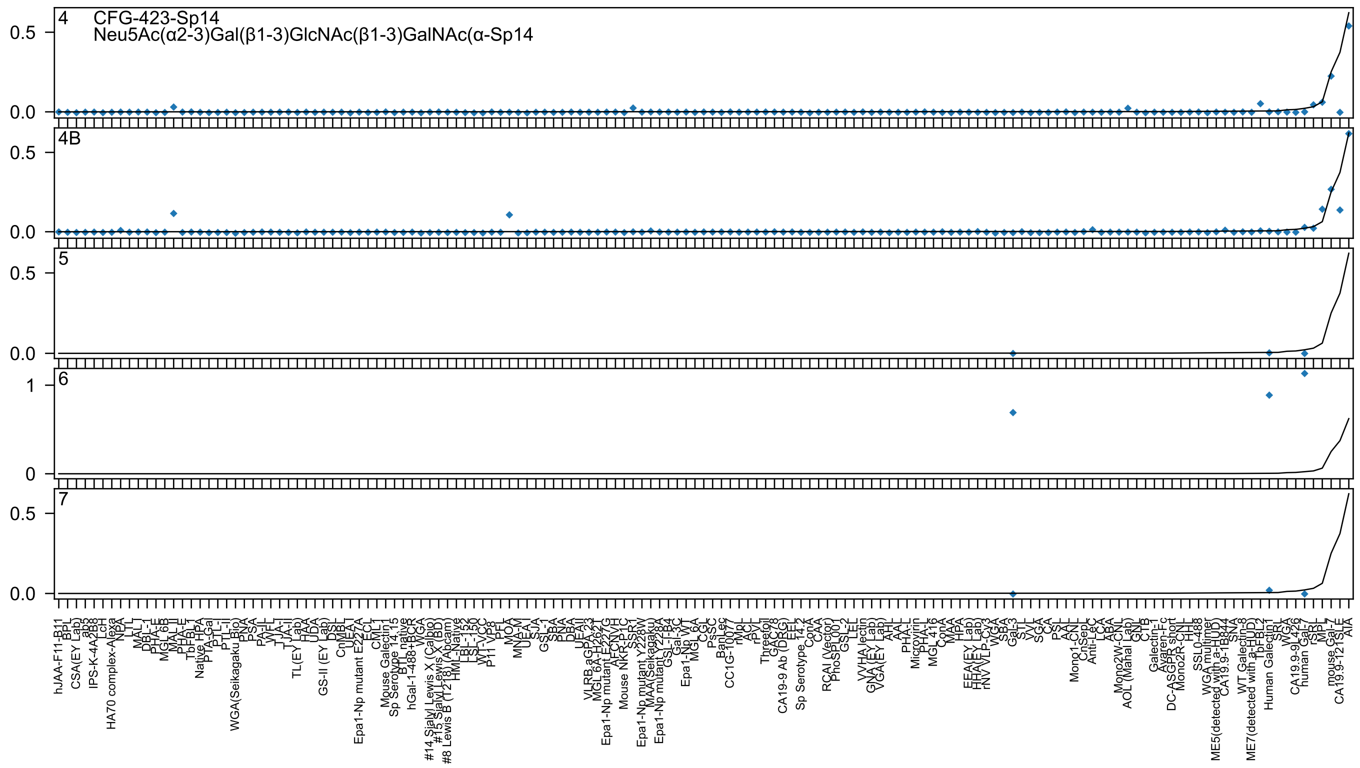
4
CFG-423-Sp14
Neu5Ac(α2-3)Gal(β1-3)GlcNAc(β1-3)GalNAc(α-Sp14
4B
5
6
7
0.5
0.0
1
0

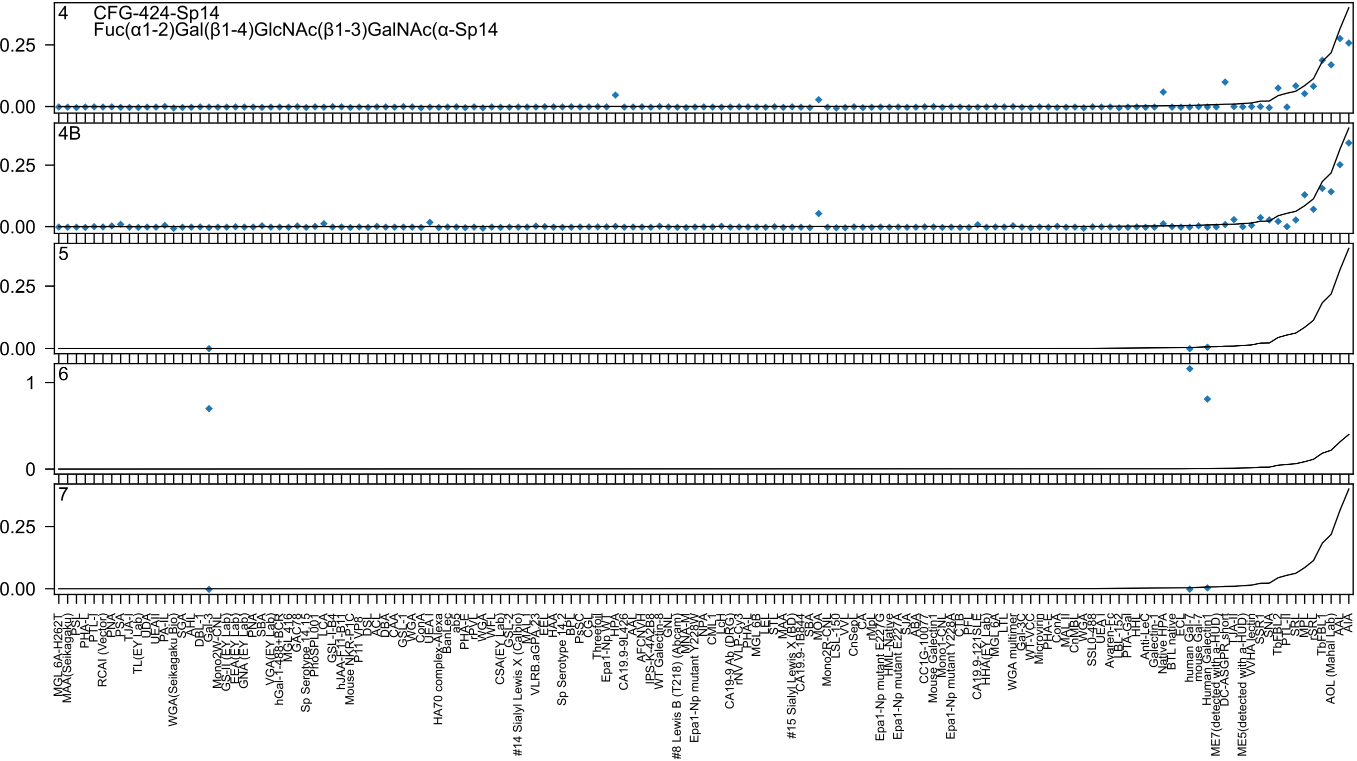
4
CFG-424-Sp14
Fuc(α1-2)Gal(β1-4)GlcNAc(β1-3)GalNAc(α-Sp14
4B
5
6
7

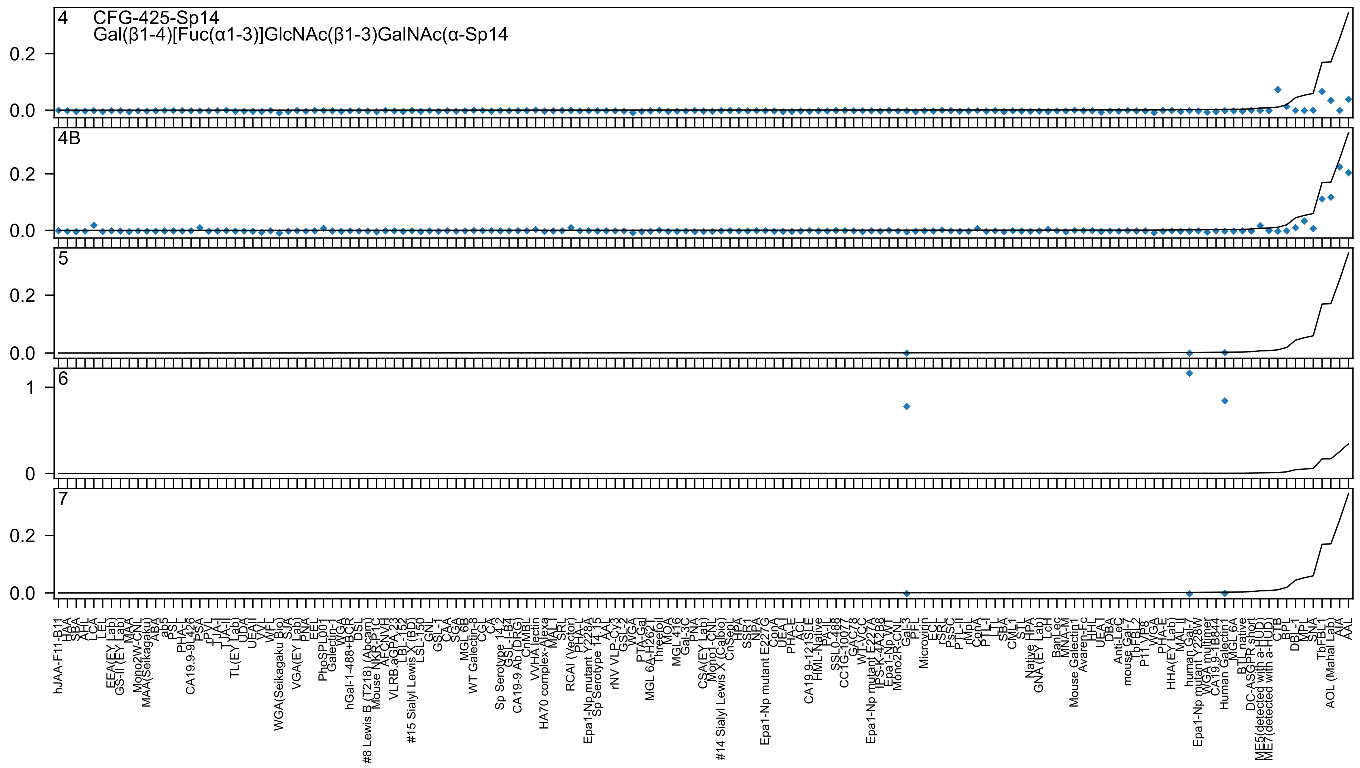

4 CFG-425-Sp14
Gal(β1-4)[Fuc(α1-3)]GlcNAc(β1-3)GalNAc(α-Sp14
4B
5
6
7

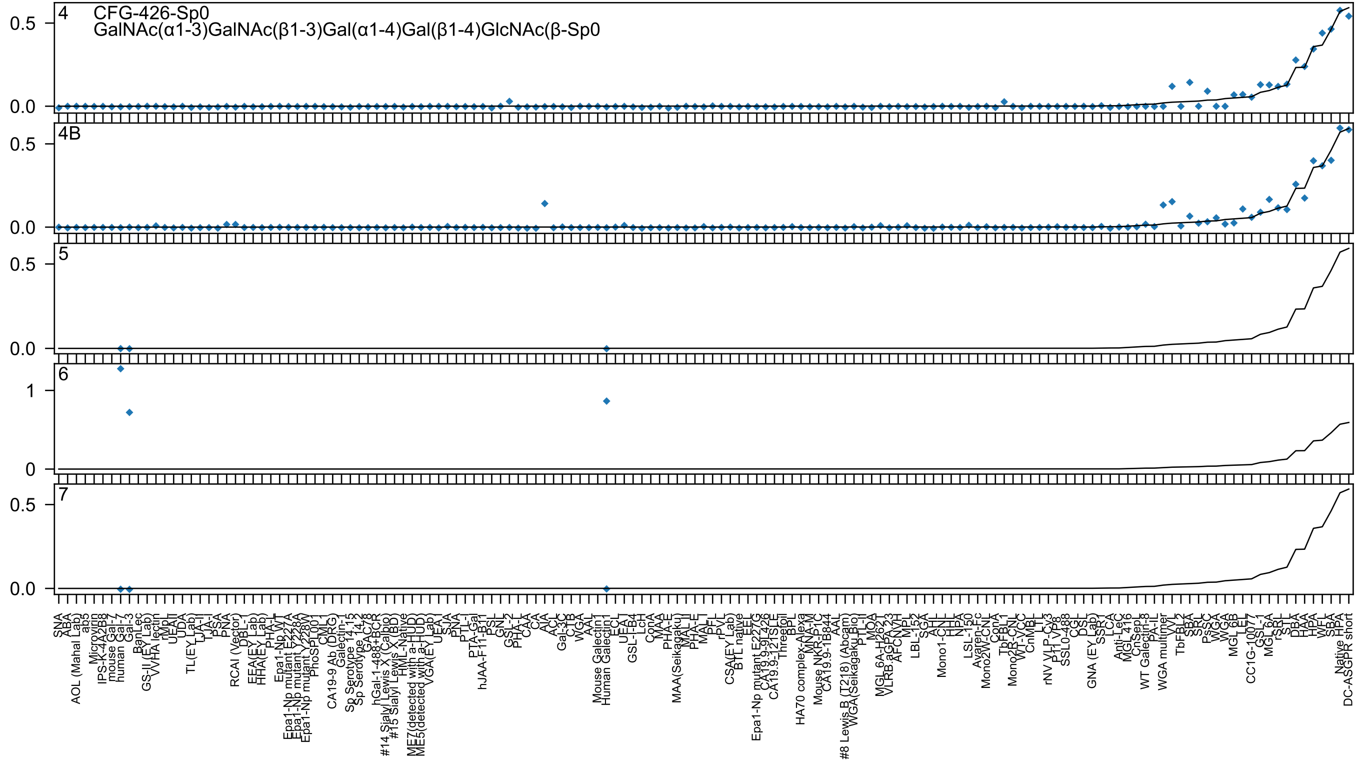

4 CFG-426-Sp0
GalNAc(α1-3)GalNAc(β1-3)Gal(α1-4)Gal(β1-4)GlcNAc(β-Sp0
4B
5
6
7

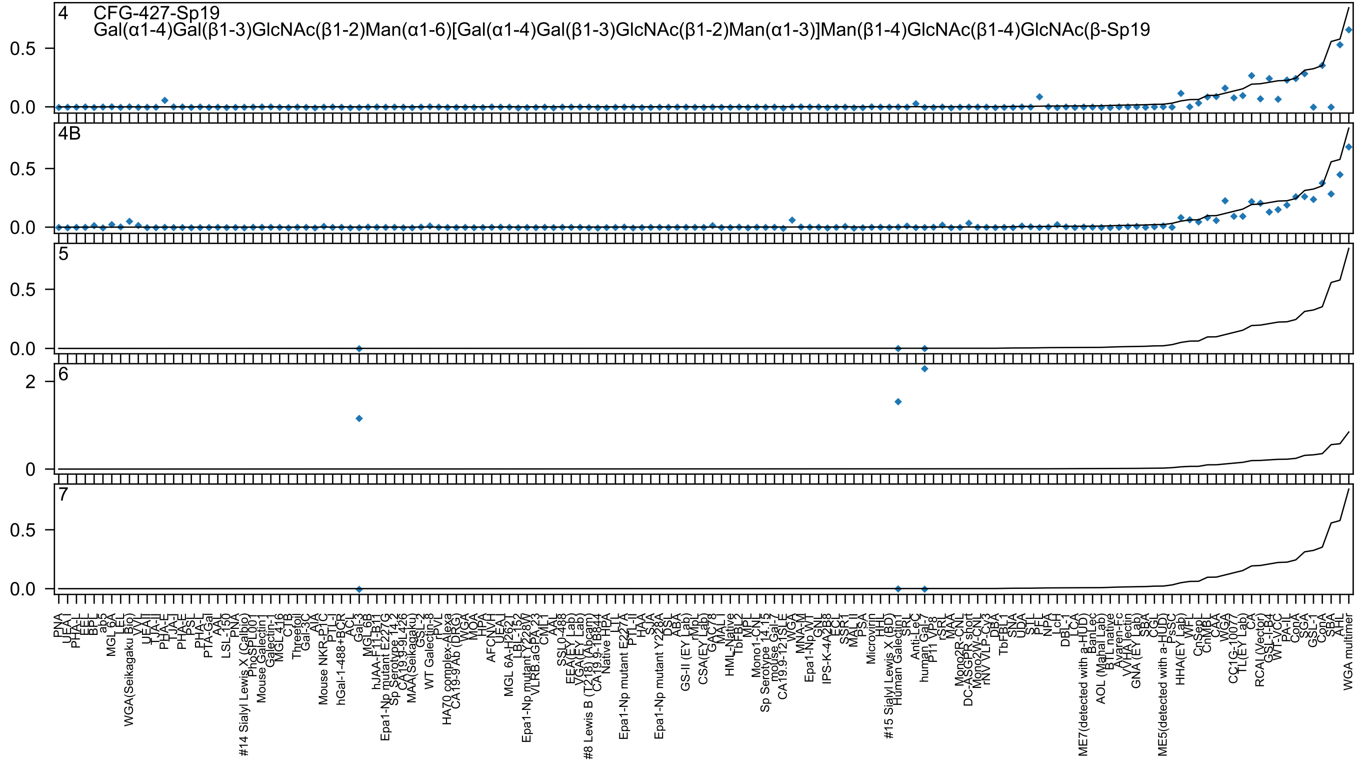

4
CFG-427-Sp19
Gal(α1-4)Gal(β1-3)GlcNAc(β1-2)Man(α1-6)[Gal(α1-4)Gal(β1-3)GlcNAc(β1-2)Man(α1-3)]Man(β1-4)GlcNAc(β1-4)GlcNAc(β-Sp19
4B
5
6
7

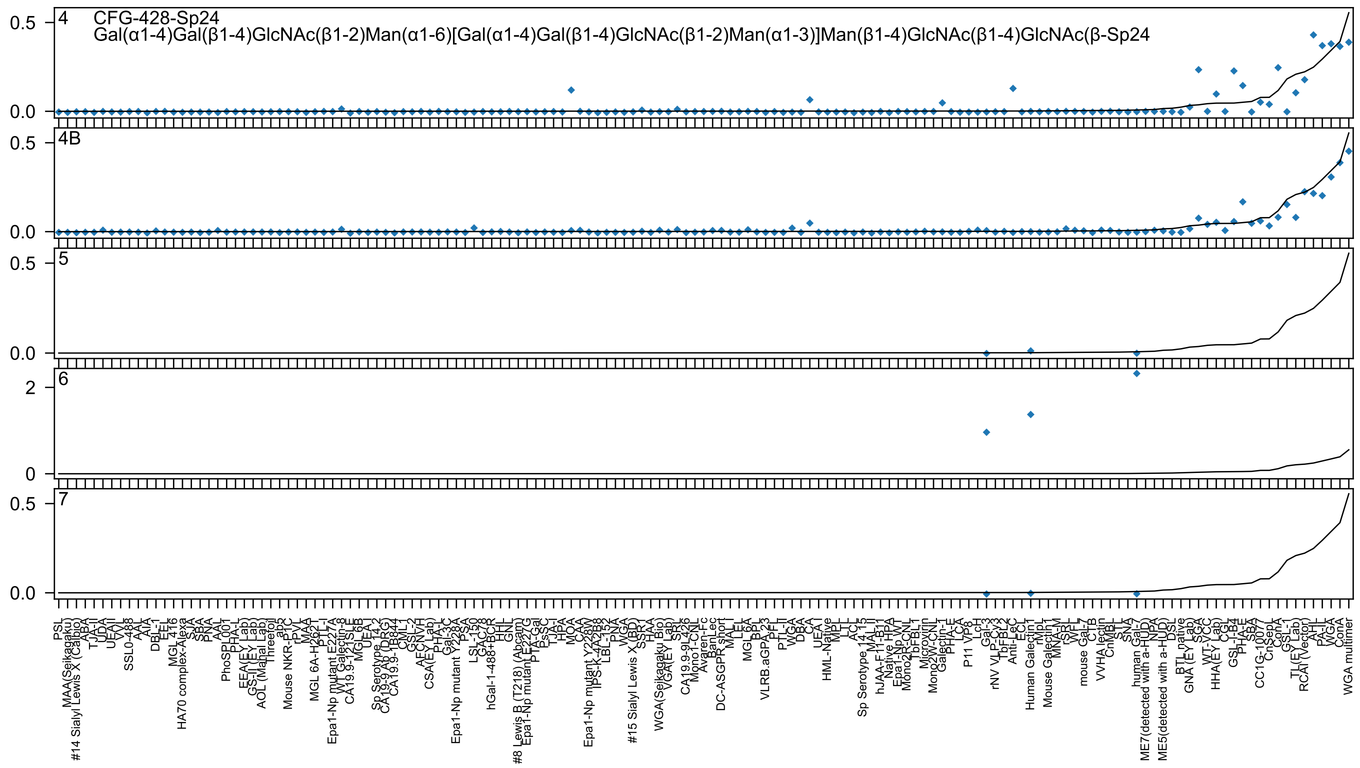

4
CFG-428-Sp24
Gal(α1-4)Gal(β1-4)GlcNAc(β1-2)Man(α1-6)[Gal(α1-4)Gal(β1-4)GlcNAc(β1-2)Man(α1-3)]Man(β1-4)GlcNAc(β1-4)GlcNAc(β-Sp24
4B
5
6
7
0.0
0.5
0
2
PSL
MAA(Seikagaku)
#14 Sialyl Lewis X (Calbio)
ABA
TJA-II
UDA
UEA-II
VVL
SSL0-488
AAL
AIA
DBL-1
EEL
MGL 416
HA70 complex-Alexa
SJA
SBA
PNA
AAL
PhoSL001
PHA-L
EEA(EY Lab)
GS-II (EY Lab)
AOL (Mahal Lab)
Threefoil
ab5
Mouse NKR-P1C
rPVL
MAA
MGL 6A-H262T
PTL-I
Epa1-Np mutant E227A
WT Galectin-8
CA19.9-121SLE
MGL 6B
UEA-I
Sp Serotype 14.2
CA19-9 Ab (DRG)
CA19.9-1B844
CML-1
GSL-2
AFCNVH
CSA(EY Lab)
PHA-L
Gal-3C
Epa1-Np mutant Y228A
PSA
LSL-150
GAC78
hGal-1-488+BCR
HHL
GNL
#8 Lewis B (T218) (Abcam)
Epa1-Np mutant E227G
PTA-Gal
PsSC
TJA-I
HPA
MOA
CAA
Epa1-Np mutant Y228W
IPS-K-4A2B8
LBL-152
PNA
WGA
#15 Sialyl Lewis X (BD)
SSR1
HAA
WGA(Seikagaku Bio)
VGA(EY Lab)
SRL
CA19.9-9L426
Mono1-CNL
Avaren-Fc
BanLec
DC-ASGPR short
MAL-I
LEL
MGL 6A
BPL
VLRB.aGPA.23
PF
PTL-II
WGA
DBA
CA
UEA-I
HML-Native
MPL
LTL
ACL
Sp Serotype 14.15
MAL-II
hJAA-F11-B11
Native HPA
Epa1-Np WT
Mono2R-CNL
TbFBL1
Microvirin
Mono2W-CNL
Galectin-1
PHA-E
LCA
P11 VP8
LcH
Gal-3
rNV VLP-Cy3
TbFBL2
Anti-LeC
ECL
Human Galectin1
rMpL
Mouse Galectin1
MNA-M
rSRL
WFL
mouse Gal-7
CTB
VVHA lectin
CnMBL
STL
SNA
human Gal-7
ME7(detected with a-HUD)
NPA
ME5(detected with a-HUD)
DSL
BTL native
GNA (EY Lab)
SGA
WT-VCC
HHA(EY Lab)
CGL
GSL-I-B4
PHA-E
SBA
CC1G-10077
CnSep
ConA
GSL-1
TL(EY Lab)
RCAI (Vector)
AHL
PA-IL
WGA
ConA
WGA multimer

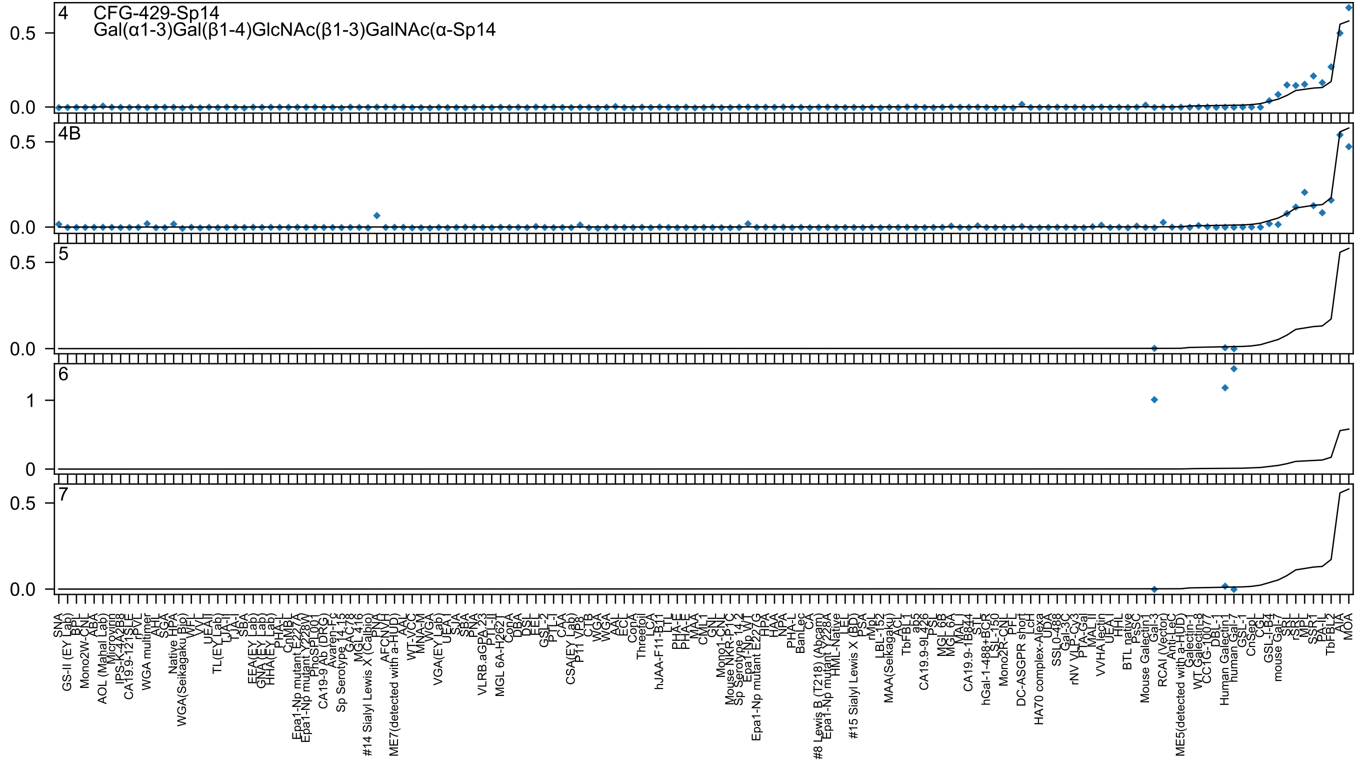

4 CFG-429-Sp14
Gal(α1-3)Gal(β1-4)GlcNAc(β1-3)GalNAc(α-Sp14
4B
5
6
7

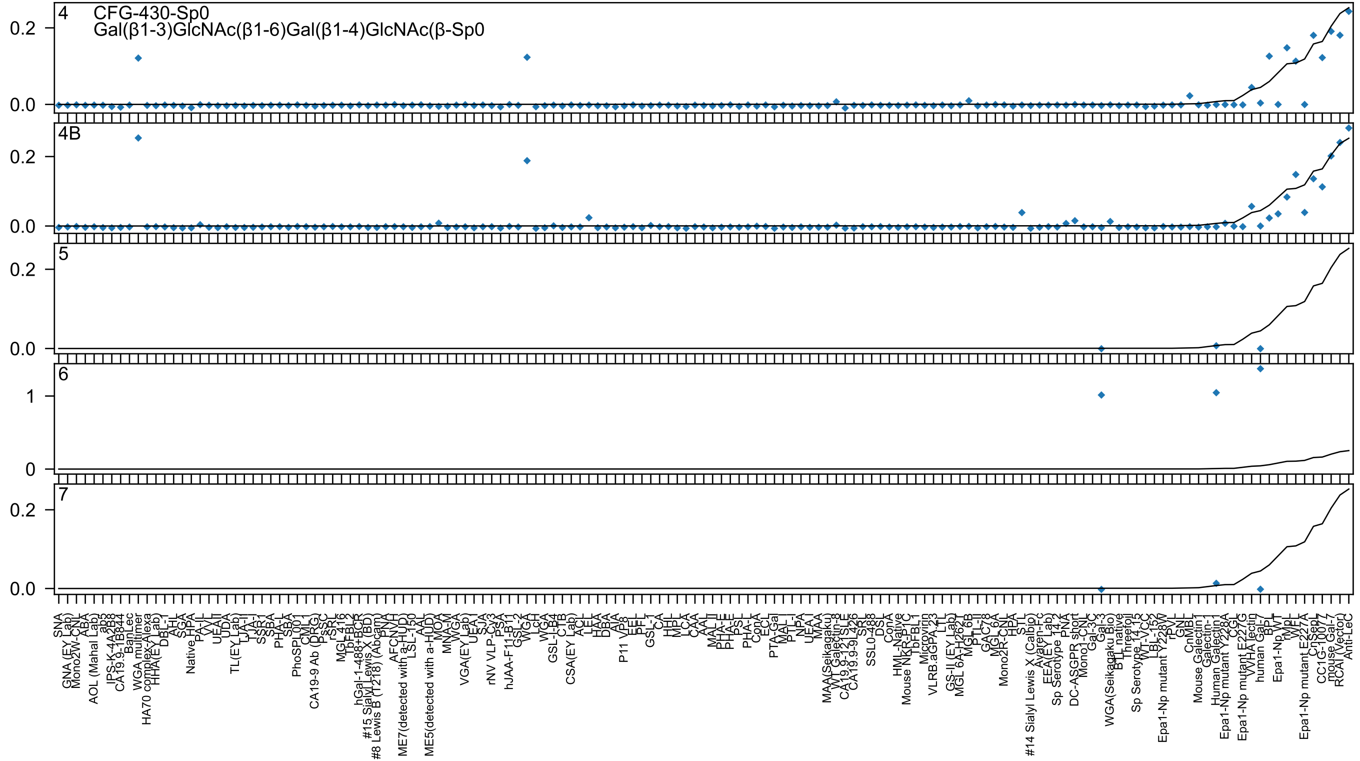

4
CFG-430-Sp0
Gal(β1-3)GlcNAc(β1-6)Gal(β1-4)GlcNAc(β-Sp0
4B
5
6
7

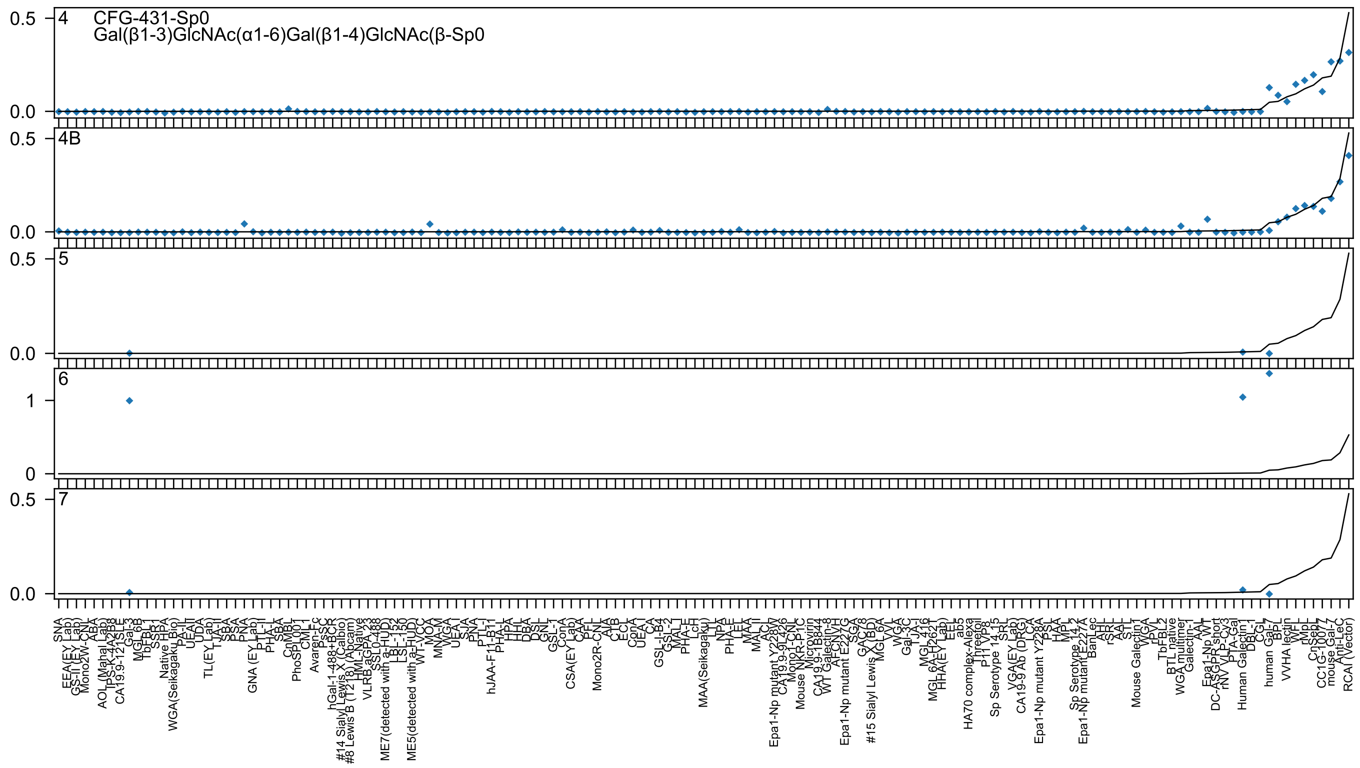

4
CFG-431-Sp0
Gal(β1-3)GlcNAc(α1-6)Gal(β1-4)GlcNAc(β-Sp0
4B
5
6
7

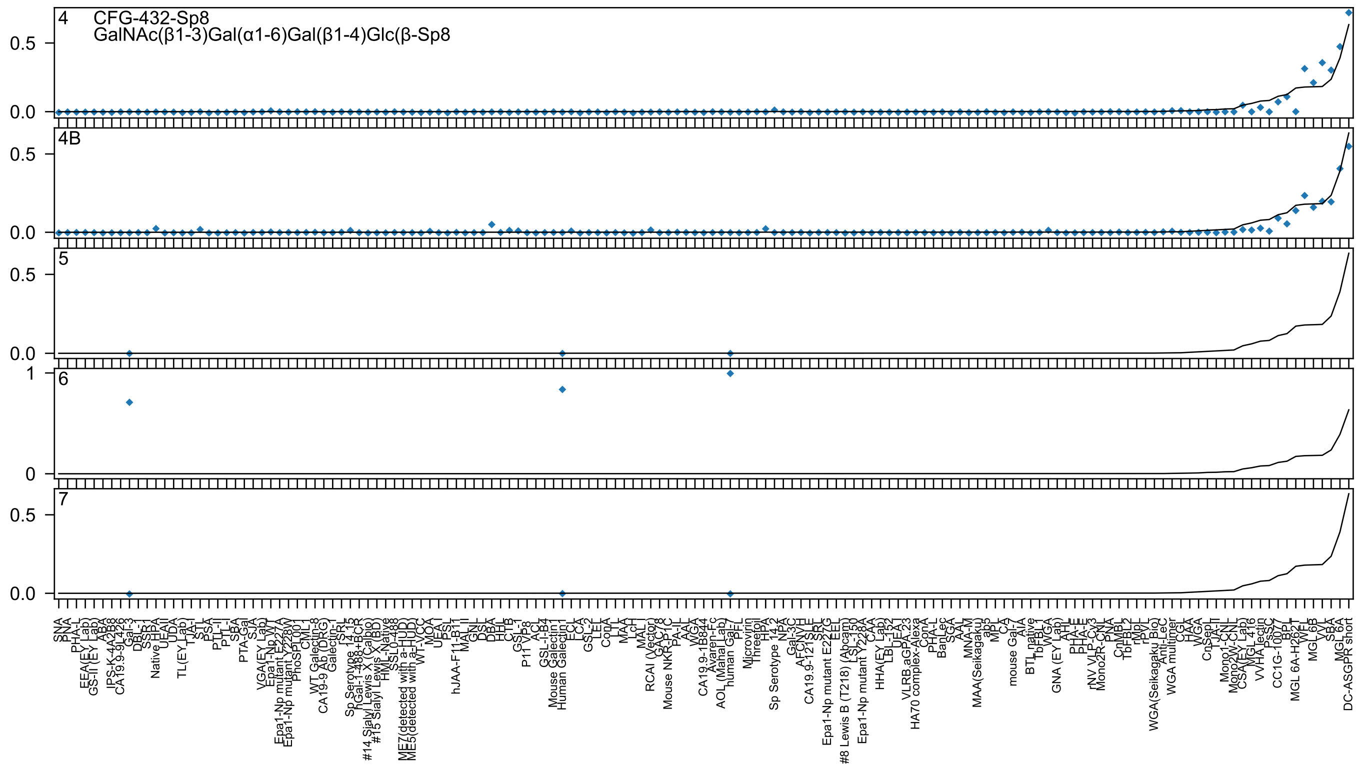

4
CFG-432-Sp8
GalNAc(β1-3)Gal(α1-6)Gal(β1-4)Glc(β-Sp8
4B
5
6
7

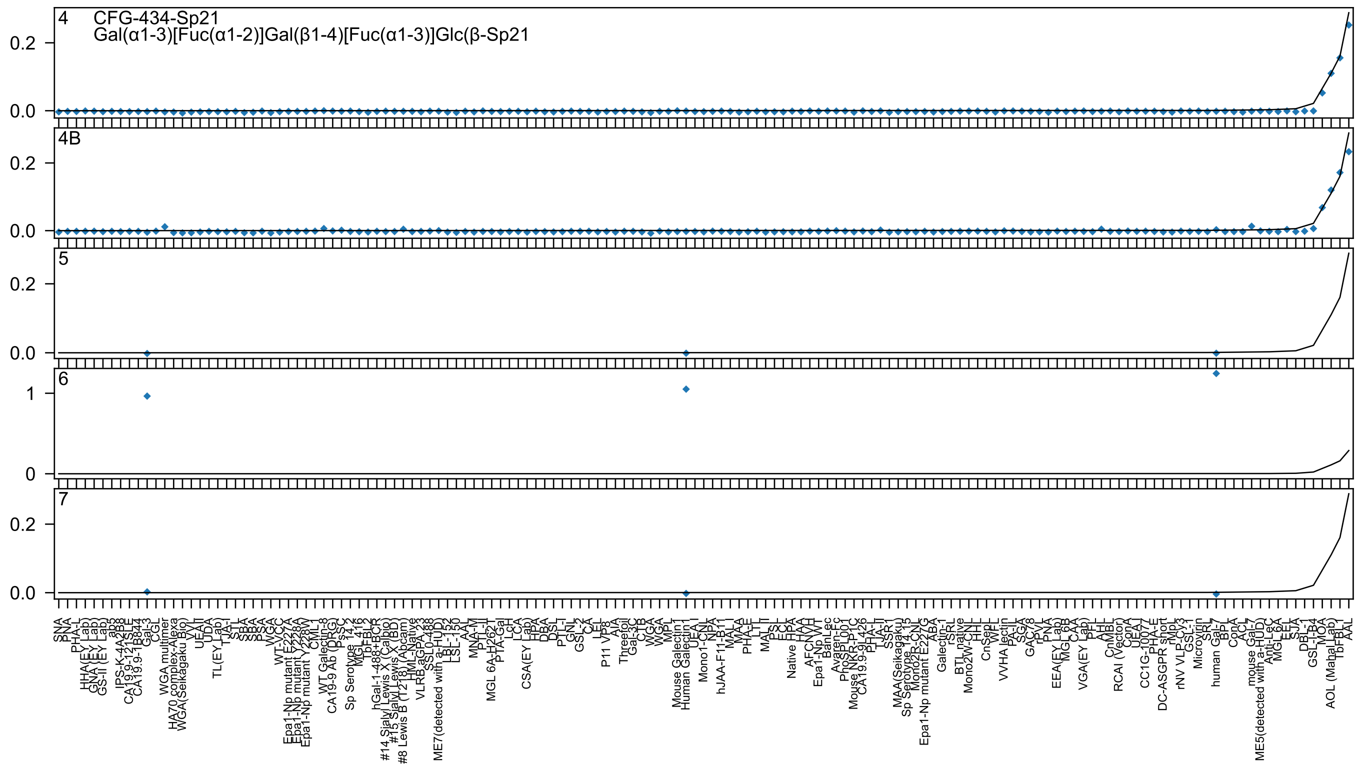
4 CFG-434-Sp21
Gal(α1-3)[Fuc(α1-2)]Gal(β1-4)[Fuc(α1-3)]Glc(β-Sp21
4B
5
6
7

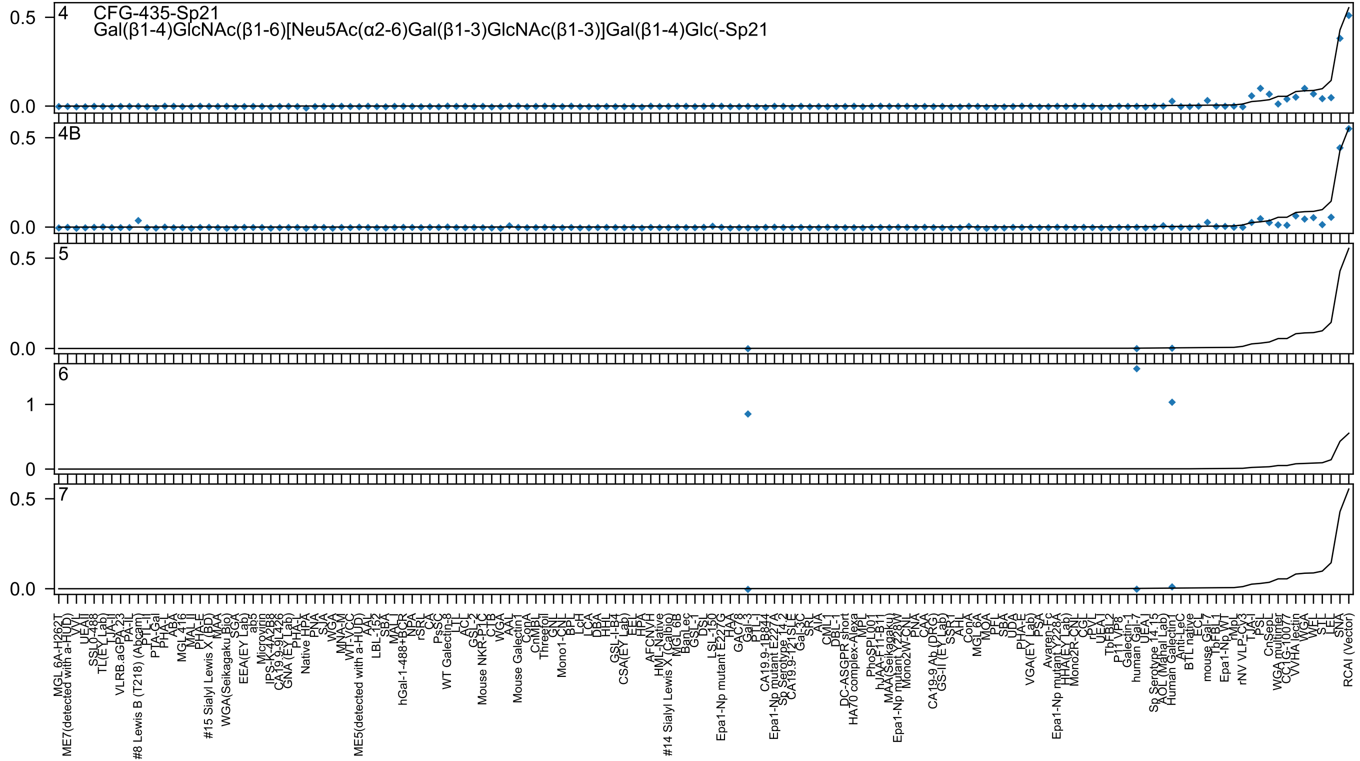
4
CFG-435-Sp21
Gal(β1-4)GlcNAc(β1-6)[Neu5Ac(α2-6)Gal(β1-3)GlcNAc(β1-3)]Gal(β1-4)Glc(-Sp21
4B
5
6
7
0.5
0.0
1
0

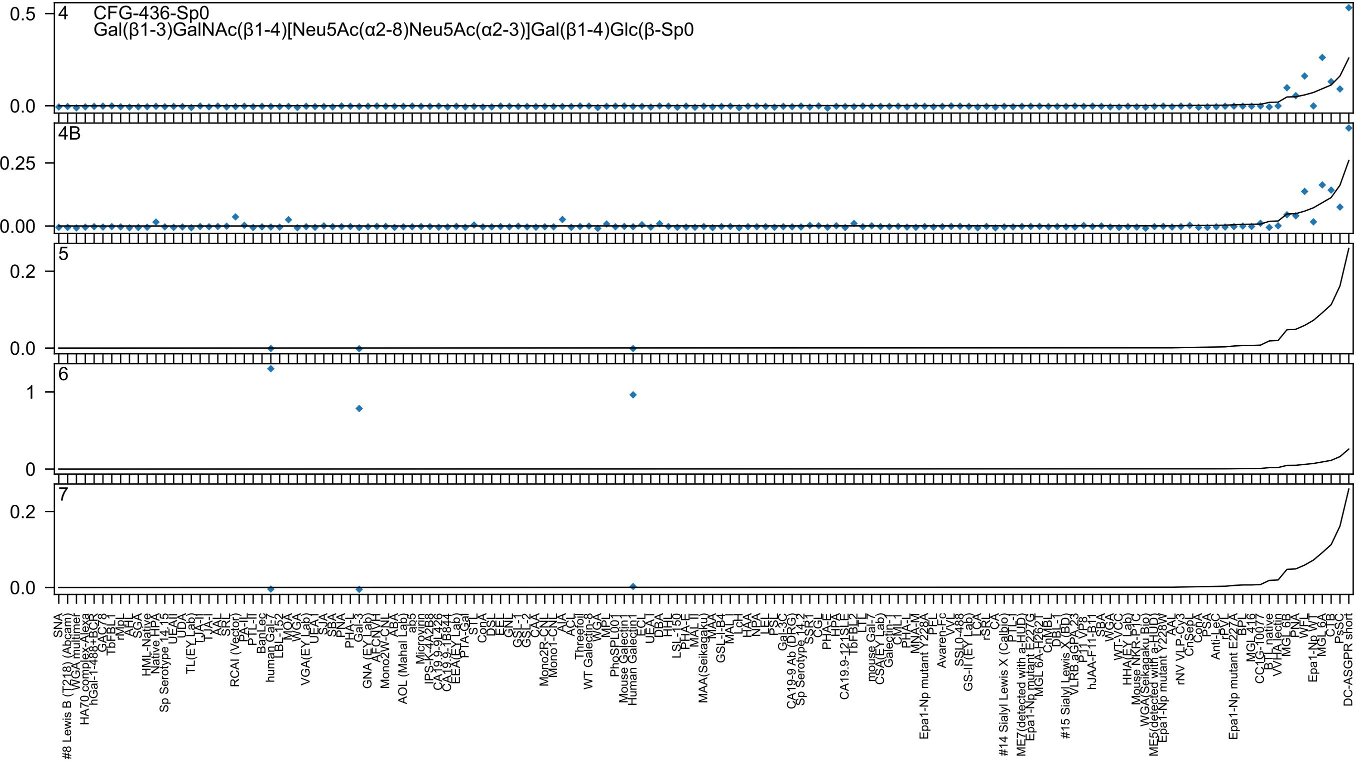

4 CFG-436-Sp0
Gal(β1-3)GalNAc(β1-4)[Neu5Ac(α2-8)Neu5Ac(α2-3)]Gal(β1-4)Glc(β-Sp0
4B
5
6
7

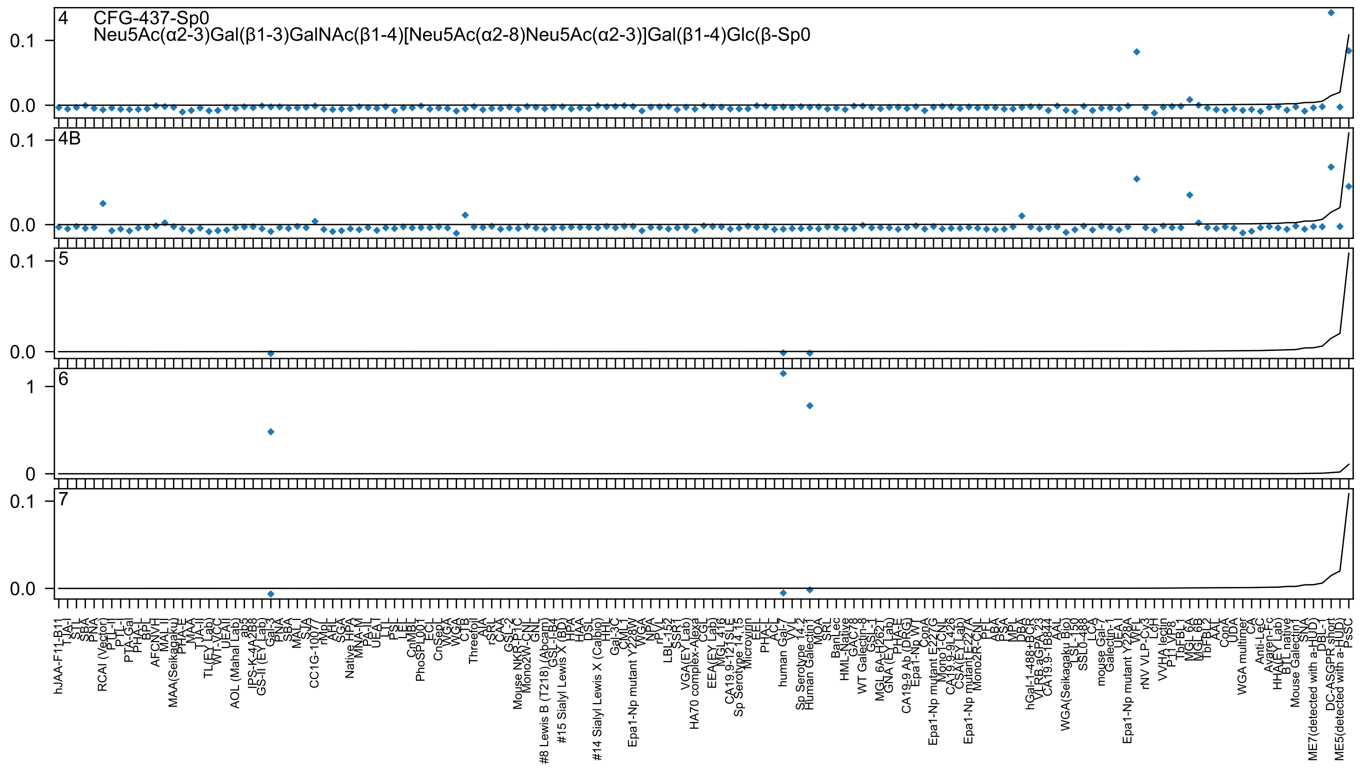
4 CFG-437-Sp0
Neu5Ac(α2-3)Gal(β1-3)GalNAc(β1-4)[Neu5Ac(α2-8)Neu5Ac(α2-3)]Gal(β1-4)Glc(β-Sp0
4B
5
6
7

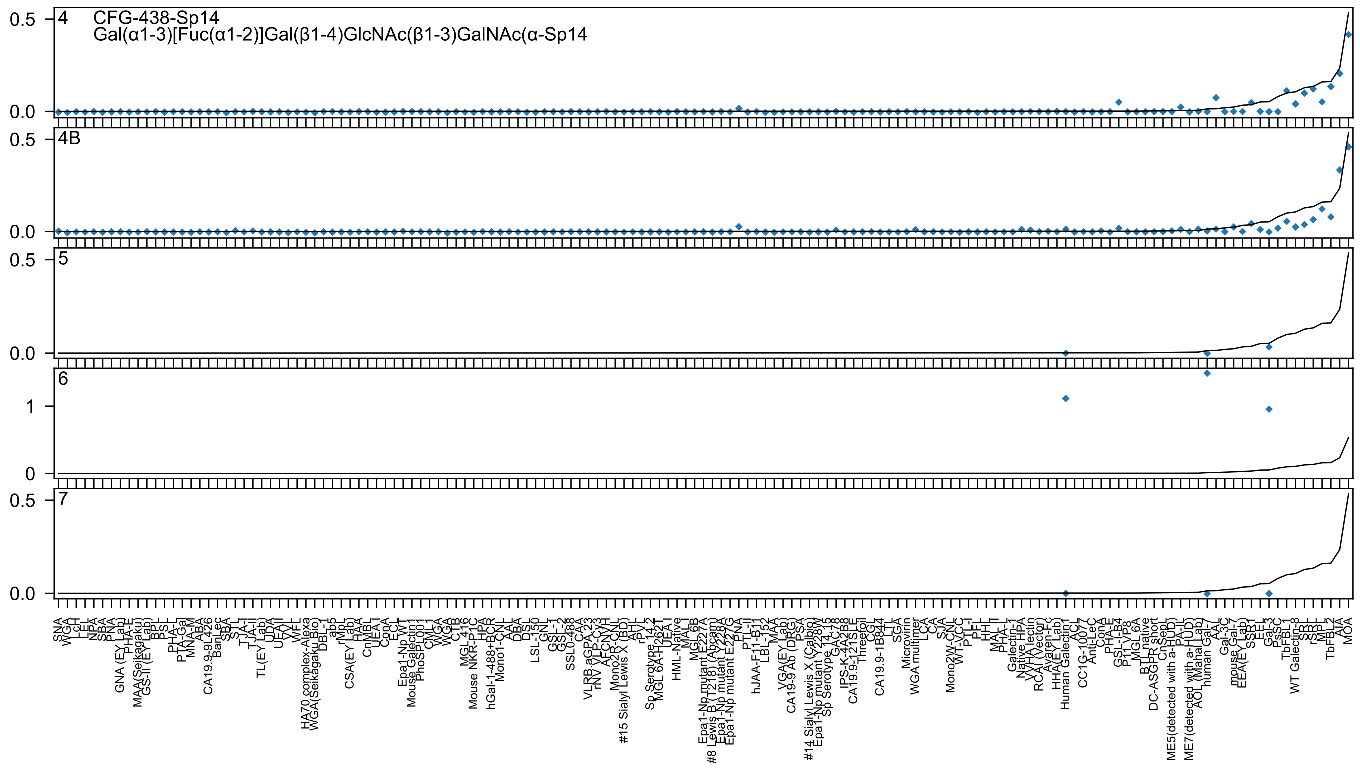
4
CFG-438-Sp14
Gal(α1-3)[Fuc(α1-2)]Gal(β1-4)GlcNAc(β1-3)GalNAc(α-Sp14
4B
5
6
7
0.5
0.0
1
0

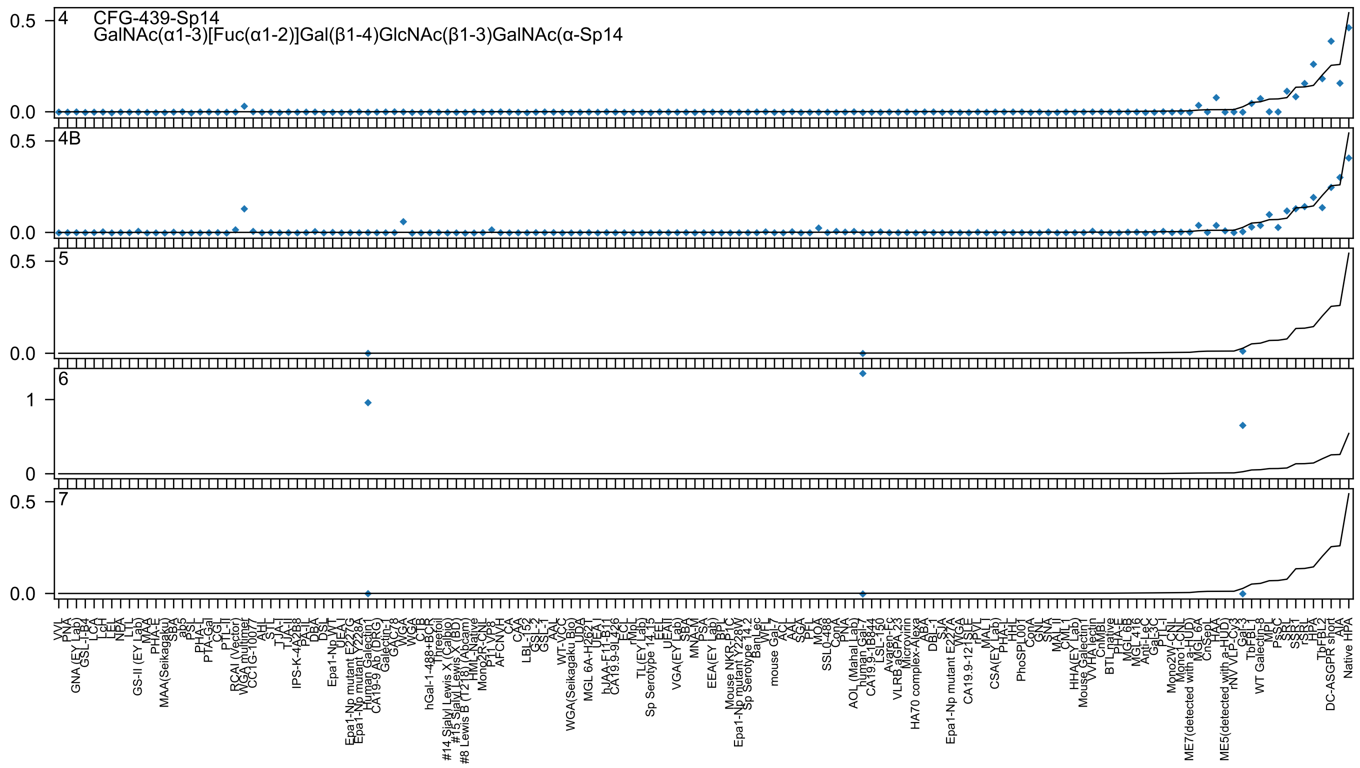

4
CFG-439-Sp14
GalNAc(α1-3)[Fuc(α1-2)]Gal(β1-4)GlcNAc(β1-3)GalNAc(α-Sp14
4B
5
6
7
0.5
0.0
1
0

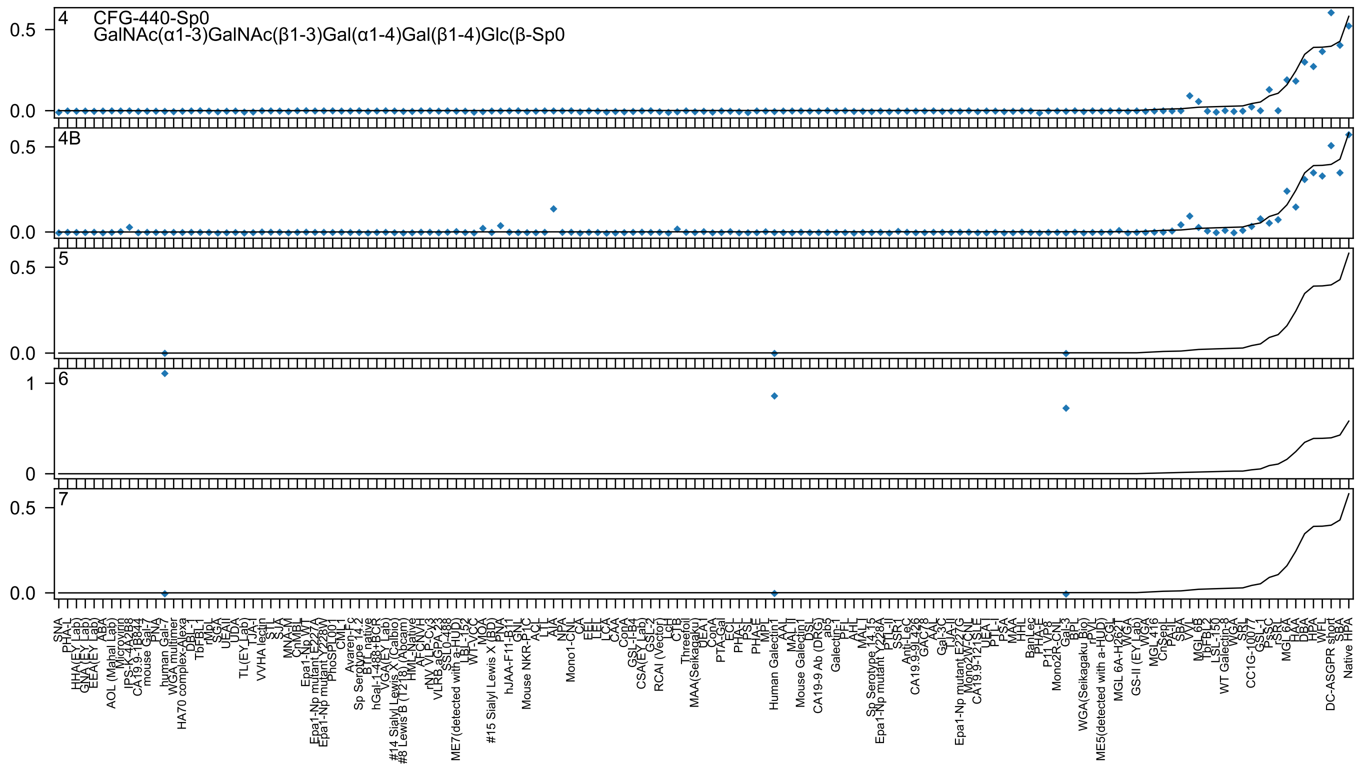

4
CFG-440-Sp0
GalNAc(α1-3)GalNAc(β1-3)Gal(α1-4)Gal(β1-4)Glc(β-Sp0
4B
5
6
7
SNA
PHA-L
HHA(EY Lab)
GNA(EY Lab)
EEA(EY Lab)
ABA
AOL (Mahal Lab)
Microvirin
IPS-K-4A2B8
CA19.9-1B844
mouse Gal-7
PNA
human Gal-7
WGA multimer
HA70 complex-Alexa
DBL-1
TbFBL1
rMpL
SGA
UEAII
UDA
TL(EY Lab)
TJA-I
VVHA lectin
STL
SJA
MNA-M
CnMBL
Epa1-Np WT
Epa1-Np mutant E227A
Epa1-Np mutant Y228W
PhoSL001
CML-I
Avaren-Fc
Sp Serotype 14.2
BTL native
hGal-1-488+BCR
VGA(EY Lab)
#14 Sialyl Lewis X (Calbio)
#8 Lewis B (T218) (Abcam)
HML-Native
AFCNVH
rNV VLP-Cy3
VLRB.aGPA.23
SSL0-488
ME7(detected with a-HUD)
LBL-152
WT-VCC
MOA
#15 Sialyl Lewis X (BD)
PNA
hJAA-F11-B11
GNL
Mouse NKR-P1C
ACL
LTL
AIA
NPA
Mono1-CNL
CA
EEL
LEL
LCA
CAA
ConA
GSL-I-B4
CSA(EY Lab)
GSL-2
RCAI (Vector)
LcH
CTB
Threefoil
MAA(Seikagaku)
UEA-I
ConA
PTA-Gal
ECL
PHA-L
PSL
PHA-E
MPL
Human Galectin1
AAL
MAL-II
Mouse Galectin1
DSL
CA19-9 Ab (DRG)
ab5
Galectin-1
PFL
AHL
MAL-I
Sp Serotype 14.5
Epa1-Np mutant Y228A
PTL-II
SSR1
Anti-LeC
CA19.9-9L426
GAC478
AAL
Gal-3C
TJA-II
Epa1-Np mutant E227G
Mono2WCNL
CA19.9-121SLE
UEA-I
PTL-I
PSA
MAA
HHL
BanLec
PHA-E
P11 VP8
Mono2R-CNL
Gal-3
BPL
WGA(Seikagaku Bio)
rPVL
ME5(detected with a-HUD)
CGL
MGL 6A-H262T
WGA
GS-II (EY Lab)
WGA
MGL 416
CnSepL
PA-IL
SBA
VVL
MGL 6B
TbFBL2
LSL-150
WT Galectin-8
WGA
SRL
CC1G-10077
GSL-1
PsSC
rSRL
MGL 6A
HAA
DBA
HPA
WFL
DC-ASGPR short
SBA
Native HPA

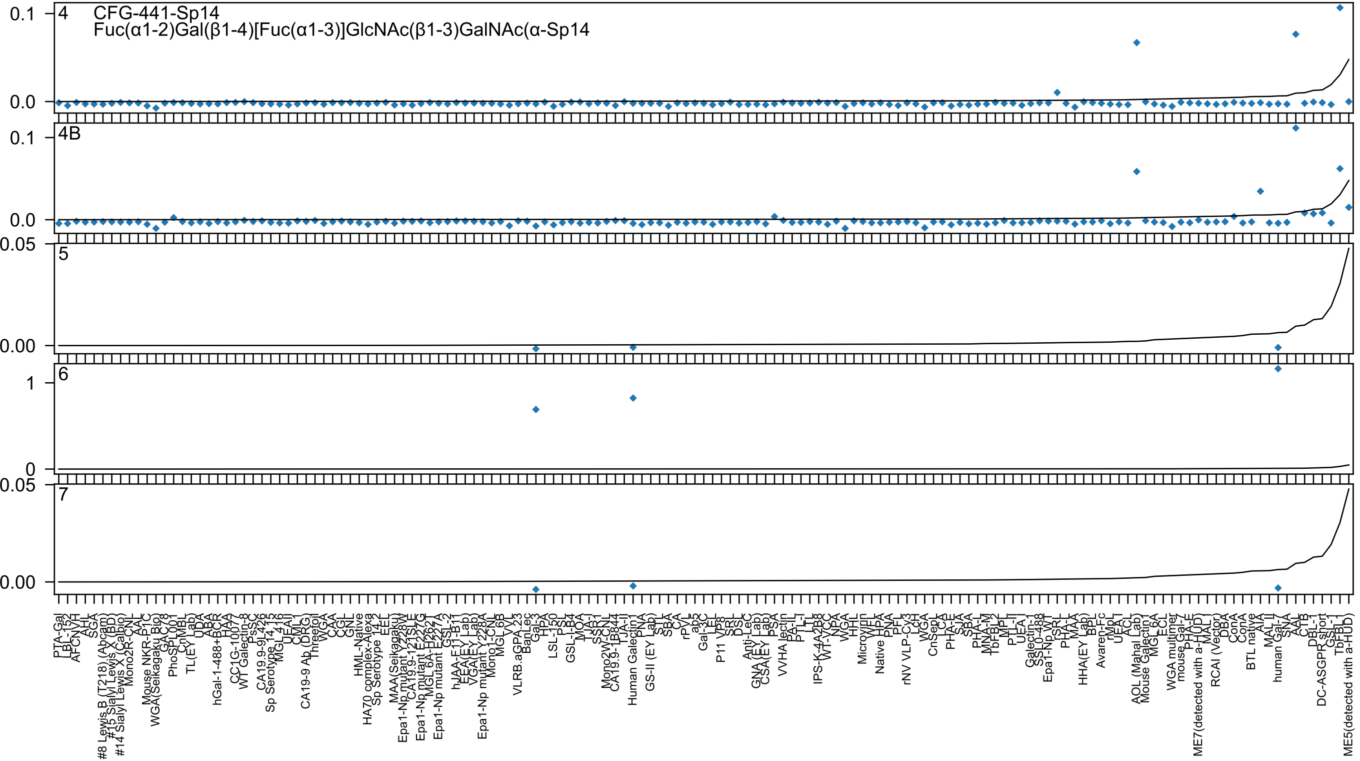

CFG-441-Sp14
Fuc(α1-2)Gal(β1-4)[Fuc(α1-3)]GlcNAc(β1-3)GalNAc(α-Sp14
4
4B
5
6
7

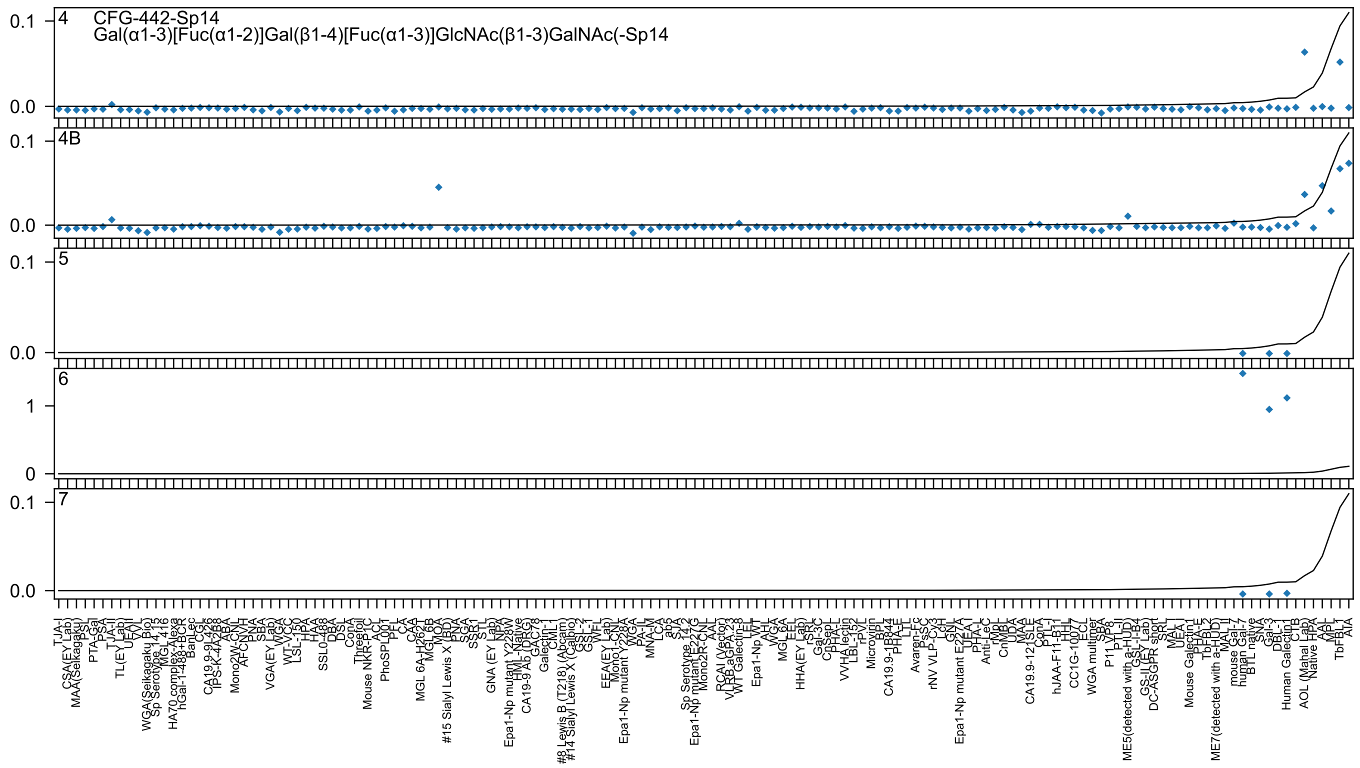

4
CFG-442-Sp14
Gal(α1-3)[Fuc(α1-2)]Gal(β1-4)[Fuc(α1-3)]GlcNAc(β1-3)GalNAc(-Sp14
4B
5
6
7

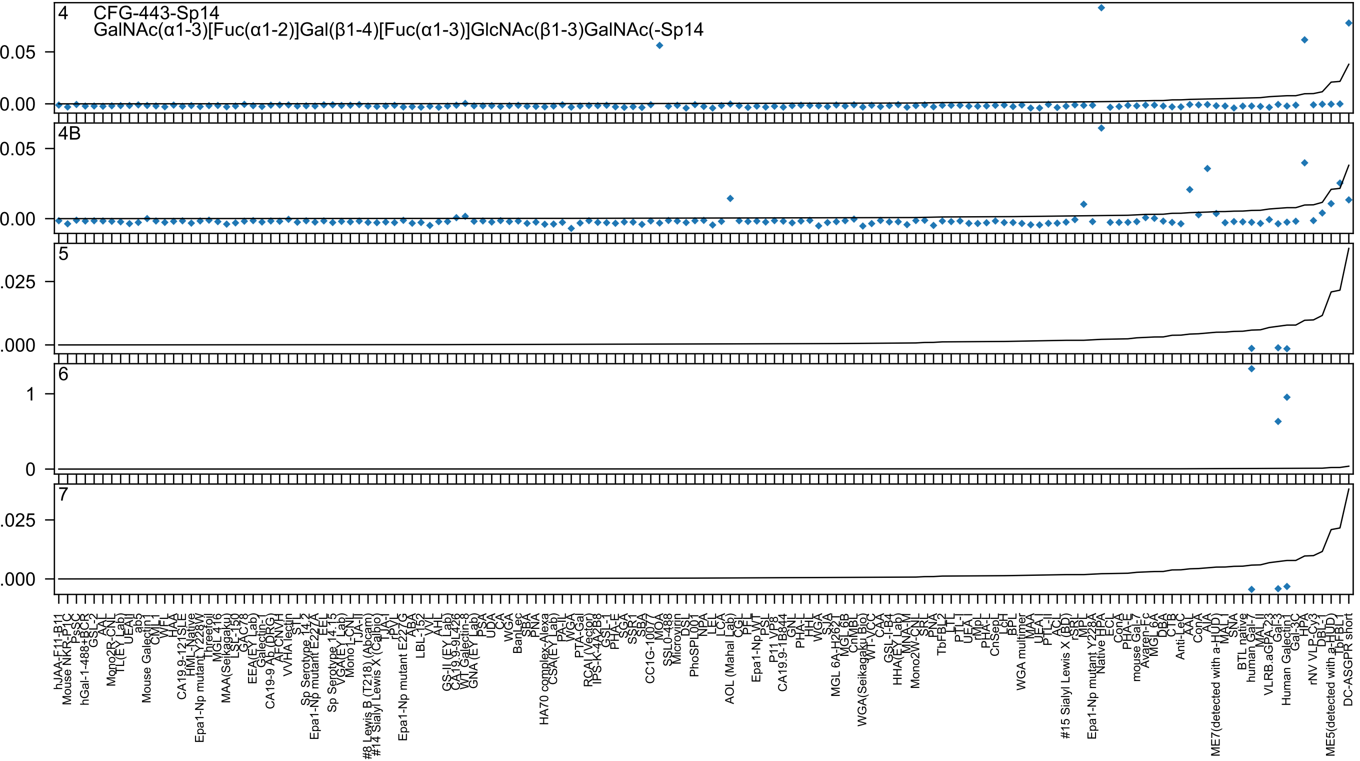

CFG-443-Sp14
GalNAc(α1-3)[Fuc(α1-2)]Gal(β1-4)[Fuc(α1-3)]GlcNAc(β1-3)GalNAc(-Sp14
4
4B
5
6
7

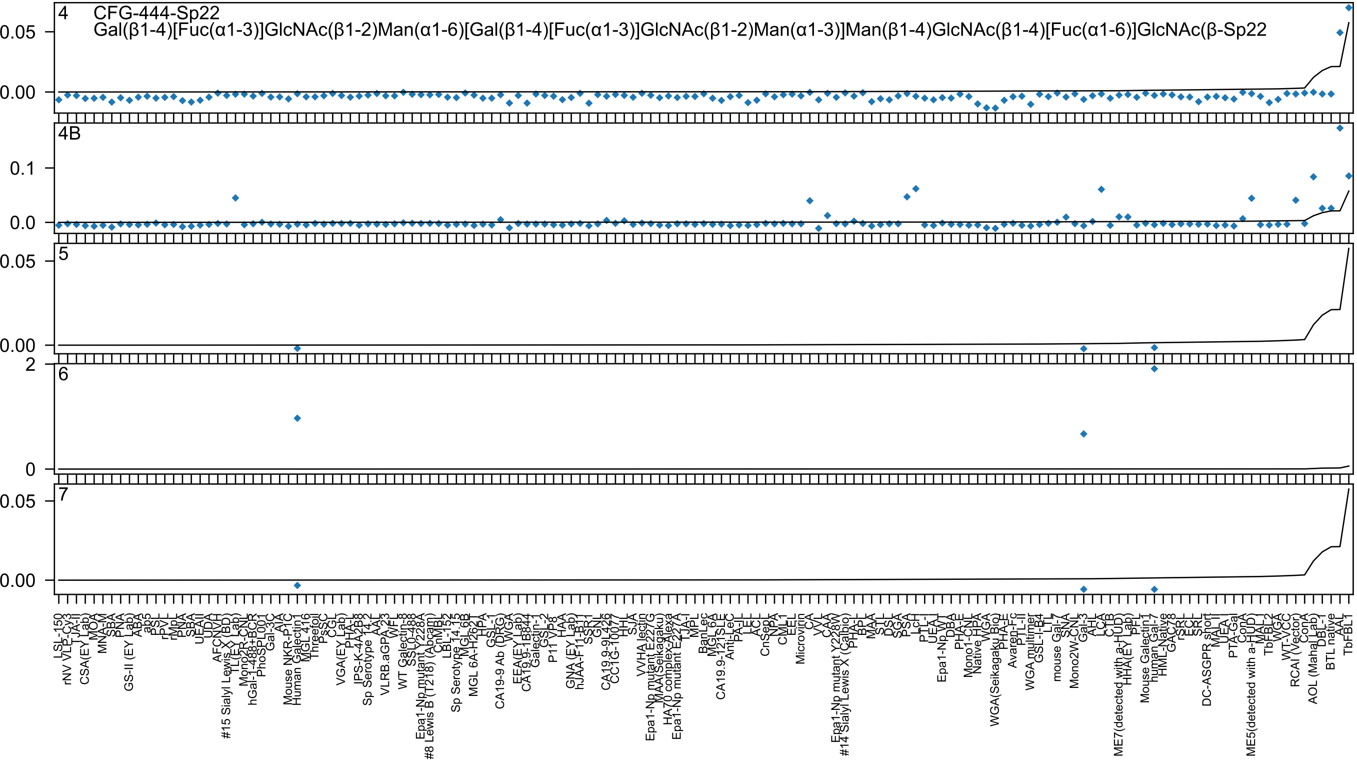
4
CFG-444-Sp22
Gal(β1-4)[Fuc(α1-3)]GlcNAc(β1-2)Man(α1-6)[Gal(β1-4)[Fuc(α1-3)]GlcNAc(β1-2)Man(α1-3)]Man(β1-4)GlcNAc(β1-4)[Fuc(α1-6)]GlcNAc(β-Sp22
0.05
0.00
4B
0.1
0.0
5
0.05
0.00
6
2
0
7
0.05
0.00
LSL-150
rNV VLP-Cy3
TJA-II
CSA(EY Lab)
MOA
MNA-M
SBA
PNA
GS-II (EY Lab)
ABA
ab5
PSL
r-PVL
rMpL
PNA
SBA
UEAII
UDA
AFCNVH
#15 Sialyl Lewis X (BD)
TL(EY Lab)
Mono2R-CNL
hGal-1-488+BCR
PhoSL
PPL001
Gal-3C
AIA
Mouse NKR-P1C
Human Galectin1
MGL 416
Threefoil
PsSC
CGL
VGA(EY Lab)
PHA-L
IPS-K-4A2B8
Sp Serotype 14.2
AAL
VLRB.aGPA.23
WFL
WT Galectin-8
SSL0-488
Epa1-Np mutant Y228A
#8 Lewis B (T218) (Abcam)
CnMBL
LBL-152
Sp Serotype 14.15
MGL 6B
MGL 6A-H262T
HPA
GSL-1
CA19-9 Ab (DRG)
WGA
EEA(EY Lab)
CA19.9-1B844
Galectin-1
GSL-2
P11 VP8
HAA
GNA (EY Lab)
hJAA-F11-B11
SSR1
GNL
CA19.9-9L426
CC1G-10077
HHL
SJA
VVHA lectin
Epa1-Np mutant E227G
MAA(Seikagaku)
HA70 complex-Alexa
Epa1-Np mutant E227A
TJA-I
MPL
BanLec
MGL 6A
CA19.9-121SLE
Anti-LeC
PA-IL
LEL
ACL
CnSep
NPA
CML1
EEL
Microvirin
CA
VVL
CAA
Epa1-Np mutant Y228W
#14 Sialyl Lewis X (Calbio)
PHA-L
BPL
MAA
STL
DSL
SGA
PSA
LcH
PTL-I
UEA-I
Epa1-Np WT
DBA
PHA-E
Mono1-CNL
Native HPA
WGA
WGA(Seikagaku Bio)
PHA-E
Avaren-Fc
PTL-II
WGA multimer
GSL-I-B4
LTL
mouse Gal-7
SNA
Mono2W-CNL
Gal-3
AAL
LCA
CTB
ME7(detected with a-HUD)
HHA(EY Lab)
PFL
Mouse Galectin1
human Gal-7
HML-Native
GAC78
rSRL
ECL
SRL
DC-ASGPR short
MAL II
UEA-I
PTA-Gal
ConA
ME5(detected with a-HUD)
MAL1
TbFBL2
WGA
WT-VCC
RCAI (Vector)
ConA
AOL (Mahal Lab)
DBL-1
BTL native
AAL
TbFBL1

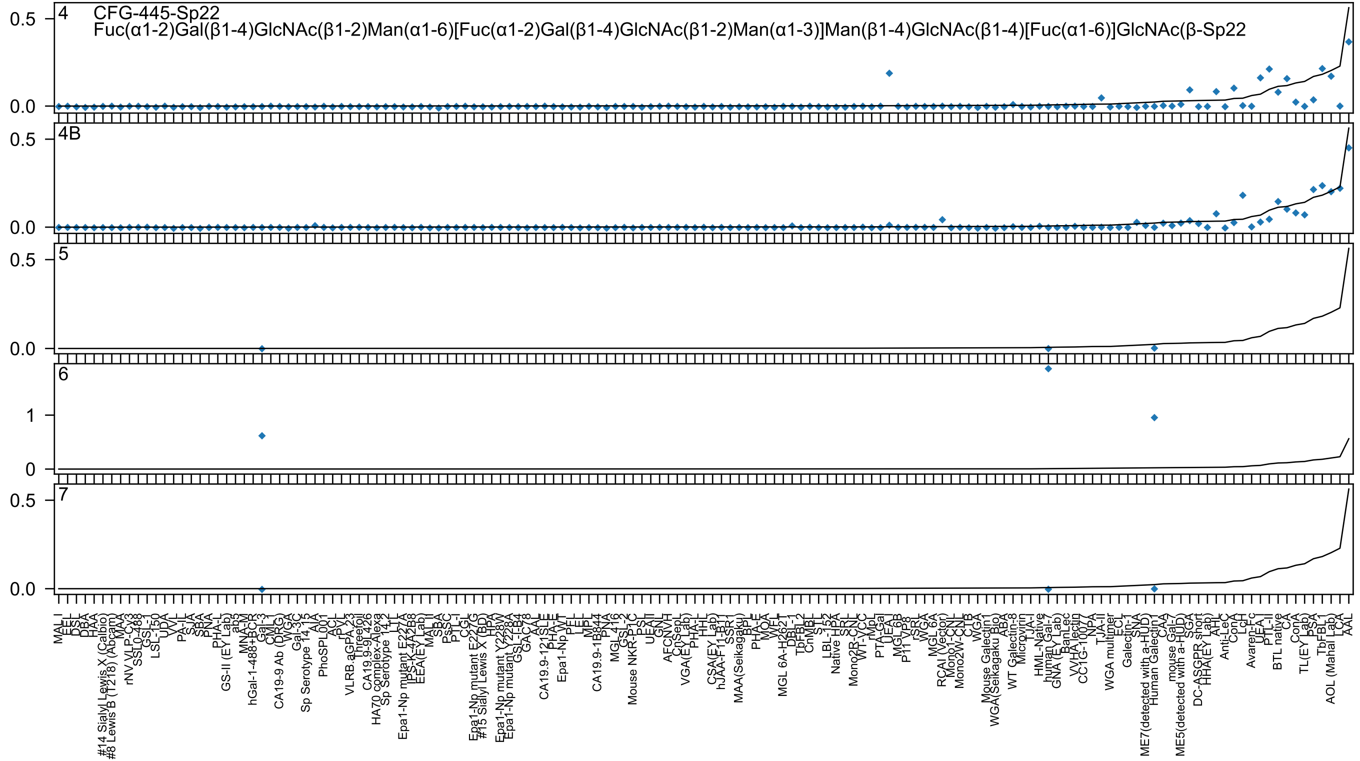

4
CFG-445-Sp22
Fuc(α1-2)Gal(β1-4)GlcNAc(β1-2)Man(α1-6)[Fuc(α1-2)Gal(β1-4)GlcNAc(β1-2)Man(α1-3)]Man(β1-4)GlcNAc(β1-4)[Fuc(α1-6)]GlcNAc(β-Sp22
4B
5
6
7

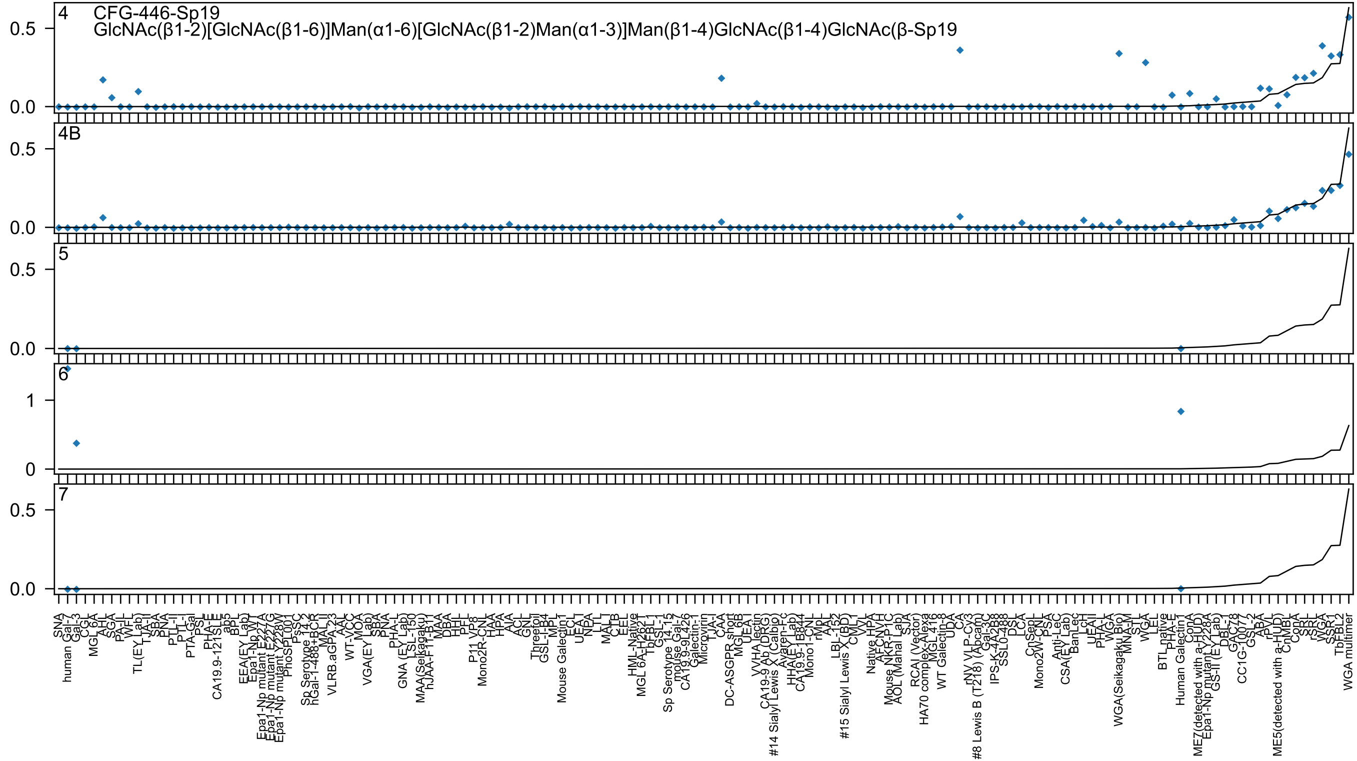
CFG-446-Sp19
GlcNAc(β1-2)[GlcNAc(β1-6)]Man(α1-6)[GlcNAc(β1-2)Man(α1-3)]Man(β1-4)GlcNAc(β1-4)GlcNAc(β-Sp19
4
4B
5
6
7

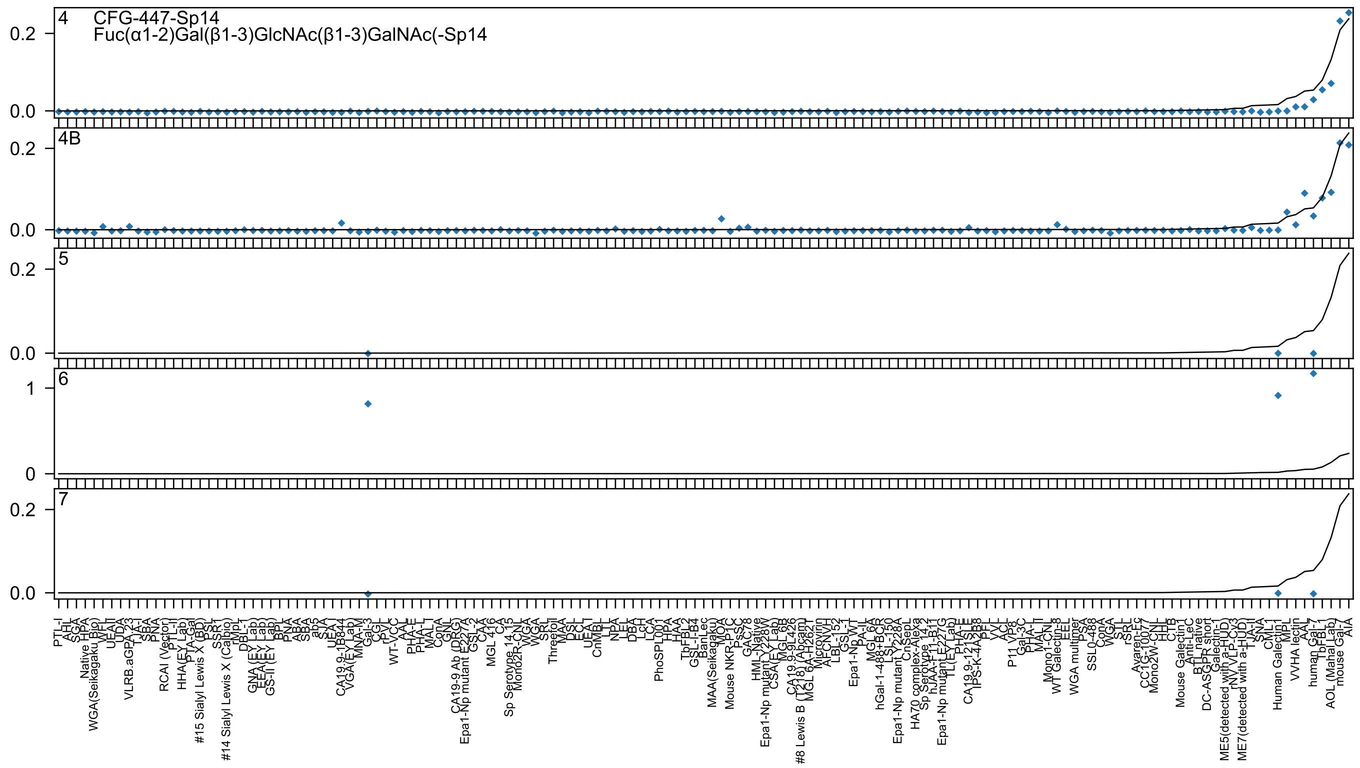

4 CFG-447-Sp14
Fuc(α1-2)Gal(β1-3)GlcNAc(β1-3)GalNAc(-Sp14
4B
5
6
7

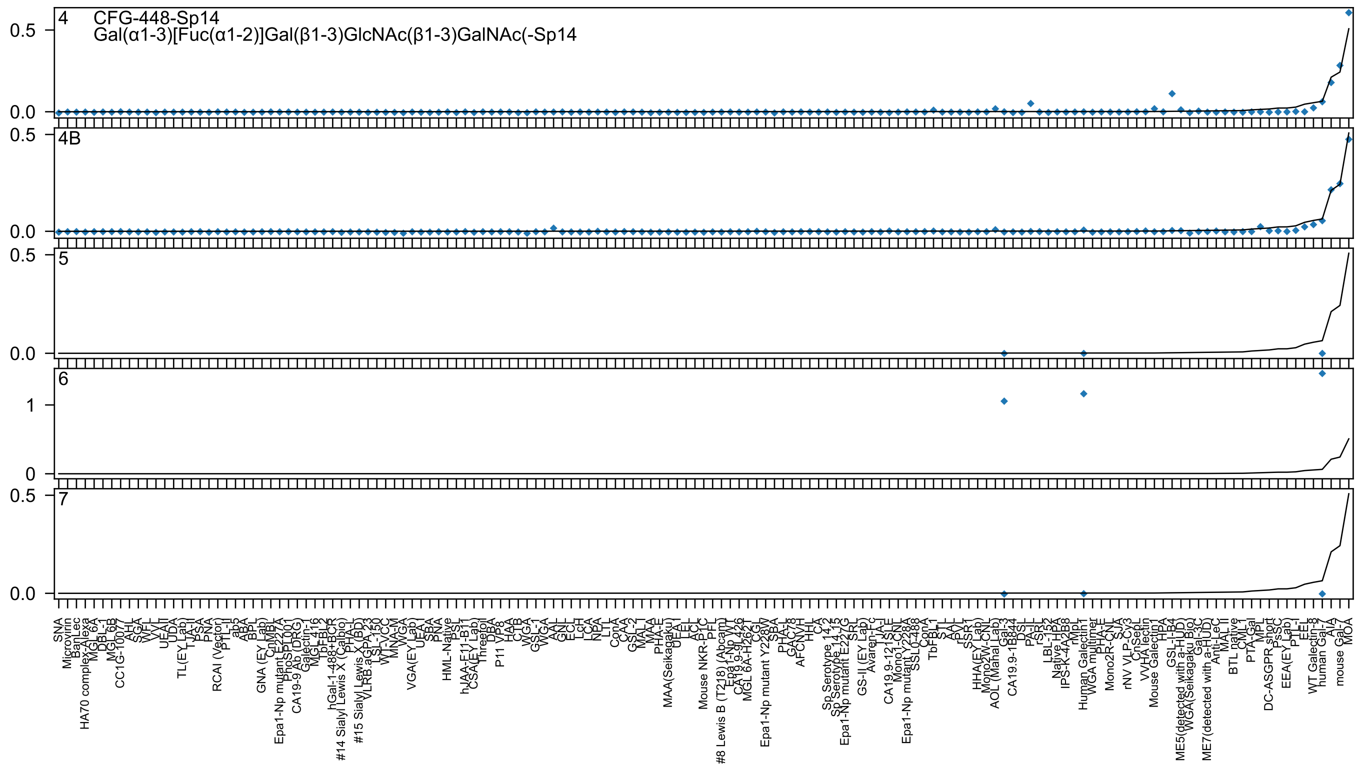
4 CFG-448-Sp14
Gal(α1-3)[Fuc(α1-2)]Gal(β1-3)GlcNAc(β1-3)GalNAc(-Sp14
4B
5
6
7

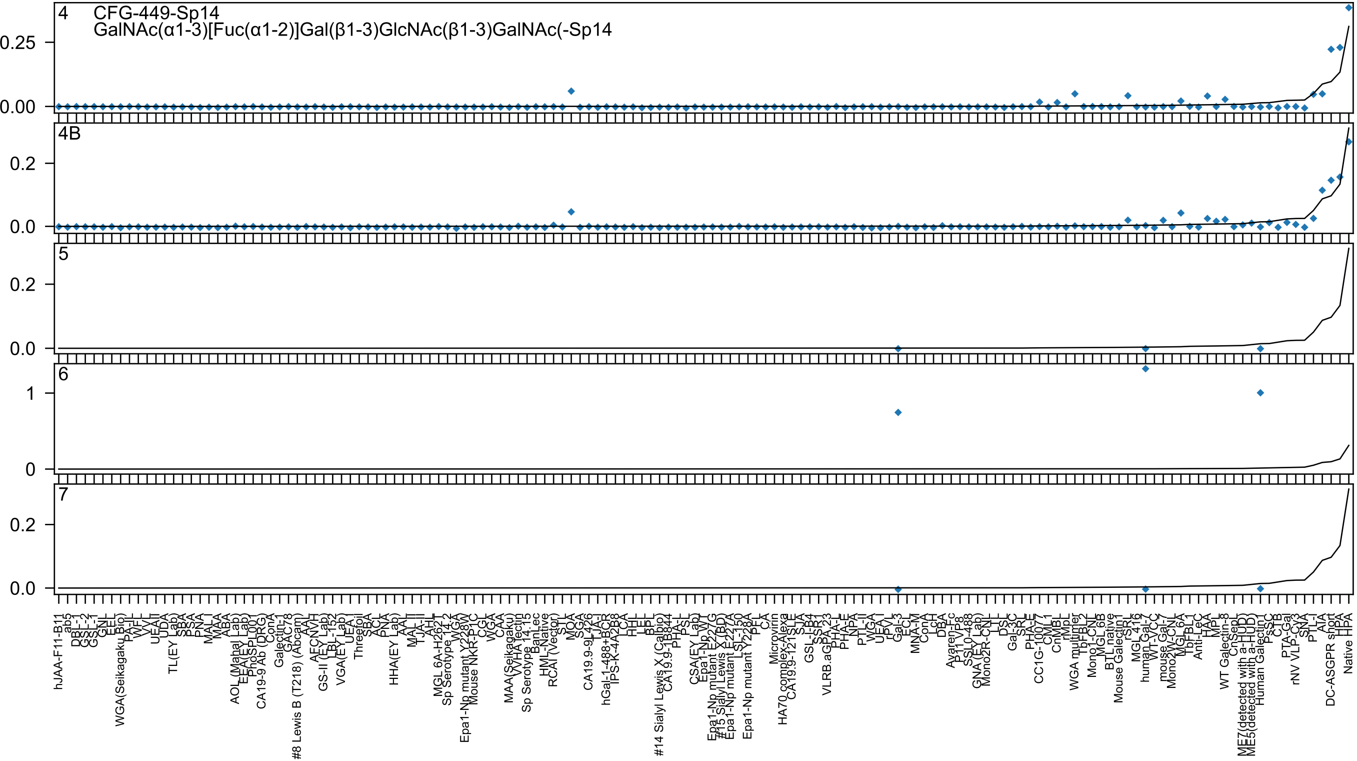

4
CFG-449-Sp14
GalNAc(α1-3)[Fuc(α1-2)]Gal(β1-3)GlcNAc(β1-3)GalNAc(-Sp14
4B
5
6
7

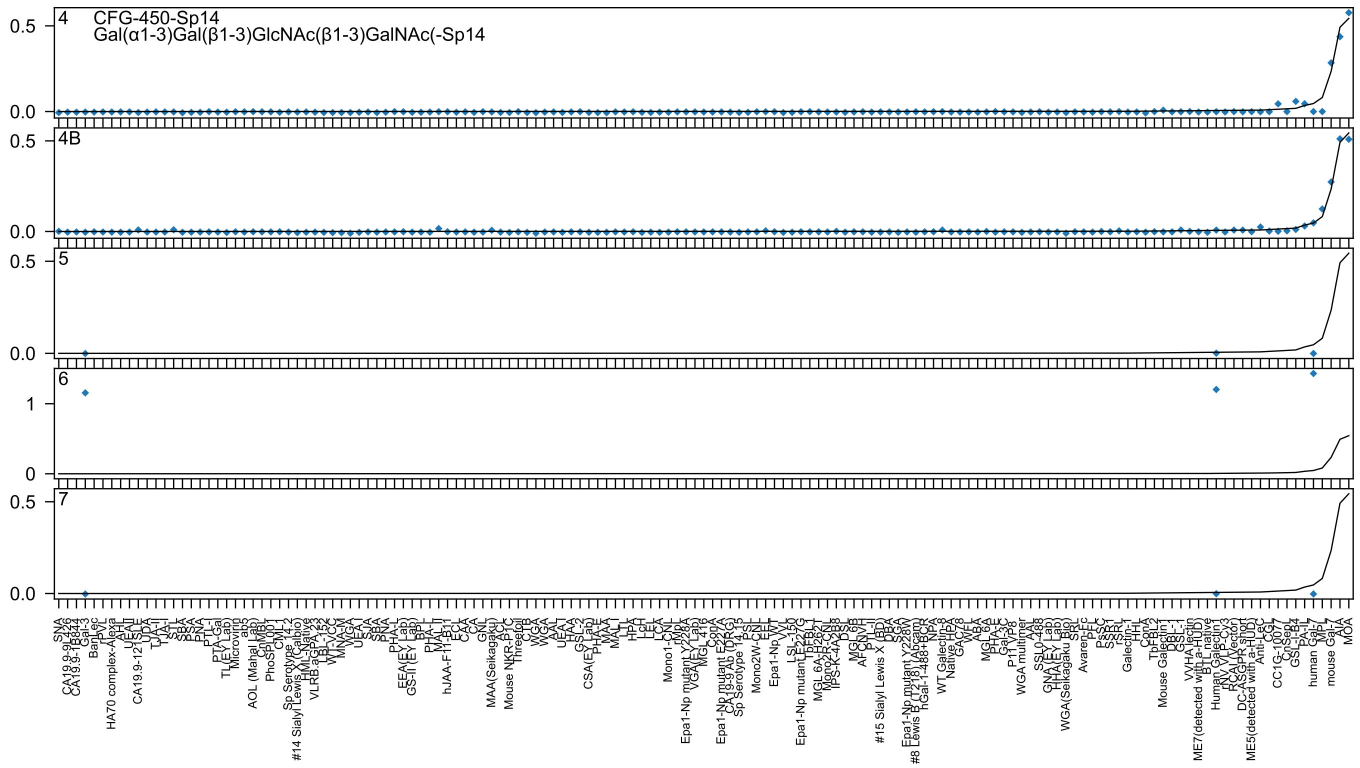

4
CFG-450-Sp14
Gal(α1-3)Gal(β1-3)GlcNAc(β1-3)GalNAc(-Sp14
4B
5
6
7

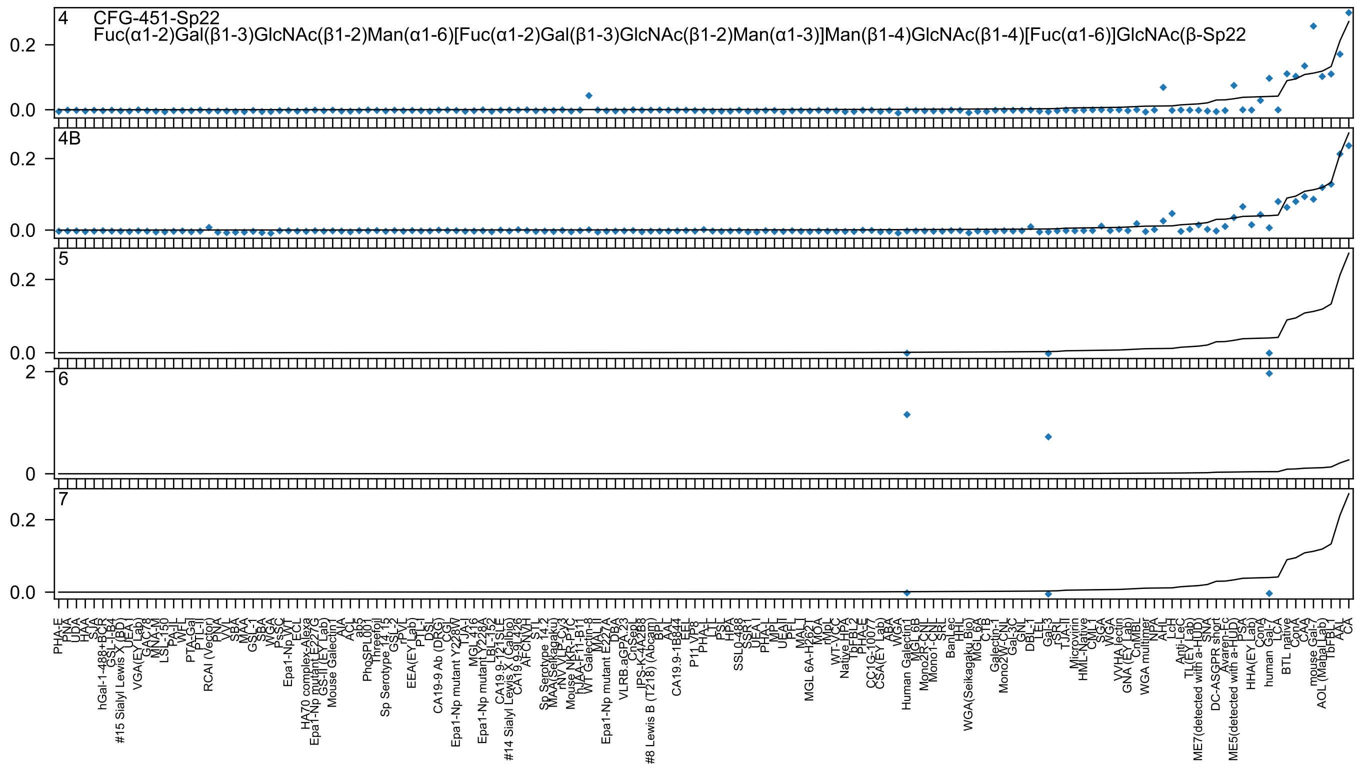

4
CFG-451-Sp22
Fuc(α1-2)Gal(β1-3)GlcNAc(β1-2)Man(α1-6)[Fuc(α1-2)Gal(β1-3)GlcNAc(β1-2)Man(α1-3)]Man(β1-4)GlcNAc(β1-4)[Fuc(α1-6)]GlcNAc(β-Sp22
4B
5
6
7

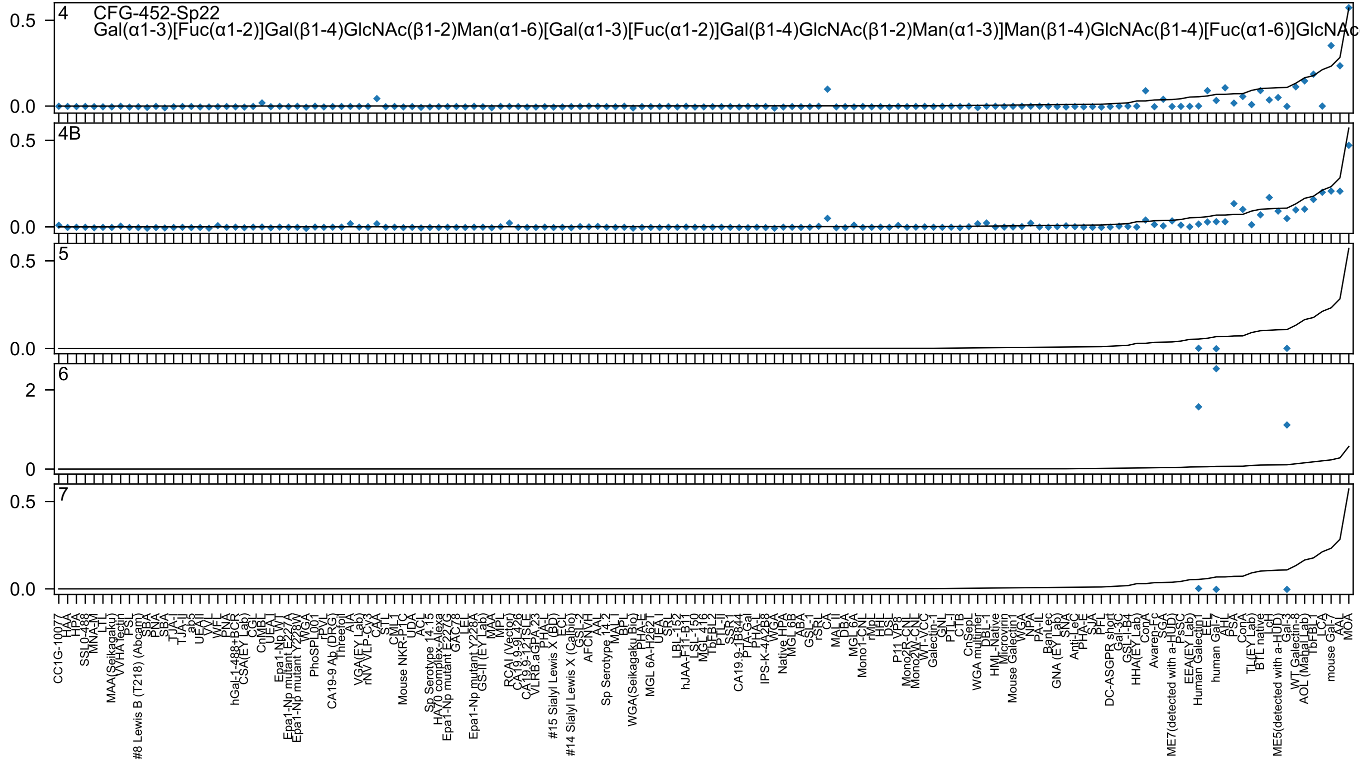

CFG-452-Sp22
Gal(α1-3)[Fuc(α1-2)]Gal(β1-4)GlcNAc(β1-2)Man(α1-6)[Gal(α1-3)[Fuc(α1-2)]Gal(β1-4)GlcNAc(β1-2)Man(α1-3)]Man(β1-4)GlcNAc(β1-4)[Fuc(α1-6)]GlcNAc
4
4B
5
6
7

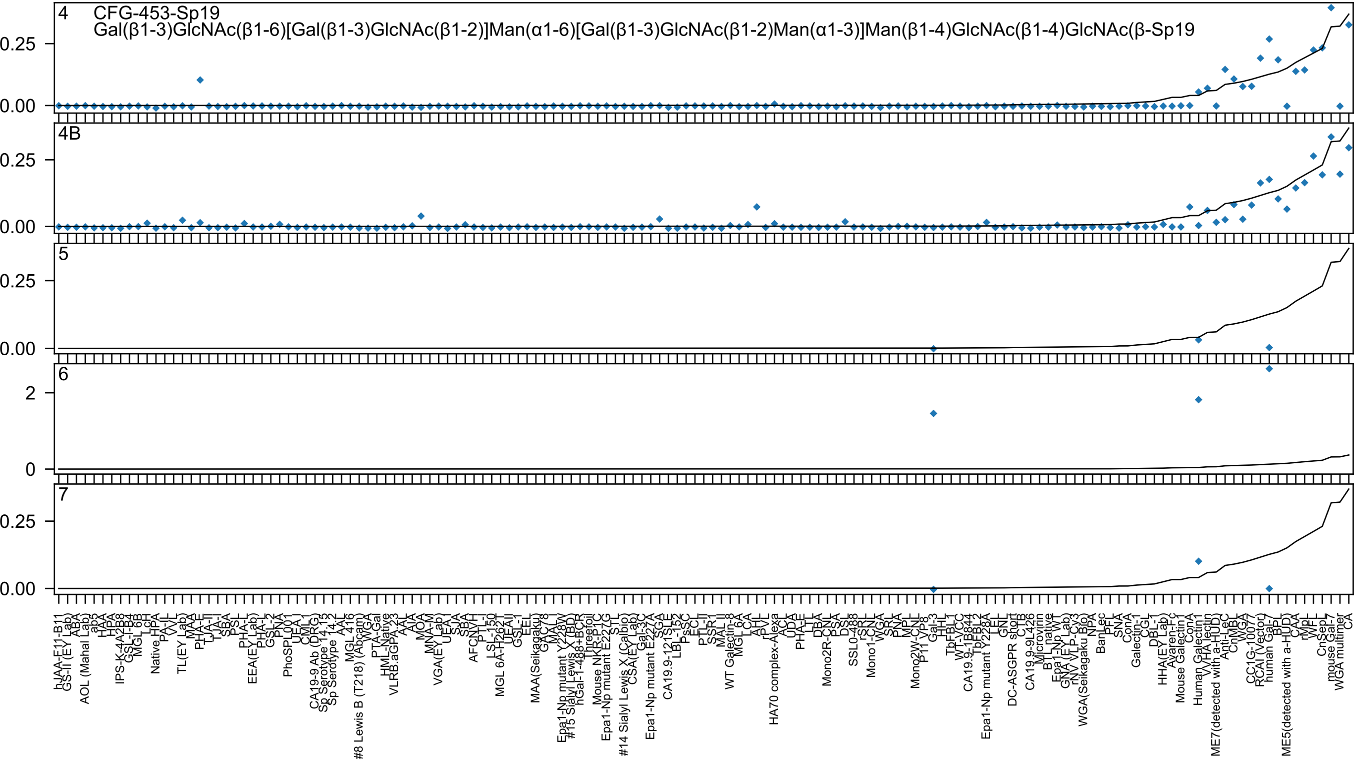
4
CFG-453-Sp19
Gal(β1-3)GlcNAc(β1-6)[Gal(β1-3)GlcNAc(β1-2)]Man(α1-6)[Gal(β1-3)GlcNAc(β1-2)Man(α1-3)]Man(β1-4)GlcNAc(β1-4)GlcNAc(β-Sp19
4B
5
6
7
0.25
0.00
2
0

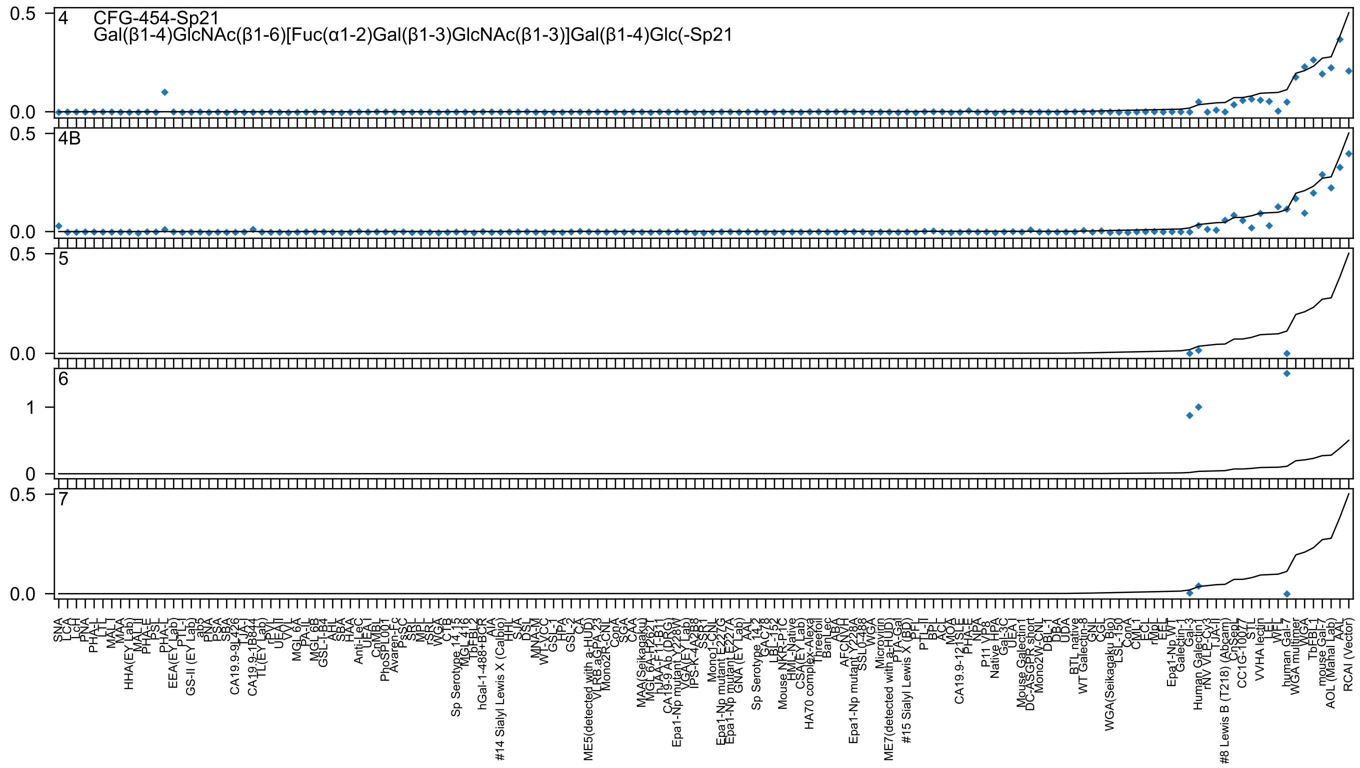

4
CFG-454-Sp21
Gal(β1-4)GlcNAc(β1-6)[Fuc(α1-2)Gal(β1-3)GlcNAc(β1-3)]Gal(β1-4)Glc(-Sp21
4B
5
6
7

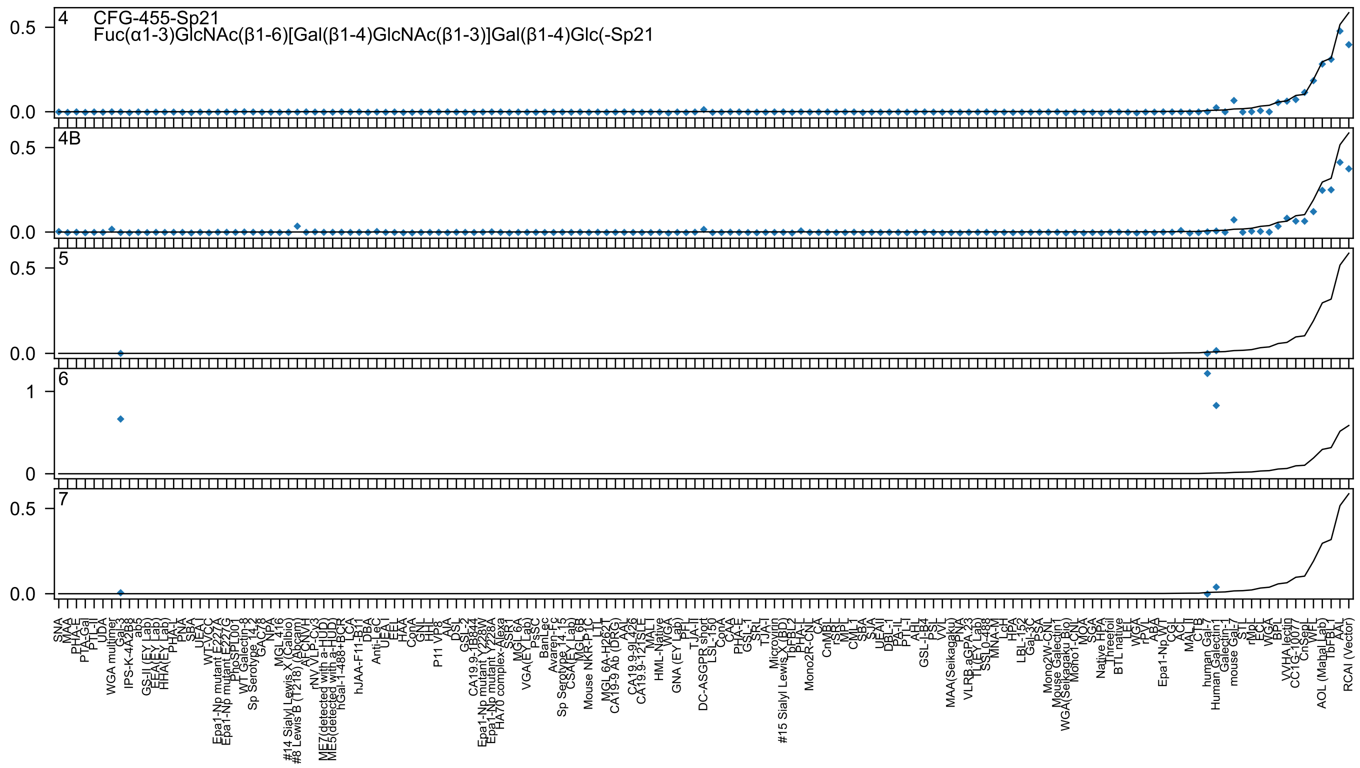
4
CFG-455-Sp21
Fuc(α1-3)GlcNAc(β1-6)[Gal(β1-4)GlcNAc(β1-3)]Gal(β1-4)Glc(-Sp21
4B
5
6
7

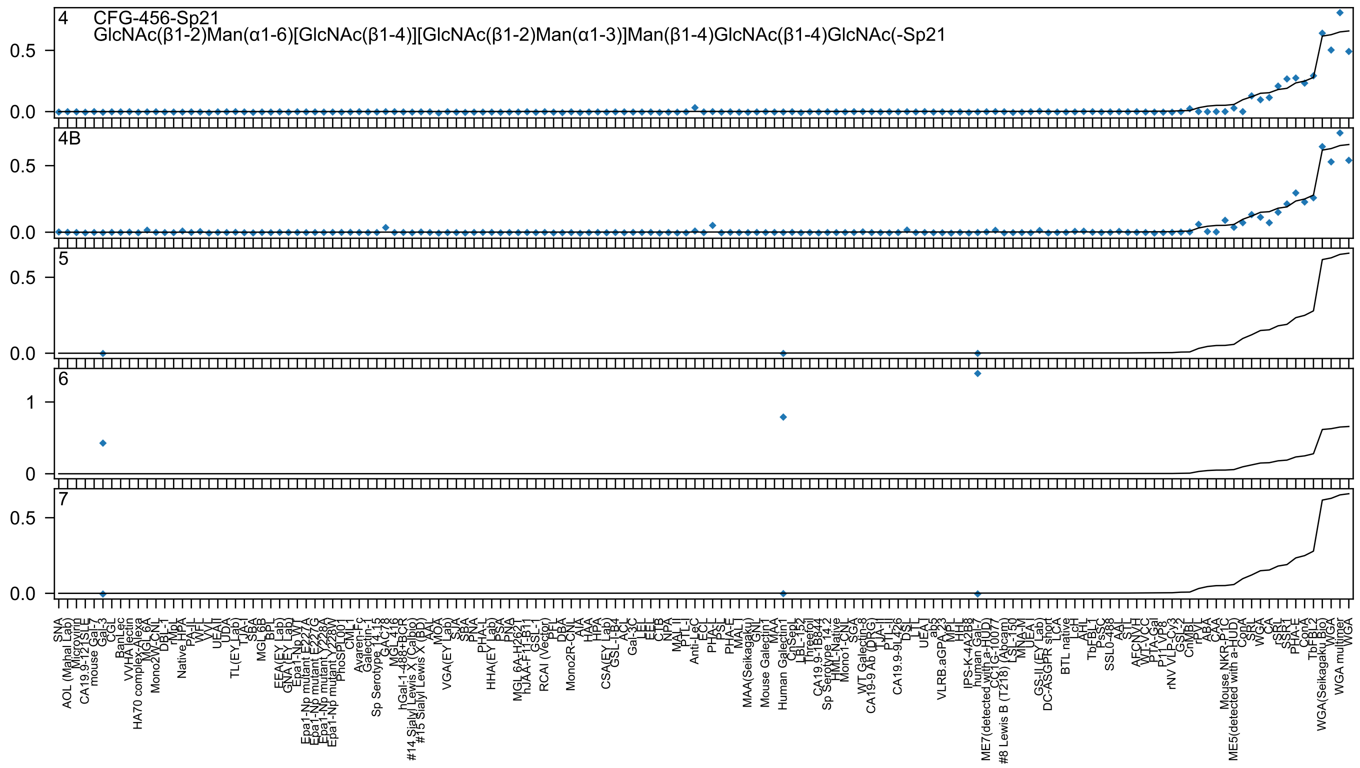
4
CFG-456-Sp21
GlcNAc(β1-2)Man(α1-6)[GlcNAc(β1-4)][GlcNAc(β1-2)Man(α1-3)]Man(β1-4)GlcNAc(β1-4)GlcNAc(-Sp21
4B
5
6
7

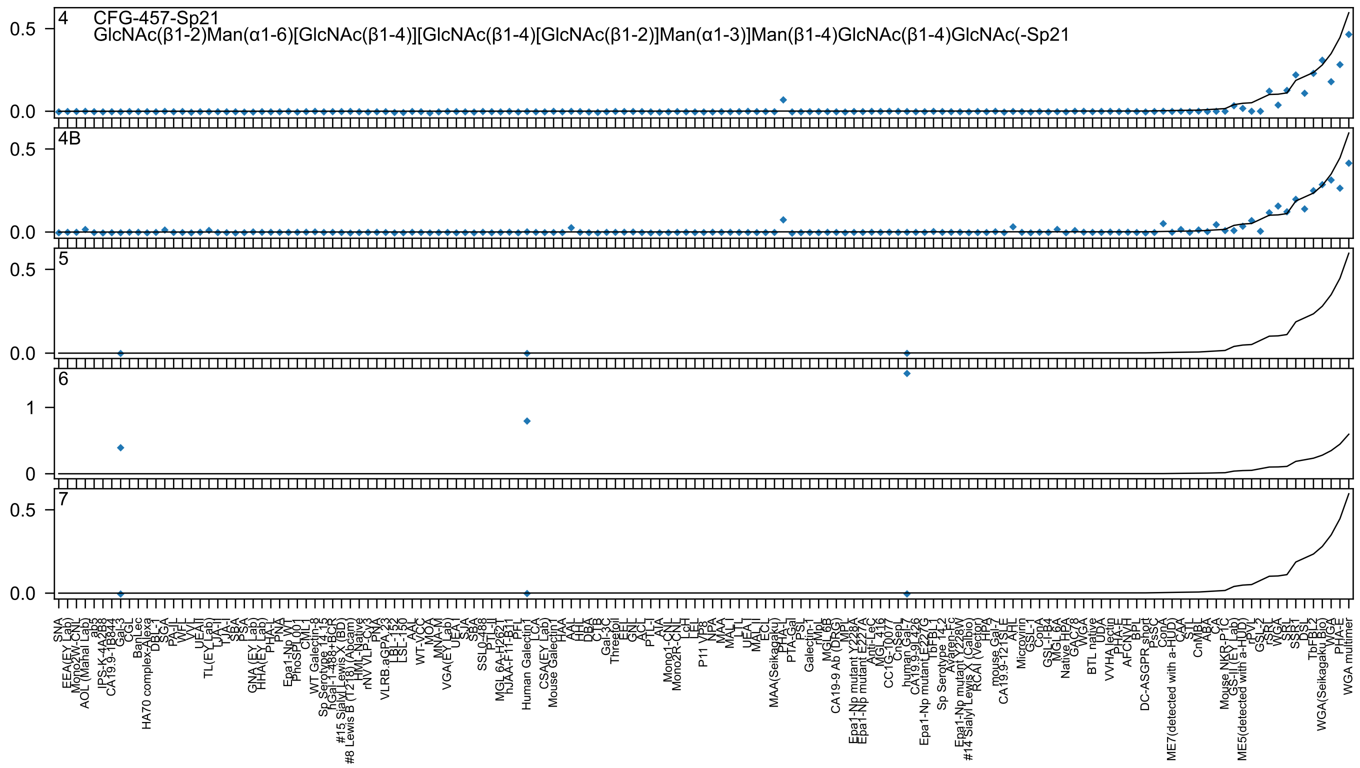

4 CFG-457-Sp21
GlcNAc(β1-2)Man(α1-6)[GlcNAc(β1-4)][GlcNAc(β1-4)[GlcNAc(β1-2)]Man(α1-3)]Man(β1-4)GlcNAc(β1-4)GlcNAc(-Sp21
4B
5
6
7

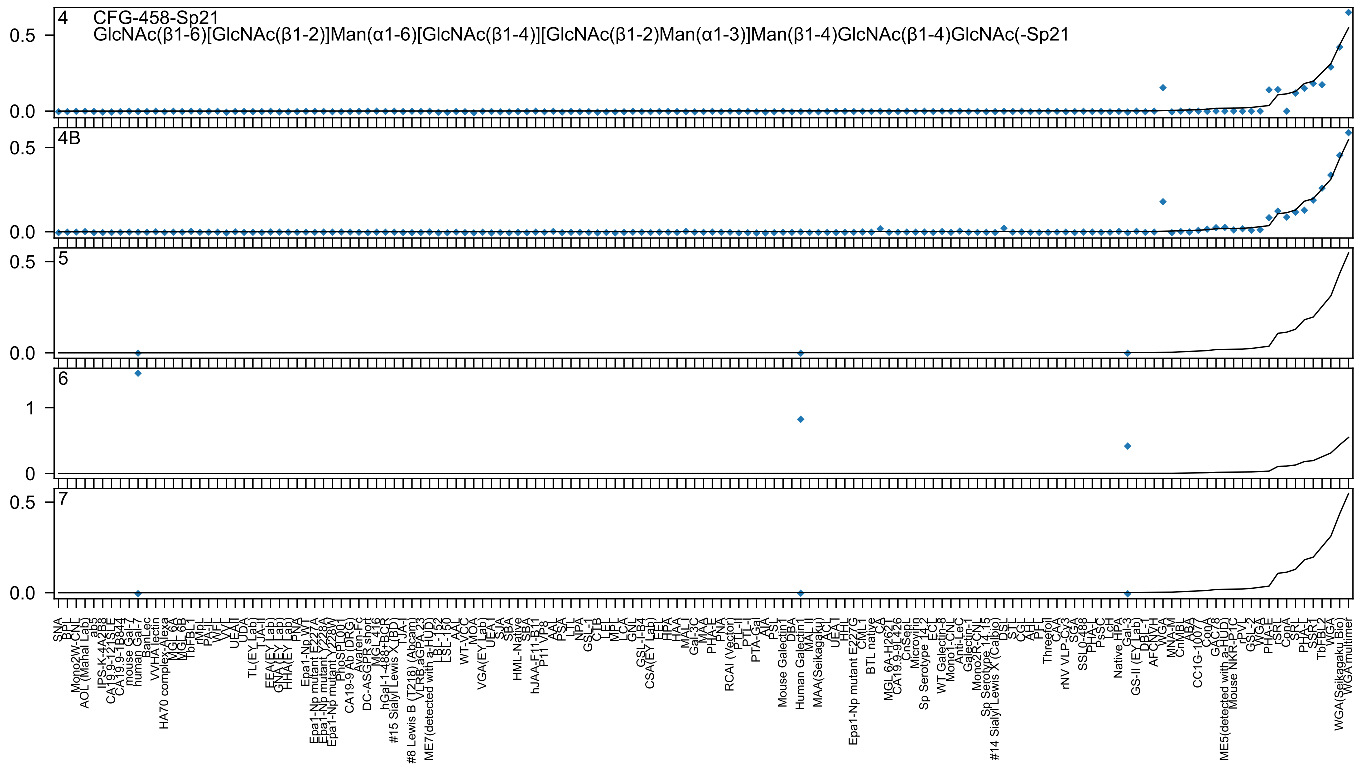

4 CFG-458-Sp21
GlcNAc(β1-6)[GlcNAc(β1-2)]Man(α1-6)[GlcNAc(β1-4)][GlcNAc(β1-2)Man(α1-3)]Man(β1-4)GlcNAc(β1-4)GlcNAc(-Sp21
4B
5
6
7

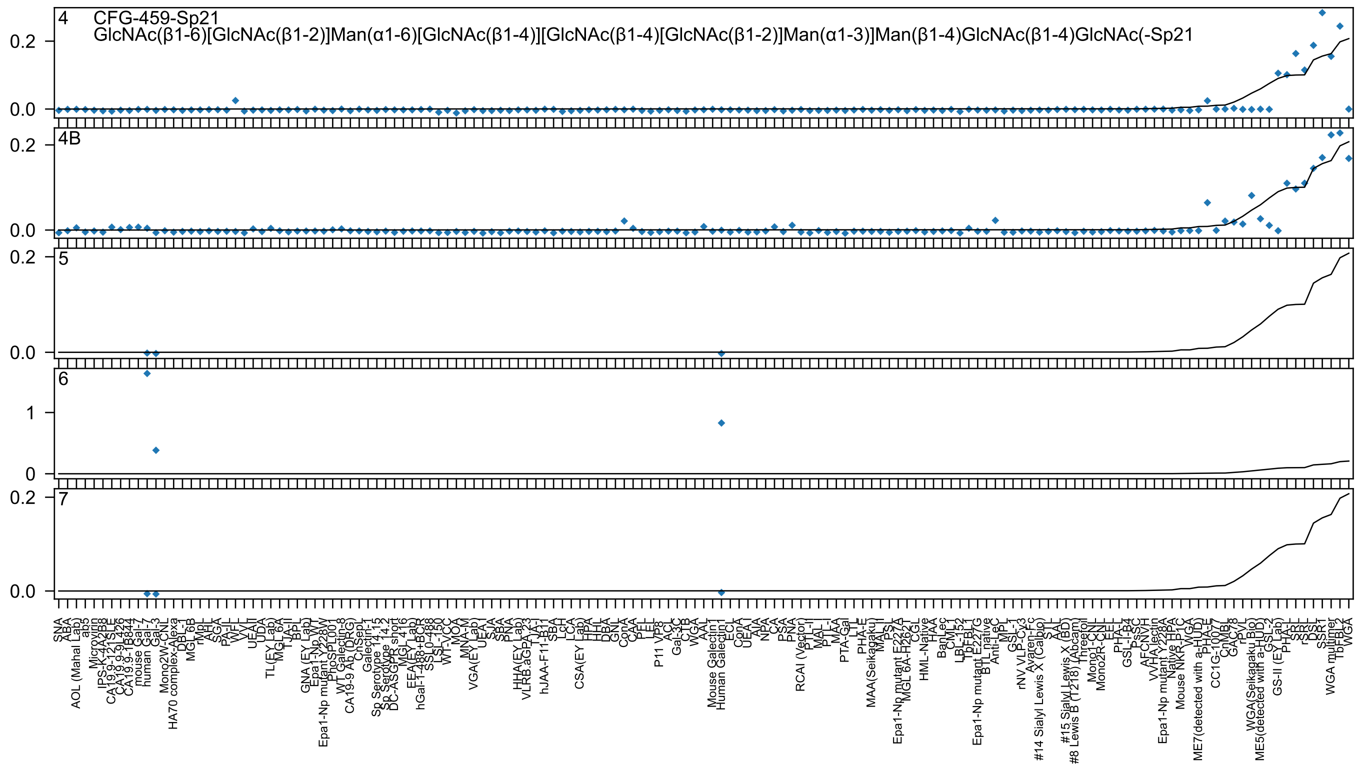

4 CFG-459-Sp21
GlcNAc(β1-6)[GlcNAc(β1-2)]Man(α1-6)[GlcNAc(β1-4)][GlcNAc(β1-4)[GlcNAc(β1-2)]Man(α1-3)]Man(β1-4)GlcNAc(β1-4)GlcNAc(-Sp21
4B
5
6
7

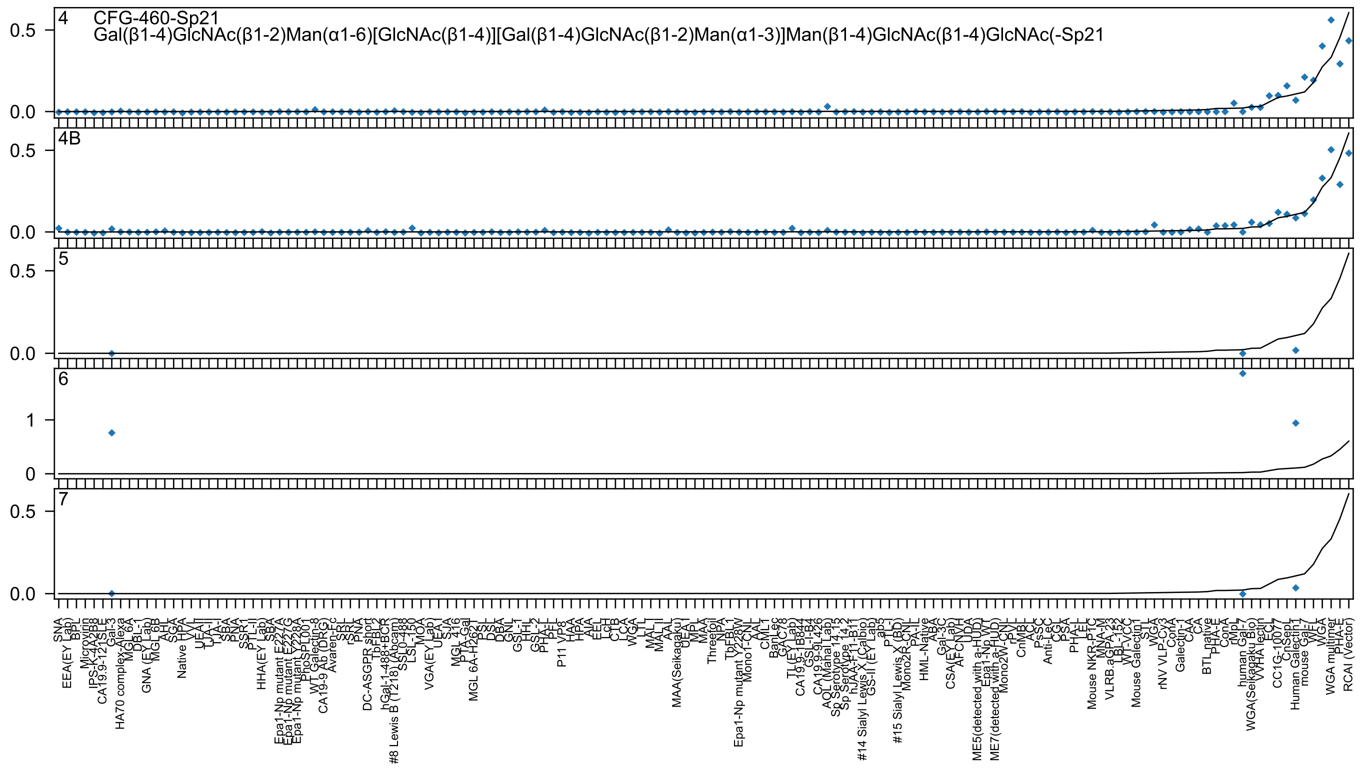

4
CFG-460-Sp21
Gal(β1-4)GlcNAc(β1-2)Man(α1-6)[GlcNAc(β1-4)][Gal(β1-4)GlcNAc(β1-2)Man(α1-3)]Man(β1-4)GlcNAc(β1-4)GlcNAc(-Sp21
4B
5
6
7

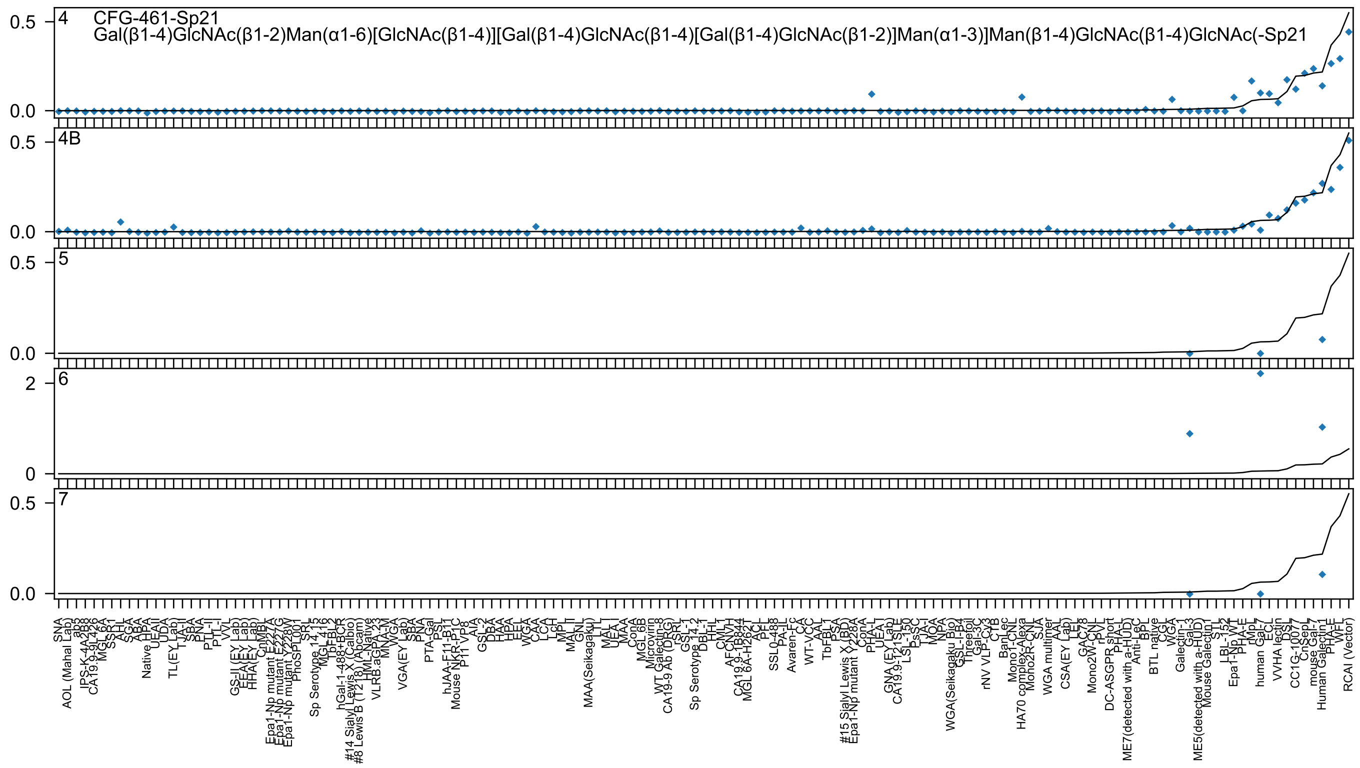
4
CFG-461-Sp21
Gal(β1-4)GlcNAc(β1-2)Man(α1-6)[GlcNAc(β1-4)][Gal(β1-4)GlcNAc(β1-4)[Gal(β1-4)GlcNAc(β1-2)]Man(α1-3)]Man(β1-4)GlcNAc(β1-4)GlcNAc(-Sp21
0.5
0.0
4B
0.5
0.0
5
0.5
0.0
6
2
0
7
0.5
0.0
SNA
AOL (Mahal Lab)
ab5
IPS-K-4A2B8
CA19.9-9L426
MGL 6A
SSR1
AHL
SGA
ABA
Native HPA
UEAII
UDA
TL(EY Lab)
TJA-II
SBA
PNA
PTL-II
PTL-I
VVL
GS-II (EY Lab)
EEA(EY Lab)
HHA(EY Lab)
CnMBL
Epa1-Np mutant E227A
Epa1-Np mutant E227G
Epa1-Np mutant Y228W
PhoSL001
SRL
Sp Serotype 14.15
MGL 416
TbFBL2
hGal-1-488+BCR
#14 Sialyl Lewis X (Calbio)
#8 Lewis B (T218) (Abcam)
HML-Native
VLRB.aGPA.23
MNA-M
WGA
VGA(EY Lab)
SBA
PNA
PTA-Gal
PSL
hJAA-F11-B11
Mouse NKR-P1C
P11 VP8
AIA
GSL-2
DBA
HAA
HPA
EEL
WGA
CAA
LCA
LcH
MPL
MAL II
GNL
MAA(Seikagaku)
LTL
MAL I
UEA I
MAA
ConA
MGL 6B
Microvirin
WT Galectin-8
CA19-9 Ab (DRG)
rSRL
GSL-I
Sp Serotype 14.2
DBL-1
HHL
CML1
AFCNVH
CA19.9-1B844
MGL 6A-H262T
ACL
PFL
SSL0-488
PA-IL
Avaren-Fc
CA
WT-VCC
AAL
TbFBL1
PSA
#15 Sialyl Lewis X (BD)
Epa1-Np mutant Y228A
ConA
PHA-L
UEA-I
GNA (EY Lab)
CA19.9-121SLE
LSL-150
PsSC
TJA-I
MOA
NPA
WGA(Seikagaku Bio)
GSL-I-B4
Threefoil
Gal-3C
rNV VLP-Cy3
CTB
BanLec
Mono1-CNL
HA70 complex-Alexa
Mono2R-CNL
SJA
WGA multimer
AAL
CSA(EY Lab)
LEL
GAC78
Mono2W-CNL
rPVL
DC-ASGPR short
PHA-L
ME7(detected with a-HUD)
Anti-LeC
BPL
BTL native
CGL
WGA
Galectin-1
Gal-3
ME5(detected with a-HUD)
Mouse Galectin1
STL
LBL-152
Epa1-Np WT
PHA-E
rMpL
human Gal-7
ECL
VVHA lectin
DSL
CC1G-10077
CnSepL
mouse Gal-7
Human Galectin1
PHA-E
WFL
RCAI (Vector)

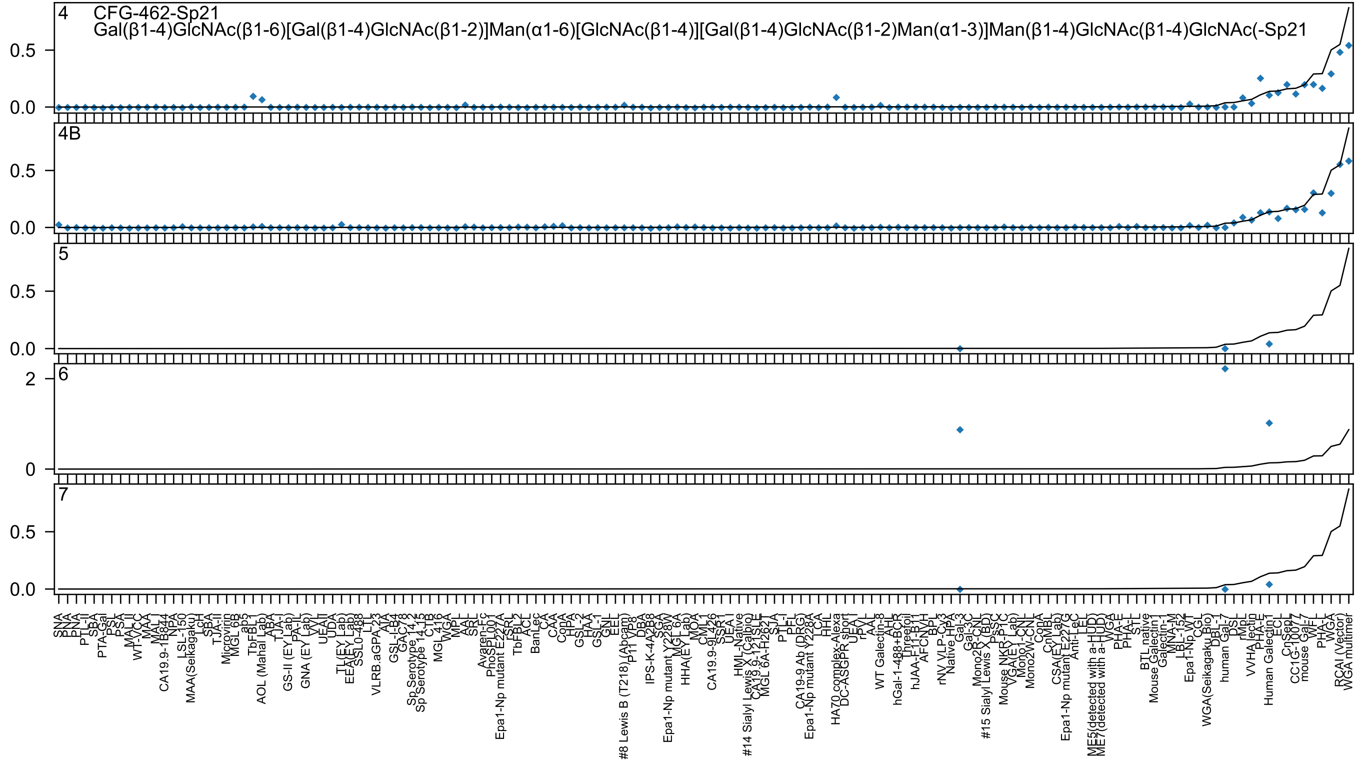

4 CFG-462-Sp21
Gal(β1-4)GlcNAc(β1-6)[Gal(β1-4)GlcNAc(β1-2)]Man(α1-6)[GlcNAc(β1-4)][Gal(β1-4)GlcNAc(β1-2)Man(α1-3)]Man(β1-4)GlcNAc(β1-4)GlcNAc(-Sp21
4B
5
6
7

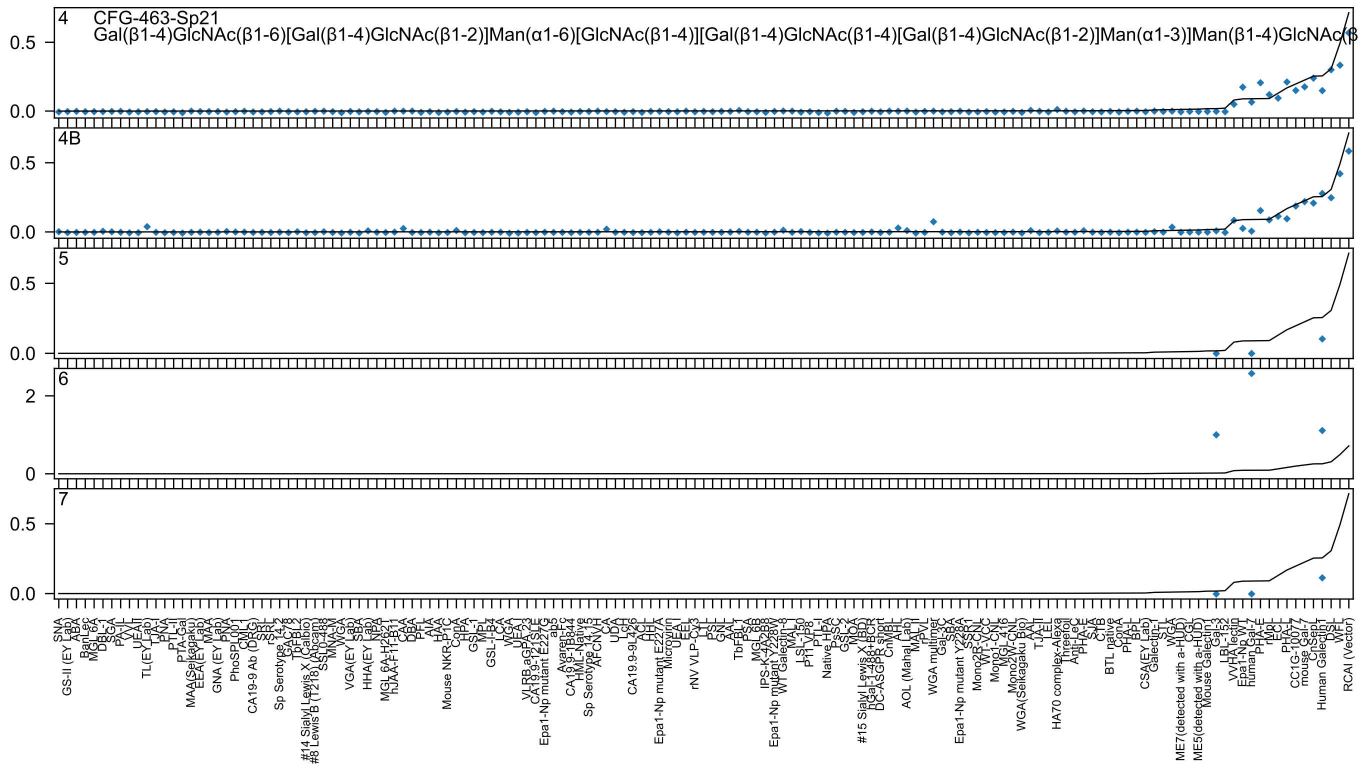
4
CFG-463-Sp21
Gal(β1-4)GlcNAc(β1-6)[Gal(β1-4)GlcNAc(β1-2)]Man(α1-6)[GlcNAc(β1-4)][Gal(β1-4)GlcNAc(β1-4)[Gal(β1-4)GlcNAc(β1-2)]Man(α1-3)]Man(β1-4)GlcNAc(β
4B
5
6
7
0.0
0.5
0
2
SNA
GS-II (EY Lab)
ABA
BanLec
MGL 6A
DBL-1
SGA
PA-IL
VVL
UEAII
TL(EY Lab)
TJA-I
PNA
PTL-II
PTA-Gal
MAA(Seikagaku)
EEA(EY Lab)
MAA
GNA (EY Lab)
PNA
PhoSL001
CML-I
CA19-9 Ab (DRG)
SRL
rSRL
Sp Serotype 14.2
GAC78
TbFBL2
#14 Sialyl Lewis X (Calbio)
#8 Lewis B (T218) (Abcam)
SSL0-488
MNA-M
WGA
VGA(EY Lab)
SBA
HHA(EY Lab)
NPA
MGL 6A-H2621
hJAA-F11-B11
CAA
DBA
PFL
AIA
HAA
Mouse NKR-P1C
ConA
HPA
GSL-1
MPL
GSL-I-B4
LCA
WGA
UEA-I
VLRB.aGPA.23
CA19.9-121SLE
Epa1-Np mutant E227G
ab5
Avaren-Fc
CA19.9-1B844
HML-Native
Sp Serotype 14.15
AFCNVH
CA
UDA
LcH
CA19.9-9L426
ACL
HHL
Epa1-Np mutant E227A
Microvirin
UEA-I
EEL
rNV VLP-Cy3
LTL
PSL
GNL
AAL
TbFBL1
PSA
MGL 6B
IPS-K-4A2B8
Epa1-Np mutant Y228W
WT Galectin-8
MAL-I
LSL-150
P11 VP8
PTL-I
Native HPA
PsSC
GSL-2
MOA
#15 Sialyl Lewis X (BD)
hGal-1-488+BCR
DC-ASGPR short
CnMBL
AHL
AOL (Mahal Lab)
MAL-II
rPVL
WGA multimer
Gal-3C
SBA
Epa1-Np mutant Y228A
SSA
Mono2R-CNL
WT-VCC
Mono1-CNL
MGL-416
Mono2W-CNL
WGA(Seikagaku Bio)
AAL
TJA-II
LEL
HA70 complex-Alexa
Threefoil
Anti-LeC
PHA-E
SJA
CTB
BTL native
ConA
PHA-L
BPL
CSA(EY Lab)
Galectin-1
STL
WGA
ME7(detected with a-HUD)
CGL
ME5(detected with a-HUD)
Mouse Galectin1
Gal-3
LBL-152
VVHA lectin
Epa1-Np WT
human Gal-7
PHA-E
rMpL
ECL
PHA-L
CC1G-10077
mouse Gal-7
CnSep1
Human Galectin1
DSL
WFL
RCAI (Vector)

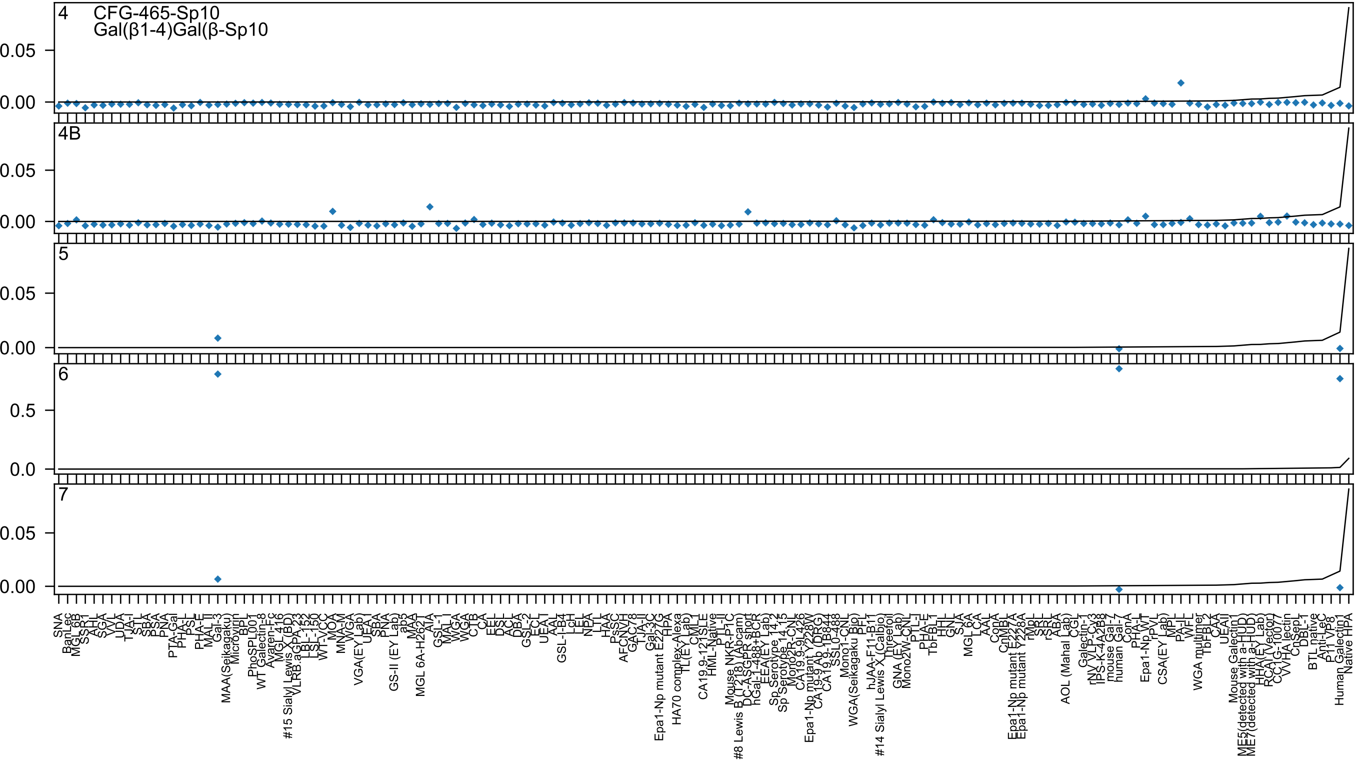

4
CFG-465-Sp10
Gal(β1-4)Gal(β-Sp10
4B
5
6
7
0.05
0.00
0.5
0.0
SNA
BanLec
MGL 6B
SSR1
AHL
SGA
VVL
UDA
TJA-I
STL
SBA
PSA
PNA
PTA-Gal
PHA-L
PSL
PHA-E
MAL II
Gal-3
MAA(Seikagaku)
Microvirin
BPL
PhoSPL001
WT Galectin-8
Avaren-Fc
MGL 416
#15 Sialyl Lewis X (BD)
VLRB.aGPA.23
LBL-152
LSL-150
WT-VCC
MOA
MNA-M
WGA
VGA(EY Lab)
UEA-I
SBA
PNA
GS-II (EY Lab)
ab5
MAA
MGL 6A-H262T
AIA
GSL-1
MAL I
WGA
WGA
CTB
CA
EEL
DSL
ACL
DBA
GSL-2
ECL
UEA-I
AAL
GSL-I-B4
LcH
LEL
NPA
LTL
HAA
PsSC
AFCNVH
GAC78
TJA-II
Gal-3C
Epa1-Np mutant E227G
HPA
HA70 complex-Alexa
TL(EY Lab)
CML
CA19.9-121SLE
HML-Native
PTL-II
Mouse NKR-P1C
#8 Lewis B (T218) (Abcam)
DC-ASGPR short
hGal-1-488+BCR
EEA(EY Lab)
Sp Serotype 14.2
Sp Serotype 14.15
Mono2R-CNL
CA19.9-9L426
Epa1-Np mutant Y228W
CA19-9 Ab (DRG)
CA19.9-1B844
SSL0-488
Mono1-CNL
WGA(Seikagaku Bio)
PFL
hJAA-F11-B1
#14 Sialyl Lewis X (Calbio)
Threefoil
GNA (EY Lab)
Mono2W-CNL
PTL-I
PHA-E
TbFBL1
HHL
GNL
SJA
MGL 6A
LCA
AAL
ConA
CnMBL
Epa1-Np mutant E227A
Epa1-Np mutant Y228A
rMpL
SRL
rSRL
ABA
AOL (Mahal Lab)
CGL
Galectin-1
rNV VLP-Cy3
IPS-K-4A2B8
mouse Gal-7
human Gal-7
ConA
PHA-L
Epa1-Np WT
rPVL
CSA(EY Lab)
MPL
PA-IL
WFL
WGA multimer
TbFBL2
CAA
UEAII
Mouse Galectin1
ME5(detected with a-HUD)
ME7(detected with a-HUD)
HHA(EY Lab)
RCA(Vector)
CC1G-10077
VVHA lectin
CnSepL
DBL-1
BTL native
Anti-LeC
P11 VP8
Human Galectin1
Native HPA

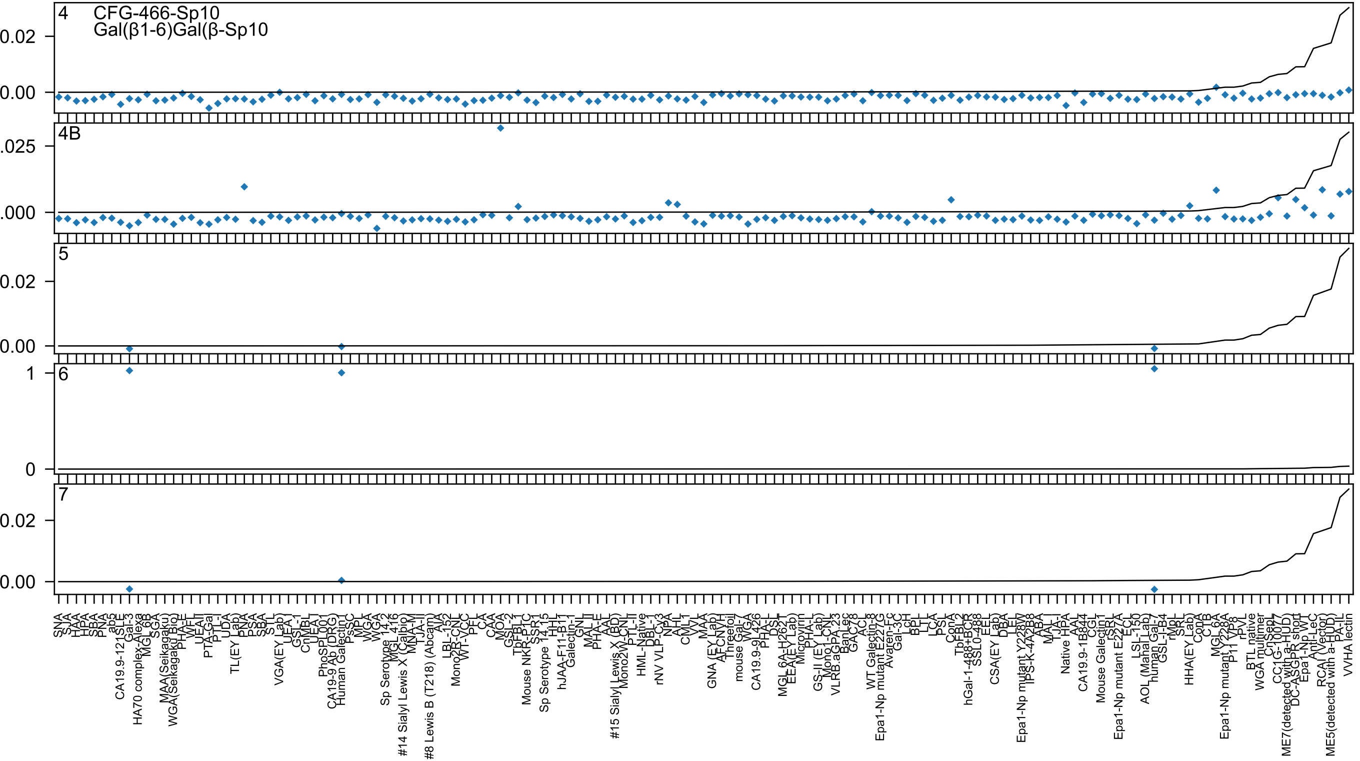

4 CFG-466-Sp10
Gal(β1-6)Gal(β-Sp10
4B
5
6
7
0.02
0.00
.025
.000
1
0

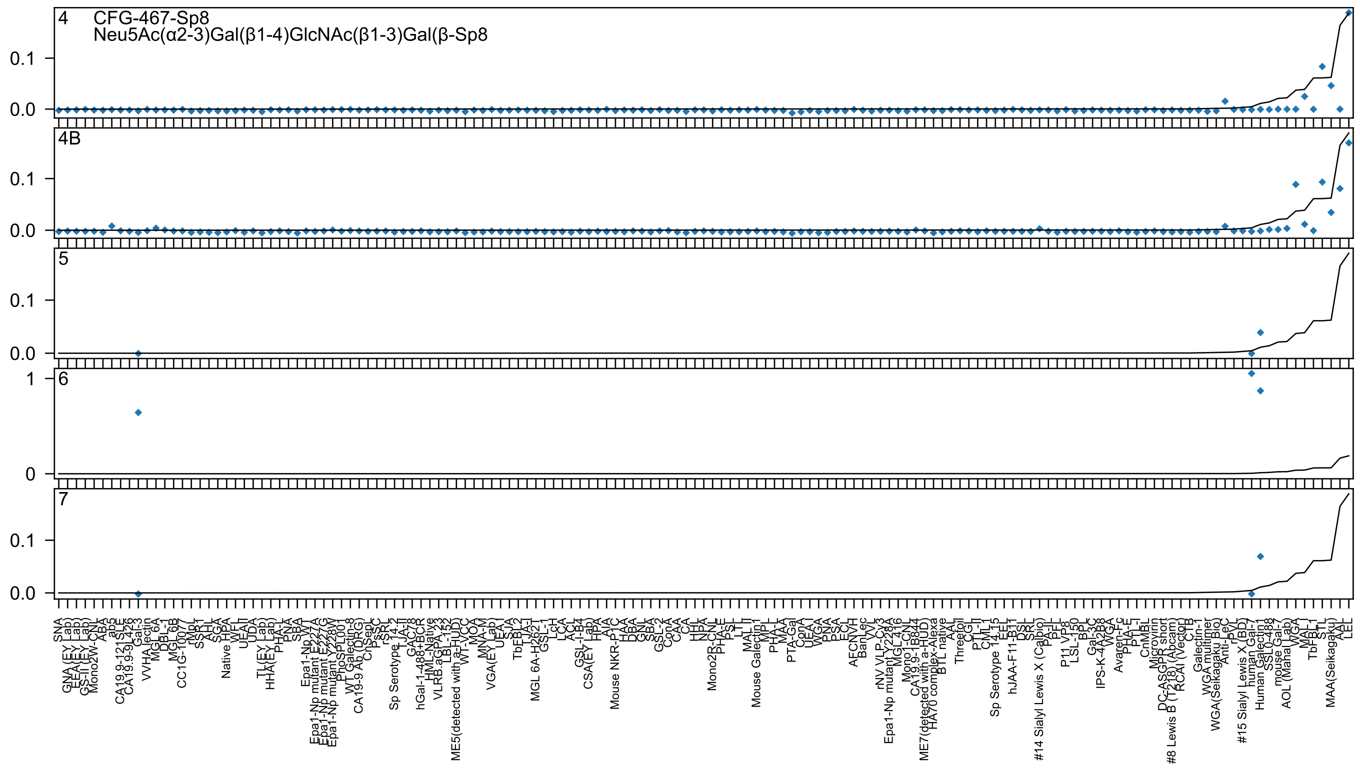
4
CFG-467-Sp8
Neu5Ac(α2-3)Gal(β1-4)GlcNAc(β1-3)Gal(β-Sp8
4B
5
6
7
0.0
0.1
0
1

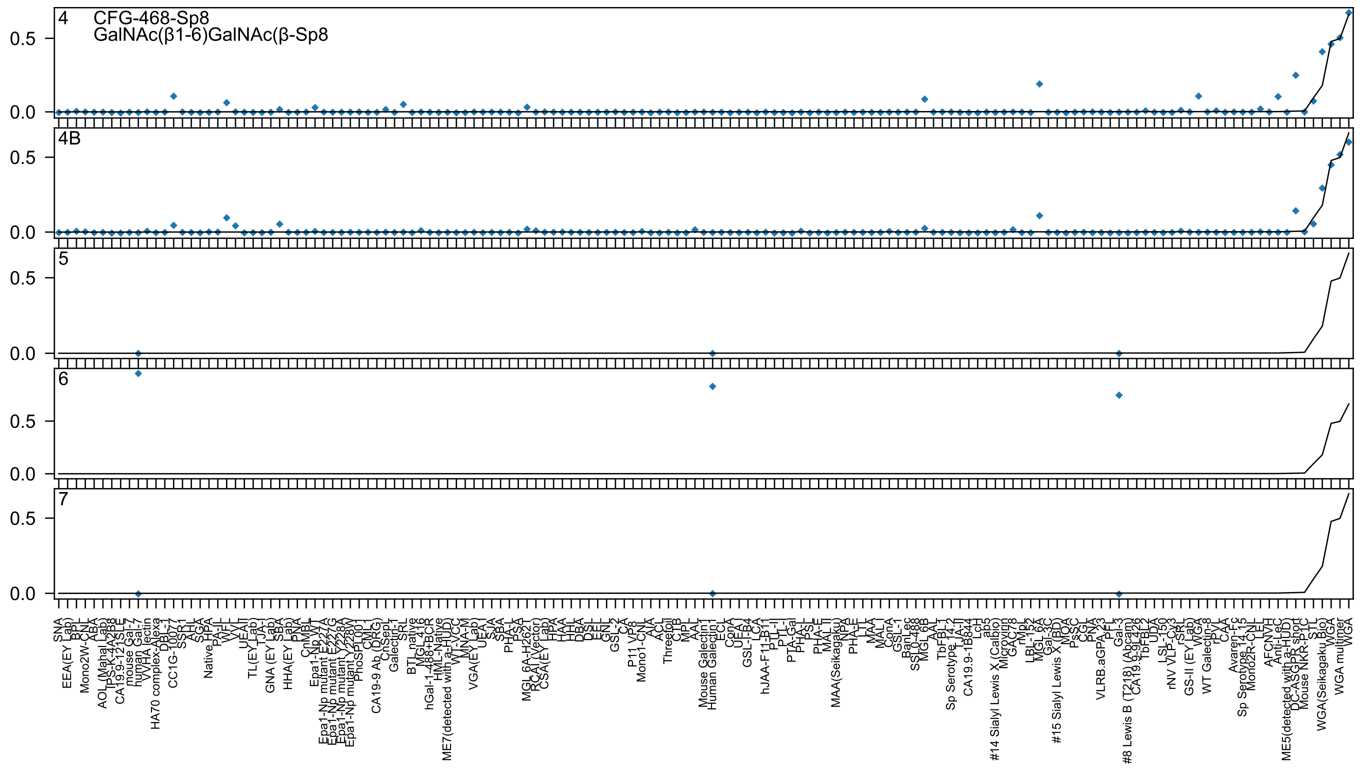

4 CFG-468-Sp8
GalNAc(β1-6)GalNAc(β-Sp8
4B
5
6
7

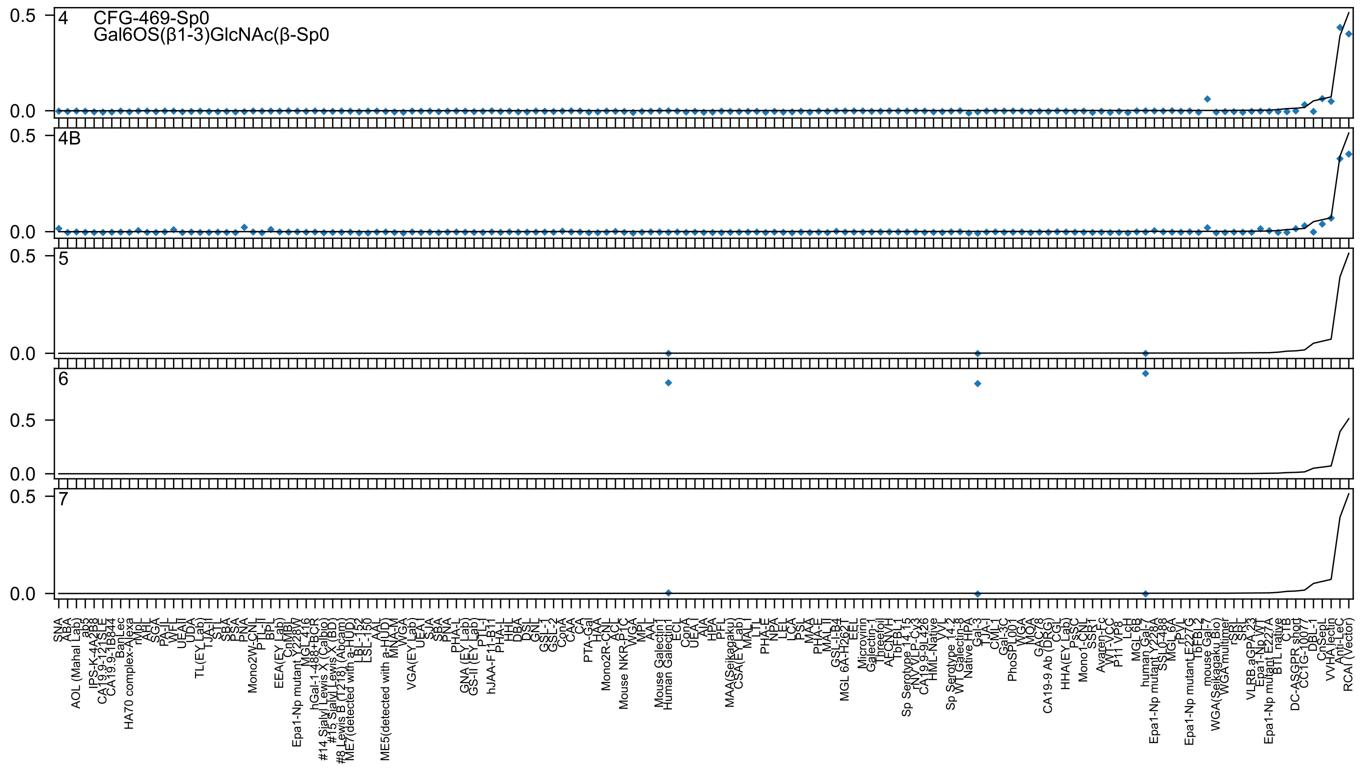

CFG-469-Sp0
Gal6OS(β1-3)GlcNAc(β-Sp0
4
4B
5
6
7

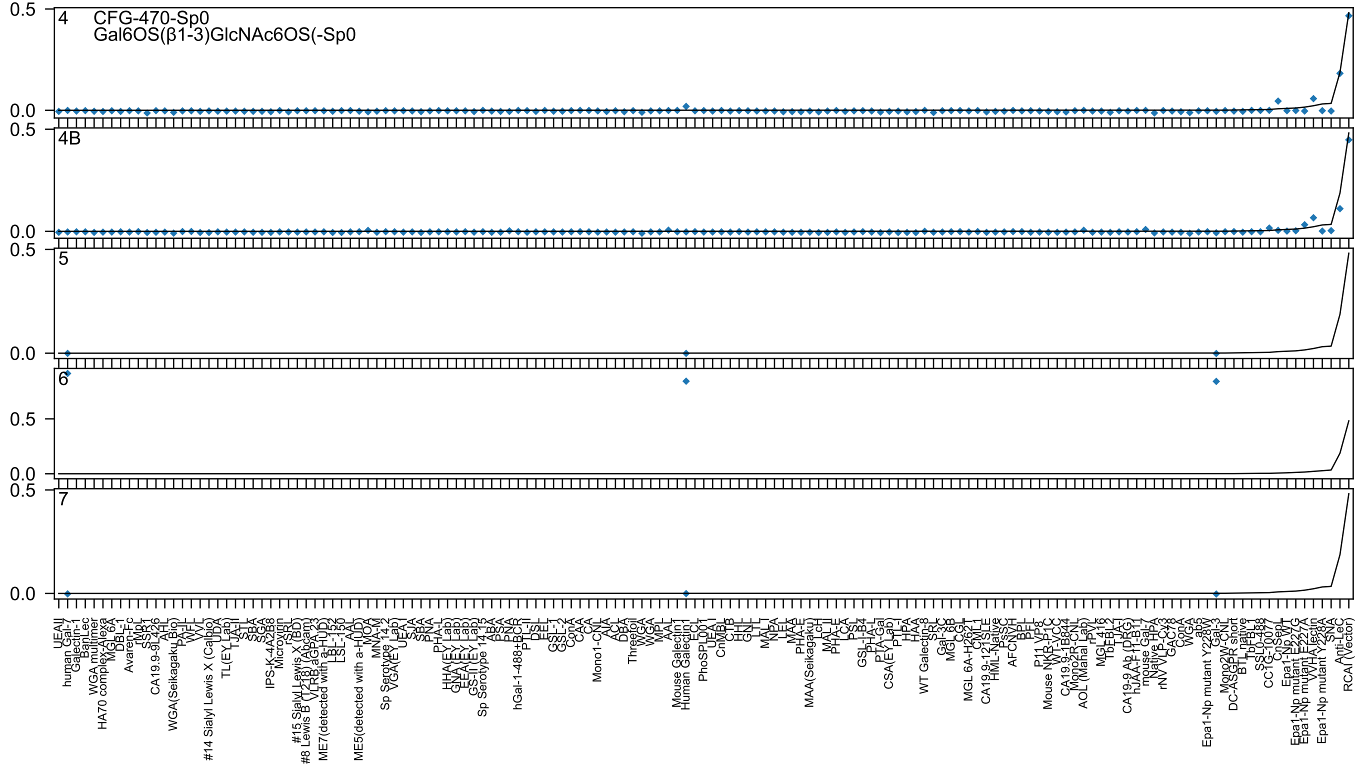
CFG-470-Sp0
Gal6OS(β1-3)GlcNAc6OS(-Sp0
4
4B
5
6
7
0.5
0.0

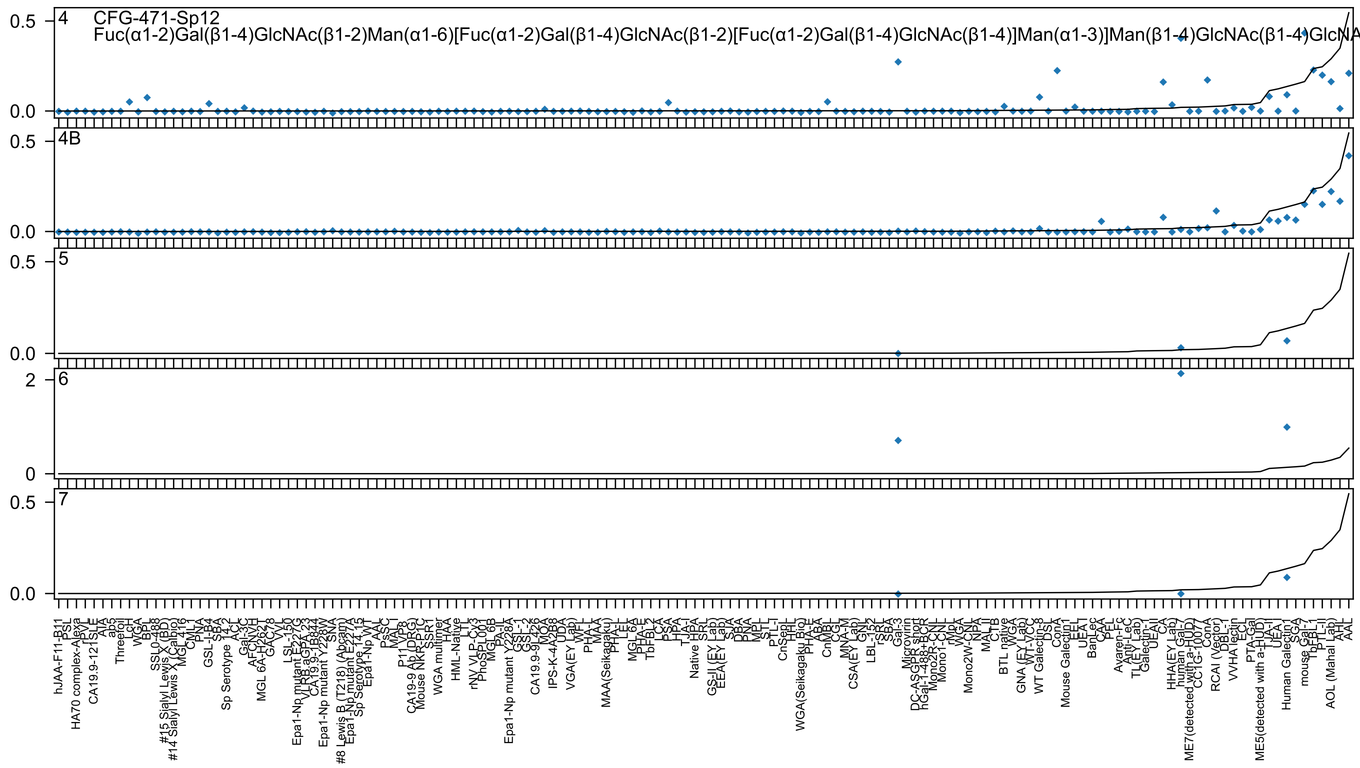
CFG-471-Sp12
Fuc(α1-2)Gal(β1-4)GlcNAc(β1-2)Man(α1-6)[Fuc(α1-2)Gal(β1-4)GlcNAc(β1-2)[Fuc(α1-2)Gal(β1-4)GlcNAc(β1-4)]Man(α1-3)]Man(β1-4)GlcNAc(β1-4)GlcNA
4
4B
5
6
7

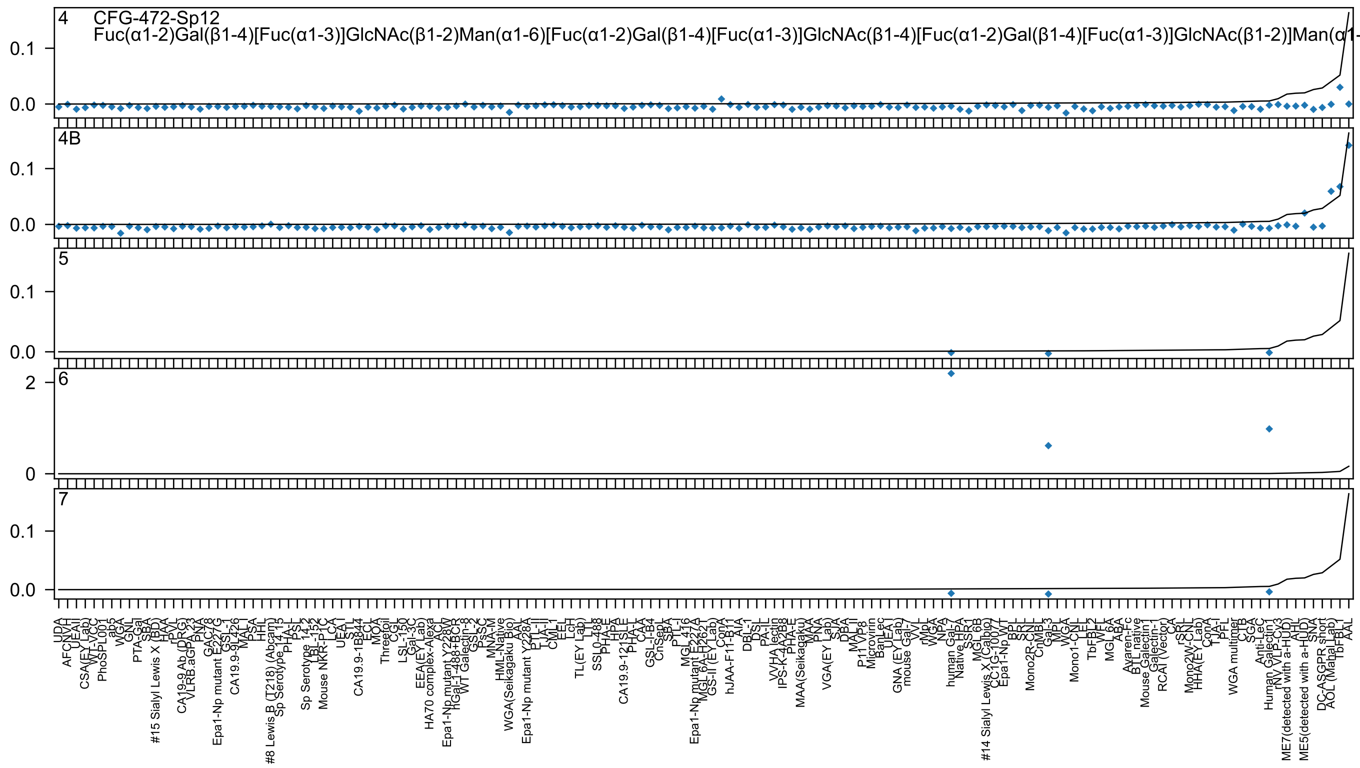
4
CFG-472-Sp12
Fuc(α1-2)Gal(β1-4)[Fuc(α1-3)]GlcNAc(β1-2)Man(α1-6)[Fuc(α1-2)Gal(β1-4)[Fuc(α1-3)]GlcNAc(β1-4)[Fuc(α1-2)Gal(β1-4)[Fuc(α1-3)]GlcNAc(β1-2)]Man(α1-
4B
5
6
7

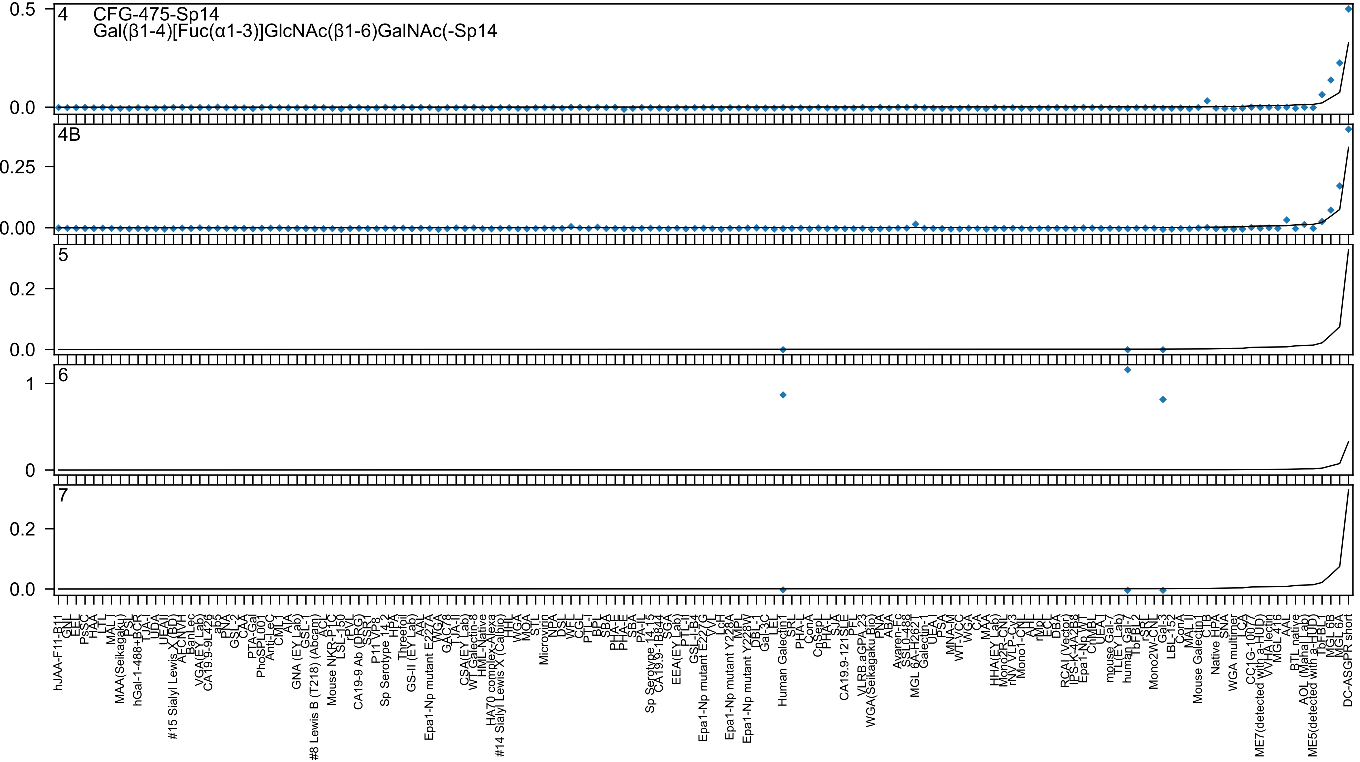
4
CFG-475-Sp14
Gal(β1-4)[Fuc(α1-3)]GlcNAc(β1-6)GalNAc(-Sp14
4B
5
6
7

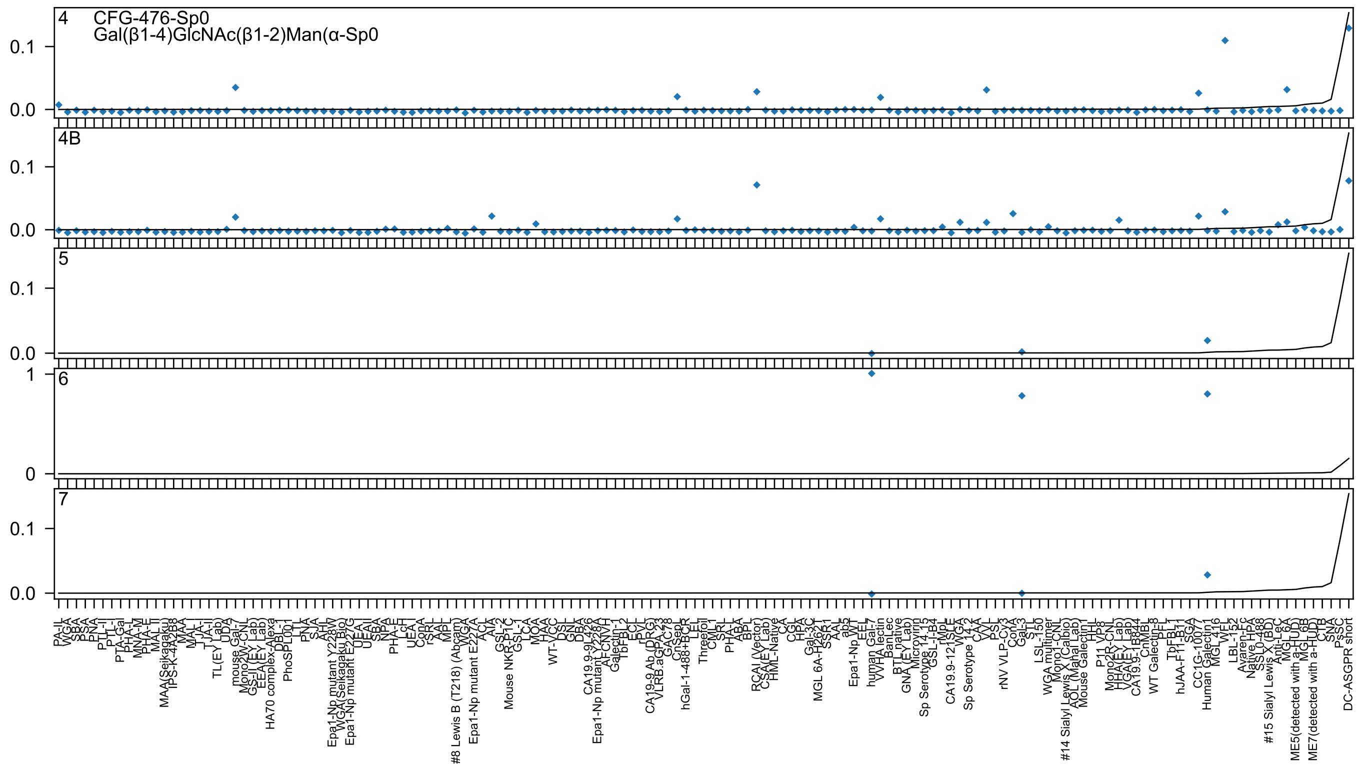
4
CFG-476-Sp0
Gal(β1-4)GlcNAc(β1-2)Man(α-Sp0
4B
5
6
7
0.1
0.0
1
0

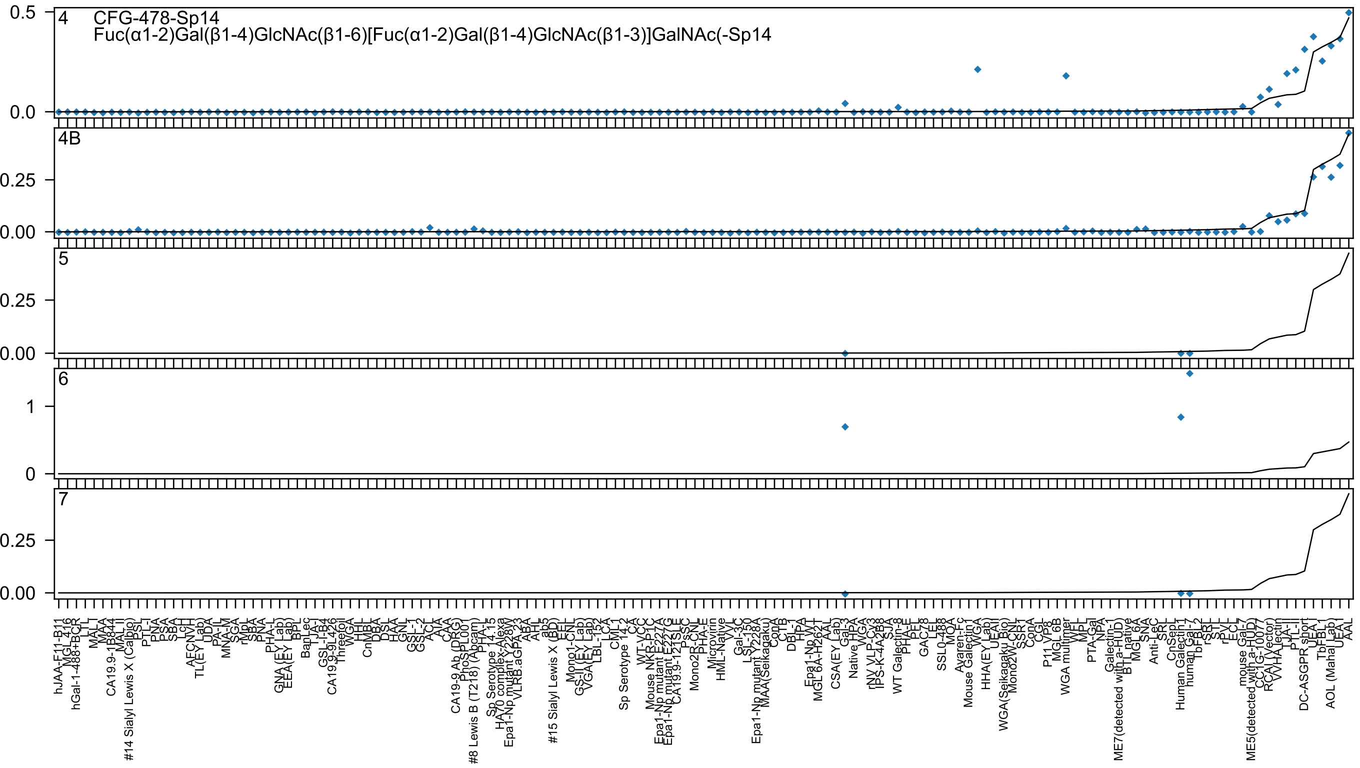

4
CFG-478-Sp14
Fuc(α1-2)Gal(β1-4)GlcNAc(β1-6)[Fuc(α1-2)Gal(β1-4)GlcNAc(β1-3)]GalNAc(-Sp14
4B
5
6
7
0.5
0.0
0.25
0.00
1
0
hJAA-F11-B11
MGL-416
hGal-1-488+BCR
LTL
MAL I
MAA
CA19.9-1B844
MAL II
#14 Sialyl Lewis X (Calbio)
PSL
PTL-I
PNA
PSA
SBA
LcH
AFCNVH
TL(EY Lab)
UDA
PA-IL
MNA-M
SGA
rMpL
SBA
PNA
PHA-L
GNA (EY Lab)
EEA(EY Lab)
BPL
BanLec
TJA-I
GSL-I-B4
CA19.9-9L426
Threefoil
WGA
HHL
CnMBL
DBA
DSL
HAA
GNL
GSL-I
GSL-2
ACL
AIA
CAA
CA19-9 Ab (DRG)
PhoSL001
#8 Lewis B (T218) (Abcam)
PHA-L
Sp Serotype 14.15
HA70 complex-Alexa
Epa1-Np mutant Y228W
VLRB.aGPA.23
ABA
AHL
ab5
#15 Sialyl Lewis X (BD)
EEL
Mono1-CNL
GS-II (EY Lab)
VGA(EY Lab)
LBL-152
LCA
CML-1
Sp Serotype 14.2
CA
WT-VCC
Mouse NKR-P1C
Epa1-Np mutant E227A
Epa1-Np mutant E227G
CA19.9-121SLE
PsSC
Mono2R-CNL
PHA-E
Microvirin
HML-Native
VV
Gal-3C
LSL-150
Epa1-Np mutant Y228A
MAA(Seikagaku)
ConA
CTB
DBL-1
HPA
Epa1-Np WT
MGL 6A-H262T
AAL
CSA(EY Lab)
Gal-3
Native HPA
WGA
rNV VLP-Cv3
IPS-K-4A2B8
SJA
WT Galectin-8
PHA-E
PEL
GAC78
LEL
SSL0-488
MOA
Avaren-Fc
Mouse Galectin1
WGA
HHA(EY Lab)
UEAII
WGA(Seikagaku Bio)
Mono2W-CNL
SSR1
ConA
CGL
P11 VP8
MGL 6B
WGA multimer
WFL
MPL
PTA-Gal
NPA
Galectin-1
ME7(detected with a-HUD)
BTL native
MGL 6A
SNA
Anti-LeC
SRL
CnSept
Human Galectin1
human Gal-7
TbFBL-2
rSRL
STL
rPVL
ECL
mouse Gal-7
ME5(detected with a-HUD)
CC1G-10077
RCAI (Vector)
VVHA lectin
TJA-II
PTL-II
DC-ASGPR short
UEA I
TbFBL1
AOL (Mahal Lab)
UEA I
AAL

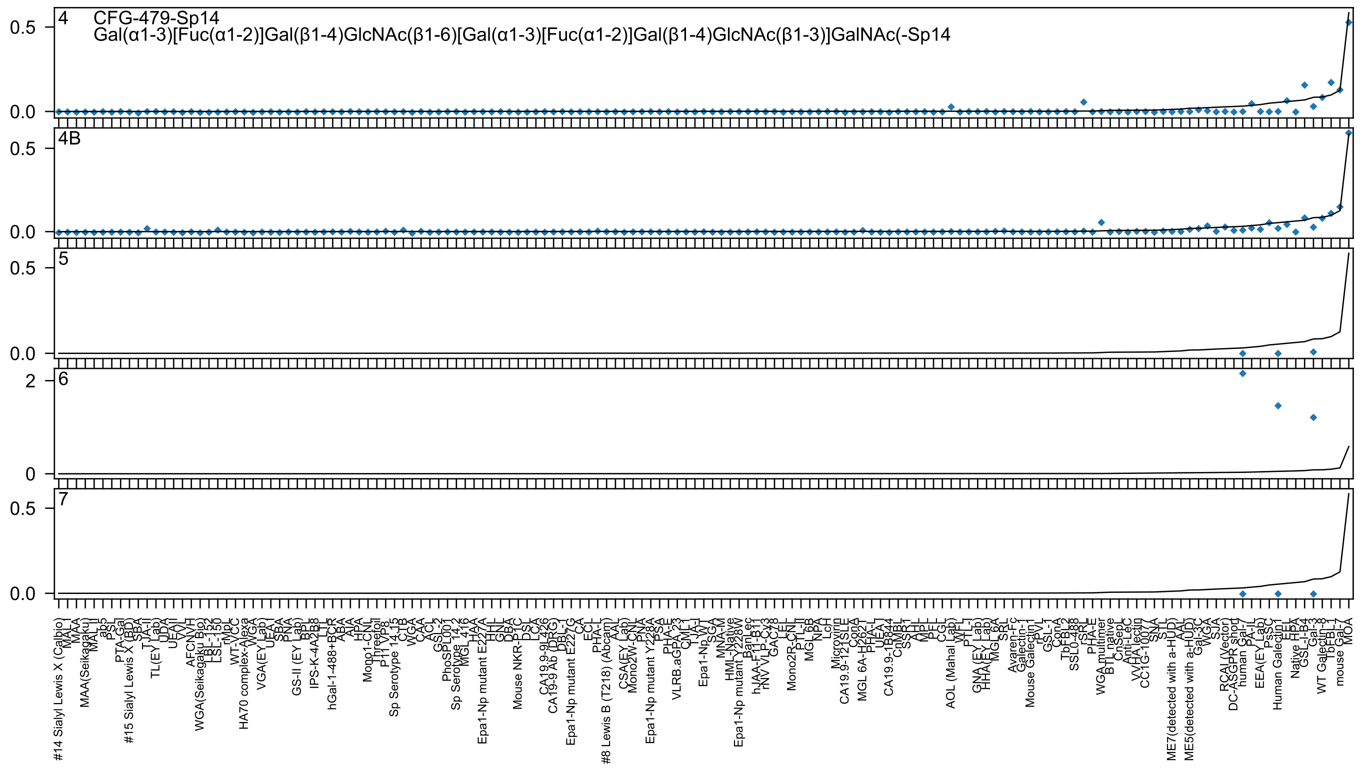

4 CFG-479-Sp14
Gal(α1-3)[Fuc(α1-2)]Gal(β1-4)GlcNAc(β1-6)[Gal(α1-3)[Fuc(α1-2)]Gal(β1-4)GlcNAc(β1-3)]GalNAc(-Sp14
4B
5
6
7

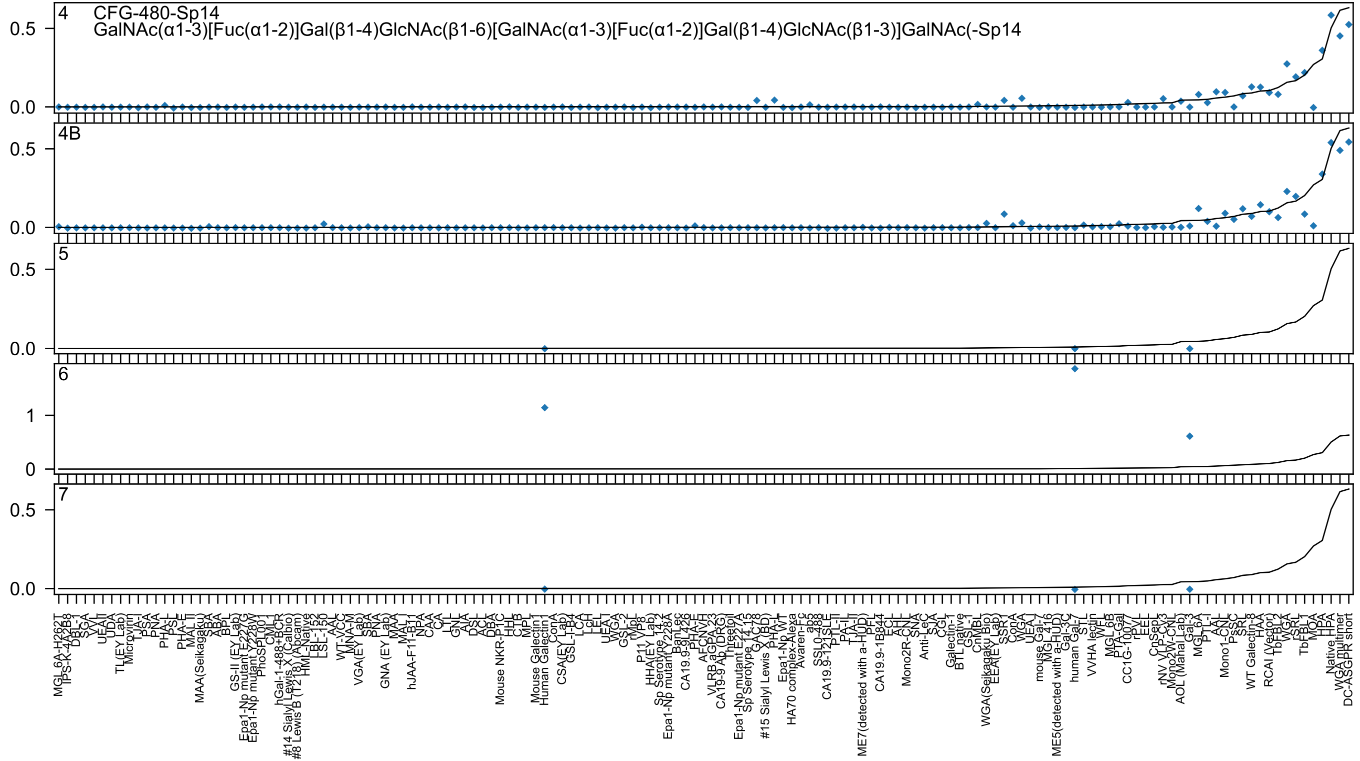

4 CFG-480-Sp14
GalNAc(α1-3)[Fuc(α1-2)]Gal(β1-4)GlcNAc(β1-6)[GalNAc(α1-3)[Fuc(α1-2)]Gal(β1-4)GlcNAc(β1-3)]GalNAc(-Sp14
4B
5
6
7

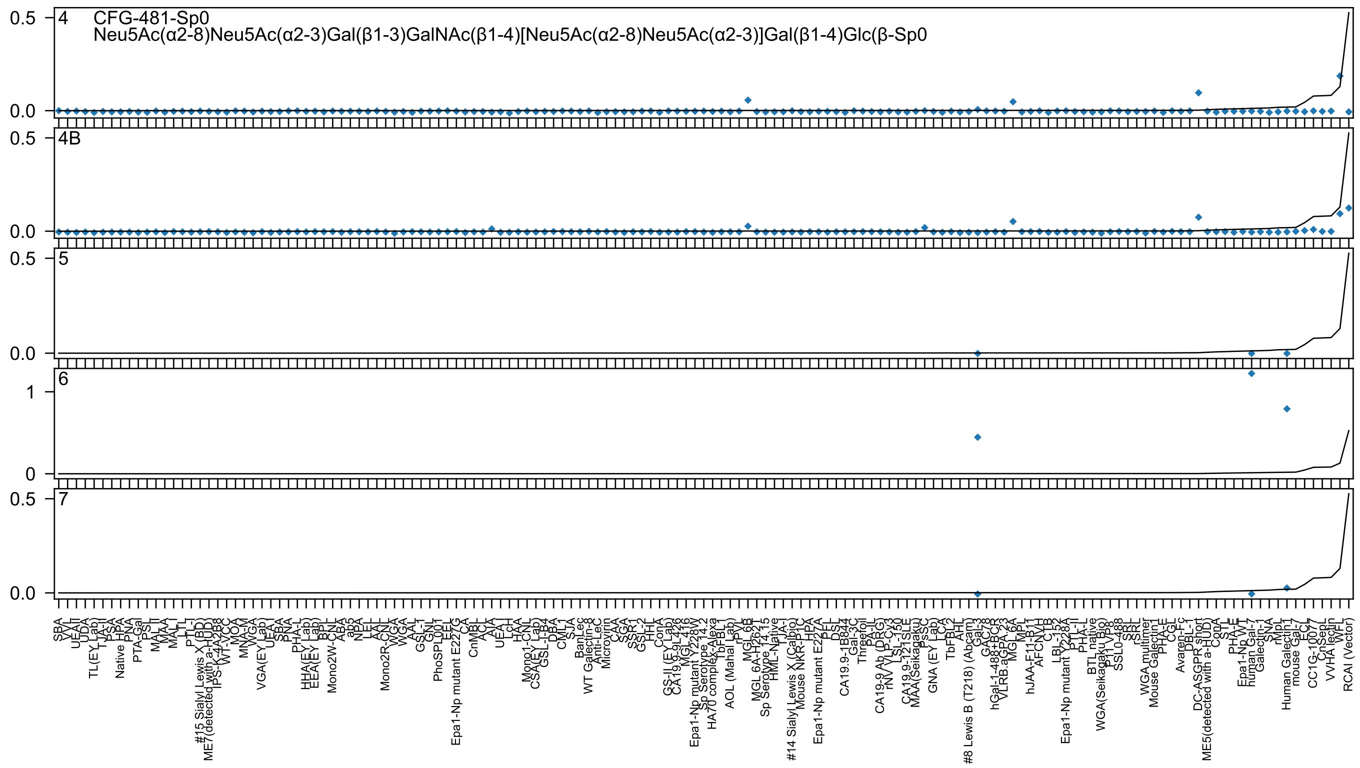

4
CFG-481-Sp0
Neu5Ac(α2-8)Neu5Ac(α2-3)Gal(β1-3)GalNAc(β1-4)[Neu5Ac(α2-8)Neu5Ac(α2-3)]Gal(β1-4)Glc(β-Sp0
4B
5
6
7

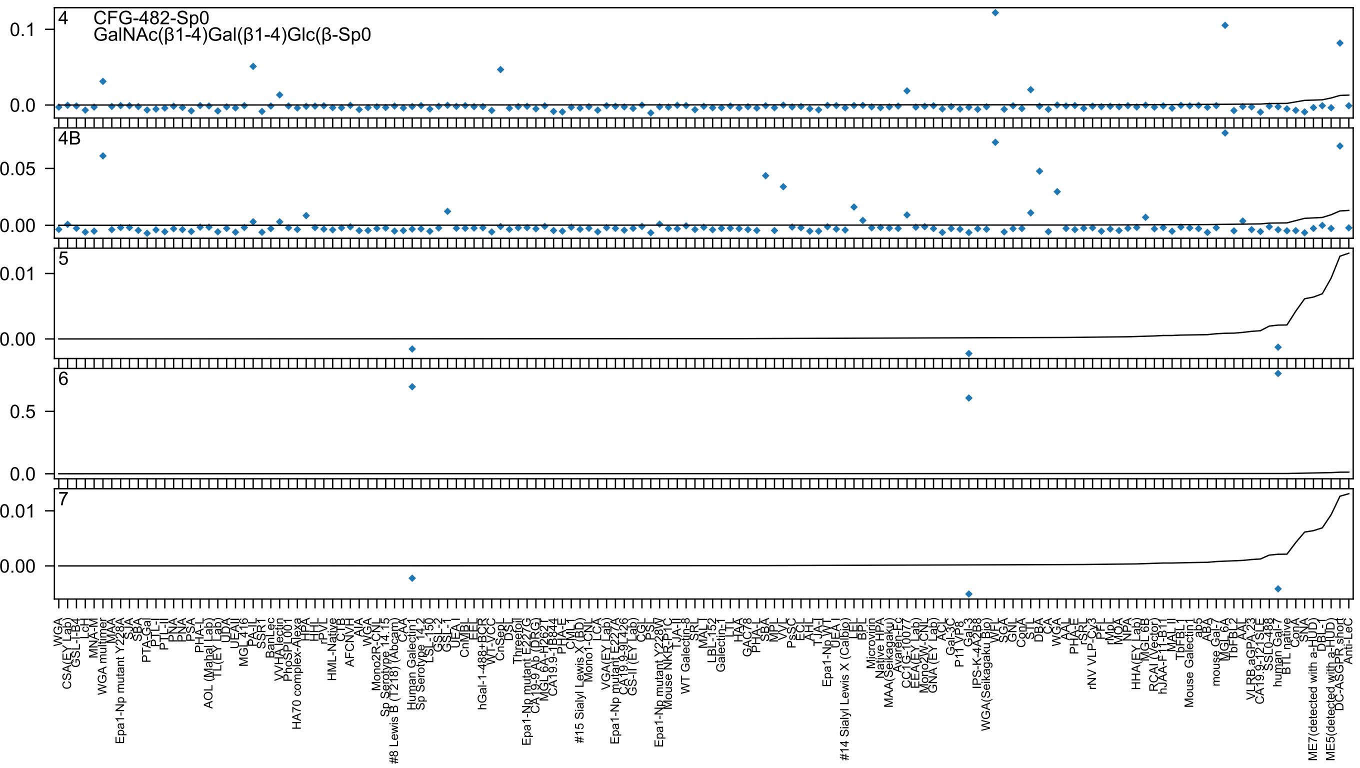
4 CFG-482-Sp0
GalNAc(β1-4)Gal(β1-4)Glc(β-Sp0
4B
5
6
7

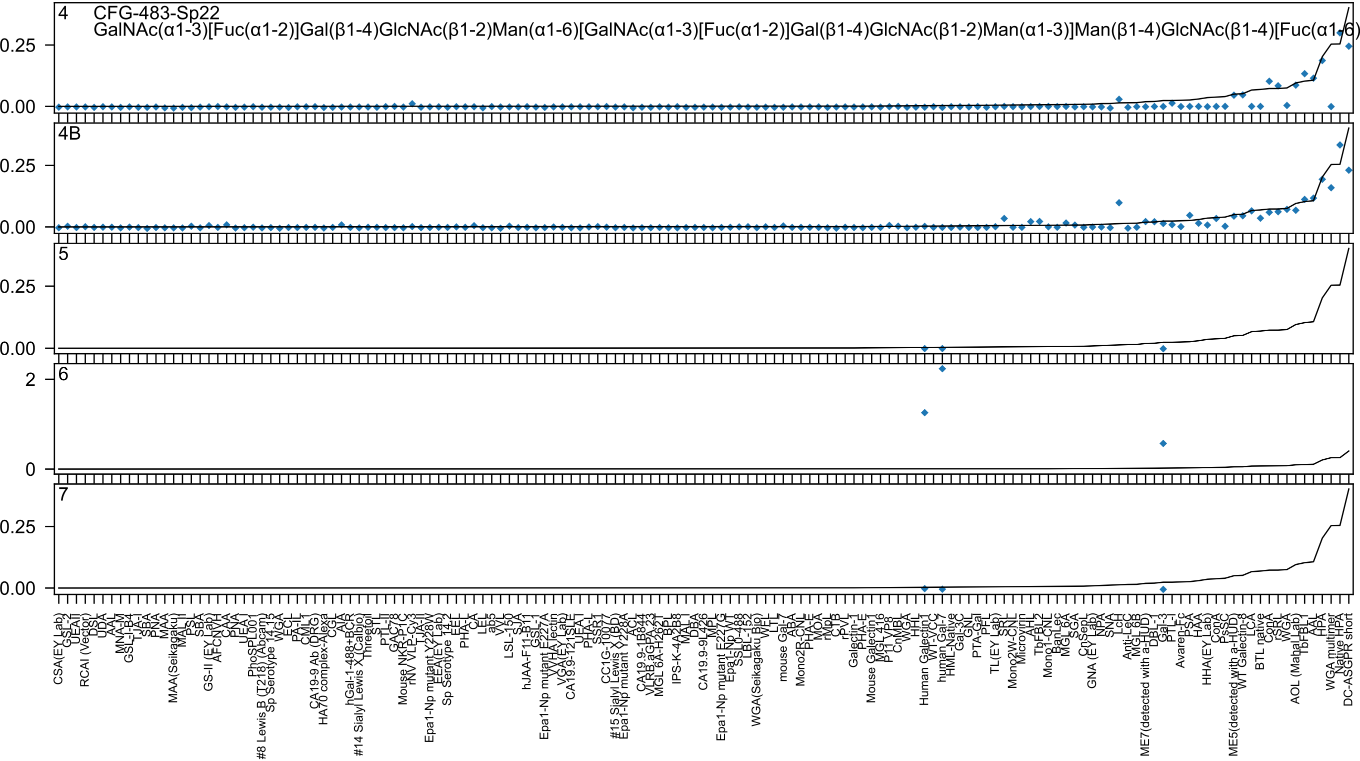

4 CFG-483-Sp22
GalNAc(α1-3)[Fuc(α1-2)]Gal(β1-4)GlcNAc(β1-2)Man(α1-6)[GalNAc(α1-3)[Fuc(α1-2)]Gal(β1-4)GlcNAc(β1-2)Man(α1-3)]Man(β1-4)GlcNAc(β1-4)[Fuc(α1-6)
4B
5
6
7

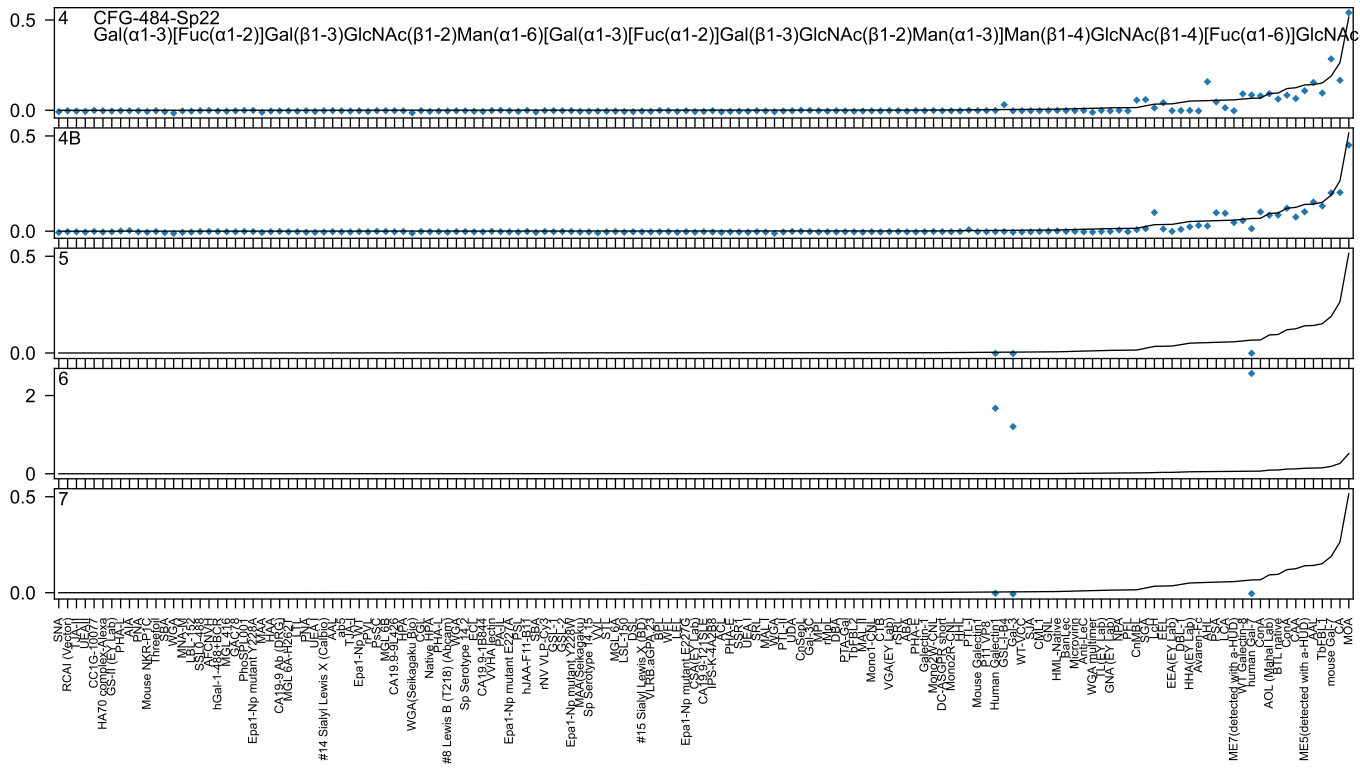

CFG-484-Sp22
Gal(α1-3)[Fuc(α1-2)]Gal(β1-3)GlcNAc(β1-2)Man(α1-6)[Gal(α1-3)[Fuc(α1-2)]Gal(β1-3)GlcNAc(β1-2)Man(α1-3)]Man(β1-4)GlcNAc(β1-4)[Fuc(α1-6)]GlcNAc
4
4B
5
6
7
0.5
0.0
2
0
SNA
RCAI (Vector)
TJA-II
UEA-I
CC1G-10077
HA70 complex-Alexa
GS-II (EY Lab)
PHA-L
AIA
PNA
Mouse NKR-P1C
Threefoil
SBA
WGA
MNA-M
LBL-152
SSL0-488
AFCNYH
hGal-1-488+BCR
MGL 416
GAC78
PhoSL001
Epa1-Np mutant Y228A
MAA
HAA
CA19-9 Ab (DRG)
MGL 6A-H262T
LTL
PNA
UEA-I
#14 Sialyl Lewis X (Calbio)
AAL
ab5
TJA-I
Epa1-Np WT
rPVL
PsSC
MGL 6B
CA19.9-9L426
HPA
WGA(Seikagaku Bio)
CGL
Native HPA
PHA-L
#8 Lewis B (T218) (Abcam)
WGA
Sp Serotype 14.2
ECL
CA19.9-1B844
VVHA lectin
PA-IL
Epa1-Np mutant E227A
PSL
hJAA-F11-B11
SBA
rNV VLP-Cv3
GSL-I
GSL-2
Epa1-Np mutant Y228W
MAA(Seikagaku)
Sp Serotype 14.15
VVL
STL
MGL 6A
LSL-150
DSL
#15 Sialyl Lewis X (BD)
VLRB.aGPA.23
BPL
WFL
LEL
Epa1-Np mutant E227G
CSA(EY Lab)
CA19.9-121SLE
IPS-K-4A2B8
ACL
PHA-E
SSR1
UEA-I
SRL
MAL-I
WGA
PTL-II
UDA
CnSept
Gal-3C
MPL
rMpL
DBA
PTA-Gal
TbFBL2
MAL-II
Mono1-CNL
CTB
VGA(EY Lab)
rSRL
ABA
PHA-E
Galectin-1
Mono2W-CNL
DC-ASGPR short
Mono2R-CNL
HHL
PTL-I
Mouse Galectin1
P11 VP8
Human Galectin1
GSL-I-B4
Gal-3
WT-VCC
SJA
CML1
GNL
HML-Native
BanLec
Microvirin
Anti-LeC
WGA multimer
TL (EY Lab)
GNA (EY Lab)
NPA
PFL
CnMBL
SGA
LcH
EEL
EEA(EY Lab)
DBL-I
HHA(EY Lab)
Avaren-Fc
AHL
PSA
LCA
ME7(detected with a-HUD)
WT Galectin-8
human Gal-7
ConA
AOL (Mahal Lab)
BTL native
ConA
CAA
ME5(detected with a-HUD)
AAL
TbFBL1
mouse Gal-7
CA
MOA

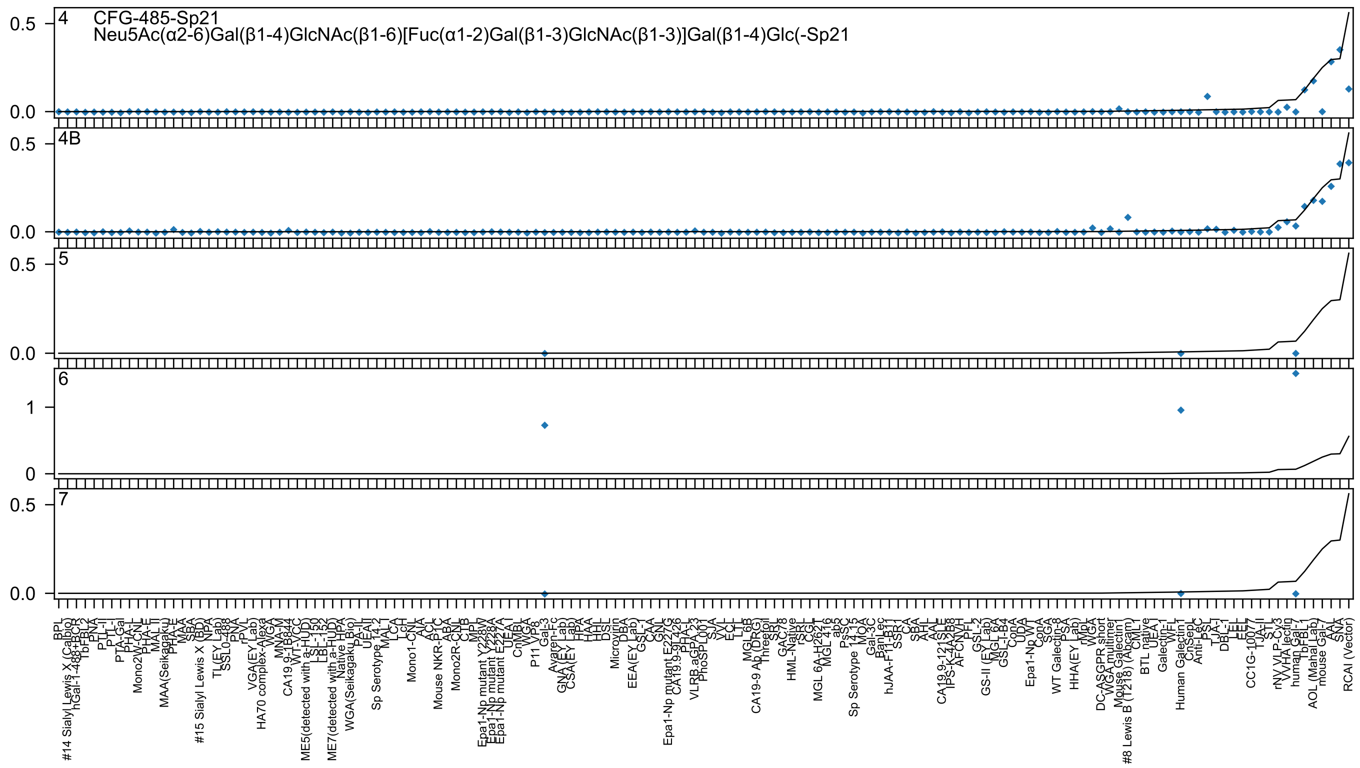
4
CFG-485-Sp21
Neu5Ac(α2-6)Gal(β1-4)GlcNAc(β1-6)[Fuc(α1-2)Gal(β1-3)GlcNAc(β1-3)]Gal(β1-4)Glc(-Sp21
4B
5
6
7

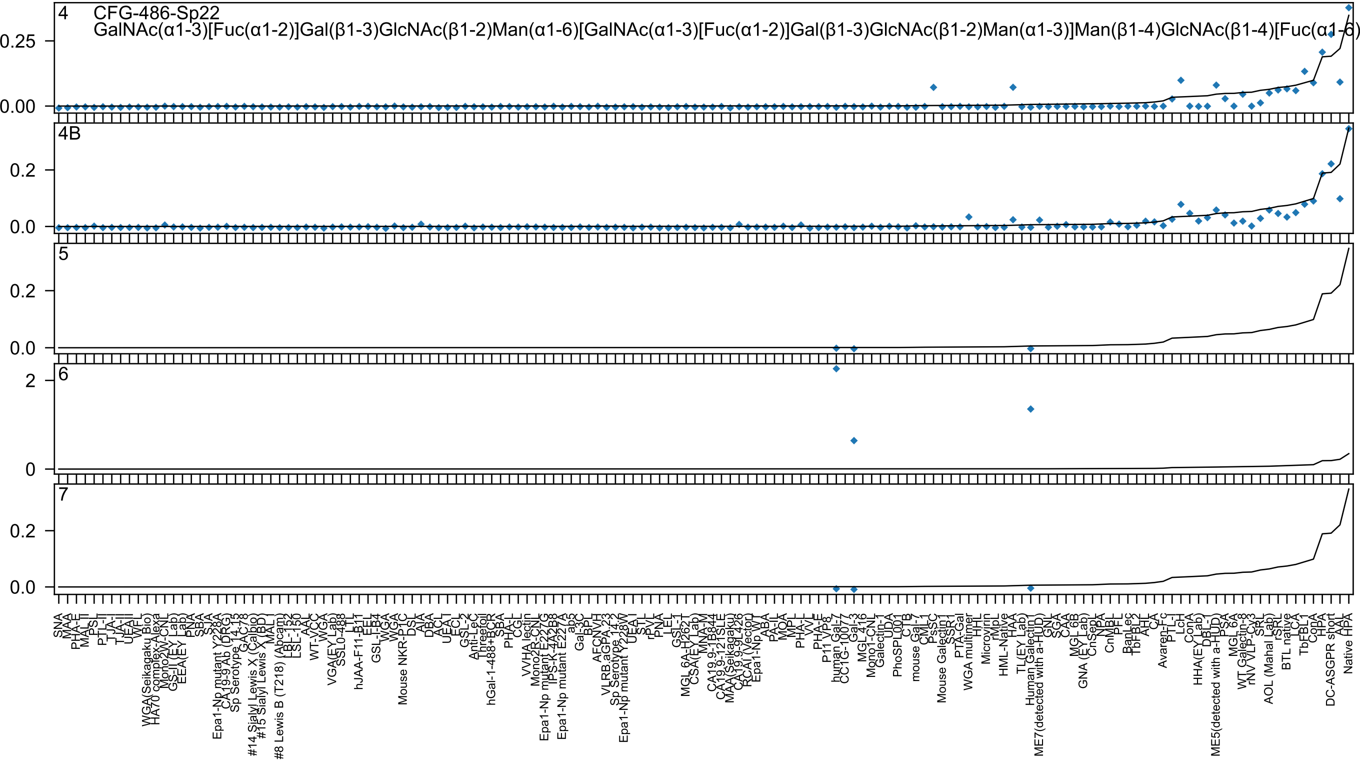

4
CFG-486-Sp22
GalNAc(α1-3)[Fuc(α1-2)]Gal(β1-3)GlcNAc(β1-2)Man(α1-6)[GalNAc(α1-3)[Fuc(α1-2)]Gal(β1-3)GlcNAc(β1-2)Man(α1-3)]Man(β1-4)GlcNAc(β1-4)[Fuc(α1-6)
4B
5
6
7

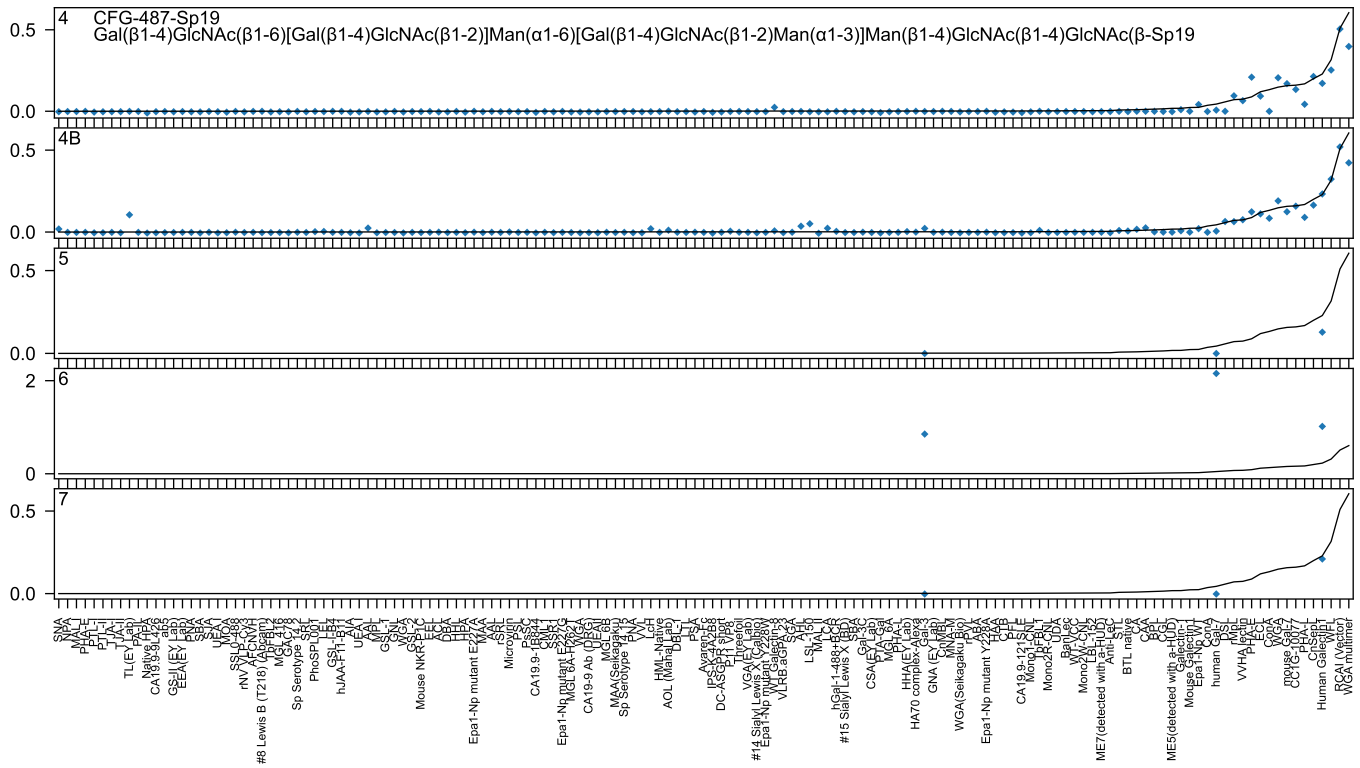

4 CFG-487-Sp19
Gal(β1-4)GlcNAc(β1-6)[Gal(β1-4)GlcNAc(β1-2)]Man(α1-6)[Gal(β1-4)GlcNAc(β1-2)Man(α1-3)]Man(β1-4)GlcNAc(β1-4)GlcNAc(β-Sp19
4B
5
6
7
0.0
0.5
0
2

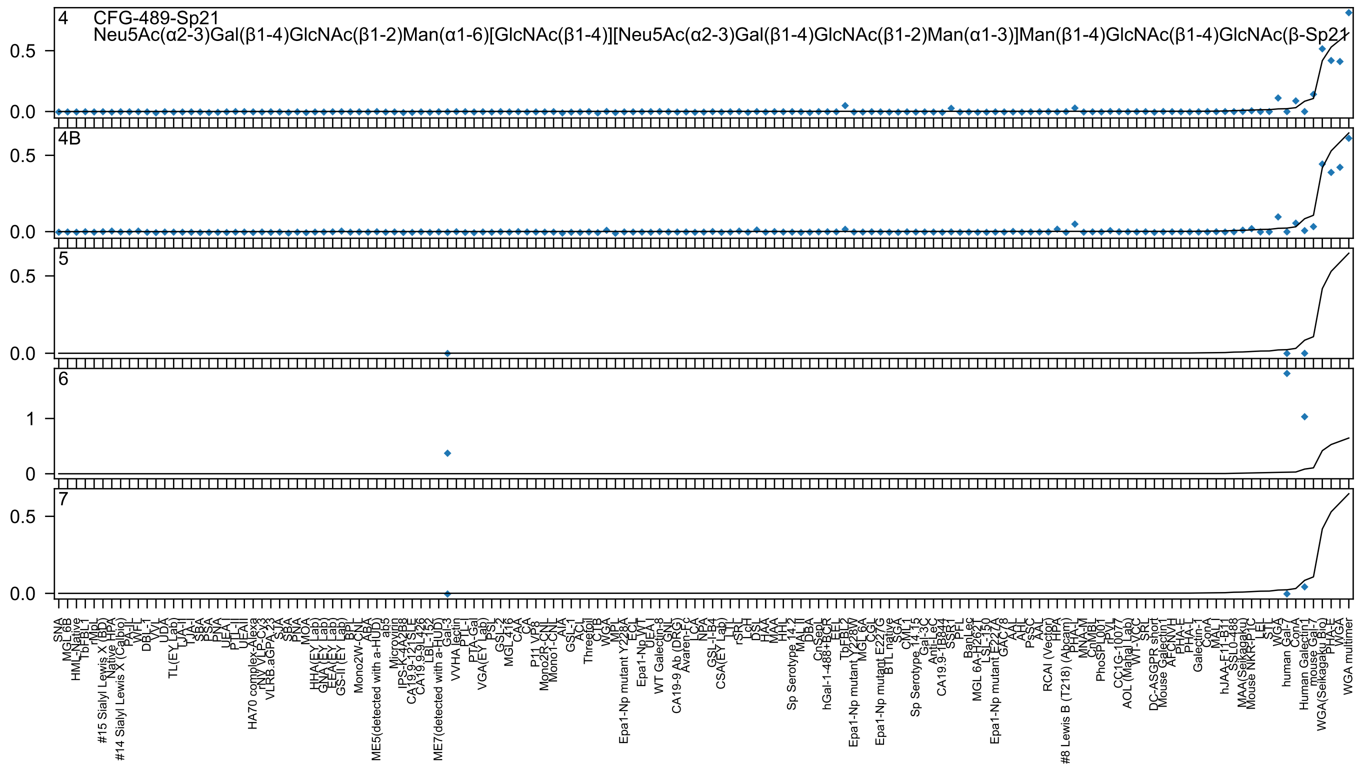

4
CFG-489-Sp21
Neu5Ac(α2-3)Gal(β1-4)GlcNAc(β1-2)Man(α1-6)[GlcNAc(β1-4)][Neu5Ac(α2-3)Gal(β1-4)GlcNAc(β1-2)Man(α1-3)]Man(β1-4)GlcNAc(β1-4)GlcNAc(β-Sp21
4B
5
6
7

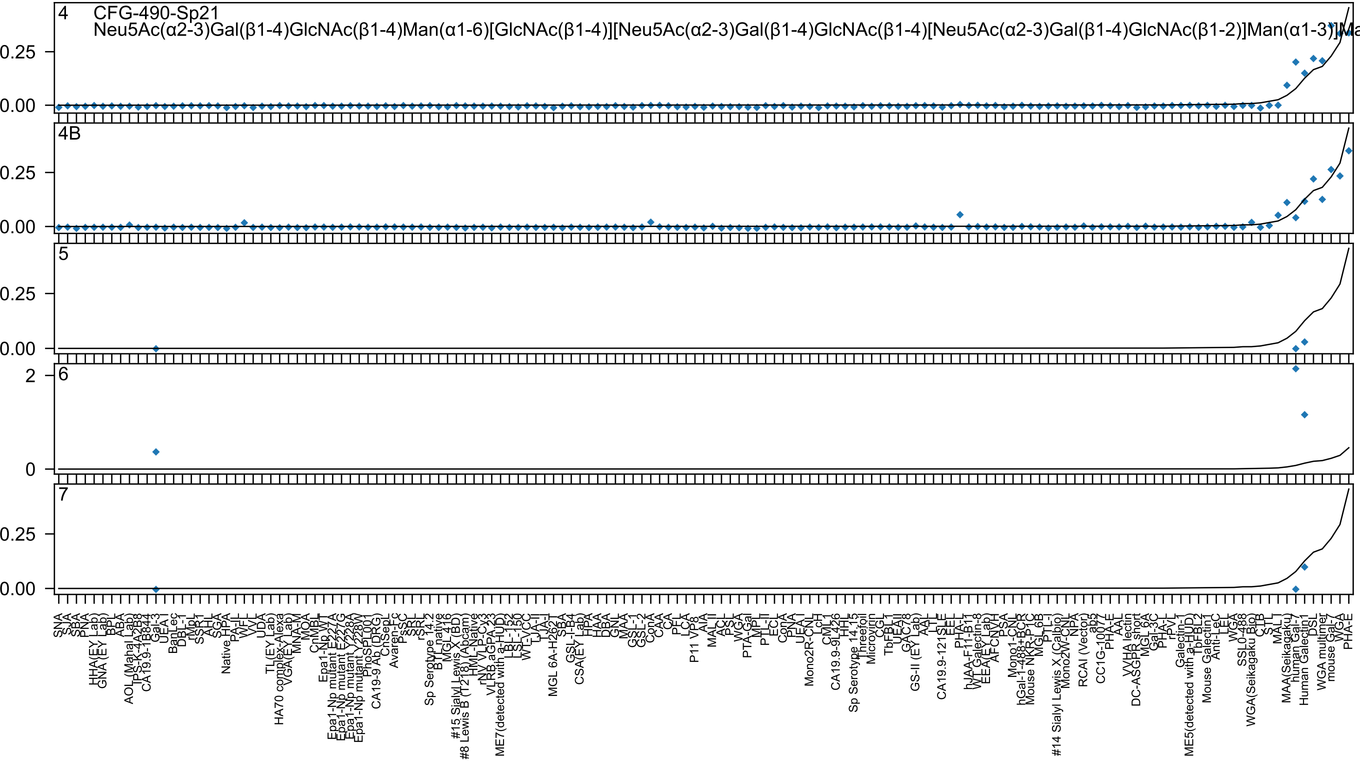

CFG-490-Sp21
Neu5Ac(α2-3)Gal(β1-4)GlcNAc(β1-4)Man(α1-6)[GlcNAc(β1-4)][Neu5Ac(α2-3)Gal(β1-4)GlcNAc(β1-4)[Neu5Ac(α2-3)Gal(β1-4)GlcNAc(β1-2)]Man(α1-3)]Ma
4
4B
5
6
7

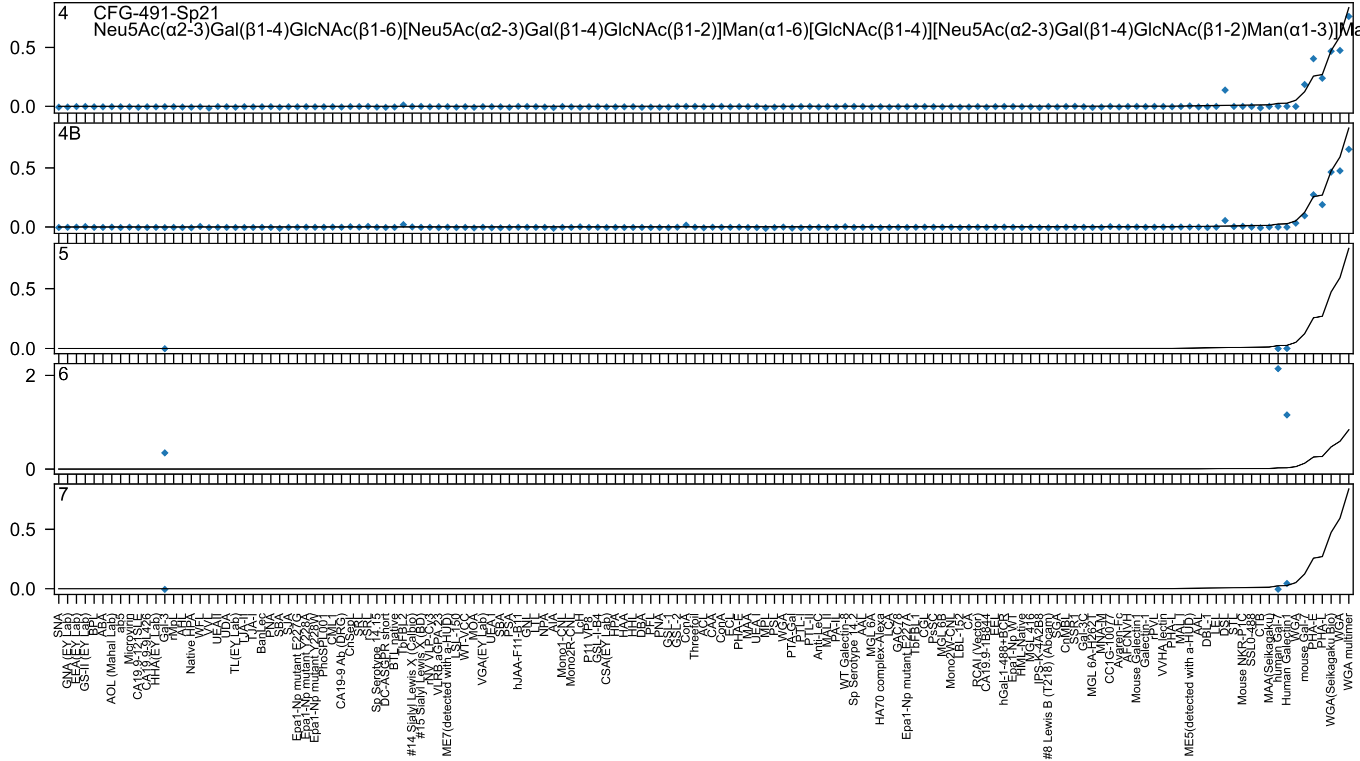
4 CFG-491-Sp21
Neu5Ac(α2-3)Gal(β1-4)GlcNAc(β1-6)[Neu5Ac(α2-3)Gal(β1-4)GlcNAc(β1-2)]Man(α1-6)[GlcNAc(β1-4)][Neu5Ac(α2-3)Gal(β1-4)GlcNAc(β1-2)Man(α1-3)]Ma
4B
5
6
7

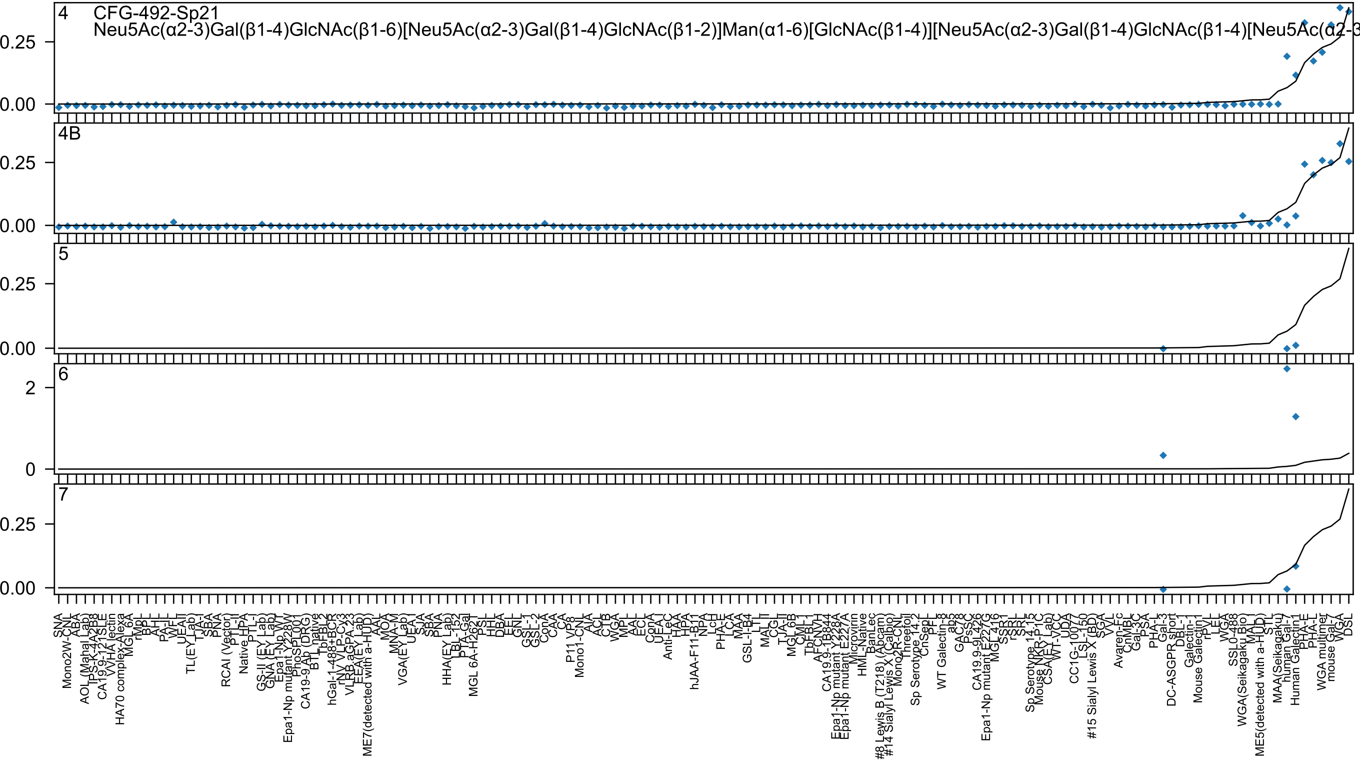

CFG-492-Sp21
Neu5Ac(α2-3)Gal(β1-4)GlcNAc(β1-6)[Neu5Ac(α2-3)Gal(β1-4)GlcNAc(β1-2)]Man(α1-6)[GlcNAc(β1-4)][Neu5Ac(α2-3)Gal(β1-4)GlcNAc(β1-4)[Neu5Ac(α2-3
4
4B
5
6
7

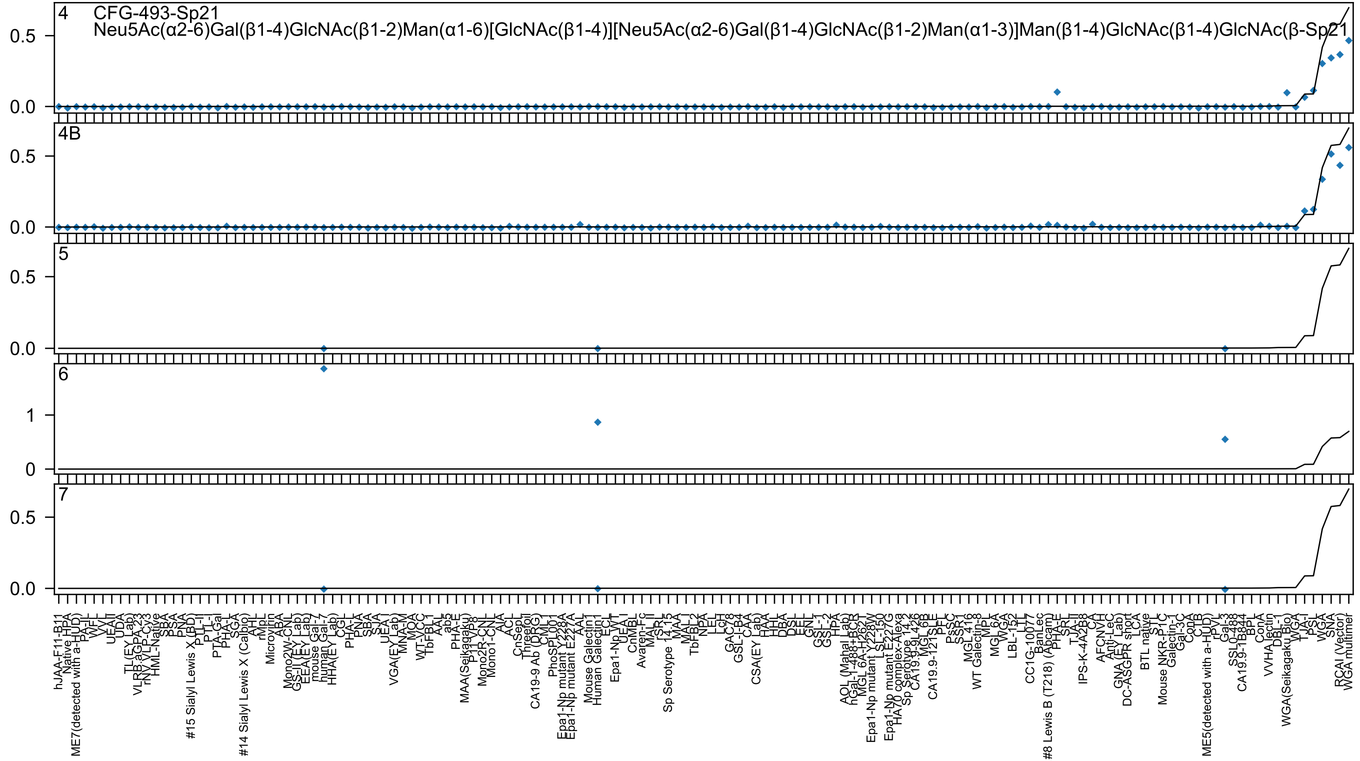

4
CFG-493-Sp21
Neu5Ac(α2-6)Gal(β1-4)GlcNAc(β1-2)Man(α1-6)[GlcNAc(β1-4)][Neu5Ac(α2-6)Gal(β1-4)GlcNAc(β1-2)Man(α1-3)]Man(β1-4)GlcNAc(β1-4)GlcNAc(β-Sp21
4B
5
6
7

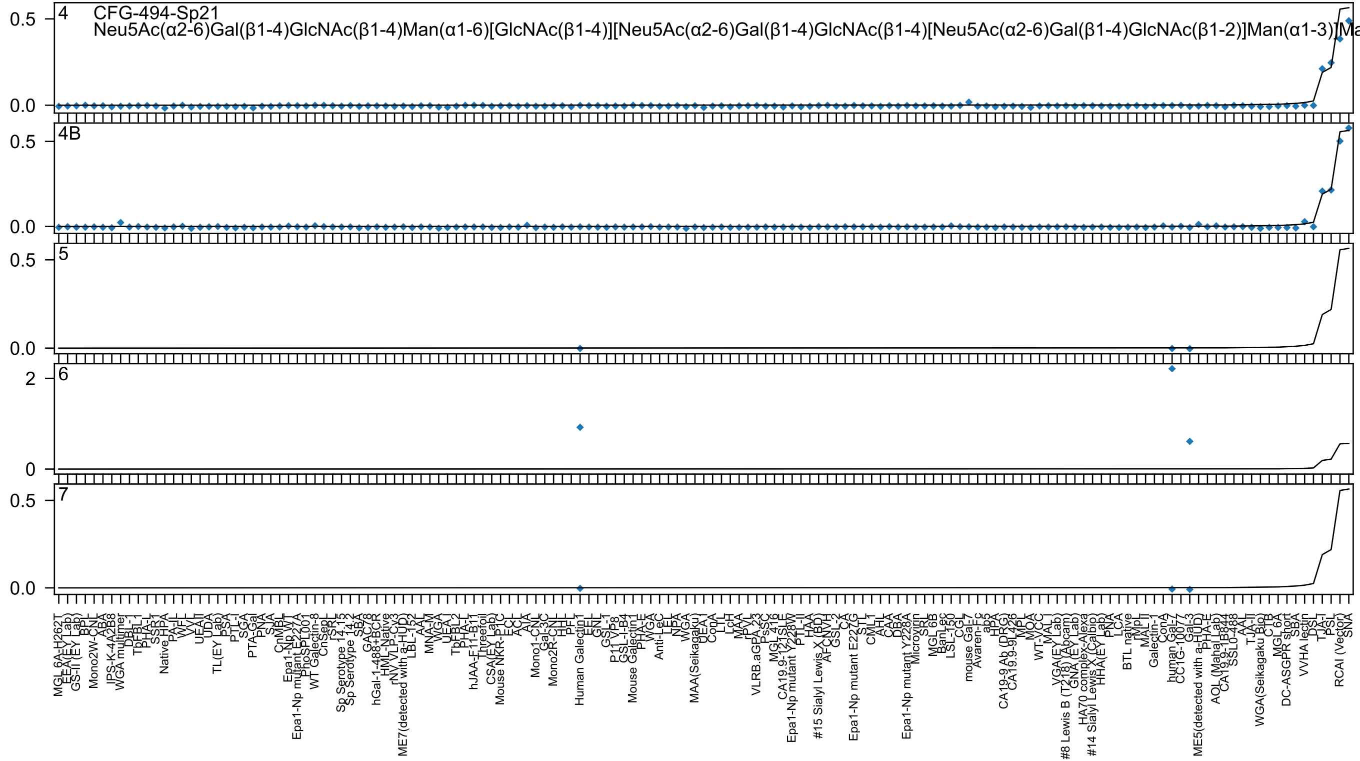
CFG-494-Sp21
Neu5Ac(α2-6)Gal(β1-4)GlcNAc(β1-4)Man(α1-6)[GlcNAc(β1-4)][Neu5Ac(α2-6)Gal(β1-4)GlcNAc(β1-4)[Neu5Ac(α2-6)Gal(β1-4)GlcNAc(β1-2)]Man(α1-3)]Ma
4
4B
5
6
7

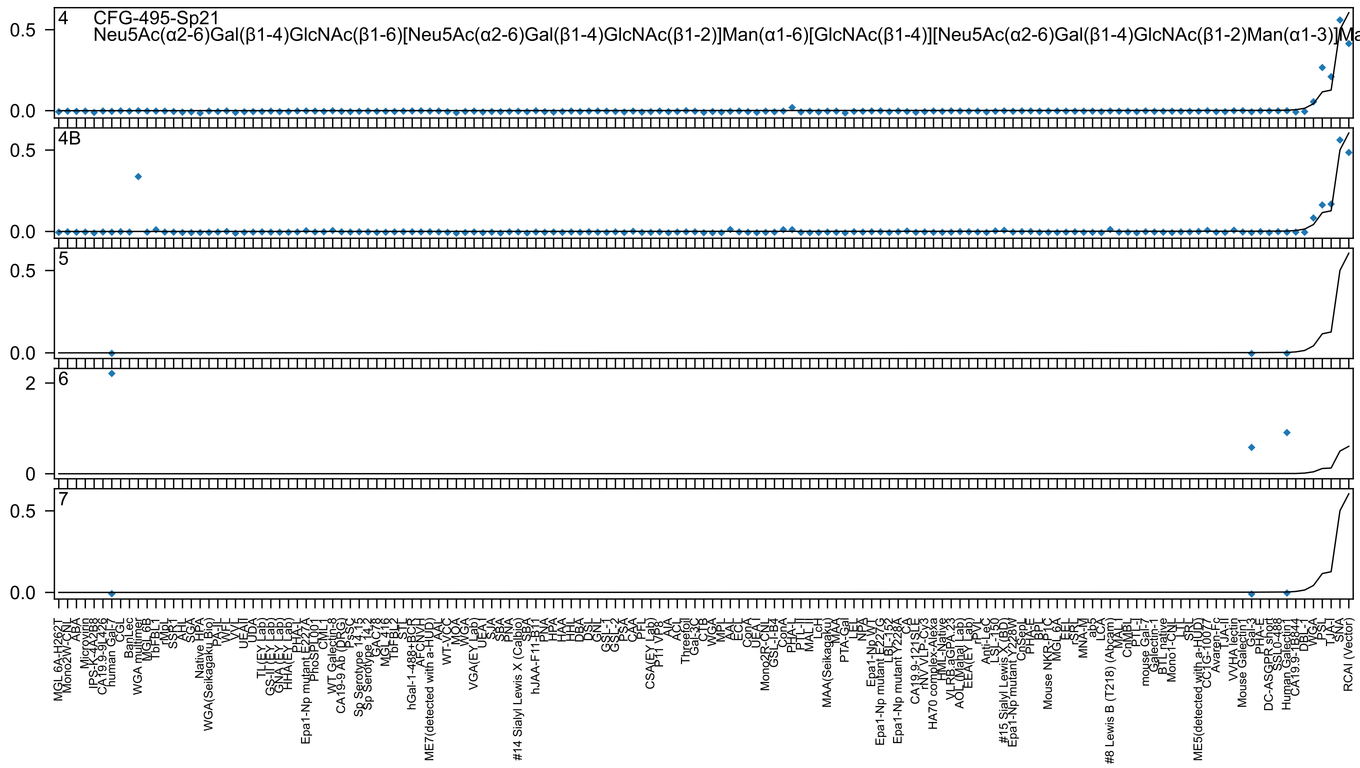

CFG-495-Sp21
Neu5Ac(α2-6)Gal(β1-4)GlcNAc(β1-6)[Neu5Ac(α2-6)Gal(β1-4)GlcNAc(β1-2)]Man(α1-6)[GlcNAc(β1-4)][Neu5Ac(α2-6)Gal(β1-4)GlcNAc(β1-2)Man(α1-3)]Ma
4
4B
5
6
7
0.0
0.5
0
2
MGL 6A-H262T
Mono2W-CNL
ABA
Microvirin
IPS-K-4A2B8
CA19.9-9L426
human Gal-7
CGL
BanLec
WGA multimer
MGL 6B
TbF-BL1
rMpL
SSR1
AHL
SGA
Native HPA
WGA(Seikagaku Bio)
PA-IL
WFL
VVL
UEAII
UDA
TL(EY Lab)
GS-II (EY Lab)
GNA (EY Lab)
HHA(EY Lab)
PHA-L
Epa1-Np mutant E227A
PhoSL001
CML1
WT Galectin-8
CA19-9 Ab (DRG)
PsSC
Sp Serotype 14.15
Sp Serotype 14.2
GAC78
MGL 416
TbF-BL2
STL
hGal-1-488+BCR
AFCNVH
ME7(detected with a-HUD)
AAL
WT-VCC
MOA
WGA
VGA(EY Lab)
UEA I
SJA
SBA
PNA
#14 Sialyl Lewis X (Calbio)
SBA
hJAA-F11-B11
PNA
HPA
HAA
HHL
DBA
DSL
GNL
GSL-I
GSL-2
PSA
CAA
PFL
CSA(EY Lab)
P11 VP8
AIA
ACL
Threefoil
Gal-3C
CTB
WGA
MPL
AAL
ECL
ConA
UEA I
Mono2R-CNL
GSL-I-B4
ConA
PHA-L
PTL-II
MAL II
LcH
MAA(Seikagaku)
MAA
PTA-Gal
LEL
NPA
Epa1-Np WT
Epa1-Np mutant E227G
LBL-152
Epa1-Np mutant Y228A
CA
CA19.9-121SLE
rNV VLP-Cy3
HA70 complex-Alexa
HML-Native
VLRB aGPA 23
AOL (Mahal Lab)
EEA(EY Lab)
rPVL
Anti-LeC
LSL-150
#15 Sialyl Lewis X (BD)
Epa1-Np mutant Y228W
CnSepL
PHA-E
BPL
Mouse NKR-P1C
MGL 6A
EEL
rSRL
MNA-M
ab5
LCA
#8 Lewis B (T218) (Abcam)
MAL I
CnMBL
PTL-I
mouse Gal-7
Galectin-1
BTL native
Mono1-CNL
LTL
SRL
ME5(detected with a-HUD)
CC1G-10077
Avaren-Fc
TJA-II
VVHA lectin
Mouse Galectin1
Gal-3
PHA-E
DC-ASGPR short
SSL0-488
Human Galectin1
CA19.9-1B844
DBL-1
WGA
PSL
TJA-I
SNA
RCAI (Vector)

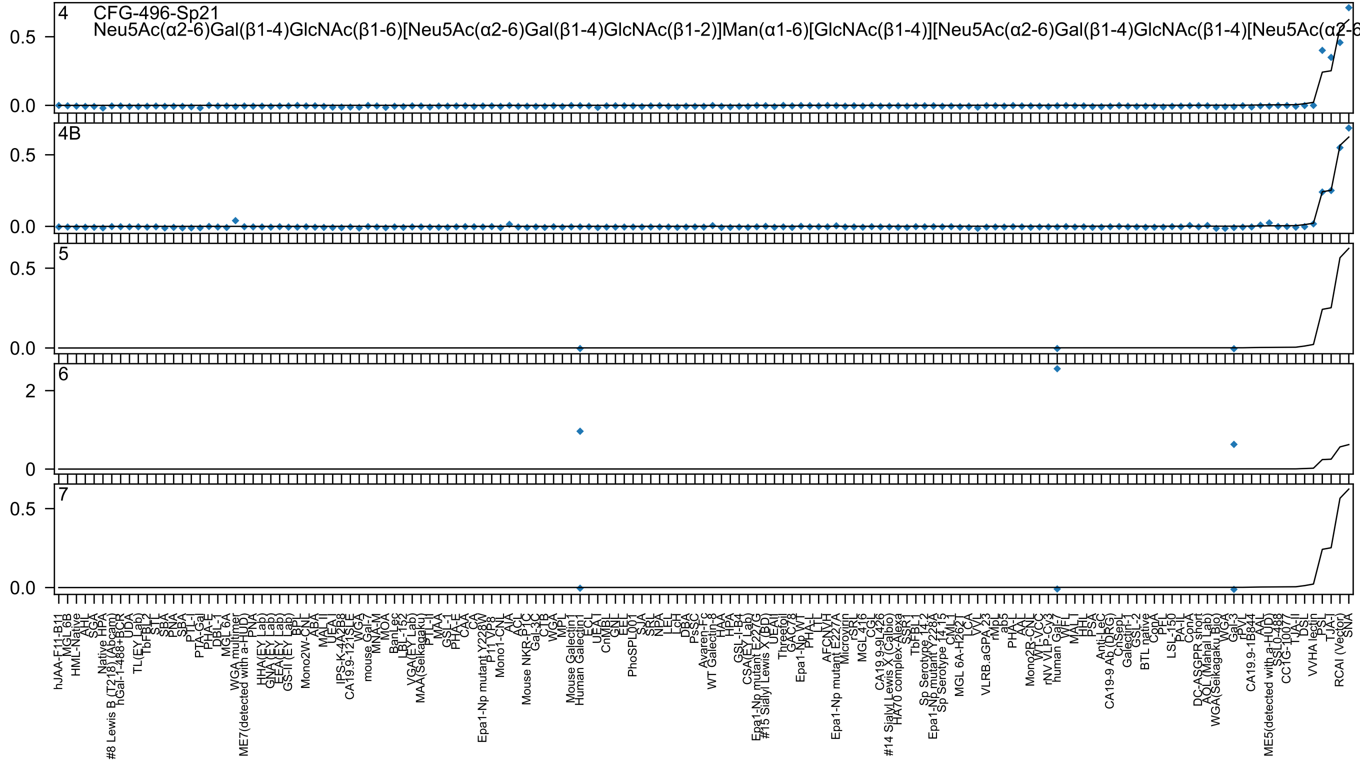
4 CFG-496-Sp21
Neu5Ac(α2-6)Gal(β1-4)GlcNAc(β1-6)[Neu5Ac(α2-6)Gal(β1-4)GlcNAc(β1-2)]Man(α1-6)[GlcNAc(β1-4)][Neu5Ac(α2-6)Gal(β1-4)GlcNAc(β1-4)[Neu5Ac(α2-6
4B
5
6
7
0.5
0.0
2
0

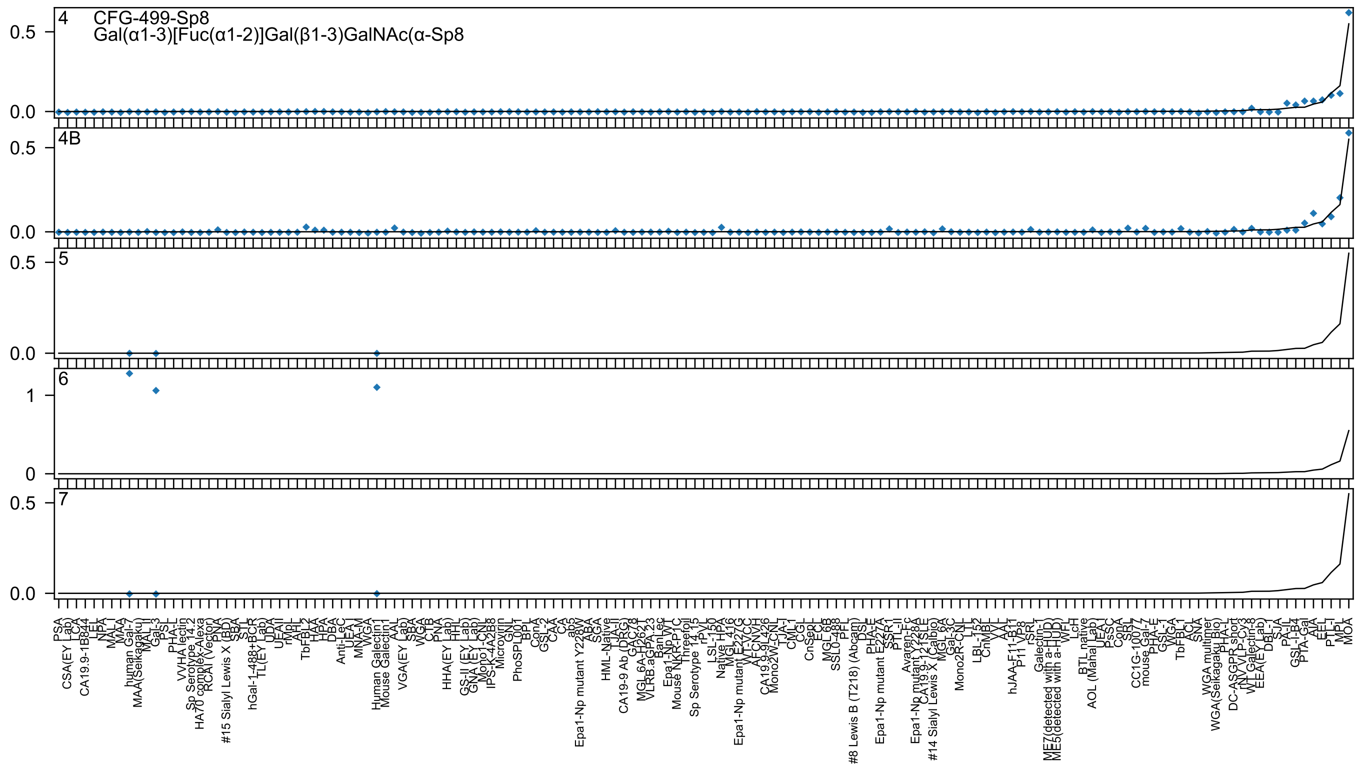
4
CFG-499-Sp8
Gal(α1-3)[Fuc(α1-2)]Gal(β1-3)GalNAc(α-Sp8
4B
5
6
7

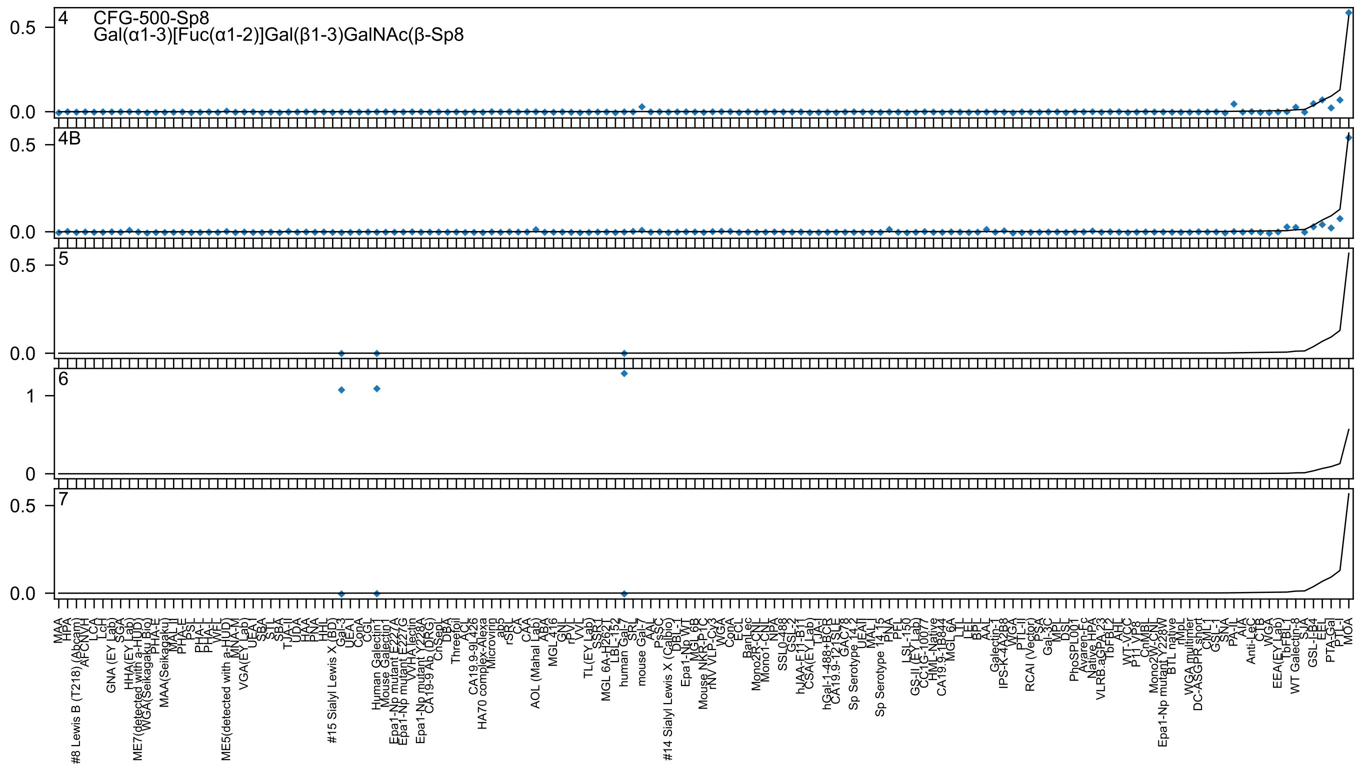
4
CFG-500-Sp8
Gal(α1-3)[Fuc(α1-2)]Gal(β1-3)GalNAc(β-Sp8
4B
5
6
7

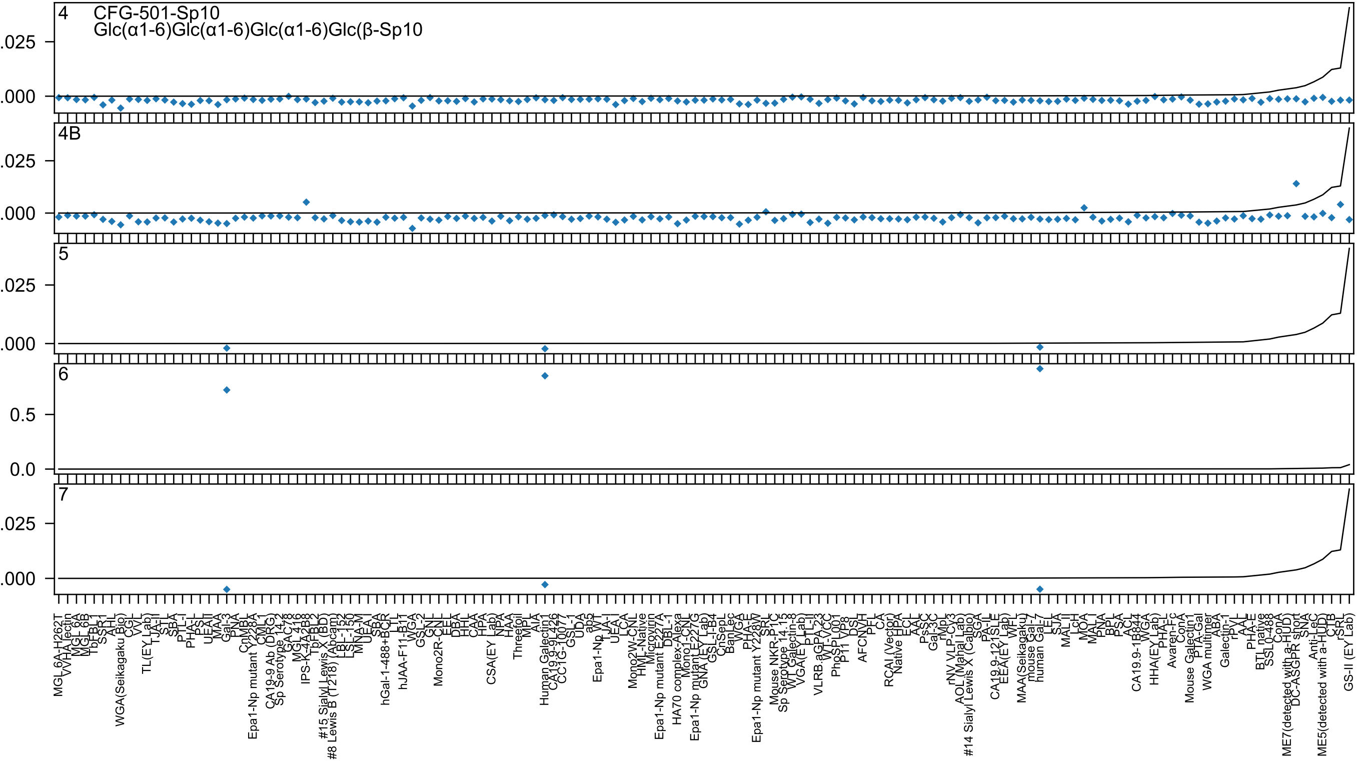
4
CFG-501-Sp10
Glc(α1-6)Glc(α1-6)Glc(α1-6)Glc(β-Sp10
4B
5
6
7
.025
.000
0.5
0.0

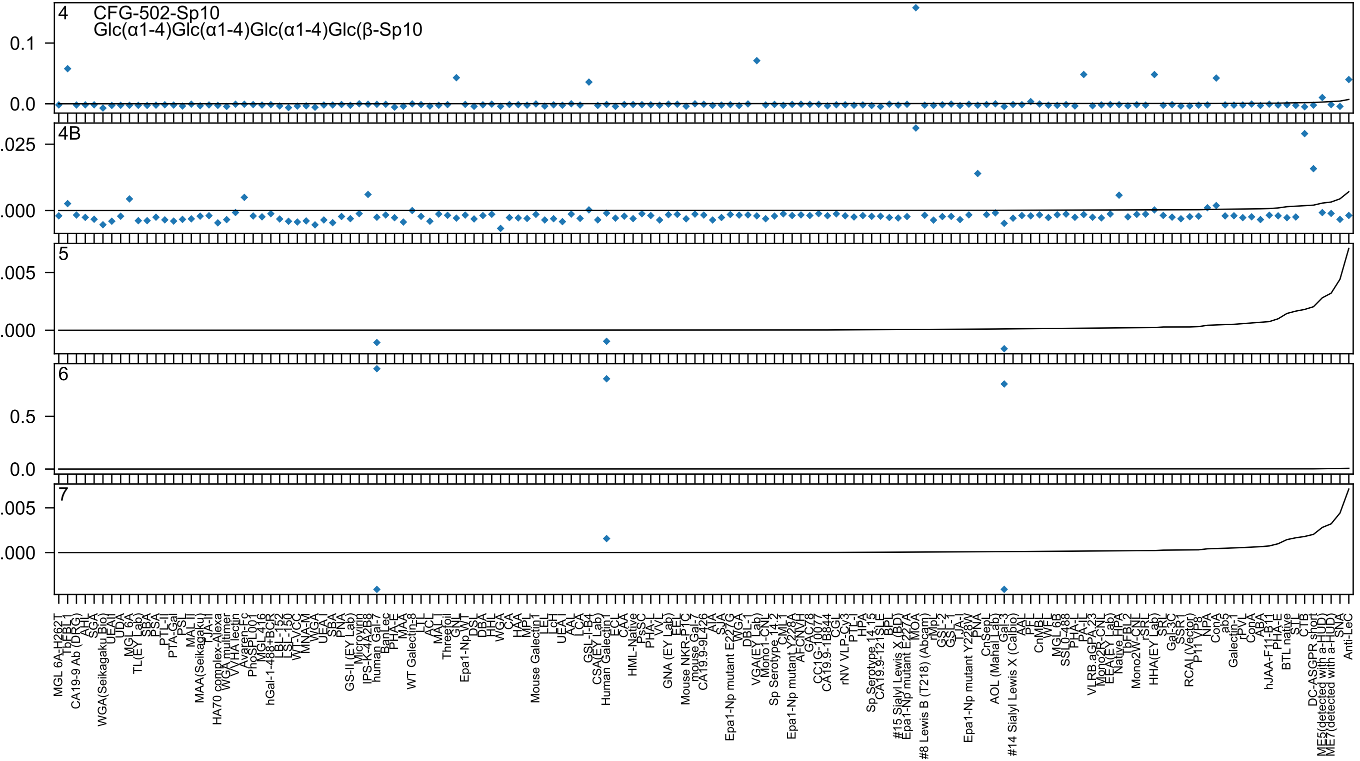
4
CFG-502-Sp10
Glc(α1-4)Glc(α1-4)Glc(α1-4)Glc(β-Sp10
4B
5
6
7

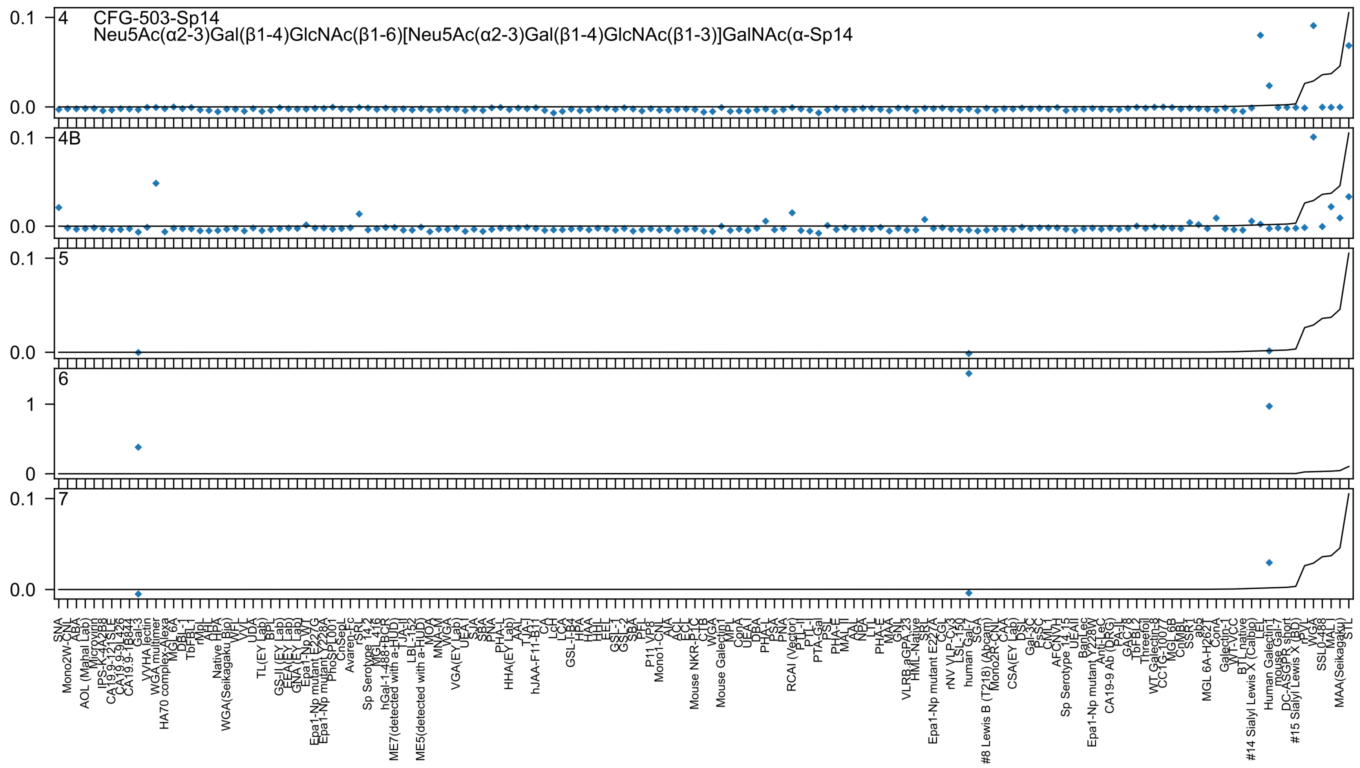

4
CFG-503-Sp14
Neu5Ac(α2-3)Gal(β1-4)GlcNAc(β1-6)[Neu5Ac(α2-3)Gal(β1-4)GlcNAc(β1-3)]GalNAc(α-Sp14
4B
5
6
7
0.1
0.0
1
0
SNA
Mono2W-CNL
ABA
AOL (Mahal Lab)
Microvirin
Gal-3
VVHA lectin
WGA multimer
HA70 complex-Alexa
MGL 6A
Native HPA
WGA(Seikagaku Bio)
TL(EY Lab)
Epa1-Np WT
Epa1-Np mutant E227G
Epa1-Np mutant Y228A
Sp Serotype 14.2
hGal-1-488+BCR
ME7(detected with a-HUD)
ME5(detected with a-HUD)
VGA(EY Lab)
HHA(EY Lab)
hJAA-F11-B11
GSL-I-B4
P11 VP8
Mono1-CNL
Mouse NKR-P1C
Mouse Galectin1
RCAI (Vector)
PTA-Gal
VLRB.aGPA.23
HML-Native
Epa1-Np mutant E227A
rNV VLP-Cy3
human Gal-7
#8 Lewis B (T218) (Abcam)
Mono2R-CNL
CSA(EY Lab)
Sp Serotype 14.15
Epa1-Np mutant Y228W
CA19-9 Ab (DRG)
WT Galectin-8
MGL 6A-H262T
#14 Sialyl Lewis X (Calbio)
Human Galectin1
mouse Gal-7
DC-ASGPR short
#15 Sialyl Lewis X (BD)
SSL0-488
MAA(Seikagaku)
STL

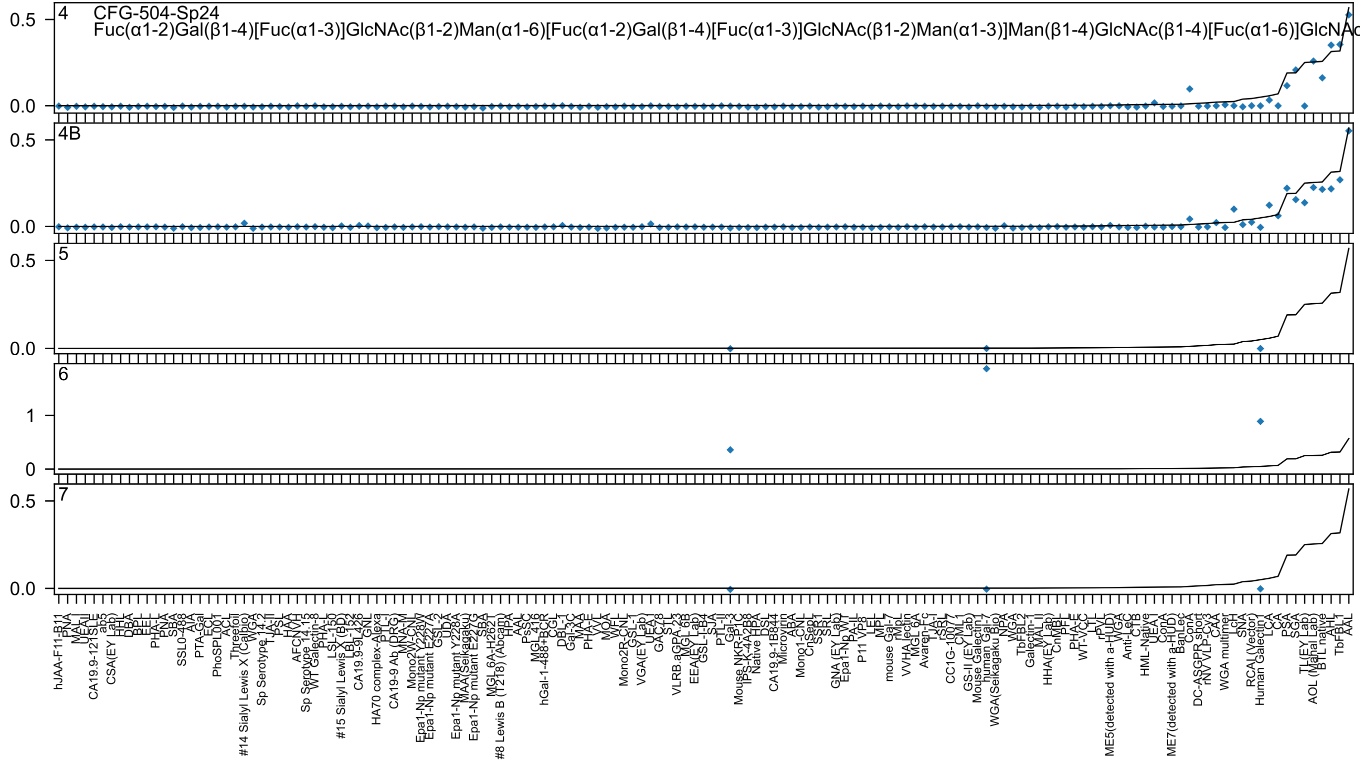

4
CFG-504-Sp24
Fuc(α1-2)Gal(β1-4)[Fuc(α1-3)]GlcNAc(β1-2)Man(α1-6)[Fuc(α1-2)Gal(β1-4)[Fuc(α1-3)]GlcNAc(β1-2)Man(α1-3)]Man(β1-4)GlcNAc(β1-4)[Fuc(α1-6)]GlcNAc
4B
5
6
7

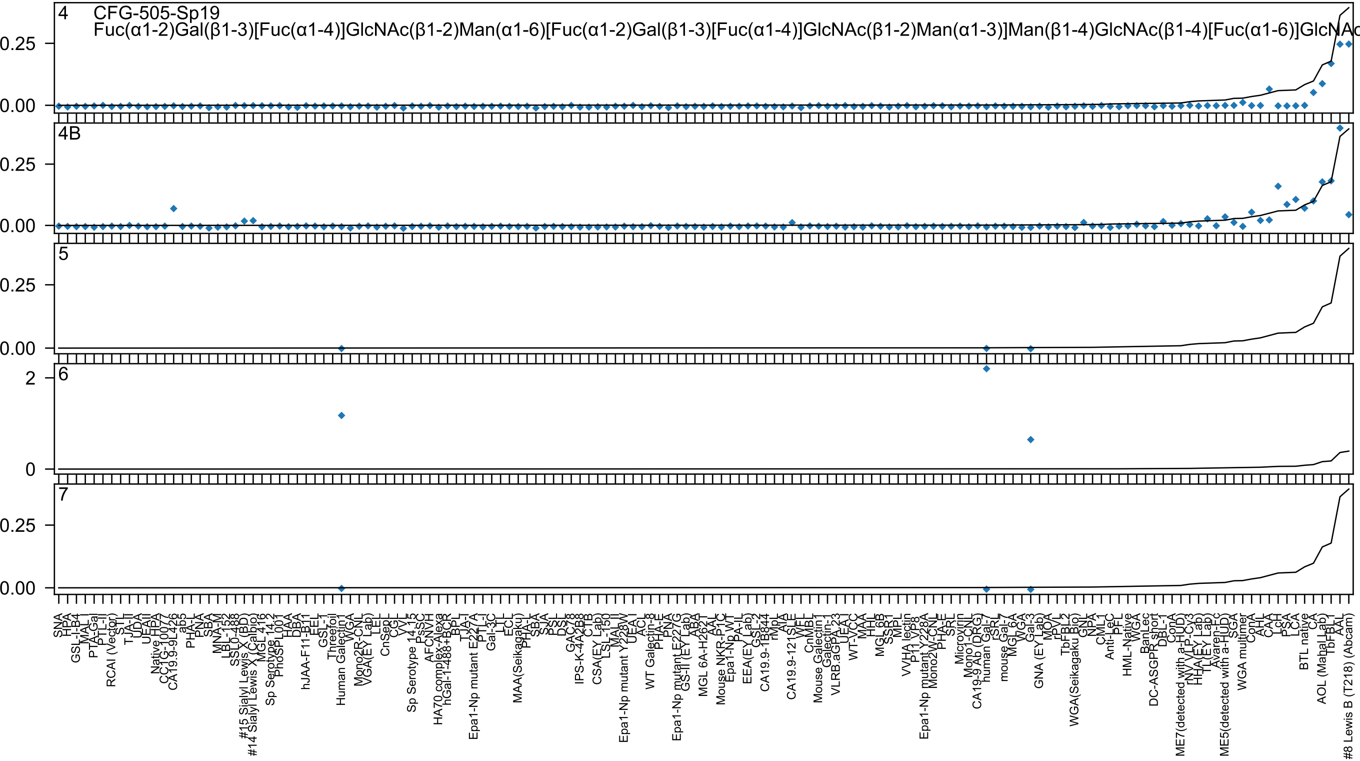

CFG-505-Sp19
Fuc(α1-2)Gal(β1-3)[Fuc(α1-4)]GlcNAc(β1-2)Man(α1-6)[Fuc(α1-2)Gal(β1-3)[Fuc(α1-4)]GlcNAc(β1-2)Man(α1-3)]Man(β1-4)GlcNAc(β1-4)[Fuc(α1-6)]GlcNAc
4
4B
5
6
7

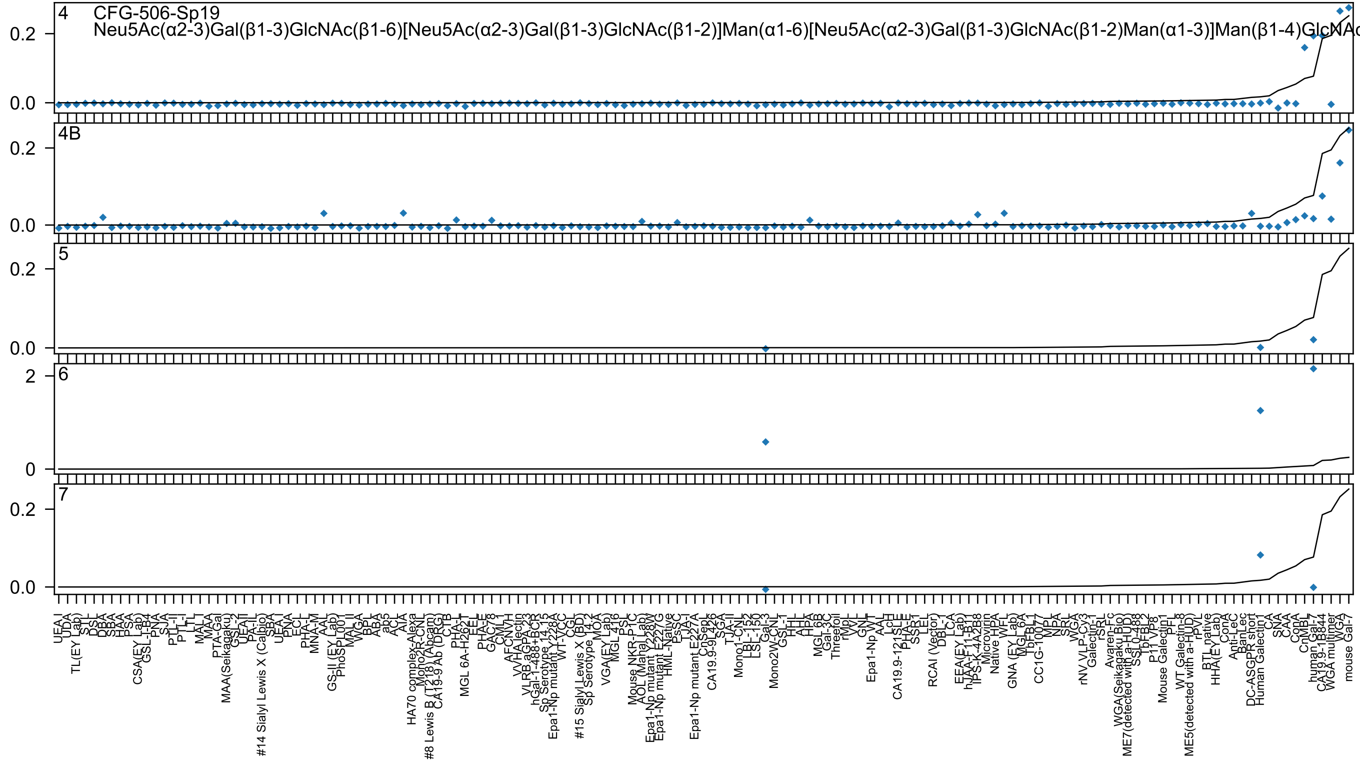
4
CFG-506-Sp19
Neu5Ac(α2-3)Gal(β1-3)GlcNAc(β1-6)[Neu5Ac(α2-3)Gal(β1-3)GlcNAc(β1-2)]Man(α1-6)[Neu5Ac(α2-3)Gal(β1-3)GlcNAc(β1-2)Man(α1-3)]Man(β1-4)GlcNAc
4B
5
6
7

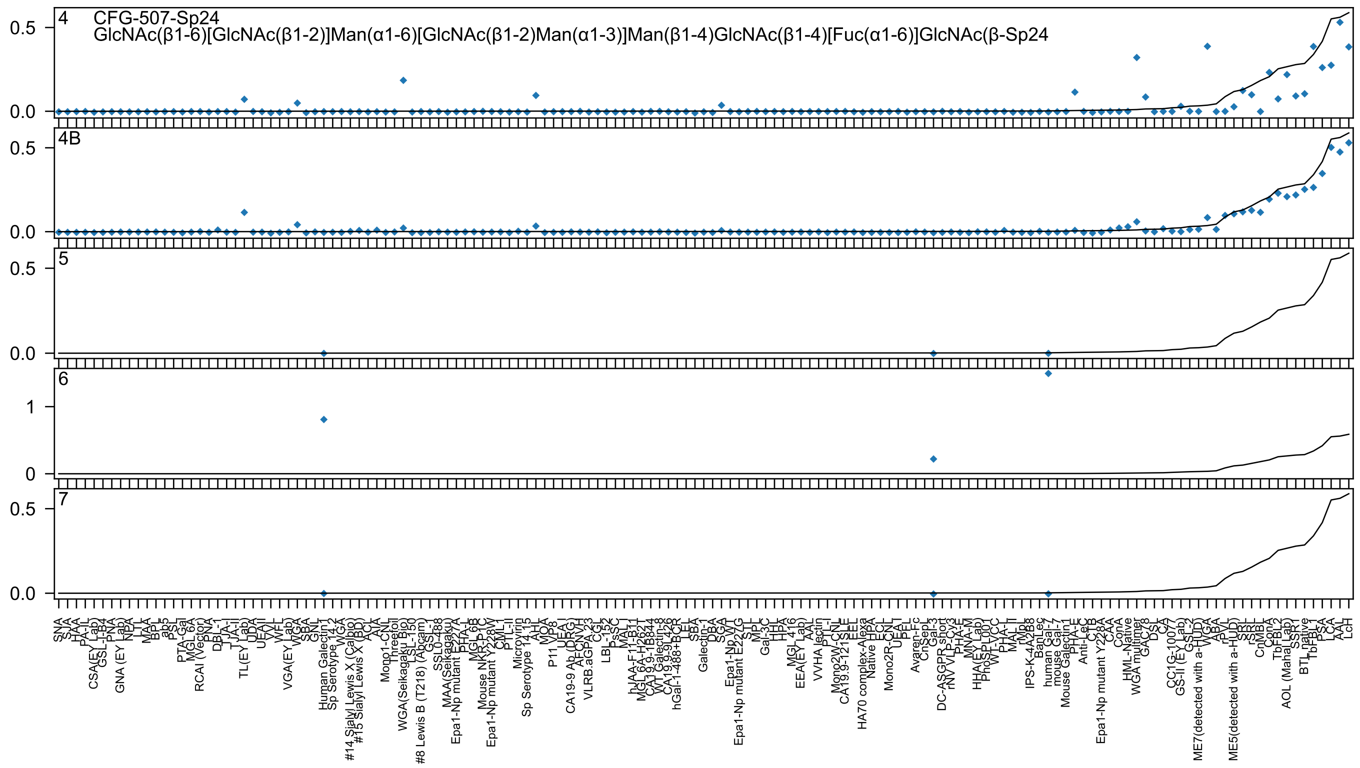

4 CFG-507-Sp24
GlcNAc(β1-6)[GlcNAc(β1-2)]Man(α1-6)[GlcNAc(β1-2)Man(α1-3)]Man(β1-4)GlcNAc(β1-4)[Fuc(α1-6)]GlcNAc(β-Sp24
4B
5
6
7

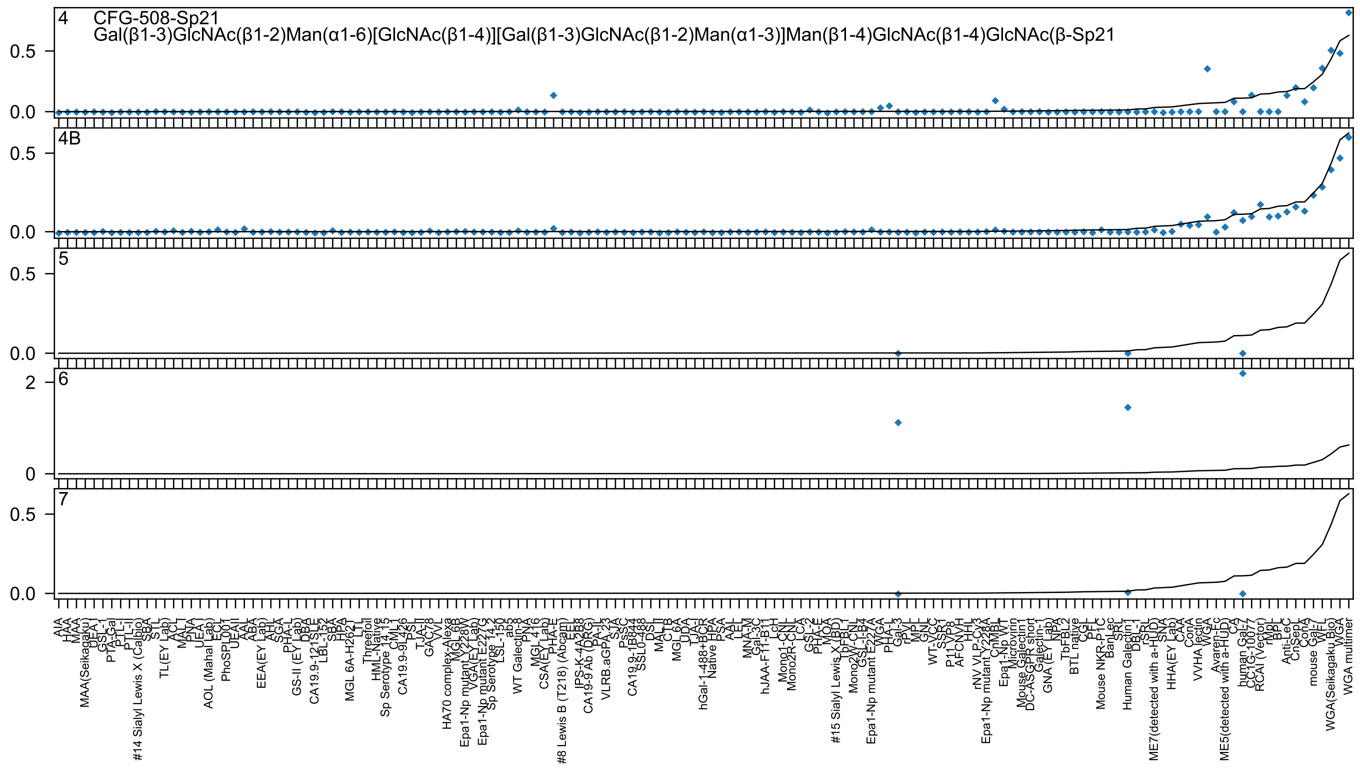

4
CFG-508-Sp21
Gal(β1-3)GlcNAc(β1-2)Man(α1-6)[GlcNAc(β1-4)][Gal(β1-3)GlcNAc(β1-2)Man(α1-3)]Man(β1-4)GlcNAc(β1-4)GlcNAc(β-Sp21
4B
5
6
7

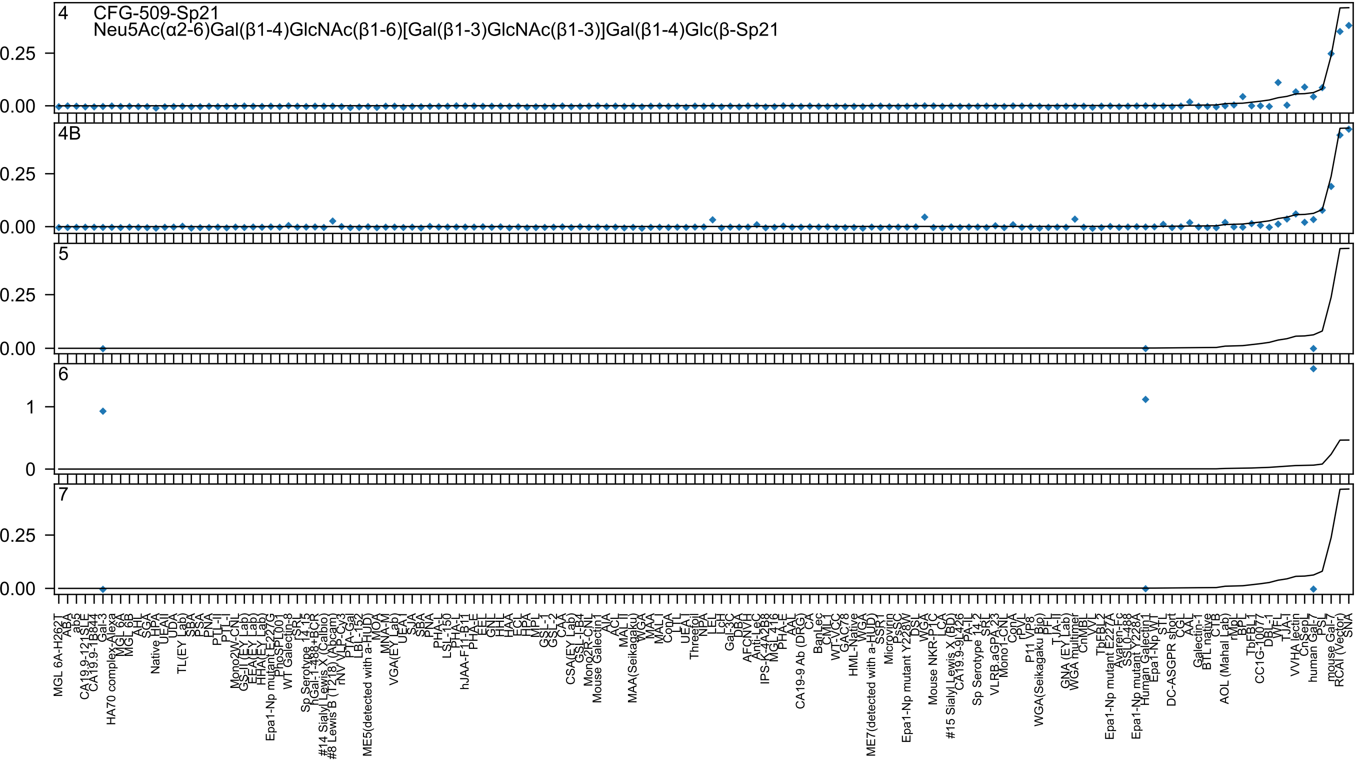

4 CFG-509-Sp21
Neu5Ac(α2-6)Gal(β1-4)GlcNAc(β1-6)[Gal(β1-3)GlcNAc(β1-3)]Gal(β1-4)Glc(β-Sp21
4B
5
6
7

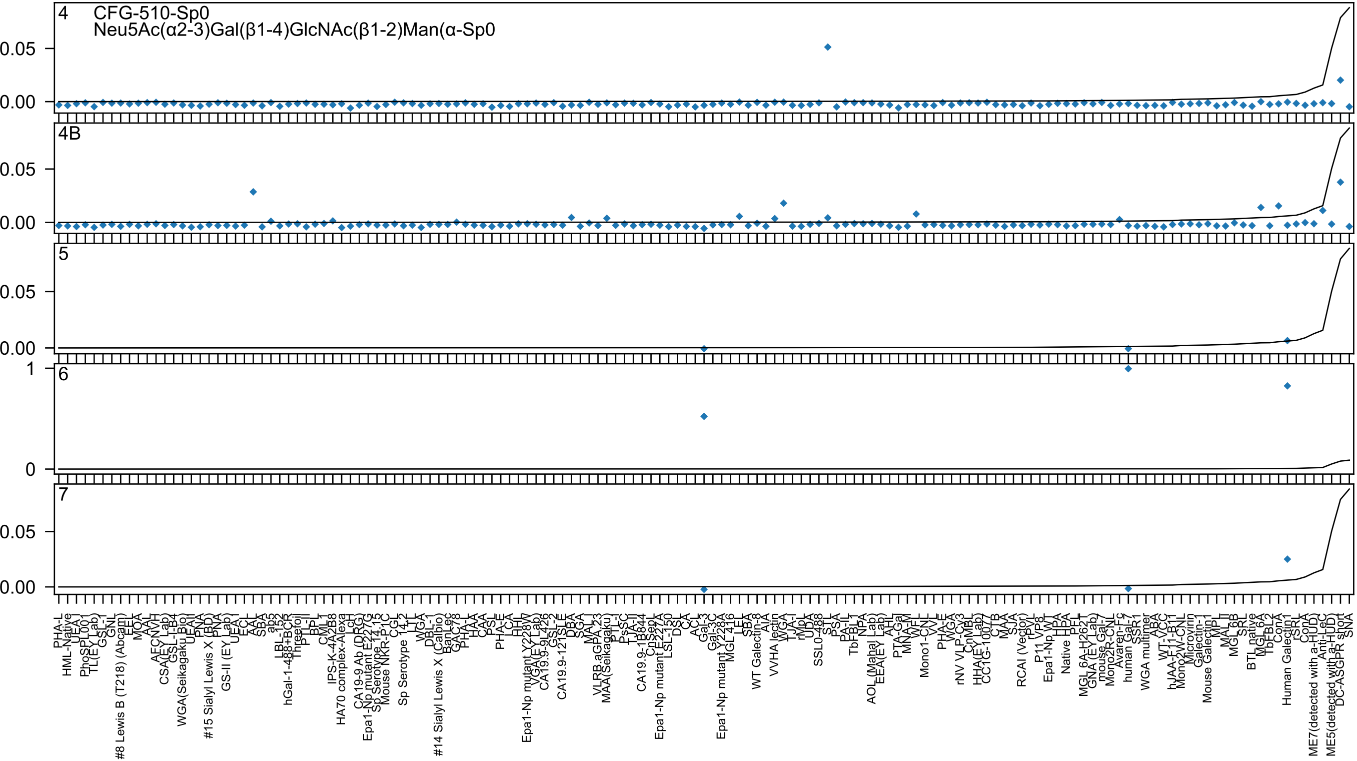
4
CFG-510-Sp0
Neu5Ac(α2-3)Gal(β1-4)GlcNAc(β1-2)Man(α-Sp0
0.05
0.00
4B
0.05
0.00
5
0.05
0.00
6
1
0
7
0.05
0.00
PHA-L
HML-Native
UEA-I
PhoSL
PL001
TL(EY Lab)
GSL-I
GNL
#8 Lewis B (T218) (Abcam)
EEL
MOA
AAL
AFCNVH
CSA(EY Lab)
GSL-I-B4
WGA(Seikagaku Bio)
UEA-I
PNA
#15 Sialyl Lewis X (BD)
PNA
GS-II (EY Lab)
UEA-I
ECL
AAL
SBA
ab5
LBL-152
hGal-1-488+BCR
Threefoil
PTL-II
BPL
CML1
IPS-K-4A2B8
HA70 complex-Alexa
LcH
CA19-9 Ab (DRG)
Epa1-Np mutant E227G
Sp Serotype 14.15
Mouse NKR-P1C
CGL
Sp Serotype 14.2
LTL
WGA
DBL-1
#14 Sialyl Lewis X (Calbio)
BanLec
GAC78
PHA-L
HAA
CAA
PSL
PHA-E
LCA
HHL
Epa1-Np mutant Y228W
VGA(EY Lab)
CA19.9-9L426
GSL-2
CA19.9-121SLE
DBA
SGA
MAL-I
VLRB.aGPA.23
MAA(Seikagaku)
PTL-I
PsSC
TJA-II
CA19.9-1B844
Epa1-Np mutant E227A
LSL-150
DSL
CA
ACL
Gal-3
Gal-3C
Epa1-Np mutant Y228A
MGL416
LEL
SBA
WT Galectin-8
AIA
VVHA lectin
WGA
TJA-I
rMpL
UDA
SSL0-488
STL
PSA
PA-IL
TbFBL1
NPA
AOL(Mahal Lab)
EEA(EY Lab)
AHL
PTA-Gal
MNA-M
WFL
Mono1-CNL
VVL
PHA-E
WGA
rNV VLP-Cy3
CnMBL
HHA(EY Lab)
CC1G-10077
CTB
MAA
SJA
RCAI (Vector)
PVL
P11 VP8
Epa1-Np WT
HPA
Native PFL
MGL 6A-H262T
GNA (EY Lab)
mouse Gal-7
Mono2R-CNL
Avaren-Fc
human Gal-7
SSR1
WGA multimer
ABA
WT-VCC
hJAA-F11-B11
Mono2W-CNL
Microvirin
Galectin-1
Mouse Galectin1
MPL
MAL-II
MGL 6B
SRL
BTL native
MGL 6A
TbFBL2
ConA
Human Galectin1
rSRL
ConA
ME7(detected with a-HUD)
Anti-LeC
ME5(detected with a-HUD)
DC-ASGPR short
SNA

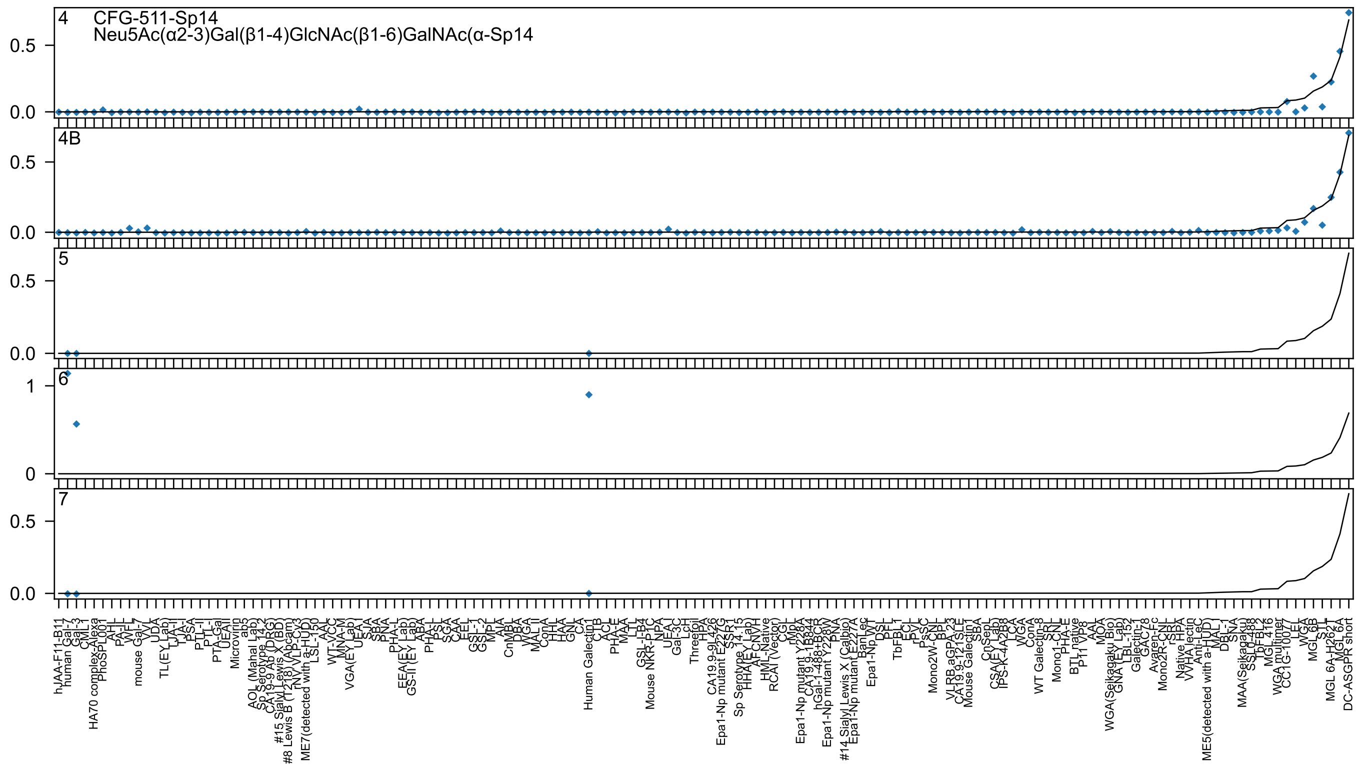
4
CFG-511-Sp14
Neu5Ac(α2-3)Gal(β1-4)GlcNAc(β1-6)GalNAc(α-Sp14
4B
5
6
7
0.5
0.0
1
0

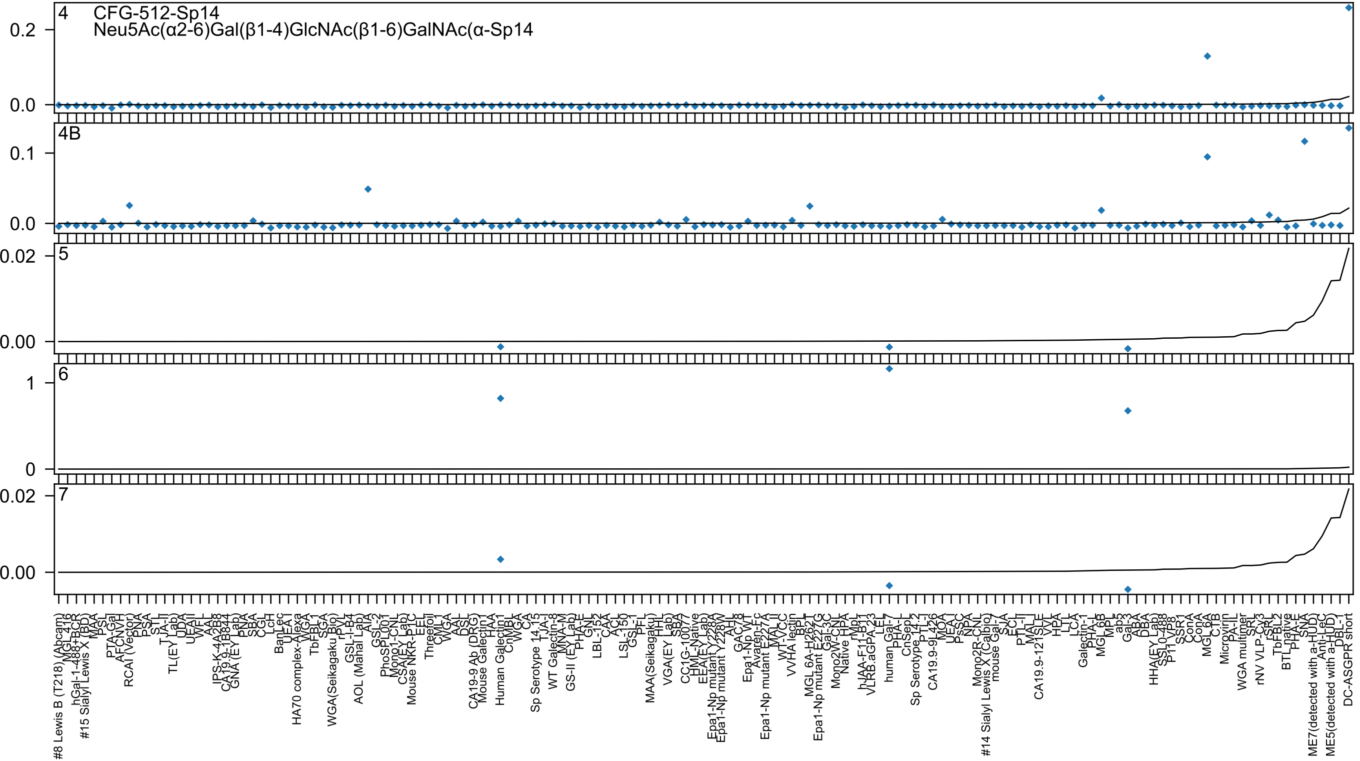

4 CFG-512-Sp14
Neu5Ac(α2-6)Gal(β1-4)GlcNAc(β1-6)GalNAc(α-Sp14
4B
5
6
7
0.2
0.1
0.0
0.02
0.00
1
0

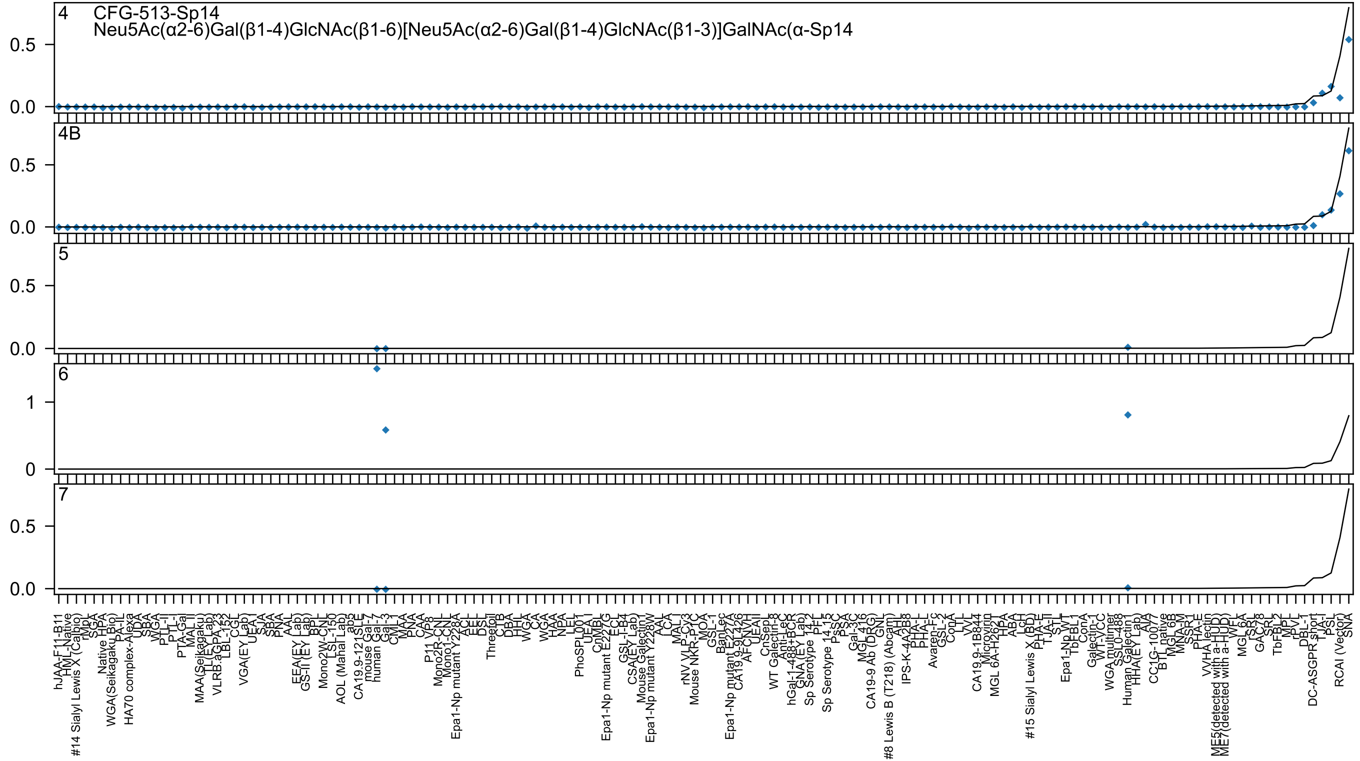
4
CFG-513-Sp14
Neu5Ac(α2-6)Gal(β1-4)GlcNAc(β1-6)[Neu5Ac(α2-6)Gal(β1-4)GlcNAc(β1-3)]GalNAc(α-Sp14
4B
5
6
7
0.5
0.0
1
0
hJAA-F11-B11
HML-Native
#14 Sialyl Lewis X (Calbio)
SNA
RCAI (Vector)
PSL
DC-ASGPR short
Human Galectin1
human Gal-7
Gal-3

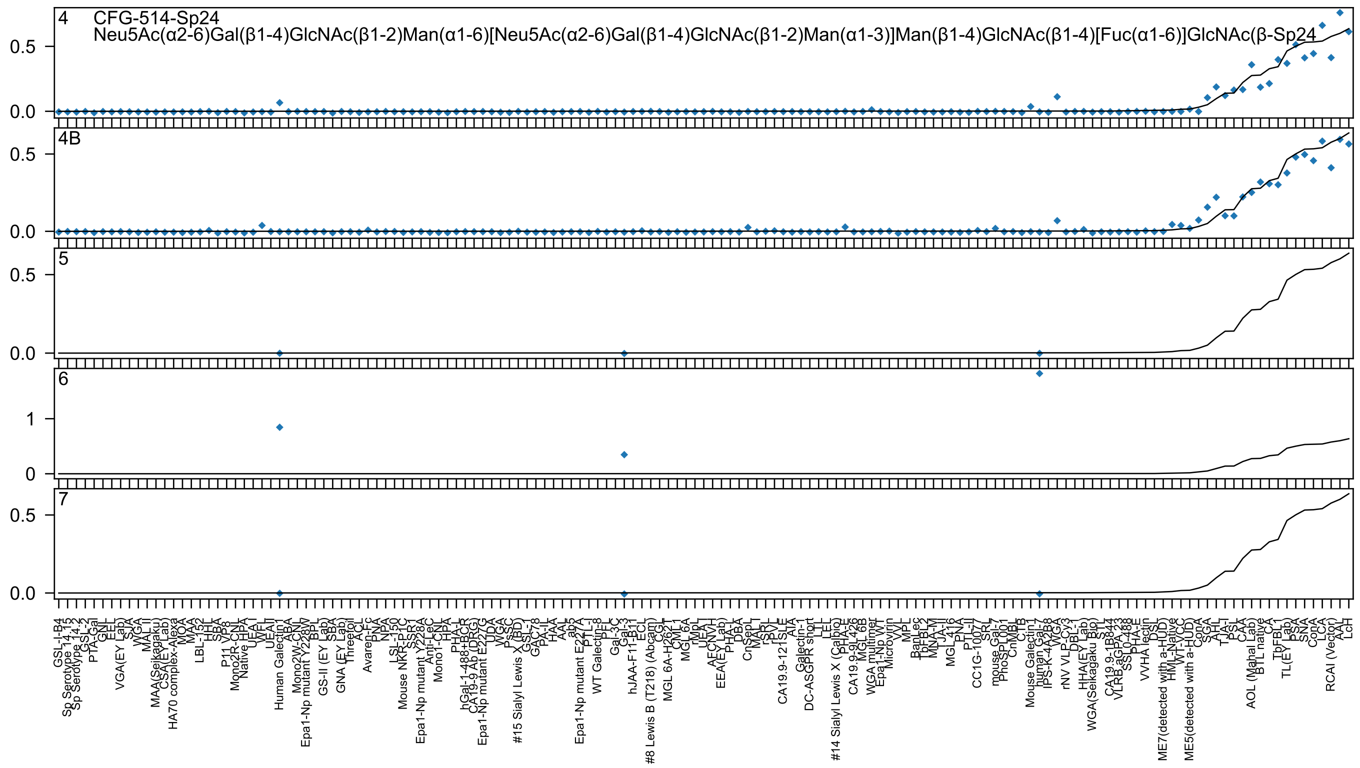
4 CFG-514-Sp24
Neu5Ac(α2-6)Gal(β1-4)GlcNAc(β1-2)Man(α1-6)[Neu5Ac(α2-6)Gal(β1-4)GlcNAc(β1-2)Man(α1-3)]Man(β1-4)GlcNAc(β1-4)[Fuc(α1-6)]GlcNAc(β-Sp24
4B
5
6
7

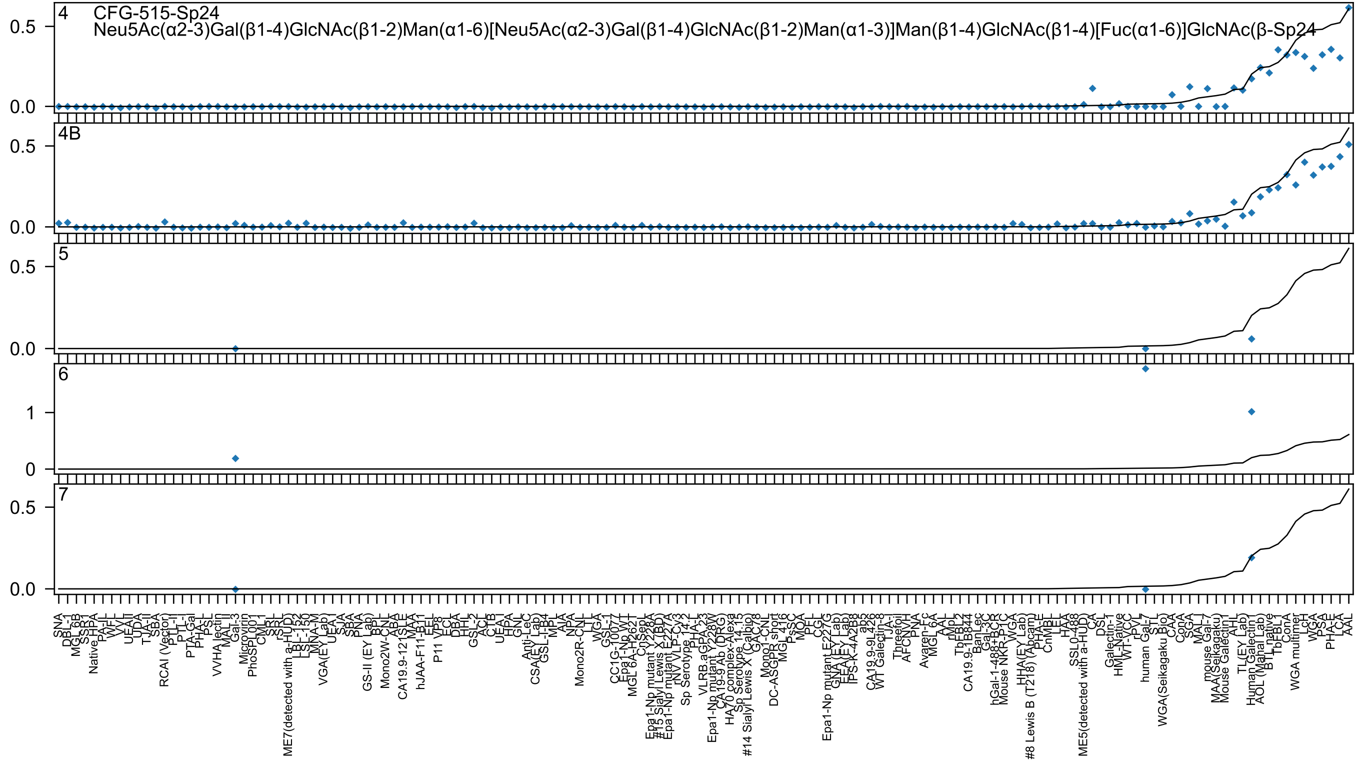
CFG-515-Sp24
Neu5Ac(α2-3)Gal(β1-4)GlcNAc(β1-2)Man(α1-6)[Neu5Ac(α2-3)Gal(β1-4)GlcNAc(β1-2)Man(α1-3)]Man(β1-4)GlcNAc(β1-4)[Fuc(α1-6)]GlcNAc(β-Sp24
4
4B
5
6
7

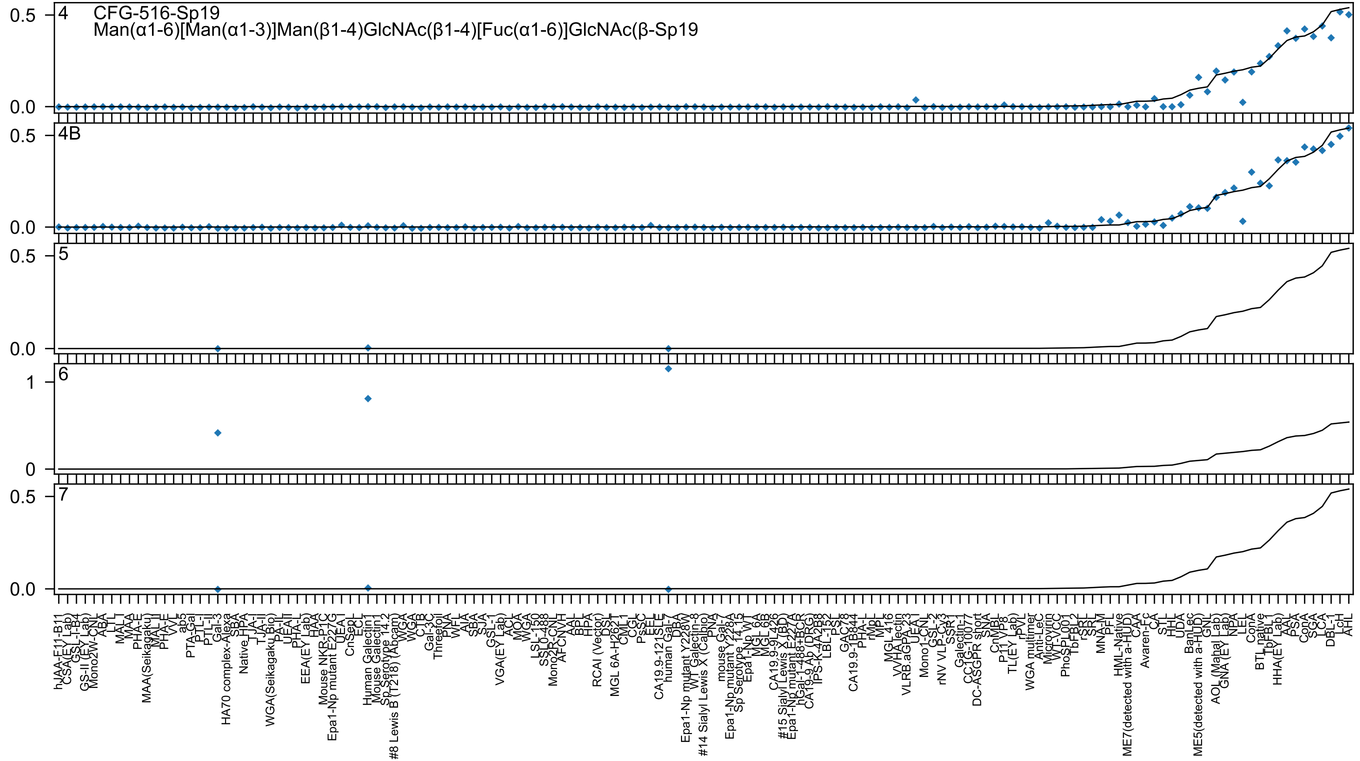

CFG-516-Sp19
Man(α1-6)[Man(α1-3)]Man(β1-4)GlcNAc(β1-4)[Fuc(α1-6)]GlcNAc(β-Sp19
4
4B
5
6
7

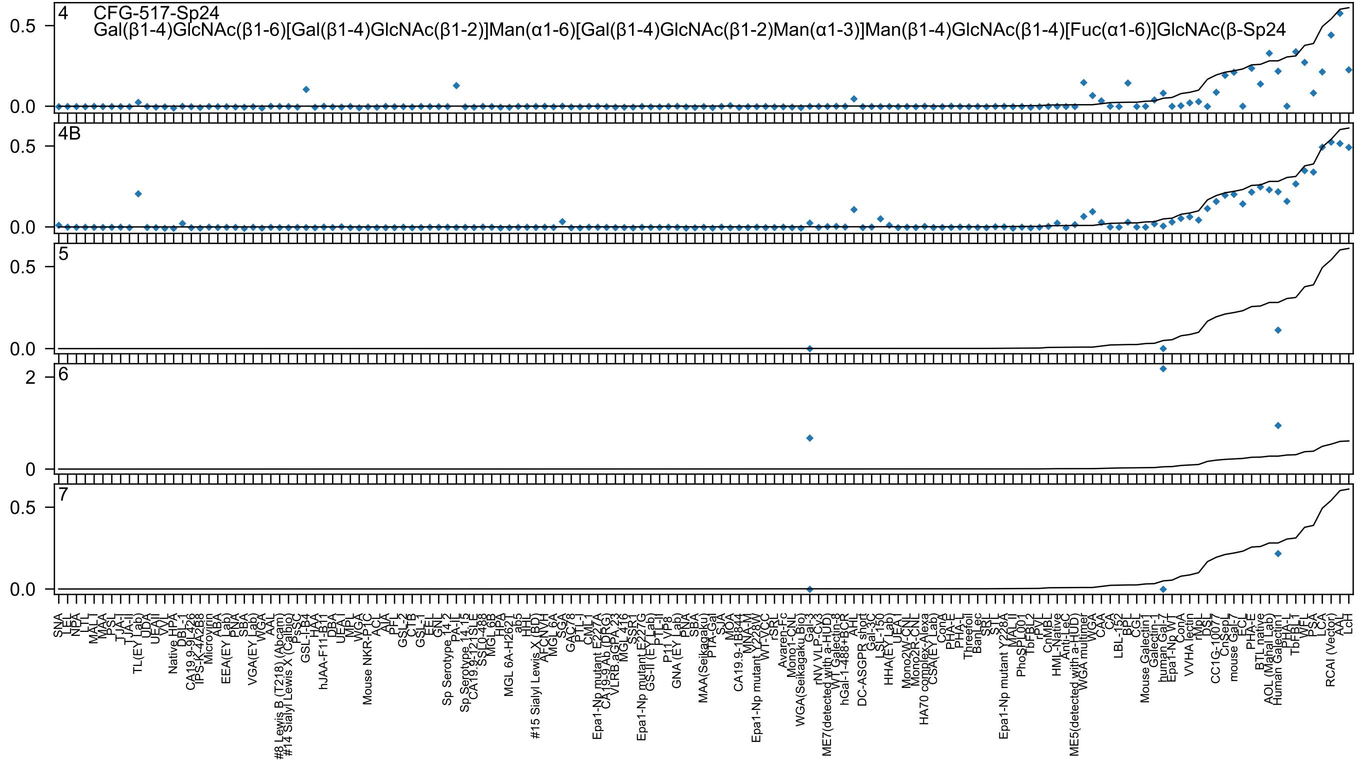

4
CFG-517-Sp24
Gal(β1-4)GlcNAc(β1-6)[Gal(β1-4)GlcNAc(β1-2)]Man(α1-6)[Gal(β1-4)GlcNAc(β1-2)Man(α1-3)]Man(β1-4)GlcNAc(β1-4)[Fuc(α1-6)]GlcNAc(β-Sp24
4B
5
6
7
0.0
0.5
0
2
SNA
LEL
NPA
LTL
MAL-I
MAA
PSL
TJA-I
TJA-II
TL(EY Lab)
UDA
UEA-II
VVL
Native HPA
DBL-1
CA19.9-9L426
IPS-K-4A2B8
Microvirin
ABA
EEA(EY Lab)
PNA
SBA
VGA(EY Lab)
WGA
AAL
#8 Lewis B (T218) (Abcam)
#14 Sialyl Lewis X (Calbio)
PsSC
GSL-I-B4
HAA
hJAA-F11-B11
DBA
UEA-I
MPL
WGA
Mouse NKR-P1C
ACL
AIA
PFL
GSL-2
CTB
GSL-1
EEL
GNL
Sp Serotype 14.2
PA-IL
Sp Serotype 14.15
CA19.9-121SLE
SSL0-488
MGL 6B
HPA
MGL 6A-H262T
ab5
HHL
#15 Sialyl Lewis X (BD)
AFCNVH
MGL 6A
SGA
GAC78
PTL-I
CML-I
Epa1-Np mutant E227A
CA19.9-Ab (DRG)
VLRB.aGPA.23
MGL 416
SSR1
Epa1-Np mutant E227G
GS-II (EY Lab)
PTL-II
P11 VP8
GNA (EY Lab)
PNA
SBA
MAA(Seikagaku)
PTA-Gal
SJA
MOA
CA19.9-1B844
MNA-M
Epa1-Np mutant Y228W
WT-VCC
rSRL
Avaren-Fc
Mono1-CNL
WGA(Seikagaku Bio)
Gal-3
rNV VLP-Cy3
ME7(detected with a-HUD)
WT Galectin-8
hGal-1-488+BCR
AHL
DC-ASGPR short
Gal-3C
LSL-150
HHA(EY Lab)
UEA-I
Mono2W-CNL
Mono2R-CNL
HA70 complex-Alexa
CSA(EY Lab)
ConA
PHA-E
PHA-L
Threefoil
BanLec
SRL
STL
Epa1-Np mutant Y228A
MAL-II
PhoSL001
TbFBL2
rPVL
CnMBL
HML-Native
Anti-LeC
ME5(detected with a-HUD)
WGA multimer
WGA
CAA
CA
LBL-152
BPL
CGL
Mouse Galectin-1
Galectin-1
human Gal-7
Epa1-Np WT
ConA
VVHA lectin
rMpL
DSL
CC1G-1007
CnSep
mouse Gal-7
ECL
PHA-E
BTL native
AOL (Mahal Lab)
Human Galectin-1
PHA-L
TbFBL1
WFL
PSA
LCA
RCAI (Vector)
AAL
LcH

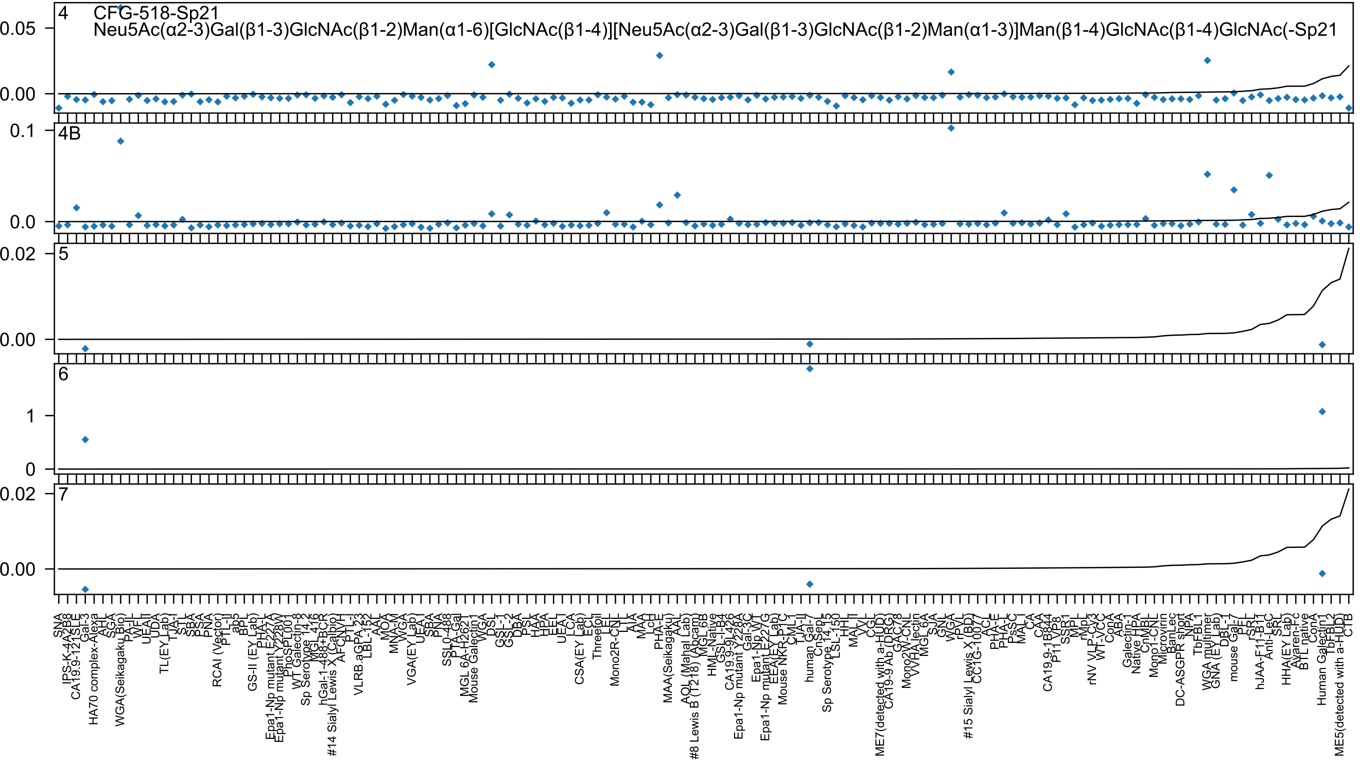

4 CFG-518-Sp21
Neu5Ac(α2-3)Gal(β1-3)GlcNAc(β1-2)Man(α1-6)[GlcNAc(β1-4)][Neu5Ac(α2-3)Gal(β1-3)GlcNAc(β1-2)Man(α1-3)]Man(β1-4)GlcNAc(β1-4)GlcNAc(-Sp21
0.05
0.00
4B
0.1
0.0
5
0.02
0.00
6
1
0
7
0.02
0.00
SNA
IPS-K-4A2B8
CA19.9-121SLE
Gal-3
HA70 complex-Alexa
AHL
SGA
WGA(Seikagaku Bio)
PA-IL
WFL
UEAII
UDA
TL(EY Lab)
TJA-I
STL
SBA
PSA
PNA
RCAI (Vector)
PTL-II
ab5
BPL
GS-II (EY Lab)
PHA-L
Epa1-Np mutant E227A
Epa1-Np mutant Y228W
PhoSL
WT Galectin-8
Sp Serotype 14_2
MGL 416
hGal-1-488+BCR
#14 Sialyl Lewis X (Calbio)
AFCNYH
PTL-I
VLRB.aGPA.23
LBL-152
AAL
MOA
MNA-M
WGA
VGA(EY Lab)
UEA-I
SBA
PNA
SSL0-488
PTA-Gal
MGL 6A-H2621
Mouse Galectin1
WGA
DSL
GSL-I
GSL-2
DBA
PSL
HAA
HPA
EEL
UEA-I
LCA
CSA(EY Lab)
ECL
Threefoil
LEL
Mono2R-CNL
LTL
AIA
MAA
LcH
PHA-E
MAA(Seikagaku)
AAL
AOL (Mahal Lab)
#8 Lewis B (T218) (Abcam)
MGL 6B
HML-Native
GSL-I-B4
CA19.9-9L426
Epa1-Np mutant Y228A
Gal-3C
Epa1-Np WT
Epa1-Np mutant E227G
EEA(EY Lab)
Mouse NKR-P1C
CML-I
TJA-II
human Gal-7
CnSep
Sp Serotype 14_5
LSL-150
HHL
MAL-II
VVL
CGL
ME7(detected with a-HUD)
CA19-9 Ab (DRG)
GAC78
Mono2W-CNL
VVHA lectin
MGL 6A
SJA
GNL
WGA
rPVL
#15 Sialyl Lewis X (BD)
CC1G-10077
ACL
PHA-E
PHA-L
PsSC
MAL-I
CA
CAA
CA19.9-1B844
P11 VP8
SSR1
MPL
rMpL
rNV VLP-Cv3
WT-VCC
ConA
ABA
Galectin-1
Native HPA
CnMBL
Mono1-CNL
Microvirin
BanLec
DC-ASGPR short
NPA
TbFBL1
WGA multimer
GNA (EY Lab)
DBL-1
mouse Gal-7
PFL
rSRL
hJAA-F11-B11
Anti-LeC
SRL
HHA(EY Lab)
Ayaren-Fc
BTL native
ConA
Human Galectin1
TbFBL2
ME5(detected with a-HUD)
CTB

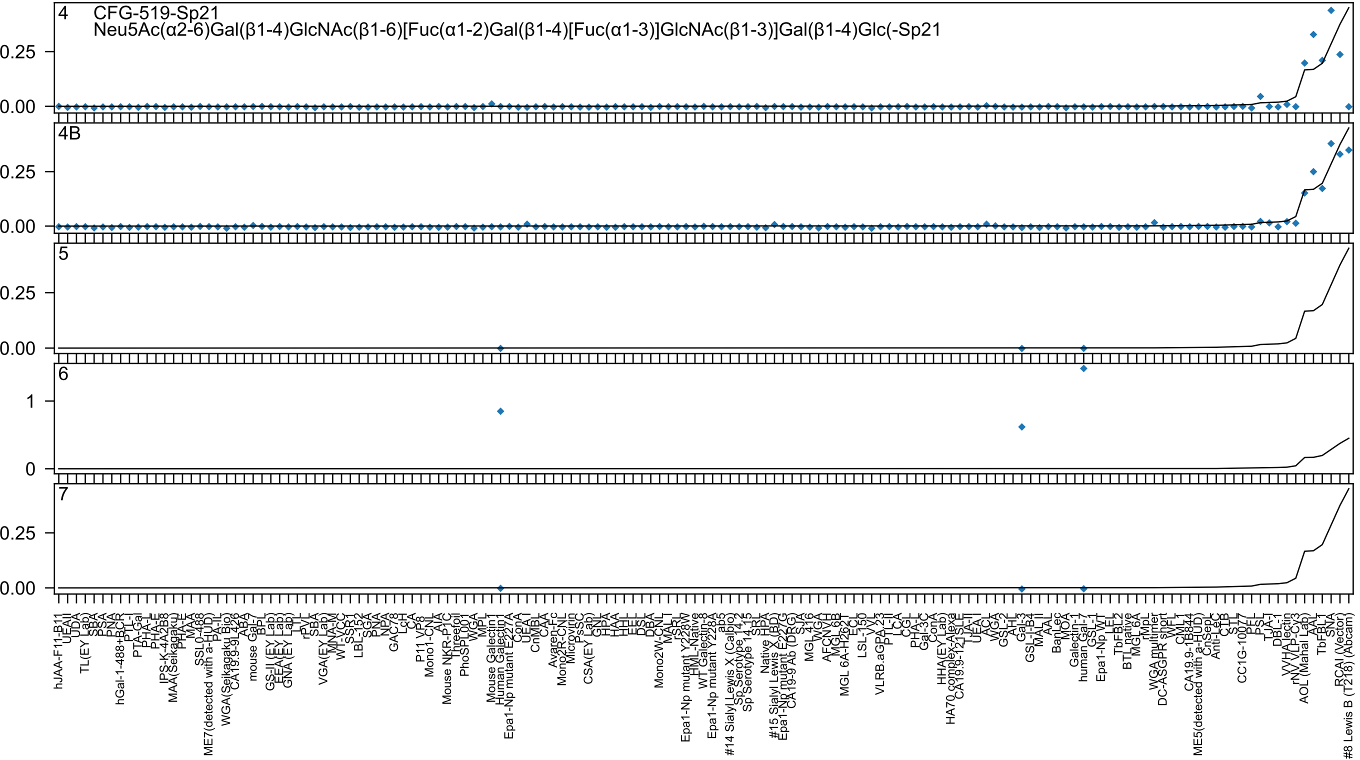
CFG-519-Sp21
Neu5Ac(α2-6)Gal(β1-4)GlcNAc(β1-6)[Fuc(α1-2)Gal(β1-4)[Fuc(α1-3)]GlcNAc(β1-3)]Gal(β1-4)Glc(-Sp21
4
4B
5
6
7

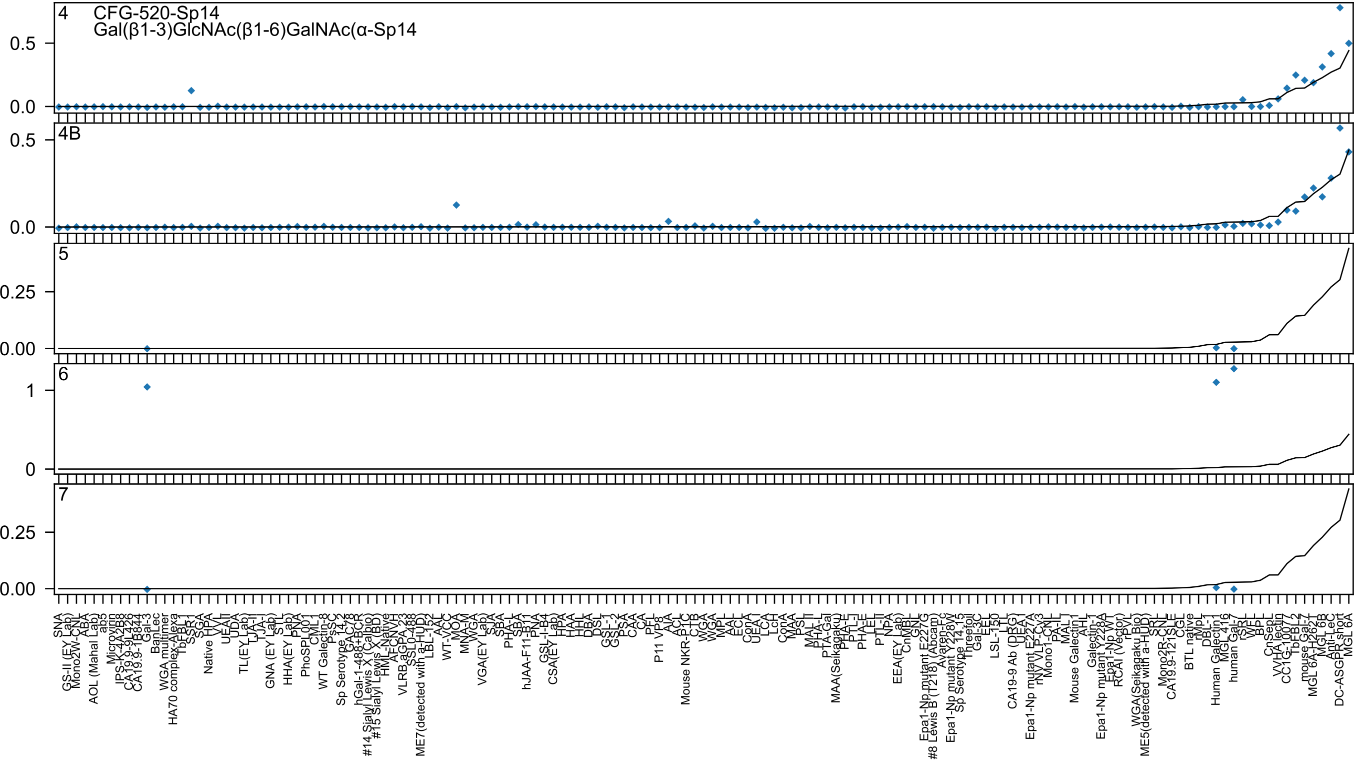
4 CFG-520-Sp14
Gal(β1-3)GlcNAc(β1-6)GalNAc(α-Sp14
4B
5
6
7

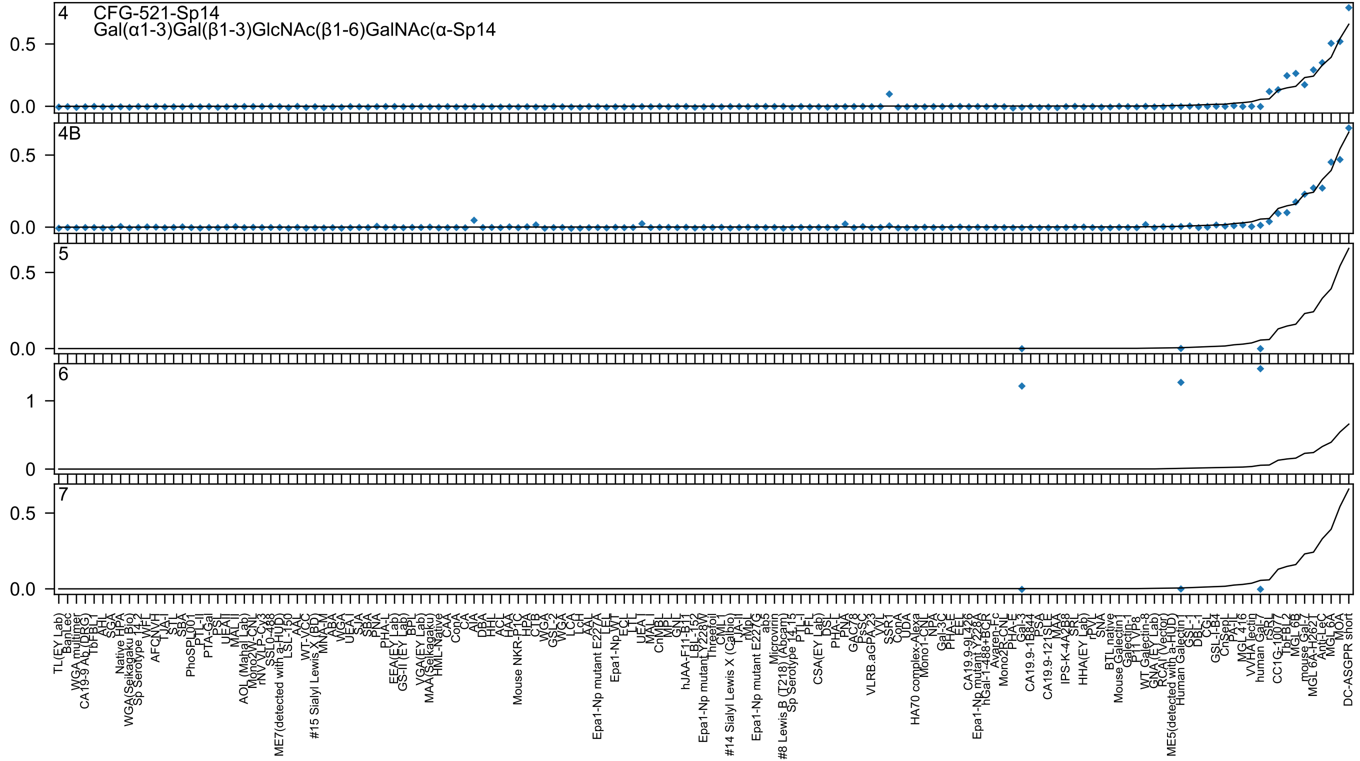
4 CFG-521-Sp14
Gal(α1-3)Gal(β1-3)GlcNAc(β1-6)GalNAc(α-Sp14
4B
5
6
7

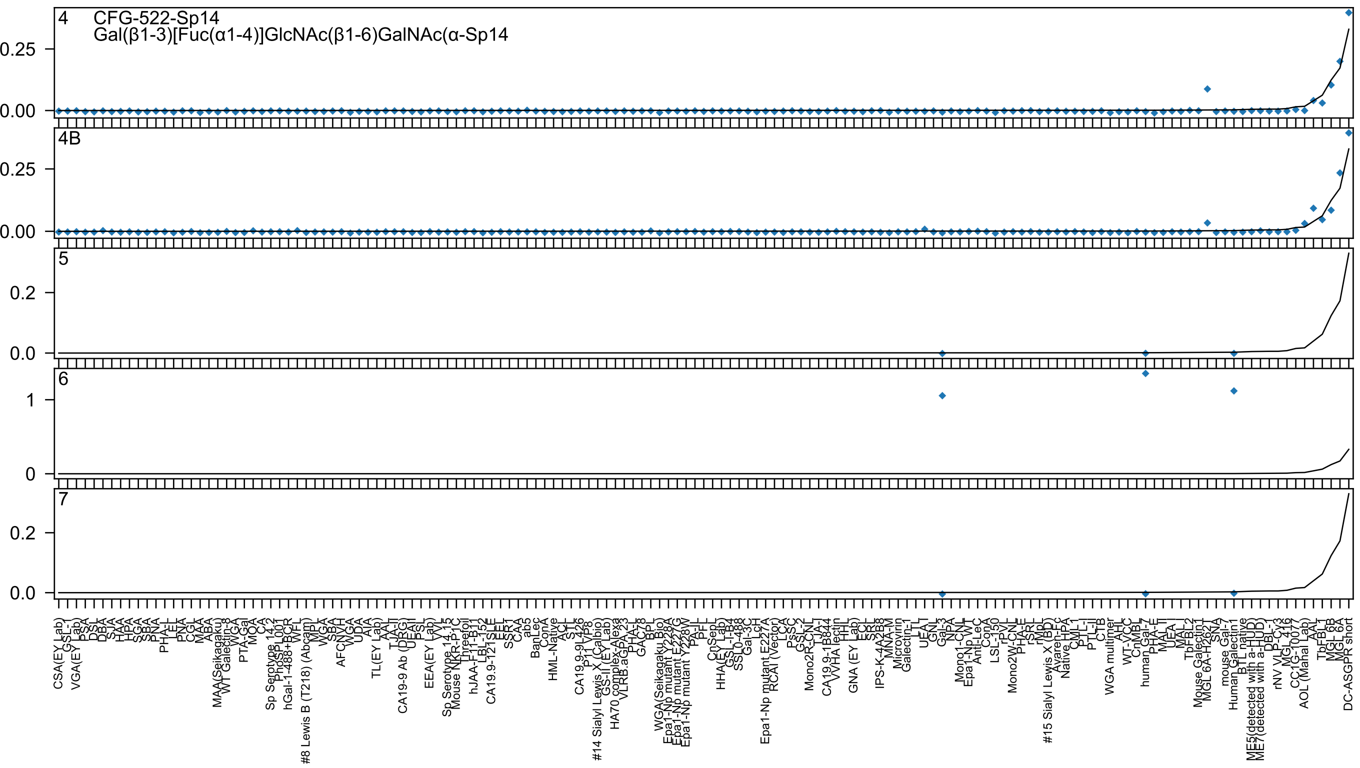

4
CFG-522-Sp14
Gal(β1-3)[Fuc(α1-4)]GlcNAc(β1-6)GalNAc(α-Sp14
4B
5
6
7

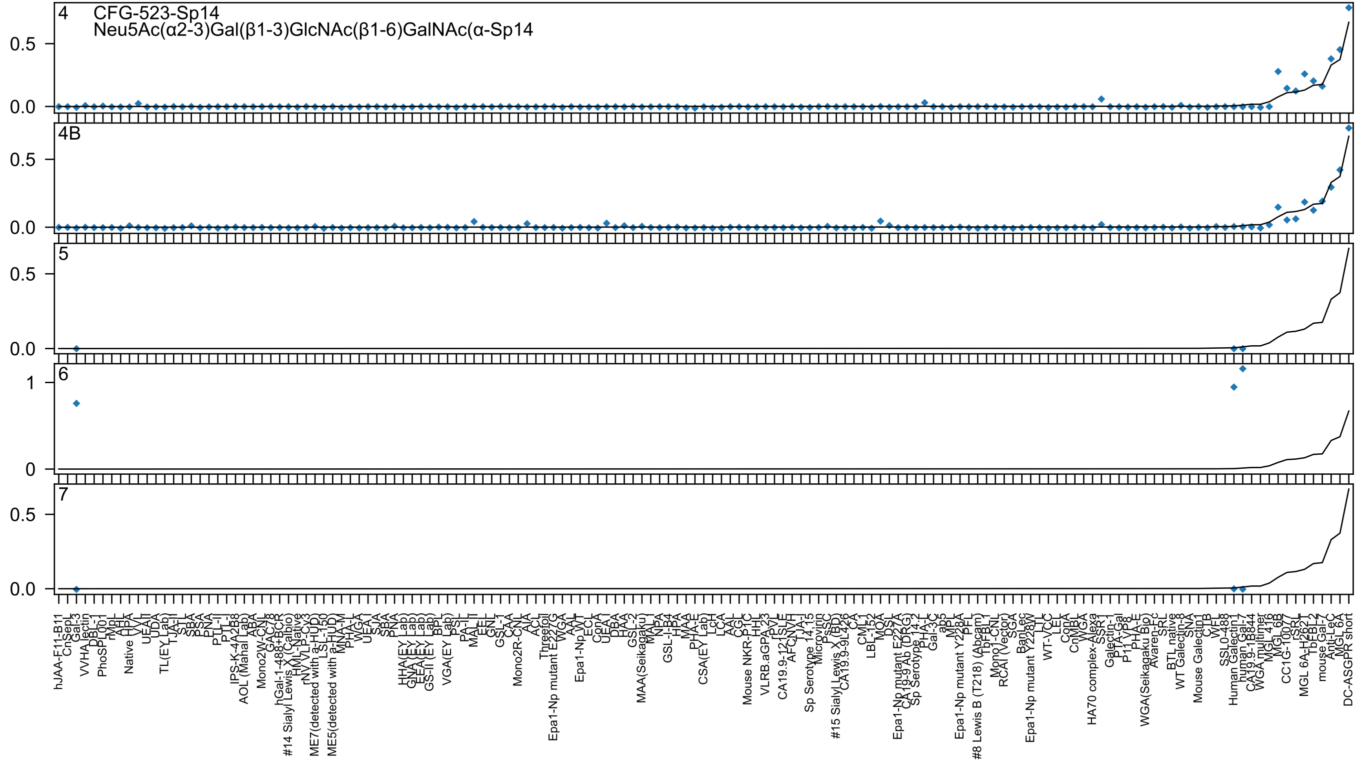

4
CFG-523-Sp14
Neu5Ac(α2-3)Gal(β1-3)GlcNAc(β1-6)GalNAc(α-Sp14
4B
5
6
7

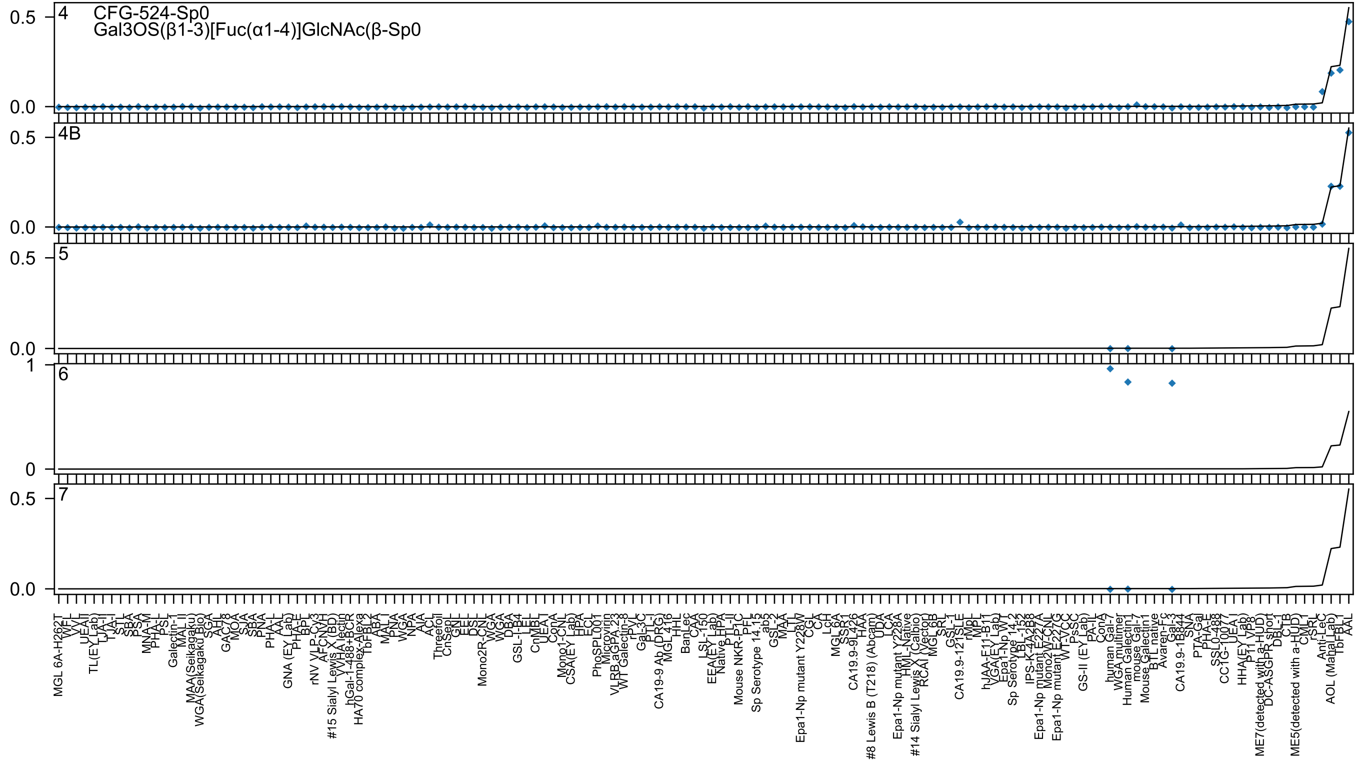

4 CFG-524-Sp0
Gal3OS(β1-3)[Fuc(α1-4)]GlcNAc(β-Sp0
4B
5
6
7

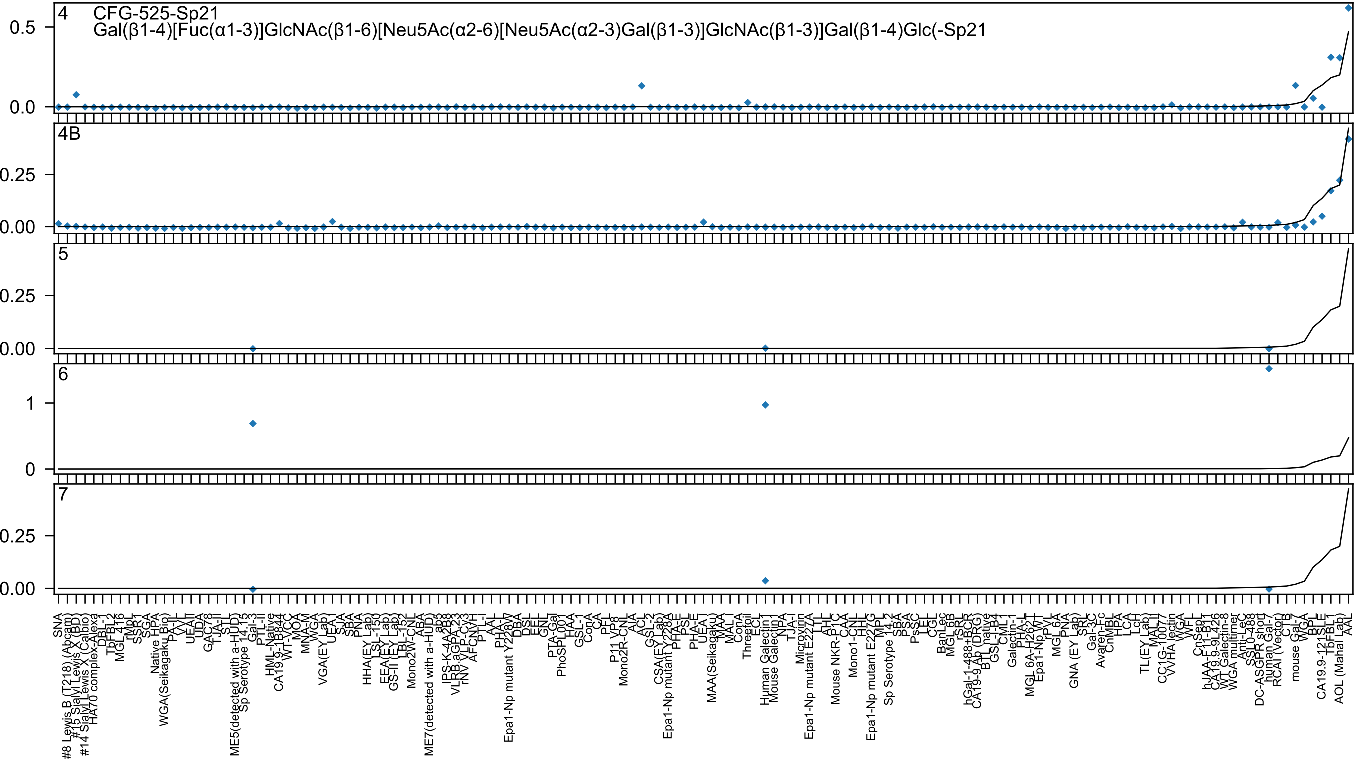

4
CFG-525-Sp21
Gal(β1-4)[Fuc(α1-3)]GlcNAc(β1-6)[Neu5Ac(α2-6)[Neu5Ac(α2-3)Gal(β1-3)]GlcNAc(β1-3)]Gal(β1-4)Glc(-Sp21
4B
5
6
7

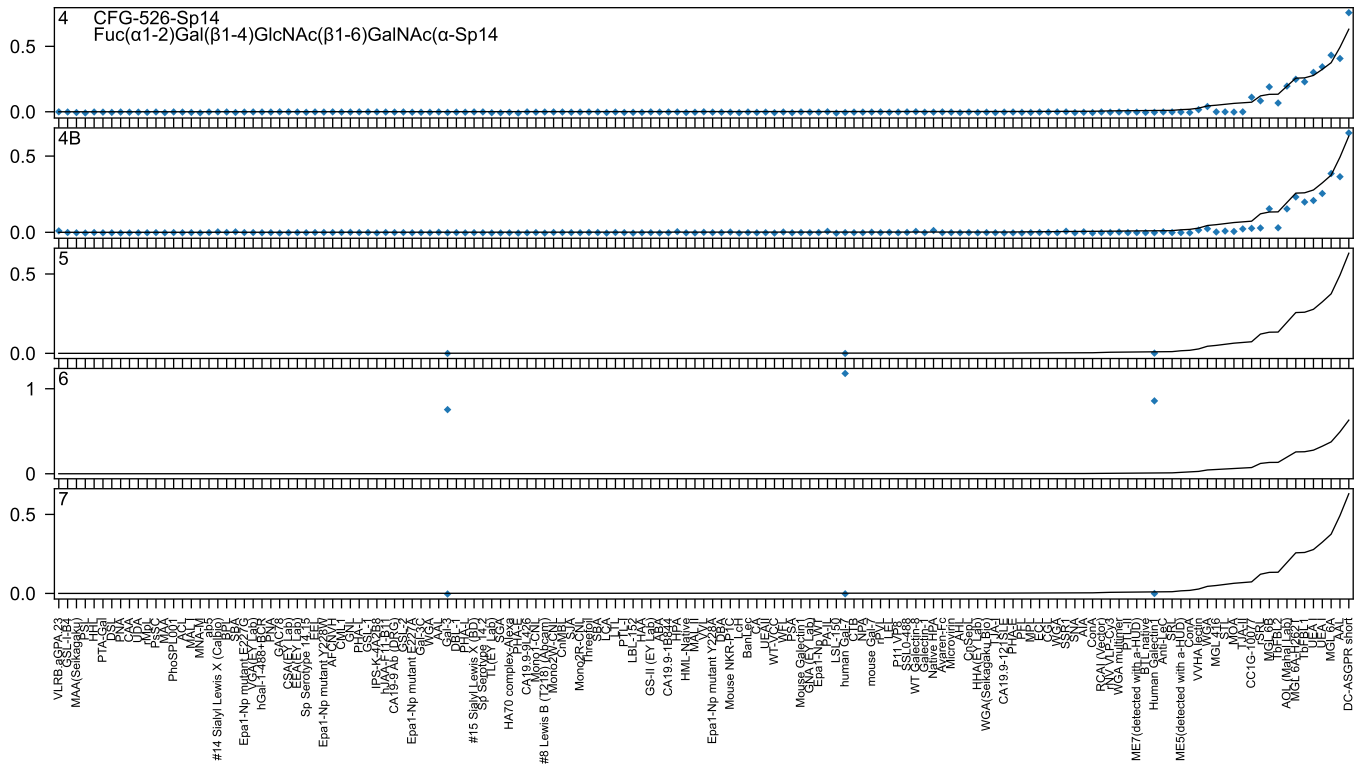
CFG-526-Sp14
Fuc(α1-2)Gal(β1-4)GlcNAc(β1-6)GalNAc(α-Sp14

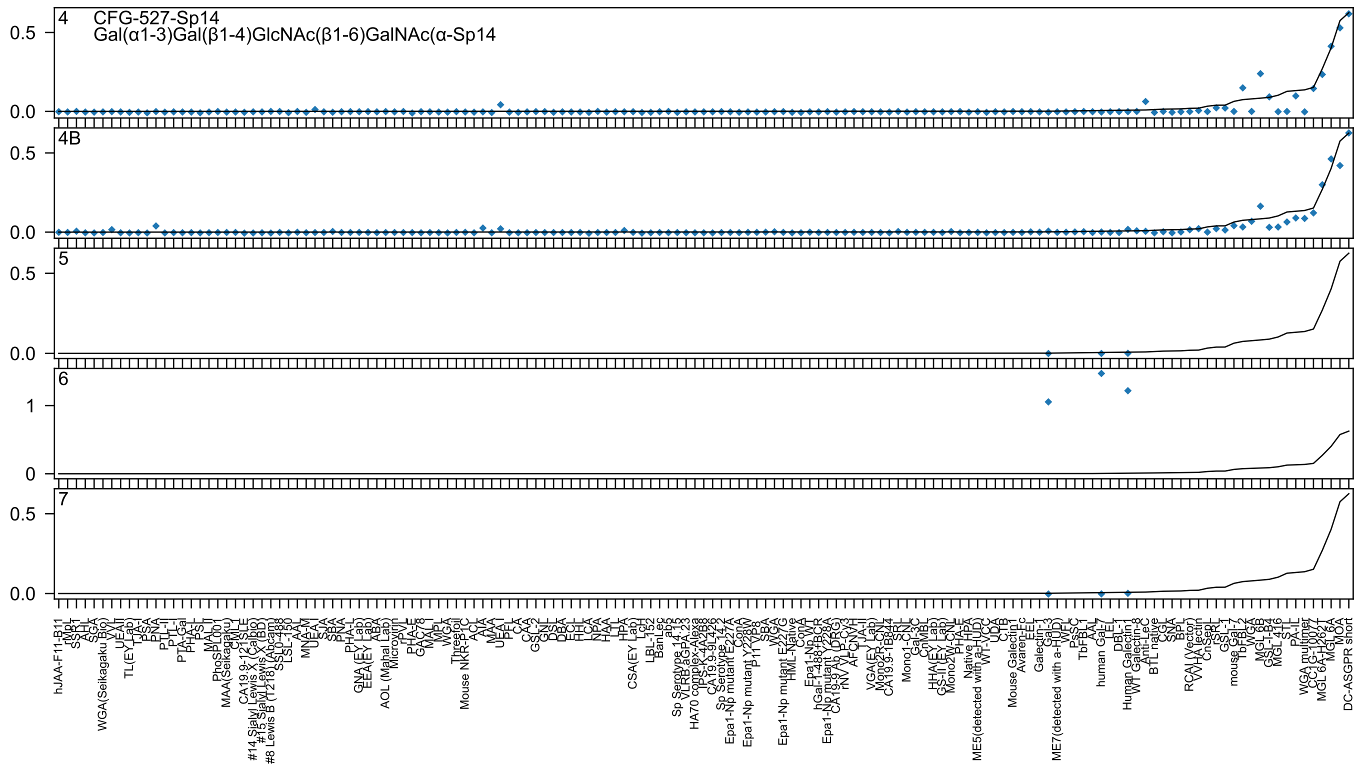
4 CFG-527-Sp14
Gal(α1-3)Gal(β1-4)GlcNAc(β1-6)GalNAc(α-Sp14
4B
5
6
7
hJAA-F11-B11
DC-ASGPR short

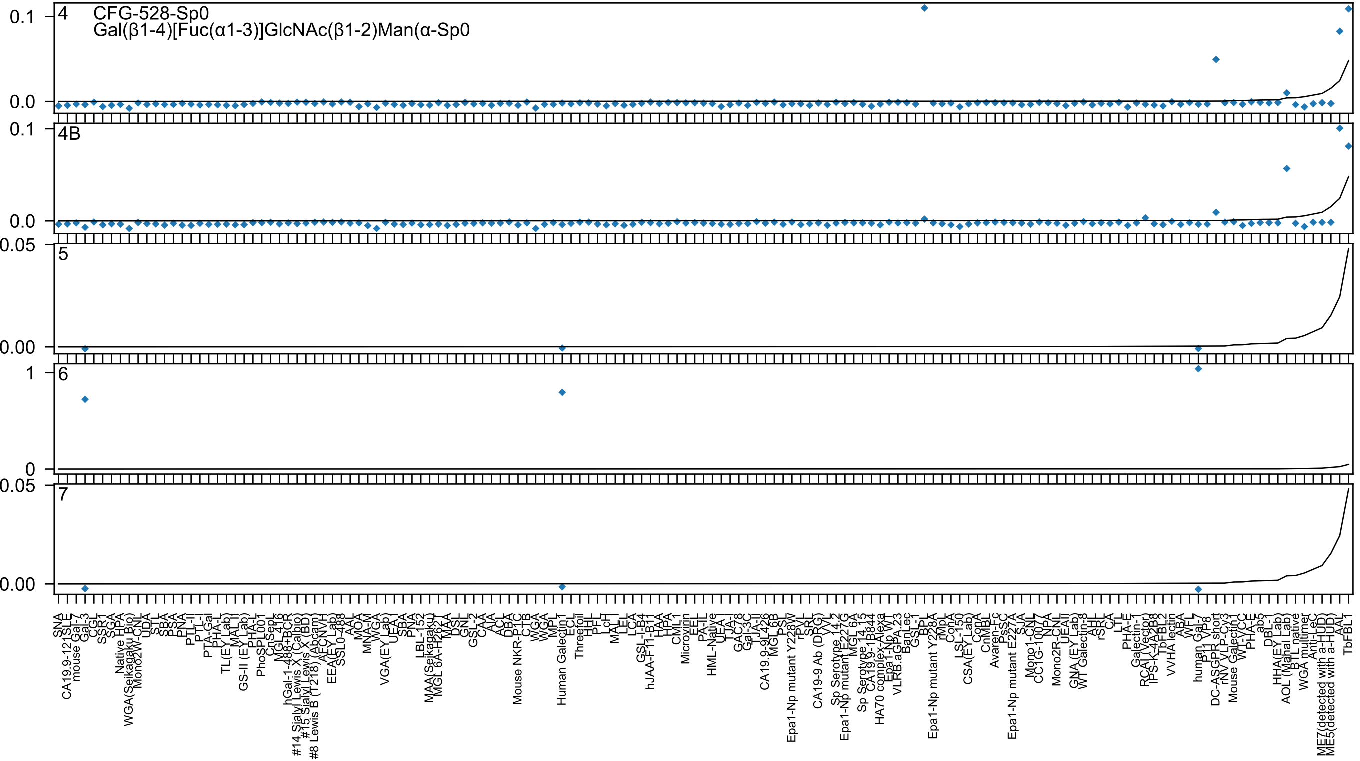

CFG-528-Sp0
Gal(β1-4)[Fuc(α1-3)]GlcNAc(β1-2)Man(α-Sp0
4
4B
5
6
7
0.1
0.0
0.05
0.00
1
0
SNA, CA19.9-121, SLE, mouse Gal-7, Gal-3, CGL, SSR1, SGA, Native HPA, WGA(Seikagaku Bio), Mono2W-CNL, UDA, STL, SBA, PSA, PNA, PTL-II, PTL-I, PTA-Gal, PHA-L, TL(EY Lab), MAL-I, GS-II (EY Lab), PHA-L, PhoSL, P001, CnSep, MGL416, hGal-1-488+BCR, #14 Sialyl Lewis X (Calbio), #15 Sialyl Lewis X (BD), #8 Lewis B (T218) (Abcam), AFCNVH, EEA(EY Lab), SSL0-488, AAL, MOA, MNA-M, WGA, VGA(EY Lab), UEA-I, SBA, PNA, LBL-152, MAA(Seikagaku), MGL 6A-H262T, MAA, DSL, GNL, GSL-2, CAA, AIA, ACL, DBA, Mouse NKR-P1C, CTB, WGA, WGA, MPL, Human Galectin1, ECL, Threefoil, HHL, PFL, LcH, MAL-I, LEL, LCA, GSL-I-B4, hJAA-F11-B11, HAA, HPA, CML1, Microvirin, EEL, PA-IL, HML-Native, UEA-I, TJA-I, GAC78, Gal-3C, TJA-II, CA19.9-9L426, MGL 6B, PSL, Epa1-Np mutant Y228W, rPVL, SRL, CA19-9 Ab (DRG), VVL, Sp Serotype 14.2, Epa1-Np mutant E227G, MGL 6A, Sp Serotype 14.15, CA19.9-1B844, HA70 complex-Alexa, Epa1-Np WT, VLRB.aGPA.23, BanLec, GSL-I, BPL, Epa1-Np mutant Y228A, rMpL, ConA, LSL-150, CSA(EY Lab), ConA, CnMBL, Avaren-Fc, PsSC, Epa1-Np mutant E227A, SJA, Mono1-CNL, CC1G-1007, NPA, Mono2R-CNL, UEA-II, GNA (EY Lab), WT Galectin-8, AHL, rSRL, CA, LTL, PHA-E, Galectin-1, RCA I (Vector), IPS-K-4A2B8, TbFBL2, VVHA lectin, ABA, WFL, human Gal-7, P11 VP8, DC-ASGPR short, rNV VLP-Cy3, Mouse Galectin1, WT-CC, PHA-E, ab5, DBL-1, HHA(EY Lab), AOL (Mahal Lab), BTL native, WGA multimer, Anti-LeC, ME7(detected with a-HUD), ME5(detected with a-HUD), AAL, TbFBL1

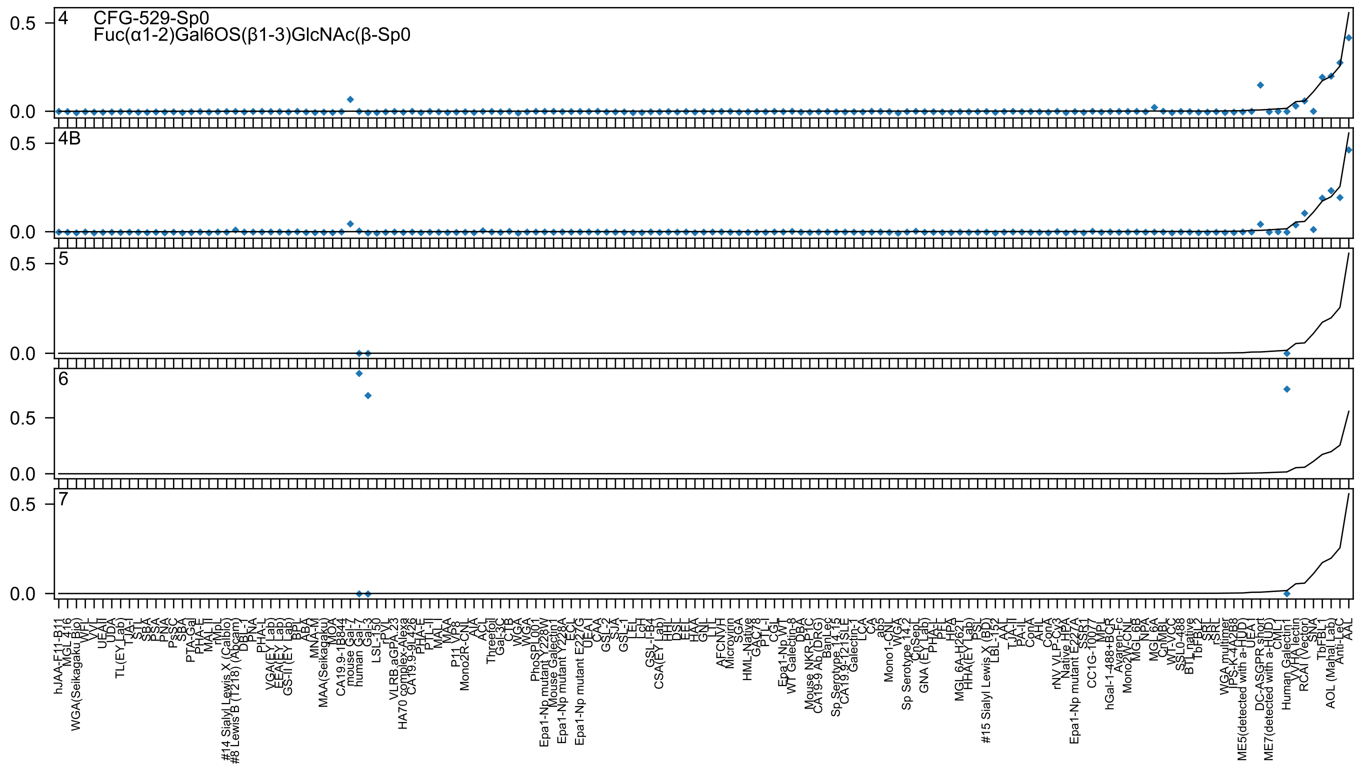
4
CFG-529-Sp0
Fuc(α1-2)Gal6OS(β1-3)GlcNAc(β-Sp0
4B
5
6
7

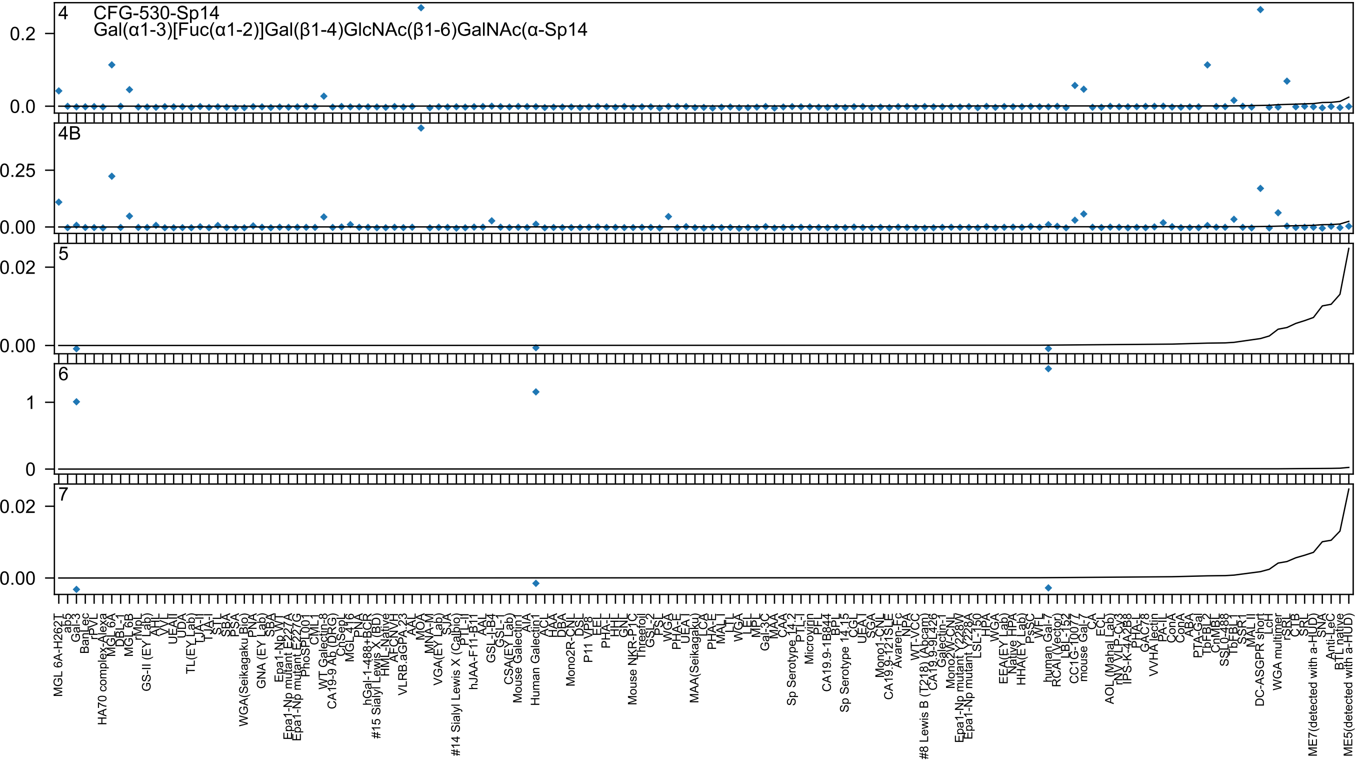

CFG-530-Sp14
Gal(α1-3)[Fuc(α1-2)]Gal(β1-4)GlcNAc(β1-6)GalNAc(α-Sp14
4
4B
5
6
7

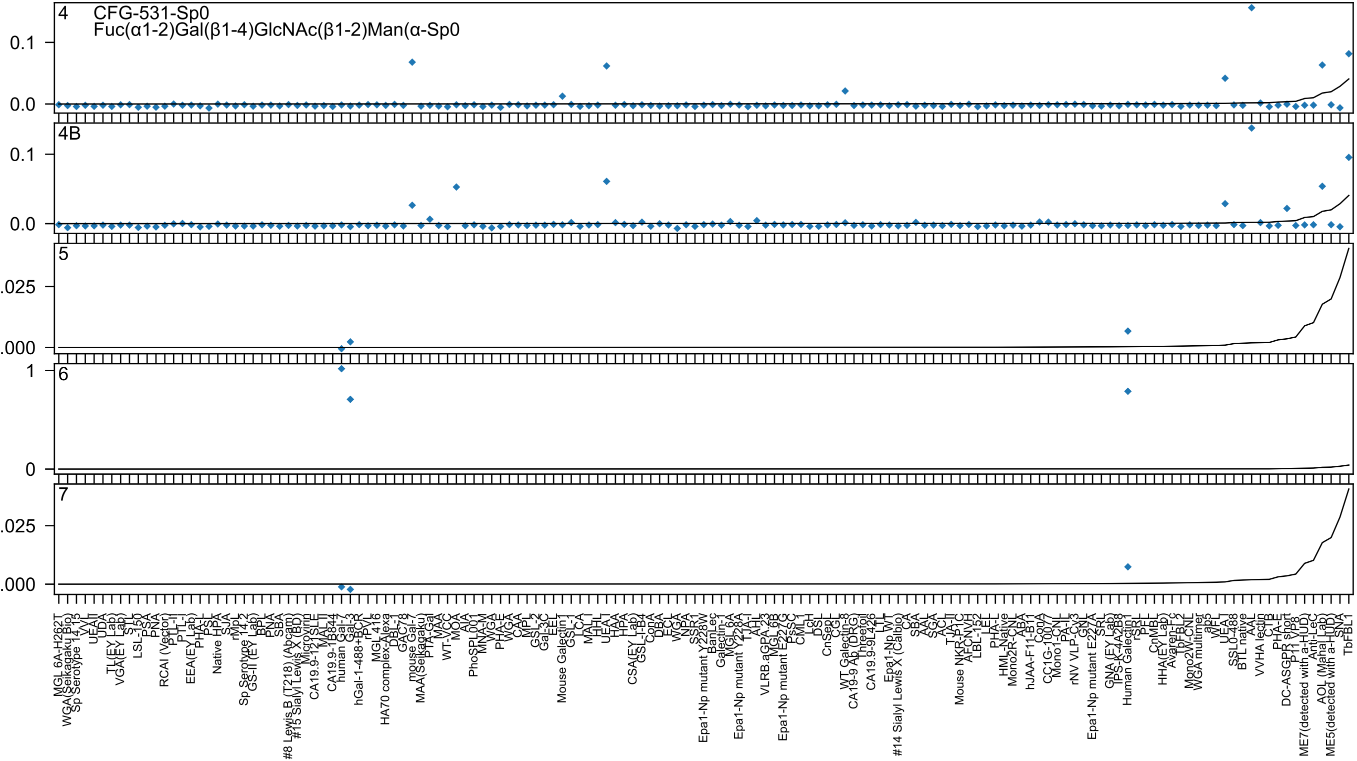

4 CFG-531-Sp0
Fuc(α1-2)Gal(β1-4)GlcNAc(β1-2)Man(α-Sp0
4B
5
6
7

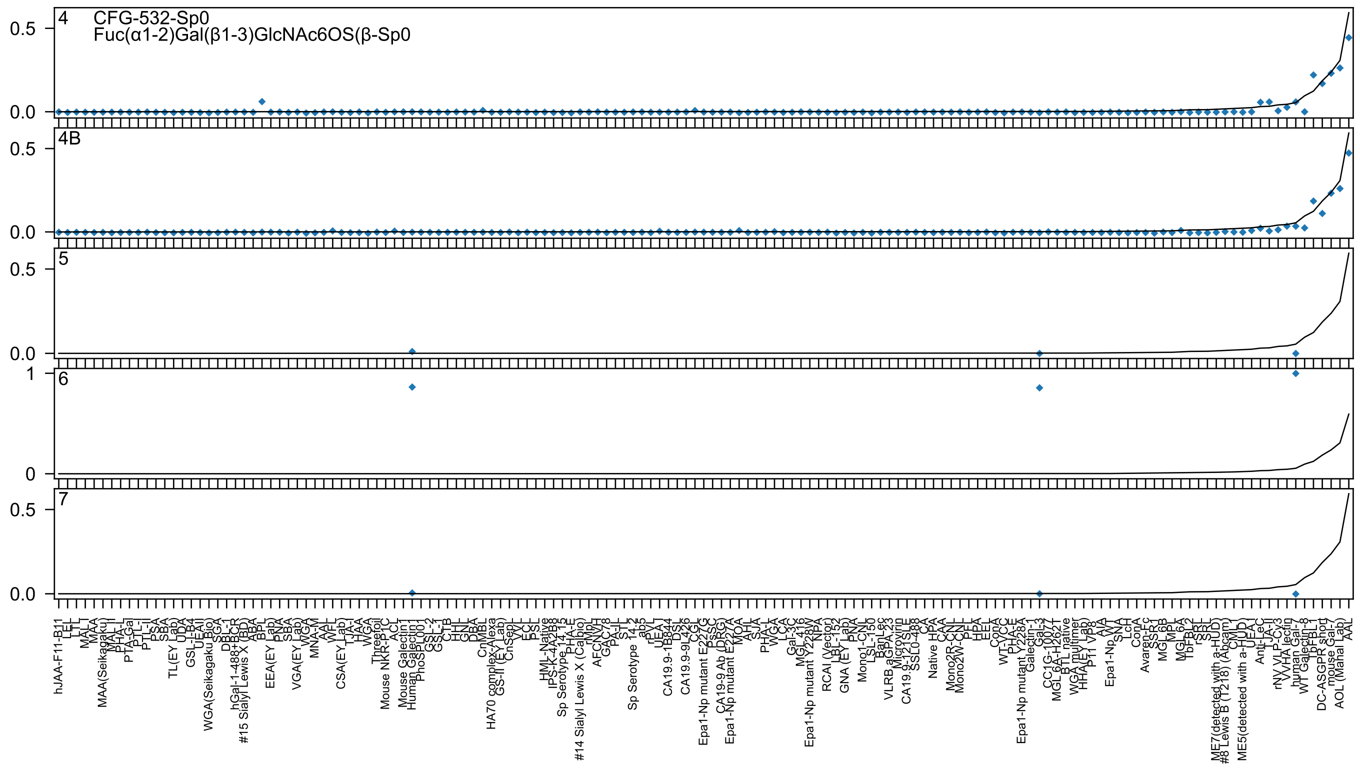

CFG-532-Sp0
Fuc(α1-2)Gal(β1-3)GlcNAc6OS(β-Sp0
4
4B
5
6
7

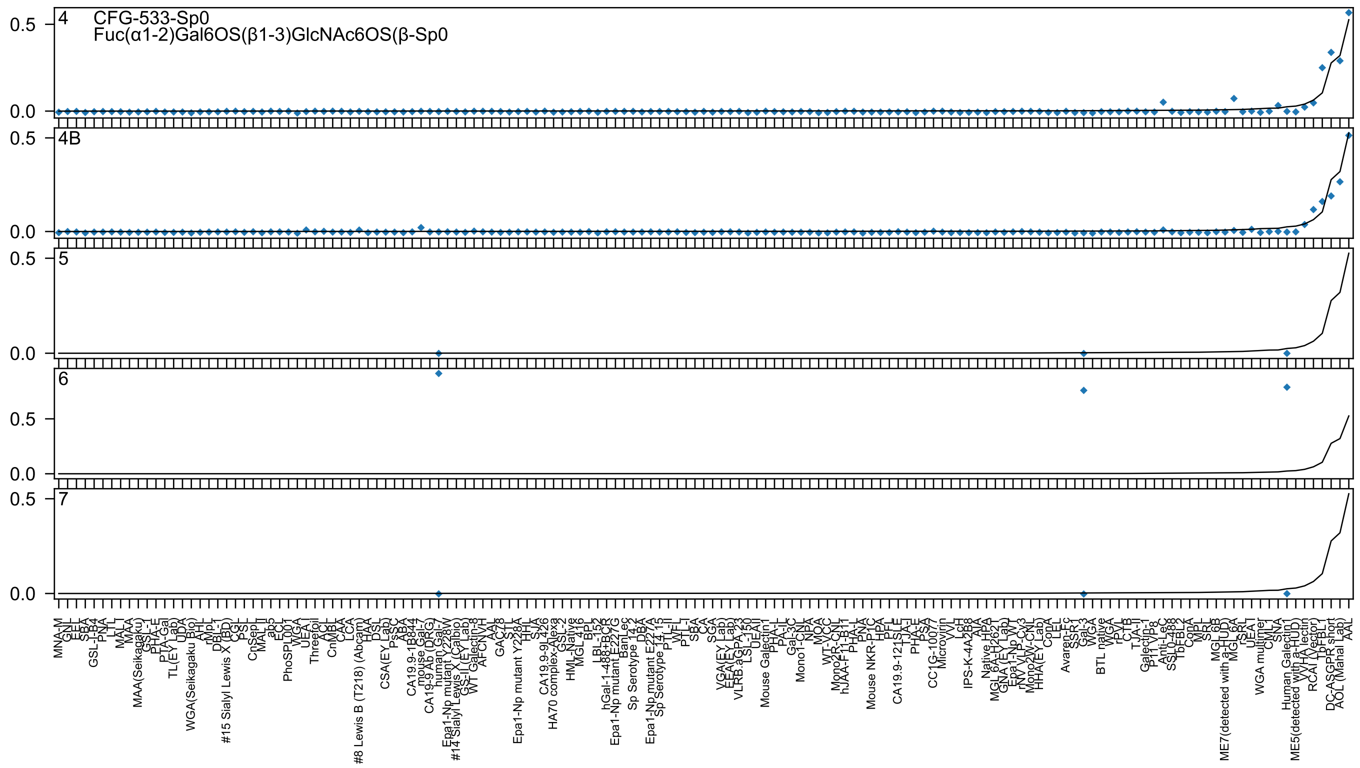

4 CFG-533-Sp0
Fuc(α1-2)Gal6OS(β1-3)GlcNAc6OS(β-Sp0
4B
5
6
7
0.5
0.0

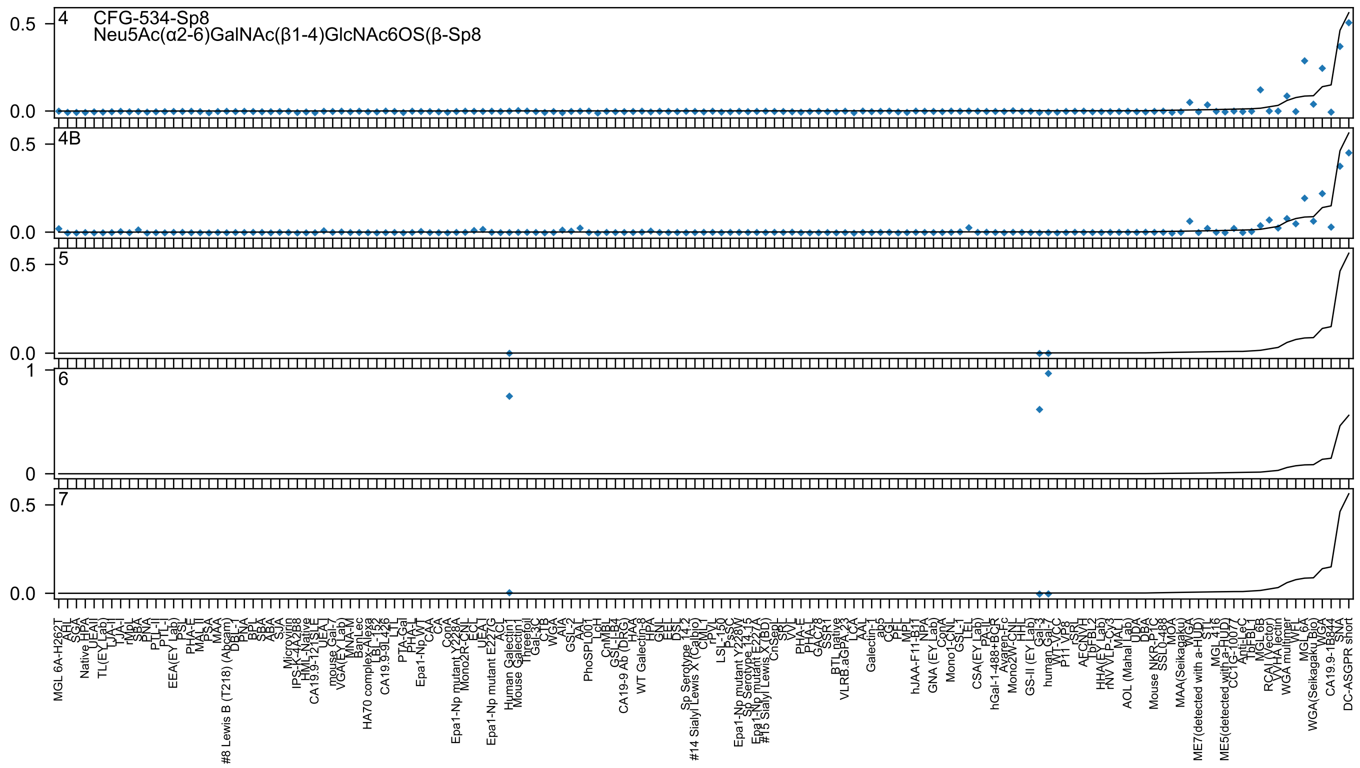

CFG-534-Sp8
Neu5Ac(α2-6)GalNAc(β1-4)GlcNAc6OS(β-Sp8
4
4B
5
6
7

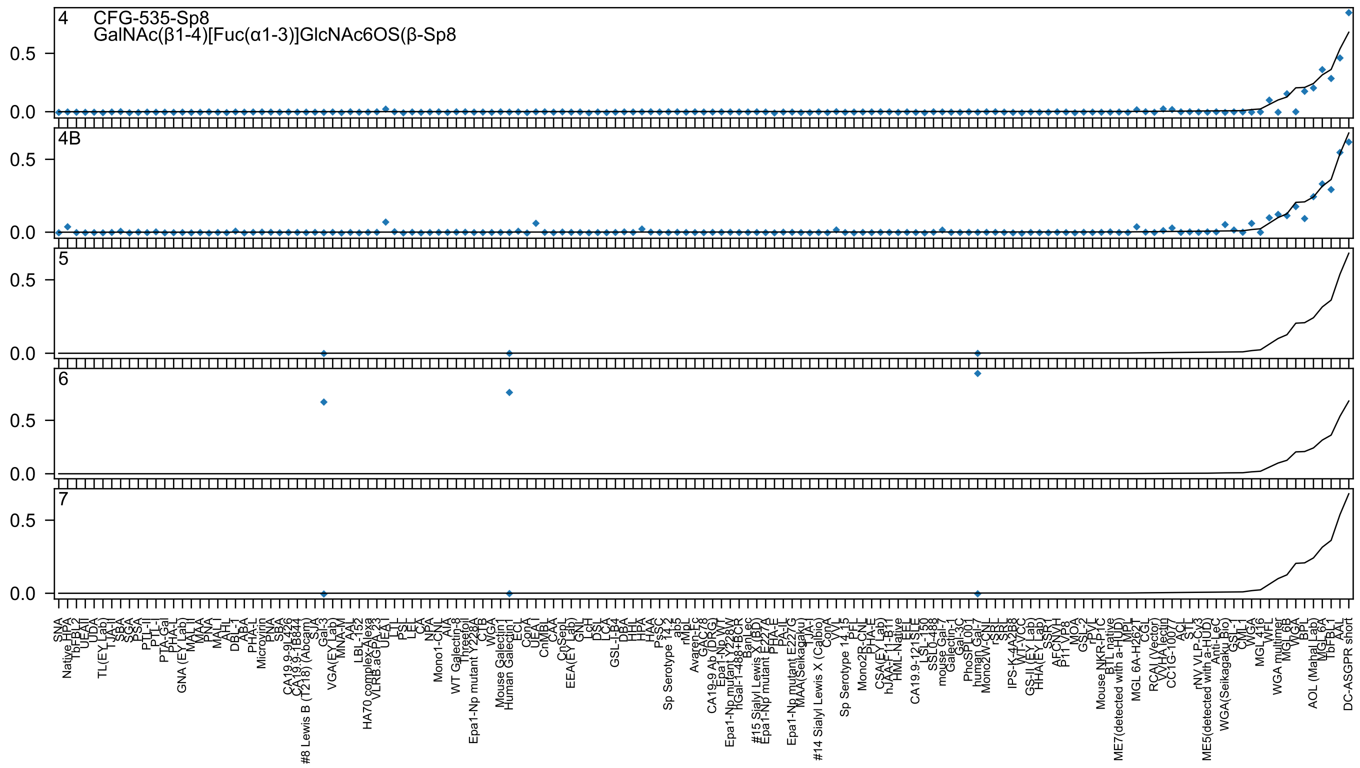

4
CFG-535-Sp8
GalNAc(β1-4)[Fuc(α1-3)]GlcNAc6OS(β-Sp8
4B
5
6
7
0.0
0.5

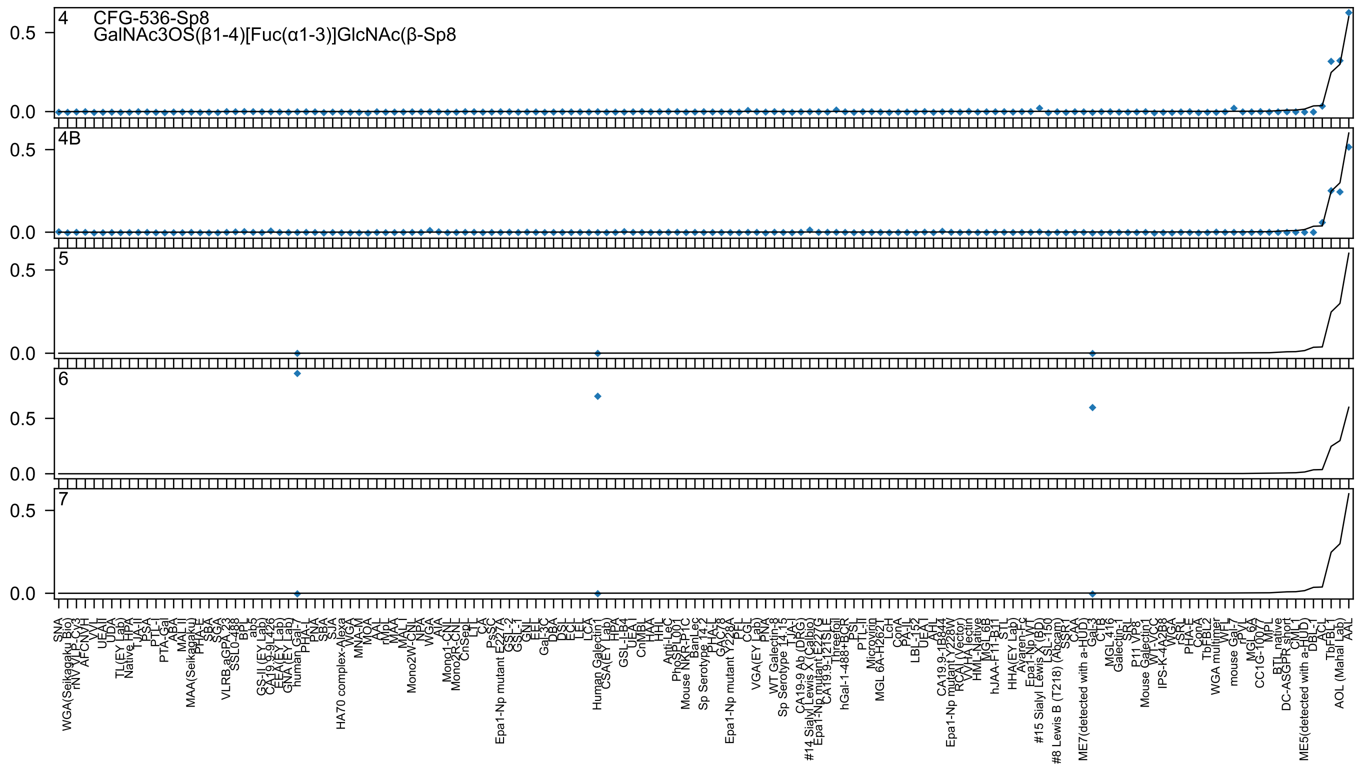
4
CFG-536-Sp8
GalNAc3OS(β1-4)[Fuc(α1-3)]GlcNAc(β-Sp8
4B
5
6
7

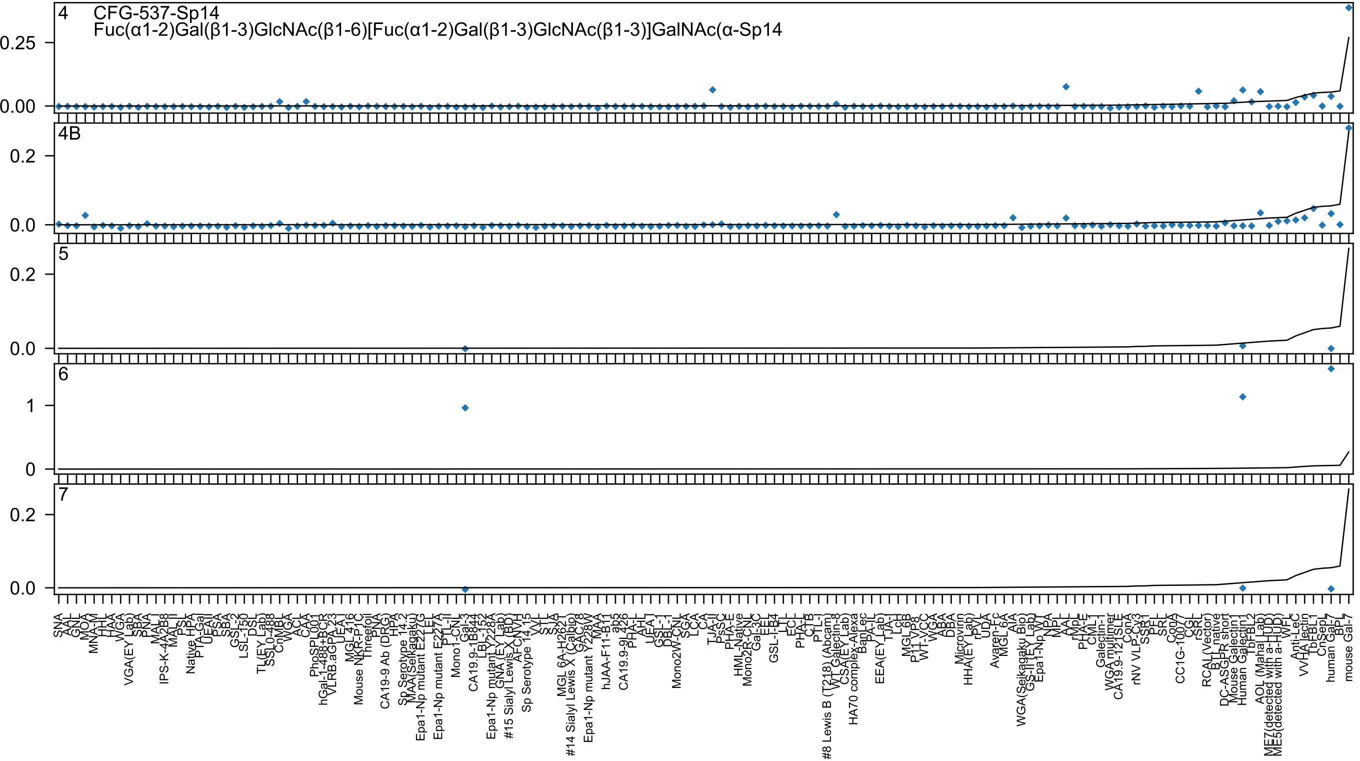

4 CFG-537-Sp14
Fuc(α1-2)Gal(β1-3)GlcNAc(β1-6)[Fuc(α1-2)Gal(β1-3)GlcNAc(β1-3)]GalNAc(α-Sp14
4B
5
6
7

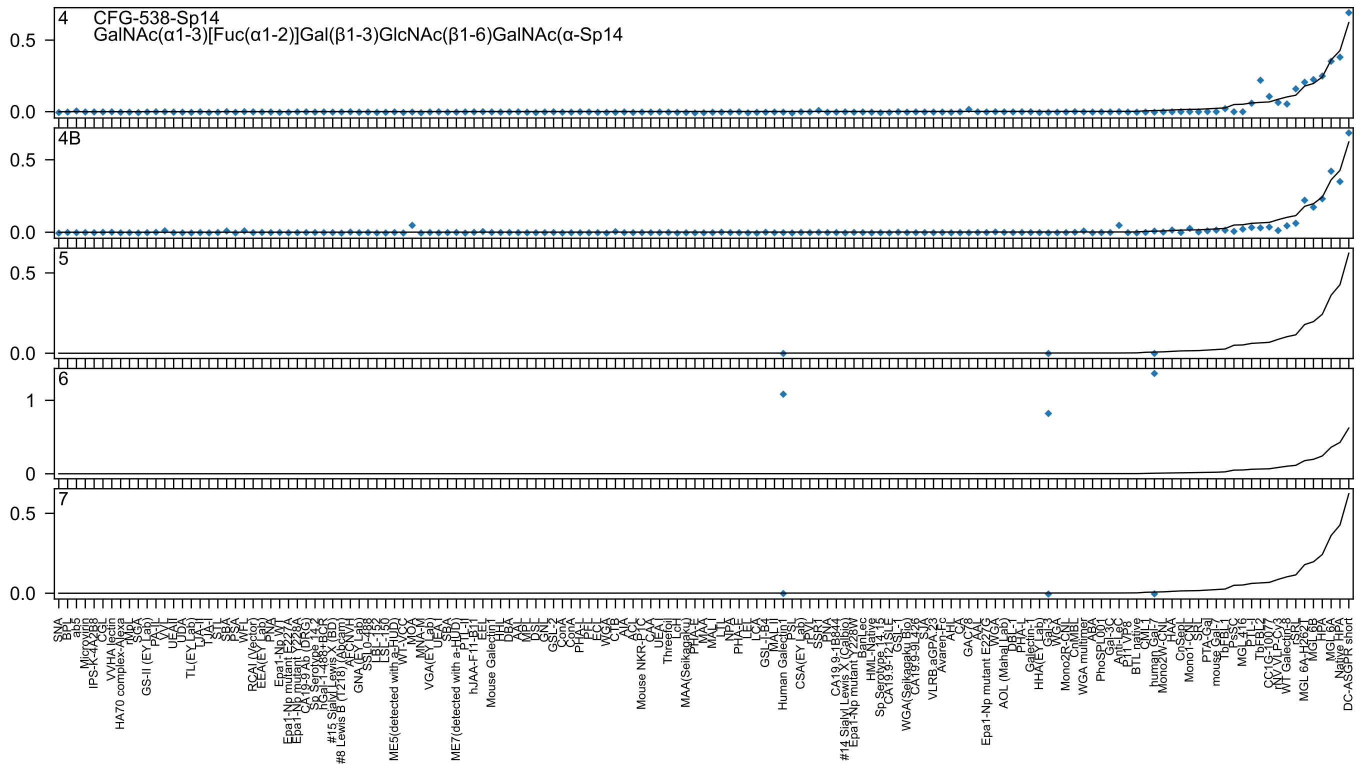
4 CFG-538-Sp14
GalNAc(α1-3)[Fuc(α1-2)]Gal(β1-3)GlcNAc(β1-6)GalNAc(α-Sp14
4B
5
6
7

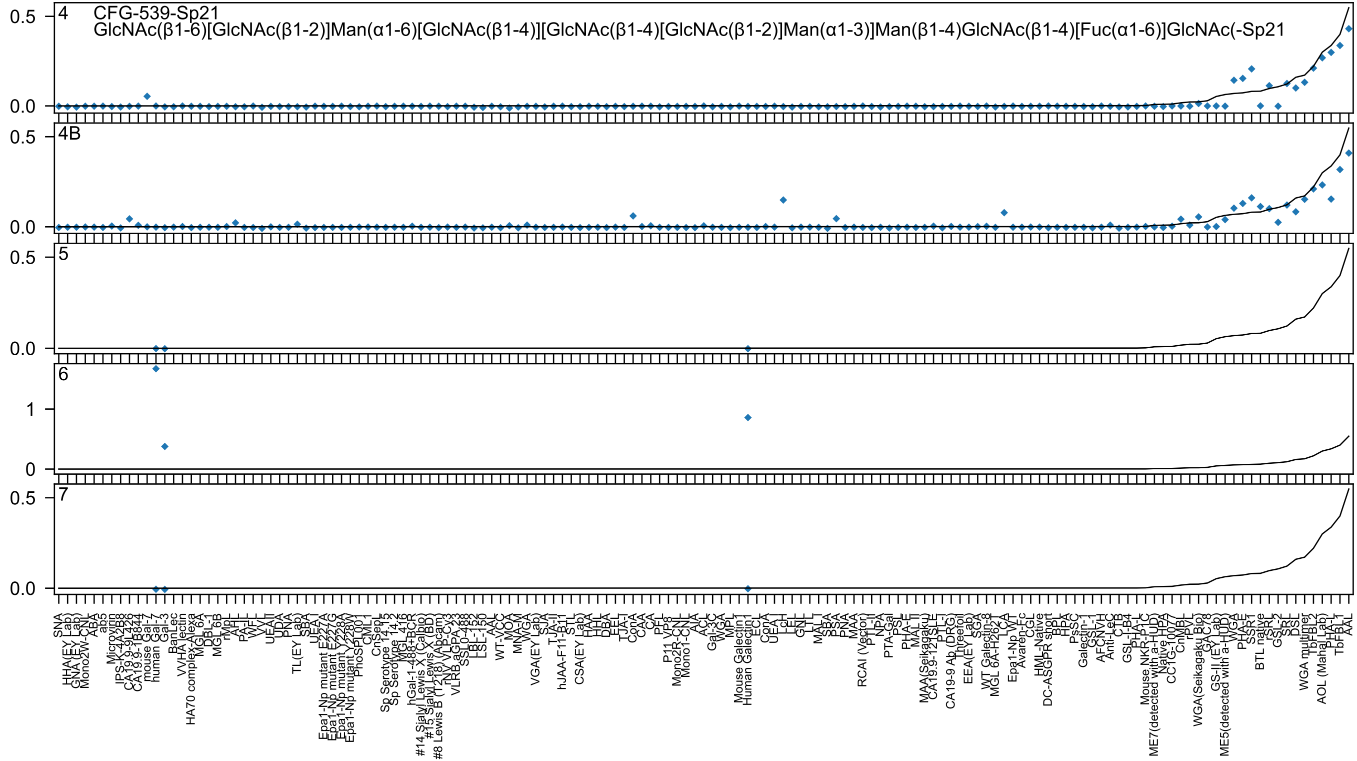

4 CFG-539-Sp21
GlcNAc(β1-6)[GlcNAc(β1-2)]Man(α1-6)[GlcNAc(β1-4)][GlcNAc(β1-4)[GlcNAc(β1-2)]Man(α1-3)]Man(β1-4)GlcNAc(β1-4)[Fuc(α1-6)]GlcNAc(-Sp21
4B
5
6
7

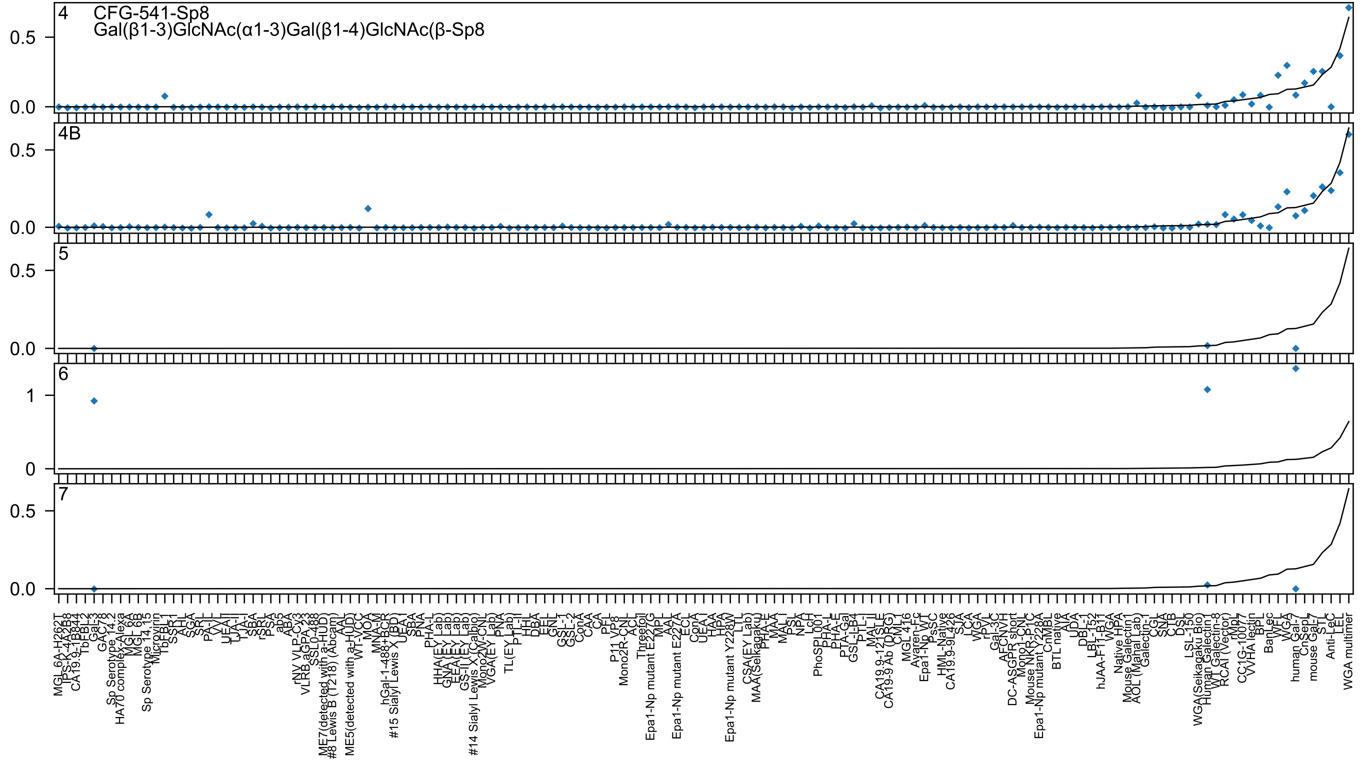

4 CFG-541-Sp8
Gal(β1-3)GlcNAc(α1-3)Gal(β1-4)GlcNAc(β-Sp8
4B
5
6
7

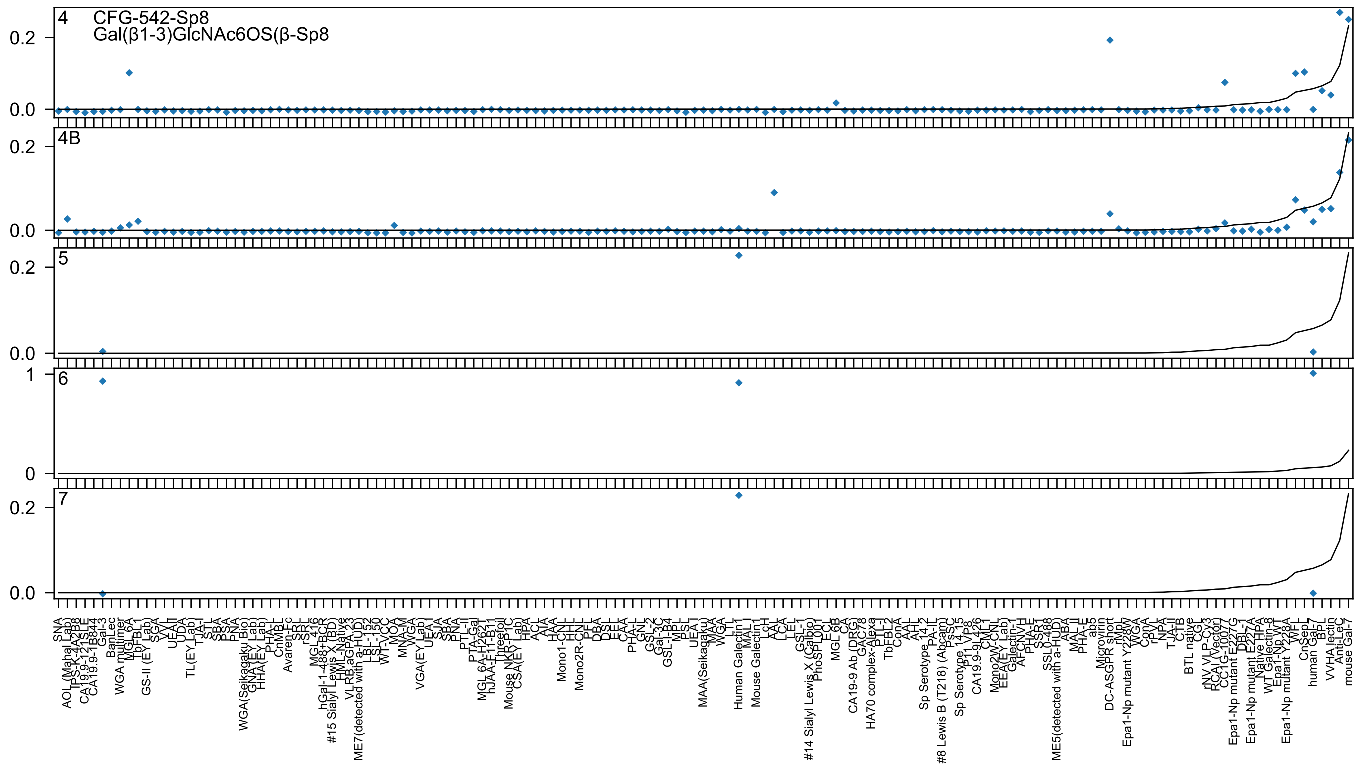

4 CFG-542-Sp8
Gal(β1-3)GlcNAc6OS(β-Sp8
4B
5
6
7

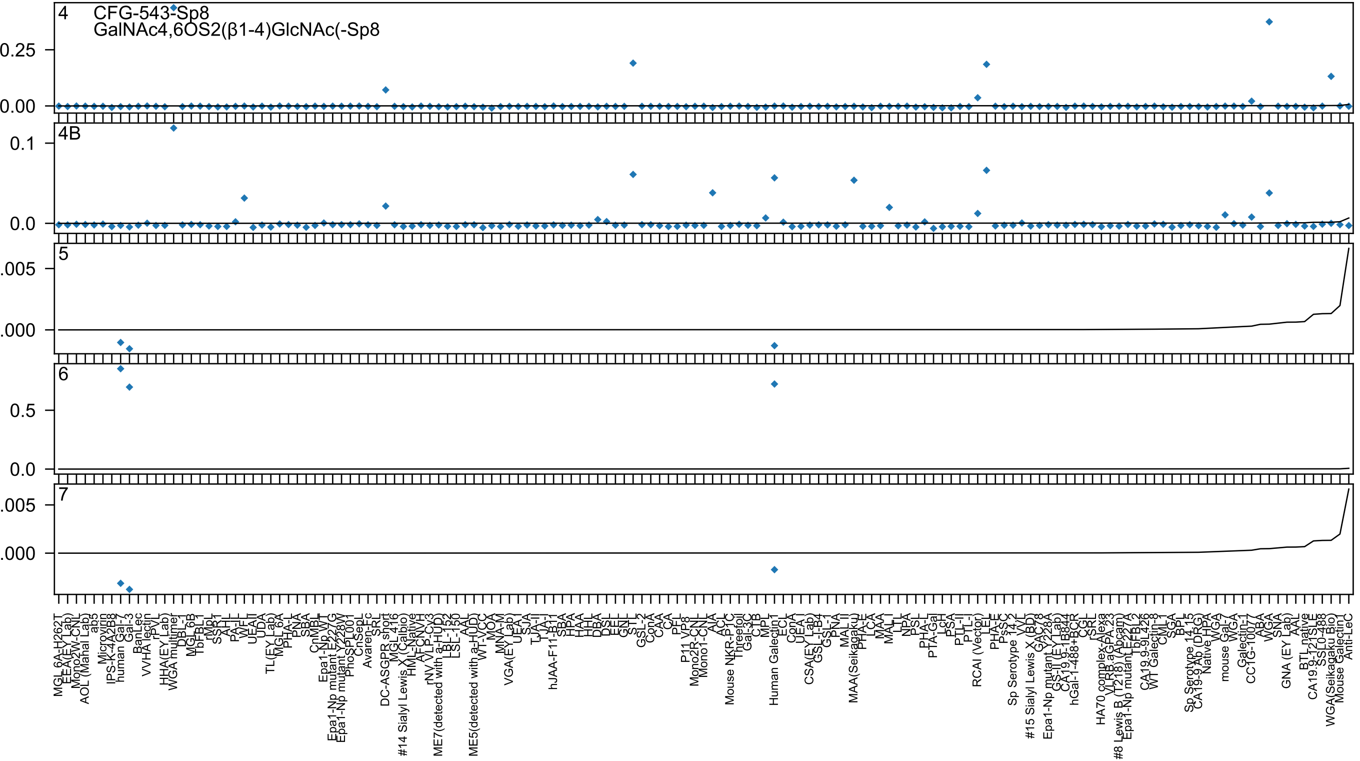

4
CFG-543-Sp8
GalNAc4,6OS2(β1-4)GlcNAc(-Sp8
4B
5
6
7

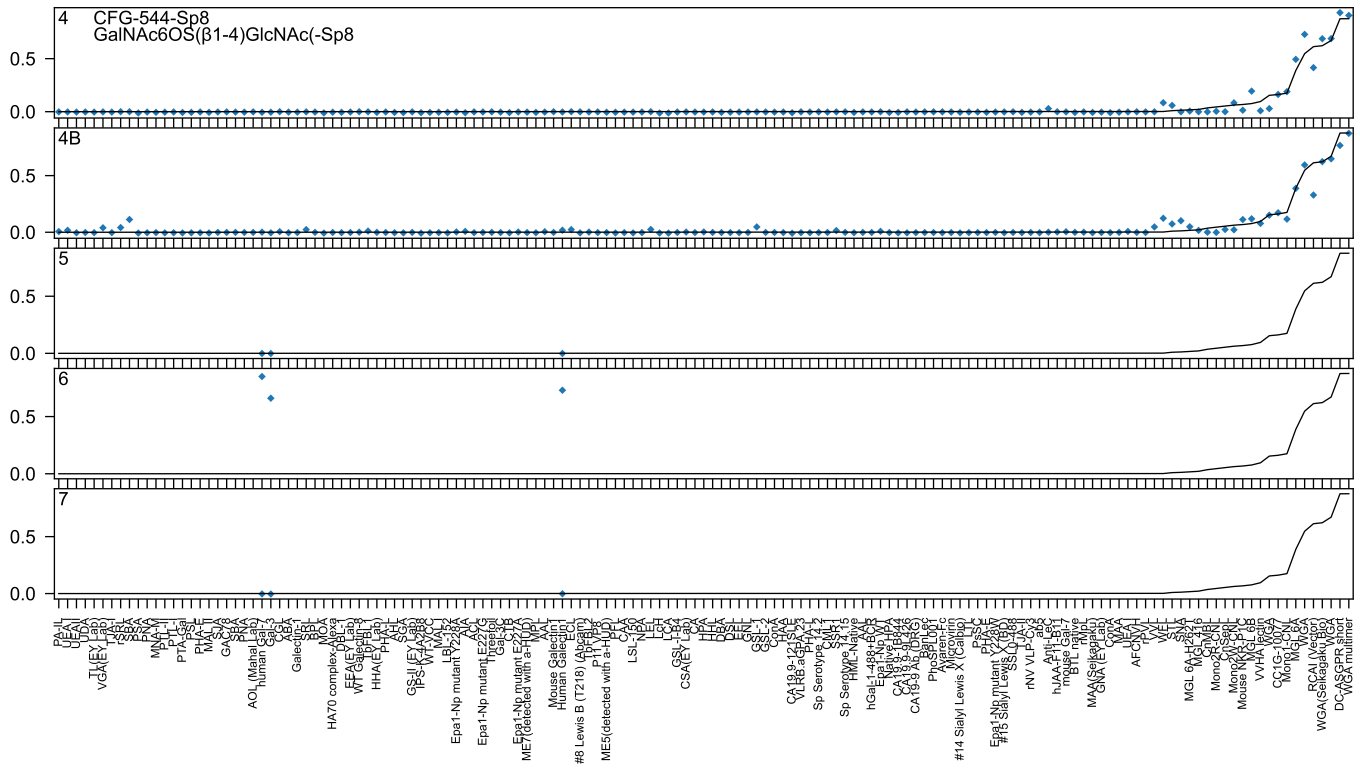

4
CFG-544-Sp8
GalNAc6OS(β1-4)GlcNAc(-Sp8
4B
5
6
7
0.5
0.0

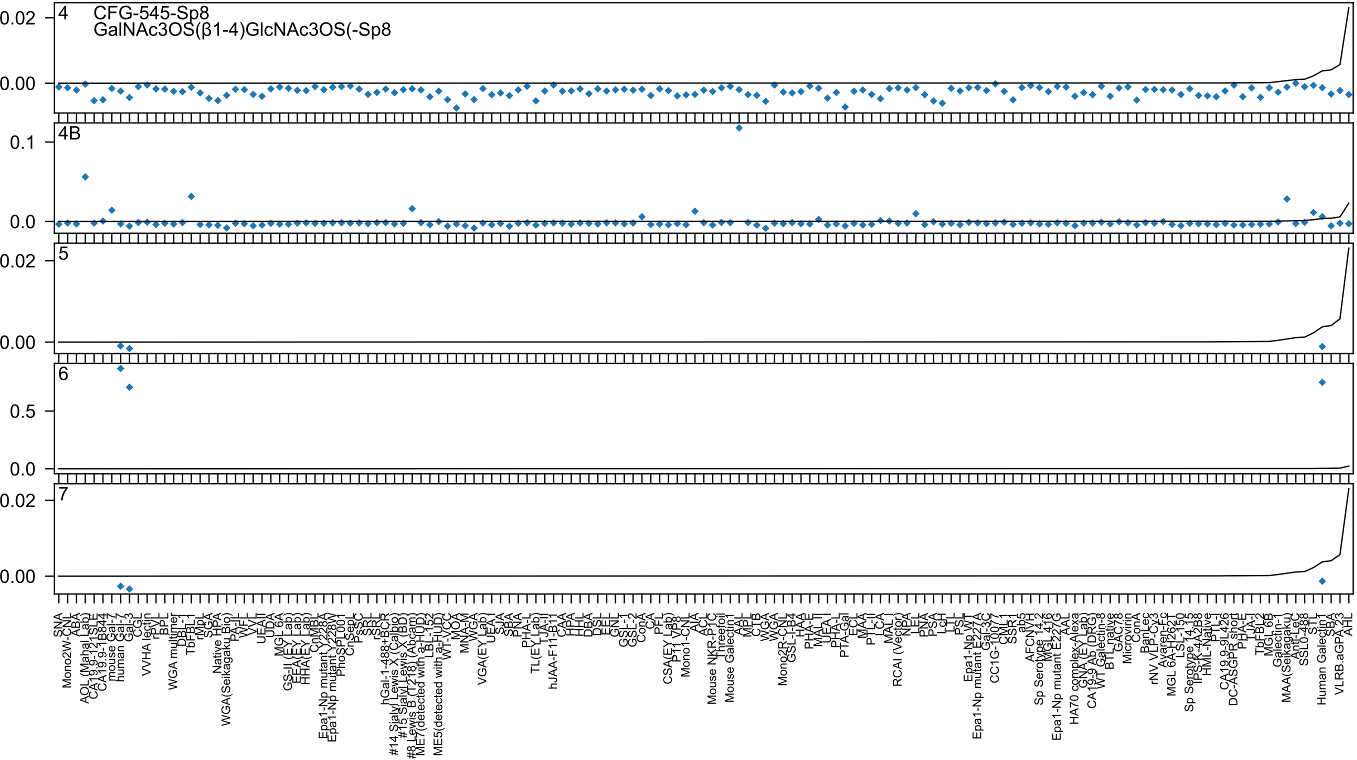
4
CFG-545-Sp8
GalNAc3OS(β1-4)GlcNAc3OS(-Sp8
4B
5
6
7

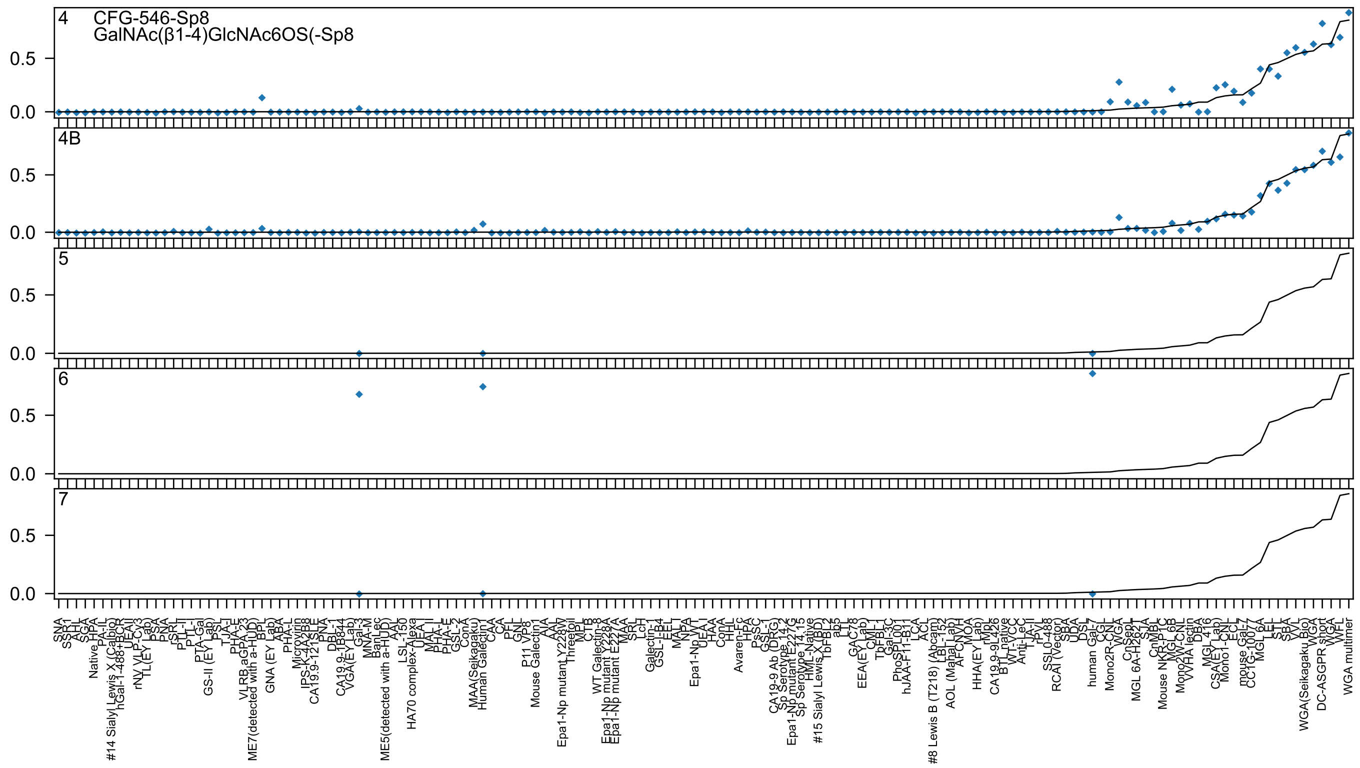
4 CFG-546-Sp8
GalNAc(β1-4)GlcNAc6OS(-Sp8
4B
5
6
7

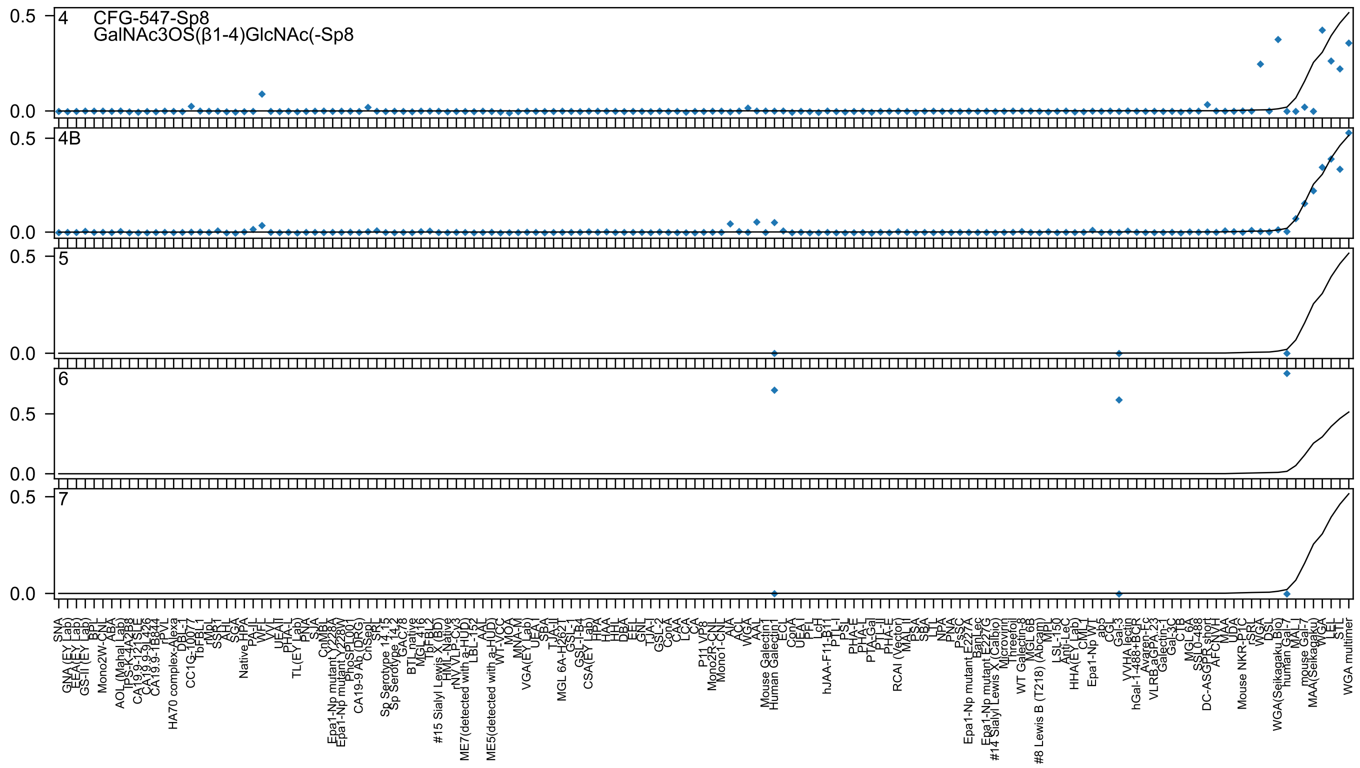

4
CFG-547-Sp8
GalNAc3OS(β1-4)GlcNAc(-Sp8
4B
5
6
7
0.5
0.0
SNA
GNA (EY Lab)
EEA(EY Lab)
GS-II (EY Lab)
BPL
Mono2W-CNL
ABA
AOL (Mahal Lab)
Human Galectin1
Mouse Galectin1
WGA multimer
STL

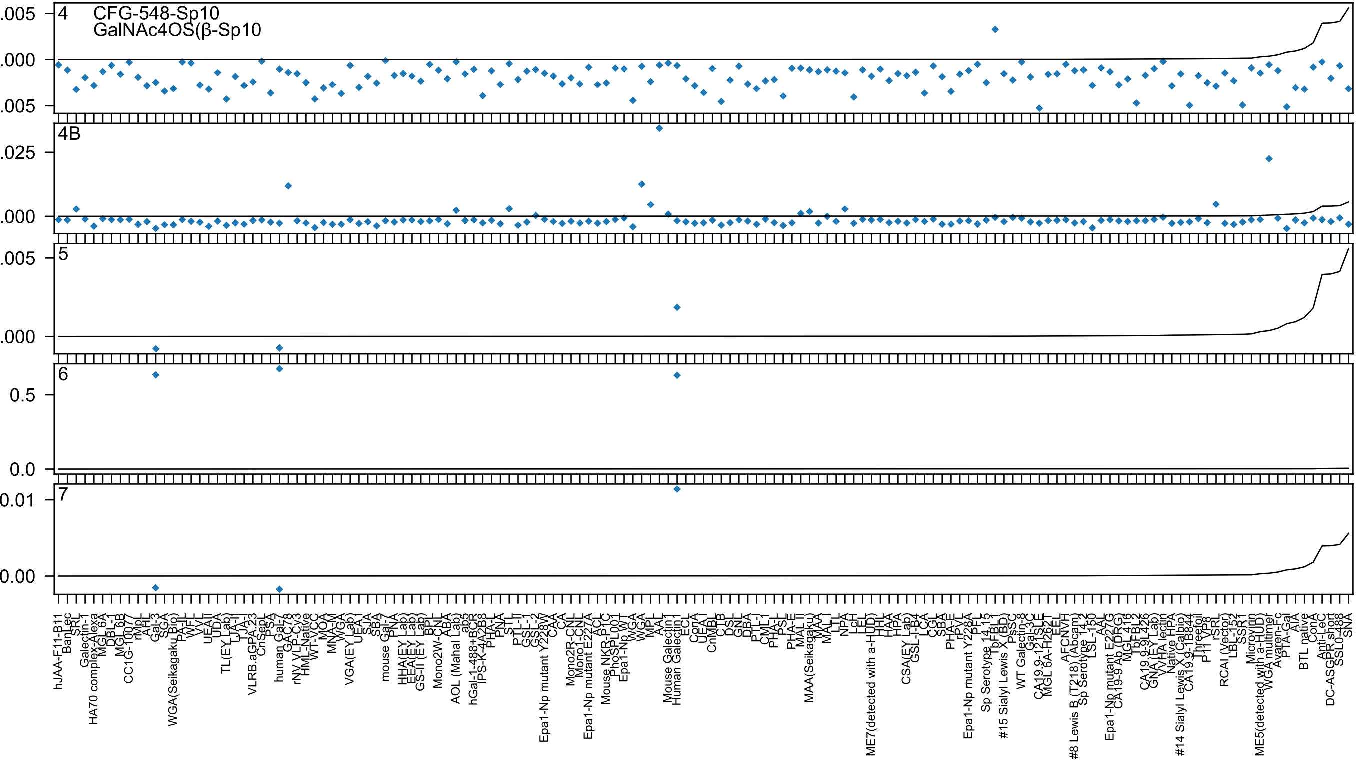

CFG-548-Sp10
GalNAc4OS(β-Sp10

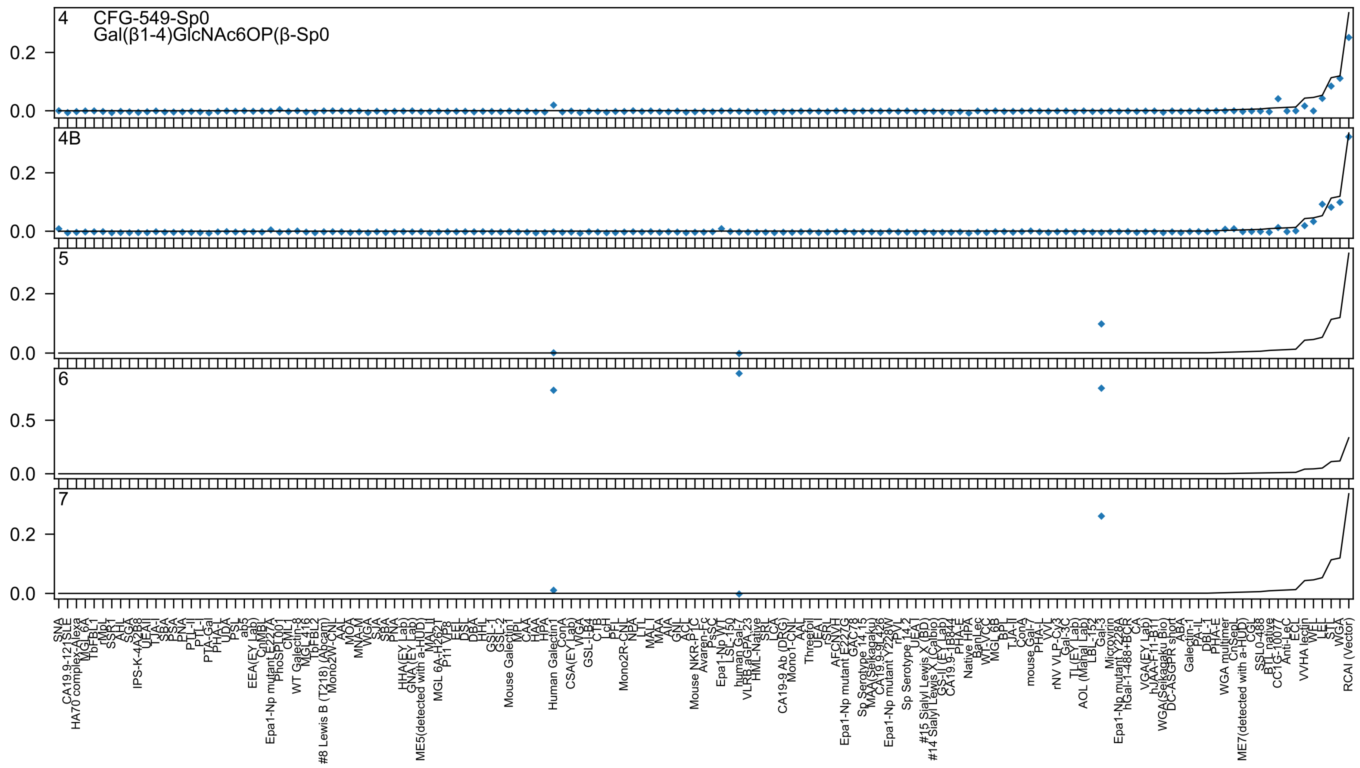
4
CFG-549-Sp0
Gal(β1-4)GlcNAc6OP(β-Sp0
4B
5
6
7

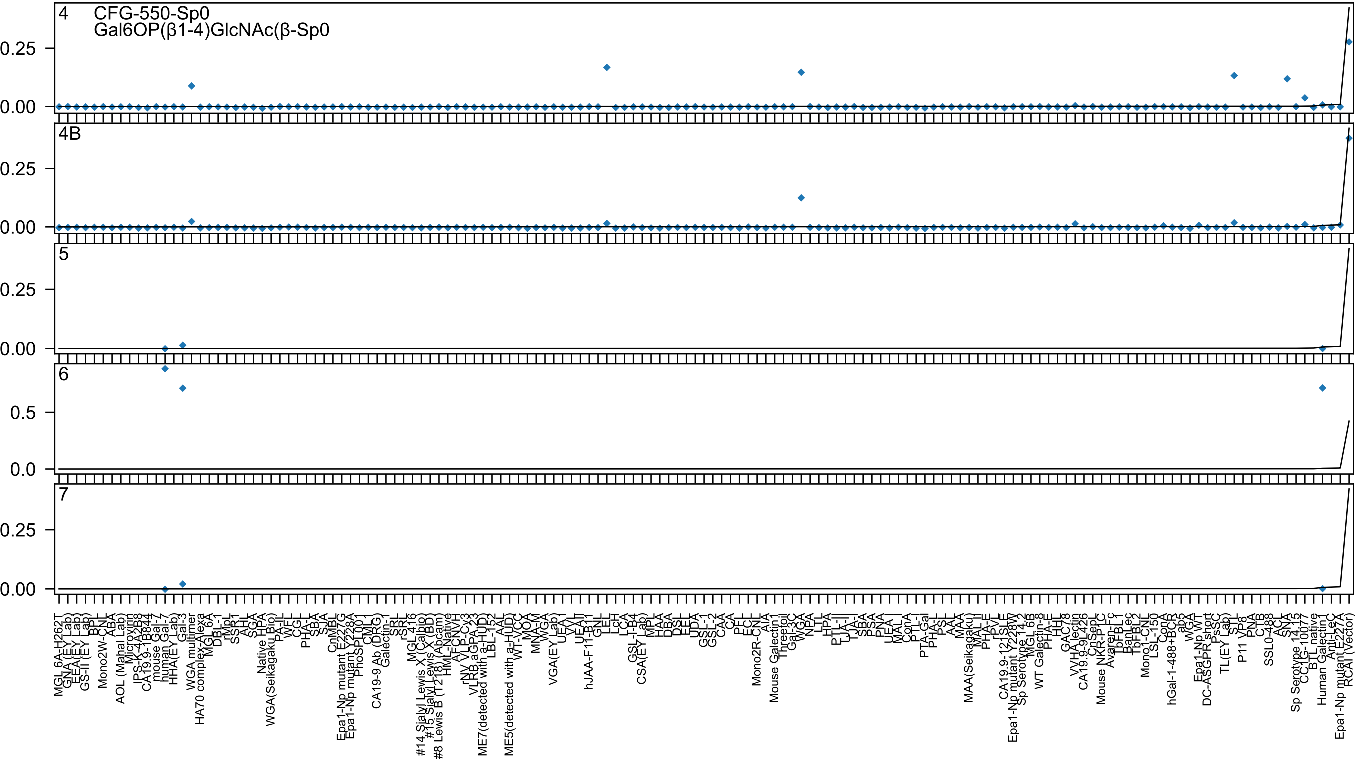
4
CFG-550-Sp0
Gal6OP(β1-4)GlcNAc(β-Sp0
4B
5
6
7

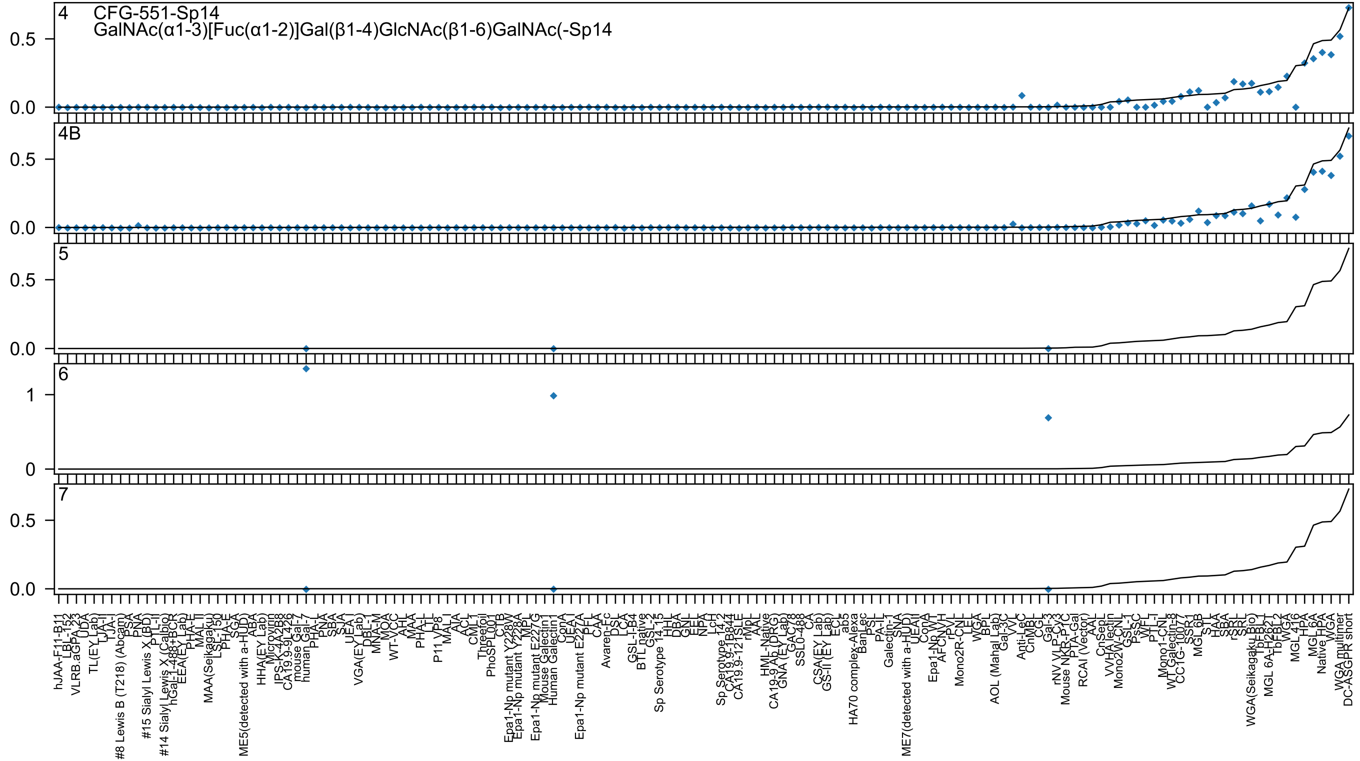

4
CFG-551-Sp14
GalNAc(α1-3)[Fuc(α1-2)]Gal(β1-4)GlcNAc(β1-6)GalNAc(-Sp14
4B
5
6
7
0.0
0.5
0
1

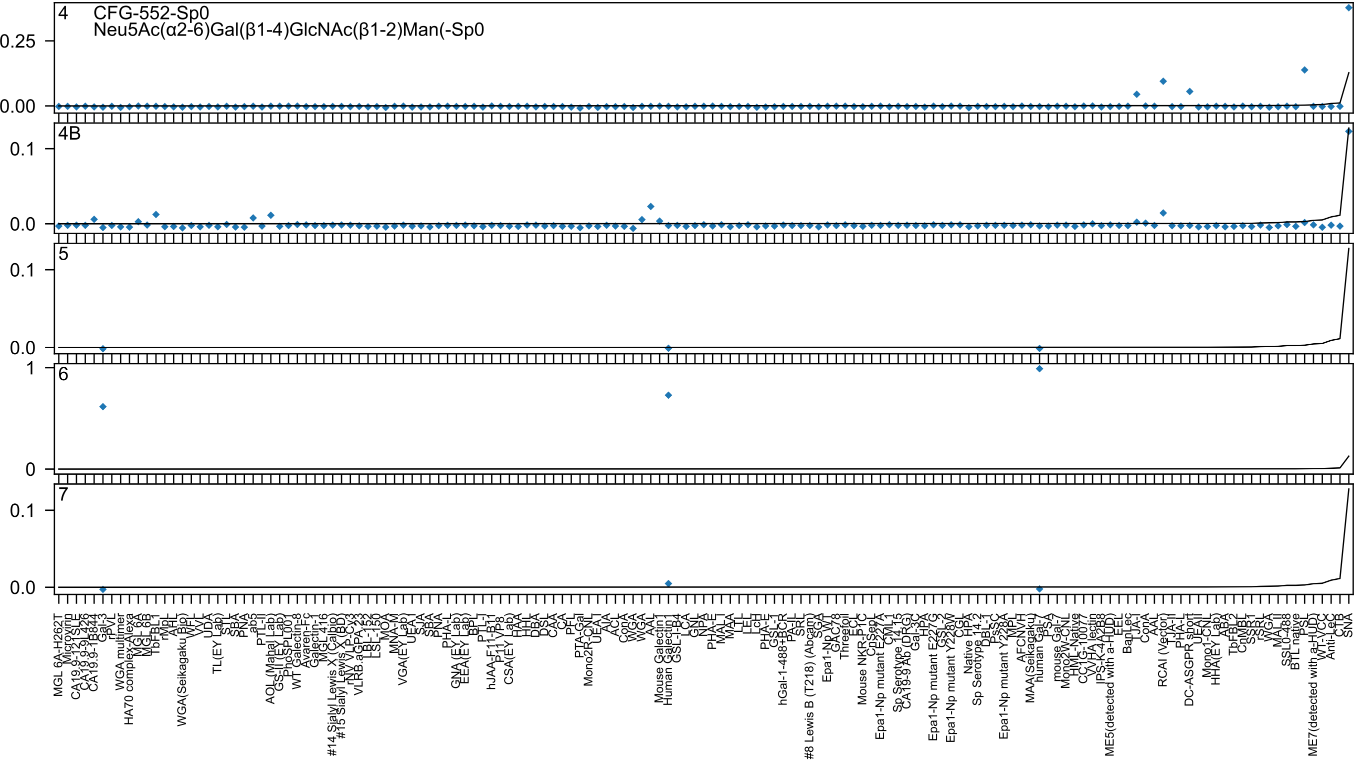
4
CFG-552-Sp0
Neu5Ac(α2-6)Gal(β1-4)GlcNAc(β1-2)Man(-Sp0
4B
5
6
7

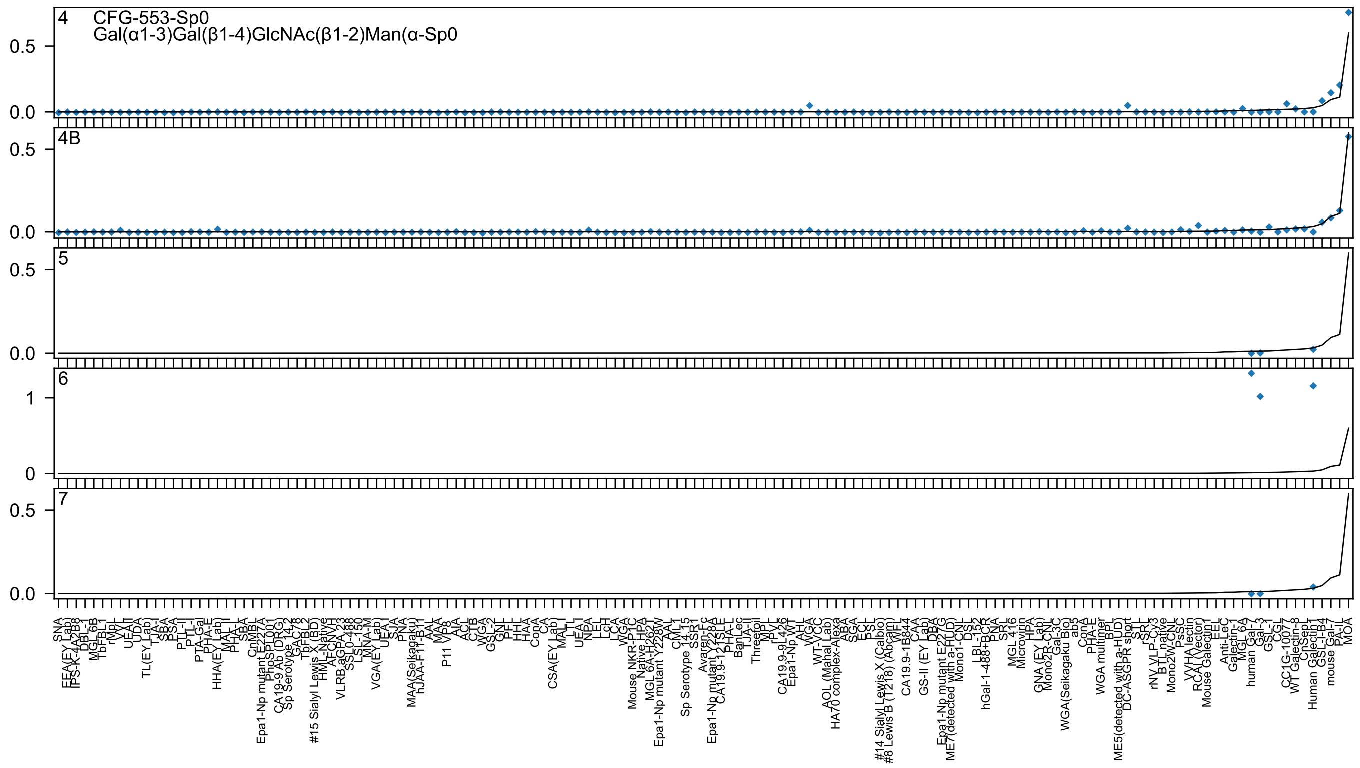
4
CFG-553-Sp0
Gal(α1-3)Gal(β1-4)GlcNAc(β1-2)Man(α-Sp0
4B
5
6
7
0.0
0.5
0
1
SNA
EEA(EY Lab)
IPS-K-4A2B8
DBL-1
MGL 6B
TbFBL1
rVPL
VVL
UEAI
UDA
TL(EY Lab)
TJA-I
SBA
PSA
PTL-II
PTL-I
PTA-Gal
PHA-E
HHA(EY Lab)
MAL-I
PHA-L
SBA
CnMBL
Epa1-Np mutant E227A
PhoSL001
CA19-9 Ab (DRG)
Sp Serotype 14-2
GAC78
TbFBL2
#15 Sialyl Lewis X (BD)
HML-Native
AFCNVH
VLRB aGPA.23
SSL0-488
LSL-150
MNA-M
VGA(EY Lab)
UEA-I
SJA
PNA
MAA(Seikagaku)
hJAA-F11-B11
AAL
MAA
P11 VP8
AIA
ACL
CTB
WGA
GSL-2
GNL
PFL
HHL
HAA
ConA
CA
CSA(EY Lab)
MAL-I
LTL
UEA-I
NPA
LEL
LcH
LCA
WGA
Mouse NKR-P1C
Native HPA
MGL 6A-H262T
Epa1-Np mutant Y228W
AAL
CML1
Sp Serotype 14-15
SSR1
Avaren-Fc
Epa1-Np mutant Y228A
CA19.9-121SLE
PHA-L
BanLec
TJA-II
Threetoil
MPL
rPVL
CA19.9-9L426
Epa1-Np WT
AHL
WGA
WT-VCC
AOL (Mahal Lab)
HA70 complex-Alexa
ABA
SGA
ECL
PSL
#14 Sialyl Lewis X (Calbio)
#8 Lewis B (T218) (Abcam)
WFL
CA19.9-1B844
CAA
GS-II (EY Lab)
DBA
Epa1-Np mutant E227G
ME7(detected with a-HUD)
Mono1-CNL
DSL
LBL-152
hGal-1-488+BCR
PNA
SRL
MGL 4.16
Microvirin
HPA
GNA (EY Lab)
Mono2R-CNL
Gal-3C
WGA(Seikagaku Bio)
ab5
ConA
PHA-E
WGA multimer
BPL
ME5(detected with a-HUD)
DC-ASGPR short
STL
rSRL
rNV VLP-Cy3
BTL native
Mono2W-CNL
PsSC
VVHA lectin
RCA I (Vector)
Mouse Galectin1
EEL
Anti-LeC
Galectin-1
MGL 6A
human Gal-7
Gal-3
GSL-1
CGL
CC1G-10077
WT Galectin-8
CnSepL
Human Galectin1
GSL-I-B4
mouse Gal-7
PA-IL
MOA

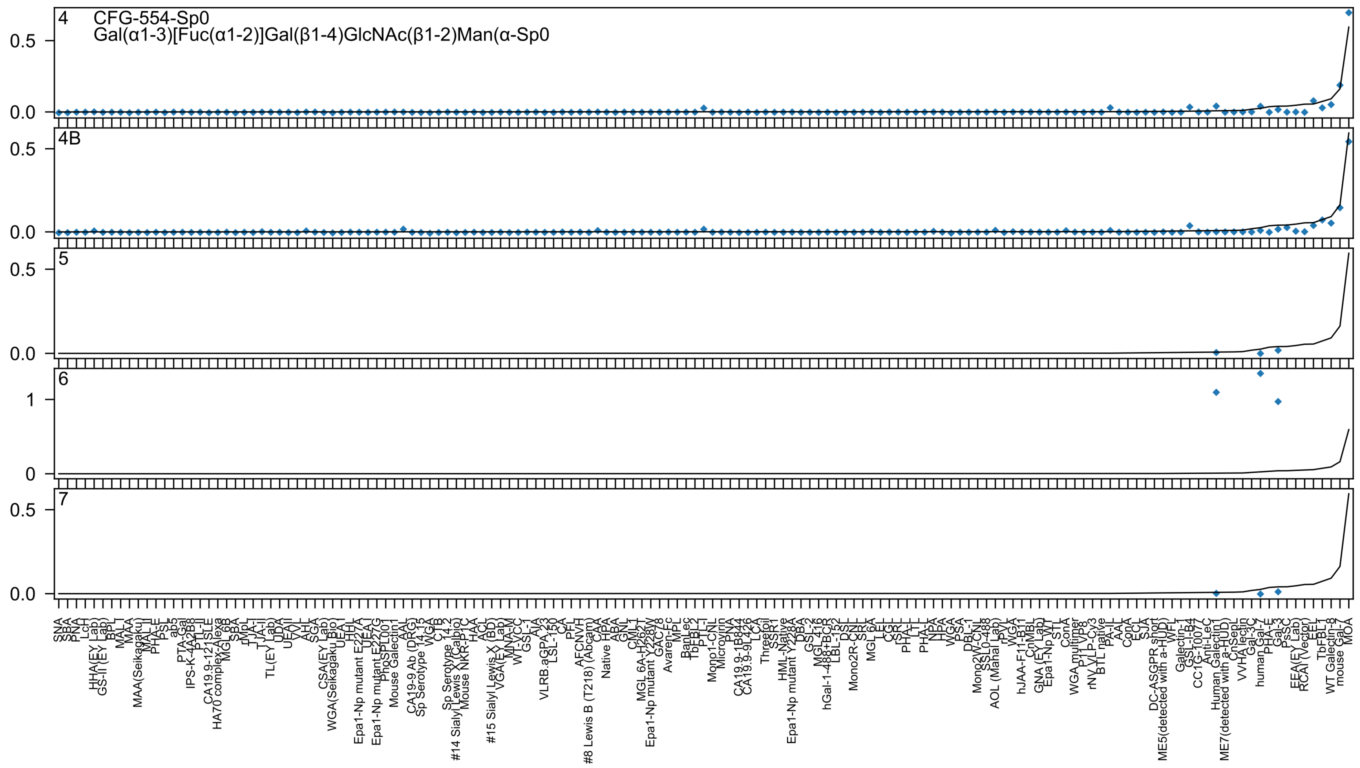
4 CFG-554-Sp0
Gal(α1-3)[Fuc(α1-2)]Gal(β1-4)GlcNAc(β1-2)Man(α-Sp0
4B
5
6
7

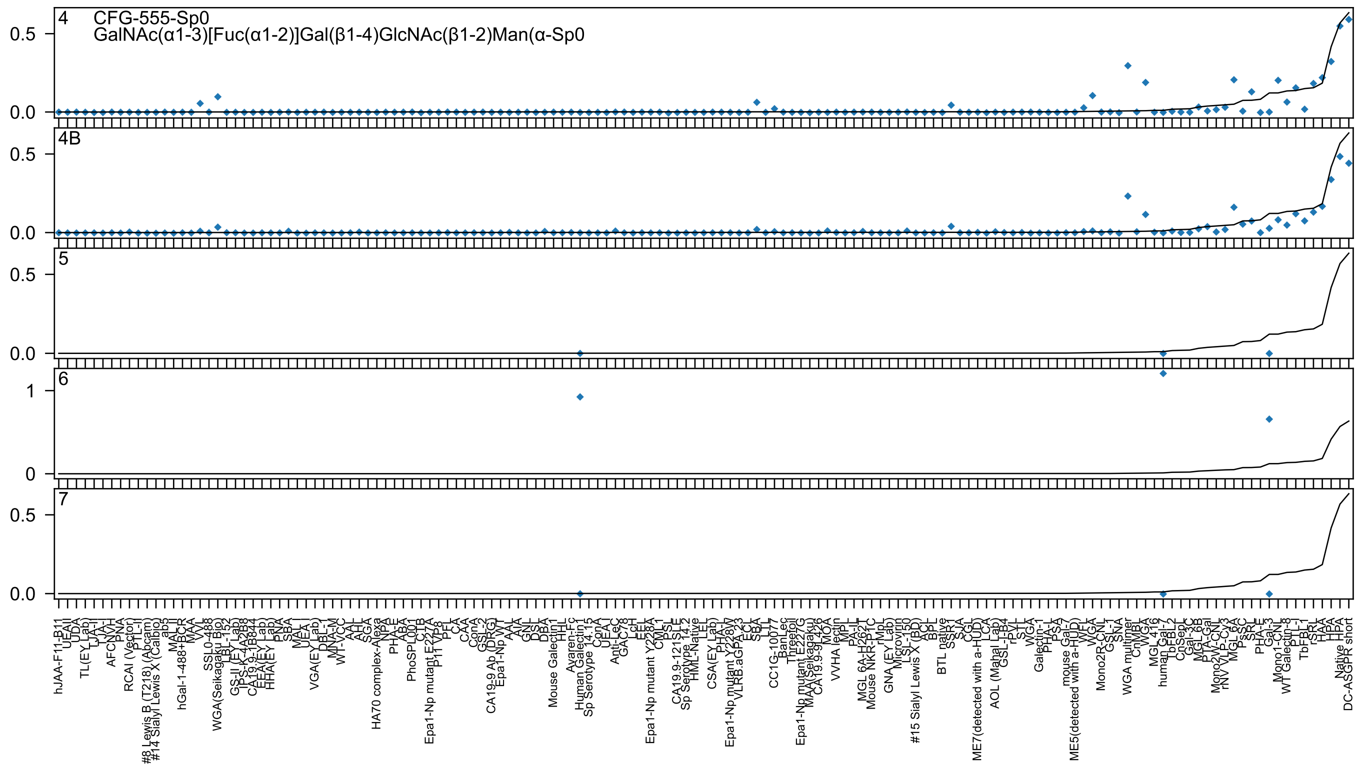

4 CFG-555-Sp0
GalNAc(α1-3)[Fuc(α1-2)]Gal(β1-4)GlcNAc(β1-2)Man(α-Sp0
4B
5
6
7

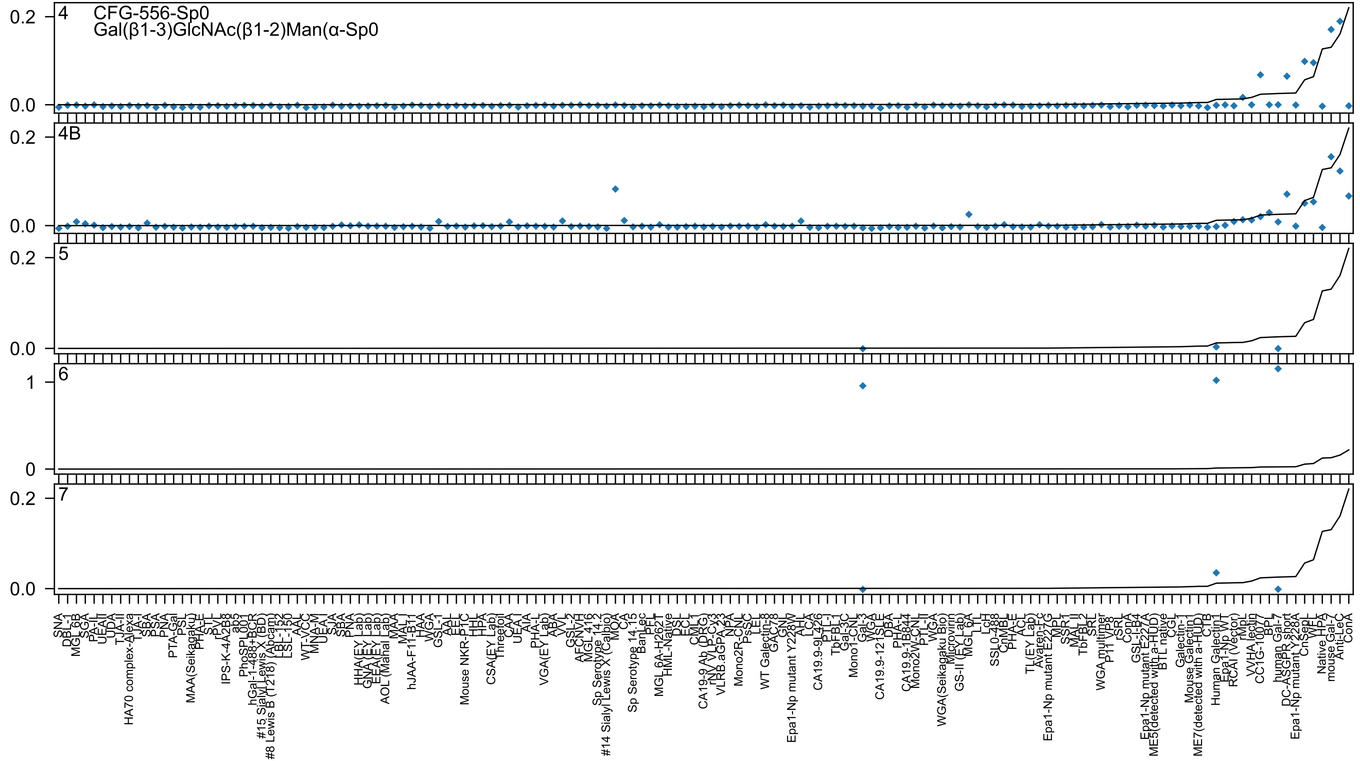

4
CFG-556-Sp0
Gal(β1-3)GlcNAc(β1-2)Man(α-Sp0
4B
5
6
7
0.2
0.0
1
0
SNA
ConA
Anti-LeC
mouse Gal-7
Native HPA

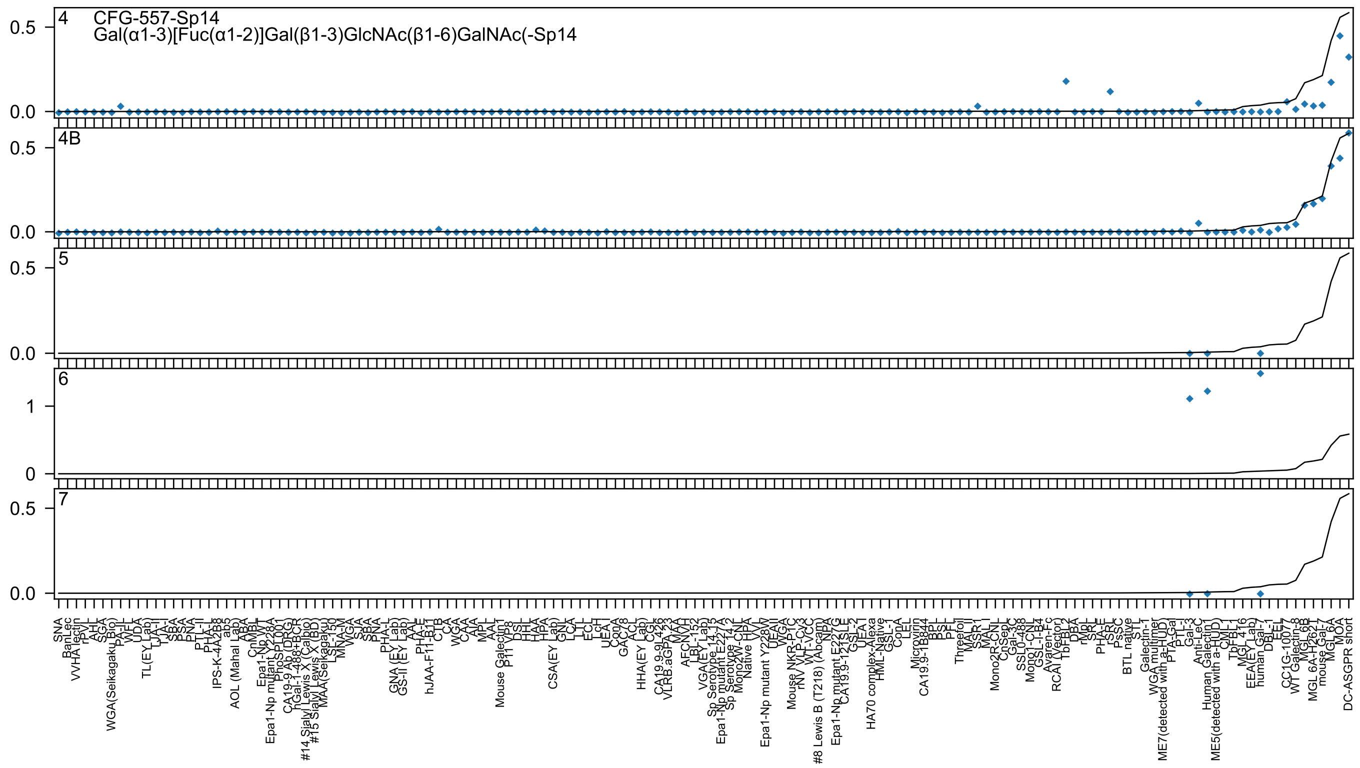
4
CFG-557-Sp14
Gal(α1-3)[Fuc(α1-2)]Gal(β1-3)GlcNAc(β1-6)GalNAc(-Sp14
4B
5
6
7

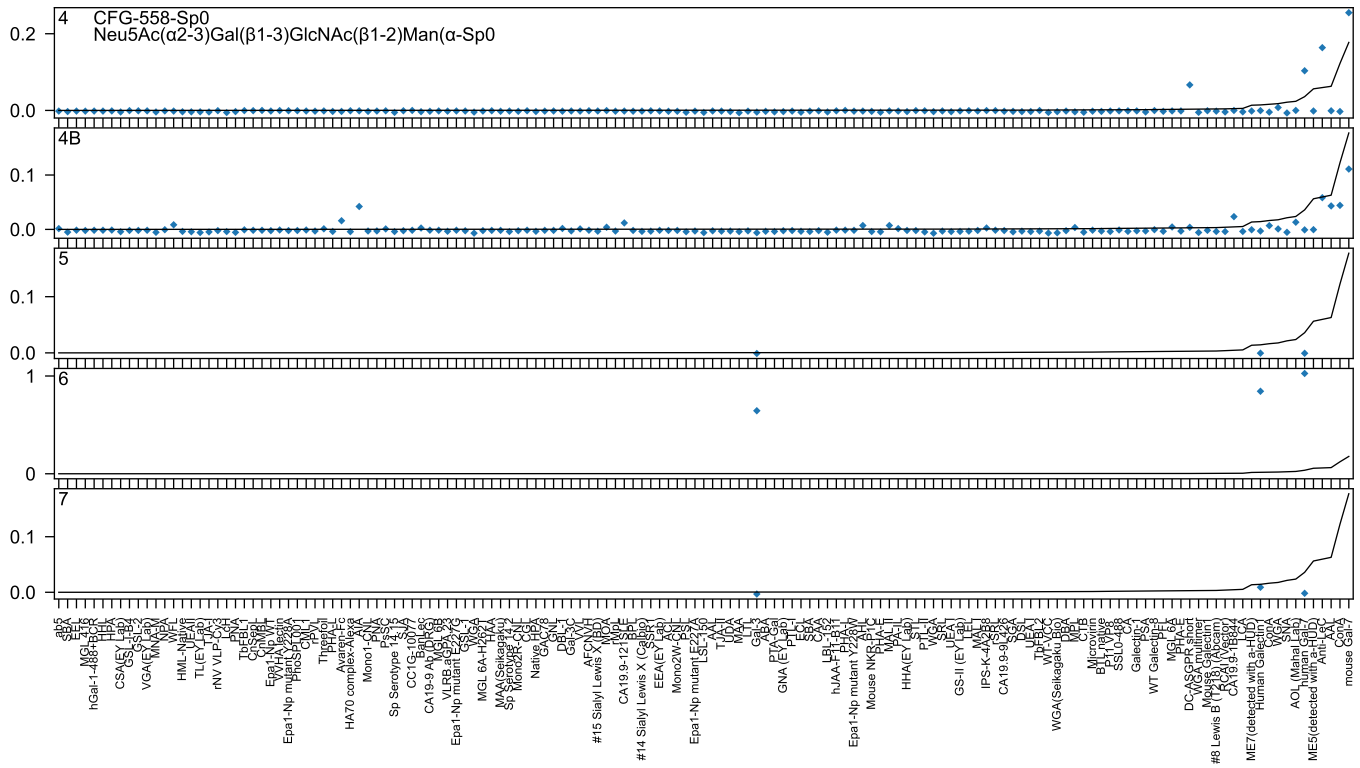

4 CFG-558-Sp0
Neu5Ac(α2-3)Gal(β1-3)GlcNAc(β1-2)Man(α-Sp0
0.2
0.0
4B
0.1
0.0
5
0.1
0.0
6
1
0
7
0.1
0.0

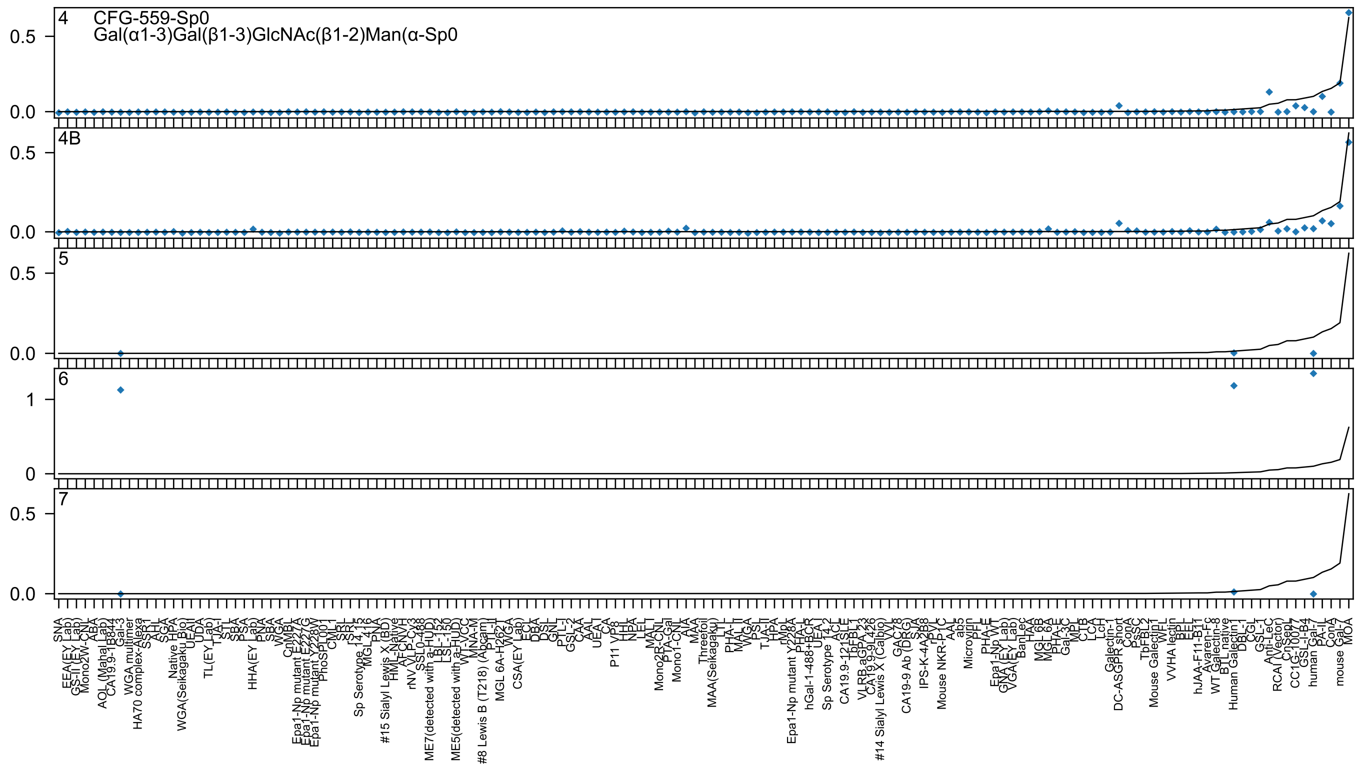

4 CFG-559-Sp0
Gal(α1-3)Gal(β1-3)GlcNAc(β1-2)Man(α-Sp0
4B
5
6
7

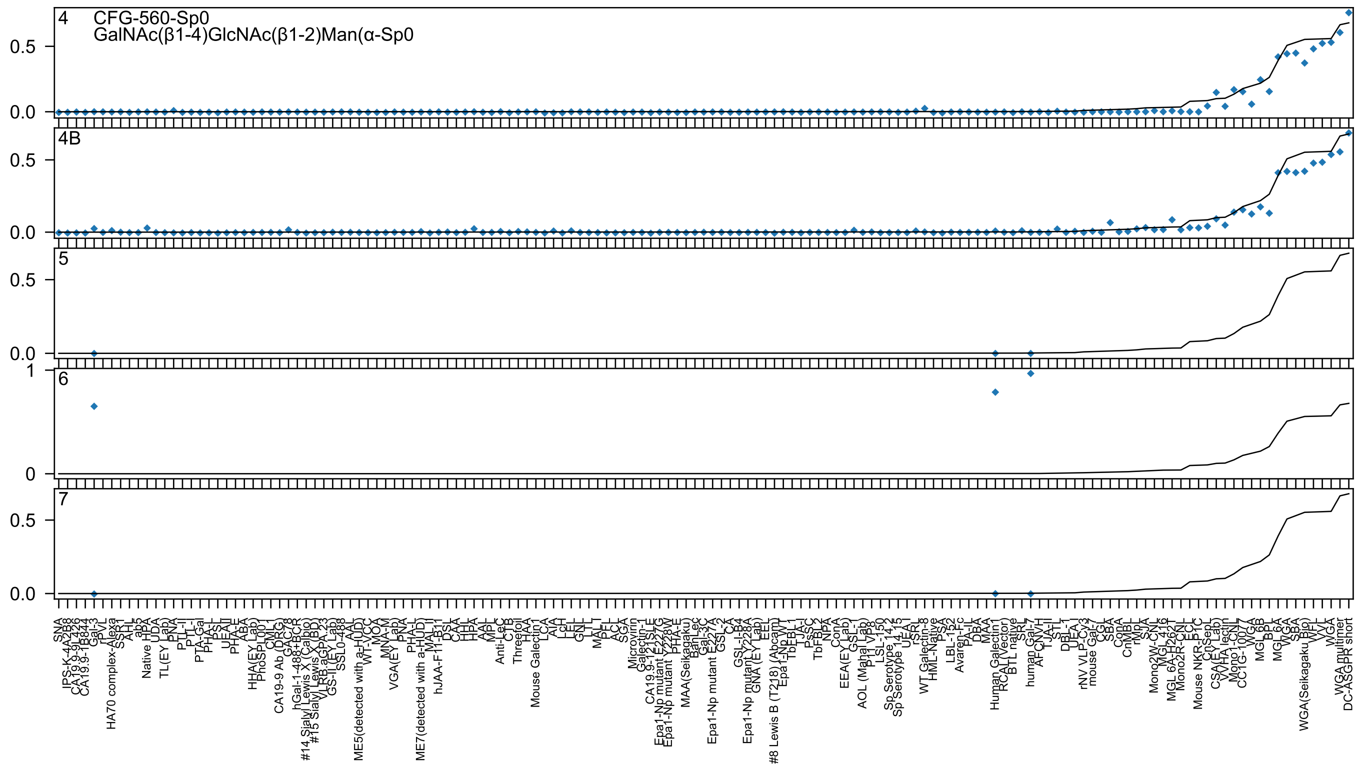
4
CFG-560-Sp0
GalNAc(β1-4)GlcNAc(β1-2)Man(α-Sp0
4B
5
6
7

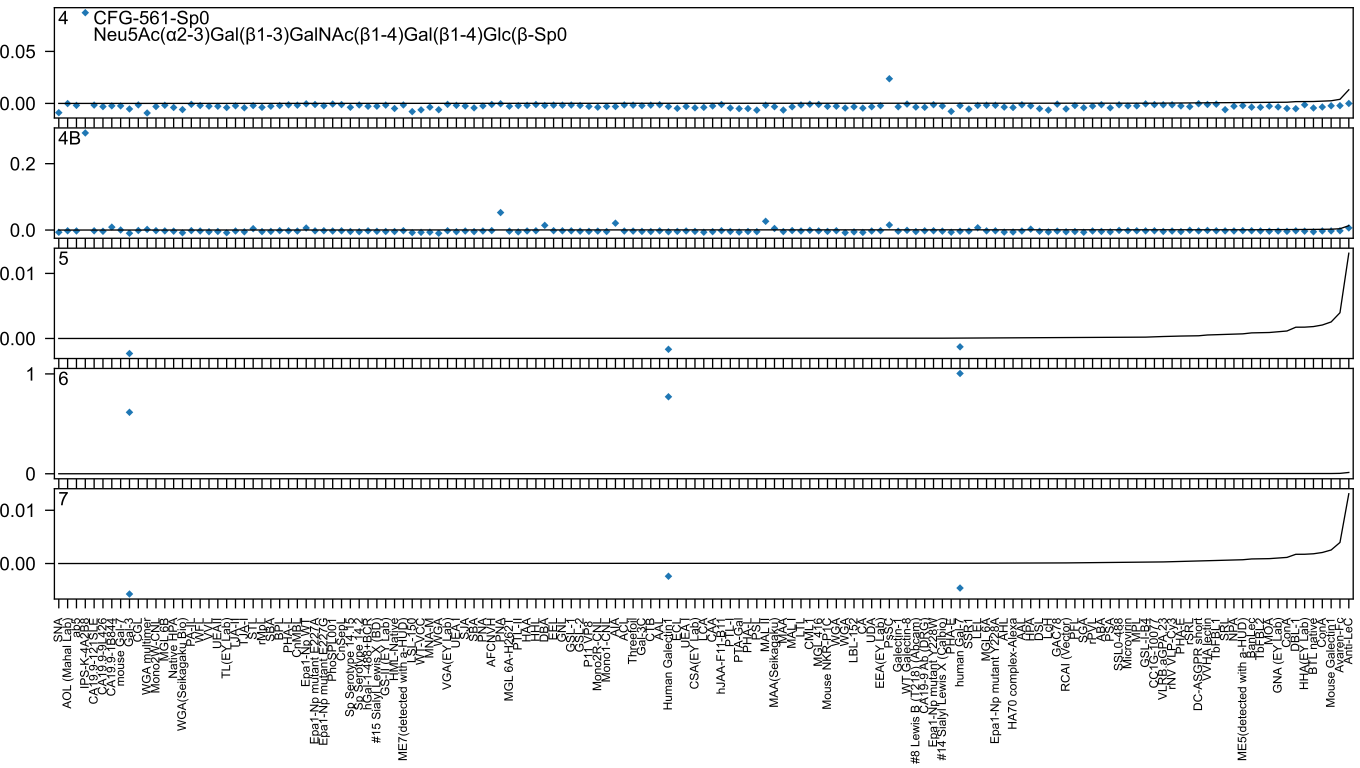

CFG-561-Sp0
Neu5Ac(α2-3)Gal(β1-3)GalNAc(β1-4)Gal(β1-4)Glc(β-Sp0
4
4B
5
6
7

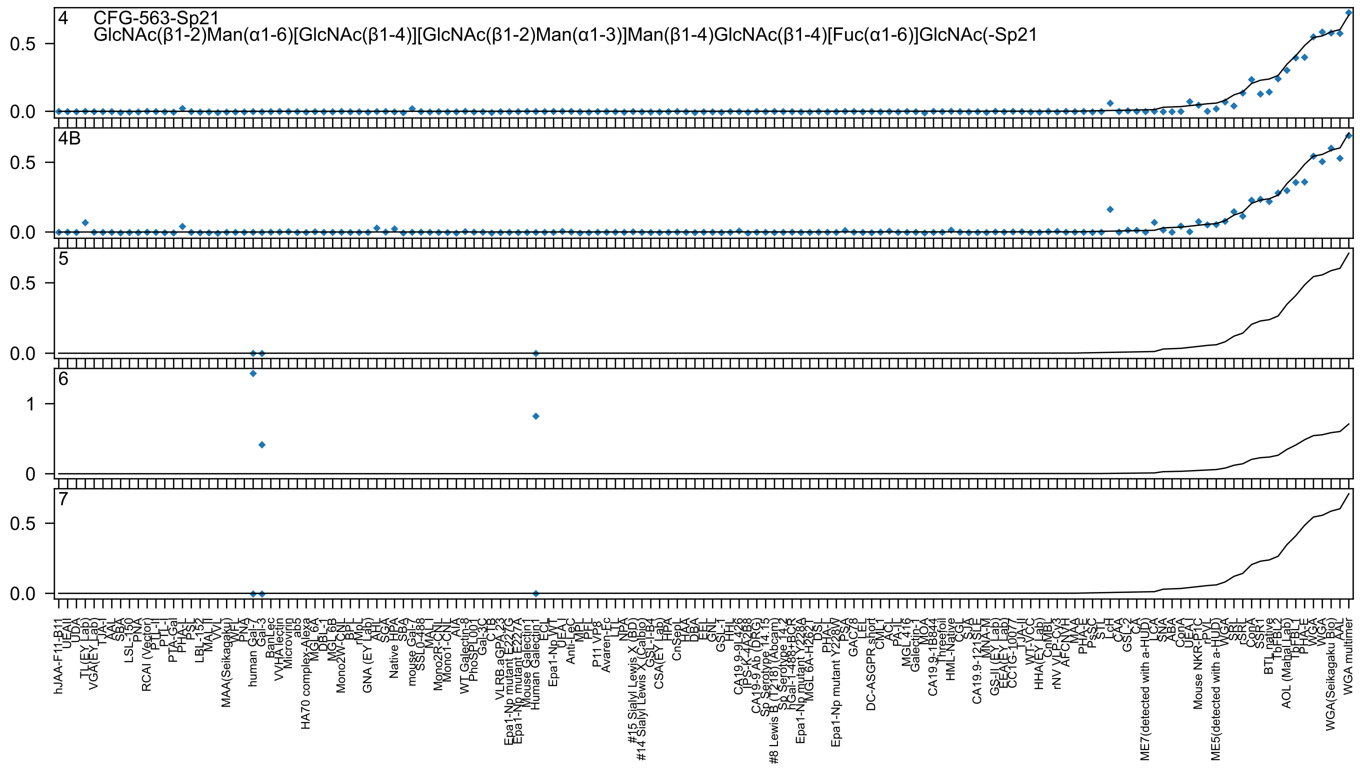

CFG-563-Sp21
GlcNAc(β1-2)Man(α1-6)[GlcNAc(β1-4)][GlcNAc(β1-2)Man(α1-3)]Man(β1-4)GlcNAc(β1-4)[Fuc(α1-6)]GlcNAc(-Sp21

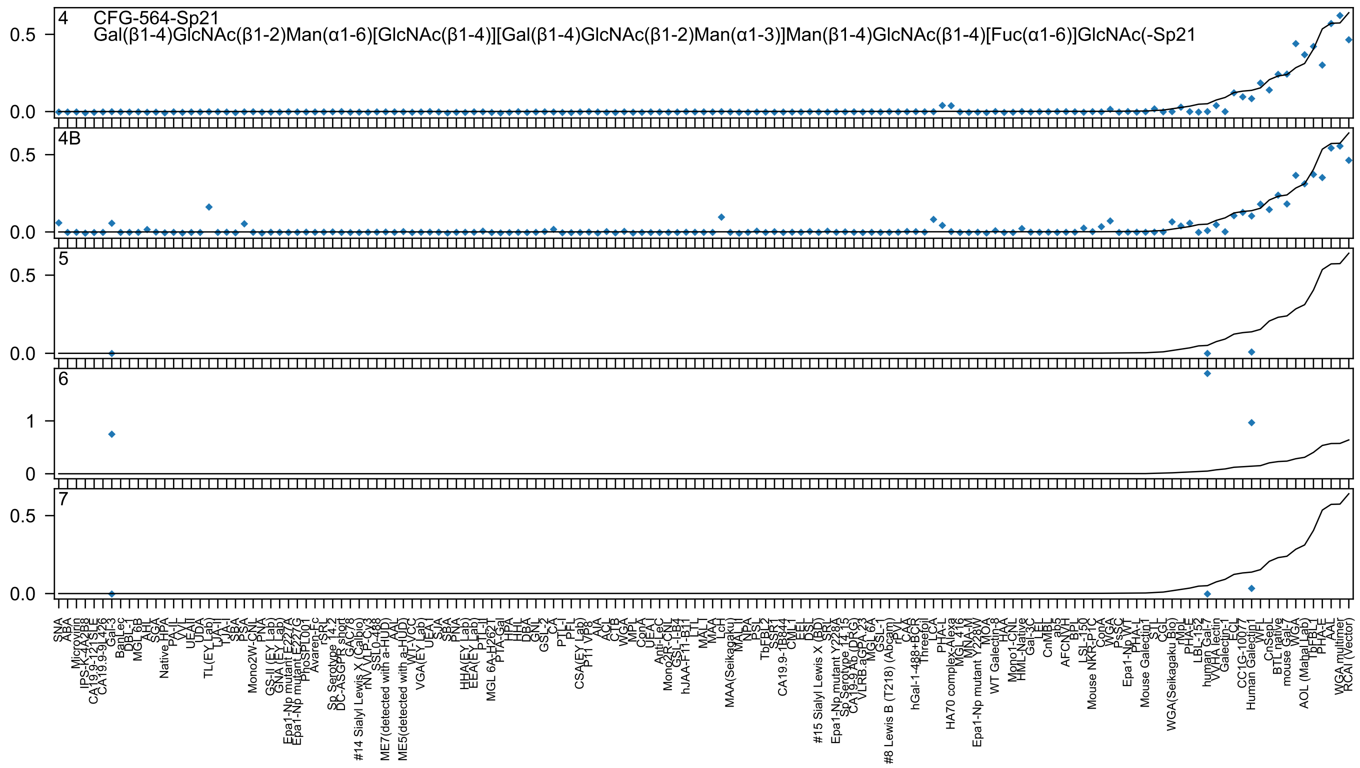
4
CFG-564-Sp21
Gal(β1-4)GlcNAc(β1-2)Man(α1-6)[GlcNAc(β1-4)][Gal(β1-4)GlcNAc(β1-2)Man(α1-3)]Man(β1-4)GlcNAc(β1-4)[Fuc(α1-6)]GlcNAc(-Sp21
4B
5
6
7
0.0
0.5
0
1

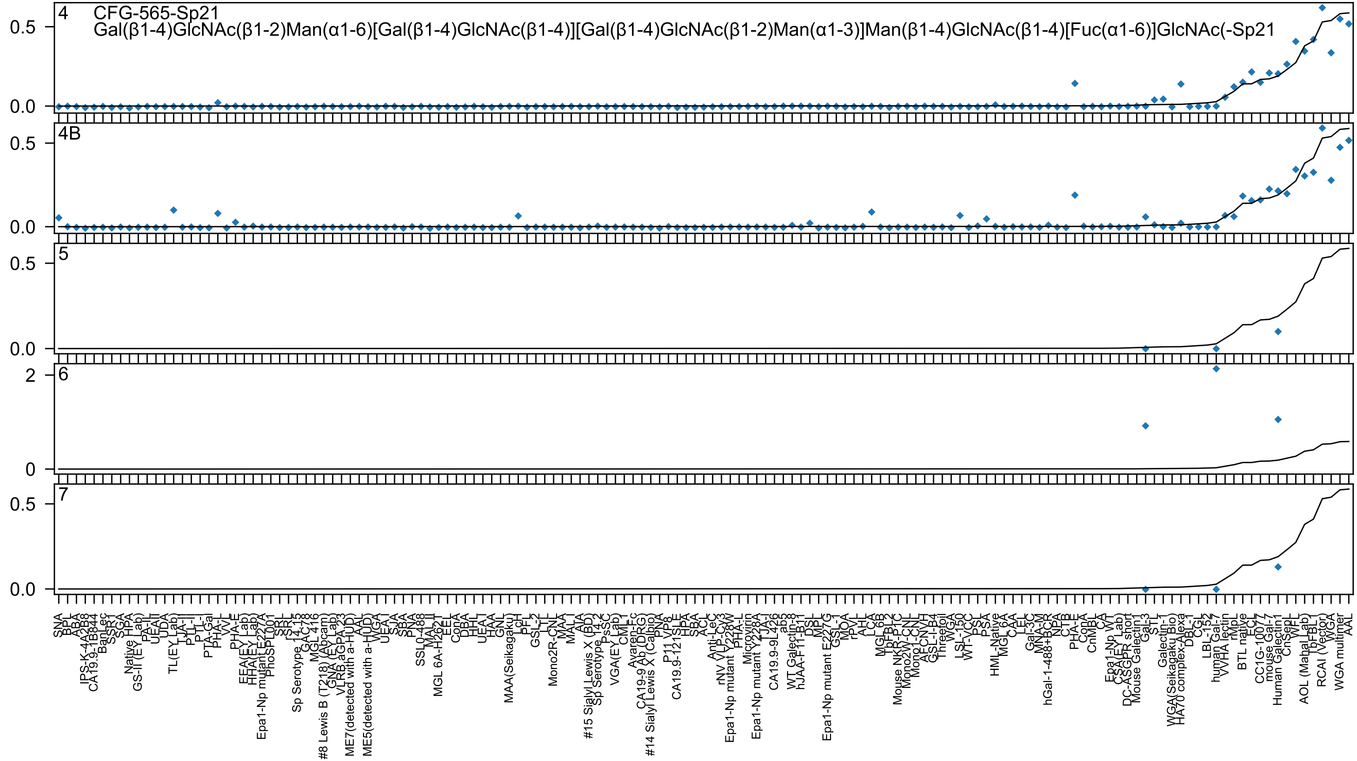
4
CFG-565-Sp21
Gal(β1-4)GlcNAc(β1-2)Man(α1-6)[Gal(β1-4)GlcNAc(β1-4)][Gal(β1-4)GlcNAc(β1-2)Man(α1-3)]Man(β1-4)GlcNAc(β1-4)[Fuc(α1-6)]GlcNAc(-Sp21
4B
5
6
7

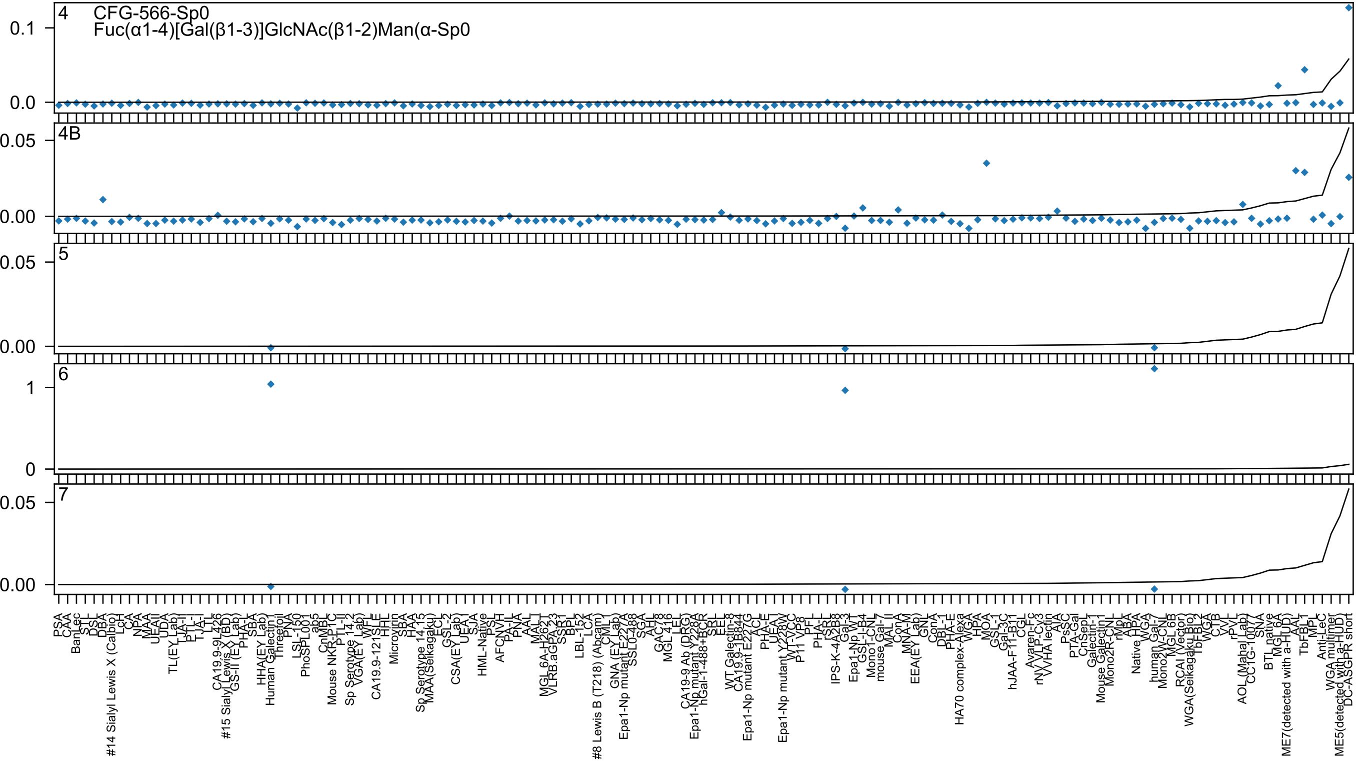

4
CFG-566-Sp0
Fuc(α1-4)[Gal(β1-3)]GlcNAc(β1-2)Man(α-Sp0
4B
5
6
7

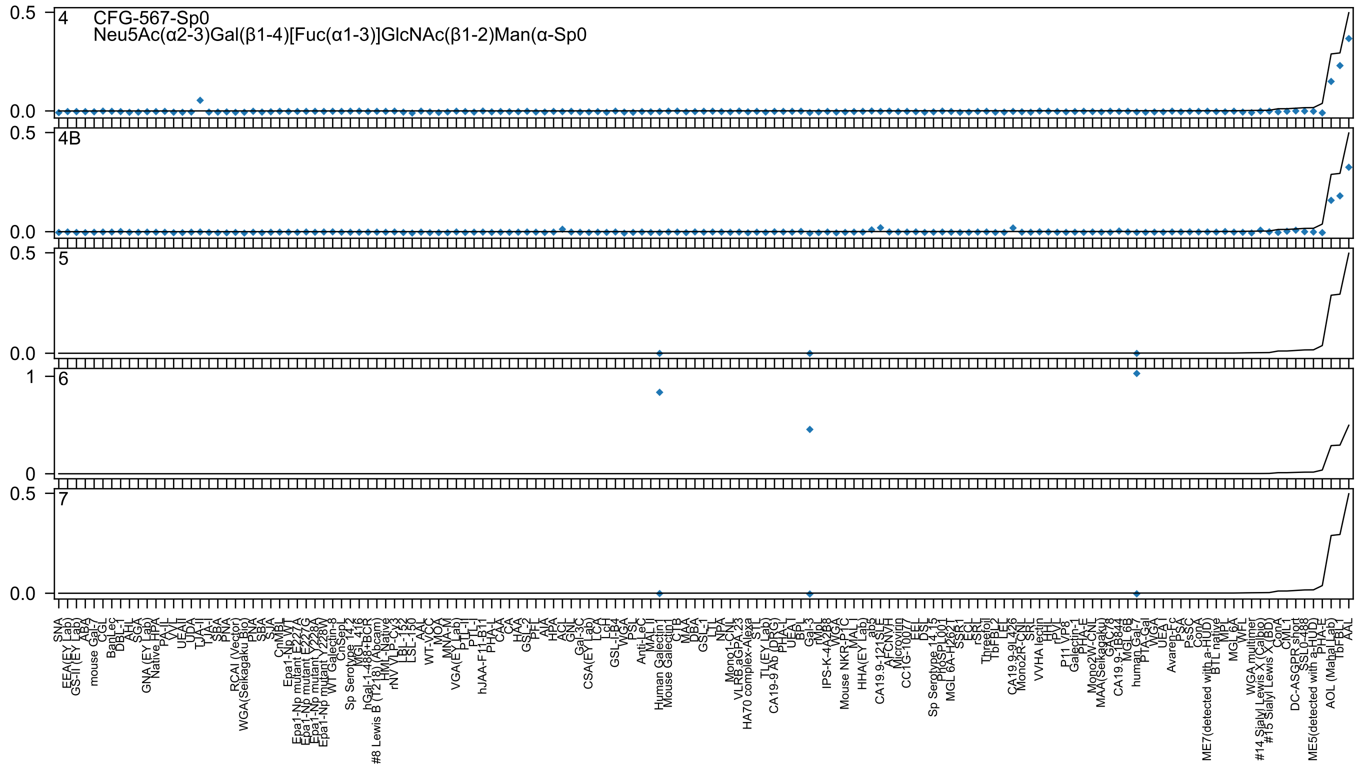
4
CFG-567-Sp0
Neu5Ac(α2-3)Gal(β1-4)[Fuc(α1-3)]GlcNAc(β1-2)Man(α-Sp0
4B
5
6
7

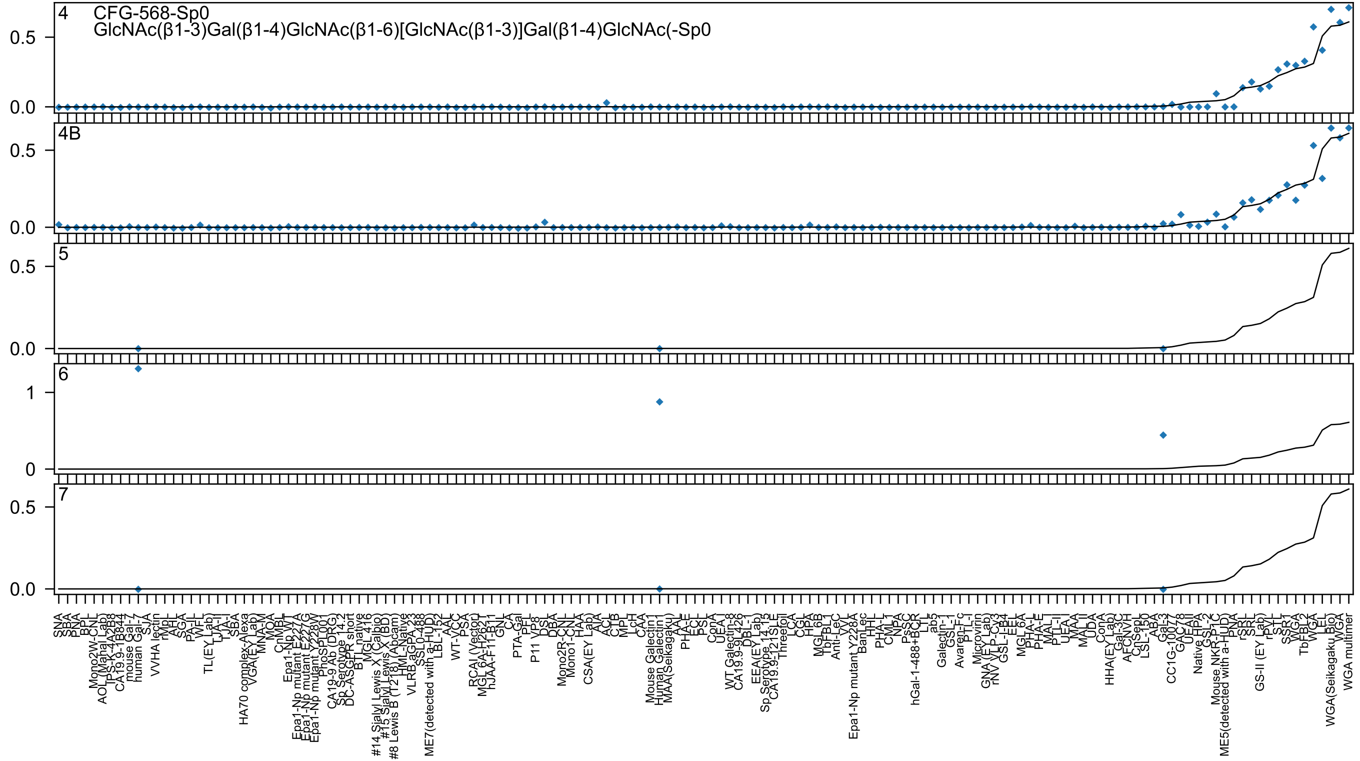

4 CFG-568-Sp0
GlcNAc(β1-3)Gal(β1-4)GlcNAc(β1-6)[GlcNAc(β1-3)]Gal(β1-4)GlcNAc(-Sp0
4B
5
6
7
0.5
0.0
1
0

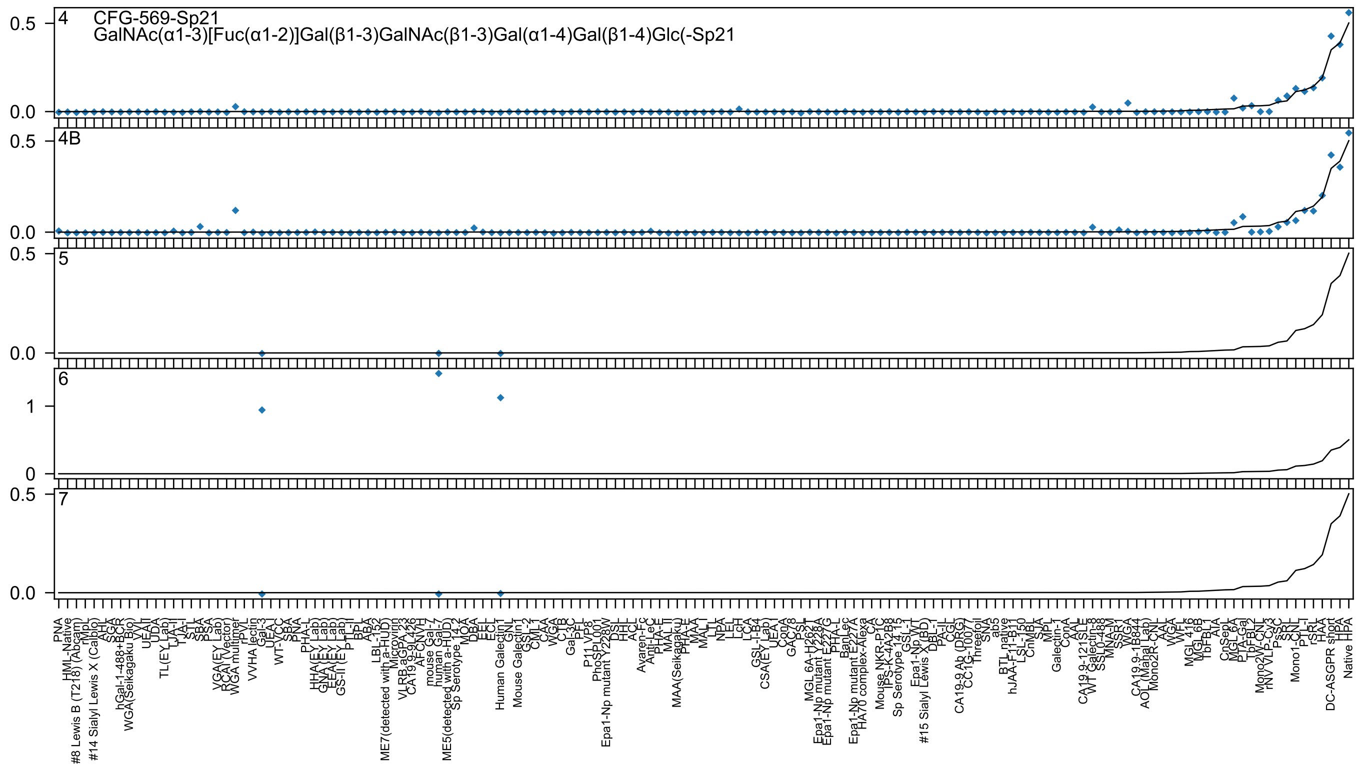

4 CFG-569-Sp21
GalNAc(α1-3)[Fuc(α1-2)]Gal(β1-3)GalNAc(β1-3)Gal(α1-4)Gal(β1-4)Glc(-Sp21
4B
5
6
7

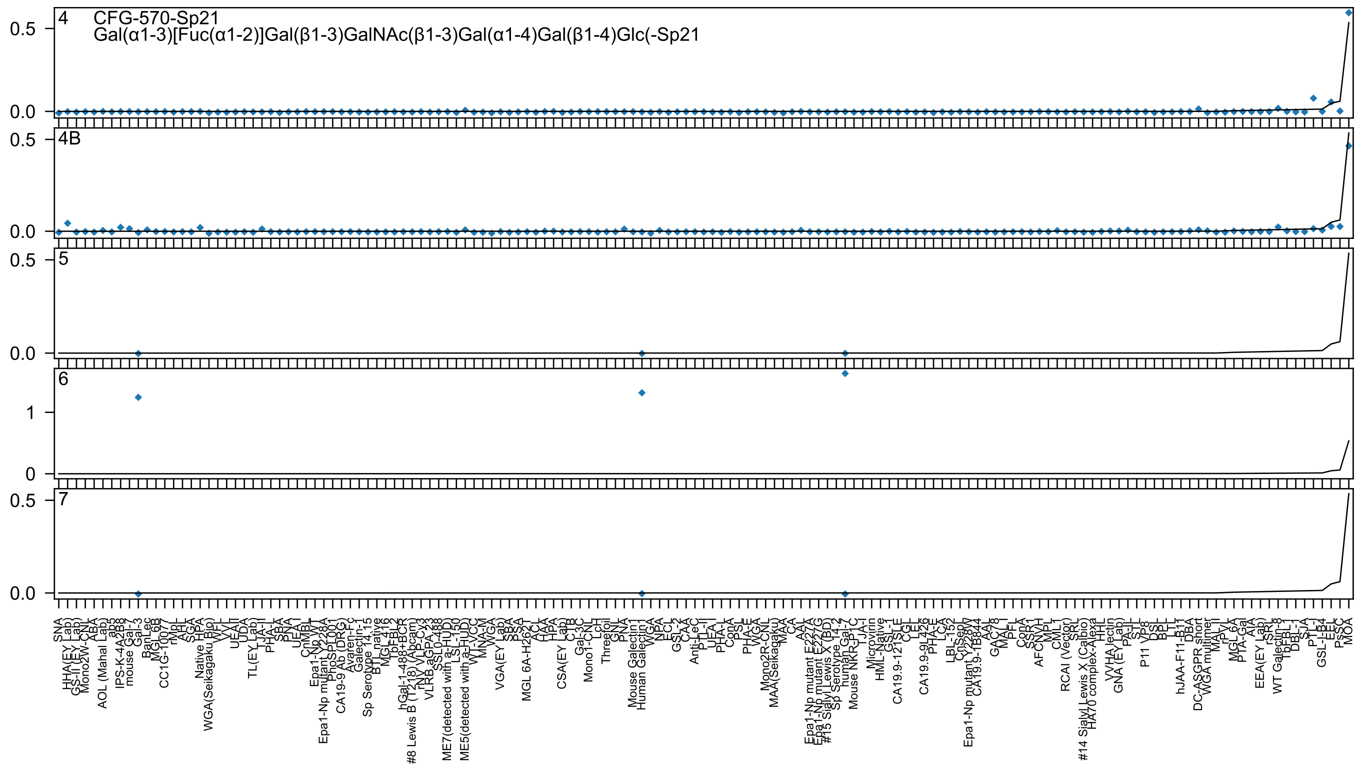

4
CFG-570-Sp21
Gal(α1-3)[Fuc(α1-2)]Gal(β1-3)GalNAc(β1-3)Gal(α1-4)Gal(β1-4)Glc(-Sp21
4B
5
6
7

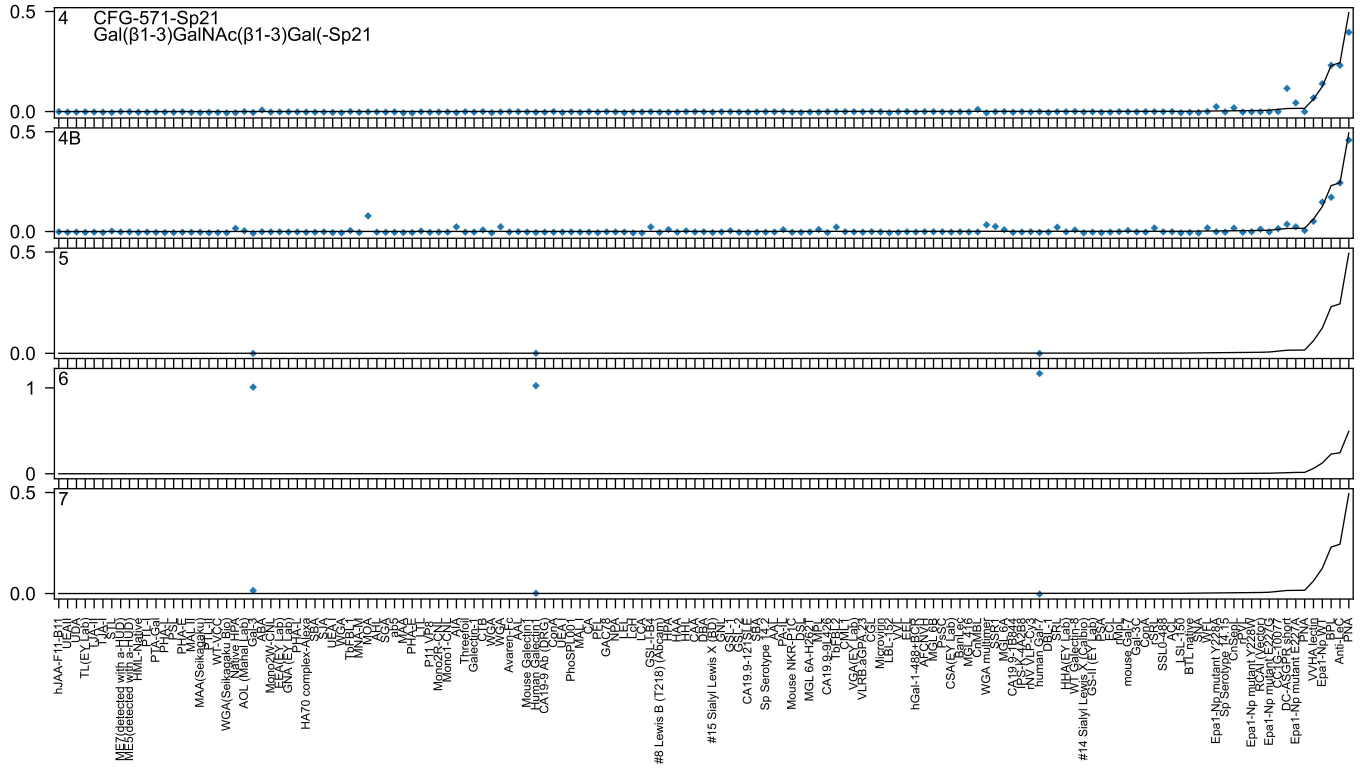

4
CFG-571-Sp21
Gal(β1-3)GalNAc(β1-3)Gal(-Sp21
4B
5
6
7

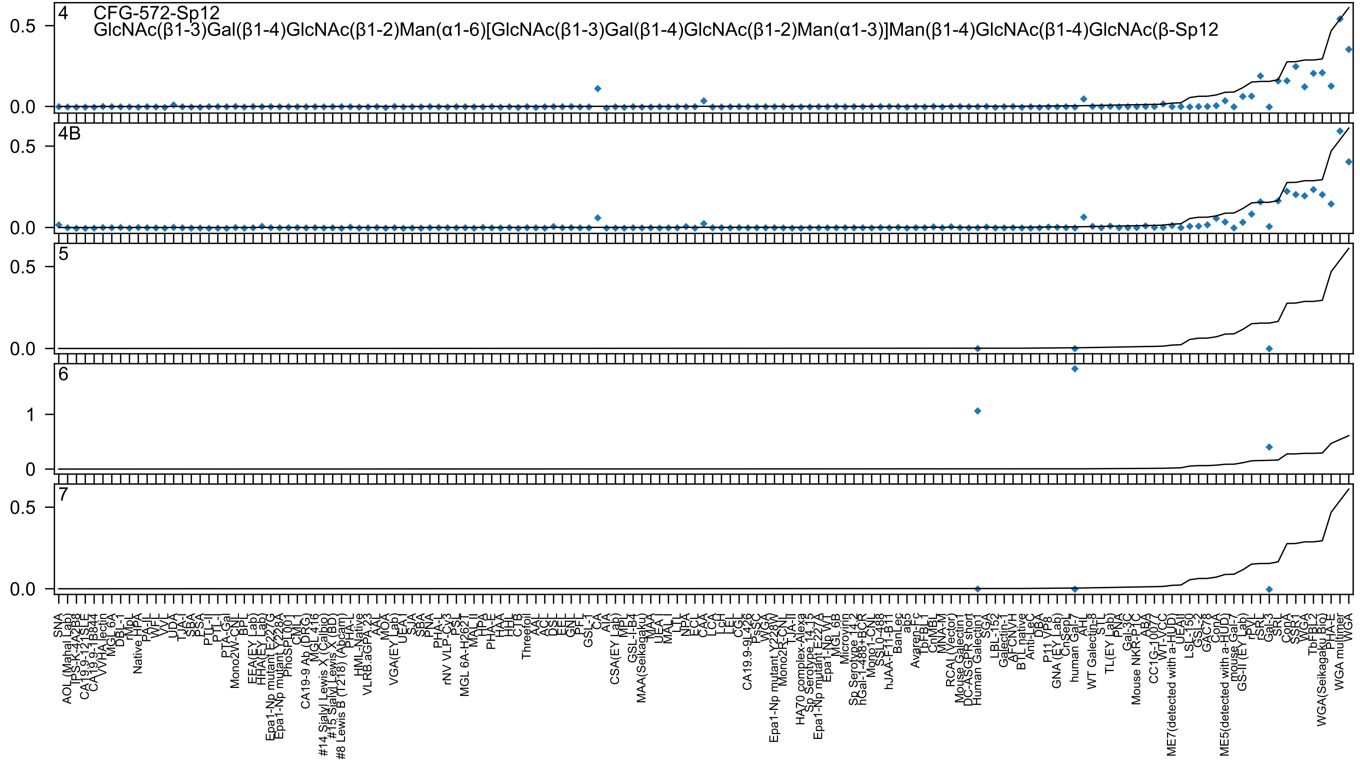
4
CFG-572-Sp12
GlcNAc(β1-3)Gal(β1-4)GlcNAc(β1-2)Man(α1-6)[GlcNAc(β1-3)Gal(β1-4)GlcNAc(β1-2)Man(α1-3)]Man(β1-4)GlcNAc(β1-4)GlcNAc(β-Sp12
4B
5
6
7

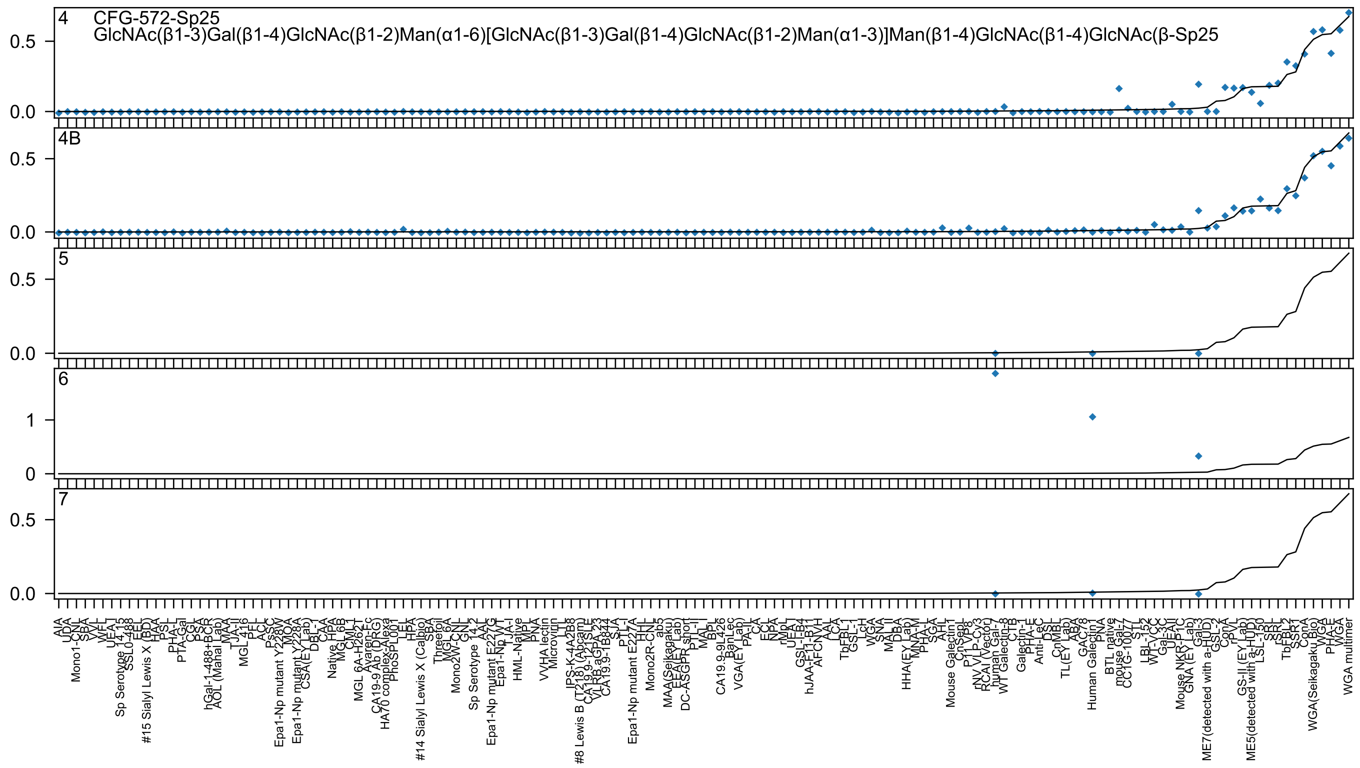
4
CFG-572-Sp25
GlcNAc(β1-3)Gal(β1-4)GlcNAc(β1-2)Man(α1-6)[GlcNAc(β1-3)Gal(β1-4)GlcNAc(β1-2)Man(α1-3)]Man(β1-4)GlcNAc(β1-4)GlcNAc(β-Sp25
4B
5
6
7
0.0
0.5
0
1

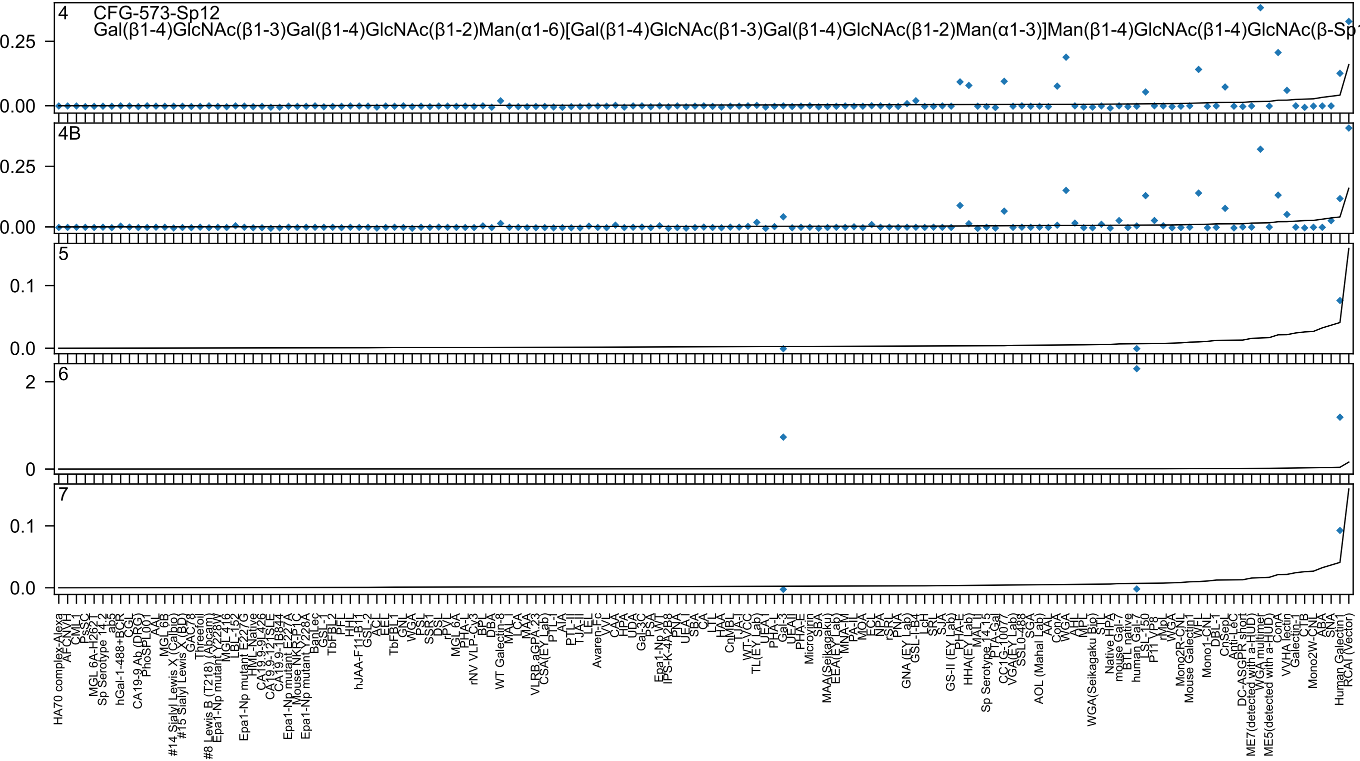

4 CFG-573-Sp12
Gal(β1-4)GlcNAc(β1-3)Gal(β1-4)GlcNAc(β1-2)Man(α1-6)[Gal(β1-4)GlcNAc(β1-3)Gal(β1-4)GlcNAc(β1-2)Man(α1-3)]Man(β1-4)GlcNAc(β1-4)GlcNAc(β-Sp
4B
5
6
7

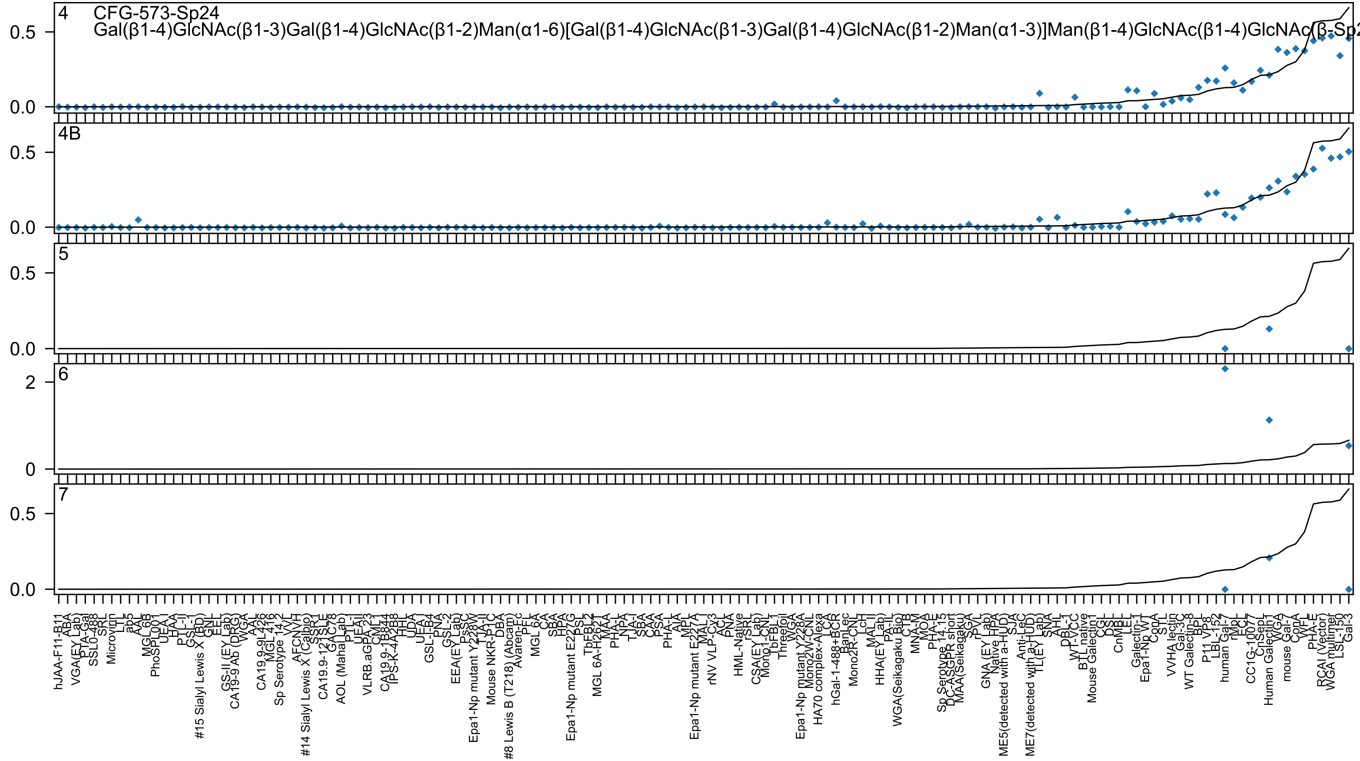

4 CFG-573-Sp24
Gal(β1-4)GlcNAc(β1-3)Gal(β1-4)GlcNAc(β1-2)Man(α1-6)[Gal(β1-4)GlcNAc(β1-3)Gal(β1-4)GlcNAc(β1-2)Man(α1-3)]Man(β1-4)GlcNAc(β1-4)GlcNAc(β-Sp2
4B
5
6
7

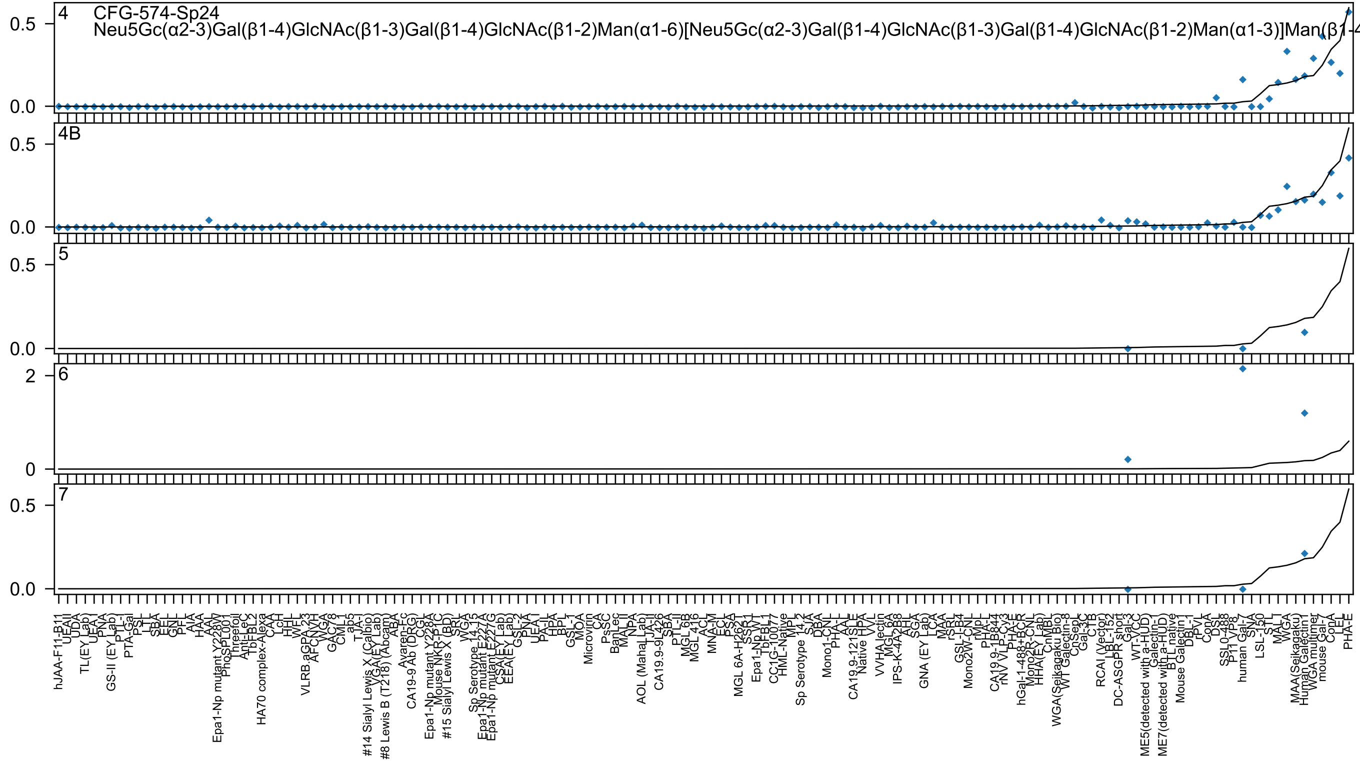

CFG-574-Sp24
Neu5Gc(α2-3)Gal(β1-4)GlcNAc(β1-3)Gal(β1-4)GlcNAc(β1-2)Man(α1-6)[Neu5Gc(α2-3)Gal(β1-4)GlcNAc(β1-3)Gal(β1-4)GlcNAc(β1-2)Man(α1-3)]Man(β1-4
4
4B
5
6
7

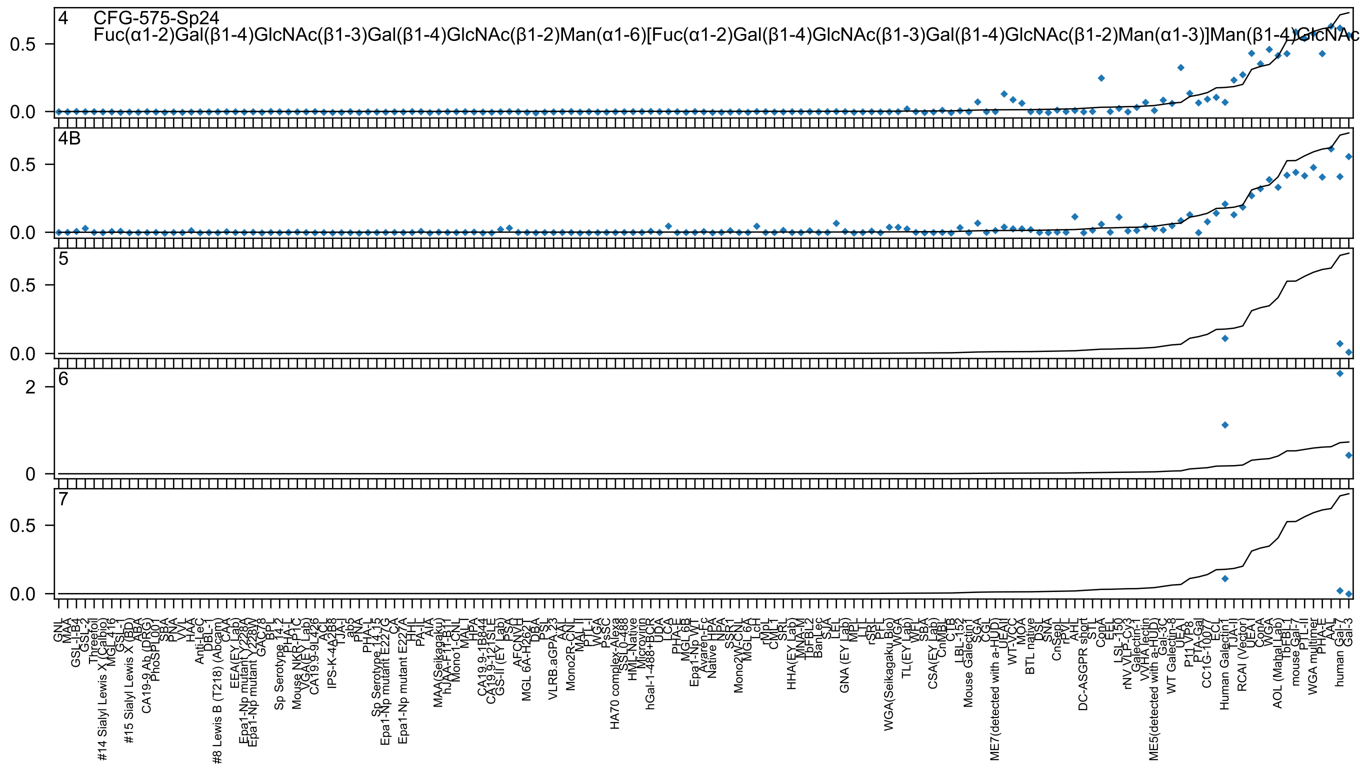
4
CFG-575-Sp24
Fuc(α1-2)Gal(β1-4)GlcNAc(β1-3)Gal(β1-4)GlcNAc(β1-2)Man(α1-6)[Fuc(α1-2)Gal(β1-4)GlcNAc(β1-3)Gal(β1-4)GlcNAc(β1-2)Man(α1-3)]Man(β1-4)GlcNAc
4B
5
6
7

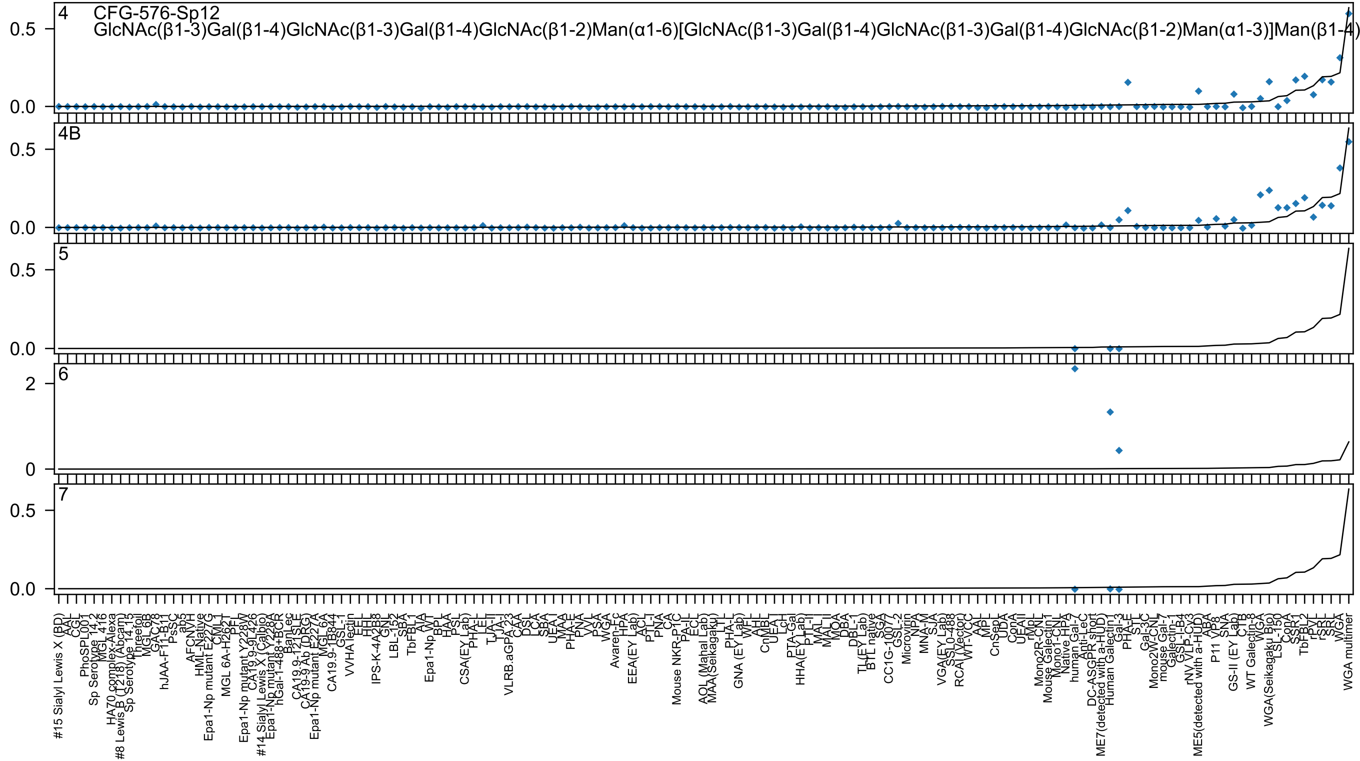
4
CFG-576-Sp12
GlcNAc(β1-3)Gal(β1-4)GlcNAc(β1-3)Gal(β1-4)GlcNAc(β1-2)Man(α1-6)[GlcNAc(β1-3)Gal(β1-4)GlcNAc(β1-3)Gal(β1-4)GlcNAc(β1-2)Man(α1-3)]Man(β1-4)
4B
5
6
7

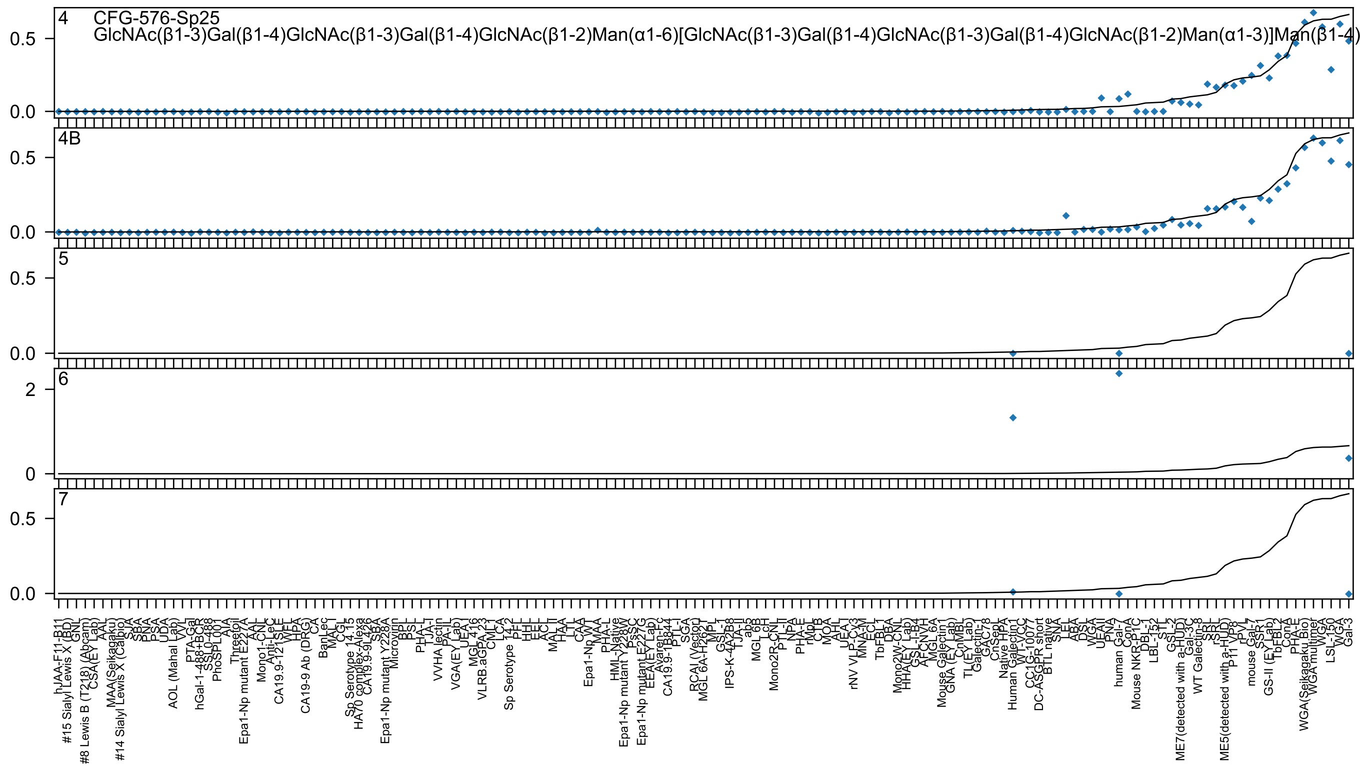

4 CFG-576-Sp25
GlcNAc(β1-3)Gal(β1-4)GlcNAc(β1-3)Gal(β1-4)GlcNAc(β1-2)Man(α1-6)[GlcNAc(β1-3)Gal(β1-4)GlcNAc(β1-3)Gal(β1-4)GlcNAc(β1-2)Man(α1-3)]Man(β1-4)
4B
5
6
7
0.0
0.5
0
2

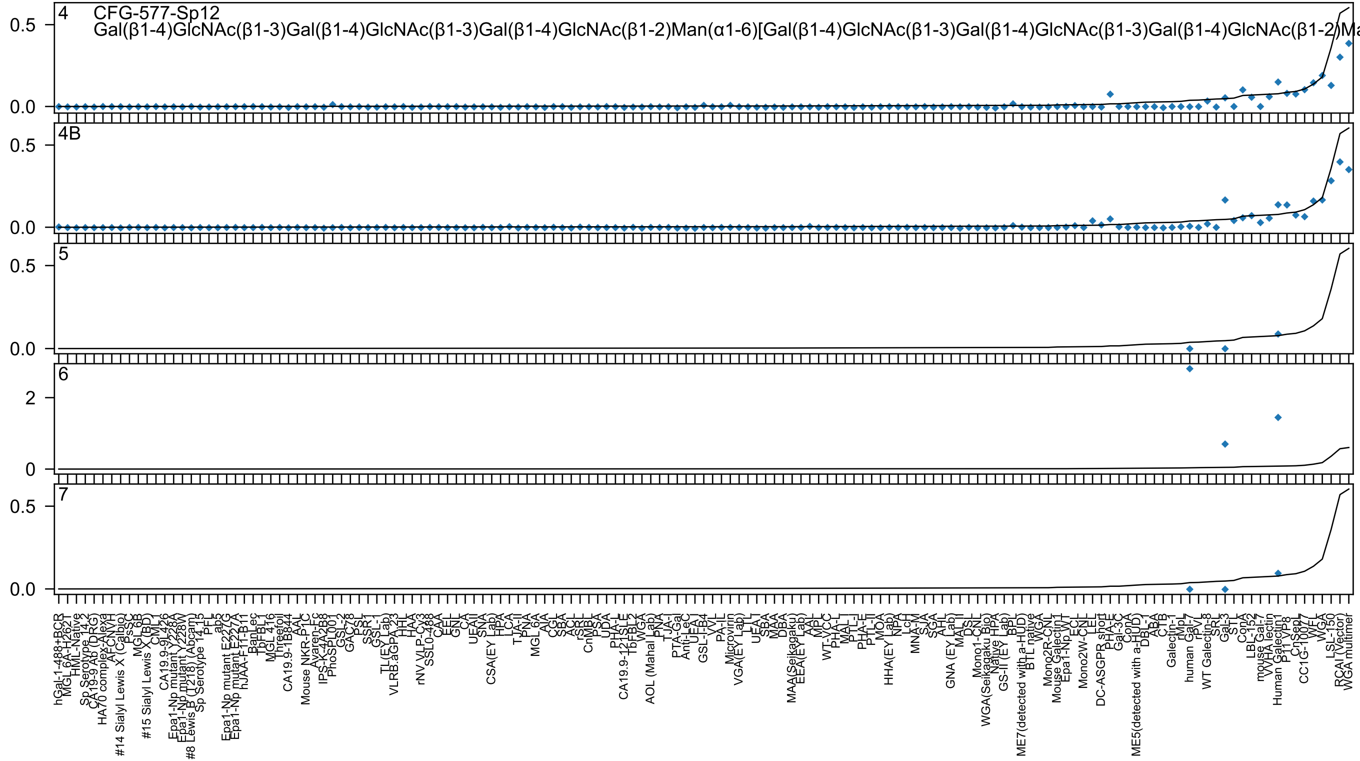
4
CFG-577-Sp12
Gal(β1-4)GlcNAc(β1-3)Gal(β1-4)GlcNAc(β1-3)Gal(β1-4)GlcNAc(β1-2)Man(α1-6)[Gal(β1-4)GlcNAc(β1-3)Gal(β1-4)GlcNAc(β1-3)Gal(β1-4)GlcNAc(β1-2)Ma
4B
5
6
7

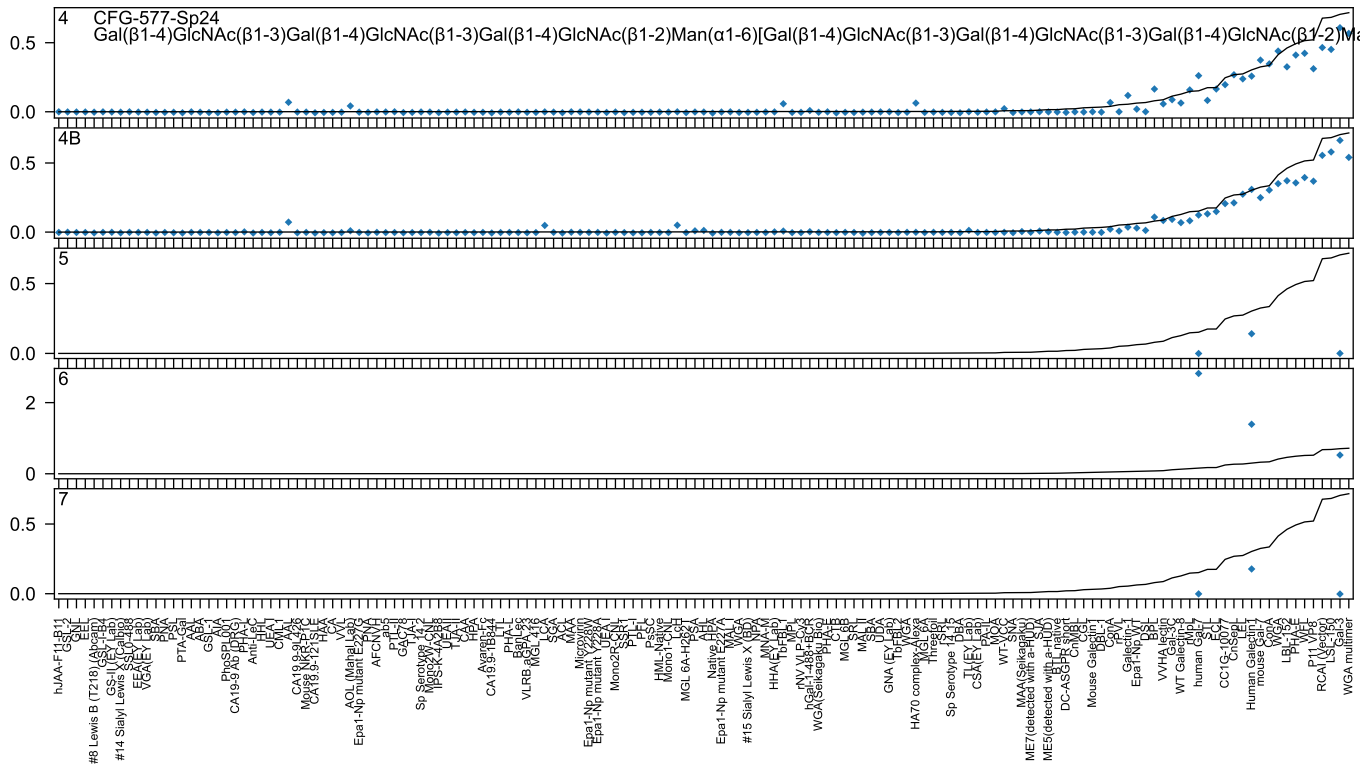
4 CFG-577-Sp24
Gal(β1-4)GlcNAc(β1-3)Gal(β1-4)GlcNAc(β1-3)Gal(β1-4)GlcNAc(β1-2)Man(α1-6)[Gal(β1-4)GlcNAc(β1-3)Gal(β1-4)GlcNAc(β1-3)Gal(β1-4)GlcNAc(β1-2)Ma
4B
5
6
7
0.0
0.5
0
2
hJAA-F11-B11
GSL-2
GNL
EEL
#8 Lewis B (T218) (Abcam)
GSL-I-B4
GS-II (EY Lab)
#14 Sialyl Lewis X (Calbio)
SSL0-488
EEA(EY Lab)
VGA(EY Lab)
SBA
PNA
PSL
PTA-Gal
AAL
ABA
GSL-1
AIA
PhoSL001
CA19-9 Ab (DRG)
PHA-L
Anti-LeC
HHL
UEA1
CML1
AAL
CA19.9-9L426
Mouse NKR-P1C
CA19.9-121SLE
HAA
CA
VVL
AOL (Manal Lab)
Epa1-Np mutant E227G
PNA
AFCNYH
ab5
PTL-II
GAC78
TJA-I
Sp Serotype 14.2
Mono2W-CNL
IPS-K-4A2B8
UEAII
TJA-II
CAA
HPA
Avaren-Fc
CA19.9-1B844
LTL
PHA-L
BanLec
VLRB.aGPA.23
MGL 416
LCA
SGA
ACL
MAA
Microvirin
Epa1-Np mutant Y228W
Epa1-Np mutant Y228A
UEA-I
Mono2R-CNL
SSR1
PTL-I
PEL
PSSC
HML-Native
Mono1-CNL
LcH
MGL 6A-H262
PSA
AHL
Native HPA
Epa1-Np mutant E227A
MAL-I
WGA
#15 Sialyl Lewis X (BD)
NPA
MNA-M
HHA(EY Lab)
TbFBL1
MPL
rNV VLP-Cy3
hGal-1-488+BCR
WGA(Seikagaku Bio)
PHA-E
CTB
MGL 6B
SRL
MAL II
SBA
UDA
GNA (EY Lab)
TbFBL2
WGA
HA70 complex-Alexa
MGL 6A
Threefoil
rSRL
Sp Serotype 14.15
DBA
TL(EY Lab)
CSA(EY Lab)
PA-IL
MOA
WT-VCC
SNA
MAA(Seikagaku)
ME7(detected with a-HUD)
SJA
ME5(detected with a-HUD)
BTL native
DC-ASGPR short
CnMBL
CGL
Mouse Galectin1
DBL-1
ConA
rPVL
Galectin-1
Epa1-Np WT
DSL
BPL
VVHA lectin
Gal-3C
WT Galectin-8
rMpL
human Gal-7
STL
ECL
CC1G-1007
CnSepL
LEL
Human Galectin1
mouse Gal-7
ConA
WGA
LBL-152
PHA-E
WFL
VP8
P11 VP8
RCAI (Vector)
LSL-150
Gal-3
WGA multimer

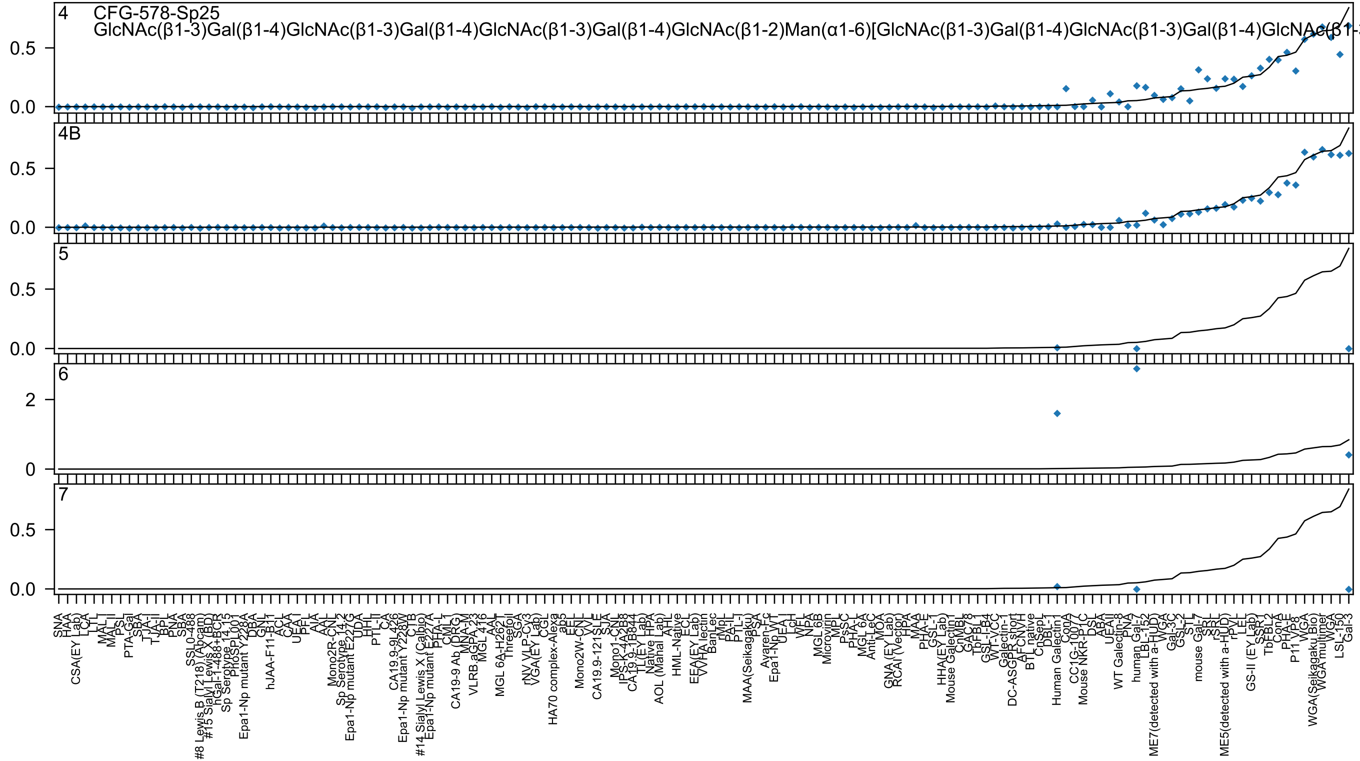
4 CFG-578-Sp25
GlcNAc(β1-3)Gal(β1-4)GlcNAc(β1-3)Gal(β1-4)GlcNAc(β1-3)Gal(β1-4)GlcNAc(β1-2)Man(α1-6)[GlcNAc(β1-3)Gal(β1-4)GlcNAc(β1-3)Gal(β1-4)GlcNAc(β1-
4B
5
6
7

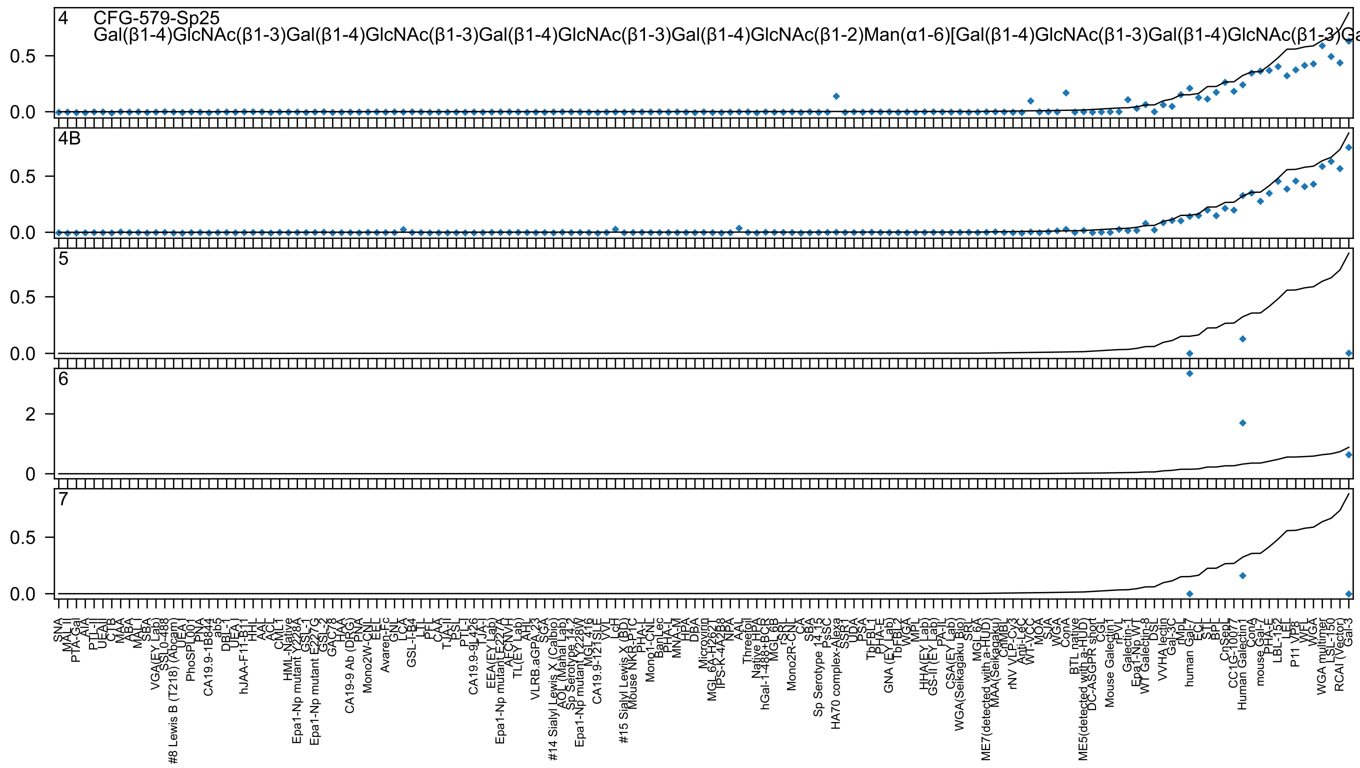
4
CFG-579-Sp25
Gal(β1-4)GlcNAc(β1-3)Gal(β1-4)GlcNAc(β1-3)Gal(β1-4)GlcNAc(β1-3)Gal(β1-4)GlcNAc(β1-2)Man(α1-6)[Gal(β1-4)GlcNAc(β1-3)Gal(β1-4)GlcNAc(β1-3)Ga
0.5
0.0
4B
0.5
0.0
5
0.5
0.0
6
2
0
7
0.5
0.0
SNA
MAL_II
PTA-Gal
AIA
PTL-II
UEA-II
CTB
MAA
ABA
MAL-I
SBA
VGA(EY Lab)
SSL0-488
#8 Lewis B (T218) (Abcam)
UEA-I
PhoSL001
PNA
CA19.9-1B844
ab5
DBL-I
UEA-I
hJAA-F11-B1
HHL
AAL
ACL
CML1
HML-Native
Epa1-Np mutant Y228A
GSL-1
Epa1-Np mutant E227G
GSL-2
GAC78
HAA
CA19-9 Ab (DRG)
PNA
Mono2W-CNL
EEL
Avaren-Fc
GNL
LCA
GSL-I-B4
LTL
PTL
CAA
TJA-II
PSL
PTL-I
CA19.9-9L426
TJA-I
EEA(EY Lab)
Epa1-Np mutant E227A
AFCNVH
TL(EY Lab)
AHL
VLRB.aGPA.23
SGA
#14 Sialyl Lewis X (Calbio)
AOL (Mahal Lab)
Sp Serotype 14_2
Epa1-Np mutant Y228W
MGL 416
CA19.9-121SLE
VVL
LcH
#15 Sialyl Lewis X (BD)
Mouse NKR-P1C
PHA-L
Mono1-CNL
BanLec
PHA-L
MNA-M
HPA
DBA
Microvirin
MGL 6A-H262T
IPS-K-4A2B8
NPA
AAL
Threefoil
Native HPA
hGal-1-488+BCR
MGL 6B
rSRL
Mono2R-CNL
CA
SBA
Sp Serotype 14_15
PsSC
HA70 complex-Alexa
SSR1
UDA
PSA
TbFBL1
PHA-E
GNA (EY Lab)
TbFBL2
WGA
MPL
HHA(EY Lab)
GS-II (EY Lab)
PA-IL
CSA(EY Lab)
WGA(Seikagaku Bio)
SRL
MGL 6A
ME7(detected with a-HUD)
MAA(Seikagaku)
CnMBL
rNV VLP-Cy3
Anti-LeC
WT-VCC
MOA
SJA
WGA
ConA
BTL native
ME5(detected with a-HUD)
DC-ASGPR short
CGL
Mouse Galectin1
rPVL
Galectin-1
Epa1-Np WT
WT Galectin-8
DSL
VVHA lectin
Gal-3C
rMpL
human Gal-7
ECL
STL
BPL
CnSep
CC1G-10077
Human Galectin1
ConA
mouse Gal-7
PHA-E
LBL-152
LEL
P11 VP8
WFL
WGA
WGA multimer
LSL-150
RCAI (Vector)
Gal-3

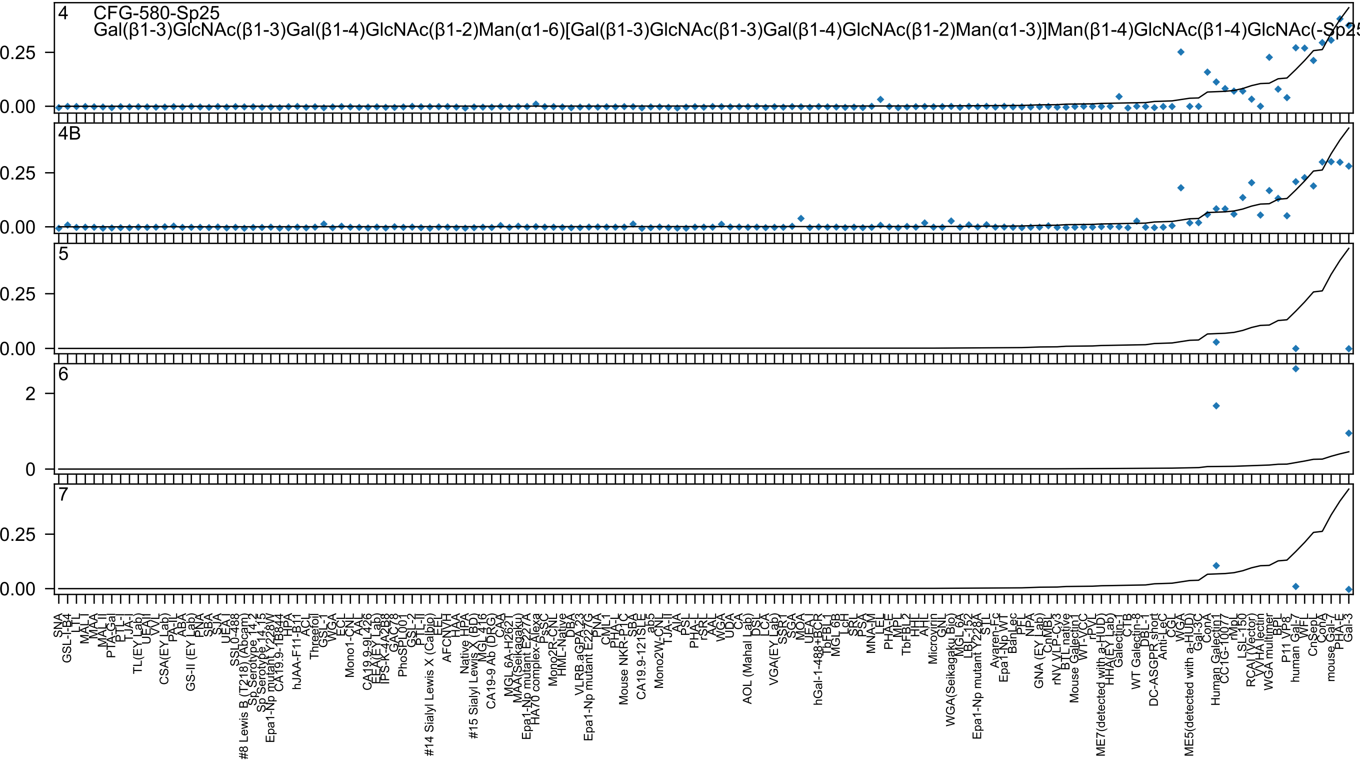

4 CFG-580-Sp25
Gal(β1-3)GlcNAc(β1-3)Gal(β1-4)GlcNAc(β1-2)Man(α1-6)[Gal(β1-3)GlcNAc(β1-3)Gal(β1-4)GlcNAc(β1-2)Man(α1-3)]Man(β1-4)GlcNAc(β1-4)GlcNAc(-Sp25
4B
5
6
7
0.25
0.00
2
0

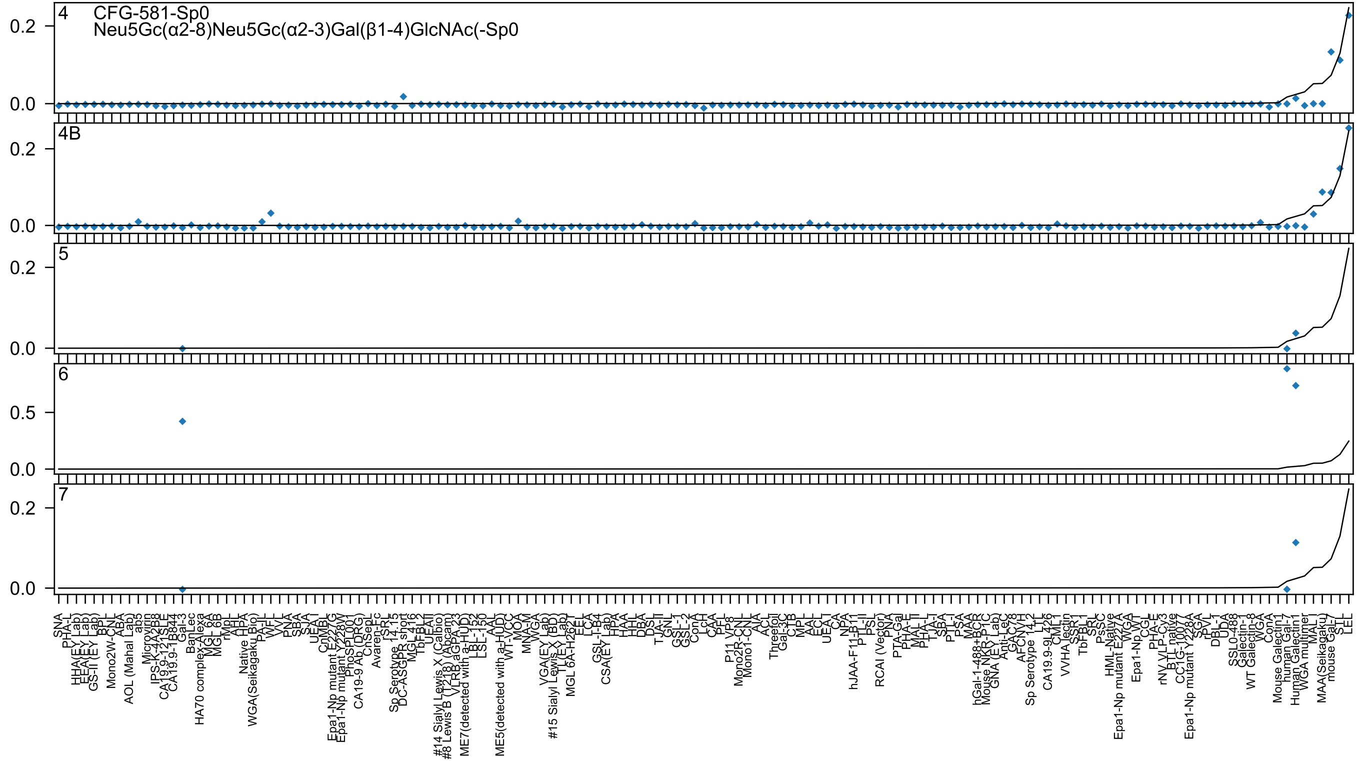
4 CFG-581-Sp0
Neu5Gc(α2-8)Neu5Gc(α2-3)Gal(β1-4)GlcNAc(-Sp0
4B
5
6
7

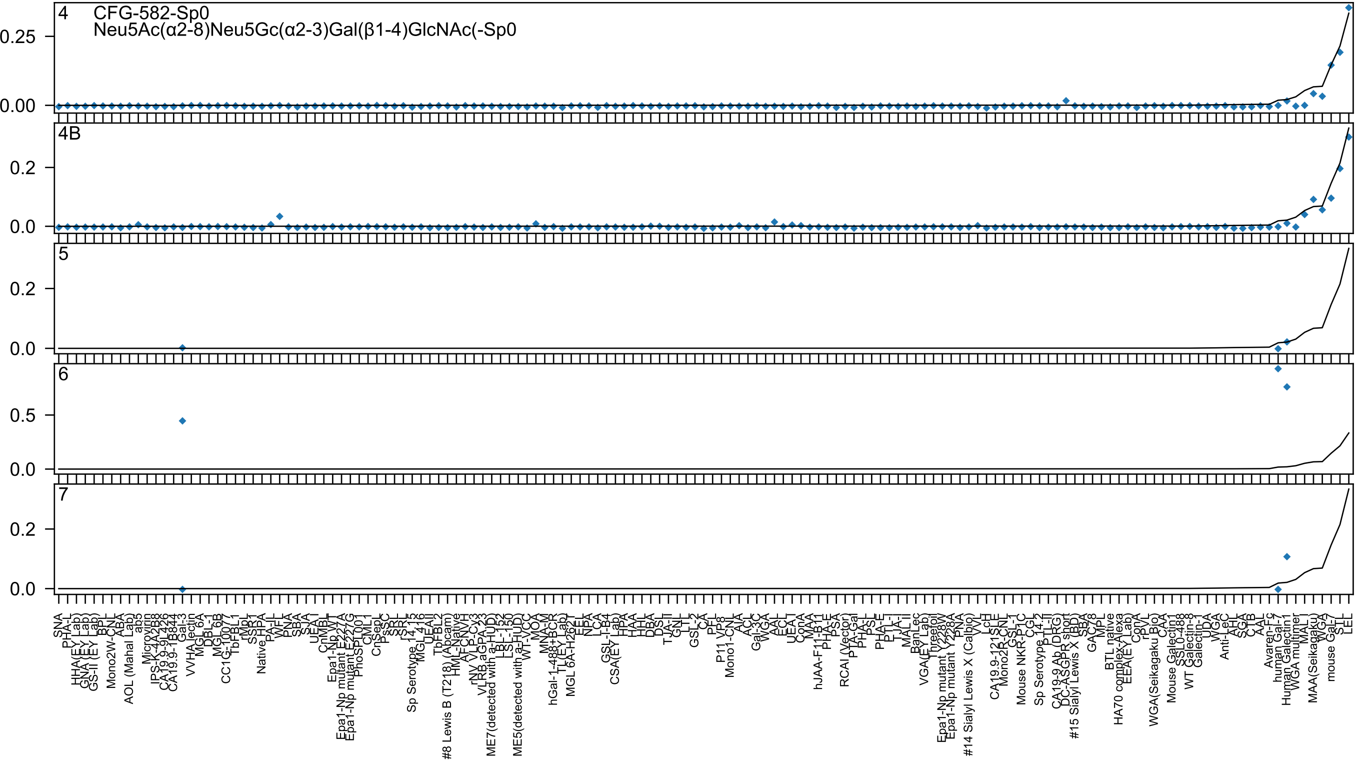

4
CFG-582-Sp0
Neu5Ac(α2-8)Neu5Gc(α2-3)Gal(β1-4)GlcNAc(-Sp0
4B
5
6
7

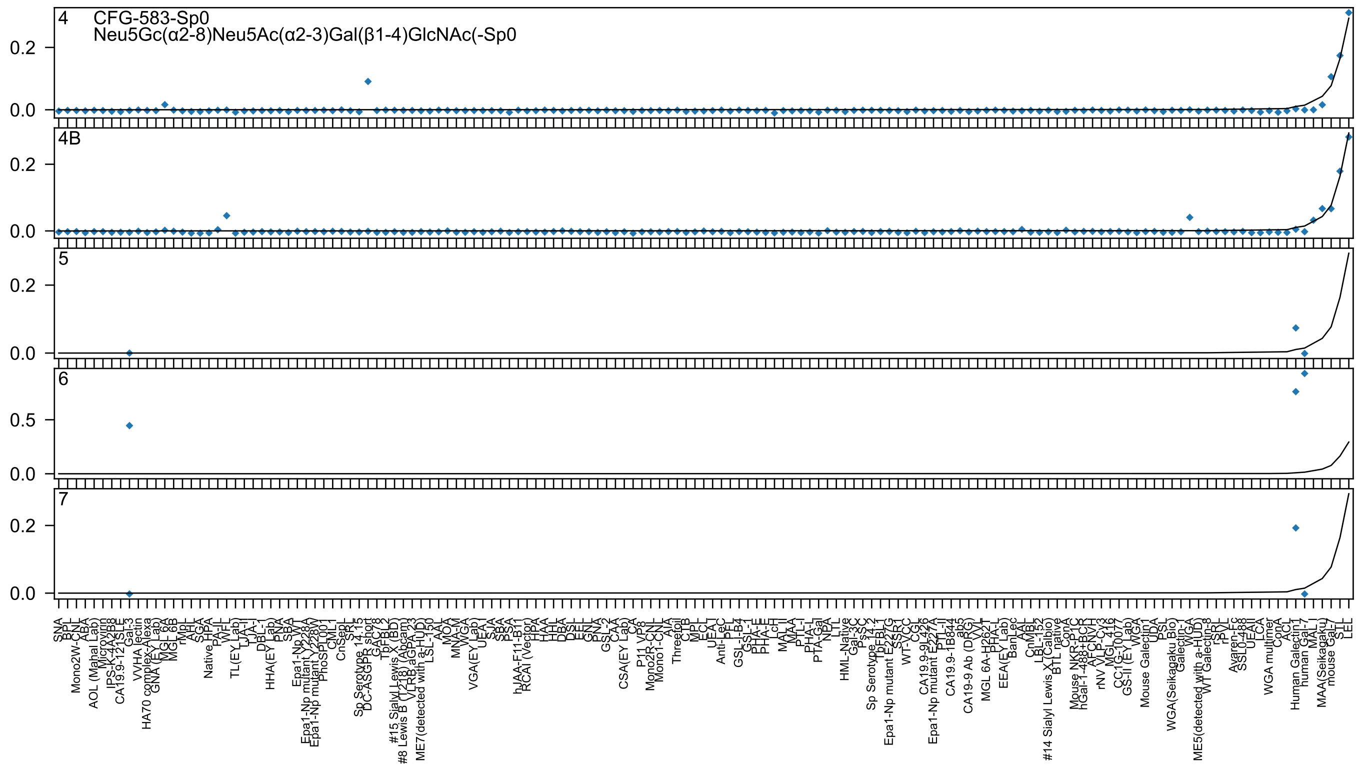

4
CFG-583-Sp0
Neu5Gc(α2-8)Neu5Ac(α2-3)Gal(β1-4)GlcNAc(-Sp0
4B
5
6
7
0.0
0.2
0.5

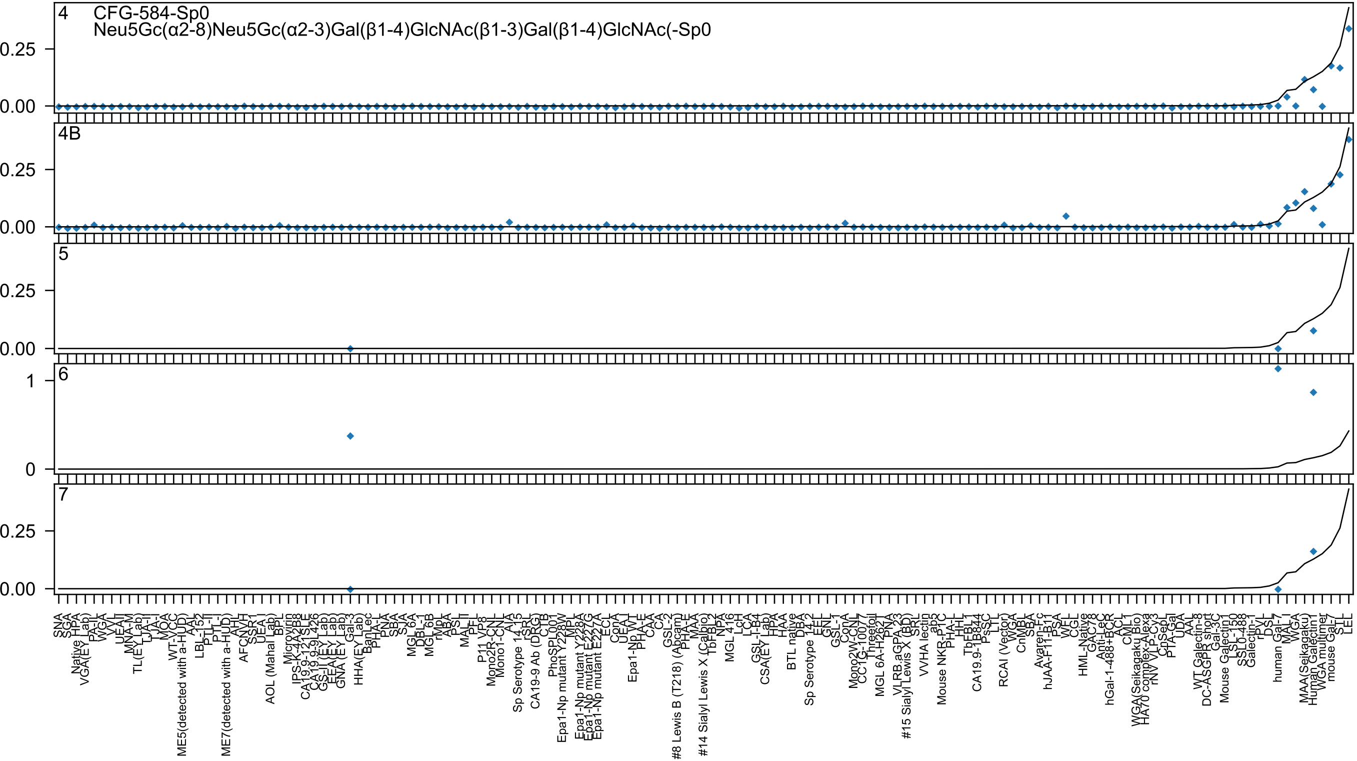
4
CFG-584-Sp0
Neu5Gc(α2-8)Neu5Gc(α2-3)Gal(β1-4)GlcNAc(β1-3)Gal(β1-4)GlcNAc(-Sp0
4B
5
6
7
0.25
0.00
1
0

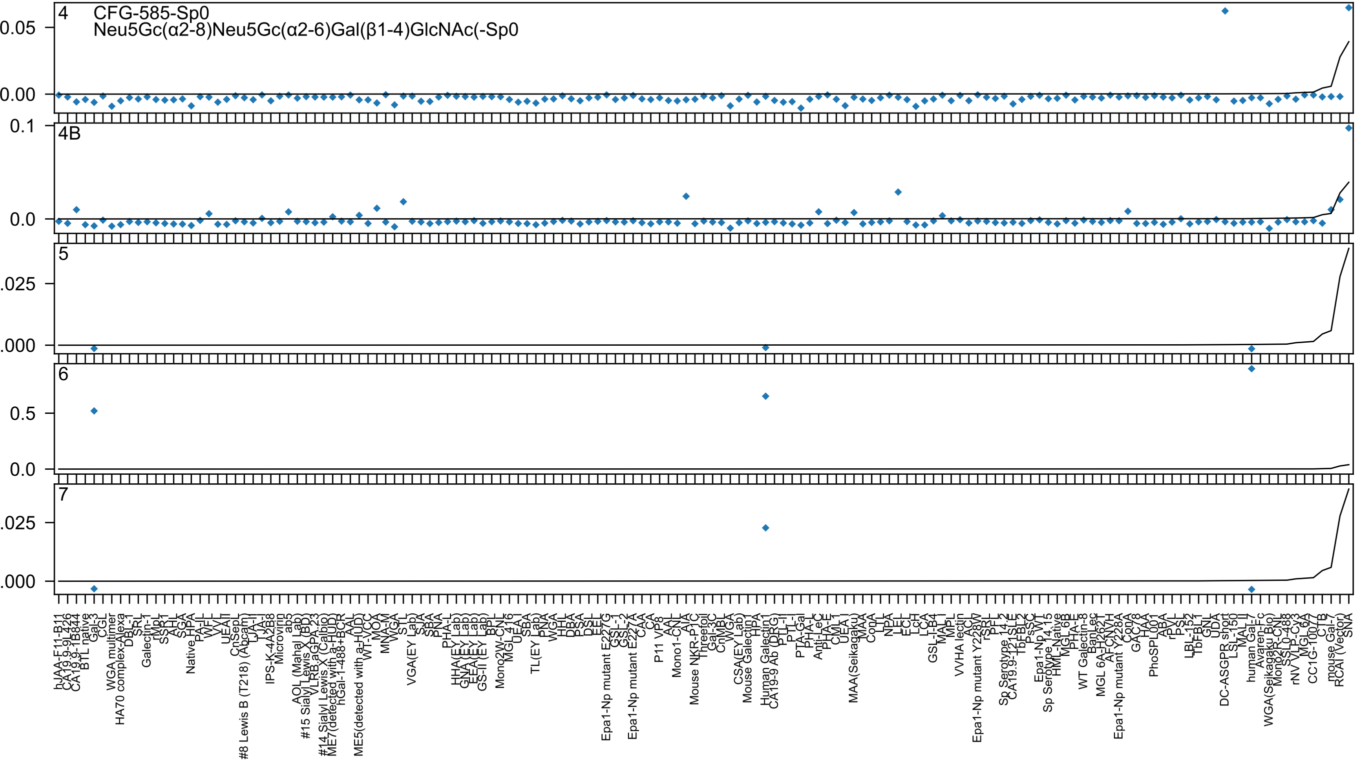

4 CFG-585-Sp0
Neu5Gc(α2-8)Neu5Gc(α2-6)Gal(β1-4)GlcNAc(-Sp0
4B
5
6
7

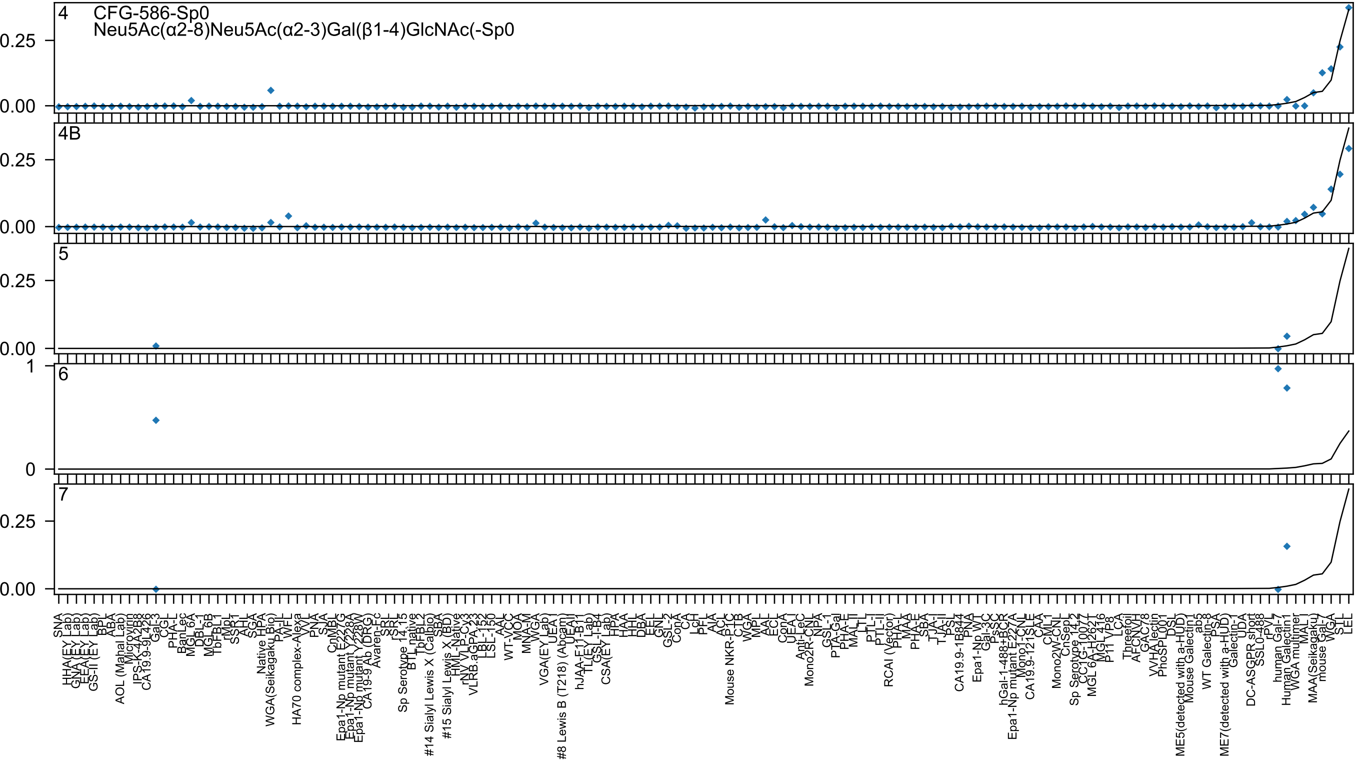

4 CFG-586-Sp0
Neu5Ac(α2-8)Neu5Ac(α2-3)Gal(β1-4)GlcNAc(-Sp0
4B
5
6
7

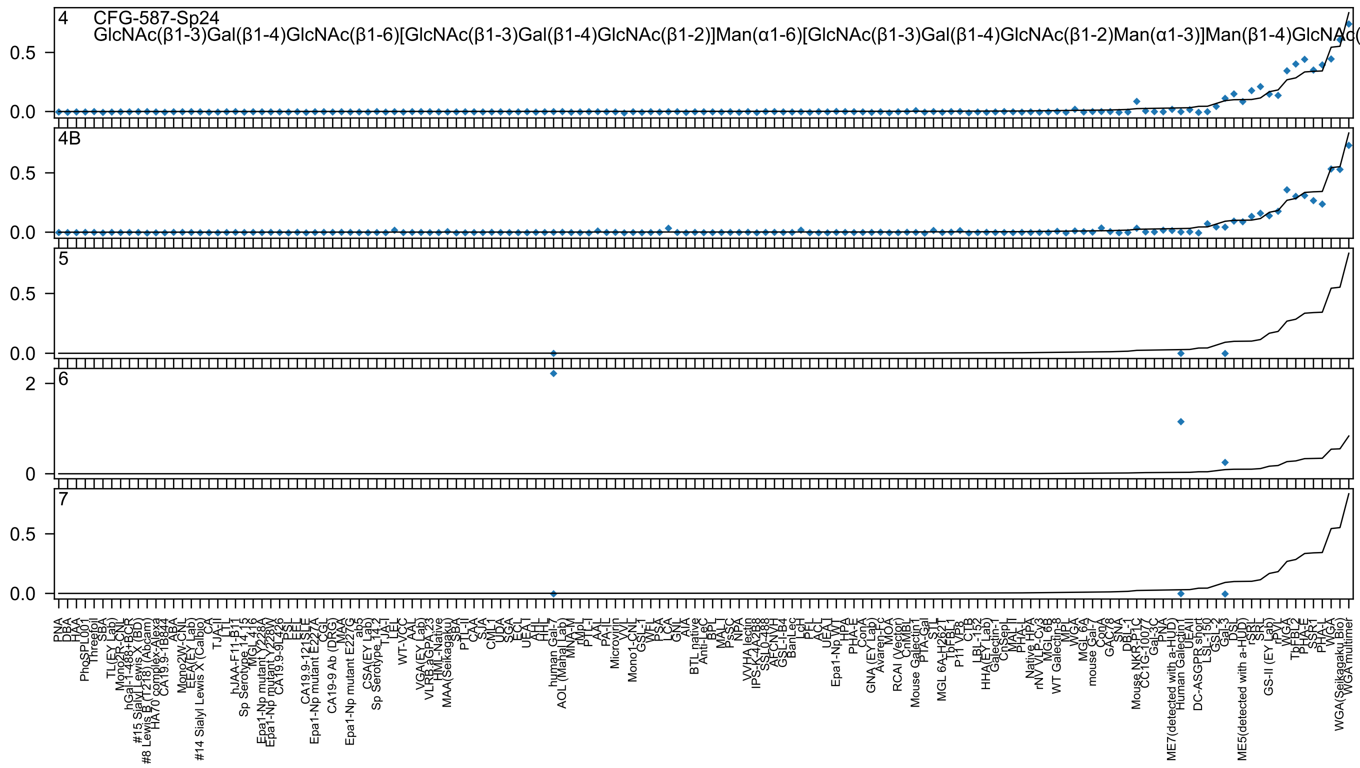
4 CFG-587-Sp24
GlcNAc(β1-3)Gal(β1-4)GlcNAc(β1-6)[GlcNAc(β1-3)Gal(β1-4)GlcNAc(β1-2)]Man(α1-6)[GlcNAc(β1-3)Gal(β1-4)GlcNAc(β1-2)Man(α1-3)]Man(β1-4)GlcNAc(
4B
5
6
7

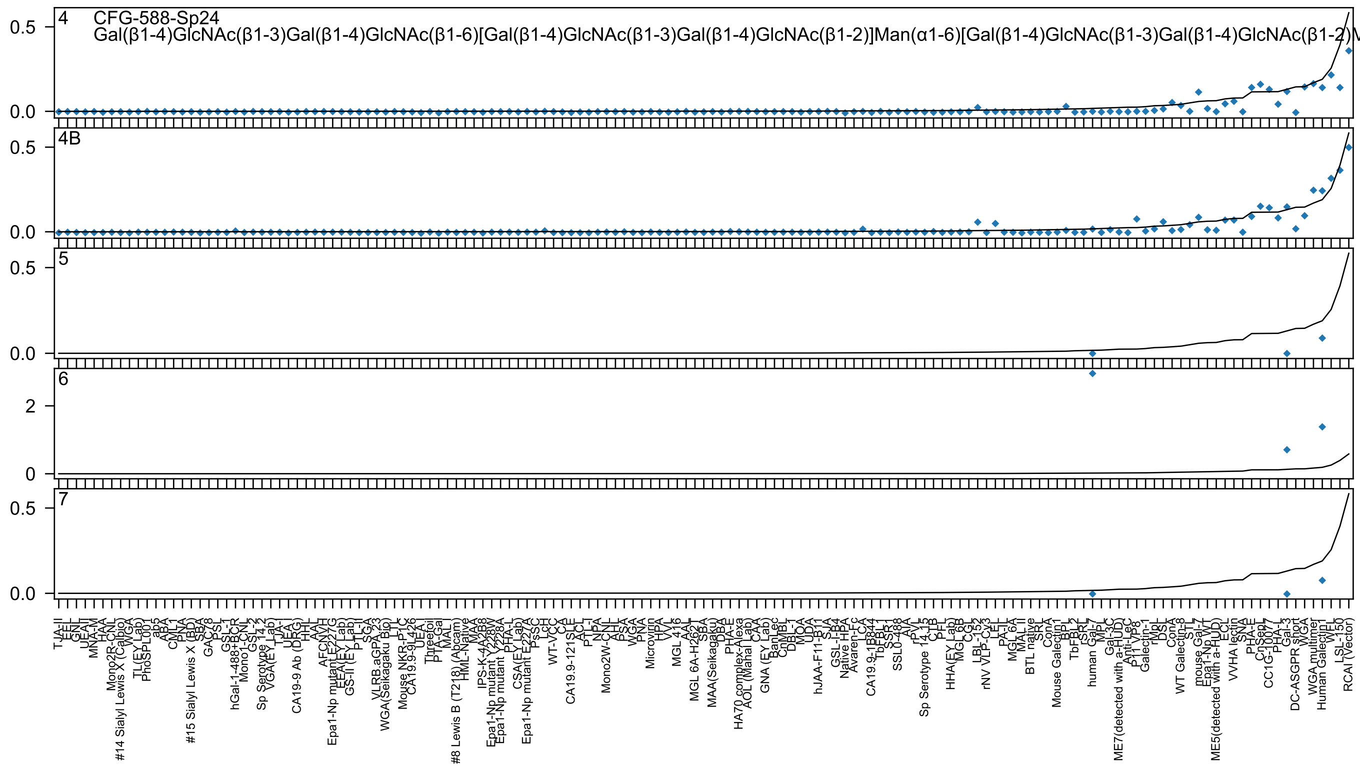
4
CFG-588-Sp24
Gal(β1-4)GlcNAc(β1-3)Gal(β1-4)GlcNAc(β1-6)[Gal(β1-4)GlcNAc(β1-3)Gal(β1-4)GlcNAc(β1-2)]Man(α1-6)[Gal(β1-4)GlcNAc(β1-3)Gal(β1-4)GlcNAc(β1-2)M
4B
5
6
7
0.0
0.5
0
2
TJA-II
EEL
GNL
UEA-I
MNA-M
HAA
Mono2R-CNL
#14 Sialyl Lewis X (Calbio)
WGA
TL(EY Lab)
PhoSL001
ab5
ABA
CML-1
PNA
#15 Sialyl Lewis X (BD)
SBA
GAC78
PSL
GSL-1
hGal-1-488+BCR
Mono1-CNL
GSL-2
Sp Serotype 14.2
VGA(EY Lab)
TJA-I
UEA-I
CA19-9 Ab (DRG)
HHL
AAL
AFCNVH
Epa1-Np mutant E227G
EEA(EY Lab)
GS-II (EY Lab)
PTL-II
SGA
VLRB.aGPA.23
WGA(Seikagaku Bio)
LTL
Mouse NKR-P1C
CA19.9-9L426
UEA-I
Threefoil
PTA-Gal
MAL-I
#8 Lewis B (T218) (Abcam)
HML-Native
MAA
IPS-K-4A2B8
Epa1-Np mutant Y228W
Epa1-Np mutant Y228A
PHA-L
CSA(EY Lab)
Epa1-Np mutant E227A
PsSC
LcH
WT-VCC
CA
CA19.9-121SLE
ACL
PTL-I
NPA
Mono2W-CNL
AHL
PSA
WGA
PNA
Microvirin
HPA
VVL
MGL 416
AAL
MGL 6A-H262T
SBA
MAA(Seikagaku)
DBA
PHA-E
HA70 complex-Alexa
AOL (Manal Lab)
CAA
GNA (EY Lab)
BanLec
CnMBL
DBL-1
MOA
UDA
hJAA-F11-B11
SJA
GSL-I-B4
Native HPA
Avaren-Fc
LCA
CA19.9-1B844
TbFBL1
SSR1
SSL0-488
AIA
rPVL
Sp Serotype 14.15
CTB
PFL
HHA(EY Lab)
MGL 6B
CGL
LBL-152
rNV VLP-Cy3
LEL
PA-IL
MGL 6A
MAL II
BTL native
SRL
ConA
Mouse Galectin1
BPL
TbFBL2
rSRL
human Gal-7
MPL
Gal-3C
ME7(detected with a-HUD)
Anti-LeC
P11 VP8
Galectin-1
rMpL
DSL
ConA
WT Galectin-8
STL
mouse Gal-7
Epa1-Np WT
ME5(detected with a-HUD)
ECL
VVHA lectin
SNA
PHA-E
CnSep1
CC1G-1007
PHA-L
Gal-3
DC-ASGPR short
WGA
WGA multimer
Human Galectin1
WFL
LSL-150
RCAI (Vector)

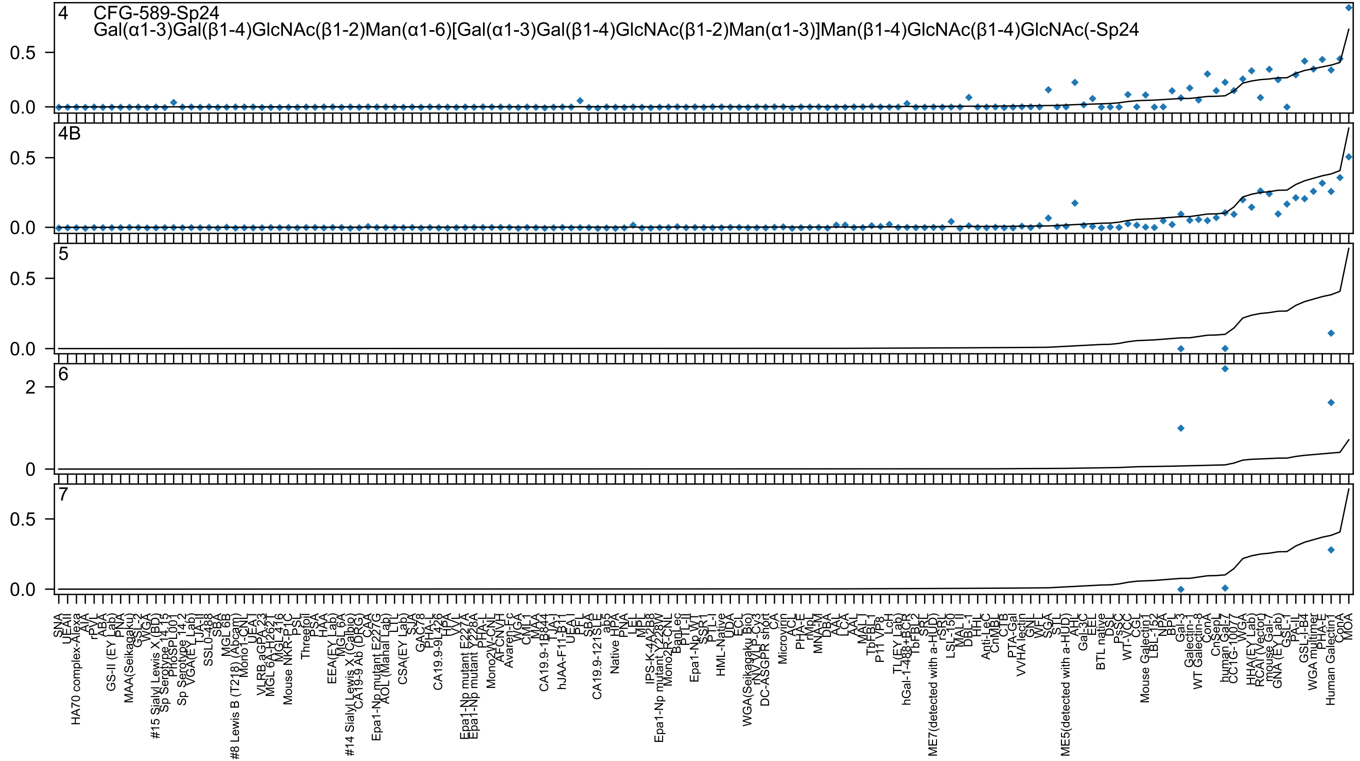
4
CFG-589-Sp24
Gal(α1-3)Gal(β1-4)GlcNAc(β1-2)Man(α1-6)[Gal(α1-3)Gal(β1-4)GlcNAc(β1-2)Man(α1-3)]Man(β1-4)GlcNAc(β1-4)GlcNAc(-Sp24
0.5
0.0
4B
0.5
0.0
5
0.5
0.0
6
2
0
7
0.5
0.0

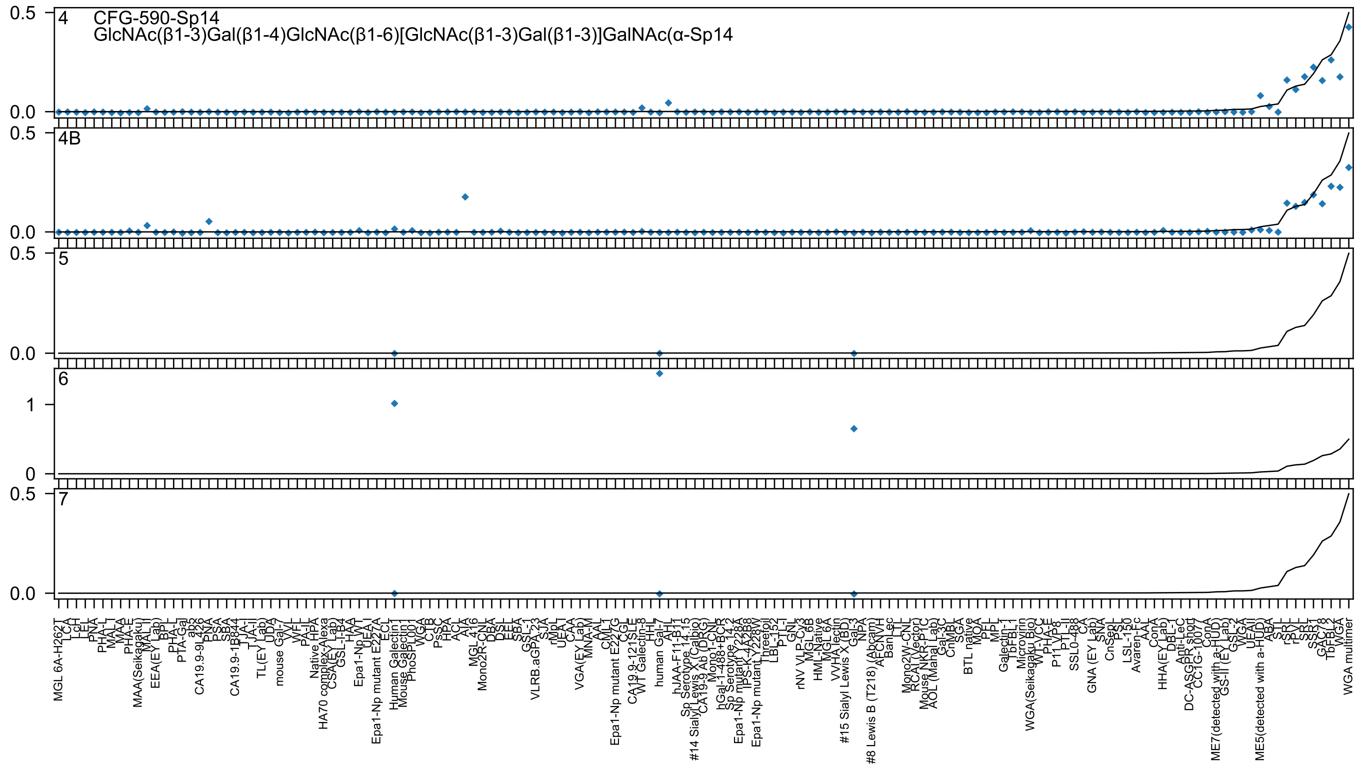
CFG-590-Sp14
GlcNAc(β1-3)Gal(β1-4)GlcNAc(β1-6)[GlcNAc(β1-3)Gal(β1-3)]GalNAc(α-Sp14
4
4B
5
6
7
0.5
0.0
1
0

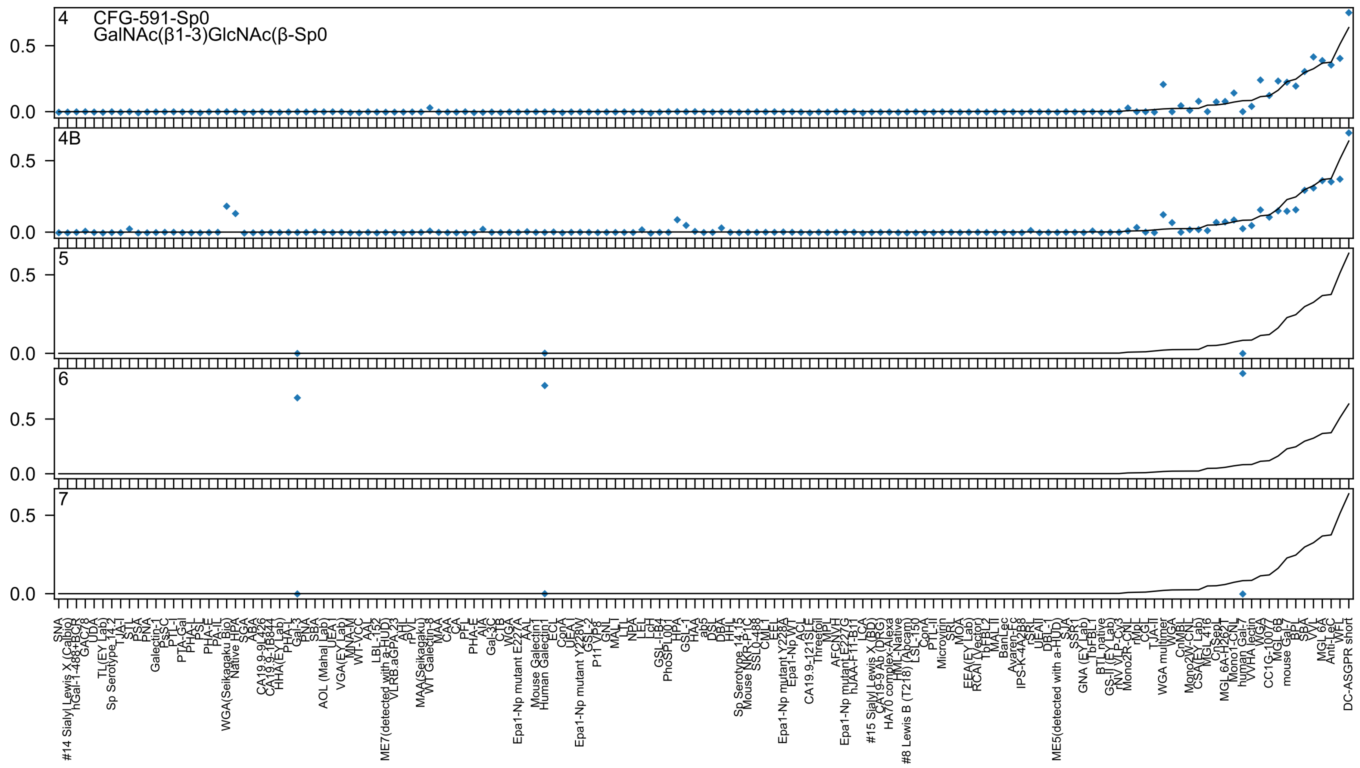
4
CFG-591-Sp0
GalNAc(β1-3)GlcNAc(β-Sp0
4B
5
6
7
0.0
0.5

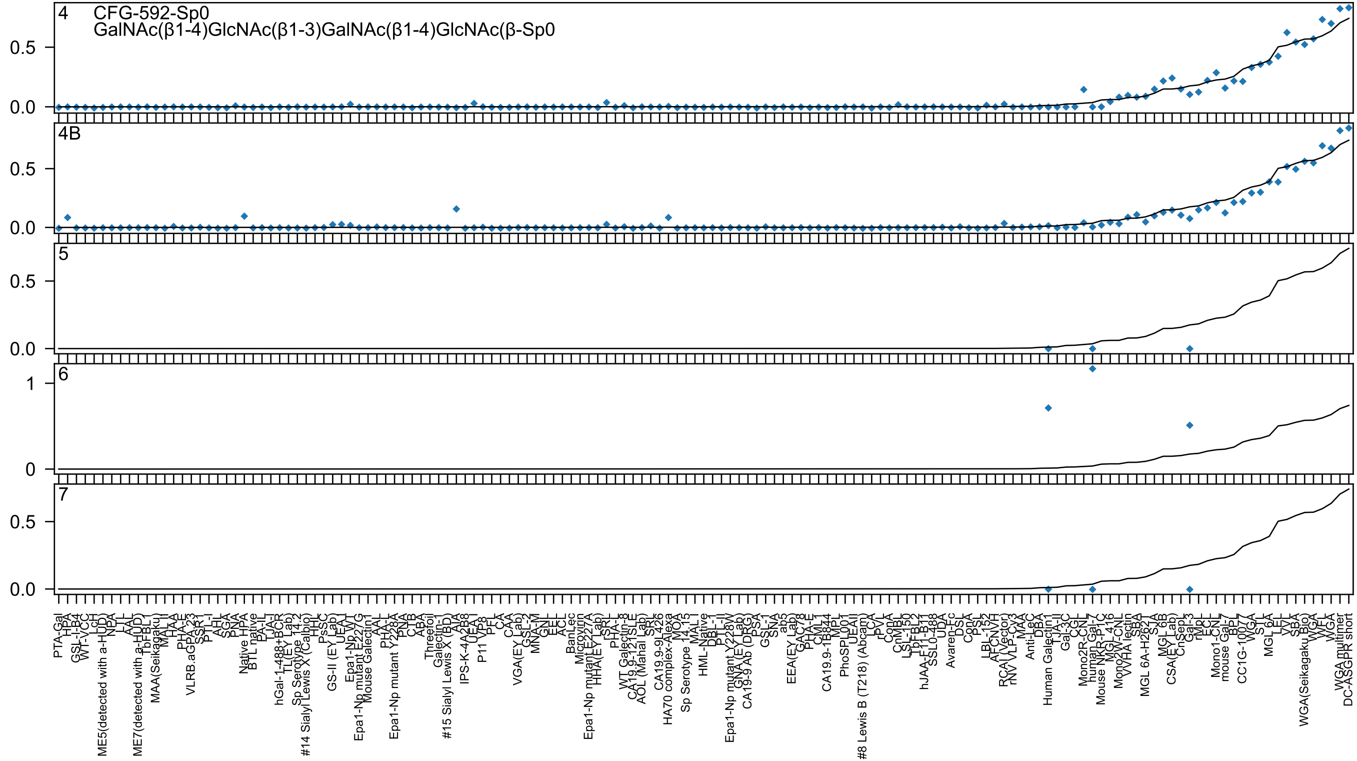
4
CFG-592-Sp0
GalNAc(β1-4)GlcNAc(β1-3)GalNAc(β1-4)GlcNAc(β-Sp0
4B
5
6
7
0.0
0.5
1
0

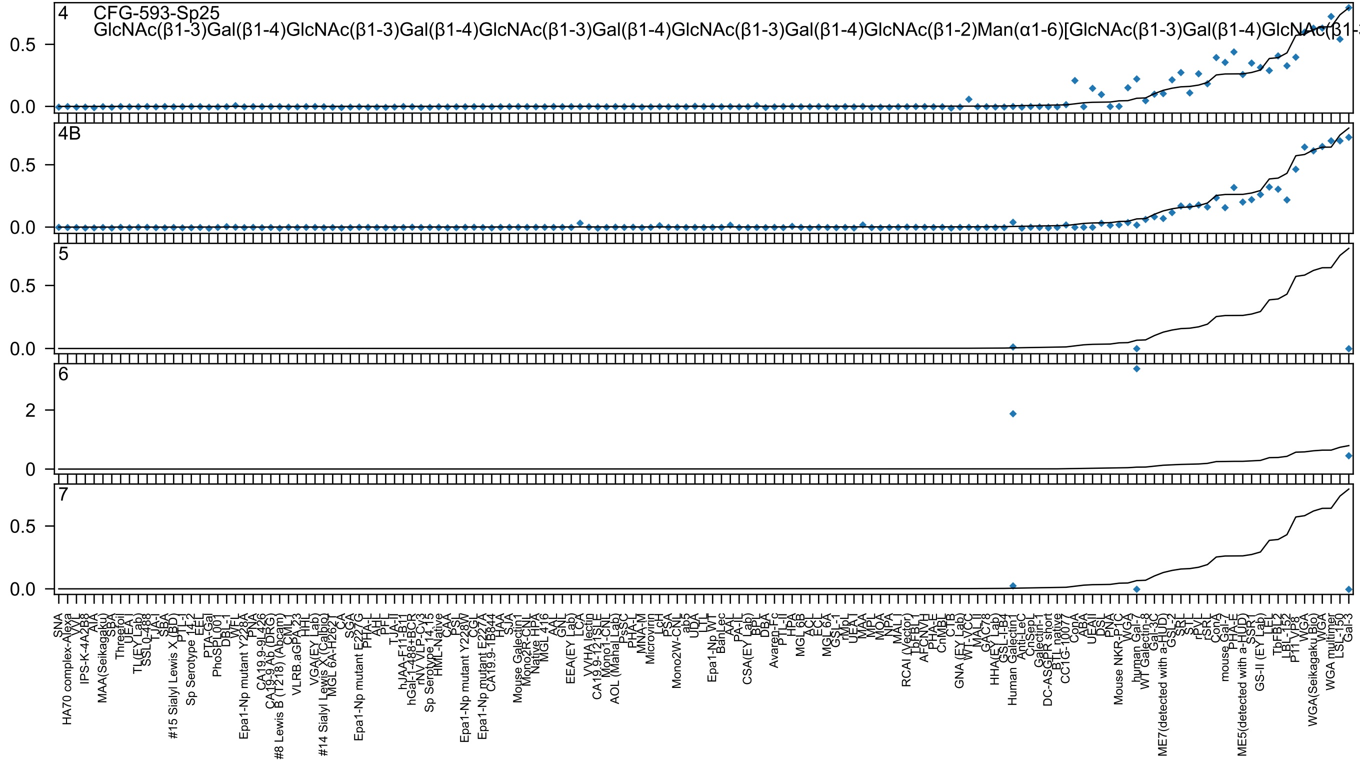
4
CFG-593-Sp25
GlcNAc(β1-3)Gal(β1-4)GlcNAc(β1-3)Gal(β1-4)GlcNAc(β1-3)Gal(β1-4)GlcNAc(β1-3)Gal(β1-4)GlcNAc(β1-2)Man(α1-6)[GlcNAc(β1-3)Gal(β1-4)GlcNAc(β1-
4B
5
6
7

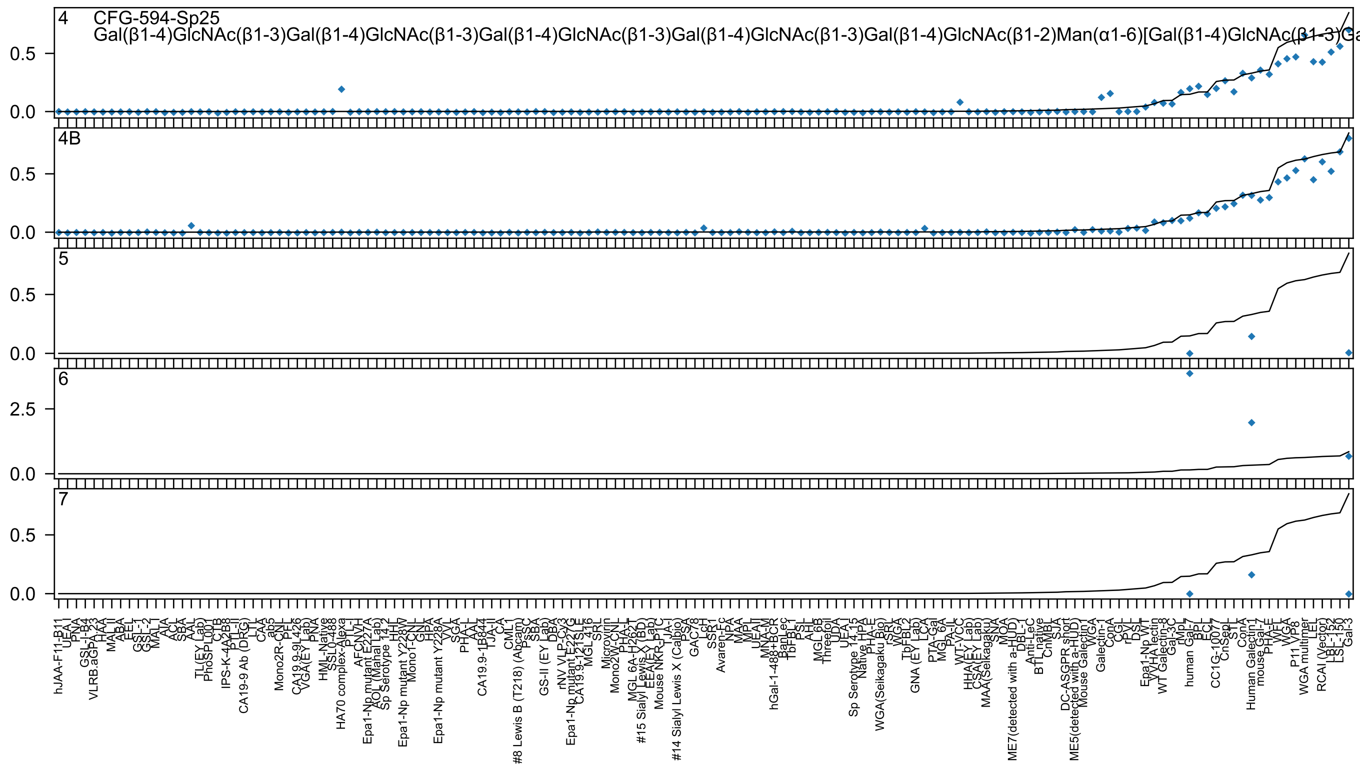
4
CFG-594-Sp25
Gal(β1-4)GlcNAc(β1-3)Gal(β1-4)GlcNAc(β1-3)Gal(β1-4)GlcNAc(β1-3)Gal(β1-4)GlcNAc(β1-3)Gal(β1-4)GlcNAc(β1-2)Man(α1-6)[Gal(β1-4)GlcNAc(β1-3)Ga
4B
5
6
7

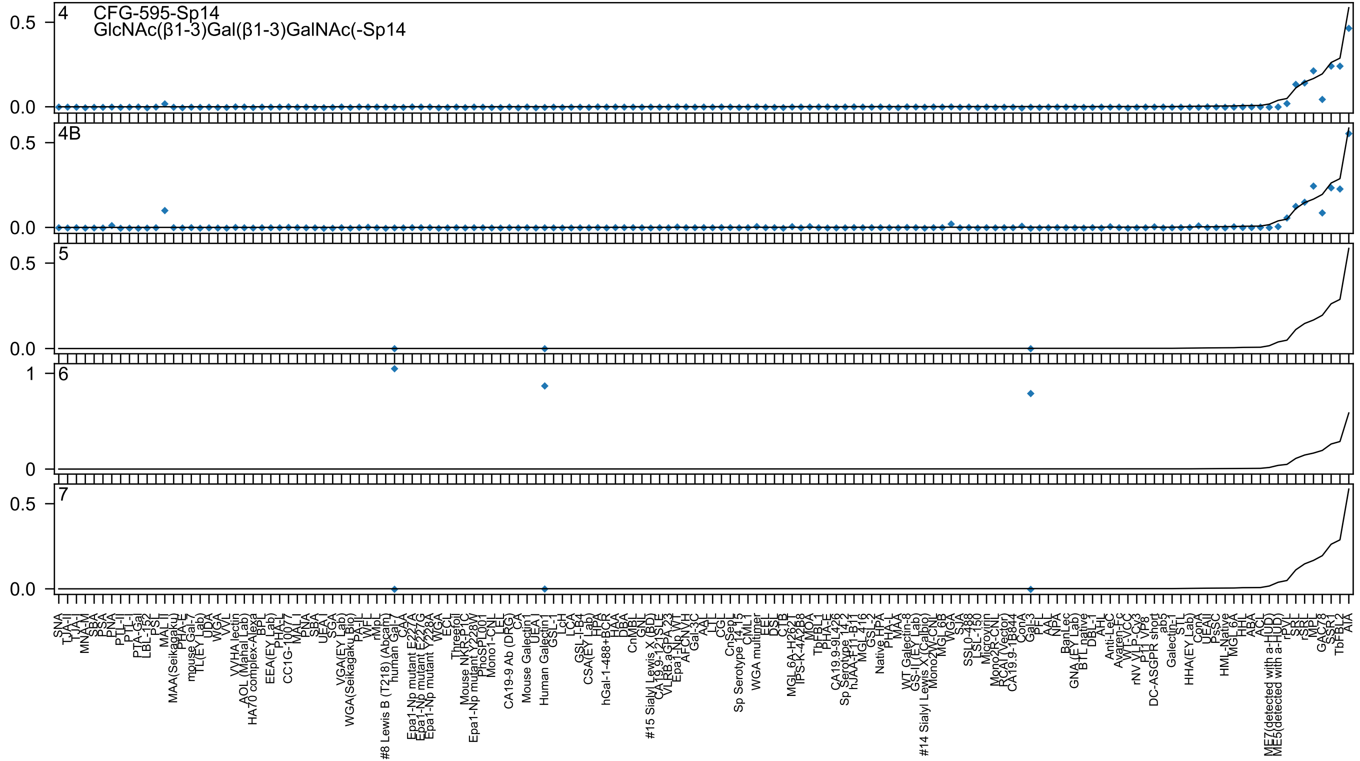
4
CFG-595-Sp14
GlcNAc(β1-3)Gal(β1-3)GalNAc(-Sp14
4B
5
6
7

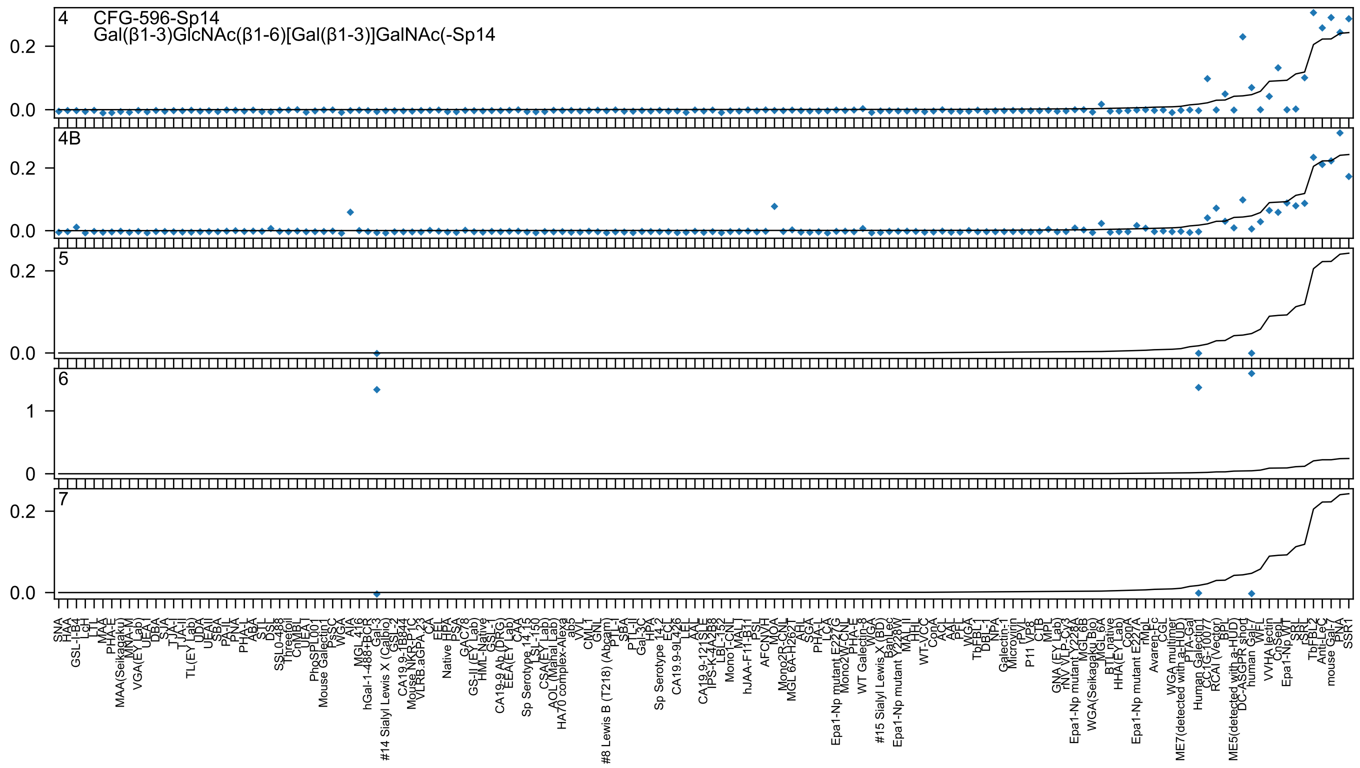

4
CFG-596-Sp14
Gal(β1-3)GlcNAc(β1-6)[Gal(β1-3)]GalNAc(-Sp14
4B
5
6
7
0.0
0.2
0
1
SNA
HAA
GSL-I-B4
LcH
LTL
MAA
PHA-E
MAA(Seikagaku)
MNA-M
VGA(EY Lab)
UEA
UEA
DBA
SJA
TJA-I
TJA-II
TL(EY Lab)
UDA
UEAII
SBA
PA-IL
PNA
PHA-L
ABA
STL
DSL
SSL0-488
Threefoil
CNMBL
UEA I
PhoSPL001
Mouse Galectin1
PsSC
WGA
AIA
MGL 416
hGal-1-488+BCR
Gal-3
#14 Sialyl Lewis X (Calbio)
GSL-2
CA19.9-1B844
Mouse NKR-P1C
VLRB.aGPA.23
CA
EEL
Native HPA
PSA
GAC78
GS-II (EY Lab)
HML-Native
GSL-1
CA19-9 Ab (DRG)
EEA(EY Lab)
CAA
Sp Serotype 14.15
LSL-150
CSA(EY Lab)
AOL (Mahal Lab)
HA70 complex-Alexa
ab5
VVL
CML1
GNL
#8 Lewis B (T218) (Abcam)
PTL-I
SBA
PTL-II
Gal-3C
HPA
Sp Serotype 14.2
ECL
CA19.9-9L426
LEL
AAL
CA19.9-121SLE
IPS-K-4A2B8
LBL-152
Mono1-CNL
MAL-I
hJAA-F11-B11
PSL
AFCNVH
MOA
Mono2R-CNL
MGL 6A-H262T
AHL
SGA
PHA-L
LCA
Epa1-Np mutant E227G
Mono2W-CNL
PHA-E
WT Galectin-8
WGA
#15 Sialyl Lewis X (BD)
BanLec
Epa1-Np mutant Y228W
MAL II
HHL
WT-VCC
ConA
ACL
AAL
PFL
WGA
TbEBL1
DBL-1
NPA
Galectin-1
Microvirin
rPVL
P11 VP8
CTB
MPL
GNA (EY Lab)
rNV VLP-Cy3
Epa1-Np mutant Y228A
MGL 6B
WGA(Seikagaku Bio)
MGL 6A
BTL native
HHA(EY Lab)
ConA
Epa1-Np mutant E227A
rMpL
Avaren-Fc
CGL
WGA multimer
ME7(detected with a-HUD)
PTA-Gal
Human Galectin1
CC1G-10077
RCAI (Vector)
BPL
ME5(detected with a-HUD)
DC-ASGPR short
human Gal-7
WFL
VVHA lectin
CnSep
Epa1-Np WT
SRL
rSRL
TbFBL2
Anti-LeC
mouse Gal-7
PNA
SSR1

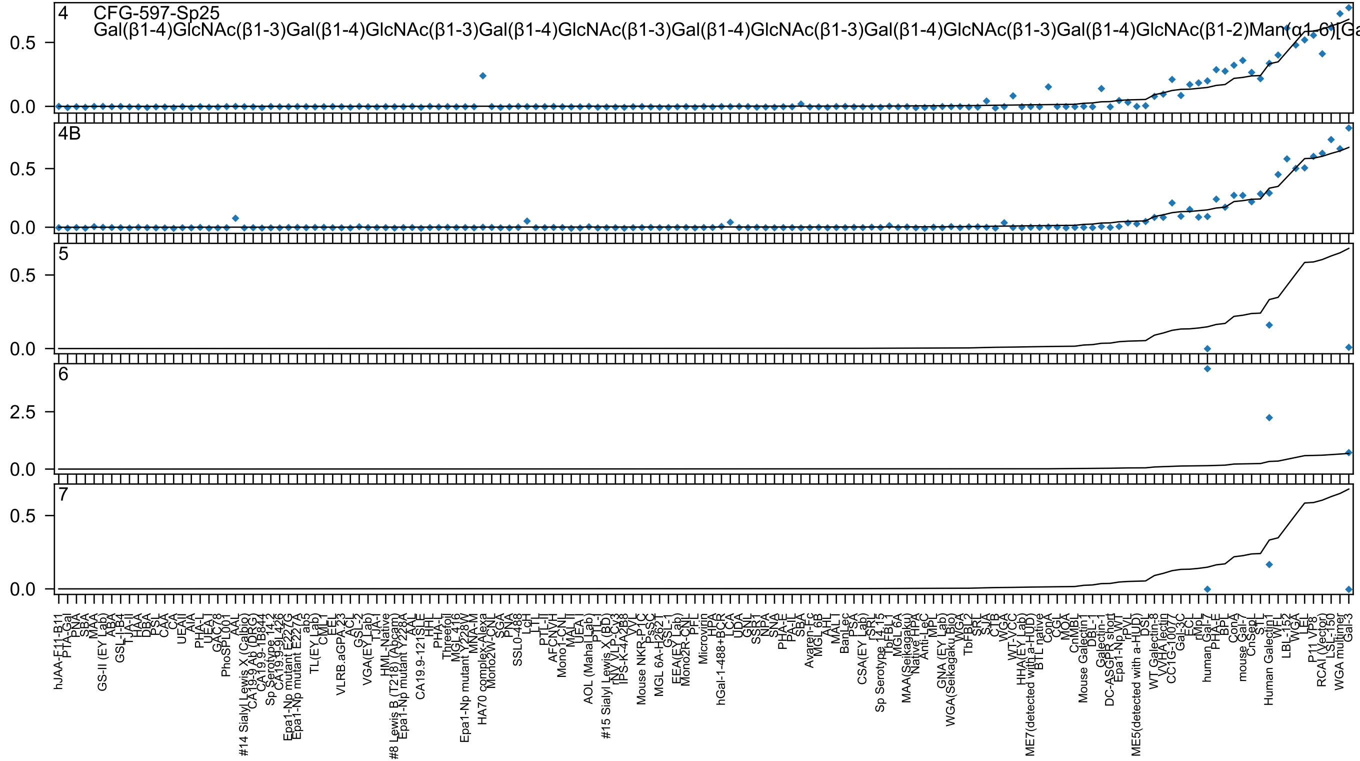

4 CFG-597-Sp25
Gal(β1-4)GlcNAc(β1-3)Gal(β1-4)GlcNAc(β1-3)Gal(β1-4)GlcNAc(β1-3)Gal(β1-4)GlcNAc(β1-3)Gal(β1-4)GlcNAc(β1-3)Gal(β1-4)GlcNAc(β1-2)Man(α1-6)[Ga
4B
5
6
7
0.0
0.5
2.5

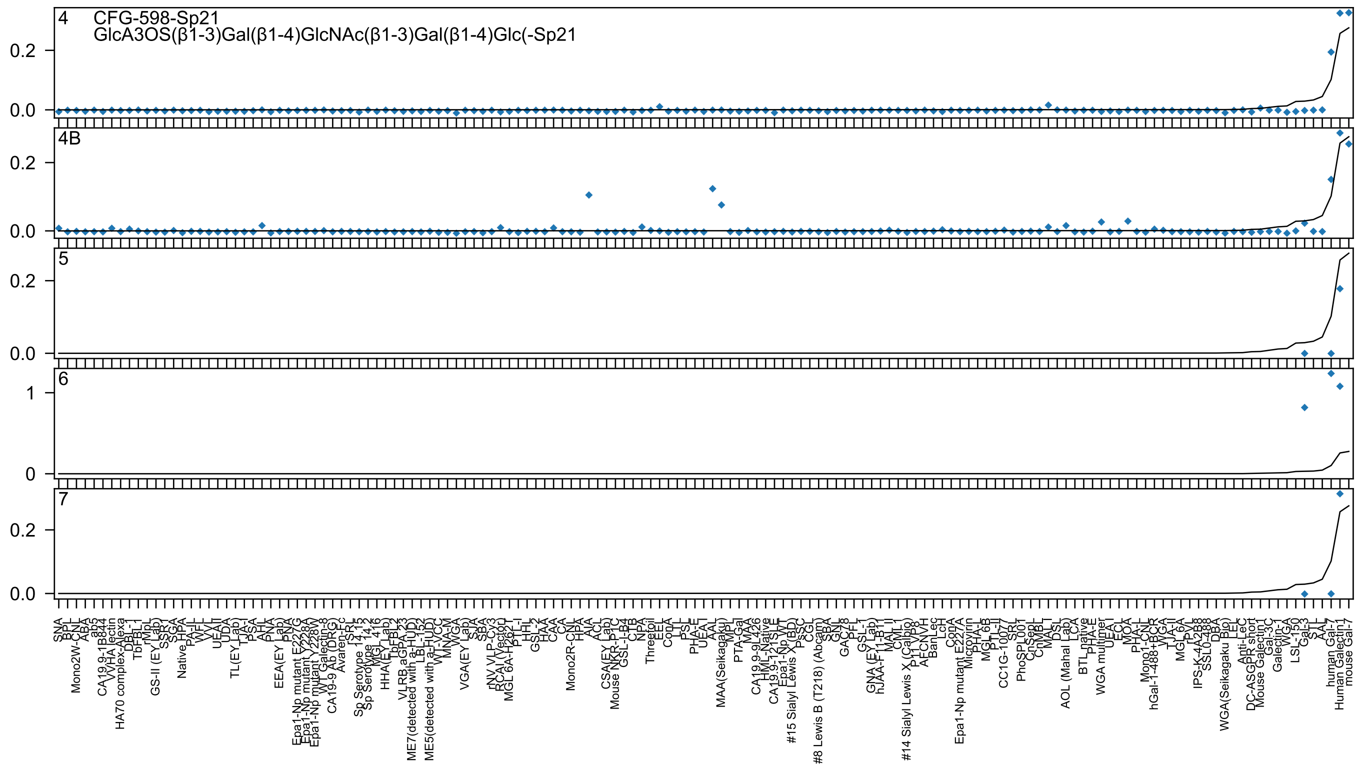
4
CFG-598-Sp21
GlcA3OS(β1-3)Gal(β1-4)GlcNAc(β1-3)Gal(β1-4)Glc(-Sp21
4B
5
6
7

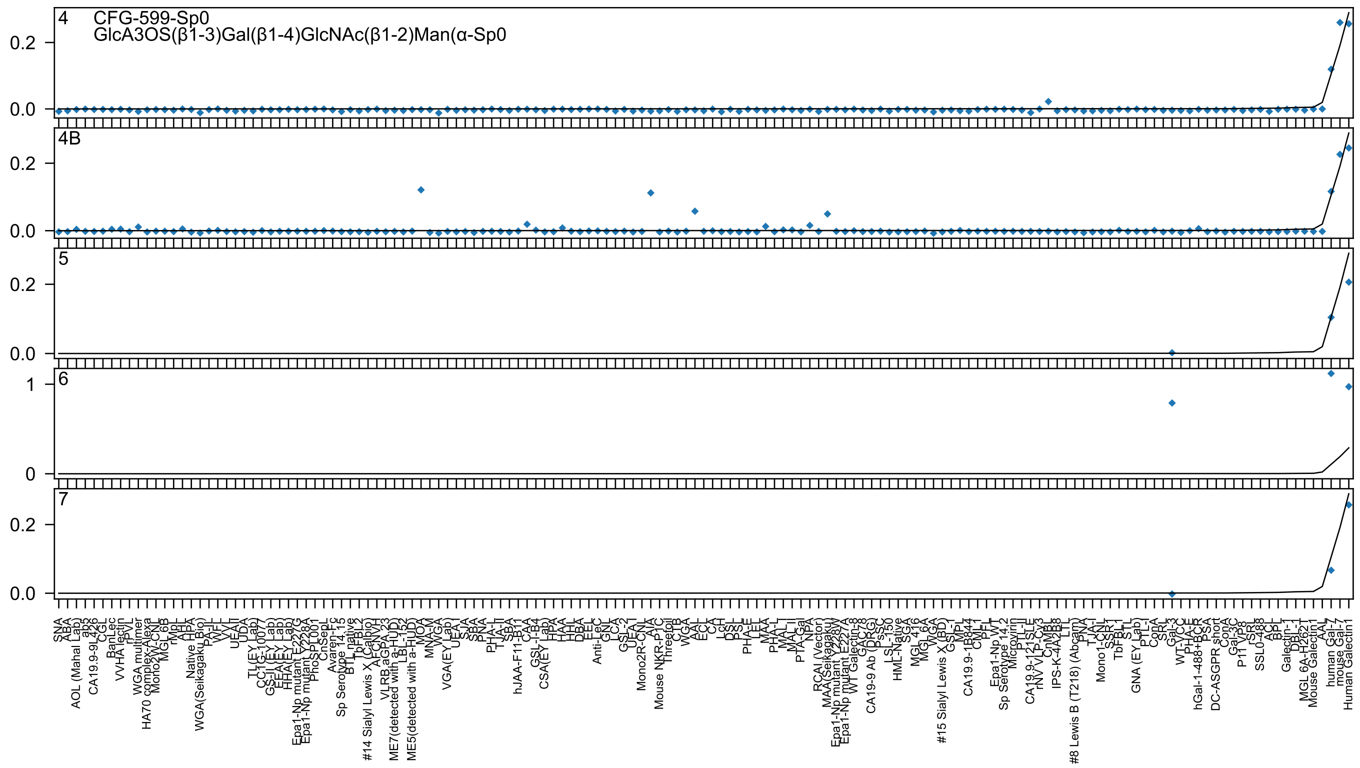

4
CFG-599-Sp0
GlcA3OS(β1-3)Gal(β1-4)GlcNAc(β1-2)Man(α-Sp0
4B
5
6
7

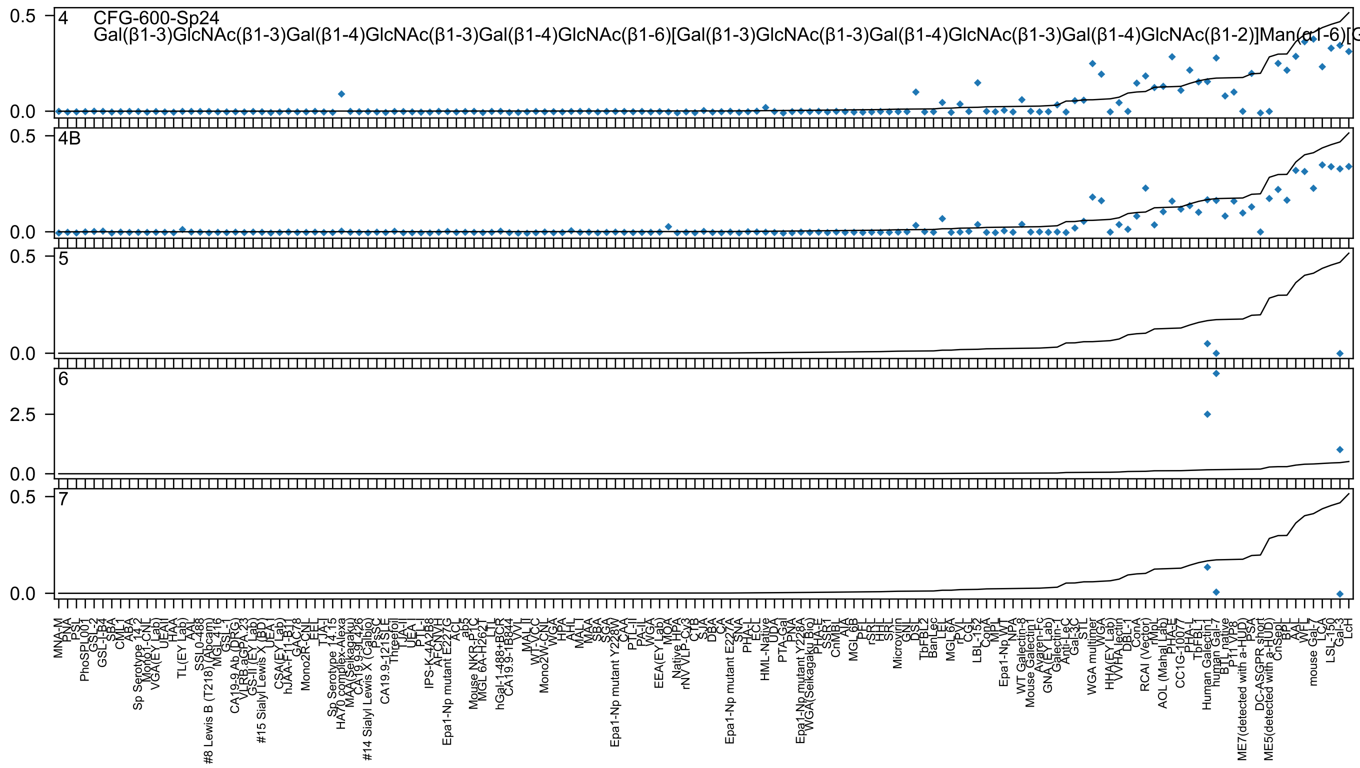
CFG-600-Sp24
Gal(β1-3)GlcNAc(β1-3)Gal(β1-4)GlcNAc(β1-3)Gal(β1-4)GlcNAc(β1-6)[Gal(β1-3)GlcNAc(β1-3)Gal(β1-4)GlcNAc(β1-3)Gal(β1-4)GlcNAc(β1-2)]Man(α1-6)[G
4
4B
5
6
7

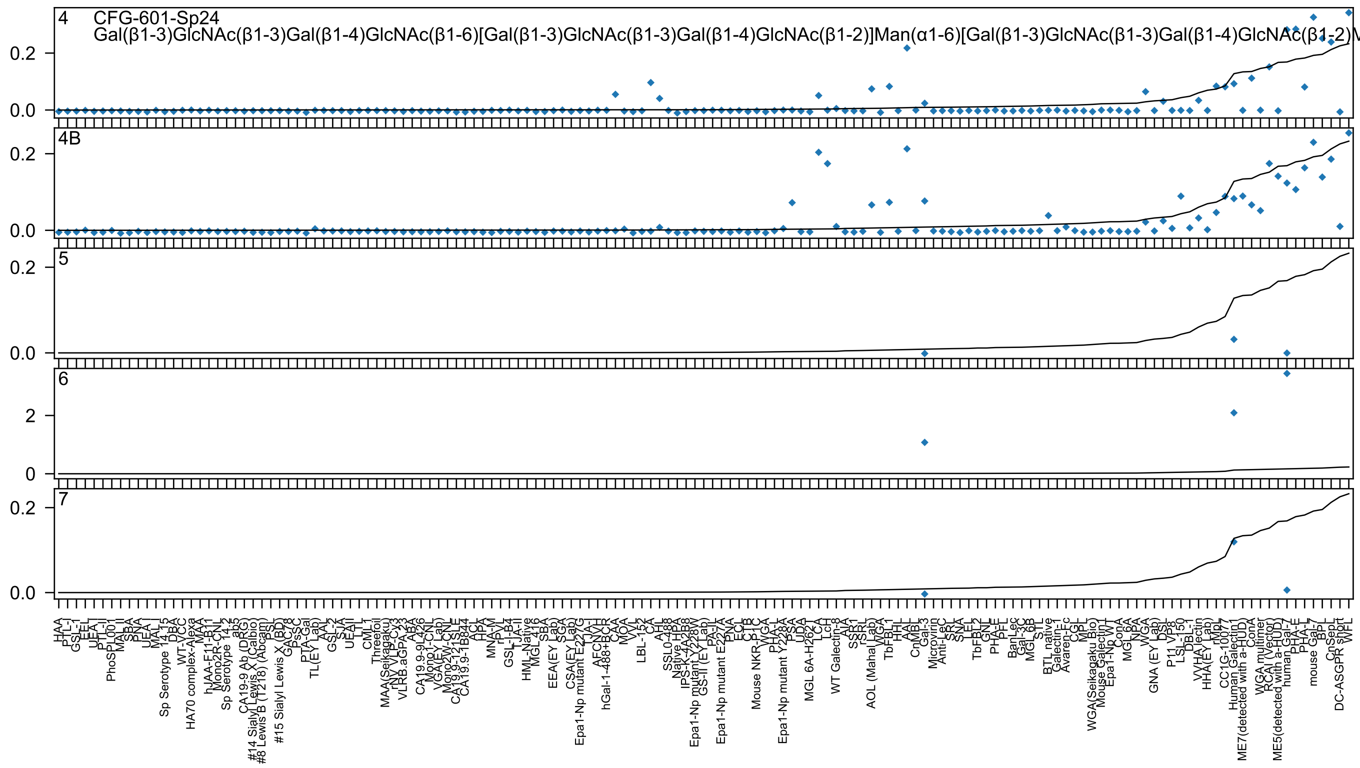

4 CFG-601-Sp24
Gal(β1-3)GlcNAc(β1-3)Gal(β1-4)GlcNAc(β1-6)[Gal(β1-3)GlcNAc(β1-3)Gal(β1-4)GlcNAc(β1-2)]Man(α1-6)[Gal(β1-3)GlcNAc(β1-3)Gal(β1-4)GlcNAc(β1-2)]M
4B
5
6
7
HAA
PTL-I
GSL-I
EEL
UEA-I
PTL-II
PhoSL
P001
MAL II
SBA
PNA
UEA-I
MAL-I
Sp Serotype 14_15
DBA
WT-VCC
HA70 complex-Alexa
MAA
hJAA-F11-B11
Mono2R-CNL
Sp Serotype 14_2
ab5
CA19-9 Ab (DRG)
#14 Sialyl Lewis X (Calbio)
#8 Lewis B (T218) (Abcam)
PSL
#15 Sialyl Lewis X (BD)
GAC78
PsSC
PTA-Gal
TL(EY Lab)
AAL
GSL-2
SJA
UEA-II
LTL
CML1
Threefoil
MAA(Seikagaku)
rNY VLP-Cy3
VLRB.aGPA.23
ABA
CA19.9-9L426
Mono1-CNL
VGA(EY Lab)
Mono2W-CNL
CA19.9-121SLE
CA19.9-1B844
ACL
HPA
MNA-M
rPVL
GSL-I-B4
TJA-II
HML-Native
MGL 416
SBA
EEA(EY Lab)
SGA
CSA(EY Lab)
Epa1-Np mutant E227G
TJA-I
AFCNYH
hGal-1-488+BCR
CAA
MOA
VVL
LBL-152
CA
AHL
SSL0-488
Native HPA
IPS-K-4A2B8
Epa1-Np mutant Y228W
GS-II (EY Lab)
PA-II
Epa1-Np mutant E227A
PNA
ECL
CTB
Mouse NKR-P1C
WGA
PHA-L
Epa1-Np mutant Y228A
PSA
UDA
MGL 6A-H262
LCA
LcH
WT Galectin-8
AIA
SSR1
rSRL
AOL (Mahal Lab)
WGA
TbFBL1
HHL
AAL
CnMBL
Gal-3
Microvirin
Anti-LeC
SRL
SNA
EEL
TbFBL2
GNL
PHA-E
PFL
BanLec
Gal-3C
MGL 6B
STL
BTL native
Galectin-1
Avaren-Fc
CGL
MPL
WGA(Seikagaku Bio)
Mouse Galectin1
Epa1-Np WT
ConA
MGL 6A
NPA
WGA
GNA (EY Lab)
DSL
P11 VP8
LSL-150
DBL-I
VVHA lectin
HHA(EY Lab)
rMpL
CC1G-10077
Human Galectin1
ME7(detected with a-HUD)
ConA
WGA multimer
RCA I (Vector)
ME5(detected with a-HUD)
human Gal-7
PHA-E
PHA-L
mouse Gal-7
BPL
CnSep1
DC-ASGPR short
WFL

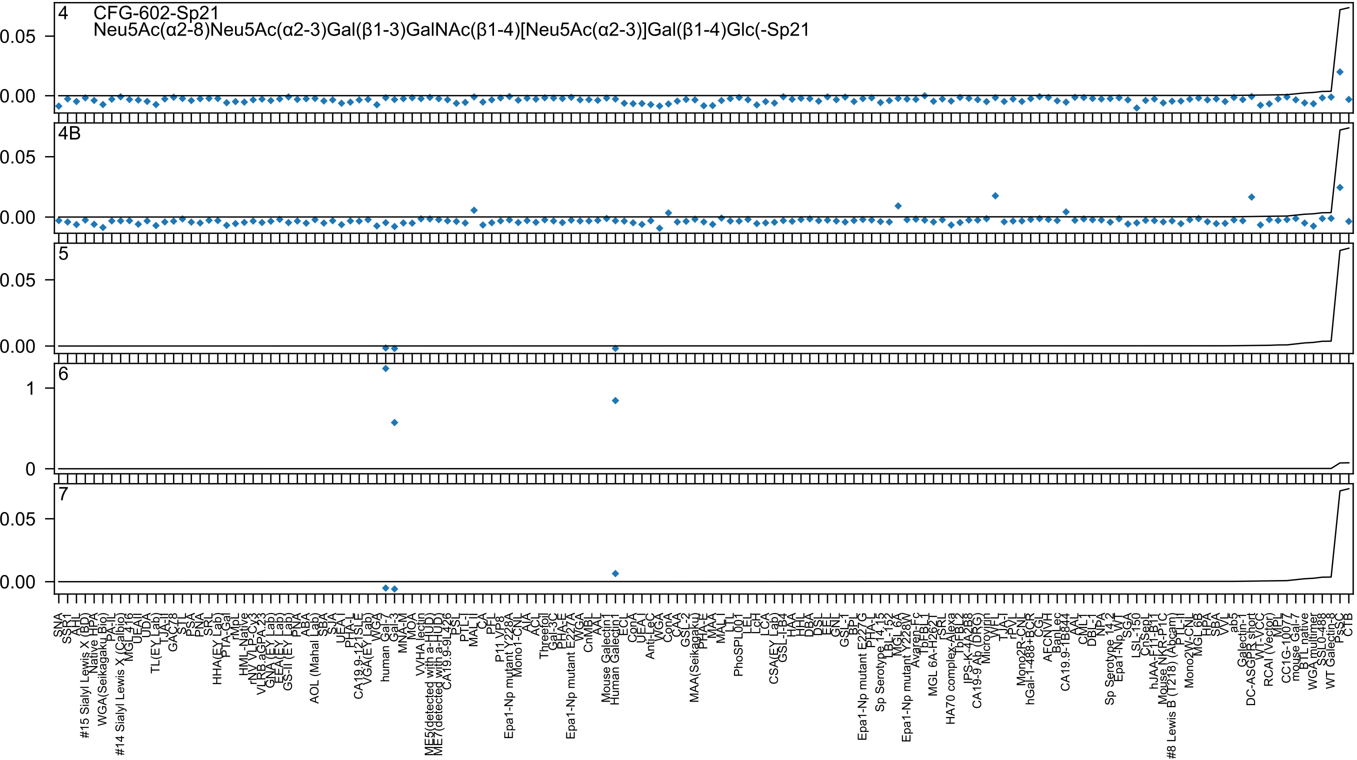

4 CFG-602-Sp21
Neu5Ac(α2-8)Neu5Ac(α2-3)Gal(β1-3)GalNAc(β1-4)[Neu5Ac(α2-3)]Gal(β1-4)Glc(-Sp21
4B
5
6
7

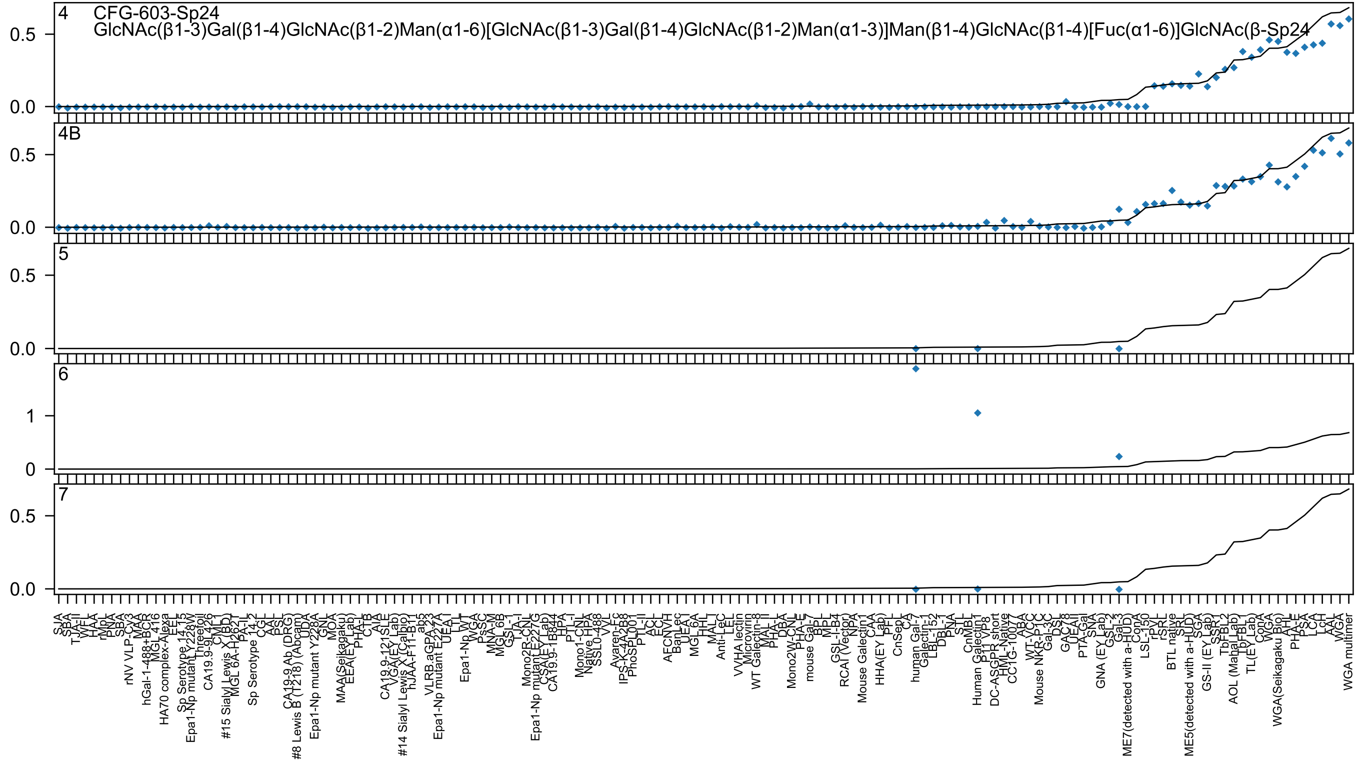
4
CFG-603-Sp24
GlcNAc(β1-3)Gal(β1-4)GlcNAc(β1-2)Man(α1-6)[GlcNAc(β1-3)Gal(β1-4)GlcNAc(β1-2)Man(α1-3)]Man(β1-4)GlcNAc(β1-4)[Fuc(α1-6)]GlcNAc(β-Sp24
4B
5
6
7

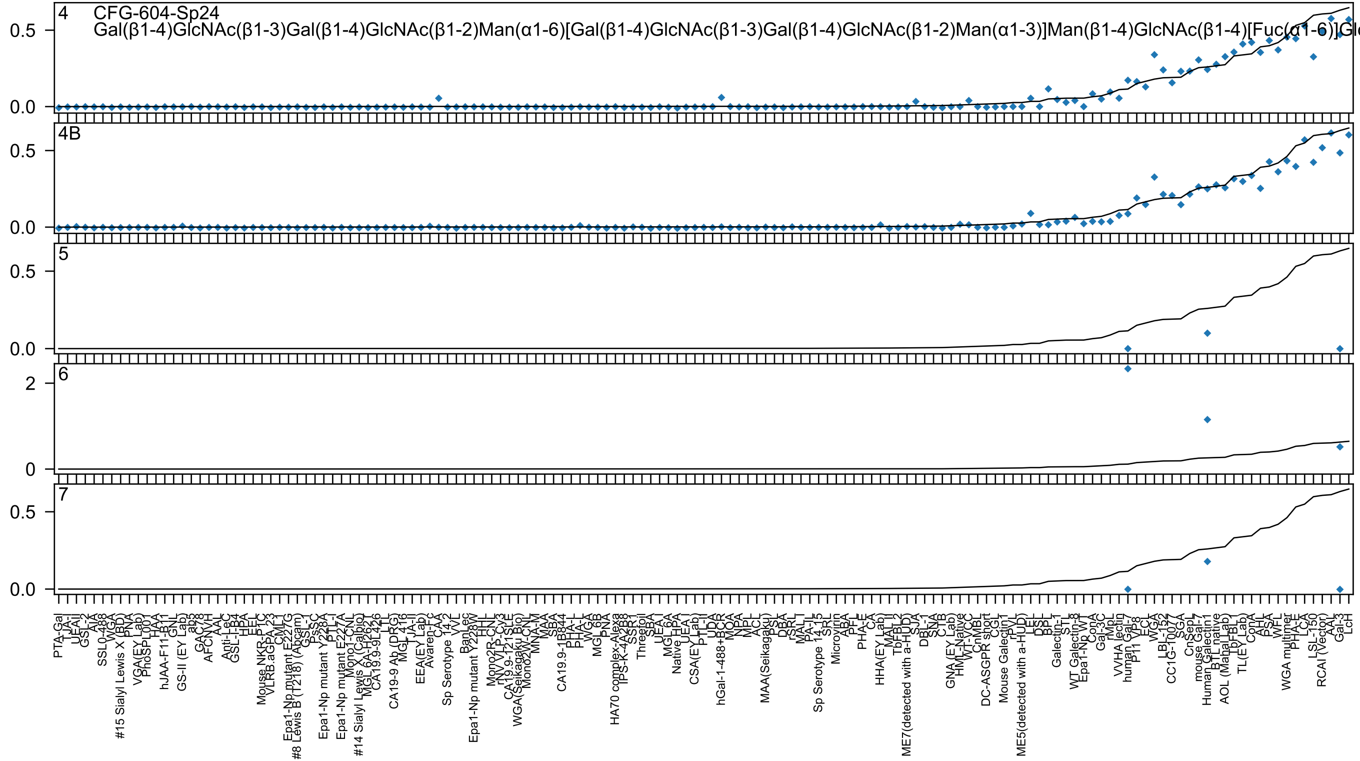

4 CFG-604-Sp24
Gal(β1-4)GlcNAc(β1-3)Gal(β1-4)GlcNAc(β1-2)Man(α1-6)[Gal(β1-4)GlcNAc(β1-3)Gal(β1-4)GlcNAc(β1-2)Man(α1-3)]Man(β1-4)GlcNAc(β1-4)[Fuc(α1-6)]Gl
4B
5
6
7
0.0
0.5
2
0

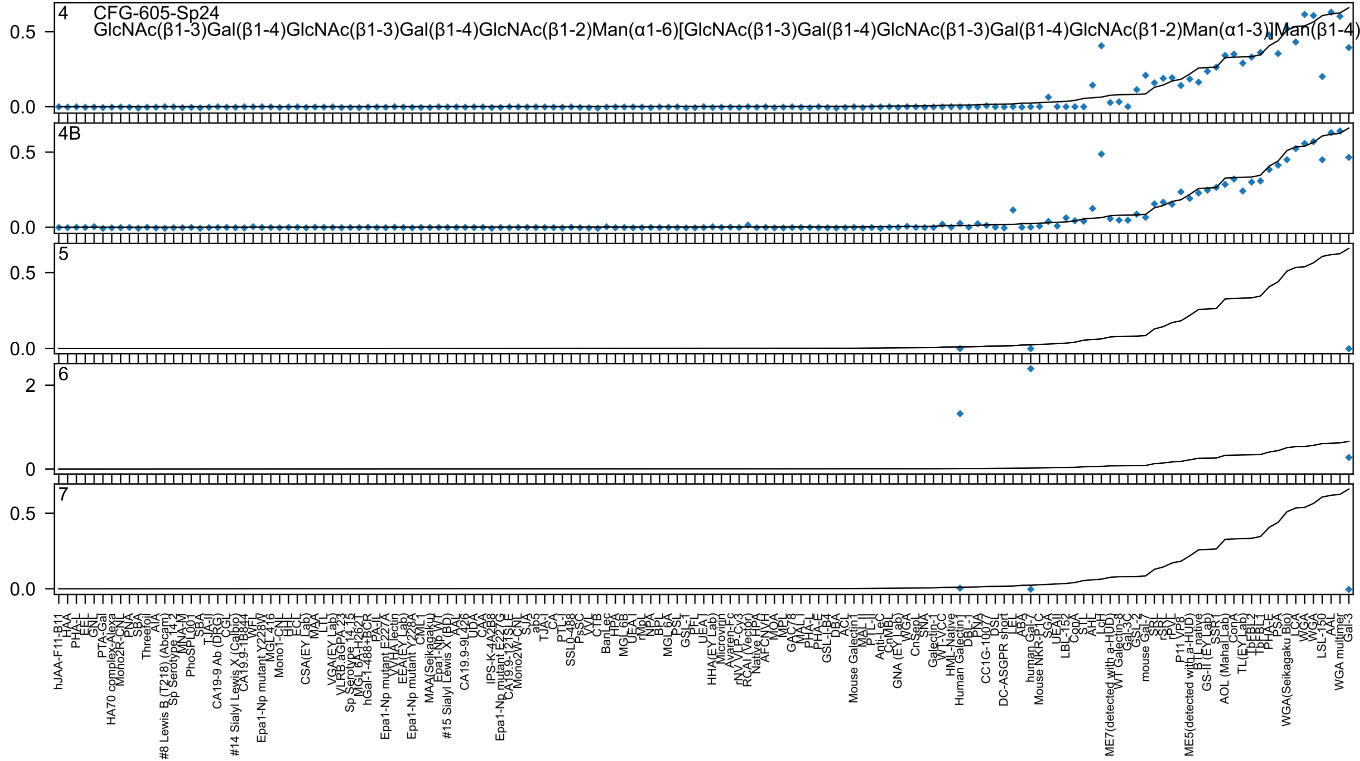
4 CFG-605-Sp24
GlcNAc(β1-3)Gal(β1-4)GlcNAc(β1-3)Gal(β1-4)GlcNAc(β1-2)Man(α1-6)[GlcNAc(β1-3)Gal(β1-4)GlcNAc(β1-3)Gal(β1-4)GlcNAc(β1-2)Man(α1-3)]Man(β1-4)
4B
5
6
7

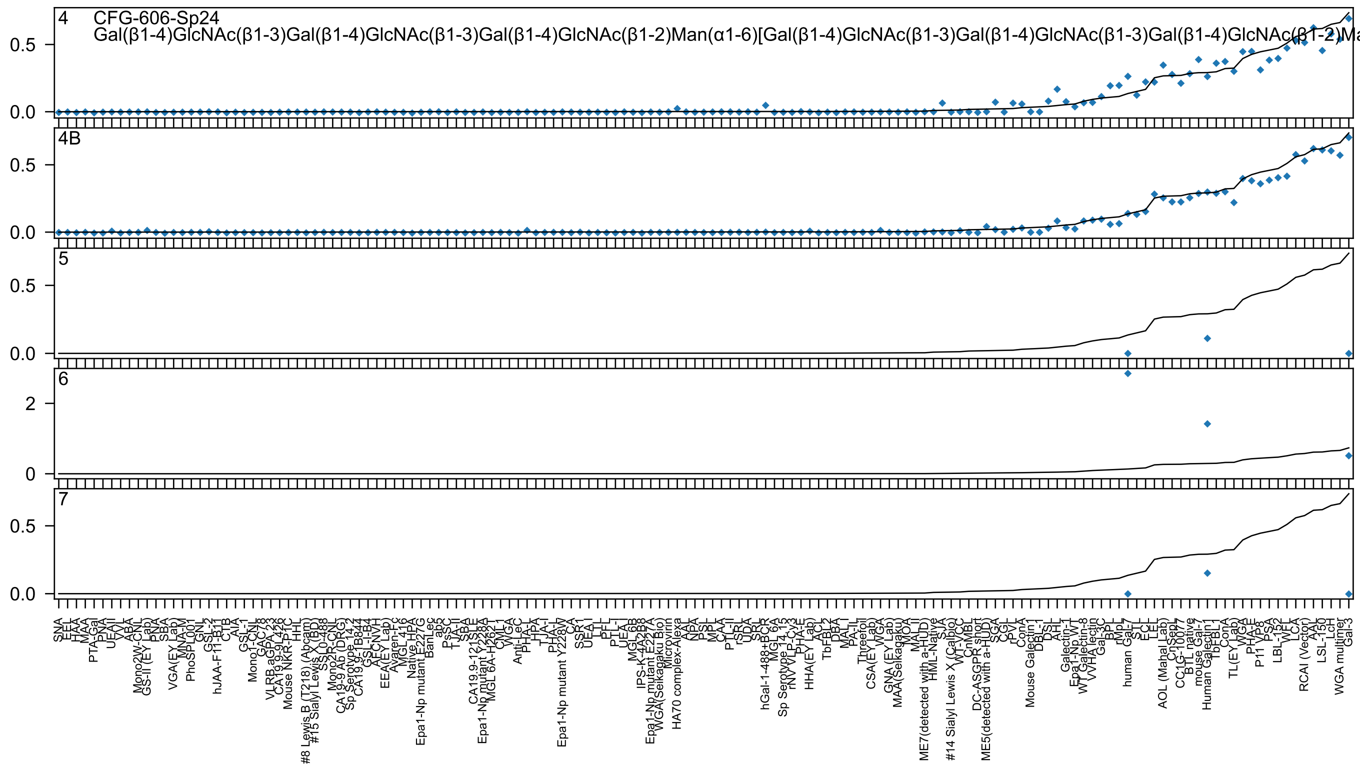
4 CFG-606-Sp24
Gal(β1-4)GlcNAc(β1-3)Gal(β1-4)GlcNAc(β1-3)Gal(β1-4)GlcNAc(β1-2)Man(α1-6)[Gal(β1-4)GlcNAc(β1-3)Gal(β1-4)GlcNAc(β1-3)Gal(β1-4)GlcNAc(β1-2)Ma
4B
5
6
7

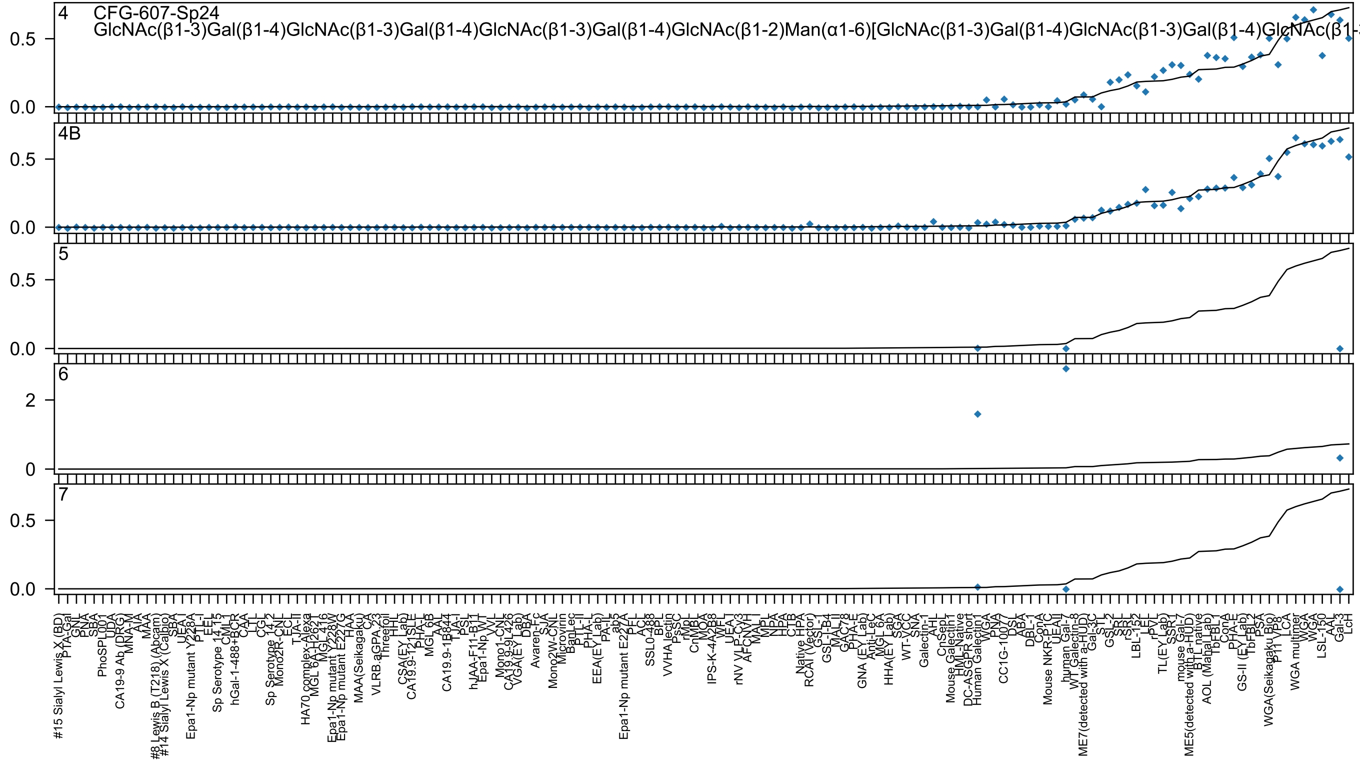

4
CFG-607-Sp24
GlcNAc(β1-3)Gal(β1-4)GlcNAc(β1-3)Gal(β1-4)GlcNAc(β1-3)Gal(β1-4)GlcNAc(β1-2)Man(α1-6)[GlcNAc(β1-3)Gal(β1-4)GlcNAc(β1-3)Gal(β1-4)GlcNAc(β1-
4B
5
6
7

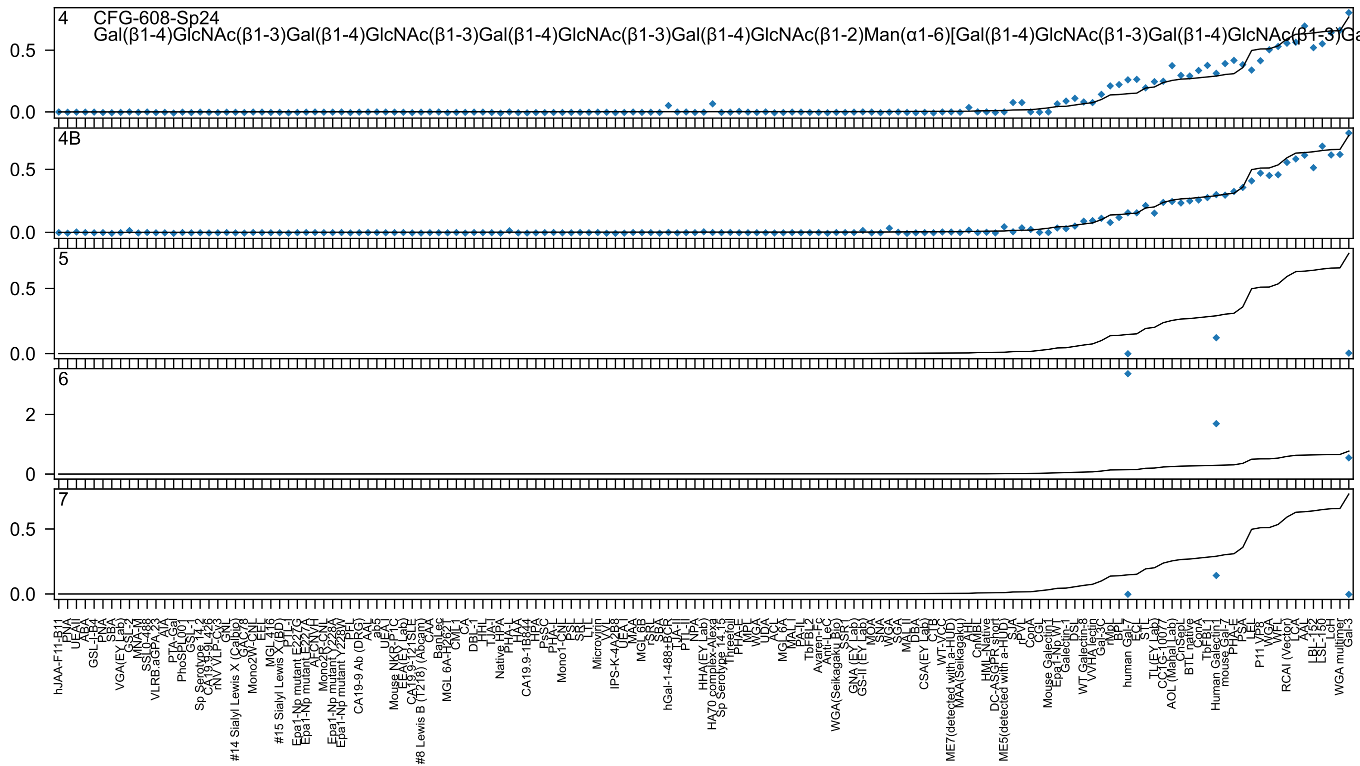

4
CFG-608-Sp24
Gal(β1-4)GlcNAc(β1-3)Gal(β1-4)GlcNAc(β1-3)Gal(β1-4)GlcNAc(β1-3)Gal(β1-4)GlcNAc(β1-2)Man(α1-6)[Gal(β1-4)GlcNAc(β1-3)Gal(β1-4)GlcNAc(β1-3)Ga
4B
5
6
7
0.0
0.5
0
2

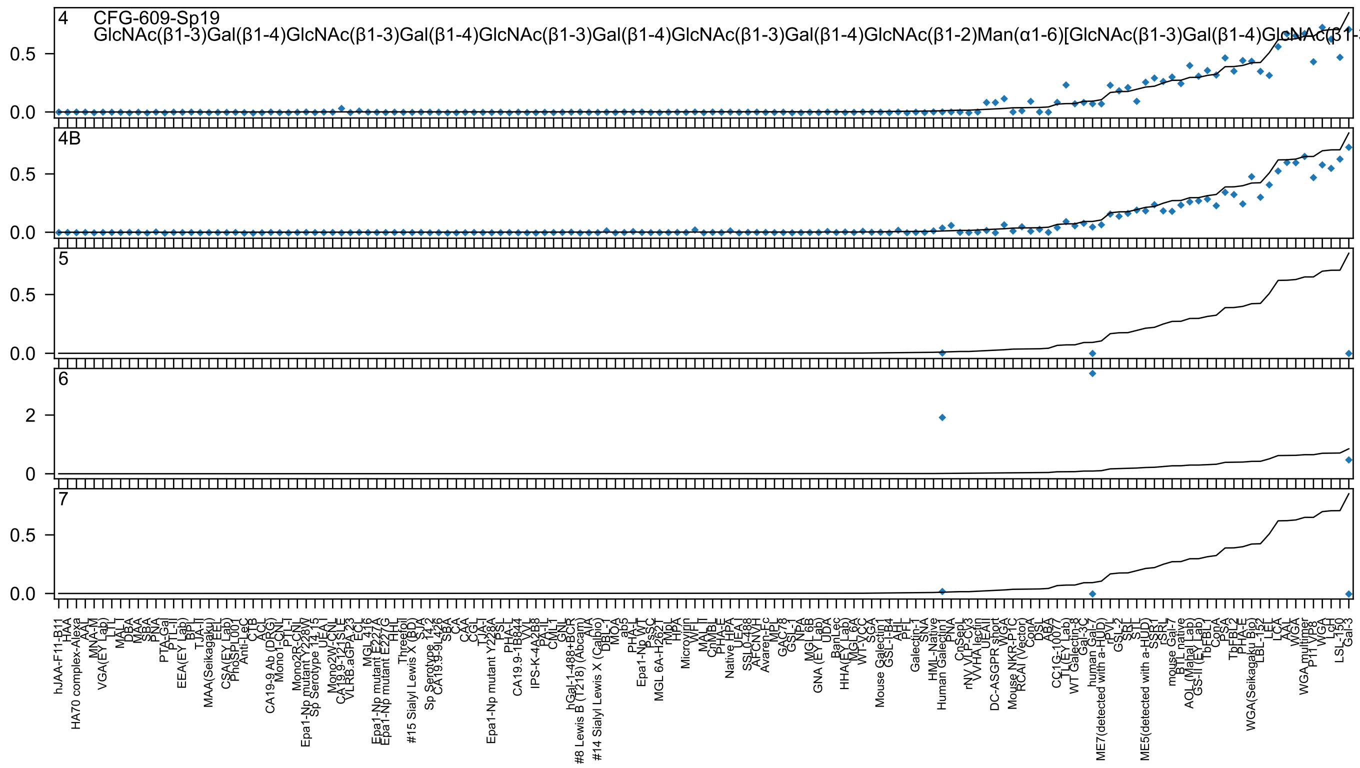

4
CFG-609-Sp19
GlcNAc(β1-3)Gal(β1-4)GlcNAc(β1-3)Gal(β1-4)GlcNAc(β1-3)Gal(β1-4)GlcNAc(β1-3)Gal(β1-4)GlcNAc(β1-2)Man(α1-6)[GlcNAc(β1-3)Gal(β1-4)GlcNAc(β1-3
4B
5
6
7

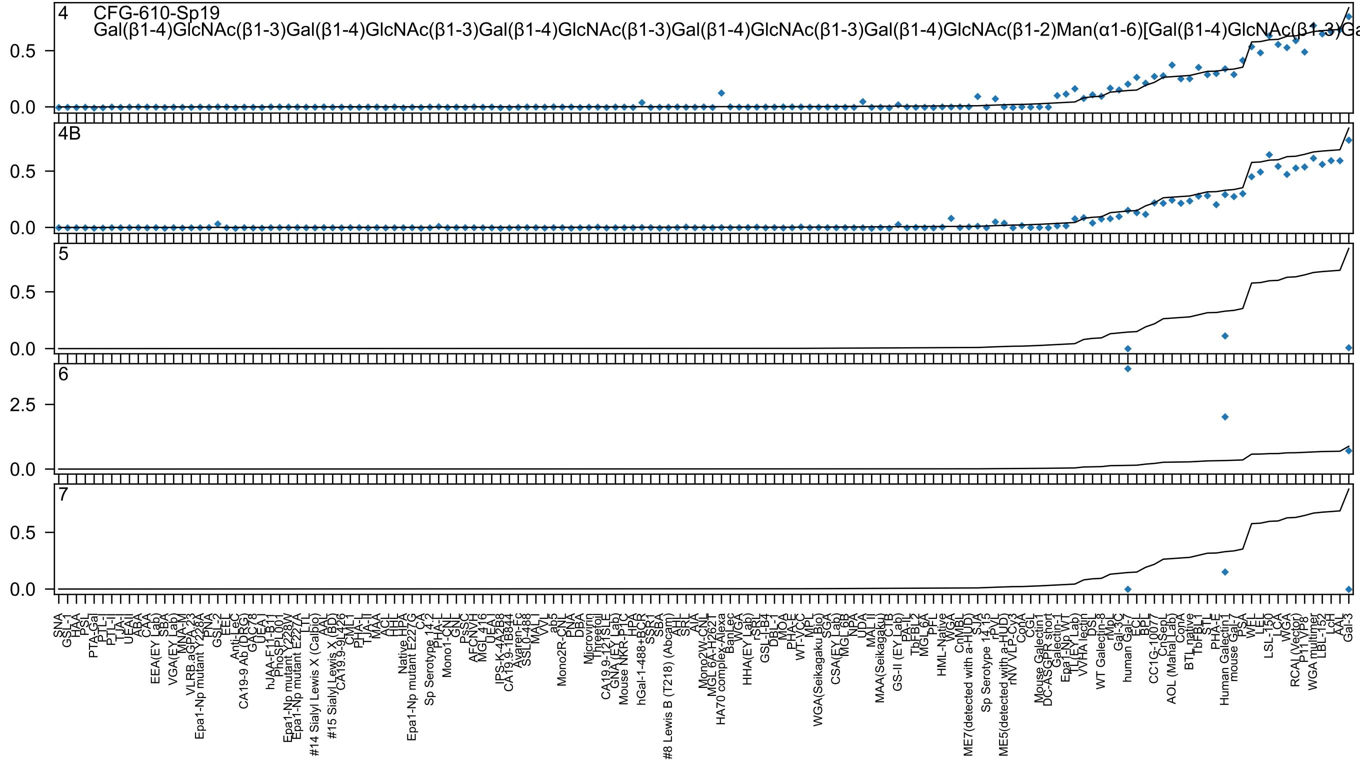

4
CFG-610-Sp19
Gal(β1-4)GlcNAc(β1-3)Gal(β1-4)GlcNAc(β1-3)Gal(β1-4)GlcNAc(β1-3)Gal(β1-4)GlcNAc(β1-3)Gal(β1-4)GlcNAc(β1-2)Man(α1-6)[Gal(β1-4)GlcNAc(β1-3)Ga
4B
5
6
7
0.0
0.5
2.5
SNA
GSL-I
HAA
PSL
PTA-Gal
PTL-I
PTL-I
TJA-I
UEAII
ABA
CAA
EEA(EY Lab)
SBA
VGA(EY Lab)
MNA-M
VLRB.aGPA.23
Epa1-Np mutant Y228A
PNA
GSL-2
EEL
Anti-LeC
CA19-9 Ab (DRG)
GAC78
UEA I
hJAA-F11-B11
PhoSL001
Epa1-Np mutant Y228W
Epa1-Np mutant E227A
LTL
#14 Sialyl Lewis X (Calbio)
AAL
#15 Sialyl Lewis X (BD)
CA19.9-9L426
CML1
PHA-L
TJA-II
MAA
ACL
HHL
Native HPA
Epa1-Np mutant E227G
CA
Sp Serotype 14.2
PHA-L
Mono1-CNL
GNL
PSSC
AFCNVH
MGL 416
UEA I
IPS-K-4A2B8
CA19.9-1B844
Avaren-Fc
SSL0-488
MAL I
VVL
ab5
Mono2R-CNL
PNA
DBA
Microvirin
Threefoil
CA19.9-121SLE
GNA (EY Lab)
Mouse NKR-P1C
HPA
hGal-1-488+BCR
SSR1
SBA
#8 Lewis B (T218) (Abcam)
AHL
SRL
AIA
Mono2W-CNL
MGL 6A-H262T
HA70 complex-Alexa
BanLec
WGA
HHA(EY Lab)
GNA
rSRL
GSL-I-B4
DBL-1
MOA
PHA-E
WT-VCC
MPL
WGA(Seikagaku Bio)
SGA
CSA(EY Lab)
MGL 6B
NPA
UDA
MAL II
MAA(Seikagaku)
CTB
GS-II (EY Lab)
PA-IL
TbFBL2
MGL 6A
PFL
HML-Native
WGA
CnMBL
ME7(detected with a-HUD)
SJA
Sp Serotype 14.15
rPVL
ME5(detected with a-HUD)
rNV VLP-Cy3
ConA
CGL
Mouse Galectin1
DC-ASGPR short
Galectin-1
Epa1-Np WT
TL(EY Lab)
VVHA lectin
DSL
WT Galectin-8
rMpL
Gal-3C
human Gal-7
ECL
BPL
CC1G-10077
CnSepL
AOL (Mahal Lab)
ConA
BTL native
TbFBL1
STL
PHA-E
Human Galectin1
mouse Gal-7
PSA
WFL
LEL
LSL-150
LCA
WGA
RCAI (Vector)
P11 VP8
WGA multimer
LBL-152
LcH
AAL
Gal-3

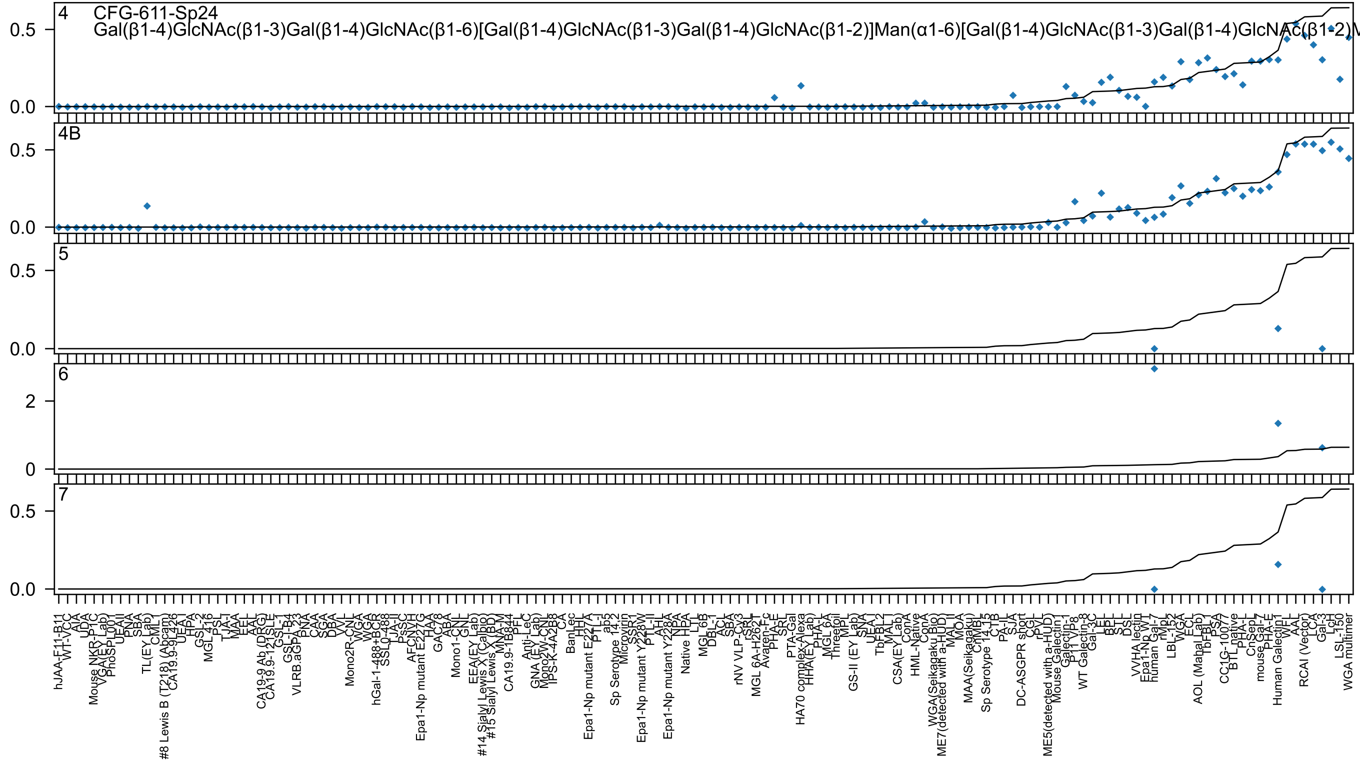
4 CFG-611-Sp24
Gal(β1-4)GlcNAc(β1-3)Gal(β1-4)GlcNAc(β1-6)[Gal(β1-4)GlcNAc(β1-3)Gal(β1-4)GlcNAc(β1-2)]Man(α1-6)[Gal(β1-4)GlcNAc(β1-3)Gal(β1-4)GlcNAc(β1-2)M
4B
5
6
7

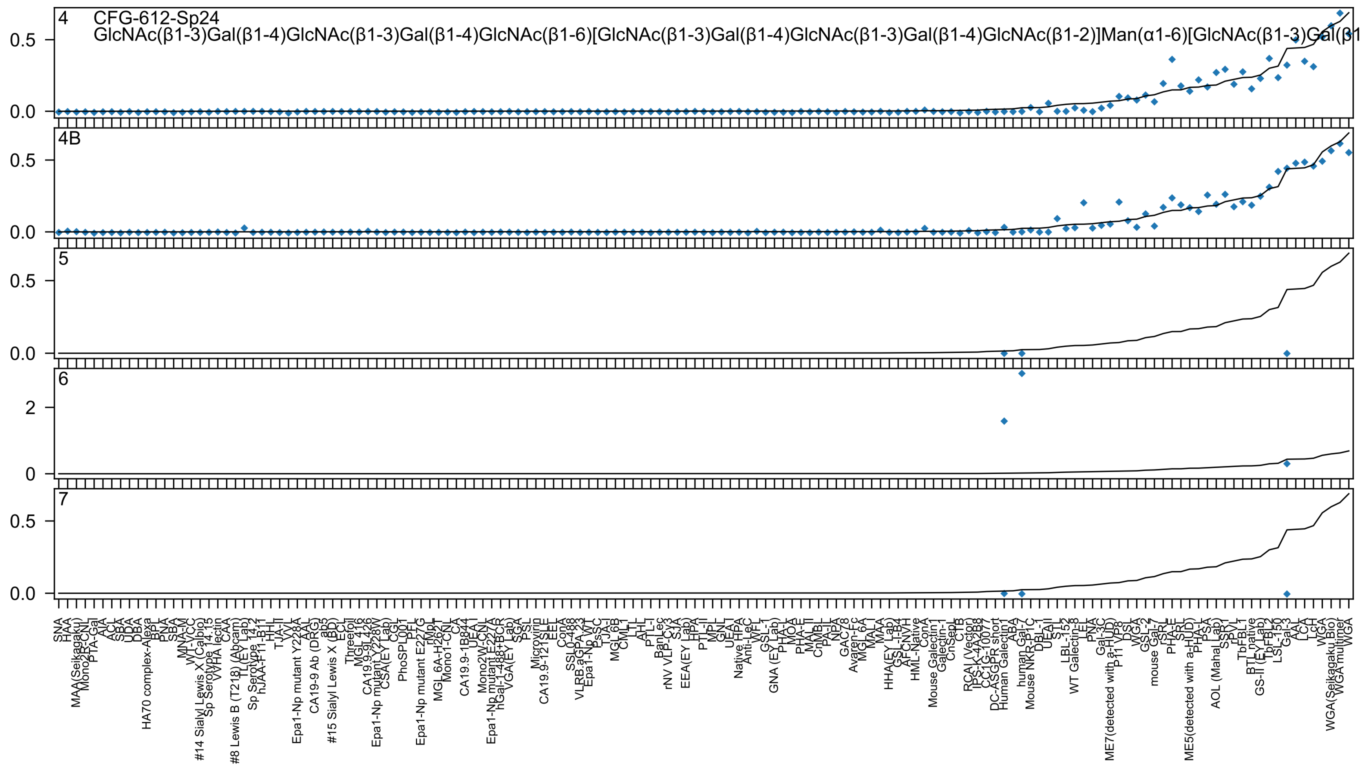

4
CFG-612-Sp24
GlcNAc(β1-3)Gal(β1-4)GlcNAc(β1-3)Gal(β1-4)GlcNAc(β1-6)[GlcNAc(β1-3)Gal(β1-4)GlcNAc(β1-3)Gal(β1-4)GlcNAc(β1-2)]Man(α1-6)[GlcNAc(β1-3)Gal(β1
4B
5
6
7

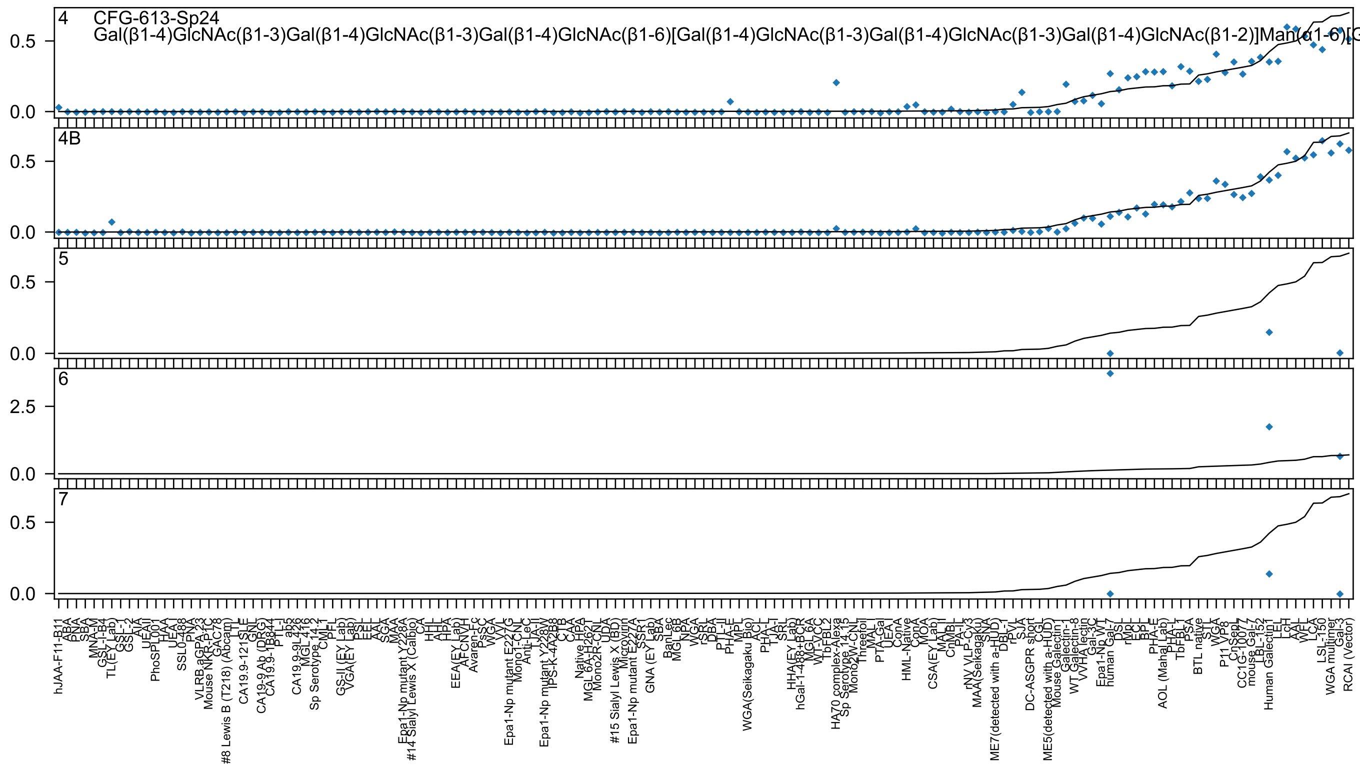

4
CFG-613-Sp24
Gal(β1-4)GlcNAc(β1-3)Gal(β1-4)GlcNAc(β1-3)Gal(β1-4)GlcNAc(β1-6)[Gal(β1-4)GlcNAc(β1-3)Gal(β1-4)GlcNAc(β1-3)Gal(β1-4)GlcNAc(β1-2)]Man(α1-6)[G
0.5
0.0
4B
0.5
0.0
5
0.5
0.0
6
2.5
0.0
7
0.5
0.0
hJAA-F11-B11
ABA
PNA
SBA
MNA-M
GSL-I-B4
TL(EY Lab)
GSL-I
GSL-2
AIA
UEAII
PhoSL
P001
HAA
UEA1
SSL0-488
PNA
VLRB.aGPA.23
Mouse NKR-P1C
GAC78
#8 Lewis B (T218) (Abcam)
LTL
CA19.9-121SLE
GNL
CA19-9 Ab (DRG)
CA19.9-1B844
PTL-I
abs
CA19.9-9L426
MGL 416
Sp Serotype 14.2
CML1
PFL
GS-II (EY Lab)
VGA(EY Lab)
PSL
EEL
AAL
SGA
MAA
Epa1-Np mutant Y228A
#14 Sialyl Lewis X (Calbio)
CA
HHL
AHL
HPA
EEA(EY Lab)
AFCNVH
Avaren-Fc
PsSC
WGA
VVL
Epa1-Np mutant E227G
Mono1-CNL
Anti-LeC
TJA-II
Epa1-Np mutant Y228W
IPS-K-4A2B8
CTB
CAA
Native HPA
MGL 6A-H262
Mono2R-CNL
UDA
#15 Sialyl Lewis X (BD)
Microvirin
Epa1-Np mutant E227A
SSR1
GNA (EY Lab)
SBA
BanLec
MGL 6B
NPA
WGA
rSRL
DBA
PTL-II
PHA-E
MPL
WGA(Seikagaku Bio)
ACL
PHA-L
TJA-I
SRL
HHA(EY Lab)
hGal-1-488+BCR
MGL 6A
WT-VCC
TbFBL2
HA70 complex-Alexa
Sp Serotype 14.15
Mono2W-CNL
Threefoil
MAL
PTA-Gal
UEA I
ConA
HML-Native
ConA
MOA
CSA(EY Lab)
MAL I
CnMBL
PA-IL
rNV VLP-Cy3
MAA(Seikagaku)
SNA
ME7(detected with a-HUD)
DBL-I
rPVL
SJA
DC-ASGPR short
CGL
ME5(detected with a-HUD)
Mouse Galectin-1
WT Galectin-8
VVHA lectin
Gal-3C
Epa1-Np WT
human Gal-7
DSL
rMpL
ECL
BPL
PHA-E
AOL (Mahal Lab)
PHA-L
TbFBL1
PSA
BTL native
STL
WGA
P11 VP8
ChSep
CC1G-10077
mouse Gal-7
LBL-152
Human Galectin1
LEL
LcH
AAL
WFL
LCA
LSL-150
WGA multimer
Gal-3
RCAI (Vector)

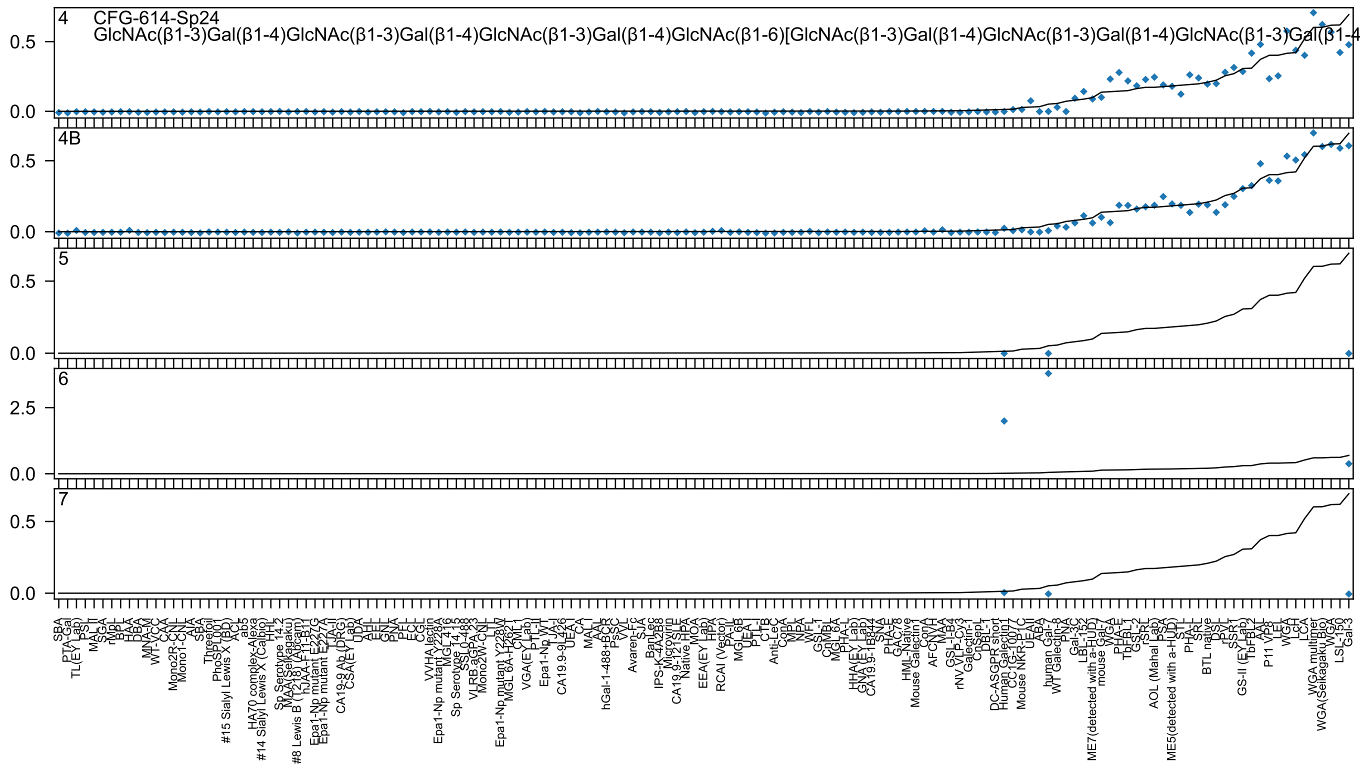
4 CFG-614-Sp24
GlcNAc(β1-3)Gal(β1-4)GlcNAc(β1-3)Gal(β1-4)GlcNAc(β1-3)Gal(β1-4)GlcNAc(β1-6)[GlcNAc(β1-3)Gal(β1-4)GlcNAc(β1-3)Gal(β1-4)GlcNAc(β1-3)Gal(β1-4
4B
5
6
7

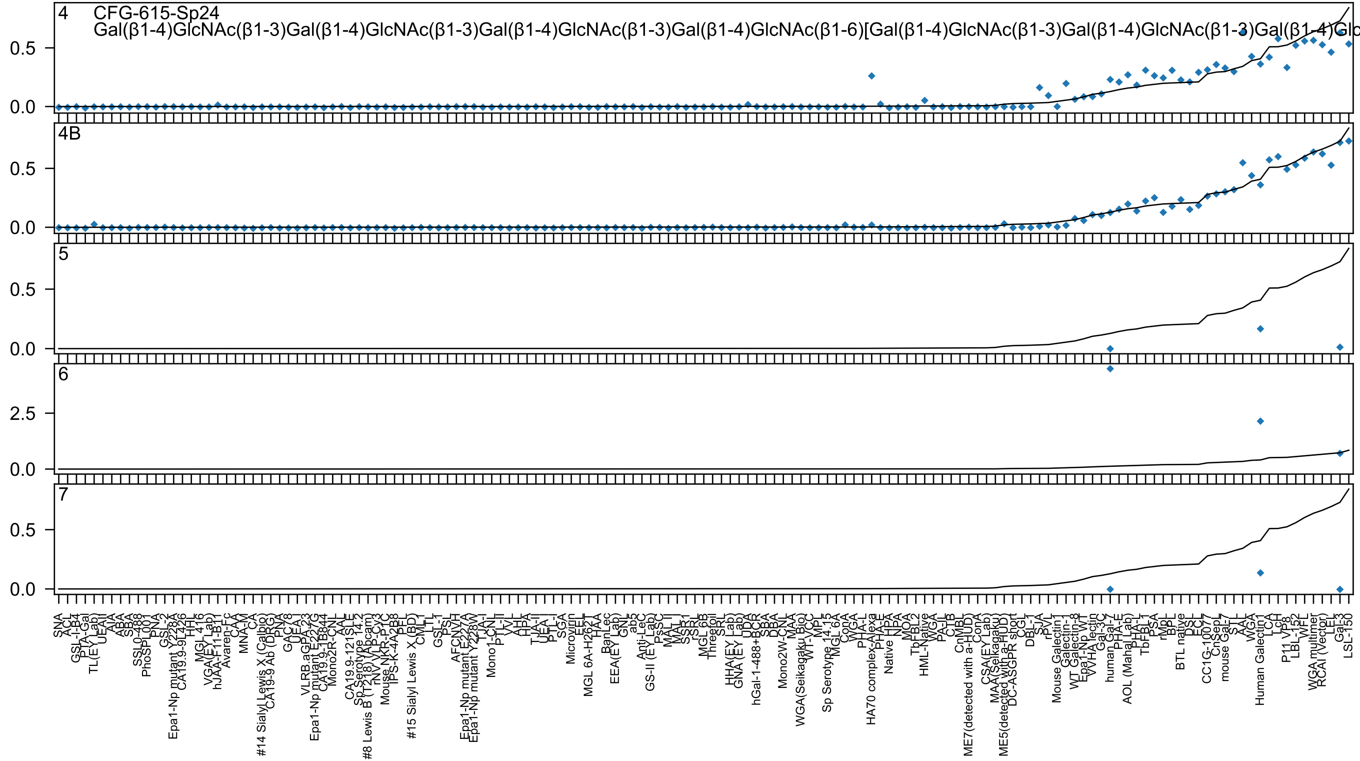
4 CFG-615-Sp24
Gal(β1-4)GlcNAc(β1-3)Gal(β1-4)GlcNAc(β1-3)Gal(β1-4)GlcNAc(β1-3)Gal(β1-4)GlcNAc(β1-6)[Gal(β1-4)GlcNAc(β1-3)Gal(β1-4)GlcNAc(β1-3)Gal(β1-4)Glc
4B
5
6
7
0.0
0.5
2.5

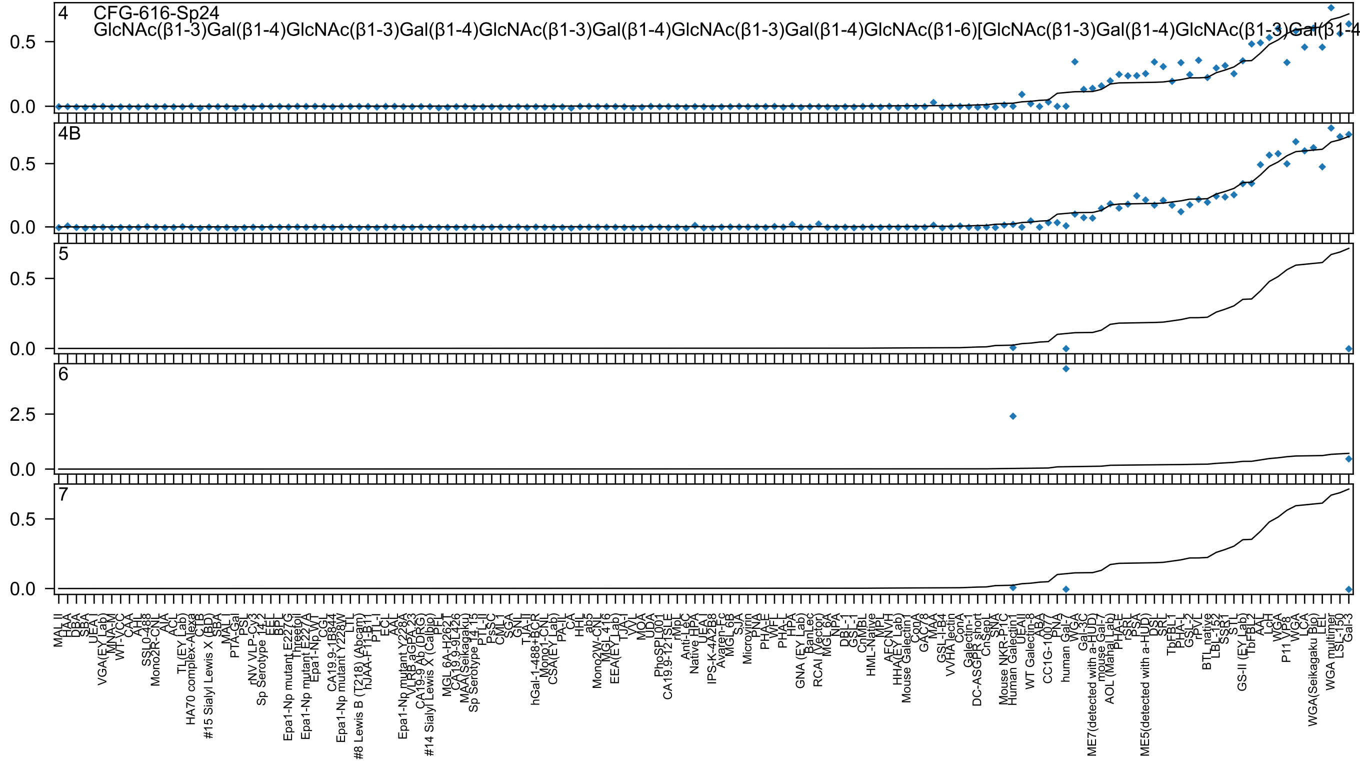
4
CFG-616-Sp24
GlcNAc(β1-3)Gal(β1-4)GlcNAc(β1-3)Gal(β1-4)GlcNAc(β1-3)Gal(β1-4)GlcNAc(β1-3)Gal(β1-4)GlcNAc(β1-6)[GlcNAc(β1-3)Gal(β1-4)GlcNAc(β1-3)Gal(β1-4
4B
5
6
7
0.0
0.5
2.5

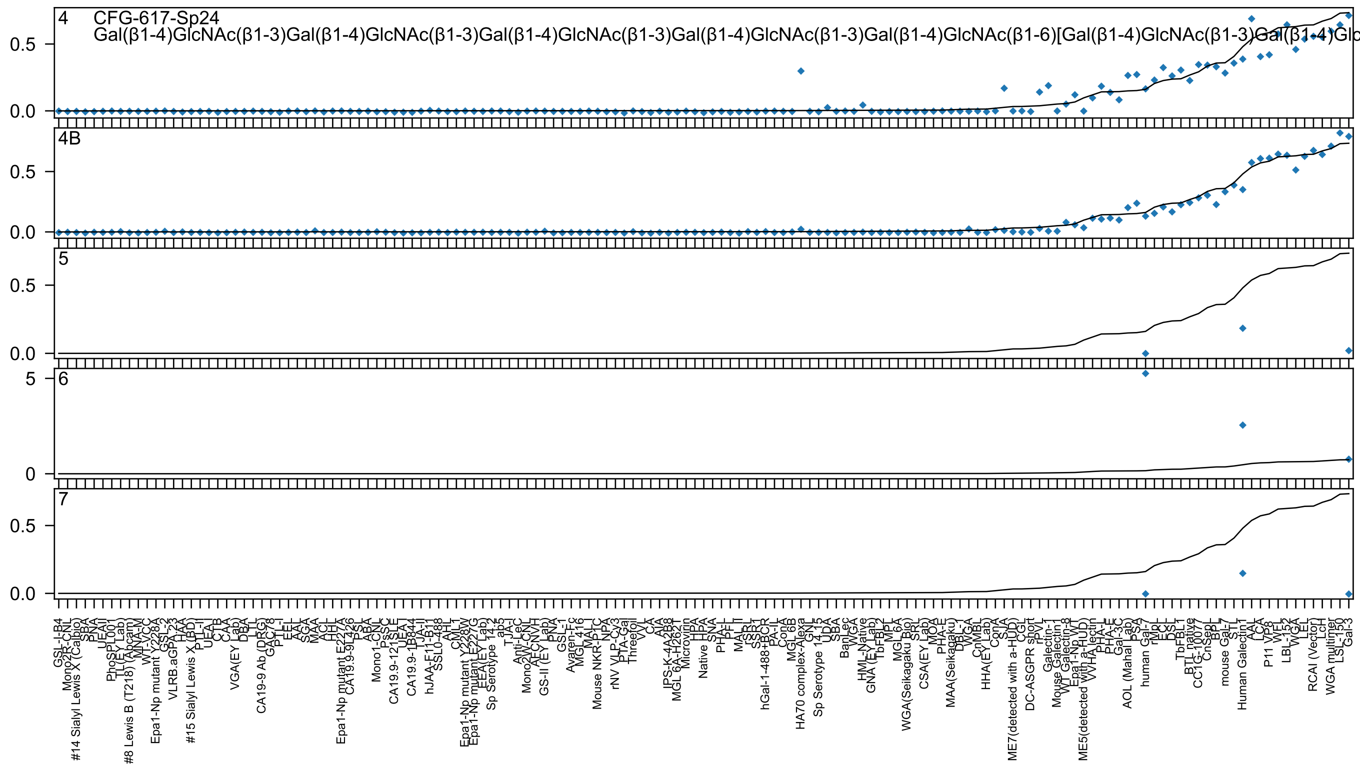

4
CFG-617-Sp24
Gal(β1-4)GlcNAc(β1-3)Gal(β1-4)GlcNAc(β1-3)Gal(β1-4)GlcNAc(β1-3)Gal(β1-4)GlcNAc(β1-3)Gal(β1-4)GlcNAc(β1-6)[Gal(β1-4)GlcNAc(β1-3)Gal(β1-4)Glc
4B
5
6
7
0.0
0.5
0
5

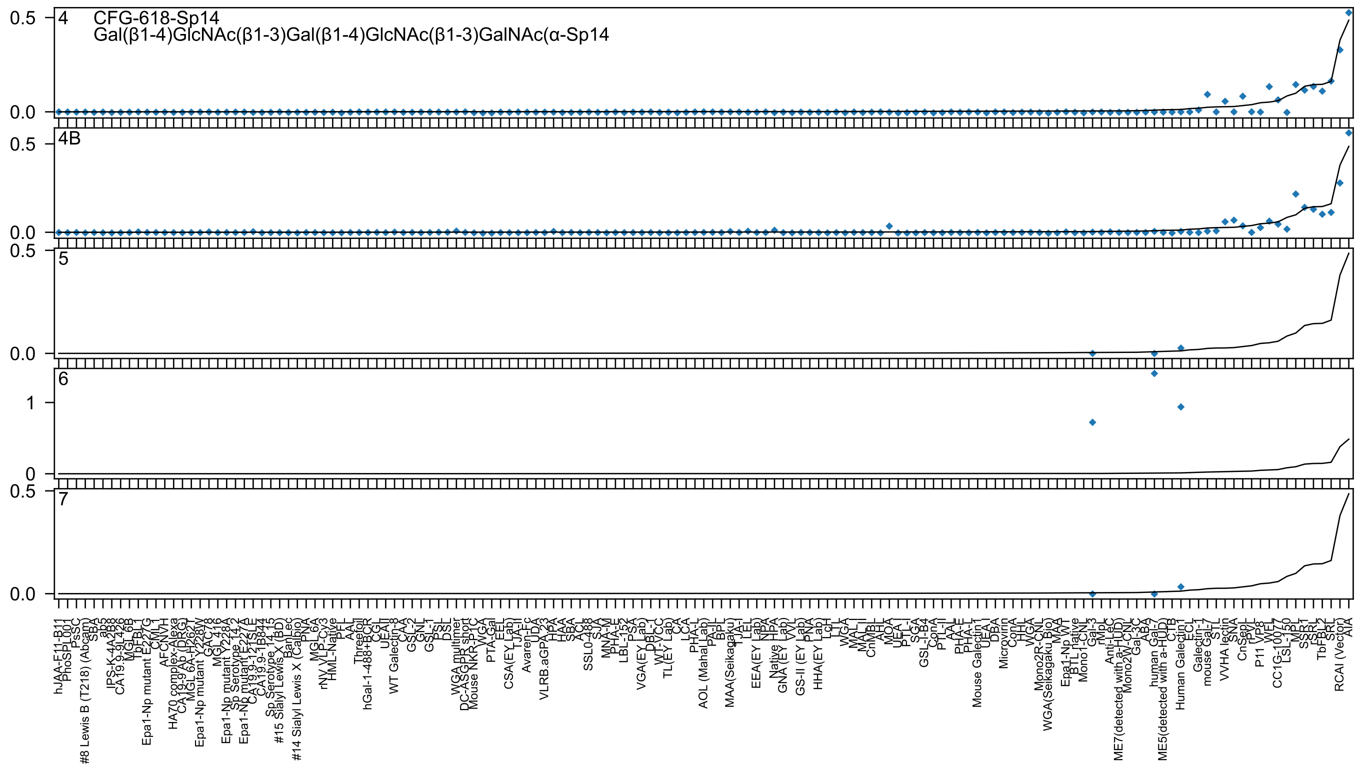

CFG-618-Sp14
Gal(β1-4)GlcNAc(β1-3)Gal(β1-4)GlcNAc(β1-3)GalNAc(α-Sp14
4
4B
5
6
7

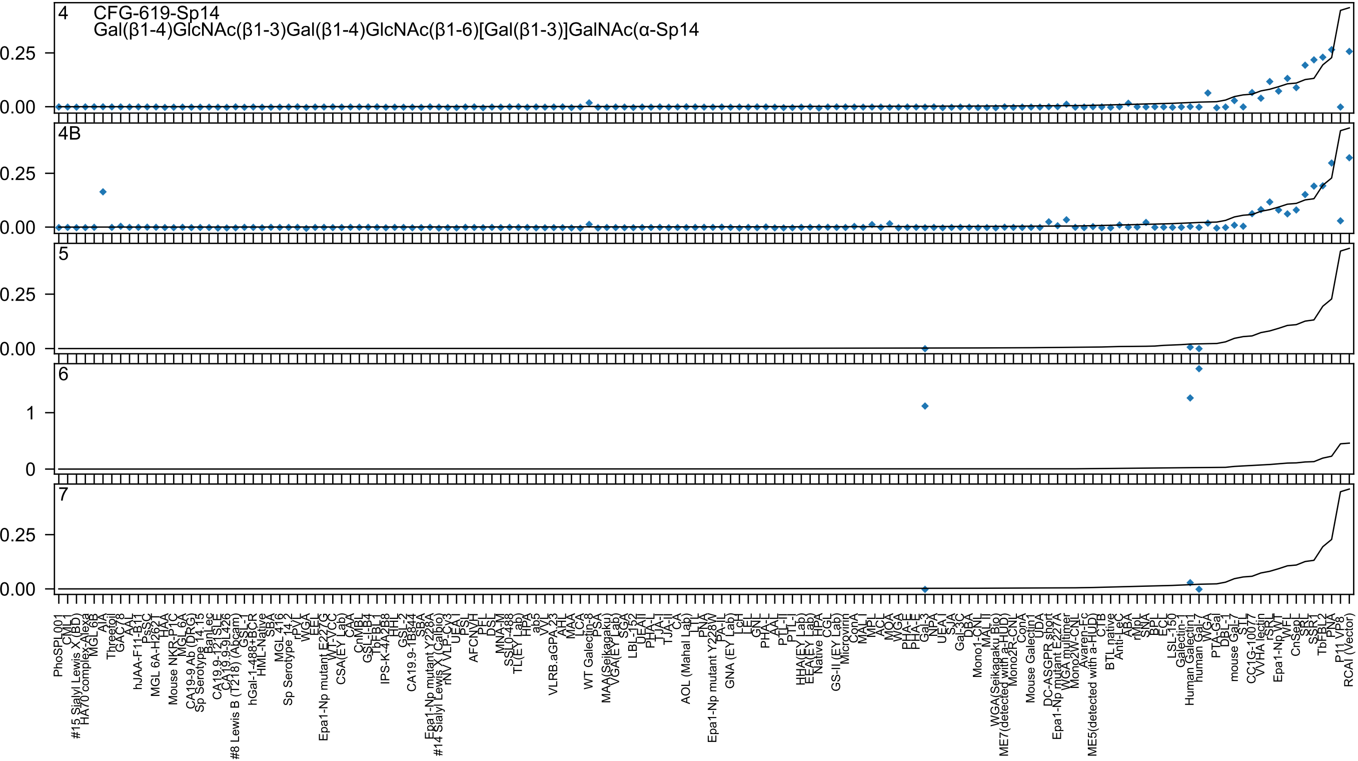
4
CFG-619-Sp14
Gal(β1-4)GlcNAc(β1-3)Gal(β1-4)GlcNAc(β1-6)[Gal(β1-3)]GalNAc(α-Sp14
4B
5
6
7
0.25
0.00
1
0

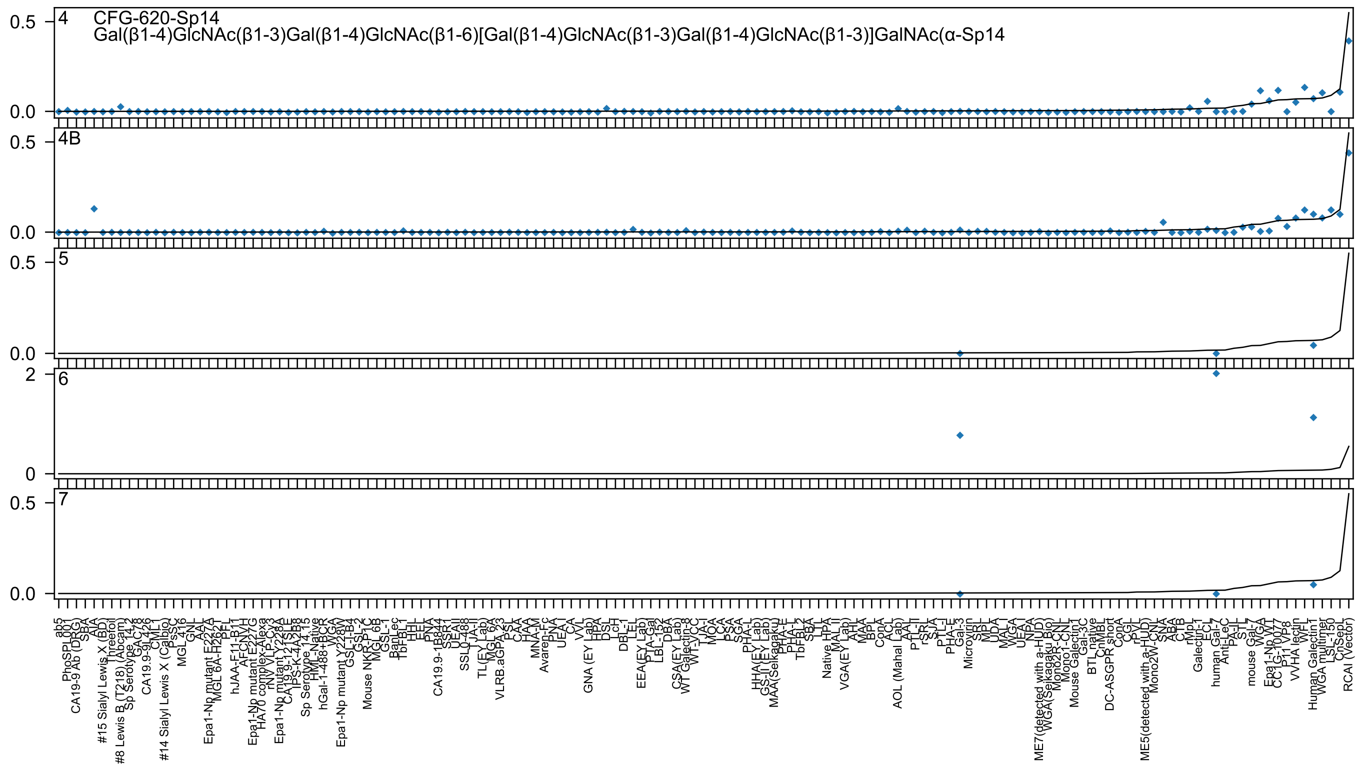

4 CFG-620-Sp14
Gal(β1-4)GlcNAc(β1-3)Gal(β1-4)GlcNAc(β1-6)[Gal(β1-4)GlcNAc(β1-3)Gal(β1-4)GlcNAc(β1-3)]GalNAc(α-Sp14
0.5
0.0
4B
0.5
0.0
5
0.5
0.0
6
2
0
7
0.5
0.0

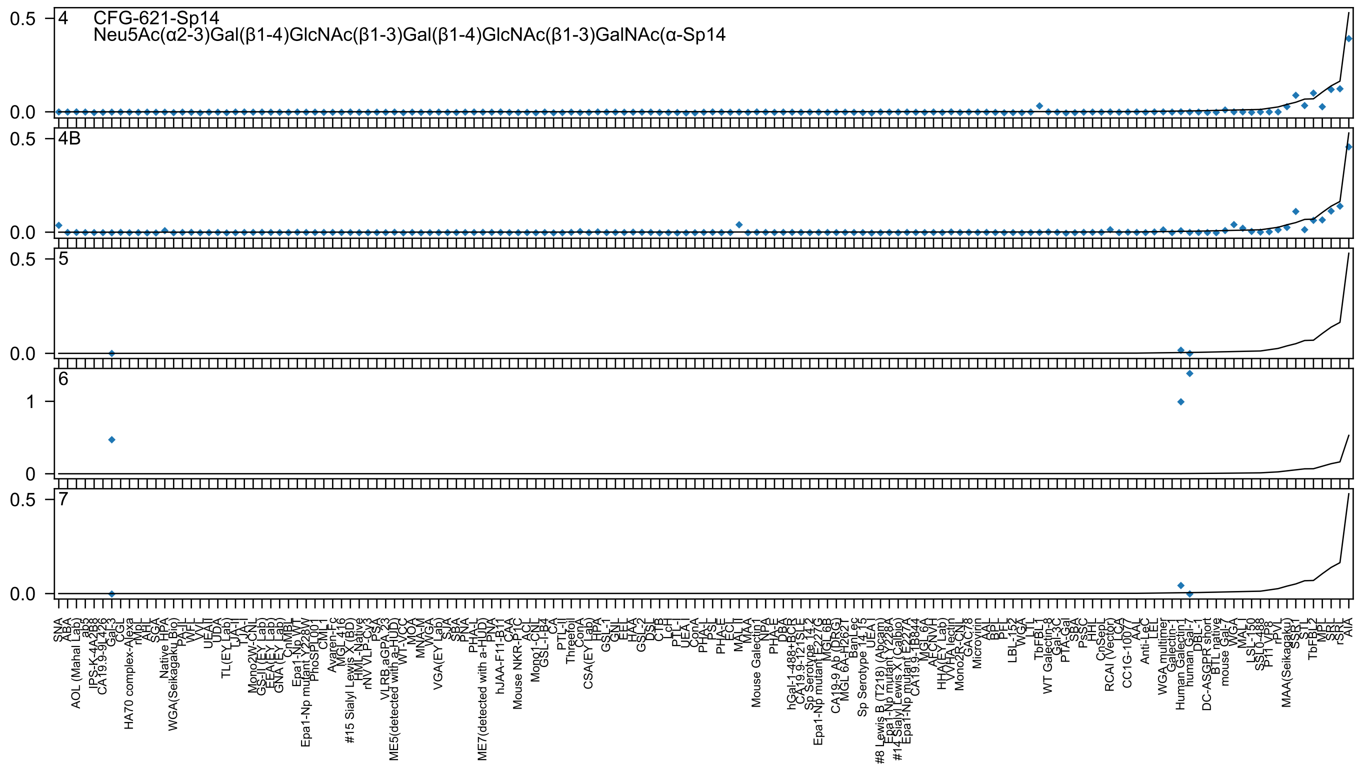

CFG-621-Sp14
Neu5Ac(α2-3)Gal(β1-4)GlcNAc(β1-3)Gal(β1-4)GlcNAc(β1-3)GalNAc(α-Sp14
4
4B
5
6
7

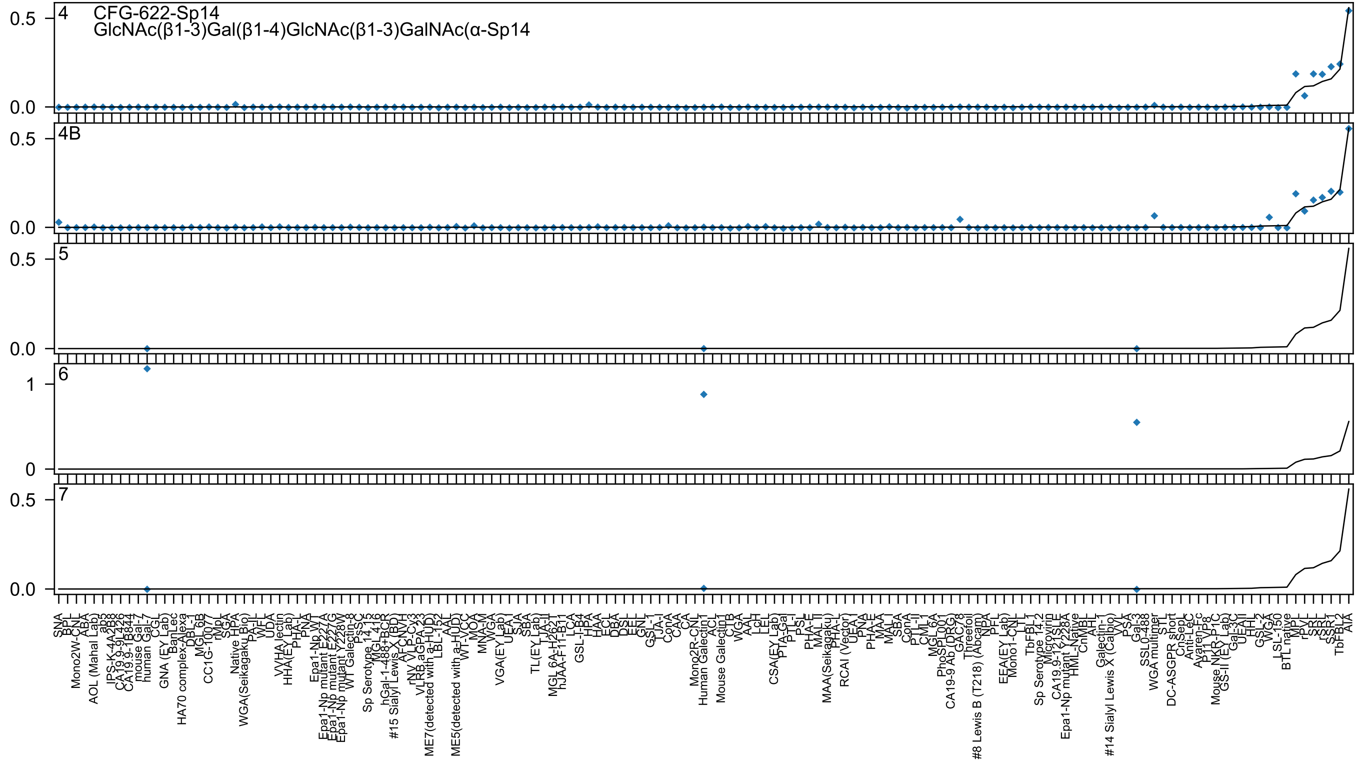

CFG-622-Sp14
GlcNAc(β1-3)Gal(β1-4)GlcNAc(β1-3)GalNAc(α-Sp14
4
4B
5
6
7
0.5
0.0
1
0

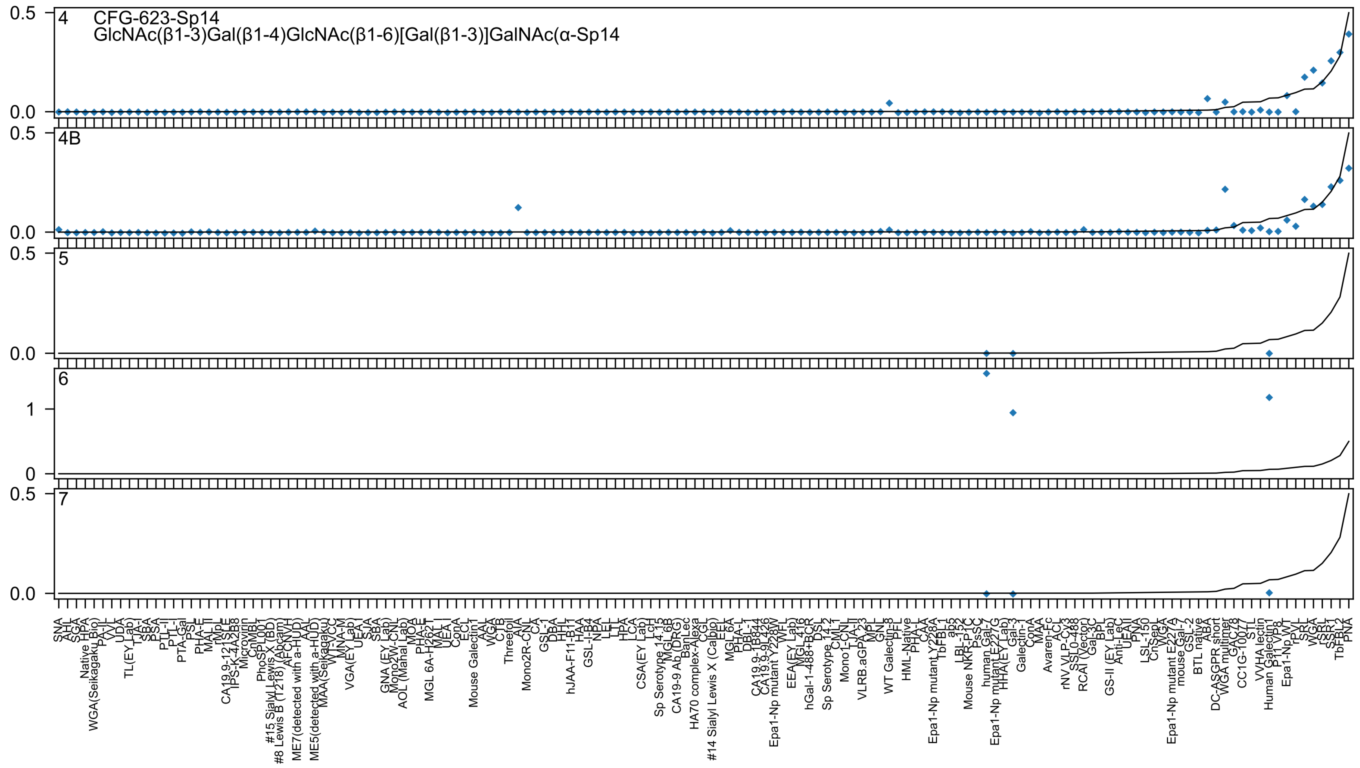
4
CFG-623-Sp14
GlcNAc(β1-3)Gal(β1-4)GlcNAc(β1-6)[Gal(β1-3)]GalNAc(α-Sp14
4B
5
6
7

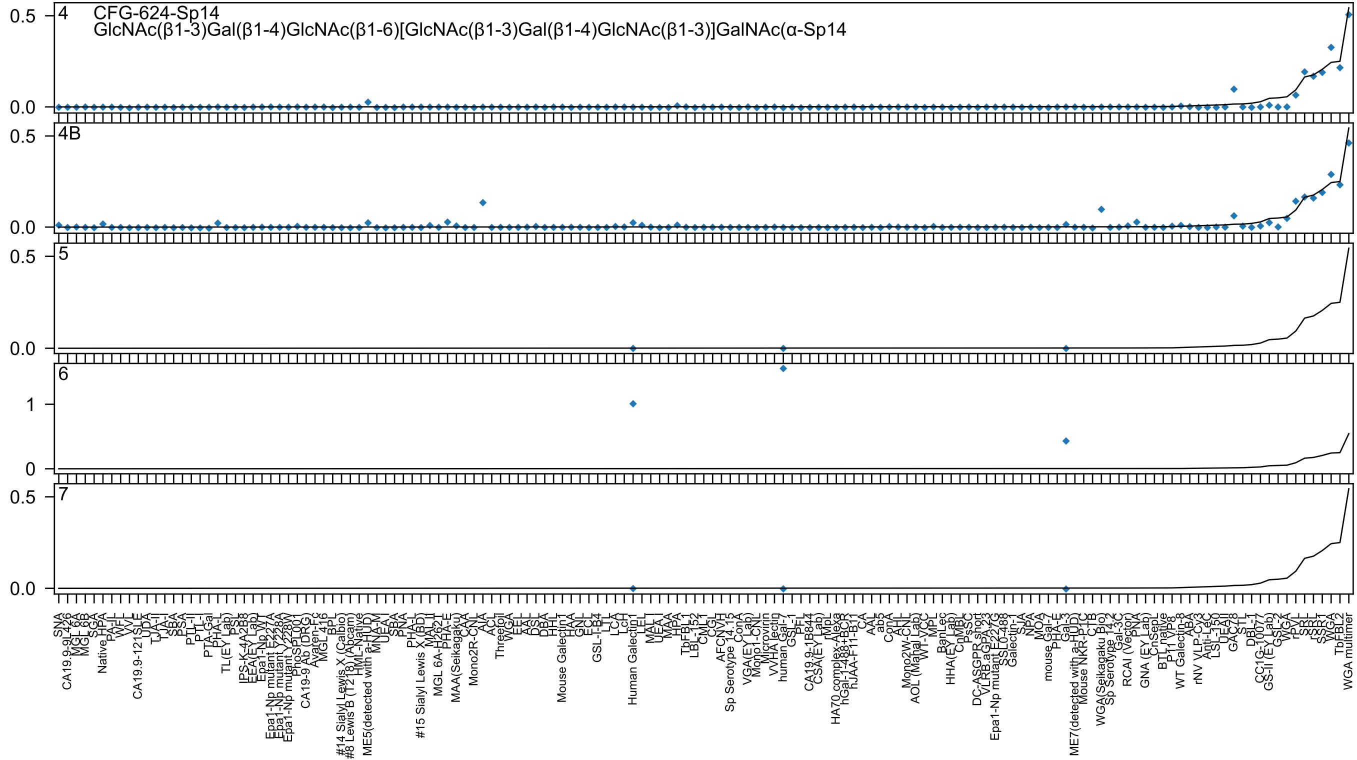
4 CFG-624-Sp14
GlcNAc(β1-3)Gal(β1-4)GlcNAc(β1-6)[GlcNAc(β1-3)Gal(β1-4)GlcNAc(β1-3)]GalNAc(α-Sp14
4B
5
6
7

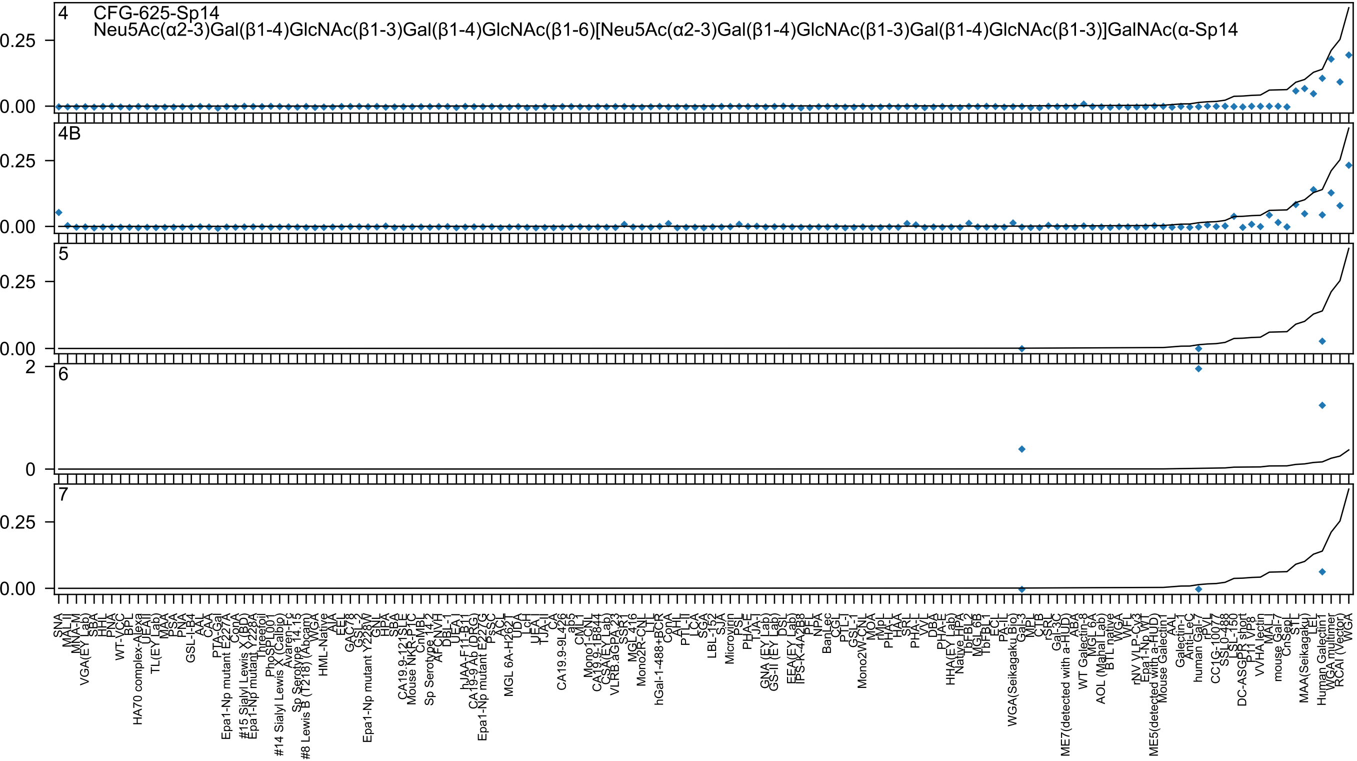

4 CFG-625-Sp14
Neu5Ac(α2-3)Gal(β1-4)GlcNAc(β1-3)Gal(β1-4)GlcNAc(β1-6)[Neu5Ac(α2-3)Gal(β1-4)GlcNAc(β1-3)Gal(β1-4)GlcNAc(β1-3)]GalNAc(α-Sp14
4B
5
6
7

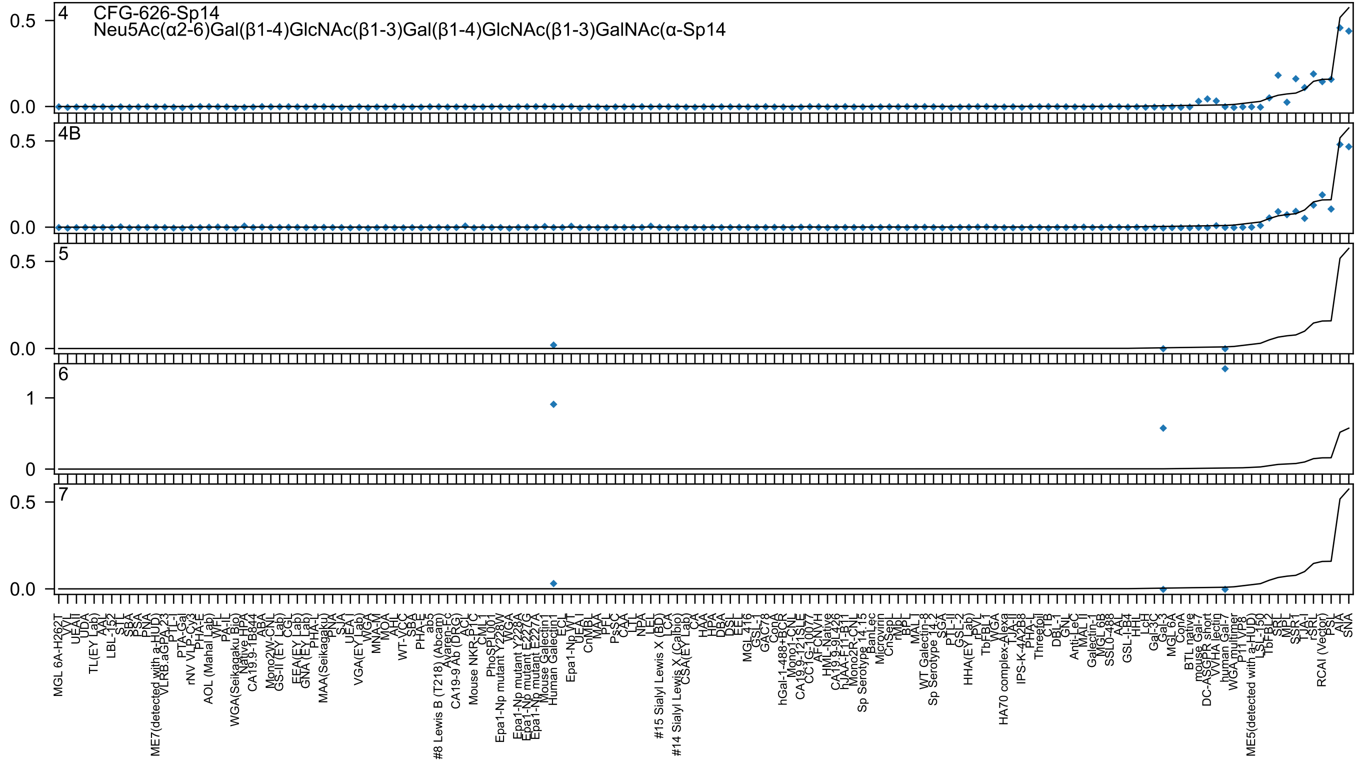
CFG-626-Sp14
Neu5Ac(α2-6)Gal(β1-4)GlcNAc(β1-3)Gal(β1-4)GlcNAc(β1-3)GalNAc(α-Sp14
4
4B
5
6
7

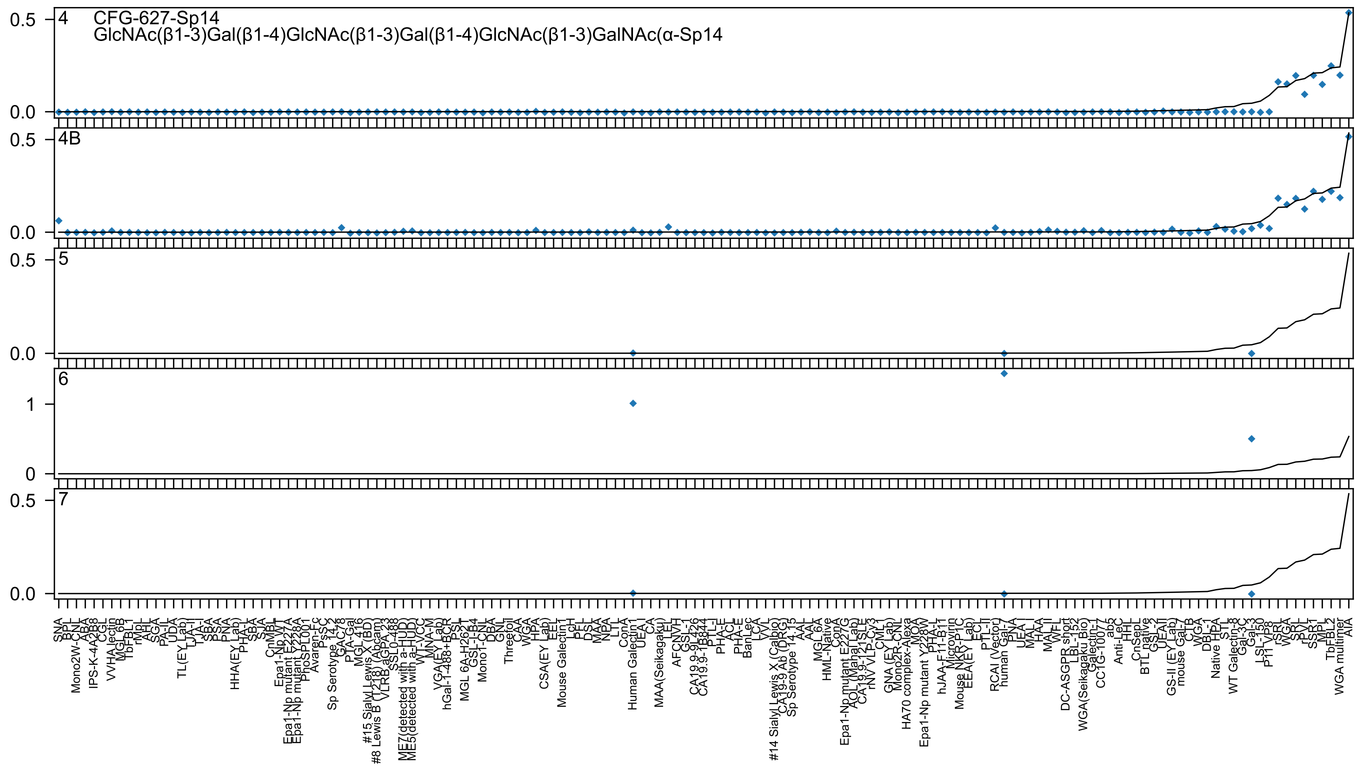

4 CFG-627-Sp14
GlcNAc(β1-3)Gal(β1-4)GlcNAc(β1-3)Gal(β1-4)GlcNAc(β1-3)GalNAc(α-Sp14
4B
5
6
7

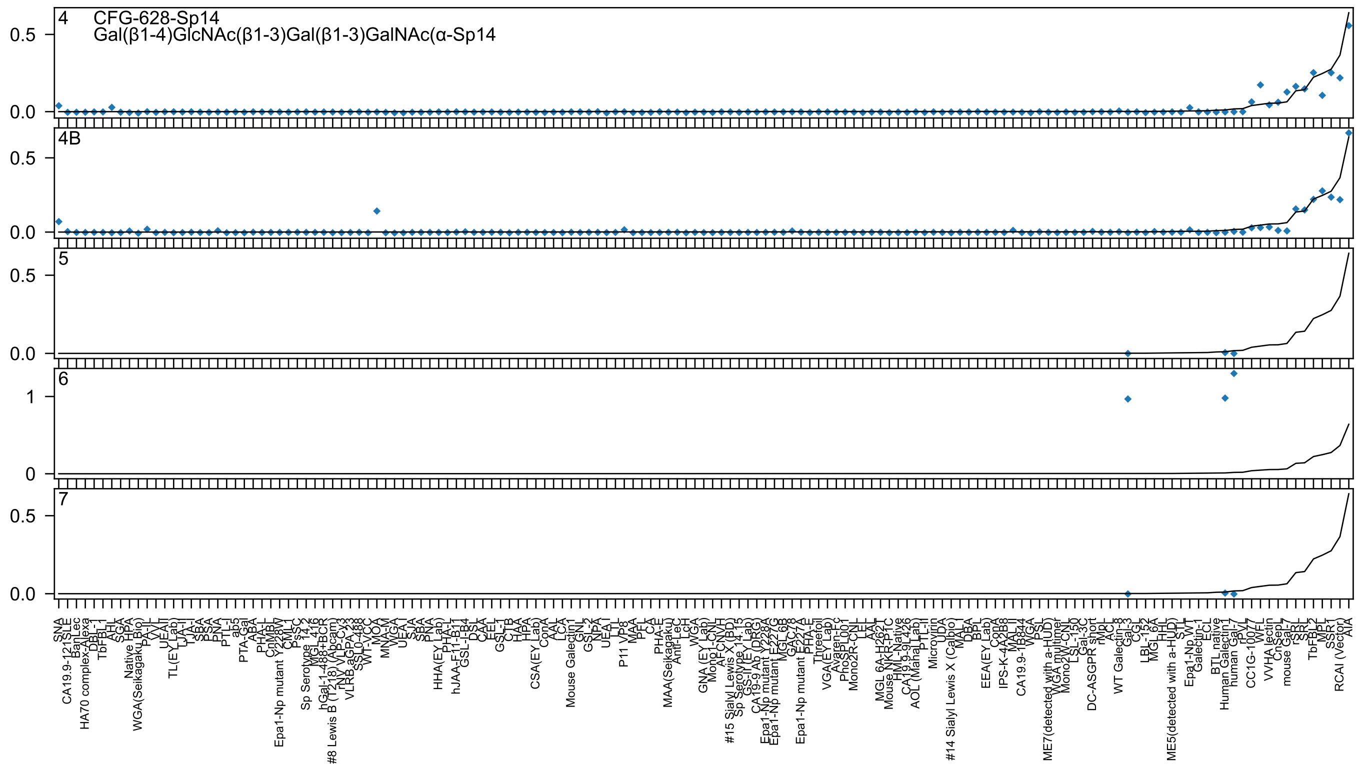

4 CFG-628-Sp14
Gal(β1-4)GlcNAc(β1-3)Gal(β1-3)GalNAc(α-Sp14
4B
5
6
7

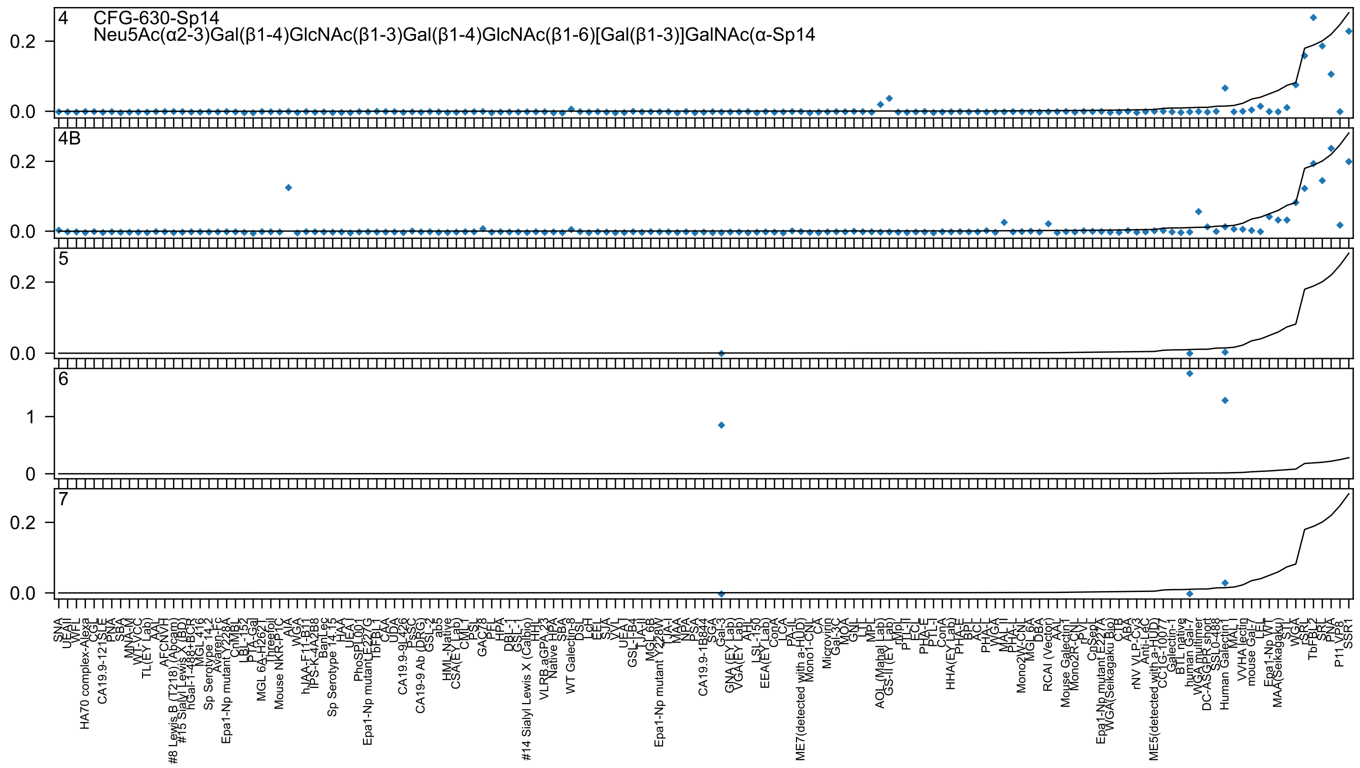
4
CFG-630-Sp14
Neu5Ac(α2-3)Gal(β1-4)GlcNAc(β1-3)Gal(β1-4)GlcNAc(β1-6)[Gal(β1-3)]GalNAc(α-Sp14
4B
5
6
7

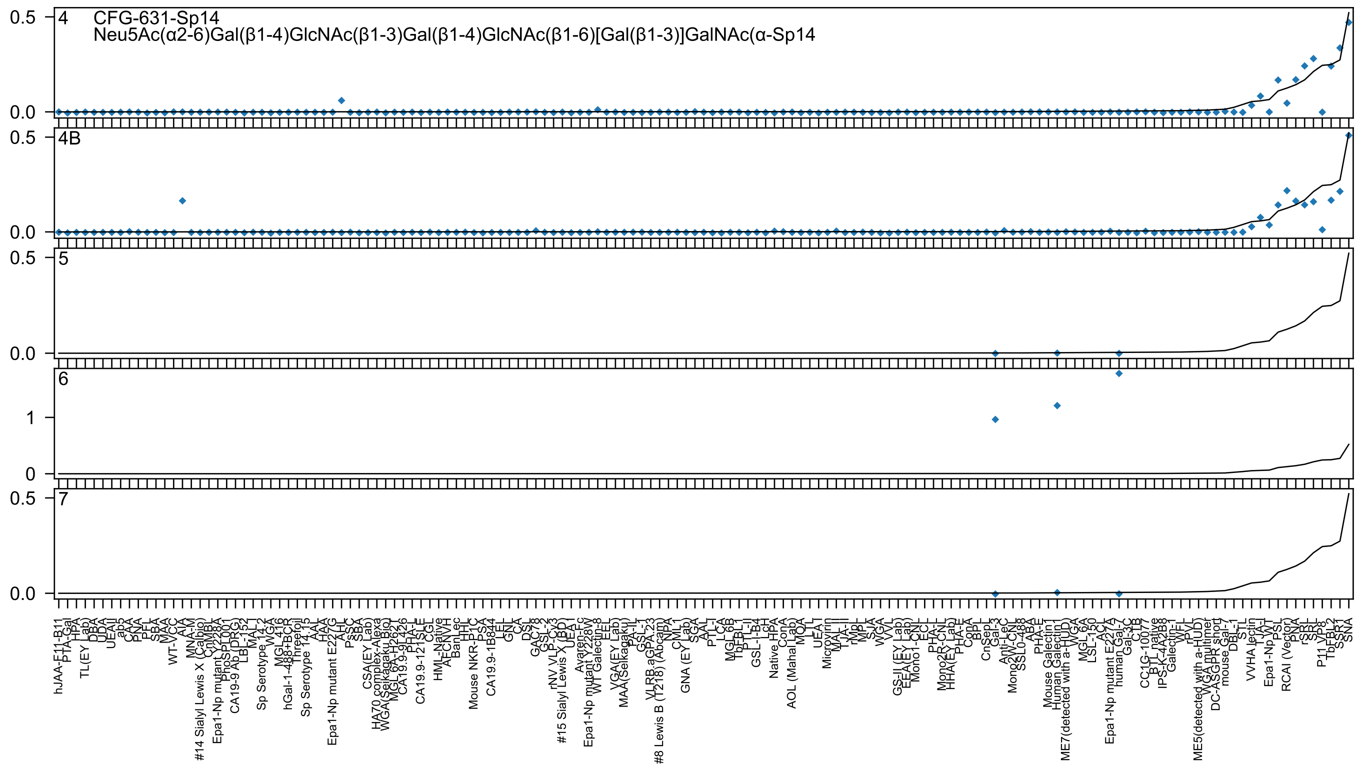

CFG-631-Sp14
Neu5Ac(α2-6)Gal(β1-4)GlcNAc(β1-3)Gal(β1-4)GlcNAc(β1-6)[Gal(β1-3)]GalNAc(α-Sp14
4
4B
5
6
7

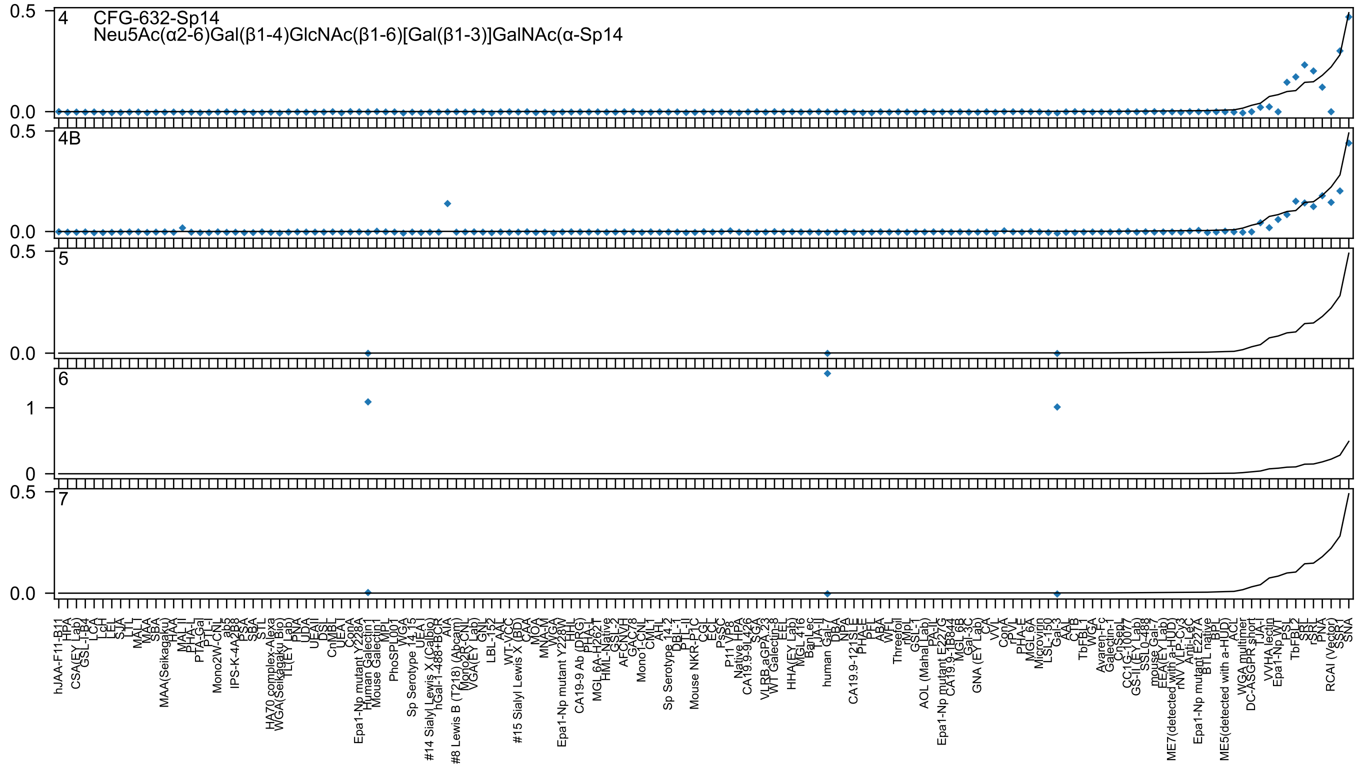

4
CFG-632-Sp14
Neu5Ac(α2-6)Gal(β1-4)GlcNAc(β1-6)[Gal(β1-3)]GalNAc(α-Sp14
4B
5
6
7

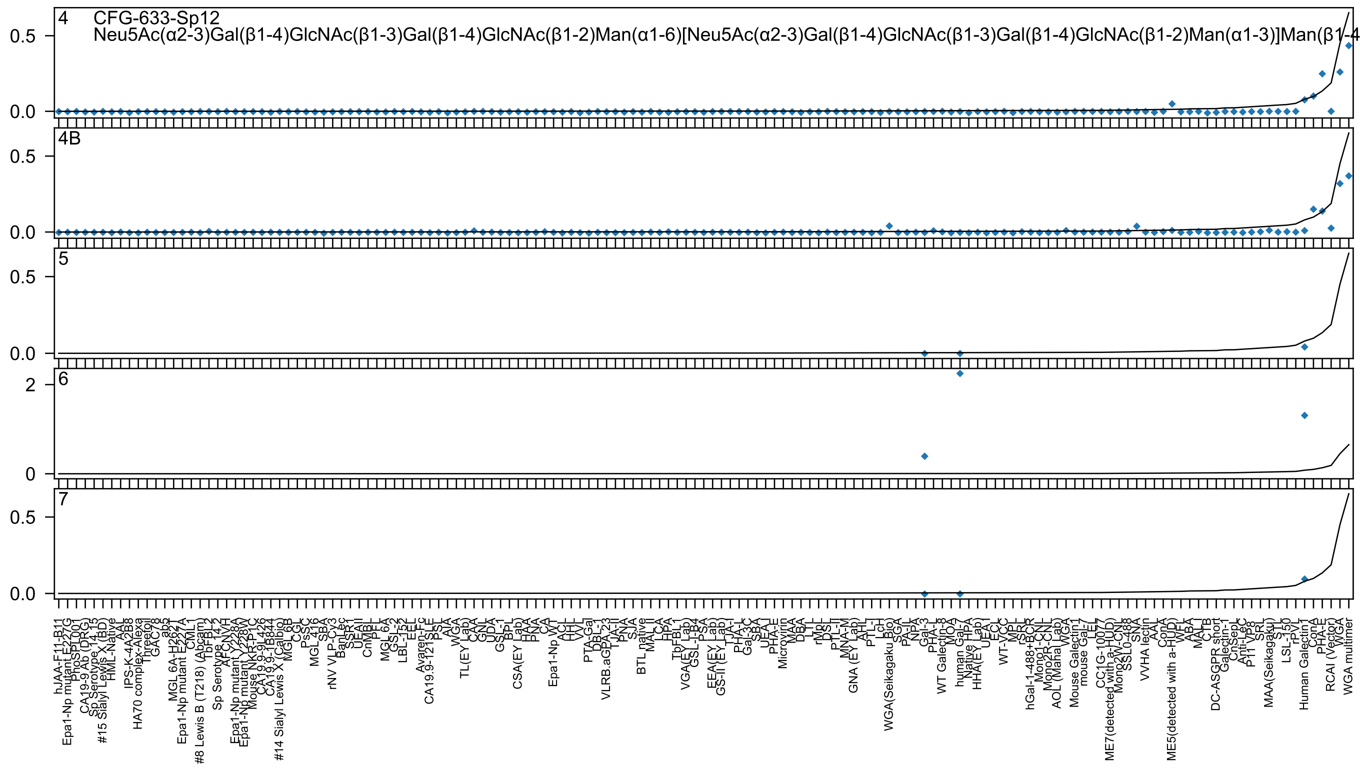

4 CFG-633-Sp12
Neu5Ac(α2-3)Gal(β1-4)GlcNAc(β1-3)Gal(β1-4)GlcNAc(β1-2)Man(α1-6)[Neu5Ac(α2-3)Gal(β1-4)GlcNAc(β1-3)Gal(β1-4)GlcNAc(β1-2)Man(α1-3)]Man(β1-4
4B
5
6
7

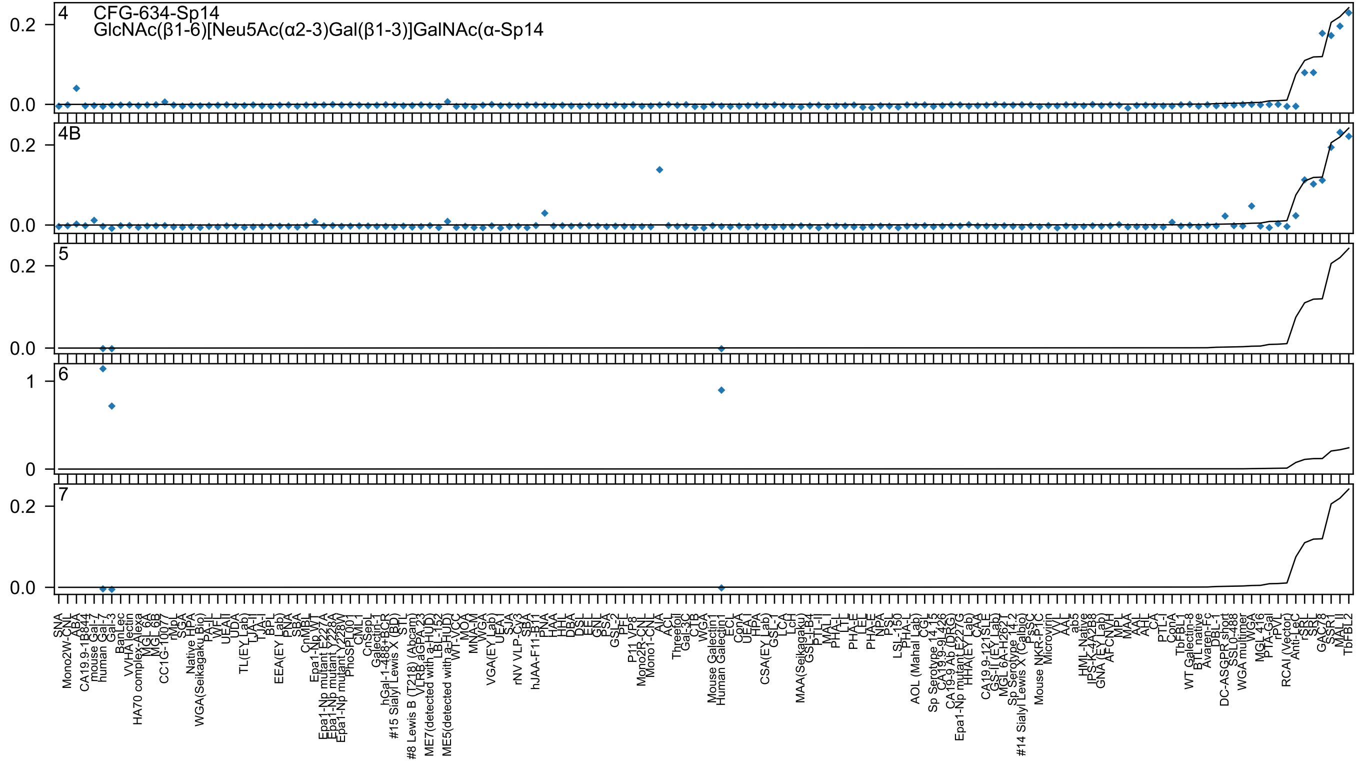

4 CFG-634-Sp14
GlcNAc(β1-6)[Neu5Ac(α2-3)Gal(β1-3)]GalNAc(α-Sp14
4B
5
6
7

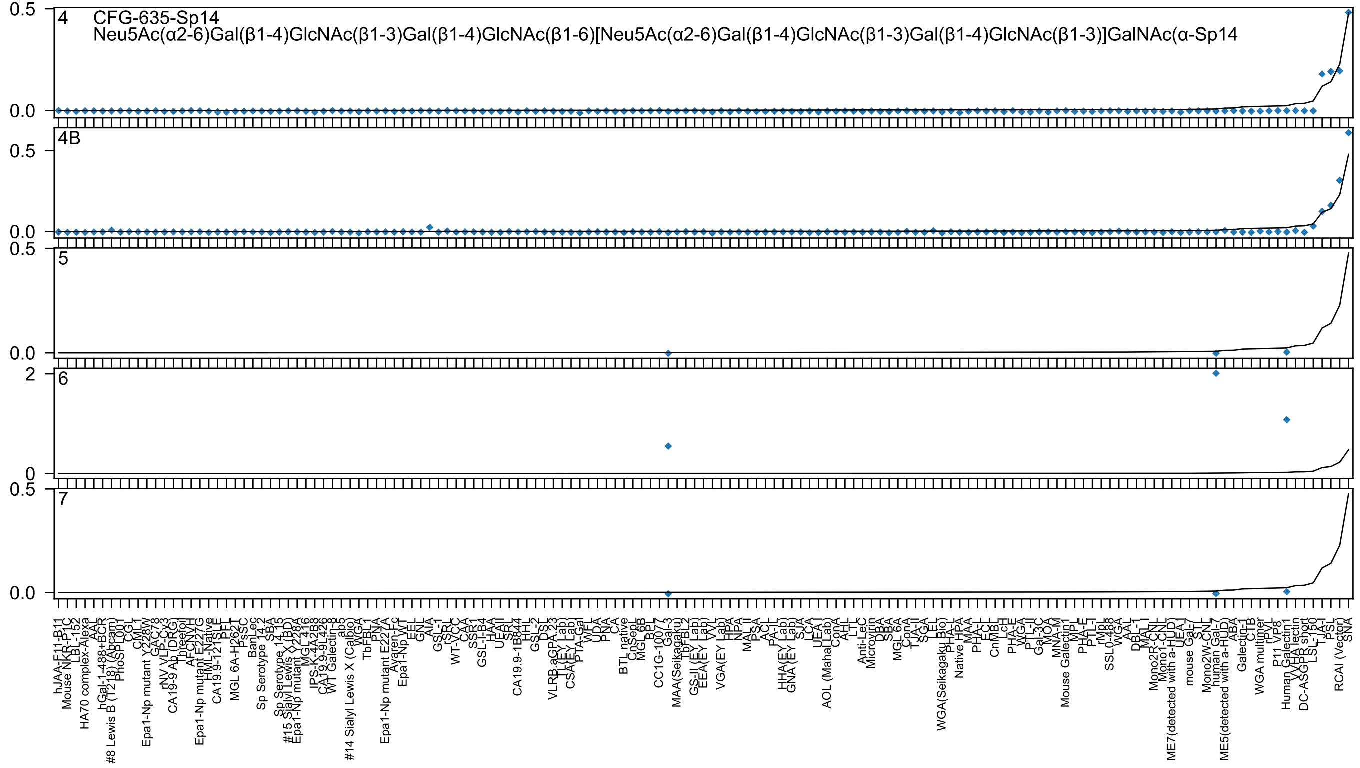

4
CFG-635-Sp14
Neu5Ac(α2-6)Gal(β1-4)GlcNAc(β1-3)Gal(β1-4)GlcNAc(β1-6)[Neu5Ac(α2-6)Gal(β1-4)GlcNAc(β1-3)Gal(β1-4)GlcNAc(β1-3)]GalNAc(α-Sp14
4B
5
6
7
0.5
0.0
2
0
hJAA-F11-B11
Mouse NKR-P1C
LBL-152
HA70 complex-Alexa
AAL
hGal-1-488+BCR
#8 Lewis B (T218) (Abcam)
PhoSL001
CGL
CML1
Epa1-Np mutant Y228W
GAC78
rNV VLP-Cy3
CA19-9 Ab (DRG)
Threefoil
AFCNVH
Epa1-Np mutant E227G
HML-Native
CA19.9-121SLE
PFL
MGL 6A-H262T
PsSC
BanLec
Sp Serotype 14.2
SBA
Sp Serotype 14.15
#15 Sialyl Lewis X (BD)
Epa1-Np mutant Y228A
MGL 416
IPS-K-4A2B8
CA19.9-9L426
WT Galectin-8
ab5
#14 Sialyl Lewis X (Calbio)
WGA
TbFBL1
PNA
Epa1-Np mutant E227A
Ayaren-Fc
Epa1-Np WT
EEL
GNL
AIA
GSL-1
rSRL
WT-VCC
CAA
SSR1
GSL-I-B4
HAA
UEAII
SRL
CA19.9-1B844
HHL
GSL-2
DSL
VLRB.aGPA.23
TL(EY Lab)
CSA(EY Lab)
PTA-Gal
WFL
UDA
PNA
CA
BTL native
CnSep
MGL 6B
BPL
CC1G-10077
Gal-3
MAA(Seikagaku)
TbFBL2
GS-II (EY Lab)
EEA(EY Lab)
VVL
VGA(EY Lab)
HPA
NPA
MAL II
PSA
ACL
PA-IL
HHA(EY Lab)
GNA (EY Lab)
SJA
LCA
UEA I
AOL (Mahal Lab)
ConA
AHL
LTL
Anti-LeC
Microvirin
DBA
SBA
MGL 6A
ConA
TJA-II
SGA
LEL
WGA(Seikagaku Bio)
PHA-L
Native HPA
MAA
PHA-L
ECL
CnMBL
LcH
PHA-E
WGA
PTL-II
Gal-3C
MOA
MNA-M
Mouse Galectin1
MPL
PHA-E
PTL-I
rMpL
SSL0-488
WGA
AAL
DBL-1
MAL-I
Mono2R-CNL
Mono1-CNL
ME7(detected with a-HUD)
UEA I
mouse Gal-7
STL
Mono2W-CNL
human Gal-7
ME5(detected with a-HUD)
ABA
Galectin-1
CTB
WGA multimer
rPVL
P11 VP8
Human Galectin1
VVA lectin
DC-ASGPR short
LSL-150
TJA-I
PSL
RCAI (Vector)
SNA

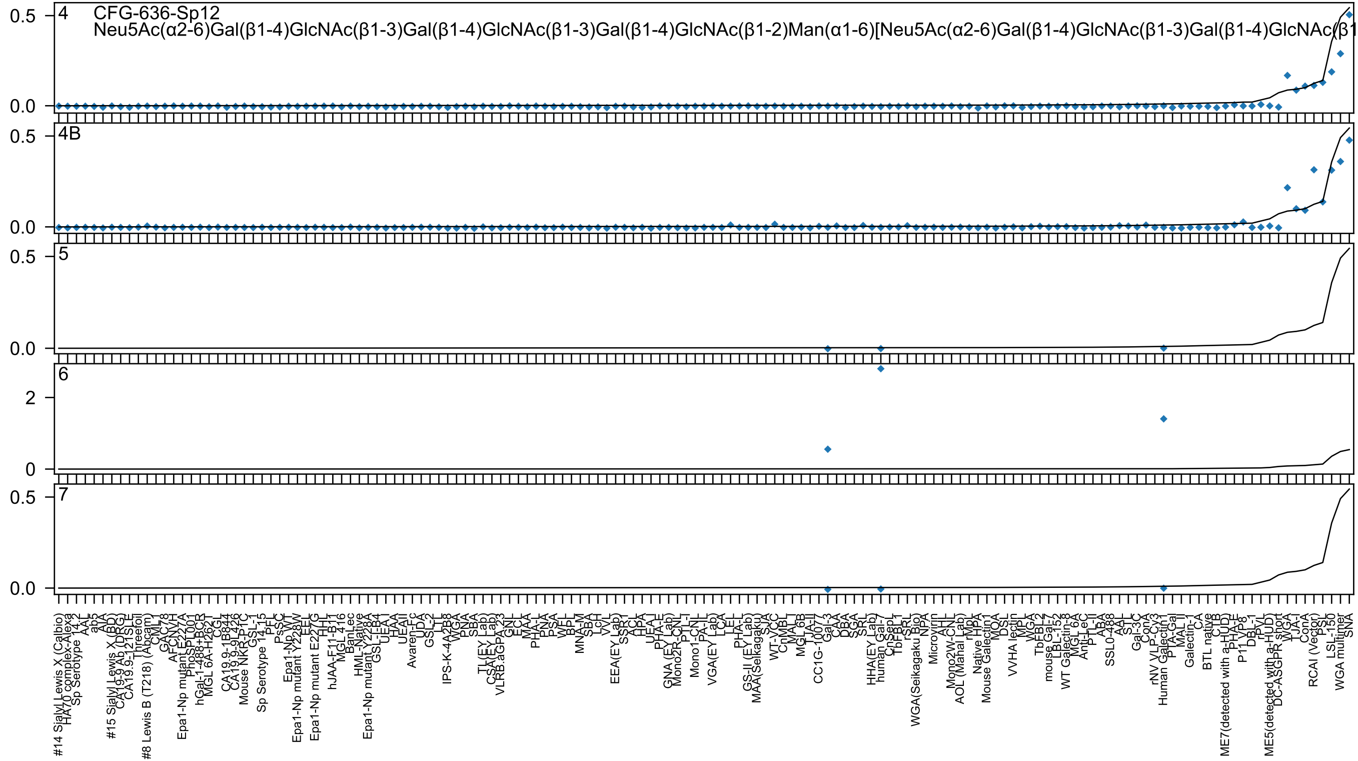
4
CFG-636-Sp12
Neu5Ac(α2-6)Gal(β1-4)GlcNAc(β1-3)Gal(β1-4)GlcNAc(β1-3)Gal(β1-4)GlcNAc(β1-2)Man(α1-6)[Neu5Ac(α2-6)Gal(β1-4)GlcNAc(β1-3)Gal(β1-4)GlcNAc(β1
4B
5
6
7
0.5
0.0
2
0

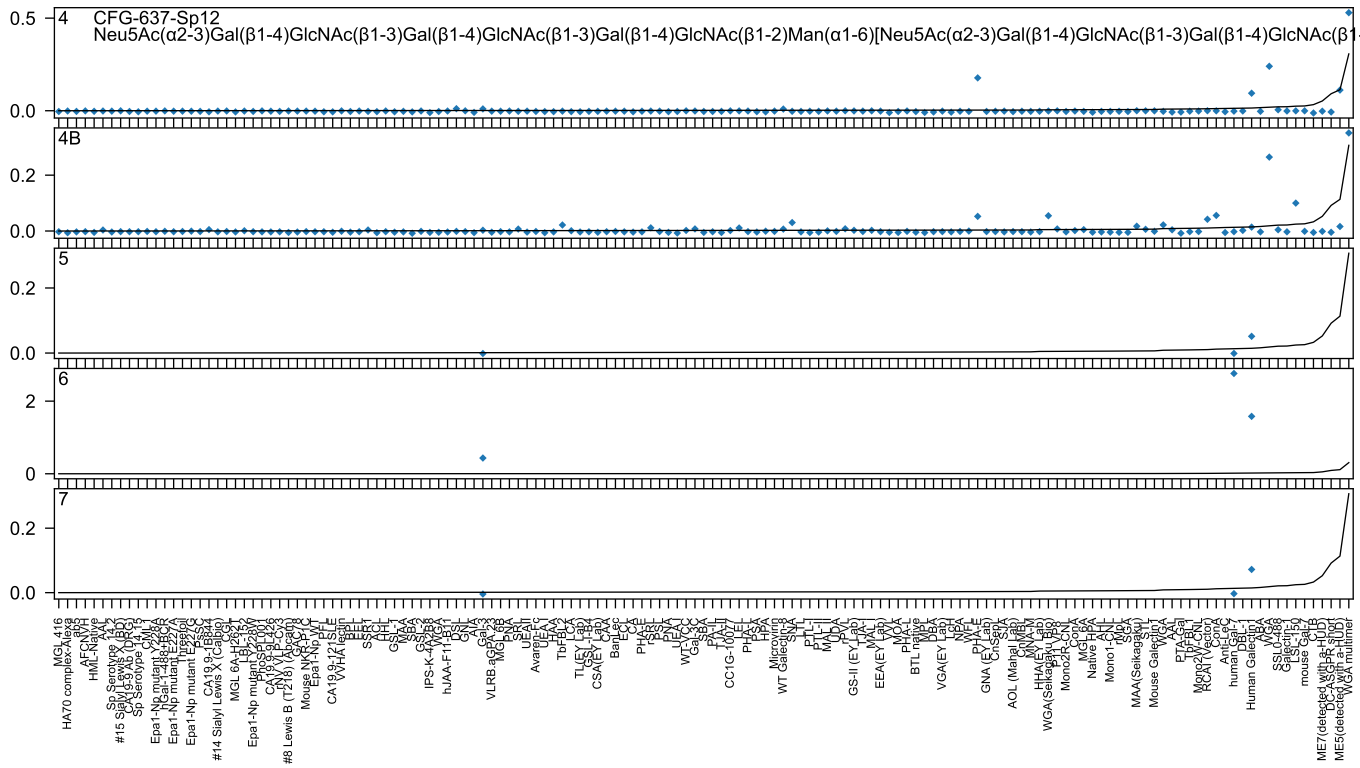
4
CFG-637-Sp12
Neu5Ac(α2-3)Gal(β1-4)GlcNAc(β1-3)Gal(β1-4)GlcNAc(β1-3)Gal(β1-4)GlcNAc(β1-2)Man(α1-6)[Neu5Ac(α2-3)Gal(β1-4)GlcNAc(β1-3)Gal(β1-4)GlcNAc(β1-
4B
5
6
7

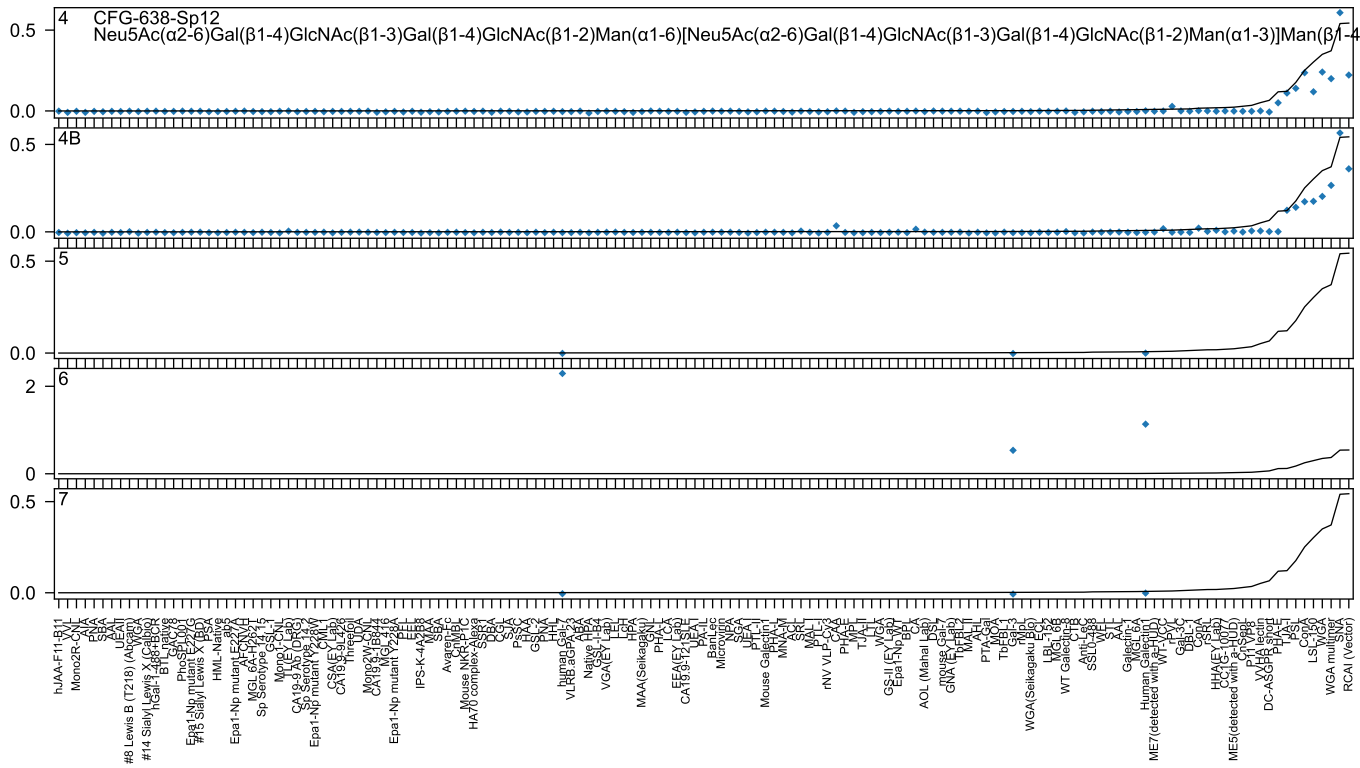
4 CFG-638-Sp12
Neu5Ac(α2-6)Gal(β1-4)GlcNAc(β1-3)Gal(β1-4)GlcNAc(β1-2)Man(α1-6)[Neu5Ac(α2-6)Gal(β1-4)GlcNAc(β1-3)Gal(β1-4)GlcNAc(β1-2)Man(α1-3)]Man(β1-4
4B
5
6
7
0.5
0.0
2
0
hJAA-F11-B11
Mono2R-CNL
#8 Lewis B (T218) (Abcam)
#14 Sialyl Lewis X (Calbio)
#15 Sialyl Lewis X (BD)
HML-Native
Epa1-Np mutant E227A
Sp Serotype 14.15
CA19-9 Ab (DRG)
Epa1-Np mutant Y228W
Epa1-Np mutant Y228A
IPS-K-4A2B8
Mouse NKR-P1C
HA70 complex-Alexa
human Gal-7
VLRB.aGPA.23
MAA(Seikagaku)
CA19.9-121SLE
Mouse Galectin1
rNV VLP-Cy3
GS-II (EY Lab)
AOL (Mahal Lab)
WGA(Seikagaku Bio)
WT Galectin-8
Human Galectin1
ME7(detected with a-HUD)
ME5(detected with a-HUD)
DC-ASGPR short
WGA multimer
RCAI (Vector)

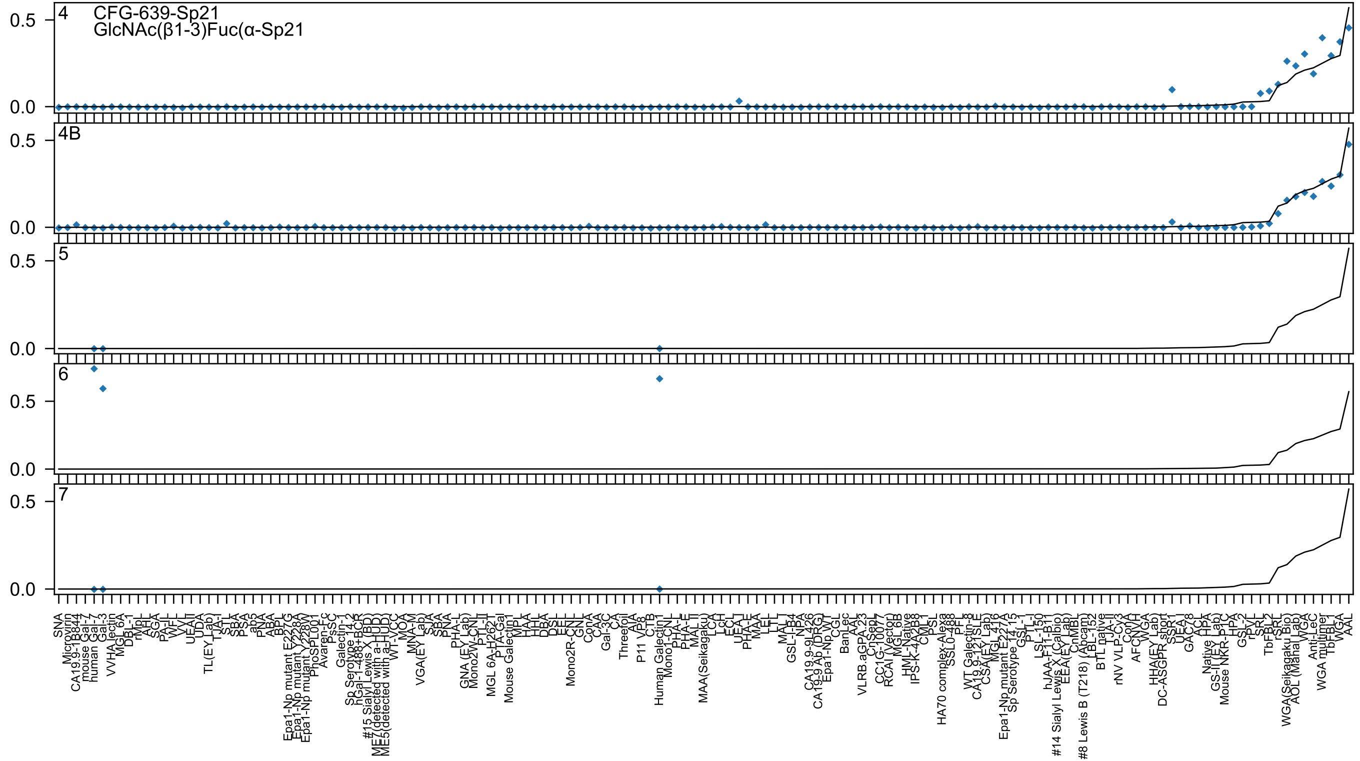

4 CFG-639-Sp21
GlcNAc(β1-3)Fuc(α-Sp21
4B
5
6
7
0.5
0.0

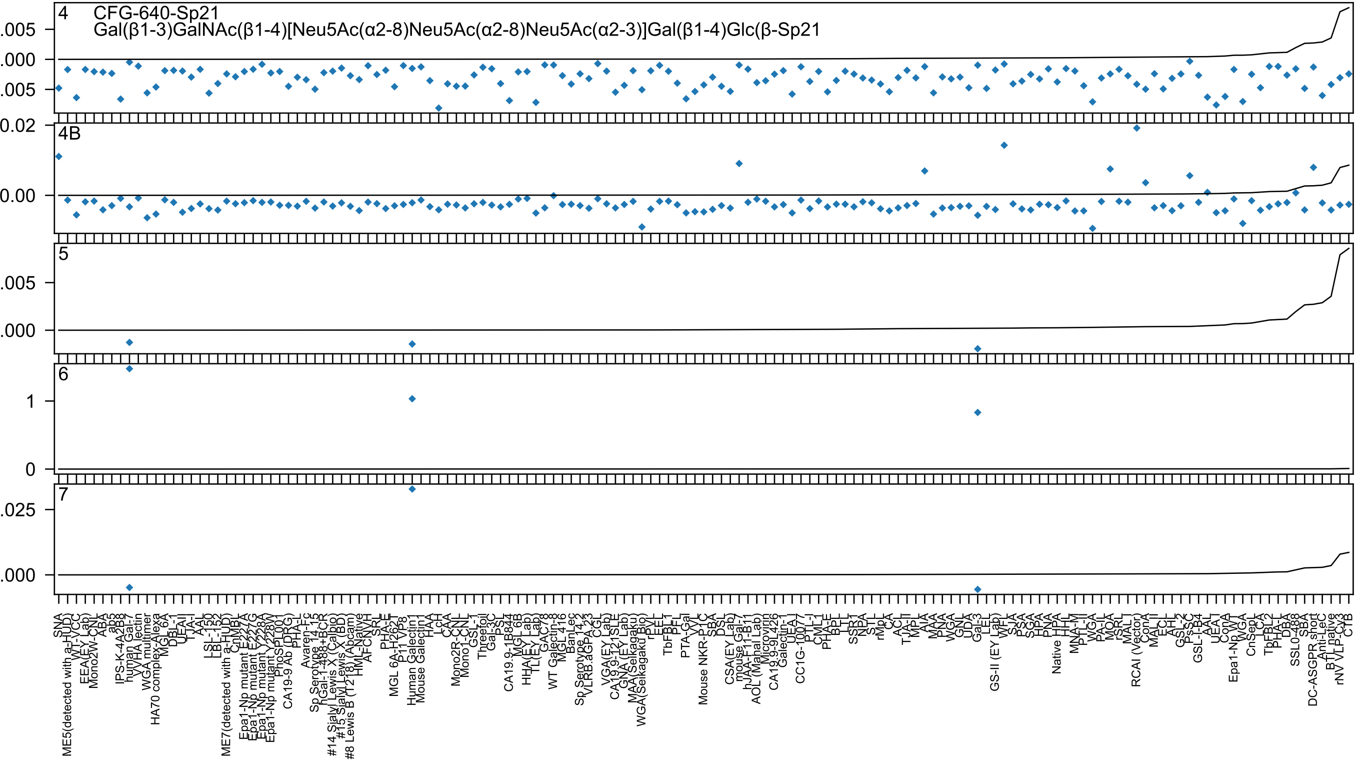

4 CFG-640-Sp21
Gal(β1-3)GalNAc(β1-4)[Neu5Ac(α2-8)Neu5Ac(α2-8)Neu5Ac(α2-3)]Gal(β1-4)Glc(β-Sp21
4B
5
6
7

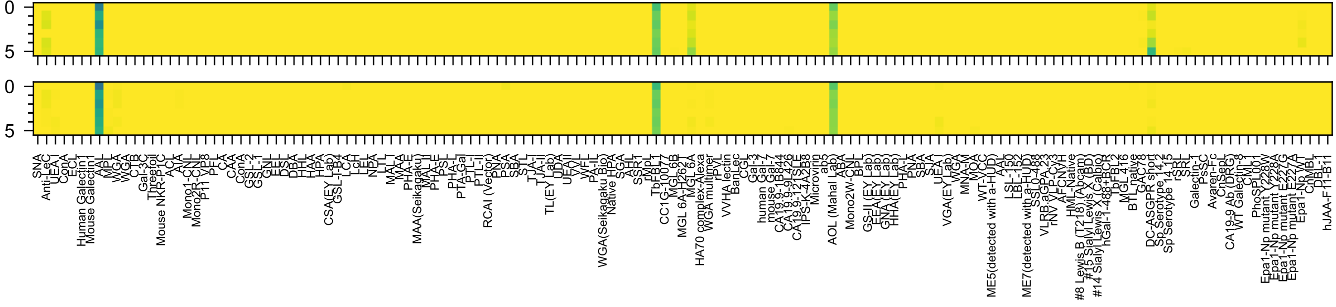
Fuc(α-Sp8

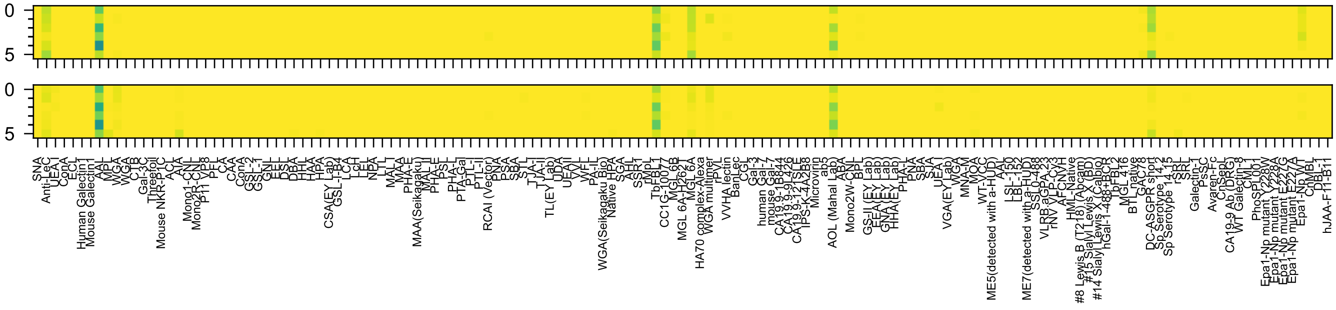
Rha(α-Sp8

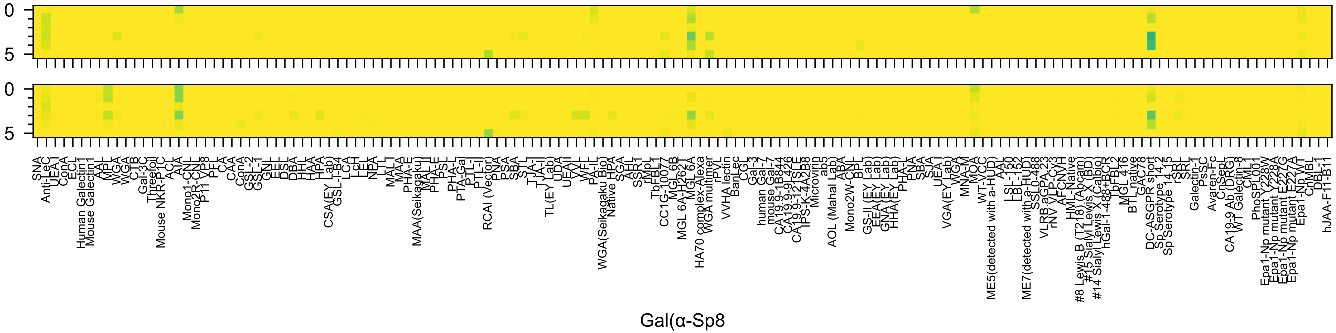
0
5
0
5
SNA
Anti-LeC
UEA-I
ConA
ECL
Human Galectin1
Mouse Galectin1
AAL
MPL
WGA
WGA
CTB
Gal-3C
Threefoil
Mouse NKR-P1C
ACL
AIA
Mono1-CNL
Mono2R-CNL
P11 VP8
PFL
CA
CAA
ConA
GSL-2
GSL-1
GNL
EEL
DSL
DBA
HHL
HAA
HPA
CSA(EY Lab)
GSL-I-B4
LCA
LcH
LEL
NPA
LTL
MAL-I
MAA
PHA-E
MAA(Seikagaku)
MAL-II
PHA-E
PSL
PHA-L
PTA-Gal
PTL-I
PTL-II
RCAI (Vector)
PNA
PSA
SBA
STL
TJA-I
TJA-II
TL(EY Lab)
UDA
UEAII
VVL
WFL
PA-IL
WGA(Seikagaku Bio)
Native HPA
SGA
AAL
SSR1
rMpL
TbFBL1
CC1G-10077
MGL 6B
MGL 6A-H262T
MGL 6A
HA70 complex-Alexa
WGA multimer
rPhL
VVHA lectin
BanLec
CGL
Gal-3
human Gal-7
mouse Gal-7
CA19.9-1B844
CA19.9-9L426
CA19.9-121SLE
IPS-K-4A2B8
Microvirin
ab5
AOL (Mahal Lab)
ABA
Mono2W-CNL
BPL
GS-II (EY Lab)
EEA(EY Lab)
GNA (EY Lab)
HHA(EY Lab)
PHA-L
PNA
SBA
SJA
UEA-I
VGA(EY Lab)
WGA
MNA-M
MOA
WT-VCC
ME5(detected with a-HUD)
AAL
LSL-150
LBL-152
ME7(detected with a-HUD)
SSL0-488
VLRB.aGPA.23
rNV VLP-Cy3
AFCNVH
HML-Native
#8 Lewis B (T218) (Abcam)
#15 Sialyl Lewis X (BD)
#14 Sialyl Lewis X (Calbio)
hGal-1-488+BCR
TbFBL2
MGL 416
BTL native
GAC78
DC-ASGPR short
Sp Serotype 14.2
Sp Serotype 14.15
rSRL
SRL
Galectin-1
PsSC
Avaren-Fc
ChSepL
CA19-9 Ab (DRG)
WT Galectin-8
CML1
PhoSPL001
Epa1-Np mutant Y228W
Epa1-Np mutant E227G
Epa1-Np mutant E227A
Epa1-Np WT
CnMBL
DBL-1
hJAA-F11-B11
Gal(α-Sp8

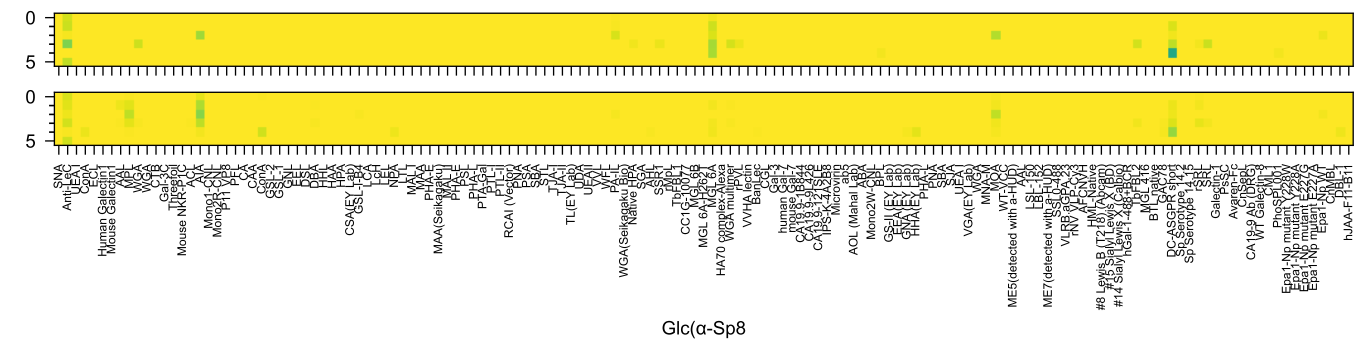
Glc(α-Sp8

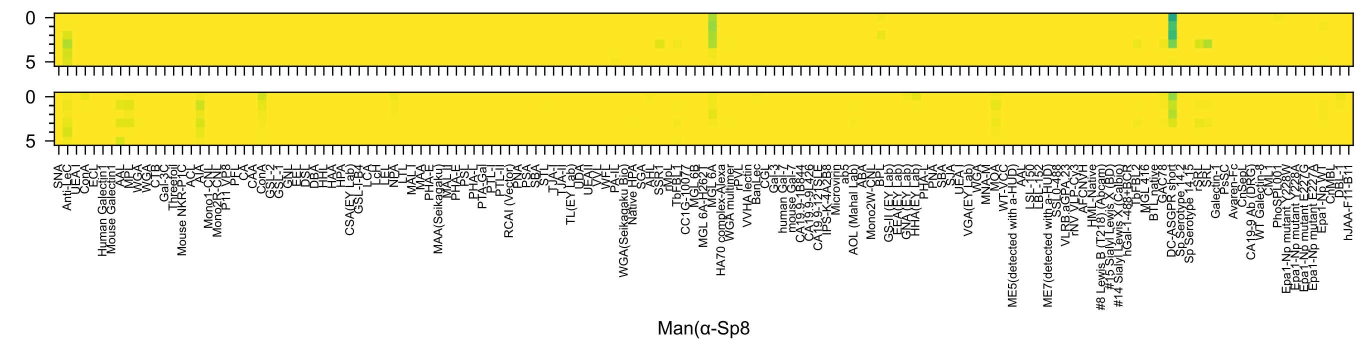

0
5
0
5
SNA
Anti-LeC
UEA-I
ConA
ECL
Human Galectin1
Mouse Galectin1
AAL
MPL
WGA
WGA
CTB
Gal-3C
Threefoil
Mouse NKR-P1C
ACL
AIA
Mono1-CNL
Mono2R-CNL
P11 VP8
PFL
CA
CAA
ConA
GSL-2
GSL-1
GNL
EEL
DSL
DBA
HHL
HAA
HPA
CSA(EY Lab)
GSL-I-B4
LCA
LcH
LEL
NPA
LTL
MAL-I
MAA
PHA-E
MAA(Seikagaku)
MAL-II
PHA-E
PSL
PHA-L
PTA-Gal
PTL-I
PTL-II
RCAI (Vector)
PNA
PSA
SBA
STL
TJA-I
TJA-II
TL(EY Lab)
UDA
UEAII
VVL
WFL
PA-IL
WGA(Seikagaku Bio)
Native HPA
SGA
AHL
SSR1
rMpL
TbFBL1
CC1G-10077
MGL6B
MGL 6A-H262T
MGL 6A
HA70 complex-Alexa
WGA multimer
rPVL
VVHA lectin
BanLec
CGL
Gal-3
human Gal-7
mouse Gal-7
CA19.9-1B844
CA19.9-9L426
CA19.9-121SLE
IPS-K-4A2B8
Microvirin
ab5
AOL (Mahal Lab)
ABA
Mono2W-CNL
BPL
GS-II (EY Lab)
EEA (EY Lab)
GNA (EY Lab)
HHA (EY Lab)
PHA-L
PNA
SBA
SJA
UEA-I
VGA(EY Lab)
WGA
MNA-M
MOA
WT-VCC
ME5(detected with a-HUD)
AAT
LSL-150
LBL-152
ME7(detected with a-HUD)
SSL0-488
VLRB.aGPA.23
rNV VLP-Cy3
AFCNVH
HML-Native
#8 Lewis B (T218) (Abcam)
#15 Sialyl Lewis X (BD)
#14 Sialyl Lewis X (Calbio)
hGal-1-488+BCR
TbFBL2
MGL 416
BTL native
GAC78
DC-ASGPR short
Sp Serotype 14_2
Sp Serotype 14_15
rSRL
SRL
Galectin-1
PsSC
Avaren-Fc
ChSepL
CA19-9 Ab (DRG)
WT Galectin-8
CML1
PhoSL001
Epa1-Np mutant Y228W
Epa1-Np mutant E227G
Epa1-Np mutant E227A
Epa1-Np WT
ChMBL
DBL-1
hJAA-F11-B11
Man(α-Sp8

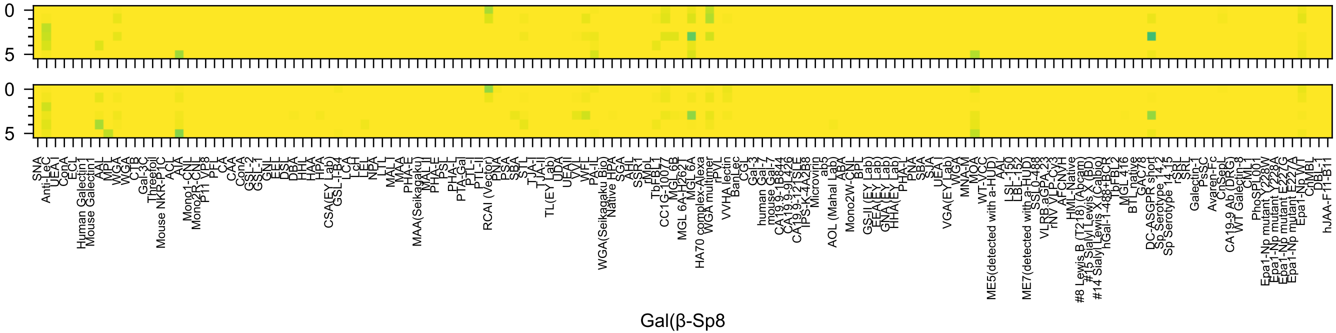
Gal(β-Sp8

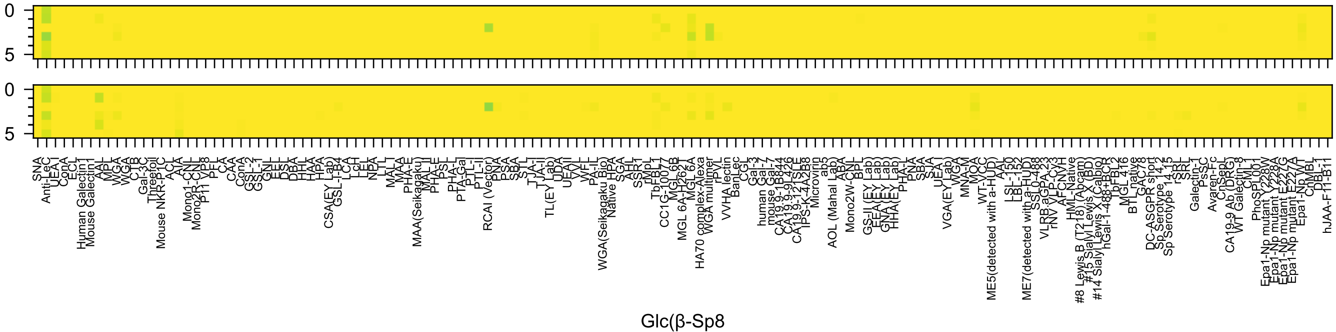

Glc(β-Sp8

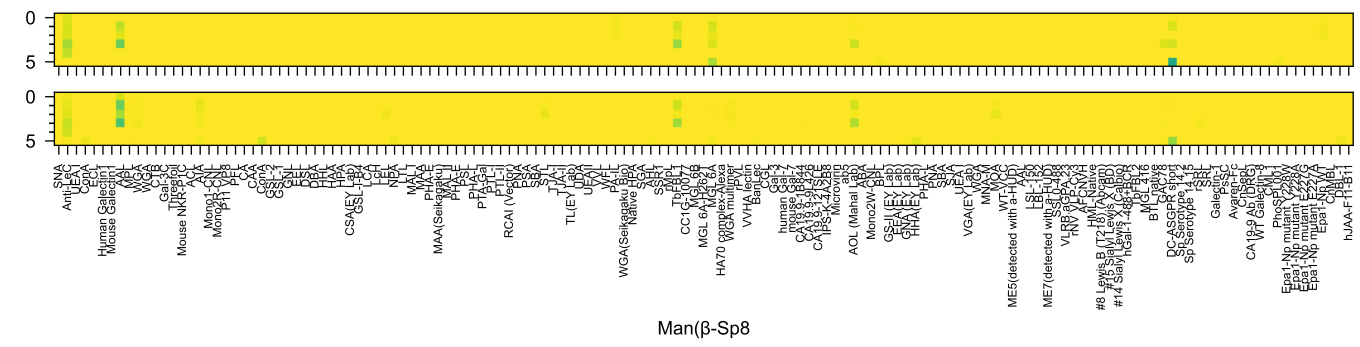
Man(β-Sp8

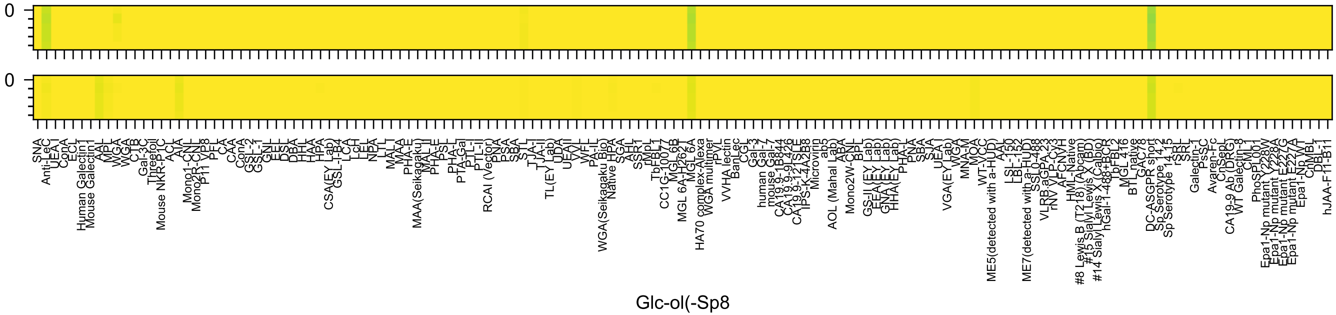

Glc-ol(-Sp8

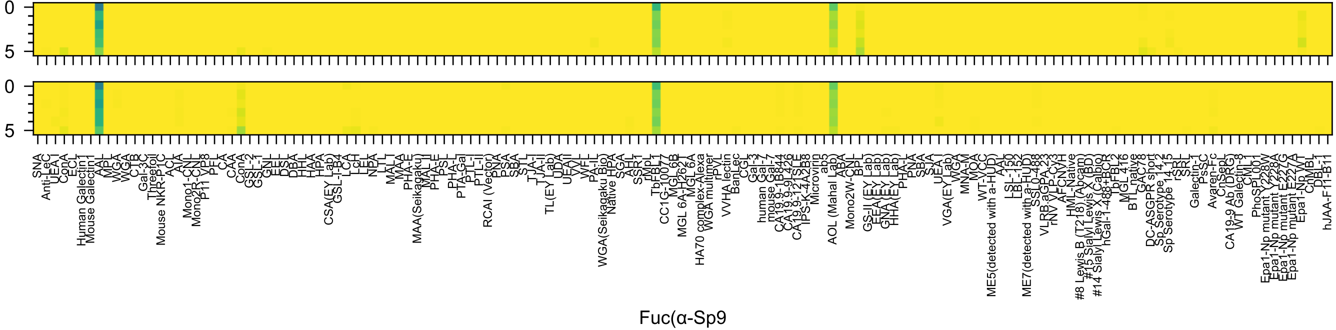
0
5
0
5
SNA
Anti-LeC
UEA-I
ConA
ECL
Human Galectin1
Mouse Galectin1
AAL
MPL
WGA
WGA
CTB
Gal-3C
Threefoil
Mouse NKR-P1C
ACL
AIA
Mono1-CNL
Mono2R-CNL
P11 VP8
PFL
CA
CAA
ConA
GSL-2
GSL-1
GNL
EEL
DSL
DBA
HHL
HAA
HPA
CSA(EY Lab)
GSL-I-B4
LCA
LcH
LEL
NPA
LTL
MAL-I
MAA
PHA-E
MAA(Seikagaku)
MAL-II
PHA-E
PSL
PHA-L
PTA-Gal
PTL-I
PTL-II
RCAI (Vector)
PNA
PSA
SBA
STL
TJA-I
TJA-II
TL(EY Lab)
UDA
UEA-II
VVL
WFL
WGA(Seikagaku Bio)
Native HPA
SGA
AAL
SSR1
rMpL
TbFBL1
CC1G-10077
MGL6B
MGL 6A-H262T
MGL 6A
HA70 complex-Alexa
WGA multimer
rPVL
VVHA lectin
BanLec
CGL
Gal-3
human Gal-7
mouse Gal-7
CA19.9-1B844
CA19.9-9L426
CA19.9-121SLE
IPS-K-4A2B8
Microvirin
ab5
AOL (Mahal Lab)
ABA
Mono2W-CNL
BPL
GS-II (EY Lab)
EEA (EY Lab)
GNA (EY Lab)
HHA (EY Lab)
PHA-L
PNA
SBA
SJA
UEA-I
VGA(EY Lab)
WGA
MNA-M
MOA
WT-VCC
ME5(detected with a-HUD)
AAL
LSL-150
LBL-152
ME7(detected with a-HUD)
SSL0-488
VLRB.aGPA.23
rNV VLP-Cy3
AFCNVH
HML-Native
#8 Lewis B (T218) (Abcam)
#15 Sialyl Lewis X (BD)
#14 Sialyl Lewis X (Calbio)
hGal-1-488+BCR
TbFBL2
MGL 416
BTL native
GAC78
DC-ASGPR short
Sp Serotype 14.2
Sp Serotype 14.15
rSRL
SRL
Galectin-1
PsSC
Avaren-Fc
ChSeqL
CA19-9 Ab (DRG)
WT Galectin-8
CML1
PhoSL001
Epa1-Np mutant Y228W
Epa1-Np mutant E227G
Epa1-Np mutant E227A
Epa1-Np WT
CnMBL
DBL-1
hJAA-F11-B11
Fuc(α-Sp9

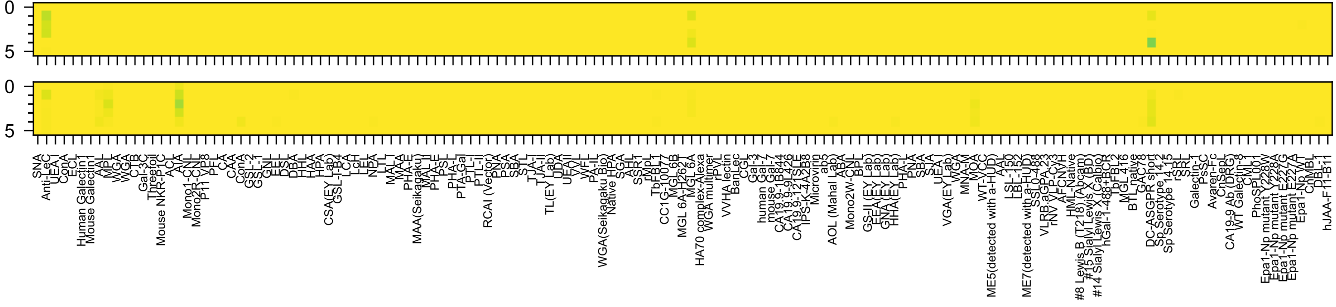
GlcA(α-Sp8

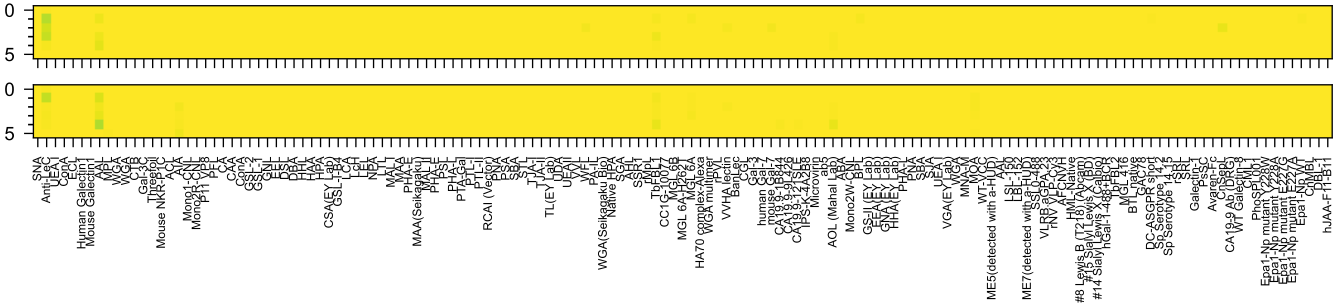

GlcA(β-Sp8

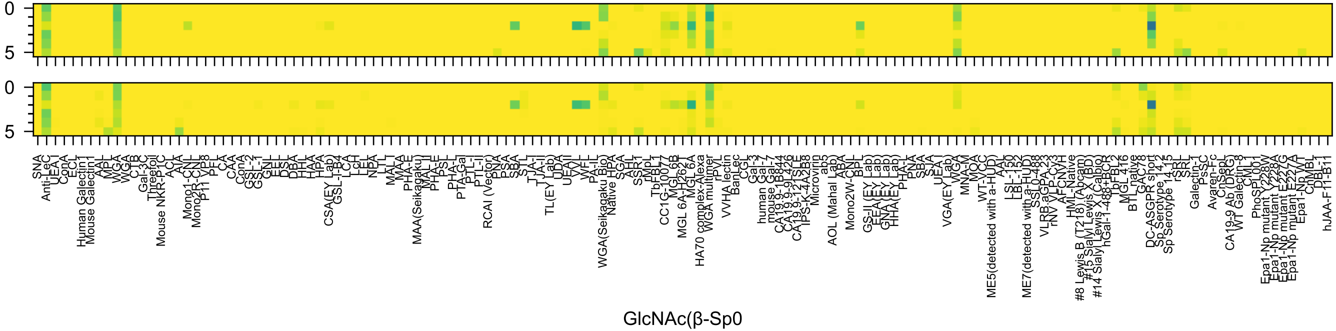
0
5
0
5
SNA
Anti-LeC
UEA-I
ConA
ECL
Human Galectin1
Mouse Galectin1
AAL
MPL
WGA
WGA
CTB
Gal-3C
Threefoil
Mouse NKR-P1C
ACL
AIA
Mono1-CNL
Mono2R-CNL
P11 VP8
PFL
CA
CAA
ConA
GSL-2
GSL-1
GNL
EEL
DSL
DBA
HHL
HAA
HPA
CSA(EY Lab)
GSL-I-B4
LCA
LcH
LEL
NPA
LTL
MAL-I
MAA
PHA-E
MAA(Seikagaku)
MAL-II
PHA-E
PSL
PHA-L
PTA-Gal
PTL-I
PTL-II
RCAI (Vector)
PNA
PSA
SBA
STL
TJA-I
TJA-II
TL(EY Lab)
UDA
UEA-II
VVL
WFL
PA-IL
WGA(Seikagaku Bio)
Native HPA
SGA
AAL
SSR1
rMpL
TbFBL1
CC1G-10077
MGL6B
MGL 6A-H262T
MGL 6A
HA70 complex-Alexa
WGA multimer
rPVL
VVHA lectin
BanLec
CGL
Gal-7
human Gal-7
mouse Gal-7
CA19.9-1B844
CA19.9-9L426
CA19.9-121SLE
IPS-K-4A2B8
Microvirin
ab5
AOL (Mahal Lab)
ABA
Mono2W-CNL
BPL
GS-II (EY Lab)
EEA (EY Lab)
GNA (EY Lab)
HHA (EY Lab)
PHA-L
PNA
SBA
SJA
UEA-I
VGA(EY Lab)
WGA
MNA-M
MOA
WT-VCC
ME5(detected with a-HUD)
AAL
LSL-150
LBL-152
ME7(detected with a-HUD)
SSL0-488
VLRB.aGPA.23
rNV VLP-Cy3
AFCNYH
HML-Native
#8 Lewis B (T218) (Abcam)
#15 Sialyl Lewis X (BD)
#14 Sialyl Lewis X (Calbio)
hGal-1-488+BCR
TbFBL2
MGL 416
BTL native
GAC78
DC-ASGPR short
Sp Serotype 14.2
Sp Serotype 14.15
rSRL
SRL
Galectin-1
PsSC
Avaren-Fc
ChSepL
CA19-9 Ab (DRG)
WT Galectin-8
CML1
PhoSPL001
Epa1-Np mutant Y228W
Epa1-Np mutant E228A
Epa1-Np mutant E227G
Epa1-Np mutant E227A
Epa1-Np WT
ChMBL
DBL-1
hJAA-F11-B11
GlcNAc(β-Sp0

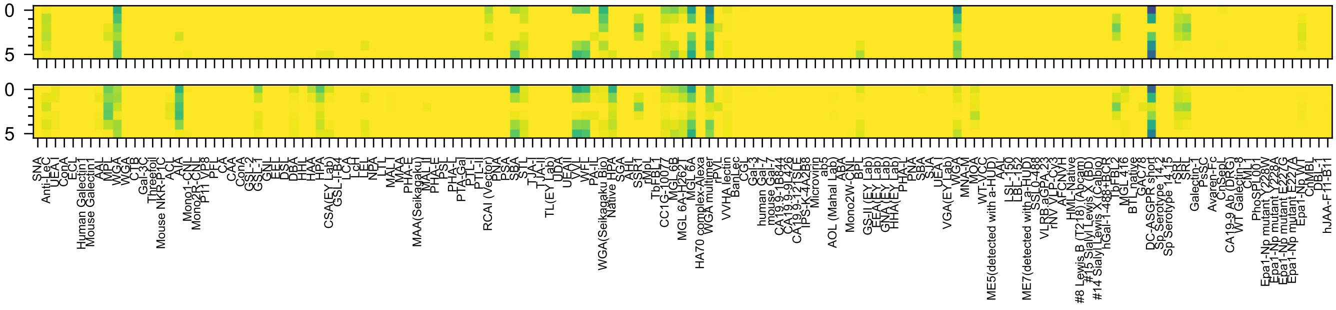
GalNAc(α-Sp8

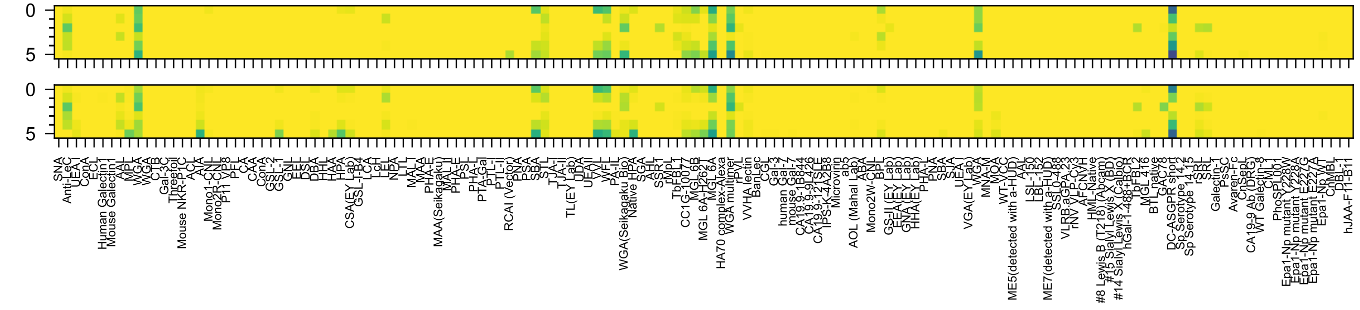
0
5
0
5
GalNAc(β-Sp8

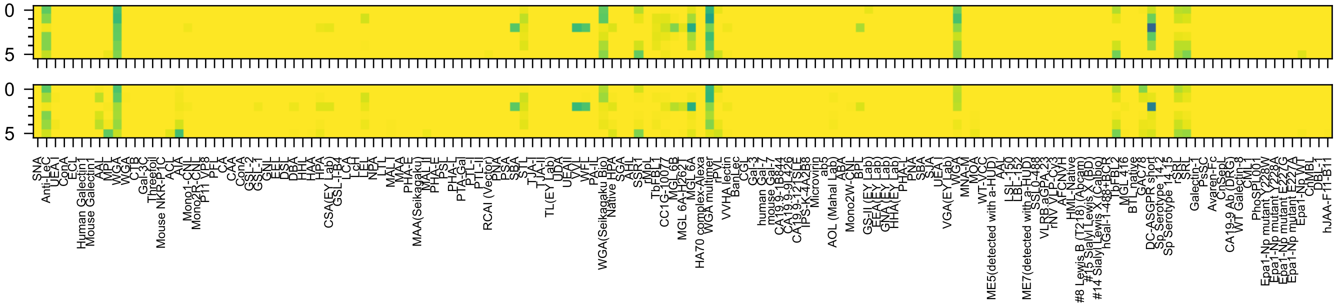

GlcNAc(β-Sp8

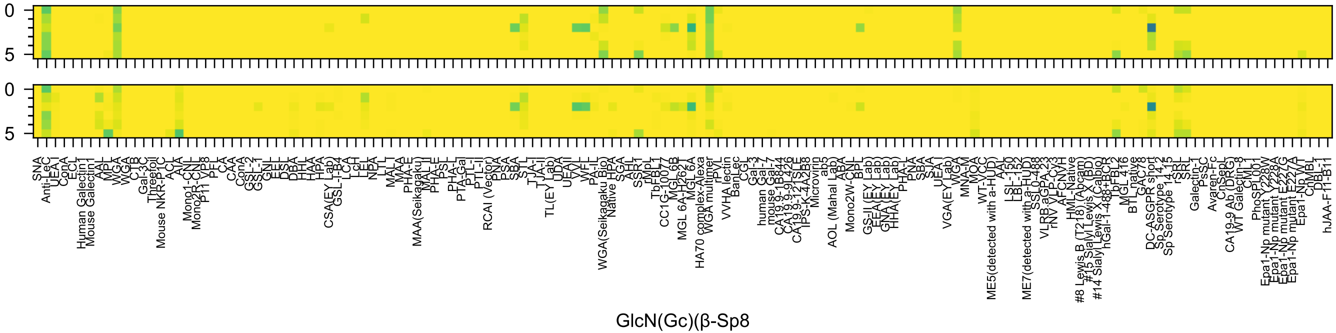
GlcN(Gc)(β-Sp8

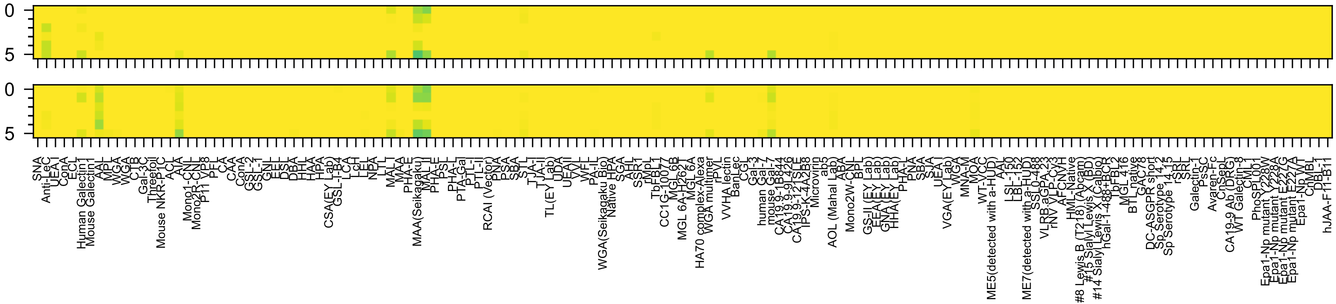

Gal3OS(β-Sp8

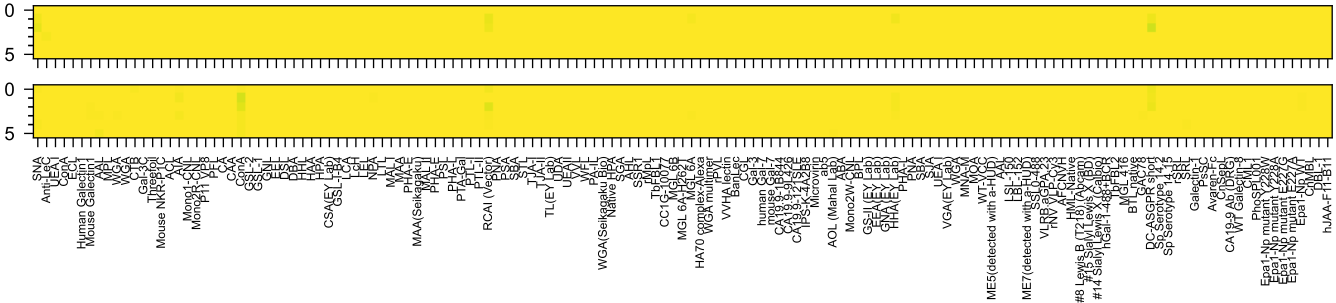
0
5
0
5
SNA
Anti-LeC
UEA-I
ConA
ECL
Human Galectin1
Mouse Galectin1
AAL
MPL
WGA
WGA
CTB
Gal-3C
Threefoil
Mouse NKR-P1C
ACL
AIA
Mono1-CNL
Mono2R-CNL
P11 VP8
PFL
CA
CAA
ConA
GSL-2
GSL-1
GNL
EEL
DSL
DBA
HHL
HAA
HPA
CSA(EY Lab)
GSL-I-B4
LCA
LcH
LEL
NPA
LTL
MAL-I
MAA
PHA-E
MAA(Seikagaku)
MAL-II
PHA-E
PSL
PHA-L
PTA-Gal
PTL-I
PTL-II
RCAI (Vector)
PNA
PSA
SBA
STL
TJA-I
TJA-II
TL(EY Lab)
UDA
UEA-II
VVL
WFL
PA-IL
WGA(Seikagaku Bio)
Native HPA
SGA
AHL
SSR1
rMpL
TbFBL1
CC1G-10077
MGL6B
MGL 6A-H262T
MGL 6A
HA70 complex-Alexa
WGA multimer
rPVL
VVHA lectin
BanLec
CGL
Gal-3
human Gal-7
mouse Gal-7
CA19.9-1B844
CA19.9-9L426
CA19.9-121SLE
IPS-K-4A2B8
Microvirin
ab5
AOL (Mahal Lab)
ABA
Mono2W-CNL
BPL
GS-II (EY Lab)
EEA (EY Lab)
GNA (EY Lab)
HHA (EY Lab)
PHA-L
PNA
SBA
SJA
UEA I
VGA(EY Lab)
VGA
MNA-M
MOA
WT-VCC
ME5(detected with a-HUD)
AAT
LSL-150
LBL-152
ME7(detected with a-HUD)
SSL0-488
VLRB.aGPA.23
rNV VLP-Cy3
AFCNVH
HML-Native
#8 Lewis B (T218) (Abcam)
#15 Sialyl Lewis X (BD)
#14 Sialyl Lewis X (Calbio)
hGal-1-488+BCR
TbFBL2
MGL 416
BTL native
GAC78
DC-ASGPR short
Sp Serotype 14.2
Sp Serotype 14.15
rSRL
SRL
Galectin-1
PsSC
Avaren-Fc
ChSepL
CA19-9 Ab (DRG)
WT Galectin-8
CML1
PhoSPL001
Epa1-Np mutant Y228W
Epa1-Np mutant E227G
Epa1-Np mutant E227A
Epa1-Np WT
CnMBL
DBL-1
hJAA-F11-B11

Man6OP(α-Sp8

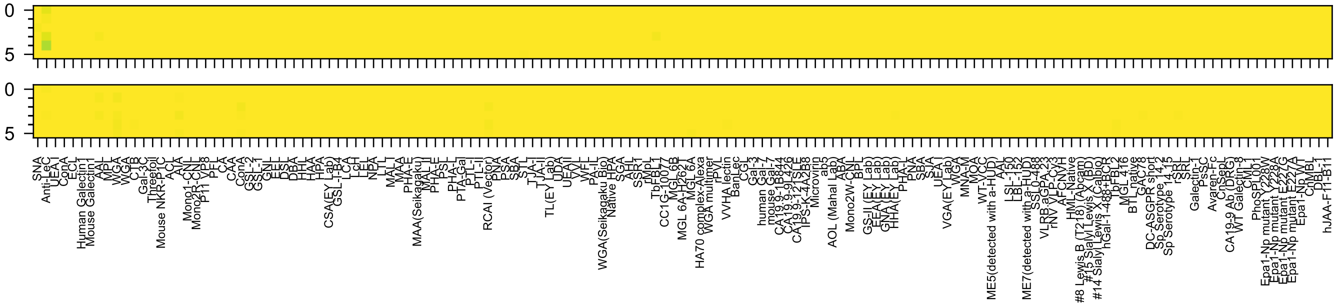

Glc6OP(β-Sp10

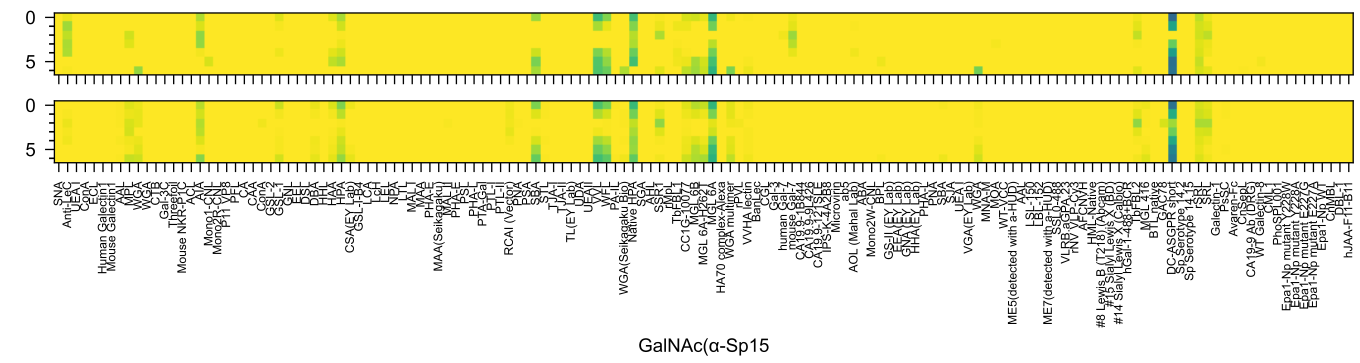
GalNAc(α-Sp15

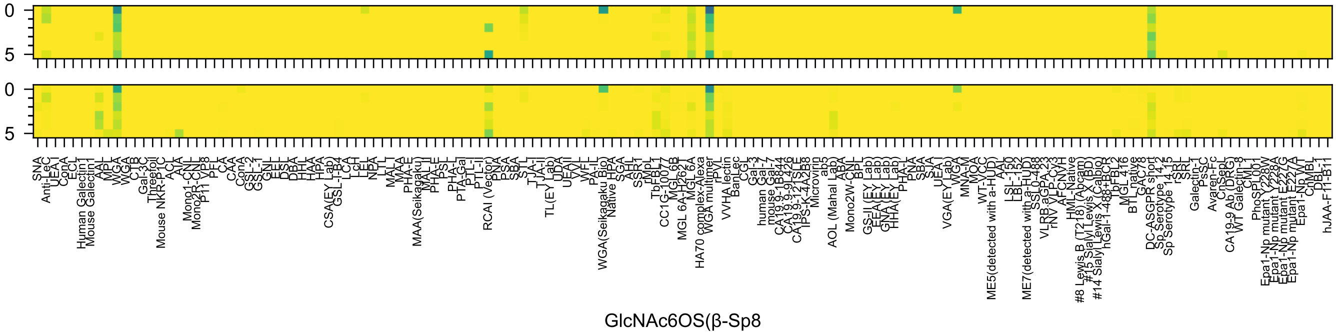
GlcNAc6OS(β-Sp8

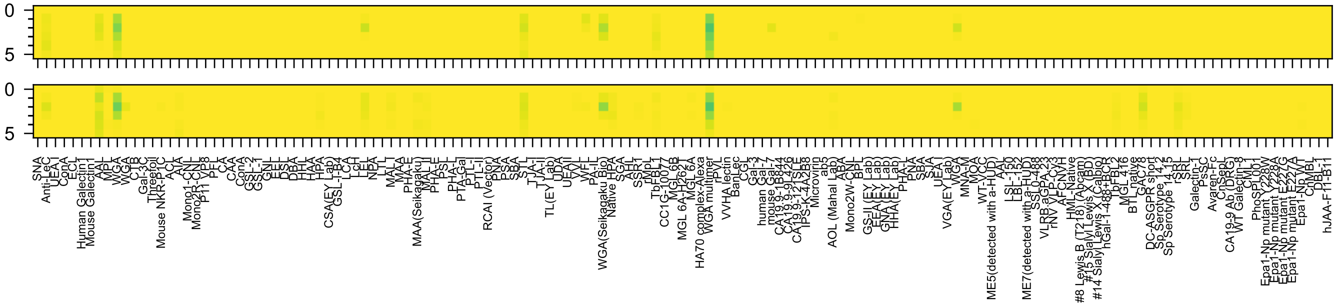

GalNAc4OS(β-Sp10

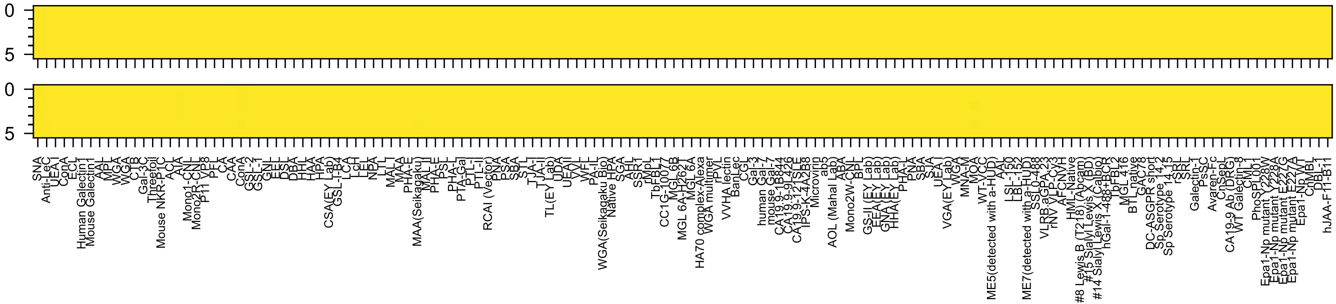
0
5
0
5
SNA
Anti-LeC
UEA I
ConA
ECL
Human Galectin1
Mouse Galectin1
AAL
MPL
WGA
WGA
CTB
Gal-3C
Threefoil
Mouse NKR-P1C
ACL
AIA
Mono1-CNL
Mono2R-CNL
P11 VP8
PFL
CA
CAA
ConA
GSL-2
GSL-1
GNL
EEL
DSL
DBA
HHL
HAA
HPA
CSA(EY Lab)
GSL-I-B4
LCA
LcH
LEL
NPA
LTL
MAL I
MAA
PHA-E
MAA(Seikagaku)
MAL II
PHA-E
PSL
PHA-L
PTA-Gal
PTL-I
PTL-II
RCAI (Vector)
PNA
PSA
SBA
STL
TJA-I
TJA-II
TL(EY Lab)
UDA
UEA II
VVL
WFL
PA-IL
WGA(Seikagaku Bio)
Native HPA
SGA
AAL
SSR1
rMpL
TbFBL1
CC1G-10077
MGL 6B
MGL 6A-H262T
MGL 6A
HA70 complex-Alexa
WGA multimer
rPVL
VVHA lectin
BanLec
CGL
Gal-3
human Gal-7
mouse Gal-7
CA19.9-1B844
CA19.9-9L426
CA19.9-121SLE
IPS-K-4A2B8
Microvirin
ab5
AOL (Mahal Lab)
ABA
Mono2W-CNL
BPL
GS-II (EY Lab)
EEA (EY Lab)
GNA (EY Lab)
HHA (EY Lab)
PHA-L
PNA
SBA
SJA
UEA I
VGA(EY Lab)
WGA
MNA-M
MOA
WT-VCC
ME5(detected with a-HUD)
AAL
LSL-150
LBL-152
ME7(detected with a-HUD)
SSL0-488
VLRB.aGPA.23
rNV VLP-Cy3
AFCNVH
HML-Native
#8 Lewis B (T218) (Abcam)
#15 Sialyl Lewis X (BD)
#14 Sialyl Lewis X (Calbio)
hGal-1-488+BCR
TbFBL2
MGL 416
BTL native
GAC78
DC-ASGPR short
Sp Serotype 14.2
Sp Serotype 14.15
rSRL
SRL
Galectin-1
PsSC
Avaren-Fc
ChSepL
CA19-9 Ab (DRG)
WT Galectin-8
CML1
PhoSL001
Epa1-Np mutant Y228W
Epa1-Np mutant E227G
Epa1-Np mutant E227A
Epa1-Np WT
ChMBL
DBL-1
hJAA-F11-B11

Neu5Ac(α-Sp8

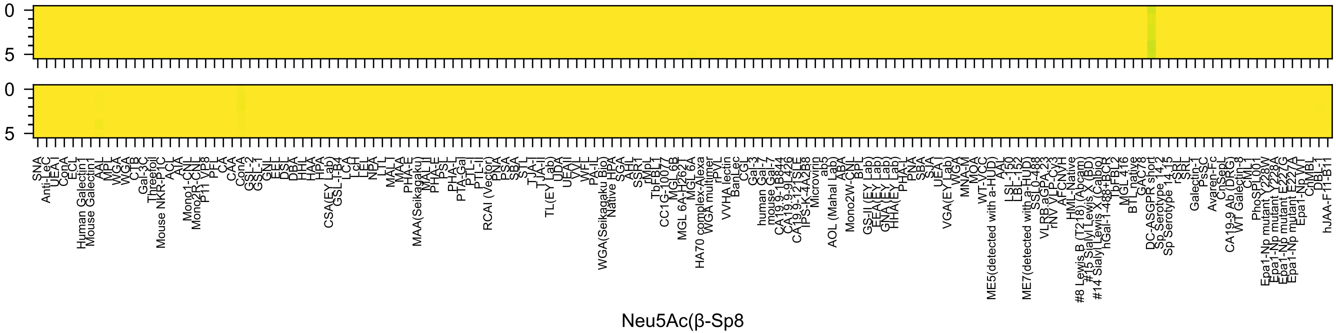
Neu5Ac(β-Sp8

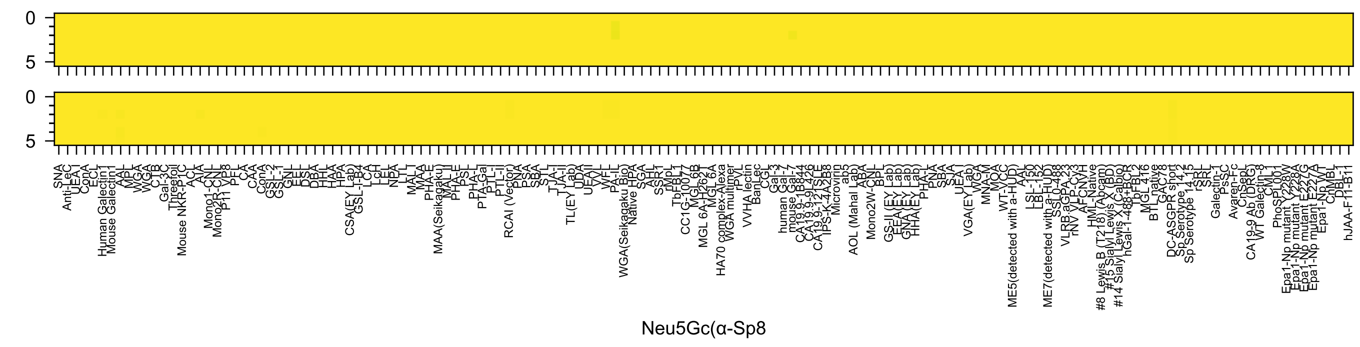

Neu5Gc(α-Sp8

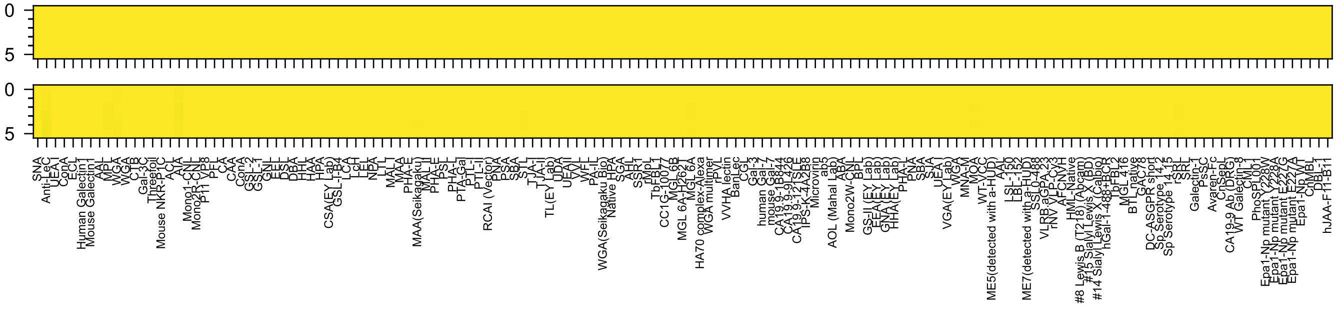
0
5
0
5
Neu5,9Ac2(α-Sp8

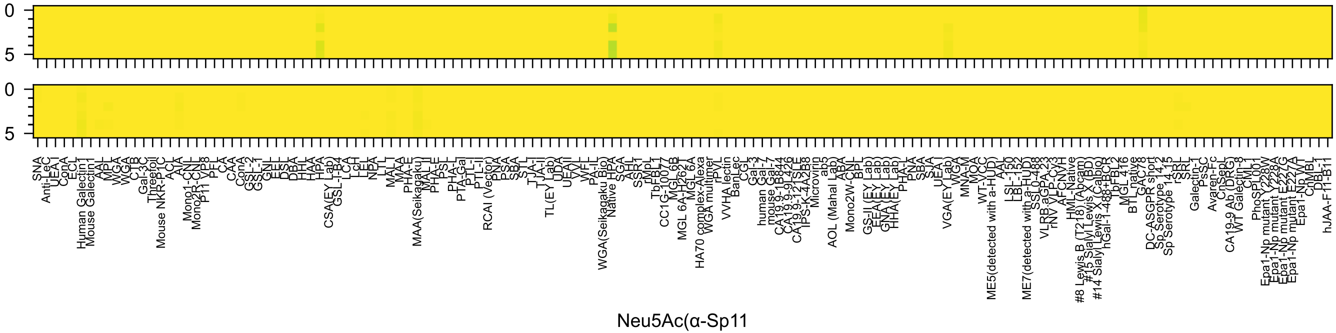
Neu5Ac(α-Sp11

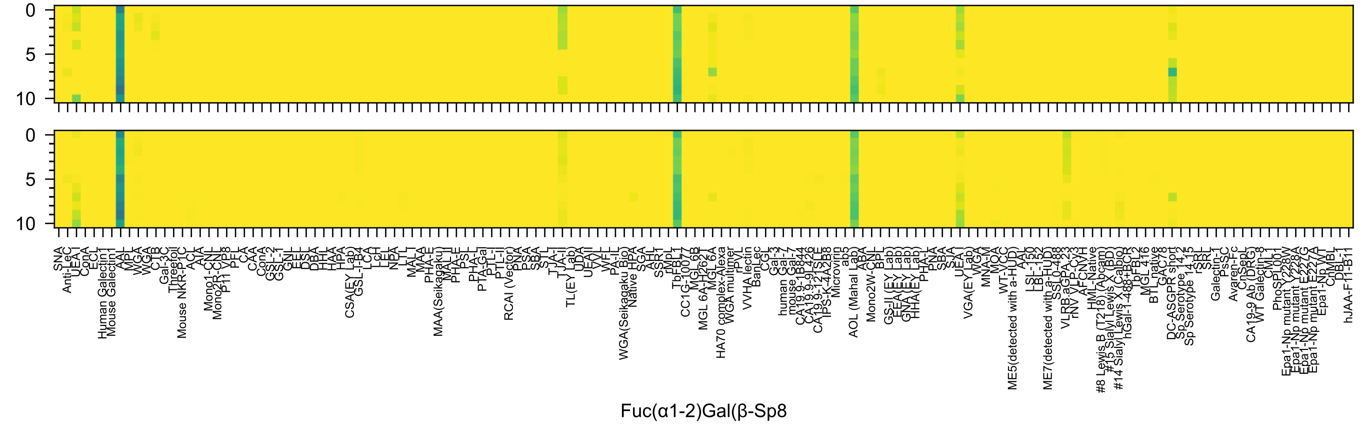
Fuc(α1-2)Gal(β-Sp8

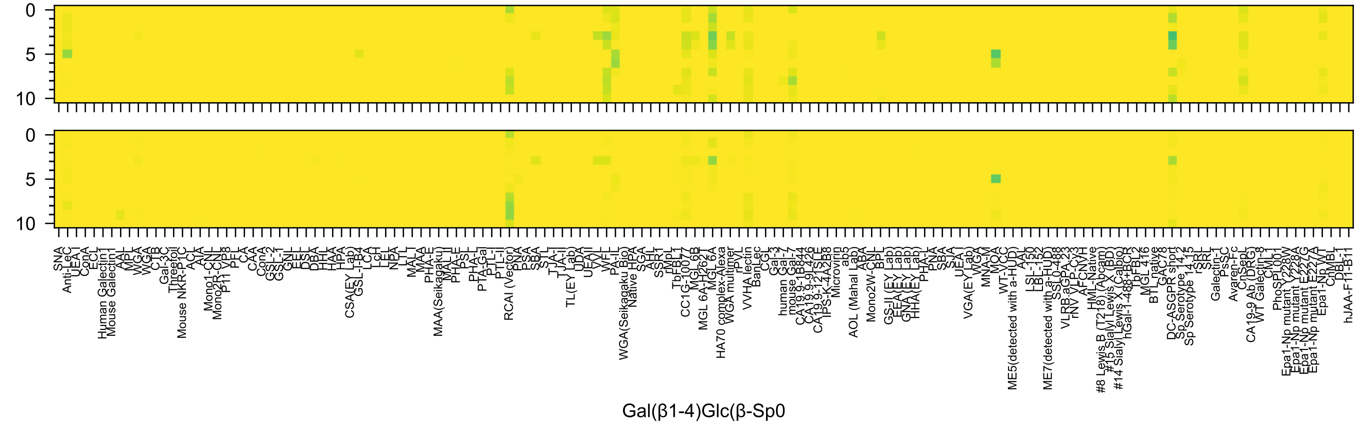

0
5
10
0
5
10
SNA
Anti-LeC
UEA I
ConA
ECL
Human Galectin1
Mouse Galectin1
AAL
MPL
WGA
WGA
CTB
Gal-3C
Threefoil
Mouse NKR-P1C
ACL
AIA
Mono1-CNL
Mono2R-CNL
P11 VP8
PFL
CA
CAA
ConA
GSL-2
GSL-1
GNL
EEL
DSL
DBA
HHL
HAA
HPA
CSA(EY Lab)
GSL-I-B4
LCA
LcH
LEL
NPA
LTL
MAL I
MAA
PHA-E
MAA(Seikagaku)
MAL II
PHA-E
PSL
PHA-L
PTA-Gal
PTL-I
PTL-II
RCAI (Vector)
PNA
PSA
SBA
STL
TJA-I
TJA-II
TL(EY Lab)
UDA
UEAII
VVL
WFL
PA-IL
WGA(Seikagaku Bio)
Native HPA
SGA
AHL
SSR1
rMpL
TbFBL1
CC1G-10077
MGL6B
MGL 6A-H262T
MGL 6A
HA70 complex-Alexa
WGA multimer
rPVL
VVHA lectin
BanLec
CGL
Gal-3
human Gal-7
mouse Gal-7
CA19.9-1B844
CA19.9-9L426
CA19.9-121SLE
IPS-K-4A2B8
Microvirin
ab5
AOL (Mahal Lab)
ABA
Mono2W-CNL
BPL
GS-II (EY Lab)
EEA(EY Lab)
GNA(EY Lab)
HHA(EY Lab)
PHA-L
PNA
SBA
SJA
UEA I
VGA(EY Lab)
WGA
MNA-M
MOA
WT-VCC
ME5(detected with a-HUD)
AAL
LSL-150
LBL-152
ME7(detected with a-HUD)
SSL0-488
VLRB.aGPA.23
rNV VLP-Cy3
AFCNYH
HML-Native
#8 Lewis B (T218) (Abcam)
#15 Sialyl Lewis X (BD)
#15 Sialyl Lewis X (Calbio)
#14 Sialyl Lewis X
hGal-1-488+BCR
TbFBL2
MGL 416
BTL native
GAC78
DC-ASGPR short
Sp Serotype 14.2
Sp Serotype 14.15
rSRL
SRL
Galectin-1
PsSC
Avaren-Fc
CnSepL
CA19-9 Ab (DRG)
WT Galectin-8
CML1
PhoSL001
Epa1-Np mutant Y228W
Epa1-Np mutant Y228A
Epa1-Np mutant E227G
Epa1-Np mutant E227A
Epa1-Np WT
ChMBL
DBL-1
hJAA-F11-B11
Gal(β1-4)Glc(β-Sp0

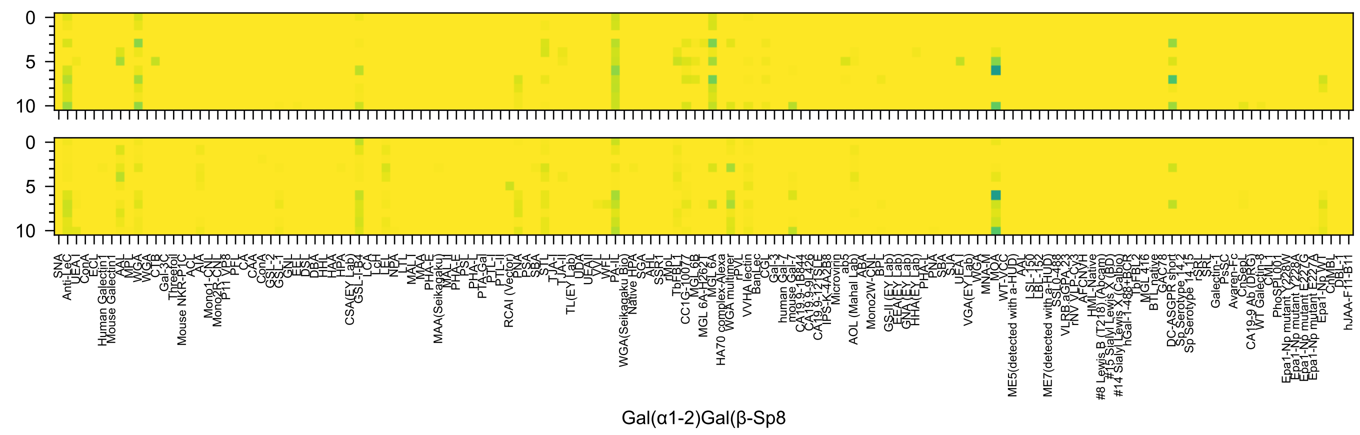
Gal(α1-2)Gal(β-Sp8

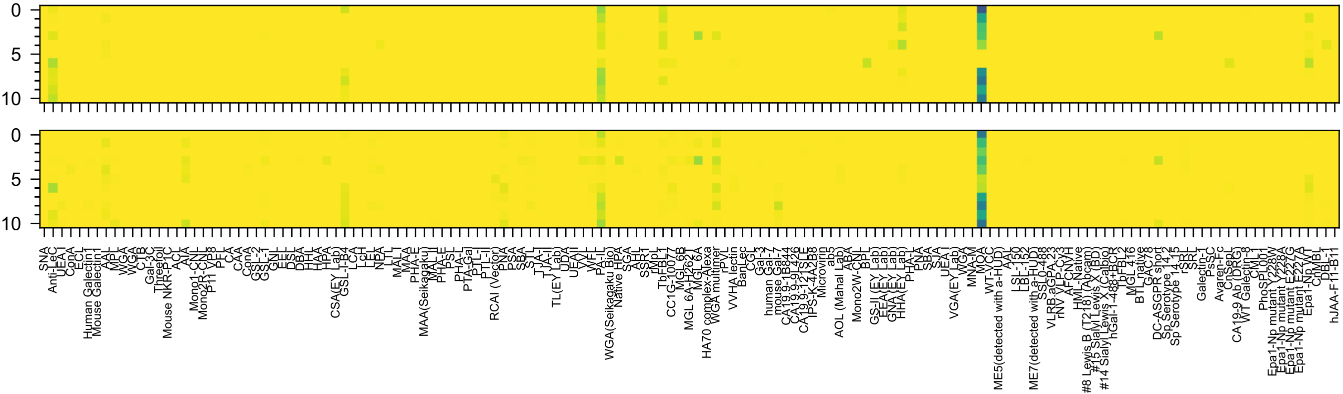

Gal(α1-3)Gal(β-Sp8

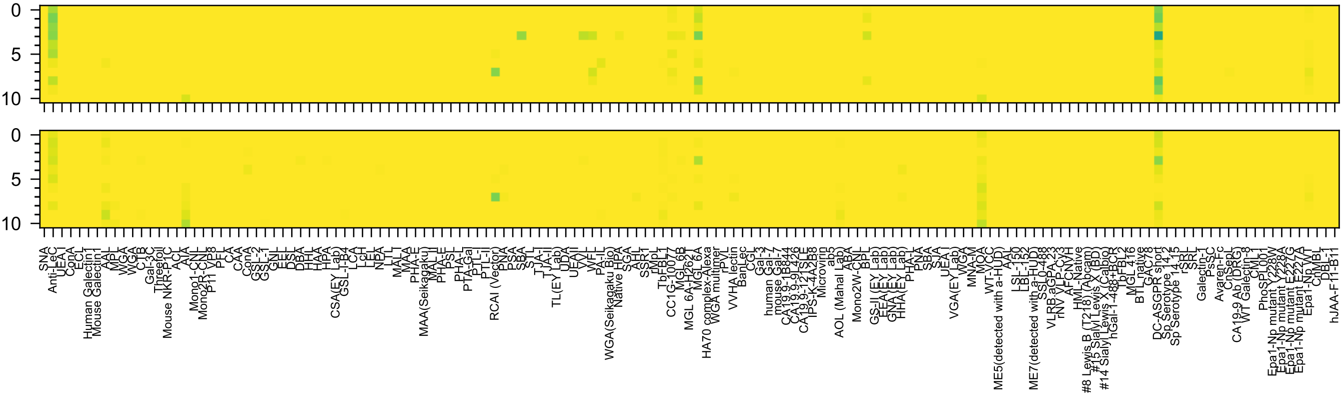

Gal(α1-6)Glc(β-Sp8

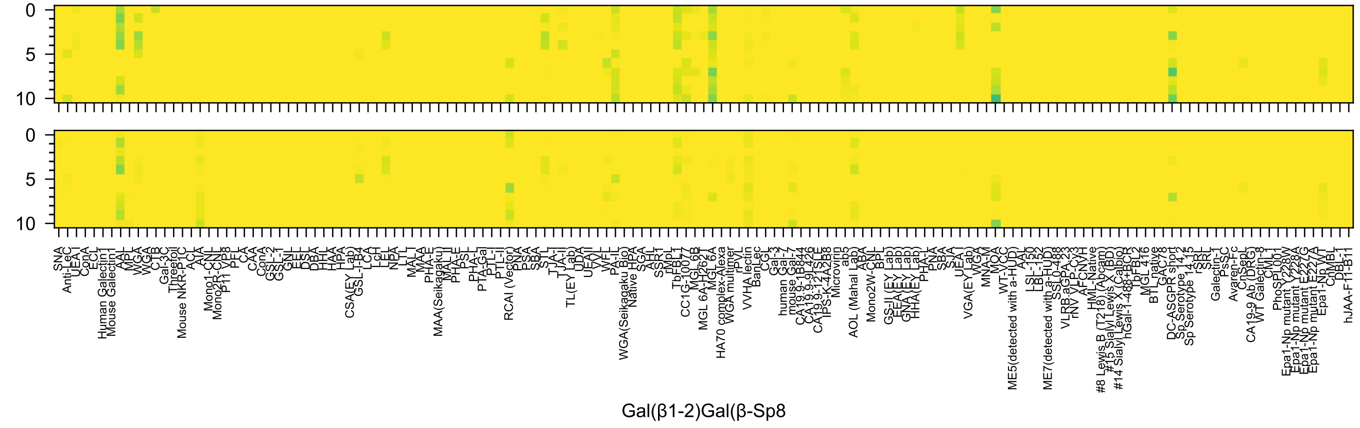
Gal(β1-2)Gal(β-Sp8

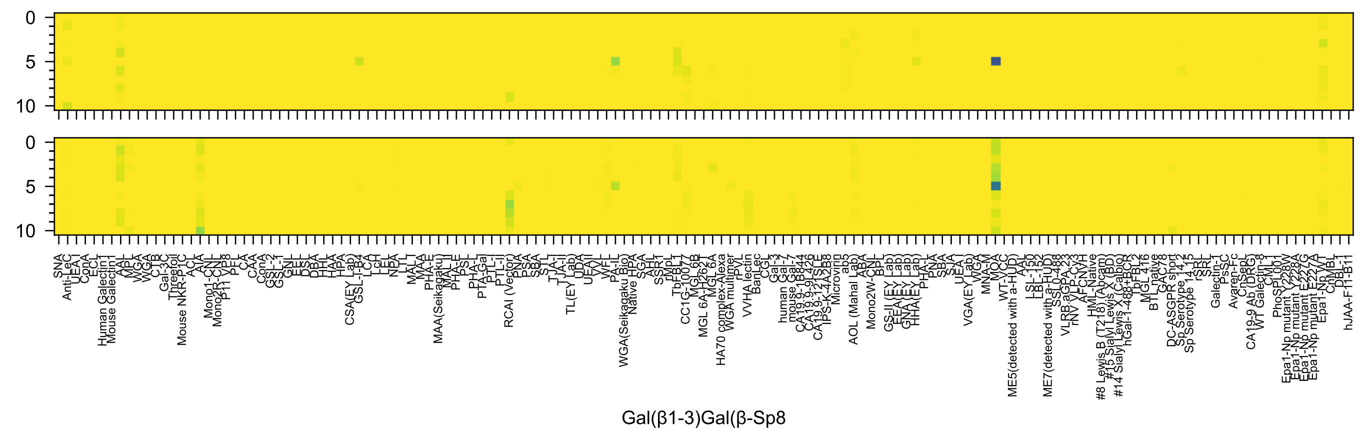
Gal(β1-3)Gal(β-Sp8

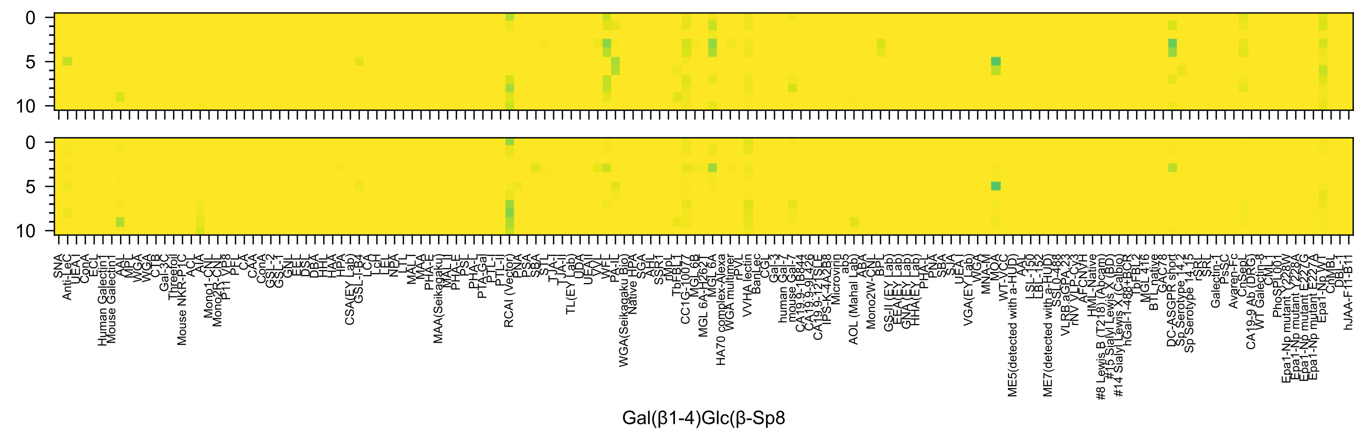

Gal(β1-4)Glc(β-Sp8

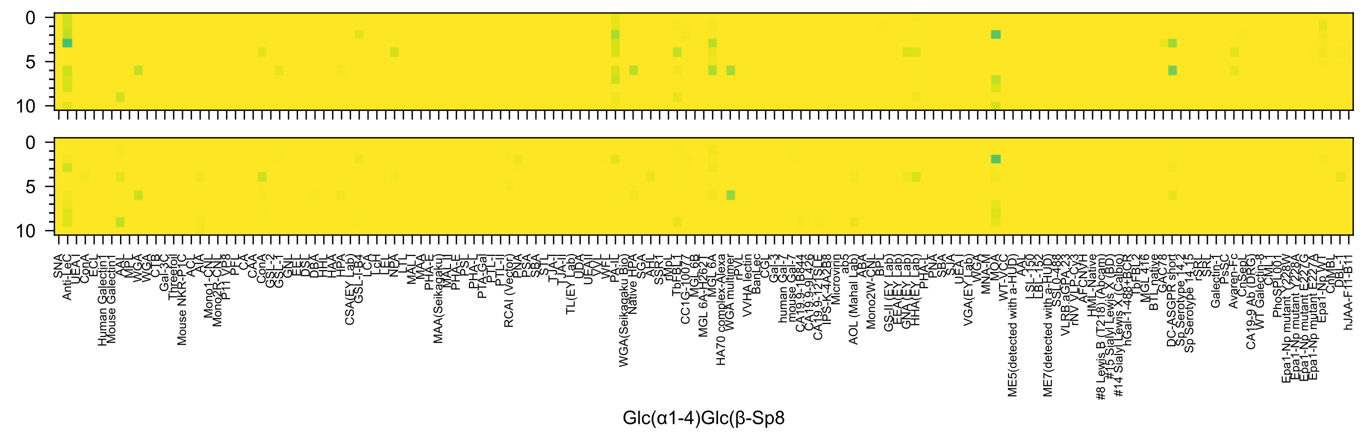

Glc(α1-4)Glc(β-Sp8

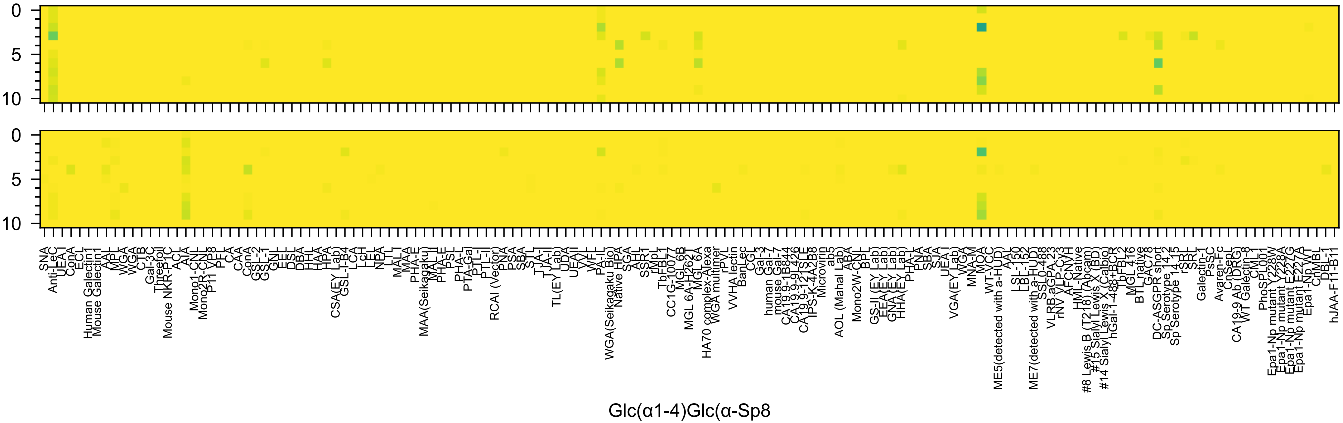
Glc(α1-4)Glc(α-Sp8

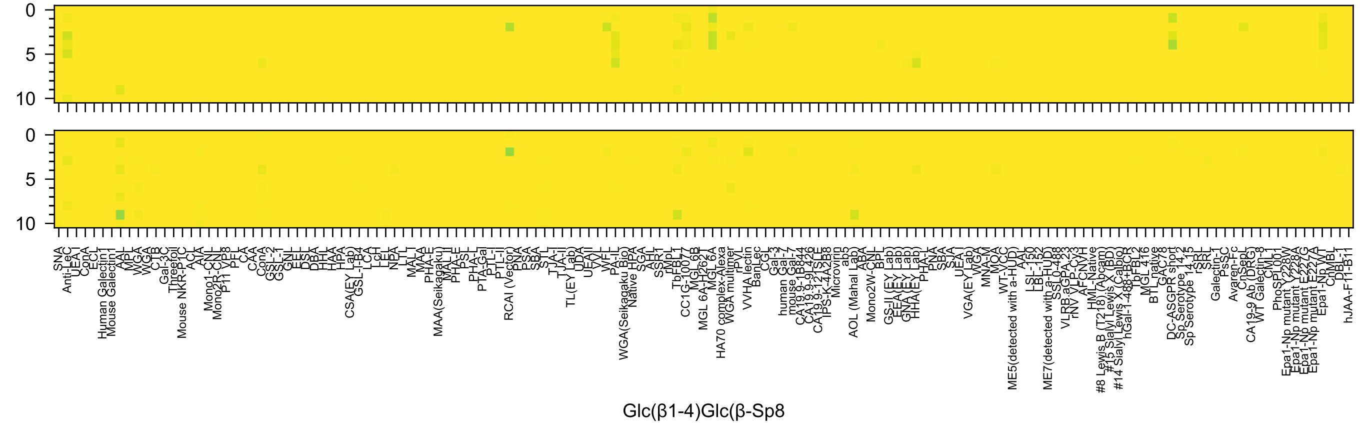
Glc(β1-4)Glc(β-Sp8

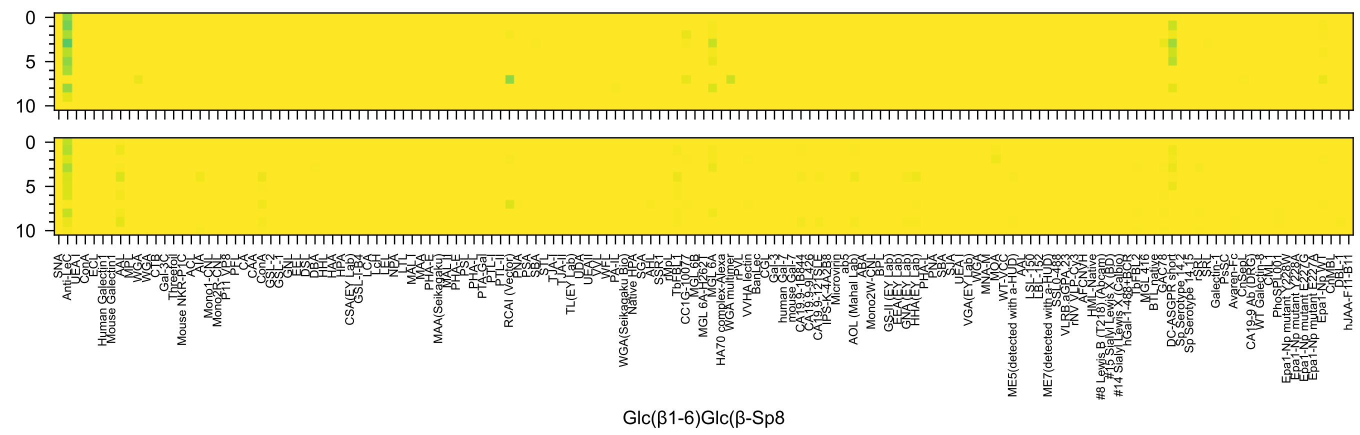
Glc(β1-6)Glc(β-Sp8

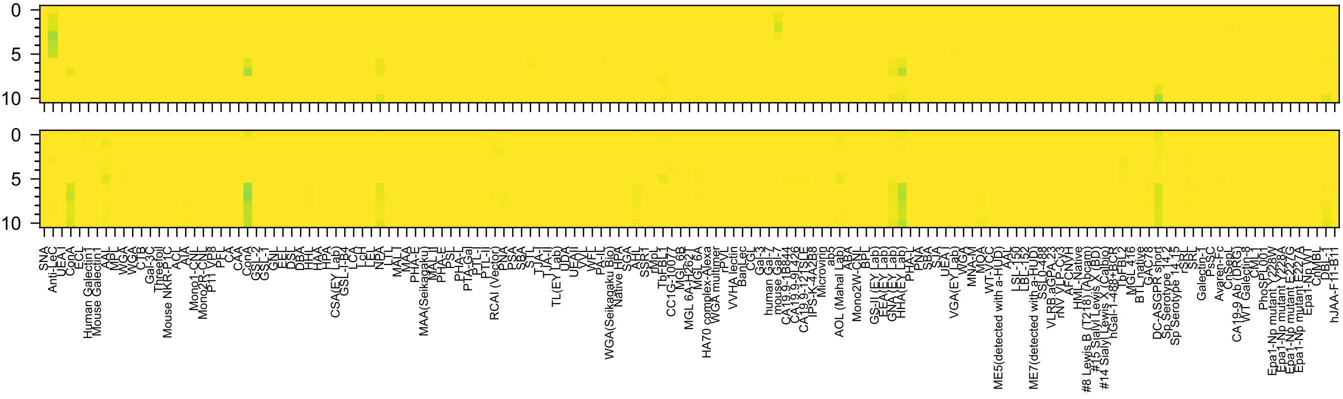

Man(α1-6)Man(β-Sp10

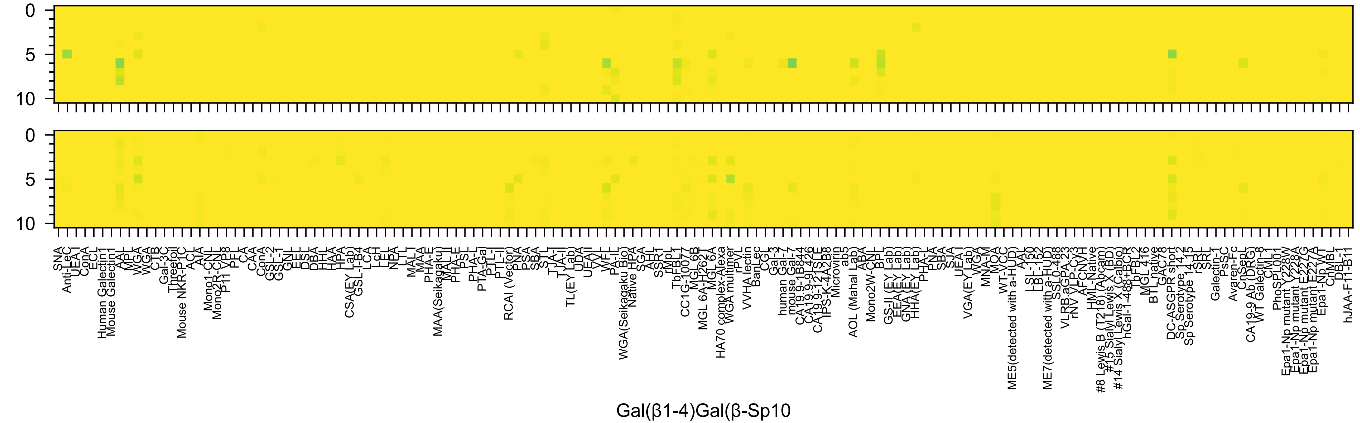
Gal(β1-4)Gal(β-Sp10

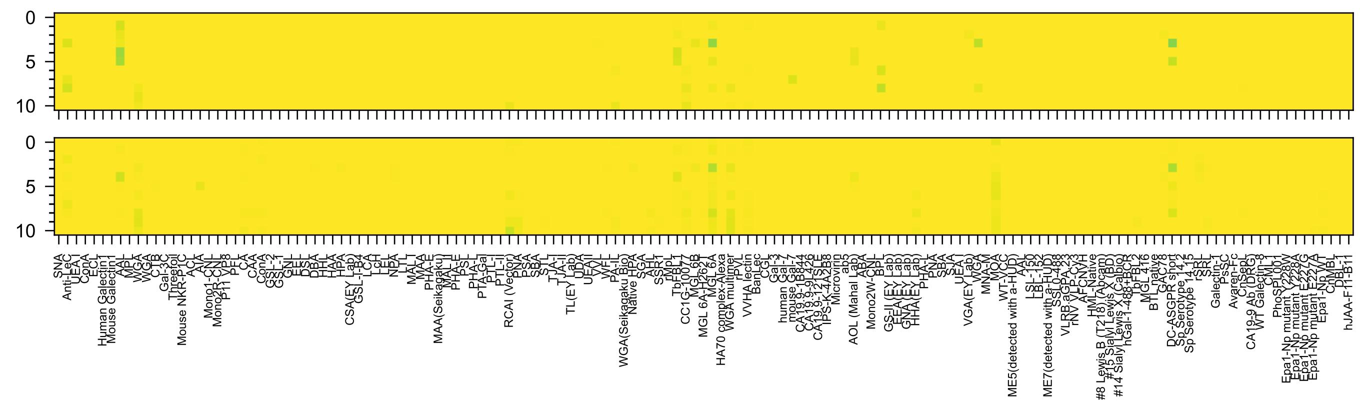

Gal(β1-6)Gal(β-Sp10

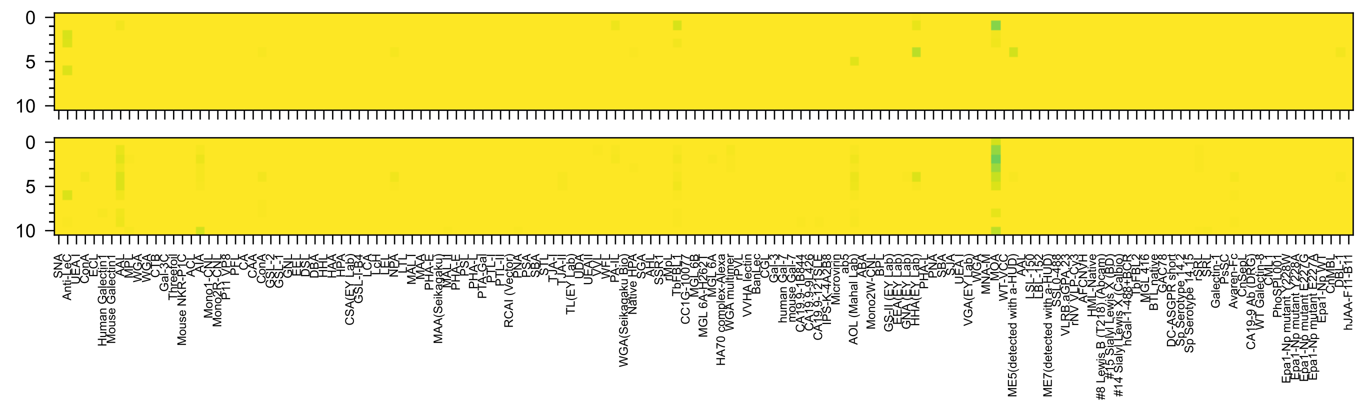

GlcA(β1-3)Gal(β-Sp8

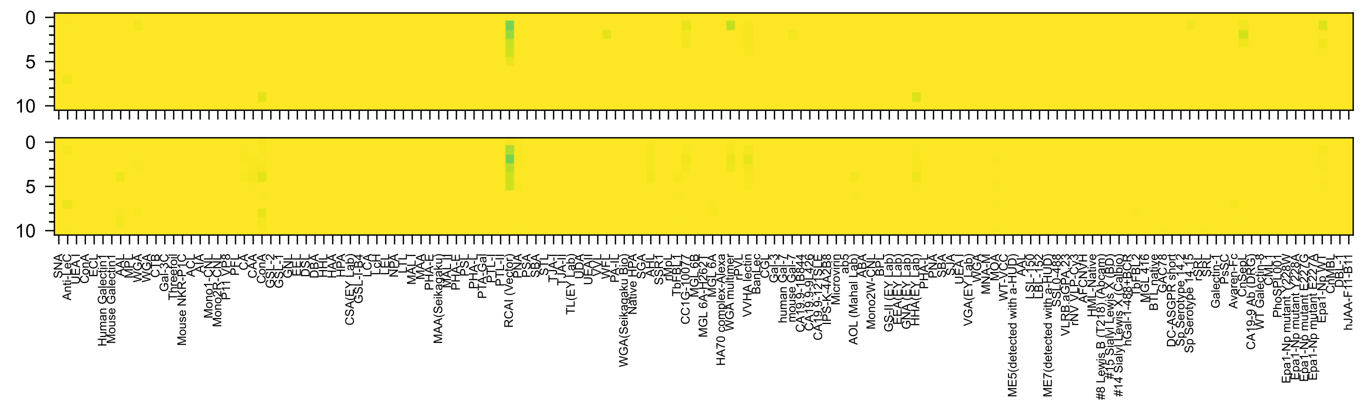

GlcA(β1-6)Gal(β-Sp8)

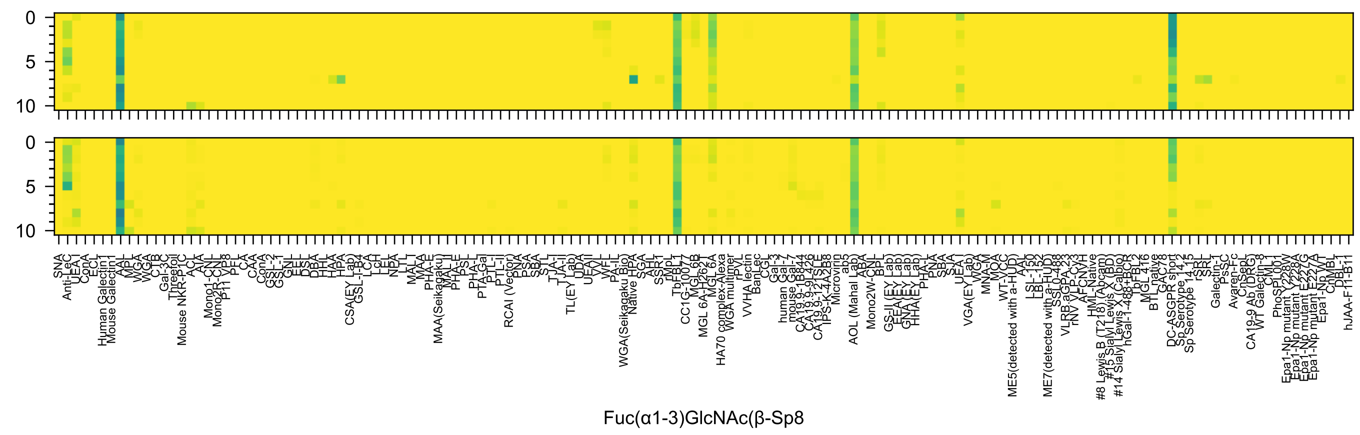
Fuc(α1-3)GlcNAc(β-Sp8

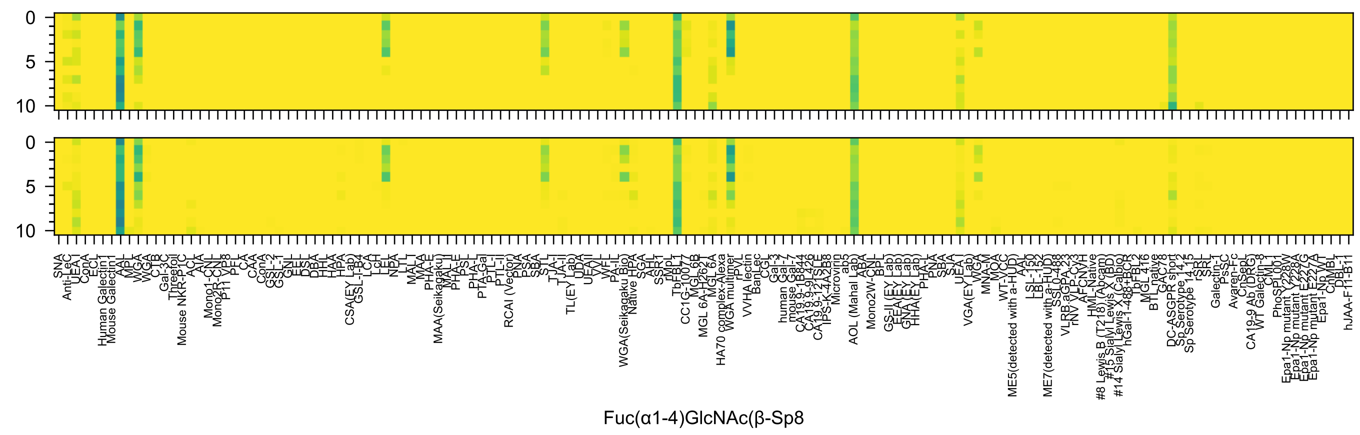
Fuc(α1-4)GlcNAc(β-Sp8

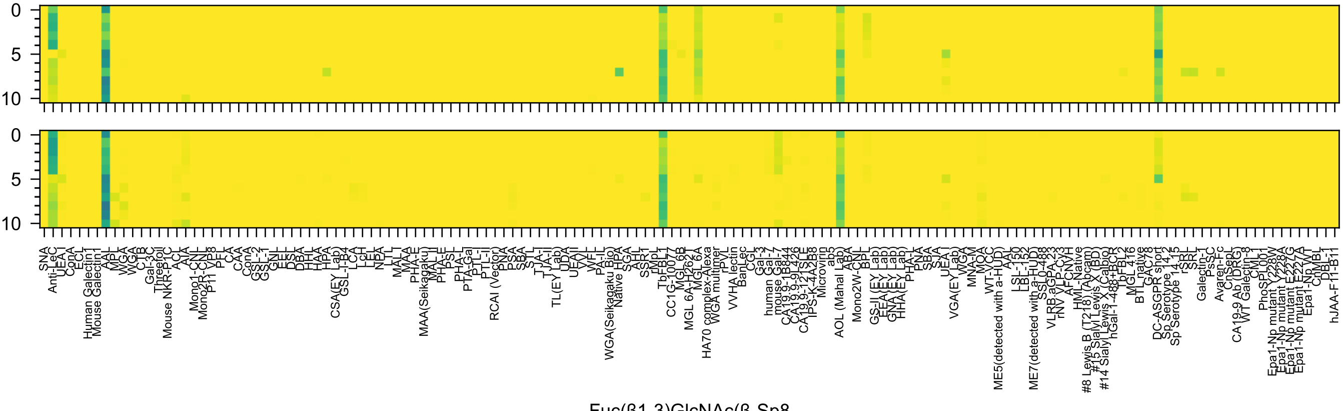
0
5
10
0
5
10
SNA
Anti-LeC
UEA I
ConA
ECL
Human Galectin1
Mouse Galectin1
AAL
MPL
WGA
WGA
CTB
Gal-3C
Threefoil
Mouse NKR-P1C
ACL
AIA
Mono1-CNL
Mono2R-CNL
P11 VP8
PFL
CA
CAA
ConA
GSL-2
GSL-1
GNL
EEL
DSL
DBA
HHL
HAA
HPA
CSA(EY Lab)
GSL-I-B4
LCA
LcH
LEL
NPA
LTL
MAL
MAA
PHA-E
MAA(Seikagaku)
MAL II
PHA-E
PSL
PHA-L
PTA-Gal
PTL-I
PTL-II
RCAI (Vector)
PNA
PSA
SBA
STL
TJA-I
TJA-II
TL(EY Lab)
UDA
UEAII
VVL
WFL
PA-IL
WGA(Seikagaku Bio)
Native HPA
SGA
AHL
SSR1
rMpL
TbFBL1
CC1G-10077
MGL 6B
MGL 6A-H262T
MGL 6A
HA70 complex-Alexa
WGA multimer
rPVL
VVHA lectin
BanLec
CGL
Gal-3
human Gal-7
mouse Gal-7
CA19.9-1B844
CA19.9-9L426
CA19.9-121SLE
IPS-K-4A2B8
Microvirin
ab5
AOL (Mahal Lab)
ABA
Mono2W-CNL
BPL
GS-II (EY Lab)
EEA(EY Lab)
GNA(EY Lab)
HHA(EY Lab)
PHA-L
PNA
SBA
SJA
UEA I
VGA(EY Lab)
WGA
MNA-M
MOA
WT-VCC
ME5(detected with a-HUD)
AAL
LSL-150
LBL-152
ME7(detected with a-HUD)
SSL0-488
VLRB.aGPA.23
rNV VLP-Cy3
AFCNYH
HML-Native
#8 Lewis B (T218) (Abcam)
#15 Sialyl Lewis X (BD)
#14 Sialyl Lewis X (Calbio)
hGal-1-488+BCR
TbFBL2
MGL 416
BTL native
GAC78
DC-ASGPR short
Sp Serotype 14.2
Sp Serotype 14.15
rSRL
SRL
Galectin-1
PsSC
Avaren-Fc
ChSeBL
CA19-9 Ab (DRG)
WT Galectin-8
CML1
PhoSL001
Epa1-Np mutant Y228W
Epa1-Np mutant E227G
Epa1-Np mutant E227A
Epa1-Np WT
ChMBL
DBL-1
hJAA-F11-B11
Fuc(β1-3)GlcNAc(β-Sp8

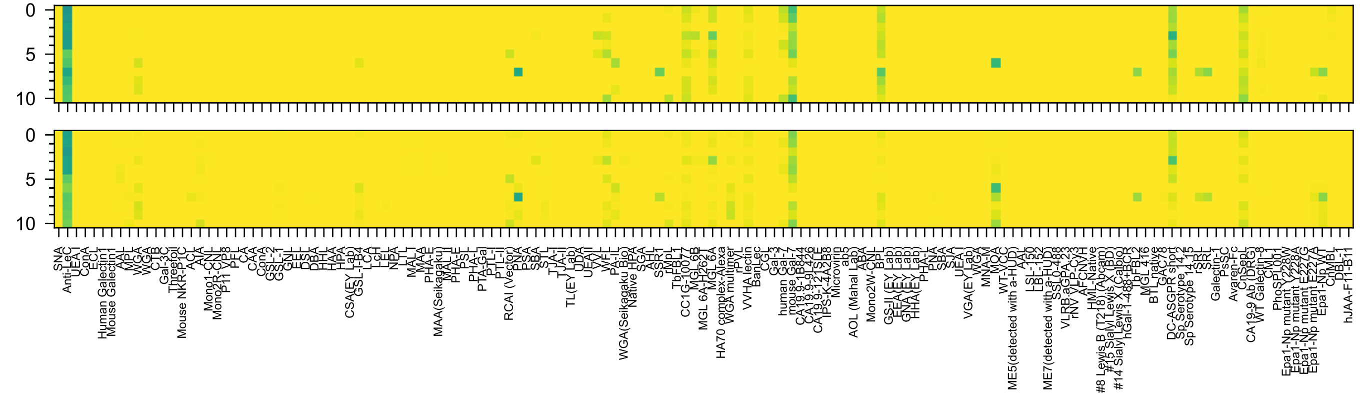

Gal(β1-3)GlcNAc(β-Sp0

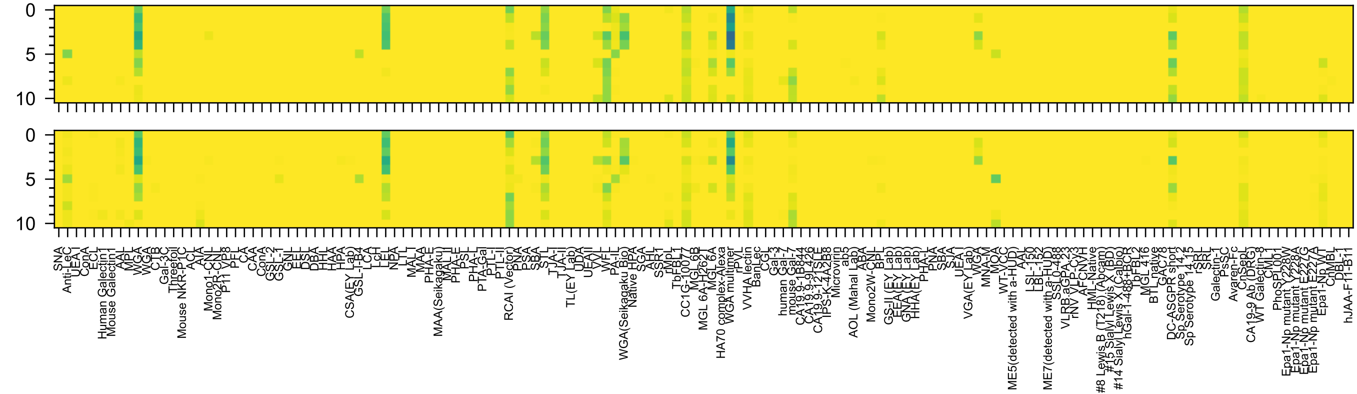

Gal(β1-4)GlcNAc(β-Sp0

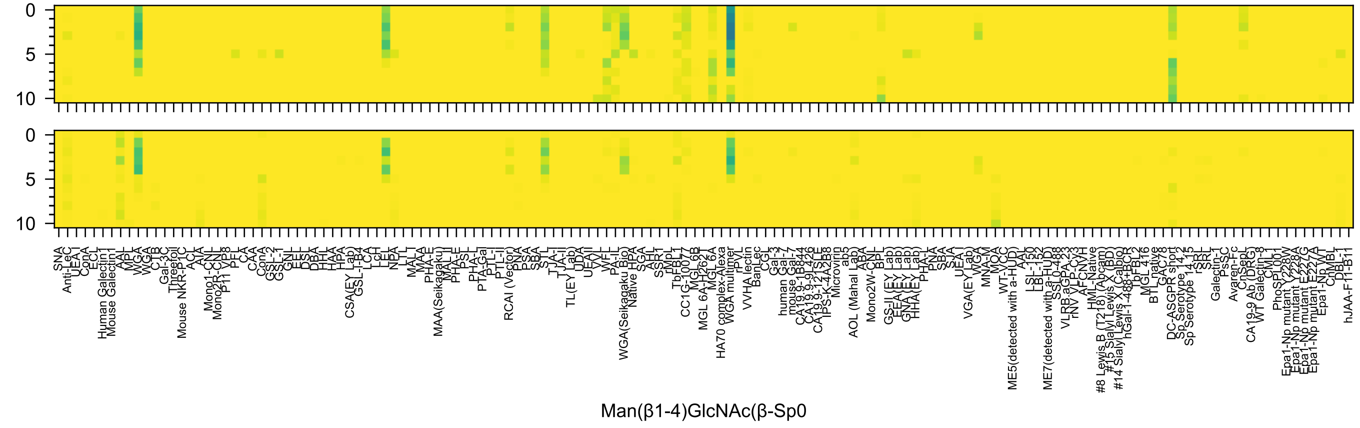
Man(β1-4)GlcNAc(β-Sp0

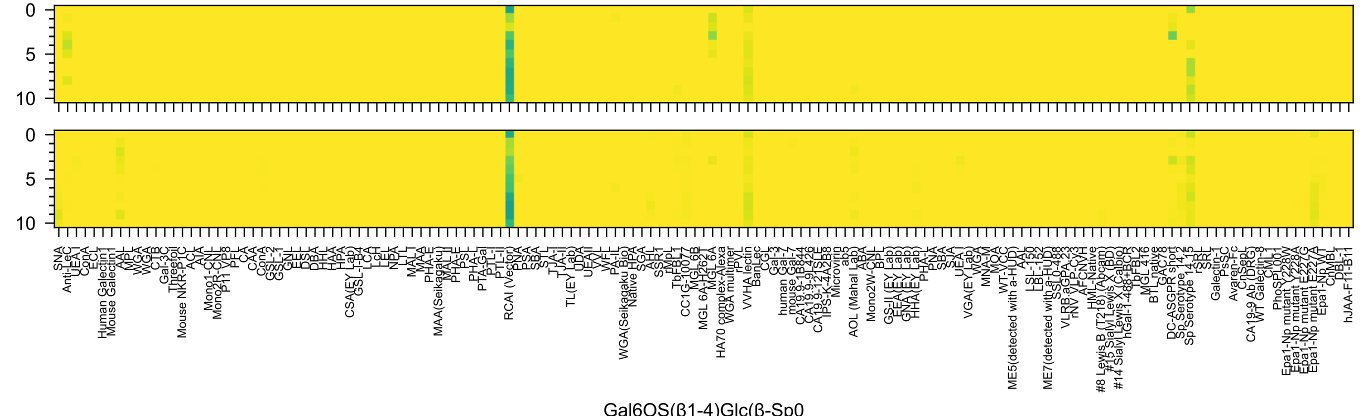
Gal6OS(β1-4)Glc(β-Sp0

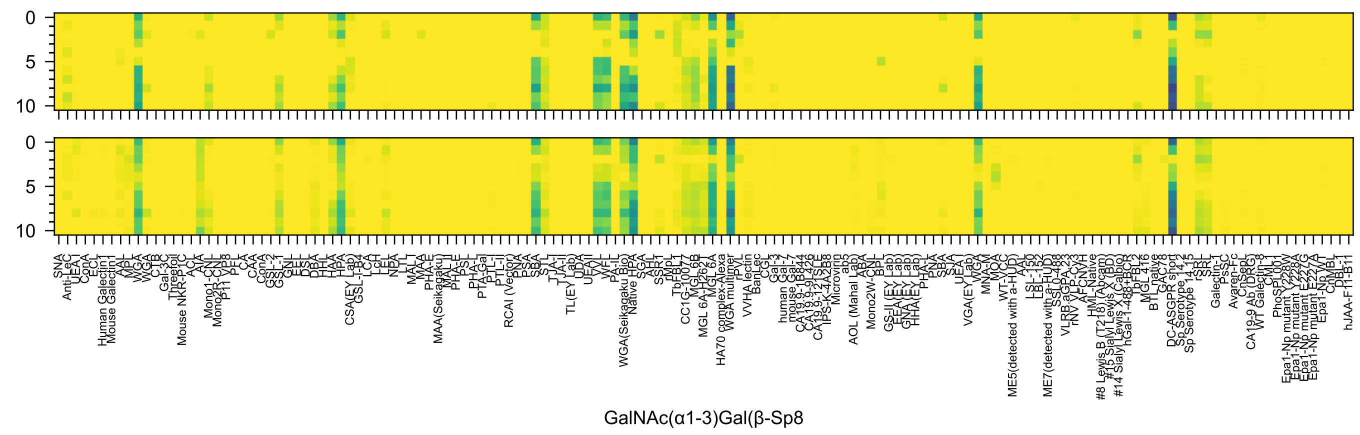
0
5
10
0
5
10
SNA
Anti-LeC
UEA-I
ConA
ECL
Human Galectin1
Mouse Galectin1
AAL
MPL
WGA
WGA
CTB
Gal-3C
Threefoil
Mouse NKR-P1C
ACL
AIA
Mono1-CNL
Mono2R-CNL
P11 VP8
PFL
CA
CAA
ConA
GSL-2
GSL-1
GNL
EEL
DSL
DBA
HHL
HAA
HPA
CSA(EY Lab)
GSL-I-B4
LCA
LcH
LEL
NPA
LTL
MAL-I
MAA
PHA-E
MAA(Seikagaku)
MAL-II
PHA-E
PSL
PHA-L
PTA-Gal
PTL-I
PTL-II
RCAI (Vector)
PNA
PSA
SBA
STL
TJA-I
TJA-II
TL(EY Lab)
UDA
UEAII
VVL
WFL
PA-IL
WGA(Seikagaku Bio)
Native HPA
SGA
AAL
SSR1
rMpL
TbFBL1
CC1G-10077
MGL6B
MGL 6A-H262T
MGL 6A
HA70 complex-Alexa
WGA multimer
rPVL
VVHA lectin
BanLec
CGL
Gal-3
human Gal-7
mouse Gal-7
CA19.9-1B844
CA19.9-9L426
CA19.9-121SLE
IPS-K-4A2B8
Microvirin
ab5
AOL (Mahal Lab)
ABA
Mono2W-CNL
BPL
GS-II (EY Lab)
EEA (EY Lab)
GNA (EY Lab)
HHA(EY Lab)
PHA-L
PNA
SBA
SJA
UEA-I
VGA(EY Lab)
WGA
MNA-M
MOA
WT-VCC
ME5(detected with a-HUD)
AAL
LSL-150
LBL-152
ME7(detected with a-HUD)
SSL0-488
VLRB.aGPA.23
rNV VLP-Cy3
AFCNVH
HML-Native
#8 Lewis B (T218) (Abcam)
#15 Sialyl Lewis X (BD)
#14 Sialyl Lewis X (Calbio)
hGal-1-488+BCR
TbFBL2
MGL 416
BTL native
GAC78
DC-ASGPR short
Sp Serotype 14.2
Sp Serotype 14.15
rSRL
SRL
Galectin-1
PsSC
Avaren-Fc
ChSeL
CA19-9 Ab (DRG)
WT Galectin-8
CML1
PhoSL001
Epa1-Np mutant Y228W
Epa1-Np mutant E227A
Epa1-Np mutant E227G
Epa1-Np WT
ChMBL
DBL-1
hJAA-F11-B11
GalNAc(α1-3)Gal(β-Sp8

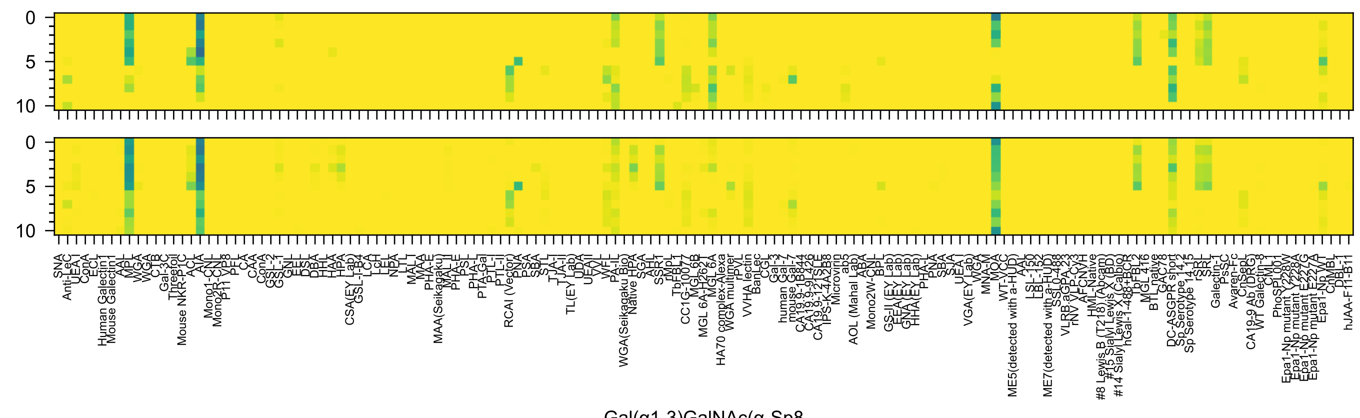
0
5
10
0
5
10
SNA
Anti-LeC
UEA-I
ConA
ECL
Human Galectin1
Mouse Galectin1
AAL
MPL
WGA
WGA
CTB
Gal-3C
Threefoil
Mouse NKR-P1C
ACL
AIA
Mono1-CNL
Mono2R-CNL
P11 VP8
PFL
CA
CAA
ConA
GSL-2
GSL-1
GNL
EEL
DSL
DBA
HHL
HAA
HPA
CSA(EY Lab)
GSL-I-B4
LCA
LcH
LEL
NPA
LTL
MAL
MAA
PHA-E
MAA(Seikagaku)
MAL II
PHA-E
PSL
PHA-L
PTA-Gal
PTL-I
PTL-II
RCAI (Vector)
PNA
PSA
SBA
STL
TJA-I
TJA-II
TL(EY Lab)
UDA
UEAII
VVL
WFL
PA-IL
WGA(Seikagaku Bio)
Native HPA
SGA
AHL
SSR1
rMpL
TbFBL1
CC1G-10077
MGL6B
MGL 6A-H262T
MGL 6A
HA70 complex-Alexa
WGA multimer
rPVL
VVHA lectin
BanLec
CGL
Gal-3
human Gal-7
mouse Gal-7
CA19.9-1B844
CA19.9-9L426
CA19.9-121SLE
IPS-K-4A2B8
Microvirin
ab5
AOL (Mahal Lab)
ABA
Mono2W-CNL
BPL
GS-II (EY Lab)
EEA(EY Lab)
GNA(EY Lab)
HHA(EY Lab)
PHA-L
PNA
SBA
SJA
UEA I
VGA(EY Lab)
WGA
MNA-M
MOA
WT-VCC
ME5(detected with a-HUD)
AAL
LSL-150
LBL-152
ME7(detected with a-HUD)
SSL0-488
VLRB.aGPA.23
rNV VLP-Cy3
AFCNYH
HML-Native
#8 Lewis B (T218) (Abcam)
#15 Sialyl Lewis X (BD)
#14 Sialyl Lewis X (Calbio)
hGal-1-488+BCR
TbFBL2
MGL 416
BTL_native
GAC78
DC-ASGPR short
Sp Serotype 14.2
Sp Serotype 14.15
rSRL
SRL
Galectin-1
PsSC
Avaren-Fc
ChSepL
CA19-9 Ab (DRG)
WT Galectin-8
CML1
PhoSPL001
Epa1-Np mutant Y228W
Epa1-Np mutant Y228A
Epa1-Np mutant E227G
Epa1-Np mutant E227A
Epa1-Np WT
CrMBL
DBL-1
hJAA-F11-B11
Gal(α1-3)GalNAc(α-Sp8

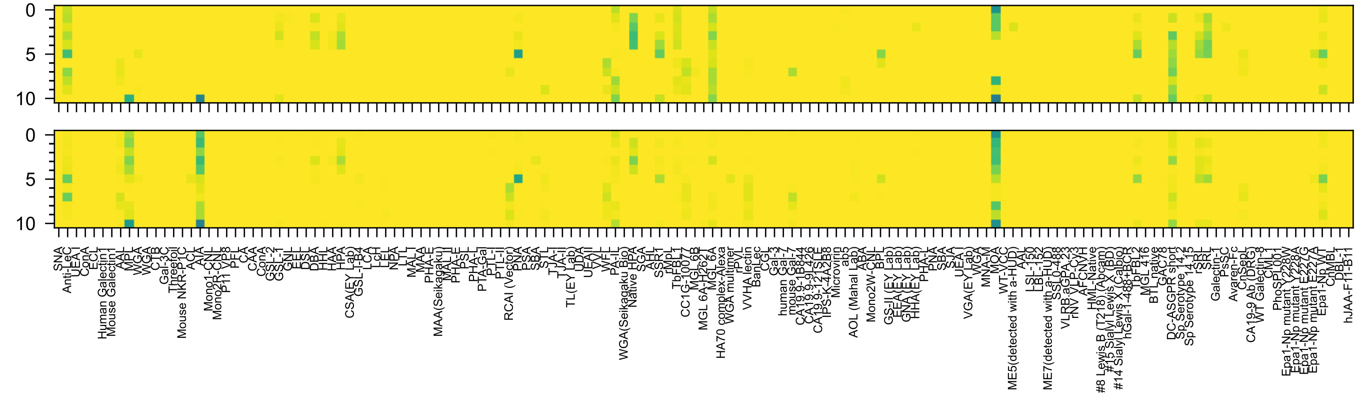

Gal(α1-3)GalNAc(β-Sp8

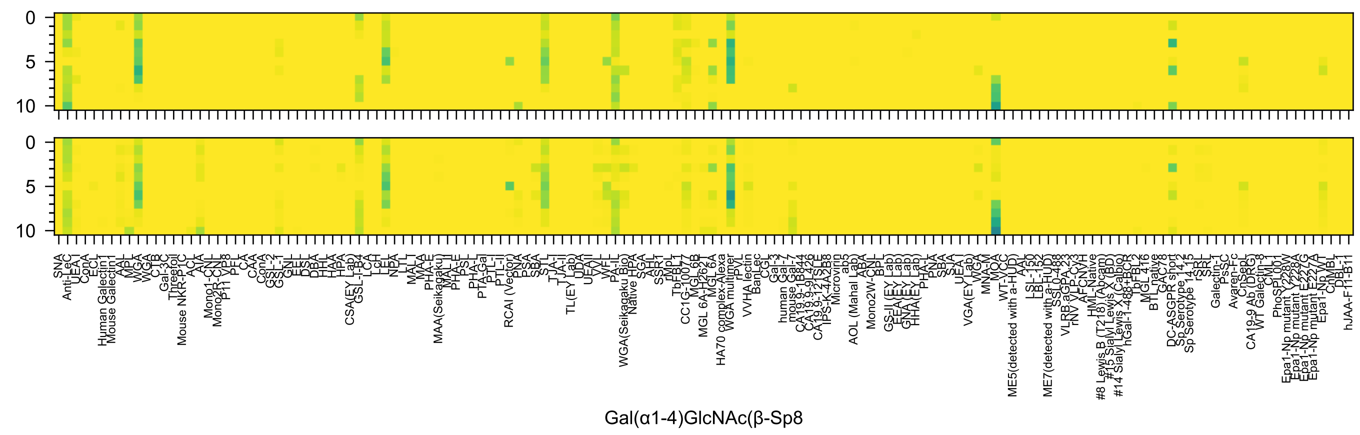
Gal(α1-4)GlcNAc(β-Sp8

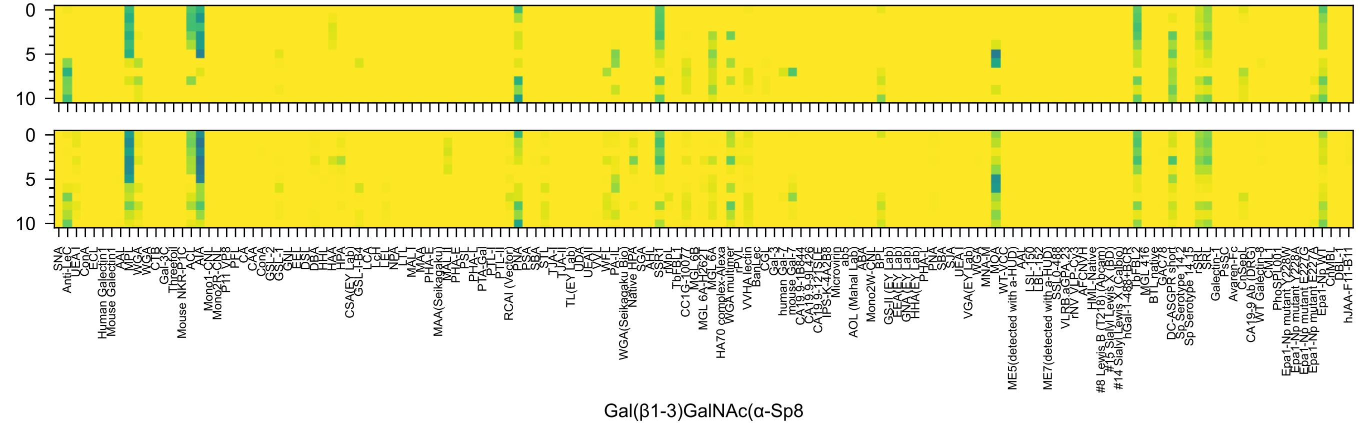
Gal(β1-3)GalNAc(α-Sp8

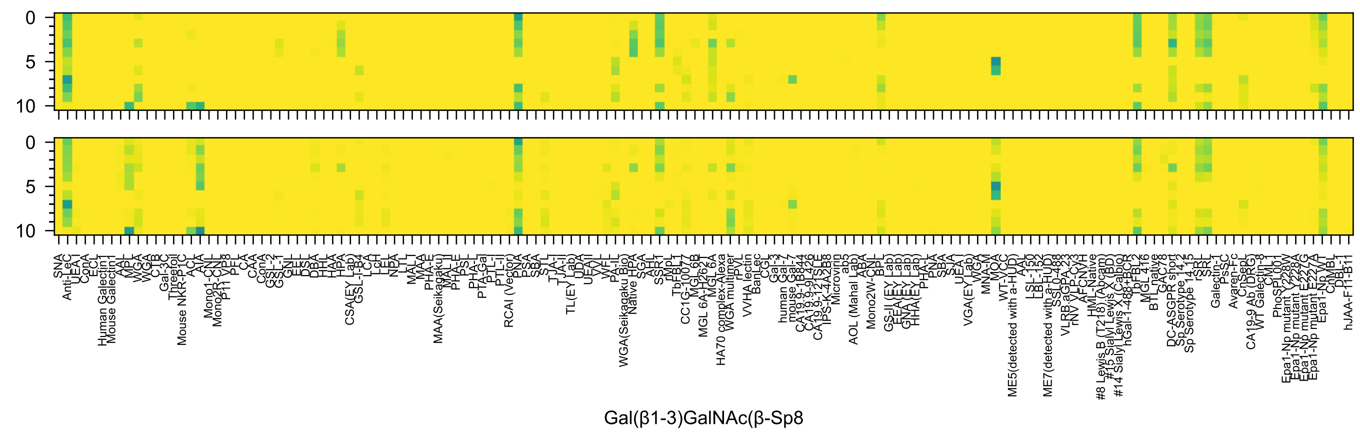
Gal(β1-3)GalNAc(β-Sp8

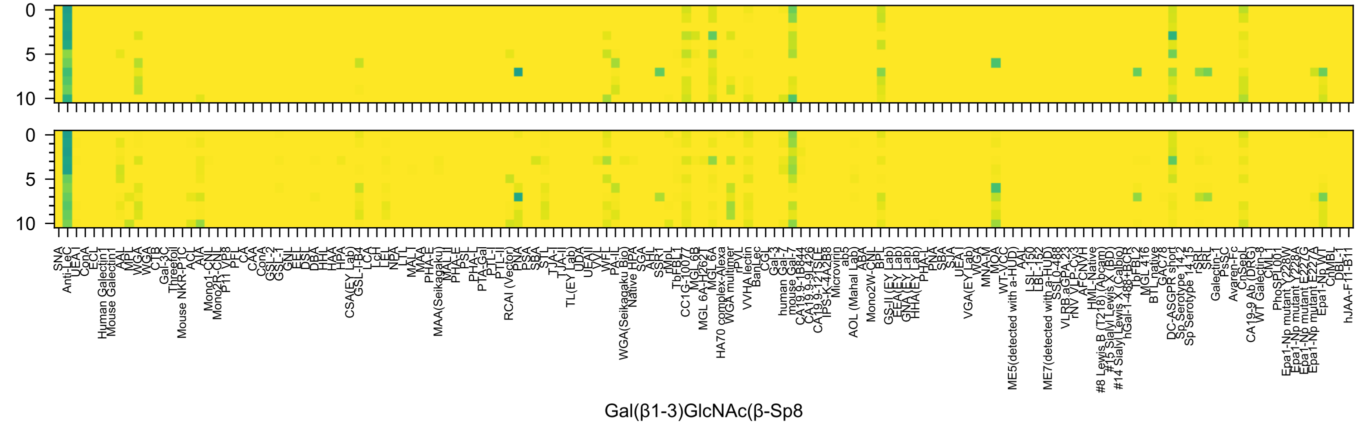
Gal(β1-3)GlcNAc(β-Sp8

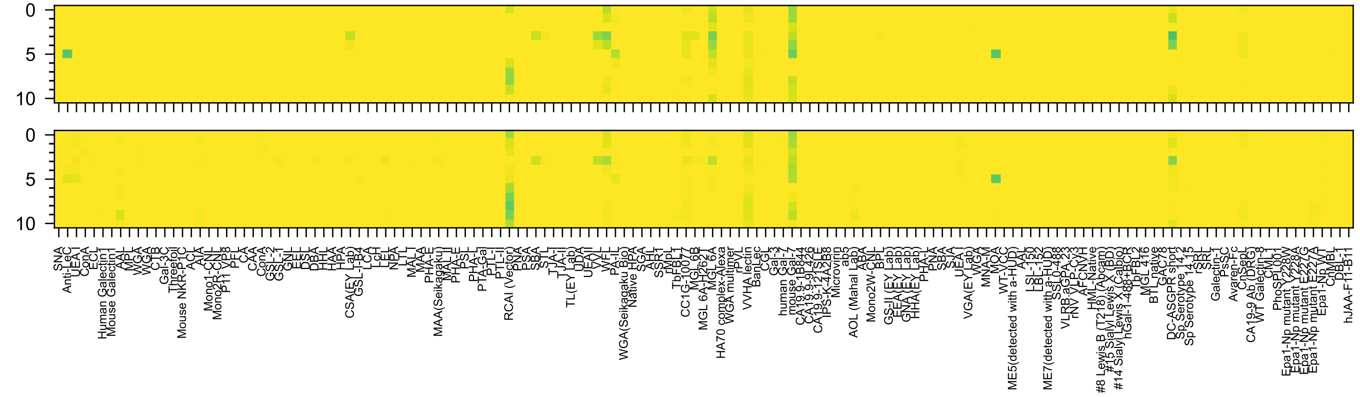

Gal(β1-4)Glc6OS(β-Sp0

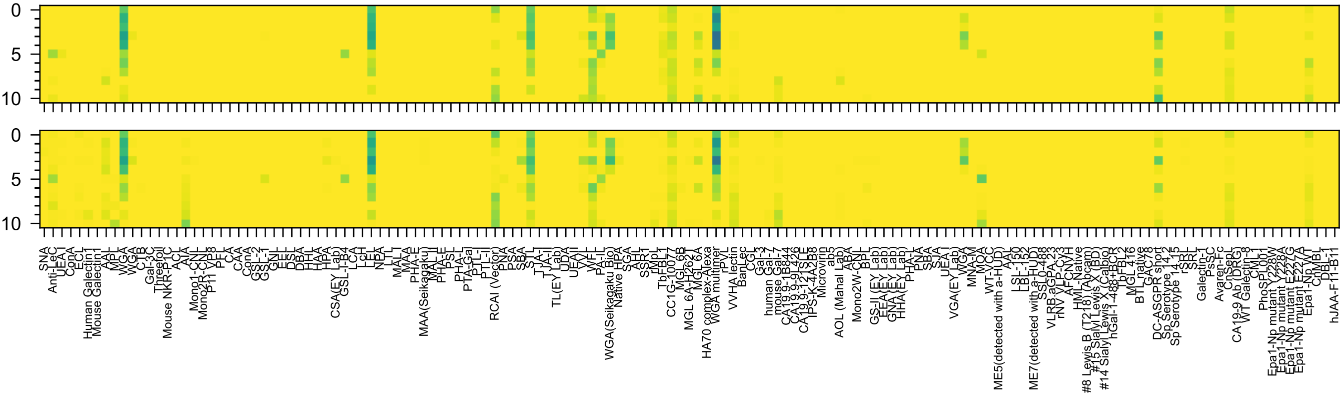

Gal(β1-4)GlcNAc(β-Sp8

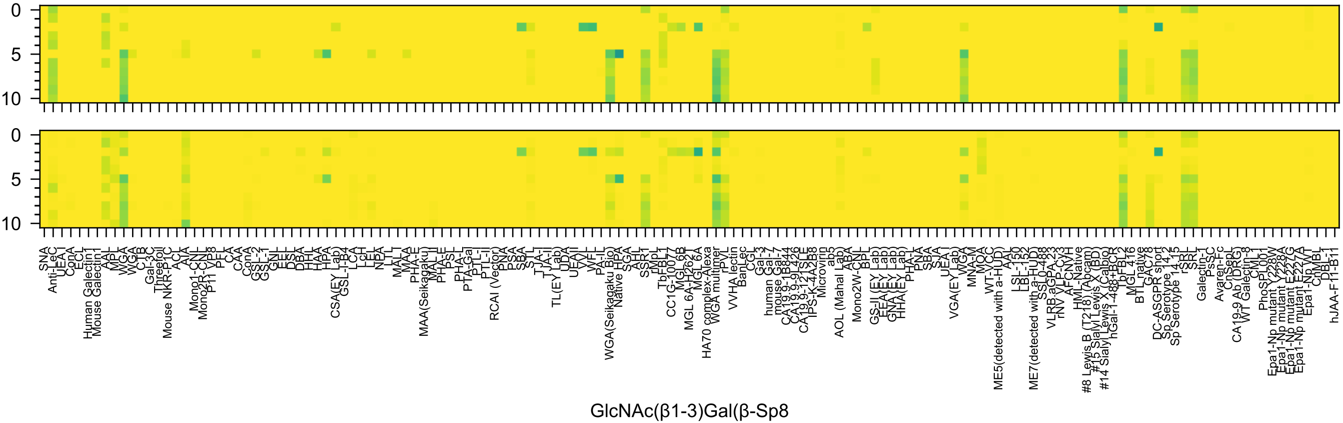
0
5
10
0
5
10
SNA
Anti-LeC
UEA-I
ConA
ECL
Human Galectin1
Mouse Galectin1
AAL
MPL
WGA
WGA
CTB
Gal-3C
Threefoil
Mouse NKR-P1C
ACL
AIA
Mono1-CNL
Mono2R-CNL
P11 VP8
PFL
CA
CAA
ConA
GSL-2
GSL-1
GNL
EEL
DSL
DBA
HHL
HAA
HPA
CSA(EY Lab)
GSL-I-B4
LCA
LcH
LEL
NPA
LTL
MAL-I
MAA
PHA-E
MAA(Seikagaku)
MAL-II
PHA-E
PSL
PHA-L
PTA-Gal
PTL-I
PTL-II
RCAI (Vector)
PNA
PSA
SBA
STL
TJA-I
TJA-II
TL(EY Lab)
UDA
UEA-II
VVL
WFL
PA-IL
WGA(Seikagaku Bio)
Native HPA
SGA
AHL
SSR1
rMpL
TbFBL1
CC1G-10077
MGL 6B
MGL 6A-H262T
MGL 6A
HA70 complex-Alexa
WGA multimer
rPVL
VVHA lectin
BanLec
CGL
Gal-3
human Gal-7
mouse Gal-7
CA19.9-1B844
CA19.9-9L426
CA19.9-121SLE
IPS-K-4A2B8
Microvirin
ab5
AOL (Mahal Lab)
ABA
Mono2W-CNL
BPL
GS-II (EY Lab)
EEA (EY Lab)
GNA (EY Lab)
HHA(EY Lab)
PHA-L
PNA
SBA
SJA
UEA-I
VGA(EY Lab)
WGA
MNA-M
MOA
WT-VCC
ME5(detected with a-HUD)
AAL
LSL-150
LBL-152
ME7(detected with a-HUD)
SSL0-488
VLRB.aGPA.23
rNV VLP-Cy3
AFCNVH
HML-Native
#8 Lewis B (T218) (Abcam)
#15 Sialyl Lewis X (BD)
#14 Sialyl Lewis X (Calbio)
hGal-1-488+BCR
TbFBL2
MGL 416
BTL native
GAC78
DC-ASGPR short
Sp Serotype 14.2
Sp Serotype 14.15
rSRL
SRL
Galectin-1
PsSC
Avaren-Fc
ChSeLL
CA19-9 Ab (DRG)
WT Galectin-8
CML1
PhoSPL001
Epa1-Np mutant Y228W
Epa1-Np mutant E227G
Epa1-Np mutant E227A
Epa1-Np WT
CnMBL
DBL-1
hJAA-F11-B11
GlcNAc(β1-3)Gal(β-Sp8

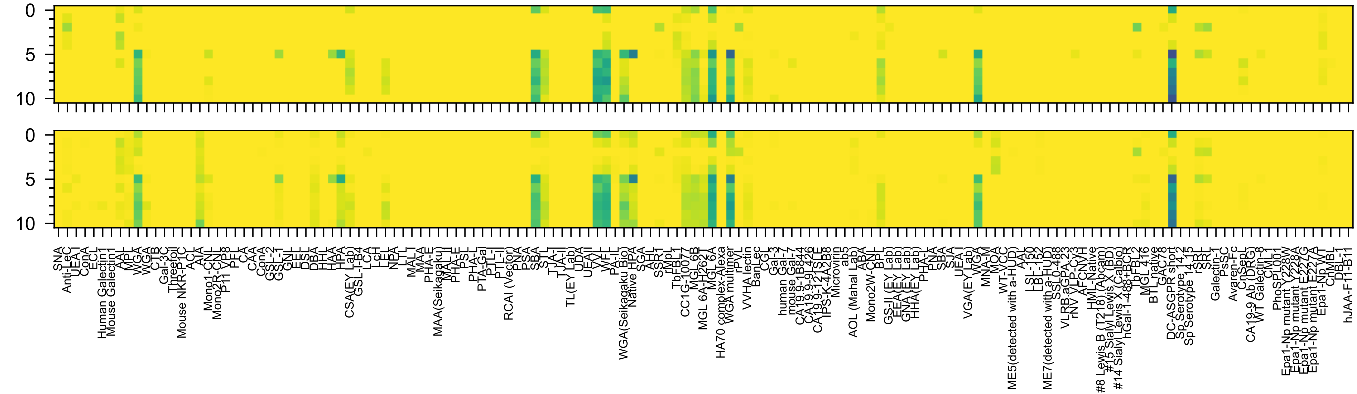

GalNAc(β1-3)Gal(β-Sp8

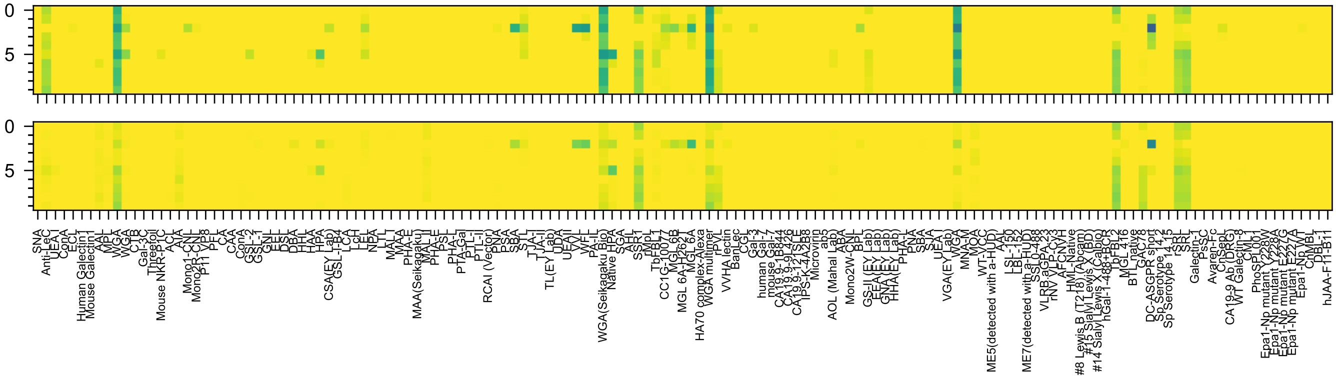

GlcNAc(β1-3)Man(-Sp10

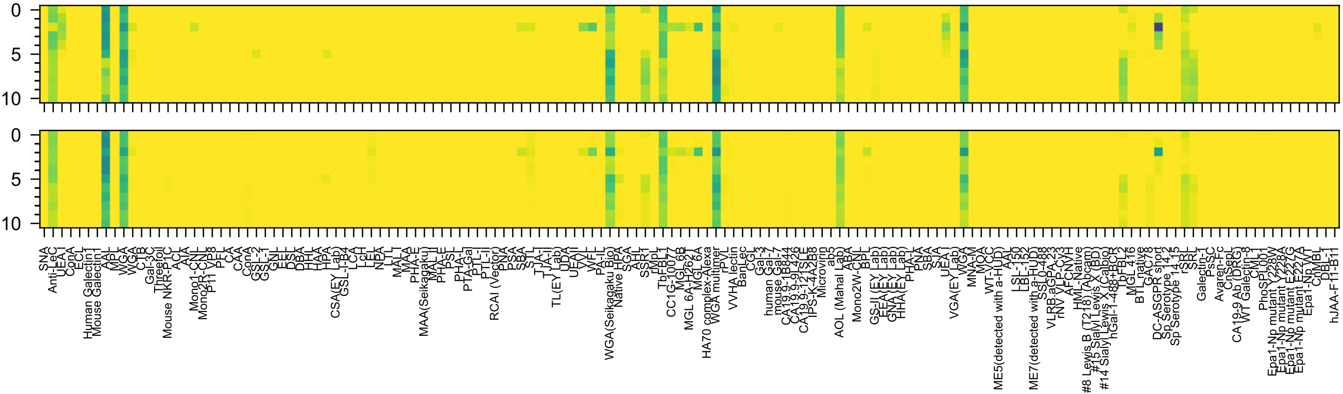

GlcNAc(β1-3)Fuc(α-Sp21

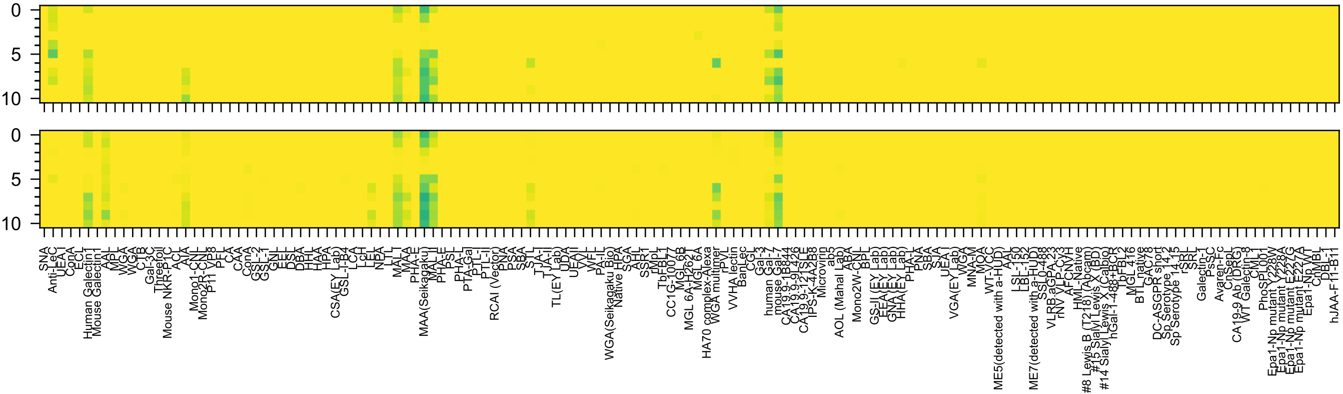
Gal3OS(β1-4)Glc(β-Sp8

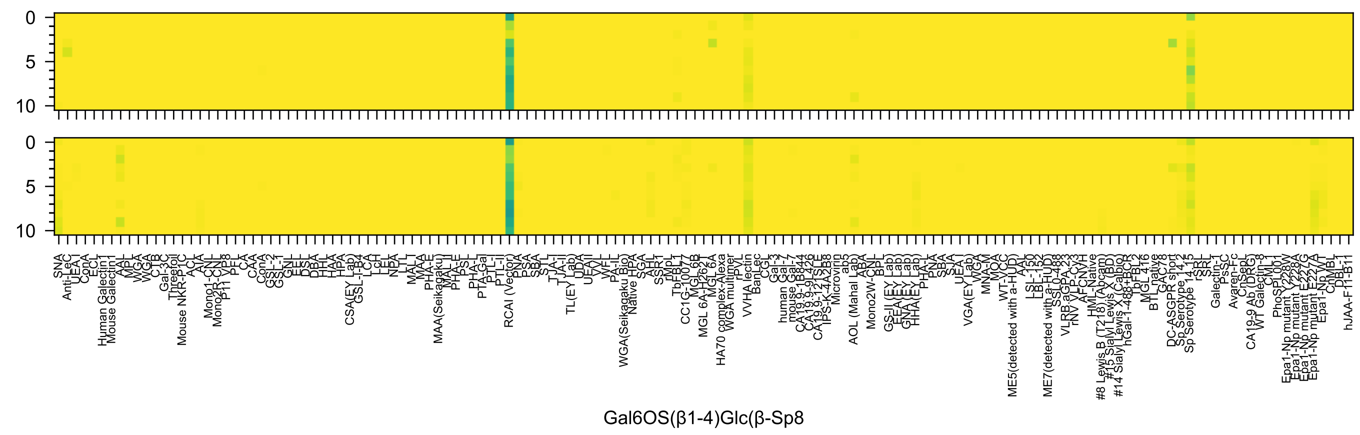
Gal6OS(β1-4)Glc(β-Sp8

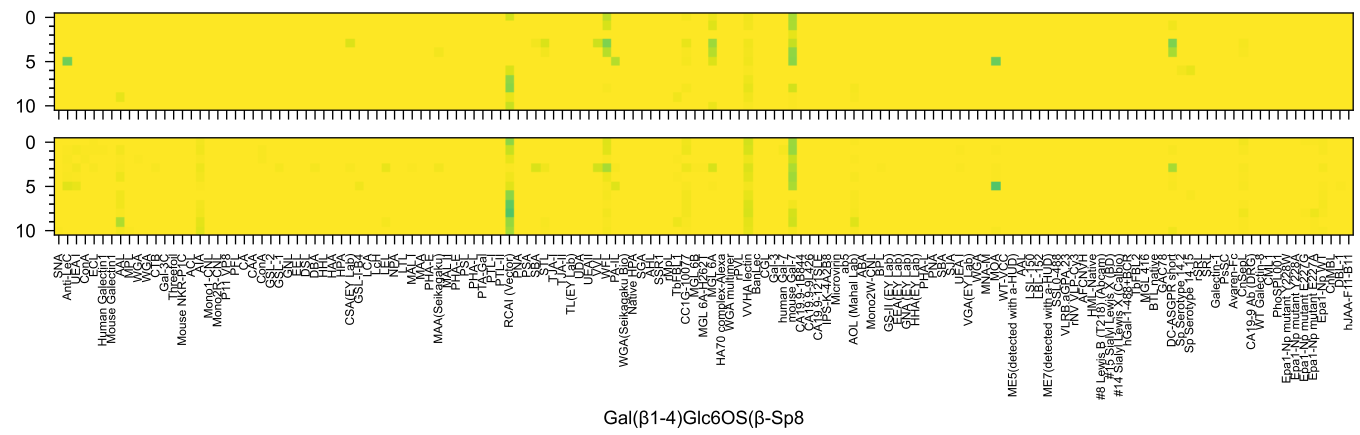
Gal(β1-4)Glc6OS(β-Sp8

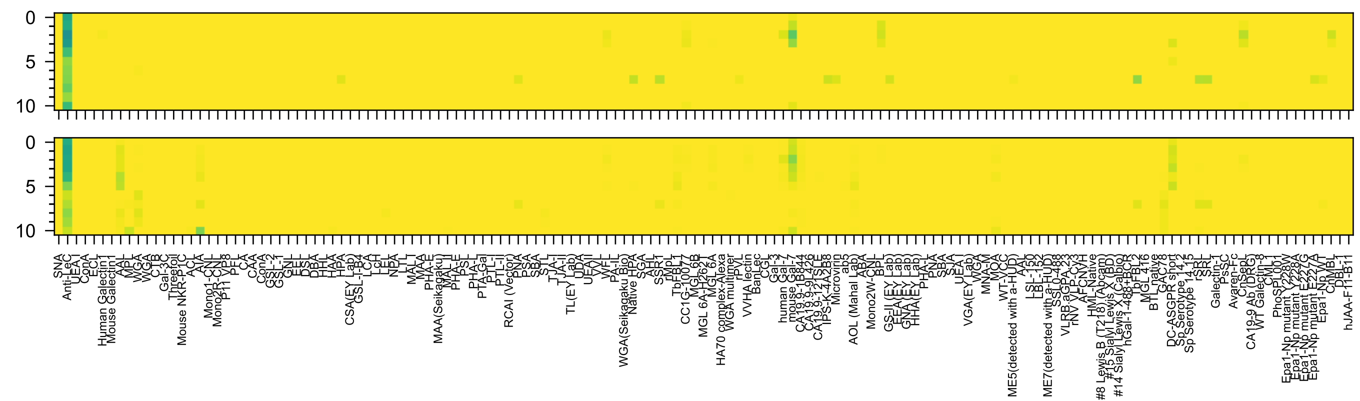

GlcA(β1-3)GlcNAc(β-Sp8

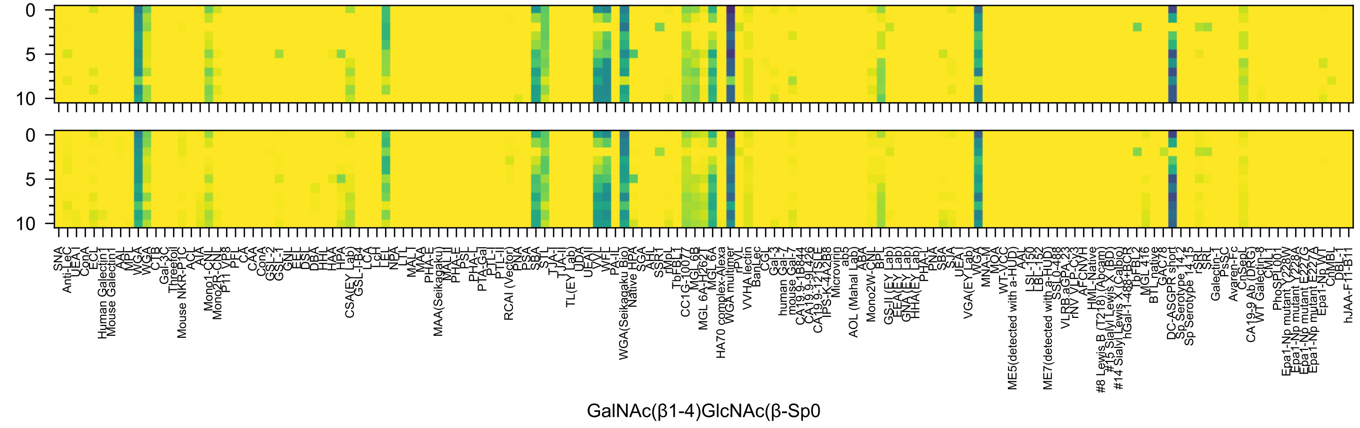
GalNAc(β1-4)GlcNAc(β-Sp0

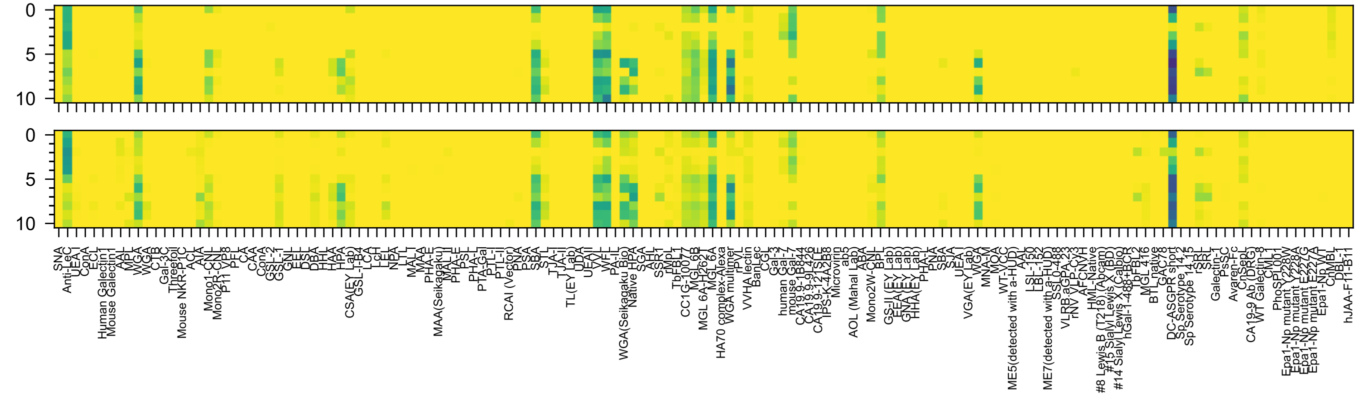

GalNAc(β1-3)GlcNAc(β-Sp0

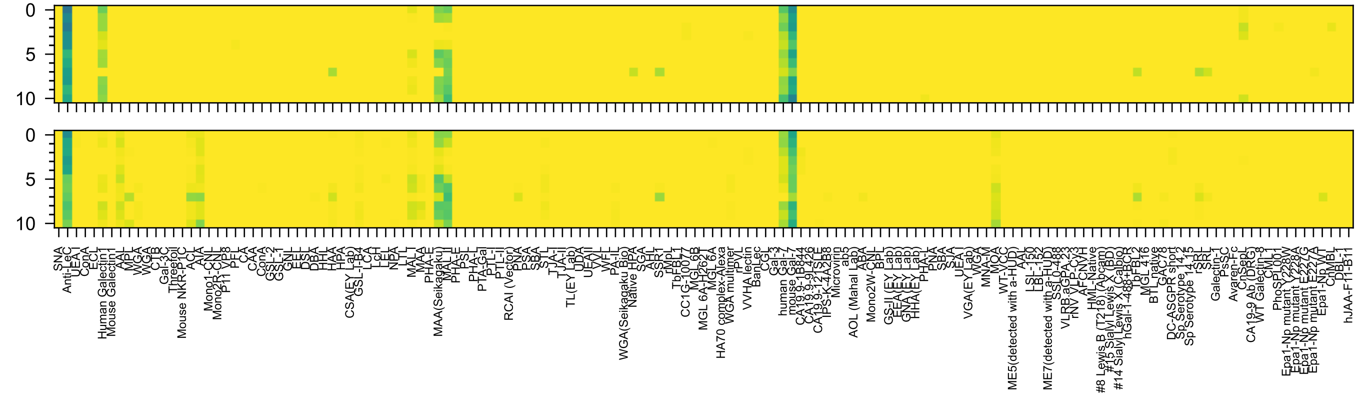

Gal3OS(β1-3)GlcNAc(β-Sp0

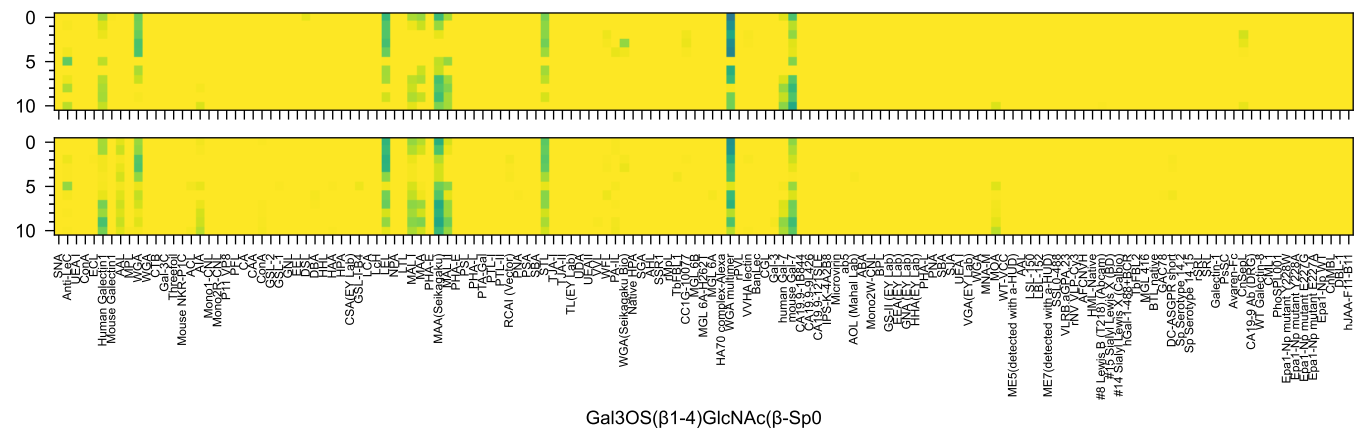
Gal3OS(β1-4)GlcNAc(β-Sp0

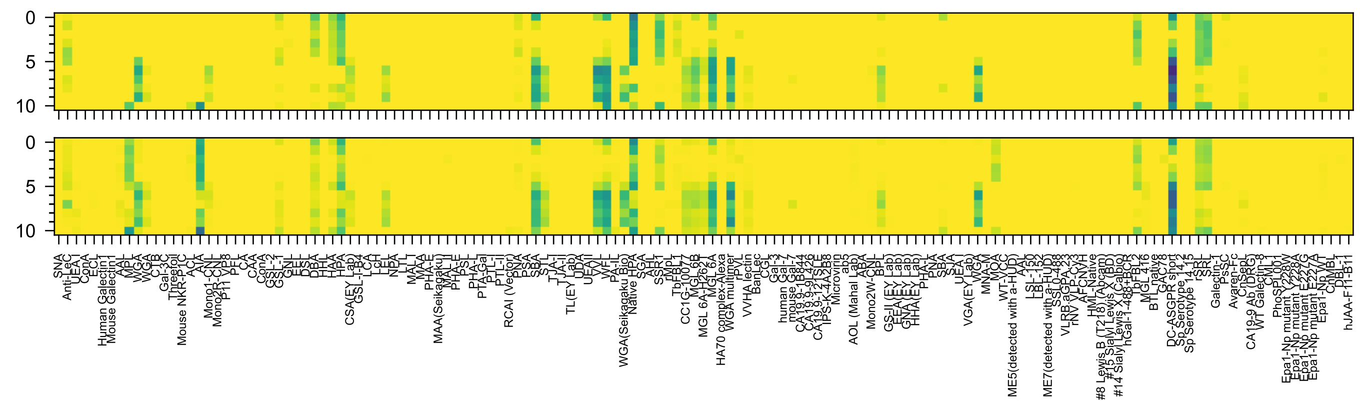

GalNAc(α1-3)GalNAc(β-Sp8

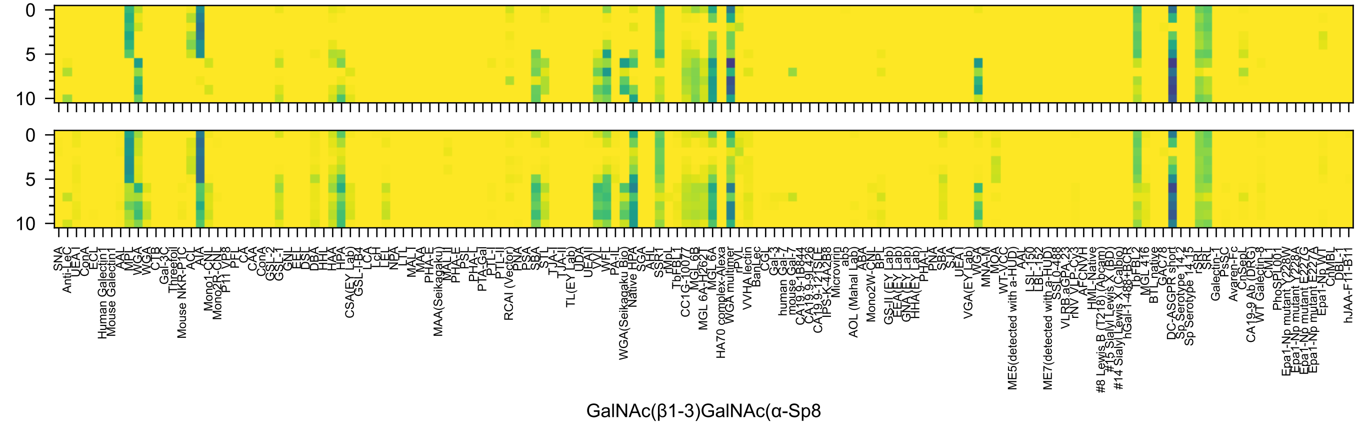

0
5
10
0
5
10
SNA
Anti-LeC
UEA-I
ConA
ECL
Human Galectin1
Mouse Galectin1
AAL
MPL
WGA
WGA
CTB
Gal-3C
Threefoil
Mouse NKR-P1C
ACL
AIA
Mono1-CNL
Mono2R-CNL
P11 VP8
PFL
CA
CAA
ConA
GSL-2
GSL-1
GNL
EEL
DSL
DBA
HHL
HAA
HPA
CSA(EY Lab)
GSL-I-B4
LCA
LcH
LEL
NPA
LTL
MAL-I
MAA
PHA-E
MAA(Seikagaku)
MAL-II
PHA-E
PSL
PHA-L
PTA-Gal
PTL-I
PTL-II
RCAI (Vector)
PNA
PSA
SBA
STL
TJA-I
TJA-II
TL(EY Lab)
UDA
UEA-II
VVL
WFL
PA-IL
WGA(Seikagaku Bio)
Native HPA
SGA
AAI
SSR1
rMpL
TbFBL1
CC1G-10077
MGL6B
MGL 6A-H262T
MGL 6A
HA70 complex-Alexa
WGA multimer
rPVL
VVHA lectin
BanLec
CGL
Gal-3
human Gal-7
mouse Gal-7
CA19.9-1B844
CA19.9-9L426
CA19.9-121SLE
IPS-K-4A2B8
Microvirin
ab5
AOL (Mahal Lab)
ABA
Mono2W-CNL
BPL
GS-II (EY Lab)
EEA(EY Lab)
GNA(EY Lab)
HHA(EY Lab)
PHA-L
PNA
SBA
SJA
UEA-I
VGA(EY Lab)
WGA
MNA-M
MOA
WT-VCC
ME5(detected with a-HUD)
AAL
LSL-150
LBL-152
ME7(detected with a-HUD)
SSL0-488
VLRB.aGPA.23
rNV VLP-Cy3
AFCNYH
HML-Native
#8 Lewis B (T218) (Abcam)
#15 Sialyl Lewis X (BD)
#14 Sialyl Lewis X (Calbio)
hGal-1-488+BCR
TbFBL2
MGL 416
BTL native
GAC78
DC-ASGPR short
Sp Serotype 14.2
Sp Serotype 14.15
rSRL
SRL
Galectin-1
PsSC
Avaren-Fc
CnSepL
CA19-9 Ab (DRG)
WT Galectin-8
CML1
PhoSL001
Epa1-Np mutant Y228V
Epa1-Np mutant Y228A
Epa1-Np mutant E227G
Epa1-Np mutant E227A
Epa1-Np WT
CnMBL
DBL-1
hJAA-F11-B11
GalNAc(β1-3)GalNAc(α-Sp8

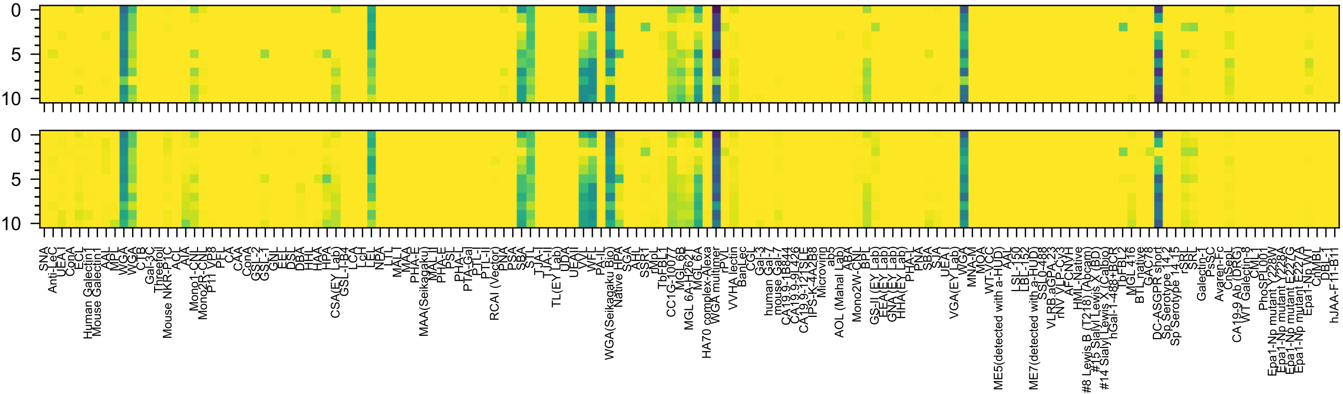

GalNAc(β1-4)GlcNAc(β-Sp8

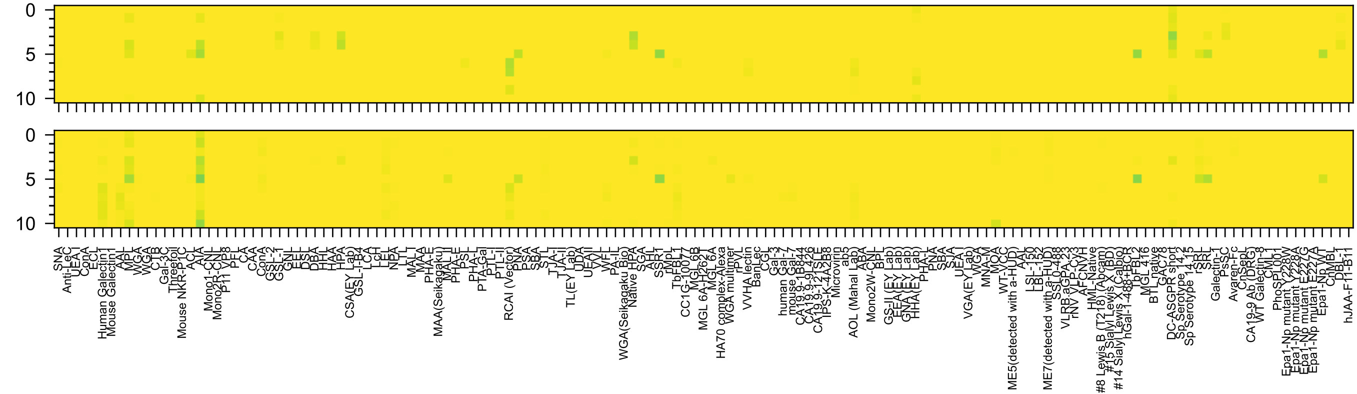

Gal(α1-3)GalNAc(α-Sp16

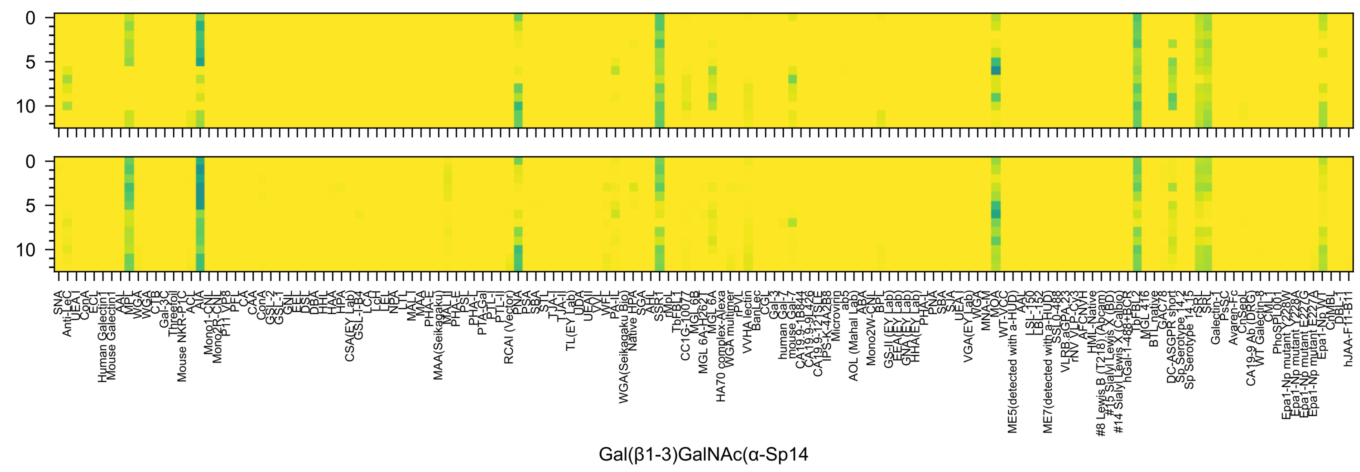
Gal(β1-3)GalNAc(α-Sp14

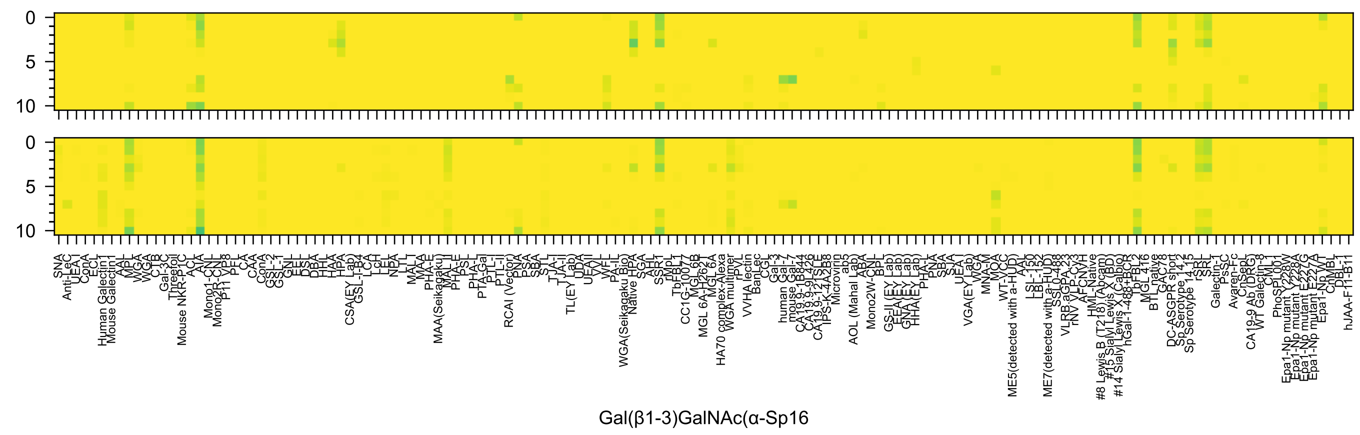
0
5
10
0
5
10
SNA
Anti-LeC
UEA I
ConA
ECL
Human Galectin1
Mouse Galectin1
AAL
MPL
WGA
WGA
CTB
Gal-3C
Threefoil
Mouse NKR-P1C
ACL
AIA
Mono1-CNL
Mono2R-CNL
P11 VP8
PFL
CA
CAA
ConA
GSL-2
GSL-1
GNL
EEL
DSL
DBA
HHL
HAA
HPA
CSA(EY Lab)
GSL-I-B4
LCA
LcH
LEL
NPA
LTL
MAL
MAA
PHA-E
MAA(Seikagaku)
MAL II
PHA-E
PSL
PHA-L
PTA-Gal
PTL-I
PTL-II
RCAI (Vector)
PNA
PSA
SBA
STL
TJA-I
TJA-II
TL(EY Lab)
UDA
UEA II
VVL
WFL
PA-IL
WGA(Seikagaku Bio)
Native HPA
SGA
AAL
SSR1
rMpL
TbFBL1
CC1G-10077
MGL6B
MGL 6A-H262T
MGL 6A
HA70 complex-Alexa
WGA multimer
rPVL
VVHA lectin
BanLec
CGL
Gal-3
human Gal-7
mouse Gal-7
CA19.9-1B844
CA19.9-9L426
CA19.9-121SLE
IPS-K-4A2B8
Microvirin
ab5
AOL (Mahal Lab)
ABA
Mono2W-CNL
BPL
GS-II (EY Lab)
EEA (EY Lab)
GNA (EY Lab)
HHA (EY Lab)
PHA-L
PNA
SBA
SJA
UEA I
VGA(EY Lab)
WGA
MNA-M
MOA
WT-VCC
ME5(detected with a-HUD)
AAL
LSL-150
LBL-152
ME7(detected with a-HUD)
SSL0-488
VLRB.aGPA.23
rNV VLP-Cy3
AFCNYH
HML-Native
#8 Lewis B (T218) (Abcam)
#15 Sialyl Lewis X (BD)
#14 Sialyl Lewis X (Calbio)
hGal-1-488+BCR
TbFBL2
MGL 416
BTL native
GAC78
DC-ASGPR short
Sp Serotype 14.2
Sp Serotype 14.15
rSRL
SRL
Galectin-1
PsSC
Avaren-Fc
ChSepL
CA19-9 Ab (DRG)
WT Galectin-8
CML1
PhoSL001
Epa1-Np mutant Y228W
Epa1-Np mutant Y228A
Epa1-Np mutant E227G
Epa1-Np mutant E227A
Epa1-Np WT
CnMBL
DBL-1
hJAA-F11-B11
Gal(β1-3)GalNAc(α-Sp16

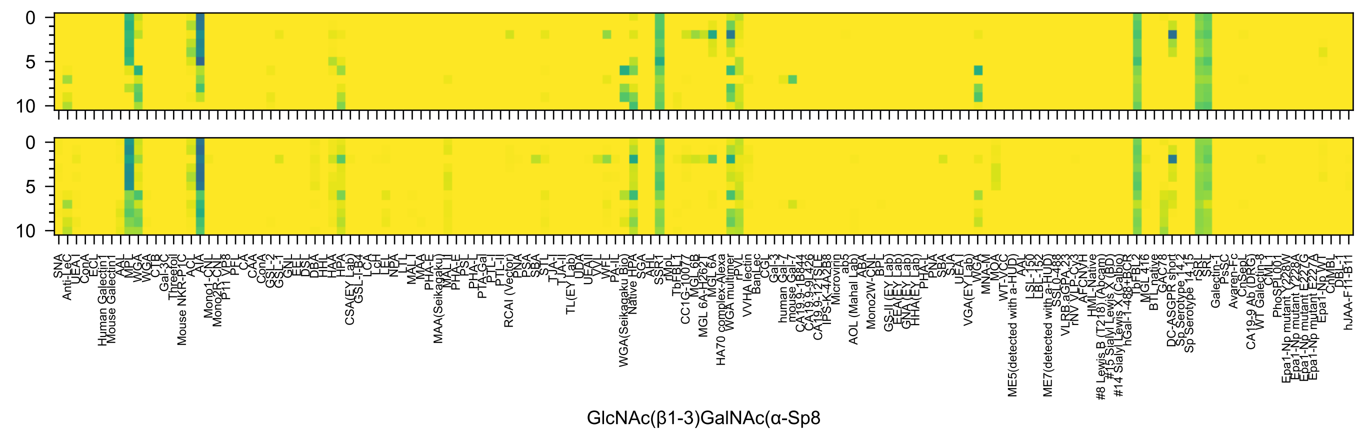
GlcNAc(β1-3)GalNAc(α-Sp8

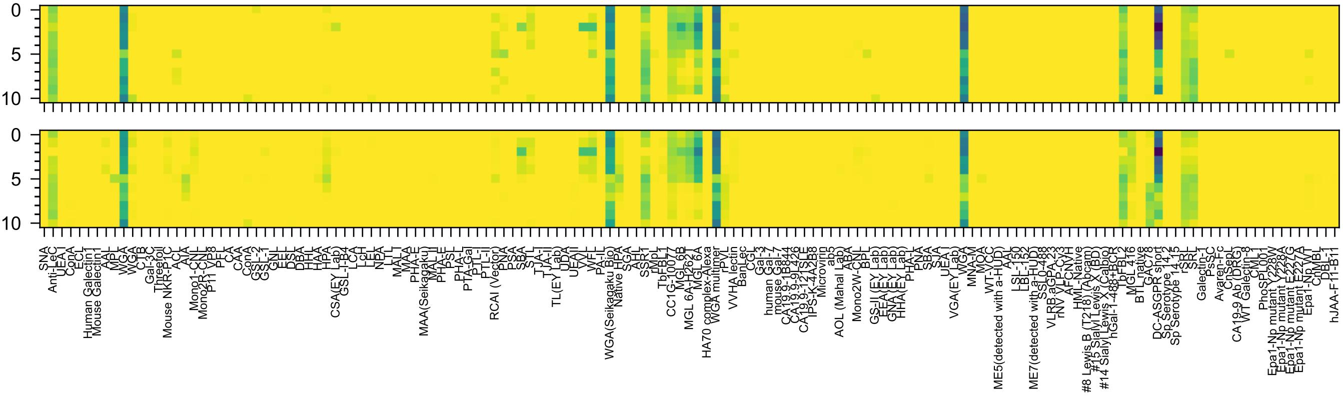

GlcNAc(β1-6)GalNAc(α-Sp8

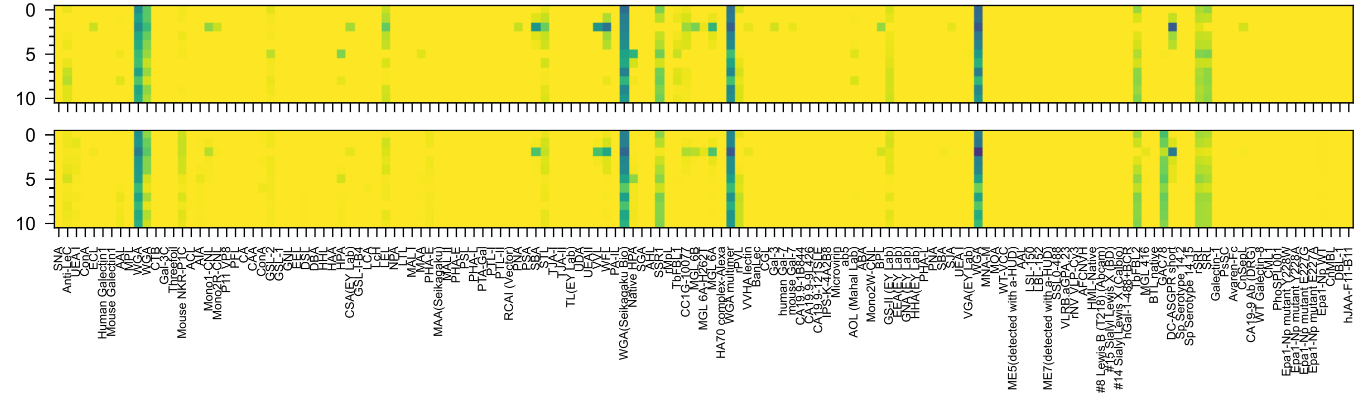

GlcNAc(β1-4)GlcNAc(β-Sp10)

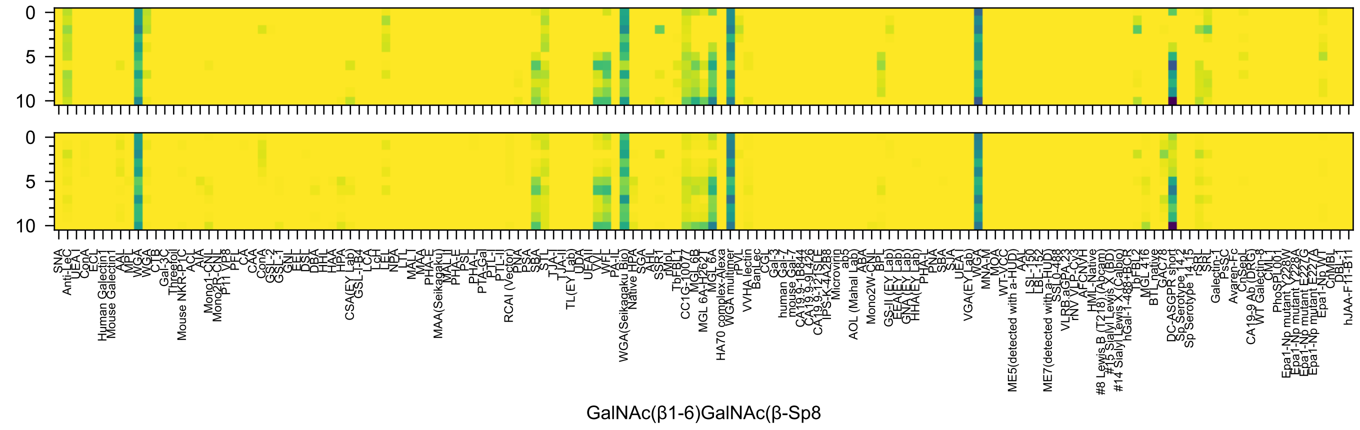
GalNAc(β1-6)GalNAc(β-Sp8

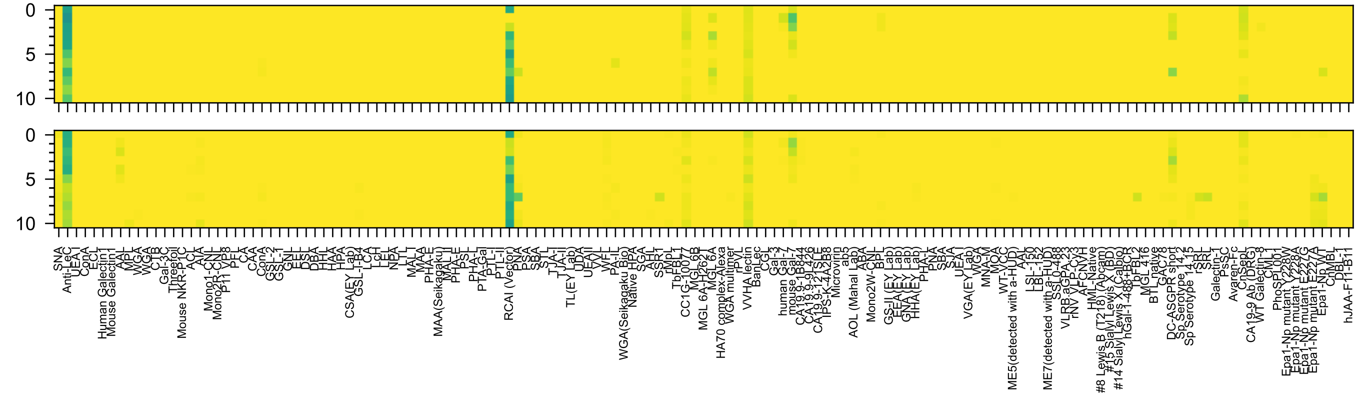

Gal6OS(β1-3)GlcNAc(β-Sp0

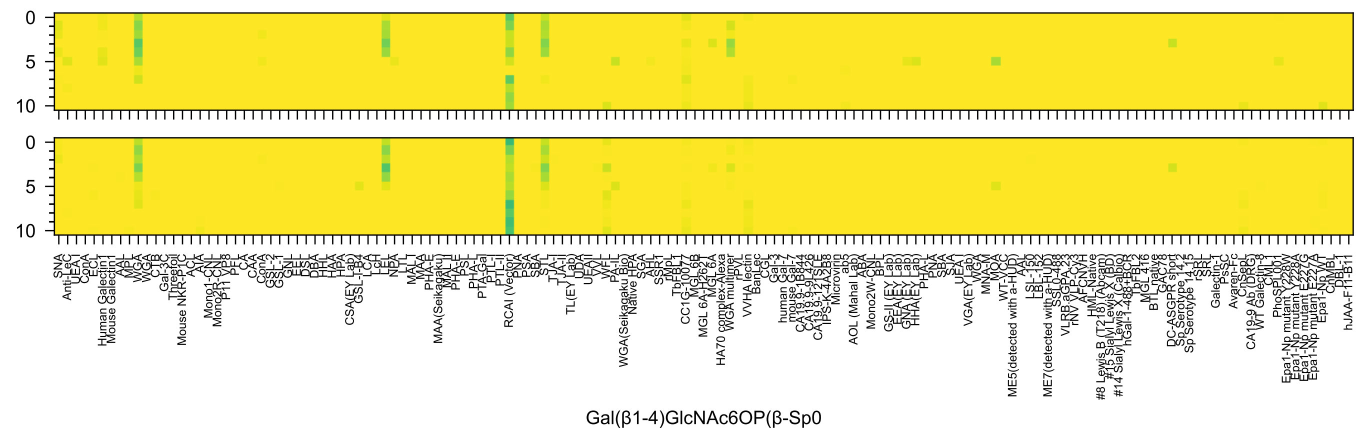
Gal(β1-4)GlcNAc6OP(β-Sp0

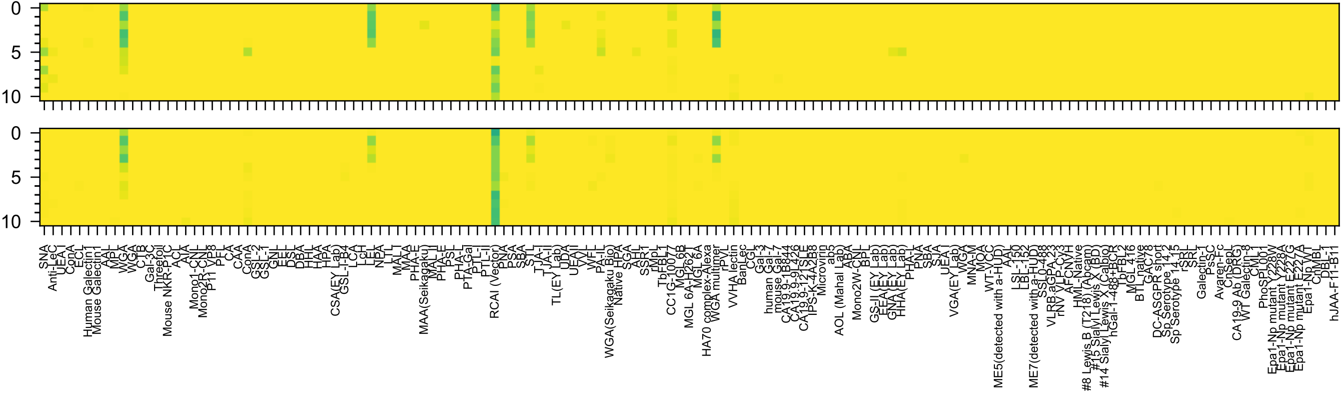

Gal6OP(β1-4)GlcNAc(β-Sp0

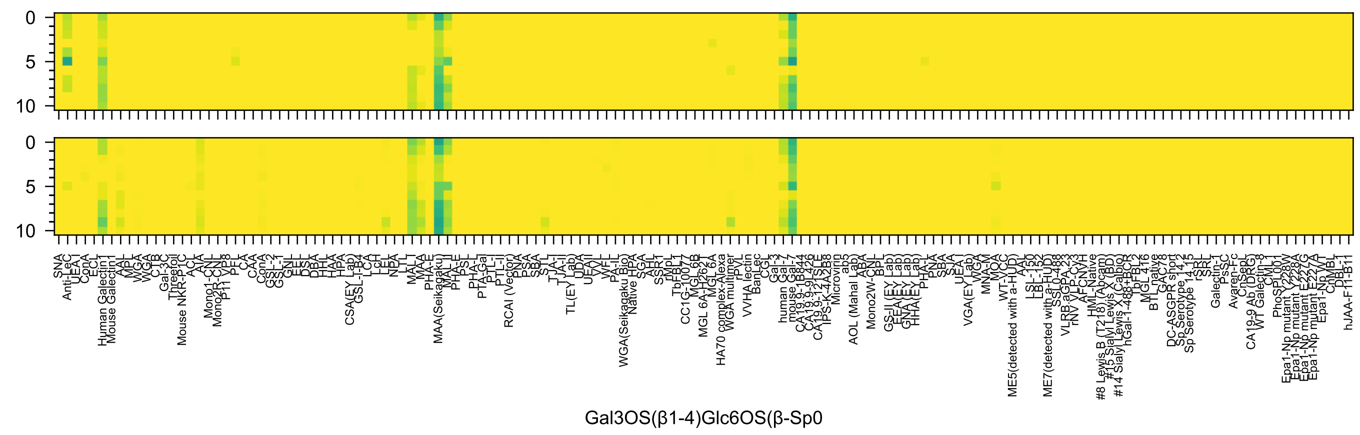

Gal3OS(β1-4)Glc6OS(β-Sp0

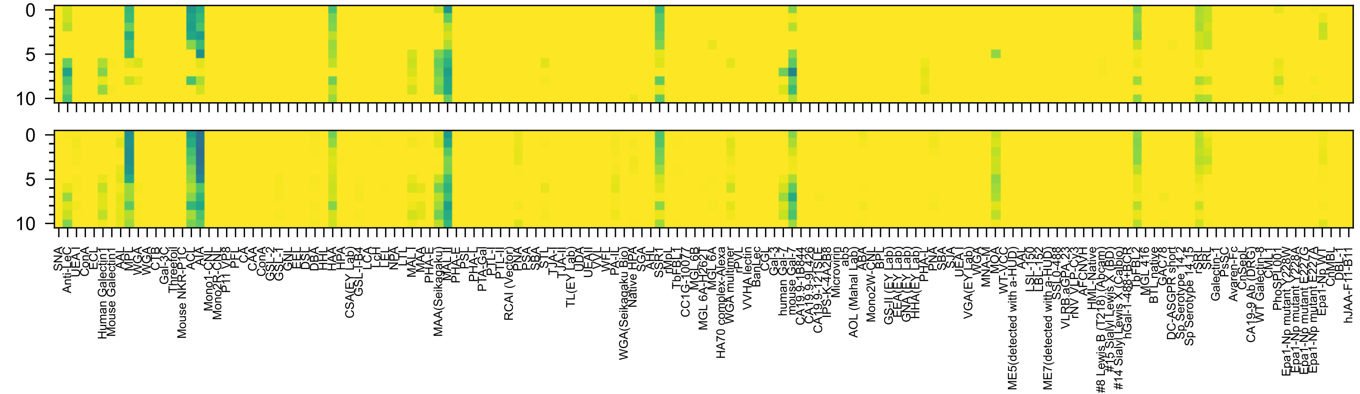

Gal3OS(β1-3)GalNAc(α-Sp8

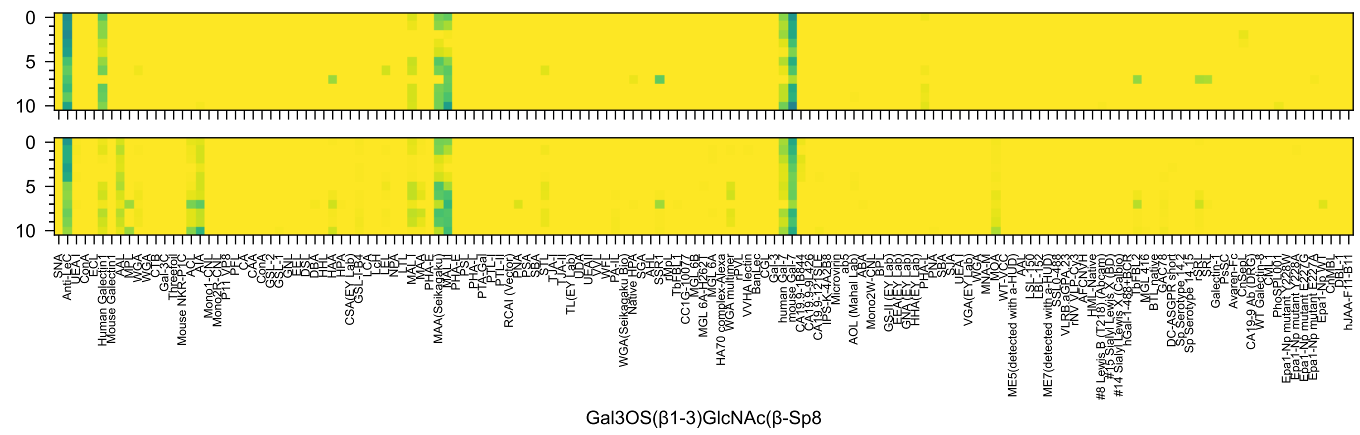
Gal3OS(β1-3)GlcNAc(β-Sp8

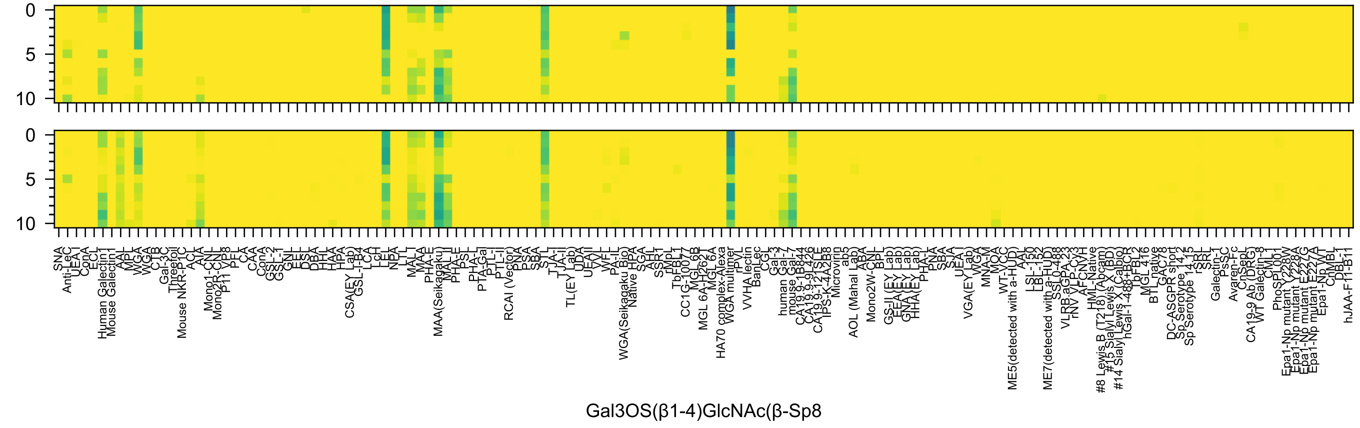
Gal3OS(β1-4)GlcNAc(β-Sp8

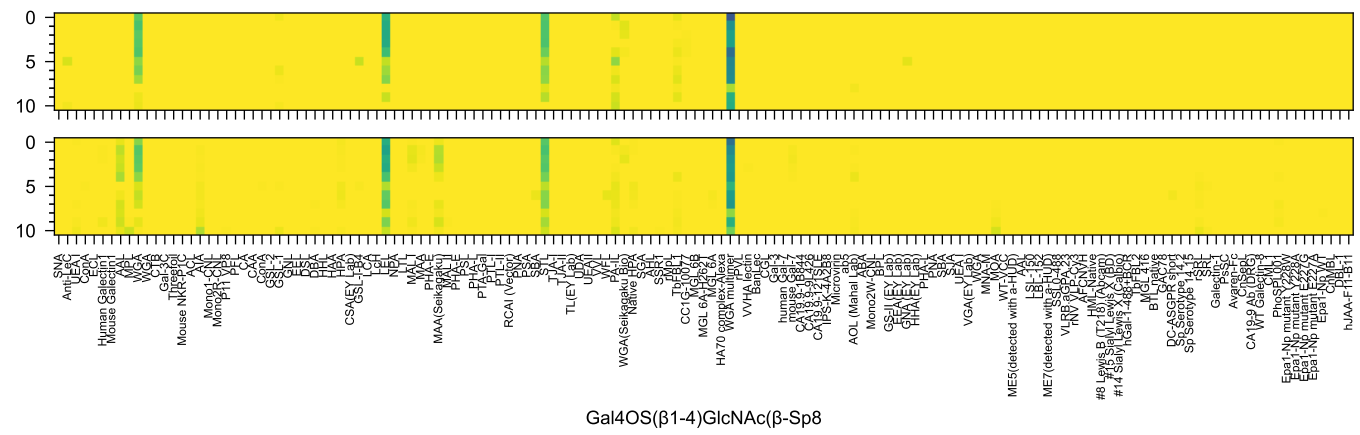
0
5
10
0
5
10
Gal4OS(β1-4)GlcNAc(β-Sp8

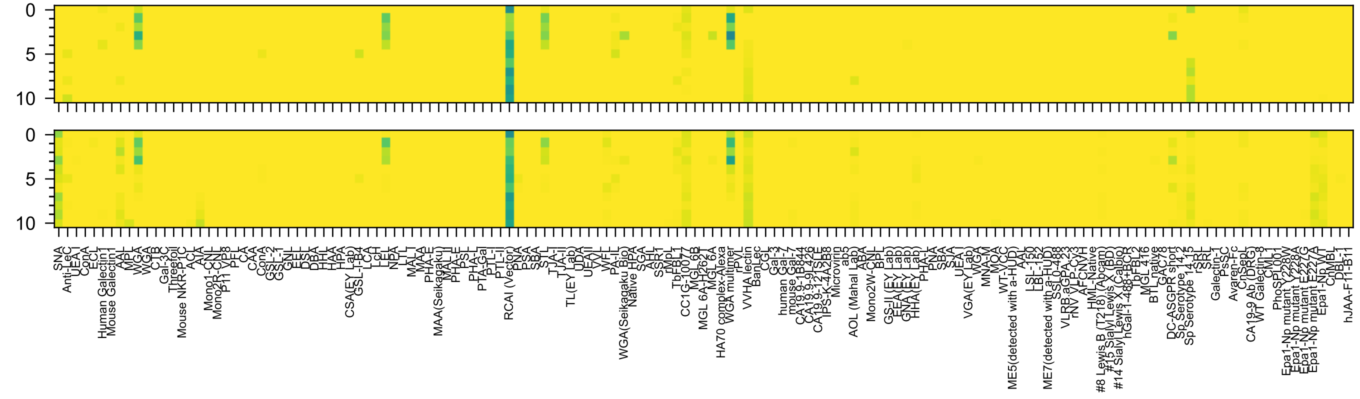

Gal6OS(β1-4)GlcNAc(β-Sp8

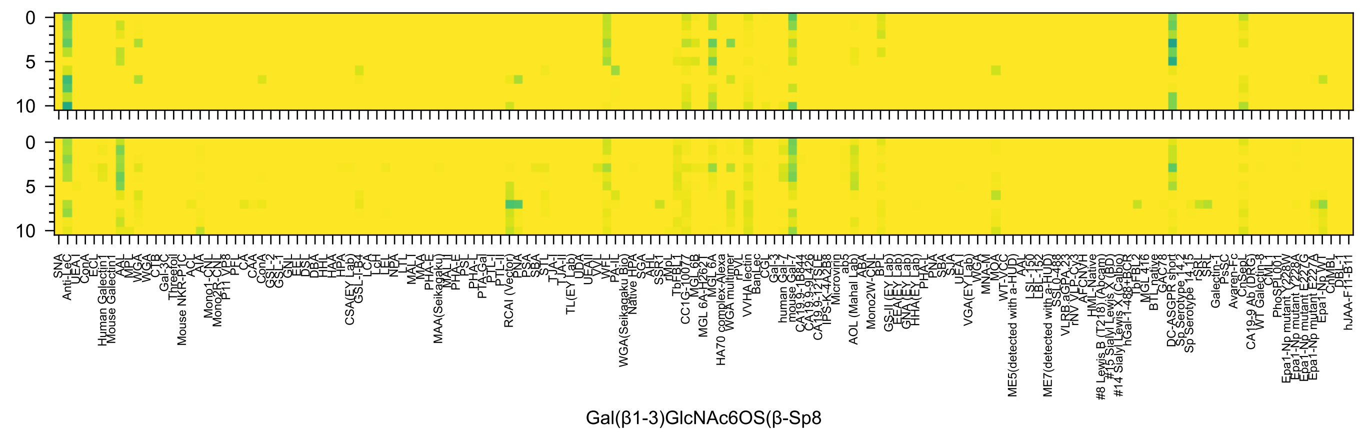
Gal(β1-3)GlcNAc6OS(β-Sp8

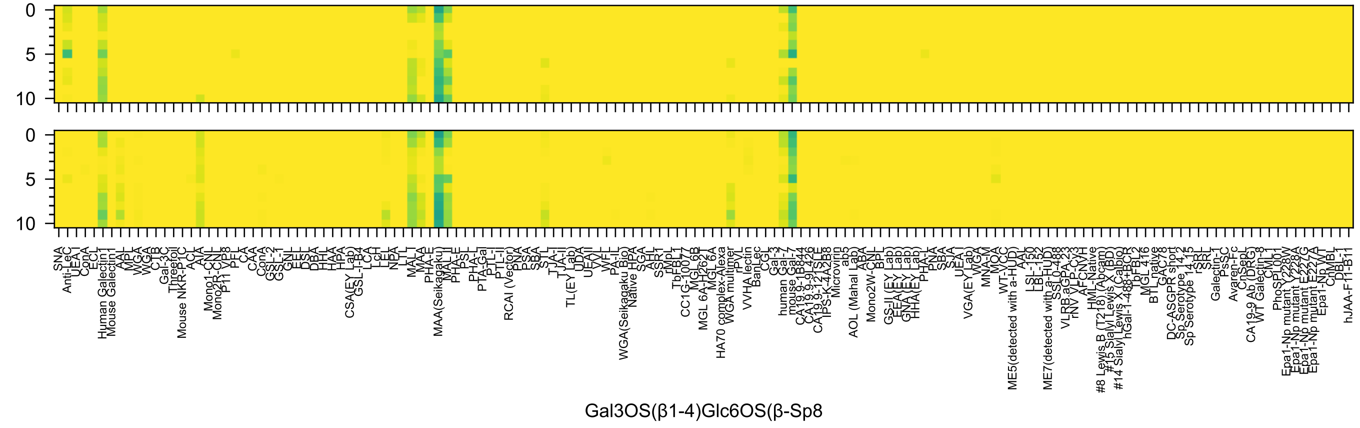

Gal3OS(β1-4)Glc6OS(β-Sp8

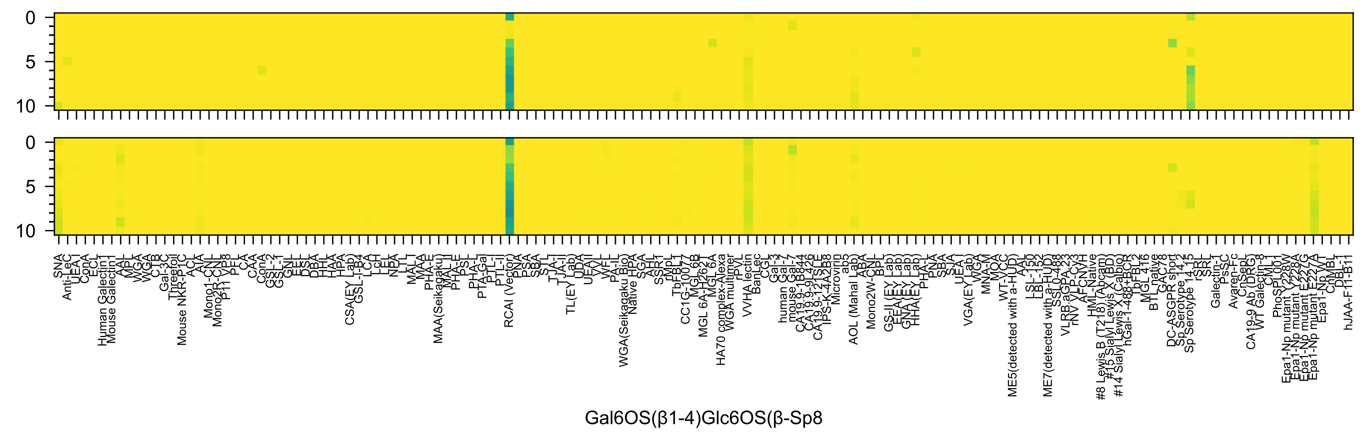
Gal6OS(β1-4)Glc6OS(β-Sp8

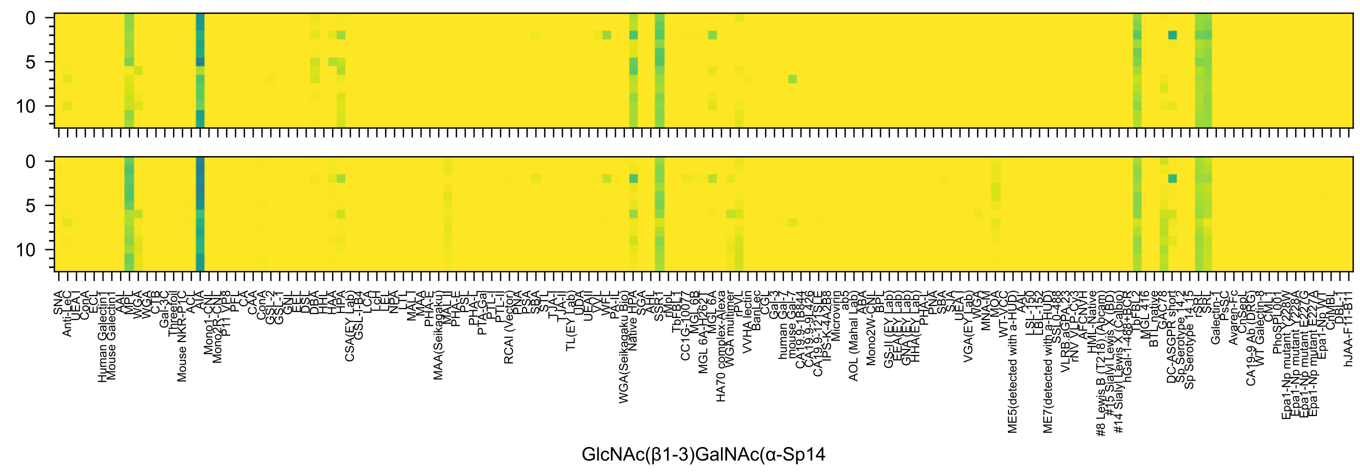
0
5
10
0
5
10
SNA
Anti-LeC
UEA-I
ConA
ECL
Human Galectin1
Mouse Galectin1
AAL
MPL
WGA
WGA
CTB
Gal-3C
Threefoil
Mouse NKR-P1C
ACL
AIA
Mono1-CNL
Mono2R-CNL
P11 VP8
PFL
CA
CAA
ConA
GSL-2
GSL-1
GNL
EEL
DSL
DBA
HHL
HAA
HPA
CSA(EY Lab)
GSL-I-B4
LCA
LcH
LEL
NPA
LTL
MAL-I
MAA
PHA-E
MAA(Seikagaku)
MAL-II
PHA-E
PSL
PHA-L
PTA-Gal
PTL-I
PTL-II
RCAI (Vector)
PNA
PSA
SBA
STL
TJA-I
TJA-II
TL(EY Lab)
UDA
UEAII
VVL
WFL
PA-IL
WGA(Seikagaku Bio)
Native HPA
SGA
AHL
SSR1
rMpL
TbFBL1
CC1G-10077
MGL6B
MGL 6A-H262T
MGL 6A
HA70 complex-Alexa
WGA multimer
rPVL
VVHA lectin
BanLec
CGL
Gal-3
human Gal-7
mouse Gal-7
CA19.9-1B844
CA19.9-9L426
CA19.9-121SLE
IPS-K-4A2B8
Microvirin
ab5
AOL (Mahal Lab)
ABA
Mono2W-CNL
BPL
GS-II (EY Lab)
EEA (EY Lab)
GNA (EY Lab)
HHA(EY Lab)
PHA-L
PNA
SBA
SJA
UEA-I
VGA(EY Lab)
WGA
MNA-M
MOA
WT-VCC
ME5(detected with a-HUD)
AAL
LSL-150
LBL-152
ME7(detected with a-HUD)
SSL0-488
VLRB.aGPA.23
rNV VLP-Cy3
AFCNYH
HML-Native
#8 Lewis B (T218) (Abcam)
#15 Sialyl Lewis X (BD)
#14 Sialyl Lewis X (Calbio)
hGal-1-488+BCR
TbFBL2
MGL 416
BTL native
GAC78
DC-ASGPR short
Sp Serotype 14-2
Sp Serotype 14-15
rSRL
SRL
Galectin-1
PsSC
Avaren-Fc
CnSepL
CA19-9 Ab (DRG)
WT Galectin-8
CML1
PhoSPL001
Epa1-Np mutant Y228W
Epa1-Np mutant Y228A
Epa1-Np mutant E227G
Epa1-Np mutant E227A
Epa1-Np WT
ChMBL
DBL-1
hJAA-F11-B11
GlcNAc(β1-3)GalNAc(α-Sp14

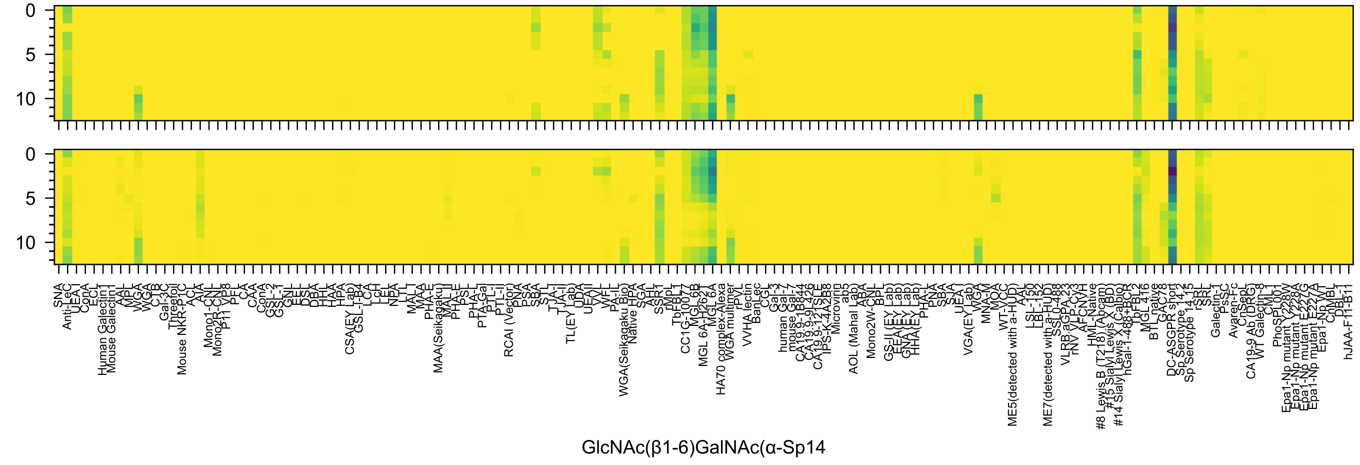
0
5
10
0
5
10
GlcNAc(β1-6)GalNAc(α-Sp14

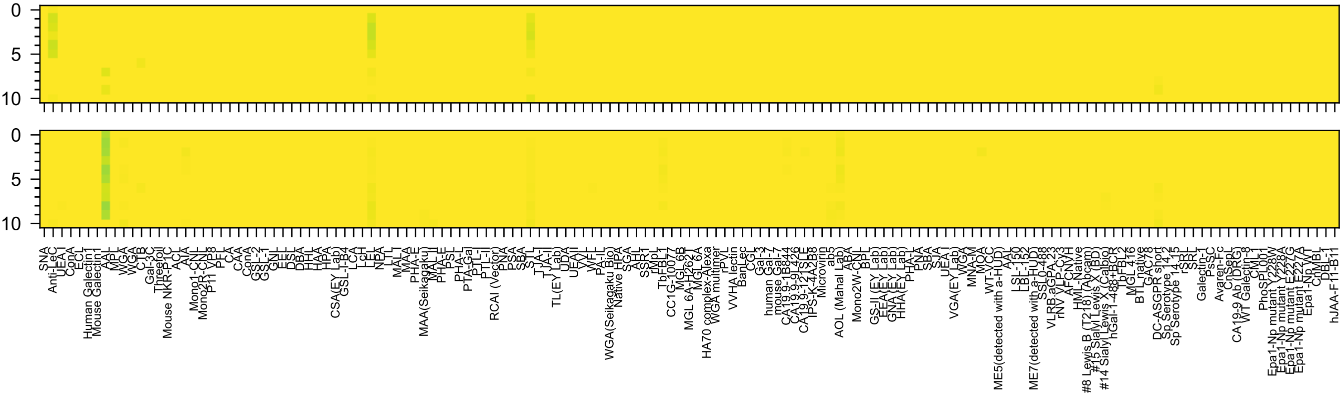

Neu5Ac(α2-3)Gal(β-Sp8

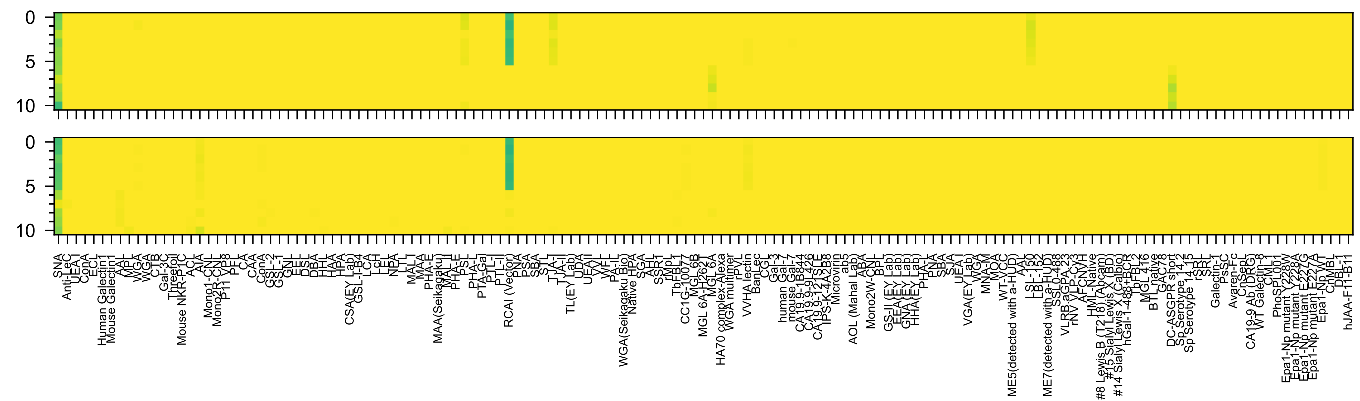

Neu5Ac(α2-6)Gal(β-Sp8

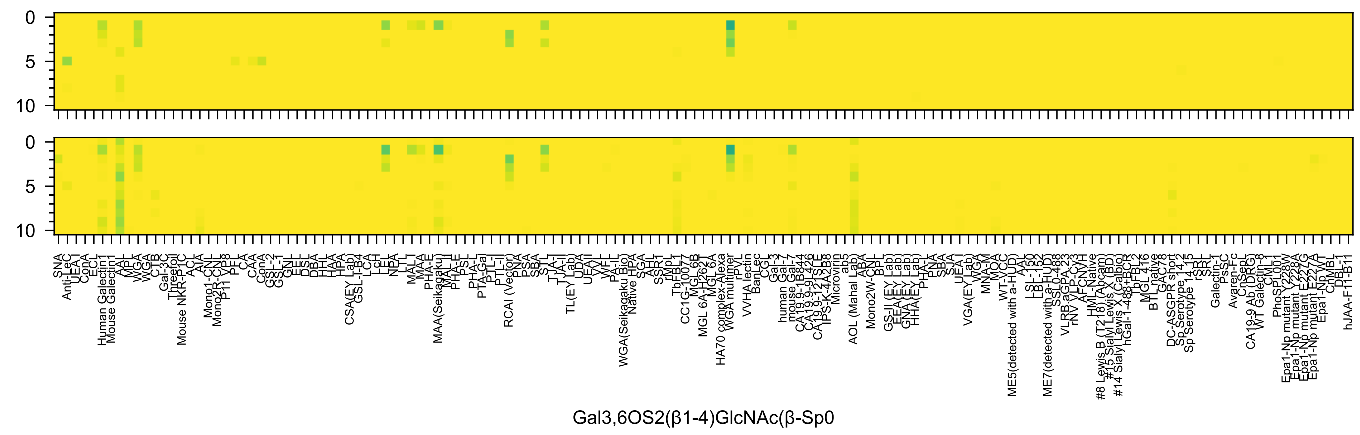
0
5
10
0
5
10
SNA
Anti-LeC
UEA I
ConA
ECL
Human Galectin1
Mouse Galectin1
AAL
MPL
WGA
WGA
CTB
Gal-3C
Threefoil
Mouse NKR-P1C
ACL
AIA
Mono1-CNL
Mono2R-CNL
P11 VP8
PFL
CA
CAA
ConA
GSL-2
GSL-1
GNL
EEL
DSL
DBA
HHL
HAA
HPA
CSA(EY Lab)
GSL-I-B4
LCA
LcH
LEL
NPA
LTL
MAL
MAA
PHA-E
MAA(Seikagaku)
MAL II
PHA-E
PSL
PHA-L
PTA-Gal
PTL-I
PTL-II
RCAI (Vector)
PNA
PSA
SBA
STL
TJA-I
TJA-II
TL(EY Lab)
UDA
UEAII
VVL
WFL
PA-IL
WGA(Seikagaku Bio)
Native HPA
SGA
AHL
SSR1
rMpL
TbFBL1
CC1G-10077
MGL-6B
MGL 6A-H262T
MGL 6A
HA70 complex-Alexa
WGA multimer
rPVL
VVHA lectin
BanLec
CGL
Gal-3
human Gal-7
mouse Gal-7
CA19.9-1B844
CA19.9-9L426
CA19.9-121SLE
IPS-K-4A2B8
Microvirin
ab5
AOL (Mahal Lab)
ABA
Mono2W-CNL
BPL
GS-II (EY Lab)
EEA(EY Lab)
GNA(EY Lab)
HHA(EY Lab)
PHA-L
PNA
SBA
SJA
UEA-I
VGA(EY Lab)
WGA
MNA-M
MOA
WT-VCC
ME5(detected with a-HUD)
AAL
LSL-150
LBL-152
ME7(detected with a-HUD)
SSL0-488
VLRB.aGPA.23
rNV VLP-Cy3
AFCNYH
HML-Native
#8 Lewis B (T218) (Abcam)
#15 Sialyl Lewis X (BD)
#14 Sialyl Lewis X (Calbio)
hGal-1-488+BCR
TbFBL2
MGL 416
BTL native
GAC78
DC-ASGPR short
Sp Serotype 14.2
Sp Serotype 14.15
rSRL
SRL
Galectin-1
PsSC
Avaren-Fc
ChSeBL
CA19-9 Ab (DRG)
WT Galectin-8
CML1
PhoSL001
Epa1-Np mutant Y228W
Epa1-Np mutant E227A
Epa1-Np mutant E227G
Epa1-Np WT
ChMBL
DBL-1
hJAA-F11-B11
Gal3,6OS2(β1-4)GlcNAc(β-Sp0

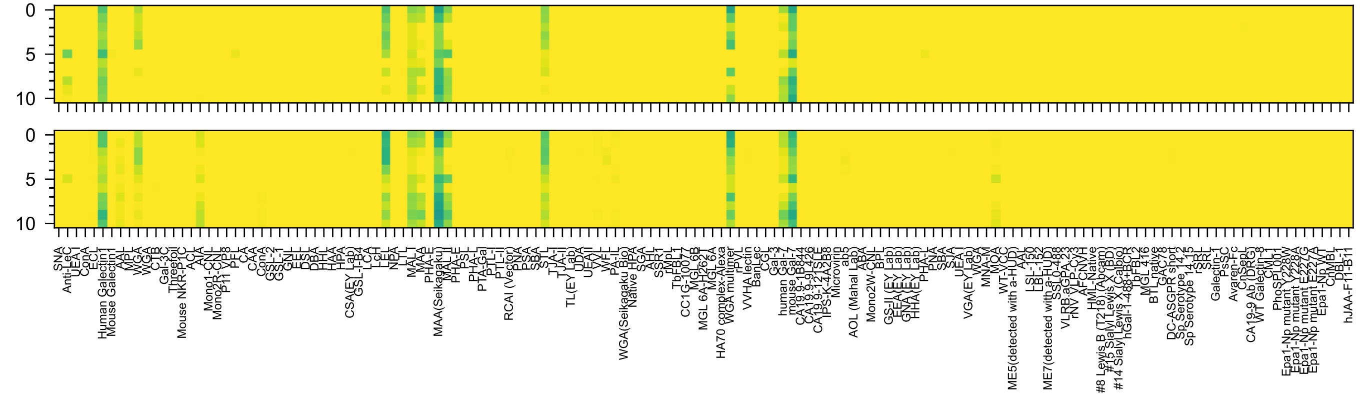

Gal3OS(β1-4)GlcNAc6OS(β-Sp0

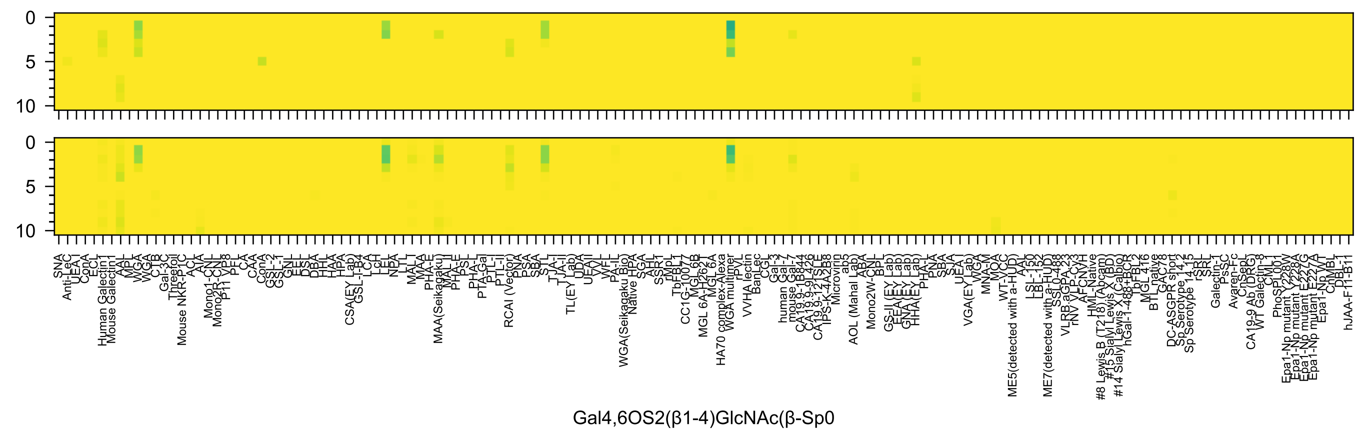

Gal4,6OS2(β1-4)GlcNAc(β-Sp0

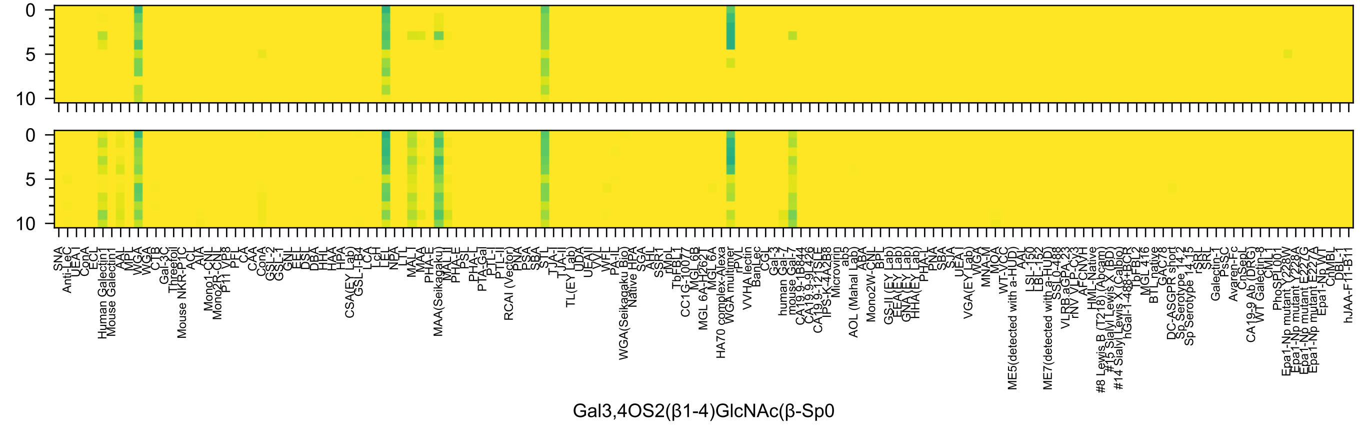
Gal3,4OS2(β1-4)GlcNAc(β-Sp0

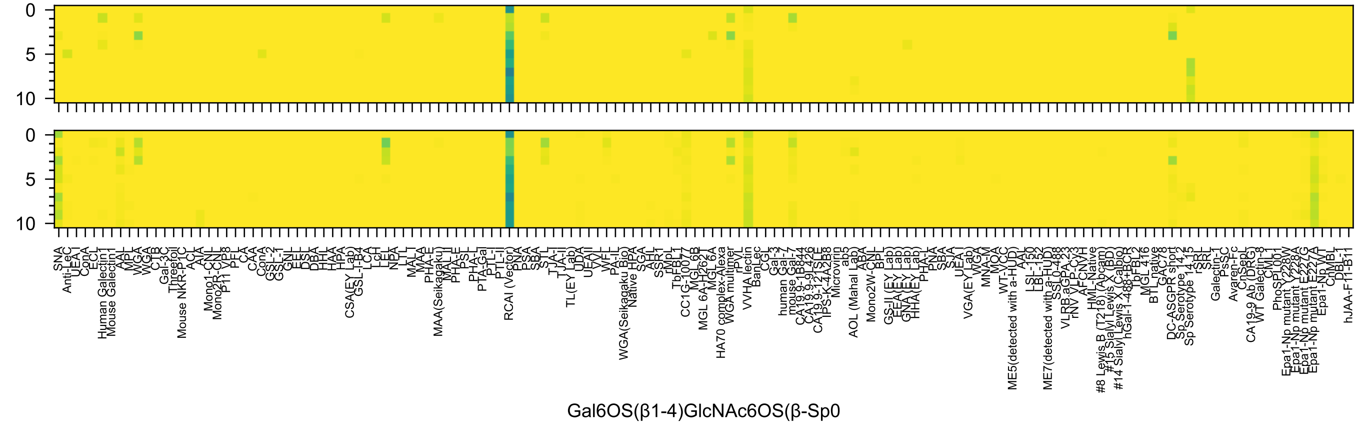
0
5
10
0
5
10
SNA
Anti-LeC
UEA I
ConA
ECL
Human Galectin1
Mouse Galectin1
AAL
MPL
WGA
WGA
CTB
Gal-3C
Threefoil
Mouse NKR-P1C
ACL
AIA
Mono1-CNL
Mono2R-CNL
P11 VP8
PFL
CA
CAA
ConA
GSL-2
GSL-1
GNL
EEL
DSL
DBA
HHL
HAA
HPA
CSA(EY Lab)
GSL-I-B4
LCA
LcH
LEL
NPA
LTL
MAL-I
MAA
PHA-E
MAA(Seikagaku)
MAL II
PHA-E
PSL
PHA-L
PTA-Gal
PTL-II
RCAI (Vector)
PNA
PSA
SBA
STL
TJA-I
TJA-II
TL(EY Lab)
UDA
UEAII
VVL
WFL
PA-IL
WGA(Seikagaku Bio)
Native HPA
SGA
AHL
SSR1
rMpL
TbFBL1
CC1G-10077
MGL 6B
MGL 6A-H262T
MGL 6A
HA70 complex-Alexa
WGA multimer
rPVL
VVHA lectin
BanLec
CGL
Gal-3
human Gal-7
mouse Gal-7
CA19.9-1B844
CA19.9-9L426
CA19.9-121SLE
IPS-K-4A2B8
Microvirin
ab5
AOL (Mahal Lab)
ABA
Mono2W-CNL
BPL
GS-II (EY Lab)
EEA (EY Lab)
GNA (EY Lab)
HHA(EY Lab)
PHA-L
PNA
SBA
SJA
UEA I
VGA(EY Lab)
WGA
MNA-M
MOA
WT-VCC
ME5(detected with a-HUD)
AAL
LSL-150
LBL-152
ME7(detected with a-HUD)
SSL0-488
VLRB.aGPA.23
rNV VLP-Cy3
AFCNVH
HML-Native
#8 Lewis B (T218) (Abcam)
#15 Sialyl Lewis X (BD)
#14 Sialyl Lewis X (Calbio)
hGal-1-488+BCR
TbFBL2
MGL 416
BTL native
GAC78
DC-ASGPR short
Sp Serotype 14.2
Sp Serotype 14.15
rSRL
SRL
Galectin-1
PsSC
Avaren-Fc
ChSepL
CA19-9 Ab (DRG)
WT Galectin-8
CML1
PhoSPL001
Epa1-Np mutant Y228W
Epa1-Np mutant Y228A
Epa1-Np mutant E227G
Epa1-Np mutant E227A
Epa1-Np WT
ChMBL
DBL-1
hJAA-F11-B11
Gal6OS(β1-4)GlcNAc6OS(β-Sp0

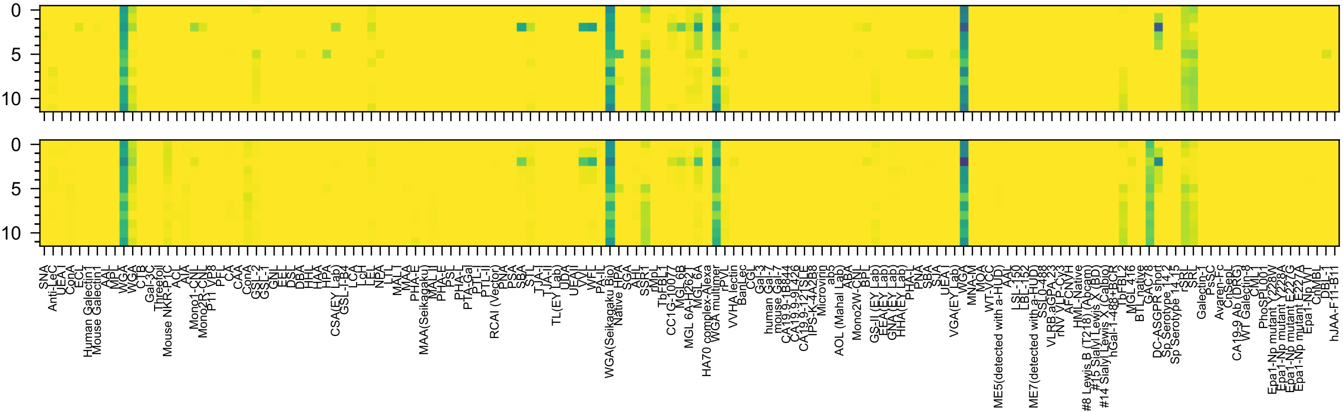

GlcNAc(β1-4)GlcNAc(β-Sp12)

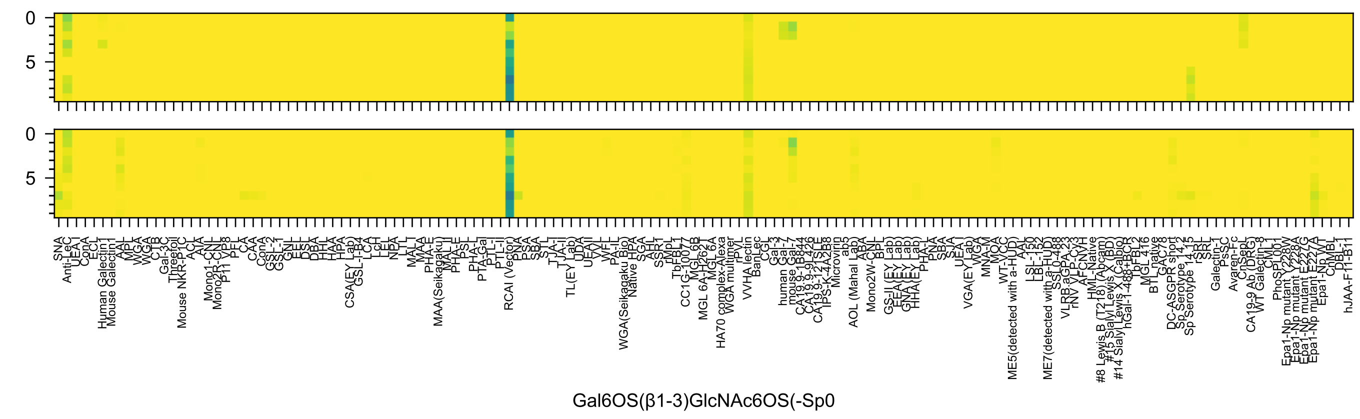
Gal6OS(β1-3)GlcNAc6OS(-Sp0

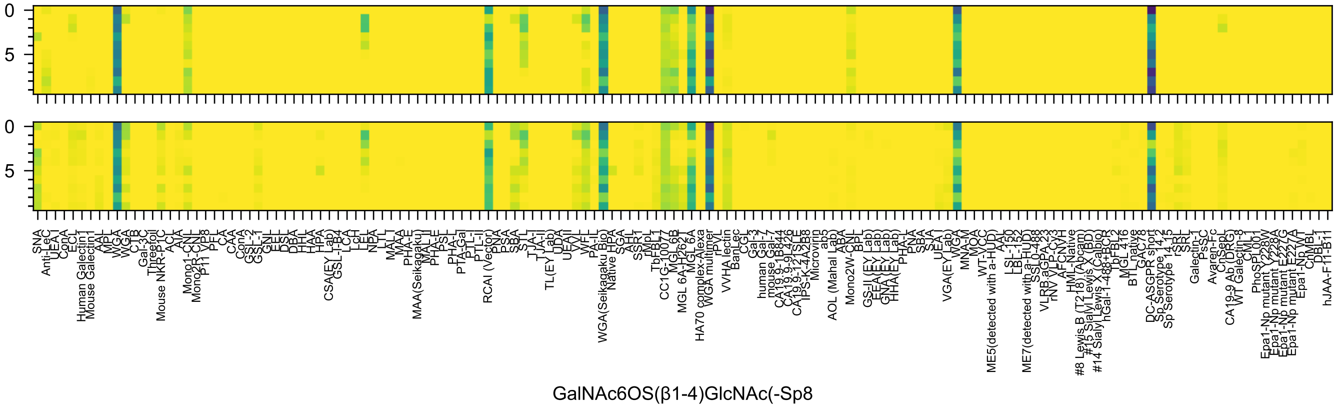
GalNAc6OS(β1-4)GlcNAc(-Sp8

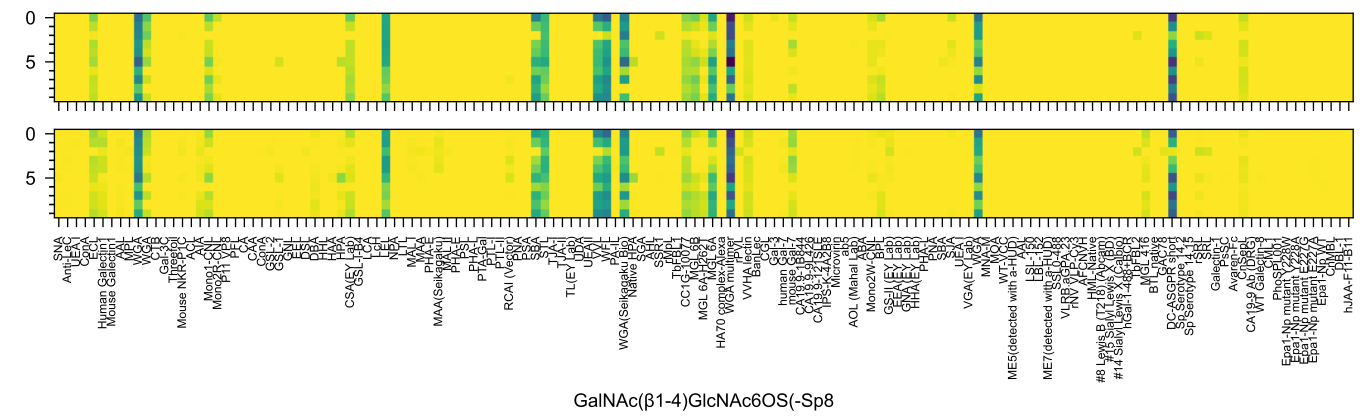
0
5
0
5
GalNAc(β1-4)GlcNAc6OS(-Sp8

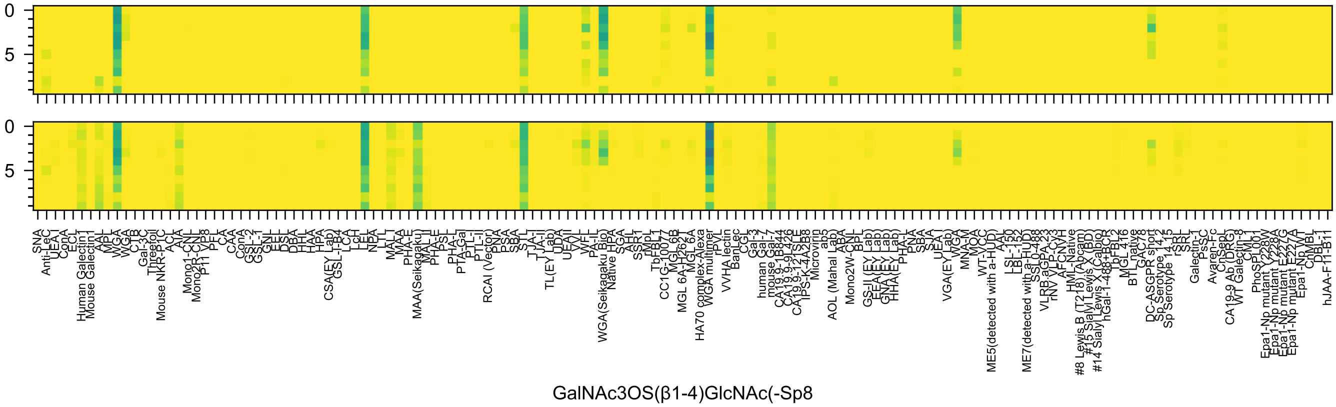
0
5
0
5
SNA
Anti-LeC
UEA I
ConA
ECL
Human Galectin1
Mouse Galectin1
AAL
MPL
WGA
WGA
CTB
Gal-3C
Threefoil
Mouse NKR-P1C
ACL
AIA
Mono1-CNL
Mono2R-CNL
P11 VP8
PFL
CA
CAA
ConA
GSL-2
GSL-1
GNL
EEL
DSL
DBA
HHL
HAA
HPA
CSA(EY Lab)
GSL-I-B4
LCA
LcH
LEL
NPA
LTL
MAL1
MAA
PHA-E
MAA(Seikagaku)
MAL-I
PHA-E
PSL
PHA-L
PTA-Gal
PTL-I
PTL-II
RCAI (Vector)
PNA
PSA
SBA
STL
TJA-I
TJA-II
TL(EY Lab)
UDA
UEAII
VVL
WFL
PA-IL
WGA(Seikagaku Bio)
Native HPA
SGA
AHL
SSR1
rMpL
TbFBL1
CC1G-10077
MGL 6B
MGL 6A-H262T
MGL 6A
HA70 complex-Alexa
WGA multimer
rPVL
VVHA lectin
BanLec
CGL
Gal-3
human Gal-7
mouse Gal-7
CA19.9-1B844
CA19.9-9L426
CA19.9-121SLE
IPS-K-4A2B8
Microvirin
ab5
AOL (Mahal Lab)
ABA
Mono2W-CNL
BPL
GS-II (EY Lab)
EEA (EY Lab)
GNA (EY Lab)
HHA(EY Lab)
PHA-L
PNA
SBA
SJA
UEA-I
VGA(EY Lab)
WGA
MNA-M
MOA
WT-VCC
ME5(detected with a-HUD)
AAL
LSL-150
LBL-152
ME7(detected with a-HUD)
SSL0-488
VLRB.aGPA.23
rNV VLP-Cy3
AFCNYH
HML-Native
#8 Lewis B (T218) (Abcam)
#15 Sialyl Lewis X (BD)
#14 Sialyl Lewis X (Calbio)
hGal-1-488+BCR
TbFBL2
MGL 416
BTL native
GAC78
DC-ASGPR short
Sp Serotype 14.2
Sp Serotype 14.15
rSRL
SRL
Galectin-1
PsSC
Avaren-Fc
CnSepL
CA19-9 Ab (DRG)
WT Galectin-8
CML1
PhoSL001
Epa1-Np mutant Y228W
Epa1-Np mutant E227G
Epa1-Np mutant E227A
Epa1-Np WT
ChMBL
DBL-1
hJAA-F11-B11
GalNAc3OS(β1-4)GlcNAc(-Sp8

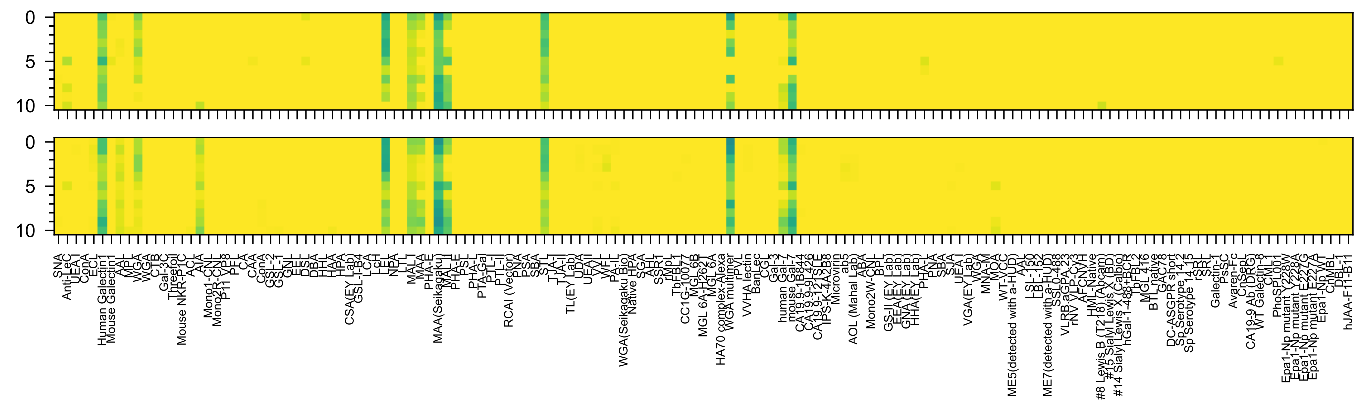

Gal3OS(β1-4)GlcNAc6OS(β-Sp8

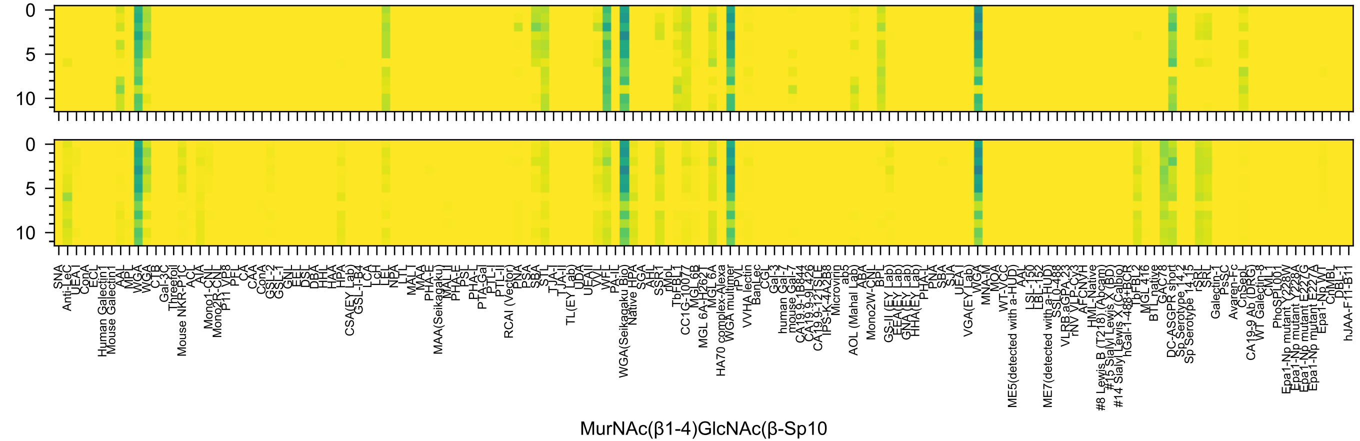
0
5
10
SNA
Anti-LeC
UEA I
ConA
ECL
Human Galectin1
Mouse Galectin1
AAL
MPL
WGA
WGA
CTB
Gal-3C
Threefoil
Mouse NKR-P1C
ACL
AIA
Mono1-CNL
Mono2R-CNL
P11 VP8
PFL
CA
CAA
ConA
GSL-2
GSL-1
GNL
EEL
DSL
DBA
HHL
HAA
HPA
CSA(EY Lab)
GSL-I-B4
LCA
LcH
LEL
NPA
LTL
MALI
MAA
PHA-E
MAA(Seikagaku)
MAL II
PHA-E
PSL
PHA-L
PTA-Gal
PTL-I
PTL-II
RCAI (Vector)
PNA
PSA
SBA
STL
TJA-I
TJA-II
TL(EY Lab)
UDA
UEAII
VVL
WFL
PA-IL
WGA(Seikagaku Bio)
Native HPA
SGA
AHL
SSR1
rMpL
TbFBL1
CC1G-10077
MGL 6B
MGL 6A-H262T
MGL 6A
HA70 complex-Alexa
WGA multimer
rPVL
VVHA lectin
BanLec
CGL
Gal-3
human Gal-7
mouse Gal-7
CA19.9-1B844
CA19.9-9L426
CA19.9-121SLE
IPS-K-4A2B8
Microvirin
ab5
AOL (Mahal Lab)
ABA
Mono2W-CNL
BPL
GS-II (EY Lab)
EEA (EY Lab)
GNA (EY Lab)
HHA (EY Lab)
PHA-L
PNA
SBA
SJA
UEA I
VGA(EY Lab)
WGA
MNA-M
MOA
WT-VCC
ME5(detected with a-HUD)
AAL
LSL-150
LBL-152
ME7(detected with a-HUD)
SSL0-488
VLRB.aGPA.23
rNV VLP-Cy3
AFCNVH
HML-Native
#8 Lewis B (T218) (Abcam)
#15 Sialyl Lewis X (BD)
#14 Sialyl Lewis X (Calbio)
hGal-1-488+BCR
TbFBL2
MGL 416
BTL native
GAC78
DC-ASGPR short
Sp Serotype 14.12
Sp Serotype 14.15
rSRL
SRL
Galectin-1
PsSC
Avaren-Fc
CnSepL
CA19-9 Ab (DRG)
WT Galectin-8
CML1
PhoSL001
Epa1-Np mutant Y228W
Epa1-Np mutant Y228A
Epa1-Np mutant E227G
Epa1-Np mutant E227A
Epa1-Np WT
ChMBL
DBL-1
hJAA-F11-B11
MurNAc(β1-4)GlcNAc(β-Sp10)

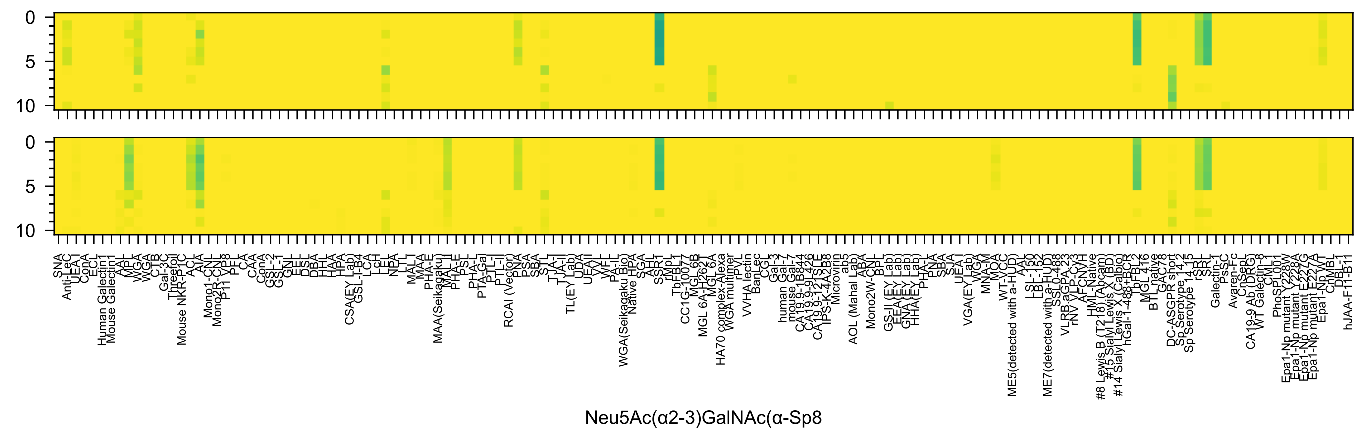

0
5
10
Neu5Ac(α2-3)GalNAc(α-Sp8

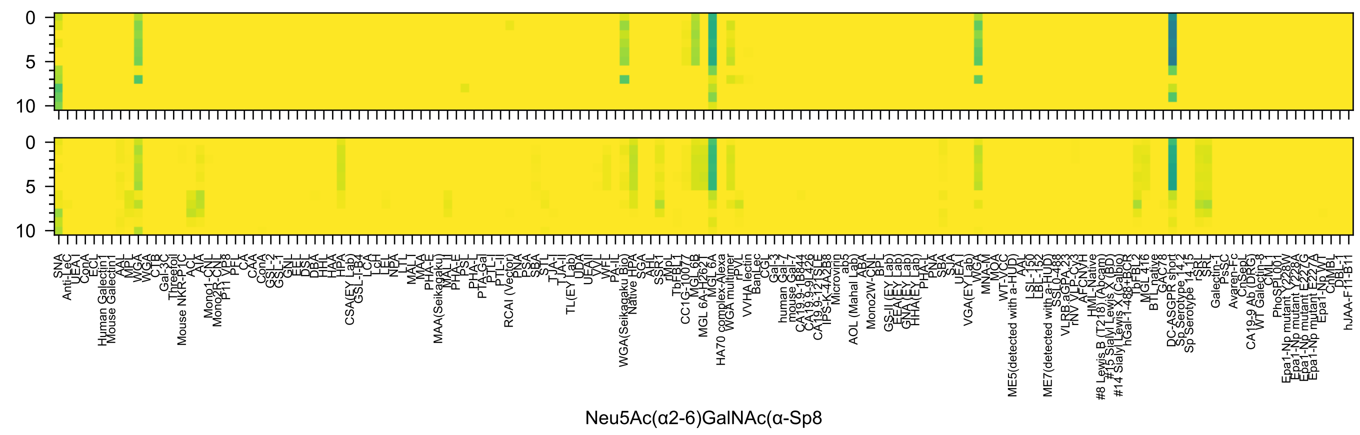

0
5
10
0
5
10
Neu5Ac(α2-6)GalNAc(α-Sp8

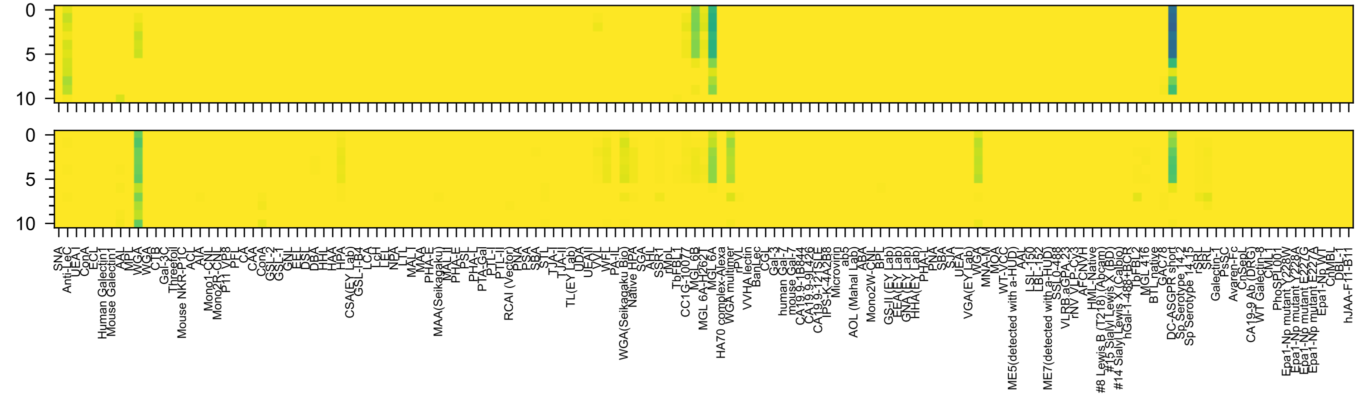

Neu5Ac(β2-6)GalNAc(α-Sp8

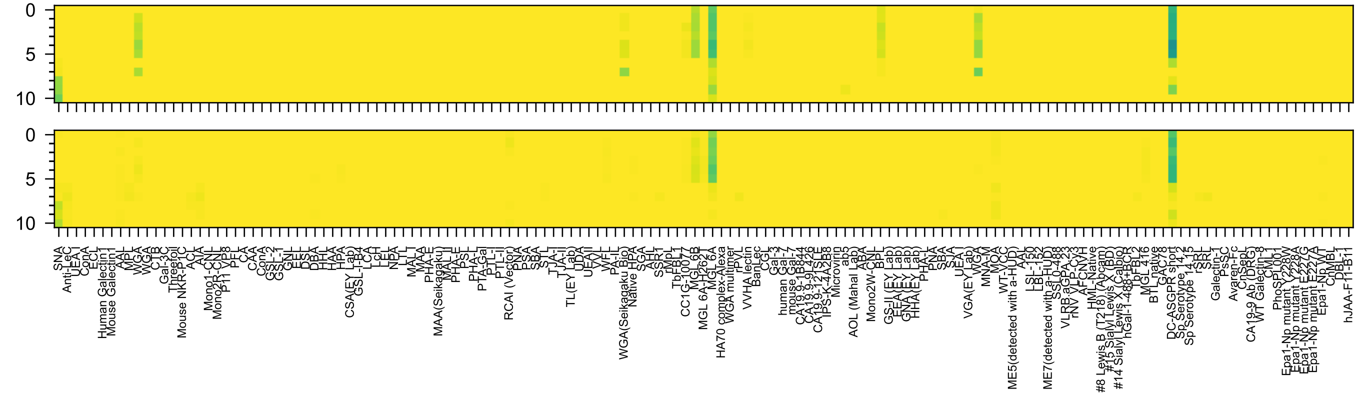

Neu5Gc(α2-6)GalNAc(α-Sp0

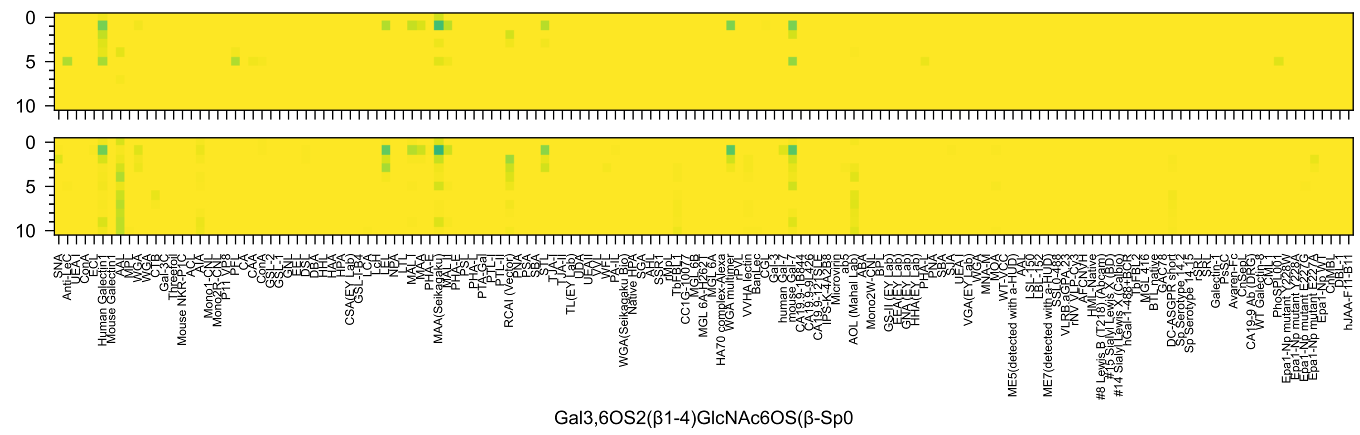
Gal3,6OS2(β1-4)GlcNAc6OS(β-Sp0

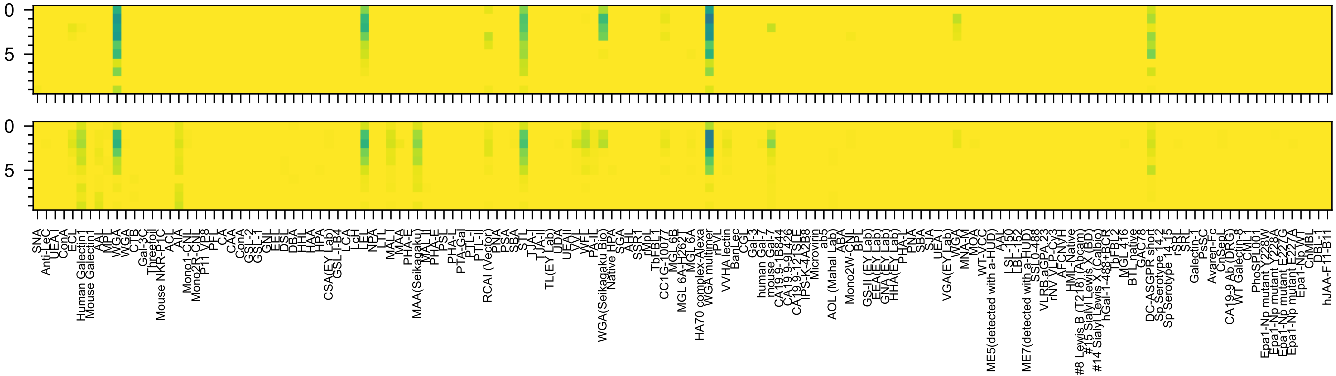

GalNAc4,6OS2(β1-4)GlcNAc(-Sp8

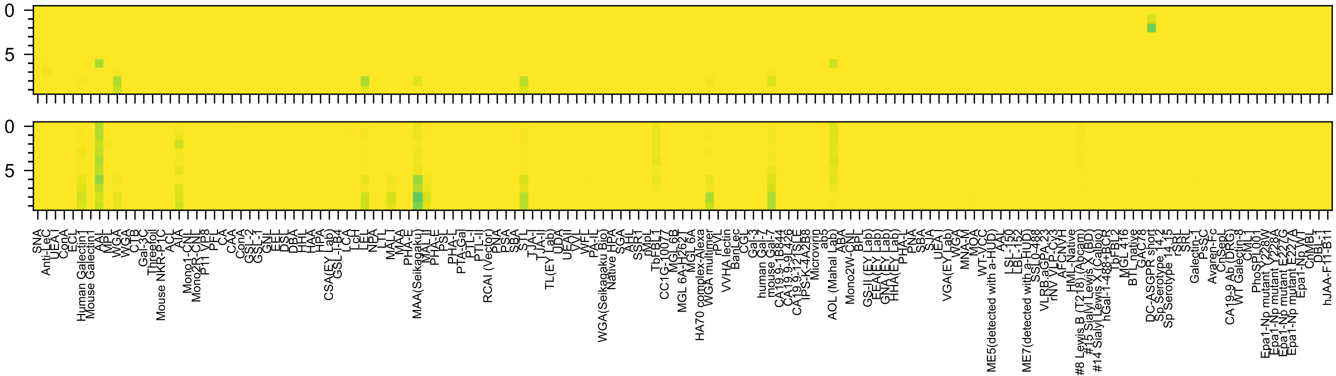

GalNAc3OS(β1-4)GlcNAc3OS(-Sp8

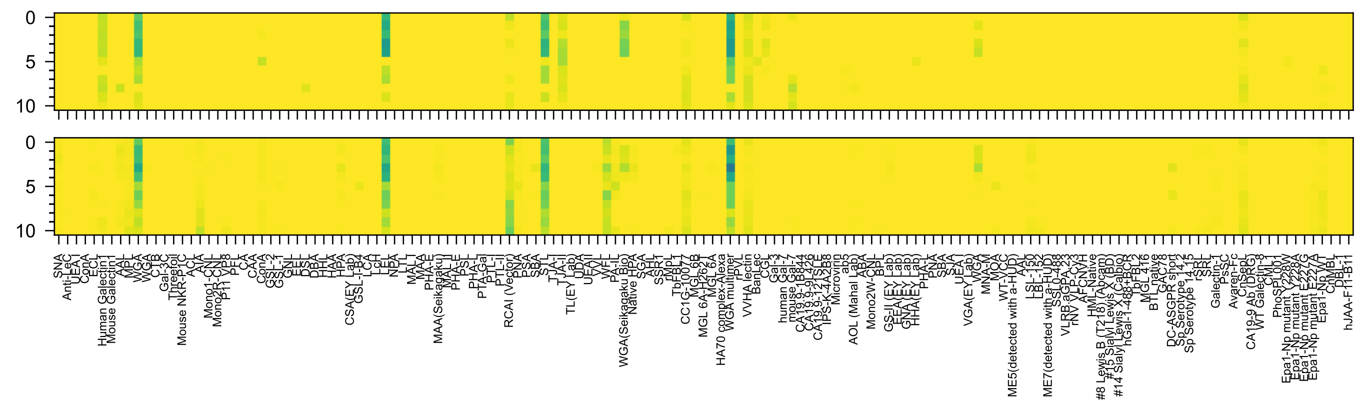

Gal(β1-4)GlcNAc(β-Sp23

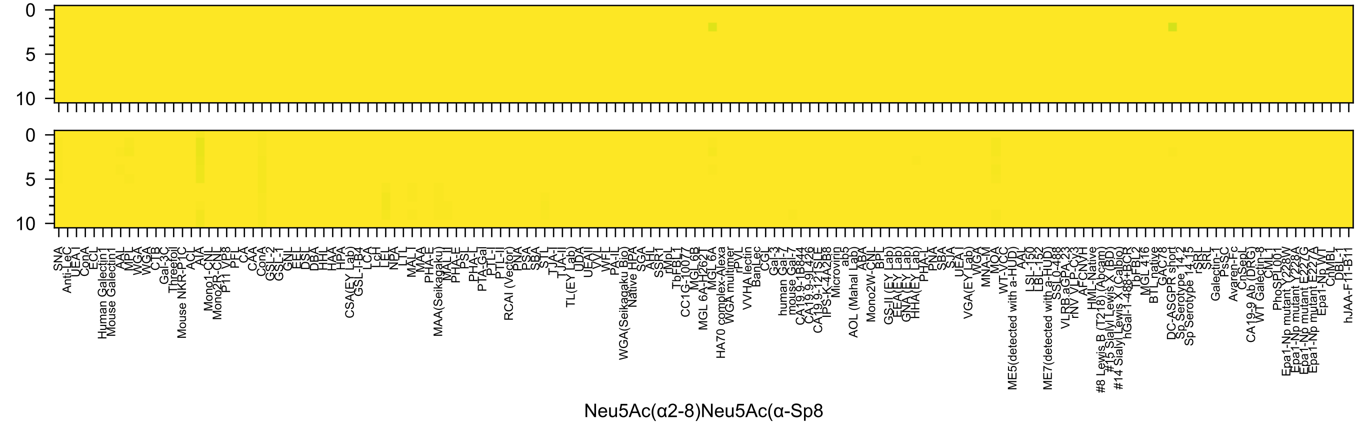
Neu5Ac(α2-8)Neu5Ac(α-Sp8

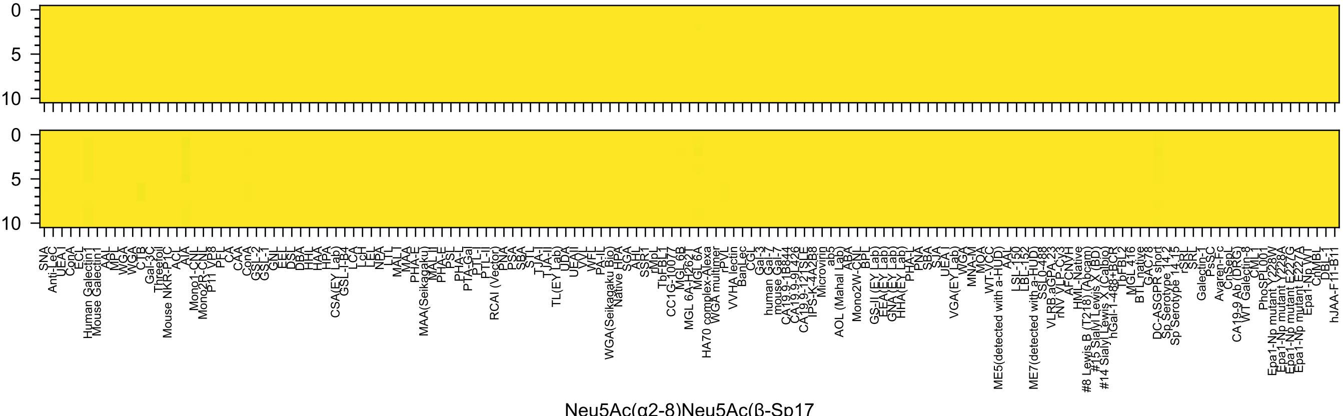
Neu5Ac(α2-8)Neu5Ac(β-Sp17

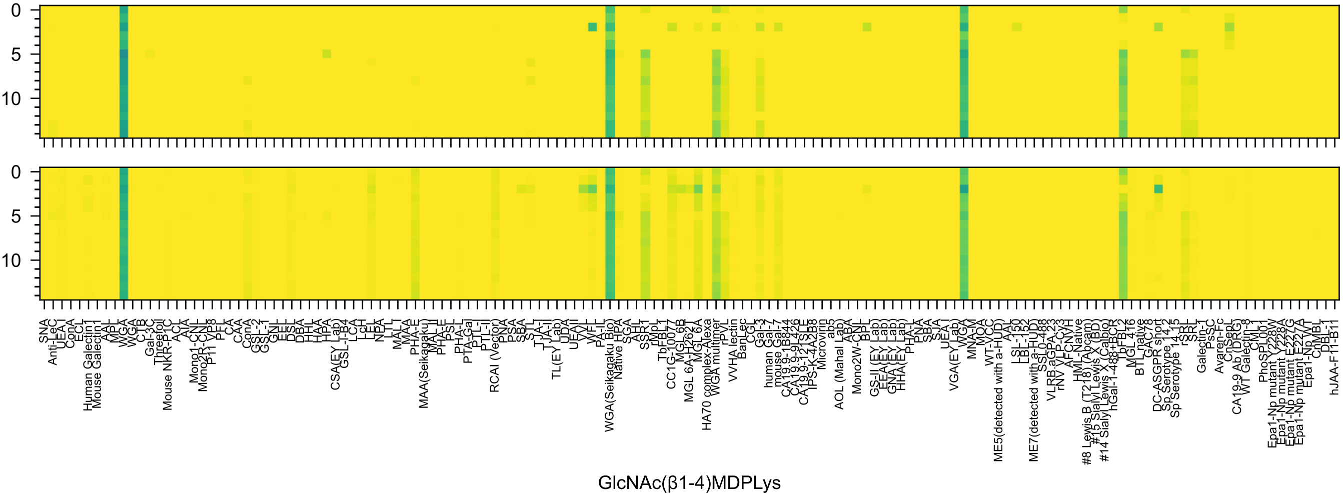
GlcNAc(β1-4)MDPLys

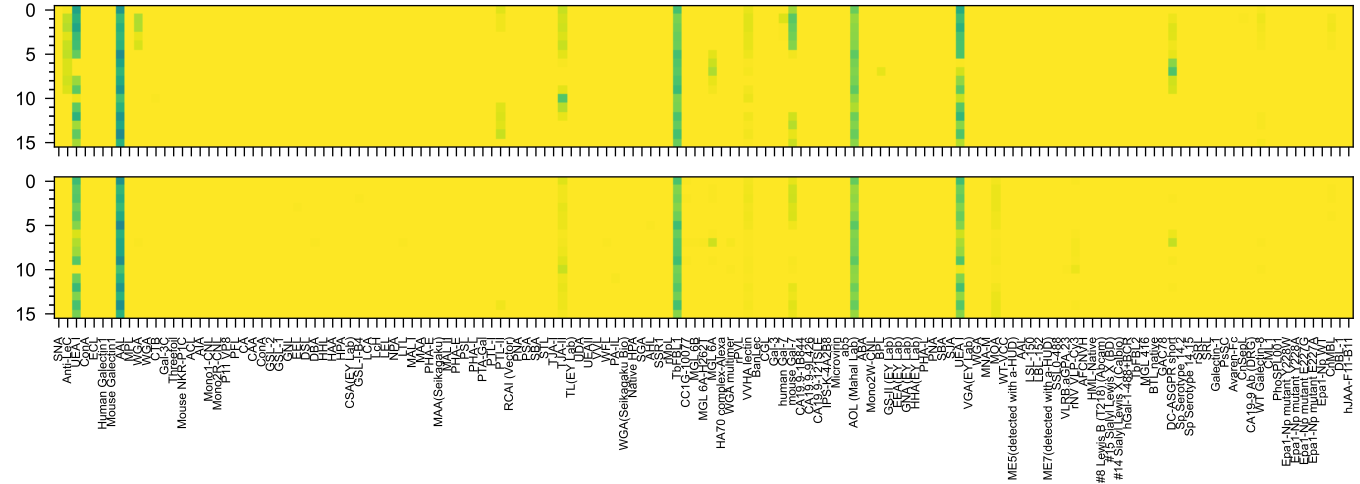

Fuc(α1-2)Gal(β1-4)Glc(β-Sp0

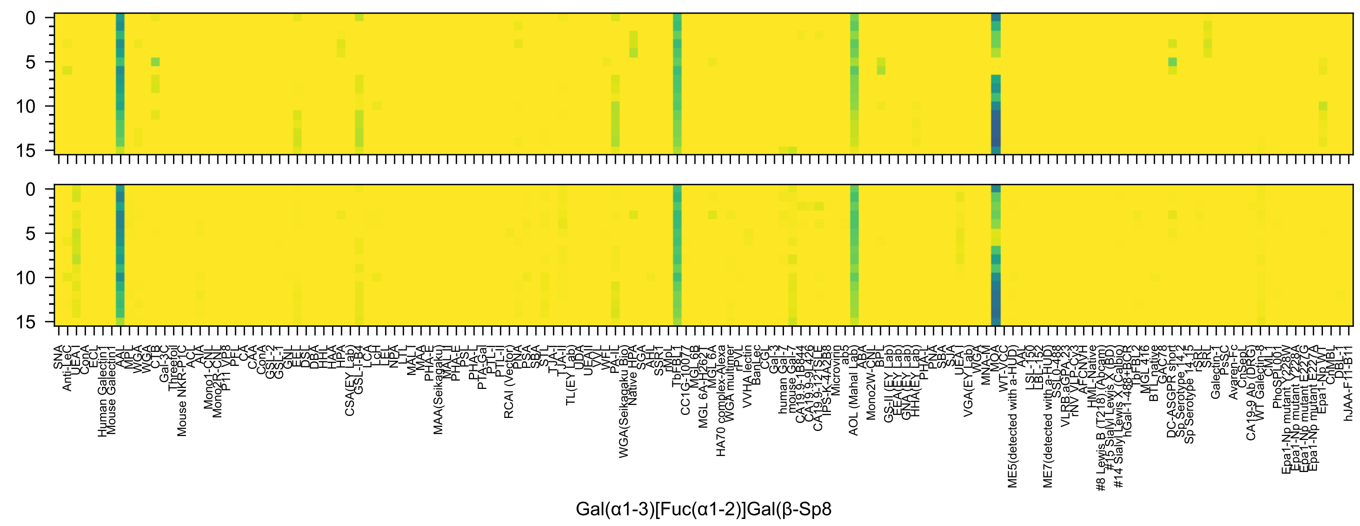

0
5
10
15
SNA
Anti-LeC
UEA I
ConA
ECL
Human Galectin1
Mouse Galectin1
AAL
MPL
WGA
WGA
CTB
Gal-3C
Threefoil
Mouse NKR-P1C
ACL
AIA
Mono1-CNL
Mono2R-CNL
P11 VP8
PFL
CA
CAA
ConA
GSL-2
GSL-1
GNL
EEL
DSL
DBA
HHL
HAA
HPA
CSA(EY Lab)
GSL-I-B4
LCA
LcH
LEL
NPA
LTL
MAL
MAA
PHA-E
MAA(Seikagaku)
MAL-II
PHA-E
PSL
PHA-L
PTA-Gal
PTL-I
PTL-II
RCAI (Vector)
PNA
PSA
SBA
STL
TJA-I
TJA-II
TL(EY Lab)
UDA
UEAII
VVL
WFL
PA-IL
WGA(Seikagaku Bio)
Native HPA
SGA
AHL
SSR1
rMPL
TbFBL1
CC1G-10077
MGL 6B
MGL 6A-H262T
MGL 6A
HA70 complex-Alexa
WGA multimer
rPVL
VVHA lectin
BanLec
CGL
Gal-3
human Gal-7
mouse Gal-7
CA19.9-1B844
CA19.9-9L426
CA19.9-121SLE
IPS-K-4A2B8
Microvirin
ab5
AOL (Mahal Lab)
ABA
Mono2W-CNL
BPL
GS-II (EY Lab)
EEA(EY Lab)
GNA(EY Lab)
HHA(EY Lab)
PHA-L
PNA
SBA
SJA
UEA I
VGA(EY Lab)
WGA
MNA-M
MOA
WT-VCC
ME5(detected with a-HUD)
AAL
LSL-150
LBL-152
ME7(detected with a-HUD)
SSL0-488
VLRB.aGPA-23
rNV VLP-Cy3
AFCNVH
HML-Native
#8 Lewis B (T218) (Abcam)
#15 Sialyl Lewis X (BD)
#14 Sialyl Lewis X (Calbio)
hGal-1-488+BCR
TbFBL2
MGL 416
BTL native
GAC78
DC-ASGPR short
Sp Serotype 14.2
Sp Serotype 14.15
rSRL
SRL
Galectin-1
PsSC
Avaren-Fc
ChSept
CA19-9 Ab (DRG)
WT Galectin-8
CML1
PhoSL001
Epa1-Np mutant Y228W
Epa1-Np mutant Y228A
Epa1-Np mutant E227G
Epa1-Np mutant E227A
Epa1-Np WT
ChMBL
DBL-1
hJAA-F11-B11
Gal(α1-3)[Fuc(α1-2)]Gal(β-Sp8

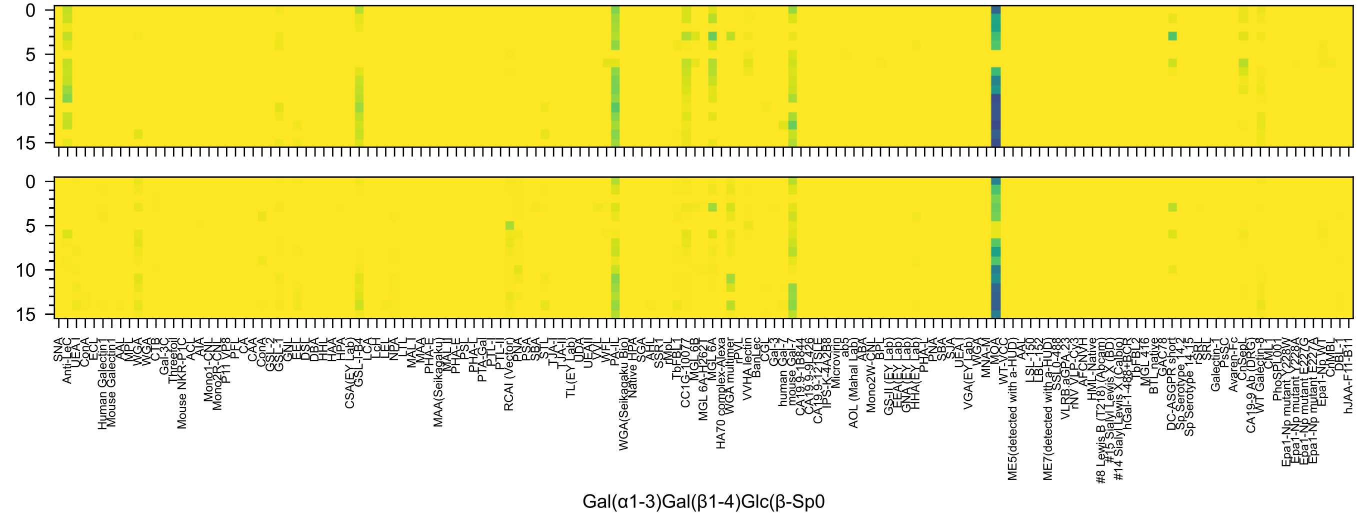

0
5
10
15
Gal(α1-3)Gal(β1-4)Glc(β-Sp0

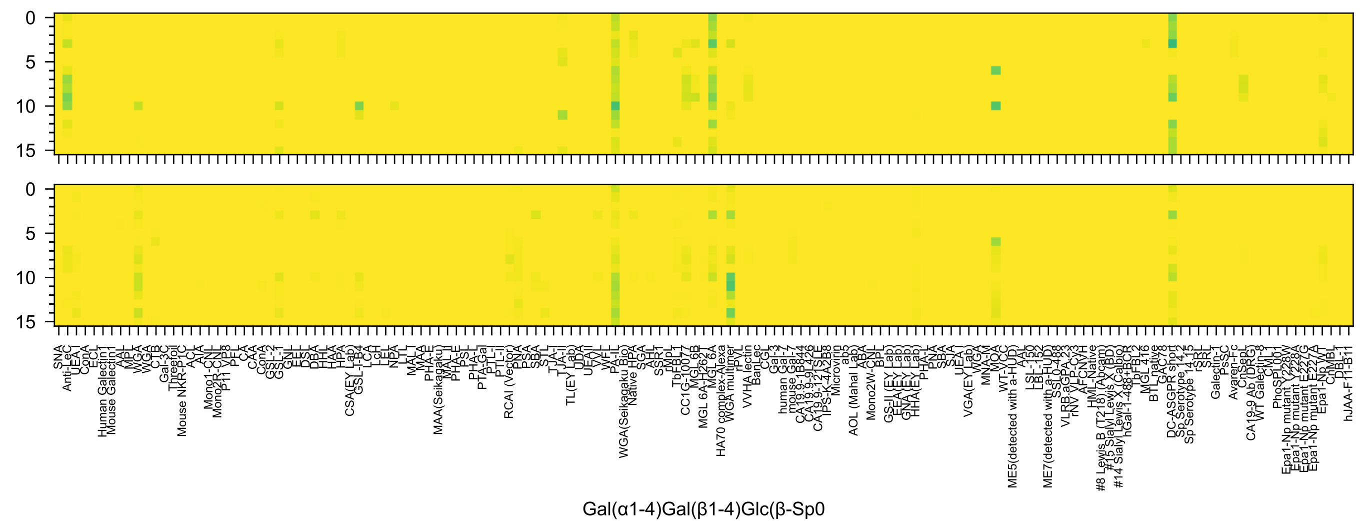

0
5
10
15
0
5
10
15
SNA
Anti-LeC
UEA I
ConA
ECL
Human Galectin1
Mouse Galectin1
AAL
MPL
WGA
WGA
CTB
Gal-3C
Threefoil
Mouse NKR-P1C
ACL
AIA
Mono1-CNL
Mono2R-CNL
P11 VP8
PFL
CA
CAA
ConA
GSL-2
GSL-1
GNL
EEL
DSL
DBA
HHL
HAA
HPA
CSA(EY Lab)
GSL-I-B4
LCA
LcH
LEL
NPA
LTL
MAL I
MAA
PHA-E
MAA(Seikagaku)
MAL II
PHA-E
PSL
PHA-L
PTA-Gal
PTL-I
PTL-II
RCAI (Vector)
PNA
PSA
SBA
STL
TJA-I
TJA-II
TL(EY Lab)
UDA
UEA II
VVL
WFL
PA-IL
WGA(Seikagaku Bio)
Native HPA
SGA
AHL
SSR1
rMpL
TbFBL1
CC1G-10077
MGL 6B
MGL 6A-H262T
MGL 6A
HA70 complex-Alexa
WGA multimer
rPVL
VVHA lectin
BanLec
CGL
Gal-3
human Gal-7
mouse Gal-7
CA19.9-1B844
CA19.9-9L426
CA19.9-121SLE
IPS-K-4A2B8
Microvirin
ab5
AOL (Mahal Lab)
ABA
Mono2W-CNL
BPL
GS-II (EY Lab)
EEA(EY Lab)
GNA(EY Lab)
HHA(EY Lab)
PHA-L
PNA
SBA
SJA
UEA I
VGA(EY Lab)
WGA
MNA-M
MOA
WT-VCC
ME5(detected with a-HUD)
AAL
LSL-150
LBL-152
ME7(detected with a-HUD)
SSL0-488
VLRB.aGPA.23
rNV VLP-Cy3
AFCNVH
HML-Native
#8 Lewis B (T218) (Abcam)
#15 Sialyl Lewis X (BD)
#14 Sialyl Lewis X (Calbio)
hGal-1-488+BCR
TbFBL2
MGL 416
BTL native
GAC78
DC-ASGPR short
Sp Serotype 14.2
Sp Serotype 14.15
rSRL
SRL
Galectin-1
PsSC
Avaren-Fc
ChSepL
CA19-9 Ab (DRG)
WT Galectin-8
CML1
PhoSL001
Epa1-Np mutant Y228W
Epa1-Np mutant Y228A
Epa1-Np mutant E227G
Epa1-Np mutant E227A
Epa1-Np WT
CnMBL
DBL-1
hJAA-F11-B11
Gal(α1-4)Gal(β1-4)Glc(β-Sp0

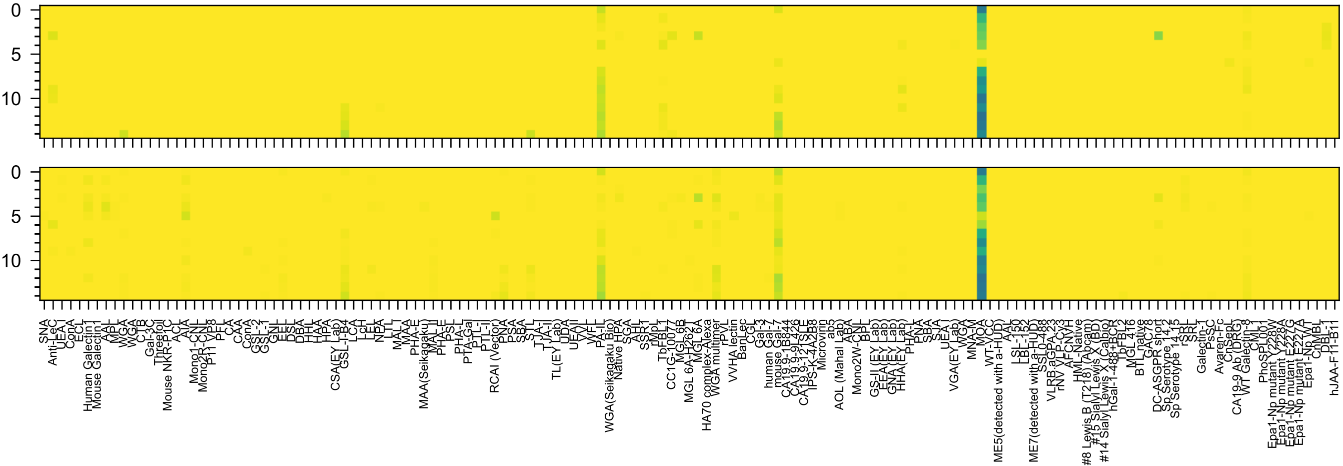

Gal(α1-3)Gal(β1-4)Glc(-Sp10

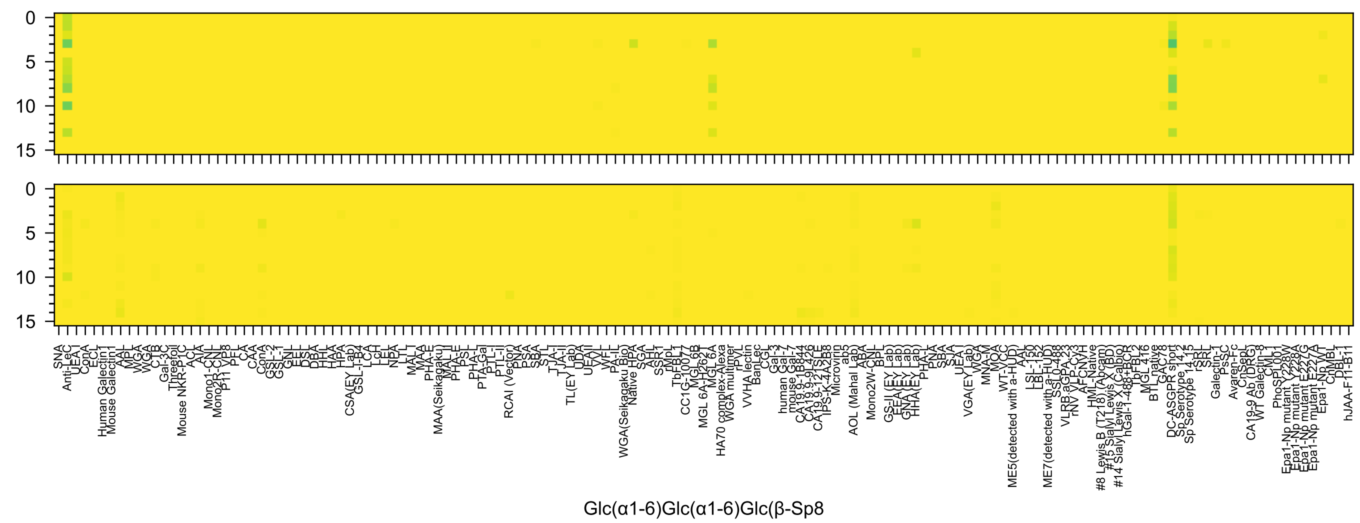
SNA
Anti-LeC
UEA-I
ConA
ECL
Human Galectin1
Mouse Galectin1
AAL
MPL
WGA
WGA
CTB
Gal-3C
Threefoil
Mouse NKR-P1C
ACL
AIA
Mono1-CNL
Mono2R-CNL
P11 VP8
PFL
CA
CAA
ConA
GSL-2
GSL-1
GNL
EEL
DSL
DBA
HHL
HAA
HPA
CSA(EY Lab)
GSL-I-B4
LCA
LcH
LEL
NPA
LTL
MAL-I
MAA
PHA-E
MAA(Seikagaku)
MAL-II
PHA-E
PSL
PHA-L
PTA-Gal
PTL-I
PTL-II
RCAI (Vector)
PNA
PSA
SBA
STL
TJA-I
TJA-II
TL(EY Lab)
UDA
UEA-II
VVL
WFL
PA-IL
WGA(Seikagaku Bio)
Native HPA
SGA
AHL
SSR1
rMpL
TbFBL1
CC1G-10077
MGL 6B
MGL 6A-H262T
MGL 6A
HA70 complex-Alexa
WGA multimer
rPVL
VVHA lectin
BanLec
CGL
Gal-3
human Gal-7
mouse Gal-7
CA19-9-1B844
CA19-9-9L426
CA19-9-121SLE
IPS-K-4A2B8
Microvirin
ab5
AOL (Mahal Lab)
ABA
Mono2W-CNL
BPL
GS-II (EY Lab)
EEA(EY Lab)
GNA(EY Lab)
HHA(EY Lab)
PHA-L
PNA
SBA
SJA
UEA-I
VGA(EY Lab)
WGA
MNA-M
MOA
WT-VCC
ME5(detected with a-HUD)
AAL
LSL-150
LBL-152
ME7(detected with a-HUD)
SSL0-488
VLRB.aGPA.23
rNV VLP-Cy3
AFCNVH
HML-Native
#8 Lewis B (T218) (Abcam)
#15 Sialyl Lewis X (BD)
#14 Sialyl Lewis X (Calbio)
hGal-1-488+BCR
TbFBL2
MGL 416
BTL native
GAC78
DC-ASGPR short
Sp Serotype 14.2
Sp Serotype 14.15
rSRL
SRL
Galectin-1
PsSC
Avaren-Fc
ChSeoL
CA19-9 Ab (DRG)
WT Galectin-8
CML1
PhoSL001
Epa1-Np mutant Y228W
Epa1-Np mutant Y228A
Epa1-Np mutant E227G
Epa1-Np mutant E227A
Epa1-Np WT
ChMBL
DBL-1
hJAA-F11-B11
Glc(α1-6)Glc(α1-6)Glc(β-Sp8

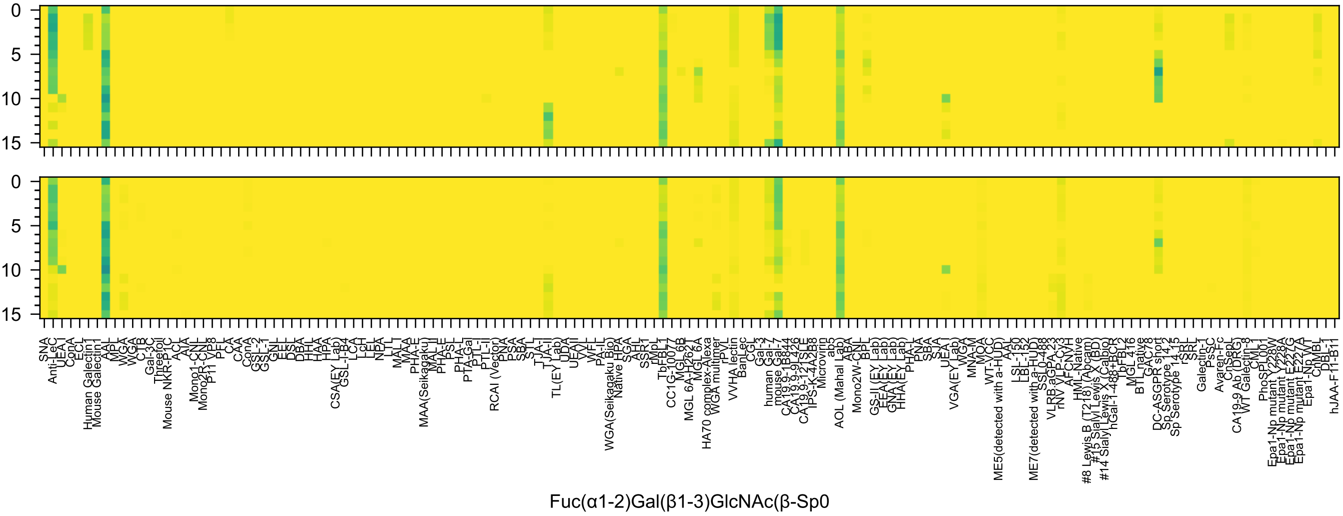
0
5
10
15
0
5
10
15
Fuc(α1-2)Gal(β1-3)GlcNAc(β-Sp0

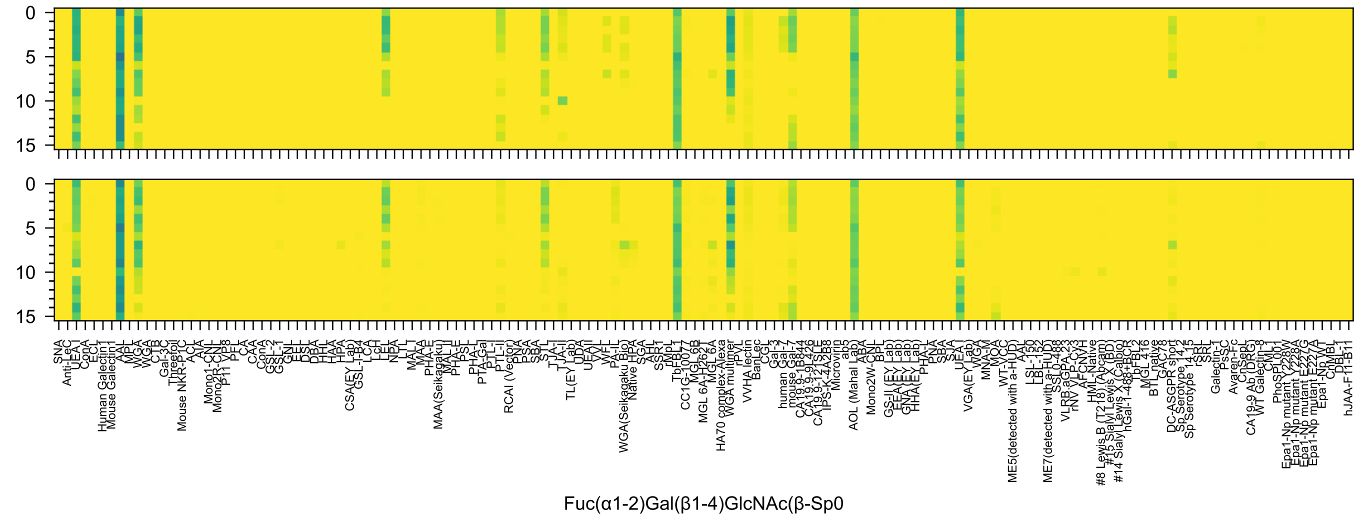
0
5
10
15
0
5
10
15
SNA
Anti-LeC
UEA I
ConA
ECL
Human Galectin1
Mouse Galectin1
AAL
MPL
WGA
WGA
CTB
Gal-3C
Threefoil
Mouse NKR-P1C
ACL
AIA
Mono1-CNL
Mono2R-CNL
P11 VP8
PFL
CA
CAA
ConA
GSL-2
GSL-1
GNL
EEL
DSL
DBA
HHL
HAA
HPA
CSA(EY Lab)
GSL-I-B4
LCA
LcH
LEL
NPA
LTL
MAL I
MAA
PHA-E
MAA(Seikagaku)
MAL II
PHA-E
PSL
PHA-L
PTA-Gal
PTL-I
PTL-II
RCAI (Vector)
PNA
PSA
SBA
STL
TJA-I
TJA-II
TL(EY Lab)
UDA
UEAII
VVL
WFL
PA-IL
WGA(Seikagaku Bio)
Native HPA
SGA
AHL
SSR1
rMpL
TbFBL1
CC1G-10077
MGL 6B
MGL 6A-H262T
MGL 6A
HA70 complex-Alexa
WGA multimer
rPVL
VVHA lectin
BanLec
CGL
Gal-3
human Gal-7
mouse Gal-7
CA19.9-1B844
CA19.9-9L426
CA19.9-121SLE
IPS-K-4A2B8
Microvirin
ab5
AOL (Mahal Lab)
ABA
Mono2W-CNL
BPL
GS-II (EY Lab)
EEA(EY Lab)
GNA(EY Lab)
HHA(EY Lab)
PHA-L
PNA
SBA
SJA
UEA I
VGA(EY Lab)
WGA
MNA-M
MOA
WT-VCC
ME5(detected with a-HUD)
AAL
LSL-150
LBL-152
ME7(detected with a-HUD)
SSL0-488
VLRB.aGPA.23
rNV VLP-Cy3
AFCNVH
HML-Native
#8 Lewis B (T218) (Abcam)
#15 Sialyl Lewis X (BD)
#14 Sialyl Lewis X (Calbio)
hGal-1-488+BCR
TbFBL2
MGL 416
BTL native
GAC78
DC-ASGPR short
Sp Serotype 14.2
Sp Serotype 14.15
rSRL
SRL
Galectin-1
PsSC
Avaren-Fc
ChSeL
CA19-9 Ab (DRG)
WT Galectin-8
CML1
PhoSL001
Epa1-Np mutant Y228W
Epa1-Np mutant Y228A
Epa1-Np mutant E227G
Epa1-Np mutant E227A
Epa1-Np WT
ChMBL
DBL-1
hJAA-F11-B11
Fuc(α1-2)Gal(β1-4)GlcNAc(β-Sp0

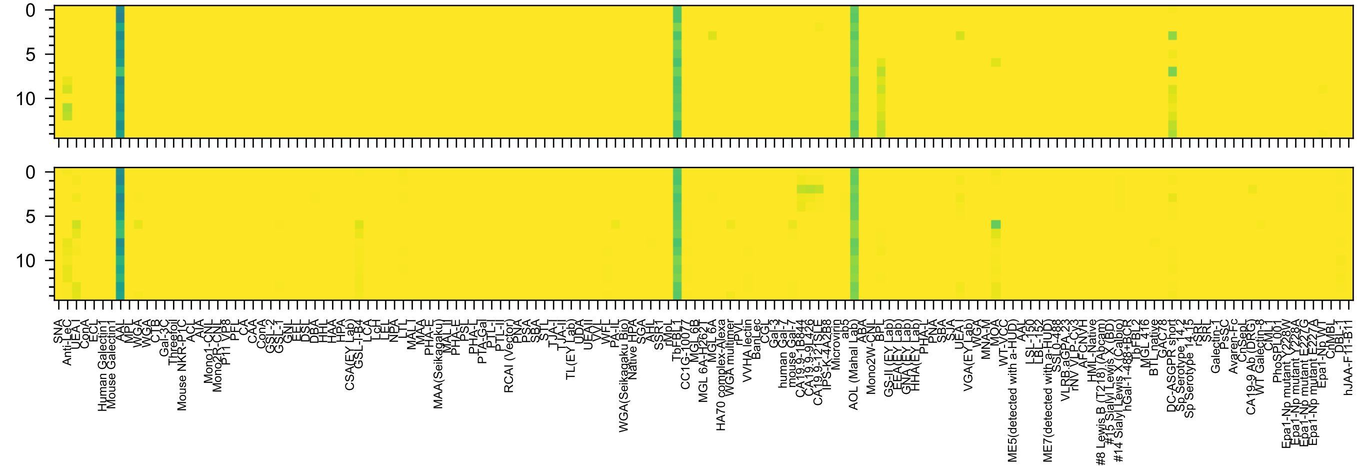

Gal(β1-3)[Fuc(α1-4)]GlcNAc(-Sp0

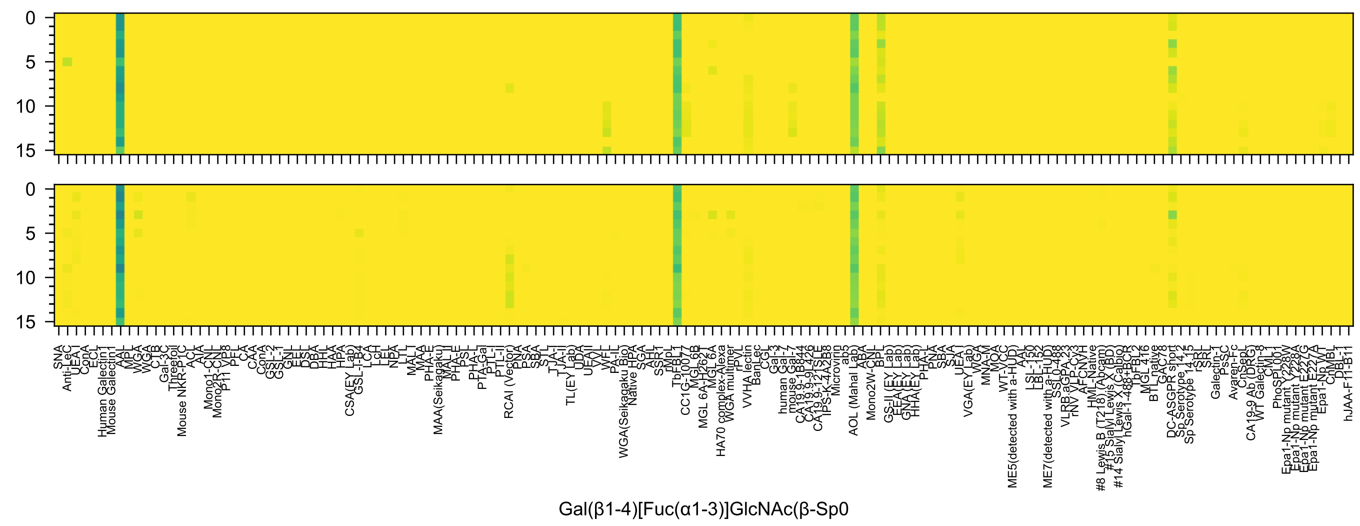
Gal(β1-4)[Fuc(α1-3)]GlcNAc(β-Sp0

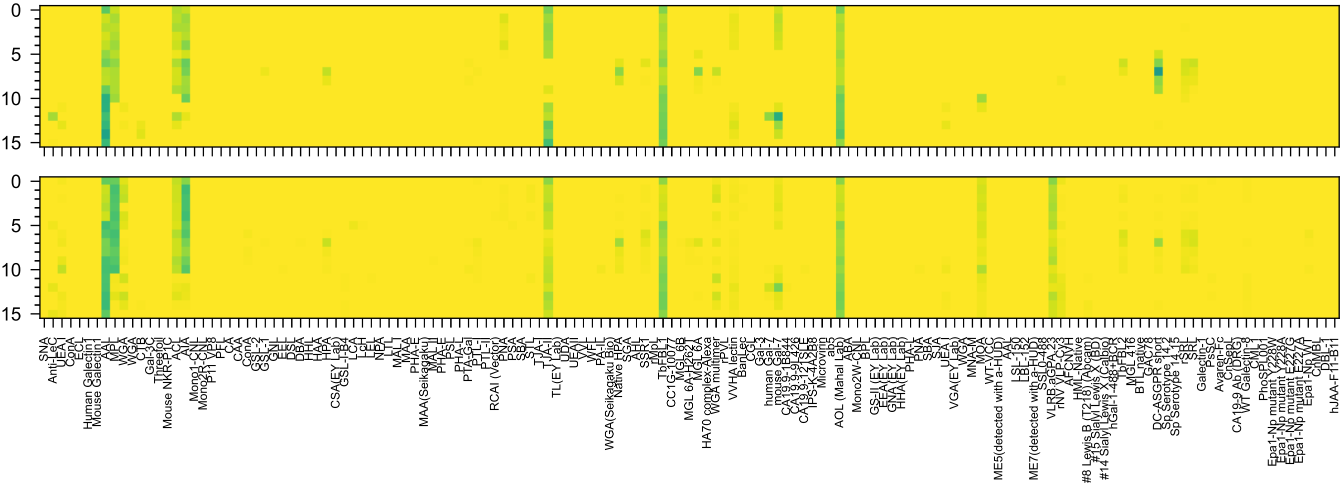
Fuc(α1-2)Gal(β1-3)GalNAc(α-Sp8

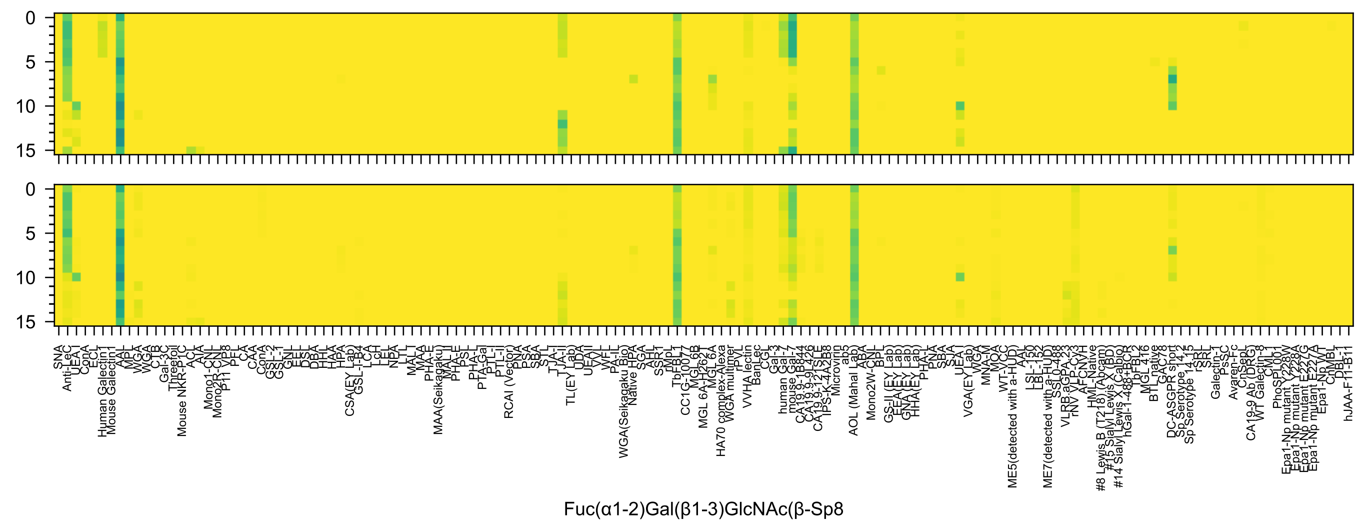
0
5
10
15
SNA
Anti-LeC
UEA-I
ConA
ECL
Human Galectin1
Mouse Galectin1
AAL
MPL
WGA
WGA
CTB
Gal-3C
Threefoil
Mouse NKR-P1C
ACL
AIA
Mono1-CNL
Mono2R-CNL
P11 VP8
PFL
CA
CAA
ConA
GSL-2
GSL-1
GNL
EEL
DSL
DBA
HHL
HAA
HPA
CSA(EY Lab)
GSL-I-B4
LCA
LcH
LEL
NPA
LTL
MAL-I
MAA
PHA-E
MAA(Seikagaku)
MAL-II
PHA-E
PSL
PHA-L
PTA-Gal
PTL-I
PTL-II
RCAI (Vector)
PNA
PSA
SBA
STL
TJA-I
TJA-II
TL(EY Lab)
UDA
UEA-II
VVL
WFL
PA-IL
WGA(Seikagaku Bio)
Native HPA
SGA
AHL
SSR1
rMpL
TbFBL1
CC1G-10077
MGL 6B
MGL 6A-H262T
MGL 6A
HA70 complex-Alexa
WGA multimer
rPVL
VVHA lectin
BanLec
CGL
Gal-3
human Gal-7
mouse Gal-7
CA19.9-1B844
CA19.9-9L426
CA19.9-121SLE
IPS-K-4A2B8
Microvirin
ab5
AOL (Mahal Lab)
ABA
Mono2W-CNL
BPL
GS-II (EY Lab)
EEA (EY Lab)
GNA (EY Lab)
HHA (EY Lab)
PHA-L
PNA
SBA
SJA
UEA-I
VGA(EY Lab)
WGA
MNA-M
MOA
WT-VCC
ME5(detected with a-HUD)
AAL
LSL-150
LBL-152
ME7(detected with a-HUD)
SSL0-488
VLRB.aGPA.23
rNV VLP-Cy3
AFCNVH
HML-Native
#8 Lewis B (T218) (Abcam)
#15 Sialyl Lewis X (BD)
#14 Sialyl Lewis X (Calbio)
hGal-1-488+BCR
TbFBL2
MGL 416
BTL native
GAC78
DC-ASGPR short
Sp Serotype 14.2
Sp Serotype 14.15
rSRL
SRL
Galectin-1
PsSC
Avaren-Fc
ChSep1
CA19-9 Ab (DRG)
WT Galectin-8
CML1
PhoSL001
Epa1-Np mutant Y228W
Epa1-Np mutant Y228A
Epa1-Np mutant E227G
Epa1-Np mutant E227A
Epa1-Np WT
ChMBL
DBL-1
hJAA-F11-B11
Fuc(α1-2)Gal(β1-3)GlcNAc(β-Sp8

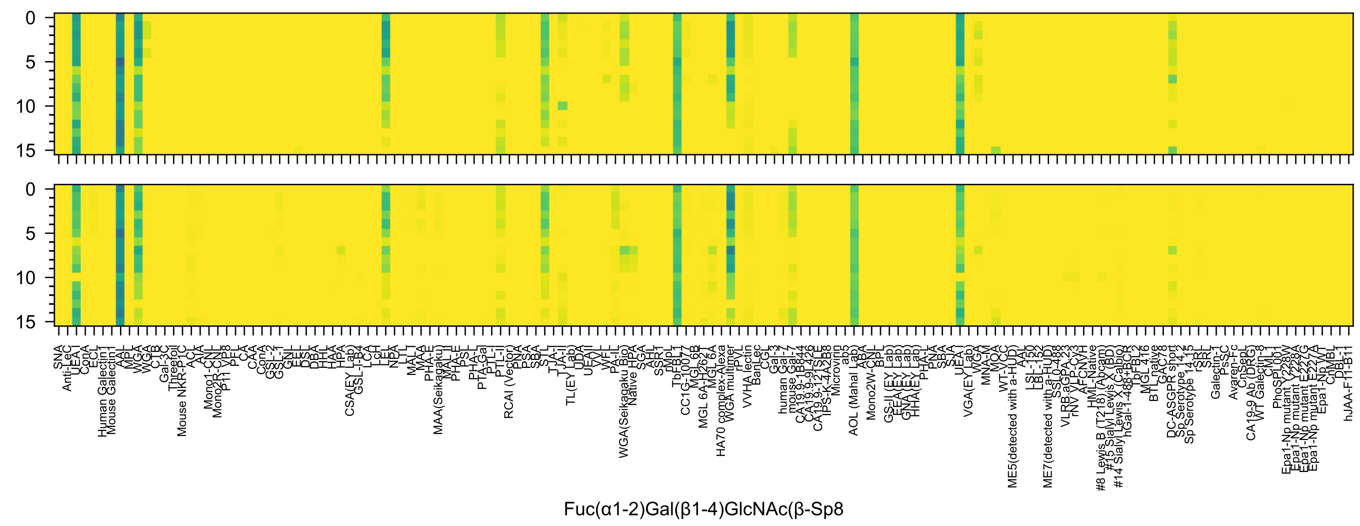

Fuc(α1-2)Gal(β1-4)GlcNAc(β-Sp8

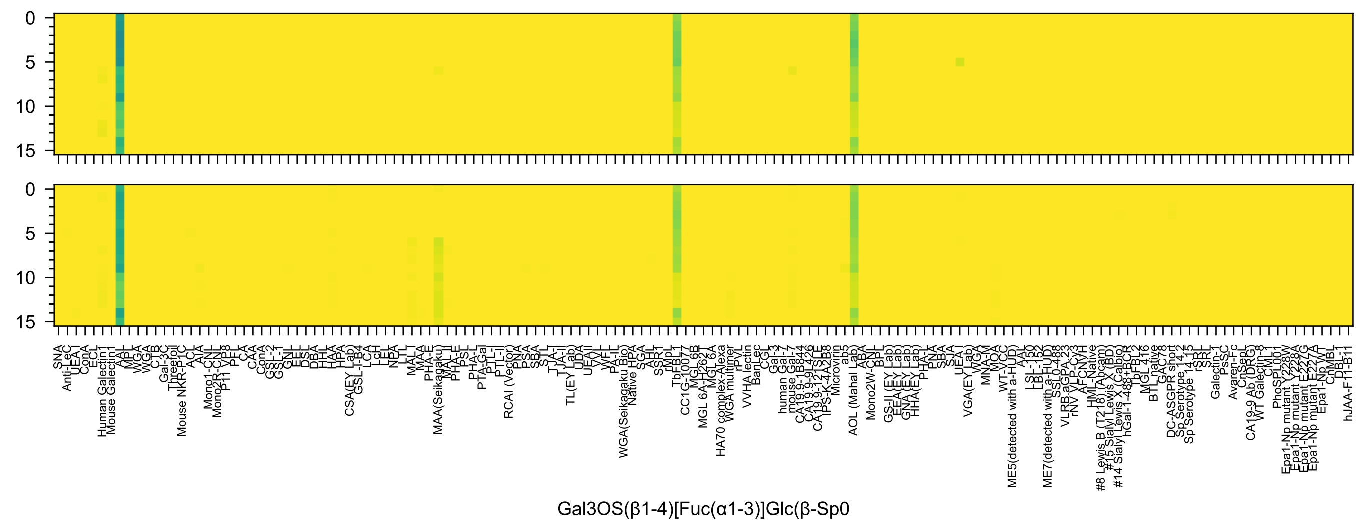
Gal3OS(β1-4)[Fuc(α1-3)]Glc(β-Sp0

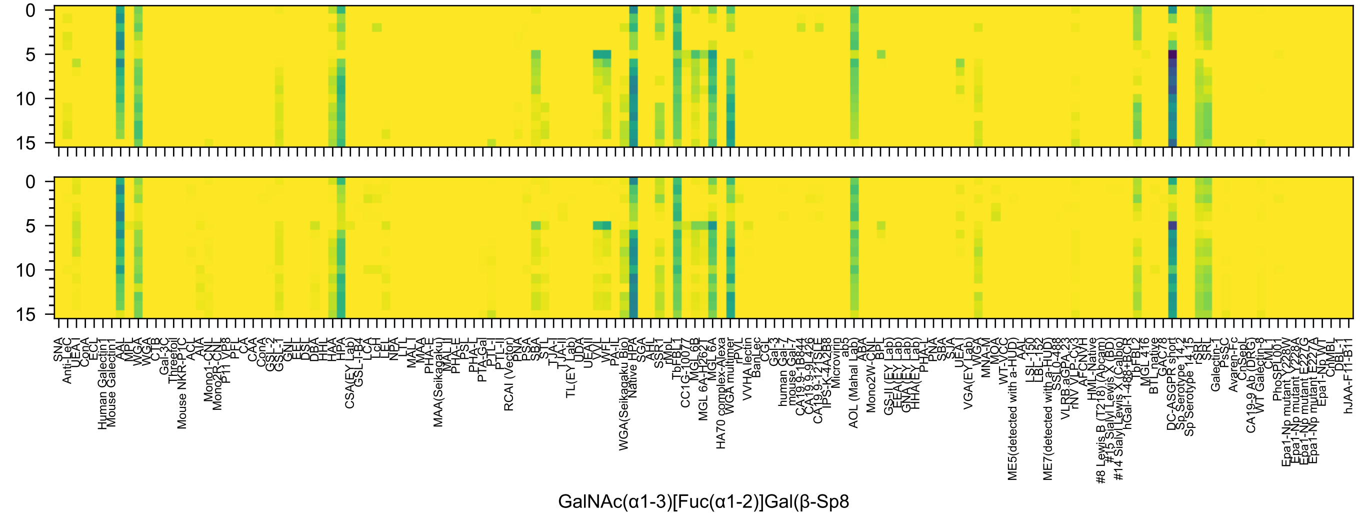
0
5
10
15
0
5
10
15
SNA
Anti-LeC
UEA-I
ConA
ECL
Human Galectin1
Mouse Galectin1
AAL
MPL
WGA
WGA
CTB
Gal-3C
Threefoil
Mouse NKR-P1C
ACL
AIA
Mono1-CNL
Mono2R-CNL
P11 VP8
PFL
CA
CAA
ConA
GSL-2
GSL-1
GNL
EEL
DSL
DBA
HHL
HAA
HPA
CSA(EY Lab)
GSL-I-B4
LCA
LcH
LEL
NPA
LTL
MAL-I
MAA
PHA-E
MAA(Seikagaku)
MAL-II
PHA-E
PSL
PHA-L
PTA-Gal
PTL-I
PTL-II
RCAI (Vector)
PNA
PSA
SBA
STL
TJA-I
TJA-II
TL(EY Lab)
UDA
UEA-II
VVL
WFL
PA-IL
WGA(Seikagaku Bio)
Native HPA
SGA
AHL
SSR1
rMpL
TbFBL1
CC1G-10077
MGL 6B
MGL 6A-H262T
MGL 6A
HA70 complex-Alexa
WGA multimer
rPVL
VVHA lectin
BanLec
CGL
Gal-3
human Gal-7
mouse Gal-7
CA19.9-1B844
CA19.9-9L426
CA19.9-121SLE
IPS-K-4A2B8
Microvirin
ab5
AOL (Mahal Lab)
ABA
Mono2W-CNL
BPL
GS-II (EY Lab)
EEA(EY Lab)
GNA(EY Lab)
HHA(EY Lab)
PHA-L
PNA
SBA
SJA
UEA-I
VGA(EY Lab)
WGA
MNA-M
MOA
WT-VCC
ME5(detected with a-HUD)
AAL
LSL-150
LBL-152
ME7(detected with a-HUD)
SsL0-488
VLRB.aGPA.23
rNV VLP-Cy3
AFCNVH
HML-Native
#8 Lewis B (T218) (Abcam)
#15 Sialyl Lewis X (BD)
#14 Sialyl Lewis X (Calbio)
hGal-1-488+BCR
TbFBL2
MGL 416
BTL native
GAC78
DC-ASGPR short
Sp Serotype 14.2
Sp Serotype 14.15
rSRL
SRL
Galectin-1
PsSC
Avaren-Fc
CnSepL
CA19-9 Ab (DRG)
WT Galectin-8
CML-1
PhoSL001
Epa1-Np mutant Y228W
Epa1-Np mutant Y228A
Epa1-Np mutant E227G
Epa1-Np mutant E227A
Epa1-Np WT
CnMBL
DBL-1
hJAA-F11-B11
GalNAc(α1-3)[Fuc(α1-2)]Gal(β-Sp8

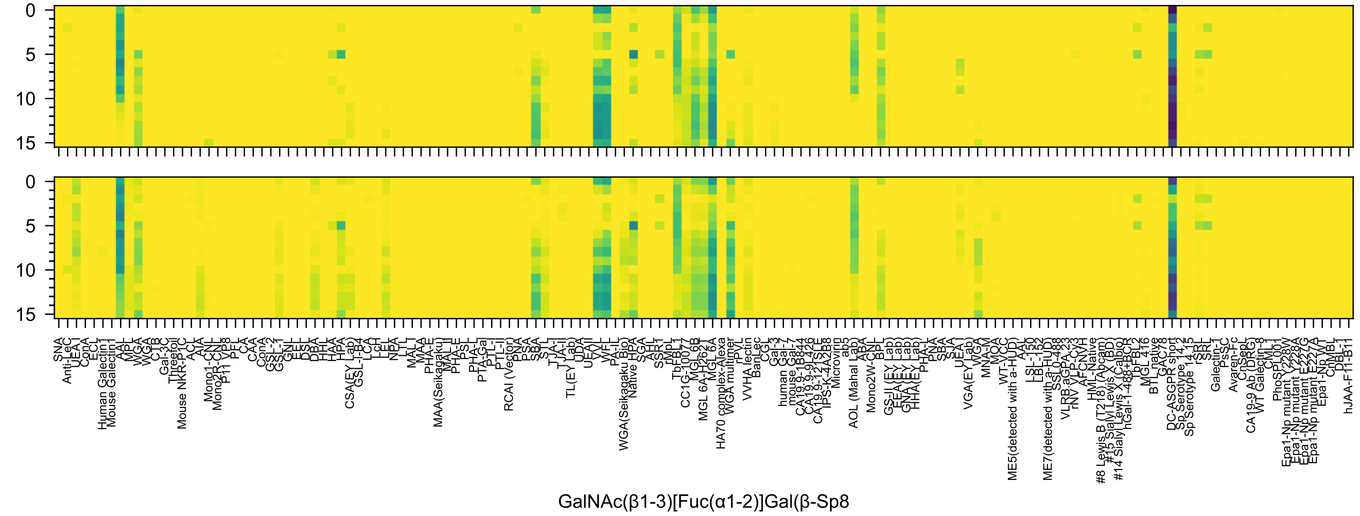
0
5
10
15
SNA
Anti-LeC
UEA-I
ConA
ECL
Human Galectin1
Mouse Galectin1
AAL
MPL
WGA
WGA
CTB
Gal-3C
Threefoil
Mouse NKR-P1C
ACL
AIA
Mono1-CNL
Mono2R-CNL
P11 VP8
PFL
CA
CAA
ConA
GSL-2
GSL-1
GNL
EEL
DSL
DBA
HHL
HAA
HPA
CSA(EY Lab)
GSL-I-B4
LCA
LCH
LEL
NPA
LTL
MAL-I
MAA
PHA-E
MAA(Seikagaku)
MAL-II
PHA-E
PSL
PHA-L
PTA-Gal
PTL-I
PTL-II
RCAI (Vector)
PNA
PSA
SBA
STL
TJA-I
TJA-II
TL(EY Lab)
UDA
UEA-II
VVL
WFL
PA-IL
WGA(Seikagaku Bio)
Native HPA
SGA
AHL
SSR1
rMpL
TbFBL1
CC1G-10077
MGL 6B
MGL 6A-H262T
MGL 6A
HA70 complex-Alexa
WGA multimer
rPVL
VVHA lectin
BanLec
CGL
Gal-3
human Gal-7
mouse Gal-7
CA19.9-1B844
CA19.9-9L426
CA19.9-121SLE
IPS-K-4A2B8
Microvirin
ab5
AOL (Mahal Lab)
ABA
Mono2W-CNL
BPL
GS-II (EY Lab)
EEA(EY Lab)
GNA(EY Lab)
HHA(EY Lab)
PHA-L
PNA
SBA
SJA
UEA-I
VGA(EY Lab)
WGA
MNA-M
MOA
WT-VCC
ME5(detected with a-HUD)
AAL
LSL-150
LBL-152
ME7(detected with a-HUD)
SSL0-488
VLRB.aGPA.23
rNV VLP-Cy3
AFCNVH
HML-Native
#8 Lewis B (T218) (Abcam)
#15 Sialyl Lewis X (BD)
#14 Sialyl Lewis X (Calbio)
hGal-1-488+BCR
TbFBL2
MGL 416
BTL native
GAC78
DC-ASGPR short
Sp Serotype 14.2
Sp Serotype 14.15
rSRL
SRL
Galectin-1
PsSC
Avaren-Fc
ChSeqL
CA19-9 Ab (DRG)
WT Galectin-8
CML1
PhoSL001
Epa1-Np mutant Y228W
Epa1-Np mutant Y228A
Epa1-Np mutant E227G
Epa1-Np mutant E227A
Epa1-Np WT
ChMBL
DBL-1
hJAA-F11-B11
GalNAc(β1-3)[Fuc(α1-2)]Gal(β-Sp8

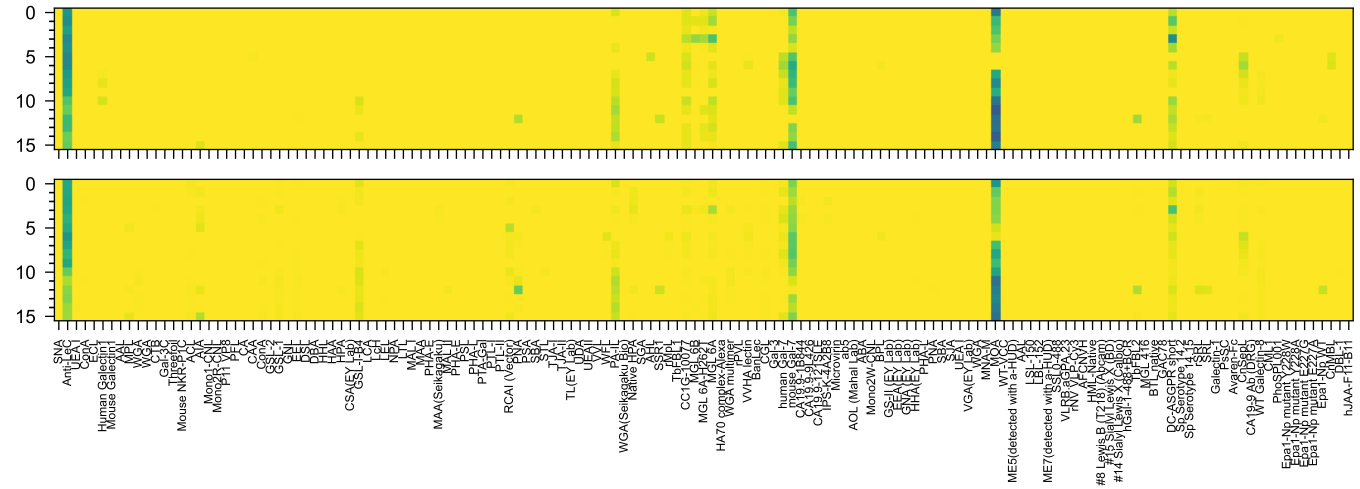

Gal(α1-3)Gal(β1-3)GlcNAc(β-Sp0

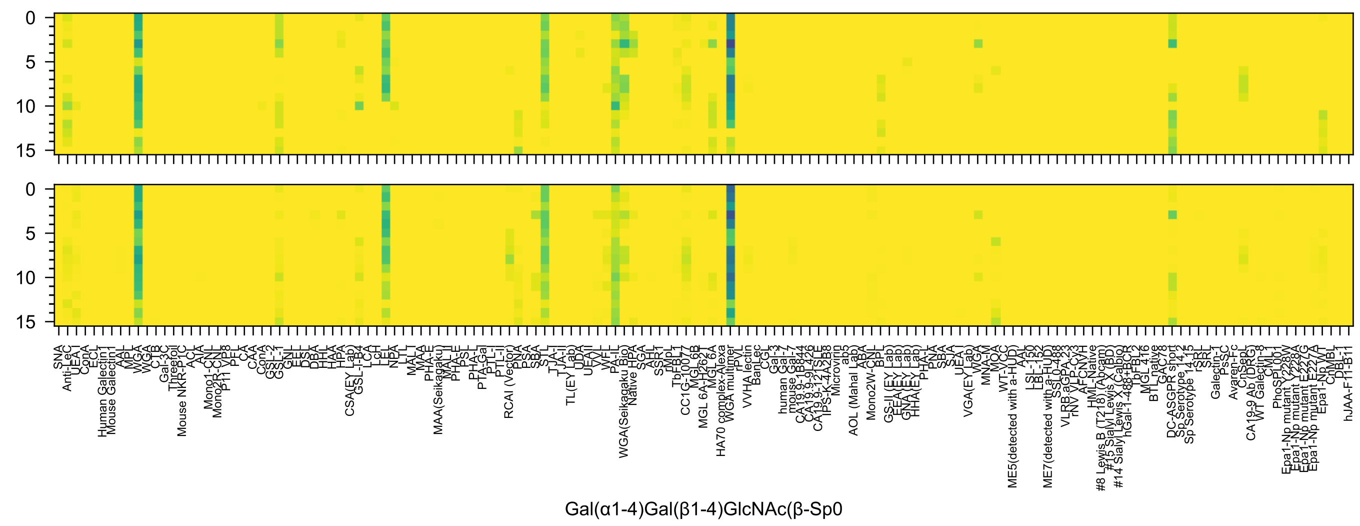
0
5
10
15
Gal(α1-4)Gal(β1-4)GlcNAc(β-Sp0

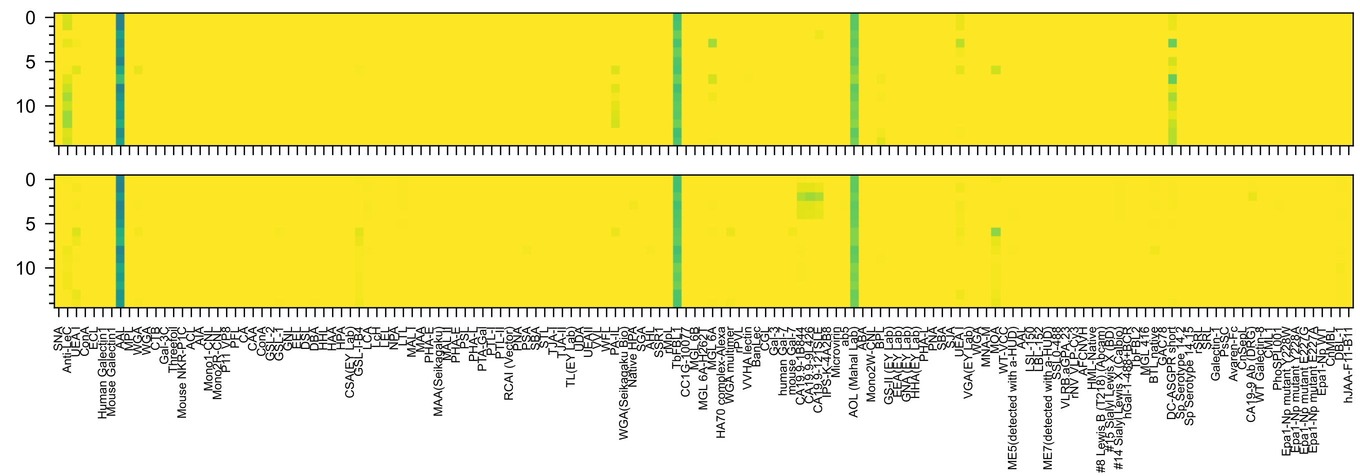

Gal(β1-3)[Fuc(α1-4)]GlcNAc(-Sp8

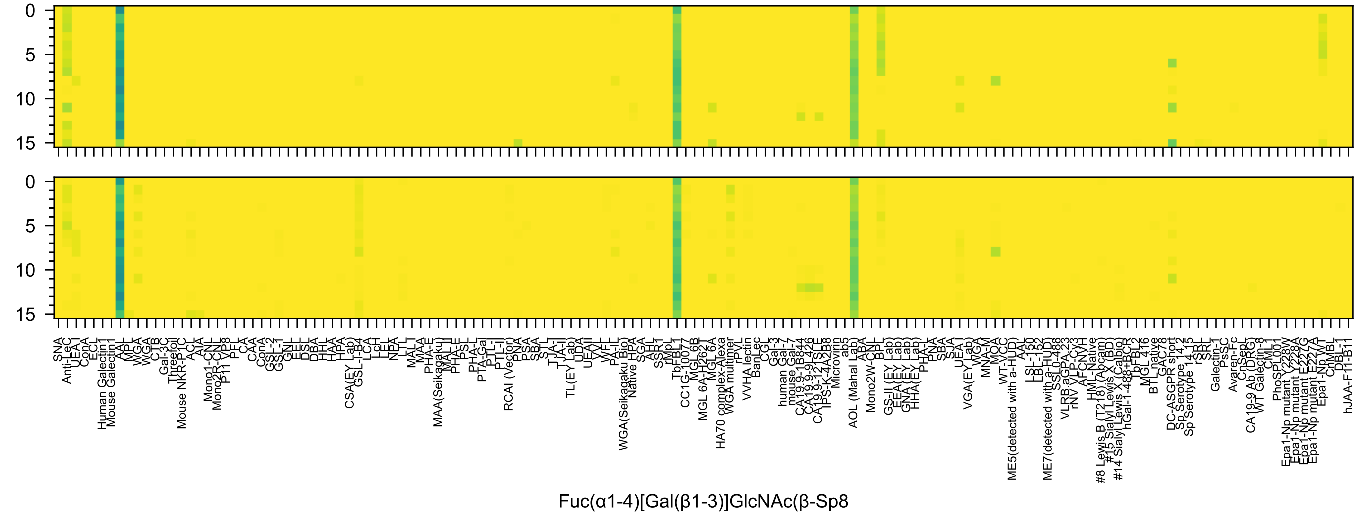
0
5
10
15
SNA
Anti-LeC
UEA I
ConA
ECL
Human Galectin1
Mouse Galectin1
AAL
MPL
WGA
WGA
CTB
Gal-3C
Threefoil
Mouse NKR-P1C
ACL
AIA
Mono1-CNL
Mono2R-CNL
P11 VP8
PFL
CA
CAA
ConA
GSL-2
GSL-1
GNL
EEL
DSL
DBA
HHL
HAA
HPA
CSA(EY Lab)
GSL-I-B4
LCA
LcH
LEL
NPA
LTL
MAL I
MAA
PHA-E
MAA(Seikagaku)
MAL II
PHA-E
PSL
PHA-L
PTA-Gal
PTL-I
PTL-II
RCAI (Vector)
PNA
PSA
SBA
STL
TJA-I
TJA-II
TL(EY Lab)
UDA
UEAII
VVL
WFL
PA-IL
WGA(Seikagaku Bio)
Native HPA
SGA
AHL
SSR1
rMpL
TbFBL1
CC1G-10077
MGL 6B
MGL 6A-H262T
MGL 6A
HA70 complex-Alexa
WGA multimer
rPVL
VVHA lectin
BanLec
CGL
Gal-3
human Gal-7
mouse Gal-7
CA19.9-1B844
CA19.9-9L426
CA19.9-121SLE
IPS-K-4A2B8
Microvirin
ab5
AOL (Mahal Lab)
ABA
Mono2W-CNL
BPL
GS-II (EY Lab)
EEA (EY Lab)
GNA (EY Lab)
HHA (EY Lab)
PHA-L
PNA
SBA
SJA
UEA I
VGA(EY Lab)
WGA
MNA-M
MOA
WT-VCC
ME5(detected with a-HUD)
AAL
LSL-150
LBL-152
ME7(detected with a-HUD)
SSL0-488
VLRB.aGPA.23
rNV VLP-Cy3
AFCNVH
HML-Native
#8 Lewis B (T218) (Abcam)
#15 Sialyl Lewis X (BD)
#14 Sialyl Lewis X (Calbio)
hGal-1-488+BCR
TbFBL2
MGL 416
BTL native
GAC78
DC-ASGPR short
Sp Serotype 14.2
Sp Serotype 14.15
rSRL
SRL
Galectin-1
PsSC
Avaren-Fc
ChSepL
CA19-9 Ab (DRG)
WT Galectin-8
CML1
PhoSL001
Epa1-Np mutant Y228W
Epa1-Np mutant Y228A
Epa1-Np mutant E227G
Epa1-Np mutant E227A
Epa1-Np WT
ChMBL
DBL-1
hJAA-F11-B11
Fuc(α1-4)[Gal(β1-3)]GlcNAc(β-Sp8

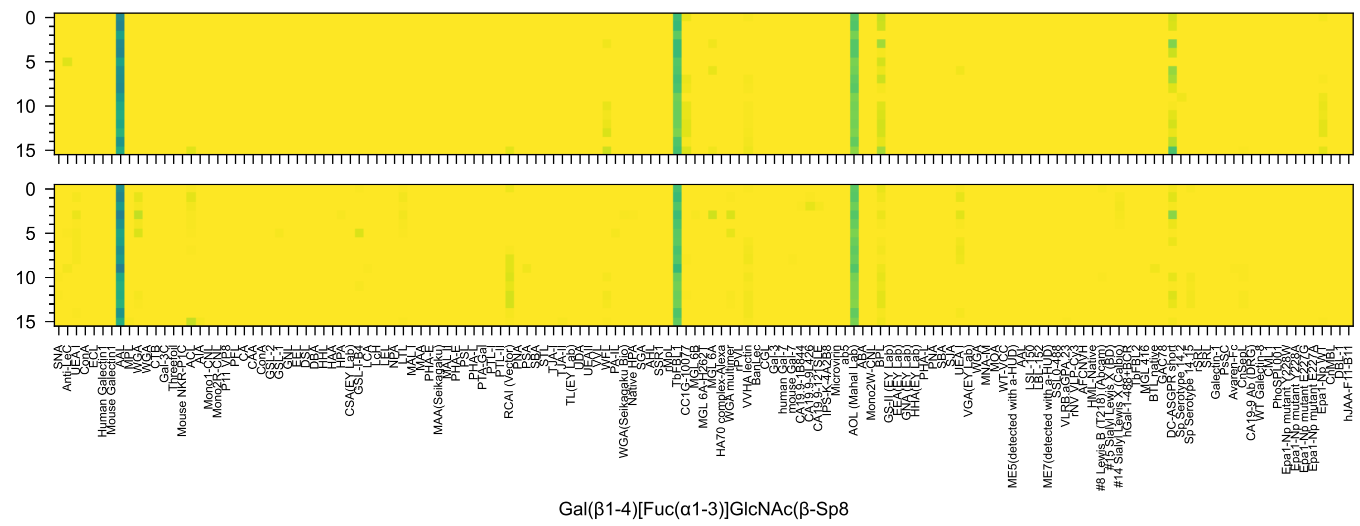
Gal(β1-4)[Fuc(α1-3)]GlcNAc(β-Sp8

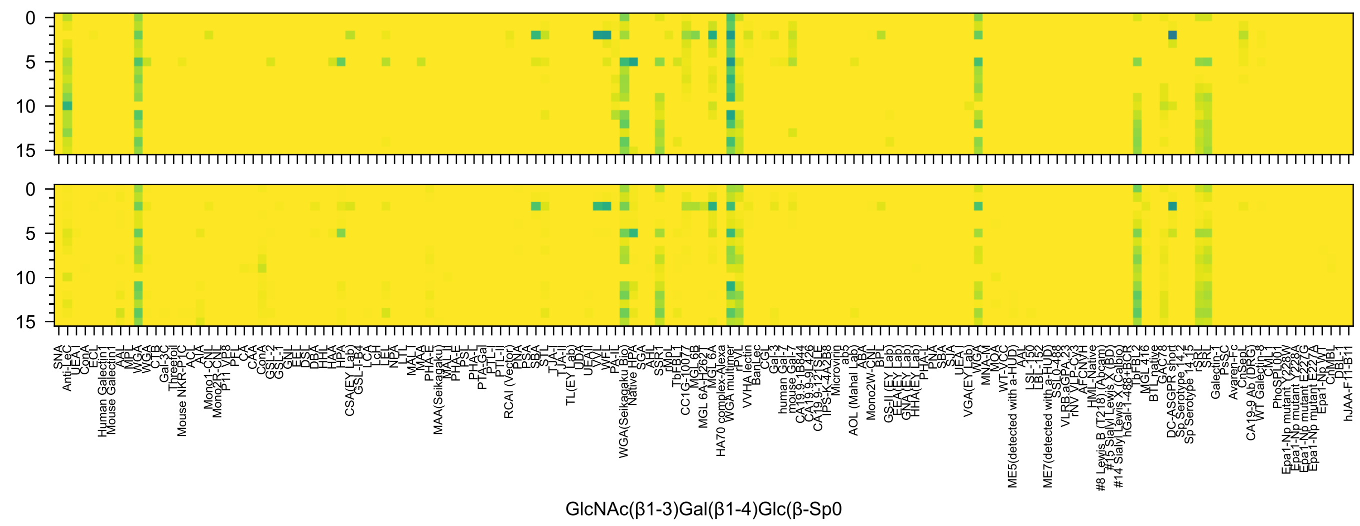

0
5
10
15
0
5
10
15
SNA
Anti-LeC
UEA I
ConA
ECL
Human Galectin1
Mouse Galectin1
AAL
MPL
WGA
WGA
CTB
Gal-3C
Threefoil
Mouse NKR-P1C
ACL
AIA
Mono1-CNL
Mono2R-CNL
P11 VP8
PFL
CA
CAA
ConA
GSL-2
GSL-1
GNL
EEL
DSL
DBA
HHL
HAA
HPA
CSA(EY Lab)
GSL-I-B4
LCA
LcH
LEL
NPA
LTL
MAL I
MAA
PHA-E
MAA(Seikagaku)
MAL II
PHA-E
PSL
PHA-L
PTA-Gal
PTL-I
PTL-II
RCAI (Vector)
PNA
PSA
SBA
STL
TJA-I
TJA-II
TL(EY Lab)
UDA
UEA II
VVL
WFL
PA-IL
WGA(Seikagaku Bio)
Native HPA
SGA
AHL
SSR1
rMpL
TbFBL1
CC1G-10077
MGL 6B
MGL 6A-H262T
MGL 6A
HA70 complex-Alexa
WGA multimer
rPVL
VVHA lectin
BanLec
CGL
Gal-3
human Gal-7
mouse Gal-7
CA19.9-1B844
CA19.9-9L426
CA19.9-121SLE
IPS-K-4A2B8
Microvirin
ab5
AOL (Mahal Lab)
ABA
Mono2W-CNL
BPL
GS-II (EY Lab)
EEA(EY Lab)
GNA(EY Lab)
HHA(EY Lab)
PHA-L
PNA
SBA
SJA
UEA I
VGA(EY Lab)
WGA
MNA-M
MOA
WT-VCC
ME5(detected with a-HUD)
AAL
LSL-150
LBL-152
ME7(detected with a-HUD)
SSL0-488
VLRB.aGPA.23
rNV VLP-Cy3
AFCNVH
HML-Native
#8 Lewis B (T218) (Abcam)
#15 Sialyl Lewis X (BD)
#14 Sialyl Lewis X (Calbio)
hGal-1-488+BCR
TbFBL2
MGL 416
BTL native
GAC78
DC-ASGPR short
Sp Serotype 14.2
Sp Serotype 14.15
rSRL
SRL
Galectin-1
PsSC
Avaren-Fc
CnSepL
CA19-9 Ab (DRG)
WT Galectin-8
CML1
PhoSL001
Epa1-Np mutant Y228W
Epa1-Np mutant Y228A
Epa1-Np mutant E227G
Epa1-Np mutant E227A
Epa1-Np WT
ChMBL
DBL-1
hJAA-F11-B11
GlcNAc(β1-3)Gal(β1-4)Glc(β-Sp0

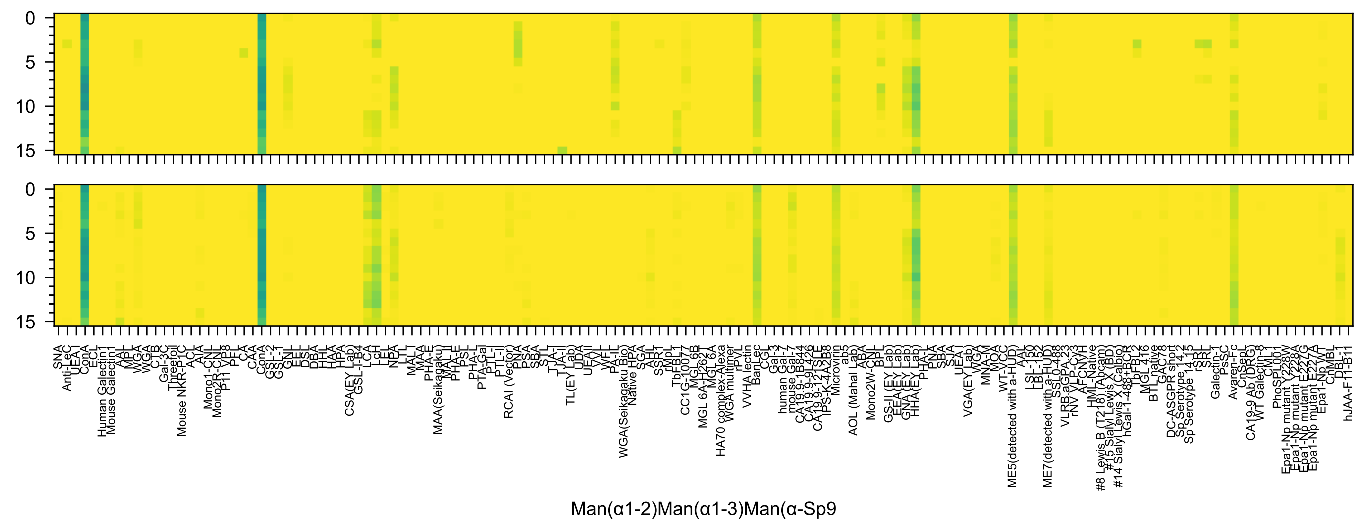
Man(α1-2)Man(α1-3)Man(α-Sp9

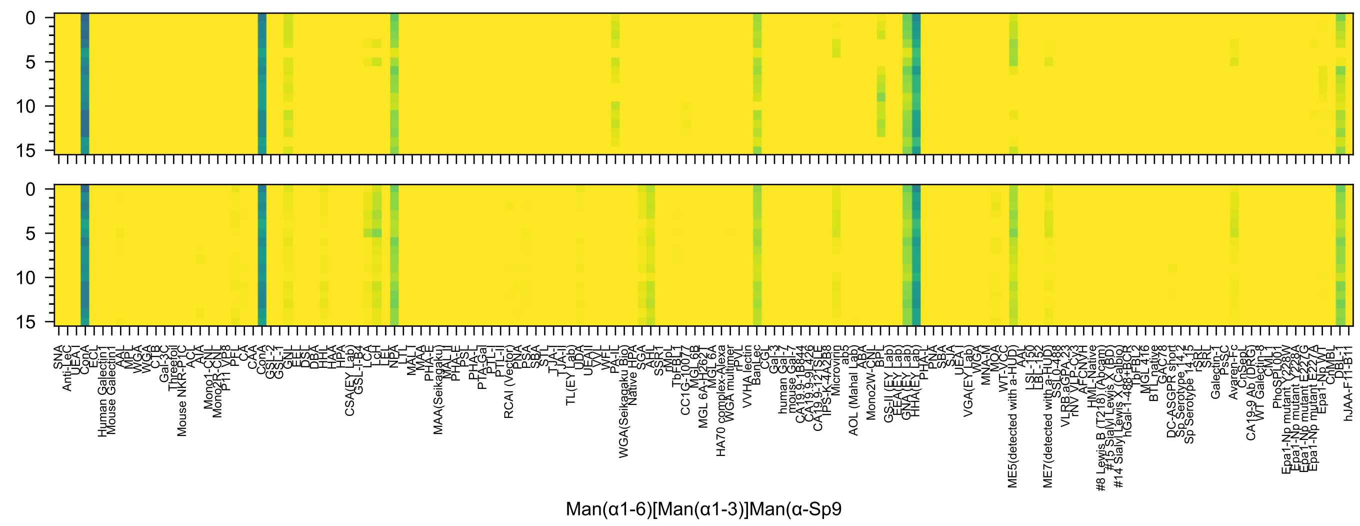
0
5
10
15
SNA
Anti-LeC
UEA-I
ConA
ECL
Human Galectin1
Mouse Galectin1
AAL
MPL
WGA
WGA
CTB
Gal-3C
Threefoil
Mouse NKR-P1C
ACL
AIA
Mono1-CNL
Mono2R-CNL
P11 VP8
PFL
CA
CAA
ConA
GSL-2
GSL-1
GNL
EEL
DSL
DBA
HHL
HAA
HPA
CSA(EY Lab)
GSL-I-B4
LCA
LcH
LEL
NPA
LTL
MAL-I
MAA
PHA-E
MAA(Seikagaku)
MAL-II
PHA-E
PSL
PHA-L
PTA-Gal
PTL-I
PTL-II
RCAI (Vector)
PNA
PSA
SBA
STL
TJA-I
TJA-II
TL(EY Lab)
UDA
UEA-II
VVL
WFL
PA-IL
WGA(Seikagaku Bio)
Native HPA
SGA
AAL
SSR1
rMpL
TbFBL1
CC1G-10077
MGL 6B
MGL 6A-H262T
MGL 6A
HA70 complex-Alexa
WGA multimer
rPVL
VVHA lectin
BanLec
CGL
Gal-3
human Gal-7
mouse Gal-7
CA19.9-1B844
CA19.9-9L426
CA19.9-121SLE
IPS-K-4A2B8
Microvirin
ab5
AOL (Mahal Lab)
ABA
Mono2W-CNL
BPL
GS-II (EY Lab)
EEA(EY Lab)
GNA(EY Lab)
HHA(EY Lab)
PHA-L
PNA
SBA
SJA
UEA-I
VGA(EY Lab)
WGA
MNA-M
MOA
WT-VCC
ME5(detected with a-HUD)
AAL
LSL-150
LBL-152
ME7(detected with a-HUD)
SSL0-488
VLRB.aGPA.23
rNV VLP-Cy3
AFCNVH
HML-Native
#8 Lewis B (T218) (Abcam)
#15 Sialyl Lewis X (BD)
#14 Sialyl Lewis X (Calbio)
hGal-1-488+BCR
TbFBL2
MGL 416
BTL native
GAC78
DC-ASGPR short
Sp Serotype 14.2
Sp Serotype 14.15
rSRL
SRL
Galectin-1
PsSC
Avaren-Fc
ChSepL
CA19-9 Ab (DRG)
WT Galectin-8
CML1
PhoSL001
Epa1-Np mutant Y228W
Epa1-Np mutant Y228A
Epa1-Np mutant E227G
Epa1-Np mutant E227A
Epa1-Np WT
ChMBL
DBL-1
hJAA-F11-B11
Man(α1-6)[Man(α1-3)]Man(α-Sp9

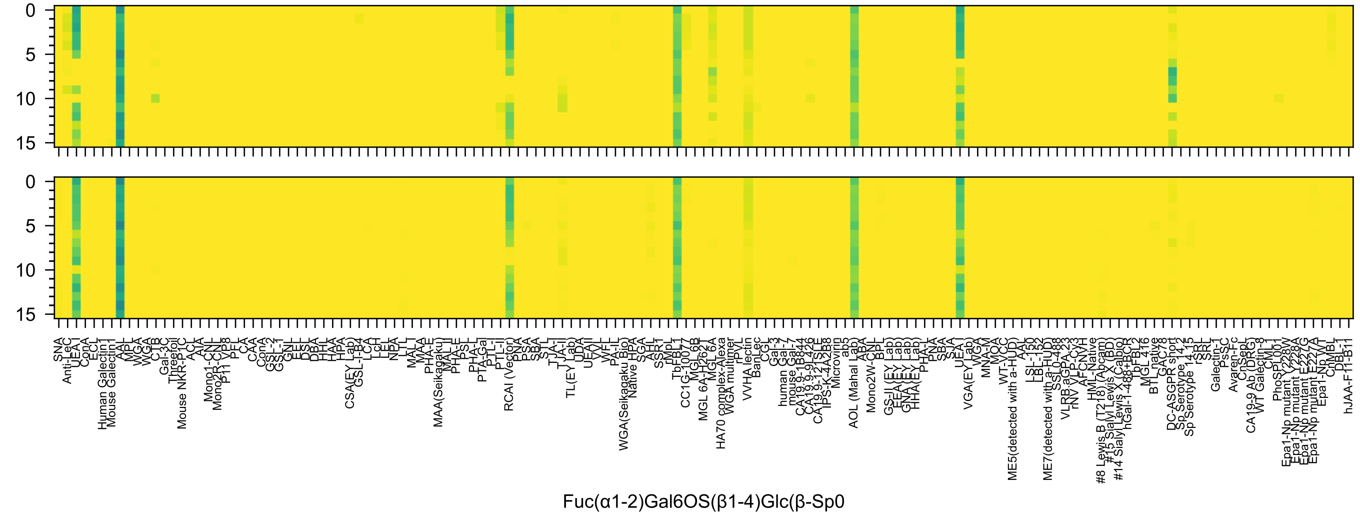

0
5
10
15
0
5
10
15
SNA
Anti-LeC
UEA-I
ConA
ECL
Human Galectin1
Mouse Galectin1
AAL
MPL
WGA
WGA
CTB
Gal-3C
Threefoil
Mouse NKR-P1C
ACL
AIA
Mono1-CNL
Mono2R-CNL
P11 VP8
PFL
CA
CAA
ConA
GSL-2
GSL-1
GNL
EEL
DSL
DBA
HHL
HAA
HPA
CSA(EY Lab)
GSL-I-B4
LCA
LcH
LEL
NPA
LTL
MAL-I
MAA
PHA-E
MAA(Seikagaku)
MAL-II
PHA-E
PSL
PHA-L
PTA-Gal
PTL-I
PTL-II
RCAI (Vector)
PNA
PSA
SBA
STL
TJA-I
TJA-II
TL(EY Lab)
UDA
UEA-II
VVL
WFL
PA-IL
WGA(Seikagaku Bio)
Native HPA
SGA
AHL
SSR1
rMpL
TbFBL1
CC1G-10077
MGL 6B
MGL 6A-H262T
MGL 6A
HA70 complex-Alexa
WGA multimer
rPVL
VVHA lectin
BanLec
CGL
Gal-3
human Gal-7
mouse Gal-7
CA19.9-1B844
CA19.9-9L426
CA19.9-121SLE
IPS-K-4A2B8
Microvirin
ab5
AOL (Mahal Lab)
ABA
Mono2W-CNL
BPL
GS-II (EY Lab)
EEA(EY Lab)
GNA(EY Lab)
HHA(EY Lab)
PHA-L
PNA
SBA
SJA
UEA-I
VGA(EY Lab)
WGA
MNA-M
MOA
WT-VCC
ME5(detected with a-HUD)
AAL
LSL-150
LBL-152
ME7(detected with a-HUD)
SS1-0488
VLRB.aGPA.23
rNV VLP-Cy3
AFCNVH
HML-Native
#8 Lewis B (T218) (Abcam)
#15 Sialyl Lewis X (BD)
#14 Sialyl Lewis X (Calbio)
hGal-1-488+BCR
TbFBL2
MGL 416
BTL native
GAC78
DC-ASGPR short
Sp Serotype 14.2
Sp Serotype 14.15
rSRL
SRL
Galectin-1
PsSC
Avaren-Fc
ChSeP
CA19-9 Ab (DRG)
WT Galectin-8
CML1
PhoSL001
Epa1-Np mutant Y228W
Epa1-Np mutant Y228A
Epa1-Np mutant E227G
Epa1-Np mutant E227A
Epa1-Np WT
ChMBL
DBL-1
hJAA-F11-B11
Fuc(α1-2)Gal6OS(β1-4)Glc(β-Sp0

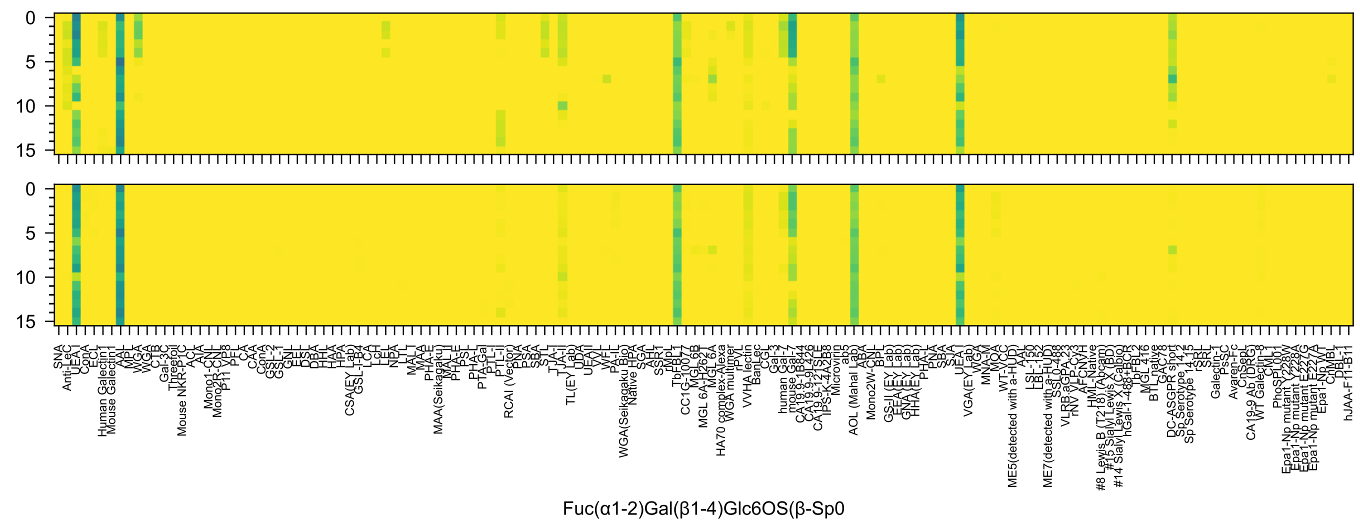
SNA
Anti-LeC
UEA I
ConA
ECL
Human Galectin1
Mouse Galectin1
AAL
MPL
WGA
WGA
CTB
Gal-3C
Threefoil
Mouse NKR-P1C
ACL
AIA
Mono1-CNL
Mono2R-CNL
P11 VP8
PFL
CA
CAA
ConA
GSL-2
GSL-1
GNL
EEL
DSL
DBA
HHL
HAA
HPA
CSA(EY Lab)
GSL-I-B4
LCA
LcH
LEL
NPA
LTL
MAL I
MAA
PHA-E
MAA(Seikagaku)
MAL II
PHA-E
PSL
PHA-L
PTA-Gal
PTL-I
PTL-II
RCAI (Vector)
PNA
PSA
SBA
STL
TJA-I
TJA-II
TL(EY Lab)
UDA
UEA II
VVL
WFL
PA-IL
WGA(Seikagaku Bio)
Native HPA
SGA
AAL
SSR1
rMpL
TbFBL1
CC1G-10077
MGL 6B
MGL 6A-H262T
MGL 6A
HA70 complex-Alexa
WGA multimer
rPVL
VVHA lectin
BanLec
CGL
Gal-3
human Gal-7
mouse Gal-7
CA19.9-1B844
CA19.9-9L426
CA19.9-121SLE
IPS-K-4A2B8
Microvirin
ab5
AOL (Mahal Lab)
ABA
Mono2W-CNL
BPL
GS-II (EY Lab)
EEA(EY Lab)
GNA(EY Lab)
HHA(EY Lab)
PHA-L
PNA
SBA
SJA
UEA I
VGA(EY Lab)
WGA
MNA-M
MOA
WT-VCC
ME5(detected with a-HUD)
AAL
LSL-150
LBL-152
ME7(detected with a-HUD)
SSL0-488
VLRB.aGPA.23
rNV VLP-Cy3
AFCNVH
HML-Native
#8 Lewis B (T218) (Abcam)
#15 Sialyl Lewis X (BD)
#14 Sialyl Lewis X (Calbio)
hGal-1-488+BCR
TbFBL2
MGL 416
BTL native
GAC78
DC-ASGPR short
Sp Serotype 14.2
Sp Serotype 14.15
rSRL
SRL
Galectin-1
PsSC
Avaren-Fc
ChSepL
CA19-9 Ab (DRG)
WT Galectin-8
CML1
PhoSL001
Epa1-Np mutant Y228W
Epa1-Np mutant Y228A
Epa1-Np mutant E227G
Epa1-Np mutant E227A
Epa1-Np WT
ChMBL
DBL-1
hJAA-F11-B11
Fuc(α1-2)Gal(β1-4)Glc6OS(β-Sp0

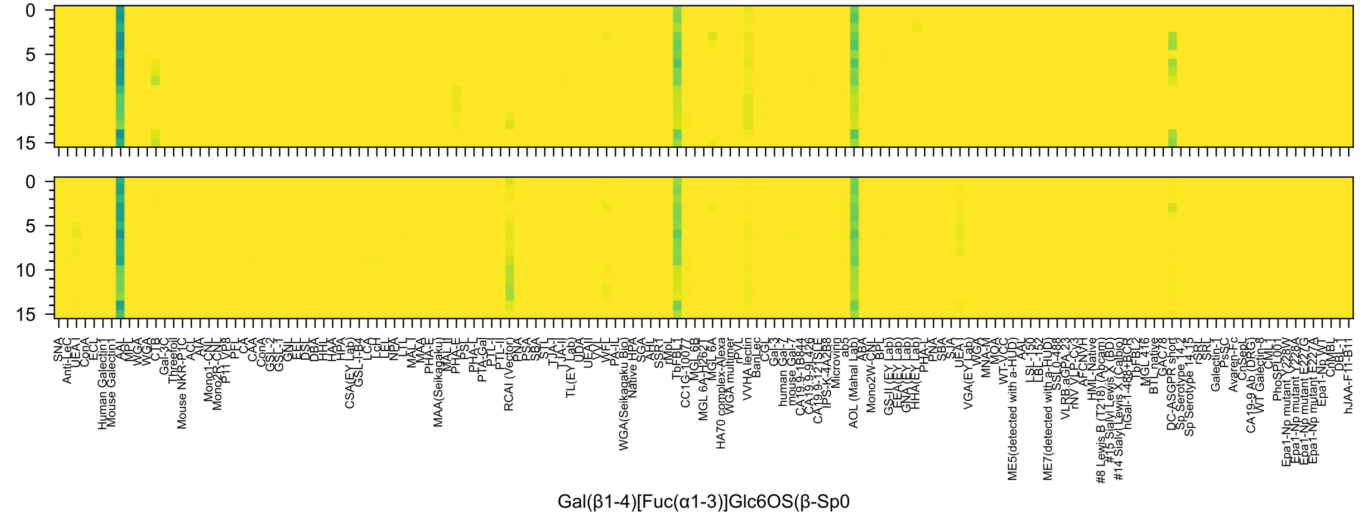
0
5
10
15
0
5
10
15
SNA
Anti-LeC
UEA I
ConA
ECL
Human Galectin1
Mouse Galectin1
AAL
MPL
WGA
WGA
CTB
Gal-3C
Threefoil
Mouse NKR-P1C
ACL
AIA
Mono1-CNL
Mono2R-CNL
P11 VP8
PFL
CA
CAA
ConA
GSL-2
GSL-1
GNL
EEL
DSL
DBA
HHL
HAA
HPA
CSA(EY Lab)
GSL-I-B4
LCA
LcH
LEL
NPA
LTL
MAL
MAA
PHA-E
MAA(Seikagaku)
MAL-II
PHA-E
PSL
PHA-L
PTA-Gal
PTL-I
PTL-II
RCAI (Vector)
PNA
PSA
SBA
STL
TJA-I
TJA-II
TL(EY Lab)
UDA
UEA-II
VVL
WFL
PA-IL
WGA(Seikagaku Bio)
Native HPA
SGA
AHL
SSR1
rMpL
TbFBL1
CC1G-10077
MGL 6B
MGL 6A-H262T
MGL 6A
HA70 complex-Alexa
WGA multimer
rPVL
VVHA lectin
BanLec
CGL
Gal-3
human Gal-7
mouse Gal-7
CA19.9-1B844
CA19.9-9L426
CA19.9-121SLE
IPS-K-4A2B8
Microvirin
ab5
AOL (Mahal Lab)
ABA
Mono2W-CNL
BPL
GS-II (EY Lab)
EEA(EY Lab)
GNA(EY Lab)
HHA(EY Lab)
PHA-L
PNA
SBA
SJA
UEA-I
VGA(EY Lab)
WGA
MNA-M
MOA
WT-VCC
ME5(detected with a-HUD)
AAL
LSL-150
LBL-152
ME7(detected with a-HUD)
SSL0-488
VLRB.aGPA-23
rNV VLP-Cy3
AFCNVH
HML-Native
#8 Lewis B (T218) (Abcam)
#15 Sialyl Lewis X (BD)
#14 Sialyl Lewis X (Calbio)
hGal-1-488+BCR
TbFBL2
MGL 416
BTL native
GAC78
DC-ASGPR short
Sp Serotype 14.2
Sp Serotype 14.15
rSRL
SRL
Galectin-1
PsSC
Avaren-Fc
ChSeprL
CA19-9 Ab (DRG)
WT Galectin-8
CML1
PhoSL001
Epa1-Np mutant Y228W
Epa1-Np mutant Y228A
Epa1-Np mutant E227G
Epa1-Np mutant E227A
Epa1-Np WT
ChMBL
DBL-1
hJAA-F11-B11
Gal(β1-4)[Fuc(α1-3)]Glc6OS(β-Sp0

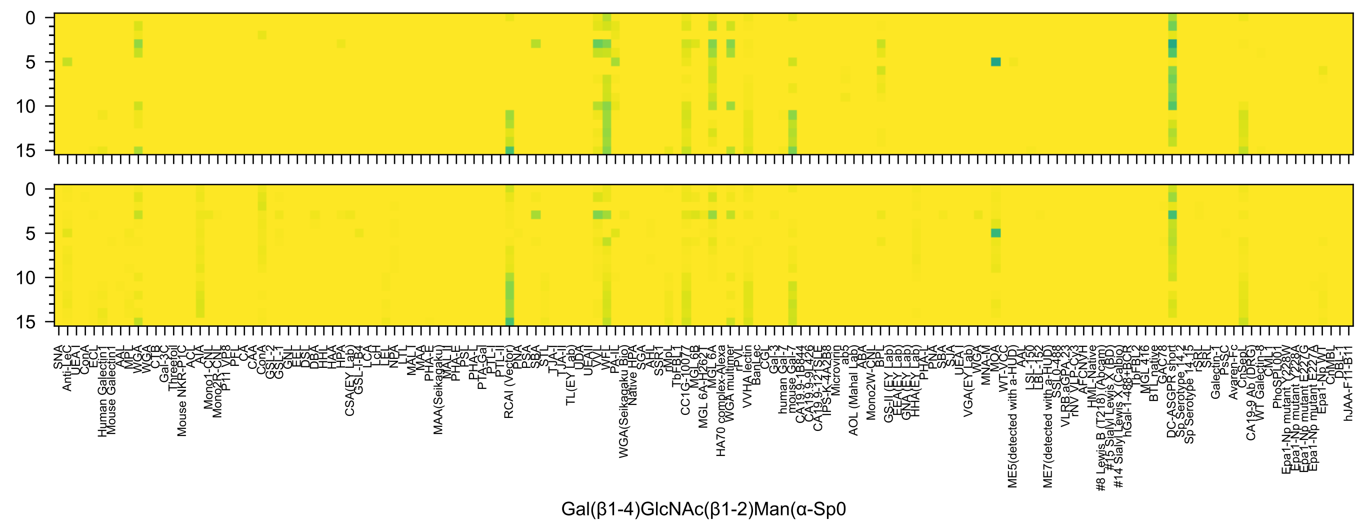
0
5
10
15
SNA
Anti-LeC
UEA-I
ConA
ECL
Human Galectin1
Mouse Galectin1
AAL
MPL
WGA
WGA
CT-B
Gal-3C
Threefoil
Mouse NKR-P1C
ACL
AIA
Mono1-CNL
Mono2R-CNL
P11 VP8
PFL
CA
CAA
ConA
GSL-2
GSL-1
GNL
EEL
DSL
DBA
HHL
HAA
HPA
CSA(EY Lab)
GSL-I-B4
LCA
LcH
LEL
NPA
LTL
MAL
MAA
PHA-E
MAA(Seikagaku)
MAL-II
PHA-E
PSL
PHA-L
PTA-Gal
PTL-I
PTL-II
RCAI (Vector)
PNA
PSA
SBA
STL
TJA-I
TJA-II
TL(EY Lab)
UDA
UEA-II
VVL
WFL
PA-IL
WGA(Seikagaku Bio)
Native HPA
SGA
AAL
SSR1
rMpL
TbFBL1
CC1G-10077
MGL 6B
MGL 6A-H262T
MGL 6A
HA70 complex-Alexa
WGA multimer
rPVL
VVHA lectin
BanLec
CGL
Gal-3
human Gal-7
mouse Gal-7
CA19.9-1B844
CA19.9-9L426
CA19.9-121SLE
IPS-K-4A2B8
Microvirin
ab5
AOL (Mahal Lab)
ABA
Mono2W-CNL
BPL
GS-II (EY Lab)
EEA(EY Lab)
GNA(EY Lab)
HHA(EY Lab)
PHA-L
PNA
SBA
SJA
UEA-I
VGA(EY Lab)
WGA
MNA-M
MOA
WT-VCC
ME5(detected with a-HUD)
AAL
LSL-150
LBL-152
ME7(detected with a-HUD)
SS1.0.488
VLRB.aGPA.23
rNV VLP-Cy3
AFCNVH
HML-Native
#8 Lewis B (T218) (Abcam)
#15 Sialyl Lewis X (BD)
#14 Sialyl Lewis X (Calbio)
hGal-1-488+BCR
TbFBL2
MGL 416
BTL native
GAC78
DC-ASGPR short
Sp Serotype 14.2
Sp Serotype 14.15
rSRL
SRL
Galectin-1
PsSC
Avaren-Fc
ChSepL
CA19-9 Ab (DRG)
WT Galectin-8
CML-1
PhoSPL001
Epa1-Np mutant Y228W
Epa1-Np mutant Y228A
Epa1-Np mutant E227G
Epa1-Np mutant E227A
Epa1-Np WT
ChMBL
DBL-1
hJAA-F11-B11
Gal(β1-4)GlcNAc(β1-2)Man(α-Sp0

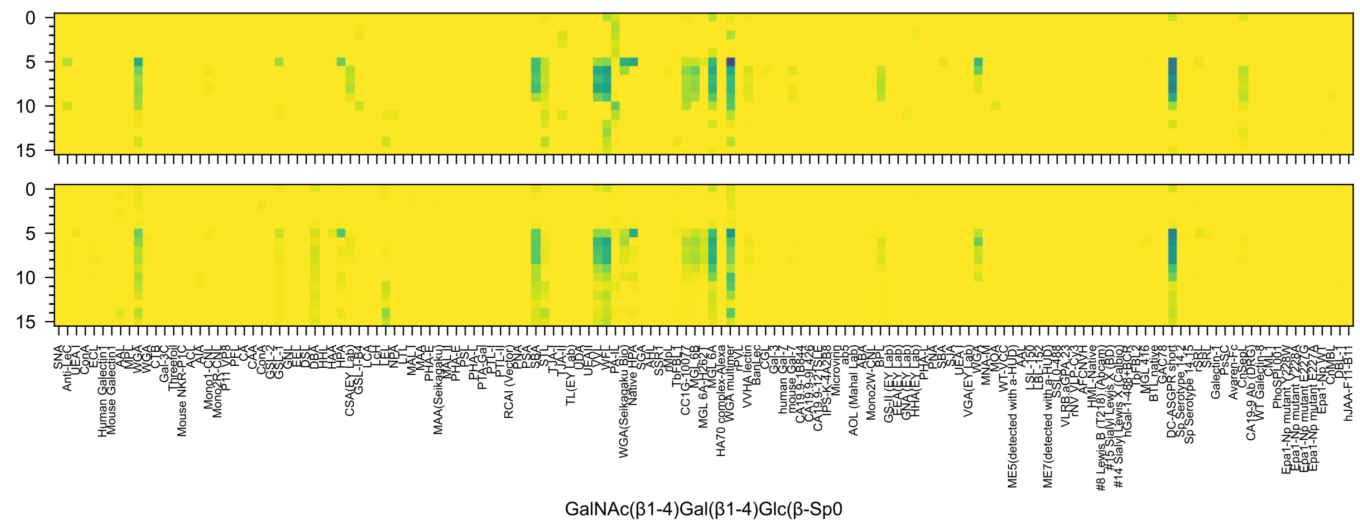
0
5
10
15
0
5
10
15
GalNAc(β1-4)Gal(β1-4)Glc(β-Sp0)

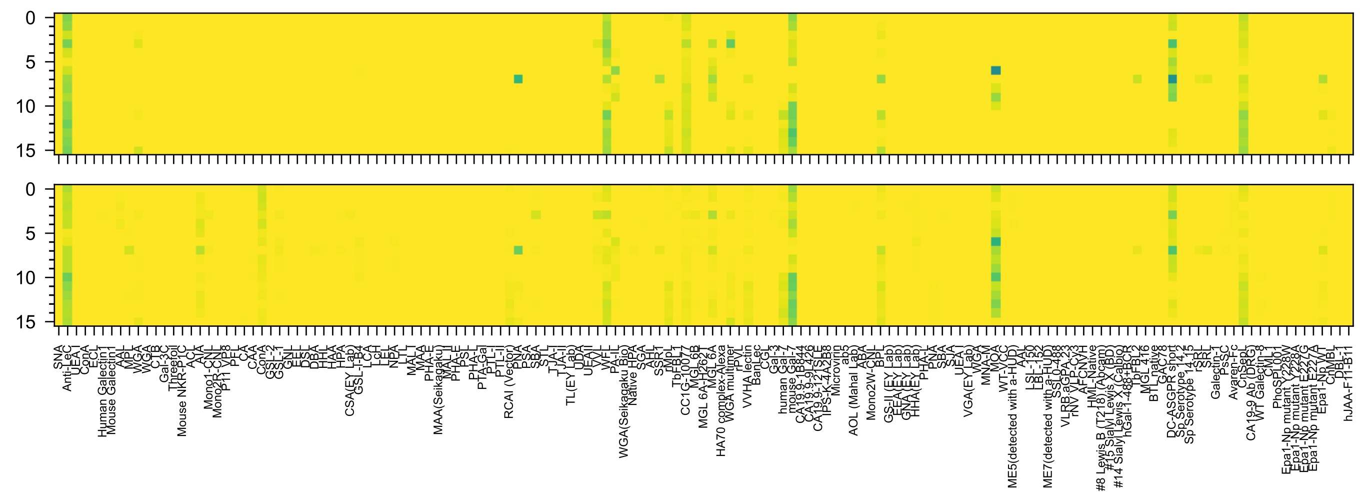

Gal(β1-3)GlcNAc(β1-2)Man(α-Sp0

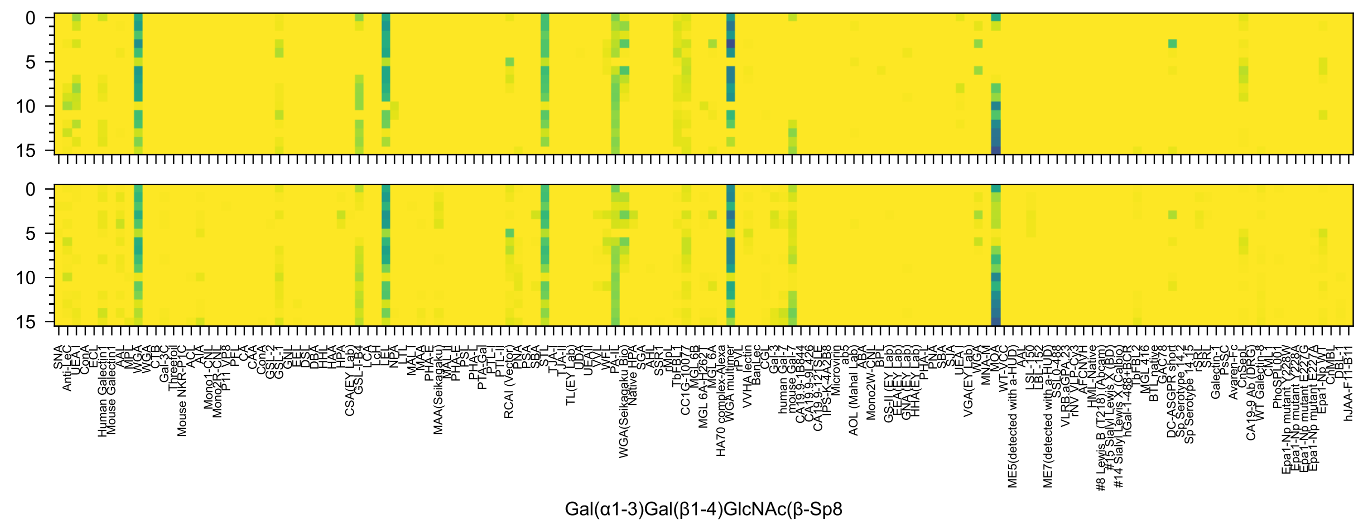
Gal(α1-3)Gal(β1-4)GlcNAc(β-Sp8

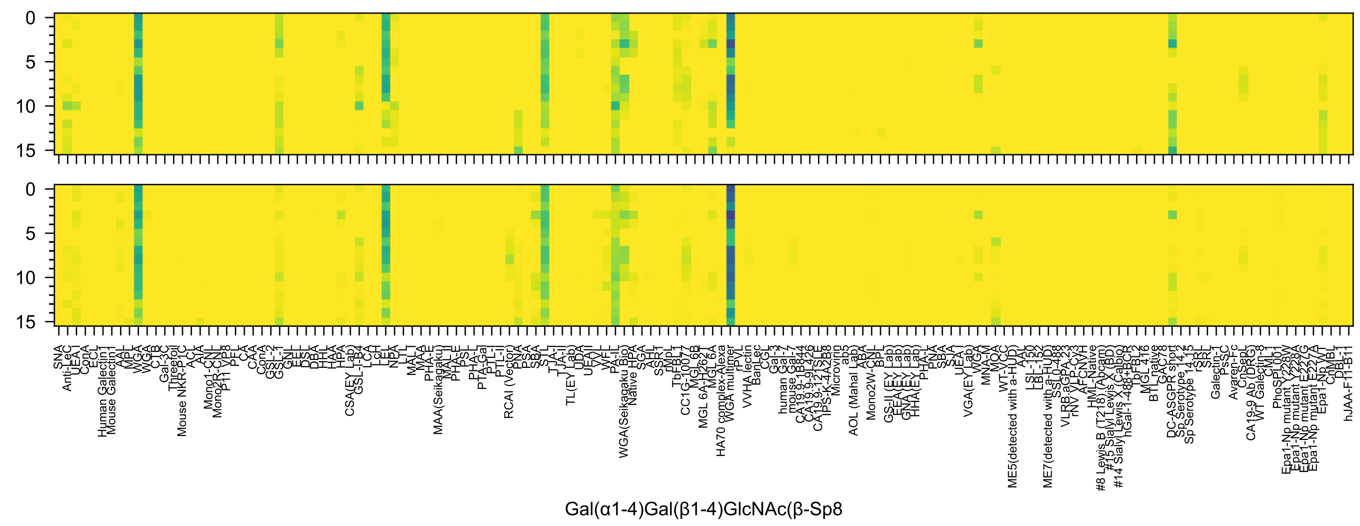

0
5
10
15
0
5
10
15
SNA
Anti-LeC
UEA-I
ConA
ECL
Human Galectin1
Mouse Galectin1
AAL
MPL
WGA
WGA
CTB
C-TB
Gal-3C
Threefoil
Mouse NKR-P1C
ACL
AIA
Mono1-CNL
Mono2R-CNL
P11 VP8
PFL
CA
CAA
ConA
GSL-2
GSL-1
GNL
EEL
DSL
DBA
HHL
HAA
HPA
CSA(EY Lab)
GSL-I-B4
LCA
LcH
LEL
NPA
LTL
MAL-I
MAA
PHA-E
MAA(Seikagaku)
MAL-II
PHA-E
PSL
PHA-L
PTA-Gal
PTL-I
PTL-II
RCAI (Vector)
PNA
PSA
SBA
STL
TJA-I
TJA-II
TL(EY Lab)
UDA
UEA-II
VVL
WFL
PA-IL
WGA(Seikagaku Bio)
Native HPA
SGA
AHL
SSR1
rMpL
TbFBL1
CC1G-10077
MGL 6B
MGL 6A-H262T
MGL 6A
HA70 complex-Alexa
WGA multimer
rPVL
VVHA lectin
BanLec
CGL
Gal-3
human Gal-7
mouse Gal-7
CA19.9-1B844
CA19.9-9L426
CA19.9-121SLE
IPS-K-4A2B8
Microvirin
ab5
AOL (Mahal Lab)
ABA
Mono2W-CNL
BPL
GS-II (EY Lab)
EEA(EY Lab)
GNA(EY Lab)
HHA(EY Lab)
PHA-L
PNA
SBA
SJA
UEA-I
VGA(EY Lab)
WGA
MNA-M
MOA
WT-VCC
ME5(detected with a-HUD)
AAL
LSL-150
LBL-152
ME7(detected with a-HUD)
SSL0-488
VLRB.aGPA.23
rNV VLP-Cy3
AFCNVH
HML-Native
#8 Lewis B (T218) (Abcam)
#15 Sialyl Lewis X (BD)
#14 Sialyl Lewis X (Calbio)
hGal-1-488+BCR
TbFBL2
MGL 416
BTL native
GAC78
DC-ASGPR short
Sp Serotype 14.2
Sp Serotype 14.15
rSRL
SRL
Galectin-1
PsSC
Avaren-Fc
ChSepL
CA19-9 Ab (DRG)
WT Galectin-8
CML1
PhoSL001
Epa1-Np mutant Y228W
Epa1-Np mutant Y228A
Epa1-Np mutant E227G
Epa1-Np mutant E227A
Epa1-Np WT
CnMBL
DBL-1
hJAA-F11-B11
Gal(α1-4)Gal(β1-4)GlcNAc(β-Sp8

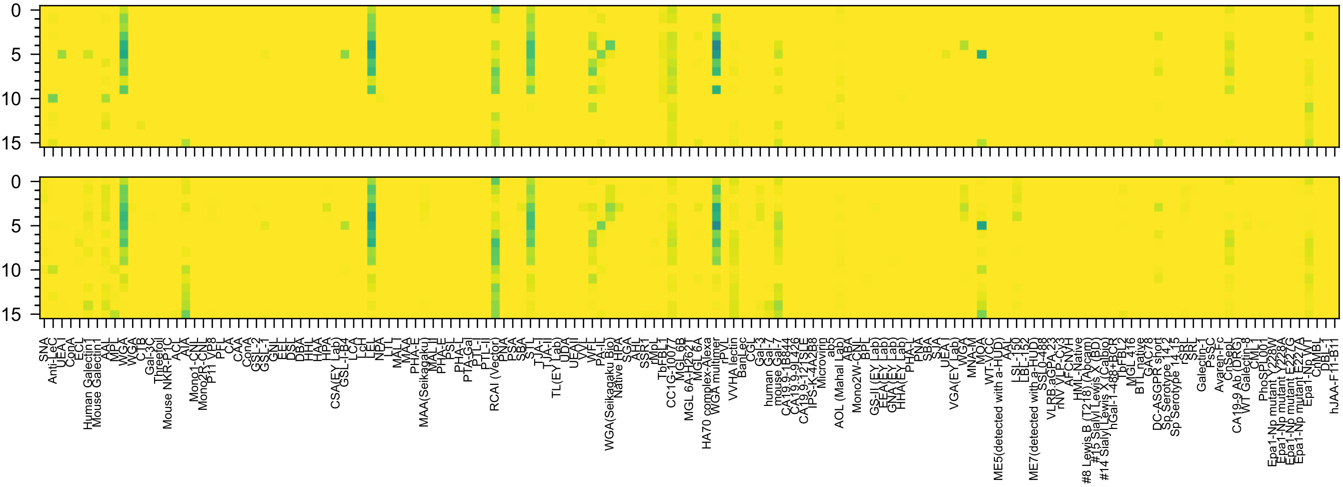

Gal(β1-3)Gal(β1-4)GlcNAc(β-Sp8

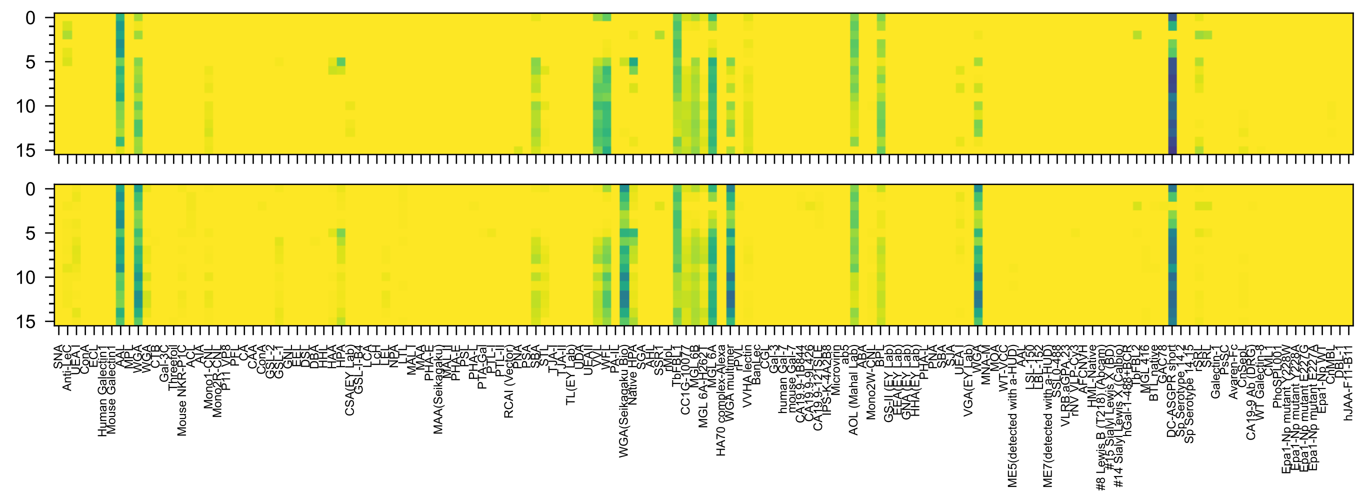

GalNAc(β1-4)[Fuc(α1-3)]GlcNAc(β-Sp0

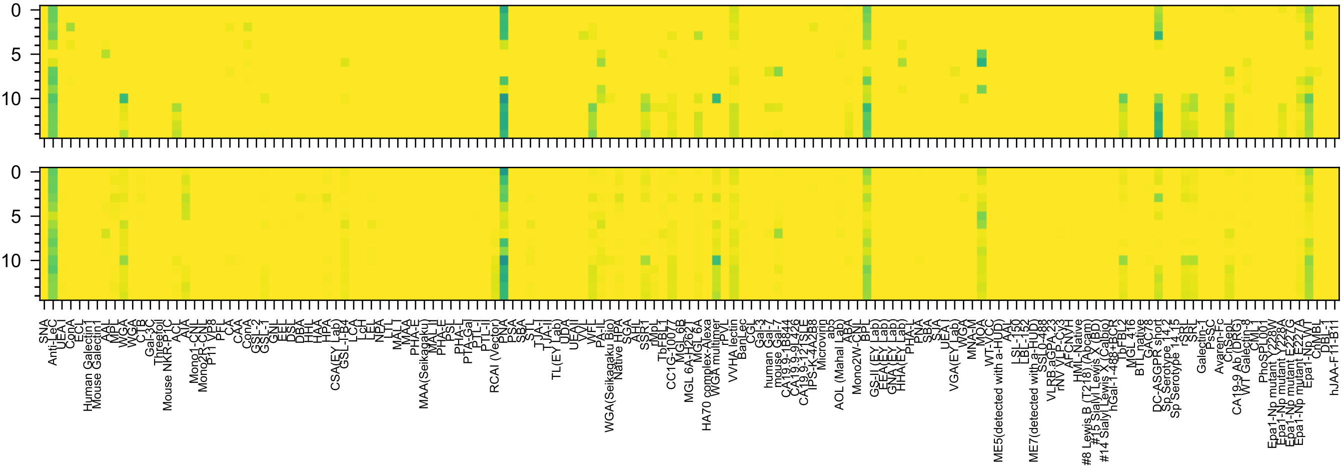

Gal(β1-3)GalNAc(β1-3)Gal(-Sp21

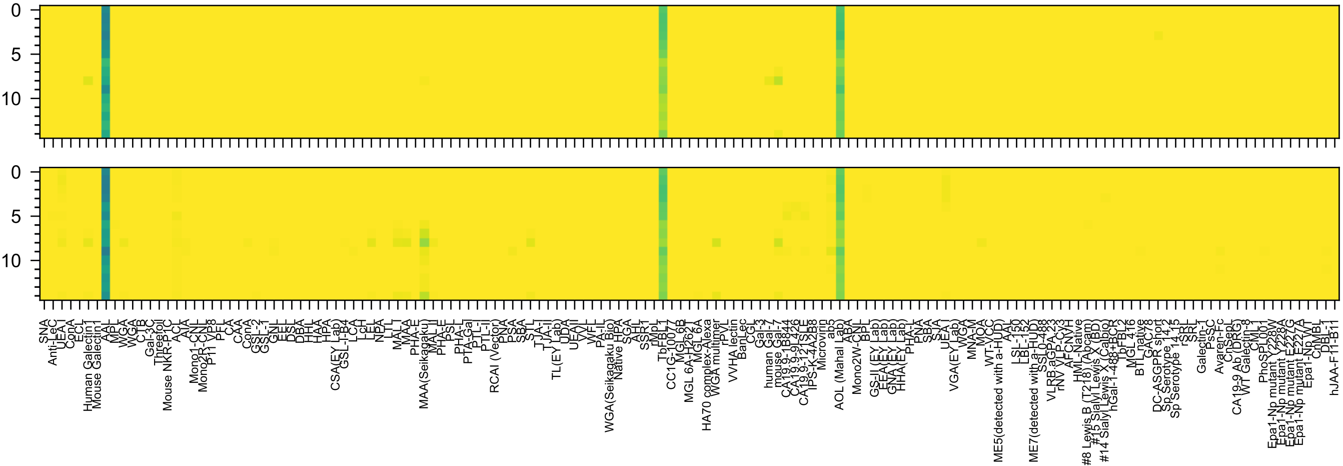

Gal3OS(β1-4)[Fuc(α1-3)]GlcNAc(-Sp0

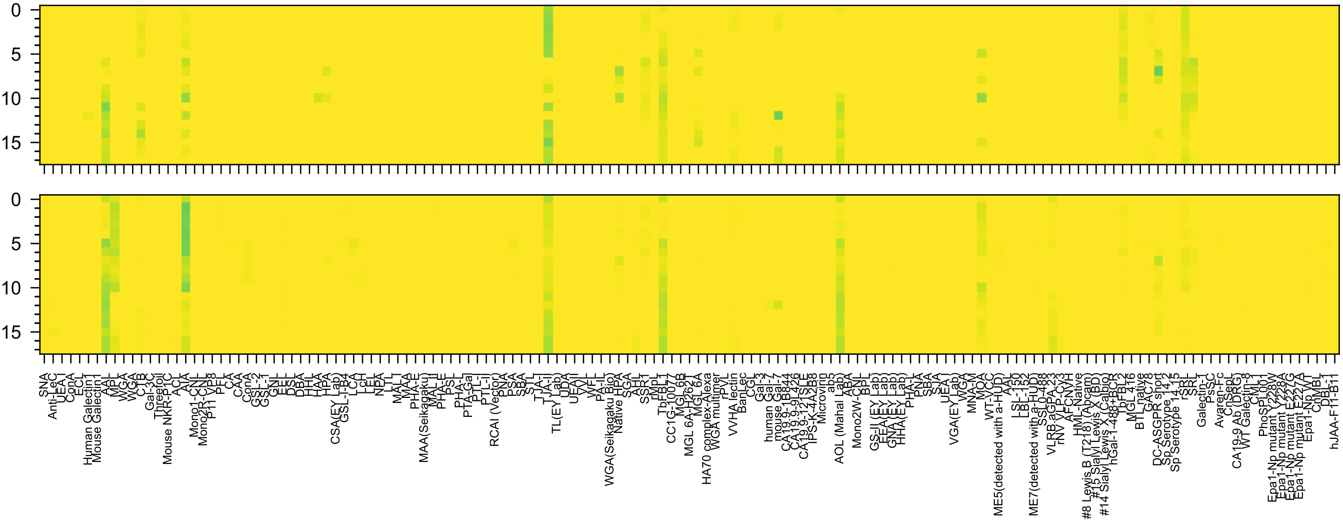

Fuc(α1-2)Gal(β1-3)GalNAc(α-Sp14

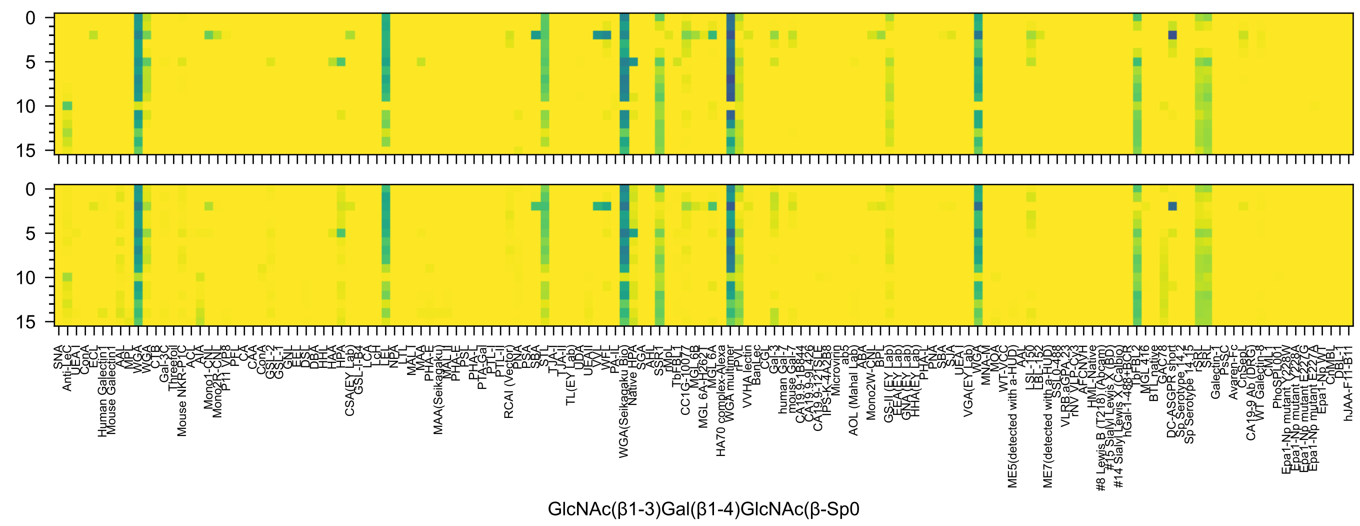
GlcNAc(β1-3)Gal(β1-4)GlcNAc(β-Sp0)

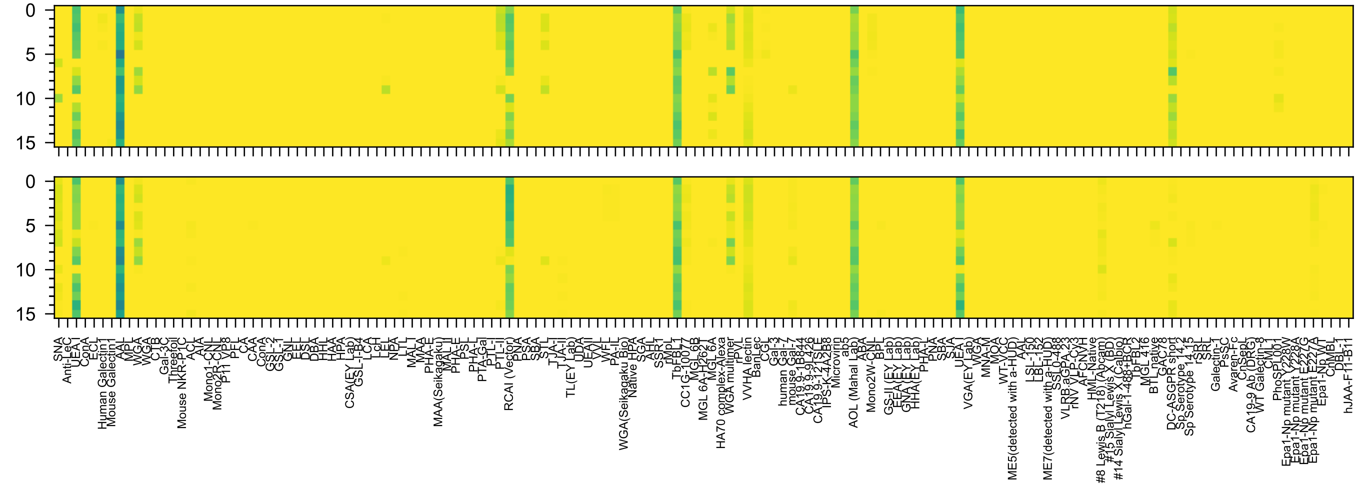

Fuc(α1-2)Gal6OS(β1-4)GlcNAc(β-Sp0

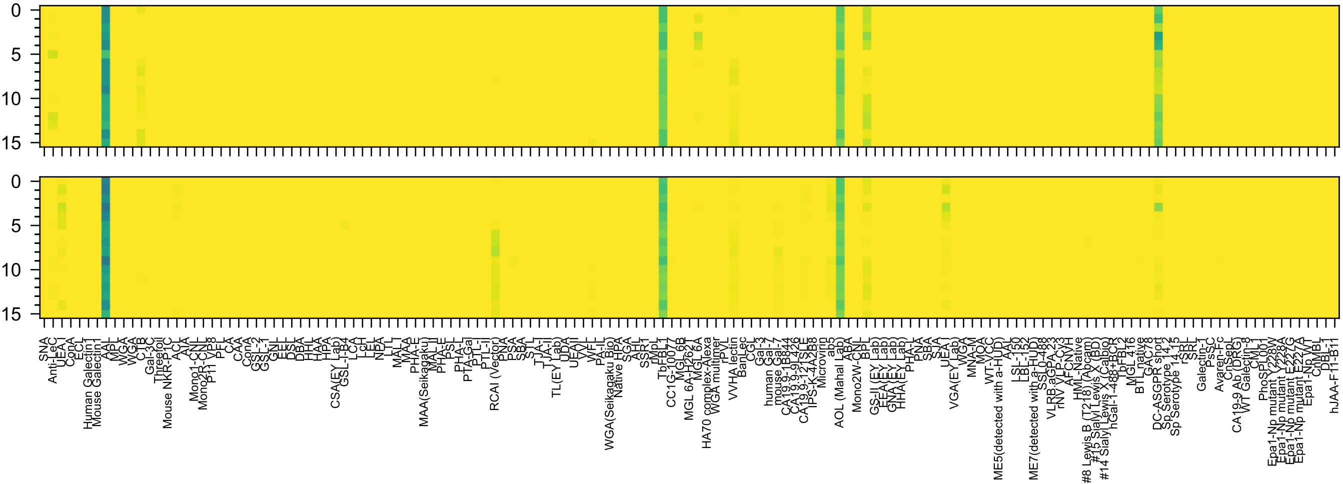

Gal(β1-4)[Fuc(α1-3)]GlcNAc6OS(β-Sp0

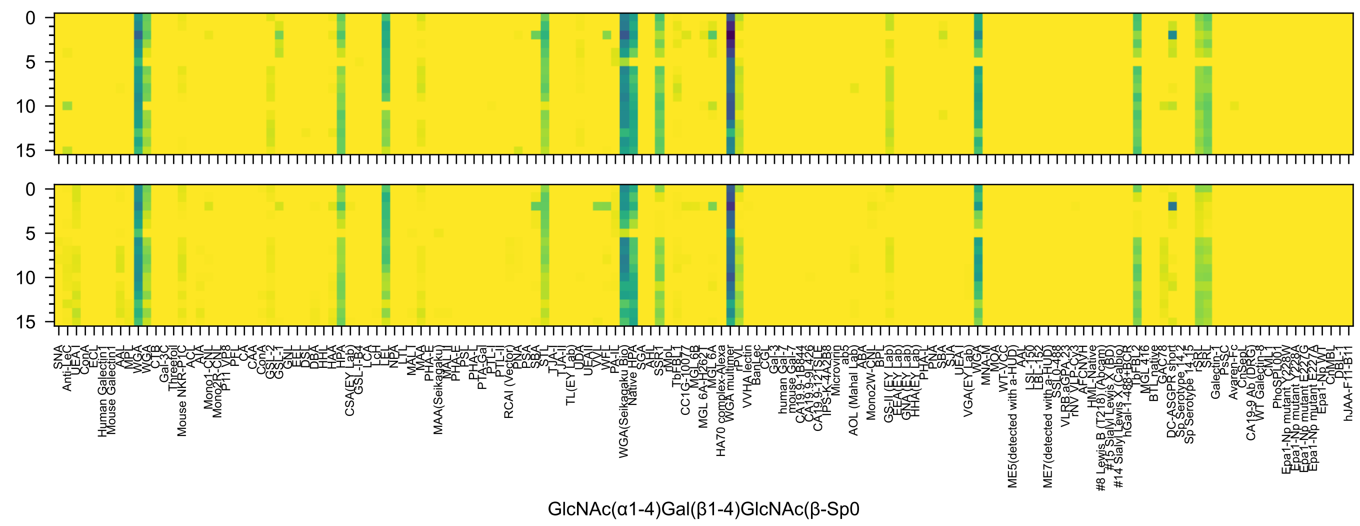
0
5
10
15
SNA
Anti-LeC
UEA-I
ConA
ECL
Human Galectin1
Mouse Galectin1
AAL
MPL
WGA
WGA
CTB
C-13
Gal-3C
Threefoil
Mouse NKR-P1C
ACL
AIA
Mono1-CNL
Mono2R-CNL
P11 VP8
PFL
CA
CAA
ConA
GSL-2
GSL-1
GNL
EEL
DSL
DBA
HHL
HAA
HPA
CSA(EY Lab)
GSL-I-B4
LCA
LcH
LEL
NPA
LTL
MAL-I
MAA
PHA-E
MAA(Seikagaku)
MAL-II
PHA-E
PSL
PHA-L
PTA-Gal
PTL-I
PTL-II
RCAI (Vector)
PNA
PSA
SBA
STL
TJA-I
TJA-II
TL(EY Lab)
UDA
UEA-II
VVL
WFL
PA-IL
WGA(Seikagaku Bio)
Native HPA
SGA
AHL
SSR1
rMpL
TbFBL1
CC1G-10077
MGL 6B
MGL 6A-H262T
MGL 6A
HA70 complex-Alexa
WGA multimer
rPVL
VVHA lectin
BanLec
CGL
Gal-3
human Gal-7
mouse Gal-7
CA19.9-1B844
CA19.9-9L426
CA19.9-121SLE
IPS-K-4A2B8
Microvirin
ab5
AOL (Mahal Lab)
ABA
Mono2W-CNL
BPL
GS-II (EY Lab)
EEA(EY Lab)
GNA(EY Lab)
HHA(EY Lab)
PHA-L
PNA
SBA
SJA
UEA-I
VGA(EY Lab)
WGA
MNA-M
MOA
WT-VCC
ME5(detected with a-HUD)
AAL
LSL-150
LBL-152
ME7(detected with a-HUD)
SSL0-488
VLRB.aGPA.23
rNV VLP-Cy3
AFCNVH
HML-Native
#8 Lewis B (T218) (Abcam)
#15 Sialyl Lewis X (BD)
#14 Sialyl Lewis X (Calbio)
hGal-1-488+BCR
TbFBL2
MGL 416
BTL native
GAC78
DC-ASGPR short
Sp Serotype 14.2
Sp Serotype 14.15
rSRL
SRL
Galectin-1
PsSC
Avaren-Fc
ChSepL
CA19-9 Ab (DRG)
WT Galectin-8
CML1
PhoSL001
Epa1-Np mutant Y228W
Epa1-Np mutant Y228A
Epa1-Np mutant E227G
Epa1-Np mutant E227A
Epa1-Np WT
ChMBL
DBL-1
hJAA-F11-B11
GlcNAc(α1-4)Gal(β1-4)GlcNAc(β-Sp0

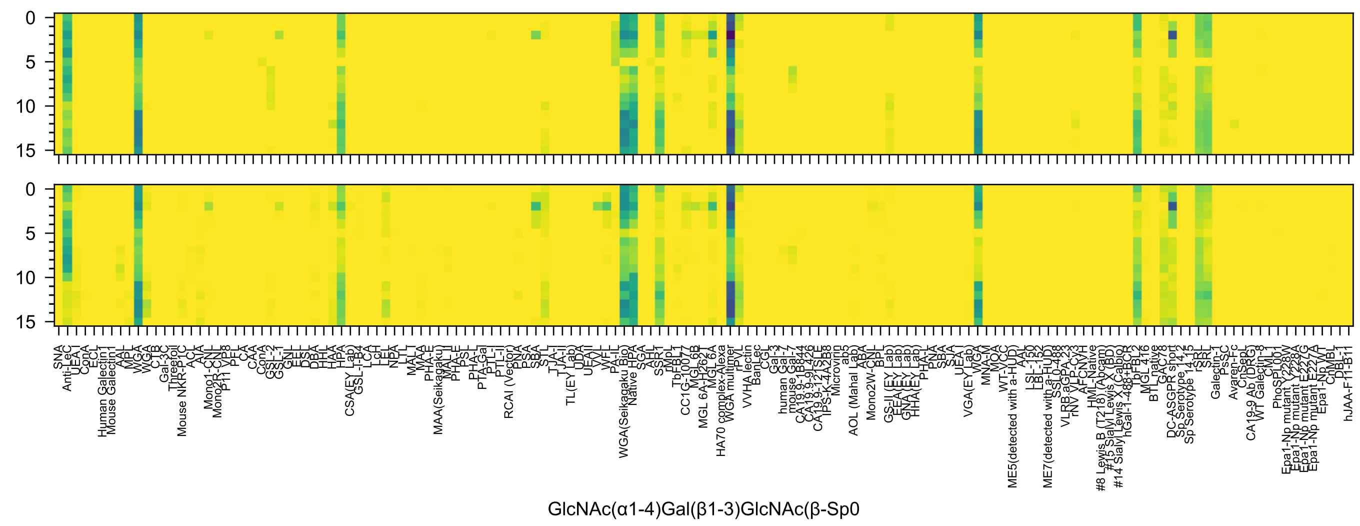
0
5
10
15
SNA
Anti-LeC
UEA-I
ConA
ECL
Human Galectin1
Mouse Galectin1
AAL
MPL
WGA
WGA
CTB
Gal-3C
Threefoil
Mouse NKR-P1C
ACL
AIA
Mono1-CNL
Mono2R-CNL
P11 VP8
PFL
CA
CAA
ConA
GSL-2
GSL-1
GNL
EEL
DSL
DBA
HHL
HAA
HPA
CSA(EY Lab)
GSL-I-B4
LCA
LcH
LEL
NPA
LTL
MAL-I
MAA
PHA-E
MAA(Seikagaku)
MAL-II
PHA-E
PSL
PHA-L
PTA-Gal
PTL-I
PTL-II
RCAI (Vector)
PNA
PSA
SBA
STL
TJA-I
TJA-II
TL(EY Lab)
UDA
UEA-II
VVL
WFL
PA-IL
WGA(Seikagaku Bio)
Native HPA
SGA
AAL
SSR1
rMpL
TbFBL1
CC1G-10077
MGL 6B
MGL 6A-H262T
MGL 6A
HA70 complex-Alexa
WGA multimer
rPVL
VVHA lectin
BanLec
CGL
Gal-3
human Gal-7
mouse Gal-7
CA19.9-1B844
CA19.9-9L426
CA19.9-121SLE
IPS-K-4A2B8
Microvirin
ab5
AOL (Mahal Lab)
ABA
Mono2W-CNL
BPL
GS-II (EY Lab)
EEA(EY Lab)
GNA(EY Lab)
HHA(EY Lab)
PHA-L
PNA
SBA
SJA
UEA-I
VGA(EY Lab)
WGA
MNA-M
MOA
WT-VCC
ME5(detected with a-HUD)
AAL
LSL-150
LBL-152
ME7(detected with a-HUD)
SSL0-488
VLRB.aGPA.23
rNV VLP-Cy3
AFCNVH
HML-Native
#8 Lewis B (T218) (Abcam)
#15 Sialyl Lewis X (BD)
#14 Sialyl Lewis X (Calbio)
hGal-1-488+BCR
TbFBL2
MGL 416
BTL native
GAC78
DC-ASGPR short
Sp Serotype 14.2
Sp Serotype 14.15
rSRL
SRL
Galectin-1
PsSC
Avaren-Fc
ChSepL
CA19-9 Ab (DRG)
WT Galectin-8
CML1
PhoSL001
Epa1-Np mutant Y228W
Epa1-Np mutant Y228A
Epa1-Np mutant E227G
Epa1-Np mutant E227A
Epa1-Np WT
ChMBL
DBL-1
hJAA-F11-B11
GlcNAc(α1-4)Gal(β1-3)GlcNAc(β-Sp0

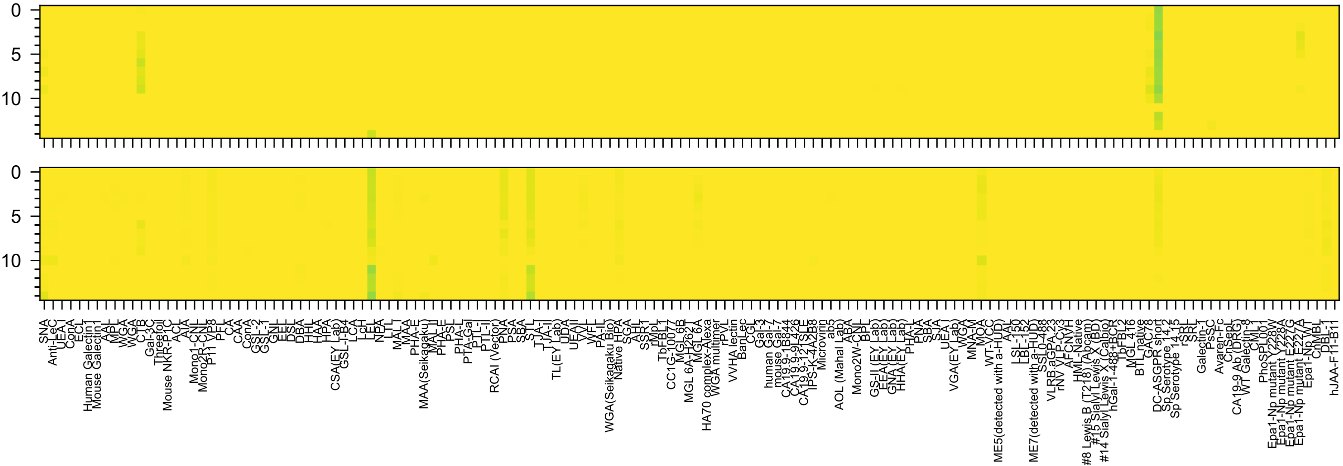

Kdn(α2-3)Gal(β1-4)Glc(-Sp0

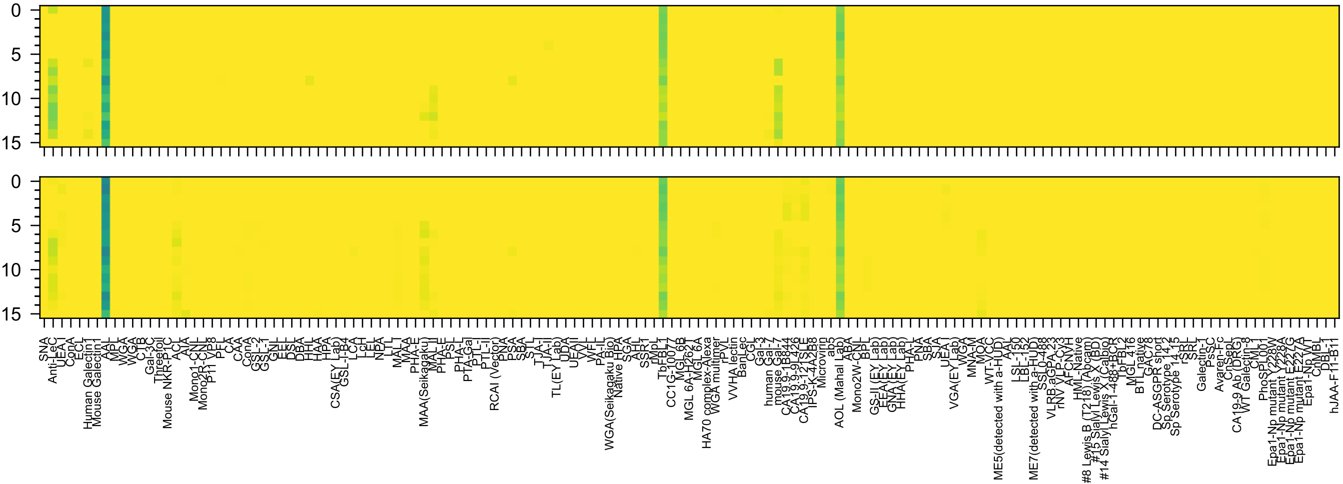

Gal3OS(β1-3)[Fuc(α1-4)]GlcNAc(β-Sp0

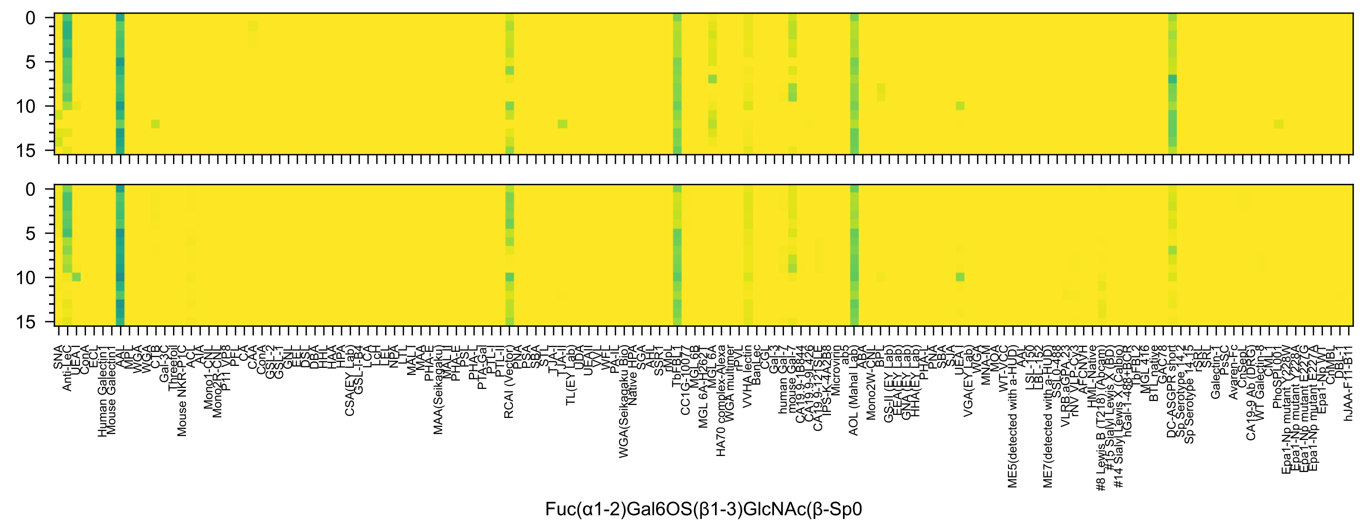

Fuc(α1-2)Gal6OS(β1-3)GlcNAc(β-Sp0

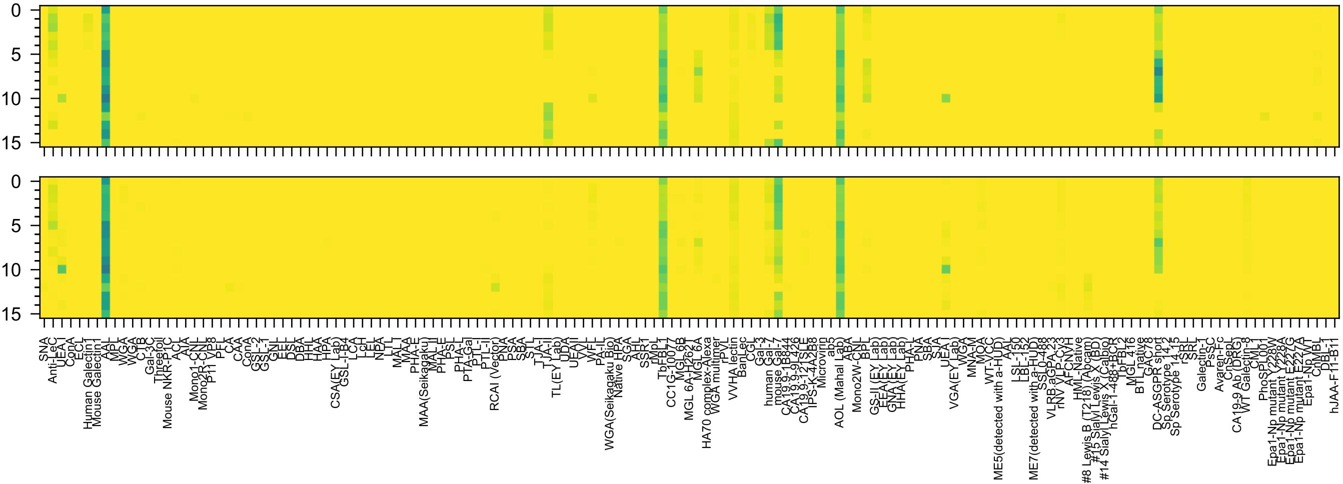
Fuc(α1-2)Gal(β1-3)GlcNAc6OS(β-Sp0

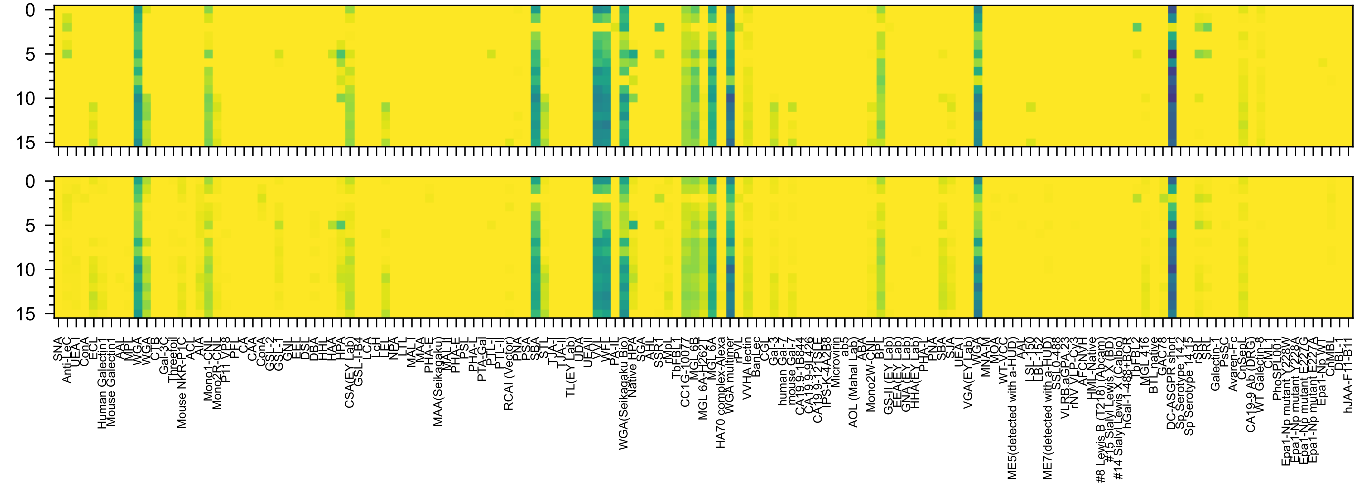

GalNAc(β1-4)GlcNAc(β1-2)Man(α-Sp0

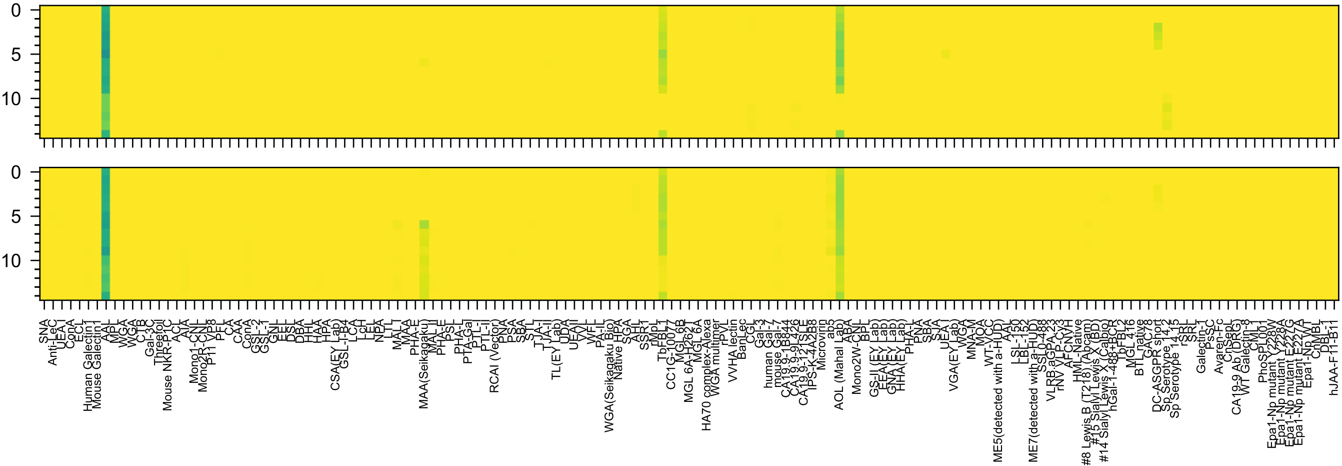

Gal3OS(β1-4)[Fuc(α1-3)]Glc6OS(-Sp0

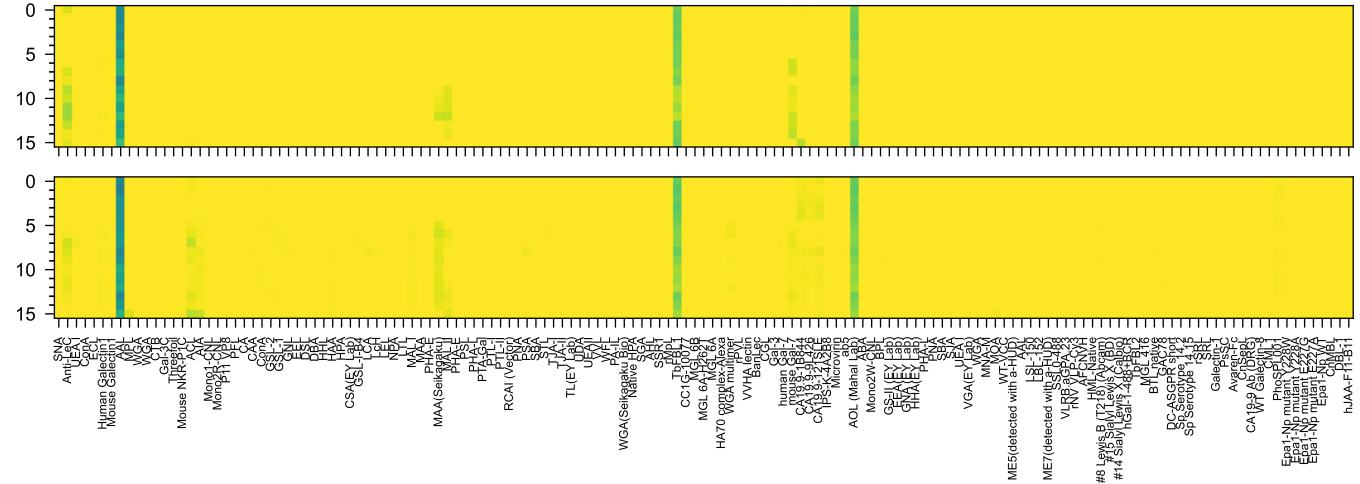

Gal3OS(β1-3)[Fuc(α1-4)]GlcNAc(β-Sp8

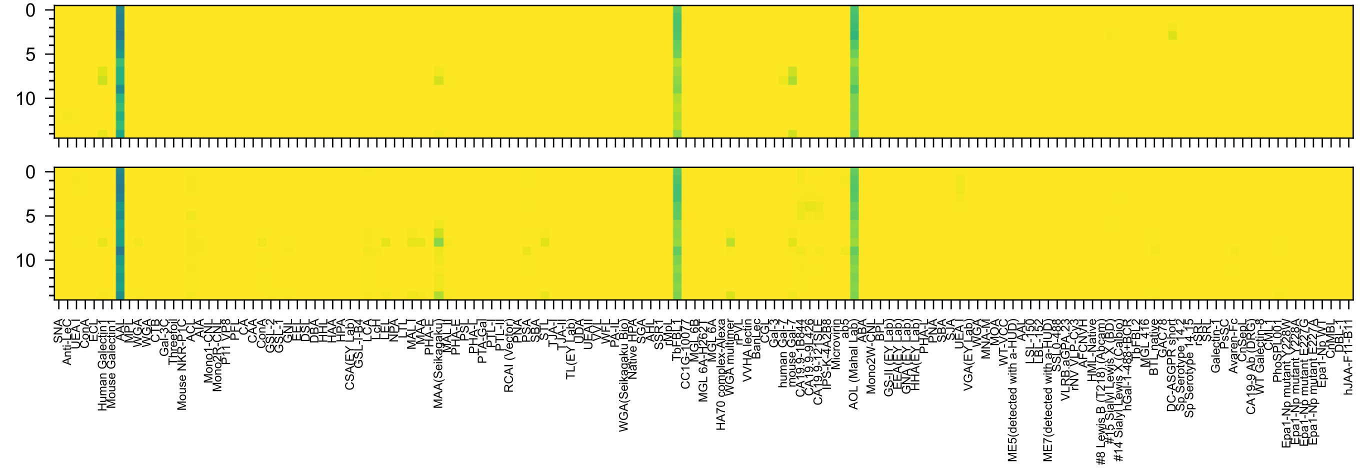

Gal3OS(β1-4)[Fuc(α1-3)]GlcNAc(-Sp8

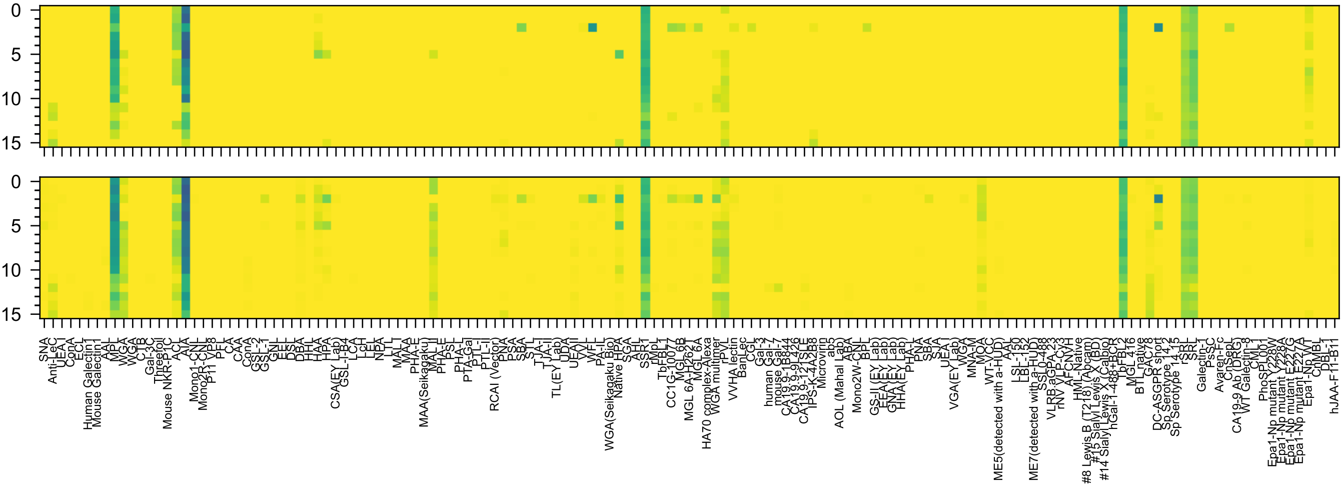
GlcNAc(β1-3)Gal(β1-3)GalNAc(α-Sp8

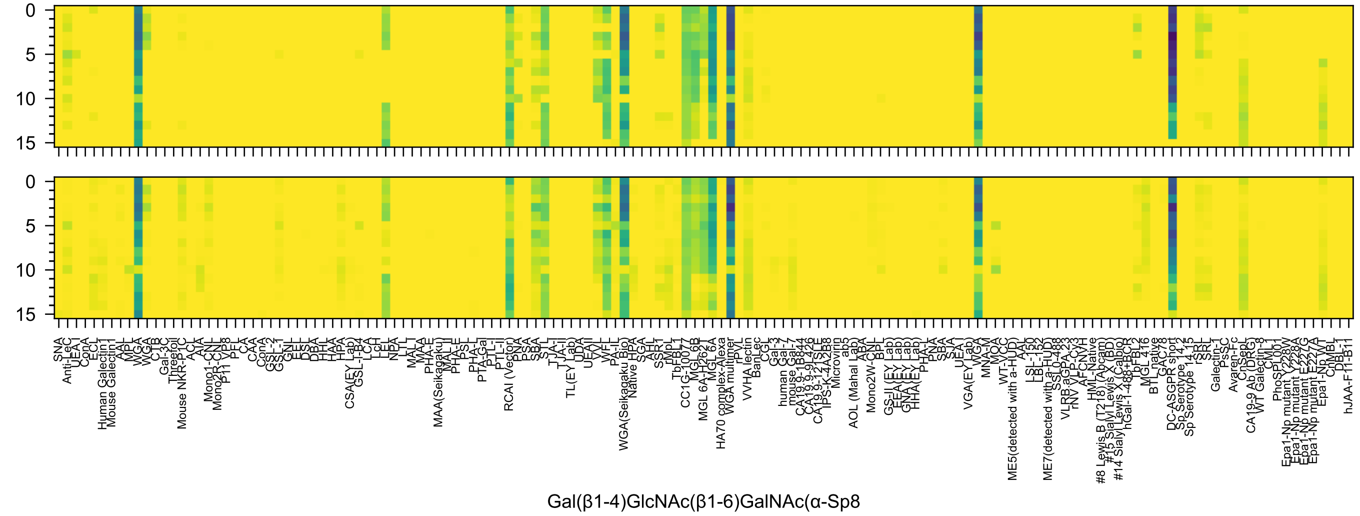
0
5
10
15
0
5
10
15
SNA
Anti-LeC
UEA-I
ConA
ECL
Human Galectin1
Mouse Galectin1
AAL
MPL
WGA
WGA
CTB
C-1B
Gal-3C
Threefoil
Mouse NKR-P1C
ACL
AIA
Mono1-CNL
Mono2R-CNL
P11 VP8
PFL
CA
CAA
ConA
GSL-2
GSL-1
GNL
EEL
DSL
DBA
HHL
HAA
HPA
CSA(EY Lab)
GSL-I-B4
LCA
LcH
LEL
NPA
LTL
MAL-I
MAA
PHA-E
MAA(Seikagaku)
MAL-II
PHA-E
PSL
PHA-L
PTA-Gal
PTL-II
PTL-I
RCAI (Vector)
PNA
PSA
SBA
STL
TJA-I
TJA-II
TL(EY Lab)
UDA
UEAII
VVL
WFL
PA-IL
WGA(Seikagaku Bio)
Native HPA
SGA
AAL
SSR1
rMpL
TbFBL1
CC1G-10077
MGL 6B
MGL 6A-H262T
MGL 6A
HA70 complex-Alexa
WGA multimer
rPVL
VVHA lectin
BanLec
CGL
Gal-3
human Gal-7
mouse Gal-7
CA19.9-1B844
CA19.9-9L426
CA19.9-121SLE
IPS-K-4A2B8
Microvirin
ab5
AOL (Mahal Lab)
ABA
Mono2W-CNL
BPL
GS-II (EY Lab)
EEA(EY Lab)
GNA(EY Lab)
HHA(EY Lab)
PHA-L
PNA
SBA
SJA
UEA-I
VGA(EY Lab)
WGA
MNA-M
MOA
WT-VCC
ME5(detected with a-HUD)
AAL
LSL-150
LBL-152
ME7(detected with a-HUD)
SSL0-488
VLRB.aGPA.23
rNV VLP-Cy3
AFCNVH
HML-Native
#8 Lewis B (T218) (Abcam)
#15 Sialyl Lewis X (BD)
#14 Sialyl Lewis X (Calbio)
hGal-1-488+BCR
TbFBL2
MGL 416
BTL native
GAC78
DC-ASGPR short
Sp Serotype 14.2
Sp Serotype 14.15
rSRL
SRL
Galectin-1
PsSC
Avaren-Fc
ChSepL
CA19-9 Ab (DRG)
WT Galectin-8
CML1
PhoSL001
Epa1-Np mutant Y228W
Epa1-Np mutant Y228A
Epa1-Np mutant E227G
Epa1-Np mutant E227A
Epa1-Np WT
ChMBL
DBL-1
hJAA-F11-B11
Gal(β1-4)GlcNAc(β1-6)GalNAc(α-Sp8

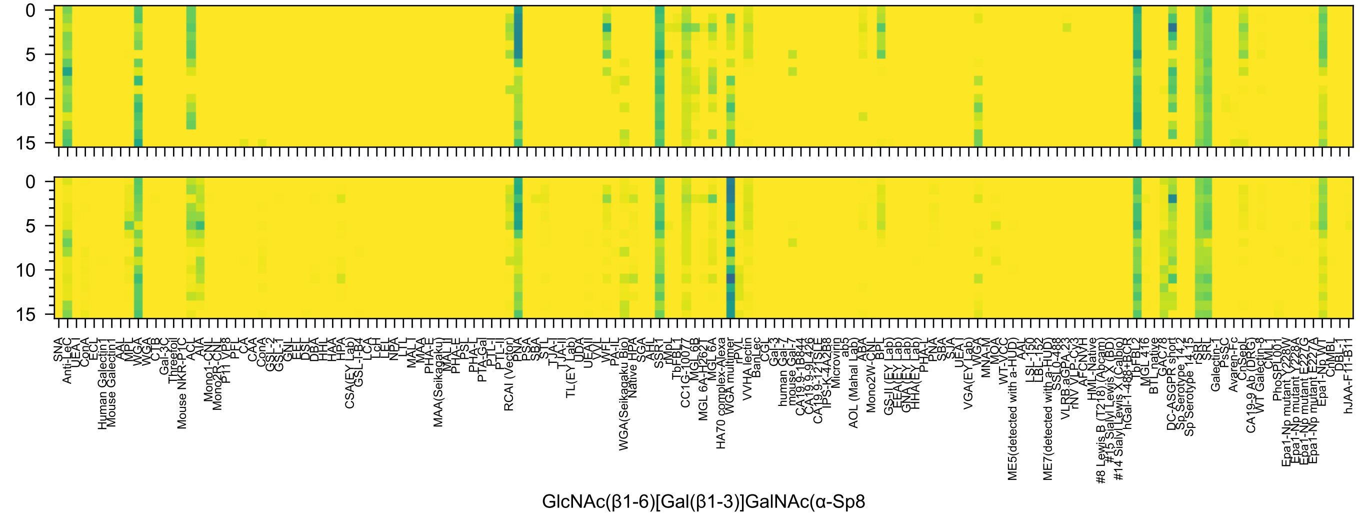

0
5
10
15
0
5
10
15
SNA
Anti-LeC
UEA I
ConA
ECL
Human Galectin1
Mouse Galectin1
AAL
MPL
WGA
WGA
CTB
Gal-3C
Threefoil
Mouse NKR-P1C
ACL
AIA
Mono1-CNL
Mono2R-CNL
P11 VP8
PFL
CA
CAA
ConA
GSL-2
GSL-1
GNL
EEL
DSL
DBA
HHL
HAA
HPA
CSA(EY Lab)
GSL-I-B4
LCA
LcH
LEL
NPA
LTL
MAL I
MAA
PHA-E
MAA(Seikagaku)
MAL II
PHA-E
PSL
PHA-L
PTA-Gal
PTL-I
PTL-II
RCAI (Vector)
PNA
PSA
SBA
STL
TJA-I
TJA-II
TL(EY Lab)
UDA
UEA II
VVL
WFL
PA-IL
WGA(Seikagaku Bio)
Native HPA
SGA
AHL
SSR1
rMpL
TbFBL1
CC1G-10077
MGL 6B
MGL 6A-H262T
MGL 6A
HA70 complex-Alexa
WGA multimer
rPVL
VVHA lectin
BanLec
CGL
Gal-3
human Gal-7
mouse Gal-7
CA19.9-1B844
CA19.9-9L426
CA19.9-121SLE
IPS-K-4A2B8
Microvirin
ab5
AOL (Mahal Lab)
ABA
Mono2W-CNL
BPL
GS-II (EY Lab)
EEA(EY Lab)
GNA(EY Lab)
HHA(EY Lab)
PHA-L
PNA
SBA
SJA
UEA I
VGA(EY Lab)
WGA
MNA-M
MOA
WT-VCC
ME5(detected with a-HUD)
AAL
LSL-150
LBL-152
ME7(detected with a-HUD)
SSL0-488
VLRB.aGPA.23
rNV VLP-Cy3
AFCNVH
HML-Native
#8 Lewis B (T218) (Abcam)
#15 Sialyl Lewis X (BD)
#14 Sialyl Lewis X (Calbio)
hGal-1-488+BCR
TbFBL2
MGL 416
BTL native
GAC78
DC-ASGPR short
Sp Serotype 14.2
Sp Serotype 14.15
rSRL
SRL
Galectin-1
PsSC
Avaren-Fc
CnSepL
CA19-9 Ab (DRG)
WT Galectin-8
CML1
PhoSPL001
Epa1-Np mutant Y228W
Epa1-Np mutant Y228A
Epa1-Np mutant E227G
Epa1-Np mutant E227A
Epa1-Np WT
ChMBL
DBL-1
hJAA-F11-B11
GlcNAc(β1-6)[Gal(β1-3)]GalNAc(α-Sp8

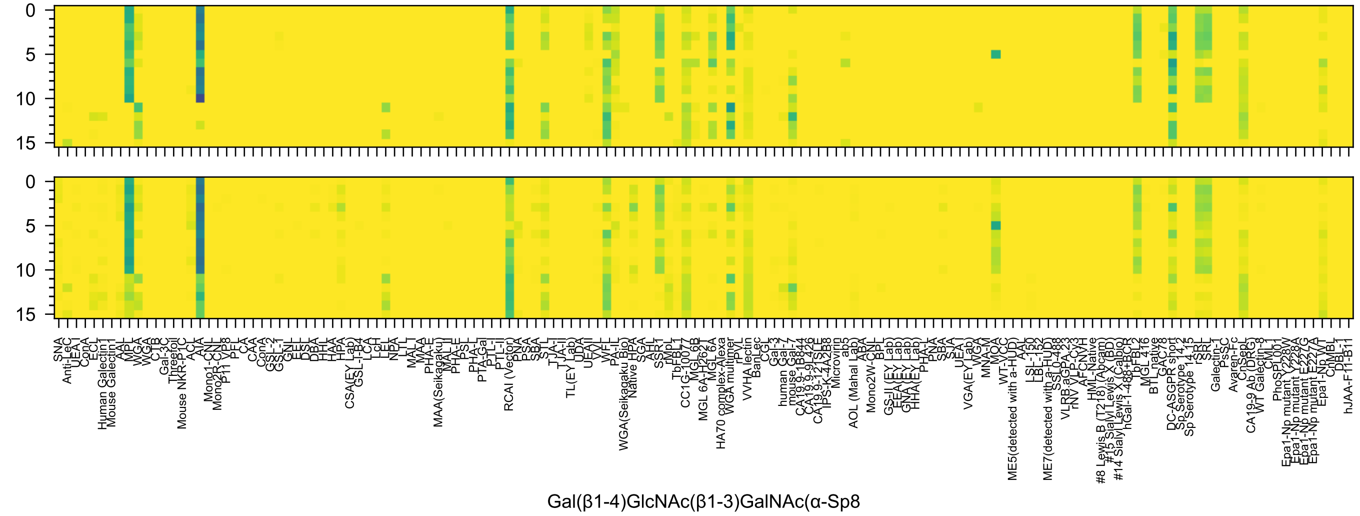
Gal(β1-4)GlcNAc(β1-3)GalNAc(α-Sp8

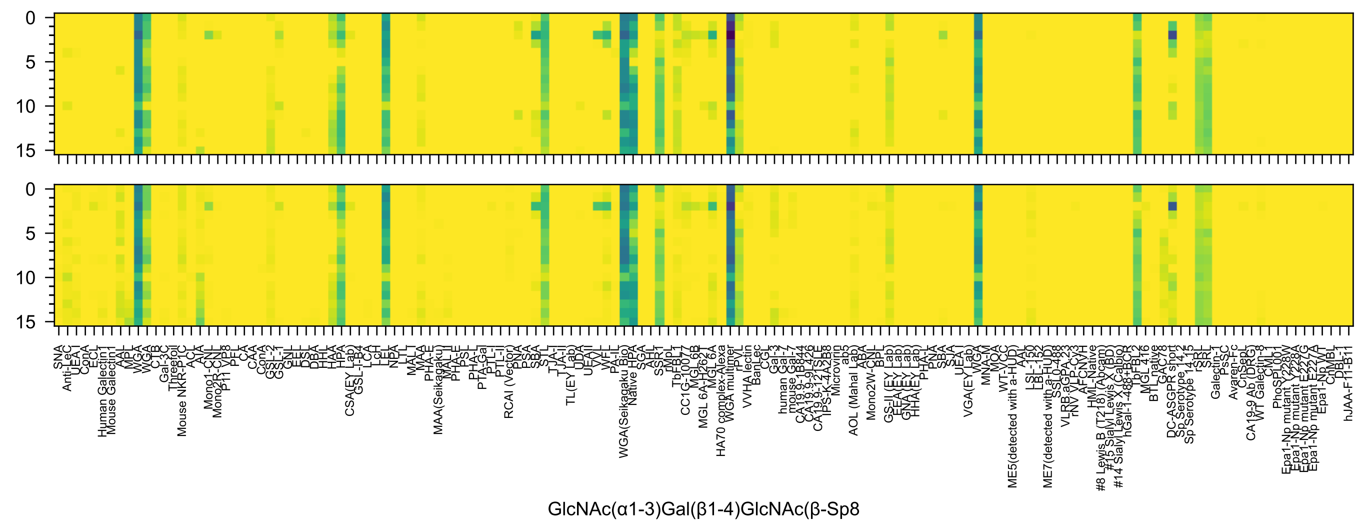
GlcNAc(α1-3)Gal(β1-4)GlcNAc(β-Sp8

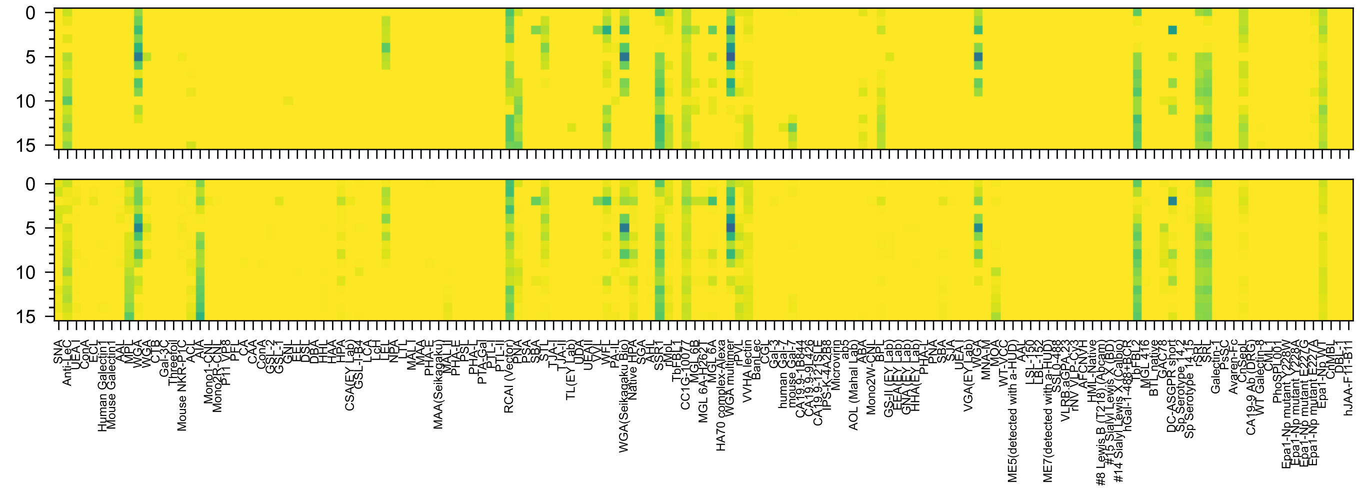

GlcNAc(α1-6)Gal(β1-4)GlcNAc(β-Sp8

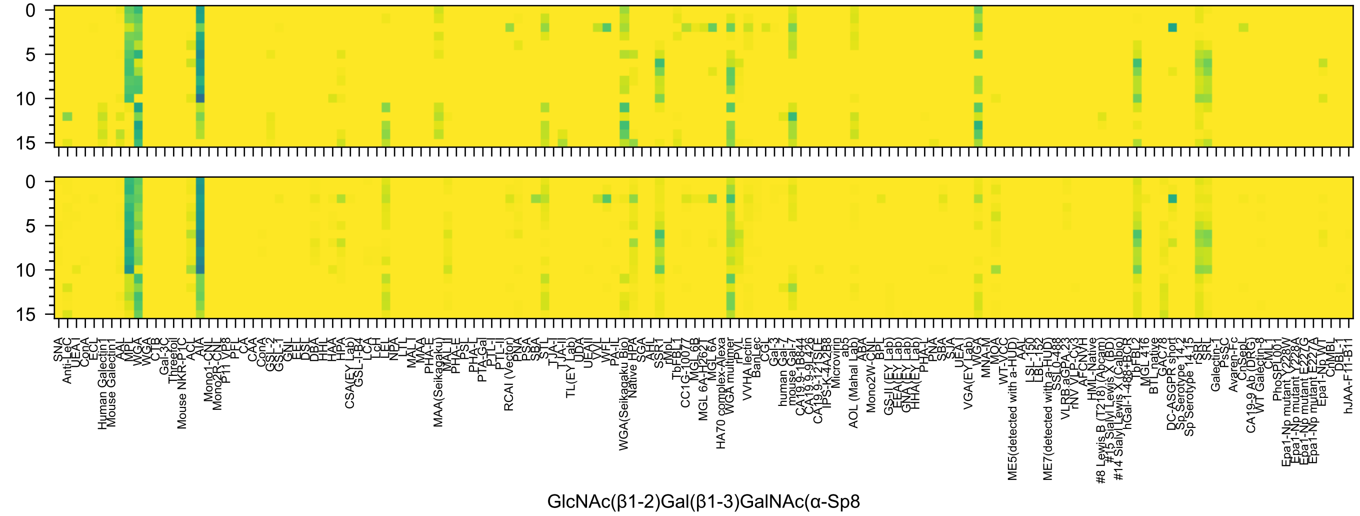

0
5
10
15
SNA
Anti-LeC
UEA-I
ConA
ECL
Human Galectin1
Mouse Galectin1
AAL
MPL
WGA
WGA
CTB
Gal-3C
Threefoil
Mouse NKR-P1C
ACL
AIA
Mono1-CNL
Mono2R-CNL
P11 VP8
PFL
CA
CAA
ConA
GSL-2
GSL-1
GNL
EEL
DSL
DBA
HHL
HAA
HPA
CSA(EY Lab)
GSL-I-B4
LCA
LcH
LEL
NPA
LTL
MAL-I
MAA
PHA-E
MAA(Seikagaku)
MAL-II
PHA-E
PSL
PHA-L
PTA-Gal
PTL-I
PTL-II
RCAI (Vector)
PNA
PSA
SBA
STL
TJA-I
TJA-II
TL(EY Lab)
UDA
UEA-II
VVL
WFL
PA-IL
WGA(Seikagaku Bio)
Native HPA
SGA
AHL
SSR1
rMpL
TbFBL1
CC1G-10077
MGL 6B
MGL 6A-H262T
MGL 6A
HA70 complex-Alexa
WGA multimer
rPVL
VVHA lectin
BanLec
CGL
Gal-3
human Gal-7
mouse Gal-7
CA19.9-1B844
CA19.9-9L426
CA19.9-121SLE
IPS-K-4A2B8
Microvirin
ab5
AOL (Mahal Lab)
ABA
Mono2W-CNL
BPL
GS-II (EY Lab)
EEA(EY Lab)
GNA(EY Lab)
HHA(EY Lab)
PHA-L
PNA
SBA
SJA
UEA-I
VGA(EY Lab)
WGA
MNA-M
MOA
WT-VCC
ME5(detected with a-HUD)
AAL
LSL-150
LBL-152
ME7(detected with a-HUD)
SSL0-488
VLRB.aGPA.23
rNV VLP-Cy3
AFCNVH
HML-Native
#8 Lewis B (T218) (Abcam)
#15 Sialyl Lewis X (BD)
#14 Sialyl Lewis X (Calbio)
hGal-1-488+BCR
TbFBL2
MGL 416
BTL native
GAC78
DC-ASGPR short
Sp Serotype 14.2
Sp Serotype 14.15
rSRL
SRL
Galectin-1
PsSC
Avaren-Fc
ChSeP
CA19-9 Ab (DRG)
WT Galectin-8
CML1
PhoSL001
Epa1-Np mutant Y228W
Epa1-Np mutant Y228A
Epa1-Np mutant E227G
Epa1-Np mutant E227A
Epa1-Np WT
ChMBL
DBL-1
hJAA-F11-B11
GlcNAc(β1-2)Gal(β1-3)GalNAc(α-Sp8

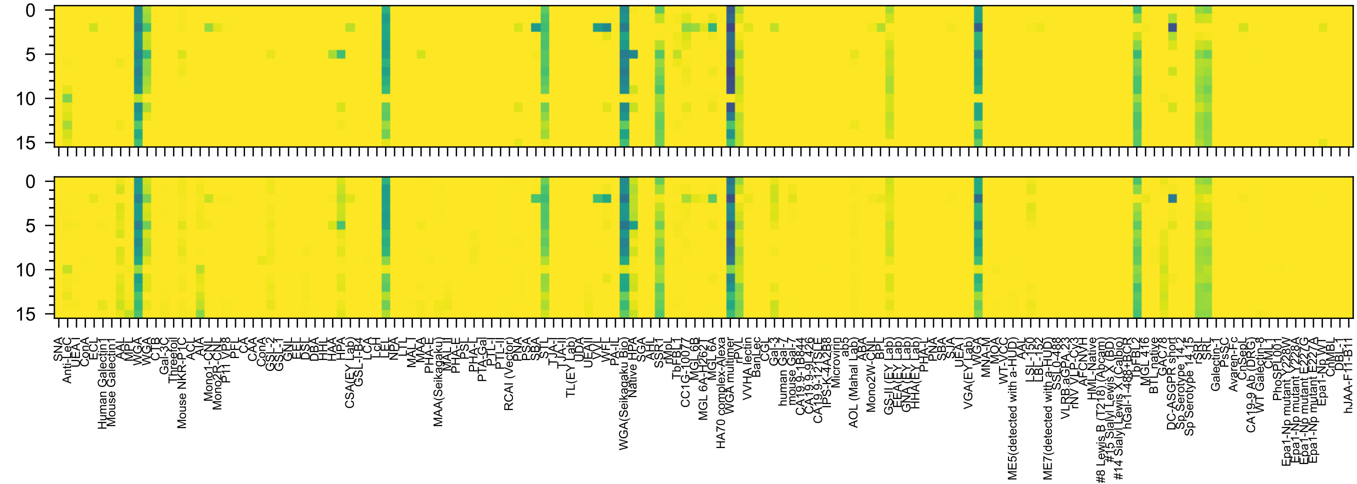

GlcNAc(β1-3)Gal(β1-4)GlcNAc(β-Sp8

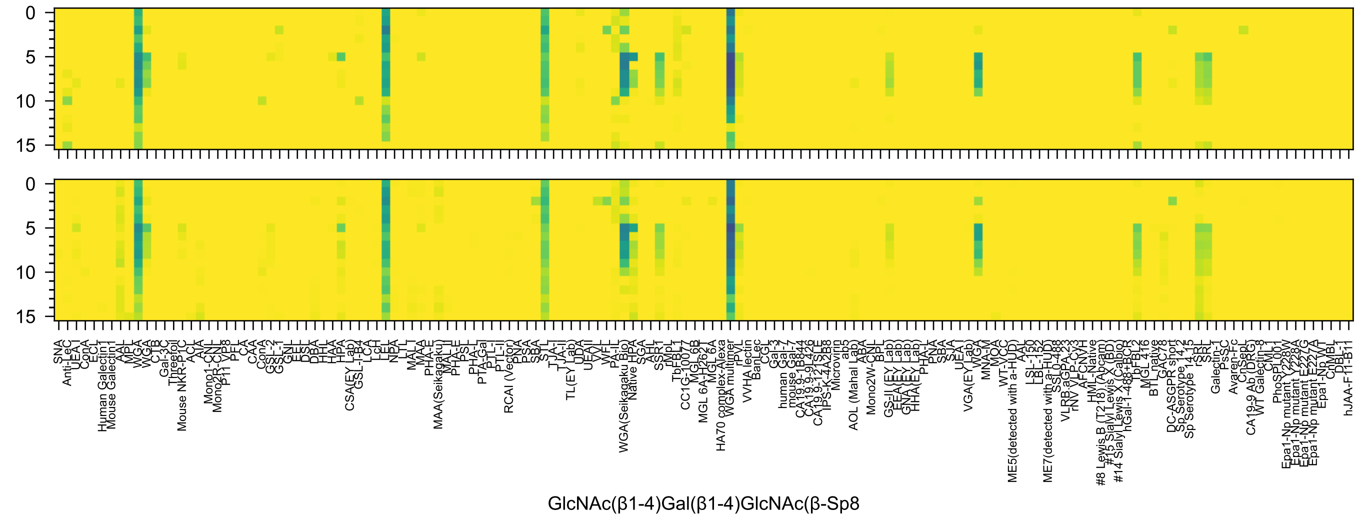
0
5
10
15
0
5
10
15
SNA
Anti-LeC
UEA I
ConA
ECL
Human Galectin1
Mouse Galectin1
AAL
MPL
WGA
WGA
CTB
C-1B
Gal-3C
Threefoil
Mouse NKR-P1C
ACL
AIA
AIA
Mono1-CNL
Mono2R-CNL
P11 VP8
PFL
CA
CAA
ConA
GSL-2
GSL-1
GNL
EEL
DSL
DBA
HHL
HAA
HPA
CSA(EY Lab)
GSL-I-B4
LCA
LcH
LEL
NPA
LTL
MAL
MAA
PHA-E
MAA(Seikagaku)
MAL-II
PHA-E
PSL
PHA-L
PTA-Gal
PTL-I
PTL-II
RCAI (Vector)
PNA
PSA
SBA
STL
TJA-I
TJA-II
TL(EY Lab)
UDA
UEAII
VVL
WFL
PA-IL
WGA(Seikagaku Bio)
Native HPA
SGA
AHL
SSR1
rMpL
TbFBL1
CC1G-10077
MGL 6B
MGL 6A-H262T
MGL 6A
HA70 complex-Alexa
WGA multimer
rPVL
VVHA lectin
BanLec
CGL
Gal-3
human Gal-7
mouse Gal-7
CA19.9-1B844
CA19.9-9L426
CA19.9-121SLE
IPS-K-4A2B8
Microvirin
ab5
AOL (Mahal Lab)
ABA
Mono2W-CNL
BPL
GS-II (EY Lab)
EEA(EY Lab)
GNA(EY Lab)
HHA(EY Lab)
PHA-L
PNA
SBA
SJA
UEA-I
VGA(EY Lab)
WGA
MNA-M
MOA
WT-VCC
ME5(detected with a-HUD)
AAL
LSL-150
LBL-152
ME7(detected with a-HUD)
SSL0-488
VLRB.aGPA.23
rNV VLP-Cy3
AFCNVH
HML-Native
#8 Lewis B (T218) (Abcam)
#15 Sialyl Lewis X (BD)
#14 Sialyl Lewis X (Calbio)
hGal-1-488+BCR
TbFBL2
MGL 416
BTL native
GAC78
DC-ASGPR short
Sp Serotype 14.2
Sp Serotype 14.15
rSRL
SRL
Galectin-1
PsSC
Avaren-Fc
ChSepL
CA19-9 Ab (DRG)
WT Galectin-8
CML1
PhoSL001
Epa1-Np mutant Y228W
Epa1-Np mutant Y228A
Epa1-Np mutant E227G
Epa1-Np mutant E227A
Epa1-Np WT
ChMBL
DBL-1
hJAA-F11-B11
GlcNAc(β1-4)Gal(β1-4)GlcNAc(β-Sp8

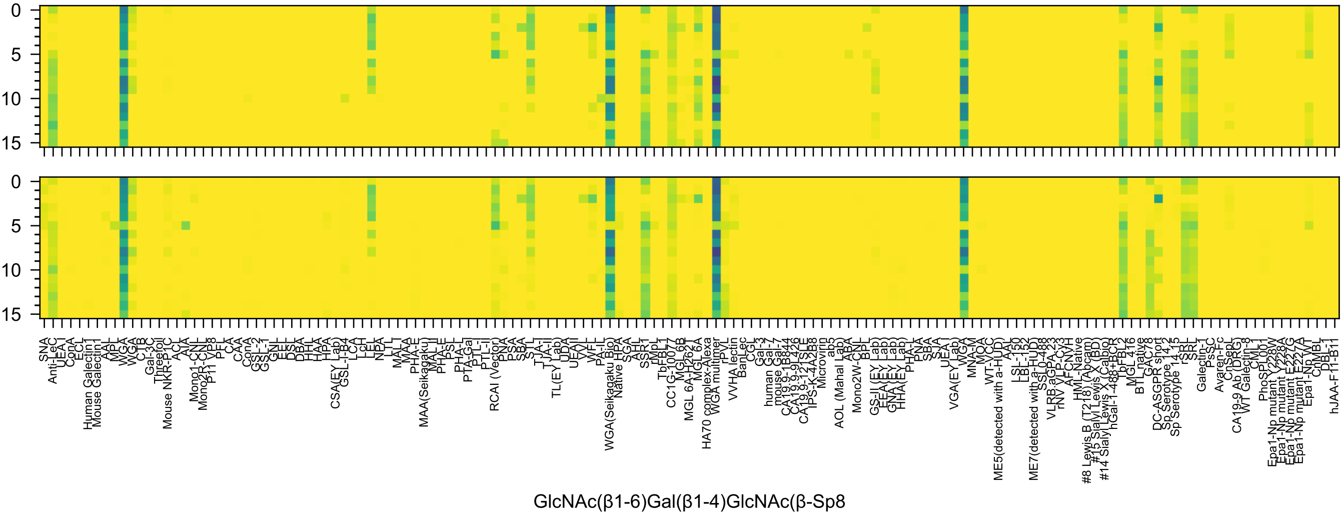
GlcNAc(β1-6)Gal(β1-4)GlcNAc(β-Sp8

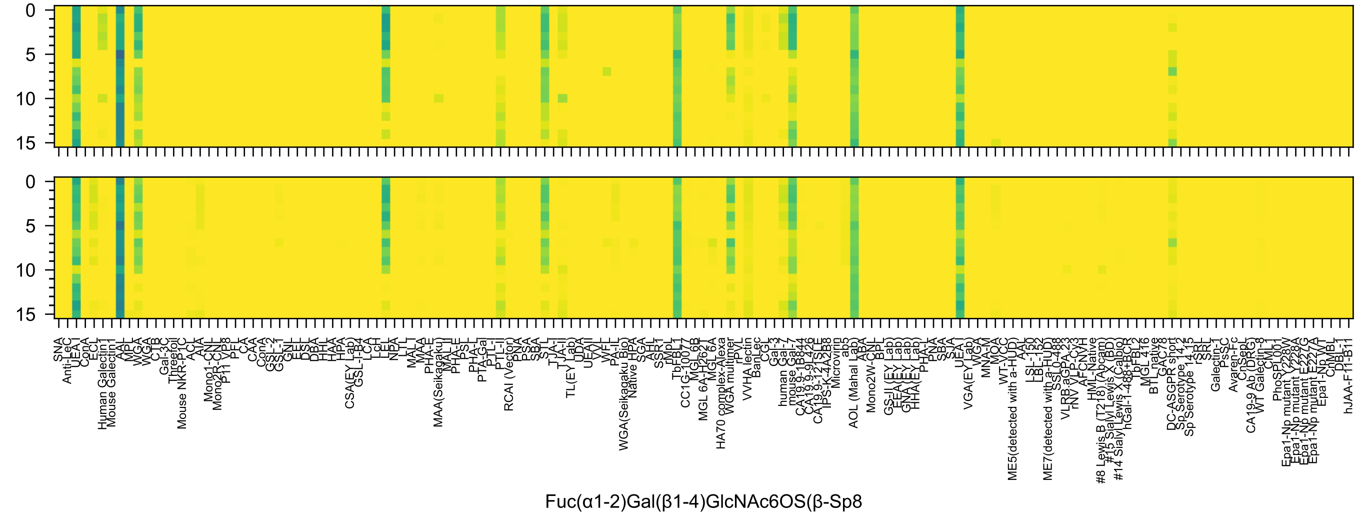
0
5
10
15
0
5
10
15
SNA
Anti-LeC
UEA I
ConA
ECL
Human Galectin1
Mouse Galectin1
AAL
MPL
WGA
WGA
CTB
C-1
Gal-3C
Threefoil
Mouse NKR-P1C
ACL
AIA
Mono1-CNL
Mono2R-CNL
P11 VP8
PFL
CA
CAA
ConA
GSL-2
GSL-1
GNL
EEL
DSL
DBA
HHL
HAA
HPA
CSA(EY Lab)
GSL-I-B4
LCA
LcH
LEL
NPA
LTL
MAL-I
MAA
PHA-E
MAA(Seikagaku)
MAL-II
PHA-E
PSL
PHA-L
PTA-Gal
PTL-I
PTL-II
RCAI (Vector)
PNA
PSA
SBA
STL
TJA-I
TJA-II
TL(EY Lab)
UDA
UEA-II
VVL
WFL
PA-IL
WGA(Seikagaku Bio)
Native HPA
SGA
AHL
SSR1
rMpL
TbFBL1
CC1G-10077
MGL 6B
MGL 6A-H262T
MGL 6A
HA70 complex-Alexa
WGA multimer
rPVL
VVHA lectin
BanLec
CGL
Gal-3
human Gal-7
mouse Gal-7
CA19.9-1B844
CA19.9-9L426
CA19.9-121SLE
IPS-K-4A2B8
Microvirin
ab5
AOL (Mahal Lab)
ABA
Mono2W-CNL
BPL
GS-II (EY Lab)
EEA(EY Lab)
GNA(EY Lab)
HHA(EY Lab)
PHA-L
PNA
SBA
SJA
UEA-I
VGA(EY Lab)
WGA
MNA-M
MOA
WT-VCC
ME5(detected with a-HUD)
AAL
LSL-150
LBL-152
ME7(detected with a-HUD)
SS10-488
VLRB.aGPA.23
rNV VLP-Cy3
AFCNVH
HML-Native
#8 Lewis B (T218) (Abcam)
#15 Sialyl Lewis X (BD)
#14 Sialyl Lewis X (Calbio)
hGal-1-488+BCR
TbFBL2
MGL 416
BTL native
GAC78
DC-ASGPR short
Sp Serotype 14.2
Sp Serotype 14.15
rSRL
SRL
Galectin-1
PsSC
Avaren-Fc
ChSepL
CA19-9 Ab (DRG)
WT Galectin-8
CML1
PhoSL001
Epa1-Np mutant Y228W
Epa1-Np mutant Y228A
Epa1-Np mutant E227G
Epa1-Np mutant E227A
Epa1-Np WT
ChMBL
DBL-1
hJAA-F11-B11
Fuc(α1-2)Gal(β1-4)GlcNAc6OS(β-Sp8

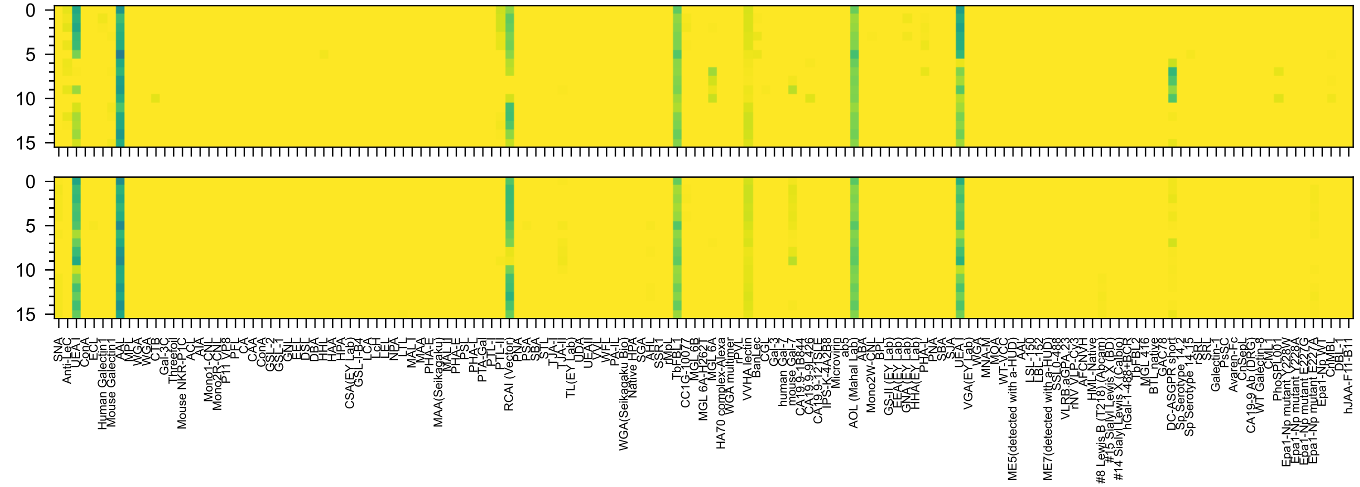

Fuc(α1-2)Gal6OS(β1-4)Glc6OS(β-Sp0

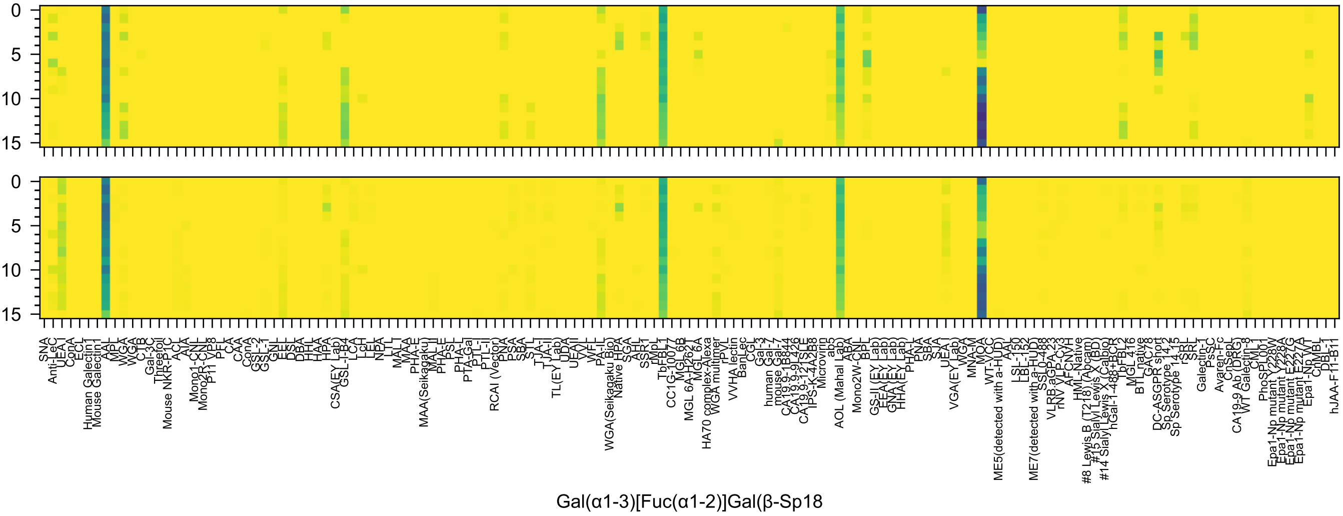
Gal(α1-3)[Fuc(α1-2)]Gal(β-Sp18

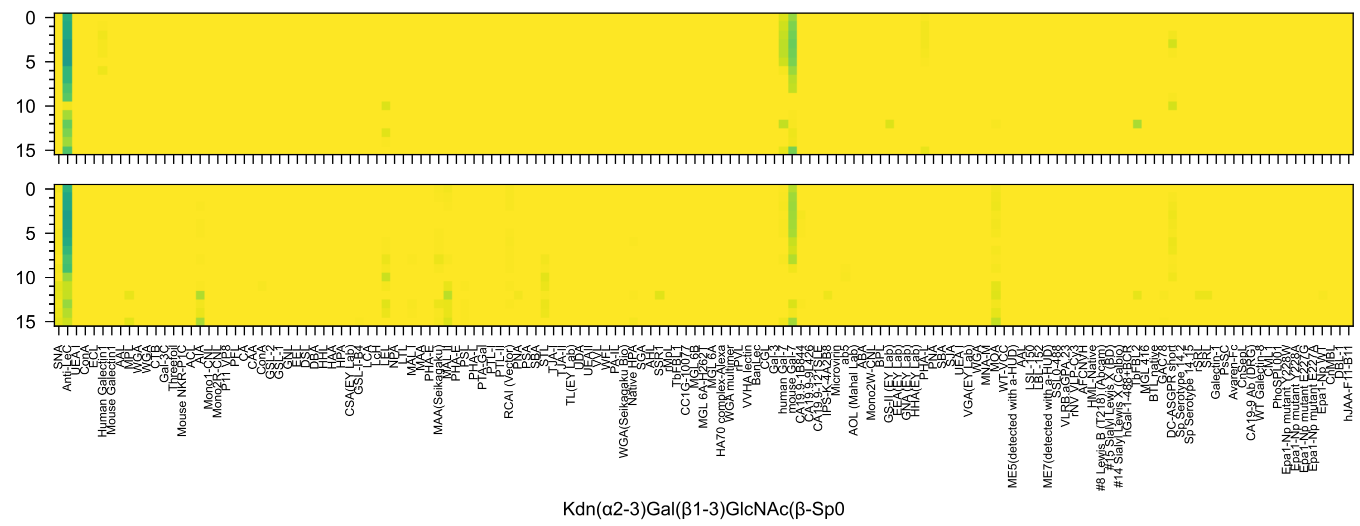
0
5
10
15
Kdn(α2-3)Gal(β1-3)GlcNAc(β-Sp0

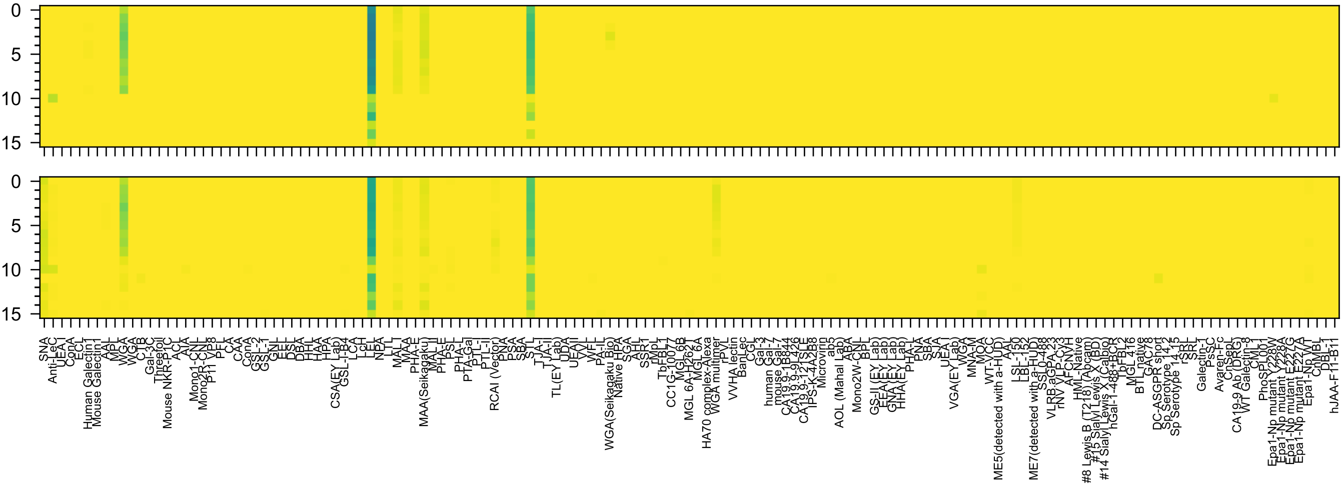

Kdn(α2-3)Gal(β1-4)GlcNAc(β-Sp0

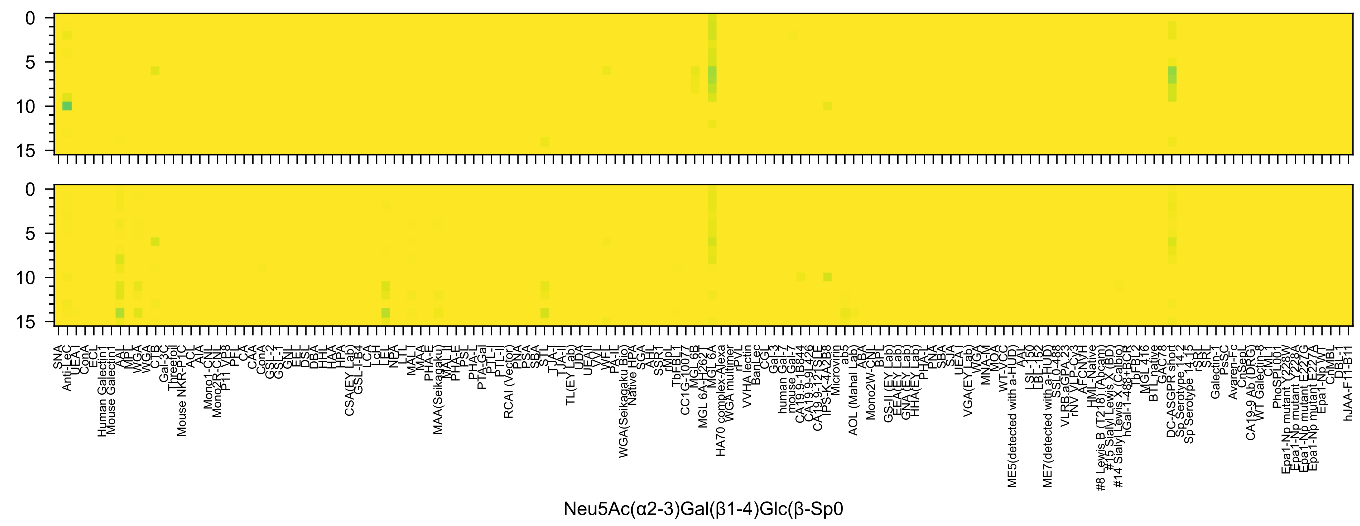
0
5
10
15
SNA
Anti-LeC
UEA-I
ConA
ECL
Human Galectin1
Mouse Galectin1
AAL
MPL
WGA
WGA
CTB
Gal-3C
Threefoil
Mouse NKR-P1C
ACL
AIA
Mono1-CNL
Mono2R-CNL
P11 VP8
PFL
CA
CAA
ConA
GSL-2
GSL-1
GNL
EEL
DSL
DBA
HHL
HAA
HPA
CSA(EY Lab)
GSL-I-B4
LCA
LcH
LEL
LEL
NPA
LTL
MAL-I
MAA
PHA-E
MAA(Seikagaku)
MAL-II
PHA-E
PSL
PHA-L
PTA-Gal
PTL-I
PTL-II
RCAI (Vector)
PNA
PSA
SBA
STL
TJA-I
TJA-II
TL(EY Lab)
UDA
UEA-II
VVL
WFL
PA-IL
WGA(Seikagaku Bio)
Native HPA
SGA
AAL
SSR1
rMpL
TbFBL1
CC1G-10077
MGL 6B
MGL 6A-H262T
MGL 6A
HA70 complex-Alexa
WGA multimer
rPVL
VVHA lectin
BanLec
CGL
Gal-3
human Gal-7
mouse Gal-7
CA19.9-1B844
CA19.9-9L426
CA19.9-121SLE
IPS-K-4A2B8
Microvirin
ab5
AOL (Mahal Lab)
ABA
Mono2W-CNL
BPL
GS-II (EY Lab)
EEA(EY Lab)
GNA(EY Lab)
HHA(EY Lab)
PHA-L
PNA
SBA
SJA
UEA-I
VGA(EY Lab)
WGA
MNA-M
MOA
WT-VCC
ME5(detected with a-HUD)
AAL
LSL-150
LBL-152
ME7(detected with a-HUD)
SSL0-488
VLRB.aGPA.23
rNV VLP-Cy3
AFCNVH
HML-Native
#8 Lewis B (T218) (Abcam)
#15 Sialyl Lewis X (BD)
#14 Sialyl Lewis X (Calbio)
hGal-1-488+BCR
TbFBL2
MGL 416
BTL native
GAC78
DC-ASGPR short
Sp Serotype 14.2
Sp Serotype 14.15
rSRL
SRL
Galectin-1
PsSC
Avaren-Fc
ChSeaL
CA19-9 Ab (DRG)
WT Galectin-8
CML1
PhoSL001
Epa1-Np mutant Y228W
Epa1-Np mutant Y228A
Epa1-Np mutant E227G
Epa1-Np mutant E227A
Epa1-Np WT
ChMBL
DBL-1
hJAA-F11-B11
Neu5Ac(α2-3)Gal(β1-4)Glc(β-Sp0

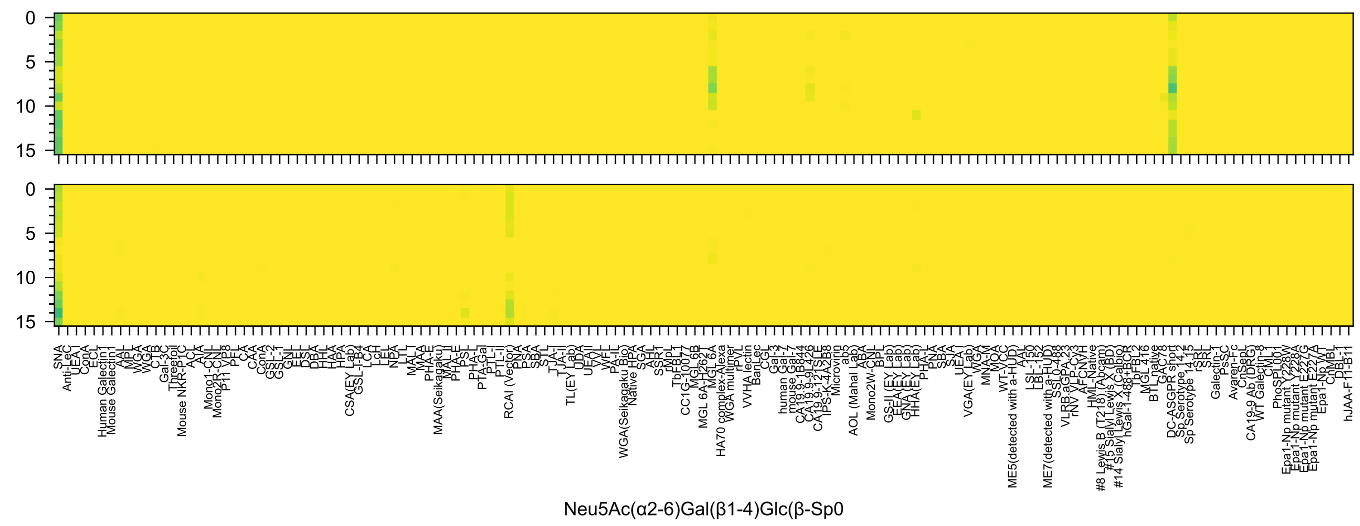
Neu5Ac(α2-6)Gal(β1-4)Glc(β-Sp0

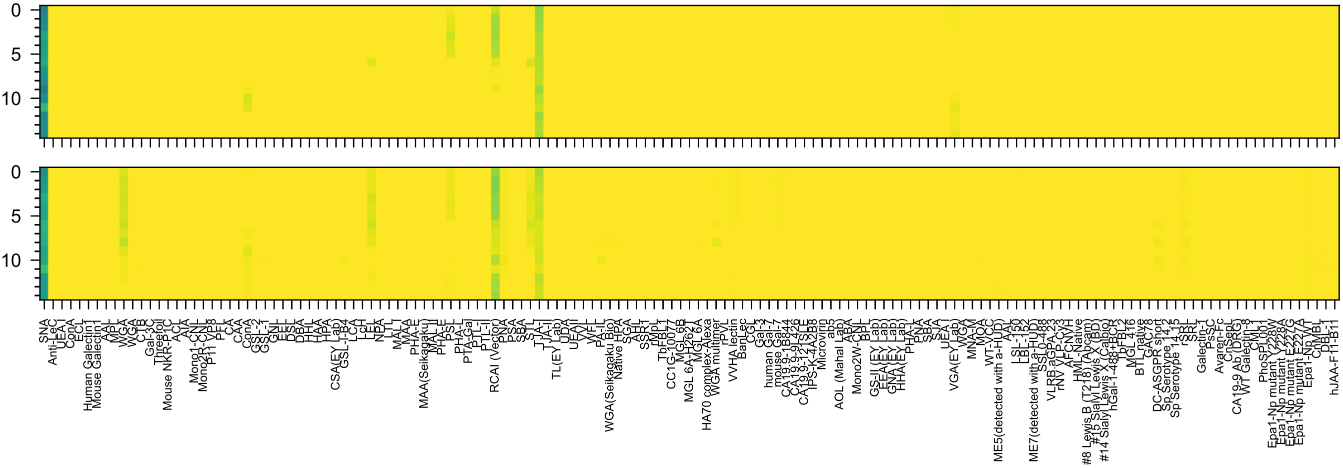
Kdn(α2-6)Gal(β1-4)GlcNAc(-Sp0

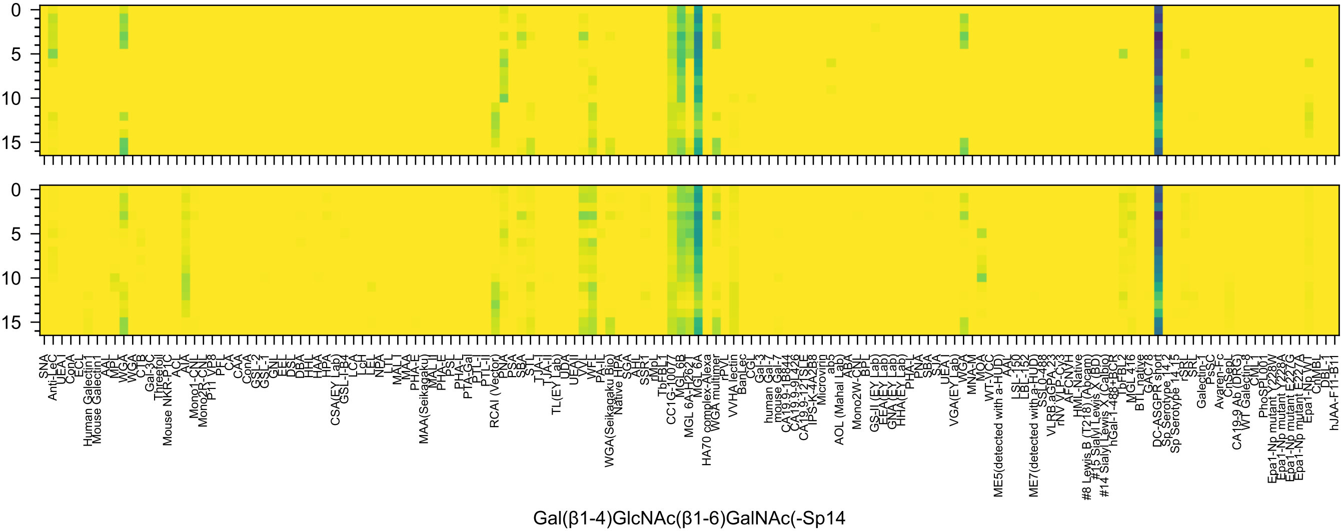
Gal(β1-4)GlcNAc(β1-6)GalNAc(-Sp14

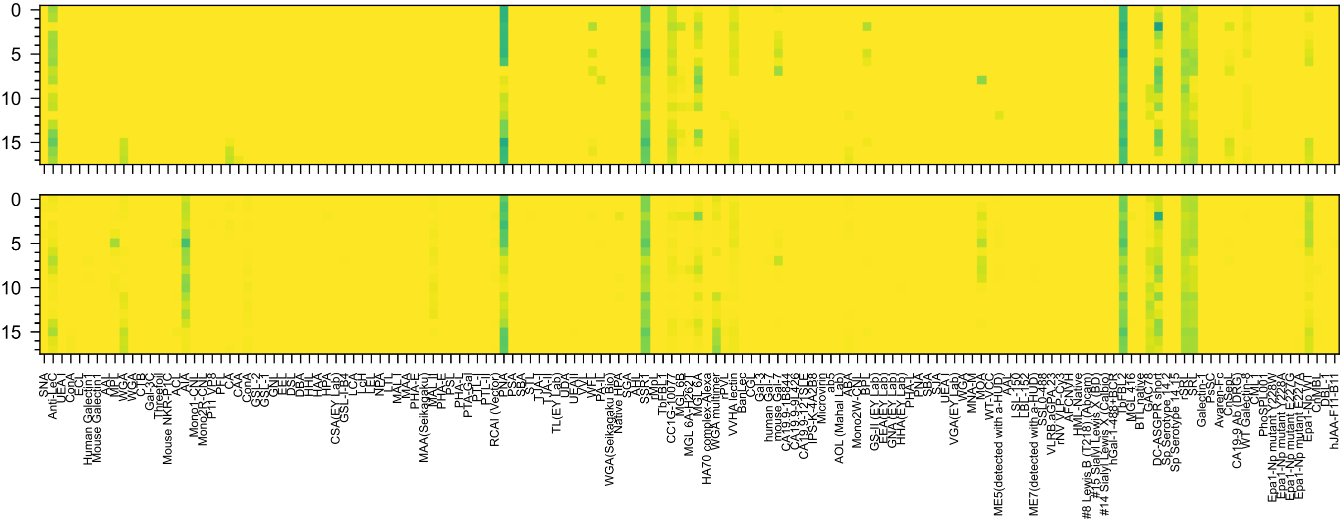
GlcNAc(β1-6)[Gal(β1-3)]GalNAc(α-Sp14

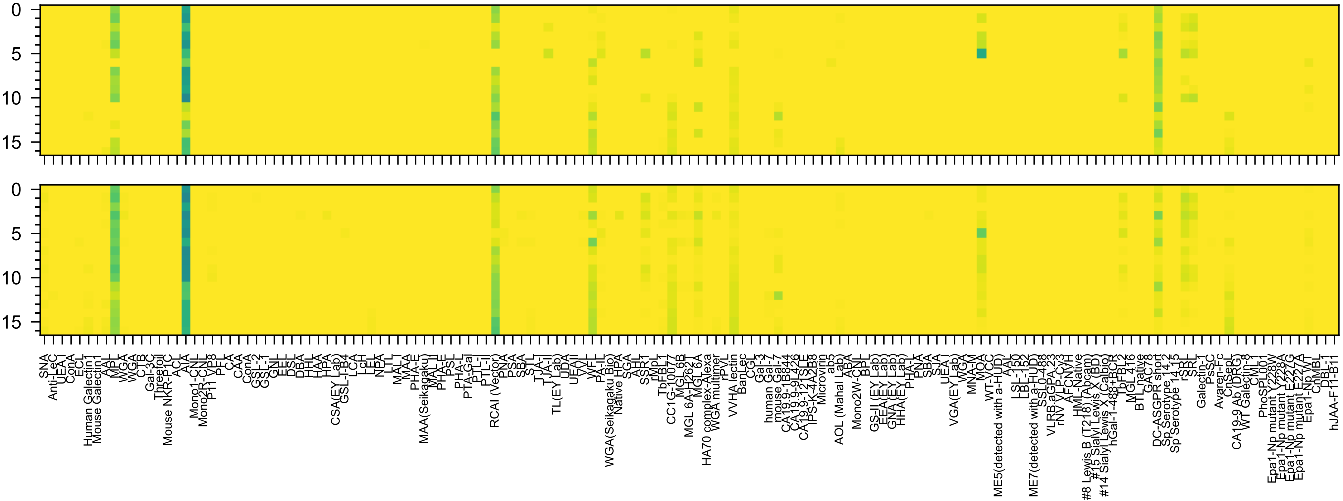

Gal(β1-4)GlcNAc(β1-3)GalNAc(-Sp14

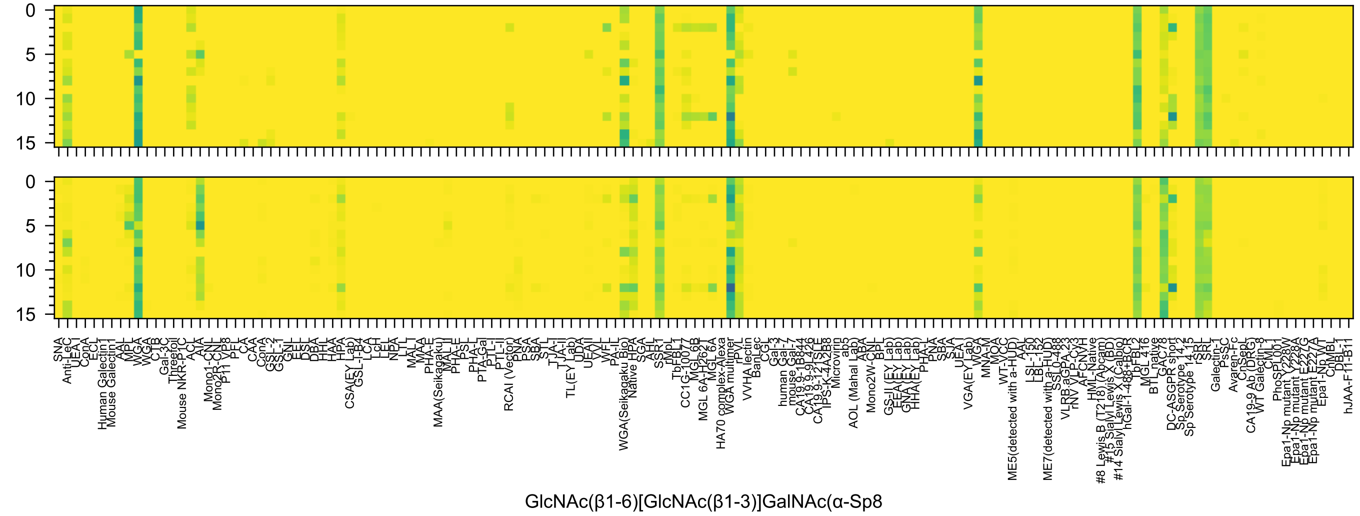
GlcNAc(β1-6)[GlcNAc(β1-3)]GalNAc(α-Sp8

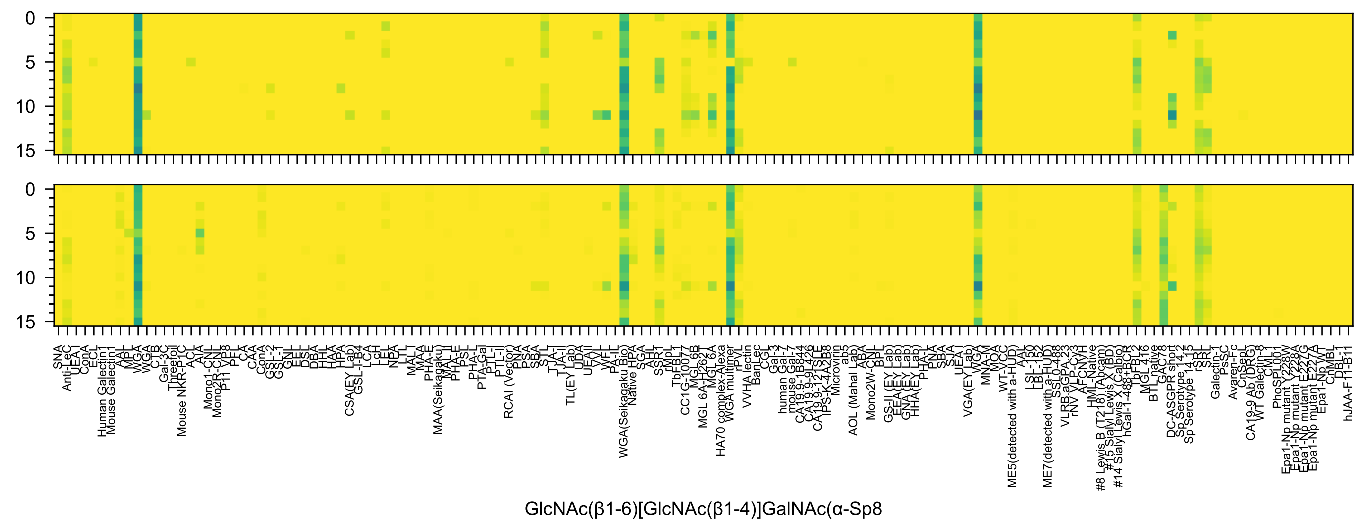
0
5
10
15
0
5
10
15
SNA
Anti-LeC
UEA-I
ConA
ECL
Human Galectin1
Mouse Galectin1
AAL
MPL
WGA
WGA
CTB
Gal-3C
Threefoil
Mouse NKR-P1C
ACL
AIA
Mono1-CNL
Mono2R-CNL
P11 VP8
PFL
CA
CAA
ConA
GSL-2
GSL-1
GNL
EEL
DSL
DBA
HHL
HAA
HPA
CSA(EY Lab)
GSL-I-B4
LCA
LcH
LEL
NPA
LTL
MAL-I
MAA
PHA-E
MAA(Seikagaku)
MAL-II
PHA-E
PSL
PHA-L
PTA-Gal
PTL-I
PTL-II
RCAI (Vector)
PNA
PSA
SBA
STL
TJA-I
TJA-II
TL(EY Lab)
UDA
UEA-II
VVL
WFL
PA-IL
WGA(Seikagaku Bio)
Native HPA
SGA
AAL
SSR1
rMpL
TbFBL1
CC1G-10077
MGL 6B
MGL 6A-H262T
MGL 6A
HA70 complex-Alexa
WGA multimer
rPVL
VVHA lectin
BanLec
CGL
Gal-3
human Gal-7
mouse Gal-7
CA19.9-1B844
CA19.9-9L426
CA19.9-121SLE
IPS-K-4A2B8
Microvirin
ab5
AOL (Mahal Lab)
ABA
Mono2W-CNL
BPL
GS-II (EY Lab)
EEA(EY Lab)
GNA(EY Lab)
HHA(EY Lab)
PHA-L
PNA
SBA
SJA
UEA-I
VGA(EY Lab)
WGA
MNA-M
MOA
WT-VCC
ME5(detected with a-HUD)
AAL
LSL-150
LBL-152
ME7(detected with a-HUD)
SSL0-488
VLRB.aGPA.23
rNV VLP-Cy3
AFCNVH
HML-Native
#8 Lewis B (T218) (Abcam)
#15 Sialyl Lewis X (BD)
#14 Sialyl Lewis X (Calbio)
hGal-1-488+BCR
TbFBL2
MGL 416
BTL native
GAC78
DC-ASGPR short
Sp Serotype 14.2
Sp Serotype 14.15
rSRL
SRL
Galectin-1
PsSC
Avaren-Fc
ChSepL
CA19-9 Ab (DRG)
WT Galectin-8
CML1
PhoSL001
Epa1-Np mutant Y228W
Epa1-Np mutant Y228A
Epa1-Np mutant E227G
Epa1-Np mutant E227A
Epa1-Np WT
ChMBL
DBL-1
hJAA-F11-B11
GlcNAc(β1-6)[GlcNAc(β1-4)]GalNAc(α-Sp8

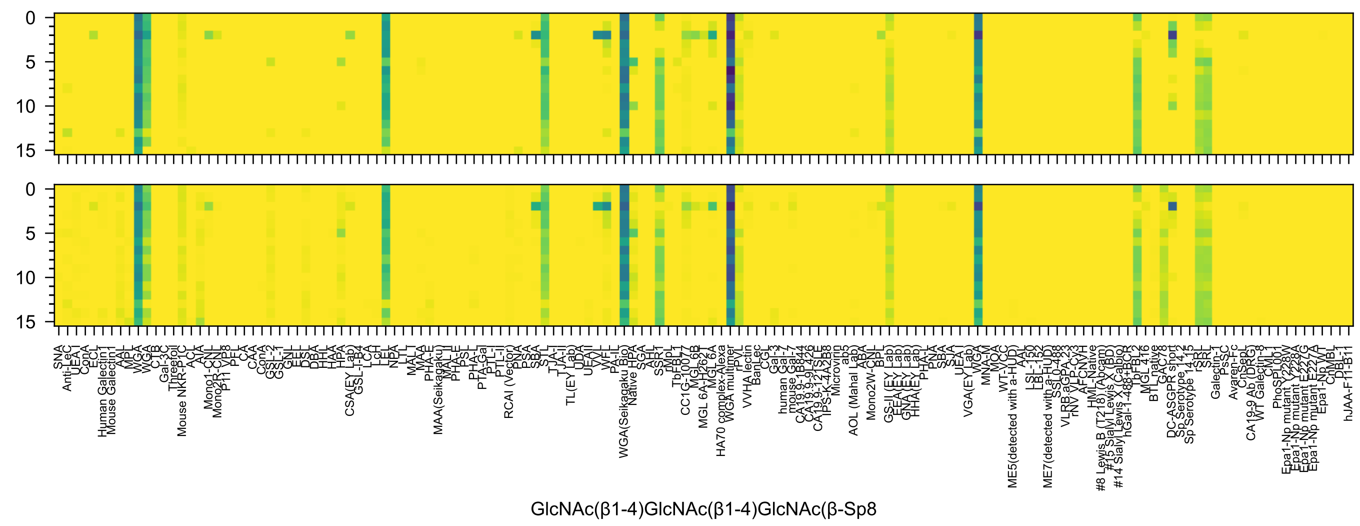
0
5
10
15
GlcNAc(β1-4)GlcNAc(β1-4)GlcNAc(β-Sp8

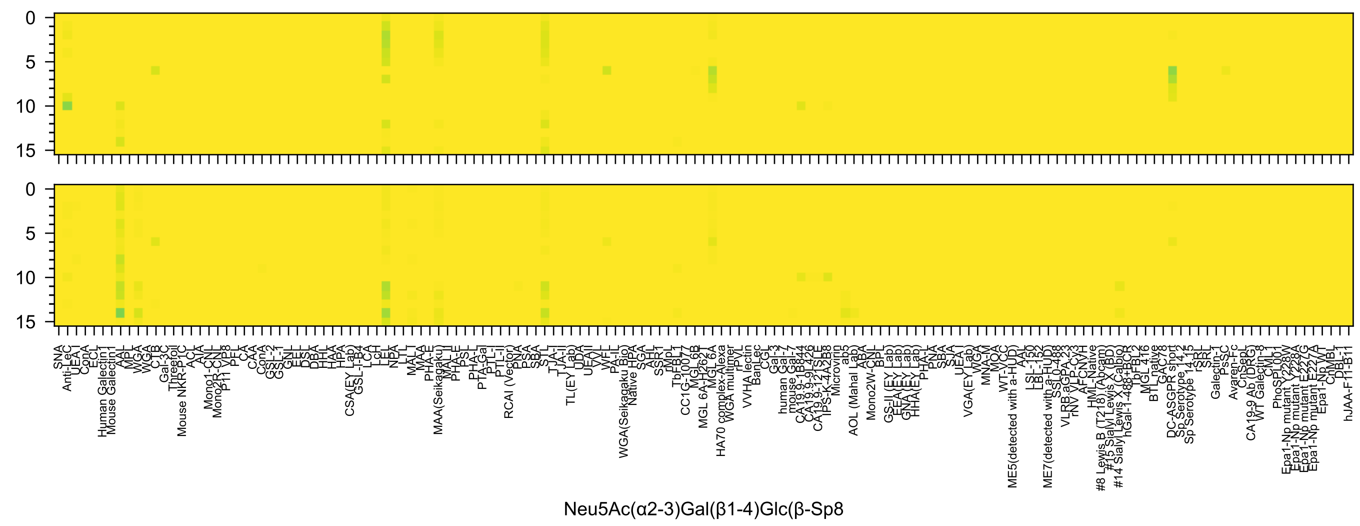
Neu5Ac(α2-3)Gal(β1-4)Glc(β-Sp8

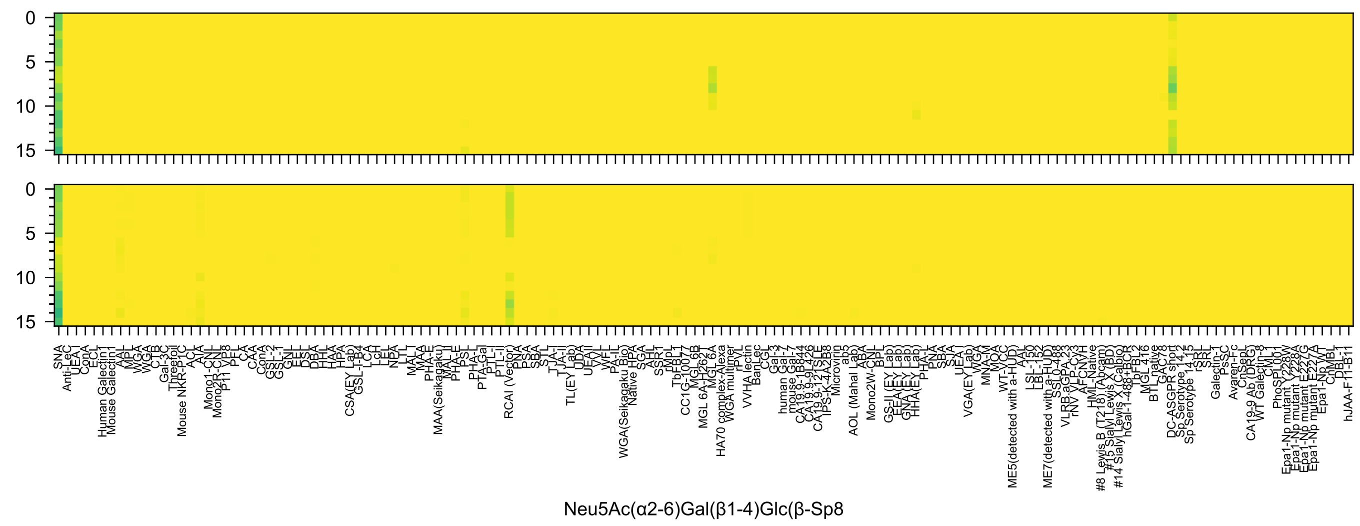

Neu5Ac(α2-6)Gal(β1-4)Glc(β-Sp8

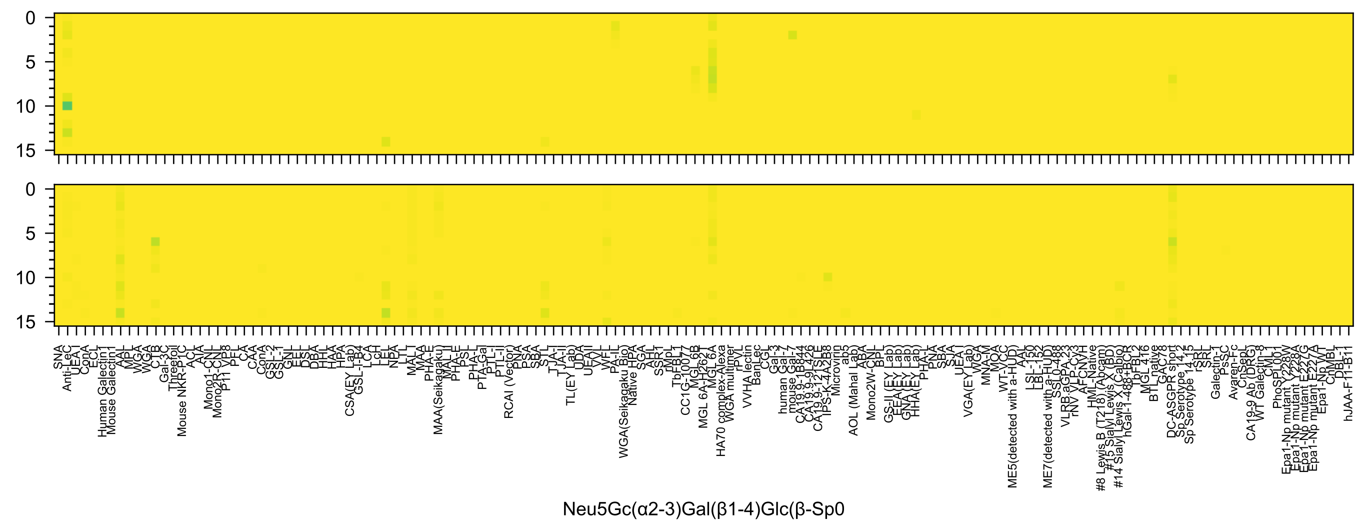

Neu5Gc(α2-3)Gal(β1-4)Glc(β-Sp0

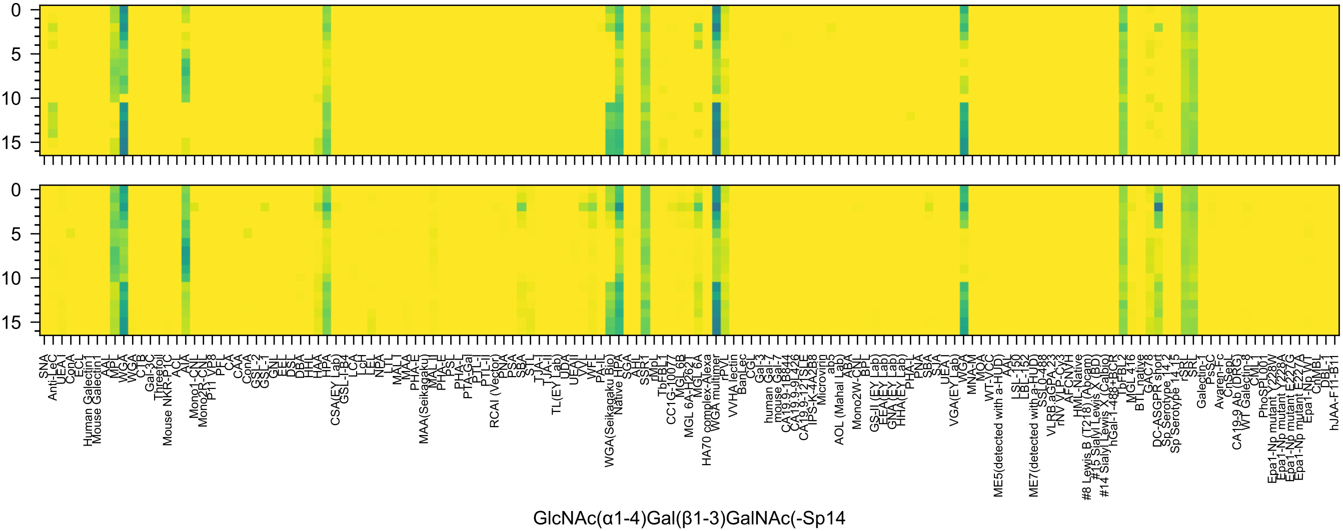
GlcNAc(α1-4)Gal(β1-3)GalNAc(-Sp14

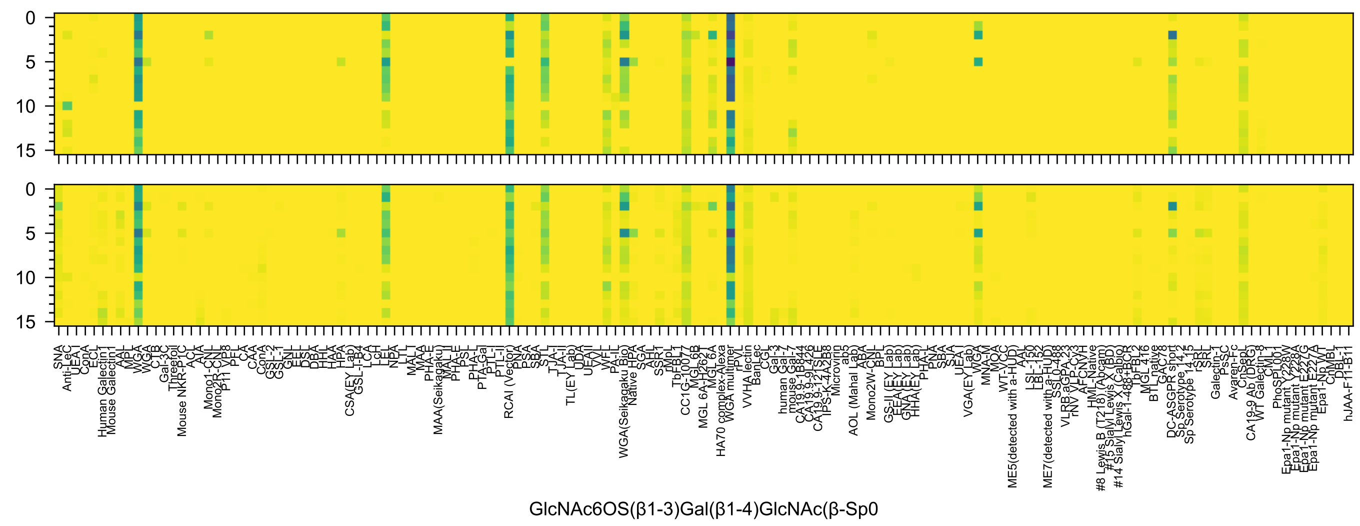
GlcNAc6OS(β1-3)Gal(β1-4)GlcNAc(β-Sp0

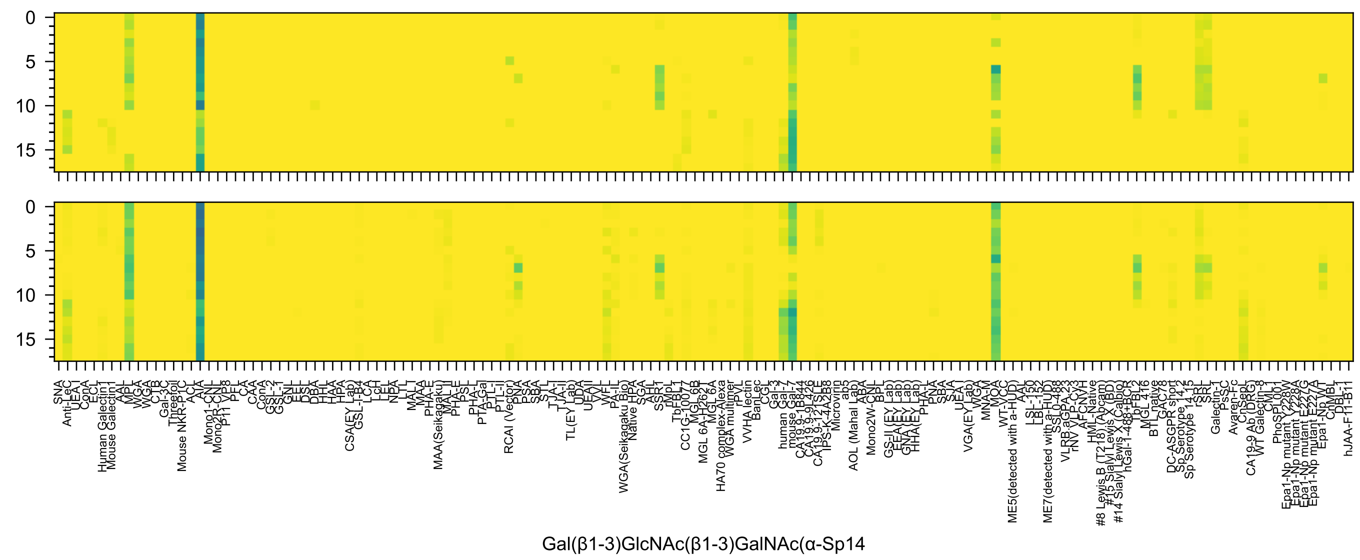

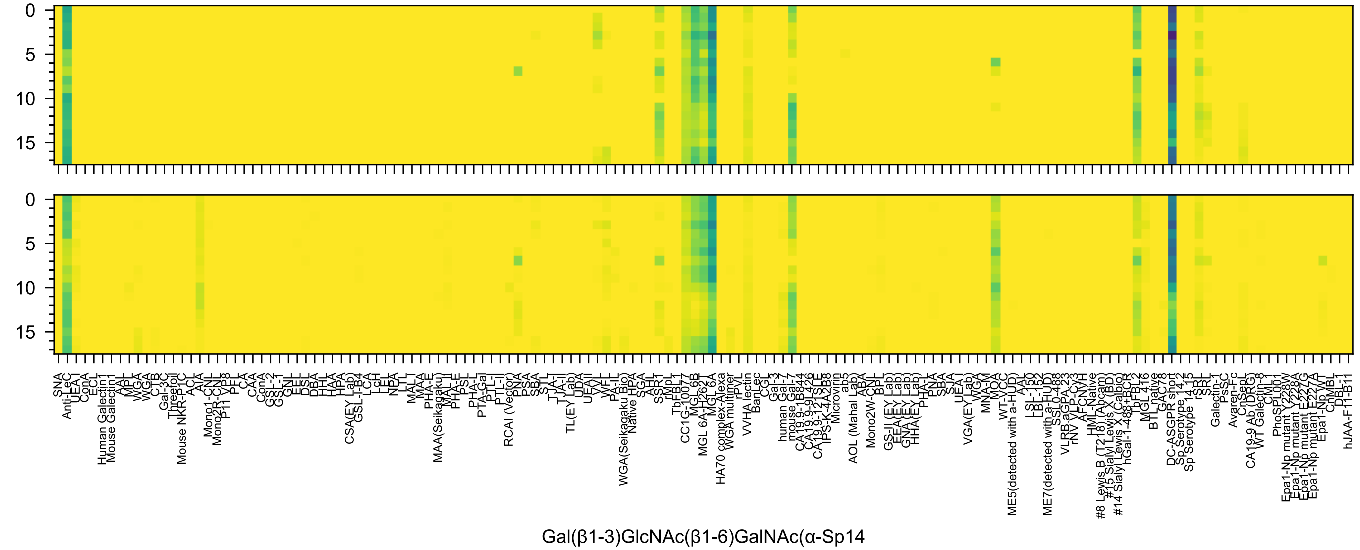
0
5
10
15
SNA
Anti-LeC
UEA-I
ConA
ECL
Human Galectin1
Mouse Galectin1
AAL
MPL
WGA
WGA
CTB
Gal-3C
Threefoil
Mouse NKR-P1C
ACL
AIA
Mono1-CNL
Mono2R-CNL
P11 VP8
PFL
CA
CAA
ConA
GSL-2
GSL-1
GNL
EEL
DSL
DBA
HHL
HAA
HPA
CSA(EY Lab)
GSL-I-B4
LCA
LcH
LEL
NPA
LTL
MAL-I
MAA
PHA-E
MAA(Seikagaku)
MAL-II
PHA-E
PSL
PHA-L
PTA-Gal
PTL-I
PTL-II
RCAI (Vector)
PNA
PSA
SBA
STL
TJA-I
TJA-II
TL(EY Lab)
UDA
UEA-II
VVL
WFL
PA-IL
WGA(Seikagaku Bio)
Native HPA
SGA
AAL
SSR1
rMpL
TbFBL1
CC1G-10077
MGL 6B
MGL 6A-H262T
MGL 6A
HA70 complex-Alexa
WGA multimer
rPVL
VVHA lectin
BanLec
CGL
Gal-3
human Gal-7
mouse Gal-7
CA19.9-1B844
CA19.9-9L426
CA19.9-121SLE
IPS-K-4A2B8
Microvirin
ab5
AOL (Mahal Lab)
ABA
Mono2W-CNL
BPL
GS-II (EY Lab)
EEA(EY Lab)
GNA(EY Lab)
HHA(EY Lab)
PHA-L
PNA
SBA
SJA
UEA-I
VGA(EY Lab)
WGA
MNA-M
MOA
WT-VCC
ME5(detected with a-HUD)
AAL
LSL-150
LBL-152
ME7(detected with a-HUD)
SSL0-488
VLRB.aGPA.23
rNV VLP-Cy3
AFCNVH
HML-Native
#8 Lewis B (T218) (Abcam)
#15 Sialyl Lewis X (BD)
#14 Sialyl Lewis X (Calbio)
hGal-1-488+BCR
TbFBL2
MGL 416
BTL native
GAC78
DC-ASGPR short
Sp Serotype 14.2
Sp Serotype 14.15
rSRL
SRL
Galectin-1
PsSC
Avaren-Fc
CnSepL
CA19-9 Ab (DRG)
WT Galectin-8
CML1
PhoSPL001
Epa1-Np mutant Y228W
Epa1-Np mutant Y228A
Epa1-Np mutant E227G
Epa1-Np mutant E227A
Epa1-Np WT
Epa1
ChMBL
DBL-1
hJAA-F11-B11
Gal(β1-3)GlcNAc(β1-6)GalNAc(α-Sp14

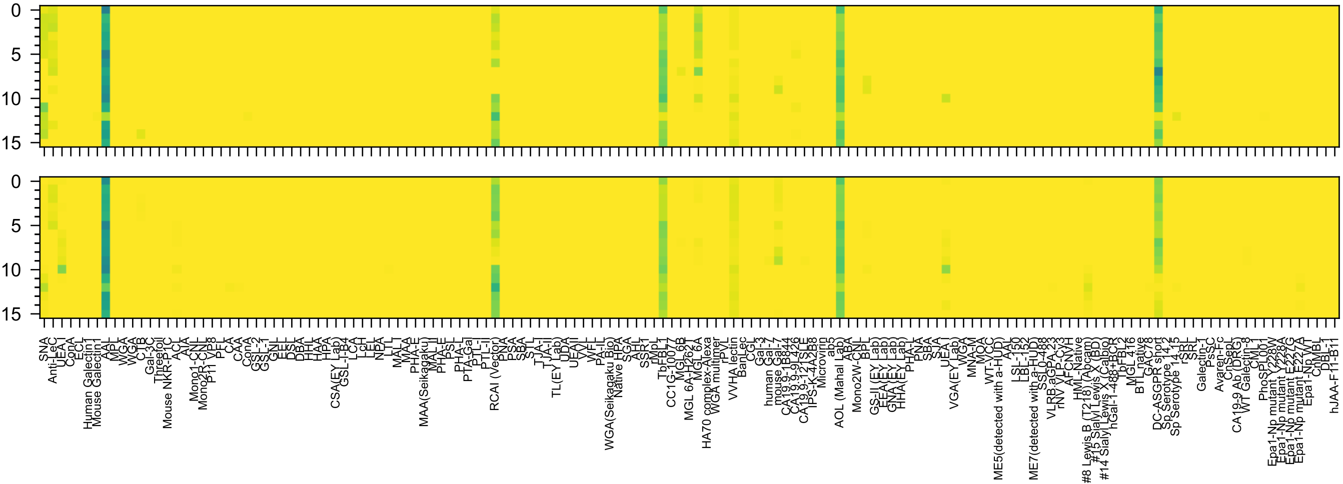

Fuc(α1-2)Gal6OS(β1-3)GlcNAc6OS(β-Sp0

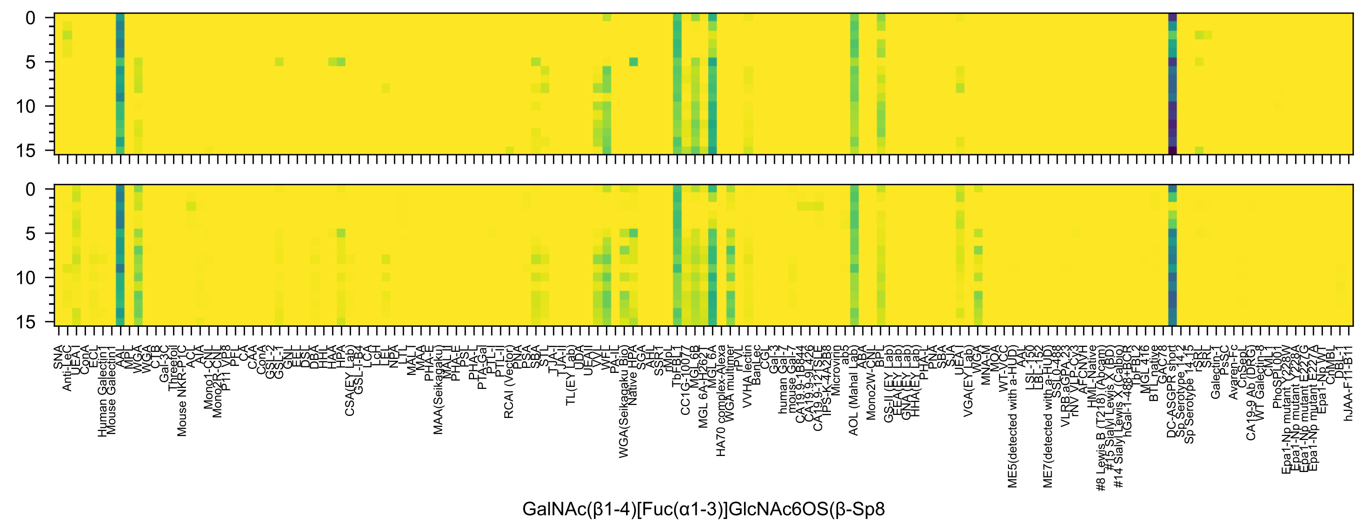
GalNAc(β1-4)[Fuc(α1-3)]GlcNAc6OS(β-Sp8

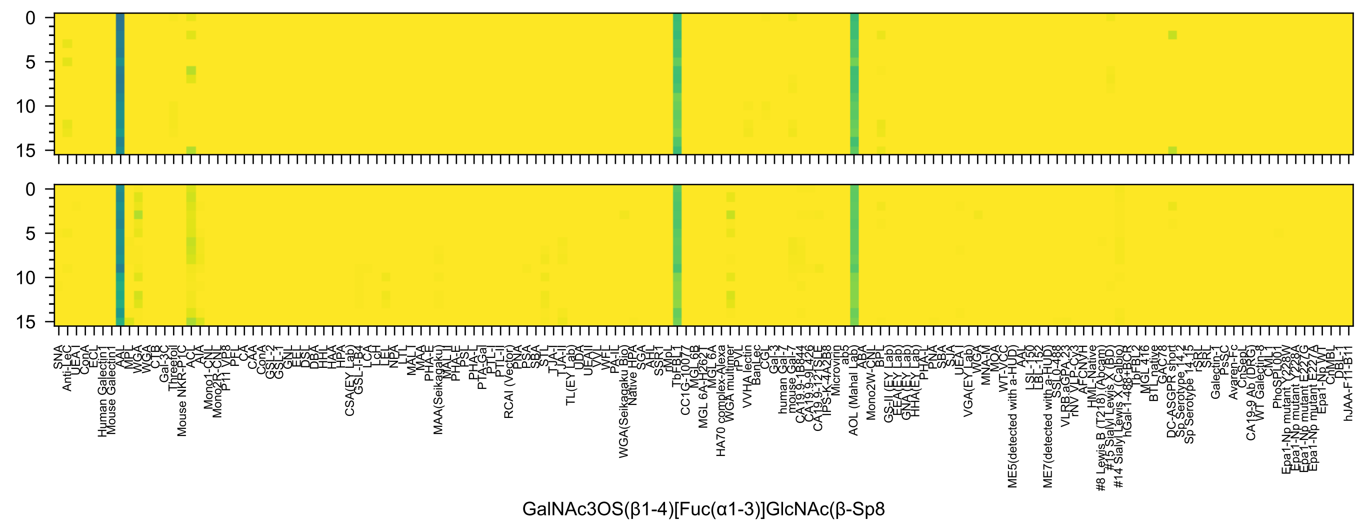
0
5
10
15
0
5
10
15
SNA
Anti-LeC
UEA-I
ConA
ECL
Human Galectin1
Mouse Galectin1
AAL
MPL
WGA
WGA
CTB
Gal-3C
Threefoil
Mouse NKR-P1C
ACL
AIA
Mono1-CNL
Mono2R-CNL
P11 VP8
PFL
CA
CAA
ConA
GSL-2
GSL-1
GNL
EEL
DSL
DBA
HHL
HAA
HPA
CSA(EY Lab)
GSL-I-B4
LCA
LcH
LEL
NPA
LTL
MAL-I
MAA
PHA-E
MAA(Seikagaku)
MAL-II
PHA-E
PSL
PHA-L
PTA-Gal
PTL-I
PTL-II
RCAI (Vector)
PNA
PSA
SBA
STL
TJA-I
TJA-II
TL(EY Lab)
UDA
UEAII
VVL
WFL
PA-IL
WGA(Seikagaku Bio)
Native HPA
SGA
AHL
SSR1
rMpL
TbFBL1
CC1G-10077
MGL 6B
MGL 6A-H262T
MGL 6A
HA70 complex-Alexa
WGA multimer
rPVL
VVHA lectin
BanLec
CGL
Gal-3
human Gal-7
mouse Gal-7
CA19.9-1B844
CA19.9-9L426
CA19.9-121SLE
IPS-K-4A2B8
Microvirin
ab5
AOL (Mahal Lab)
ABA
Mono2W-CNL
BPL
GS-II (EY Lab)
EEA(EY Lab)
GNA(EY Lab)
HHA(EY Lab)
PHA-L
PNA
SBA
SJA
UEA-I
VGA(EY Lab)
WGA
MNA-M
MOA
WT-VCC
ME5(detected with a-HUD)
AAL
LSL-150
LBL-152
ME7(detected with a-HUD)
SSL0-488
VLRB.aGPA.23
rNV VLP-Cy3
AFCNVH
HML-Native
#8 Lewis B (T218) (Abcam)
#15 Sialyl Lewis X (BD)
#14 Sialyl Lewis X (Calbio)
hGal-1-488+BCR
TbFBL2
MGL 416
BTL native
GAC78
DC-ASGPR short
Sp Serotype 14.2
Sp Serotype 14.15
rSRL
SRL
Galectin-1
PsSC
Avaren-Fc
ChSep1
CA19-9 Ab (DRG)
WT Galectin-8
CML1
PhoSL001
Epa1-Np mutant Y228W
Epa1-Np mutant Y228A
Epa1-Np mutant E227G
Epa1-Np mutant E227A
Epa1-Np WT
ChMBL
DBL-1
hJAA-F11-B11
GalNAc3OS(β1-4)[Fuc(α1-3)]GlcNAc(β-Sp8

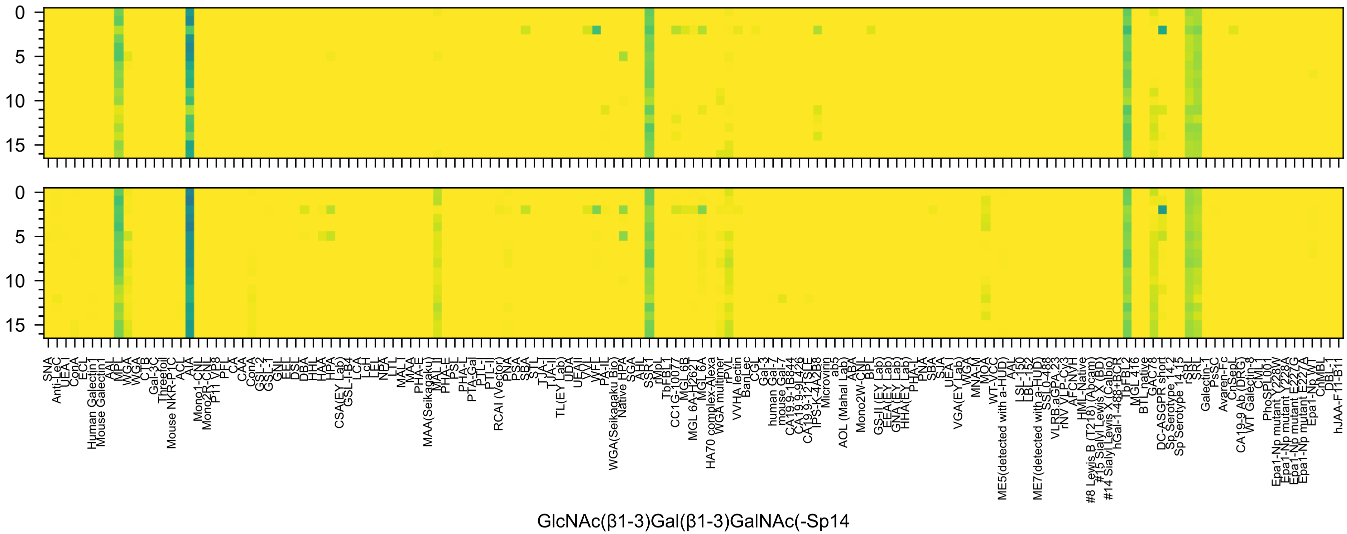
0
5
10
15
0
5
10
15
GlcNAc(β1-3)Gal(β1-3)GalNAc(-Sp14

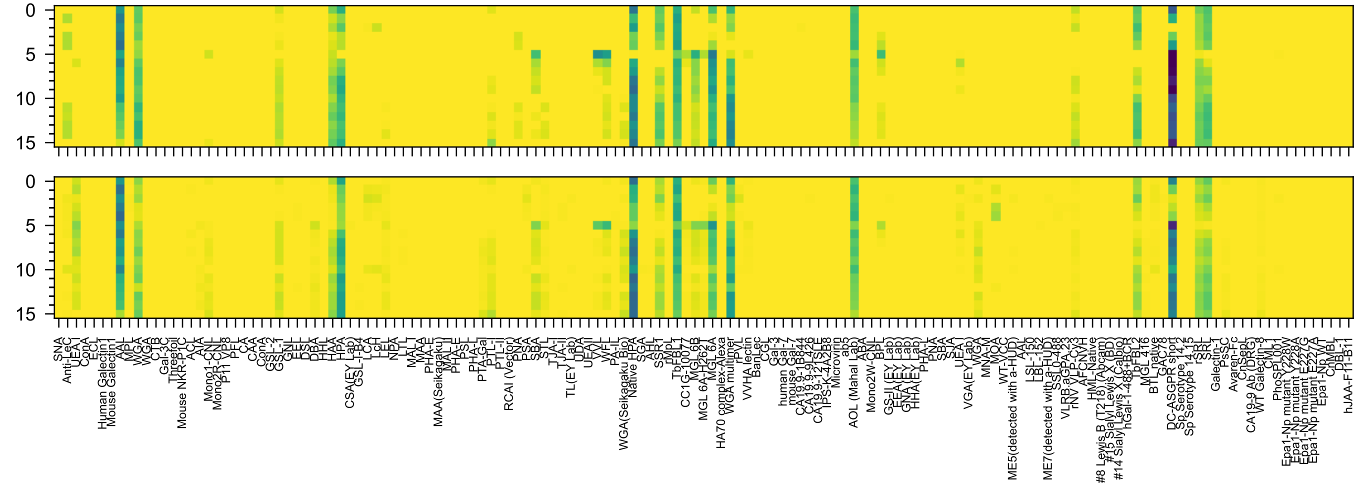

GalNAc(α1-3)[Fuc(α1-2)]Gal(β-Sp18

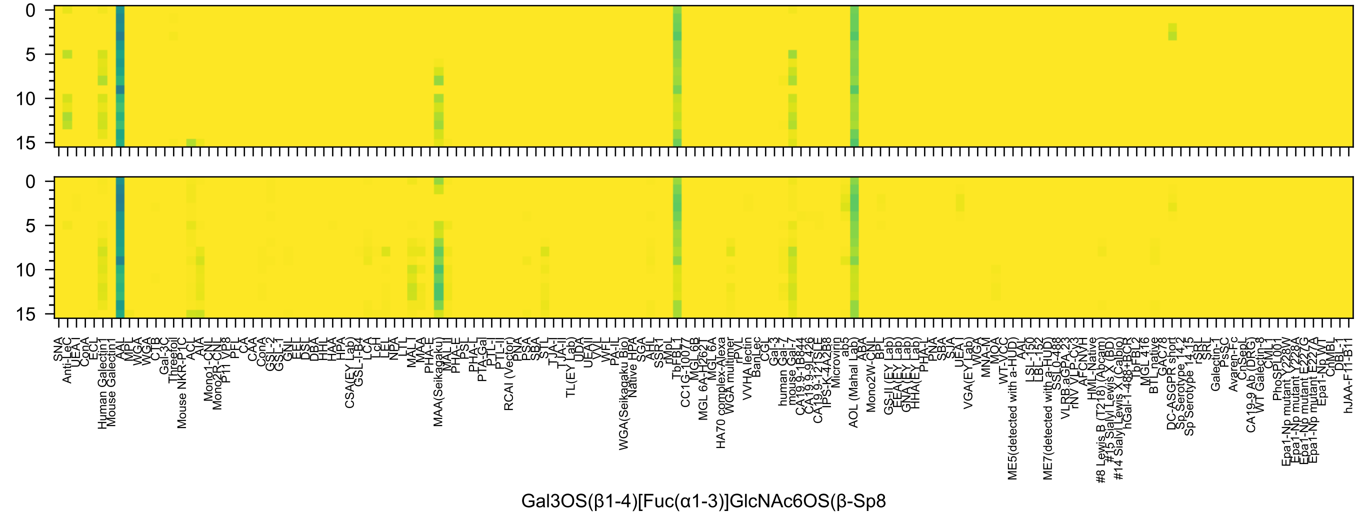
Gal3OS(β1-4)[Fuc(α1-3)]GlcNAc6OS(β-Sp8

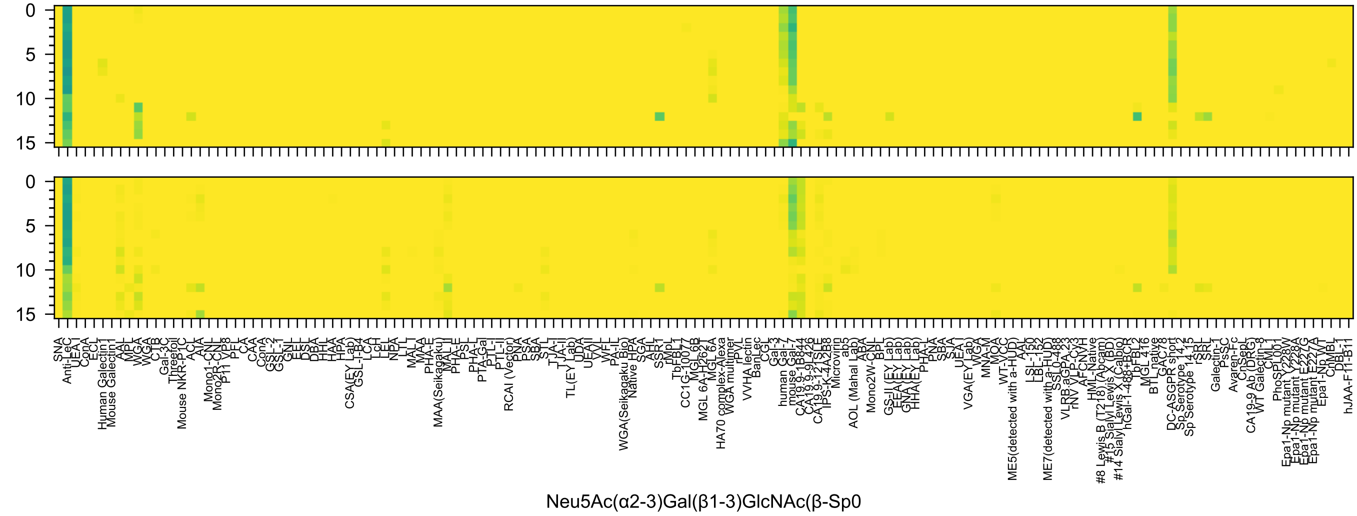
0
5
10
15
Neu5Ac(α2-3)Gal(β1-3)GlcNAc(β-Sp0

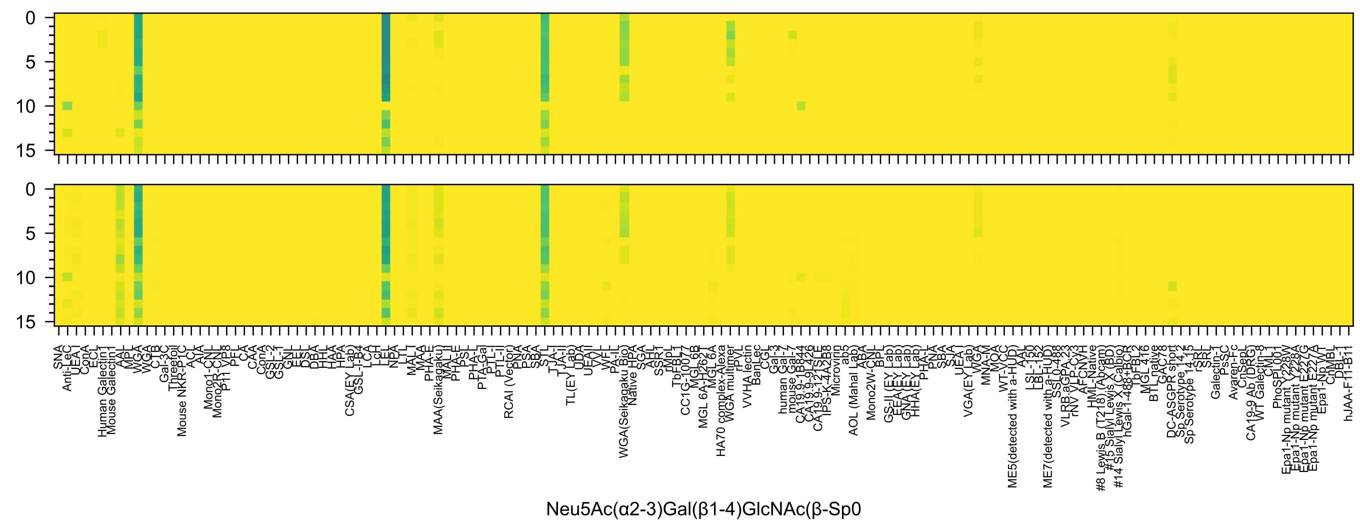
Neu5Ac(α2-3)Gal(β1-4)GlcNAc(β-Sp0

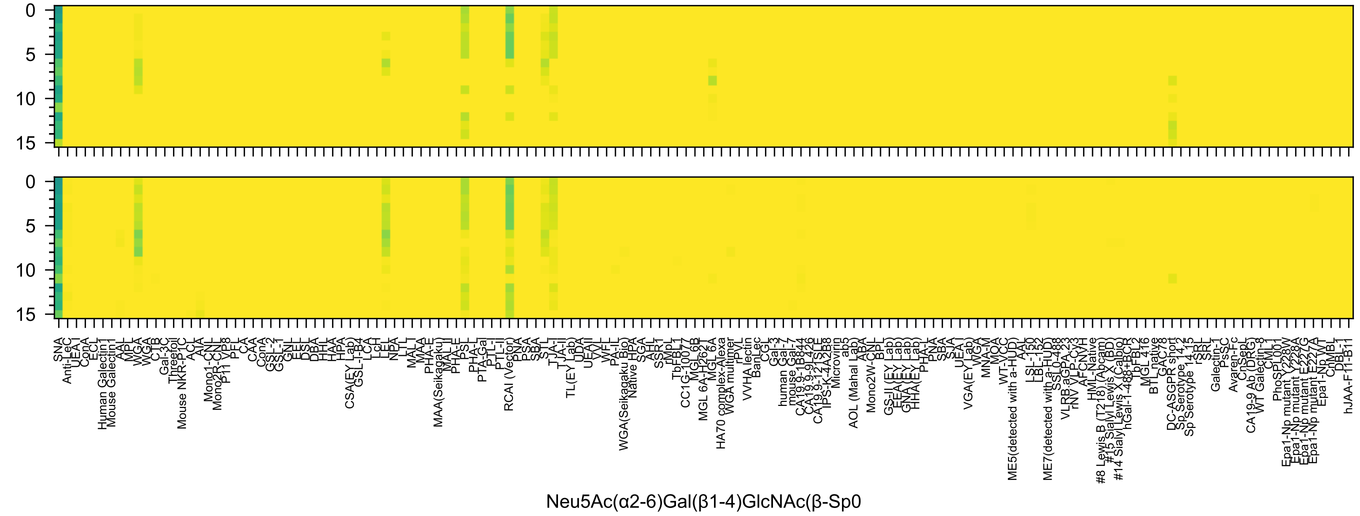
0
5
10
15
0
5
10
15
SNA
Anti-LeC
UEA-I
ConA
ECL
Human Galectin1
Mouse Galectin1
AAL
MPL
WGA
WGA
CTB
Gal-3C
Threefoil
Mouse NKR-P1C
ACL
AIA
Mono1-CNL
Mono2R-CNL
P11 VP8
PFL
CA
CAA
ConA
GSL-2
GSL-1
GNL
EEL
DSL
DBA
HHL
HAA
HPA
CSA(EY Lab)
GSL-I-B4
LCA
LcH
LEL
NPA
LTL
MAL-I
MAA
PHA-E
MAA(Seikagaku)
MAL-II
PHA-E
PSL
PHA-L
PTA-Gal
PTL-I
PTL-II
RCAI (Vector)
PNA
PSA
SBA
STL
TJA-I
TJA-II
TL(EY Lab)
UDA
UEA-II
VVL
WFL
PA-IL
WGA(Seikagaku Bio)
Native HPA
SGA
AHL
SSR1
rMpL
TbFBL1
CC1G-10077
MGL 6B
MGL 6A-H262T
MGL 6A
HA70 complex-Alexa
WGA multimer
rPVL
VVHA lectin
BanLec
CGL
Gal-3
human Gal-7
mouse Gal-7
CA19.9-1B844
CA19.9-9L426
CA19.9-121SLE
IPS-K-4A2B8
Microvirin
ab5
AOL (Mahal Lab)
ABA
Mono2W-CNL
BPL
GS-II (EY Lab)
EEA (EY Lab)
GNA (EY Lab)
HHA (EY Lab)
PHA-L
PNA
SBA
SJA
UEA-I
VGA (EY Lab)
WGA
MNA-M
MOA
WT-VCC
ME5(detected with a-HUD)
AAL
LSL-150
LBL-152
ME7(detected with a-HUD)
SSL0.488
VLRB.aGPA.23
rNV VLP-Cy3
AFCNVH
HML-Native
#8 Lewis B (T218) (Abcam)
#15 Sialyl Lewis X (BD)
#14 Sialyl Lewis X (Calbio)
hGal-1-488+BCR
TbFBL2
MGL 416
BTL native
GAC78
DC-ASGPR short
Sp Serotype 14.2
Sp Serotype 14.15
rSRL
SRL
Galectin-1
PsSC
Avaren-Fc
ChSepL
CA19-9 Ab (DRG)
WT Galectin-8
CML-1
PhoSL001
Epa1-Np mutant Y228W
Epa1-Np mutant Y228A
Epa1-Np mutant E227G
Epa1-Np mutant E227A
Epa1-Np WT
ChMBL
DBL-1
hJAA-F11-B11
Neu5Ac(α2-6)Gal(β1-4)GlcNAc(β-Sp0

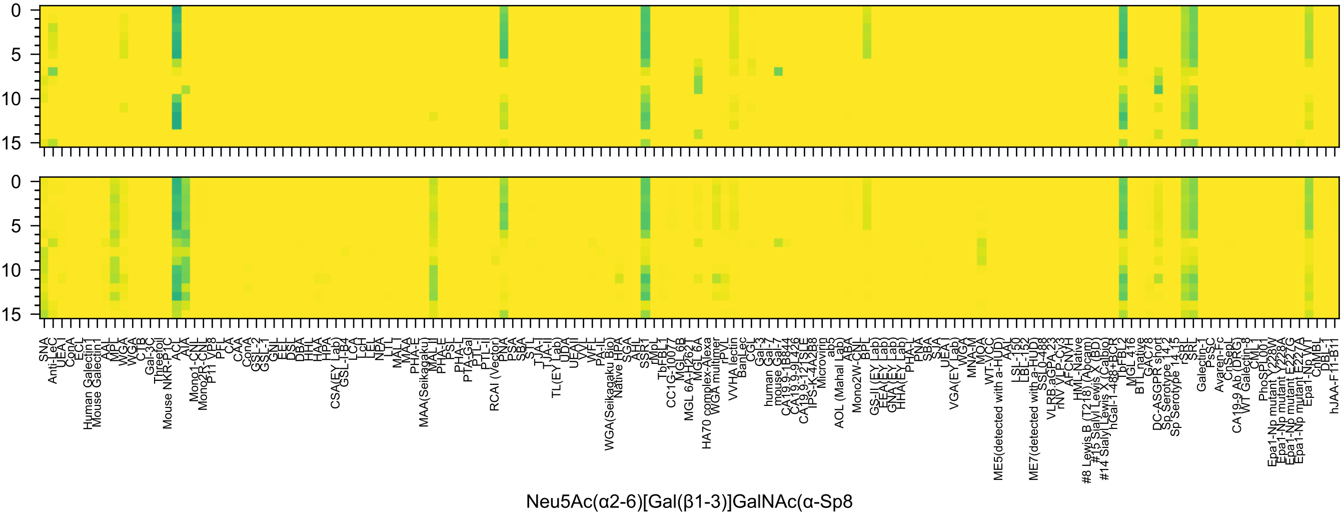
0
5
10
15
0
5
10
15
SNA
Anti-LeC
UEA I
ConA
ECL
Human Galectin1
Mouse Galectin1
AAL
MPL
WGA
WGA
CTB
Gal-3C
Threefoil
Mouse NKR-P1C
ACL
AIA
Mono1-CNL
Mono2R-CNL
P11 VP8
PFL
CA
CAA
ConA
GSL-2
GSL-1
GNL
EEL
DSL
DBA
HHL
HAA
HPA
CSA(EY Lab)
GSL-I-B4
LCA
LcH
LEL
NPA
LTL
MAL-I
MAA
PHA-E
MAA(Seikagaku)
MAL-II
PHA-E
PSL
PHA-L
PTA-Gal
PTL-I
PTL-II
RCAI (Vector)
PNA
PSA
SBA
STL
TJA-I
TJA-II
TL(EY Lab)
UDA
UEA-II
VVL
WFL
PA-IL
WGA(Seikagaku Bio)
Native HPA
SGA
AAL
SSR1
rMpL
TbFBL1
CC1G-10077
MGL 6B
MGL 6A-H262T
MGL 6A
HA70 complex-Alexa
WGA multimer
rPVL
VVHA lectin
BanLec
CGL
Gal-3
human Gal-7
mouse Gal-7
CA19.9-1B844
CA19.9-9L426
CA19.9-121SLE
IPS-K-4A2B8
Microvirin
ab5
AOL (Mahal Lab)
ABA
Mono2W-CNL
BPL
GS-II (EY Lab)
EEA (EY Lab)
GNA (EY Lab)
HHA (EY Lab)
PHA-L
PNA
SBA
SJA
UEA-I
VGA(EY Lab)
WGA
MNA-M
MOA
WT-VCC
ME5(detected with a-HUD)
AAL
LSL-150
LBL-152
ME7(detected with a-HUD)
SSL0-488
VLRB.aGPA.23
rNV VLP-Cy3
AFCNVH
HML-Native
#8 Lewis B (T218) (Abcam)
#15 Sialyl Lewis X (BD)
#14 Sialyl Lewis X (Calbio)
hGal-1-488+BCR
TbFBL2
MGL 416
BTL native
GAC78
DC-ASGPR short
Sp Serotype 14.2
Sp Serotype 14.15
rSRL
SRL
Galectin-1
PsSC
Avaren-Fc
ChSeP
CA19-9 Ab (DRG)
WT Galectin-8
CML1
PhoSL001
Epa1-Np mutant Y228W
Epa1-Np mutant Y228A
Epa1-Np mutant E227G
Epa1-Np mutant E227A
Epa1-Np WT
CnMBL
DBL-1
hJAA-F11-B11
Neu5Ac(α2-6)[Gal(β1-3)]GalNAc(α-Sp8

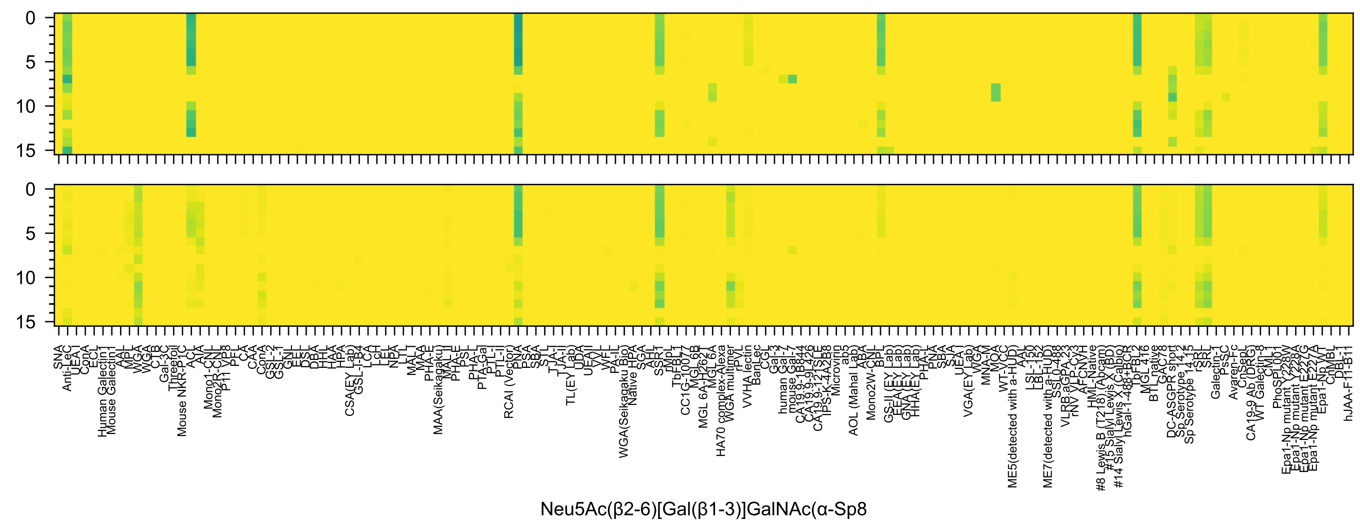
Neu5Ac(β2-6)[Gal(β1-3)]GalNAc(α-Sp8

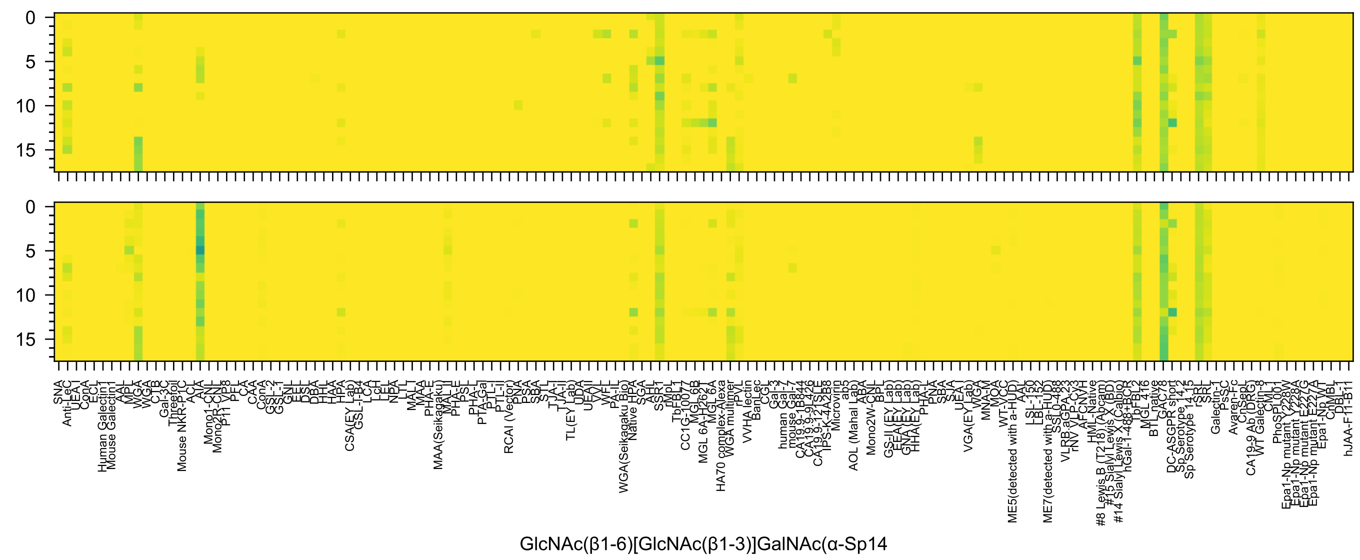
GlcNAc(β1-6)[GlcNAc(β1-3)]GalNAc(α-Sp14

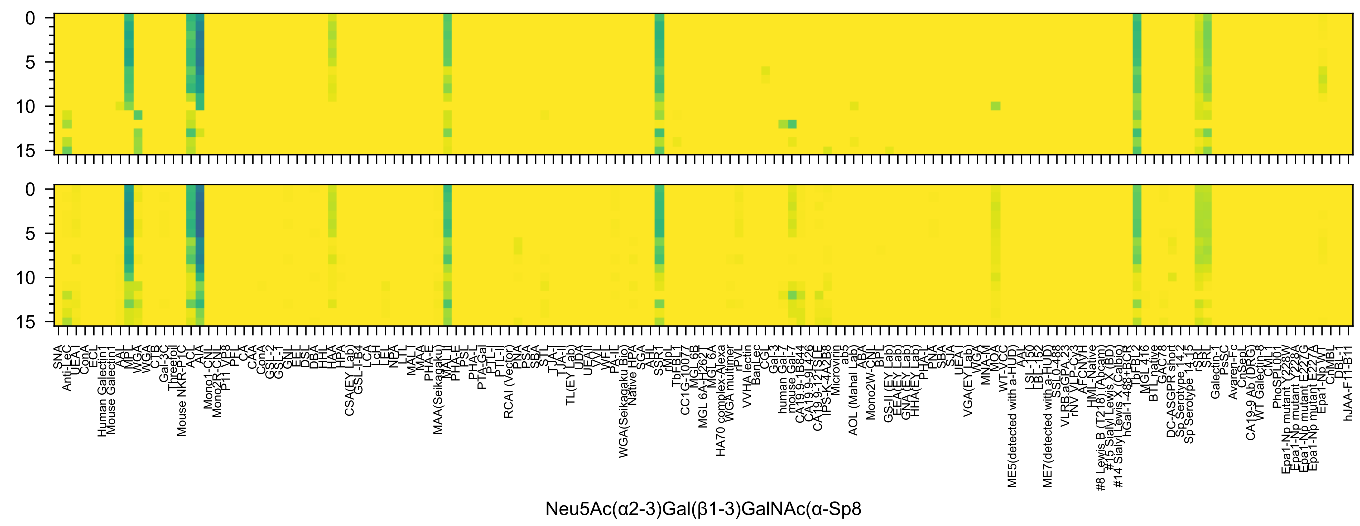
0
5
10
15
0
5
10
15
Neu5Ac(α2-3)Gal(β1-3)GalNAc(α-Sp8

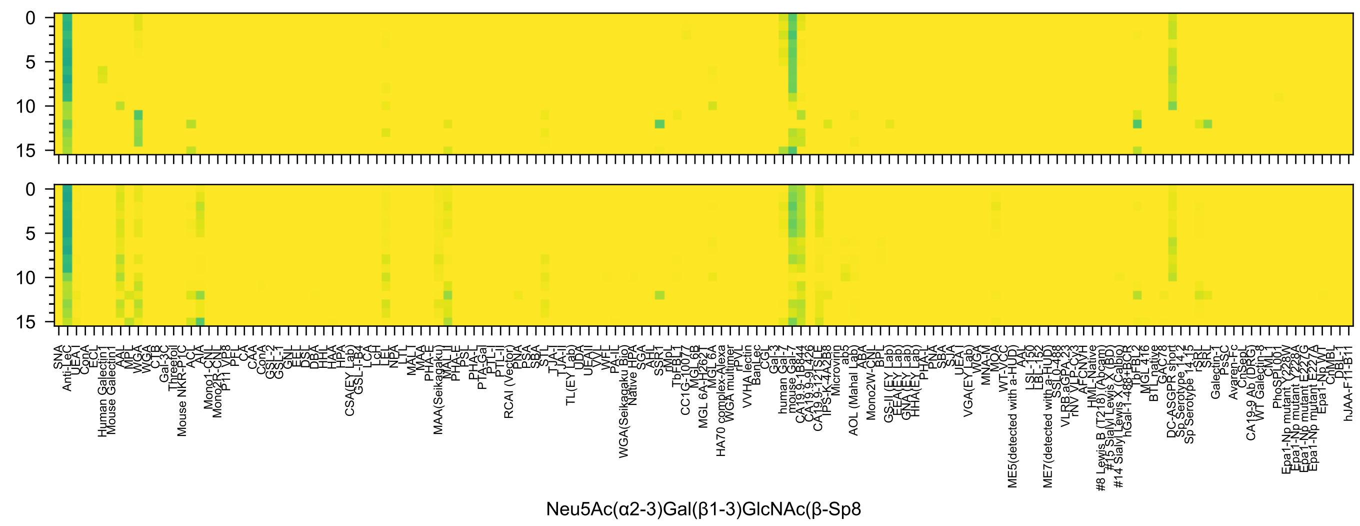

Neu5Ac(α2-3)Gal(β1-3)GlcNAc(β-Sp8

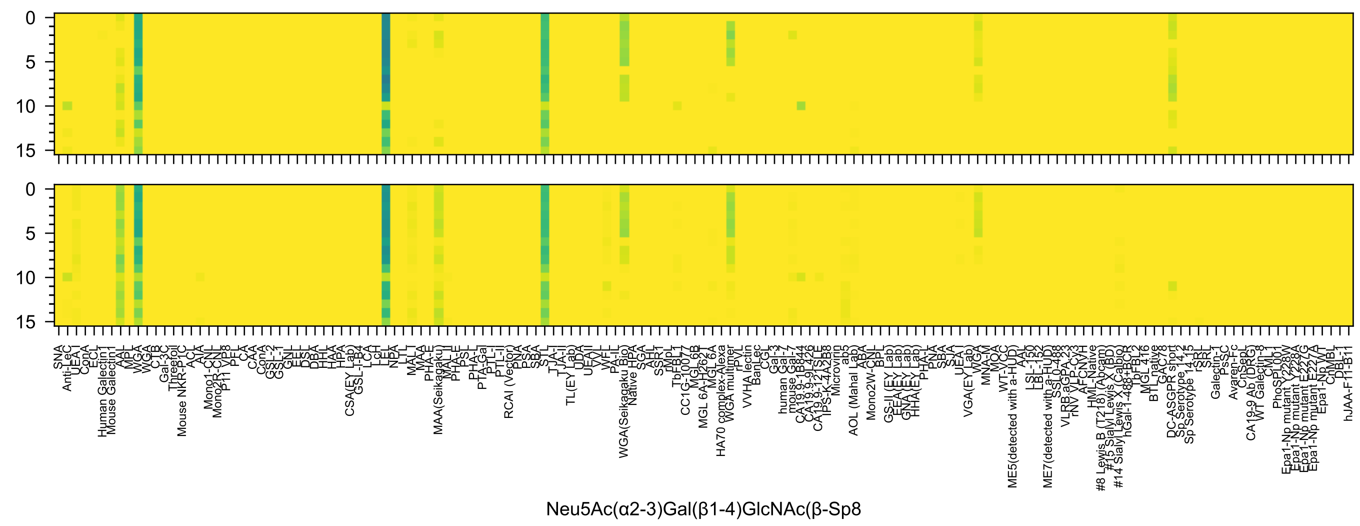
Neu5Ac(α2-3)Gal(β1-4)GlcNAc(β-Sp8

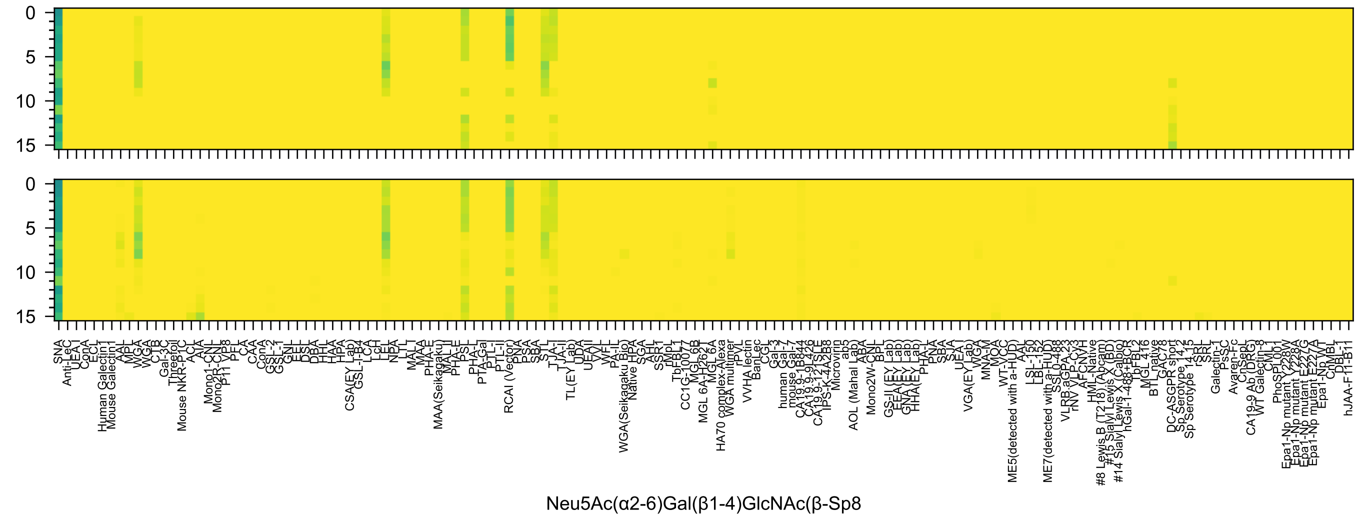
Neu5Ac(α2-6)Gal(β1-4)GlcNAc(β-Sp8

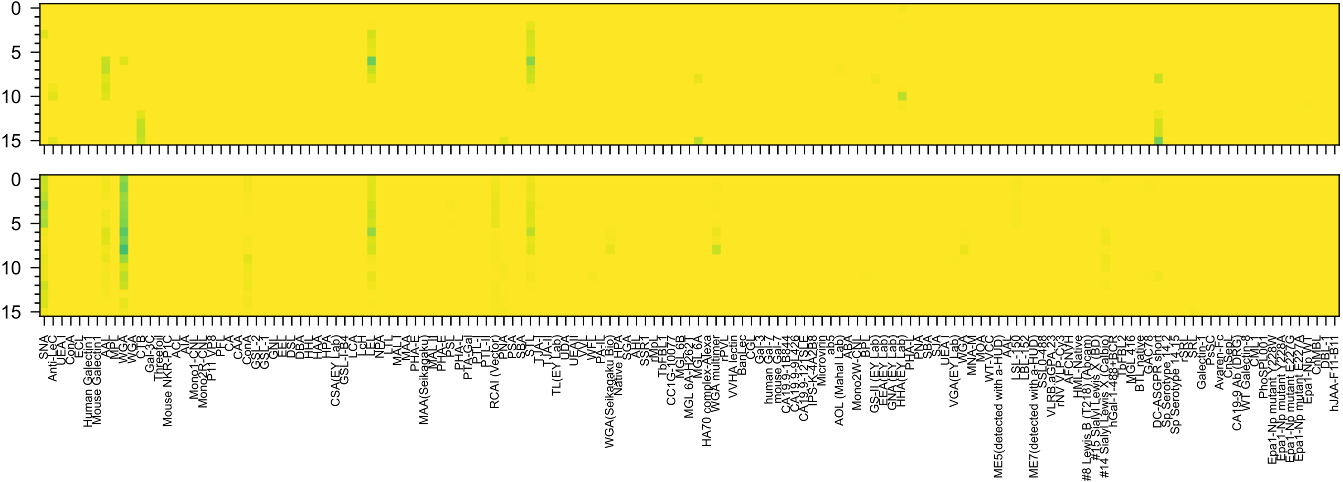

Neu5Ac(β2-6)Gal(β1-4)GlcNAc(β-Sp8

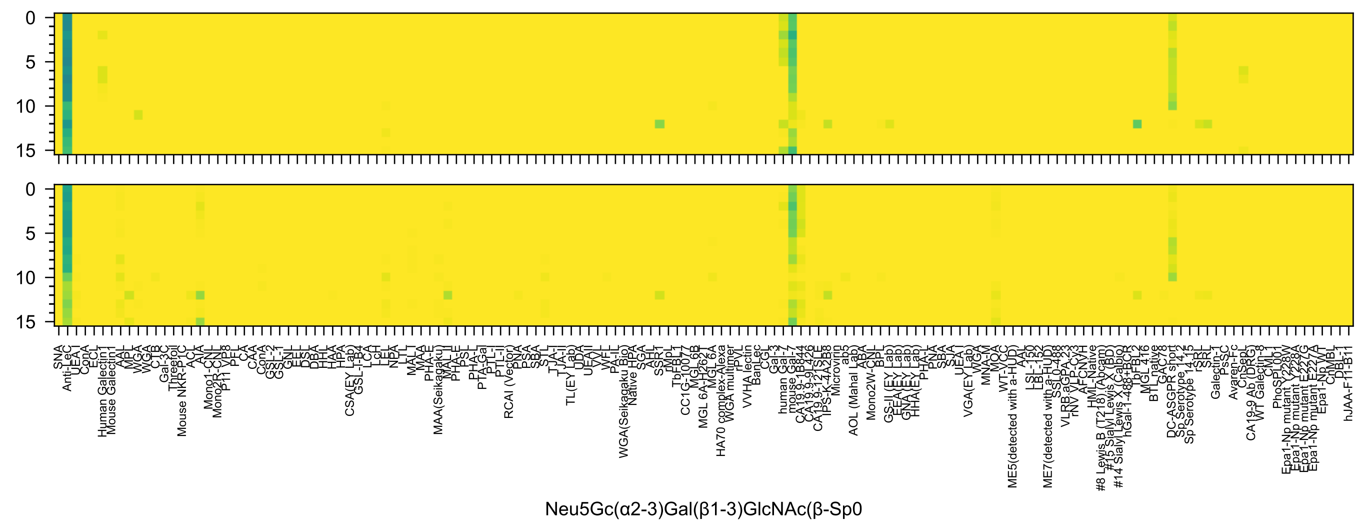
0
5
10
15
0
5
10
15
SNA
Anti-LeC
UEA-I
ConA
ECL
Human Galectin1
Mouse Galectin1
AAL
MPL
WGA
WGA
CTB
Gal-3C
Threefoil
Mouse NKR-P1C
ACL
AIA
Mono1-CNL
Mono2R-CNL
P11 VP8
PFL
CA
CAA
ConA
GSL-2
GSL-1
GNL
EEL
DSL
DBA
HHL
HAA
HPA
CSA(EY Lab)
GSL-I-B4
LCA
LcH
LEL
NPA
LTL
MAL-I
MAA
PHA-E
MAA(Seikagaku)
MAL-II
PHA-E
PSL
PHA-L
PTA-Gal
PTL-I
PTL-II
RCAI (Vector)
PNA
PSA
SBA
STL
TJA-I
TJA-II
TL(EY Lab)
UDA
UEAII
VVL
WFL
PA-IL
WGA(Seikagaku Bio)
Native HPA
SGA
AHL
SSR1
rMpL
TbFBL1
CC1G-10077
MGL 6B
MGL 6A-H262T
MGL 6A
HA70 complex-Alexa
WGA multimer
rPVL
VVHA lectin
BanLec
CGL
Gal-3
human Gal-7
mouse Gal-7
CA19.9-1B844
CA19.9-9L426
CA19.9-121SLE
IPS-K-4A2B8
Microvirin
ab5
AOL (Mahal Lab)
ABA
Mono2W-CNL
BPL
GS-II (EY Lab)
EEA(EY Lab)
GNA(EY Lab)
HHA(EY Lab)
PHA-L
PNA
SBA
SJA
UEA-I
VGA(EY Lab)
WGA
MNA-M
MOA
WT-VCC
ME5(detected with a-HUD)
AAL
LSL-150
LBL-152
ME7(detected with a-HUD)
SSL0-488
VLRB.aGPA.23
rNV VLP-Cy3
AFCNVH
HML-Native
#8 Lewis B (T218) (Abcam)
#15 Sialyl Lewis X (BD)
#14 Sialyl Lewis X (Calbio)
hGal-1-488+BCR
TbFBL2
MGL 416
BTL native
GAC78
DC-ASGPR short
Sp Serotype 14.2
Sp Serotype 14.15
rSRL
SRL
Galectin-1
PsSC
Avaren-Fc
ChSeaL
CA19-9 Ab (DRG)
WT Galectin-8
CML1
PhoSL001
Epa1-Np mutant Y228W
Epa1-Np mutant Y228A
Epa1-Np mutant E227G
Epa1-Np mutant E227A
Epa1-Np WT
Epa1
ChMBL
DBL-1
hJAA-F11-B11
Neu5Gc(α2-3)Gal(β1-3)GlcNAc(β-Sp0

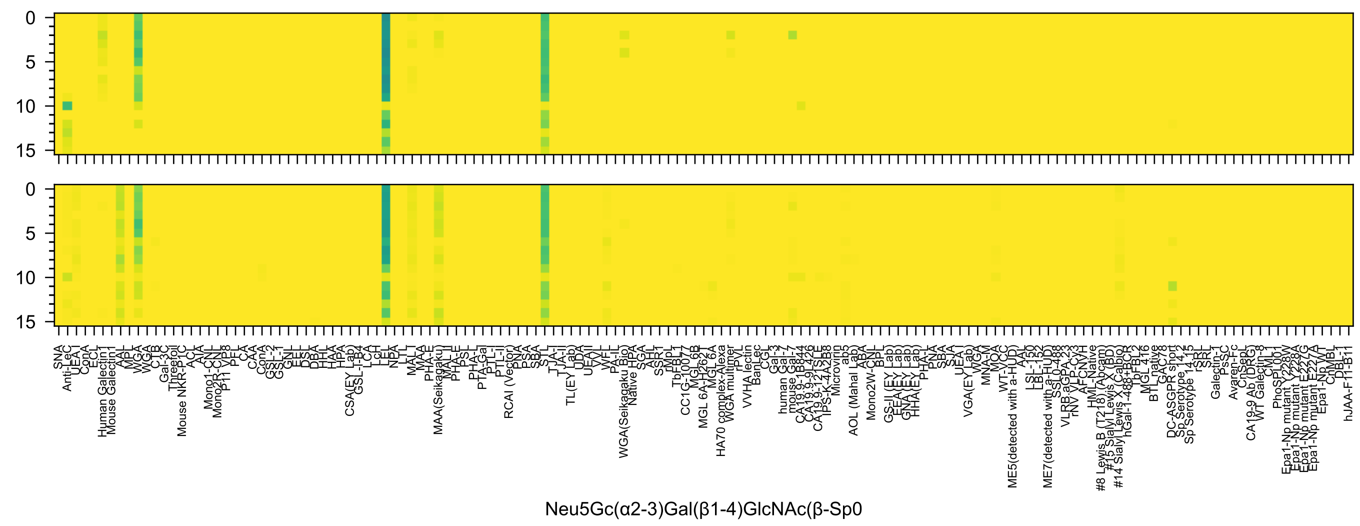
0
5
10
15
0
5
10
15
SNA
Anti-LeC
UEA I
ConA
ECL
Human Galectin1
Mouse Galectin1
AAL
MPL
WGA
WGA
CTB
C-1B
Gal-3C
Threefoil
Mouse NKR-P1C
ACL
AIA
Mono1-CNL
Mono2R-CNL
P11 VP8
PFL
CA
CAA
ConA
GSL-2
GSL-1
GNL
EEL
DSL
DBA
HHL
HAA
HPA
CSA(EY Lab)
GSL-I-B4
LCA
LcH
LEL
NPA
LTL
MAL
MAA
PHA-E
MAA(Seikagaku)
MAL II
PHA-E
PSL
PHA-L
PTA-Gal
PTL-I
PTL-II
RCAI (Vector)
PNA
PSA
SBA
STL
TJA-I
TJA-II
TL(EY Lab)
UDA
UEAII
VVL
WFL
PA-IL
WGA(Seikagaku Bio)
Native HPA
SGA
AHL
SSR1
rMpL
TbFBL1
CC1G-10077
MGL6B
MGL 6A-H262T
MGL 6A
HA70 complex-Alexa
WGA multimer
rPVL
VVHA lectin
BanLec
CGL
Gal-3
human Gal-7
mouse Gal-7
CA19.9-1B844
CA19.9-9L426
CA19.9-121SLE
IPS-K-4A2B8
Microvirin
ab5
AOL (Mahal Lab)
ABA
Mono2W-CNL
BPL
GS-II (EY Lab)
EEA(EY Lab)
GNA(EY Lab)
HHA(EY Lab)
PHA-L
PNA
SBA
SJA
UEA-I
VGA(EY Lab)
WGA
MNA-M
MOA
WT-VCC
ME5(detected with a-HUD)
AAL
LSL-150
LBL-152
ME7(detected with a-HUD)
SSL0-488
VLRB.aGPA.23
rNV VLP-Cy3
AFCNVH
HML-Native
#8 Lewis B (T218) (Abcam)
#15 Sialyl Lewis X (BD)
#14 Sialyl Lewis X (Calbio)
hGal-1-488+BCR
TbFBL2
MGL 416
BTL native
GAC78
DC-ASGPR short
Sp Serotype 14.2
Sp Serotype 14.15
rSRL
SRL
Galectin-1
PsSC
Avaren-Fc
ChSep1
CA19-9 Ab (DRG)
WT Galectin-8
CML1
PhoSL001
Epa1-Np mutant Y228W
Epa1-Np mutant Y228A
Epa1-Np mutant E227G
Epa1-Np mutant E227A
Epa1-Np WT
ChMBL
DBL-1
hJAA-F11-B11
Neu5Gc(α2-3)Gal(β1-4)GlcNAc(β-Sp0

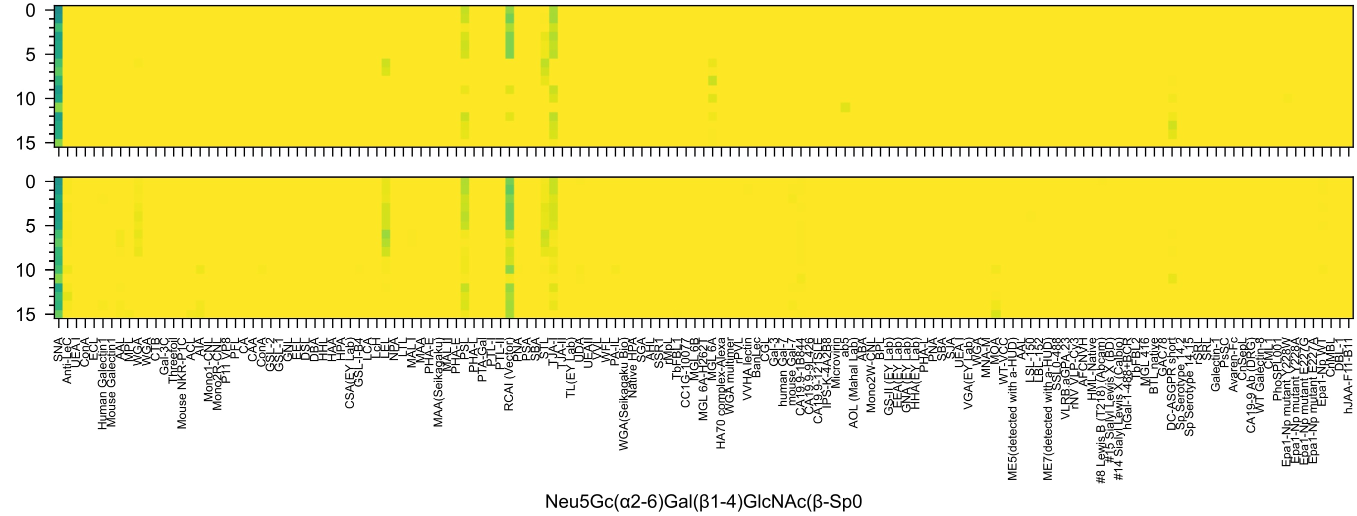
0
5
10
15
0
5
10
15
Neu5Gc(α2-6)Gal(β1-4)GlcNAc(β-Sp0

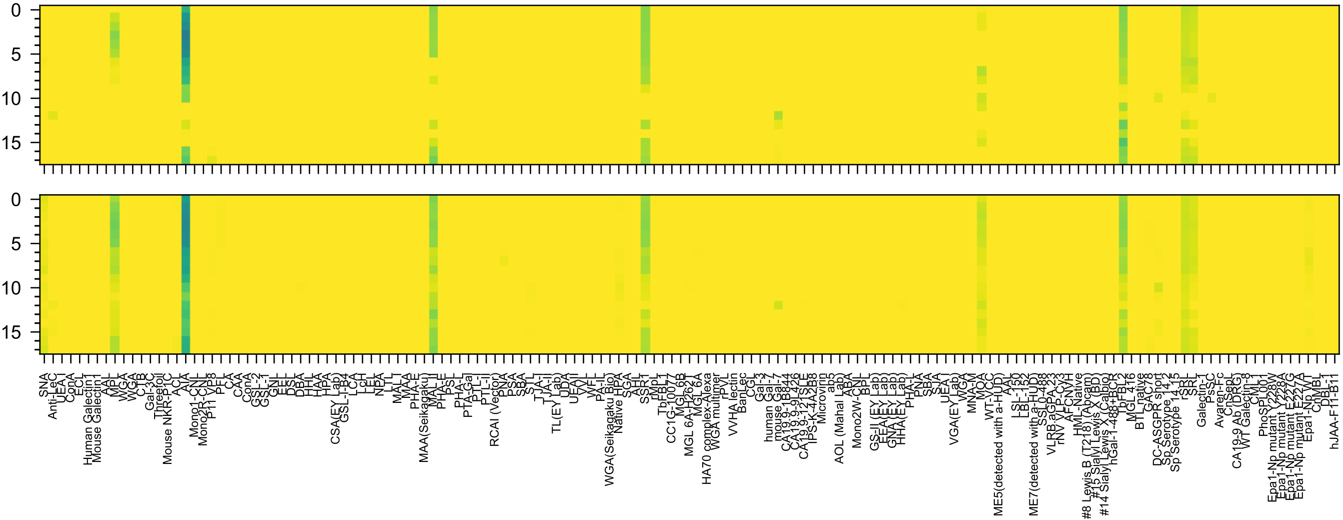

Kdn(α2-3)Gal(β1-3)GalNAc(α-Sp14

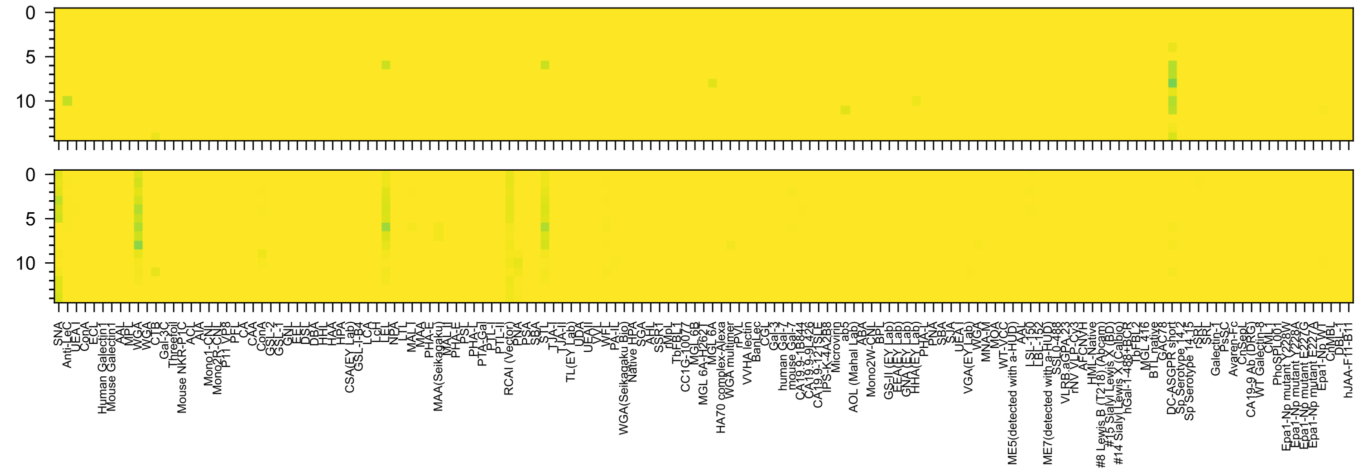
Neu5Gc(β2-6)Gal(β1-4)GlcNAc(-Sp8

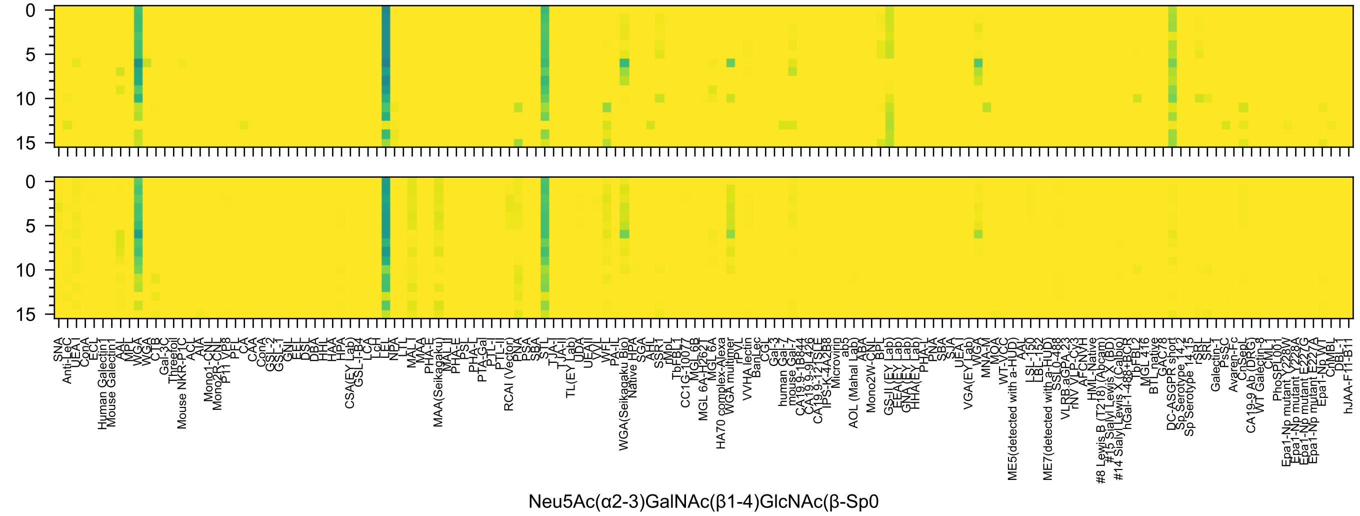
0
5
10
15
0
5
10
15
SNA
Anti-LeC
UEA-I
ConA
ECL
Human Galectin1
Mouse Galectin1
AAL
MPL
WGA
WGA
CTB
Gal-3C
Threefoil
Mouse NKR-P1C
ACL
AIA
Mono1-CNL
Mono2R-CNL
P11 VP8
PFL
CA
CAA
ConA
GSL-2
GSL-1
GNL
EEL
DSL
DBA
HHL
HAA
HPA
CSA(EY Lab)
GSL-I-B4
LCA
LcH
LEL
NPA
LTL
MAL-I
MAA
PHA-E
MAA(Seikagaku)
MAL-II
PHA-E
PSL
PHA-L
PTA-Gal
PTL-I
PTL-II
RCAI (Vector)
PNA
PSA
SBA
STL
TJA-I
TJA-II
TL(EY Lab)
UDA
UEA-II
VVL
WFL
PA-IL
WGA(Seikagaku Bio)
Native HPA
SGA
AAL
SSR1
rMpL
TbFBL1
CC1G-10077
MGL 6B
MGL 6A-H262T
MGL 6A
HA70 complex-Alexa
WGA multimer
rPVL
VVHA lectin
BanLec
CGL
Gal-3
human Gal-7
mouse Gal-7
CA19.9-1B844
CA19.9-9L426
CA19.9-121SLE
IPS-K-4A2B8
Microvirin
ab5
AOL (Mahal Lab)
ABA
Mono2W-CNL
BPL
GS-II (EY Lab)
EEA(EY Lab)
GNA(EY Lab)
HHA(EY Lab)
PHA-L
PNA
SBA
SJA
UEA-I
VGA(EY Lab)
WGA
MNA-M
MOA
WT-VCC
ME5(detected with a-HUD)
AAL
LSL-150
LBL-152
ME7(detected with a-HUD)
SSL0-488
VLRB.aGPA.23
rNV VLP-Cy3
AFCNVH
HML-Native
#8 Lewis B (T218) (Abcam)
#15 Sialyl Lewis X (BD)
#14 Sialyl Lewis X (Calbio)
hGal-1-488+BCR
TbFBL2
MGL 416
BTL native
GAC78
DC-ASGPR short
Sp Serotype 14.2
Sp Serotype 14.15
rSRL
SRL
Galectin-1
PsSC
Avaren-Fc
ChSepL
CA19-9 Ab (DRG)
WT Galectin-8
CML
PhoSL001
Epa1-Np mutant Y228W
Epa1-Np mutant Y228A
Epa1-Np mutant E227G
Epa1-Np mutant E227A
Epa1-Np WT
ChMBL
DBL-1
hJAA-F11-B11
Neu5Ac(α2-3)GalNAc(β1-4)GlcNAc(β-Sp0

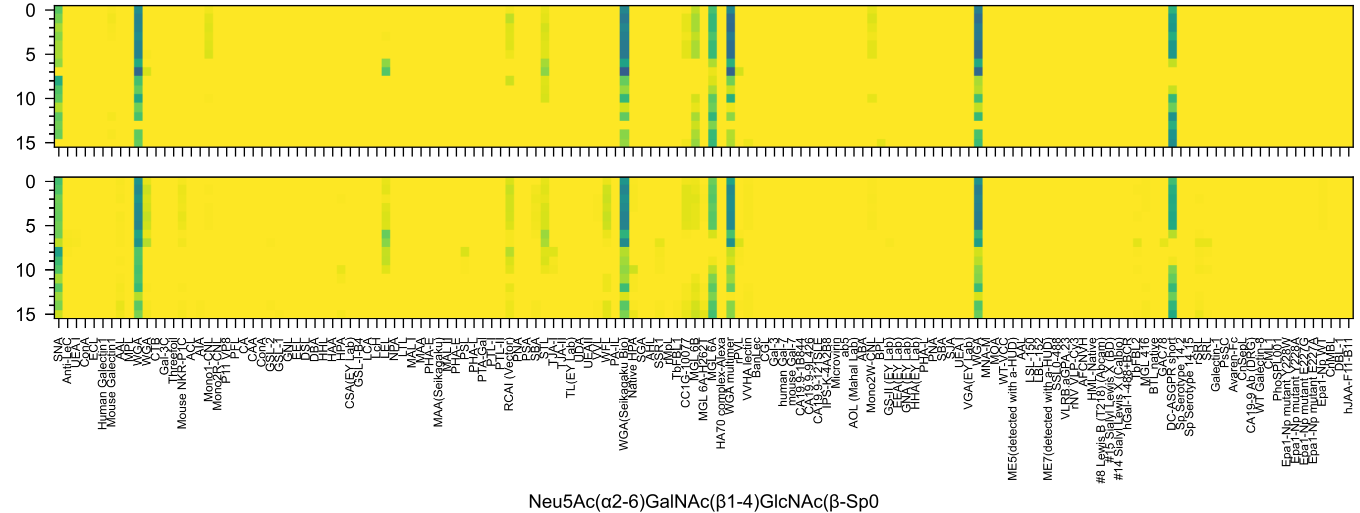
0
5
10
15
0
5
10
15
SNA
Anti-LeC
UEA I
ConA
ECL
Human Galectin1
Mouse Galectin1
AAL
MPL
WGA
WGA
CTB
C-1B
Gal-3C
Threefoil
Mouse NKR-P1C
ACL
AIA
Mono1-CNL
Mono2R-CNL
P11 VP8
PFL
CA
CAA
ConA
GSL-2
GSL-1
GNL
EEL
DSL
DBA
HHL
HAA
HPA
CSA(EY Lab)
GSL-I-B4
LCA
LcH
LEL
NPA
LTL
MAL-I
MAA
PHA-E
MAA(Seikagaku)
MAL-II
PHA-E
PSL
PHA-L
PTA-Gal
PTL-I
PTL-II
RCAI (Vector)
PNA
PSA
SBA
STL
TJA-I
TJA-II
TL(EY Lab)
UDA
UEA-II
VVL
WFL
PA-IL
WGA(Seikagaku Bio)
Native HPA
SGA
AAHL
SSR1
rMpL
TbFBL1
CC1G-10077
MGL 6B
MGL 6A-H262T
MGL 6A
HA70 complex-Alexa
WGA multimer
rPVL
VVHA lectin
BanLec
CGL
Gal-3
human Gal-7
mouse Gal-7
CA19.9-1B844
CA19.9-9L426
CA19.9-121SLE
IPS-K-4A2B8
Microvirin
ab5
AOL (Mahal Lab)
ABA
Mono2W-CNL
BPL
GS-II (EY Lab)
EEA (EY Lab)
GNA (EY Lab)
HHA(EY Lab)
PHA-L
PNA
SBA
SJA
UEA-I
VGA(EY Lab)
WGA
MNA-M
MOA
WT-VCC
ME5(detected with a-HUD)
AAL
LSL-150
LBL-152
ME7(detected with a-HUD)
SSL0-488
VLRB.aGPA.23
rNV VLP-Cy3
AFCNVH
HML-Native
#8 Lewis B (T218) (Abcam)
#15 Sialyl Lewis X (BD)
#14 Sialyl Lewis X (Calbio)
hGal-1-488+BCR
TbFBL2
MGL 416
BTL native
GAC78
DC-ASGPR short
Sp Serotype 14.2
Sp Serotype 14.15
rSRL
SRL
Galectin-1
PsSC
Avaren-Fc
ChSeaL
CA19-9 Ab (DRG)
WT Galectin-8
CML1
PhoSL001
Epa1-Np mutant Y228W
Epa1-Np mutant Y228A
Epa1-Np mutant E227G
Epa1-Np mutant E227A
Epa1-Np WT
ChMBL
DBL-1
hJAA-F11-B11
Neu5Ac(α2-6)GalNAc(β1-4)GlcNAc(β-Sp0

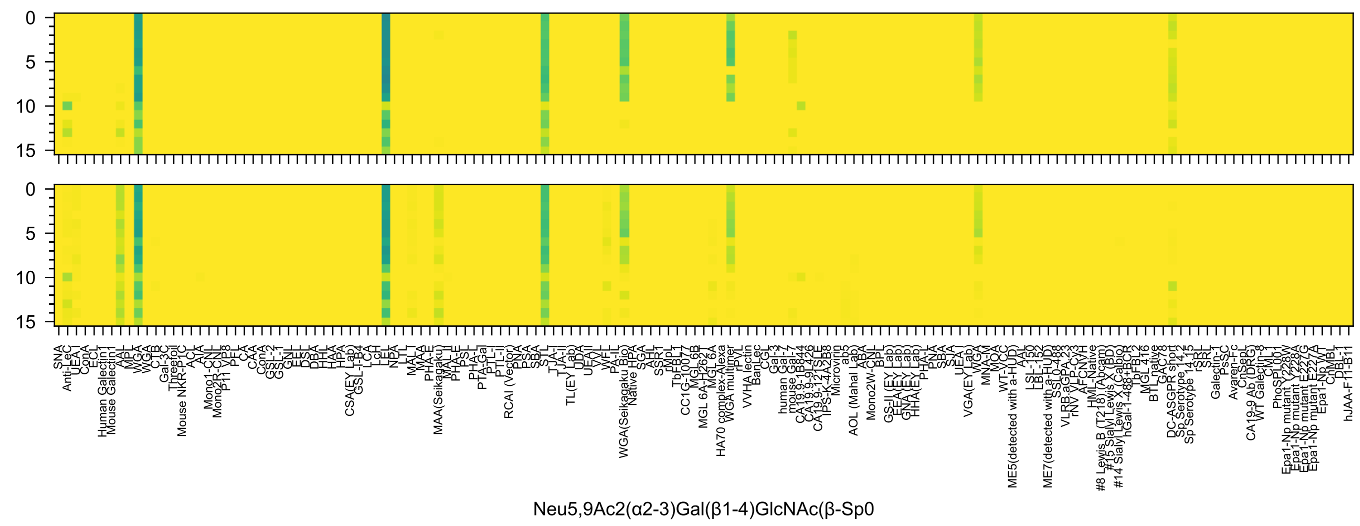
0
5
10
15
Neu5,9Ac2(α2-3)Gal(β1-4)GlcNAc(β-Sp0

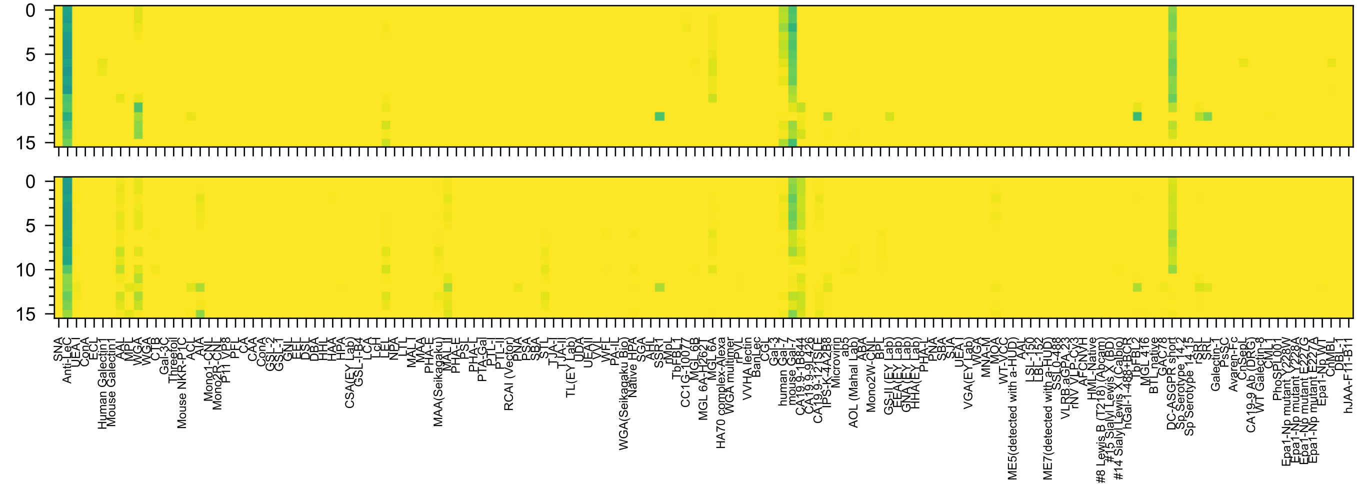

Neu5,9Ac2(α2-3)Gal(β1-3)GlcNAc(β-Sp0

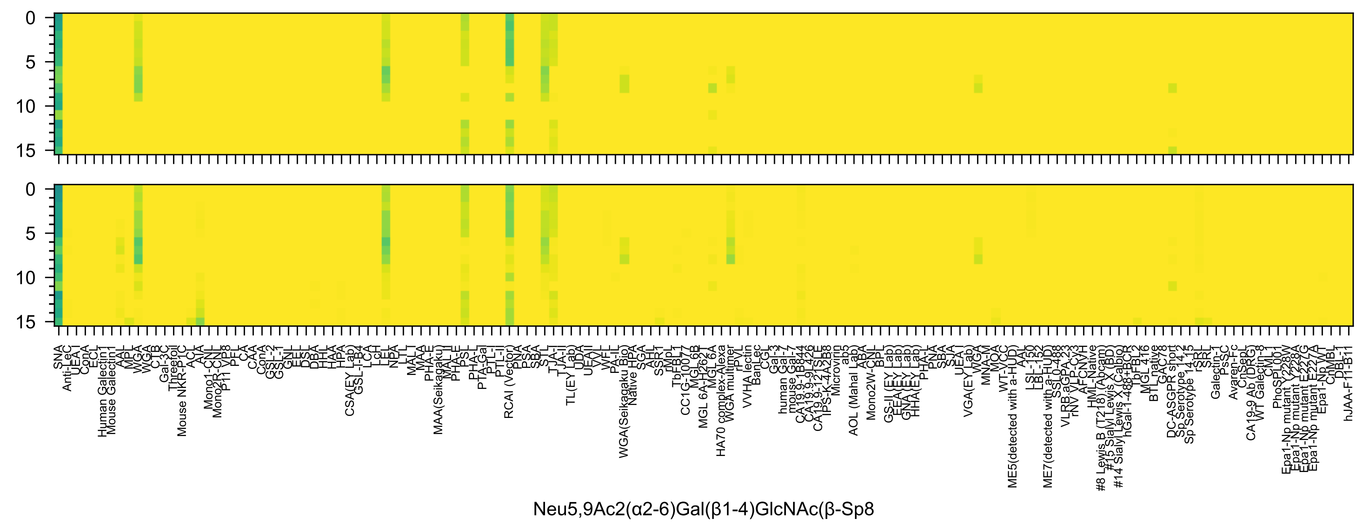

0
5
10
15
SNA
Anti-LeC
UEA-I
ConA
ECL
Human Galectin1
Mouse Galectin1
AAL
MPL
WGA
WGA
CTB
Gal-3C
Threefoil
Mouse NKR-P1C
ACL
AIA
Mono1-CNL
Mono2R-CNL
P11 VP8
PFL
CA
CAA
ConA
GSL-2
GSL-1
GNL
EEL
DSL
DBA
HHL
HAA
HPA
CSA(EY Lab)
GSL-I-B4
LCA
LcH
LEL
NPA
LTL
MAL-I
MAA
PHA-E
MAA(Seikagaku)
MAL-II
PHA-E
PSL
PHA-L
PTA-Gal
PTL-I
PTL-II
RCAI (Vector)
PNA
PSA
SBA
STL
TJA-I
TJA-II
TL(EY Lab)
UDA
UEA-II
VVL
WFL
PA-IL
WGA(Seikagaku Bio)
Native HPA
SGA
AHL
SSR1
rMpL
TbFBL1
CC1G-10077
MGL 6B
MGL 6A-H262T
MGL 6A
HA70 complex-Alexa
WGA multimer
rPVL
VVHA lectin
BanLec
CGL
Gal-3
human Gal-7
mouse Gal-7
CA19.9-1B844
CA19.9-9L426
CA19.9-121SLE
IPS-K-4A2B8
Microvirin
ab5
AOL (Mahal Lab)
ABA
Mono2W-CNL
BPL
GS-II (EY Lab)
EEA(EY Lab)
GNA(EY Lab)
HHA(EY Lab)
PHA-L
PNA
SBA
SJA
UEA-I
VGA(EY Lab)
WGA
MNA-M
MOA
WT-VCC
ME5(detected with a-HUD)
AAL
LSL-150
LBL-152
ME7(detected with a-HUD)
SSL0-488
VLRB.aGPA.23
rNV VLP-Cy3
AFCNVH
HML-Native
#8 Lewis B (T218) (Abcam)
#15 Sialyl Lewis X (BD)
#14 Sialyl Lewis X (Calbio)
hGal-1-488+BCR
TbFBL2
MGL 416
BTL native
GAC78
DC-ASGPR short
Sp Serotype 14.2
Sp Serotype 14.15
rSRL
SRL
Galectin-1
PsSC
Avaren-Fc
ChSepL
CA19-9 Ab (DRG)
WT Galectin-8
CML1
PhoSL001
Epa1-Np mutant Y228W
Epa1-Np mutant Y228A
Epa1-Np mutant E227G
Epa1-Np mutant E227A
Epa1-Np WT
ChMBL
DBL-1
hJAA-F11-B11
Neu5,9Ac2(α2-6)Gal(β1-4)GlcNAc(β-Sp8

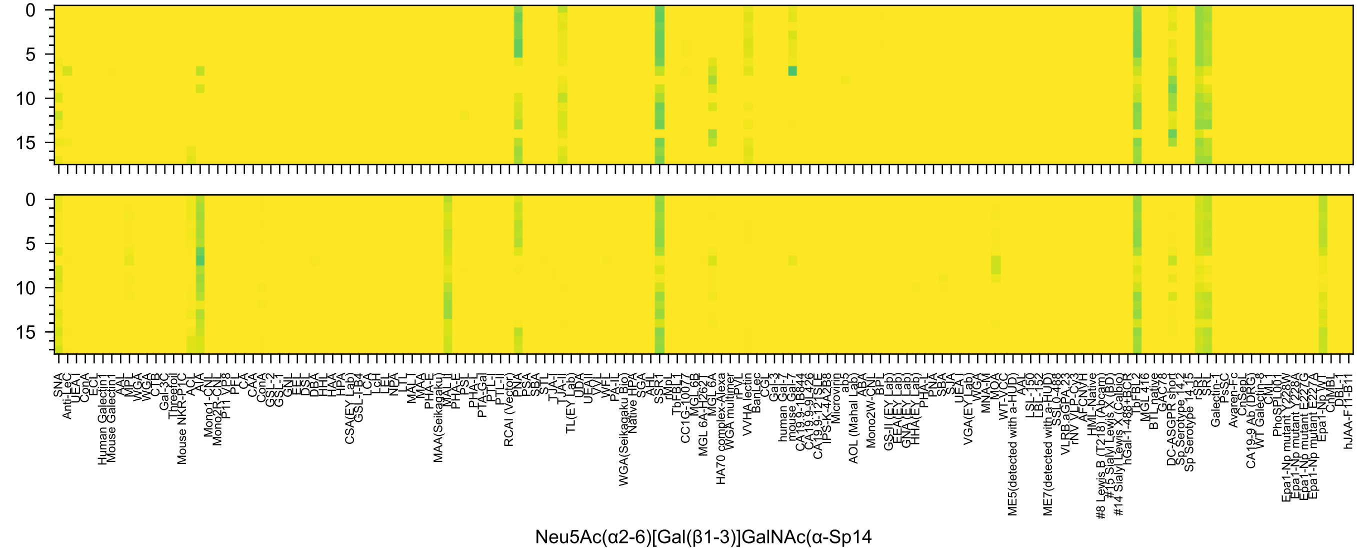
0
5
10
15
SNA
Anti-LeC
UEA-I
ConA
ECL
Human Galectin1
Mouse Galectin1
AAL
MPL
WGA
WGA
CTB
Gal-3C
Threefoil
Mouse NKR-P1C
ACL
AIA
Mono1-CNL
Mono2R-CNL
P11 VP8
PFL
CA
CAA
ConA
GSL-2
GSL-1
GNL
EEL
DSL
DBA
HHL
HAA
HPA
CSA(EY Lab)
GSL-I-B4
LCA
LcH
LEL
NPA
LTL
MAL-I
MAA
PHA-E
MAA(Seikagaku)
MAL-II
PHA-E
PSL
PHA-L
PTA-Gal
PTL-I
PTL-II
RCAI (Vector)
PNA
PSA
SBA
STL
TJA-I
TJA-II
TL(EY Lab)
UDA
UEA-II
VVL
WFL
PA-IL
WGA(Seikagaku Bio)
Native HPA
SGA
AAL
SSR1
rMpL
TbFBL1
CC1G-10077
MGL 6B
MGL 6A-H262T
MGL 6A
HA70 complex-Alexa
WGA multimer
rPVL
VVHA lectin
BanLec
CGL
Gal-3
human Gal-7
mouse Gal-7
CA19.9-1B844
CA19.9-9L426
CA19.9-121SLE
IPS-K-4A2B8
Microvirin
ab5
AOL (Mahal Lab)
ABA
Mono2W-CNL
BPL
GS-II (EY Lab)
EEA(EY Lab)
GNA(EY Lab)
HHA(EY Lab)
PHA-L
PNA
SBA
SJA
UEA-I
VGA(EY Lab)
WGA
MNA-M
MOA
WT-VCC
ME5(detected with a-HUD)
AAT
LSL-150
LBL-152
ME7(detected with a-HUD)
SSL0-488
VLRB.aGPA.23
rNV VLP-Cy3
AFCNVH
HML-Native
#8 Lewis B (T218) (Abcam)
#15 Sialyl Lewis X (BD)
#14 Sialyl Lewis X (Calbio)
hGal-1-488+BCR
TbFBL2
MGL 416
BTL native
GAC78
DC-ASGPR short
Sp Serotype 14.2
Sp Serotype 14.15
rSRL
SRL
Galectin-1
PsSC
Avaren-Fc
CnSepL
CA19-9 Ab (DRG)
WT Galectin-8
CML1
PhoSL001
Epa1-Np mutant Y228V
Epa1-Np mutant Y228A
Epa1-Np mutant E227G
Epa1-Np mutant E227A
Epa1-Np WT
CnMBL
DBL-1
hJAA-F11-B11
Neu5Ac(α2-6)[Gal(β1-3)]GalNAc(α-Sp14

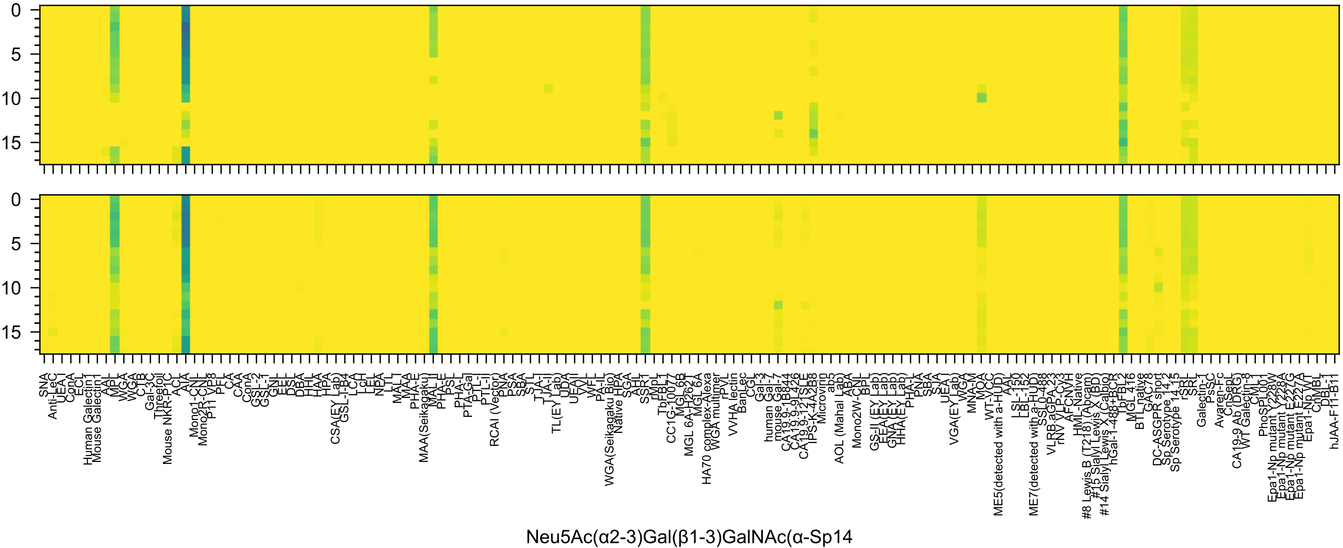
0
5
10
15
SNA
Anti-LeC
UEA I
ConA
ECL
Human Galectin1
Mouse Galectin1
AAL
MPL
WGA
WGA
CTB
Gal-3C
Threefoil
Mouse NKR-P1C
ACL
AIA
Mono1-CNL
Mono2R-CNL
P11 VP8
PFL
CA
CAA
ConA
GSL-2
GSL-1
GNL
EEL
DSL
DBA
HHL
HAA
HPA
CSA(EY Lab)
GSL-I-B4
LCA
LcH
LEL
NPA
LTL
MAL I
MAA
PHA-E
MAA(Seikagaku)
MAL II
PHA-E
PSL
PHA-L
PTA-Gal
PTL-I
PTL-II
RCAI (Vector)
PNA
PSA
SBA
STL
TJA-I
TJA-II
TL(EY Lab)
UDA
UEA II
VVL
WFL
PA-IL
WGA(Seikagaku Bio)
Native HPA
SGA
AAH
SSR1
rMpL
TbFBL1
CC1G-10077
MGL 6B
MGL 6A-H262T
MGL 6A
HA70 complex-Alexa
WGA multimer
rPVL
VVHA lectin
BanLec
CGL
Gal-3
Gal-7
human Gal-7
mouse Gal-7
CA19.9-1B844
CA19.9-9L426
CA19.9-121SLE
IPS-K-4A2B8
Microvirin
ab5
AOL (Mahal Lab)
ABA
Mono2W-CNL
BPL
GS-II (EY Lab)
EEA(EY Lab)
GNA(EY Lab)
HHA(EY Lab)
PHA-L
PNA
SBA
SJA
UEA I
VGA(EY Lab)
WGA
MNA-M
MOA
WT-VCC
ME5(detected with a-HUD)
AAL
LSL-150
LBL-152
ME7(detected with a-HUD)
SSL0-488
VLRB.aGPA.23
rNV VLP-Cy3
AFCNVH
HML-Native
#8 Lewis B (T218) (Abcam)
#15 Sialyl Lewis X (BD)
#14 Sialyl Lewis X (Calbio)
hGal-1-488+BCR
TbFBL2
MGL 416
BTL_native
GAC78
DC-ASGPR short
Sp Serotype 14.2
Sp Serotype 14.15
rSRL
SRL
Galectin-1
PsSC
Avaren-Fc
ChSeL
CA19-9 Ab (DRG)
WT Galectin-8
CML1
PhoSL001
Epa1-Np mutant Y228W
Epa1-Np mutant Y228A
Epa1-Np mutant E227G
Epa1-Np mutant E227A
Epa1-Np WT
Epa1
ChMBL
DBL-1
hJAA-F11-B11
Neu5Ac(α2-3)Gal(β1-3)GalNAc(α-Sp14

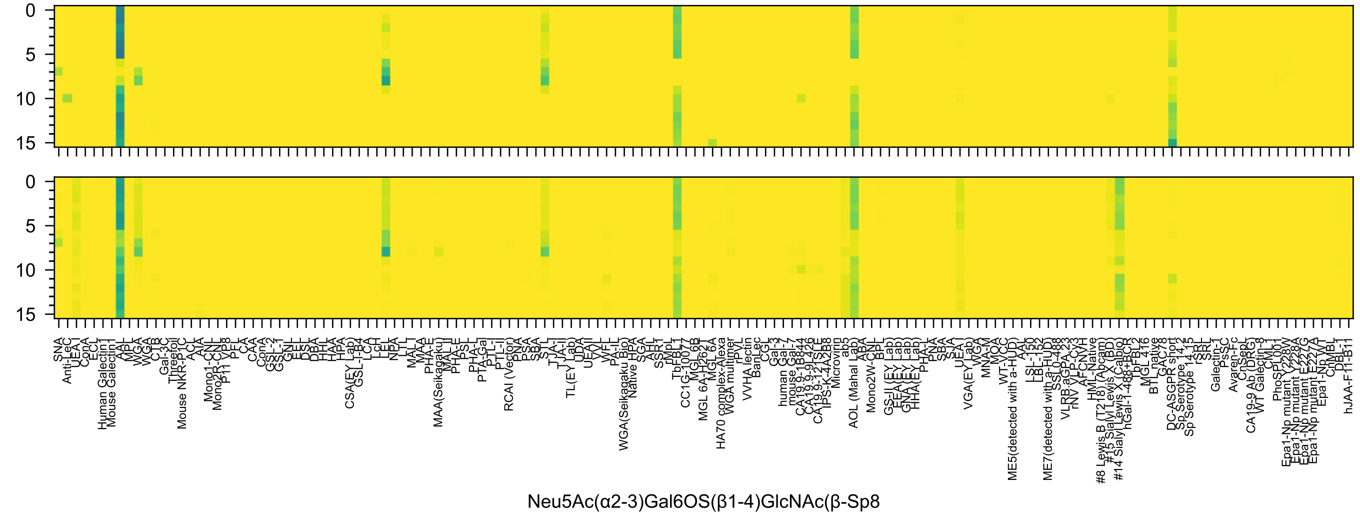

0
5
10
15
SNA
Anti-LeC
UEA-I
ConA
ECL
Human Galectin1
Mouse Galectin1
AAL
MPL
WGA
WGA
CTB
C-3
Gal-3C
Threefoil
Mouse NKR-P1C
ACL
AIA
Mono1-CNL
Mono2R-CNL
P11 VP8
PFL
CA
CAA
ConA
GSL-2
GSL-1
GNL
EEL
DSL
DBA
HHL
HAA
HPA
CSA(EY Lab)
GSL-I-B4
LCA
LcH
LEL
LEL
NPA
LTL
MAL-I
MAA
PHA-E
MAA(Seikagaku)
MAL-II
PHA-E
PSL
PHA-L
PTA-Gal
PTL-I
PTL-II
RCAI (Vector)
PNA
PSA
SBA
STL
TJA-I
TJA-II
TL(EY Lab)
UDA
UEA-II
VVL
WFL
PA-IL
WGA(Seikagaku Bio)
Native HPA
SGA
AAL
SSR1
rMpL
TbFBL1
CC1G-10077
MGL 6B
MGL 6A-H262T
MGL 6A
HA70 complex-Alexa
WGA multimer
rPVL
VVHA lectin
BanLec
CGL
Gal-3
human Gal-7
mouse Gal-7
CA19.9-1B844
CA19.9-9L426
CA19.9-121SLE
IPS-K-4A2B8
Microvirin
ab5
AOL (Mahal Lab)
ABA
Mono2W-CNL
BPL
GS-II (EY Lab)
EEA(EY Lab)
GNA(EY Lab)
HHA(EY Lab)
PHA-L
PNA
SBA
SJA
UEA-I
VGA(EY Lab)
WGA
MNA-M
MOA
WT-VCC
ME5(detected with a-HUD)
AAL
LSL-150
LBL-152
ME7(detected with a-HUD)
SSL0-488
VLRB.aGPA.23
rNV VLP-Cy3
AFCNVH
HML-Native
#8 Lewis B (T218) (Abcam)
#15 Sialyl Lewis X (BD)
#14 Sialyl Lewis X (Calbio)
hGal-1-488+BCR
TbFBL2
MGL 416
BTL native
GAC78
DC-ASGPR short
Sp Serotype 14.2
Sp Serotype 14.15
rSRL
SRL
Galectin-1
PsSC
Avaren-Fc
ChSepL
CA19-9 Ab (DRG)
WT Galectin-8
CML1
PhoSL001
Epa1-Np mutant Y228W
Epa1-Np mutant Y228A
Epa1-Np mutant E227G
Epa1-Np mutant E227A
Epa1-Np WT
ChMBL
DBL-1
hJAA-F11-B11
Neu5Ac(α2-3)Gal6OS(β1-4)GlcNAc(β-Sp8

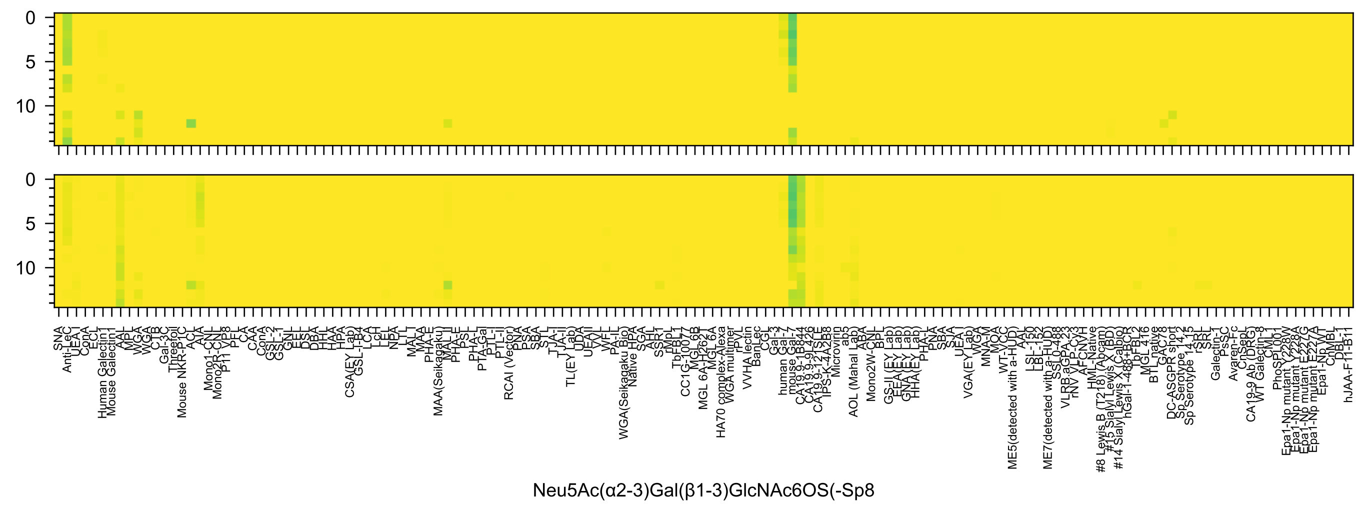
Neu5Ac(α2-3)Gal(β1-3)GlcNAc6OS(-Sp8

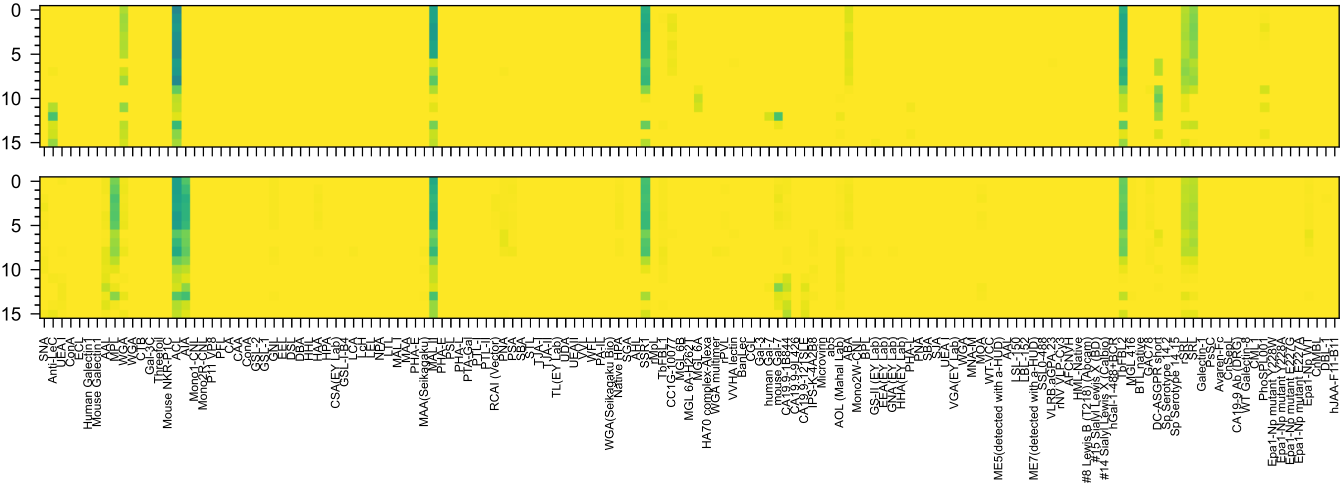

Neu5Ac(α2-3)Gal(β1-3)GalNAc6OS(α-Sp8

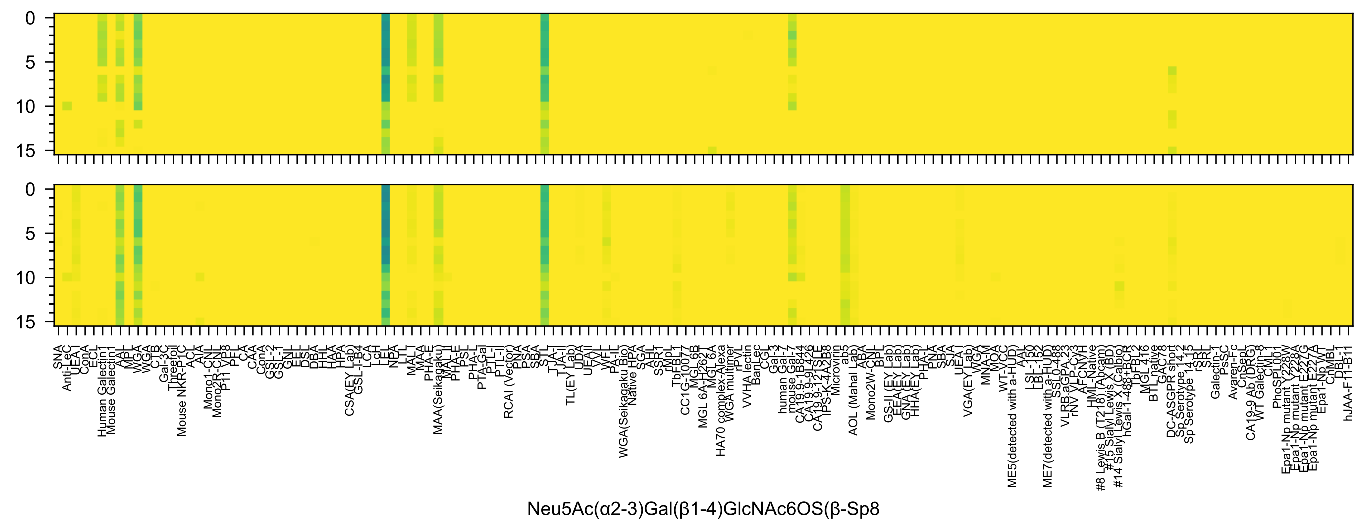
0
5
10
15
0
5
10
15
SNA
Anti-LeC
UEA-I
ConA
ECL
Human Galectin1
Mouse Galectin1
AAL
MPL
WGA
WGA
CTB
Gal-3C
Threefoil
Mouse NKR-P1C
ACL
AIA
Mono1-CNL
Mono2R-CNL
P11 VP8
PFL
CA
CAA
ConA
GSL-2
GSL-1
GNL
EEL
DSL
DBA
HHL
HAA
HPA
CSA(EY Lab)
GSL-I-B4
LCA
LcH
LEL
NPA
LTL
MALT
MAA
PHA-E
MAA(Seikagaku)
MAL-II
PHA-E
PSL
PHA-L
PTA-Gal
PTL-I
PTL-II
RCAI (Vector)
PNA
PSA
SBA
STL
TJA-I
TJA-II
TL(EY Lab)
UDA
UEAII
VVL
WFL
PA-IL
WGA(Seikagaku Bio)
Native HPA
SGA
AHL
SSR1
rMpL
TbFBL1
CC1G-10077
MGL 6B
MGL 6A-H262T
MGL 6A
HA70 complex-Alexa
WGA multimer
rPVL
VVHA lectin
BanLec
CGL
Gal-3
human Gal-7
mouse Gal-7
CA19.9-1B844
CA19.9-9L426
CA19.9-121SLE
IPS-K-4A2B8
Microvirin
ab5
AOL (Mahal Lab)
ABA
Mono2W-CNL
BPL
GS-II (EY Lab)
EEA (EY Lab)
GNA (EY Lab)
HHA (EY Lab)
PHA-L
PNA
SBA
SJA
UEA-I
VGA(EY Lab)
WGA
MNA-M
MOA
WT-VCC
ME5(detected with a-HUD)
AAL
LSL-150
LBL-152
ME7(detected with a-HUD)
SSL0-488
VLRB.aGPA.23
rNV VLP-Cy3
AFCNVH
HML-Native
#8 Lewis B (T218) (Abcam)
#15 Sialyl Lewis X (BD)
#14 Sialyl Lewis X (Calbio)
hGal-1-488+BCR
TbFBL2
MGL 416
BTL native
GAC78
DC-ASGPR short
Sp Serotype 14.2
Sp Serotype 14.15
rSRL
SRL
Galectin-1
PsSC
Avaren-Fc
ChSeNL
CA19-9 Ab (DRG)
WT Galectin-8
CML1
PhoSL001
Epa1-Np mutant Y228W
Epa1-Np mutant Y228A
Epa1-Np mutant E227G
Epa1-Np mutant E227A
Epa1-Np WT
ChMBL
DBL-1
hJAA-F11-B11
Neu5Ac(α2-3)Gal(β1-4)GlcNAc6OS(β-Sp8

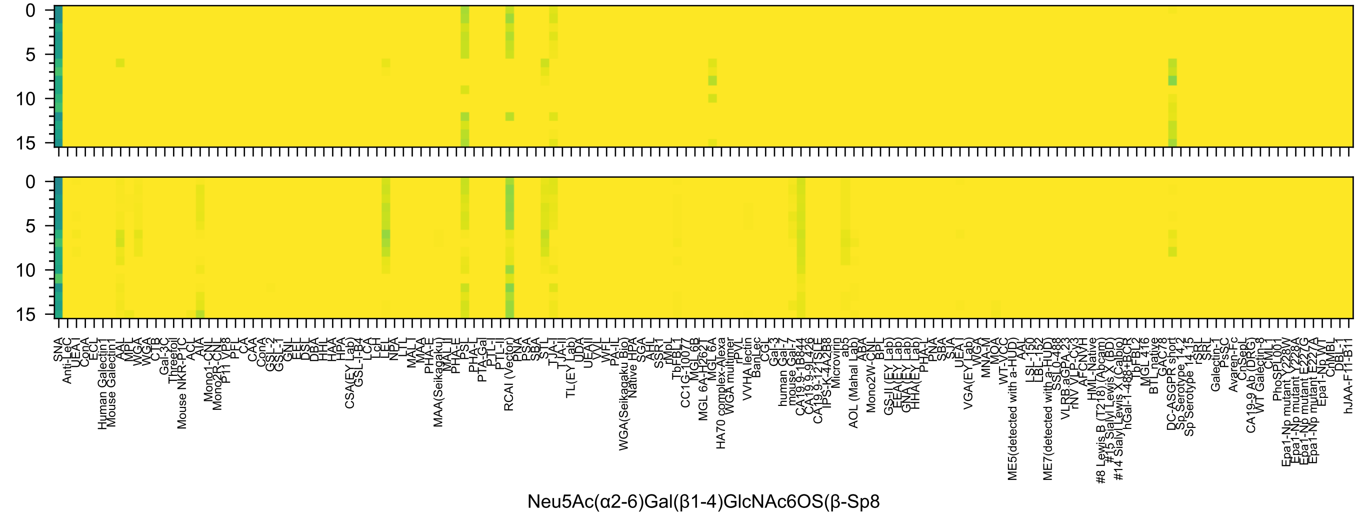
0
5
10
15
0
5
10
15
SNA
Anti-LeC
UEA I
ConA
ECL
Human Galectin1
Mouse Galectin1
AAL
MPL
WGA
WGA
CTB
Gal-3C
Threefoil
Mouse NKR-P1C
ACL
AIA
Mono1-CNL
Mono2R-CNL
P11 VP8
PFL
CA
CAA
ConA
GSL-2
GSL-1
GNL
EEL
DSL
DBA
HHL
HAA
HPA
CSA(EY Lab)
GSL-I-B4
LCA
LcH
LEL
NPA
LTL
MAL
MAA
PHA-E
MAA(Seikagaku)
MAL-II
PHA-E
PSL
PHA-L
PTA-Gal
PTL-I
PTL-II
RCAI (Vector)
PNA
PSA
SBA
STL
TJA-I
TJA-II
TL(EY Lab)
UDA
UEAII
VVL
WFL
PA-IL
WGA(Seikagaku Bio)
Native HPA
SGA
AHL
SSR1
rMpL
TbFBL1
CC1G-10077
MGL 6B
MGL 6A-H262T
MGL 6A
HA70 complex-Alexa
WGA multimer
rPVL
VVHA lectin
BanLec
CGL
Gal-3
human Gal-7
mouse Gal-7
CA19.9-1B844
CA19.9-9L426
CA19.9-121SLE
IPS-K-4A2B8
Microvirin
ab5
AOL (Mahal Lab)
ABA
Mono2W-CNL
BPL
GS-II (EY Lab)
EEA(EY Lab)
GNA(EY Lab)
HHA(EY Lab)
PHA-L
PNA
SBA
SJA
UEA-I
VGA(EY Lab)
WGA
MNA-M
MOA
WT-VCC
ME5(detected with a-HUD)
AAL
LSL-150
LBL-152
ME7(detected with a-HUD)
SSL0-488
VLRB.aGPA.23
rNV VLP-Cy3
AFCNVH
HML-Native
#8 Lewis B (T218) (Abcam)
#15 Sialyl Lewis X (BD)
#14 Sialyl Lewis X (Calbio)
hGal-1-488+BCR
TbFBL2
MGL 416
BTL native
GAC78
DC-ASGPR short
Sp Serotype 14.2
Sp Serotype 14.15
rSRL
SRL
Galectin-1
PsSC
Avaren-Fc
ChSep1
CA19-9 Ab (DRG)
WT Galectin-8
CML1
PhoSL001
Epa1-Np mutant Y228W
Epa1-Np mutant Y228A
Epa1-Np mutant E227G
Epa1-Np mutant E227A
Epa1-Np WT
ChMBL
DBL-1
hJAA-F11-B11
Neu5Ac(α2-6)Gal(β1-4)GlcNAc6OS(β-Sp8

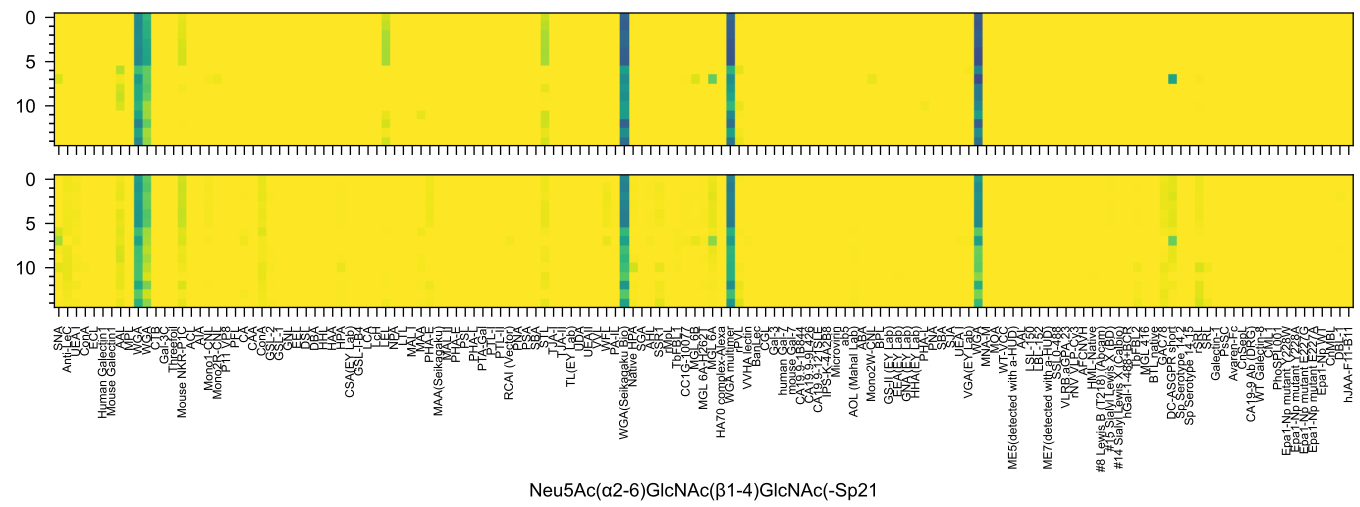

Neu5Ac(α2-6)GlcNAc(β1-4)GlcNAc(-Sp21

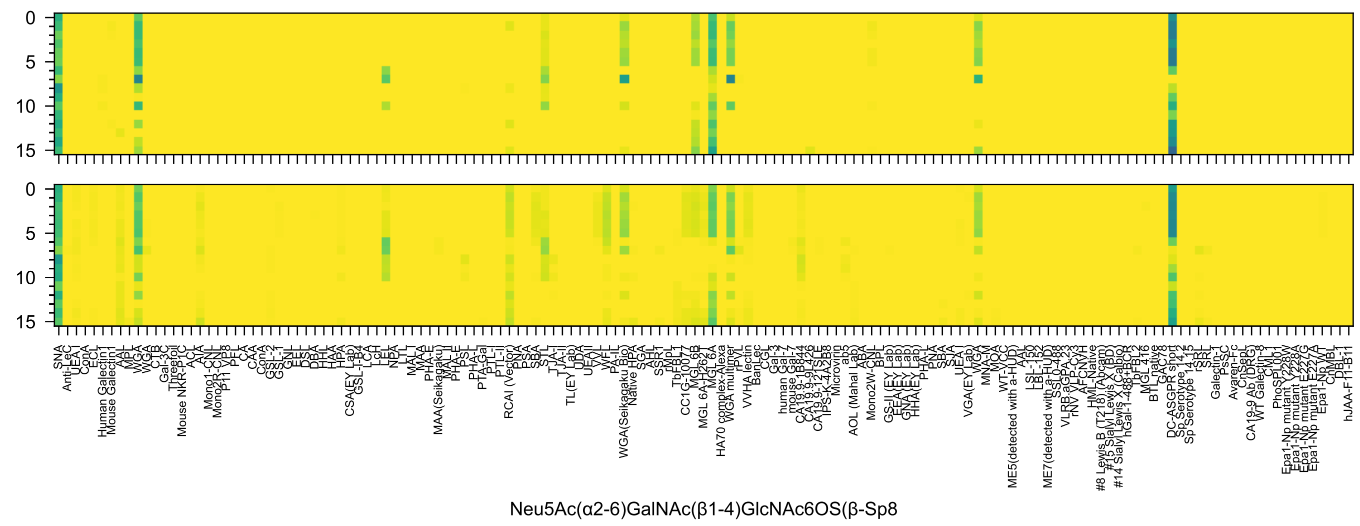
0
5
10
15
0
5
10
15
SNA
Anti-LeC
UEA-I
ConA
ECL
Human Galectin1
Mouse Galectin1
AAL
MPL
WGA
WGA
CTB
C-18
Gal-3C
Threefoil
Mouse NKR-P1C
ACL
AIA
Mono1-CNL
Mono2R-CNL
P11 VP8
PFL
CA
CAA
ConA
GSL-2
GSL-1
GNL
EEL
DSL
DBA
HHL
HAA
HPA
CSA(EY Lab)
GSL-I-B4
LCA
LcH
LEL
NPA
LTL
MAL-I
MAA
PHA-E
MAA(Seikagaku)
MAL-II
PSL
PHA-L
PTA-Gal
PTL-I
PTL-II
RCAI (Vector)
PNA
PSA
SBA
STL
TJA-I
TJA-II
TL(EY Lab)
UDA
UEA-II
VVL
WFL
PA-IL
WGA(Seikagaku Bio)
Native HPA
SGA
AHL
SSR1
rMpL
TbFBL1
CC1G-10077
MGL 6B
MGL 6A-H262T
MGL 6A
HA70 complex-Alexa
WGA multimer
rPVL
VVHA lectin
BanLec
CGL
Gal-3
human Gal-7
mouse Gal-7
CA19.9-1B844
CA19.9-9L426
CA19.9-121SLE
IPS-K-4A2B8
Microvirin
ab5
AOL (Mahal Lab)
ABA
Mono2W-CNL
BPL
GS-II (EY Lab)
EEA(EY Lab)
GNA(EY Lab)
HHA(EY Lab)
PHA-L
PNA
SBA
SJA
UEA-I
VGA(EY Lab)
WGA
MNA-M
MOA
WT-VCC
ME5(detected with a-HUD)
AAL
LSL-150
LBL-152
ME7(detected with a-HUD)
SSL0-488
VLRB.aGPA.23
rNV VLP-Cy3
AFCNVH
HML-Native
#8 Lewis B (T218) (Abcam)
#15 Sialyl Lewis X (BD)
#14 Sialyl Lewis X (Calbio)
hGal-1-488+BCR
TbFBL2
MGL 416
BTL native
GAC78
DC-ASGPR short
Sp Serotype 14.2
Sp Serotype 14.15
rSRL
SRL
Galectin-1
PsSC
Avaren-Fc
ChSeI
CA19-9 Ab (DRG)
WT Galectin-8
CML1
PhoSL001
Epa1-Np mutant Y228W
Epa1-Np mutant Y228A
Epa1-Np mutant E227G
Epa1-Np mutant E227A
Epa1-Np WT
CnMBL
DBL-1
hJAA-F11-B11
Neu5Ac(α2-6)GalNAc(β1-4)GlcNAc6OS(β-Sp8

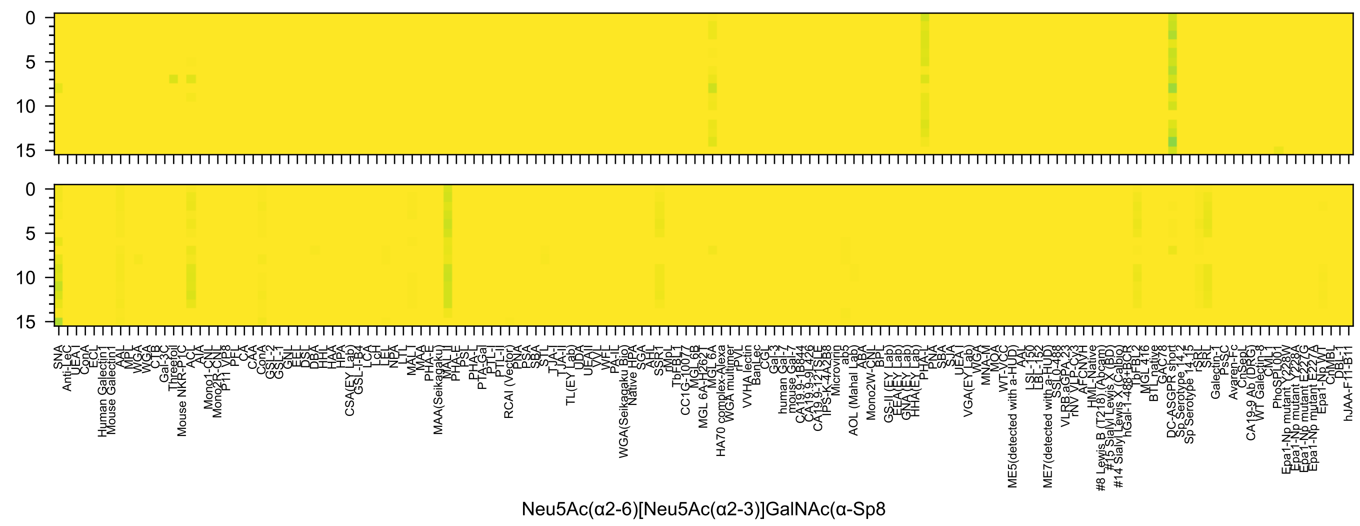
Neu5Ac(α2-6)[Neu5Ac(α2-3)]GalNAc(α-Sp8

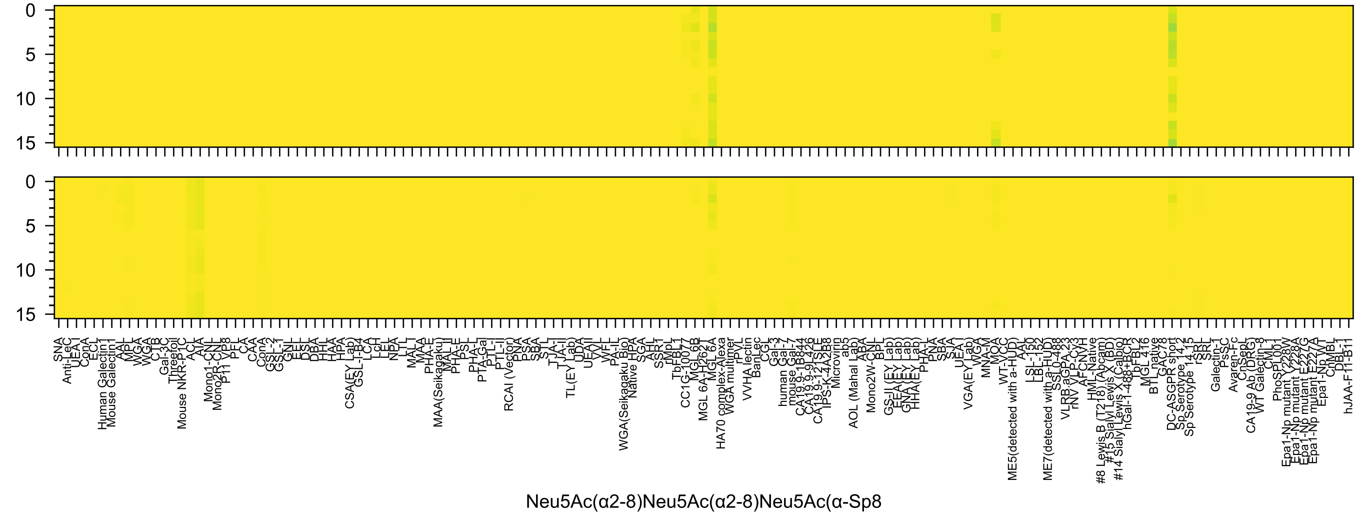
0
5
10
15
SNA
Anti-LeC
UEA I
ConA
ECL
Human Galectin1
Mouse Galectin1
AAL
MPL
WGA
WGA
CTB
Gal-3C
Threefoil
Mouse NKR-P1C
ACL
AIA
Mono1-CNL
Mono2R-CNL
P11 VP8
PFL
CA
CAA
ConA
GSL-2
GSL-1
GNL
EEL
DSL
DBA
HHL
HAA
HPA
CSA(EY Lab)
GSL-I-B4
LCA
LcH
LEL
NPA
LTL
MAL I
MAA
PHA-E
MAA(Seikagaku)
MAL II
PHA-E
PSL
PHA-L
PTA-Gal
PTL-I
PTL-II
RCAI (Vector)
PNA
PSA
SBA
STL
TJA-I
TJA-II
TL(EY Lab)
UDA
UEA II
VVL
WFL
PA-IL
WGA(Seikagaku Bio)
Native HPA
SGA
AHL
SSR1
rMpL
TbFBL1
CC1G-10077
MGL 6B
MGL 6A-H262T
MGL 6A
HA70 complex-Alexa
WGA multimer
rPVL
VVHA lectin
BanLec
CGL
Gal-3
human Gal-7
mouse Gal-7
CA19.9-1B844
CA19.9-9L426
CA19.9-121SLE
IPS-K-4A2B8
Microvirin
ab5
AOL (Mahal Lab)
ABA
Mono2W-CNL
BPL
GS-II (EY Lab)
EEA (EY Lab)
GNA (EY Lab)
HHA (EY Lab)
PHA-L
PNA
SBA
SJA
UEA I
VGA(EY Lab)
WGA
MNA-M
MOA
WT-VCC
ME5(detected with a-HUD)
AAL
LSL-150
LBL-152
ME7(detected with a-HUD)
SSL0-488
VLRB.aGPA.23
rNV VLP-Cy3
AFCNVH
HML-Native
#8 Lewis B (T218) (Abcam)
#15 Sialyl Lewis X (BD)
#14 Sialyl Lewis X (Calbio)
hGal-1-488+BCR
TbFBL2
MGL 416
BTL native
GAC78
DC-ASGPR short
Sp Serotype 14.2
Sp Serotype 14.15
rSRL
SRL
Galectin-1
PsSC
Avaren-Fc
ChSeeL
CA19-9 Ab (DRG)
WT Galectin-8
CML1
PhoSL001
Epa1-Np mutant Y228W
Epa1-Np mutant Y228A
Epa1-Np mutant E227G
Epa1-Np mutant E227A
Epa1-Np WT
ChMBL
DBL-1
hJAA-F11-B11
Neu5Ac(α2-8)Neu5Ac(α2-8)Neu5Ac(α-Sp8

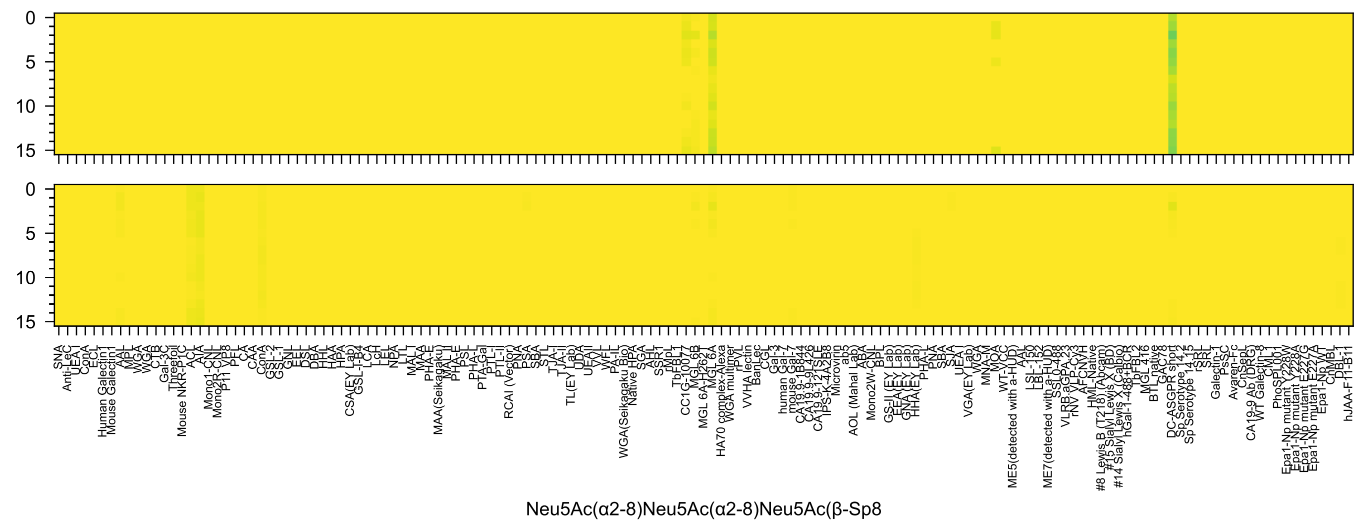
Neu5Ac(α2-8)Neu5Ac(α2-8)Neu5Ac(β-Sp8

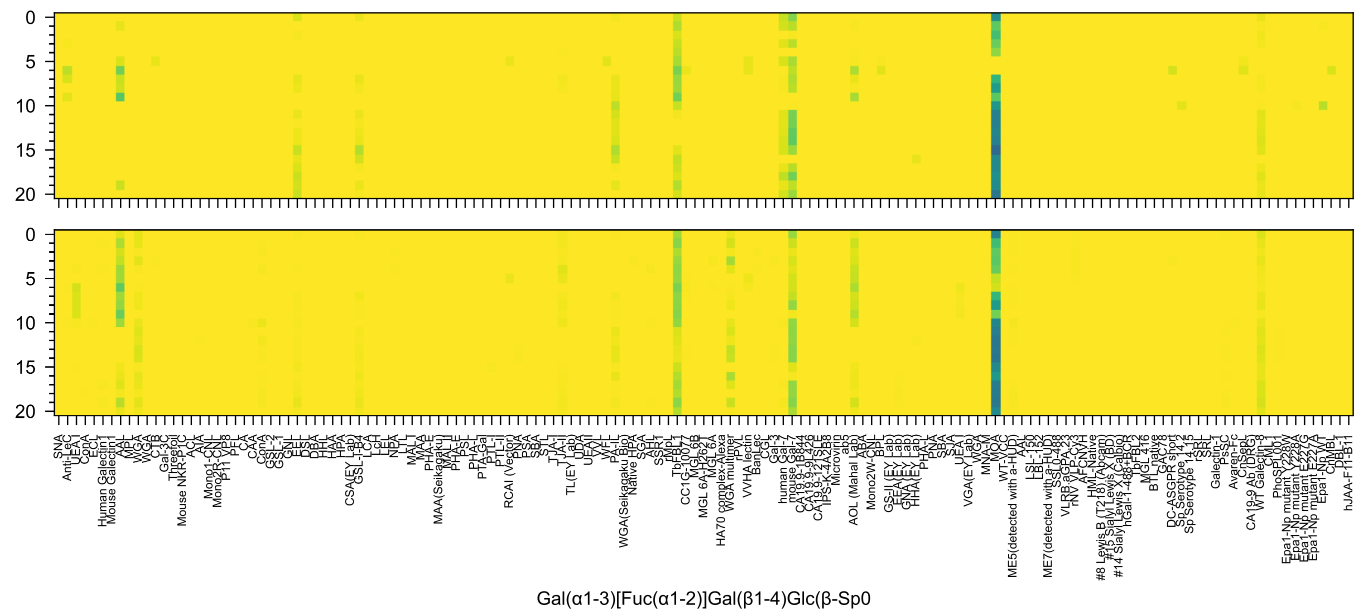
Gal(α1-3)[Fuc(α1-2)]Gal(β1-4)Glc(β-Sp0

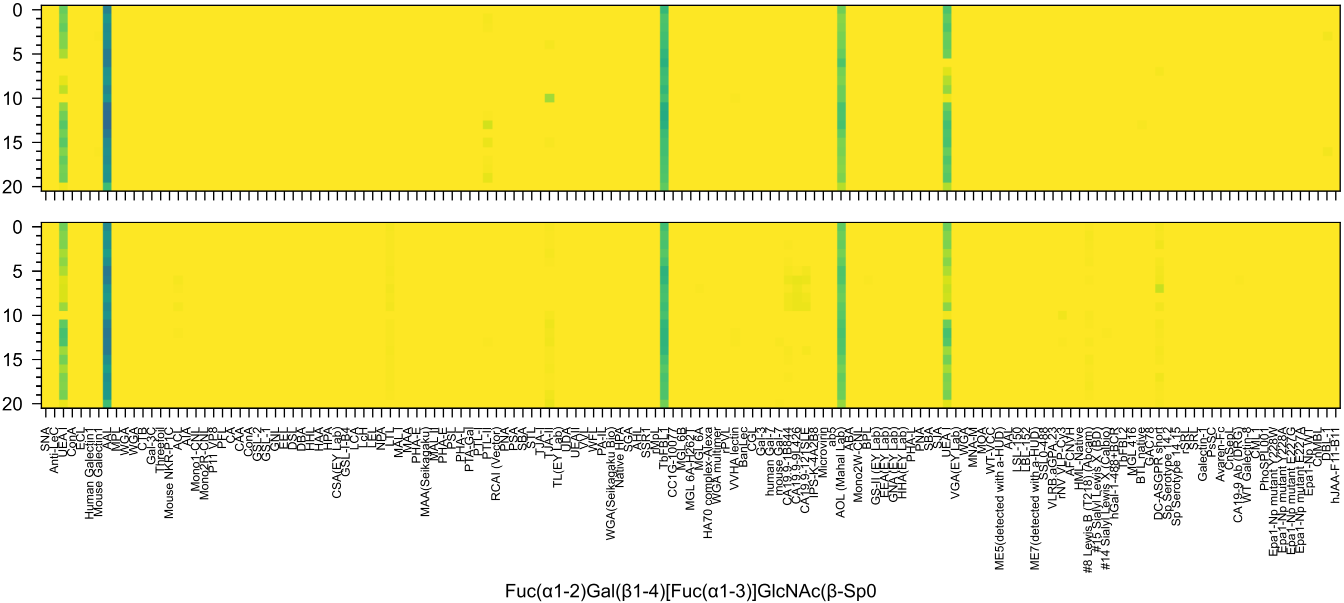
Fuc(α1-2)Gal(β1-4)[Fuc(α1-3)]GlcNAc(β-Sp0

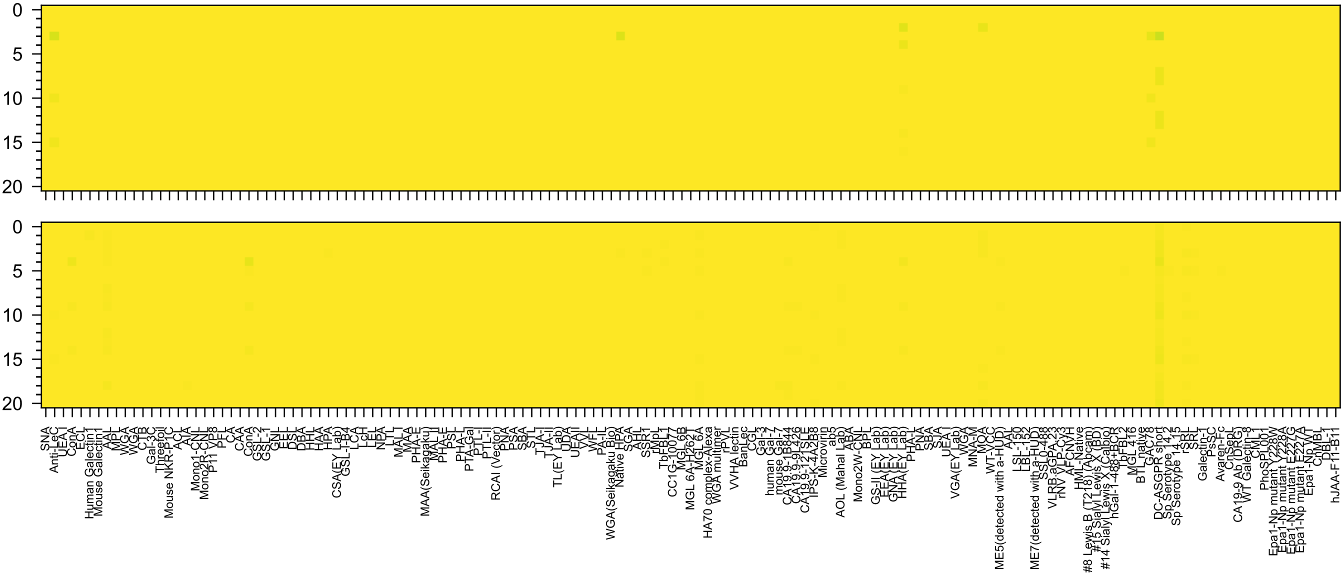

Glc(α1-6)Glc(α1-6)Glc(α1-6)Glc(β-Sp10

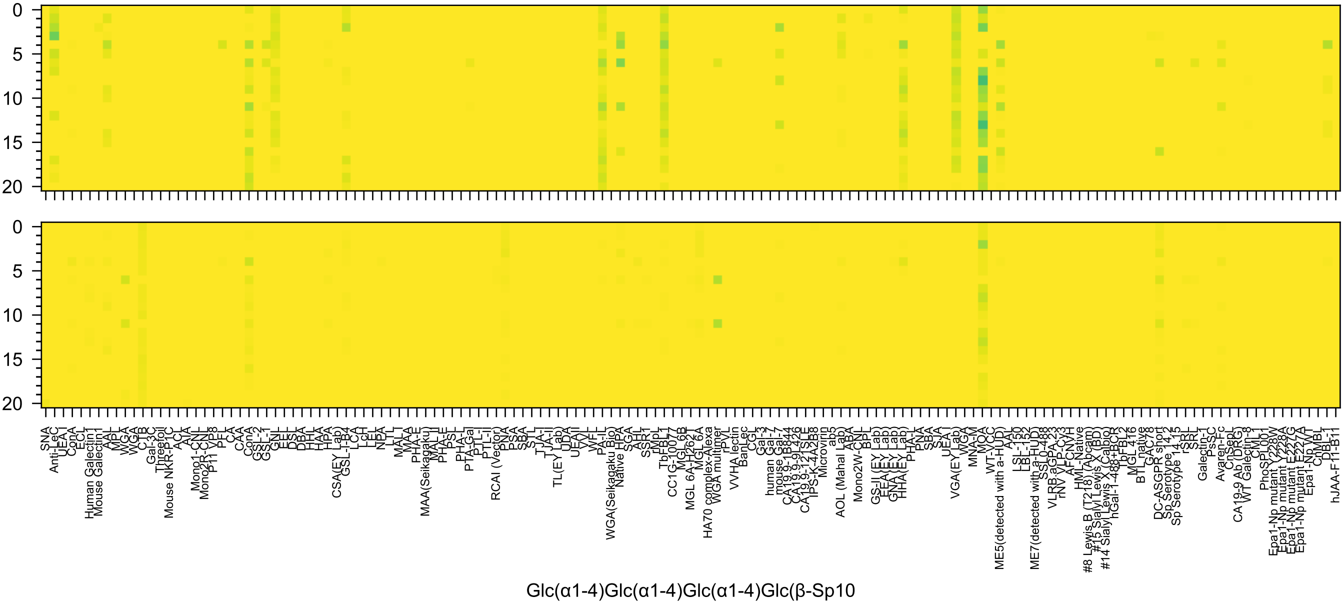

Glc(α1-4)Glc(α1-4)Glc(α1-4)Glc(β-Sp10

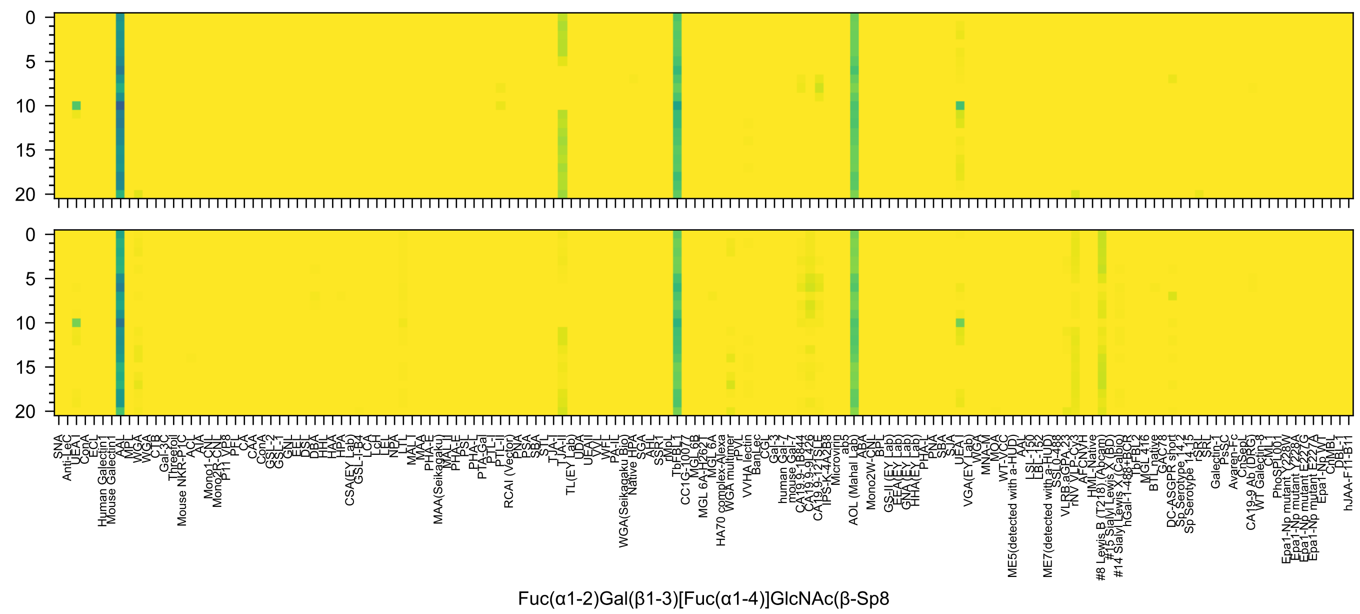
Fuc(α1-2)Gal(β1-3)[Fuc(α1-4)]GlcNAc(β-Sp8

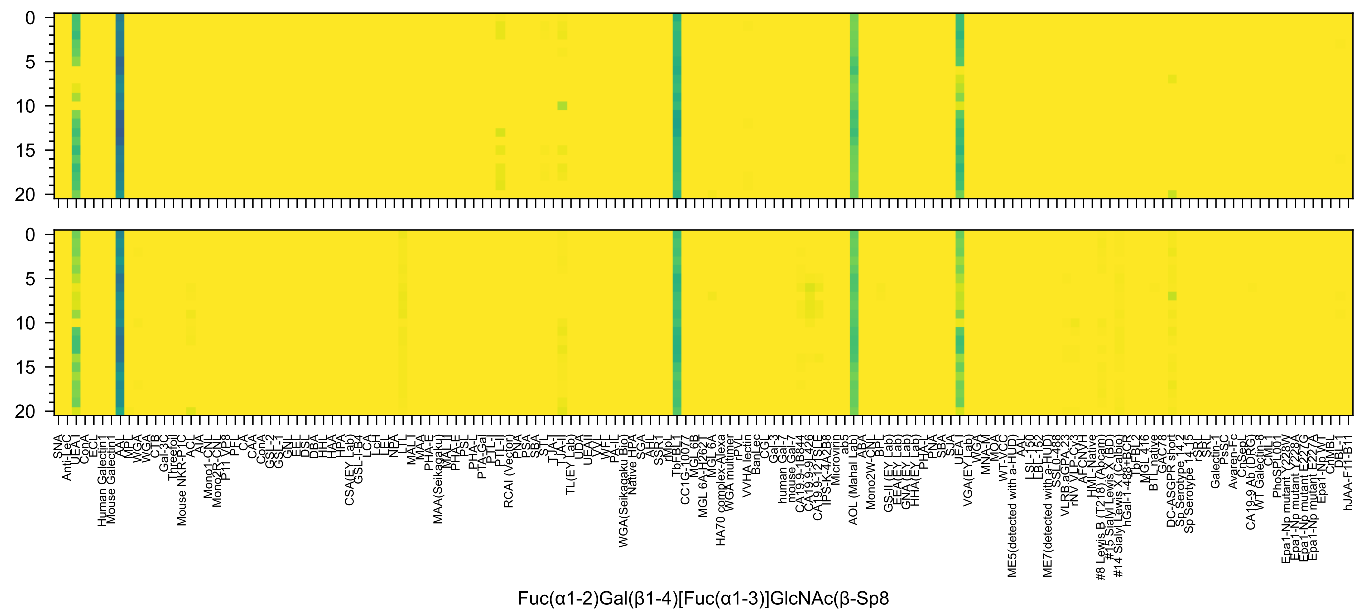

Fuc(α1-2)Gal(β1-4)[Fuc(α1-3)]GlcNAc(β-Sp8

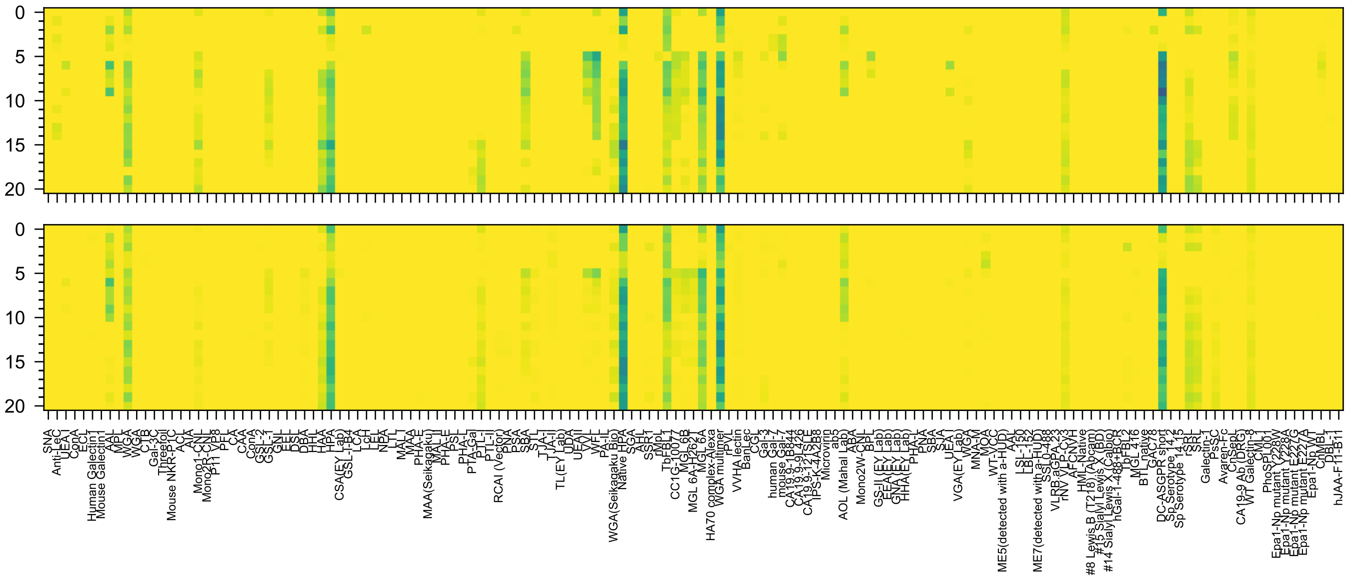

GalNAc(α1-3)[Fuc(α1-2)]Gal(β1-4)Glc(β-Sp0

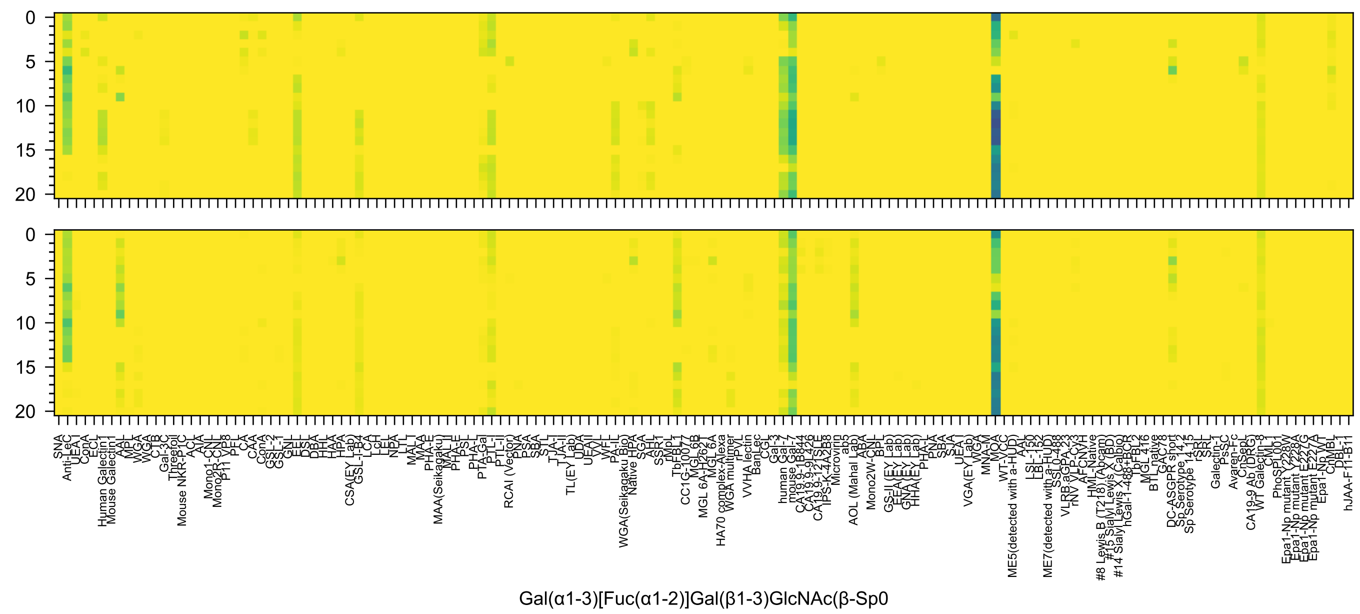
Gal(α1-3)[Fuc(α1-2)]Gal(β1-3)GlcNAc(β-Sp0

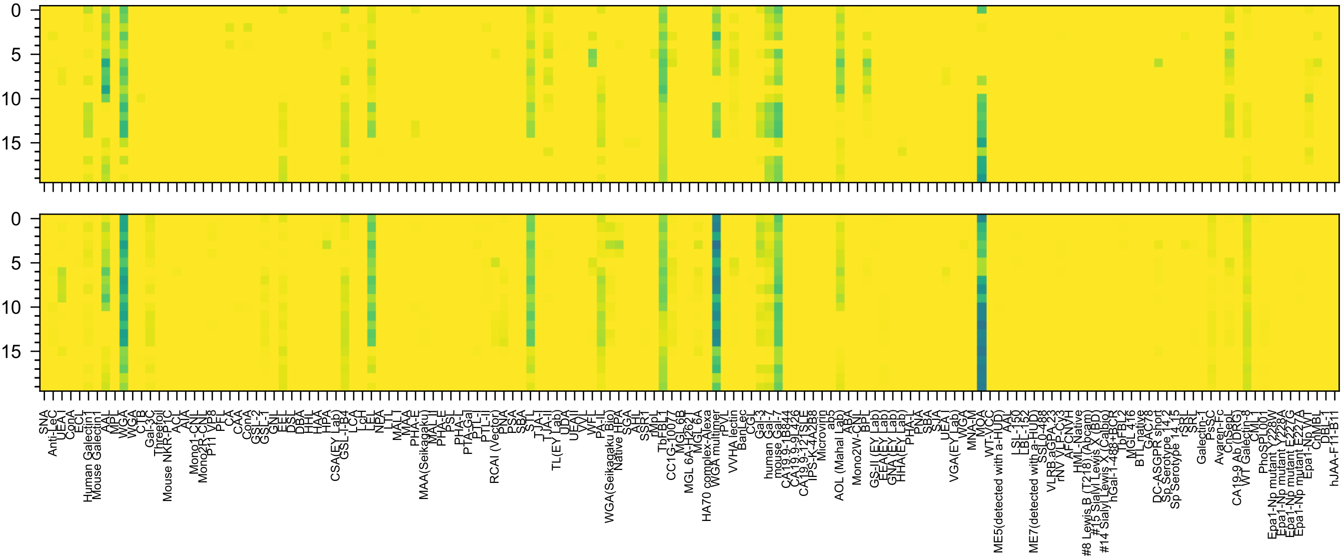

Gal(α1-3)[Fuc(α1-2)]Gal(β1-4)GlcNAc(-Sp0

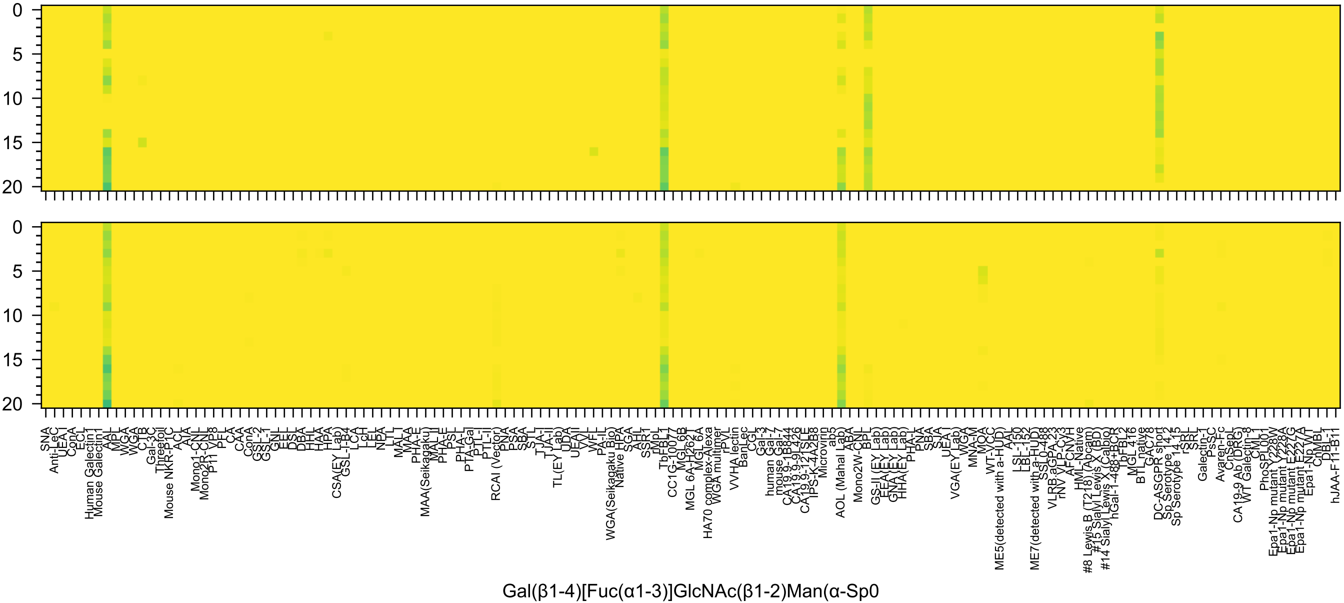

Gal(β1-4)[Fuc(α1-3)]GlcNAc(β1-2)Man(α-Sp0

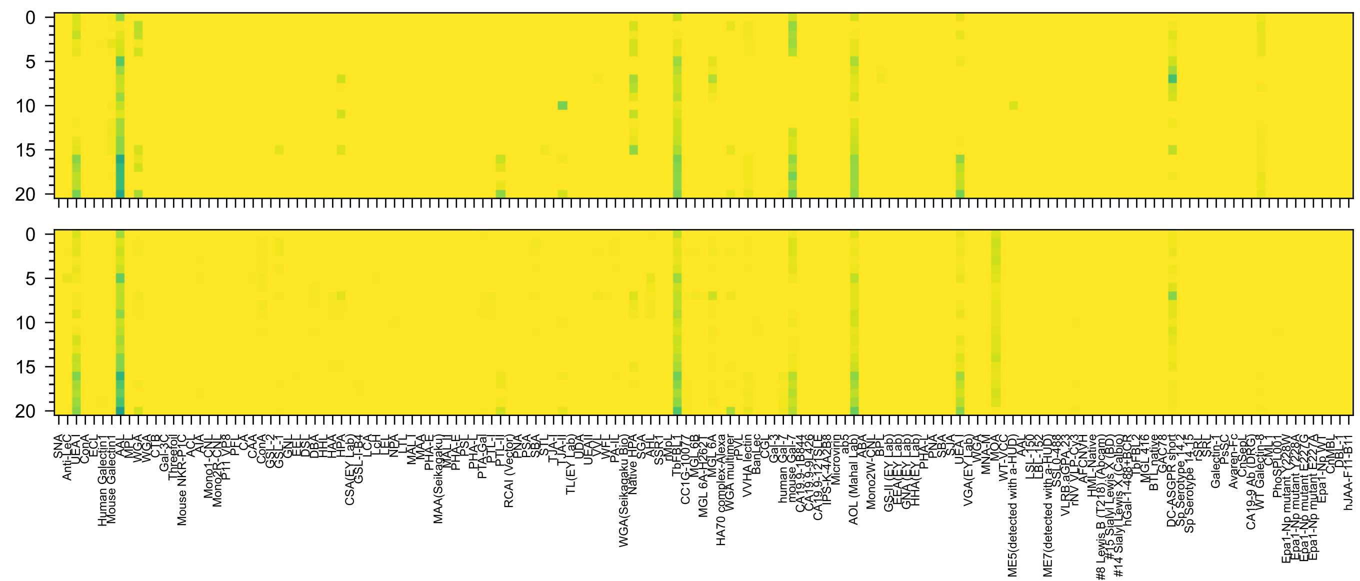

Fuc(α1-2)Gal(β1-4)GlcNAc(β1-2)Man(α-Sp0

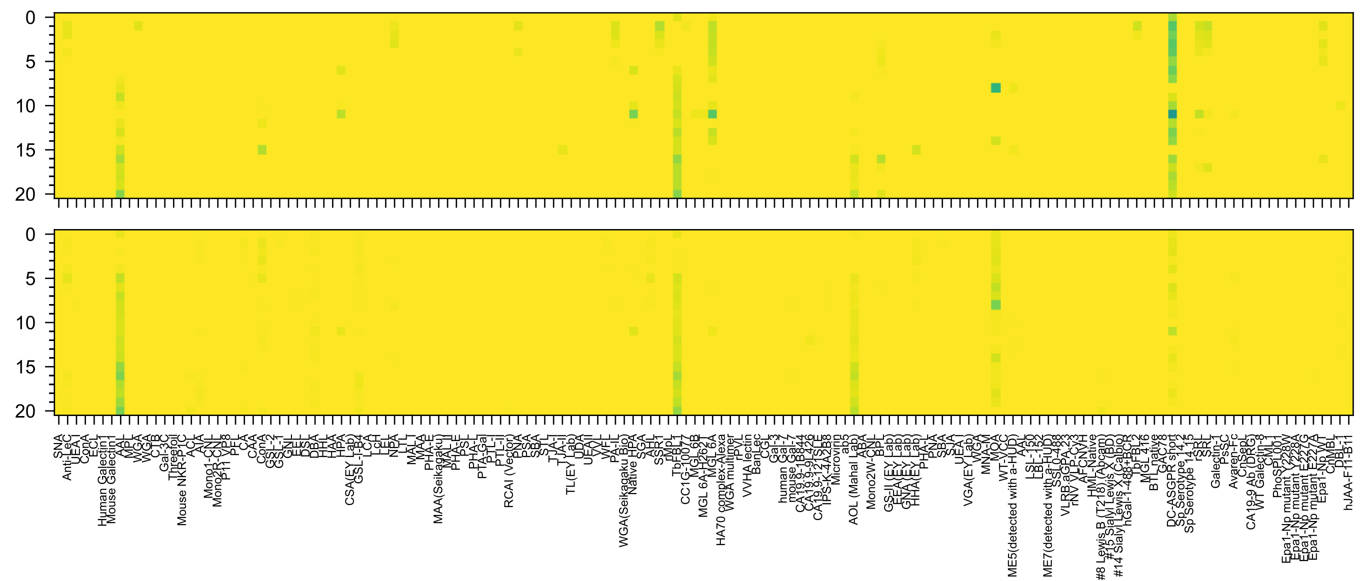

Fuc(α1-4)[Gal(β1-3)]GlcNAc(β1-2)Man(α-Sp0

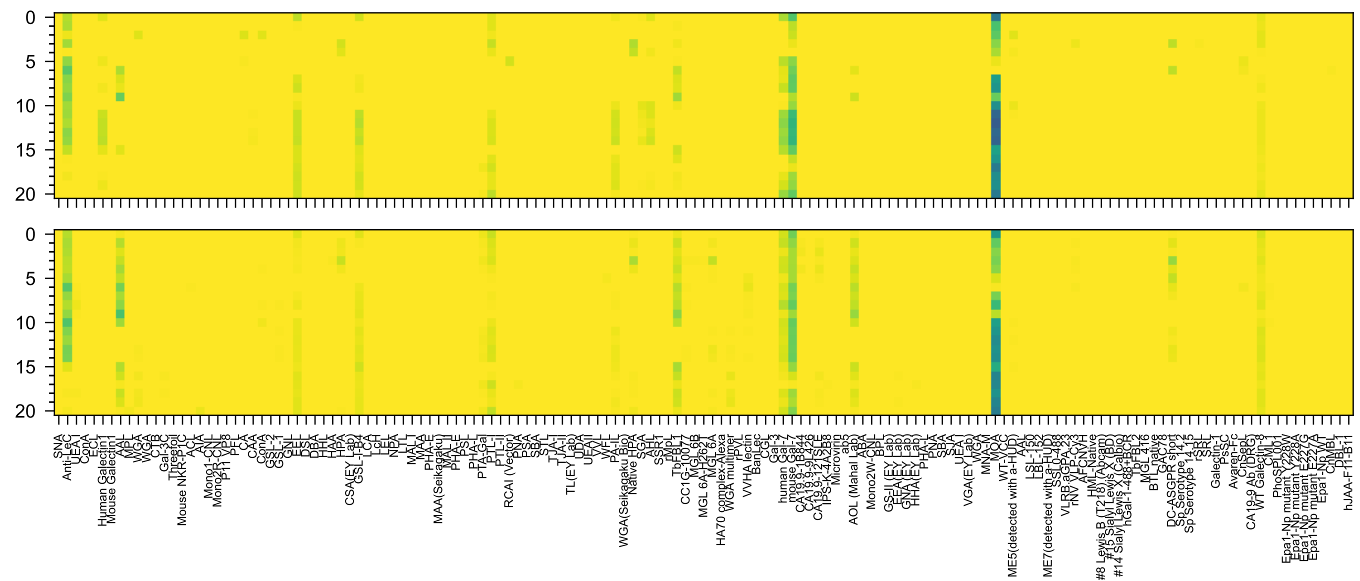

Gal(α1-3)[Fuc(α1-2)]Gal(β1-3)GlcNAc(β-Sp8

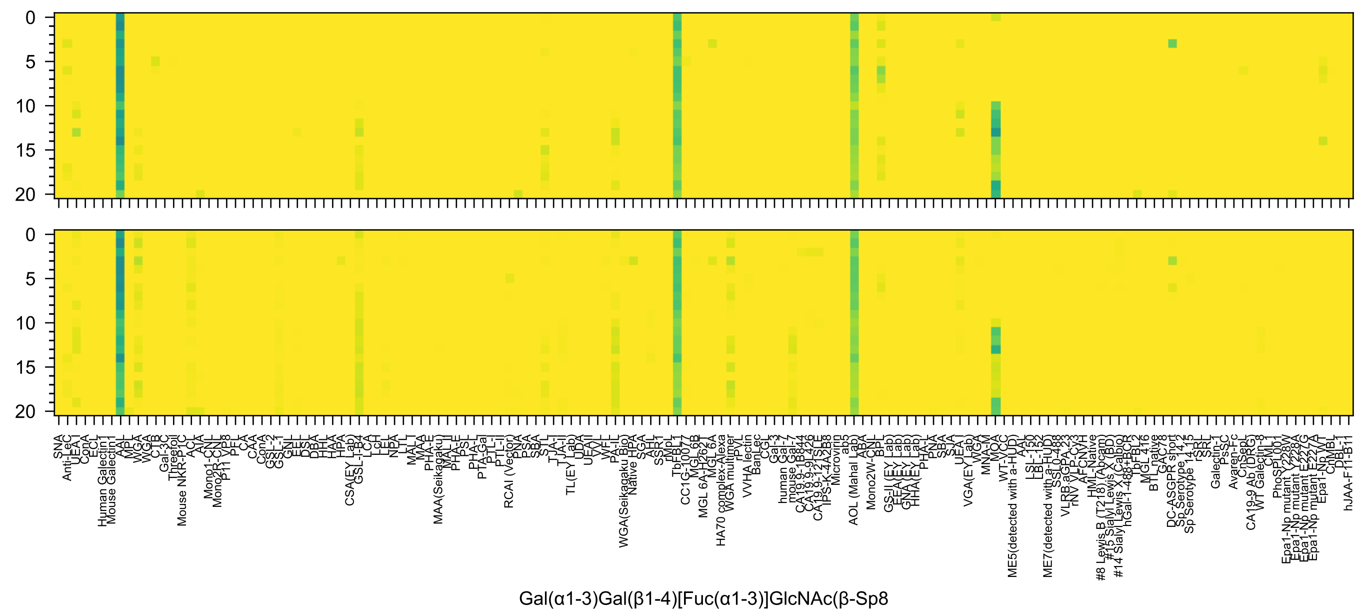
0
5
10
15
20
SNA
Anti-LeC
UEA I
ConA
ECL
Human Galectin1
Mouse Galectin1
AAL
MPL
WGA
WGA
CTB
Gal-3C
Threefoil
Mouse NKR-P1C
ACL
AIA
Mono1-CNL
Mono2R-CNL
P11 VP8
PFL
CA
CAA
ConA
GSL-2
GSL-1
GNL
EEL
DSL
DBA
HHL
HAA
HPA
CSA(EY Lab)
GSL-I-B4
LCA
LcH
LEL
NPA
LTL
MAL I
MAA
PHA-E
MAA(Seikagaku)
MAL II
PHA-E
PSL
PHA-L
PTA-Gal
PTL-I
PTL-II
RCAI (Vector)
PNA
PSA
SBA
STL
TJA-I
TJA-II
TL(EY Lab)
UDA
UEA II
VVL
WFL
PA-IL
WGA(Seikagaku Bio)
Native HPA
SGA
AHL
SSR1
rMpL
TbFBL1
CC1G-10077
MGL 6B
MGL 6A-H262T
MGL 6A
HA70 complex-Alexa
WGA multimer
rPVL
VVHA lectin
BanLec
CGL
Gal-3
human Gal-7
mouse Gal-7
CA19.9-1B844
CA19.9-9L426
CA19.9-121SLE
IPS-K-4A2B8
Microvirin
ab5
AOL (Mahal Lab)
ABA
Mono2W-CNL
BPL
GS-II (EY Lab)
EEA(EY Lab)
GNA(EY Lab)
HHA(EY Lab)
PHA-L
PNA
SBA
SJA
UEA I
VGA(EY Lab)
WGA
MNA-M
MOA
WT-VCC
ME5(detected with a-HUD)
AAL
LSL-150
LBL-152
ME7(detected with a-HUD)
SSL0-488
VLRB.aGPA.23
rNV VLP-Cy3
AFCNVH
HML-Native
#8 Lewis B (T218) (Abcam)
#15 Sialyl Lewis X (BD)
#14 Sialyl Lewis X (Calbio)
hGal-1-488+BCR
TbFBL2
MGL 416
BTL native
GAC78
DC-ASGPR short
Sp Serotype 14.2
Sp Serotype 14.15
rSRL
SRL
Galectin-1
PsSC
Avaren-Fc
CnSepL
CA19-9 Ab (DRG)
WT Galectin-8
CML
PhoSPL001
Epa1-Np mutant Y228W
Epa1-Np mutant Y228A
Epa1-Np mutant E227G
Epa1-Np mutant E227A
Epa1-Np WT
CnMBL
DBL-1
hJAA-F11-B11
Gal(α1-3)Gal(β1-4)[Fuc(α1-3)]GlcNAc(β-Sp8

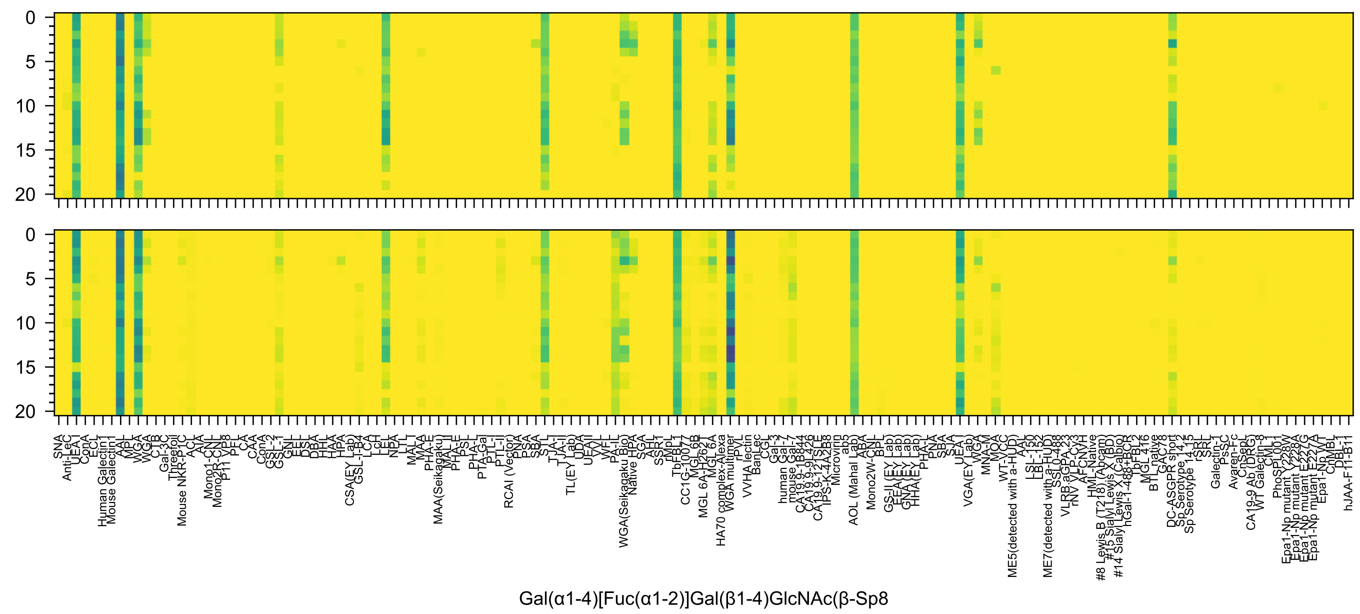
Gal(α1-4)[Fuc(α1-2)]Gal(β1-4)GlcNAc(β-Sp8

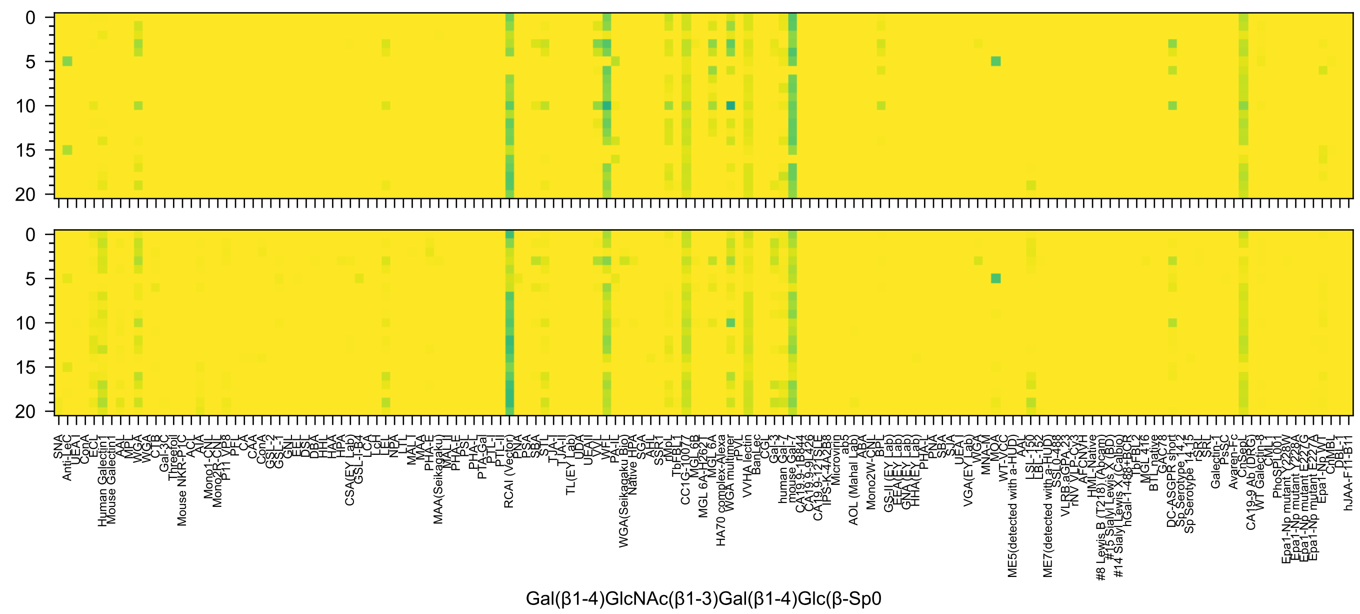
Gal(β1-4)GlcNAc(β1-3)Gal(β1-4)Glc(β-Sp0

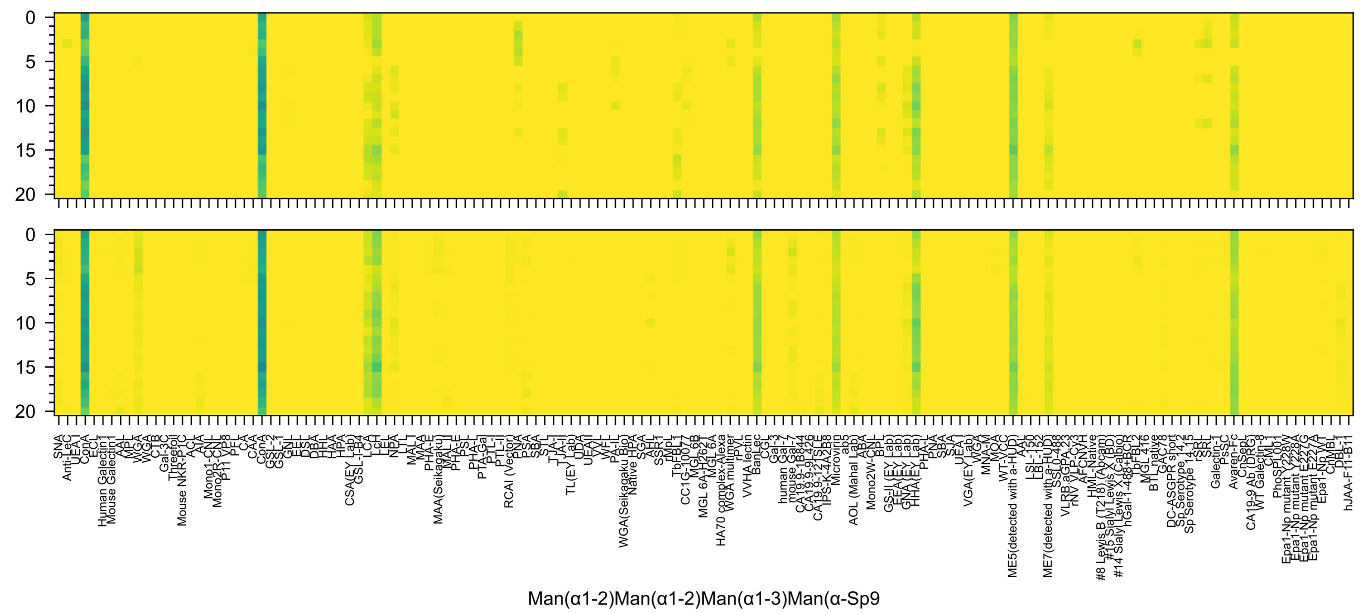

Man(α1-2)Man(α1-2)Man(α1-3)Man(α-Sp9)

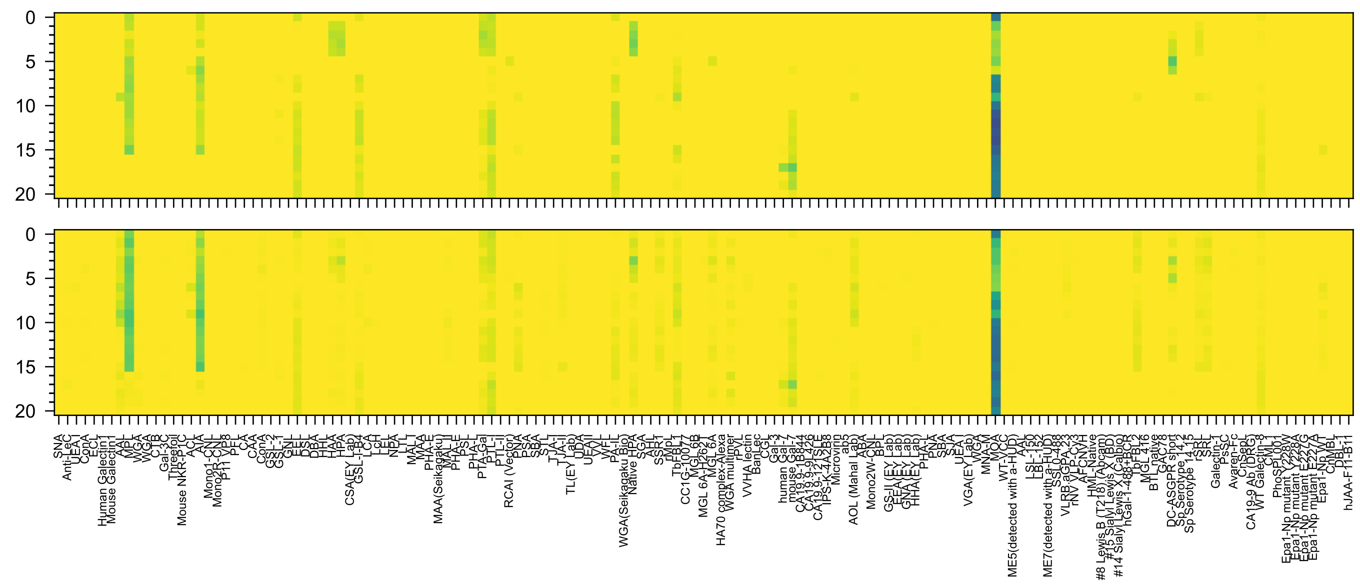

Gal(α1-3)[Fuc(α1-2)]Gal(β1-3)GalNAc(α-Sp8

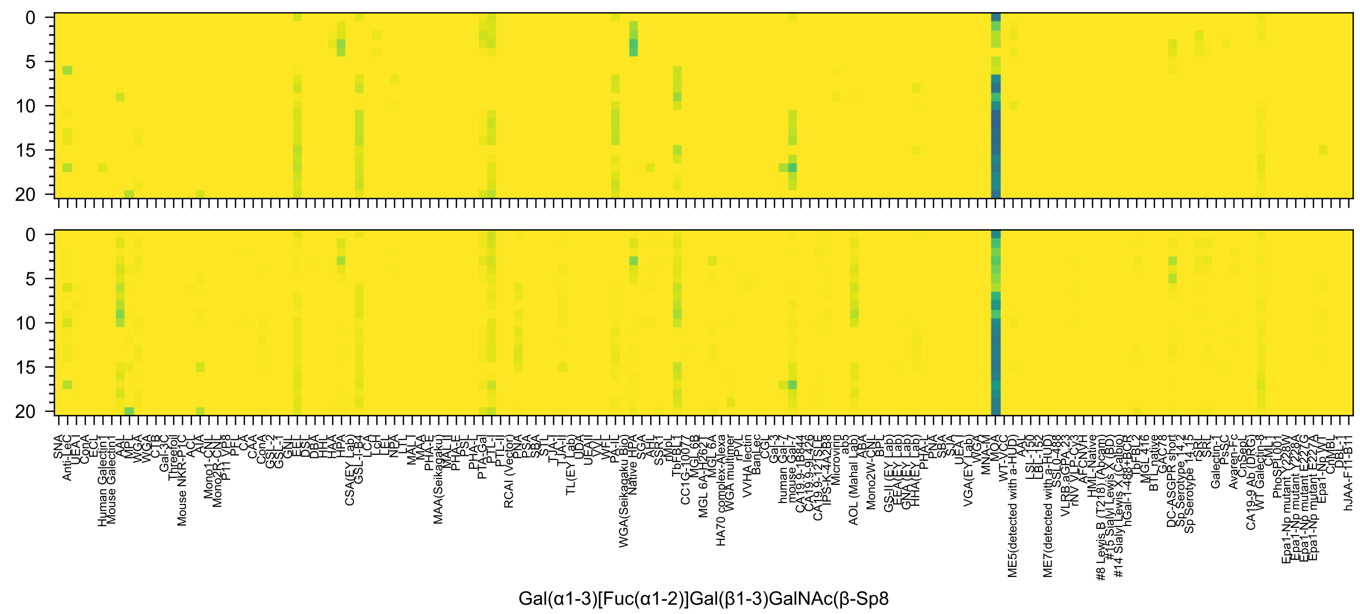
Gal(α1-3)[Fuc(α1-2)]Gal(β1-3)GalNAc(β-Sp8

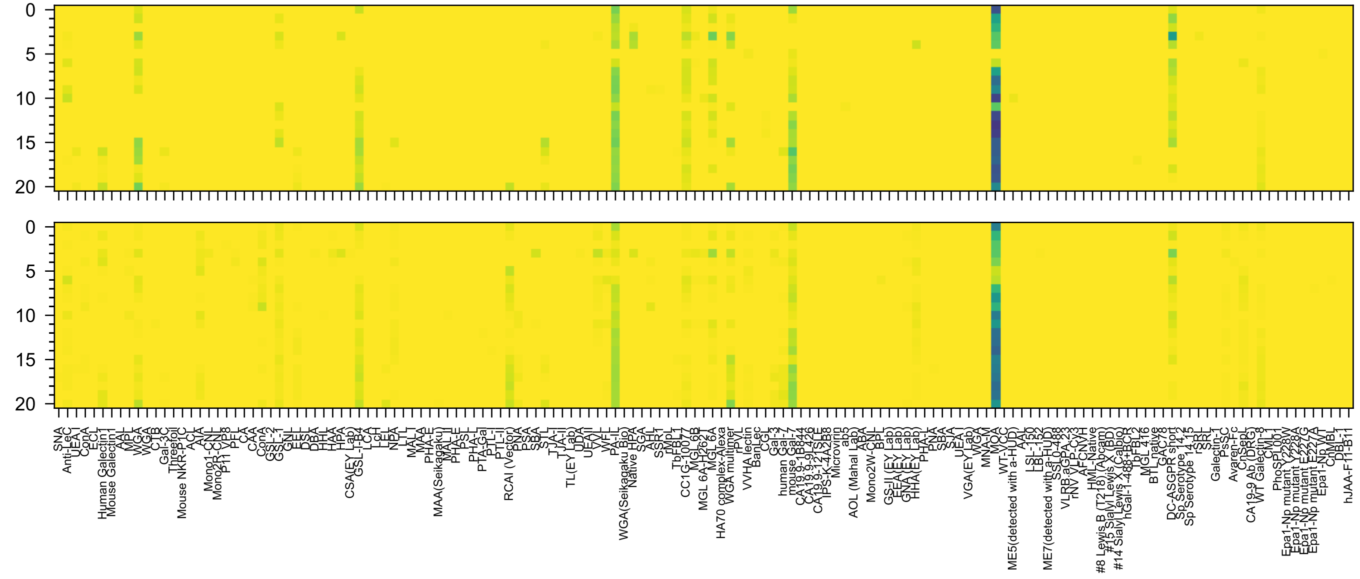

Gal(α1-3)Gal(β1-4)GlcNAc(β1-2)Man(α-Sp0

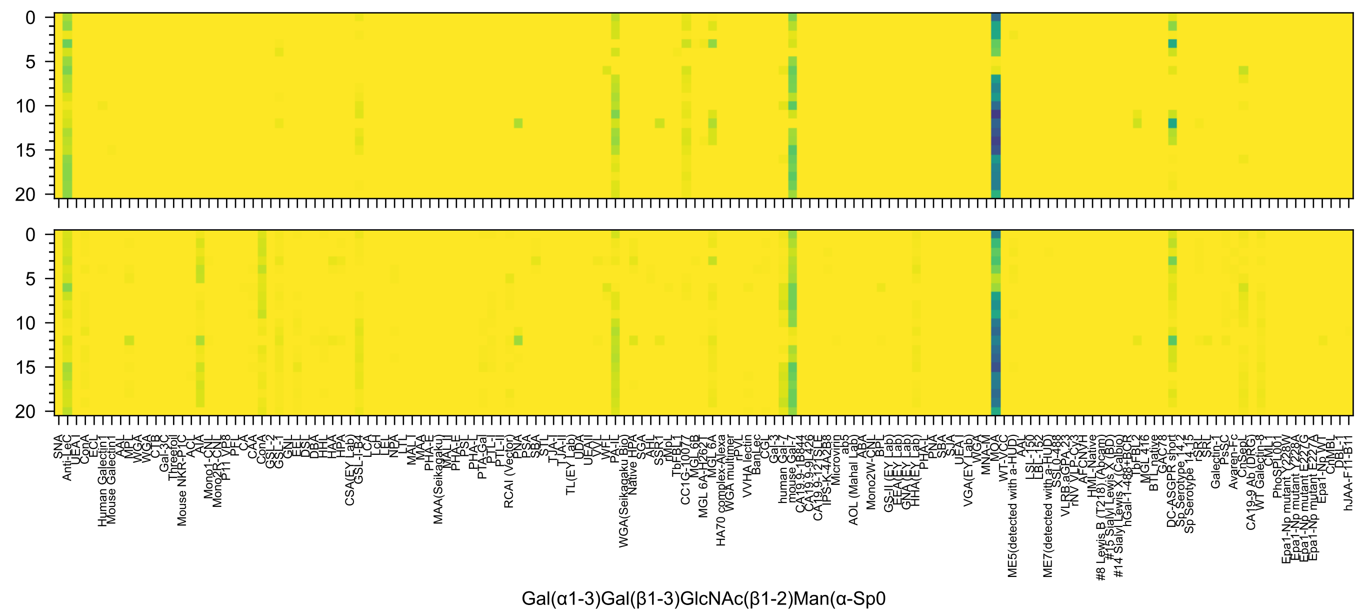

0
5
10
15
20
SNA
Anti-LeC
UEA-I
ConA
ECL
Human Galectin1
Mouse Galectin1
AAL
MPL
WGA
WGA
CTB
Gal-3C
Threefoil
Mouse NKR-P1C
ACL
AIA
Mono1-CNL
Mono2R-CNL
P11 VP8
PFL
CA
CAA
ConA
GSL-2
GSL-1
GNL
EEL
DSL
DBA
HHL
HAA
HPA
CSA(EY Lab)
GSL-I-B4
LCA
LcH
LEL
NPA
LTL
MAL-I
MAA
PHA-E
MAA(Seikagaku)
MAL-II
PHA-E
PSL
PHA-L
PTA-Gal
PTL-I
PTL-II
RCAI (Vector)
PNA
PSA
SBA
STL
TJA-I
TJA-II
TL(EY Lab)
UDA
UEA-II
VVL
WFL
PA-IL
WGA(Seikagaku Bio)
Native HPA
SGA
AHL
SSR1
rMpL
TbFBL1
CC1G-10077
MGL 6B
MGL 6A-H262T
MGL 6A
HA70 complex-Alexa
WGA multimer
rPVL
VVHA lectin
BanLec
CGL
Gal-3
human Gal-7
mouse Gal-7
CA19.9-1B844
CA19.9-9L426
CA19.9-121SLE
IPS-K-4A2B8
Microvirin
ab5
AOL (Mahal Lab)
ABA
Mono2W-CNL
BPL
GS-II (EY Lab)
EEA(EY Lab)
GNA(EY Lab)
HHA(EY Lab)
PHA-L
PNA
SBA
SJA
UEA-I
VGA(EY Lab)
WGA
MNA-M
MOA
WT-VCC
ME5(detected with a-HUD)
AAL
LSL-150
LBL-152
ME7(detected with a-HUD)
SSL0-488
VLRB.aGPA.23
rNV VLP-Cy3
AFCN-H
HML-Native
#8 Lewis B (T218) (Abcam)
#15 Sialyl Lewis X (BD)
#14 Sialyl Lewis X (Calbio)
hGal-1-488+BCR
bFBL2
MGL 416
BTL native
GAC78
DC-ASGPR short
Sp Serotype 14
Sp Serotype 14.15
rSRL
SRL
Galectin-1
PsSC
Avaren-Fc
CnSepL
CA19-9 Ab (DRG)
WT Galectin-8
CML
PhoSL001
Epa1-Np mutant Y228W
Epa1-Np mutant Y228A
Epa1-Np mutant E227G
Epa1-Np mutant E227A
Epa1-Np WT
ChMBL
DBL-1
hJAA-F11-B11
Gal(α1-3)Gal(β1-3)GlcNAc(β1-2)Man(α-Sp0

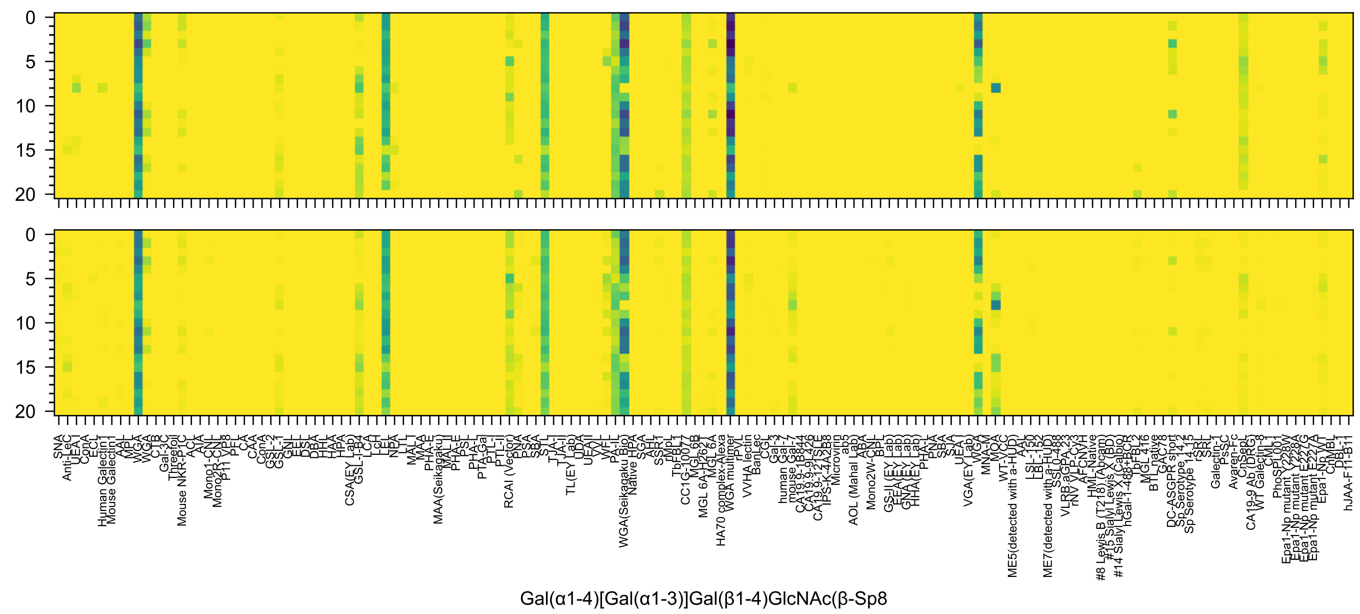

Gal(α1-4)[Gal(α1-3)]Gal(β1-4)GlcNAc(β-Sp8

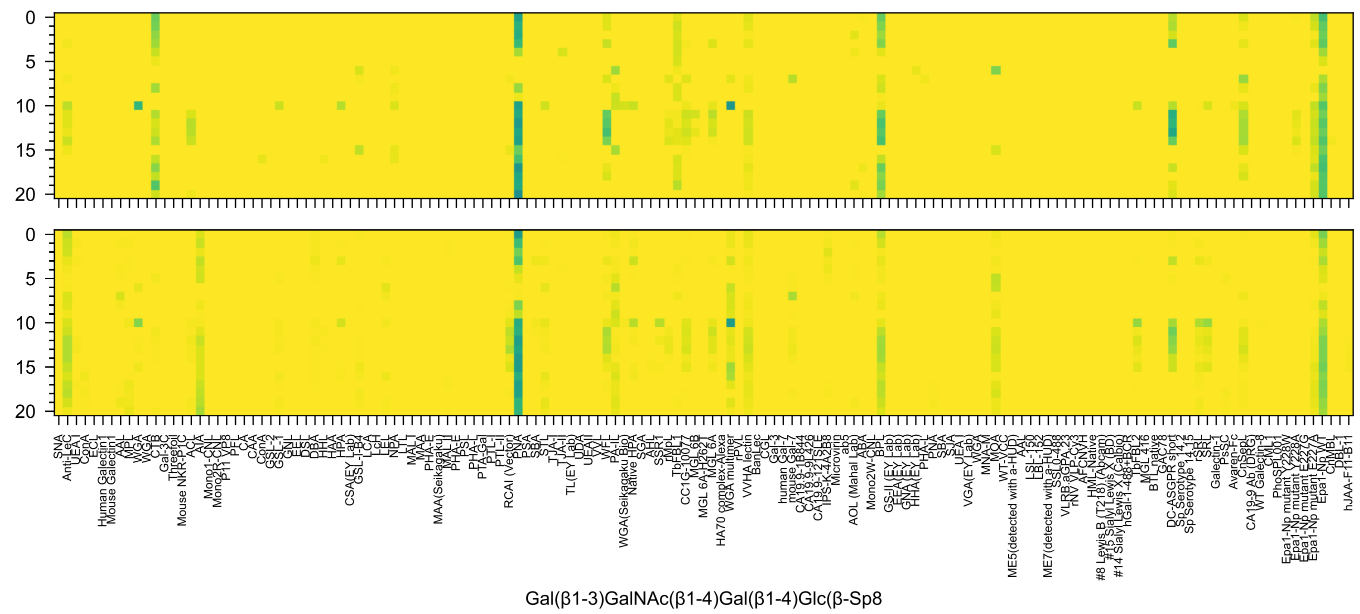

0
5
10
15
20
Gal(β1-3)GalNAc(β1-4)Gal(β1-4)Glc(β-Sp8

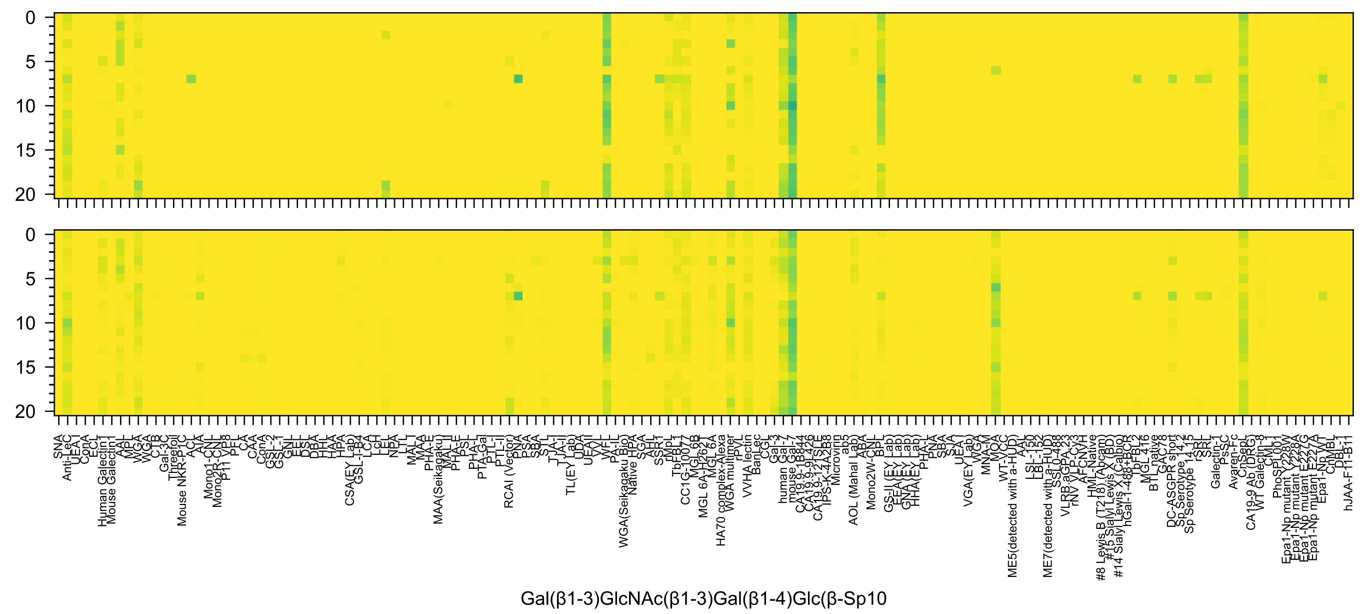
Gal(β1-3)GlcNAc(β1-3)Gal(β1-4)Glc(β-Sp10

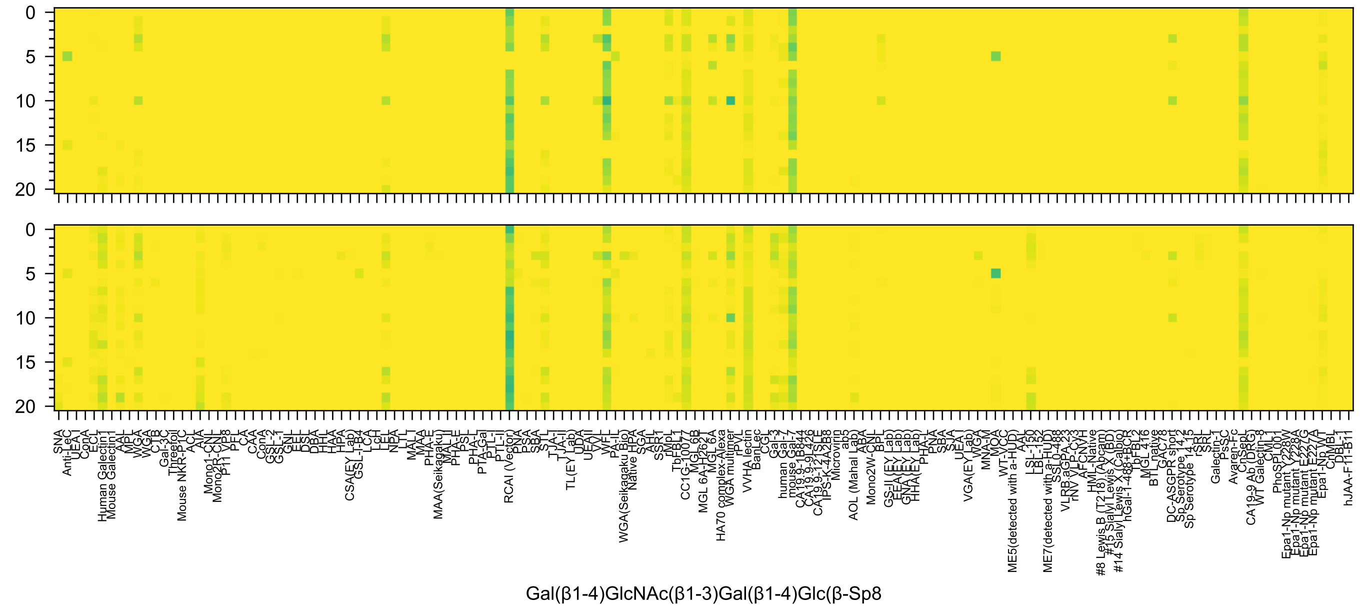
Gal(β1-4)GlcNAc(β1-3)Gal(β1-4)Glc(β-Sp8

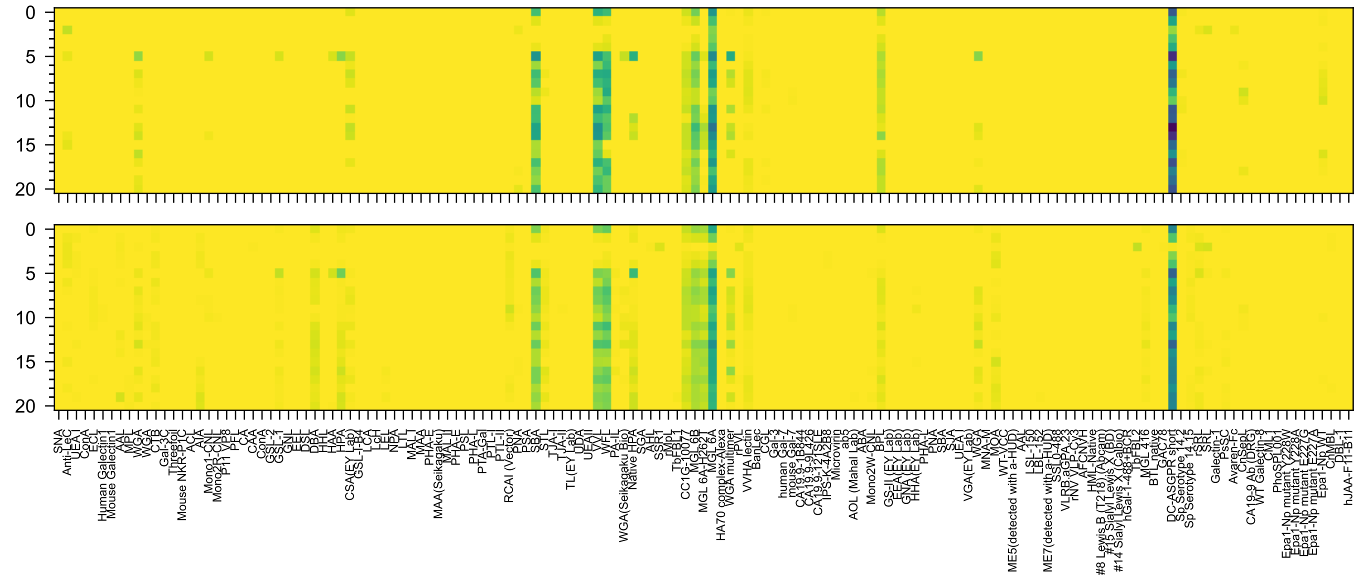

GalNAc(β1-3)Gal(α1-6)Gal(β1-4)Glc(β-Sp8

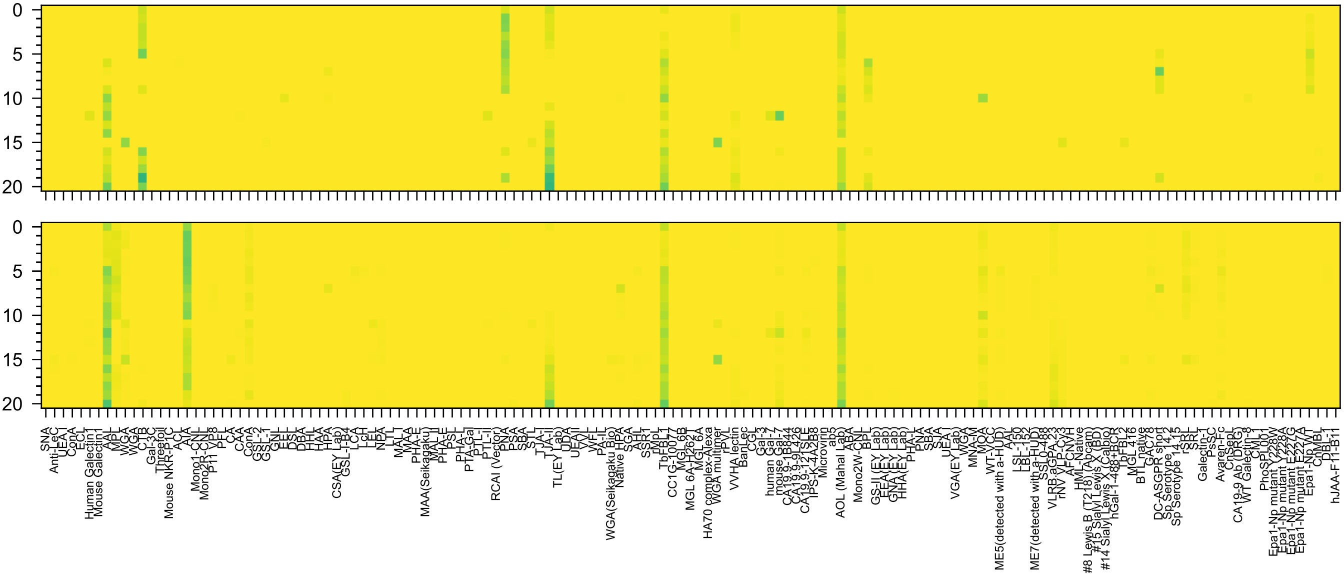

Fuc(α1-2)Gal(β1-3)GalNAc(β1-3)Gal(α-Sp9

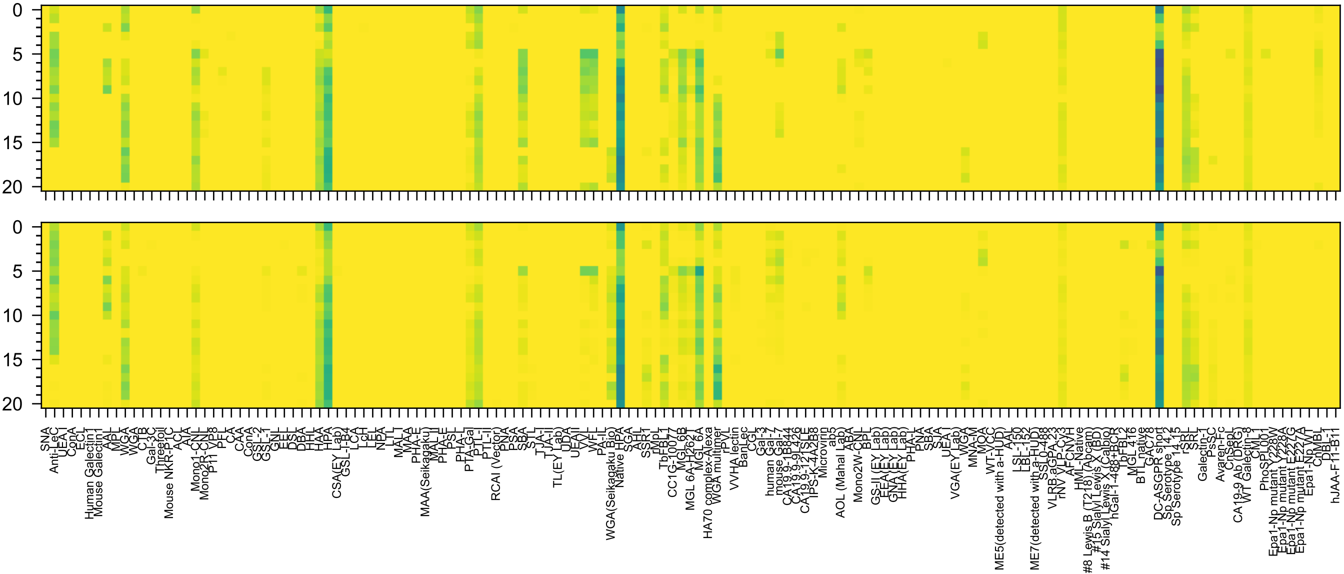

GalNAc(α1-3)[Fuc(α1-2)]Gal(β1-3)GlcNAc(β-Sp0

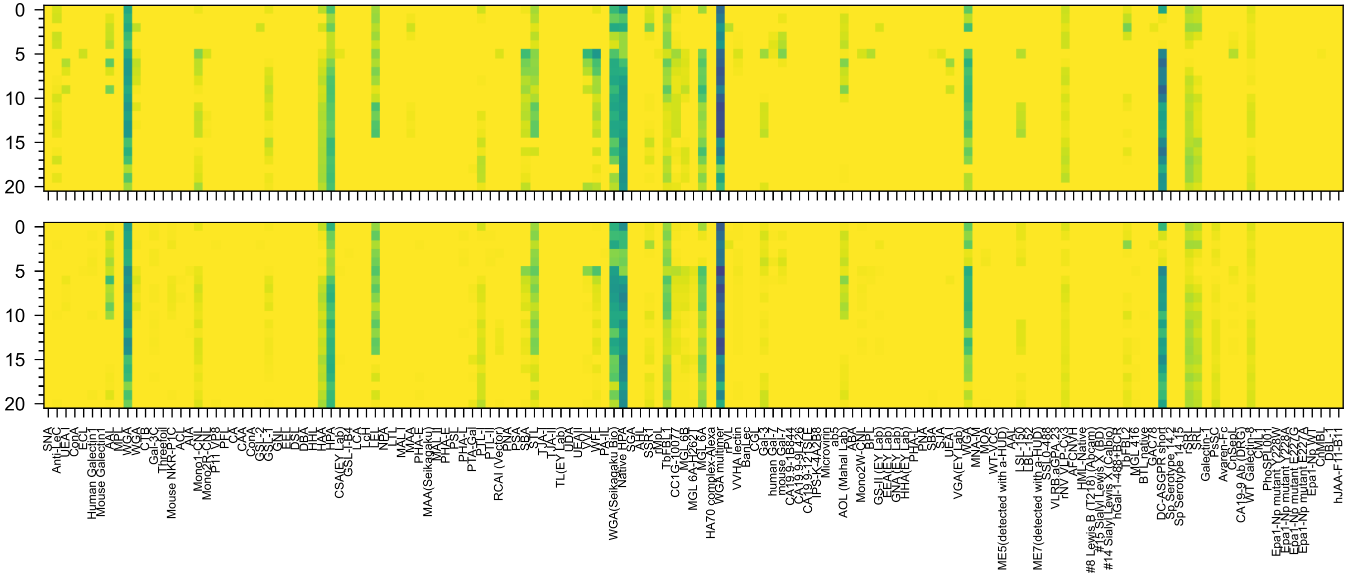

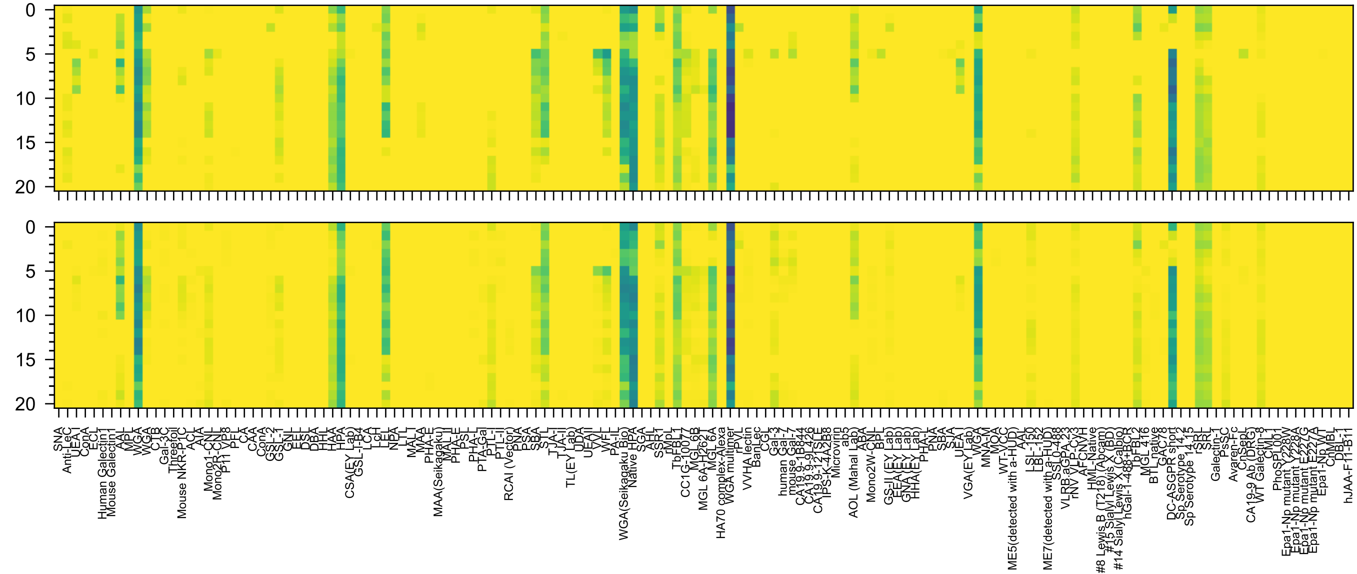

GalNAc(α1-3)[Fuc(α1-2)]Gal(β1-4)GlcNAc(β-Sp8

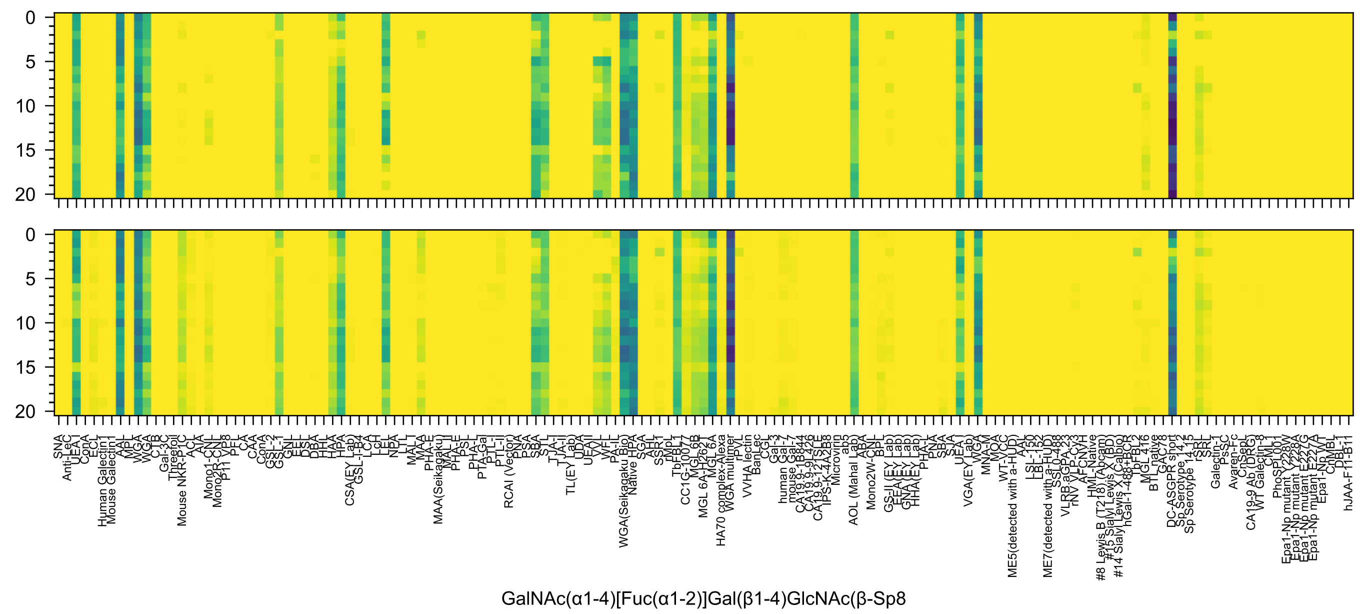
GalNAc(α1-4)[Fuc(α1-2)]Gal(β1-4)GlcNAc(β-Sp8

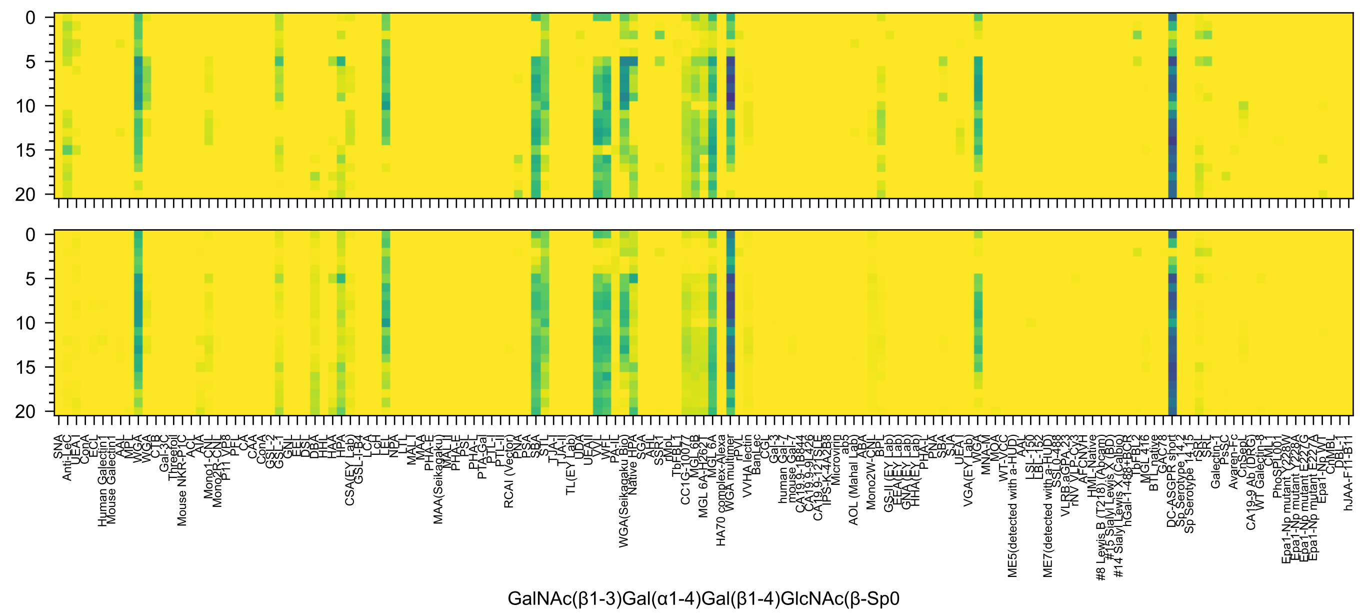
0
5
10
15
20
0
5
10
15
20
SNA
Anti-LeC
UEA-I
ConA
ECL
Human Galectin1
Mouse Galectin1
AAL
MPL
WGA
WGA
CTB
Gal-3C
Threefoil
Mouse NKR-P1C
ACL
AIA
Mono1-CNL
Mono2R-CNL
P11 VP8
PFL
CA
CAA
ConA
GSL-2
GSL-1
GNL
EEL
DSL
DBA
HHL
HAA
HPA
CSA(EY Lab)
GSL-I-B4
LCA
LcH
LEL
NPA
LTL
MAL-I
MAA
PHA-E
MAA(Seikagaku)
MAL-II
PHA-E
PSL
PHA-L
PTA-Gal
PTL-I
PTL-II
RCAI (Vector)
PNA
PSA
SBA
STL
TJA-I
TJA-II
TL(EY Lab)
UDA
UEA-II
VVL
WFL
PA-IL
WGA(Seikagaku Bio)
Native HPA
SGA
AHL
SSR1
rMpL
TbFBL1
CC1G-10077
MGL 6B
MGL 6A-H262T
MGL 6A
HA70 complex-Alexa
WGA multimer
rPVL
VVHA lectin
BanLec
CGL
Gal-3
human Gal-7
mouse Gal-7
CA19.9-1B844
CA19.9-9L426
CA19.9-121SLE
IPS-K-4A2B8
Microvirin
ab5
AOL (Mahal Lab)
ABA
Mono2W-CNL
BPL
GS-II (EY Lab)
EEA(EY Lab)
GNA(EY Lab)
HHA(EY Lab)
PHA-L
PNA
SBA
SJA
UEA-I
VGA(EY Lab)
WGA
MNA-M
MOA
WT-VCC
ME5(detected with a-HUD)
AAL
LSL-150
LBL-152
ME7(detected with a-HUD)
SSL0-488
VLRB.aGPA.23
rNV VLP-Cy3
AFCN-H
HML-Native
#8 Lewis B (T218) (Abcam)
#15 Sialyl Lewis X (BD)
#14 Sialyl Lewis X (Calbio)
hGal-1-488+BCR
TbFBL2
MGL 416
BTL native
GAC78
DC-ASGPR short
Sp Serotype 14.15
Sp Serotype 14.15
rSRL
SRL
Galectin-1
PsSC
Avaren-Fc
CnSepL
CA19-9 Ab (DRG)
WT Galectin-8
CML1
PhoSL001
Epa1-Np mutant Y228W
Epa1-Np mutant Y228A
Epa1-Np mutant E227G
Epa1-Np mutant E227A
Epa1-Np WT
ChMBL
DBL-1
hJAA-F11-B11
GalNAc(β1-3)Gal(α1-4)Gal(β1-4)GlcNAc(β-Sp0

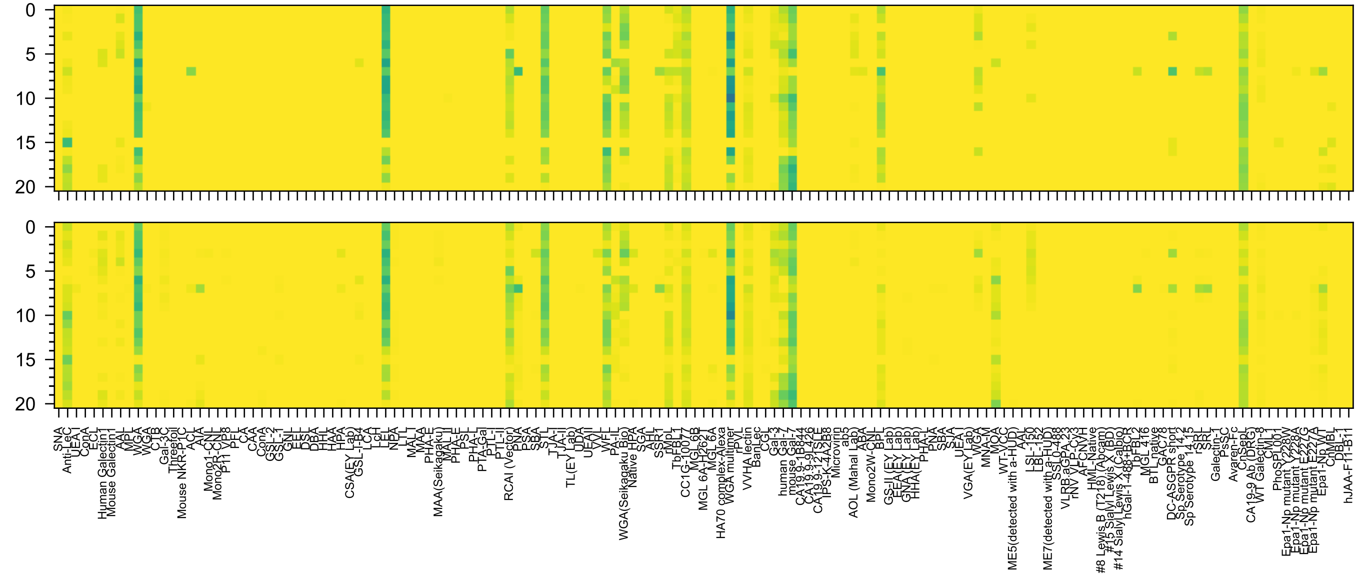

Gal(β1-3)GlcNAc(β1-3)Gal(β1-4)GlcNAc(β-Sp0

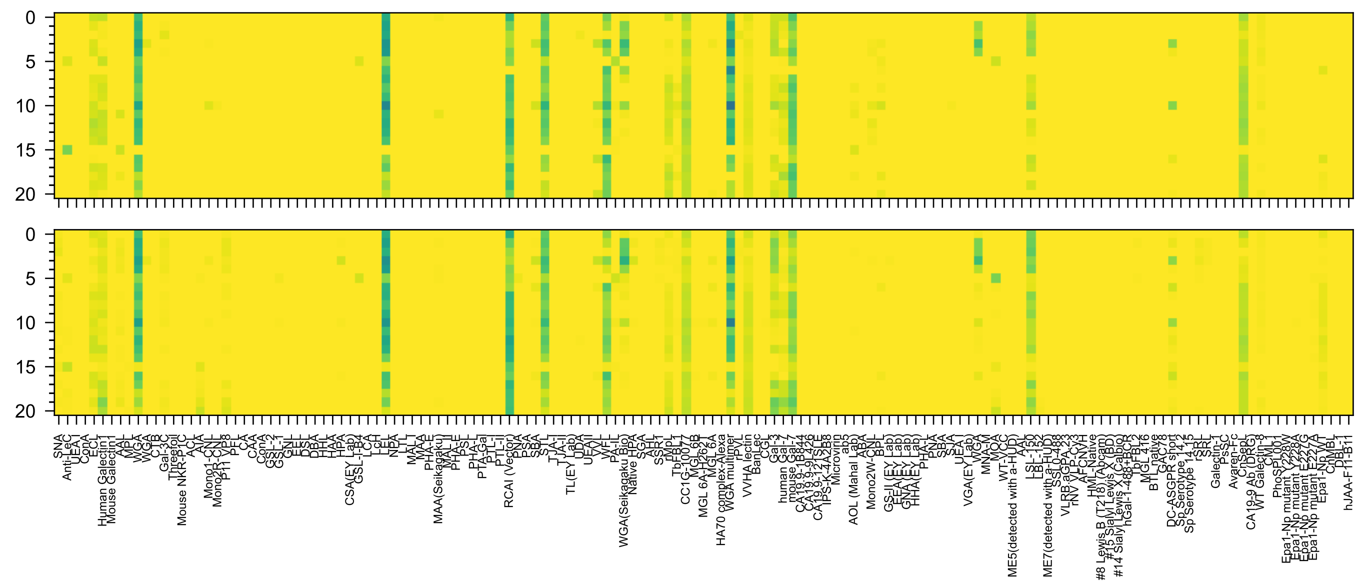

Gal(β1-4)GlcNAc(β1-3)Gal(β1-4)GlcNAc(β-Sp0

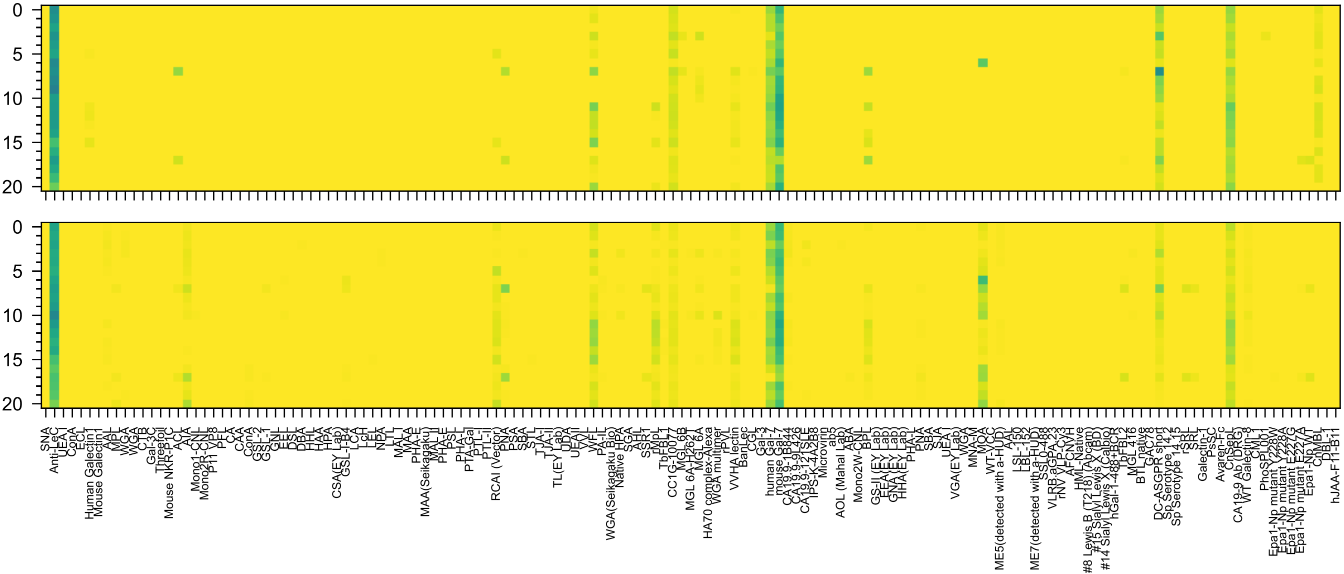

Gal(β1-3)GlcNAc(β1-3)Gal(β1-3)GlcNAc(β-Sp0

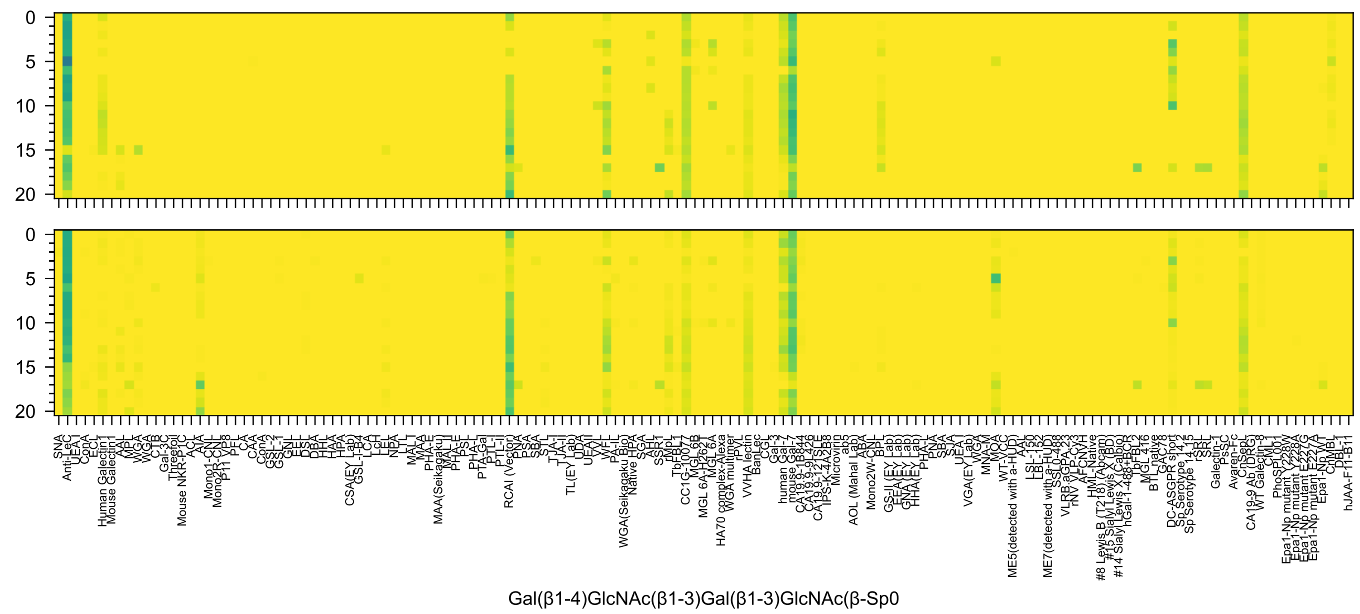

Gal(β1-4)GlcNAc(β1-3)Gal(β1-3)GlcNAc(β-Sp0

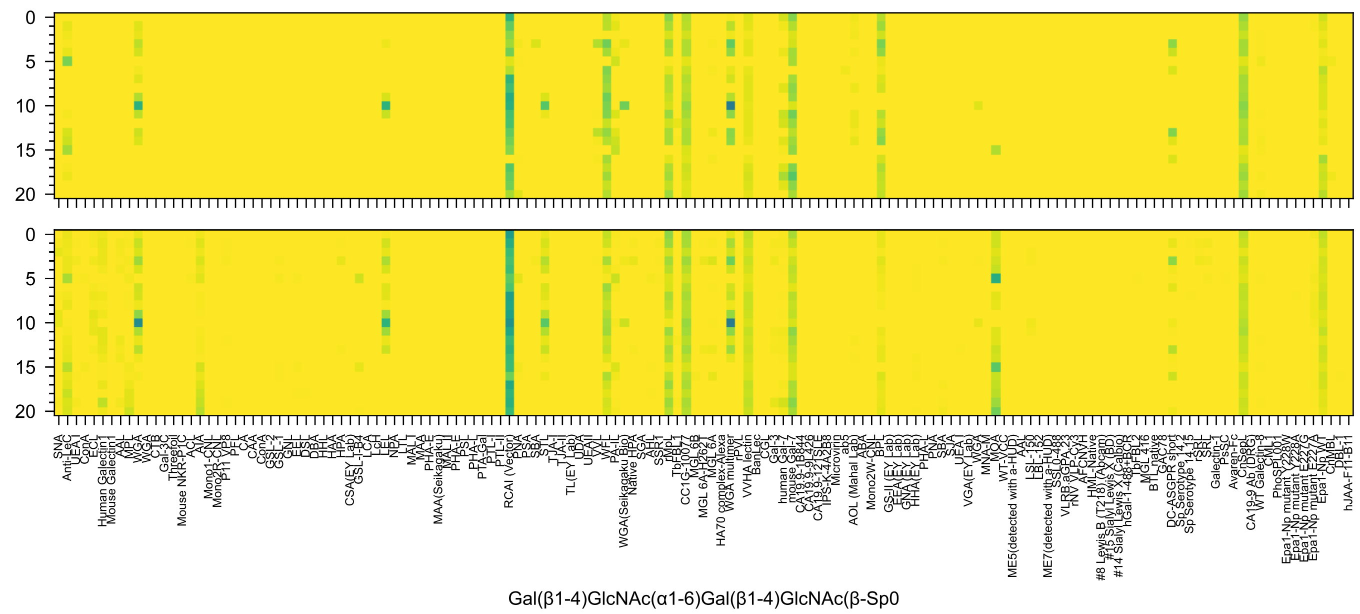
Gal(β1-4)GlcNAc(α1-6)Gal(β1-4)GlcNAc(β-Sp0)

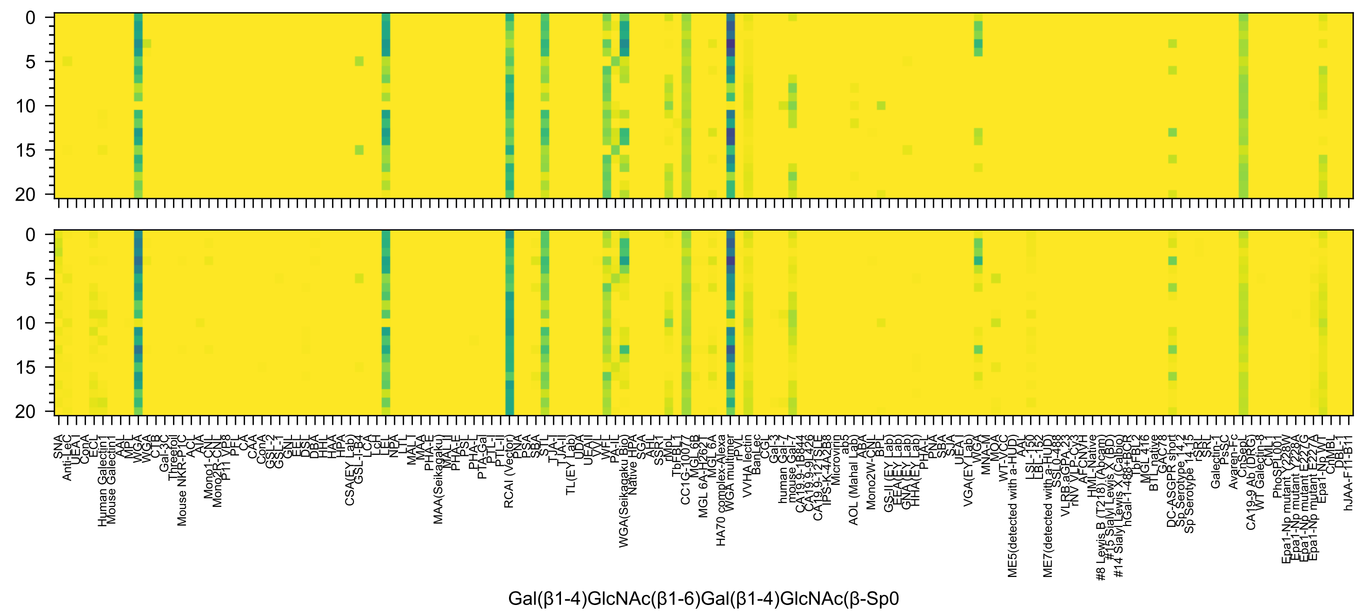

Gal(β1-4)GlcNAc(β1-6)Gal(β1-4)GlcNAc(β-Sp0

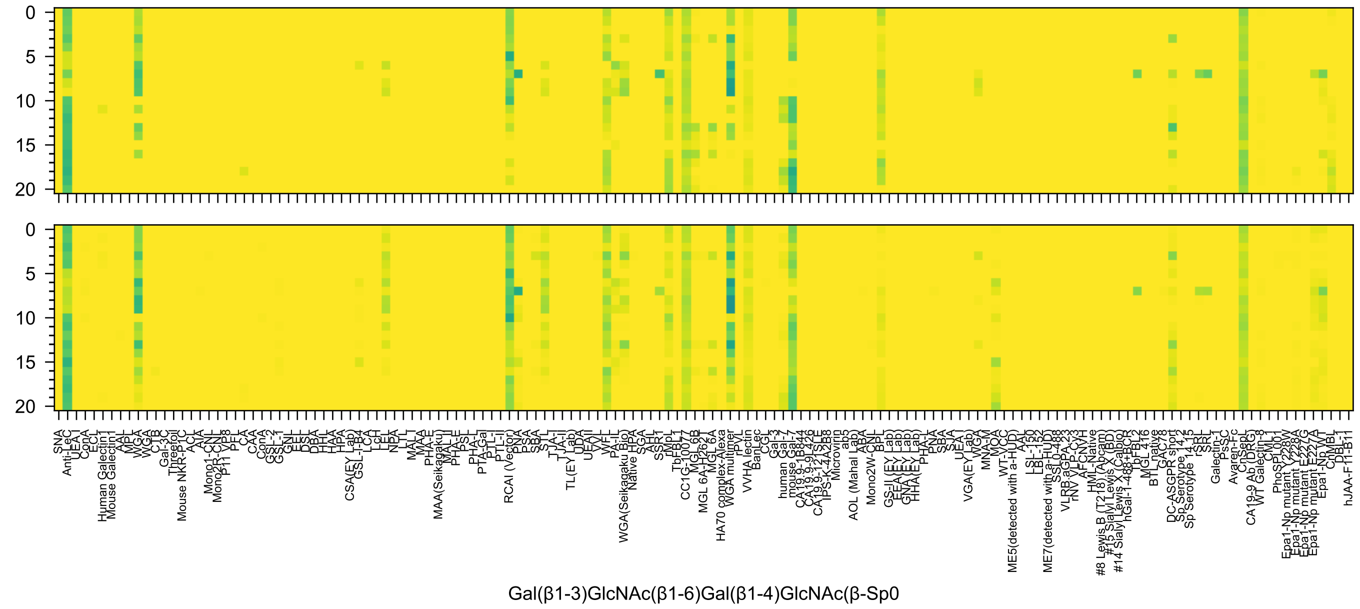
Gal(β1-3)GlcNAc(β1-6)Gal(β1-4)GlcNAc(β-Sp0

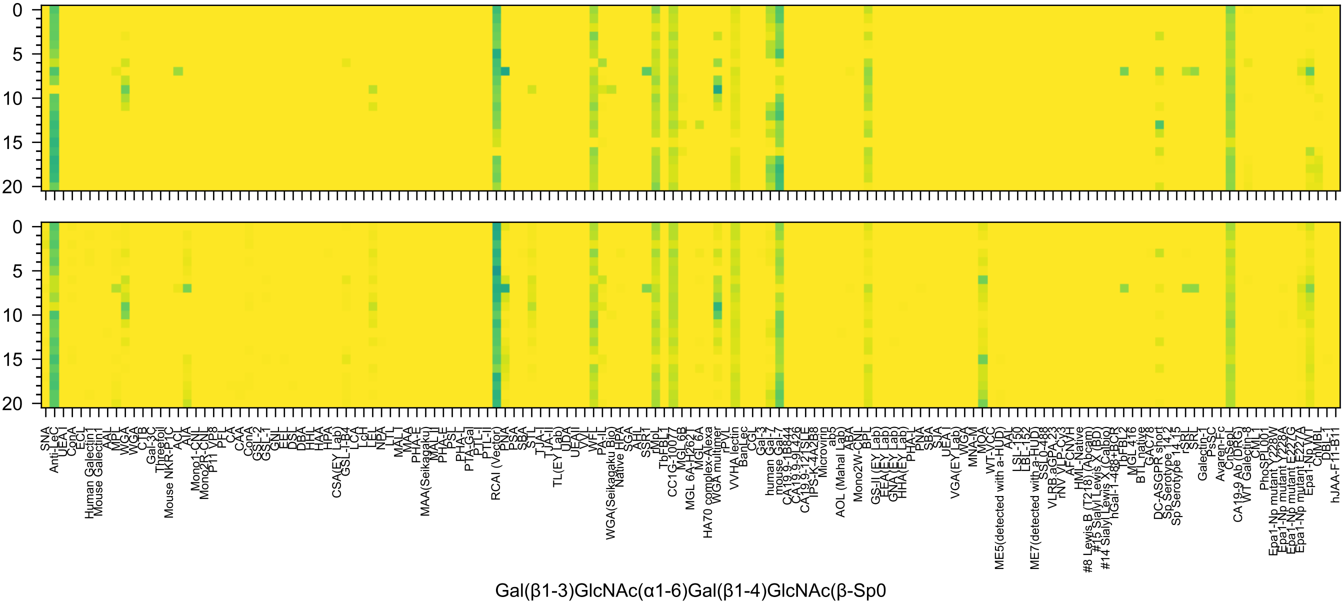
Gal(β1-3)GlcNAc(α1-6)Gal(β1-4)GlcNAc(β-Sp0

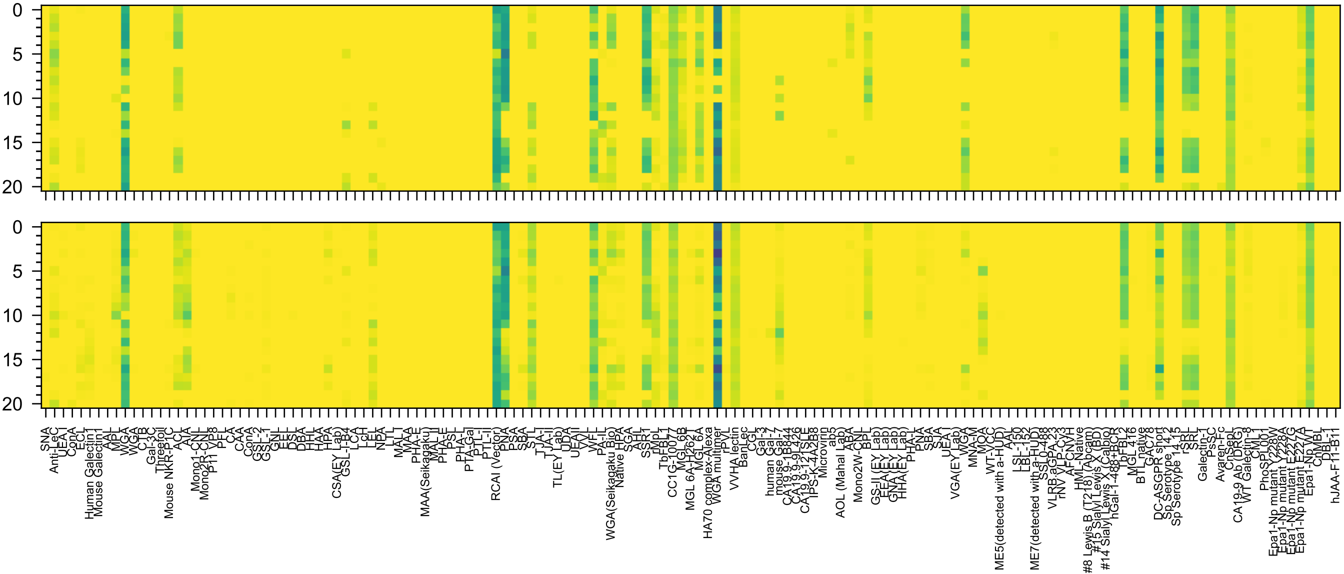

Gal(β1-4)GlcNAc(β1-6)[Gal(β1-3)]GalNAc(α-Sp8

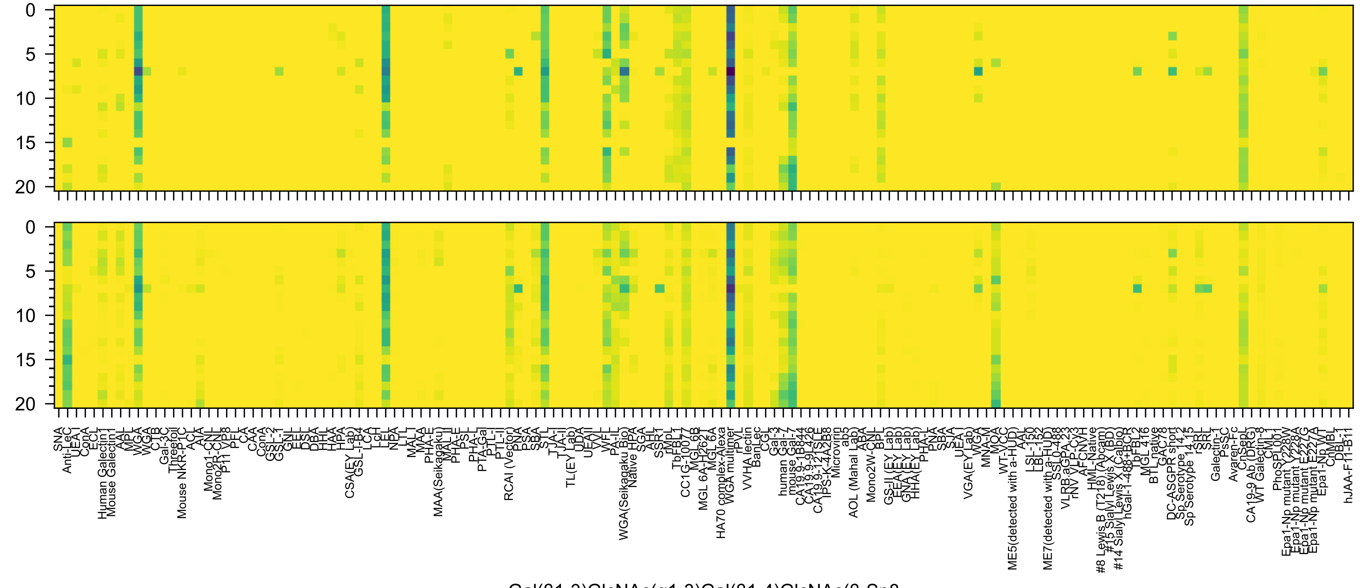
Gal(β1-3)GlcNAc(α1-3)Gal(β1-4)GlcNAc(β-Sp8

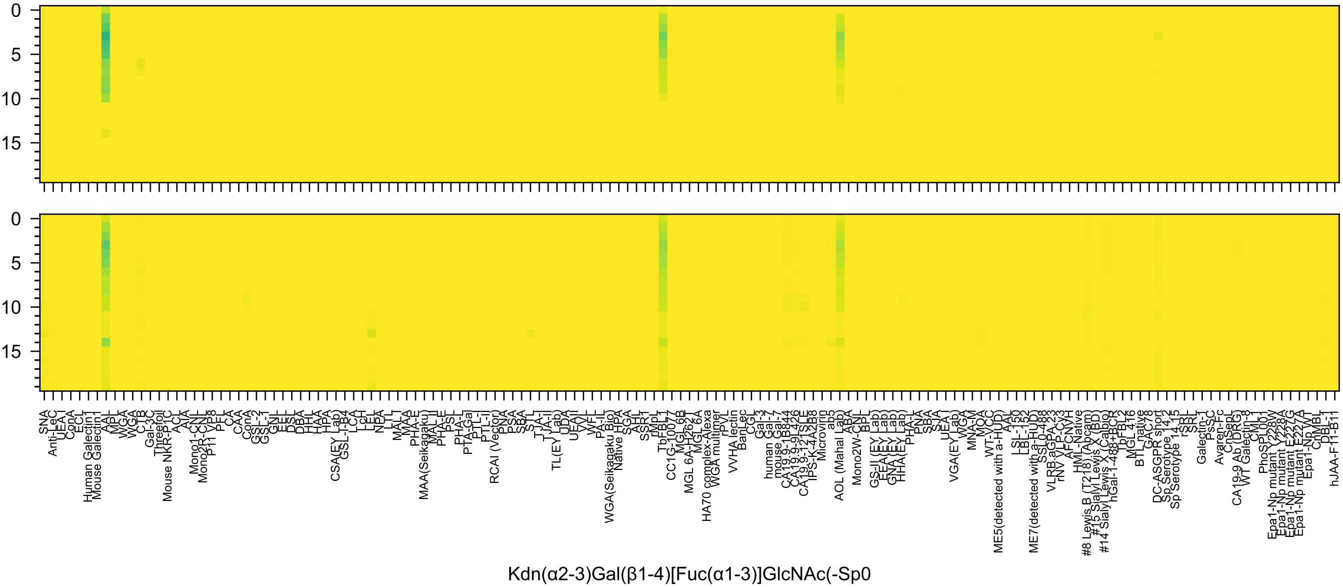
Kdn(α2-3)Gal(β1-4)[Fuc(α1-3)]GlcNAc(-Sp0

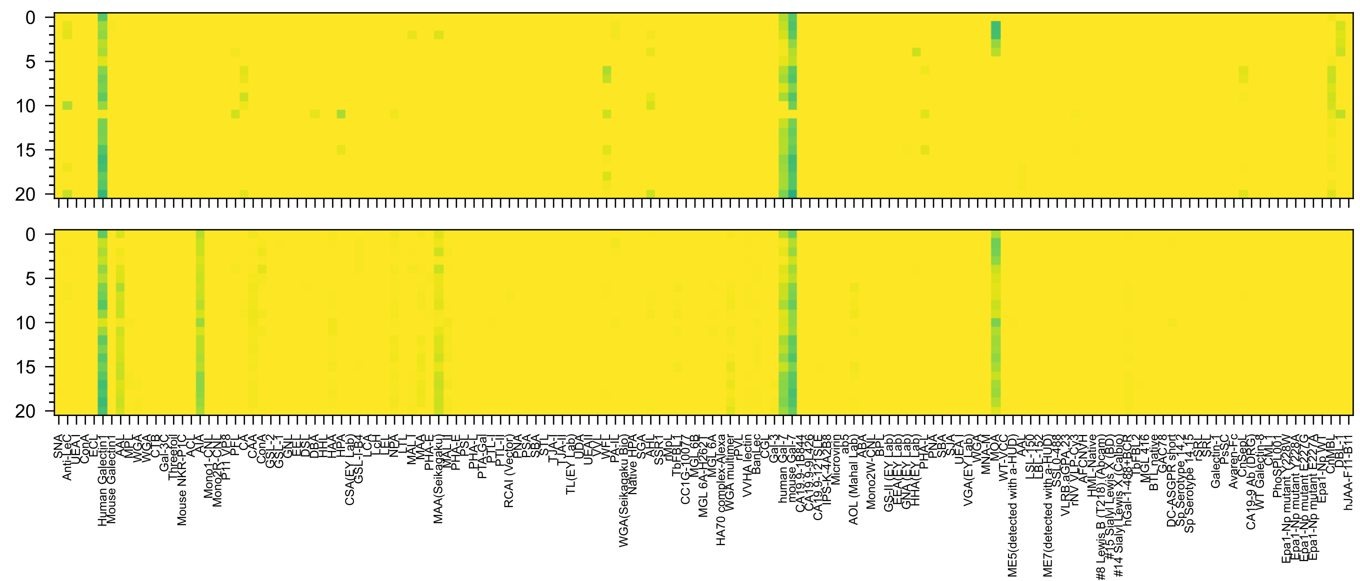

GlcA3OS(β1-3)Gal(β1-4)GlcNAc(β1-2)Man(α-Sp0

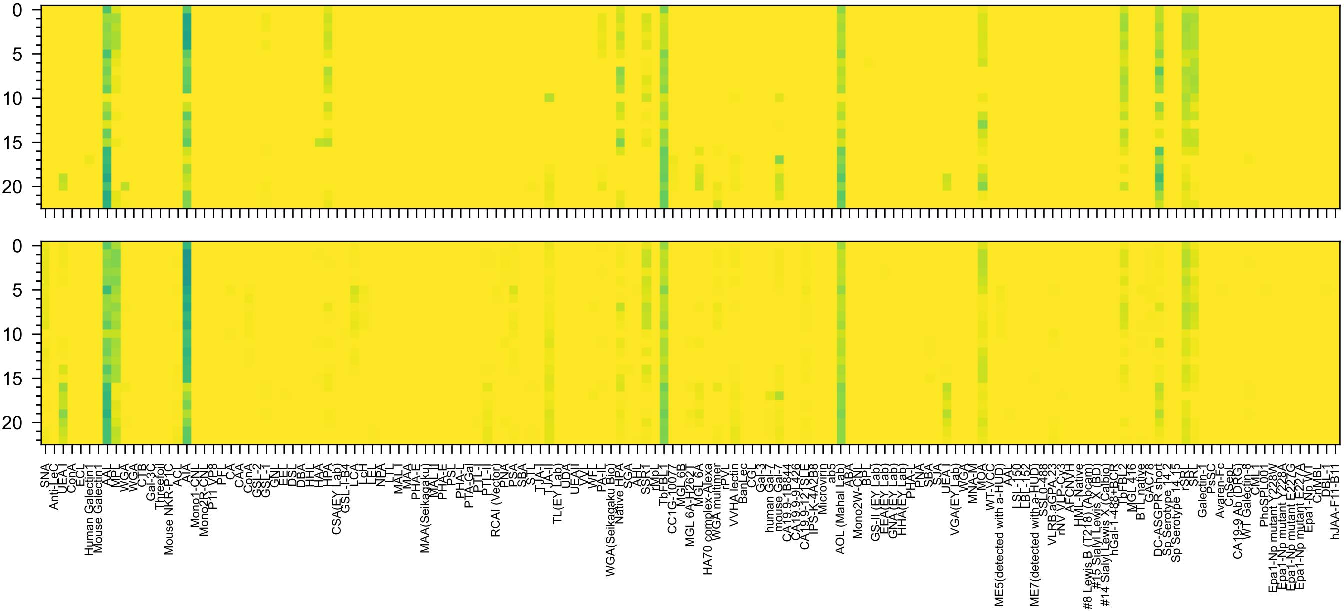

Fuc(α1-2)Gal(β1-4)GlcNAc(β1-3)GalNAc(α-Sp14

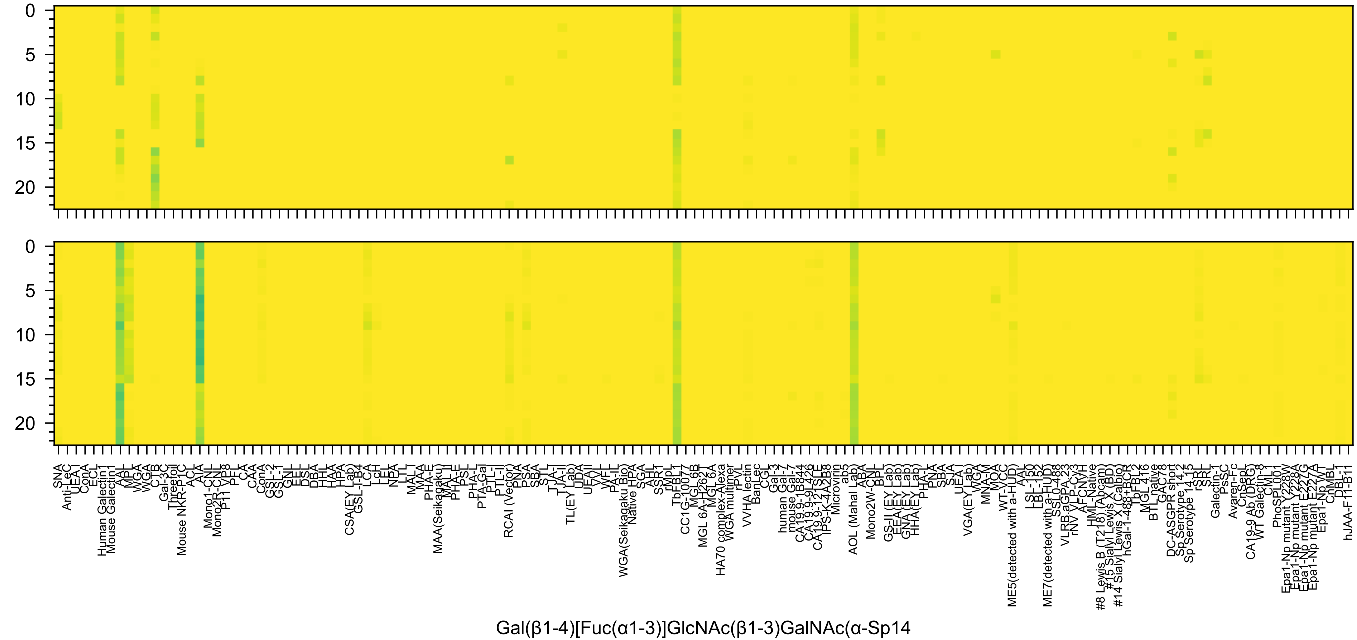

Gal(β1-4)[Fuc(α1-3)]GlcNAc(β1-3)GalNAc(α-Sp14

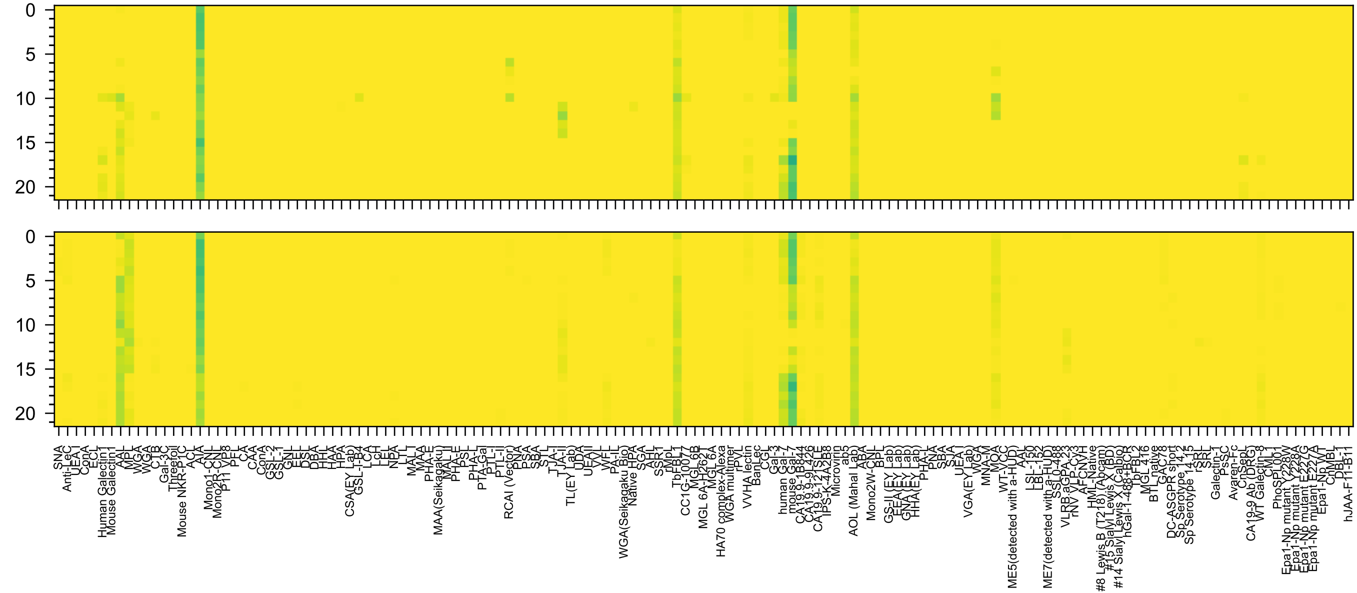

Fuc(α1-2)Gal(β1-3)GlcNAc(β1-3)GalNAc(-Sp14

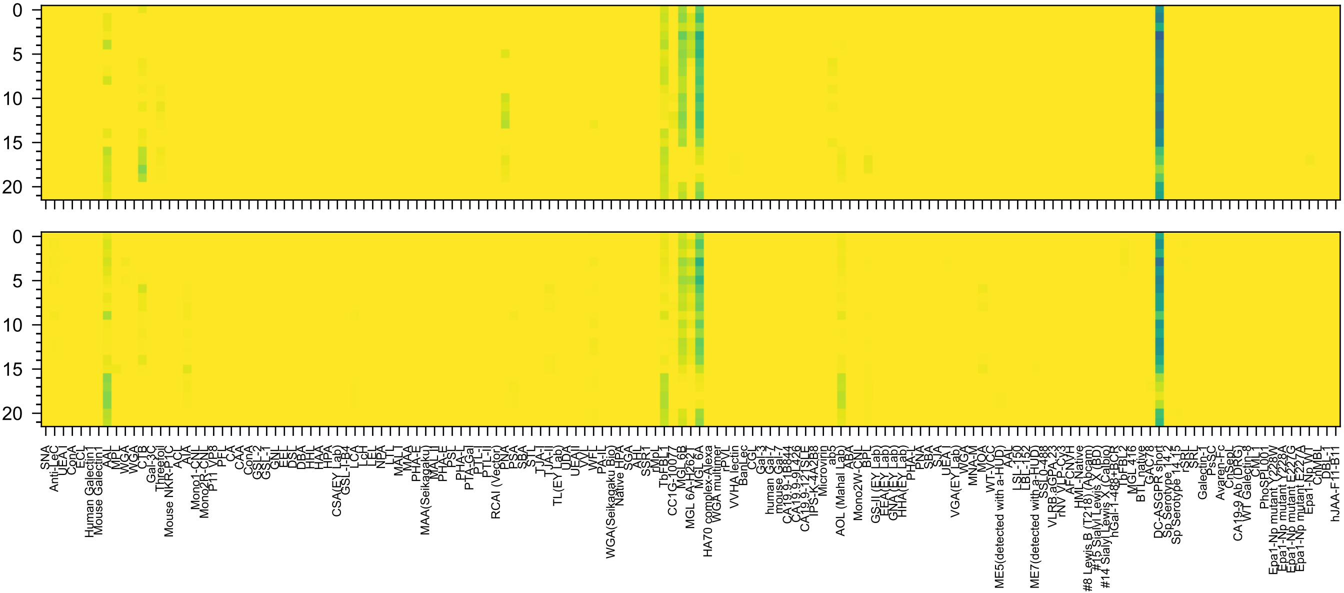

Gal(β1-4)[Fuc(α1-3)]GlcNAc(β1-6)GalNAc(-Sp14

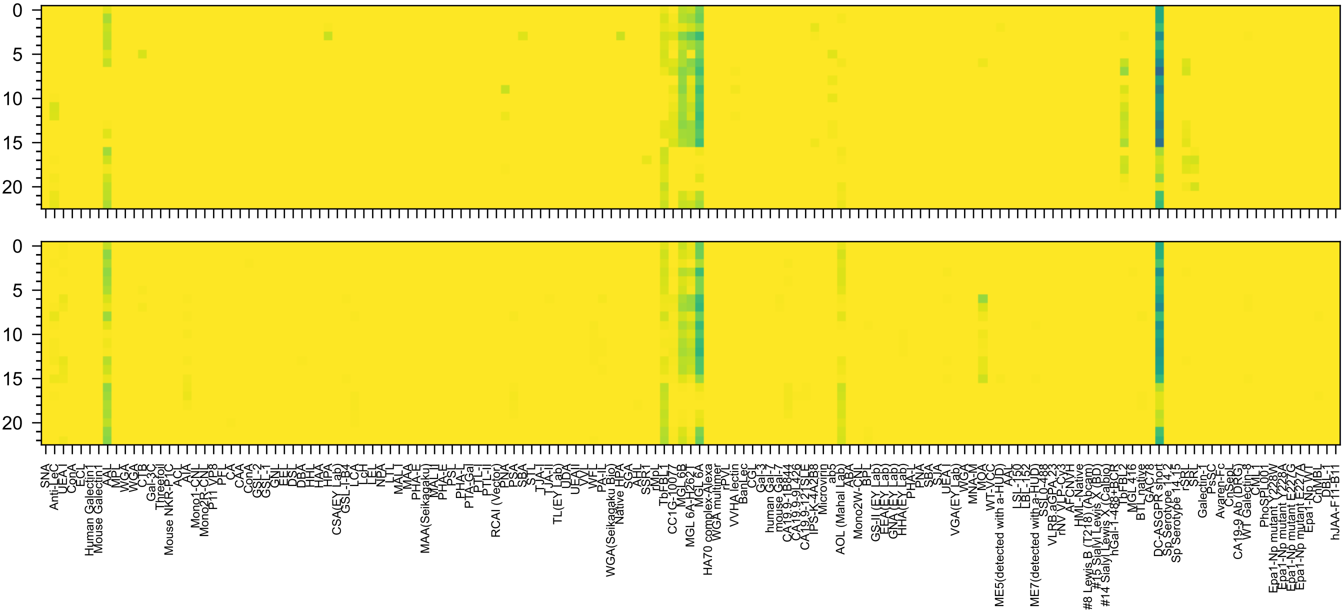

Gal(β1-3)[Fuc(α1-4)]GlcNAc(β1-6)GalNAc(α-Sp14

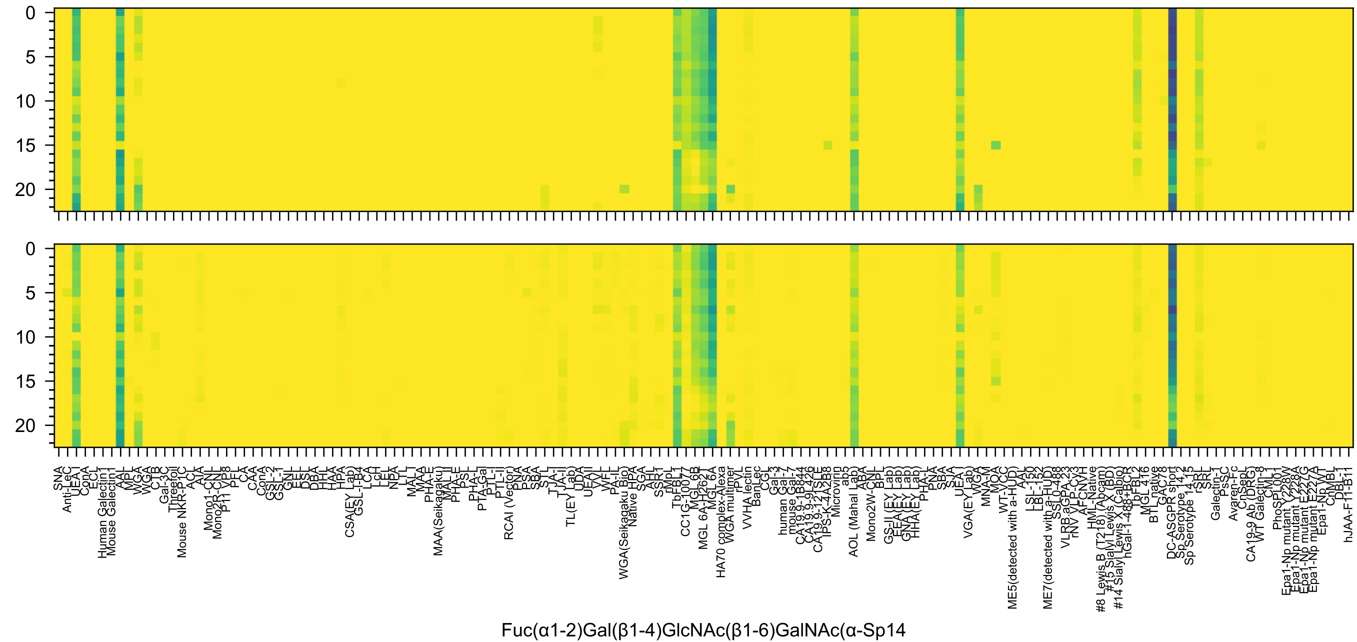
0
5
10
15
20
Fuc(α1-2)Gal(β1-4)GlcNAc(β1-6)GalNAc(α-Sp14

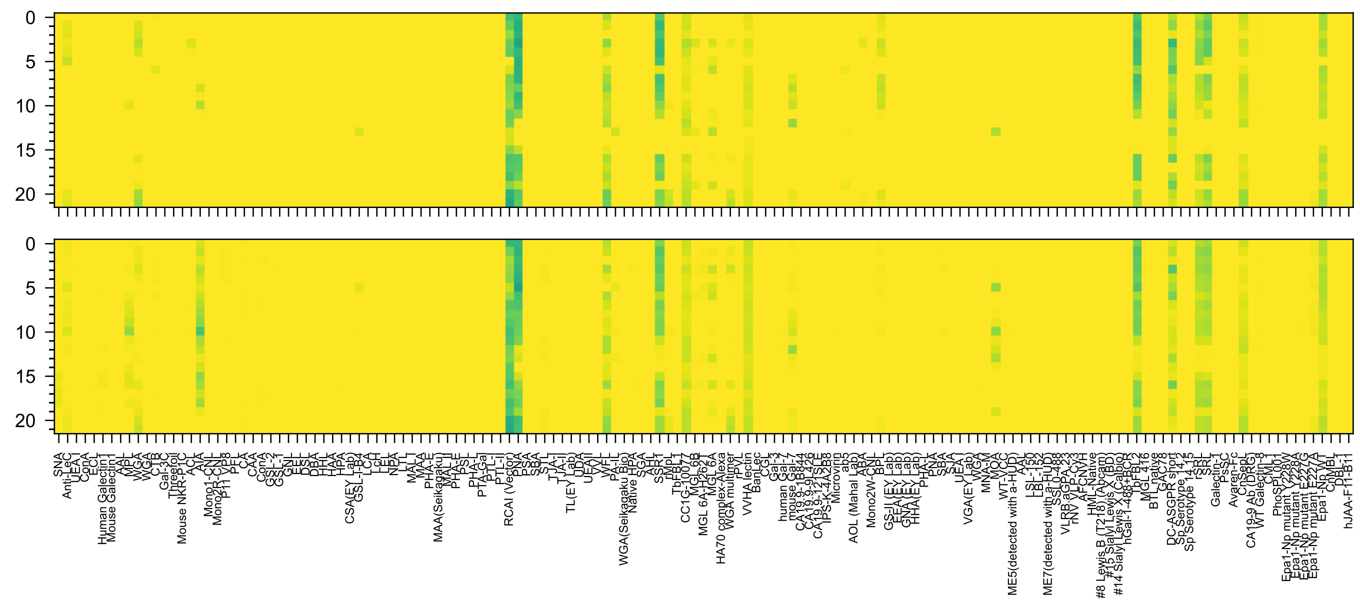

Gal(β1-4)GlcNAc(β1-6)[Gal(β1-3)]GalNAc(-Sp14

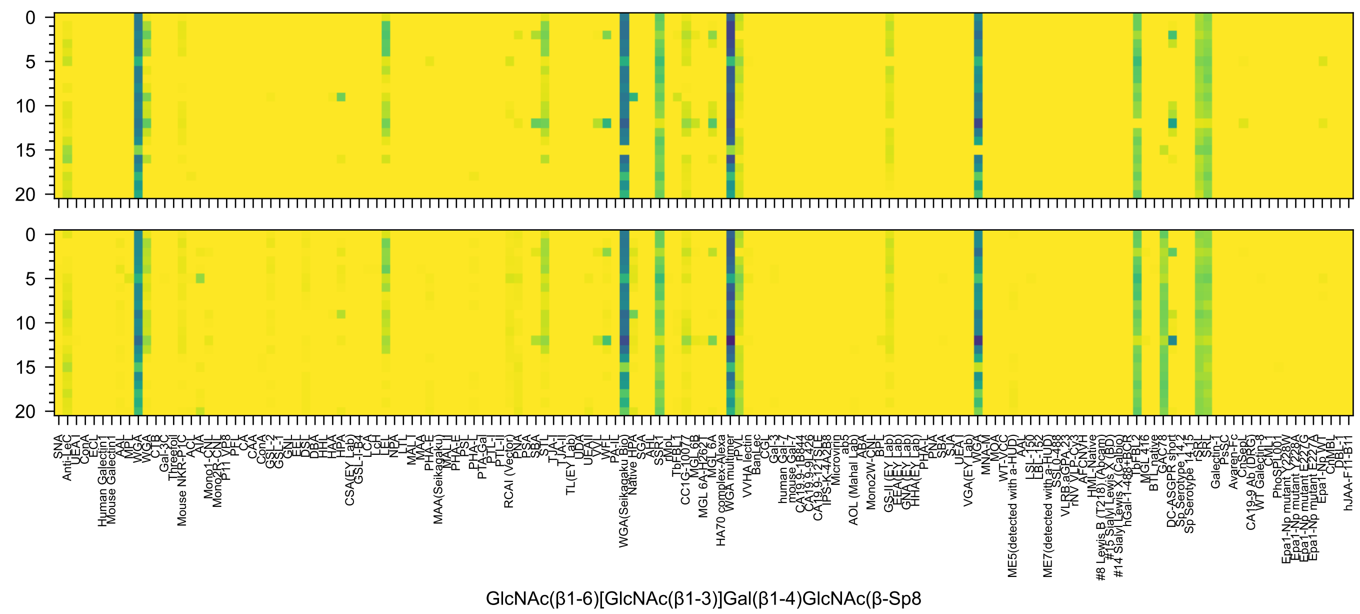
GlcNAc(β1-6)[GlcNAc(β1-3)]Gal(β1-4)GlcNAc(β-Sp8

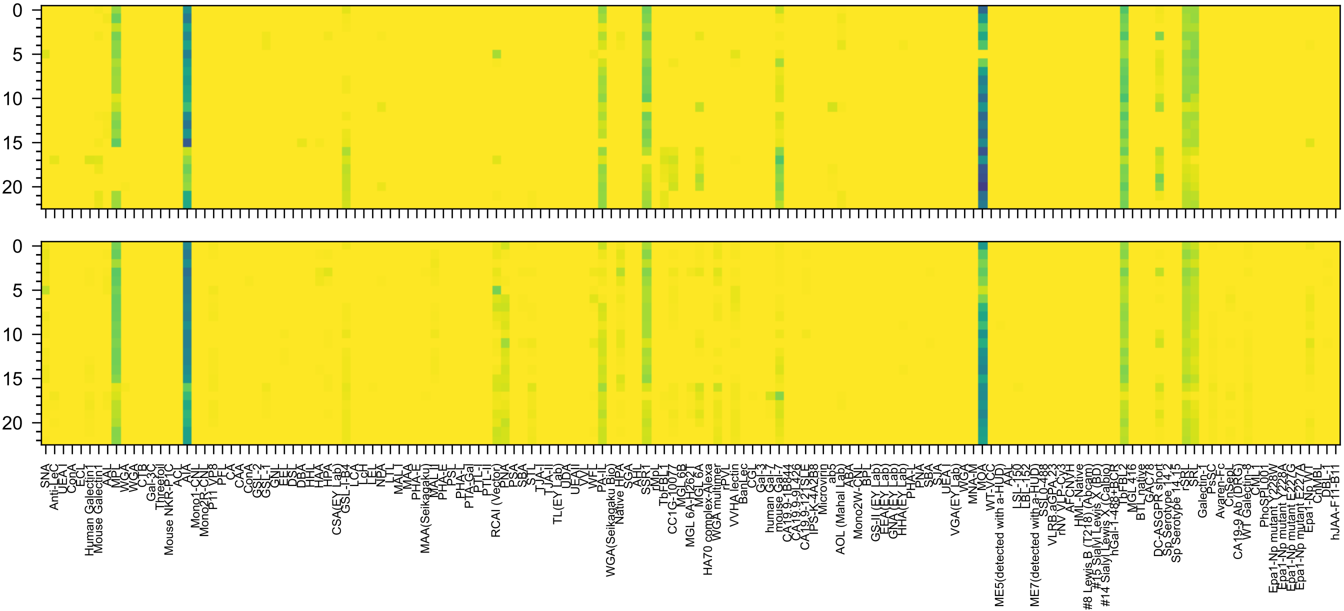

Gal(α1-3)Gal(β1-4)GlcNAc(β1-3)GalNAc(α-Sp14

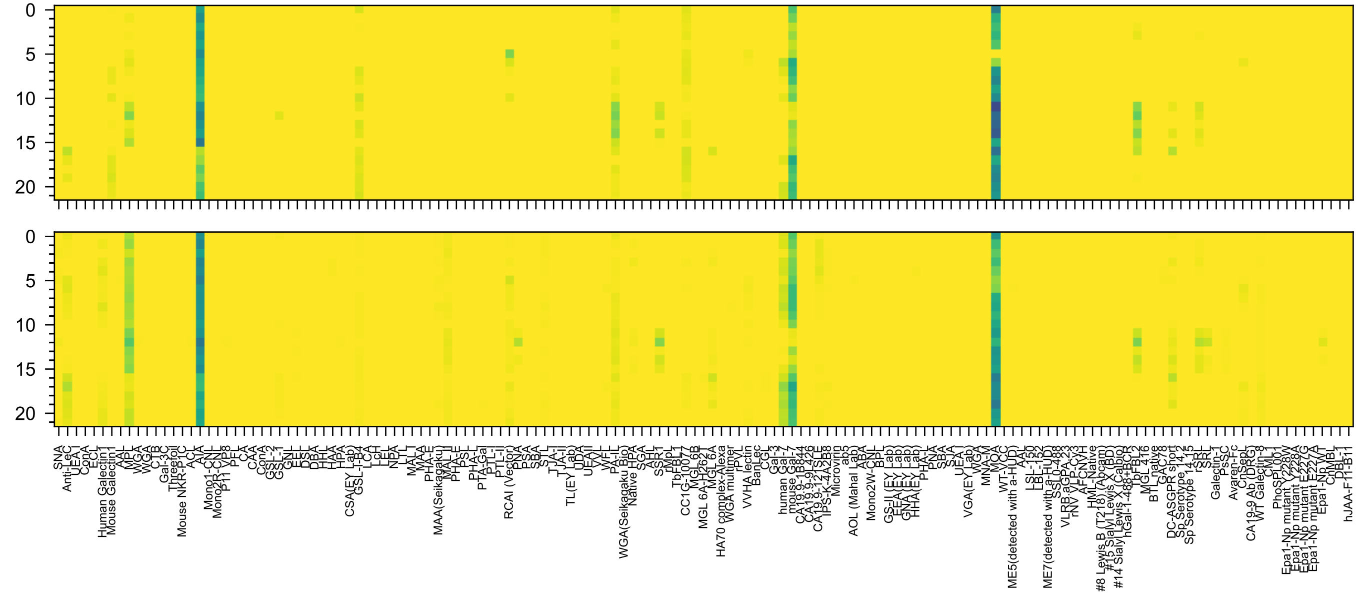

Gal(α1-3)Gal(β1-3)GlcNAc(β1-3)GalNAc(-Sp14

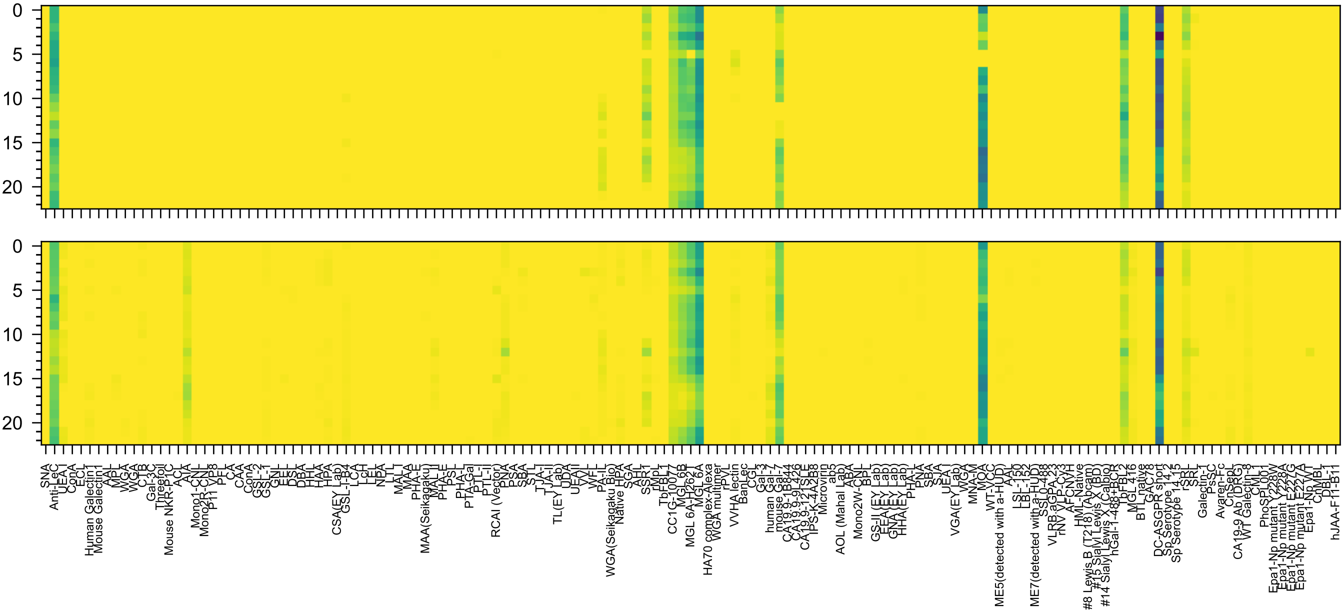

Gal(α1-3)Gal(β1-3)GlcNAc(β1-6)GalNAc(α-Sp14

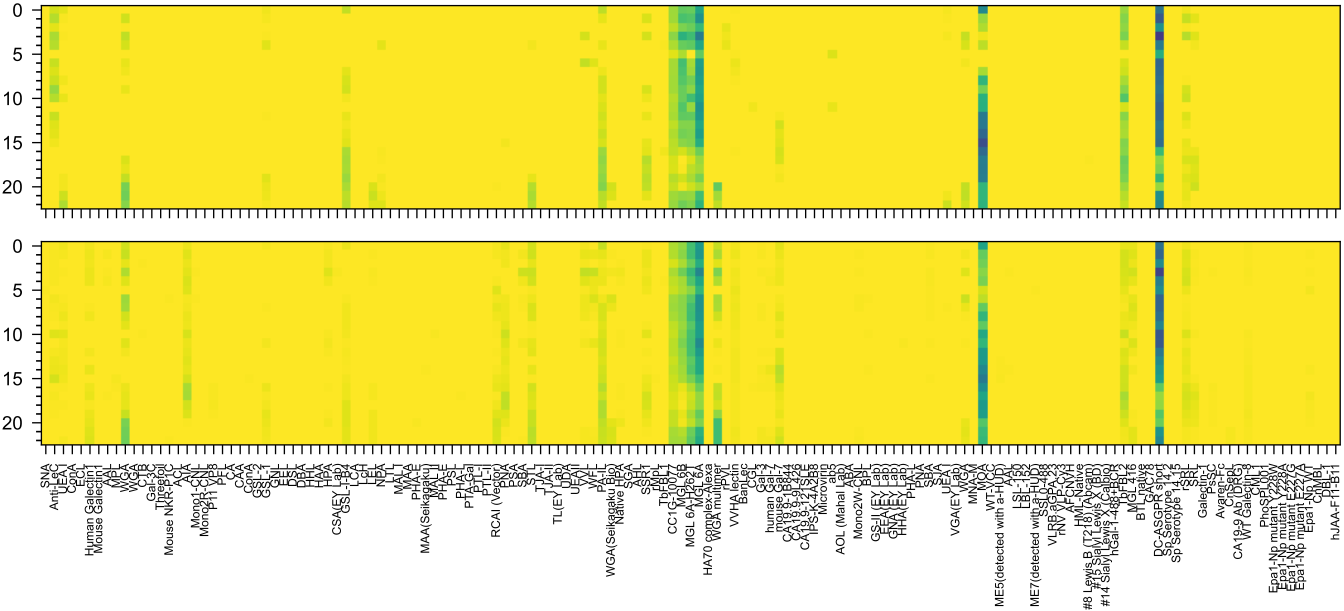

Gal(α1-3)Gal(β1-4)GlcNAc(β1-6)GalNAc(α-Sp14

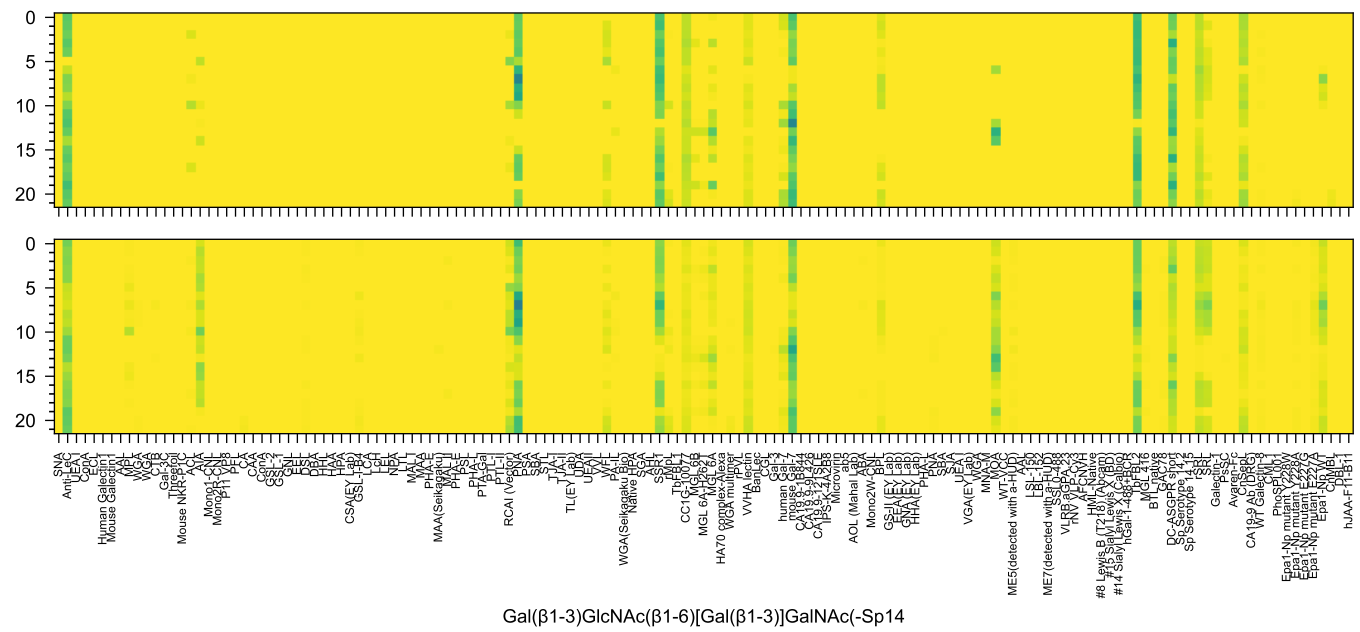
SNA
Anti-LeC
UEA-I
ConA
ECL
Human Galectin1
Mouse Galectin1
AAL
MPL
WGA
WGA
CTB
Gal-3C
Threefoil
Mouse NKR-P1C
ACL
AIA
Mono1-CNL
Mono2R-CNL
P11 VP8
PFL
CA
CAA
ConA
GSL-2
GSL-1
GNL
EEL
DSL
DBA
HHL
HAA
HPA
CSA(EY Lab)
GSL-I-B4
LCA
LcH
LEL
NPA
LTL
MAL-I
MAA
PHA-E
MAA(Seikagaku)
MAL-II
PHA-E
PSL
PHA-L
PTA-Gal
PTL-I
PTL-II
RCAI (Vector)
PNA
PSA
SBA
STL
TJA-I
TJA-II
TL(EY Lab)
UDA
UEAII
VVL
WFL
PA-IL
WGA(Seikagaku Bio)
Native HPA
SGA
AHL
SSR1
rMpL
TbFBL1
CC1G-10077
MGL6B
MGL 6A-H262T
MGL_6A
HA70 complex-Alexa
WGA multimer
rPVL
VVHA lectin
BanLec
CGL
Gal-3
human Gal-7
mouse Gal-7
CA19.9-1B844
CA19.9-9L426
CA19.9-121SLE
IPS-K-4A2B8
Microvirin
ab5
AOL (Mahal Lab)
ABA
Mono2W-CNL
BPL
GS-II (EY Lab)
EEA (EY Lab)
GNA (EY Lab)
HHA (EY Lab)
PHA-L
PNA
SBA
SJA
UEA-I
VGA(EY Lab)
WGA
MNA-M
MOA
WT-VCC
ME5(detected with a-HUD)
AAL
LSL-150
LBL-152
ME7(detected with a-HUD)
SSL0-488
VLRB.aGPA.23
rNV VLP-Cy3
AFCNVH
HML-Native
#8 Lewis B (T218) (Abcam)
#15 Sialyl Lewis X (BD)
#14 Sialyl Lewis X (Calbio)
hGal-1-488+BCR
TbFBL2
MGL 416
BTL native
GAC78
DC-ASGPR short
Sp Serotype 14.2
Sp Serotype 14.15
rSRL
SRL
Galectin-1
PsSC
Avaren-Fc
ChSepL
CA19-9 Ab (DRG)
WT Galectin-8
CML1
PhoSL001
Epa1-Np mutant Y228W
Epa1-Np mutant Y228A
Epa1-Np mutant E227G
Epa1-Np mutant E227A
Epa1-Np WT
ChMBL
DBL-1
hJAA-F11-B11
Gal(β1-3)GlcNAc(β1-6)[Gal(β1-3)]GalNAc(-Sp14

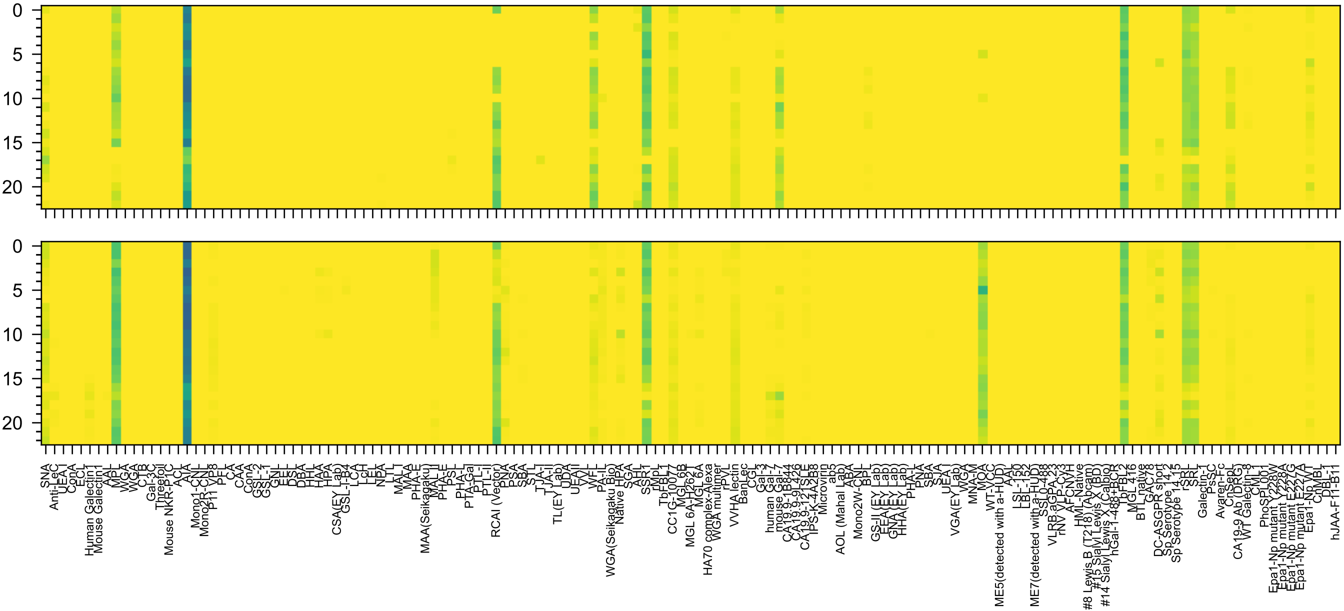

Gal(β1-4)GlcNAc(β1-3)Gal(β1-3)GalNAc(α-Sp14

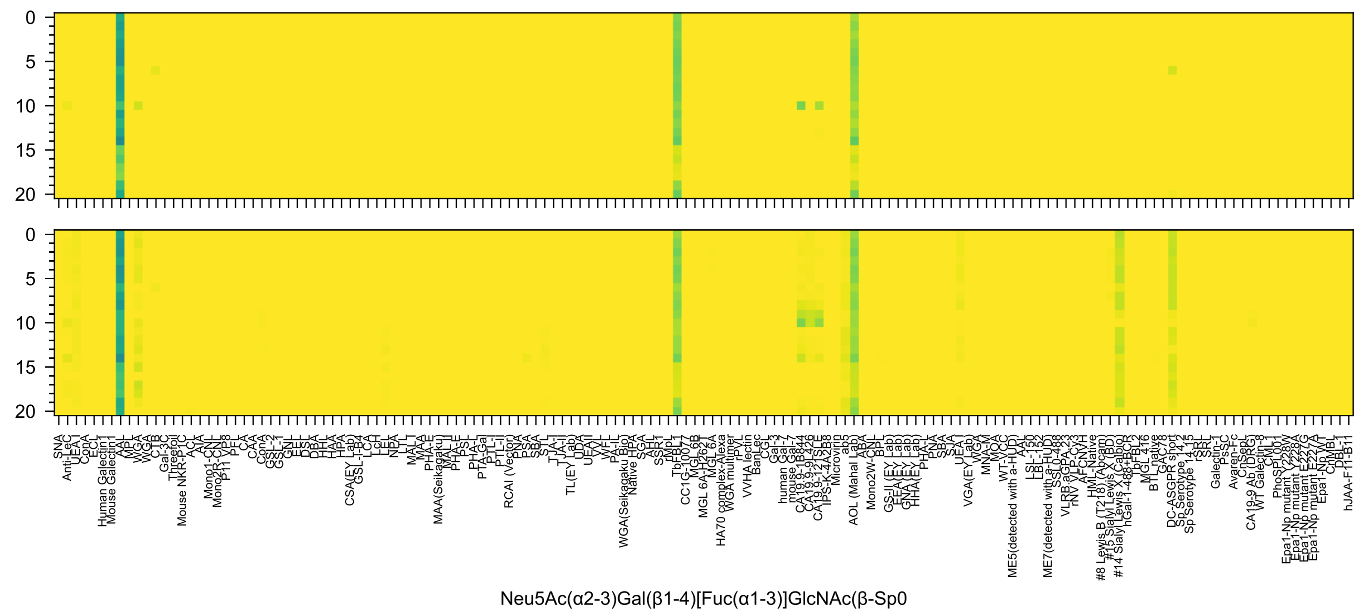
Neu5Ac(α2-3)Gal(β1-4)[Fuc(α1-3)]GlcNAc(β-Sp0

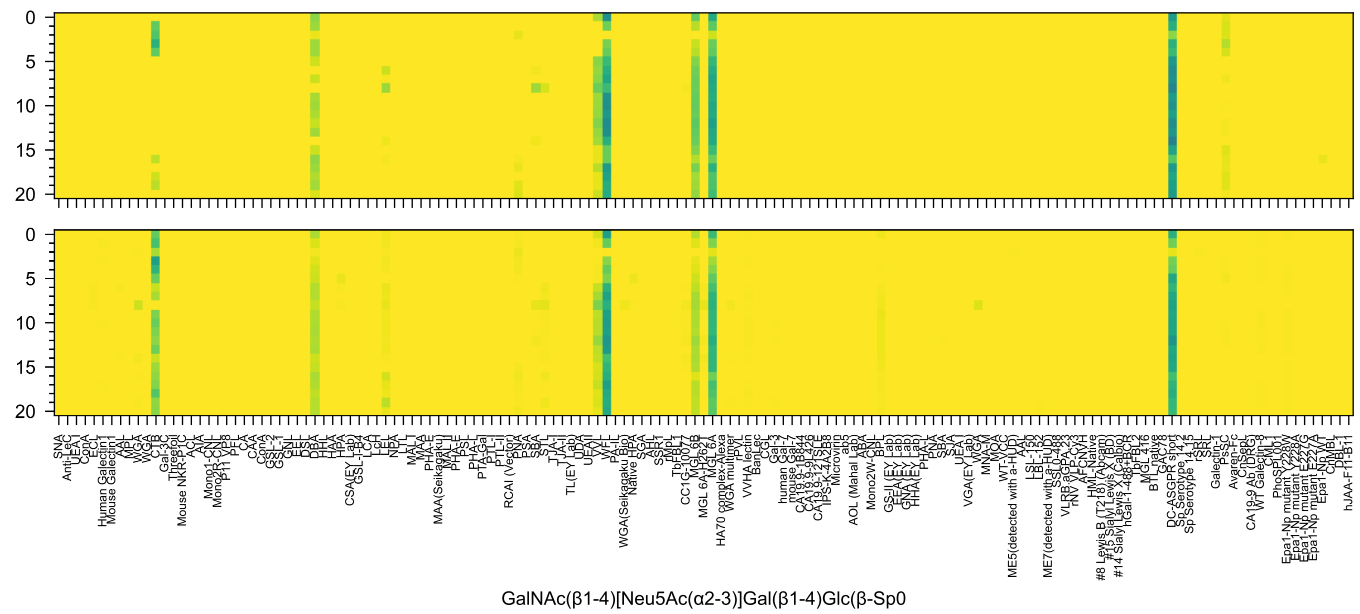
GalNAc(β1-4)[Neu5Ac(α2-3)]Gal(β1-4)Glc(β-Sp0

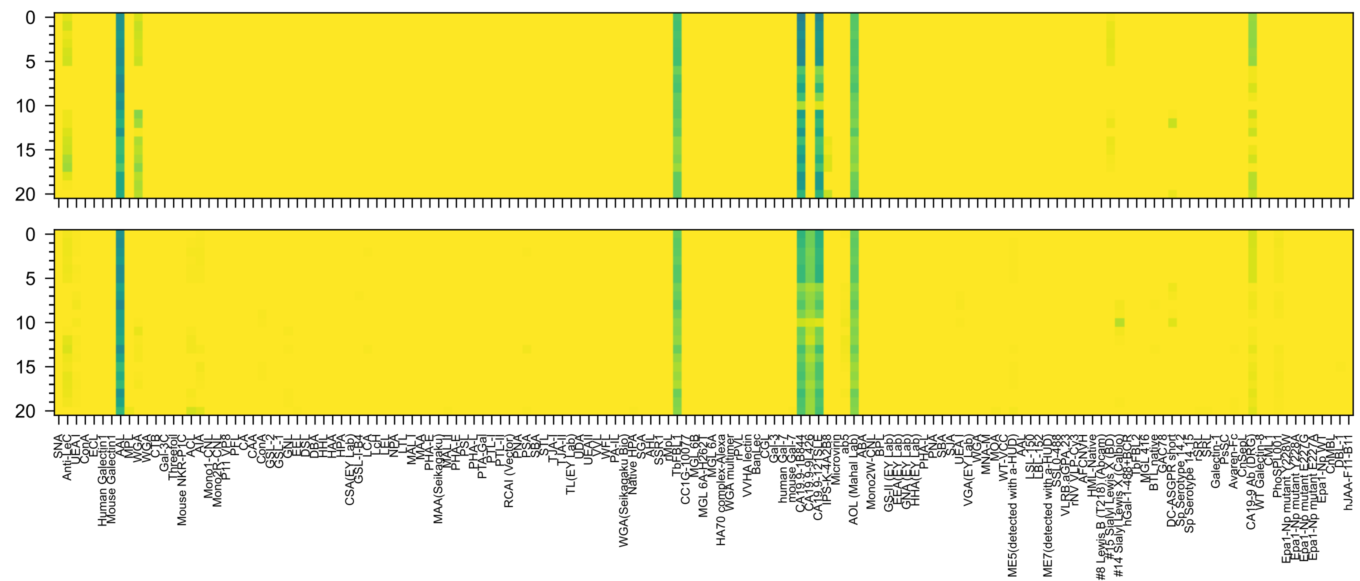

Neu5Ac(α2-3)Gal(β1-3)[Fuc(α1-4)]GlcNAc(β-Sp8

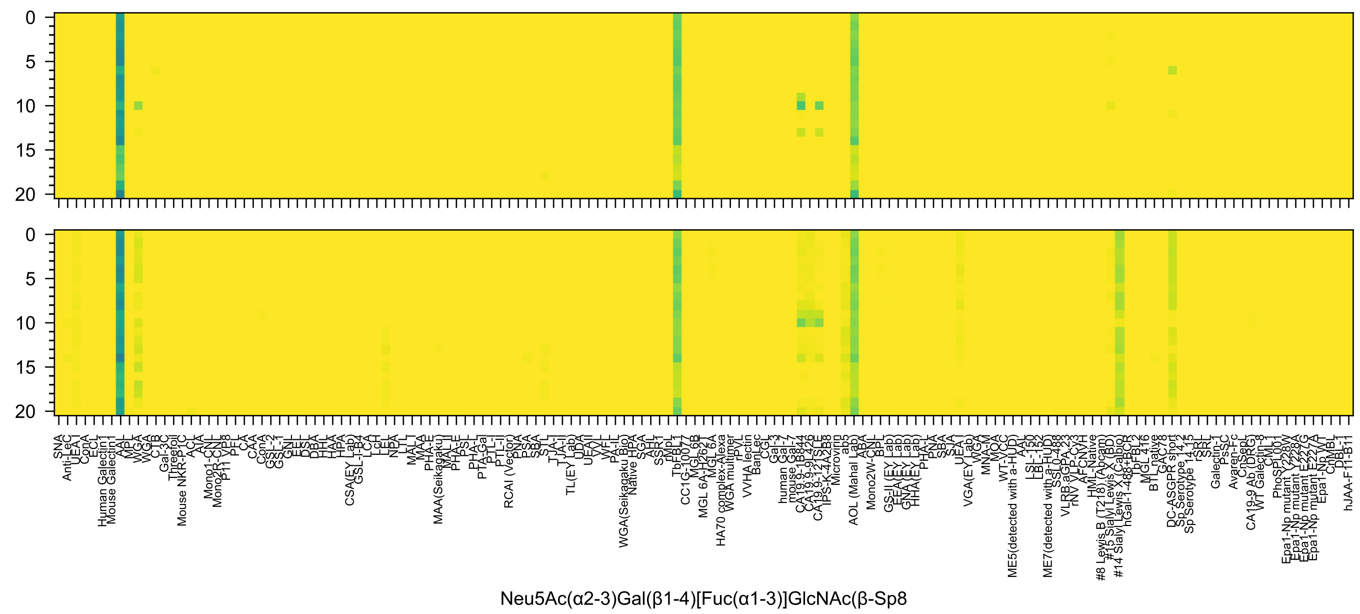
Neu5Ac(α2-3)Gal(β1-4)[Fuc(α1-3)]GlcNAc(β-Sp8

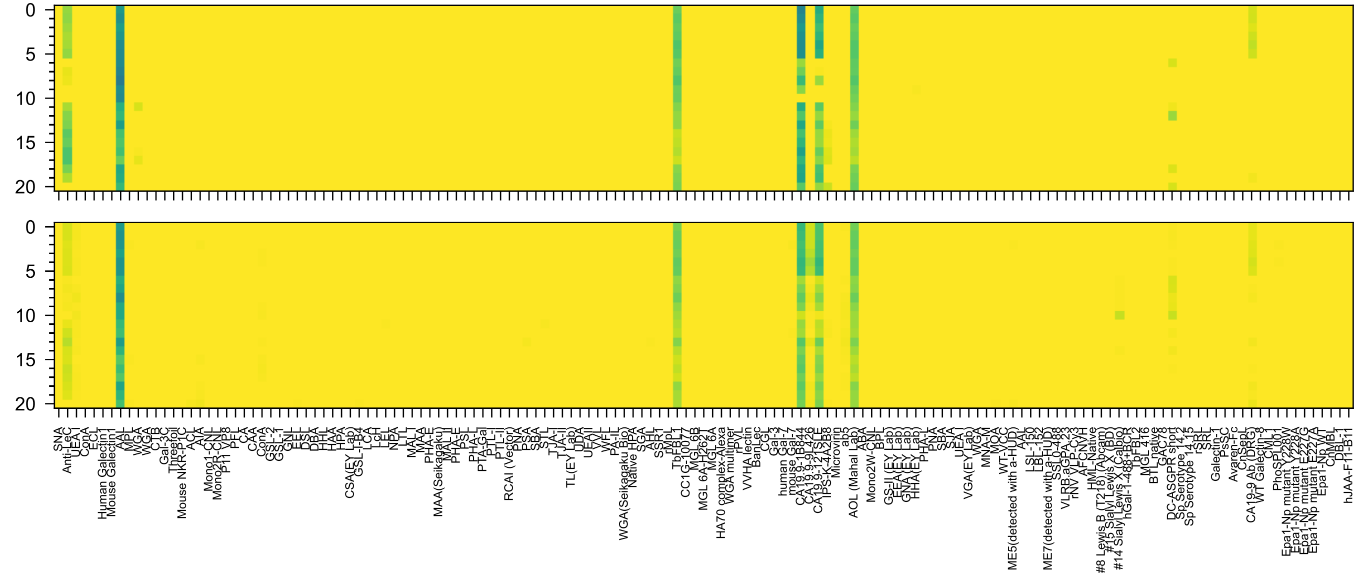

Neu5Gc(α2-3)Gal(β1-3)[Fuc(α1-4)]GlcNAc(β-Sp0

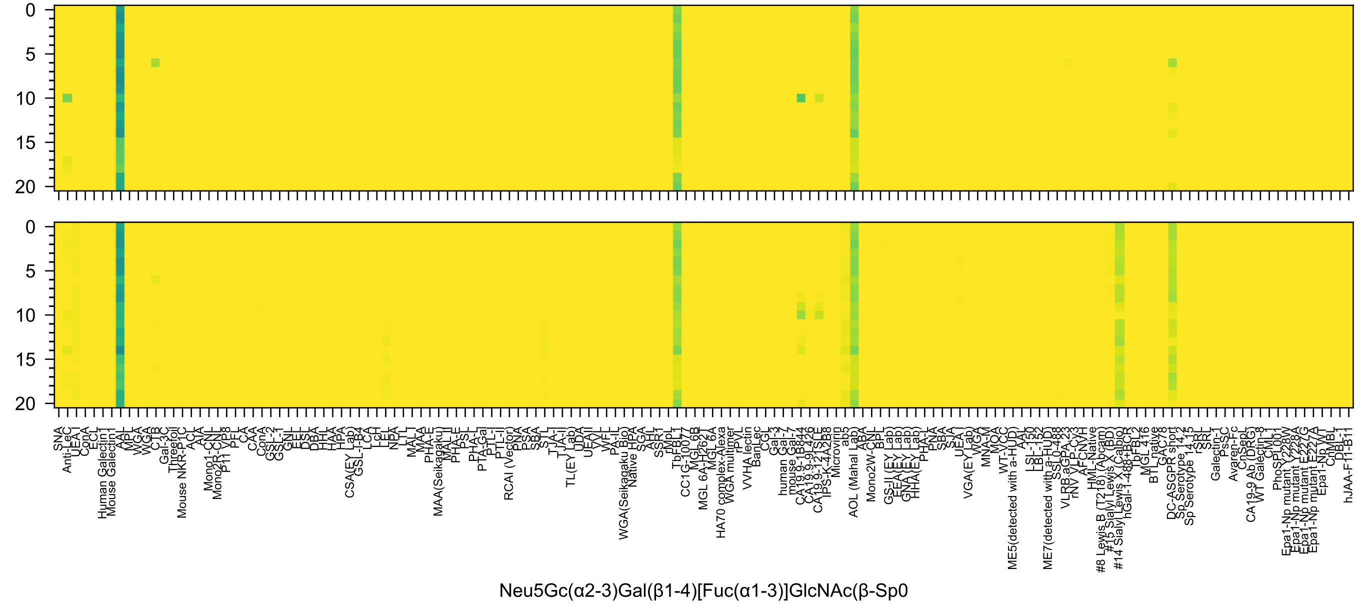
Neu5Gc(α2-3)Gal(β1-4)[Fuc(α1-3)]GlcNAc(β-Sp0

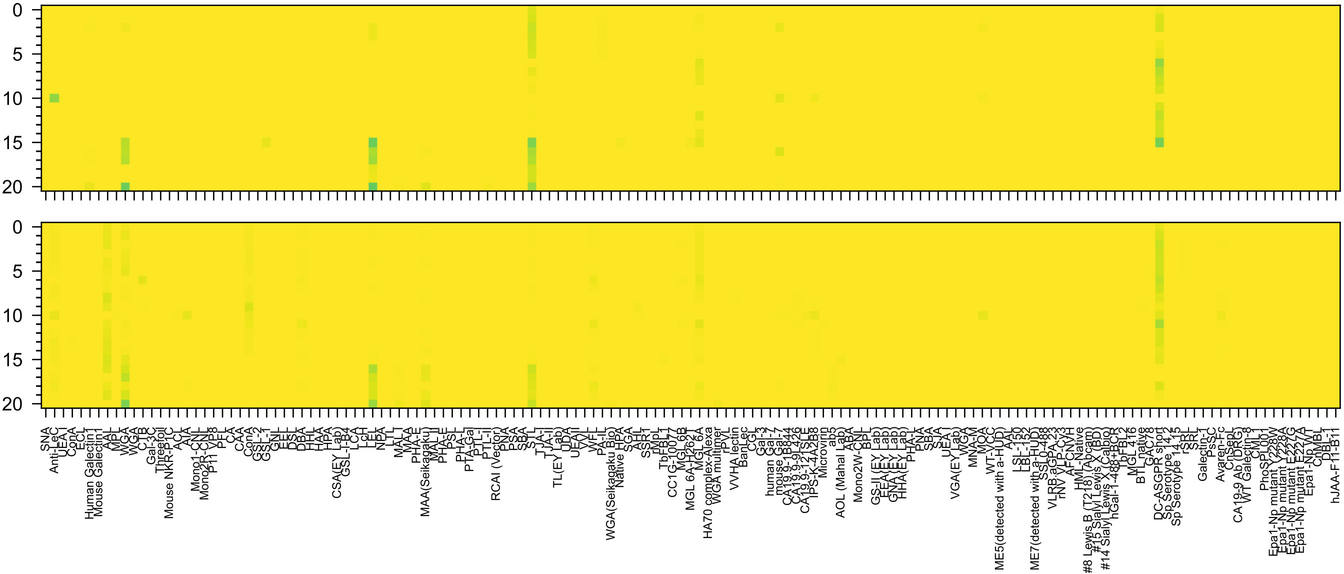

Neu5Ac(α2-3)Gal(β1-4)GlcNAc(β1-2)Man(α-Sp0

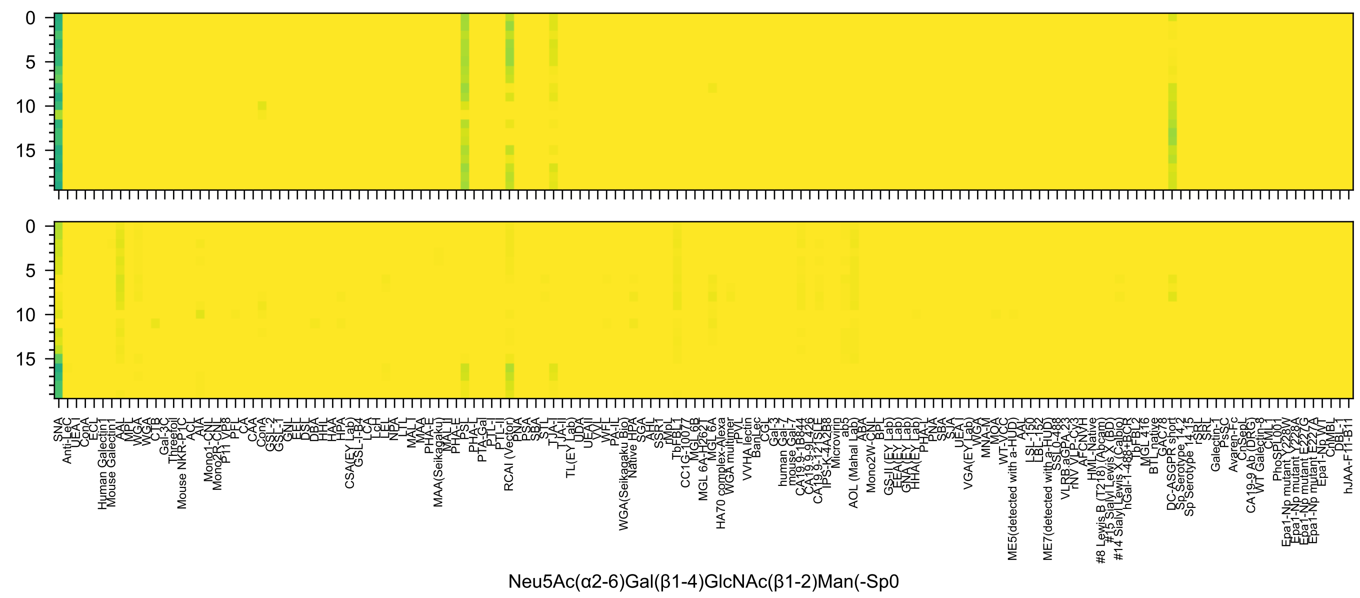

0
5
10
15
SNA
Anti-LeC
UEA-I
ConA
ECL
Human Galectin1
Mouse Galectin1
AAL
MPL
WGA
WGA
CTB
Gal-3C
Threefoil
Mouse NKR-P1C
ACL
AIA
Mono1-CNL
Mono2R-CNL
P11 VP8
PFL
CA
CAA
ConA
GSL-2
GSL-1
GNL
EEL
DSL
DBA
HHL
HAA
HPA
CSA(EY Lab)
GSL-I-B4
LCA
LcH
LEL
NPA
LTL
MAL-I
MAA
PHA-E
MAA(Seikagaku)
MAL-II
PHA-E
PSL
PHA-L
PTA-Gal
PTL-I
PTL-II
RCAI (Vector)
PNA
PSA
SBA
STL
TJA-I
TJA-II
TL(EY Lab)
UDA
UEAII
VVL
WFL
PA-IL
WGA(Seikagaku Bio)
Native HPA
SGA
AAI
SSR1
rMpL
TbFBL1
CC1G-10077
MGL 6B
MGL 6A-H262T
MGL 6A
HA70 complex-Alexa
WGA multimer
rPVL
VVHA lectin
BanLec
CGL
Gal-3
human Gal-7
mouse Gal-7
CA19.9-1B844
CA19.9-9L426
CA19.9-121SLE
IPS-K-4A2B8
Microvirin
ab5
AOL (Mahal Lab)
ABA
Mono2W-CNL
BPL
GS-II (EY Lab)
EEA(EY Lab)
GNA(EY Lab)
HHA(EY Lab)
PHA-L
PNA
SBA
SJA
UEA-I
VGA(EY Lab)
WGA
MNA-M
MOA
WT-VCC
ME5(detected with a-HUD)
AAL
LSL-150
LBL-152
ME7(detected with a-HUD)
SSL0-488
VLRB.aGPA.23
rNV VLP-Cy3
AFCNVH
HML-Native
#8 Lewis B (T218) (Abcam)
#15 Sialyl Lewis X (BD)
#14 Sialyl Lewis X (Calbio)
hGal-1-488+BCR
TbFBL2
MGL 416
BTL native
GAC78
DC-ASGPR short
Sp Serotype 14.2
Sp Serotype 14.15
rSRL
SRL
Galectin-1
PsSC
Avaren-Fc
CnSepL
CA19-9 Ab (DRG)
WT Galectin-8
CML1
PhoSL001
Epa1-Np mutant Y228V
Epa1-Np mutant Y228A
Epa1-Np mutant E227G
Epa1-Np mutant E227A
Epa1-Np WT
ChMBL
DBL-1
hJAA-F11-B11
Neu5Ac(α2-6)Gal(β1-4)GlcNAc(β1-2)Man(-Sp0

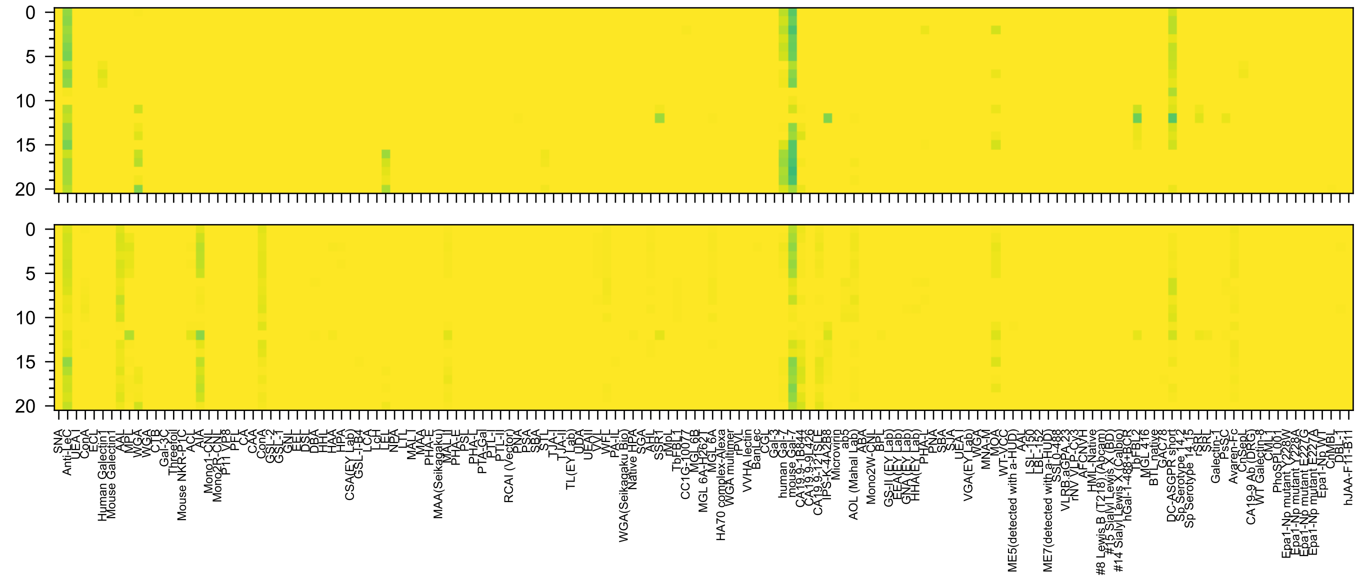

Neu5Ac(α2-3)Gal(β1-3)GlcNAc(β1-2)Man(α-Sp0

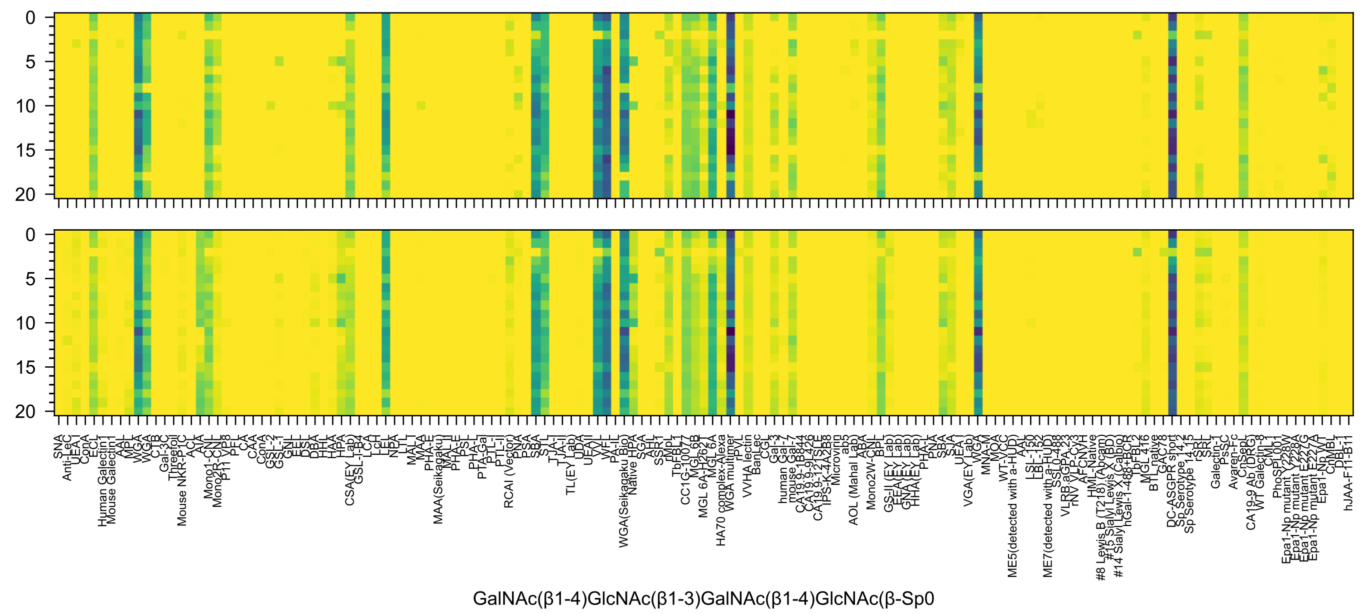
0
5
10
15
20
0
5
10
15
20
SNA
Anti-LeC
UEA I
ConA
ECL
Human Galectin1
Mouse Galectin1
AAL
MPL
WGA
WGA
CTB
Gal-3C
Threefoil
Mouse NKR-P1C
ACL
AIA
Mono1-CNL
Mono2R-CNL
P11 VP8
PFL
CA
CAA
ConA
GSL-2
GSL-1
GNL
EEL
DSL
DBA
HHL
HAA
HPA
CSA(EY Lab)
GSL-I-B4
LCA
LcH
LEL
NPA
LTL
MAL-I
MAA
PHA-E
MAA(Seikagaku)
MAL II
PHA-E
PSL
PHA-L
PTA-Gal
PTL-I
PTL-II
RCAI (Vector)
PNA
PSA
SBA
STL
TJA-I
TJA-II
TL(EY Lab)
UDA
UEA II
VVL
WFL
PA-IL
WGA(Seikagaku Bio)
Native HPA
SGA
AHL
SSR1
rMpL
TbFBL1
CC1G-10077
MGL 6B
MGL 6A-H262T
MGL 6A
HA70 complex-Alexa
WGA multimer
rPVL
VVHA lectin
BanLec
CGL
Gal-3
human Gal-7
mouse Gal-7
CA19.9-1B844
CA19.9-9L426
CA19.9-121SLE
IPS-K-4A2B8
Microvirin
ab5
AOL (Mahal Lab)
ABA
Mono2W-CNL
BPL
GS-II (EY Lab)
EEA(EY Lab)
GNA(EY Lab)
HHA(EY Lab)
PHA-L
PNA
SBA
SJA
UEA I
VGA(EY Lab)
WGA
MNA-M
MOA
WT-VCC
ME5(detected with a-HUD)
AAL
LSL-150
LBL-152
ME7(detected with a-HUD)
SSL0-488
VLRB.aGPA.23
rNV VLP-Cy3
AFCNVH
HML-Native
#8 Lewis B (T218) (Abcam)
#15 Sialyl Lewis X (BD)
#14 Sialyl Lewis X (Calbio)
hGal-1-488+BCR
TbFBL2
MGL 416
BTL native
GAC78
DC-ASGPR short
Sp Serotype 14.2
Sp Serotype 14.15
rSRL
SRL
Galectin-1
PsSC
Avaren-Fc
CnSepL
CA19-9 Ab (DRG)
WT Galectin-8
CML1
PhoSPL001
Epa1-Np mutant Y228W
Epa1-Np mutant Y228A
Epa1-Np mutant E227G
Epa1-Np mutant E227A
Epa1-Np WT
CnMBL
DBL-1
hJAA-F11-B11
GalNAc(β1-4)GlcNAc(β1-3)GalNAc(β1-4)GlcNAc(β-Sp0

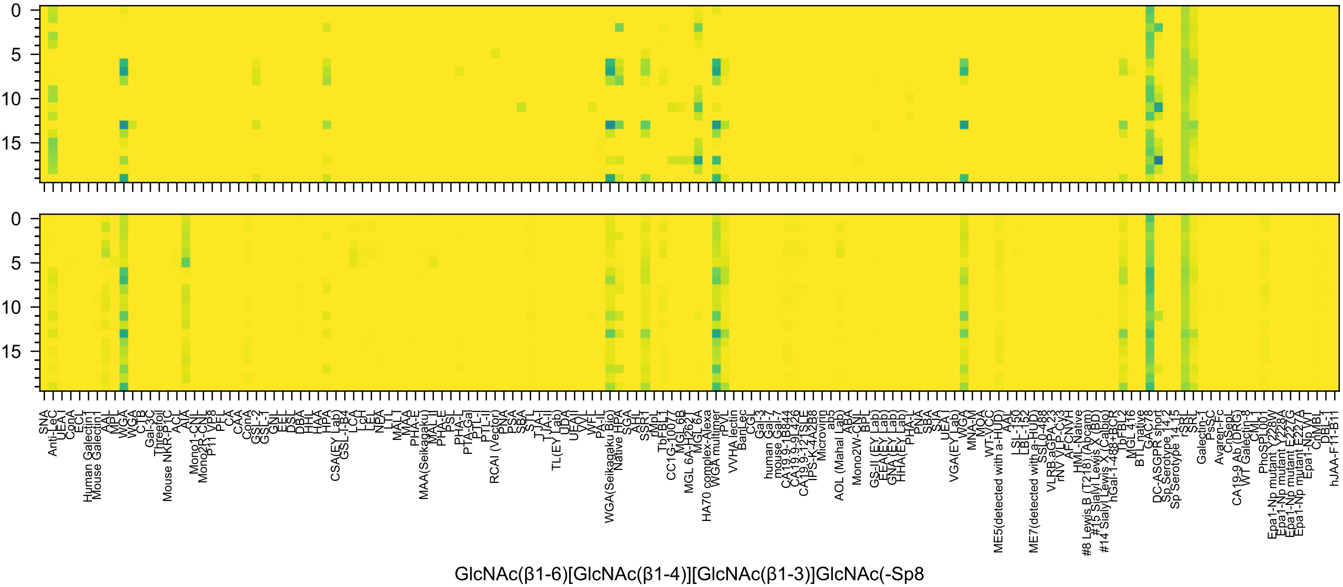
0
5
10
15
0
5
10
15
SNA
Anti-LeC
UEA-I
ConA
ECL
Human Galectin1
Mouse Galectin1
AAL
MPL
WGA
WGA
CTB
Gal-3C
Threefoil
Mouse NKR-P1C
ACL
AIA
Mono1-CNL
Mono2R-CNL
P11 VP8
PFL
CA
CAA
ConA
GSL-2
GSL-1
GNL
EEL
DSL
DBA
HHL
HAA
HPA
CSA(EY Lab)
GSL-I-B4
LCA
LcH
LEL
NPA
LTL
MAL-I
MAA
PHA-E
MAA(Seikagaku)
MAL-II
PHA-E
PSL
PHA-L
PTA-Gal
PTL-I
PTL-II
RCAI (Vector)
PNA
PSA
SBA
STL
TJA-I
TJA-II
TL(EY Lab)
UDA
UEA-II
VVL
WFL
PA-IL
WGA(Seikagaku Bio)
Native HPA
SGA
AAL
SSR1
rMpL
TbFBL1
CC1G-10077
MGL 6B
MGL 6A-H262T
MGL 6A
HA70 complex-Alexa
WGA multimer
rPVL
VVHA lectin
BanLec
CGL
Gal-3
human Gal-7
mouse Gal-7
CA19.9-1B844
CA19.9-9L426
CA19.9-121SLE
IPS-K-4A2B8
Microvirin
ab5
AOL (Mahal Lab)
ABA
Mono2W-CNL
BPL
GS-II (EY Lab)
EEA(EY Lab)
GNA(EY Lab)
HHA(EY Lab)
PHA-L
PNA
SBA
SJA
UEA-I
VGA(EY Lab)
WGA
MNA-M
MOA
WT-VCC
ME5(detected with a-HUD)
AAL
LSL-150
LBL-152
ME7(detected with a-HUD)
SSL0-488
VLRB.aGPA.23
rNV VLP-Cy3
AFCNVH
HML-Native
#8 Lewis B (T218) (Abcam)
#15 Sialyl Lewis X (BD)
#14 Sialyl Lewis X (Calbio)
hGal-1-488+BCR
TbFBL2
MGL 416
BTL native
GAC78
DC-ASGPR short
Sp Serotype 14.2
Sp Serotype 14.15
rSRL
SRL
Galectin-1
PsSC
Avaren-Fc
CnSepL
CA19-9 Ab (DRG)
WT Galectin-8
CML
PhoSL001
Epa1-Np mutant Y228W
Epa1-Np mutant Y228A
Epa1-Np mutant E227G
Epa1-Np mutant E227A
Epa1-Np WT
ChMBL
DBL-1
hJAA-F11-B11
GlcNAc(β1-6)[GlcNAc(β1-4)][GlcNAc(β1-3)]GlcNAc(-Sp8

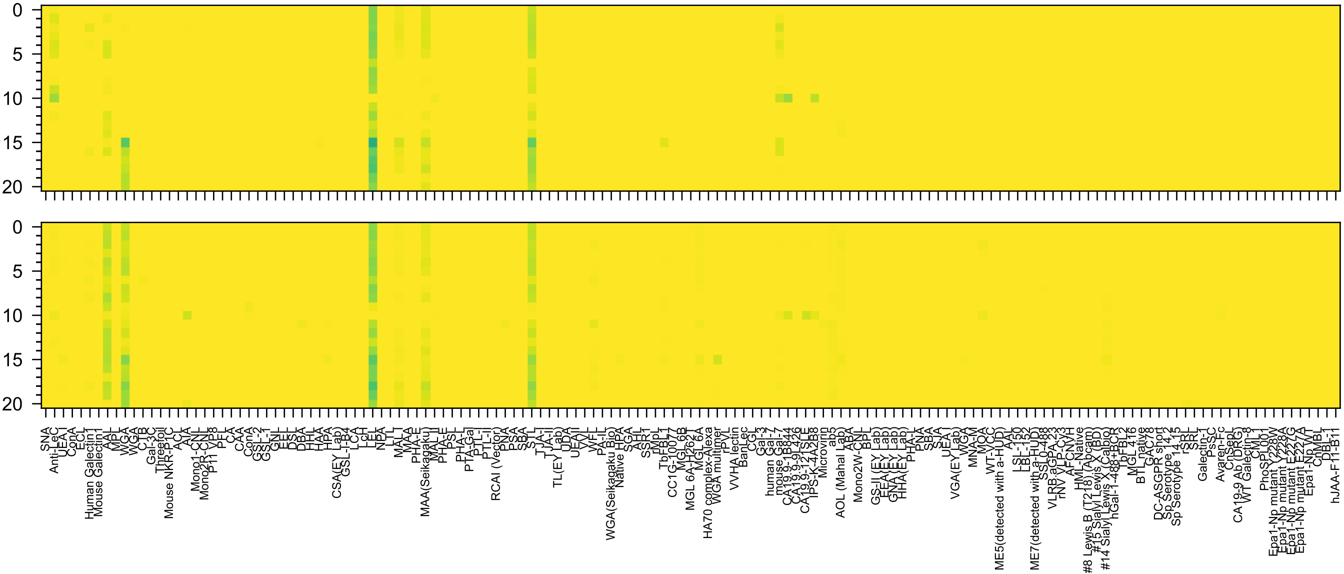

Neu5Ac(α2-3)Gal(β1-4)GlcNAc(β1-3)Gal(β-Sp8

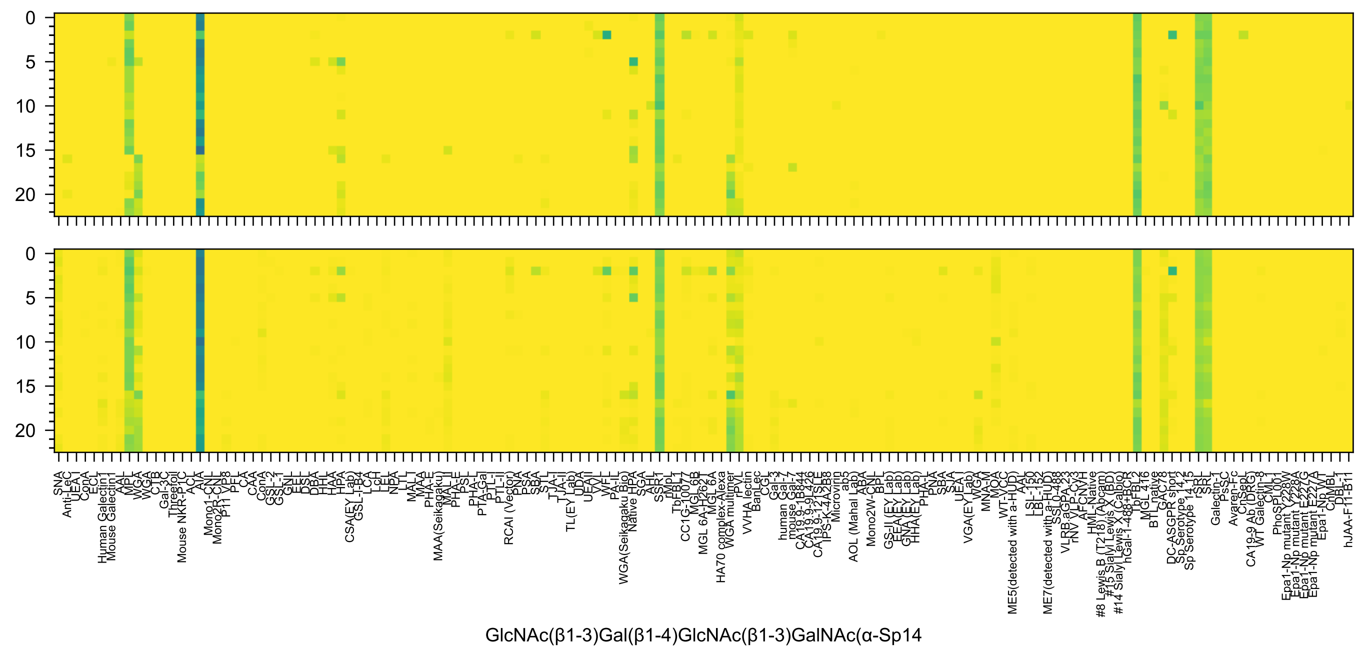
GlcNAc(β1-3)Gal(β1-4)GlcNAc(β1-3)GalNAc(α-Sp14

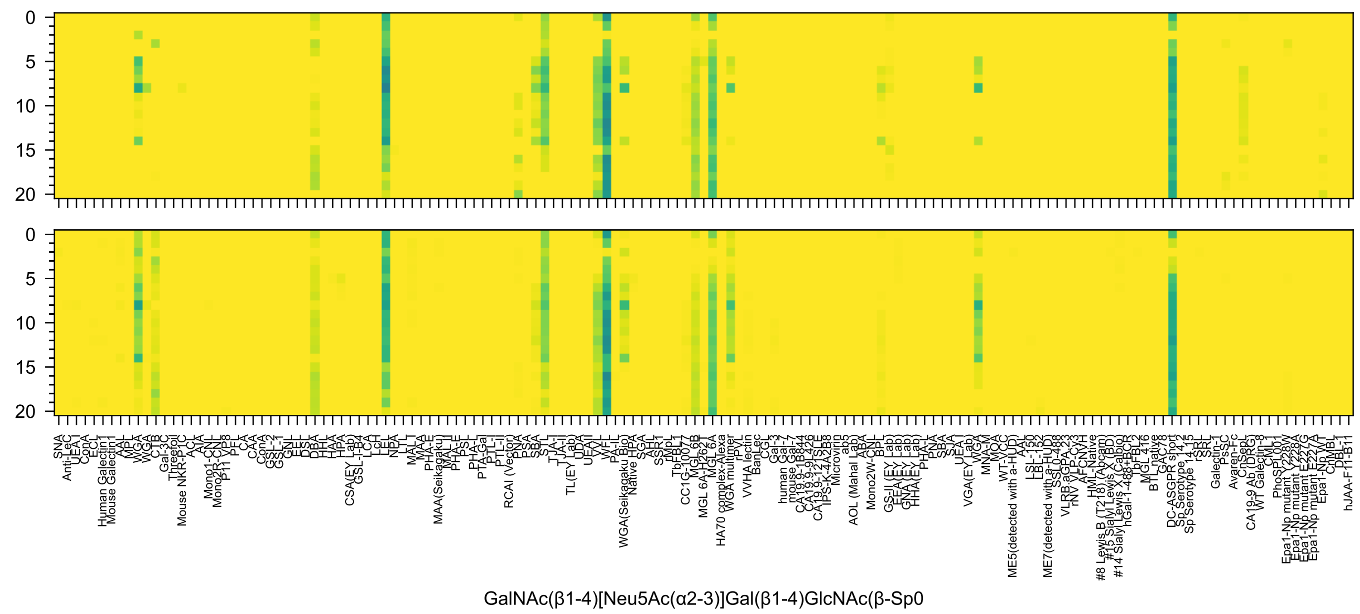
GalNAc(β1-4)[Neu5Ac(α2-3)]Gal(β1-4)GlcNAc(β-Sp0

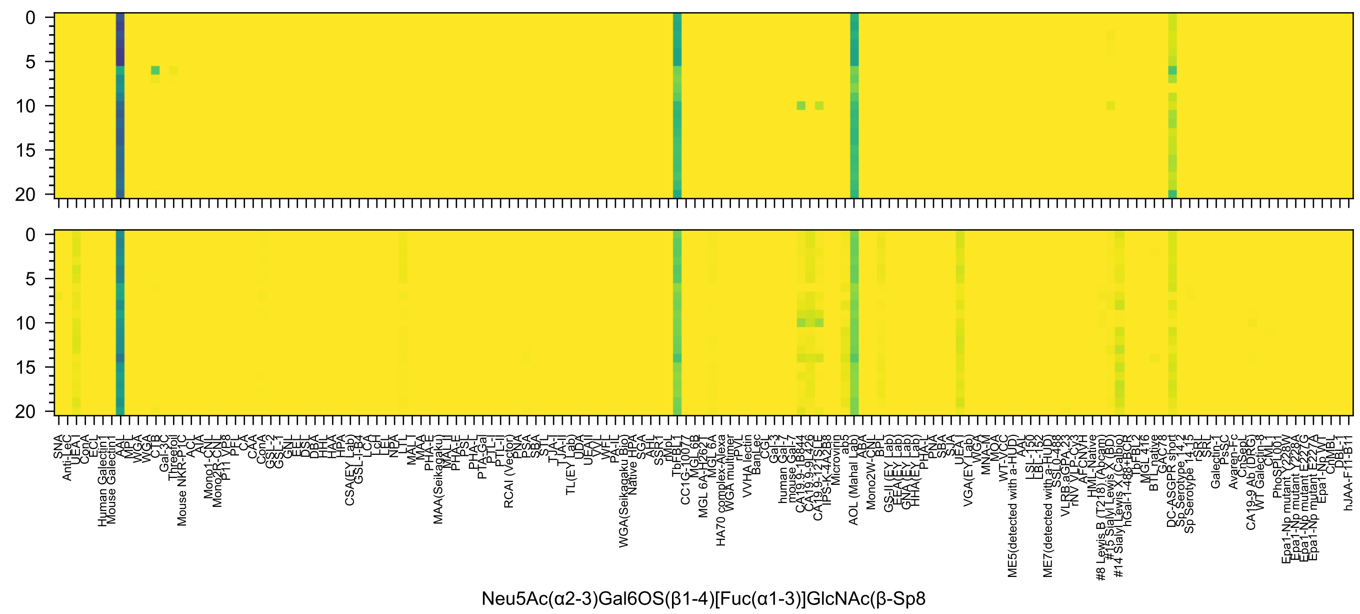
Neu5Ac(α2-3)Gal6OS(β1-4)[Fuc(α1-3)]GlcNAc(β-Sp8

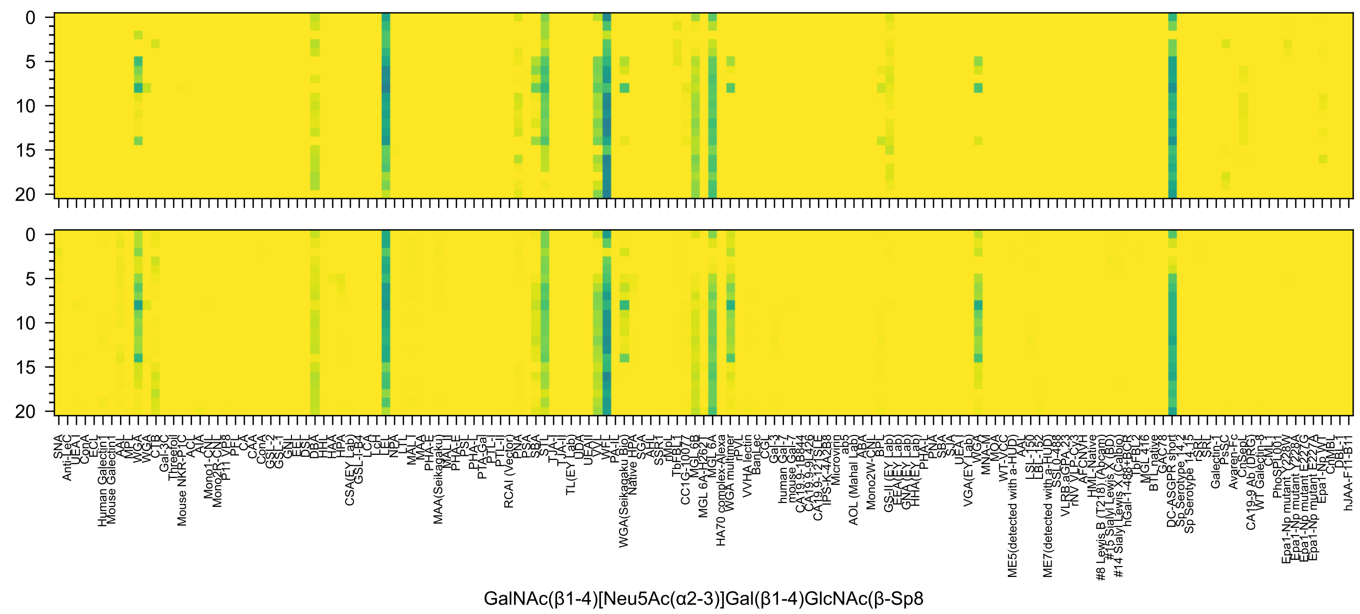
GalNAc(β1-4)[Neu5Ac(α2-3)]Gal(β1-4)GlcNAc(β-Sp8

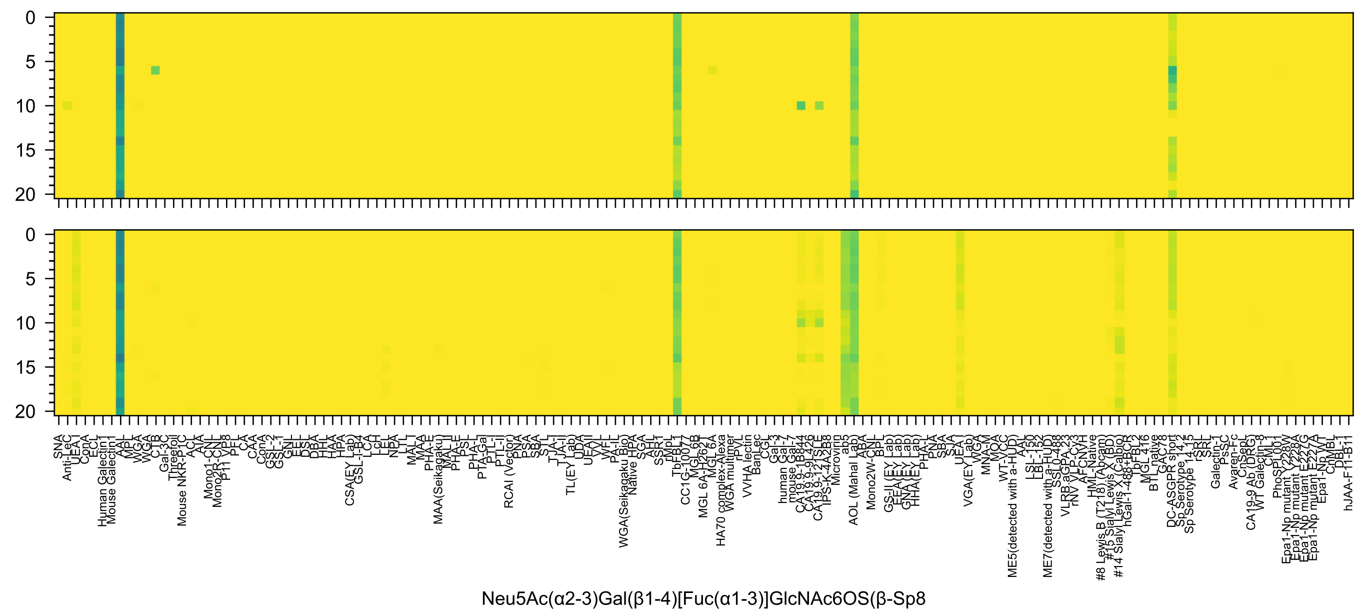
Neu5Ac(α2-3)Gal(β1-4)[Fuc(α1-3)]GlcNAc6OS(β-Sp8

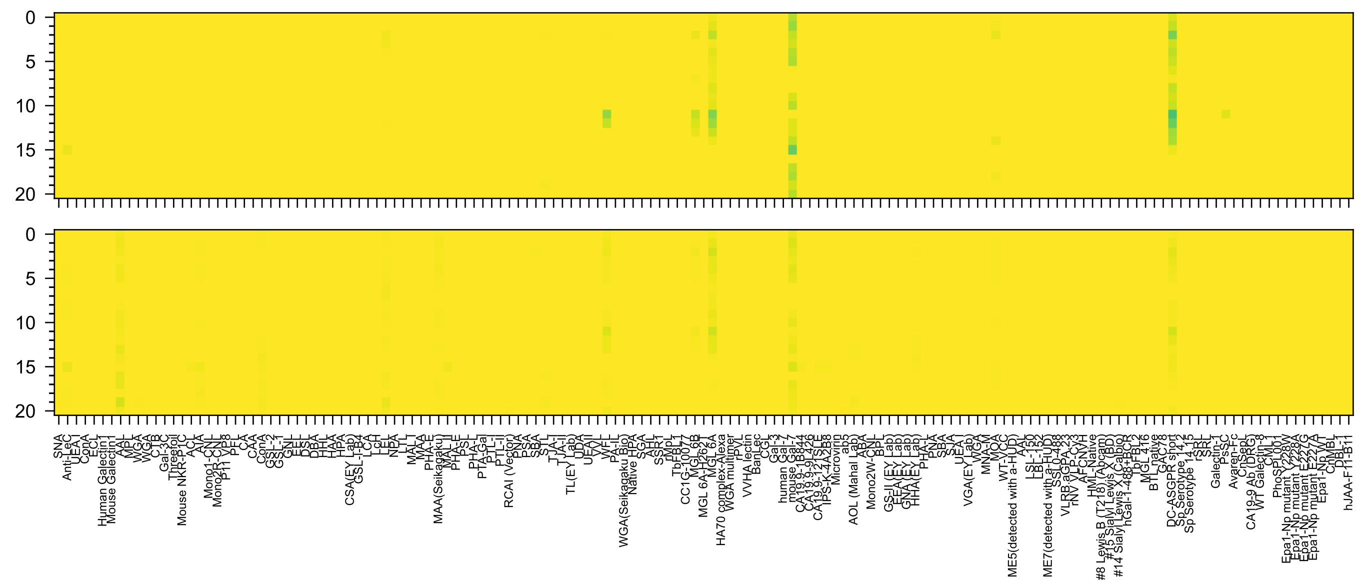
Neu5Ac(α2-8)Neu5Ac(α2-3)Gal(β1-4)Glc(β-Sp0

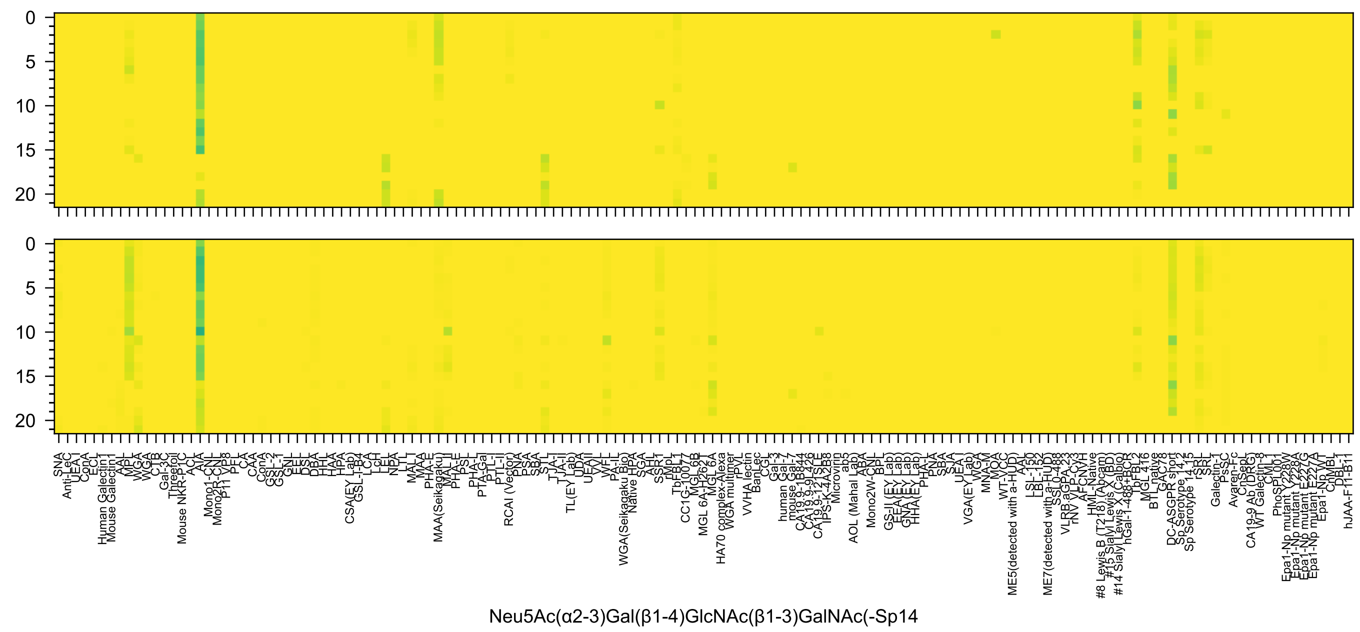
0
5
10
15
20
Neu5Ac(α2-3)Gal(β1-4)GlcNAc(β1-3)GalNAc(-Sp14

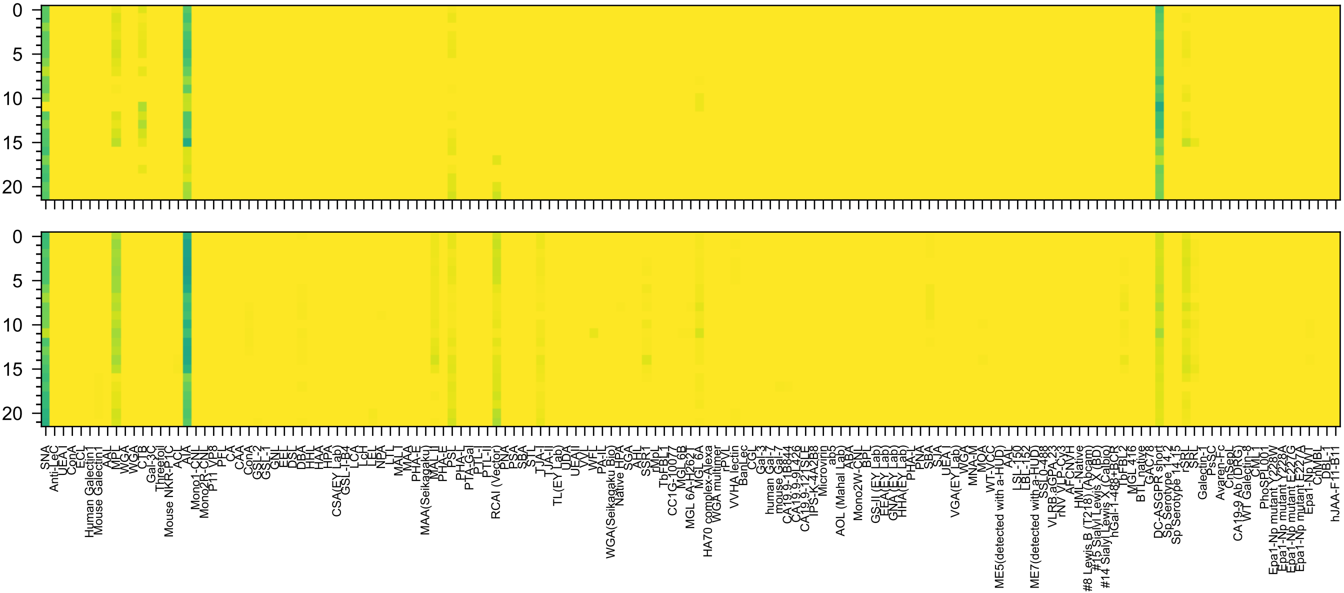

Neu5Ac(α2-6)Gal(β1-4)GlcNAc(β1-3)GalNAc(-Sp14

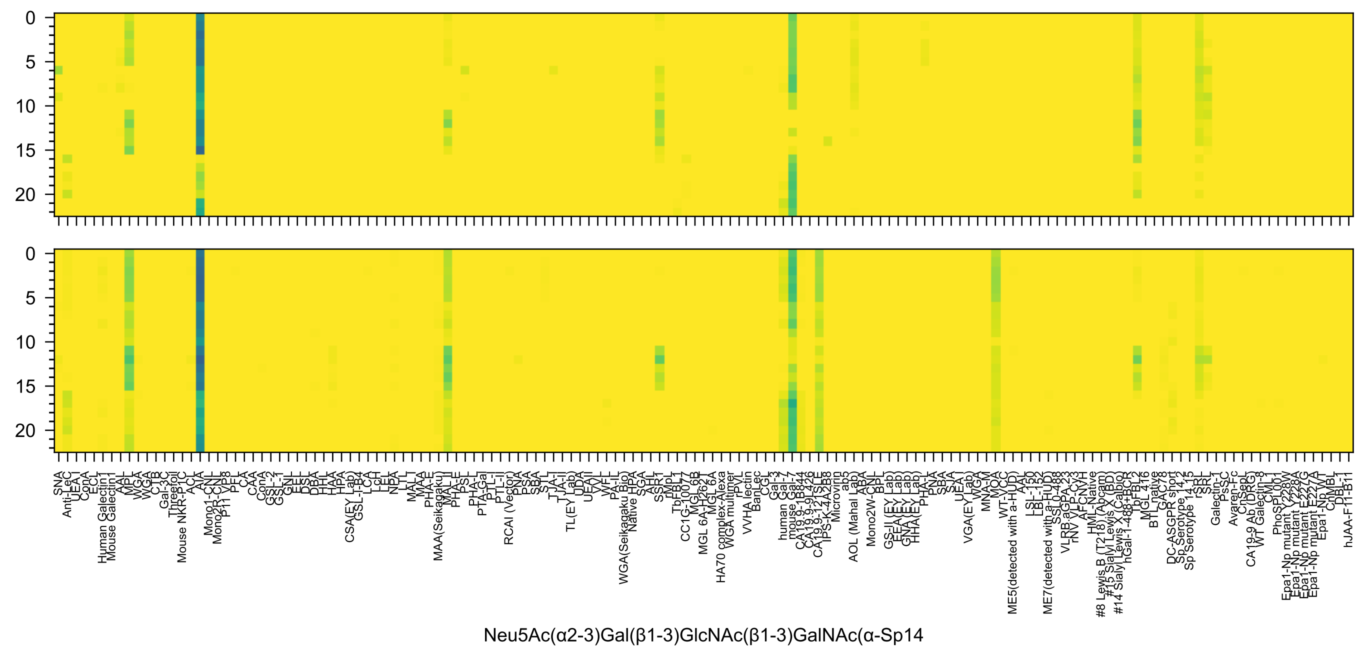
0
5
10
15
20
Neu5Ac(α2-3)Gal(β1-3)GlcNAc(β1-3)GalNAc(α-Sp14

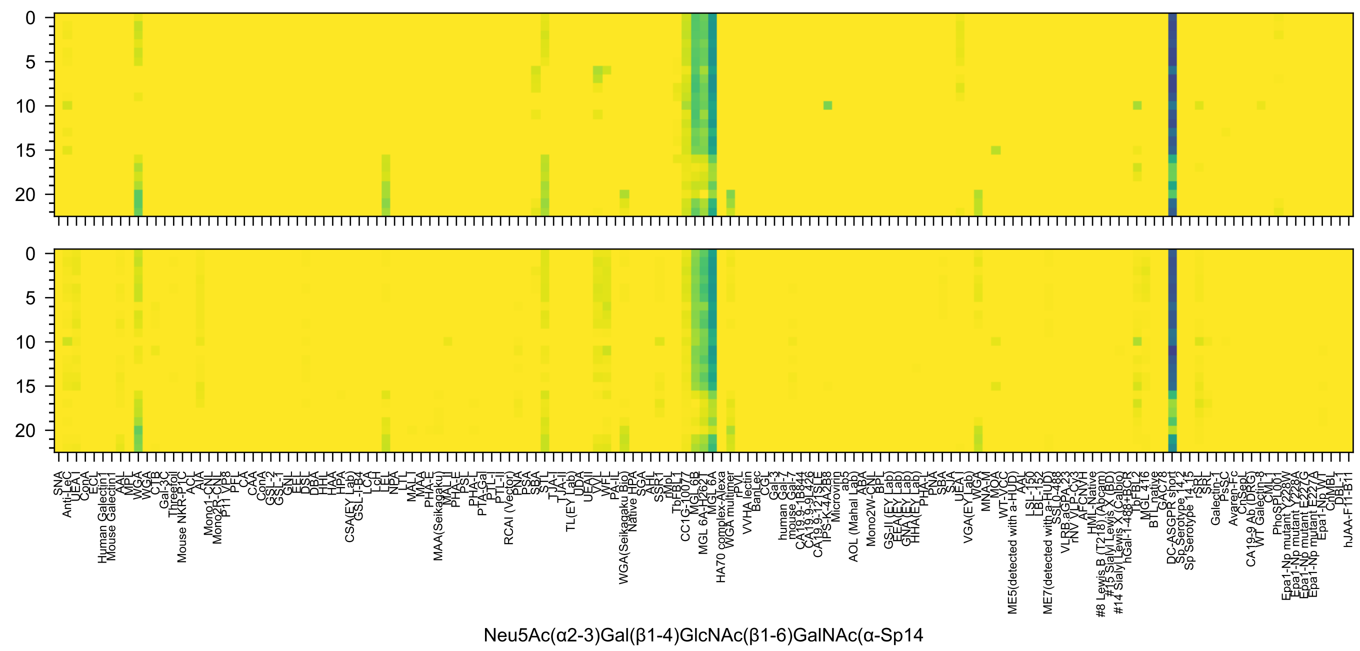
Neu5Ac(α2-3)Gal(β1-4)GlcNAc(β1-6)GalNAc(α-Sp14

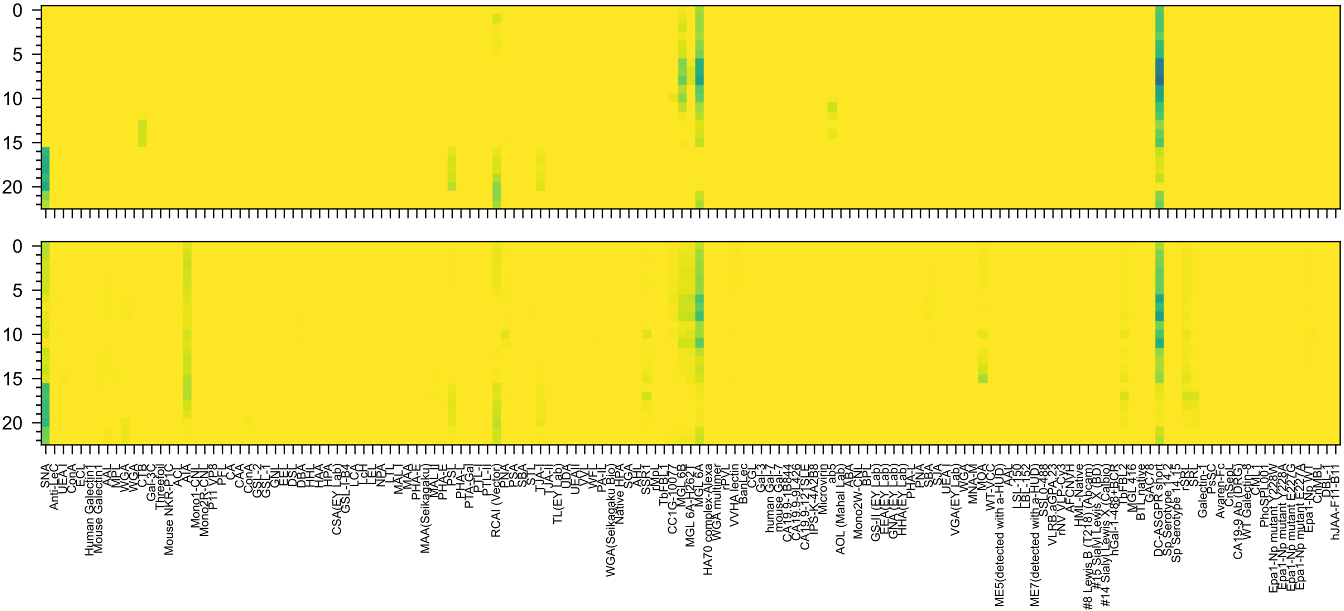

Neu5Ac(α2-6)Gal(β1-4)GlcNAc(β1-6)GalNAc(α-Sp14

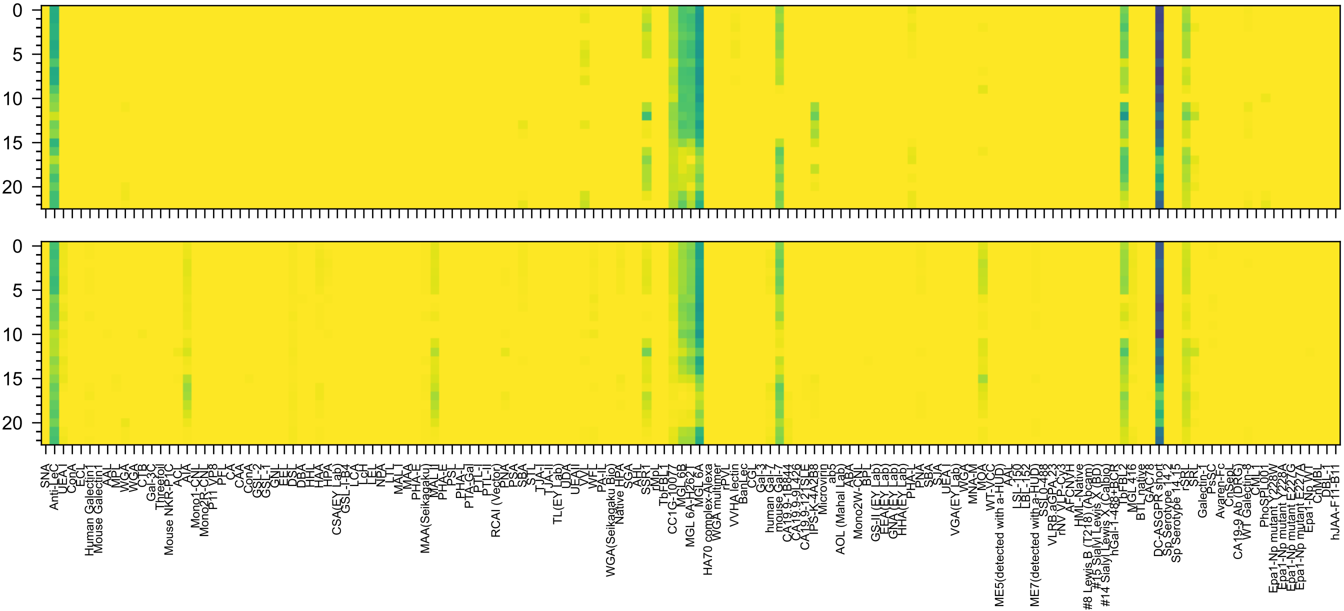

Neu5Ac(α2-3)Gal(β1-3)GlcNAc(β1-6)GalNAc(α-Sp14

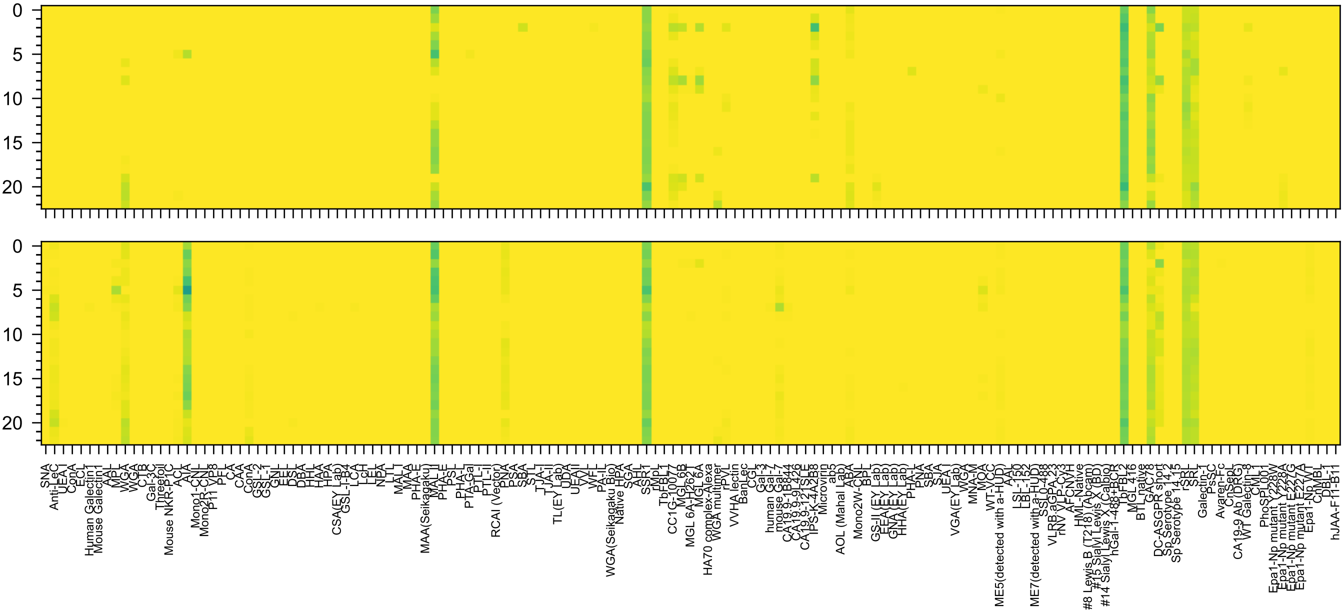

GlcNAc(β1-6)[Neu5Ac(α2-3)Gal(β1-3)]GalNAc(α-Sp14

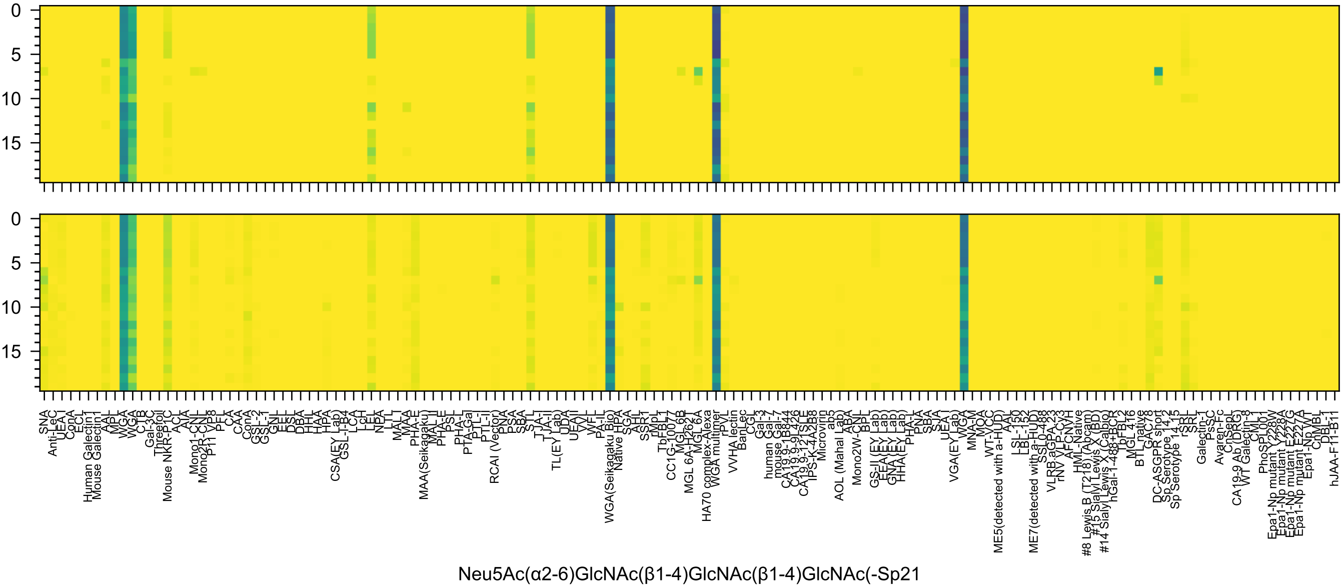
0
5
10
15
Neu5Ac(α2-6)GlcNAc(β1-4)GlcNAc(β1-4)GlcNAc(-Sp21

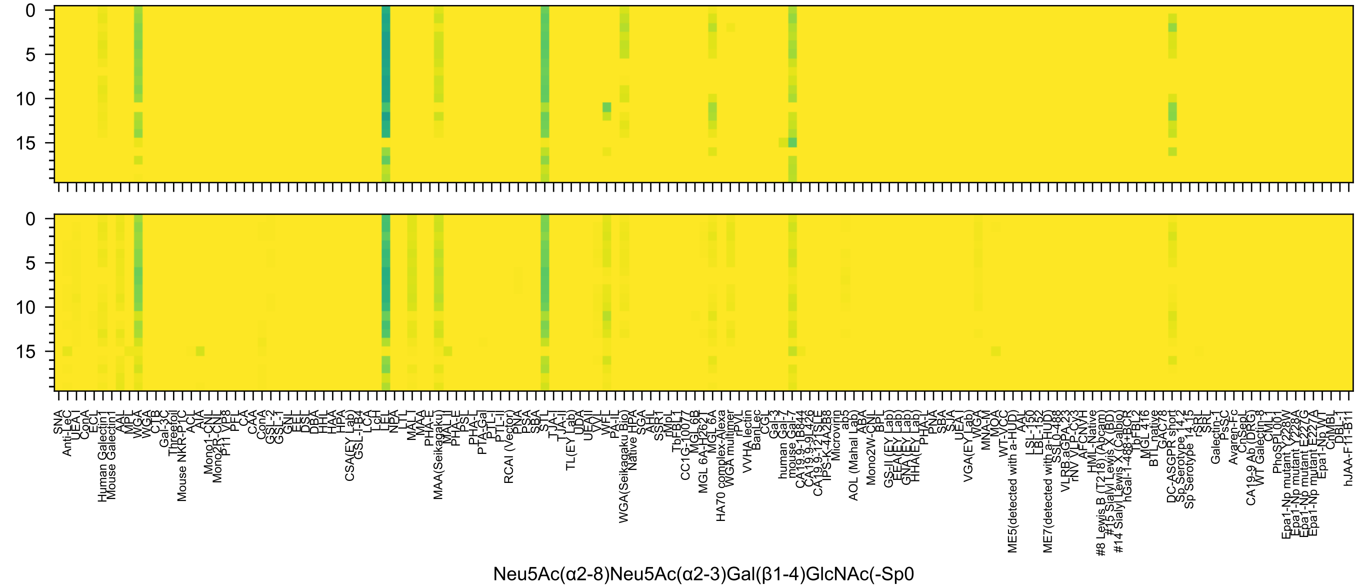
0
5
10
15
0
5
10
15
SNA
Anti-LeC
UEA I
ConA
ECL
Human Galectin1
Mouse Galectin1
AAL
MPL
WGA
WGA
CTB
Gal-3C
Threefoil
Mouse NKR-P1C
ACL
AIA
Mono1-CNL
Mono2R-CNL
P11 VP8
PFL
CA
CAA
ConA
GSL-2
GSL-1
GNL
EEL
DSL
DBA
HHL
HAA
HPA
CSA(EY Lab)
GSL-I-B4
LCA
LcH
LEL
NPA
LTL
MAL I
MAA
PHA-E
MAA(Seikagaku)
MAL II
PHA-E
PSL
PHA-L
PTA-Gal
PTL-I
PTL-II
RCAI (Vector)
PNA
PSA
SBA
STL
TJA-I
TJA-II
TL(EY Lab)
UDA
UEA II
VVL
WFL
PA-IL
WGA(Seikagaku Bio)
Native HPA
SGA
AAH
SSR1
rMpL
TbFBL1
CC1G-10077
MGL 6B
MGL 6A-H262T
MGL 6A
HA70 complex-Alexa
WGA multimer
rPVL
VVHA lectin
BanLec
CGL
Gal-3
human Gal-7
mouse Gal-7
CA19.9-1B844
CA19.9-9L426
CA19.9-121SLE
IPS-K-4A2B8
Microvirin
ab5
AOL (Mahal Lab)
ABA
Mono2W-CNL
BPL
GS-II (EY Lab)
EEA(EY Lab)
GNA(EY Lab)
HHA(EY Lab)
PHA-L
PNA
SBA
SJA
UEA I
VGA(EY Lab)
WGA
MNA-M
MOA
WT-VCC
ME5(detected with a-HUD)
AAL
LSL-150
LBL-152
ME7(detected with a-HUD)
SSL0-488
VLRB.aGPA.23
rNV VLP-Cy3
AFCNVH
HML-Native
#8 Lewis B (T218) (Abcam)
#15 Sialyl Lewis X (BD)
#14 Sialyl Lewis X (Calbio)
hGal-1-488+BCR
TbFBL2
MGL 416
BTL native
GAC78
DC-ASGPR short
Sp Serotype 14.2
Sp Serotype 14.15
rSRL
SRL
Galectin-1
PsSC
Avaren-Fc
ChSept
CA19-9 Ab (DRG)
WT Galectin-8
CML1
PhoSPL001
Epa1-Np mutant Y228W
Epa1-Np mutant Y228A
Epa1-Np mutant E227G
Epa1-Np mutant E227A
Epa1-Np WT
CnMBL
DBL-1
hJAA-F11-B11
Neu5Ac(α2-8)Neu5Ac(α2-3)Gal(β1-4)GlcNAc(-Sp0

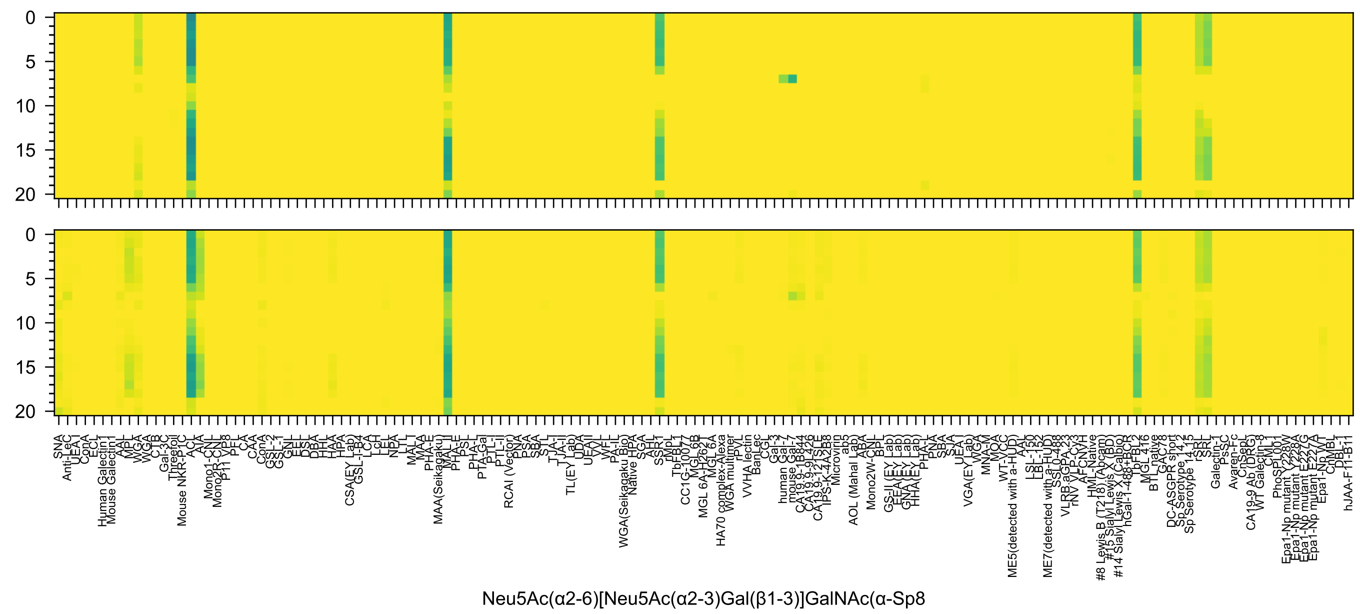
0
5
10
15
20
SNA
Anti-LeC
UEA I
ConA
ECL
Human Galectin1
Mouse Galectin1
AAL
MPL
WGA
WGA
CTB
Gal-3C
Threefoil
Mouse NKR-P1C
ACL
AIA
Mono1-CNL
Mono2R-CNL
P11 VP8
PFL
CA
CAA
ConA
GSL-2
GSL-1
GNL
EEL
DSL
DBA
HHL
HAA
HPA
CSA(EY Lab)
GSL-I-B4
LCA
LcH
LEL
NPA
LTL
MAL
MAA
PHA-E
MAA(Seikagaku)
MAL-II
PHA-E
PSL
PHA-L
PTA-Gal
PTL-I
PTL-II
RCAI (Vector)
PNA
PSA
SBA
STL
TJA-I
TJA-II
TL(EY Lab)
UDA
UEAII
VVL
WFL
PA-IL
WGA(Seikagaku Bio)
Native HPA
SGA
AHL
SSR1
rMpL
TbFBL1
CC1G-10077
MGL 6B
MGL 6A-H262T
MGL 6A
HA70 complex-Alexa
WGA multimer
rPVL
VVHA lectin
BanLec
CGL
Gal-3
human Gal-7
mouse Gal-7
CA19.9-1B844
CA19.9-9L426
CA19.9-121SLE
IPS-K-4A2B8
Microvirin
ab5
AOL (Mahal Lab)
ABA
Mono2W-CNL
BPL
GS-II (EY Lab)
EEA(EY Lab)
GNA(EY Lab)
HHA(EY Lab)
PHA-L
PNA
SBA
SJA
UEA-I
VGA(EY Lab)
WGA
MNA-M
MOA
WT-VCC
ME5(detected with a-HUD)
AAL
LSL-150
LBL-152
ME7(detected with a-HUD)
SSL0-488
VLRB.aGPA.23
rNV VLP-Cy3
AFCNVH
HML-Native
#8 Lewis B (T218) (Abcam)
#15 Sialyl Lewis X (BD)
#14 Sialyl Lewis X (Calbio)
hGal-1-488+BCR
TbFBL2
MGL 416
BTL native
GAC78
DC-ASGPR short
Sp Serotype 14.2
Sp Serotype 14.15
rSRL
SRL
Galectin-1
PsSC
Avaren-Fc
ChSeoL
CA19-9 Ab (DRG)
WT Galectin-8
CML1
PhoSL001
Epa1-Np mutant Y228W
Epa1-Np mutant Y228A
Epa1-Np mutant E227G
Epa1-Np mutant E227A
Epa1-Np WT
CnMBL
DBL-1
hJAA-F11-B11
Neu5Ac(α2-6)[Neu5Ac(α2-3)Gal(β1-3)]GalNAc(α-Sp8

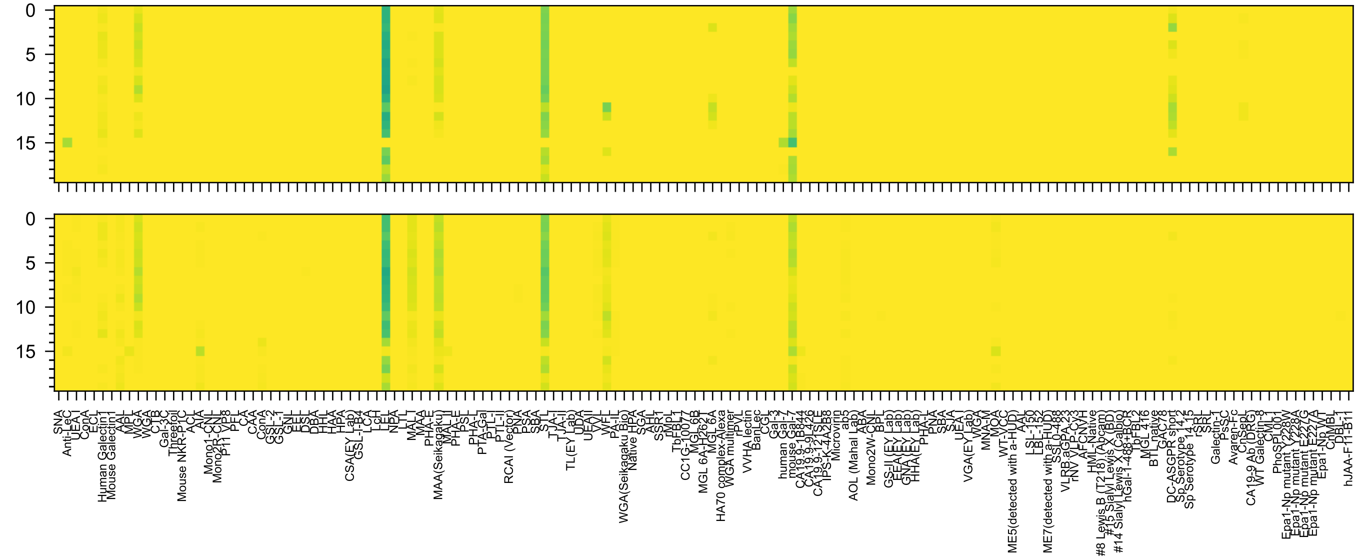

Neu5Ac(α2-8)Neu5Gc(α2-3)Gal(β1-4)GlcNAc(-Sp0

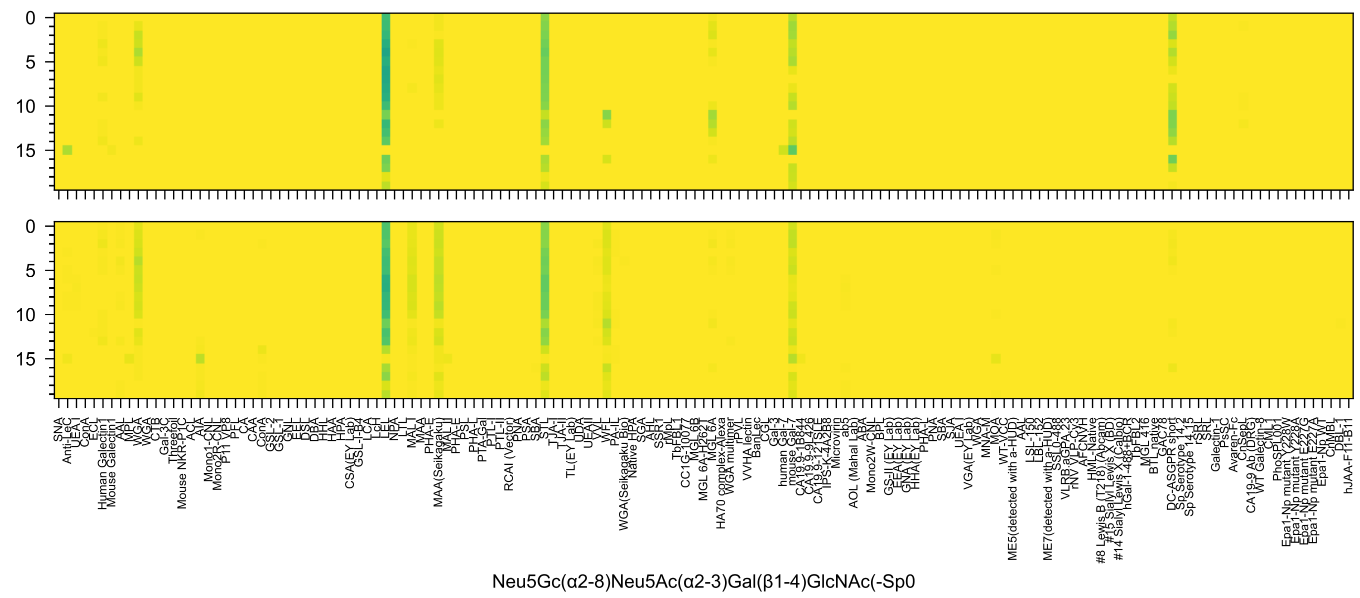
0
5
10
15
SNA
Anti-LeC
UEA-I
ConA
ECL
Human Galectin-1
Mouse Galectin-1
AAL
MPL
WGA
WGA
CTB
Gal-3C
Threefoil
Mouse NKR-P1C
ACL
AIA
Mono1-CNL
Mono2R-CNL
P11 VP8
PFL
CA
CAA
ConA
GSL-2
GSL-1
GNL
EEL
DSL
DBA
HHL
HAA
HPA
CSA(EY Lab)
GSL-I-B4
LCA
LcH
LEL
NPA
LTL
MAL-I
MAA
PHA-E
MAA(Seikagaku)
MAL-II
PHA-E
PSL
PHA-L
PTA-Gal
PTL-I
PTL-II
RCAI (Vector)
PNA
PSA
SBA
STL
TJA-I
TJA-II
TL(EY Lab)
UDA
UEA-II
VVL
WFL
PA-IL
WGA(Seikagaku Bio)
Native HPA
SGA
AAL
SSR1
rMpL
TbFBL1
CC1G-10077
MGL 6B
MGL 6A-H262T
MGL 6A
HA70 complex-Alexa
WGA multimer
rPVL
VVHA lectin
BanLec
CGL
Gal-3
human Gal-7
mouse Gal-7
CA19.9-1B844
CA19.9-9L426
CA19.9-121SLE
IPS-K-4A2B8
Microvirin
ab5
AOL (Mahal Lab)
ABA
Mono2W-CNL
BPL
GS-II (EY Lab)
EEA(EY Lab)
GNA(EY Lab)
HHA(EY Lab)
PHA-L
PNA
SBA
SJA
UEA-I
VGA(EY Lab)
WGA
MNA-M
MOA
WT-VCC
ME5(detected with a-HUD)
AAL
LSL-150
LBL-152
ME7(detected with a-HUD)
SSL0-488
VLRB.aGPA.23
rNV VLP-Cy3
AFCNVH
HML-Native
#8 Lewis B (T218) (Abcam)
#15 Sialyl Lewis X (BD)
#14 Sialyl Lewis X (Calbio)
hGal-1-488+BCR
TbFBL2
MGL 416
BTL native
GAC78
DC-ASGPR short
Sp Serotype 14.2
Sp Serotype 14.15
rSRL
SRL
Galectin-1
PsSC
Avaren-Fc
CnSepL
CA19-9 Ab (DRG)
WT Galectin-8
CML1
PhoSPL001
Epa1-Np mutant Y228W
Epa1-Np mutant Y228A
Epa1-Np mutant E227G
Epa1-Np mutant E227A
Epa1-Np WT
ChMBL
DBL-1
hJAA-F11-B11
Neu5Gc(α2-8)Neu5Ac(α2-3)Gal(β1-4)GlcNAc(-Sp0

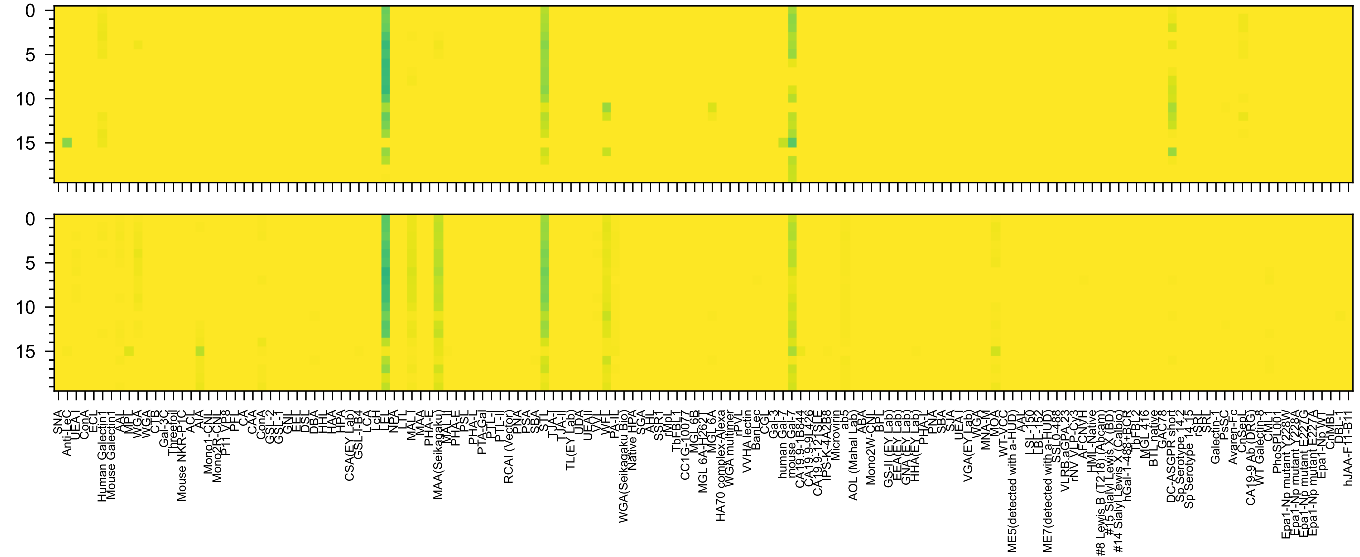

Neu5Gc(α2-8)Neu5Gc(α2-3)Gal(β1-4)GlcNAc(-Sp0

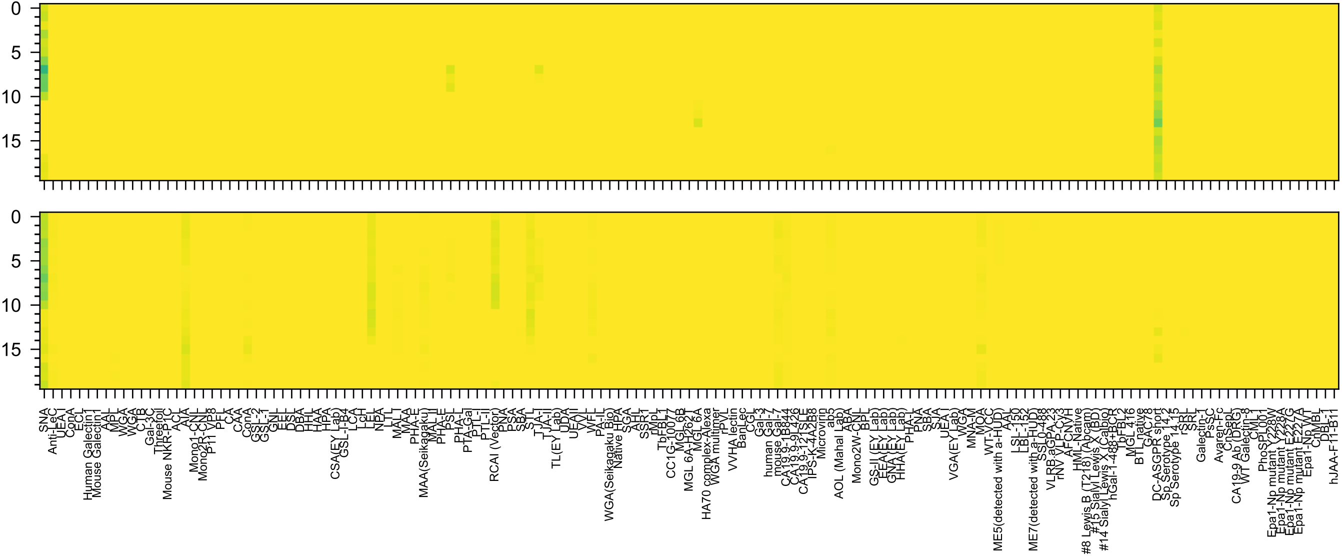
Neu5Gc(α2-8)Neu5Gc(α2-6)Gal(β1-4)GlcNAc(-Sp0

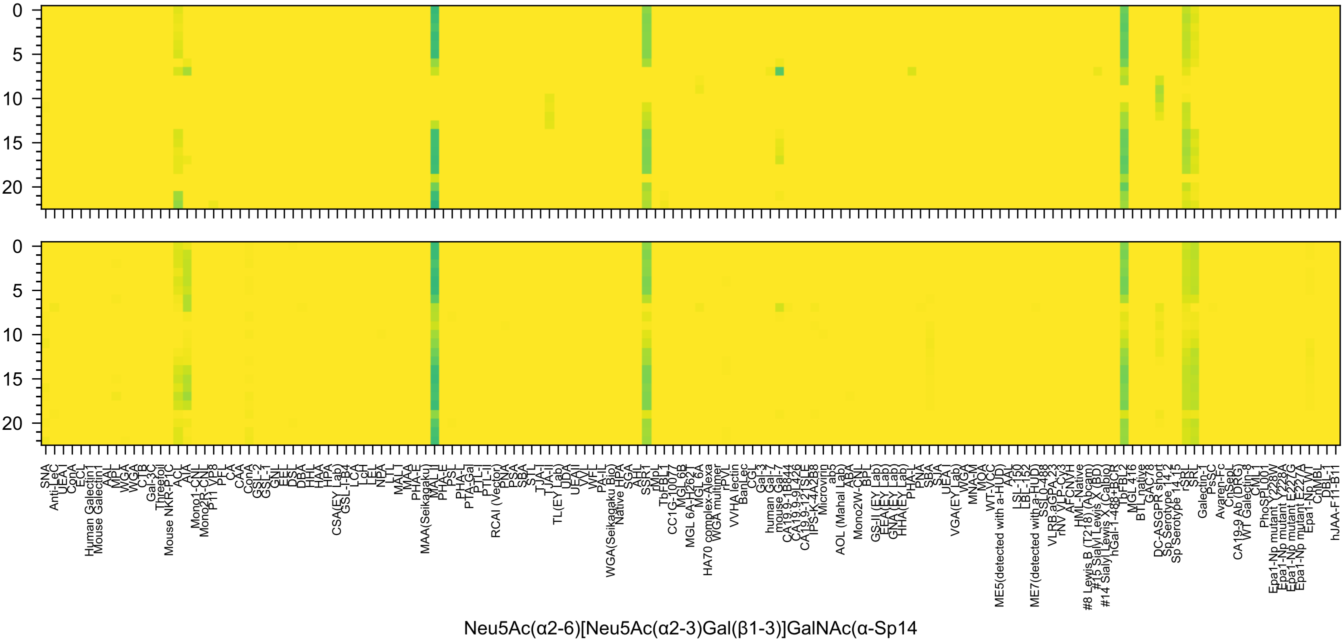
Neu5Ac(α2-6)[Neu5Ac(α2-3)Gal(β1-3)]GalNAc(α-Sp14

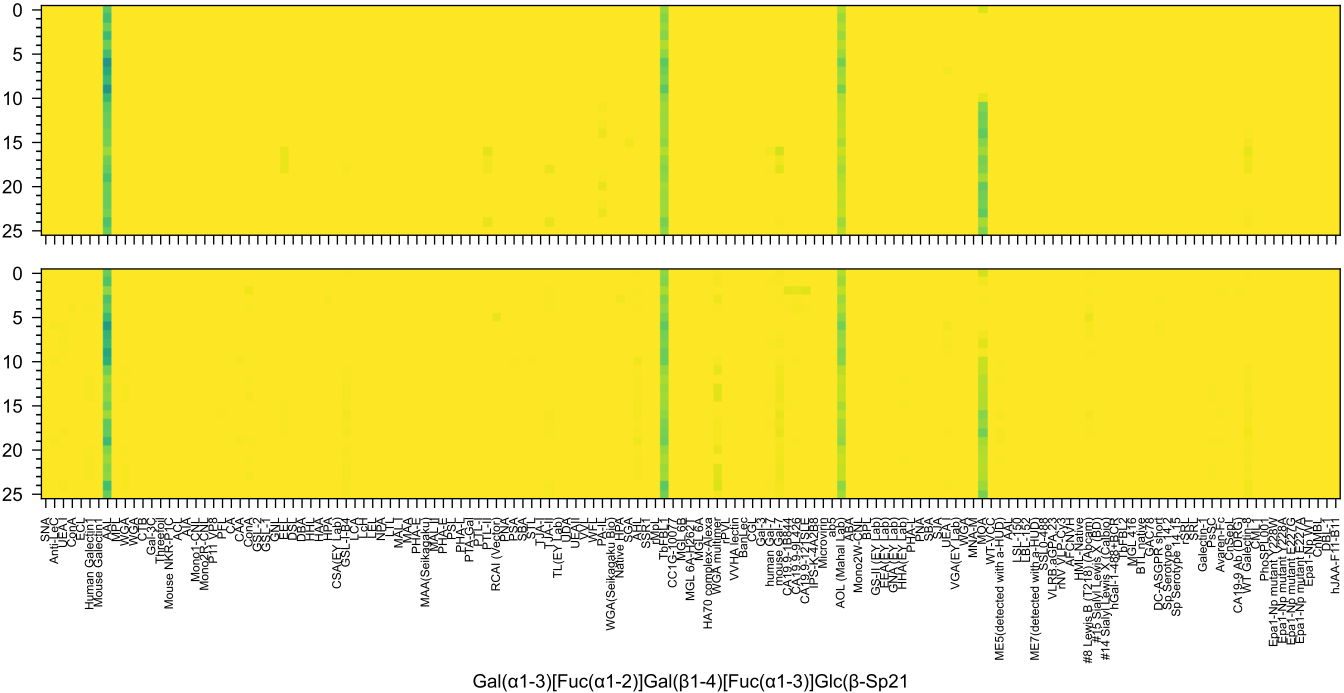
Gal(α1-3)[Fuc(α1-2)]Gal(β1-4)[Fuc(α1-3)]Glc(β-Sp21

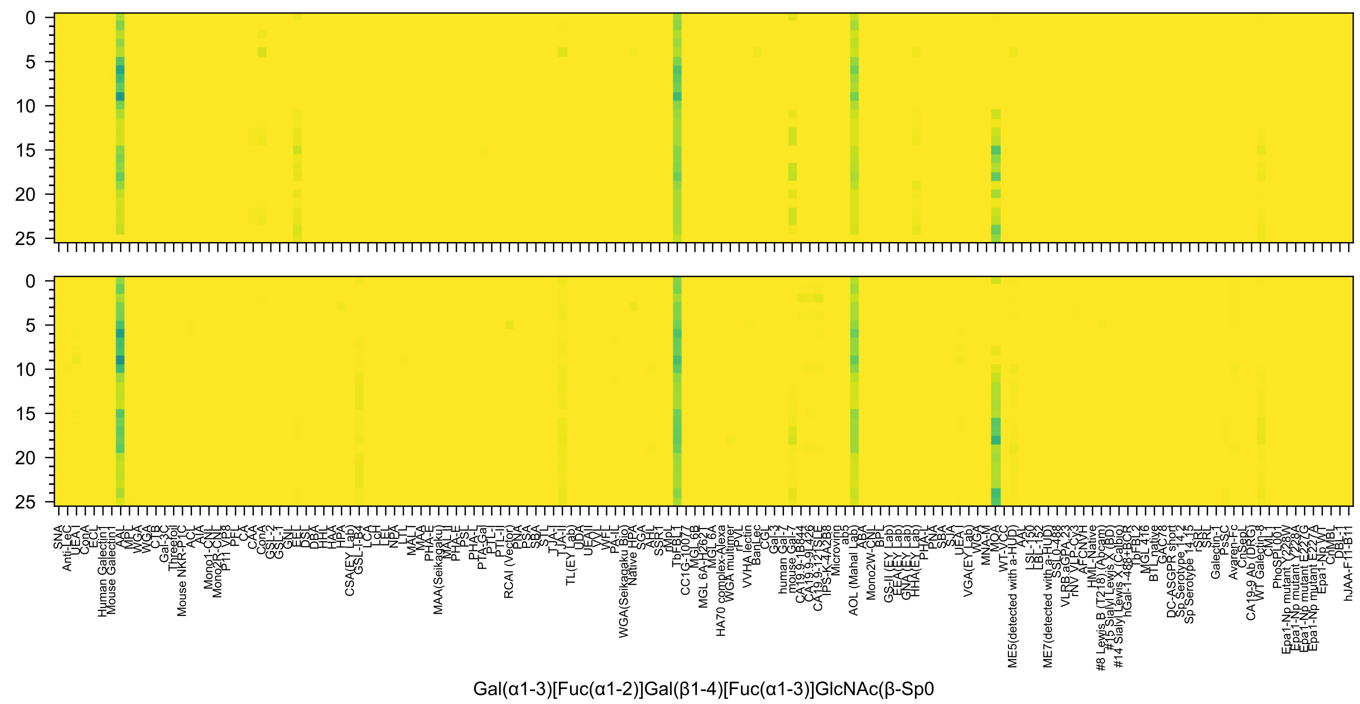
SNA
Anti-LeC
UEA I
ConA
ECL
Human Galectin1
Mouse Galectin1
AAL
MPL
WGA
WGA
CTB
Gal-3C
Threefoil
Mouse NKR-P1C
ACL
AIA
Mono1-CNL
Mono2R-CNL
P11 VP8
PFL
CA
CAA
ConA
GSL-2
GSL-1
GNL
EEL
DSL
DBA
HHL
HAA
HPA
CSA(EY Lab)
GSL-I-B4
LCA
LcH
LEL
NPA
LTL
MAL1
MAA
PHA-E
MAA(Seikagaku)
MAL II
PHA-E
PSL
PHA-L
PTA-Gal
PTL-I
PTL-II
RCAI (Vector)
PNA
PSA
SBA
STL
TJA-I
TJA-II
TL(EY Lab)
UDA
UEAII
VVL
WFL
PA-IL
WGA(Seikagaku Bio)
Native HPA
SGA
AHL
SSR1
rMpL
TbFBL1
CC1G-10077
MGL 6B
MGL 6A-H262T
MGL 6A
HA70 complex-Alexa
WGA multimer
rPVL
VVHA lectin
BanLec
CGL
Gal-3
human Gal-7
mouse Gal-7
CA19.9-1B844
CA19.9-9L426
CA19.9-121SLE
IPS-K-4A2B8
Microvirin
ab5
AOL (Mahal Lab)
ABA
Mono2W-CNL
BPL
GS-II (EY Lab)
EEA (EY Lab)
GNA (EY Lab)
HHA (EY Lab)
PHA-L
PNA
SBA
SJA
UEA1
VGA(EY Lab)
WGA
MNA-M
MOA
WT-VCC
ME5(detected with a-HUD)
AAL
LSL-150
LBL-152
ME7(detected with a-HUD)
SSL0-488
VLRB.aGPA.23
rNV VLP-Cy3
AFCNVH
HML-Native
#8 Lewis B (T218) (Abcam)
#15 Sialyl Lewis X (BD)
#14 Sialyl Lewis X (Calbio)
hGal-1-488+BCR
TbFBL2
MGL 416
BTL native
GAC78
DC-ASGPR short
Sp Serotype 14.2
Sp Serotype 14.15
rSRL
SRL
Galectin-1
PsSC
Avaren-Fc
ChSeoL
CA19-9 Ab (DRG)
WT Galectin-8
CML
PhoSL001
Epa1-Np mutant Y228W
Epa1-Np mutant Y228A
Epa1-Np mutant E227G
Epa1-Np mutant E227A
Epa1-Np WT
ChMBL
DBL-1
hJAA-F11-B11
Gal(α1-3)[Fuc(α1-2)]Gal(β1-4)[Fuc(α1-3)]GlcNAc(β-Sp0

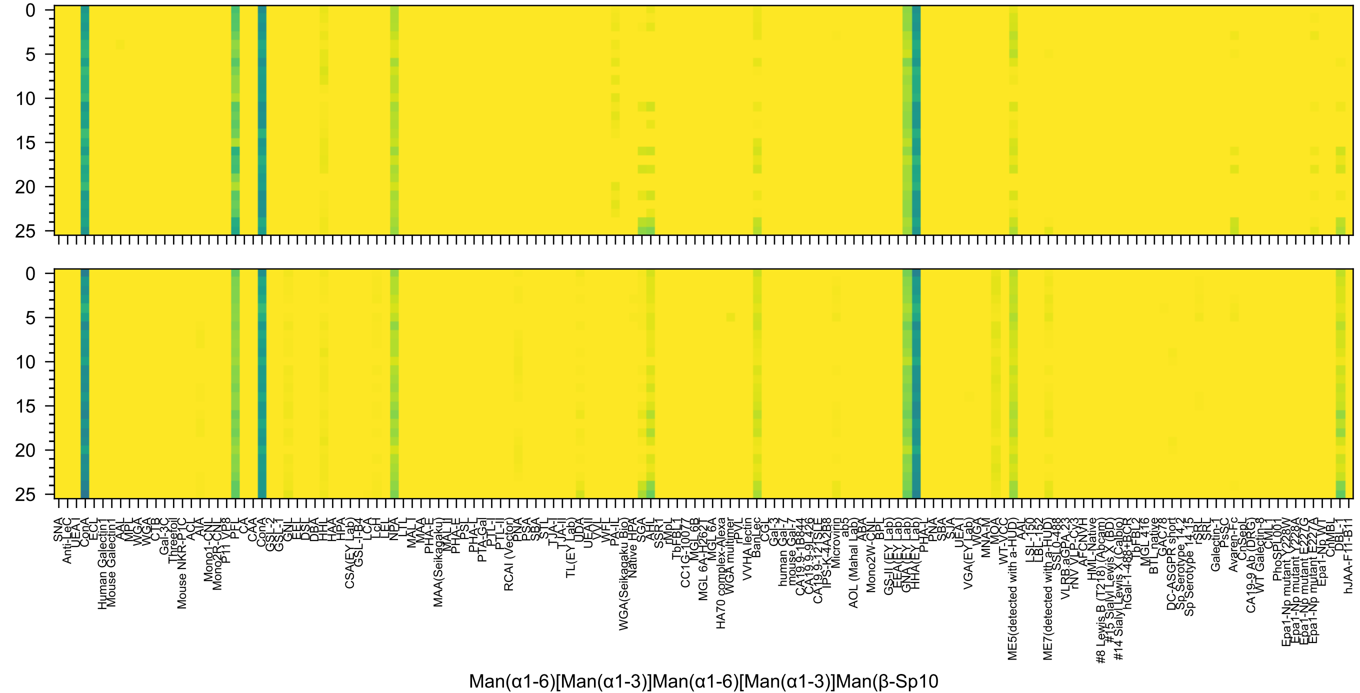

0
5
10
15
20
25
0
5
10
15
20
25
SNA
Anti-LeC
UEA I
ConA
ECL
Human Galectin1
Mouse Galectin1
AAL
MPL
WGA
WGA
CTB
Gal-3C
Threefoil
Mouse NKR-P1C
ACL
AIA
Mono1-CNL
Mono2R-CNL
P11 VP8
PFL
CA
CAA
ConA
GSL-2
GSL-1
GNL
EEL
DSL
DBA
HHL
HAA
HPA
CSA(EY Lab)
GSL-I-B4
LCA
LcH
LEL
NPA
LTL
MAL I
MAA
PHA-E
MAA(Seikagaku)
MAL II
PHA-E
PSL
PHA-L
PTA-Gal
PTL-I
PTL-II
RCAI (Vector)
PNA
PSA
SBA
STL
TJA-I
TJA-II
TL(EY Lab)
UDA
UEAII
VVL
WFL
PA-IL
WGA(Seikagaku Bio)
Native HPA
SGA
AAL
SSR1
rMpL
TbFBL1
CC1G-10077
MGL 6B
MGL 6A-H262T
MGL 6A
HA70 complex-Alexa
WGA multimer
rPVL
VVHA lectin
BanLec
CGL
Gal-3
human Gal-7
mouse Gal-7
CA19.9-1B844
CA19.9-9L426
CA19.9-121SLE
IPS-K-4A2B8
Microvirin
ab5
AOL (Mahal Lab)
ABA
Mono2W-CNL
BPL
GS-II (EY Lab)
EEA (EY Lab)
GNA (EY Lab)
HHA (EY Lab)
PHA-L
PNA
SBA
SJA
UEA1
VGA(EY Lab)
WGA
MNA-M
MOA
WT-VCC
ME5(detected with a-HUD)
AAL
LSL-150
LBL-152
ME7(detected with a-HUD)
SSL0-488
VLRB.aGPA.23
rNV VLP-Cy3
AFCNVH
HML-Native
#8 Lewis B (T218) (Abcam)
#15 Sialyl Lewis X (BD)
#14 Sialyl Lewis X (Calbio)
hGal-1-488+BCR
TbFBL2
MGL 416
BTL native
GAC78
DC-ASGPR short
Sp Serotype 14.2
Sp Serotype 14.15
rSRL
SRL
Galectin-1
PsSC
Avaren-Fc
ChSepL
CA19-9 Ab (DRG)
WT Galectin-8
CML
PhoSL001
Epa1-Np mutant Y228W
Epa1-Np mutant Y228A
Epa1-Np mutant E227G
Epa1-Np mutant E227A
Epa1-Np WT
ChMBL
DBL-1
hJAA-F11-B11
Man(α1-6)[Man(α1-3)]Man(α1-6)[Man(α1-3)]Man(β-Sp10

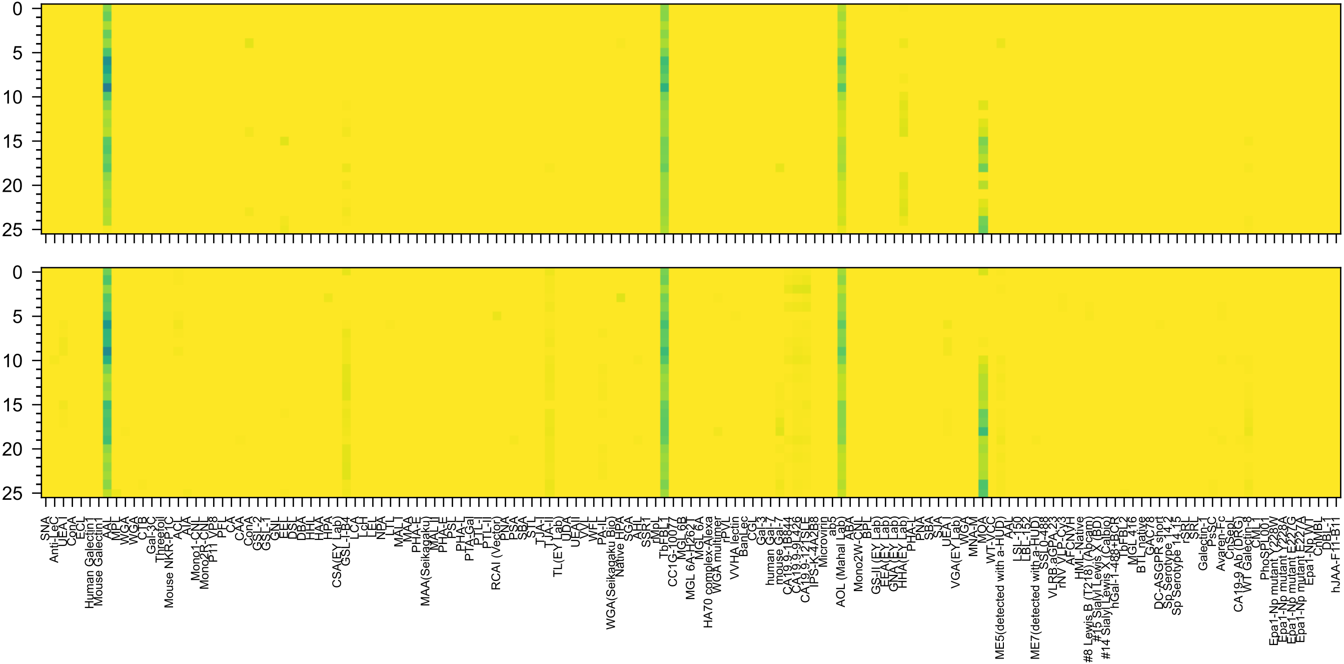

Gal(α1-3)[Fuc(α1-2)]Gal(β1-4)[Fuc(α1-3)]GlcNAc(β-Sp8

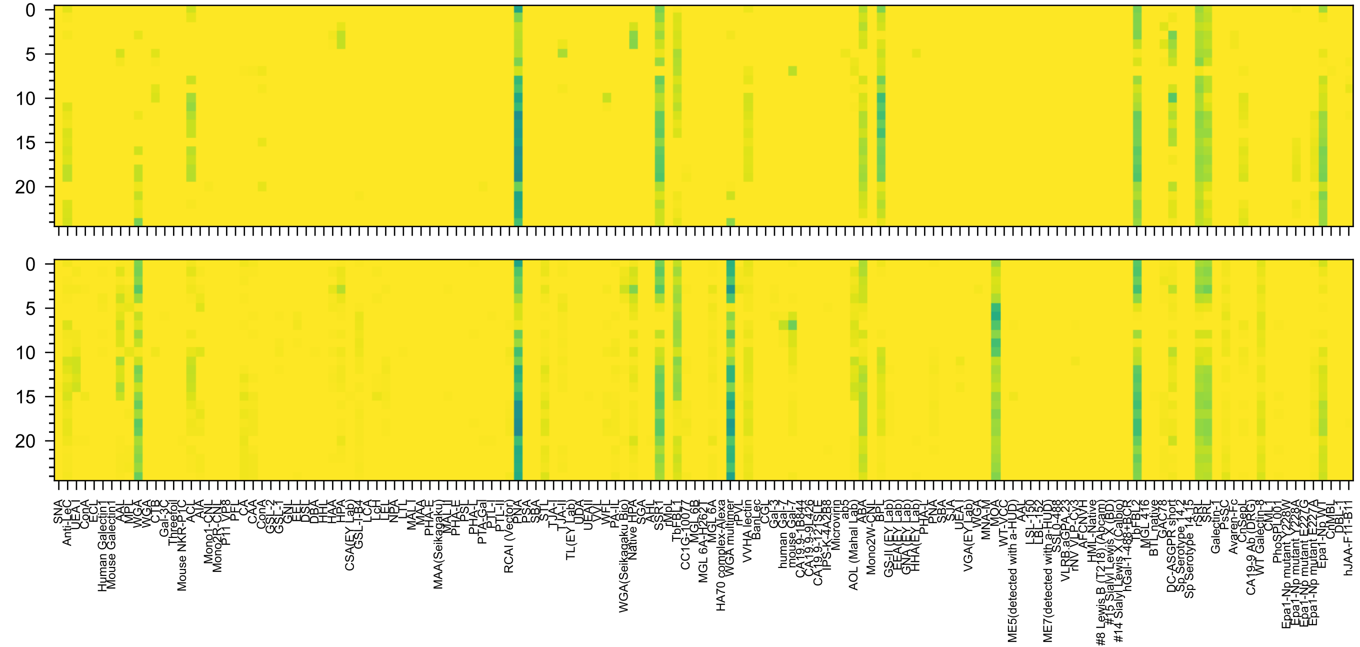

Gal(β1-3)GalNAc(α1-3)[Fuc(α1-2)]Gal(β1-4)Glc(-Sp0

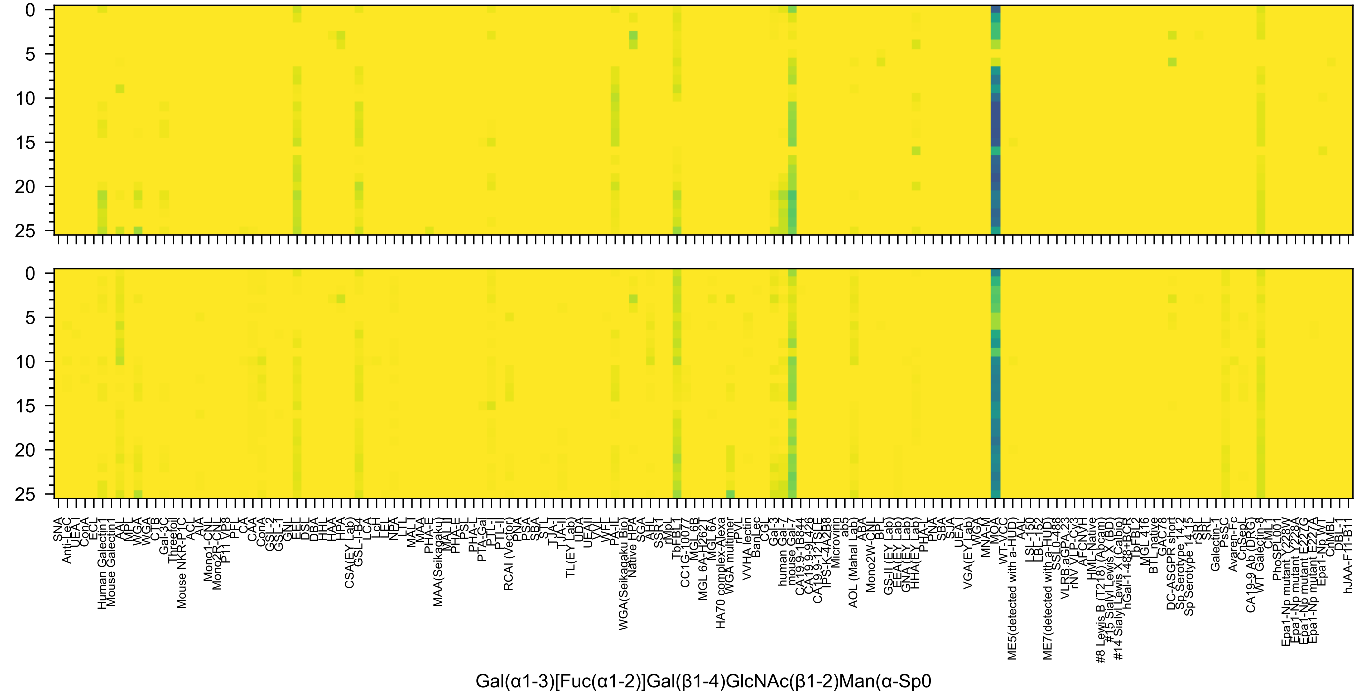
0
5
10
15
20
25
SNA
Anti-LeC
UEA I
ConA
ECL
Human Galectin1
Mouse Galectin1
AAL
MPL
WGA
WGA
CTB
Gal-3C
Threefoil
Mouse NKR-P1C
ACL
AIA
Mono1-CNL
Mono2R-CNL
P11 VP8
PFL
CA
CAA
ConA
GSL-2
GSL-1
GNL
EEL
DSL
DBA
HHL
HAA
HPA
CSA(EY Lab)
GSL-I-B4
LCA
LcH
LEL
NPA
LTL
MAL1
MAA
PHA-E
MAA(Seikagaku)
MAL II
PHA-E
PSL
PHA-L
PTA-Gal
PTL-I
PTL-II
RCAI (Vector)
PNA
PSA
SBA
STL
TJA-I
TJA-II
TL(EY Lab)
UDA
UEAII
VVL
WFL
PA-IL
WGA(Seikagaku Bio)
Native HPA
SGA
AHL
SSR1
rMpL
TbFBL1
CC1G-10077
MGL 6B
MGL 6A-H262T
MGL 6A
HA70 complex-Alexa
WGA multimer
rPVL
VVHA lectin
BanLec
CGL
Gal-3
human Gal-7
mouse Gal-7
CA19.9-1B844
CA19.9-9L426
CA19.9-121SLE
IPS-K-4A2B8
Microvirin
ab5
AOL (Mahal Lab)
ABA
Mono2W-CNL
BPL
GS-II (EY Lab)
EEA (EY Lab)
GNA (EY Lab)
HHA (EY Lab)
PHA-L
PNA
SBA
SJA
UEA I
VGA(EY Lab)
WGA
MNA-M
MOA
WT-VCC
ME5(detected with a-HUD)
AAL
LSL-150
LBL-152
ME7(detected with a-HUD)
SSL0-488
VLRB.aGPA.23
rNV VLP-Cy3
AFCNVH
HML-Native
#8 Lewis B (T218) (Abcam)
#15 Sialyl Lewis X (BD)
#14 Sialyl Lewis X (Calbio)
hGal-1-488+BCR
TbFBL2
MGL 416
BTL native
GAC78
DC-ASGPR short
Sp Serotype 14.2
Sp Serotype 14.15
rSRL
SRL
Galectin-1
PsSC
Avaren-Fc
CnSepL
CA19-9 Ab (DRG)
WT Galectin-8
CML1
PhoSL001
Epa1-Np mutant Y228W
Epa1-Np mutant Y228A
Epa1-Np mutant E227G
Epa1-Np mutant E227A
Epa1-Np WT
ChMBL
DBL-1
hJAA-F11-B11
Gal(α1-3)[Fuc(α1-2)]Gal(β1-4)GlcNAc(β1-2)Man(α-Sp0

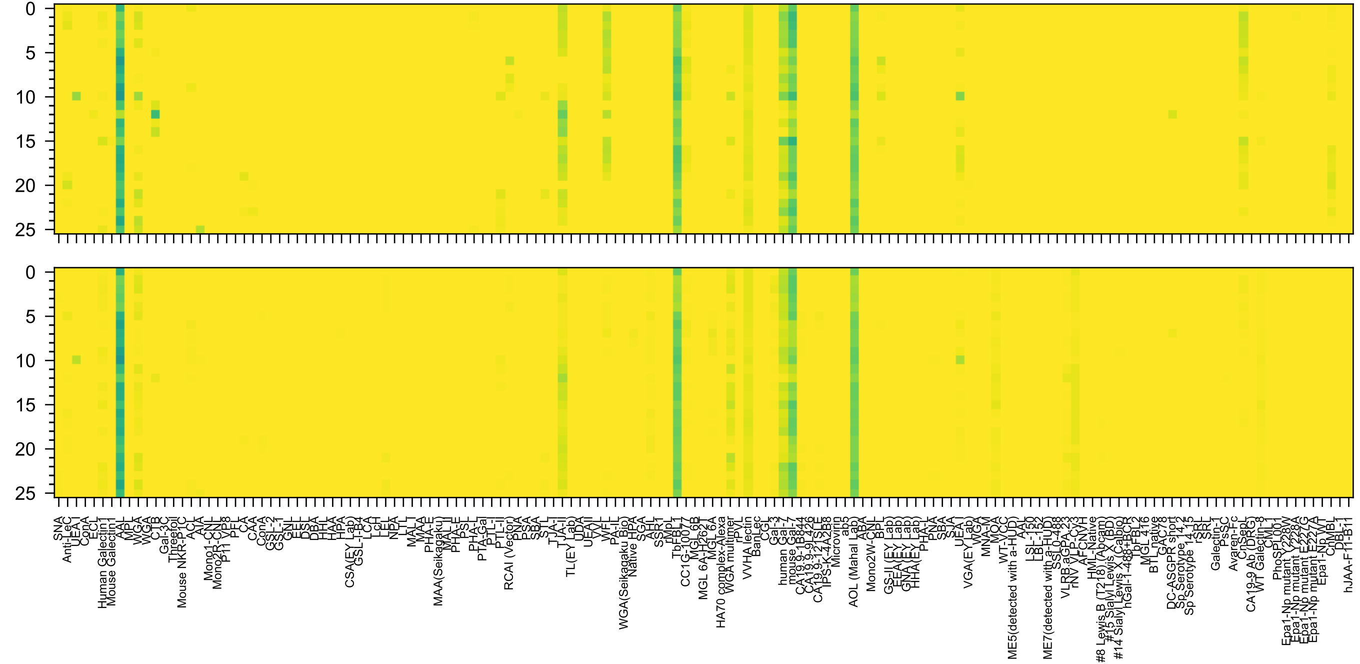
Fuc(α1-2)Gal(β1-3)GlcNAc(β1-3)Gal(β1-4)Glc(β-Sp8

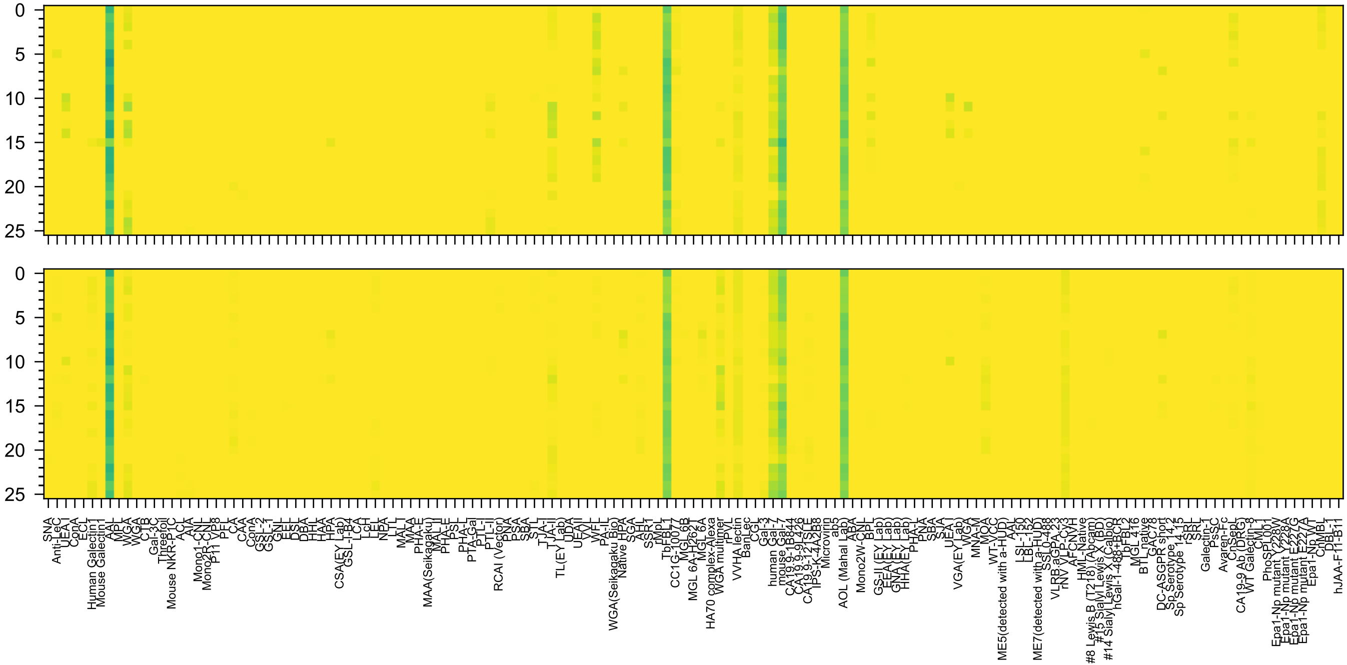

Fuc(α1-2)Gal(β1-3)GlcNAc(β1-3)Gal(β1-4)Glc(β-Sp10

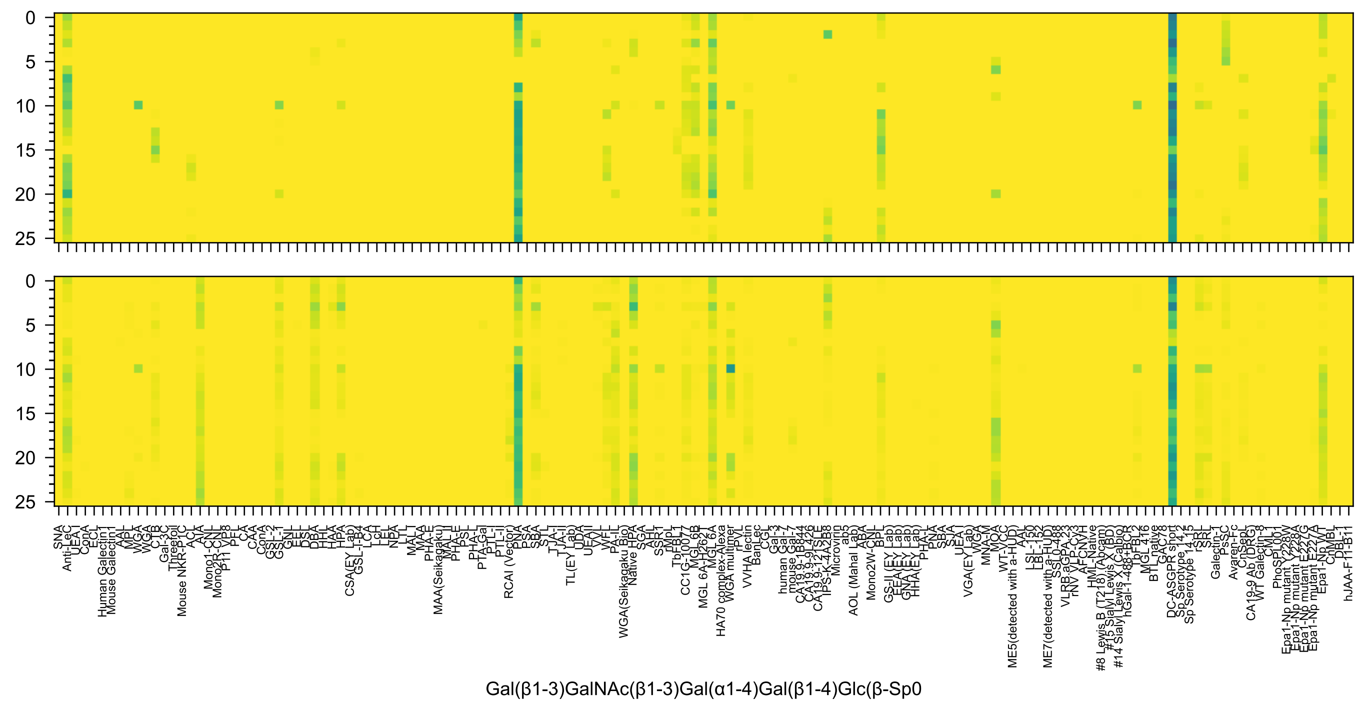

0
5
10
15
20
25
SNA
Anti-LeC
UEA I
ConA
ECL
Human Galectin1
Mouse Galectin1
AAL
MPL
WGA
WGA
CTB
Gal-3C
Threefoil
Mouse NKR-P1C
ACL
AIA
Mono1-CNL
Mono2R-CNL
P11 VP8
PFL
CA
CAA
ConA
GSL-2
GSL-1
GNL
EEL
DSL
DBA
HHL
HAA
HPA
CSA(EY Lab)
GSL-I-B4
LCA
LcH
LEL
NPA
LTL
MAL I
MAA
PHA-E
MAA(Seikagaku)
MAL II
PHA-E
PSL
PHA-L
PTA-Gal
PTL-I
PTL-II
RCAI (Vector)
PNA
PSA
SBA
STL
TJA-I
TJA-II
TL(EY Lab)
UDA
UEAII
VVL
WFL
PA-IL
WGA(Seikagaku Bio)
Native HPA
SGA
AHL
SSR1
rMpL
TbFBL1
CC1G-10077
MGL 6B
MGL 6A-H262T
MGL 6A
HA70 complex-Alexa
WGA multimer
rPVL
VVHA lectin
BanLec
CGL
Gal-3
human Gal-7
mouse Gal-7
CA19.9-1B844
CA19.9-9L426
CA19.9-121SLE
IPS-K-4A2B8
Microvirin
ab5
AOL (Mahal Lab)
ABA
Mono2W-CNL
BPL
GS-II (EY Lab)
EEA(EY Lab)
GNA (EY Lab)
HHA(EY Lab)
PHA-L
PNA
SBA
SJA
UEA-I
VGA(EY Lab)
WGA
MNA-M
MOA
WT-VCC
ME5(detected with a-HUD)
AAL
LSL-150
LBL-152
ME7(detected with a-HUD)
SSL0-488
VLRB.aGPA.23
rNV VLP-Cy3
AFCNVH
HML-Native
#8 Lewis B (T218) (Abcam)
#15 Sialyl Lewis X (BD)
#14 Sialyl Lewis X (Calbio)
hGal-1-488+BCR
TbFBL2
MGL 416
BTL native
GAC78
DC-ASGPR short
Sp Serotype 14.2
Sp Serotype 14.15
rSRL
SRL
Galectin-1
PsSC
Avaren-Fc
CnSepL
CA19-9 Ab (DRG)
WT Galectin-8
CML1
PhoSL001
Epa1-Np mutant Y228W
Epa1-Np mutant Y228A
Epa1-Np mutant E227G
Epa1-Np mutant E227A
Epa1-Np WT
ChMBL
DBL-1
hJAA-F11-B11
Gal(β1-3)GalNAc(β1-3)Gal(α1-4)Gal(β1-4)Glc(β-Sp0

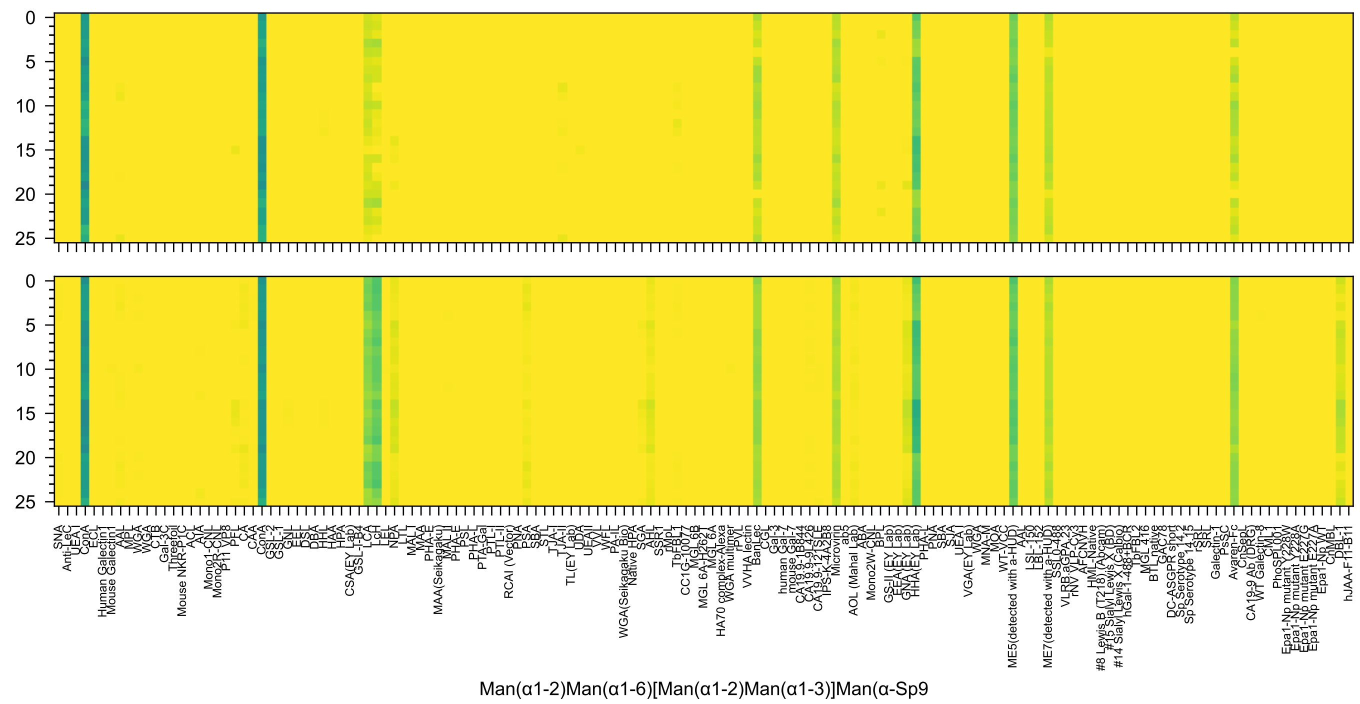
Man(α1-2)Man(α1-6)[Man(α1-2)Man(α1-3)]Man(α-Sp9

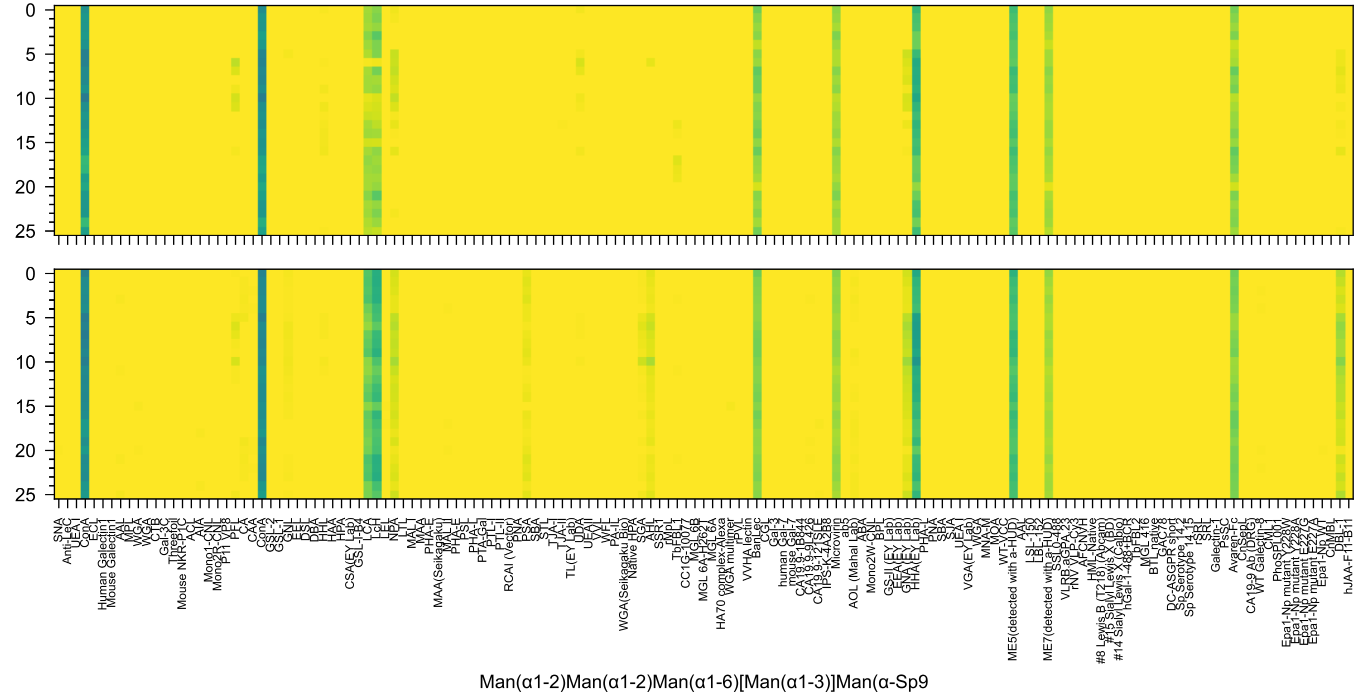
0
5
10
15
20
25
SNA
Anti-LeC
UEA I
ConA
ECL
Human Galectin1
Mouse Galectin1
AAL
MPL
WGA
WGA
CTB
Gal-3C
Threefoil
Mouse NKR-P1C
ACL
AIA
Mono1-CNL
Mono2R-CNL
P11 VP8
PFL
CA
CAA
ConA
GSL-2
GSL-1
GNL
EEL
DSL
DBA
HHL
HAA
HPA
CSA(EY Lab)
GSL-I-B4
LCA
LcH
LEL
NPA
LTL
MAL1
MAA
PHA-E
MAA(Seikagaku)
MAL-II
PHA-E
PSL
PHA-L
PTA-Gal
PTL-I
PTL-II
RCAI (Vector)
PNA
PSA
SBA
STL
TJA-I
TJA-II
TL(EY Lab)
UDA
UEAII
VVL
WFL
PA-IL
WGA(Seikagaku Bio)
Native HPA
SGA
AAL
SSR1
rMpL
TbFBL1
CC1G-10077
MGL 6B
MGL 6A-H262T
MGL 6A
HA70 complex-Alexa
WGA multimer
rPVL
VVHA lectin
BanLec
CGL
Gal-3
human Gal-7
mouse Gal-7
CA19.9-1B844
CA19.9-9L426
CA19.9-121SLE
IPS-K-4A2B8
Microvirin
ab5
AOL (Mahal Lab)
ABA
Mono2W-CNL
BPL
GS-II (EY Lab)
EEA (EY Lab)
GNA (EY Lab)
HHA (EY Lab)
PHA-L
PNA
SBA
SJA
UEA1
VGA(EY Lab)
WGA
MNA-M
MOA
WT-VCC
ME5(detected with a-HUD)
AAL
LSL-150
LBL-152
ME7(detected with a-HUD)
SSL0-488
VLRB.aGPA.23
rNV VLP-Cy3
AFCNVH
HML-Native
#8 Lewis B (T218) (Abcam)
#15 Sialyl Lewis X (BD)
#14 Sialyl Lewis X (Calbio)
hGal-1-488+BCR
TbFBL2
MGL 416
BTL native
GAC78
DC-ASGPR short
Sp Serotype 14.2
Sp Serotype 14.15
rSRL
SRL
Galectin-1
PsSC
Avaren-Fc
ChSepL
CA19-9 Ab (DRG)
WT Galectin-8
CML1
PhoSL001
Epa1-Np mutant Y228W
Epa1-Np mutant Y228A
Epa1-Np mutant E227G
Epa1-Np mutant E227A
Epa1-Np WT
ChMBL
DBL-1
hJAA-F11-B11
Man(α1-2)Man(α1-2)Man(α1-6)[Man(α1-3)]Man(α-Sp9

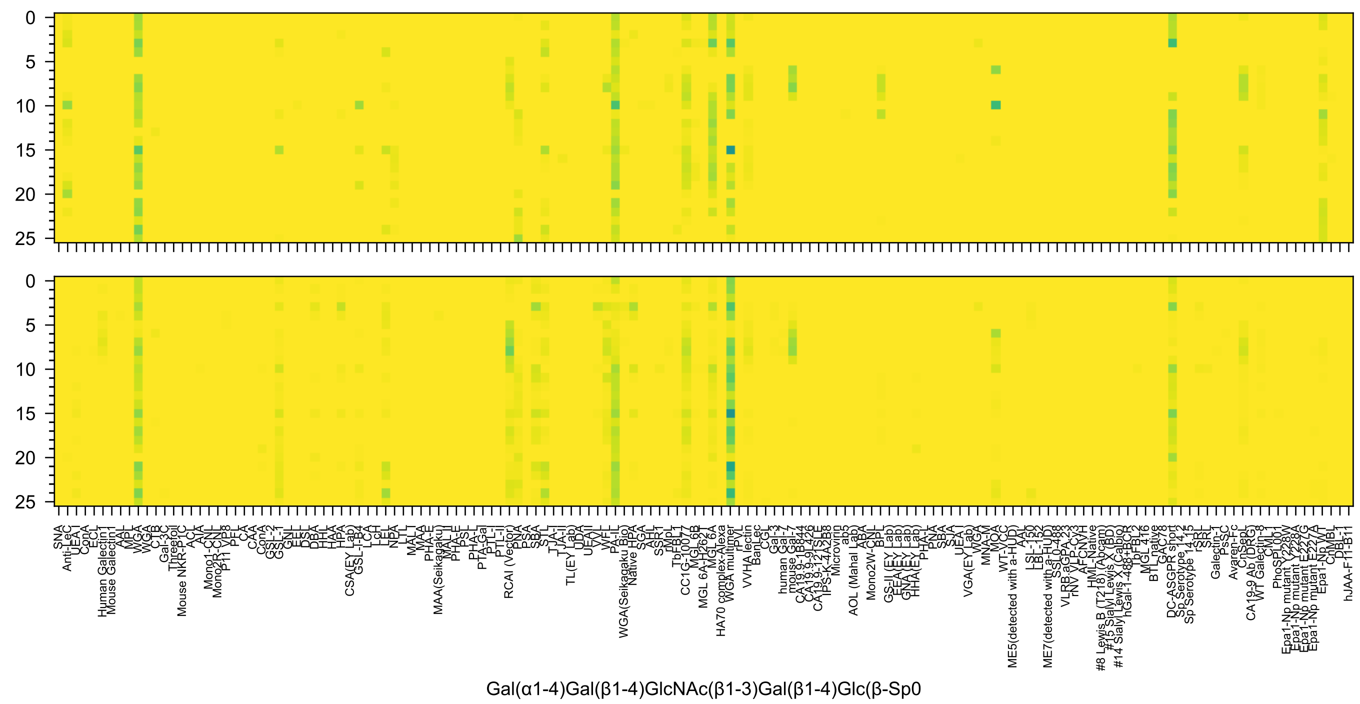

0
5
10
15
20
25
SNA
Anti-LeC
UEA-I
ConA
ECL
Human Galectin1
Mouse Galectin1
AAL
MPL
WGA
WGA
CTB
Gal-3C
Threefoil
Mouse NKR-P1C
ACL
AIA
Mono1-CNL
Mono2R-CNL
P11 VP8
PFL
CA
CAA
ConA
GSL-2
GSL-1
GNL
EEL
ESL
DSL
DBA
HHL
HAA
HPA
CSA(EY Lab)
GSL-I-B4
LCA
LcH
LEL
NPA
LTL
MAL-I
MAA
PHA-E
MAA(Seikagaku)
MAL-II
PHA-E
PSL
PHA-L
PTA-Gal
PTL-I
PTL-II
RCAI (Vector)
PNA
PSA
SBA
STL
TJA-I
TJA-II
TL(EY Lab)
UDA
UEAII
VVL
WFL
PA-IL
WGA(Seikagaku Bio)
Native HPA
SGA
AAL
SSR1
rMpL
TbFBL1
CC1G-10077
MGL 6B
MGL 6A-H262T
MGL 6A
HA70 complex-Alexa
WGA multimer
rPVL
VVHA lectin
BanLec
CGL
Gal-3
human Gal-7
mouse Gal-7
CA19.9-1B844
CA19.9-9L426
CA19.9-121SLE
IPS-K-4A2B8
Microvirin
ab5
AOL (Mahal Lab)
ABA
Mono2W-CNL
BPL
GS-II (EY Lab)
EEA (EY Lab)
GNA (EY Lab)
HHA (EY Lab)
PHA-L
PNA
SBA
SJA
UEA-I
VGA(EY Lab)
WGA
MNA-M
MOA
WT-VCC
ME5(detected with a-HUD)
AAL
LSL-150
LBL-152
ME7(detected with a-HUD)
SSL0-488
VLRB.aGPA.23
rNV VLP-Cy3
AFCNVH
HML-Native
#8 Lewis B (T218) (Abcam)
#15 Sialyl Lewis X (BD)
#14 Sialyl Lewis X (Calbio)
hGal-1-488+BCR
TbFBL2
MGL 416
BTL native
GAC78
DC-ASGPR short
Sp Serotype 14.2
Sp Serotype 14.15
rSRL
SRL
Galectin-1
PsSC
Avaren-Fc
CnSepL
CA19-9 Ab (DRG)
WT Galectin-8
CML1
PhoSPL001
Epa1-Np mutant Y228W
Epa1-Np mutant Y228A
Epa1-Np mutant E227G
Epa1-Np mutant E227A
Epa1-Np WT
ChMBL
DBL-1
hJAA-F11-B11
Gal(α1-4)Gal(β1-4)GlcNAc(β1-3)Gal(β1-4)Glc(β-Sp0

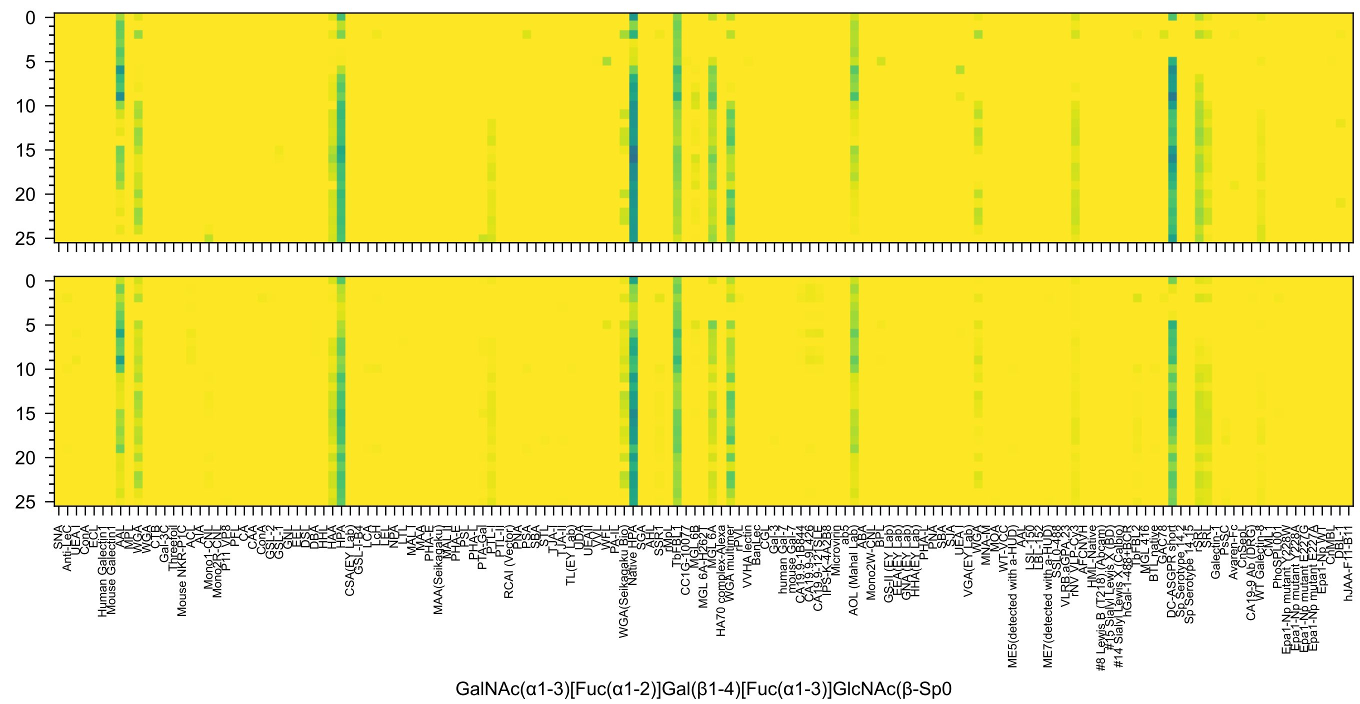
GalNAc(α1-3)[Fuc(α1-2)]Gal(β1-4)[Fuc(α1-3)]GlcNAc(β-Sp0

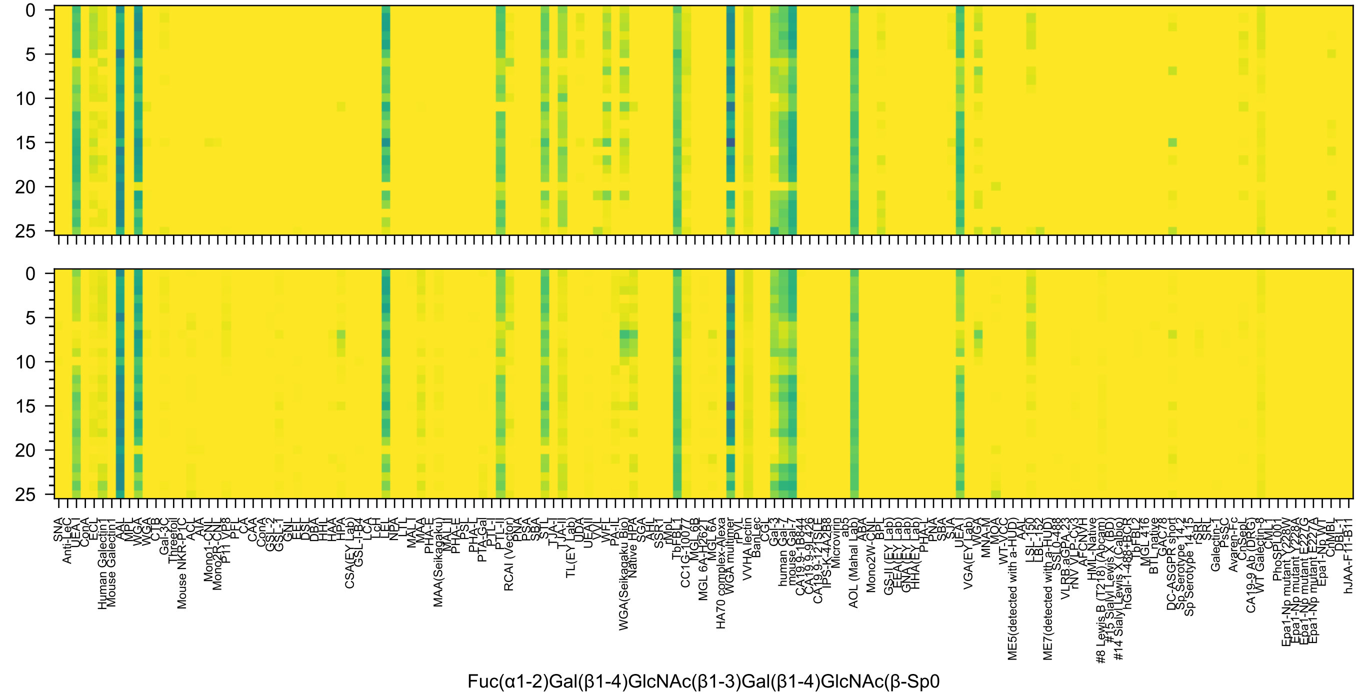

0
5
10
15
20
25
SNA
Anti-LeC
UEA-I
ConA
ECL
Human Galectin1
Mouse Galectin1
AAL
MPL
WGA
CTB
Gal-3C
Threefoil
Mouse NKR-P1C
ACL
AIA
Mono1-CNL
Mono2R-CNL
P11 VP8
PFL
CA
CAA
ConA
GSL-2
GSL-1
GNL
EEL
DSL
DBA
HHL
HAA
HPA
CSA(EY Lab)
GSL-I-B4
LCA
LcH
LEL
NPA
LTL
MAL-I
MAA
PHA-E
MAA(Seikagaku)
PSL
PHA-L
PTA-Gal
PTL-I
PTL-II
RCAI (Vector)
PNA
PSA
SBA
STL
TJA-I
TJA-II
TL(EY Lab)
UDA
UEA-II
VVL
WFL
PA-IL
WGA(Seikagaku Bio)
Native HPA
SGA
AHL
SSR1
rMpL
TbFBL1
CC1G-10077
MGL 6B
MGL 6A-H262T
MGL 6A
HA70 complex-Alexa
WGA multimer
rPVL
VVHA lectin
BanLec
CGL
Gal-3
human Gal-7
mouse Gal-7
CA19.9-1B844
CA19.9-9L426
CA19.9-121SLE
IPS-K-4A2B8
Microvirin
ab5
AOL (Mahal Lab)
ABA
Mono2W-CNL
BPL
GS-II (EY Lab)
EEA (EY Lab)
GNA (EY Lab)
HHA (EY Lab)
SJA
VGA(EY Lab)
MNA-M
MOA
WT-VCC
ME5(detected with a-HUD)
LSL-150
LBL-152
ME7(detected with a-HUD)
SSL0-488
VLRB aGPA.23
rNV VLP-Cy3
AFCNVH
HML-Native
#8 Lewis B (T218) (Abcam)
#15 Sialyl Lewis X (BD)
hGal-1-488+BCR
TbFBL2
MGL 416
BTL native
GAC78
DC-ASGPR short
Sp Serotype 14.2
Sp Serotype 14.15
rSRL
SRL
Galectin-1
PsSC
Avaren-Fc
ChSepL
CA19-9 Ab (DRG)
WT Galectin-8
CML
PhoSPL001
Epa1-Np mutant Y228W
Epa1-Np mutant Y228A
Epa1-Np mutant E227G
Epa1-Np mutant E227A
Epa1-Np WT
ChMBL
DBL-1
hJAA-F11-B11
Fuc(α1-2)Gal(β1-4)GlcNAc(β1-3)Gal(β1-4)GlcNAc(β-Sp0

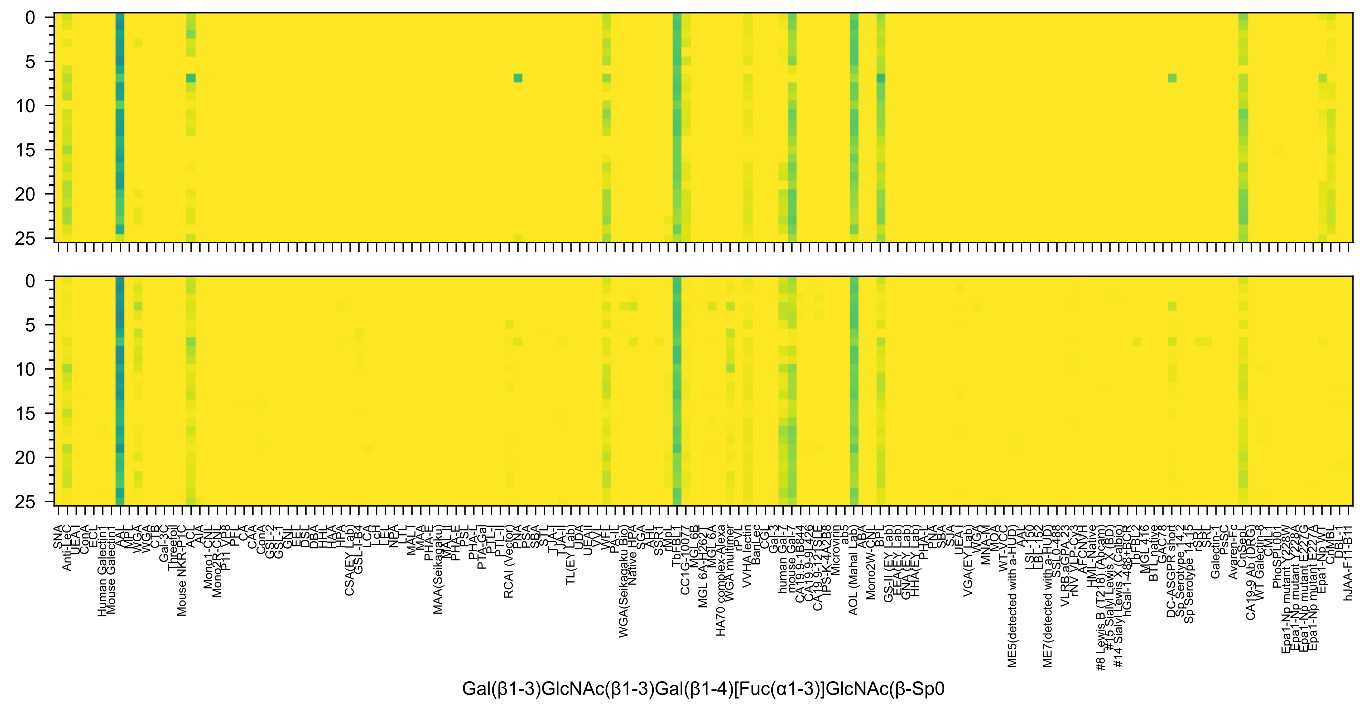
Gal(β1-3)GlcNAc(β1-3)Gal(β1-4)[Fuc(α1-3)]GlcNAc(β-Sp0

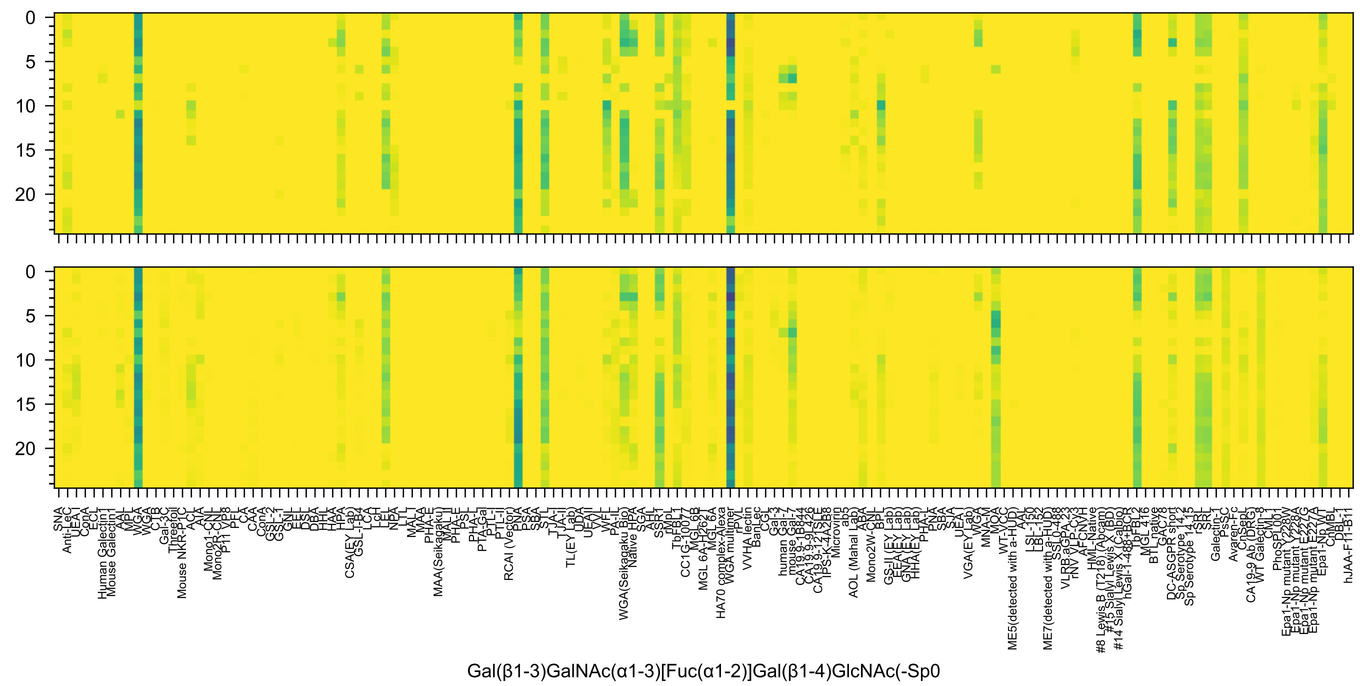
0
5
10
15
20
SNA
Anti-LeC
UEA-I
ConA
ECL
Human Galectin1
Mouse Galectin1
AAL
MPL
WGA
WGA
CTB
Gal-3C
Threefoil
Mouse NKR-P1C
ACL
AIA
Mono1-CNL
Mono2R-CNL
P11 VP8
PFL
CA
CAA
ConA
GSL-2
GSL-1
GNL
EEL
DSL
DBA
HHL
HAA
HPA
CSA(EY Lab)
GSL-I-B4
LCA
LcH
LEL
NPA
LTL
MAL-I
MAA
PHA-E
MAA(Seikagaku)
MAL-II
PHA-E
PSL
PHA-L
PTA-Gal
PTL-I
PTL-II
RCAI (Vector)
PNA
PSA
SBA
STL
TJA-I
TJA-II
TL(EY Lab)
UDA
UEAII
VVL
WFL
PA-IL
WGA(Seikagaku Bio)
Native HPA
SGA
AHL
SSR1
rMpL
TbFBL1
CC1G-10077
MGL 6B
MGL 6A-H262T
MGL 6A
HA70 complex-Alexa
WGA multimer
rPVL
VVHA lectin
BanLec
CGL
Gal-3
human Gal-7
mouse Gal-7
CA19.9-1B844
CA19.9-9L426
CA19.9-121SLE
IPS-K-4A2B8
Microvirin
ab5
AOL (Mahal Lab)
ABA
Mono2W-CNL
BPL
GS-II (EY Lab)
EEA(EY Lab)
GNA(EY Lab)
HHA(EY Lab)
PHA-L
PNA
SBA
SJA
UEA-I
VGA(EY Lab)
WGA
MNA-M
MOA
WT-VCC
ME5(detected with a-HUD)
AAL
LSL-150
LBL-152
ME7(detected with a-HUD)
SSL0-488
VLRB.aGPA.23
rNV_VLP-Cy3
AFCNVH
HML-Native
#8 Lewis B (T218) (Abcam)
#15 Sialyl Lewis X (BD)
#14 Sialyl Lewis X (Calbio)
hGal-1-488+BCR
TbFBL2
MGL 416
BTL native
GAC78
DC-ASGPR short
Sp Serotype 14.2
Sp Serotype 14.15
rSRL
SRL
Galectin-1
PsSC
Avaren-Fc
CnSepL
CA19-9 Ab (DRG)
WT Galectin-8
CML1
PhoSL001
Epa1-Np mutant Y228W
Epa1-Np mutant Y228A
Epa1-Np mutant E227G
Epa1-Np mutant E227A
Epa1-Np WT
ChMBL
DBL-1
hJAA-F11-B11
Gal(β1-3)GalNAc(α1-3)[Fuc(α1-2)]Gal(β1-4)GlcNAc(-Sp0

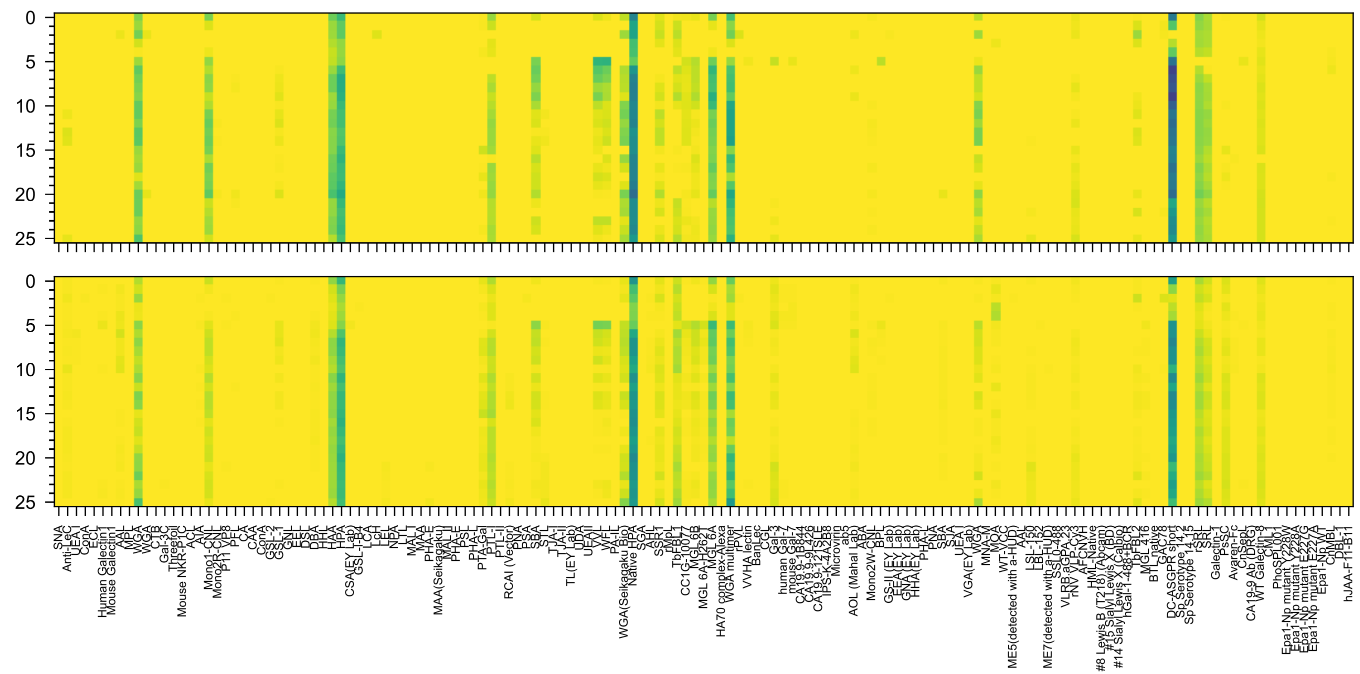

GalNAc(α1-3)[Fuc(α1-2)]Gal(β1-4)GlcNAc(β1-2)Man(α-Sp0

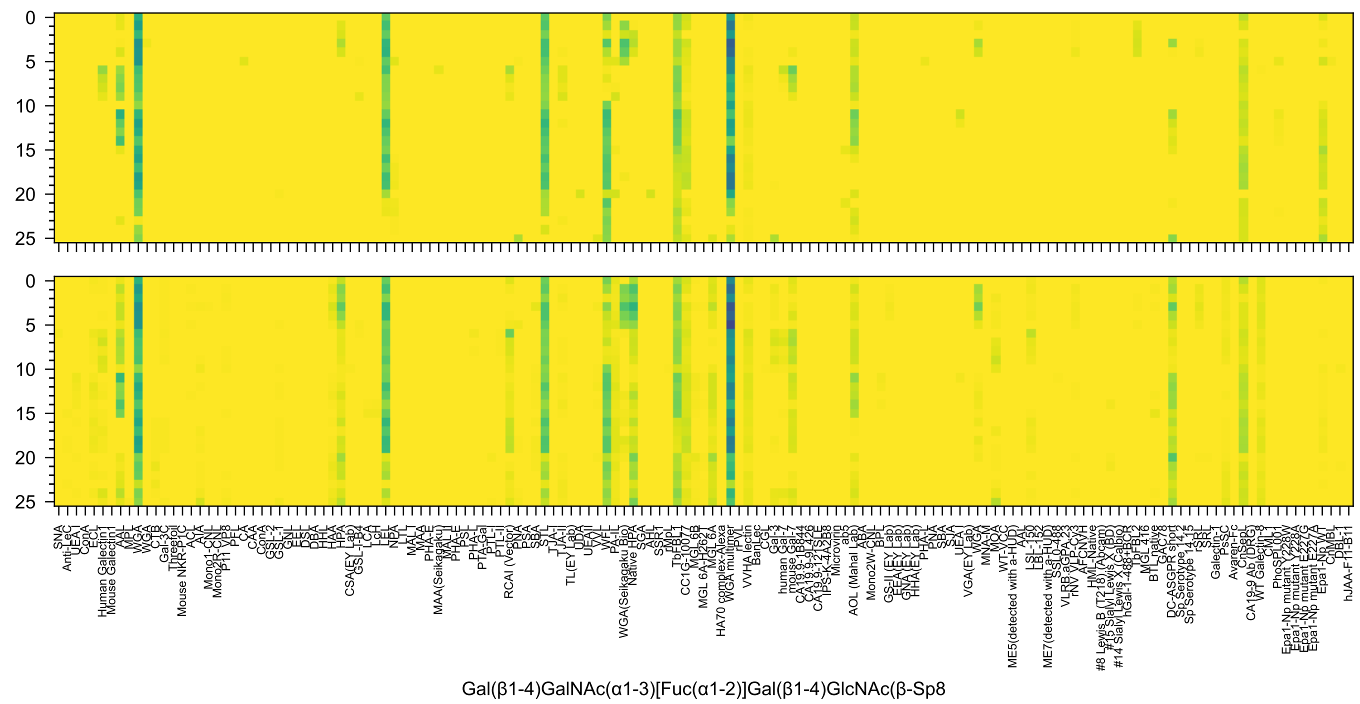
Gal(β1-4)GalNAc(α1-3)[Fuc(α1-2)]Gal(β1-4)GlcNAc(β-Sp8

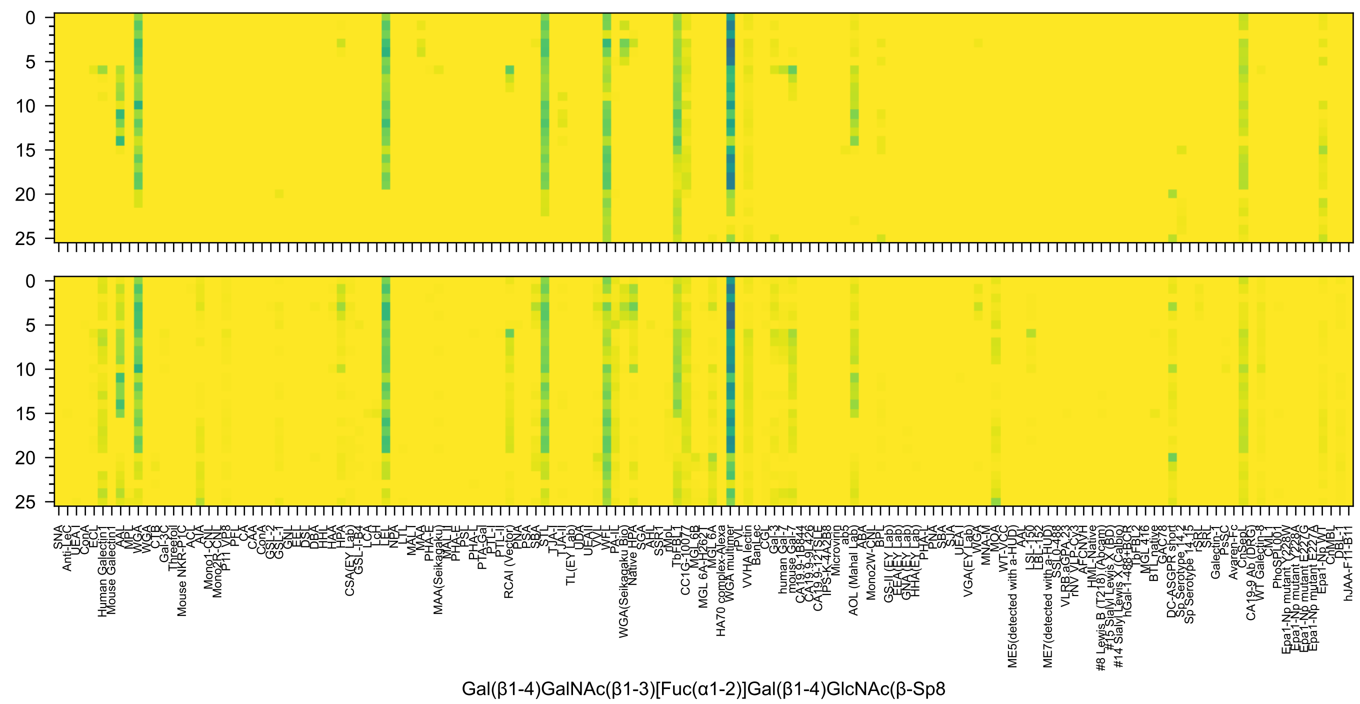

0
5
10
15
20
25
SNA
Anti-LeC
UEA-I
ConA
ECL
Human Galectin1
Mouse Galectin1
AAL
MPL
WGA
WGA
CTB
Gal-3C
Threefoil
Mouse NKR-P1C
ACL
AIA
Mono1-CNL
Mono2R-CNL
P11 VP8
PFL
CA
CAA
ConA
GSL-2
GSL-1
GNL
EEL
DSL
DBA
HHL
HAA
HPA
CSA(EY Lab)
GSL-I-B4
LCA
LcH
LEL
NPA
LTL
MAL-I
MAA
PHA-E
MAA(Seikagaku)
MAL-II
PHA-E
PSL
PHA-L
PTA-Gal
PTL-I
PTL-II
RCAI (Vector)
PNA
PSA
SBA
STL
TJA-I
TJA-II
TL(EY Lab)
UDA
UEAII
VVL
WFL
PA-IL
WGA(Seikagaku Bio)
Native HPA
SGA
AAL
SSA
rMpL
TbFBL1
CC1G-10077
MGL 6B
MGL 6A-H262T
MGL 6A
HA70 complex-Alexa
WGA multimer
rPVL
VVHA lectin
BanLec
CGL
Gal-3
human Gal-7
mouse Gal-7
CA19.9-1B844
CA19.9-9L426
CA19.9-121SLE
IPS-K-4A2B8
Microvirin
ab5
AOL (Mahal Lab)
ABA
Mono2W-CNL
BPL
GS-II (EY Lab)
EEA (EY Lab)
GNA (EY Lab)
HHA (EY Lab)
PHA-L
PNA
SBA
SJA
UEA-I
VGA(EY Lab)
WGA
MNA-M
MOA
WT-VCC
ME5(detected with a-HUD)
AAL
LSL-150
LBL-152
ME7(detected with a-HUD)
SSL0-488
VLRB.aGPA.23
rNV VLP-Cy3
AFCNVH
HML-Native
#8 Lewis B (T218) (Abcam)
#15 Sialyl Lewis X (BD)
#14 Sialyl Lewis X (Calbio)
hGal-1-488+BCR
TbFBL2
MGL 416
BTL native
GAC78
DC-ASGPR short
Sp Serotype 14.2
Sp Serotype 14.15
rSRL
SRL
Galectin-1
PsSC
Avaren-Fc
CnSepL
CA19-9 Ab (DRG)
WT Galectin-8
CML1
PhoSL001
Epa1-Np mutant Y228W
Epa1-Np mutant Y228A
Epa1-Np mutant E227G
Epa1-Np mutant E227A
Epa1-Np WT
ChMBL
DBL-1
hJAA-F11-B11
Gal(β1-4)GalNAc(β1-3)[Fuc(α1-2)]Gal(β1-4)GlcNAc(β-Sp8

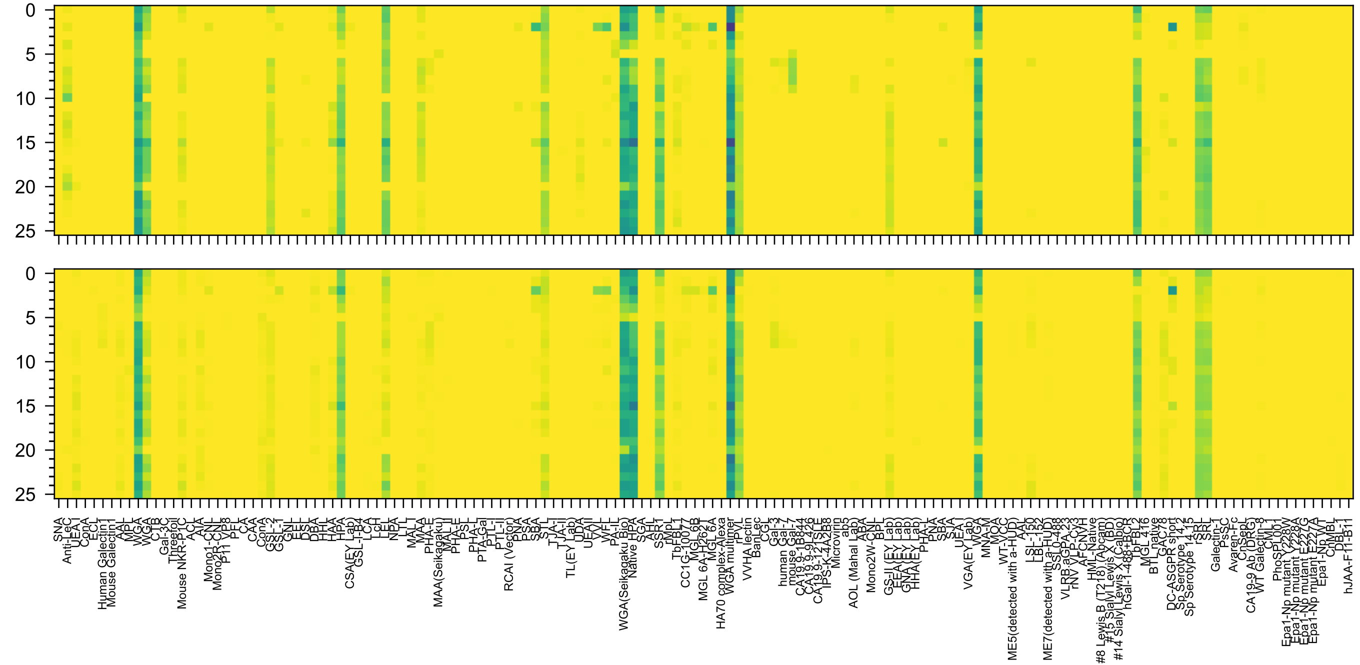

GlcNAc(α1-4)Gal(β1-4)GlcNAc(β1-3)Gal(β1-4)Glc(β-Sp0

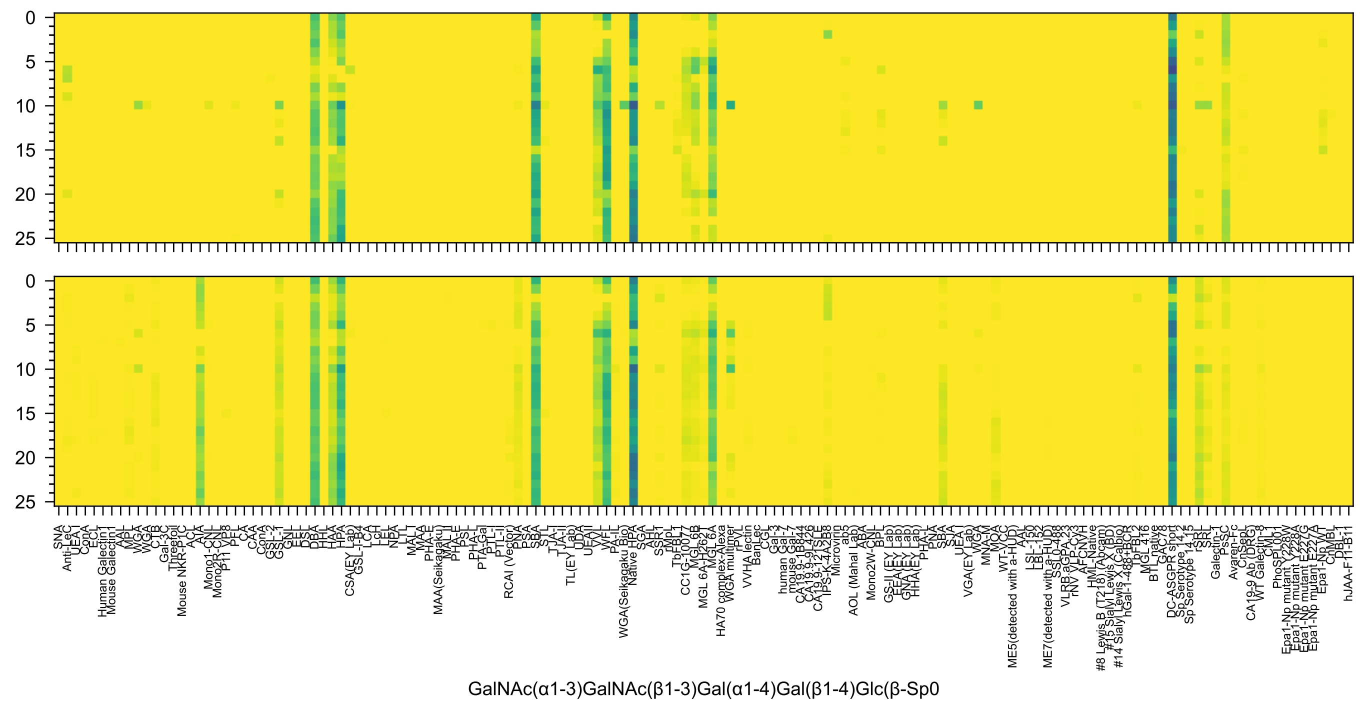
0
5
10
15
20
25
SNA
Anti-LeC
UEA I
ConA
ECL
Human Galectin1
Mouse Galectin1
AAL
MPL
WGA
WGA
CTB
Gal-3C
Threefoil
Mouse NKR-P1C
ACL
AIA
Mono1-CNL
Mono2R-CNL
P11 VP8
PFL
CA
CAA
ConA
GSL-2
GSL-1
GNL
EEL
DSL
DBA
HHL
HAA
HPA
CSA(EY Lab)
GSL-I-B4
LCA
LcH
LEL
NPA
LTL
MAL-I
MAA
PHA-E
MAA(Seikagaku)
MAL-I
PHA-E
PSL
PHA-L
PTA-Gal
PTL-I
PTL-II
RCAI (Vector)
PNA
PSA
SBA
STL
TJA-I
TJA-II
TL(EY Lab)
UDA
UEAII
VVL
WFL
PA-IL
WGA(Seikagaku Bio)
Native HPA
SGA
AHL
SSR1
rMpL
TbFBL1
CC1G-10077
MGL 6B
MGL 6A-H262T
MGL 6A
HA70 complex-Alexa
WGA multimer
rPVL
VVHA lectin
BanLec
CGL
Gal-3
human Gal-7
mouse Gal-7
CA19.9-1B844
CA19.9-9L426
CA19.9-121SLE
IPS-K-4A2B8
Microvirin
ab5
AOL (Mahal Lab)
ABA
Mono2W-CNL
BPL
GS-II (EY Lab)
EEA (EY Lab)
GNA (EY Lab)
HHA (EY Lab)
PHA-L
PNA
SBA
SJA
UEA-I
VGA(EY Lab)
WGA
MNA-M
MOA
WT-VCC
ME5(detected with a-HUD)
AAL
LSL-150
LBL-152
ME7(detected with a-HUD)
SSL0-488
VLRB.aGPA.23
rNV VLP-Cy3
AFCNVH
HML-Native
#8 Lewis B (T218) (Abcam)
#15 Sialyl Lewis X (BD)
#14 Sialyl Lewis X (Calbio)
hGal-1-488+BCR
TbFBL2
MGL 416
BTL native
GAC78
DC-ASGPR short
Sp Serotype 14.2
Sp Serotype 14.15
rSRL
SRL
Galectin-1
PsSC
Avaren-Fc
ChSepL
CA19-9 Ab (DRG)
WT Galectin-8
CML1
PhoSL001
Epa1-Np mutant Y228W
Epa1-Np mutant Y228A
Epa1-Np mutant E227G
Epa1-Np mutant E227A
Epa1-Np WT
ChMBL
DBL-1
hJAA-F11-B11
GalNAc(α1-3)GalNAc(β1-3)Gal(α1-4)Gal(β1-4)Glc(β-Sp0

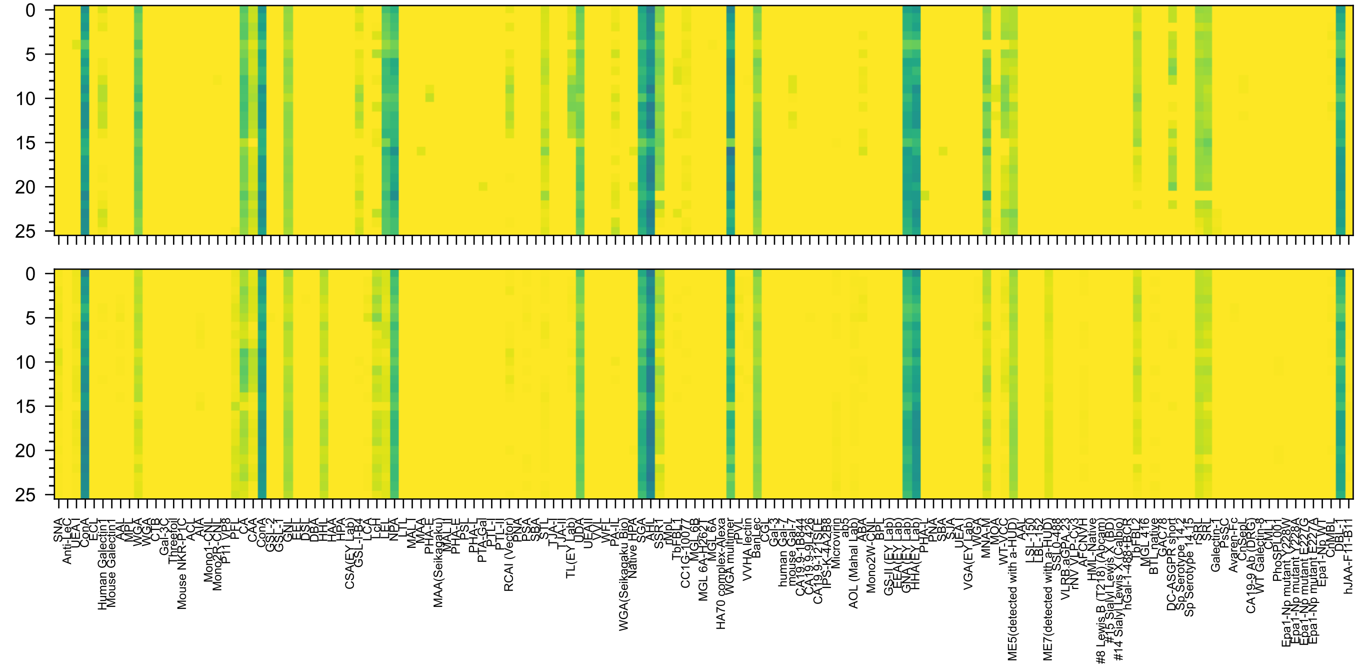
Man(α1-6)[Man(α1-3)]Man(β1-4)GlcNAc(β1-4)GlcNAc(β-Sp13

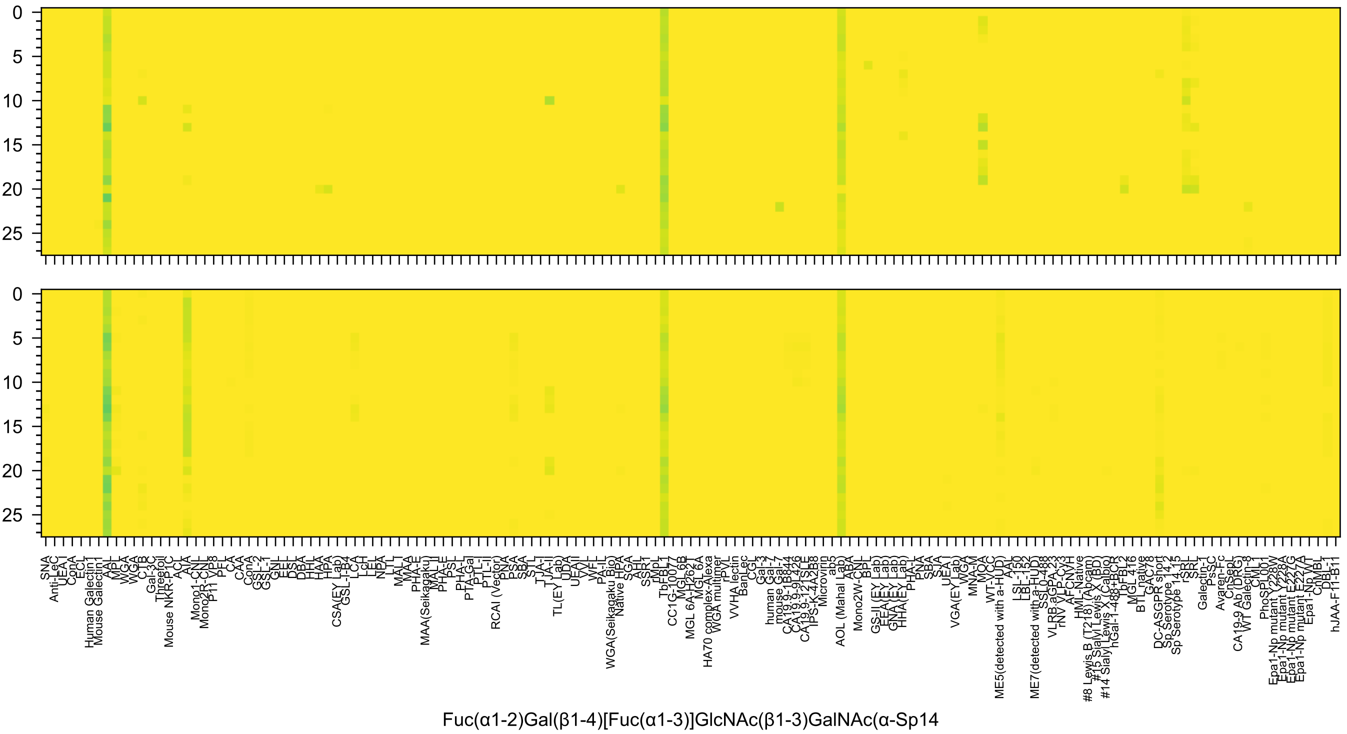
Fuc(α1-2)Gal(β1-4)[Fuc(α1-3)]GlcNAc(β1-3)GalNAc(α-Sp14

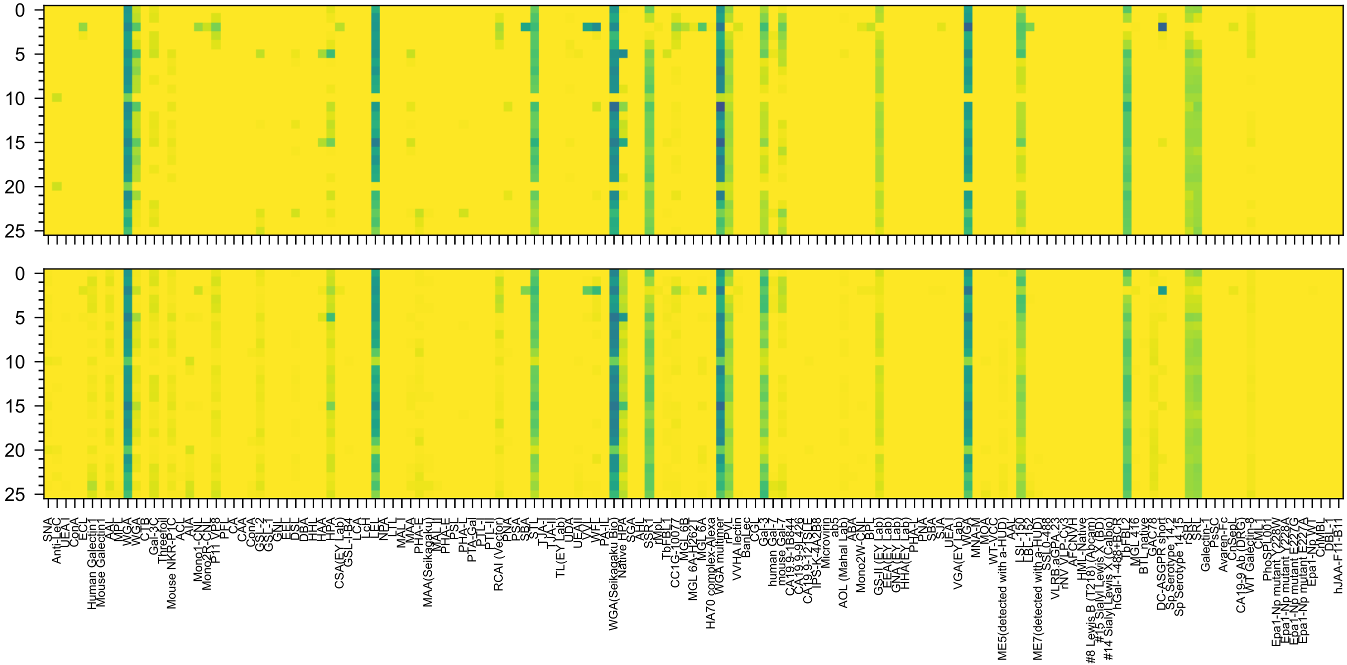

GlcNAc(β1-3)Gal(β1-4)GlcNAc(β1-3)Gal(β1-4)GlcNAc(β-Sp0

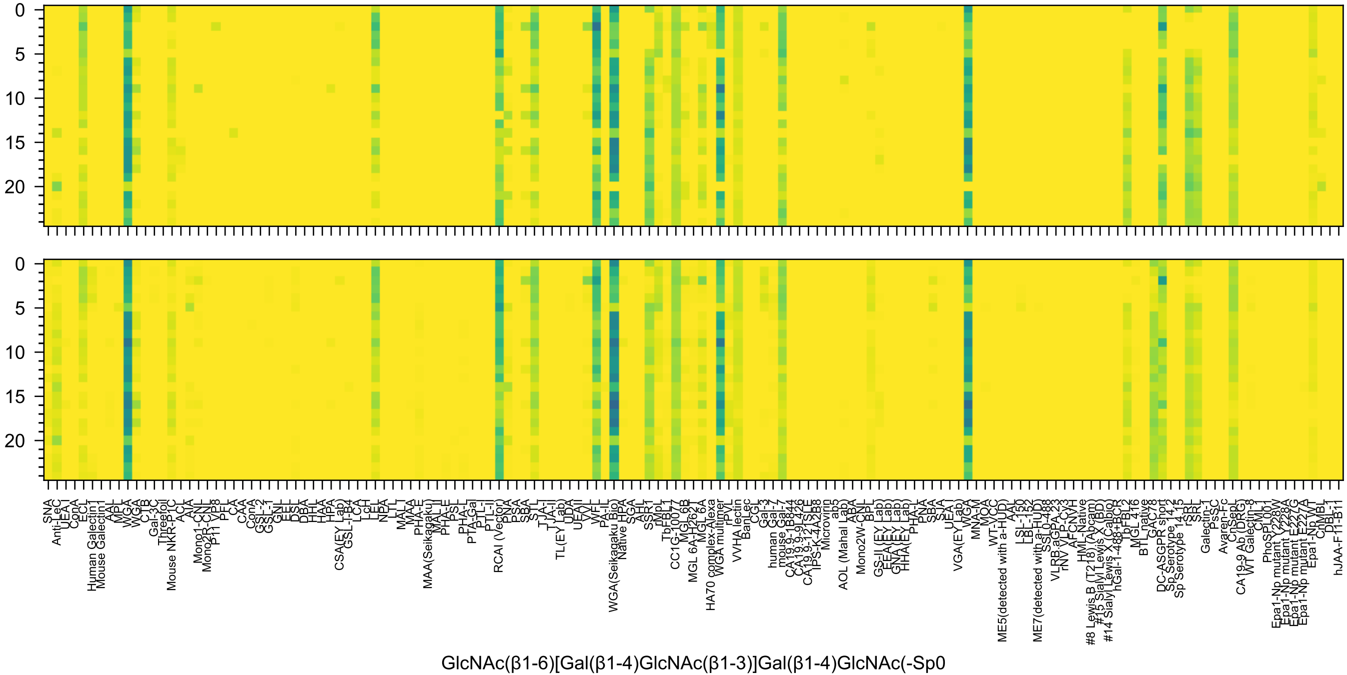
0
5
10
15
20
SNA
Anti-LeC
UEA I
ConA
ECL
Human Galectin1
Mouse Galectin1
AAL
MPL
WGA
WGA
CTB
Gal-3C
Threefoil
Mouse NKR-P1C
ACL
AIA
AIA
Mono1-CNL
Mono2R-CNL
P11 VP8
PFL
CA
CAA
ConA
GSL-2
GSL-1
GNL
EEL
DSL
DBA
HHL
HAA
HPA
CSA(EY Lab)
GSL-I-B4
LCA
LcH
LEL
NPA
LTL
MAL-I
MAA
PHA-E
MAA(Seikagaku)
MAL-II
PHA-E
PSL
PHA-L
PTA-Gal
PTL-I
PTL-II
RCAI (Vector)
PNA
PSA
SBA
STL
TJA-I
TJA-II
TL(EY Lab)
UDA
UEA-II
VVL
WFL
PA-IL
WGA(Seikagaku Bio)
Native HPA
SGA
AAL
SSR1
rMpL
TbFBL1
CC1G-10077
MGL 6B
MGL 6A-H262T
MGL 6A
HA70 complex-Alexa
WGA multimer
rPVL
VVHA lectin
BanLec
CGL
Gal-3
human Gal-7
mouse Gal-7
CA19.9-1B844
CA19.9-9L426
CA19.9-121SLE
IPS-K-4A2B8
Microvirin
ab5
AOL (Mahal Lab)
ABA
Mono2W-CNL
BPL
GS-II (EY Lab)
EEA(EY Lab)
GNA(EY Lab)
HHA(EY Lab)
PHA-L
PNA
SBA
SJA
UEA-I
VGA(EY Lab)
WGA
MNA-M
MOA
WT-VCC
ME5(detected with a-HUD)
AAL
LSL-150
LBL-152
ME7(detected with a-HUD)
SSL0-488
VLRB aGPA.3
rNV VLP-Cy3
AFCNVH
HML-Native
#8 Lewis B (T218) (Abcam)
#15 Sialyl Lewis X (BD)
#14 Sialyl Lewis X (Calbio)
hGal-1-488+BCR
TbFBL2
MGL 416
BTL native
GAC78
DC-ASGPR short
Sp Serotype 14.2
Sp Serotype 14.15
rSRL
SRL
Galectin-1
PsSC
Avaren-Fc
CnSepL
CA19-9 Ab (DRG)
WT Galectin-8
CML1
PhoSL001
Epa1-Np mutant Y228W
Epa1-Np mutant Y228A
Epa1-Np mutant E227G
Epa1-Np mutant E227A
Epa1-Np WT
ChMBL
DBL-1
hJAA-F11-B11
GlcNAc(β1-6)[Gal(β1-4)GlcNAc(β1-3)]Gal(β1-4)GlcNAc(-Sp0

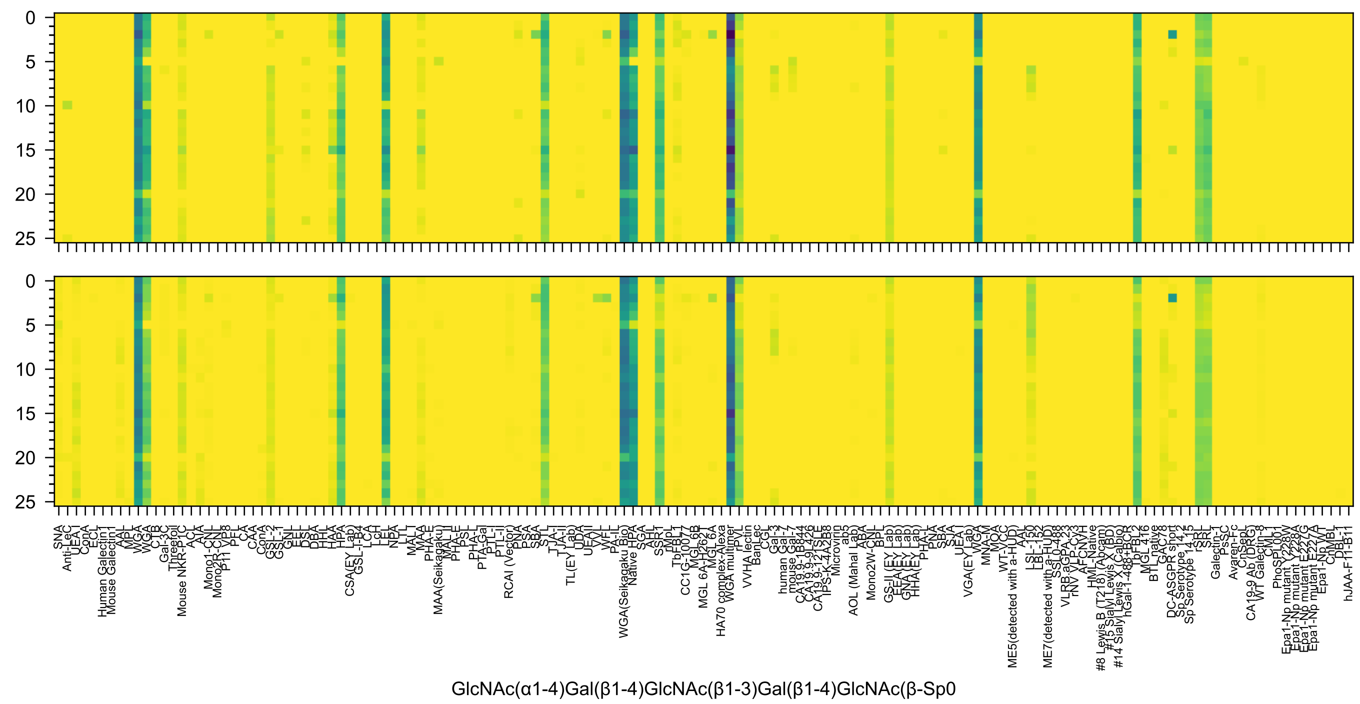
GlcNAc(α1-4)Gal(β1-4)GlcNAc(β1-3)Gal(β1-4)GlcNAc(β-Sp0

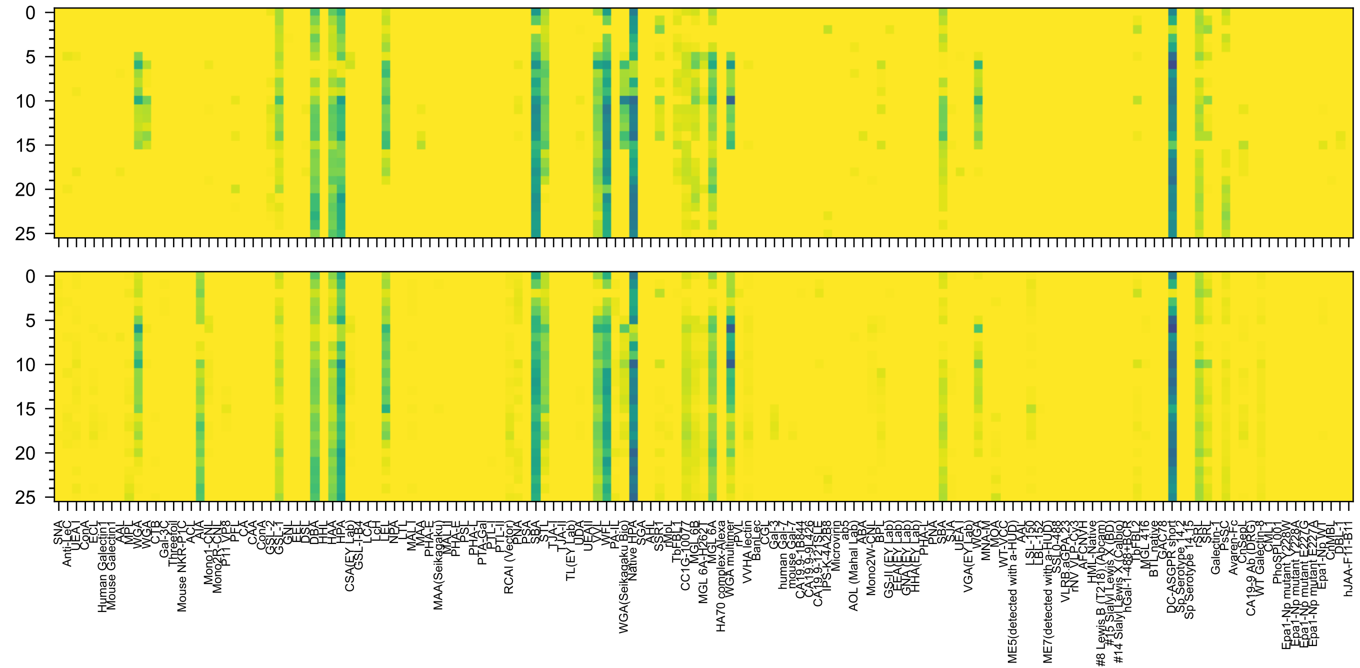

GalNAc(α1-3)GalNAc(β1-3)Gal(α1-4)Gal(β1-4)GlcNAc(β-Sp0

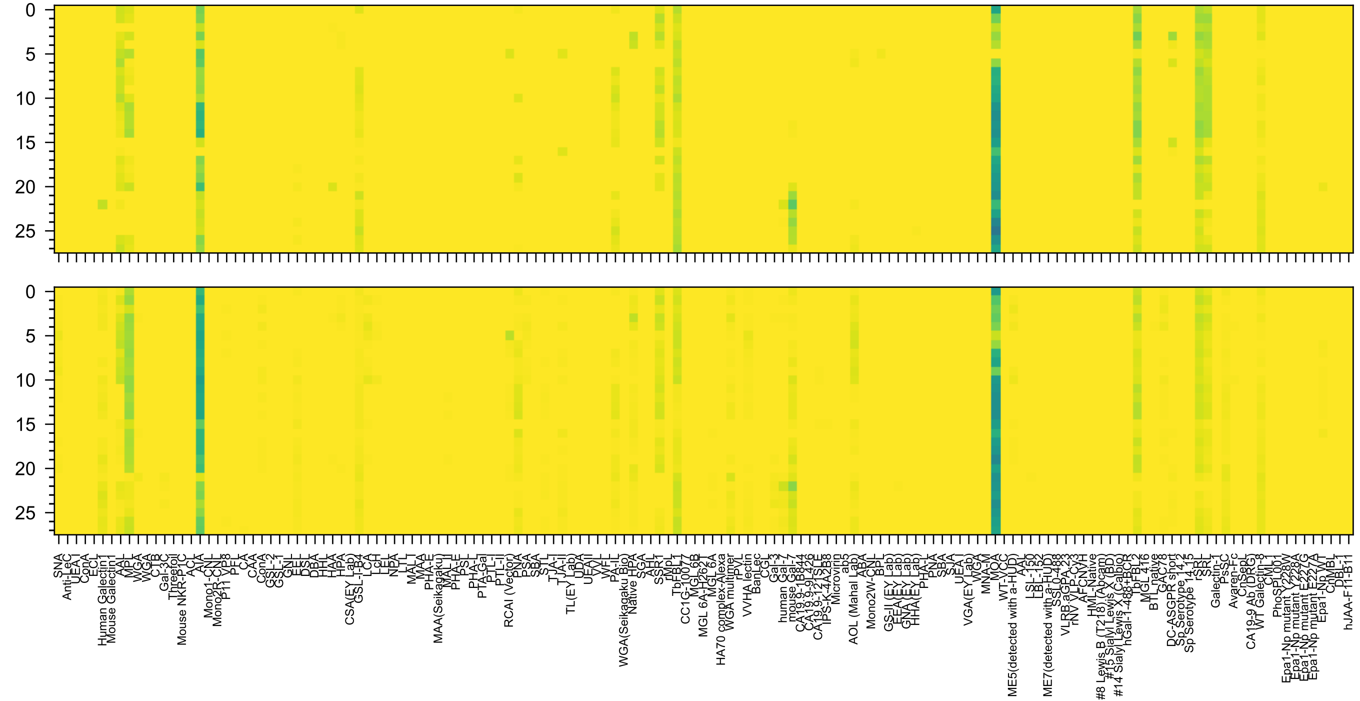

Gal(α1-3)[Fuc(α1-2)]Gal(β1-4)GlcNAc(β1-3)GalNAc(α-Sp14

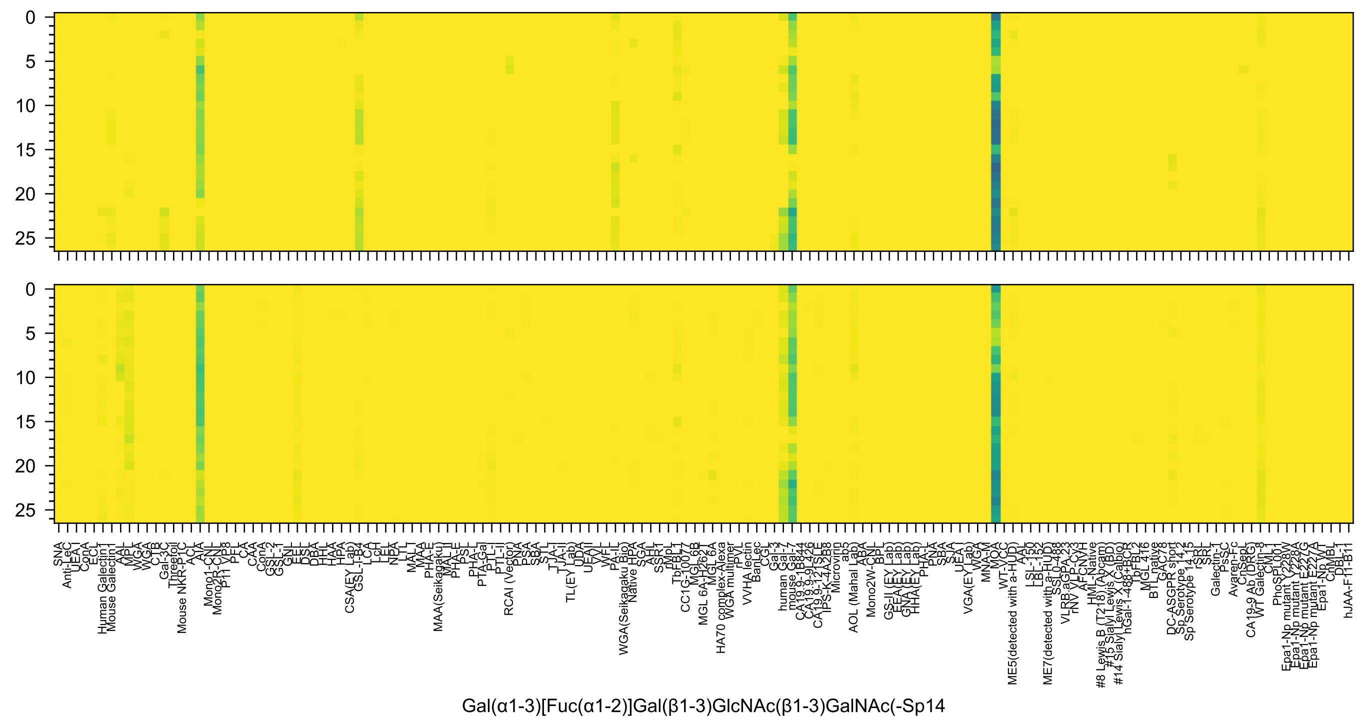
0
5
10
15
20
25
0
5
10
15
20
25
SNA
Anti-LeC
UEA-I
ConA
ECL
Human Galectin1
Mouse Galectin1
AAL
MPL
WGA
WGA
CTB
Gal-3C
Threefoil
Mouse NKR-P1C
ACL
AIA
Mono1-CNL
Mono2R-CNL
P11 VP8
PFL
CA
CAA
ConA
GSL-2
GSL-1
GNL
EEL
DSL
DBA
HHL
HAA
HPA
CSA(EY Lab)
GSL-I-B4
LCA
LcH
LEL
NPA
LTL
MAL
MAA
PHA-E
MAA(Seikagaku)
MAL II
PHA-E
PSL
PHA-L
PTA-Gal
PTL-I
PTL-II
RCAI (Vector)
PNA
PSA
SBA
STL
TJA-I
TJA-II
TL(EY Lab)
UDA
UEAII
VVL
WFL
PA-IL
WGA(Seikagaku Bio)
Native HPA
SGA
AHL
SSR1
rMpL
TbFBL1
CC1G-10077
MGL6B
MGL 6A-H262T
MGL 6A
HA70 complex-Alexa
WGA multimer
rPVL
VVHA lectin
BanLec
CGL
Gal-3
human Gal-7
mouse Gal-7
CA19.9-1B844
CA19.9-9L426
CA19.9-121SLE
IPS-K-4A2B8
Microvirin
ab5
AOL (Mahal Lab)
ABA
Mono2W-CNL
BPL
GS-II (EY Lab)
EEA(EY Lab)
GNA(EY Lab)
HHA(EY Lab)
PHA-L
PNA
SBA
SJA
UEA-I
VGA(EY Lab)
WGA
MNA-M
MOA
WT-VCC
ME5(detected with a-HUD)
AAL
LSL-150
LBL-152
ME7(detected with a-HUD)
SSL0-488
VLRB.aGPA.23
rNV VLP-Cy3
AFCNVH
HML-Native
#8 Lewis B (T218) (Abcam)
#15 Sialyl Lewis X (BD)
#14 Sialyl Lewis X (Calbio)
hGal-1-488+BCR
TbFBL2
MGL 416
BTL native
GAC78
DC-ASGPR short
Sp Serotype 14.2
Sp Serotype 14.15
rSRL
SRL
Galectin-1
PsSC
Avaren-Fc
ChSepL
CA19-9 Ab (DRG)
WT Galectin-8
CML1
PhoSL001
Epa1-Np mutant Y228W
Epa1-Np mutant Y228A
Epa1-Np mutant E227G
Epa1-Np mutant E227A
Epa1-Np WT
ChMBL
DBL-1
hJAA-F11-B11
Gal(α1-3)[Fuc(α1-2)]Gal(β1-3)GlcNAc(β1-3)GalNAc(-Sp14

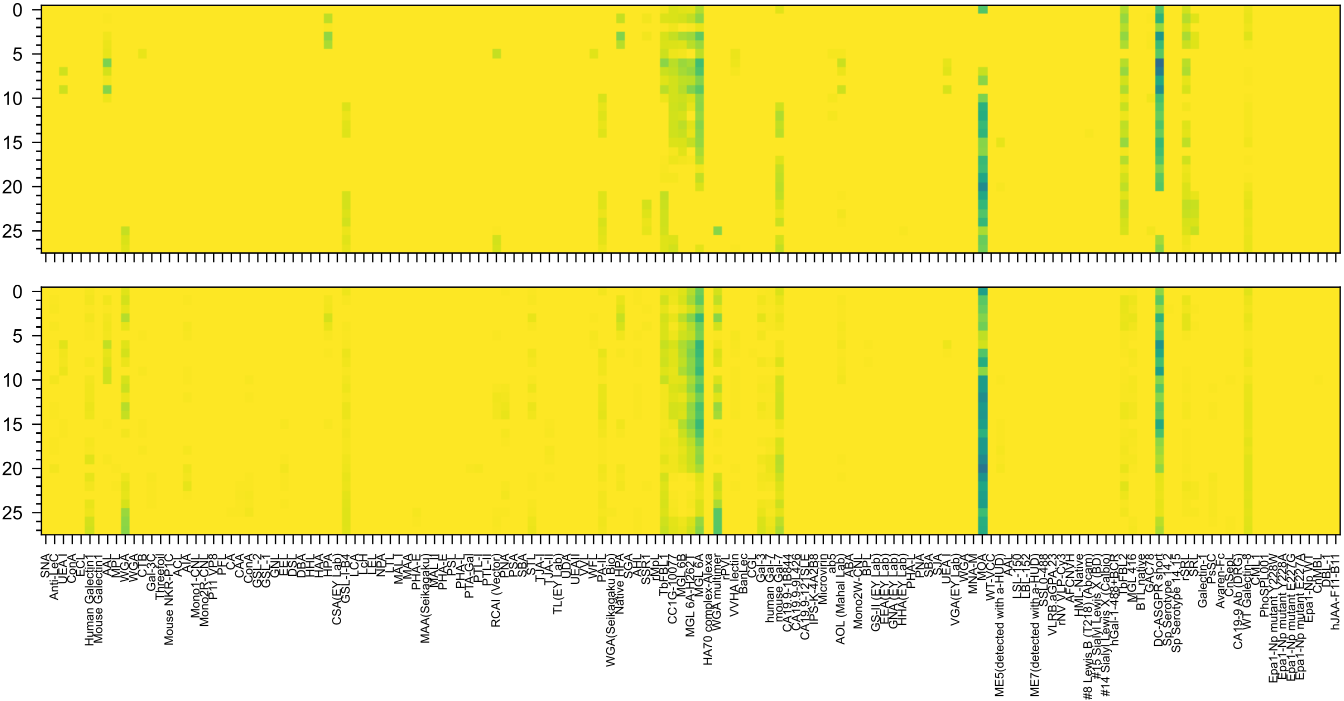

Gal(α1-3)[Fuc(α1-2)]Gal(β1-4)GlcNAc(β1-6)GalNAc(α-Sp14

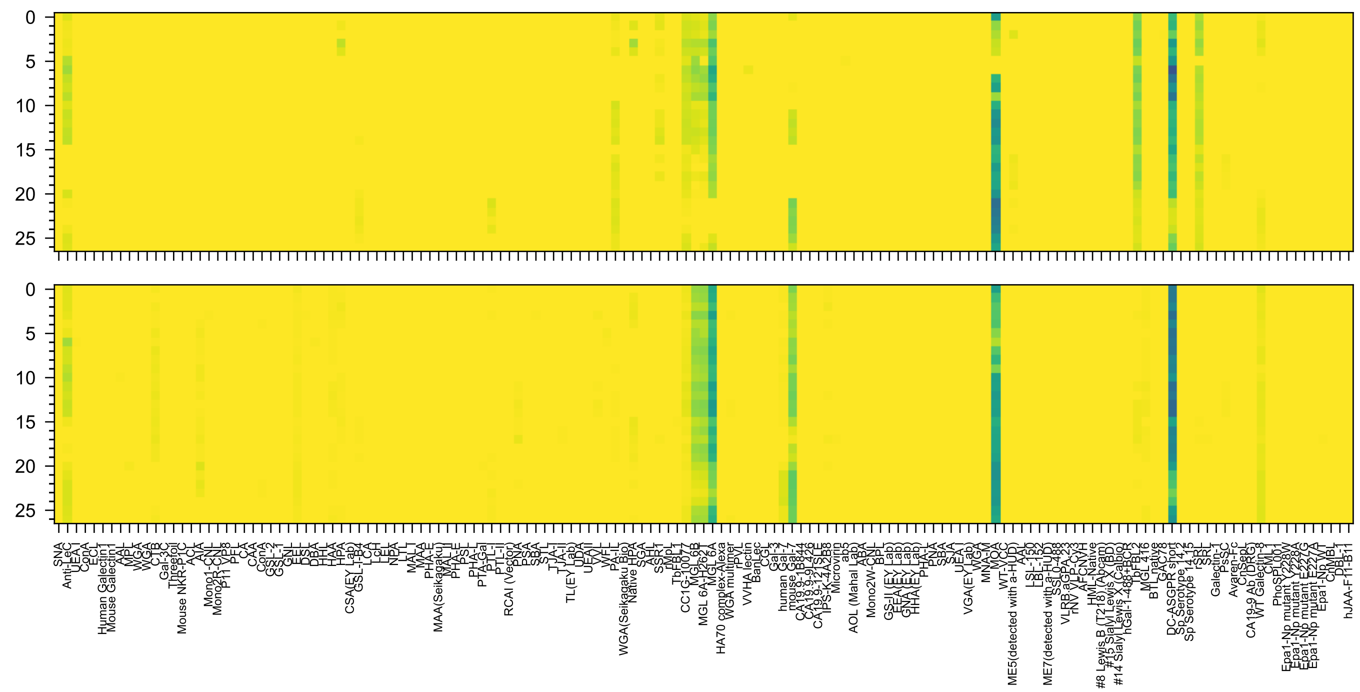

Gal(α1-3)[Fuc(α1-2)]Gal(β1-3)GlcNAc(β1-6)GalNAc(-Sp14

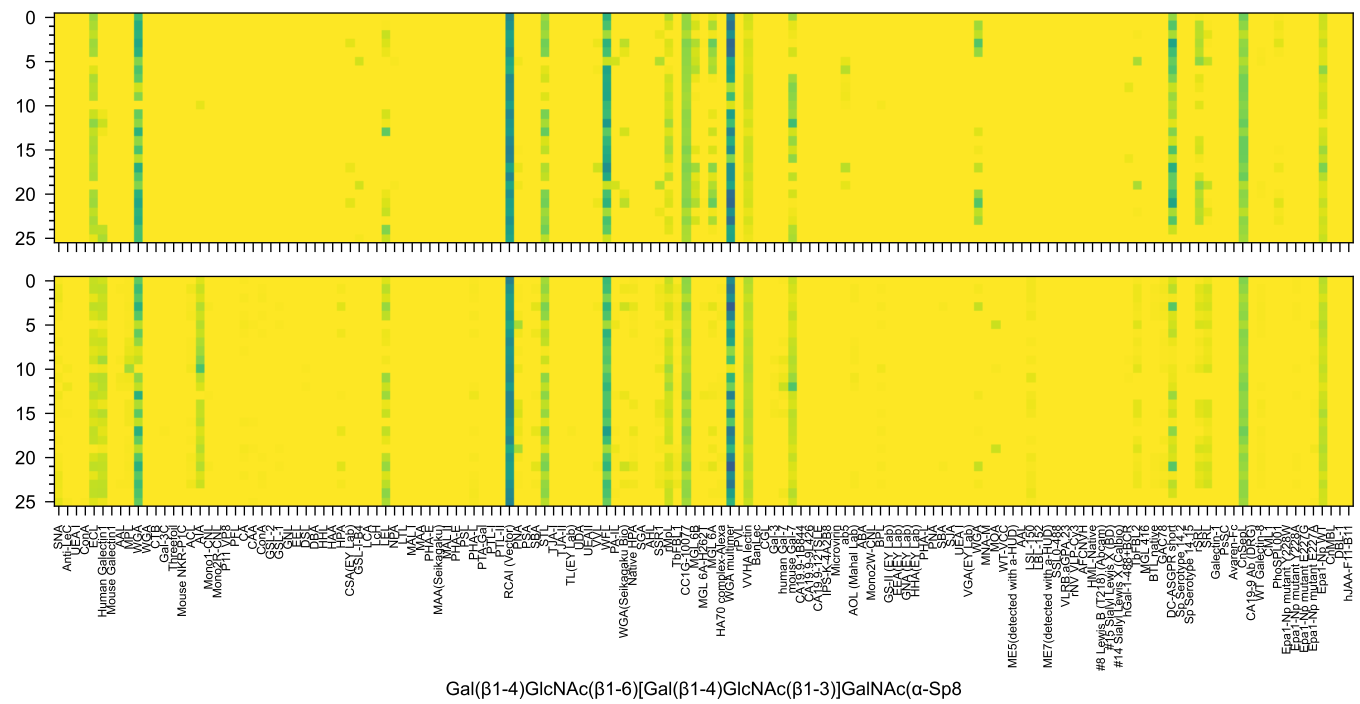
Gal(β1-4)GlcNAc(β1-6)[Gal(β1-4)GlcNAc(β1-3)]GalNAc(α-Sp8

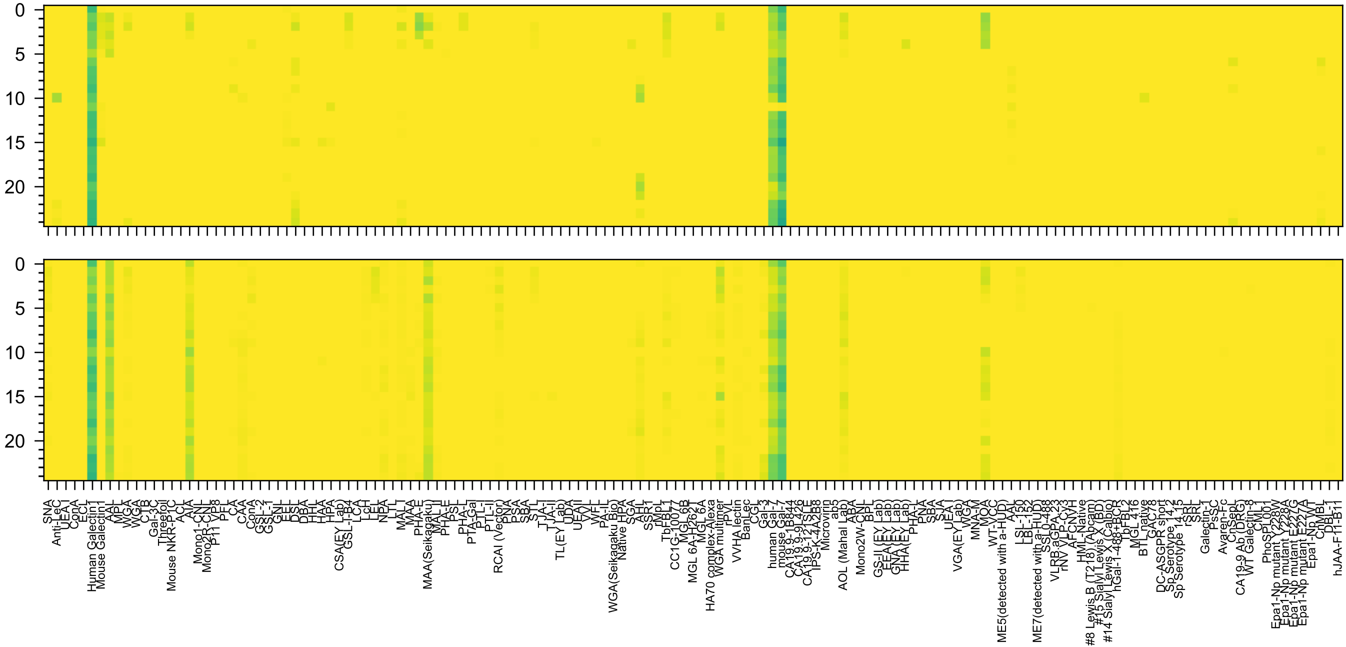

GlcA3OS(β1-3)Gal(β1-4)GlcNAc(β1-3)Gal(β1-4)Glc(-Sp21

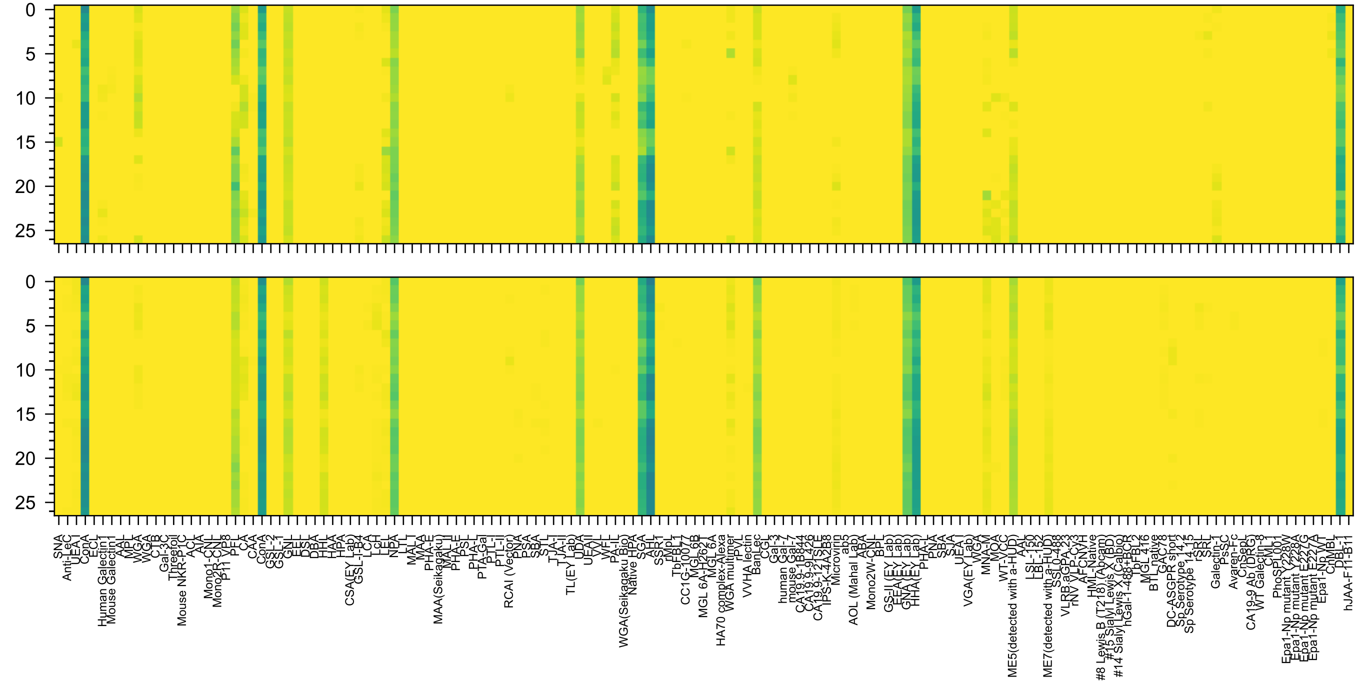

Man(α1-6)[Man(α1-3)]Man(β1-4)GlcNAc(β1-4)GlcNAc(β-Sp12

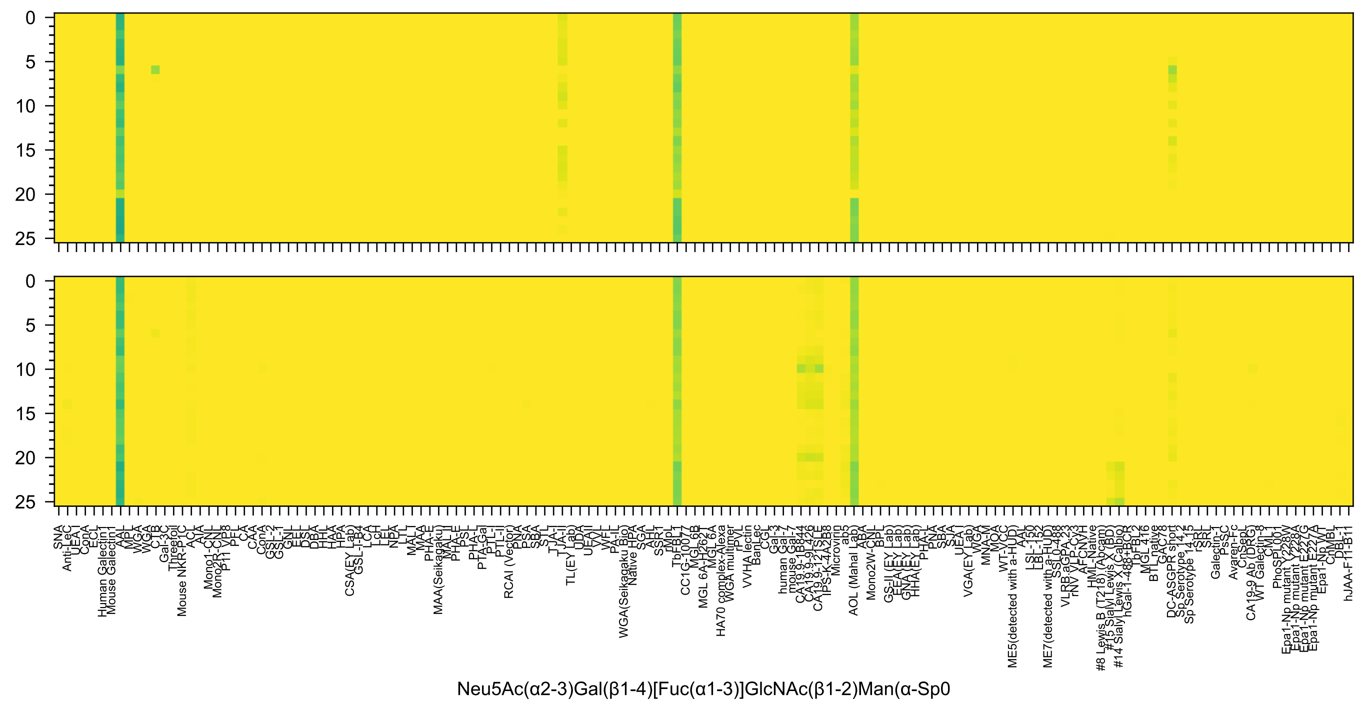
Neu5Ac(α2-3)Gal(β1-4)[Fuc(α1-3)]GlcNAc(β1-2)Man(α-Sp0

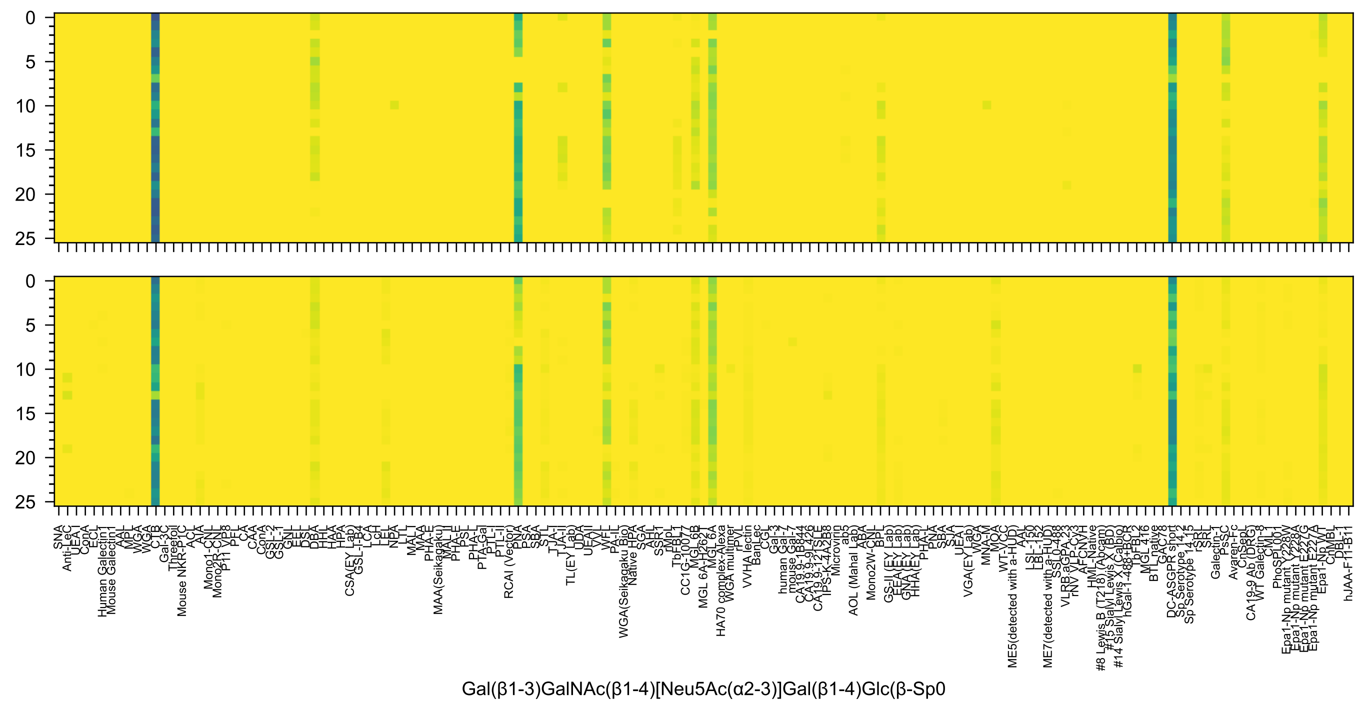

Gal(β1-3)GalNAc(β1-4)[Neu5Ac(α2-3)]Gal(β1-4)Glc(β-Sp0

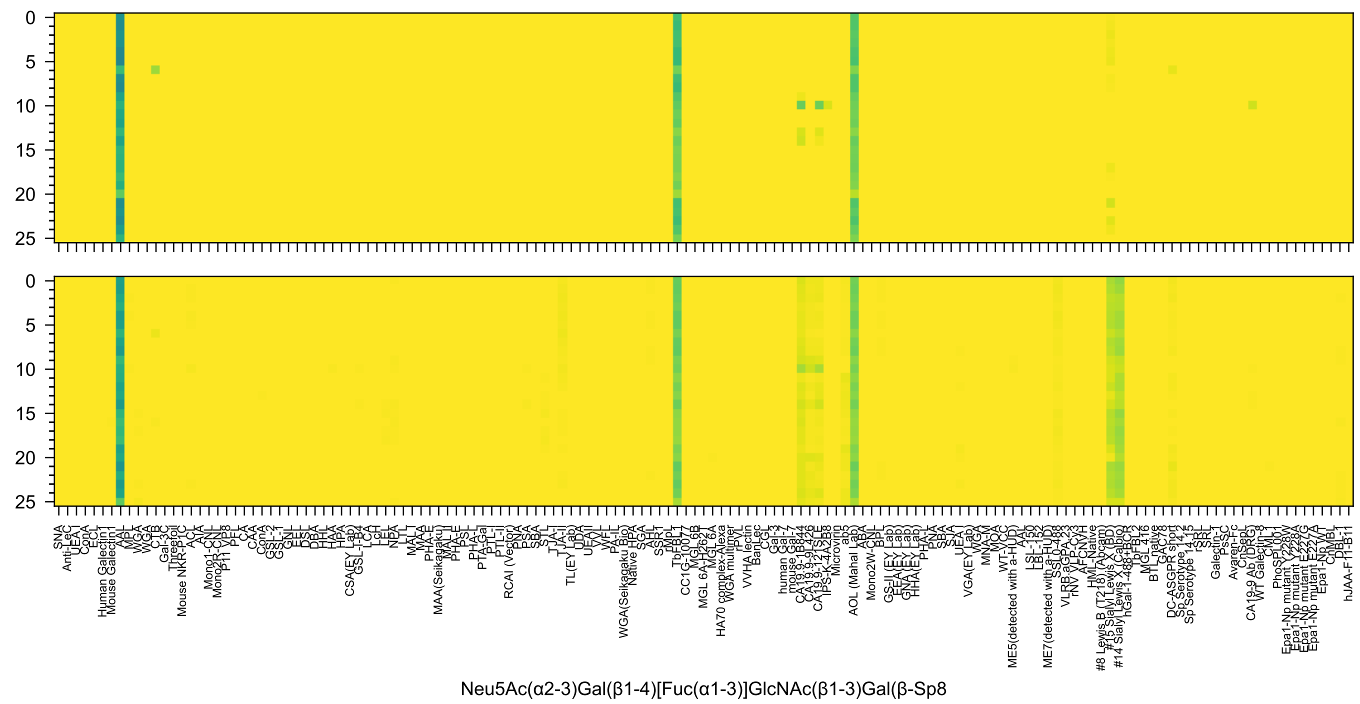
Neu5Ac(α2-3)Gal(β1-4)[Fuc(α1-3)]GlcNAc(β1-3)Gal(β-Sp8

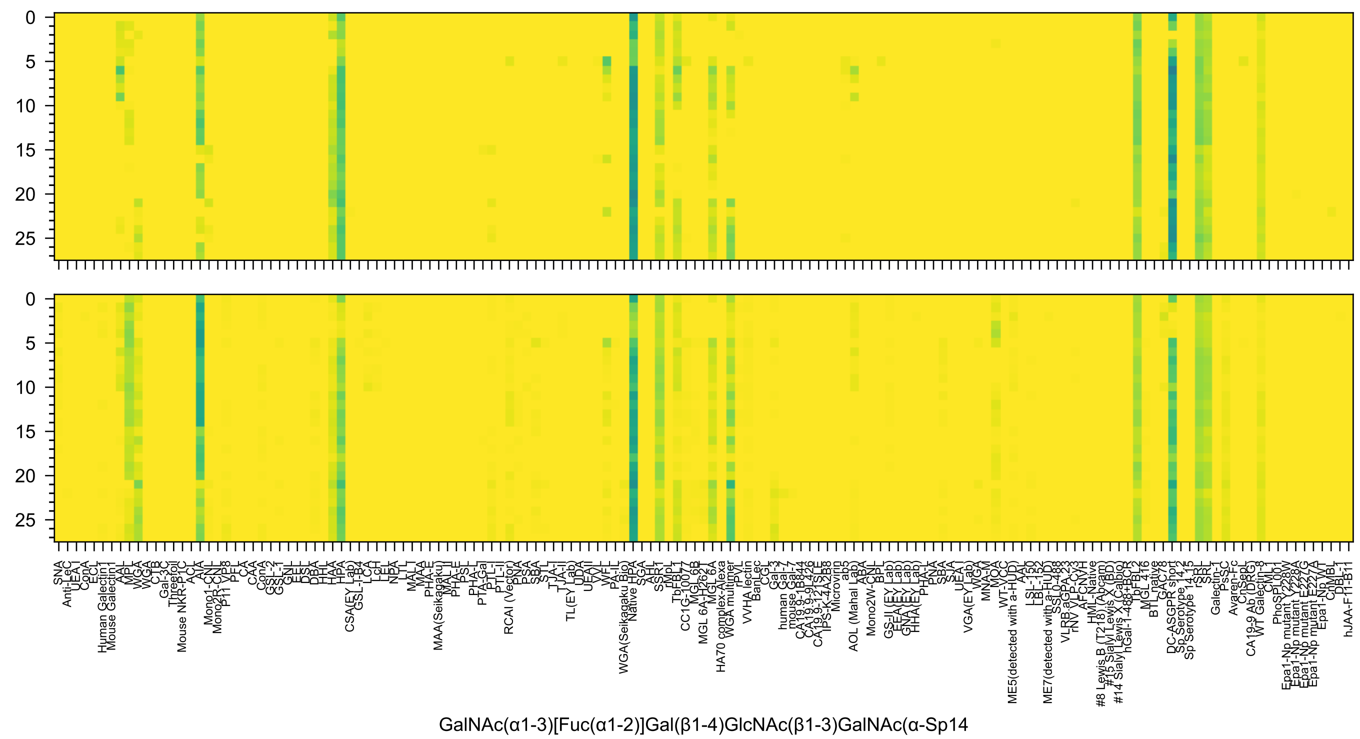
GalNAc(α1-3)[Fuc(α1-2)]Gal(β1-4)GlcNAc(β1-3)GalNAc(α-Sp14

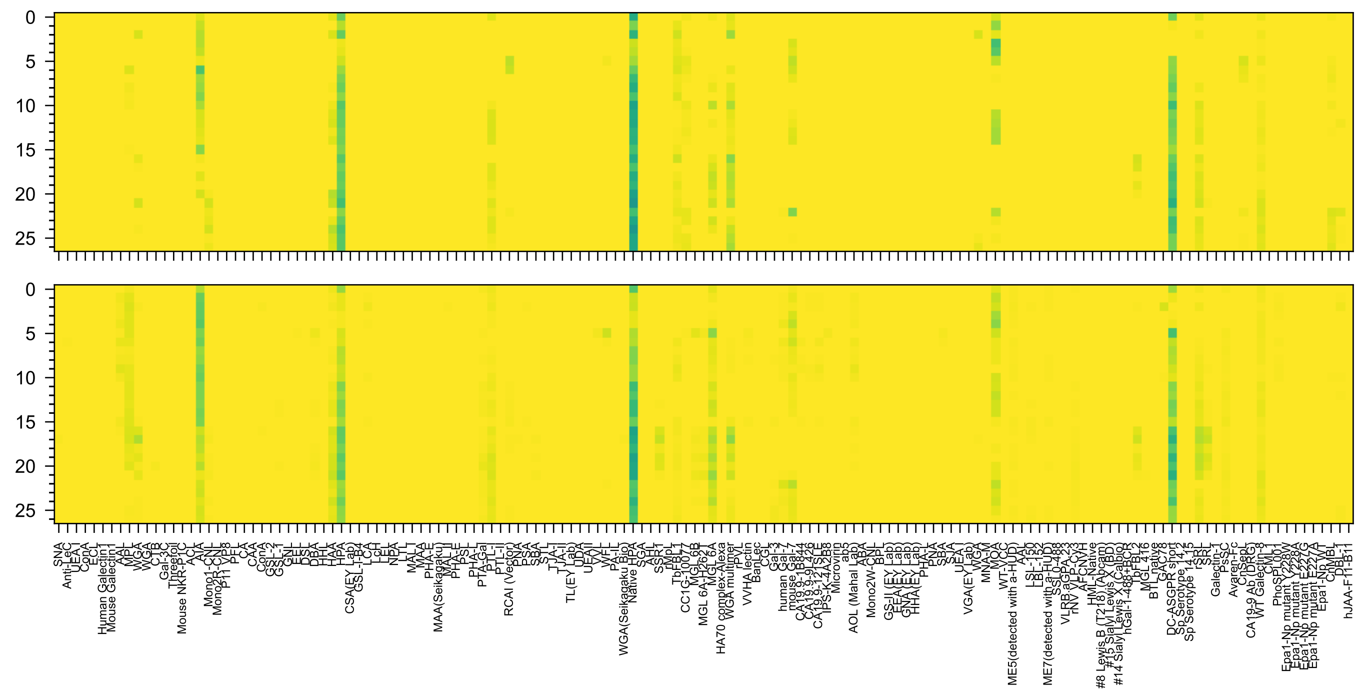

GalNAc(α1-3)[Fuc(α1-2)]Gal(β1-3)GlcNAc(β1-3)GalNAc(-Sp14

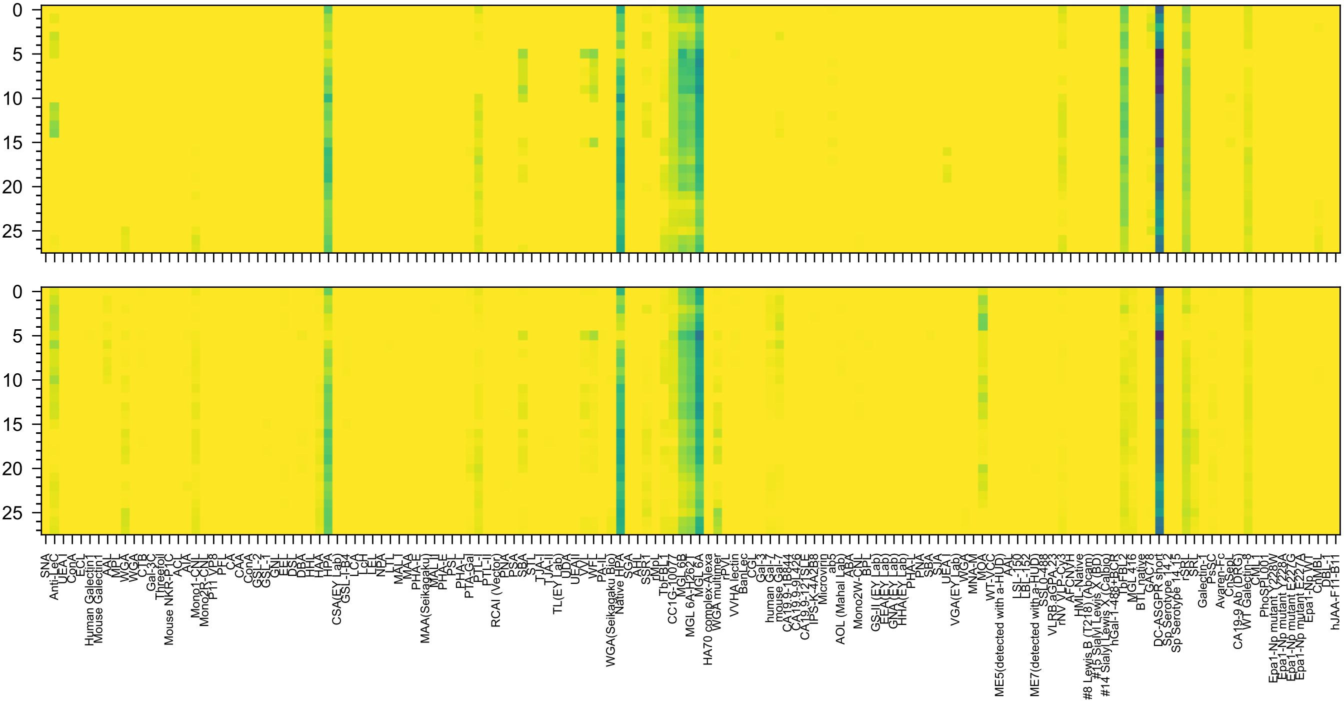

GalNAc(α1-3)[Fuc(α1-2)]Gal(β1-3)GlcNAc(β1-6)GalNAc(α-Sp14

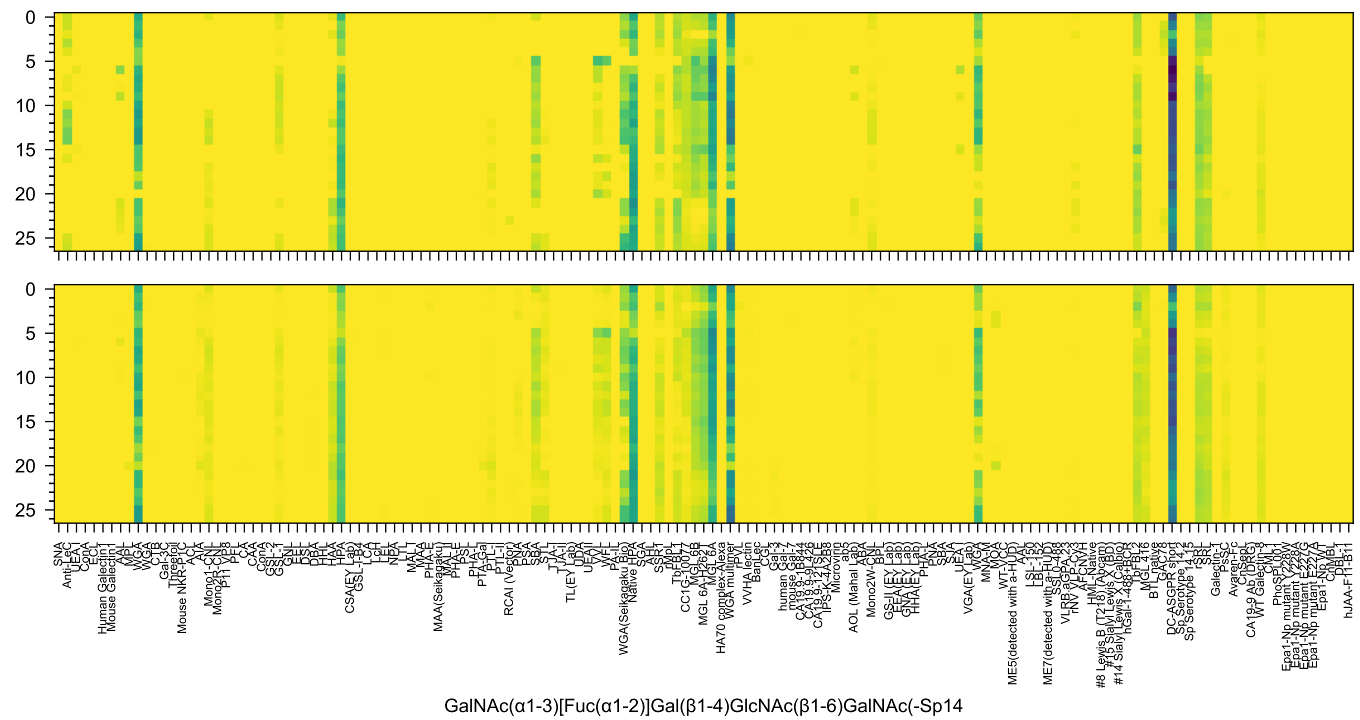
GalNAc(α1-3)[Fuc(α1-2)]Gal(β1-4)GlcNAc(β1-6)GalNAc(-Sp14

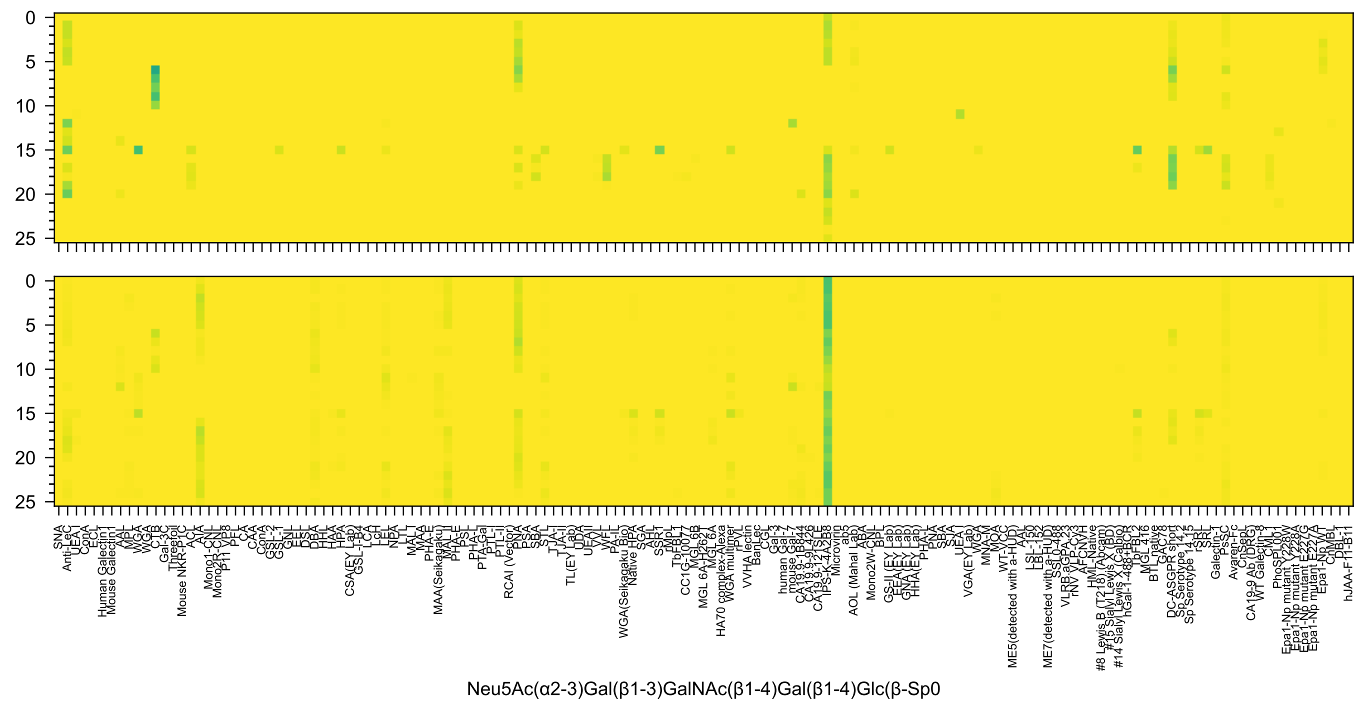

0
5
10
15
20
25
SNA
Anti-LeC
UEA-I
ConA
ECL
Human Galectin1
Mouse Galectin1
AAL
MPL
WGA
WGA
CTB
Gal-3C
Threefoil
Mouse NKR-P1C
ACL
AIA
Mono1-CNL
Mono2R-CNL
P11 VP8
PFL
CA
CAA
ConA
GSL-2
GSL-1
GNL
EEL
DSL
DBA
HHL
HAA
HPA
CSA(EY Lab)
GSL-I-B4
LCA
LcH
LEL
NPA
LTL
MAL-I
MAA
PHA-E
MAA(Seikagaku)
MAL-I
PHA-E
PSL
PHA-L
PTA-Gal
PTL-I
PTL-II
RCAI (Vector)
PNA
PSA
SBA
STL
TJA-I
TJA-II
TL(EY Lab)
UDA
UEA-II
VVL
WFL
PA-IL
WGA(Seikagaku Bio)
Native HPA
SGA
AHL
SSR1
rMpL
TbFBL1
CC1G-10077
MGL 6B
MGL 6A-H262T
MGL 6A
HA70 complex-Alexa
WGA multimer
rPVL
VVHA lectin
BanLec
CGL
Gal-3
Gal-7
human Gal-7
mouse Gal-7
CA19.9-1B844
CA19.9-9L426
CA19.9-121SLE
IPS-K-4A2B8
Microvirin
ab5
AOL (Mahal Lab)
ABA
Mono2W-CNL
BPL
GS-II (EY Lab)
EEA (EY Lab)
GNA (EY Lab)
HHA (EY Lab)
PHA-L
PNA
SBA
SJA
UEA-I
VGA(EY Lab)
WGA
MNA-M
MOA
WT-VCC
ME5(detected with a-HUD)
AAL
LSL-150
LBL-152
ME7(detected with a-HUD)
SSL0-488
VLRB.aGPA.23
rNV VLP-Cy3
AFCNVH
HML-Native
#8 Lewis B (T218) (Abcam)
#15 Sialyl Lewis X (BD)
#14 Sialyl Lewis X (Calbio)
hGal-1-488+BCR
TbFBL2
MGL 416
BTL native
GAC78
DC-ASGPR short
Sp Serotype 14.2
Sp Serotype 14.15
rSRL
SRL
Galectin-1
PsSC
Avaren-Fc
ChSepL
CA19-9 Ab (DRG)
WT Galectin-8
CML1
PhoSPL001
Epa1-Np mutant Y228W
Epa1-Np mutant Y228A
Epa1-Np mutant E227G
Epa1-Np mutant E227A
Epa1-Np WT
ChMBL
DBL-1
hJAA-F11-B11
Neu5Ac(α2-3)Gal(β1-3)GalNAc(β1-4)Gal(β1-4)Glc(β-Sp0

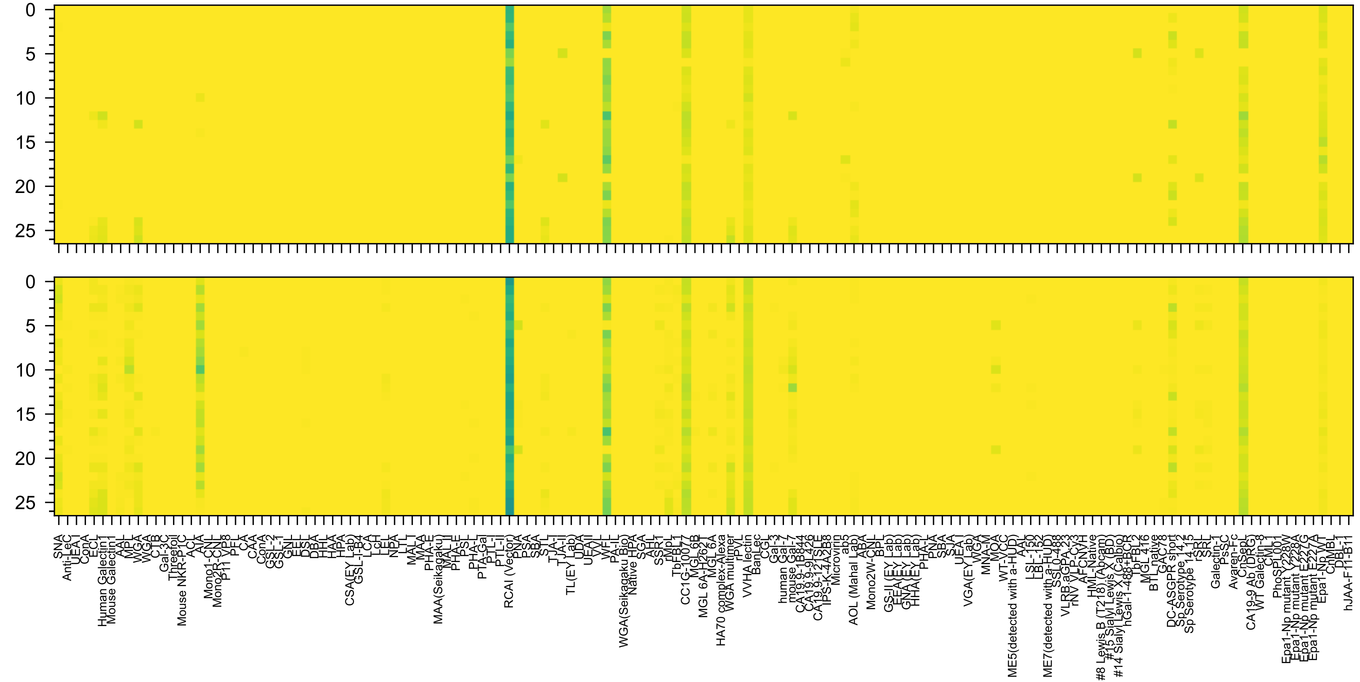

Gal(β1-4)GlcNAc(β1-6)[Gal(β1-4)GlcNAc(β1-3)]GalNAc(-Sp14

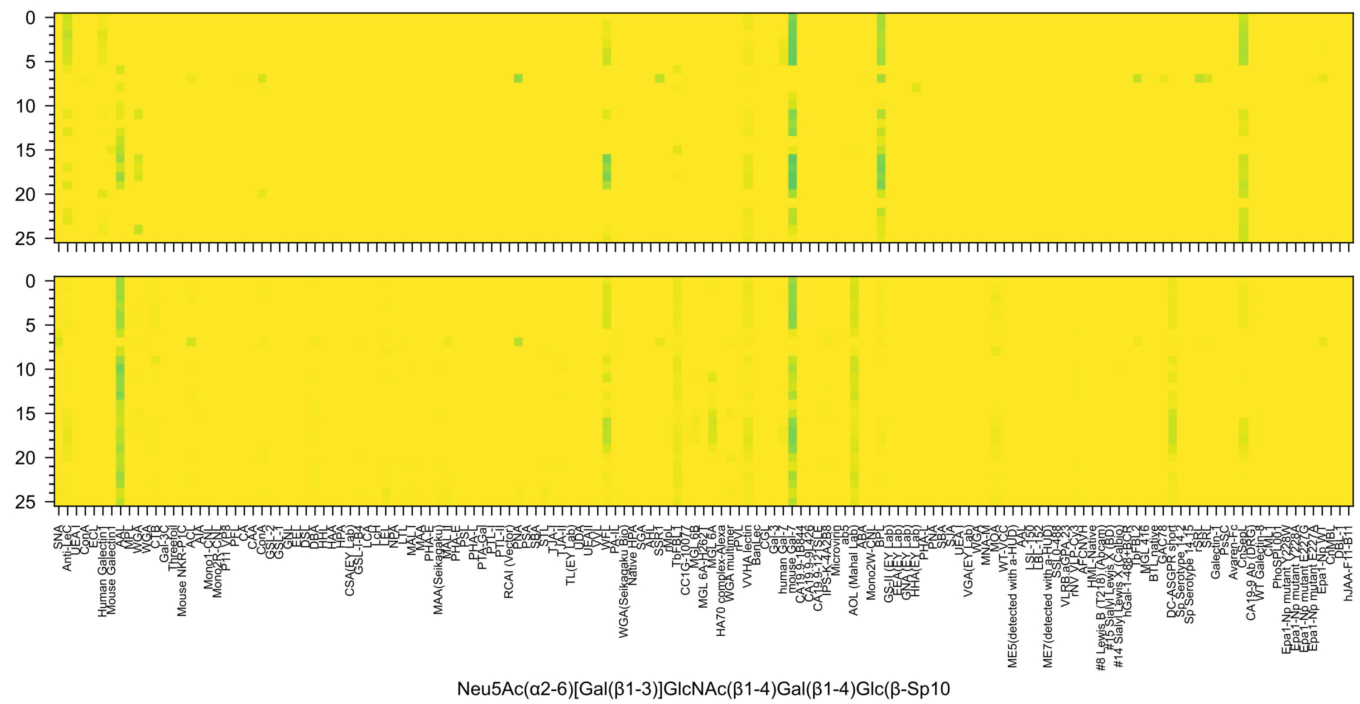

0
5
10
15
20
25
0
5
10
15
20
25
SNA
Anti-LeC
UEA-I
ConA
ECL
Human Galectin1
Mouse Galectin1
AAL
MPL
WGA
WGA
CTB
Gal-3C
Threefoil
Mouse NKR-P1C
ACL
AIA
Mono1-CNL
Mono2R-CNL
P11 VP8
PFL
CA
CAA
ConA
GSL-2
GSL-1
GNL
EEL
DSL
DBA
HHL
HAA
HPA
CSA(EY Lab)
GSL-I-B4
LCA
LcH
LEL
NPA
LTL
MAL-I
MAA
PHA-E
MAA(Seikagaku)
MAL-II
PHA-E
PSL
PHA-L
PTA-Gal
PTL-I
PTL-II
RCAI (Vector)
PNA
PSA
SBA
STL
TJA-I
TJA-II
TL(EY Lab)
UDA
UEA-II
VVL
WFL
PA-IL
WGA(Seikagaku Bio)
Native HPA
SGA
AAL
SSR-1
rMpL
TbFBL1
CC1G-10077
MGL 6B
MGL 6A-H262T
MGL 6A
HA70 complex-Alexa
WGA multimer
rPVL
VVHA lectin
BanLec
CGL
Gal-3
human Gal-7
mouse Gal-7
CA19.9-1B844
CA19.9-9L426
CA19.9-121SLE
IPS-K-4A2B8
Microvirin
ab5
AOL (Mahal Lab)
ABA
Mono2W-CNL
BPL
GS-II (EY Lab)
EEA (EY Lab)
GNA (EY Lab)
HHA (EY Lab)
PHA-L
PNA
SBA
SJA
UEA-I
VGA(EY Lab)
WGA
MNA-M
MOA
WT-VCC
ME5(detected with a-HUD)
AAL
LSL-150
LBL-152
ME7(detected with a-HUD)
SSL0-488
VLRB.aGPA.23
rNV VLP-Cy3
AFCNVH
HML-Native
#8 Lewis B (T218) (Abcam)
#15 Sialyl Lewis X (BD)
#14 Sialyl Lewis X (Calbio)
hGal-1-488+BCR
TbFBL2
MGL 416
BTL native
GAC78
DC-ASGPR short
Sp Serotype 14.2
Sp Serotype 14.15
rSRL
SRL
Galectin-1
PsSC
Avaren-Fc
CnSepL
CA19-9 Ab (DRG)
WT Galectin-8
CML
PhoSL001
Epa1-Np mutant Y228W
Epa1-Np mutant Y228A
Epa1-Np mutant E227G
Epa1-Np mutant E227A
Epa1-Np WT
ChMBL
DBL-1
hJAA-F11-B11
Neu5Ac(α2-6)[Gal(β1-3)]GlcNAc(β1-4)Gal(β1-4)Glc(β-Sp10

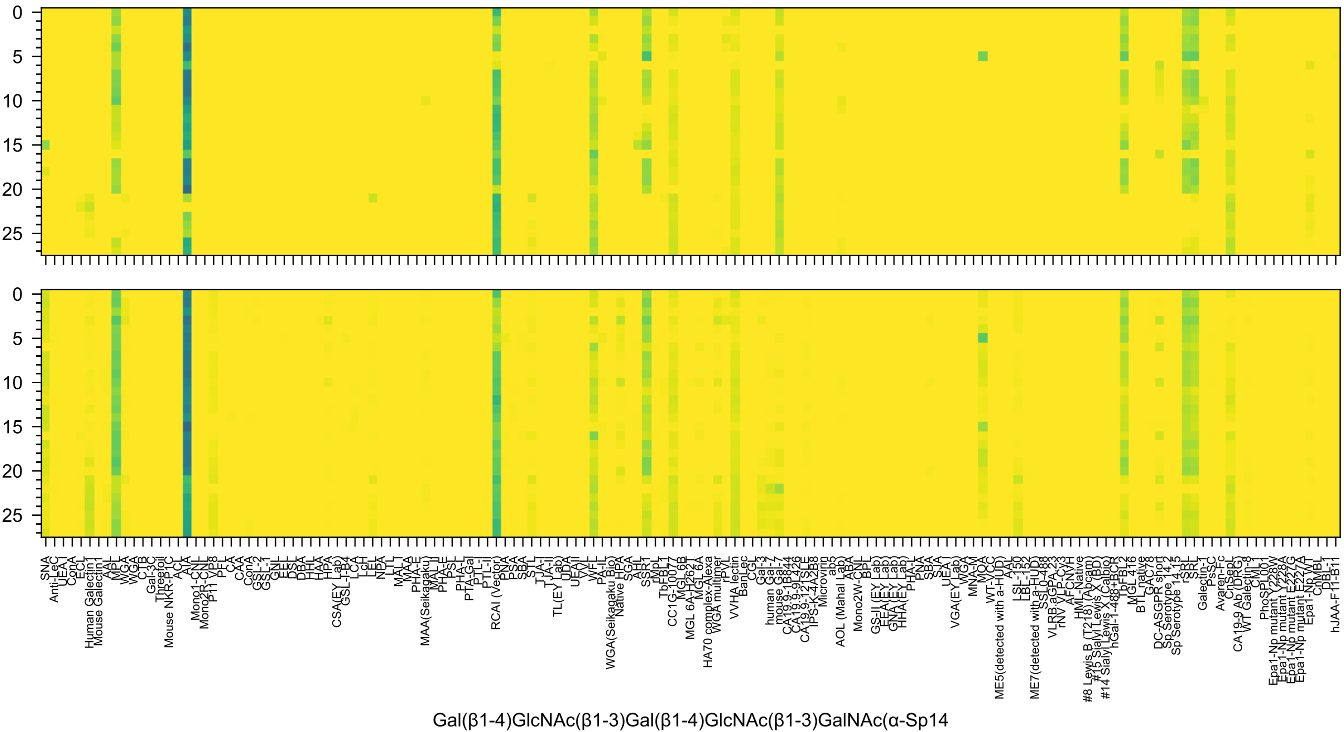
Gal(β1-4)GlcNAc(β1-3)Gal(β1-4)GlcNAc(β1-3)GalNAc(α-Sp14

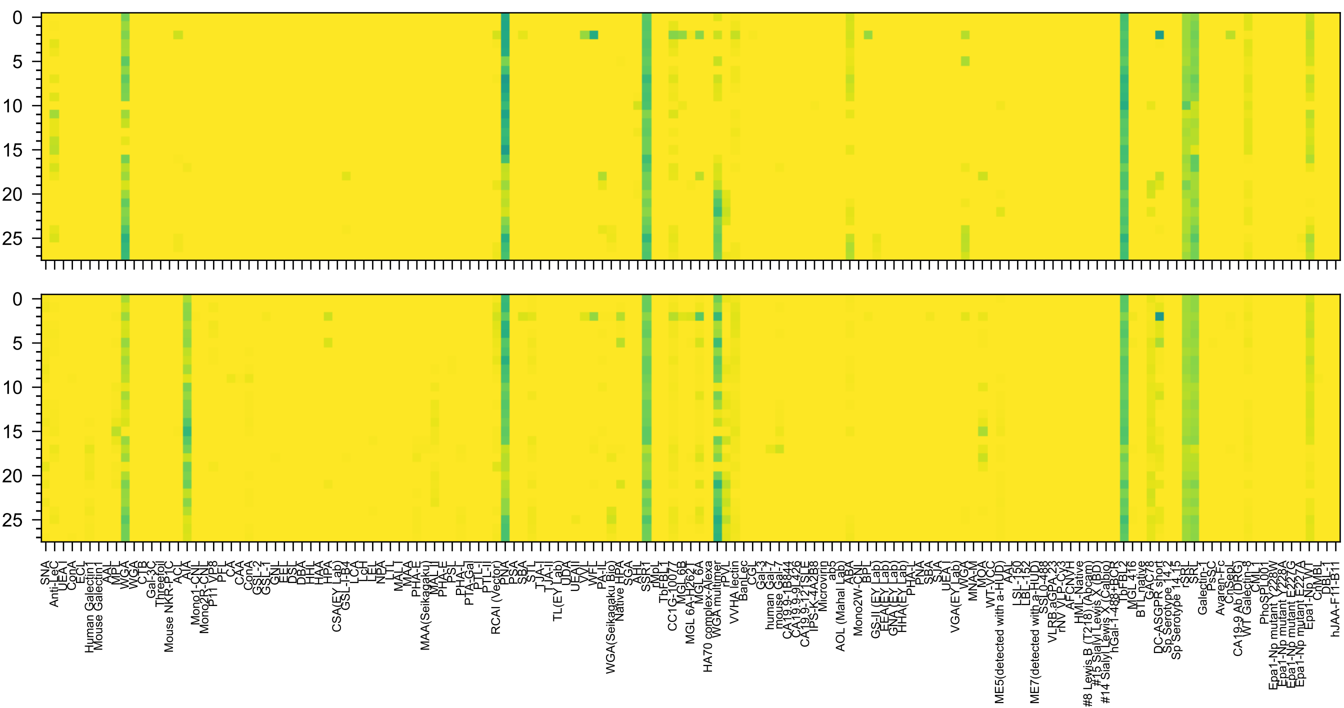

GlcNAc(β1-3)Gal(β1-4)GlcNAc(β1-6)[Gal(β1-3)]GalNAc(α-Sp14

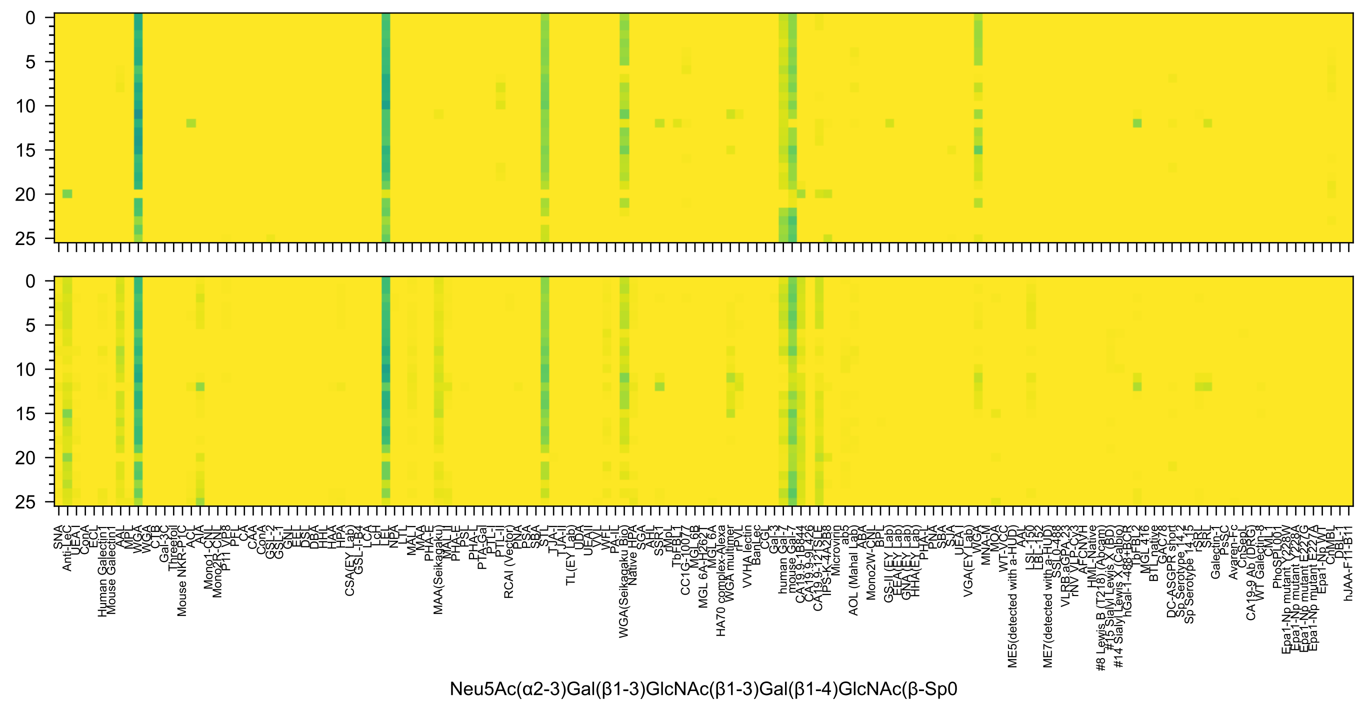

0
5
10
15
20
25
SNA
Anti-LeC
UEA-I
ConA
ECL
Human Galectin1
Mouse Galectin1
AAL
MPL
WGA
WGA
CTB
Gal-3C
Threefoil
Mouse NKR-P1C
ACL
AIA
Mono1-CNL
Mono2R-CNL
P11 VP8
PFL
CA
CAA
ConA
GSL-2
GSL-1
GNL
EEL
DSL
DBA
HHL
HAA
HPA
CSA(EY Lab)
GSL-I-B4
LCA
LcH
LEL
NPA
LTL
MAL-I
MAA
PHA-E
MAA(Seikagaku)
MAL-II
PHA-E
PSL
PHA-L
PTA-Gal
PTL-I
PTL-II
RCAI (Vector)
PNA
PSA
SBA
STL
TJA-I
TJA-II
TL(EY Lab)
UDA
UEA-II
VVL
WFL
PA-IL
PA-IIL
WGA(Seikagaku Bio)
Native HPA
SGA
AHL
SSR1
rMpL
TbFBL1
CC1G-10077
MGL 6B
MGL 6A-H262T
MGL 6A
HA70 complex-Alexa
WGA multimer
rPVL
VVHA lectin
BanLec
CGL
Gal-3
human Gal-7
mouse Gal-7
CA19.9-1B844
CA19.9-9L426
CA19.9-121SLE
IPS-K-4A2B8
Microvirin
ab5
AOL (Mahal Lab)
ABA
Mono2W-CNL
BPL
GS-II (EY Lab)
EEA (EY Lab)
GNA (EY Lab)
HHA (EY Lab)
PHA-L
PNA
SBA
SJA
UEA-I
VGA(EY Lab)
WGA
MNA-M
MOA
WT-VCC
ME5(detected with a-HUD)
AAL
LSL-150
LBL-152
ME7(detected with a-HUD)
SSL0-488
VLRB.aGPA.23
rNV VLP-Cy3
AFCNVH
HML-Native
#8 Lewis B (T218) (Abcam)
#15 Sialyl Lewis X (BD)
#14 Sialyl Lewis X (Calbio)
hGal-1-488+BCR
TbFBL2
MGL 416
BTL native
GAC78
DC-ASGPR short
Sp Serotype 14.2
Sp Serotype 14.15
rSRL
SRL
Galectin-1
PsSC
Avaren-Fc
ChSepL
CA19-9 Ab (DRG)
WT Galectin-8
CML
PhoSL001
Epa1-Np mutant Y228W
Epa1-Np mutant Y228A
Epa1-Np mutant E227G
Epa1-Np mutant E227A
Epa1-Np WT
ChMBL
DBL-1
hJAA-F11-B11
Neu5Ac(α2-3)Gal(β1-3)GlcNAc(β1-3)Gal(β1-4)GlcNAc(β-Sp0

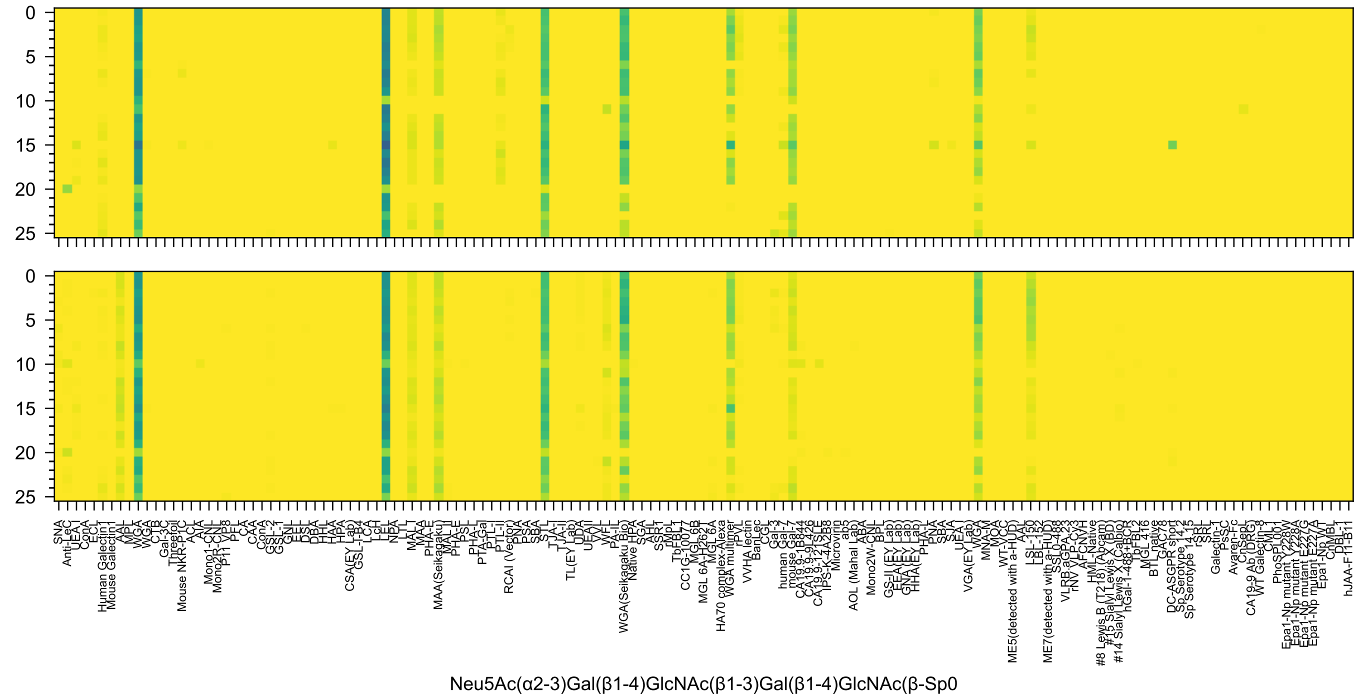

0
5
10
15
20
25
SNA
Anti-LeC
UEA-I
ConA
ECL
Human Galectin1
Mouse Galectin1
AAL
MPL
WGA
WGA
CTB
Gal-3C
Threefoil
Mouse NKR-P1C
ACL
AIA
Mono1-CNL
Mono2R-CNL
P11 VP8
PFL
CA
CAA
ConA
GSL-2
GSL-1
GNL
EEL
DSL
DBA
HHL
HAA
HPA
CSA(EY Lab)
GSL-I-B4
LCA
LcH
LEL
NPA
LTL
MAL-I
MAA
PHA-E
MAA(Seikagaku)
MAL-II
PHA-E
PSL
PHA-L
PTA-Gal
PTL-I
PTL-II
RCAI (Vector)
PNA
PSA
SBA
STL
TJA-I
TJA-II
TL(EY Lab)
UDA
UEA-II
VVL
WFL
PA-IL
WGA(Seikagaku Bio)
Native HPA
SGA
AHL
SSR1
rMpL
TbFBL1
CC1G-10077
MGL 6B
MGL 6A-H262T
MGL 6A
HA70 complex-Alexa
WGA multimer
rPVL
VVHA lectin
BanLec
CGL
Gal-3
human Gal-7
mouse Gal-7
CA19.9-1B844
CA19.9-9L426
CA19.9-121SLE
IPS-K-4A2B8
Microvirin
ab5
AOL (Mahal Lab)
ABA
Mono2W-CNL
BPL
GS-II (EY Lab)
EEA (EY Lab)
GNA (EY Lab)
HHA (EY Lab)
PHA-L
PNA
SBA
SJA
UEA-I
VGA(EY Lab)
WGA
MNA-M
MOA
WT-VCC
ME5(detected with a-HUD)
AAL
LSL-150
LBL-152
ME7(detected with a-HUD)
SSL0-488
VLRB.aGPA.23
rNV VLP-Cy3
AFCNYH
HML-Native
#8 Lewis B (T218) (Abcam)
#15 Sialyl Lewis X (BD)
#14 Sialyl Lewis X (Calbio)
hGal-1-488+BCR
TbFBL2
MGL 416
BTL native
GAC78
DC-ASGPR short
Sp Serotype 14.2
Sp Serotype 14.15
rSRL
SRL
Galectin-1
PsSC
Avaren-Fc
CnSepL
CA19-9 Ab (DRG)
WT Galectin-8
CML1
PhoSPL001
Epa1-Np mutant Y228W
Epa1-Np mutant Y228A
Epa1-Np mutant E227G
Epa1-Np mutant E227A
Epa1-Np WT
ChMBL
DBL-1
hJAA-F11-B11
Neu5Ac(α2-3)Gal(β1-4)GlcNAc(β1-3)Gal(β1-4)GlcNAc(β-Sp0

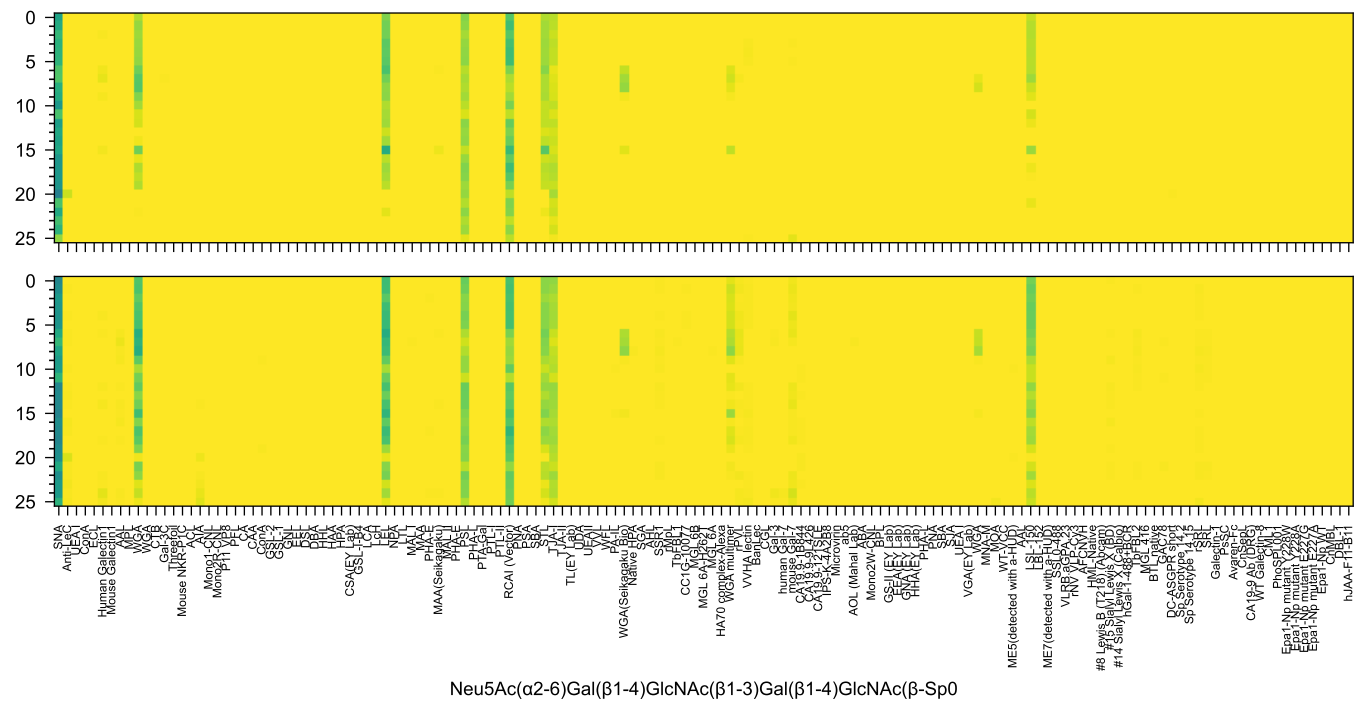

0
5
10
15
20
25
SNA
Anti-LeC
UEA I
ConA
ECL
Human Galectin1
Mouse Galectin1
AAL
MPL
WGA
WGA
CTB
Gal-3C
Threefoil
Mouse NKR-P1C
ACL
AIA
Mono1-CNL
Mono2R-CNL
P11 VP8
PFL
CA
CAA
ConA
GSL-2
GSL-1
GNL
EEL
DSL
DBA
HHL
HAA
HPA
CSA(EY Lab)
GSL-I-B4
LCA
LcH
LEL
NPA
LTL
MAL I
MAA
PHA-E
MAA(Seikagaku)
MAL II
PHA-E
PSL
PHA-L
PTA-Gal
PTL-I
PTL-II
RCAI (Vector)
PNA
PSA
SBA
STL
TJA-I
TJA-II
TL(EY Lab)
UDA
UEAII
VVL
WFL
PA-IL
WGA(Seikagaku Bio)
Native HPA
SGA
AHL
SSR1
rMpL
TbFBL1
CC1G-10077
MGL 6B
MGL 6A-H262T
MGL 6A
HA70 complex-Alexa
WGA multimer
rPVL
VVHA lectin
BanLec
CGL
Gal-3
human Gal-7
mouse Gal-7
CA19.9-1B844
CA19.9-9L426
CA19.9-121SLE
IPS-K-4A2B8
Microvirin
ab5
AOL (Mahal Lab)
ABA
Mono2W-CNL
BPL
GS-II (EY Lab)
EEA (EY Lab)
GNA (EY Lab)
HHA (EY Lab)
PHA-L
PNA
SBA
SJA
UEA I
VGA(EY Lab)
WGA
MNA-M
MOA
WT-VCC
ME5(detected with a-HUD)
AAL
LSL-150
LBL-152
ME7(detected with a-HUD)
SSL0-488
VLRB.aGPA.23
rNV VLP-Cy3
AFCNVH
HML-Native
#8 Lewis B (T218) (Abcam)
#15 Sialyl Lewis X (BD)
#14 Sialyl Lewis X (Calbio)
hGal-1-488+BCR
TbFBL2
MGL 416
BTL native
GAC78
DC-ASGPR short
Sp Serotype 14.2
Sp Serotype 14.15
rSRL
SRL
Galectin-1
PsSC
Avaren-Fc
CnSepL
CA19-9 Ab (DRG)
WT Galectin-8
CML
PhoSL001
Epa1-Np mutant Y228W
Epa1-Np mutant Y228A
Epa1-Np mutant E227G
Epa1-Np mutant E227A
Epa1-Np WT
ChMBL
DBL-1
hJAA-F11-B11
Neu5Ac(α2-6)Gal(β1-4)GlcNAc(β1-3)Gal(β1-4)GlcNAc(β-Sp0

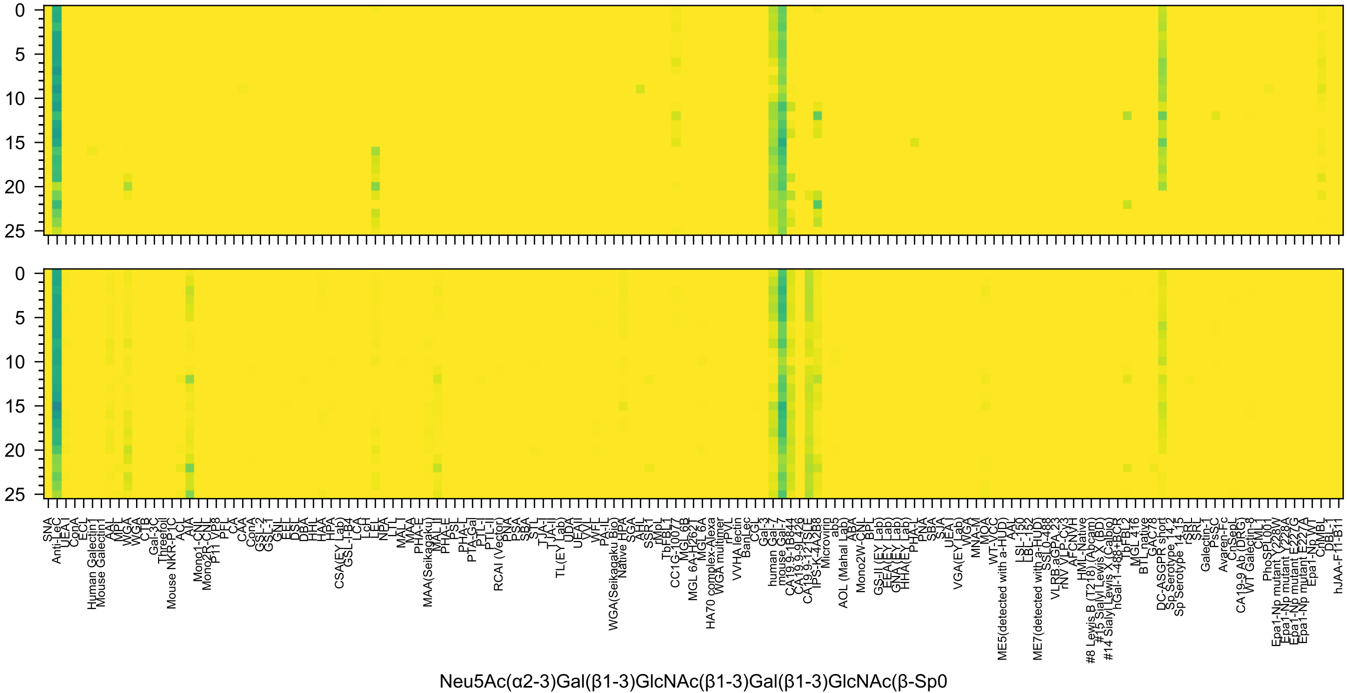
0
5
10
15
20
25
0
5
10
15
20
25
SNA
Anti-LeC
UEA I
ConA
ECL
Human Galectin1
Mouse Galectin1
AAL
MPL
WGA
WGA
CTB
Gal-3C
Threefoil
Mouse NKR-P1C
ACL
AIA
Mono1-CNL
Mono2R-CNL
P11 VP8
PFL
CA
CAA
ConA
GSL-2
GSL-1
GNL
EEL
DSL
DBA
HHL
HAA
HPA
CSA(EY Lab)
GSL-I-B4
LCA
LcH
LEL
NPA
LTL
MAL I
MAA
PHA-E
MAA(Seikagaku)
MAL II
PHA-E
PSL
PHA-L
PTA-Gal
PTL-I
PTL-II
RCAI (Vector)
PNA
PSA
SBA
STL
TJA-I
TJA-II
TL(EY Lab)
UDA
UEA II
VVL
WFL
PA-IL
WGA(Seikagaku Bio)
Native HPA
SGA
AHL
SSR1
rMpL
TbFBL1
CC1G-10077
MGL 6B
MGL 6A-H262T
MGL 6A
HA70 complex-Alexa
WGA multimer
rPVL
VVHA lectin
BanLec
CGL
Gal-3
human Gal-7
mouse Gal-7
CA19.9-1B844
CA19.9-9L426
CA19.9-121SLE
IPS-K-4A2B8
Microvirin
ab5
AOL (Mahal Lab)
ABA
Mono2W-CNL
BPL
GS-II (EY Lab)
EEA (EY Lab)
GNA (EY Lab)
HHA(EY Lab)
PHA-L
PNA
SBA
SJA
UEA I
VGA(EY Lab)
WGA
MNA-M
MOA
WT-VCC
ME5(detected with a-HUD)
AAL
LSL-150
LBL-152
ME7(detected with a-HUD)
SSL0-488
VLRB.aGPA.23
rNV VLP-Cy3
AFCNVH
HML-Native
#8 Lewis B (T218) (Abcam)
#15 Sialyl Lewis X (BD)
#14 Sialyl Lewis X (Calbio)
hGal-1-488+BCR
TbFBL2
MGL 416
BTL native
GAC78
DC-ASGPR short
Sp Serotype 14.2
Sp Serotype 14.15
rSRL
SRL
Galectin-1
PsSC
Avaren-Fc
CnSepL
CA19-9 Ab (DRG)
WT Galectin-8
CML1
PhoSPL001
Epa1-Np mutant Y228W
Epa1-Np mutant Y228A
Epa1-Np mutant E227G
Epa1-Np mutant E227A
Epa1-Np WT
ChMBL
DBL-1
hJAA-F11-B11
Neu5Ac(α2-3)Gal(β1-3)GlcNAc(β1-3)Gal(β1-3)GlcNAc(β-Sp0

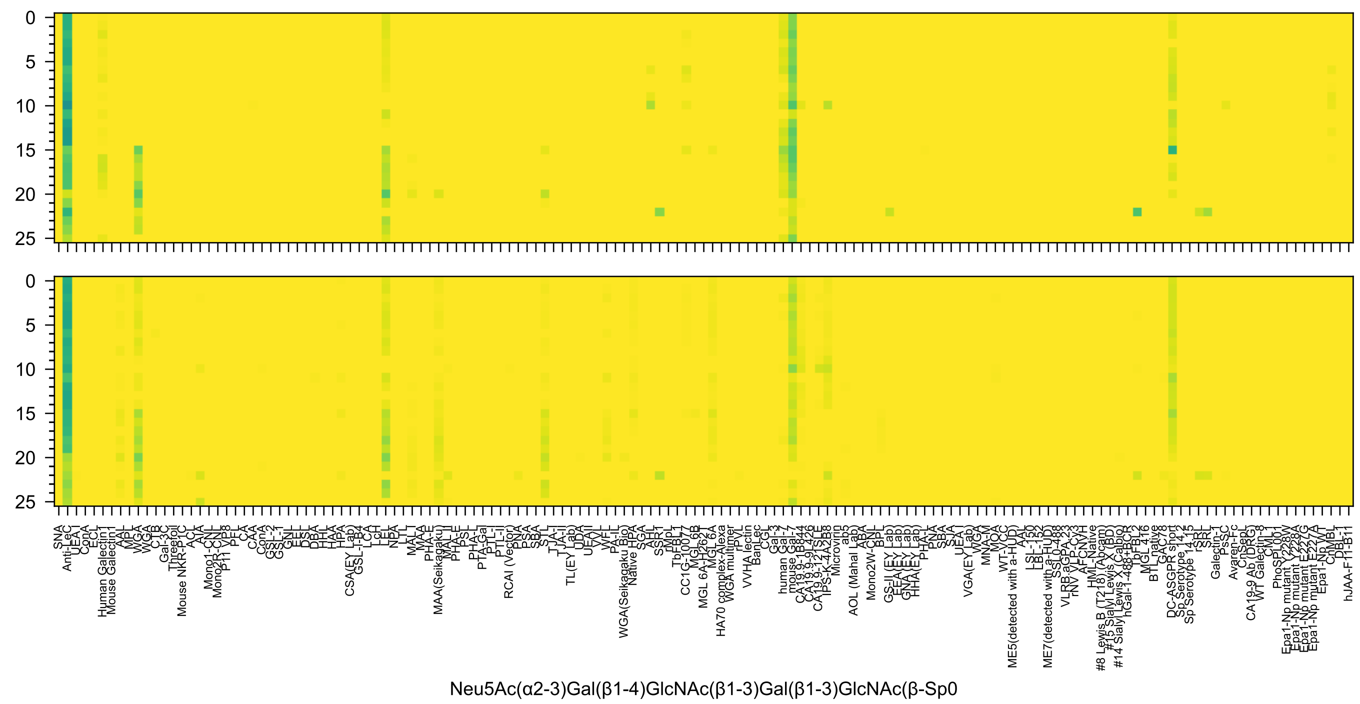
Neu5Ac(α2-3)Gal(β1-4)GlcNAc(β1-3)Gal(β1-3)GlcNAc(β-Sp0)

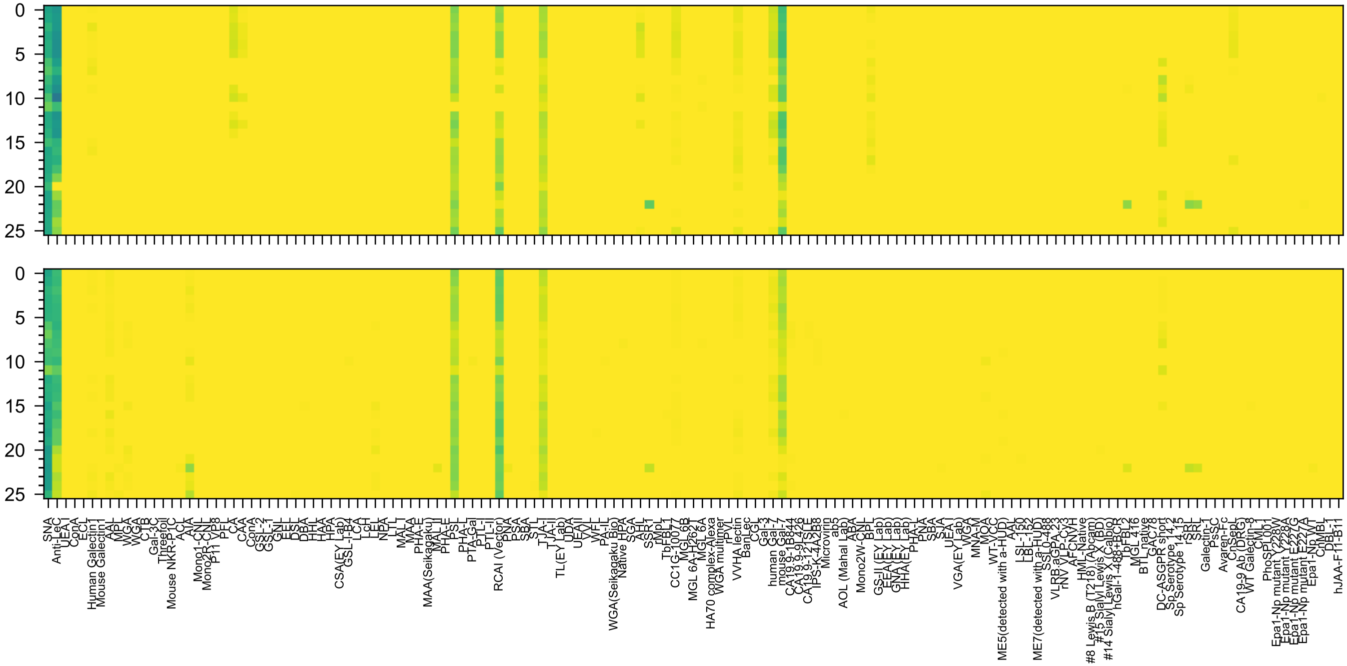

Neu5Ac(α2-6)Gal(β1-4)GlcNAc(β1-3)Gal(β1-3)GlcNAc(β-Sp0

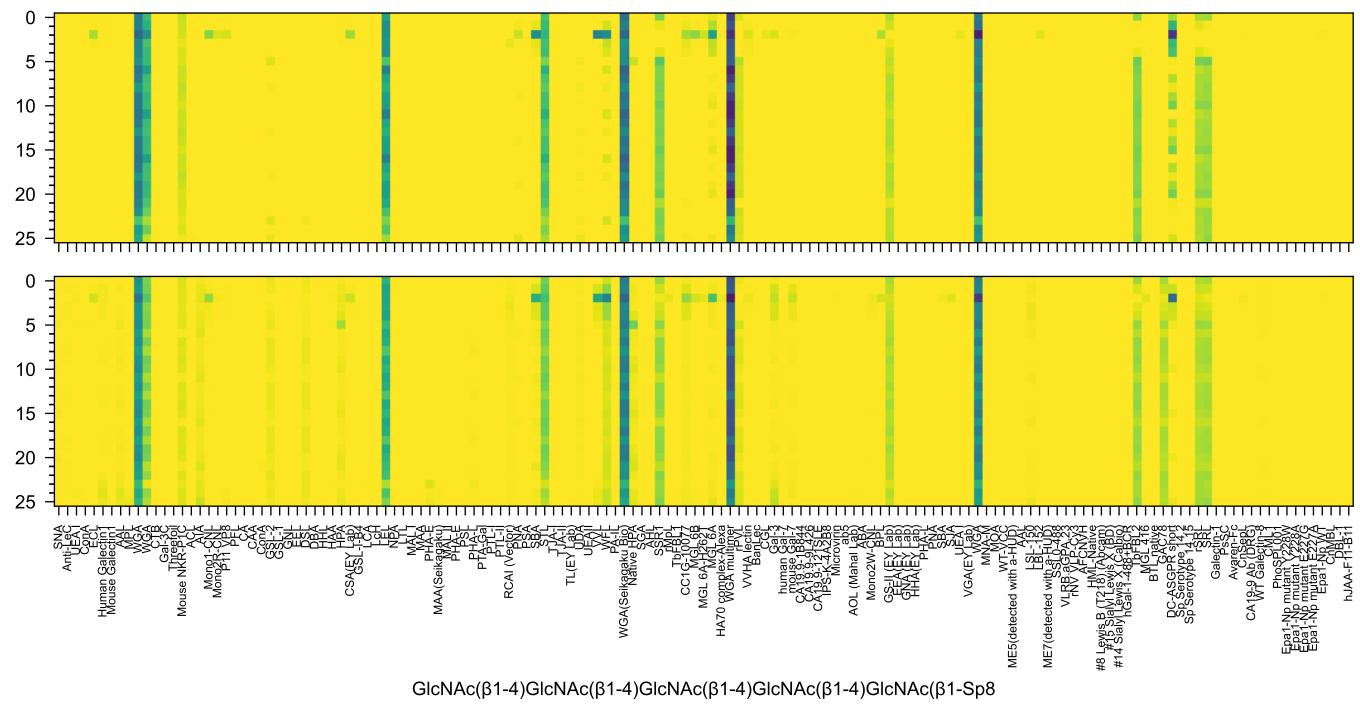
0
5
10
15
20
25
SNA
Anti-LeC
UEA I
ConA
ECL
Human Galectin1
Mouse Galectin1
AAL
MPL
WGA
WGA
CTB
Gal-3C
Threefoil
Mouse NKR-P1C
ACL
AIA
Mono1-CNL
Mono2R-CNL
P11 VP8
PFL
CA
CAA
ConA
GSL-2
GSL-1
GNL
EEL
DSL
DBA
HHL
HAA
HPA
CSA(EY Lab)
GSL-I-B4
LCA
LcH
LEL
NPA
LTL
MAL1
MAA
PHA-E
MAA(Seikagaku)
MAL-I
PHA-E
PSL
PHA-L
PTA-Gal
PTL-I
PTL-II
RCAI (Vector)
PNA
PSA
SBA
STL
TJA-I
TJA-II
TL(EY Lab)
UDA
UEAII
VVL
WFL
PA-IL
WGA(Seikagaku Bio)
Native HPA
SGA
AHL
SSR1
rMpL
TbFBL1
CC1G-10077
MGL 6B
MGL 6A-H262T
MGL 6A
HA70 complex-Alexa
WGA multimer
rPVL
VVHA lectin
BanLec
CGL
Gal-3
human Gal-7
mouse Gal-7
CA19.9-1B844
CA19.9-9L426
CA19.9-121SLE
IPS-K-4A2B8
Microvirin
ab5
AOL (Mahal Lab)
ABA
Mono2W-CNL
BPL
GS-II (EY Lab)
EEA(EY Lab)
GNA(EY Lab)
HHA(EY Lab)
PHA-L
PNA
SBA
SJA
UEA1
VGA(EY Lab)
WGA
MNA-M
MOA
WT-VCC
ME5(detected with a-HUD)
AAL
LSL-150
LBL-152
ME7(detected with a-HUD)
SSL0-488
VLRB.aGPA.23
rNV VLP-Cy3
AFCNVH
HML-Native
#8 Lewis B (T218) (Abcam)
#15 Sialyl Lewis X (BD)
#14 Sialyl Lewis X (Calbio)
hGal-1-488+BCR
TbFBL2
MGL 416
BTL native
GAC78
DC-ASGPR short
Sp Serotype 14.2
Sp Serotype 14.15
rSRL
SRL
Galectin-1
PsSC
Avaren-Fc
CnSepL
CA19-9 Ab (DRG)
WT Galectin-8
CML1
PhoSL001
Epa1-Np mutant Y228W
Epa1-Np mutant Y228A
Epa1-Np mutant E227G
Epa1-Np mutant E227A
Epa1-Np WT
ChMBL
DBL-1
hJAA-F11-B11
GlcNAc(β1-4)GlcNAc(β1-4)GlcNAc(β1-4)GlcNAc(β1-4)GlcNAc(β1-Sp8

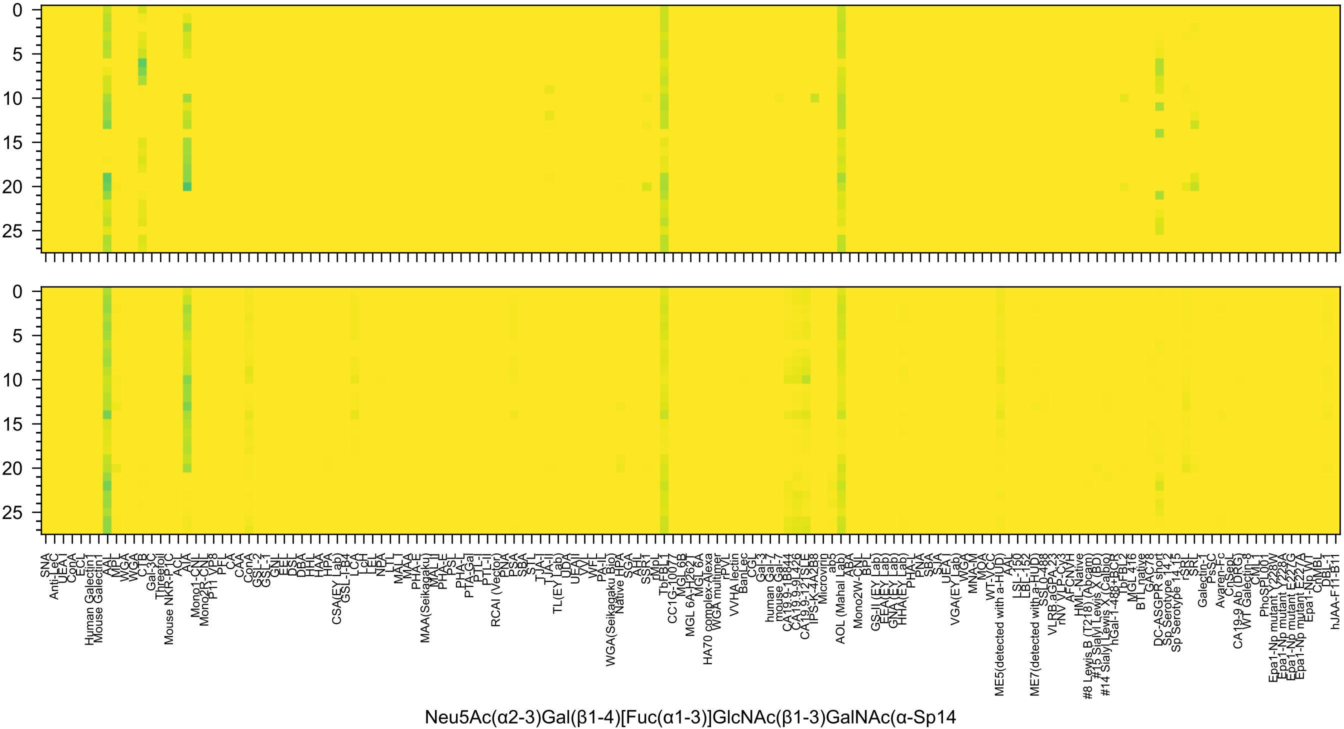

0
5
10
15
20
25
Neu5Ac(α2-3)Gal(β1-4)[Fuc(α1-3)]GlcNAc(β1-3)GalNAc(α-Sp14

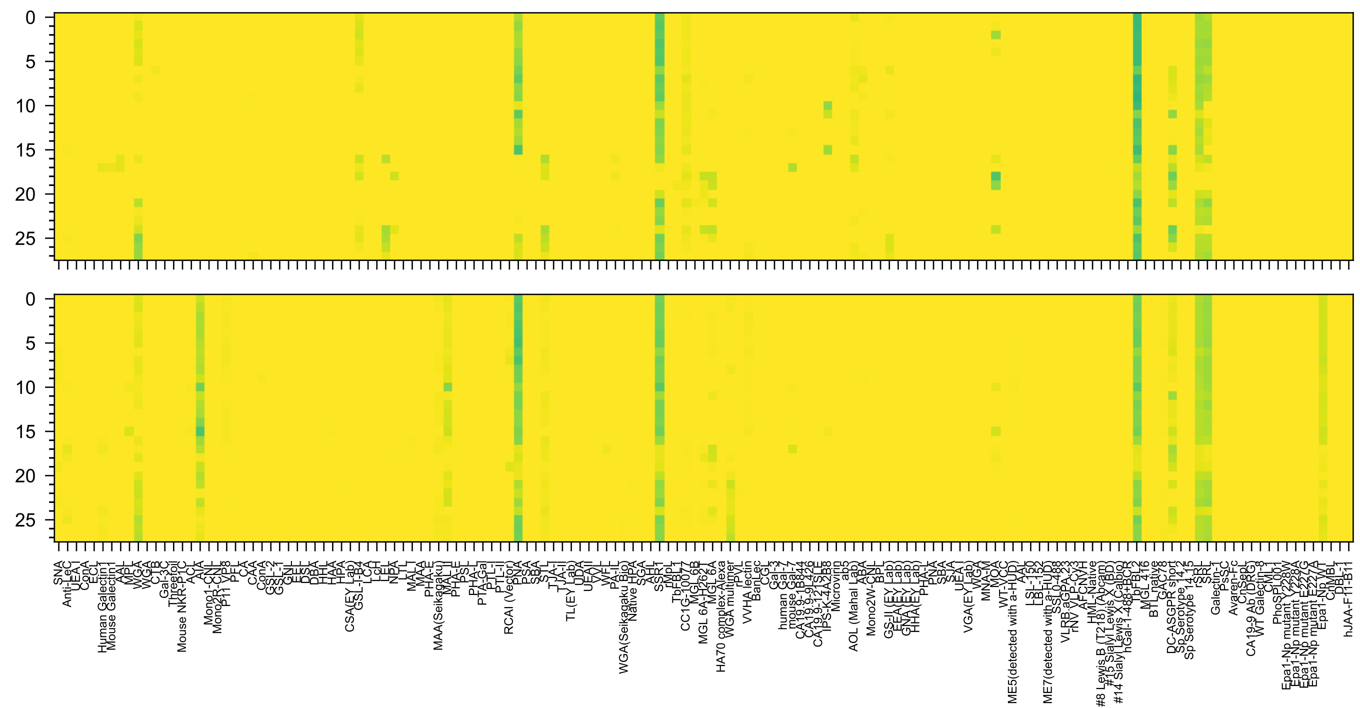

Neu5Ac(α2-3)Gal(β1-4)GlcNAc(β1-6)[Gal(β1-3)]GalNAc(α-Sp14

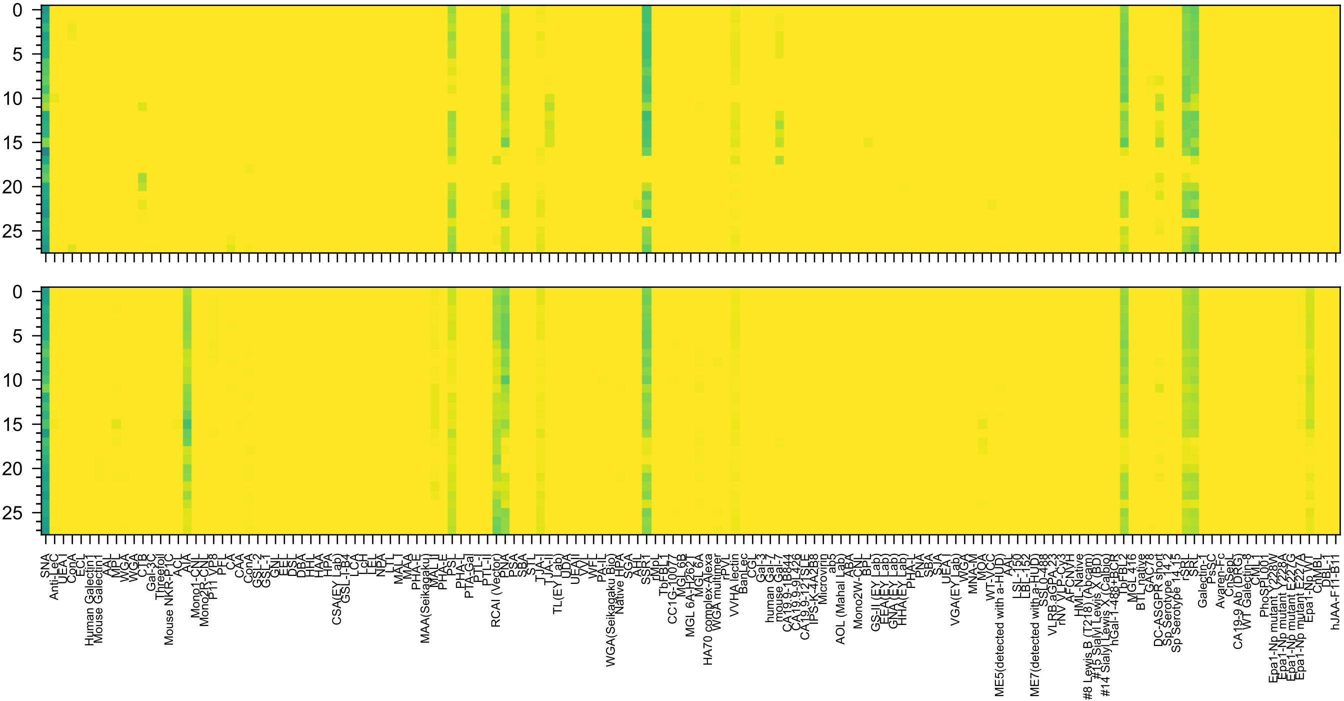

Neu5Ac(α2-6)Gal(β1-4)GlcNAc(β1-6)[Gal(β1-3)]GalNAc(α-Sp14

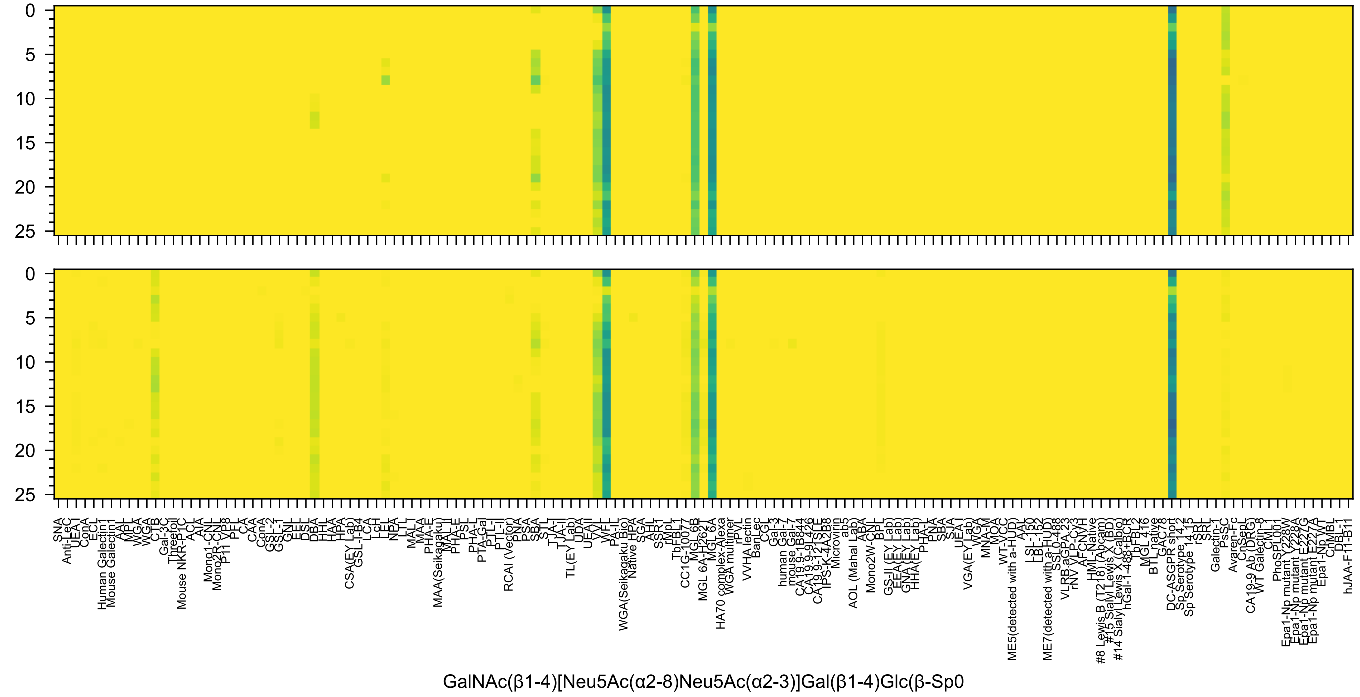
GalNAc(β1-4)[Neu5Ac(α2-8)Neu5Ac(α2-3)]Gal(β1-4)Glc(β-Sp0

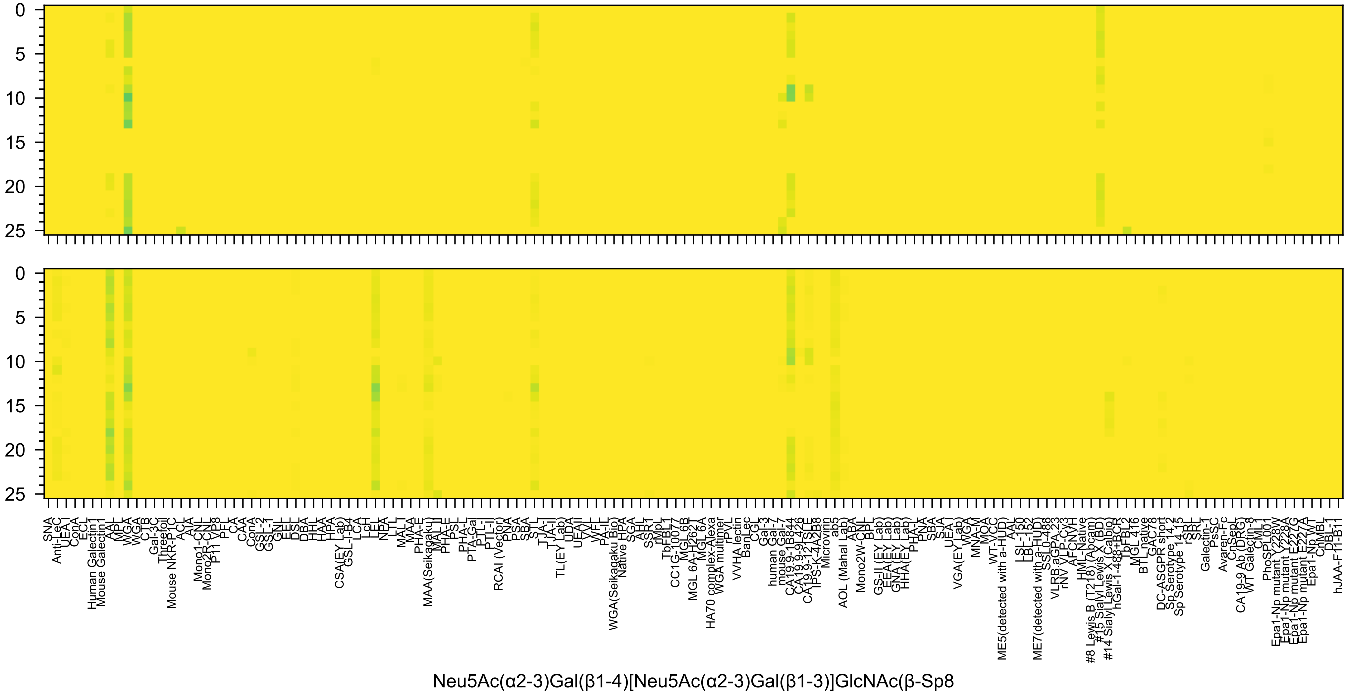
Neu5Ac(α2-3)Gal(β1-4)[Neu5Ac(α2-3)Gal(β1-3)]GlcNAc(β-Sp8

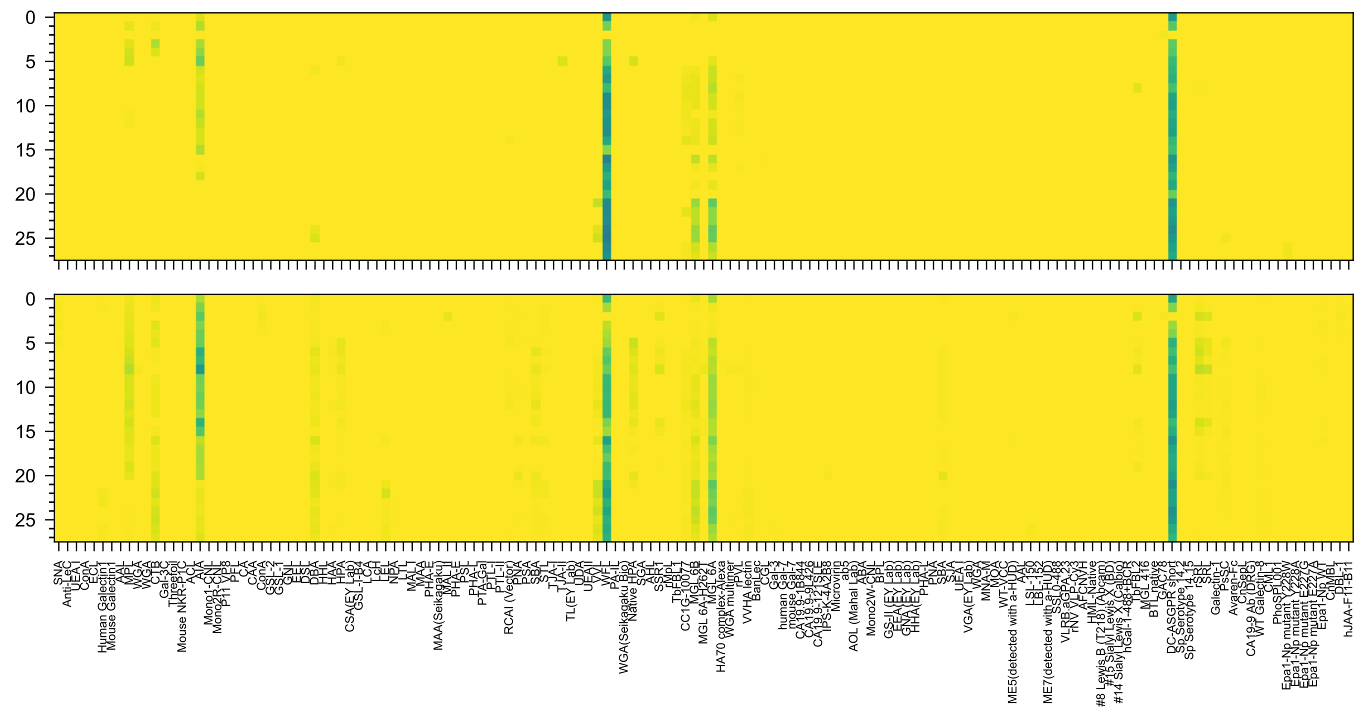
GalNAc(β1-4)[Neu5Ac(α2-3)]Gal(β1-4)GlcNAc(β1-3)GalNAc(α-Sp14

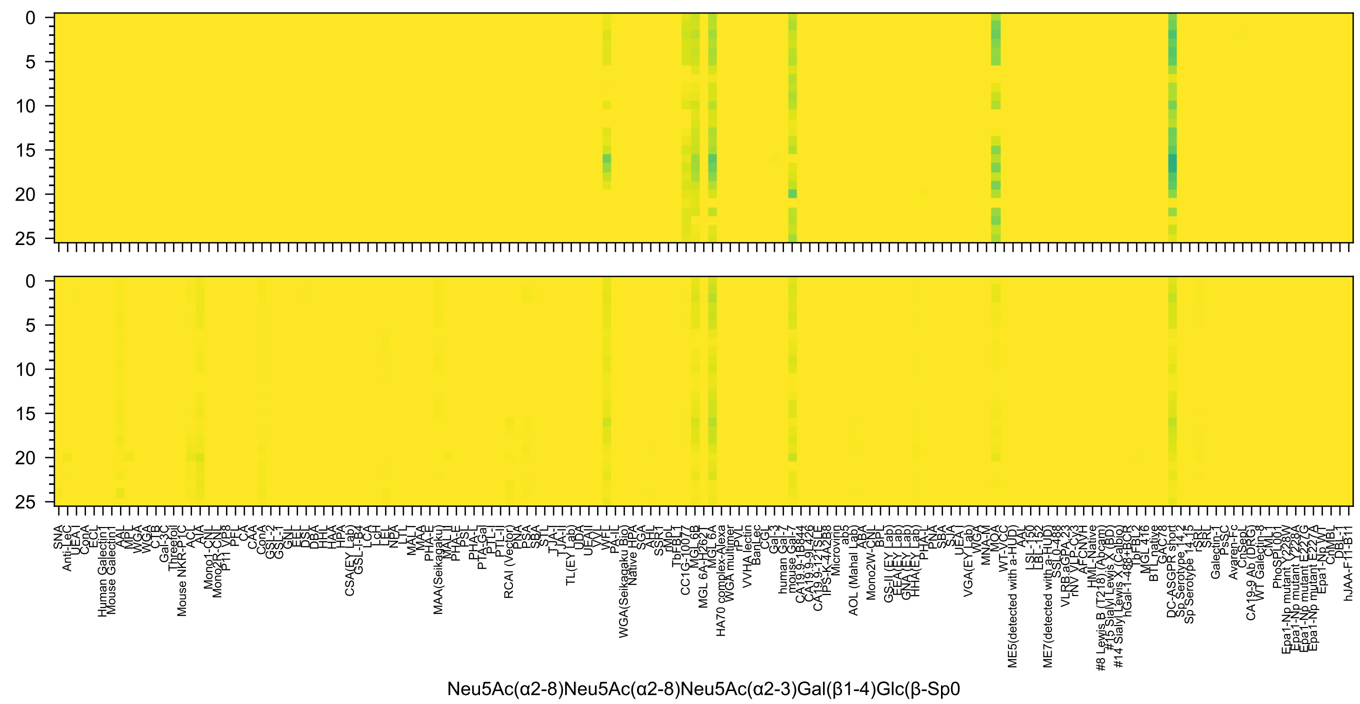

0
5
10
15
20
25
SNA
Anti-LeC
UEA I
ConA
ECL
Human Galectin1
Mouse Galectin1
AAL
MPL
WGA
WGA
CTB
Gal-3C
Threefoil
Mouse NKR-P1C
ACL
AIA
Mono1-CNL
Mono2R-CNL
P11 VP8
PFL
CA
CAA
ConA
GSL-2
GSL-1
GNL
EEL
DSL
DBA
HHL
HAA
HPA
CSA(EY Lab)
GSL-I-B4
LCA
LcH
LEL
NPA
LTL
MAL I
MAA
PHA-E
MAA(Seikagaku)
MAL II
PHA-E
PSL
PHA-L
PTA-Gal
PTL-I
PTL-II
RCAI (Vector)
PNA
PSA
SBA
STL
TJA-I
TJA-II
TL(EY Lab)
UDA
UEA II
VVL
WFL
PA-IL
WGA(Seikagaku Bio)
Native HPA
SGA
AAL
SSR1
rMpL
TbFBL1
CC1G-10077
MGL 6B
MGL 6A-H262T
MGL 6A
HA70 complex-Alexa
WGA multimer
rPVL
VVHA lectin
BanLec
CGL
Gal-3
human Gal-7
mouse Gal-7
CA19.9-1B844
CA19.9-9L426
CA19.9-121SLE
IPS-K-4A2B8
Microvirin
ab5
AOL (Mahal Lab)
ABA
Mono2W-CNL
BPL
GS-II (EY Lab)
EEA (EY Lab)
GNA (EY Lab)
HHA (EY Lab)
PHA-L
PNA
SBA
SJA
UEA I
VGA(EY Lab)
WGA
MNA-M
MOA
WT-VCC
ME5(detected with a-HUD)
AAL
LSL-150
LBL-152
ME7(detected with a-HUD)
SSL0-488
VLRB.aGPA.23
rNV VLP-Cy3
AFCNVH
HML-Native
#8 Lewis B (T218) (Abcam)
#15 Sialyl Lewis X (BD)
#14 Sialyl Lewis X (Calbio)
hGal-1-488+BCR
TbFBL2
MGL 416
BTL native
GAC78
DC-ASGPR short
Sp Serotype 14.2
Sp Serotype 14.15
rSRL
SRL
Galectin-1
PsSC
Avaren-Fc
ChSepL
CA19-9 Ab (DRG)
WT Galectin-8
CML1
PhoSL001
Epa1-Np mutant Y228W
Epa1-Np mutant Y228A
Epa1-Np mutant E227G
Epa1-Np mutant E227A
Epa1-Np WT
ChMBL
DBL-1
hJAA-F11-B11
Neu5Ac(α2-8)Neu5Ac(α2-8)Neu5Ac(α2-3)Gal(β1-4)Glc(β-Sp0

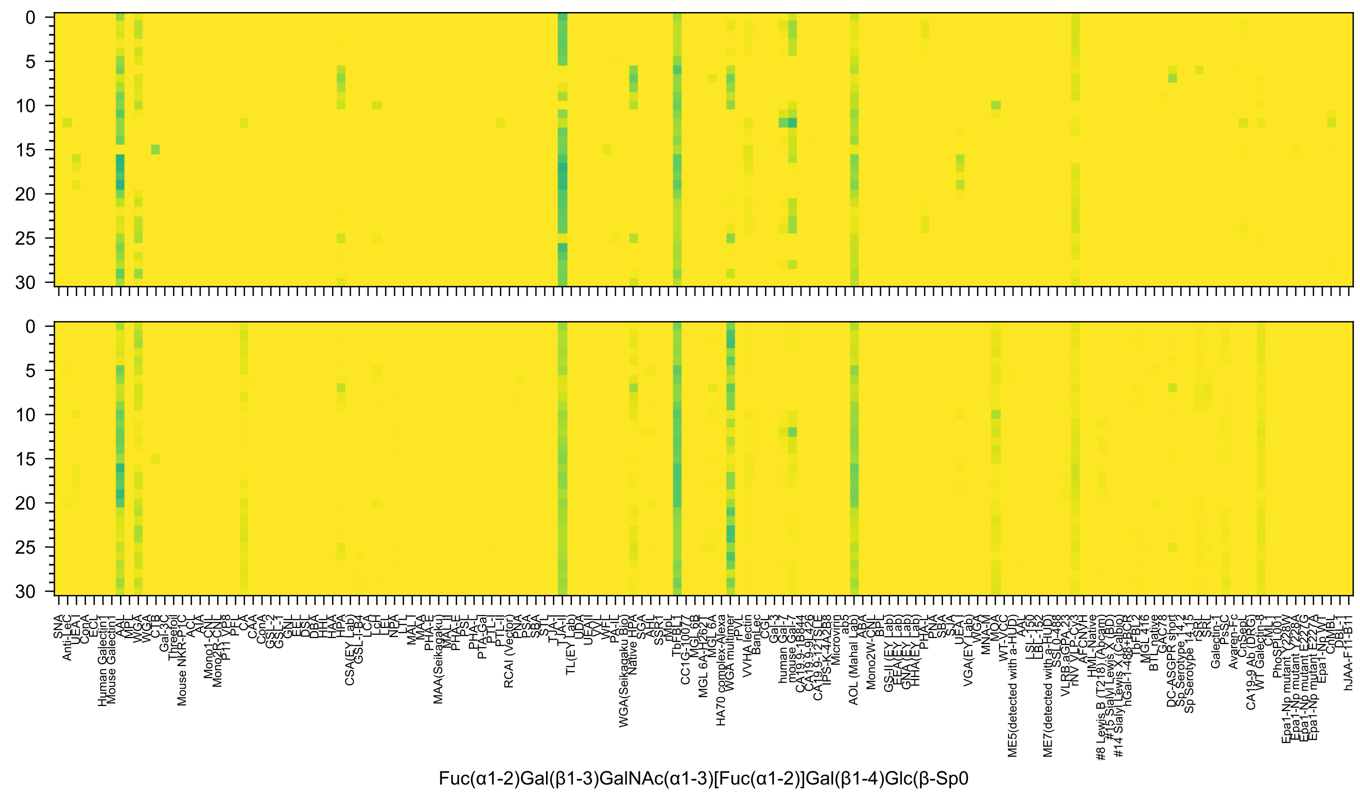
Fuc(α1-2)Gal(β1-3)GalNAc(α1-3)[Fuc(α1-2)]Gal(β1-4)Glc(β-Sp0

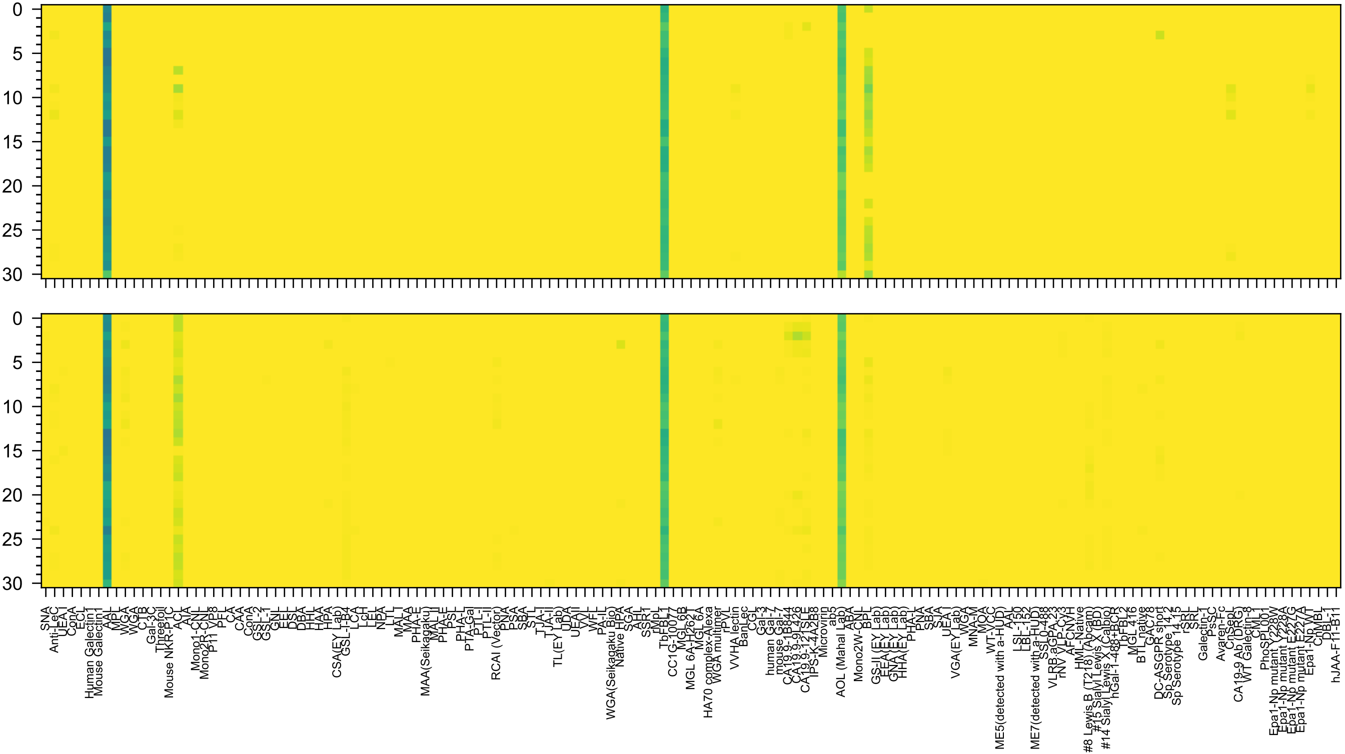

Gal(β1-3)[Fuc(α1-4)]GlcNAc(β1-3)Gal(β1-4)[Fuc(α1-3)]GlcNAc(β-Sp0

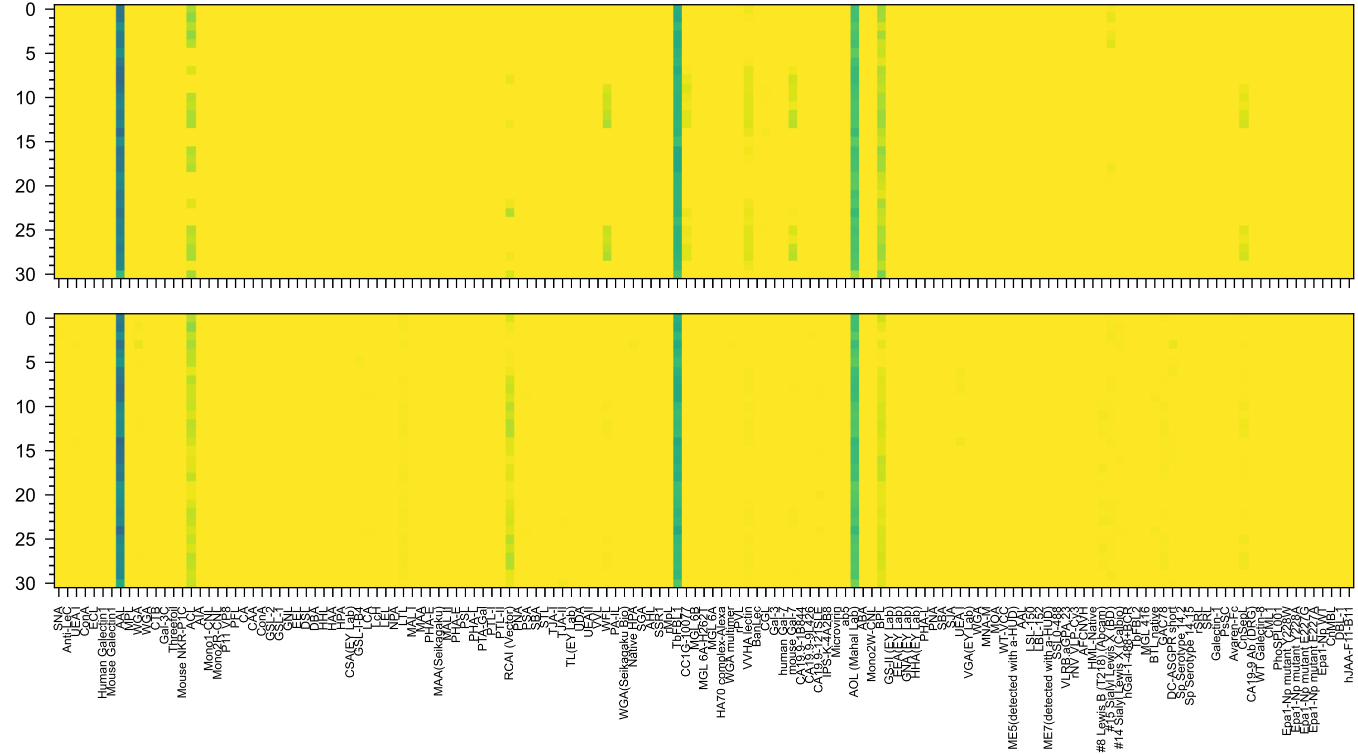

Gal(β1-4)[Fuc(α1-3)]GlcNAc(β1-3)Gal(β1-4)[Fuc(α1-3)]GlcNAc(β-Sp0

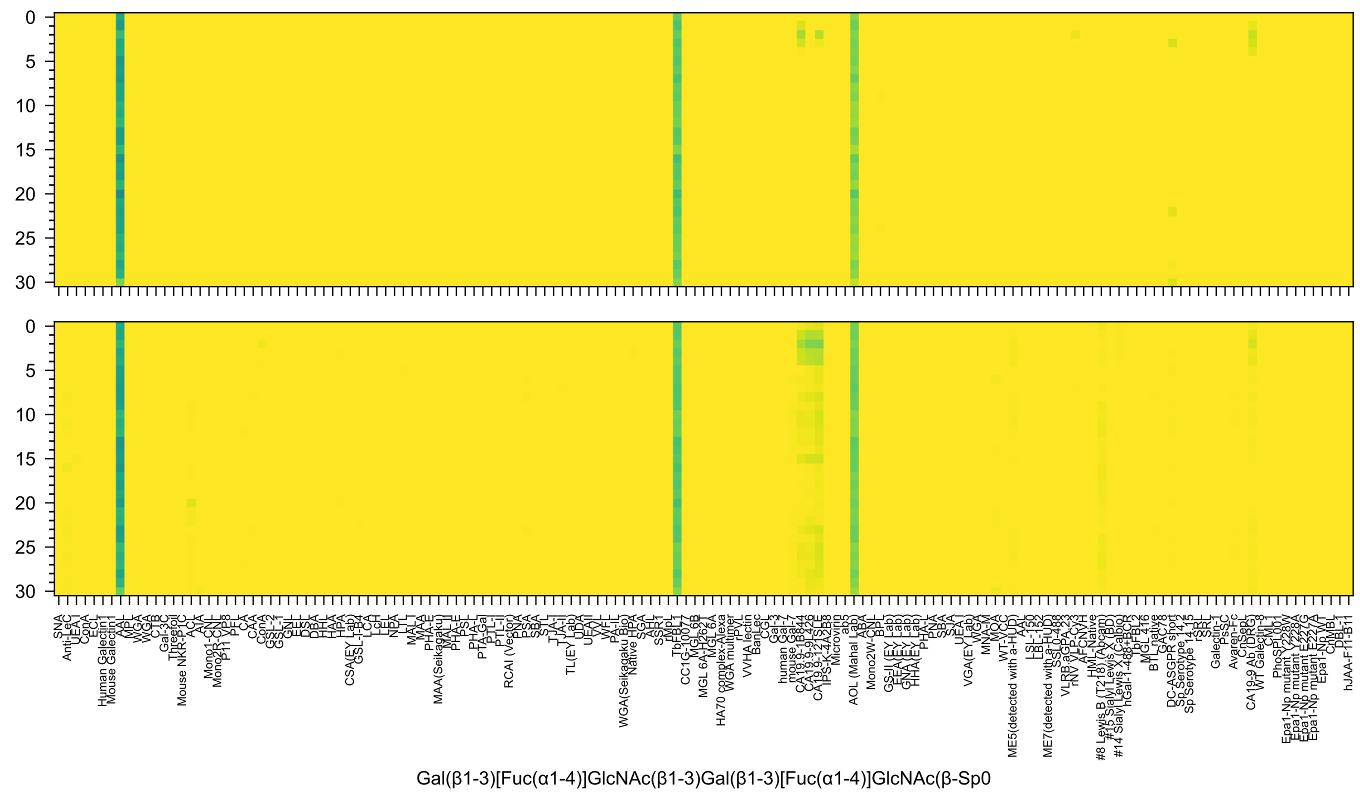
Gal(β1-3)[Fuc(α1-4)]GlcNAc(β1-3)Gal(β1-3)[Fuc(α1-4)]GlcNAc(β-Sp0

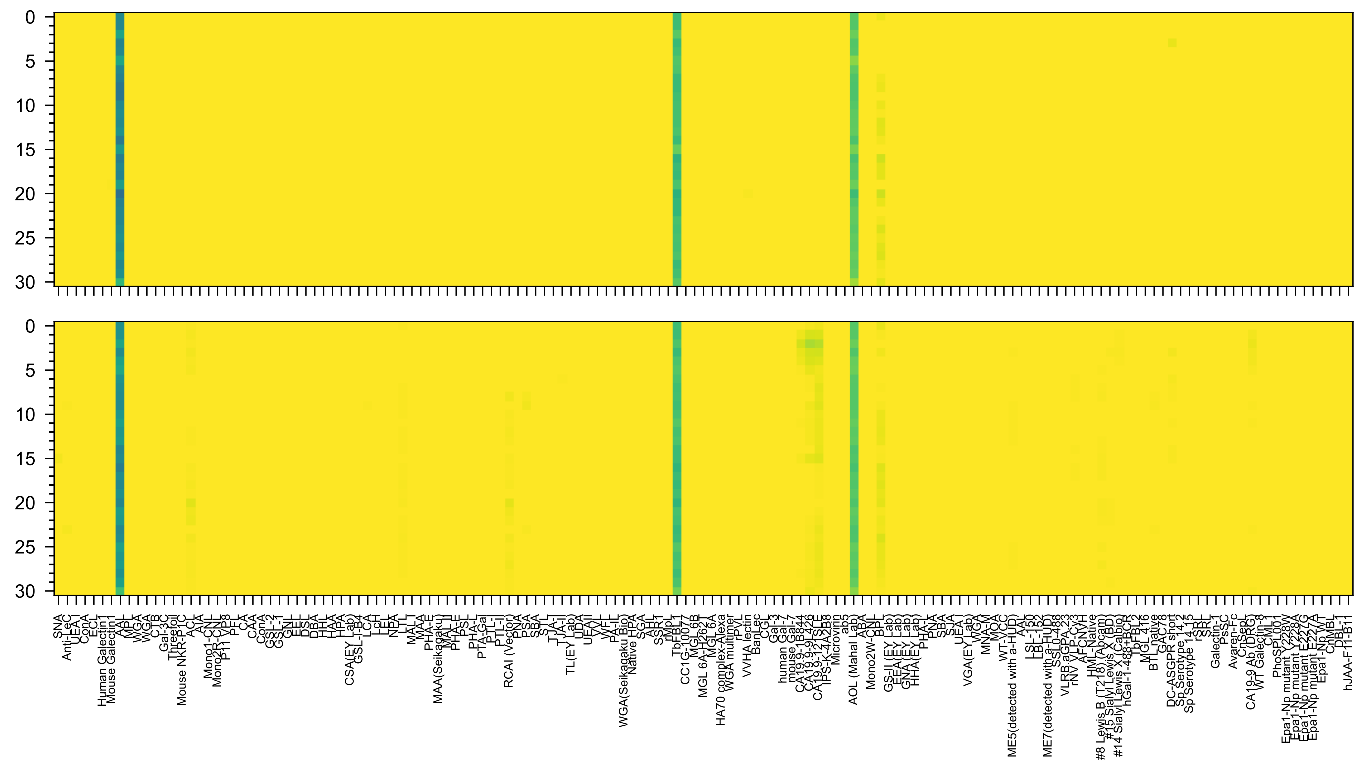

Gal(β1-4)[Fuc(α1-3)]GlcNAc(β1-3)Gal(β1-3)[Fuc(α1-4)]GlcNAc(β-Sp0

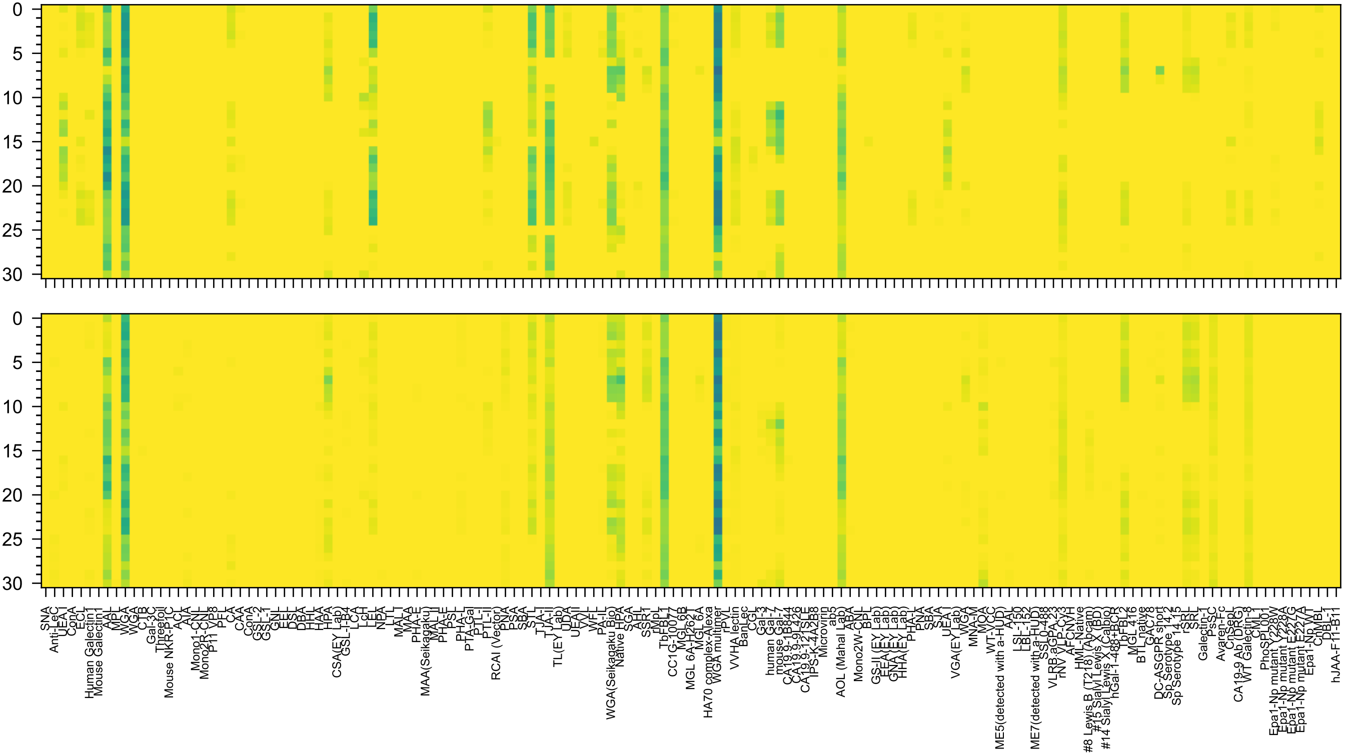

Fuc(α1-2)Gal(β1-3)GalNAc(α1-3)[Fuc(α1-2)]Gal(β1-4)GlcNAc(β-Sp0

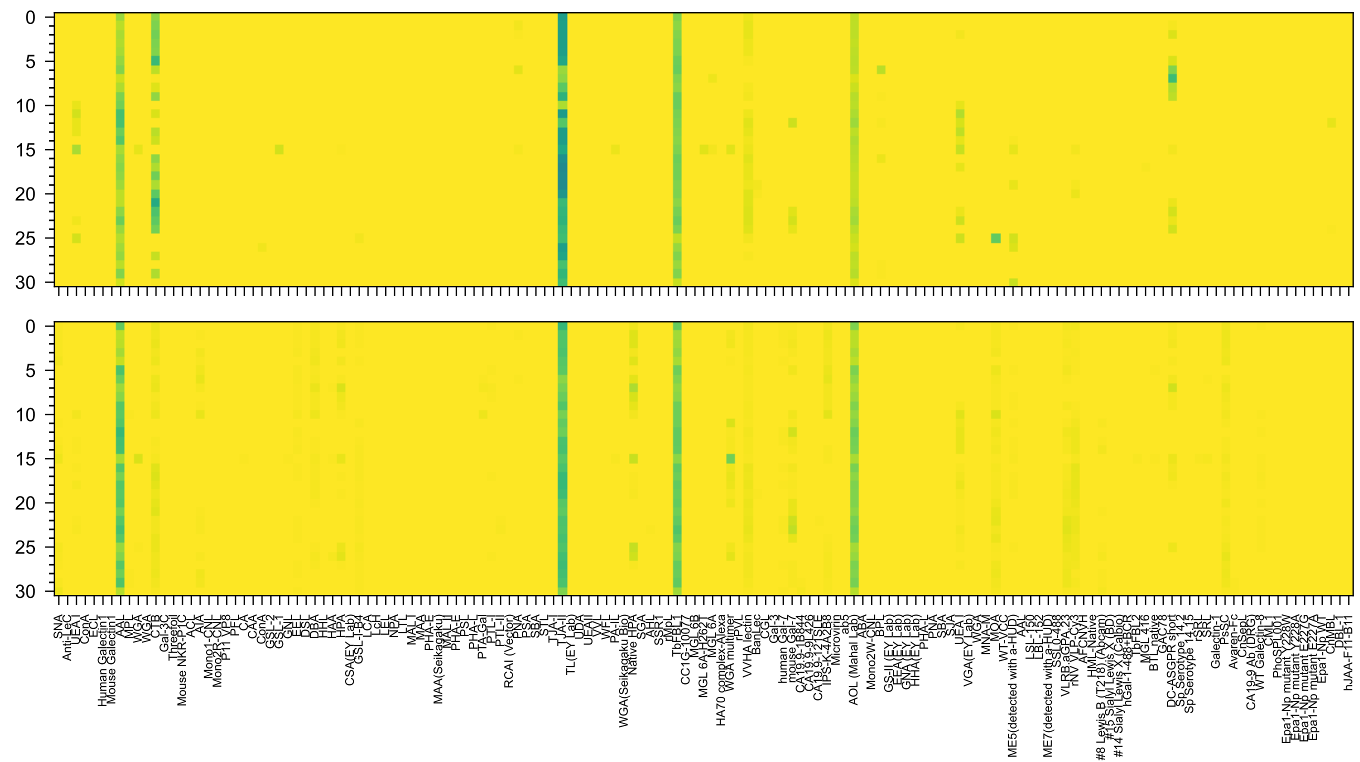

Fuc(α1-2)Gal(β1-3)GalNAc(β1-3)Gal(α1-4)Gal(β1-4)Glc(β-Sp9

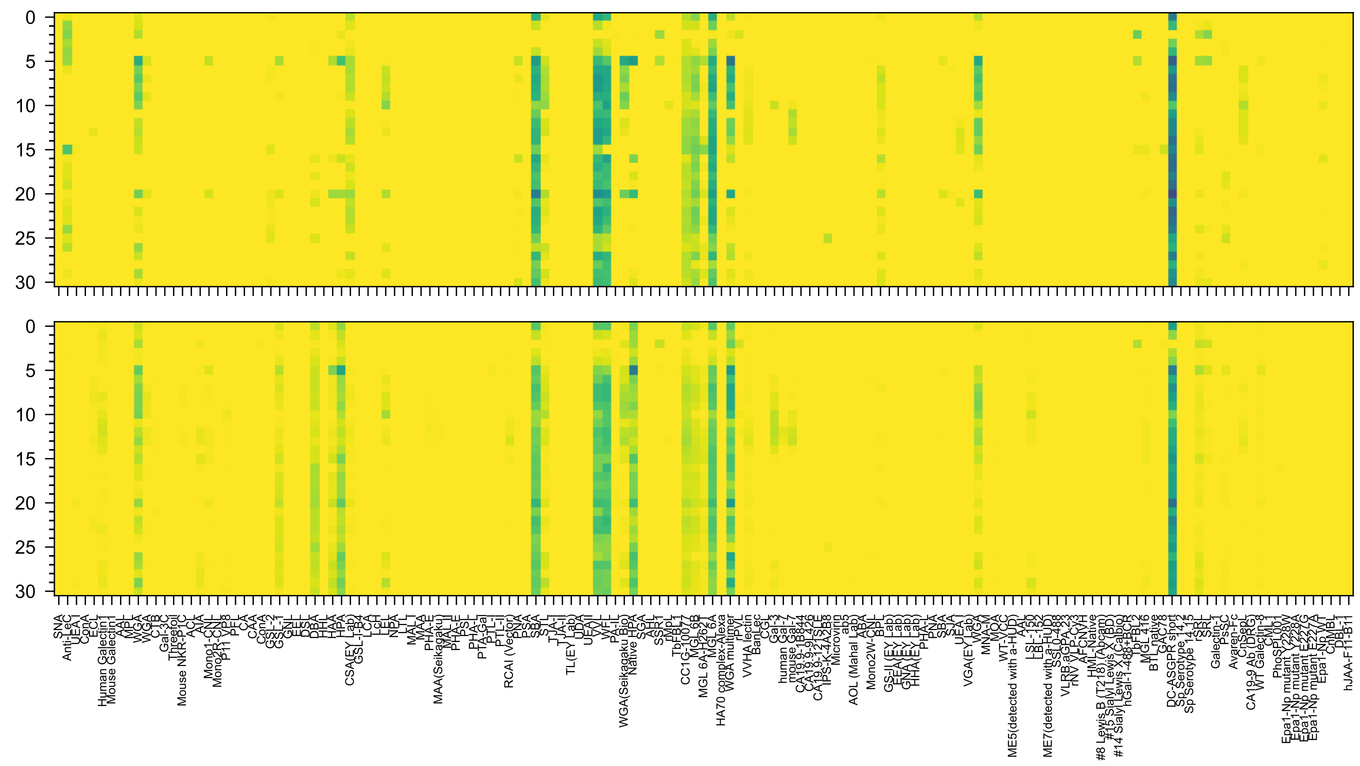

GalNAc(β1-3)Gal(α1-4)Gal(β1-4)GlcNAc(β1-3)Gal(β1-4)Glc(β-Sp0

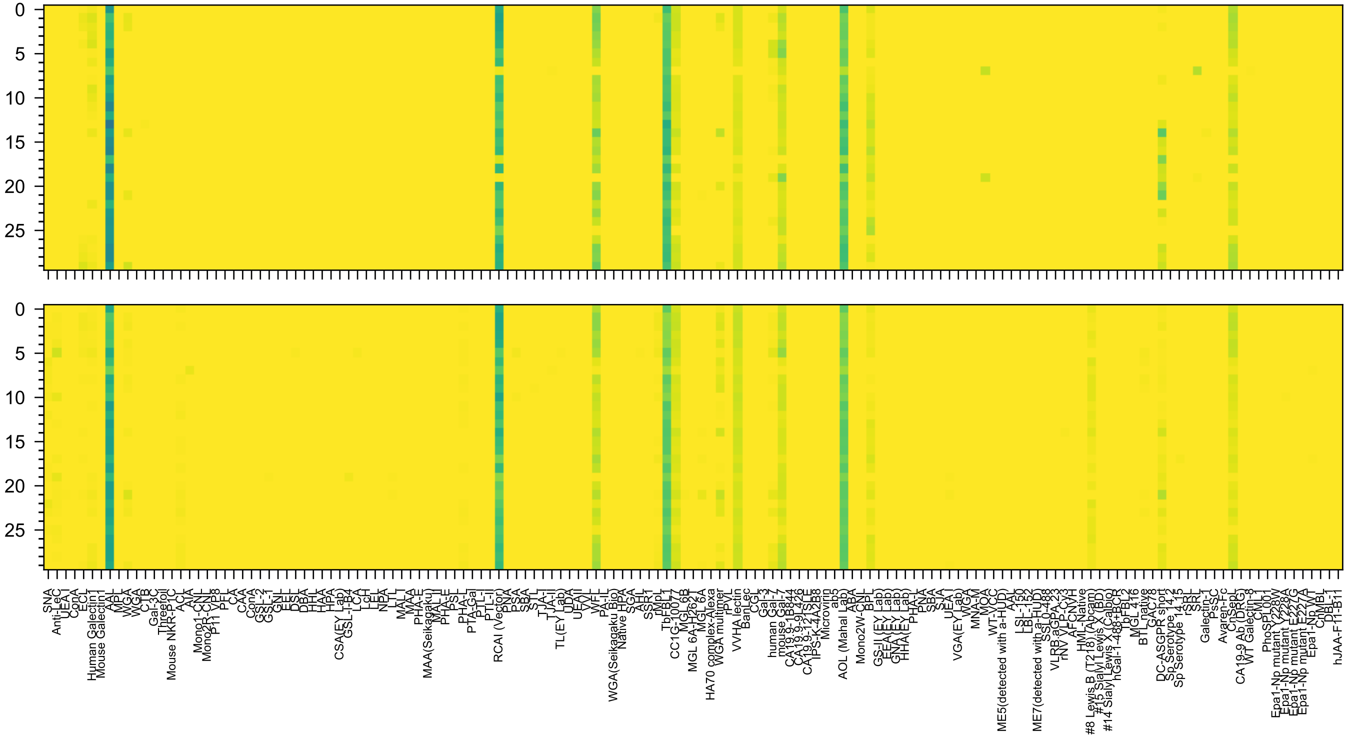

Fuc(α1-3)GlcNAc(β1-6)[Gal(β1-4)GlcNAc(β1-3)]Gal(β1-4)Glc(-Sp21

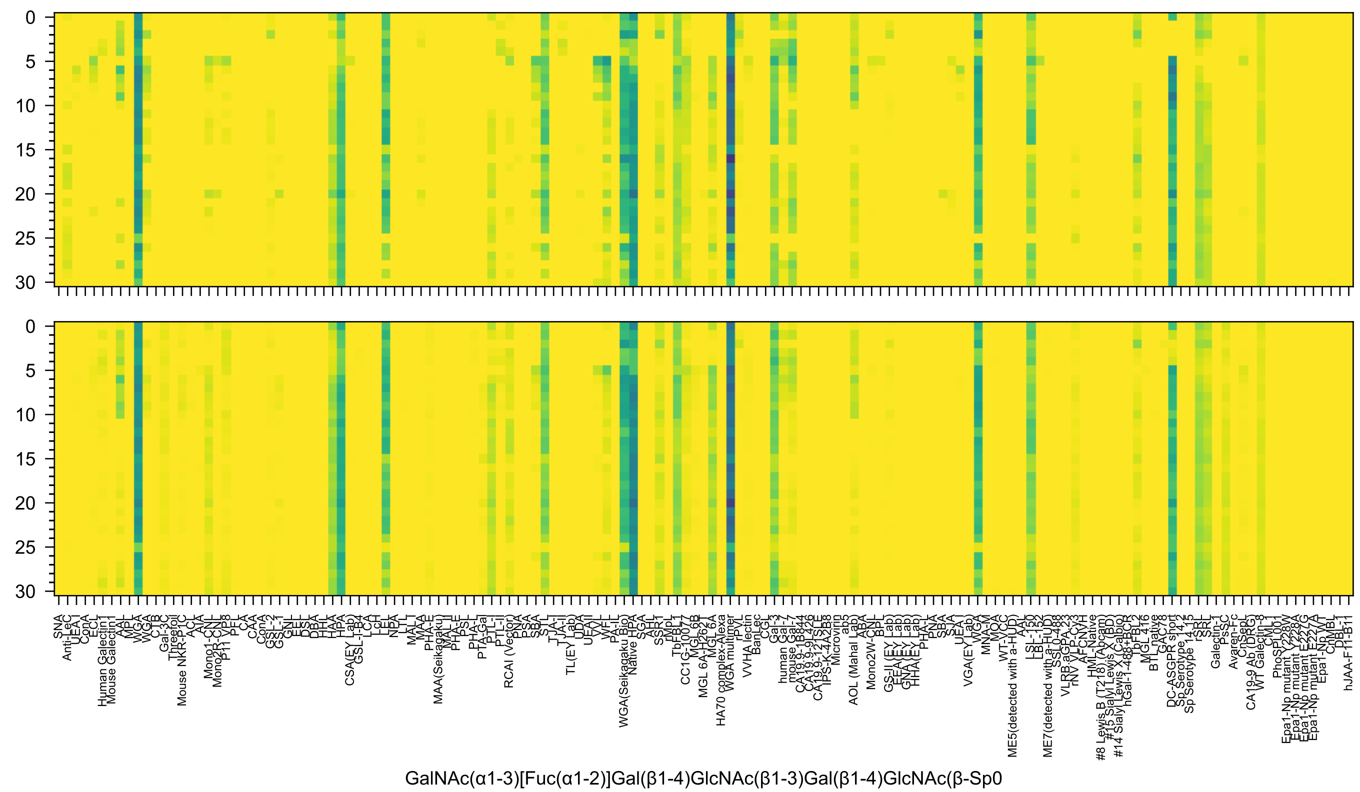
GalNAc(α1-3)[Fuc(α1-2)]Gal(β1-4)GlcNAc(β1-3)Gal(β1-4)GlcNAc(β-Sp0

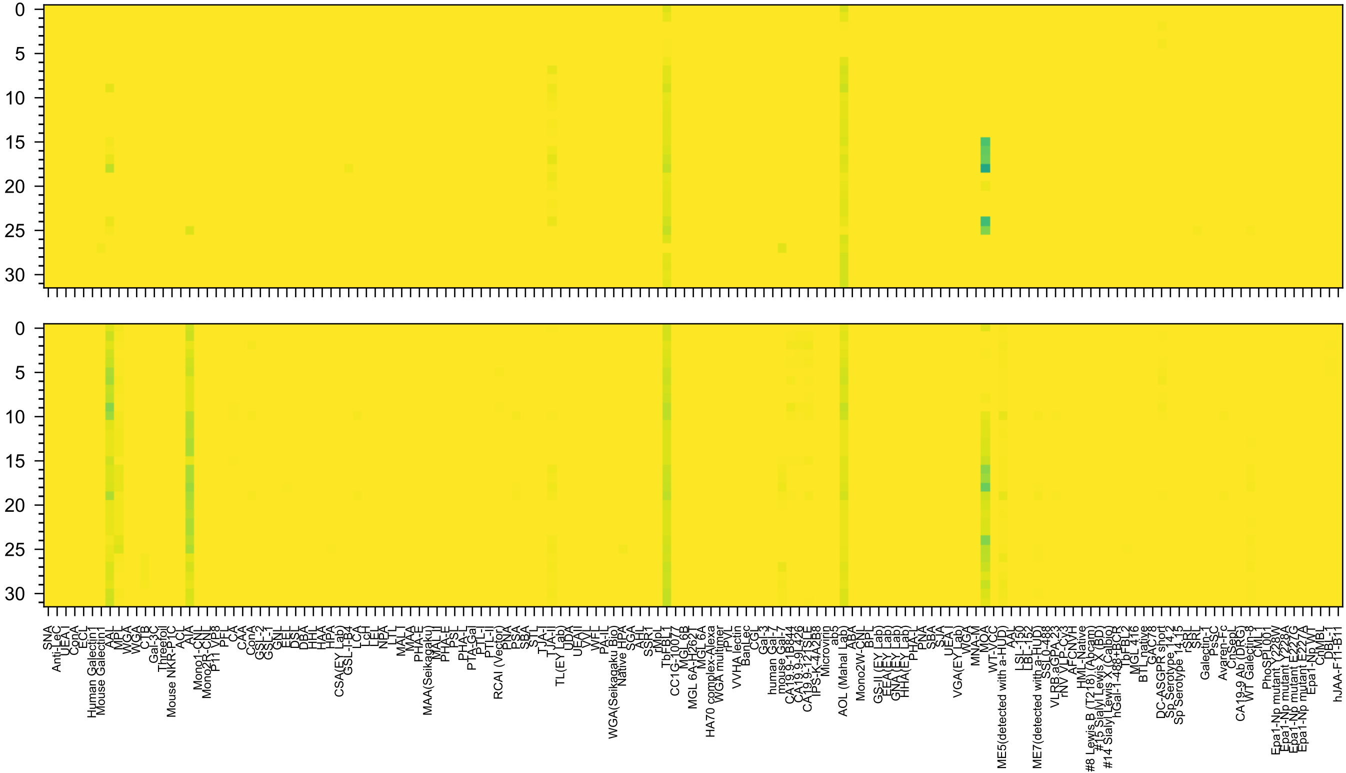

Gal(α1-3)[Fuc(α1-2)]Gal(β1-4)[Fuc(α1-3)]GlcNAc(β1-3)GalNAc(-Sp14

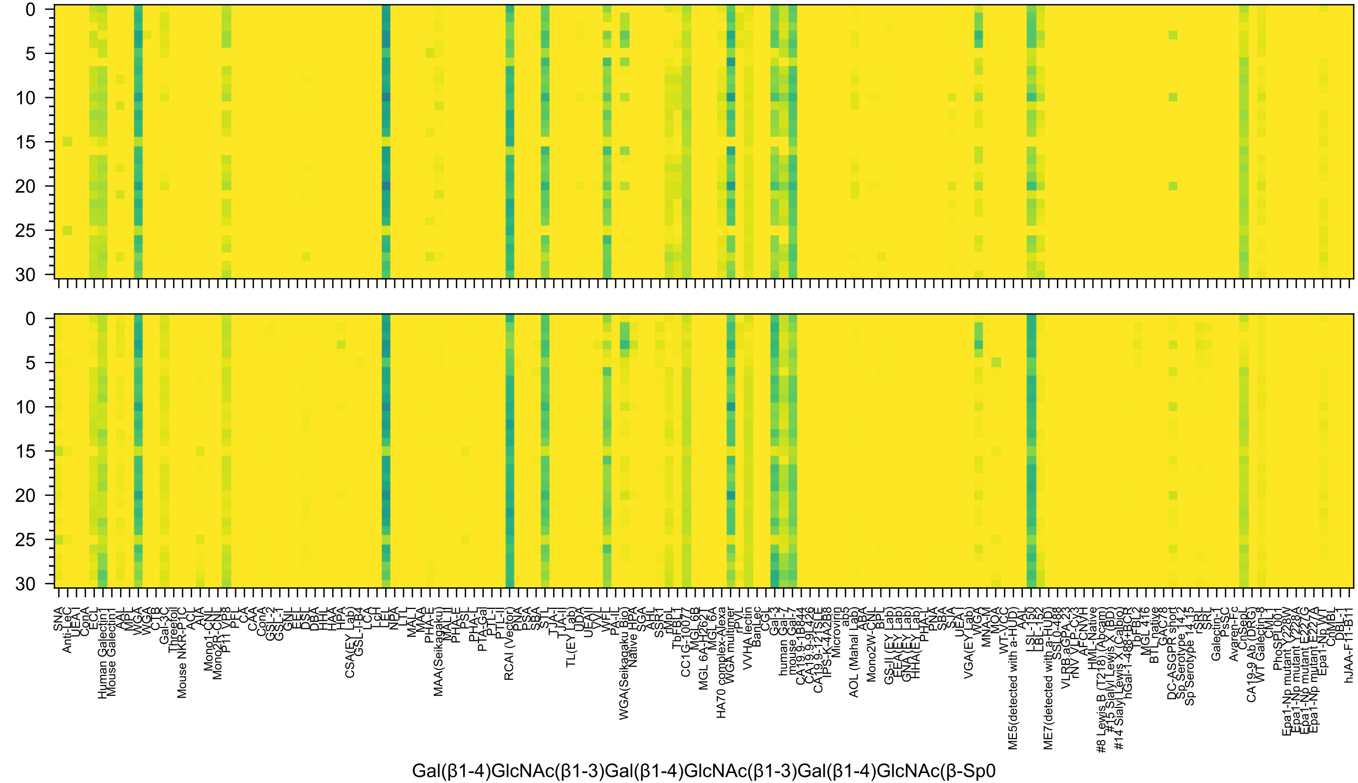
0
5
10
15
20
25
30
SNA
Anti-LeC
UEA-I
ConA
ECL
Human Galectin1
Mouse Galectin1
AAL
MPL
WGA
WGA
CTB
Gal-3C
Threefoil
Mouse NKR-P1C
ACL
AIA
Mono1-CNL
Mono2R-CNL
P11 VP8
PFL
CA
CAA
ConA
GSL-2
GSL-1
GNL
EEL
DSL
DBA
HHL
HAA
HPA
CSA(EY Lab)
GSL-I-B4
LCA
LcH
LEL
NPA
LTL
MAL-I
MAA
PHA-E
MAA(Seikagaku)
MAL_II
PHA-E
PSL
PHA-L
PTA-Gal
PTL-I
PTL-II
RCAI (Vector)
PNA
PSA
SBA
STL
TJA-I
TJA-II
TL(EY Lab)
UDA
UEAII
VVL
WFL
PA-IL
WGA(Seikagaku Bio)
Native HPA
SGA
AHL
SSR1
rMpL
TbFBL1
CC1G-10077
MGL6B
MGL 6A-H262T
MGL 6A
HA70 complex-Alexa
WGA multimer
rPVL
VVHA lectin
BanLec
CGL
Gal-3
human Gal-7
mouse Gal-7
Microvirin
ab5
AOL (Mahal Lab)
ABA
Mono2W-CNL
BPL
GS-II (EY Lab)
EEA(EY Lab)
GNA(EY Lab)
HHA(EY Lab)
PHA-L
PNA
SBA
SJA
UEA-I
VGA(EY Lab)
WGA
MNA-M
MOA
WT-VCC
ME5(detected with a-HUD)
AAL
LSL-150
LBL-152
ME7(detected with a-HUD)
rNV VLP-Cy3
HML-Native
#8 Lewis B (T218) (Abcam)
TbFBL2
MGL 416
BTL native
DC-ASGPR short
Sp Serotype 14.2
Sp Serotype 14.15
rSRL
SRL
Galectin-1
PsSC
Avaren-Fc
CnSepL
CA19-9 Ab (DRG)
WT Galectin-8
Epa1-Np mutant Y228W
Epa1-Np mutant Y228A
Epa1-Np mutant E227G
Epa1-Np mutant E227A
Epa1-Np WT
ChMBL
DBL-1
hJAA-F11-B1
Gal(β1-4)GlcNAc(β1-3)Gal(β1-4)GlcNAc(β1-3)Gal(β1-4)GlcNAc(β-Sp0

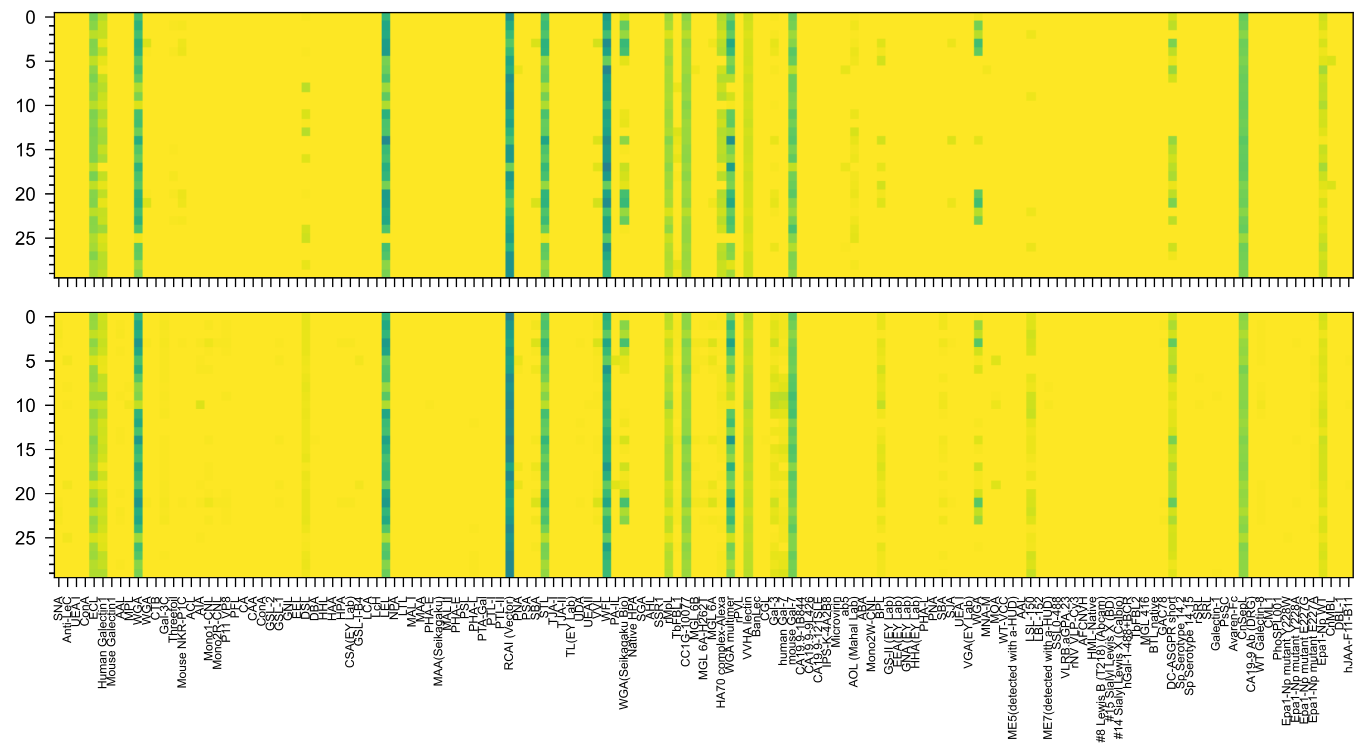

Gal(β1-4)GlcNAc(β1-6)[Gal(β1-4)GlcNAc(β1-3)]Gal(β1-4)GlcNAc(-Sp0

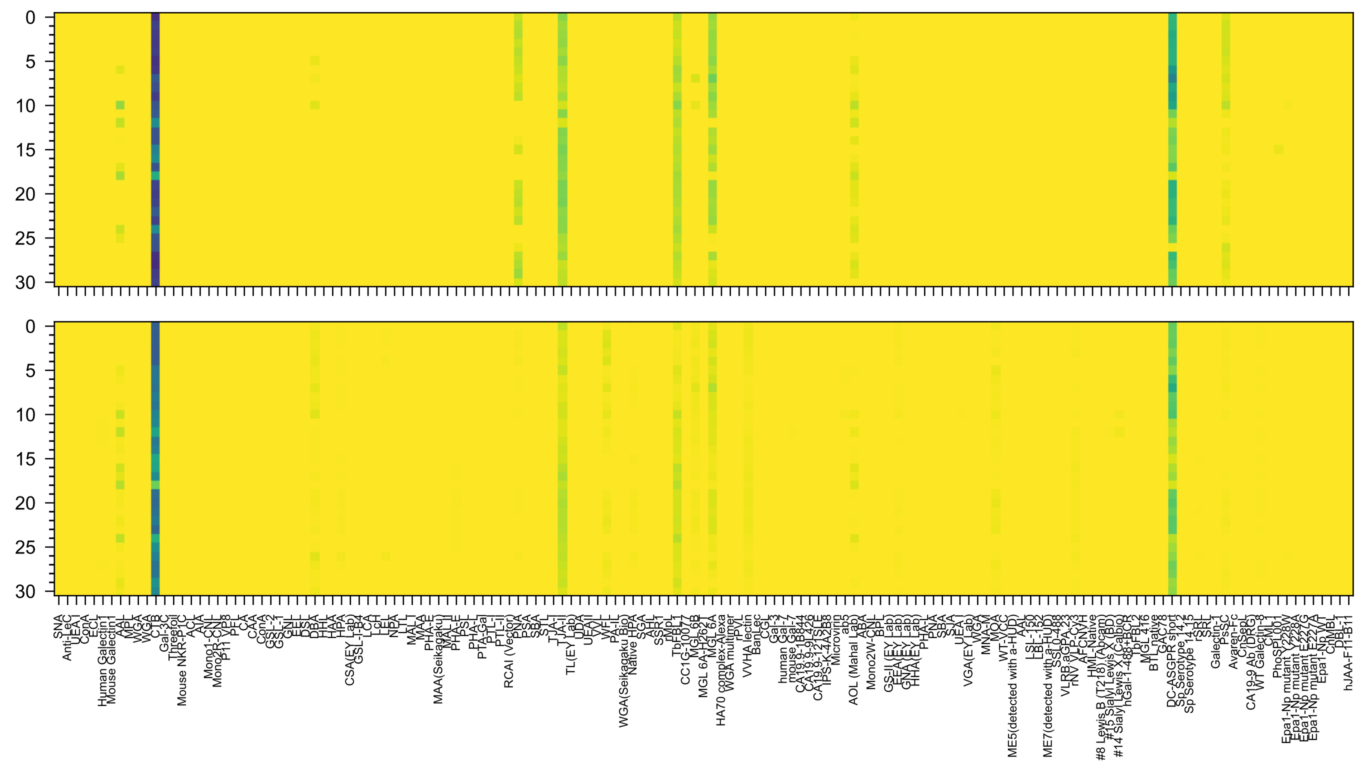

Fuc(α1-2)Gal(β1-3)GalNAc(β1-4)[Neu5Ac(α2-3)]Gal(β1-4)Glc(β-Sp0

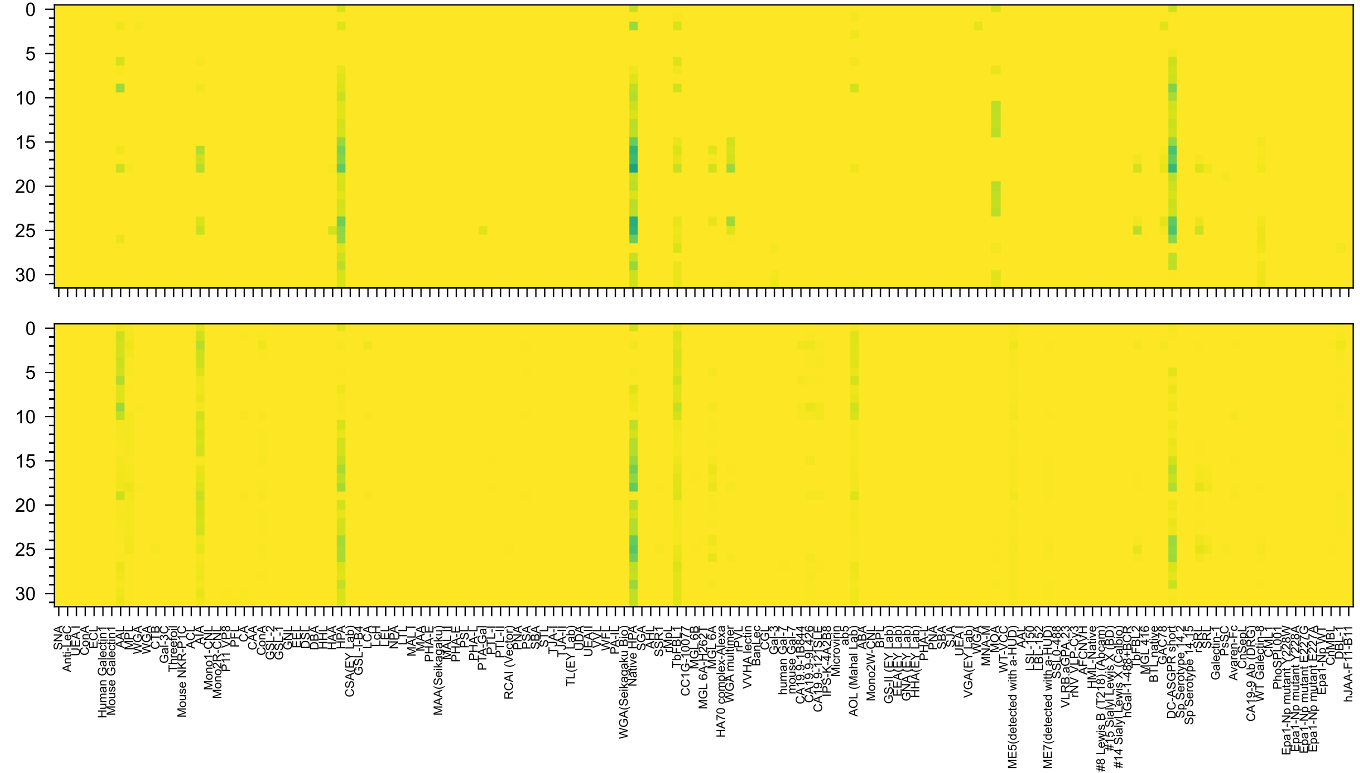

GalNAc(α1-3)[Fuc(α1-2)]Gal(β1-4)[Fuc(α1-3)]GlcNAc(β1-3)GalNAc(-Sp14

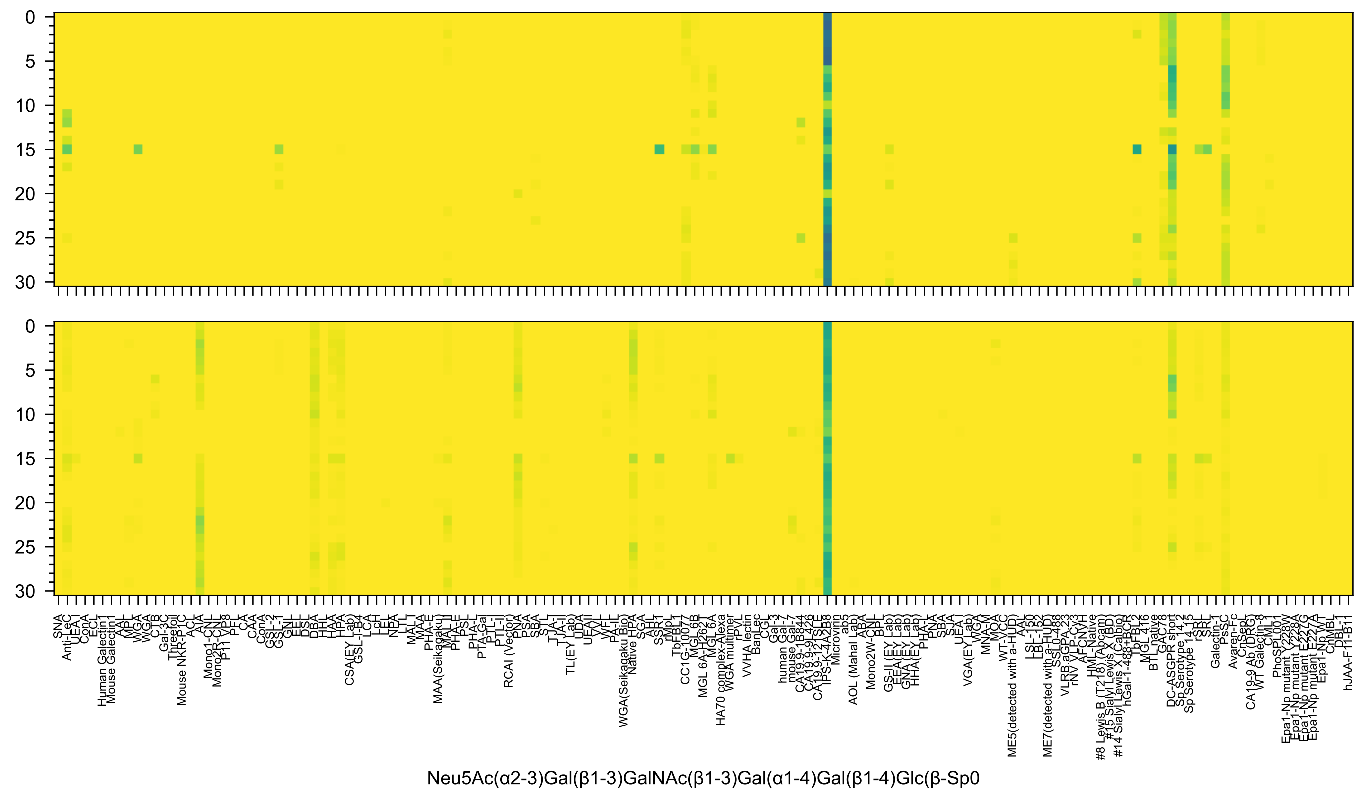
Neu5Ac(α2-3)Gal(β1-3)GalNAc(β1-3)Gal(α1-4)Gal(β1-4)Glc(β-Sp0

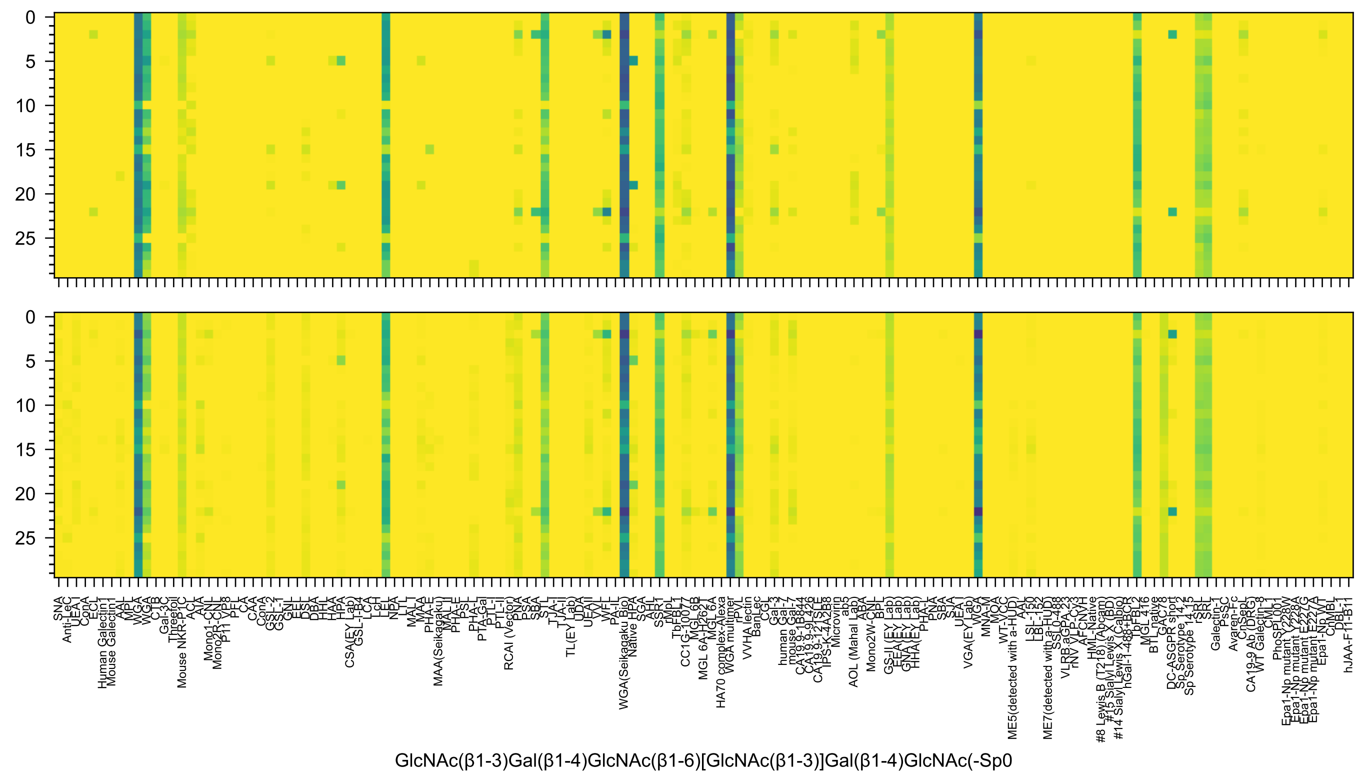
0
5
10
15
20
25
SNA
Anti-LeC
UEA-I
ConA
ECL
Human Galectin1
Mouse Galectin1
AAL
MPL
WGA
WGA
CTB
Gal-3C
Threefoil
Mouse NKR-P1C
ACL
AIA
Mono1-CNL
Mono2R-CNL
P11 VP8
PFL
CA
CAA
ConA
GSL-2
GSL-1
GNL
EEL
DSL
DBA
HHL
HAA
HPA
CSA(EY Lab)
GSL-I-B4
LCA
LcH
LEL
NPA
LTL
MAL-I
MAA
PHA-E
MAA(Seikagaku)
MAL-I
PHA-E
PSL
PHA-L
PTA-Gal
PTL-I
PTL-II
RCAI (Vector)
PNA
PSA
SBA
STL
TJA-I
TJA-II
TL(EY Lab)
UDA
UEA-II
VVL
WFL
PA-IL
WGA(Seikagaku Bio)
Native HPA
SGA
AHL
SSR1
rMpL
TbFBL1
CC1G-10077
MGL 6B
MGL 6A-H262T
MGL 6A
HA70 complex-Alexa
WGA multimer
rPVL
VVHA lectin
BanLec
CGL
Gal-3
human Gal-7
mouse Gal-7
CA19.9-1B844
CA19.9-9L426
CA19.9-121SLE
IPS-K-4A2B8
Microvirin
ab5
AOL (Mahal Lab)
ABA
Mono2W-CNL
BPL
GS-II (EY Lab)
EEA (EY Lab)
GNA (EY Lab)
HHA (EY Lab)
PHA-L
PNA
SBA
SJA
UEA-I
VGA(EY Lab)
WGA
MNA-M
MOA
WT-VCC
ME5(detected with a-HUD)
AAL
LSL-150
LBL-152
ME7(detected with a-HUD)
SSL0-488
VLRB.aGPA.23
rNV VLP-Cy3
AFCNVH
HML-Native
#8 Lewis B (T218) (Abcam)
#15 Sialyl Lewis X (BD)
#14 Sialyl Lewis X (Calbio)
hGal-1-488+BCR
TbFBL2
MGL 416
BTL native
GAC78
DC-ASGPR short
Sp Serotype 14.2
Sp Serotype 14.15
rSRL
SRL
Galectin-1
PsSC
Avaren-Fc
ChSepL
CA19-9 Ab (DRG)
WT Galectin-8
CML1
PhoSPL001
Epa1-Np mutant Y228W
Epa1-Np mutant Y228A
Epa1-Np mutant E227G
Epa1-Np mutant E227A
Epa1-Np WT
Epa1-Np
CnMBL
DBL-1
hJAA-F11-B1
GlcNAc(β1-3)Gal(β1-4)GlcNAc(β1-6)[GlcNAc(β1-3)]Gal(β1-4)GlcNAc(-Sp0

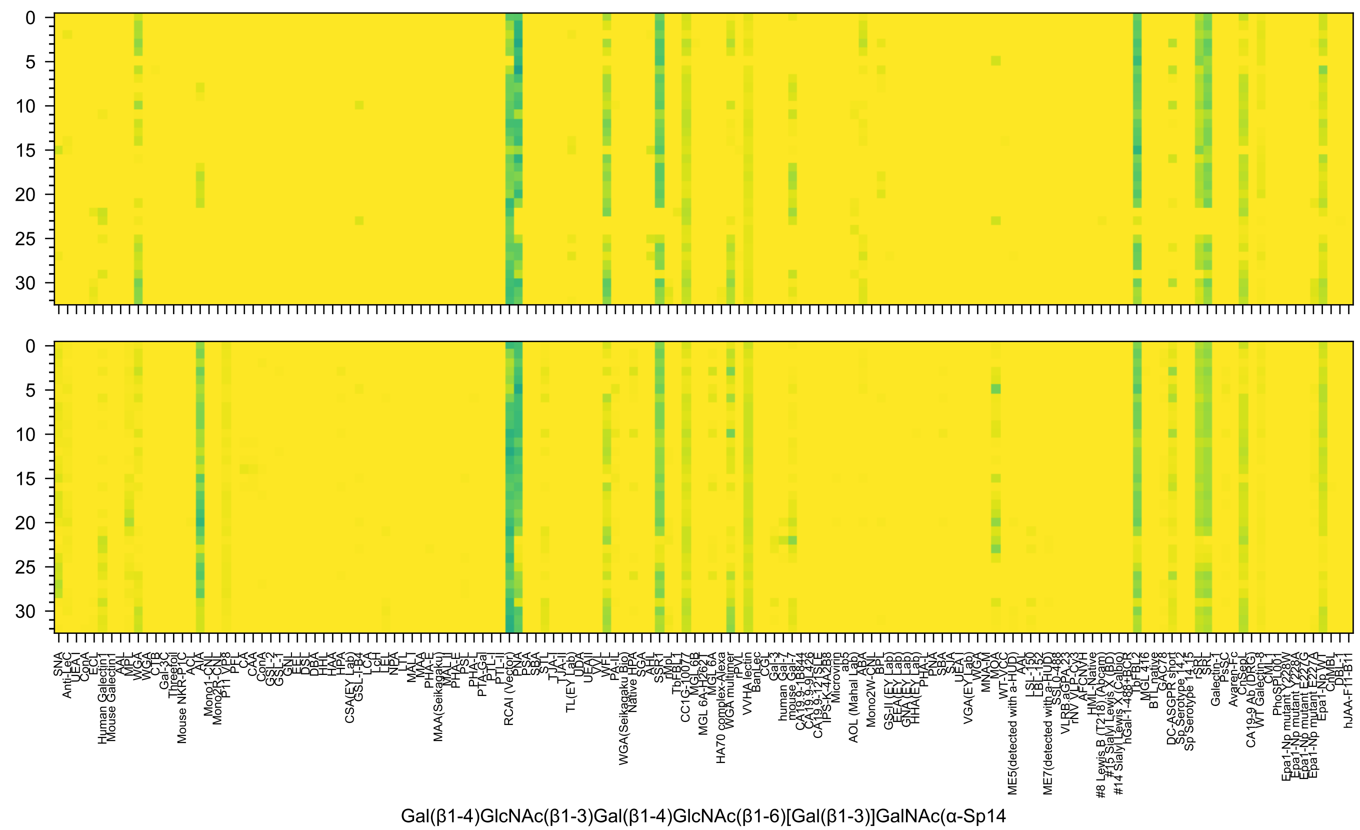
0
5
10
15
20
25
30
SNA
Anti-LeC
UEA-I
ConA
ECL
Human Galectin1
Mouse Galectin1
AAL
MPL
WGA
WGA
CTB
Gal-3C
Threefoil
Mouse NKR-P1C
ACL
AIA
Mono1-CNL
Mono2R-CNL
P11 VP8
PFL
CA
CAA
ConA
GSL-2
GSL-1
GNL
EEL
DSL
DBA
HHL
HAA
HPA
CSA(EY Lab)
GSL-I-B4
LCA
LcH
LEL
NPA
LTL
MAL-I
MAA
PHA-E
MAA(Seikagaku)
MAL-II
PHA-E
PSL
PHA-L
PTA-Gal
PTL-I
PTL-II
RCAI (Vector)
PNA
PSA
SBA
STL
TJA-I
TJA-II
TL(EY Lab)
UDA
UEA-II
VVL
WFL
PA-IL
WGA(Seikagaku Bio)
Native HPA
SGA
AHL
SSR1
rMpL
TbFBL1
CC1G-10077
MGL 6B
MGL 6A-H262T
MGL 6A
HA70 complex-Alexa
WGA multimer
rPVL
VVHA lectin
BanLec
CGL
Gal-3
human Gal-7
mouse Gal-7
CA19.9-1B844
CA19.9-9L426
CA19.9-121SLE
IPS-K-4A2B8
Microvirin
ab5
AOL (Mahal Lab)
ABA
Mono2W-CNL
BPL
GS-II (EY Lab)
EEA(EY Lab)
GNA(EY Lab)
HHA(EY Lab)
PHA-L
PNA
SBA
SJA
UEA-I
VGA(EY Lab)
WGA
MNA-M
MOA
WT-VCC
ME5(detected with a-HUD)
AAL
LSL-150
LBL-152
ME7(detected with a-HUD)
SSL0-488
VLRB.aGPA.23
rNV VLP-Cy3
AFC-NVH
HML-Native
#8 Lewis B (T218) (Abcam)
#15 Sialyl Lewis X (BD)
#14 Sialyl Lewis X (Calbio)
hGal-1-488+BCR
TbFBL2
MGL 416
BTL native
GAC78
DC-ASGPR short
Sp Serotype 14-2
Sp Serotype 14-15
rSRL
SRL
Galectin-1
PsSC
Avaren-Fc
CnSepL
CA19-9 Ab (DRG)
WT Galectin-8
CML1
PhoSPL001
Epa1-Np mutant Y228W
Epa1-Np mutant Y228A
Epa1-Np mutant E227G
Epa1-Np mutant E227A
Epa1-Np WT
CnMBL
DBL-1
hJAA-F11-B1
Gal(β1-4)GlcNAc(β1-3)Gal(β1-4)GlcNAc(β1-6)[Gal(β1-3)]GalNAc(α-Sp14

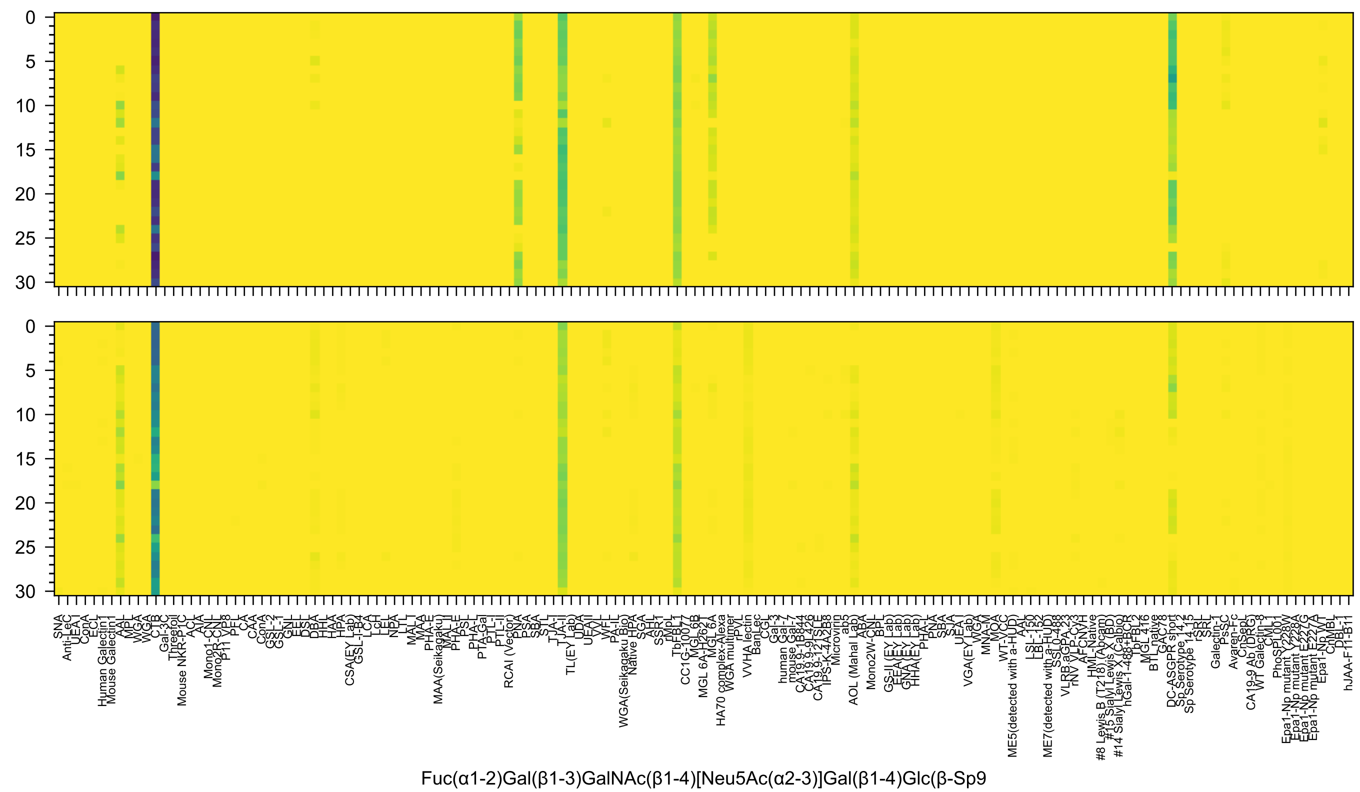

Fuc(α1-2)Gal(β1-3)GalNAc(β1-4)[Neu5Ac(α2-3)]Gal(β1-4)Glc(β-Sp9

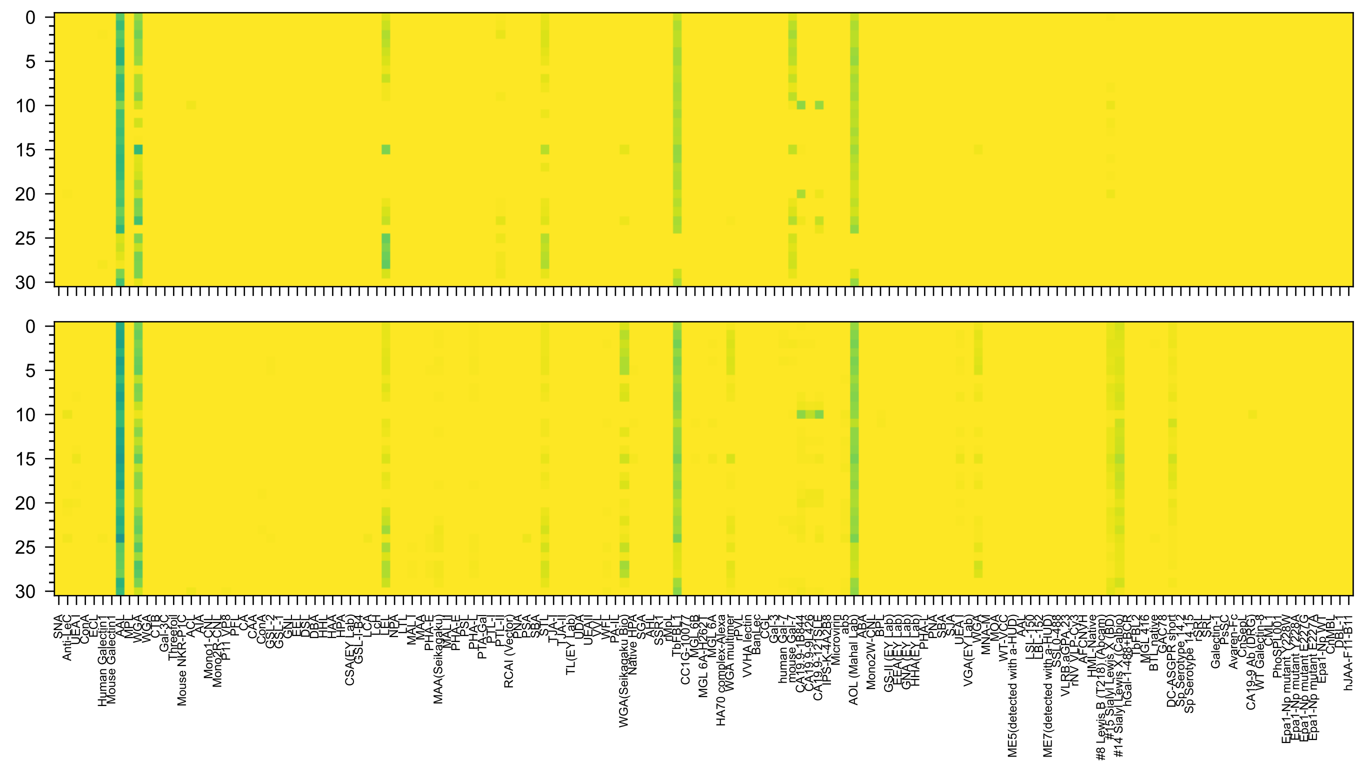
Neu5Ac(α2-3)Gal(β1-4)GlcNAc(β1-3)Gal(β1-4)[Fuc(α1-3)]GlcNAc(β-Sp0

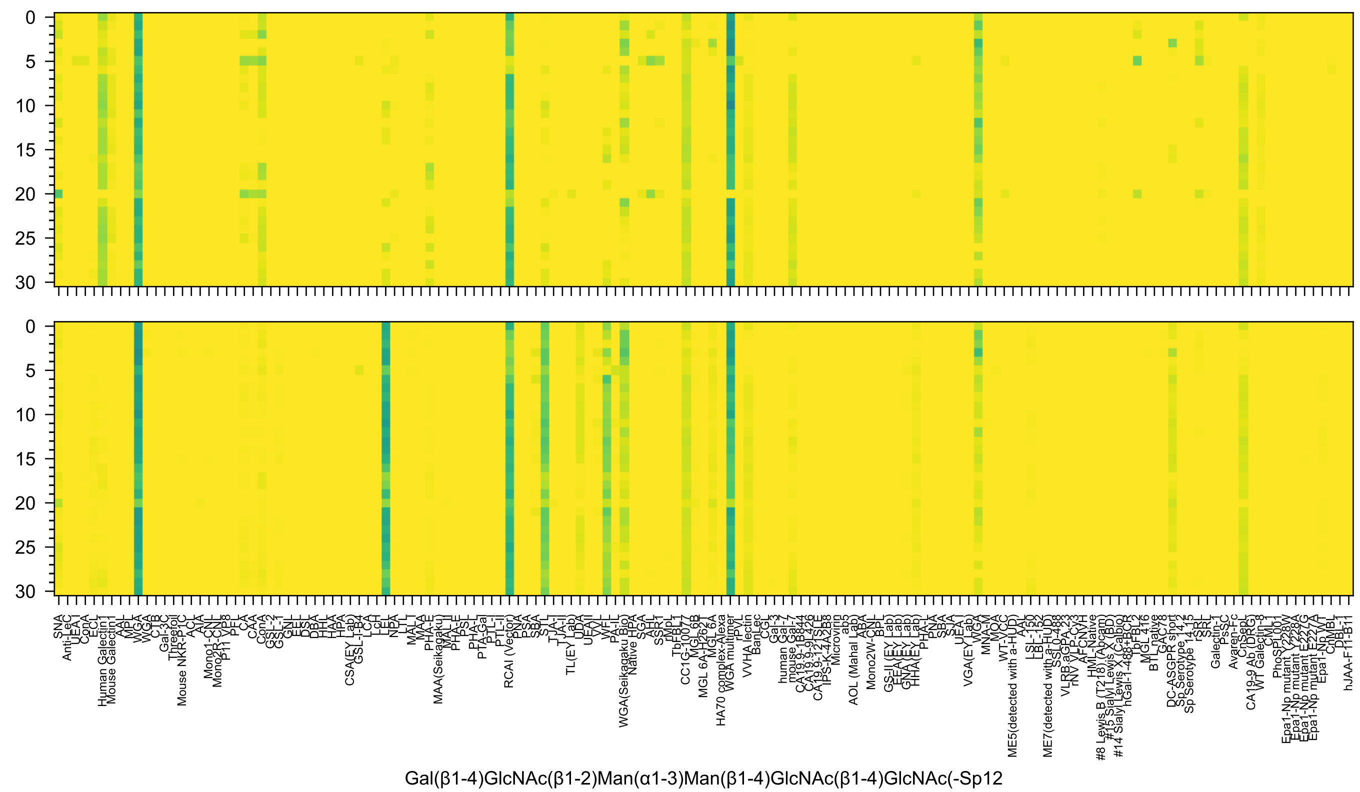
Gal(β1-4)GlcNAc(β1-2)Man(α1-3)Man(β1-4)GlcNAc(β1-4)GlcNAc(-Sp12

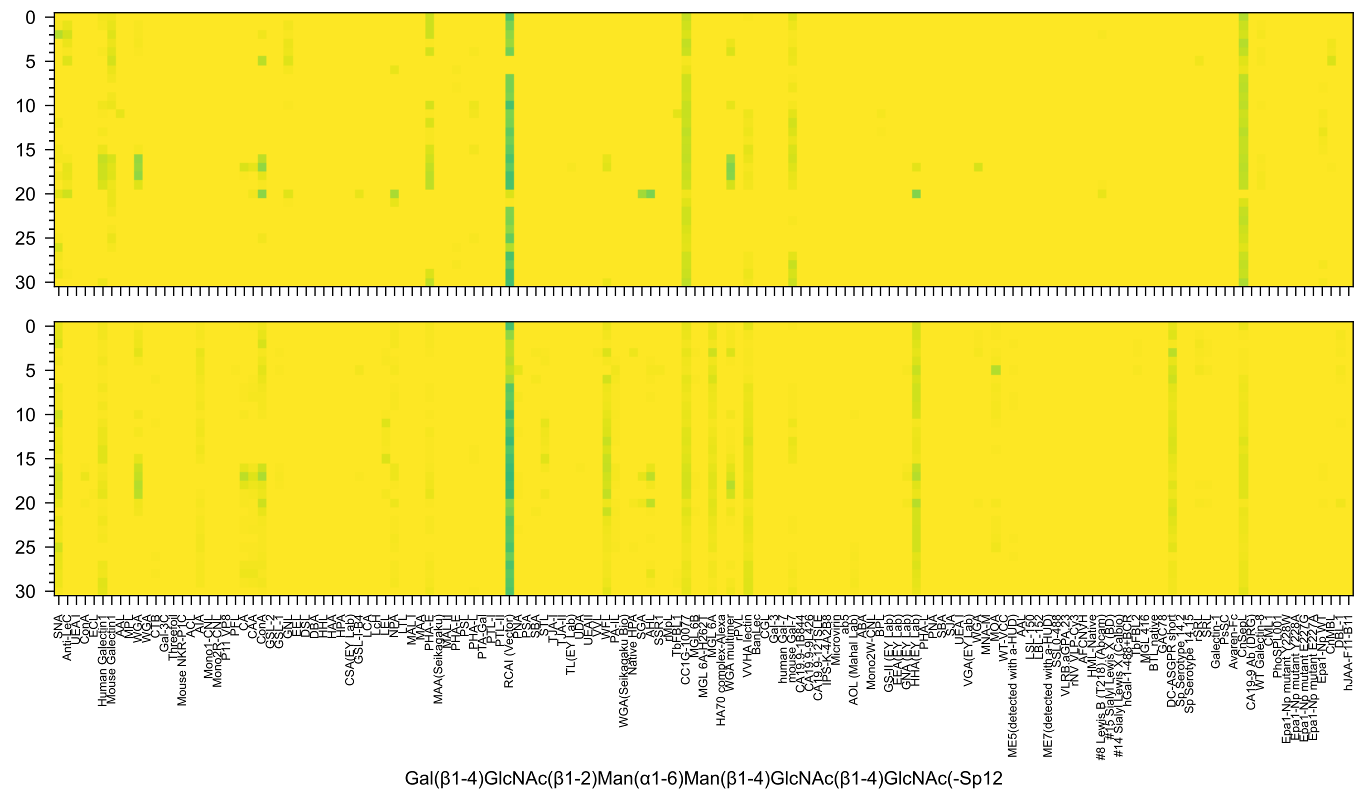
Gal(β1-4)GlcNAc(β1-2)Man(α1-6)Man(β1-4)GlcNAc(β1-4)GlcNAc(-Sp12

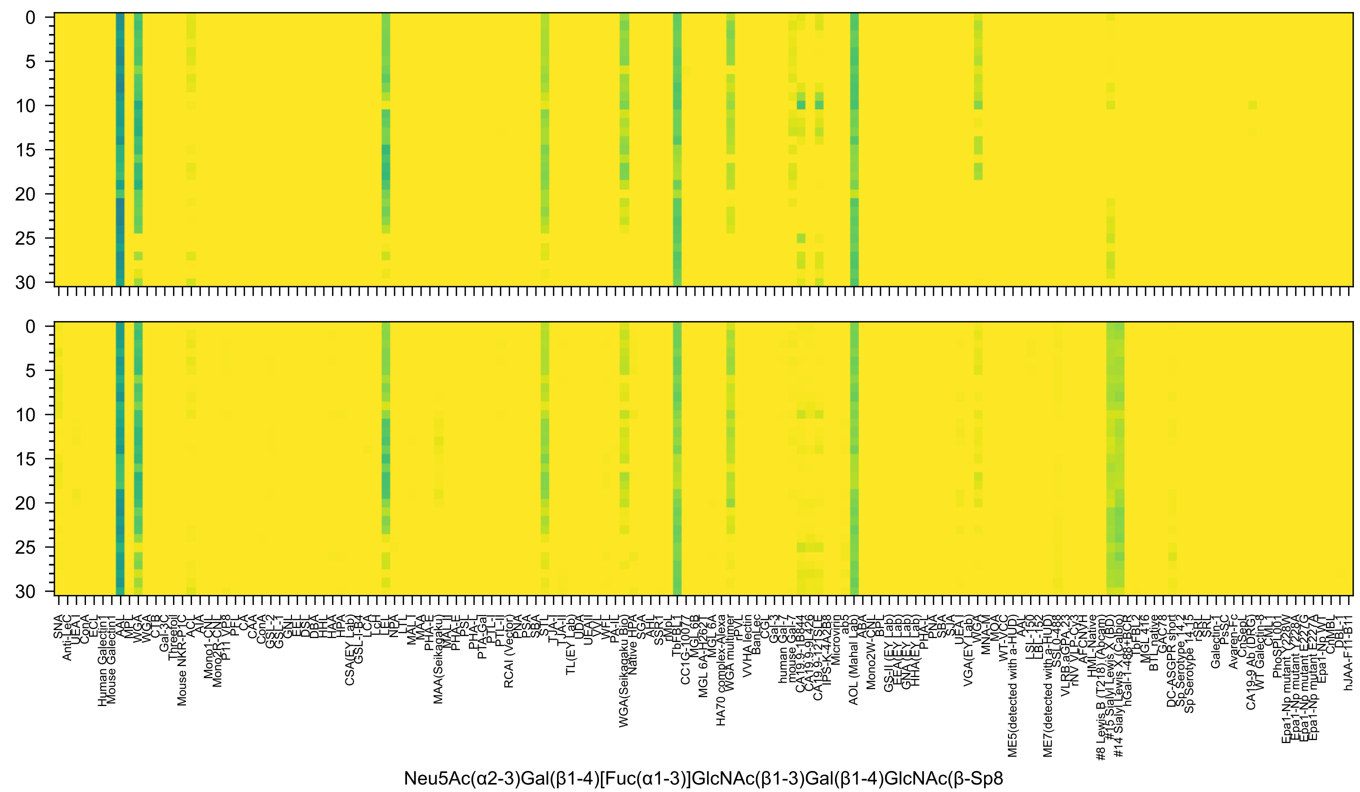
Neu5Ac(α2-3)Gal(β1-4)[Fuc(α1-3)]GlcNAc(β1-3)Gal(β1-4)GlcNAc(β-Sp8

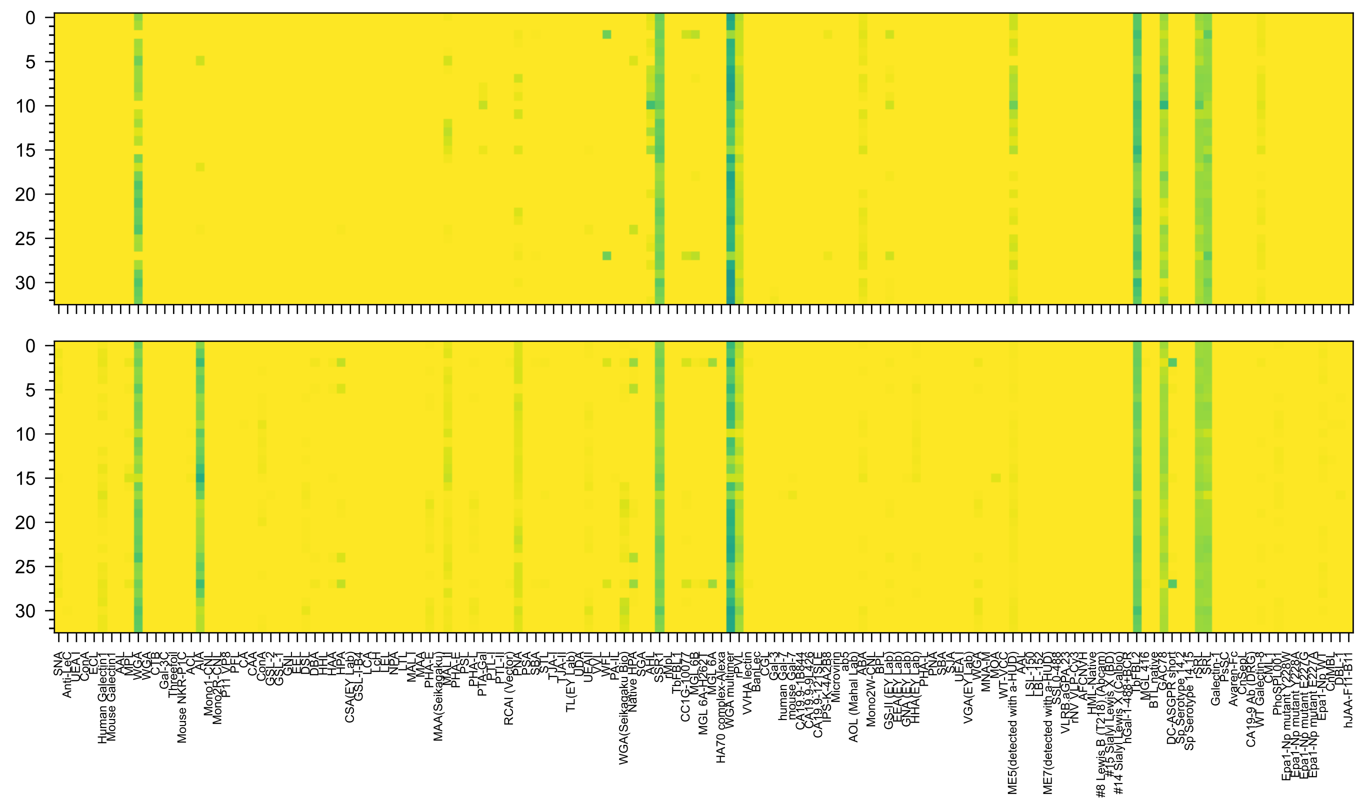

GlcNAc(β1-3)Gal(β1-4)GlcNAc(β1-6)[GlcNAc(β1-3)Gal(β1-3)]GalNAc(α-Sp14

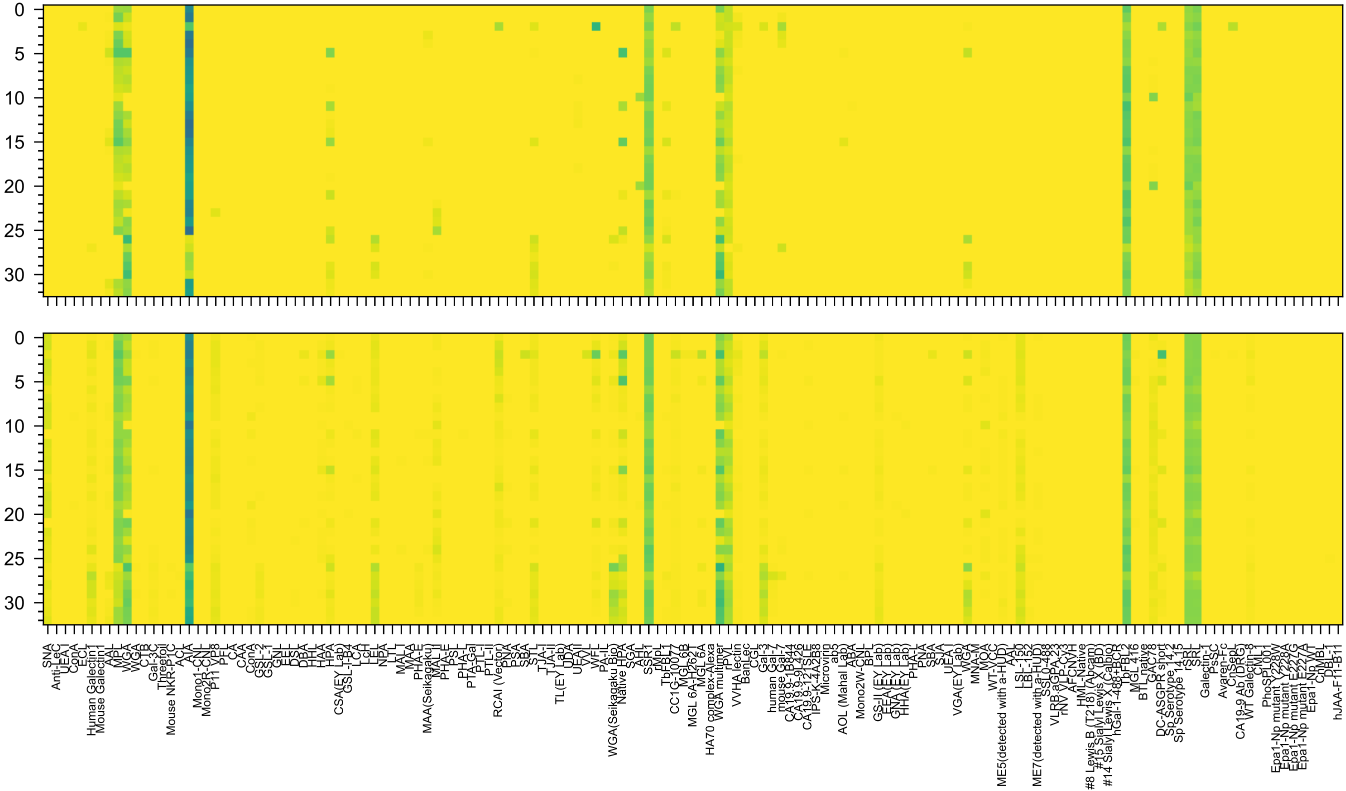

GlcNAc(β1-3)Gal(β1-4)GlcNAc(β1-3)Gal(β1-4)GlcNAc(β1-3)GalNAc(α-Sp14

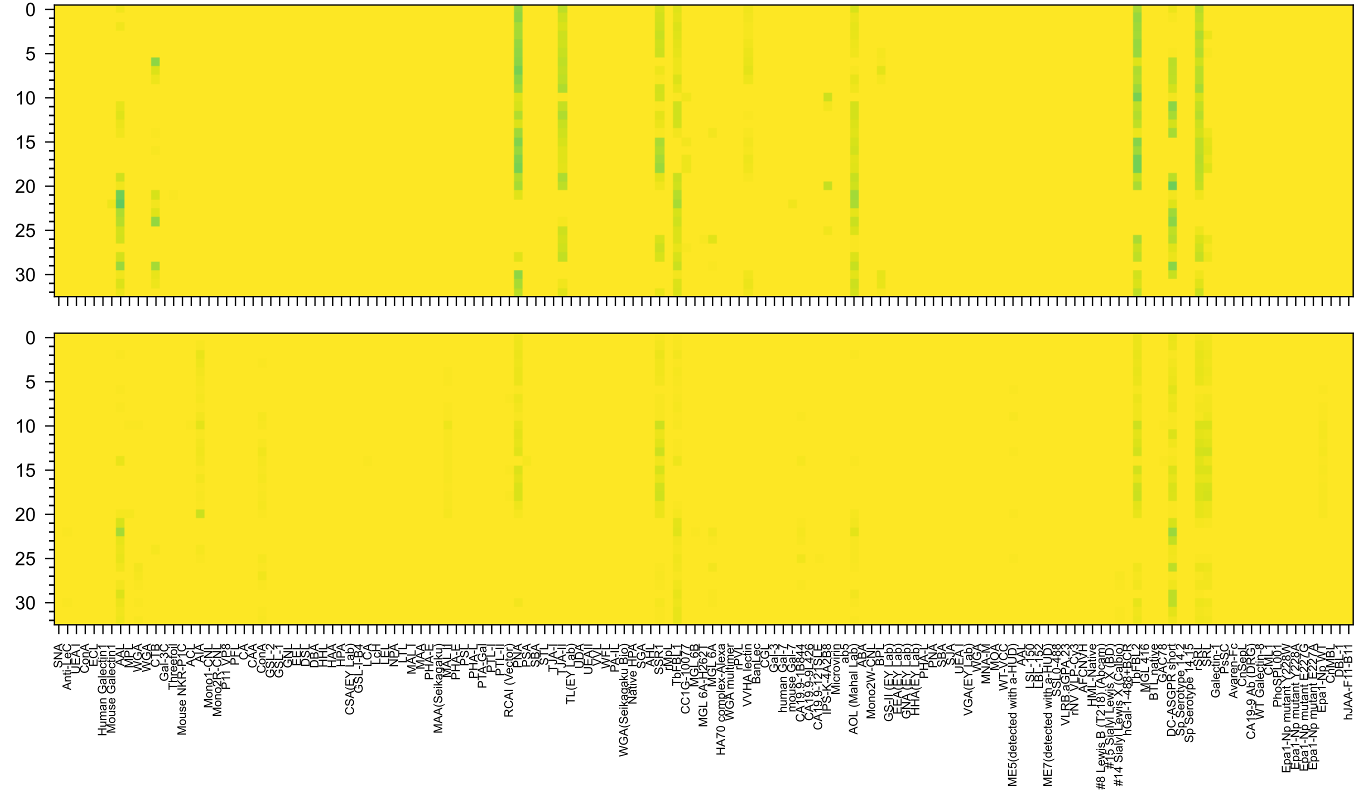

Neu5Ac(α2-3)Gal(β1-4)[Fuc(α1-3)]GlcNAc(β1-6)[Gal(β1-3)]GalNAc(α-Sp14

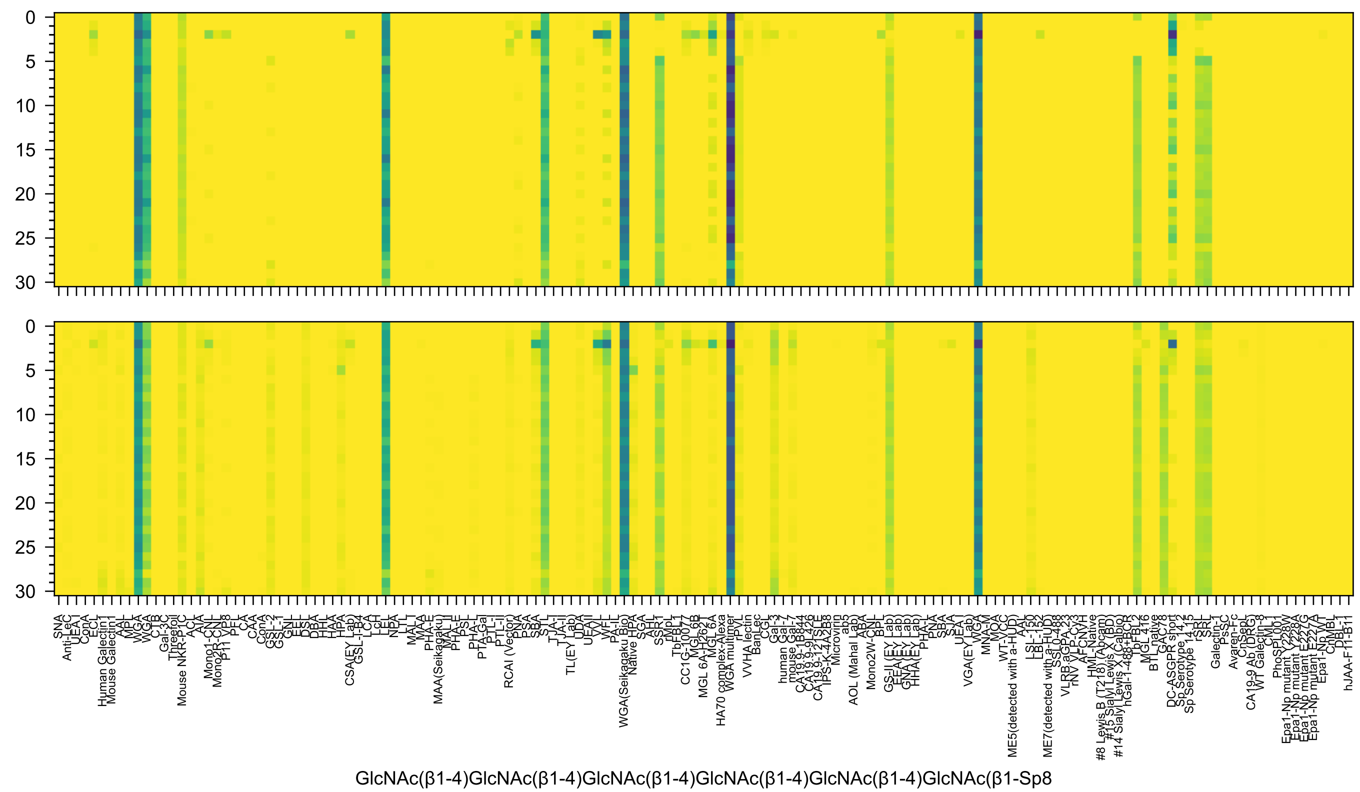

SNA
Anti-LeC
UEA-I
ConA
ECL
Human Galectin1
Mouse Galectin1
AAL
MPL
WGA
WGA
CTB
Gal-3C
Threefoil
Mouse NKR-P1C
ACL
AIA
Mono1-CNL
Mono2R-CNL
P11 VP8
PFL
CAA
ConA
GSL-2
GSL-1
GNL
EEL
DSL
DBA
HHL
HAA
HPA
CSA(EY Lab)
GSL-I-B4
LCA
LcH
LEL
NPA
LTL
MAL-I
MAA
PHA-E
MAA(Seikagaku)
MAL_II
PHA-E
PSL
PHA-L
PTA-Gal
PTL-I
PTL-II
RCAI (Vector)
PNA
PSA
SBA
STL
TJA-I
TJA-II
TL(EY Lab)
UDA
UEA-II
VVL
WFL
PA-IL
WGA(Seikagaku Bio)
Native HPA
SGA
AHL
SSR1
rMpL
TbFBL1
CC1G-10077
MGL 6B
MGL 6A-H262T
MGL 6A
HA70 complex-Alexa
WGA multimer
rPVL
VVHA lectin
BanLec
CGL
Gal-3
human Gal-7
mouse Gal-7
CA19.9-1B844
CA19.9-9L426
CA19.9-121SLE
IPS-K-4A2B8
Microvirin
ab5
AOL (Mahal Lab)
ABA
Mono2W-CNL
BPL
GS-II (EY Lab)
EEA (EY Lab)
GNA (EY Lab)
HHA (EY Lab)
PHA-L
PNA
SBA
SJA
UEA-I
VGA(EY Lab)
WGA
MNA-M
MOA
ME5(detected with a-HUD)
WT-VCC
AAL
LSL-150
LBL-152
ME7(detected with a-HUD)
SSL0-488
VLRB.aGPA.23
rNV VLP-Cy3
AFCNVH
HML-Native
#8 Lewis B (T218) (Abcam)
#15 Sialyl Lewis X (BD)
#5 Sialyl Lewis X (Calbio)
#14 Sialyl Lewis
hGal-1-488+BCR
TbFBL2
MGL 416
BTL native
GAC78
DC-ASGPR short
Sp Serotype 14.2
Sp Serotype 14.15
rSRL
SRL
Galectin-1
PssC
Avaren-Fc
CnSepL
CA19-9 Ab (DRG)
WT Galectin-8
CML-1
PhoSL001
Epa1-Np mutant Y228W
Epa1-Np mutant Y228A
Epa1-Np mutant E227G
Epa1-Np mutant E227A
Epa1-Np WT
ChMBL
DBL-1
hJAA-F11-B1
GlcNAc(β1-4)GlcNAc(β1-4)GlcNAc(β1-4)GlcNAc(β1-4)GlcNAc(β1-4)GlcNAc(β1-Sp8

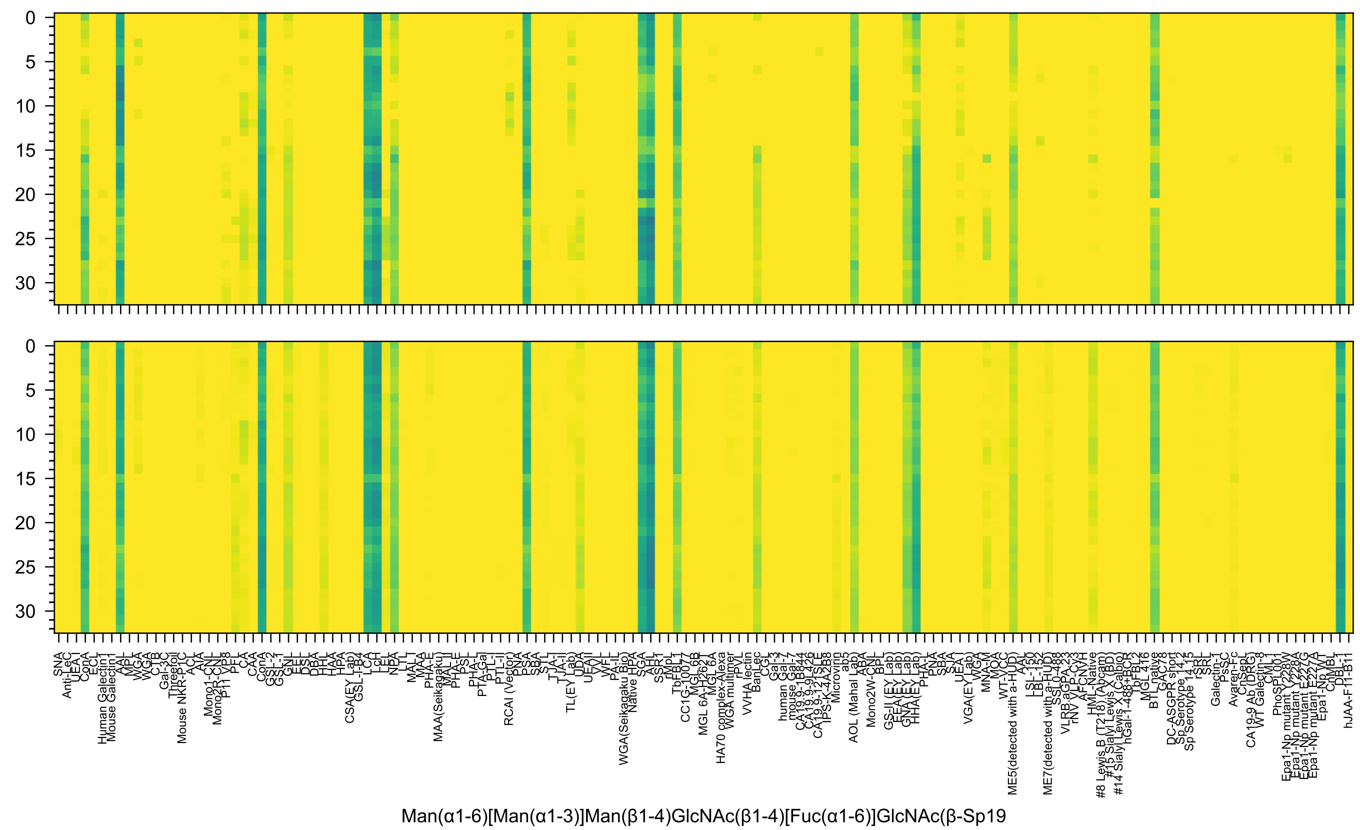

0
5
10
15
20
25
30
0
5
10
15
20
25
30
SNA
Anti-LeC
UEA-I
ConA
ECL
Human Galectin1
Mouse Galectin1
AAL
MPL
WGA
WGA
CTB
Gal-3C
Threefoil
Mouse NKR-P1C
ACL
AIA
Mono1-CNL
Mono2R-CNL
P11 VP8
PFL
CA
CAA
ConA
GSL-2
GSL-1
GNL
EEL
DSL
DBA
HHL
HAA
HPA
CSA(EY Lab)
GSL-I-B4
LCA
LcH
LEL
NPA
LTL
MAL-I
MAA
PHA-E
MAA(Seikagaku)
MAL-II
PHA-E
PSL
PHA-L
PTA-Gal
PTL-I
PTL-II
RCAI (Vector)
PNA
PSA
SBA
STL
TJA-I
TJA-II
TL(EY Lab)
UDA
UEA-II
VVL
WFL
PA-IL
WGA(Seikagaku Bio)
Native HPA
SGA
AHL
SSR1
rMpL
TbFBL1
CC1G-10077
MGL 6B
MGL 6A-H262T
MGL 6A
HA70 complex-Alexa
WGA multimer
rPVL
VVHA lectin
BanLec
CGL
Gal-3
human Gal-7
mouse Gal-7
CA19.9-1B844
CA19.9-9L426
CA19.9-121SLE
IPS-K-4A2B8
Microvirin
ab5
AOL (Mahal Lab)
ABA
Mono2W-CNL
BPL
GS-II (EY Lab)
EEA(EY Lab)
GNA(EY Lab)
HHA(EY Lab)
PHA-L
PNA
SBA
SJA
UEA-I
VGA(EY Lab)
WGA
MNA-M
MOA
WT-VCC
ME5(detected with a-HUD)
AAL
LSL-150
LBL-152
ME7(detected with a-HUD)
SSL0-488
VLRB.aGPA.23
rNV VLP-Cy3
AFL-NVH
HML-Native
#8 Lewis B (T218) (Abcam)
#15 Sialyl Lewis X (BD)
#14 Sialyl Lewis X (Calbio)
hGal-1-488+BCP
TbFBL2
MGL 416
BTL native
GAC78
DC-ASGPR short
Sp Serotype 14-2
Sp Serotype 14-15
rSRL
SRL
Galectin-1
PsSC
Avaren-Fc
CnSepL
CA19-9 Ab (DRG)
WT Galectin-8
CML-1
PhoSL001
Epa1-Np mutant Y228W
Epa1-Np mutant Y228A
Epa1-Np mutant E227G
Epa1-Np mutant E227A
Epa1-Np WT
ChMBL
DBL-1
hJAA-F11-B11
Man(α1-6)[Man(α1-3)]Man(β1-4)GlcNAc(β1-4)[Fuc(α1-6)]GlcNAc(β-Sp19

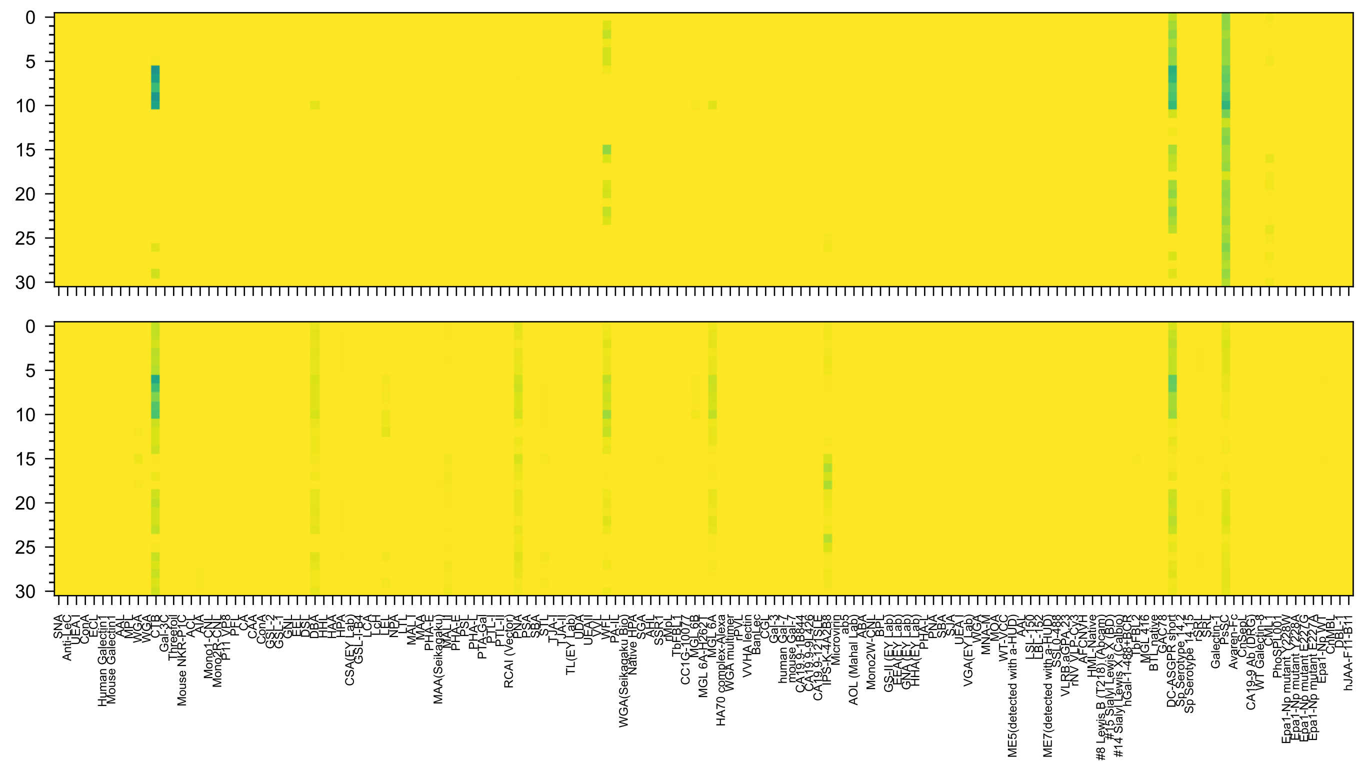

Neu5Ac(α2-3)Gal(β1-3)GalNAc(β1-4)[Neu5Ac(α2-3)]Gal(β1-4)Glc(β-Sp0

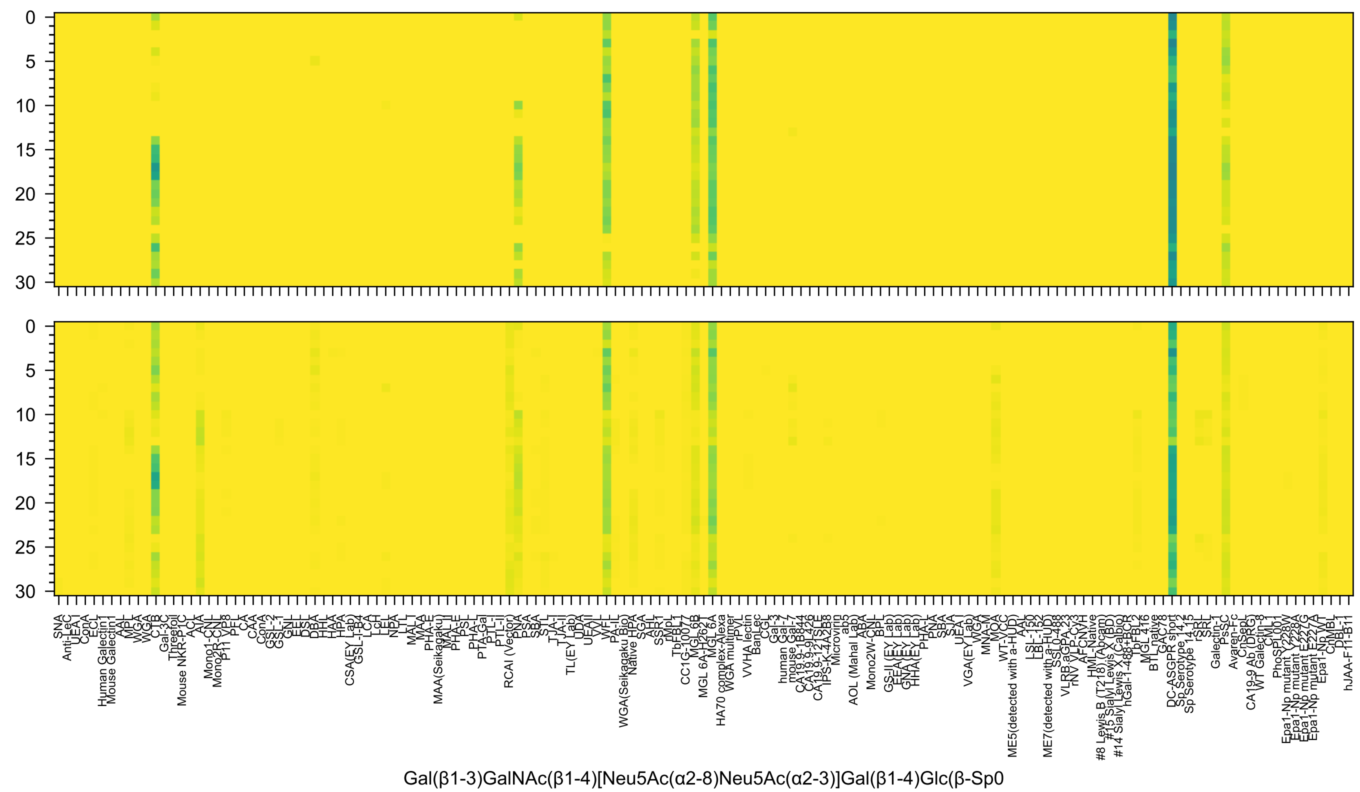

Gal(β1-3)GalNAc(β1-4)[Neu5Ac(α2-8)Neu5Ac(α2-3)]Gal(β1-4)Glc(β-Sp0

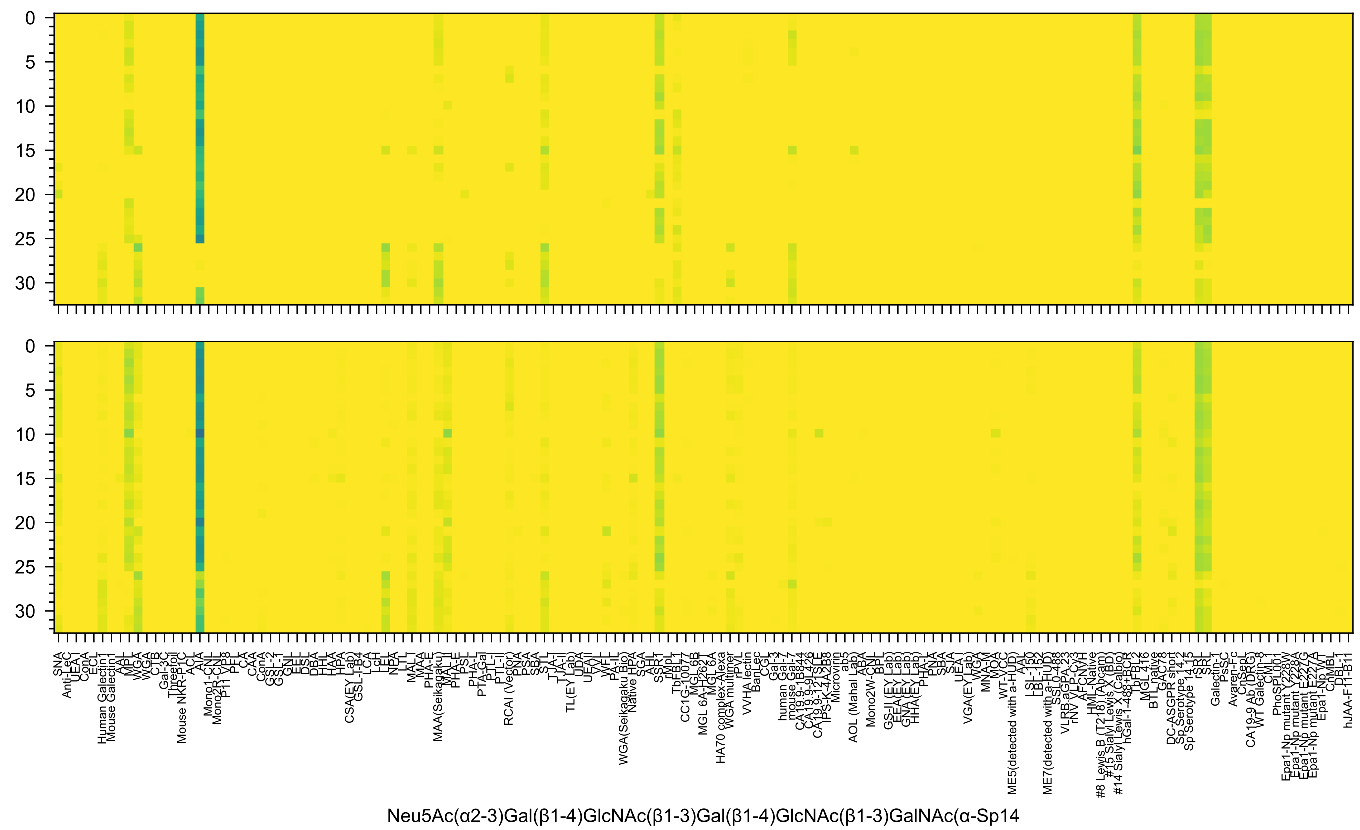
Neu5Ac(α2-3)Gal(β1-4)GlcNAc(β1-3)Gal(β1-4)GlcNAc(β1-3)GalNAc(α-Sp14

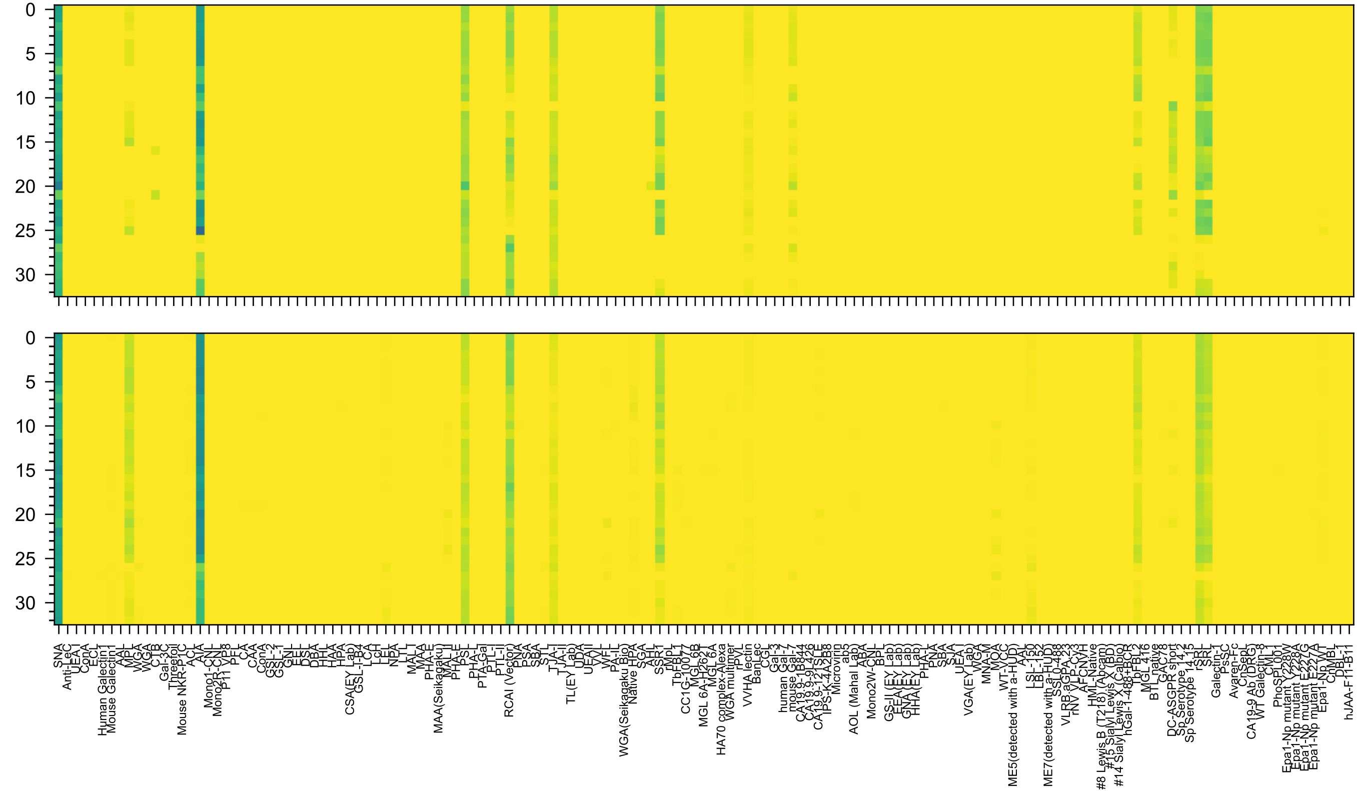

Neu5Ac(α2-6)Gal(β1-4)GlcNAc(β1-3)Gal(β1-4)GlcNAc(β1-3)GalNAc(α-Sp14

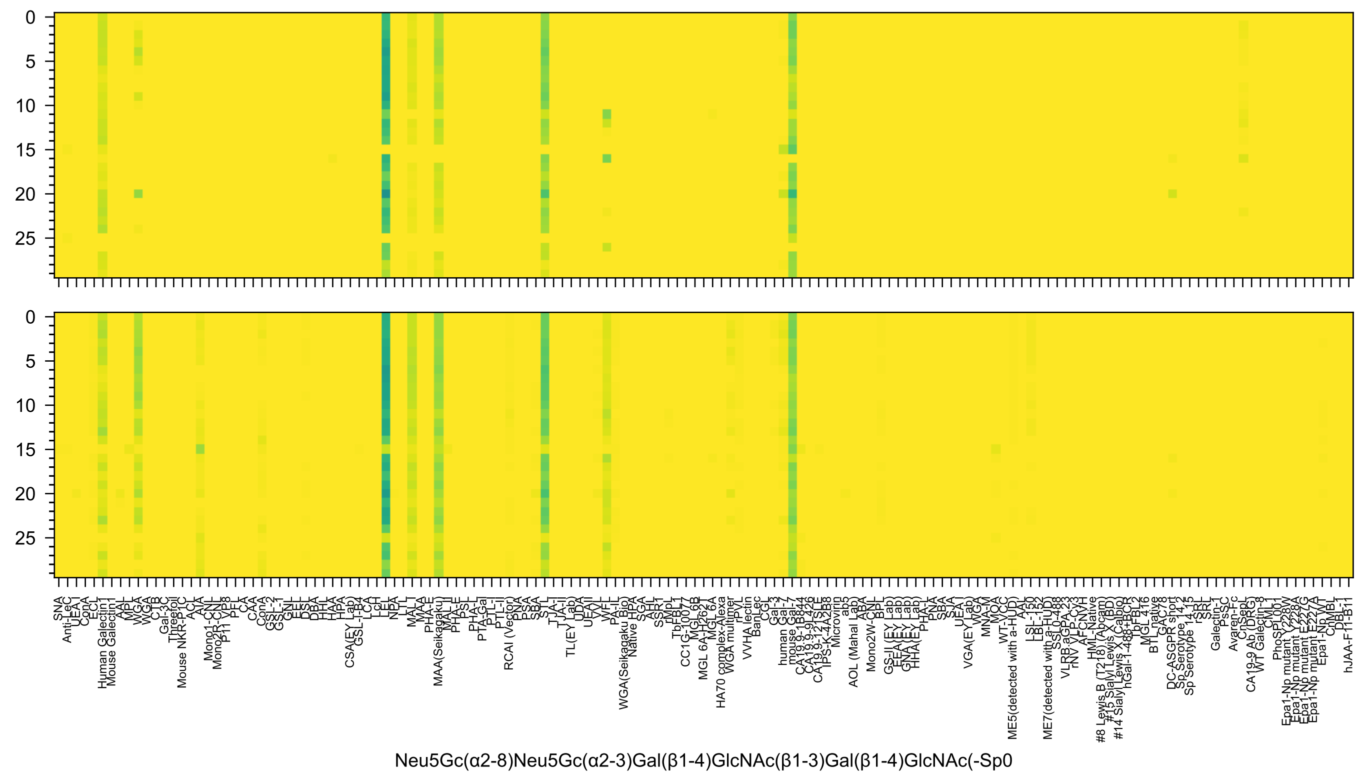

0
5
10
15
20
25
0
5
10
15
20
25
SNA
Anti-LeC
UEA-I
ConA
ECL
Human Galectin1
Mouse Galectin1
AAL
MPL
WGA
WGA
CTB
Gal-3C
Threefoil
Mouse NKR-P1C
ACL
AIA
Mono1-CNL
Mono2R-CNL
P11 VP8
PFL
CA
CAA
ConA
GSL-2
GSL-1
GNL
EEL
DSL
DBA
HHL
HAA
HPA
CSA(EY Lab)
GSL-I-B4
LCA
LcH
LEL
NPA
LTL
MAL-I
MAA
PHA-E
MAA(Seikagaku)
MAL-I
PHA-E
PSL
PHA-L
PTA-Gal
PTL-I
PTL-II
RCAI (Vector)
PNA
PSA
SBA
STL
TJA-I
TJA-II
TL(EY Lab)
UDA
UEA-II
VVL
WFL
PA-IL
WGA(Seikagaku Bio)
Native HPA
SGA
AHL
SSR1
rMpL
TbFBL1
CC1G-10077
MGL 6B
MGL 6A-H262T
MGL 6A
HA70 complex-Alexa
WGA multimer
rPVL
VVHA lectin
BanLec
CGL
Gal-3
human Gal-7
mouse Gal-7
CA19.9-1B844
CA19.9-9L426
CA19.9-121SLE
IPS-K-4A2B8
Microvirin
ab5
AOL (Mahal Lab)
ABA
Mono2W-CNL
BPL
GS-II (EY Lab)
EEA (EY Lab)
GNA (EY Lab)
HHA (EY Lab)
PHA-L
PNA
SBA
SJA
UEA-I
VGA(EY Lab)
WGA
MNA-M
MOA
WT-VCC
ME5(detected with a-HUD)
AAL
LSL-150
LBL-152
ME7(detected with a-HUD)
SSL0-488
VLRB.aGPA.23
rNV VLP-Cy3
AFCNVH
HML-Native
#8 Lewis B (T218) (Abcam)
#15 Sialyl Lewis X (BD)
#14 Sialyl Lewis X (Calbio)
hGal-1-488+BCR
TbFBL2
MGL 416
BTL native
GAC78
DC-ASGPR short
Sp Serotype 14.2
Sp Serotype 14.15
rSRL
SRL
Galectin-1
PsSC
Avaren-Fc
ChSepL
CA19-9 Ab (DRG)
WT Galectin-8
CML1
PhoSPL001
Epa1-Np mutant Y228W
Epa1-Np mutant E228A
Epa1-Np mutant E227G
Epa1-Np mutant E227A
Epa1-Np WT
CnMBL
DBL-1
hJAA-F11-B1
Neu5Gc(α2-8)Neu5Gc(α2-3)Gal(β1-4)GlcNAc(β1-3)Gal(β1-4)GlcNAc(-Sp0

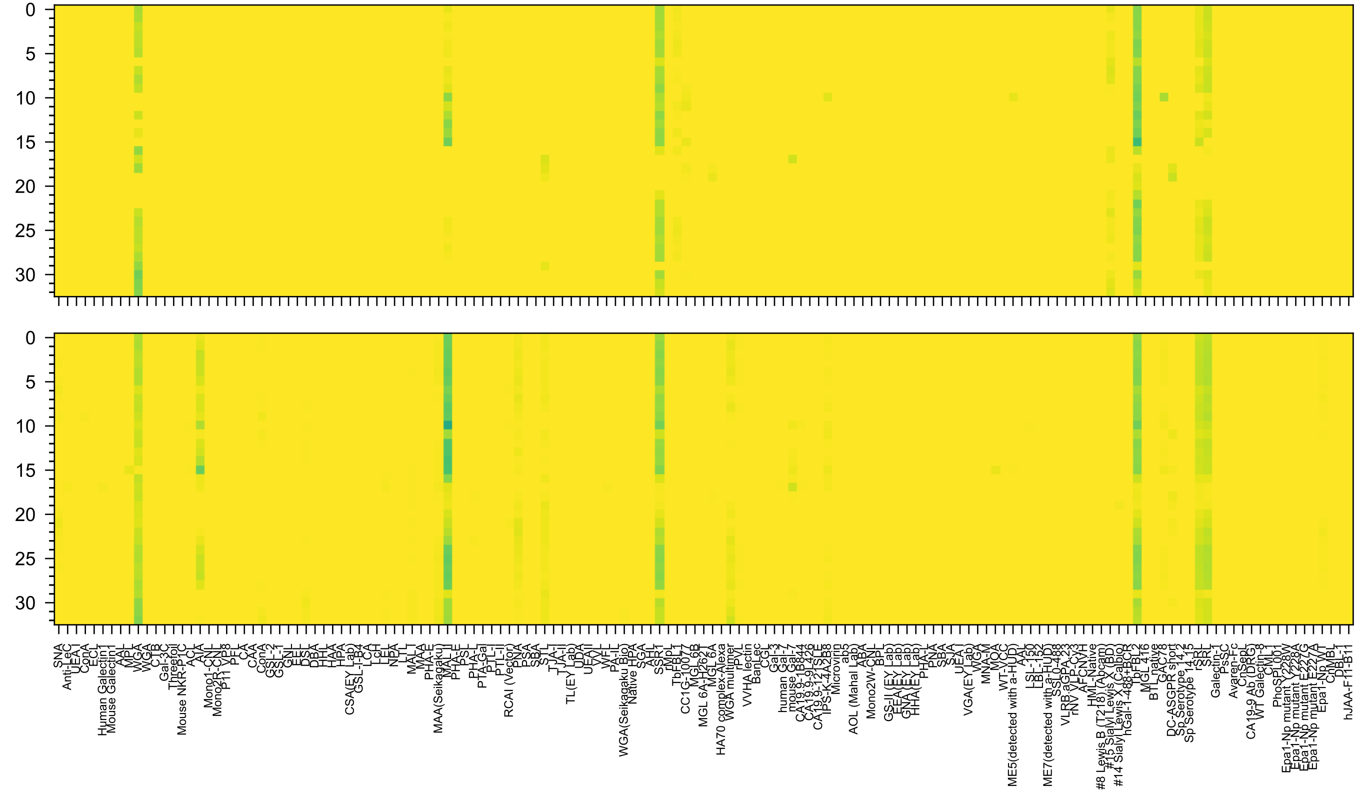

Neu5Ac(α2-3)Gal(β1-4)GlcNAc(β1-6)[Neu5Ac(α2-3)Gal(β1-3)]GalNAc(α-Sp14

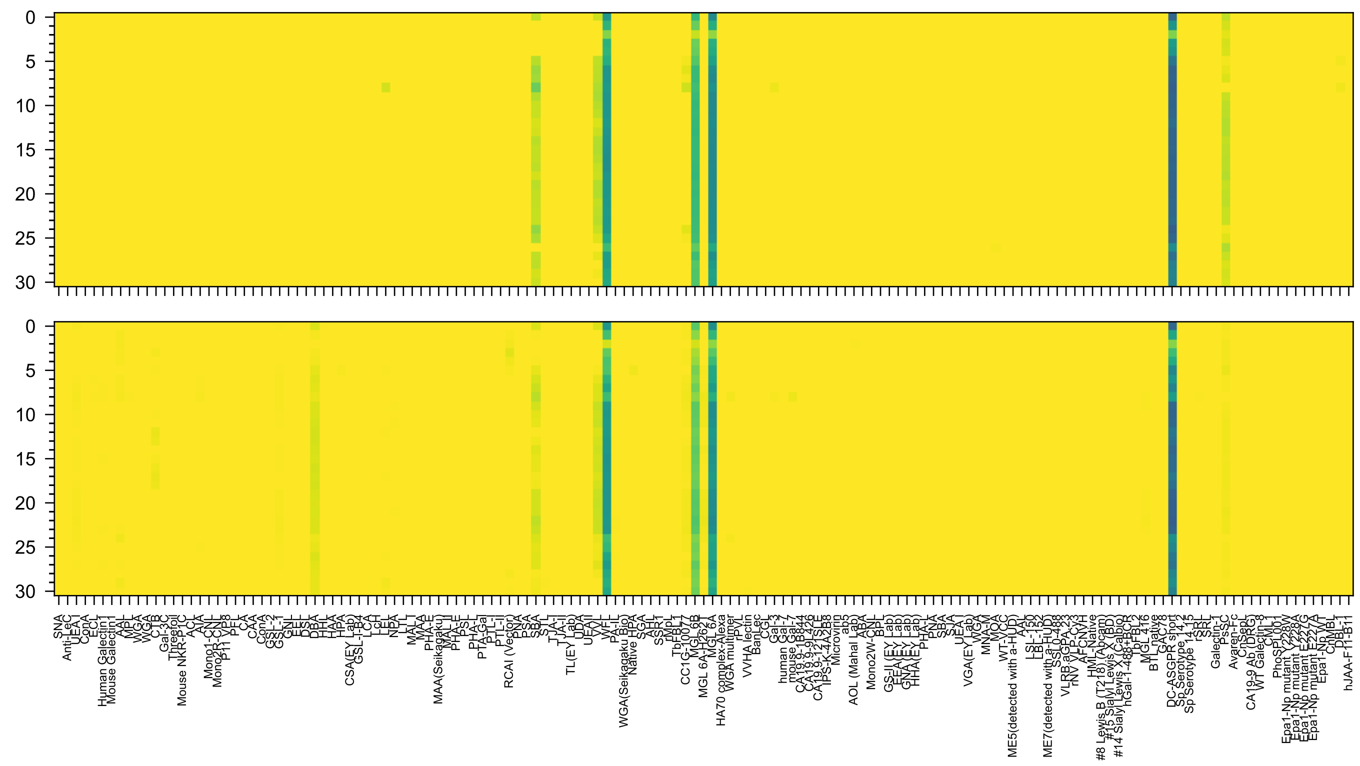

GalNAc(β1-4)[Neu5Ac(α2-8)Neu5Ac(α2-8)Neu5Ac(α2-3)]Gal(β1-4)Glc(β-Sp0

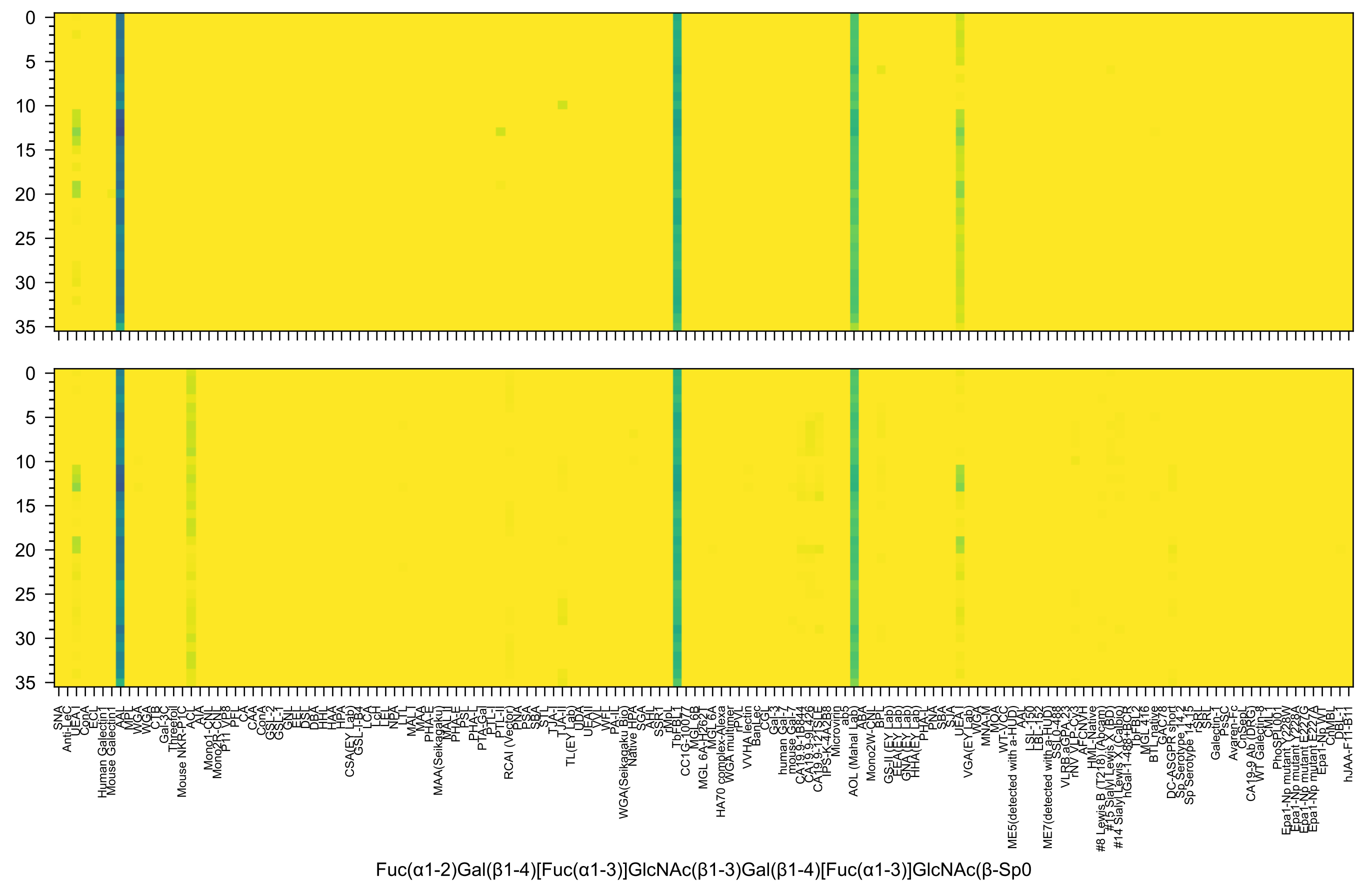
Fuc(α1-2)Gal(β1-4)[Fuc(α1-3)]GlcNAc(β1-3)Gal(β1-4)[Fuc(α1-3)]GlcNAc(β-Sp0

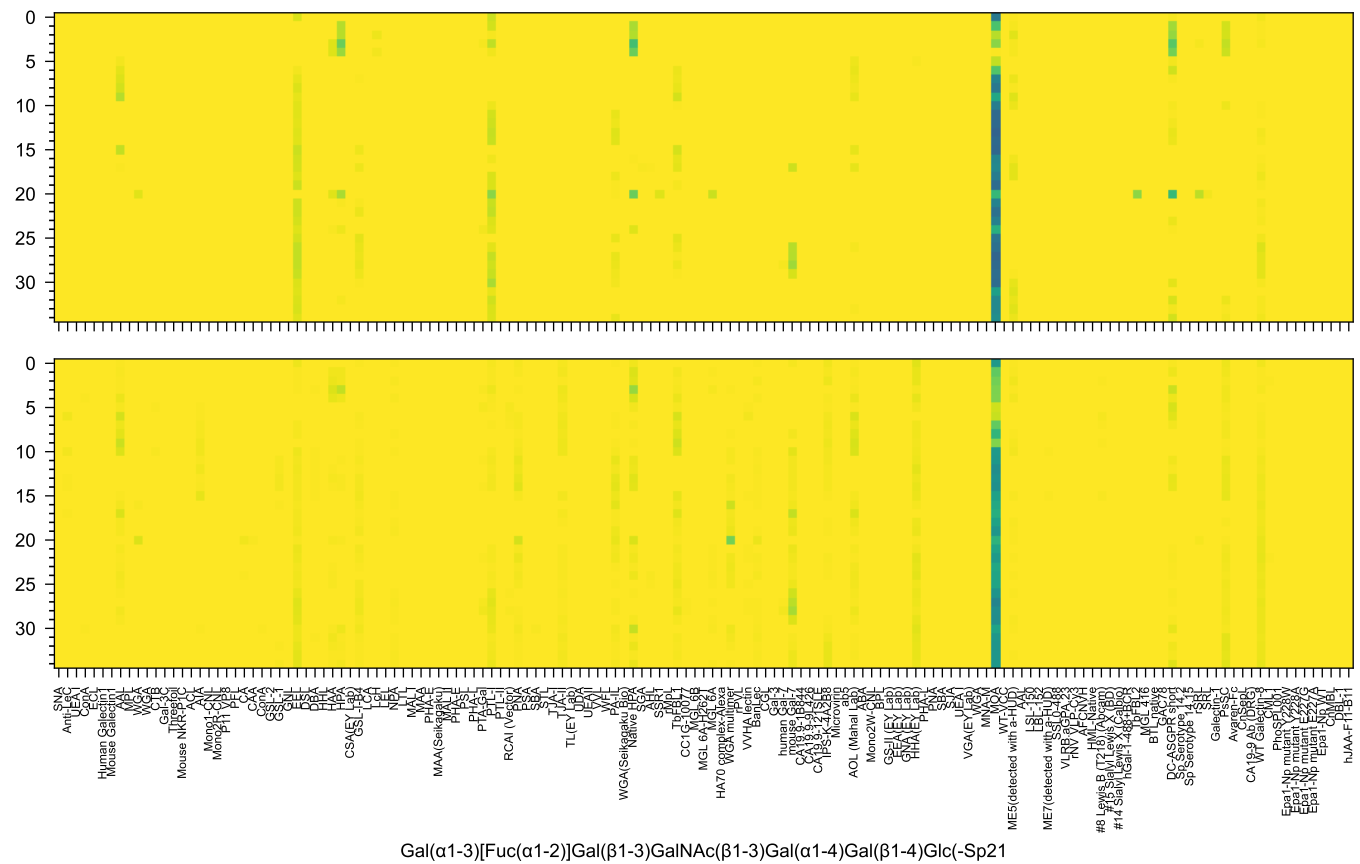
0
5
10
15
20
25
30
Gal(α1-3)[Fuc(α1-2)]Gal(β1-3)GalNAc(β1-3)Gal(α1-4)Gal(β1-4)Glc(-Sp21

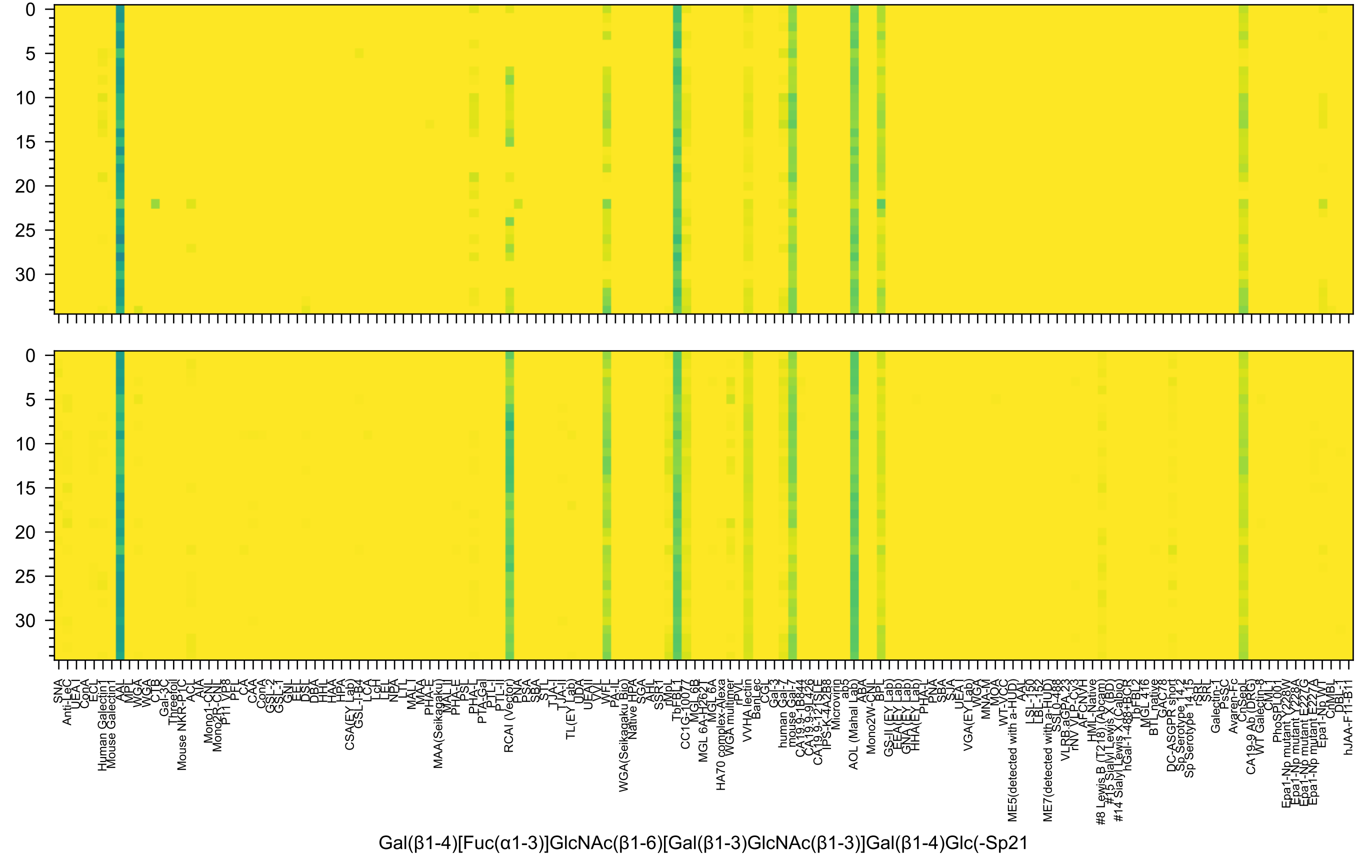

0
5
10
15
20
25
30
SNA
Anti-LeC
UEA I
ConA
ECL
Human Galectin1
Mouse Galectin1
AAL
MPL
WGA
WGA
CTB
Gal-3C
Threefoil
Mouse NKR-P1C
ACL
AIA
Mono1-CNL
Mono2R-CNL
P11 VP8
PFL
CA
CAA
ConA
GSL-2
GSL-1
GNL
EEL
DSL
DBA
HHL
HAA
HPA
CSA(EY Lab)
GSL-I-B4
LCA
LcH
LEL
NPA
LTL
MAL
MAA
PHA-E
MAA(Seikagaku)
MAL-I
PHA-E
PSL
PHA-L
PTA-Gal
PTL-I
PTL-II
RCAI (Vector)
PNA
PSA
SBA
STL
TJA-I
TJA-II
TL(EY Lab)
UDA
UEAII
VVL
WFL
PA-IL
WGA(Seikagaku Bio)
Native HPA
SGA
AHL
SSR1
rMpL
TbFBL1
CC1G-10077
MGL 6B
MGL 6A-H262T
MGL 6A
HA70 complex-Alexa
WGA multimer
rPVL
VVHA lectin
BanLec
CGL
Gal-3
human Gal-7
mouse Gal-7
CA19.9-1B844
CA19.9-9L426
CA19.9-121SLE
IPS-K-4A2B8
Microvirin
ab5
AOL (Mahal Lab)
ABA
Mono2W-CNL
BPL
GS-II (EY Lab)
EEA(EY Lab)
GNA(EY Lab)
HHA(EY Lab)
PHA-L
PNA
SBA
SJA
UEA I
VGA(EY Lab)
WGA
MNA-M
MOA
WT-VCC
ME5(detected with a-HUD)
AAL
LSL-150
LBL-152
ME7(detected with a-HUD)
SSL0-488
VLRB.aGPA.23
rNV VLP-Cy3
AFCNVH
HML-Native
#8 Lewis B (T218) (Abcam)
#15 Sialyl Lewis X (BD)
#14 Sialyl Lewis X (Calbio)
hGal-1-488+BCR
TbFBL2
MGL416
BTL native
GAC78
DC-ASGPR short
Sp Serotype 14.2
Sp Serotype 14.15
rSRL
SRL
Galectin-1
PsSC
Avaren-Fc
CnSepL
CA19-9 Ab (DRG)
WT Galectin-8
CML
PhoSPL001
Epa1-Np mutant Y228W
Epa1-Np mutant Y228A
Epa1-Np mutant E227G
Epa1-Np mutant E227A
Epa1-Np WT
ChMBL
DBL-1
hJAA-F11-B11
Gal(β1-4)[Fuc(α1-3)]GlcNAc(β1-6)[Gal(β1-3)GlcNAc(β1-3)]Gal(β1-4)Glc(-Sp21

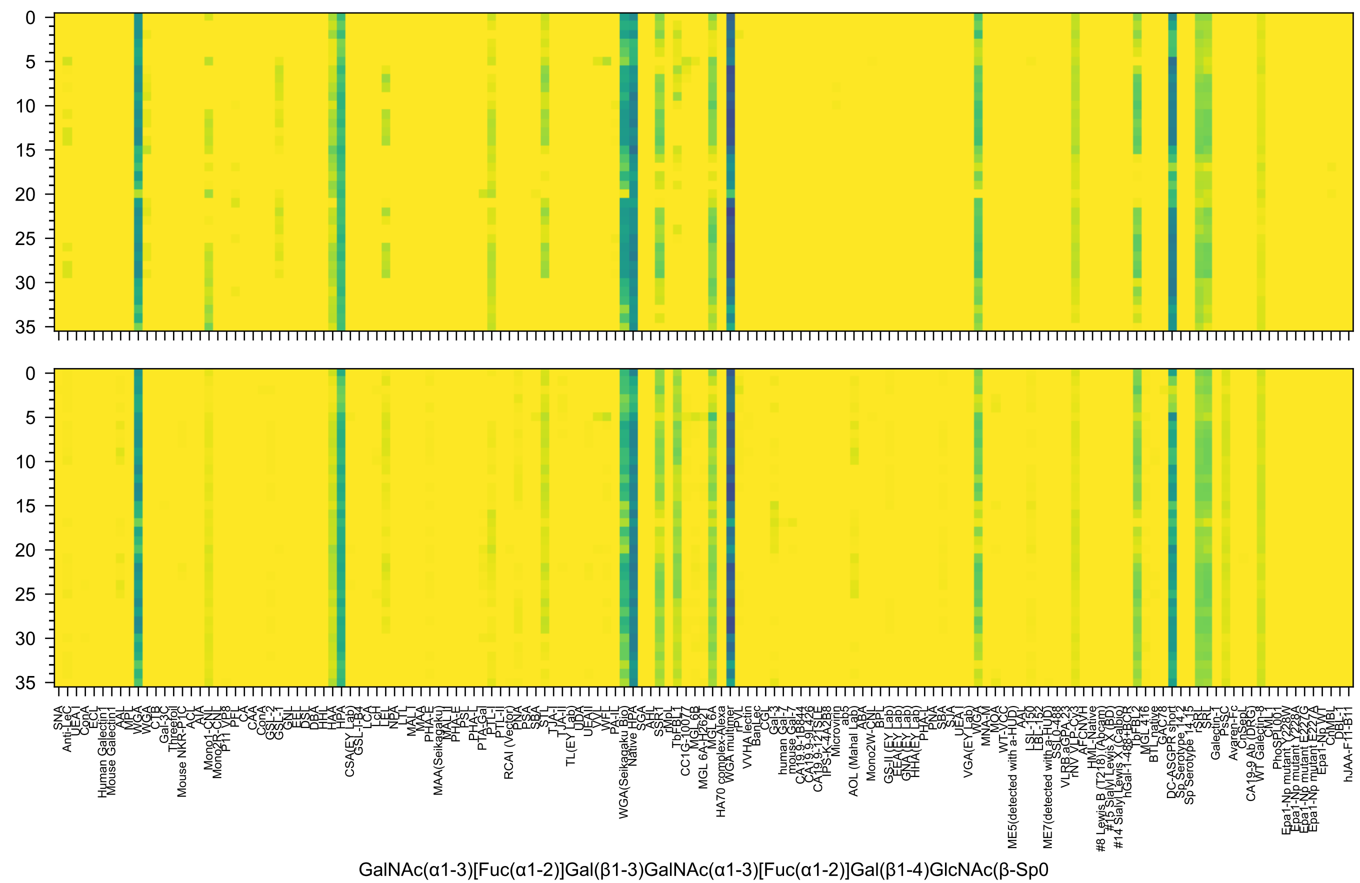
GalNAc(α1-3)[Fuc(α1-2)]Gal(β1-3)GalNAc(α1-3)[Fuc(α1-2)]Gal(β1-4)GlcNAc(β-Sp0

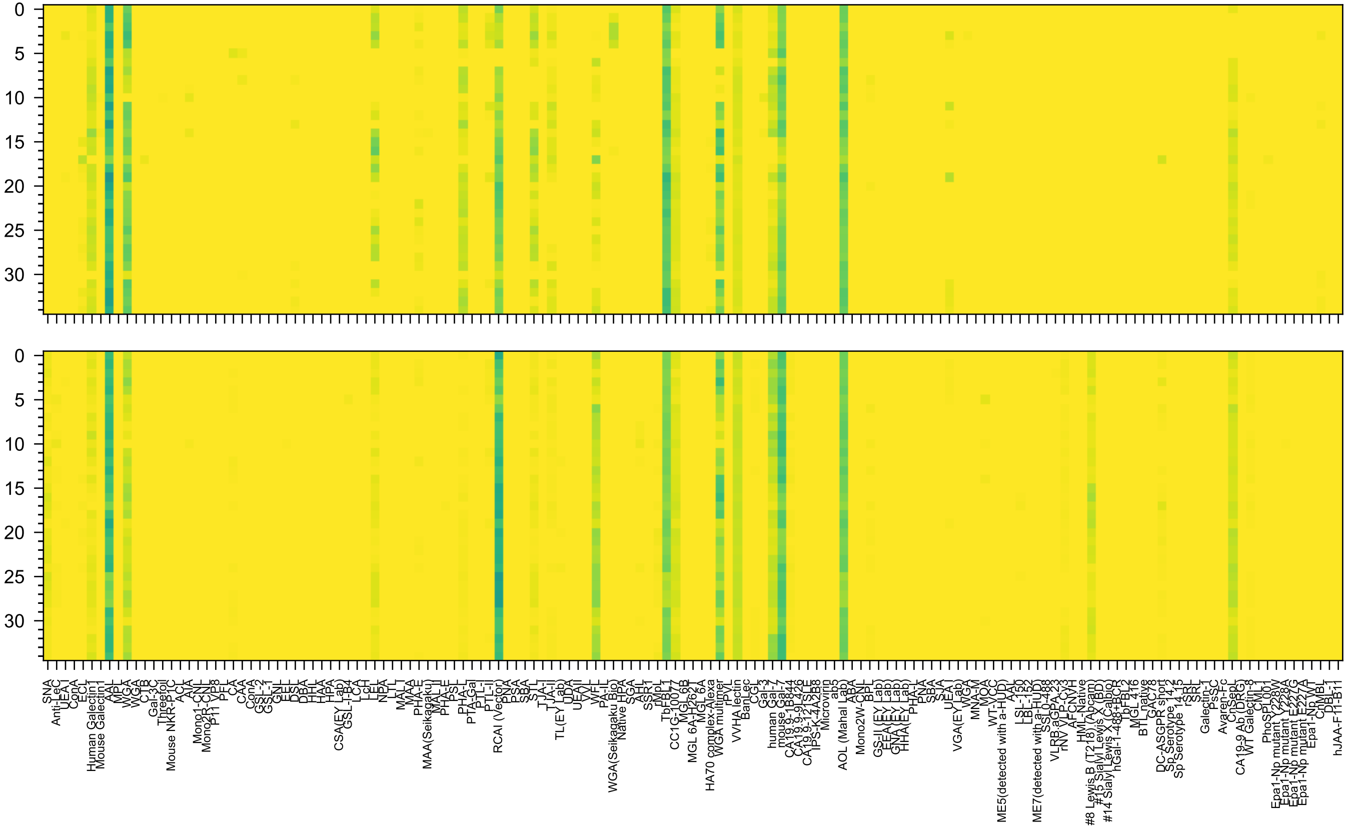

Gal(β1-4)GlcNAc(β1-6)[Fuc(α1-2)Gal(β1-3)GlcNAc(β1-3)]Gal(β1-4)Glc(-Sp21

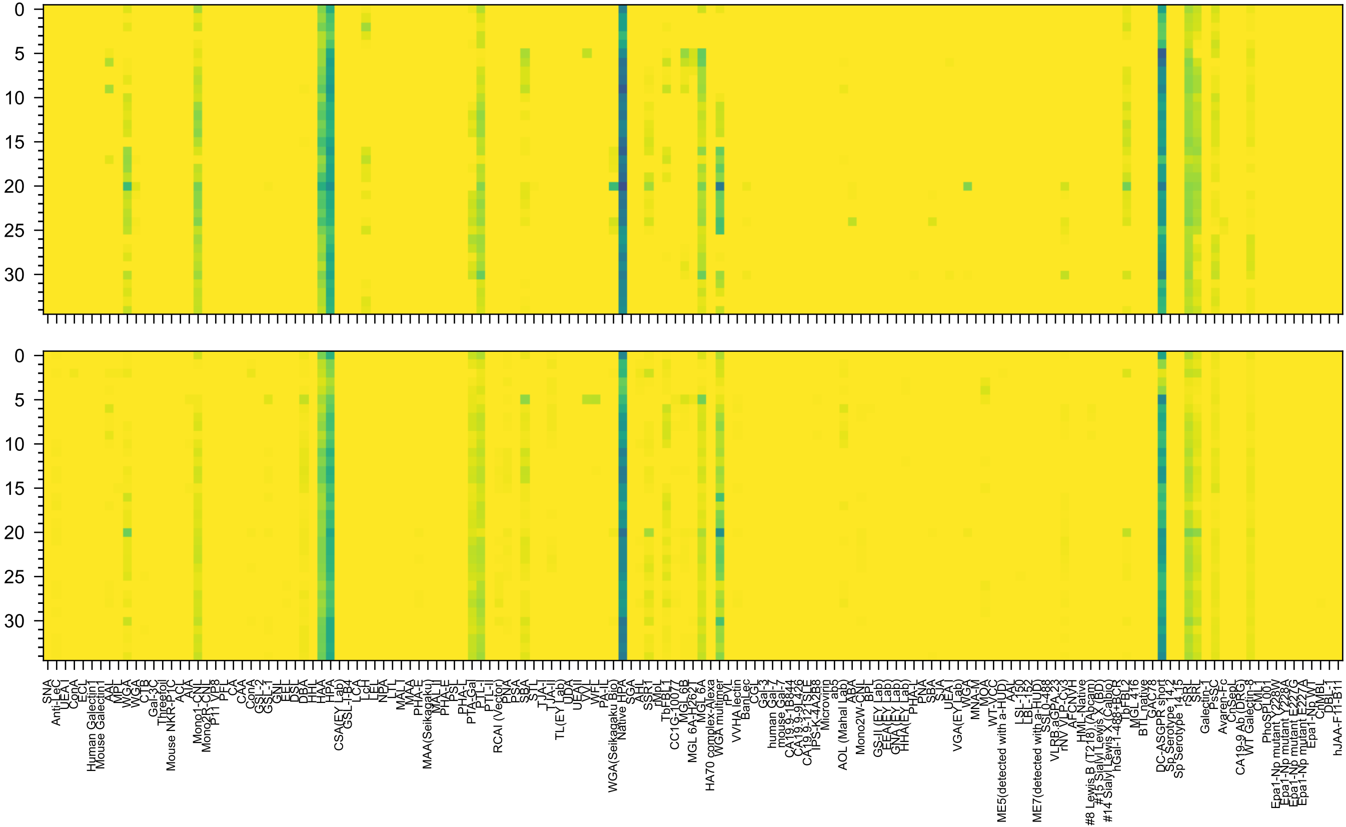

GalNAc(α1-3)[Fuc(α1-2)]Gal(β1-3)GalNAc(β1-3)Gal(α1-4)Gal(β1-4)Glc(-Sp21

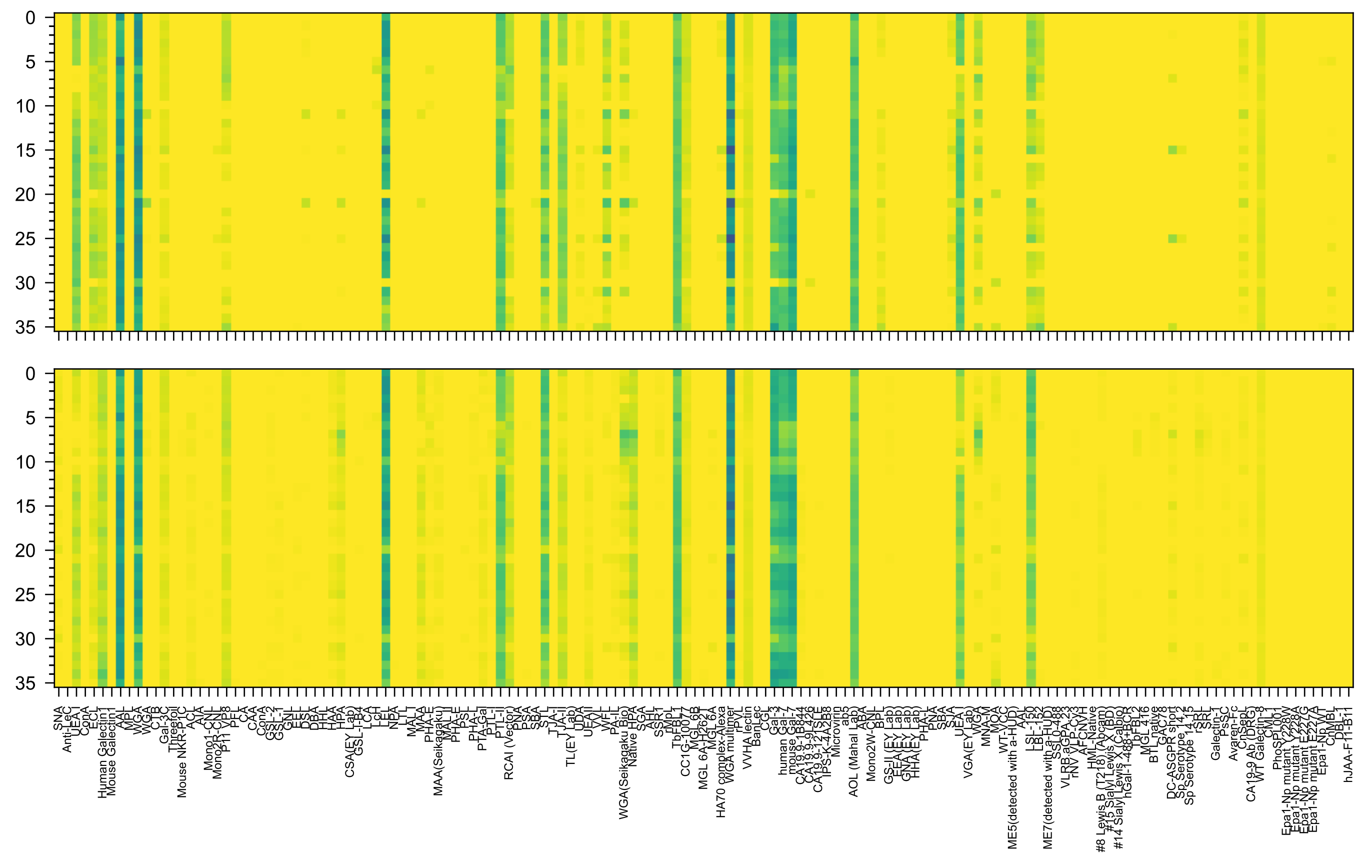

Fuc(α1-2)Gal(β1-4)GlcNAc(β1-3)Gal(β1-4)GlcNAc(β1-3)Gal(β1-4)GlcNAc(β-Sp0

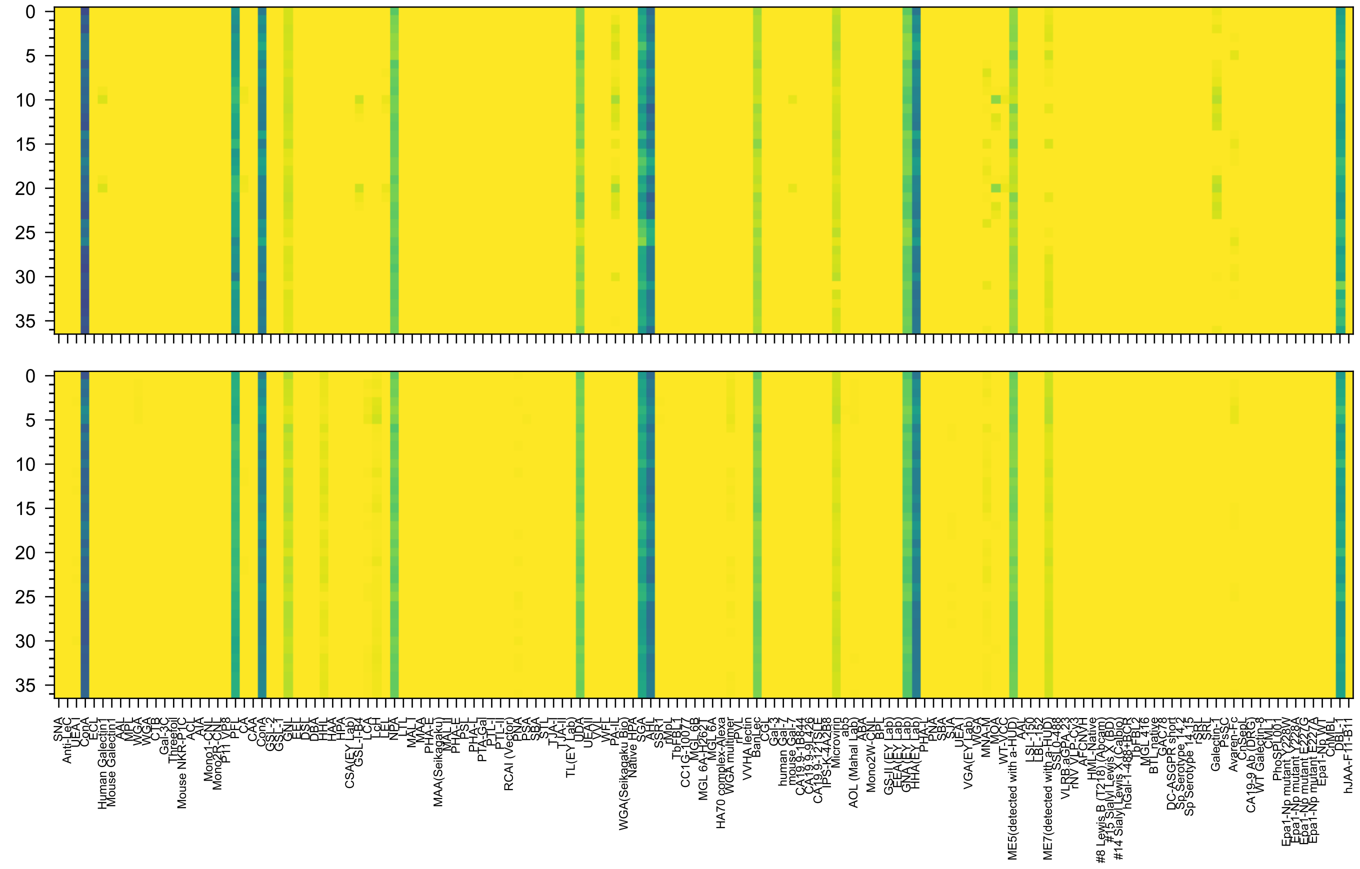

Man(α1-6)[Man(α1-3)]Man(α1-6)[Man(α1-3)]Man(β1-4)GlcNAc(β1-4)GlcNAc(β-Sp12

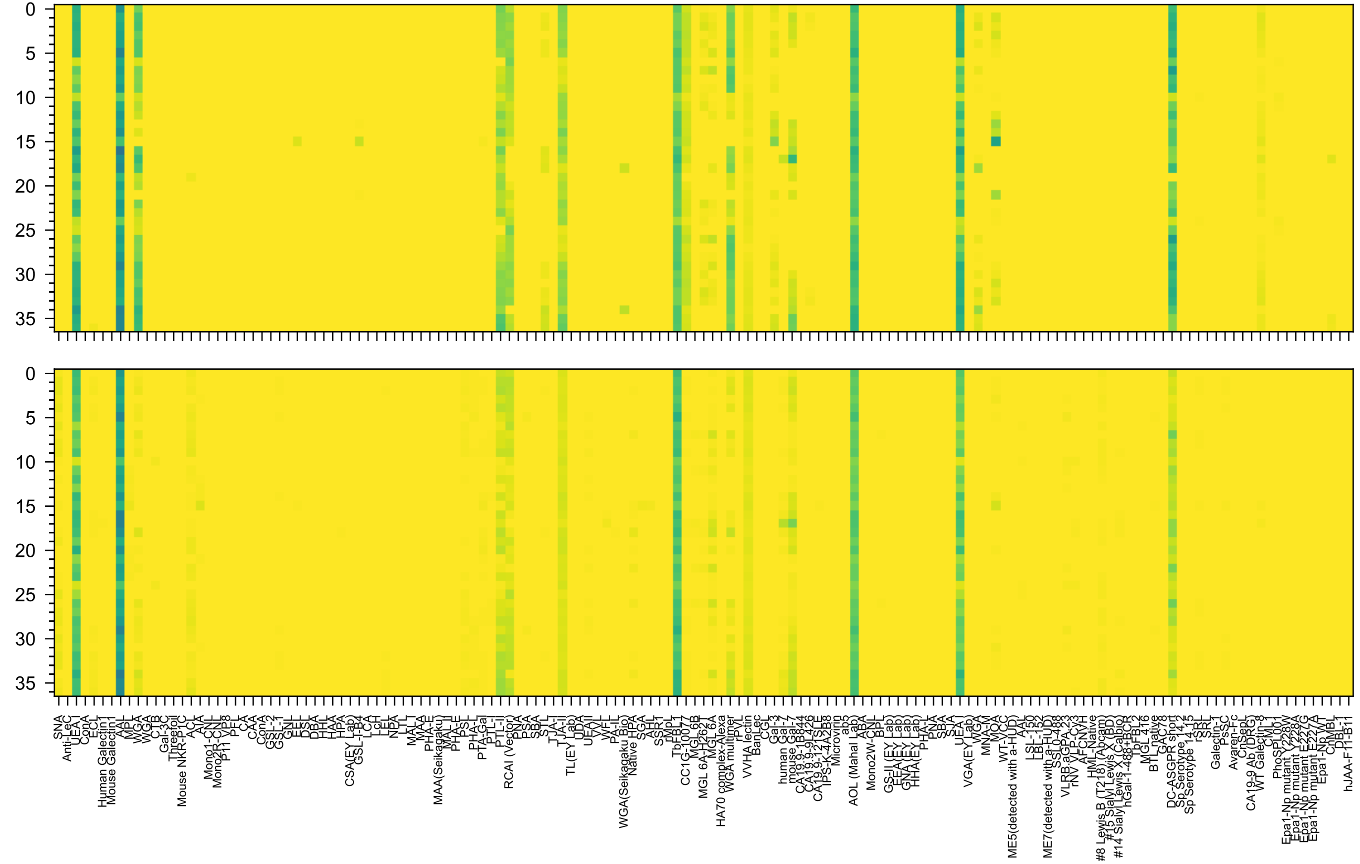

Fuc(α1-2)Gal(β1-4)GlcNAc(β1-6)[Fuc(α1-2)Gal(β1-4)GlcNAc(β1-3)]GalNAc(-Sp14

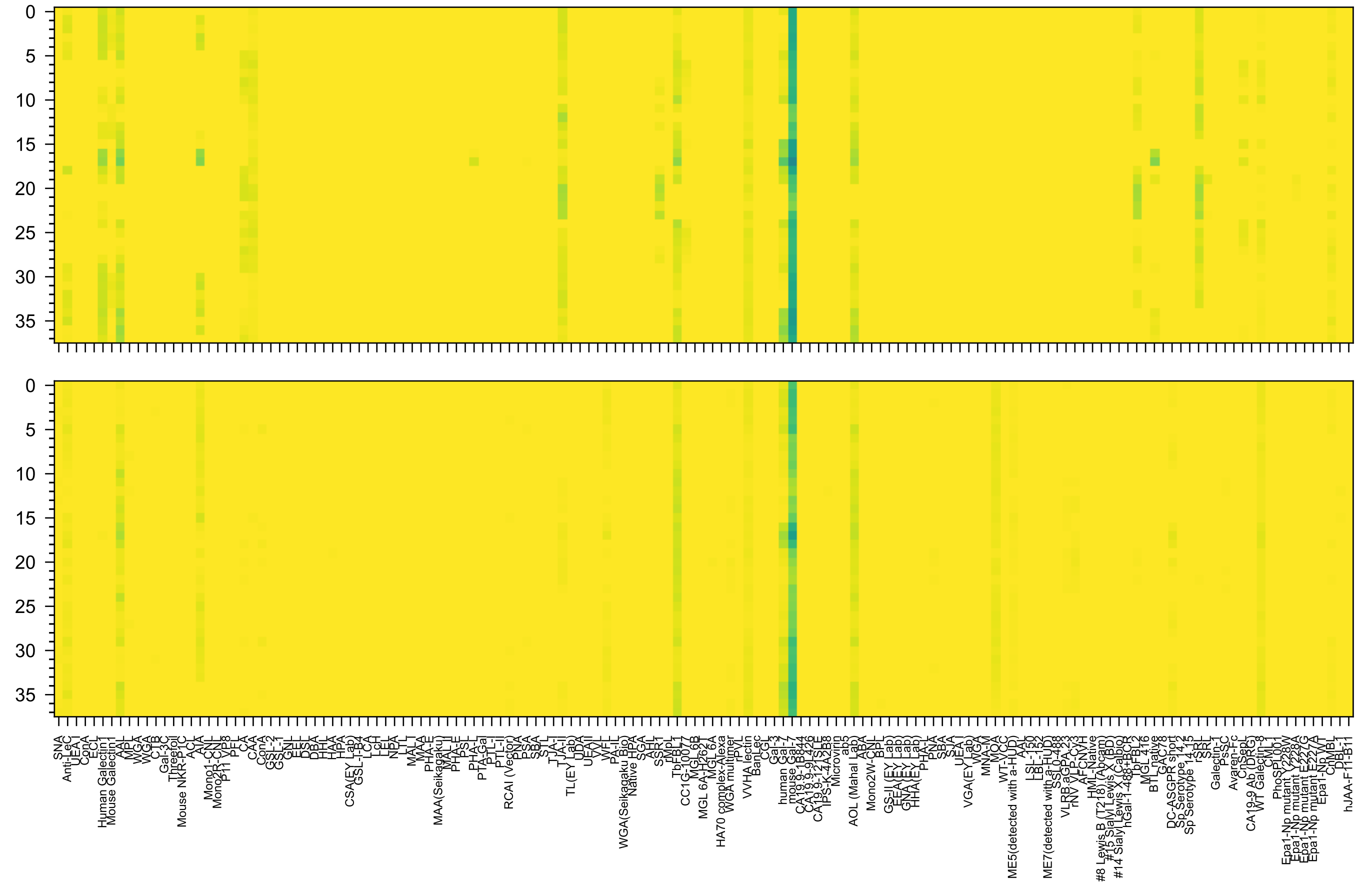

Fuc(α1-2)Gal(β1-3)GlcNAc(β1-6)[Fuc(α1-2)Gal(β1-3)GlcNAc(β1-3)]GalNAc(α-Sp14

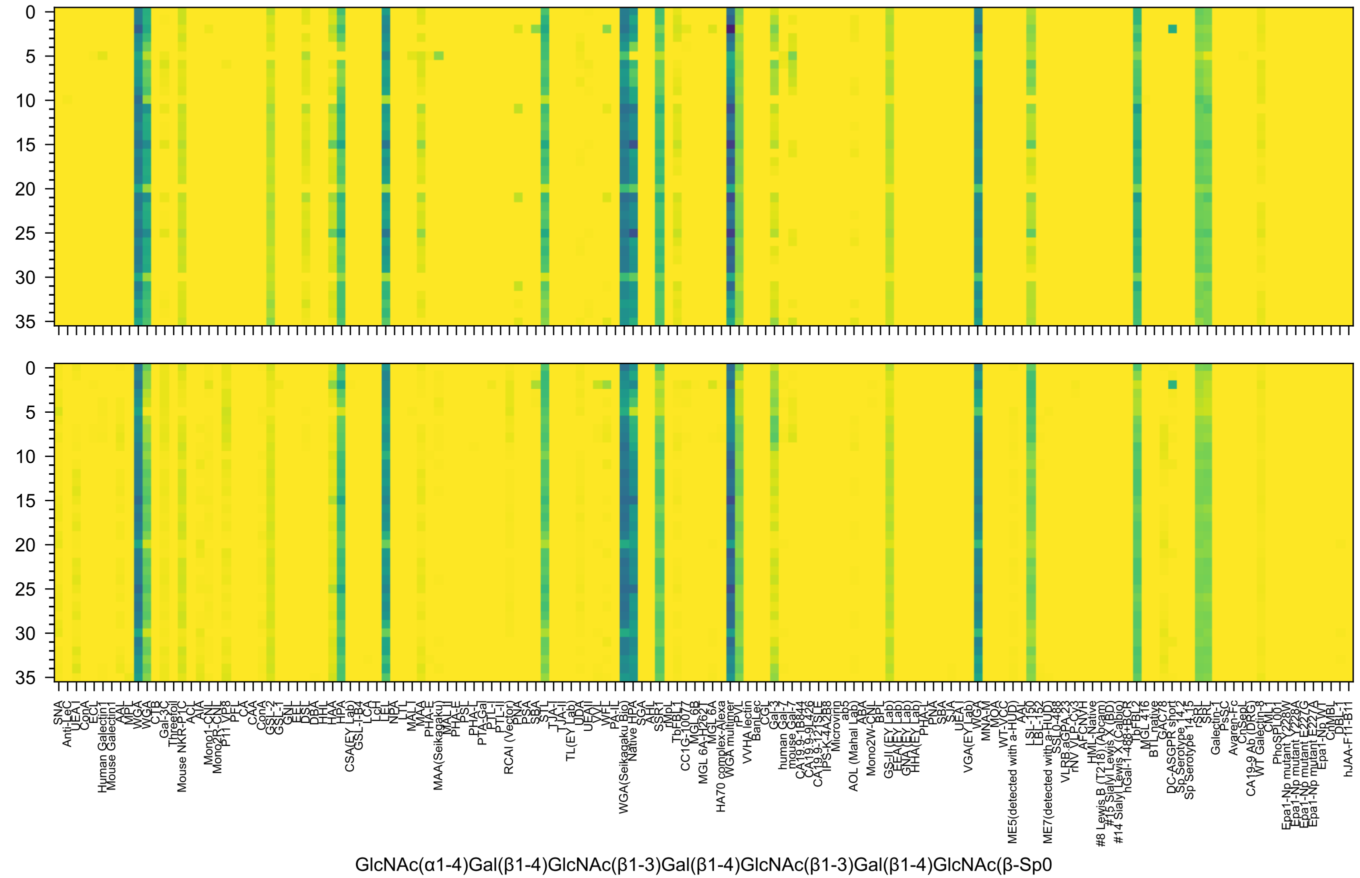
0
5
10
15
20
25
30
35
GlcNAc(α1-4)Gal(β1-4)GlcNAc(β1-3)Gal(β1-4)GlcNAc(β1-3)Gal(β1-4)GlcNAc(β-Sp0

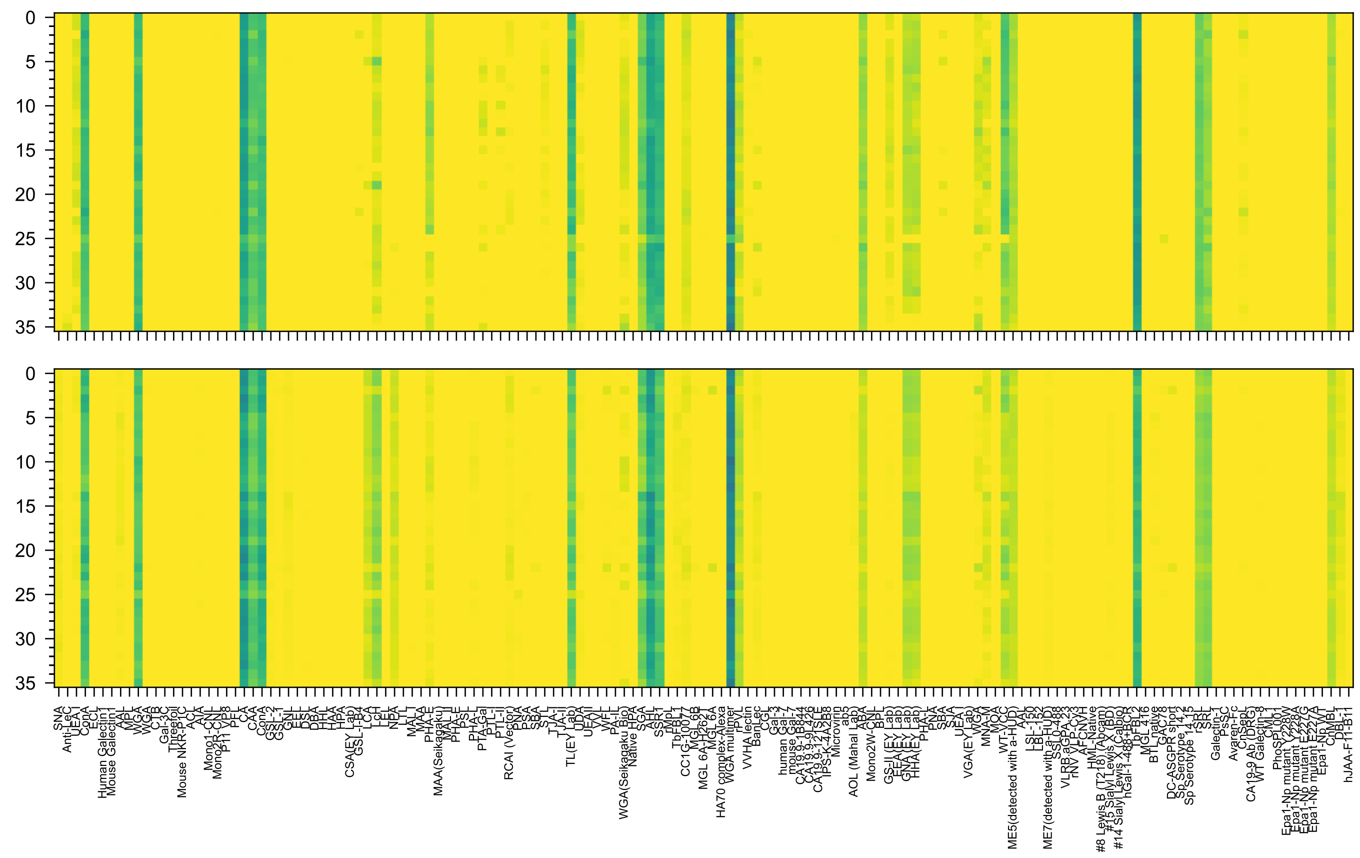

GlcNAc(β1-2)Man(α1-6)[GlcNAc(β1-2)Man(α1-3)]Man(β1-4)GlcNAc(β1-4)GlcNAc(β-Sp13

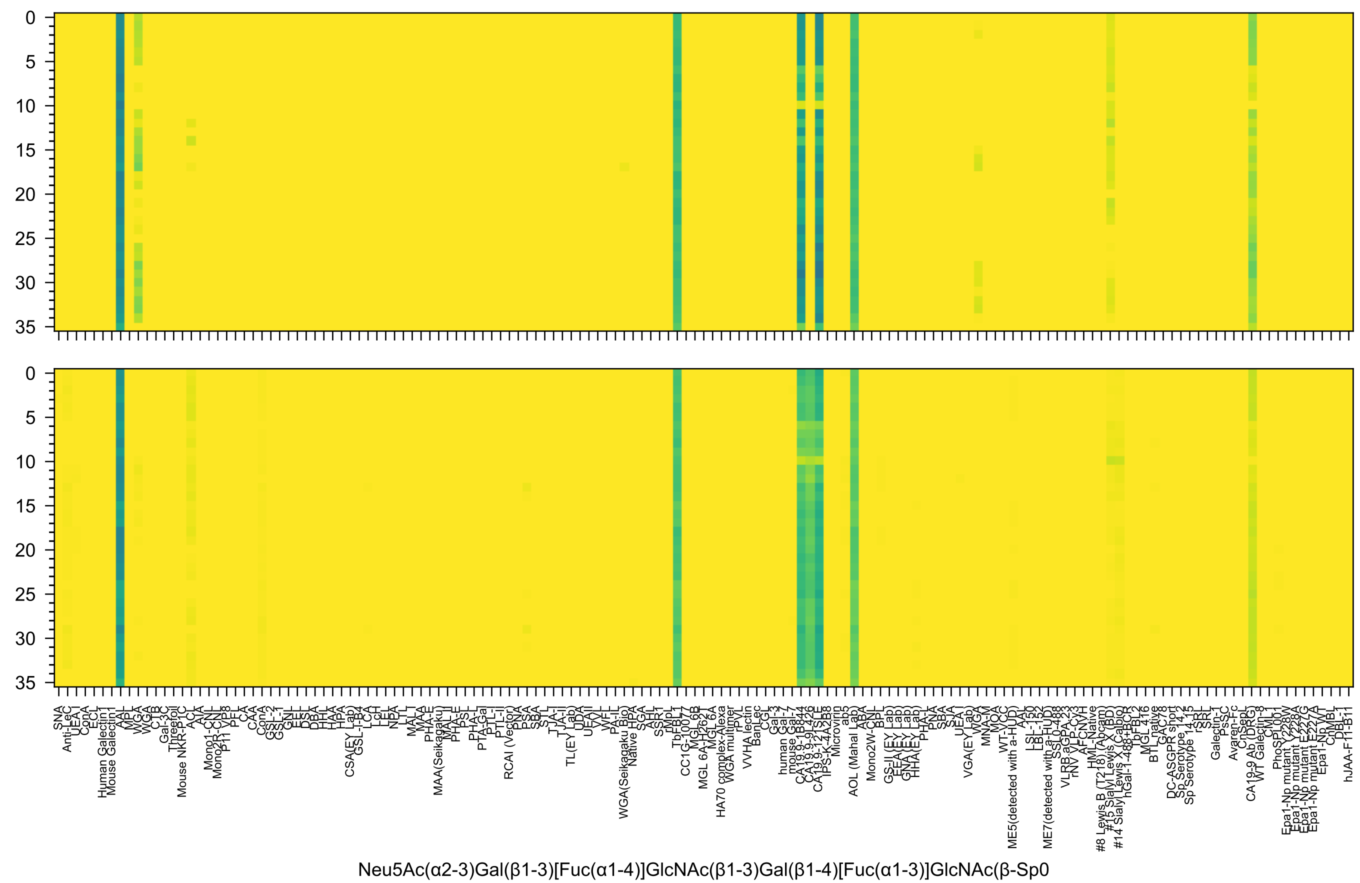
Neu5Ac(α2-3)Gal(β1-3)[Fuc(α1-4)]GlcNAc(β1-3)Gal(β1-4)[Fuc(α1-3)]GlcNAc(β-Sp0

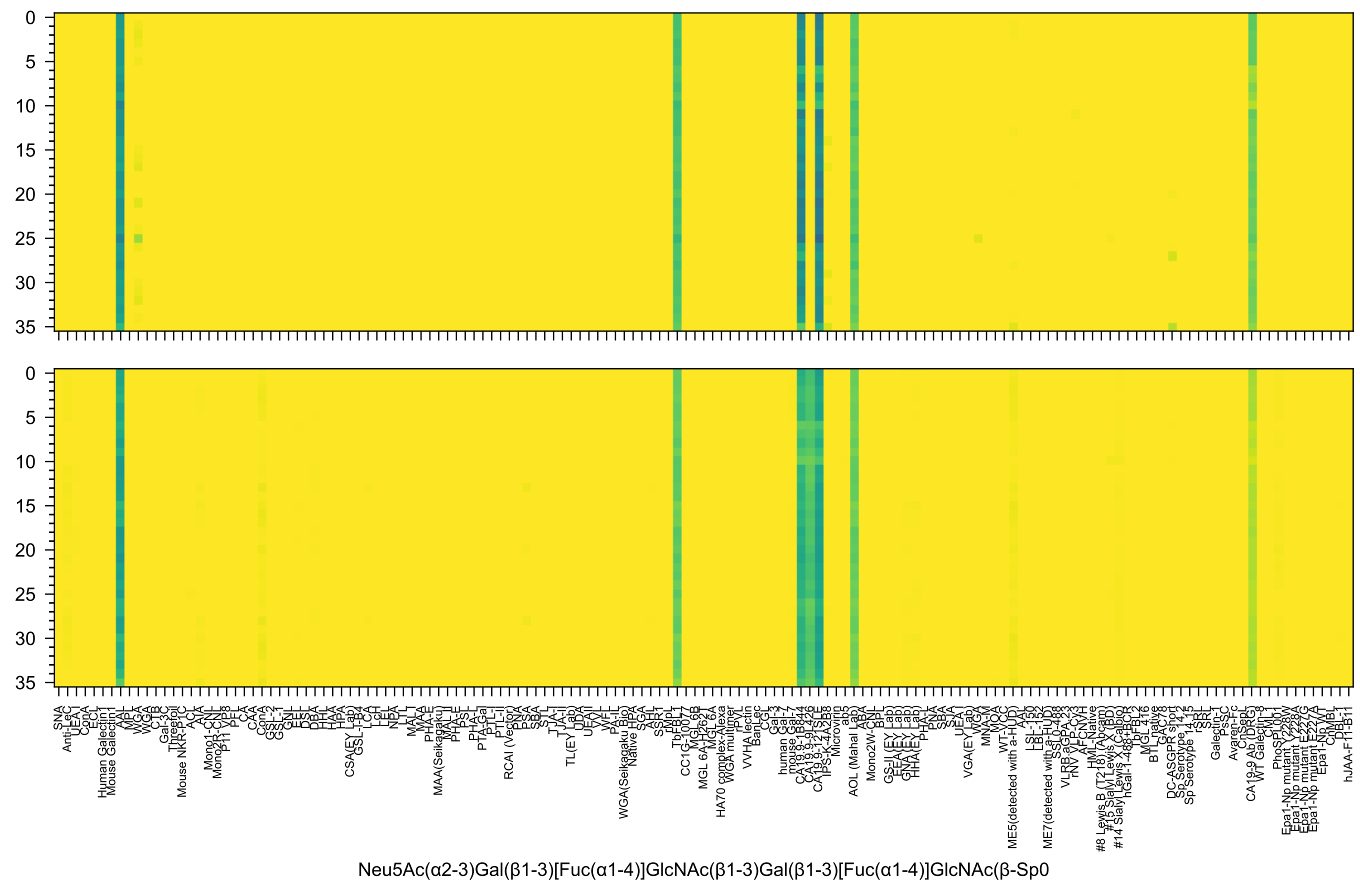
Neu5Ac(α2-3)Gal(β1-3)[Fuc(α1-4)]GlcNAc(β1-3)Gal(β1-3)[Fuc(α1-4)]GlcNAc(β-Sp0

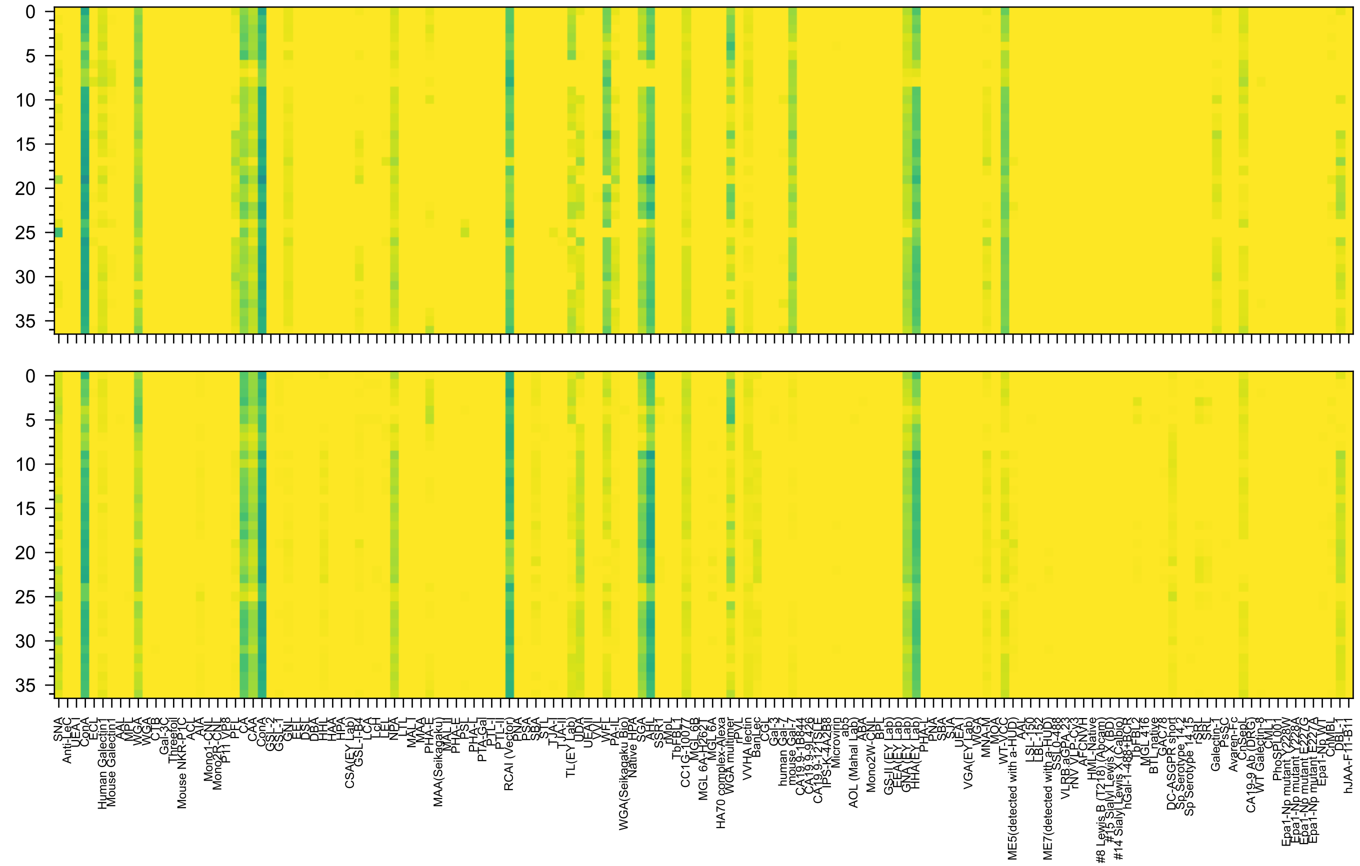

Man(α1-6)[Gal(β1-4)GlcNAc(β1-2)Man(α1-3)]Man(β1-4)GlcNAc(β1-4)GlcNAc(β-Sp12

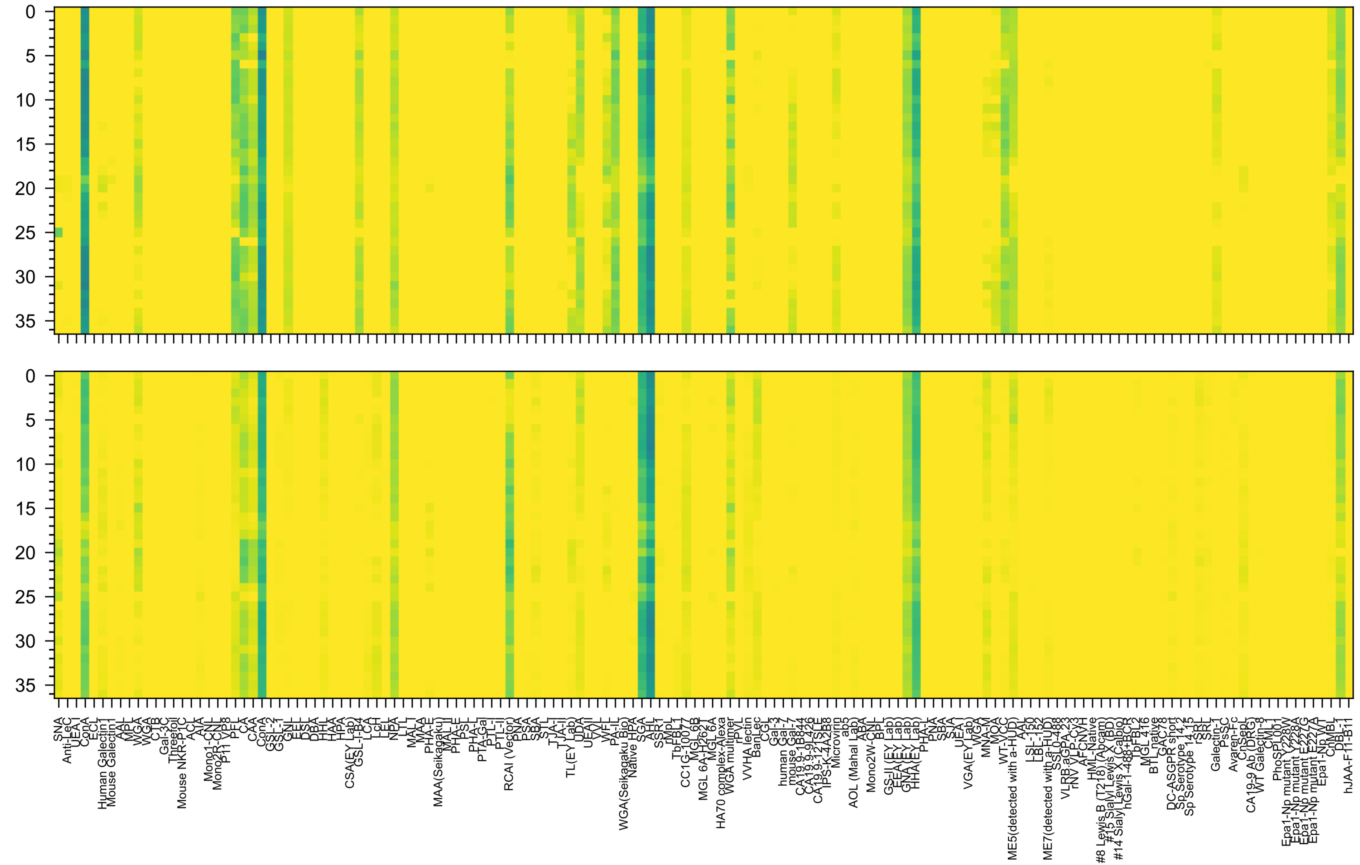

Gal(β1-4)GlcNAc(β1-2)Man(α1-6)[Man(α1-3)]Man(β1-4)GlcNAc(β1-4)GlcNAc(β-Sp12

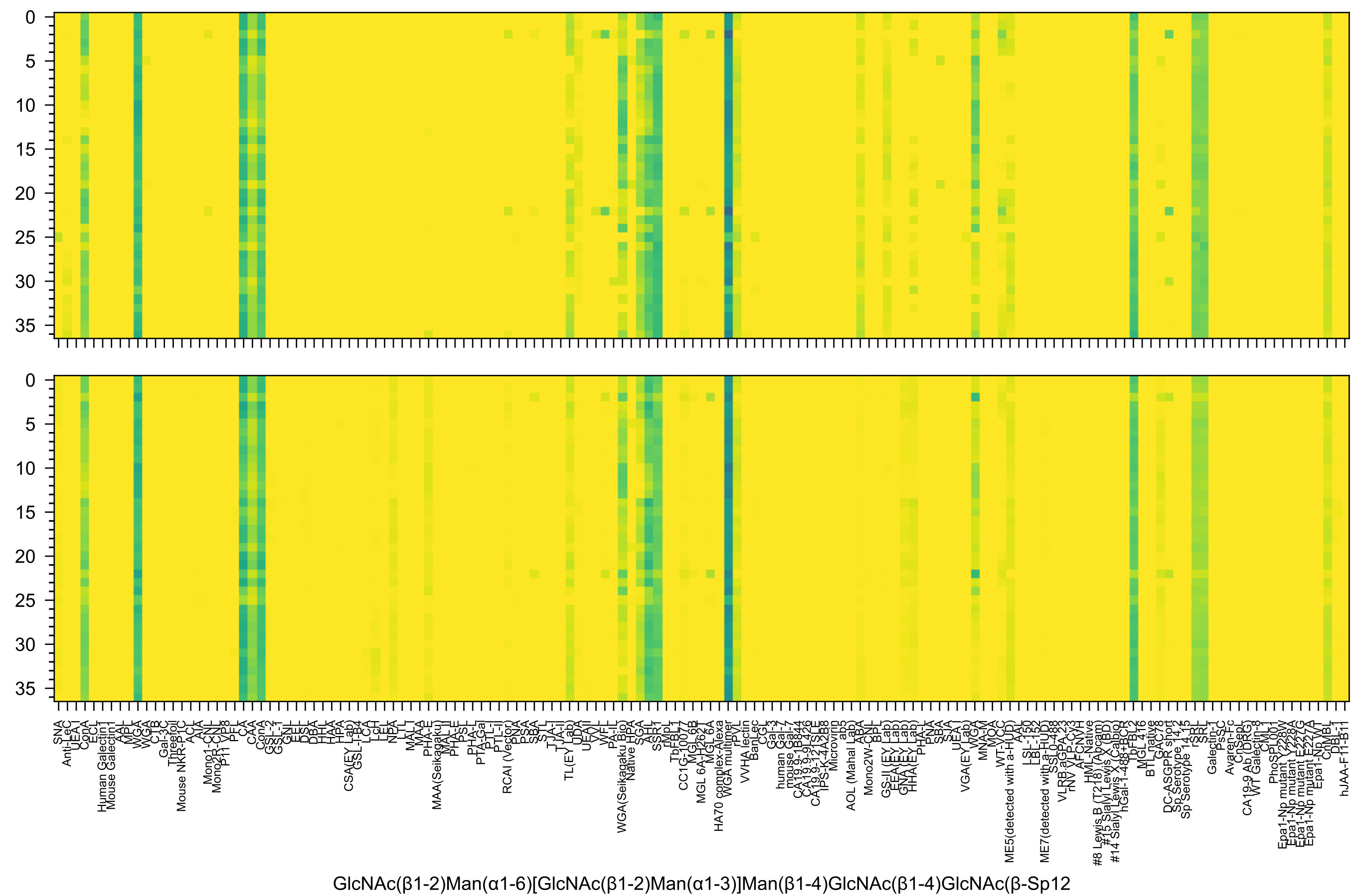
GlcNAc(β1-2)Man(α1-6)[GlcNAc(β1-2)Man(α1-3)]Man(β1-4)GlcNAc(β1-4)GlcNAc(β-Sp12

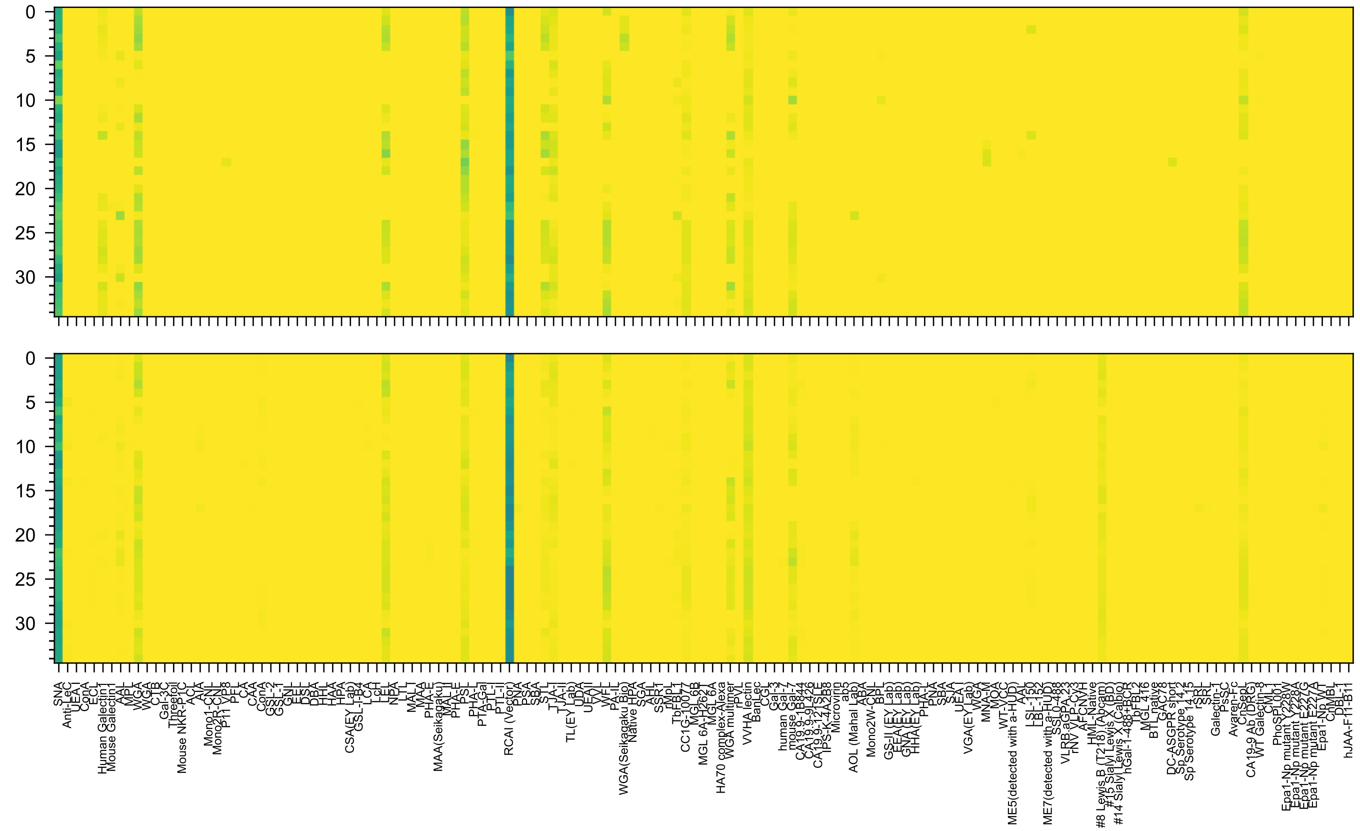

Gal(β1-4)GlcNAc(β1-6)[Neu5Ac(α2-6)Gal(β1-3)GlcNAc(β1-3)]Gal(β1-4)Glc(-Sp21

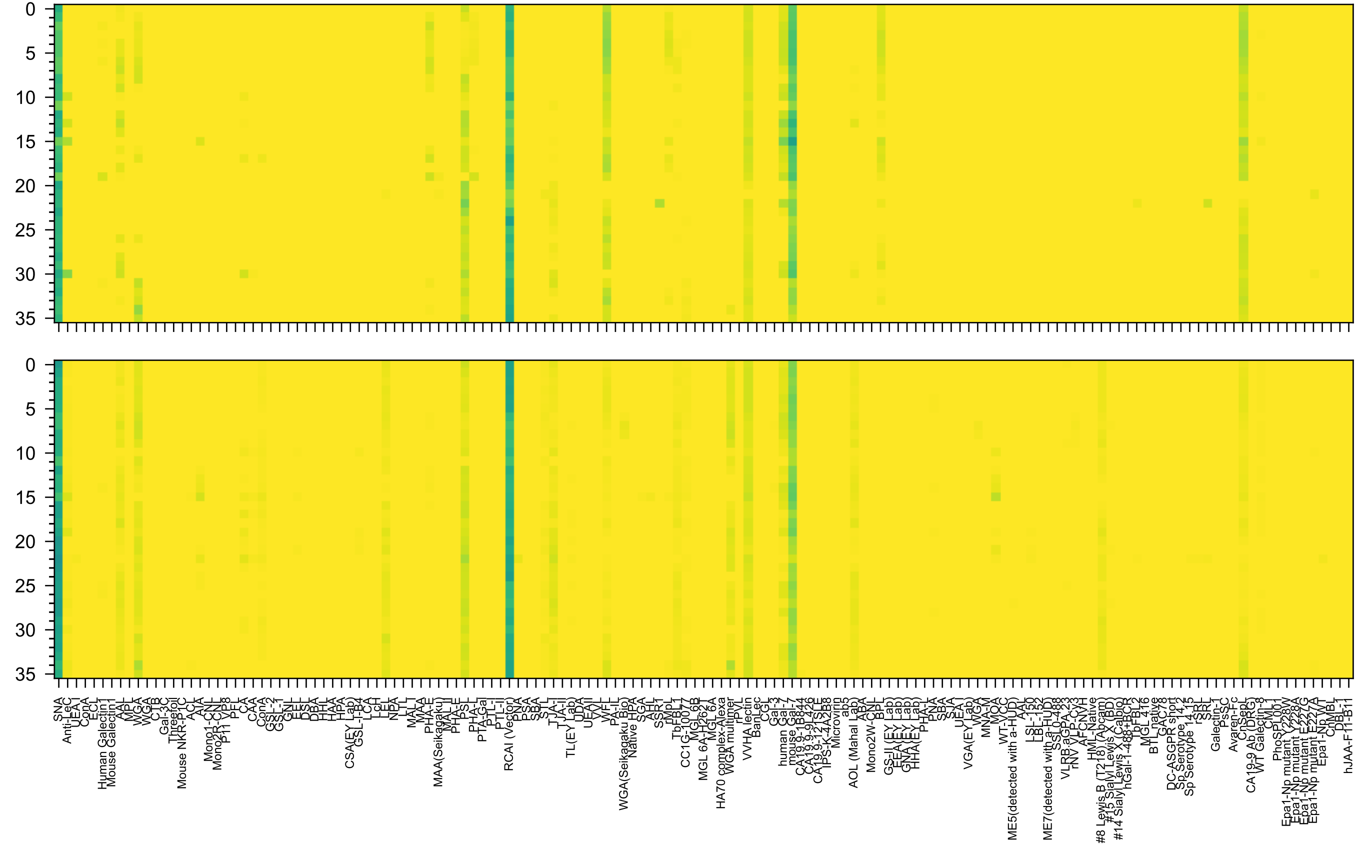

Neu5Ac(α2-6)Gal(β1-4)GlcNAc(β1-6)[Gal(β1-3)GlcNAc(β1-3)]Gal(β1-4)Glc(β-Sp21

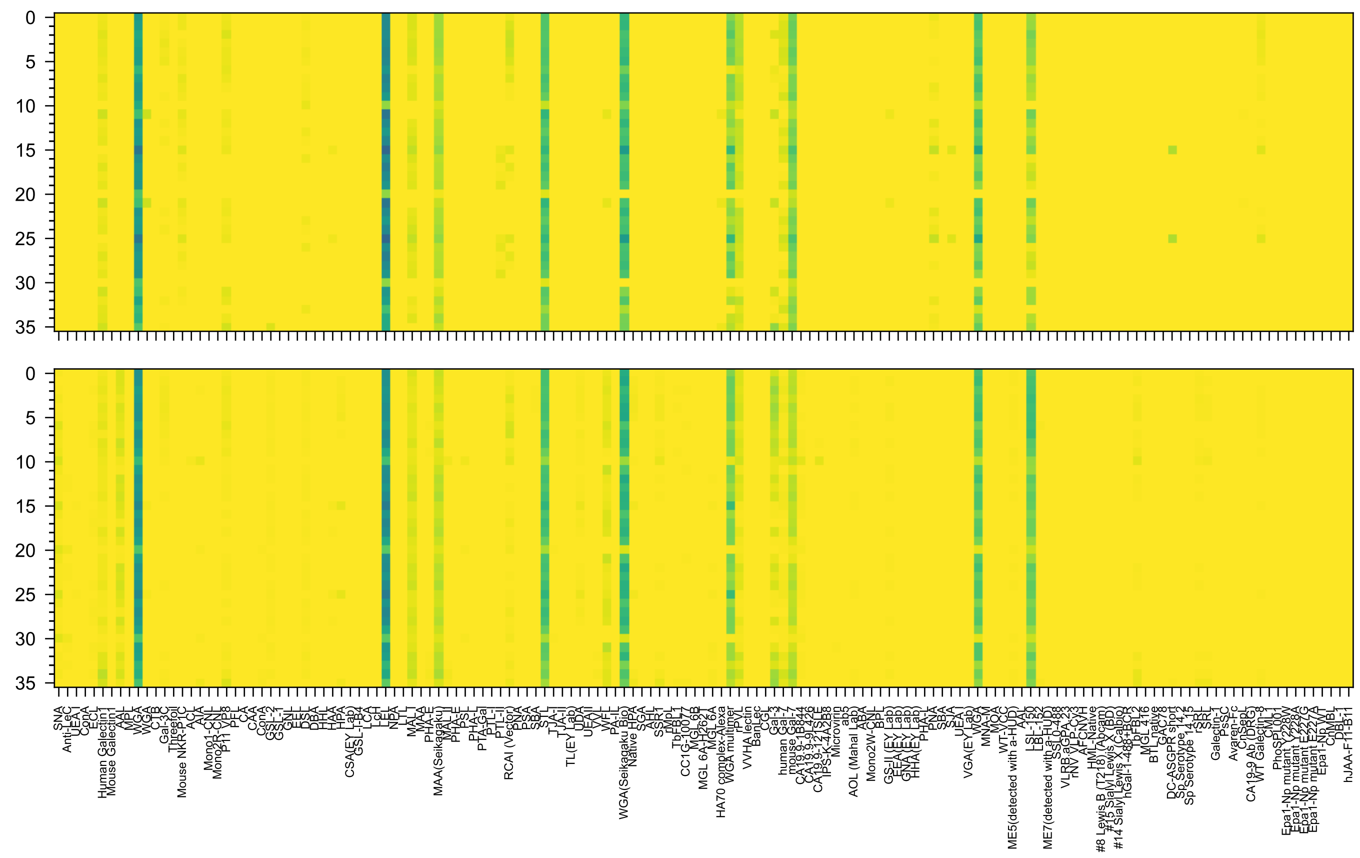

Neu5Ac(α2-3)Gal(β1-4)GlcNAc(β1-3)Gal(β1-4)GlcNAc(β1-3)Gal(β1-4)GlcNAc(β-Sp0

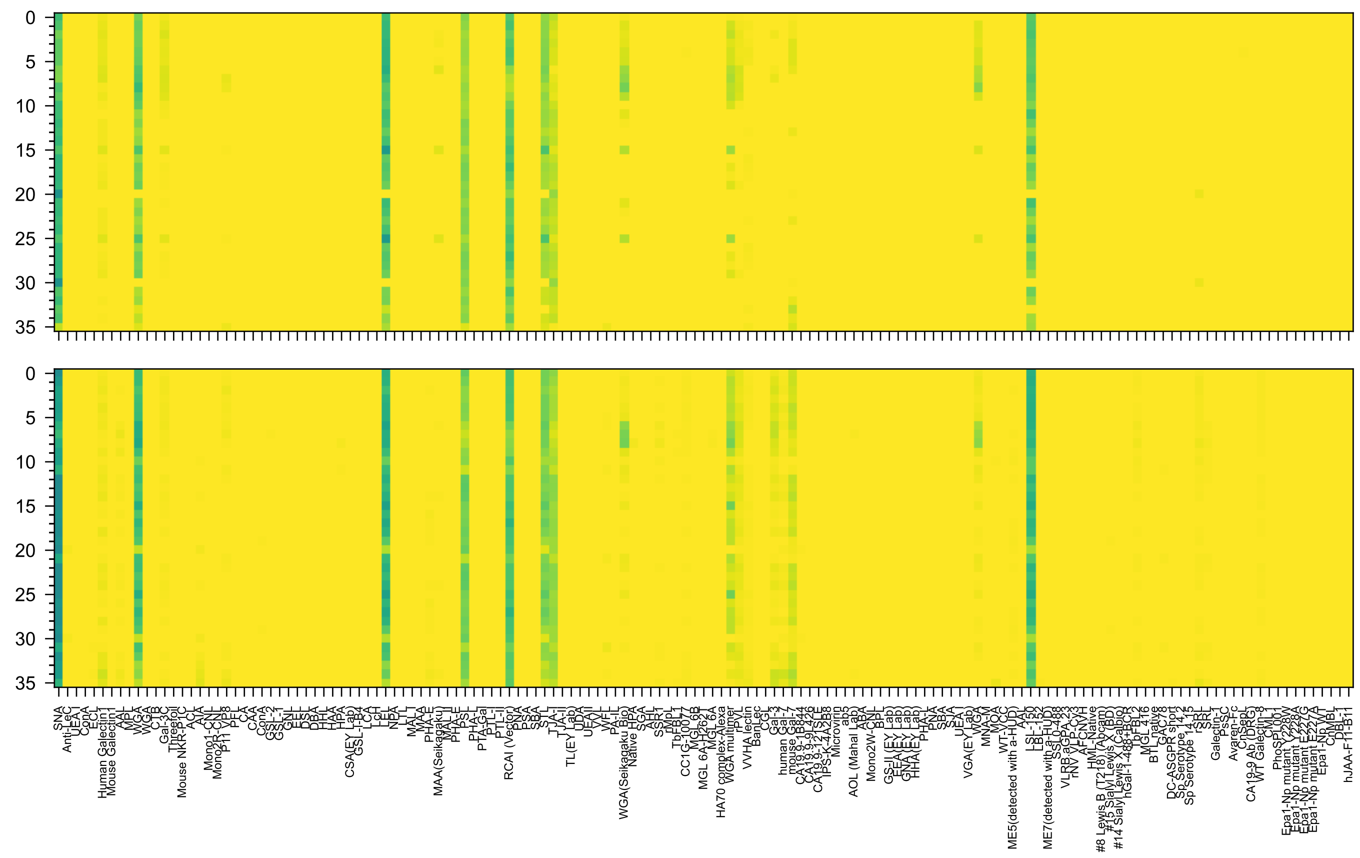

Neu5Ac(α2-6)Gal(β1-4)GlcNAc(β1-3)Gal(β1-4)GlcNAc(β1-3)Gal(β1-4)GlcNAc(β-Sp0

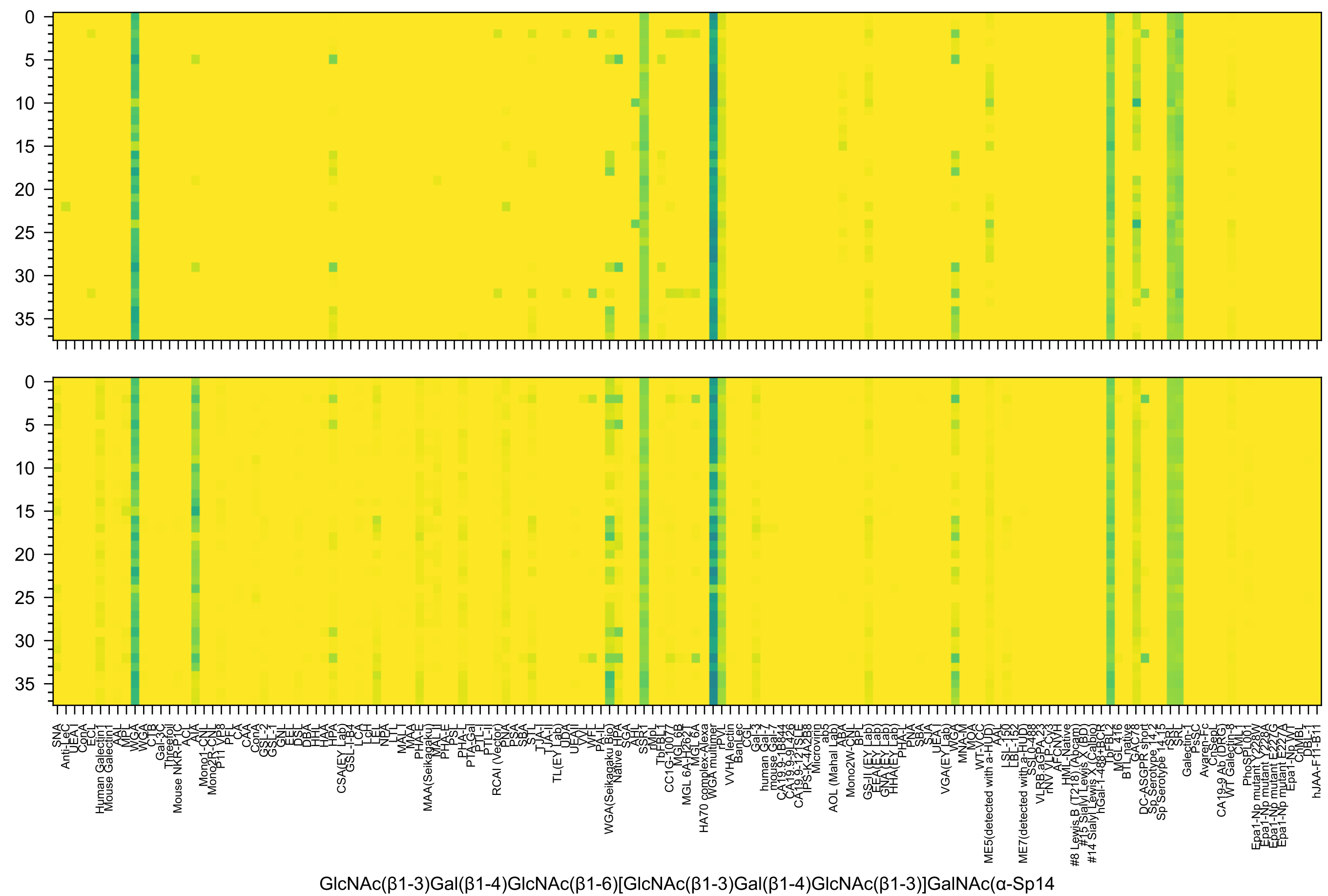

GlcNAc(β1-3)Gal(β1-4)GlcNAc(β1-6)[GlcNAc(β1-3)Gal(β1-4)GlcNAc(β1-3)]GalNAc(α-Sp14

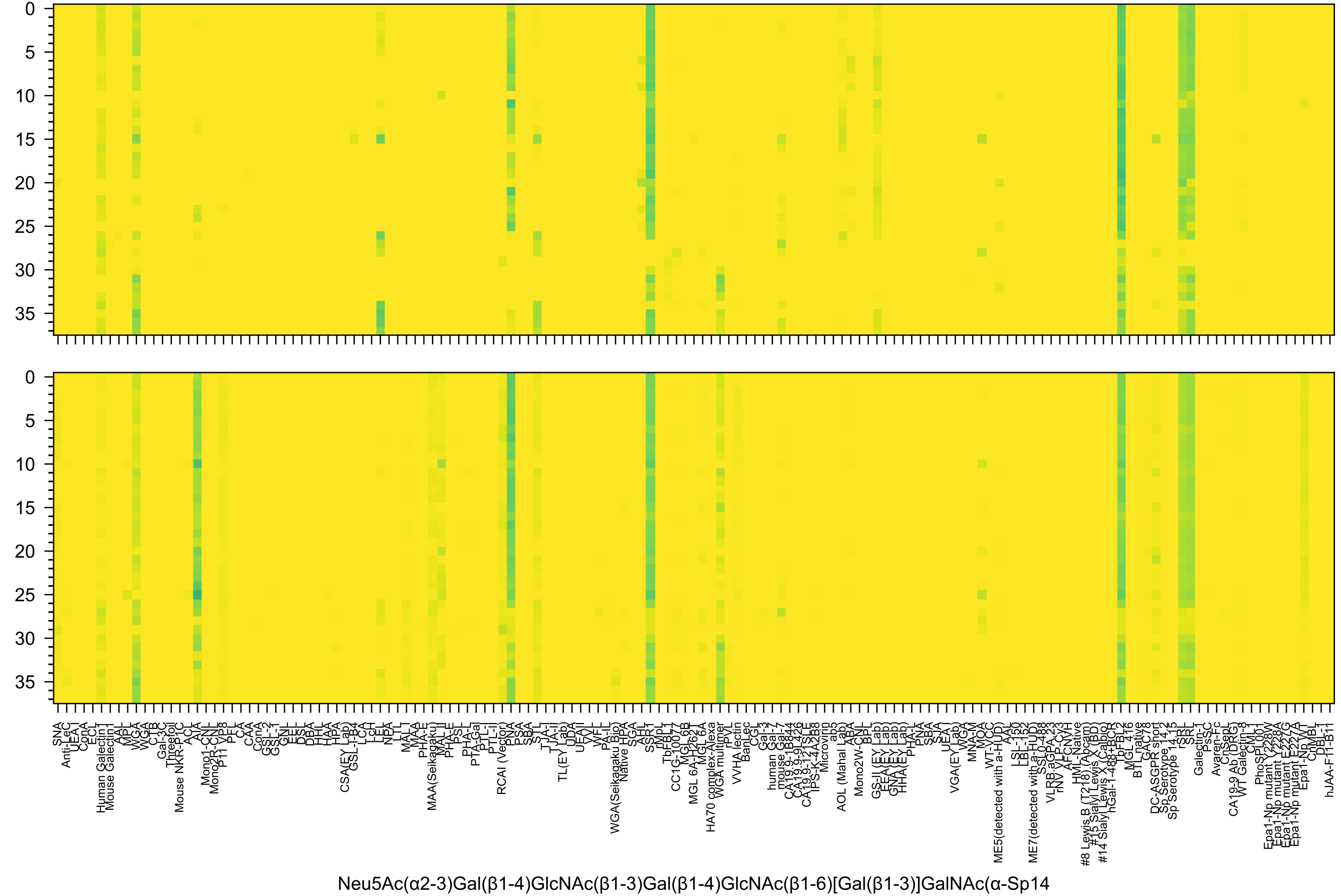
0
5
10
15
20
25
30
35
SNA
Anti-LeC
UEA I
ConA
ECL
Human Galectin1
Mouse Galectin1
AAL
MPL
WGA
WGA
CTB
Gal-3C
Threefoil
Mouse NKR-P1C
ACL
AIA
Mono1-CNL
Mono2R-CNL
P11 VP8
PFL
CA
CAA
ConA
GSL-2
GSL-1
GNL
EEL
DSL
DBA
HHL
HAA
HPA
CSA(EY Lab)
GSL-I-B4
LCA
LcH
LEL
NPA
LTL
MAL-I
MAA
PHA-E
MAA(Seikagaku)
MAL-II
PHA-E
PSL
PHA-L
PTA-Gal
PTL-I
PTL-II
RCAI (Vector)
PNA
PSA
SBA
STL
TJA-I
TJA-II
TL(EY Lab)
UDA
UEA-II
VVL
WFL
PA-IL
WGA(Seikagaku Bio)
Native HPA
SGA
AHL
SSR1
rMpL
TbFBL1
CC1G-10077
MGL 6B
MGL 6A-H262T
MGL 6A
HA70 complex-Alexa
WGA multimer
rPVL
VVHA lectin
BanLec
CGL
Gal-3
human Gal-7
mouse Gal-7
CA19.9-1B844
CA19.9-9L426
CA19.9-121SLE
IPS-K-4A2B8
Microvirin
ab5
AOL (Mahal Lab)
ABA
Mono2W-CNL
BPL
GS-II (EY Lab)
EEA(EY Lab)
GNA(EY Lab)
HHA(EY Lab)
PHA-L
PNA
SBA
SJA
UEA-1
VGA(EY Lab)
WGA
MNA-M
MOA
WT-VCC
ME5(detected with a-HUD)
AAL
LSL-150
LBL-152
ME7(detected with a-HUD)
SSL0-488
VLRB.aGPA.23
rNV
VLP-Cy3
AFCNVH
HML-Native
#8 Lewis B (T218) (Abcam)
#15 Sialyl Lewis X (BD)
#14 Sialyl Lewis X (Calbio)
hGal-1-488+BCR
TbFBL2
MGL 416
BTL native
GAC78
DC-ASGPR short
Sp Serotype 14.2
Sp Serotype 14.15
rSRL
SRL
Galectin-1
PsSC
Avaren-Fc
CnSpL
CA19-9 Ab (DRG)
WT Galectin-8
CVL-1
PhoSL001
Epa1-Np mutant Y228W
Epa1-Np mutant E228A
Epa1-Np mutant E227G
Epa1-Np mutant E227A
Epa1-Np WT
ChMBL
DBL-1
hJAA-F11-B11
Neu5Ac(α2-3)Gal(β1-4)GlcNAc(β1-3)Gal(β1-4)GlcNAc(β1-6)[Gal(β1-3)]GalNAc(α-Sp14

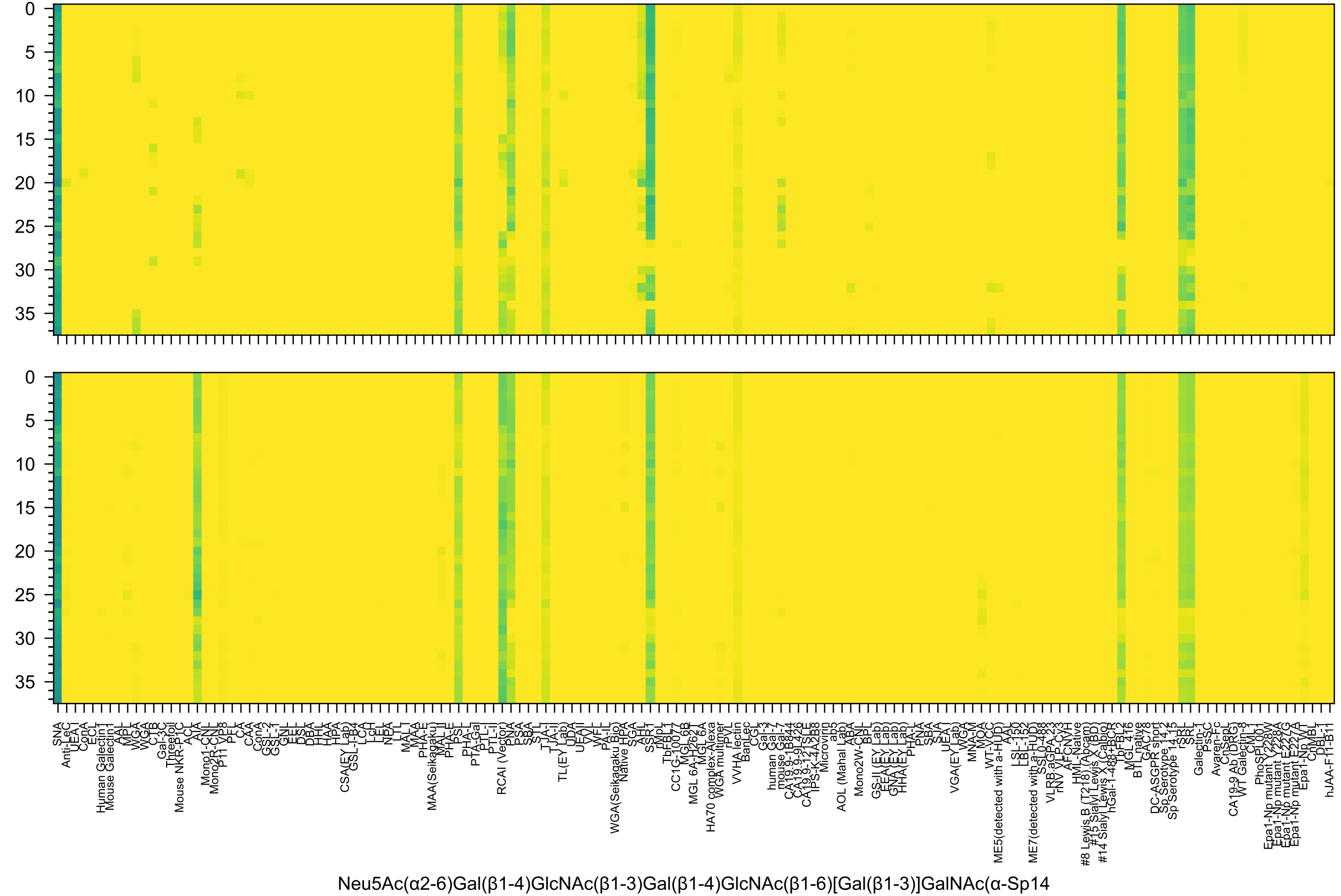
0
5
10
15
20
25
30
35
SNA
Anti-LeC
UEA-I
ConA
ECL
Human Galectin1
Mouse Galectin1
AAL
MPL
WGA
WGA
CTB
Gal-3C
Threefoil
Mouse NKR-P1C
ACL
AIA
Mono1-CNL
Mono2R-CNL
P11 VP8
PFL
CA
CAA
ConA
GSL-2
GSL-1
GNL
EEL
DSL
DBA
HHL
HAA
HPA
CSA(EY Lab)
GSL-I-B4
LCA
LcH
LEL
NPA
LTL
MAL-I
MAA
PHA-E
MAA(Seikagaku)
MAL-II
PHA-E
PSL
PHA-L
PTA-Gal
PTL-I
PTL-II
RCAI (Vector)
PNA
PSA
SBA
STL
TJA-I
TJA-II
TL(EY Lab)
UDA
UEA-I
VVL
WFL
PA-IL
WGA(Seikagaku Bio)
Native HPA
SGA
AHL
SSR1
rMpL
TbFBL1
CC1G-10077
MGL 6B
MGL 6A-H262I
MGL 6A
HA70 complex-Alexa
WGA multimer
rPVL
VVHA lectin
BanLec
CGL
Gal-3
human Gal-7
mouse Gal-7
CA19.9-1B844
CA19.9-9L426
CA19.9-121SLE
IPS-K-4A2B8
Microvirin
ab5
AOL (Mahal Lab)
ABA
Mono2W-CNL
BPL
GS-II (EY Lab)
EEA(EY Lab)
GNA(EY Lab)
HHA(EY Lab)
PHA-L
PNA
SBA
SJA
UEA-I
VGA(EY Lab)
WGA
MNA-M
MOA
WT-VCC
ME5(detected with a-HUD)
AAL
LSL-150
LBL-152
ME7(detected with a-HUD)
SSL0-488
VLRB.aGPA.23
rNV_VLP-Cy3
AFC-NVH
HML-Native
#8 Lewis B (T218) (Abcam)
#15 Sialyl Lewis X (BD)
#14 Sialyl Lewis X (Calbio)
hGal-1-488+BCR
TbFBL2
MGL 416
BTL native
GAC78
DC-ASGPR short
Sp Serotype 14.2
Sp Serotype 14.15
rSRL
SRL
Galectin-1
PsSC
Avaren-Fc
CnSepL
CA19-9 Ab (DRG)
WT Galectin-8
CML-1
PhoSL001
Epa1-Np mutant Y228W
Epa1-Np mutant E228A
Epa1-Np mutant E227G
Epa1-Np mutant E227A
Epa1-Np WT
ChMBL
DBL-1
hJAA-F11-B11
Neu5Ac(α2-6)Gal(β1-4)GlcNAc(β1-3)Gal(β1-4)GlcNAc(β1-6)[Gal(β1-3)]GalNAc(α-Sp14

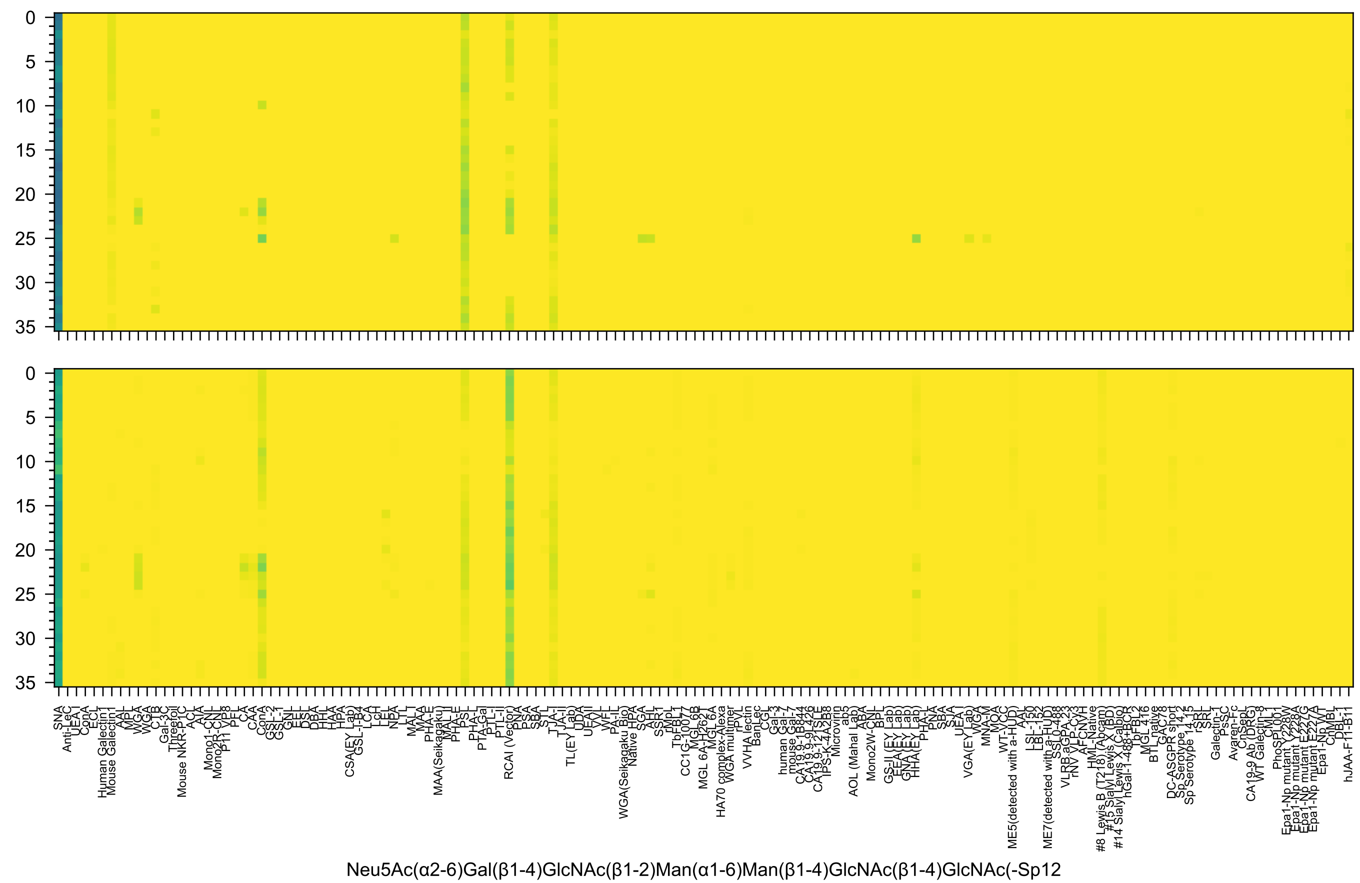
SNA
Anti-LeC
UEA1
ConA
ECL
Human Galectin1
Mouse Galectin1
AAL
MPL
WGA
WGA
CTB
Gal-3C
Threefoil
Mouse NKR-P1C
ACL
AIA
Mono1-CNL
Mono2R-CNL
P11 VP8
PFL
CA
CAA
ConA
GSL-2
GSL-1
GNL
EEL
DSL
DBA
HHL
HAA
HPA
CSA(EY Lab)
GSL-I-B4
LCA
LcH
LEL
NPA
LTL
MAL-I
MAA
PHA-E
MAA(Seikagaku)
MAL-II
PHA-E
PSL
PHA-L
PTA-Gal
PTL-I
PTL-II
RCAI (Vector)
PNA
PSA
SBA
STL
TJA-I
TJA-II
TL(EY Lab)
UDA
UEAII
VVL
WFL
PA-IL
WGA(Seikagaku Bio)
Native HPA
SGA
AHL
SSR1
rMpL
TbFBL1
CC1G-10077
MGL 6B
MGL 6A-H262T
MGL 6A
HA70 complex-Alexa
WGA multimer
rPVL
VVHA lectin
BanLec
CGL
Gal-3
Gal-7
human Gal-8
mouse Gal-8
CA19.9-1B844
CA19.9-9L426
CA19.9-121SLE
IPS-K-4A2B8
Microvirin
ab5
AOL (Mahal Lab)
ABA
Mono2W-CNL
BPL
GS-II (EY Lab)
EEA(EY Lab)
GNA(EY Lab)
HHA(EY Lab)
PHA-L
PNA
SBA
SJA
UEA-I
VGA(EY Lab)
WGA
MNA-M
MOA
WT-VCC
ME5(detected with a-HUD)
AAL
LSL-150
LBL-152
ME7(detected with a-HUD)
SSL0-488
VLRB.aGPA.23
rNV VLP-Cy3
AFCNVH
HML-Native
#8 Lewis B (T218) (Abcam)
#15 Sialyl Lewis X (BD)
#14 Sialyl Lewis X (Calbio)
hGal-1-488+BCR
TbFBL2
MGL 416
BTL native
GAC78
DC-ASGPR short
Sp Serotype 14.2
Sp Serotype 14.15
rSRL
SRL
Galectin-1
PsSC
Avaren-Fc
CnSepL
CA19-9 Ab (DRG)
WT Galectin-8
CML1
PhoSPL001
Epa1-Np mutant Y228W
Epa1-Np mutant Y228A
Epa1-Np mutant E227G
Epa1-Np mutant E227A
Epa1-Np WT
ChMBL
DBL-1
hJAA-F11-B1
Neu5Ac(α2-6)Gal(β1-4)GlcNAc(β1-2)Man(α1-6)Man(β1-4)GlcNAc(β1-4)GlcNAc(-Sp12

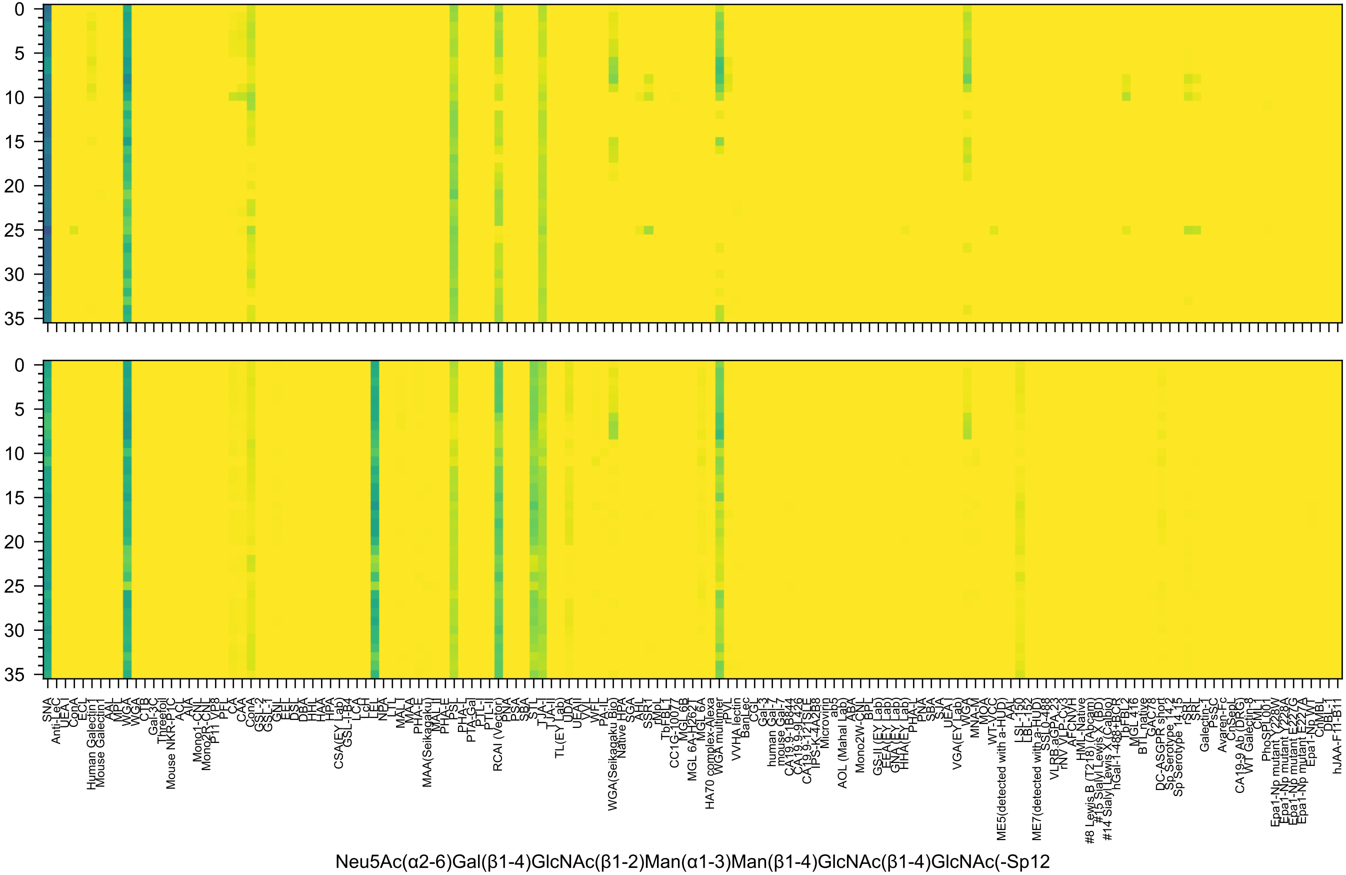
0
5
10
15
20
25
30
35
0
5
10
15
20
25
30
35
SNA
Anti-LeC
UEA1
ConA
ECL
Human Galectin1
Mouse Galectin1
AAL
MPL
WGA
WGA
CTB
Gal-3C
Threefoil
Mouse NKR-P1C
ACL
AIA
Mono1-CNL
Mono2R-CNL
P11 VP8
PFL
CA
CAA
ConA
GSL-2
GSL-1
GNL
EEL
DSL
DBA
HHL
HAA
HPA
CSA(EY Lab)
GSL-I-B4
LCA
LcH
LEL
NPA
LTL
MAL1
MAA
PHA-E
MAA(Seikagaku)
MAL-II
PHA-E
PSL
PHA-L
PTA-Gal
PTL-I
PTL-II
RCAI (Vector)
PNA
PSA
SBA
SJA
STL
TJA-I
TJA-II
TL(EY Lab)
UDA
UEAII
VVL
WFL
PA-IL
WGA(Seikagaku Bio)
Native HPA
SGA
AHL
SSR1
rMpL
TbFBL1
CC1G-10077
MGL 6B
MGL 6A-H262T
MGL 6A
HA70 complex-Alexa
WGA multimer
rPVL
VVHA lectin
BanLec
CGL
Gal-3
human Gal-7
mouse Gal-7
CA19.9-1B844
CA19.9-9L426
CA19.9-121SLE
IPS-K-4A2B8
Microvirin
ab5
AOL (Mahal Lab)
ABA
Mono2W-CNL
BPL
GS-II (EY Lab)
EEA(EY Lab)
GNA (EY Lab)
HHA(EY Lab)
PHA-L
PNA
SBA
SJA
UEA-I
VGA(EY Lab)
WGA
MNA-M
MOA
WT-VCC
ME5(detected with a-HUD)
AAL
LSL-150
LBL-152
ME7(detected with a-HUD)
SSL0-488
VLRB.aGPA.23
rNV VLP-Cy3
AFCNVH
HML-Native
#8 Lewis B (T218) (Abcam)
#15 Sialyl Lewis X (BD)
#14 Sialyl Lewis X (Calbio)
hGal-1-488+BCR
TbFBL2
MGL 416
BTL native
GAC78
DC-ASGPR short
Sp Serotype 14.2
Sp Serotype 14.15
rSRL
SRL
Galectin-1
PsSC
Avaren-Fc
CnSepL
CA19-9 Ab (DRG)
WT Galectin-8
CML1
PhoSL001
Epa1-Np mutant Y228W
Epa1-Np mutant Y228A
Epa1-Np mutant E227G
Epa1-Np mutant E227A
Epa1-Np WT
ChMBL
DBL-1
hJAA-F11-B11
Neu5Ac(α2-6)Gal(β1-4)GlcNAc(β1-2)Man(α1-3)Man(β1-4)GlcNAc(β1-4)GlcNAc(-Sp12

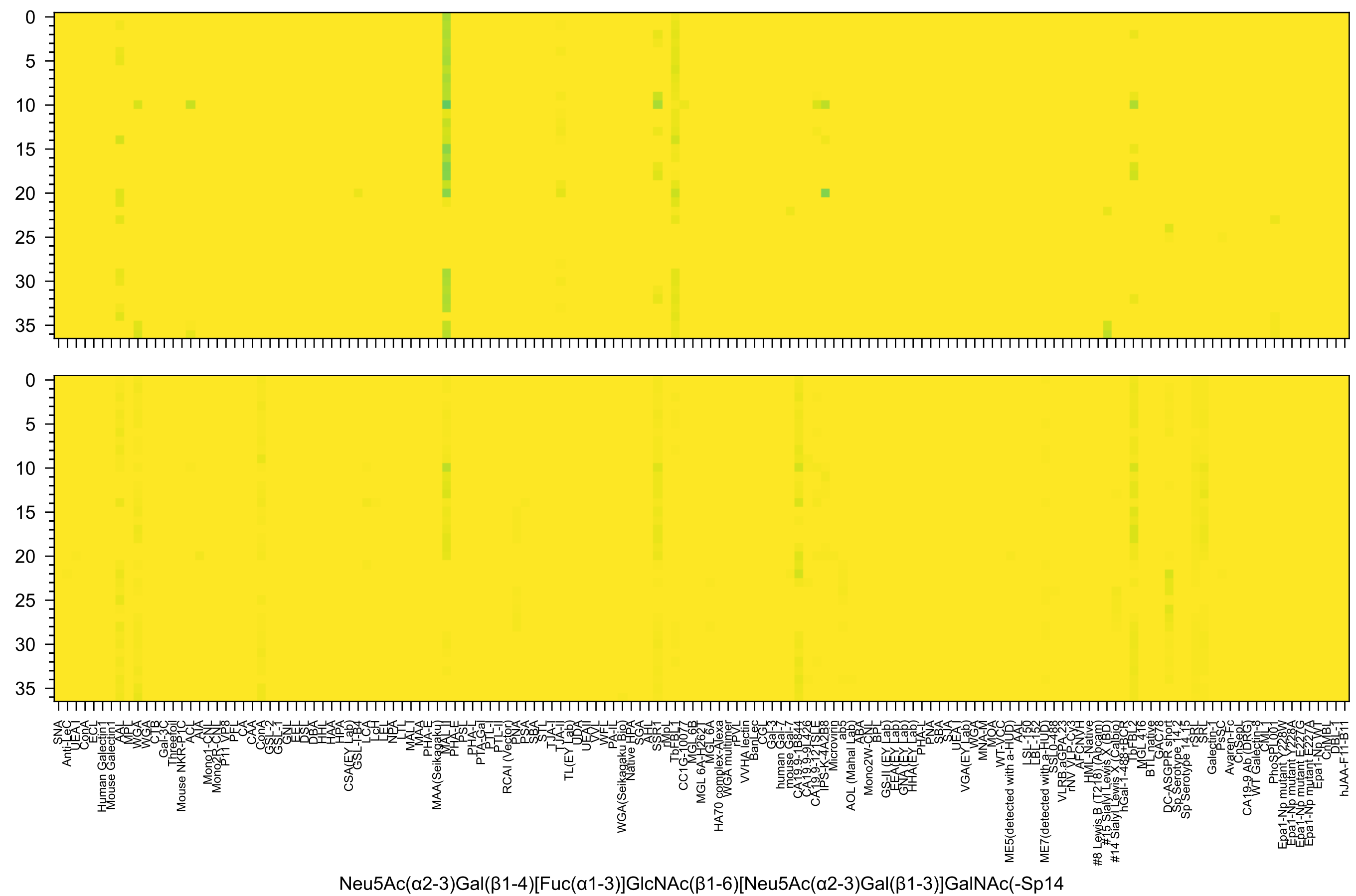
Neu5Ac(α2-3)Gal(β1-4)[Fuc(α1-3)]GlcNAc(β1-6)[Neu5Ac(α2-3)Gal(β1-3)]GalNAc(-Sp14

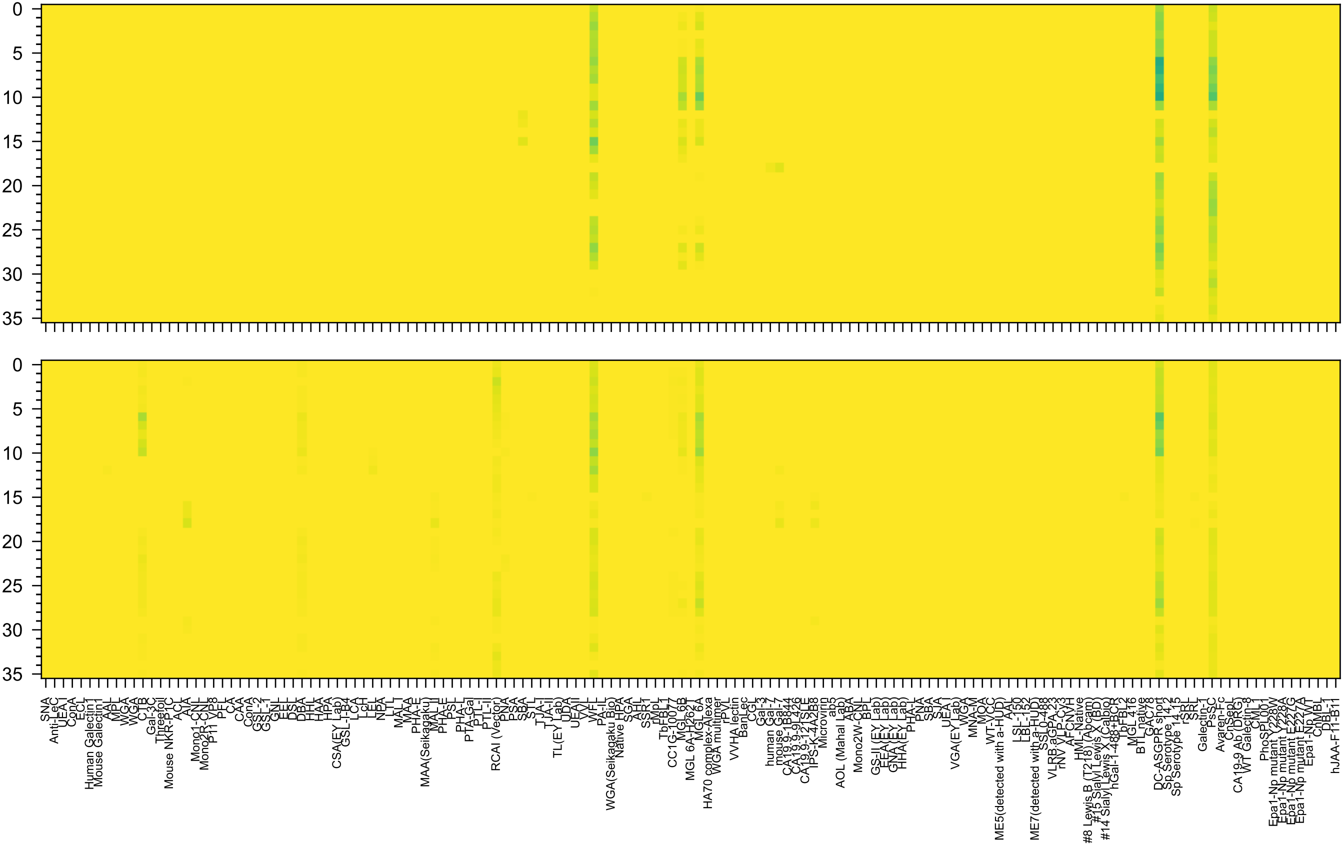

Neu5Ac(α2-3)Gal(β1-3)GalNAc(β1-4)[Neu5Ac(α2-8)Neu5Ac(α2-3)]Gal(β1-4)Glc(β-Sp0

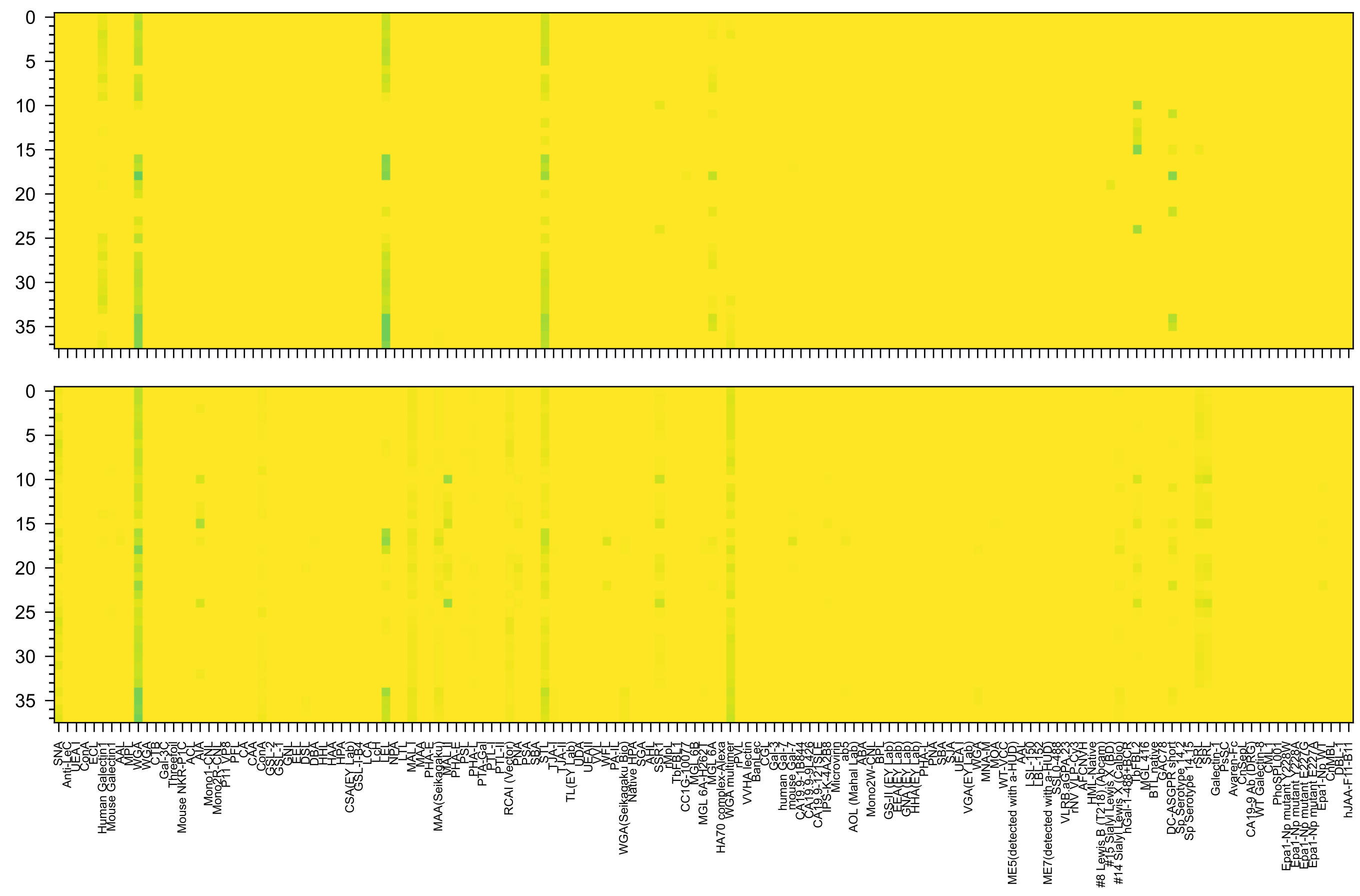

Neu5Ac(α2-3)Gal(β1-4)GlcNAc(β1-6)[Neu5Ac(α2-3)Gal(β1-4)GlcNAc(β1-3)]GalNAc(α-Sp14

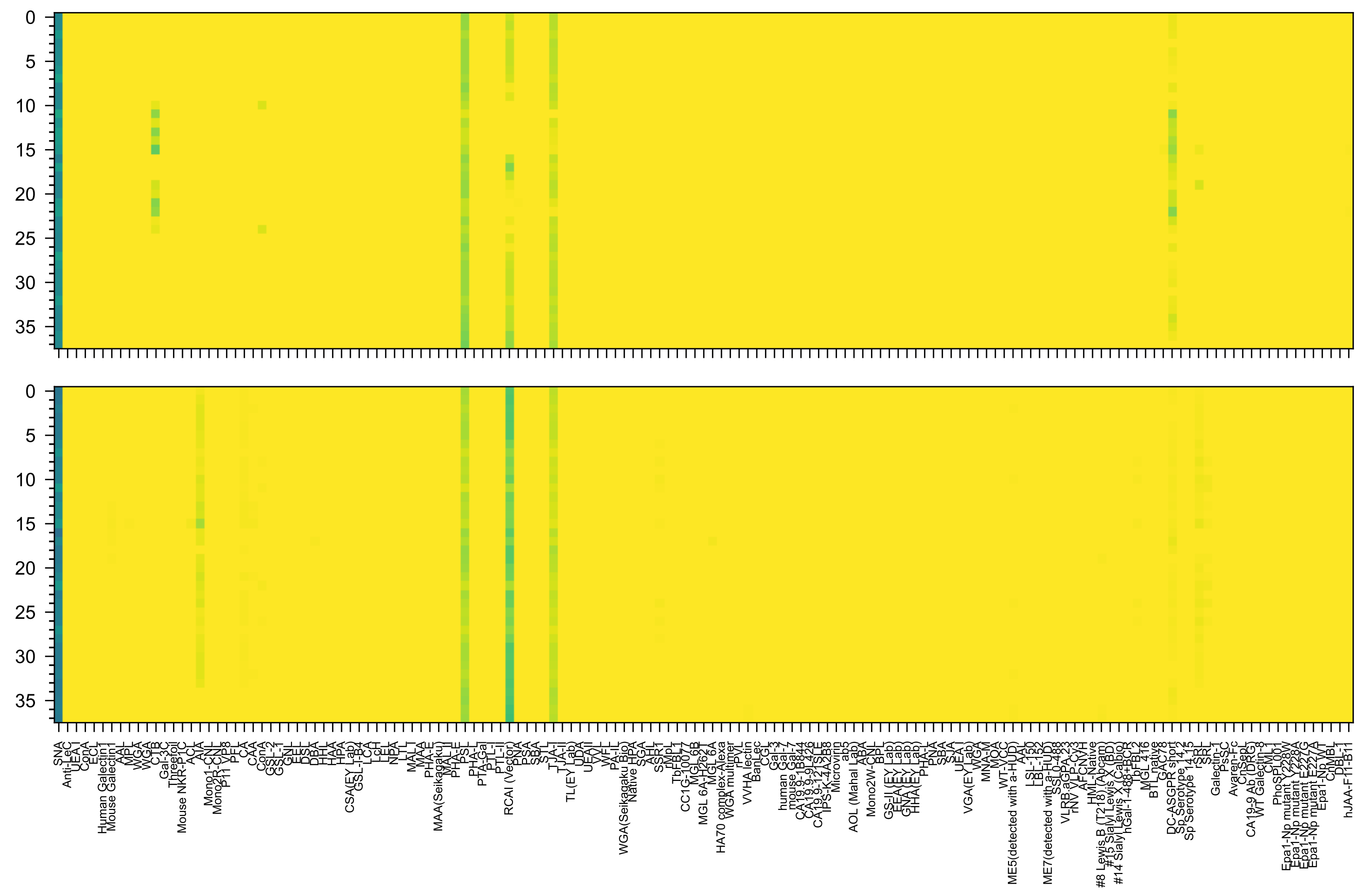

Neu5Ac(α2-6)Gal(β1-4)GlcNAc(β1-6)[Neu5Ac(α2-6)Gal(β1-4)GlcNAc(β1-3)]GalNAc(α-Sp14

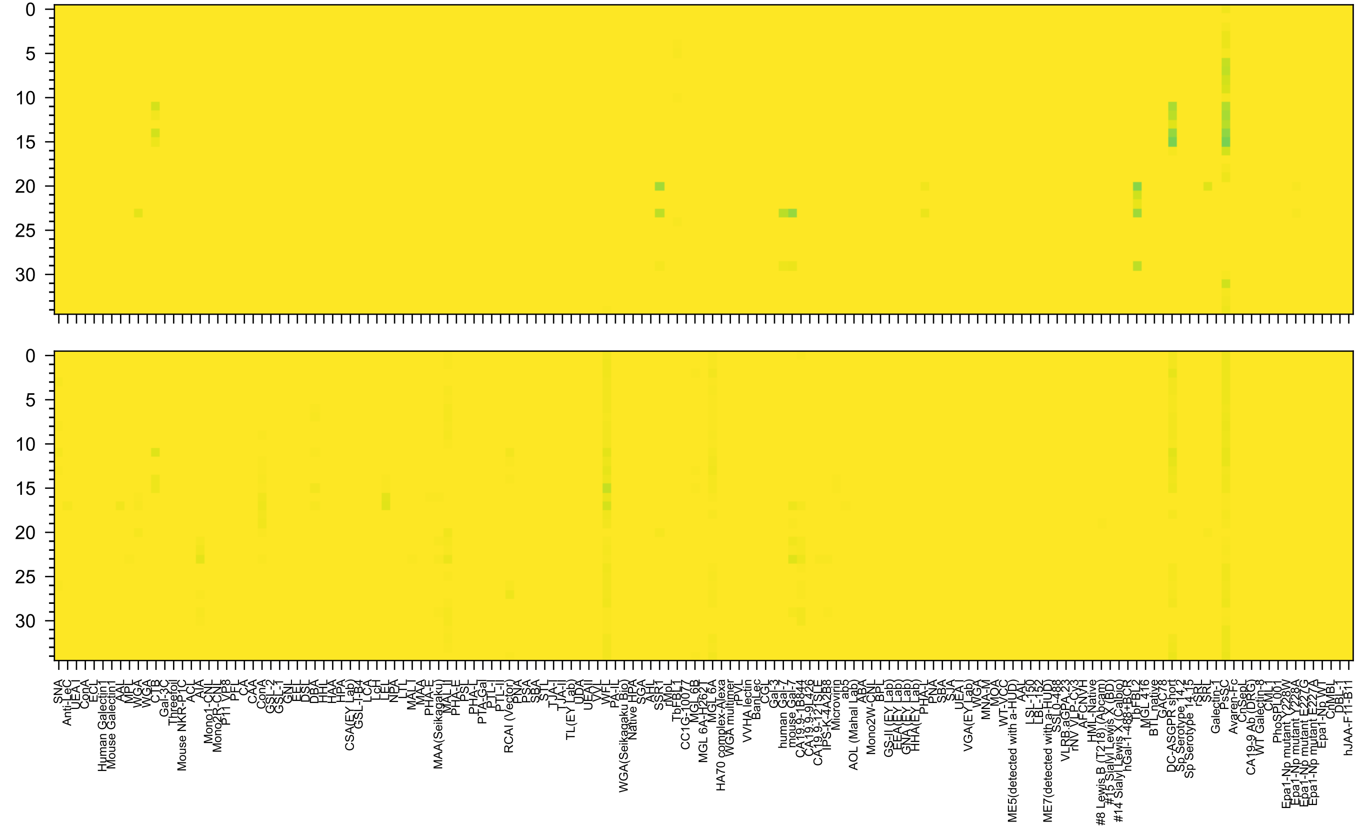

Neu5Ac(α2-8)Neu5Ac(α2-3)Gal(β1-3)GalNAc(β1-4)[Neu5Ac(α2-3)]Gal(β1-4)Glc(-Sp21

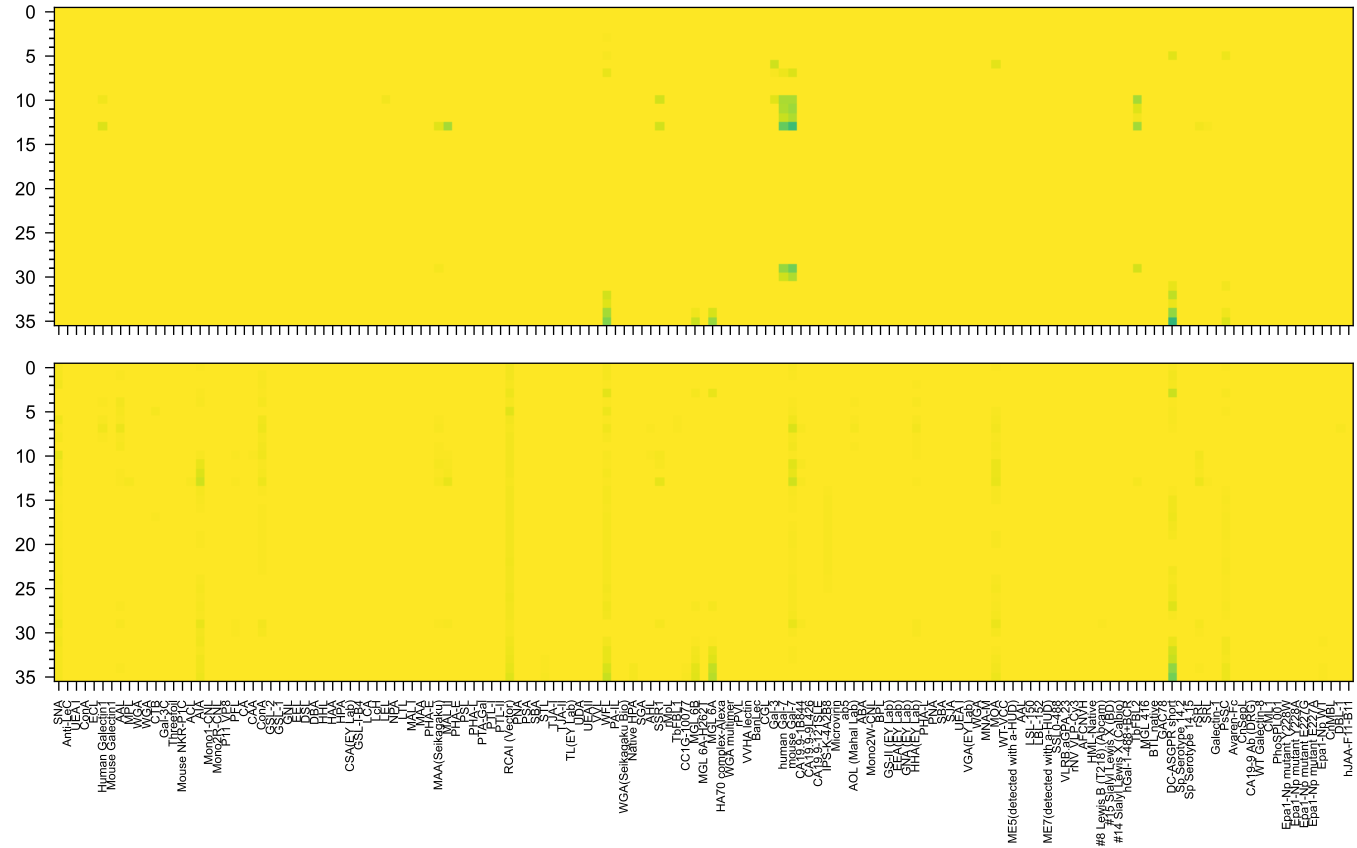

Gal(β1-3)GalNAc(β1-4)[Neu5Ac(α2-8)Neu5Ac(α2-8)Neu5Ac(α2-3)]Gal(β1-4)Glc(β-Sp21

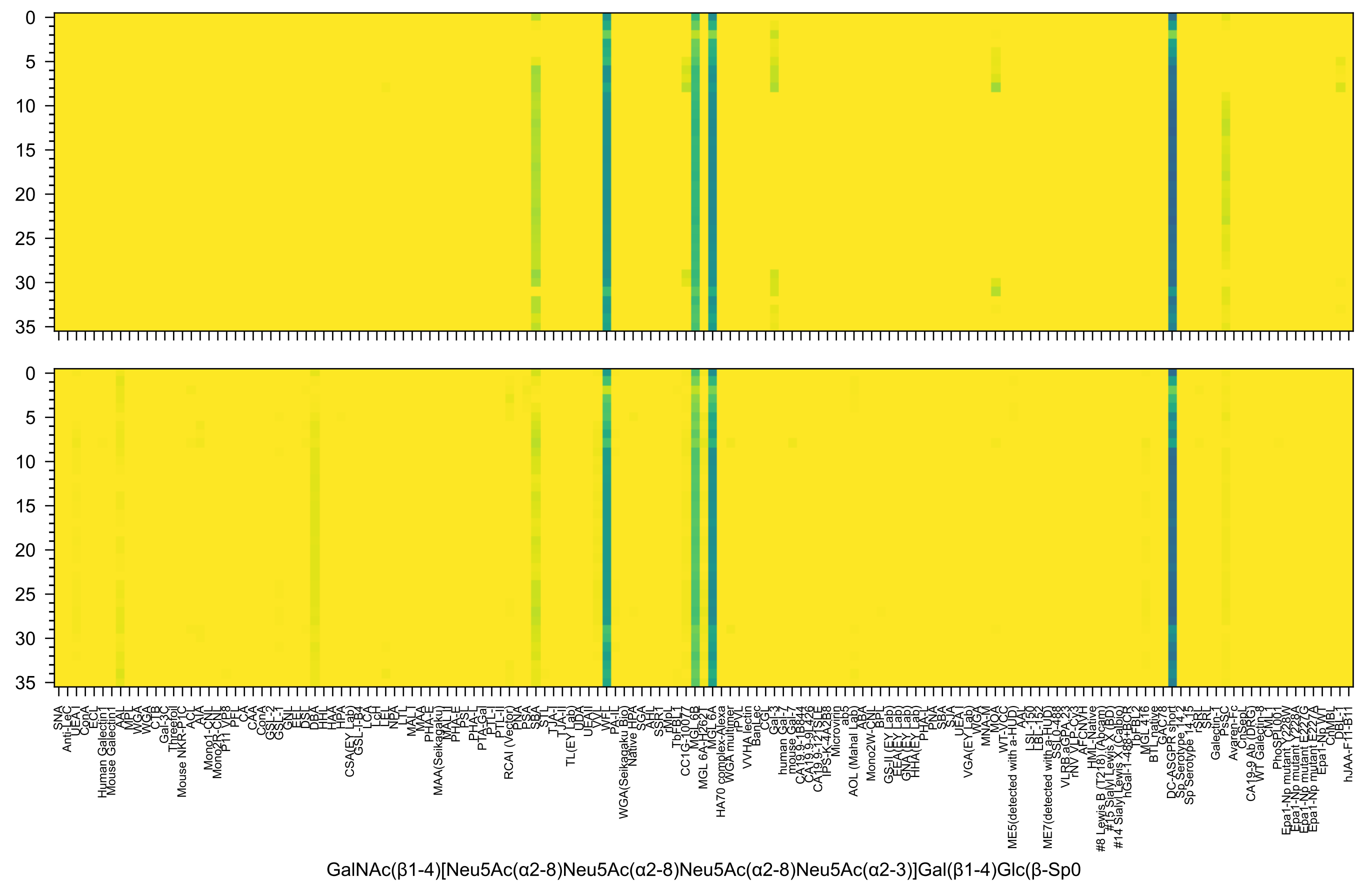
GalNAc(β1-4)[Neu5Ac(α2-8)Neu5Ac(α2-8)Neu5Ac(α2-8)Neu5Ac(α2-3)]Gal(β1-4)Glc(β-Sp0

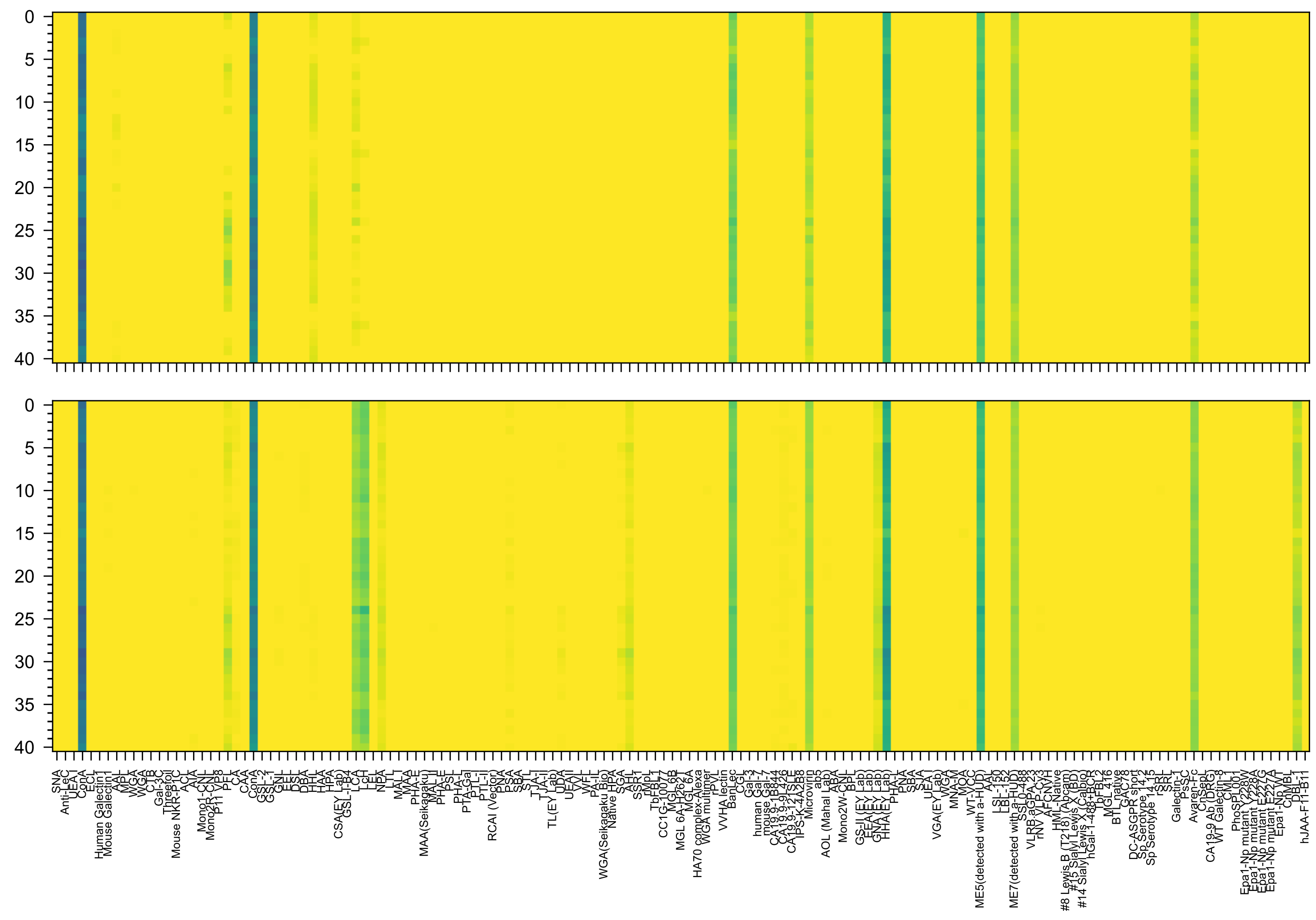

Man(α1-2)Man(α1-6)[Man(α1-3)]Man(α1-6)[Man(α1-2)Man(α1-2)Man(α1-3)]Man(α-Sp9

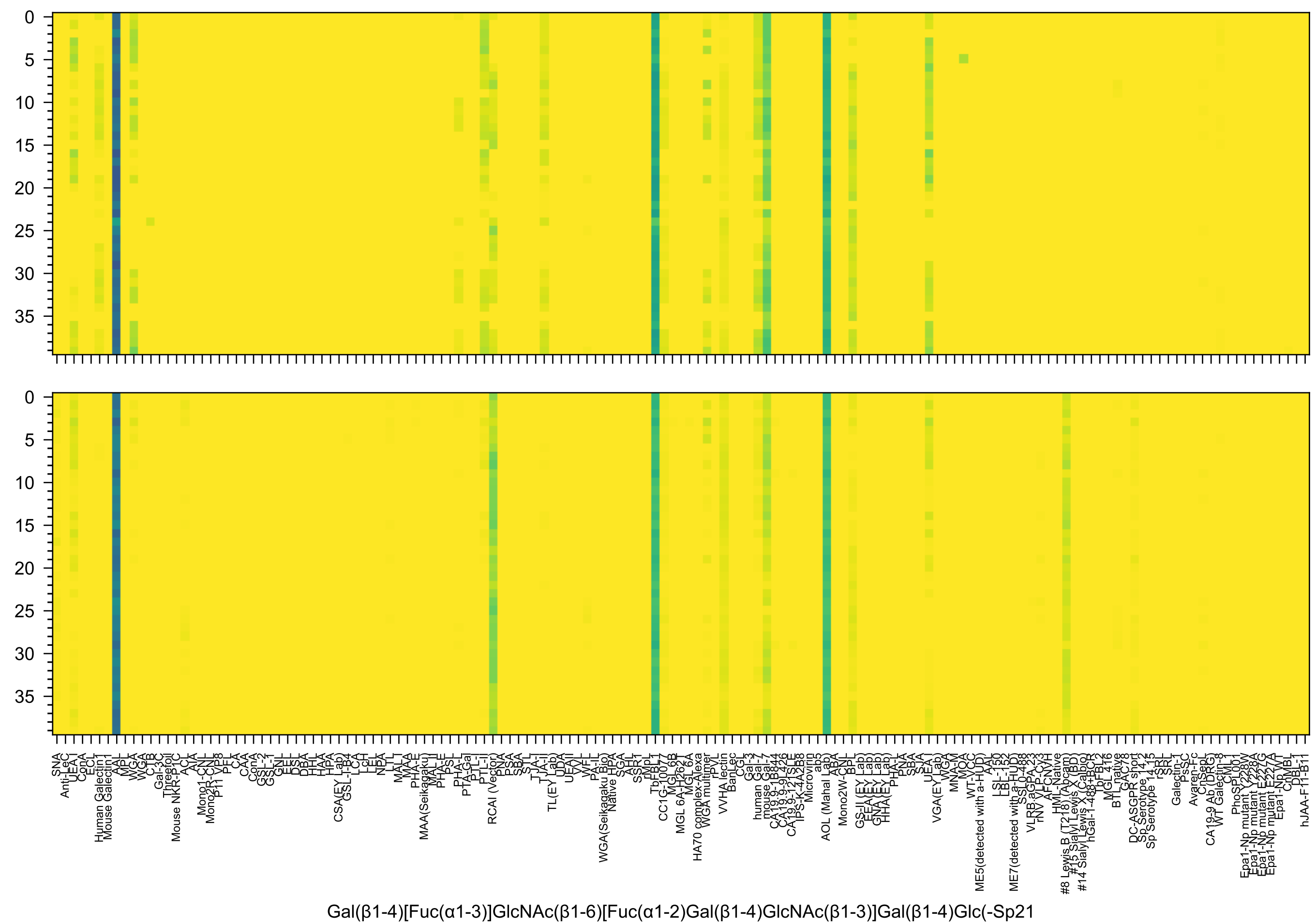
0
5
10
15
20
25
30
35
Gal(β1-4)[Fuc(α1-3)]GlcNAc(β1-6)[Fuc(α1-2)Gal(β1-4)GlcNAc(β1-3)]Gal(β1-4)Glc(-Sp21

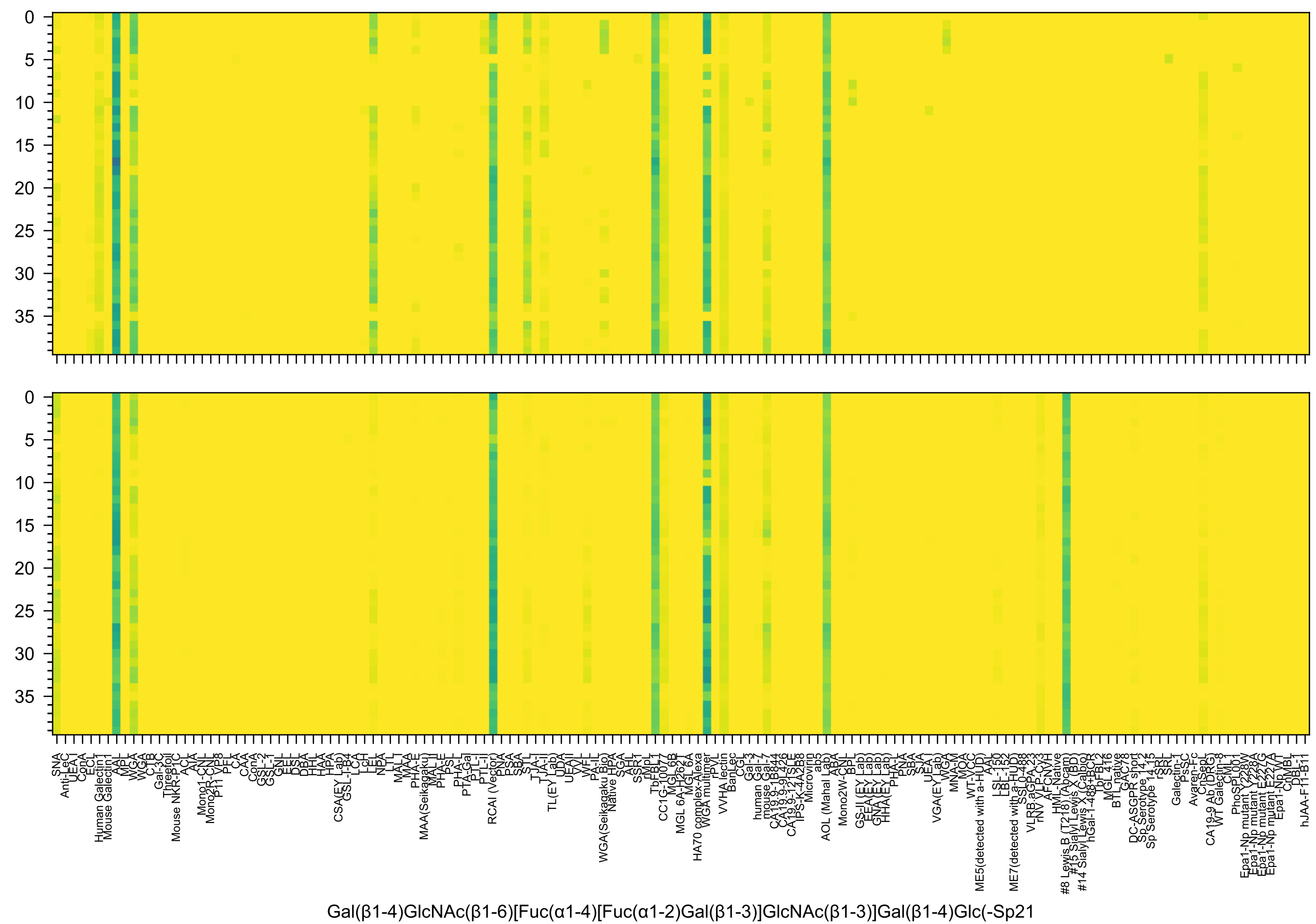
Gal(β1-4)GlcNAc(β1-6)[Fuc(α1-4)[Fuc(α1-2)Gal(β1-3)]GlcNAc(β1-3)]Gal(β1-4)Glc(-Sp21

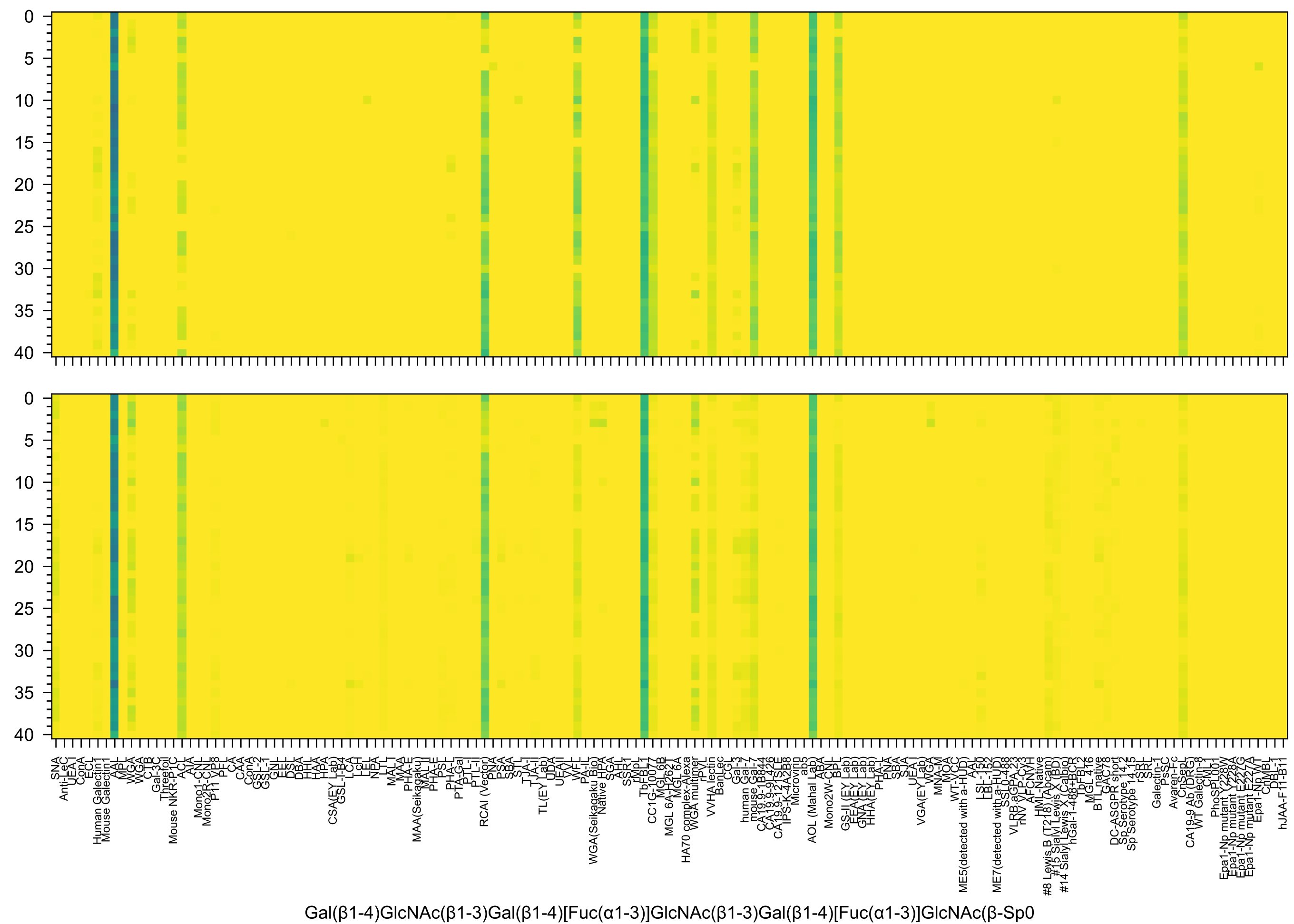

0
5
10
15
20
25
30
35
40
SNA
Anti-LeC
UEA I
ConA
ECL
Human Galectin1
Mouse Galectin1
AAL
MPL
WGA
WGA
CTB
Gal-3C
Threefoil
Mouse NKR-P1C
ACL
AIA
Mono1-CNL
Mono2R-CNL
P11 VP8
PEL
CA
CAA
ConA
GSL-2
GSL-1
GNL
EEL
DSL
DBA
HHL
HAA
HPA
CSA(EY Lab)
GSL-I-B4
LCA
LcH
LEL
NPA
LTL
MAL I
MAA
PHA-E
MAA(Seikagaku)
MAL II
PHA-E
PSL
PHA-L
PTA-Gal
PTL-I
PTL-II
RCAI (Vector)
PNA
PSA
SBA
SJL
STL
TJA-I
TJA-II
TL(EY Lab)
UDA
UEA II
VVL
WFL
PA-IL
WGA(Seikagaku Bio)
Native HPA
SGA
AHL
SSR1
rMpL
TbFBL1
CC1G-10077
MGL6B
MGL 6A-H262T
MGL 6A
HA70 complex-Alexa
WGA multimer
rPVL
VVHA lectin
BanLec
CGL
Gal-3
human Gal-7
mouse Gal-7
CA19.9-1B844
CA19.9-9L426
CA19.9-121SLE
IPS-K-4A2B8
Microvirin
ab5
AOL (Mahal Lab)
ABA
Mono2W-CNL
BPL
GS-II (EY Lab)
EEA (EY Lab)
GNA (EY Lab)
HHA (EY Lab)
PHA-L
PNA
SBA
SJA
UEA I
VGA(EY Lab)
WGA
MNA-M
MOA
WT-VCC
ME5(detected with a-HUD)
AAL
LSL-150
LBL-152
ME7(detected with a-HUD)
SSL0-488
VLRB aGPA.23
rNV VLP-Cy3
AFCNVH
HML-Native
#8 Lewis B (T218) (Abcam)
#15 Sialyl Lewis X (BD)
#14 Sialyl Lewis X (Calbio)
hGal-1-488+BCR
TbFBL2
MGL 416
BTL native
GAC78
DC-ASGPR short
Sp Serotype 14.2
Sp Serotype 14.15
rSRL
SRL
Galectin-1
PsSC
Avaren-Fc
CnSepL
CA19-9 Ab (DRG)
WT Galectin-8
CML1
PhoSL001
Epa1-Np mutant Y228W
Epa1-Np mutant Y228A
Epa1-Np mutant E227G
Epa1-Np mutant E227A
Epa1-Np WT
CnMBL
DBL1
hJAA-F11-B11
Gal(β1-4)GlcNAc(β1-3)Gal(β1-4)[Fuc(α1-3)]GlcNAc(β1-3)Gal(β1-4)[Fuc(α1-3)]GlcNAc(β-Sp0

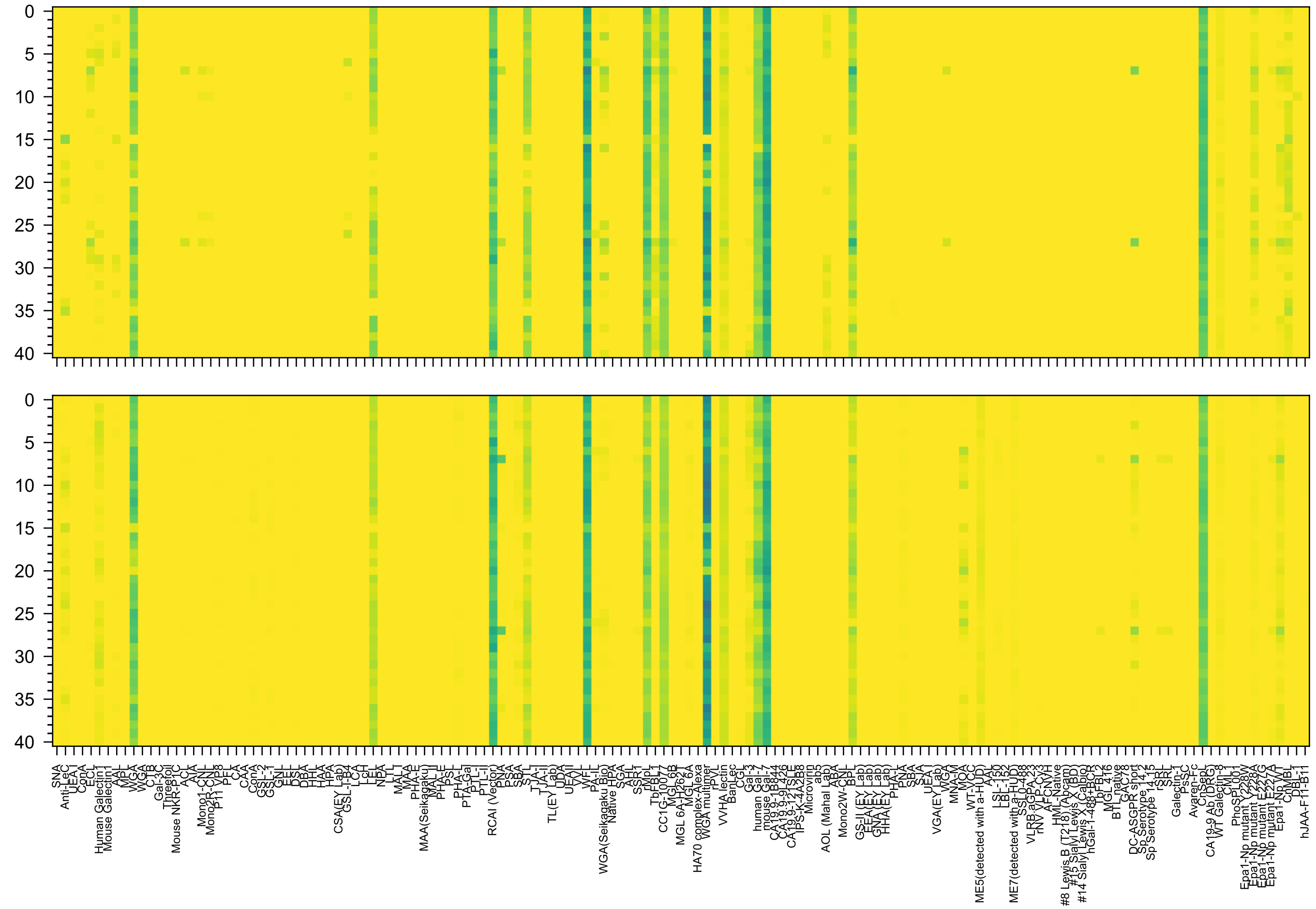

Gal(β1-3)GlcNAc(β1-3)Gal(β1-4)GlcNAc(β1-6)[Gal(β1-3)GlcNAc(β1-3)]Gal(β1-4)Glc(β-Sp0

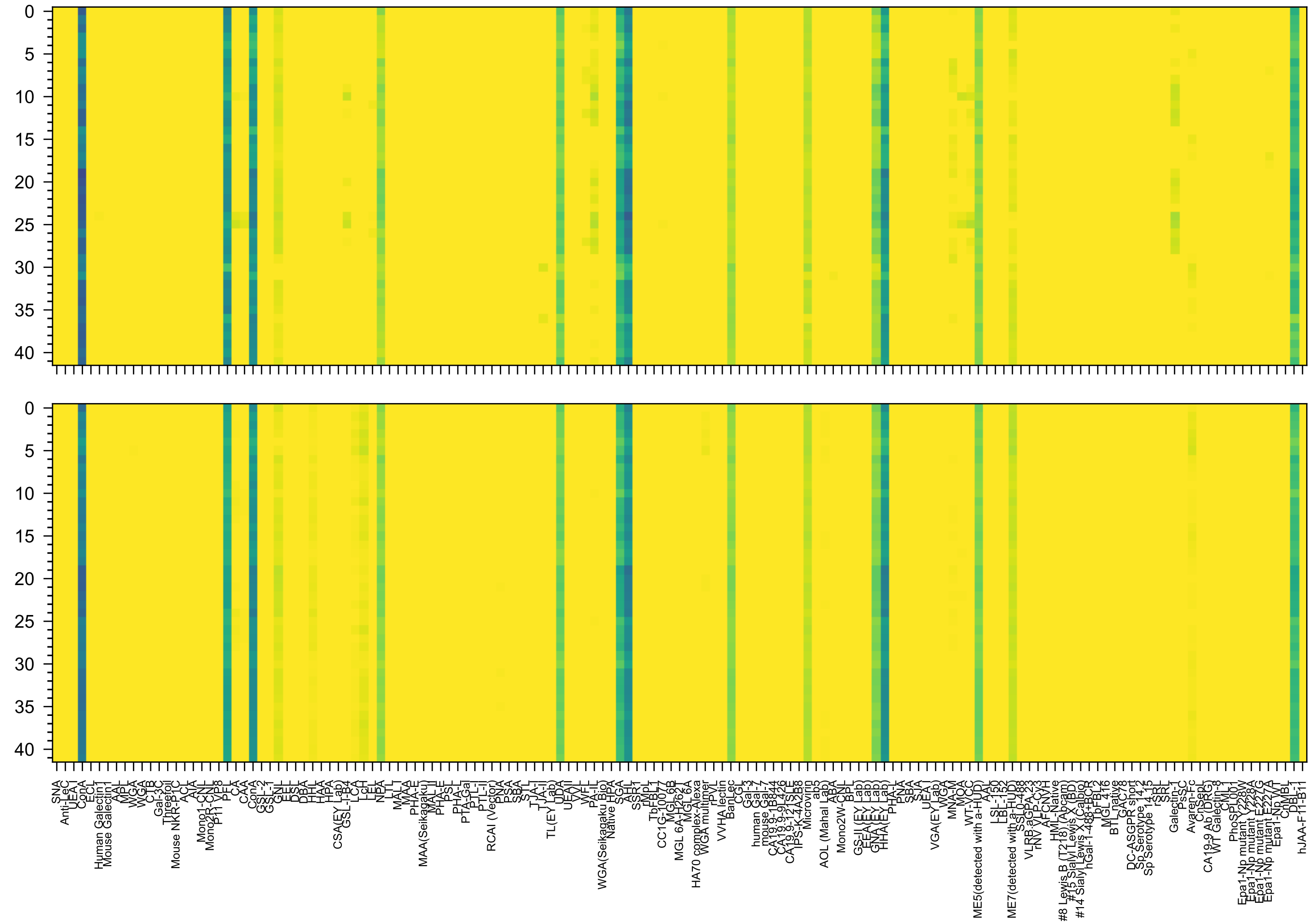

Man(α1-6)[Man(α1-3)]Man(α1-6)[Man(α1-2)Man(α1-3)]Man(β1-4)GlcNAc(β1-4)GlcNAc(β-Sp12

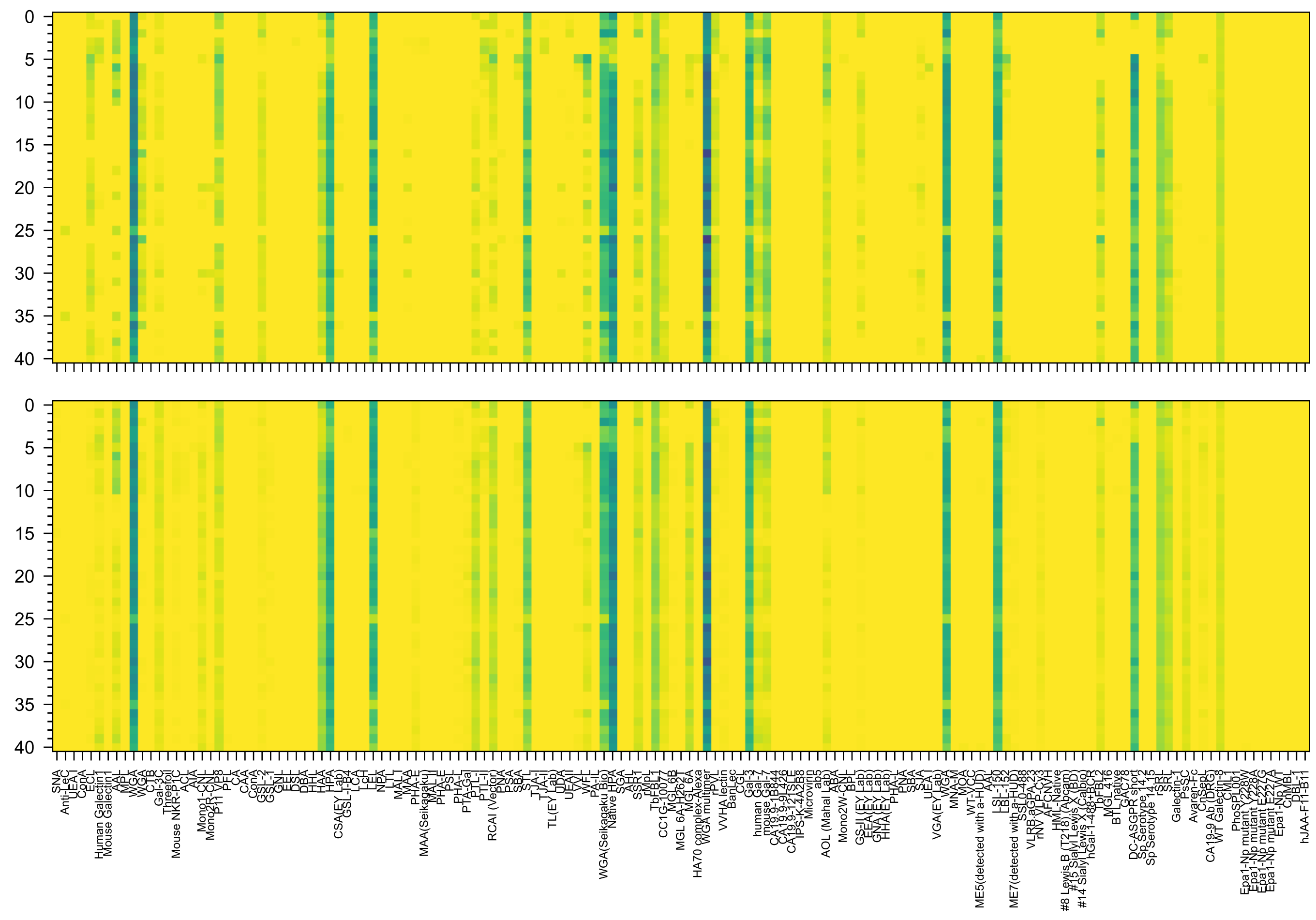
GalNAc(α1-3)[Fuc(α1-2)]Gal(β1-4)GlcNAc(β1-3)Gal(β1-4)GlcNAc(β1-3)Gal(β1-4)GlcNAc(β-Sp0

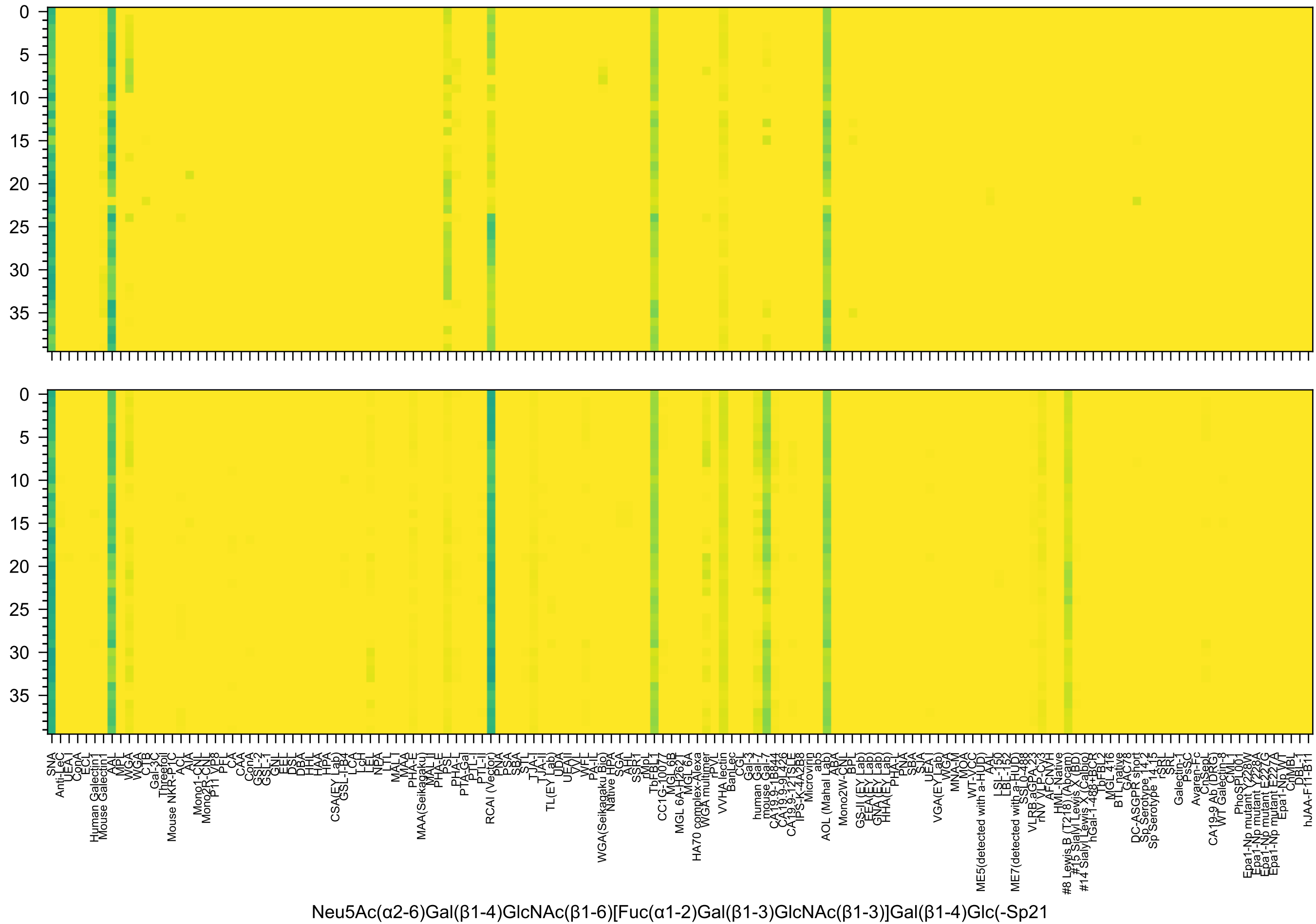
Neu5Ac(α2-6)Gal(β1-4)GlcNAc(β1-6)[Fuc(α1-2)Gal(β1-3)GlcNAc(β1-3)]Gal(β1-4)Glc(-Sp21

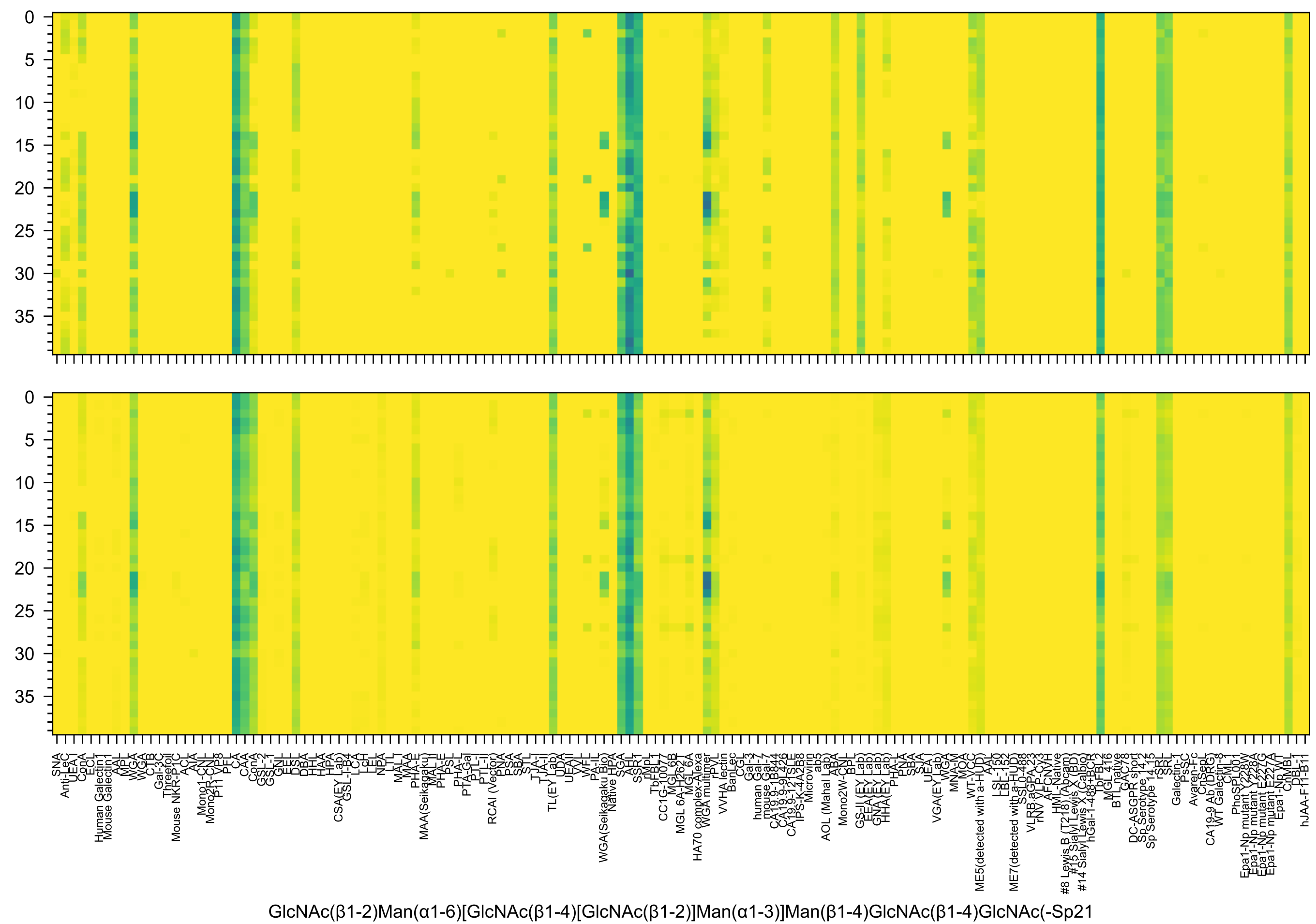
GlcNAc(β1-2)Man(α1-6)[GlcNAc(β1-4)[GlcNAc(β1-2)]Man(α1-3)]Man(β1-4)GlcNAc(β1-4)GlcNAc(-Sp21

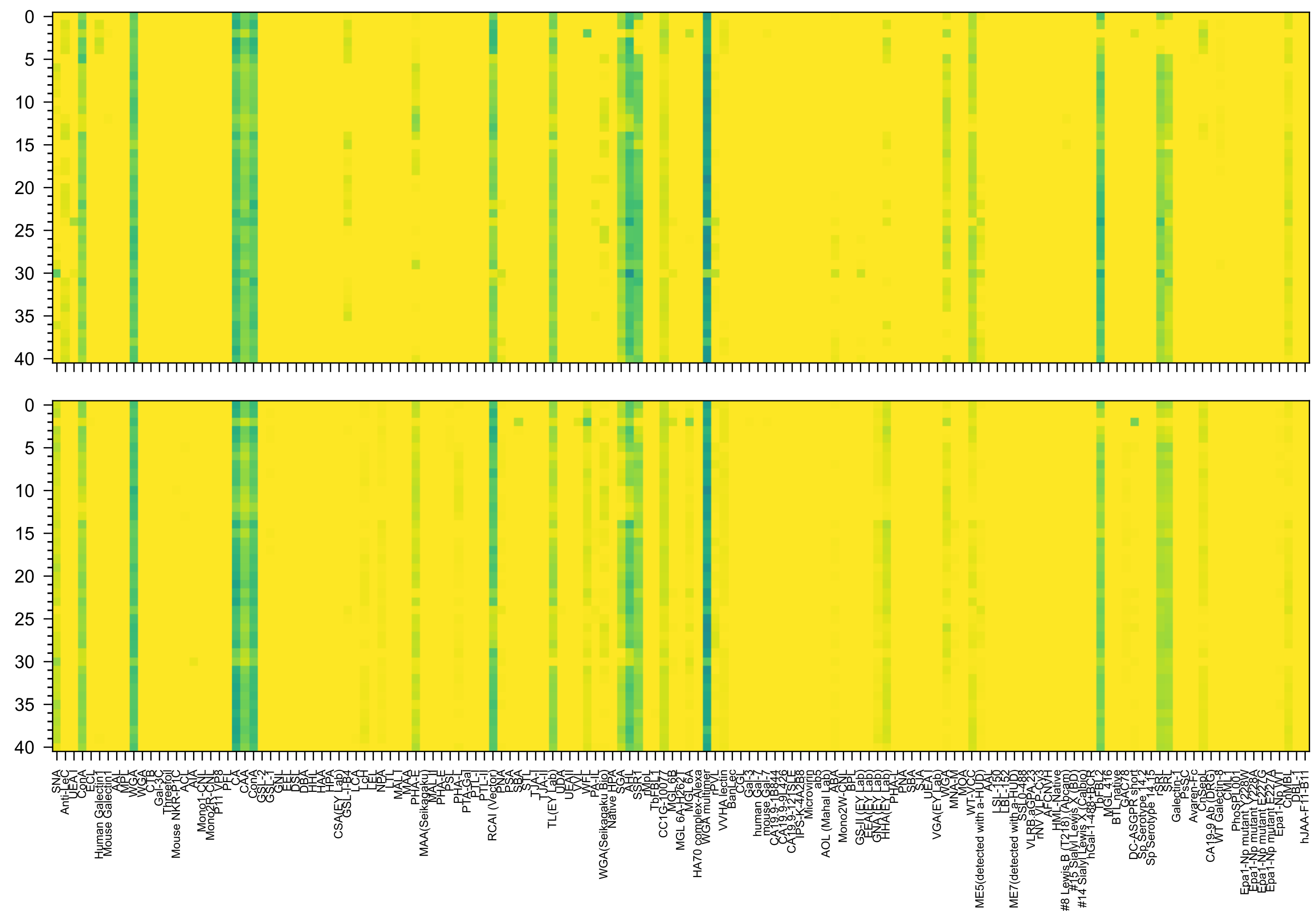
GlcNAc(β1-2)Man(α1-6)[Gal(β1-4)GlcNAc(β1-2)Man(α1-3)]Man(β1-4)GlcNAc(β1-4)GlcNAc(-Sp12

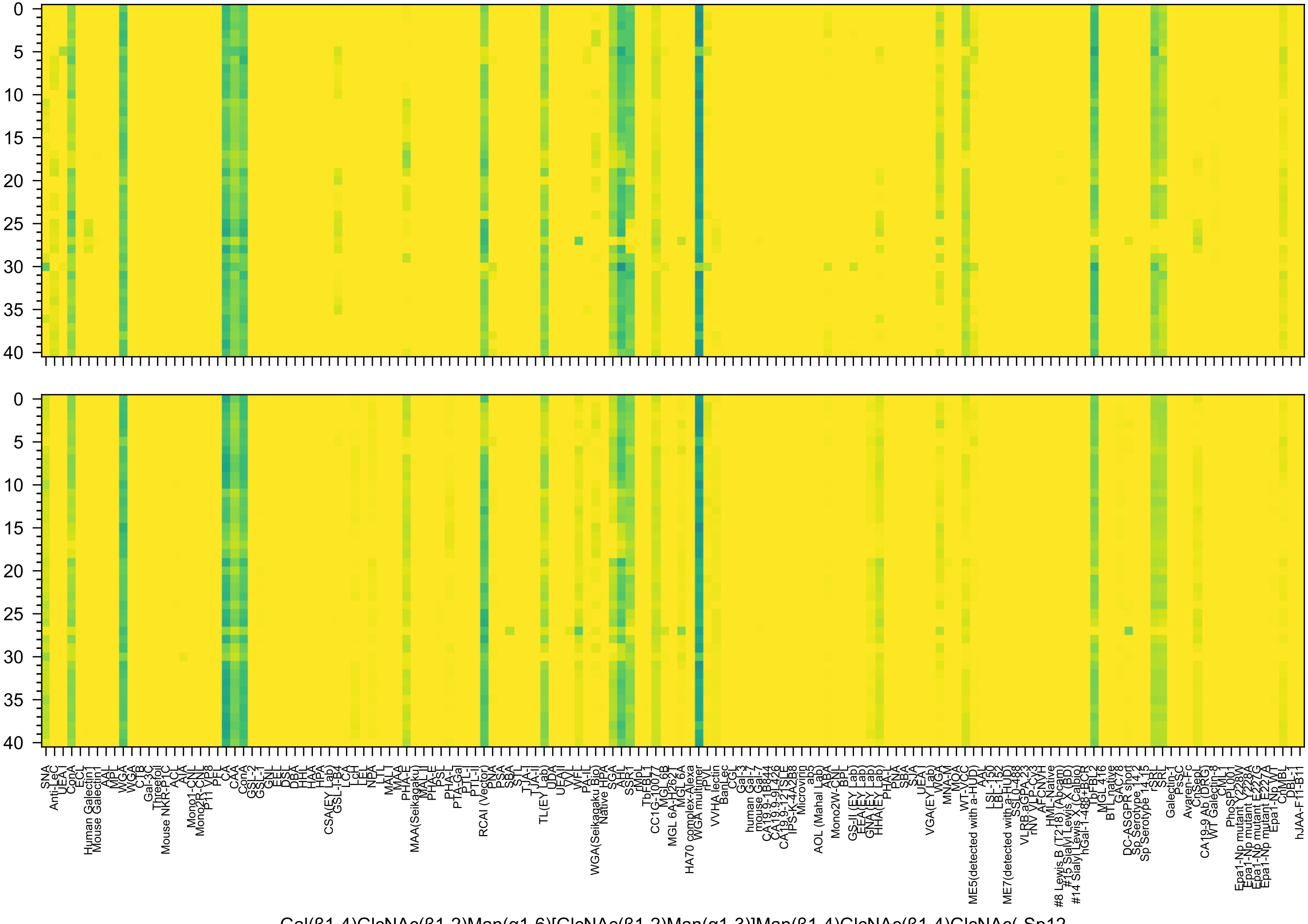
Gal(β1-4)GlcNAc(β1-2)Man(α1-6)[GlcNAc(β1-2)Man(α1-3)]Man(β1-4)GlcNAc(β1-4)GlcNAc(-Sp12

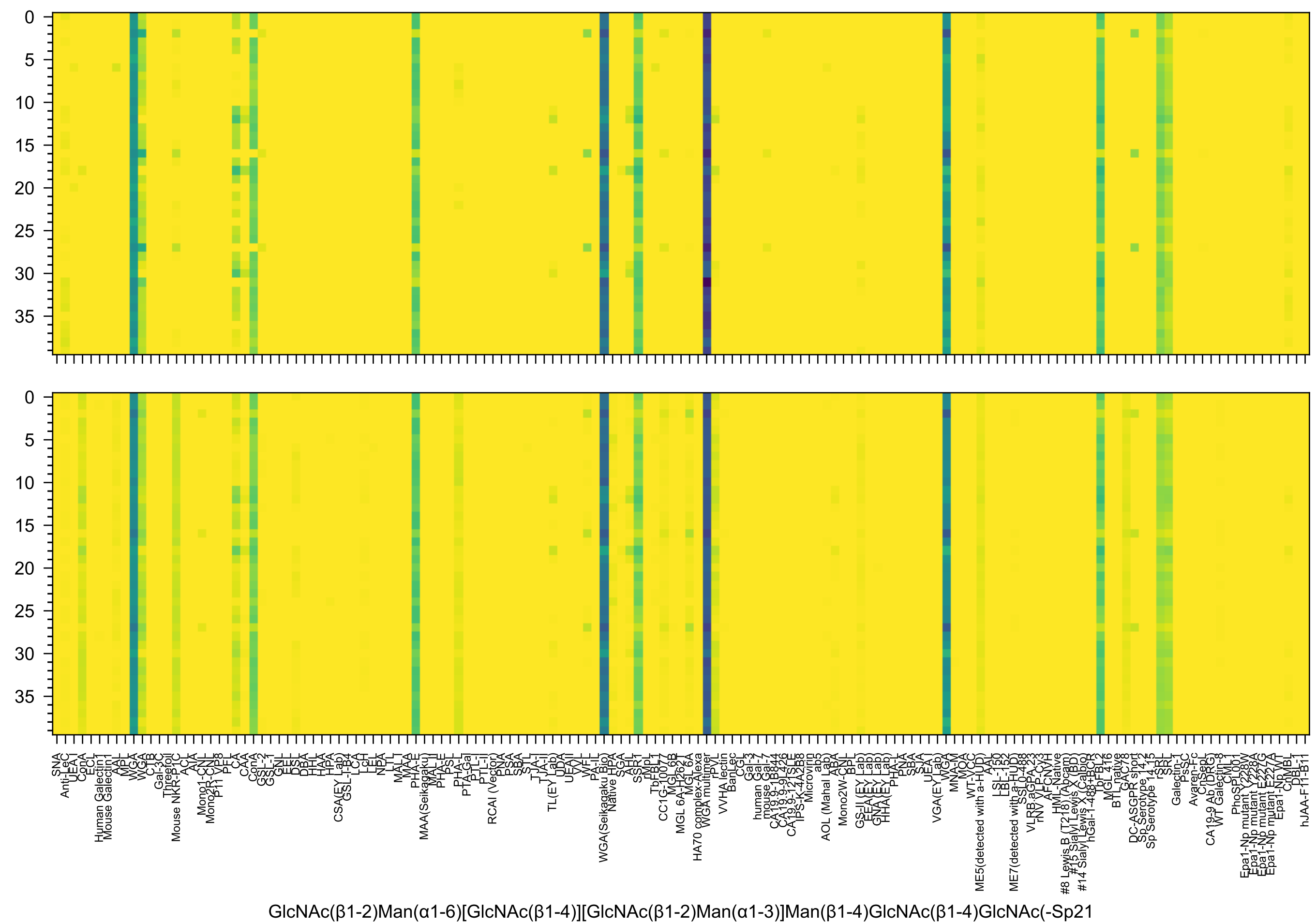
GlcNAc(β1-2)Man(α1-6)[GlcNAc(β1-4)][GlcNAc(β1-2)Man(α1-3)]Man(β1-4)GlcNAc(β1-4)GlcNAc(-Sp21

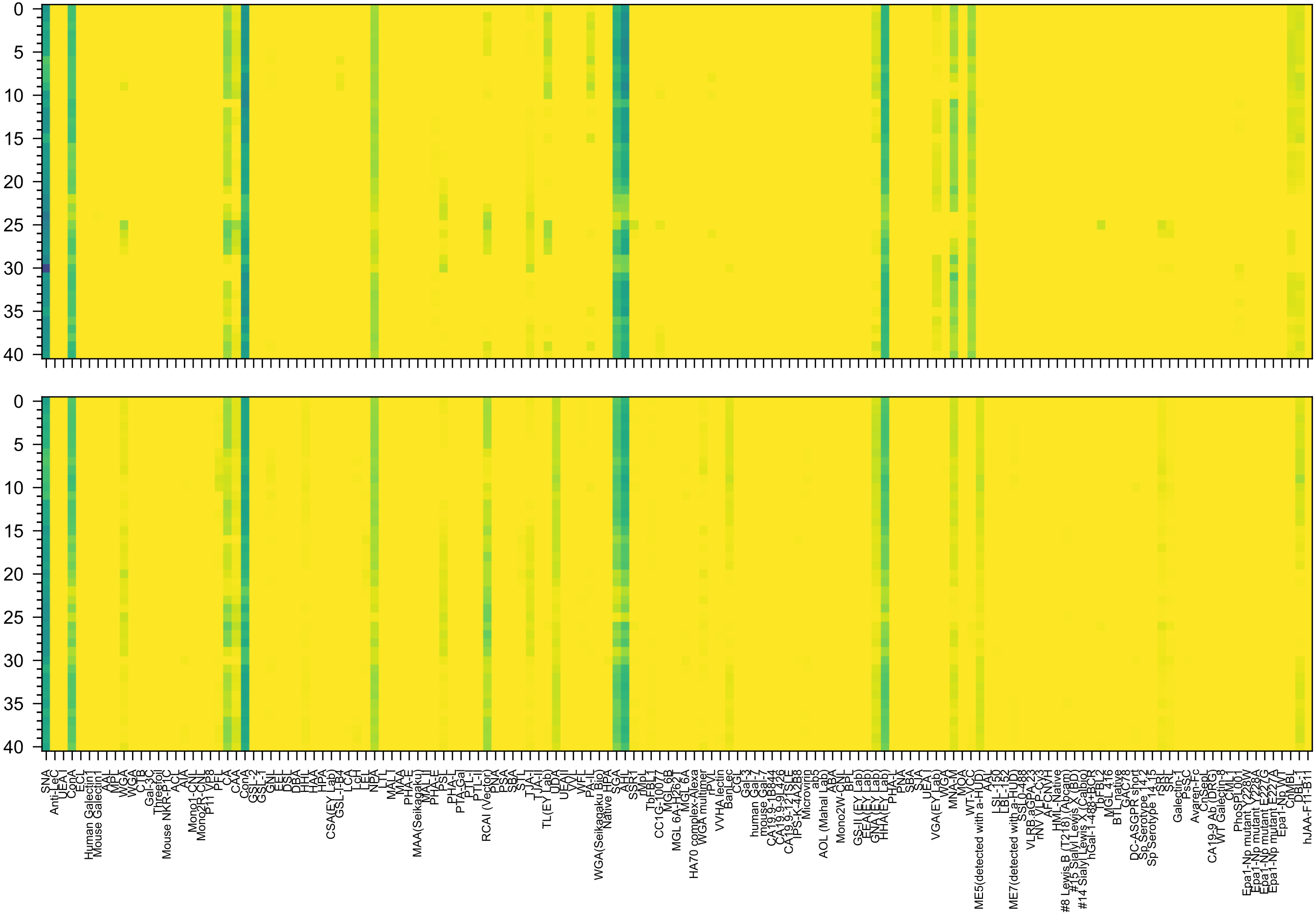

Neu5Ac(α2-6)Gal(β1-4)GlcNAc(β1-2)Man(α1-6)[Man(α1-3)]Man(β1-4)GlcNAc(β1-4)GlcNAc(-Sp12

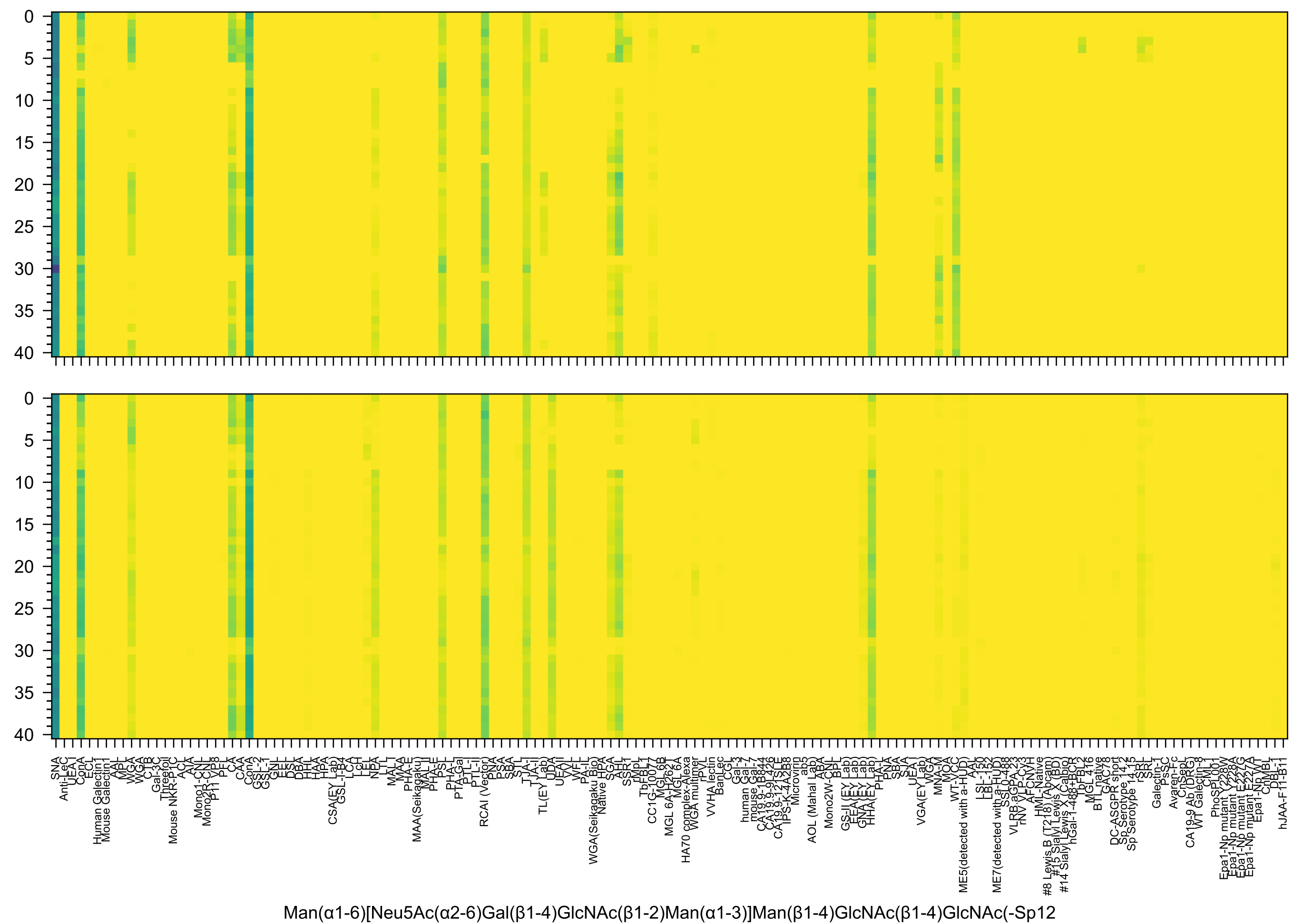
Man(α1-6)[Neu5Ac(α2-6)Gal(β1-4)GlcNAc(β1-2)Man(α1-3)]Man(β1-4)GlcNAc(β1-4)GlcNAc(-Sp12

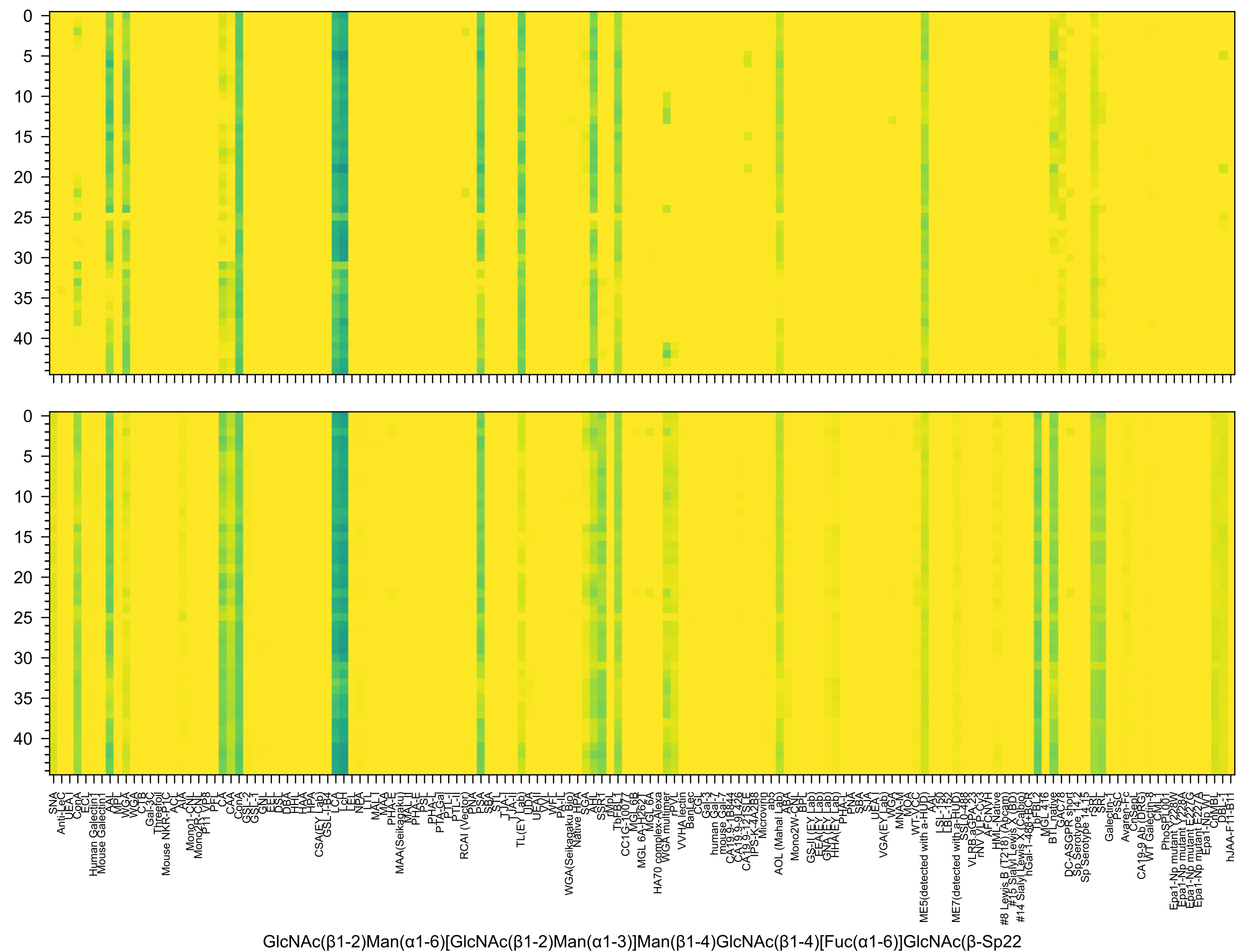
GlcNAc(β1-2)Man(α1-6)[GlcNAc(β1-2)Man(α1-3)]Man(β1-4)GlcNAc(β1-4)[Fuc(α1-6)]GlcNAc(β-Sp22

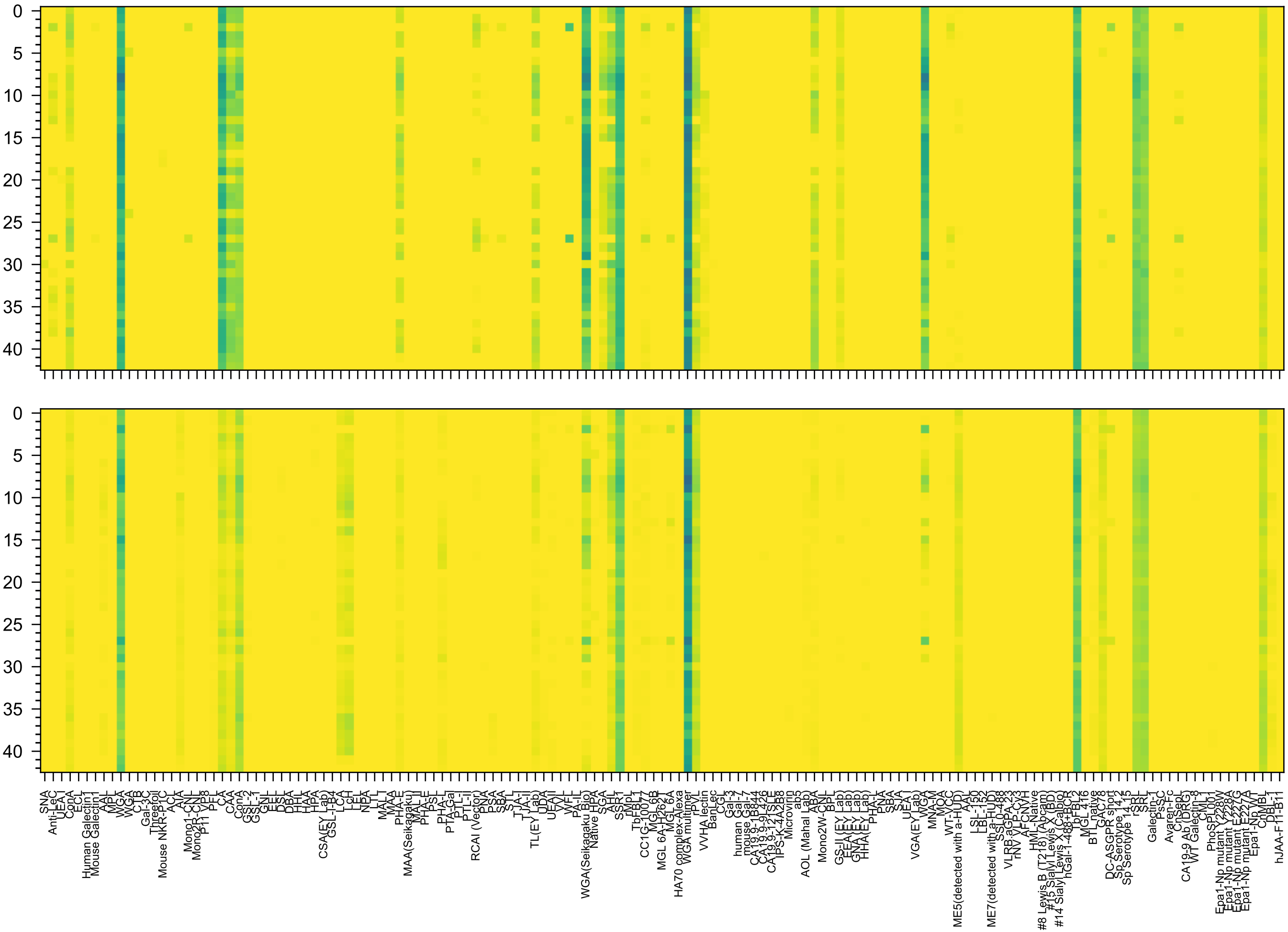

GlcNAc(β1-2)[GlcNAc(β1-6)]Man(α1-6)[GlcNAc(β1-2)Man(α1-3)]Man(β1-4)GlcNAc(β1-4)GlcNAc(β-Sp19

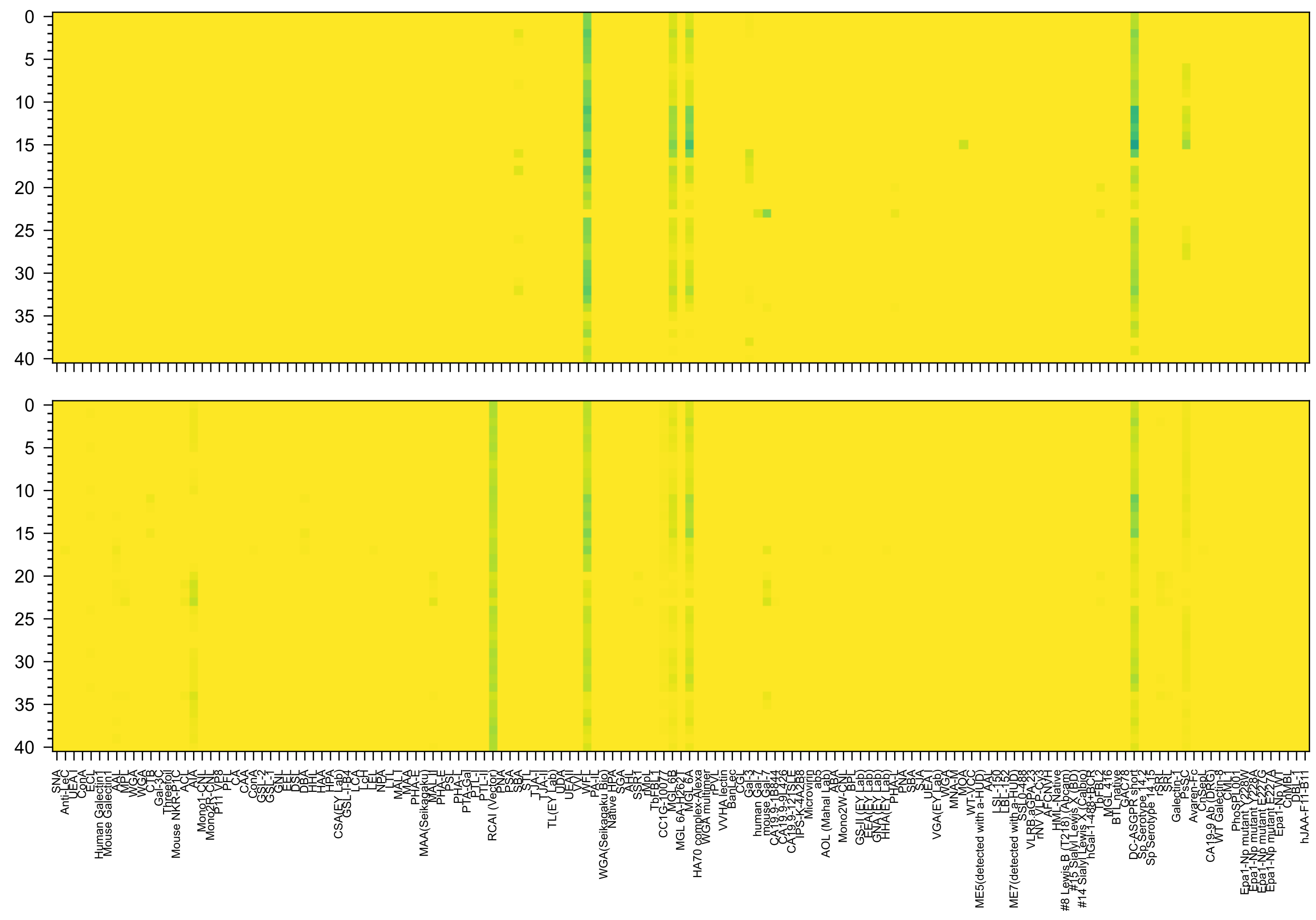

Neu5Ac(α2-8)Neu5Ac(α2-3)Gal(β1-3)GalNAc(β1-4)[Neu5Ac(α2-8)Neu5Ac(α2-3)]Gal(β1-4)Glc(β-Sp0

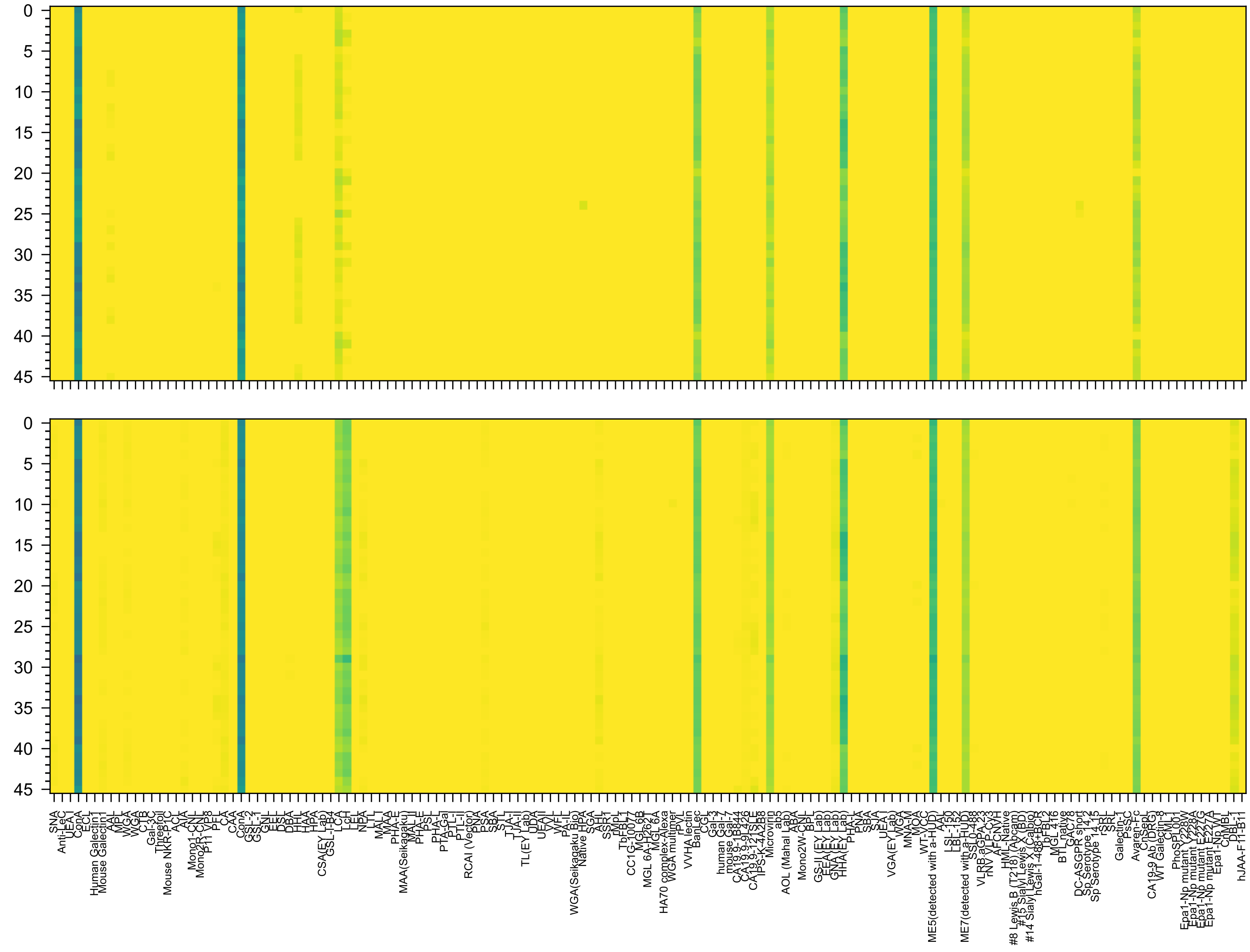

Man(α1-2)Man(α1-6)[Man(α1-2)Man(α1-3)]Man(α1-6)[Man(α1-2)Man(α1-2)Man(α1-3)]Man(α-Sp9

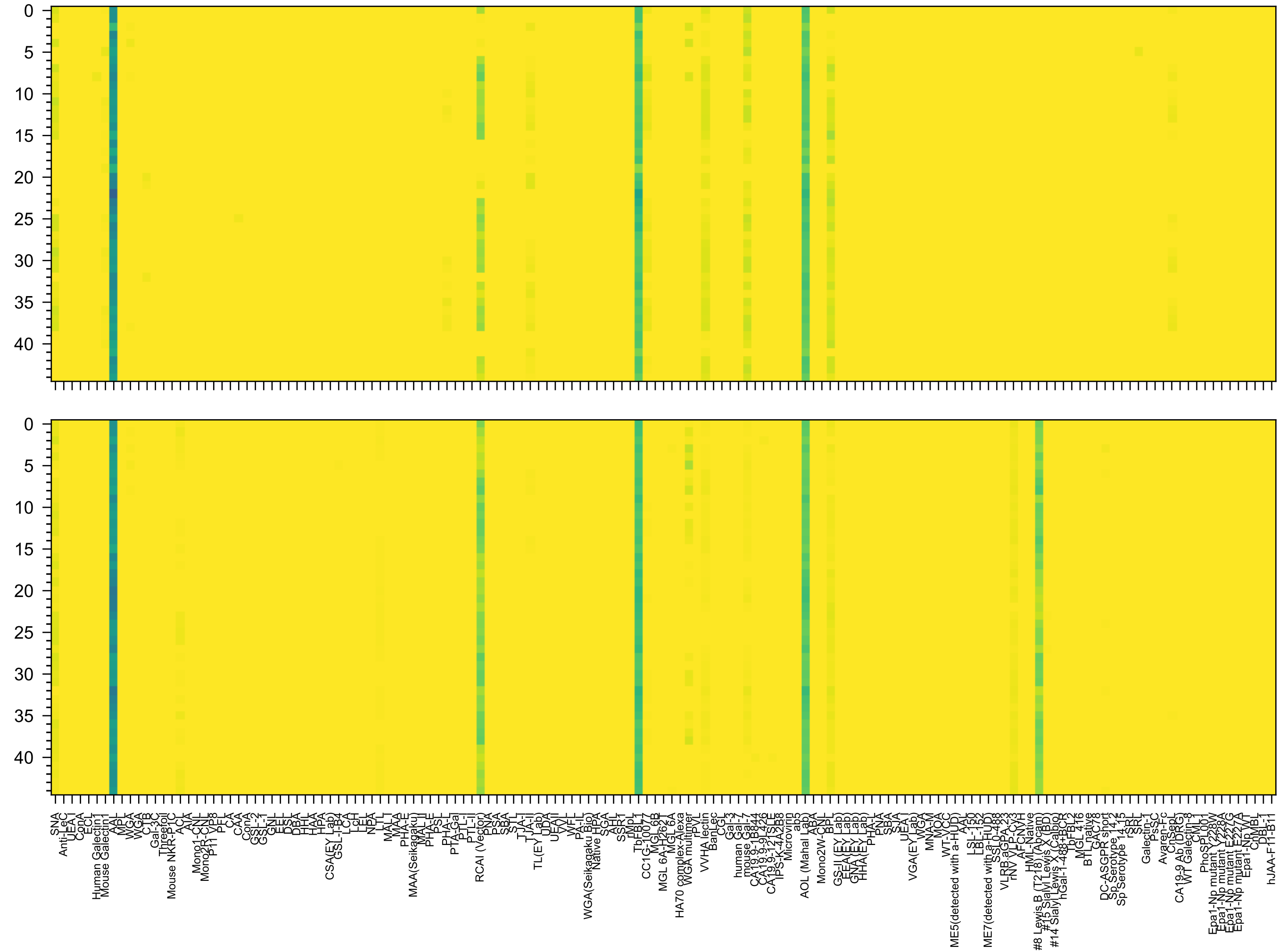

Gal(β1-4)[Fuc(α1-3)]GlcNAc(β1-6)[Fuc(α1-4)[Fuc(α1-2)Gal(β1-3)]GlcNAc(β1-3)]Gal(β1-4)Glc(-Sp21

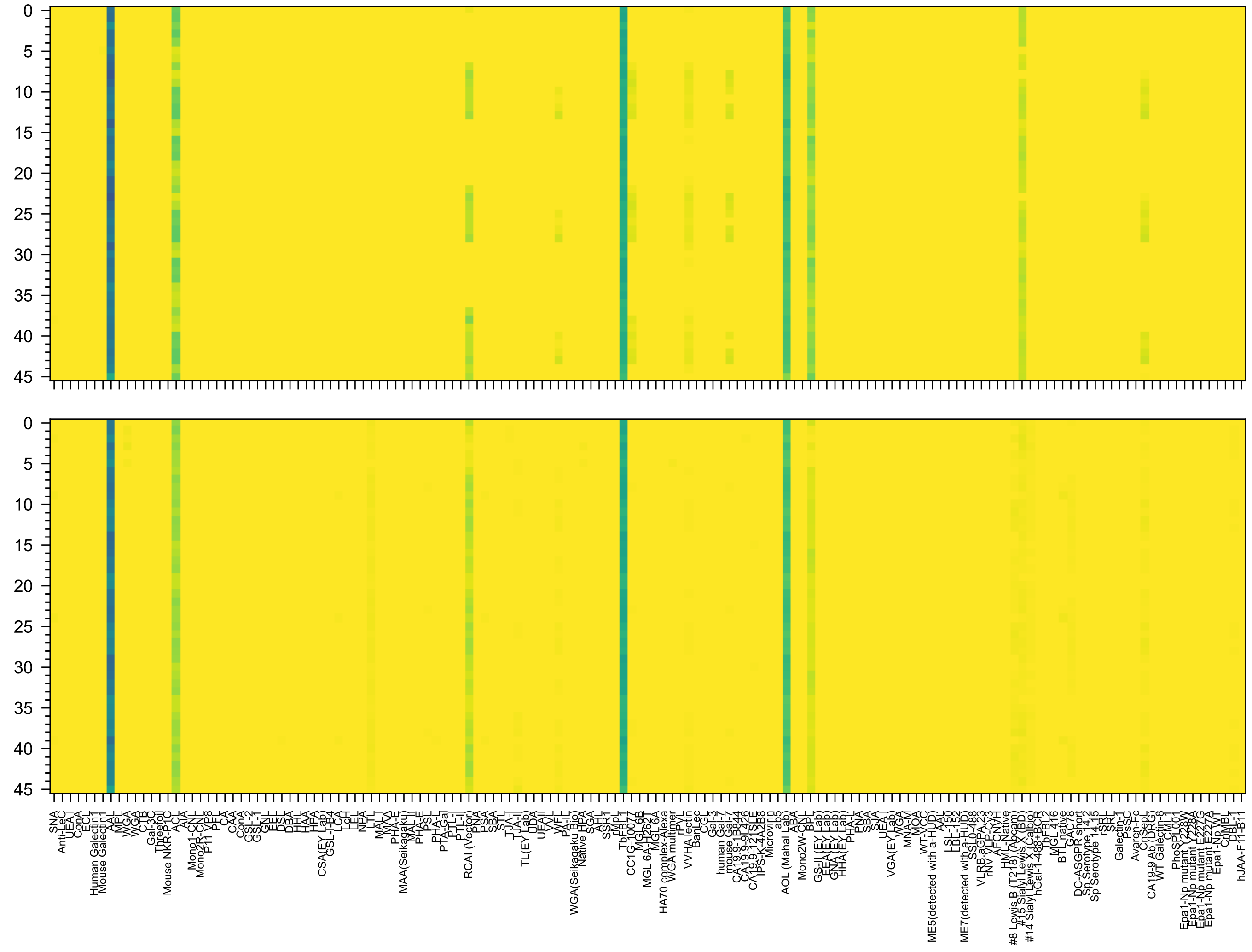

Gal(β1-4)[Fuc(α1-3)]GlcNAc(β1-3)Gal(β1-4)[Fuc(α1-3)]GlcNAc(β1-3)Gal(β1-4)[Fuc(α1-3)]GlcNAc(β-Sp0

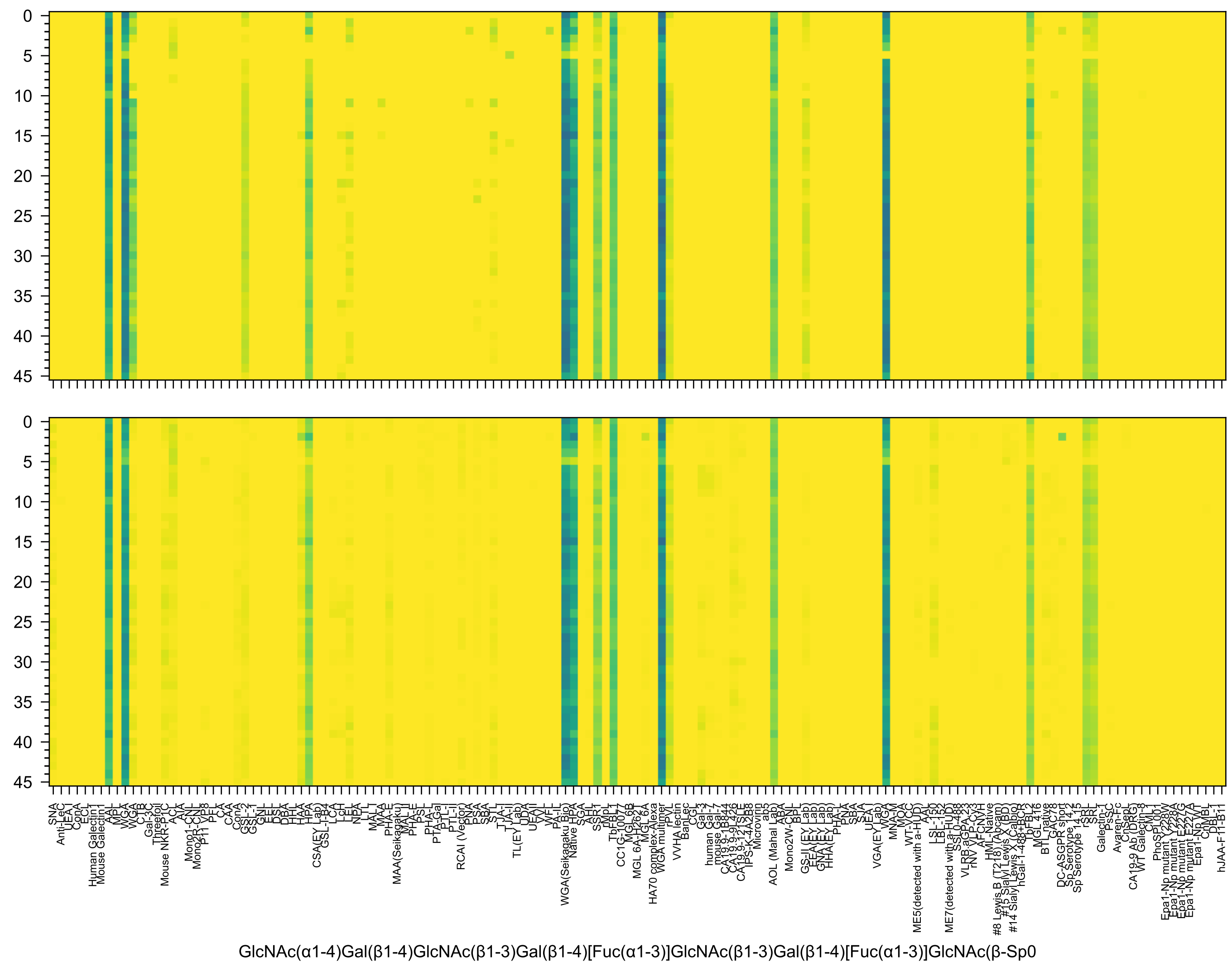
GlcNAc(α1-4)Gal(β1-4)GlcNAc(β1-3)Gal(β1-4)[Fuc(α1-3)]GlcNAc(β1-3)Gal(β1-4)[Fuc(α1-3)]GlcNAc(β-Sp0

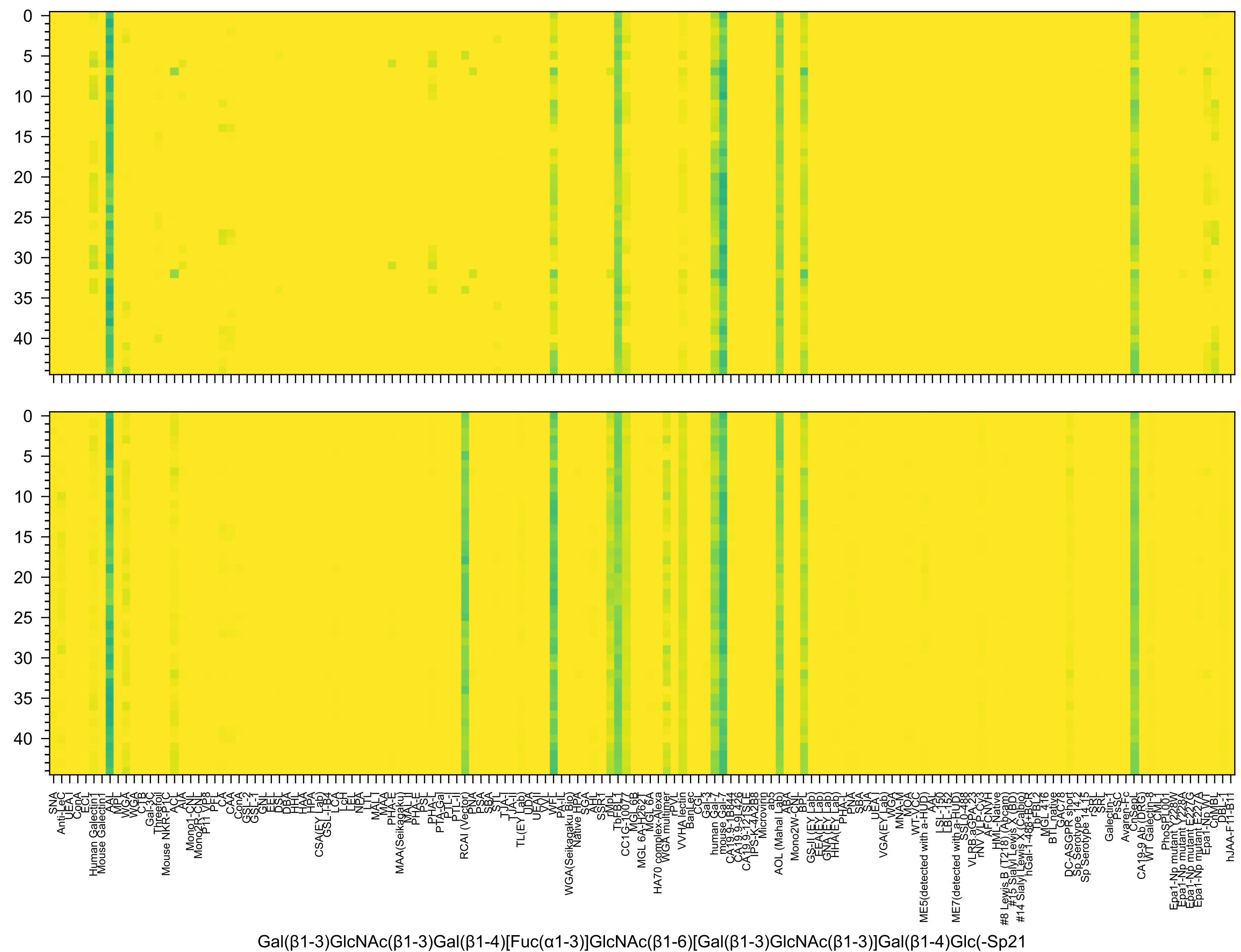
Gal(β1-3)GlcNAc(β1-3)Gal(β1-4)[Fuc(α1-3)]GlcNAc(β1-6)[Gal(β1-3)GlcNAc(β1-3)]Gal(β1-4)Glc(-Sp21

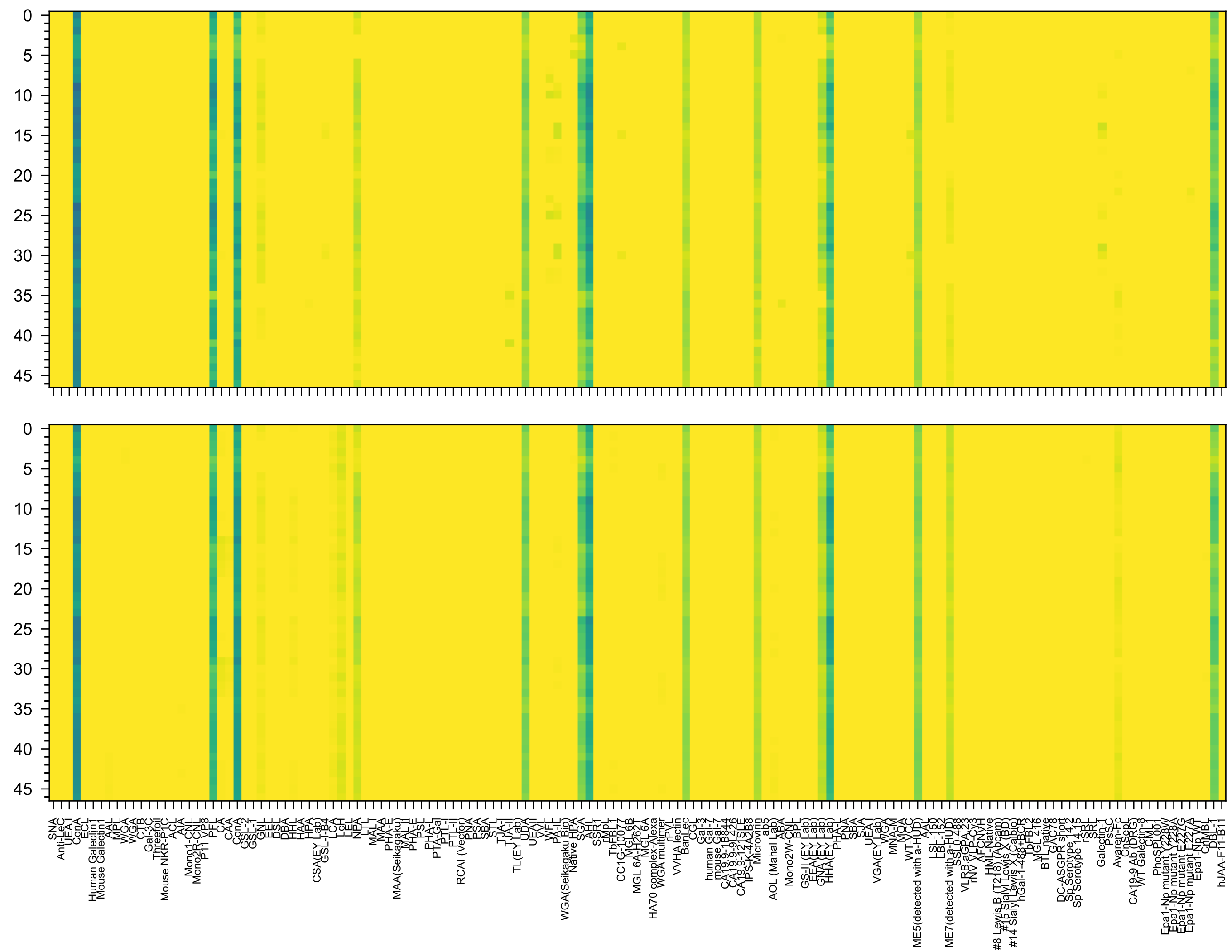

Man(α1-6)[Man(α1-2)Man(α1-3)]Man(α1-6)[Man(α1-2)Man(α1-3)]Man(β1-4)GlcNAc(β1-4)GlcNAc(β-Sp12

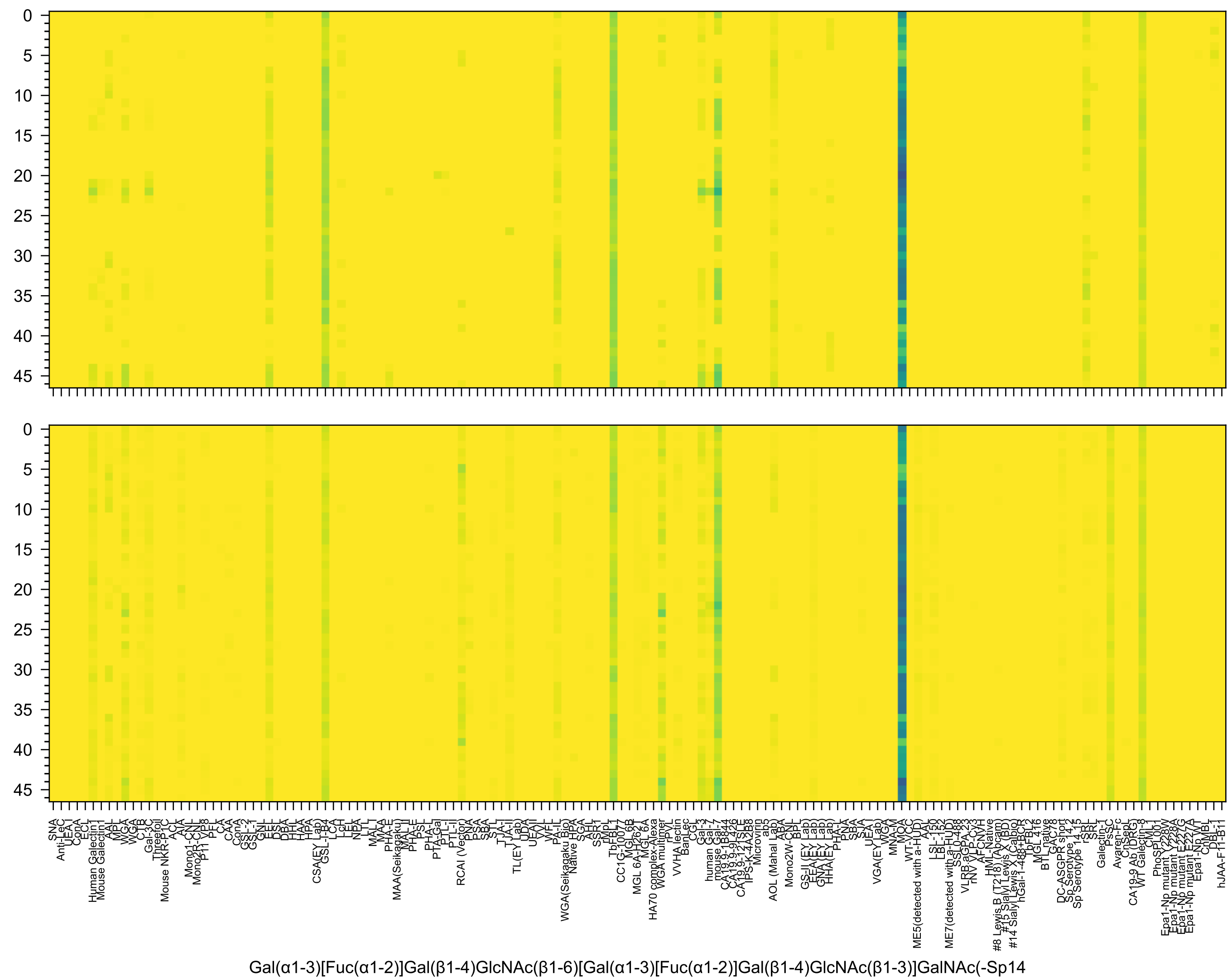
Gal(α1-3)[Fuc(α1-2)]Gal(β1-4)GlcNAc(β1-6)[Gal(α1-3)[Fuc(α1-2)]Gal(β1-4)GlcNAc(β1-3)]GalNAc(-Sp14

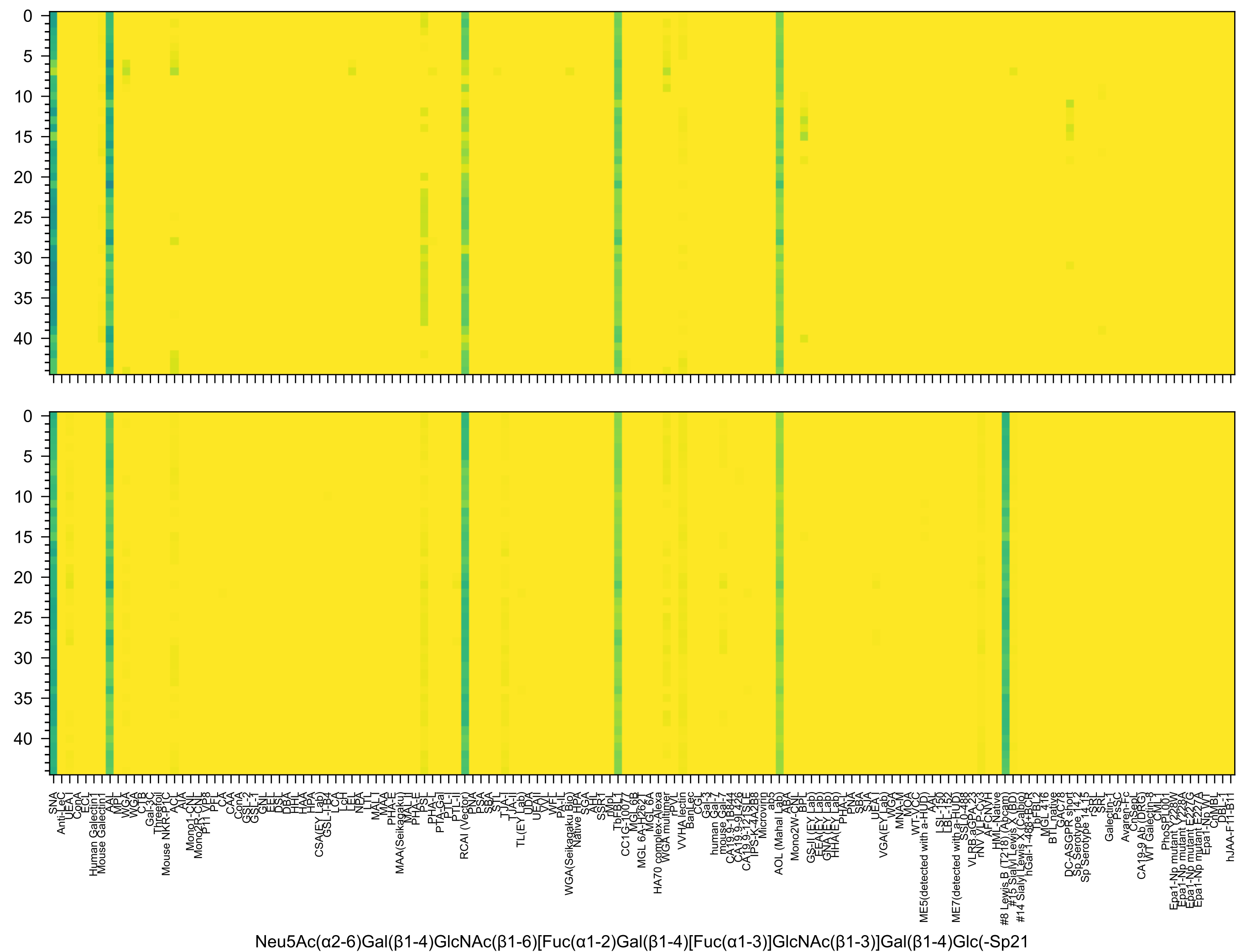
0
5
10
15
20
25
30
35
40
SNA
Anti-LeC
UEA-I
ConA
ECL
Human Galectin1
Mouse Galectin1
AAL
MPL
WGA
WGA
CTB
Gal-3C
Threefoil
Mouse NKR-P1C
ACL
AIA
CNL
Mono1-CNL
Mono2R-CNL
P11-VP8
PFL
CA
CAA
ConA
GSL-2
GSL-1
GNL
EEL
DSL
DBA
HHL
HAA
HPA
CSA(EY Lab)
GSL-I-B4
LCA
LcH
LEL
NPA
LTL
MAL-I
MAA
PHA-E
MAA(Seikagaku)
MAL-II
PHA-E
PSL
PHA-L
PTA-Gal
PTL-I
PTL-II
RCAI (Vector)
PNA
PSA
SBA
STL
TJA-I
TJA-II
TL(EY Lab)
UDA
UEAII
VVL
WFL
PA-IL
WGA(Seikagaku Bio)
Native HPA
SGA
AHL
SSR1
rMpL
TbFBL7
CC1G-10077
MGL-6B
MGL 6A-H262T
MGL6A
HA70 complex-Alexa
WGA multimer
PVL
VVHA lectin
BanLec
CGL
Gal-3
human Gal-7
mouse Gal-7
CA19.9-1B844
CA19.9-9L426
CA19.9-121SLE
IPS-K-4A2B8
Microvirin
ab5
AOL (Mahal Lab)
ABA
Mono2W-CNL
BPL
GS-II (EY Lab)
EEA(EY Lab)
GNA(EY Lab)
HHA(EY Lab)
PHA-L
PNA
SBA
SJA
UEA-I
VGA(EY Lab)
WGA
MNA-M
MOA
WT-VCC
ME5(detected with a-HUD)
AAL
LSL-150
LBL-152
ME7(detected with a-HUD)
SSL0-488
VLRB.aGPA.23
rNV VLP-Cy3
AFCNVH
HML-Native
#8 Lewis B (T218) (Abcam)
#15 Sialyl Lewis X (BD)
#14 Sialyl Lewis X (Calbio)
hGal-1-488+BCR
TbFBL2
MGL 416
BTL native
GAC78
DC-ASGPR short
Sp Serotype 14.2
Sp Serotype 14.15
rSRL
SRL
Galectin-1
PssC
Avaren-Fc
ChSep1
CA19-9 Ab (DRG)
WT Galectin-8
PhoSL
Epa1-Np mutant Y228W
Epa1-Np mutant Y228A
Epa1-Np mutant E227G
Epa1-Np mutant E227A
Epa1-Np WT
Epa1
ChMBL
DBL
hJAA-F11-B11
Neu5Ac(α2-6)Gal(β1-4)GlcNAc(β1-6)[Fuc(α1-2)Gal(β1-4)[Fuc(α1-3)]GlcNAc(β1-3)]Gal(β1-4)Glc(-Sp21

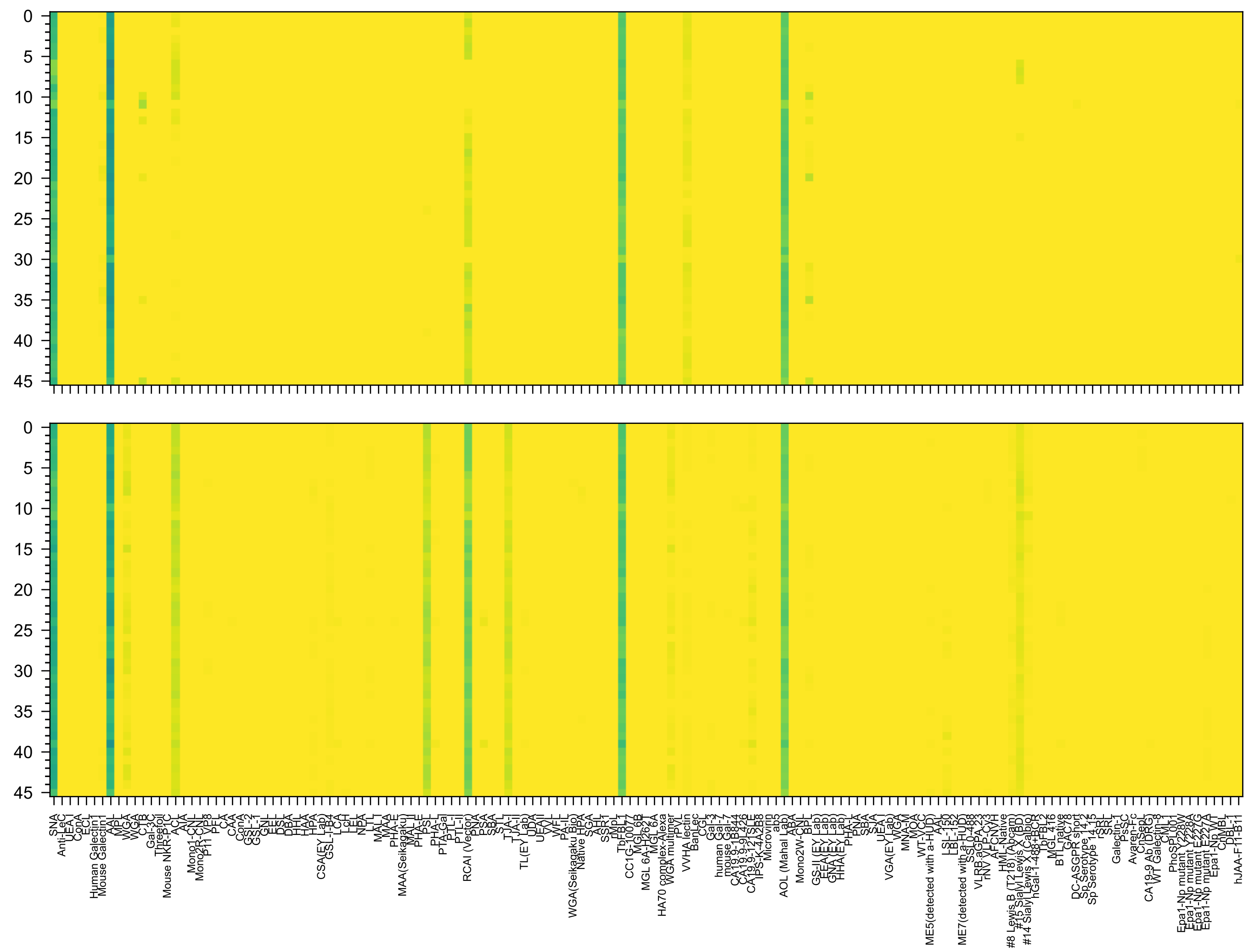

Neu5Ac(α2-6)Gal(β1-4)GlcNAc(β1-3)Gal(β1-4)[Fuc(α1-3)]GlcNAc(β1-3)Gal(β1-4)[Fuc(α1-3)]GlcNAc(β-Sp0

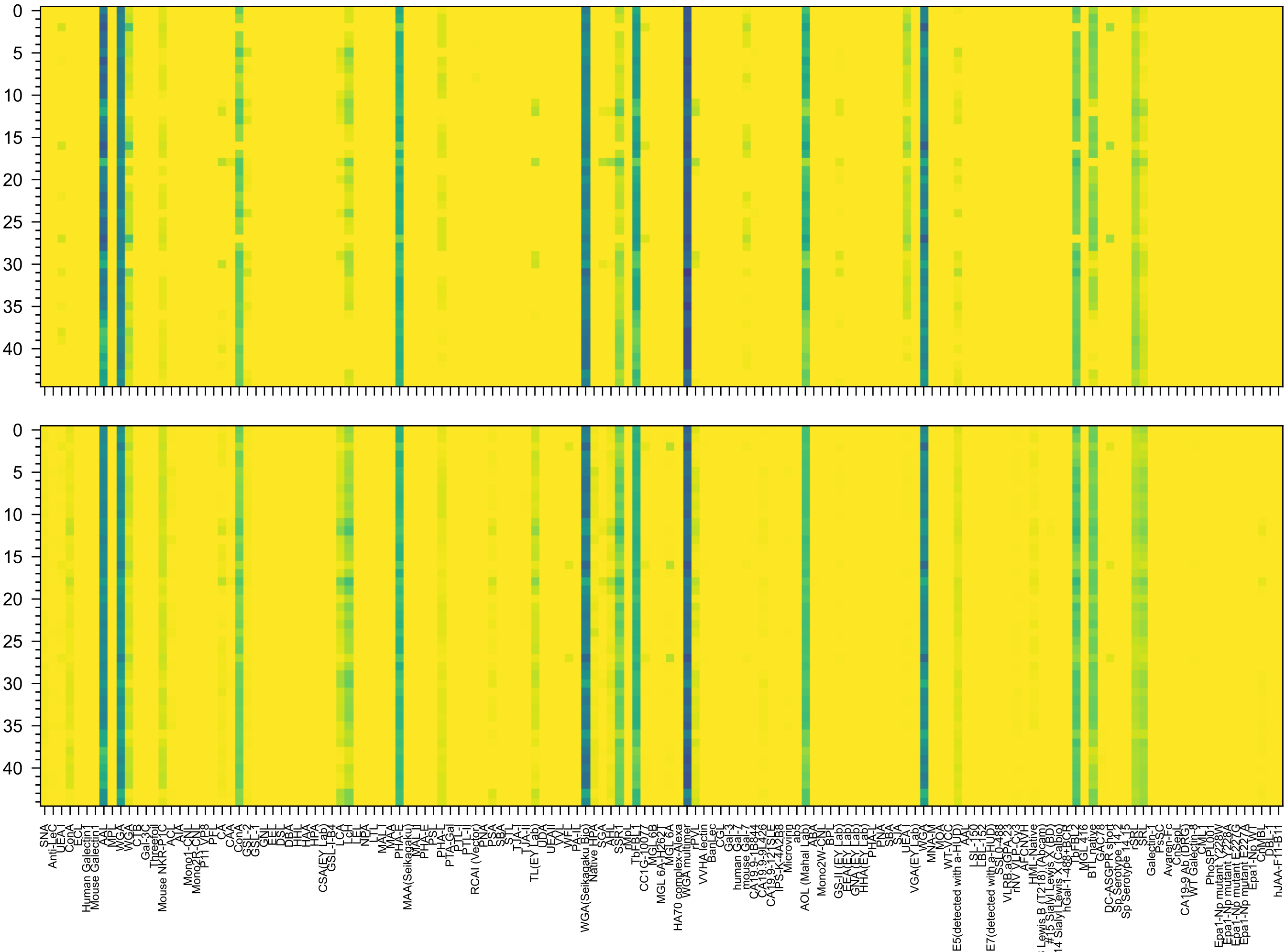

GlcNAc(β1-2)Man(α1-6)[GlcNAc(β1-4)][GlcNAc(β1-2)Man(α1-3)]Man(β1-4)GlcNAc(β1-4)[Fuc(α1-6)]GlcNAc(-Sp21

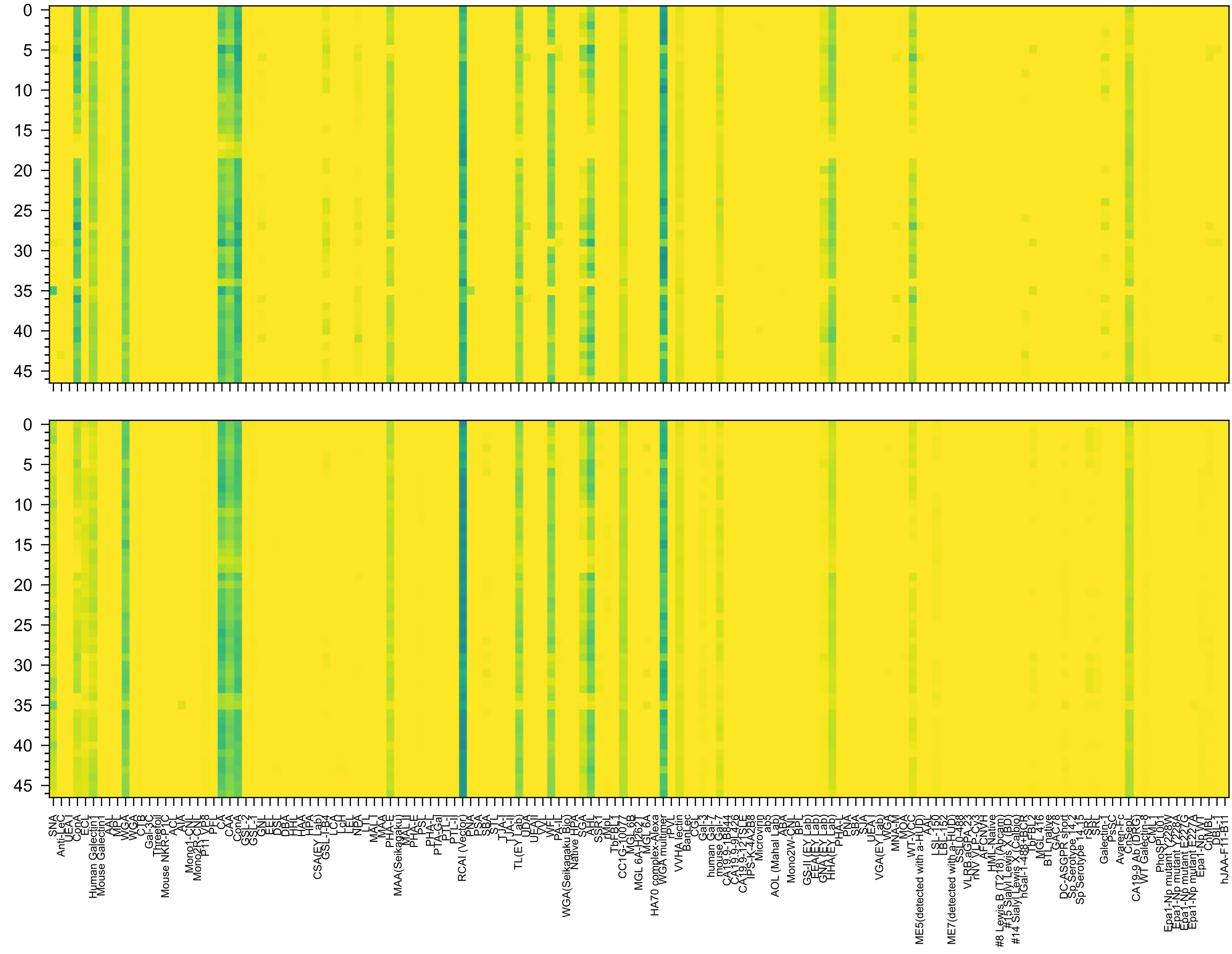

Gal(β1-4)GlcNAc(β1-2)Man(α1-6)[Gal(β1-4)GlcNAc(β1-2)Man(α1-3)]Man(β1-4)GlcNAc(β1-4)GlcNAc(β-Sp12

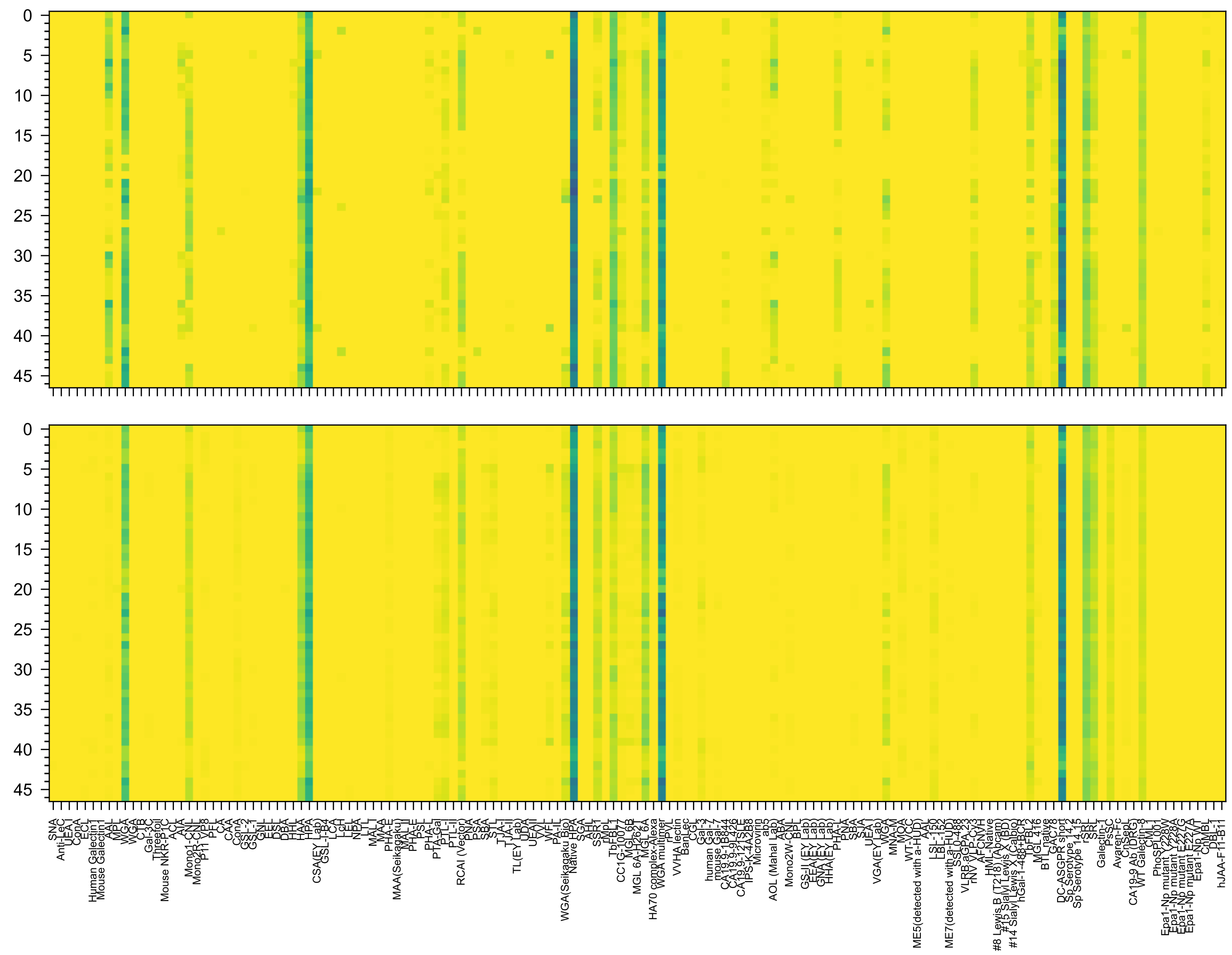

GalNAc(α1-3)[Fuc(α1-2)]Gal(β1-4)GlcNAc(β1-6)[GalNAc(α1-3)[Fuc(α1-2)]Gal(β1-4)GlcNAc(β1-3)]GalNAc(-Sp14

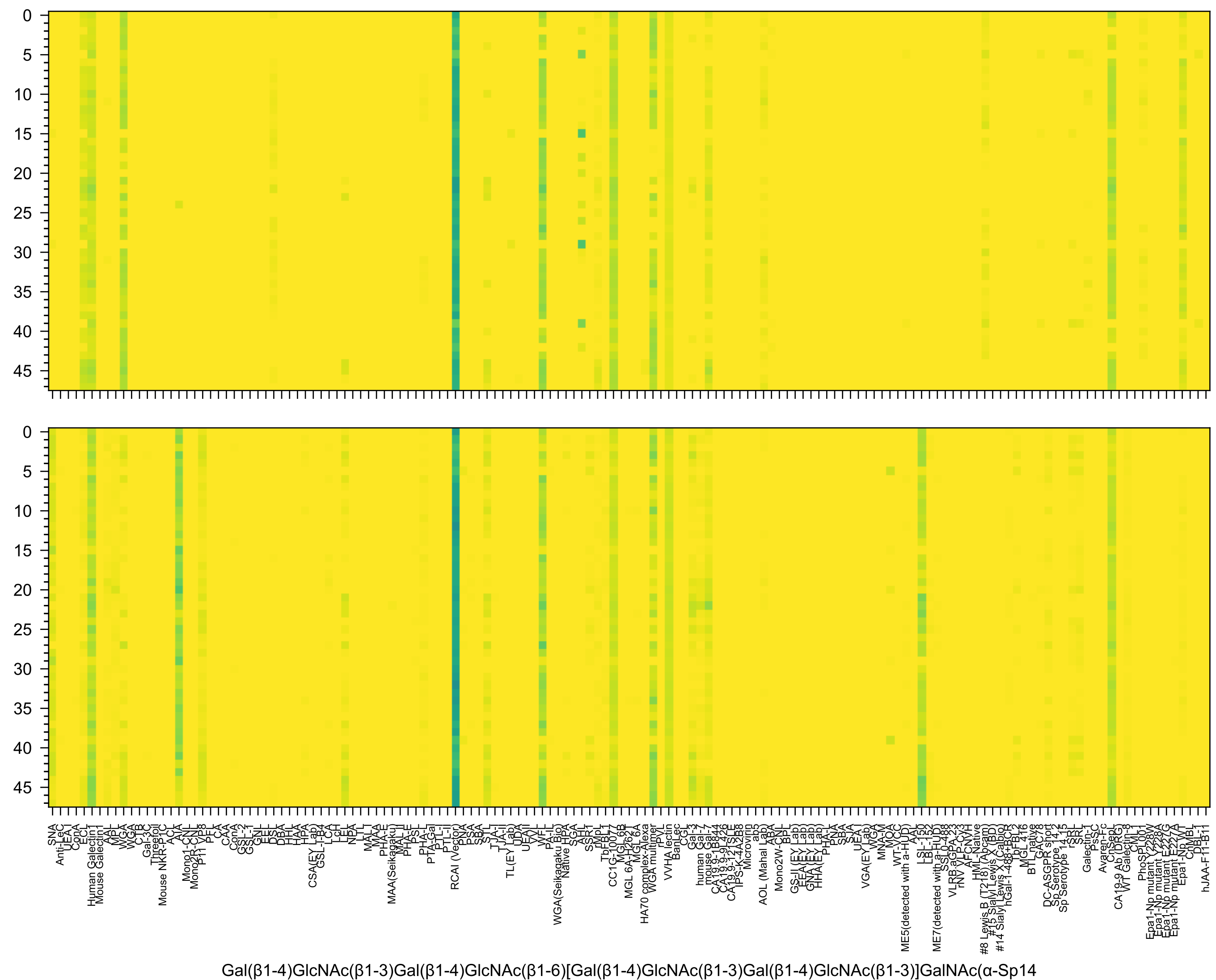
0
5
10
15
20
25
30
35
40
45
SNA
Anti-LeC
UEA I
ConA
ECL
Human Galectin1
Mouse Galectin1
AAL
MPL
WGA
WGA
CTB
Gal-3C
Threefoil
Mouse NKR-P1C
ACL
AIA
Mono1-CNL
Mono2R-CNL
P11 VP8
PFL
CA
CAA
ConA
GSL-2
GSL-1
GNL
EEL
DSL
DBA
HHL
HAA
HPA
CSA(EY Lab)
GSL-I-B4
LCA
LcH
LEL
NPA
LTL
MAL I
MAA
MAA(Seikagaku)
PHA-E
MAL II
PHA-E
PSL
PHA-L
PTA-Gal
PTL-I
PTL-II
RCAI (Vector)
PNA
PSA
SBA
STL
TJA-I
TJA-II
TL(EY Lab)
UDA
UEAII
VVL
WFL
WGA(Seikagaku Bio)
PA-IL
Native HPA
SGA
AHL
SSR1
rMpL
TbFBL1
CC1G-10077
MGL 6B
MGL 6A-H262T
MGL 6A
HA70 complex-Alexa
WGA multimer
rPVL
VVHA lectin
BanLec
CGL
Gal-3
human Gal-7
mouse Gal-7
CA19.9-1B844
CA19.9-9L426
CA19.9-121SLE
IPS-K-4A2B8
Microvirin
ab5
AOL (Mahal Lab)
ABA
Mono2W-CNL
BPL
GS-II (EY Lab)
EEA (EY Lab)
GNA (EY Lab)
HHA (EY Lab)
PHA-L
PNA
SBA
SJA
UEA I
VGA(EY Lab)
WGA
MNA-M
MOA
WT-VCC
ME5(detected with a-HUD)
AAL
LSL-150
LBL-152
ME7(detected with a-HUD)
SSL0-488
VLRB.aGPA.23
rNV VLP-Cy3
AFCNVH
HML-Native
#8 Lewis B (T218) (Abcam)
#15 Sialyl Lewis X (BD)
#14 Sialyl Lewis X (Calbio)
hGal-1-488+BCR
TbFBL2
MGL 416
BTL native
GAC78
DC-ASGPR short
Sp Serotype 14.2
Sp Serotype 14.15
rSRL
SRL
Galectin-1
PsSC
Avaren-Fc
CnSepL
CA19-9 Ab (DRG)
WT Galectin-8
CML
PhoSL001
Epa1-Np mutant Y228W
Epa1-Np mutant Y228A
Epa1-Np mutant E227G
Epa1-Np mutant E227A
Epa1-Np WT
ChMBL
DBL-1
hJAA-F11-B11
Gal(β1-4)GlcNAc(β1-3)Gal(β1-4)GlcNAc(β1-6)[Gal(β1-4)GlcNAc(β1-3)Gal(β1-4)GlcNAc(β1-3)]GalNAc(α-Sp14

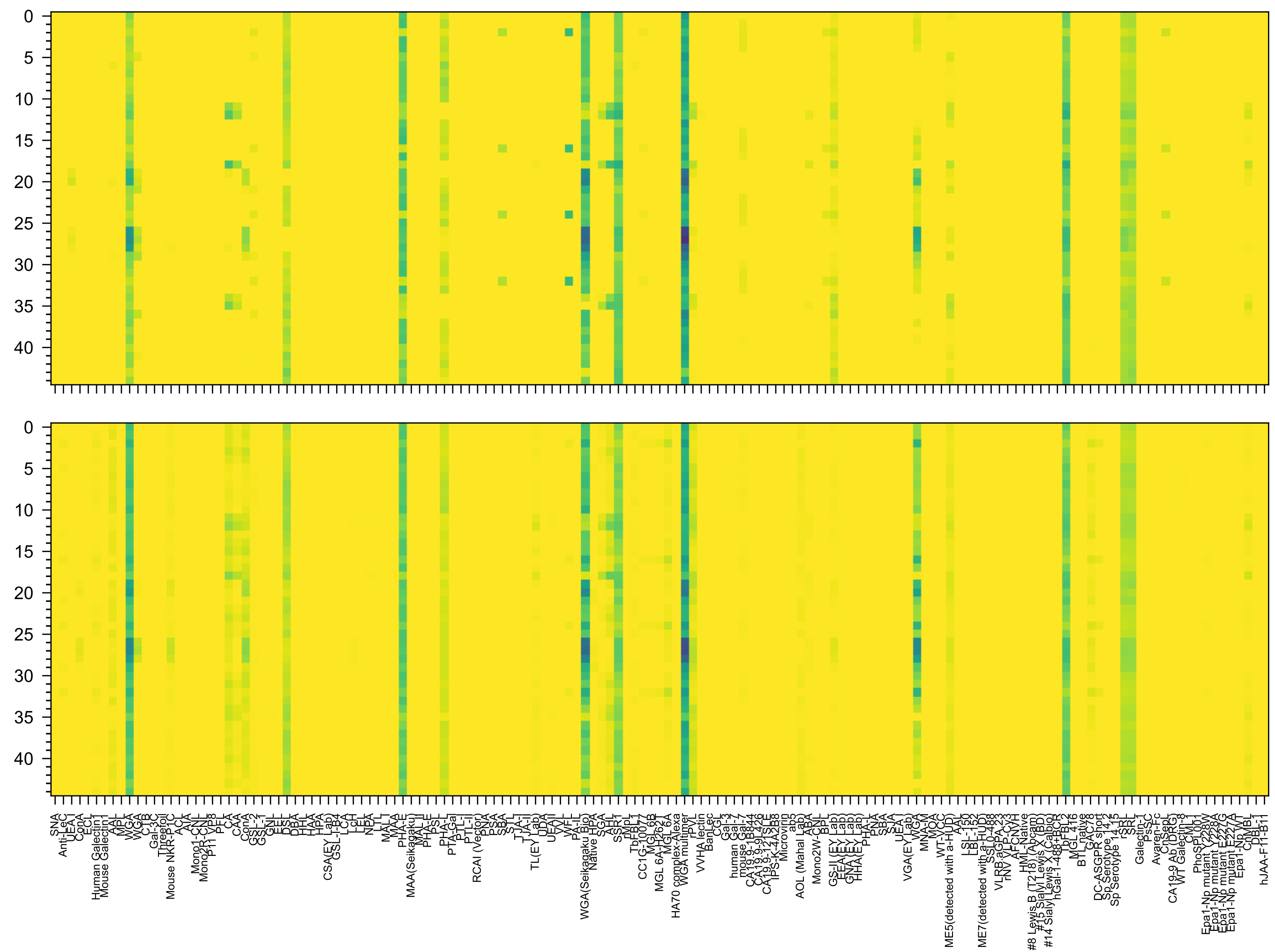

GlcNAc(β1-2)Man(α1-6)[GlcNAc(β1-4)][GlcNAc(β1-4)[GlcNAc(β1-2)]Man(α1-3)]Man(β1-4)GlcNAc(β1-4)GlcNAc(-Sp21

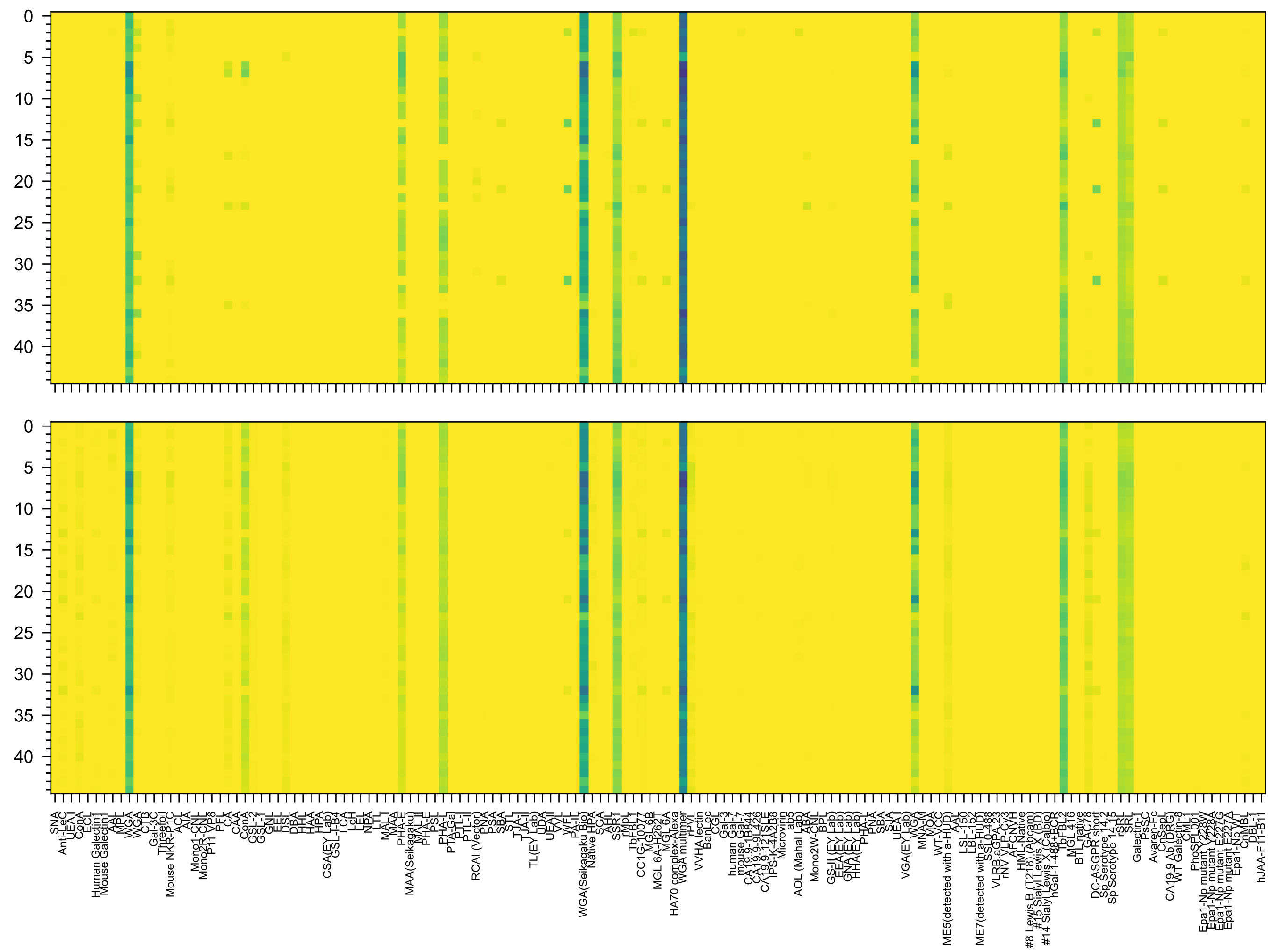

GlcNAc(β1-6)[GlcNAc(β1-2)]Man(α1-6)[GlcNAc(β1-4)][GlcNAc(β1-2)Man(α1-3)]Man(β1-4)GlcNAc(β1-4)GlcNAc(-Sp21

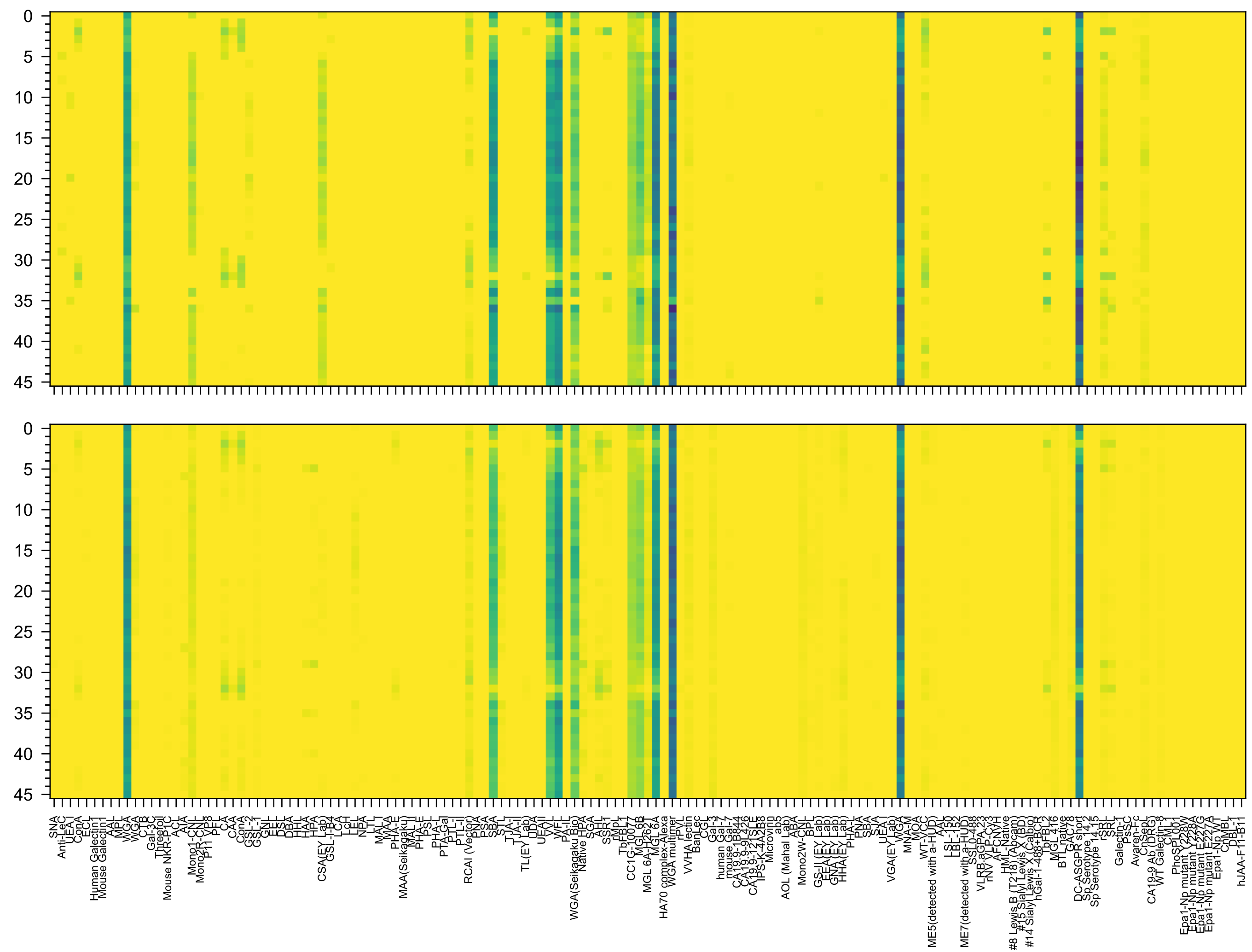

GalNAc(β1-4)GlcNAc(β1-2)Man(α1-6)[GalNAc(β1-4)GlcNAc(β1-2)Man(α1-3)]Man(β1-4)GlcNAc(β1-4)GlcNAc(-Sp12

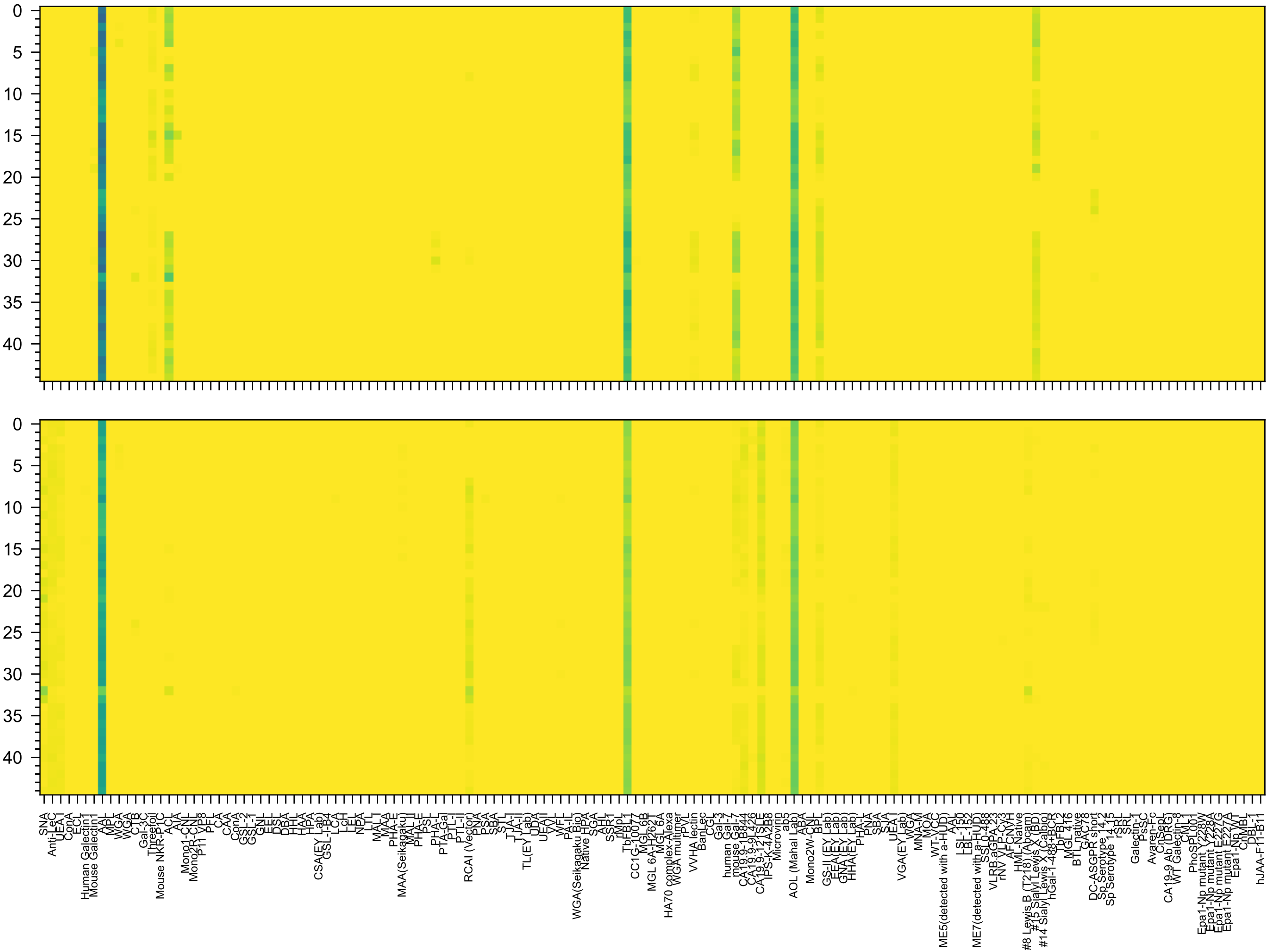

Gal(β1-4)[Fuc(α1-3)]GlcNAc(β1-6)[Neu5Ac(α2-6)[Neu5Ac(α2-3)Gal(β1-3)]GlcNAc(β1-3)]Gal(β1-4)Glc(-Sp21

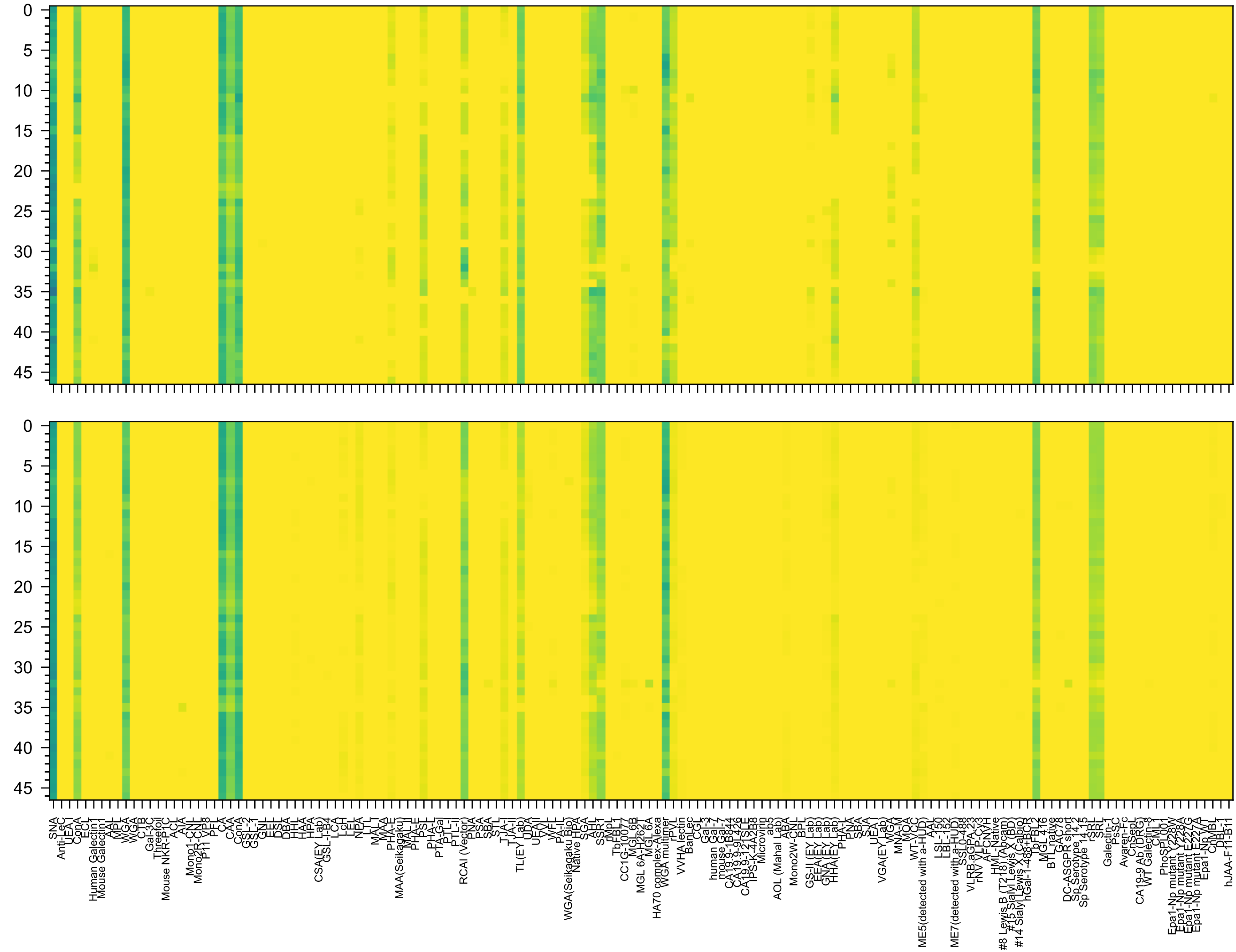

Neu5Ac(α2-6)Gal(β1-4)GlcNAc(β1-2)Man(α1-6)[GlcNAc(β1-2)Man(α1-3)]Man(β1-4)GlcNAc(β1-4)GlcNAc(β-Sp12

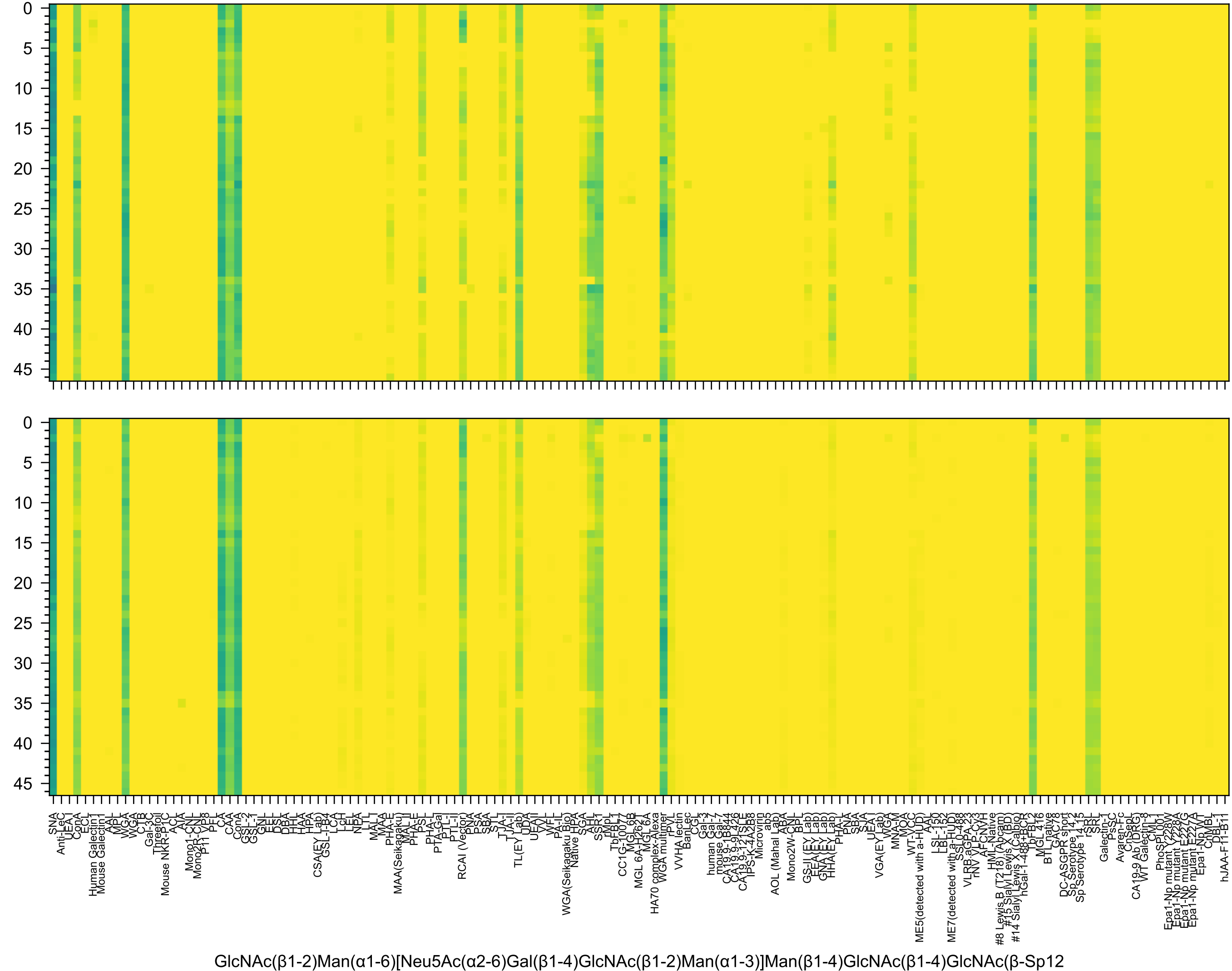

0
5
10
15
20
25
30
35
40
45
SNA
Anti-LeC
UEA-I
ConA
ECL
Human Galectin1
Mouse Galectin1
AAL
MPL
WGA
WGA
CTB
Gal-3C
Threefoil
Mouse NKR-P1C
ACL
AIA
Mono1-CNL
Mono2R-CNL
P11 VP8
PFL
CA
CAA
ConA
GSL-2
GSL-1
GNL
EEL
DSL
DBA
HHL
HAA
HPA
CSA(EY Lab)
GSL-I-B4
LCA
LcH
LEL
NPA
LTL
MAL-I
MAA
PHA-E
MAA(Seikagaku)
MAL-II
PHA-E
PSL
PHA-L
PTA-Gal
PTL-I
PTL-II
RCAI (Vector)
PNA
PSA
SBA
STL
TJA-I
TJA-II
TL(EY Lab)
UDA
UEA-II
VVL
WFL
PA-IL
WGA(Seikagaku Bio)
Native HPA
SGA
AHL
SSR1
rMpL
TbFBL1
CC1G-10077
MGL 6B
MGL 6A-H262T
MGL 6A
HA70 complex-Alexa
WGA multimer
rPVL
VVHA lectin
BanLec
CGL
Gal-3
human Gal-7
mouse Gal-7
CA19.9-1B844
CA19.9-9L426
CA19.9-121SLE
IPS-K-4A2B8
Microvirin
ab5
AOL (Mahal Lab)
ABA
Mono2W-CNL
BPL
GS-II (EY Lab)
EEA (EY Lab)
GNA (EY Lab)
HHA (EY Lab)
PHA-L
PNA
SBA
SJA
UEA-I
VGA(EY Lab)
WGA
MNA-M
MOA
WT-VCC
ME5(detected with a-HUD)
AAL
LSL-150
LBL-152
ME7(detected with a-HUD)
SSL0-488
VLRB.aGPA.23
rNV VLP-Cy3
AFCN/H
HML-Native
#8 Lewis B (T218) (Abcam)
#15 Sialyl Lewis X (BD)
#14 Sialyl Lewis X (Calbio)
hGal-1-488+BCR
TbFBL2
MGL 416
BTL native
GAC78
DC-ASGPR short
Sp Serotype 14.2
Sp Serotype 14.15
rSRL
SRL
Galectin-1
PsSC
Avaren-Fc
CnSep
CA19-9 Ab (DRG)
WT Galectin-8
CML1
PhoSPL001
Epa1-Np mutant Y228W
Epa1-Np mutant Y228A
Epa1-Np mutant E227G
Epa1-Np mutant E227A
Epa1-Np WT
CnMBL
DBL-1
hJAA-F11-B1
GlcNAc(β1-2)Man(α1-6)[Neu5Ac(α2-6)Gal(β1-4)GlcNAc(β1-2)Man(α1-3)]Man(β1-4)GlcNAc(β1-4)GlcNAc(β-Sp12

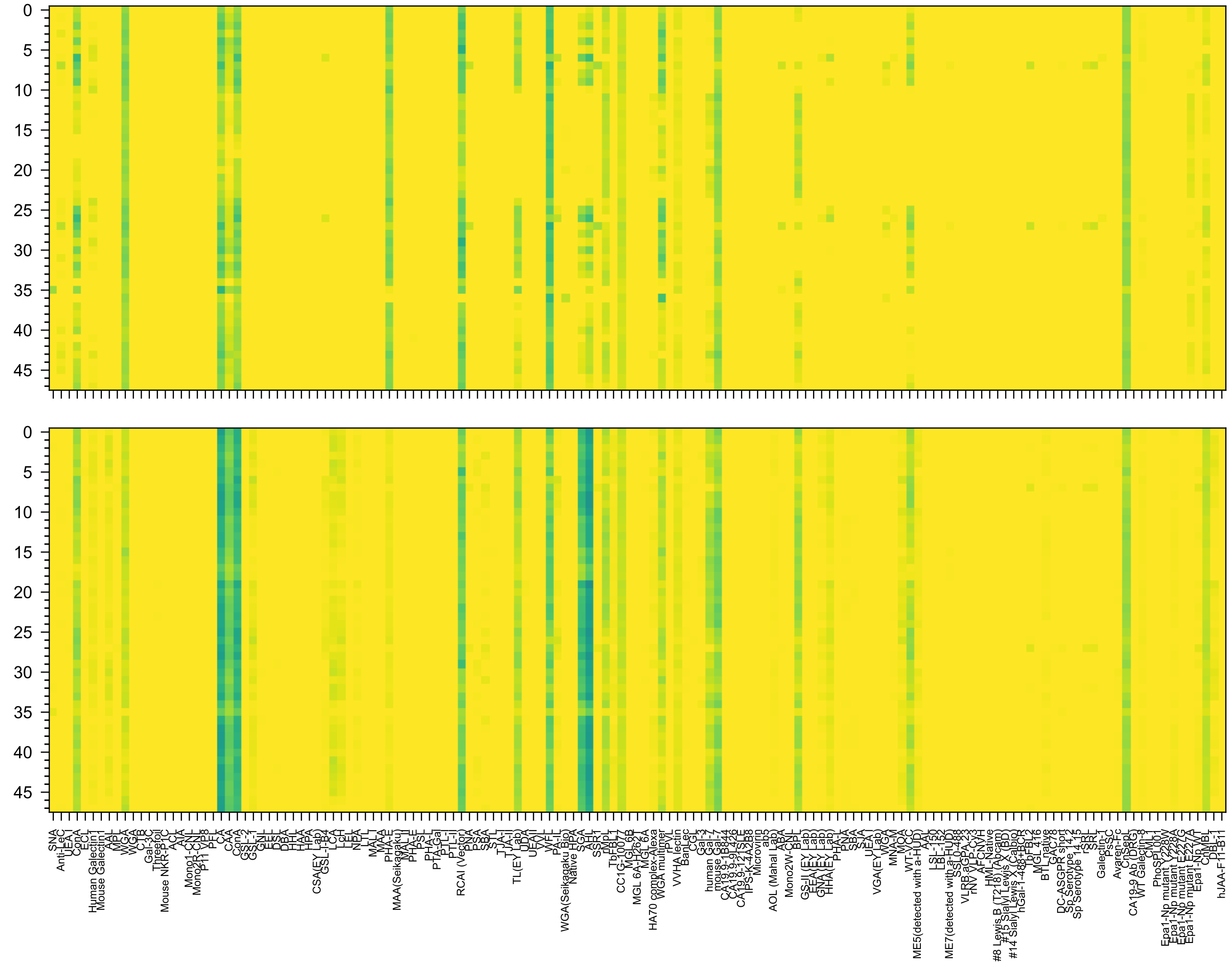

Gal(β1-3)GlcNAc(β1-2)Man(α1-6)[Gal(β1-3)GlcNAc(β1-2)Man(α1-3)]Man(β1-4)GlcNAc(β1-4)GlcNAc(β-Sp19

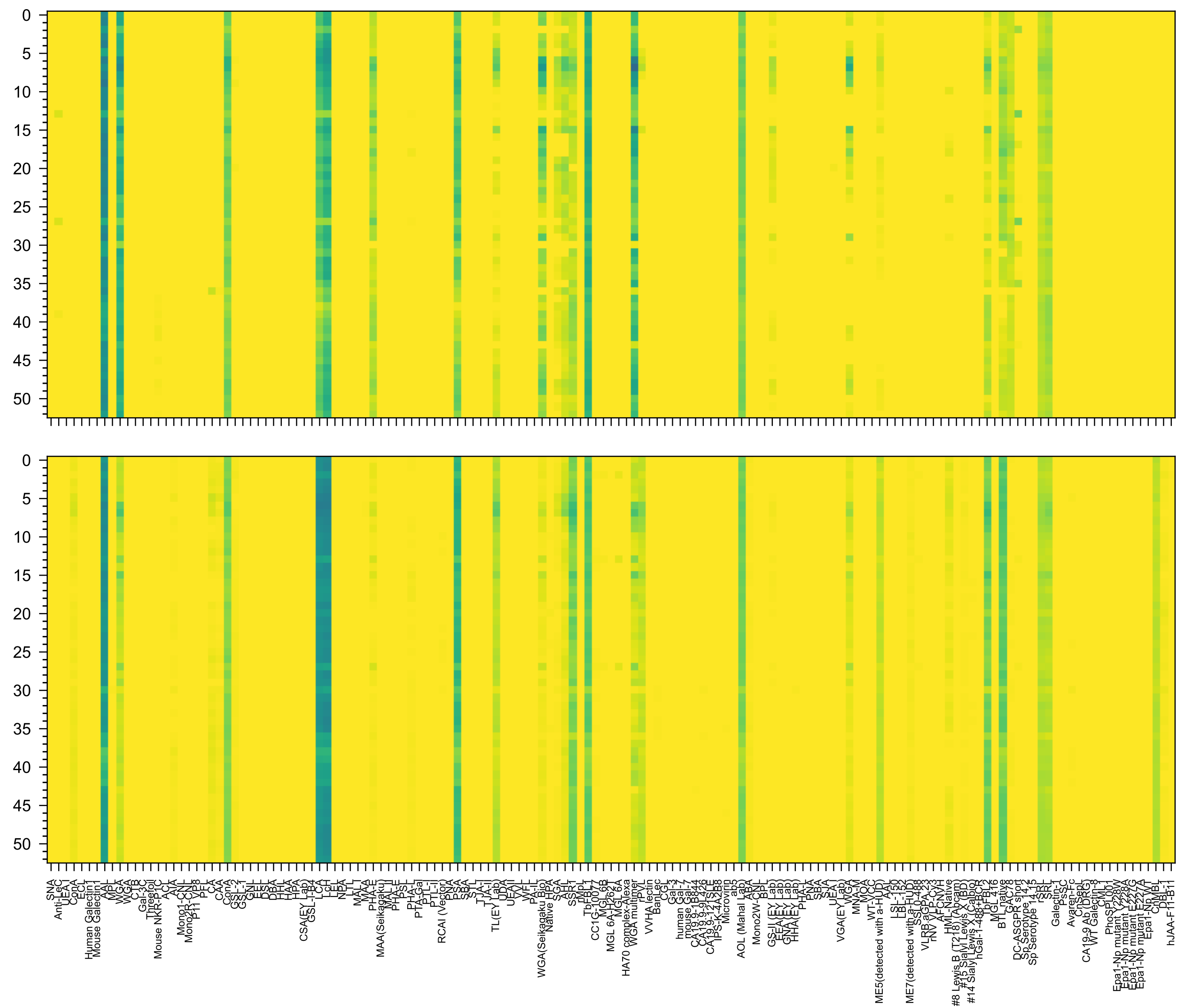

GlcNAc(β1-6)[GlcNAc(β1-2)]Man(α1-6)[GlcNAc(β1-2)Man(α1-3)]Man(β1-4)GlcNAc(β1-4)[Fuc(α1-6)]GlcNAc(β-Sp24

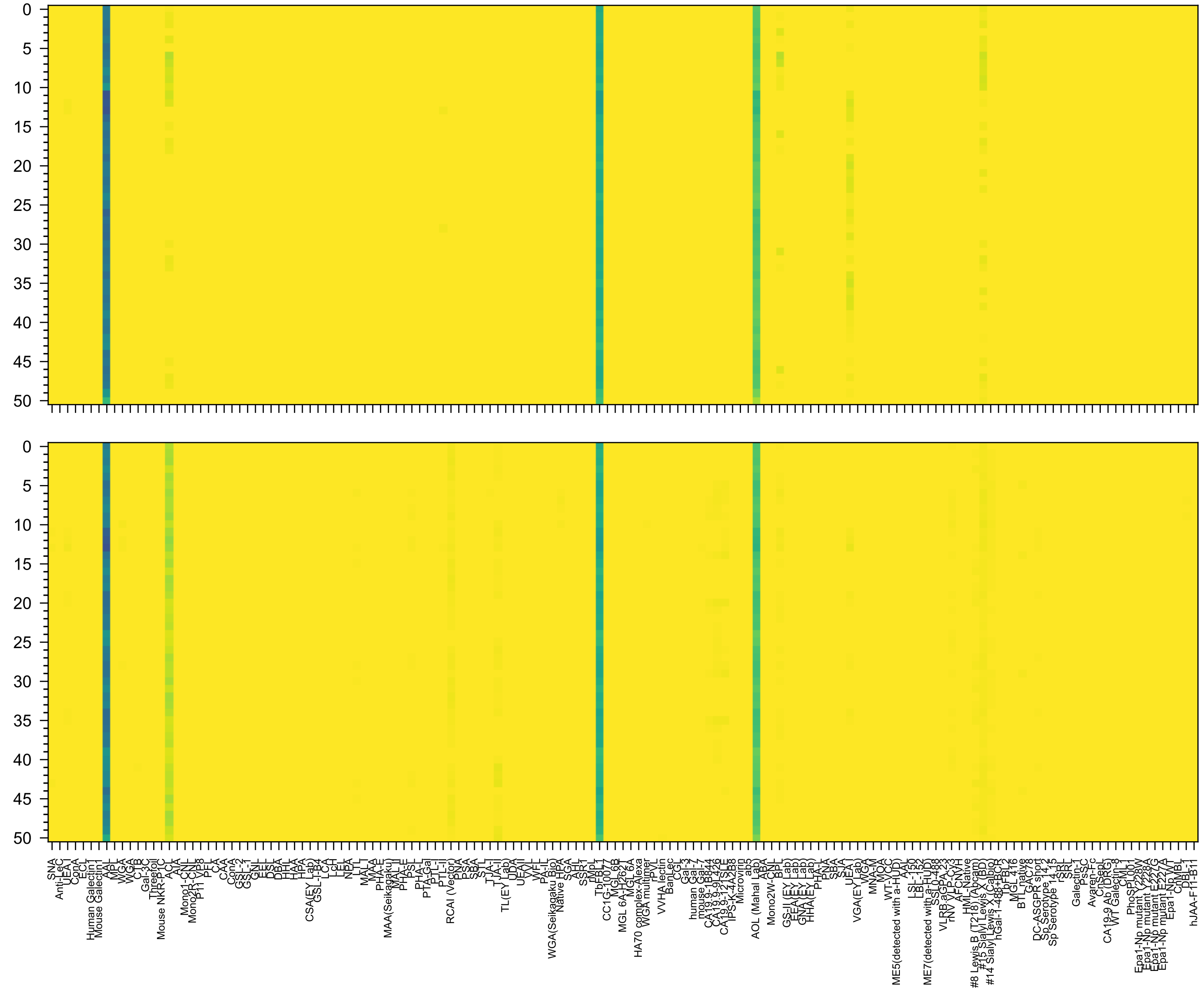

Fuc(α1-2)Gal(β1-4)[Fuc(α1-3)]GlcNAc(β1-3)Gal(β1-4)[Fuc(α1-3)]GlcNAc(β1-3)Gal(β1-4)[Fuc(α1-3)]GlcNAc(β-Sp0

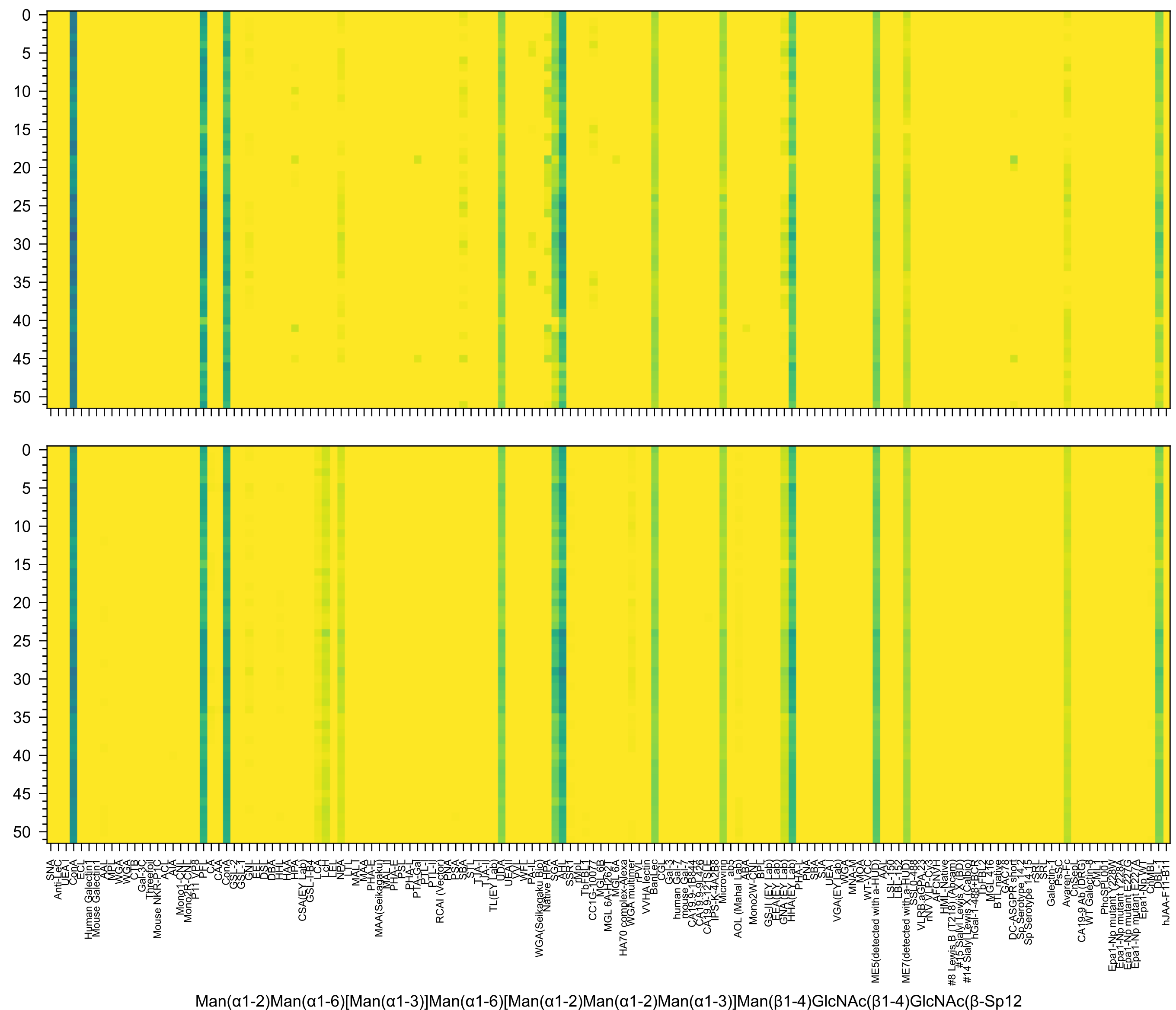
Man(α1-2)Man(α1-6)[Man(α1-3)]Man(α1-6)[Man(α1-2)Man(α1-2)Man(α1-3)]Man(β1-4)GlcNAc(β1-4)GlcNAc(β-Sp12

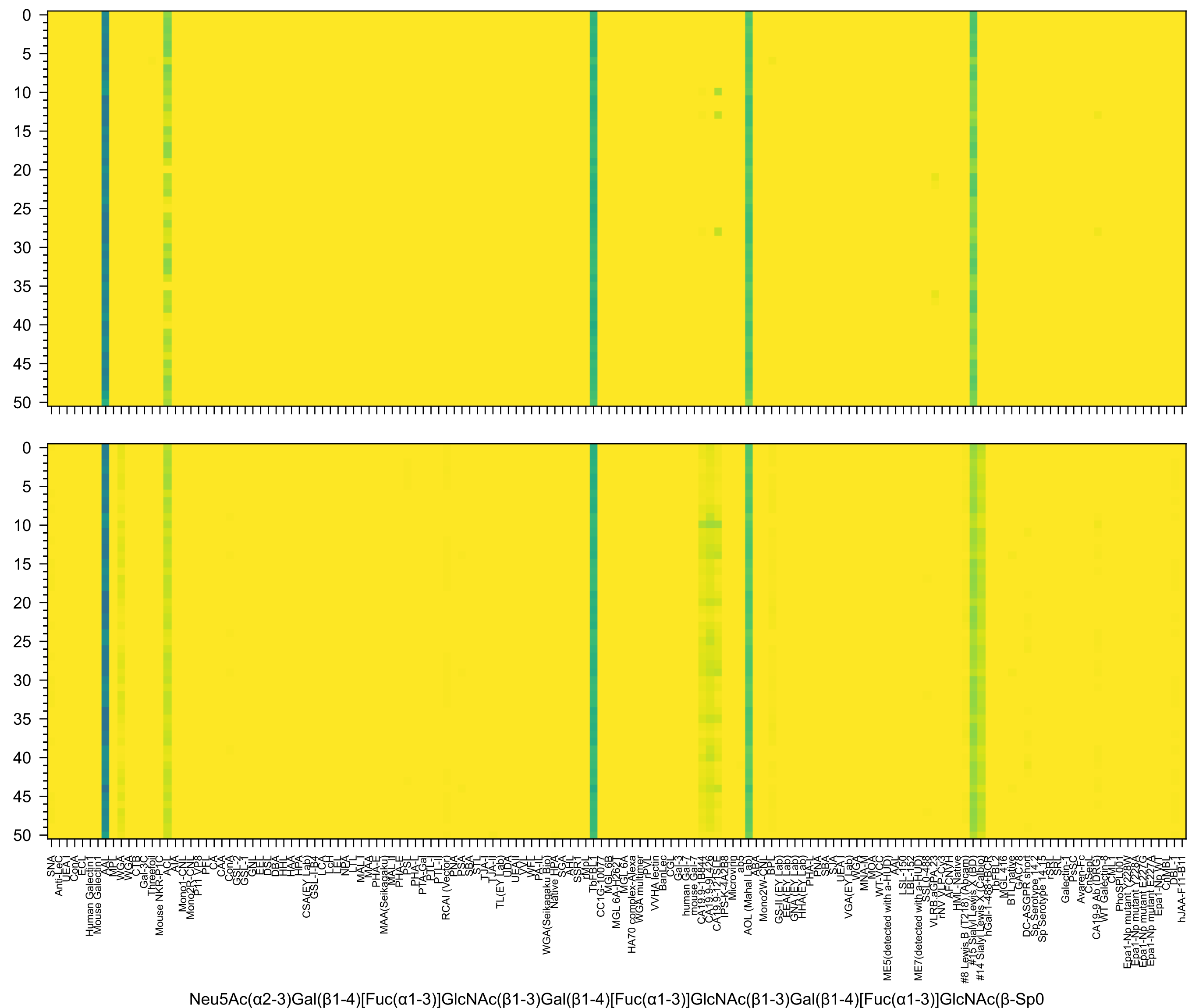
Neu5Ac(α2-3)Gal(β1-4)[Fuc(α1-3)]GlcNAc(β1-3)Gal(β1-4)[Fuc(α1-3)]GlcNAc(β1-3)Gal(β1-4)[Fuc(α1-3)]GlcNAc(β-Sp0

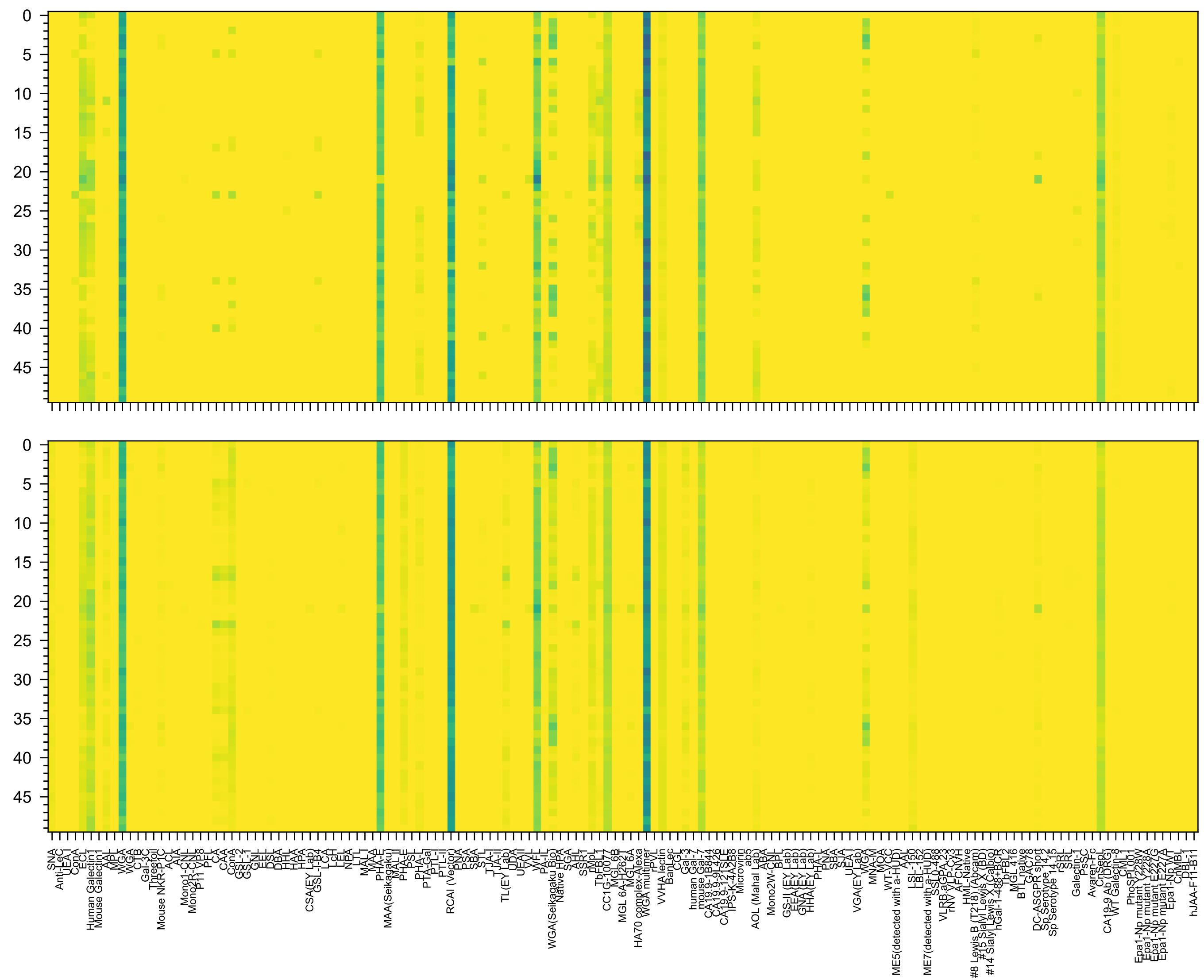
Gal(β1-4)GlcNAc(β1-2)Man(α1-6)[GlcNAc(β1-4)][Gal(β1-4)GlcNAc(β1-2)Man(α1-3)]Man(β1-4)GlcNAc(β1-4)GlcNAc(-Sp21

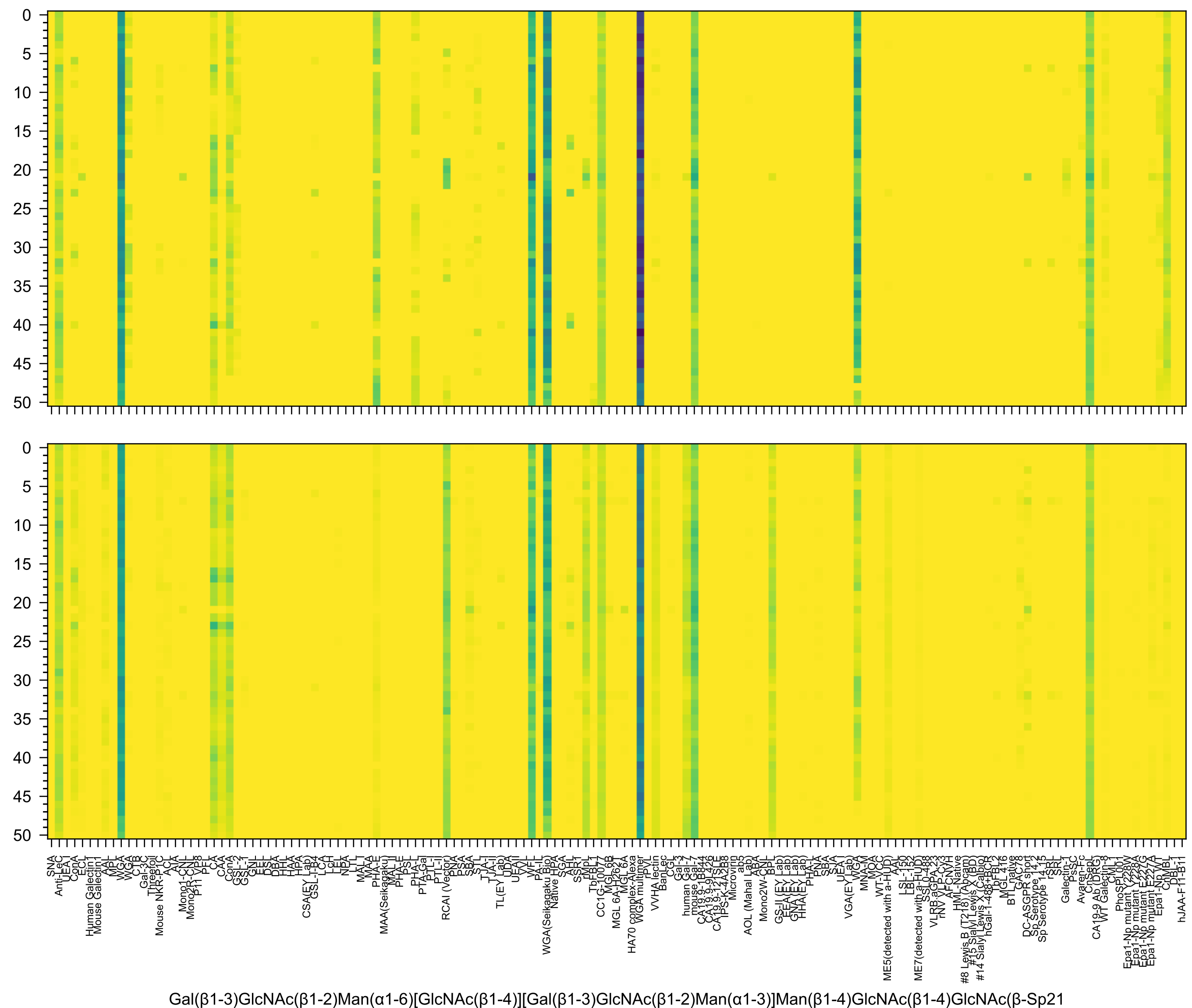
Gal(β1-3)GlcNAc(β1-2)Man(α1-6)[GlcNAc(β1-4)][Gal(β1-3)GlcNAc(β1-2)Man(α1-3)]Man(β1-4)GlcNAc(β1-4)GlcNAc(β-Sp21

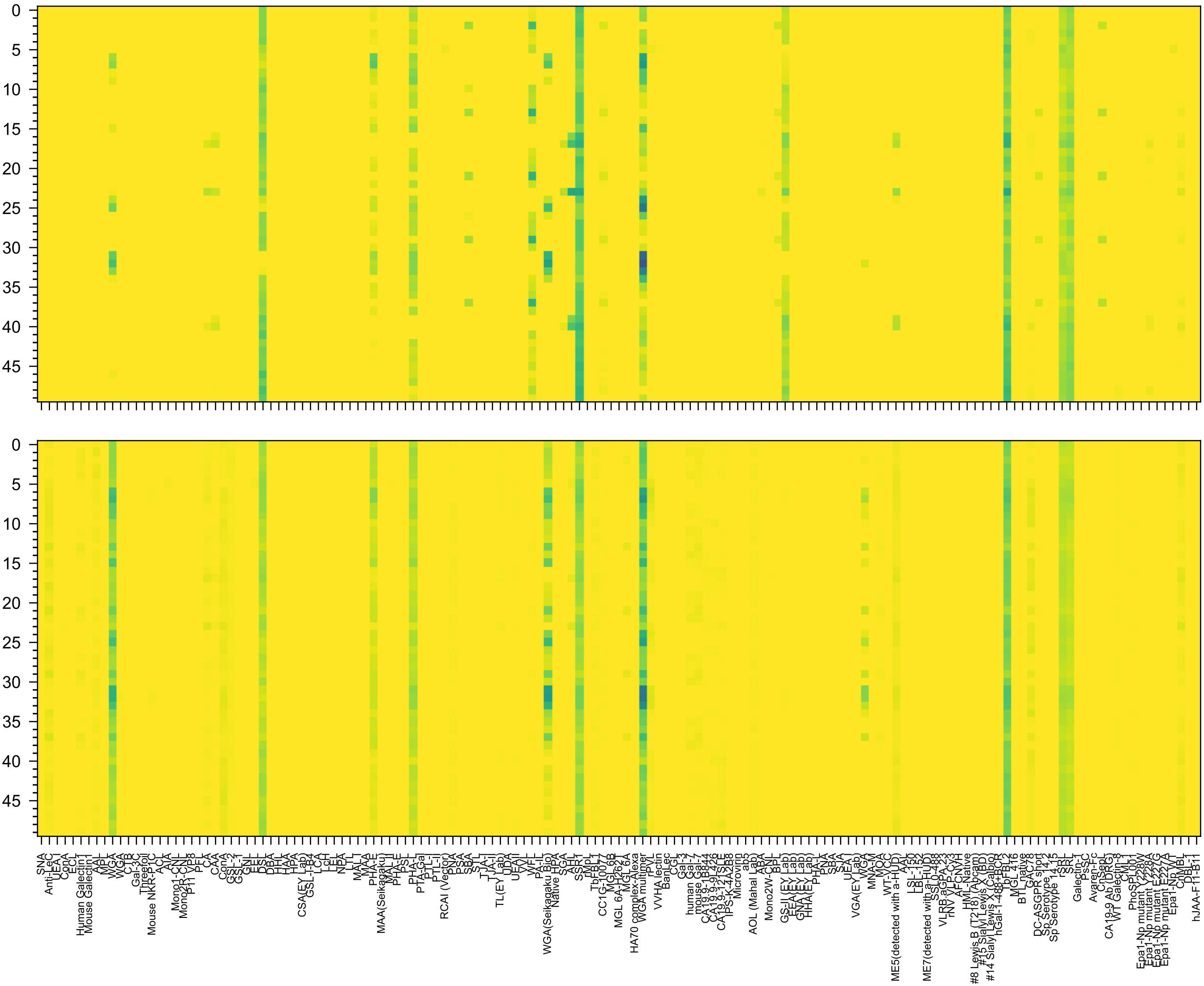

GlcNAc(β1-6)[GlcNAc(β1-2)]Man(α1-6)[GlcNAc(β1-4)][GlcNAc(β1-4)[GlcNAc(β1-2)]Man(α1-3)]Man(β1-4)GlcNAc(β1-4)GlcNAc(-Sp21

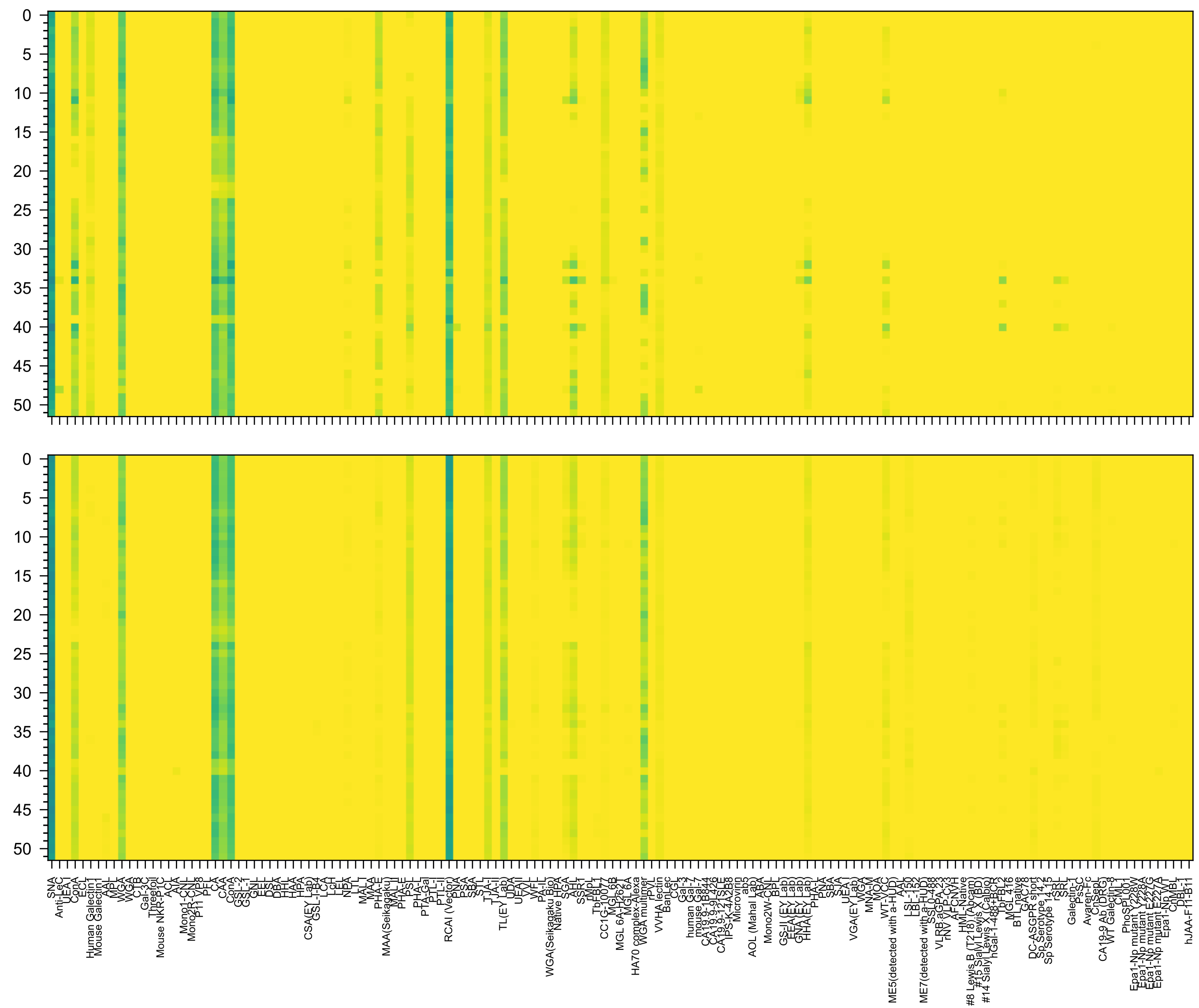

Neu5Ac(α2-6)Gal(β1-4)GlcNAc(β1-2)Man(α1-6)[Gal(β1-4)GlcNAc(β1-2)Man(α1-3)]Man(β1-4)GlcNAc(β1-4)GlcNAc(β-Sp12

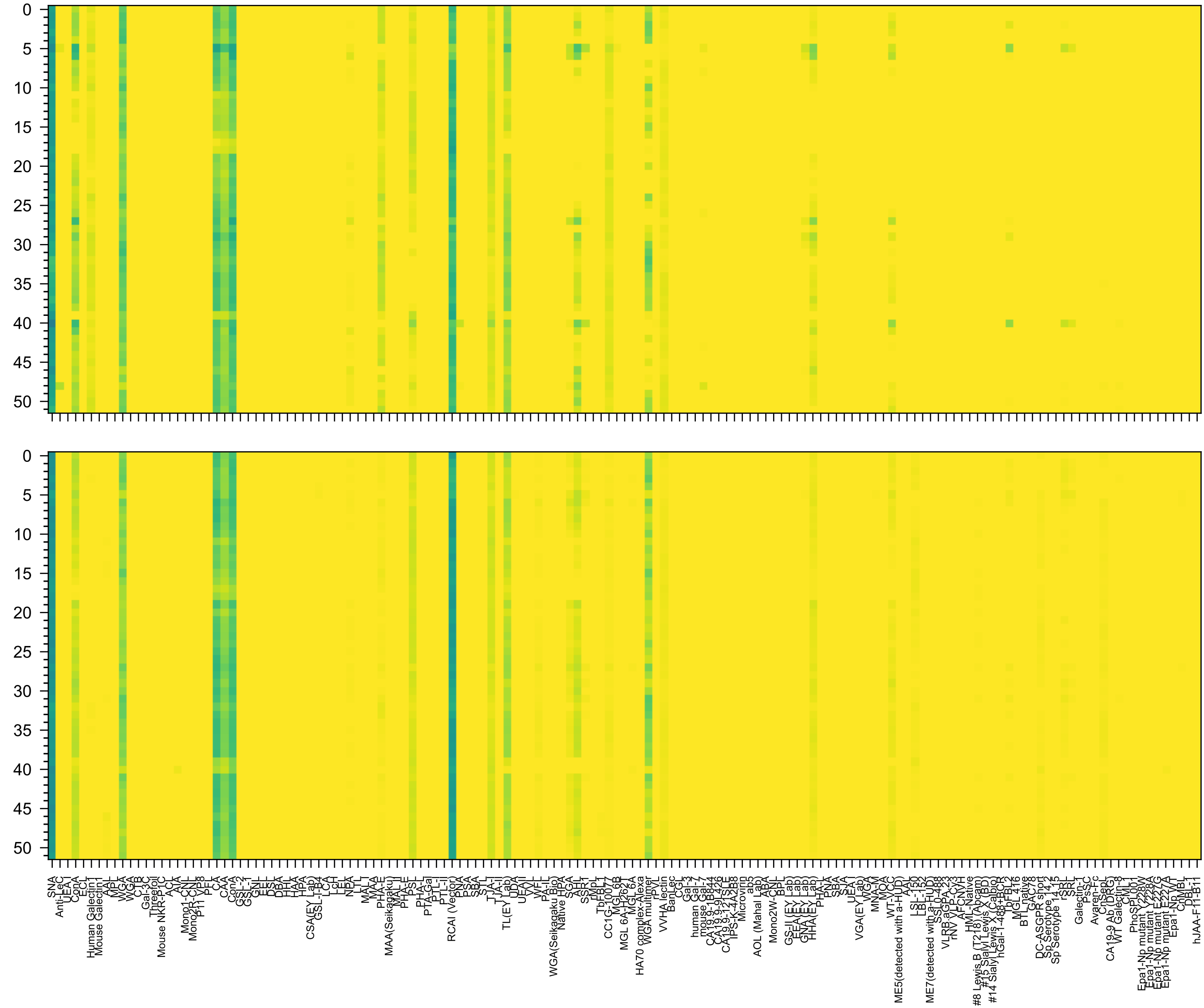

Gal(β1-4)GlcNAc(β1-2)Man(α1-6)[Neu5Ac(α2-6)Gal(β1-4)GlcNAc(β1-2)Man(α1-3)]Man(β1-4)GlcNAc(β1-4)GlcNAc(β-Sp12

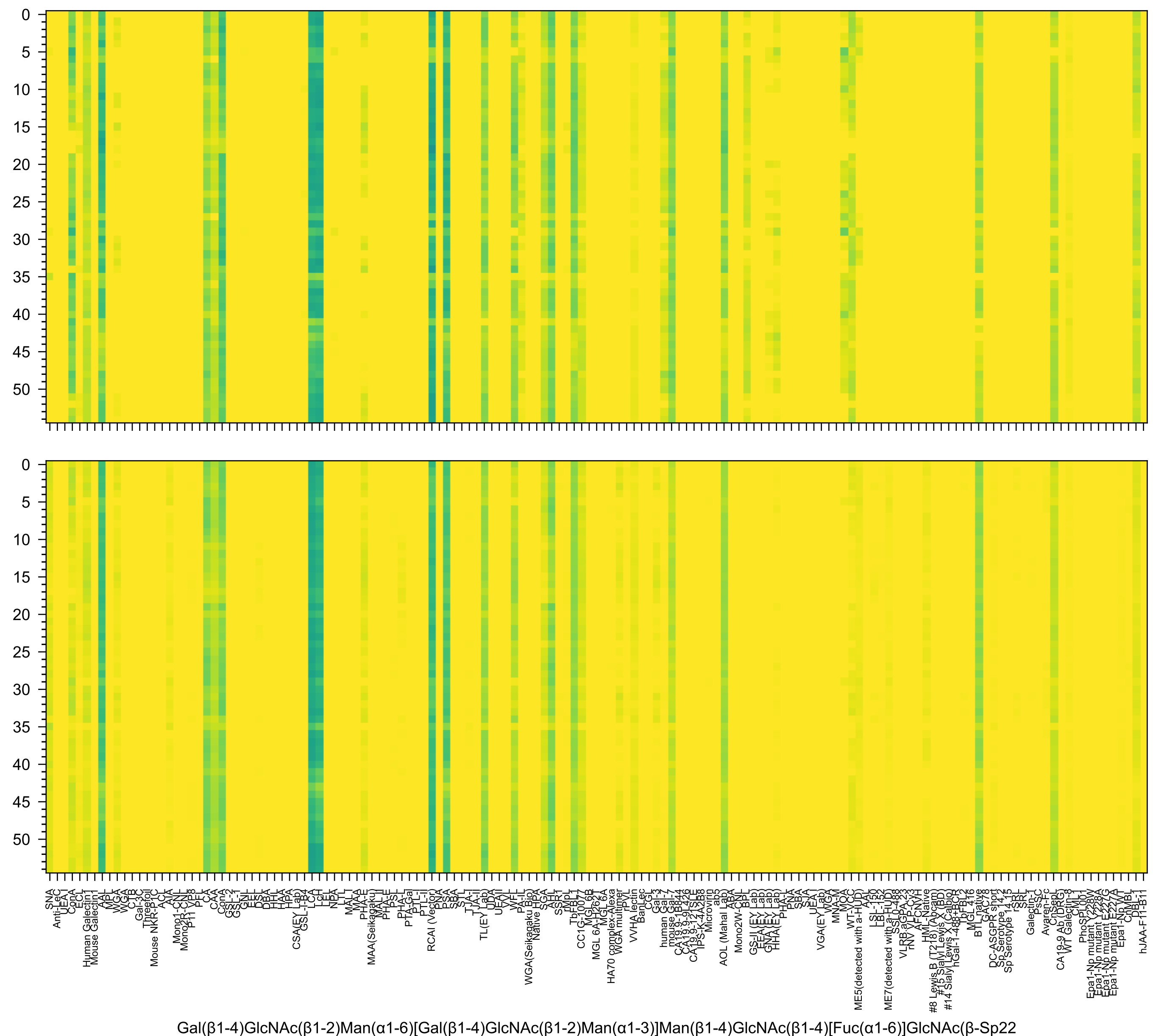
Gal(β1-4)GlcNAc(β1-2)Man(α1-6)[Gal(β1-4)GlcNAc(β1-2)Man(α1-3)]Man(β1-4)GlcNAc(β1-4)[Fuc(α1-6)]GlcNAc(β-Sp22

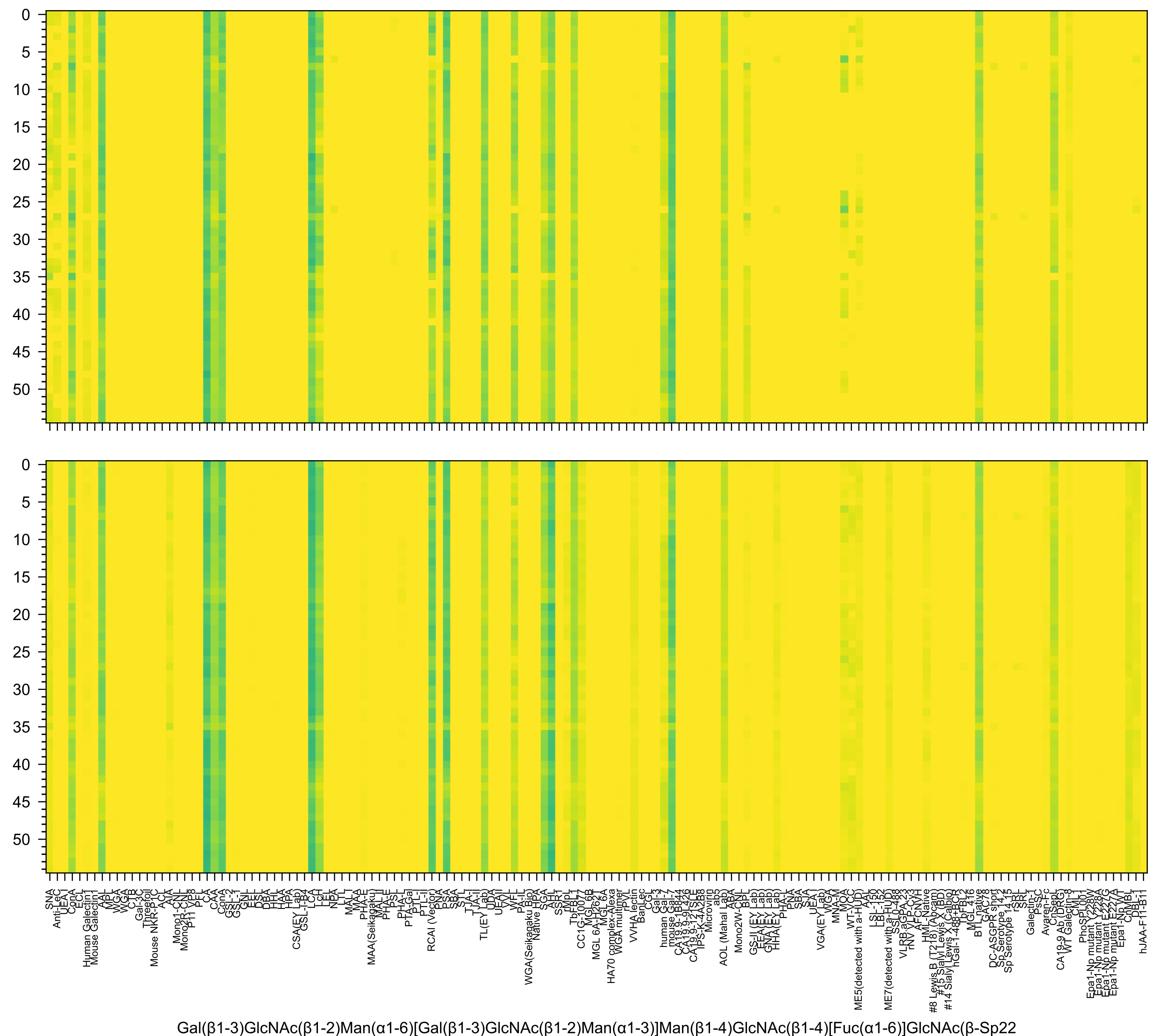
Gal(β1-3)GlcNAc(β1-2)Man(α1-6)[Gal(β1-3)GlcNAc(β1-2)Man(α1-3)]Man(β1-4)GlcNAc(β1-4)[Fuc(α1-6)]GlcNAc(β-Sp22

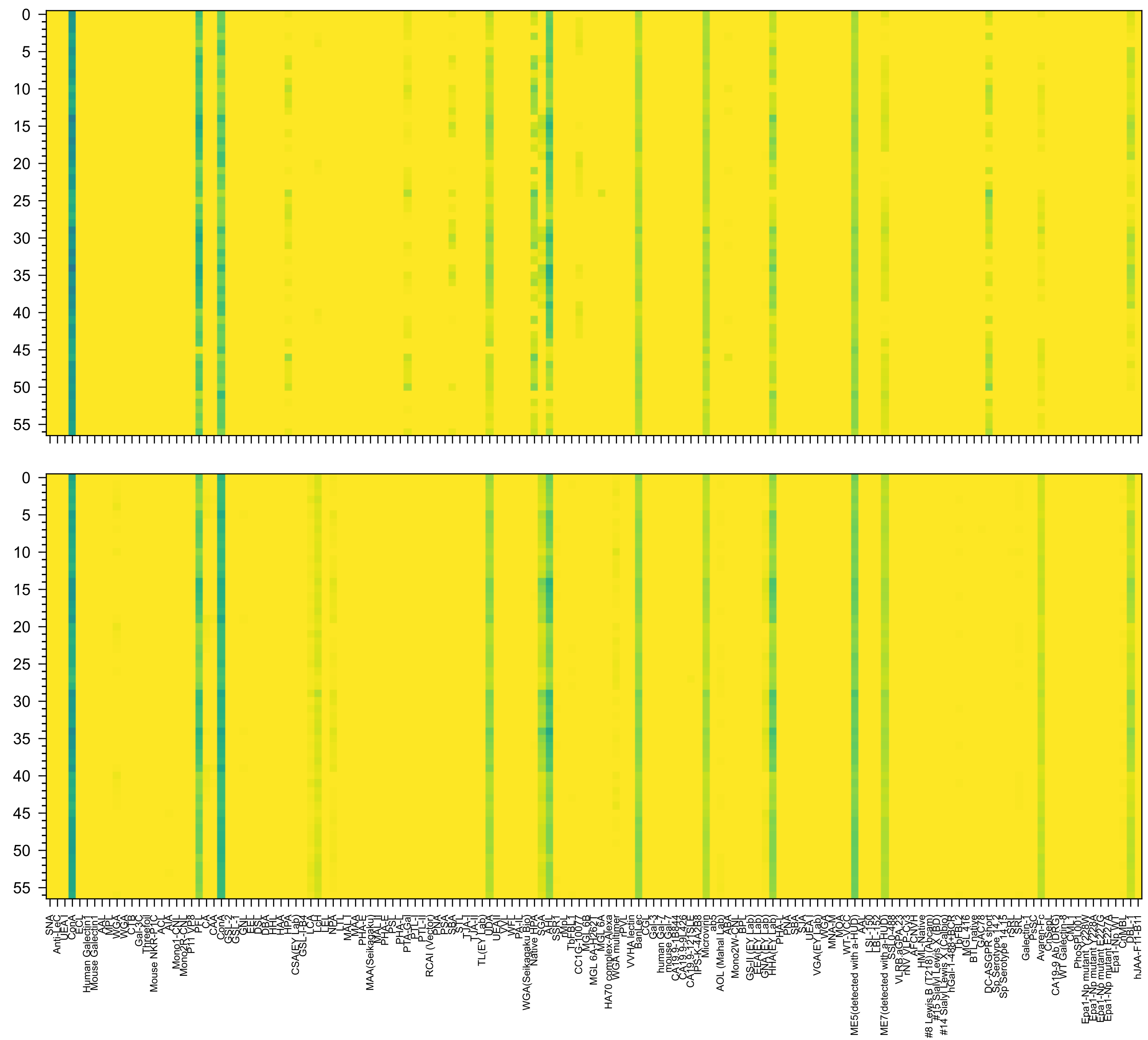

Man(α1-2)Man(α1-6)[Man(α1-2)Man(α1-3)]Man(α1-6)[Man(α1-2)Man(α1-2)Man(α1-3)]Man(β1-4)GlcNAc(β1-4)GlcNAc(β-Sp12

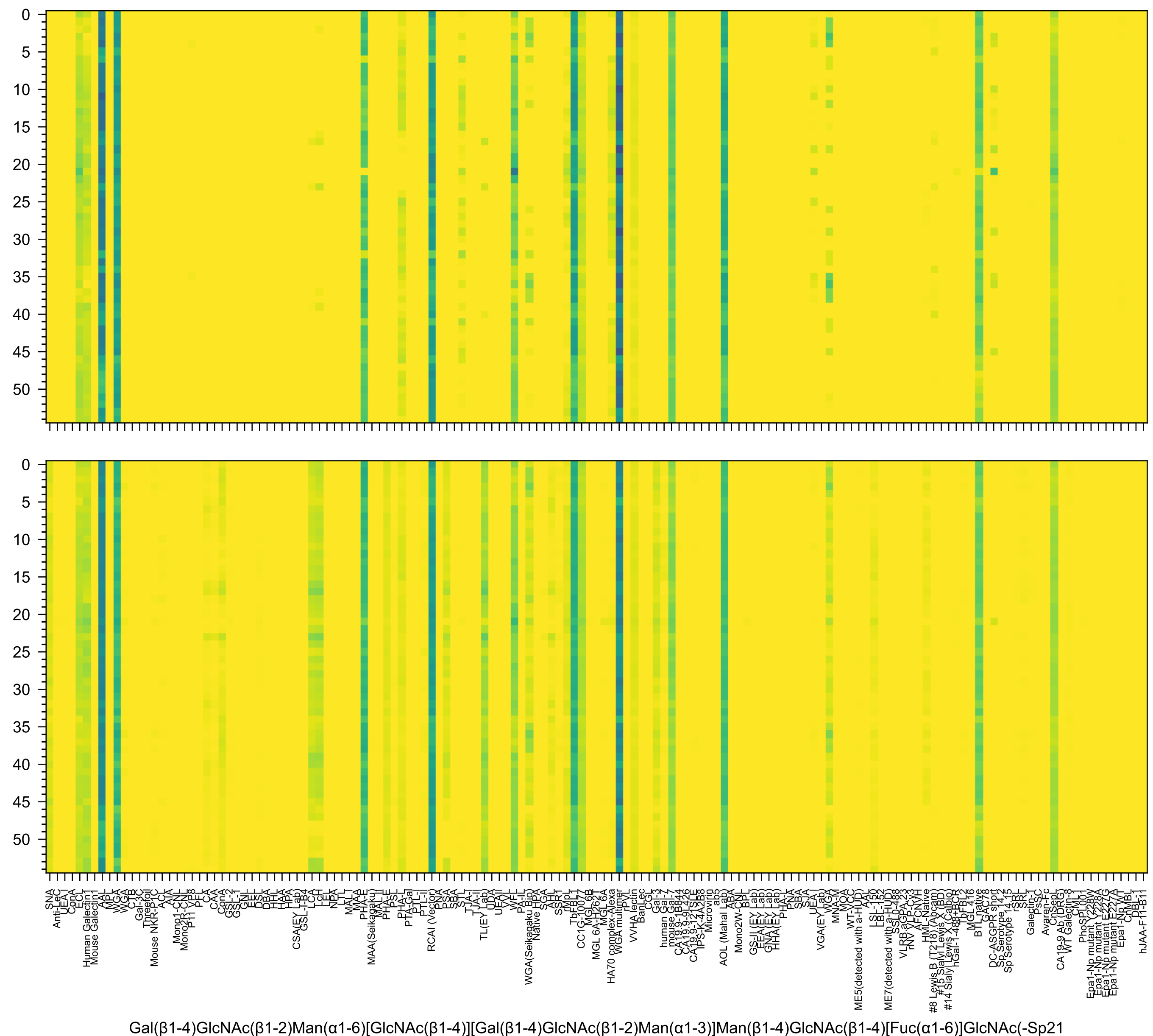
Gal(β1-4)GlcNAc(β1-2)Man(α1-6)[GlcNAc(β1-4)][Gal(β1-4)GlcNAc(β1-2)Man(α1-3)]Man(β1-4)GlcNAc(β1-4)[Fuc(α1-6)]GlcNAc(-Sp21

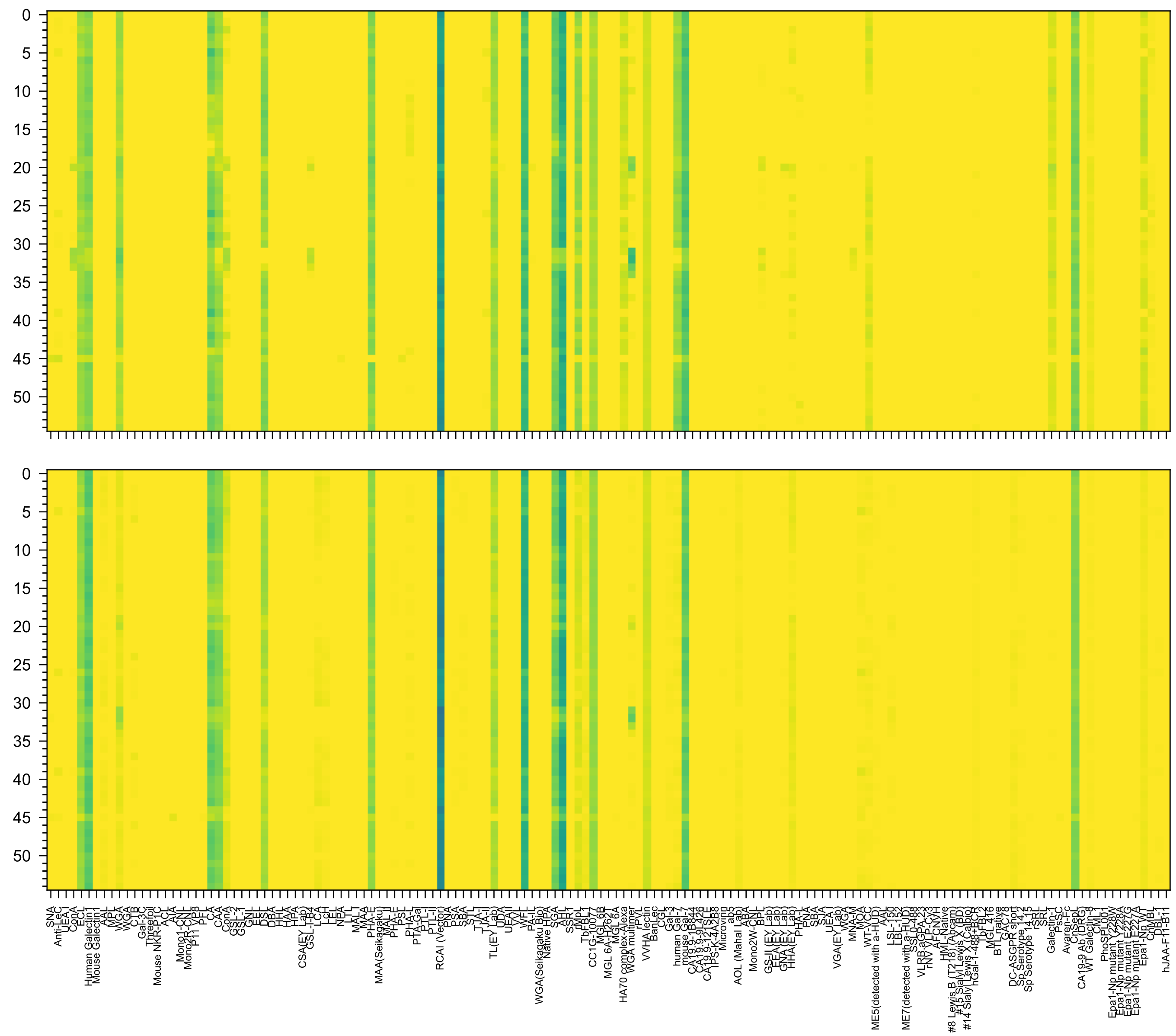

Gal(β1-4)GlcNAc(β1-2)Man(α1-6)[Gal(β1-4)GlcNAc(β1-4)[Gal(β1-4)GlcNAc(β1-2)]Man(α1-3)]Man(β1-4)GlcNAc(β1-4)GlcNAc(-Sp21

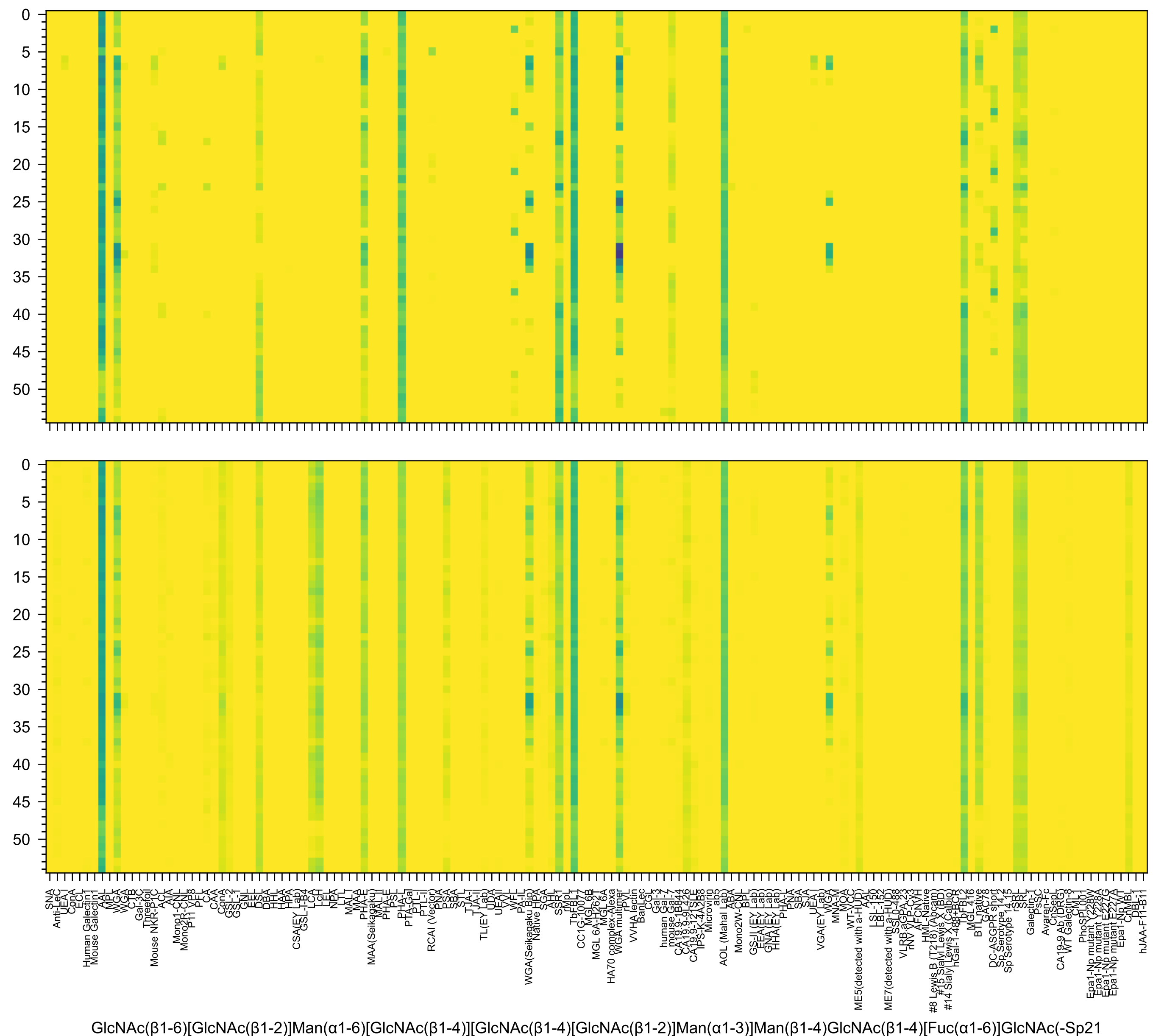
GlcNAc(β1-6)[GlcNAc(β1-2)]Man(α1-6)[GlcNAc(β1-4)][GlcNAc(β1-4)[GlcNAc(β1-2)]Man(α1-3)]Man(β1-4)GlcNAc(β1-4)[Fuc(α1-6)]GlcNAc(-Sp21

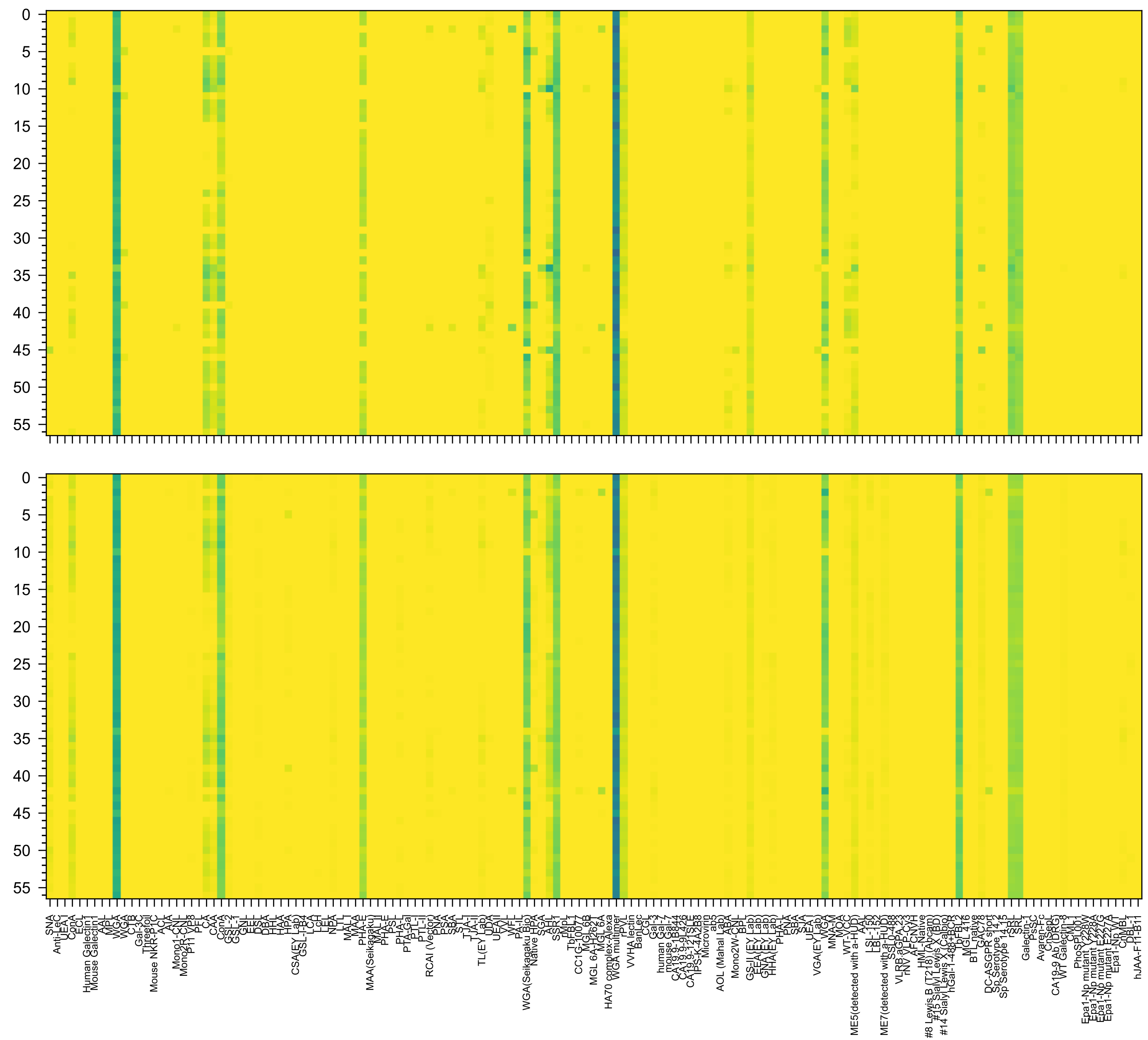

GlcNAc(β1-3)Gal(β1-4)GlcNAc(β1-2)Man(α1-6)[GlcNAc(β1-3)Gal(β1-4)GlcNAc(β1-2)Man(α1-3)]Man(β1-4)GlcNAc(β1-4)GlcNAc(β-Sp12

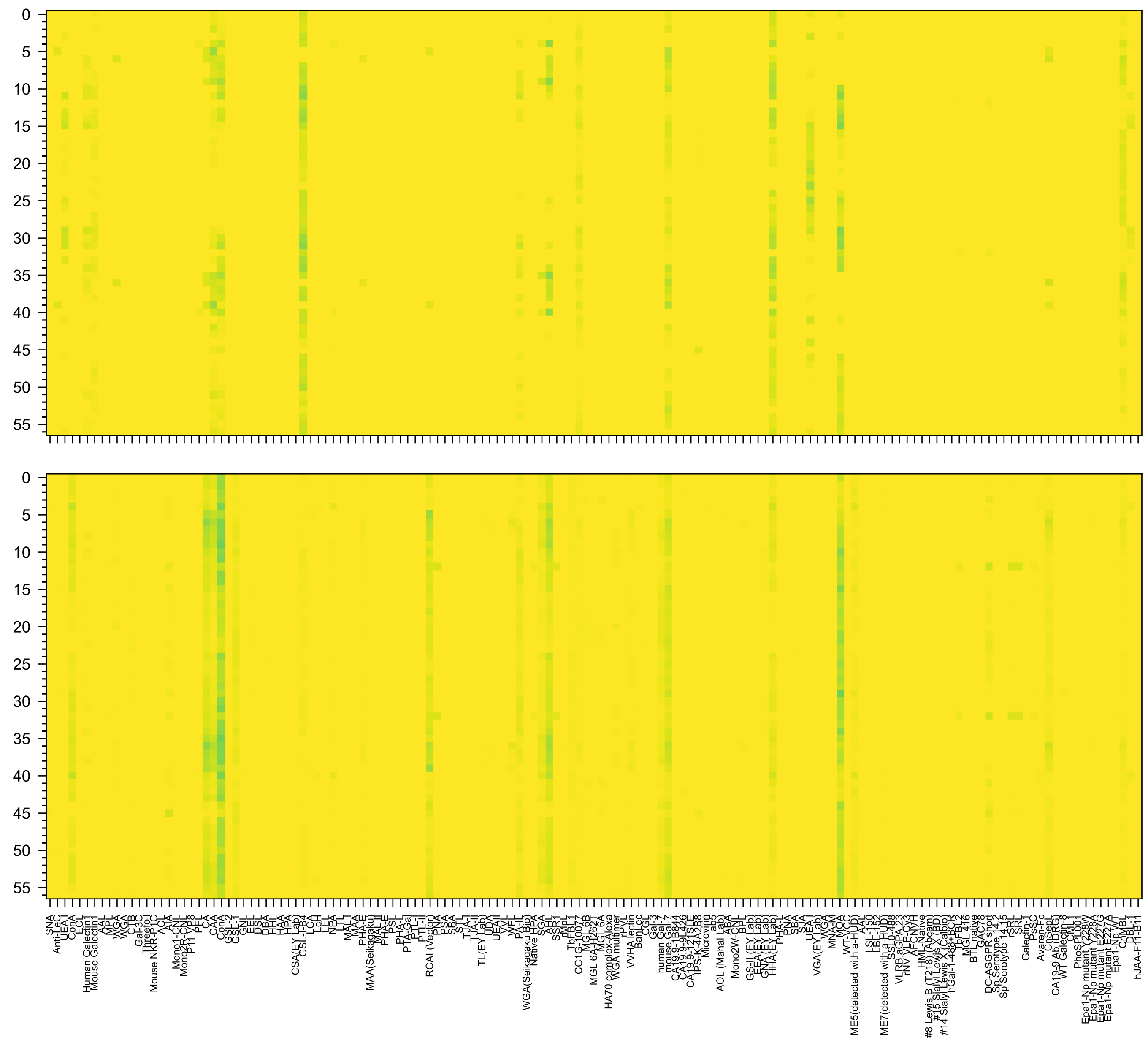

Gal(α1-3)Gal(β1-3)GlcNAc(β1-2)Man(α1-6)[Gal(α1-3)Gal(β1-3)GlcNAc(β1-2)Man(α1-3)]Man(β1-4)GlcNAc(β1-4)GlcNAc(-Sp19

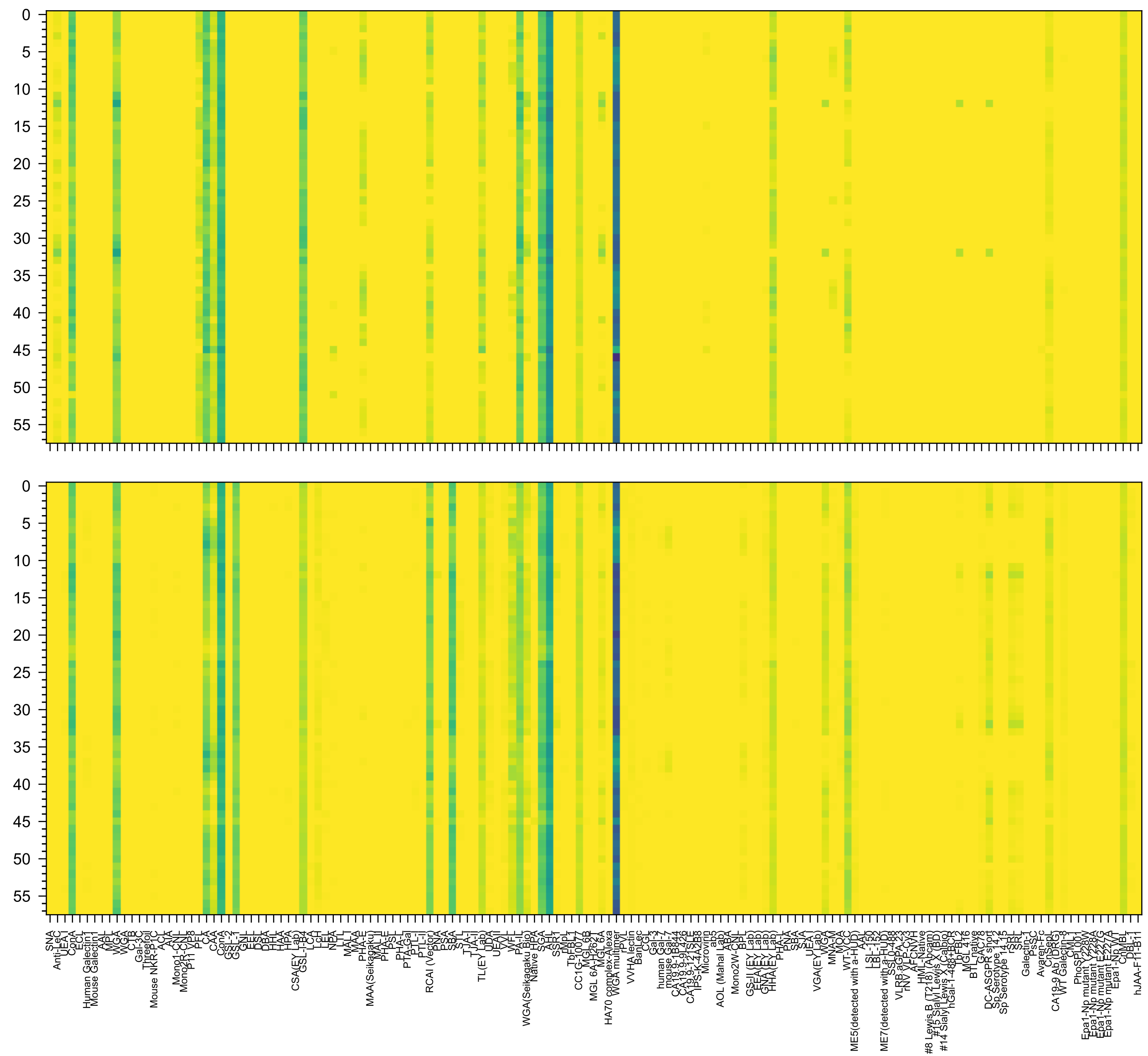

Gal(α1-4)Gal(β1-3)GlcNAc(β1-2)Man(α1-6)[Gal(α1-4)Gal(β1-3)GlcNAc(β1-2)Man(α1-3)]Man(β1-4)GlcNAc(β1-4)GlcNAc(β-Sp19

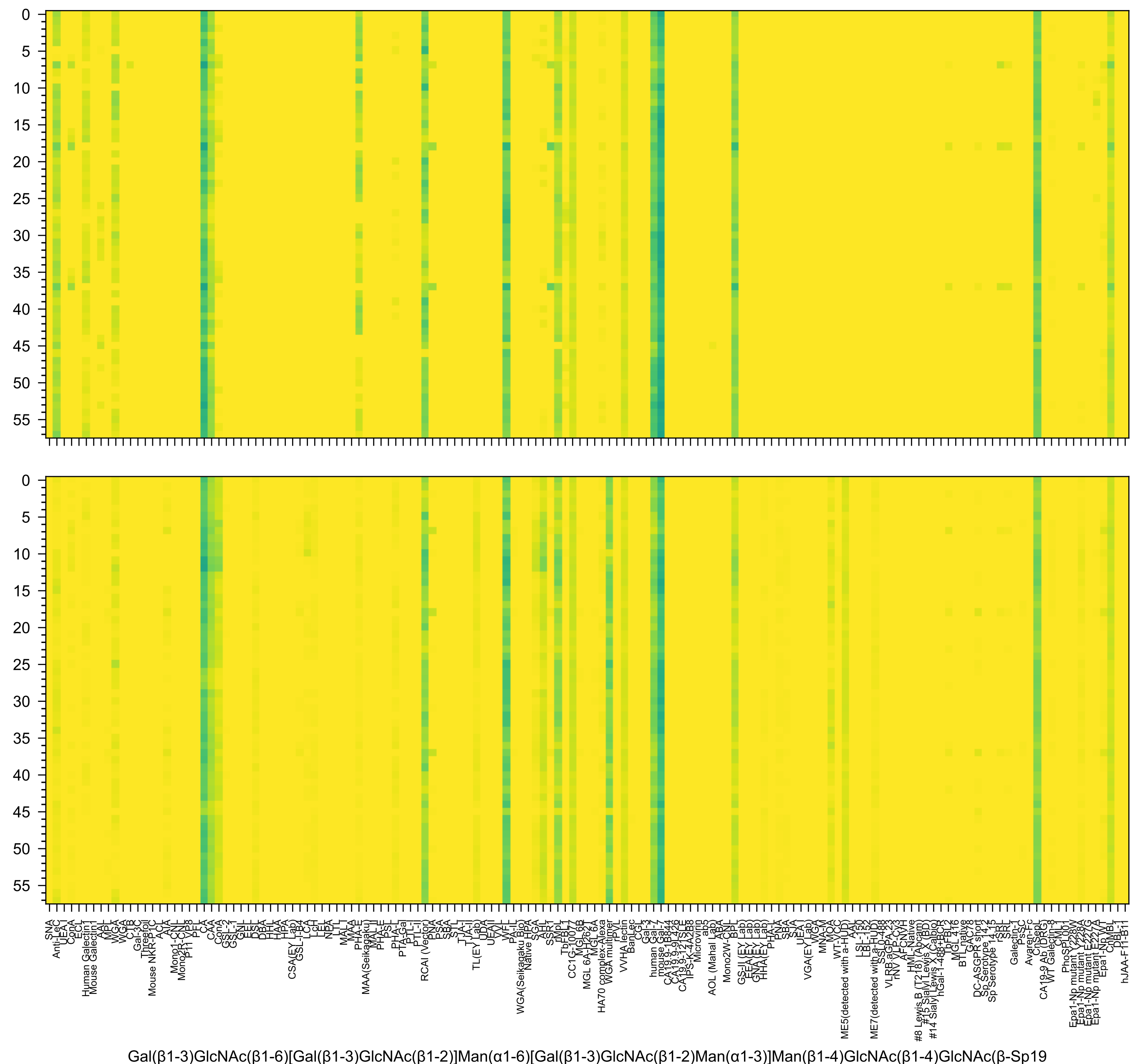
Gal(β1-3)GlcNAc(β1-6)[Gal(β1-3)GlcNAc(β1-2)]Man(α1-6)[Gal(β1-3)GlcNAc(β1-2)Man(α1-3)]Man(β1-4)GlcNAc(β1-4)GlcNAc(β-Sp19

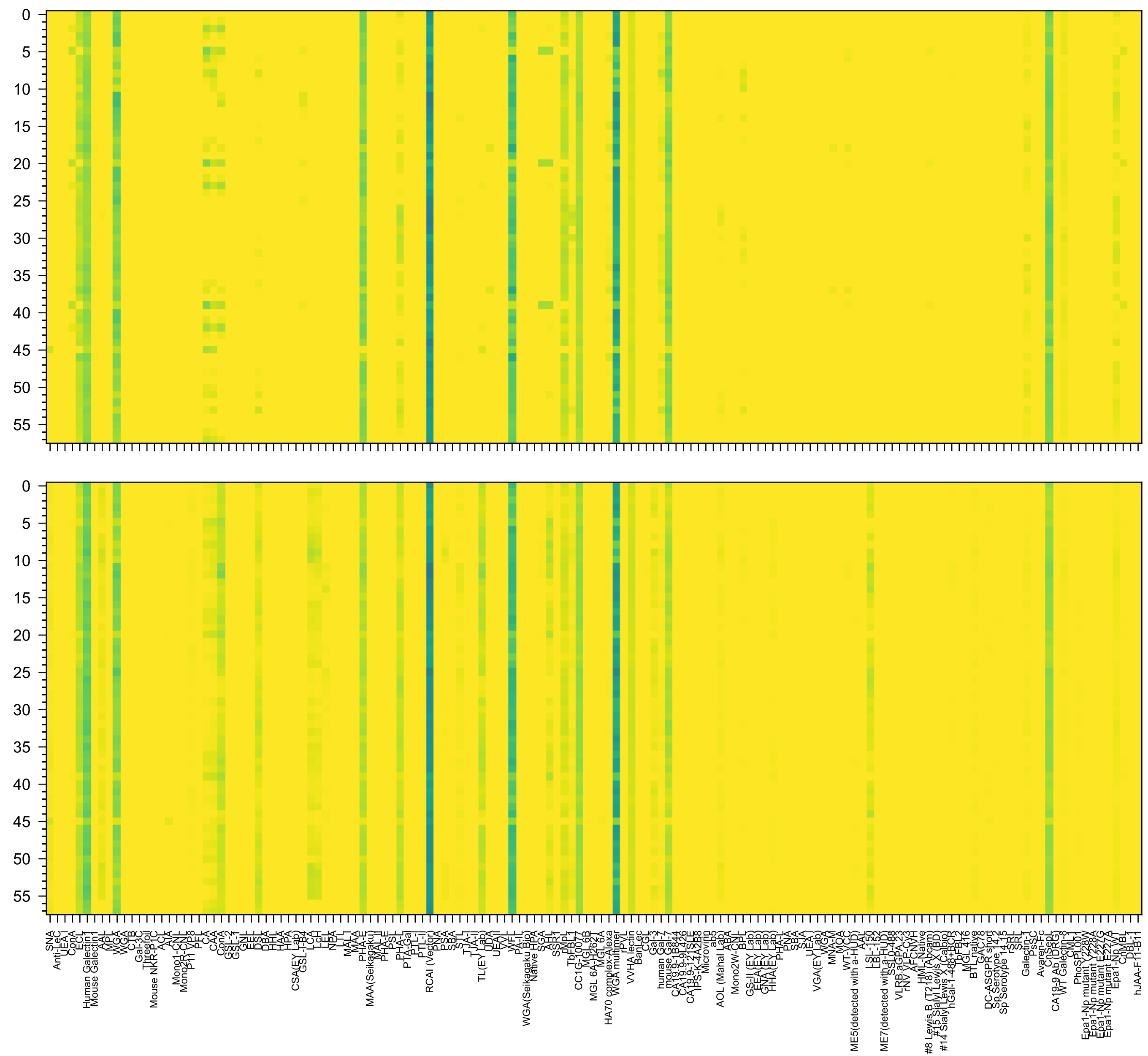

Gal(β1-4)GlcNAc(β1-6)[Gal(β1-4)GlcNAc(β1-2)]Man(α1-6)[Gal(β1-4)GlcNAc(β1-2)Man(α1-3)]Man(β1-4)GlcNAc(β1-4)GlcNAc(β-Sp19

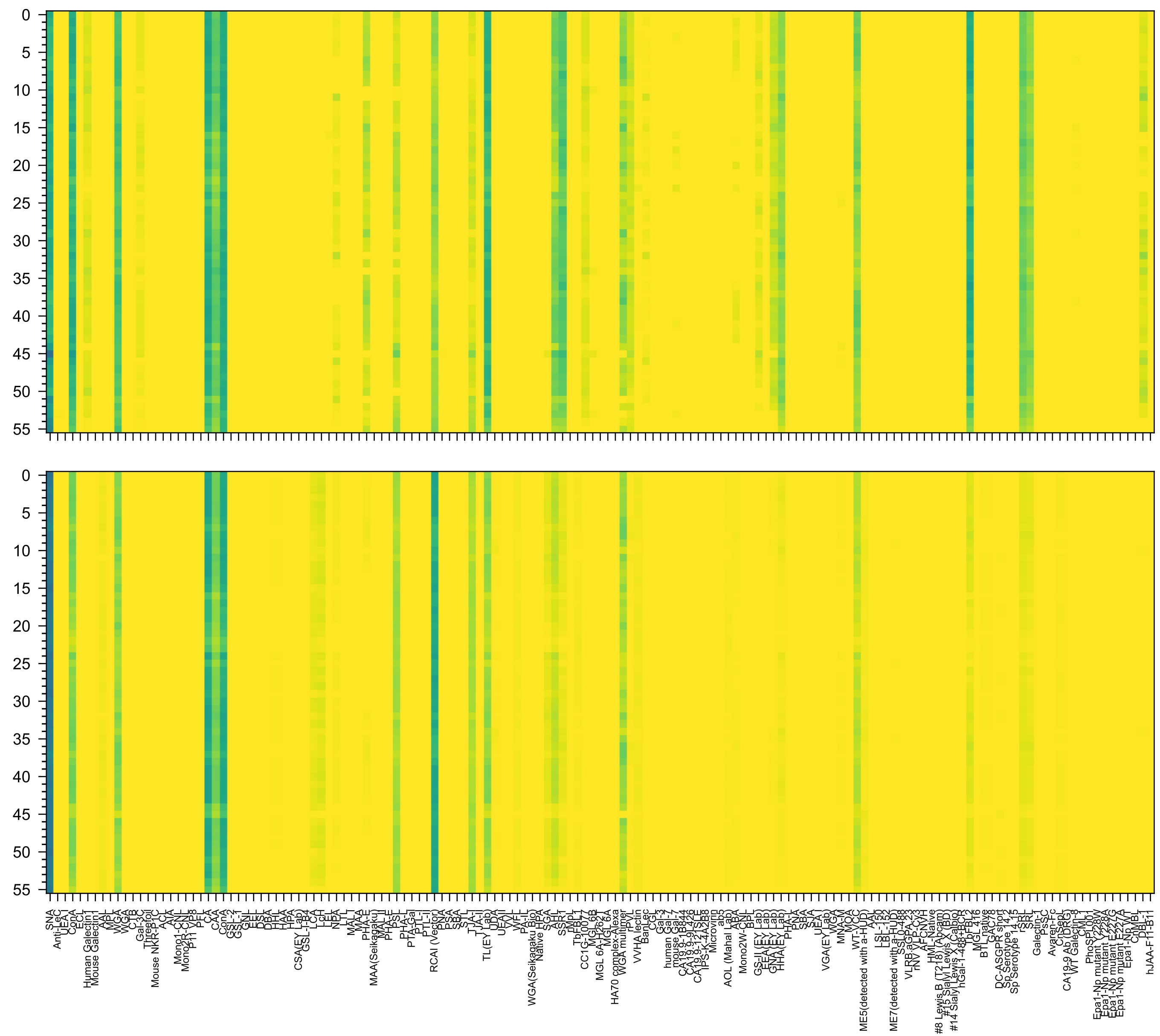

Neu5Ac(α2-6)Gal(β1-4)GlcNAc(β1-2)Man(α1-6)[Neu5Ac(α2-6)Gal(β1-4)GlcNAc(β1-2)Man(α1-3)]Man(β1-4)GlcNAc(β1-4)GlcNAc(β-Sp13

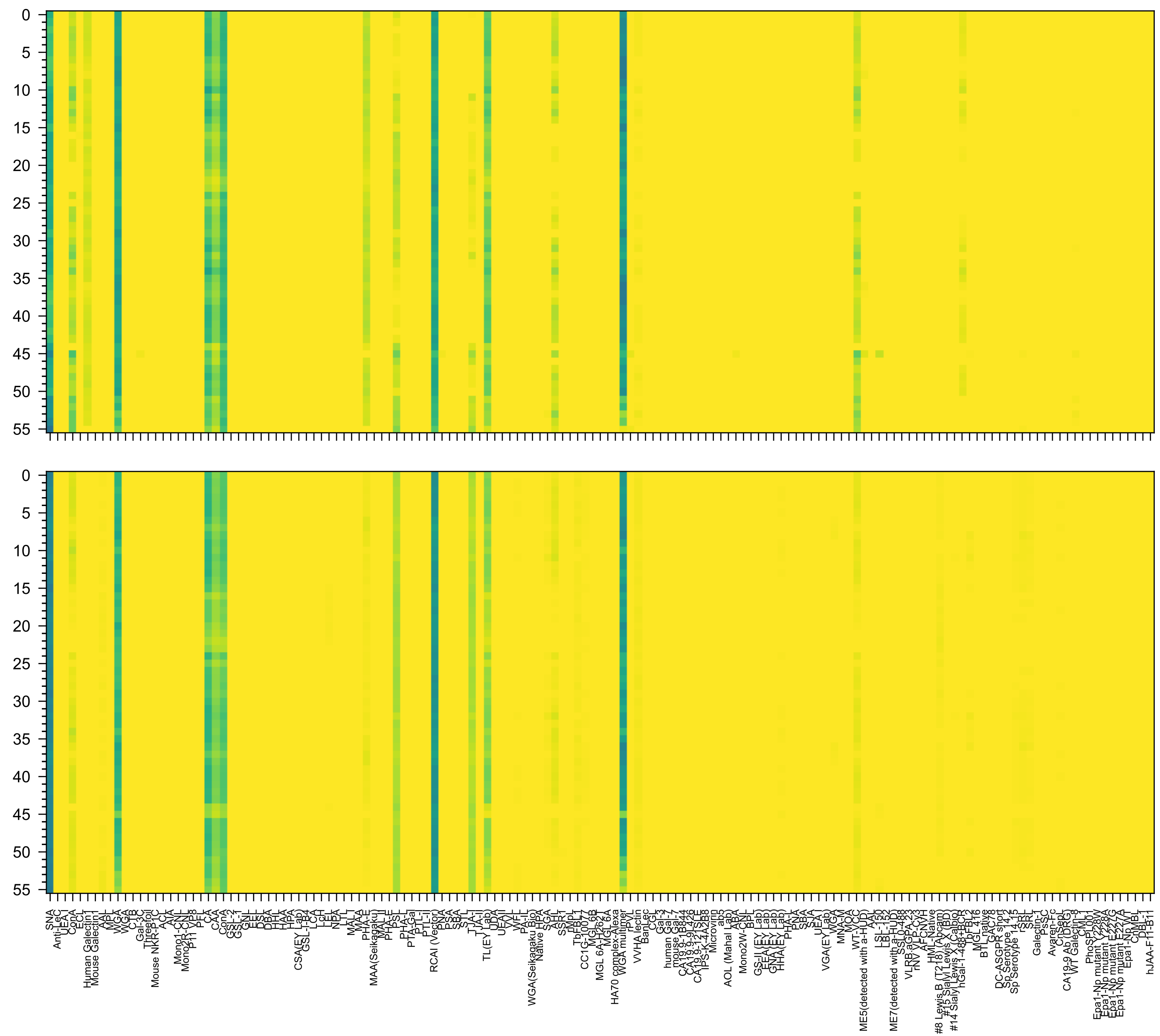
Neu5Ac(α2-6)Gal(β1-4)GlcNAc(β1-2)Man(α1-6)[Neu5Ac(α2-6)Gal(β1-4)GlcNAc(β1-2)Man(α1-3)]Man(β1-4)GlcNAc(β1-4)GlcNAc(β-Sp21

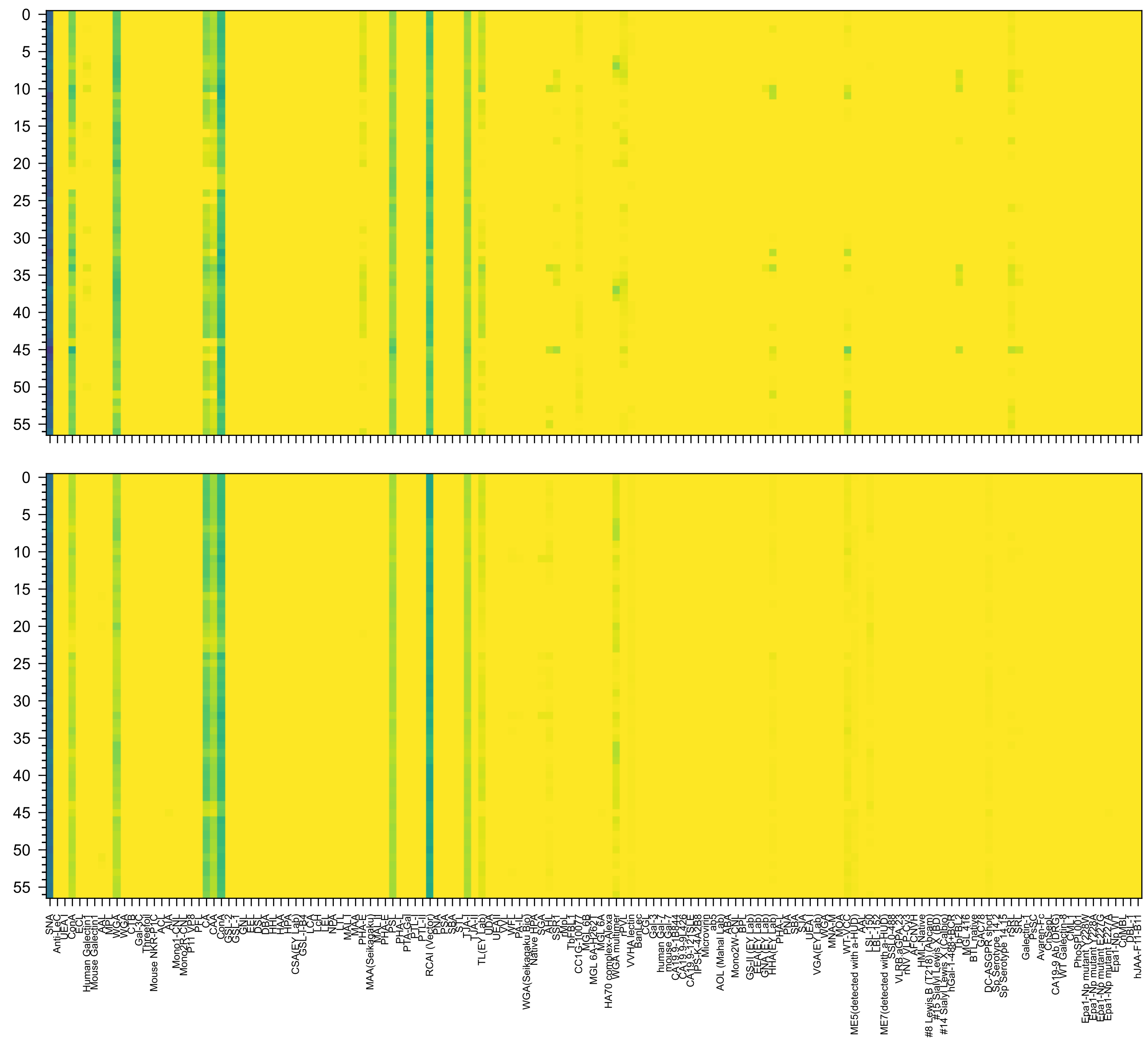

Neu5Ac(α2-6)Gal(β1-4)GlcNAc(β1-2)Man(α1-6)[Neu5Ac(α2-6)Gal(β1-4)GlcNAc(β1-2)Man(α1-3)]Man(β1-4)GlcNAc(β1-4)GlcNAc(β-Sp12

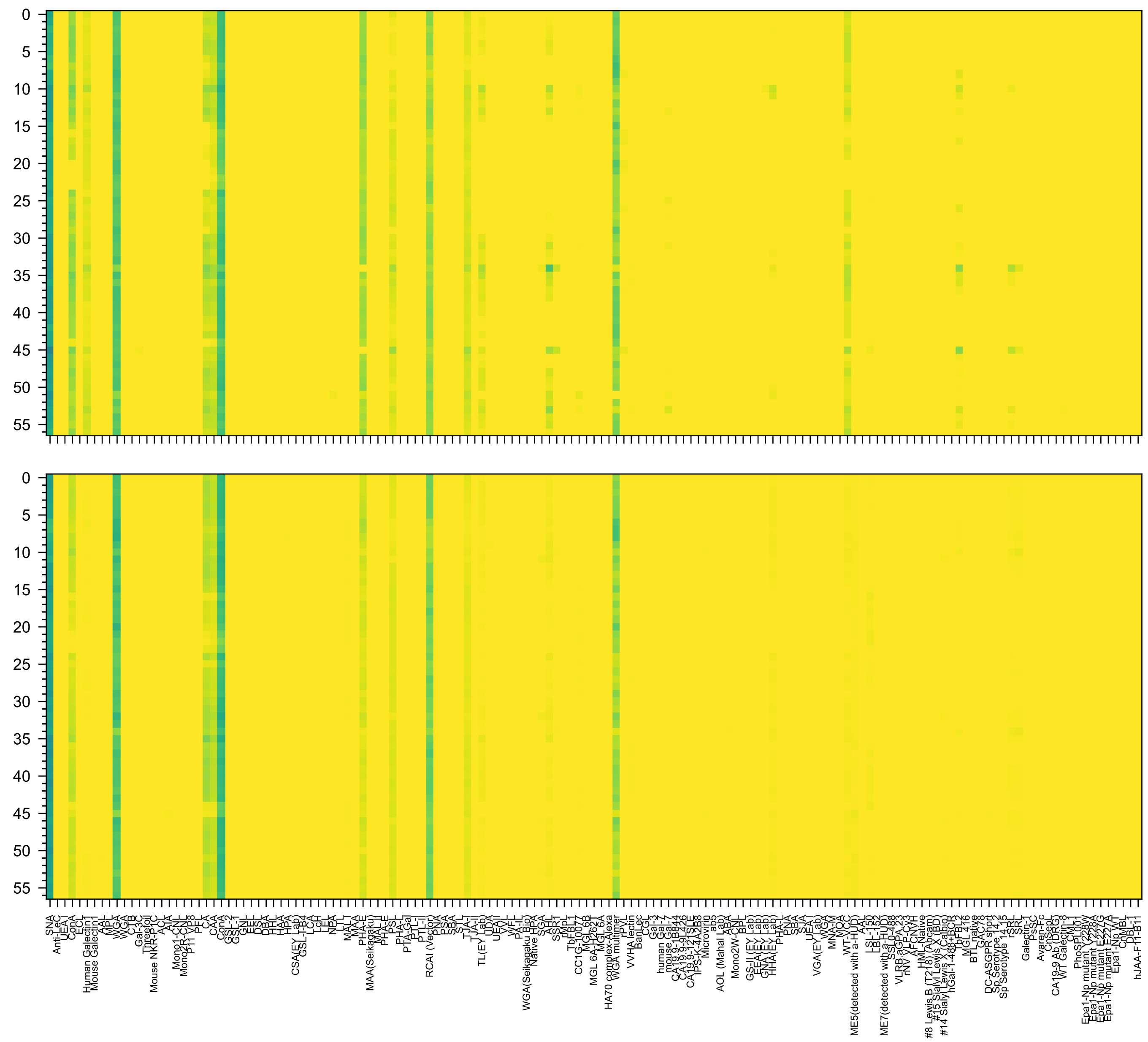

Neu5Ac(α2-6)Gal(β1-4)GlcNAc(β1-2)Man(α1-6)[Neu5Ac(α2-3)Gal(β1-4)GlcNAc(β1-2)Man(α1-3)]Man(β1-4)GlcNAc(β1-4)GlcNAc(β-Sp12

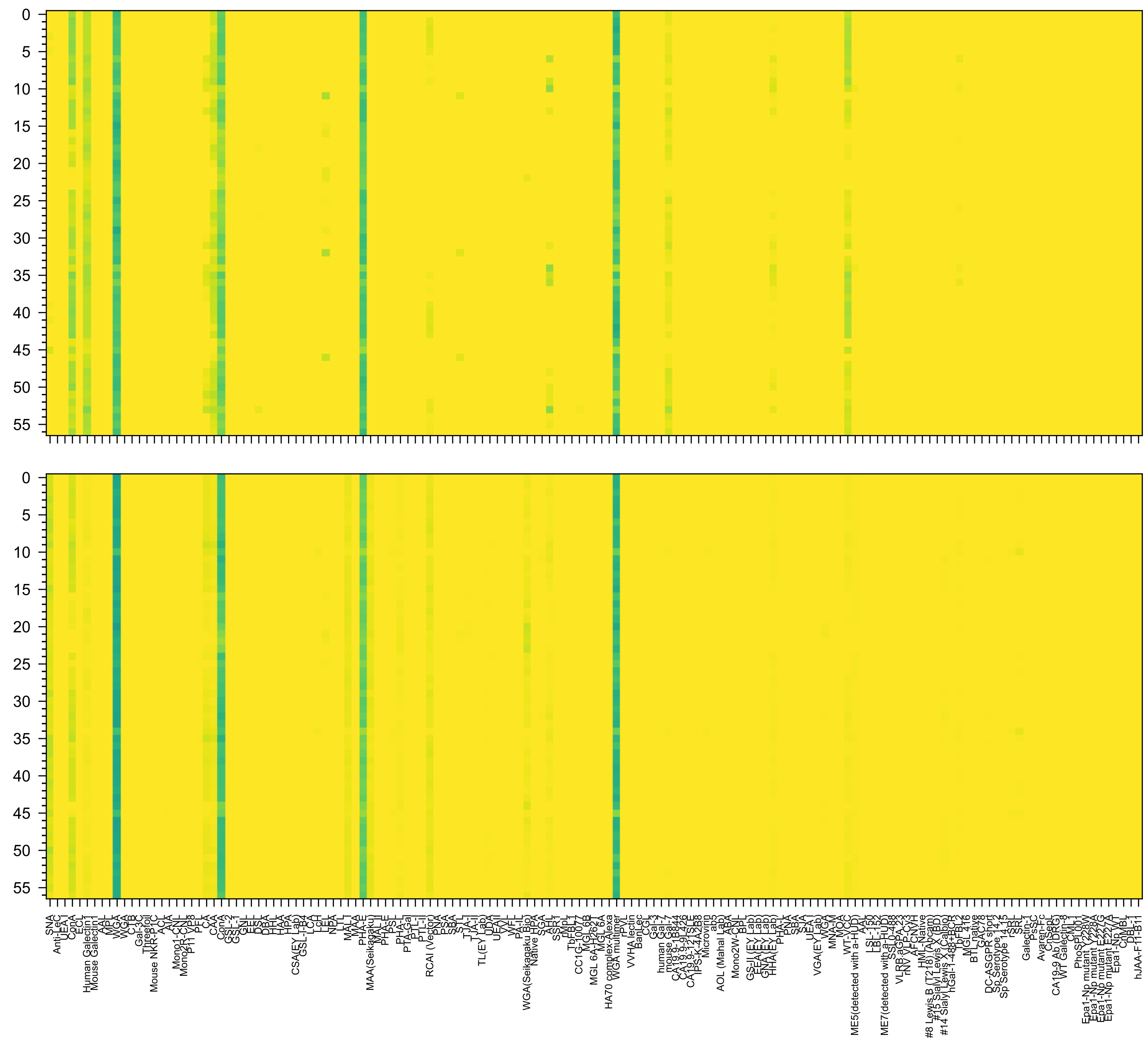
Neu5Ac(α2-3)Gal(β1-4)GlcNAc(β1-2)Man(α1-6)[Neu5Ac(α2-3)Gal(β1-4)GlcNAc(β1-2)Man(α1-3)]Man(β1-4)GlcNAc(β1-4)GlcNAc(β-Sp12

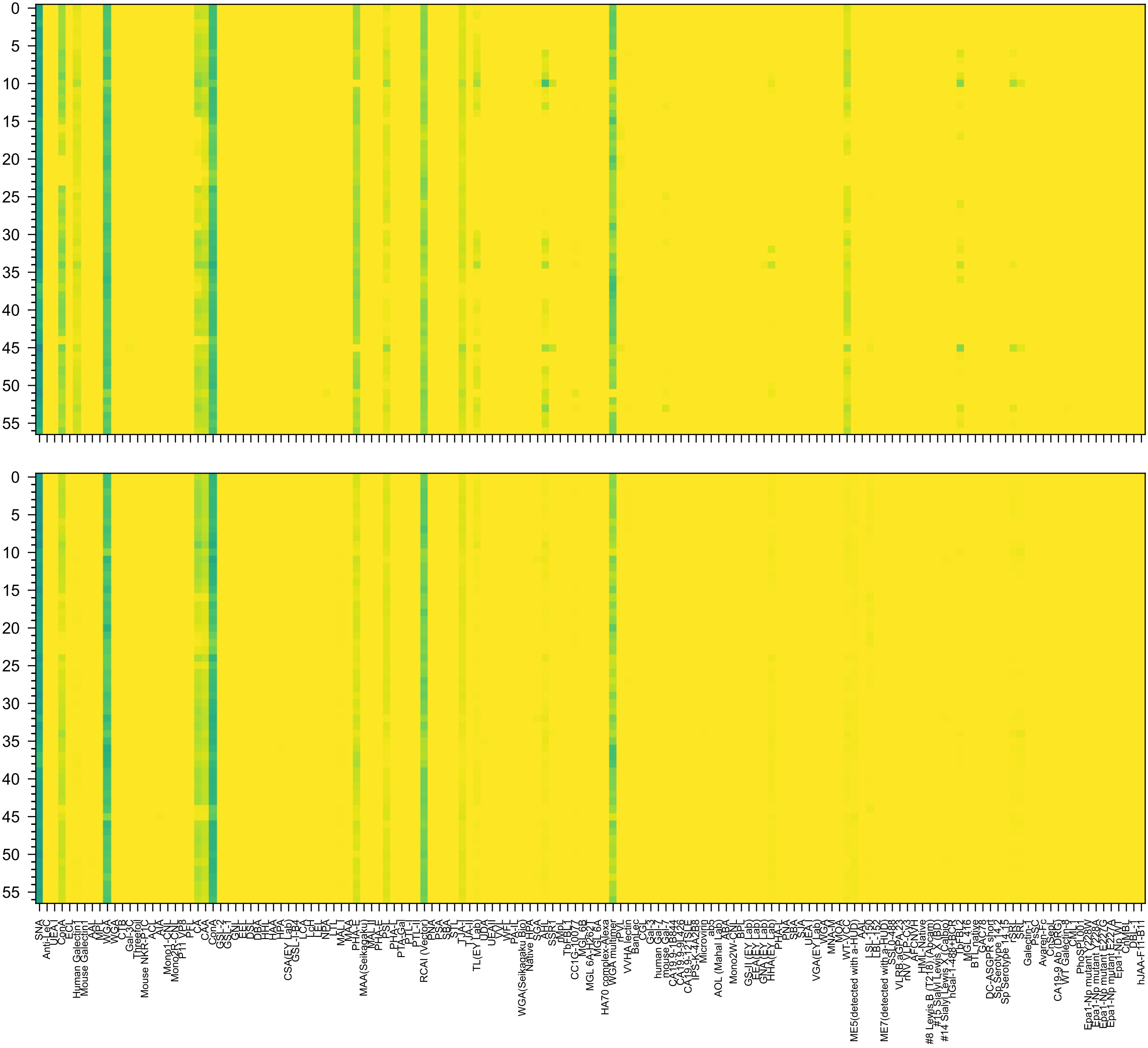

Neu5Ac(α2-3)Gal(β1-4)GlcNAc(β1-2)Man(α1-6)[Neu5Ac(α2-6)Gal(β1-4)GlcNAc(β1-2)Man(α1-3)]Man(β1-4)GlcNAc(β1-4)GlcNAc(β-Sp12

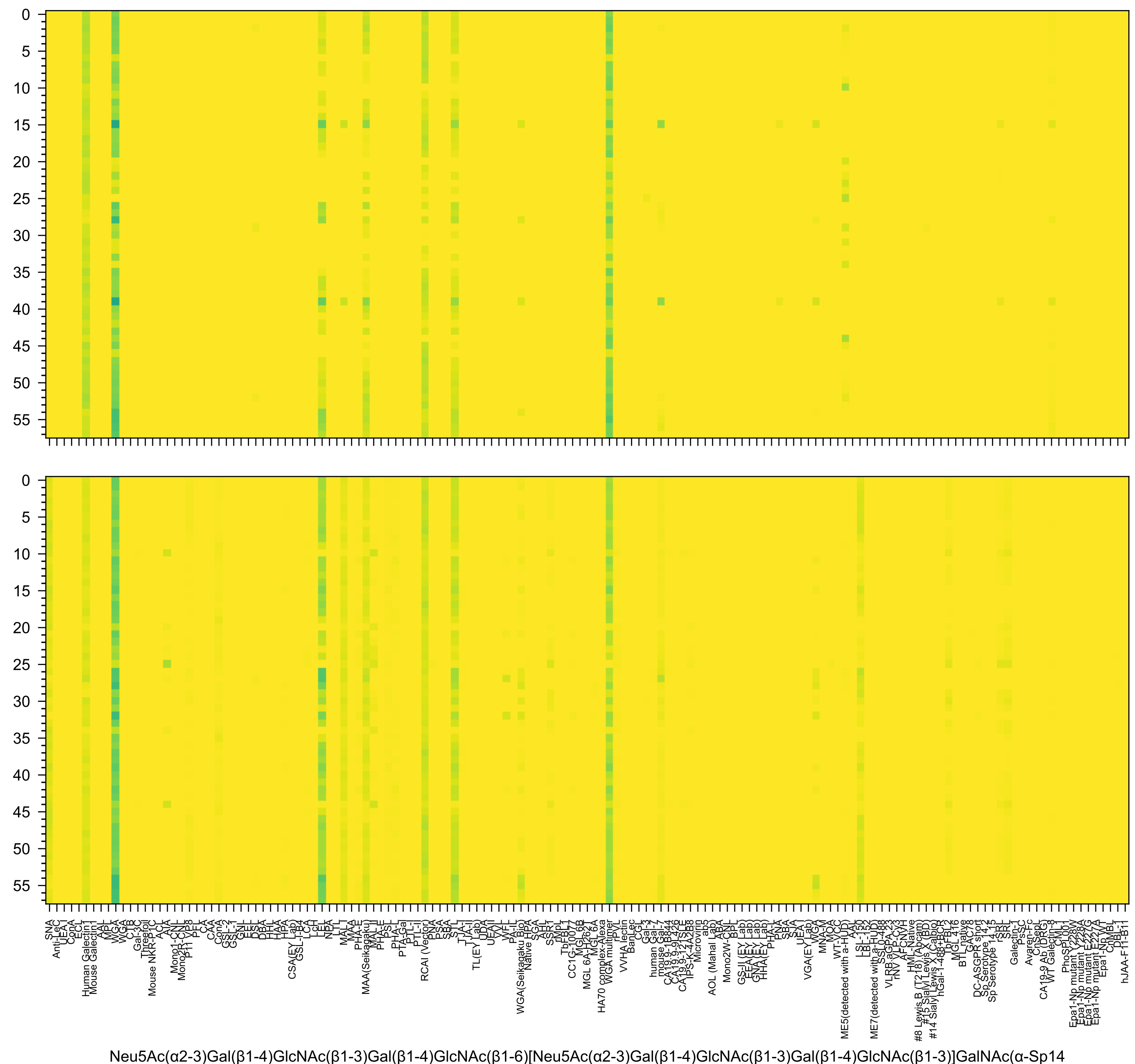
Neu5Ac(α2-3)Gal(β1-4)GlcNAc(β1-3)Gal(β1-4)GlcNAc(β1-6)[Neu5Ac(α2-3)Gal(β1-4)GlcNAc(β1-3)Gal(β1-4)GlcNAc(β1-3)]GalNAc(α-Sp14

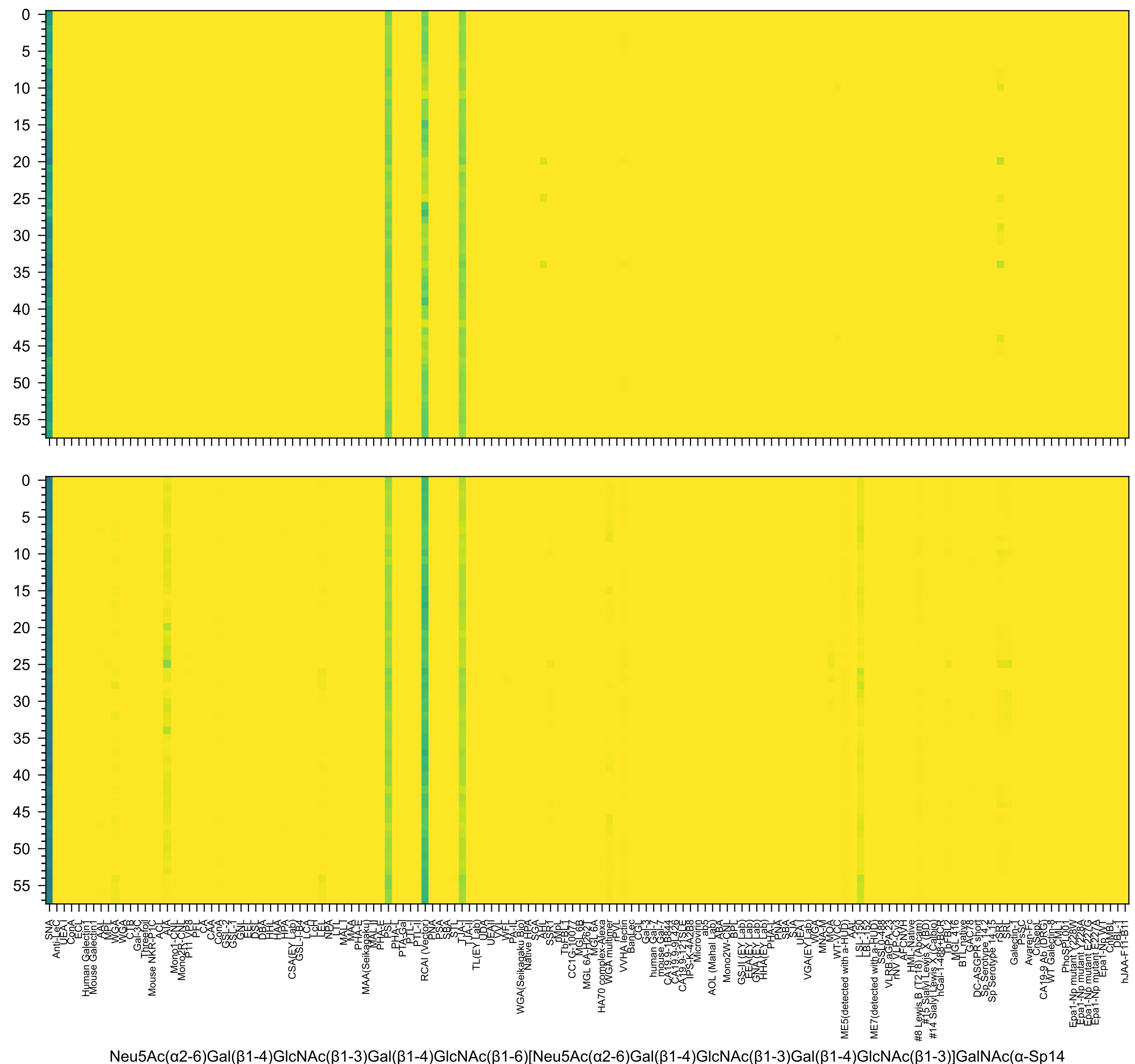
Neu5Ac(α2-6)Gal(β1-4)GlcNAc(β1-3)Gal(β1-4)GlcNAc(β1-6)[Neu5Ac(α2-6)Gal(β1-4)GlcNAc(β1-3)Gal(β1-4)GlcNAc(β1-3)]GalNAc(α-Sp14

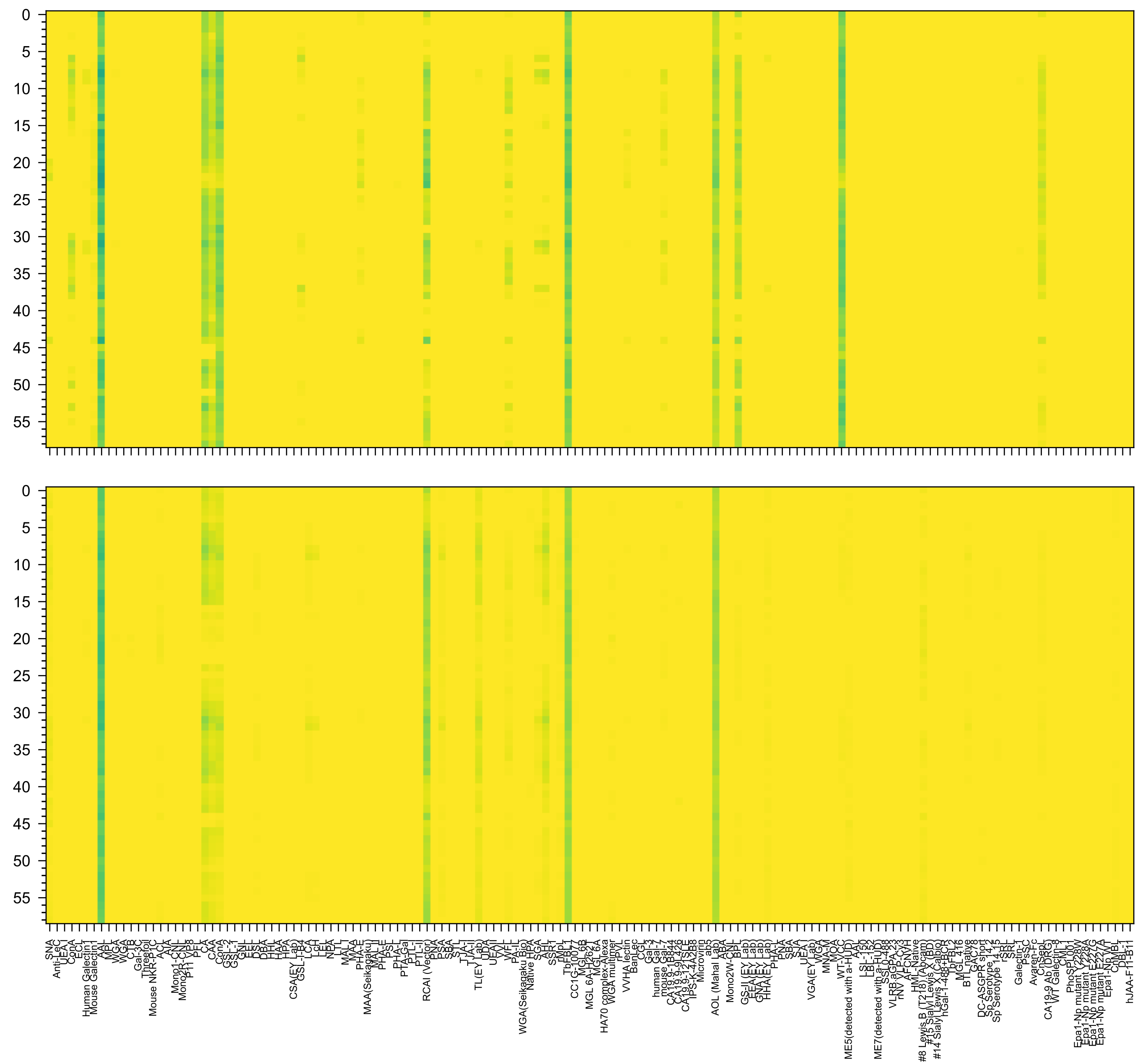

Gal(β1-4)[Fuc(α1-3)]GlcNAc(β1-2)Man(α1-6)[Gal(β1-4)[Fuc(α1-3)]GlcNAc(β1-2)Man(α1-3)]Man(β1-4)GlcNAc(β1-4)GlcNAc(β-Sp20

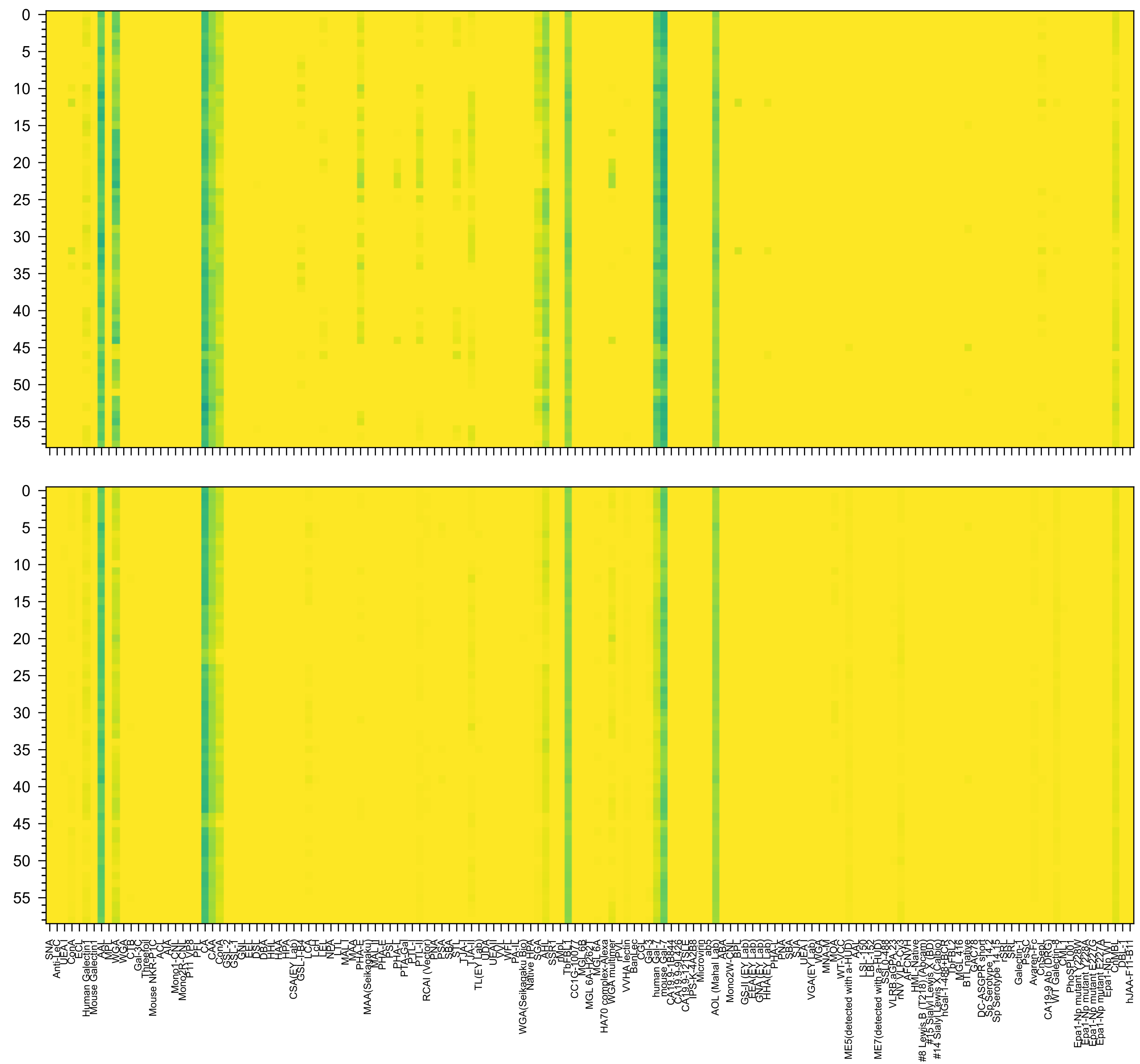

Fuc(α1-2)Gal(β1-3)GlcNAc(β1-2)Man(α1-6)[Fuc(α1-2)Gal(β1-3)GlcNAc(β1-2)Man(α1-3)]Man(β1-4)GlcNAc(β1-4)GlcNAc(β-Sp20

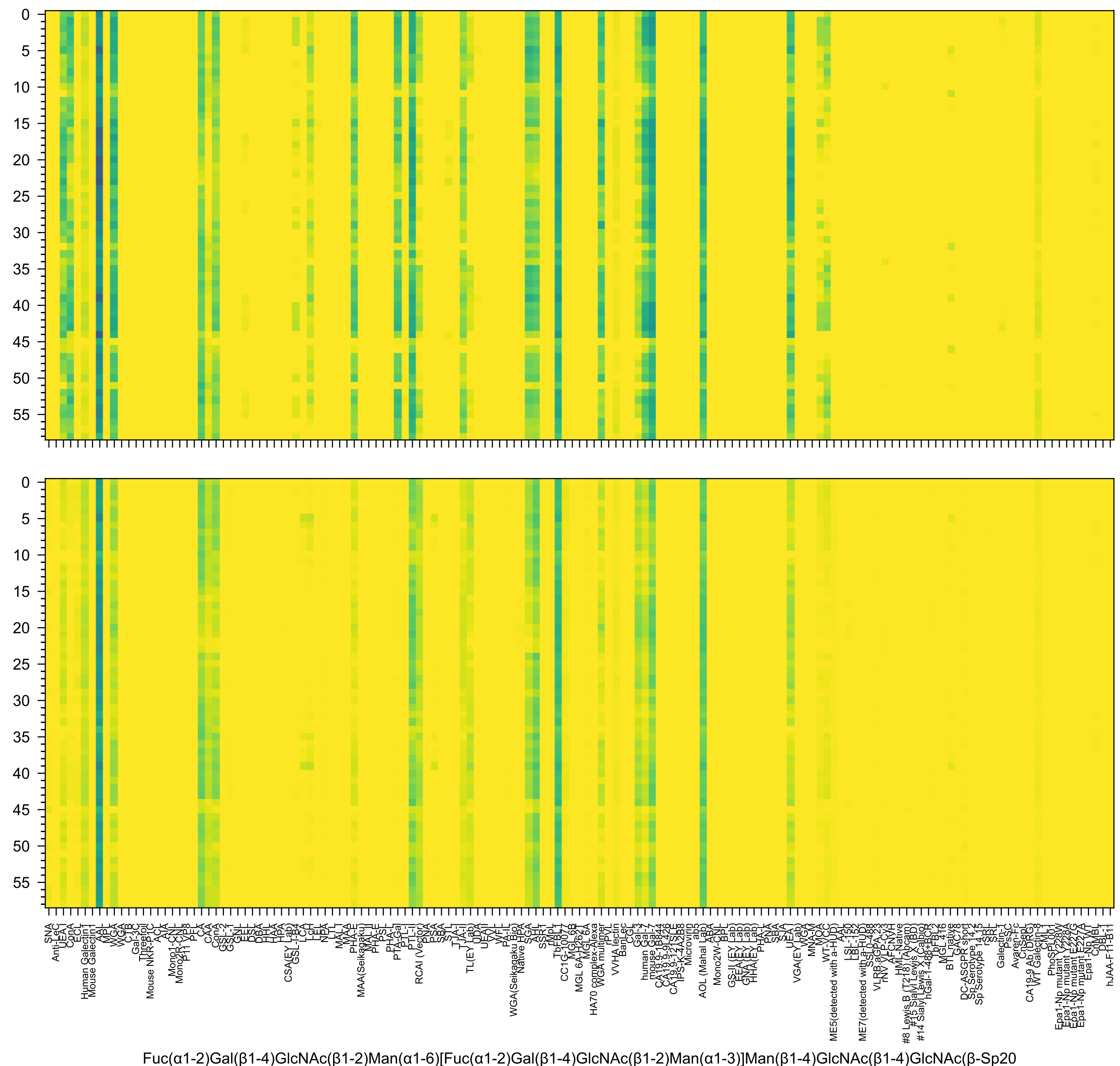
Fuc(α1-2)Gal(β1-4)GlcNAc(β1-2)Man(α1-6)[Fuc(α1-2)Gal(β1-4)GlcNAc(β1-2)Man(α1-3)]Man(β1-4)GlcNAc(β1-4)GlcNAc(β-Sp20

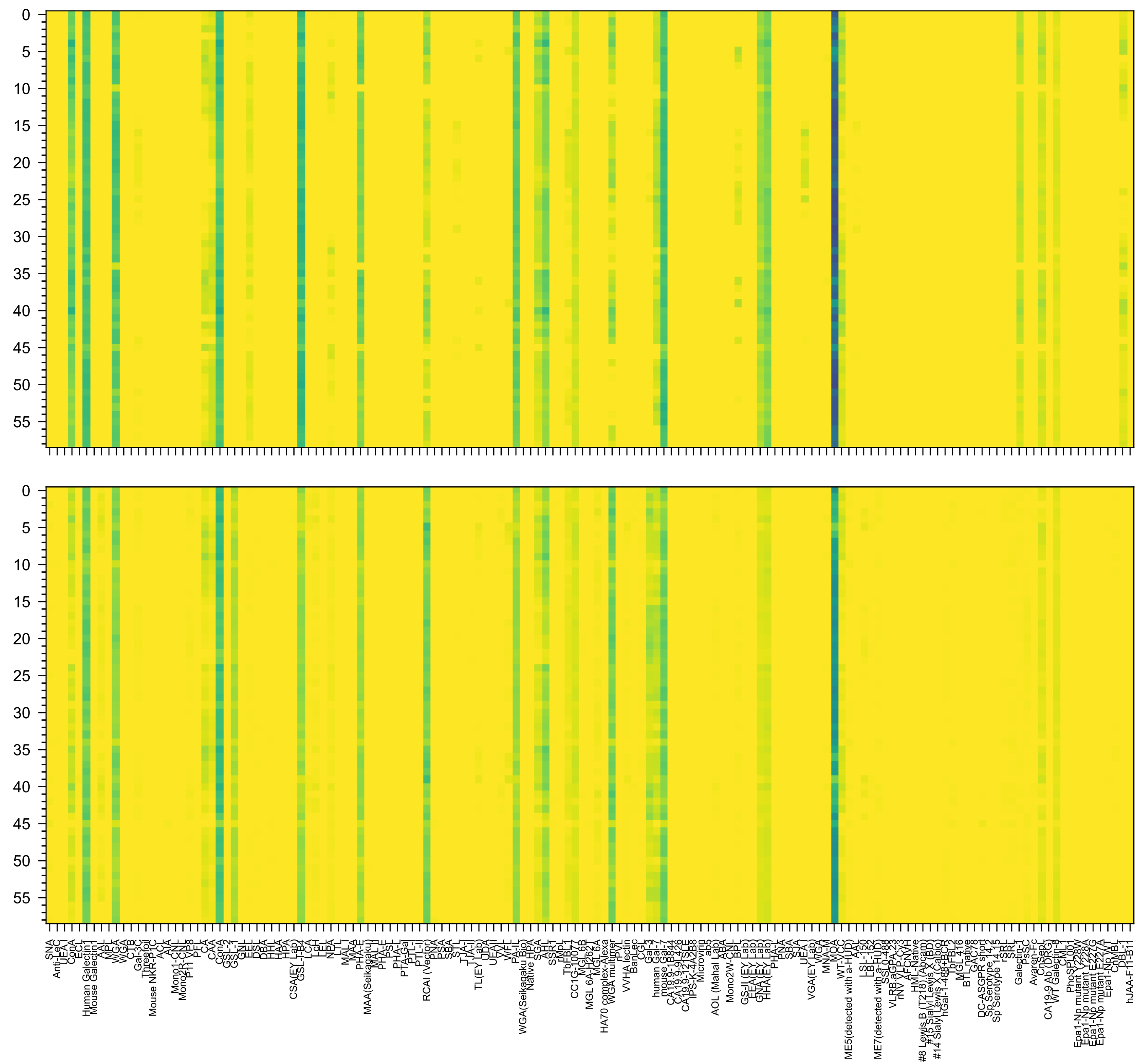

Gal(α1-3)Gal(β1-4)GlcNAc(β1-2)Man(α1-6)[Gal(α1-3)Gal(β1-4)GlcNAc(β1-2)Man(α1-3)]Man(β1-4)GlcNAc(β1-4)GlcNAc(β-Sp20

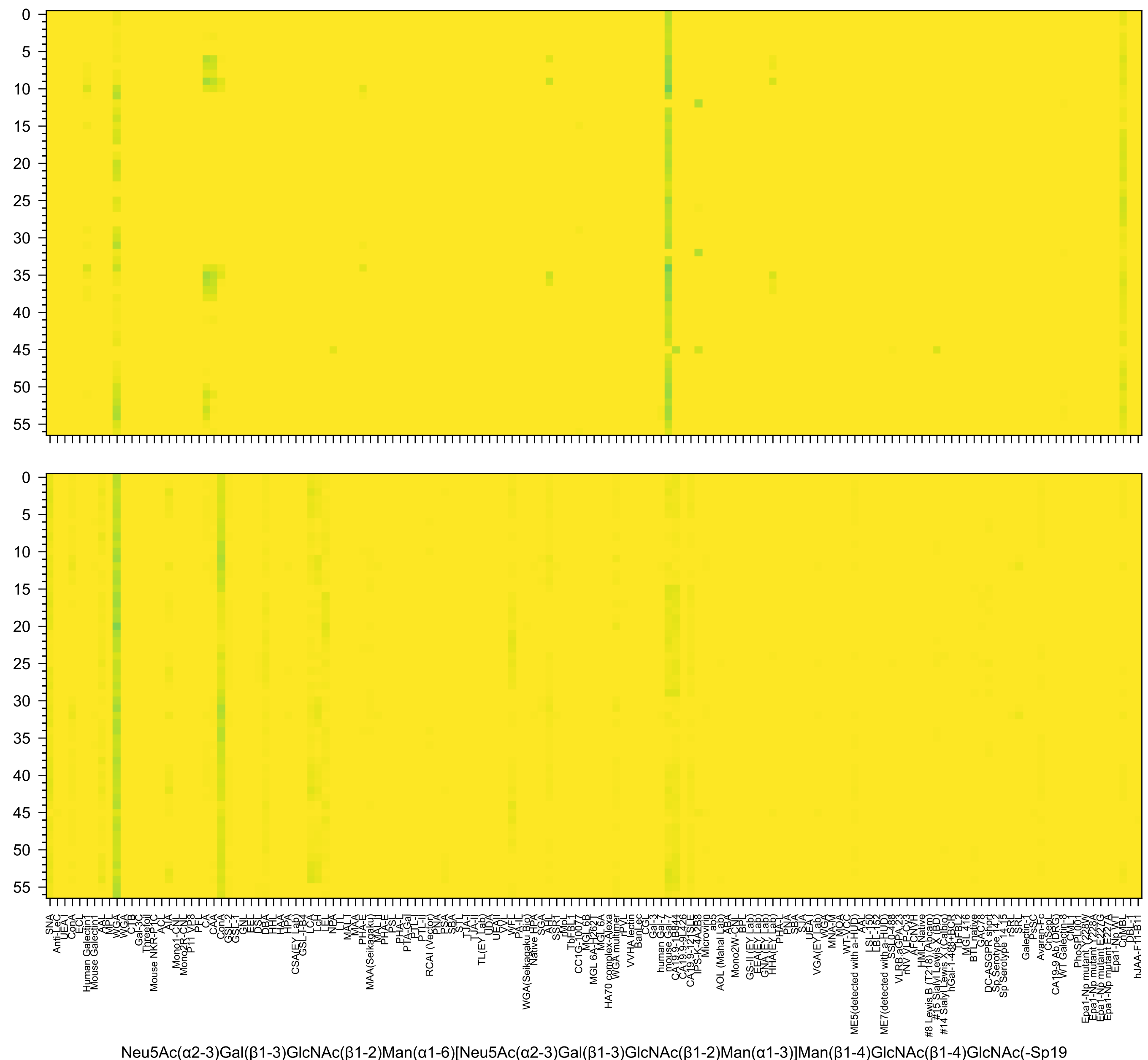
0
5
10
15
20
25
30
35
40
45
50
55
SNA
Anti-LeC
UEA-I
ConA
ECL
Human Galectin1
Mouse Galectin1
AAL
MPL
WGA
WGA
CTB
Gal-3C
Threefoil
Mouse NKR-P1C
ACL
AIA
Mono1-CNL
Mono2R-CNL
P11 VP8
PFL
CA
CAA
ConA
GSL-2
GSL-1
GNL
EEL
DSL
DBA
HHL
HAA
HPA
CSA(EY Lab)
GSL-I-B4
LCA
LcH
LEL
NPA
LTL
MAL I
MAA
PHA-E
MAA(Seikagaku)
MAL II
PHA-E
PSL
PHA-L
PTA-Gal
PTL-I
PTL-II
RCAI (Vector)
PNA
PSA
SBA
STL
TJA-I
TJA-II
TL(EY Lab)
UDA
UEA II
VVL
WFL
PA-IL
WGA(Seikagaku Bio)
Native HPA
SGA
AHL
SSR1
rMpL
TbFBL1
CC1G-10077
MGL 6B
MGL 6A-H262T
MGL 6A
HA70 complex-Alexa
WGA multimer
rPVL
VVHA lectin
BanLec
CGL
Gal-3
human Gal-7
mouse Gal-7
CA19.9-1B844
CA19.9-9L426
CA19.9-121SLE
IPS-K-4A2B8
Microvirin
ab5
AOL (Mahal Lab)
ABA
Mono2W-CNL
BPL
GS-II (EY Lab)
EEA(EY Lab)
GNA(EY Lab)
HHA(EY Lab)
PHA-L
PNA
SBA
SJA
UEA I
VGA(EY Lab)
WGA
MNA-M
MOA
WT-VCC
ME5(detected with a-HUD)
AAL
LSL-150
LBL-152
ME7(detected with a-HUD)
SSL0-488
VLRB.aGPA.23
rNV VLP-Cy3
AFCNVH
HML-Native
#8 Lewis B (T218) (Abcam)
#15 Sialyl Lewis X (BD)
#14 Sialyl Lewis X (Calbio)
hGal-1-488+BCR
TbFBL2
MGL 416
BTL native
GAC78
DC-ASGPR short
Sp Serotype 14.2
Sp Serotype 14.15
rSRL
SRL
Galectin-1
PsSC
Avaren-Fc
CnSepL
CA19-9 Ab (DRG)
WT Galectin-8
CML1
PhoSPL001
Epa1-Np mutant Y228W
Epa1-Np mutant Y228A
Epa1-Np mutant E227G
Epa1-Np mutant E227A
Epa1-Np WT
CnMBL
DBL-1
hJAA-F11-B11
Neu5Ac(α2-3)Gal(β1-3)GlcNAc(β1-2)Man(α1-6)[Neu5Ac(α2-3)Gal(β1-3)GlcNAc(β1-2)Man(α1-3)]Man(β1-4)GlcNAc(β1-4)GlcNAc(-Sp19

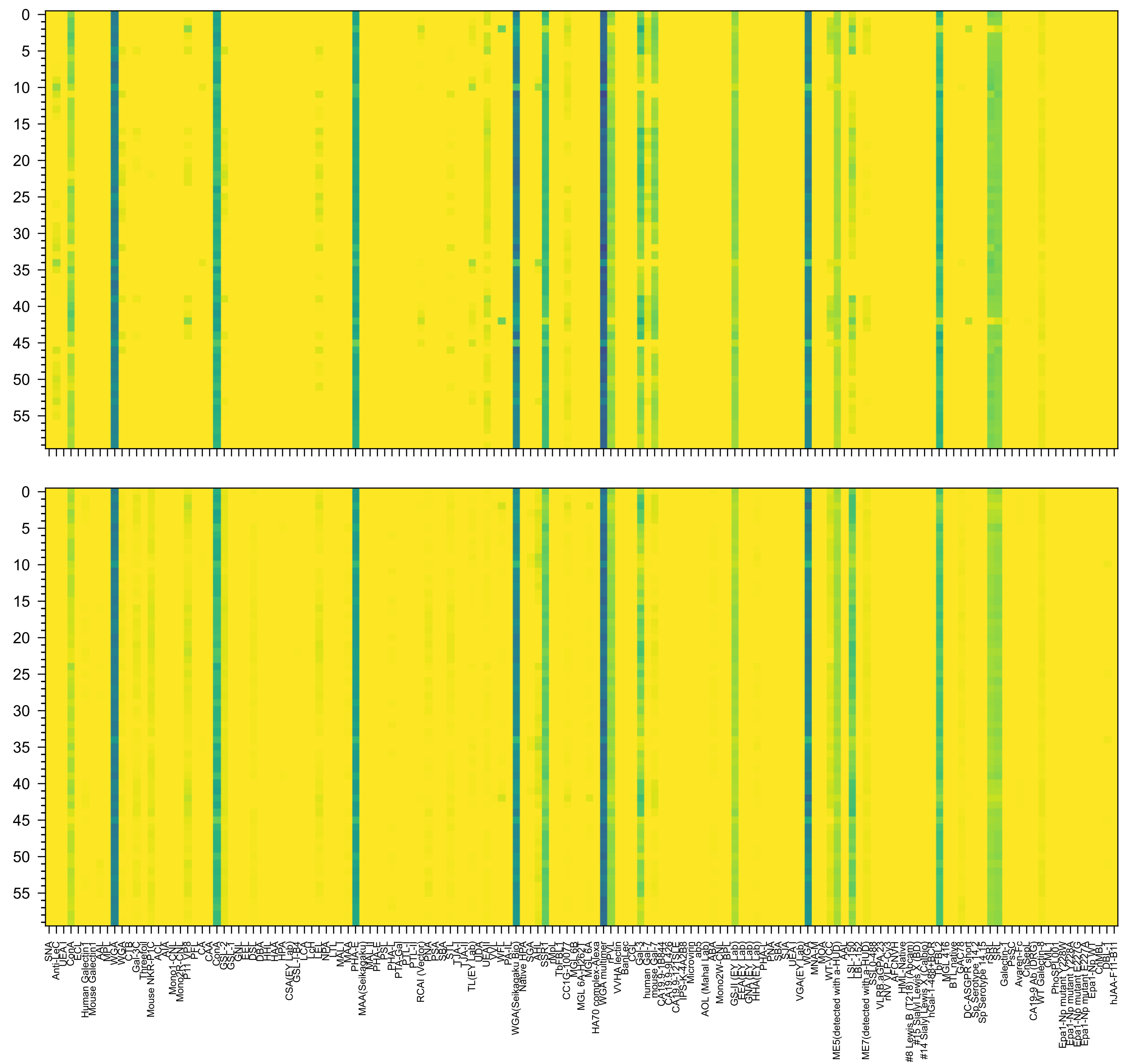

GlcNAc(β1-3)Gal(β1-4)GlcNAc(β1-2)Man(α1-6)[GlcNAc(β1-3)Gal(β1-4)GlcNAc(β1-2)Man(α1-3)]Man(β1-4)GlcNAc(β1-4)GlcNAc(β-Sp25

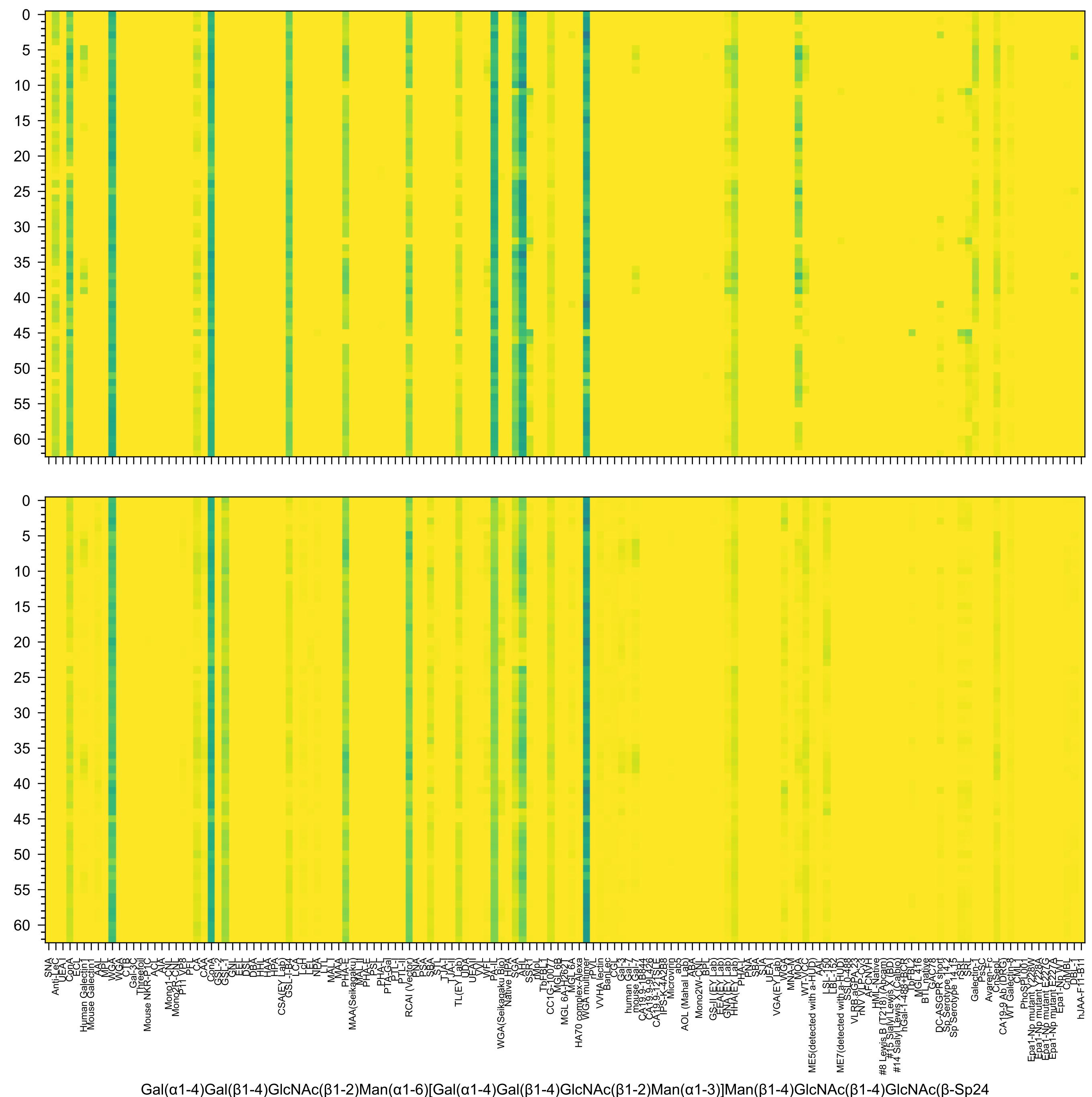
Gal(α1-4)Gal(β1-4)GlcNAc(β1-2)Man(α1-6)[Gal(α1-4)Gal(β1-4)GlcNAc(β1-2)Man(α1-3)]Man(β1-4)GlcNAc(β1-4)GlcNAc(β-Sp24

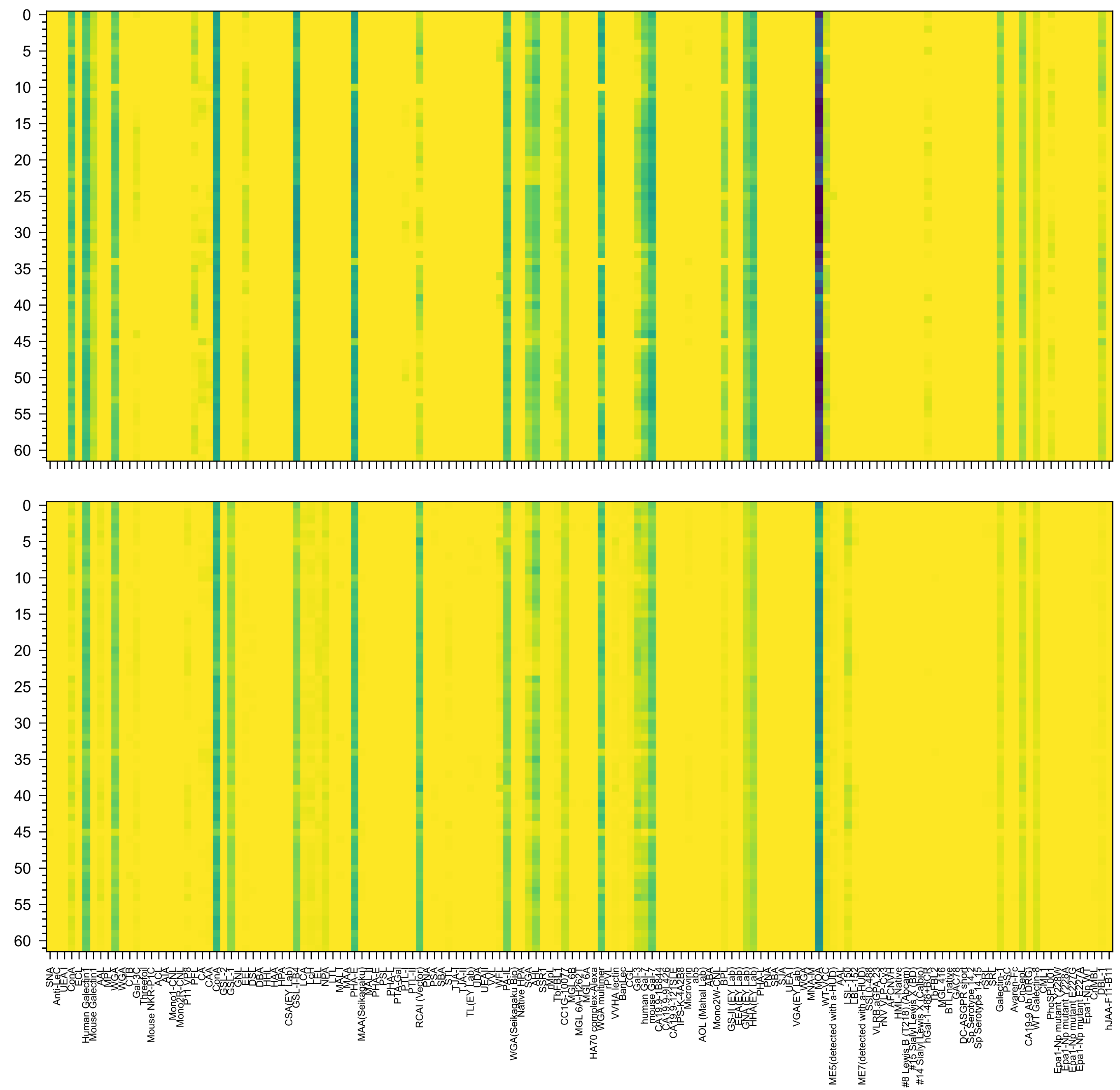

Gal(α1-3)Gal(β1-4)GlcNAc(β1-2)Man(α1-6)[Gal(α1-3)Gal(β1-4)GlcNAc(β1-2)Man(α1-3)]Man(β1-4)GlcNAc(β1-4)GlcNAc(-Sp24

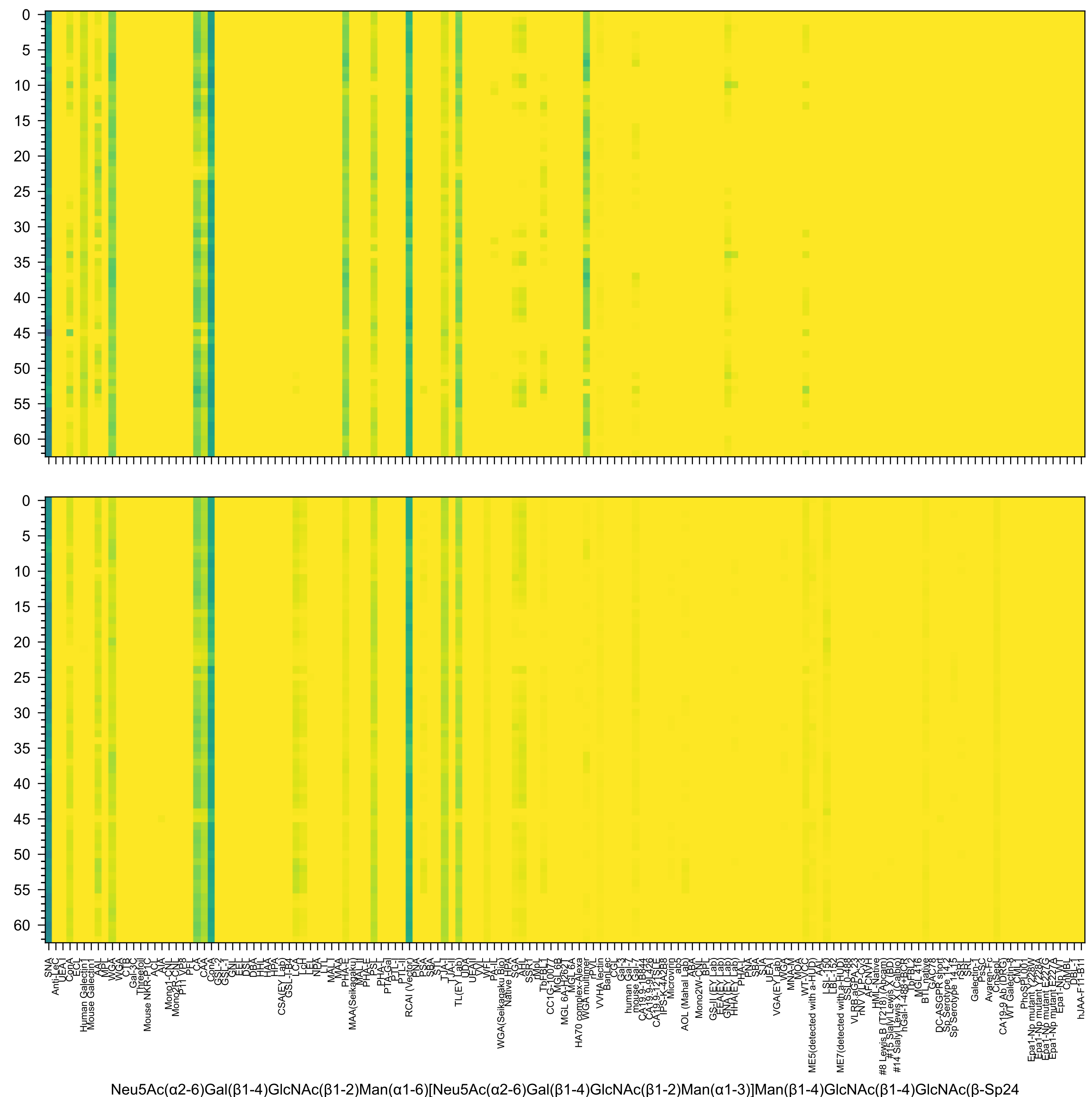
Neu5Ac(α2-6)Gal(β1-4)GlcNAc(β1-2)Man(α1-6)[Neu5Ac(α2-6)Gal(β1-4)GlcNAc(β1-2)Man(α1-3)]Man(β1-4)GlcNAc(β1-4)GlcNAc(β-Sp24

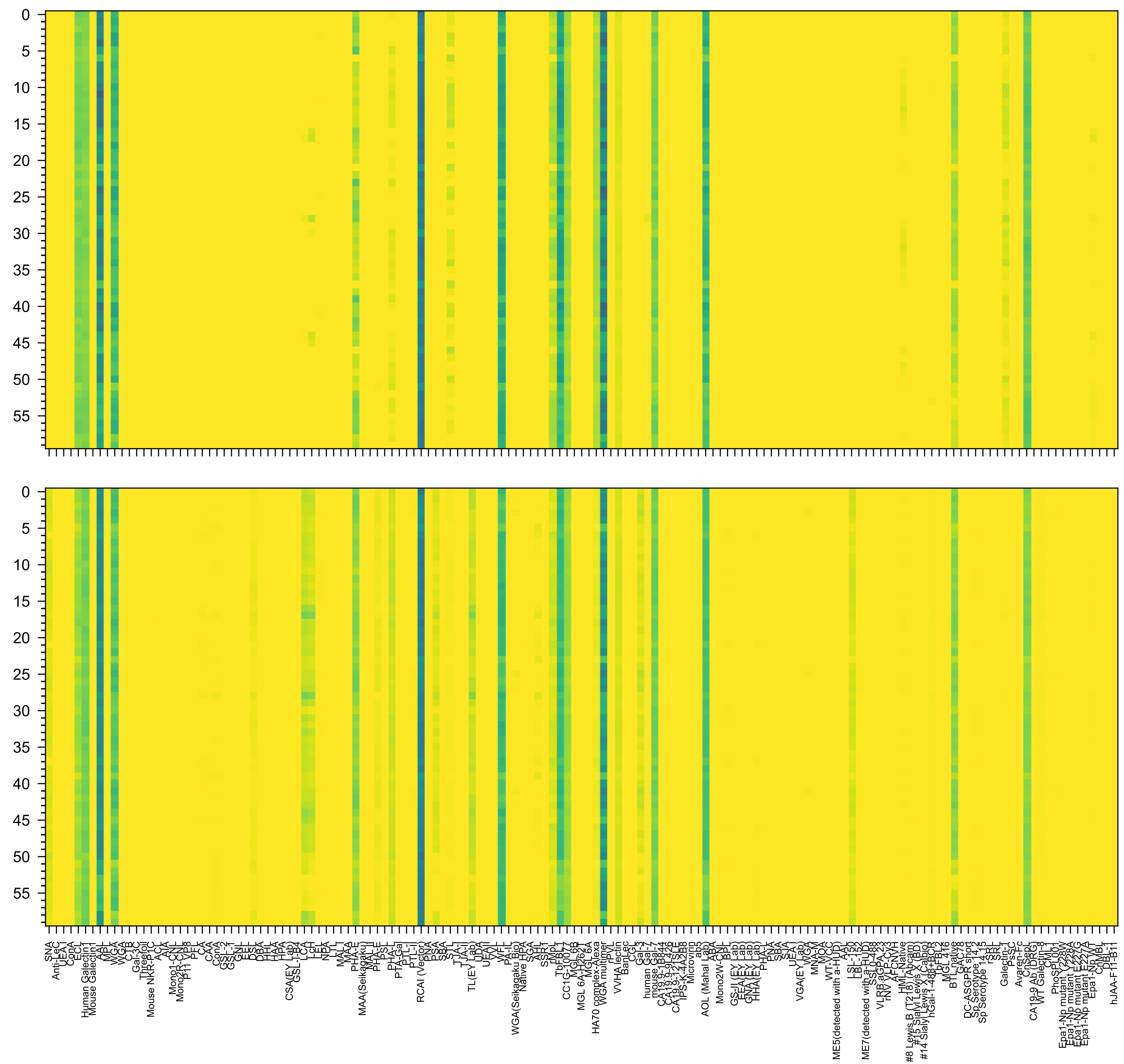

Gal(β1-4)GlcNAc(β1-2)Man(α1-6)[Gal(β1-4)GlcNAc(β1-4)][Gal(β1-4)GlcNAc(β1-2)Man(α1-3)]Man(β1-4)GlcNAc(β1-4)[Fuc(α1-6)]GlcNAc(-Sp21

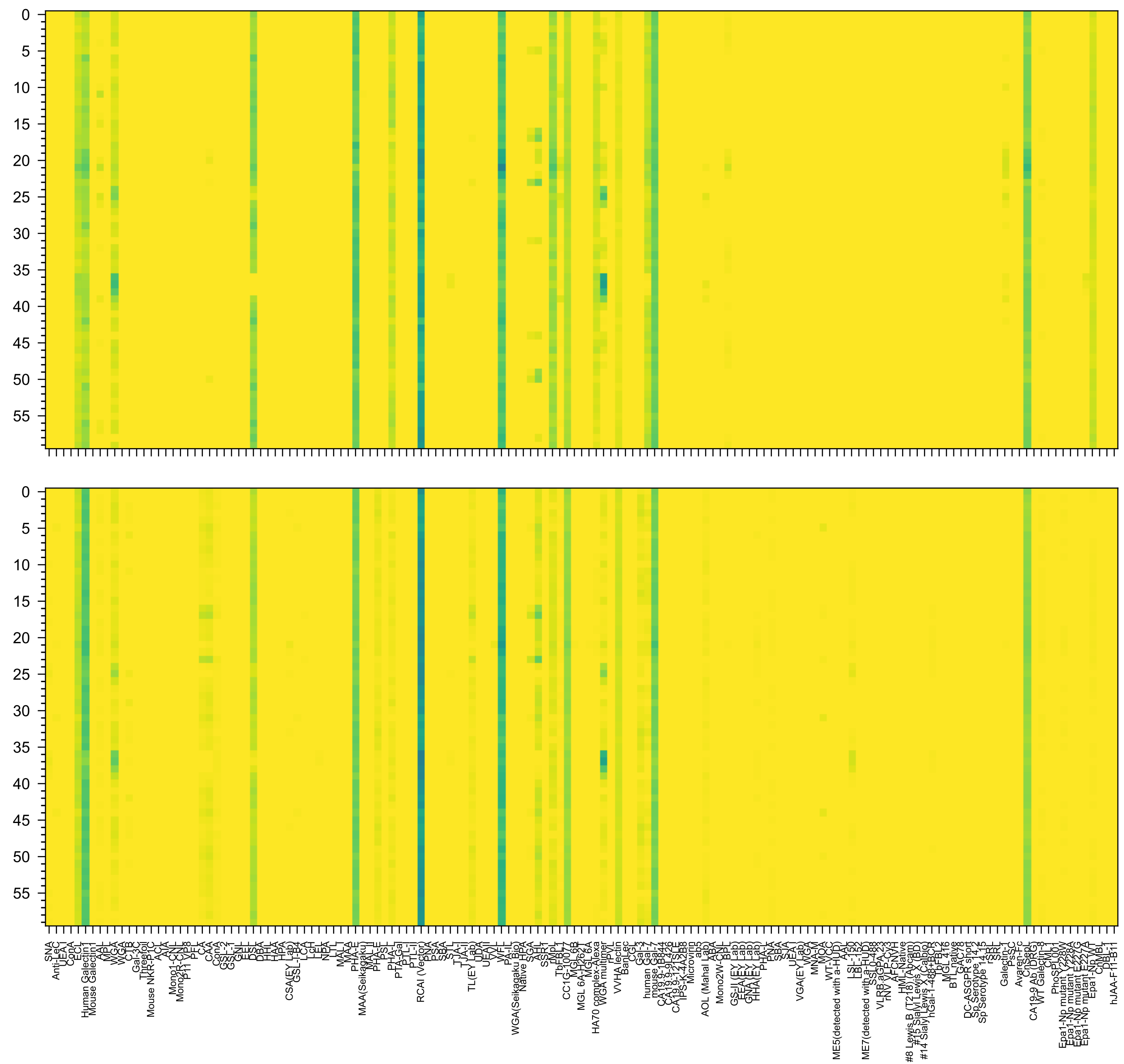

Gal(β1-4)GlcNAc(β1-2)Man(α1-6)[GlcNAc(β1-4)][Gal(β1-4)GlcNAc(β1-4)[Gal(β1-4)GlcNAc(β1-2)]Man(α1-3)]Man(β1-4)GlcNAc(β1-4)GlcNAc(-Sp21

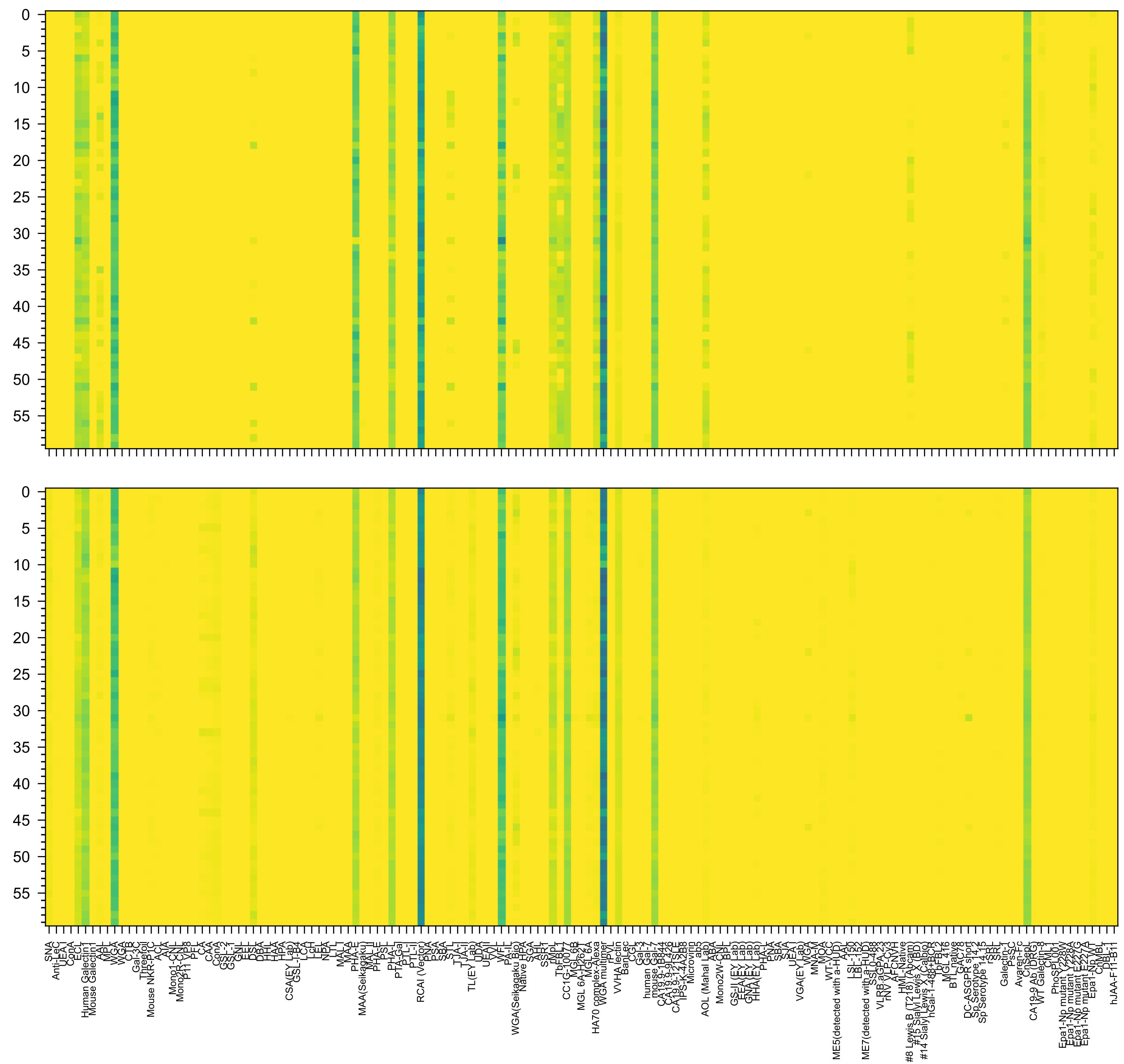

Gal(β1-4)GlcNAc(β1-6)[Gal(β1-4)GlcNAc(β1-2)]Man(α1-6)[GlcNAc(β1-4)][Gal(β1-4)GlcNAc(β1-2)Man(α1-3)]Man(β1-4)GlcNAc(β1-4)GlcNAc(-Sp21

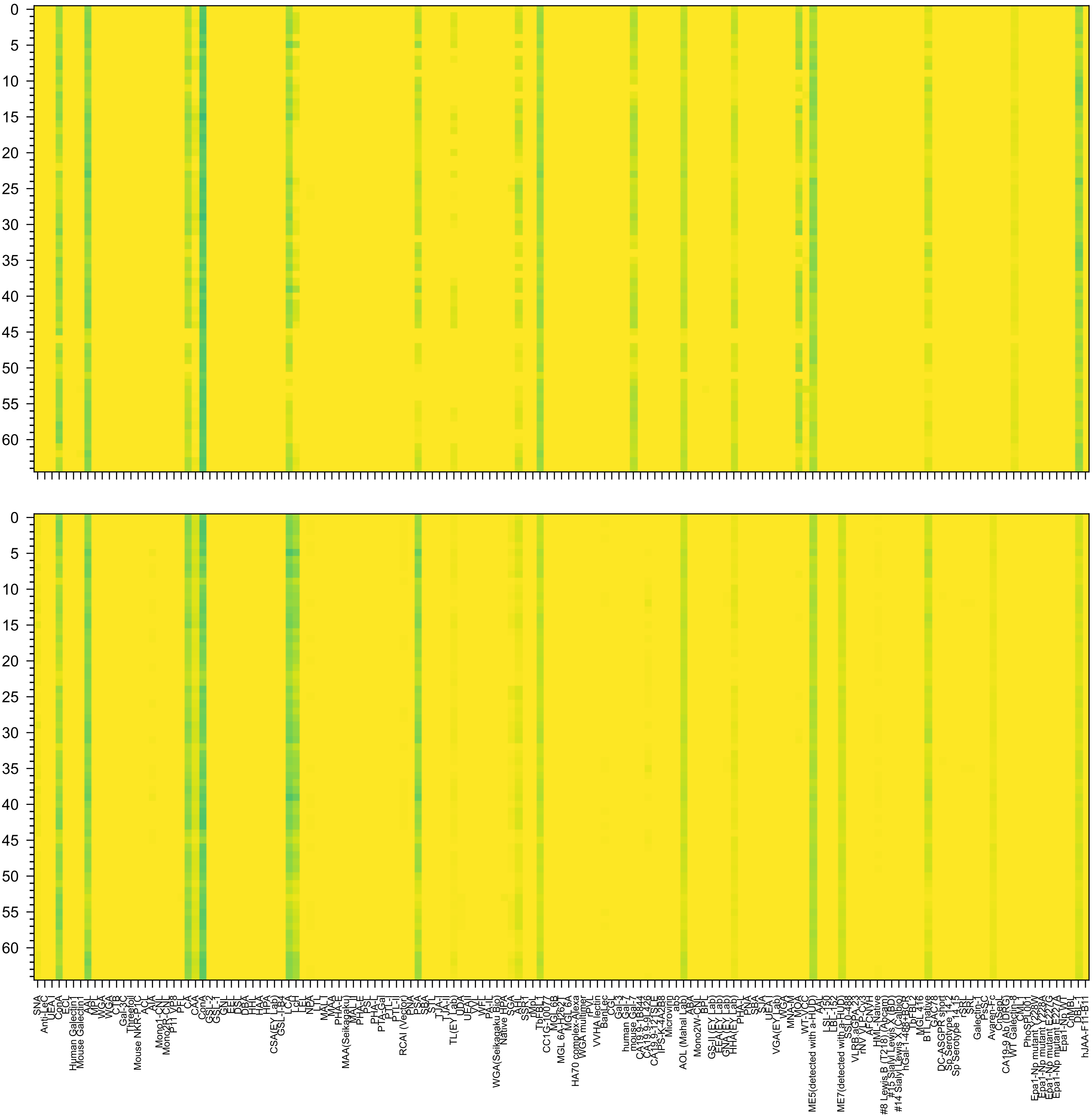

Fuc(α1-4)[Gal(β1-3)]GlcNAc(β1-2)Man(α1-6)[Fuc(α1-4)[Gal(β1-3)]GlcNAc(β1-2)Man(α1-3)]Man(β1-4)GlcNAc(β1-4)[Fuc(α1-6)]GlcNAc(β-Sp22

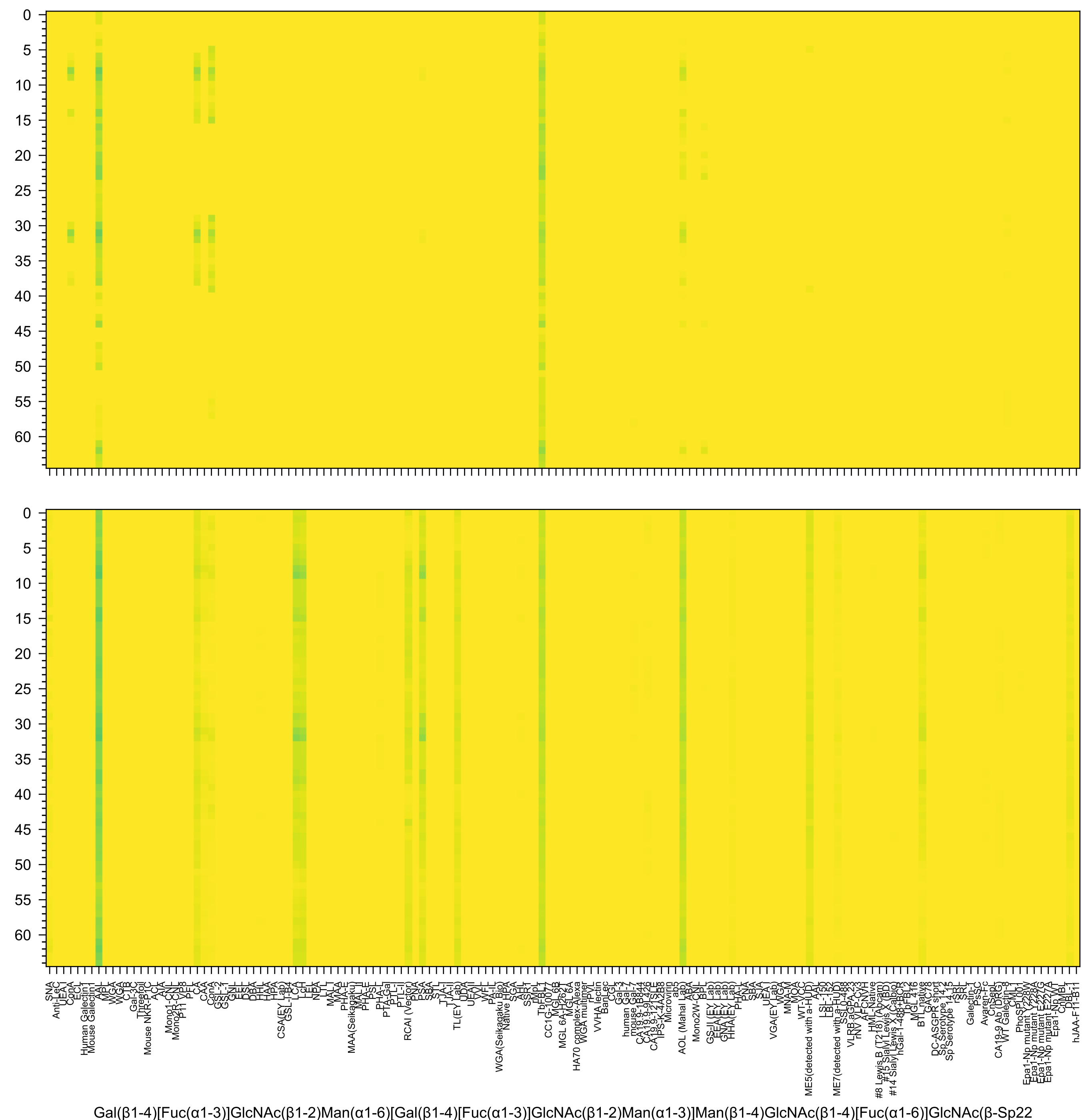
Gal(β1-4)[Fuc(α1-3)]GlcNAc(β1-2)Man(α1-6)[Gal(β1-4)[Fuc(α1-3)]GlcNAc(β1-2)Man(α1-3)]Man(β1-4)GlcNAc(β1-4)[Fuc(α1-6)]GlcNAc(β-Sp22

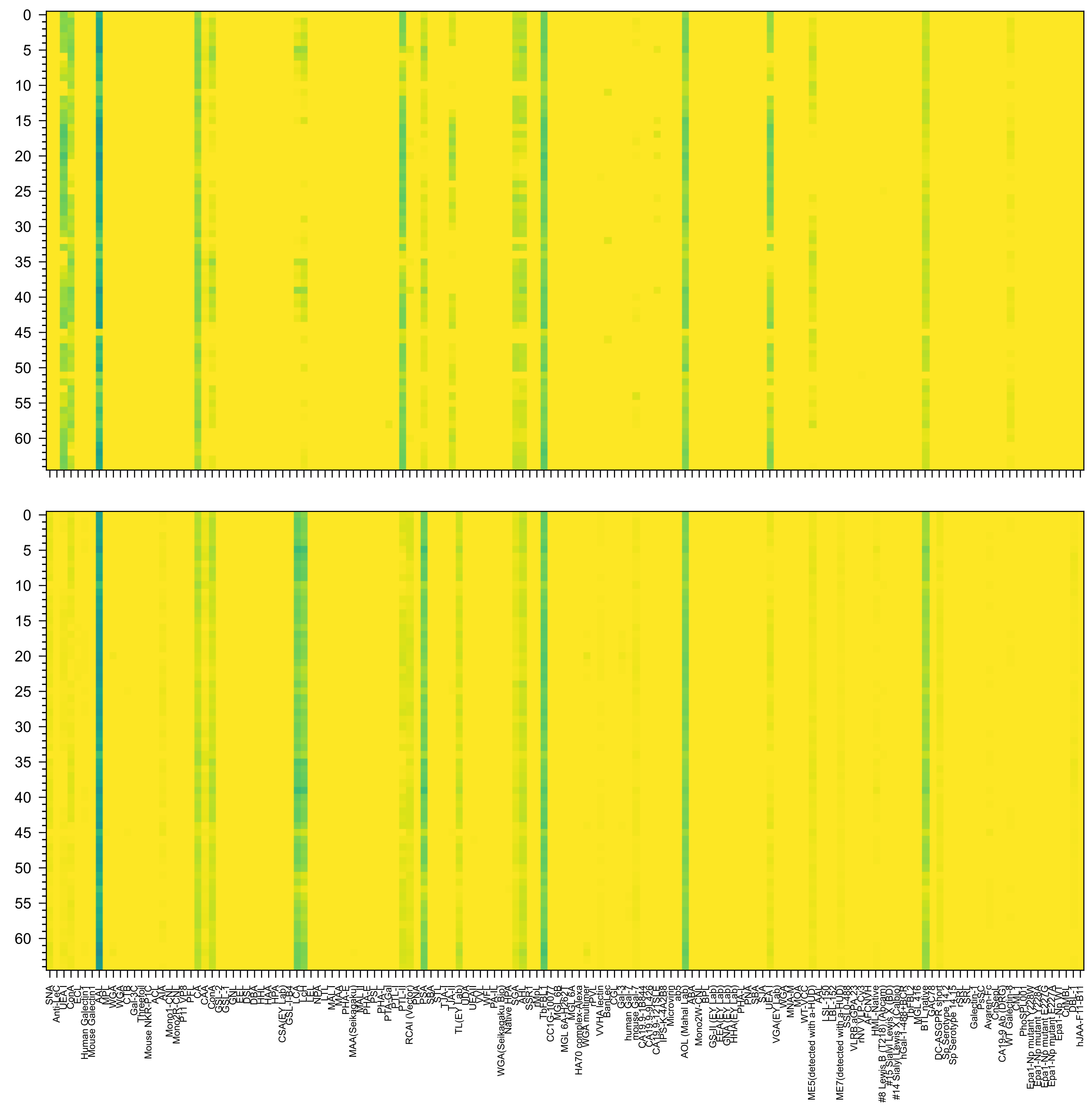

Fuc(α1-2)Gal(β1-4)GlcNAc(β1-2)Man(α1-6)[Fuc(α1-2)Gal(β1-4)GlcNAc(β1-2)Man(α1-3)]Man(β1-4)GlcNAc(β1-4)[Fuc(α1-6)]GlcNAc(β-Sp22

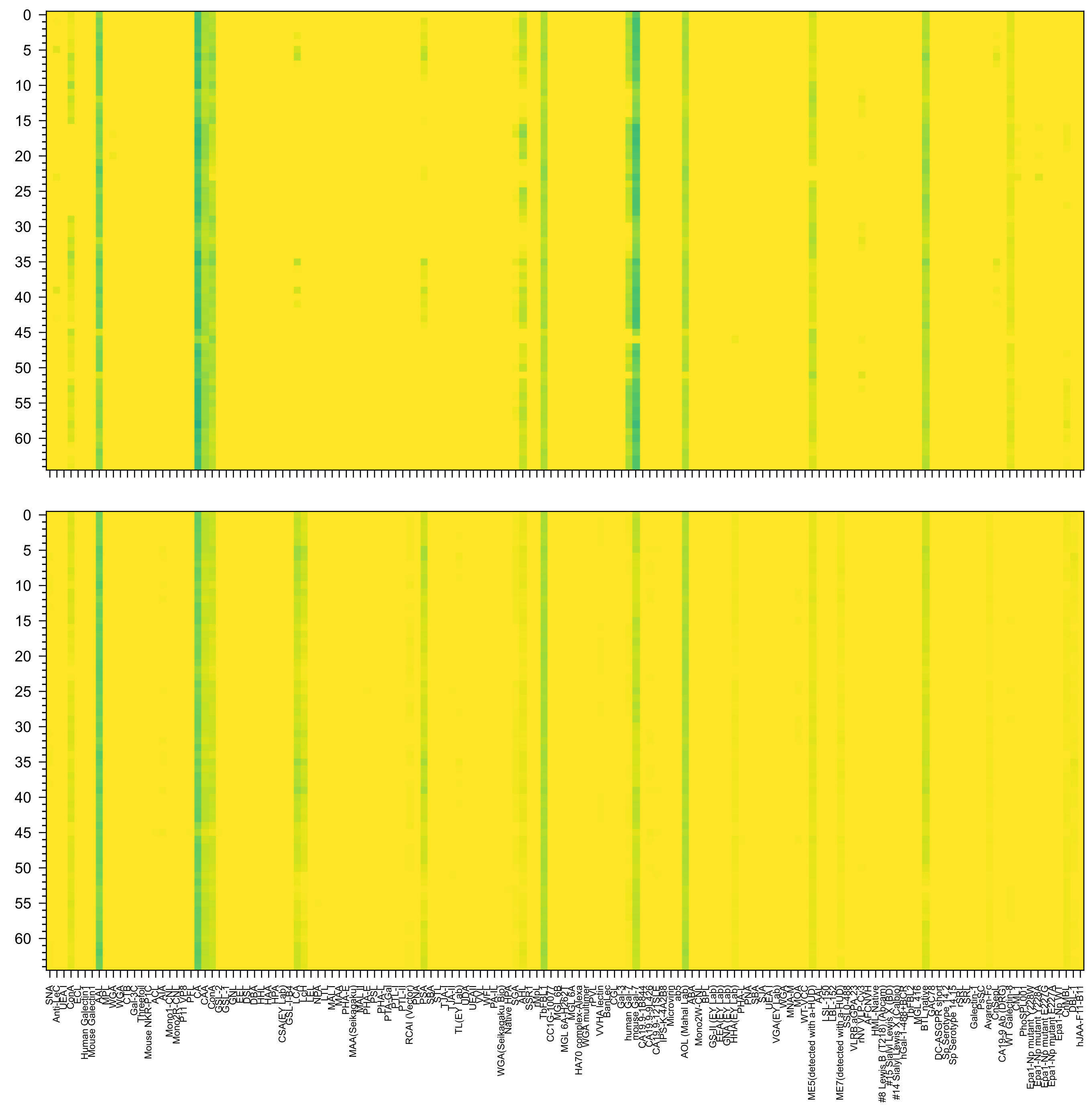

Fuc(α1-2)Gal(β1-3)GlcNAc(β1-2)Man(α1-6)[Fuc(α1-2)Gal(β1-3)GlcNAc(β1-2)Man(α1-3)]Man(β1-4)GlcNAc(β1-4)[Fuc(α1-6)]GlcNAc(β-Sp22

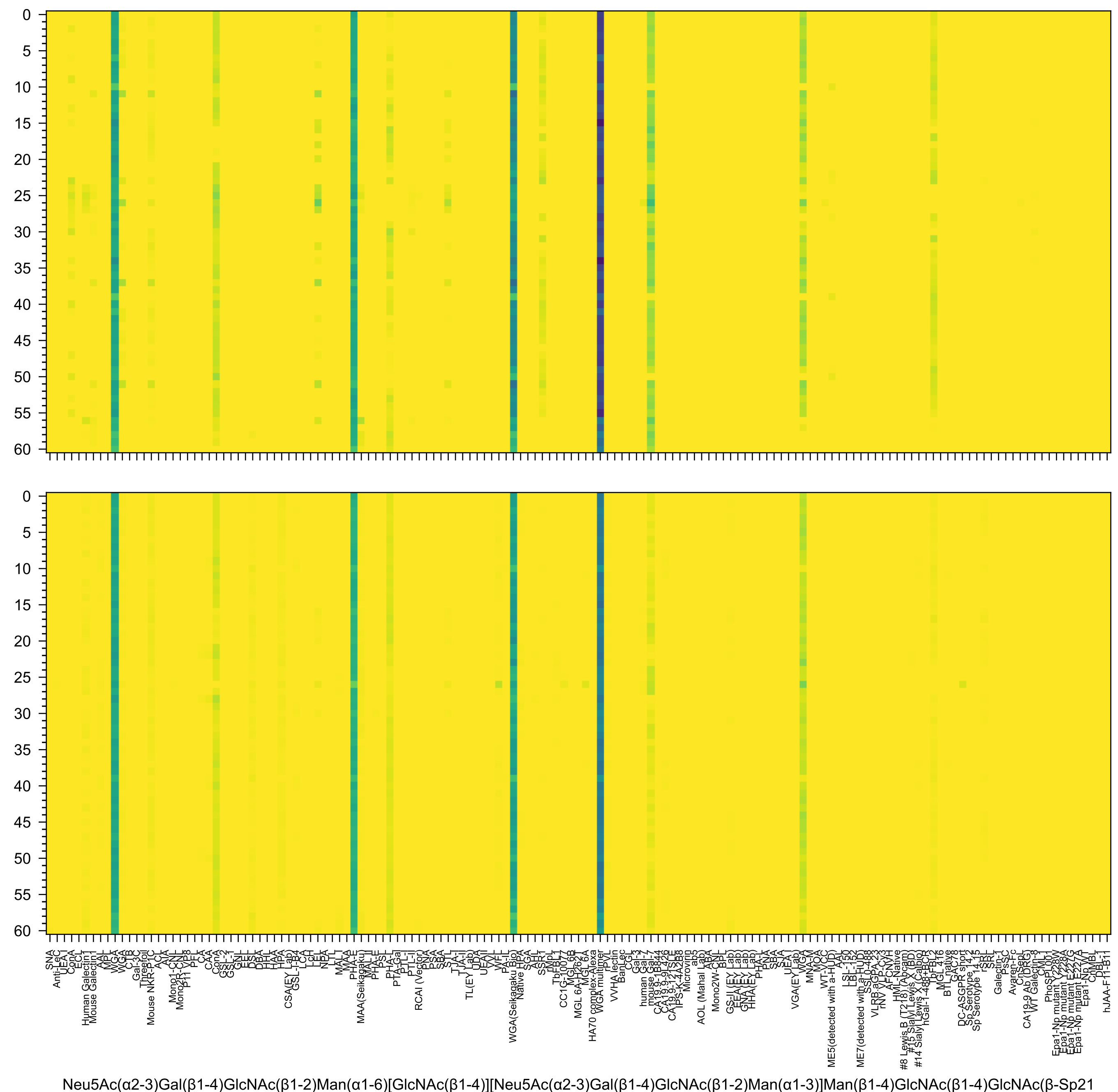
Neu5Ac(α2-3)Gal(β1-4)GlcNAc(β1-2)Man(α1-6)[GlcNAc(β1-4)][Neu5Ac(α2-3)Gal(β1-4)GlcNAc(β1-2)Man(α1-3)]Man(β1-4)GlcNAc(β1-4)GlcNAc(β-Sp21

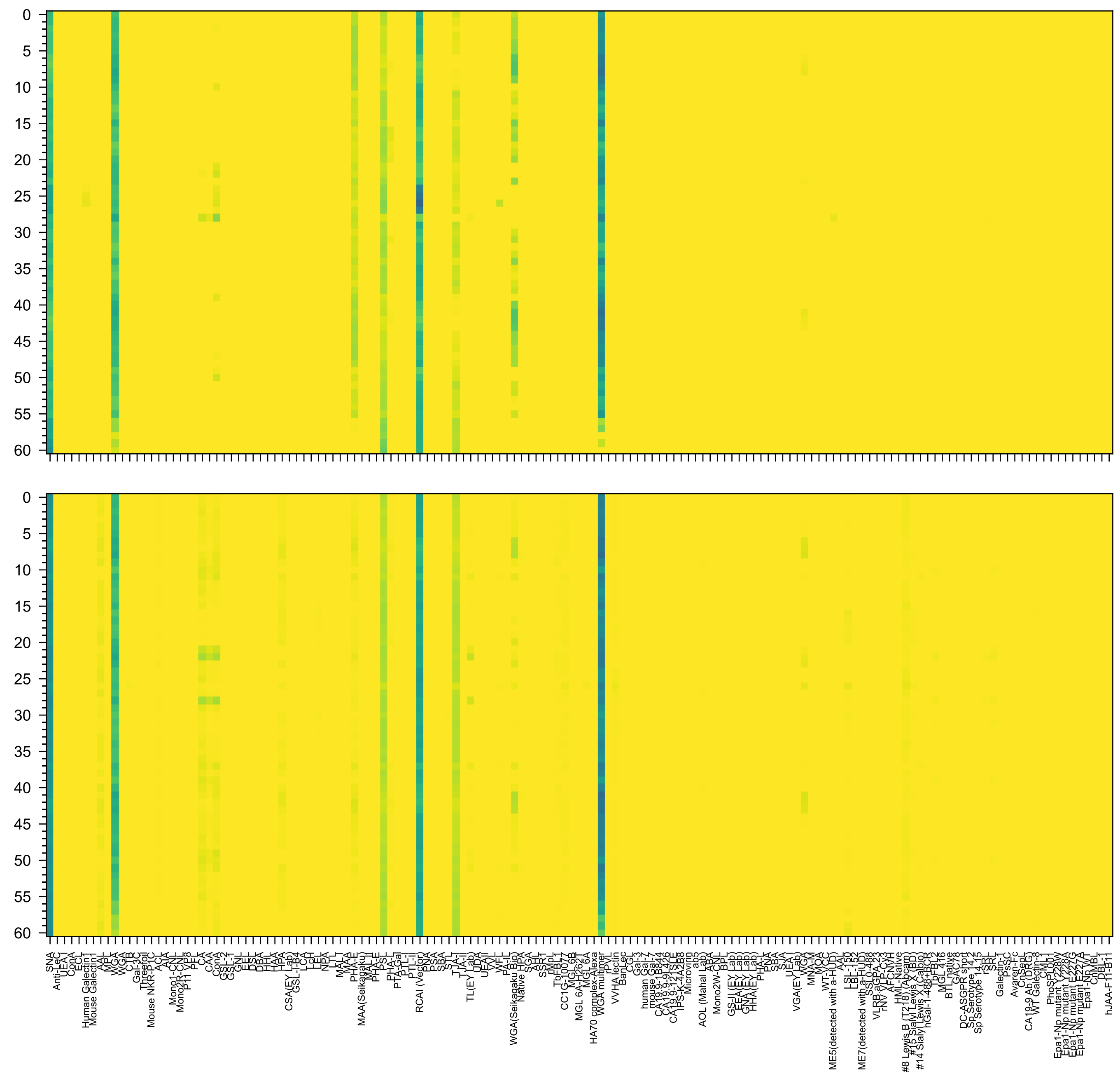

Neu5Ac(α2-6)Gal(β1-4)GlcNAc(β1-2)Man(α1-6)[GlcNAc(β1-4)][Neu5Ac(α2-6)Gal(β1-4)GlcNAc(β1-2)Man(α1-3)]Man(β1-4)GlcNAc(β1-4)GlcNAc(β-Sp21

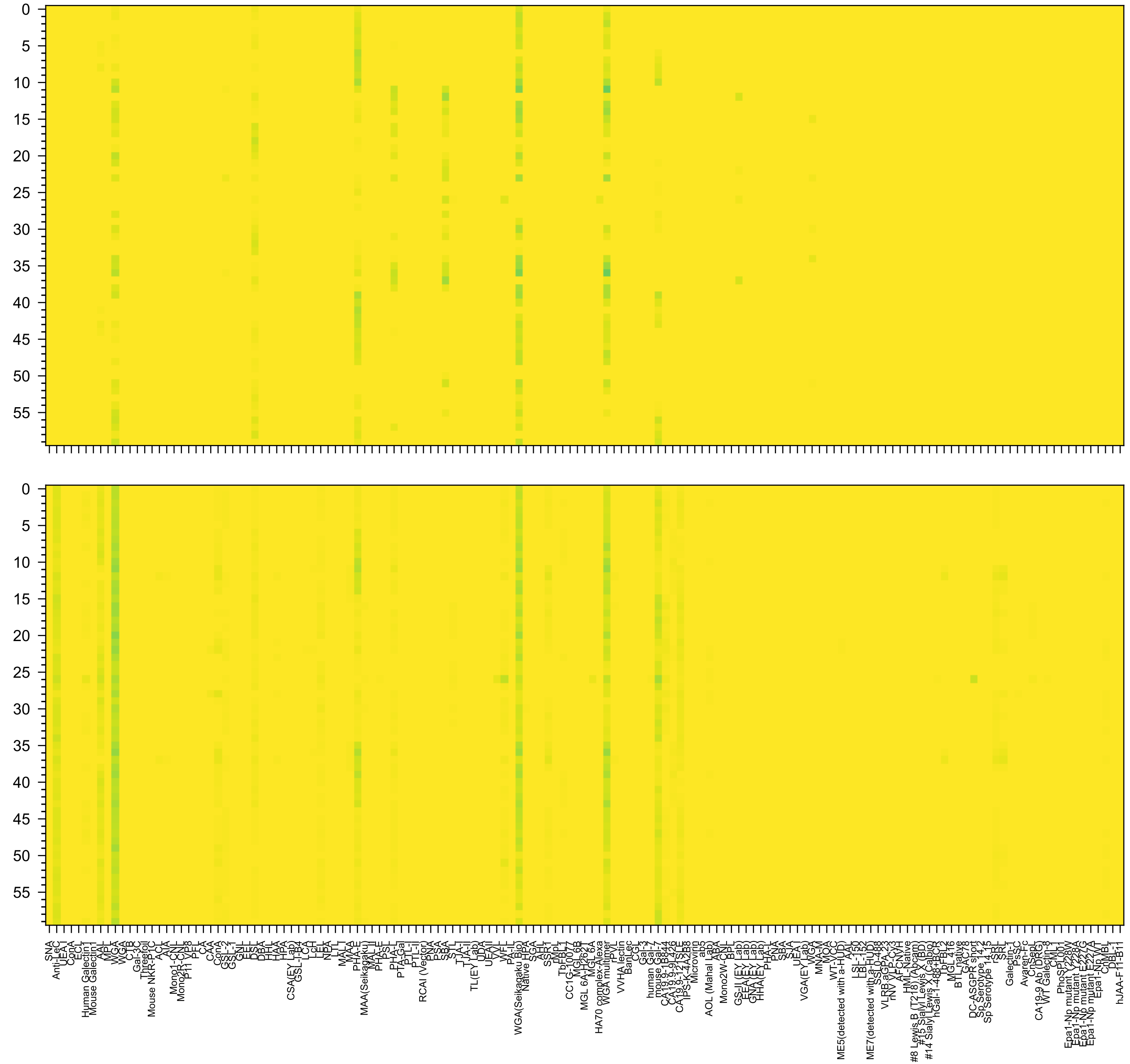

Neu5Ac(α2-3)Gal(β1-3)GlcNAc(β1-2)Man(α1-6)[GlcNAc(β1-4)][Neu5Ac(α2-3)Gal(β1-3)GlcNAc(β1-2)Man(α1-3)]Man(β1-4)GlcNAc(β1-4)GlcNAc(-Sp21

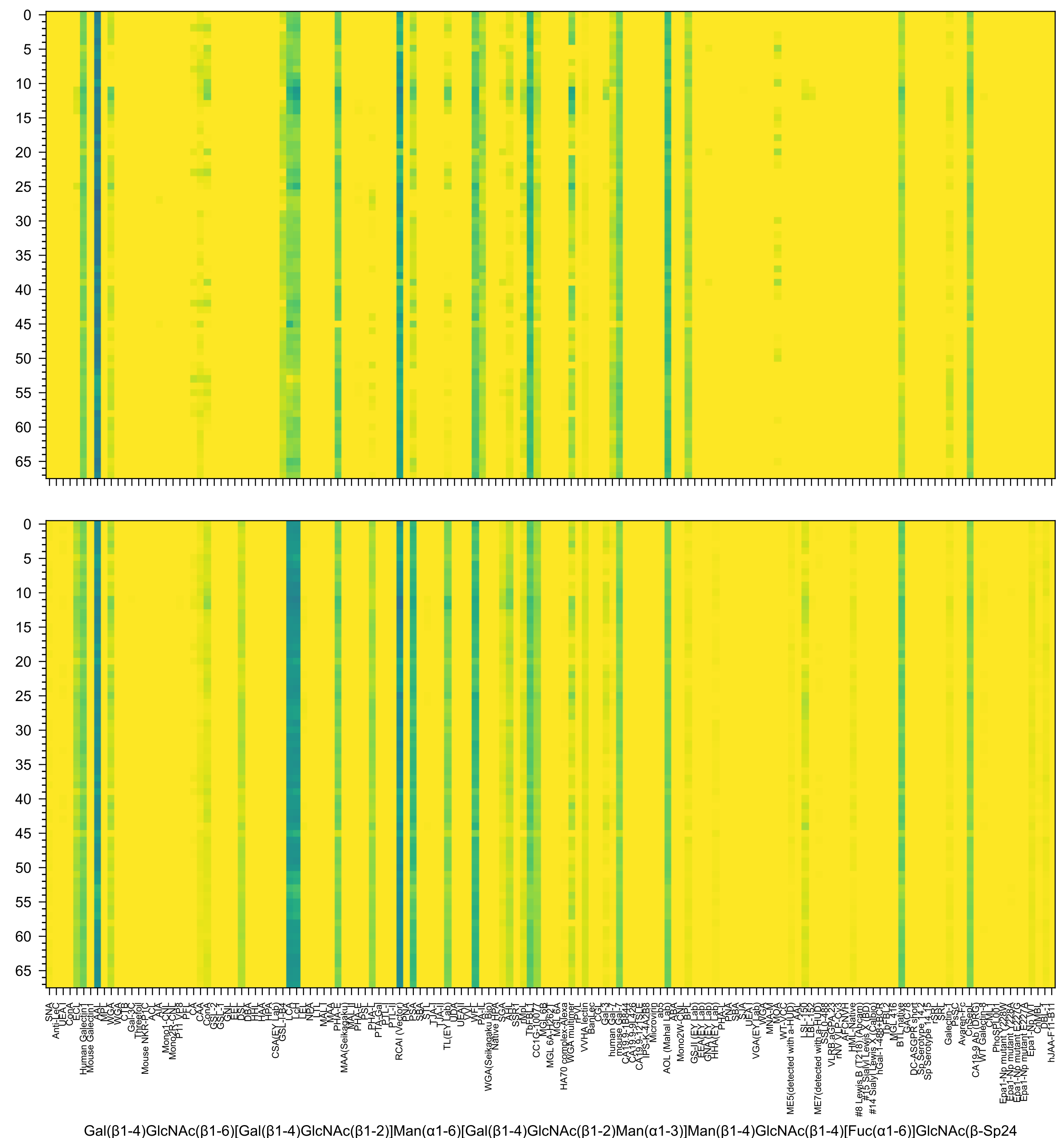

Gal(β1-4)GlcNAc(β1-6)[Gal(β1-4)GlcNAc(β1-2)]Man(α1-6)[Gal(β1-4)GlcNAc(β1-2)Man(α1-3)]Man(β1-4)GlcNAc(β1-4)[Fuc(α1-6)]GlcNAc(β-Sp24

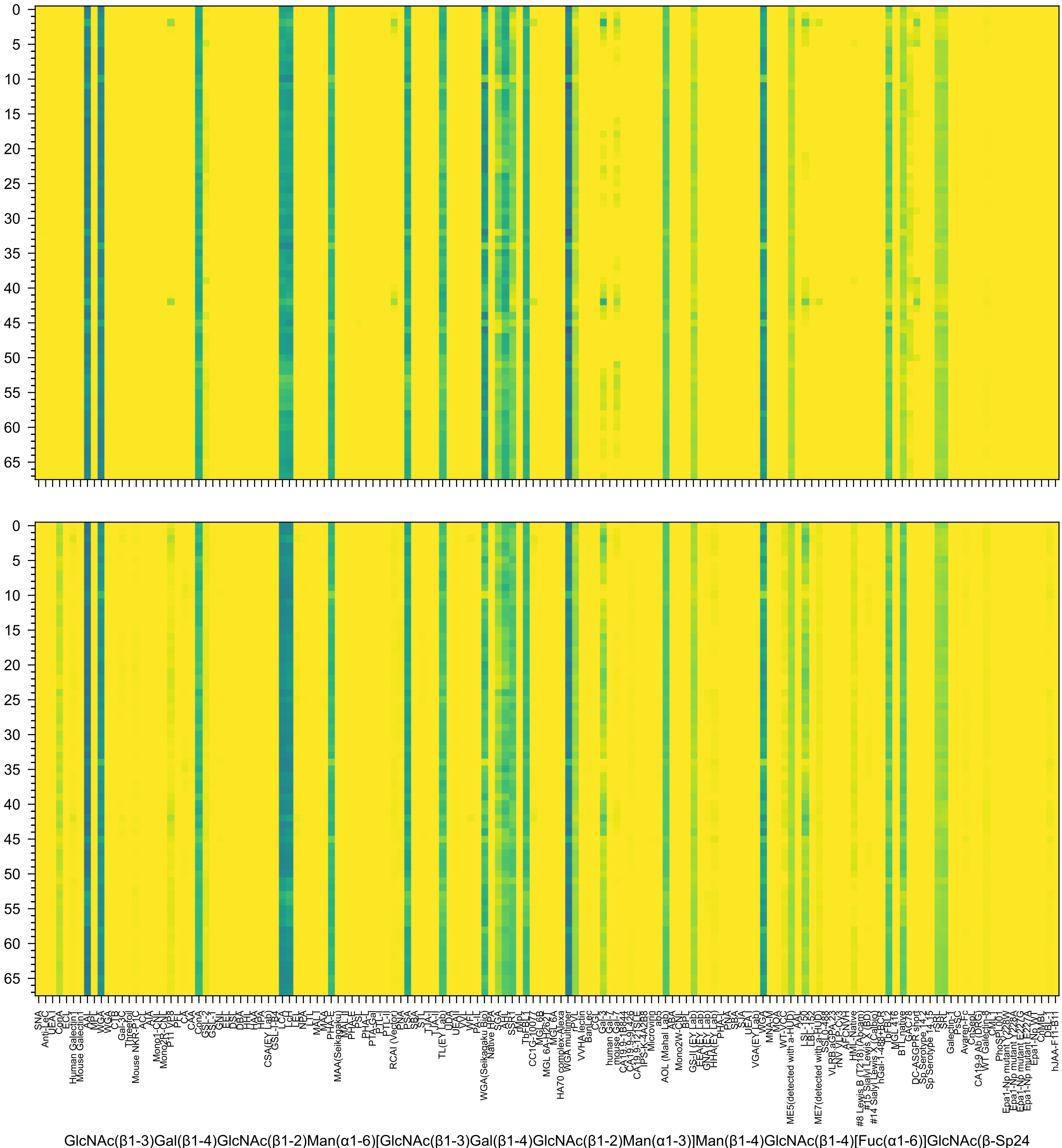
0
5
10
15
20
25
30
35
40
45
50
55
60
65
SNA
Anti-LeC
UEA I
ConA
ECL
Human Galectin1
Mouse Galectin1
AAL
MPL
WGA
WGA
CTB
Gal-3C
Threefoil
Mouse NKR-P1C
ACL
AIA
Mono1-CNL
Mono2R-CNL
P11 VP8
PFL
CA
CAA
ConA
GSL-2
GSL-1
GNL
EEL
DSL
DBA
HHL
HAA
HPA
CSA(EY Lab)
GSL-I-B4
LCA
LcH
LEL
NPA
LTL
MAL I
MAA
PHA-E
MAA(Seikagaku)
MAL II
PHA-E
PSL
PHA-L
PTA-Gal
PTL-I
PTL-II
RCAI (Vector)
PNA
PSA
SBA
STL
TJA-I
TJA-II
TL(EY Lab)
UDA
UEA II
VVL
WFL
PA-IL
WGA(Seikagaku Bio)
Native HPA
SGA
AHL
SSR1
rMpL
TbFBL1
CC1G-10077
MGL 6B
MGL 6A-H262T
MGL 6A
HA70 complex-Alexa
WGA multimer
rPVL
VVHA lectin
BanLec
CGL
Gal-3
human Gal-7
mouse Gal-7
CA19.9-1B844
CA19.9-9L426
CA19.9-121SLE
IPS-K-4A2B8
Microvirin
ab5
AOL (Mahal Lab)
ABA
Mono2W-CNL
BPL
GS-II(EY Lab)
EEA(EY Lab)
GNA(EY Lab)
HHA(EY Lab)
PHA-L
PNA
SBA
SJA
UEA I
VGA(EY Lab)
WGA
MNA-M
MOA
WT-VCC
ME5(detected with a-HUD)
AAL
LSL-150
LBL-152
ME7(detected with a-HUD)
SSL0-488
VLRB.aGPA.23
rNV VLP-Cy3
AFCNVH
HML-Native
#8 Lewis B (T218) (Abcam)
#15 Sialyl Lewis X (BD)
#14 Sialyl Lewis X (Calbio)
hGal-1-488+BCR
TbFBL2
MGL 416
BTL native
GAC78
DC-ASGPR short
Sp Serotype 14.2
Sp Serotype 14.15
rSRL
SRL
Galectin-1
PsSC
Avaren-Fc
CnSepL
CA19-9 Ab (DRG)
WT Galectin-8
CML1
PhoSL001
Epa1-Np mutant Y228W
Epa1-Np mutant Y228A
Epa1-Np mutant E227G
Epa1-Np mutant E227A
Epa1-Np WT
CnMBL
DBL-1
hJAA-F11-B11
GlcNAc(β1-3)Gal(β1-4)GlcNAc(β1-2)Man(α1-6)[GlcNAc(β1-3)Gal(β1-4)GlcNAc(β1-2)Man(α1-3)]Man(β1-4)GlcNAc(β1-4)[Fuc(α1-6)]GlcNAc(β-Sp24

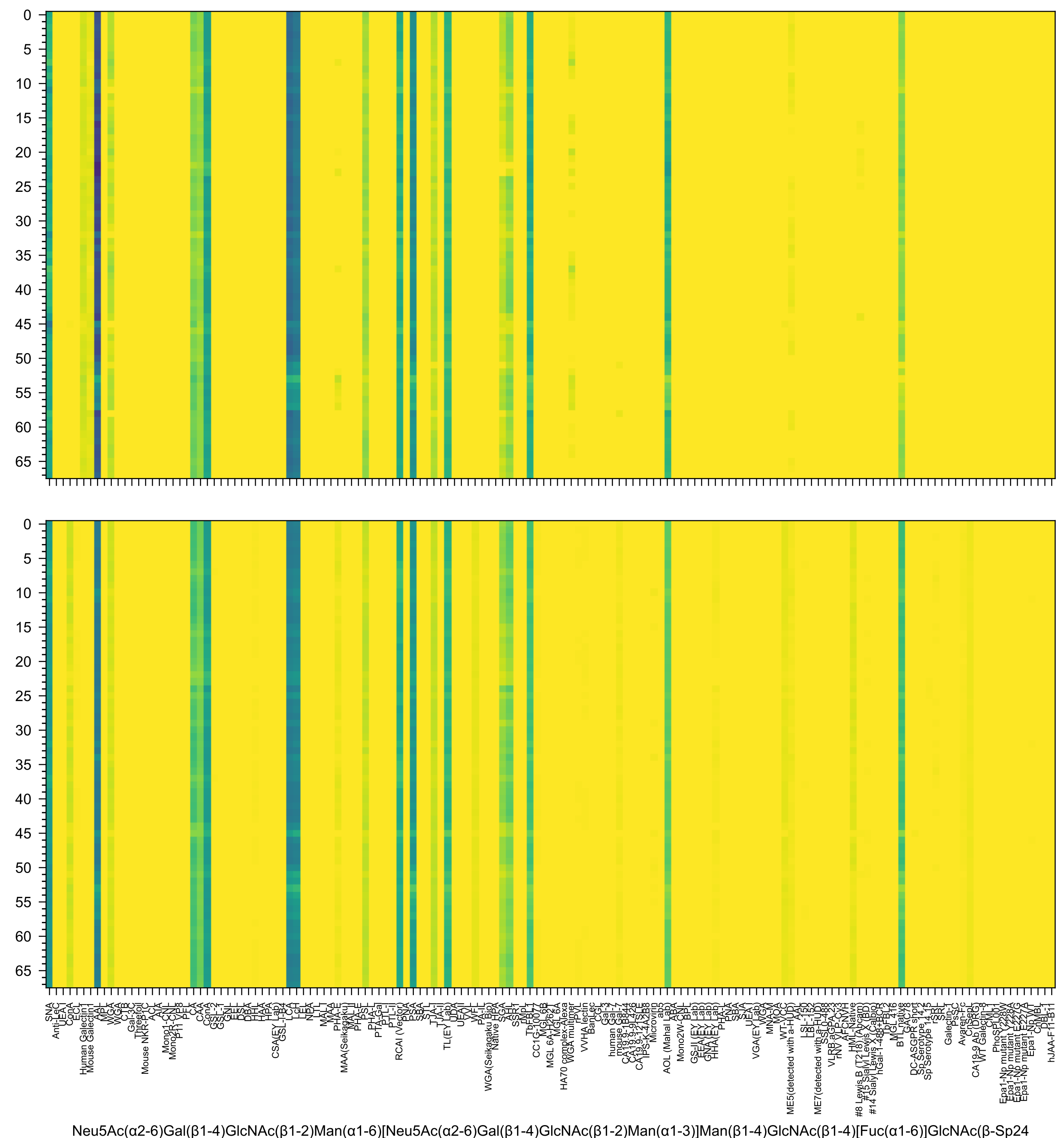
0
5
10
15
20
25
30
35
40
45
50
55
60
65
SNA
Anti-LeC
ECL
ConA
Human Galectin1
Mouse Galectin1
ABL
WGA
WGA
CTB
Gal-3C
Threefoil
Mouse NKR-P1C
ACL
AIA
Mono1-CNL
Mono2R-CNL
P11 VP8
PET
CA
CA
ConA
GSL-2
GSL-1
GNL
EEL
DSL
DBA
DHL
HAA
HPA
CSA(EY Lab)
GSL-I-B4
LCA
LcH
LEL
NPA
LTL
MAL I
MAA
PHA-E
MAA(Seikagaku)
MAL I
PHA-E
PSL
PHA-L
PTA-Gal
PTL-I
PTL-II
RCAI (Vector)
PNA
PSA
SBA
STL
TJA-I
TJA-II
TL(EY Lab)
UDA
UEA I
VVL
WFL
PA-IL
PA-IIL
WGA(Seikagaku Bio)
Native HPA
SGA
AHL
SSR1
rMPL
Tb-10L1
CC1G-10317
MGL 6B
MGL 6A-H262
MGL 6A
HA70 complex-Alexa
WGA multimer
rPVL
VVHA lectin
BanLec
CGL
Gal-3
human Gal-7
mouse Gal-7
Microvirin
ab5
AOL (Mahal Lab)
ABA
Mono2W-CNL
BPL
GS-II(EY Lab)
EEA(EY Lab)
GNA(EY Lab)
HHA(EY Lab)
PHA-L
PNA
SBA
SJA
UEA I
VGA(EY Lab)
WGA
MNA-M
MOA
WT-VCC
ME5(detected with a-HUD)
AAL
LSL-150
LBL-152
ME7(detected with a-HUD)
HML-Native
#8 Lewis B (T218) (Abcam)
#15 Sialyl Lewis X (BD)
#14 Sialyl Lewis A (Calbio)
MGL 416
BTL native
GAC78
DC-ASGPR short
Sp Serotype 14 2
Sp Serotype 14 15
rSRL
SRL
Galectin-1
PsSC
Avaren-Fc
CA19-9 Ab (DRG)
WT Galectin-8
CML1
PhoSPL001
Epa1-Np mutant Y228W
Epa1-Np mutant E227A
Epa1-Np mutant E227G
Epa1-Np WT
CnMBL
DBL-1
hJAA-F11-B11
Neu5Ac(α2-6)Gal(β1-4)GlcNAc(β1-2)Man(α1-6)[Neu5Ac(α2-6)Gal(β1-4)GlcNAc(β1-2)Man(α1-3)]Man(β1-4)GlcNAc(β1-4)[Fuc(α1-6)]GlcNAc(β-Sp24

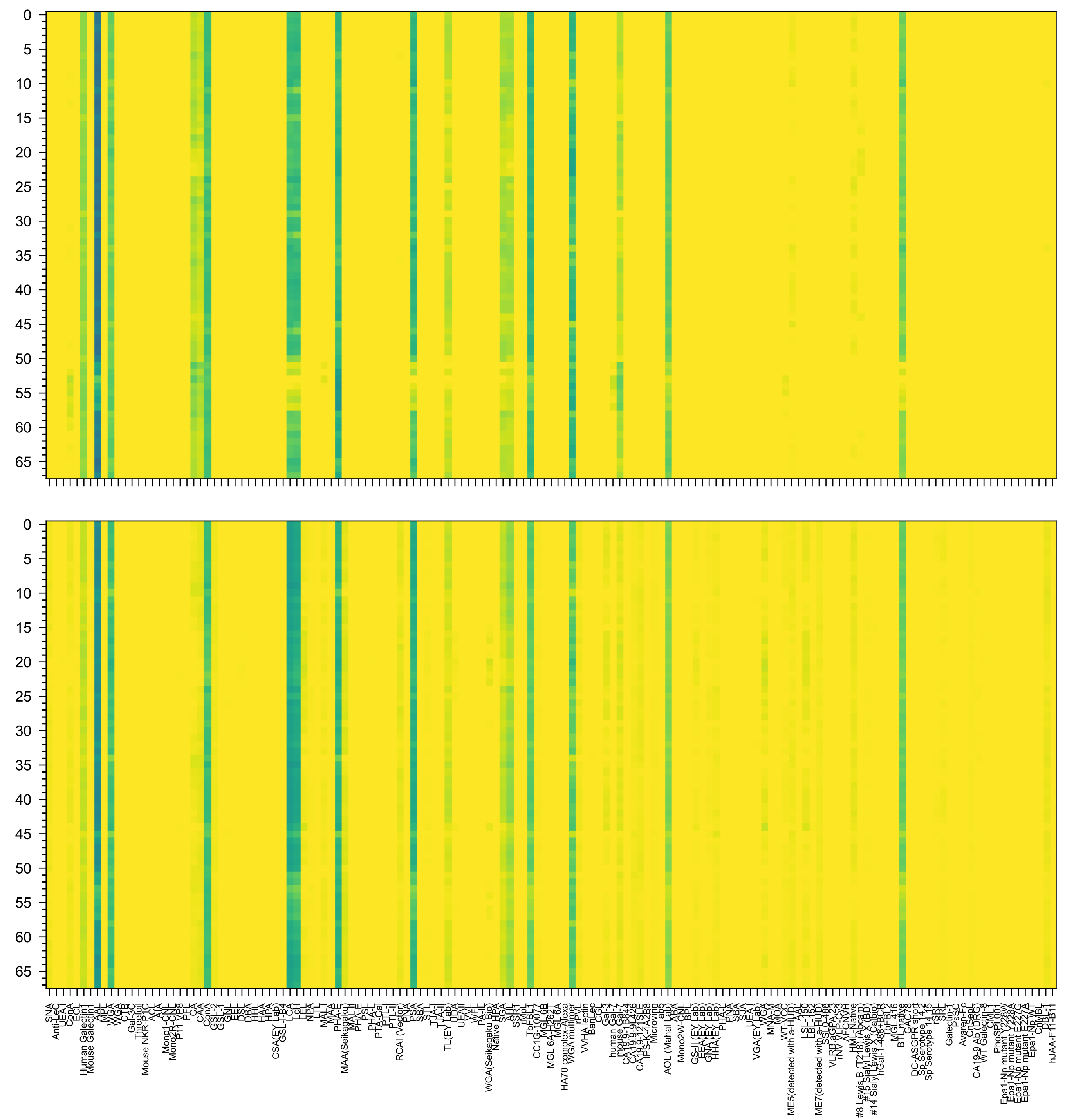

Neu5Ac(α2-3)Gal(β1-4)GlcNAc(β1-2)Man(α1-6)[Neu5Ac(α2-3)Gal(β1-4)GlcNAc(β1-2)Man(α1-3)]Man(β1-4)GlcNAc(β1-4)[Fuc(α1-6)]GlcNAc(β-Sp24

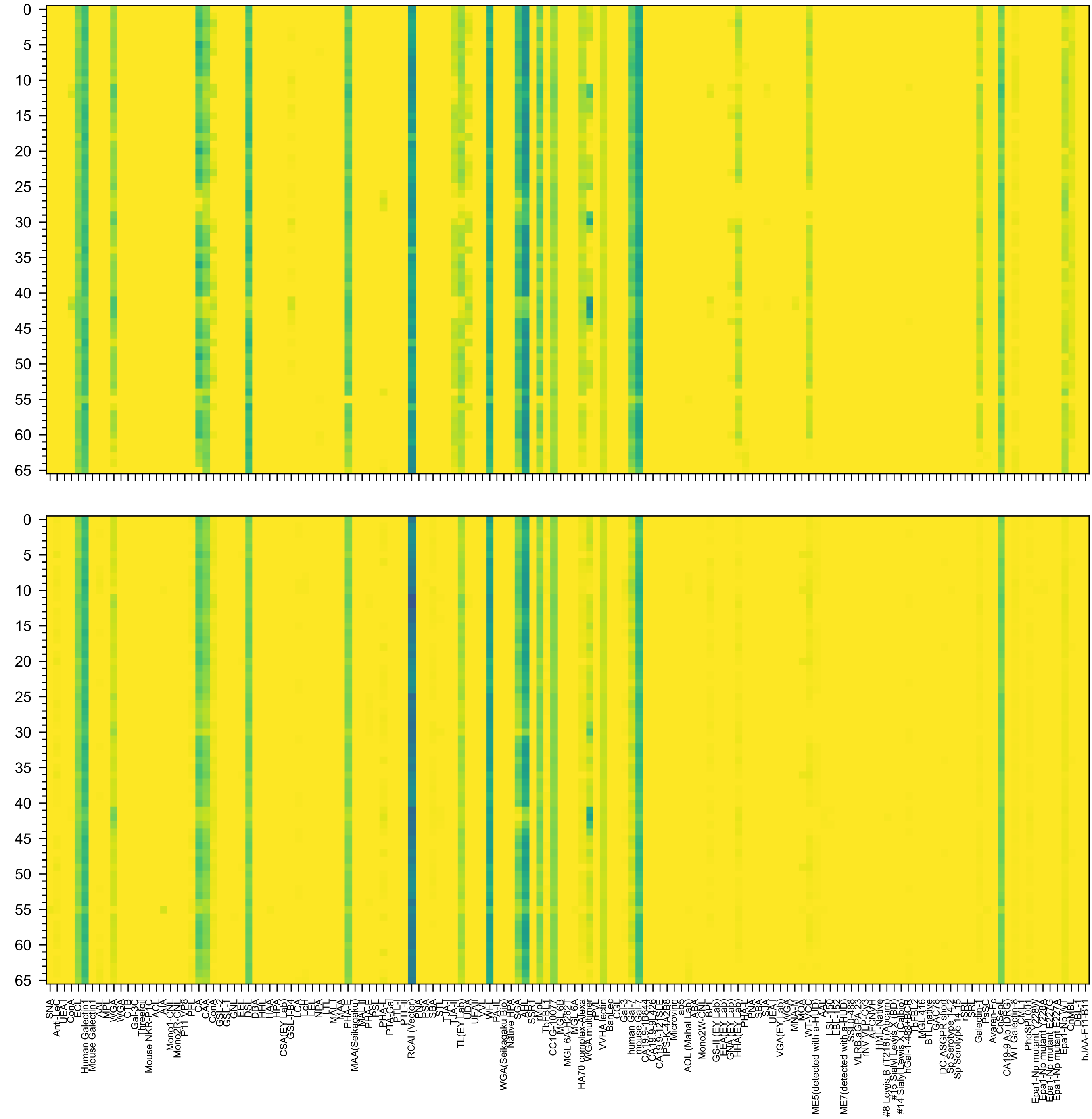

Gal(β1-4)GlcNAc(β1-6)[Gal(β1-4)GlcNAc(β1-2)]Man(α1-6)[Gal(β1-4)GlcNAc(β1-4)[Gal(β1-4)GlcNAc(β1-2)]Man(α1-3)]Man(β1-4)GlcNAc(β1-4)GlcNAc(β-Sp21

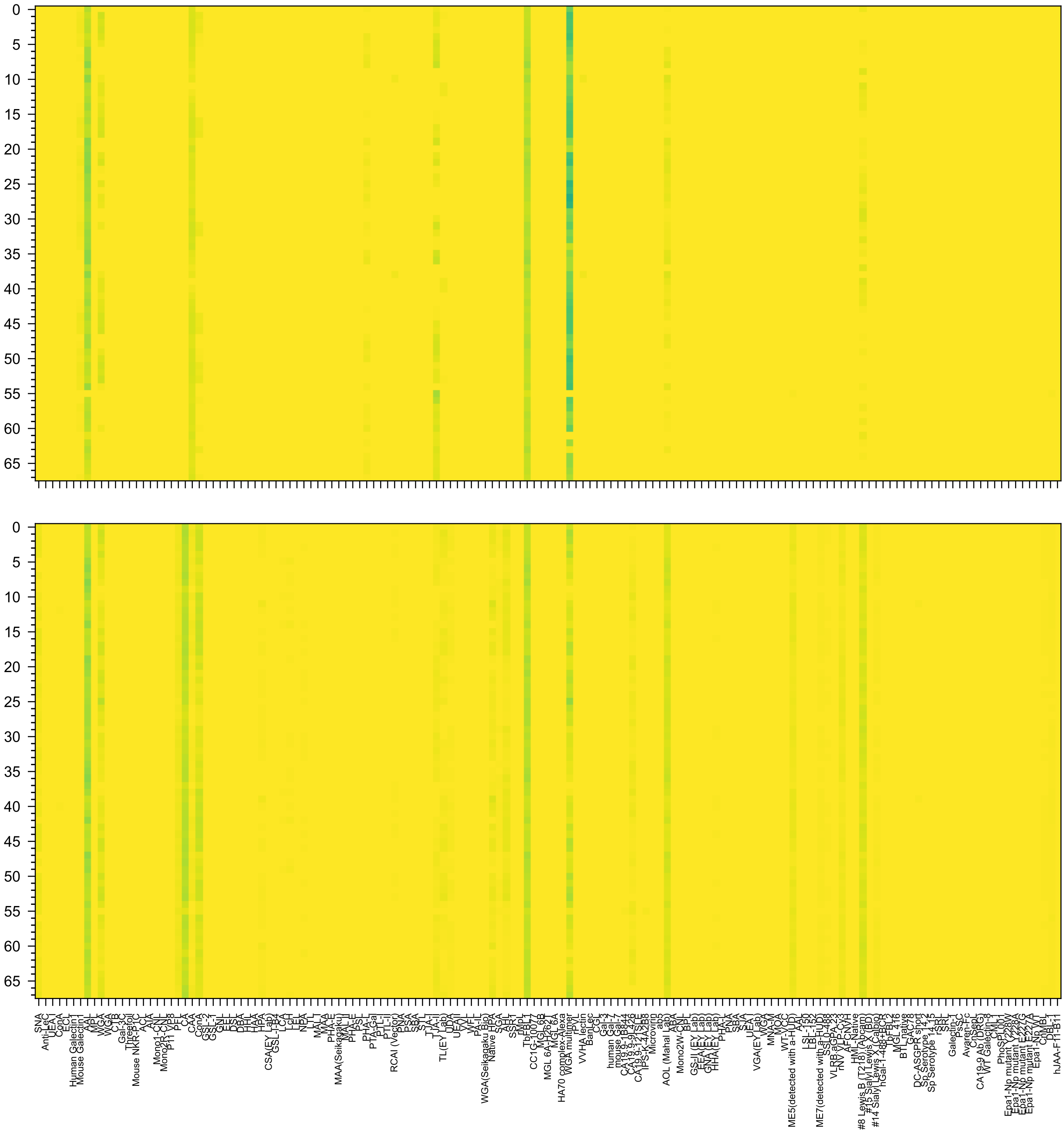

Fuc(α1-4)[Fuc(α1-2)Gal(β1-3)]GlcNAc(β1-2)Man(α1-6)[Fuc(α1-4)[Fuc(α1-2)Gal(β1-3)]GlcNAc(β1-2)Man(α1-3)]Man(β1-4)GlcNAc(β1-4)GlcNAc(β-Sp19

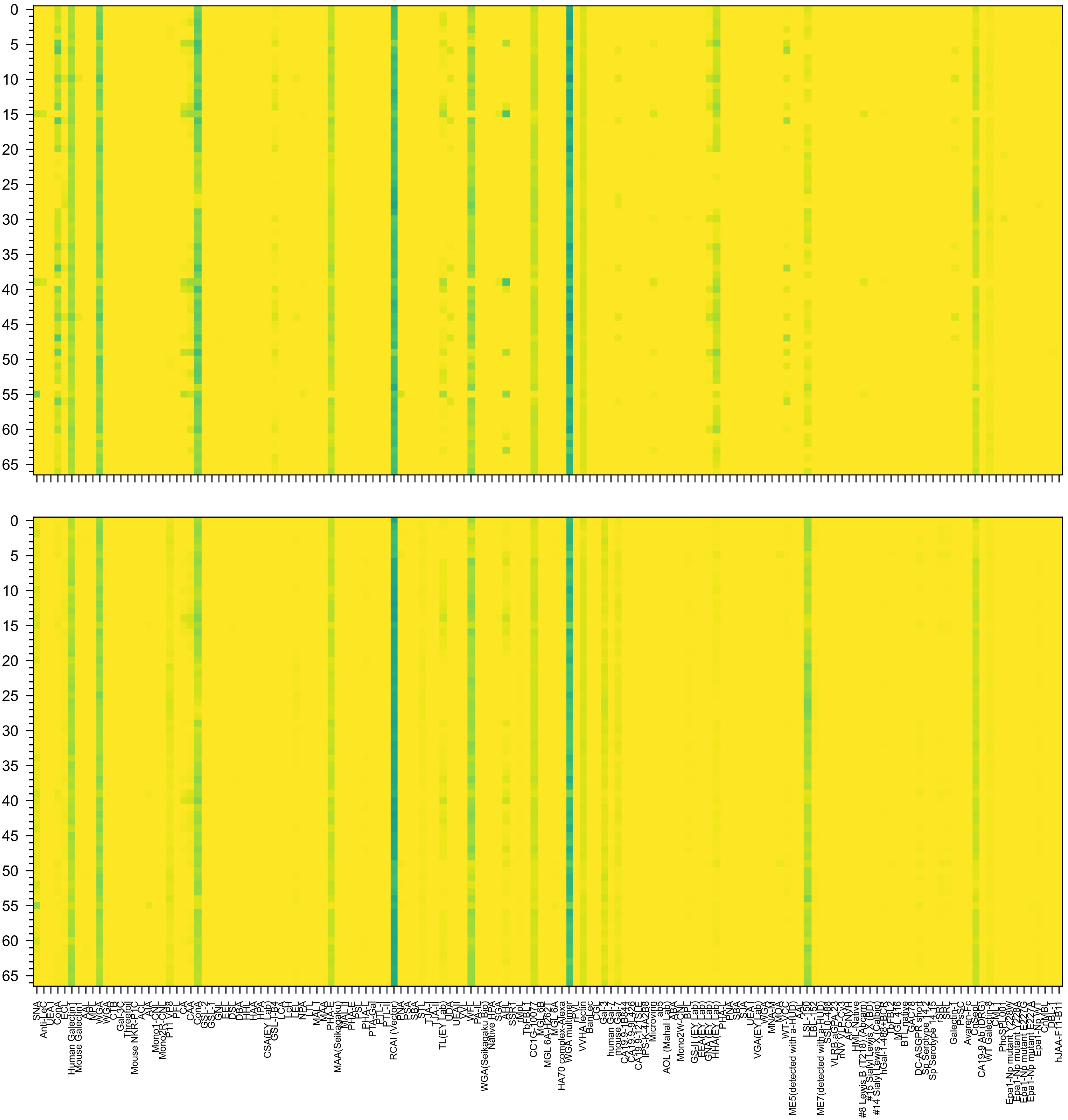

Gal(β1-4)GlcNAc(β1-3)Gal(β1-4)GlcNAc(β1-2)Man(α1-6)[Gal(β1-4)GlcNAc(β1-3)Gal(β1-4)GlcNAc(β1-2)Man(α1-3)]Man(β1-4)GlcNAc(β1-4)GlcNAc(β-Sp12

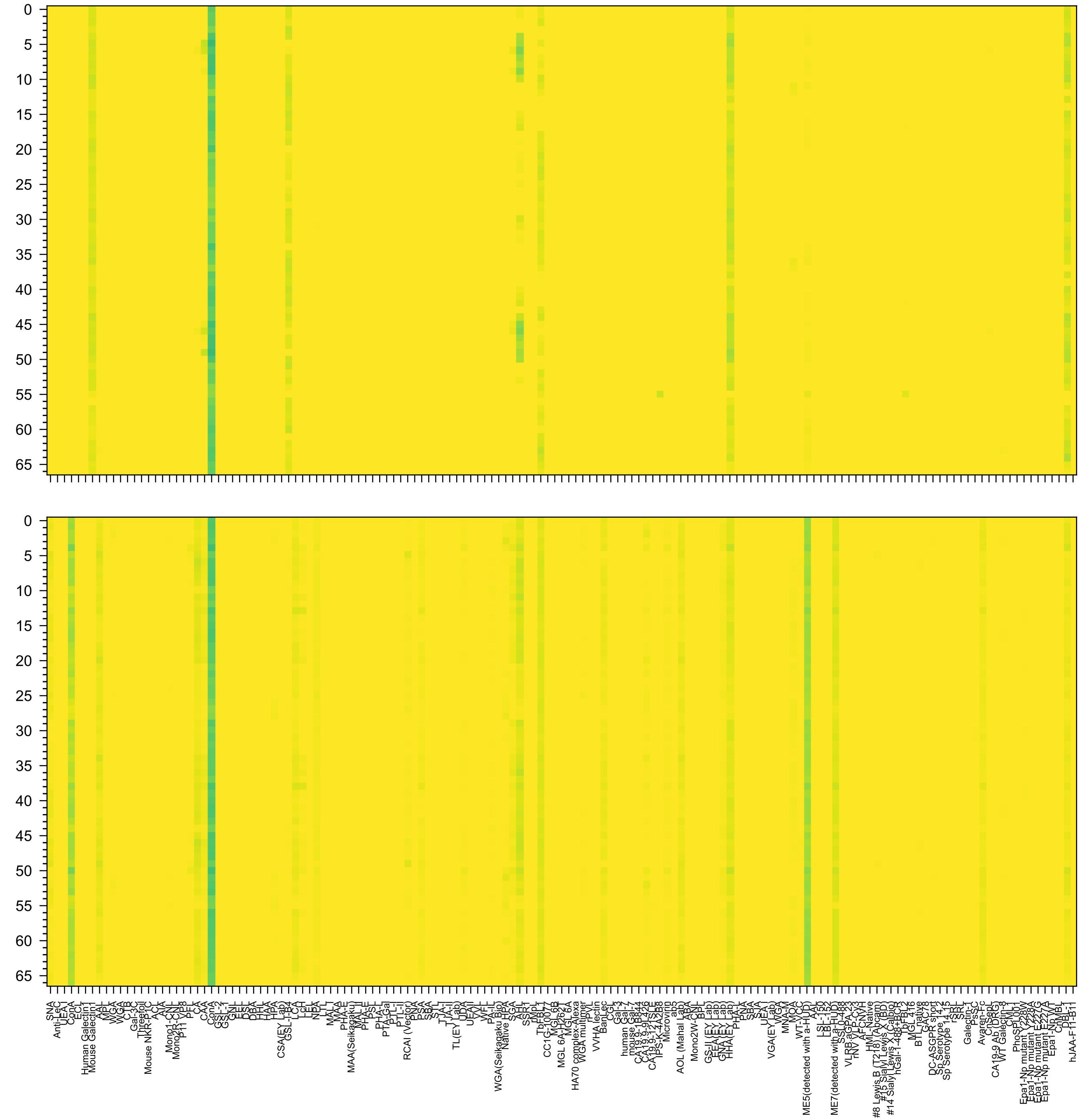

Gal(α1-3)Gal(β1-3)[Fuc(α1-4)]GlcNAc(β1-2)Man(α1-6)[Gal(α1-3)Gal(β1-3)[Fuc(α1-4)]GlcNAc(β1-2)Man(α1-3)]Man(β1-4)GlcNAc(β1-4)GlcNAc(-Sp19

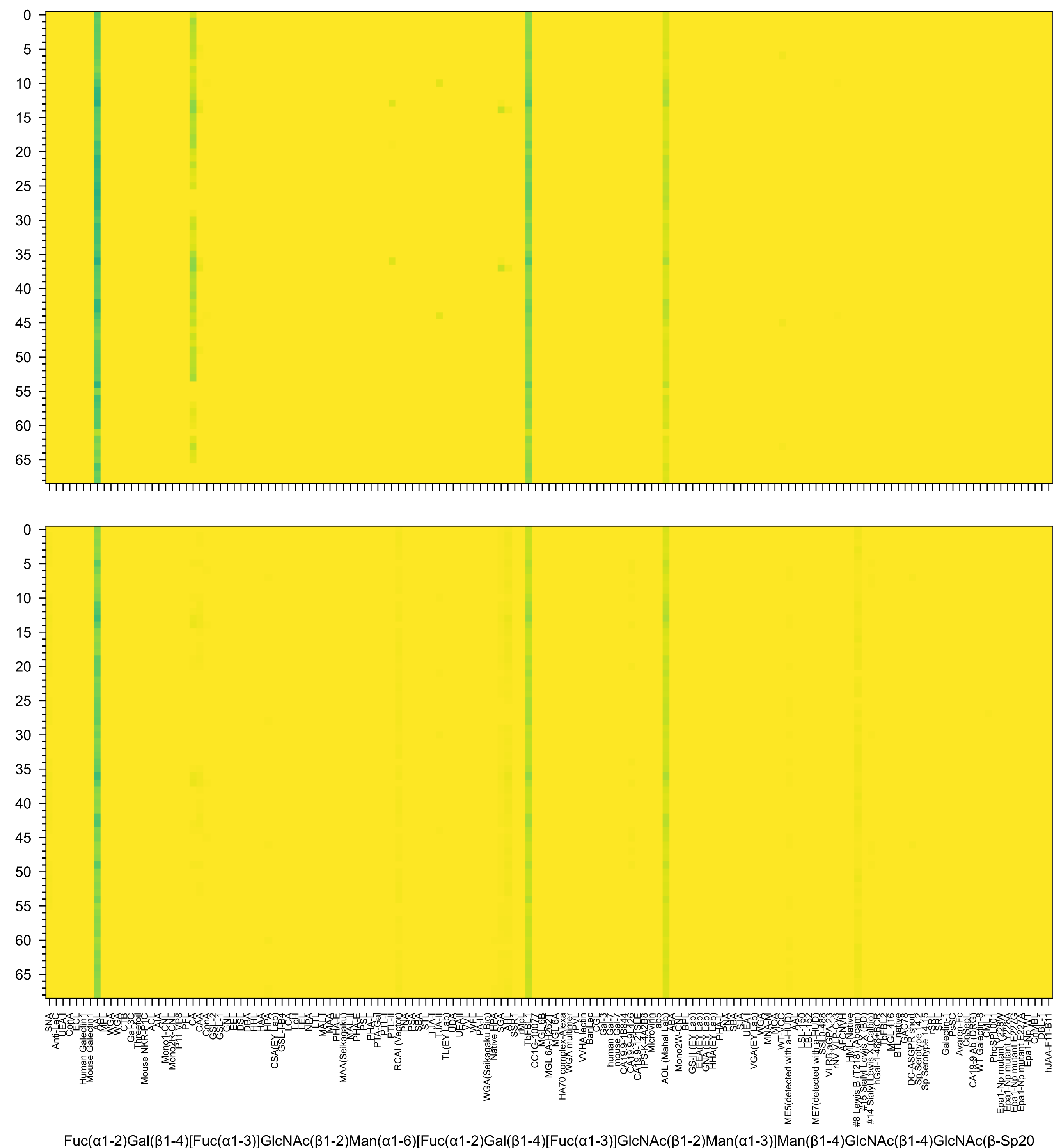
Fuc(α1-2)Gal(β1-4)[Fuc(α1-3)]GlcNAc(β1-2)Man(α1-6)[Fuc(α1-2)Gal(β1-4)[Fuc(α1-3)]GlcNAc(β1-2)Man(α1-3)]Man(β1-4)GlcNAc(β1-4)GlcNAc(β-Sp20

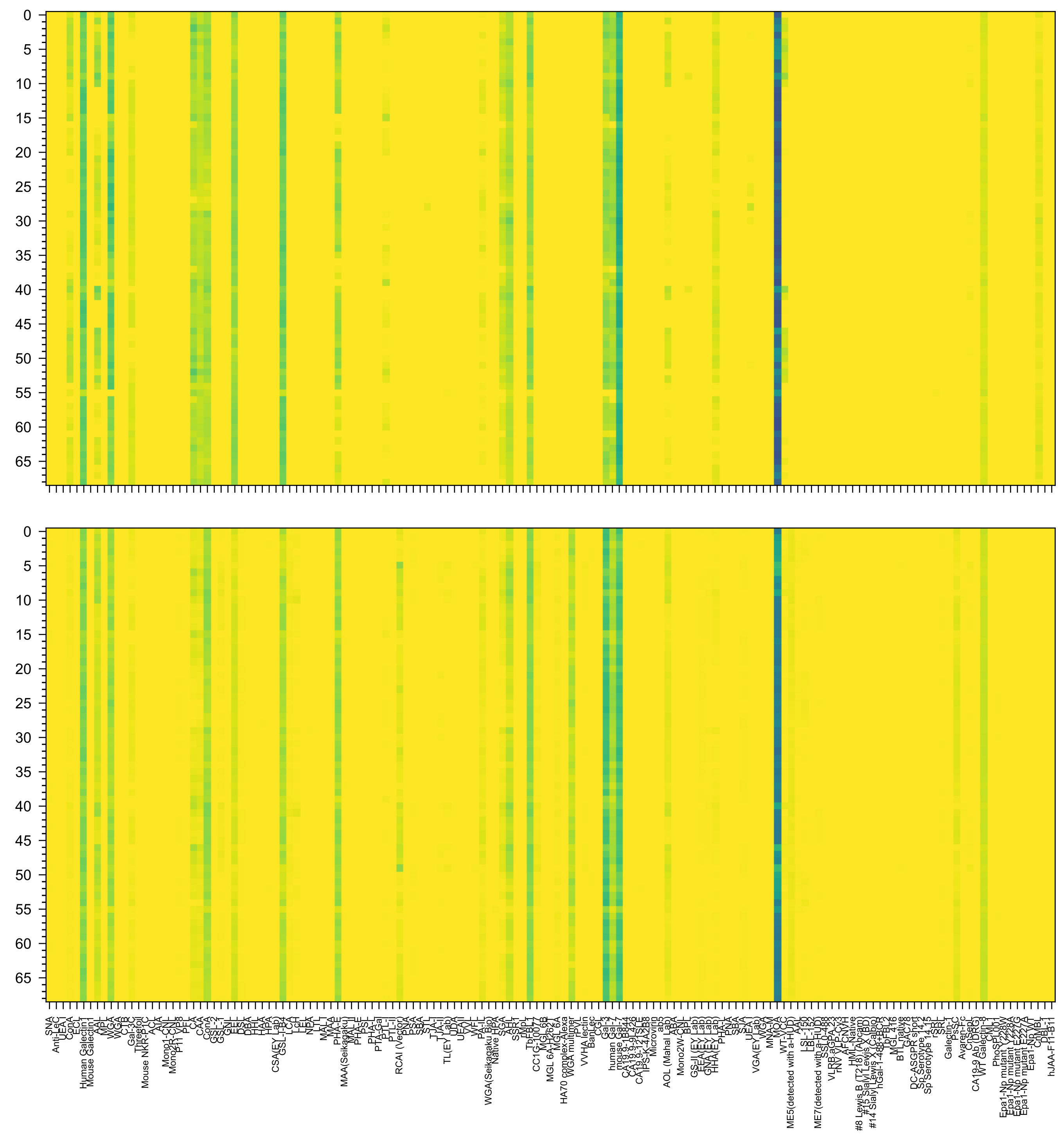

Gal(α1-3)[Fuc(α1-2)]Gal(β1-4)GlcNAc(β1-2)Man(α1-6)[Gal(α1-3)[Fuc(α1-2)]Gal(β1-4)GlcNAc(β1-2)Man(α1-3)]Man(β1-4)GlcNAc(β1-4)GlcNAc(β-Sp20

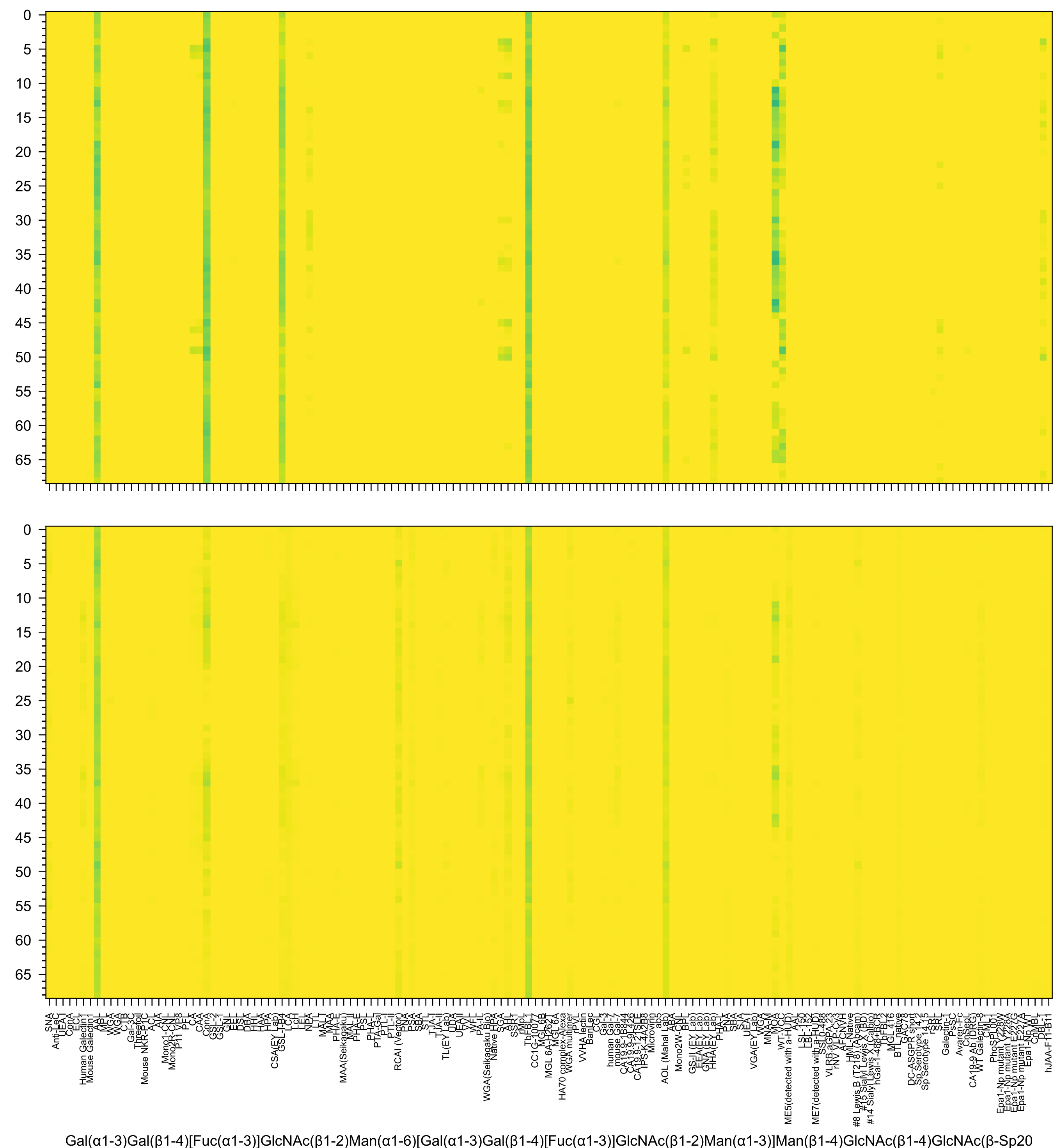
Gal(α1-3)Gal(β1-4)[Fuc(α1-3)]GlcNAc(β1-2)Man(α1-6)[Gal(α1-3)Gal(β1-4)[Fuc(α1-3)]GlcNAc(β1-2)Man(α1-3)]Man(β1-4)GlcNAc(β1-4)GlcNAc(β-Sp20

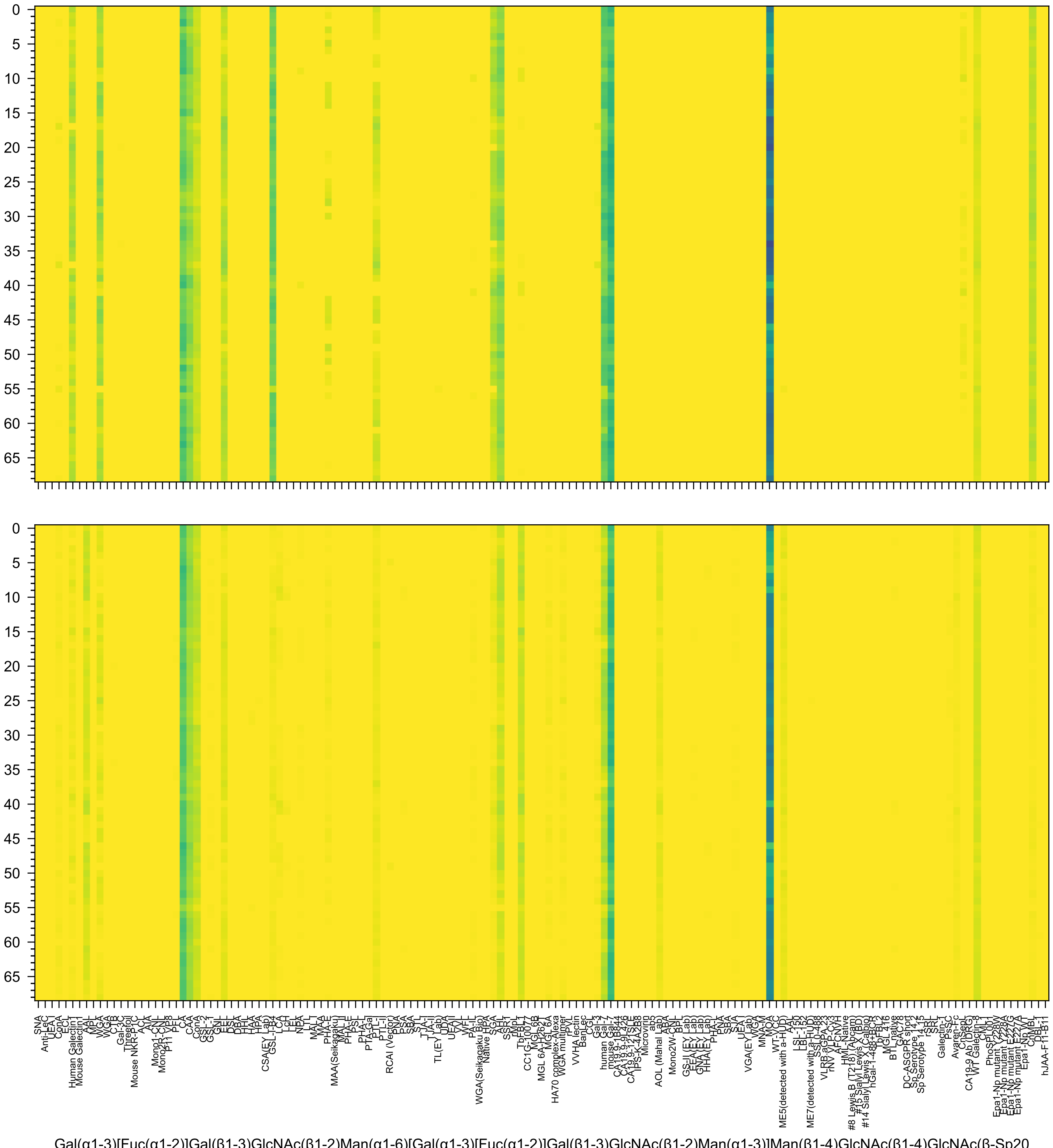
Gal(α1-3)[Fuc(α1-2)]Gal(β1-3)GlcNAc(β1-2)Man(α1-6)[Gal(α1-3)[Fuc(α1-2)]Gal(β1-3)GlcNAc(β1-2)Man(α1-3)]Man(β1-4)GlcNAc(β1-4)GlcNAc(β-Sp20

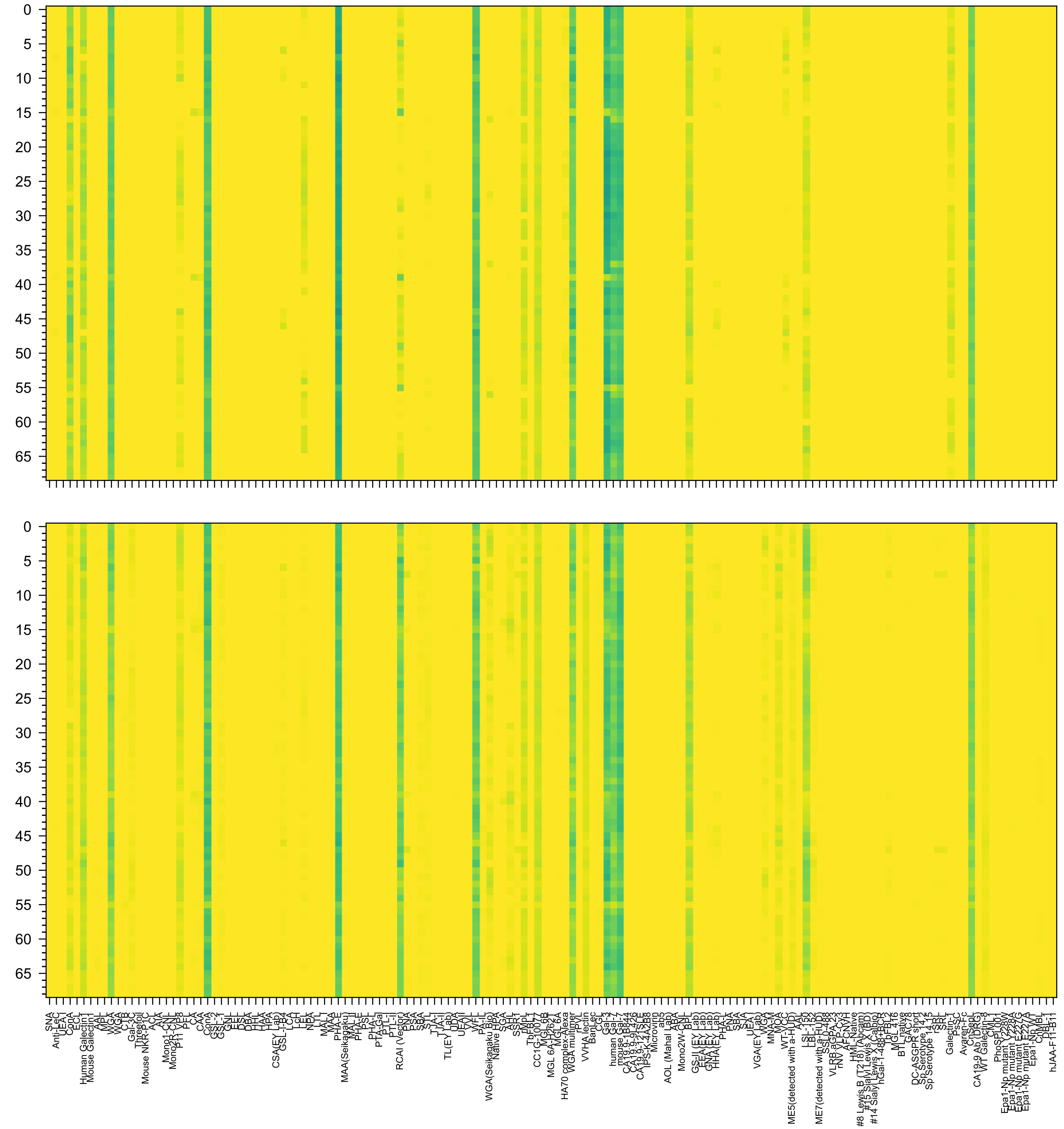

Gal(β1-3)GlcNAc(β1-3)Gal(β1-4)GlcNAc(β1-2)Man(α1-6)[Gal(β1-3)GlcNAc(β1-3)Gal(β1-4)GlcNAc(β1-2)Man(α1-3)]Man(β1-4)GlcNAc(β1-4)GlcNAc(-Sp25

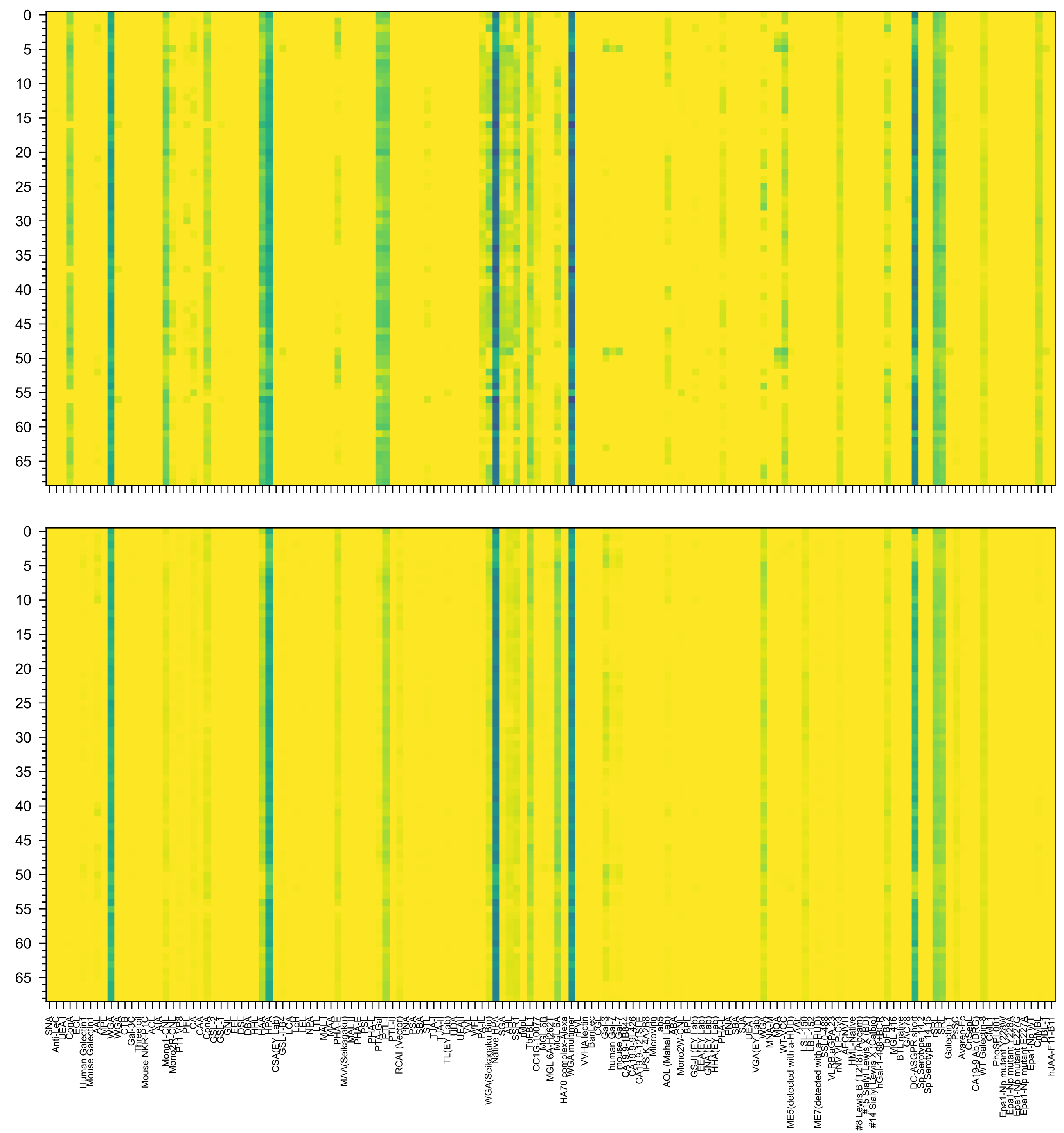

GalNAc(α1-3)[Fuc(α1-2)]Gal(β1-4)GlcNAc(β1-2)Man(α1-6)[GalNAc(α1-3)[Fuc(α1-2)]Gal(β1-4)GlcNAc(β1-2)Man(α1-3)]Man(β1-4)GlcNAc(β1-4)GlcNAc(β-Sp20

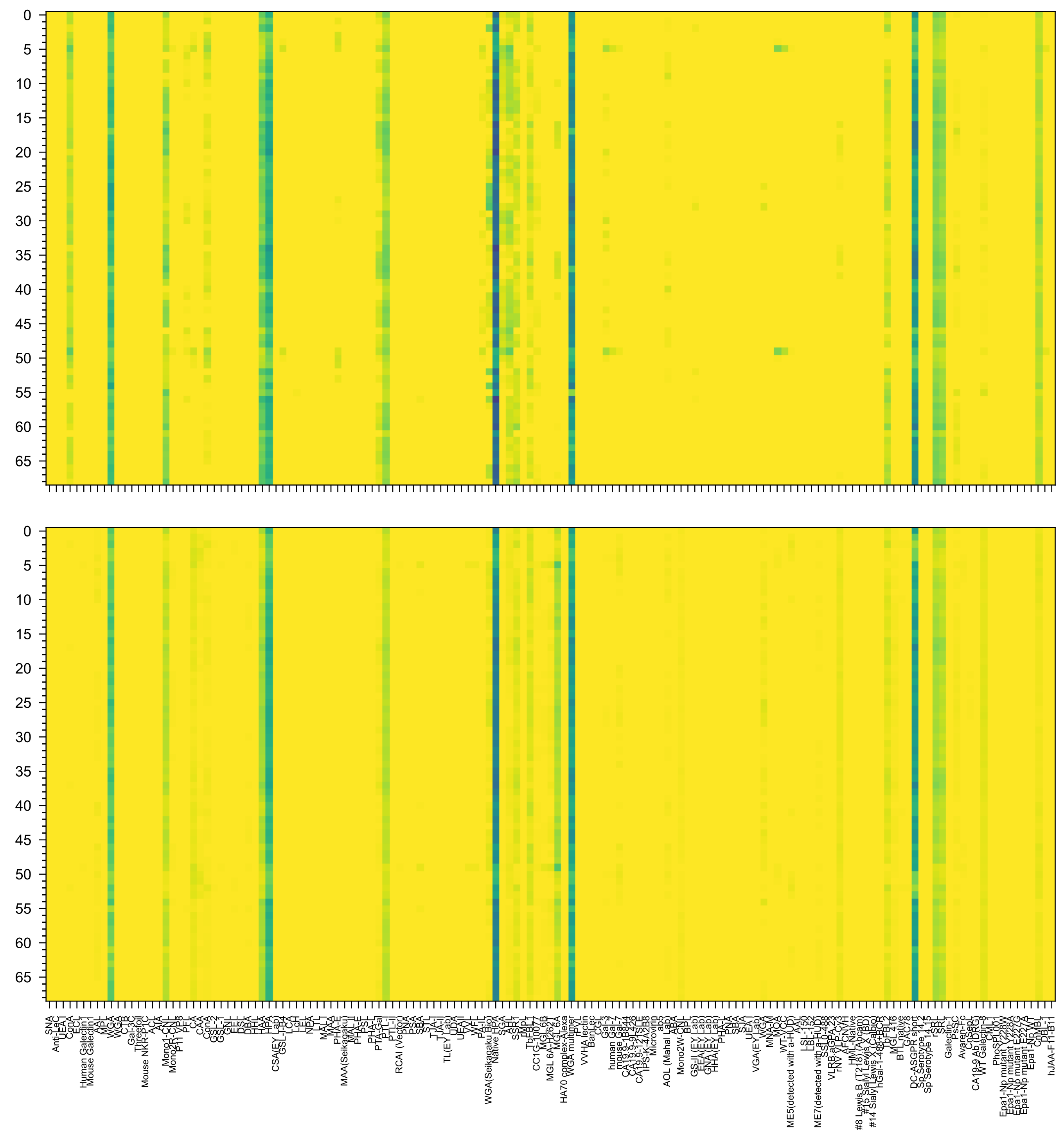

GalNAc(α1-3)[Fuc(α1-2)]Gal(β1-3)GlcNAc(β1-2)Man(α1-6)[GalNAc(α1-3)[Fuc(α1-2)]Gal(β1-3)GlcNAc(β1-2)Man(α1-3)]Man(β1-4)GlcNAc(β1-4)GlcNAc(β-Sp20

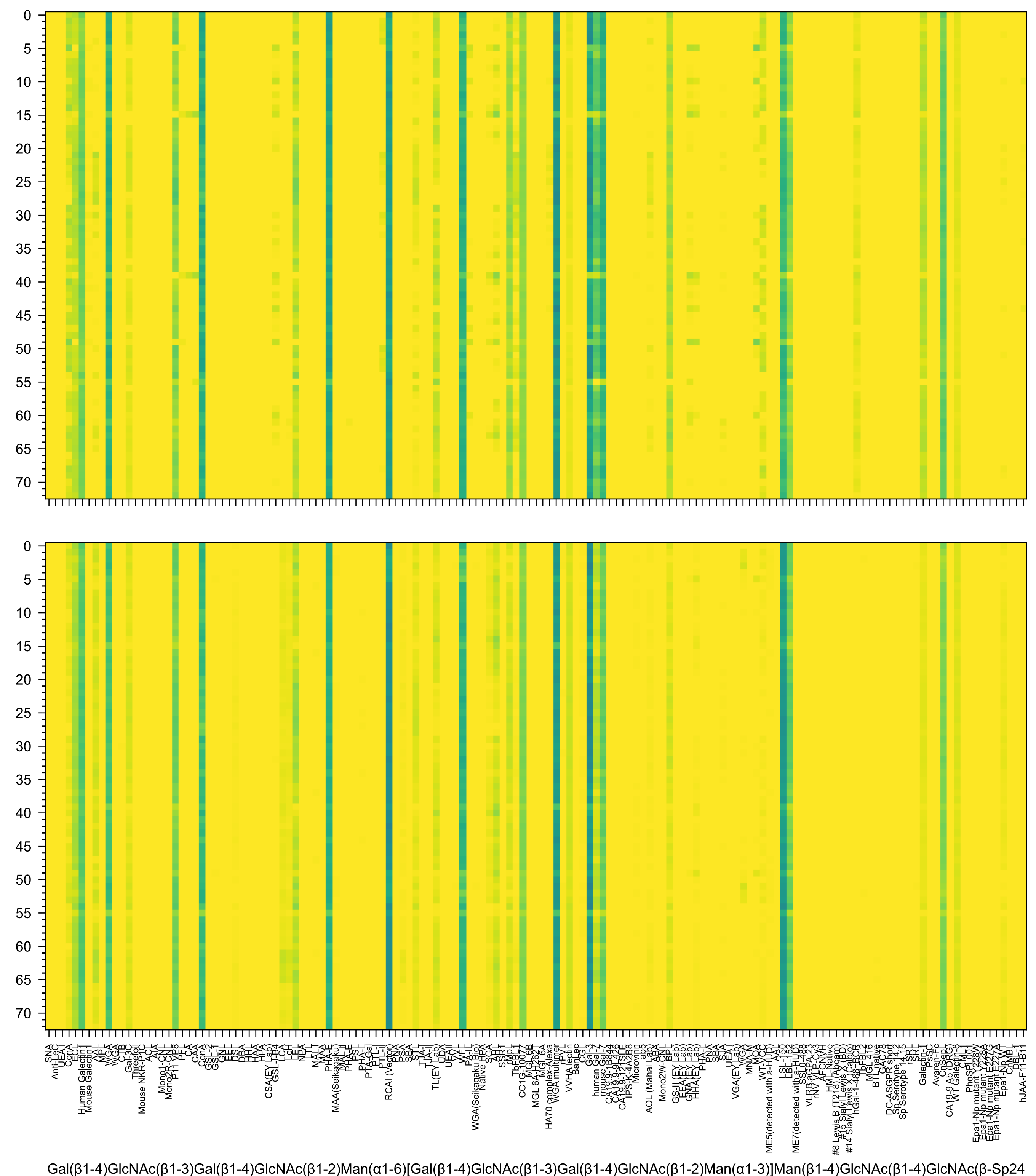
Gal(β1-4)GlcNAc(β1-3)Gal(β1-4)GlcNAc(β1-2)Man(α1-6)[Gal(β1-4)GlcNAc(β1-3)Gal(β1-4)GlcNAc(β1-2)Man(α1-3)]Man(β1-4)GlcNAc(β1-4)GlcNAc(β-Sp24

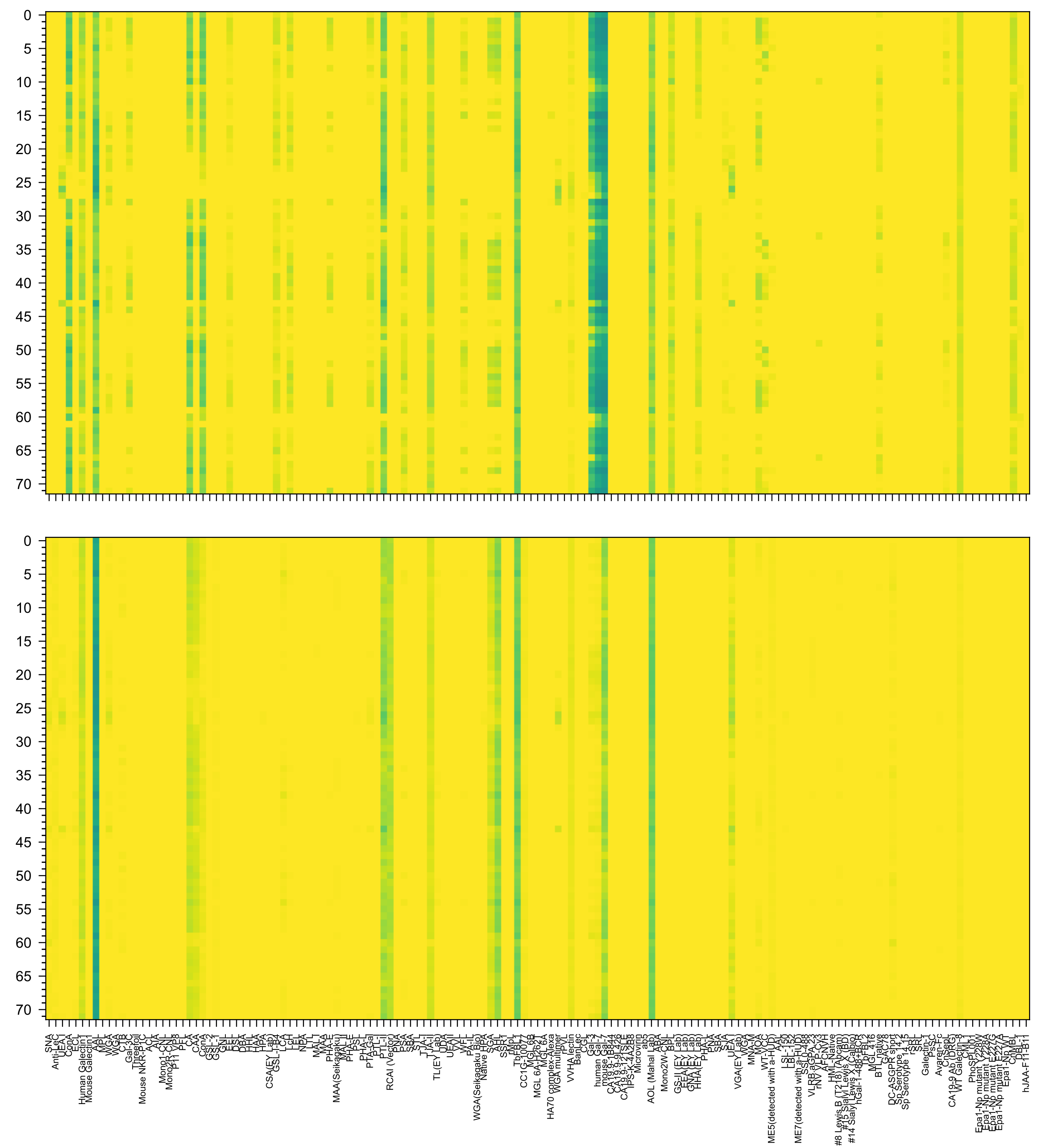

Fuc(α1-2)Gal(β1-4)GlcNAc(β1-2)Man(α1-6)[Fuc(α1-2)Gal(β1-4)GlcNAc(β1-2)[Fuc(α1-2)Gal(β1-4)GlcNAc(β1-4)]Man(α1-3)]Man(β1-4)GlcNAc(β1-4)GlcNAc(β-Sp1

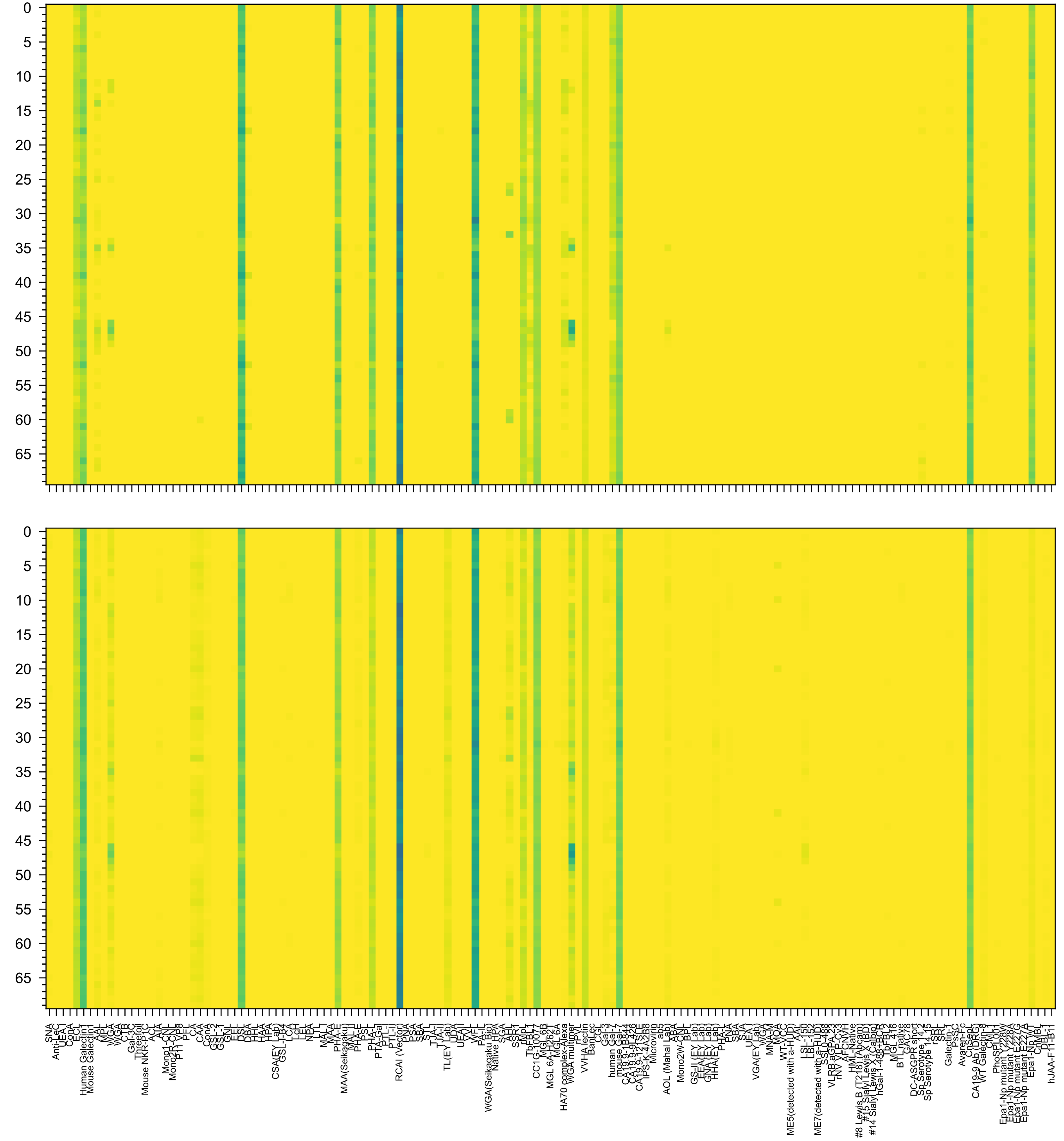

Gal(β1-4)GlcNAc(β1-6)[Gal(β1-4)GlcNAc(β1-2)]Man(α1-6)[GlcNAc(β1-4)][Gal(β1-4)GlcNAc(β1-4)[Gal(β1-4)GlcNAc(β1-2)]Man(α1-3)]Man(β1-4)GlcNAc(β1-4)GlcNAc(

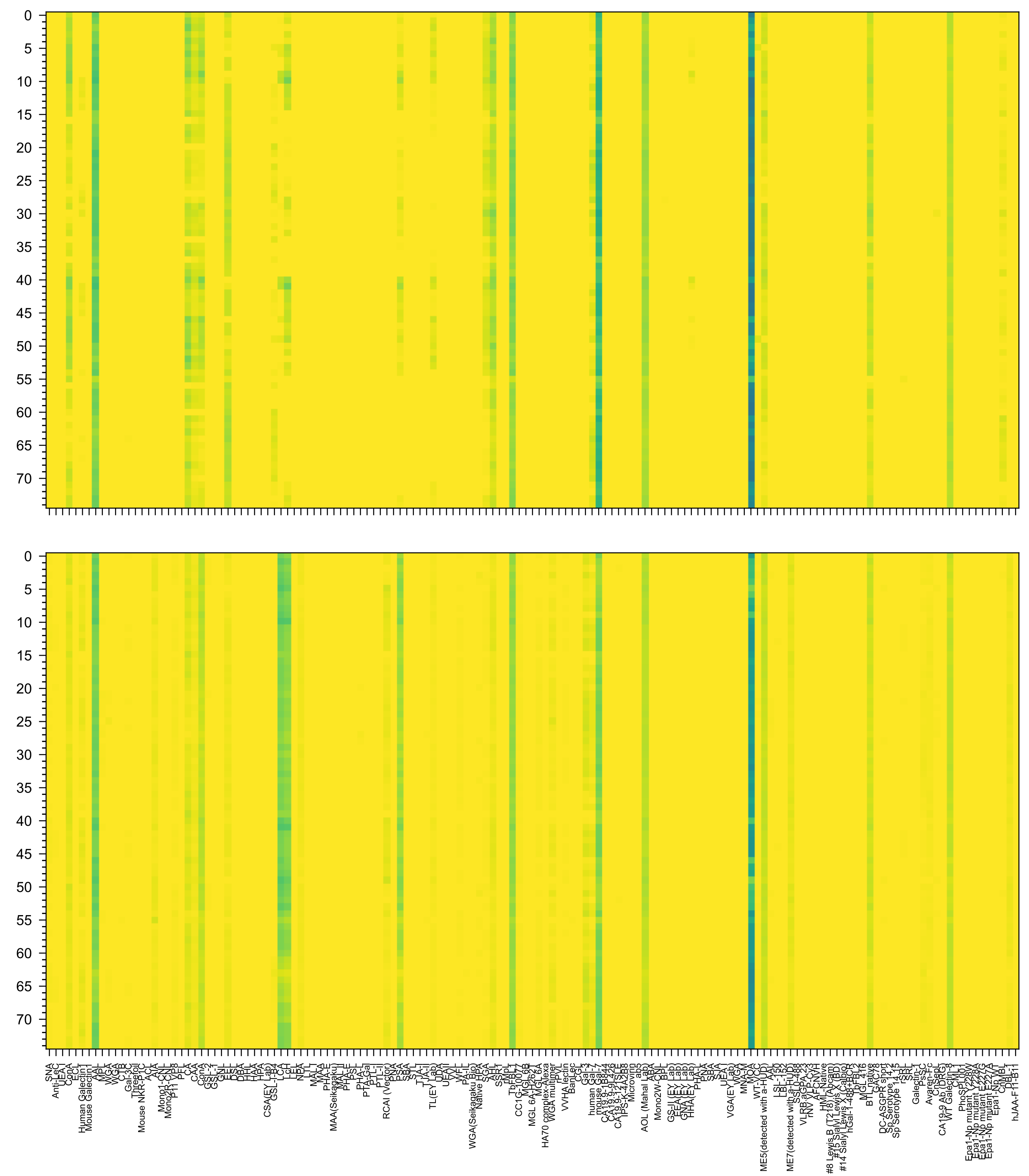

Gal(α1-3)[Fuc(α1-2)]Gal(β1-4)GlcNAc(β1-2)Man(α1-6)[Gal(α1-3)[Fuc(α1-2)]Gal(β1-4)GlcNAc(β1-2)Man(α1-3)]Man(β1-4)GlcNAc(β1-4)[Fuc(α1-6)]GlcNAc(β-Sp2

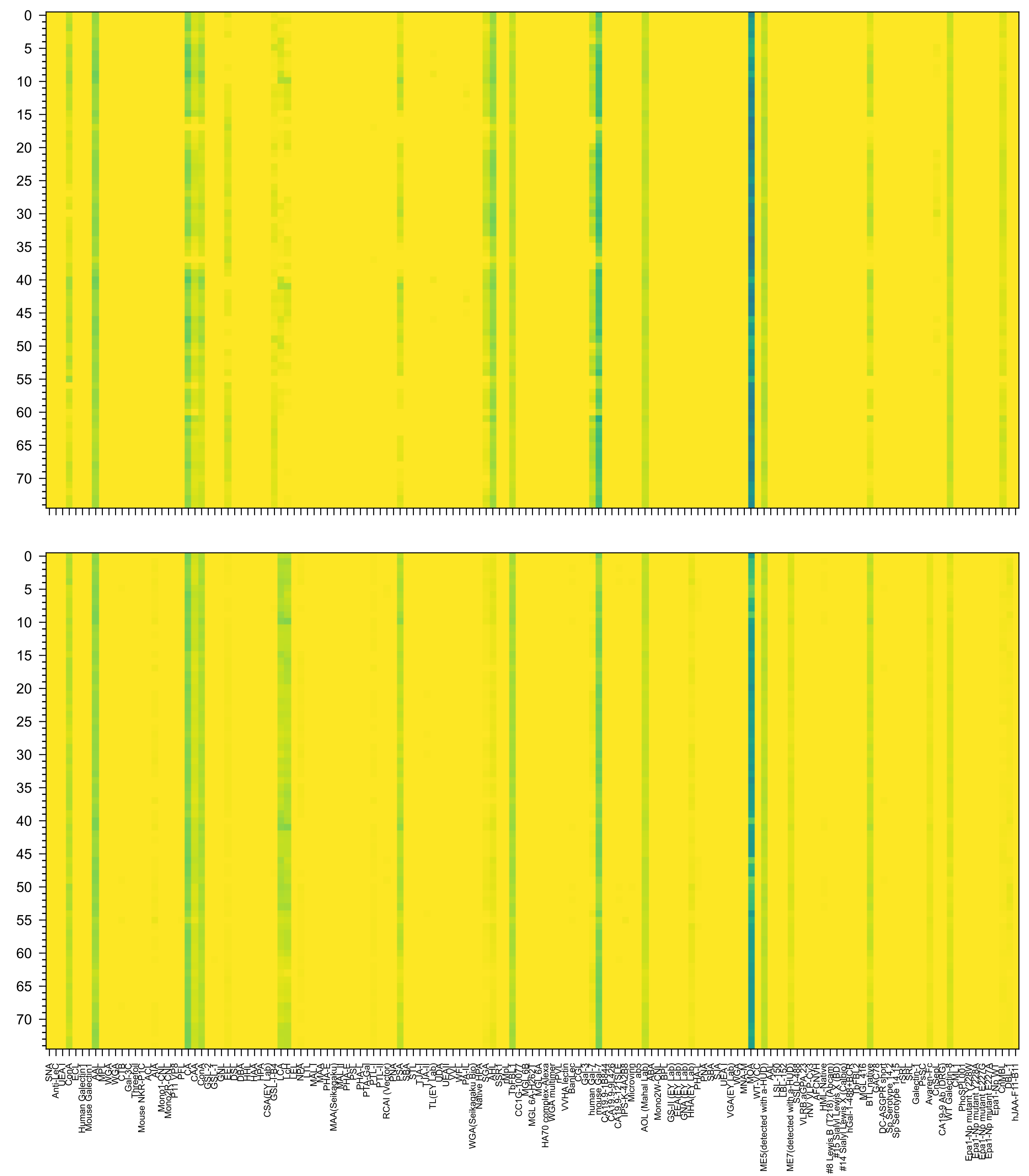

Gal(α1-3)[Fuc(α1-2)]Gal(β1-3)GlcNAc(β1-2)Man(α1-6)[Gal(α1-3)[Fuc(α1-2)]Gal(β1-3)GlcNAc(β1-2)Man(α1-3)]Man(β1-4)GlcNAc(β1-4)[Fuc(α1-6)]GlcNAc(β-Sp2

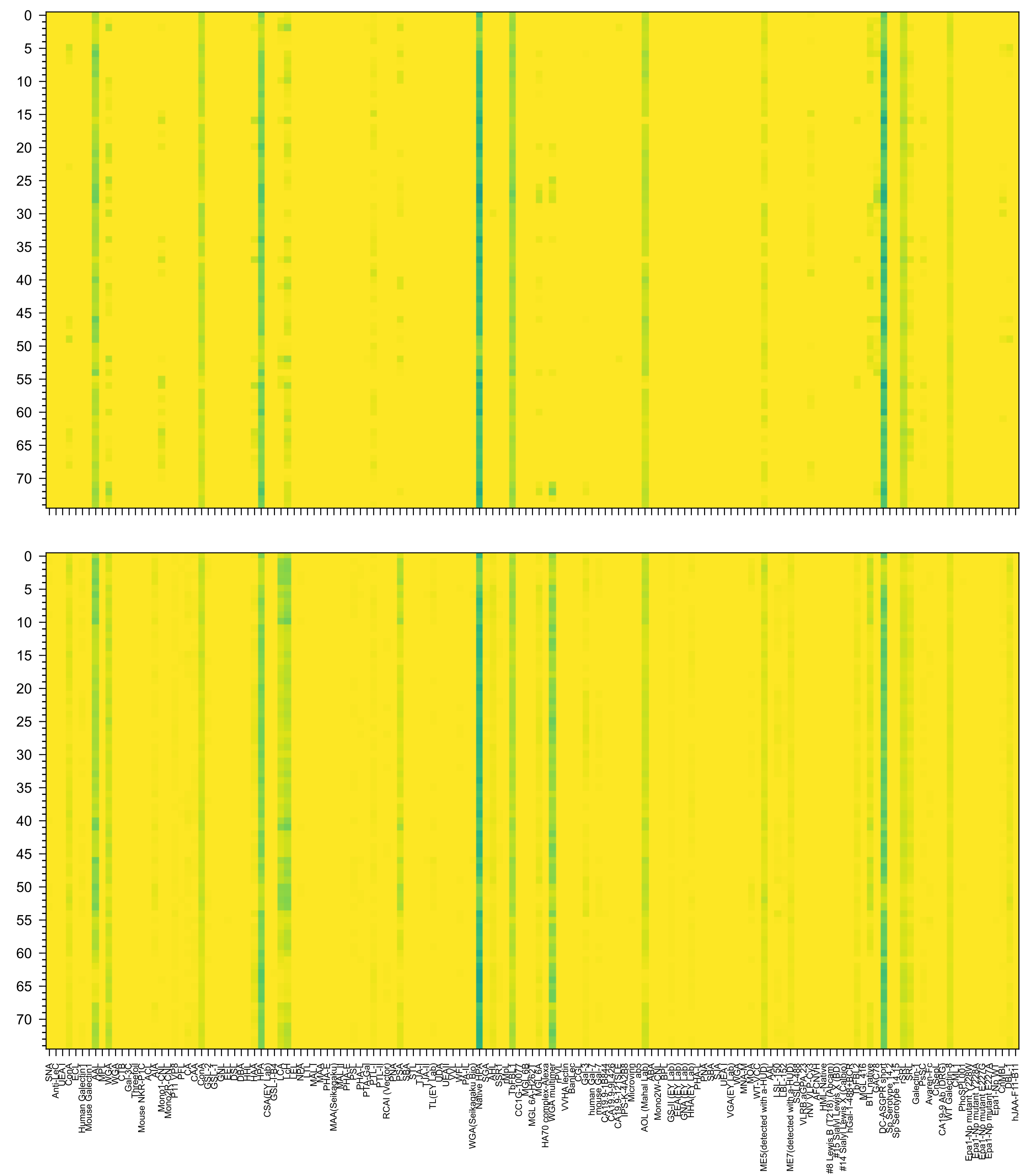

GalNAc(α1-3)[Fuc(α1-2)]Gal(β1-4)GlcNAc(β1-2)Man(α1-6)[GalNAc(α1-3)[Fuc(α1-2)]Gal(β1-4)GlcNAc(β1-2)Man(α1-3)]Man(β1-4)GlcNAc(β1-4)[Fuc(α1-6)]GlcNAc(β-

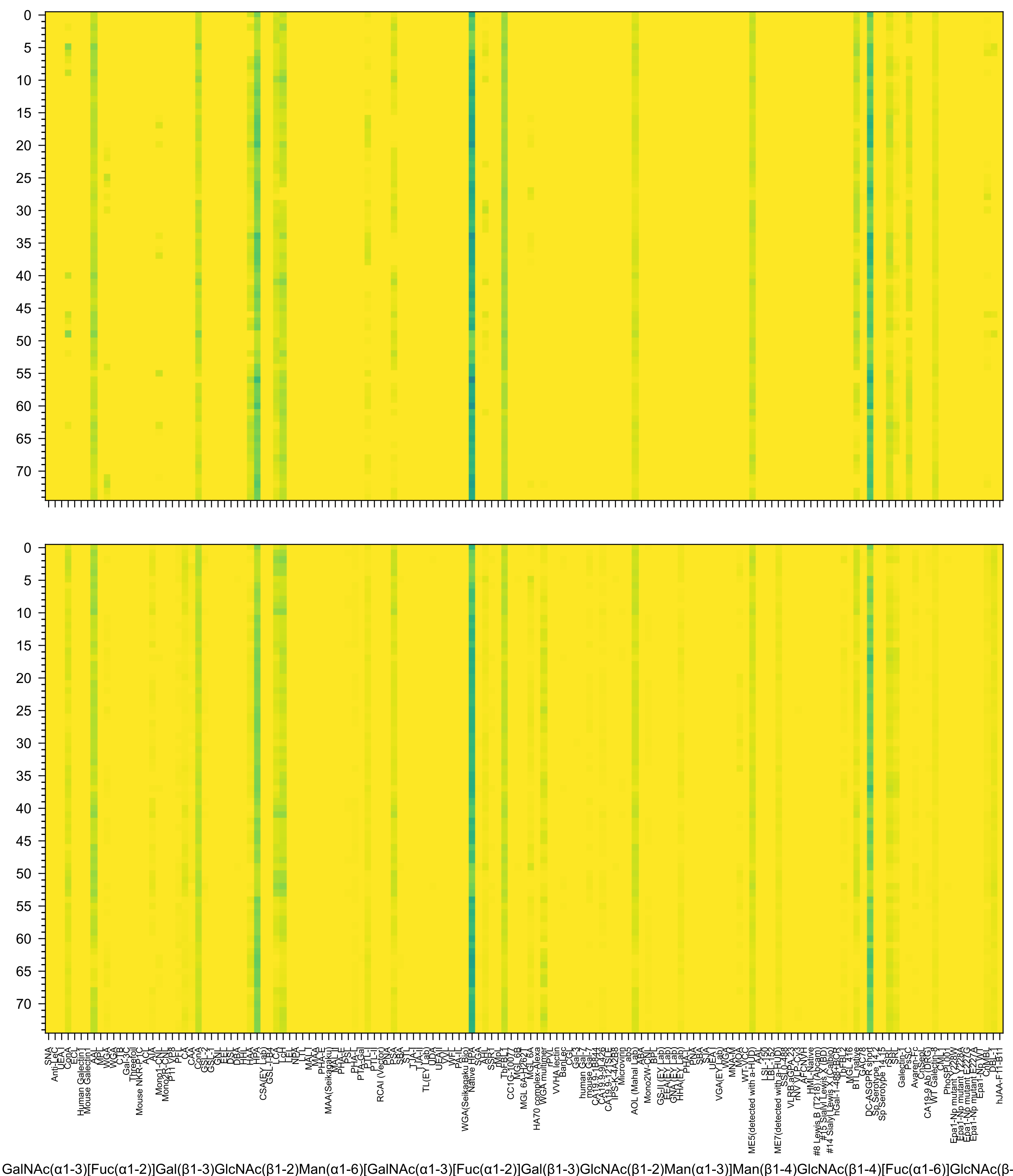
GalNAc(α1-3)[Fuc(α1-2)]Gal(β1-3)GlcNAc(β1-2)Man(α1-6)[GalNAc(α1-3)[Fuc(α1-2)]Gal(β1-3)GlcNAc(β1-2)Man(α1-3)]Man(β1-4)GlcNAc(β1-4)[Fuc(α1-6)]GlcNAc(β-

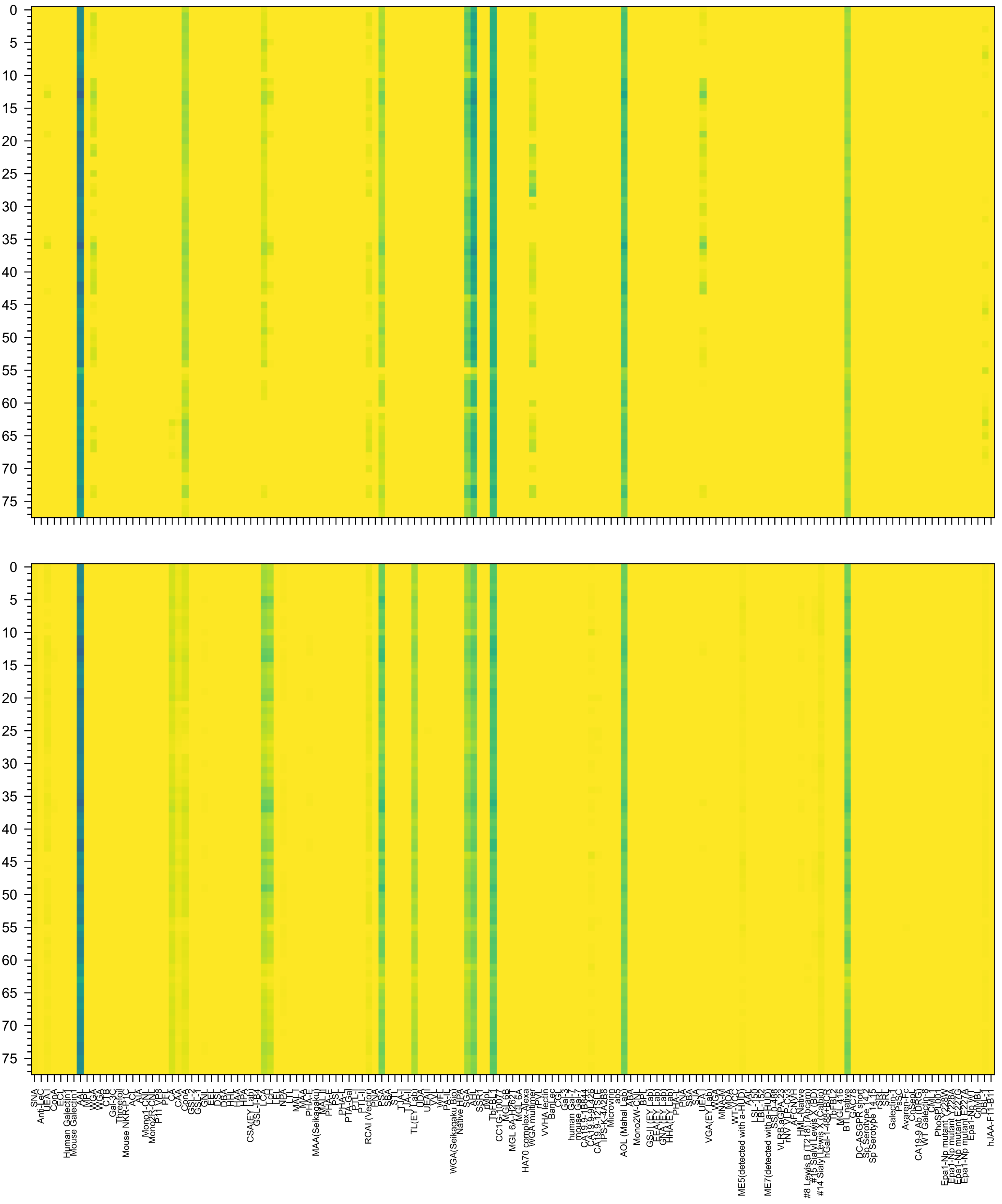
Fuc(α1-2)Gal(β1-4)[Fuc(α1-3)]GlcNAc(β1-2)Man(α1-6)[Fuc(α1-2)Gal(β1-4)[Fuc(α1-3)]GlcNAc(β1-2)Man(α1-3)]Man(β1-4)GlcNAc(β1-4)[Fuc(α1-6)]GlcNAc(β-Sp2

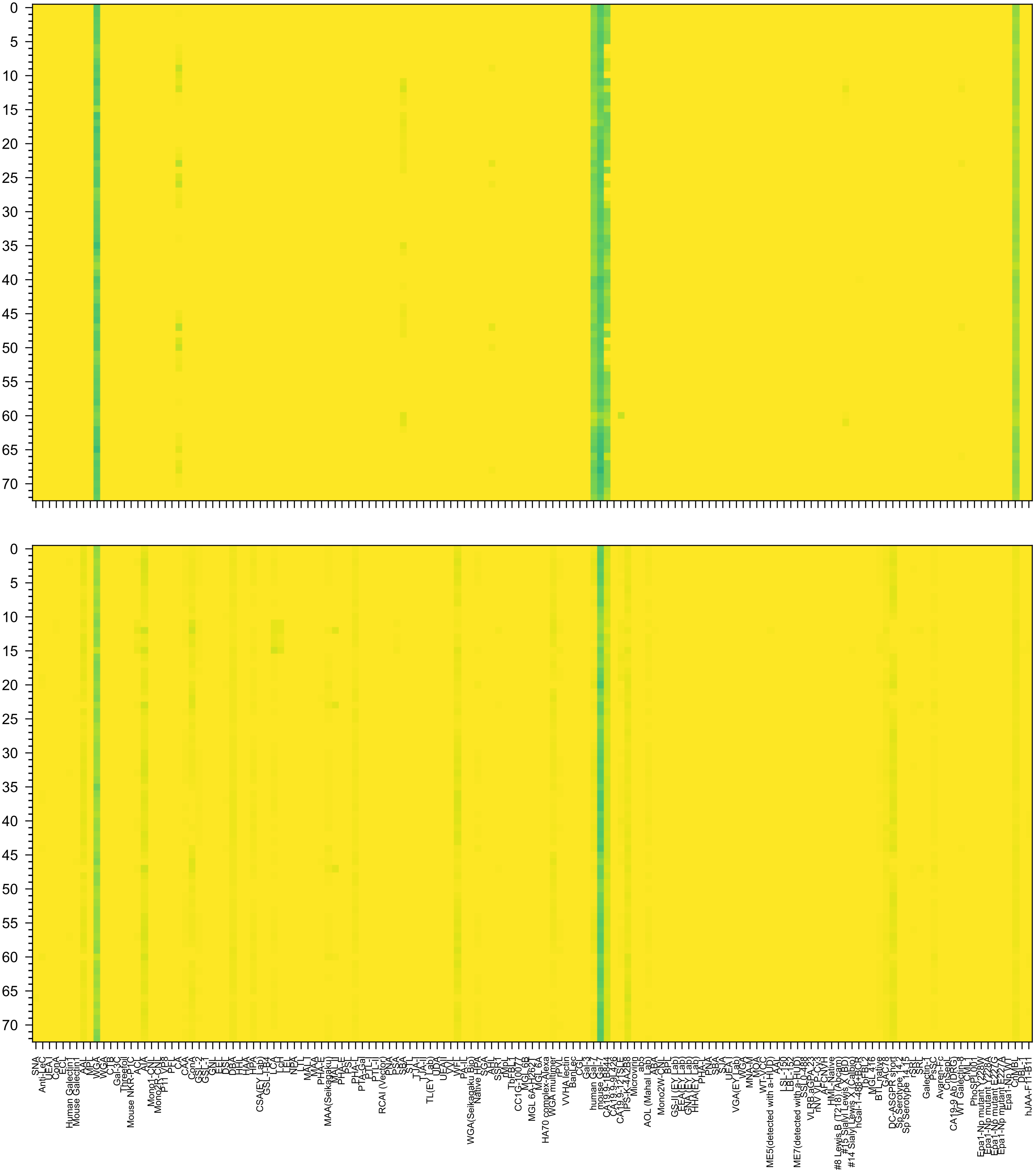

eu5Ac(α2-3)Gal(β1-3)GlcNAc(β1-6)[Neu5Ac(α2-3)Gal(β1-3)GlcNAc(β1-2)]Man(α1-6)[Neu5Ac(α2-3)Gal(β1-3)GlcNAc(β1-2)Man(α1-3)]Man(β1-4)GlcNAc(β1-4)GlcNAc

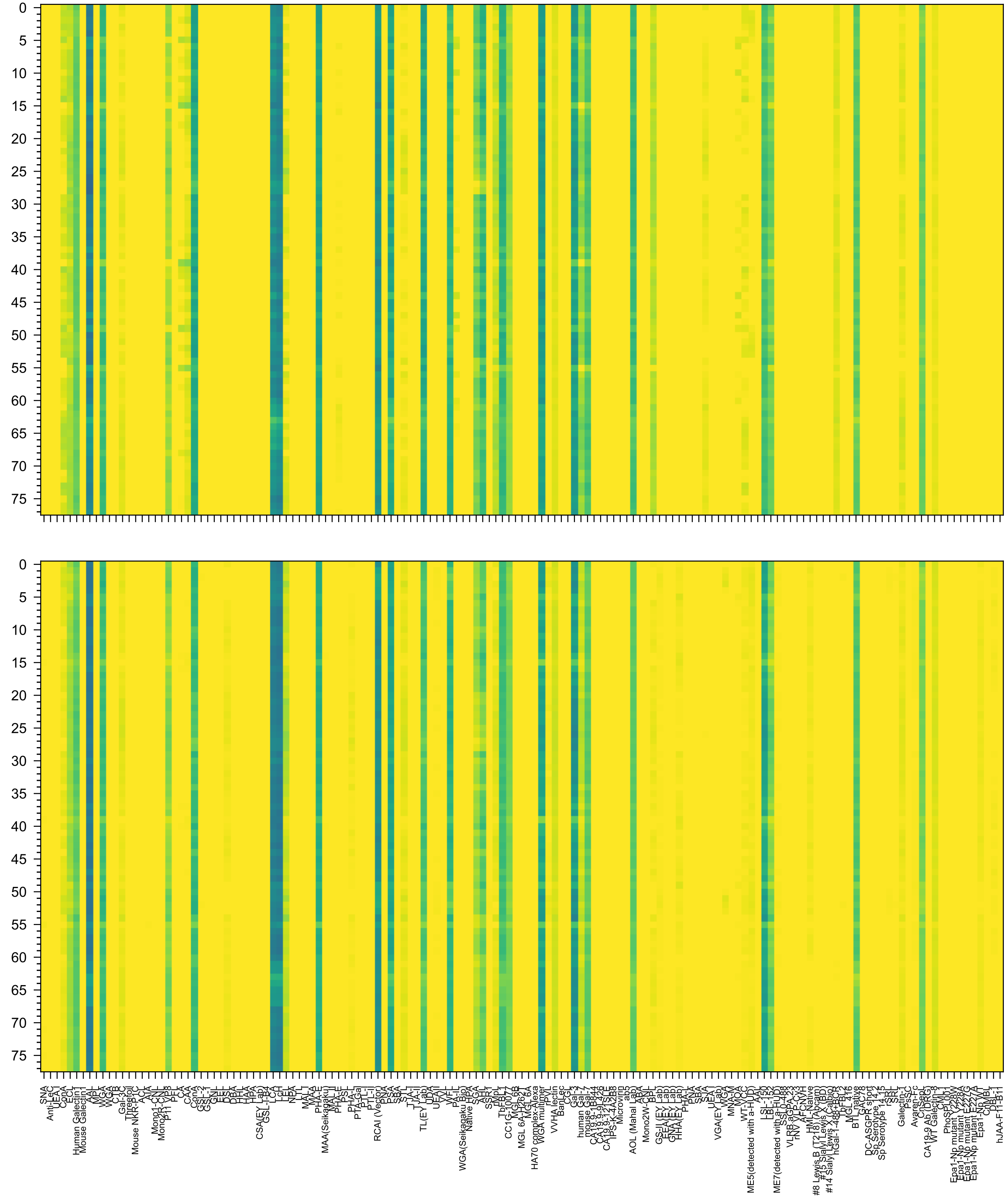

Gal(β1-4)GlcNAc(β1-3)Gal(β1-4)GlcNAc(β1-2)Man(α1-6)[Gal(β1-4)GlcNAc(β1-3)Gal(β1-4)GlcNAc(β1-2)Man(α1-3)]Man(β1-4)GlcNAc(β1-4)[Fuc(α1-6)]GlcNAc(β-S

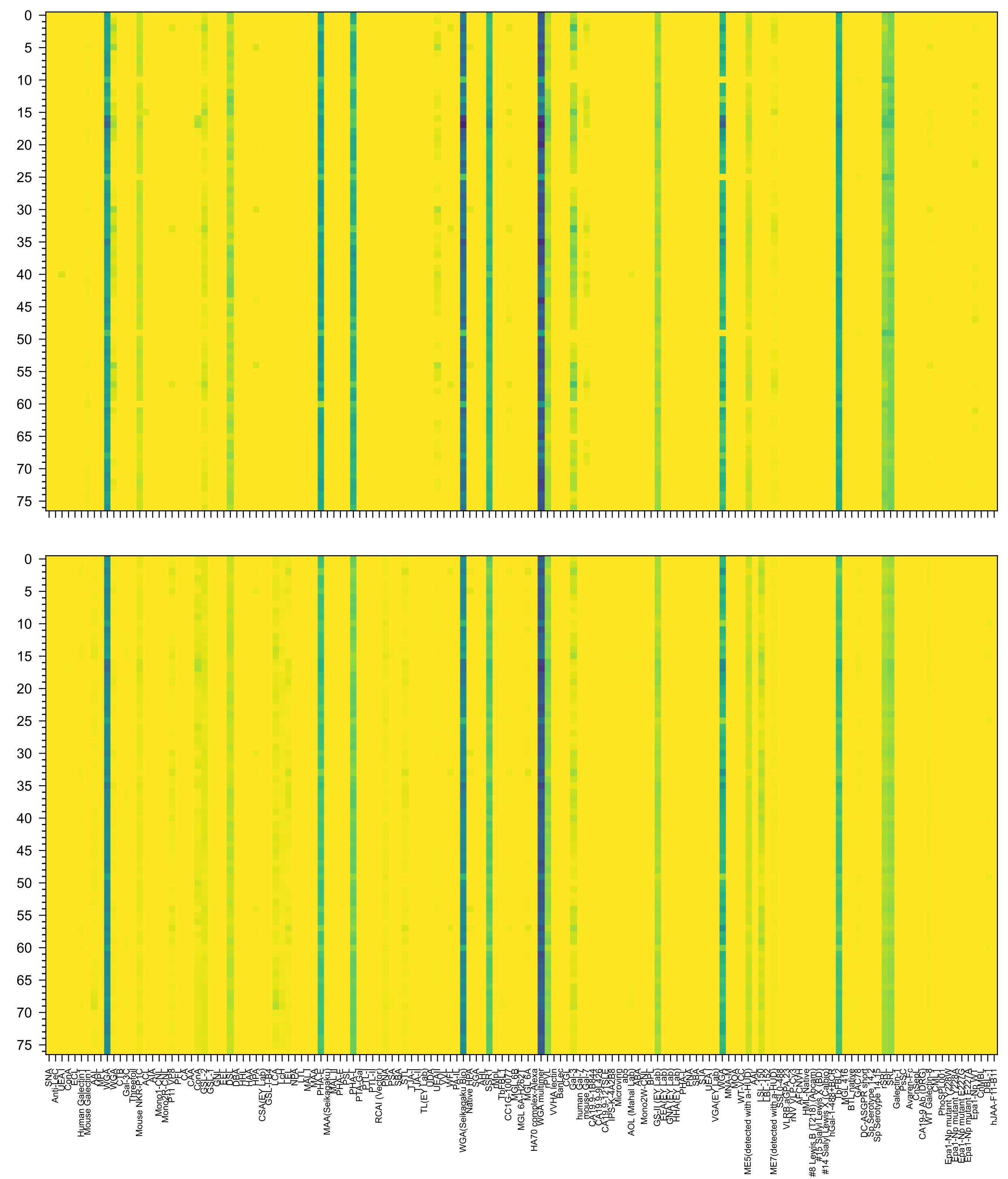

lcNAc(β1-3)Gal(β1-4)GlcNAc(β1-6)[GlcNAc(β1-3)Gal(β1-4)GlcNAc(β1-2)]Man(α1-6)[GlcNAc(β1-3)Gal(β1-4)GlcNAc(β1-2)Man(α1-3)]Man(β1-4)GlcNAc(β1-4)GlcNAc

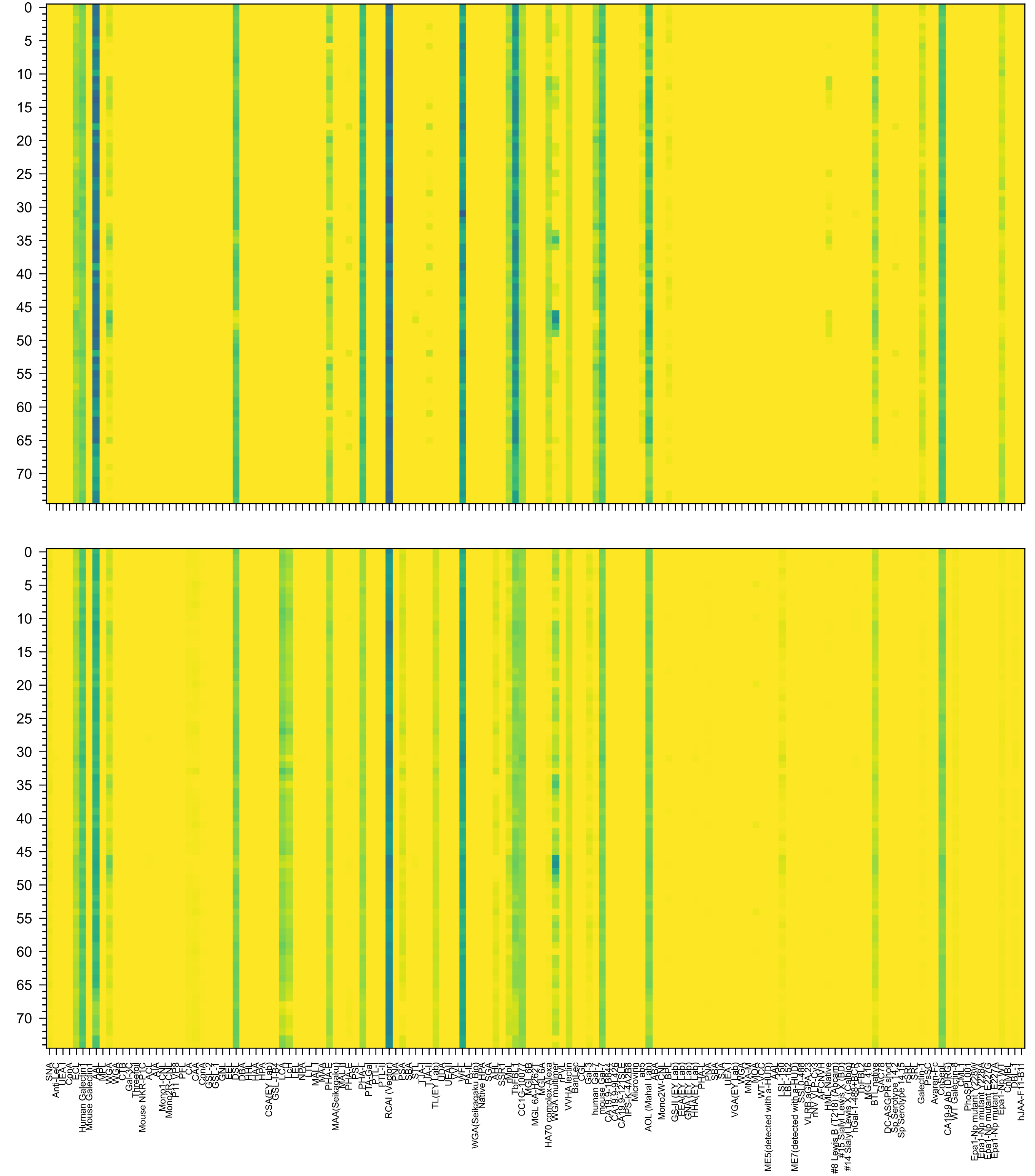

-4)GlcNAc(β1-6)[Gal(β1-4)GlcNAc(β1-2)]Man(α1-6)[GlcNAc(β1-4)][Gal(β1-4)GlcNAc(β1-4)[Gal(β1-4)GlcNAc(β1-2)]Man(α1-3)]Man(β1-4)GlcNAc(β1-4)[Fuc(α1-6)]Gl

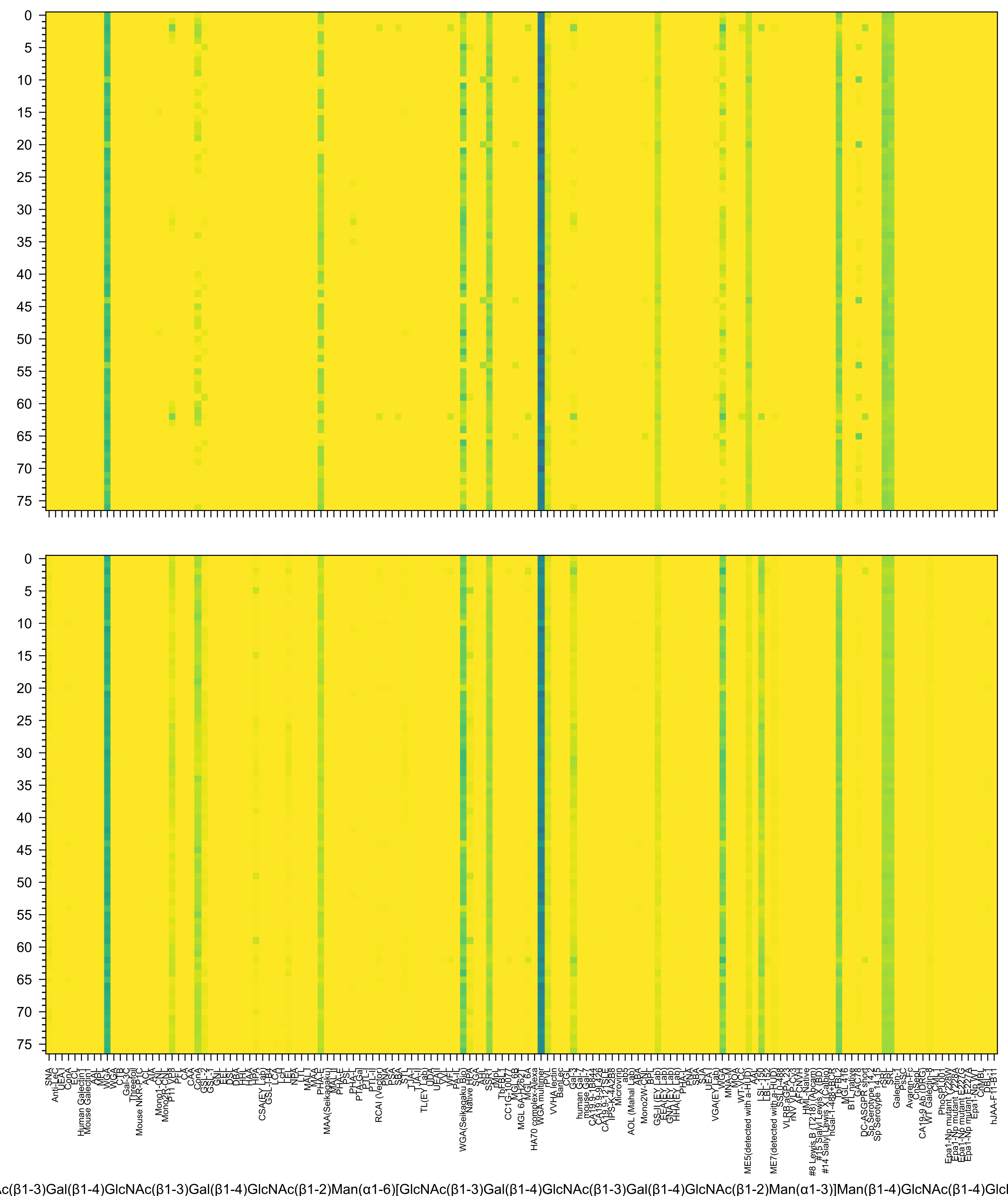
c(β1-3)Gal(β1-4)GlcNAc(β1-3)Gal(β1-4)GlcNAc(β1-2)Man(α1-6)[GlcNAc(β1-3)Gal(β1-4)GlcNAc(β1-3)Gal(β1-4)GlcNAc(β1-2)Man(α1-3)]Man(β1-4)GlcNAc(β1-4)Glc

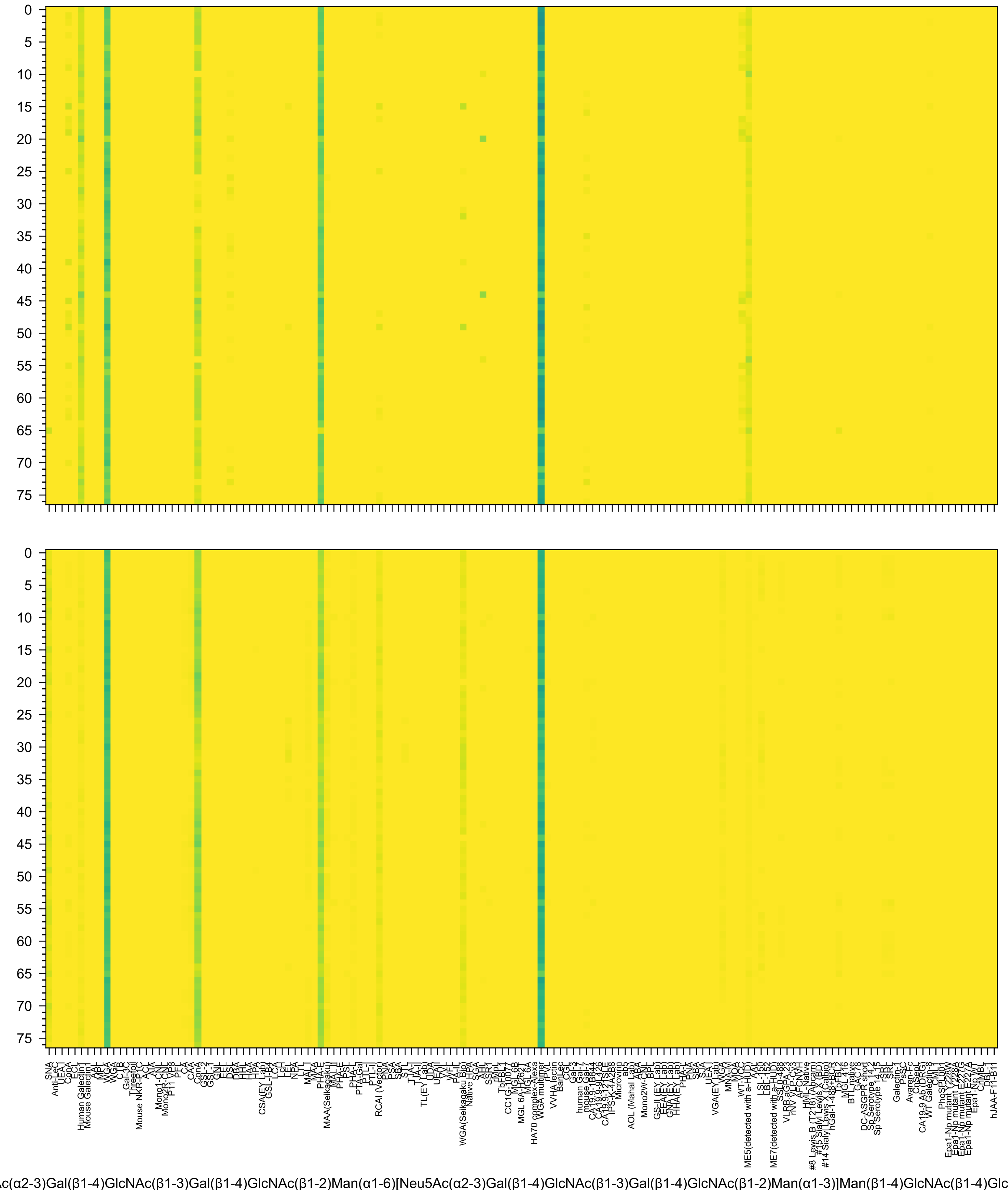
c(α2-3)Gal(β1-4)GlcNAc(β1-3)Gal(β1-4)GlcNAc(β1-2)Man(α1-6)[Neu5Ac(α2-3)Gal(β1-4)GlcNAc(β1-3)Gal(β1-4)GlcNAc(β1-2)Man(α1-3)]Man(β1-4)GlcNAc(β1-4)Glc

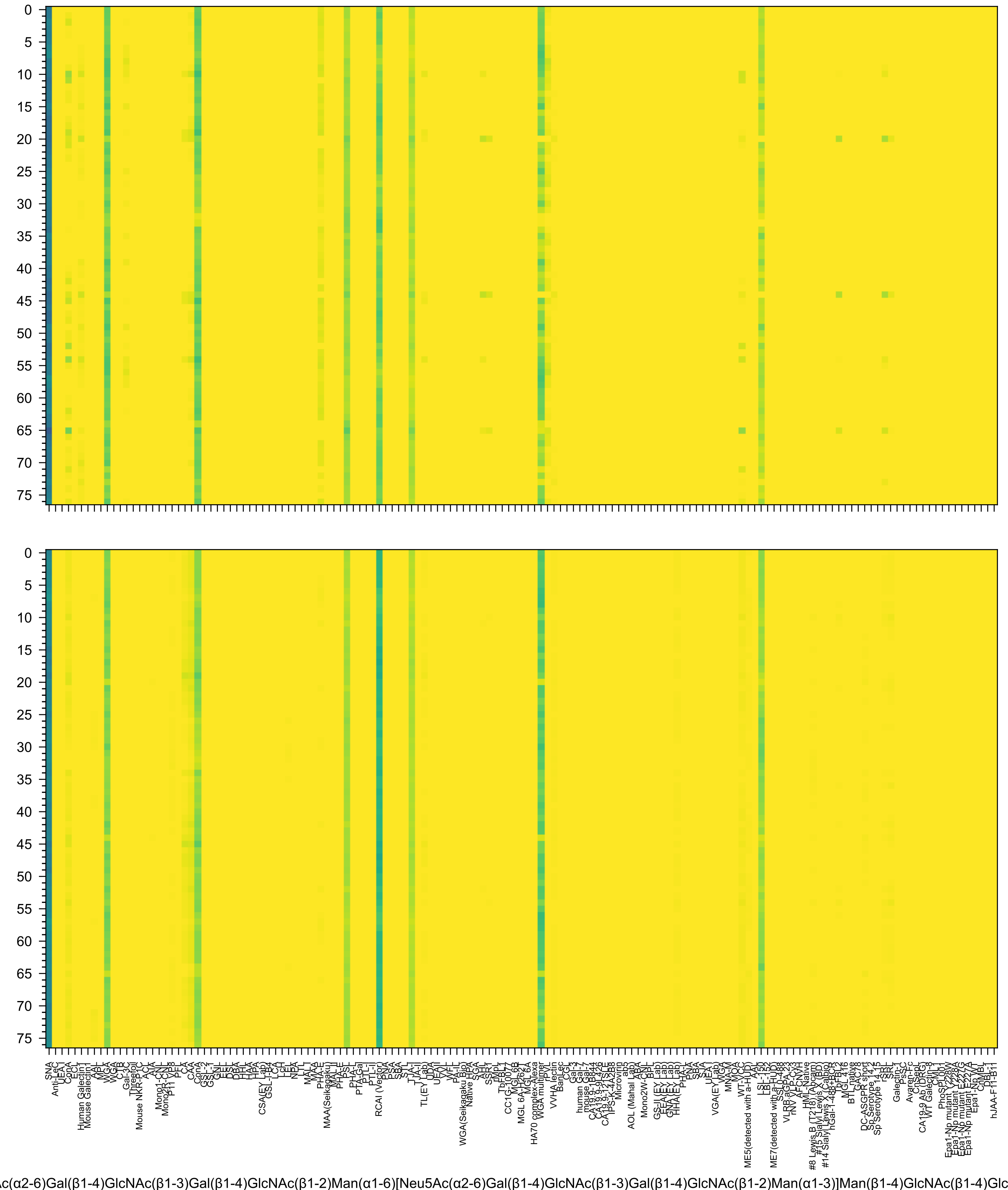
Ac(α2-6)Gal(β1-4)GlcNAc(β1-3)Gal(β1-4)GlcNAc(β1-2)Man(α1-6)[Neu5Ac(α2-6)Gal(β1-4)GlcNAc(β1-3)Gal(β1-4)GlcNAc(β1-2)Man(α1-3)]Man(β1-4)GlcNAc(β1-4)Glc

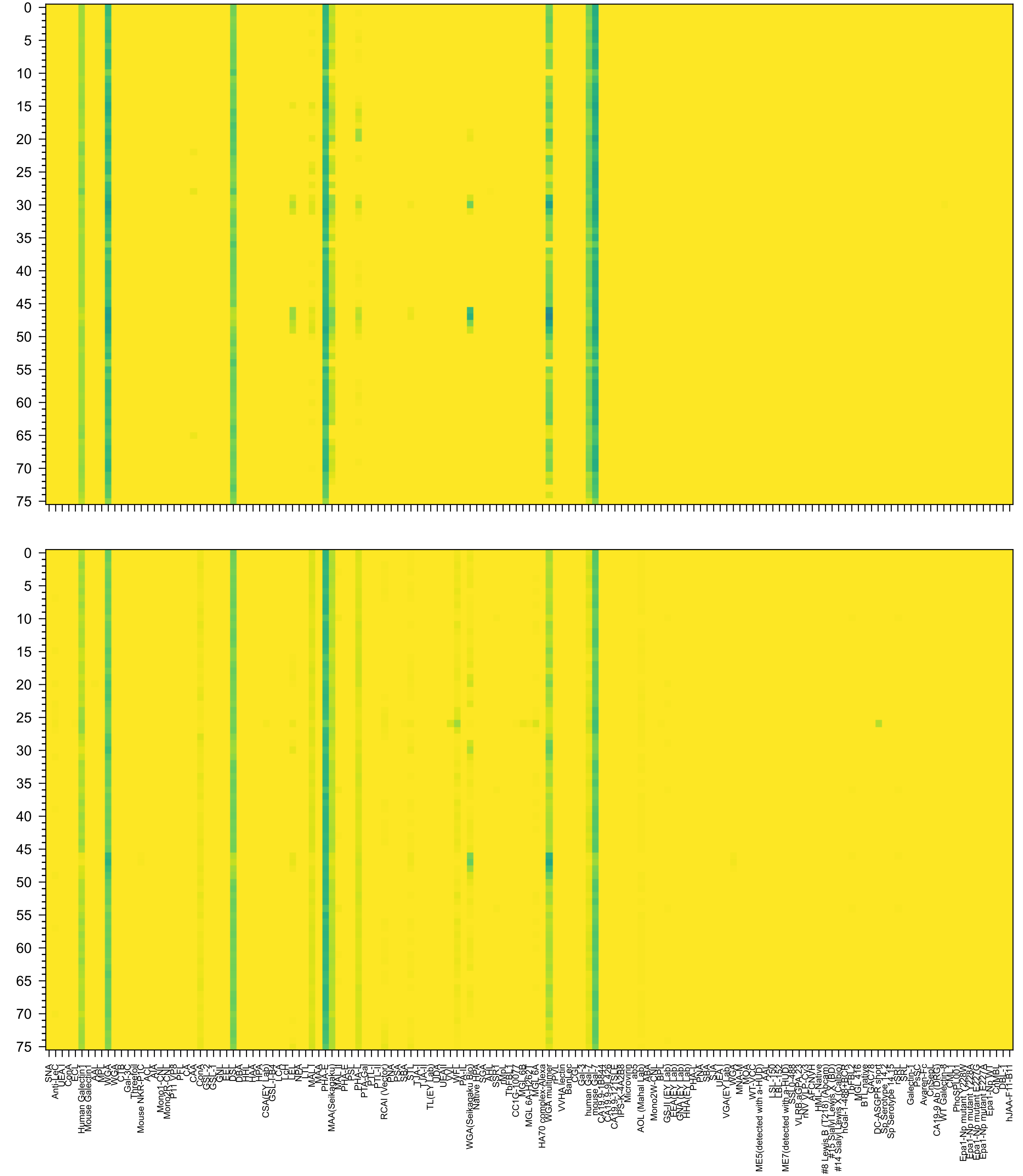

2-3)Gal(β1-4)GlcNAc(β1-4)Man(α1-6)[GlcNAc(β1-4)][Neu5Ac(α2-3)Gal(β1-4)GlcNAc(β1-4)[Neu5Ac(α2-3)Gal(β1-4)GlcNAc(β1-2)]Man(α1-3)]Man(β1-4)GlcNAc(β1-4)

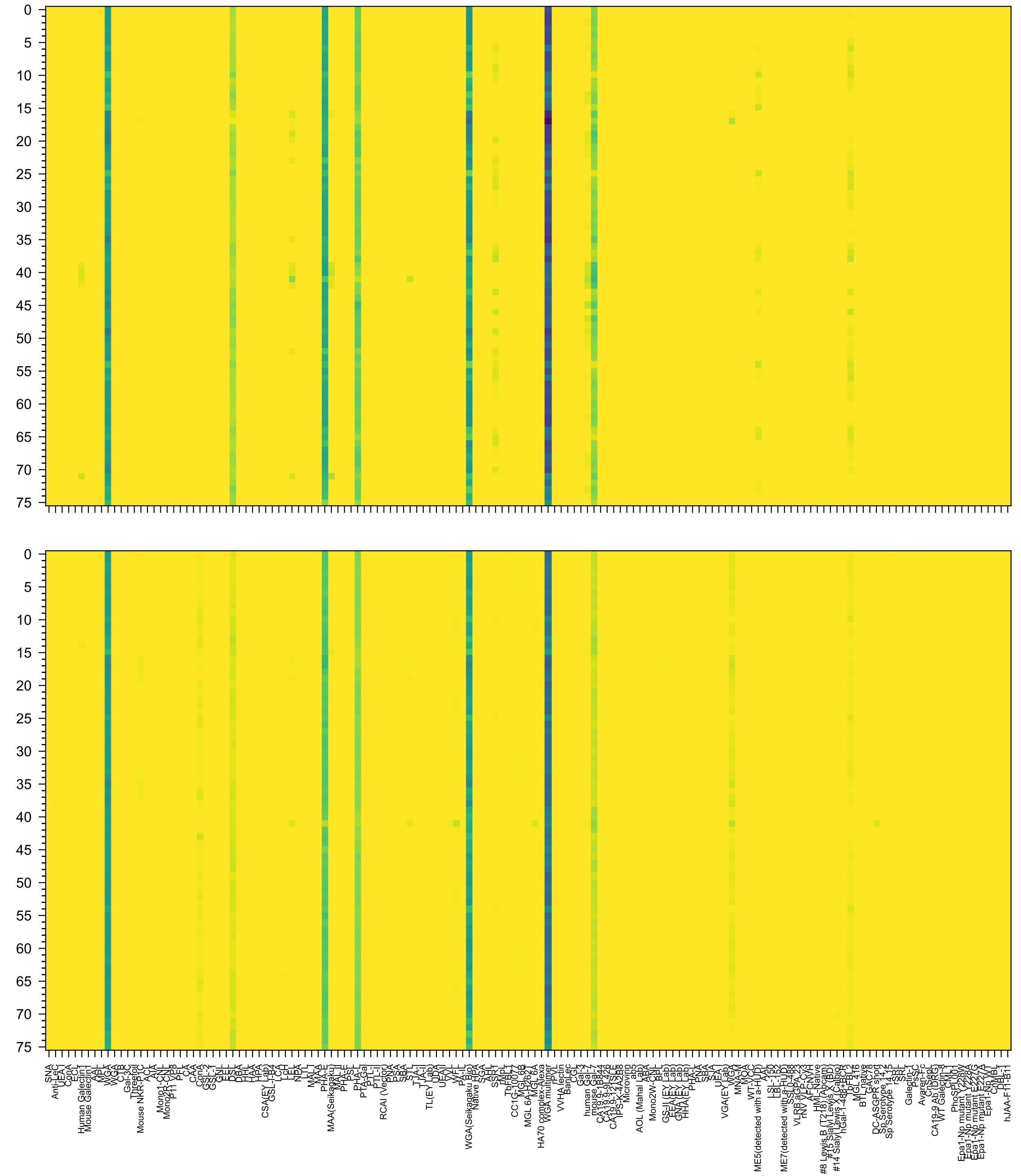

2-3)Gal(β1-4)GlcNAc(β1-6)[Neu5Ac(α2-3)Gal(β1-4)GlcNAc(β1-2)]Man(α1-6)[GlcNAc(β1-4)][Neu5Ac(α2-3)Gal(β1-4)GlcNAc(β1-2)Man(α1-3)]Man(β1-4)GlcNAc(β1-4)

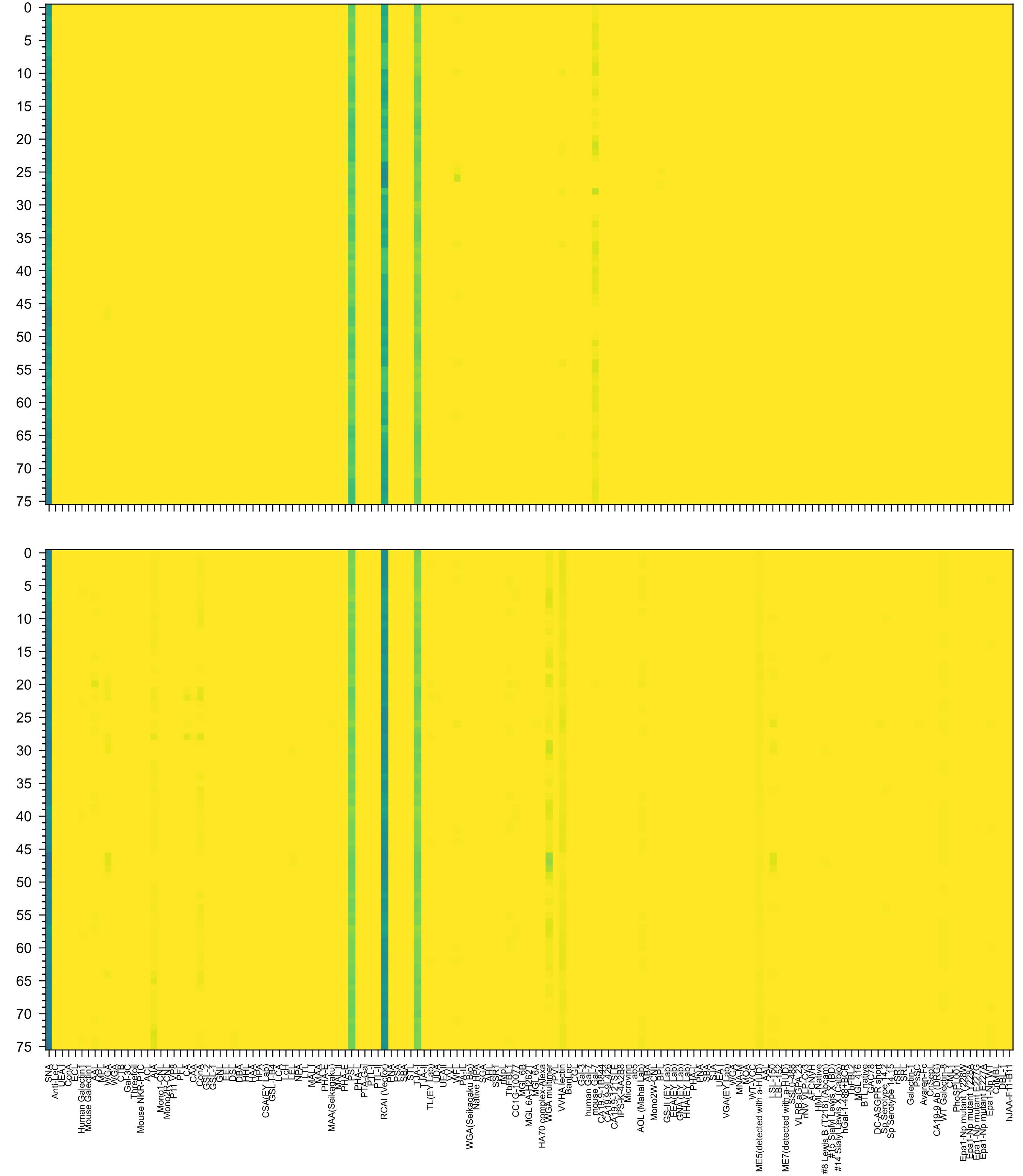

2-6)Gal(β1-4)GlcNAc(β1-4)Man(α1-6)[GlcNAc(β1-4)][Neu5Ac(α2-6)Gal(β1-4)GlcNAc(β1-4)[Neu5Ac(α2-6)Gal(β1-4)GlcNAc(β1-2)]Man(α1-3)]Man(β1-4)GlcNAc(β1-4)

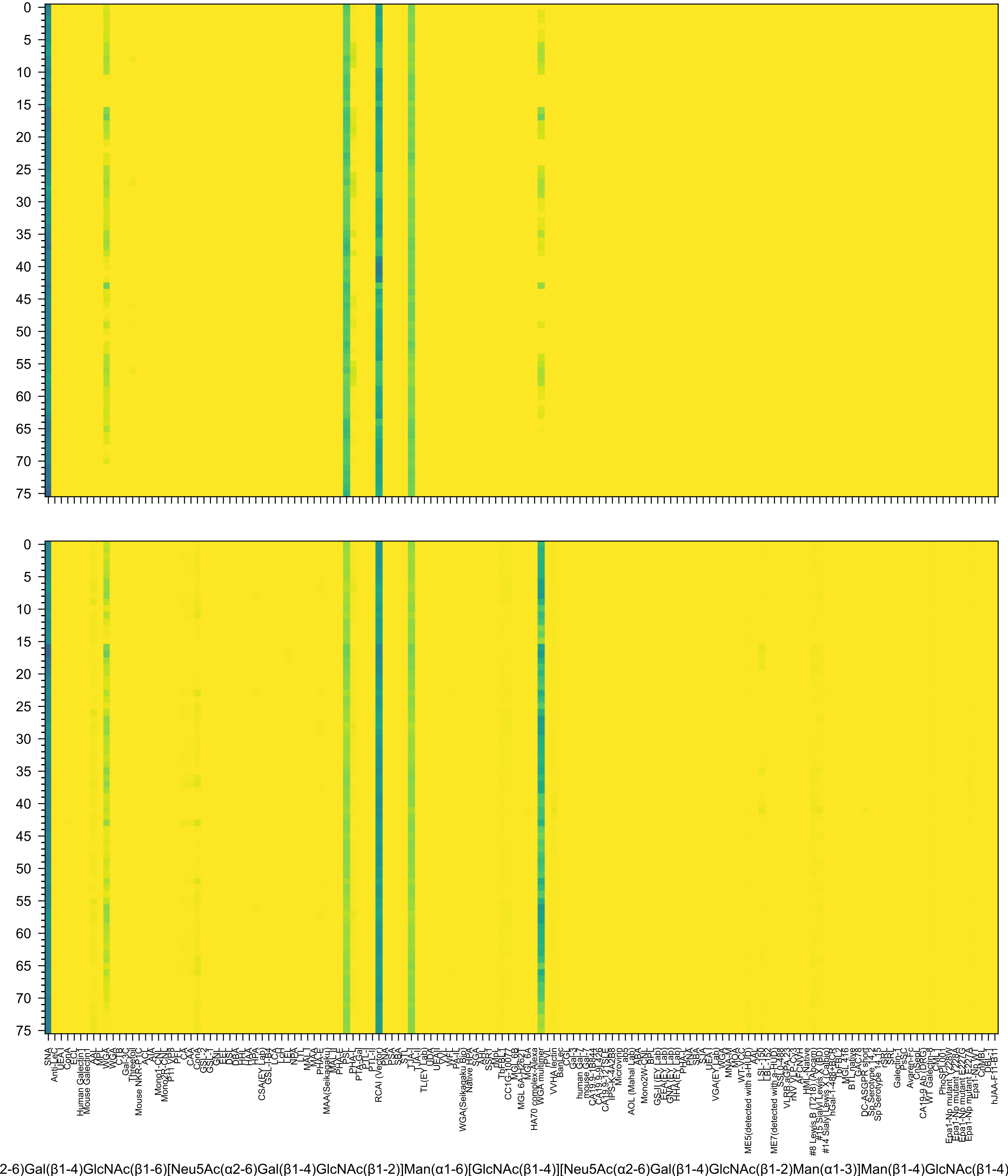
2-6)Gal(β1-4)GlcNAc(β1-6)[Neu5Ac(α2-6)Gal(β1-4)GlcNAc(β1-2)]Man(α1-6)[GlcNAc(β1-4)][Neu5Ac(α2-6)Gal(β1-4)GlcNAc(β1-2)Man(α1-3)]Man(β1-4)GlcNAc(β1-4)

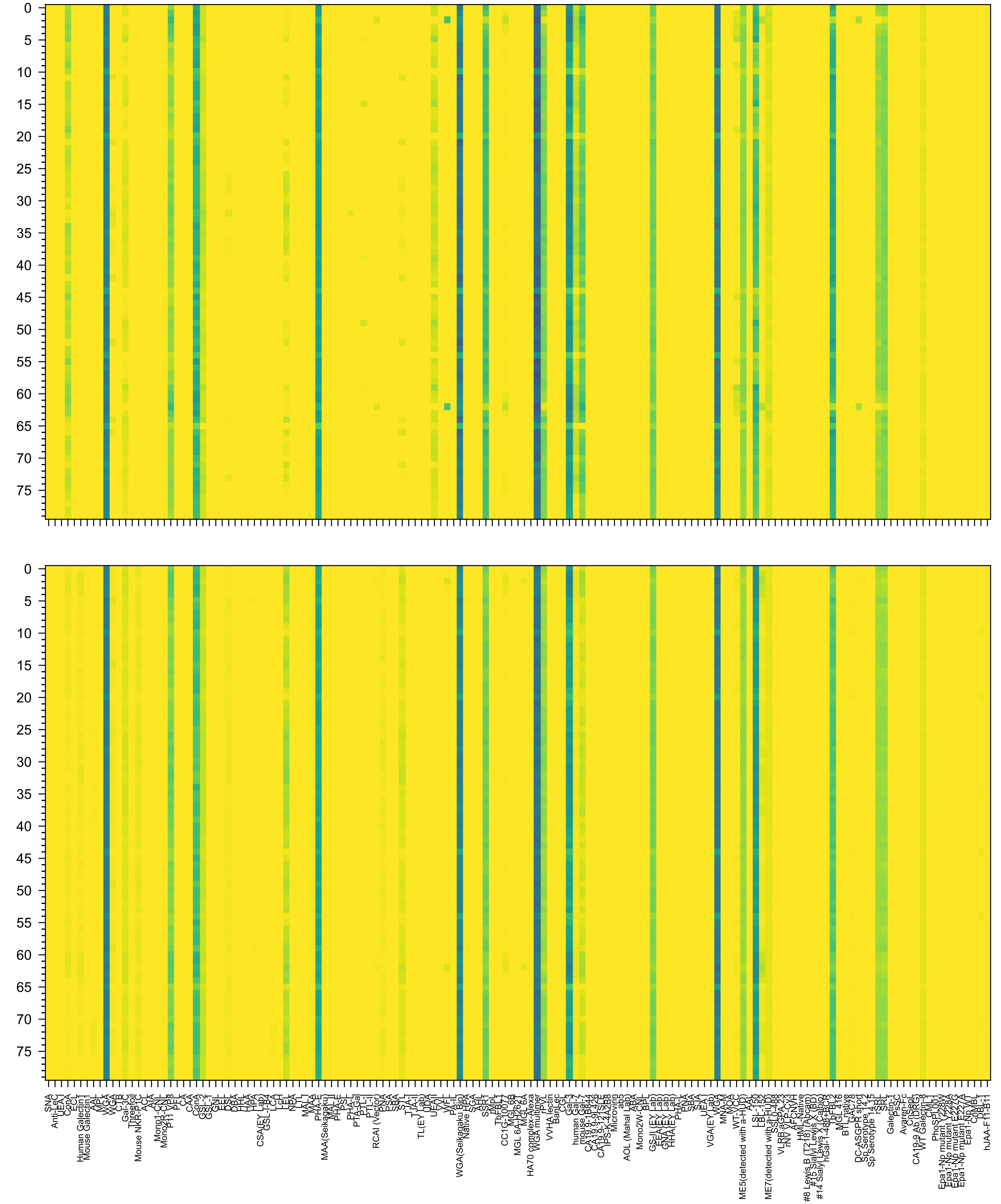

Ac(β1-3)Gal(β1-4)GlcNAc(β1-3)Gal(β1-4)GlcNAc(β1-2)Man(α1-6)[GlcNAc(β1-3)Gal(β1-4)GlcNAc(β1-3)Gal(β1-4)GlcNAc(β1-2)Man(α1-3)]Man(β1-4)GlcNAc(β1-4)Glc

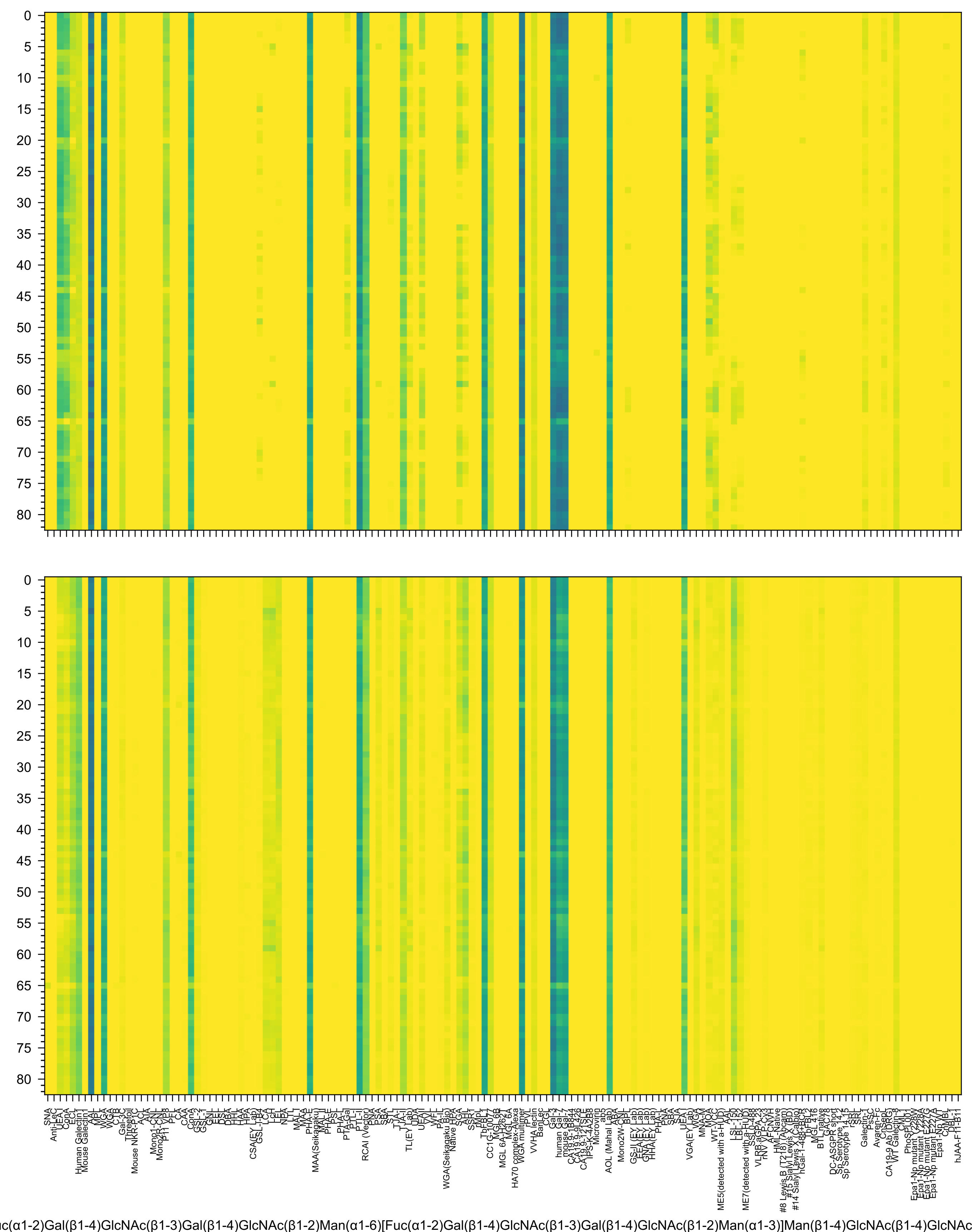
uc(α1-2)Gal(β1-4)GlcNAc(β1-3)Gal(β1-4)GlcNAc(β1-2)Man(α1-6)[Fuc(α1-2)Gal(β1-4)GlcNAc(β1-3)Gal(β1-4)GlcNAc(β1-2)Man(α1-3)]Man(β1-4)GlcNAc(β1-4)GlcNAc

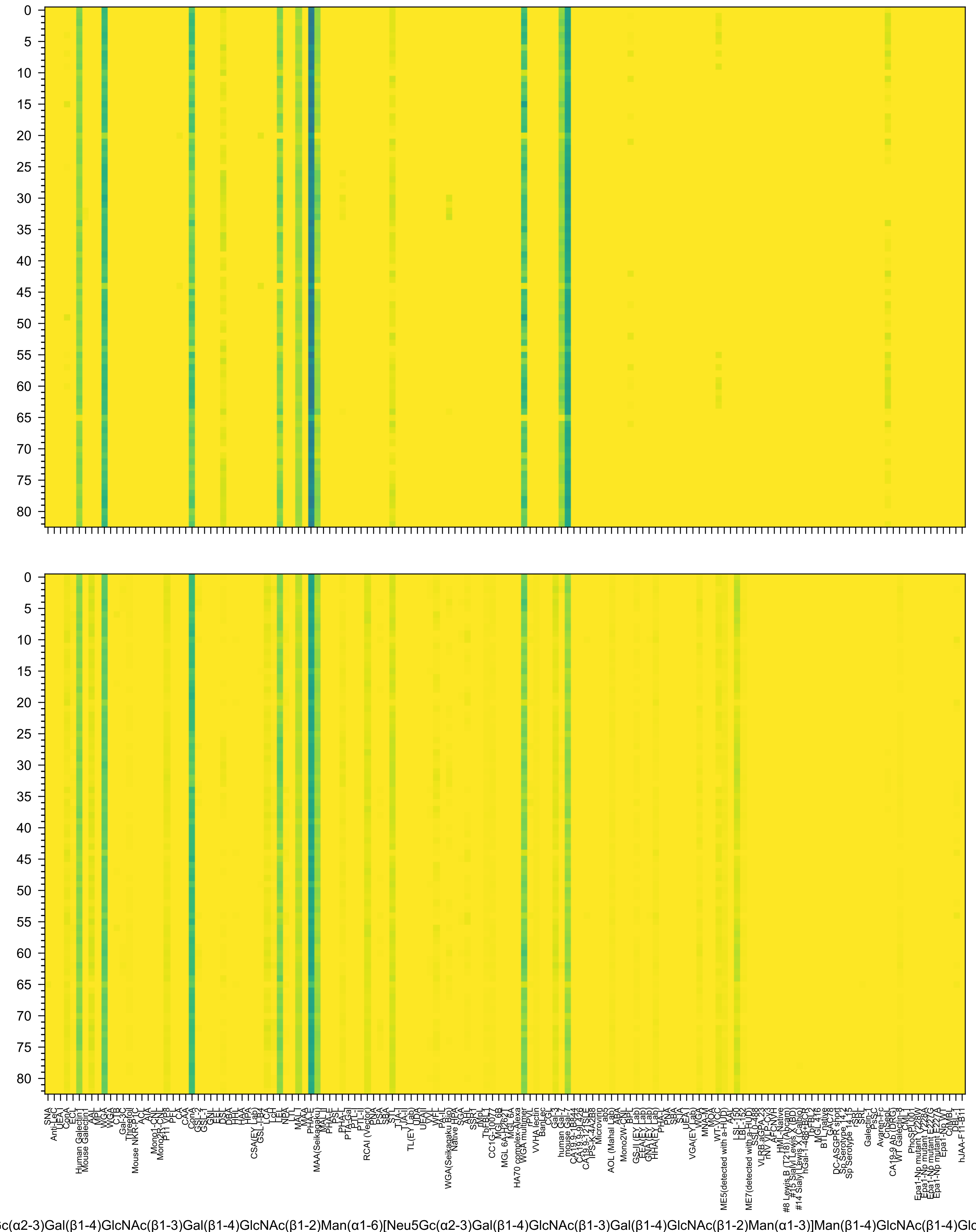
c(α2-3)Gal(β1-4)GlcNAc(β1-3)Gal(β1-4)GlcNAc(β1-2)Man(α1-6)[Neu5Gc(α2-3)Gal(β1-4)GlcNAc(β1-3)Gal(β1-4)GlcNAc(β1-2)Man(α1-3)]Man(β1-4)GlcNAc(β1-4)Glc

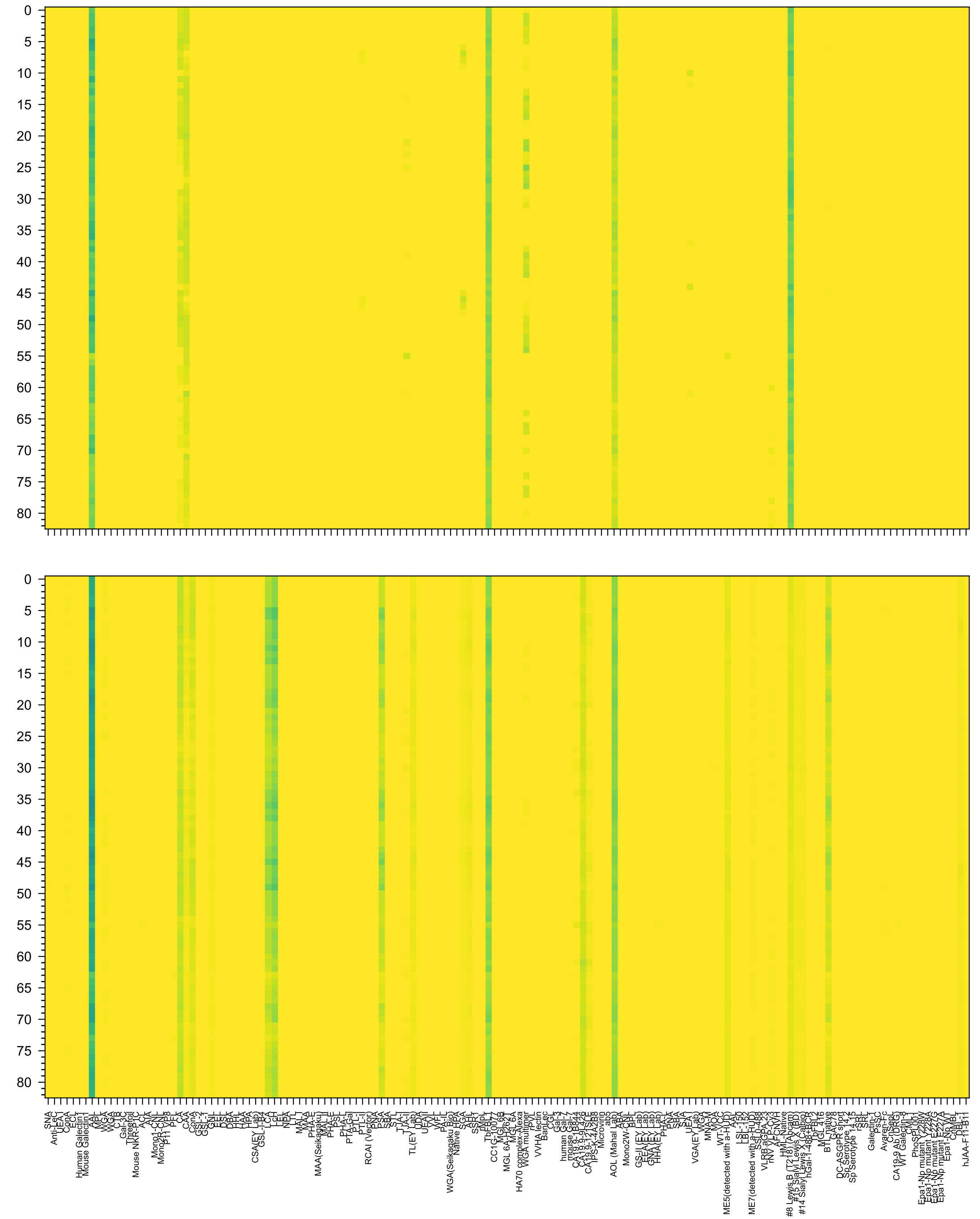

2)Gal(β1-3)[Fuc(α1-4)]GlcNAc(β1-2)Man(α1-6)[Fuc(α1-2)Gal(β1-3)[Fuc(α1-4)]GlcNAc(β1-2)Man(α1-3)]Man(β1-4)GlcNAc(β1-4)[Fuc(α1-6)]GlcNAc(β1-4)[Fuc(α1-6)]G

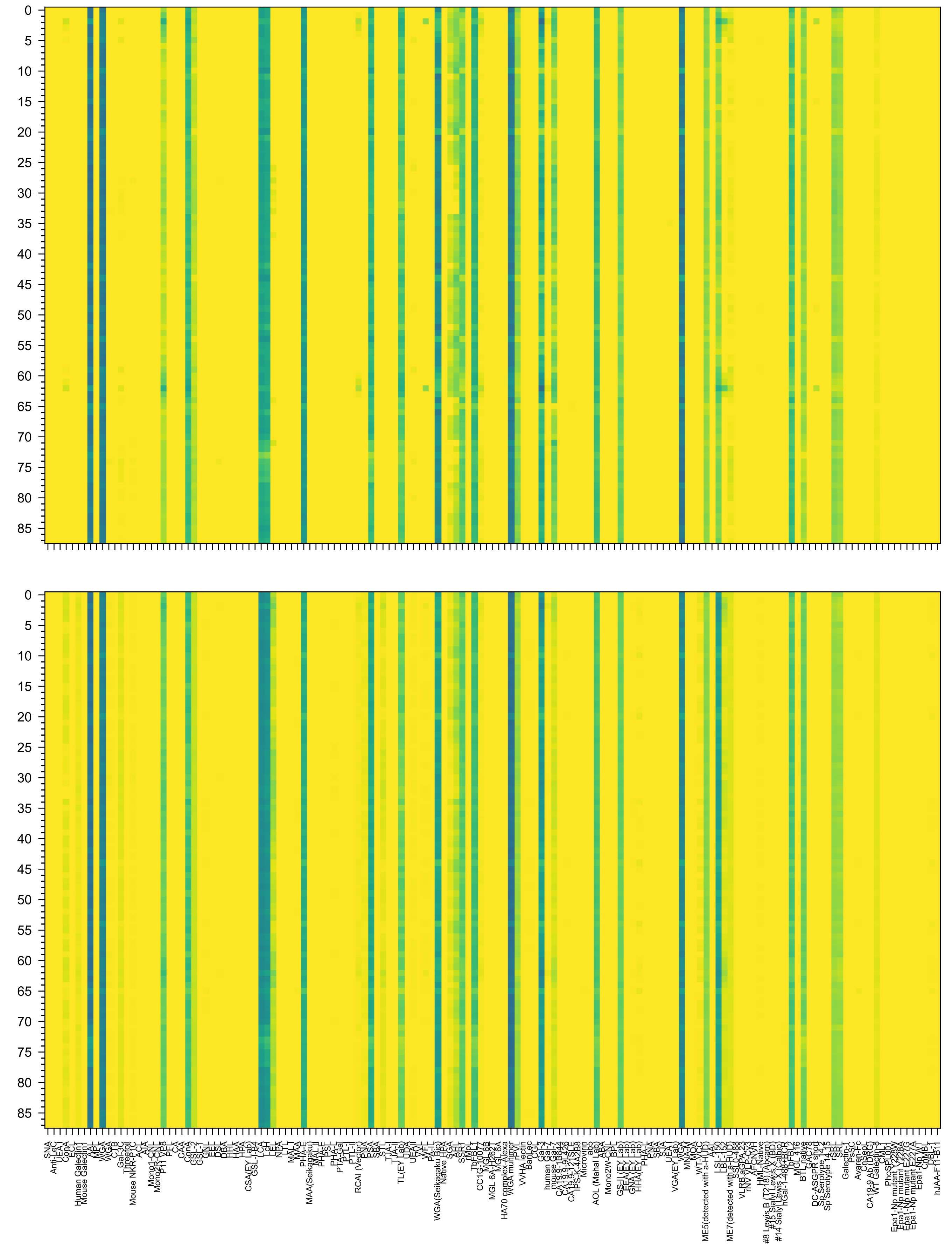

3)Gal(β1-4)GlcNAc(β1-3)Gal(β1-4)GlcNAc(β1-2)Man(α1-6)[GlcNAc(β1-3)Gal(β1-4)GlcNAc(β1-3)Gal(β1-4)GlcNAc(β1-2)Man(α1-3)]Man(β1-4)GlcNAc(β1-4)[Fuc(α1-6

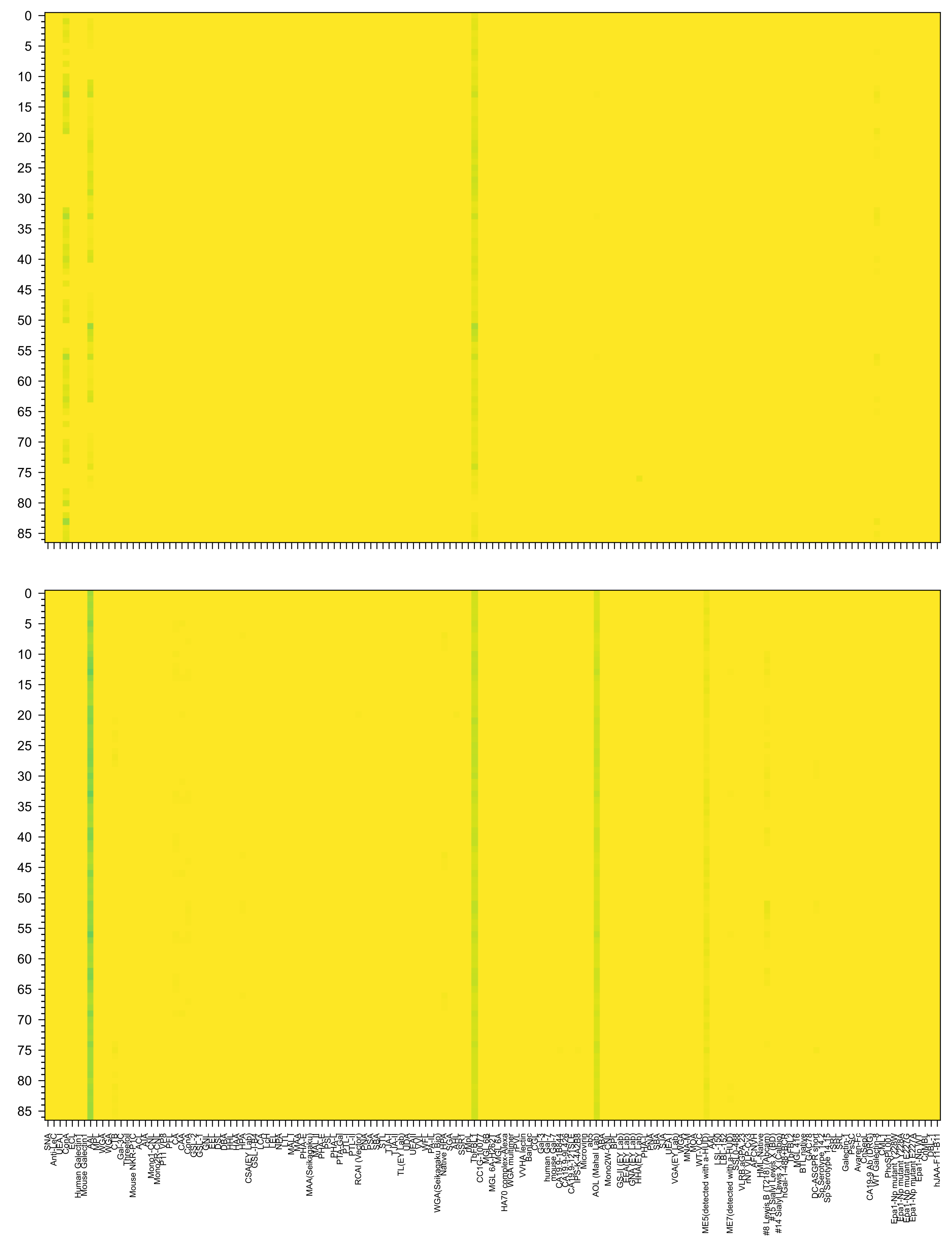

l(β1-4)[Fuc(α1-3)]GlcNAc(β1-2)Man(α1-6)[Fuc(α1-2)Gal(β1-4)[Fuc(α1-3)]GlcNAc(β1-4)[Fuc(α1-2)Gal(β1-4)[Fuc(α1-3)]GlcNAc(β1-2)]Man(α1-3)]Man(β1-4)GlcNAc(β1

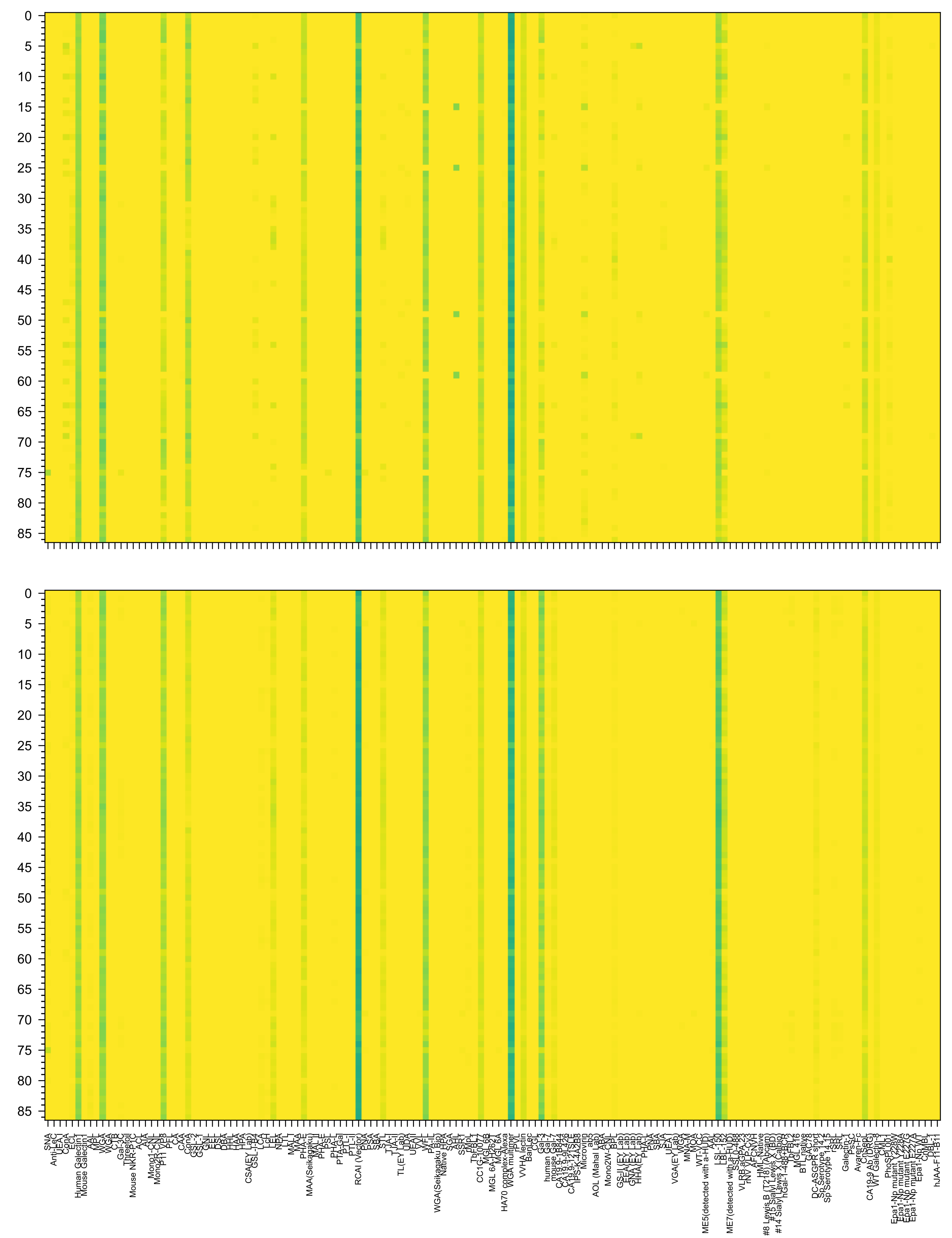

Ac(β1-3)Gal(β1-4)GlcNAc(β1-3)Gal(β1-4)GlcNAc(β1-2)Man(α1-6)[Gal(β1-4)GlcNAc(β1-3)Gal(β1-4)GlcNAc(β1-3)Gal(β1-4)GlcNAc(β1-2)Man(α1-3)]Man(β1-4)GlcNAc

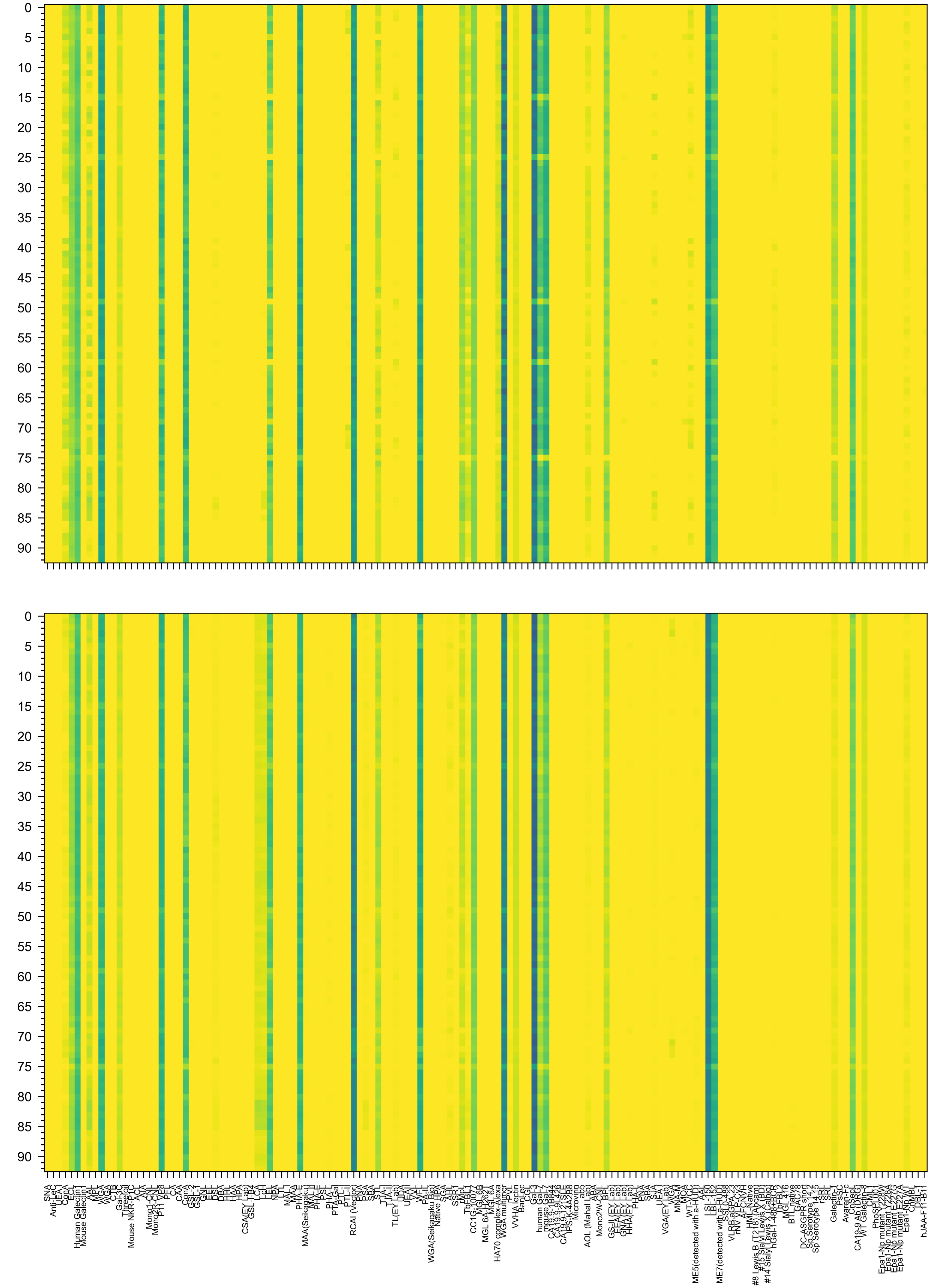

Ac(β1-3)Gal(β1-4)GlcNAc(β1-3)Gal(β1-4)GlcNAc(β1-2)Man(α1-6)[Gal(β1-4)GlcNAc(β1-3)Gal(β1-4)GlcNAc(β1-3)Gal(β1-4)GlcNAc(β1-2)Man(α1-3)]Man(β1-4)GlcNAc

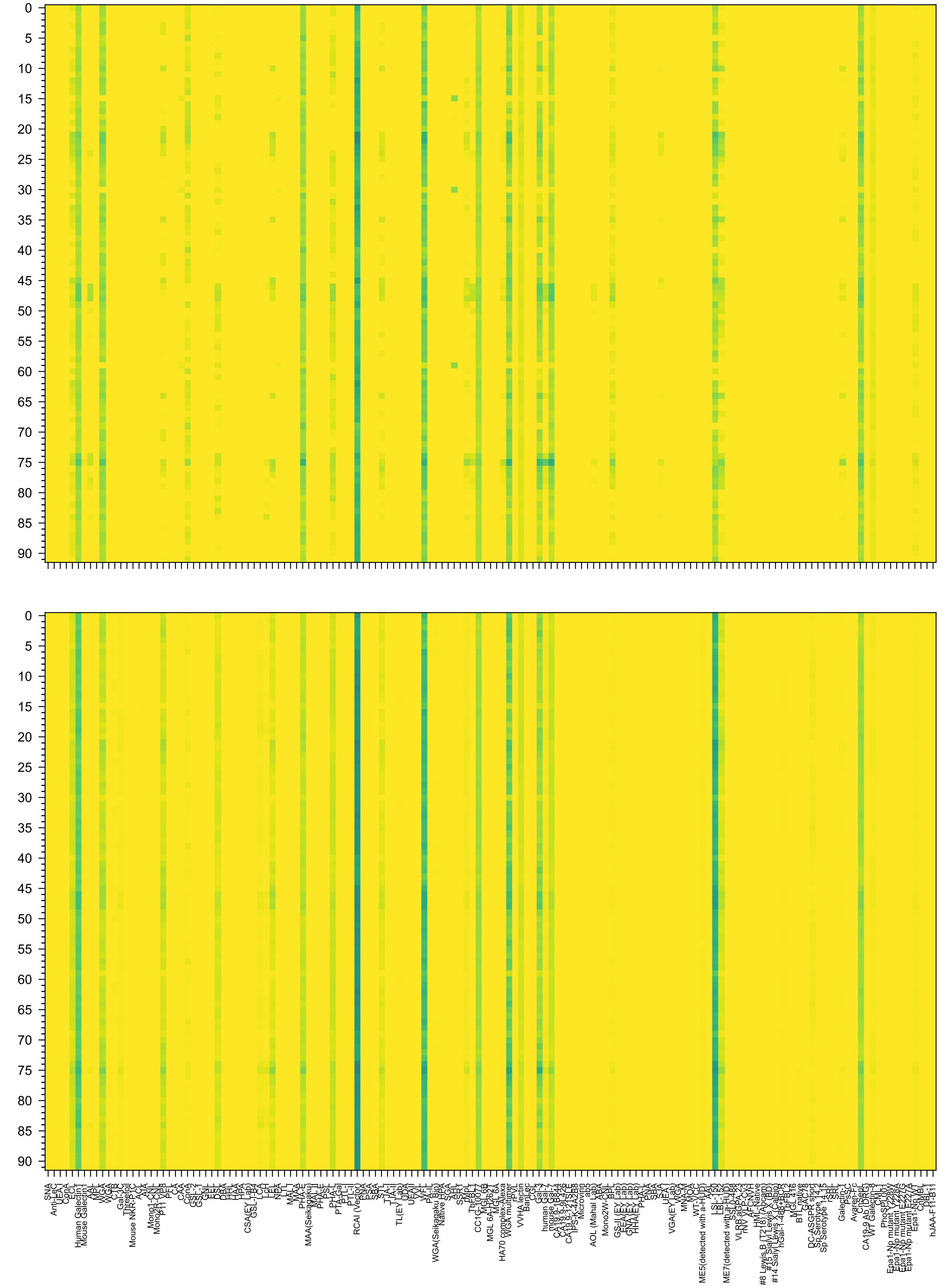
c(β1-3)Gal(β1-4)GlcNAc(β1-6)[Gal(β1-4)GlcNAc(β1-3)Gal(β1-4)GlcNAc(β1-2)]Man(α1-6)[Gal(β1-4)GlcNAc(β1-3)Gal(β1-4)GlcNAc(β1-2)Man(α1-3)]Man(α1-4)GlcNA

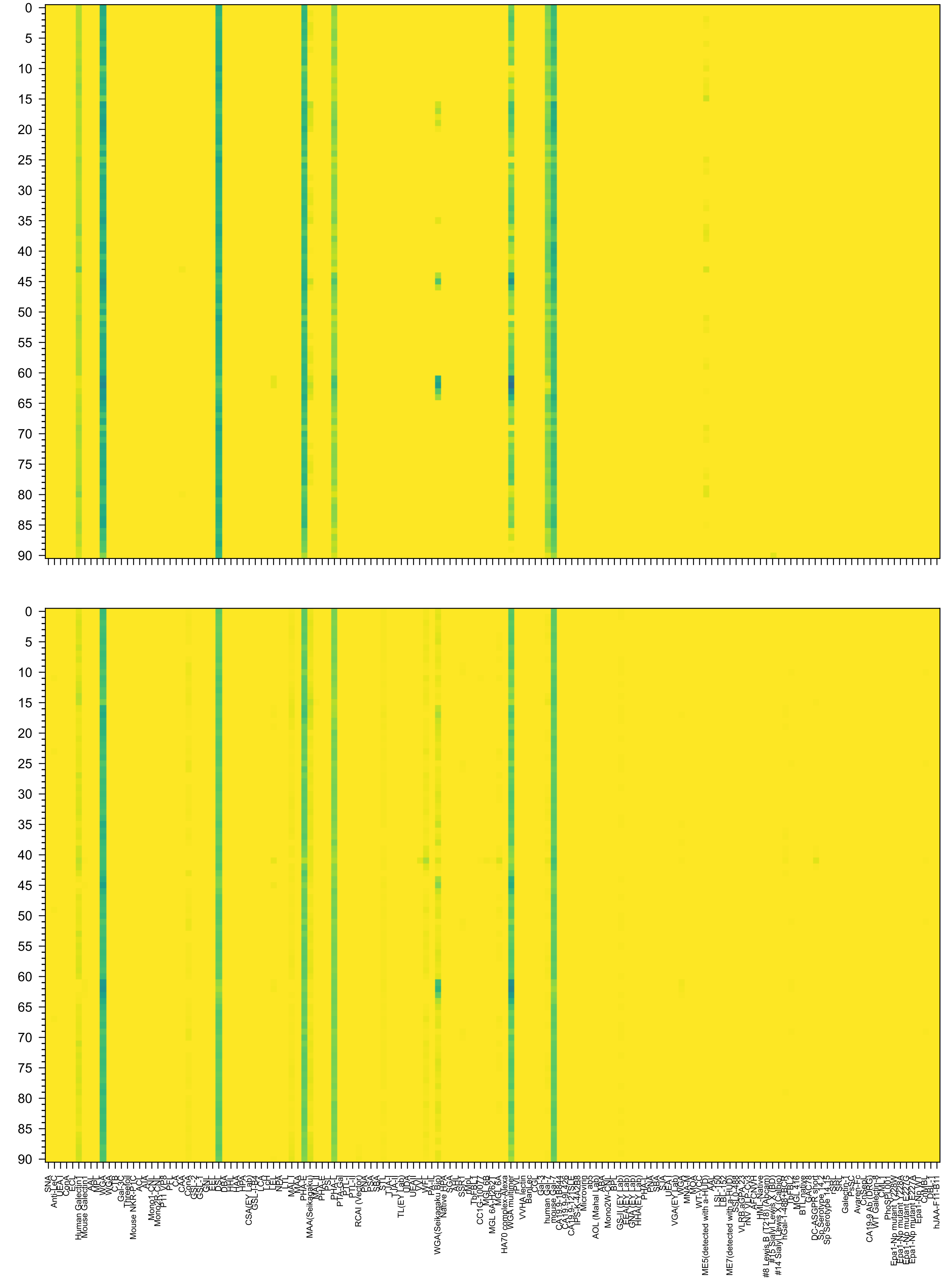

Ac(β1-6)[Neu5Ac(α2-3)Gal(β1-4)GlcNAc(β1-2)]Man(α1-6)[GlcNAc(β1-4)][Neu5Ac(α2-3)Gal(β1-4)GlcNAc(β1-4)][Neu5Ac(α2-3)Gal(β1-4)GlcNAc(β1-2)]Man(α1-3)]Man(

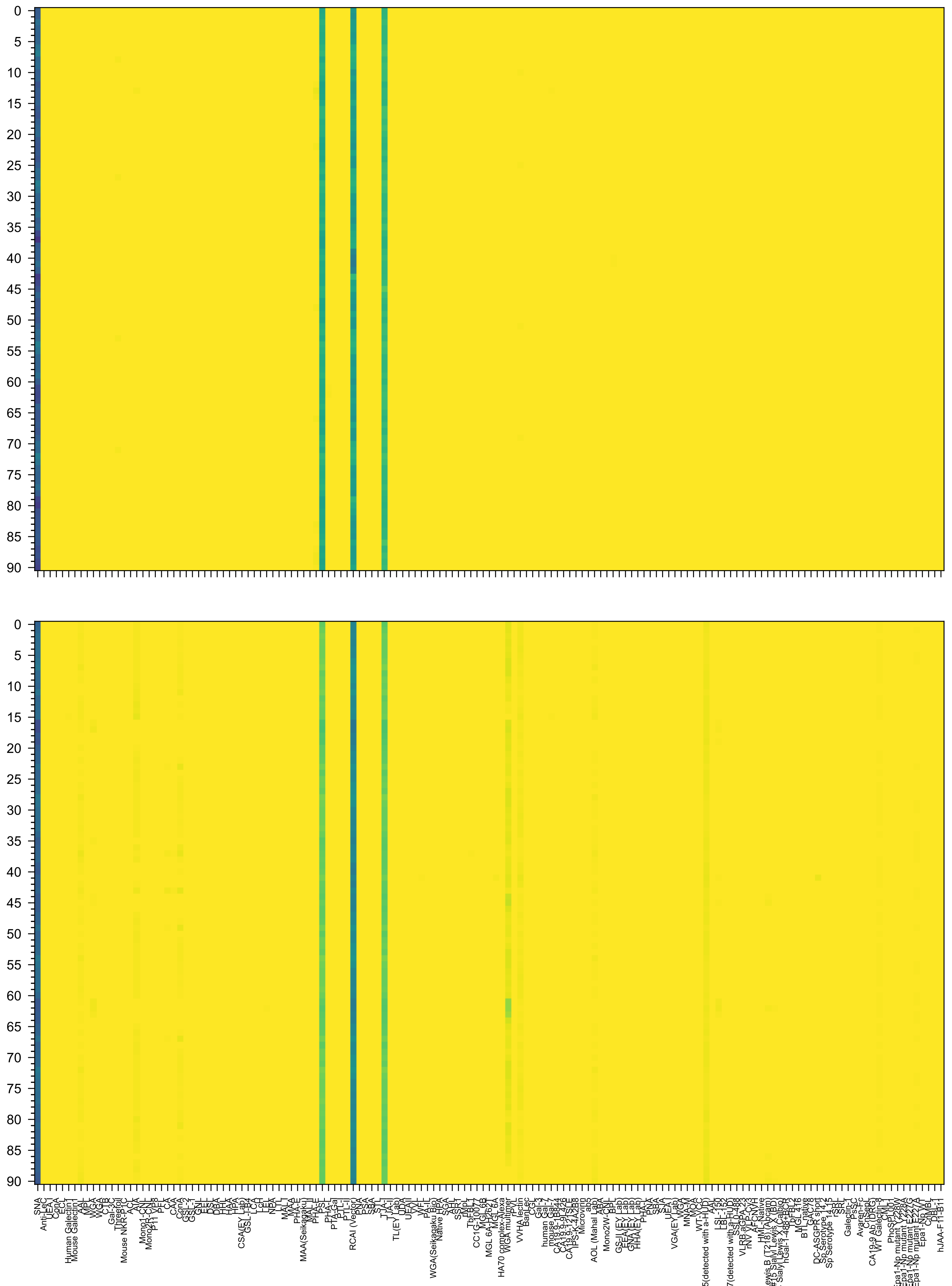

Ac(β1-6)[Neu5Ac(α2-6)Gal(β1-4)GlcNAc(β1-2)]Man(α1-6)[GlcNAc(β1-4)][Neu5Ac(α2-6)Gal(β1-4)GlcNAc(β1-4)][Neu5Ac(α2-6)Gal(β1-4)GlcNAc(β1-2)]Man(α1-3)]Man

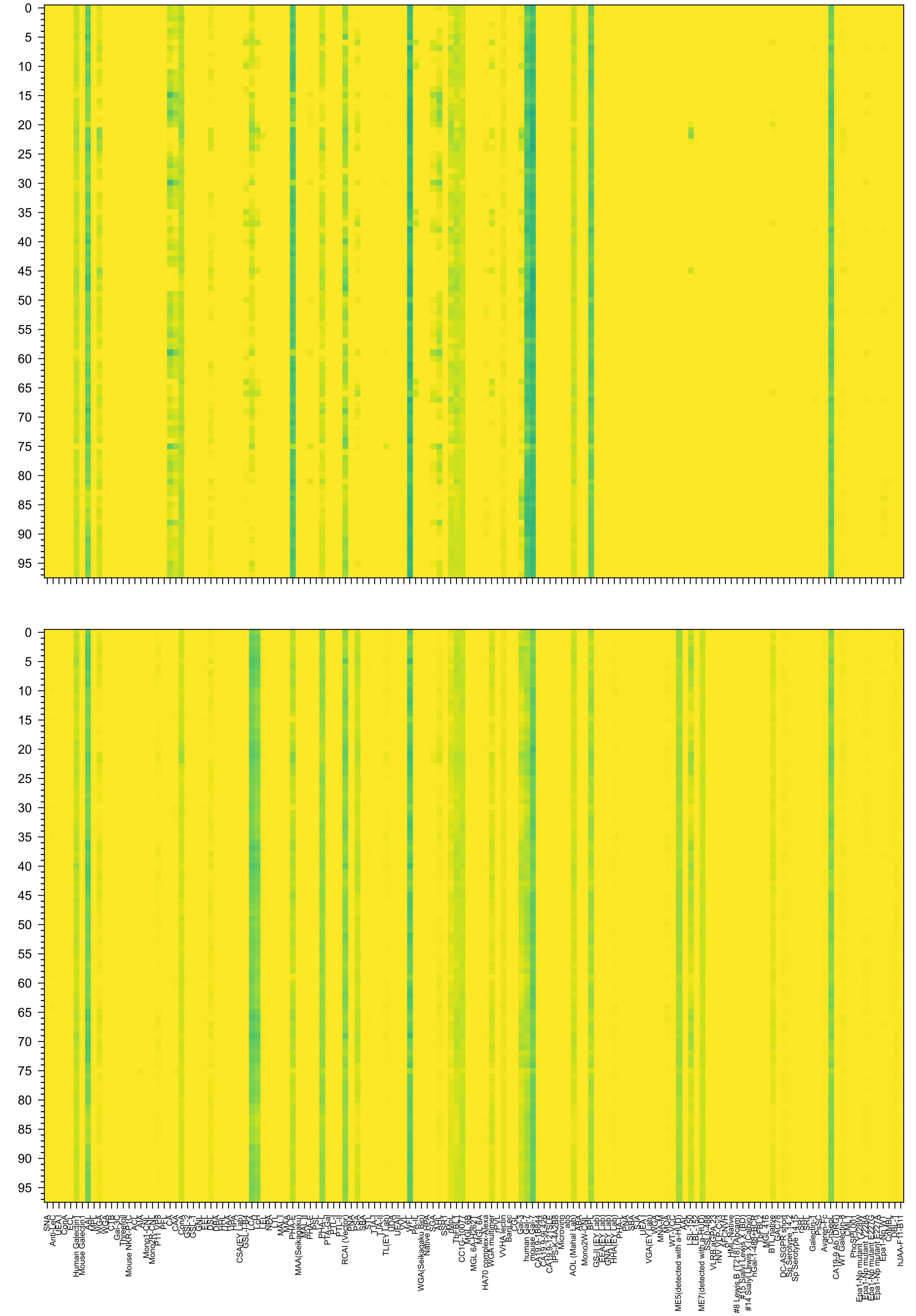

)Gal(β1-4)GlcNAc(β1-6)[Gal(β1-3)GlcNAc(β1-3)Gal(β1-4)GlcNAc(β1-2)]Man(α1-6)[Gal(β1-3)GlcNAc(β1-3)Gal(β1-4)GlcNAc(β1-2)Man(α1-3)]Man(β1-4)GlcNAc(β1-4

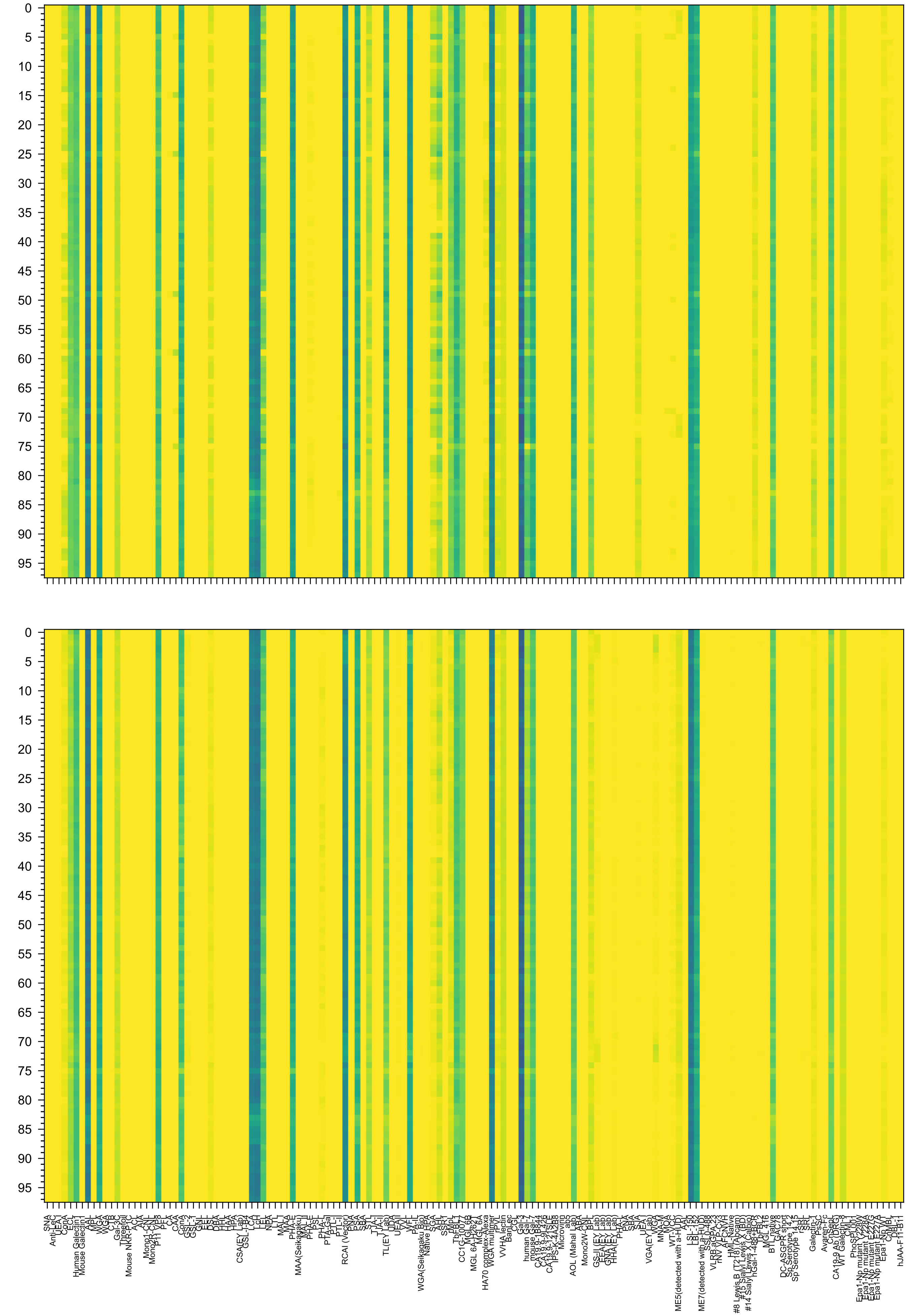

3)Gal(β1-4)GlcNAc(β1-3)Gal(β1-4)GlcNAc(β1-2)Man(α1-6)[Gal(β1-4)GlcNAc(β1-3)Gal(β1-4)GlcNAc(β1-3)Gal(β1-4)GlcNAc(β1-2)Man(α1-3)]Man(β1-4)GlcNAc(β1-4)

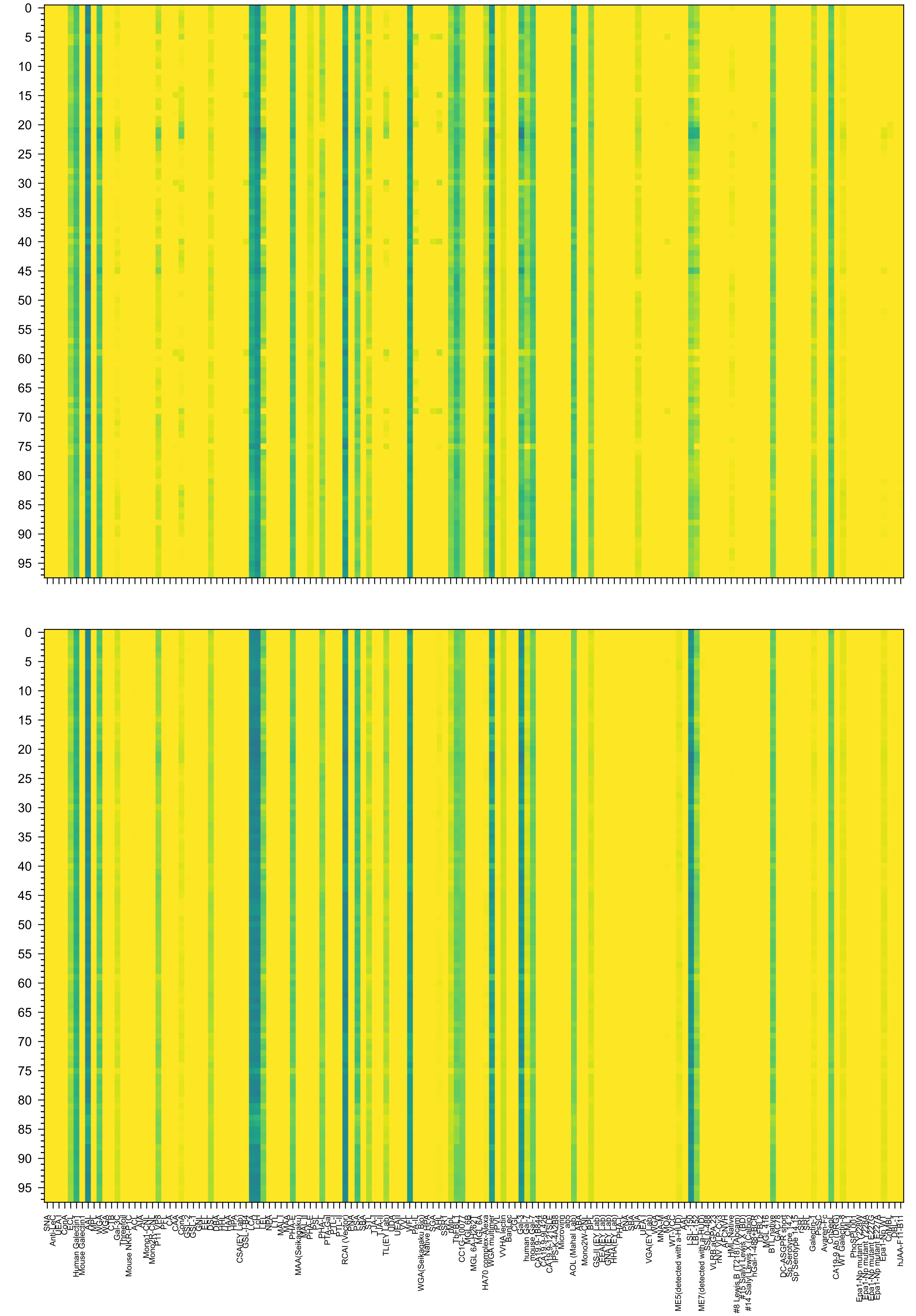

3)Gal(β1-4)GlcNAc(β1-6)[Gal(β1-4)GlcNAc(β1-3)Gal(β1-4)GlcNAc(β1-2)]Man(α1-6)[Gal(β1-4)GlcNAc(β1-3)Gal(β1-4)GlcNAc(β1-2)Man(α1-3)]Man(β1-4)GlcNAc(β1-4

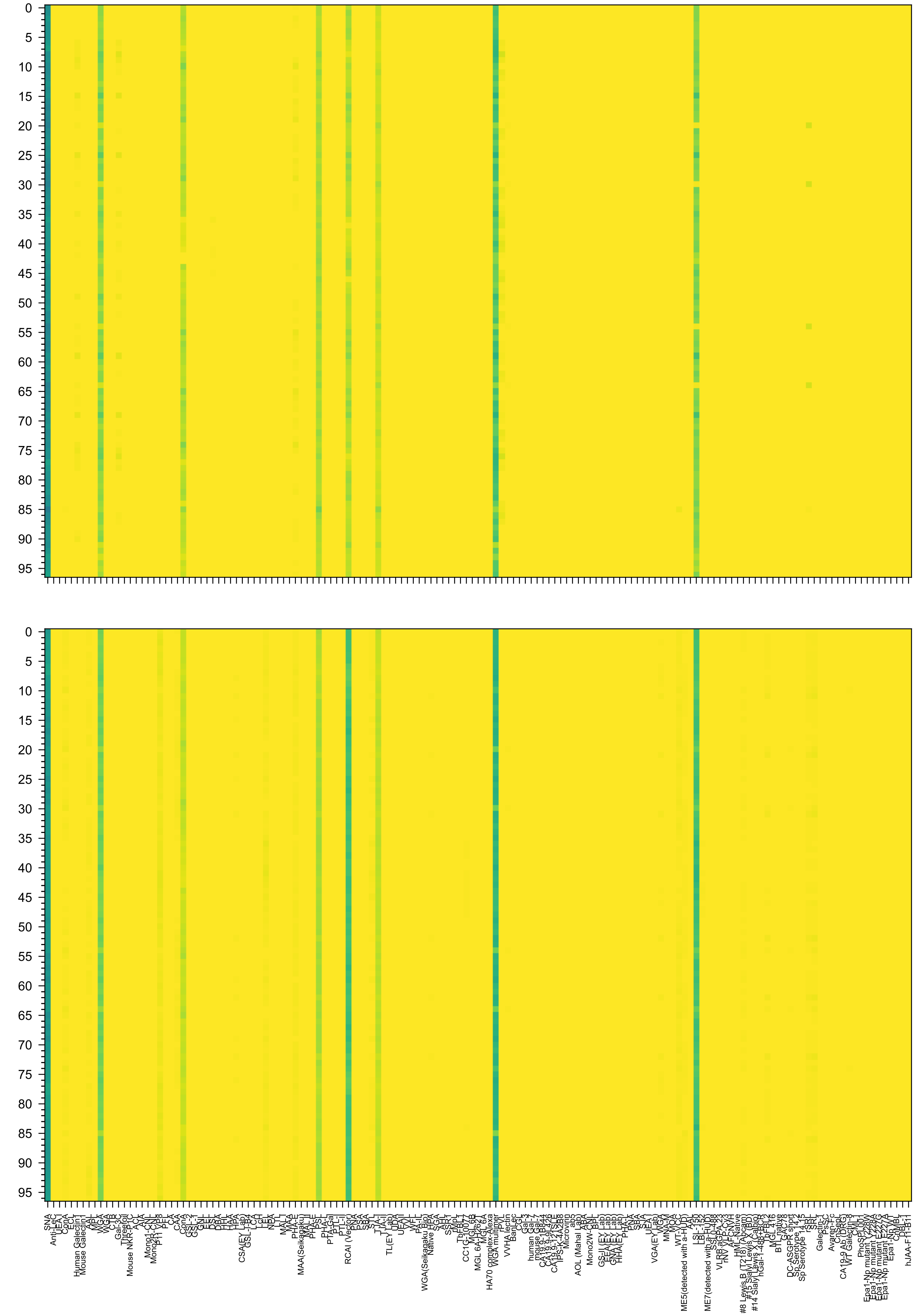

Ac(β1-3)Gal(β1-4)GlcNAc(β1-3)Gal(β1-4)GlcNAc(β1-2)Man(α1-6)[Neu5Ac(α2-6)Gal(β1-4)GlcNAc(β1-3)Gal(β1-4)GlcNAc(β1-3)Gal(β1-4)GlcNAc(β1-2)Man(α1-3)]Man

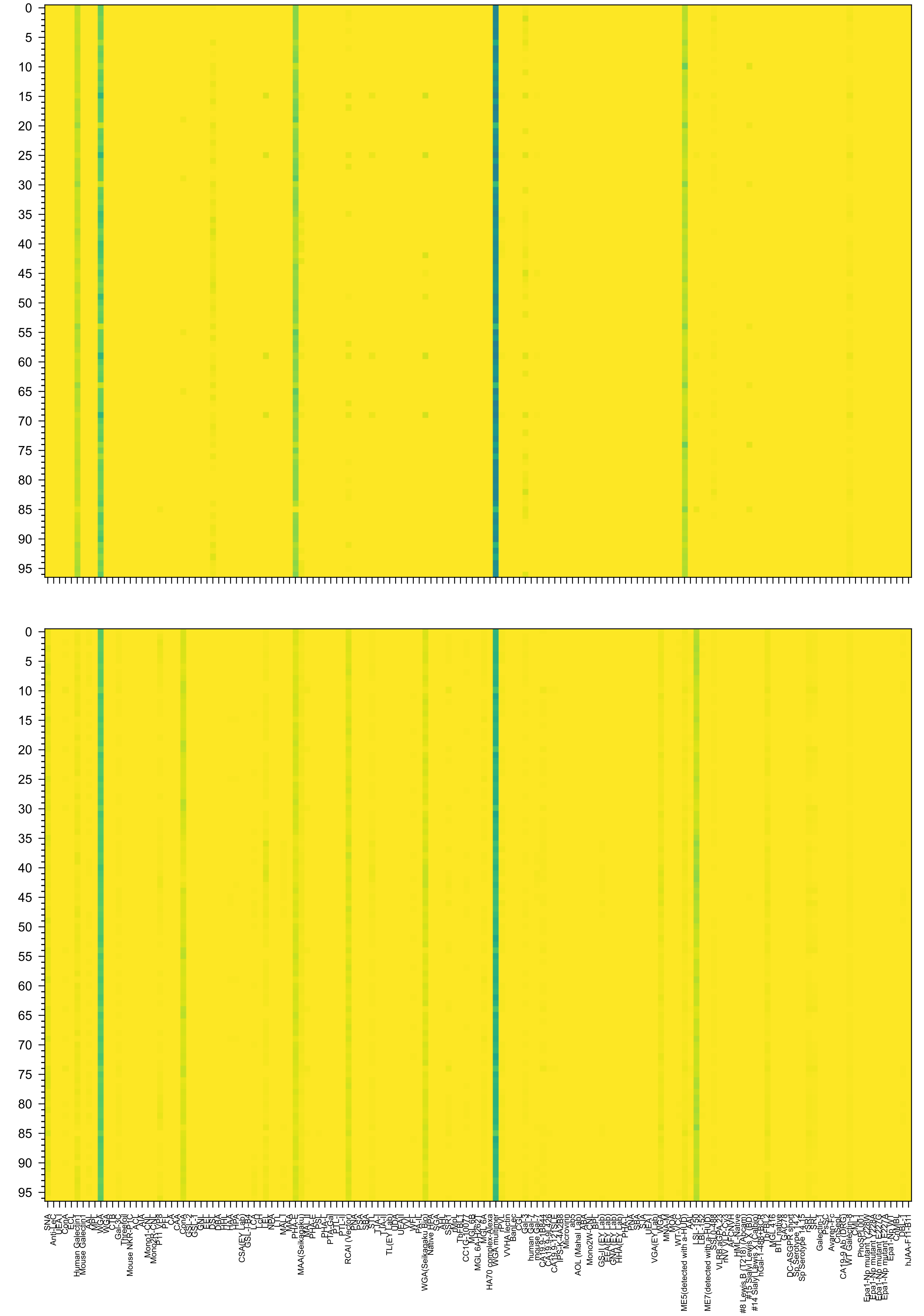

Ac(β1-3)Gal(β1-4)GlcNAc(β1-3)Gal(β1-4)GlcNAc(β1-2)Man(α1-6)[Neu5Ac(α2-3)Gal(β1-4)GlcNAc(β1-3)Gal(β1-4)GlcNAc(β1-3)Gal(β1-4)GlcNAc(β1-2)Man(α1-3)]Man

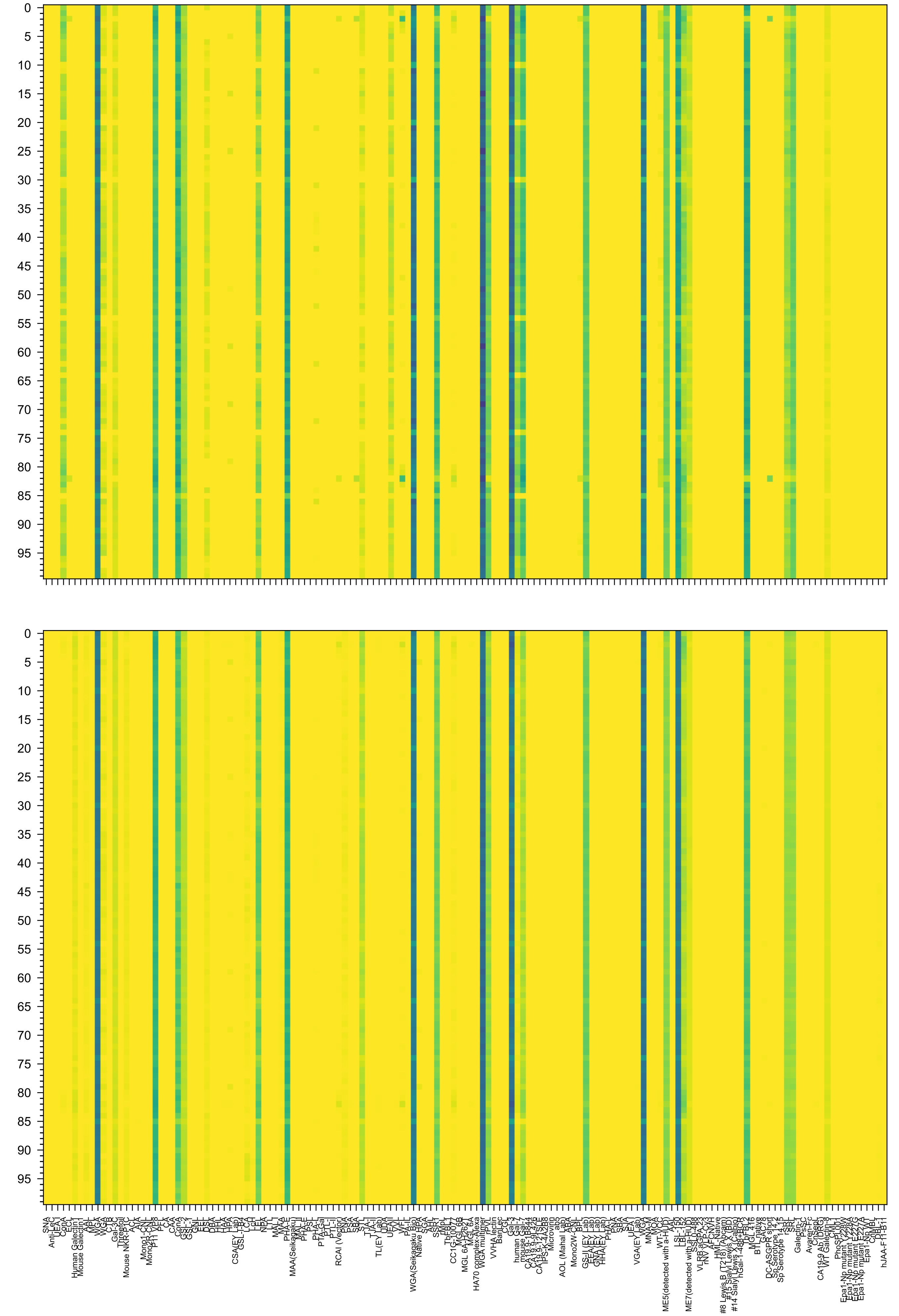

c(β1-3)Gal(β1-4)GlcNAc(β1-3)Gal(β1-4)GlcNAc(β1-2)Man(α1-6)[GlcNAc(β1-3)Gal(β1-4)GlcNAc(β1-3)Gal(β1-4)GlcNAc(β1-3)Gal(β1-4)GlcNAc(β1-2)Man(α1-3)]Man

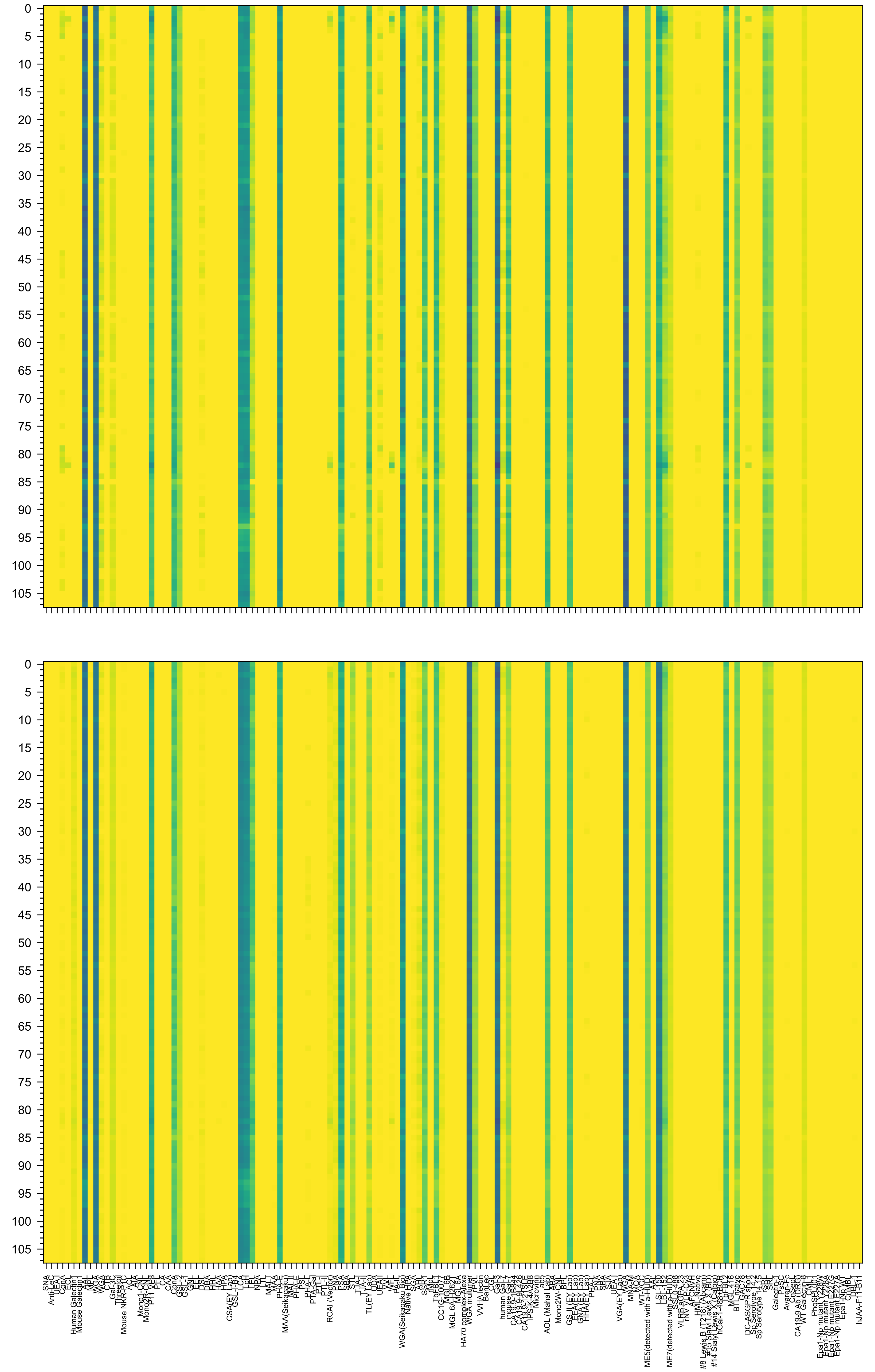

3)Gal(β1-4)GlcNAc(β1-3)Gal(β1-4)GlcNAc(β1-2)Man(α1-6)[GlcNAc(β1-3)Gal(β1-4)GlcNAc(β1-3)Gal(β1-4)GlcNAc(β1-3)Gal(β1-4)GlcNAc(β1-2)Man(α1-3)]Man(β1-4)

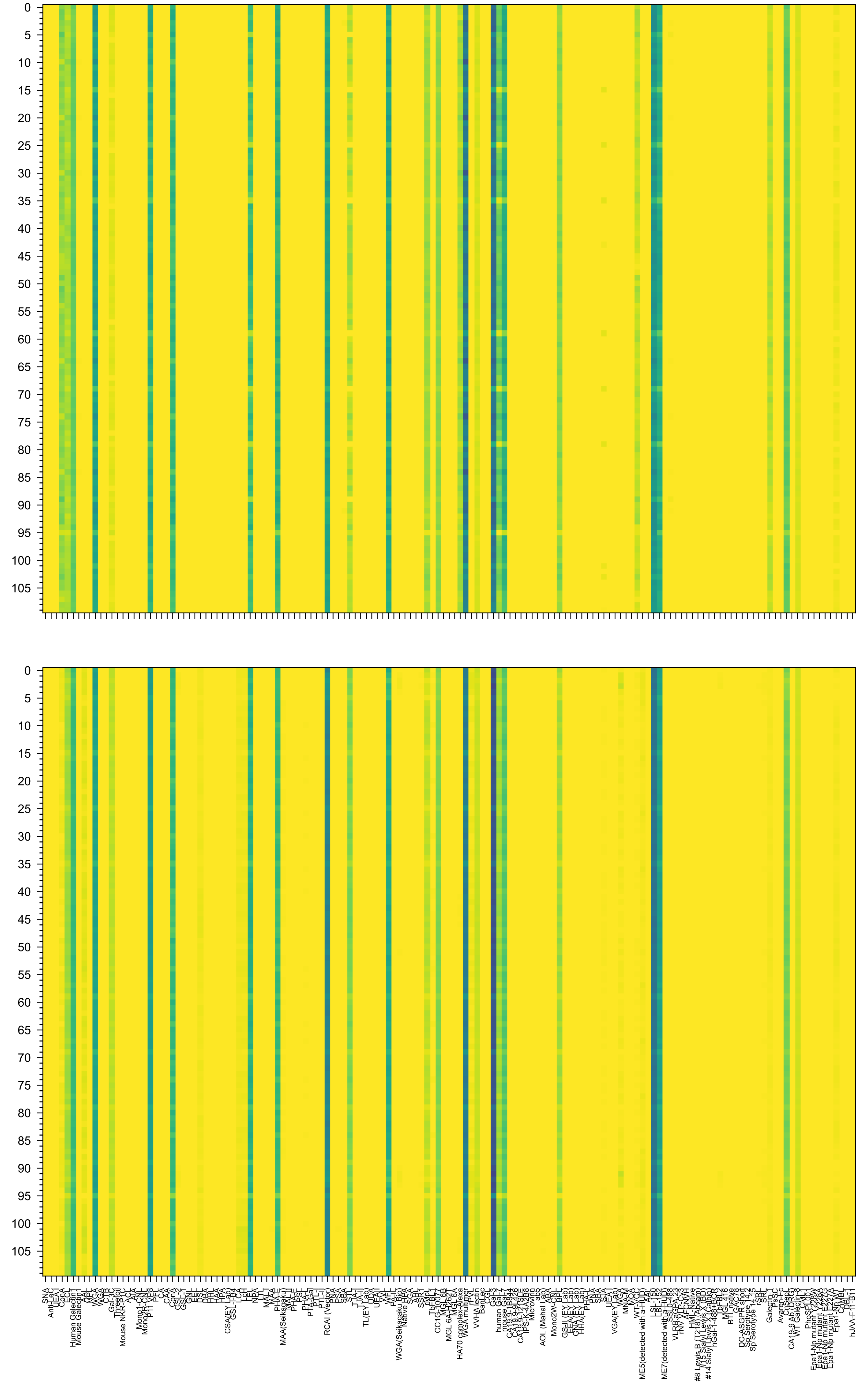

c(β1-3)Gal(β1-4)GlcNAc(β1-3)Gal(β1-4)GlcNAc(β1-2)Man(α1-6)[Gal(β1-4)GlcNAc(β1-3)Gal(β1-4)GlcNAc(β1-3)Gal(β1-4)GlcNAc(β1-3)Gal(β1-4)GlcNAc(β1-2)Man(α

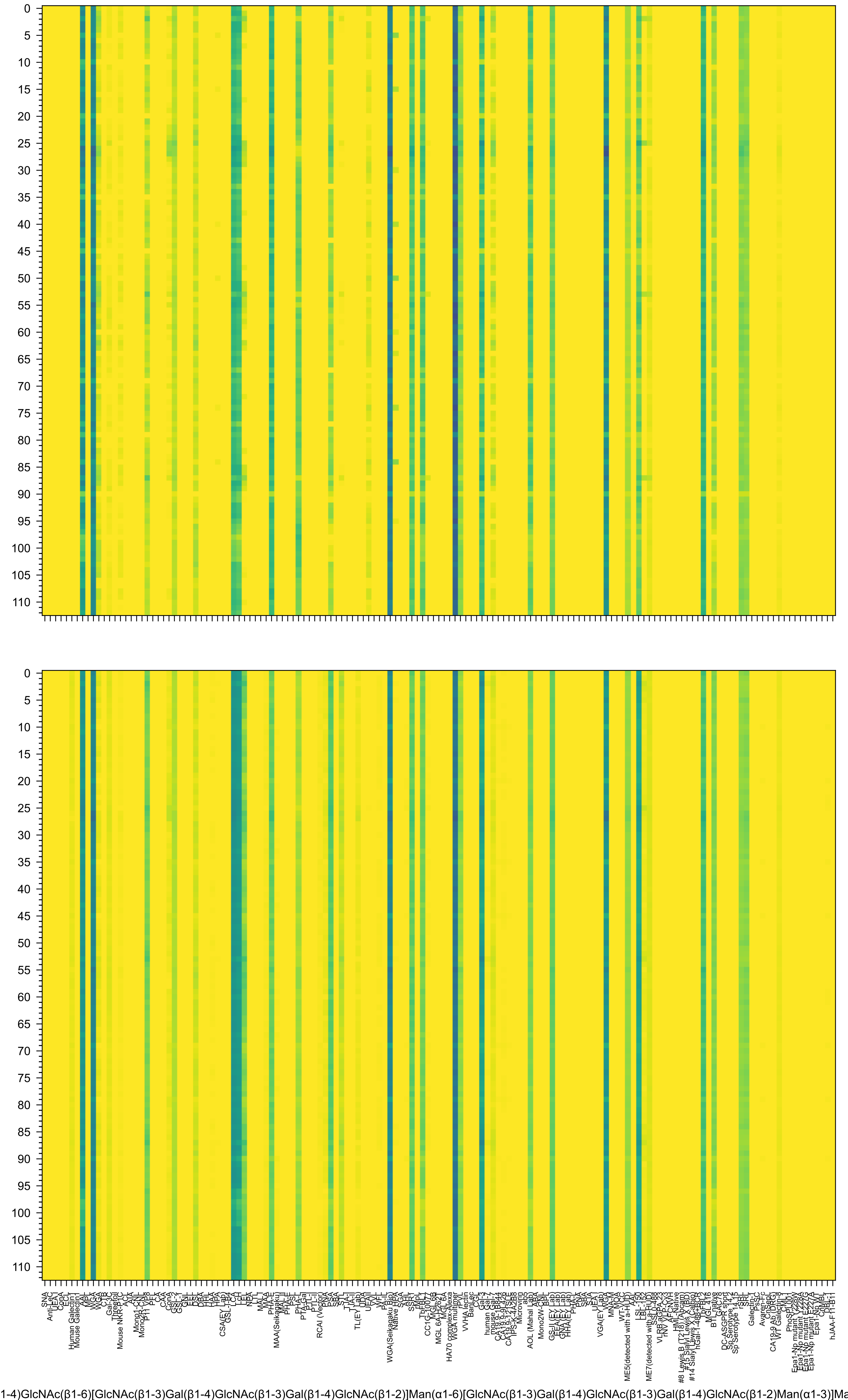
1-4)GlcNAc(β1-6)[GlcNAc(β1-3)Gal(β1-4)GlcNAc(β1-3)Gal(β1-4)GlcNAc(β1-2)]Man(α1-6)[GlcNAc(β1-3)Gal(β1-4)GlcNAc(β1-3)Gal(β1-4)GlcNAc(β1-2)Man(α1-3)]Ma

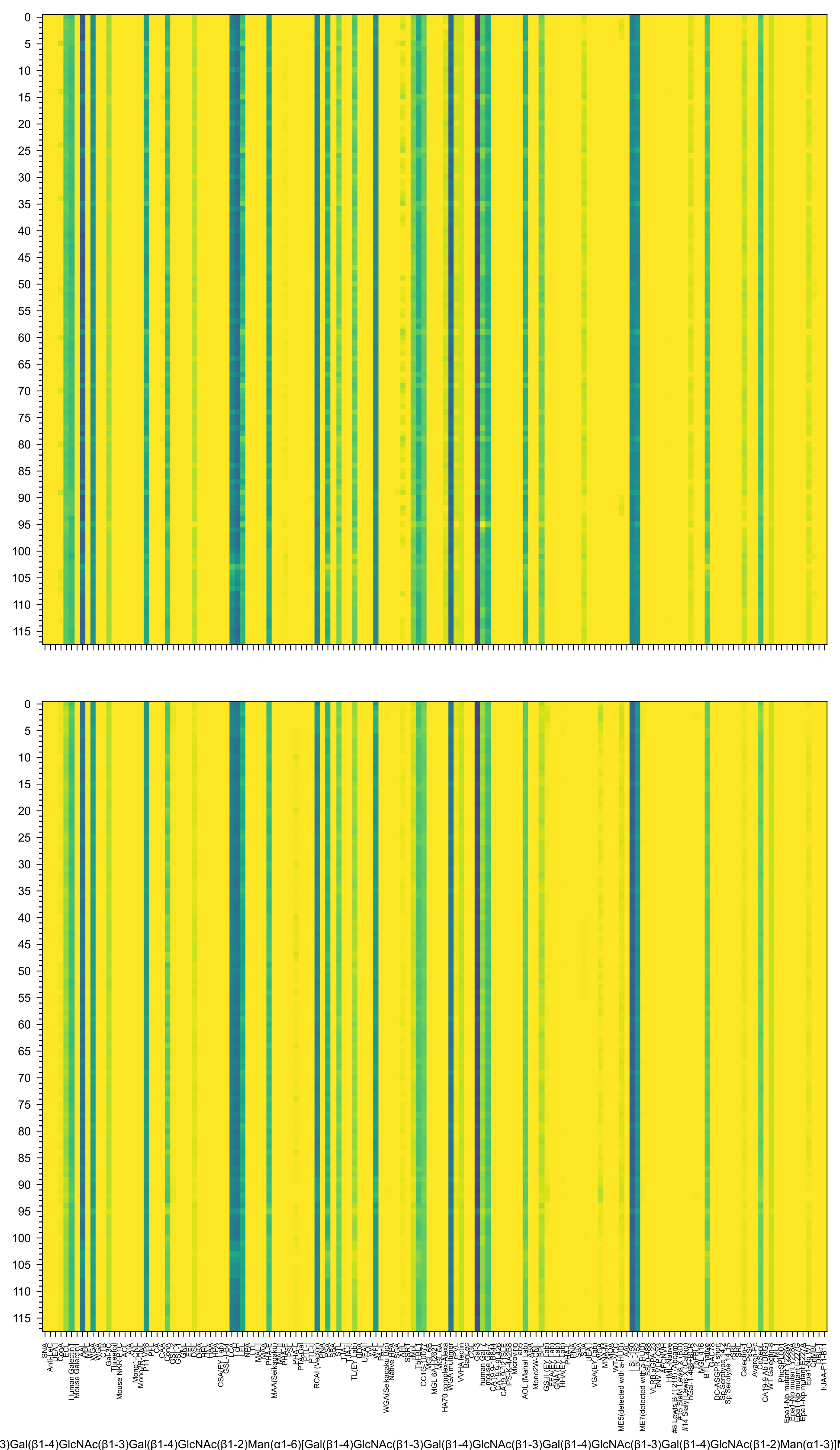
3)Gal(β1-4)GlcNAc(β1-3)Gal(β1-4)GlcNAc(β1-2)Man(α1-6)[Gal(β1-4)GlcNAc(β1-3)Gal(β1-4)GlcNAc(β1-3)Gal(β1-4)GlcNAc(β1-3)Gal(β1-4)GlcNAc(β1-2)Man(α1-3)]M

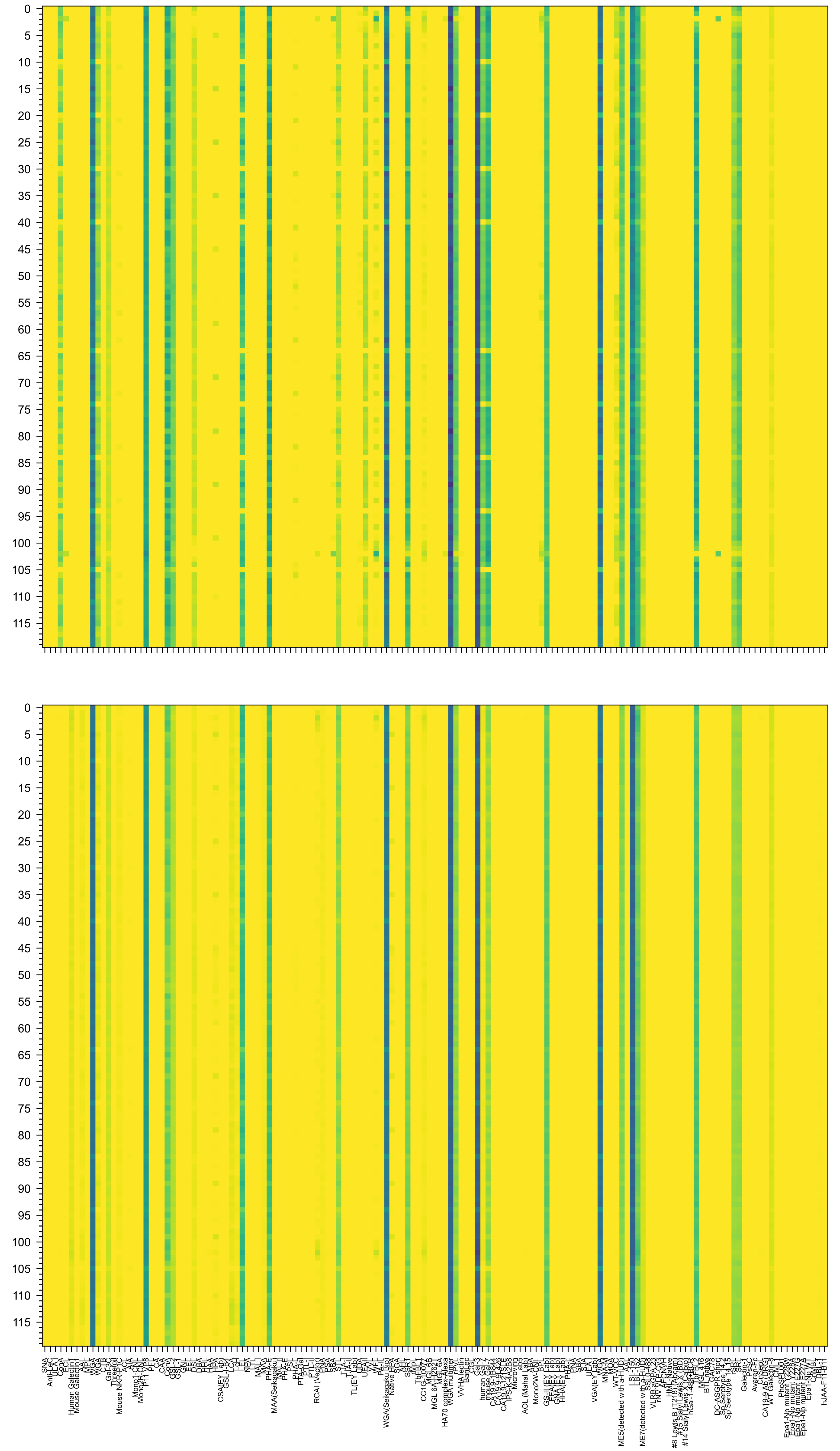

c(β1-3)Gal(β1-4)GlcNAc(β1-3)Gal(β1-4)GlcNAc(β1-2)Man(α1-6)[GlcNAc(β1-3)Gal(β1-4)GlcNAc(β1-3)Gal(β1-4)GlcNAc(β1-3)Gal(β1-4)GlcNAc(β1-3)Gal(β1-4)GlcNA

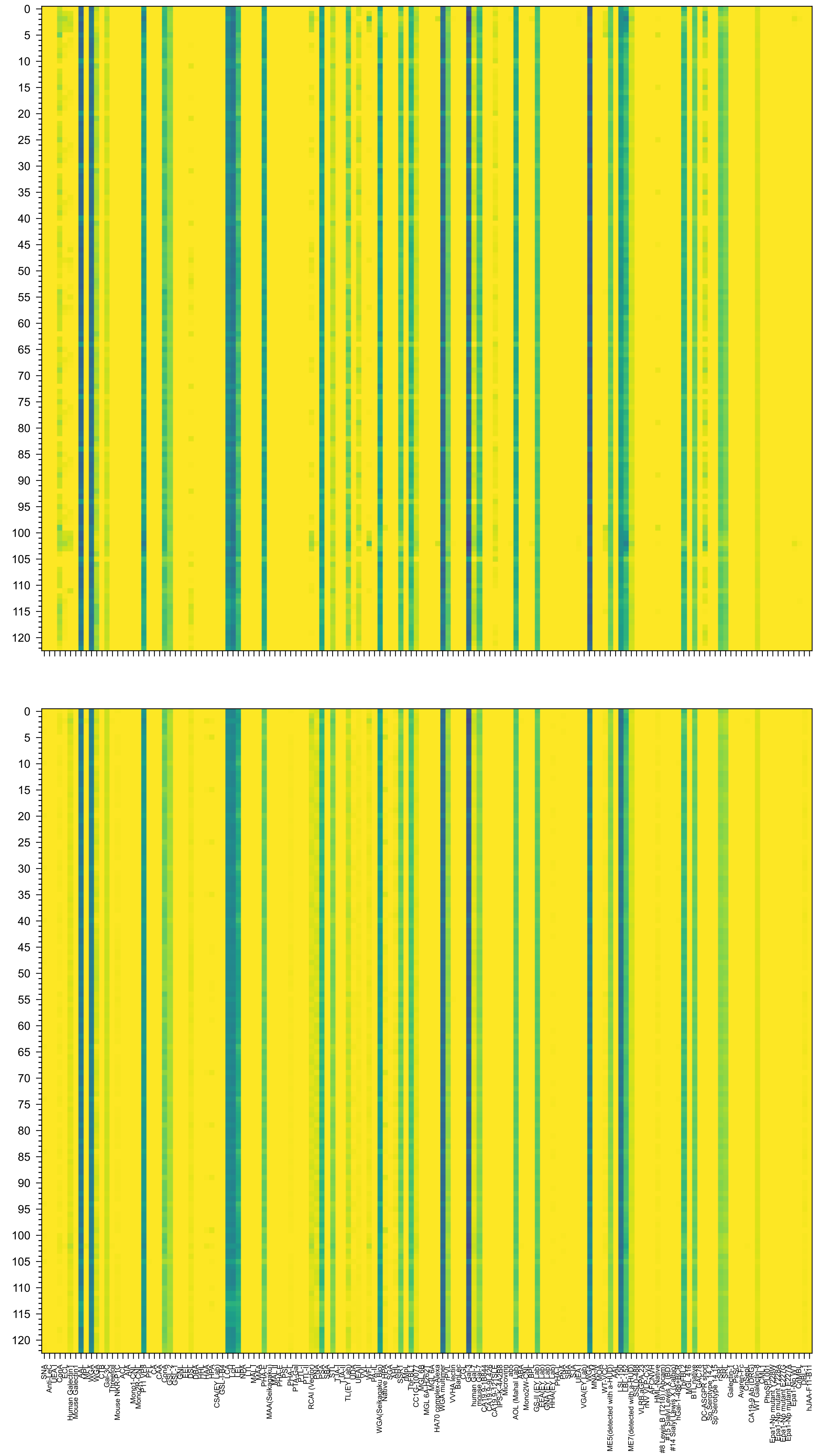

3)Gal(β1-4)GlcNAc(β1-3)Gal(β1-4)GlcNAc(β1-2)Man(α1-6)[GlcNAc(β1-3)Gal(β1-4)GlcNAc(β1-3)Gal(β1-4)GlcNAc(β1-3)Gal(β1-4)GlcNAc(β1-3)Gal(β1-4)GlcNAc(β1-

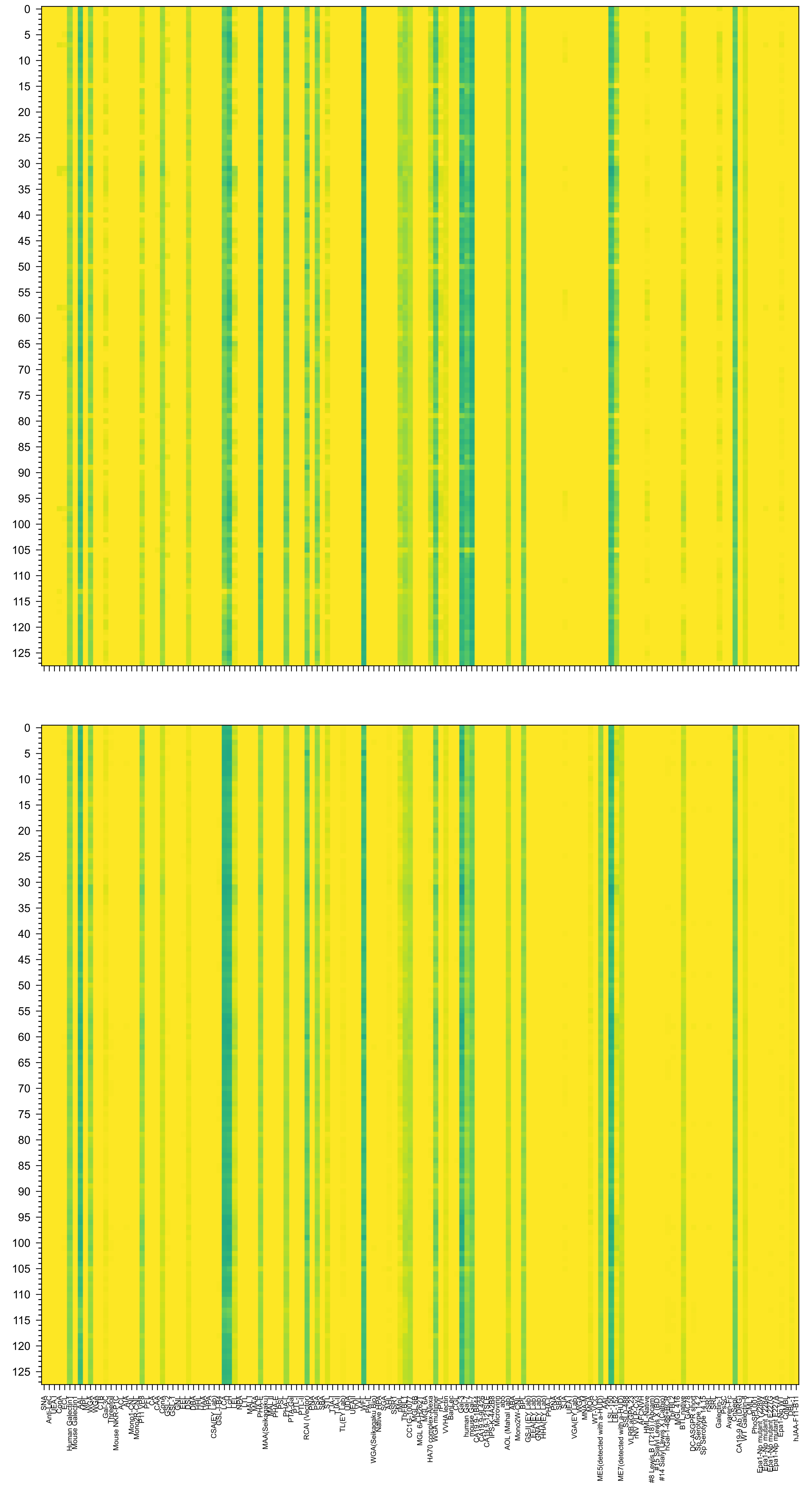

GlcNAc(β1-6)[Gal(β1-3)GlcNAc(β1-3)Gal(β1-4)GlcNAc(β1-3)Gal(β1-4)GlcNAc(β1-2)]Man(α1-6)[Gal(β1-3)GlcNAc(β1-3)Gal(β1-4)GlcNAc(β1-3)Gal(β1-4)GlcNAc(β1-2

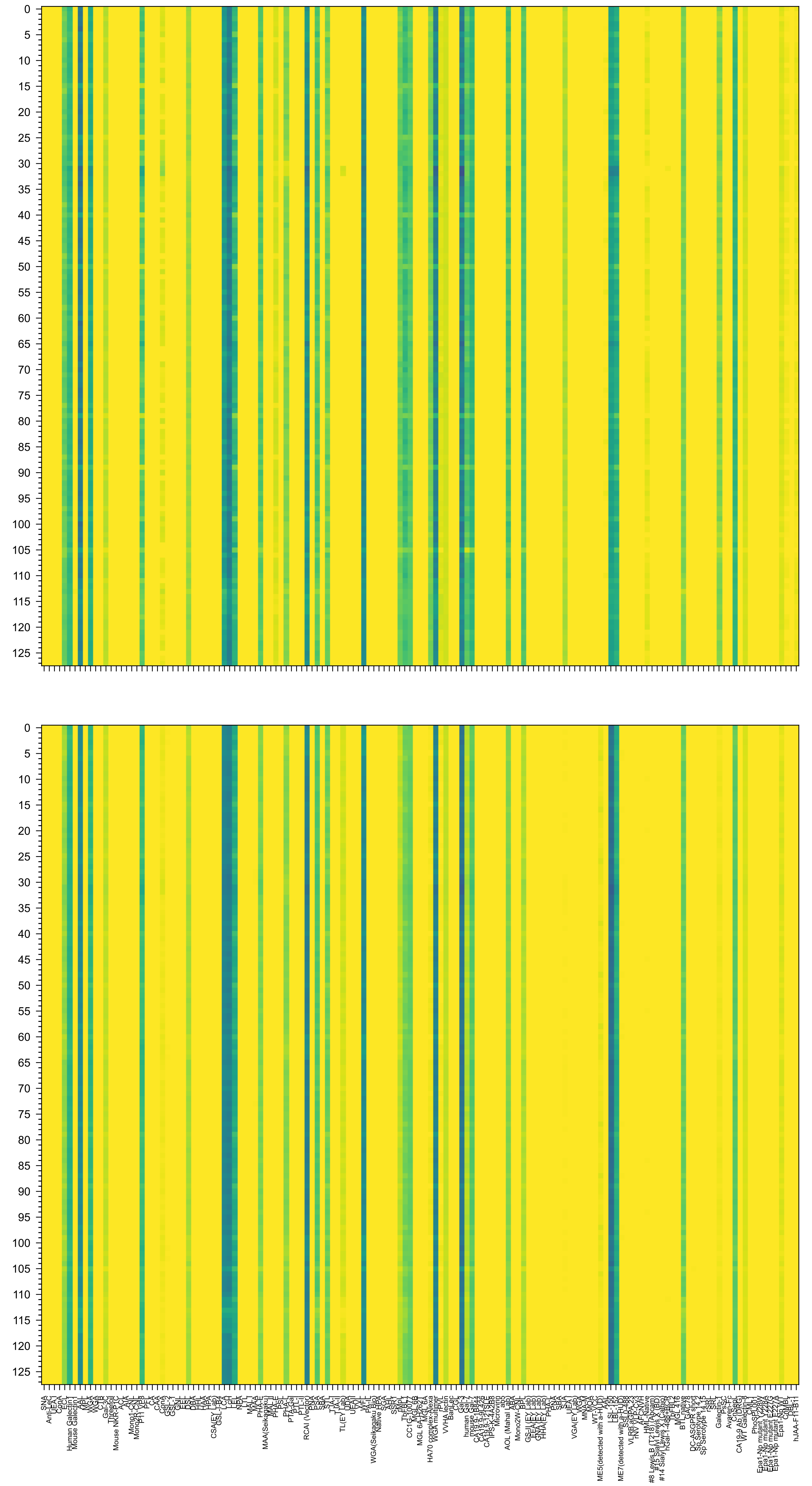

GlcNAc(β1-6)[Gal(β1-4)GlcNAc(β1-3)Gal(β1-4)GlcNAc(β1-3)Gal(β1-4)GlcNAc(β1-2)]Man(α1-6)[Gal(β1-4)GlcNAc(β1-3)Gal(β1-4)GlcNAc(β1-3)Gal(β1-4)GlcNAc(β1-2

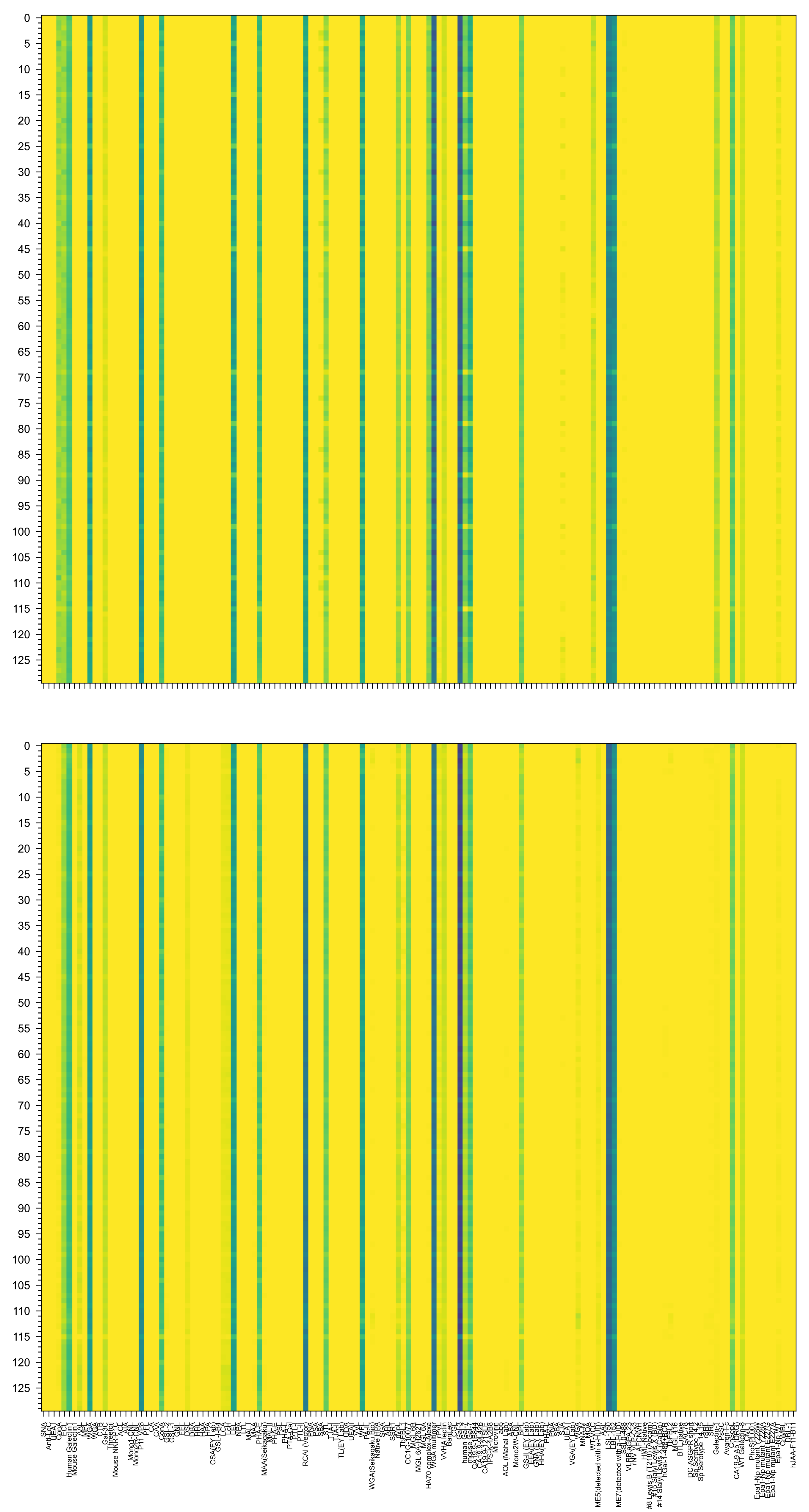

c(β1-3)Gal(β1-4)GlcNAc(β1-3)Gal(β1-4)GlcNAc(β1-2)Man(α1-6)[Gal(β1-4)GlcNAc(β1-3)Gal(β1-4)GlcNAc(β1-3)Gal(β1-4)GlcNAc(β1-3)Gal(β1-4)GlcNAc(β1-3)Gal(β

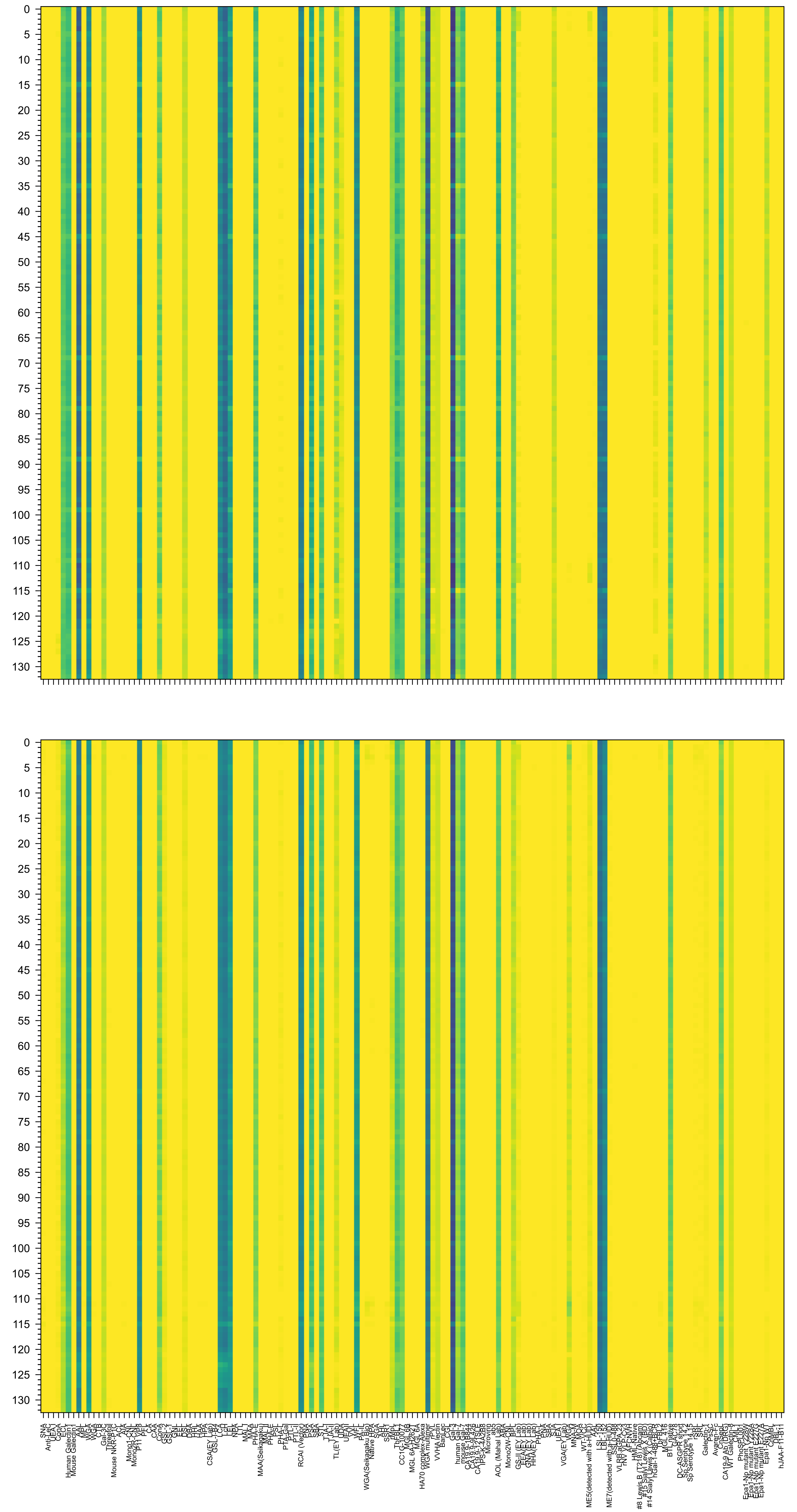

3)Gal(β1-4)GlcNAc(β1-3)Gal(β1-4)GlcNAc(β1-2)Man(α1-6)[Gal(β1-4)GlcNAc(β1-3)Gal(β1-4)GlcNAc(β1-3)Gal(β1-4)GlcNAc(β1-3)Gal(β1-4)GlcNAc(β1-3)Gal(β1-4)GlcNAc(β1-3)Gal(β1-4)Gl

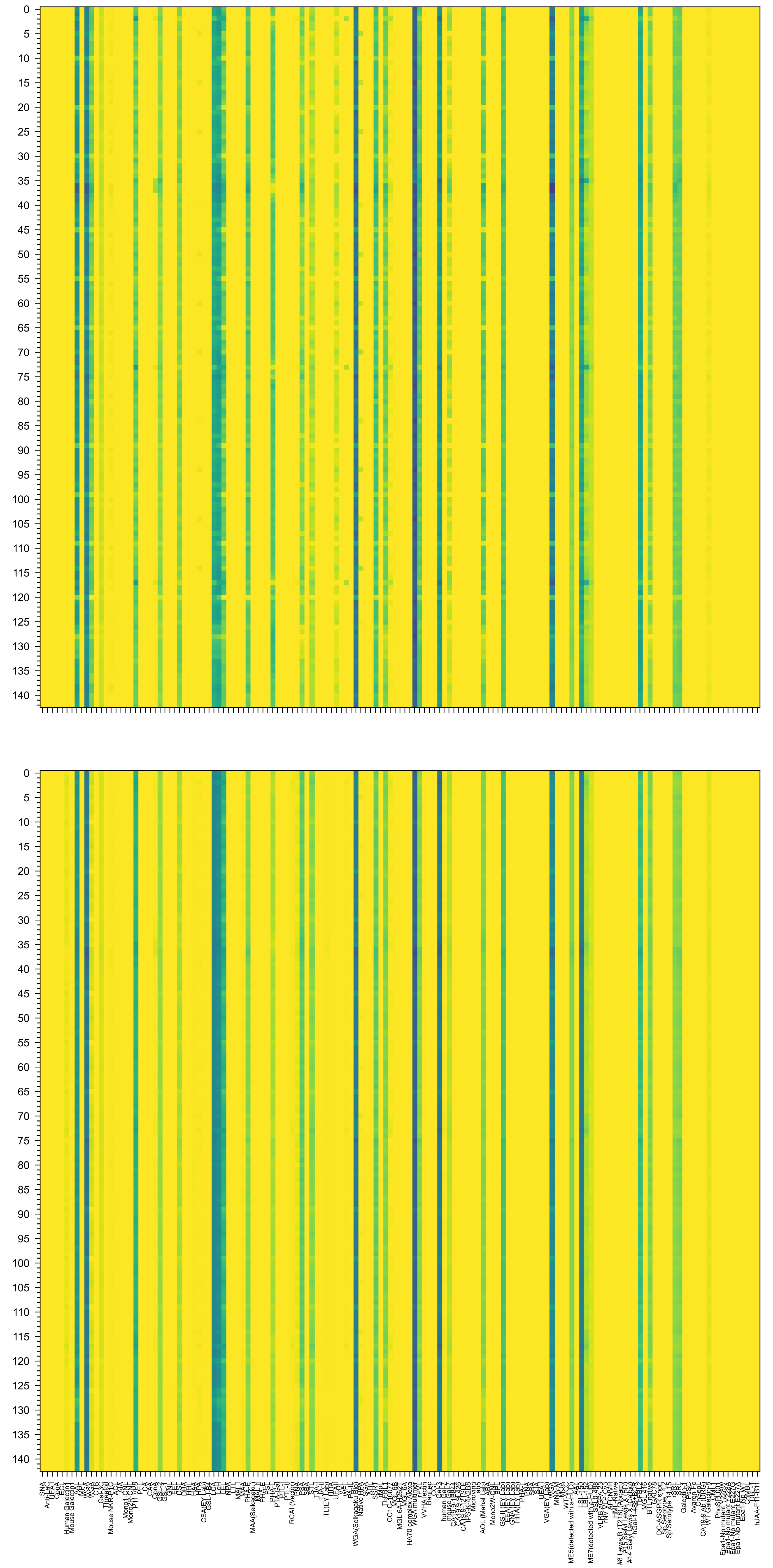

(β1-6)[GlcNAc(β1-3)Gal(β1-4)GlcNAc(β1-3)Gal(β1-4)GlcNAc(β1-3)Gal(β1-4)GlcNAc(β1-2)]Man(α1-6)[GlcNAc(β1-3)Gal(β1-4)GlcNAc(β1-3)Gal(β1-4)GlcNAc(β1-3)Ga

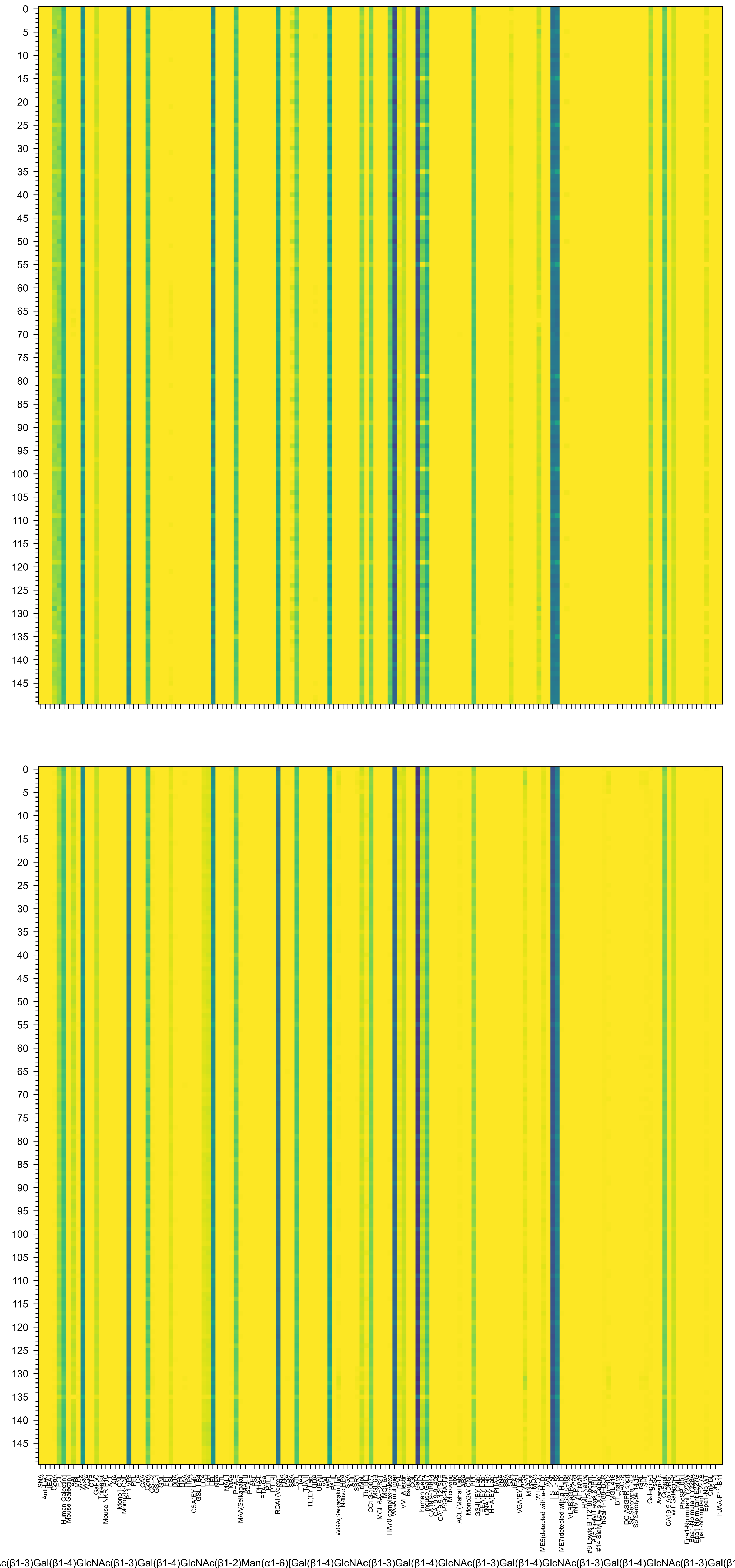
c(β1-3)Gal(β1-4)GlcNAc(β1-3)Gal(β1-4)GlcNAc(β1-2)Man(α1-6)[Gal(β1-4)GlcNAc(β1-3)Gal(β1-4)GlcNAc(β1-3)Gal(β1-4)GlcNAc(β1-3)Gal(β1-4)GlcNAc(β1-3)Gal(β

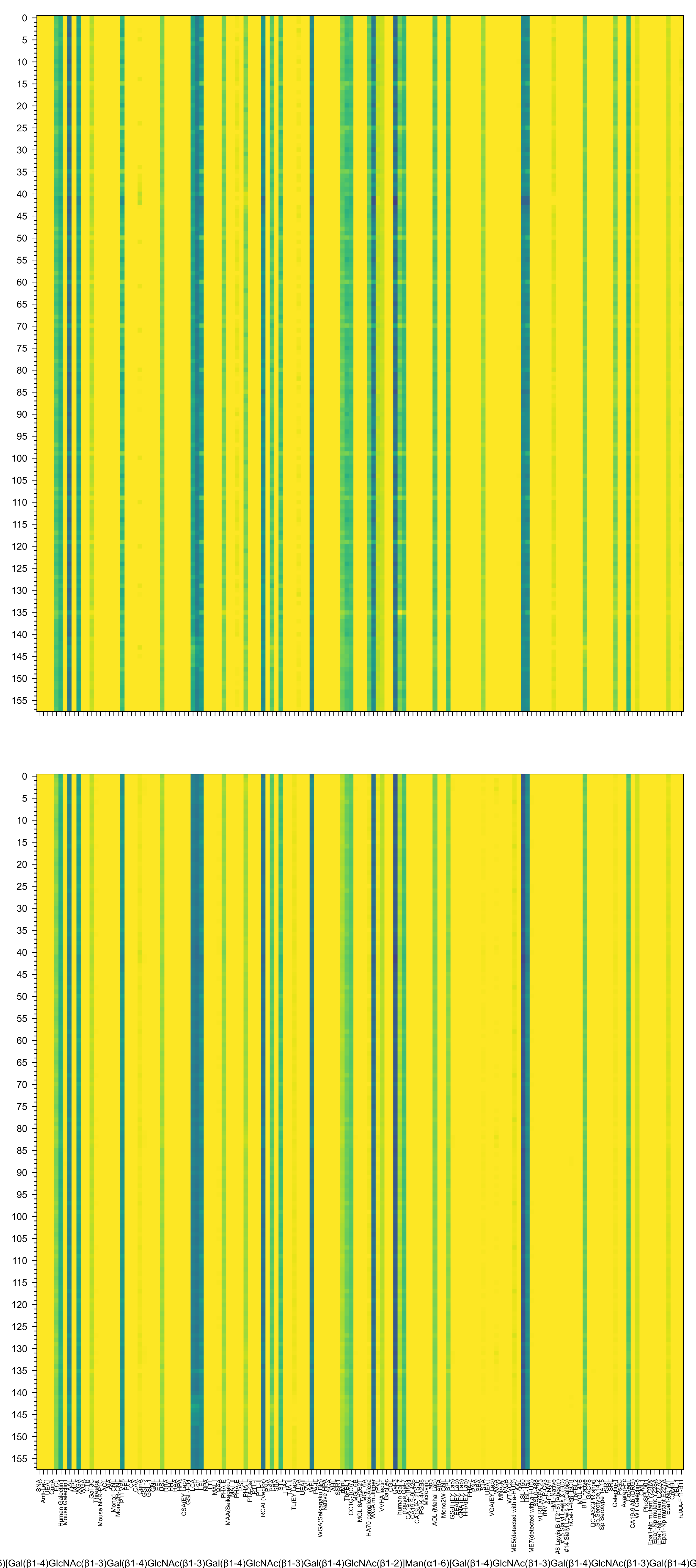
6)[Gal(β1-4)GlcNAc(β1-3)Gal(β1-4)GlcNAc(β1-3)Gal(β1-4)GlcNAc(β1-3)Gal(β1-4)GlcNAc(β1-2)]Man(α1-6)[Gal(β1-4)GlcNAc(β1-3)Gal(β1-4)GlcNAc(β1-3)Gal(β1-4)G

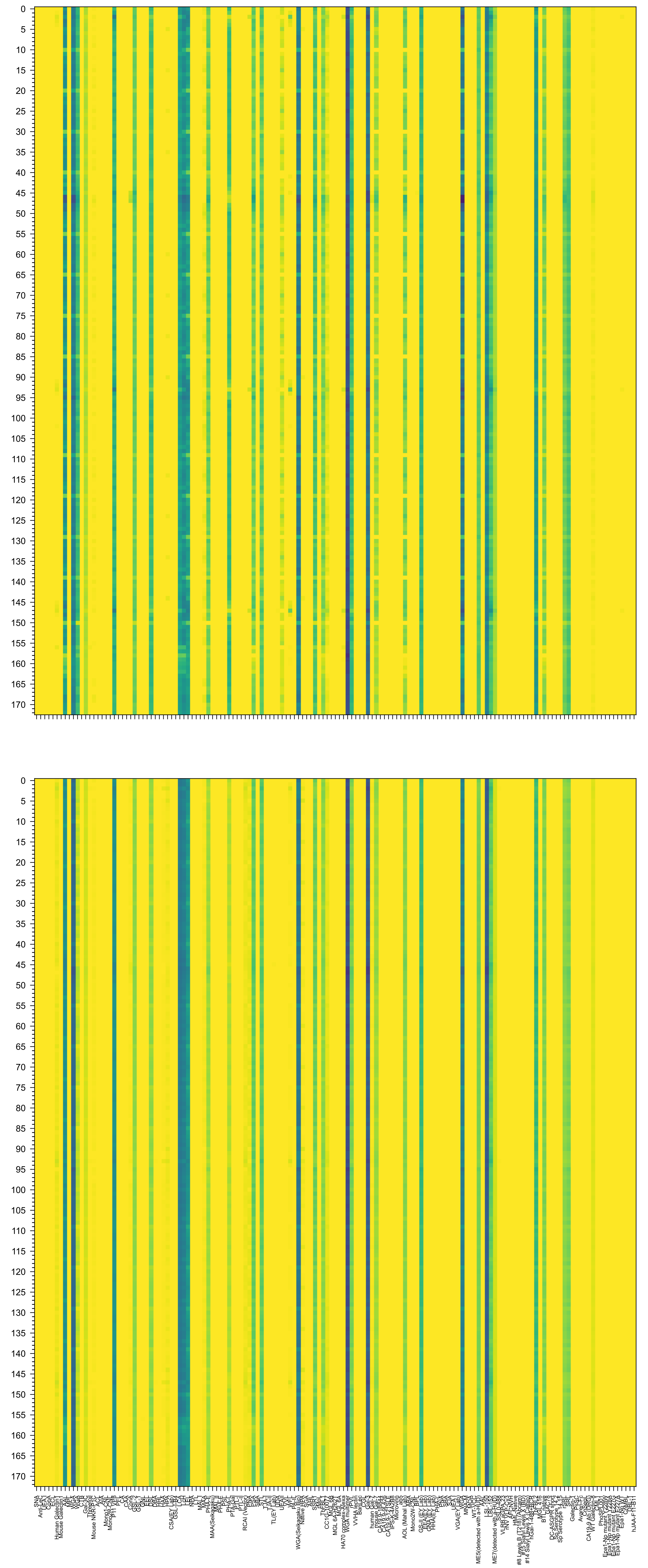

Ac(β1-3)Gal(β1-4)GlcNAc(β1-3)Gal(β1-4)GlcNAc(β1-3)Gal(β1-4)GlcNAc(β1-3)Gal(β1-4)GlcNAc(β1-2)]Man(α1-6)[GlcNAc(β1-3)Gal(β1-4)GlcNAc(β1-3)Gal(β1-4)GlcN

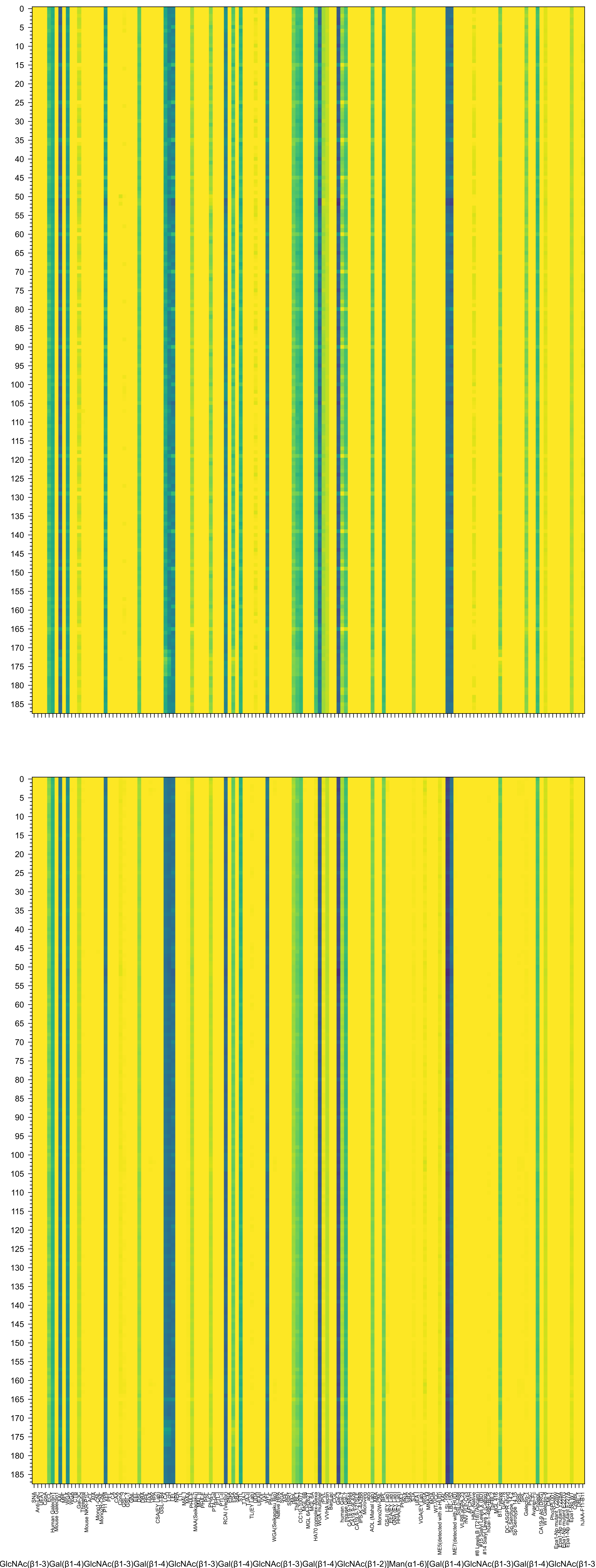

REFERENCES AND NOTES

1. *Essentials of Glycobiology*, A. Varki, R. D. Cummings, J. D. Esko, P. Stanley, G. W. Hart, M. Aebi, D. Mohnen, T. Kinoshita, N. H. Packer, J. H. Prestegard, R. L. Schnaar, P. H. Seeberger, Eds. (Cold Spring Harbor Laboratory Press, NY, ed. 4, 2022).

2. R. Amon, E. M. Reuven, S. Leviatan Ben-Arye, V. Padler-Karavani, Glycans in immune recognition and response. *Carbohydr. Res.* **389**, 115–122 (2014).

3. E. Tecle, P. Gagneux, Sugar-coated sperm: Unraveling the functions of the mammalian sperm glycocalyx. *Mol. Reprod. Dev.* **82**, 635–650 (2015).

4. L. Abril-Parreno, H. Wilkinson, A. Krogenaes, J. Morgan, M. E. Gallagher, C. Reid, X. Druart, S. Fair, R. Saldova, Identification and characterization of O-linked glycans in cervical mucus as biomarkers of sperm transport: A novel sheep model. *Glycobiology* **32**, 23–35 (2022).

5. M. Guberman, P. H. Seeberger, Automated glycan assembly: A perspective. *J. Am. Chem. Soc.* **141**, 5581–5592 (2019).

6. R. A. Laine, A calculation of all possible oligosaccharide isomers both branched and linear yields 1.05 x 10^{12} structures for a reducing hexasaccharide: The isomer barrier to development of single-method saccharide sequencing or synthesis systems. *Glycobiology* **4**, 759–767 (1994).

7. R. A. Laine, Information capacity of the carbohydrate code. *Pure Appl. Chem.* **69**, 1867–1874 (1997).

8. R. D. Cummings, The repertoire of glycan determinants in the human glycome. *Mol. Biosyst.* **5**, 1087–1104 (2009).

9. D. Bojar, L. Meche, G. Meng, W. Eng, D. F. Smith, R. D. Cummings, L. K. Mahal, A useful guide to lectin binding: Machine-learning directed annotation of 57 unique lectin specificities. *ACS Chem. Biol.* **17**, 2993–3012 (2022).

10. D. Bojar, F. Lisacek, Glycoinformatics in the artificial intelligence era. *Chem. Rev.* **122**, 15971–15988 (2022).

11. T. Kuhn, *The Structure of Scientific Revolutions* (The Univ. of Chicago Press, 4th ed., 2012).

12. G. L. Zhang, M. J. Porter, A. K. Awol, B. C. Orsburn, S. W. Canner, J. J. Gray, R. N. O'Meally, R. N. Cole, R. L. Schnaar, The human ganglioside interactome in live cells revealed using clickable photoaffinity ganglioside probes. *J. Am. Chem. Soc.* **146**, 17801–17816 (2024).

13. S. W. Canner, J. J. Gray, paper presented at the *Society for Glycobiology Annual Meeting* (Amelia Island, Florida, 2024).

14. S. W. Canner, R. L. Schnaar, J. J. Gray, Predictions from deep learning propose substantial protein-carbohydrate interplay. bioRxiv 2025.03.07.641884 [Preprint] (2025); https://biorxiv.org/content/10.1101/2025.03.07.641884v2.

15. A. N. Zelensky, J. E. Gready, The C-type lectin-like domain superfamily. *FEBS J.* **272**, 6179–6217 (2005).

16. G. D. Brown, J. A. Willment, L. Whitehead, C-type lectins in immunity and homeostasis. *Nat. Rev. Immunol.* **18**, 374–389 (2018).

17. K. Harrison, A. S. Mackay, L. Kambanis, J. W. C. Maxwell, R. J. Payne, Synthesis and applications of mirror-image proteins. *Nat. Rev. Chem.* **7**, 383–404 (2023).

18. Y. Xu, T. F. Zhu, Mirror-image T7 transcription of chirally inverted ribosomal and functional RNAs. *Science* **378**, 405–412 (2022).

19. Z. Wang, W. Xu, L. Liu, T. F. Zhu, A synthetic molecular system capable of mirror-image genetic replication and transcription. *Nat. Chem.* **8**, 698–704 (2016).

20. M. Wang, W. J. Jiang, X. Y. Liu, J. X. Wang, B. C. Zhang, C. Y. Fan, L. Liu, G. Pena-Alcantara, J. J. Ling, J. Chen, T. F. Zhu, Mirror-image gene transcription and reverse transcription. *Chem. Us* **5**, 848–857 (2019).

21. J. Weidmann, M. Schnolzer, P. E. Dawson, J. D. Hoheisel, Copying life: Synthesis of an enzymatically active mirror-image DNA-ligase made of D-amino acids. *Cell Chem. Biol.* **26**, 645–651.e3 (2019).

22. R. C. Milton, S. C. Milton, S. B. Kent, Total chemical synthesis of a D-enzyme: The enantiomers of HIV-1 protease show reciprocal chiral substrate specificity [corrected]. *Science* **256**, 1445–1448 (1992).

23. C. Liu, P. R. Jalagam, J. Feng, W. Wang, T. Raja, M. R. Sura, R. Manepalli, B. R. Aliphedi, S. Medavarapu, S. K. Nair, V. Muthalagu, R. Natesan, A. Gupta, B. Beno, M. Panda, K. Ghosh, J. K. Shukla, H. Sale, P. Haldar, N. Kalidindi, D. Shah, D. Patel, A. Mathur, B. A. Ellsworth, D. Cheng, A. Regueiro-Ren, Identification of monosaccharide derivatives as potent, selective, and orally bioavailable inhibitors of human and mouse galectin-3. *J. Med. Chem.* **65**, 11084–11099 (2022).

24. P. S. Marinec, K. E. Landgraf, M. Uppalapati, G. Chen, D. Xie, Q. Jiang, Y. Zhao, A. Petriello, K. Deshayes, S. B. H. Kent, D. Ault-Riche, S. S. Sidhu, A non-immunogenic bivalent D-protein potently inhibits retinal vascularization and tumor growth. *ACS Chem. Biol.* **16**, 548–556 (2021).

25. K. P. Adamala, D. Agashe, Y. Belkaid, D. M. de C. Bittencourt, Y. Cai, M. W. Chang, I. A. Chen, G. M. Church, V. S. Cooper, M. M. Davis, N. K. Devaraj, D. Endy, K. M. Esvelt, J. I. Glass, T. W. Hand, T. V. Inglesby, F. J. Isaacs, W. G. James, J. D. G. Jones, M. S. Kay, R. E. Lenski, C. Liu, R. Medzhitov, M. L. Nicotra, S. B. Oehm, J. Pannu, D. A. Relman, P. Schwille, J. A. Smith, H. Suga, J. W. Szostak, N. J. Talbot, J. M. Tiedje, J. C. Venter, G. Winter, W. Zhang, X. Zhu, M. T. Zuber, Confronting risks of mirror life. *Science* **386**, 1351–1353 (2024).

26. R. Derda, K. F. Aoki-Kinoshita, C. S. Bennett, C. R. Bertozzi, D. Bojar, C. D. Castro, T. Feizi, E. D. Goddard-Borger, A. Imberty, B. Imperiali, J. Jimenez-Barbero, D. L. Kasper, L. L. Kiessling, R. A. Laine, N. E. Lewis, F. Lisacek, Y. Liu, T. L. Lowary, M. S. Macauley, L. K. Mahal, A. Molinaro, N. H. Packer, J. C. Paulson, R. J. Payne, M. R. Pratt, C. Rademacher, P. H. Seeberger, S. J. Williams, R. J. Woods, I. Yamada, Remember The Glycans: Consideration of Glycans in Evaluating the Threat of Mirror-Image Life Forms. eLetter reply to "Confronting

Risks of mirror life" K. P. Adamala, D. Agashe, Y. Belkaid, D. M. de C. Bittencourt, Y. Cai, M. W. Chang, I. A. Chen, G. M. Church, V. S. Cooper, M. M. Davis, N. K. Devaraj, D. Endy, K. M. Esvelt, J. I. Glass, T. W. Hand, T. V. Inglesby, F. J. Isaacs, W. G. James, J. D. G. Jones, M. S. Kay, R. E. Lenski, C. Liu, R. Medzhitov, M. L. Nicotra, S. B. Oehm, J. Pannu, D. A. Relman, P. Schwille, J. A. Smith, H. Suga, J. W. Szostak, N. J. Talbot, J. M. Tiedje, J. C. Venter, G. Winter, W. Zhang, X. Zhu, M. T. Zuber. *Science* **386**, (2024).

27. J. Lundstrom, E. Korhonen, F. Lisacek, D. Bojar, LectinOracle: A generalizable deep learning model for lectin-glycan binding prediction. *Adv. Sci. (Weinh)* **9**, e2103807 (2022).

28. S. Mohapatra, J. An, R. Gomez-Bombarelli, Chemistry-informed macromolecule graph representation for similarity computation, unsepervised and supervised learning. *Mach. Learn. Sci. Technol.* **3**, 015028 (2022).

29. M. Xu, Y. Geng, Y. Zhang, L. Yang, GlycanML: A multi-task and multi-structure benchmark for glycan machine learning. arXiv 2405.16206, 1–14 [cs.LG] (2024).

30. M. Hosono, H. Kawauchi, K. Nitta, Y. Takayanagi, H. Shiokawa, R. Mineki, K. Murayama, Three rhamnose-binding lectins from *Osmerus eperlanus mordax* (olive rainbow smelt) roe. *Biol. Pharm. Bull.* **16**, 239–243 (1993).

31. V. Horejsi, V. Matousek, M. Ticha, T. Trnka, in *Sixth International Lectin Meeting* T. C. Bog-Hansen, J. Breborowicz, Eds. (De Gruyter, Poznan, 1984), vol. 4, pp. 297–306.

32. D. Sudakevitz, A. Imberty, N. Gilboa-Garber, Production, properties and specificity of a new bacterial L-fucose- and D-arabinose-binding lectin of the plant aggressive pathogen *Ralstonia solanacearum,* and its comparison to related plant and microbial lectins. *J. Biochem.* **132**, 353–358 (2002).

33. H. Ohashi, T. Ohashi, R. Misaki, K. Fujiyama, Arabidopsis thaliana alpha1,2-l-fucosyltransferase catalyzes the transfer of L-galactose to xyloglucan oligosaccharides. *FEBS Lett.* **593**, 187–194 (2019).

34. R. Shishiuchi, H. Kang, T. Tagami, Y. Ueda, W. Lang, A. Kimura, M. Okuyama, Discovery of α-L-glucosidase raises the possibility of α-L-glucosides in nature. *ACS Omega* **7**, 47411–47423 (2022).

35. C. Zong, A. Venot, X. Li, W. Lu, W. Xiao, J. L. Wilkes, C. L. Salanga, T. M. Handel, L. Wang, M. A. Wolfert, G. J. Boons, Heparan sulfate microarray reveals that heparan sulfate-protein binding exhibits different ligand requirements. *J. Am. Chem. Soc.* **139**, 9534–9543 (2017).

36. L. Wang, A. W. Sorum, B. S. Huang, M. K. Kern, G. Su, N. Pawar, X. Huang, J. Liu, N. L. B. Pohl, L. C. Hsieh-Wilson, Efficient platform for synthesizing comprehensive heparan sulfate oligosaccharide libraries for decoding glycosaminoglycan-protein interactions. *Nat. Chem.* **15**, 1108–1117 (2023).

37. C. Ruprecht, M. P. Bartetzko, D. Senf, A. Lakhina, P. J. Smith, M. J. Soto, H. Oh, J. Y. Yang, D. Chapla, D. Varon Silva, M. H. Clausen, M. G. Hahn, K. W. Moremen, B. R. Urbanowicz, F. Pfrengle, A glycan array-based assay for the identification and characterization of plant glycosyltransferases. *Angew. Chem. Int. Ed. Engl.* **59**, 12493–12498 (2020).

38. D. A. Wesener, K. Wangkanont, R. McBride, X. Song, M. B. Kraft, H. L. Hodges, L. C. Zarling, R. A. Splain, D. F. Smith, R. D. Cummings, J. C. Paulson, K. T. Forest, L. L. Kiessling, Recognition of microbial glycans by human intelectin-1. *Nat. Struct. Mol. Biol.* **22**, 603–610 (2015).

39. S. R. Stowell, C. M. Arthur, R. McBride, O. Berger, N. Razi, J. Heimburg-Molinaro, L. C. Rodrigues, J. P. Gourdine, A. J. Noll, S. von Gunten, D. F. Smith, Y. A. Knirel, J. C. Paulson, R. D. Cummings, Microbial glycan microarrays define key features of host-microbial interactions. *Nat. Chem. Biol.* **10**, 470–476 (2014).

40. R. B. Zheng, S. A. F. Jegouzo, M. Joe, Y. Bai, H. A. Tran, K. Shen, J. Saupe, L. Xia, M. F. Ahmed, Y. H. Liu, P. S. Patil, A. Tripathi, S. C. Hung, M. E. Taylor, T. L. Lowary, K. Drickamer, Insights into interactions of mycobacteria with the host innate immune system from a novel array of synthetic mycobacterial glycans. *ACS Chem. Biol.* **12**, 2990–3002 (2017).

41. A. Geissner, A. Reinhardt, C. Rademacher, T. Johannssen, J. Monteiro, B. Lepenies, M. Thepaut, F. Fieschi, J. Mrazkova, M. Wimmerova, F. Schuhmacher, S. Gotze, D. Grunstein, X. Guo, H. S. Hahm, J. Kandasamy, D. Leonori, C. E. Martin, S. G. Parameswarappa, S. Pasari, M. K. Schlegel, H. Tanaka, G. Xiao, Y. Yang, C. L. Pereira, C. Anish, P. H. Seeberger, Microbe-focused glycan array screening platform. *Proc. Natl. Acad. Sci. U.S.A.* **116**, 1958–1967 (2019).

42. S. V. Benjamin, S. A. F. Jegouzo, C. Lieng, C. Daniels, M. Coispeau, R. J. Lau, S. Kim, Y. Metaxa, J. Philpott, T. Li, C. Dai, X. Wang, M. L. Newby, G. B. Pier, M. Crispin, A. Clements, M. E. Taylor, K. Drickamer, A human lectin array for characterizing host-pathogen interactions. *J. Biol. Chem.* **300**, 107869 (2024).

43. S. Ghosh, R. Chorghade, R. C. Diehl, G. J. Dodge, S. Bae, A. E. Dugan, M. Halim, M. G. Wuo, H. Bartlett, L. Herndon, L. L. Kiessling, B. Imperiali, Glycan analysis probes inspired by human lectins for investigating host-microbe crosstalk. bioRxiv 2024.12.24.630132, 1–39 [Preprint] (2024); https://pubmed.ncbi.nlm.nih.gov/39763805/.

44. J. Eichler, N-glycosylation in Archaea-New roles for an ancient posttranslational modification. *Mol. Microbiol.* **114**, 735–741 (2020).

45. R. Burkholz, J. Quackenbush, D. Bojar, Using graph convolutional neural networks to learn a representation for glycans. *Cell Rep.* **35**, 109251 (2021).

46. A. Alkuhlani, W. Gad, M. Roushdy, A.-B. M. Salem, GNNGLY: Graph neural networks for glycan classification. *IEEE Access* **11**, 51838–51847 (2023).

47. R. Joeres, D. Bojar, Higher-order message passing for glycan representation Learning. arXiv.org 2409.13467, 1–14 [cs.LG] (2024).

48. T. Liu, Y. Lin, X. Wen, R. N. Jorissen, M. K. Gilson, BindingDB: A web-accessible database of experimentally determined protein-ligand binding affinities. *Nucleic Acids Res.* **35**, D198–D201 (2007).

49. B. Zdrazil, E. Felix, F. Hunter, E. J. Manners, J. Blackshaw, S. Corbett, M. de Veij, H. Ioannidis, D. M. Lopez, J. F. Mosquera, M. P. Magarinos, N. Bosc, R. Arcila, T. Kiziloren, A. Gaulton, A. P. Bento, M. F. Adasme, P. Monecke, G. A. Landrum, A. R. Leach, The ChEMBL Database in 2023: A drug discovery platform spanning multiple bioactivity data types and time periods. *Nucleic Acids Res.* **52**, D1180–D1192 (2024).

50. R. J. Woods, Predicting the structures of glycans, glycoproteins, and their complexes. *Chem. Rev.* **118**, 8005–8024 (2018).

51. M. Agostino, E. Yuriev, P. A. Ramsland, A computational approach for exploring carbohydrate recognition by lectins in innate immunity. *Front. Immunol.* **2**, 23 (2011).

52. A. Ranaudo, M. Giulini, A. Pelissou Ayuso, A. Bonvin, Modeling protein-glycan interactions with HADDOCK. *J. Chem. Inf. Model.* **64**, 7816–7825 (2024).

53. C. B. Anfinsen, Principles that govern the folding of protein chains. *Science* **181**, 223–230 (1973).

54. J. Jumper, R. Evans, A. Pritzel, T. Green, M. Figurnov, O. Ronneberger, K. Tunyasuvunakool, R. Bates, A. Zidek, A. Potapenko, A. Bridgland, C. Meyer, S. A. A. Kohl, A. J. Ballard, A. Cowie, B. Romera-Paredes, S. Nikolov, R. Jain, J. Adler, T. Back, S. Petersen, D. Reiman, E. Clancy, M. Zielinski, M. Steinegger, M. Pacholska, T. Berghammer, S. Bodenstein, D. Silver, O. Vinyals, A. W. Senior, K. Kavukcuoglu, P. Kohli, D. Hassabis, Highly accurate protein structure prediction with AlphaFold. *Nature* **596**, 583–589 (2021).

55. P. Bryant, A. Elofsson, Peptide binder design with inverse folding and protein structure prediction. *Commun. Chem.* **6**, 229 (2023).

56. Z. L. Klamer, C. M. Harris, J. M. Beirne, J. E. Kelly, J. Zhang, B. B. Haab, CarboGrove: A resource of glycan-binding specificities through analyzed glycan-array datasets from all platforms. *Glycobiology* **32**, 679–690 (2022).

57. K. Hashimoto, I. Takigawa, M. Shiga, M. Kanehisa, H. Mamitsuka, Mining significant tree patterns in carbohydrate sugar chains. *Bioinformatics* **24**, i167–i173 (2008).

58. K. F. Aoki-Kinoshita, N. Ueda, H. Mamitsuka, M. Kanehisa, ProfilePSTMM: Capturing tree-structure motifs in carbohydrate sugar chains. *Bioinformatics* **22**, e25–e34 (2006).

59. E. J. Carpenter, S. Seth, N. Yue, R. Greiner, R. Derda, GlyNet: A multi-task neural network for predicting protein-glycan interactions. *Chem. Sci.* **13**, 6669–6686 (2022).

60. D. Bojar, R. K. Powers, D. M. Camacho, J. J. Collins, Deep-learning resources for studying glycan-mediated host-microbe interactions. *Cell Host Microbe* **29**, 132–144.e3 (2021).

61. A. Chatterjee, R. Walters, Z. Shafi, O. S. Ahmed, M. Sebek, D. Gysi, R. Yu, T. Eliassi-Rad, A. L. Barabasi, G. Menichetti, Improving the generalizability of protein-ligand binding predictions with AI-Bind. *Nat. Commun.* **14**, 1989 (2023).

62. M. Xu, Z. Zhang, J. Lu, Z. Zhu, Y. Zhang, M. Chang, R. Liu, J. Tang, PEER: A Comprehensive and Multi-Task Benchmark for Protein Understanding. *Advances in Neural Information Processing Systems 35 (NeurIPS 2022)*, [cs.LG] (2022); https://arxiv.org/abs/2206.02096.

63. Y. Akune, M. Hosoda, S. Kaiya, D. Shinmachi, K. F. Aoki-Kinoshita, The RINGS resource for glycome informatics analysis and data mining on the Web. *OMICS* **14**, 475–486 (2010).

64. K. F. Aoki-Kinoshita, Mining frequent subtrees in glycan data using the RINGS glycan miner tool. *Methods Mol. Biol.* **939**, 87–95 (2013).

65. Z. Klamer, B. Staal, A. R. Prudden, L. Liu, D. F. Smith, G. J. Boons, B. Haab, Mining high-complexity motifs in glycans: A new language to uncover the fine specificities of lectins and glycosidases. *Anal. Chem.* **89**, 12342–12350 (2017).

66. S. Vinga, J. Almeida, Alignment-free sequence comparison–A review. *Bioinformatics* **19**, 513–523 (2003).

67. Y. Qian, Y. Zhang, J. Zhang, Alignment-free sequence comparison with multiple k values. *IEEE/ACM Trans. Comput. Biol. Bioinform.* **18**, 1841–1849 (2021).

68. D. P. Kingma, J. Ba, Adam: A Method for Stochastic Optimization. *Proceedings of the 3rd International Conference on Learning Representations (ICLR)*, [cs.LG] (2014); https://arxiv.org/abs/1412.6980.

69. A. D. Ho, S. C. Wu, N. A. Kamili, A. V. Blenda, R. D. Cummings, S. R. Stowell, C. M. Arthur, An automated approach to assess relative galectin-glycan affinity following glycan microarray analysis. *Front. Mol. Biosci.* **9**, 893185 (2022).

70. Z. Klamer, B. Haab, Combined analysis of multiple glycan-array datasets: New explorations of protein-glycan interactions. *Anal. Chem.* **93**, 10925–10933 (2021).

71. T. Kalliokoski, C. Kramer, A. Vulpetti, P. Gedeck, Comparability of mixed IC_{50} data–A statistical analysis. *PLOS ONE* **8**, e61007 (2013).

72. G. A. Landrum, S. Riniker, Combining IC_{50} or K_i values from different sources is a source of significant noise. *J. Chem. Inf. Model.* **64**, 1560–1567 (2024).

73. A. Horovitz, A. Levitzki, An accurate method for determination of receptor-ligand and enzyme-inhibitor dissociation constants from displacement curves. *Proc. Natl. Acad. Sci. U.S.A.* **84**, 6654–6658 (1987).

74. T. Y. Wang, H. Ji, D. Everton, A. T. H. Le, S. M. Krylova, R. Fournier, S. N. Krylov, Fundamental determinants of the accuracy of equilibrium constants for affinity complexes. *Anal. Chem.* **95**, 15826–15832 (2023).

75. C. Kramer, T. Kalliokoski, P. Gedeck, A. Vulpetti, The experimental uncertainty of heterogeneous public K_i data. *J. Med. Chem.* **55**, 5165–5173 (2012).

76. K. Shams-Ud-Doha, E. N. Kitova, P. I. Kitov, Y. St-Pierre, J. S. Klassen, Human milk oligosaccharide specificities of human galectins. Comparison of electrospray ionization mass spectrometry and glycan microarray screening results. *Anal. Chem.* **89**, 4914–4921 (2017).

77. K. Bachhawat-Sikder, C. J. Thomas, A. Surolia, Thermodynamic analysis of the binding of galactose and poly-*N*-acetyllactosamine derivatives to human galectin-3. *FEBS Lett.* **500**, 75–79 (2001).

78. T. Q. Chen, C. Guestrin, XGBoost: A scalable tree boosting system. *KDD '16: Proceedings of the 22nd ACM SIGKDD International Conference on Knowledge Discovery and Data Mining*, 785–794 (2016).

79. S. Ghosh, R. Chorghade, R. C. Diehl, G. J. Dodge, S. Bae, A. E. Dugan, M. Halim, M. G. Wuo, H. Bartlett, L. Herndon, L. L. Kiessling, B. Imperiali, Tools for investigating host-microbe crosstalk using glycan analysis probes inspired by human lectins. *Glycobiology* **35**, cwaf031 (2025).

80. S. C. Wu, A. D. Ho, N. A. Kamili, J. Wang, K. L. Murdock, R. D. Cummings, C. M. Arthur, S. R. Stowell, Full-length galectin-3 is required for high affinity microbial interactions and antimicrobial activity. *Front. Microbiol.* **12**, 731026 (2021).

81. S. C. Wu, H. M. Jan, M. L. Vallecillo-Zuniga, M. F. Rathgeber, C. S. Stowell, K. L. Murdock, K. R. Patel, H. Nakahara, C. J. Stowell, M. H. Nahm, C. M. Arthur, R. D. Cummings, S. R. Stowell, Whole microbe arrays accurately predict interactions and overall antimicrobial activity of galectin-8 toward distinct strains of *Streptococcus pneumoniae*. *Sci. Rep.* **13**, 5324 (2023).

82. Y. Yoshikai, T. Mizuno, S. Nemoto, H. Kusuhara, Difficulty in chirality recognition for Transformer architectures learning chemical structures from string representations. *Nat. Commun.* **15**, 1197 (2024).

83. T. N. Schumacher, L. M. Mayr, D. L. Minor Jr., M. A. Milhollen, M. W. Burgess, P. S. Kim, Identification of D-peptide ligands through mirror-image phage display. *Science* **271**, 1854–1857 (1996).

84. M. Sojitra, S. Sarkar, J. Maghera, E. Rodrigues, E. J. Carpenter, S. Seth, D. Ferrer Vinals, N. J. Bennett, R. Reddy, A. Khalil, X. Xue, M. R. Bell, R. B. Zheng, P. Zhang, C. Nycholat, J. J. Bailey, C. C. Ling, T. L. Lowary, J. C. Paulson, M. S. Macauley, R. Derda, Genetically encoded multivalent liquid glycan array displayed on M13 bacteriophage. *Nat. Chem. Biol.* **17**, 806–816 (2021).

85. K. T. Pilobello, D. E. Slawek, L. K. Mahal, A ratiometric lectin microarray approach to analysis of the dynamic mammalian glycome. *Proc. Natl. Acad. Sci. U.S.A.* **104**, 11534–11539 (2007).

86. C. R. Woese, G. E. Fox, Phylogenetic structure of the prokaryotic domain: The primary kingdoms. *Proc. Natl. Acad. Sci. U.S.A.* **74**, 5088–5090 (1977).

87. F. Rohden, J. D. Hoheisel, H. J. Wieden, Through the looking glass: Milestones on the road towards mirroring life. *Trends Biochem. Sci.* **46**, 931–943 (2021).

88. I. J. Goldstein, C. E. Hollerman, E. E. Smith, Protein-carbohydrate interaction. II. Inhibition studies on the interaction of concanavalin a with polysaccharides. *Biochemistry* **4**, 876–883 (1965).

89. T. Terada, Y. Watanabe, H. Tateno, T. Naganuma, T. Ogawa, K. Muramoto, H. Kamiya, Structural characterization of a rhamnose-binding glycoprotein (lectin) from Spanish mackerel (*Scomberomorous niphonius*) eggs. *Biochim. Biophys. Acta* **1770**, 617–629 (2007).

90. T. O. Mizgina, I. V. Chikalovets, T. A. Bulanova, V. I. Molchanova, A. P. Filshtein, R. H. Ziganshin, E. A. Rogozhin, N. V. Shilova, O. V. Chernikov, New L-rhamnose-binding lectin from the bivalve *Glycymeris yessoensis*: Purification, partial structural characterization and antibacterial activity. *Mar. Drugs* **22**, 27 (2024).

91. Y. Watanabe, H. Tateno, S. Nakamura-Tsuruta, J. Kominami, J. Hirabayashi, O. Nakamura, T. Watanabe, H. Kamiya, T. Naganuma, T. Ogawa, R. J. Naude, K. Muramoto, The function of rhamnose-binding lectin in innate immunity by restricted binding to Gb3. *Dev. Comp. Immunol.* **33**, 187–197 (2009).

92. J. H. Wong, T. B. Ng, Isolation and characterization of a glucose/mannose/rhamnose-specific lectin from the knife bean Canavalia gladiata. *Arch. Biochem. Biophys.* **439**, 91–98 (2005).

93. G. Glashagen, S. de Vries, U. Uciechowska-Kaczmarzyk, S. A. Samsonov, S. Murail, P. Tuffery, M. Zacharias, Coarse-grained and atomic resolution biomolecular docking with the ATTRACT approach. *Proteins* **88**, 1018–1028 (2020).

94. M. L. Nance, J. W. Labonte, J. Adolf-Bryfogle, J. J. Gray, Development and evaluation of GlycanDock: A protein-glycoligand docking refinement algorithm in Rosetta. *J. Phys. Chem. B* **125**, 6807–6820 (2021).

95. S. E. Mottarella, D. Beglov, N. Beglova, M. A. Nugent, D. Kozakov, S. Vajda, Docking server for the identification of heparin binding sites on proteins. *J. Chem. Inf. Model.* **54**, 2068–2078 (2014).

96. E. D. Boittier, J. M. Burns, N. S. Gandhi, V. Ferro, GlycoTorch Vina: Docking designed and tested for glycosaminoglycans. *J. Chem. Inf. Model.* **60**, 6328–6343 (2020).

97. J. W. Labonte, J. Adolf-Bryfogle, W. R. Schief, J. J. Gray, Residue-centric modeling and design of saccharide and glycoconjugate structures. *J. Comput. Chem.* **38**, 276–287 (2017).

98. A. K. Nivedha, D. F. Thieker, S. Makeneni, H. Hu, R. J. Woods, Vina-Carb: Improving glycosidic angles during carbohydrate docking. *J. Chem. Theory Comput.* **12**, 892–901 (2016).

99. M. S. Ardejani, L. Noodleman, E. T. Powers, J. W. Kelly, Stereoelectronic effects in stabilizing protein-*N*-glycan interactions revealed by experiment and machine learning. *Nat. Chem.* **13**, 480–487 (2021).

100. K. L. Hudson, G. J. Bartlett, R. C. Diehl, J. Agirre, T. Gallagher, L. L. Kiessling, D. N. Woolfson, Carbohydrate-aromatic interactions in proteins. *J. Am. Chem. Soc.* **137**, 15152–15160 (2015).

101. F. Pedregosa, G. Varoquaux, A. Gramfort, V. Michel, B. Thirion, O. Grisel, M. Blondel, P. Prettenhofer, R. Weiss, V. Dubourg, J. Vanderplas, A. Passos, D. Cournapeau, M. Brucher, M. Perrot, E. Duchesnay, Scikit-learn: Machine learning in python. *J. Mach. Learn. Res.* **12**, 2825–2830 (2011).

102. L. Breiman, Random forests. *Mach. Learn.* **45**, 5–32 (2001).

103. M. Sojitra, E. N. Schmidt, G. M. Lima, E. J. Carpenter, K. A. McCord, A. Atrazhev, M. S. Macauley, R. Derda, Measuring carbohydrate recognition profile of lectins on live cells using liquid glycan array (LiGA). *Nat. Protoc.* **20**, 989–1019 (2025).

104. C. L. Lin, M. Sojitra, E. J. Carpenter, E. S. Hayhoe, S. Sarkar, E. A. Volker, C. Wang, D. T. Bui, L. Yang, J. S. Klassen, P. Wu, M. S. Macauley, T. L. Lowary, R. Derda, Chemoenzymatic synthesis of genetically-encoded multivalent liquid *N*-glycan arrays. *Nat. Commun.* **14**, 5237 (2023).

105. J. C. Manimala, Z. Li, A. Jain, S. VedBrat, J. C. Gildersleeve, Carbohydrate array analysis of anti-Tn antibodies and lectins reveals unexpected specificities: Implications for diagnostic and vaccine development. *Chembiochem* **6**, 2229–2241 (2005).

106. B. Becker, R. H. Furneaux, F. Reck, O. A. Zubkov, A simple synthesis of 8-(methoxycarbonyl)octyl 3,6-di-O-(alpha-D-mannopyranosyl)-alpha-D-mannopyranoside and derivatives and their use in the preparation of neoglycoconjugates. *Carbohydr. Res.* **315**, 148–158 (1999).

107. M. Ohta, F. Matsuura, Y. Kobayashi, S. Shigeta, K. Ono, S. Oka, Further characterization of allergenically active oligosaccharitols isolated from a sea squirt H-antigen. *Arch. Biochem. Biophys.* **290**, 474–483 (1991).

108. W. L. Matochko, S. Cory Li, S. K. Tang, R. Derda, Prospective identification of parasitic sequences in phage display screens. *Nucleic Acids Res.* **42**, 1784–1798 (2014).

109. B. A. Salameh, I. Cumpstey, A. Sundin, H. Leffler, U. J. Nilsson, 1*H*-1,2,3-triazol-1-yl thiodigalactoside derivatives as high affinity galectin-3 inhibitors. *Bioorg. Med. Chem.* **18**, 5367–5378 (2010).

110. M. Mahanti, K. B. Pal, A. P. Sundin, H. Leffler, U. J. Nilsson, Epimers switch galectin-9 domain selectivity: 3*N*-aryl galactosides bind the C-terminal and gulosides bind the N-terminal. *ACS Med. Chem. Lett.* **11**, 34–39 (2020).

111. K. Peterson, R. Kumar, O. Stenstrom, P. Verma, P. R. Verma, M. Hakansson, B. Kahl-Knutsson, F. Zetterberg, H. Leffler, M. Akke, D. T. Logan, U. J. Nilsson, Systematic tuning of fluoro-galectin-3 interactions provides thiodigalactoside derivatives with single-digit nM affinity and high selectivity. *J. Med. Chem.* **61**, 1164–1175 (2018).

112. T. Delaine, I. Cumpstey, L. Ingrassia, M. Le Mercier, P. Okechukwu, H. Leffler, R. Kiss, U. J. Nilsson, Galectin-inhibitory thiodigalactoside ester derivatives have antimigratory effects in cultured lung and prostate cancer cells. *J. Med. Chem.* **51**, 8109–8114 (2008).

113. V. K. Rajput, H. Leffler, U. J. Nilsson, B. Mukhopadhyay, Synthesis and evaluation of iminocoumaryl and coumaryl derivatized glycosides as galectin antagonists. *Bioorg. Med. Chem. Lett.* **24**, 3516–3520 (2014).

114. A. Dahlqvist, F. R. Zetterberg, H. Leffler, U. J. Nilsson, Aminopyrimidine-galactose hybrids are highly selective galectin-3 inhibitors. *Medchemcomm* **10**, 913–925 (2019).

115. V. K. Rajput, A. MacKinnon, S. Mandal, P. Collins, H. Blanchard, H. Leffler, T. Sethi, H. Schambye, B. Mukhopadhyay, U. J. Nilsson, A selective galactose-coumarin-derived galectin-3 inhibitor demonstrates involvement of galectin-3-glycan interactions in a pulmonary fibrosis model. *J. Med. Chem.* **59**, 8141–8147 (2016).

116. T. Murakami, K. Yoshioka, Y. Sato, M. Tanaka, O. Niwa, S. Yabuki, Synthesis and galectin-binding activities of mercaptododecyl glycosides containing a terminal beta-galactosyl group. *Bioorg. Med. Chem. Lett.* **21**, 1265–1269 (2011).

117. C. T. Oberg, H. Blanchard, H. Leffler, U. J. Nilsson, Protein subtype-targeting through ligand epimerization: Talose-selectivity of galectin-4 and galectin-8. *Bioorg. Med. Chem. Lett.* **18**, 3691–3694 (2008).

118. H. van Hattum, H. M. Branderhorst, E. E. Moret, U. J. Nilsson, H. Leffler, R. J. Pieters, Tuning the preference of thiodigalactoside- and lactosamine-based ligands to galectin-3 over galectin-1. *J. Med. Chem.* **56**, 1350–1354 (2013).

119. P. Sorme, Y. Qian, P. G. Nyholm, H. Leffler, U. J. Nilsson, Low micromolar inhibitors of galectin-3 based on 3′-derivatization of N-acetyllactosamine. *Chembiochem* **3**, 183–189 (2002).

120. F. R. Zetterberg, K. Peterson, R. E. Johnsson, T. Brimert, M. Hakansson, D. T. Logan, H. Leffler, U. J. Nilsson, Monosaccharide derivatives with low-nanomolar lectin affinity and high selectivity based on combined fluorine-amide, phenyl-arginine, sulfur-π, and halogen bond interactions. *ChemMedChem* **13**, 133–137 (2018).

121. D. Giguere, M. A. Bonin, P. Cloutier, R. Patnam, C. St-Pierre, S. Sato, R. Roy, Synthesis of stable and selective inhibitors of human galectins-1 and -3. *Bioorg. Med. Chem.* **16**, 7811–7823 (2008).

122. M. F. Marchiori, D. E. Souto, L. O. Bortot, J. F. Pereira, L. T. Kubota, R. D. Cummings, M. Dias-Baruffi, I. Carvalho, V. L. Campo, Synthetic 1,2,3-triazole-linked glycoconjugates bind with high affinity to human galectin-3. *Bioorg. Med. Chem.* **23**, 3414–3425 (2015).

123. C. T. Oberg, H. Leffler, U. J. Nilsson, Arginine binding motifs: Design and synthesis of galactose-derived arginine tweezers as galectin-3 inhibitors. *J. Med. Chem.* **51**, 2297–2301 (2008).

124. T. Delaine, P. Collins, A. MacKinnon, G. Sharma, J. Stegmayr, V. K. Rajput, S. Mandal, I. Cumpstey, A. Larumbe, B. A. Salameh, B. Kahl-Knutsson, H. van Hattum, M. van Scherpenzeel, R. J. Pieters, T. Sethi, H. Schambye, S. Oredsson, H. Leffler, H. Blanchard, U. J. Nilsson, Galectin-3-binding glycomimetics that strongly reduce bleomycin-induced lung fibrosis and modulate intracellular glycan recognition. *Chembiochem* **17**, 1759–1770 (2016).

125. A. C. Gibbs, Elements and modulation of functional dynamics. *J. Med. Chem.* **57**, 7819–7837 (2014).

126. S. Carlsson, C. T. Oberg, M. C. Carlsson, A. Sundin, U. J. Nilsson, D. Smith, R. D. Cummings, J. Almkvist, A. Karlsson, H. Leffler, Affinity of galectin-8 and its carbohydrate

recognition domains for ligands in solution and at the cell surface. *Glycobiology* **17**, 663–676 (2007).

127. R. P. Dings, M. C. Miller, I. Nesmelova, L. Astorgues-Xerri, N. Kumar, M. Serova, X. Chen, E. Raymond, T. R. Hoye, K. H. Mayo, Antitumor agent calixarene 0118 targets human galectin-1 as an allosteric inhibitor of carbohydrate binding. *J. Med. Chem.* **55**, 5121–5129 (2012).
